# Multi-Omics Analysis to Characterize Cigarette Smoke Induced Molecular Alterations in Esophageal Cells

**DOI:** 10.3389/fonc.2020.01666

**Published:** 2020-11-05

**Authors:** Aafaque Ahmad Khan, Krishna Patel, Shankargouda Patil, Niraj Babu, Kiran K. Mangalaparthi, Hitendra Singh Solanki, Vishalakshi Nanjappa, Anjali Kumari, Malini Manoharan, Coral Karunakaran, Saktivel Murugan, Bipin Nair, Rekha V. Kumar, Manjusha Biswas, David Sidransky, Ravi Gupta, Rohit Gupta, Arati Khanna-Gupta, Prashant Kumar, Aditi Chatterjee, Harsha Gowda

**Affiliations:** ^1^Institute of Bioinformatics, International Technology Park, Bangalore, India; ^2^Cell Biology Program, The Hospital for Sick Children, Toronto, ON, Canada; ^3^Amrita School of Biotechnology, Amrita Vishwa Vidyapeetham, Kollam, India; ^4^Division of Oral Pathology, Department of Maxillofacial Surgery and Diagnostic Sciences, College of Dentistry, Jazan University, Jazan, Saudi Arabia; ^5^Department of Medical Biotechnologies, School of Dental Medicine, University of Siena, Siena, Italy; ^6^Manipal Academy of Higher Education, Manipal, India; ^7^Medgenome Labs Pvt. Ltd., Bangalore, India; ^8^Department of Pathology, Kidwai Memorial Institute of Oncology, Bangalore, India; ^9^Department of Molecular Pathology, Mitra Biotech, Bangalore, India; ^10^Department of Otolaryngology-Head and Neck Surgery, Johns Hopkins University School of Medicine, Baltimore, MD, United States; ^11^Genetics and Computational Biology, QIMR Berghofer Medical Research Institute, Brisbane, QLD, Australia

**Keywords:** esophageal squamous cell carcinoma, cigarette smoke, mass spectrometry, tandem mass tag, next generation sequencing, DNA repair

## Abstract

Though smoking remains one of the established risk factors of esophageal squamous cell carcinoma, there is limited data on molecular alterations associated with cigarette smoke exposure in esophageal cells. To investigate molecular alterations associated with chronic exposure to cigarette smoke, non-neoplastic human esophageal epithelial cells were treated with cigarette smoke condensate (CSC) for up to 8 months. Chronic treatment with CSC increased cell proliferation and invasive ability of non-neoplastic esophageal cells. Whole exome sequence analysis of CSC treated cells revealed several mutations and copy number variations. This included loss of high mobility group nucleosomal binding domain 2 (HMGN2) and a missense variant in mediator complex subunit 1 (MED1). Both these genes play an important role in DNA repair. Global proteomic and phosphoproteomic profiling of CSC treated cells lead to the identification of 38 differentially expressed and 171 differentially phosphorylated proteins. Bioinformatics analysis of differentially expressed proteins and phosphoproteins revealed that most of these proteins are associated with DNA damage response pathway. Proteomics data revealed decreased expression of HMGN2 and hypophosphorylation of MED1. Exogenous expression of HMGN2 and MED1 lead to decreased proliferative and invasive ability of smoke exposed cells. Immunohistochemical labeling of HMGN2 in primary ESCC tumor tissue sections (from smokers) showed no detectable expression while strong to moderate staining of HMGN2 was observed in normal esophageal tissues. Our data suggests that cigarette smoke perturbs expression of proteins associated with DNA damage response pathways which might play a vital role in development of ESCC.

## Introduction

In United States, more than 50% of esophageal cancer deaths are attributed to smoking (1). Cigarette smoke contains a mixture of thousands of compounds, including more than 70 known carcinogens (2–4). Studies have shown that carcinogens in cigarette smoke lead to p53 mutations, aberrant cell cycle regulation and are associated with DNA damage, which plays key role in development of cancer (2, 5, 6). Most carcinogens in cigarette smoke require metabolic activation process that enable them to bind to DNA and form DNA adducts (7). Normally, cellular repair systems remove these adducts and revert DNA to its normal state (8). However, persistent miscoding of adducts compromises DNA repair systems resulting in accumulation of mutations in the genome. Multiple studies have indicated that cigarette smoke derived DNA adducts are responsible for miscoding mutations (9–11). Mutations in tumor suppressor genes or oncogenes enables uncontrolled growth and aid in development of cancer (12). Alterations at the genomic, transcriptomic and proteomic level has been reported in both smokers and non-smokers (13–15). Despite these early studies, molecular alterations associated with cigarette smoke exposure that leads to malignancy are poorly understood.

In this study, we developed an *in vitro* cellular model where non-neoplastic esophageal cell line Het1A was chronically treated with cigarette smoke condensate (CSC). To understand molecular alterations associated with chronic treatment with CSC, we carried out whole exome sequencing and mass spectrometry based quantitative proteomic and phosphoproteomic analysis.

## Materials and Methods

### Cell Culture

Non-neoplastic esophageal epithelial cells, Het-1A, were procured from ATCC (ATCC CRL-2692). Cells were cultured and maintained in keratinocyte serum free medium (KSFM) supplemented with bovine pituitary extract (25 μg/ml), epidermal growth factor (EGF) (0.2 ng/ml) (ThermoFisher Scientific, MA), 1% penicillin/streptomycin and CaCl_2_ (0.4 mM). The cells were cultured at 37°C in a humidified air incubator with 5% CO_2_.

### Preparation of Cigarette Smoke Condensate and Treatment of Cells

Cigarette smoke condensate (CSC) was purchased from Murty Pharmaceuticals, Inc., KY. To study the effect, Het1A cells were chronically treated with 0.1% CSC (Murty Pharmaceuticals, Inc., KY) for 8 months. Cells cultured without any treatment with CSC were labeled as Het-1A-Parental. Cells chronically treated with CSC were maintained and passaged in a dedicated incubator for 2, 4, 6, and 8 months (Het-1A-Smoke-2M, Het-1A-Smoke-4M, Het-1A-Smoke-6M, and Het-1A-Smoke-8M). Both parental and CSC treated cells were cultured and passaged simultaneously for the same duration. Henceforth, Het-1A cells treated with CSC will be referred as Het-1A-Smoke or CSC treated cells while parental Het-1A cells will be referred as Het-1A-Parental or untreated cells.

### Cell Proliferation Assays

Het-1A-Parental, Het-1A-Smoke-2M, Het-1A-Smoke-4M, Het-1A-Smoke-6M, and Het-1A-Smoke-8M cells were seeded at a density of 20 × 10^3^ cells per well in 6-well plates. Cell proliferation was monitored for 8 days and the cells were counted every 48 h using trypan blue exclusion method. All assays were performed in replicates and repeated thrice.

### Cell Invasion Assays

Invasion assays were carried out as described previously (16). Cell invasion assays were performed in a transwell system (BD Biosciences, San Jose, CA) with Matrigel (BD Biosciences, San Jose, CA) coated filters for Het-1A-Parental and Het-1A CSC treated cells (2M, 4M, 6M, and 8 M). Cell invasion was evaluated after 48 h. Briefly, invasiveness of cells was assayed in membrane invasion culture system using polyethylene terephthalate (PET) membrane (8-μm pore size) (BD Biosciences, San Jose, CA) coated with Matrigel. The cells were seeded at a density of 20 × 10^3^ in 500 μl of serum free media on the Matrigel-coated PET membrane in the upper compartment and placed in a compartment filled with complete growth media. All plates were incubated at 37°C for 48 h. Post-incubation, upper surface of the membrane was wiped with a cotton-tip applicator to remove non-migratory cells. Cells that migrated to the lower side of the membrane were fixed and stained using 4% methylene blue. All experiments were performed in triplicate and repeated thrice. Ten random fields were counted and average was plotted.

### Western Blot Analysis

Cells were grown to 80% confluence and were harvested in RIPA lysis buffer (10 mM Tris pH 7.4, 150 mM NaCl, 5 mM EDTA, 1% Triton-X-100, 0.1% SDS containing protease and phosphatase inhibitor cocktails) and sonicated. Briefly, 30 μg of cell lysate was resolved by SDS-PAGE and transferred onto nitrocellulose membrane. The membrane was blocked with 5% non-fat dry milk in PBS-T and incubated overnight with primary antibody followed by secondary antibody incubation for 1 h. Proteins on the membrane were visualized using enhanced chemiluminescence detection kit as per manufacturer's instructions. β-Actin was used as loading control for all Western blots. Antibodies for Bcl-2 and Bcl-xL, Bax, caspase-3, caspase-7, and caspase-9 were purchased from Cell Signaling Technology (Cell Signaling Technology, Danvers, MA). β-actin antibody was procured from Sigma (St. Louis, MO).

### Whole Exome Sequence Analysis

Het-1A-Parental and Het-1A-Smoke-8M cells were grown and harvested at 80% confluence and genomic DNA was extracted. The DNA library for exome sequencing was prepared using Agilent SureSelectXT Human All Exon V5 kit as per manufacturer's instructions. Paired-end sequencing was performed on the Illumina HiSeq 2500 with read length of 100 bp and data was acquired in FASTQ format. The quality of raw reads was assessed using FastQC (17). The paired end clean reads were aligned against reference genome hg19 (GRCh37) using Burrows-Wheeler alignment (BWA, version 0.7.12) (18). Post-alignment, we processed binary map alignment file using MarkDuplicates of Picard tools, indel realignment using IndelRealigner, and base recalibration using BaseRecalibrator of GATK tool suite (the Genome Analysis Toolkit, Broad Institute) (19, 20). Somatic variants were identified using Strelka with default parameters (21). The somatic variants identified were then annotated using *in-house* tool VariMAT and OncoMD. VariMAT is a comprehensive annotation tool that provides variant annotation and integrates multiple databases, such as dbSNP, ExAC, 1,000-genome-phase3, OncoMD, and COSMIC. OncoMD is a database of somatic mutations collected from published literature and large databases, such as TCGA (22). VariMAT also integrates multiple variant consequence predictors, such as SIFT, CONDEL, LRT, and others (23–25). OncoCNV was used to identify copy number alterations using a *P*-value cutoff of 0.000001 with a ≤1-fold change for deletion events and ≥3-fold for amplification.

### Sample Preparation

Het-1A-Parental, Het-1A-Smoke-2M, Het-1A-Smoke-4M, Het-1A-Smoke-6M, and Het-1A-Smoke-8M cells were grown to 80% confluence and serum starved for 12 h. Cells were washed with 1× PBS thrice and harvested in lysis buffer [2% SDS, 5 mM sodium fluoride, 1 mM β-glycerophosphate, 1 mM sodium orthovanadate in 50 mM Triethyl ammonium bicarbonate (TEABC)]. The cell lysates were sonicated, centrifuged and protein concentration was measured using BCA method.

### In-Solution Digestion and TMT Labeling

In-solution trypsin digestion of samples from both conditions was carried out as described previously (26). Equal amounts of protein lysate from all conditions were reduced using 5 mM dithiothreitol (DTT) and incubated at 60°C for 45 min. The reduced protein lysate was alkylated using iodoacetamide (IAA) (20 mM) and incubated for 15 min in dark at room temperature. To remove SDS, samples were buffer exchanged with 8M urea followed by 50 mM TEABC. Proteins were digested using sequencing grade trypsin (Promega, Madison, WI) at an enzyme to substrate ratio of 1:20. Trypsin digestion was carried out at 37°C for 16 h. The digested peptides were lyophilized and labeled with Tandem Mass Tag (TMT) reagents as per manufacturers' instructions. Briefly, peptide samples were dissolved in 50 mM TEABC (pH 8.0) and added to TMT reagents dissolved in anhydrous acetonitrile. Het-1A-Parental, Het-1A-Smoke-2M, Het-1A-Smoke-4M, Het-1A-Smoke-6M, and Het-1A-Smoke-8M cells were labeled with TMT tags as detailed in Supplementary Table 1. After incubation at room temperature for 1 h, the reaction was quenched with 5% hydroxylamine. Ten percent of each labeled sample was used for global proteomic profiling and remaining sample was used for phosphoproteomic analysis. All the labeled samples were pooled and subjected to fractionation.

### Basic pH Reversed Phase Liquid Chromatography (bRPLC)

Pooled samples were fractionated on 1,290 infinity High Pressure Liquid Chromatography system (Agilent, Santa Clara, CA, USA) by injecting 900 μl of sample reconstituted in solvent A (10 mM TEABC in water; pH 8.5) on Xbridge (4.6 × 250 mm, 5 μm; Waters, Milford, MA, USA) column. The peptides were separated by using a gradient of 2% solvent A (10 mM TEABC in water; pH 8.5) to 40% solvent B (10 mM TEABC in 90% acetonitrile, pH 8.5) over 40 min and further taken to 100% solvent B where it was held for 5 min before coming back to 2% solvent A for re-equilibration (27). A total of 96 fractions were collected and concatenated to 10 fractions for total proteomic and 13 fractions for phosphoproteomic analysis. All the fractions were dried under vacuum, desalted using C18 StageTips and stored at −20°C till further analysis.

### Phosphopeptide Enrichment

Titanium dioxide (TiO_2_) based phosphopeptide enrichment was carried out as described previously (28). Briefly, TiO_2_ beads (Titansphere; GL Sciences, Inc.) were suspended in DHB solution (80% ACN, 1% TFA, and 5% 2,5-Dihydroxybenzoic acid) at room temperature for 1 h. Each of the fractions were then resuspended in 5% DHB solution and incubated with TiO_2_ beads for 30 min at room temperature with gentle rotation. TiO_2_ beads enriched with phosphopeptides were washed thrice with DHB solution and then with 40% ACN twice. The enriched phosphopeptides were eluted thrice into tubes containing 20% TFA on ice using 2% ammonia solution. The peptides were concentrated by vacuum centrifugation and subjected to C18 cleanup before mass spectrometry analysis.

### LC-MS/MS Analysis

For total proteomics and phosphoproteomics, LC-MS/MS analysis was carried out on Orbitrap Fusion tribrid mass spectrometer connected to Proxeon Easy nLC 1000 system. Each fraction was run in duplicate. The nano spray flex ion source was maintained at 1,950 V. Peptides were reconstituted in 0.1% formic acid and *in-house* packed trap column (75 μm × 2 cm 3 μm Magic C18 AQ, MichromBioresources, Inc., Auburn, CA, USA) was used for initial loading of peptides which were then separated on the *in-house* packed analytical column (75 μm × 25 cm 3 μm Magic C18 AQ, MichromBioresources, Inc., Auburn, CA, USA) using a gradient of 5–35% solvent B (95% Acetonitrile, 0.1% Formic acid) for 100 min. The mass spectrometer was operated in data dependent top speed mode with a cycle time of 3 s. Both the survey scan (MS) and MS/MS scans were acquired in Orbitrap mass analyzer. MS scans were acquired with a mass range of 400–1,600 m/z at 120 k resolution at 200 m/z using 55 ms injection time and AGC target of 2^*^10^5^. Charge state filter and mono isotopic precursor selection were enabled. Based on the intensity, precursor ions were isolated in Quadrupole using an isolation width of 2 m/z and then subjected to fragmentation using high energy collision induced dissociation with 35% normalized collision energy. The fragment ion spectra were acquired with a mass range of 100–2,000 at 30,000 resolution. Injection time of 200 ms and 5^*^10^4^ AGC target settings were used for fragment ion spectra. Dynamic exclusion of precursor ions was enabled with 30 s exclusion time. Lock mass of 445.12002 m/z from ambient air was used for internal calibration.

### Proteomic Data Analysis

Proteome Discoverer (version 1.4.0.288) software suite (Thermo Fisher Scientific, Bremen, Germany) was used for MS/MS searches and protein quantitation. SEQUEST and Mascot (version 2.4.1, Matrix Science, London, UK) search algorithms were used for database searches with NCBI RefSeq human protein database (Version 65, containing 36,211 protein entries along with common contaminants). The search parameters included trypsin as the protease with maximum of two missed cleavages allowed; oxidation of methionine was set as a dynamic modification while static modifications included carbamidomethylation (alkylation) at cysteine and TMT modification at N-terminus of the peptide and at lysine (K). Precursor mass tolerance was set to 20 ppm and fragment mass tolerance was set to 0.05 Da. The false discovery rate (FDR) was calculated by carrying out decoy database searches and peptides scoring better than 1% FDR score cut-off were considered for further analysis. All abundance ratios were calculated by the quantitation node under the consensus workflow.

### Plasmids and Transfection

Human HMGN2 ORF mammalian expression plasmid (catalog: HG16545-ACG) and human MED1 ORF mammalian expression plasmid (catalog:HG13221-ACG) were purchased from Sino Biological Inc. (Beijing, BJ). Het-1A-Smoke-8M cells were transiently transfected with the indicated expression plasmids using X-tremeGENE HP DNA Transfection Reagent (Roche Molecular Biochemicals, Mannheim, BW) in accordance with manufacturer's specifications.

### Immunohistochemical Assays

Immunohistochemical analysis was done using paraffin embedded tissue sections. Tissue sections for esophageal squamous cell carcinoma (ESCC) and adjacent normal cases were obtained from Kidwai Memorial Institute of Oncology, Bangalore. An ethical clearance was obtained from the Ethics Committee of KMIO, Bangalore. Immunohistochemical staining was carried out as described previously (29). Briefly, formalin fixed paraffin embedded tissue sections were deparaffinized prior to antigen retrieval by addition of antigen retrieval buffer (citrate buffer) for 20 min. Action of endogenous peroxidases was quenched using blocking solution (methanol and H_2_O_2_ mixed at 3:1 ratio) and then washed with wash buffer (phosphate buffered saline). Monoclonal rabbit anti-HMGN2 antibody (Cat#[EPR7091] ab124997, Abcam) was used as a primary antibody at a dilution of 1:200. The sections were incubated overnight with primary antibody at 4°C. The sections were rinsed with wash buffer followed by incubation with rabbit secondary antibody conjugated with horseradish peroxidase (Merck-Banglore Genei, India). The staining was developed for 5 min using DAB chromogen (Dako, Glostrup, Denmark), followed by counterstaining with hematoxylin (Nice Chemicals, Kochi, India). The tissue sections were then examined under the microscope by pathologist and immunohistochemical labeling was assessed to score the intensity of staining. The staining was scored on a scale of 0–3, where 0 represented no staining, 2+ represented moderate staining, and 3+ represented intense staining.

### Statistical Analysis

All cellular assays (proliferation and invasion) were done in triplicate. Statistical significance between smoke exposed and untreated or HMGN2/MED1 overexpressed groups was calculated using un-paired Student's *t*-test. Differences with a *p*-value ≤0.05 were considered significant.

## Results

### Chronic Treatment With Cigarette Smoke Condensate Increases Proliferation, Invasion, and Expression of Survival Proteins in Esophageal Cells

**Figure 1:**
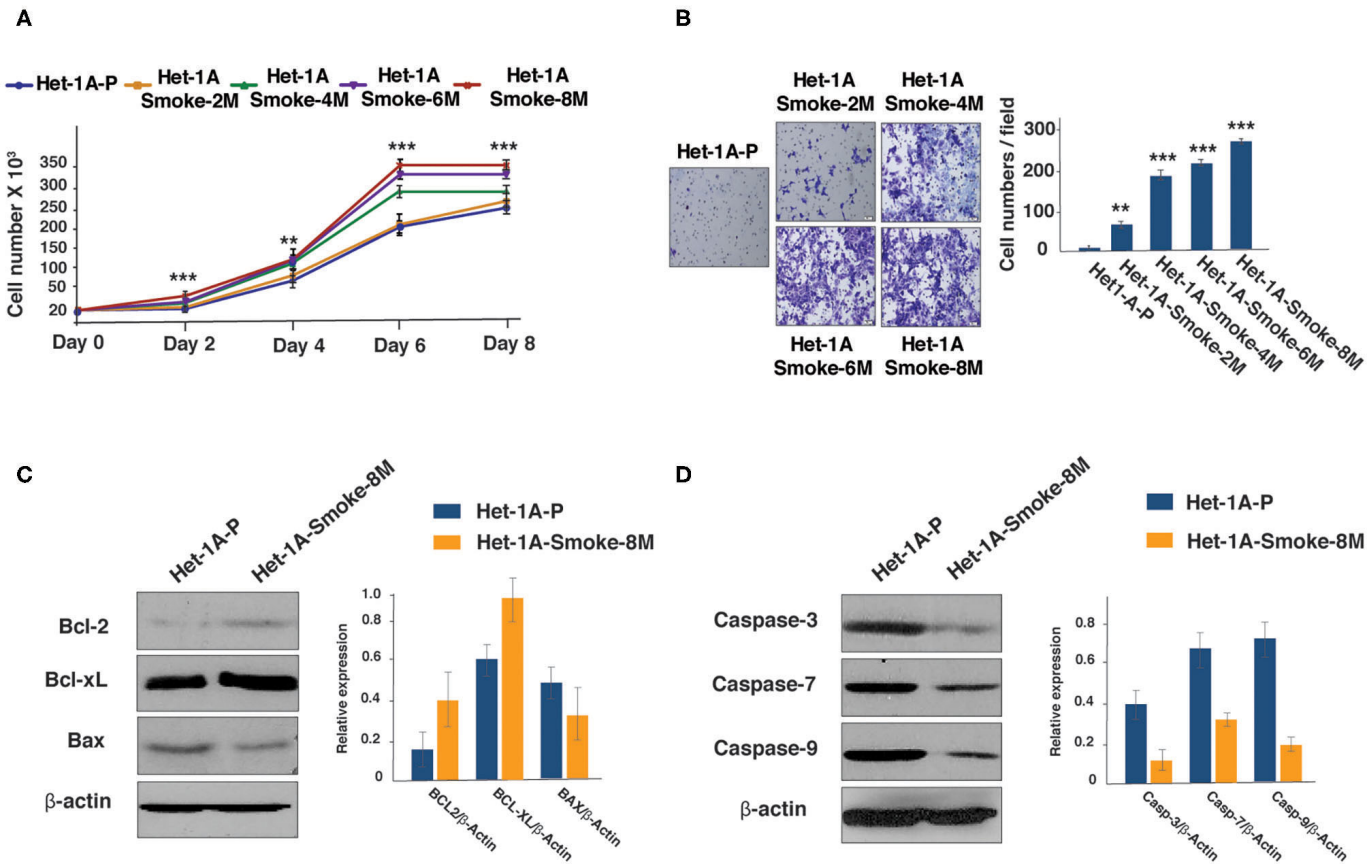
Chronic exposure to CSC treatment increases proliferative and invasive ability of esophageal cells. **(A)** Growth curve depicting cellular proliferation rates of Het-1A-Parental and Het-1A-Smoke (2M, 4M, 6M, and 8M) cells (***p < 0.0001, **p < 0.01). **(B)** Invasion assays were carried out using Het-1A-Parental, Het-1A-Smoke-2M, Het-1A-Smoke-4M, Het-1A-Smoke-6M, and Het-1A-Smoke-8M cells. Representative images were photographed for each time point at 10× magnification. Graphical representation of invasive ability of indicated cells (***p < 0.0001, **p < 0.01). **(C)** Western blot analysis of Bcl2, Bcl-xL and Bax in Het-1A-Parental and Het-1A-Smoke-8M cells. β-actin was used as loading control. **(D)** Western blot analysis of caspase-3, caspase-7, and caspase-9 in Het-1A-Parental and Het-1A-Smoke-8M cells. β-actin was used as loading control.

Het-1A cells were chronically treated with CSC for 2, 4, 6, and 8 months. Proliferative and invasive ability of CSC treated cells were evaluated. We observed progressive increase in proliferative ability of the cells (Figure 1A). *In vitro* invasion assay using Matrigel showed that Het-1A cells showed an increase in their invasive ability after chronic treatment with CSC (Figure 1B).

It is known that cancer cells escape apoptosis by regulating the expression of survival proteins (30, 31). Since chronic treatment with CSC resulted in increased proliferation and invasive ability of non-neoplastic esophageal cells, we examined the expression of Bcl-2 family proteins in cells treated with CSC for a period of 8 months (Het-1A-Smoke-8M). Western blot analysis showed increased expression of both Bcl2 and Bcl-xL but a decrease in Bax expression in Het-1A-Smoke-8M cells (Figure 1C). In addition, we also observed decreased expression of apoptotic marker proteins, such as Caspase-3, Caspase-7, and Caspase-9 in Het-1A-Smoke-8M cells (Figure 1D).

### Whole Exome Sequencing Reveals Genomic Alterations Associated With Cigarette Smoke Condensate Treatment

**Figure 2:**
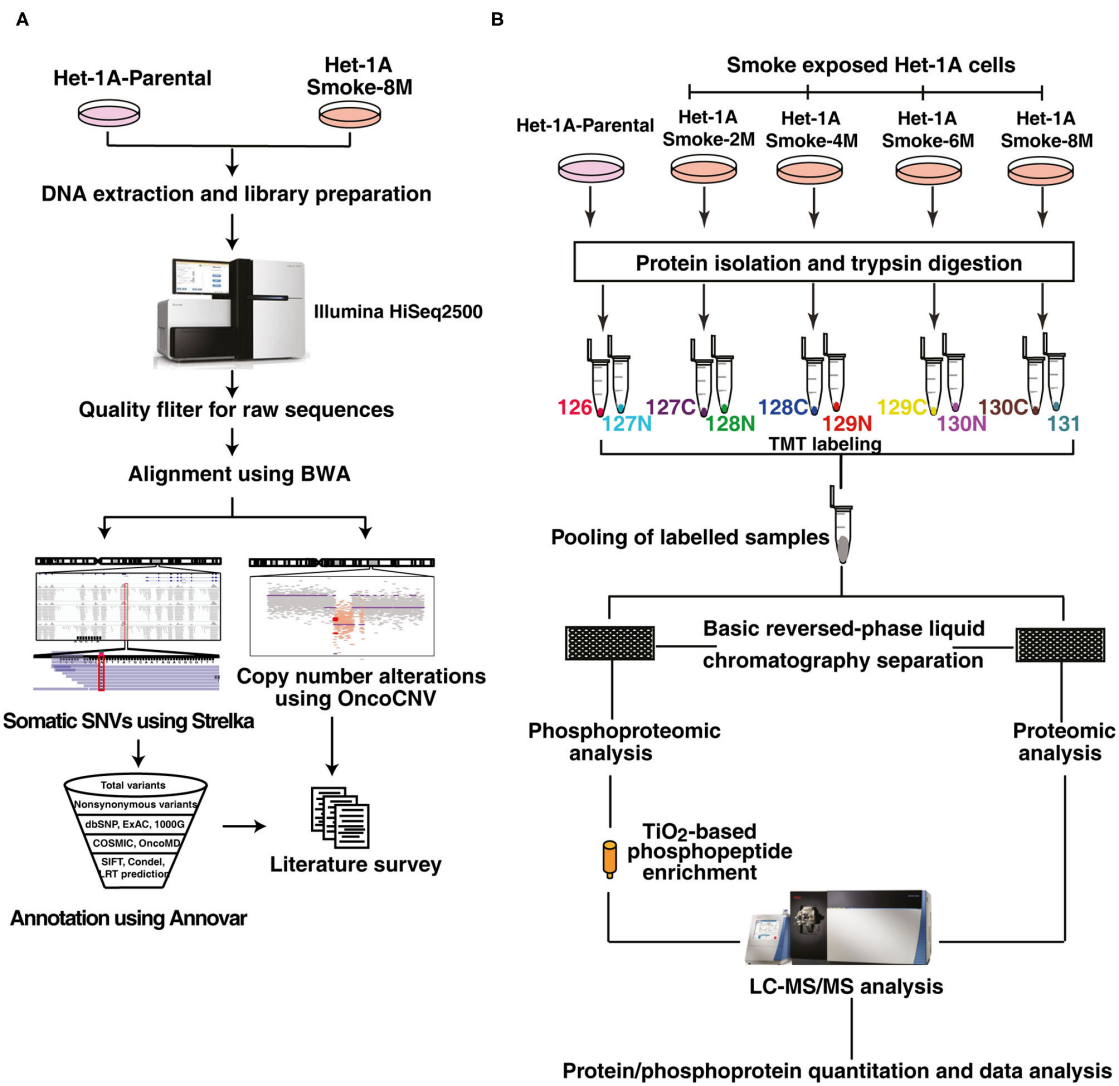
Workflow employed for exome sequencing, proteomic and phosphoproteomic analysis. **(A)** Workflow employed for whole exome sequencing of CSC treated and untreated Het-1A cells. Het-1A-Parental and Het-1A-Smoke-8M cells were harvested at 80% confluence and genomic DNA was extracted. Whole exome sequencing was done using Illumina HiSeq2500. Raw reads obtained were filtered and computational analysis was done for single nucleotide variants and copy number variants. **(B)** Workflow employed for proteomic and phosphoproteomic analysis. Proteins were extracted and quantified from Het-1A-Parental, Het-1A-Smoke-2M, Het-1A-Smoke-4M, Het-1A-Smoke-6M, and Het-1A-Smoke-8M cells. In-solution trypsin digestion of equal amount of proteins from each condition was performed and peptides were labeled with TMT reagents. Ten percent of each sample was pooled and fractionated using basic reversed phase liquid chromatography for proteomic analysis. TiO_2_ based phosphophopeptide enrichment was done for phosphproteomic analysis. All fractions were analyzed by mass spectrometer in duplicate.

These observations indicate that chronic treatment of CSC induces oncogenic transformation in normal esophageal epithelial cells. To identify mutations and copy number alterations associated with cell transformation, we did whole exome sequencing (WES) of Het-1A-Smoke-8M and Het-1A-Parental cells. The workflow employed for exome sequencing is represented in Figure 2A. We acquired ~106 million reads for Het-1A-Parental and ~110 million reads for Het-1A-Smoke-8M cells by whole exome sequencing. Using BWA, we achieved 99.9% alignment for Het-1A-Parental and Het-1A-Smoke-8M cells (Supplementary Table 2). Using Strelka, we identified 213 single nucleotide variants (SNVs) in cells treated with CSC. Among these, 89 (~42%) were C:G->T:A transversions (Supplementary Figure 1A). We observed 102 non-synonymous SNVs in CSC treated cells (Supplementary Figure 1B). Complete list of SNVs with variant and gene annotation is provided in Supplementary Table 3. Previous studies on lung cancers have shown that cigarette smoke increases copy number alterations (32). By analyzing exome data from CSC treated cells, we identified genomic regions that show copy number variations. Copy number variation (CNV) analysis lead to identification of 64 affected genes including deletions in cytoband p36.11 and p35.3, which affected 41 genes and copy number gain in 13q34, 16q11.2, and 16q12.1 affecting 23 genes. Genes that showed copy number loss include high mobility group nucleosomal binding domain 2 (HMGN2), ribosomal protein S6 kinase A1 (RPS6KA1), mitogen-activated protein kinase kinase kinase 6 (MAP3K6), WASP family member 2 (WASF2), FGR proto-oncogene, Src family tyrosine kinase (FGR), syntaxin 12 (STX12), and DnaJ heat shock protein family (Hsp40) member C8 (DNAJC8). The genes that showed copy number gain include RAS P21 protein activator 3 (RASA3), cell division cycle 16 (CDC16), myosin light chain kinase 3 (MYLK3), glutamic-pyruvic Transaminase 2 (GPT2), and Siah E3 ubiquitin protein ligase 1 (SIAH1). A complete list of affected genes with associated details is provided in Supplementary Table 4.

### Chronic Treatment With Cigarette Smoke Condensate Alters Cellular Proteome of Esophageal Cells

**Figure 3:**
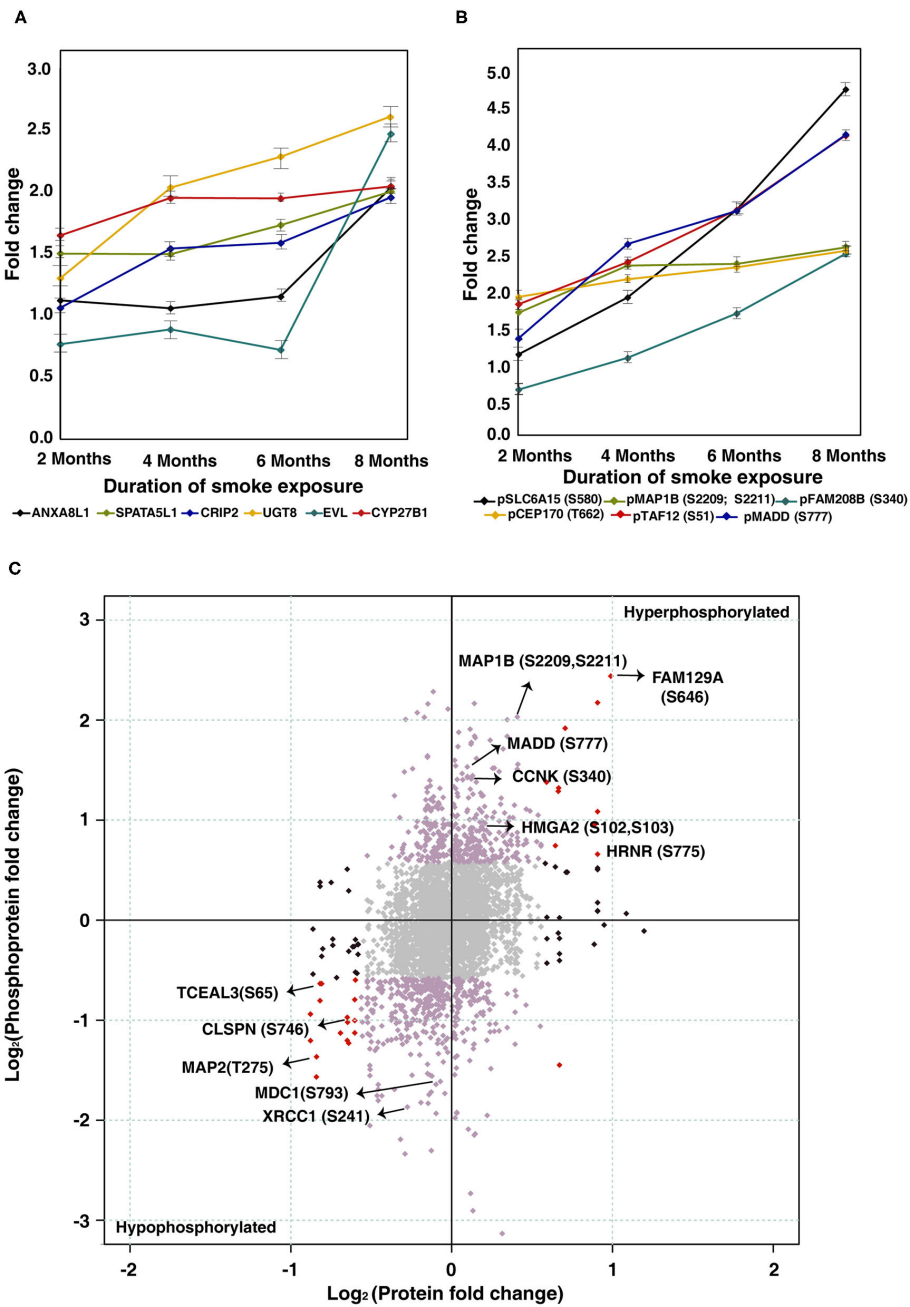
Chronic treatment with cigarette smoke condensate alters cellular proteome of esophageal cells. **(A)** Expression profile of proteins that showed overexpression in CSC treated cells as a function of duration of exposure. **(B)** Expression profile of proteins that showed hyperphosphorylation in CSC treated cells as a function of duration of treatment. **(C)** Global representation of proteomic and phosphoproteomic data. Purple dots indicate differentially phosphorylated proteins that are unchanged in proteomic data. Red dots indicate proteins and phosphoproteins that showed similar dysregulation pattern in both datasets (upregulated/hyperphosphorylated and/or downregulated/hypophosphorylated). Black dots represent molecules that are differentially expressed at proteome level but unchanged in phosphoproteomic data. Gray dots indicate molecules that are unchanged in both datasets.

To characterize protein expression changes and signaling alterations associated with CSC treatment in Het1A cells, we carried out quantitative proteomic and phosphoproteomic profiling. The workflow employed for proteomic analysis is depicted in Figure 2B. We identified 6,530 proteins from both replicates of which 38 proteins were differentially expressed in CSC treated cells (8 months) compared to untreated parental cells (fold change ≥2). A subset of proteins showed progressive increase or decrease in expression across all time points in CSC treated cells. Cysteine-rich protein 2 (CRIP2, 2-fold), spermatogenesis-associated protein 5-like protein 1 (SPATA5L1, 2-fold), annexin A8-like protein 1 (ANXA8L1, 2-fold), myeloid derived growth factor (MYDGF, 2.1-fold), 2-hydroxyacylsphingosine 1-beta-galactosyltransferase (UGT8, 2.6-fold), and 25-hydroxyvitamin D-1 alpha hydroxylase (CYP27B1, 2-fold) are amongst proteins that showed increase in expression with increased duration of CSC treatment (Figure 3A).

Proteins like high mobility group nucleosomal binding domain 2 (HMGN2, 0.5-fold), transcription elongation factor A protein-like 1 (TCEAL1, 0.5-fold), transcription elongation factor A protein-like 3 (TCEAL3, 0.5-fold), transcription elongation factor A protein-like 4 (TCEAL4, 0.4-fold), myosin-13 (MYH13, 0.5-fold) were downregulated with increased duration of CSC treatment (Supplementary Figure 2A). A complete list of quantified proteins and number of dysregulated proteins at each timepoint of CSC treatment from both replicates is provided in Supplementary Table 5. A complete list of identified peptides and proteins from both replicates is provided in Supplementary Table 6.

### Chronic Treatment With Cigarette Smoke Condensate Induces Widespread Perturbation of Signaling Pathways in Esophageal Cells

Altered kinase signaling is associated with multiple cancers (33, 34). Since we observed altered protein expression in response to CSC treatment, we sought to study associated signaling alterations in CSC treated cells in the same temporal setting using phosphoproteomic approach. We have employed TMT labeling followed by TiO_2_ based phosphopeptide enrichment and analyzed the enriched phosphoproteome using Orbitrap Fusion mass spectrometer (Figure 2B). We identified 4,137 phosphosites from both replicates encoded by 1,894 proteins of which 195 phosphosites corresponding to 171 proteins were differentially phosphorylated (fold change ≥2) in Het-1A-Smoke-8M cells. Complete list of phosphorylated peptides identified across all time points of CSC treated cells is provided in Supplementary Table 7.

Among differentially phosphorylated proteins, we identified a subset of proteins that were hyperphosphorylated across all time points of CSC treatment. This includes sodium-dependent neutral amino acid transporter B(0)AT2 (SLC6A15) at Ser580 (4.8-fold), microtubule-associated protein 1B (MAP1B) at Ser2209 and Ser2211 (2.7-fold), centrosomal protein of 170 kDa (CEP170) at Thr662 (2.6-fold), transcription initiation factor TFIID subunit 12 (TAF12) at Ser51 (4.2-fold), and MAP kinase-activating death domain protein (MADD) at Ser777 (4.2-fold) (Figure 3B).

Proteins which were hypophosphorylated at all time points included MKI67 FHA domain-interacting nucleolar phosphoprotein (NIFK) at Thr223 (0.3-fold), Fibroblast Growth Factor Receptor 1 Oncogene Partner (FGFR1OP) at Thr70 (0.4-fold), Eukaryotic Translation Elongation Factor 2 (EEF2) at Thr435 (0.4-fold), Methyl-CpG Binding Protein 2 (MECP2) at Ser229 (0.7-fold) echinoderm microtubule-associated protein-like 1 (EML1) at Ser160 (0.4-fold), and mediator of RNA polymerase II transcription subunit 1 (MED1) at Ser1156 (0.4-fold) (Supplementary Figure 2B). A global representation of total proteome and phosphoproteome data is presented in Figure 3C.

### Alteration in DNA Repair Mechanism in Esophageal Cells Chronically Treated With Cigarette Smoke Condensate

**Figure 4:**
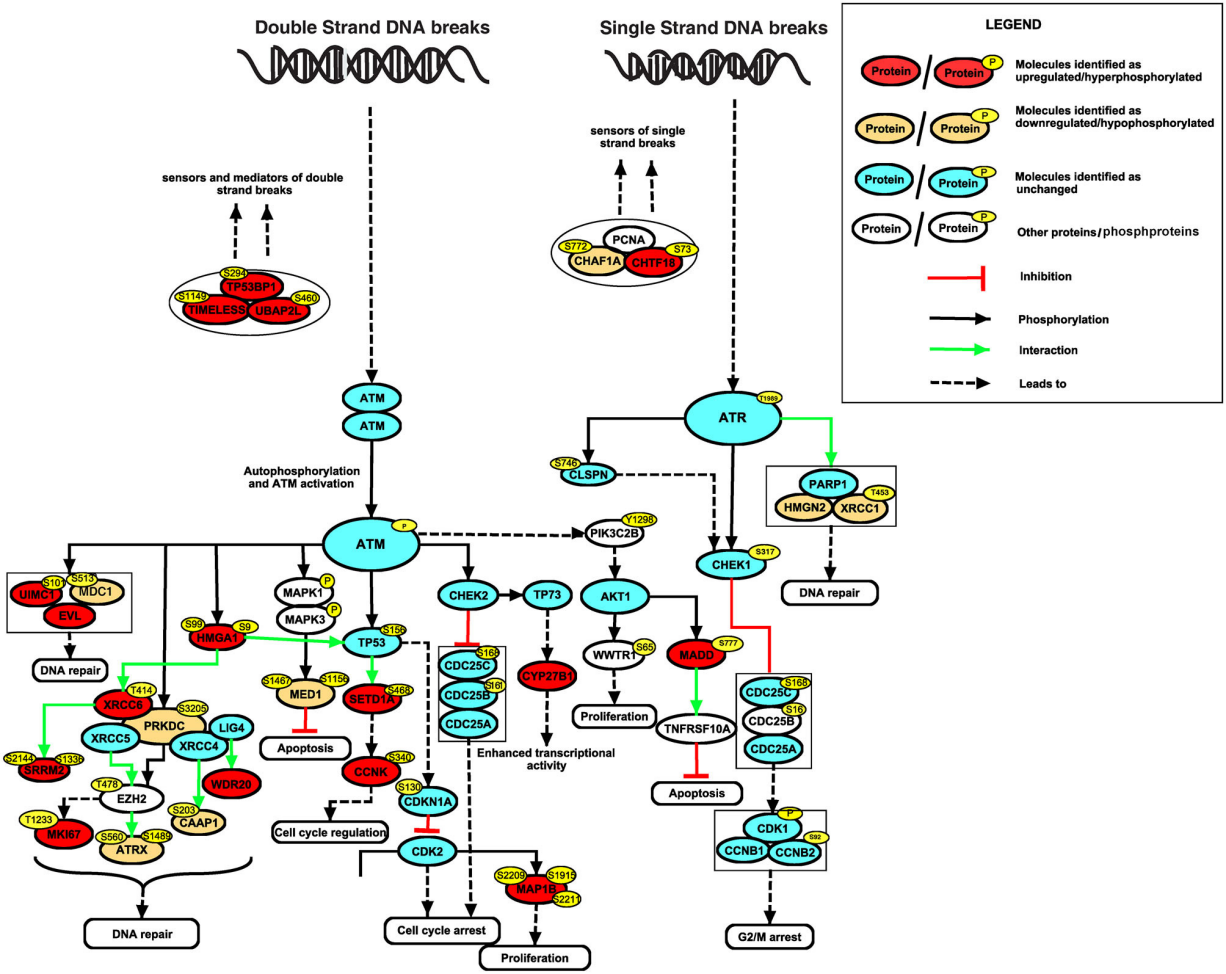
Alteration in DNA repair mechanism in esophageal cells chronically treated with cigarette smoke condensate. Signaling network of DNA damage and repair pathway identified based on differentially expressed proteins and phosphoproteins in CSC treated cells.

To identify altered signaling pathways and networks in response to CSC treatment, we performed Ingenuity Pathway Analysis (IPA) using proteins and phosphoproteins which were differentially expressed in Het-1A-Smoke-8M cells. Our data indicates enrichment of molecules involved in DNA damage response and repair (Supplementary Table 8). This included high mobility group AT-hook 1 (HMGA1 at Ser9, 2.1-fold), MAP kinase activating death domain (MADD at Ser777, 4.2-fold), microtubule-associated protein 1B (MAP1B at Ser2209 and Ser2211, 2.7-fold), cyclin-K (CCNK at Ser340, 2.5-fold), Enah/Vasp-like (EVL, 2.5-fold), and cytochrome P450 family 27 subfamily B member 1 (CYP27B1, 2-fold). In addition, using Human Protein Reference Database (HPRD) (35) as background, we did bioinformatics analysis of differentially expressed proteins and phosphoproteins to categorize them based on their biological function and cellular localization using FunRich (36). Subcellular localization-based classification revealed that around 39% of proteins were localized to nucleus (Supplementary Figure 3A). Functional enrichment analysis showed that 27% of proteins are involved in regulation of nucleobase, nucleotide and nucleic acid metabolism (Supplementary Figure 3B). Several differentially expressed proteins and phosphoproteins that are localized to nucleus or regulate nucleic acid metabolism are known to be involved in DNA damage response. These include cyclin Y (CCNY, hyperphosphorylated at Ser193 by 4.3-fold), chromosome transmission fidelity factor 18 (CHTF18, hyperphosphorylated at Ser73 by 2.5-fold), phosphatidylinositol-4-phosphate 3-kinase catalytic subunit type 2 beta (PIK3C2B, hyperphosphorylated at Tyr1298 by 2.3-fold), WD repeat domain 20 (WDR20, upregulated by 2.1-fold) and ring finger protein 168 (RNF168, downregulated by 0.4-fold). A pathway map of differentially expressed proteins and phosphoproteins, drawn using “PathVisio” (http://www.PathVisio.org) (37), depicting DNA damage response pathway is shown in Figure 4.

**Figure 5:**
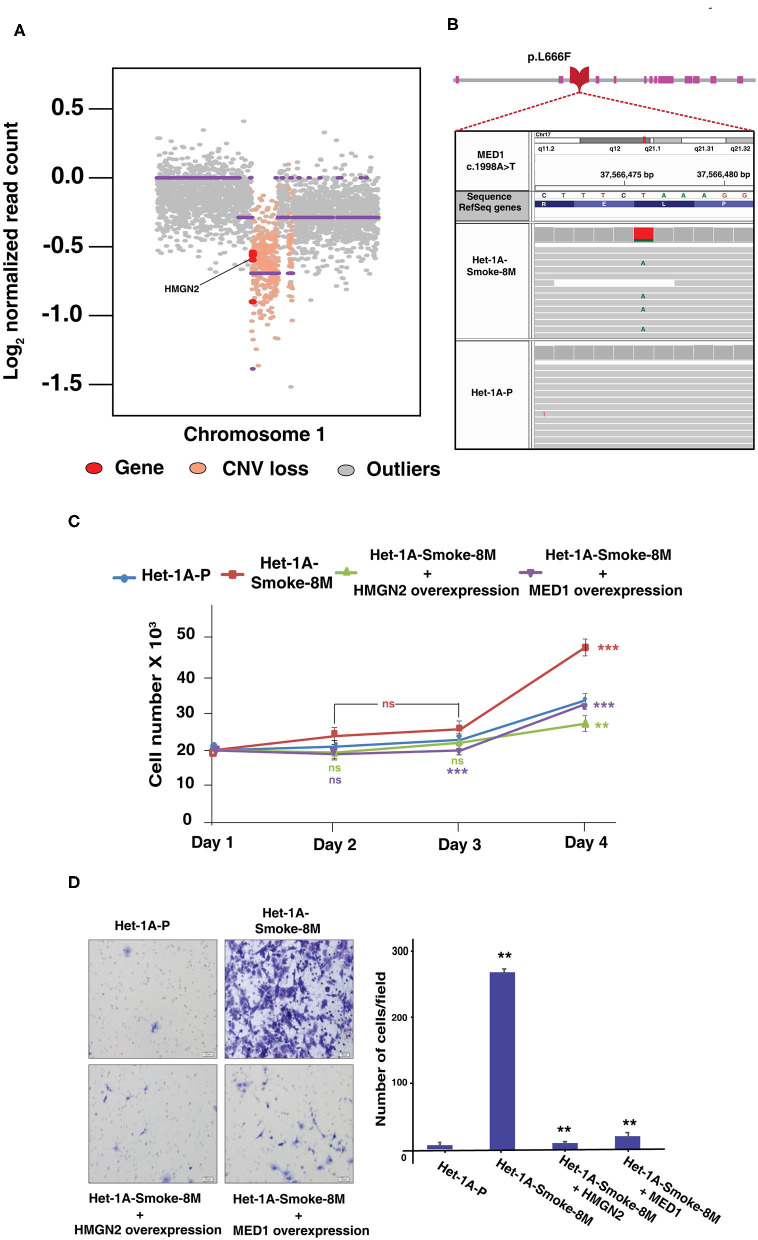
Genomic alterations based on whole exome sequencing data. **(A)** Copy number loss of *HMGN2* in Het-1A-Smoke-8M cells. **(B)** An IGV snapshot of *MED1* gene representing non-synonymous SNV (pL666F). **(C)** Proliferation curve of Het-1A-Smoke-8M cells with and without exogenous overexpression of HMGN2 and MED1 (***p < 0.0001, **p < 0.01, ns, statistically not significant). **(D)** Invasive potential of Het-1A-Smoke-8M cells with and without exogenous overexpression of HMGN2 and MED1 (**p < 0.01).

With the integration of proteomic, phosphoproteomic and exome sequencing datasets, we observed a loss in copy number of high mobility group nucleosomal binding domain 2 (HMGN2) (Figure 5A). HMGN2 was downregulated 2-fold in CSC treated cells (Het1A-Smoke-8M). In addition to HMGN2, copy number variation analysis revealed loss of other genes that are known to play a role in DNA damage response and chromatin remodeling. These genes include DnaJ heat shock protein family (Hsp40) member C8 (DNAJC8), WAS protein family member 2 (WASF2), ribosomal protein S6 kinase A1 (RPS6KA1), AT-rich interaction domain 1A (ARID1A), GPN-loop GTPase 2 (GPN2), and mitogen-activated protein kinase kinase kinase 6 (MAP3K6). A representative snapshot of genes which show copy number loss is provided in Supplementary Figure 4. Somatic SNV analysis of Het1A-Smoke-8M cells revealed mediator of RNA polymerase II transcription subunit 1 (MED1) was mutated at exon17 (c.A1998T:p.L666F) (Figure 5B). MED1 was observed to be hypophosphorylated at Ser1156 (0.4-fold) in our phosphoproteomic data set. In addition, we identified mutation in genes that are associated with DNA damage response including WW domain containing oxidoreductase (WWOX, mutated at exon5:c.A425T:p.K142M), baculoviral IAP repeat-containing 6 (BIRC6, mutated at exon73:c.A14270G:p.K4757R), nuclear receptor binding SET domain protein 1 (NSD1, mutated at exon5:c.C1688G:p.T563S and exon6:c.C881G:p.T294S), growth arrest-specific 2 (GAS2, mutated at exon6:c.C548A:p.S183Y), aldehyde dehydrogenase 1 family, member L1 (ALDH1L1, mutated at exon2:c.A74G:p.E25G), and epidermal growth factor receptor (EGFR, mutated at exon16:c.C1897T:p.L633F (Supplementary Figure 5).

Both HMGN2 and MED1 are reported as potential tumor suppressors and are known to play an important role in DNA damage response. Analysis of publicly available datasets in cBioPortal indicates copy number loss in HMGN2 in multiple cancers (38) (Supplementary Figure 6A). Similarly, data from BioMuta database (39, 40) indicates higher mutation rate of MED1 in smoking associated cancers (Supplementary Figure 6B). A representative MS/MS spectrum for HMGN2 and MED1 from our proteomic and phosphoproteomic data sets is shown in Supplementary Figures 7A,B, respectively.

### Overexpression of HMGN2 and MED1 Decreases Proliferative and Invasive Ability of Esophageal Cells Chronically Treated With Cigarette Smoke Condensate

HMGN2 and MED1 are known to be potential tumor suppressors. We evaluated role of both HMGN2 and MED1 on proliferation and invasion ability of CSC treated esophageal cells. Exogenous expression of HMGN2 and MED1 in CSC treated cells (Het-1A-Smoke-8M) resulted in decrease in both proliferative and invasive ability of Het-1A-Smoke-8M cells (Figures 5C,D). These observations suggest HMGN2 and MED1 might play a potential tumor suppressor role in esophageal cells.

### Immunohistochemical Validation of HMGN2 in ESCC Tissue From Smokers

**Figure 6:**
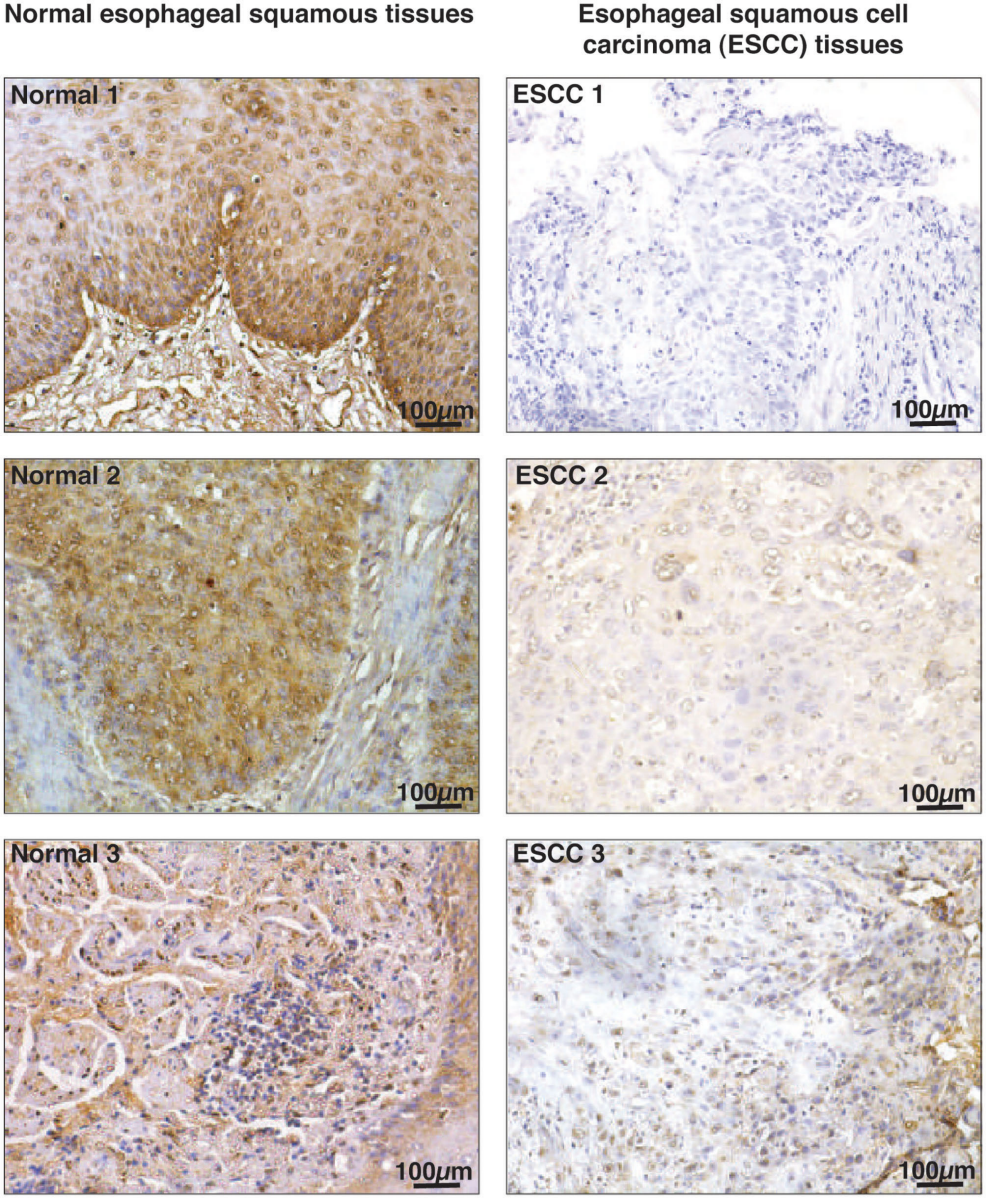
Immunohistochemical validation of HMGN2 in ESCC tissue sections and adjacent normal. Tissue sections from ESCC and adjacent normal were stained with anti-HMGN2 antibody. Scale bar represents 100 μm.

We evaluated expression of HMGN2 in primary ESCC tissue sections from smokers. Immunohistochemical validation was carried out using 10 paired tissue sections from ESCC and adjacent normal. All ESCC cases (10 of 10) showed no staining for HMGN2 while 60% adjacent normal tissue (6 of 10) showed strong (3+) and 40% (4 of 10) showed moderate staining (2+). The results of immunohistochemical validation are provided in Supplementary Table 9. Representative staining patterns for HMGN2 in ESCC and adjacent normal tissues is depicted in Figure 6.

## Discussion

Cigarette smoke is a well-known risk factor for various cancers including ESCC. Molecular alterations associated with cigarette smoke exposure have been investigated in some detail in lung cancers. However, molecular alterations associated with cigarette smoke exposure in esophageal cells remains poorly understood. We and others have shown that *in vitro* cell models with chronic cigarette smoke extract/condensate are helpful in understanding oncogenic transformation of non-neoplastic esophageal cells (41), lung epithelial type-II cells (42), urothelial cells (43), breast epithelial cells (44), and oral cells (45, 46). Cigarette smoke contains more than 70 carcinogenic compounds that are responsible for development of cancer. However, underlying molecular mechanisms associated with development of cancer are poorly understood. In this study, we developed an *in vitro* cell model where non-neoplastic esophageal cells were chronically treated with CSC and genomic, proteomic and phosphoproteomic alterations were characterized.

Our data indicates that CSC treatment induces oncogenic transformation in normal esophageal cells. This is supported by increase in proliferative and invasive ability of CSC treated esophageal cells. In addition, we observed an increase in the ratio of Bcl-2/BAX which are important regulators of cell survival and play an important role in development of cancer (47–50).

Our integrated analysis of exome, proteome and phosphoproteome revealed enrichment of genes and proteins involved in pathways that regulate DNA damage response, cell growth and/maintenance and regulation of nucleic acid metabolism. Altered expression of DNA damage response genes have been associated with development and progression of cancer (51). DNA damage response pathway proteins that showed altered expression in our study included tumor suppressor p53-binding protein 1 (TP53BP1), an important regulator of the cellular response to DSBs that promotes end-joining of distal DNA ends (52). X-ray repair cross-complementing proteins (XRCC1 and XRCC6), which recruit several DNA damage response proteins and plays key role in DNA repair and cancer progression (53–56). Another dysregulated protein in our data, high mobility group AT-hook 2 (HMGA2), promotes ATM expression and promotes cancer cell resistance to genotoxic agents (57).

In agreement with previous reports (58), we observed high C:G->T:A transversions in CSC treated cells. Single nucleotide variant analysis revealed non-synonymous deleterious variants in genes involved in DNA damage response. Epidermal growth factor receptor (*EGFR*) harbors C>T transversion leading to non-synonymous mutations. EGFR is known to phosphorylate ATM upon double strand DNA breaks (59) and mutation burden of *EGFR* (C>T) is reported to be high in smokers (60, 61). WW domain containing oxidoreductase (WWOX) is known to interact with ATM upon DNA damage. Loss of WWOX is associated with defective G2/M cell cycle checkpoint (32). Genetic alterations in WWOX and its tumor suppressor function is known in esophageal cancer (62, 63). We report a missense mutation in *MED1* gene (exon17:c.A1998T:p.L666F) which is predicted to be deleterious by SIFT and CONDEL. MED1 is a central molecule involved in base excision repair and mismatch repair mechanisms (64). Inactivation of *MED1* increases C>T transition mutations and promotes gastrointestinal tumor formation (65). Our data indicates ~42% of identified SNVs and 20% of annotated non-synonymous SNVs as C>T transversion. Mutations and inactivation of *MED1* is reported to activate tumorigenesis in colorectal cancer and various carcinomas (66, 67). In addition, downregulation and loss of expression of MED1 is reported to promote invasion and metastasis in many cancers (68–70). Taken together, it shows that MED1 can act as a potential tumor suppressor gene and further investigation is needed to validate its role in pathobiology of ESCC.

We observed downregulation and copy number loss in *HMGN2*. Loss of *HMGN2* is observed in several cancers where cigarette smoking is a risk factor. Loss of *HMGN2* is frequently observed in lung squamous cell carcinoma patients with history of smoking as compared to non-smokers (38, 59). HMGN2 is also reported as potential tumor suppressor and plays an important role in DNA damage response. HMGN2 helps other DNA repair proteins to bind to damaged DNA and modulate genome repair (18). It is also reported to act as anti-tumor molecule and reduces proliferation and invasion in oral squamous cell carcinoma and osteosarcoma cell lines (71, 72). These observations suggest HMGN2 as a potential tumor suppressor. Loss of HMGN2 might play a vital role in development and progression of ESCC.

## Conclusion

In this study, we chronically treated non-neoplastic human esophageal epithelial cells (Het1A) with cigarette smoke condensate. Esophageal cells showed increased proliferative and invasive property suggesting potential oncogenic phenotype. We observed elevated expression of anti-apoptotic proteins Bcl2 and Bcl-xL and reduced expression of apoptotic protein Bax in cigarette smoke treated esophageal cells. Integrative analysis by employing whole exome sequencing, proteomic and phosphoproteomic profiling revealed molecular alterations affecting DNA repair pathways. We observed loss of HMGN2 and MED1 in cigarette smoke treated cells. HMGN2 and MED1 have been previously reported as potential tumor suppressors and are known to play important role in DNA damage response. Immunohistochemistry revealed loss of HMGN2 expression in ESCC tissue sections compared to adjacent normal. Overexpression of HMGN2 and MED1 lead to decreased proliferative and invasive ability in CSC treated cells. These findings suggest that cigarette smoke affects genes and proteins associated with DNA damage response pathways which might play a vital role in development of ESCC.

## Data Availability Statement

The Mass spectrometry data has been deposited to ProteomeXchange Consortium via the PRIDE partner repository with the dataset identifiers PXD007799 and PXD007814. Raw sequencing data are available in Sequence Read Archive hosted by National Center for Biotechnology Information (NCBI) search database with accession number SRP117798.

## Author Contributions

HG, AC, and SP participated in the study conception and study design. AAK, NB, HS, and VN were involved in cell culture and performed all assays and experiments. AAK and KM carried out the fractionation and mass spectrometric analysis of samples. CK and SM were involved in the DNA extraction, quality control, and next generation sequencing. RK and MB did the immunohistochemical scoring and imaging for tissues. AAK and KP prepared the manuscript and manuscript figures. AAK, KP, AK, MM, RaG, and RoG were involved in the genomic data analyses and interpretation. AAK, KP, and NB were involved in the proteomic and phosphoproteomic data analyses and interpretation. AC, HG, AK-G, SP, DS, BN, and PK edited, critically read, and revised the manuscript. All authors have read and approved the final manuscript.

## Conflict of Interest

AK, MM, CK, SM, RaG, RoG, and AK-G were employed by the company Medgenome Lab Pvt. Ltd. MB was employed by the company Mitra Biotech. The remaining authors declare that the research was conducted in the absence of any commercial or financial relationships that could be construed as a potential conflict of interest.

## Supplementary Material

Supplementary Figure 1 Whole exome sequence analysis of Het-1A-8M. **(A)** Summary of transitions and transversions identified in cigarette smoke condensate treated cells. **(B)** Summary of somatic variants identified in response to cigarette smoke condensate treatment.

Supplementary Figure 2 Chronic treatment with cigarette smoke condensate alters cellular proteome of esophageal cells. **(A)** Expression profile of proteins that showed downregulation in cigarette smoke condensate treated cells as a function of duration of exposure. **(B)** Expression profile of proteins that showed hyperphosphorylation in cigarette smoke condensate treated cells, as a function of duration of treatment.

Supplementary Figure 3 Gene ontology-based annotation of differentially phosphorylated proteins identified in Het-1A-Smoke-8M cells. Classification of proteins based on **(A)** subcellular localization. **(B)** Molecular function.

Supplementary Figure 4 Genes affected by copy number loss in cells chronically treated with cigarette smoke condensate (Het1A-Smoke-8M). *DNAJC8, ARID1A, GPN2, MAP3K6, WASF2, RPS6KA1*.

Supplementary Figure 5 Representative IGV snapshot of SNV bearing genes. *WWOX* (exon5:c.A425T:p.K142M), *BIRC6* (exon73:c.A14270G:p.K4757R), *NSD1* (exon5:c.C1688G:p.T563S and exon6:c.C881G:p.T294S), *GAS2* (exon6:c.C548A:p.S183Y), *ALDH1L1* (exon2:c.A74G:p.E25G), and *EGFR* (exon16:c.C1897T:p.L633F).

Supplementary Figure 6 *HMGN2* and *MED1* genomic aberration report from publicly available datasets. **(A)** Data used from cBioPortal depicting copy number alteration of *HMGN2* in multiple cancers associated with cigarette smoke. **(B)** Data used from BioMuta depicting mutation load of *MED1* in cancers associated with cigarette smoke.

Supplementary Figure 7 Representative MS/MS spectrum of **(A)** high mobility group nucleosomal binding domain 2 (HMGN2, 0.5-fold downregulated, **(B)** mediator complex subunit 1 (MED1, 0.5-fold hypophosphorylated).

Supplementary Table 1 TMT labels used for labeling cigarette smoke condensate treated and untreated Het-1A cells.

Supplementary Table 2 Summary of exome sequencing analysis of untreated and treated Het1A cells with cigarette smoke condensate for 8 months.

Supplementary Table 3 List of somatic single nucleotide variants identified in Het-1A cells chronically treated with cigarette smoke condensate for 8 months.

Supplementary Table 4 List of somatic copy number alterations and affected genes in Het-1A cells chronically treated with cigarette smoke condensate for 8 months.

Supplementary Table 5 List of proteins quantified in untreated and chronically treated Het1A cells with cigarette smoke condensate treated for 8 months.

Supplementary Table 6 List of all proteins and peptides identified in untreated and chronically treated Het1A cells with cigarette smoke condensate treated for 8 months.

Supplementary Table 7 List of differentially expressed proteins identified in smoke exposed Het-1A cells across both replicates.

Supplementary Table 8 List of phosphopeptides identified and quantified in untreated and chronically treated Het1A cells with cigarette smoke condensate for 8 months using SequestHT and Mascot search algorithms with a PhosphoRS probability score ≥75%.

Supplementary Table 9 List of differentially phosphorylated protein in smoke exposed Het-1A cells across both replicates.

Supplementary Table 10 Top six biological networks identified by IPA.

Supplementary Table 11 Summary of the immunohistochemical validation for HMGN2 in ESCC tissues and adjacent normal tissues from smokers.
