# Supplementary material for: Multi-Omics Analysis to Characterize Cigarette Smoke Induced Molecular Alterations in Esophageal Cells
Source: Front Oncol. 2020 Nov 5;10:1666. doi: 10.3389/fonc.2020.01666 (PMC7675040; doi:10.3389/fonc.2020.01666)
Supplement: Supplementary Table 1 — TMT labels used for labeling cigarette smoke condensate treated and untreated Het-1A cells. [file Table_1.pdf]

Khan *et al.* , 2019. Multi-omics analysis to characterize cigarette smoke induced molecular alterations in esophageal cells
Supplementary Table 1. TMT labels used for labeling cigarette smoke condensate treated and untreated Het-1A cells

Sample	TMT label
Het-1A-untreated	126
	127N
Het-1A-Smoke-2M	127C
	128N
Het-1A-Smoke-4M	128C
	129N
Het-1A-Smoke-6M	129C
	130N
Het-1A-Smoke-8M	130C
	131
