# Supplementary material for: Multi-Omics Analysis to Characterize Cigarette Smoke Induced Molecular Alterations in Esophageal Cells
Source: Front Oncol. 2020 Nov 5;10:1666. doi: 10.3389/fonc.2020.01666 (PMC7675040; doi:10.3389/fonc.2020.01666)
Supplement: Supplementary Table 2 — Summary of exome sequencing analysis of untreated and treated Het1A cells with cigarette smoke condensate for 8 months. [file Table_2.pdf]

Khan *et al.* , 2019. Multi-omics analysis to characterize cigarette smoke induced molecular alterations in esophageal cells

Supplementary Table 2. Summary of exome sequencing analysis of untreated and treated Het1A cells with cigarette smoke condensate for 8 months

	Fastq metrics			Trimming metrics			Alignment metrics	Target region coverage statistics						Mutations		
Condition	Total reads	Average read length	Average base quality	Total reads	Average read length	Average base quality	Alignment percentage	1x- 10x	11x – 30x	31x – 50x	51x – 100x	>100x	On-target region average depth	somatic SNVs (with allele frequency >= 10%)	somatic non-silent SNVs (with allele frequency >= 10%)	Genes affected by CNAs with $\log_2$(fold change) ≤ 1 and $\log_2$(fold change) ≥ 3
**Het-1A-Parental**	10,65,54,180	100	38.84	10,65,53,760	99.87	38.69	99.89%	1.45	9.08	13.01	32.6	43.63	107.81	-	-	-
**Het-1A-Smoke-8M**	11,06,83,654	100	39.1	11,06,83,022	99.88	38.94	99.91%	2.24	13.32	17.3	36.7	30.22	84.05	124	56	64
