# Supplementary material for: Multi-Omics Analysis to Characterize Cigarette Smoke Induced Molecular Alterations in Esophageal Cells
Source: Front Oncol. 2020 Nov 5;10:1666. doi: 10.3389/fonc.2020.01666 (PMC7675040; doi:10.3389/fonc.2020.01666)
Supplement: Supplementary Table 3 — List of somatic single nucleotide variants identified in Het-1A cells chronically treated with cigarette smoke condensate for 8 months. [file Table_3.pdf]

Khan *et al.*, 2020. Multi-omics analysis to characterize cigarette smoke induced molecular alterations in esophageal cells
Supplementary Table 3. List of somatic single nucleotide variants identified in Het-1A cells chronically treated with cigarette smoke condensate for 8 months

																													Protein quantification	
Chromosome	Genomic position	Reference allele	Alternate allele	Mutation type	Gene symbol	Nucleotide change	Description	OncoMD	COSMIC	dbSNP	ExAC	1000G	SIFT	CONDEL	LRT	VEP	FATHMM	METASVM	METALR	PhastCons7way	DP in Het-1A-Parental	Reference read in Het-1A-Parental	Alternative read in Het-1A-Parental	Alternate allele frequency in Het-1A-Parental (%)	DP in Het-1A-Smoke-8M	Reference read in Het-1A-Smoke-8M	Alternate read in Het-1A-Smoke-8M	Alternate allele frequency in Het-1A-Smoke-8M (%)	Het-1A-Smoke - 8M/Parental (130C+131)/(126+127N) Replicate 1	Het-1A-Smoke - 8M/Parental (130C+131)/(126+127N) Replicate 2
chr11	22770740	C	A	MISSENSE	GAS2	c.548C>A,c.548C>A,c.548C>A	GAS2:NM_005256:exon5:c.C548A:p.S183Y,GAS2:NM_001143830:exon6:c.C548A:p.S183Y,GAS2:NM_177553:exon6:c.C548A:p.S183Y	NA	NA	NA	NA	NA	D	N	D	MODERATE	T	T	T	1	68	68	0	0	77	64	13	16.9	NA	NA
chr16	78198095	A	T	MISSENSE	WWOX	c.425A>T,c.425A>T	WWOX:NM_001291997:exon4:c.A86T:p.K29M,WWOX:NM_016373:exon5:c.A425T:p.K142M,WWOX:NM_130791:exon5:c.A425T:p.K142M	chr16*78198095*78198095*A*T*1*1*Head_and_neck_cancer	chr16*78198095*78198095*COSM121057*A*T*1*upper_aerodigestive_tract*1	NA	NA	NA	D	D	D	MODERATE	D	D	D	0.999	116	116	0	0	83	69	14	16.9	NA	NA
chr5	176637088	C	G	MISSENSE	NSD1	c.881C>G,c.1688C>G	NSD1:NM_022455:exon5:c.C1688G:p.T563S,NSD1:NM_172349:exon6:c.C881G:p.T294S	NA	NA	NA	NA	NA	T_lc	N	N	MODERATE	D	T	D	0.729	164	164	0	0	143	113	27	18.9	NA	NA
chr2	32836525	A	G	MISSENSE	BIRC6	c.14270A>G	BIRC6:NM_016252:exon73:c.A14270G:p.K4757R	NA	NA	NA	NA	NA	NA	NA	D	MODERATE	T	T	T	1	45	45	0	0	32	26	6	18.8	1.18	1.15
chr3	125879749	T	C	MISSENSE	ALDH1L1	c.74A>G,c.104A>G,c.74A>G	ALDH1L1:NM_001270364:exon2:c.A104G:p.E35G,ALDH1L1:NM_001270365:exon2:c.A74G:p.E25G,ALDH1L1:NM_012190:exon2:c.A74G:p.E25G	NA	NA	NA	NA	NA	D_lc	N	N	MODERATE	T	T	T	0.99	82	82	0	0	73	56	17	23.3	NA	NA
chr7	55238884	C	T	MISSENSE	EGFR	c.1897C>T	EGFR:NM_005228:exon16:c.C1897T:p.L633F	chr7*55238884*55238884*C*T*1*1*Lung_cancer	chr7*55238884*55238884*COSM379422*C*T*1*lung*1	NA	NA	NA	T	N	D	MODERATE	T	T	T	0.976	113	113	0	0	82	71	11	13.4	0.76	0.77
chr1	168168138	T	C	INTRONIC	TIPRL	c.613-19T>C	NM_152902.3	NA	NA	NA	NA	NA	NA	NA	NA	MODIFIER	NA	NA	NA	NA	19	19	0	0	25	18	7	28.0	1.01	1.19
chr7	5372340	G	A	SILENT	TNRC18	c.6060C>T	TNRC18:NM_001080495:exon19:c.C6060T:p.A2020A	NA	NA	NA	NA	NA	NA	NA	NA	LOW	NA	NA	NA	NA	39	39	0	0	25	18	7	28.0	NA	NA
chr2	179615412	C	T	INTRONIC	TTN	c.10597+2439G>A,c.10360+2439G>A,c.10798+2439G>A,c.11311+2439G>A,c.10222+2439G>A	TTN:NM_133379:exon46:c.G11715A:p.L3905L	NA	NA	NA	NA	NA	NA	NA	NA	MODIFIER	NA	NA	NA	NA	47	46	0	0	36	26	10	27.8	1.03	0.96
chr2	179615412	C	T	SILENT	TTN	c.11715G>A	TTN:NM_133379:exon46:c.G11715A:p.L3905L	NA	NA	NA	NA	NA	NA	NA	NA	LOW	NA	NA	NA	NA	47	46	0	0	36	26	10	27.8	1.03	0.96
chr12	58002568	C	G	INTRONIC	DTX3	c.968+48C>G,c.968+48C>G,c.977+48C>G	NM_001286245.1	NA	NA	NA	NA	NA	NA	NA	NA	MODIFIER	NA	NA	NA	NA	167	167	0	0	132	95	36	27.3	NA	NA
chr4	186598299	G	A	INTRONIC	SORBS2	c.162+1278C>T,c.-46+1278C>T,c.255+1278C>T,c.213+1278C>T,c.93+1278C>T,c.213+1278C>T	SORBS2:NM_001145673:exon5:c.C344T:p.S115F	NA	NA	NA	NA	NA	NA	NA	NA	MODIFIER	NA	NA	NA	NA	24	24	0	0	26	19	7	26.9	NA	NA
chr4	186598299	G	A	MISSENSE	SORBS2	c.344C>T	NM_001145673.1	NA	NA	NA	NA	NA	D_lc	D	NA	MODERATE	NA	NA	NA	NA	24	24	0	0	26	19	7	26.9	NA	NA
chr19	48613788	T	G	5UTR	PLA2G4C	c.-77A>C,c.-16A>C	NM_003706.2,NM_001159322.1	NA	NA	rs1654377	0.000637574	NA	NA	NA	NA	MODIFIER	NA	NA	NA	NA	19	19	0	0	41	30	11	26.8	NA	NA
chr17	5185720	C	T	5UTR	RABEP1	c.-62C>T,c.-62C>T	NM_004703.4	NA	NA	NA	NA	NA	NA	NA	NA	MODIFIER	NA	NA	NA	NA	58	58	0	0	57	42	15	26.3	0.98	0.95
chr1	158735837	C	A	SILENT	OR6N1	c.636G>T	OR6N1:NM_001005185:exon1:c.G636T:p.L212L	NA	NA	NA	NA	NA	NA	NA	NA	LOW	NA	NA	NA	NA	67	67	0	0	55	41	14	25.5	NA	NA
chr20	13850840	T	A	MISSENSE	SEL1L2	c.1114A>T	SEL1L2:NM_001271539:exon13:c.A1114T:p.I372F	NA	NA	NA	NA	NA	T	D	D	MODERATE	T	T	T	0.999	53	53	0	0	60	45	15	25.0	NA	NA
chr2	179577068	G	T	MISSENSE	TTN	c.23849C>A,c.27581C>A	TTN:NM_133378:exon92:c.C23849A:p.S7950Y,TTN:NM_001256850:exon93:c.C26630A:p.S8877Y,TTN:NM_001267550:exon95:c.C27581A:p.S9194Y	NA	NA	NA	NA	NA	NA	NA	NA	MODERATE	NA	NA	NA	NA	264	263	0	0	160	121	39	24.4	1.03	0.96
chr2	179577068	G	T	INTRONIC	TTN	c.13657+25741C>A,c.13858+25741C>A,c.13282+25741C>A	TTN:NM_133378:exon92:c.C23849A:p.S7950Y,TTN:NM_001256850:exon93:c.C26630A:p.S8877Y,TTN:NM_001267550:exon95:c.C27581A:p.S9194Y	NA	NA	NA	NA	NA	NA	NA	NA	MODIFIER	NA	NA	NA	NA	264	263	0	0	160	121	39	24.4	1.03	0.96
chr18	34349288	C	G	MISSENSE	FHOD3	c.4136C>G,c.4685C>G,c.4085C>G	FHOD3:NM_001281739:exon23:c.C4085G:p.A1362G,FHOD3:NM_025135:exon24:c.C4136G:p.A1379G,FHOD3:NM_001281740:exon28:c.C4685G:p.A1562G	NA	NA	NA	NA	NA	D	D	N	MODERATE	T	T	T	0.999	128	128	0	0	99	75	24	24.2	NA	NA
chr7	94928423	G	A	INTRONIC-SS-ACR-PRX	PON1	c.910-9C>T	NM_000446.5	NA	NA	NA	NA	NA	NA	NA	NA	MODIFIER	NA	NA	NA	NA	82	82	0	0	79	59	19	24.1	NA	NA
chr8	124358994	T	A	INTRONIC-SS-ACR-PRX	ATAD2	c.2213-4A>T	NM_014109.3	NA	chr8*124358988*124359005*COSM1623572*TATCTATAAAACCAAAAA*T*1*liver*1	NA	NA	NA	NA	NA	NA	LOW	NA	NA	NA	NA	38	36	1	2.63	34	26	8	23.5	0.95	0.93
chr7	124404566	A	T	SILENT	GPR37	c.465T>A	GPR37:NM_005302:exon1:c.T465A:p.G155G	NA	NA	NA	NA	NA	NA	NA	NA	LOW	NA	NA	NA	NA	67	66	0	0	50	39	11	22.0	NA	NA
chr2	15557821	A	C	MISSENSE	NBAS	c.2593T>G	NBAS:NM_015909:exon24:c.T2593G:p.S865A	NA	NA	NA	NA	NA	D	D	D	MODERATE	T	T	T	0.993	76	76	0	0	42	33	9	21.4	1.10	1.08
chr1	245849581	C	A	MISSENSE	KIF26B	c.3296C>A	KIF26B:NM_018012:exon12:c.C3296A:p.P1099Q	NA	NA	NA	NA	NA	D	NA	NA	MODERATE	NA	NA	NA	NA	182	182	0	0	202	158	43	21.3	NA	NA
chr17	37566476	T	A	MISSENSE	MED1	c.1998A>T	MED1:NM_004774:exon17:c.A1998T:p.L666F	NA	NA	NA	NA	NA	D_lc	D	NA	MODERATE	NA	NA	NA	NA	188	187	0	0	124	98	26	21.0	0.96	1.01
chr3	2928801	G	T	MISSENSE	CNTN4	c.833G>T,c.833G>T	CNTN4:NM_001206955:exon9:c.G833T:p.G278V,CNTN4:NM_175607:exon10:c.G833T:p.G278V	NA	NA	NA	NA	NA	D	D	D	MODERATE	T	T	T	1	111	111	0	0	93	73	19	20.4	NA	NA
chr5	132267859	A	G	INTRONIC	AFF4	c.963+11T>C	NM_014423.3	NA	NA	NA	NA	NA	NA	NA	NA	MODIFIER	NA	NA	NA	NA	34	34	0	0	30	23	6	20.0	1.11	0.99
chr9	15744487	C	T	NONSENSE	CCDC171	c.2266C>T	CCDC171:NM_173550:exon17:c.C2266T:p.Q756X	NA	NA	NA	NA	NA	NA	NA	NA	HIGH	NA	NA	NA	0.998	66	66	0	0	65	52	13	20.0	NA	NA
chr19	18368148	T	C	MISSENSE	KIAA1683	c.3247A>G,c.3946A>G	KIAA1683:NM_001145304:exon4:c.A3946G:p.I1316V,KIAA1683:NM_001145305:exon4:c.A3247G:p.I1083V,KIAA1683:NM_025249:exon4:c.A3385G:p.I1129V	NA	NA	NA	NA	NA	T	N	N	MODERATE	T	T	T	0.001	144	144	0	0	120	96	24	20.0	NA	NA
chr9	107299006	T	G	MISSENSE	OR13C3	c.89A>C	OR13C3:NM_001001961:exon1:c.A89C:p.D30A	NA	NA	NA	NA	NA	T	N	NA	MODERATE	NA	NA	NA	NA	55	55	0	0	52	41	10	19.2	NA	NA
chr1	152276762	T	G	MISSENSE	FLG	c.10600A>C	FLG:NM_002016:exon3:c.A10600C:p.N3534H	chr1*152276762*152276762*T*A*1*1*Brain_cancer	NA	rs12732870	2.47E-05	0.00179712	NA	NA	NA	MODERATE	NA	NA	NA	NA	163	158	1	0.61	148	120	28	18.9	NA	NA
chr11	57578884	A	C	MISSENSE	CTNND1	c.2546A>C,c.2243A>C,c.2384A>C,c.2546A>C,c.2384A>C,c.2261A>C,c.2384A>C,c.2402A>C,c.2243A>C,c.2261A>C,c.2243A>C,c.2243A>C,c.2243A>C,c.2402A>C,c.2384A>C,c.2546A>C,c.2564A>C,c.2384A>C,c.2546A>C,c.2243A>C,c.2546A>C	CTNND1:NM_001085469:exon13:c.A2243C:p.N748T,CTNND1:NM_001085464:exon14:c.A2243C:p.N748T,CTNND1:NM_001085465:exon14:c.A2243C:p.N748T,CTNND1:NM_001085467:exon14:c.A2243C:p.N748T,CTNND1:NM_001085468:exon14:c.A2243C:p.N748T,CTNND1:NM_001206890:exon14:c.A2243C:p.N748T,CTNND1:NM_001085462:exon15:c.A2546C:p.N849T,CTNND1:NM_001085463:exon15:c.A2261C:p.N754T,CTNND1:NM_001085466:exon15:c.A2261C:p.N754T,CTNND1:NM_001206886:exon15:c.A2384C:p.N795T,CTNND1:NM_001206887:exon15:c.A2384C:p.N795T,CTNND1:NM_001206888:exon15:c.A2384C:p.N795T,CTNND1:NM_001085459:exon16:c.A2546C:p.N849T,CTNND1:NM_001085460:exon16:c.A2546C:p.N849T,CTNND1:NM_001085461:exon16:c.A2546C:p.N849T,CTNND1:NM_001206883:exon16:c.A2402C:p.N801T,CTNND1:NM_001206884:exon16:c.A2402C:p.N801T,CTNND1:NM_001206889:exon16:c.A2384C:p.N795T,CTNND1:NM_001206891:exon16:c.A2384C:p.N795T,CTNND1:NM_001331:exon16:c.A2546C:p.N849T,CTNND1:NM_001085458:exon17:c.A2564C:p.N855T,CTNND1:NM_001206885:exon17:c.A2564C:p.N855T	NA	NA	NA	NA	NA	T	N	N	MODERATE	T	T	T	1	127	126	0	0	91	74	17	18.7	0.96	1.03
chr3	43344685	G	T	5UTR	SNRK	c.-11G>T,c.-11G>T	NM_001100594.1	NA	NA	NA	NA	NA	NA	NA	NA	MODIFIER	NA	NA	NA	NA	114	109	1	0.88	82	62	15	18.3	0.91	0.85
chr4	147215159	T	C	MISSENSE	SLC10A7	c.644A>G	SLC10A7:NM_001029998:exon8:c.A644G:p.Y215C,SLC10A7:NM_001300842:exon8:c.A644G:p.Y215C	NA	NA	NA	NA	NA	D	D	D	MODERATE	0	D	D	1	40	40	0	0	55	45	10	18.2	NA	NA
chr9	134006249	A	G	INTRONIC	NUP214	c.663+26A>G	NM_005085.3	NA	NA	NA	NA	NA	NA	NA	NA	MODIFIER	NA	NA	NA	NA	48	47	0	0	22	18	4	18.2	1.08	1.15
chr17	3629375	G	A	INTRONIC	ITGAE	c.3084+1838C>T	GSG2:NM_031965:exon1:c.G2146A:p.D716N	NA	NA	NA	NA	NA	NA	NA	NA	MODIFIER	NA	NA	NA	NA	260	259	0	0	157	129	28	17.8	NA	NA
chr17	3629375	G	A	MISSENSE	GSG2	c.2146G>A	GSG2:NM_031965:exon1:c.G2146A:p.D716N	NA	NA	NA	NA	NA	D	D	D	MODERATE	T	T	T	0.941	260	259	0	0	157	129	28	17.8	0.87	-
chr18	19079947	C	T	MISSENSE	GREB1L	c.3649C>T	GREB1L:NM_001142966:exon22:c.C3649T:p.P1217S	NA	NA	NA	NA	NA	T	N	N	MODERATE	T	T	T	0.047	73	73	0	0	68	56	12	17.7	NA	NA
chr14	77691855	G	A	INTRONIC	TMEM63C	c.350+51G>A	NM_020431.2	NA	NA	NA	NA	NA	NA	NA	NA	MODIFIER	NA	NA	NA	NA	188	186	0	0	120	99	21	17.5	NA	NA
chr4	1987575	C	T	SILENT	NELFA	c.852G>A	NELFA:NM_005663:exon7:c.G885A:p.E295E	NA	NA	NA	NA	NA	NA	NA	NA	LOW	NA	NA	NA	0.076	63	63	0	0	65	54	11	16.9	0.90	1.03
chr20	3870129	C	A	SILENT	PANK2	c.382C>A	PANK2:NM_153638:exon1:c.C382A:p.R128R	NA	NA	NA	NA	NA	NA	NA	NA	LOW	NA	NA	NA	0.988	69	69	0	0	65	53	11	16.9	1.13	0.99
chr20	3870129	C	A	INTRONIC	PANK2	c.-246+578C>A	PANK2:NM_153638:exon1:c.C382A:p.R128R	NA	NA	NA	NA	NA	NA	NA	NA	MODIFIER	NA	NA	NA	NA	69	69	0	0	65	53	11	16.9	1.13	0.99
chr12	125263041	C	T	3UTR	SCARB1	c.*92G>A	SCARB1:NM_001082959:exon12:c.G1493A:p.S498N	NA	NA	NA	NA	NA	NA	NA	NA	MODIFIER	NA	NA	NA	NA	36	35	0	0	30	22	5	16.7	1.07	1.03
chr12	125263041	C	T	MISSENSE	SCARB1	c.1493G>A	SCARB1:NM_001082959:exon12:c.G1493A:p.S498N	NA	NA	NA	NA	NA	T_lc	N	NA	MODERATE	NA	NA	NA	NA	36	35	0	0	30	22	5	16.7	1.07	1.03
chr6	35261591	G	A	MISSENSE	ZNF76	c.1393G>A	ZNF76:NM_003427:exon12:c.G1393A:p.G465S	NA	NA	rs758339207	9.07E-05	NA	T	N	N	MODERATE	T	T	T	0.872	130	126	1	0.77	96	80	16	16.7	NA	NA
chr2	220432184	T	A	MISSENSE	OBSL1	c.1648A>T,c.1648A>T,c.1648A>T	OBSL1:NM_001173408:exon4:c.A1648T:p.I550F,OBSL1:NM_001173431:exon4:c.A1648T:p.I550F,OBSL1:NM_015311:exon4:c.A1648T:p.I550F	NA	NA	NA	NA	NA	T	N	NA	MODERATE	NA	NA	NA	NA	110	108	0	0	60	50	10	16.7	NA	NA
chr2	69555385	C	T	INTRONIC	GFPT1	c.1839+18G>A,c.1893+18G>A	NM_001244710.1	NA	NA	NA	NA	NA	NA	NA	NA	MODIFIER	NA	NA	NA	NA	77	75	1	1.3	73	61	12	16.4	1.18	1.16
chr3	121386480	A	C	INTRONIC	GOLGB1	c.9435-23T>G,c.9405-23T>G	NM_001256487.1,NM_004487.4,NM_001256488.1	NA	NA	NA	NA	NA	NA	NA	NA	MODIFIER	NA	NA	NA	NA	48	48	0	0	31	25	5	16.1	1.09	1.04
chr16	3294096	C	T	INTRONIC	MEFV	c.1759+158G>A,c.1127-29G>A	NM_001198536.1	NA	NA	rs572084690	5.09E-05	0.000199681	NA	NA	NA	MODIFIER	NA	NA	NA	NA	112	110	0	0	75	62	12	16.0	NA	NA
chr16	76482075	A	G	SILENT	CNTNAP4	c.630A>G,c.702A>G	CNTNAP4:NM_138994:exon5:c.A630G:p.Q210Q	NA	NA	rs374636743	8.29E-05	0.000998403	NA	NA	NA	LOW	NA	NA	NA	0.996	71	71	0	0	77	65	12	15.6	NA	NA
chr12	76816325	A	G	INTRONIC	OSBPL8	c.218-11911T>C,c.92-11911T>C	NM_001003712.1	NA	NA	NA	NA	NA	NA	NA	NA	MODIFIER	NA	NA	NA	NA	69	69	0	0	71	60	11	15.5	1.30	1.26
chr9	140110759	C	T	MISSENSE	NDOR1	c.1561C>T,c.1712C>T,c.1685C>T	NDOR1:NM_001144027:exon13:c.C1561T:p.P521S,NDOR1:NM_001144026:exon14:c.C1712T:p.A571V,NDOR1:NM_001144028:exon14:c.C1664T:p.A555V,NDOR1:NM_014434:exon14:c.C1685T:p.A562V	NA	NA	rs767503630	8.32E-06	NA	D	N	N	MODERATE	T	T	T	0.137	254	253	1	0.39	183	154	28	15.3	-	0.94
chr6	76712572	C	T	INTRONIC	IMPG1	c.1291+63G>A	NM_001563.2,NM_001282368.1	NA	NA	NA	NA	NA	NA	NA	NA	MODIFIER	NA	NA	NA	NA	56	56	0	0	59	50	9	15.3	NA	NA
chr1	155920969	C	A	MISSENSE	ARHGEF2	c.2354G>T,c.2270G>T	ARHGEF2:NM_001162383:exon20:c.G2354T:p.G785V,ARHGEF2:NM_001162384:exon20:c.G2351T:p.G784V,ARHGEF2:NM_004723:exon20:c.G2270T:p.G757V	NA	NA	NA	NA	NA	T	D	D	MODERATE	T	T	T	0.771	63	62	0	0	53	45	8	15.1	1.10	1.19
chr1	74941281	C	A	INTRONIC	TNNI3K	c.2424+12047C>A,c.2121+12047C>A	NM_015978.2	NA	NA	NA	NA	NA	NA	NA	NA	MODIFIER	NA	NA	NA	NA	288	288	0	0	213	181	32	15.0	NA	NA
chr1	74941282	T	A	INTRONIC	TNNI3K	c.2424+12048T>A,c.2121+12048T>A	NM_015978.2	NA	NA	NA	NA	NA	NA	NA	NA	MODIFIER	NA	NA	NA	NA	287	287	0	0	213	181	32	15.0	NA	NA
chr10	72288971	C	A	INTRONIC	PALD1	c.186-14C>A	NM_014431.2	NA	NA	rs781687420	0.281410556	NA	NA	NA	NA	MODIFIER	NA	NA	NA	NA	36	36	0	0	40	34	6	15.0	NA	NA
chr11	17631837	C	T	MISSENSE	OTOG	c.5026C>T	OTOG:NM_001277269:exon35:c.C5026T:p.H1676Y,OTOG:NM_001292063:exon36:c.C4990T:p.H1664Y	NA	NA	NA	NA	NA	NA	NA	N	MODERATE	T	T	T	0.256	106	106	0	0	87	74	13	14.9	NA	NA
chr12	6939768	G	A	INTRONIC	LEPREL2	c.299-35G>A	NM_014262.3	NA	NA	NA	NA	NA	NA	NA	NA	MODIFIER	NA	NA	NA	NA	66	66	0	0	74	62	11	14.9	NA	NA
chr1	228475930	G	A	MISSENSE	OBSCN	c.9980G>A,c.11267G>A,c.9980G>A	OBSCN:NM_001098623:exon37:c.G9980A:p.C3327Y,OBSCN:NM_052843:exon37:c.G9980A:p.C3327Y,OBSCN:NM_001271223:exon42:c.G11267A:p.C3756Y	NA	NA	NA	NA	NA	NA	NA	N	MODERATE	T	T	T	0.985	194	192	0	0	182	155	27	14.8	0.88	-
chr3	174974253	G	C	MISSENSE	NAALADL2	c.873G>C	NAALADL2:NM_207015:exon4:c.G873C:p.R291S	NA	NA	NA	NA	NA	D	N	N	MODERATE	T	T	T	0.261	36	36	0	0	27	23	4	14.8	NA	NA
chr3	129389548	T	A	MISSENSE	TMCC1	c.794A>T,c.1136A>T	TMCC1:NM_001017395:exon4:c.A1136T:p.K379I,TMCC1:NM_001128224:exon4:c.A794T:p.K265I	NA	NA	NA	NA	NA	D	D	D	MODERATE	T	T	T	1	178	178	0	0	149	126	22	14.8	NA	NA
chr5	74643098	G	T	MISSENSE	HMGCR	c.520G>T,c.520G>T	HMGCR:NM_000859:exon6:c.G520T:p.V174F,HMGCR:NM_001130996:exon6:c.G520T:p.V174F	NA	NA	NA	NA	NA	D	D	D	MODERATE	D	D	D	0.991	118	117	0	0	109	92	16	14.7	1.11	-
chr19	52376062	C	T	MISSENSE	ZNF577	c.1004G>A,c.1181G>A	ZNF577:NM_001135590:exon7:c.G1004A:p.S335N,ZNF577:NM_032679:exon7:c.G1181A:p.S394N	NA	NA	NA	NA	NA	T	N	NA	MODERATE	NA	NA	NA	NA	129	129	0	0	110	94	16	14.6	NA	NA
chr1	150531144	G	A	MISSENSE	ADAMTSL4	c.2578G>A	ADAMTSL4:NM_025008:exon15:c.G2578A:p.A860T	NA	NA	NA	NA	NA	T_lc	N	NA	MODERATE	NA	NA	NA	NA	148	147	1	0.68	124	106	18	14.5	NA	NA
chr1	150531144	G	A	INTRONIC	ADAMTSL4	c.2559+19G>A,c.2628+19G>A	ADAMTSL4:NM_025008:exon15:c.G2578A:p.A860T	NA	NA	NA	NA	NA	NA	NA	NA	MODIFIER	NA	NA	NA	NA	148	147	1	0.68	124	106	18	14.5	NA	NA
chr10	79595570	C	A	SILENT	DLG5	c.1548G>T	DLG5:NM_004747:exon8:c.G1548T:p.A516A	NA	NA	NA	NA	NA	NA	NA	NA	LOW	NA	NA	NA	NA	92	92	0	0	90	77	13	14.4	NA	NA
chr3	97631140	G	T	INTRONIC	CRYBG3	c.2270+20G>T	NM_153605.3	NA	NA	NA	NA	NA	NA	NA	NA	MODIFIER	NA	NA	NA	NA	34	34	0	0	28	24	4	14.3	1.07	0.95
chr5	102249587	G	T	INTRONIC	PAM	c.269-51G>T,c.269-51G>T,c.269-51G>T	NM_001177306.1,NM_000919.3,NM_138766.2	NA	NA	rs112746759	0.014625396	0.00738818	NA	NA	NA	MODIFIER	NA	NA	NA	NA	103	103	0	0	99	84	14	14.1	NA	NA
chr7	4900818	G	A	SILENT	PAPOLB	c.621C>T	PAPOLB:NM_020144:exon1:c.C624T:p.T208T	NA	NA	rs767678590	3.31E-05	NA	NA	NA	NA	LOW	NA	NA	NA	NA	158	158	0	0	142	122	20	14.1	NA	NA
chr7	4900818	G	A	INTRONIC	RADIL	c.535+16418C>T	PAPOLB:NM_020144:exon1:c.C624T:p.T208T	NA	NA	rs767678590	3.31E-05	NA	NA	NA	NA	MODIFIER	NA	NA	NA	NA	158	158	0	0	142	122	20	14.1	NA	NA
chr8	77761771	C	A	MISSENSE	ZFHX4	c.3669C>A	ZFHX4:NM_024721:exon8:c.C3669A:p.N1223K	NA	NA	NA	NA	NA	NA	NA	U	MODERATE	T	T	T	1	68	68	0	0	57	49	8	14.0	NA	NA
chr17	72846759	G	A	MISSENSE	GRIN2C	c.1261C>T,c.1261C>T	GRIN2C:NM_000835:exon5:c.C1261T:p.P421S,GRIN2C:NM_001278553:exon5:c.C1261T:p.P421S	NA	NA	rs755801106	1.66E-05	NA	T	N	D	MODERATE	T	T	T	0.991	181	179	0	0	144	124	20	13.9	NA	NA
chr1	213414169	T	C	SILENT	RPS6KC1	c.1314T>C,c.1350T>C,c.714T>C	RPS6KC1:NM_001136138:exon10:c.T1314C:p.P438P,RPS6KC1:NM_001287221:exon10:c.T807C:p.P269P,RPS6KC1:NM_001287218:exon11:c.T714C:p.P238P,RPS6KC1:NM_001287219:exon11:c.T714C:p.P238P,RPS6KC1:NM_012424:exon11:c.T1350C:p.P450P	NA	NA	NA	NA	NA	NA	NA	NA	LOW	NA	NA	NA	NA	113	113	0	0	124	107	17	13.7	NA	NA
chr2	27801951	C	T	MISSENSE	C2orf16	c.2512C>T	C2orf16:NM_032266:exon1:c.C2512T:p.L838F	NA	NA	NA	NA	NA	T	N	NA	MODERATE	NA	NA	NA	NA	226	226	0	0	183	157	25	13.7	NA	NA
chr1	147410812	G	T	INTRONIC	GPR89B	c.103-16G>T	NM_016334.3	NA	NA	NA	NA	NA	NA	NA	NA	MODIFIER	NA	NA	NA	NA	57	57	0	0	59	51	8	13.6	NA	NA
chr7	150762053	G	A	MISSENSE-SS-PRX	SLC4A2	c.578G>A,c.551G>A,c.536G>A,c.578G>A	SLC4A2:NM_001199693:exon4:c.G551A:p.G184E,SLC4A2:NM_001199694:exon4:c.G536A:p.G179E,SLC4A2:NM_001199692:exon5:c.G578A:p.G193E,SLC4A2:NM_003040:exon5:c.G578A:p.G193E	chr7*150762052*150762053*GG*TT*1*1*Lung_cancer	chr7*150762051*150762053*COSM380672*TGG*TTT*1*lung*1	NA	NA	NA	T_lc	N	N	MODERATE	T	T	T	0.83	82	82	0	0	74	64	10	13.5	-	0.86
chr12	109865414	G	A	INTRONIC	MYO1H	c.1931+23G>A	NM_001101421.3	NA	NA	NA	NA	NA	NA	NA	NA	MODIFIER	NA	NA	NA	NA	122	122	0	0	89	76	12	13.5	NA	NA
chr9	8500876	G	A	INTRONIC	PTPRD	c.1792+3385C>T,c.1822+3385C>T,c.1813+3385C>T	PTPRD:NM_002839:exon24:c.C2006T:p.T669I	NA	NA	NA	NA	NA	NA	NA	NA	MODIFIER	NA	NA	NA	NA	82	81	0	0	67	58	9	13.4	0.77	-
chr9	8500876	G	A	MISSENSE	PTPRD	c.2006C>T	PTPRD:NM_002839:exon24:c.C2006T:p.T669I	NA	NA	NA	NA	NA	T	N	N	MODERATE	T	T	T	1	82	81	0	0	67	58	9	13.4	0.77	-
chr11	129756247	G	A	MISSENSE	NFRKB	c.430C>T,c.430C>T	NFRKB:NM_006165:exon3:c.C469T:p.H157Y,NFRKB:NM_001143835:exon5:c.C430T:p.H144Y	NA	NA	NA	NA	NA	T	N	N	MODERATE	0	T	T	0.999	145	144	0	0	67	57	9	13.4	-	1.17
chrX	68725754	G	T	MISSENSE	FAM155B	c.629G>T	FAM155B:NM_015686:exon1:c.G629T:p.G210V	NA	NA	NA	NA	NA	D	D	N	MODERATE	T	T	T	1	201	197	2	1	82	71	11	13.4	NA	NA
chrX	82763991	G	A	MISSENSE	POU3F4	c.659G>A	POU3F4:NM_000307:exon1:c.G659A:p.G220D	NA	NA	NA	NA	NA	D	D	D	MODERATE	T	D	D	1	66	66	0	0	45	39	6	13.3	NA	NA
chr22	44645481	C	A	3UTR	KIAA1644	c.*84G>T	NM_001099294.1	NA	NA	NA	NA	NA	NA	NA	NA	MODIFIER	NA	NA	NA	NA	27	27	0	0	30	26	4	13.3	NA	NA
chr1	196918681	A	C	MISSENSE	CFHR2	c.155A>C	CFHR2:NM_005666:exon2:c.A155C:p.Y52S	NA	NA	NA	NA	NA	T	D	NA	MODERATE	NA	NA	NA	0.023	111	111	0	0	115	100	15	13.0	NA	NA
chr11	65632574	C	A	SILENT	MUS81	c.1359C>A	MUS81:NM_025128:exon13:c.C1359A:p.L453L	NA	NA	NA	NA	NA	NA	NA	NA	LOW	NA	NA	NA	NA	284	283	1	0.35	177	154	23	13.0	NA	NA
chr5	175111348	C	T	INTRONIC	HRH2	c.1076+36C>T	NM_001131055.1	NA	NA	NA	NA	NA	NA	NA	NA	MODIFIER	NA	NA	NA	NA	102	100	0	0	63	55	8	12.7	NA	NA
chr5	175111348	C	T	3UTR	HRH2	c.*32C>T	NM_022304.2	NA	NA	NA	NA	NA	NA	NA	NA	MODIFIER	NA	NA	NA	NA	102	100	0	0	63	55	8	12.7	NA	NA
chr3	119155768	T	G	MISSENSE	TMEM39A	c.982A>C	TMEM39A:NM_018266:exon7:c.A982C:p.N328H	NA	NA	NA	NA	NA	D	D	D	MODERATE	T	T	T	1	91	91	0	0	79	69	10	12.7	NA	NA
chr16	47117463	G	T	NONSENSE	NETO2	c.1247C>A,c.1226C>A	NETO2:NM_001201477:exon9:c.C1226A:p.S409X,NETO2:NM_018092:exon9:c.C1247A:p.S416X	NA	NA	NA	NA	NA	NA	NA	NA	HIGH	NA	NA	NA	0.999	146	144	0	0	159	139	20	12.6	NA	NA
chr12	13103073	G	A	SILENT	GPRC5D	c.246C>T	GPRC5D:NM_018654:exon1:c.C246T:p.I82I	NA	NA	rs370143091	8.24E-06	NA	NA	NA	NA	LOW	NA	NA	NA	0.795	68	65	1	1.47	64	56	8	12.5	NA	NA
chr2	179408883	G	T	INTRONIC	TTN	c.69209-41C>A,c.96029-41C>A,c.69410-41C>A,c.88325-41C>A,c.68834-41C>A	NM_003319.4	NA	NA	NA	NA	NA	NA	NA	NA	MODIFIER	NA	NA	NA	NA	133	133	0	0	80	69	10	12.5	1.03	0.96
chr2	179417685	G	T	MISSENSE	TTN	c.63122C>A,c.89942C>A,c.63323C>A,c.82238C>A,c.62747C>A	TTN:NM_003319:exon163:c.C62747A:p.S20916Y,TTN:NM_133432:exon164:c.C63122A:p.S21041Y,TTN:NM_133437:exon164:c.C63323A:p.S21108Y,TTN:NM_133378:exon284:c.C82238A:p.S27413Y,TTN:NM_001256850:exon285:c.C85019A:p.S28340Y,TTN:NM_001267550:exon335:c.C89942A:p.S29981Y	NA	NA	NA	NA	NA	NA	NA	NA	MODERATE	NA	NA	NA	NA	169	169	0	0	88	76	11	12.5	1.03	0.96
chr18	31226270	A	G	MISSENSE	ASXL3	c.308A>G	ASXL3:NM_030632:exon4:c.A308G:p.D103G	NA	NA	rs764906167	3.33E-05	NA	NA	NA	NA	MODERATE	NA	NA	NA	NA	35	35	0	0	56	49	7	12.5	NA	NA
chr1	118494571	G	C	INTRONIC	WDR3	c.1804-28G>C	NM_006784.2	NA	NA	NA	NA	NA	NA	NA	NA	MODIFIER	NA	NA	NA	NA	69	69	0	0	49	43	6	12.2	1.08	1.13
chr19	11618613	C	T	INTRONIC	ECSIT	c.796+193G>A,c.797-69G>A	ECSIT:NM_001142465:exon4:c.G207A:p.R69R,ECSIT:NM_016581:exon6:c.G849A:p.R283R	NA	NA	NA	NA	NA	NA	NA	NA	MODIFIER	NA	NA	NA	NA	268	262	2	0.75	180	157	22	12.2	0.95	1.02
chr19	11618613	C	T	SILENT	ECSIT	c.849G>A,c.207G>A	ECSIT:NM_001142465:exon4:c.G207A:p.R69R,ECSIT:NM_016581:exon6:c.G849A:p.R283R	NA	NA	NA	NA	NA	NA	NA	NA	LOW	NA	NA	NA	NA	268	262	2	0.75	180	157	22	12.2	0.95	1.02
chr16	101657	A	C	INTRONIC	POLR3K	c.112-12T>G	NM_016310.3	NA	NA	NA	NA	NA	NA	NA	NA	MODIFIER	NA	NA	NA	NA	100	98	1	1	100	88	12	12.0	NA	NA
chr15	68438237	G	A	MISSENSE	PIAS1	c.686G>A	PIAS1:NM_016166:exon5:c.G686A:p.S229N	NA	NA	NA	NA	NA	T	N	D	MODERATE	T	T	T	1	89	89	0	0	67	59	8	11.9	0.70	0.91
chr9	85640702	A	G	MISSENSE	RASEF	c.566T>C	RASEF:NM_152573:exon2:c.T566C:p.I189T	NA	NA	NA	NA	NA	T	N	D	MODERATE	T	T	T	1	158	156	1	0.63	126	111	15	11.9	NA	NA
chr3	143101016	G	C	INTRONIC	SLC9A9	c.1470-60C>G	NM_173653.3	NA	NA	NA	NA	NA	NA	NA	NA	MODIFIER	NA	NA	NA	NA	89	89	0	0	76	66	9	11.8	NA	NA
chr5	140572289	C	T	MISSENSE	PCDHB10	c.164C>T	PCDHB10:NM_018930:exon1:c.C164T:p.A55V	NA	NA	NA	NA	NA	D_lc	N	NA	MODERATE	NA	NA	NA	NA	66	66	0	0	51	45	6	11.8	NA	NA
chr6	170639522	A	T	INTRONIC	FAM120B	c.1916-15A>T,c.-89-15A>T	NM_001286381.1	NA	NA	NA	NA	NA	NA	NA	NA	MODIFIER	NA	NA	NA	NA	229	227	0	0	197	170	23	11.7	1.07	1.11
chr10	50533062	C	A	INTRONIC	C10orf71	c.2074+398C>A	C10orf71:NM_001135196:exon3:c.C2472A:p.G824G	NA	NA	NA	NA	NA	NA	NA	NA	MODIFIER	NA	NA	NA	NA	110	108	0	0	120	106	14	11.7	NA	NA
chr2	207516534	A	C	3UTR	DYTN	c.*8T>G	NM_001093730.1	NA	NA	NA	NA	NA	NA	NA	NA	MODIFIER	NA	NA	NA	NA	45	45	0	0	52	45	6	11.5	NA	NA
chr17	6596438	C	T	SILENT	SLC13A5	c.1071G>A,c.1149G>A,c.1200G>A,c.1200G>A	SLC13A5:NM_001284510:exon8:c.G1071A:p.K357K,SLC13A5:NM_001143838:exon9:c.G1200A:p.K400K,SLC13A5:NM_001284509:exon9:c.G1149A:p.K383K,SLC13A5:NM_177550:exon9:c.G1200A:p.K400K	NA	NA	NA	NA	NA	NA	NA	NA	LOW	NA	NA	NA	0.019	180	176	1	0.56	123	109	14	11.4	NA	NA
chrX	82763990	G	A	MISSENSE	POU3F4	c.658G>A	POU3F4:NM_000307:exon1:c.G658A:p.G220S	chrX*82763990*82763990*G*T*1*1*Head_and_neck_cancer	chrX*82763990*82763990*COSM125508*G*T*1*upper_aerodigestive_tract*1	NA	NA	NA	D	D	D	MODERATE	T	D	T	1	65	65	0	0	44	39	5	11.4	NA	NA
chr9	103109150	T	C	MISSENSE	TEX10	c.719A>G,c.728A>G	TEX10:NM_001161584:exon3:c.A728G:p.D243G,TEX10:NM_017746:exon3:c.A719G:p.D240G	NA	NA	NA	NA	NA	D	N	D	MODERATE	0	T	T	0.999	164	164	0	0	142	125	16	11.3	1.00	1.01
chr17	10312949	A	G	INTRONIC	MYH8	c.1588-44T>C	NM_002472.2	NA	NA	NA	NA	NA	NA	NA	NA	MODIFIER	NA	NA	NA	NA	163	162	0	0	90	80	10	11.1	NA	NA
chr19	15580404	G	A	INTRONIC	PGLYRP2	c.1641+39C>T	NM_052890.3	NA	NA	NA	NA	NA	NA	NA	NA	MODIFIER	NA	NA	NA	NA	172	169	2	1.16	108	95	12	11.1	NA	NA
chr11	64622012	G	A	SILENT	EHD1	c.1398C>T,c.1398C>T	EHD1:NM_006795:exon5:c.C1398T:p.N466N,EHD1:NM_001282445:exon6:c.C1440T:p.N480N,EHD1:NM_001282444:exon7:c.C1398T:p.N466N	NA	NA	rs761231096	0.000008241	NA	NA	NA	NA	LOW	NA	NA	NA	0.995	592	591	1	0.17	350	311	38	10.9	0.84	0.83
chr1	249211609	T	G	MISSENSE	PGBD2	c.826T>G,c.73T>G	PGBD2:NM_001017434:exon3:c.T73G:p.F25V,PGBD2:NM_170725:exon3:c.T826G:p.F276V	NA	NA	NA	NA	NA	D	D	N	MODERATE	T	T	T	0.997	235	234	1	0.43	194	171	21	10.8	NA	NA
chr7	35707122	T	A	MISSENSE	HERPUD2	c.416A>T	HERPUD2:NM_022373:exon5:c.A416T:p.E139V	NA	NA	NA	NA	NA	D	N	D	MODERATE	T	T	T	0.987	61	61	0	0	47	41	5	10.6	NA	NA
chr15	66625179	T	C	SILENT	DIS3L	c.2538T>C,c.2787T>C	DIS3L:NM_001143688:exon16:c.T2787C:p.F929F,DIS3L:NM_133375:exon16:c.T2538C:p.F846F	NA	NA	NA	NA	NA	NA	NA	NA	LOW	NA	NA	NA	1	202	202	0	0	113	101	12	10.6	1.11	1.02
chr7	142919188	C	T	MISSENSE	TAS2R40	c.17C>T	TAS2R40:NM_176882:exon1:c.C17T:p.T6I	NA	NA	NA	NA	NA	T	N	U	MODERATE	T	T	T	0.038	71	71	0	0	57	49	6	10.5	NA	NA
chr14	31535590	A	T	INTRONIC	AP4S1	c.138+50A>T,c.138+50A>T,c.138+50A>T,c.138+50A>T,c.138+50A>T	NM_001254729.1	NA	NA	NA	NA	NA	NA	NA	NA	MODIFIER	NA	NA	NA	NA	128	127	0	0	67	60	7	10.5	0.87	0.91
chr15	88476422	A	G	INTRONIC-SS-ACR-PRX	NTRK3	c.1717-7T>C,c.1717-7T>C	NM_001012338.2	NA	NA	NA	NA	NA	NA	NA	NA	LOW	NA	NA	NA	NA	72	72	0	0	58	52	6	10.3	NA	NA
chr3	20043402	C	G	MISSENSE	PP2D1	c.210G>C	PP2D1:NM_001252657:exon2:c.G210C:p.K70N	NA	NA	NA	NA	NA	T	N	N	MODERATE	T	T	T	0.021	162	160	1	0.62	117	105	12	10.3	NA	NA
chr11	113085125	T	A	SILENT	NCAM1	c.1084T>A,c.597T>A,c.1128T>A,c.921T>A	NCAM1:NM_000615:exon9:c.T951A:p.T317T,NCAM1:NM_001076682:exon9:c.T951A:p.T317T,NCAM1:NM_001242607:exon9:c.T951A:p.T317T,NCAM1:NM_001242608:exon9:c.T951A:p.T317T,NCAM1:NM_181351:exon9:c.T951A:p.T317T	NA	NA	NA	NA	NA	NA	NA	NA	LOW	NA	NA	NA	NA	166	165	0	0	117	105	12	10.3	NA	NA
chr19	53553042	C	G	MISSENSE	ERVV-2	c.538C>G	ERVV-2:NM_001191055:exon2:c.C538G:p.Q180E	NA	NA	NA	NA	NA	T	N	NA	MODERATE	NA	NA	NA	NA	196	196	0	0	148	133	15	10.1	NA	NA
chr9	139617638	T	C	SILENT	FAM69B	c.708T>C	FAM69B:NM_152421:exon5:c.T708C:p.H236H	NA	NA	rs755047860	1.68E-05	NA	NA	NA	NA	LOW	NA	NA	NA	0.035	177	177	0	0	129	116	13	10.1	NA	NA
chr12	6925448	C	T	SILENT	CD4	c.834C>T	CD4:NM_001195014:exon4:c.C297T:p.L99L,CD4:NM_001195015:exon4:c.C15T:p.L5L,CD4:NM_001195016:exon4:c.C15T:p.L5L,CD4:NM_001195017:exon4:c.C15T:p.L5L,CD4:NM_000616:exon6:c.C834T:p.L278L	NA	NA	NA	NA	NA	NA	NA	NA	LOW	NA	NA	NA	NA	112	112	0	0	100	89	10	10.0	NA	NA
chr19	5789181	G	A	INTRONIC	DUS3L	c.900+37C>T,c.175-772C>T	NM_001161619.1	NA	NA	rs767759381	9.47E-06	NA	NA	NA	NA	MODIFIER	NA	NA	NA	NA	204	201	0	0	120	107	12	10.0	1.00	1.14
chr9	117852865	C	T	MISSENSE	TNC	c.433G>A	TNC:NM_002160:exon2:c.G433A:p.G145S	NA	NA	NA	NA	NA	T	N	N	MODERATE	T	T	T	0.83	152	150	2	1.32	141	127	14	9.9	0.81	0.65
chr1	146683952	G	A	INTRONIC-SS-DNR-PRX	FMO5	c.630+9C>T,c.630+9C>T,c.630+9C>T	NM_001144829.2	NA	NA	NA	NA	NA	NA	NA	NA	MODIFIER	NA	NA	NA	NA	86	85	0	0	62	56	6	9.7	NA	NA

Khan *et al.* . 2020. Multi-omics analysis to characterize cigarette smoke induced molecular alterations in esophageal cells
Supplementary Table 3. List of somatic single nucleotide variants identified in Het-1A cells chronically treated with cigarette smoke condensate for 8 months

																													Protein quantification	
Chromosome	Genomic position	Reference allele	Alternate allele	Mutation type	Gene symbol	Nucleotide change	Description	OncoMD	COSMIC	dbSNP	ExAC	1000G	SIFT	CONDEL	LRT	VEP	FATHMM	METASVM	METALR	PhastCons7way	DP in Het-1A-Parental	Reference read in Het-1A-Parental	Alternative read in Het-1A-Parental	Alternate allele frequency in Het-1A-Parental (%)	DP in Het-1A-Smoke-8M	Reference read in Het-1A-Smoke-8M	Alternate read in Het-1A-Smoke-8M	Alternate allele frequency in Het-1A-Smoke-8M (%)	Het-1A-Smoke - 8M/Parental (130C+131)/(126+127N) Replicate 1	Het-1A-Smoke - 8M/Parental (130C+131)/(126+127N) Replicate 2
chr12	57865376	C	A	SILENT	***GLI1***	c.2853C>A,c.2469C>A,c.2730C>A	GLI1:NM_001160045:exon10:c.C2469A:p.G823G,GLI1:NM_001167609:exon11:c.C2730A:p.G910G,GLI1:NM_005269:exon12:c.C2853A:p.G951G	NA	NA	NA	NA	NA	NA	NA	NA	LOW	NA	NA	NA	NA	100	99	1	1	73	66	7	9.6	NA	NA
chr9	135947077	G	A	MISSENSE	***CEL***	c.2197G>A	CEL:NM_001807:exon11:c.G2197A:p.D733N	NA	NA	NA	NA	NA	D_lc	D	NA	MODERATE	NA	NA	NA	NA	92	90	0	0	74	67	7	9.5	NA	NA
chr2	37543524	T	C	SILENT	***PRKD3***	c.144A>G	PRKD3:NM_005813:exon1:c.A144G:p.P48P	NA	NA	NA	NA	NA	NA	NA	NA	LOW	NA	NA	NA	NA	137	136	0	0	117	106	11	9.4	NA	NA
chr12	57432751	G	A	MISSENSE	***MYO1A***	c.1375C>T,c.1375C>T	MYO1A:NM_005379:exon16:c.C1375T:p.R459W,MYO1A:NM_001256041:exon17:c.C1375T:p.R459W	NA	NA	NA	NA	NA	D	D	D	MODERATE	T	D	D	0.892	79	79	0	0	75	68	7	9.3	-	0.76
chr8	41397381	C	T	INTRONIC	***GINS4***	c.396-54C>T	NM_032336.2	NA	NA	NA	NA	NA	NA	NA	NA	MODIFIER	NA	NA	NA	NA	111	109	0	0	75	68	7	9.3	0.91	0.89
chr14	53098848	A	G	INTRONIC	***GPR137C***	c.718-30A>G	NM_001099652.1	NA	NA	NA	NA	NA	NA	NA	NA	MODIFIER	NA	NA	NA	NA	133	133	0	0	77	70	7	9.1	NA	NA
chr16	2329054	C	T	MISSENSE	***ABCA3***	c.4437G>A	ABCA3:NM_001089:exon29:c.G4437A:p.M1479I	NA	NA	rs776272923	0.000008297	NA	D	D	D	MODERATE	D	D	D	0.999	98	98	0	0	88	80	8	9.1	NA	NA
chrX	101091715	C	T	INTRONIC	***NXF5***	c.1082+749G>A	NM_032946.2	chrX*101091715*101091715*G*A*1*1*Rectal_cancer|chrX*101091715*101091715*C*T*1*1*Colorectal_cancer	chrX*101091715*101091715*COSM171856*C*T*1*large_intestine*1	rs373035757	4.56E-05	NA	NA	NA	NA	MODIFIER	NA	NA	NA	NA	124	123	0	0	56	51	5	8.9	NA	NA
chr6	12120318	A	G	MISSENSE	***HIVEP1***	c.290A>G	HIVEP1:NM_002114:exon4:c.A290G:p.Q97R	NA	NA	NA	NA	NA	T	N	N	MODERATE	T	T	T	0.346	179	177	0	0	90	82	8	8.9	NA	NA
chr15	74282628	T	C	INTRONIC	***STOML1***	c.240+64A>G,c.240+64A>G,c.240+64A>G,c.-22+64A>G	NM_001256676.1	NA	NA	NA	NA	NA	NA	NA	NA	MODIFIER	NA	NA	NA	NA	112	111	0	0	79	71	7	8.9	NA	NA
chr11	2593289	C	T	NONSENSE	***KCNQ1***	c.730C>T	KCNQ1:NM_000218:exon5:c.C730T:p.Q244X,KCNQ1:NM_181798:exon5:c.C349T:p.Q117X	NA	NA	NA	NA	NA	NA	NA	NA	HIGH	NA	NA	NA	0.855	70	68	0	0	68	62	6	8.8	NA	NA
chr6	38840564	C	T	INTRONIC-SS-DNR-PRX	***DNAH8***	c.7201+6C>T	NM_001206927.1	NA	NA	NA	NA	NA	NA	NA	NA	LOW	NA	NA	NA	NA	124	122	0	0	80	72	7	8.8	-	0.81
chr20	44517555	G	A	INTRONIC	***NEURL2***	c.743-43C>T	NM_001278535.1,NM_080749.3	NA	NA	rs781461236	0.000008723	NA	NA	NA	NA	MODIFIER	NA	NA	NA	NA	113	112	0	0	80	73	7	8.8	NA	NA
chr1	113253678	G	C	MISSENSE	***PPM1J***	c.1062C>G	PPM1J:NM_005167:exon7:c.C1062G:p.I354M	NA	NA	NA	8.24E-06	NA	D	D	D	MODERATE	T	T	T	0.992	122	122	0	0	81	72	7	8.6	NA	NA
chr6	26087821	G	C	INTRONIC	***HFE***	c.76+77G>C,c.76+77G>C,c.76+77G>C,c.76+77G>C,c.76+77G>C,c.76+77G>C	NM_139011.2	NA	NA	NA	NA	NA	NA	NA	NA	MODIFIER	NA	NA	NA	NA	136	133	0	0	81	74	7	8.6	NA	NA
chr1	78478844	C	T	SILENT	***DNAJB4***	c.321C>T	DNAJB4:NM_007034:exon2:c.C321T:p.P107P	NA	NA	NA	NA	NA	NA	NA	NA	LOW	NA	NA	NA	NA	217	216	0	0	151	138	13	8.6	1.11	1.07
chr5	74442988	T	G	MISSENSE	***ANKRD31***	c.2248A>C	ANKRD31:NM_001164443:exon14:c.A2248C:p.I750L	NA	NA	NA	NA	NA	D	N	NA	MODERATE	NA	NA	NA	NA	208	207	0	0	153	140	13	8.5	NA	NA
chr5	147594531	T	C	3UTR	***SPINK6***	c.*18T>C	NM_205841.3,NM_001195290.1	NA	NA	NA	NA	NA	NA	NA	NA	MODIFIER	NA	NA	NA	NA	113	112	0	0	119	109	10	8.4	NA	NA
chr8	133141566	C	A	SILENT	***KCNQ3***	c.2202G>T,c.2562G>T	KCNQ3:NM_001204824:exon15:c.G2202T:p.S734S,KCNQ3:NM_004519:exon15:c.G2562T:p.S854S	NA	NA	NA	NA	NA	NA	NA	NA	LOW	NA	NA	NA	NA	119	118	0	0	111	102	9	8.1	NA	NA
chr1	209806453	A	C	MISSENSE	***LAMB3***	c.590T>G,c.590T>G,c.590T>G	LAMB3:NM_001017402:exon6:c.T590G:p.V197G,LAMB3:NM_000228:exon7:c.T590G:p.V197G,LAMB3:NM_001127641:exon7:c.T590G:p.V197G	NA	NA	NA	NA	NA	T	N	D	MODERATE	T	T	T	0.995	116	115	0	0	75	69	6	8.0	NA	NA
chr2	70919633	G	C	MISSENSE	***ADD2***	c.607C>G,c.607C>G,c.655C>G,c.607C>G,c.607C>G	ADD2:NM_001185055:exon6:c.C655G:p.P219A,ADD2:NM_001185054:exon7:c.C607G:p.P203A,ADD2:NM_001617:exon7:c.C607G:p.P203A,ADD2:NM_017482:exon7:c.C607G:p.P203A,ADD2:NM_017488:exon7:c.C607G:p.P203A	NA	NA	NA	NA	NA	T	N	N	MODERATE	T	T	T	0.999	110	110	0	0	101	93	8	7.9	1.23	0.81
chr14	59931069	G	T	SILENT	***GPR135***	c.876C>A,c.876C>A	GPR135:NM_022571:exon1:c.C876A:p.L292L	NA	NA	NA	NA	NA	NA	NA	NA	LOW	NA	NA	NA	NA	182	182	0	0	127	115	10	7.9	NA	NA
chr15	52897357	T	G	MISSENSE	***FAM214A***	c.2433A>C,c.2454A>C	FAM214A:NM_001286495:exon6:c.A2454C:p.Q818H,FAM214A:NM_019600:exon7:c.A2433C:p.Q811H	NA	NA	NA	NA	NA	T	D	N	MODERATE	T	T	T	0.987	93	93	0	0	89	82	7	7.9	NA	NA
chr5	131676351	T	C	MISSENSE	***SLC22A4***	c.1538T>C	SLC22A4:NM_003059:exon9:c.T1538C:p.M513T	NA	NA	NA	NA	NA	T	N	N	MODERATE	T	T	T	0.997	148	146	1	0.68	156	142	12	7.7	NA	NA
chr7	139829336	G	A	SILENT	***KDM7A***	c.516C>T	KDM7A:NM_030647:exon4:c.C516T:p.L172L	NA	NA	NA	NA	NA	NA	NA	NA	LOW	NA	NA	NA	NA	151	151	0	0	130	120	10	7.7	NA	NA
chr7	139829335	G	A	MISSENSE	***KDM7A***	c.517C>T	KDM7A:NM_030647:exon4:c.C517T:p.P173S	chr7*139829335*139829335*C*T*2*2*Melanoma	chr7*139829335*139829335*COSM224335*G*A*1*skin*1	NA	NA	NA	D	D	D	MODERATE	T	D	T	0.998	150	149	0	0	132	121	10	7.6	NA	NA
chr6	154360803	T	C	INTRONIC	***OPRM1***	c.47+29109T>C	OPRM1:NM_000914:exon1:c.T124C:p.S42P,OPRM1:NM_001008503:exon1:c.T124C:p.S42P,OPRM1:NM_001008504:exon1:c.T124C:p.S42P,OPRM1:NM_001008505:exon1:c.T124C:p.S42P,OPRM1:NM_001145282:exon1:c.T124C:p.S42P,OPRM1:NM_001145283:exon1:c.T124C:p.S42P,OPRM1:NM_001145284:exon1:c.T124C:p.S42P,OPRM1:NM_001145285:exon1:c.T124C:p.S42P,OPRM1:NM_001145286:exon1:c.T124C:p.S42P,OPRM1:NM_001285522:exon1:c.T124C:p.S42P,OPRM1:NM_001285523:exon1:c.T124C:p.S42P,OPRM1:NM_001285524:exon2:c.T403C:p.S135P,OPRM1:NM_001145279:exon3:c.T403C:p.S135P	NA	NA	NA	NA	NA	NA	NA	NA	MODIFIER	NA	NA	NA	NA	169	169	0	0	187	172	14	7.5	NA	NA
chr6	154360803	T	C	MISSENSE	***OPRM1***	c.124T>C,c.124T>C,c.124T>C,c.124T>C,c.124T>C,c.124T>C,c.124T>C,c.124T>C,c.403T>C,c.124T>C,c.124T>C	OPRM1:NM_000914:exon1:c.T124C:p.S42P,OPRM1:NM_001008503:exon1:c.T124C:p.S42P,OPRM1:NM_001008504:exon1:c.T124C:p.S42P,OPRM1:NM_001008505:exon1:c.T124C:p.S42P,OPRM1:NM_001145282:exon1:c.T124C:p.S42P,OPRM1:NM_001145283:exon1:c.T124C:p.S42P,OPRM1:NM_001145284:exon1:c.T124C:p.S42P,OPRM1:NM_001145285:exon1:c.T124C:p.S42P,OPRM1:NM_001145286:exon1:c.T124C:p.S42P,OPRM1:NM_001285522:exon1:c.T124C:p.S42P,OPRM1:NM_001285523:exon1:c.T124C:p.S42P,OPRM1:NM_001285524:exon2:c.T403C:p.S135P,OPRM1:NM_001145279:exon3:c.T403C:p.S135P	NA	NA	NA	NA	NA	D	D	N	MODERATE	T	T	T	1	169	169	0	0	187	172	14	7.5	NA	NA
chr14	52187115	G	A	INTRONIC-SS-DNR-PRX	***FRMD6***	c.1336+7G>A,c.1336+7G>A,c.286+7G>A	NM_001267047.1	NA	NA	NA	NA	NA	NA	NA	NA	LOW	NA	NA	NA	NA	231	230	0	0	107	99	8	7.5	-	0.85
chr8	144733577	G	T	MISSENSE	***ZNF623***	c.1535G>T,c.1415G>T,c.1415G>T	ZNF623:NM_014789:exon1:c.G1535T:p.G512V,ZNF623:NM_001082480:exon2:c.G1415T:p.G472V,ZNF623:NM_001261843:exon2:c.G1415T:p.G472V	NA	NA	NA	NA	NA	D	D	NA	MODERATE	NA	NA	NA	NA	143	142	0	0	136	125	10	7.4	NA	NA
chr19	46124831	G	A	SILENT	***EML2***	c.1509C>T,c.1347C>T,c.906C>T	EML2:NM_012155:exon10:c.C906T:p.A302A,EML2:NM_001193268:exon13:c.C1509T:p.A503A,EML2:NM_001193269:exon13:c.C1347T:p.A449A	NA	NA	rs762909126	1.67E-05	NA	NA	NA	NA	LOW	NA	NA	NA	NA	98	98	0	0	96	89	7	7.3	1.21	1.43
chr19	46124830	G	A	SILENT	***EML2***	c.1510C>T,c.1348C>T,c.907C>T	EML2:NM_012155:exon10:c.C907T:p.L303L,EML2:NM_001193268:exon13:c.C1510T:p.L504L,EML2:NM_001193269:exon13:c.C1348T:p.L450L	NA	NA	NA	NA	NA	NA	NA	NA	LOW	NA	NA	NA	1	98	97	0	0	96	89	7	7.3	1.21	1.43
chr11	70218409	T	C	MISSENSE	***PPFIA1***	c.2954T>C,c.2954T>C	PPFIA1:NM_003626:exon22:c.T2954C:p.M985T,PPFIA1:NM_177423:exon22:c.T2954C:p.M985T	NA	NA	NA	NA	NA	D	D	U	MODERATE	T	T	T	1	215	215	0	0	152	141	11	7.2	0.96	0.92
chrX	5811101	C	A	MISSENSE	***NLGN4X***	c.2208G>T,c.2208G>T	NLGN4X:NM_001282146:exon6:c.G2208T:p.Q736H,NLGN4X:NM_020742:exon6:c.G2208T:p.Q736H,NLGN4X:NM_181332:exon6:c.G2208T:p.Q736H,NLGN4X:NM_001282145:exon7:c.G2208T:p.Q736H	chrX*5811101*5811101*G*C*2*2*Lung_cancer	chrX*5811101*5811101*COSM356282*C*G*1*lung*1	NA	NA	NA	T_lc	N	NA	MODERATE	NA	NA	NA	NA	389	389	0	0	296	275	20	6.8	NA	NA
chr11	104874073	T	C	SILENT	***CASP5***	c.297A>G,c.45A>G,c.510A>G	CASP5:NM_001136110:exon2:c.A45G:p.A15A,CASP5:NM_001136109:exon3:c.A297G:p.A99A,CASP5:NM_001136112:exon4:c.A510G:p.A170A,CASP5:NM_004347:exon4:c.A471G:p.A157A	NA	NA	rs145780625	0.000239104	0.000599042	NA	NA	NA	LOW	NA	NA	NA	NA	129	129	0	0	89	83	6	6.7	NA	NA
chr8	144660393	C	A	SILENT	***NAPRT1***	c.96G>T	NAPRT:NM_001286829:exon1:c.G96T:p.A32A,NAPRT:NM_145201:exon1:c.G96T:p.A32A	NA	NA	NA	NA	NA	NA	NA	NA	LOW	NA	NA	NA	NA	138	138	0	0	120	112	8	6.7	NA	NA
chr11	76750601	T	G	SILENT	***B3GNT6***	c.6T>G	NM_138706.4	NA	NA	NA	NA	NA	NA	NA	NA	LOW	NA	NA	NA	NA	128	128	0	0	105	97	7	6.7	NA	NA
chr11	44069723	A	C	MISSENSE	***ACCSL***	c.137A>C	ACCSL:NM_001031854:exon1:c.A137C:p.Q46P	NA	NA	NA	NA	NA	D	D	N	MODERATE	T	T	T	0.075	102	100	0	0	106	99	7	6.6	NA	NA
chr11	117045715	G	T	INTRONIC	***PAFAH1B2***	c.537-907G>T,c.412-907G>T	NM_001184747.1	NA	NA	NA	NA	NA	NA	NA	NA	MODIFIER	NA	NA	NA	NA	133	133	0	0	91	85	6	6.6	1.01	1.03
chr11	117045715	G	T	3UTR	***PAFAH1B2***	c.*101G>T	NM_001184748.1	NA	NA	NA	NA	NA	NA	NA	NA	MODIFIER	NA	NA	NA	NA	133	133	0	0	91	85	6	6.6	1.01	1.03
chr1	237896939	G	T	INTRONIC	***RYR2***	c.11040-66G>T	NM_001035.2	NA	NA	NA	NA	NA	NA	NA	NA	MODIFIER	NA	NA	NA	NA	77	77	0	0	76	71	5	6.6	NA	NA
chr11	129794861	C	T	SILENT	***PRDM10***	c.1794G>A,c.1806G>A,c.1548G>A,c.1536G>A	PRDM10:NM_199439:exon8:c.G1536A:p.L512L,PRDM10:NM_199438:exon9:c.G1548A:p.L516L,PRDM10:NM_199437:exon12:c.G1794A:p.L598L,PRDM10:NM_020228:exon13:c.G1806A:p.L602L	NA	NA	NA	NA	NA	NA	NA	NA	LOW	NA	NA	NA	1	215	215	0	0	152	142	10	6.6	NA	NA
chr10	118390940	C	T	INTRONIC	***PNLIPRP2***	c.817+72C>T	NM_005396.4	NA	NA	NA	NA	NA	NA	NA	NA	MODIFIER	NA	NA	NA	NA	90	89	0	0	93	87	6	6.5	NA	NA
chr20	42242516	G	T	MISSENSE	***IFT52***	c.512G>T	IFT52:NM_001303458:exon7:c.G512T:p.G171V,IFT52:NM_001303459:exon7:c.G512T:p.G171V,IFT52:NM_016004:exon7:c.G512T:p.G171V	NA	NA	NA	NA	NA	D	D	D	MODERATE	0	D	D	1	350	347	0	0	281	262	18	6.4	1.31	1.58
chr14	105180951	G	T	MISSENSE	***INF2***	c.3452G>T,c.3452G>T	INF2:NM_001031714:exon21:c.G3452T:p.G1151V,INF2:NM_022489:exon21:c.G3452T:p.G1151V	NA	NA	NA	NA	NA	D_lc	NA	N	MODERATE	D	T	T	0.109	256	249	0	0	189	174	12	6.4	0.92	0.87
chr1	20141191	C	T	MISSENSE	***RNF186***	c.404G>A	RNF186:NM_019062:exon1:c.G404A:p.S135N	NA	NA	NA	NA	NA	T	N	N	MODERATE	T	T	T	0.013	202	202	0	0	191	179	12	6.3	NA	NA
chr2	179432185	A	G	MISSENSE	***TTN***	c.51854T>C,c.78674T>C,c.52055T>C,c.70970T>C,c.51479T>C	TTN:NM_003319:exon154:c.T51479C:p.I17160T,TTN:NM_133432:exon155:c.T51854C:p.I17285T,TTN:NM_133437:exon155:c.T52055C:p.I17352T,TTN:NM_133378:exon275:c.T70970C:p.I23657T,TTN:NM_001256850:exon276:c.T73751C:p.I24584T,TTN:NM_001267550:exon326:c.T78674C:p.I26225T	NA	NA	rs12463674	0.236804897	0.129992	NA	NA	NA	MODERATE	NA	NA	NA	NA	182	181	0	0	112	105	7	6.3	1.03	0.96
chr6	161512556	A	T	MISSENSE	***MAP3K4***	c.3119A>T,c.3119A>T	MAP3K4:NM_001301072:exon12:c.A3119T:p.Y1040F,MAP3K4:NM_005922:exon12:c.A3119T:p.Y1040F,MAP3K4:NM_006724:exon12:c.A3119T:p.Y1040F,MAP3K4:NM_001291958:exon13:c.A1478T:p.Y493F	NA	NA	NA	NA	NA	T	D	D	MODERATE	T	T	T	0.999	203	203	0	0	195	183	12	6.2	0.90	0.94
chr9	33960876	C	T	MISSENSE-SS-PRX	***UBAP2***	c.13G>A,c.746G>A	UBAP2:NM_001282529:exon7:c.G13A:p.G5R,UBAP2:NM_018449:exon10:c.G746A:p.G249E	NA	NA	NA	NA	NA	D	D	N	MODERATE	T	T	T	0.684	117	117	0	0	100	94	6	6.0	0.93	1.29
chr7	37052967	G	A	SILENT	***ELMO1***	c.1374C>T,c.1374C>T	ELMO1:NM_001206480:exon16:c.C1374T:p.I458I,ELMO1:NM_001206482:exon16:c.C1374T:p.I458I,ELMO1:NM_014800:exon16:c.C1374T:p.I458I	NA	NA	rs763822878	8.25E-06	NA	NA	NA	NA	LOW	NA	NA	NA	0.998	141	140	1	0.71	100	94	6	6.0	-	1.17
chr8	95953160	T	C	MISSENSE	***TP53INP1***	c.10A>G,c.10A>G	TP53INP1:NM_001135733:exon2:c.A10G:p.R4G,TP53INP1:NM_033285:exon2:c.A10G:p.R4G	NA	NA	NA	NA	NA	D	D	D	MODERATE	T	T	T	0.998	102	102	0	0	101	95	6	5.9	NA	NA
chr20	44526992	A	T	INTRONIC	***CTSA***	c.1414-14A>T,c.1363-14A>T	NM_001167594.1	NA	NA	NA	NA	NA	NA	NA	NA	MODIFIER	NA	NA	NA	NA	292	289	0	0	203	191	12	5.9	1.10	0.99
chr2	74129610	G	A	MISSENSE	***ACTG2***	c.250G>A	ACTG2:NM_001615:exon3:c.G250A:p.E84K	NA	NA	NA	NA	NA	D_lc	D	D	MODERATE	D	D	D	1	244	244	0	0	188	175	11	5.9	NA	NA
chr2	74129610	G	A	INTRONIC	***ACTG2***	c.126+1046G>A	ACTG2:NM_001615:exon3:c.G250A:p.E84K	NA	NA	NA	NA	NA	NA	NA	NA	MODIFIER	NA	NA	NA	NA	244	244	0	0	188	175	11	5.9	NA	NA
chr16	55362889	G	A	SILENT	***IRX6***	c.999G>A	IRX6:NM_024335:exon5:c.G999A:p.E333E	NA	NA	NA	NA	NA	NA	NA	NA	LOW	NA	NA	NA	0.003	197	193	0	0	154	144	9	5.8	NA	NA
chr9	127570152	G	A	MISSENSE	***OLFML2A***	c.619G>A,c.1261G>A	OLFML2A:NM_001282715:exon4:c.G619A:p.D207N,OLFML2A:NM_182487:exon7:c.G1261A:p.D421N	chr9*127570152*127570152*G*A*1*1*Lung_cancer	NA	NA	NA	NA	D	D	D	MODERATE	D	D	D	0.972	110	110	0	0	138	130	8	5.8	NA	NA
chr3	43753304	G	T	MISSENSE	***ABHD5***	c.610G>T	ABHD5:NM_016006:exon4:c.G610T:p.A204S	NA	NA	NA	NA	NA	D	N	N	MODERATE	D	T	T	0.996	151	150	0	0	105	99	6	5.7	1.05	1.13
chr10	134740060	G	A	INTRONIC	***TTC40***	c.1065+12C>T	NM_001200049.2	NA	NA	rs113601557	7.00E-05	NA	NA	NA	NA	MODIFIER	NA	NA	NA	NA	87	87	0	0	108	102	6	5.6	NA	NA
chr19	16640722	G	C	INTRONIC	***CHERP***	c.877-11C>G	NM_006387.5	NA	NA	NA	NA	NA	NA	NA	NA	MODIFIER	NA	NA	NA	NA	124	124	0	0	90	84	5	5.6	0.94	0.95
chr8	144358773	C	A	SILENT	***GLI4***	c.930C>A	GLI4:NM_138465:exon4:c.C930A:p.L310L	chr8*144358772*144358774*TCA*del*1*1*Colorectal_cancer|chr8*144358773*144358773*C*T*1*1*Head_and_neck_cancer	chr8*144358771*144358774*COSM1455310*CTCA*C*1*large_intestine*1	NA	NA	NA	NA	NA	NA	LOW	NA	NA	NA	NA	263	255	1	0.38	199	182	11	5.5	NA	NA
chr19	33167782	G	C	MISSENSE	***RGS9BP***	c.613G>C	RGS9BP:NM_207391:exon1:c.G613C:p.G205R	NA	NA	NA	NA	NA	T	N	N	MODERATE	T	T	T	0.003	132	132	0	0	109	103	6	5.5	NA	NA
chr7	100676498	G	A	MISSENSE	***MUC17***	c.1801G>A	MUC17:NM_001040105:exon3:c.G1801A:p.V601M	NA	NA	NA	NA	NA	NA	NA	NA	MODERATE	NA	NA	NA	NA	823	816	1	0.12	747	706	40	5.4	NA	NA
chr2	80530337	T	G	INTRONIC	***CTNNA2***	c.1057-89999T>G,c.1057-89999T>G	LRRTM1:NM_178839:exon2:c.A608C:p.N203T	NA	NA	NA	NA	NA	NA	NA	NA	MODIFIER	NA	NA	NA	NA	192	191	0	0	169	158	9	5.3	1.03	-
chr2	80530337	T	G	MISSENSE	***LRRTM1***	c.608A>C	LRRTM1:NM_178839:exon2:c.A608C:p.N203T	NA	NA	NA	NA	NA	T	D	U	MODERATE	T	D	D	1	192	191	0	0	169	158	9	5.3	NA	NA
chr17	43174686	C	T	INTRONIC	***NMT1***	c.713+74C>T	NM_021079.3	NA	NA	NA	NA	NA	NA	NA	NA	MODIFIER	NA	NA	NA	NA	146	144	1	0.68	133	126	7	5.3	0.95	0.96
chr1	152636655	C	T	MISSENSE	***LCE2D***	c.74C>T	LCE2D:NM_178430:exon2:c.C74T:p.P25L	NA	NA	NA	NA	NA	T_lc	NA	NA	MODERATE	NA	NA	NA	NA	297	296	0	0	286	269	15	5.2	NA	NA
chr6	170862328	C	T	STARTLOSS	***PSMB1***	c.3G>A	PSMB1:NM_002793:exon1:c.G3A:p.M1I	NA	NA	NA	NA	NA	T_lc	N	N	HIGH	T	T	T	0.64	120	120	0	0	134	127	7	5.2	1.06	1.04
chr17	38859414	T	A	NONSENSE	***KRT24***	c.532A>T	KRT24:NM_019016:exon1:c.A532T:p.K178X	NA	NA	NA	NA	NA	NA	NA	NA	HIGH	NA	NA	NA	NA	156	154	1	0.64	98	92	5	5.1	NA	NA
chr5	154179369	C	T	INTRONIC	***LARP1***	c.1315+50C>T	NM_015315.4	NA	NA	NA	NA	NA	NA	NA	NA	MODIFIER	NA	NA	NA	NA	113	113	0	0	119	113	6	5.0	1.01	1.00
chr22	39001341	T	G	MISSENSE	***FAM227A***	c.1139A>C	FAM227A:NM_001013647:exon13:c.A1139C:p.Q380P,FAM227A:NM_001291030:exon13:c.A860C:p.Q287P	NA	NA	NA	NA	NA	T	N	U	MODERATE	0	T	T	0.002	224	223	0	0	179	170	9	5.0	NA	NA
chr15	73615387	G	A	MISSENSE	***HCN4***	c.3047C>T	HCN4:NM_005477:exon8:c.C3047T:p.P1016L	NA	NA	rs761909019	7.65E-05	NA	D_lc	NA	NA	MODERATE	NA	NA	NA	NA	163	163	0	0	180	171	9	5.0	NA	NA
chr20	62421865	G	T	SILENT	***ZBTB46***	c.246C>A	ZBTB46:NM_025224:exon2:c.C246A:p.G82G	NA	NA	NA	NA	NA	NA	NA	NA	LOW	NA	NA	NA	NA	667	666	0	0	558	528	27	4.8	NA	NA
chr16	77913102	T	A	MISSENSE	***VAT1L***	c.863T>A	VAT1L:NM_020927:exon6:c.T863A:p.F288Y	NA	NA	rs762359663	8.24E-06	NA	D	D	D	MODERATE	T	T	T	1	219	218	1	0.46	209	199	10	4.8	1.12	-
chr6	168312139	G	T	MISSENSE	***MLLT4***	c.2007G>T,c.1959G>T	MLLT4:NM_001207008:exon14:c.G1959T:p.K653N,MLLT4:NM_001040000:exon15:c.G2007T:p.K669N,MLLT4:NM_001291964:exon15:c.G1884T:p.K628N	NA	NA	NA	NA	NA	T	D	D	MODERATE	T	T	T	1	212	212	0	0	148	141	7	4.7	1.19	1.10
chr9	140508612	C	T	SILENT	***ARRDC1***	c.564C>T	ARRDC1:NM_152285:exon5:c.C564T:p.D188D	NA	NA	rs148857250	0.000066893	0.000199681	NA	NA	NA	LOW	NA	NA	NA	0.934	183	182	0	0	149	141	7	4.7	-	0.96
chr14	78397982	C	T	MISSENSE	***ADCK1***	c.1328C>T,c.1124C>T	ADCK1:NM_001142545:exon9:c.C1124T:p.A375V,ADCK1:NM_020421:exon10:c.C1328T:p.A443V	NA	NA	NA	NA	NA	T	N	N	MODERATE	T	T	T	0.853	267	266	0	0	151	144	7	4.6	1.06	-
chr20	60793546	C	T	INTRONIC-SS-DNR	***HRH3***	c.417+1G>A	NM_007232.2	NA	NA	NA	NA	NA	NA	NA	NA	HIGH	NA	NA	NA	NA	176	176	0	0	154	147	7	4.6	NA	NA
chr7	143807247	C	T	MISSENSE	***OR2A2***	c.572C>T	OR2A2:NM_001005480:exon1:c.C572T:p.T191I	chr7*143807247*143807247*C*T*1*1*Lung_cancer	chr7*143807247*143807247*COSM1548838*C*T*1*lung*1	NA	NA	NA	T	N	N	MODERATE	T	T	T	0.121	196	195	0	0	176	167	8	4.6	NA	NA
chr16	31154900	C	T	INTRONIC-SS-DNR-PRX	***PRSS36***	c.970+9G>A,c.970+9G>A	NM_001258291.1	NA	NA	NA	NA	NA	NA	NA	NA	MODIFIER	NA	NA	NA	NA	434	433	0	0	345	330	15	4.4	NA	NA
chr6	165808737	C	G	MISSENSE	***PDE10A***	c.1438G>C	PDE10A:NM_001130690:exon15:c.G1438C:p.G480R	chr6*165808737*165808737*C*A*1*1*Lung_cancer	chr6*165808737*165808737*COSM741205*C*A*2*lung*2	NA	NA	NA	T	N	N	MODERATE	T	T	T	0.998	173	173	0	0	139	133	6	4.3	NA	NA
chr6	165808736	C	A	MISSENSE	***PDE10A***	c.1439G>T	PDE10A:NM_001130690:exon15:c.G1439T:p.G480V	chr6*165808736*165808736*C*A*1*1*Lung_cancer	chr6*165808736*165808736*COSM595048*C*A*1*lung*1	NA	NA	NA	T	N	N	MODERATE	T	T	T	0.999	174	174	0	0	139	133	6	4.3	NA	NA
chr1	16260251	G	A	MISSENSE	***SPEN***	c.7516G>A	SPEN:NM_015001:exon11:c.G7516A:p.E2506K	NA	NA	NA	NA	NA	NA	NA	NA	MODERATE	NA	NA	NA	NA	364	362	1	0.27	258	247	11	4.3	0.97	1.06
chr16	48594846	G	C	MISSENSE	***N4BP1***	c.1708C>G	N4BP1:NM_153029:exon2:c.C1708G:p.L570V	NA	NA	NA	NA	NA	T	N	N	MODERATE	T	T	T	0.988	134	134	0	0	176	169	7	4.0	0.87	0.98
chr20	45354194	A	T	SILENT	***SLC2A10***	c.519A>T	SLC2A10:NM_030777:exon2:c.A519T:p.A173A	NA	NA	NA	NA	NA	NA	NA	NA	LOW	NA	NA	NA	NA	470	464	1	0.21	338	323	13	3.9	1.33	-
chr20	62371472	C	G	INTRONIC	***SLC2A4RG***	c.126+81C>G	NM_020062.3	NA	NA	NA	NA	NA	NA	NA	NA	MODIFIER	NA	NA	NA	NA	124	124	0	0	203	195	7	3.5	NA	NA
chr20	45354352	A	G	MISSENSE	***SLC2A10***	c.677A>G	SLC2A10:NM_030777:exon2:c.A677G:p.D226G	NA	NA	NA	NA	NA	D	N	D	MODERATE	T	T	T	0.099	430	429	0	0	349	333	11	3.2	1.33	-
chr11	1016801	G	C	MISSENSE	***MUC6***	c.6000C>G	MUC6:NM_005961:exon31:c.C6000G:p.H2000Q	chr11*1016801*1016801*C*G*1*1*Breast_cancer	NA	rs761958882	0.00480309	NA	NA	NA	NA	MODERATE	NA	NA	NA	NA	1210	1113	15	1.24	1068	953	26	2.4	NA	NA
