# Supplementary material for: Multi-Omics Analysis to Characterize Cigarette Smoke Induced Molecular Alterations in Esophageal Cells
Source: Front Oncol. 2020 Nov 5;10:1666. doi: 10.3389/fonc.2020.01666 (PMC7675040; doi:10.3389/fonc.2020.01666)
Supplement: Supplementary Table 4 — List of somatic copy number alterations and affected genes in Het-1A cells chronically treated with cigarette smoke condensate for 8 months. [file Table_4.pdf]

Khan *et al.* , 2020. Multi-omics analysis to characterize cigarette smoke induced molecular alterations in esophageal cells
Supplementary Table 4. List of somatic copy number alterations and affected genes in Het-1A- cells chronically treated with cigarette smoke condensate for 8 months

								Protein quantification	
Gene	Chromosome	Cytoband	Start co-ordinate	End co-ordinate	Copy number	P-value	Q-value	Het-1A-Smoke - 8M/Parental (130C+131)/(126+127N) Replicate 1	Het-1A-Smoke - 8M/Parental (130C+131)/(126+127N) Replicate 2
**HMGN2**	chr1	p36.11	26799099	26801598	1	3.52E-169	3.80E-167	0.50	0.47
**DHDDS**	chr1	p36.11	26759360	26795627	1	3.52E-169	3.80E-167	NA	NA
**RPS6KA1**	chr1	p36.11	26856360	26901027	1	3.52E-169	3.80E-167	0.84	1.06
**ARID1A**	chr1	p36.11	27022864	27107252	1	3.52E-169	3.80E-167	1.03	-
**PIGV**	chr1	p36.11	27117189	27124403	1	3.52E-169	3.80E-167	NA	NA
**ZDHHC18**	chr1	p36.11	27153272	27182775	1	3.52E-169	3.80E-167	NA	NA
**SFN**	chr1	p36.11	27189732	27190519	1	3.52E-169	3.80E-167	0.72	0.69
**GPN2**	chr1	p36.11	27206129	27216574	1	3.52E-169	3.80E-167	0.95	-
**GPATCH3**	chr1	p36.11	27217182	27226906	1	3.52E-169	3.80E-167	NA	NA
**NUDC**	chr1	p36.11	27237556	27272617	1	3.52E-169	3.80E-167	0.79	0.82
**NR0B2**	chr1	p36.11	27238282	27240454	1	3.52E-169	3.80E-167	NA	NA
**C1orf172**	chr1	p36.11	27276558	27278843	1	3.52E-169	3.80E-167	NA	NA
**TRNP1**	chr1	p36.11	27320314	27321027	1	3.52E-169	3.80E-167	NA	NA
**FAM46B**	chr1	p36.11	27332404	27339119	1	3.52E-169	3.80E-167	NA	NA
**SLC9A1**	chr1	p36.11	27426743	27480885	1	3.52E-169	3.80E-167	0.69	-
**WDTC1**	chr1	p36.11	27587436	27632861	1	3.52E-169	3.80E-167	NA	NA
**TMEM222**	chr1	p36.11	27648742	27662037	1	3.52E-169	3.80E-167	0.88	1.00
**SYTL1**	chr1	p36.11	27671844	27680428	1	3.52E-169	3.80E-167	1.26	-
**MAP3K6**	chr1	p36.11	27681800	27693001	1	3.52E-169	3.80E-167	-	0.95
**FCN3**	chr1	p36.11	27695683	27701396	1	3.52E-169	3.80E-167	NA	NA
**CD164L2**	chr1	p36.11	27705916	27709736	1	3.52E-169	3.80E-167	NA	NA
**GPR3**	chr1	p36.11	27720252	27721343	1	3.52E-169	3.80E-167	NA	NA
**WASF2**	chr1	p36.11	27734763	27755312	1	3.52E-169	3.80E-167	0.84	0.91
**AHDC1**	chr1	p35.3	27873826	27878643	1	3.52E-169	3.80E-167	NA	NA
**FGR**	chr1	p35.3	27939472	27950372	1	3.52E-169	3.80E-167	NA	NA
**IFI6**	chr1	p35.3	27992811	27995820	1	3.52E-169	3.80E-167	NA	NA
**FAM76A**	chr1	p35.3	28052614	28087207	1	3.52E-169	3.80E-167	NA	NA
**STX12**	chr1	p35.3	28099724	28148913	1	3.52E-169	3.80E-167	0.76	0.68
**PPP1R8**	chr1	p35.3	28157291	28178232	1	3.52E-169	3.80E-167	0.88	0.81
**THEMIS2**	chr1	p35.3	28199052	28212498	1	3.52E-169	3.80E-167	NA	NA
**RPA2**	chr1	p35.3	28218718	28241316	1	3.52E-169	3.80E-167	1.00	0.94
**SMPDL3B**	chr1	p35.3	28261564	28285316	1	3.52E-169	3.80E-167	NA	NA
**XKR8**	chr1	p35.3	28286579	28293679	1	3.52E-169	3.80E-167	NA	NA
**EYA3**	chr1	p35.3	28300973	28384680	1	3.52E-169	3.80E-167	-	0.72
**PTAFR**	chr1	p35.3	28476501	28477552	1	3.52E-169	3.80E-167	NA	NA
**DNAJC8**	chr1	p35.3	28527854	28559612	1	3.52E-169	3.80E-167	0.64	0.65
**ATPIF1**	chr1	p35.3	28562665	28573210	1	3.52E-169	3.80E-167	NA	NA
**SESN2**	chr1	p35.3	28586280	28607321	1	3.52E-169	3.80E-167	NA	NA
**PTPRU**	chr1	p35.3	29563148	29652184	1	2.08E-20	1.48E-18	NA	NA
**MATN1**	chr1	p35.2	31186350	31196470	1	2.08E-20	1.48E-18	NA	NA
**LAPTM5**	chr1	p35.2	31206330	31230625	1	2.08E-20	1.48E-18	NA	NA
**LINC00452**	chr13	q34	114618849	114624078	3	4.44E-16	2.97E-14	NA	NA
**RASA3**	chr13	q34	114748751	114897999	3	4.44E-16	2.97E-14	NA	NA
**CDC16**	chr13	q34	115000492	115037969	3	4.44E-16	2.97E-14	1.02	1.14
**UPF3A**	chr13	q34	115047123	115070390	3	4.44E-16	2.97E-14	NA	NA
**ANKRD26P1**	chr16	q11.2	46508168	46602905	3	6.86E-103	6.79E-101	NA	NA
**SHCBP1**	chr16	q11.2	46615596	46655369	3	6.86E-103	6.79E-101	0.96	1.09
**VPS35**	chr16	q11.2	46694317	46723116	3	6.86E-103	6.79E-101	0.91	0.96
**ORC6**	chr16	q11.2	46723489	46731586	3	6.86E-103	6.79E-101	0.91	1.00
**MYLK3**	chr16	q11.2	46741536	46782120	3	6.86E-103	6.79E-101	NA	NA
**C16orf87**	chr16	q11.2	46836760	46865118	3	6.86E-103	6.79E-101	NA	NA
**GPT2**	chr16	q11.2	46918564	46962913	3	6.86E-103	6.79E-101	1.23	1.18
**DNAJA2**	chr16	q11.2	46990889	47007567	3	6.86E-103	6.79E-101	1.12	1.09
**NETO2**	chr16	q12.1	47117116	47177628	3	6.86E-103	6.79E-101	NA	NA
**ITFG1**	chr16	q12.1	47189549	47497957	3	6.86E-103	6.79E-101	1.08	-
**PHKB**	chr16	q12.1	47495233	47733349	3	6.86E-103	6.79E-101	0.94	1.14
**ABCC12**	chr16	q12.1	48117561	48180421	3	6.86E-103	6.79E-101	NA	NA
**ABCC11**	chr16	q12.1	48201226	48265926	3	6.86E-103	6.79E-101	NA	NA
**LONP2**	chr16	q12.1	48278213	48396417	3	6.86E-103	6.79E-101	0.94	-
**SIAH1**	chr16	q12.1	48399220	48419207	3	6.86E-103	6.79E-101	NA	NA
**N4BP1**	chr16	q12.1	48576840	48643868	3	6.86E-103	6.79E-101	0.87	0.98
**CBLN1**	chr16	q12.1	49313311	49315395	3	6.86E-103	6.79E-101	NA	NA
**C16orf78**	chr16	q12.1	49407846	49433159	3	6.86E-103	6.79E-101	NA	NA
**ZNF423**	chr16	q12.1	49525038	49856676	3	6.86E-103	6.79E-101	NA	NA
