# Supplementary material for: Multi-Omics Analysis to Characterize Cigarette Smoke Induced Molecular Alterations in Esophageal Cells
Source: Front Oncol. 2020 Nov 5;10:1666. doi: 10.3389/fonc.2020.01666 (PMC7675040; doi:10.3389/fonc.2020.01666)
Supplement: Supplementary Table 5 — List of proteins quantified in untreated and chronically treated Het1A cells with cigarette smoke condensate treated for 8 months. [file Table_5.pdf]

Khan *et al.* , 2019. Multi-omics analysis to characterize cigarette smoke induced molecular alterations in esophageal cells
Supplementary Table 5. List of proteins quantified in untreated and chronically treated Het1A cells with cigarette smoke condensate for 8 months

NP_Accession	Protein group Accession	Gene ID	Description	Het-1A-Smoke - 2M/Parental	Het-1A-Smoke - 4M/Parental	Het-1A-Smoke - 6M/Parental	Het-1A-Smoke - 8M/Parental	Het-1A-Smoke - 2M/Parental	Het-1A-Smoke - 4M/Parental	Het-1A-Smoke - 6M/Parental	Het-1A-Smoke - 8M/Parental	Het-1A-Smoke - 2M/Parental	Het-1A-Smoke - 4M/Parental	Het-1A-Smoke - 6M/Parental	Het-1A-Smoke - 8M/Parental	Coverage (%)	Proteins	Unique Peptides	# Peptides	PSM	# AAs	MW [kDa]	calc. pI
				Replicate 1				Replicate 2				Average of replicates											
NP_002766.1	4506141	HTRA1	serine protease HTRA1 precursor	1.4	2.8	2.4	3.6	1.3	2.7	2.1	3.4	1.4	2.7	2.2	3.5	36.0	1	11	11	53	480	51.3	7.8
NP_000294.1	4557839	PMM2	phosphomannomutase 2	5.2	4.4	3.7	4.3	1.1	1.1	1.2	1.1	3.2	2.8	2.4	2.7	17.5	1	4	5	5	246	28.1	6.8
NP_000415.2	119395754	KRT5	keratin, type II cytoskeletal 5	1.1	2.1	1.0	2.6	1.1	2.0	1.1	2.5	1.1	2.0	1.0	2.6	19.7	7	6	14	78	590	62.3	7.7
NP_000414.2	47132620	KRT2	keratin, type II cytoskeletal 2 epidermal	1.1	2.1	1.0	2.5	1.0	2.0	1.0	2.5	1.1	2.0	1.0	2.5	59.8	3	22	29	190	639	65.4	8.0
NP_000412.3	195972866	KRT10	keratin, type I cytoskeletal 10	1.1	2.1	1.0	2.6	1.1	1.7	1.0	2.2	1.1	1.9	1.0	2.4	52.7	18	26	29	461	584	58.8	5.2
NP_006112.3	119395750	KRT1	keratin, type II cytoskeletal 1	1.1	1.8	1.0	2.2	1.0	1.8	1.0	2.4	1.0	1.8	1.0	2.3	57.9	1	26	33	263	644	66.0	8.1
NP_056535.1	7657465	PODXL2	podocalyxin-like protein 2 precursor	1.6	3.2	2.0	3.4	0.9	1.0	1.0	1.2	1.3	2.1	1.5	2.3	2.3	1	1	1	8	605	65.0	4.3
NP_444513.1	16751921	DCD	dermcidin preproprotein	1.1	1.9	0.9	2.3	1.1	1.7	0.8	2.3	1.1	1.8	0.9	2.3	26.4	1	3	3	8	110	11.3	6.5
NP_000217.2	55956899	KRT9	keratin, type I cytoskeletal 9	1.0	1.7	1.0	2.2	1.0	1.8	1.0	2.3	1.0	1.7	1.0	2.2	72.6	2	25	27	217	623	62.0	5.2
NP_001009931.1	57864582	HRNR	hornerin	1.1	2.6	1.1	3.1	1.0	1.1	0.9	1.3	1.0	1.9	1.0	2.2	4.3	1	4	4	9	2850	282.2	10.0
NP_851825.1	31317278	WDR20	WD repeat-containing protein 20 isoform 3	3.3	4.4	3.2	3.4	1.0	1.0	1.1	0.9	2.2	2.7	2.1	2.1	5.5	6	1	1	1	508	56.2	8.1
NP_061980.1	33457348	MYDGF	UPF0556 protein C19orf10 precursor	1.1	1.4	1.2	2.2	1.1	1.3	1.2	1.9	1.1	1.4	1.2	2.1	8.7	1	1	1	1	173	18.8	6.7
NP_001092315.2	524552373	ANXA8L1	annexin A8-like protein 1 isoform 1	1.0	0.9	1.0	1.9	1.2	1.2	1.3	2.2	1.1	1.1	1.2	2.0	5.2	6	2	2	2	327	36.9	5.8
NP_076968.2	229577436	SPATA5L1	spermatogenesis-associated protein 5-like protein 1	1.9	2.0	2.4	2.8	1.1	1.0	1.0	1.2	1.5	1.5	1.7	2.0	2.1	1	1	1	2	753	80.7	8.1
NP_001303.1	4503049	CRIP2	cysteine-rich protein 2 isoform 1	1.1	1.6	1.8	2.1	1.0	1.5	1.4	1.8	1.1	1.5	1.6	2.0	11.1	3	2	2	3	208	22.5	8.7
NP_742101.2	98991763	EYA4	eyes absent homolog 4 isoform b	1.1	1.2	1.2	1.2	1.4	2.5	2.1	2.5	1.3	1.8	1.6	1.9	6.5	3	2	2	2	616	67.0	5.3
NP_002909.4	238859557	RFX1	MHC class II regulatory factor RFX1	1.1	0.9	1.0	1.1	2.1	2.2	0.8	2.6	1.6	1.5	0.9	1.8	3.1	1	2	2	3	979	104.7	6.3
NP_003361.1	4507869	VASP	vasodilator-stimulated phosphoprotein	0.9	1.5	1.2	1.6	1.2	1.9	1.5	2.0	1.0	1.7	1.3	1.8	5.5	1	2	2	3	380	39.8	8.9
NP_001180499.1	302191611	CSRP1	cysteine and glycine-rich protein 1 isoform 3	1.4	1.7	1.7	1.8	1.4	1.6	1.6	1.8	1.4	1.6	1.7	1.8	61.0	2	9	9	36	187	20.0	8.5
NP_001171563.1	296010902	BCAT1	branched-chain-amino-acid aminotransferase, cytosolic isoform 3	1.8	1.8	1.4	1.9	1.6	1.9	1.8	1.6	1.7	1.9	1.6	1.7	3.4	5	1	1	1	325	36.3	5.1
NP_000117.1	4503607	ETFA	electron transfer flavoprotein subunit alpha, mitochondrial isoform a	1.3	1.7	1.4	1.7	1.2	1.7	1.5	1.8	1.3	1.7	1.5	1.7	33.6	2	9	9	29	333	35.1	8.4
NP_000517.2	15431310	KRT14	keratin, type I cytoskeletal 14	1.2	1.7	1.2	1.8	1.2	1.5	1.0	1.7	1.2	1.6	1.1	1.7	33.7	12	5	13	100	472	51.6	5.2
NP_077007.1	13129110	WDR77	methylosome protein 50	1.1	1.1	1.1	1.7	1.2	1.2	1.2	1.8	1.2	1.1	1.1	1.7	2.1	1	1	1	2	342	36.7	5.2
NP_004083.3	194097323	ECHS1	enoyl-CoA hydratase, mitochondrial	1.3	1.6	1.4	1.8	1.3	1.5	1.4	1.6	1.3	1.5	1.4	1.7	26.6	1	5	5	19	290	31.4	8.1
NP_005548.2	24430192	KRT16	keratin, type I cytoskeletal 16	1.1	1.9	1.2	1.7	1.3	1.5	1.1	1.6	1.2	1.7	1.2	1.7	34.0	12	7	14	94	473	51.2	5.0
NP_002887.2	93277122	RBM4	RNA-binding protein 4 isoform 1	1.1	1.3	1.2	1.4	1.1	1.6	1.4	1.9	1.1	1.4	1.3	1.7	14.8	5	4	4	9	364	40.3	7.1
NP_005909.2	21735621	MDH2	malate dehydrogenase, mitochondrial isoform 1 precursor	1.3	1.5	1.3	1.7	1.3	1.5	1.3	1.6	1.3	1.5	1.3	1.7	58.0	4	16	16	83	338	35.5	8.7
NP_665807.1	21956645	MTPN	myotrophin	1.3	2.0	1.8	1.6	1.3	1.5	1.6	1.6	1.3	1.7	1.7	1.6	14.4	1	1	1	3	118	12.9	5.5
NP_001003785.1	51479152	ATP5H	ATP synthase subunit d, mitochondrial isoform b	1.2	1.3	1.3	2.0	1.0	1.0	0.9	1.3	1.1	1.2	1.1	1.6	11.7	2	1	1	2	137	15.8	7.2
NP_077305.2	20149675	EFHD2	EF-hand domain-containing protein D2	1.2	1.3	1.4	1.5	1.5	1.5	2.0	1.8	1.3	1.4	1.7	1.6	19.2	3	4	5	10	240	26.7	5.2
NP_001243439.1	374671775;4507231	SSBP1	single-stranded DNA-binding protein, mitochondrial precursor	1.1	1.2	1.2	1.5	1.2	1.1	1.2	1.7	1.2	1.2	1.2	1.6	41.2	1	6	6	18	148	17.2	9.6
NP_115716.1	14150128	UQCC2	ubiquinol-cytochrome-c reductase complex assembly factor 2	1.4	1.3	1.4	1.8	1.3	1.2	1.3	1.5	1.3	1.2	1.3	1.6	28.6	1	4	4	9	126	14.9	7.4
NP_443198.1	16757970	FAM129A	protein Niban	1.3	1.5	1.4	1.3	1.9	2.2	2.1	1.8	1.6	1.8	1.8	1.6	9.3	1	7	7	14	928	103.1	4.8
NP_001035879.1	109240550	PSPC1	paraspeckle component 1	1.0	1.4	1.1	1.5	1.0	1.5	1.2	1.6	1.0	1.4	1.1	1.6	13.2	1	4	5	18	523	58.7	6.7
NP_000058.1	4557395	CA2	carbonic anhydrase 2	1.0	0.8	0.9	1.4	0.9	0.9	1.0	1.7	0.9	0.8	1.0	1.6	8.5	1	2	2	3	260	29.2	7.4
NP_778206.1	669250853	SERPINB4	serpin B4 isoform 2	0.9	1.0	0.9	1.4	0.8	0.8	0.7	1.7	0.8	0.9	0.8	1.6	1.9	3	1	1	1	369	42.3	6.0
NP_689620.2	40255109	GRPEL2	grpE protein homolog 2, mitochondrial precursor	1.5	1.3	1.1	1.4	1.5	1.6	1.2	1.7	1.5	1.4	1.2	1.6	11.6	1	2	2	3	225	25.4	7.7
NP_001139632.1	225903439	TATDN1	putative deoxyribonuclease TATDN1 isoform b	1.2	1.3	1.3	1.4	1.5	1.6	1.4	1.7	1.3	1.5	1.4	1.6	13.6	2	4	4	8	250	28.1	6.5
NP_000427.1	4557817	OXCT1	succinyl-CoA:3-ketoacid coenzyme A transferase 1, mitochondrial precursor	1.1	1.6	1.2	1.6	1.2	1.4	1.3	1.5	1.2	1.5	1.2	1.5	9.8	2	3	3	12	520	56.1	7.5
NP_060897.3	93102377	RRN3	RNA polymerase I-specific transcription initiation factor RRN3 isoform 1	1.3	1.2	1.3	1.5	1.6	1.4	1.4	1.6	1.5	1.3	1.4	1.5	1.7	1	1	1	4	651	74.1	5.6
NP_612453.1	19923973	KCTD12	BTB/POZ domain-containing protein KCTD12	1.1	1.1	1.2	1.4	1.2	1.3	1.3	1.7	1.2	1.2	1.3	1.5	5.9	2	2	2	3	325	35.7	5.6
NP_000445.1	4507149	SOD1	superoxide dismutase [Cu-Zn]	1.4	1.8	1.5	1.6	1.3	1.6	1.4	1.5	1.4	1.7	1.5	1.5	45.5	1	5	5	10	154	15.9	6.1
NP_004441.1	4758302	ERH	enhancer of rudimentary homolog	1.0	1.0	1.1	1.1	1.5	1.6	1.4	2.0	1.3	1.3	1.3	1.5	16.4	1	1	1	2	104	12.3	5.9
NP_001116849.1	183227678	PARK7	protein DJ-1	1.1	1.4	1.2	1.4	1.3	1.7	1.4	1.7	1.2	1.5	1.3	1.5	17.5	1	2	2	4	189	19.9	6.8
NP_002789.1	23110925	PSMB6	proteasome subunit beta type-6 isoform 1 proproteín	1.0	1.1	1.0	1.3	1.3	1.3	1.3	1.7	1.2	1.2	1.2	1.5	12.6	1	3	3	6	239	25.3	4.9
NP_001137254.1	219842220	FKBP11	peptidyl-prolyl cis-trans isomerase FKBP11 isoform 3 precursor	1.3	1.6	1.6	1.5	1.4	1.6	1.6	1.5	1.3	1.6	1.6	1.5	32.2	3	4	4	19	146	15.8	6.2
NP_775109.2	155969697	KRT6C	keratin, type II cytoskeletal 6C	0.9	1.3	0.9	1.4	1.1	1.5	1.0	1.6	1.0	1.4	1.0	1.5	32.3	8	2	18	100	564	60.0	8.0
NP_003748.1	4503513	EIF3I	eukaryotic translation initiation factor 3 subunit I	1.2	1.3	1.2	1.6	1.1	1.2	1.1	1.5	1.1	1.2	1.2	1.5	16.0	1	5	5	11	325	36.5	5.6
NP_000684.2	153266822	ALDH1A3	aldehyde dehydrogenase family 1 member A3 isoform 1	1.4	1.3	1.3	1.5	1.4	1.3	1.3	1.5	1.4	1.3	1.3	1.5	46.1	3	19	20	110	512	56.1	7.3
NP_001157260.1	255003738	PTBP3	polypyrimidine tract-binding protein 3 isoform 2	1.5	1.7	1.5	2.0	1.0	0.9	1.0	1.0	1.2	1.3	1.2	1.5	13.0	6	2	5	28	524	56.8	9.2
NP_001188411.1	319996739	OSBPL6	oxysterol-binding protein-related protein 6 isoform e	2.7	1.7	1.5	1.5	1.7	1.5	1.5	1.5	2.2	1.6	1.5	1.5	1.2	5	1	1	1	898	102.2	7.1
NP_003356.2	46593007	UQCRC1	cytochrome b-c1 complex subunit 1, mitochondrial precursor	1.4	1.6	1.6	1.7	1.3	1.3	1.2	1.3	1.3	1.4	1.4	1.5	20.8	1	6	6	22	480	52.6	6.4
NP_009005.1	5902134	CORO1A	coronin-1A	1.7	1.4	1.4	1.8	1.1	1.1	1.1	1.2	1.4	1.3	1.2	1.5	16.9	1	4	4	8	461	51.0	6.7
NP_004640.3	296531406	C21orf33	ES1 protein homolog, mitochondrial isoform Ia precursor	1.5	1.7	1.5	1.7	1.2	1.4	1.3	1.3	1.4	1.6	1.4	1.5	12.3	14	3	3	5	268	28.1	8.3
NP_057011.2	21361591	TGFB1I1	transforming growth factor beta-1-induced transcript 1 protein isoform 2	2.1	1.5	1.7	1.8	1.2	1.0	1.1	1.2	1.6	1.2	1.4	1.5	3.6	2	1	1	4	444	47.9	7.6
NP_000137.2	20149498	FTL	ferritin light chain	1.3	1.4	1.3	1.4	1.0	1.2	1.1	1.6	1.1	1.3	1.2	1.5	3.4	1	1	1	1	175	20.0	5.8
NP_115729.1	14150155	VPS25	vacuolar protein-sorting-associated protein 25	1.0	1.0	1.1	1.2	1.6	1.3	1.7	1.8	1.3	1.1	1.4	1.5	18.2	1	4	4	8	176	20.7	6.3
NP_001822.3	355594753	CLU	clusterin preproprotein	1.2	1.5	1.4	1.6	1.2	1.4	1.4	1.4	1.2	1.4	1.4	1.5	3.8	1	1	1	2	449	52.5	6.3
NP_057068.1	7706753	UCHL5	ubiquitin carboxyl-terminal hydrolase isozyme L5 isoform 1	1.2	1.2	1.3	1.5	1.1	1.1	1.3	1.5	1.2	1.2	1.3	1.5	38.6	1	1	9	25	329	37.6	5.3
NP_006704.3	217330646	SUB1	activated RNA polymerase II transcriptional coactivator p15	1.0	1.0	1.1	1.5	1.0	1.2	1.1	1.5	1.0	1.1	1.1	1.5	44.9	1	5	5	17	127	14.4	9.6
NP_079472.1	24308295	GRPEL1	grpE protein homolog 1, mitochondrial precursor	1.8	1.3	1.3	1.3	1.2	1.6	1.5	1.6	1.5	1.4	1.4	1.5	12.4	1	3	3	5	217	24.3	8.1
NP_004855.2	153792495	GDF15	growth/differentiation factor 15 precursor	1.2	1.2	1.4	1.4	2.0	1.6	1.3	1.5	1.6	1.4	1.4	1.5	5.2	1	1	1	4	308	34.1	9.7
NP_000062.1	4557415;295821202	CBS	cystathionine beta-synthase	1.4	1.5	1.3	1.4	1.5	1.6	1.3	1.5	1.4	1.5	1.3	1.5	12.5	2	5	5	14	551	60.5	6.7
NP_000893.2	116256329	MME	neprilysin	1.5	1.7	2.3	1.7	1.0	1.1	1.3	1.2	1.2	1.4	1.8	1.5	3.7	1	2	2	4	750	85.5	5.7
NP_001124499.1	196162711	PDLIM4	PDZ and LIM domain protein 4 isoform 2	1.1	1.4	1.1	1.6	1.0	1.2	1.1	1.4	1.1	1.3	1.1	1.5	8.5	2	2	2	5	246	25.8	7.6
NP_001172023.1	297632350	NIT1	nitrilase homolog 1 isoform 4	1.9	1.9	2.1	1.9	0.9	1.0	0.9	1.1	1.4	1.4	1.5	1.5	37.8	4	7	7	10	291	31.8	6.9
NP_001155046.1	239048390	MAFF	transcription factor MafF isoform b	1.5	2.4	2.0	1.7	1.2	1.7	1.4	1.3	1.3	2.0	1.7	1.5	11.9	2	1	1	5	135	14.6	10.2
NP_009109.3	148727341	STRAP	serine-threonine kinase receptor-associated protein	1.0	1.0	1.2	1.5	1.0	1.0	1.2	1.4	1.0	1.0	1.2	1.5	45.4	1	11	11	37	350	38.4	5.1
NP_001856.2	262118227	COX7A2	cytochrome c oxidase subunit 7A2, mitochondrial precursor	1.1	1.4	1.2	1.2	1.3	1.3	1.5	1.7	1.2	1.4	1.3	1.5	11.3	1	1	1	1	115	12.8	9.1
NP_003968.2	201860300	AIP	AH receptor-interacting protein	1.3	1.3	1.4	1.4	1.3	1.3	1.2	1.5	1.3	1.3	1.3	1.5	16.4	1	3	4	8	330	37.6	6.3
NP_060682.2	39930375	ENAH	protein enabled homolog isoform b	1.0	1.1	1.0	1.2	0.9	1.5	1.1	1.7	1.0	1.3	1.0	1.5	3.7	2	2	2	3	570	63.9	6.4
NP_079510.1	13376840	WDR61	WD repeat-containing protein 61 isoform a	1.2	1.0	1.2	1.6	1.0	1.1	1.2	1.4	1.1	1.0	1.2	1.5	11.8	2	2	2	6	305	33.6	5.5
NP_005101.1	4826742	GFPT2	glutamine--fructose-6-phosphate aminotransferase [isomerizing] 2	1.3	1.3	1.4	1.5	1.2	1.3	1.3	1.4	1.2	1.3	1.3	1.5	30.8	1	10	15	44	682	76.9	7.4
NP_001035517.1	94538320	HAGH	hydroxyacylglutathione hydrolase, mitochondrial isoform 2	2.2	1.5	1.1	1.6	2.0	1.5	1.1	1.3	2.1	1.5	1.1	1.5	6.2	2	1	1	2	260	28.8	7.3
NP_055410.1	7657033	NT5C	5'(3')-deoxyribonucleotidase, cytosolic type isoform 1	1.0	1.1	1.0	1.1	1.8	1.7	1.4	1.8	1.4	1.4	1.2	1.4	23.4	2	3	3	5	201	23.4	6.6
NP_057088.2	56676379	IFT52	intraflagellar transport protein 52 homolog	1.0	0.8	1.0	1.3	1.1	1.1	1.1	1.6	1.1	0.9	1.0	1.4	4.6	1	1	1	4	437	49.7	5.3
NP_001278623.1	619329027	NR2C2	nuclear receptor subfamily 2 group C member 2 isoform 2	1.1	1.1	1.0	1.1	0.8	2.2	2.1	1.8	1.0	1.7	1.5	1.4	2.5	2	1	1	1	596	65.4	6.3
NP_444278.1	16596694	MRPL53	39S ribosomal protein L53, mitochondrial	1.0	1.1	1.0	1.3	1.4	1.2	1.4	1.6	1.2	1.2	1.2	1.4	7.1	1	1	1	1	112	12.1	8.8
NP_057139.1	7706333	MRPL48	39S ribosomal protein L48, mitochondrial	1.1	1.1	1.0	1.2	1.0	1.3	1.1	1.7	1.1	1.2	1.0	1.4	17.0	1	3	3	7	212	23.9	9.0
NP_003086.1	4507131	SNRPF	small nuclear ribonucleoprotein F	0.9	1.1	1.3	1.4	1.0	1.2	1.3	1.5	1.0	1.1	1.3	1.4	55.8	1	2	2	5	86	9.7	4.7
NP_005800.3	32189392	PRDX2	peroxiredoxin-2	1.1	1.2	1.1	1.4	1.1	1.3	1.2	1.5	1.1	1.2	1.1	1.4	61.6	1	7	8	25	198	21.9	6.0
NP_057637.2	24497605;301069416	NUP62	nuclear pore glycoprotein p62	1.0	1.1	1.1	1.3	1.1	1.2	1.2	1.5	1.0	1.1	1.1	1.4	7.5	1	3	3	11	522	53.2	5.3
NP_005828.2	153791431	POP7	ribonuclease P protein subunit p20	1.0	1.5	1.0	1.5	1.1	1.2	1.0	1.4	1.0	1.3	1.0	1.4	10.7	1	1	1	4	140	15.6	8.9
NP_001020420.1	70906444	DUT	deoxyuridine 5'-triphosphate nucleotidohydrolase, mitochondrial isoform 3	1.2	1.4	1.2	1.3	1.2	1.5	1.3	1.5	1.2	1.5	1.3	1.4	18.4	3	2	2	4	141	15.4	6.6
NP_002296.1	4504981	LGALS1	galectin-1	1.4	1.9	1.8	1.4	1.4	1.9	1.8	1.4	1.4	1.9	1.8	1.4	37.0	1	5	5	21	135	14.7	5.5
NP_001268464.1	528281423	EPB41L3	band 4.1-like protein 3 isoform 4	1.0	1.1	1.7	1.5	1.0	1.1	1.5	1.3	1.0	1.1	1.6	1.4	20.9	4	9	13	27	756	84.7	5.4
NP_002782.1	23110944	PSMA6	proteasome subunit alpha type-6 isoform a	1.1	1.3	1.1	1.5	1.1	1.2	1.1	1.4	1.1	1.3	1.1	1.4	43.9	3	10	10	40	246	27.4	6.8

Khan *et al.* , 2019. Multi-omics analysis to characterize cigarette smoke induced molecular alterations in esophageal cells
Supplementary Table 5. List of proteins quantified in untreated and chronically treated Het1A cells with cigarette smoke condensate for 8 months

NP_Accession	Protein group Accession	Gene ID	Description	Het-1A-Smoke - 2M/Parental	Het-1A-Smoke - 4M/Parental	Het-1A-Smoke - 6M/Parental	Het-1A-Smoke - 8M/Parental	Het-1A-Smoke - 2M/Parental	Het-1A-Smoke - 4M/Parental	Het-1A-Smoke - 6M/Parental	Het-1A-Smoke - 8M/Parental	Het-1A-Smoke - 2M/Parental	Het-1A-Smoke - 4M/Parental	Het-1A-Smoke - 6M/Parental	Het-1A-Smoke - 8M/Parental	Coverage (%)	Proteins	Unique Peptides	# Peptides	PSM	# AAs	MW [kDa]	calc. pI
				Replicate 1				Replicate 2				Average of replicates											
NP_001287670.1	663429597	NUDT12	peroxisomal NADH pyrophosphatase NUDT12 isoform 2	1.4	1.2	1.2	1.4	1.0	1.2	1.0	1.4	1.2	1.2	1.1	1.4	2.0	2	1	1	2	444	50.0	6.8
NP_001013860.1	66392157	RLTPR	leucine-rich repeat-containing protein 16C	1.2	1.4	1.6	1.4	1.3	1.5	1.7	1.4	1.2	1.4	1.7	1.4	0.8	1	1	1	2	1435	154.6	6.8
NP_444252.1	16753215	PFN2	profilin-2 isoform a	1.2	1.3	1.2	1.5	1.3	1.3	1.2	1.4	1.3	1.3	1.2	1.4	20.0	2	2	2	4	140	15.0	7.0
NP_061158.1	8923904	BIN3	bridging integrator 3	1.3	1.3	1.3	1.6	1.4	1.2	1.1	1.2	1.3	1.2	1.2	1.4	5.1	1	1	1	1	253	29.6	7.5
NP_001269.3	62241001	CHUK	inhibitor of nuclear factor kappa-B kinase subunit alpha	1.8	1.8	1.9	1.7	1.1	1.0	1.1	1.1	1.4	1.4	1.5	1.4	14.8	1	9	9	11	745	84.6	6.7
NP_000512.1	4504373	HEXB	beta-hexosaminidase subunit beta isoform 1 preproprotein	1.2	1.3	1.3	1.5	1.1	1.3	1.3	1.4	1.1	1.3	1.3	1.4	11.2	2	6	6	12	556	63.1	6.8
NP_055077.2	28466983	P3H3	prolyl 3-hydroxylase 3 precursor	1.0	1.5	1.8	1.2	1.2	1.5	1.5	1.6	1.1	1.5	1.6	1.4	2.7	1	1	1	2	736	81.8	6.3
NP_001238979.1	354983495	PCMT1	protein-L-isoaspartate(D-aspartate) O-methyltransferase isoform 3	1.3	1.5	1.3	1.5	1.2	1.3	1.2	1.3	1.2	1.4	1.3	1.4	32.4	5	5	5	9	250	26.6	8.2
NP_005779.1	44771198	PRMT3	protein arginine N-methyltransferase 3 isoform 1	1.2	1.3	1.2	1.6	1.1	1.1	1.1	1.3	1.2	1.2	1.2	1.4	16.6	3	7	7	18	531	59.9	5.4
NP_004823.1	4758484	GSTO1	glutathione S-transferase omega-1 isoform 1	1.2	1.2	1.3	1.5	1.2	1.1	1.3	1.4	1.2	1.1	1.3	1.4	49.0	3	10	10	52	241	27.5	6.6
NP_114381.1	14702180	EIF4H	eukaryotic translation initiation factor 4H isoform 2	1.0	1.3	0.9	1.4	1.0	1.4	1.0	1.4	1.0	1.3	1.0	1.4	25.9	2	4	4	11	228	25.2	8.1
NP_001186904.1	316983136	UQCRB	cytochrome b-c1 complex subunit 7 isoform 2	1.9	1.5	1.5	1.5	1.6	1.4	1.4	1.4	1.7	1.4	1.5	1.4	39.2	3	3	3	12	79	10.0	5.6
NP_653164.2	94536842	RPIA	ribose-5-phosphate isomerase	1.3	1.3	1.3	1.5	1.1	1.2	1.2	1.3	1.2	1.3	1.2	1.4	2.9	1	1	1	2	311	33.2	8.5
NP_001907.2	21359867	CYC1	cytochrome c1, heme protein, mitochondrial precursor	1.0	1.1	1.1	1.2	2.2	2.4	2.9	1.6	1.6	1.8	2.0	1.4	41.9	1	7	7	34	325	35.4	9.0
NP_001129674.1	209969697	ISOC2	isochorismatase domain-containing protein 2, mitochondrial isoform 3	1.2	1.0	1.0	1.2	1.1	1.5	1.2	1.6	1.2	1.3	1.1	1.4	31.1	3	2	2	4	135	14.8	9.0
NP_001740.1	4502565	CAPNS1	calpain small subunit 1 isoform 1	1.4	1.4	1.6	1.4	1.3	1.4	1.4	1.4	1.3	1.4	1.5	1.4	56.7	3	7	7	50	268	28.3	5.2
NP_002795.2	21361144	PSMC3	26S protease regulatory subunit 6A	1.0	1.0	1.0	1.4	1.0	1.0	1.0	1.4	1.0	1.0	1.0	1.4	43.1	1	14	14	45	439	49.2	5.2
NP_001240805.1	359751465	UXS1	UDP-glucuronic acid decarboxylase 1 isoform 3	1.2	1.1	1.2	1.1	2.0	1.2	1.6	1.7	1.6	1.1	1.4	1.4	6.8	3	1	1	2	252	28.3	9.0
NP_689657.1	22748929	PTGR2	prostaglandin reductase 2	1.3	1.4	1.2	1.3	1.7	1.1	1.4	1.5	1.5	1.3	1.3	1.4	26.5	1	5	5	7	351	38.5	5.4
NP_002055.1	4504025;343887374	GLRX	glutaredoxin-1	1.4	1.4	1.5	1.3	1.5	1.5	1.3	1.5	1.4	1.5	1.4	1.4	37.7	1	2	2	6	106	11.8	8.1
NP_000128.1	4557587	FAH	fumarylacetoacetase	1.2	1.1	1.2	1.4	1.3	1.2	1.0	1.4	1.2	1.2	1.1	1.4	20.3	1	7	7	22	419	46.3	6.9
NP_004748.2	45006986	AIMP1	aminoacyl tRNA synthase complex-interacting multifunctional protein 1 isoform a precursor	1.2	1.2	1.2	1.3	1.1	1.2	1.1	1.4	1.2	1.2	1.2	1.4	30.1	2	5	7	20	312	34.3	8.4
NP_060354.4	148747556	PINX1	PIN2/TERF1-interacting telomerase inhibitor 1 isoform 1	1.6	2.1	1.9	1.8	0.9	0.9	1.0	1.0	1.2	1.5	1.4	1.4	26.2	2	6	6	12	328	37.0	9.6
NP_055112.2	41327741	ETHE1	persulfide dioxygenase ETHE1, mitochondrial	1.2	1.4	1.5	1.4	1.1	1.3	1.3	1.4	1.2	1.4	1.4	1.4	3.9	1	1	1	2	254	27.9	6.8
NP_001007.2	14277700	RPS12	40S ribosomal protein S12	1.1	1.4	1.3	1.4	1.1	1.4	1.2	1.3	1.1	1.4	1.3	1.4	25.0	1	3	3	7	132	14.5	7.2
NP_001243893.1	379698839	CCDC51	coiled-coil domain-containing protein 51 isoform 1	1.3	1.3	1.3	1.5	1.3	1.1	1.1	1.3	1.3	1.2	1.2	1.4	6.8	2	2	2	3	411	45.8	8.2
NP_000999.1	4506727	RPS4Y1	40S ribosomal protein S4, Y isoform 1	1.1	1.4	1.5	1.4	1.1	1.3	1.4	1.4	1.1	1.3	1.5	1.4	38.4	1	7	13	56	263	29.4	10.2
NP_066953.1	10863927	PPIA	peptidyl-prolyl cis-trans isomerase A isoform 1	1.2	1.6	1.3	1.4	1.2	1.6	1.2	1.4	1.2	1.6	1.3	1.4	59.4	9	9	11	105	165	18.0	7.8
NP_003312.3	34147630	TUFM	elongation factor Tu, mitochondrial precursor	1.1	1.2	1.2	1.4	1.1	1.2	1.1	1.4	1.1	1.2	1.2	1.4	51.9	1	21	21	85	455	49.8	7.6
NP_001933.2	119703744	DSG1	desmoglein-1 preproprotein	1.0	1.2	0.9	1.3	1.7	1.5	1.1	1.5	1.3	1.4	1.0	1.4	1.5	1	1	1	2	1049	113.7	5.0
NP_003960.1	4507791	UBE2M	NEDD8-conjugating enzyme Ubc12	1.3	1.4	1.3	1.4	1.3	1.4	1.3	1.4	1.3	1.4	1.3	1.4	39.3	1	7	7	31	183	20.9	7.7
NP_001182533.1	307219240	SEPHS1	selenide, water dikinase 1 isoform 3	1.1	1.2	1.2	1.5	1.0	1.0	1.0	1.2	1.0	1.1	1.1	1.4	1.9	4	1	1	1	321	35.5	5.2
NP_115963.1	14211889	DPY30	protein dpy-30 homolog	0.9	0.6	0.9	1.4	1.0	0.7	1.1	1.3	1.0	0.7	1.0	1.4	31.3	2	3	3	5	99	11.2	4.9
NP_003777.2	9955970	ABCC3	canalicular multispecific organic anion transporter 2 isoform 1	1.7	1.3	1.3	1.5	1.2	1.3	1.2	1.3	1.4	1.3	1.2	1.4	0.7	1	1	1	2	1527	169.2	7.2
NP_001120954.1	188595645	SPRYD7	SPRY domain-containing protein 7 isoform 2	1.3	1.5	1.4	1.7	1.1	1.2	1.1	1.1	1.2	1.4	1.3	1.4	5.1	2	1	1	4	157	17.5	5.9
NP_003350.1	4507813	UGDH	UDP-glucose 6-dehydrogenase isoform 1	1.3	1.2	1.2	1.3	1.3	1.5	1.3	1.5	1.3	1.3	1.2	1.4	40.9	3	12	12	30	494	55.0	7.1
NP_059997.3	237820711	LY6K	lymphocyte antigen 6K isoform 1 precursor	1.1	1.5	1.7	1.3	1.1	1.5	1.9	1.5	1.1	1.5	1.8	1.4	15.2	1	2	2	6	165	18.7	7.4
NP_057383.2	82880658	HSPA14	heat shock 70 kDa protein 14 isoform 1	1.2	1.1	1.2	1.4	1.2	1.1	1.2	1.4	1.2	1.1	1.2	1.4	23.0	2	10	10	28	509	54.8	5.6
NP_055146.1	7657683	SLC7A11	cystine/glutamate transporter	1.6	1.5	1.2	1.3	1.5	1.6	1.3	1.4	1.6	1.6	1.2	1.4	7.2	1	3	3	5	501	55.4	9.2
NP_660183.1	22035620	PYCARD	apoptosis-associated speck-like protein containing a CARD isoform b	1.0	1.0	1.2	1.0	1.9	2.0	2.5	1.8	1.4	1.5	1.9	1.4	6.3	2	1	1	2	176	20.0	5.9
NP_001011885.1	59814146	BTBD1	BTB/POZ domain-containing protein 1 isoform 2	1.3	1.0	1.1	1.5	1.0	1.0	1.1	1.3	1.1	1.0	1.1	1.4	5.7	2	1	1	2	385	42.1	7.0
NP_955474.1	40805825	COPE	coatomer subunit epsilon isoform b	1.2	1.5	1.5	1.3	1.1	1.0	1.2	1.4	1.2	1.2	1.4	1.4	25.7	3	4	4	8	257	28.8	5.3
NP_006551.1	5729794	CELF1	CUGBP Elav-like family member 1 isoform 1	1.0	1.3	1.1	1.4	1.0	1.2	1.1	1.3	1.0	1.3	1.1	1.4	14.5	9	4	4	10	482	51.5	8.5
NP_003839.2	157779135	SUCLG2	succinyl-CoA ligase [GDP-forming] subunit beta, mitochondrial isoform 2 precursor	1.2	1.2	1.2	1.4	1.3	1.2	1.3	1.3	1.2	1.2	1.2	1.4	26.6	2	9	9	30	432	46.5	6.4
NP_001034556.1	86990435	RGS19	regulator of G-protein signaling 19	1.1	1.4	1.4	1.5	1.3	1.1	1.3	1.2	1.2	1.2	1.3	1.4	6.0	1	1	1	2	217	24.6	5.6
NP_001657.3	156071465	ARHGAP4	rho GTPase-activating protein 4 isoform 2	1.1	1.5	1.0	1.5	1.2	1.2	1.1	1.3	1.1	1.4	1.1	1.4	2.9	2	1	1	2	946	105.0	6.3
NP_005611.1	5032057	S100A11	protein S100-A11	1.3	1.4	1.6	1.3	1.1	1.6	1.3	1.4	1.2	1.5	1.4	1.4	23.8	1	2	2	3	105	11.7	7.1
NP_116120.1	14249348	TXNDC17	thioredoxin domain-containing protein 17	1.2	1.2	1.1	1.3	1.5	1.4	1.3	1.4	1.3	1.3	1.2	1.4	11.4	1	1	1	1	123	13.9	5.5
NP_001165375.1	284813529	VPRBP	protein VPRBP isoform 2	1.2	1.5	1.1	1.6	1.1	1.2	1.1	1.1	1.1	1.3	1.1	1.4	1.7	2	2	2	2	1506	168.8	5.1
NP_001172026.1	297374821	INS	insulin preproprotein	1.1	1.6	1.3	1.1	1.5	2.7	2.0	1.6	1.3	2.1	1.7	1.4	6.4	2	1	1	1	110	12.0	5.3
NP_000280.1	4505749	PFKM	ATP-dependent 6-phosphofructokinase, muscle type isoform 2	1.2	1.3	1.4	1.4	1.2	1.2	1.2	1.3	1.2	1.2	1.3	1.4	21.7	2	10	14	33	780	85.1	8.0
NP_006422.1	5453603	CCT2	T-complex protein 1 subunit beta isoform 1	1.1	1.2	1.1	1.4	1.1	1.2	1.1	1.4	1.1	1.2	1.1	1.4	70.8	2	30	30	168	535	57.5	6.5
NP_002194.2	116295258	ITGA2	integrin alpha-2 precursor	1.3	1.4	1.4	1.4	1.2	1.4	1.5	1.3	1.3	1.4	1.5	1.4	8.1	1	6	6	15	1181	129.2	5.2
NP_065728.1	10190720	NMRAL1	nmrA-like family domain-containing protein 1	1.3	1.3	1.3	1.4	1.2	1.3	1.4	1.3	1.2	1.3	1.3	1.4	23.8	1	7	7	18	299	33.3	7.5
NP_001166143.1	291045302	PNPLA4	patatin-like phospholipase domain-containing protein 4 isoform 2	1.6	1.5	1.8	1.4	1.6	1.4	1.5	1.3	1.6	1.4	1.6	1.3	17.5	2	2	2	3	166	18.6	9.2
NP_001976.1	4503609	ETFB	electron transfer flavoprotein subunit beta isoform 1	1.1	1.3	1.3	1.4	1.1	1.2	1.2	1.3	1.1	1.3	1.3	1.3	42.0	2	11	11	38	255	27.8	8.1
NP_001005336.1	56549117	DNM1	dynamin-1 isoform 2	1.2	1.3	1.4	1.4	1.2	1.2	1.3	1.3	1.2	1.2	1.4	1.3	26.7	4	11	17	39	851	96.0	7.0
NP_001273632.1	557878699	UFM1	ubiquitin-fold modifier 1 isoform 2	1.5	1.5	1.1	1.4	1.5	1.1	1.1	1.3	1.5	1.3	1.1	1.3	33.7	3	1	1	2	83	8.9	9.3
NP_003084.1	4507127	SNRPC	U1 small nuclear ribonucleoprotein C	1.0	0.9	0.9	1.2	0.9	1.0	0.8	1.5	0.9	1.0	0.9	1.3	13.2	1	2	2	7	159	17.4	9.7
NP_001013269.1	61744483	SLC3A2	4F2 cell-surface antigen heavy chain isoform f	1.3	1.4	1.1	1.3	1.3	1.4	1.2	1.4	1.3	1.4	1.1	1.3	56.0	4	28	28	212	529	57.9	5.4
NP_001900.1	4503143	CTSD	cathepsin D preproprotein	1.3	1.7	1.4	1.4	1.2	1.5	1.4	1.3	1.2	1.6	1.4	1.3	25.5	1	8	8	34	412	44.5	6.5
NP_001010891.4	267844826	MTX3	metaxin-3 isoform 2	1.5	1.3	1.2	1.5	1.1	1.1	1.2	1.2	1.3	1.2	1.2	1.3	4.0	2	1	1	3	248	27.9	7.8
NP_001166939.1	291084858	PDHB	pyruvate dehydrogenase E1 component subunit beta, mitochondrial isoform 2 precursor	1.2	1.3	1.4	1.3	1.2	1.3	1.2	1.4	1.2	1.3	1.3	1.3	27.0	2	7	7	35	341	37.5	6.4
NP_061830.3	85815829	SH3BP1	SH3 domain-binding protein 1	1.1	1.3	1.2	1.3	1.1	1.1	1.2	1.4	1.1	1.2	1.2	1.3	12.8	1	5	5	14	701	75.7	6.8
NP_001129477.1	209869993;4503915	GART	trifunctional purine biosynthetic protein adenosine-3 isoform 1	1.1	1.2	1.2	1.4	1.1	1.2	1.2	1.3	1.1	1.2	1.2	1.3	50.2	2	38	38	182	1010	107.7	6.7
NP_056338.2	224496106	REXO2	oligoribonuclease, mitochondrial precursor	1.4	1.2	1.3	1.4	1.4	1.2	1.3	1.3	1.4	1.2	1.3	1.3	7.6	1	1	1	7	237	26.8	6.9
NP_005057.1	4826998	SFPQ	splicing factor, proline- and glutamine-rich	1.0	1.0	1.0	1.3	0.9	1.1	1.0	1.3	0.9	1.1	1.0	1.3	33.7	1	18	19	76	707	76.1	9.4
NP_001264152.1	469608407	TAGLN2	transgelin-2 isoform b	1.0	1.2	1.2	1.3	1.1	1.1	1.1	1.3	1.1	1.2	1.1	1.3	14.1	2	2	2	4	199	22.4	8.3
NP_003698.1	4506753	RUVBL1	ruvB-like 1	1.1	1.1	1.1	1.3	1.2	1.2	1.2	1.4	1.1	1.2	1.1	1.3	50.0	1	16	16	73	456	50.2	6.4
NP_006462.1	5453740	MYL12A	myosin regulatory light chain 12A isoform 1	1.4	1.9	1.8	1.5	1.3	1.3	1.3	1.2	1.3	1.6	1.5	1.3	72.5	5	1	11	159	171	19.8	4.8
NP_002442.2	47132622	MTAP	S-methyl-5'-thioadenosine phosphorylase	1.2	1.5	1.4	1.3	1.4	1.5	1.6	1.4	1.3	1.5	1.5	1.3	40.3	1	8	8	27	283	31.2	7.2
NP_005013.1	4826898	PFN1	profilin-1	1.1	1.3	1.2	1.4	1.1	1.2	1.1	1.3	1.1	1.3	1.1	1.3	45.7	1	5	5	33	140	15.0	8.3
NP_001271318.1	548923795	NOLC1	nucleolar and coiled-body phosphoprotein 1 isoform 3	1.0	1.1	1.2	1.2	1.3	1.3	1.3	1.4	1.2	1.2	1.3	1.3	27.0	3	19	19	68	700	73.7	9.5
NP_861516.1	32967285	UBE2C	ubiquitin-conjugating enzyme E2 C isoform 3	1.0	1.2	1.1	1.3	1.0	1.2	1.2	1.3	1.0	1.2	1.2	1.3	33.3	6	3	3	9	150	16.7	8.0
NP_006448.4	374092020	PDLIM5	PDZ and LIM domain protein 5 isoform a	1.0	1.3	1.2	1.5	1.0	1.0	1.0	1.2	1.0	1.1	1.1	1.3	15.4	7	7	7	11	596	63.9	8.2
NP_000593.1	10835159	SERPINE1	plasminogen activator inhibitor 1 precursor	1.5	1.8	2.1	1.2	2.2	2.8	4.0	1.5	1.8	2.3	3.0	1.3	14.2	1	3	3	5	402	45.0	7.2
NP_001273358.1	556695454	THSD4	thrombospondin type-1 domain-containing protein 4 isoform 2 precursor	1.4	1.7	1.8	1.7	1.4	1.3	1.4	1.0	1.4	1.5	1.6	1.3	1.7	2	1	1	1	658	73.4	6.0
NP_002850.2	170932516	PXN	paxillin isoform 1	1.2	1.3	1.1	1.6	1.0	1.1	1.0	1.1	1.1	1.2	1.0	1.3	10.8	4	3	3	3	557	60.9	6.4
NP_001186184.1	312922343	SGOL1	shugoshin-like 1 isoform 1AB	1.1	1.2	1.1	1.1	1.4	2.2	2.8	1.5	1.3	1.7	1.9	1.3	4.7	6	1	1	1	258	29.4	9.6
NP_005318.3	296179429	HADH	hydroxyacyl-coenzyme A dehydrogenase, mitochondrial isoform 2 precursor	1.4	1.4	1.3	1.3	1.2	1.3	1.1	1.3	1.3	1.3	1.2	1.3	26.8	2	6	6	21	314	34.3	8.9
NP_036524.1	6912582	PEF1	peflin	1.3	1.2	1.2	1.4	1.1	1.1	1.0	1.3	1.2	1.1	1.1	1.3	8.5	1	2	2	7	284	30.4	6.5
NP_038470.1	7305503	STOML2	stomatin-like protein 2, mitochondrial isoform a	1.2	1.2	1.2	1.3	1.2	1.2	1.3	1.3	1.2	1.2	1.2	1.3	44.7	4	11	11	44	356	38.5	7.4
NP_001273000.1	554506511	C11orf54	ester hydrolase C11orf54 isoform d	1.2	1.2	1.3	1.4	1.1	1.2	1.2	1.3	1.1	1.2	1.2	1.3	18.4	4	3	3	7	256	28.7	7.0
NP_001035095.1	91208418	TCERG1	transcription elongation regulator 1 isoform 2	1.0	1.2	1.0	1.3	1.0	1.2	1.1	1.3	1.0	1.2	1.1	1.3	9.8	2	9	9	18	1077	121.6	8.5
NP_064550.3	256818821	RAD18	E3 ubiquitin-protein ligase RAD18	1.5	1.3	1.3	1.3	1.1	1.2	1.3	1.3	1.3	1.2	1.3	1.3	9.5	1	5	5	8	495	56.2	7.6
NP_036212.3	197382691	HDHD1	pseudouridine-5'-phosphatase isoform b	1.8	1.5	1.2	1.6	1.0	0.9	1.0	1.1	1.4	1.2	1.1	1.3	16.7	4	2	2	3	228	25.2	5.3
NP_620164.1	20270371	CMBL	carboxymethylenebutenolidase homolog	1.2	1.2	1.4	1.3	1.1	1.2	1.4	1.3	1.1	1.2	1.4	1.3	19.6	1	5	5	11	245	28.0	7.2
NP_001093141.1	153792354;153792327	C8orf59	uncharacterized protein C8orf59	0.9	1.1	0.9	1.3	1.1	1.2	1.1	1.4	1.0	1.1	1.0	1.3	18.0	2	2	2	2	100	11.4	10.5

Khan *et al.* , 2019. Multi-omics analysis to characterize cigarette smoke induced molecular alterations in esophageal cells
Supplementary Table 5. List of proteins quantified in untreated and chronically treated Het1A cells with cigarette smoke condensate for 8 months

NP_Accession	Protein group Accession	Gene ID	Description	Het-1A-Smoke - 2M/Parental	Het-1A-Smoke - 4M/Parental	Het-1A-Smoke - 6M/Parental	Het-1A-Smoke - 8M/Parental	Het-1A-Smoke - 2M/Parental	Het-1A-Smoke - 4M/Parental	Het-1A-Smoke - 6M/Parental	Het-1A-Smoke - 8M/Parental	Het-1A-Smoke - 2M/Parental	Het-1A-Smoke - 4M/Parental	Het-1A-Smoke - 6M/Parental	Het-1A-Smoke - 8M/Parental	Coverage (%)	Proteins	Unique Peptides	# Peptides	PSM	# AAs	MW [kDa]	calc. pI
				Replicate 1				Replicate 2				Average of replicates											
NP_036287.1	6912356	EML2	echinoderm microtubule-associated protein-like 2 isoform 2	1.2	1.4	1.3	1.2	1.5	1.5	1.6	1.4	1.4	1.4	1.4	1.3	8.0	3	5	5	8	649	70.6	6.3
NP_110409.2	21359965	CLPTM1L	cleft lip and palate transmembrane protein 1-like protein	1.1	1.2	1.1	1.3	1.1	1.2	1.2	1.3	1.1	1.2	1.2	1.3	4.1	1	2	2	5	538	62.2	8.6
NP_004568.2	46249388	PSPH	phosphoserine phosphatase	1.2	1.1	1.1	1.2	1.4	1.3	1.4	1.4	1.3	1.2	1.3	1.3	17.3	1	3	3	4	225	25.0	5.7
NP_002406.1	4505185	MIF	macrophage migration inhibitory factor	1.3	1.3	1.2	1.2	1.4	1.3	1.3	1.4	1.4	1.3	1.3	1.3	7.8	1	1	1	8	115	12.5	7.9
NP_001131074.1	212286159	MEMO1	protein MEMO1 isoform 2	1.1	1.2	1.1	1.4	0.8	1.0	0.9	1.3	0.9	1.1	1.0	1.3	13.5	3	3	3	5	274	31.3	7.1
NP_036226.1	6912238	PRDX5	peroxiredoxin-5, mitochondrial isoform a precursor	1.4	1.6	1.3	1.3	1.4	1.5	1.3	1.3	1.4	1.6	1.3	1.3	46.3	3	6	6	30	214	22.0	8.6
NP_001273991.1	559098432	RABGEF1	rab5 GDP/GTP exchange factor isoform c	1.2	1.3	1.2	1.4	0.9	1.1	1.0	1.2	1.1	1.2	1.1	1.3	8.8	3	2	2	2	491	56.9	7.0
NP_004795.1	4757988	CIAO1	probable cytosolic iron-sulfur protein assembly protein CIAO1	1.1	1.0	1.1	1.3	0.9	1.0	1.2	1.3	1.0	1.0	1.1	1.3	10.6	1	2	2	3	339	37.8	5.0
NP_036246.1	6912286	CASP14	caspase-14 precursor	0.9	0.9	1.0	1.0	1.2	1.4	1.0	1.6	1.1	1.2	1.0	1.3	6.2	1	1	1	1	242	27.7	5.6
NP_001191742.1	325651886	NT5E	5'-nucleotidase isoform 2 preproprotein	1.1	1.4	1.6	1.3	1.1	1.5	1.8	1.3	1.1	1.4	1.7	1.3	3.2	2	1	1	2	524	57.9	7.0
NP_001229784.1	338827748	ARFIP2	arfaptin-2 isoform 3	2.5	1.9	2.3	1.5	0.9	1.1	0.9	1.2	1.7	1.5	1.6	1.3	17.8	4	3	4	8	303	33.8	7.2
NP_001254552.1	390517020	ITPA	inosine triphosphate pyrophosphatase isoform c	1.1	1.2	1.1	1.3	1.2	1.3	1.2	1.4	1.2	1.2	1.1	1.3	9.2	3	1	1	4	153	16.8	7.2
NP_000550.2	28302131	HBG1	hemoglobin subunit gamma-1	1.2	1.6	1.4	1.3	1.0	1.4	1.3	1.3	1.1	1.5	1.4	1.3	17.0	4	2	3	48	147	16.1	7.2
NP_001240756.1	359465585	GALNT14	polypeptide N-acetylgalactosaminyltransferase 14 isoform 3	1.2	1.2	1.4	1.4	1.2	1.0	1.0	1.2	1.2	1.1	1.2	1.3	3.0	3	1	1	1	532	61.5	6.8
NP_001171546.1	296010848	ASNS	asparagine synthetase [glutamine-hydrolyzing] isoform b	1.5	1.3	1.0	1.3	1.6	1.4	1.1	1.3	1.6	1.4	1.1	1.3	25.2	3	11	11	42	540	62.1	7.1
NP_002363.2	51477714	MAN2A1	alpha-mannosidase 2	1.3	1.3	1.3	1.4	1.0	1.1	1.1	1.2	1.2	1.2	1.2	1.3	2.8	1	3	3	5	1144	131.1	7.6
NP_006139.1	5453710	LASP1	LIM and SH3 domain protein 1 isoform a	1.0	1.1	1.2	1.3	1.0	1.1	1.2	1.3	1.0	1.1	1.2	1.3	24.9	2	6	6	14	261	29.7	7.0
NP_001188295.1	318984125	DNAL1	dynein light chain 1, axonemal isoform 2	1.3	0.8	1.0	1.3	1.0	1.0	1.0	1.3	1.1	0.9	1.0	1.3	13.3	2	1	1	4	151	17.1	5.4
NP_073720.1	12408658	CDC25C	M-phase inducer phosphatase 3 isoform b	0.9	1.1	1.0	1.3	0.8	1.1	1.0	1.3	0.8	1.1	1.0	1.3	7.8	3	2	2	4	400	45.6	7.1
NP_001138416.1	222144324	MYL12B	myosin regulatory light chain 12B	1.2	1.3	1.5	1.5	1.2	1.1	1.1	1.1	1.2	1.2	1.3	1.3	72.1	4	1	11	167	172	19.8	4.8
NP_003341.1	4507797	UBE2V2	ubiquitin-conjugating enzyme E2 variant 2	1.2	1.1	1.1	1.3	1.2	1.1	1.1	1.3	1.2	1.1	1.1	1.3	48.3	2	2	6	26	145	16.4	8.1
NP_060705.2	271398239	CNDP2	cytosolic non-specific dipeptidase isoform 1	1.2	1.2	1.1	1.3	1.3	1.2	1.2	1.4	1.3	1.2	1.1	1.3	37.3	2	13	13	48	475	52.8	6.0
NP_001011546.1	58530848	DSTN	destrin isoform b	1.2	1.3	1.3	1.3	1.1	1.2	1.3	1.3	1.1	1.3	1.3	1.3	35.8	5	4	5	19	148	16.6	8.3
NP_942593.2	298228964	ATG16L1	autophagy-related protein 16-1 isoform 3	0.9	0.9	0.9	0.9	1.2	1.1	1.1	1.7	1.1	1.0	1.0	1.3	10.4	5	4	4	5	444	49.5	7.9
NP_003336.1	4507785;35494003	UBE2I	SUMO-conjugating enzyme UBC9	1.0	1.1	1.1	1.3	1.0	1.1	1.0	1.3	1.0	1.1	1.1	1.3	73.4	1	8	8	28	158	18.0	8.7
NP_001975.1	33413400	ESD	S-formylglutathione hydrolase	1.1	1.1	1.1	1.3	1.2	1.3	1.2	1.3	1.1	1.2	1.2	1.3	61.7	1	12	12	38	282	31.4	7.0
NP_115683.3	110815802	C9orf64	UPF0553 protein C9orf64	1.5	1.5	1.4	1.4	1.2	1.4	1.2	1.2	1.3	1.4	1.3	1.3	13.5	1	4	4	16	341	39.0	5.9
NP_001231867.1	349732256	TXN	thioredoxin isoform 2	1.6	1.4	1.6	1.3	1.4	1.4	1.4	1.3	1.5	1.4	1.5	1.3	56.5	2	4	4	28	85	9.4	6.0
NP_001266289.1	525342616	LYPLA1	acyl-protein thioesterase 1 isoform 6	1.2	1.2	1.2	1.4	1.0	1.1	1.0	1.2	1.1	1.1	1.1	1.3	19.3	6	3	3	8	166	18.0	5.1
NP_000010.1	4557237	ACAT1	acetyl-CoA acetyltransferase, mitochondrial precursor	1.3	1.5	1.4	1.3	1.3	1.5	1.4	1.3	1.3	1.5	1.4	1.3	21.8	1	7	7	39	427	45.2	8.9
NP_000680.2	21361176	ALDH1A1	retinal dehydrogenase 1	1.9	2.4	2.5	1.3	1.6	1.7	1.8	1.3	1.7	2.0	2.1	1.3	11.4	1	4	6	35	501	54.8	6.7
NP_001949.1	4503475	EEF1A2	elongation factor 1-alpha 2	1.2	1.3	1.7	1.2	1.2	1.5	1.6	1.4	1.2	1.4	1.7	1.3	54.2	1	7	18	600	463	50.4	9.0
NP_061153.1	8923898	RNF114	E3 ubiquitin-protein ligase RNF114	1.1	1.1	1.2	1.3	1.2	1.1	1.0	1.3	1.2	1.1	1.1	1.3	10.1	1	2	2	3	228	25.7	7.3
NP_001136065.1	217272844	ITPK1	inositol-tetrakisphosphate 1-kinase isoform a	1.1	1.2	1.3	1.4	1.1	1.0	1.2	1.2	1.1	1.1	1.2	1.3	3.1	1	1	1	2	414	45.6	6.2
NP_056031.2	41281583	PPIP5K2	inositol hexakisphosphate and diphosphoinositol-pentakisphosphate kinase 2 isoform 2	1.2	1.4	1.2	1.2	1.3	1.3	1.3	1.4	1.2	1.4	1.3	1.3	5.3	7	4	4	6	1222	138.0	8.1
NP_005619.1	5032093	SLC1A5	neutral amino acid transporter B(0) isoform 1	1.5	1.5	1.3	1.3	1.4	1.5	1.3	1.3	1.4	1.5	1.3	1.3	20.2	3	8	8	37	541	56.6	5.5
NP_061819.2	12056473	NANS	sialic acid synthase	1.2	1.5	1.3	1.4	1.1	1.3	1.2	1.2	1.2	1.4	1.3	1.3	18.4	1	5	5	13	359	40.3	6.7
NP_008882.2	21071067	TAF5	transcription initiation factor TFIID subunit 5	1.1	1.4	1.2	1.4	1.3	1.2	1.1	1.2	1.2	1.3	1.1	1.3	4.0	1	1	1	6	800	86.8	5.6
NP_694997.2	110227615	EML3	echinoderm microtubule-associated protein-like 3 isoform 3	1.0	1.1	1.0	1.3	1.2	1.3	1.3	1.3	1.1	1.2	1.2	1.3	3.0	3	2	2	4	896	95.1	7.1
NP_002070.1	4504067	GOT1	aspartate aminotransferase, cytoplasmic	1.3	1.3	1.3	1.3	1.4	1.3	1.3	1.3	1.4	1.3	1.3	1.3	46.0	1	13	13	55	413	46.2	7.0
NP_001276086.1	574584840	MPI	mannose-6-phosphate isomerase isoform 4	1.2	1.3	1.2	1.3	1.2	1.2	1.2	1.3	1.2	1.2	1.2	1.3	24.0	4	5	5	12	362	39.8	5.4
NP_001277283.1	594140703	DHFR	dihydrofolate reductase isoform 2	1.0	1.0	1.1	1.3	1.0	0.9	0.9	1.3	1.0	1.0	1.0	1.3	37.8	4	4	4	11	135	15.7	6.3
NP_061982.3	41350216	ALG1	chitobiosyldiphosphodolichol beta-mannosyltransferase	1.0	1.2	1.1	1.2	1.3	1.3	1.1	1.4	1.1	1.2	1.1	1.3	8.4	1	3	3	8	464	52.5	7.2
NP_733829.1	25470890	DAZAP1	DAZ-associated protein 1 isoform a	1.0	1.1	1.1	1.3	0.9	1.0	1.0	1.3	1.0	1.1	1.0	1.3	12.4	2	3	3	17	378	40.5	8.3
NP_001255968.1	392841224	SHOC2	leucine-rich repeat protein SHOC-2 isoform 2	1.2	1.4	1.3	1.4	1.1	1.2	1.2	1.2	1.2	1.3	1.2	1.3	6.2	2	2	2	2	536	59.7	9.1
NP_037364.1	7019485	PDCD6	programmed cell death protein 6 isoform 1	1.2	1.2	1.2	1.3	1.2	1.1	1.2	1.3	1.2	1.1	1.2	1.3	30.9	4	4	4	12	191	21.9	5.4
NP_009176.2	27262647	ATXN2L	ataxin-2-like protein isoform A	0.9	1.1	0.9	1.3	1.0	1.2	1.0	1.3	1.0	1.2	1.0	1.3	15.1	7	13	13	22	1075	113.3	8.6
NP_003214.1	4507447	TFAP4	transcription factor AP-4	1.0	1.1	1.2	1.1	0.8	1.2	1.2	1.5	0.9	1.2	1.2	1.3	5.3	1	1	1	2	338	38.7	5.9
NP_060417.2	157388923	MOCOS	molybdenum cofactor sulfurase	1.7	1.7	1.4	1.6	1.0	1.1	1.1	1.0	1.3	1.4	1.2	1.3	8.2	1	4	4	7	888	98.1	6.7
NP_003477.4	71979932	SLC7A5	large neutral amino acids transporter small subunit 1	1.4	1.4	1.1	1.4	1.2	1.1	1.1	1.2	1.3	1.2	1.1	1.3	17.4	3	8	8	43	507	55.0	7.7
NP_001248753.1	387849314	PSMG1	proteasome assembly chaperone 1 isoform 3	1.3	1.2	1.1	1.3	1.1	1.2	1.2	1.3	1.2	1.2	1.2	1.3	32.5	3	7	7	22	283	32.2	7.2
NP_071375.1	27501458	CHTF18	chromosome transmission fidelity protein 18 homolog	0.9	0.9	0.9	1.0	1.3	1.1	1.2	1.6	1.1	1.0	1.1	1.3	3.6	1	2	2	6	975	107.3	7.2
NP_001147.1	4502111	ANXA7	annexin A7 isoform 1	1.2	1.1	1.3	1.3	1.2	1.3	1.3	1.3	1.2	1.2	1.3	1.3	23.6	2	9	9	36	466	50.3	6.6
NP_001171904.1	296923763;19224660	PACSIN3	protein kinase C and casein kinase substrate in neurons protein 3	1.0	1.1	1.1	1.2	1.0	1.1	1.1	1.3	1.0	1.1	1.1	1.3	12.3	1	4	4	7	424	48.5	6.2
NP_006611.1	5729864	HBS1L	HBS1-like protein isoform 1	1.1	1.3	1.3	1.3	1.1	1.2	1.3	1.2	1.1	1.2	1.3	1.3	38.6	2	17	21	61	684	75.4	6.6
NP_003818.2	47933379	NAPA	alpha-soluble NSF attachment protein	1.2	1.3	1.2	1.4	1.0	1.1	1.1	1.2	1.1	1.2	1.1	1.3	33.9	5	6	6	14	295	33.2	5.4
NP_009106.1	6005721	ERLIN2	erlin-2 isoform 1	1.1	1.2	1.0	1.3	1.2	1.1	1.0	1.2	1.1	1.2	1.0	1.3	21.5	2	4	7	22	339	37.8	5.6
NP_203520.1	15718708;122056474	CASP8	caspase-8 isoform C precursor	1.1	1.1	1.2	1.4	1.1	1.1	1.2	1.2	1.1	1.1	1.2	1.3	13.2	5	4	4	6	464	53.7	5.2
NP_001157281.1	255759952	WDR81	WD repeat-containing protein 81 isoform 1	1.5	1.1	1.4	1.4	1.1	1.0	1.0	1.2	1.3	1.1	1.2	1.3	1.4	1	1	1	1	1941	211.6	5.6
NP_006102.2	167614485	ACAA2	3-ketoacyl-CoA thiolase, mitochondrial	1.1	1.2	1.2	1.3	1.3	1.1	1.0	1.3	1.2	1.2	1.1	1.3	29.0	1	6	6	17	397	41.9	8.1
NP_001240318.1	358438328	ISYNA1	inositol-3-phosphate synthase 1 isoform 4	1.1	1.3	1.3	1.4	0.9	0.9	1.1	1.2	1.0	1.1	1.2	1.3	14.7	3	4	4	8	430	47.1	5.4
NP_001027451.1	73760407	TFAP2A	transcription factor AP-2-alpha isoform b	1.1	1.1	1.3	1.5	1.1	1.2	1.1	1.1	1.1	1.2	1.2	1.3	6.5	5	2	2	6	431	47.2	8.4
NP_859076.3	165932358	METTL2A	methyltransferase-like protein 2A	1.1	1.2	1.2	1.3	1.2	1.2	1.0	1.2	1.2	1.2	1.1	1.3	5.3	2	1	1	2	378	43.5	6.0
NP_002686.2	14589951	POLR2E	DNA-directed RNA polymerases I, II, and III subunit RPABC1	0.9	1.1	1.0	1.2	1.0	1.1	1.1	1.4	1.0	1.1	1.0	1.3	6.2	1	1	1	1	210	24.6	5.9
NP_775882.2	109452587	FAM98B	protein FAM98B	1.0	1.0	1.0	1.1	1.1	1.1	1.1	1.5	1.1	1.1	1.1	1.3	32.3	1	9	9	29	433	45.5	8.7
NP_775788.2	46309463	VKORC1L1	vitamin K epoxide reductase complex subunit 1-like protein 1 isoform 1	1.0	1.3	1.3	1.4	1.2	1.1	1.4	1.2	1.1	1.2	1.3	1.3	11.9	2	2	2	3	176	19.8	9.1
NP_001177369.1	298919181	NCOR1	nuclear receptor corepressor 1 isoform 3	1.1	1.4	1.5	1.5	0.9	1.0	0.9	1.1	1.0	1.2	1.2	1.3	1.7	2	2	2	3	2337	258.4	6.9
NP_001003712.1	51243032	OSBPL8	oxysterol-binding protein-related protein 8 isoform b	1.3	1.2	1.5	1.3	1.1	1.2	1.3	1.3	1.2	1.2	1.4	1.3	5.3	2	4	4	8	847	96.9	7.5
NP_057117.2	116875831	RMDN1	regulator of microtubule dynamics protein 1 isoform 1	1.1	1.1	1.2	1.2	1.3	1.3	1.3	1.3	1.2	1.2	1.3	1.3	6.7	3	2	2	3	314	35.8	8.5
NP_006824.2	34147637	COPS6	COP9 signalosome complex subunit 6	1.1	1.2	1.1	1.3	1.2	1.2	1.2	1.3	1.1	1.2	1.2	1.3	12.5	1	4	4	9	327	36.1	5.7
NP_938074.2	151301219	PRMT1	protein arginine N-methyltransferase 1 isoform 3	1.1	1.2	1.3	1.3	1.1	1.1	1.3	1.2	1.1	1.2	1.3	1.3	47.6	5	14	14	80	353	40.5	5.5
NP_057038.2	116063554	DERA	deoxyribose-phosphate aldolase isoform 1	1.1	1.2	1.2	1.3	1.1	1.2	1.2	1.3	1.1	1.2	1.2	1.3	11.0	2	2	2	3	318	35.2	8.9
NP_003184.1	4507375;118442828	TBCE	tubulin-specific chaperone E isoform a	1.1	1.2	1.2	1.3	1.2	1.2	1.2	1.3	1.2	1.2	1.2	1.3	23.7	3	8	8	25	527	59.3	6.8
NP_006695.1	5730041	SUGT1	suppressor of G2 allele of SKP1 homolog isoform SGT1A	1.6	1.1	1.5	1.4	1.1	1.1	1.3	1.2	1.4	1.1	1.4	1.3	6.9	2	1	1	2	333	37.8	5.2
NP_055955.1	11056036	TTLL12	tubulin--tyrosine ligase-like protein 12	1.2	1.2	1.3	1.3	1.2	1.2	1.3	1.2	1.2	1.2	1.3	1.3	22.5	1	11	11	27	644	74.4	5.5
NP_219482.1	15529978	ZNF622	zinc finger protein 622	1.1	1.1	1.2	1.3	1.2	1.2	1.2	1.3	1.1	1.2	1.2	1.3	21.0	1	8	8	18	477	54.2	6.2
NP_036220.1	6912586	PGLS	6-phosphogluconolactonase	1.1	1.3	1.0	1.4	1.1	1.1	1.0	1.2	1.1	1.2	1.0	1.3	36.8	1	6	6	18	258	27.5	6.0
NP_001273013.1	554506526	TRMT112	tRNA methyltransferase 112 homolog isoform 3	1.4	1.0	1.3	1.3	1.0	1.0	1.2	1.3	1.2	1.0	1.3	1.3	13.2	2	1	1	2	106	12.0	8.1
NP_150597.1	15150811	MRPS36	28S ribosomal protein S36, mitochondrial	0.8	1.2	1.0	1.4	1.1	1.1	1.2	1.2	1.0	1.1	1.1	1.3	17.5	1	1	1	1	103	11.5	10.0
NP_006395.2	34335272	PROCR	endothelial protein C receptor precursor	1.1	1.0	1.2	1.2	1.1	1.1	1.2	1.3	1.1	1.1	1.2	1.3	14.3	1	3	3	8	238	26.7	7.2
NP_066952.1	11056044	PPA1	inorganic pyrophosphatase	1.2	1.1	1.2	1.2	1.1	1.2	1.2	1.3	1.1	1.1	1.2	1.3	61.3	1	14	16	73	289	32.6	5.9
NP_001243425.1	374532779	MAN2C1	alpha-mannosidase 2C1 isoform 4	1.2	1.1	1.1	1.2	1.2	1.3	1.2	1.4	1.2	1.2	1.2	1.3	8.0	4	6	6	12	941	104.6	6.5
NP_001389.2	70995211	ECH1	delta(3,5)-Delta(2,4)-dienoyl-CoA isomerase, mitochondrial precursor	1.2	1.4	1.3	1.3	1.2	1.3	1.3	1.3	1.2	1.4	1.3	1.3	41.5	1	11	11	41	328	35.8	8.0
NP_789845.1	29171702	PPA2	inorganic pyrophosphatase 2, mitochondrial isoform 1 precursor	1.1	1.2	1.3	1.2	1.2	1.4	1.3	1.3	1.2	1.3	1.3	1.3	29.6	4	6	8	24	334	37.9	7.4
NP_001159830.1	261862350;261862352	SHMT2	serine hydroxymethyltransferase, mitochondrial isoform 3	1.3	1.4	1.2	1.3	1.2	1.3	1.2	1.2	1.2	1.3	1.2	1.3	55.1	3	19	20	153	483	53.4	8.1
NP_689953.1	23308751	HIBADH	3-hydroxyisobutyrate dehydrogenase, mitochondrial precursor	1.2	1.3	1.1	1.3	1.2	1.2	1.1	1.3	1.2	1.2	1.1	1.3	24.1	1	5	5	14	336	35.3	8.1
NP_612449.2	88702793	VASN	vasorin precursor	1.3	1.4	1.4	1.4	1.2	1.1	1.1	1.1	1.3	1.3	1.2	1.3	9.1	1	3	3	4	673	71.7	7.4
NP_112092.1	13562090	APOL2	apolipoprotein L2	1.1	1.0	1.1	1.5	1.0	1.1	1.0	1.1	1.0	1.0	1.0	1.3	5.6	1	2	2	3	337	37.1	6.7

Khan *et al.* , 2019. Multi-omics analysis to characterize cigarette smoke induced molecular alterations in esophageal cells
Supplementary Table 5. List of proteins quantified in untreated and chronically treated Het1A cells with cigarette smoke condensate for 8 months

NP_Accession	Protein group Accession	Gene ID	Description	Het-1A-Smoke - 2M/Parental	Het-1A-Smoke - 4M/Parental	Het-1A-Smoke - 6M/Parental	Het-1A-Smoke - 8M/Parental	Het-1A-Smoke - 2M/Parental	Het-1A-Smoke - 4M/Parental	Het-1A-Smoke - 6M/Parental	Het-1A-Smoke - 8M/Parental	Het-1A-Smoke - 2M/Parental	Het-1A-Smoke - 4M/Parental	Het-1A-Smoke - 6M/Parental	Het-1A-Smoke - 8M/Parental	Coverage (%)	Proteins	Unique Peptides	# Peptides	PSM	# AAs	MW [kDa]	calc. pI
				Replicate 1				Replicate 2				Average of replicates											
NP_005029.1	4826932	PPID	peptidyl-prolyl cis-trans isomerase D	1.0	1.1	1.1	1.2	1.0	1.1	1.0	1.3	1.0	1.1	1.1	1.3	31.4	1	10	11	19	370	40.7	7.2
NP_001015885.1	62739173	RAE1	mRNA export factor	1.0	1.0	1.1	1.3	1.0	1.1	1.1	1.3	1.0	1.1	1.1	1.3	29.1	1	8	8	19	368	40.9	7.8
NP_003339.1	4507793	UBE2N	ubiquitin-conjugating enzyme E2 N	1.2	1.2	1.3	1.4	1.1	1.2	1.2	1.2	1.2	1.2	1.3	1.3	42.1	2	5	5	16	152	17.1	6.6
NP_002781.2	23110942	PSMA5	proteasome subunit alpha type-5 isoform 1	1.0	0.9	1.1	1.3	1.0	0.9	1.0	1.2	1.0	0.9	1.0	1.3	51.9	2	9	9	48	241	26.4	4.8
NP_065093.2	31543417	PLSCR3	phospholipid scramblase 3	1.2	1.2	1.4	1.5	1.4	1.0	1.1	1.0	1.3	1.1	1.2	1.3	6.8	1	1	1	2	295	31.6	6.7
NP_006326.1	5454120	TIMM17A	mitochondrial import inner membrane translocase subunit Tim17-A	1.1	1.3	1.2	1.3	1.1	1.3	1.2	1.2	1.1	1.3	1.2	1.3	26.3	1	2	2	6	171	18.0	7.9
NP_001165084.1	284172431	PREPL	prolyl endopeptidase-like isoform 4	1.3	1.3	1.2	1.3	1.2	1.3	1.2	1.2	1.2	1.3	1.2	1.3	16.0	9	6	6	10	638	73.3	5.4
NP_001269136.1	532524986	SIGMAR1	sigma non-opioid intracellular receptor 1 isoform 8	1.1	1.0	1.1	1.1	1.0	1.2	1.2	1.4	1.0	1.1	1.1	1.3	17.7	7	3	3	15	203	22.8	5.9
NP_113673.3	237820620	GRWD1	glutamate-rich WD repeat-containing protein 1	1.0	0.9	1.0	1.2	1.0	1.0	1.1	1.3	1.0	1.0	1.1	1.3	20.2	1	6	6	15	446	49.4	4.9
NP_004620.1	4759336	FANCG	Fanconi anemia group G protein	0.7	0.8	0.8	0.9	1.1	1.7	1.2	1.6	0.9	1.2	1.0	1.3	1.1	3	1	1	1	622	68.5	5.5
NP_000261.2	157168362	PNP	purine nucleoside phosphorylase	1.1	1.0	1.1	1.2	1.2	1.0	1.1	1.3	1.1	1.0	1.1	1.3	47.1	1	9	9	54	289	32.1	6.9
NP_004159.2	156416003	SDHA	succinate dehydrogenase [ubiquinone] flavoprotein subunit, mitochondrial isoform 1	1.3	1.2	1.3	1.3	1.2	1.2	1.3	1.3	1.3	1.2	1.3	1.3	38.4	2	15	15	45	664	72.6	7.4
NP_005520.4	126012571	HSPG2	basement membrane-specific heparan sulfate proteoglycan core protein isoform b precursor	1.4	2.0	1.7	1.5	0.9	1.0	1.0	1.0	1.1	1.5	1.3	1.3	0.4	2	1	1	6	4391	468.5	6.5
NP_001138826.1	223890246	MKLN1	muskelin isoform 1	1.0	1.1	1.0	1.2	0.8	2.1	0.7	1.4	0.9	1.6	0.9	1.3	2.7	2	1	1	1	712	82.4	6.3
NP_898871.1	34101272	BCKDHB	2-oxoisovalerate dehydrogenase subunit beta, mitochondrial precursor	1.3	1.3	1.3	1.3	1.2	1.2	1.1	1.3	1.2	1.3	1.2	1.3	2.6	1	1	1	1	392	43.1	6.3
NP_001163935.1	281427168	CDK17	cyclin-dependent kinase 17 isoform 2	1.2	1.2	1.2	1.2	1.2	1.3	1.4	1.3	1.2	1.2	1.3	1.3	10.1	55	3	4	11	523	59.5	8.8
NP_060282.1	8923390	CHCHD3	MICOS complex subunit MIC19	1.0	1.0	1.0	1.3	1.1	1.0	1.1	1.3	1.0	1.0	1.1	1.3	22.0	1	5	5	14	227	26.1	8.3
NP_005130.1	4826643	ANXA3	annexin A3	1.2	1.1	1.2	1.2	1.2	1.2	1.2	1.3	1.2	1.1	1.2	1.3	32.5	1	9	9	28	323	36.4	5.9
NP_065393.1	24431935	RTN4	reticulon-4 isoform A	1.4	1.0	1.2	1.4	1.1	1.1	1.1	1.1	1.3	1.0	1.1	1.3	19.8	3	1	11	75	1192	129.9	4.5
NP_478102.2	300863096	CDKN2A	cyclin-dependent kinase inhibitor 2A isoform p14ARF	1.4	1.5	1.6	1.6	1.1	1.2	1.1	0.9	1.2	1.3	1.4	1.3	20.5	1	2	2	6	132	13.9	12.4
NP_001139786.1	226443152;14249382	ABHD14B	alpha/beta hydrolase domain-containing protein 14B isoform 1	1.5	1.4	1.4	1.3	1.3	1.2	1.3	1.2	1.4	1.3	1.3	1.3	26.2	2	4	4	7	210	22.3	6.4
NP_003866.1	4504035	GMPS	GMP synthase [glutamine-hydrolyzing]	1.2	1.3	1.2	1.3	1.2	1.2	1.2	1.2	1.2	1.2	1.2	1.3	52.5	1	28	28	115	693	76.7	6.9
NP_071909.1	11968009	SIL1	nucleotide exchange factor SIL1 precursor	1.2	1.2	1.2	1.3	1.3	1.3	1.2	1.2	1.2	1.2	1.2	1.3	3.0	1	1	1	2	461	52.1	5.4
NP_115700.1	14150100	NTPCR	cancer-related nucleoside-triphosphatase	1.1	1.1	0.9	1.2	1.1	1.2	1.1	1.3	1.1	1.1	1.0	1.3	12.6	1	2	2	5	190	20.7	9.5
NP_872319.1	32698866	SPC24	kinetochore protein Spc24	1.0	1.0	1.1	1.1	1.3	1.3	1.3	1.5	1.2	1.1	1.2	1.3	12.7	1	2	2	5	197	22.4	4.7
NP_110437.2	42794771	TXNDC5	thioredoxin domain-containing protein 5 isoform 1 precursor	1.0	1.2	1.1	1.2	1.1	1.2	1.1	1.3	1.0	1.2	1.1	1.3	50.7	2	16	16	54	432	47.6	6.0
NP_001015055.1	62739179	RTKN	rhotekin isoform a	1.2	1.4	1.3	1.2	1.2	1.2	1.1	1.3	1.2	1.3	1.2	1.3	2.7	1	1	1	4	563	62.6	7.4
NP_976227.1	42741675	PDLIM7	PDZ and LIM domain protein 7 isoform 2	1.0	1.3	1.2	1.3	1.1	1.1	1.1	1.2	1.0	1.2	1.2	1.3	23.2	3	7	7	17	423	46.5	8.3
NP_036394.1	6912420	HS2ST1	heparan sulfate 2-O-sulfotransferase 1 isoform 1	1.2	1.5	1.5	1.6	1.0	0.9	1.0	0.9	1.1	1.2	1.3	1.3	4.8	1	1	1	2	356	41.9	8.7
NP_036205.1	24307939	CCT5	T-complex protein 1 subunit epsilon isoform a	1.1	1.0	1.1	1.3	1.1	1.0	1.0	1.2	1.1	1.0	1.1	1.3	64.9	5	28	29	140	541	59.6	5.7
NP_001153239.1	229577398;7661744	BZW2	basic leucine zipper and W2 domain-containing protein 2	1.1	1.1	1.2	1.3	1.1	1.1	1.1	1.2	1.1	1.1	1.2	1.3	15.5	1	6	8	15	419	48.1	6.7
NP_612472.1	19923999	TLCD1	TLC domain-containing protein 1 isoform 1 precursor	1.0	1.1	1.1	0.9	1.7	1.5	2.0	1.6	1.3	1.3	1.6	1.3	6.9	1	1	1	2	247	28.5	9.5
NP_003890.1	4505573	ARHGEF7	rho guanine nucleotide exchange factor 7 isoform a	1.0	1.1	1.0	1.3	1.0	1.0	1.0	1.2	1.0	1.1	1.0	1.3	5.4	6	3	3	4	646	73.1	7.2
NP_056036.1	39930351	DNMBP	dynamin-binding protein	1.2	1.3	1.5	1.3	1.1	1.3	1.4	1.2	1.1	1.3	1.4	1.3	11.3	1	11	11	24	1577	177.2	5.4
NP_877419.1	33519428	TXNRD1	thioredoxin reductase 1, cytoplasmic isoform 2	1.5	1.5	1.3	1.3	1.4	1.5	1.3	1.2	1.4	1.5	1.3	1.3	56.3	5	21	22	98	499	54.7	6.5
NP_061176.3	38788380	DHTKD1	probable 2-oxoglutarate dehydrogenase E1 component DHKTD1, mitochondrial	1.2	1.2	1.2	1.3	1.3	1.2	1.4	1.2	1.3	1.2	1.3	1.3	7.6	1	7	7	10	919	103.0	6.9
NP_006319.1	5454064	RBM14	RNA-binding protein 14 isoform 1	1.0	1.1	1.1	1.3	1.0	1.0	1.0	1.2	1.0	1.0	1.1	1.2	23.8	4	14	14	39	669	69.4	9.7
NP_068831.1	12056468;4504811	JUP	junction plakoglobin	1.5	1.2	1.1	1.3	1.0	1.1	1.0	1.2	1.3	1.2	1.0	1.2	13.0	1	7	9	21	745	81.7	6.1
NP_001128661.1	206597509	AGFG1	arf-GAP domain and FG repeat-containing protein 1 isoform 4	1.3	1.0	0.9	1.2	1.1	1.1	1.0	1.3	1.2	1.1	1.0	1.2	7.9	4	3	3	4	522	54.1	8.9
NP_005014.2	167860116	PGGT1B	geranylgeranyl transferase type-1 subunit beta	1.1	1.3	1.2	1.2	1.1	1.2	1.2	1.3	1.1	1.3	1.2	1.2	13.3	1	4	4	8	377	42.3	6.8
NP_001268644.1	528281407;527498279	PHB	prohibitin isoform 1	1.3	1.3	1.3	1.2	1.2	1.3	1.3	1.3	1.2	1.3	1.3	1.2	66.5	2	13	13	94	272	29.8	5.8
NP_004270.2	94538354	PMPCB	mitochondrial-processing peptidase subunit beta precursor	1.1	1.1	1.0	1.2	1.3	1.2	1.2	1.3	1.2	1.1	1.1	1.2	15.5	1	6	6	16	489	54.3	6.8
NP_001753.1	4502643	CCT6A	T-complex protein 1 subunit zeta isoform a	1.1	1.1	1.1	1.3	1.1	1.1	1.1	1.2	1.1	1.1	1.1	1.2	49.5	5	21	21	152	531	58.0	6.7
NP_000476.1	4502171	APRT	adenine phosphoribosyltransferase isoform a	1.2	1.1	1.1	1.3	1.1	1.0	1.1	1.2	1.2	1.1	1.1	1.2	45.6	2	6	6	54	180	19.6	6.0
NP_006494.1	5729991	PSMC4	26S protease regulatory subunit 6B isoform 1	1.1	1.0	1.1	1.3	1.0	1.0	1.0	1.2	1.0	1.0	1.1	1.2	29.4	2	11	11	32	418	47.3	5.2
NP_001263271.1	444741681	C18orf8	uncharacterized protein C18orf8 isoform 2	1.1	1.1	1.1	1.2	1.1	1.2	1.1	1.3	1.1	1.2	1.1	1.2	1.8	2	1	1	2	561	63.6	8.5
NP_653171.1	21700763	HN1L	hematological and neurological expressed 1-like protein	1.1	1.1	1.2	1.2	1.3	1.6	1.3	1.3	1.2	1.4	1.3	1.2	7.4	1	1	1	2	190	20.1	9.3
NP_862821.1	33188456	UBE2D2	ubiquitin-conjugating enzyme E2 D2 isoform 2	1.1	1.3	1.3	1.1	1.2	1.3	1.1	1.4	1.1	1.3	1.2	1.2	29.7	6	2	2	13	118	13.6	8.3
NP_004228.1	11321607	TRIP13	pachytene checkpoint protein 2 homolog isoform 1	1.2	1.2	1.3	1.3	1.1	1.2	1.4	1.2	1.2	1.2	1.4	1.2	39.1	2	14	14	49	432	48.5	6.1
NP_003745.1	4503519	EIF3F	eukaryotic translation initiation factor 3 subunit F	1.2	1.1	1.1	1.2	1.2	1.1	1.2	1.2	1.2	1.1	1.1	1.2	50.4	1	12	12	28	357	37.5	5.5
NP_036606.2	18699734	UCK2	uridine-cytidine kinase 2	1.4	1.2	1.3	1.2	1.2	1.2	1.2	1.3	1.3	1.2	1.2	1.2	11.5	1	2	3	7	261	29.3	6.7
NP_003293.2	91208423	TRIP6	thyroid receptor-interacting protein 6	1.0	1.2	1.0	1.1	1.0	1.2	1.2	1.3	1.0	1.2	1.1	1.2	3.8	1	1	1	2	476	50.3	7.4
NP_001138994.1	224809474	RAI14	ankycorbin isoform b	1.2	1.1	1.3	1.3	1.1	1.1	1.2	1.2	1.2	1.1	1.3	1.2	12.1	4	8	8	18	951	106.8	6.0
NP_001265532.1	518831687	NUP54	nucleoporin p54 isoform 2	1.0	1.1	1.0	1.2	1.2	1.1	1.1	1.3	1.1	1.1	1.0	1.2	6.1	2	2	2	3	459	50.7	6.8
NP_005774.2	18104959	TXNDC9	thioredoxin domain-containing protein 9	1.0	1.0	1.2	1.4	0.9	1.0	1.0	1.1	1.0	1.0	1.1	1.2	4.0	1	1	1	2	226	26.5	5.9
NP_060310.1	8923448	MRPL16	39S ribosomal protein L16, mitochondrial	1.0	0.9	1.0	1.0	1.4	1.4	1.6	1.5	1.2	1.2	1.3	1.2	13.6	1	2	2	4	251	28.4	10.1
NP_057037.1	7705716	NOSIP	nitric oxide synthase-interacting protein	0.9	1.0	1.0	1.2	1.0	1.2	1.0	1.3	0.9	1.1	1.0	1.2	6.0	1	2	2	3	301	33.2	8.8
NP_004448.2	42794752	ACSL3	long-chain-fatty-acid--CoA ligase 3	1.2	1.2	1.3	1.3	1.1	1.1	1.1	1.2	1.1	1.2	1.2	1.2	22.1	1	11	12	44	720	80.4	8.4
NP_060773.3	21361625	EXOC2	exocyst complex component 2	1.2	1.3	1.3	1.3	1.2	1.2	1.3	1.2	1.2	1.3	1.3	1.2	11.0	1	6	6	12	924	104.0	6.9
NP_001254733.1	392513654;392513652	PAAF1	proteasomal ATPase-associated factor 1 isoform 1	1.4	1.3	1.4	1.2	1.2	1.3	1.4	1.3	1.3	1.3	1.4	1.2	16.0	3	3	3	5	375	40.4	6.7
NP_060640.2	21361709	RPRD1A	regulation of nuclear pre-mRNA domain-containing protein 1A	0.8	1.1	0.9	1.1	1.0	1.3	1.1	1.3	0.9	1.2	1.0	1.2	10.9	1	2	3	9	312	35.7	7.5
NP_976035.1	42560246	POLR1C	DNA-directed RNA polymerases I and III subunit RPAC1	1.2	1.2	1.2	1.4	1.1	1.1	1.1	1.1	1.1	1.1	1.2	1.2	9.8	1	2	2	20	346	39.2	5.5
NP_001074010.2	256017159	MGA	MAX gene-associated protein isoform 2	0.9	1.0	0.9	1.0	1.4	1.5	1.3	1.5	1.2	1.2	1.1	1.2	2.0	2	3	3	3	2856	315.0	6.8
NP_000215.1	4557888;40354195	KRT18	keratin, type I cytoskeletal 18	1.0	1.0	0.9	1.3	1.0	1.0	0.9	1.2	1.0	1.0	0.9	1.2	66.1	10	22	23	132	430	48.0	5.5
NP_006223.2	14589953	POLR2H	DNA-directed RNA polymerases I, II, and III subunit RPABC3 isoform 2	1.0	0.9	1.0	1.2	0.8	0.6	0.9	1.3	0.9	0.8	0.9	1.2	31.3	4	3	3	9	150	17.1	4.7
NP_001168.1	4502227	ARL1	ADP-ribosylation factor-like protein 1 isoform 1	1.1	1.1	1.1	1.3	1.1	1.2	1.1	1.2	1.1	1.2	1.1	1.2	46.4	2	6	6	30	181	20.4	5.7
NP_001010853.1	58082085	PM20D2	peptidase M20 domain-containing protein 2	1.0	1.0	1.0	1.3	1.0	1.0	1.0	1.2	1.0	1.0	1.0	1.2	9.9	1	4	4	6	436	47.7	5.8
NP_000691.1	4502101	ANXA1	annexin A1	1.1	1.2	1.6	1.3	1.1	1.3	1.6	1.2	1.1	1.3	1.6	1.2	60.4	1	16	16	84	346	38.7	7.0
NP_060713.1	8922712	Sep-11	septin-11 isoform 2	1.1	1.3	1.3	1.3	1.1	1.1	1.1	1.2	1.1	1.2	1.2	1.2	26.3	5	5	8	14	429	49.4	6.8
NP_001684.2	19913428	ATP6V1B2	V-type proton ATPase subunit B, brain isoform	1.1	1.3	1.2	1.3	1.1	1.2	1.1	1.2	1.1	1.3	1.2	1.2	17.6	2	6	6	15	511	56.5	5.8
NP_001003681.1	51173873	HMGXB4	HMG domain-containing protein 4	1.0	1.3	1.0	1.3	1.1	1.2	1.1	1.2	1.1	1.3	1.1	1.2	6.0	1	2	2	2	601	65.7	9.3
NP_006431.2	22035672	TXNRD2	thioredoxin reductase 2, mitochondrial isoform 1 precursor	1.3	1.2	1.2	1.4	1.3	1.1	1.1	1.1	1.3	1.1	1.2	1.2	18.9	2	4	5	13	524	56.5	7.5
NP_001230076.1	341865581	NVL	nuclear valosin-containing protein-like isoform 4	1.3	1.5	1.2	1.3	0.9	1.0	1.1	1.2	1.1	1.3	1.1	1.2	5.2	4	3	3	3	765	85.0	6.2
NP_001269455.1	542133141	POLR3F	DNA-directed RNA polymerase III subunit RPC6 isoform 2	1.5	1.2	1.3	1.2	1.1	1.3	1.3	1.2	1.3	1.2	1.3	1.2	4.4	2	1	1	1	275	31.0	7.3
NP_001091987.1	148664211	CADM1	cell adhesion molecule 1 isoform E precursor	1.0	0.9	0.9	0.8	1.3	1.2	1.6	1.6	1.2	1.1	1.2	1.2	8.9	5	2	2	8	414	45.6	5.1
NP_036255.2	74024895	MTO1	protein MTO1 homolog, mitochondrial isoform a	4.4	1.1	1.2	1.1	1.3	1.3	1.2	1.3	2.8	1.2	1.2	1.2	3.5	3	2	2	3	692	77.3	8.4
NP_001244943.1	384381466	FDXR	NADPH:adrenodoxin oxidoreductase, mitochondrial isoform 5 precursor	1.1	1.1	1.1	1.2	0.8	0.8	0.7	1.3	0.9	1.0	0.9	1.2	10.4	6	2	5	8	483	52.9	8.4
NP_003366.2	42476281;296317339	VDAC2	voltage-dependent anion-selective channel protein 2 isoform 2	1.2	1.3	1.3	1.3	1.2	1.2	1.2	1.2	1.2	1.3	1.3	1.2	58.2	2	13	13	103	294	31.5	7.6
NP_001018048.1	66346676	VPS13A	vacuolar protein sorting-associated protein 13A isoform D	1.3	1.2	1.4	1.3	1.3	1.3	1.2	1.2	1.3	1.3	1.3	1.2	2.7	4	5	5	7	3069	347.5	6.2
NP_004406.2	58530840	DSP	desmoplakin isoform I	0.9	1.0	0.9	1.1	1.0	1.2	1.0	1.3	1.0	1.1	1.0	1.2	4.7	2	12	12	22	2871	331.6	6.8
NP_054861.2	37594464	NUDT5	ADP-sugar pyrophosphatase	1.2	0.9	1.1	1.2	1.1	0.9	1.2	1.2	1.1	0.9	1.2	1.2	25.1	1	4	4	10	219	24.3	4.9
NP_055872.4	291190787	MYCBP2	E3 ubiquitin-protein ligase MYCBP2	1.1	1.2	1.0	1.5	0.8	0.9	1.0	0.9	0.9	1.1	1.0	1.2	0.7	1	2	2	2	4678	513.3	7.0
NP_066272.1	13994151	PDLIM1	PDZ and LIM domain protein 1	0.9	1.2	1.1	1.4	0.9	1.0	1.0	1.1	0.9	1.1	1.0	1.2	41.0	2	9	9	16	329	36.0	7.0
NP_000544.2	110735439	WRN	Werner syndrome ATP-dependent helicase	1.2	1.2	1.2	1.2	1.2	1.2	1.1	1.2	1.2	1.2	1.1	1.2	1.6	1	2	2	2	1432	162.4	6.3

Khan *et al.* , 2019. Multi-omics analysis to characterize cigarette smoke induced molecular alterations in esophageal cells
Supplementary Table 5. List of proteins quantified in untreated and chronically treated Het1A cells with cigarette smoke condensate for 8 months

NP_Accession	Protein group Accession	Gene ID	Description	Het-1A-Smoke - 2M/Parental	Het-1A-Smoke - 4M/Parental	Het-1A-Smoke - 6M/Parental	Het-1A-Smoke - 8M/Parental	Het-1A-Smoke - 2M/Parental	Het-1A-Smoke - 4M/Parental	Het-1A-Smoke - 6M/Parental	Het-1A-Smoke - 8M/Parental	Het-1A-Smoke - 2M/Parental	Het-1A-Smoke - 4M/Parental	Het-1A-Smoke - 6M/Parental	Het-1A-Smoke - 8M/Parental	Coverage (%)	Proteins	Unique Peptides	# Peptides	PSM	# AAs	MW [kDa]	calc. pI
				Replicate 1				Replicate 2				Average of replicates											
NP_060950.3	302129698	TMEM126B	complex I assembly factor TMEM126B, mitochondrial isoform a	1.0	1.1	1.0	1.3	1.2	1.1	1.3	1.2	1.1	1.1	1.1	1.2	12.2	4	2	2	5	230	25.9	8.8
NP_005304.3	21361657	PDIA3	protein disulfide-isomerase A3 precursor	1.1	1.3	1.1	1.2	1.1	1.3	1.1	1.2	1.1	1.3	1.1	1.2	55.6	1	26	26	177	505	56.7	6.4
NP_001248332.1	387157878	NFKB2	nuclear factor NF-kappa-B p100 subunit isoform b	1.0	1.0	1.4	1.3	1.2	1.1	1.2	1.2	1.1	1.0	1.3	1.2	9.1	2	5	5	9	899	96.6	6.3
NP_001966.1	5803011	ENO2	gamma-enolase	1.0	1.1	1.2	1.3	1.0	1.0	1.2	1.2	1.0	1.1	1.2	1.2	36.9	1	6	11	279	434	47.2	5.0
NP_938149.2	88900491	GANAB	neutral alpha-glucosidase AB isoform 3 precursor	1.0	1.2	1.1	1.2	1.1	1.1	1.1	1.3	1.0	1.1	1.1	1.2	63.6	2	1	43	273	966	109.4	6.2
NP_001128523.1	205277429	FAM160B1	protein FAM160B1 isoform b	1.1	1.2	1.3	1.2	1.1	1.2	1.2	1.2	1.1	1.2	1.3	1.2	3.7	2	1	1	2	738	83.7	5.3
NP_001216.1	4502577	CASP4	caspase-4 isoform alpha precursor	1.0	1.0	1.1	1.2	1.0	1.1	1.0	1.3	1.0	1.1	1.0	1.2	17.8	2	3	3	5	377	43.2	6.0
NP_001265553.1	520261838	NFXL1	NF-X1-type zinc finger protein NFXL1	1.1	1.2	1.1	1.3	1.2	1.2	1.3	1.2	1.1	1.2	1.2	1.2	5.9	1	3	3	4	911	101.3	8.4
NP_006103.1	5174637	PPIE	peptidyl-prolyl cis-trans isomerase E isoform 1	1.1	1.1	1.0	1.2	1.0	1.1	0.9	1.2	1.0	1.1	1.0	1.2	17.3	4	3	5	11	301	33.4	5.6
NP_055738.3	118918397	FNDC3A	fibronectin type-III domain-containing protein 3A isoform 2	1.0	1.1	1.2	1.2	1.0	1.2	1.3	1.3	1.0	1.1	1.2	1.2	0.8	2	1	1	2	1142	125.7	7.1
NP_001182502.1	307133775	DICER1	endoribonuclease Dicer isoform 2	1.1	1.0	1.1	1.0	1.9	1.5	1.4	1.4	1.5	1.3	1.2	1.2	1.1	2	1	1	2	1829	208.3	5.7
NP_057044.2	148596990	CUTC	copper homeostasis protein cutC homolog	1.1	1.2	1.1	1.2	1.1	1.1	1.1	1.2	1.1	1.2	1.1	1.2	26.4	1	4	4	8	273	29.3	8.2
NP_057152.2	151108473	FIS1	mitochondrial fission 1 protein	0.9	1.0	1.1	1.1	1.0	1.1	1.1	1.3	0.9	1.1	1.1	1.2	20.4	1	3	3	11	152	16.9	8.8
NP_073733.1	12383062	KLC2	kinesin light chain 2 isoform 1	1.2	1.1	1.2	1.2	1.1	1.3	1.2	1.2	1.2	1.2	1.2	1.2	10.0	2	3	6	9	622	68.9	7.2
NP_005527.1	5031789	IMPA1	inositol monophosphatase 1 isoform 1	1.1	1.2	1.1	1.2	1.2	1.2	1.2	1.2	1.2	1.2	1.2	1.2	18.8	3	5	5	17	277	30.2	5.3
NP_001035251.1	93102393	C16orf13	UPF0585 protein C16orf13 isoform c	1.2	1.2	1.2	1.2	1.2	1.4	1.3	1.3	1.2	1.3	1.3	1.2	9.4	4	1	1	2	107	12.0	7.4
NP_001273066.1	554790420	NQO1	NAD(P)H dehydrogenase [quinone] 1 isoform d	1.4	1.5	1.2	1.2	1.4	1.6	1.2	1.2	1.4	1.5	1.2	1.2	55.5	4	11	11	90	202	22.8	8.5
NP_031383.1	6678676	COG2	conserved oligomeric Golgi complex subunit 2 isoform 1	1.0	1.1	1.2	1.2	1.2	1.3	1.1	1.3	1.1	1.2	1.2	1.2	8.7	2	3	3	5	738	83.2	6.6
NP_003095.2	156627571	SORD	sorbitol dehydrogenase	1.2	1.1	1.2	1.2	1.1	1.2	1.2	1.2	1.2	1.1	1.2	1.2	36.1	1	10	10	24	357	38.3	8.0
NP_116584.2	32481209	MAPKAPK2	MAP kinase-activated protein kinase 2 isoform 2	1.0	1.2	1.3	1.2	1.0	1.2	1.2	1.3	1.0	1.2	1.2	1.2	5.8	2	2	2	6	400	45.5	8.7
NP_057030.3	31880783	PELO	protein pelota homolog	1.0	1.1	1.1	1.1	1.2	1.2	1.2	1.3	1.1	1.1	1.1	1.2	20.8	1	6	6	13	385	43.3	6.3
NP_060726.3	217330644	WDR12	ribosome biogenesis protein WDR12	1.3	1.3	1.3	1.3	1.3	1.2	1.2	1.1	1.3	1.3	1.2	1.2	31.4	1	10	10	22	423	47.7	5.9
NP_001880.2	13236495	CRYZ	quinone oxidoreductase isoform a	1.0	1.1	1.1	1.3	1.0	1.1	1.1	1.2	1.0	1.1	1.1	1.2	32.2	3	8	8	27	329	35.2	8.4
NP_001258866.1	429836856	RMND1	required for meiotic nuclear division protein 1 homolog isoform 2	1.1	1.2	1.4	1.4	1.0	1.1	1.1	1.1	1.1	1.1	1.3	1.2	7.9	2	1	1	2	279	32.2	5.9
NP_005106.2	19913412;19913410	MVP	major vault protein isoform 1	1.2	1.2	1.4	1.2	1.3	1.2	1.3	1.2	1.2	1.2	1.3	1.2	15.6	3	8	8	19	893	99.3	5.5
NP_937838.1	38045944	UBA3	NEDD8-activating enzyme E1 catalytic subunit isoform 2	1.2	1.3	1.4	1.3	1.2	1.2	1.1	1.1	1.2	1.2	1.3	1.2	25.8	2	7	7	19	449	50.0	5.3
NP_009011.1	5901998	LSM6	U6 snRNA-associated Sm-like protein LSm6	1.1	1.0	1.0	1.1	1.2	1.3	1.3	1.3	1.2	1.1	1.2	1.2	23.8	1	2	2	2	80	9.1	9.6
NP_001138503.1	222537759	PLAU	urokinase-type plasminogen activator isoform 2	1.1	1.5	1.6	1.1	1.1	1.6	1.8	1.4	1.1	1.5	1.7	1.2	3.6	2	1	1	2	414	46.9	8.4
NP_002636.2	157671929	PIK3C2A	phosphatidylinositol 4-phosphate 3-kinase C2 domain-containing subunit alpha	1.2	1.2	1.2	1.1	1.4	1.2	1.2	1.3	1.3	1.2	1.2	1.2	2.0	1	3	3	5	1686	190.6	8.0
NP_859048.1	32455266;4505591	PRDX1	peroxiredoxin-1	1.1	1.3	1.0	1.3	1.1	1.2	1.0	1.2	1.1	1.3	1.0	1.2	60.8	1	9	12	93	199	22.1	8.1
NP_002627.1	4505777	PHF1	PHD finger protein 1 isoform a	1.2	1.2	1.1	1.1	1.1	1.6	1.1	1.4	1.1	1.4	1.1	1.2	5.0	2	1	1	1	457	49.6	7.9
NP_004367.2	17921987	COX15	cytochrome c oxidase assembly protein COX15 homolog isoform 2	1.3	1.3	1.4	1.3	1.3	1.2	1.2	1.2	1.3	1.2	1.3	1.2	12.9	2	3	3	11	388	43.8	9.6
NP_861448.2	224809432	TMTC3	transmembrane and TPR repeat-containing protein 3	1.3	1.3	1.2	1.3	1.2	1.0	1.1	1.2	1.2	1.2	1.2	1.2	1.1	1	1	1	2	914	103.8	8.8
NP_003123.2	63253298	SRM	spermidine synthase	1.1	1.1	1.1	1.3	1.1	1.0	1.1	1.2	1.1	1.1	1.1	1.2	36.1	1	7	7	19	302	33.8	5.5
NP_001186363.1	313661502	NIP7	60S ribosome subunit biogenesis protein NIP7 homolog isoform 2	1.1	1.1	1.2	1.1	1.1	1.2	1.2	1.3	1.1	1.2	1.2	1.2	44.4	2	4	4	7	133	15.2	8.3
NP_006800.2	21361356	TOMM34	mitochondrial import receptor subunit TOM34	1.0	1.0	1.0	1.3	1.0	0.9	0.9	1.2	1.0	1.0	0.9	1.2	40.5	1	13	13	40	309	34.5	9.0
NP_001167568.1	291575128	LDHB	L-lactate dehydrogenase B chain	1.1	1.2	1.2	1.2	1.1	1.2	1.3	1.2	1.1	1.2	1.3	1.2	70.7	1	18	21	291	334	36.6	6.0
NP_003671.1	4507947	YARS	tyrosine--tRNA ligase, cytoplasmic	1.4	1.3	1.1	1.2	1.3	1.3	1.1	1.3	1.3	1.3	1.1	1.2	59.5	1	28	28	107	528	59.1	7.0
NP_001408.2	50053795	EIF4B	eukaryotic translation initiation factor 4B isoform 2	0.8	1.0	1.0	1.1	0.9	1.2	1.0	1.3	0.9	1.1	1.0	1.2	31.8	2	16	16	35	611	69.1	5.7
NP_006272.2	103471999	STK3	serine/threonine-protein kinase 3 isoform 1	1.1	1.1	1.1	1.2	1.1	1.1	1.1	1.2	1.1	1.1	1.1	1.2	13.2	3	2	5	11	491	56.3	5.2
NP_001155253.1	239985428	PDP1	pyruvate dehyrogenase phosphatase catalytic subunit 1 isoform 3	1.1	1.1	1.2	1.1	1.4	1.5	1.8	1.3	1.2	1.3	1.5	1.2	9.3	2	3	3	4	537	61.0	6.7
NP_005970.1	5174659;66737374	S100A13	protein S100-A13	1.1	0.8	1.2	1.2	1.1	0.7	1.3	1.2	1.1	0.7	1.3	1.2	40.8	1	5	5	11	98	11.5	6.2
NP_001677.2	32189394	ATP5B	ATP synthase subunit beta, mitochondrial precursor	1.0	1.0	1.0	1.2	1.0	1.0	1.1	1.2	1.0	1.0	1.0	1.2	68.2	1	23	23	225	529	56.5	5.4
NP_003840.2	109452591	SUCLG1	succinyl-CoA ligase [ADP/GDP-forming] subunit alpha, mitochondrial precursor	1.0	1.0	1.0	1.3	1.0	1.0	1.0	1.1	1.0	1.0	1.0	1.2	9.0	1	2	2	7	346	36.2	8.8
NP_001278868.1	633257781	MTR	methionine synthase isoform 2	1.1	1.2	1.1	1.3	1.1	1.2	1.1	1.1	1.1	1.2	1.1	1.2	8.5	3	6	7	12	1214	134.7	5.5
NP_005787.1	5031985	NUTF2	nuclear transport factor 2	0.7	0.9	0.8	1.2	1.0	1.1	1.2	1.3	0.9	1.0	1.0	1.2	24.4	3	2	2	2	127	14.5	5.4
NP_002532.2	51873036	OGDH	2-oxoglutarate dehydrogenase, mitochondrial isoform 1 precursor	1.3	1.2	1.3	1.2	1.3	1.2	1.3	1.2	1.3	1.2	1.3	1.2	38.6	6	30	30	92	1023	115.9	6.9
NP_000032.1	4557325	APOE	apolipoprotein E isoform b precursor	1.1	1.1	1.2	1.1	1.4	1.3	1.4	1.3	1.3	1.2	1.3	1.2	8.5	2	3	3	3	317	36.1	5.7
NP_055330.1	7657385;313482820	CNOT2	CCR4-NOT transcription complex subunit 2	1.1	1.1	1.0	1.3	1.0	1.1	1.1	1.2	1.0	1.1	1.1	1.2	12.2	1	4	4	4	540	59.7	7.7
NP_001010867.1	58197556	IBA57	putative transferase CAF17, mitochondrial precursor	1.0	1.1	0.9	1.0	1.6	1.1	1.0	1.4	1.3	1.1	1.0	1.2	33.7	1	6	6	12	356	38.1	9.8
NP_065823.1	32698730	NUFIP2	nuclear fragile X mental retardation-interacting protein 2	1.1	1.1	1.0	1.3	1.0	1.0	1.0	1.1	1.1	1.1	1.0	1.2	16.0	1	7	7	11	695	76.1	8.7
NP_003925.1	100816392	FUBP3	far upstream element-binding protein 3	1.2	1.2	1.1	1.2	1.1	1.2	1.1	1.2	1.2	1.2	1.1	1.2	31.3	1	10	12	24	572	61.6	8.4
NP_219483.1	15529984	ARMC6	armadillo repeat-containing protein 6 isoform 2	1.1	1.2	1.1	1.3	1.0	1.1	1.0	1.1	1.1	1.1	1.0	1.2	7.6	2	3	3	4	476	51.5	6.0
NP_001152980.1	226958414	IVD	isovaleryl-CoA dehydrogenase, mitochondrial isoform 2 precursor	1.2	1.1	1.2	1.2	1.2	1.2	1.4	1.2	1.2	1.2	1.3	1.2	21.5	2	4	4	10	396	43.0	7.7
NP_002152.2	94721241	IARS	isoleucine--tRNA ligase, cytoplasmic	1.3	1.3	1.2	1.2	1.3	1.2	1.2	1.2	1.3	1.3	1.2	1.2	30.3	1	29	29	102	1262	144.4	6.2
NP_116119.2	47519420	RTN4IP1	reticulon-4-interacting protein 1, mitochondrial precursor	1.1	1.0	1.0	1.1	1.2	1.2	1.3	1.4	1.2	1.1	1.1	1.2	11.6	1	3	3	5	396	43.6	9.1
NP_009031.1	6005717	ATP5I	ATP synthase subunit e, mitochondrial	1.2	1.2	1.2	1.2	1.1	1.0	1.1	1.2	1.1	1.1	1.2	1.2	60.9	4	4	4	20	69	7.9	9.3
NP_000467.1	4502011	AK1	adenylate kinase isoenzyme 1	1.3	1.2	1.3	1.3	1.0	1.1	1.0	1.1	1.1	1.2	1.1	1.2	24.2	1	3	3	4	194	21.6	8.6
NP_872270.1	33286420	PKM	pyruvate kinase PKM isoform b	1.2	1.4	1.3	1.2	1.2	1.3	1.3	1.3	1.2	1.4	1.3	1.2	69.5	8	2	33	961	531	58.0	7.7
NP_067077.1	11037061	C21orf59	UPF0769 protein C21orf59	1.2	1.0	1.1	1.1	**2.0**	1.2	1.2	1.3	1.6	1.1	1.2	1.2	7.6	1	1	1	1	290	33.2	7.4
NP_004542.1	4758788	NDUFS3	NADH dehydrogenase [ubiquinone] iron-sulfur protein 3, mitochondrial precursor	1.2	1.3	1.3	1.3	1.1	1.1	1.1	1.1	1.1	1.2	1.2	1.2	42.1	1	9	9	20	264	30.2	7.5
NP_001027464.1	73808090	ZNF207	BUB3-interacting and GLEBS motif-containing protein ZNF207 isoform b	1.2	1.1	1.0	1.3	0.8	1.0	0.9	1.2	1.0	1.1	1.0	1.2	7.1	3	3	3	7	463	49.7	9.1
NP_002930.2	21361547;42822872	RNH1	ribonuclease inhibitor	1.1	1.1	1.2	1.3	1.1	1.0	1.1	1.1	1.1	1.1	1.2	1.2	44.7	1	12	12	57	461	49.9	4.8
NP_001128496.1	203098816	PDHX	pyruvate dehydrogenase protein X component, mitochondrial isoform 2	1.1	1.1	1.1	1.2	1.1	1.2	1.1	1.2	1.1	1.2	1.1	1.2	7.2	3	3	3	9	486	51.4	6.3
NP_001295287.1	816197626	CIAPIN1	anamorsin isoform 3	1.1	1.2	1.3	1.2	1.1	1.2	1.2	1.2	1.1	1.2	1.2	1.2	12.0	3	2	3	4	266	28.9	7.0
NP_079509.5	110227603	COASY	bifunctional coenzyme A synthase isoform a precursor	1.1	1.1	1.2	1.2	1.2	1.2	1.3	1.2	1.2	1.2	1.2	1.2	34.4	2	8	8	36	564	62.3	7.0
NP_001171131.1	295054188	HABP2	hyaluronan-binding protein 2 isoform 2	1.1	1.2	1.1	1.2	1.1	1.2	1.1	1.2	1.1	1.2	1.1	1.2	1.7	2	1	1	4	534	59.8	6.4
NP_004719.2	50659095	DDX21	nucleolar RNA helicase 2 isoform 1	1.1	1.3	1.4	1.2	1.1	1.2	1.3	1.2	1.1	1.3	1.3	1.2	49.2	4	30	32	191	783	87.3	9.3
NP_064576.1	9910244	MRPS22	28S ribosomal protein S22, mitochondrial	1.1	1.1	1.1	1.2	1.1	1.2	1.2	1.2	1.1	1.1	1.2	1.2	15.8	1	5	5	12	360	41.3	7.9
NP_006401.3	148728164;148728168	HTATIP2	oxidoreductase HTATIP2 isoform b	1.0	1.3	1.3	1.3	1.3	1.1	1.5	1.1	1.2	1.2	1.4	1.2	6.6	3	2	2	4	242	27.0	8.4
NP_001814.2	21536286	CKB	creatine kinase B-type	1.2	1.0	1.3	1.2	1.3	1.1	1.4	1.2	1.3	1.0	1.4	1.2	65.6	2	16	16	108	381	42.6	5.6
NP_000230.1	4557894	LYZ	lysozyme C precursor	**3.3**	1.4	1.0	1.4	**2.6**	1.3	0.8	1.0	3.0	1.4	0.9	1.2	6.1	1	1	1	2	148	16.5	9.2
NP_714913.1	24308456	ELMOD2	ELMO domain-containing protein 2	1.2	1.2	1.2	1.2	1.3	1.2	1.2	1.2	1.2	1.2	1.2	1.2	6.5	1	2	2	3	293	34.9	8.0
NP_057123.1	7706322	C14orf166	UPF0568 protein C14orf166	1.3	1.2	1.4	1.2	1.3	1.2	1.4	1.3	1.3	1.2	1.4	1.2	32.4	1	7	7	25	244	28.1	6.7
NP_001089.1	4501867	ACO2	aconitate hydratase, mitochondrial precursor	1.3	1.3	1.4	1.2	1.3	1.3	1.3	1.2	1.3	1.3	1.4	1.2	38.2	1	23	23	124	780	85.4	7.6
NP_055958.2	164420681	METAP1	methionine aminopeptidase 1	1.1	1.1	1.2	1.1	1.1	1.2	1.3	1.3	1.1	1.1	1.2	1.2	16.3	1	4	4	8	386	43.2	7.2
NP_001120668.1	187608750	CNBP	cellular nucleic acid-binding protein isoform 6	1.0	1.2	1.1	1.3	1.1	1.0	1.0	1.1	1.0	1.1	1.0	1.2	18.8	6	3	3	4	170	18.7	7.7
NP_115690.3	116063536	COQ5	2-methoxy-6-polyprenyl-1,4-benzoquinol methylase, mitochondrial precursor	1.2	1.2	1.1	1.2	1.0	1.1	1.0	1.2	1.1	1.1	1.1	1.2	17.7	1	4	4	7	327	37.1	6.9
NP_001182447.1	306922382	MICU1	calcium uptake protein 1, mitochondrial isoform 2	1.2	1.3	1.3	1.2	1.1	1.4	1.5	1.2	1.2	1.4	1.4	1.2	5.7	3	2	2	5	476	54.3	8.4
NP_002303.2	23199993	LIG4	DNA ligase 4	1.2	1.2	1.2	1.2	1.2	1.1	1.2	1.2	1.2	1.2	1.2	1.2	2.5	1	2	2	4	911	103.9	8.0
NP_061887.2	33469941	POLR1B	DNA-directed RNA polymerase I subunit RPA2 isoform 1	1.1	1.0	1.2	1.2	1.0	1.1	1.2	1.2	1.1	1.0	1.2	1.2	3.5	6	3	3	4	1135	128.1	7.8
NP_066972.1	21624607	COTL1	coactosin-like protein	1.3	1.2	1.4	1.2	1.4	1.4	1.5	1.2	1.4	1.3	1.4	1.2	73.9	1	10	10	52	142	15.9	5.7
NP_001032411.1	82880645	USP14	ubiquitin carboxyl-terminal hydrolase 14 isoform b	1.0	1.1	1.0	1.2	1.0	1.1	1.0	1.2	1.0	1.1	1.0	1.2	51.0	2	18	18	64	459	52.4	5.8

Khan *et al.* , 2019. Multi-omics analysis to characterize cigarette smoke induced molecular alterations in esophageal cells

Supplementary Table 5. List of proteins quantified in untreated and chronically treated Het1A cells with cigarette smoke condensate for 8 months

NP_Accession	Protein group Accession	Gene ID	Description	Het-1A-Smoke - 2M/Parental	Het-1A-Smoke - 4M/Parental	Het-1A-Smoke - 6M/Parental	Het-1A-Smoke - 8M/Parental	Het-1A-Smoke - 2M/Parental	Het-1A-Smoke - 4M/Parental	Het-1A-Smoke - 6M/Parental	Het-1A-Smoke - 8M/Parental	Het-1A-Smoke - 2M/Parental	Het-1A-Smoke - 4M/Parental	Het-1A-Smoke - 6M/Parental	Het-1A-Smoke - 8M/Parental	Coverage (%)	Proteins	Unique Peptides	# Peptides	PSM	# AAs	MW [kDa]	calc. pI
				Replicate 1				Replicate 2				Average of replicates											
NP_001035536.1	94721263	MTMR12	myotubularin-related protein 12	1.1	1.1	1.0	1.1	1.1	1.3	1.2	1.3	1.1	1.2	1.1	1.2	1.3	1	1	1	4	747	86.1	6.6
NP_057329.3	613410229	HSD17B11	estradiol 17-beta-dehydrogenase 11 precursor	1.3	1.1	1.3	1.2	0.9	1.1	1.4	1.2	1.1	1.1	1.3	1.2	11.0	1	2	2	6	300	32.9	9.2
NP_006147.1	5453760	NEDD8	NEDD8 precursor	1.0	1.0	1.1	1.2	1.9	1.2	1.2	1.2	1.4	1.1	1.1	1.2	34.6	2	3	3	11	81	9.1	8.4
NP_005923.2	156105687	MIPEP	mitochondrial intermediate peptidase	1.2	1.3	1.2	1.3	1.0	1.1	1.1	1.1	1.1	1.2	1.2	1.2	3.0	1	1	1	1	713	80.6	7.0
NP_597700.1	19263340	GPT2	alanine aminotransferase 2 isoform 1	1.5	1.5	1.2	1.2	1.2	1.3	1.1	1.2	1.4	1.4	1.1	1.2	6.9	2	2	2	3	523	57.9	7.7
NP_115547.1	149274651	CEP78	centrosomal protein of 78 kDa isoform b	1.0	1.3	1.1	1.1	1.1	1.1	1.1	1.3	1.0	1.2	1.1	1.2	1.7	2	1	1	1	706	78.3	8.3
NP_060829.2	227498137	UFSP2	ufm1-specific protease 2	1.0	1.1	1.1	1.1	1.1	1.2	1.2	1.3	1.1	1.1	1.2	1.2	10.2	1	2	2	4	469	53.2	7.0
NP_001161803.1	270288780	NDUFB4	NADH dehydrogenase [ubiquinone] 1 beta subcomplex subunit 4 isoform 2	1.2	1.3	1.2	1.4	1.1	1.1	1.1	1.0	1.1	1.2	1.2	1.2	25.0	2	2	2	8	120	14.0	9.8
NP_055051.1	7657134	GNPAT	dihydroxyacetone phosphate acyltransferase	1.0	1.1	1.1	1.2	1.1	1.2	1.2	1.2	1.1	1.1	1.2	1.2	9.1	1	6	6	8	680	77.1	6.6
NP_001026890.2	149588660	GSTCD	glutathione S-transferase C-terminal domain-containing protein isoform 1	1.2	1.1	1.1	1.2	1.4	1.2	1.1	1.2	1.3	1.2	1.1	1.2	3.8	2	2	2	3	633	71.0	7.8
NP_114366.1	14141166	PCBP2	poly(rC)-binding protein 2 isoform b	1.1	1.0	1.1	1.1	1.2	1.3	1.3	1.3	1.1	1.1	1.2	1.2	69.3	11	10	15	81	362	38.2	6.8
NP_002519.1	4505471	NTHL1	endonuclease III-like protein 1	1.1	1.0	1.3	1.2	1.2	1.4	1.3	1.2	1.1	1.2	1.3	1.2	5.5	1	1	1	2	312	34.4	9.7
NP_064502.9	108773810	LARS	leucine--tRNA ligase, cytoplasmic	1.3	1.3	1.2	1.2	1.2	1.2	1.2	1.2	1.2	1.2	1.2	1.2	41.9	1	37	37	217	1176	134.4	7.3
NP_001598.1	4501853	ACAA1	3-ketoacyl-CoA thiolase, peroxisomal isoform a	1.1	1.0	1.0	1.2	1.1	1.1	1.0	1.2	1.1	1.1	1.0	1.2	26.9	2	6	6	11	424	44.3	8.4
NP_001265300.1	507834103;507834113	MPP2	MAGUK p55 subfamily member 2 isoform 2	1.2	1.2	1.0	1.2	1.0	1.1	1.0	1.2	1.1	1.1	1.0	1.2	12.8	6	4	5	7	541	60.5	6.6
NP_001191229.1	324021730	ARHGAP19	rho GTPase-activating protein 19 isoform 2	1.4	1.4	1.4	1.2	1.2	1.2	1.2	1.2	1.3	1.3	1.3	1.2	4.1	3	1	1	2	465	52.4	9.3
NP_067640.2	54607091	SENP2	sentrin-specific protease 2	1.3	1.1	1.4	1.3	1.0	1.1	1.1	1.1	1.2	1.1	1.3	1.2	2.7	1	1	1	2	589	67.8	9.5
NP_001265338.1	508772599	ANXA11	annexin A11 isoform 2	0.9	1.1	1.1	1.2	1.0	1.1	1.1	1.2	1.0	1.1	1.1	1.2	17.8	4	7	7	25	472	51.2	7.7
NP_009121.1	6005824	SEC23IP	SEC23-interacting protein	1.1	1.1	1.1	1.2	1.0	1.0	1.1	1.2	1.0	1.0	1.1	1.2	12.4	1	9	10	26	1000	111.0	5.5
NP_060367.1	8923559	OXSM	3-oxoacyl-[acyl-carrier-protein] synthase, mitochondrial isoform 1 precursor	1.1	1.1	1.1	1.1	1.2	1.1	1.1	1.3	1.1	1.1	1.1	1.2	2.0	1	1	1	1	459	48.8	7.7
NP_002282.2	167614504	LAMB1	laminin subunit beta-1 precursor	1.3	1.6	1.6	1.2	1.3	1.5	1.5	1.2	1.3	1.6	1.6	1.2	9.7	1	14	14	37	1786	197.9	4.9
NP_003162.2	31543667	SUPV3L1	ATP-dependent RNA helicase SUPV3L1, mitochondrial isoform 1	1.1	1.1	1.1	1.1	1.3	1.2	1.2	1.3	1.2	1.1	1.2	1.2	15.5	2	9	9	23	786	87.9	8.0
NP_001910.2	62530384	ECI1	enoyl-CoA delta isomerase 1, mitochondrial isoform 1 precursor	1.2	1.3	1.3	1.2	1.1	1.2	1.2	1.2	1.1	1.3	1.2	1.2	35.4	2	8	8	35	302	32.8	8.5
NP_612639.2	104485446	TAF8	transcription initiation factor TFIID subunit 8	1.9	1.3	1.1	1.3	0.8	0.8	0.9	1.1	1.4	1.1	1.0	1.2	6.1	1	1	1	4	310	34.2	6.5
NP_001014451.1	62241024	DDX19B	ATP-dependent RNA helicase DDX19B isoform 2	1.0	1.0	1.0	1.1	1.3	1.2	1.3	1.3	1.1	1.1	1.1	1.2	49.3	6	18	18	47	448	50.5	6.1
NP_003365.1	4507879	VDAC1	voltage-dependent anion-selective channel protein 1	1.2	1.2	1.2	1.2	1.1	1.2	1.2	1.2	1.2	1.2	1.2	1.2	74.2	1	16	17	258	283	30.8	8.5
NP_005852.2	56181387	STUB1	E3 ubiquitin-protein ligase CHIP isoform a	1.1	1.1	1.0	1.2	1.0	1.1	1.0	1.2	1.0	1.1	1.0	1.2	31.0	2	7	7	16	303	34.8	5.9
NP_004150.1	4758970	PSMB8	proteasome subunit beta type-8 isoform E1 proprotein	1.0	1.0	0.9	1.2	1.0	1.1	1.1	1.2	1.0	1.1	1.0	1.2	15.1	2	3	3	4	272	29.8	5.8
NP_009185.2	31543419	PNKP	bifunctional polynucleotide phosphatase/kinase	2.0	1.0	1.1	1.2	1.2	1.1	1.2	1.2	1.6	1.1	1.1	1.2	6.7	1	3	3	11	521	57.0	8.5
NP_057403.1	7705330;255760066	COPS7A	COP9 signalosome complex subunit 7a	1.0	1.3	1.2	1.2	1.1	1.2	1.1	1.1	1.1	1.2	1.1	1.2	22.6	1	4	4	12	275	30.3	8.2
NP_000169.1	4504169	GSS	glutathione synthetase	1.0	1.0	0.9	1.2	1.1	1.0	0.9	1.2	1.1	1.0	0.9	1.2	30.6	1	11	11	56	474	52.4	5.9
NP_115544.1	29789283	WDR75	WD repeat-containing protein 75 isoform 1	1.1	1.2	1.3	1.2	1.1	1.1	1.2	1.2	1.1	1.1	1.2	1.2	17.6	2	11	11	29	830	94.4	6.0
NP_067026.3	284795266	SRPRB	signal recognition particle receptor subunit beta	1.2	1.3	1.2	1.2	1.3	1.4	1.2	1.2	1.3	1.3	1.2	1.2	66.4	1	15	15	46	271	29.7	9.0
NP_687033.1	23110939	PSMA3	proteasome subunit alpha type-3 isoform 2	1.1	1.1	1.1	1.2	1.1	1.0	1.0	1.2	1.1	1.1	1.0	1.2	35.9	2	11	11	35	248	27.6	5.3
NP_055946.1	157743245	WDR43	WD repeat-containing protein 43	1.1	1.1	1.3	1.2	1.0	1.0	1.2	1.2	1.1	1.1	1.2	1.2	22.3	1	11	11	25	677	74.8	5.6
NP_787117.3	51896031	C5orf51	UPF0600 protein C5orf51	1.1	1.0	1.1	1.3	1.0	1.1	1.1	1.1	1.0	1.1	1.1	1.2	29.3	1	5	5	12	294	33.6	5.3
NP_006784.1	5802974	PRDX3	thioredoxin-dependent peroxide reductase, mitochondrial isoform a precursor	1.2	1.2	1.2	1.2	1.2	1.0	1.2	1.2	1.2	1.1	1.2	1.2	19.5	3	4	4	15	256	27.7	7.8
NP_078837.3	33356128	NAA16	N-alpha-acetyltransferase 16, NatA auxiliary subunit isoform 1	1.0	1.0	1.1	1.0	1.0	1.1	1.3	1.4	1.0	1.0	1.2	1.2	4.2	5	2	4	23	864	101.4	7.9
NP_001269607.1	544186104	PEX1	peroxisome biogenesis factor 1 isoform 3	1.1	1.2	1.2	1.2	1.2	1.2	1.2	1.2	1.2	1.2	1.2	1.2	3.0	3	2	2	3	1075	119.5	6.1
NP_001276333.1	574957012	MMS19	MMS19 nucleotide excision repair protein homolog isoform 3	1.2	1.2	1.3	1.3	1.2	1.1	1.2	1.1	1.2	1.1	1.3	1.2	4.9	3	4	4	7	871	96.1	6.2
NP_001138767.1	223941808	NGLY1	peptide-N(4)-(N-acetyl-beta- glucosaminyl)asparagine amidase isoform 4	1.2	1.2	1.2	1.3	0.9	0.9	0.9	1.1	1.0	1.1	1.0	1.2	4.1	4	2	2	4	558	63.8	8.1
NP_055454.1	7662078	TTC37	tetratricopeptide repeat protein 37	1.2	1.2	1.3	1.2	1.1	1.2	1.2	1.2	1.2	1.2	1.2	1.2	9.3	1	11	11	21	1564	175.4	7.5
NP_001009552.1	57222565	PPP2CB	serine/threonine-protein phosphatase 2A catalytic subunit beta isoform	1.2	1.0	1.1	1.2	1.0	1.2	1.2	1.2	1.1	1.1	1.2	1.2	40.1	1	1	8	41	309	35.6	5.4
NP_001274514.1	567316262	NUP35	nucleoporin NUP53 isoform c	1.2	1.1	1.1	1.2	1.1	1.1	1.1	1.2	1.2	1.1	1.1	1.2	12.0	3	2	2	8	208	22.8	9.0
NP_002790.1	4506203	PSMB7	proteasome subunit beta type-7 proprotein	1.1	1.1	1.1	1.2	1.2	1.2	1.1	1.2	1.1	1.2	1.1	1.2	43.3	1	8	8	48	277	29.9	7.7
NP_067639.1	11055992	SCPEP1	retinoid-inducible serine carboxypeptidase precursor	1.0	1.1	1.0	1.2	1.2	1.3	1.1	1.2	1.1	1.2	1.1	1.2	13.9	1	5	5	9	452	50.8	5.8
NP_008860.4	67782311	SKIV2L	helicase SKI2W	1.1	1.1	1.3	1.2	1.1	1.2	1.2	1.2	1.1	1.2	1.2	1.2	20.4	1	17	17	36	1246	137.7	6.1
NP_079494.1	13376826	ULBP1	NKG2D ligand 1 precursor	1.3	1.3	1.2	1.1	1.4	2.2	1.5	1.3	1.3	1.8	1.4	1.2	16.8	1	3	3	4	244	28.0	7.4
NP_004537.1	4758778	NDUFB2	NADH dehydrogenase [ubiquinone] 1 beta subcomplex subunit 2, mitochondrial precursor	1.0	1.1	0.9	1.2	1.1	1.2	1.2	1.2	1.0	1.2	1.1	1.2	31.4	1	1	1	1	105	12.1	5.7
NP_001026882.2	94420683	TRMT11	tRNA (guanine(10)-N2)-methyltransferase homolog	1.1	1.1	1.2	1.2	1.1	1.0	1.0	1.2	1.1	1.1	1.1	1.2	2.6	1	1	1	2	463	53.4	7.8
NP_689508.3	38202255	TARS	threonine--tRNA ligase, cytoplasmic isoform 1	1.2	1.3	1.2	1.2	1.3	1.3	1.3	1.2	1.3	1.3	1.3	1.2	50.5	4	29	32	147	723	83.4	6.7
NP_061862.1	168229174	RBM27	RNA-binding protein 27	1.1	1.1	1.1	1.3	1.0	1.0	1.0	1.1	1.0	1.1	1.0	1.2	7.4	1	7	7	17	1060	118.6	9.2
NP_065777.1	14149738	NLN	neurolysin, mitochondrial	1.2	1.1	1.1	1.1	1.3	1.1	1.1	1.2	1.2	1.1	1.1	1.2	11.4	1	6	6	16	704	80.6	6.6
NP_003106.3	156627575	UAP1	UDP-N-acetylhexosamine pyrophosphorylase	1.4	1.2	1.4	1.2	1.3	1.4	1.4	1.2	1.3	1.3	1.4	1.2	28.5	1	10	11	28	505	57.0	6.4
NP_001277136.1	589269226	DCP1A	mRNA-decapping enzyme 1A isoform e	1.3	1.1	1.9	1.3	0.9	1.0	1.0	1.0	1.1	1.1	1.4	1.2	10.6	5	2	2	2	423	45.3	6.5
NP_004438.3	56682953	EPS8	epidermal growth factor receptor kinase substrate 8	1.9	1.2	1.6	1.3	1.0	1.0	1.0	1.0	1.4	1.1	1.3	1.2	6.1	1	3	3	5	822	91.8	7.5
NP_000705.2	74275350;375065825	TSPO	translocator protein	1.1	1.2	1.2	1.3	0.9	2.9	2.3	1.1	1.0	2.1	1.7	1.2	18.3	1	2	2	9	169	18.8	9.4
NP_056541.2	30089954	DCAF8	DDB1- and CUL4-associated factor 8	1.2	1.4	1.3	1.2	1.1	1.1	1.2	1.2	1.2	1.3	1.2	1.2	2.0	1	1	1	4	597	66.8	5.4
NP_055952.2	154146218	EFR3A	protein EFR3 homolog A	1.0	1.0	1.0	1.2	1.0	1.0	1.1	1.1	1.0	1.0	1.0	1.2	5.1	1	2	2	4	821	92.9	6.7
NP_037415.1	11034825	MAT2B	methionine adenosyltransferase 2 subunit beta isoform 1	1.0	1.0	0.9	1.1	1.0	1.1	1.0	1.3	1.0	1.0	1.0	1.2	7.2	2	2	2	5	334	37.5	7.4
NP_006279.2	19923362	THY1	thy-1 membrane glycoprotein preproprotein	1.4	1.5	1.6	1.2	1.4	1.4	1.4	1.1	1.4	1.5	1.5	1.2	24.2	1	3	3	9	161	17.9	8.7
NP_055209.2	118200356	GHITM	growth hormone-inducible transmembrane protein	1.3	1.4	1.3	1.2	1.3	1.3	1.3	1.1	1.3	1.3	1.3	1.2	9.3	1	3	3	19	345	37.2	9.9
NP_009166.2	8051636	XPOT	exportin-T	1.2	1.2	1.1	1.1	1.3	1.3	1.2	1.3	1.2	1.2	1.2	1.2	30.2	1	20	20	64	962	109.9	5.4
NP_073581.2	257900512	C16orf58	UPF0420 protein C16orf58	1.2	1.2	1.1	1.2	1.3	1.1	1.1	1.2	1.3	1.2	1.1	1.2	14.5	1	4	4	6	468	51.0	6.9
NP_055331.1	7657387	CNOT3	CCR4-NOT transcription complex subunit 3	1.1	1.1	1.0	1.1	1.1	1.2	1.1	1.3	1.1	1.1	1.1	1.2	1.2	1	1	1	1	753	81.8	6.2
NP_054737.1	7661730	MRPS28	28S ribosomal protein S28, mitochondrial	0.9	1.2	1.1	1.2	1.3	1.2	1.3	1.2	1.1	1.2	1.2	1.2	4.3	1	1	1	2	187	20.8	9.1
NP_005958.1	5174607	NAB2	NGFI-A-binding protein 2	1.1	1.2	1.0	1.2	1.0	1.1	1.2	1.2	1.1	1.2	1.1	1.2	4.8	1	1	1	2	525	56.6	7.0
NP_078941.2	19913371	TBL1XR1	F-box-like/WD repeat-containing protein TBL1XR1	1.0	1.0	1.0	1.2	1.0	1.0	1.0	1.2	1.0	1.0	1.0	1.2	9.9	4	4	4	7	514	55.6	5.6
NP_003492.2	156104864	ACOX3	peroxisomal acyl-coenzyme A oxidase 3 isoform a	1.2	1.5	1.3	1.4	1.0	0.9	1.0	1.0	1.1	1.2	1.2	1.2	3.6	1	1	1	2	700	77.6	7.3
NP_002991.2	115387094	SDHB	succinate dehydrogenase [ubiquinone] iron-sulfur subunit, mitochondrial precursor	1.2	1.2	1.3	1.2	1.2	1.1	1.3	1.2	1.2	1.2	1.3	1.2	27.9	1	8	8	18	280	31.6	8.8
NP_000843.1	4504183	GSTP1	glutathione S-transferase P	1.5	1.2	1.3	1.2	1.3	1.2	1.2	1.2	1.4	1.2	1.3	1.2	53.3	1	9	9	65	210	23.3	5.6
NP_031401.1	6678271	TARDBP	TAR DNA-binding protein 43	1.0	1.0	1.0	1.2	1.0	1.0	0.9	1.2	1.0	1.0	1.0	1.2	36.2	1	9	9	51	414	44.7	6.2
NP_003079.1	4507115	FSCN1	fascin	1.0	1.0	1.1	1.2	0.9	1.0	1.1	1.2	1.0	1.0	1.1	1.2	54.6	1	18	18	82	493	54.5	7.2
NP_001177392.1	299473759	CTH	cystathionine gamma-lyase isoform 3	1.5	1.5	1.1	1.2	1.8	1.5	1.1	1.2	1.6	1.5	1.1	1.2	25.5	3	6	6	24	373	41.2	6.9
NP_002475.2	118572611	NUBP1	cytosolic Fe-S cluster assembly factor NUBP1 isoform 1	1.0	1.1	1.1	1.2	1.0	1.0	0.9	1.1	1.0	1.0	1.0	1.2	38.1	2	6	6	17	320	34.5	5.3
NP_001171659.1	296080714	PIGT	GPI transamidase component PIG-T isoform 4 precursor	1.0	1.2	1.0	1.2	1.1	1.1	1.2	1.1	1.1	1.1	1.1	1.2	8.6	4	3	3	5	476	54.1	8.2
NP_001327.2	22538442	CTSZ	cathepsin Z preproprotein	0.9	0.9	0.9	1.2	0.9	0.8	0.8	1.1	0.9	0.8	0.9	1.2	8.9	1	3	3	4	303	33.8	7.1
NP_001078880.1	146134341	NADK2	NAD kinase 2, mitochondrial isoform 1	1.1	1.1	1.1	1.2	1.4	1.1	1.1	1.2	1.3	1.1	1.1	1.2	11.3	3	4	4	11	442	49.4	8.2
NP_073609.2	21264616	EPS8L2	epidermal growth factor receptor kinase substrate 8-like protein 2	1.1	1.3	1.4	1.3	1.2	1.2	1.3	1.1	1.1	1.2	1.3	1.2	12.7	1	5	5	10	715	80.6	6.8
NP_006434.1	5454002	DNPH1	2'-deoxynucleoside 5'-phosphate N-hydrolase 1 isoform 1	1.2	1.1	1.1	1.1	1.2	1.1	1.0	1.3	1.2	1.1	1.1	1.2	29.3	2	2	2	28	174	19.1	5.0
NP_004359.1	4758018	CNN2	calponin-2 isoform a	1.0	1.0	1.1	1.1	1.1	1.2	1.3	1.2	1.0	1.1	1.2	1.2	14.2	4	3	4	13	309	33.7	7.3
NP_001020015.1	67782354	CD82	CD82 antigen isoform 2	0.7	0.8	0.8	1.2	1.0	1.0	1.0	1.2	0.8	0.9	0.9	1.2	6.6	2	1	1	2	242	26.8	5.1
NP_055690.1	7661878	KIF14	kinesin-like protein KIF14 isoform 1	1.1	1.3	1.4	1.3	1.2	1.2	1.1	1.1	1.1	1.2	1.3	1.2	4.3	2	6	6	8	1648	186.4	7.9
NP_001269209.1	533112488;24797097	PYCR1	pyrroline-5-carboxylate reductase 1, mitochondrial isoform 1	1.1	1.2	1.1	1.2	1.2	1.2	1.1	1.2	1.2	1.2	1.1	1.2	17.2	4	4	5	15	319	33.3	7.6
NP_001243034.1	371502087	WNT5A	protein Wnt-5a isoform 2 precursor	1.0	1.3	1.1	1.3	1.0	1.1	1.2	1.1	1.0	1.2	1.2	1.2	4.4	2	1	1	3	365	40.9	8.3
NP_004173.1	4759298	UXT	protein UXT isoform 2	1.2	1.1	1.2	1.2	1.1	1.0	1.1	1.2	1.1	1.1	1.2	1.2	19.8	2	3	3	6	157	18.2	7.6

Khan *et al.* , 2019. Multi-omics analysis to characterize cigarette smoke induced molecular alterations in esophageal cells
Supplementary Table 5. List of proteins quantified in untreated and chronically treated Het1A cells with cigarette smoke condensate for 8 months

NP_Accession	Protein group Accession	Gene ID	Description	Het-1A-Smoke - 2M/Parental	Het-1A-Smoke - 4M/Parental	Het-1A-Smoke - 6M/Parental	Het-1A-Smoke - 8M/Parental	Het-1A-Smoke - 2M/Parental	Het-1A-Smoke - 4M/Parental	Het-1A-Smoke - 6M/Parental	Het-1A-Smoke - 8M/Parental	Het-1A-Smoke - 2M/Parental	Het-1A-Smoke - 4M/Parental	Het-1A-Smoke - 6M/Parental	Het-1A-Smoke - 8M/Parental	Coverage (%)	Proteins	Unique Peptides	# Peptides	PSM	# AAs	MW [kDa]	calc. pI
				Replicate 1				Replicate 2				Average of replicates											
NP_001265401.1	510937041	**MON2**	protein MON2 homolog isoform 5	1.2	1.2	1.2	1.2	1.1	1.2	1.1	1.2	**1.2**	**1.2**	**1.1**	**1.2**	2.6	5	4	4	4	1645	182.4	6.0
NP_055661.3	120952851	**KIAA0196**	WASH complex subunit strumpellin	1.2	1.2	1.2	1.2	1.4	1.2	1.2	1.2	**1.3**	**1.2**	**1.2**	**1.2**	11.3	1	9	9	17	1159	134.2	7.0
NP_076956.1	13129018	**GGCT**	gamma-glutamylcyclotransferase isoform 1	1.0	1.0	1.1	1.1	1.2	1.1	1.2	1.3	**1.1**	**1.1**	**1.1**	**1.2**	17.6	3	3	3	7	188	21.0	5.1
NP_001193356.1	330340389	**USMG5**	up-regulated during skeletal muscle growth protein 5	1.0	1.3	1.3	1.3	1.0	1.0	1.1	1.1	**1.0**	**1.2**	**1.2**	**1.2**	25.9	1	1	1	3	58	6.5	9.8
NP_002964.3	171543895	**ATXN2**	ataxin-2	1.1	1.2	1.1	1.3	1.2	1.1	1.0	1.1	**1.2**	**1.2**	**1.1**	**1.2**	4.9	1	4	5	8	1313	140.2	9.6
NP_001136088.1	217330574	**EHBP1**	EH domain-binding protein 1 isoform 3	0.9	1.0	1.0	1.3	1.0	1.0	1.0	1.1	**0.9**	**1.0**	**1.0**	**1.2**	7.0	3	4	4	6	1160	132.2	5.8
NP_005521.1	5031777	**IDH3A**	isocitrate dehydrogenase [NAD] subunit alpha, mitochondrial precursor	1.2	1.1	1.0	1.2	1.1	1.1	1.1	1.2	**1.1**	**1.1**	**1.0**	**1.2**	18.6	1	7	7	17	366	39.6	6.9
NP_001488.2	13929462	**B4GALT1**	beta-1,4-galactosyltransferase 1	0.9	1.1	0.9	1.0	1.1	0.8	0.9	1.3	**1.0**	**0.9**	**0.9**	**1.2**	7.0	1	2	2	3	398	43.9	8.6
NP_002792.1	4506191	**PSMB10**	proteasome subunit beta type-10 precursor	1.1	1.0	1.2	1.1	1.0	1.0	1.1	1.3	**1.1**	**1.0**	**1.1**	**1.2**	7.3	1	1	1	2	273	28.9	7.8
NP_003787.2	19924159	**URI1**	unconventional prefoldin RPB5 interactor 1 isoform a	1.1	1.2	1.1	1.2	1.1	1.2	1.2	1.2	**1.1**	**1.2**	**1.1**	**1.2**	13.3	2	6	6	13	535	59.8	5.0
NP_954646.1	40317624	**MRRF**	ribosome-recycling factor, mitochondrial isoform 2 precursor	1.0	1.1	1.2	1.2	1.1	1.1	1.2	1.1	**1.0**	**1.1**	**1.2**	**1.2**	25.9	3	3	3	6	201	22.4	9.7
NP_055093.2	31541941	**HSPA4L**	heat shock 70 kDa protein 4L	1.1	1.2	1.2	1.3	1.1	1.1	1.0	1.1	**1.1**	**1.1**	**1.1**	**1.2**	30.0	3	14	19	104	839	94.5	5.9
NP_001227.1	4502601	**CBR3**	carbonyl reductase [NADPH] 3	1.0	1.1	1.1	1.1	1.1	1.2	1.2	1.2	**1.1**	**1.2**	**1.1**	**1.2**	22.7	1	2	5	18	277	30.8	6.2
NP_149100.2	188528628	**PNPT1**	polyribonucleotide nucleotidyltransferase 1, mitochondrial precursor	1.2	1.1	1.2	1.2	1.1	1.1	1.1	1.2	**1.1**	**1.1**	**1.1**	**1.2**	31.6	1	18	18	43	783	85.9	7.8
NP_742125.1	26667186;26667183	**CAMK2D**	calcium/calmodulin-dependent protein kinase type II subunit delta isoform 1	1.1	1.0	1.2	1.3	0.9	1.1	1.2	1.1	**1.0**	**1.0**	**1.2**	**1.2**	13.8	15	2	5	13	478	54.1	7.3
NP_061156.1	8923900	**CMAS**	N-acylneuraminate cytidylyltransferase	1.1	1.2	1.2	1.1	1.3	1.5	1.6	1.2	**1.2**	**1.3**	**1.4**	**1.2**	24.4	1	8	8	20	434	48.3	7.9
NP_003680.2	41327764	**AKR7A2**	aflatoxin B1 aldehyde reductase member 2	1.2	1.0	1.0	1.2	1.0	1.0	0.9	1.2	**1.1**	**1.0**	**1.0**	**1.2**	26.5	1	6	6	12	359	39.6	7.2
NP_001186792.1	315434251	**KCNAB2**	voltage-gated potassium channel subunit beta-2 isoform 4	1.2	1.2	1.1	1.2	1.1	1.4	1.3	1.2	**1.2**	**1.3**	**1.2**	**1.2**	18.0	9	4	4	5	300	33.6	8.4
NP_005763.3	157426877	**RCL1**	RNA 3'-terminal phosphate cyclase-like protein isoform a	1.0	0.9	1.1	1.0	1.0	1.2	1.1	1.3	**1.0**	**1.1**	**1.1**	**1.2**	9.9	3	2	2	5	373	40.8	9.3
NP_004986.1	4826834	**MMP14**	matrix metalloproteinase-14 preproprotein	1.0	1.2	1.1	1.1	1.1	1.3	1.1	1.2	**1.1**	**1.2**	**1.1**	**1.2**	1.4	1	1	1	2	582	65.9	7.8
NP_064508.3	190194386	**TM9SF3**	transmembrane 9 superfamily member 3 precursor	1.1	1.1	1.2	1.2	1.1	1.2	1.2	1.2	**1.1**	**1.2**	**1.2**	**1.2**	9.3	1	5	5	12	589	67.8	7.2
NP_071505.2	188219591	**TIA1**	nucleolysin TIA-1 isoform p40 isoform 2	1.0	0.9	0.9	1.2	1.0	0.9	1.0	1.2	**1.0**	**0.9**	**1.0**	**1.2**	10.6	2	4	5	12	386	42.9	7.7
NP_115725.1	14150147	**NUDT16L1**	protein syndesmos isoform 1	1.0	1.1	1.1	1.2	1.1	1.1	1.1	1.2	**1.1**	**1.1**	**1.1**	**1.2**	10.4	1	1	2	4	211	23.3	8.9
NP_001008895.1	57165424	**CUL4A**	cullin-4A isoform 1	1.0	1.0	1.1	1.1	0.9	1.1	1.0	1.3	**1.0**	**1.1**	**1.1**	**1.2**	21.3	3	7	15	55	759	87.6	8.1
NP_001123203.1	193211603	**PLEKHG4**	puratrophin-1 isoform 2	1.1	1.0	1.1	1.2	1.0	1.0	1.1	1.2	**1.0**	**1.0**	**1.1**	**1.2**	1.2	2	1	1	2	1110	122.8	6.0
NP_001139411.1	225543097	**PAFAH1B3**	platelet-activating factor acetylhydrolase IB subunit gamma	1.2	1.1	1.2	1.2	1.2	1.1	1.2	1.2	**1.2**	**1.1**	**1.2**	**1.2**	16.5	1	3	3	16	231	25.7	6.8
NP_001030611.1	79750824	**FAM129B**	niban-like protein 1 isoform 2	1.0	1.1	1.2	1.2	1.1	1.1	1.3	1.2	**1.1**	**1.1**	**1.2**	**1.2**	29.7	2	14	14	48	733	82.6	6.2
NP_056144.3	133922609	**MAU2**	MAU2 chromatid cohesion factor homolog	1.0	1.3	1.1	1.3	1.0	0.9	0.9	1.0	**1.0**	**1.1**	**1.0**	**1.2**	1.5	1	1	1	1	613	69.0	7.3
NP_001193929.1	332801090	**HNRNPDL**	heterogeneous nuclear ribonucleoprotein D-like isoform b	0.9	1.1	1.1	1.2	0.9	1.0	1.1	1.1	**0.9**	**1.1**	**1.1**	**1.2**	35.5	2	10	12	82	363	40.0	10.0
NP_056991.2	41393561	**LAP3**	cytosol aminopeptidase	1.0	1.2	1.1	1.2	1.1	1.1	1.0	1.2	**1.1**	**1.1**	**1.0**	**1.2**	51.1	1	20	20	54	519	56.1	7.9
NP_150253.2	67089160	**PML**	protein PML isoform 11	1.2	1.2	1.2	1.4	1.0	1.0	1.0	1.0	**1.1**	**1.1**	**1.1**	**1.2**	6.8	3	1	4	6	781	85.7	7.0
NP_113619.2	325197106	**COG3**	conserved oligomeric Golgi complex subunit 3	1.2	1.2	1.4	1.2	1.2	1.2	1.2	1.2	**1.2**	**1.2**	**1.3**	**1.2**	4.4	1	3	3	6	828	94.0	5.6
NP_000356.1	4507645	**TPI1**	triosephosphate isomerase isoform 1	1.0	1.3	1.2	1.2	1.0	1.2	1.2	1.2	**1.0**	**1.3**	**1.2**	**1.2**	78.3	3	16	16	150	249	26.7	6.9
NP_852480.1	31415882	**EXOSC8**	exosome complex component RRP43	1.0	1.1	1.1	1.2	1.0	1.0	1.0	1.1	**1.0**	**1.1**	**1.1**	**1.2**	8.0	1	2	2	3	276	30.0	5.3
NP_001008662.1	56713256	**CCBL2**	kynurenine--oxoglutarate transaminase 3 isoform 2	1.1	1.2	1.2	1.2	1.0	1.1	1.1	1.2	**1.1**	**1.1**	**1.1**	**1.2**	30.7	2	8	9	25	420	47.7	6.8
NP_004125.3	24234688	**HSPA9**	stress-70 protein, mitochondrial precursor	1.1	1.1	1.0	1.2	1.1	1.1	1.0	1.2	**1.1**	**1.1**	**1.0**	**1.2**	62.9	1	39	40	373	679	73.6	6.2
NP_002718.2	45935371	**SRGN**	serglycin precursor	1.2	1.7	1.6	1.3	1.4	1.6	**2.0**	1.1	**1.3**	**1.7**	**1.8**	**1.2**	8.2	1	1	1	4	158	17.6	5.0
YP_003024033.1	251831114	**ND3**	NADH dehydrogenase subunit 3	1.0	1.0	1.0	1.3	1.1	1.1	1.1	1.0	**1.1**	**1.0**	**1.0**	**1.2**	13.0	1	1	1	1	115	13.2	4.4
NP_001273725.1	557948057;56676399	**NTMT1**	N-terminal Xaa-Pro-Lys N-methyltransferase 1 isoform a	1.3	1.0	1.1	1.1	1.2	1.1	1.1	1.2	**1.3**	**1.0**	**1.1**	**1.2**	35.4	3	6	6	15	223	25.4	5.5
NP_004309.2	14589866	**ASPH**	aspartyl/asparaginyl beta-hydroxylase isoform a	1.1	1.5	1.3	1.2	1.1	1.4	1.3	1.2	**1.1**	**1.4**	**1.3**	**1.2**	33.3	12	17	17	83	758	85.8	5.0
NP_071348.3	38348727	**THADA**	thyroid adenoma-associated protein isoform a	1.1	1.1	1.1	1.1	1.2	1.1	1.2	1.2	**1.1**	**1.1**	**1.1**	**1.2**	3.9	3	6	6	10	1953	219.5	6.1
NP_057034.2	21265070	**MRPL2**	39S ribosomal protein L2, mitochondrial isoform 1	1.2	1.2	1.2	1.2	1.2	1.1	1.1	1.2	**1.2**	**1.2**	**1.2**	**1.2**	19.3	2	5	5	8	305	33.3	11.3
NP_060286.1	8923398;224591430	**LYAR**	cell growth-regulating nucleolar protein	1.0	1.1	1.1	1.2	1.0	1.1	1.0	1.2	**1.0**	**1.1**	**1.1**	**1.2**	40.1	1	13	13	44	379	43.6	9.6
NP_003194.3	44890065	**GCFC2**	GC-rich sequence DNA-binding factor 2 isoform 1	1.0	1.0	1.1	1.2	1.3	1.1	1.2	1.2	**1.1**	**1.1**	**1.1**	**1.2**	10.2	3	5	5	10	781	89.3	6.0
NP_006001.4	618857509	**MANF**	mesencephalic astrocyte-derived neurotrophic factor precursor	1.4	1.4	1.3	1.2	1.3	1.3	1.2	1.1	**1.3**	**1.4**	**1.2**	**1.2**	25.4	1	4	4	12	185	21.1	8.9
NP_004981.2	14043022	**MARS**	methionine--tRNA ligase, cytoplasmic	1.2	1.3	1.2	1.2	1.2	1.2	1.1	1.1	**1.2**	**1.2**	**1.1**	**1.2**	39.2	1	24	24	138	900	101.1	6.2
NP_001008938.1	57164942	**CKAP5**	cytoskeleton-associated protein 5 isoform a	1.0	1.1	1.1	1.2	1.1	1.1	1.2	1.2	**1.1**	**1.1**	**1.2**	**1.2**	28.5	2	47	48	132	2032	225.4	7.8
NP_060831.2	61743952	**AGPAT5**	1-acyl-sn-glycerol-3-phosphate acyltransferase epsilon	1.0	1.1	1.1	1.2	1.0	1.2	1.0	1.2	**1.0**	**1.1**	**1.1**	**1.2**	16.5	1	4	4	9	364	42.0	9.1
NP_808592.2	189011548	**ASAH1**	acid ceramidase isoform a preproprotein	1.2	1.2	1.2	1.2	1.1	1.1	1.1	1.1	**1.2**	**1.2**	**1.1**	**1.2**	4.8	3	2	2	4	395	44.6	7.6
NP_006105.1	5174723;193083122	**TOMM40**	mitochondrial import receptor subunit TOM40 homolog	1.1	1.2	1.2	1.2	1.0	1.1	1.1	1.1	**1.1**	**1.2**	**1.1**	**1.2**	30.2	1	8	8	28	361	37.9	7.3
NP_036611.2	21464101	**YWHAG**	14-3-3 protein gamma	1.0	1.2	1.1	1.2	1.0	1.2	1.1	1.2	**1.0**	**1.2**	**1.1**	**1.2**	80.6	1	12	18	214	247	28.3	4.9
NP_006657.1	5730023	**RUVBL2**	ruvB-like 2	1.1	1.1	1.1	1.2	1.0	1.1	1.1	1.1	**1.1**	**1.1**	**1.1**	**1.2**	44.3	1	20	20	73	463	51.1	5.6
NP_006244.2	19923359	**PRKAB1**	5'-AMP-activated protein kinase subunit beta-1	0.9	0.9	0.9	1.1	1.3	1.2	1.1	1.2	**1.1**	**1.1**	**1.0**	**1.2**	20.7	1	2	3	6	270	30.4	6.4
NP_001419.1	4503571	**ENO1**	alpha-enolase isoform 1	1.0	1.0	1.0	1.2	1.0	1.0	1.0	1.2	**1.0**	**1.0**	**1.0**	**1.2**	63.6	2	19	24	1110	434	47.1	7.4
NP_005894.3	47778925;47778931	**SMAD5**	mothers against decapentaplegic homolog 5	1.0	1.2	1.0	1.2	1.1	1.1	1.0	1.2	**1.1**	**1.1**	**1.0**	**1.2**	12.0	3	3	4	7	465	52.2	7.7
NP_000875.2	66933016	**IMPDH2**	inosine-5'-monophosphate dehydrogenase 2	1.1	1.1	1.1	1.2	1.1	1.1	1.1	1.2	**1.1**	**1.1**	**1.1**	**1.2**	45.1	9	17	17	110	514	55.8	6.9
NP_002309.1	4505011	**LOXL2**	lysyl oxidase homolog 2 precursor	1.0	1.1	1.2	1.2	1.3	1.6	1.5	1.2	**1.2**	**1.3**	**1.3**	**1.2**	2.6	1	1	1	2	774	86.7	6.4
NP_075385.1	13491174	**MARCKSL1**	MARCKS-related protein	0.8	1.0	1.0	1.1	0.9	0.9	0.9	1.2	**0.9**	**1.0**	**0.9**	**1.2**	53.3	1	5	5	25	195	19.5	4.7
NP_001273586.1	557786153	**TMEM68**	transmembrane protein 68 isoform 1	1.0	1.1	1.1	1.3	1.1	1.1	1.1	1.1	**1.1**	**1.1**	**1.1**	**1.2**	4.0	1	1	1	2	324	37.4	7.9
NP_078831.4	525342666	**FBXL6**	F-box/LRR-repeat protein 6 isoform 2	1.0	0.8	0.7	0.9	1.1	1.3	1.1	1.4	**1.0**	**1.1**	**0.9**	**1.2**	3.6	2	1	1	1	533	57.8	9.2
NP_037470.1	7019323	**ALG5**	dolichyl-phosphate beta-glucosyltransferase isoform 1	1.3	1.1	1.1	1.1	1.2	1.3	1.2	1.2	**1.2**	**1.2**	**1.2**	**1.2**	8.0	2	2	2	4	324	36.9	9.3
NP_006467.4	51479156	**ATP5L**	ATP synthase subunit g, mitochondrial	1.1	1.0	1.1	1.2	1.1	1.1	1.1	1.2	**1.1**	**1.1**	**1.1**	**1.2**	39.8	1	5	5	11	103	11.4	9.6
NP_073580.1	12232401	**SMYD3**	histone-lysine N-methyltransferase SMYD3 isoform 2	1.1	1.1	1.0	1.2	1.0	1.0	1.0	1.1	**1.0**	**1.1**	**1.0**	**1.2**	8.4	2	2	2	4	369	42.6	6.3
NP_955468.1	40806170	**SPAST**	spastin isoform 2	1.4	1.1	0.8	1.3	1.0	1.1	1.1	1.1	**1.2**	**1.1**	**0.9**	**1.2**	6.0	2	2	2	3	584	63.6	9.7
NP_071413.1	11545859	**GOLPH3**	Golgi phosphoprotein 3	1.2	1.2	1.3	1.2	1.0	1.1	1.1	1.2	**1.1**	**1.2**	**1.2**	**1.2**	35.2	1	5	7	20	298	33.8	6.4
NP_001008530.1	56682964	**LGMN**	legumain preproprotein	1.2	1.2	1.2	1.2	1.1	1.1	1.0	1.2	**1.2**	**1.1**	**1.1**	**1.2**	9.7	1	3	3	11	433	49.4	6.6
NP_001186211.2	345498499	**LRBA**	lipopolysaccharide-responsive and beige-like anchor protein isoform 1	1.1	1.1	1.2	1.2	1.1	1.1	1.2	1.1	**1.1**	**1.1**	**1.2**	**1.2**	8.5	2	15	15	25	2851	317.5	5.6
NP_078772.1	33457336	**IRF2BPL**	interferon regulatory factor 2-binding protein-like	1.0	1.1	1.0	1.2	1.0	1.1	1.0	1.1	**1.0**	**1.1**	**1.0**	**1.2**	2.4	3	2	2	3	796	82.6	8.2
NP_003747.1	4503515	**EIF3H**	eukaryotic translation initiation factor 3 subunit H	1.1	1.1	1.1	1.2	1.1	1.1	1.1	1.2	**1.1**	**1.1**	**1.1**	**1.2**	46.9	1	13	13	50	352	39.9	6.5
NP_001034624.1	88014625	**SKA1**	spindle and kinetochore-associated protein 1	0.8	1.1	0.9	1.2	0.8	1.0	0.9	1.1	**0.8**	**1.0**	**0.9**	**1.2**	12.9	1	2	2	3	255	29.5	7.1
NP_001231496.1	347300410;4557361	**BID**	BH3-interacting domain death agonist isoform 2	1.6	1.3	1.2	1.1	1.2	1.3	1.3	1.2	**1.4**	**1.3**	**1.3**	**1.2**	35.4	3	4	4	12	195	22.0	5.4
NP_001153566.1	231567183	**ACOT13**	acyl-coenzyme A thioesterase 13 isoform 2	1.1	1.1	1.0	1.2	1.1	1.0	1.0	1.1	**1.1**	**1.1**	**1.0**	**1.2**	25.6	2	2	2	3	117	12.4	8.5
NP_001024.1	4506749	**RRM1**	ribonucleoside-diphosphate reductase large subunit	1.0	1.0	1.1	1.2	1.2	1.1	1.3	1.1	**1.1**	**1.0**	**1.2**	**1.2**	36.9	1	22	22	94	792	90.0	7.2
NP_077001.1	13129100	**DCTPP1**	dCTP pyrophosphatase 1	1.2	1.1	1.2	1.3	1.0	0.9	1.1	1.1	**1.1**	**1.0**	**1.1**	**1.2**	61.8	1	7	7	21	170	18.7	5.0
NP_001036199.1	112382214	**SEC24B**	protein transport protein Sec24B isoform b	0.9	1.0	1.0	1.2	1.2	1.1	1.1	1.2	**1.1**	**1.1**	**1.0**	**1.2**	1.8	2	2	2	3	1233	133.5	7.4
NP_079140.2	40068479	**MRM1**	rRNA methyltransferase 1, mitochondrial precursor	1.1	1.1	1.0	1.2	1.0	1.0	0.9	1.2	**1.0**	**1.0**	**1.0**	**1.2**	17.0	1	3	3	6	353	38.6	7.9
NP_001098.1	4557245	**ACYP1**	acylphosphatase-1 isoform a	1.1	1.0	1.0	1.2	1.2	1.1	1.0	1.1	**1.1**	**1.1**	**1.0**	**1.2**	7.1	2	1	1	1	99	11.3	9.3
NP_004993.1	6681764	**NDUFA9**	NADH dehydrogenase [ubiquinone] 1 alpha subcomplex subunit 9, mitochondrial precursor	1.2	1.3	1.3	1.2	1.1	1.2	1.2	1.1	**1.1**	**1.2**	**1.3**	**1.2**	29.2	1	9	9	23	377	42.5	9.8
NP_054880.2	21265080	**MRPL18**	39S ribosomal protein L18, mitochondrial	1.6	1.5	1.6	1.4	1.2	1.0	1.1	0.9	**1.4**	**1.3**	**1.3**	**1.2**	7.2	1	1	1	1	180	20.6	9.5
NP_115717.3	62955833	**DDI2**	protein DDI1 homolog 2	1.1	0.9	1.0	1.1	1.2	1.0	1.1	1.2	**1.2**	**1.0**	**1.0**	**1.2**	10.5	1	3	3	5	399	44.5	5.0
NP_001272.2	50428925	**TBCB**	tubulin-folding cofactor B isoform 1	1.4	1.2	1.0	1.1	1.2	1.2	1.1	1.2	**1.3**	**1.2**	**1.1**	**1.2**	28.7	2	5	5	12	244	27.3	5.1
NP_079172.2	55749804	**ERMP1**	endoplasmic reticulum metallopeptidase 1	1.1	1.1	1.2	1.2	1.0	1.0	1.1	1.2	**1.1**	**1.1**	**1.2**	**1.2**	6.2	1	4	4	5	904	100.2	7.5
NP_001026859.1	72534670	**PLAA**	phospholipase A-2-activating protein	1.1	1.1	1.1	1.2	1.2	1.2	1.2	1.2	**1.2**	**1.1**	**1.2**	**1.2**	17.6	1	10	10	29	795	87.1	6.4
NP_004437.2	62241042	**EPRS**	bifunctional glutamate/proline--tRNA ligase	1.2	1.3	1.2	1.2	1.2	1.2	1.1	1.2	**1.2**	**1.2**	**1.2**	**1.2**	40.2	1	43	44	210	1512	170.5	7.3
NP_001029345.2	238814322	**ALDH1L2**	mitochondrial 10-formyltetrahydrofolate dehydrogenase precursor	1.7	1.7	1.5	1.2	1.8	1.7	1.5	1.2	**1.8**	**1.7**	**1.5**	**1.2**	21.2	3	14	14	38	923	101.7	6.5
NP_001136271.1	218563739	**C17orf49**	chromatin complexes subunit BAP18 isoform 3	1.2	1.2	0.9	1.3	1.1	0.9	1.0	1.0	**1.1**	**1.1**	**0.9**	**1.2**	21.7	3	1	1	2	138	13.9	7.2
NP_001272971.1	554506554	**ACADM**	medium-chain specific acyl-CoA dehydrogenase, mitochondrial isoform c	1.3	1.4	1.3	1.2	1.2	1.2	1.2	1.1	**1.2**	**1.3**	**1.2**	**1.2**	12.5	5	5	5	10	385	42.4	6.4
NP_005759.4	42542394	**LPCAT3**	lysophospholipid acyltransferase 5	1.6	1.5	1.6	1.2	1.4	1.3	1.4	1.1	**1.5**	**1.4**	**1.5**	**1.2**	5.1	1	2	2	6	487	56.0	8.7
NP_059142.2	27886582	**MRPL39**	39S ribosomal protein L39, mitochondrial isoform a	1.1	1.1	1.1	1.1	1.2	1.3	1.2	1.2	**1.1**	**1.2**	**1.2**	**1.2**	17.5	2	6	6	13	338	38.7	7.6

NP_Accession	Protein group Accession	Gene ID	Description	Het-1A-Smoke - 2M/Parental	Het-1A-Smoke - 4M/Parental	Het-1A-Smoke - 6M/Parental	Het-1A-Smoke - 8M/Parental	Het-1A-Smoke - 2M/Parental	Het-1A-Smoke - 4M/Parental	Het-1A-Smoke - 6M/Parental	Het-1A-Smoke - 8M/Parental	Het-1A-Smoke - 2M/Parental	Het-1A-Smoke - 4M/Parental	Het-1A-Smoke - 6M/Parental	Het-1A-Smoke - 8M/Parental	Coverage (%)	Proteins	Unique Peptides	# Peptides	PSM	# AAs	MW [kDa]	calc. pI
				Replicate 1				Replicate 2				Average of replicates											
NP_073572.2	15826852	ACBD3	Golgi resident protein GCP60	1.5	1.2	1.0	1.3	1.3	1.0	1.1	1.1	1.4	1.1	1.1	1.2	19.9	1	5	5	9	528	60.6	5.1
NP_001001563.1	48526509	TIMM50	mitochondrial import inner membrane translocase subunit TIM50	1.2	1.1	1.2	1.2	1.2	1.1	1.2	1.2	1.2	1.1	1.2	1.2	21.3	1	8	8	31	456	50.4	9.4
NP_002047.2	205277386	GFPT1	glutamine--fructose-6-phosphate aminotransferase [isomerizing] 1 isoform 2	1.1	1.1	1.0	1.2	1.1	1.1	1.0	1.2	1.1	1.1	1.0	1.2	45.2	2	18	23	72	681	76.7	6.8
NP_068579.3	82546830	EXOC4	exocyst complex component 4 isoform a	1.1	1.0	1.0	1.1	1.2	1.1	1.2	1.2	1.2	1.1	1.1	1.2	11.9	2	7	7	11	974	110.4	6.5
NP_149131.1	15149465	CALD1	caldesmon isoform 5	1.1	1.4	1.3	1.2	0.9	1.3	1.3	1.2	1.0	1.3	1.3	1.2	34.6	5	14	14	32	532	61.2	6.7
NP_036425.1	109150416	PXDN	peroxidasin homolog precursor	1.0	1.2	1.1	0.9	1.3	1.3	1.2	1.4	1.2	1.3	1.1	1.2	3.5	1	3	3	5	1479	165.2	7.2
NP_001668.1	4502277	ATP1B1	sodium/potassium-transporting ATPase subunit beta-1	1.1	1.1	1.3	1.1	1.0	1.2	1.3	1.2	1.1	1.2	1.3	1.2	24.4	1	5	5	19	303	35.0	8.5
NP_002102.4	90903231	HTT	huntingtin	1.1	1.3	1.1	1.2	1.1	1.2	1.1	1.1	1.1	1.2	1.1	1.2	0.3	1	1	1	1	3144	347.6	6.2
NP_001258978.1	440309857	TRAP1	heat shock protein 75 kDa, mitochondrial isoform 2	1.1	1.1	1.0	1.2	1.1	1.1	1.1	1.2	1.1	1.1	1.1	1.2	58.5	2	28	29	203	651	74.2	7.9
NP_001138495.1	222537730	SCRN2	secernin-2 isoform 2	1.2	1.3	1.1	1.4	1.0	1.0	1.0	1.0	1.1	1.2	1.0	1.2	4.2	2	1	1	1	378	41.3	6.2
NP_001136150.1	218156303	ANO6	anoctamin-6 isoform b	1.0	1.1	1.4	1.2	1.0	1.2	1.3	1.2	1.0	1.1	1.4	1.2	2.5	4	1	1	6	892	103.9	8.2
NP_060016.3	188497630	CNOT11	CCR4-NOT transcription complex subunit 11	1.0	1.0	1.0	1.1	1.1	1.1	1.2	1.2	1.0	1.0	1.1	1.2	5.9	1	2	2	4	510	55.2	6.4
NP_001116143.1	169881279;95147551	CCBL1	kynurenine--oxoglutarate transaminase 1 isoform a	1.2	1.2	1.2	1.1	1.2	1.2	1.2	1.2	1.2	1.2	1.2	1.2	14.0	3	4	5	6	422	47.8	6.5
NP_055439.1	7657532	S100A6	protein S100-A6	1.6	1.2	1.9	1.2	1.4	1.1	1.6	1.2	1.5	1.2	1.8	1.2	34.4	1	4	4	18	90	10.2	5.5
NP_002026.1	4503817	KDSR	3-ketodihydrosphingosine reductase precursor	1.1	1.1	1.1	1.2	1.1	1.2	1.2	1.2	1.1	1.2	1.2	1.2	4.2	1	1	1	1	332	36.2	7.1
NP_001106207.1	163838631	FAM208A	protein FAM208A isoform a	0.9	1.1	1.2	1.2	1.1	1.1	1.1	1.1	1.0	1.1	1.2	1.2	3.0	2	3	3	5	1512	170.7	6.3
NP_006280.3	223029410	TLN1	talin-1	1.1	1.2	1.1	1.2	1.1	1.2	1.1	1.2	1.1	1.2	1.1	1.2	49.3	1	79	81	288	2541	269.6	6.1
NP_005362.3	167004078	METTL1	tRNA (guanine-N(7)-)-methyltransferase isoform a	1.1	1.2	1.2	1.2	2.0	1.2	1.0	1.1	1.5	1.2	1.1	1.2	24.3	2	5	5	14	276	31.5	7.6
NP_004841.2	41872583	ROCK2	rho-associated protein kinase 2	1.3	1.2	1.2	1.1	1.1	1.3	1.2	1.2	1.2	1.2	1.2	1.2	1.9	1	2	3	5	1388	160.8	6.0
NP_057336.3	153792694	BIRC6	baculoviral IAP repeat-containing protein 6	1.1	1.1	1.2	1.2	1.2	1.1	1.2	1.1	1.1	1.1	1.2	1.2	5.6	1	18	18	40	4857	529.9	6.0
NP_001161807.1	270265879	ME2	NAD-dependent malic enzyme, mitochondrial isoform 2 precursor	1.2	1.2	1.2	1.1	1.1	1.1	1.0	1.2	1.2	1.1	1.1	1.2	10.7	2	4	4	9	479	53.6	8.5
NP_079012.3	83320070	GSDMD	gasdermin-D	1.1	1.1	1.1	1.1	1.0	1.0	1.0	1.2	1.1	1.0	1.0	1.2	23.4	1	7	7	14	484	52.8	5.1
NP_005156.1	4885063	ALDOC	fructose-bisphosphate aldolase C	1.0	1.1	1.0	1.2	1.1	1.2	1.1	1.2	1.0	1.2	1.1	1.2	56.3	2	12	15	119	364	39.4	6.9
NP_001230314.1	343432584	PARVB	beta-parvin isoform c	1.1	1.0	1.1	1.1	1.2	1.0	1.3	1.3	1.1	1.0	1.2	1.2	2.5	3	1	1	1	327	37.5	5.7
NP_060225.4	39995082	NSUN2	tRNA (cytosine(34)-C(5))-methyltransferase isoform 1	1.1	1.1	1.2	1.1	1.1	1.1	1.2	1.2	1.1	1.1	1.2	1.2	60.0	2	31	31	84	767	86.4	6.8
NP_001229707.1	338753358	IKBKB	inhibitor of nuclear factor kappa-B kinase subunit beta isoform 5	1.0	1.0	1.1	1.1	1.2	1.3	1.3	1.2	1.1	1.2	1.2	1.2	6.3	3	3	3	3	697	79.5	5.5
NP_056018.2	183396804	RPRD2	regulation of nuclear pre-mRNA domain-containing protein 2 isoform 1	1.0	1.0	1.0	1.2	1.0	1.1	1.0	1.1	1.0	1.0	1.0	1.2	4.6	3	5	5	16	1461	155.9	7.4
NP_001114.2	32484973	ADK	adenosine kinase isoform a	1.1	1.2	1.3	1.2	1.1	1.2	1.2	1.1	1.1	1.2	1.3	1.2	48.7	4	11	11	64	345	38.7	6.7
NP_055637.2	190343021	SEC24D	protein transport protein Sec24D	1.2	1.2	1.1	1.1	1.1	1.3	1.2	1.2	1.1	1.2	1.2	1.2	2.7	1	3	3	3	1032	112.9	7.3
NP_002780.1	4506185	PSMA4	proteasome subunit alpha type-4 isoform 1	1.1	1.0	1.1	1.1	1.1	1.1	1.1	1.2	1.1	1.1	1.1	1.2	54.4	2	10	10	55	261	29.5	7.7
NP_000145.1	4503895	GALK1	galactokinase	1.1	1.2	1.1	1.3	1.0	1.0	0.9	1.0	1.1	1.1	1.0	1.2	14.3	1	3	3	7	392	42.2	6.5
NP_064632.2	34147522	ADCK3	chaperone activity of bc1 complex-like, mitochondrial	1.2	1.3	1.1	1.2	1.1	1.2	1.1	1.2	1.1	1.2	1.1	1.2	2.9	1	1	1	1	647	71.9	7.0
NP_000276.2	149589008	PEPD	xaa-Pro dipeptidase isoform 1	1.2	1.1	1.0	1.2	1.1	1.0	1.1	1.1	1.2	1.0	1.1	1.2	18.7	3	6	6	12	493	54.5	6.0
NP_001271153.1	545746266;545746350	ELP3	elongator complex protein 3 isoform 4	1.0	1.1	1.1	1.2	1.2	1.2	1.1	1.2	1.1	1.2	1.1	1.2	17.3	5	6	6	10	428	49.4	8.7
NP_056050.1	14149675	CSTF2T	cleavage stimulation factor subunit 2 tau variant	1.0	1.1	1.1	1.1	1.2	1.3	1.1	1.3	1.1	1.2	1.1	1.2	4.6	1	1	3	3	616	64.4	7.3
NP_002694.3	29570798	PPAT	amidophosphoribosyltransferase proprotein	1.1	1.1	1.1	1.2	1.0	1.0	1.1	1.2	1.1	1.1	1.1	1.2	32.7	1	11	11	22	517	57.4	6.8
NP_008938.2	162329583	CPSF6	cleavage and polyadenylation specificity factor subunit 6	0.9	1.0	0.9	1.1	0.9	1.0	1.0	1.2	0.9	1.0	0.9	1.2	10.3	2	4	4	8	551	59.2	7.2
NP_036311.3	74229027	FBXO7	F-box only protein 7 isoform 1	1.3	1.3	1.3	1.3	1.2	1.2	1.2	1.1	1.2	1.2	1.2	1.2	5.4	3	2	2	8	522	58.5	6.6
NP_065863.2	157426887	DOCK6	dedicator of cytokinesis protein 6	1.0	1.1	1.2	1.0	1.1	1.4	1.3	1.3	1.0	1.2	1.3	1.2	0.8	1	1	1	1	2047	229.4	6.7
NP_060224.3	134152667	UHRF1BP1	UHRF1-binding protein 1	1.2	1.2	1.1	1.1	1.4	1.0	0.7	1.2	1.3	1.1	0.9	1.2	3.4	1	2	3	7	1440	159.4	6.1
NP_005262.1	4885281	GLUD1	glutamate dehydrogenase 1, mitochondrial precursor	1.2	1.3	1.4	1.2	1.1	1.2	1.2	1.1	1.2	1.2	1.3	1.2	35.3	2	17	17	80	558	61.4	7.8
NP_060263.2	93277074	RPP25	ribonuclease P protein subunit p25	1.0	1.1	0.9	1.2	0.9	0.9	0.9	1.1	1.0	1.0	0.9	1.2	13.6	1	1	1	1	199	20.6	9.6
NP_003279.2	40805860	TPD52L2	tumor protein D54 isoform e	1.0	1.0	1.0	1.1	1.1	1.1	1.1	1.2	1.1	1.0	1.1	1.2	35.4	10	7	7	34	206	22.2	5.4
NP_733792.1	25306287	GFM2	ribosome-releasing factor 2, mitochondrial isoform 2	1.2	1.1	1.1	1.1	1.1	1.2	1.1	1.3	1.1	1.1	1.1	1.2	9.0	4	5	5	9	732	81.1	6.2
NP_001015001.1	66346730	CKMT1A	creatine kinase U-type, mitochondrial precursor	1.0	1.0	0.9	1.2	1.1	1.0	1.2	1.1	1.0	1.0	1.1	1.2	8.9	2	2	2	3	417	47.0	8.3
NP_060285.2	261824035	HAUS4	HAUS augmin-like complex subunit 4 isoform 1	1.0	1.0	0.9	1.1	1.1	1.0	1.1	1.2	1.0	1.0	1.0	1.2	4.7	1	1	1	1	363	42.4	5.7
NP_006076.4	116812595	BPNT1	3'(2'),5'-bisphosphate nucleotidase 1 isoform 1	1.1	1.1	1.2	1.1	1.1	1.2	1.2	1.2	1.1	1.1	1.2	1.2	36.0	3	7	7	16	308	33.4	5.7
NP_612208.1	19923881	GNPDA2	glucosamine-6-phosphate isomerase 2 isoform 1	1.3	1.3	1.4	1.3	1.1	0.8	1.1	1.0	1.2	1.0	1.3	1.2	37.0	3	3	6	20	276	31.1	6.9
NP_006829.1	5803092	METAP2	methionine aminopeptidase 2	1.1	1.1	1.1	1.2	1.0	1.1	1.0	1.1	1.1	1.1	1.1	1.2	23.9	1	8	8	21	478	52.9	5.8
NP_478059.1	17402893	PSAT1	phosphoserine aminotransferase isoform 1	1.6	1.5	1.1	1.2	1.5	1.4	1.1	1.2	1.5	1.5	1.1	1.2	58.9	2	16	18	91	370	40.4	7.7
NP_055706.1	7657441	PDAP1	28 kDa heat- and acid-stable phosphoprotein	1.0	1.0	1.1	1.1	1.0	1.1	1.1	1.2	1.0	1.0	1.1	1.2	29.3	1	6	6	24	181	20.6	8.9
NP_006815.2	237649015	EBNA1BP2	probable rRNA-processing protein EBP2 isoform 2	0.9	1.2	1.1	1.2	1.0	1.2	1.1	1.1	1.0	1.2	1.1	1.2	34.0	2	9	9	28	306	34.8	10.1
NP_055527.1	55741677	SETD1A	histone-lysine N-methyltransferase SETD1A	1.1	1.2	1.0	1.1	0.9	1.0	1.0	1.2	1.0	1.1	1.0	1.2	4.9	1	4	4	9	1707	185.9	5.1
NP_059830.1	9257257	WDR1	WD repeat-containing protein 1 isoform 1	1.2	1.3	1.4	1.2	1.1	1.1	1.2	1.1	1.1	1.2	1.3	1.2	45.2	2	22	22	64	606	66.2	6.7
NP_006576.2	48762932	CCT8	T-complex protein 1 subunit theta isoform 1	1.0	1.1	1.0	1.2	1.0	1.1	1.0	1.1	1.0	1.1	1.0	1.2	69.9	4	33	33	275	548	59.6	5.6
NP_057111.1	7705793	LACTB2	beta-lactamase-like protein 2	1.2	1.1	1.1	1.2	1.2	1.0	1.0	1.1	1.2	1.0	1.1	1.2	35.4	1	7	7	18	288	32.8	6.8
NP_001012632.1	61743967	CTBP1	C-terminal-binding protein 1 isoform 2	1.1	1.1	1.1	1.2	1.2	1.0	1.1	1.2	1.1	1.0	1.1	1.2	14.5	2	4	5	9	429	46.4	6.7
NP_006182.2	124494254	PA2G4	proliferation-associated protein 2G4	1.0	1.1	1.1	1.2	1.0	1.1	1.0	1.2	1.0	1.1	1.1	1.2	50.0	3	15	17	84	394	43.8	6.6
NP_000099.2	91199540	DLD	dihydrolipoyl dehydrogenase, mitochondrial isoform 1 precursor	1.3	1.2	1.2	1.2	1.3	1.1	1.1	1.1	1.3	1.1	1.2	1.2	36.4	4	13	13	50	509	54.1	7.9
NP_817126.1	30089997	ACTL6A	actin-like protein 6A isoform 2	1.0	1.1	1.1	1.1	1.1	1.1	1.1	1.2	1.1	1.1	1.1	1.2	20.2	3	5	5	14	387	43.2	6.1
NP_001186738.1	315259084	INTS7	integrator complex subunit 7 isoform 4	0.9	1.1	1.0	1.3	1.0	1.0	1.0	1.1	1.0	1.1	1.0	1.2	2.3	4	2	2	2	913	101.1	7.8
NP_006443.1	5453539	PAICS	multifunctional protein ADE2 isoform 2	1.2	1.2	1.3	1.2	1.1	1.1	1.1	1.1	1.1	1.1	1.2	1.2	45.2	2	16	16	68	425	47.0	7.2
NP_057366.2	19923437	AK3	GTP:AMP phosphotransferase AK3, mitochondrial isoform a	1.0	1.1	1.1	1.1	1.1	1.1	1.1	1.2	1.1	1.1	1.1	1.2	35.2	3	8	8	20	227	25.5	9.2
NP_001266284.1	525342536	VPS45	vacuolar protein sorting-associated protein 45 isoform 4	1.2	1.1	1.1	1.1	1.2	1.1	1.0	1.2	1.2	1.1	1.1	1.2	18.3	4	6	6	8	447	50.7	8.9
NP_001181.2	50658084	BCAT2	branched-chain-amino-acid aminotransferase, mitochondrial isoform a precursor	1.1	1.1	1.1	1.1	1.1	1.1	1.1	1.2	1.1	1.1	1.1	1.2	18.1	3	6	6	8	392	44.3	8.6
NP_002574.2	55769533	PAWR	PRKC apoptosis WT1 regulator protein	1.0	1.1	1.1	1.2	1.0	1.2	1.1	1.2	1.0	1.1	1.1	1.2	25.6	1	5	5	9	340	36.5	5.4
NP_001138303.1	221307584	PHB2	prohibitin-2 isoform 1	1.2	1.3	1.3	1.1	1.2	1.3	1.3	1.2	1.2	1.3	1.3	1.2	69.6	2	20	20	96	299	33.3	9.8
NP_006364.2	18379349	VAT1	synaptic vesicle membrane protein VAT-1 homolog	1.2	1.3	1.4	1.2	1.1	1.2	1.3	1.1	1.2	1.3	1.3	1.2	35.9	1	9	9	56	393	41.9	6.3
NP_002960.2	48255970	MAPK12	mitogen-activated protein kinase 12	0.9	1.2	1.1	1.1	1.2	1.2	1.3	1.2	1.0	1.2	1.2	1.2	12.8	2	3	3	4	367	41.9	6.4
NP_001138868.1	223972653	ALDH16A1	aldehyde dehydrogenase family 16 member A1 isoform 2	1.1	1.1	1.1	1.2	1.4	1.3	0.8	1.1	1.3	1.2	1.0	1.2	10.1	2	5	5	16	751	79.9	7.6
NP_001278532.1	618468299	DYNLT1	dynein light chain Tctex-type 1 isoform 3	1.2	1.2	1.2	1.2	1.3	1.0	1.2	1.1	1.3	1.1	1.2	1.2	41.5	3	2	2	4	82	9.4	4.8
NP_116199.2	31377644	ATAD1	ATPase family AAA domain-containing protein 1	1.2	1.2	1.2	1.1	0.9	1.0	1.0	1.2	1.1	1.1	1.1	1.2	16.1	1	4	4	10	361	40.7	6.9
NP_001188408.1	319918873	CORO7-PAM1	CORO7-PAM16 protein	1.1	1.2	1.1	1.2	1.2	1.2	1.1	1.1	1.2	1.2	1.1	1.2	10.0	5	6	6	9	1048	114.1	6.3
NP_001166217.1	289629267	SEC23B	protein transport protein Sec23B isoform 2	1.3	1.2	1.1	1.2	1.2	1.2	1.2	1.2	1.2	1.2	1.1	1.2	14.7	2	6	8	20	749	84.4	7.0
NP_036339.1	14141157	HNRNPH3	heterogeneous nuclear ribonucleoprotein H3 isoform a	1.0	1.1	1.1	1.0	1.1	1.1	1.1	1.3	1.1	1.1	1.1	1.2	61.6	7	11	13	72	346	36.9	6.9
NP_005253.3	54112432	GFER	FAD-linked sulfhydryl oxidase ALR	1.2	1.2	1.0	1.1	1.1	1.1	1.0	1.2	1.2	1.1	1.0	1.2	12.2	1	1	1	2	205	23.4	7.6
NP_000134.2	19743875	FH	fumarate hydratase, mitochondrial	1.1	1.2	1.1	1.2	1.1	1.1	1.0	1.1	1.1	1.2	1.1	1.2	40.4	5	16	16	58	510	54.6	8.8
NP_001001795.1	49169841	C8orf82	UPF0598 protein C8orf82	1.1	1.1	1.1	1.1	1.1	1.0	1.0	1.2	1.1	1.0	1.1	1.2	8.3	1	1	1	1	216	23.9	9.1
NP_001273128.1	555290018	SCAF8	protein SCAF8 isoform d	1.0	1.1	1.2	1.2	1.1	1.0	1.0	1.1	1.1	1.1	1.1	1.2	6.2	4	3	5	12	1271	140.4	8.3
NP_075015.1	13259531	SMN2	survival motor neuron protein isoform c	1.1	1.0	1.1	1.2	1.1	1.1	1.1	1.1	1.1	1.0	1.1	1.2	23.2	4	3	3	14	250	27.1	5.2
NP_872634.1	33188449	SRPK2	SRSF protein kinase 2 isoform a	1.0	0.9	1.0	1.1	1.0	1.0	1.1	1.2	1.0	1.0	1.0	1.2	8.9	5	4	6	11	699	79.0	5.2
NP_067653.3	145275185	C12orf10	UPF0160 protein MYG1, mitochondrial precursor	1.1	1.0	1.0	1.1	1.1	1.0	1.1	1.2	1.1	1.0	1.1	1.2	30.3	1	8	8	19	376	42.4	6.7
NP_005737.1	5031977	NAMPT	nicotinamide phosphoribosyltransferase precursor	1.0	0.9	1.0	1.2	1.0	0.9	1.0	1.1	1.0	0.9	1.0	1.2	51.1	1	21	21	82	491	55.5	7.2
NP_009294.1	6466450	COMT	catechol O-methyltransferase isoform S-COMT	1.3	1.2	1.2	1.2	1.1	1.2	1.2	1.1	1.2	1.2	1.2	1.2	46.6	2	9	9	27	221	24.4	5.3
NP_057104.2	156415992	TFB1M	dimethyladenosine transferase 1, mitochondrial	1.1	1.1	1.2	1.2	1.0	1.2	1.2	1.1	1.0	1.1	1.2	1.2	13.3	1	4	4	5	346	39.5	9.3
NP_000511.2	189181666	HEXA	beta-hexosaminidase subunit alpha preproprotein	1.1	1.0	1.0	1.2	1.1	1.0	0.9	1.1	1.1	1.0	1.0	1.2	15.7	1	8	8	14	529	60.7	5.2
NP_002083.3	149193321	GRSF1	G-rich sequence factor 1 isoform 1	0.8	0.9	0.9	1.1	0.9	0.9	1.0	1.2	0.9	0.9	1.0	1.2	24.4	2	9	9	32	480	53.1	6.2
NP_001257449.1	395394053	DAAM1	disheveled-associated activator of morphogenesis 1 isoform 2	1.0	1.1	1.0	1.1	0.9	0.9	1.1	1.2	1.0	1.0	1.0	1.2	1.1	2	1	1	2	1068	122.2	7.2
NP_004332.2	18105007	CAD	CAD protein isoform 1	1.0	1.0	1.0	1.2	1.1	1.0	1.0	1.1	1.0	1.0	1.0	1.2	30.2	2	44	46	136	2225	242.8	6.5
NP_074035.1	13124875	MYH11	myosin-11 isoform SM2A	3.4	1.1	1.0	1.0	1.3	1.2	1.4	1.3	2.3	1.2	1.2	1.2	10.3	13	1	19	167	1938	223.4	5.5
NP_570853.1	19718762	UBE3A	ubiquitin-protein ligase E3A isoform 1	1.1	1.1	1.0	1.2	0.9	1.0	0.9	1.1	1.0	1.1	1.0	1.2	8.5	3	5	5	8	852	97.9	5.2
NP_006039.2	38327034	UBE4B	ubiquitin conjugation factor E4 B isoform 2	1.0	1.1	1.0	1.3	1.0	1.0	1.0	1.0	1.0	1.1	1.0	1.2	3.1	2	4	4	6	1173	133.2	6.1
NP_006030.2	110624774	MRC2	C-type mannose receptor 2 precursor	1.3	1.1	1.0	1.1	1.1	1.2	1.1	1.2	1.2	1.1	1.1	1.2	4.9	1	4	4	8	1479	166.6	5.8
NP_878256.1	33469951	RABGGTA	geranylgeranyl transferase type-2 subunit alpha	1.2	1.0	1.0	1.1	1.1	1.2	1.1	1.2	1.1	1.1	1.0	1.2	8.5	1	5	5	7	567	65.0	5.7

Khan *et al.*, 2019. Multi-omics analysis to characterize cigarette smoke induced molecular alterations in esophageal cells
Supplementary Table 5. List of proteins quantified in untreated and chronically treated Het1A cells with cigarette smoke condensate for 8 months

NP_Accession	Protein group Accession	Gene ID	Description	Het-1A-Smoke - 2M/Parental	Het-1A-Smoke - 4M/Parental	Het-1A-Smoke - 6M/Parental	Het-1A-Smoke - 8M/Parental	Het-1A-Smoke - 2M/Parental	Het-1A-Smoke - 4M/Parental	Het-1A-Smoke - 6M/Parental	Het-1A-Smoke - 8M/Parental	Het-1A-Smoke - 2M/Parental	Het-1A-Smoke - 4M/Parental	Het-1A-Smoke - 6M/Parental	Het-1A-Smoke - 8M/Parental	Coverage (%)	Proteins	Unique Peptides	# Peptides	PSM	# AAs	MW [kDa]	calc. pI
				Replicate 1				Replicate 2				Average of replicates											
NP_003642.3	224586882	YBX3	Y-box-binding protein 3 isoform a	1.4	1.7	1.6	1.1	1.5	1.5	2.0	1.2	1.4	1.6	1.8	1.2	38.7	1	3	10	57	372	40.1	9.8
NP_006848.1	24307919	SNRNP27	U4/U6.U5 small nuclear ribonucleoprotein 27 kDa protein	0.9	1.2	1.1	1.2	1.1	1.0	1.1	1.2	1.0	1.1	1.1	1.2	12.3	1	1	1	1	155	18.8	11.6
NP_110407.2	21314739	SLC25A32	mitochondrial folate transporter/carrier	1.1	1.2	1.2	1.1	1.5	1.2	1.4	1.2	1.3	1.2	1.3	1.2	9.8	1	2	2	3	315	35.4	9.5
NP_057226.1	7705855	HSD17B12	very-long-chain 3-oxoacyl-CoA reductase	1.4	1.3	1.3	1.1	1.2	1.3	1.3	1.2	1.3	1.3	1.3	1.2	46.5	1	10	10	37	312	34.3	9.3
NP_891552.1	41281996	HEATR3	HEAT repeat-containing protein 3	1.0	1.1	1.0	1.2	1.0	1.1	1.0	1.1	1.0	1.1	1.0	1.2	6.6	1	2	2	2	680	74.5	5.1
NP_009039.1	6005890	TCEB2	transcription elongation factor B polypeptide 2 isoform a	1.2	0.9	1.1	1.2	1.1	0.8	1.1	1.1	1.1	0.9	1.1	1.2	60.2	2	5	5	12	118	13.1	4.9
NP_002499.2	115298674	NID1	nidogen-1 precursor	1.1	1.1	1.3	1.3	1.0	1.0	0.9	1.0	1.1	1.0	1.1	1.2	5.0	1	2	2	3	1247	136.3	5.3
NP_060891.3	222352143	TBC1D2	TBC1 domain family member 2A isoform 2	1.0	0.9	0.9	1.2	1.0	1.0	1.0	1.1	1.0	0.9	0.9	1.2	7.2	4	4	5	7	917	104.0	6.5
NP_001034267.1	85794908	ATP5J2	ATP synthase subunit f, mitochondrial isoform 2d	1.2	1.2	1.3	1.2	1.1	1.2	1.3	1.1	1.2	1.2	1.3	1.2	61.2	5	2	3	18	49	5.7	9.7
NP_004543.1	4758790	NDUFS5	NADH dehydrogenase [ubiquinone] iron-sulfur protein 5	0.9	1.2	1.1	1.2	1.0	1.1	1.1	1.1	0.9	1.1	1.1	1.2	20.8	1	2	2	2	106	12.5	9.1
NP_001245321.1	385719194	CLPB	caseinolytic peptidase B protein homolog isoform 2	1.0	1.1	1.1	1.1	1.1	1.2	1.2	1.2	1.1	1.2	1.1	1.2	18.9	4	12	12	27	677	75.4	9.0
NP_001274360.1	566006168;71040094	ARFIP1	arfaptin-1 isoform 1	1.1	1.1	1.0	1.1	1.2	1.1	1.1	1.2	1.1	1.1	1.1	1.2	24.9	2	7	8	22	373	41.7	6.7
NP_005484.2	39812378	RANBP9	ran-binding protein 9	1.1	1.2	1.1	1.3	1.1	1.0	1.0	1.1	1.1	1.1	1.0	1.2	5.4	1	2	2	3	729	77.8	6.8
NP_064587.1	9910460	NIT2	omega-amidase NIT2	1.2	1.2	1.0	1.2	1.2	1.2	1.1	1.1	1.2	1.2	1.1	1.2	37.3	1	8	8	25	276	30.6	7.2
NP_056014.2	68131557	ANKRD28	serine/threonine-protein phosphatase 6 regulatory ankyrin repeat subunit A isoform a	1.1	1.1	1.1	1.2	1.0	1.0	1.0	1.1	1.1	1.1	1.0	1.1	7.5	2	4	4	9	1053	112.9	6.3
NP_004271.1	4758862	EEF1E1	eukaryotic translation elongation factor 1 epsilon-1 isoform 1	1.1	1.2	1.2	1.1	1.1	1.2	1.2	1.2	1.1	1.2	1.2	1.1	17.2	2	3	3	6	174	19.8	8.5
NP_060517.1	8922328	RBM22	pre-mRNA-splicing factor RBM22	0.9	0.9	0.9	1.2	0.9	1.0	0.9	1.1	0.9	0.9	0.9	1.1	21.4	1	9	9	25	420	46.9	8.5
NP_001257481.1	396080285	MDK	midkine isoform b precursor	1.1	1.3	1.3	1.2	1.2	1.2	1.4	1.1	1.1	1.2	1.4	1.1	27.6	2	2	2	2	87	9.5	10.1
NP_001146.2	71773329	ANXA6	annexin A6 isoform 1	1.1	1.2	1.3	1.1	1.1	1.2	1.3	1.1	1.1	1.2	1.3	1.1	61.7	2	37	37	149	673	75.8	5.6
NP_005561.1	5031873	LMAN1	protein ERGIC-53 precursor	1.3	1.3	1.3	1.2	1.2	1.3	1.2	1.1	1.3	1.3	1.2	1.1	25.5	1	9	10	52	510	57.5	6.8
NP_859531.1	32454754	ORC5	origin recognition complex subunit 5 isoform 2	1.2	1.2	1.1	1.2	1.0	1.0	1.0	1.1	1.1	1.1	1.1	1.1	5.6	2	2	2	2	324	37.4	6.5
NP_001352.2	45006951	DHODH	dihydroorotate dehydrogenase (quinone), mitochondrial	1.1	1.1	1.1	1.1	1.0	1.1	1.1	1.2	1.1	1.1	1.1	1.1	20.8	1	6	6	10	395	42.8	9.7
NP_001267718.1	526118153	SURF4	surfeit locus protein 4 isoform 3	1.0	1.0	1.0	1.1	1.4	1.3	1.2	1.2	1.2	1.2	1.1	1.1	23.3	6	3	3	21	159	18.0	6.0
NP_003817.1	4505331	NAPG	gamma-soluble NSF attachment protein	1.0	1.1	0.9	1.2	1.1	1.0	1.1	1.1	1.1	1.1	1.0	1.1	3.2	1	1	1	2	312	34.7	5.4
NP_003741.1	4503509	EIF3A	eukaryotic translation initiation factor 3 subunit A	1.1	1.1	1.1	1.2	1.1	1.1	1.1	1.1	1.1	1.1	1.1	1.1	31.8	1	40	40	180	1382	166.5	6.8
NP_073742.2	124248539	AIDA	axin interactor, dorsalization-associated protein	1.1	1.1	1.1	1.2	0.9	1.1	1.1	1.1	1.0	1.1	1.1	1.1	23.9	1	3	3	10	306	35.0	6.6
NP_001273561.1	557636651	RBM26	RNA-binding protein 26 isoform 2	1.1	1.1	1.1	1.1	1.2	1.2	1.1	1.2	1.1	1.1	1.1	1.1	15.7	3	14	14	31	983	111.0	9.1
NP_060596.2	224589127	TMEM33	transmembrane protein 33	1.1	1.1	1.2	1.1	1.2	1.2	1.2	1.2	1.1	1.1	1.2	1.1	22.3	1	6	6	14	247	28.0	9.7
NP_000095.2	189491763	CYP1B1	cytochrome P450 1B1	1.2	1.2	1.2	1.1	1.3	1.5	1.3	1.2	1.2	1.4	1.2	1.1	31.9	1	13	13	44	543	60.8	9.0
NP_001271345.1	548923675	HACL1	2-hydroxyacyl-CoA lyase 1 isoform d	1.2	1.1	1.2	1.1	1.2	1.2	1.2	1.2	1.2	1.1	1.2	1.1	22.4	4	8	8	14	496	54.7	7.4
NP_000182.2	62198232	HMGCL	hydroxymethylglutaryl-CoA lyase, mitochondrial isoform 1 precursor	1.1	1.2	1.1	1.1	1.2	1.3	1.2	1.2	1.1	1.2	1.1	1.1	26.2	2	6	6	14	325	34.3	8.5
NP_001857.1	4502991	COX7B	cytochrome c oxidase subunit 7B, mitochondrial precursor	1.0	1.1	1.1	1.1	1.0	1.2	1.2	1.2	1.0	1.2	1.2	1.1	8.8	1	1	1	1	80	9.2	10.3
NP_054781.1	7661532	NOB1	RNA-binding protein NOB1	1.0	1.0	1.0	1.1	1.1	1.1	1.2	1.2	1.0	1.0	1.1	1.1	16.5	1	4	4	7	412	46.6	7.2
NP_000777.1	4503243	CYP51A1	lanosterol 14-alpha demethylase isoform 1 precursor	1.8	1.6	1.1	1.2	1.7	1.5	1.1	1.1	1.8	1.5	1.1	1.1	18.3	2	7	7	39	509	57.2	8.5
NP_036561.1	7110715	SEC14L2	SEC14-like protein 2 isoform 1	1.0	0.9	1.0	1.1	1.2	1.2	1.2	1.2	1.1	1.0	1.1	1.1	21.3	5	5	5	19	403	46.1	7.8
NP_569122.1	18765756	DYRK1A	dual specificity tyrosine-phosphorylation-regulated kinase 1A isoform 5	1.0	1.2	1.2	1.2	1.1	1.2	1.1	1.1	1.1	1.2	1.1	1.1	3.2	9	2	2	2	529	60.3	9.1
NP_652766.1	41281715	PQBP1	polyglutamine-binding protein 1 isoform 3	0.8	1.1	0.9	1.1	1.0	1.2	1.0	1.2	0.9	1.1	1.0	1.1	30.0	5	2	2	3	170	18.8	5.0
NP_859052.3	145580631	QSOX2	sulfhydryl oxidase 2 precursor	1.1	1.2	1.2	1.2	1.1	1.1	1.1	1.1	1.1	1.2	1.1	1.1	13.0	1	7	7	17	698	77.5	7.7
NP_037368.1	7106299	ATXN10	ataxin-10 isoform 1	1.1	1.0	1.2	1.1	1.2	1.1	1.2	1.2	1.2	1.1	1.2	1.1	27.8	3	8	9	23	475	53.5	5.2
NP_116052.2	164607167	USP30	ubiquitin carboxyl-terminal hydrolase 30	0.9	0.9	1.0	1.0	1.8	1.3	1.0	1.3	1.3	1.1	1.0	1.1	3.5	1	1	1	1	517	58.5	8.3
NP_004291.1	4757714	ACP1	low molecular weight phosphotyrosine protein phosphatase isoform c	1.2	1.1	1.0	1.1	1.1	1.3	1.1	1.2	1.1	1.2	1.0	1.1	18.4	3	2	2	5	158	18.0	6.7
NP_660208.2	157671927	SPATA5	spermatogenesis-associated protein 5	1.2	1.2	1.2	1.2	0.8	0.9	1.0	1.1	1.0	1.0	1.1	1.1	6.5	1	4	5	7	893	97.8	5.7
NP_001252541.1	388454220	CNOT1	CCR4-NOT transcription complex subunit 1 isoform c	1.1	1.1	1.1	1.2	1.1	1.1	1.1	1.1	1.1	1.1	1.1	1.1	7.6	3	16	16	34	2371	266.2	7.0
NP_006547.1	5729980	PMVK	phosphomevalonate kinase	1.1	1.0	1.0	1.1	1.1	1.0	1.1	1.2	1.1	1.0	1.0	1.1	22.9	1	5	5	9	192	22.0	5.7
NP_060440.2	39932583	NRDE2	protein NRDE2 homolog	1.0	1.2	1.1	1.2	1.1	1.1	1.2	1.1	1.1	1.1	1.1	1.1	2.9	1	3	3	4	1164	132.6	7.7
NP_001189485.1	321400140	CD44	CD44 antigen isoform 7 precursor	1.4	1.7	1.7	1.2	1.2	1.6	1.6	1.1	1.3	1.6	1.7	1.1	22.4	8	7	7	24	340	37.3	5.5
NP_002687.1	4505947	POLR2G	DNA-directed RNA polymerase II subunit RPB7	1.3	1.1	1.2	1.2	1.2	1.0	1.0	1.1	1.2	1.1	1.1	1.1	21.5	1	3	3	7	172	19.3	5.5
NP_009145.1	6005872	SEC63	translocation protein SEC63 homolog	1.2	1.1	1.1	1.1	1.1	1.2	1.1	1.1	1.2	1.2	1.1	1.1	24.6	1	14	14	33	760	87.9	5.3
NP_001273746.1	558472824	TNFAIP8	tumor necrosis factor alpha-induced protein 8 isoform d	1.0	0.8	1.2	1.1	1.1	1.2	1.1	1.2	1.0	1.0	1.1	1.1	12.1	4	1	1	3	166	19.5	7.9
NP_004714.2	15011974	ARHGEF2	rho guanine nucleotide exchange factor 2 isoform 3	1.2	1.2	1.1	1.1	1.4	1.4	1.3	1.2	1.3	1.3	1.2	1.1	21.5	3	14	14	35	958	108.2	6.9
NP_003897.2	19923191	MCM3AP	germinal-center associated nuclear protein	1.2	1.3	1.1	1.3	1.0	1.0	1.0	1.0	1.1	1.1	1.0	1.1	1.0	1	1	1	2	1980	218.3	6.4
NP_705618.1	24430149	NUP155	nuclear pore complex protein Nup155 isoform 1	1.1	1.1	1.1	1.1	1.1	1.1	1.2	1.2	1.1	1.1	1.2	1.1	18.1	3	18	18	43	1391	155.1	6.2
NP_001030324.2	241982785	UFD1L	ubiquitin fusion degradation protein 1 homolog isoform B	1.2	1.1	1.1	1.2	1.5	1.1	1.1	1.1	1.4	1.1	1.1	1.1	25.9	2	5	5	9	266	29.9	5.4
NP_056255.2	36796743	MTHFD1L	monofunctional C1-tetrahydrofolate synthase, mitochondrial isoform 2 precursor	1.0	1.0	1.0	1.2	1.0	1.0	1.0	1.1	1.0	1.0	1.0	1.1	27.5	20	20	21	64	978	105.7	8.1
NP_006627.2	94721354	MTHFD2	bifunctional methylenetetrahydrofolate dehydrogenase/cyclohydrolase, mitochondrial precursor	1.2	1.2	1.0	1.2	1.1	1.2	1.0	1.1	1.1	1.2	1.0	1.1	30.6	1	7	7	12	350	37.9	8.7
NP_006261.1	5454028	RRAS	ras-related protein R-Ras precursor	1.0	1.0	1.1	1.2	1.2	1.0	1.3	1.1	1.1	1.0	1.2	1.1	33.5	1	4	5	9	218	23.5	6.9
NP_002700.1	4506005	PPP1CB	serine/threonine-protein phosphatase PP1-beta catalytic subunit isoform 1	1.1	1.0	1.0	1.2	1.1	1.1	1.1	1.1	1.1	1.1	1.1	1.1	62.1	1	6	15	107	327	37.2	6.2
NP_567823.1	18379332	WAC	WW domain-containing adapter protein with coiled-coil isoform 3	1.2	1.1	1.1	1.1	1.0	1.1	1.1	1.2	1.1	1.1	1.1	1.1	3.9	2	1	1	1	544	59.5	9.5
NP_002699.1	4506003	PPP1CA	serine/threonine-protein phosphatase PP1-alpha catalytic subunit isoform 1	1.1	1.1	1.1	1.2	1.1	1.0	1.0	1.1	1.1	1.1	1.1	1.1	56.7	3	4	14	114	330	37.5	6.3
NP_005145.3	190684690;190684692	USP8	ubiquitin carboxyl-terminal hydrolase 8 isoform a	1.1	1.1	1.1	1.2	1.0	1.0	1.0	1.1	1.1	1.1	1.1	1.1	15.2	2	15	15	26	1118	127.4	8.5
NP_620124.1	20270303	RHOT2	mitochondrial Rho GTPase 2	1.0	1.1	1.0	1.0	1.2	1.1	1.2	1.2	1.1	1.1	1.1	1.1	22.2	1	7	8	31	618	68.1	5.9
NP_005498.1	5031635	CFL1	cofilin-1	1.1	1.1	1.2	1.1	1.0	1.1	1.1	1.2	1.1	1.1	1.2	1.1	78.9	1	12	16	183	166	18.5	8.1
NP_057028.2	21361513	AMDHD2	putative N-acetylglucosamine-6-phosphate deacetylase isoform 1	1.2	1.0	1.0	1.1	1.1	1.1	1.0	1.2	1.1	1.1	1.0	1.1	4.8	2	1	1	4	439	46.9	6.7
NP_001245308.1	385648268	SLC4A7	sodium bicarbonate cotransporter 3 isoform b	1.1	1.1	1.1	1.1	1.3	1.1	1.2	1.2	1.2	1.1	1.2	1.1	20.7	6	13	13	36	1131	127.3	6.8
NP_004987.2	134142337	ABCC1	multidrug resistance-associated protein 1	1.2	1.2	1.1	1.2	1.1	1.1	1.1	1.1	1.1	1.1	1.1	1.1	8.8	1	8	8	20	1531	171.5	7.1
NP_277050.2	39932589	ARHGAP18	rho GTPase-activating protein 18	0.9	1.4	1.6	1.2	1.0	1.2	1.3	1.1	1.0	1.3	1.5	1.1	1.2	1	1	1	2	663	74.9	6.4
NP_001005242.2	148664186	PKP2	plakophilin-2 isoform 2a	1.5	1.1	1.1	1.2	1.2	1.2	1.4	1.1	1.3	1.2	1.3	1.1	1.6	2	1	1	2	837	92.7	9.3
NP_001278893.1	634743290	MLLT4	afadin isoform 4	1.1	1.3	1.1	1.2	1.1	1.1	1.2	1.1	1.1	1.2	1.1	1.1	10.5	1	2	14	23	1781	201.7	6.5
NP_004277.2	82546879	GTPBP1	GTP-binding protein 1	1.1	1.2	1.2	1.1	1.2	1.3	1.3	1.2	1.2	1.2	1.3	1.1	22.7	1	10	10	17	669	72.4	8.3
NP_003208.2	148746209	TMBIM6	bax inhibitor 1 isoform 1	1.1	1.1	1.2	1.2	0.9	1.0	1.0	1.1	1.0	1.0	1.1	1.1	3.4	2	1	1	1	237	26.5	8.5
NP_075060.1	12597655	CENPH	centromere protein H	1.1	1.0	1.1	1.1	1.1	1.0	1.0	1.2	1.1	1.0	1.0	1.1	7.7	1	2	2	4	247	28.5	5.3
NP_054896.2	156142176	TIMM21	mitochondrial import inner membrane translocase subunit Tim21 precursor	1.2	1.0	1.1	1.0	1.4	1.2	1.2	1.3	1.3	1.1	1.2	1.1	10.9	1	3	3	6	248	28.2	9.7
NP_733839.1	25121993	MSI2	RNA-binding protein Musashi homolog 2 isoform b	0.9	0.9	0.9	1.0	0.9	1.0	0.9	1.2	0.9	0.9	0.9	1.1	33.1	2	4	7	15	251	28.4	8.5
NP_056110.2	148839305	SMCHD1	structural maintenance of chromosomes flexible hinge domain-containing protein 1	1.1	1.2	1.2	1.2	1.0	1.1	1.1	1.1	1.1	1.1	1.1	1.1	5.2	1	7	8	16	2005	226.2	7.3
NP_001258848.1	429836835	SEC11A	signal peptidase complex catalytic subunit SEC11A isoform 6	1.1	1.0	1.0	1.1	1.3	1.2	1.3	1.2	1.2	1.1	1.2	1.1	20.1	6	3	3	4	139	16.1	9.0
NP_071400.1	11545835	TSPYL2	testis-specific Y-encoded-like protein 2	1.5	1.3	1.1	1.1	1.3	1.3	1.1	1.2	1.4	1.3	1.1	1.1	6.2	1	3	3	4	693	79.4	4.6
NP_001078816.1	145386578	TOR2A	prosalusin isoform a precursor	0.9	0.9	1.0	1.2	1.1	1.1	1.2	1.1	1.0	1.0	1.1	1.1	7.2	4	2	2	4	321	35.7	7.6
NP_659419.3	87116683	ZFC3H1	zinc finger C3H1 domain-containing protein	1.1	1.1	1.0	1.1	1.2	1.1	1.1	1.2	1.2	1.1	1.0	1.1	3.1	1	4	4	5	1989	226.2	8.1
NP_057731.1	7706200	THEM6	protein THEM6 precursor	1.1	1.2	1.2	1.2	1.1	1.1	1.1	1.1	1.1	1.1	1.1	1.1	37.5	1	7	7	20	208	23.8	9.6
NP_060864.1	8923001	ABHD10	mycophenolic acid acyl-glucuronide esterase, mitochondrial isoform 1 precursor	1.4	1.2	1.2	1.1	1.6	1.4	1.3	1.2	1.5	1.3	1.3	1.1	28.8	2	8	8	16	306	33.9	8.6
NP_001275664.1	570359590	ATE1	arginyl-tRNA--protein transferase 1 isoform 4	1.0	1.2	1.1	1.1	1.0	1.1	1.1	1.1	1.0	1.1	1.1	1.1	20.6	5	5	5	9	422	47.8	5.8
NP_115675.1	14150015	DCUN1D5	DCN1-like protein 5	1.0	1.1	1.1	1.1	1.3	1.3	1.2	1.1	1.1	1.2	1.2	1.1	42.2	5	8	8	13	237	27.5	5.6
NP_055641.3	30089960	CDC42BPA	serine/threonine-protein kinase MRCK alpha isoform A	1.1	1.2	1.1	1.0	1.4	1.3	1.5	1.3	1.2	1.3	1.3	1.1	1.1	9	1	2	2	1638	186.0	6.3
NP_710162.1	24233572	ZNF367	zinc finger protein 367	1.0	1.4	1.0	1.3	1.0	1.0	1.0	1.0	1.0	1.2	1.0	1.1	8.9	1	1	1	1	350	38.4	7.9
NP_001268354.1	526479822	DCTN3	dynactin subunit 3 isoform 3	1.1	1.0	1.0	1.1	1.0	1.0	1.0	1.1	1.0	1.0	1.0	1.1	35.4	5	5	5	7	158	18.0	6.0
NP_690856.1	23312364	UHRF2	E3 ubiquitin-protein ligase UHRF2	1.0	1.0	1.0	1.0	1.3	1.2	1.5	1.3	1.1	1.1	1.3	1.1	3.9	1	2	3	5	802	89.9	8.2
NP_036513.2	32483367	ORC3	origin recognition complex subunit 3 isoform 2	1.0	1.0	1.1	1.2	1.0	1.0	1.1	1.1	1.0	1.0	1.1	1.1	4.2	3	2	3	7	711	82.2	7.6

Khan *et al.* , 2019. Multi-omics analysis to characterize cigarette smoke induced molecular alterations in esophageal cells
Supplementary Table 5. List of proteins quantified in untreated and chronically treated Het1A cells with cigarette smoke condensate for 8 months

NP_Accession	Protein group Accession	Gene ID	Description	Het-1A-Smoke - 2M/Parental	Het-1A-Smoke - 4M/Parental	Het-1A-Smoke - 6M/Parental	Het-1A-Smoke - 8M/Parental	Het-1A-Smoke - 2M/Parental	Het-1A-Smoke - 4M/Parental	Het-1A-Smoke - 6M/Parental	Het-1A-Smoke - 8M/Parental	Het-1A-Smoke - 2M/Parental	Het-1A-Smoke - 4M/Parental	Het-1A-Smoke - 6M/Parental	Het-1A-Smoke - 8M/Parental	Coverage (%)	Proteins	Unique Peptides	# Peptides	PSM	# AAs	MW [kDa]	calc. pI
				Replicate 1				Replicate 2				Average of replicates											
NP_065171.2	30794376	DOLPP1	dolichyldiphosphatase 1 isoform a	1.2	1.2	1.1	1.3	1.4	0.9	1.1	1.0	1.3	1.0	1.1	1.1	12.6	2	2	2	3	238	27.0	9.4
NP_001276897.1	584593193	PPP3CB	serine/threonine-protein phosphatase 2B catalytic subunit beta isoform isoform d	1.1	1.2	1.4	1.2	1.1	1.1	1.3	1.1	1.1	1.1	1.3	1.1	11.5	8	1	6	17	514	58.0	5.6
NP_060933.3	271397437	ITFG2	integrin-alpha FG-GAP repeat-containing protein 2	1.0	1.1	1.2	1.2	1.1	1.2	1.2	1.1	1.0	1.1	1.2	1.1	11.4	1	2	2	2	447	49.3	5.2
NP_003706.2	586798161	USO1	general vesicular transport factor p115 isoform 2	1.2	1.0	1.1	1.1	1.2	1.2	1.2	1.1	1.2	1.1	1.2	1.1	27.6	2	17	17	50	962	107.8	4.9
NP_006699.2	118402586	GLO1	lactoylglutathione lyase	1.4	1.2	1.2	1.2	1.3	1.1	1.2	1.1	1.3	1.1	1.2	1.1	51.1	1	8	8	35	184	20.8	5.3
NP_991330.1	45387955	C19orf70	protein QIL1 isoform 2 precursor	1.1	1.2	1.1	1.2	1.2	1.0	1.2	1.1	1.2	1.1	1.2	1.1	74.6	2	5	5	17	118	13.1	9.4
NP_064631.2	31881740	SLC12A9	solute carrier family 12 member 9 isoform 1	1.1	1.2	1.3	1.1	1.1	1.3	1.2	1.2	1.1	1.2	1.3	1.1	1.8	1	1	1	2	914	96.0	8.1
NP_055313.1	7657138	GOLIM4	Golgi integral membrane protein 4 isoform 1	1.2	1.4	1.4	1.3	1.0	1.1	1.0	1.0	1.1	1.3	1.2	1.1	8.1	2	5	5	12	696	81.8	4.8
NP_006428.2	112789550	PARP4	poly [ADP-ribose] polymerase 4	1.1	1.1	1.3	1.1	1.2	1.2	1.3	1.2	1.2	1.2	1.3	1.1	3.7	1	5	5	8	1724	192.5	5.7
NP_036249.1	6912292;188035908	CBX5	chromobox protein homolog 5	1.1	1.0	1.1	1.2	1.0	0.9	1.0	1.1	1.0	1.0	1.1	1.1	35.6	1	5	5	18	191	22.2	5.9
NP_443157.1	24308382	LENG8	leukocyte receptor cluster member 8	0.9	1.1	1.0	1.2	1.2	1.1	1.0	1.0	1.0	1.1	1.0	1.1	3.6	1	2	2	3	800	88.1	9.1
NP_001129109.1	208431776	PIP5K1A	phosphatidylinositol 4-phosphate 5-kinase type-1 alpha isoform 4	1.0	1.1	1.2	1.2	1.0	1.0	1.0	1.1	1.0	1.1	1.1	1.1	12.6	6	2	5	8	500	56.0	7.5
NP_079229.2	38679914	NAA25	N-alpha-acetyltransferase 25, NatB auxiliary subunit	1.1	1.1	1.1	1.1	1.1	1.2	1.2	1.2	1.1	1.1	1.1	1.1	22.0	1	15	15	35	972	112.2	6.6
NP_055318.2	120587023	UTP20	small subunit processome component 20 homolog	1.1	1.1	1.1	1.1	1.0	1.0	1.0	1.2	1.0	1.1	1.0	1.1	6.8	1	14	14	28	2785	318.2	7.4
NP_001892.1	4503123	CTGF	connective tissue growth factor precursor	1.7	1.7	1.5	1.2	1.4	1.3	1.3	1.1	1.5	1.5	1.4	1.1	6.6	1	2	2	4	349	38.0	7.9
NP_886552.2	155030240	TRNT1	CCA tRNA nucleotidyltransferase 1, mitochondrial isoform 1	1.1	1.0	1.1	1.1	1.1	1.0	1.0	1.1	1.1	1.0	1.1	1.1	13.4	2	5	5	13	434	50.1	8.1
NP_006058.1	5174615	EMC8	ER membrane protein complex subunit 8 isoform 1	1.4	1.1	1.2	1.2	1.1	0.9	1.0	1.1	1.3	1.0	1.1	1.1	12.4	2	2	2	6	210	23.8	6.4
NP_061936.2	19923497	EML4	echinoderm microtubule-associated protein-like 4 isoform a	1.0	1.0	1.0	1.1	1.0	1.1	1.0	1.2	1.0	1.1	1.0	1.1	21.8	2	16	16	52	981	108.8	6.3
NP_942136.1	38679977	ACACA	acetyl-CoA carboxylase 1 isoform 2	1.2	1.2	1.2	1.2	1.2	1.2	1.2	1.1	1.2	1.2	1.2	1.1	28.1	5	45	45	115	2346	265.4	6.4
NP_037366.1	10801345	EIF3K	eukaryotic translation initiation factor 3 subunit K isoform 1	1.2	1.0	1.1	1.2	1.0	1.1	1.1	1.1	1.1	1.1	1.1	1.1	48.2	3	8	8	52	218	25.0	4.9
NP_065111.1	23943862	NAT14	N-acetyltransferase 14	0.9	1.0	1.0	1.3	1.1	1.1	1.0	1.0	1.0	1.1	1.0	1.1	5.8	1	1	1	1	206	21.6	10.7
NP_001153774.1	237757312	SYNJ1	synaptojanin-1 isoform c	1.1	1.3	1.3	1.2	1.0	1.2	1.1	1.1	1.1	1.2	1.2	1.1	2.3	4	2	2	3	1295	143.2	7.3
NP_001258941.1	436408724;436408726	ITPRIP	inositol 1,4,5-trisphosphate receptor-interacting protein precursor	1.2	1.3	1.3	1.2	1.0	1.1	1.2	1.1	1.1	1.2	1.3	1.1	8.6	1	3	3	5	547	62.0	5.9
NP_001269098.1	532164692	VPS36	vacuolar protein-sorting-associated protein 36 isoform 3	1.1	1.1	1.1	1.1	1.1	1.0	1.1	1.1	1.1	1.1	1.1	1.1	14.9	3	4	4	9	328	36.9	7.5
NP_001001396.1	48255959	ATP2B4	plasma membrane calcium-transporting ATPase 4 isoform 4a	0.9	1.1	1.0	1.0	1.1	1.2	1.1	1.3	1.0	1.1	1.1	1.1	10.5	6	2	8	17	1170	129.3	7.5
NP_005056.3	19923669	SEL1L	protein sel-1 homolog 1 isoform 1 precursor	1.6	1.7	1.4	1.3	1.1	1.1	1.1	1.0	1.3	1.4	1.3	1.1	5.2	2	2	2	3	794	88.7	5.4
NP_060759.2	21361728	VPS53	vacuolar protein sorting-associated protein 53 homolog isoform 2	1.1	1.2	1.2	1.2	1.2	1.1	1.1	1.1	1.1	1.1	1.1	1.1	9.0	2	3	3	7	670	76.3	6.0
NP_005040.2	48762926	PWP2	periodic tryptophan protein 2 homolog	1.0	1.0	1.1	1.1	1.1	1.0	1.1	1.2	1.0	1.0	1.1	1.1	20.7	2	13	13	52	919	102.4	6.2
NP_001265164.1	506325748	LMCD1	LIM and cysteine-rich domains protein 1 isoform 4	1.7	1.5	1.1	1.3	1.0	1.0	1.0	1.0	1.3	1.3	1.0	1.1	6.1	4	1	1	1	247	27.4	8.4
NP_055385.3	214829673	ARFGAP3	ADP-ribosylation factor GTPase-activating protein 3 isoform 1	1.1	1.0	1.0	1.0	1.3	1.3	1.1	1.2	1.2	1.1	1.0	1.1	14.5	2	6	6	7	516	56.9	7.4
NP_055814.1	7661922	RAB21	ras-related protein Rab-21	1.0	1.2	1.2	1.2	1.0	1.2	1.1	1.1	1.0	1.2	1.1	1.1	19.6	1	4	4	8	225	24.3	7.9
NP_001485.2	6598323	GDI2	rab GDP dissociation inhibitor beta isoform 1	1.0	1.0	1.1	1.1	1.1	1.1	1.2	1.1	1.1	1.0	1.1	1.1	54.2	2	14	20	153	445	50.6	6.5
NP_056070.1	27597061	UBR2	E3 ubiquitin-protein ligase UBR2 isoform 1	1.3	1.2	1.1	1.1	1.1	1.2	1.2	1.2	1.2	1.2	1.1	1.1	4.1	2	4	4	6	1755	200.4	6.2
NP_004148.1	4758958	PRKAR2A	cAMP-dependent protein kinase type II-alpha regulatory subunit	1.1	1.0	1.1	1.1	1.1	1.0	1.1	1.2	1.1	1.0	1.1	1.1	41.1	1	13	14	51	404	45.5	5.1
NP_005689.2	16445419	SCAMP3	secretory carrier-associated membrane protein 3 isoform 1	1.1	1.1	1.0	1.1	1.3	1.2	1.2	1.2	1.2	1.1	1.1	1.1	32.3	2	7	7	15	347	38.3	7.6
NP_006801.1	5803121	PDIA5	protein disulfide-isomerase A5 precursor	1.2	1.2	1.1	1.2	1.2	1.1	1.1	1.1	1.2	1.1	1.1	1.1	13.9	1	5	5	14	519	59.6	7.9
NP_079107.6	151301096	TGS1	trimethylguanosine synthase	1.1	1.0	1.0	1.2	1.0	1.0	1.0	1.0	1.0	1.0	1.0	1.1	1.1	1	1	1	2	853	96.6	4.9
NP_059140.1	8393116	CHRAC1	chromatin accessibility complex protein 1	0.9	0.8	0.9	1.0	1.0	1.0	1.1	1.3	1.0	0.9	1.0	1.1	31.3	1	3	3	4	131	14.7	5.1
NP_877963.1	33598946	PLCG1	1-phosphatidylinositol 4,5-bisphosphate phosphodiesterase gamma-1 isoform b	1.1	1.0	1.0	1.1	1.0	0.9	0.9	1.1	1.0	1.0	0.9	1.1	23.5	2	20	20	68	1290	148.4	6.0
NP_037511.2	62420888	DPP7	dipeptidyl peptidase 2 preproprotein	1.1	1.0	1.0	1.1	1.3	1.0	1.1	1.2	1.2	1.0	1.0	1.1	18.1	1	6	6	8	492	54.3	6.3
NP_055268.1	7656922;38372933	CHMP2A	charged multivesicular body protein 2a	1.0	1.0	0.9	1.1	1.0	1.1	1.0	1.1	1.0	1.0	1.0	1.1	11.7	1	2	2	5	222	25.1	6.0
NP_061881.2	22129774	MIEF1	mitochondrial dynamics protein MID51	1.1	1.0	1.3	1.1	1.0	1.4	1.1	1.2	1.0	1.2	1.2	1.1	3.9	1	1	1	2	463	51.3	7.6
NP_000662.3	71565154	ADH5	alcohol dehydrogenase class-3	1.1	1.2	1.2	1.2	1.0	1.1	1.1	1.1	1.1	1.1	1.2	1.1	25.7	1	10	10	32	374	39.7	7.5
NP_115488.2	28872734	MRPL43	39S ribosomal protein L43, mitochondrial isoform a	1.1	1.0	1.0	1.1	1.1	1.2	1.1	1.2	1.1	1.1	1.0	1.1	22.0	5	3	3	10	159	17.8	9.9
NP_056240.2	103471997	POLR1A	DNA-directed RNA polymerase I subunit RPA1	1.0	1.0	1.1	1.1	1.1	1.2	1.2	1.2	1.1	1.1	1.2	1.1	11.1	1	12	12	23	1720	194.7	7.0
NP_001986.2	40807491	ACSL1	long-chain-fatty-acid--CoA ligase 1 isoform a	1.1	1.2	1.3	1.1	1.1	1.2	1.3	1.1	1.1	1.2	1.3	1.1	21.6	12	12	12	35	698	77.9	7.2
NP_055153.1	13489112	PISD	phosphatidylserine decarboxylase proenzyme	1.4	1.1	1.1	1.0	1.4	1.3	1.2	1.2	1.4	1.2	1.1	1.1	6.1	1	3	3	6	375	43.0	9.7
NP_001119594.1	186928860	SLC25A19	mitochondrial thiamine pyrophosphate carrier	1.0	1.1	1.1	1.1	1.0	1.2	1.0	1.2	1.0	1.1	1.1	1.1	2.8	1	1	1	2	320	35.5	9.6
NP_001600.1	4501859	ACADSB	short/branched chain specific acyl-CoA dehydrogenase, mitochondrial precursor	1.5	1.2	1.1	1.2	1.0	1.1	1.2	1.0	1.3	1.1	1.2	1.1	3.0	1	1	1	2	432	47.5	7.0
NP_060542.4	73695475	HEATR1	HEAT repeat-containing protein 1	1.1	1.0	1.1	1.1	1.1	1.1	1.2	1.2	1.1	1.0	1.1	1.1	17.0	1	30	30	82	2144	242.2	6.5
NP_064628.1	9910382	TOMM22	mitochondrial import receptor subunit TOM22 homolog	1.0	1.0	1.1	1.2	1.0	1.1	1.1	1.1	1.0	1.1	1.1	1.1	62.0	1	5	5	32	142	15.5	4.3
NP_056104.2	54234024	VPS39	vam6/Vps39-like protein	0.8	0.9	1.0	1.2	1.0	1.0	1.0	1.0	0.9	1.0	1.0	1.1	1.3	1	1	1	2	875	100.7	7.1
NP_525127.1	17933772	S100A16	protein S100-A16	1.1	1.0	1.4	1.2	1.1	1.1	1.3	1.1	1.1	1.0	1.4	1.1	52.4	1	4	4	31	103	11.8	6.8
NP_003901.2	32171175	BUD31	protein BUD31 homolog	1.0	1.0	1.0	1.1	1.2	1.2	1.1	1.2	1.1	1.1	1.0	1.1	39.6	1	5	5	16	144	17.0	8.8
NP_004805.2	115298668	SNRNP40	U5 small nuclear ribonucleoprotein 40 kDa protein	1.0	0.9	1.1	1.1	1.0	1.1	1.0	1.2	1.0	1.0	1.0	1.1	26.6	1	7	7	10	357	39.3	8.1
NP_002620.1	4505753	PGAM1	phosphoglycerate mutase 1	1.1	1.1	1.2	1.1	1.0	1.1	1.2	1.1	1.1	1.1	1.2	1.1	81.9	2	10	16	237	254	28.8	7.2
NP_004512.1	4758648	KIF5B	kinesin-1 heavy chain	1.1	1.0	1.1	1.1	1.1	1.0	1.1	1.1	1.1	1.0	1.1	1.1	42.0	1	28	34	113	963	109.6	6.5
NP_001162089.1	281182834	IFT46	intraflagellar transport protein 46 homolog isoform 2	0.9	1.3	1.1	1.3	0.9	1.0	0.9	1.0	0.9	1.1	1.0	1.1	6.6	2	1	1	2	304	34.3	4.5
NP_002259.1	4504901	KPNA4	importin subunit alpha-3	1.1	1.1	1.2	1.1	1.1	1.1	1.2	1.2	1.1	1.1	1.2	1.1	32.1	1	8	11	34	521	57.9	5.0
NP_009215.1	6005846	TWF2	twinfilin-2	1.2	1.2	1.2	1.1	1.2	1.2	1.2	1.1	1.2	1.2	1.2	1.1	46.1	1	10	10	31	349	39.5	6.8
NP_055638.2	296939604	WNK1	serine/threonine-protein kinase WNK1 isoform 2	1.1	1.1	1.1	1.1	1.1	1.1	1.1	1.1	1.1	1.1	1.1	1.1	8.3	5	10	10	15	2134	225.4	6.4
NP_000886.1	4505029	LTA4H	leukotriene A-4 hydrolase isoform 1	1.1	1.1	1.1	1.2	1.1	1.1	1.1	1.1	1.1	1.1	1.1	1.1	36.2	3	18	18	82	611	69.2	6.2
NP_061330.2	32171238	BAIAP2L1	brain-specific angiogenesis inhibitor 1-associated protein 2-like protein 1	1.1	0.8	1.0	1.0	1.2	1.1	1.1	1.3	1.2	0.9	1.1	1.1	9.2	1	2	2	3	511	56.8	8.7
NP_001790.1	4502743	CDK7	cyclin-dependent kinase 7	0.9	0.9	1.0	1.0	1.1	1.1	1.2	1.3	1.0	1.0	1.1	1.1	19.4	1	5	5	13	346	39.0	8.5
NP_001156758.1	253970504	EWSR1	RNA-binding protein EWS isoform 4	1.0	1.1	1.1	1.2	1.0	1.1	1.0	1.1	1.0	1.1	1.0	1.1	25.2	5	9	9	61	600	62.5	9.4
NP_005727.1	5031569	ACTR1A	alpha-centractin	1.0	1.1	1.2	1.2	1.1	1.1	1.1	1.1	1.0	1.1	1.2	1.1	39.9	3	6	13	102	376	42.6	6.6
NP_056380.2	231573214	LTN1	E3 ubiquitin-protein ligase listerin	1.1	1.2	1.1	1.1	1.1	1.1	1.2	1.1	1.1	1.1	1.1	1.1	5.7	1	8	8	14	1812	205.0	6.5
NP_002297.2	115430223	LGALS3	galectin-3 isoform 1	1.0	1.2	1.1	1.1	1.1	1.3	1.1	1.1	1.0	1.3	1.1	1.1	10.0	1	3	3	8	250	26.1	8.6
NP_056138.1	24308039	UFL1	E3 UFM1-protein ligase 1	1.0	1.1	1.0	1.1	1.1	1.1	1.0	1.1	1.1	1.1	1.0	1.1	25.2	1	17	18	39	794	89.5	6.8
NP_620149.1	20270341	LYPLAL1	lysophospholipase-like protein 1	1.2	1.2	1.2	1.2	1.1	1.1	1.0	1.1	1.1	1.1	1.1	1.1	22.8	5	4	4	7	237	26.3	7.8
NP_057490.2	156416000	UFC1	ubiquitin-fold modifier-conjugating enzyme 1	0.9	1.0	1.0	1.1	1.0	1.0	1.1	1.1	1.0	1.0	1.0	1.1	6.0	1	1	1	1	167	19.4	7.4
NP_112180.4	31742501	SNX27	sorting nexin-27	1.1	1.1	1.2	1.1	1.1	1.0	1.0	1.1	1.1	1.1	1.1	1.1	12.5	1	5	5	10	528	59.8	6.3
NP_067000.1	10863977	LSM2	U6 snRNA-associated Sm-like protein LSm2	1.2	1.0	1.1	1.2	1.0	1.0	0.9	1.1	1.1	1.0	1.0	1.1	39.0	1	3	3	6	95	10.8	6.5
NP_056312.2	31377765	TMEM87A	transmembrane protein 87A isoform 1 precursor	0.9	1.0	0.9	1.1	0.8	1.0	0.9	1.2	0.8	1.0	0.9	1.1	13.0	3	5	5	14	555	63.4	6.7
NP_149078.1	14861836	ALG2	alpha-1,3/1,6-mannosyltransferase ALG2	1.2	1.2	1.2	1.1	1.2	1.2	1.1	1.1	1.2	1.2	1.2	1.1	7.5	1	3	3	6	416	47.1	7.0
NP_002990.2	38201675	SDC4	syndecan-4 precursor	0.9	1.1	1.2	1.0	1.1	1.2	1.3	1.2	1.0	1.1	1.2	1.1	27.3	1	5	5	14	198	21.6	4.5
NP_002861.1	4506363	RAB13	ras-related protein Rab-13 isoform 1	0.8	1.0	0.9	1.2	0.8	0.9	1.0	1.1	0.8	1.0	1.0	1.1	24.6	2	2	5	12	203	22.8	9.2
NP_599031.1	19913396	SMTN	smoothelin isoform b	1.3	1.3	1.4	1.1	1.2	1.3	1.3	1.1	1.2	1.3	1.4	1.1	18.6	5	11	11	41	915	98.9	8.9
NP_001129490.1	209862837;4503583	EPHX1	epoxide hydrolase 1	1.1	1.0	1.0	1.1	1.0	1.2	1.0	1.2	1.0	1.1	1.0	1.1	11.2	1	5	5	11	455	52.9	7.3
NP_060743.2	32171254	TMEM143	transmembrane protein 143	1.1	1.3	1.4	1.1	1.1	1.4	1.1	1.1	1.1	1.4	1.3	1.1	3.3	1	1	1	2	459	51.7	9.7
NP_002638.2	34761064	PIK3C3	phosphatidylinositol 3-kinase catalytic subunit type 3 isoform 1	1.0	1.0	1.1	1.1	1.1	1.2	1.2	1.1	1.1	1.1	1.1	1.1	9.8	2	7	7	14	887	101.5	6.8
NP_057303.2	218749883	MAN1B1	endoplasmic reticulum mannosyl-oligosaccharide 1,2-alpha-mannosidase	1.1	1.2	1.1	1.2	1.1	1.1	1.2	1.1	1.1	1.2	1.1	1.1	2.4	1	1	1	4	699	79.5	7.7
NP_004648.1	4759082	SDPR	serum deprivation-response protein	0.8	0.7	1.0	1.1	0.8	0.7	1.1	1.1	0.8	0.7	1.0	1.1	17.2	1	6	6	20	425	47.1	5.2
NP_001271430.1	548962166	NUBP2	cytosolic Fe-S cluster assembly factor NUBP2 isoform 2	1.2	1.1	1.2	1.2	1.0	1.1	1.1	1.1	1.1	1.1	1.2	1.1	28.4	2	3	3	5	211	22.8	5.1

Khan *et al.* , 2019. Multi-omics analysis to characterize cigarette smoke induced molecular alterations in esophageal cells
Supplementary Table 5. List of proteins quantified in untreated and chronically treated Het1A cells with cigarette smoke condensate for 8 months

NP_Accession	Protein group Accession	Gene ID	Description	Het-1A-Smoke - 2M/Parental	Het-1A-Smoke - 4M/Parental	Het-1A-Smoke - 6M/Parental	Het-1A-Smoke - 8M/Parental	Het-1A-Smoke - 2M/Parental	Het-1A-Smoke - 4M/Parental	Het-1A-Smoke - 6M/Parental	Het-1A-Smoke - 8M/Parental	Het-1A-Smoke - 2M/Parental	Het-1A-Smoke - 4M/Parental	Het-1A-Smoke - 6M/Parental	Het-1A-Smoke - 8M/Parental	Coverage (%)	Proteins	Unique Peptides	# Peptides	PSM	# AAs	MW [kDa]	calc. pI
				Replicate 1				Replicate 2				Average of replicates											
NP_003487.1	4507691	TRRAP	transformation/transcription domain-associated protein isoform 2	1.0	1.1	1.1	1.1	1.1	1.1	1.1	1.2	1.1	1.1	1.1	1.1	1.8	2	7	7	11	3830	434.1	8.2
NP_001020774.1	71040090	RFX5	DNA-binding protein RFX5	1.1	1.4	1.2	1.2	1.1	1.0	0.9	1.0	1.1	1.2	1.0	1.1	4.6	1	2	2	2	616	65.3	9.3
NP_203744.1	31324577	MICALL1	MICAL-like protein 1	1.0	1.1	1.0	1.1	1.2	1.2	1.1	1.2	1.1	1.2	1.1	1.1	3.5	1	2	2	3	863	93.4	7.3
NP_001123291.1	193794826;6552328	CYB5R3	NADH-cytochrome b5 reductase 3 isoform 2	1.1	1.2	1.3	1.1	1.1	1.2	1.3	1.1	1.1	1.2	1.3	1.1	61.2	3	12	12	50	278	31.6	7.6
NP_001193639.1	332000017	PRKAG1	5'-AMP-activated protein kinase subunit gamma-1 isoform 4	1.0	1.1	1.0	1.1	1.1	1.0	1.0	1.1	1.0	1.0	1.0	1.1	38.1	8	8	8	31	299	34.1	8.5
NP_001138904.1	224177528	SMIM20	small integral membrane protein 20	1.2	1.2	1.3	1.2	1.5	1.4	1.4	1.1	1.3	1.3	1.3	1.1	20.9	1	1	1	6	67	7.7	9.9
NP_001739.2	157389005	CAPN2	calpain-2 catalytic subunit isoform 1	1.1	1.3	1.3	1.2	1.2	1.2	1.4	1.1	1.1	1.2	1.3	1.1	47.7	2	27	27	189	700	80.0	5.0
NP_001073888.2	331028525	UNK	RING finger protein unkempt homolog	1.2	1.1	0.9	1.0	1.0	1.1	1.2	1.3	1.1	1.1	1.0	1.1	5.6	1	3	3	5	810	88.0	6.9
NP_004652.2	118402596	CDC23	cell division cycle protein 23 homolog	1.0	1.1	1.1	1.1	1.0	1.2	1.1	1.2	1.0	1.1	1.1	1.1	9.7	1	4	4	4	597	68.8	7.0
NP_001159574.1	260656005	NDUFV1	NADH dehydrogenase [ubiquinone] flavoprotein 1, mitochondrial isoform 2 precursor	1.1	1.2	1.2	1.1	1.1	1.2	1.2	1.1	1.1	1.2	1.2	1.1	37.6	2	10	10	28	455	49.8	8.2
NP_001558.3	222136583	INPPL1	phosphatidylinositol 3,4,5-trisphosphate 5-phosphatase 2	1.2	1.1	1.0	1.1	1.1	1.1	1.1	1.1	1.1	1.1	1.1	1.1	7.7	1	6	6	10	1258	138.5	6.5
NP_001265139.1	503774357	NUP153	nuclear pore complex protein Nup153 isoform 3	1.2	0.9	1.1	1.0	1.1	1.2	1.2	1.3	1.2	1.1	1.1	1.1	7.1	3	7	7	17	1433	149.3	8.8
NP_001120690.1	187828568;379056383	POLD2	DNA polymerase delta subunit 2 isoform 1	1.0	1.2	1.1	1.2	1.0	1.0	1.0	1.1	1.0	1.1	1.1	1.1	23.9	2	6	6	11	469	51.3	5.6
NP_001265143.1	501354657	LRRC20	leucine-rich repeat-containing protein 20 isoform 4	1.1	1.1	1.1	1.2	1.1	1.0	1.1	1.0	1.1	1.0	1.1	1.1	36.5	4	2	2	2	115	12.9	5.2
NP_005424.1	4885661	YES1	tyrosine-protein kinase Yes	1.2	1.3	1.4	1.2	1.0	1.0	1.0	1.0	1.1	1.1	1.2	1.1	6.3	58	1	3	8	543	60.8	6.7
NP_066957.3	223468571	MRPL23	39S ribosomal protein L23, mitochondrial	1.1	1.1	1.1	1.1	1.1	1.1	1.3	1.2	1.1	1.1	1.2	1.1	10.5	2	2	2	5	153	17.8	9.7
NP_001277154.1	589811544	PTDSS1	phosphatidylserine synthase 1 isoform 2	1.2	1.0	1.1	1.1	1.3	1.2	1.2	1.2	1.2	1.1	1.2	1.1	14.4	2	4	4	10	327	38.2	8.7
NP_009103.2	24497451	NUP50	nuclear pore complex protein Nup50 isoform b	1.0	1.1	1.0	1.1	1.0	1.1	1.0	1.1	1.0	1.1	1.0	1.1	12.0	2	4	4	13	468	50.1	7.1
NP_001268446.1	528078329	CEP85	centrosomal protein of 85 kDa isoform 2	1.2	0.8	0.9	1.1	1.5	1.0	1.3	1.2	1.4	0.9	1.1	1.1	2.8	2	1	1	1	711	80.3	6.2
NP_057575.2	22547134	MRPL37	39S ribosomal protein L37, mitochondrial	1.1	1.1	1.2	1.2	1.1	1.1	1.1	1.0	1.1	1.1	1.2	1.1	24.1	1	7	7	21	423	48.1	8.6
NP_001243513.1	375268693	GYPC	glycophorin-C isoform 3	1.3	1.2	1.1	1.2	1.0	1.1	1.1	1.1	1.2	1.1	1.1	1.1	24.3	3	1	1	2	107	11.5	4.9
NP_001185844.1	312032471	PPFIBP1	liprin-beta-1 isoform 3	1.2	1.3	1.2	1.1	1.5	1.3	1.6	1.1	1.3	1.3	1.4	1.1	3.3	5	2	2	3	858	96.9	6.5
NP_006382.1	5453998	IPO7	importin-7	1.1	1.1	1.2	1.1	1.1	1.1	1.2	1.1	1.1	1.1	1.2	1.1	44.1	1	29	30	182	1038	119.4	4.8
NP_478061.1	54607072	YBEY	putative ribonuclease isoform 1	1.0	1.0	1.0	1.2	1.0	0.9	1.0	1.1	1.0	0.9	1.0	1.1	21.0	2	3	3	3	167	19.3	7.5
NP_001273758.1	558472750	NAPRT	nicotinate phosphoribosyltransferase isoform 2	1.0	1.1	1.1	1.1	1.1	1.1	1.0	1.1	1.0	1.1	1.0	1.1	17.0	2	6	6	11	525	56.1	5.7
NP_001244292.1	383209673	CRAT	carnitine O-acetyltransferase isoform 2	1.1	1.2	1.2	1.1	1.1	1.2	1.3	1.1	1.1	1.2	1.3	1.1	22.2	2	12	12	31	605	68.5	8.1
NP_777549.1	29789409	NDUFAF2	mimitin, mitochondrial	1.2	1.1	1.3	1.1	1.2	1.1	1.2	1.2	1.2	1.1	1.3	1.1	42.6	1	6	6	28	169	19.8	9.0
NP_001171501.1	295842330	SF1	splicing factor 1 isoform 6	1.0	1.1	1.0	1.1	0.9	1.0	1.0	1.1	1.0	1.0	1.0	1.1	22.3	6	10	10	47	673	71.7	9.6
NP_001186797.1	315467838;95113651	GLRX3	glutaredoxin-3	1.2	1.1	1.3	1.1	1.3	1.2	1.4	1.1	1.3	1.2	1.4	1.1	23.6	1	6	6	26	335	37.4	5.4
NP_697021.1	24497577;320202986	AKR1A1	alcohol dehydrogenase [NADP(+)]	1.0	1.1	0.9	1.1	1.1	1.0	0.9	1.1	1.1	1.1	0.9	1.1	43.1	1	12	13	42	325	36.5	6.8
NP_001073867.1	122937211	KIAA0368	proteasome-associated protein ECM29 homolog	1.1	1.1	1.1	1.1	1.1	1.1	1.1	1.1	1.1	1.1	1.1	1.1	16.6	1	23	23	60	2017	223.6	8.7
NP_001656.2	46249393	RHOG	rho-related GTP-binding protein RhoG precursor	1.1	1.1	1.1	1.0	1.1	1.3	1.2	1.2	1.1	1.2	1.2	1.1	33.5	3	4	5	16	191	21.3	8.1
NP_115551.2	50980309	UTP15	U3 small nucleolar RNA-associated protein 15 homolog isoform 1	1.1	1.2	1.2	1.2	1.1	1.1	1.2	1.1	1.1	1.1	1.2	1.1	18.5	3	8	8	19	518	58.4	9.1
NP_071904.4	148886661	XPO4	exportin-4	1.1	1.1	1.1	1.0	1.2	1.2	1.2	1.2	1.2	1.2	1.2	1.1	9.0	1	6	7	16	1151	130.1	5.0
NP_055101.2	17738308	NCS1	neuronal calcium sensor 1 isoform 1	1.1	1.0	1.0	1.1	1.0	1.0	1.0	1.1	1.0	1.0	1.0	1.1	12.6	2	2	2	2	190	21.9	4.8
NP_073613.2	19923592	OSBPL11	oxysterol-binding protein-related protein 11	1.0	1.1	0.9	1.1	1.2	1.0	0.9	1.2	1.1	1.0	0.9	1.1	7.0	3	4	4	18	747	83.6	7.1
NP_057396.1	7706501	WBP11	WW domain-binding protein 11	0.9	1.1	0.9	1.1	1.0	1.2	1.0	1.1	0.9	1.1	1.0	1.1	21.5	1	8	8	28	641	70.0	8.4
NP_001166159.1	289577053	GGA1	ADP-ribosylation factor-binding protein GGA1 isoform 5	1.0	1.0	0.9	1.1	1.3	1.2	1.0	1.1	1.1	1.1	1.0	1.1	11.8	4	3	3	7	566	62.1	5.7
NP_001171313.1	295424842	SRPR	signal recognition particle receptor subunit alpha isoform 2	1.1	1.1	1.0	1.1	1.2	1.2	1.1	1.2	1.2	1.2	1.1	1.1	26.7	2	13	13	29	610	66.5	9.0
NP_001135.3	21071001	AMFR	E3 ubiquitin-protein ligase AMFR	1.1	1.1	1.0	1.1	1.1	1.2	1.1	1.1	1.1	1.1	1.1	1.1	3.6	1	1	1	4	643	72.9	6.4
NP_001536.1	4557657	ICT1	peptidyl-tRNA hydrolase ICT1, mitochondrial isoform 1 precursor	1.1	1.1	1.1	1.2	1.1	1.0	1.0	1.1	1.1	1.1	1.0	1.1	27.2	2	5	5	9	206	23.6	10.1
NP_001275912.1	572880605	SDAD1	protein SDA1 homolog isoform 2	0.8	1.0	1.0	1.1	0.9	1.0	1.0	1.1	0.9	1.0	1.0	1.1	4.6	3	3	3	4	650	75.4	9.2
NP_852478.1	31657142	ITGA1	integrin alpha-1 precursor	1.0	1.0	1.0	1.3	0.9	0.9	0.9	1.0	0.9	1.0	1.0	1.1	0.5	1	1	1	1	1179	130.8	6.3
NP_055529.2	41281447	IFT140	intraflagellar transport protein 140 homolog	1.1	1.2	1.1	1.1	1.0	1.1	1.1	1.1	1.1	1.1	1.1	1.1	0.8	1	1	1	2	1462	165.1	6.0
NP_002568.2	32483399	PAK2	serine/threonine-protein kinase PAK 2	0.9	1.0	1.0	1.1	1.0	1.0	1.0	1.1	1.0	1.0	1.0	1.1	31.1	7	11	11	22	524	58.0	6.0
NP_005994.2	163644321	UQCRFS1	cytochrome b-c1 complex subunit Rieske, mitochondrial	1.2	1.3	1.3	1.2	1.1	1.1	1.2	1.1	1.1	1.2	1.3	1.1	30.7	1	8	8	81	274	29.6	8.3
NP_001138934.1	224451038	YIF1B	protein YIF1B isoform 2	1.1	1.0	1.1	1.2	1.0	1.0	1.0	1.1	1.0	1.0	1.0	1.1	8.8	6	2	2	5	283	31.2	7.4
NP_064621.3	143770802	MRPL1	39S ribosomal protein L1, mitochondrial precursor	1.1	1.3	1.2	1.2	1.1	1.2	1.1	1.1	1.1	1.2	1.2	1.1	33.9	1	9	9	24	325	36.9	8.8
NP_005491.1	4885585	SAE1	SUMO-activating enzyme subunit 1 isoform a	1.1	0.9	1.0	1.1	1.1	1.0	1.0	1.1	1.1	0.9	1.0	1.1	62.7	3	15	15	76	346	38.4	5.3
NP_055205.2	77404397	SND1	staphylococcal nuclease domain-containing protein 1	1.1	1.1	1.2	1.1	1.1	1.1	1.1	1.1	1.1	1.1	1.2	1.1	44.5	1	33	33	145	910	101.9	7.2
NP_004913.2	38373669	SEC24C	protein transport protein Sec24C	1.2	1.2	1.2	1.1	1.2	1.2	1.2	1.2	1.2	1.2	1.2	1.1	23.4	1	15	15	46	1094	118.2	7.1
NP_001758.2	156071472	CD2	T-cell surface antigen CD2 precursor	0.9	1.2	1.0	1.0	1.2	1.0	1.0	1.2	1.0	1.1	1.0	1.1	8.3	1	1	1	1	351	39.4	9.6
NP_808227.1	29570791;4503095	CSNK2A1	casein kinase II subunit alpha isoform a	1.1	1.1	1.2	1.1	1.1	1.1	1.2	1.2	1.1	1.1	1.2	1.1	40.2	3	12	12	53	391	45.1	7.7
NP_009207.2	15082258	CBX3	chromobox protein homolog 3	1.1	1.2	1.7	1.1	1.3	1.0	1.7	1.1	1.2	1.1	1.7	1.1	44.8	1	5	6	20	183	20.8	5.3
NP_068800.2	133922562	PGM5	phosphoglucomutase-like protein 5	1.1	1.1	1.0	1.1	1.2	1.3	1.3	1.1	1.2	1.2	1.2	1.1	1.6	1	1	1	1	567	62.2	7.2
NP_000636.2	116734849	AGL	glycogen debranching enzyme isoform 2	1.0	1.0	1.0	1.0	1.1	1.1	1.1	1.2	1.0	1.1	1.1	1.1	3.9	3	4	4	5	1515	172.5	6.7
NP_067052.2	93204871	NTN4	netrin-4 precursor	1.5	1.4	1.4	1.1	1.5	1.5	1.9	1.2	1.5	1.5	1.6	1.1	2.4	1	1	1	1	628	70.0	8.0
NP_006458.2	115529475	POLR3G	DNA-directed RNA polymerase III subunit RPC7	1.1	1.0	1.1	1.1	1.0	1.2	1.2	1.1	1.1	1.1	1.2	1.1	10.8	1	2	2	3	223	25.9	4.6
NP_000364.1	4507835	UMPS	uridine 5'-monophosphate synthase	1.1	1.1	1.0	1.1	1.1	1.2	1.1	1.1	1.1	1.2	1.1	1.1	22.9	1	9	9	24	480	52.2	7.2
NP_003749.2	83281438	EIF3J	eukaryotic translation initiation factor 3 subunit J isoform 1	1.0	1.2	1.1	1.1	1.0	1.1	1.1	1.1	1.0	1.1	1.1	1.1	39.5	3	10	10	40	258	29.0	4.8
NP_008835.5	13654237	PRKDC	DNA-dependent protein kinase catalytic subunit isoform 1	1.1	1.1	1.2	1.1	1.1	1.1	1.2	1.1	1.1	1.1	1.2	1.1	47.0	2	159	160	904	4128	468.8	7.1
NP_001171820.1	296434309	FLAD1	FAD synthase isoform 3	1.0	0.9	1.0	1.1	1.1	1.2	1.1	1.1	1.1	1.1	1.0	1.1	12.3	4	4	4	15	446	49.2	6.0
NP_001054.1	4557871	TF	serotransferrin precursor	1.4	1.9	1.7	1.1	1.3	1.6	1.5	1.1	1.3	1.8	1.6	1.1	14.6	1	8	8	15	698	77.0	7.1
NP_001157666.1	255958306	PLIN3	perilipin-3 isoform 3	1.1	0.9	1.1	1.1	1.1	1.0	1.1	1.1	1.1	0.9	1.1	1.1	59.0	3	16	16	62	422	45.8	5.4
NP_060918.2	21361794	CAND1	cullin-associated NEDD8-dissociated protein 1	1.1	1.1	1.2	1.1	1.1	1.1	1.2	1.1	1.1	1.1	1.2	1.1	37.4	3	37	37	150	1230	136.3	5.8
NP_001974.1	4503599	ERCC1	DNA excision repair protein ERCC-1 isoform 2	1.1	1.1	1.0	1.1	1.3	1.1	1.1	1.1	1.2	1.1	1.0	1.1	15.2	3	3	3	7	297	32.5	6.3
NP_077808.1	13435375	GMEB1	glucocorticoid modulatory element-binding protein 1 isoform 2	1.1	1.1	1.0	1.1	1.1	1.3	1.0	1.2	1.1	1.2	1.0	1.1	11.4	3	4	5	7	563	61.3	4.8
NP_001001132.1	47717125	ITSN1	intersectin-1 isoform ITSN-s	1.4	1.3	1.1	1.1	1.0	1.0	1.0	1.1	1.2	1.1	1.1	1.1	1.3	2	1	1	2	1220	137.6	7.3
NP_001271220.1	546230945	CBLL1	E3 ubiquitin-protein ligase Hakai isoform 2	1.1	1.2	1.1	1.1	1.0	1.2	1.1	1.1	1.0	1.2	1.1	1.1	2.9	2	1	1	2	490	54.4	8.4
NP_001490.1	4557627	GLE1	nucleoporin GLE1 isoform 2	0.9	1.2	1.1	1.1	1.1	1.1	1.1	1.1	1.0	1.1	1.1	1.1	1.1	2	1	1	1	659	75.4	7.0
NP_001230405.1	343478281	STARD13	stAR-related lipid transfer protein 13 isoform 4	1.6	1.0	0.9	1.2	1.0	1.0	1.1	1.0	1.3	1.0	1.0	1.1	2.0	3	1	2	2	1078	120.6	6.9
NP_110416.1	23308607	HM13	minor histocompatibility antigen H13 isoform 1	1.1	1.1	1.0	1.1	1.2	1.2	1.1	1.1	1.2	1.1	1.1	1.1	17.0	4	6	6	64	377	41.5	6.4
NP_002124.1	4504437	HMOX1	heme oxygenase 1	1.6	1.4	1.0	1.1	1.5	1.5	1.1	1.1	1.6	1.4	1.1	1.1	13.9	1	3	3	10	288	32.8	8.3
NP_005467.1	4885285	GNE	bifunctional UDP-N-acetylglucosamine 2-epimerase/N-acetylmannosamine kinase isoform 2	1.2	1.2	1.2	1.1	1.1	1.1	1.1	1.1	1.1	1.2	1.1	1.1	12.2	5	6	6	11	722	79.2	6.8
NP_001006600.1	55770895	ERBB2IP	protein LAP2 isoform 7	1.1	1.2	1.3	1.1	1.1	1.2	1.3	1.1	1.1	1.2	1.3	1.1	12.8	6	10	11	25	1302	146.0	5.3
NP_940978.2	54873613	AGRN	agrin isoform 2 precursor	1.3	1.4	1.4	1.2	1.4	1.3	1.2	1.1	1.4	1.3	1.3	1.1	3.5	2	5	5	11	2045	214.7	6.4
NP_003581.1	4503165	CUL3	cullin-3 isoform 1	1.1	1.1	1.1	1.1	1.1	1.1	1.1	1.1	1.1	1.1	1.1	1.1	11.1	3	7	8	27	768	88.9	8.5
NP_057126.2	50511943	EXOSC3	exosome complex component RRP40 isoform 1	1.0	1.0	1.0	1.0	1.1	1.1	1.2	1.2	1.1	1.0	1.1	1.1	38.9	2	6	6	19	275	29.6	8.1
NP_068817.1	116174780	SEC24A	protein transport protein Sec24A isoform 1	1.1	1.1	1.0	1.1	1.0	1.1	1.0	1.1	1.1	1.1	1.0	1.1	9.9	2	6	6	17	1093	119.7	7.7
NP_689753.2	31542785	SCFD2	sec1 family domain-containing protein 2	1.0	1.2	1.1	1.1	1.1	1.1	1.1	1.1	1.1	1.2	1.1	1.1	1.5	1	1	1	2	684	75.1	6.7
NP_060297.1	8923421	SARS2	serine--tRNA ligase, mitochondrial isoform b precursor	1.1	1.1	1.0	1.1	1.1	1.1	1.1	1.2	1.1	1.1	1.0	1.1	26.1	2	10	10	16	518	58.2	8.1
NP_079390.3	109255234	CEP290	centrosomal protein of 290 kDa	1.0	1.1	1.0	1.0	1.1	1.2	1.2	1.2	1.1	1.2	1.1	1.1	0.9	2	2	2	2	2479	290.2	5.9
NP_001093900.1	155030202	RAP1GDS1	rap1 GTPase-GDP dissociation stimulator 1 isoform 6	1.1	1.1	1.1	1.2	0.9	1.0	1.0	1.1	1.0	1.1	1.1	1.1	17.4	6	6	7	23	516	56.5	5.4
NP_758455.1	27436908	MRPL54	39S ribosomal protein L54, mitochondrial	1.0	1.2	1.2	1.1	1.1	1.1	1.0	1.1	1.1	1.1	1.1	1.1	18.8	1	1	1	3	138	15.8	9.6
NP_055227.1	7656952	CACYBP	calcyclin-binding protein isoform 1	1.0	1.1	1.1	1.1	1.0	1.0	1.0	1.1	1.0	1.1	1.1	1.1	48.3	3	10	10	57	228	26.2	8.3
NP_057213.2	38373690	COPS4	COP9 signalosome complex subunit 4 isoform 1	1.2	1.1	1.2	1.1	1.2	1.2	1.2	1.1	1.2	1.2	1.2	1.1	23.4	2	7	7	14	406	46.2	5.8
NP_031398.2	45446743;45446747	DDX42	ATP-dependent RNA helicase DDX42	1.1	1.1	1.1	1.2	1.0	1.1	1.0	1.1	1.0	1.1	1.1	1.1	22.2	1	15	15	54	938	102.9	7.0
NP_006538.2	30795212	IGF2BP3	insulin-like growth factor 2 mRNA-binding protein 3	1.0	1.1	1.1	1.1	1.1	1.2	1.2	1.2	1.1	1.1	1.1	1.1	29.7	3	12	13	33	579	63.7	8.9
NP_113608.1	13899231	MRPL9	39S ribosomal protein L9, mitochondrial isoform 1	1.2	1.1	1.1	1.1	1.1	1.0	1.0	1.1	1.1	1.1	1.1	1.1	13.5	2	3	3	12	267	30.2	10.1
NP_004031.1	4757764	RHOB	rho-related GTP-binding protein RhoB precursor	1.0	1.1	1.1	1.0	1.0	1.1	1.4	1.2	1.0	1.1	1.3	1.1	16.8	1	1	2	6	196	22.1	5.2
NP_001257339.1	394582075	LIN9	protein lin-9 homolog isoform 3	0.9	0.9	0.9	1.0	1.2	1.4	1.3	1.3	1.0	1.2	1.1	1.1	4.9	3	1	1	1	490	56.2	9.2

Supplementary Table 5. List of proteins quantified in untreated and chronically treated Het1A cells with cigarette smoke condensate for 8 months

NP_Accession	Protein group Accession	Gene ID	Description	Het-1A-Smoke - 2M/Parental	Het-1A-Smoke - 4M/Parental	Het-1A-Smoke - 6M/Parental	Het-1A-Smoke - 8M/Parental	Het-1A-Smoke - 2M/Parental	Het-1A-Smoke - 4M/Parental	Het-1A-Smoke - 6M/Parental	Het-1A-Smoke - 8M/Parental	Het-1A-Smoke - 2M/Parental	Het-1A-Smoke - 4M/Parental	Het-1A-Smoke - 6M/Parental	Het-1A-Smoke - 8M/Parental	Coverage (%)	Proteins	Unique Peptides	# Peptides	PSM	# AAs	MW [kDa]	calc. pI
				Replicate 1				Replicate 2				Average of replicates											
NP_060238.3	21361633	LRRC40	leucine-rich repeat-containing protein 40	1.1	1.0	1.0	1.1	1.1	1.1	1.1	1.1	1.1	1.0	1.0	1.1	34.6	1	14	14	23	602	68.2	6.4
NP_055449.1	7661862	PPM1F	protein phosphatase 1F	1.4	1.1	1.1	1.1	1.1	1.1	1.1	1.1	1.2	1.1	1.1	1.1	12.1	1	3	3	15	454	49.8	5.1
NP_001273578.1	557786221	STK24	serine/threonine-protein kinase 24 isoform c precursor	1.0	1.0	1.0	1.0	1.1	1.1	1.1	1.2	1.0	1.0	1.1	1.1	26.0	3	8	10	24	412	45.8	5.6
NP_001265574.1	520975339	NDUFB9	NADH dehydrogenase [ubiquinone] 1 beta subcomplex subunit 9 isoform 2	1.1	1.0	1.2	1.1	1.1	1.1	1.0	1.1	1.1	1.1	1.1	1.1	27.6	3	2	2	8	123	14.9	5.6
NP_079239.3	157426875	FBXL18	F-box/LRR-repeat protein 18	1.0	1.1	1.0	1.1	1.0	1.1	1.0	1.2	1.0	1.1	1.0	1.1	5.9	1	3	3	5	718	78.9	8.3
NP_006420.1	5453607	CCT7	T-complex protein 1 subunit eta isoform a	1.0	1.0	1.0	1.1	1.0	1.0	1.0	1.1	1.0	1.0	1.0	1.1	68.9	4	30	30	241	543	59.3	7.6
NP_056199.2	47578107	NIPBL	nipped-B-like protein isoform B	1.2	1.1	1.2	1.1	1.1	1.1	1.1	1.1	1.1	1.1	1.1	1.1	3.9	2	8	8	16	2697	304.2	7.8
NP_057346.1	7705915	TUBE1	tubulin epsilon chain	1.0	1.0	1.0	1.0	1.1	1.1	1.2	1.2	1.0	1.1	1.1	1.1	4.2	1	1	1	1	475	52.9	6.6
NP_659547.2	94721324	NSMCE1	non-structural maintenance of chromosomes element 1 homolog	1.1	1.0	1.0	1.1	1.0	1.1	1.0	1.1	1.0	1.0	1.0	1.1	3.0	1	1	1	2	266	30.8	7.5
NP_078994.3	186700623	RABL6	rab-like protein 6 isoform 1	1.0	1.0	0.9	1.1	0.9	0.9	0.9	1.1	1.0	0.9	0.9	1.1	11.1	3	5	5	14	729	79.5	5.2
NP_001077083.1	134288884	EARS2	probable glutamate--tRNA ligase, mitochondrial isoform 1	1.1	1.1	1.1	1.1	1.0	1.1	1.1	1.1	1.1	1.1	1.1	1.1	23.3	2	10	10	27	523	58.7	8.8
NP_065142.2	29826287	MRPL47	39S ribosomal protein L47, mitochondrial isoform a	1.1	1.2	1.1	1.1	1.1	1.1	1.2	1.1	1.1	1.2	1.2	1.1	18.4	2	5	5	12	250	29.4	10.4
NP_036218.1	6912398	GTF3C3	general transcription factor 3C polypeptide 3 isoform 1	1.1	1.0	1.0	1.2	0.9	0.9	1.0	1.1	1.0	0.9	1.0	1.1	17.8	2	10	10	33	886	101.2	5.1
NP_001247403.1	386781221	WDR4	tRNA (guanine-N(7)-)-methyltransferase non-catalytic subunit WDR4 isoform 2	1.1	0.9	0.9	1.0	1.1	1.2	1.2	1.2	1.1	1.1	1.1	1.1	7.1	2	2	2	2	411	45.3	6.9
NP_001034437.1	86788015;86788132	EFEMP1	EGF-containing fibulin-like extracellular matrix protein 1 precursor	1.4	1.4	1.2	1.1	1.4	1.4	1.2	1.1	1.4	1.4	1.2	1.1	14.6	1	6	6	14	493	54.6	5.1
NP_877496.1	33469947	ELMO2	engulfment and cell motility protein 2	1.0	1.0	1.1	1.1	1.0	1.1	1.1	1.1	1.0	1.0	1.1	1.1	20.0	3	11	11	25	720	82.6	5.9
NP_001679.2	21361565	ATP5F1	ATP synthase F(0) complex subunit B1, mitochondrial precursor	1.1	1.1	1.1	1.1	1.1	1.0	1.2	1.1	1.1	1.1	1.1	1.1	37.9	1	9	9	39	256	28.9	9.4
NP_001531.1	4504517	HSPB1	heat shock protein beta-1	0.8	1.0	1.0	1.1	0.8	1.1	1.2	1.2	0.8	1.0	1.1	1.1	36.1	1	5	5	16	205	22.8	6.4
NP_001658.2	148612885	ARL2	ADP-ribosylation factor-like protein 2 isoform 1	1.1	1.0	1.1	1.1	1.1	1.0	1.1	1.1	1.1	1.0	1.1	1.1	40.8	2	6	6	15	184	20.9	6.3
NP_005076.3	33946327	NUP214	nuclear pore complex protein Nup214	1.1	1.0	1.1	1.1	1.1	1.1	1.2	1.1	1.1	1.1	1.1	1.1	11.7	1	13	13	29	2090	213.5	7.5
NP_056655.2	70166944	ADAR	double-stranded RNA-specific adenosine deaminase isoform b	1.1	1.1	1.1	1.1	1.1	1.0	1.0	1.1	1.1	1.0	1.0	1.1	15.8	4	14	14	35	1200	133.1	8.5
NP_899152.1	34452730	PAIP1	polyadenylate-binding protein-interacting protein 1 isoform 3	1.1	1.0	1.1	1.1	1.1	1.1	1.1	1.1	1.1	1.1	1.1	1.1	17.2	3	4	4	6	367	42.0	4.5
NP_510868.1	17402871	BCCIP	BRCA2 and CDKN1A-interacting protein isoform BCCIPbeta	1.0	1.1	1.1	1.1	1.1	1.0	1.1	1.1	1.0	1.0	1.1	1.1	56.1	3	11	11	51	314	36.0	4.6
NP_002931.2	108773782;108773784	ABCE1	ATP-binding cassette sub-family E member 1	1.0	1.0	1.0	1.1	1.1	1.1	1.2	1.1	1.0	1.1	1.1	1.1	40.4	1	17	17	57	599	67.3	8.3
NP_659486.2	193083197	UBLCP1	ubiquitin-like domain-containing CTD phosphatase 1	0.9	1.0	0.9	1.1	0.9	0.9	0.8	1.1	0.9	1.0	0.8	1.1	33.0	1	7	7	18	318	36.8	6.5
NP_112233.2	31621303	SFXN3	sideroflexin-3	1.0	1.1	1.4	1.0	1.2	1.3	1.6	1.2	1.1	1.2	1.5	1.1	18.8	1	3	4	6	325	36.0	9.1
NP_003082.1	4507125	SNRPB	small nuclear ribonucleoprotein-associated proteins B and B' isoform B	1.1	1.0	1.1	1.2	1.0	0.9	1.0	1.1	1.0	1.0	1.0	1.1	16.5	3	4	4	31	231	23.6	10.9
NP_076988.1	13129078	ASPSCR1	tether containing UBX domain for GLUT4 isoform 1	1.0	1.0	1.0	1.1	1.1	1.1	1.2	1.1	1.1	1.0	1.1	1.1	15.0	2	5	5	13	553	60.1	6.6
NP_064543.3	47174864	EXOSC5	exosome complex component RRP46	1.0	1.0	1.0	1.1	1.1	1.3	1.1	1.1	1.0	1.2	1.0	1.1	5.1	1	1	1	4	235	25.2	7.6
NP_005780.2	30410794	PSME3	proteasome activator complex subunit 3 isoform 1	1.3	1.1	1.1	1.2	1.1	0.9	1.1	1.1	1.2	1.0	1.1	1.1	45.7	3	8	8	30	254	29.5	5.9
NP_004304.1	4757780	ARRB2	beta-arrestin-2 isoform 1	0.9	1.0	1.0	1.0	0.9	1.2	1.1	1.2	0.9	1.1	1.0	1.1	16.6	6	5	5	9	409	46.1	7.7
NP_001273007.1	554506522	UBXN1	UBX domain-containing protein 1 isoform 3	0.9	0.9	0.9	1.1	0.8	0.8	0.9	1.2	0.9	0.9	0.9	1.1	21.9	3	3	3	8	238	27.0	8.0
NP_775899.3	256542310	DNAH17	dynein heavy chain 17, axonemal	1.1	1.0	1.0	1.1	1.1	1.4	1.4	1.1	1.1	1.2	1.2	1.1	0.3	2	1	2	2	4462	509.0	5.7
NP_001271219.1	546230986	GUSB	beta-glucuronidase isoform 2 precursor	1.0	0.9	0.8	1.1	0.8	1.0	0.8	1.1	0.9	0.9	0.8	1.1	7.7	4	4	4	9	505	58.3	7.0
NP_001258980.1	440309863	C16orf91	protein CCSMST1 precursor	1.0	1.1	1.1	1.1	1.0	1.0	1.1	1.1	1.0	1.1	1.1	1.1	15.9	1	1	1	2	132	15.0	7.0
NP_002037.2	7669492	GAPDH	glyceraldehyde-3-phosphate dehydrogenase isoform 1	1.0	1.1	1.2	1.1	1.0	1.1	1.2	1.1	1.0	1.1	1.2	1.1	77.0	3	18	18	783	335	36.0	8.5
NP_059998.2	23199995	WBSCR22	probable 18S rRNA (guanine-N(7))-methyltransferase isoform 2	1.0	1.1	1.1	1.1	0.9	1.1	1.0	1.1	1.0	1.1	1.1	1.1	12.5	2	3	3	6	281	31.9	8.7
NP_060919.3	542133148	UBAP2	ubiquitin-associated protein 2 isoform 1	1.0	0.9	0.9	0.9	0.9	1.2	1.0	1.3	0.9	1.0	0.9	1.1	5.5	3	4	4	9	1119	117.1	7.3
NP_005566.2	61742777	LNPEP	leucyl-cystinyl aminopeptidase isoform 1	1.1	1.2	1.1	1.1	1.0	1.1	1.1	1.1	1.1	1.2	1.1	1.1	13.0	2	8	8	16	1025	117.3	5.7
NP_004388.2	164664518;380692342	DDX6	probable ATP-dependent RNA helicase DDX6	1.1	1.1	1.0	1.1	1.1	1.1	1.1	1.1	1.1	1.1	1.1	1.1	39.1	2	13	13	44	483	54.4	8.7
NP_001099015.1	157694522	ECHDC1	ethylmalonyl-CoA decarboxylase isoform 4	1.0	0.9	0.9	1.0	1.1	1.2	1.1	1.2	1.0	1.1	1.0	1.1	18.6	5	1	1	2	70	7.7	9.0
NP_476433.1	17149844	FKBP2	peptidyl-prolyl cis-trans isomerase FKBP2 precursor	1.1	1.1	1.2	1.1	1.2	1.2	1.4	1.1	1.2	1.1	1.3	1.1	8.5	1	1	1	1	142	15.6	9.1
NP_005044.1	4826964	RAD23A	UV excision repair protein RAD23 homolog A isoform 1	1.2	1.3	1.4	1.4	1.0	0.8	1.0	0.8	1.1	1.1	1.2	1.1	60.9	2	1	11	52	363	39.6	4.6
NP_000405.1	4504505	HSD17B4	peroxisomal multifunctional enzyme type 2 isoform 2	1.1	1.1	1.0	1.1	1.1	1.1	1.0	1.1	1.1	1.1	1.0	1.1	45.9	5	24	24	92	736	79.6	8.8
NP_056449.1	24308113	KIAA1279	KIF1-binding protein	1.2	1.1	1.1	1.1	1.1	1.1	1.1	1.1	1.1	1.1	1.1	1.1	17.9	1	6	6	12	621	71.8	5.5
NP_076962.2	34222121	NUP37	nucleoporin Nup37	1.1	1.1	1.1	1.1	1.0	1.0	1.1	1.1	1.1	1.0	1.1	1.1	21.2	1	5	5	14	326	36.7	5.9
NP_919261.2	51702222	SPTY2D1	protein SPT2 homolog	1.1	1.1	1.2	1.1	1.1	1.0	1.1	1.1	1.1	1.1	1.1	1.1	3.1	1	2	2	2	685	75.6	9.8
NP_054786.2	13569840	MACROD1	O-acetyl-ADP-ribose deacetylase MACROD1	1.1	1.1	1.1	1.1	1.2	1.2	1.2	1.2	1.1	1.2	1.1	1.1	30.8	1	8	8	17	325	35.5	9.5
NP_000628.2	50301238	GSR	glutathione reductase, mitochondrial isoform 1 precursor	1.1	1.1	1.1	1.1	1.1	1.2	1.1	1.1	1.1	1.2	1.1	1.1	38.5	4	11	11	25	522	56.2	8.5
NP_001128662.1	206597515	ARAP1	arf-GAP with Rho-GAP domain, ANK repeat and PH domain-containing protein 1 isoform d	1.2	1.0	1.4	1.0	1.2	1.2	1.3	1.3	1.2	1.1	1.3	1.1	1.2	3	1	1	1	1133	127.6	6.1
NP_060176.2	38327642	WDR55	WD repeat-containing protein 55	1.2	1.2	1.1	1.2	1.1	1.1	1.1	1.0	1.2	1.1	1.1	1.1	26.6	1	5	5	11	383	42.1	4.9
NP_001275510.1	568384725	BROX	BRO1 domain-containing protein BROX isoform c	0.9	1.0	1.1	1.1	1.0	1.1	1.2	1.2	1.0	1.0	1.1	1.1	1.8	3	1	1	1	338	38.2	8.6
NP_005008.2	31543385	PCYT1A	choline-phosphate cytidylyltransferase A	1.2	1.2	1.2	1.1	1.1	1.2	1.1	1.1	1.2	1.2	1.2	1.1	13.1	4	4	4	14	367	41.7	7.3
NP_060789.2	20127586	TDP1	tyrosyl-DNA phosphodiesterase 1	1.0	1.0	1.1	1.1	1.0	1.0	1.0	1.1	1.0	1.0	1.0	1.1	3.5	1	1	1	2	608	68.4	7.6
NP_114131.1	13994300	TMEM120A	transmembrane protein 120A	1.1	1.1	1.2	1.0	1.2	1.2	1.3	1.2	1.1	1.1	1.2	1.1	5.0	1	1	1	1	343	40.6	9.0
NP_003085.1	4507129	SNRPE	small nuclear ribonucleoprotein E isoform 1	1.2	1.1	1.2	1.2	1.0	1.1	1.0	1.1	1.1	1.1	1.1	1.1	39.1	2	2	2	28	92	10.8	9.4
NP_998754.1	47132519	RAPH1	ras-associated and pleckstrin homology domains-containing protein 1 isoform 1	1.1	1.1	1.1	1.0	1.2	1.1	1.3	1.2	1.1	1.1	1.2	1.1	3.4	1	1	2	2	1250	135.2	8.9
NP_115500.1	14149777	HDHD2	haloacid dehalogenase-like hydrolase domain-containing protein 2	1.1	1.1	1.1	1.1	1.2	1.3	1.2	1.1	1.2	1.2	1.2	1.1	35.9	1	5	5	13	259	28.5	6.2
NP_057156.1	7705636	GOLT1B	vesicle transport protein GOT1B	1.4	1.2	1.1	1.2	1.3	1.1	1.1	1.1	1.3	1.2	1.1	1.1	10.9	1	1	1	3	138	15.4	10.4
NP_001688.1	4502303	ATP5O	ATP synthase subunit O, mitochondrial precursor	1.1	1.1	1.1	1.1	1.0	1.0	1.1	1.1	1.1	1.0	1.1	1.1	44.1	1	9	9	32	213	23.3	10.0
NP_001273588.1	557786174	TBC1D4	TBC1 domain family member 4 isoform 3	1.1	1.1	1.1	1.0	1.2	1.3	1.6	1.2	1.1	1.2	1.3	1.1	1.1	3	1	1	1	1235	139.5	6.9
NP_075066.1	12597661	MRPL44	39S ribosomal protein L44, mitochondrial	1.1	1.1	1.2	1.1	1.1	1.1	1.1	1.2	1.1	1.1	1.2	1.1	33.1	1	8	8	26	332	37.5	8.4
NP_003282.2	186972143	TPP2	tripeptidyl-peptidase 2	1.1	1.2	1.2	1.1	1.0	1.1	1.1	1.1	1.1	1.1	1.2	1.1	20.9	1	21	21	49	1249	138.3	6.3
NP_004541.1	4758786	NDUFS2	NADH dehydrogenase [ubiquinone] iron-sulfur protein 2, mitochondrial isoform 1 precursor	1.1	1.1	1.1	1.1	1.1	1.0	1.1	1.1	1.1	1.0	1.1	1.1	32.2	2	9	9	31	463	52.5	7.5
NP_788276.1	28872792	CDK5RAP3	CDK5 regulatory subunit-associated protein 3 isoform b	1.0	1.0	0.9	1.1	1.1	1.1	1.0	1.1	1.1	1.1	1.0	1.1	28.5	7	12	12	31	506	56.9	4.8
NP_612632.1	20357556	CTTN	src substrate cortactin isoform b	1.3	1.3	1.7	1.2	1.2	1.2	1.3	1.0	1.2	1.2	1.5	1.1	8.4	3	4	4	10	513	57.4	5.3
NP_004918.1	4826649	MRPL49	39S ribosomal protein L49, mitochondrial	1.2	1.3	1.4	1.3	0.9	1.0	1.0	0.9	1.1	1.1	1.2	1.1	15.7	1	2	2	5	166	19.2	9.5
NP_714963.1	24430135	DPM3	dolichol-phosphate mannosyltransferase subunit 3 isoform 2	1.2	1.2	1.0	1.1	1.3	1.1	1.1	1.1	1.2	1.1	1.1	1.1	10.9	2	1	1	6	92	10.1	5.9
NP_001293020.1	807045926	INTS6	integrator complex subunit 6 isoform 4	1.4	1.2	1.1	1.2	1.0	1.1	1.1	1.1	1.2	1.1	1.1	1.1	2.1	3	1	1	4	709	80.5	8.7
NP_001018063.1	64762445	PFKFB2	6-phosphofructo-2-kinase/fructose-2, 6-bisphosphatase 2 isoform b	1.1	1.1	1.1	1.1	1.1	1.1	1.0	1.1	1.1	1.1	1.1	1.1	5.9	2	2	2	3	471	54.4	7.6
NP_001138917.1	224451135	SCAF4	splicing factor, arginine/serine-rich 15 isoform 3	0.9	1.0	0.9	1.2	0.9	1.0	1.1	1.1	0.9	1.0	1.0	1.1	7.0	3	3	5	18	1125	123.6	9.6
NP_004348.2	21237748;87159822	CD151	CD151 antigen	1.1	1.2	1.5	1.2	0.9	1.0	1.4	1.1	1.0	1.1	1.5	1.1	6.7	1	2	2	5	253	28.3	7.5
NP_005861.2	215490089	SAP18	histone deacetylase complex subunit SAP18	1.1	1.1	1.0	1.2	1.0	1.0	1.0	1.1	1.0	1.0	1.0	1.1	40.1	1	6	6	15	172	19.5	9.8
NP_001008564.1	56788377	NUPL1	nucleoporin p58/p45 isoform b	1.1	1.1	1.1	1.1	1.1	1.1	1.1	1.1	1.1	1.1	1.1	1.1	6.5	2	3	3	7	587	59.7	9.3
NP_071415.1	11545863	MCCC2	methylcrotonoyl-CoA carboxylase beta chain, mitochondrial	2.3	1.2	1.2	1.2	1.1	1.1	1.1	1.1	1.7	1.2	1.1	1.1	23.3	1	10	10	27	563	61.3	7.7
NP_009171.1	6005956	DUSP12	dual specificity protein phosphatase 12	1.1	1.0	1.1	1.0	1.1	1.1	1.2	1.2	1.1	1.1	1.2	1.1	2.4	1	1	1	2	340	37.7	6.8
NP_004175.2	47419914	WARS	tryptophan--tRNA ligase, cytoplasmic isoform a	1.2	1.1	1.0	1.1	1.3	1.2	1.1	1.1	1.2	1.1	1.0	1.1	59.5	2	20	20	141	471	53.1	6.2
NP_005993.1	5174741	UCHL3	ubiquitin carboxyl-terminal hydrolase isozyme L3 isoform 2	1.0	1.0	1.1	1.1	1.0	1.1	1.2	1.1	1.0	1.1	1.1	1.1	50.4	2	8	8	28	230	26.2	4.9
NP_919268.1	35038601	SASS6	spindle assembly abnormal protein 6 homolog isoform 1	1.1	1.0	0.9	1.1	1.0	1.1	1.0	1.1	1.0	1.0	0.9	1.1	3.7	1	1	1	2	657	74.4	7.5
NP_060501.3	197927448	WDR6	WD repeat-containing protein 6	1.1	1.0	1.0	1.1	1.0	1.0	1.0	1.1	1.1	1.0	1.0	1.1	1.8	1	2	2	2	1151	124.9	6.9
NP_001137261.1	219842250	PPHLN1	periphilin-1 isoform 8	0.9	1.1	1.1	1.1	0.9	1.1	1.1	1.1	0.9	1.1	1.1	1.1	14.7	8	4	4	7	293	33.9	6.6
NP_055670.1	82546847	AP5Z1	AP-5 complex subunit zeta-1	1.1	1.1	1.0	1.1	1.0	1.0	1.1	1.1	1.1	1.1	1.0	1.1	2.4	1	1	1	2	807	88.5	7.0
NP_005871.1	5031741	DNAJA2	dnaJ homolog subfamily A member 2	1.1	1.4	1.3	1.1	1.2	1.4	1.3	1.1	1.2	1.4	1.3	1.1	51.5	1	13	13	62	412	45.7	6.5

Khan *et al.* , 2019. Multi-omics analysis to characterize cigarette smoke induced molecular alterations in esophageal cells
Supplementary Table 5. List of proteins quantified in untreated and chronically treated Het1A cells with cigarette smoke condensate for 8 months

NP_Accession	Protein group Accession	Gene ID	Description	Het-1A-Smoke - 2M/Parental	Het-1A-Smoke - 4M/Parental	Het-1A-Smoke - 6M/Parental	Het-1A-Smoke - 8M/Parental	Het-1A-Smoke - 2M/Parental	Het-1A-Smoke - 4M/Parental	Het-1A-Smoke - 6M/Parental	Het-1A-Smoke - 8M/Parental	Het-1A-Smoke - 2M/Parental	Het-1A-Smoke - 4M/Parental	Het-1A-Smoke - 6M/Parental	Het-1A-Smoke - 8M/Parental	Coverage (%)	Proteins	Unique Peptides	# Peptides	PSM	# AAs	MW [kDa]	calc. pI
				Replicate 1				Replicate 2				Average of replicates											
NP_060592.2	40789249	**DARS2**	aspartate--tRNA ligase, mitochondrial	1.1	1.1	1.1	1.1	1.2	1.1	1.2	1.1	**1.1**	**1.1**	**1.1**	**1.1**	17.4	1	10	10	25	645	73.5	8.0
NP_430255.2	67188550	**CTDP1**	RNA polymerase II subunit A C-terminal domain phosphatase isoform 2	1.1	1.0	0.9	1.0	1.0	1.0	1.1	1.2	**1.1**	**1.0**	**1.0**	**1.1**	3.1	3	2	2	3	867	93.4	6.4
NP_001036083.1	110825963	**PARP2**	poly [ADP-ribose] polymerase 2 isoform 2	1.0	1.0	0.9	1.2	1.0	0.8	0.8	1.0	**1.0**	**0.9**	**0.9**	**1.1**	19.7	2	8	8	21	570	64.8	8.7
NP_036595.2	42741679	**ATP6V0A2**	V-type proton ATPase 116 kDa subunit a isoform 2	1.0	1.2	1.1	1.2	0.9	0.8	0.8	1.0	**1.0**	**1.0**	**1.0**	**1.1**	4.1	1	2	2	6	856	98.0	6.6
NP_000170.1	4504191	**MSH6**	DNA mismatch repair protein Msh6 isoform 1	1.1	1.0	0.9	1.1	1.0	1.0	1.0	1.1	**1.0**	**1.0**	**1.0**	**1.1**	21.3	3	22	22	62	1360	152.7	6.9
NP_005990.1	5174731	**TSNAX**	translin-associated protein X	1.0	1.0	1.0	1.1	1.0	1.0	1.0	1.1	**1.0**	**1.0**	**1.0**	**1.1**	29.0	1	7	7	23	290	33.1	6.6
NP_004777.1	4759274	**TXNL1**	thioredoxin-like protein 1	0.8	0.9	0.9	1.1	0.9	0.9	1.0	1.1	**0.9**	**0.9**	**1.0**	**1.1**	25.3	1	4	4	8	289	32.2	5.0
NP_004857.4	116256358	**SCAMP1**	secretory carrier-associated membrane protein 1 isoform 1	0.9	1.1	1.0	1.1	1.0	1.2	1.2	1.2	**1.0**	**1.1**	**1.1**	**1.1**	11.0	2	3	3	6	338	37.9	7.4
NP_001188306.1	319655561	**ALDH7A1**	alpha-aminoadipic semialdehyde dehydrogenase isoform 2	1.1	1.0	1.0	1.1	1.1	1.0	1.0	1.1	**1.1**	**1.0**	**1.0**	**1.1**	54.2	3	18	18	123	511	55.3	6.9
NP_065134.1	9966881	**NUP107**	nuclear pore complex protein Nup107	1.0	1.0	1.1	1.1	1.1	1.1	1.1	1.1	**1.1**	**1.0**	**1.1**	**1.1**	29.2	1	17	17	62	925	106.3	5.4
NP_000192.2	167466198	**ICAM1**	intercellular adhesion molecule 1 precursor	1.1	1.2	1.2	1.0	1.3	1.4	1.4	1.2	**1.2**	**1.3**	**1.3**	**1.1**	18.6	1	6	6	21	532	57.8	8.0
NP_056083.3	112421122	**DNAJC13**	dnaJ homolog subfamily C member 13	1.2	1.2	1.2	1.1	1.2	1.1	1.1	1.1	**1.2**	**1.2**	**1.2**	**1.1**	5.0	1	11	11	20	2243	254.3	6.7
NP_003791.3	134142828	**RNGTT**	mRNA-capping enzyme isoform a	1.0	1.1	1.1	1.1	1.1	1.0	1.0	1.1	**1.0**	**1.1**	**1.1**	**1.1**	6.7	3	3	3	8	597	68.5	8.1
NP_057710.3	148229134	**MEX3C**	RNA-binding E3 ubiquitin-protein ligase MEX3C	1.1	1.1	1.1	1.1	1.1	1.1	1.0	1.1	**1.1**	**1.1**	**1.0**	**1.1**	5.3	1	2	2	3	659	69.3	5.0
NP_001269201.1	533112473	**ASH2L**	set1/Ash2 histone methyltransferase complex subunit ASH2 isoform d	1.0	1.1	1.1	1.1	1.3	1.1	1.1	1.1	**1.2**	**1.1**	**1.1**	**1.1**	10.8	4	3	3	10	489	55.3	8.1
NP_005227.1	4885217	**ERCC4**	DNA repair endonuclease XPF	1.1	1.0	1.1	1.1	1.3	1.2	1.2	1.1	**1.2**	**1.1**	**1.1**	**1.1**	8.3	1	5	5	9	916	104.4	6.9
NP_116258.2	189571677	**NUDCD1**	nudC domain-containing protein 1 isoform 1	1.1	1.1	1.2	1.1	1.1	1.1	1.2	1.1	**1.1**	**1.1**	**1.2**	**1.1**	45.8	2	18	18	54	583	66.7	5.1
NP_443091.1	16418361	**RFT1**	protein RFT1 homolog	1.1	1.2	1.1	1.0	1.3	1.2	1.2	1.2	**1.2**	**1.2**	**1.2**	**1.1**	5.6	1	3	3	5	541	60.3	8.9
NP_001258538.1	410110900	**STX10**	syntaxin-10 isoform 2	1.0	0.9	0.9	1.0	1.1	0.8	1.7	1.2	**1.0**	**0.9**	**1.3**	**1.1**	42.3	4	5	5	7	220	24.8	4.8
NP_001117.2	34577063	**ADSS**	adenylosuccinate synthetase isozyme 2	1.1	1.1	1.2	1.1	1.1	1.1	1.1	1.1	**1.1**	**1.1**	**1.1**	**1.1**	27.6	1	11	11	34	456	50.1	6.6
NP_071896.8	20806097	**NOC3L**	nucleolar complex protein 3 homolog	1.1	1.1	1.1	1.1	1.0	1.0	1.1	1.1	**1.0**	**1.1**	**1.1**	**1.1**	18.9	1	12	12	41	800	92.5	9.2
NP_852664.1	32455248	**PIK3R1**	phosphatidylinositol 3-kinase regulatory subunit alpha isoform 1	1.0	1.1	1.1	1.2	1.0	1.0	1.1	1.0	**1.0**	**1.0**	**1.1**	**1.1**	5.5	4	2	2	8	724	83.5	6.2
NP_006421.2	38455427	**CCT4**	T-complex protein 1 subunit delta isoform a	1.0	1.0	1.0	1.1	1.0	1.0	1.0	1.1	**1.0**	**1.0**	**1.0**	**1.1**	62.9	2	26	27	156	539	57.9	7.8
NP_001070674.1	116256336	**SEC31A**	protein transport protein Sec31A isoform 4	1.2	1.1	1.1	1.1	1.1	1.1	1.1	1.1	**1.1**	**1.1**	**1.1**	**1.1**	20.6	7	15	15	41	1106	121.6	6.9
NP_057581.2	16950607	**MRPL51**	39S ribosomal protein L51, mitochondrial	1.0	1.0	0.9	1.0	1.1	1.0	1.1	1.2	**1.1**	**1.0**	**1.0**	**1.1**	13.3	1	1	1	4	128	15.1	11.3
NP_003963.1	27477070	**BTAF1**	TATA-binding protein-associated factor 172	1.1	1.1	1.1	1.1	1.1	1.1	1.2	1.1	**1.1**	**1.1**	**1.1**	**1.1**	5.1	1	5	5	12	1849	206.8	6.5
NP_002100.2	6996014	**HARS**	histidine--tRNA ligase, cytoplasmic isoform 1	1.1	1.0	1.1	1.1	1.1	1.0	1.1	1.1	**1.1**	**1.0**	**1.1**	**1.1**	64.6	7	20	27	131	509	57.4	5.9
NP_054774.2	32526901	**IFT81**	intraflagellar transport protein 81 homolog isoform 1	1.0	1.0	1.1	1.0	1.0	1.2	1.1	1.2	**1.0**	**1.1**	**1.1**	**1.1**	3.1	1	2	2	4	676	79.7	8.8
NP_644810.1	21281677	**WDR36**	WD repeat-containing protein 36	1.1	1.1	1.1	1.1	1.1	1.0	1.1	1.1	**1.1**	**1.0**	**1.1**	**1.1**	12.6	1	10	10	24	951	105.3	7.5
NP_001276104.1	574584850	**VPS52**	vacuolar protein sorting-associated protein 52 homolog isoform 3	1.0	0.9	1.0	1.0	**2.0**	1.2	1.7	1.2	**1.5**	**1.1**	**1.3**	**1.1**	7.2	4	3	3	7	598	68.5	8.4
NP_056175.3	193211480	**SKIV2L2**	superkiller viralicidic activity 2-like 2	1.1	1.1	1.1	1.2	1.0	1.1	1.1	1.1	**1.1**	**1.1**	**1.1**	**1.1**	33.3	1	28	29	77	1042	117.7	6.5
NP_056090.1	40018629	**KIAA1033**	WASH complex subunit 7 isoform 2	1.2	1.3	1.2	1.2	1.1	1.1	1.2	1.1	**1.2**	**1.2**	**1.2**	**1.1**	8.6	2	9	9	16	1173	136.3	7.4
NP_004227.1	4759264	**COPS2**	COP9 signalosome complex subunit 2 isoform 1	1.1	1.1	1.1	1.2	1.0	1.0	1.1	1.1	**1.1**	**1.1**	**1.1**	**1.1**	33.0	2	11	11	38	443	51.6	5.5
NP_067022.1	10864011	**SQRDL**	sulfide:quinone oxidoreductase, mitochondrial	1.1	1.0	1.1	1.2	1.1	0.9	1.0	1.1	**1.1**	**1.0**	**1.1**	**1.1**	14.7	1	5	5	12	450	49.9	9.1
NP_056348.2	20149621	**TKFC**	bifunctional ATP-dependent dihydroxyacetone kinase/FAD-AMP lyase (cyclizing)	1.2	1.2	1.2	1.1	1.1	1.2	1.2	1.1	**1.2**	**1.2**	**1.2**	**1.1**	22.8	1	9	9	22	575	58.9	7.5
NP_060823.3	42415492	**MIS18BP1**	mis18-binding protein 1	1.0	0.9	0.9	1.0	1.1	1.2	1.1	1.2	**1.0**	**1.0**	**1.0**	**1.1**	2.2	1	3	3	4	1132	129.0	9.2
NP_001001790.1	49169828	**TOMM5**	mitochondrial import receptor subunit TOM5 homolog isoform 1	1.1	1.0	1.1	1.1	1.1	0.9	1.2	1.1	**1.1**	**1.0**	**1.1**	**1.1**	13.7	3	1	1	2	51	6.0	9.7
NP_060827.2	37537710	**LARP6**	la-related protein 6 isoform 1	1.1	1.3	1.1	1.3	1.4	1.0	1.0	0.9	**1.3**	**1.2**	**1.0**	**1.1**	5.7	2	2	2	3	491	54.7	8.3
NP_005925.2	21361272	**MLLT1**	protein ENL	1.1	1.2	1.1	1.2	1.0	1.0	1.0	1.0	**1.0**	**1.1**	**1.0**	**1.1**	3.9	1	2	2	4	559	62.0	8.6
NP_005462.1	13027378	**GNPDA1**	glucosamine-6-phosphate isomerase 1	1.1	1.0	1.0	1.1	1.1	1.1	1.0	1.1	**1.1**	**1.0**	**1.0**	**1.1**	66.4	1	11	14	63	289	32.6	6.9
NP_001918.3	55749932	**DES**	desmin	**8.9**	1.7	1.1	1.1	**4.9**	1.3	0.9	1.1	**6.9**	**1.5**	**1.0**	**1.1**	8.5	5	1	5	80	470	53.5	5.3
NP_001559.1	4503521	**EIF3E**	eukaryotic translation initiation factor 3 subunit E	1.1	1.0	1.1	1.1	1.1	1.0	1.1	1.1	**1.1**	**1.0**	**1.1**	**1.1**	62.3	1	22	22	108	445	52.2	6.0
NP_115545.3	38505218	**ACAD11**	acyl-CoA dehydrogenase family member 11	1.2	1.2	1.2	1.2	1.1	1.1	1.2	1.1	**1.2**	**1.2**	**1.2**	**1.1**	13.0	1	7	7	10	780	87.2	8.0
NP_006322.4	194328699	**EMG1**	ribosomal RNA small subunit methyltransferase NEP1	1.0	1.0	1.0	1.0	1.0	1.1	1.1	1.2	**1.0**	**1.1**	**1.1**	**1.1**	34.4	1	7	7	16	244	26.7	9.2
NP_001265855.1	523967094	**PPWD1**	peptidylprolyl isomerase domain and WD repeat-containing protein 1 isoform 2	1.0	1.0	1.0	1.1	1.0	1.1	1.0	1.1	**1.0**	**1.0**	**1.0**	**1.1**	13.3	4	7	7	17	616	70.2	8.3
NP_002794.1	4506209	**PSMC2**	26S protease regulatory subunit 7 isoform 1	1.1	1.1	1.1	1.1	1.1	1.1	1.1	1.1	**1.1**	**1.1**	**1.1**	**1.1**	55.4	2	22	22	106	433	48.6	5.9
NP_006464.1	5453844	**TAF6L**	TAF6-like RNA polymerase II p300/CBP-associated factor-associated factor 65 kDa subunit 6L	1.0	1.0	1.1	1.0	1.0	1.1	1.0	1.2	**1.0**	**1.1**	**1.1**	**1.1**	9.5	1	3	3	6	622	67.8	9.0
NP_003306.3	262206280	**DNAJC7**	dnaJ homolog subfamily C member 7 isoform 1	1.0	0.9	1.0	1.1	0.9	1.0	0.9	1.1	**1.0**	**1.0**	**1.0**	**1.1**	38.3	2	16	16	52	494	56.4	7.0
NP_002140.2	19913441;19913443	**HPCAL1**	hippocalcin-like protein 1	1.1	1.1	1.3	1.0	1.3	1.5	1.7	1.2	**1.2**	**1.3**	**1.5**	**1.1**	32.6	2	5	5	10	193	22.3	5.4
NP_001959.1	4503535	**EIF4E**	eukaryotic translation initiation factor 4E isoform 1	1.0	1.0	1.0	1.1	1.1	1.1	1.2	1.1	**1.0**	**1.0**	**1.1**	**1.1**	21.7	3	4	4	6	217	25.1	6.2
NP_060504.1	8922301	**WDR70**	WD repeat-containing protein 70	1.2	1.1	1.2	1.2	0.8	1.1	0.9	1.0	**1.0**	**1.1**	**1.0**	**1.1**	8.4	1	3	3	6	654	73.2	6.3
NP_055317.1	7657381	**PRPF19**	pre-mRNA-processing factor 19	1.0	1.1	1.0	1.1	1.0	1.0	1.0	1.1	**1.0**	**1.1**	**1.0**	**1.1**	55.6	1	14	14	57	504	55.1	6.6
NP_000262.2	255652944	**NPC1**	Niemann-Pick C1 protein precursor	1.1	1.2	1.1	1.1	1.1	1.2	1.1	1.1	**1.1**	**1.2**	**1.1**	**1.1**	7.8	1	7	7	13	1278	142.1	5.4
NP_001123478.1	194097354	**GSPT1**	eukaryotic peptide chain release factor GTP-binding subunit ERF3A isoform 2	1.0	1.1	1.1	1.1	1.0	1.1	1.0	1.1	**1.0**	**1.1**	**1.1**	**1.1**	42.8	4	23	23	75	636	68.5	5.3
NP_060791.3	55770900	**BRIX1**	ribosome biogenesis protein BRX1 homolog	0.9	1.0	1.1	1.1	1.0	1.0	1.0	1.1	**0.9**	**1.0**	**1.1**	**1.1**	28.6	1	8	8	18	353	41.4	9.9
NP_001104571.1	161783807	**CNRIP1**	CB1 cannabinoid receptor-interacting protein 1 isoform CRIP1b	0.9	0.9	0.9	1.1	0.9	0.9	0.9	1.1	**0.9**	**0.9**	**0.9**	**1.1**	30.5	2	2	2	4	128	14.2	5.1
NP_001164274.1	283046706	**LARP4**	la-related protein 4 isoform d	0.9	1.2	1.2	1.2	1.0	1.0	1.1	1.1	**1.0**	**1.1**	**1.2**	**1.1**	13.1	6	5	5	13	582	64.5	6.0
NP_001230889.1	345478677	**NEDD4L**	E3 ubiquitin-protein ligase NEDD4-like isoform 7	1.0	1.2	1.3	1.2	1.0	1.1	1.2	1.0	**1.0**	**1.1**	**1.3**	**1.1**	7.9	7	2	5	18	911	104.9	5.6
NP_001273705.1	557948022	**TEX30**	testis-expressed sequence 30 protein isoform 3	1.0	1.0	1.0	1.2	1.0	1.0	0.9	1.0	**1.0**	**1.0**	**1.0**	**1.1**	12.9	2	2	2	3	186	21.2	8.6
NP_006775.1	5803221	**WDR3**	WD repeat-containing protein 3	1.0	1.0	1.0	1.1	1.0	1.0	1.1	1.1	**1.0**	**1.0**	**1.0**	**1.1**	40.5	1	26	26	69	943	106.0	6.6
NP_078794.1	13375656	**ULBP3**	NKG2D ligand 3 precursor	0.9	0.9	1.1	1.1	1.0	1.1	1.0	1.1	**1.0**	**1.0**	**1.0**	**1.1**	6.2	1	1	1	1	244	27.9	8.0
NP_001018146.1	66392203	**NME1-NME2**	NME1-NME2 protein	1.3	0.9	1.1	1.1	1.3	1.0	1.1	1.1	**1.3**	**0.9**	**1.1**	**1.1**	77.2	3	6	14	128	267	30.1	8.9
NP_078974.1	13375983	**SLC25A22**	mitochondrial glutamate carrier 1	1.1	1.2	1.3	1.1	1.2	1.2	1.3	1.1	**1.2**	**1.2**	**1.3**	**1.1**	12.1	2	4	4	7	323	34.4	9.3
NP_065108.1	9966849	**TIGAR**	fructose-2,6-bisphosphatase TIGAR	1.0	1.1	1.1	1.1	1.1	1.1	1.1	1.2	**1.1**	**1.1**	**1.1**	**1.1**	25.9	1	4	4	13	270	30.0	7.7
NP_055845.1	119874201	**FRYL**	protein furry homolog-like	1.1	1.3	1.3	1.2	1.1	1.1	1.1	1.0	**1.1**	**1.2**	**1.2**	**1.1**	4.2	1	9	9	16	3013	339.4	5.6
NP_001737.1	10716563	**CANX**	calnexin precursor	1.0	1.3	1.0	1.1	1.0	1.3	1.0	1.1	**1.0**	**1.3**	**1.0**	**1.1**	65.7	1	33	34	344	592	67.5	4.6
NP_001185825.1	312032405	**ACY1**	aminoacylase-1 isoform b	1.2	1.0	1.1	1.3	0.7	0.9	0.8	0.9	**0.9**	**0.9**	**0.9**	**1.1**	7.1	4	2	2	3	336	37.6	5.9
NP_001020471.2	106507164	**RAB12**	ras-related protein Rab-12	1.1	1.1	1.2	1.1	1.1	1.1	1.1	1.1	**1.1**	**1.1**	**1.2**	**1.1**	11.9	9	2	3	7	244	27.2	8.4
NP_055650.1	7662298	**OSBPL2**	oxysterol-binding protein-related protein 2 isoform 1	1.1	1.1	1.1	1.0	1.2	1.1	1.1	1.2	**1.1**	**1.1**	**1.1**	**1.1**	5.3	3	2	2	5	468	53.9	6.6
NP_001243639.1	375493587;375493589	**ANAPC10**	anaphase-promoting complex subunit 10 isoform 3	1.1	1.1	1.2	1.2	1.0	1.0	1.0	1.0	**1.0**	**1.1**	**1.1**	**1.1**	7.9	3	1	1	4	114	12.8	8.5
NP_055557.2	164519076	**TM9SF4**	transmembrane 9 superfamily member 4 precursor	1.1	1.0	1.0	1.1	1.2	1.1	1.2	1.1	**1.1**	**1.1**	**1.1**	**1.1**	7.8	1	4	5	8	642	74.5	6.5
NP_008824.2	56699485	**EIF2D**	eukaryotic translation initiation factor 2D isoform 1	1.0	1.0	0.9	1.1	1.0	1.0	1.0	1.1	**1.0**	**1.0**	**0.9**	**1.1**	5.1	1	2	2	2	584	64.7	7.6
NP_006412.2	51479145	**ARFGEF1**	brefeldin A-inhibited guanine nucleotide-exchange protein 1	1.1	1.1	1.0	1.1	1.0	1.0	1.0	1.1	**1.0**	**1.0**	**1.0**	**1.1**	9.9	1	10	12	21	1849	208.6	5.8
NP_001154992.1	240849567	**COG5**	conserved oligomeric Golgi complex subunit 5 isoform 3	1.2	1.2	1.2	1.2	1.1	1.0	1.1	1.0	**1.2**	**1.1**	**1.1**	**1.1**	8.1	3	5	5	8	823	90.8	6.5
NP_055066.1	7657581	**SLC25A13**	calcium-binding mitochondrial carrier protein Aralar2 isoform 2	1.1	1.1	1.1	1.1	1.1	1.0	1.1	1.1	**1.1**	**1.1**	**1.1**	**1.1**	20.9	2	6	9	25	675	74.1	8.6
NP_001311.3	23503295	**CSNK2B**	casein kinase II subunit beta isoform 1	1.0	0.9	1.1	1.1	1.1	1.0	1.1	1.1	**1.0**	**1.0**	**1.1**	**1.1**	47.9	2	5	5	20	215	24.9	5.6
NP_115865.1	16554616	**MRPS6**	28S ribosomal protein S6, mitochondrial	0.9	0.9	0.9	1.1	1.0	1.0	1.0	1.1	**0.9**	**1.0**	**1.0**	**1.1**	16.8	1	2	2	4	125	14.2	9.3
NP_064551.3	116805327	**MCCC1**	methylcrotonoyl-CoA carboxylase subunit alpha, mitochondrial isoform 1 precursor	1.1	1.1	1.1	1.1	1.2	1.1	1.2	1.1	**1.1**	**1.1**	**1.1**	**1.1**	17.7	2	7	7	14	725	80.4	7.8
NP_036602.1	6912734	**TNPO3**	transportin-3 isoform 1	1.0	1.1	1.1	1.1	1.1	1.1	1.2	1.1	**1.1**	**1.1**	**1.1**	**1.1**	19.4	2	13	13	34	923	104.1	5.6
NP_004672.2	33356163	**EIF1AY**	eukaryotic translation initiation factor 1A, Y-chromosomal isoform 1	1.1	1.2	1.2	1.1	1.1	1.2	1.1	1.1	**1.1**	**1.2**	**1.2**	**1.1**	70.1	2	1	10	101	144	16.4	5.2
NP_001244271.1	383087730;383087732	**BCS1L**	mitochondrial chaperone BCS1	1.1	0.9	1.1	1.1	1.2	1.0	1.1	1.1	**1.2**	**1.0**	**1.1**	**1.1**	9.6	1	3	3	5	419	47.5	8.5
NP_001242952.1	365733560	**CLIC5**	chloride intracellular channel protein 5 isoform c	1.0	1.0	1.2	1.0	1.2	1.2	1.6	1.2	**1.1**	**1.1**	**1.4**	**1.1**	11.2	5	1	2	3	205	22.8	5.7
NP_001144.1	4502105	**ANXA4**	annexin A4	**2.8**	1.1	1.0	1.1	0.9	0.9	0.9	1.1	**1.9**	**1.0**	**0.9**	**1.1**	44.2	1	9	12	40	321	36.1	6.1
NP_001274.1	4557471	**AP1S1**	AP-1 complex subunit sigma-1A	1.0	1.0	1.0	1.0	1.1	1.1	1.1	1.2	**1.0**	**1.1**	**1.0**	**1.1**	24.1	1	3	3	9	158	18.7	5.7
NP_002464.1	12667788	**MYH9**	myosin-9	1.2	1.2	1.2	1.1	1.2	1.2	1.3	1.1	**1.2**	**1.2**	**1.3**	**1.1**	63.0	1	98	122	1165	1960	226.4	5.6
NP_056480.1	12962937	**AAAS**	aladin isoform 1	1.2	1.1	1.1	1.1	1.0	1.0	1.0	1.1	**1.1**	**1.1**	**1.1**	**1.1**	13.4	2	4	4	11	546	59.5	7.5

Khan *et al.* , 2019. Multi-omics analysis to characterize cigarette smoke induced molecular alterations in esophageal cells
Supplementary Table 5. List of proteins quantified in untreated and chronically treated Het1A cells with cigarette smoke condensate for 8 months

NP_Accession	Protein group Accession	Gene ID	Description	Het-1A-Smoke - 2M/Parental	Het-1A-Smoke - 4M/Parental	Het-1A-Smoke - 6M/Parental	Het-1A-Smoke - 8M/Parental	Het-1A-Smoke - 2M/Parental	Het-1A-Smoke - 4M/Parental	Het-1A-Smoke - 6M/Parental	Het-1A-Smoke - 8M/Parental	Het-1A-Smoke - 2M/Parental	Het-1A-Smoke - 4M/Parental	Het-1A-Smoke - 6M/Parental	Het-1A-Smoke - 8M/Parental	Coverage (%)	Proteins	Unique Peptides	# Peptides	PSM	# AAs	MW [kDa]	calc. pI
				Replicate 1				Replicate 2				Average of replicates											
NP_690866.1	23097250	**TIPRL**	TIP41-like protein isoform 1	1.0	1.0	1.0	1.0	1.1	1.1	1.2	1.2	**1.1**	**1.0**	**1.1**	**1.1**	41.9	2	9	9	16	272	31.4	5.9
NP_114143.1	13994322	**TBC1D10A**	TBC1 domain family member 10A isoform 2	1.2	1.2	1.1	1.1	1.2	1.2	1.1	1.1	**1.2**	**1.2**	**1.1**	**1.1**	3.5	2	2	2	2	508	57.1	8.4
NP_004636.1	4758024	**COIL**	coilin	1.0	1.1	1.1	1.1	1.0	1.0	1.0	1.1	**1.0**	**1.0**	**1.0**	**1.1**	12.7	1	6	6	12	576	62.6	9.1
NP_001157718.1	256000760	**C1orf27**	protein odr-4 homolog isoform 3	1.1	1.1	1.2	1.1	1.0	1.1	1.1	1.1	**1.0**	**1.1**	**1.1**	**1.1**	8.8	3	4	4	4	422	47.6	5.8
NP_001189431.1	321267511	**NIPSNAP1**	protein NipSnap homolog 1 isoform 2	1.2	1.2	1.2	1.2	1.2	1.1	1.2	1.0	**1.2**	**1.2**	**1.2**	**1.1**	40.9	2	6	7	31	264	31.4	9.1
NP_000311.2	208973246	**QDPR**	dihydropteridine reductase isoform 1	0.9	1.0	0.9	1.0	1.3	1.1	0.9	1.2	**1.1**	**1.1**	**0.9**	**1.1**	20.9	2	3	3	8	244	25.8	7.4
NP_690852.1	23097240	**LRWD1**	leucine-rich repeat and WD repeat-containing protein 1	1.0	1.0	1.0	1.1	1.0	1.0	1.0	1.1	**1.0**	**1.0**	**1.0**	**1.1**	16.7	1	7	7	15	647	70.8	7.2
NP_002481.2	51317370	**NDUFA6**	NADH dehydrogenase [ubiquinone] 1 alpha subcomplex subunit 6	1.0	1.1	1.2	1.1	1.1	1.2	1.2	1.1	**1.1**	**1.1**	**1.2**	**1.1**	11.7	1	2	2	3	154	17.9	10.1
NP_149082.1	15011941	**TRIM4**	tripartite motif-containing protein 4 isoform beta	1.1	1.3	1.1	1.2	1.0	1.0	1.0	1.0	**1.1**	**1.1**	**1.0**	**1.1**	3.2	2	1	1	1	474	54.1	8.0
NP_055644.2	41327773	**DDX46**	probable ATP-dependent RNA helicase DDX46 isoform 2	1.0	1.1	1.1	1.1	1.1	1.0	1.0	1.1	**1.0**	**1.0**	**1.1**	**1.1**	31.8	2	28	28	61	1031	117.3	9.3
NP_065816.2	82659109	**UBR4**	E3 ubiquitin-protein ligase UBR4	1.1	1.2	1.2	1.1	1.1	1.1	1.2	1.1	**1.1**	**1.1**	**1.2**	**1.1**	20.9	1	71	72	243	5183	573.5	6.0
NP_057086.2	55770836	**SCCPDH**	saccharopine dehydrogenase-like oxidoreductase	1.0	0.9	1.0	1.1	1.0	1.1	1.1	1.1	**1.0**	**1.0**	**1.1**	**1.1**	19.8	1	6	6	10	429	47.1	9.1
NP_114109.1	13994261	**MRPL32**	39S ribosomal protein L32, mitochondrial precursor	1.1	1.1	1.1	1.2	1.0	1.1	1.0	1.0	**1.1**	**1.1**	**1.0**	**1.1**	11.7	1	1	1	2	188	21.4	9.7
NP_062817.2	256818794	**EIF4ENIF1**	eukaryotic translation initiation factor 4E transporter isoform a	1.0	1.1	1.0	1.2	1.0	1.0	0.9	1.0	**1.0**	**1.1**	**1.0**	**1.1**	4.4	2	3	3	4	985	108.1	8.3
NP_003243.1	4507499	**TIAL1**	nucleolysin TIAR isoform 1	1.0	1.0	1.0	1.1	1.0	1.0	1.0	1.1	**1.0**	**1.0**	**1.0**	**1.1**	16.0	2	4	5	17	375	41.6	7.7
NP_002185.1	4504703	**INPP1**	inositol polyphosphate 1-phosphatase	1.1	1.1	1.0	1.2	1.1	1.0	1.0	1.0	**1.1**	**1.0**	**1.0**	**1.1**	3.0	1	1	1	2	399	44.0	5.3
NP_056473.2	157694511	**NOC2L**	nucleolar complex protein 2 homolog	0.9	0.9	0.9	1.1	1.0	0.9	1.0	1.1	**0.9**	**0.9**	**1.0**	**1.1**	14.4	1	10	10	18	749	84.9	5.6
NP_061184.1	21237783	**COG1**	conserved oligomeric Golgi complex subunit 1	1.1	1.2	1.0	1.1	1.1	1.0	1.0	1.1	**1.1**	**1.1**	**1.0**	**1.1**	5.7	1	3	3	6	980	108.9	7.3
NP_001186385.1	313747419	**BRD2**	bromodomain-containing protein 2 isoform 3	1.0	1.0	1.1	1.2	0.9	0.9	0.9	1.0	**0.9**	**1.0**	**1.0**	**1.1**	7.6	4	5	5	12	754	83.1	9.1
NP_060555.2	21361659	**IPO9**	importin-9	1.2	1.1	1.2	1.1	1.1	1.1	1.1	1.1	**1.1**	**1.1**	**1.2**	**1.1**	24.1	1	18	18	73	1041	115.9	4.8
NP_000687.3	115387104	**ALDH9A1**	4-trimethylaminobutyraldehyde dehydrogenase	1.1	1.1	1.1	1.2	1.1	1.0	1.0	1.0	**1.1**	**1.1**	**1.1**	**1.1**	34.8	1	14	14	42	518	56.3	6.6
NP_001032354.1	83700220	**GGPS1**	geranylgeranyl pyrophosphate synthase	1.2	1.1	1.3	1.2	1.2	1.3	1.2	1.0	**1.2**	**1.2**	**1.2**	**1.1**	7.7	1	2	2	3	300	34.8	6.1
NP_001273086.1	555290083	**DYNC1LI2**	cytoplasmic dynein 1 light intermediate chain 2 isoform 2	1.0	1.0	1.1	1.1	1.0	1.0	1.1	1.1	**1.0**	**1.0**	**1.1**	**1.1**	35.7	2	8	8	23	415	45.0	6.1
NP_001274744.1	568214245	**UBAP2L**	ubiquitin-associated protein 2-like isoform c	0.8	1.0	0.9	1.1	0.9	1.0	0.9	1.1	**0.9**	**1.0**	**0.9**	**1.1**	21.2	4	13	13	43	976	103.1	6.9
NP_008955.1	5902022	**TMEM115**	transmembrane protein 115	1.1	1.2	1.1	1.2	1.0	1.0	1.1	1.0	**1.1**	**1.1**	**1.1**	**1.1**	6.0	1	2	2	2	351	38.2	8.2
NP_002284.3	145309326	**LAMC1**	laminin subunit gamma-1 precursor	1.2	1.2	1.2	1.0	1.3	1.1	1.2	1.2	**1.2**	**1.2**	**1.2**	**1.1**	5.7	1	6	6	14	1609	177.5	5.1
NP_073617.1	12232469	**RMND5A**	protein RMD5 homolog A	1.1	1.1	1.2	1.1	0.9	0.9	1.0	1.1	**1.0**	**1.0**	**1.1**	**1.1**	9.2	1	2	2	2	391	44.0	6.1
NP_001186912.1	316983156	**NDUFS1**	NADH-ubiquinone oxidoreductase 75 kDa subunit, mitochondrial isoform 4	1.2	1.1	1.2	1.1	1.2	1.0	1.2	1.1	**1.2**	**1.1**	**1.2**	**1.1**	32.4	5	12	12	34	670	73.5	5.9
NP_001616.1	4502013	**AK2**	adenylate kinase 2, mitochondrial isoform a	1.1	1.2	1.1	1.1	1.1	1.1	0.9	1.1	**1.1**	**1.1**	**1.0**	**1.1**	49.0	3	9	9	49	239	26.5	7.8
NP_000048.1	4557365	**BLM**	Bloom syndrome protein isoform 1	0.9	1.0	0.8	1.1	1.1	1.0	1.0	1.1	**1.0**	**1.0**	**0.9**	**1.1**	7.1	3	7	7	10	1417	158.9	7.5
NP_001273300.1	556503352	**VTA1**	vacuolar protein sorting-associated protein VTA1 homolog isoform b	1.1	1.1	1.0	1.1	1.1	1.1	1.2	1.1	**1.1**	**1.1**	**1.1**	**1.1**	35.0	3	8	8	32	280	31.1	6.4
NP_005019.2	6857820	**PIP4K2A**	phosphatidylinositol 5-phosphate 4-kinase type-2 alpha	1.1	1.1	1.1	1.1	0.9	1.1	1.1	1.1	**1.0**	**1.1**	**1.1**	**1.1**	4.9	1	1	2	4	406	46.2	7.0
NP_001632.2	18375501;18375503	**APEX1**	DNA-(apurinic or apyrimidinic site) lyase	0.9	0.8	0.9	1.1	0.9	0.9	0.9	1.1	**0.9**	**0.9**	**0.9**	**1.1**	50.3	1	13	13	66	318	35.5	8.1
NP_001252510.1	387942388;156151411	**TTI2**	TELO2-interacting protein 2	1.2	1.1	1.0	1.2	1.1	1.0	1.0	1.0	**1.1**	**1.1**	**1.0**	**1.1**	7.5	1	3	3	3	508	56.9	7.1
NP_057724.2	58331150	**MRPS30**	28S ribosomal protein S30, mitochondrial	1.2	1.2	1.1	1.1	1.1	1.1	1.1	1.1	**1.1**	**1.1**	**1.1**	**1.1**	22.1	1	6	6	18	439	50.3	8.0
NP_055428.1	24797106	**FAF2**	FAS-associated factor 2	1.1	1.2	1.1	1.1	1.1	1.1	1.1	1.1	**1.1**	**1.2**	**1.1**	**1.1**	49.0	1	11	11	60	445	52.6	5.6
NP_115727.5	633257785	**MRPL45**	39S ribosomal protein L45, mitochondrial isoform 1	1.0	1.0	1.0	1.0	1.1	1.2	1.2	1.2	**1.1**	**1.1**	**1.1**	**1.1**	37.6	2	10	10	29	306	35.3	9.0
NP_003582.2	19482174;311771637	**CUL2**	cullin-2 isoform c	1.1	1.1	1.2	1.1	1.1	1.1	1.2	1.1	**1.1**	**1.1**	**1.2**	**1.1**	14.8	3	9	9	18	745	86.9	6.9
NP_037486.2	85067507	**CNOT7**	CCR4-NOT transcription complex subunit 7 isoform 1	1.2	1.1	1.0	1.1	1.2	1.0	1.1	1.1	**1.2**	**1.0**	**1.0**	**1.1**	13.0	2	3	3	7	285	32.7	4.8
NP_006779.1	5803145	**RALBP1**	ralA-binding protein 1	1.0	1.1	1.0	1.1	1.0	1.1	1.0	1.1	**1.0**	**1.1**	**1.0**	**1.1**	4.9	1	2	2	3	655	76.0	5.9
NP_037506.2	22027538	**PDCD6IP**	programmed cell death 6-interacting protein isoform 1	1.1	1.1	1.1	1.1	1.1	1.1	1.1	1.1	**1.1**	**1.1**	**1.1**	**1.1**	46.8	3	33	33	122	868	96.0	6.5
NP_065843.3	68051721	**NCEH1**	neutral cholesterol ester hydrolase 1 isoform b	1.3	1.4	1.6	1.1	1.3	1.4	1.7	1.1	**1.3**	**1.4**	**1.7**	**1.1**	26.1	3	8	8	31	440	49.0	7.9
NP_004957.1	4826760	**HNRNPF**	heterogeneous nuclear ribonucleoprotein F	0.9	0.9	1.1	1.1	1.0	1.0	1.0	1.1	**1.0**	**0.9**	**1.0**	**1.1**	37.8	1	9	11	83	415	45.6	5.6
NP_003364.1	4507877	**VCL**	vinculin isoform VCL	1.2	1.3	1.4	1.1	1.1	1.2	1.3	1.1	**1.2**	**1.3**	**1.4**	**1.1**	57.9	2	50	50	285	1066	116.6	6.1
NP_001346.1	4503291	**DDT**	D-dopachrome decarboxylase	1.6	1.7	1.5	1.4	1.2	1.1	1.1	0.8	**1.4**	**1.4**	**1.3**	**1.1**	16.1	1	2	2	8	118	12.7	7.3
NP_003253.1	4507525	**SEC62**	translocation protein SEC62	1.3	1.5	1.4	1.1	1.2	1.3	1.2	1.1	**1.3**	**1.4**	**1.3**	**1.1**	11.0	1	5	5	12	399	45.8	7.1
NP_001239605.1	358248211	**ACSS1**	acetyl-coenzyme A synthetase 2-like, mitochondrial isoform 3	1.1	0.9	0.9	1.0	1.0	1.0	1.0	1.2	**1.1**	**0.9**	**0.9**	**1.1**	1.9	4	1	1	1	568	62.7	7.3
NP_620156.1	20270355	**GALM**	aldose 1-epimerase	1.0	1.0	1.0	1.1	0.8	0.8	0.8	1.1	**0.9**	**0.9**	**0.9**	**1.1**	9.4	1	2	2	2	342	37.7	6.7
NP_002005.1	4503729	**FKBP4**	peptidyl-prolyl cis-trans isomerase FKBP4	1.1	1.2	1.2	1.1	1.2	1.2	1.2	1.1	**1.1**	**1.2**	**1.2**	**1.1**	68.6	1	26	27	166	459	51.8	5.4
NP_060853.3	56243590	**WDR33**	pre-mRNA 3' end processing protein WDR33 isoform 1	0.9	1.2	1.2	1.2	0.9	1.0	0.9	1.0	**0.9**	**1.1**	**1.0**	**1.1**	6.7	2	7	7	19	1336	145.8	9.2
NP_002068.1	4504063	**GOLGA1**	Golgin subfamily A member 1	1.0	1.2	1.1	1.1	1.2	1.2	1.0	1.1	**1.1**	**1.2**	**1.0**	**1.1**	2.2	1	1	1	2	767	88.1	5.3
NP_055578.2	94557305	**MRPL19**	39S ribosomal protein L19, mitochondrial	1.3	1.1	1.1	1.1	1.0	1.0	1.1	1.1	**1.2**	**1.0**	**1.1**	**1.1**	32.9	1	8	8	20	292	33.5	9.5
NP_057327.2	49574502	**CYB5R1**	NADH-cytochrome b5 reductase 1	1.1	1.3	1.2	1.1	1.1	1.2	1.3	1.1	**1.1**	**1.3**	**1.3**	**1.1**	32.8	1	7	7	14	305	34.1	9.4
NP_853553.1	31712030	**AUP1**	ancient ubiquitous protein 1	0.9	0.9	1.0	1.0	1.1	1.1	1.0	1.2	**1.0**	**1.0**	**1.0**	**1.1**	9.5	1	4	4	6	410	45.8	8.6
NP_004960.2	155969707	**IDE**	insulin-degrading enzyme isoform 1	1.0	1.1	1.1	1.1	1.1	1.0	1.1	1.1	**1.1**	**1.1**	**1.1**	**1.1**	14.5	2	12	12	34	1019	117.9	6.6
NP_060703.3	94536836	**OGFOD1**	2-oxoglutarate and iron-dependent oxygenase domain-containing protein 1	1.0	1.0	1.1	1.1	0.9	1.0	1.0	1.1	**1.0**	**1.0**	**1.1**	**1.1**	18.5	1	6	6	9	542	63.2	5.1
NP_071409.3	269847098	**LHPP**	phospholysine phosphohistidine inorganic pyrophosphate phosphatase isoform 1	1.1	1.0	0.9	1.1	1.0	1.0	0.9	1.1	**1.1**	**1.0**	**0.9**	**1.1**	30.0	2	4	4	15	270	29.1	6.2
NP_068746.2	157426893	**GUF1**	translation factor GUF1, mitochondrial	1.1	1.1	1.2	1.0	0.9	1.0	1.0	1.1	**1.0**	**1.0**	**1.1**	**1.1**	2.2	1	1	1	2	669	74.3	8.6
NP_001120869.1	188528696	**ERLEC1**	endoplasmic reticulum lectin 1 isoform 2 precursor	1.1	1.1	1.1	1.1	1.1	1.0	1.1	1.0	**1.1**	**1.1**	**1.1**	**1.1**	10.7	3	3	3	6	457	51.9	6.1
NP_060130.3	164519146	**GATAD2A**	transcriptional repressor p66-alpha	0.9	0.9	0.9	1.1	1.0	1.0	1.0	1.1	**1.0**	**0.9**	**0.9**	**1.1**	8.9	1	2	4	7	633	68.0	9.9
NP_995319.1	45447090	**GMPPA**	mannose-1-phosphate guanyltransferase alpha	1.2	1.2	1.2	1.1	1.4	1.2	1.2	1.1	**1.3**	**1.2**	**1.2**	**1.1**	15.0	1	5	5	9	420	46.3	7.2
NP_037380.1	7019471	**NXT1**	NTF2-related export protein 1	1.1	1.0	1.3	1.0	1.1	1.3	1.3	1.2	**1.1**	**1.2**	**1.3**	**1.1**	5.7	3	1	1	1	140	15.8	5.0
NP_006294.2	11125770	**AIMP2**	aminoacyl tRNA synthase complex-interacting multifunctional protein 2	1.1	1.1	1.1	1.1	1.2	1.1	1.2	1.1	**1.2**	**1.1**	**1.1**	**1.1**	46.3	1	10	10	44	320	35.3	8.2
NP_005810.1	5032131	**STX6**	syntaxin-6 isoform 1	1.1	1.1	1.1	1.2	1.0	0.9	0.9	1.0	**1.0**	**1.0**	**1.0**	**1.1**	18.0	2	3	3	6	255	29.2	4.9
NP_997647.1	47132557	**FN1**	fibronectin isoform 1 preproprotein	1.2	1.4	**2.1**	1.0	1.3	1.2	1.2	1.2	**1.2**	**1.3**	**1.7**	**1.1**	29.2	10	42	42	145	2477	272.2	5.5
NP_001099000.1	157649073	**CAP1**	adenylyl cyclase-associated protein 1	1.1	1.1	1.1	1.1	1.1	1.1	1.1	1.1	**1.1**	**1.1**	**1.1**	**1.1**	62.5	2	22	22	159	475	51.6	8.0
NP_001026873.2	89145415	**ELP6**	elongator complex protein 6	1.0	1.0	1.1	1.1	0.9	1.0	1.1	1.1	**1.0**	**1.0**	**1.1**	**1.1**	7.1	1	2	2	7	266	29.8	6.8
NP_057210.2	118343647	**HSPB11**	intraflagellar transport protein 25 homolog	0.9	1.0	0.9	1.2	0.9	0.8	0.8	1.0	**0.9**	**0.9**	**0.9**	**1.1**	17.4	1	1	1	2	144	16.3	5.0
NP_056147.2	122939165	**NUDCD3**	nudC domain-containing protein 3	1.1	1.1	1.0	1.0	1.3	1.3	1.2	1.2	**1.2**	**1.2**	**1.1**	**1.1**	31.0	1	8	8	13	361	40.8	5.2
NP_057022.2	19923796	**NMD3**	60S ribosomal export protein NMD3	1.2	1.1	1.1	1.1	1.1	1.1	1.1	1.1	**1.1**	**1.1**	**1.1**	**1.1**	31.2	1	10	10	24	503	57.6	7.1
NP_001230.1	4502623	**CCNH**	cyclin-H isoform 1	1.0	1.1	1.0	1.2	1.0	1.0	0.9	1.0	**1.0**	**1.1**	**0.9**	**1.1**	9.3	2	2	2	3	323	37.6	7.2
NP_001229430.1	335334993	**MIF4GD**	MIF4G domain-containing protein isoform 4	1.0	1.0	1.0	1.1	1.0	1.1	1.1	1.1	**1.0**	**1.0**	**1.0**	**1.1**	10.8	3	2	2	3	222	25.4	5.3
NP_115737.1	14150171	**THOC3**	THO complex subunit 3	1.1	1.2	1.0	1.3	1.3	0.9	0.8	0.9	**1.2**	**1.0**	**0.9**	**1.1**	6.0	1	1	1	2	351	38.7	6.1
NP_001243821.1	379317149	**SRI**	sorcin isoform D	1.0	1.0	1.0	1.1	1.1	0.8	0.9	1.1	**1.1**	**0.9**	**0.9**	**1.1**	6.1	4	1	1	3	180	20.0	5.6
NP_653182.1	21389361	**L3HYPDH**	trans-3-hydroxy-L-proline dehydratase	1.1	1.0	1.1	1.0	1.2	1.2	1.3	1.2	**1.2**	**1.1**	**1.2**	**1.1**	18.4	1	5	5	13	354	38.1	6.7
NP_060220.3	203098013	**RETSAT**	all-trans-retinol 13,14-reductase precursor	1.2	1.1	1.1	1.1	1.2	1.2	1.2	1.1	**1.2**	**1.2**	**1.1**	**1.1**	20.3	1	9	9	16	610	66.8	8.3
NP_612390.1	19923907	**OXNAD1**	oxidoreductase NAD-binding domain-containing protein 1 precursor	1.1	1.1	1.1	1.2	1.3	1.0	1.1	1.0	**1.2**	**1.1**	**1.1**	**1.1**	5.8	1	2	2	2	312	34.8	8.4
NP_065392.1	24308201	**APMAP**	adipocyte plasma membrane-associated protein	1.0	1.1	1.1	1.1	1.1	1.1	1.1	1.1	**1.1**	**1.1**	**1.1**	**1.1**	38.2	1	11	11	52	416	46.5	6.2
NP_001009183.1	57165352	**GEMIN2**	gem-associated protein 2 isoform gamma	1.1	1.0	1.0	1.2	1.0	0.9	1.0	1.0	**1.0**	**1.0**	**1.0**	**1.1**	26.0	3	3	3	8	250	28.1	6.8
NP_001368.2	283806679	**DYNC2H1**	cytoplasmic dynein 2 heavy chain 1 isoform 1	1.1	1.1	1.2	1.1	1.1	1.0	1.1	1.1	**1.1**	**1.1**	**1.2**	**1.1**	1.4	2	4	4	5	4307	492.3	6.5
NP_006835.2	21361361	**ILVBL**	acetolactate synthase-like protein	1.0	1.1	1.0	1.0	0.9	0.9	0.9	1.1	**1.0**	**1.0**	**1.0**	**1.1**	16.3	1	5	5	18	632	67.8	8.1
NP_057688.2	38372909	**KDM3B**	lysine-specific demethylase 3B	1.0	1.1	1.0	1.1	1.0	1.1	1.0	1.1	**1.0**	**1.1**	**1.0**	**1.1**	11.5	1	12	12	33	1761	191.5	7.2
NP_001489.1	4557625	**GCLC**	glutamate--cysteine ligase catalytic subunit isoform a	1.4	1.3	1.1	1.1	1.3	1.2	1.1	1.1	**1.4**	**1.3**	**1.1**	**1.1**	28.4	2	13	13	50	637	72.7	6.1
NP_056311.2	33946282	**KIAA1429**	protein virilizer homolog isoform 1	1.1	1.1	1.2	1.2	1.2	1.0	1.2	1.0	**1.2**	**1.1**	**1.2**	**1.1**	3.8	2	6	6	12	1812	201.9	5.0
NP_006459.3	21359969	**POLR3C**	DNA-directed RNA polymerase III subunit RPC3 isoform 1	0.9	1.0	1.0	1.1	1.1	1.1	1.0	1.1	**1.0**	**1.0**	**1.0**	**1.1**	10.3	2	4	4	9	534	60.6	7.3
NP_057090.2	31542303	**ABHD5**	1-acylglycerol-3-phosphate O-acyltransferase ABHD5	1.1	1.0	1.0	1.1	1.3	1.3	1.2	1.1	**1.2**	**1.1**	**1.1**	**1.1**	4.6	1	1	1	2	349	39.1	6.6
NP_004037.1	4757810;50345984	**ATP5A1**	ATP synthase subunit alpha, mitochondrial isoform a precursor	1.1	1.1	1.1	1.1	1.1	1.1	1.1	1.1	**1.1**	**1.1**	**1.1**	**1.1**	67.6	3	31	32	281	553	59.7	9.1
NP_057147.2	116875826	**HDDC2**	HD domain-containing protein 2	1.0	1.0	0.9	1.3	1.0	0.9	0.9	0.9	**1.0**	**1.0**	**0.9**	**1.1**	35.3	1	5	5	14	204	23.4	5.5
NP_057422.3	39725950	**IPO11**	importin-11 isoform 2	1.1	1.1	1.1	1.1	1.1	1.1	1.2	1.1	**1.1**	**1.1**	**1.1**	**1.1**	12.0	2	8	8	18	975	112.5	5.2

Khan *et al.* , 2019. Multi-omics analysis to characterize cigarette smoke induced molecular alterations in esophageal cells
Supplementary Table 5. List of proteins quantified in untreated and chronically treated Het1A cells with cigarette smoke condensate for 8 months

NP_Accession	Protein group Accession	Gene ID	Description	Het-1A-Smoke - 2M/Parental	Het-1A-Smoke - 4M/Parental	Het-1A-Smoke - 6M/Parental	Het-1A-Smoke - 8M/Parental	Het-1A-Smoke - 2M/Parental	Het-1A-Smoke - 4M/Parental	Het-1A-Smoke - 6M/Parental	Het-1A-Smoke - 8M/Parental	Het-1A-Smoke - 2M/Parental	Het-1A-Smoke - 4M/Parental	Het-1A-Smoke - 6M/Parental	Het-1A-Smoke - 8M/Parental	Coverage (%)	Proteins	Unique Peptides	# Peptides	PSM	# AAs	MW [kDa]	calc. pI
				Replicate 1				Replicate 2				Average of replicates											
NP_055429.2	163644283	PSME4	proteasome activator complex subunit 4	1.2	1.1	1.2	1.1	1.0	1.0	1.1	1.1	1.1	1.1	1.1	1.1	3.0	1	4	4	8	1843	211.2	6.9
NP_056325.2	20149619	DHRS7B	dehydrogenase/reductase SDR family member 7B	1.2	1.2	1.0	1.2	1.1	1.3	1.0	1.0	1.2	1.3	1.0	1.1	11.7	1	3	3	7	325	35.1	9.6
NP_570711.1	18860847	MEN1	menin isoform 2	1.2	1.0	1.0	1.1	1.1	1.1	1.3	1.1	1.1	1.1	1.2	1.1	12.5	2	4	4	7	610	67.4	6.6
NP_056993.2	41393547	NBAS	neuroblastoma-amplified sequence	1.1	1.2	1.1	1.1	1.2	1.2	1.2	1.1	1.2	1.2	1.2	1.1	4.0	1	7	7	10	2371	268.4	6.0
NP_060404.3	34996489	PHIP	PH-interacting protein	1.0	1.1	1.1	1.1	1.0	1.1	1.1	1.0	1.0	1.1	1.1	1.1	14.6	1	17	18	44	1821	206.5	8.9
NP_055157.1	7657347	MTCH2	mitochondrial carrier homolog 2	1.0	1.1	1.1	1.1	1.0	1.0	1.1	1.1	1.0	1.1	1.1	1.1	24.8	1	5	5	8	303	33.3	8.0
NP_665739.3	302699211	POGZ	pogo transposable element with ZNF domain isoform 3	1.1	1.1	1.1	1.1	1.1	1.1	1.2	1.1	1.1	1.1	1.1	1.1	9.7	5	8	8	17	1315	145.2	7.2
NP_055756.1	7662340	MORC2	MORC family CW-type zinc finger protein 2	1.4	1.1	1.1	1.1	1.1	1.2	1.1	1.1	1.3	1.1	1.1	1.1	10.8	3	7	7	10	970	110.7	8.6
NP_001138392.1	222136605	TTC26	intraflagellar transport protein 56 isoform 2	1.0	1.0	1.1	1.1	0.9	1.0	1.0	1.1	1.0	1.0	1.0	1.1	8.4	5	3	3	8	487	56.3	6.8
NP_006293.2	149999606	MOGS	mannosyl-oligosaccharide glucosidase isoform 1	1.0	1.0	1.0	1.1	1.1	1.0	1.0	1.1	1.0	1.0	1.0	1.1	35.2	2	20	20	58	837	91.9	8.9
NP_055987.2	115298682	PRRC2C	protein PRRC2C	0.9	1.0	1.0	1.1	0.9	1.0	1.0	1.1	0.9	1.0	1.0	1.1	10.7	1	20	21	36	2817	308.4	9.1
NP_000523.2	119943100	PCCB	propionyl-CoA carboxylase beta chain, mitochondrial isoform 1 precursor	1.1	1.1	1.1	1.1	1.2	1.1	1.1	1.1	1.2	1.1	1.1	1.1	12.6	2	4	4	12	539	58.2	7.6
NP_066923.3	32307132	NFS1	cysteine desulfurase, mitochondrial isoform a precursor	1.2	1.0	1.0	1.1	1.0	0.9	1.0	1.1	1.1	1.0	1.0	1.1	25.8	2	7	7	16	457	50.2	8.3
NP_008965.2	6631085	DNAJB4	dnaJ homolog subfamily B member 4	0.9	1.1	1.0	1.1	1.0	1.1	1.1	1.1	1.0	1.1	1.0	1.1	13.1	1	2	3	11	337	37.8	8.5
NP_000373.1	4557303	ALDH3A2	fatty aldehyde dehydrogenase isoform 2	1.0	1.0	1.0	1.1	1.1	1.1	1.1	1.1	1.1	1.1	1.0	1.1	12.6	2	5	5	14	485	54.8	7.9
NP_001265557.1	520975432	CRNKL1	crooked neck-like protein 1 isoform d	1.0	1.0	1.1	1.1	1.0	1.1	1.0	1.1	1.0	1.1	1.0	1.1	12.7	4	7	7	15	687	83.1	6.8
NP_001026847.1	72534833	RAB24	ras-related protein Rab-24	1.0	1.0	1.0	1.1	0.9	0.9	0.9	1.1	1.0	1.0	0.9	1.1	15.8	1	2	2	2	203	23.1	6.2
NP_000565.1	10835143	CD55	complement decay-accelerating factor isoform 1 preproprotein	1.4	1.2	1.3	1.1	1.3	1.3	1.6	1.1	1.3	1.2	1.4	1.1	4.5	5	2	2	3	381	41.4	7.6
NP_476516.1	17149828	NAA15	N-alpha-acetyltransferase 15, NatA auxiliary subunit	1.0	1.1	1.1	1.1	1.0	1.0	1.1	1.0	1.0	1.1	1.1	1.1	31.5	1	23	24	104	866	101.2	7.4
NP_004422.2	32967311	EPHA2	ephrin type-A receptor 2 precursor	1.1	1.2	1.2	1.1	1.0	1.1	1.2	1.1	1.1	1.1	1.2	1.1	28.4	1	19	19	63	976	108.2	6.2
NP_064544.2	190358534	SMARCAD1	SWI/SNF-related matrix-associated actin-dependent regulator of chromatin subfamily A containing DEAD/H box 1 isoform b	1.1	1.0	1.1	1.0	1.0	1.1	1.1	1.1	1.1	1.1	1.1	1.1	7.1	3	6	6	11	1026	117.3	5.6
NP_079010.2	19923599	CLMN	calmin	1.1	0.9	1.0	1.1	1.0	1.0	1.0	1.0	1.1	1.0	1.0	1.1	6.7	1	5	5	7	1002	111.6	4.9
NP_061943.2	31542656	DDX49	probable ATP-dependent RNA helicase DDX49	0.9	1.0	1.0	1.1	1.1	1.2	1.1	1.1	1.0	1.1	1.1	1.1	22.6	1	7	7	12	483	54.2	9.1
NP_443097.1	16506297	MGME1	mitochondrial genome maintenance exonuclease 1	1.1	1.1	1.2	1.1	1.1	0.9	1.1	1.1	1.1	1.0	1.1	1.1	18.6	1	5	5	5	344	39.4	7.7
NP_006273.1	5454096	STK4	serine/threonine-protein kinase 4	0.9	0.8	0.9	1.1	0.9	0.9	0.9	1.1	0.9	0.8	0.9	1.1	24.6	1	6	9	24	487	55.6	5.1
NP_004658.3	126032348	HERC2	E3 ubiquitin-protein ligase HERC2	1.0	1.1	1.1	1.1	1.0	1.1	1.2	1.1	1.0	1.1	1.1	1.1	0.9	1	4	5	8	4834	526.9	6.3
NP_071942.1	14028877	MRPS25	28S ribosomal protein S25, mitochondrial	1.0	1.1	1.0	1.1	1.0	1.1	1.1	1.1	1.0	1.1	1.0	1.1	28.3	1	3	3	9	173	20.1	8.8
NP_002487.1	4505371	NDUFS8	NADH dehydrogenase [ubiquinone] iron-sulfur protein 8, mitochondrial precursor	1.1	0.9	1.0	1.1	1.2	1.1	1.1	1.1	1.2	1.0	1.1	1.1	23.8	1	5	5	11	210	23.7	6.3
NP_001830.1	4502923	CNN3	calponin-3 isoform 1	1.0	1.1	1.1	1.1	1.0	1.1	1.1	1.0	1.0	1.1	1.1	1.1	21.0	3	3	4	13	329	36.4	6.0
NP_942592.1	38683816	ANKRD17	ankyrin repeat domain-containing protein 17 isoform b	1.1	1.1	1.1	1.1	1.1	1.0	1.0	1.1	1.1	1.1	1.1	1.1	3.9	3	2	7	16	2352	246.6	6.7
NP_001243716.1	377652346	PLEKHA5	pleckstrin homology domain-containing family A member 5 isoform 5	1.0	1.1	1.2	1.0	1.1	1.0	1.2	1.2	1.1	1.1	1.2	1.1	4.0	4	2	2	3	1098	125.4	7.4
NP_115824.1	24308334	FAM120B	constitutive coactivator of peroxisome proliferator-activated receptor gamma isoform c	1.1	1.0	1.1	1.1	1.1	1.1	1.0	1.1	1.1	1.0	1.0	1.1	9.9	4	7	7	11	910	103.7	5.7
NP_004095.4	41872631	FASN	fatty acid synthase	1.2	1.3	1.3	1.1	1.2	1.3	1.2	1.1	1.2	1.3	1.2	1.1	60.4	1	108	109	988	2511	273.3	6.4
NP_001263987.1	457867145	ERCC6-PGBD	Cockayne syndrome B-piggyBac fusion protein	0.9	1.0	1.1	0.9	1.3	1.4	1.4	1.2	1.1	1.2	1.3	1.1	0.7	2	1	1	1	1061	119.4	6.6
NP_060052.3	31543906	UBE2Q1	ubiquitin-conjugating enzyme E2 Q1	1.1	1.1	1.1	1.1	1.0	1.0	1.1	1.0	1.0	1.0	1.1	1.1	21.3	4	6	6	10	422	46.1	5.1
NP_001013454.1	61835204	MPST	3-mercaptopyruvate sulfurtransferase isoform 2	1.0	1.0	1.1	1.1	1.2	1.1	1.1	1.1	1.1	1.1	1.1	1.1	26.3	2	5	5	14	297	33.2	6.6
NP_002963.2	239735519	SBF1	myotubularin-related protein 5	1.1	1.0	1.2	1.1	1.1	1.1	1.1	1.1	1.1	1.0	1.1	1.1	3.2	1	3	3	4	1893	210.9	7.1
NP_002430.3	284813531	MSH3	DNA mismatch repair protein Msh3	1.0	1.0	1.0	1.0	1.2	1.2	1.2	1.2	1.1	1.1	1.1	1.1	10.7	1	9	9	14	1137	127.3	8.0
NP_005984.3	41350333	TBCD	tubulin-specific chaperone D	1.0	1.0	1.1	1.1	1.1	1.0	1.1	1.1	1.0	1.0	1.1	1.1	15.8	1	14	14	35	1192	132.5	6.2
NP_060324.1	8923477	TMEM160	transmembrane protein 160 precursor	1.1	1.1	1.2	1.0	1.2	1.2	1.3	1.2	1.1	1.2	1.3	1.1	3.7	1	1	1	2	188	19.6	8.0
NP_001018171.1	66529396	PON2	serum paraoxonase/arylesterase 2 isoform 2	1.0	0.9	1.1	1.1	1.2	1.0	1.0	1.1	1.1	0.9	1.0	1.1	13.2	2	3	3	12	342	38.0	5.7
NP_001093120.1	153791276	GXYLT1	glucoside xylosyltransferase 1 isoform 2	0.8	1.0	0.9	1.1	0.8	0.9	0.9	1.1	0.8	0.9	0.9	1.1	5.9	2	2	2	2	409	46.8	9.0
NP_821158.2	109148539	GGT7	gamma-glutamyltransferase 7	1.0	1.0	0.9	1.0	1.2	1.2	1.1	1.1	1.1	1.1	1.0	1.1	6.7	1	3	3	4	662	70.4	5.1
NP_001157685.1	256017180	LRCH1	leucine-rich repeat and calponin homology domain-containing protein 1 isoform 3	1.0	1.2	1.1	1.1	1.1	1.1	1.1	1.0	1.1	1.1	1.1	1.1	5.6	3	3	3	7	696	77.2	5.9
NP_056171.3	355390315	SCRIB	protein scribble homolog isoform b	1.1	1.1	1.0	1.1	1.0	1.1	1.0	1.1	1.1	1.1	1.0	1.1	13.4	3	13	13	30	1630	174.8	5.1
NP_892022.2	122939153;122939155	NNT	NAD(P) transhydrogenase, mitochondrial	1.1	1.2	1.2	1.1	1.1	1.1	1.2	1.1	1.1	1.2	1.2	1.1	4.9	1	5	5	11	1086	113.8	8.1
NP_001271175.1	545746375	SIPA1L1	signal-induced proliferation-associated 1-like protein 1 isoform 3	1.0	1.1	1.0	1.1	1.1	1.1	1.0	1.1	1.0	1.1	1.0	1.1	2.5	6	4	4	8	1782	197.3	8.3
NP_065750.1	21218438	GATAD2B	transcriptional repressor p66-beta	1.0	1.0	1.0	1.1	1.0	1.0	1.0	1.1	1.0	1.0	1.0	1.1	9.3	1	3	4	6	593	65.2	9.7
NP_694858.1	23510381	TNPO1	transportin-1 isoform 2	1.1	1.1	1.2	1.1	1.1	1.1	1.1	1.1	1.1	1.1	1.2	1.1	36.7	2	17	22	105	890	101.2	5.0
NP_001182147.1	304571975	DCXR	L-xylulose reductase isoform 2	1.1	1.1	0.9	1.1	1.1	1.1	1.0	1.1	1.1	1.1	1.0	1.1	50.8	2	7	7	17	242	25.7	8.1
NP_006653.2	146219843	SRCAP	helicase SRCAP	1.1	1.3	1.1	1.2	1.0	1.1	0.9	1.0	1.0	1.2	1.0	1.1	5.1	1	8	8	18	3230	343.3	6.0
NP_002778.1	4506181	PSMA2	proteasome subunit alpha type-2	1.0	1.0	1.1	1.1	1.1	1.0	1.1	1.1	1.0	1.0	1.1	1.1	47.9	1	6	6	28	234	25.9	7.4
NP_001127949.1	197333695	PARN	poly(A)-specific ribonuclease PARN isoform 2	1.0	1.0	1.0	1.1	1.1	1.0	1.0	1.1	1.0	1.0	1.0	1.1	16.4	3	8	8	20	578	66.5	6.4
NP_006397.1	5453549	PRDX4	peroxiredoxin-4 precursor	1.1	1.1	1.1	1.2	0.8	0.9	0.9	1.0	1.0	1.0	1.0	1.1	38.8	1	6	8	44	271	30.5	6.3
NP_004930.1	4826686	DDX1	ATP-dependent RNA helicase DDX1	1.1	1.1	1.1	1.1	1.1	1.1	1.1	1.1	1.1	1.1	1.1	1.1	43.4	1	25	25	82	740	82.4	7.2
NP_060481.3	134152708	ARGLU1	arginine and glutamate-rich protein 1	0.9	1.0	1.0	1.1	0.9	1.0	1.0	1.1	0.9	1.0	1.0	1.1	2.9	1	1	1	2	273	33.2	10.3
YP_003024031.1	251831112	ATP6	ATP synthase F0 subunit 6	1.1	1.1	1.2	1.1	1.1	1.0	1.0	1.0	1.1	1.0	1.1	1.1	4.4	1	1	1	10	226	24.8	10.1
NP_001032412.2	156105693	HELZ2	helicase with zinc finger domain 2 isoform 1	1.1	1.1	1.1	1.1	1.1	1.2	1.2	1.1	1.1	1.2	1.1	1.1	2.0	2	4	4	6	2649	294.5	7.5
NP_536338.1	17978481	VPS16	vacuolar protein sorting-associated protein 16 homolog isoform 3	1.1	1.0	0.9	1.2	1.0	0.9	0.9	1.0	1.1	1.0	0.9	1.1	4.3	2	2	2	4	695	78.3	7.9
NP_001269888.1	545479138	CCAR1	cell division cycle and apoptosis regulator protein 1 isoform b	1.0	1.0	1.1	1.1	1.0	1.0	1.1	1.1	1.0	1.0	1.1	1.1	14.2	2	12	12	14	1135	131.0	5.8
NP_001092879.1	150378549	EHBP1L1	EH domain-binding protein 1-like protein 1	1.1	1.0	1.1	1.1	1.0	1.1	1.1	1.1	1.1	1.0	1.1	1.1	4.1	1	4	4	6	1523	161.8	4.8
NP_001135822.1	215272341	THOC6	THO complex subunit 6 homolog isoform 2	1.1	1.1	1.0	1.1	1.0	1.1	1.1	1.1	1.0	1.1	1.0	1.1	19.6	2	4	4	12	296	32.9	7.2
NP_055121.1	7657015	RTCB	tRNA-splicing ligase RtcB homolog	1.1	1.1	1.1	1.1	1.0	1.0	1.0	1.1	1.0	1.0	1.1	1.1	48.5	1	17	17	63	505	55.2	7.2
NP_002446.3	597955330	MTX1	metaxin-1 isoform 1	1.1	1.0	1.0	1.0	1.2	1.2	1.2	1.2	1.2	1.1	1.1	1.1	18.5	2	7	7	16	466	51.4	9.8
NP_076424.1	13027602	DDRGK1	DDRGK domain-containing protein 1 precursor	1.1	1.0	0.9	1.1	1.1	1.0	1.0	1.1	1.1	1.0	1.0	1.1	46.5	1	7	7	26	314	35.6	5.1
NP_060579.3	190194365	MTPAP	poly(A) RNA polymerase, mitochondrial precursor	1.0	1.1	1.2	1.1	1.0	1.1	1.1	1.1	1.0	1.1	1.1	1.1	8.6	1	4	4	9	582	66.1	9.0
NP_001022.1	4506715	RPS28	40S ribosomal protein S28	1.2	1.2	1.2	1.1	1.0	1.2	1.0	1.1	1.1	1.2	1.1	1.1	30.4	1	2	2	3	69	7.8	10.7
NP_001073881.2	148276990	ZBTB38	zinc finger and BTB domain-containing protein 38	1.0	1.1	1.3	1.1	1.1	1.3	1.4	1.1	1.1	1.2	1.3	1.1	1.8	1	2	2	3	1195	134.2	8.0
NP_004276.2	52145310	H6PD	GDH/6PGL endoplasmic bifunctional protein isoform 2 precursor	0.9	1.0	1.0	1.0	1.2	1.1	1.2	1.2	1.0	1.1	1.1	1.1	3.8	2	3	3	3	791	88.8	7.3
NP_079523.3	48976061	ACAD10	acyl-CoA dehydrogenase family member 10 isoform b	1.1	1.0	1.1	1.0	1.2	0.8	1.0	1.1	1.2	0.9	1.1	1.1	11.2	2	7	7	10	1059	118.8	8.1
NP_002060.4	33946324	GNAI1	guanine nucleotide-binding protein G(i) subunit alpha-1 isoform 1	1.3	1.1	1.1	1.2	1.0	0.9	1.0	1.0	1.1	1.0	1.1	1.1	23.2	9	2	8	26	354	40.3	6.0
NP_002878.2	15149476	RARS	arginine--tRNA ligase, cytoplasmic	1.0	1.0	1.0	1.1	1.0	1.0	1.0	1.1	1.0	1.0	1.0	1.1	48.9	1	27	27	160	660	75.3	6.7
NP_963906.1	42491362	IKBIP	inhibitor of nuclear factor kappa-B kinase-interacting protein isoform 2	1.2	1.1	1.2	1.1	1.2	1.1	1.1	1.1	1.2	1.1	1.1	1.1	16.9	2	3	6	12	350	39.3	9.2
NP_001395.1	4503481	EEF1G	elongation factor 1-gamma	1.1	1.1	1.1	1.1	1.1	1.1	1.1	1.1	1.1	1.1	1.1	1.1	76.0	1	27	27	357	437	50.1	6.7
NP_000932.3	127139033	POR	NADPH--cytochrome P450 reductase	1.0	1.1	1.0	1.1	1.1	1.1	1.1	1.1	1.1	1.1	1.0	1.1	29.7	1	14	14	42	680	77.0	5.6
NP_055362.1	7657649	TMOD3	tropomodulin-3	1.0	1.0	1.1	1.1	1.1	1.1	1.2	1.1	1.0	1.1	1.2	1.1	9.1	1	3	3	5	352	39.6	5.2
NP_060762.3	222831590	QRSL1	glutamyl-tRNA(Gln) amidotransferase subunit A, mitochondrial	1.0	0.9	0.9	0.9	0.9	1.0	0.9	1.3	1.0	1.0	0.9	1.1	3.4	1	1	1	2	528	57.4	5.7
NP_036265.3	109134349	COPG2	coatomer subunit gamma-2 isoform 1	1.0	1.0	1.0	1.1	1.0	1.0	1.0	1.1	1.0	1.0	1.0	1.1	36.6	2	20	23	64	871	97.6	5.8
NP_001153221.1	229331971	RIOK2	serine/threonine-protein kinase RIO2 isoform 2	1.0	1.1	1.1	1.0	1.0	1.1	1.0	1.1	1.0	1.1	1.0	1.1	8.4	2	3	3	4	474	54.5	5.4
NP_001631.3	23510451	APEH	acylamino-acid-releasing enzyme	1.1	1.0	1.0	1.1	1.1	1.0	1.0	1.1	1.1	1.0	1.0	1.1	26.0	1	11	11	30	732	81.2	5.5
NP_056016.1	21327667	BOP1	ribosome biogenesis protein BOP1	1.1	1.1	1.2	1.1	1.1	1.1	1.1	1.1	1.1	1.1	1.1	1.1	26.5	1	13	13	33	746	83.6	6.2
NP_006787.2	300192933	AFG3L2	AFG3-like protein 2	1.2	1.1	1.1	1.1	1.1	1.1	1.1	1.1	1.1	1.1	1.1	1.1	30.0	1	20	20	56	797	88.5	8.7
NP_001273762.1	558472758	GAK	cyclin-G-associated kinase isoform 2	1.2	1.1	1.1	1.1	1.0	1.0	1.1	1.0	1.1	1.1	1.1	1.1	12.0	2	7	7	13	1213	132.1	5.7
NP_722560.1	24476013	PTK2	focal adhesion kinase 1 isoform a	1.0	1.1	1.1	1.2	1.1	1.0	1.0	1.0	1.1	1.0	1.0	1.1	11.9	3	9	9	20	1052	119.2	6.6
NP_002147.2	31542947	HSPD1	60 kDa heat shock protein, mitochondrial	1.0	1.0	1.0	1.1	1.0	1.0	1.0	1.1	1.0	1.0	1.0	1.1	73.5	1	33	33	403	573	61.0	5.9
NP_060422.4	38683855	PTCD3	pentatricopeptide repeat domain-containing protein 3, mitochondrial precursor	1.2	1.1	1.1	1.1	1.1	1.0	1.1	1.1	1.2	1.0	1.1	1.1	29.3	3	14	15	35	689	78.5	6.4
NP_006251.1	5453980	DNAJC3	dnaJ homolog subfamily C member 3 precursor	1.2	1.1	1.1	1.1	1.2	1.2	1.1	1.1	1.2	1.1	1.1	1.1	21.0	1	6	7	18	504	57.5	6.2

Khan *et al.* , 2019. Multi-omics analysis to characterize cigarette smoke induced molecular alterations in esophageal cells
Supplementary Table 5. List of proteins quantified in untreated and chronically treated Het1A cells with cigarette smoke condensate for 8 months

NP_Accession	Protein group Accession	Gene ID	Description	Het-1A-Smoke - 2M/Parental	Het-1A-Smoke - 4M/Parental	Het-1A-Smoke - 6M/Parental	Het-1A-Smoke - 8M/Parental	Het-1A-Smoke - 2M/Parental	Het-1A-Smoke - 4M/Parental	Het-1A-Smoke - 6M/Parental	Het-1A-Smoke - 8M/Parental	Het-1A-Smoke - 2M/Parental	Het-1A-Smoke - 4M/Parental	Het-1A-Smoke - 6M/Parental	Het-1A-Smoke - 8M/Parental	Coverage (%)	Proteins	Unique Peptides	# Peptides	PSM	# AAs	MW [kDa]	calc. pI
				Replicate 1				Replicate 2				Average of replicates											
NP_054894.1	7661806	MRPL15	39S ribosomal protein L15, mitochondrial	1.2	1.1	1.1	1.1	1.1	1.0	1.0	1.0	1.1	1.0	1.1	1.1	17.2	6	5	5	9	296	33.4	10.0
NP_006363.4	228008291	SYNCRIP	heterogeneous nuclear ribonucleoprotein Q isoform 1	0.9	1.0	1.0	1.2	1.0	1.0	1.0	1.0	0.9	1.0	1.0	1.1	52.3	2	1	31	457	623	69.6	8.6
NP_002213.5	269954692	ITPR1	inositol 1,4,5-trisphosphate receptor type 1 isoform 2	1.0	1.2	1.2	1.1	1.1	1.2	1.1	1.1	1.1	1.2	1.2	1.1	0.6	13	1	2	2	2695	306.6	6.3
NP_006612.2	21361647	AHCYL1	putative adenosylhomocysteinase 2 isoform a	1.1	1.0	1.1	1.1	1.1	1.1	1.0	1.1	1.1	1.1	1.1	1.1	24.7	2	5	10	32	530	58.9	6.9
NP_056997.1	7705696	TXNDC12	thioredoxin domain-containing protein 12 precursor	1.1	1.1	1.1	1.1	1.2	1.0	1.2	1.1	1.2	1.0	1.1	1.1	14.0	1	2	2	3	172	19.2	5.4
NP_536316.1	18104971	PTP4A2	protein tyrosine phosphatase type IVA 2 isoform 1	1.2	1.1	1.2	1.0	1.2	1.2	1.2	1.1	1.2	1.2	1.2	1.1	13.8	3	2	2	4	167	19.1	8.4
NP_001244316.1	383792178	CHEK2	serine/threonine-protein kinase Chk2 isoform d	1.1	1.2	1.2	1.1	1.3	1.1	1.4	1.1	1.2	1.1	1.3	1.1	14.0	4	3	3	7	322	36.1	7.0
NP_001261.2	68299795	CHD1	chromodomain-helicase-DNA-binding protein 1	1.1	1.1	1.0	1.0	1.1	1.2	1.3	1.1	1.1	1.1	1.2	1.1	3.3	1	3	6	8	1710	196.6	7.1
NP_006828.2	38027923	COPS5	COP9 signalosome complex subunit 5	1.0	1.0	1.1	1.1	1.1	1.1	1.2	1.1	1.1	1.1	1.2	1.1	15.3	1	5	5	12	334	37.6	6.5
NP_001137532.1	221139926	NHSL1	NHS-like protein 1 isoform 2	0.8	1.0	0.9	0.9	1.2	1.1	1.1	1.3	1.0	1.0	1.0	1.1	1.0	2	1	1	2	1606	170.4	7.5
NP_055994.2	223278379	RRP12	RRP12-like protein isoform 1	1.0	1.1	1.1	1.1	1.1	1.1	1.1	1.1	1.0	1.1	1.1	1.1	13.1	3	13	13	41	1297	143.6	8.7
NP_000242.1	4557761	MSH2	DNA mismatch repair protein Msh2 isoform 1	1.0	1.0	1.0	1.1	1.1	1.0	1.0	1.1	1.0	1.0	1.0	1.1	30.1	2	24	24	54	934	104.7	5.8
NP_005244.1	4885245	FOSL2	fos-related antigen 2	1.2	1.5	1.8	1.2	1.0	1.1	1.0	1.0	1.1	1.3	1.4	1.1	10.1	1	2	2	4	326	35.2	7.5
NP_001129503.2	222144227	ATG7	ubiquitin-like modifier-activating enzyme ATG7 isoform b	1.1	1.0	1.0	1.1	1.1	1.0	1.1	1.1	1.1	1.0	1.0	1.1	17.3	3	9	9	23	676	75.0	6.0
NP_110379.2	57863257	TCP1	T-complex protein 1 subunit alpha isoform a	1.1	1.1	1.1	1.1	1.0	1.0	1.0	1.1	1.1	1.1	1.1	1.1	67.6	2	30	30	272	556	60.3	6.1
NP_005558.1	5031863	LGALS3BP	galectin-3-binding protein precursor	1.3	1.3	1.3	1.1	1.2	1.3	1.3	1.1	1.3	1.3	1.3	1.1	19.5	1	10	10	30	585	65.3	5.3
NP_001245298.1	385719169	DIAPH3	protein diaphanous homolog 3 isoform f	1.2	1.1	1.2	1.1	1.1	1.1	1.2	1.1	1.2	1.1	1.2	1.1	9.5	7	7	7	12	1112	127.8	6.9
NP_001185940.1	312261189	DCTN5	dynactin subunit 5 isoform 2	1.0	1.0	1.0	1.3	0.7	0.7	0.9	0.8	0.8	0.9	0.9	1.1	13.1	3	1	1	2	84	9.7	9.1
NP_001034702.1	88853865	IAH1	isoamyl acetate-hydrolyzing esterase 1 homolog precursor	1.0	1.0	1.2	1.1	1.1	1.1	1.1	1.1	1.0	1.1	1.1	1.1	12.1	1	2	2	3	248	27.6	5.3
NP_705831.1	23957690	COG7	conserved oligomeric Golgi complex subunit 7	1.0	1.0	1.0	1.0	1.1	1.1	1.1	1.1	1.0	1.0	1.0	1.1	10.1	1	5	5	9	770	86.3	5.5
NP_056528.2	42544136	RRM2B	ribonucleoside-diphosphate reductase subunit M2 B isoform 1	1.0	1.0	1.0	1.0	1.1	1.0	1.0	1.1	1.1	1.0	1.0	1.1	28.5	3	7	8	19	351	40.7	5.0
NP_613075.1	20336746	H2AFY	core histone macro-H2A.1 isoform 1	1.0	1.0	1.0	1.0	0.9	1.2	1.1	1.1	1.0	1.1	1.1	1.1	67.2	3	16	18	109	369	39.2	9.8
NP_004975.2	45446749	KIF5A	kinesin heavy chain isoform 5A	1.0	0.9	0.8	1.0	1.0	1.1	1.0	1.2	1.0	1.0	0.9	1.1	10.5	1	3	9	37	1032	117.3	5.9
NP_005434.4	46094058	PAPSS1	bifunctional 3'-phosphoadenosine 5'-phosphosulfate synthase 1	1.0	1.0	1.0	1.1	1.0	1.1	1.0	1.1	1.0	1.0	1.0	1.1	15.5	1	7	7	12	624	70.8	6.9
NP_115866.1	21265093	MRPL41	39S ribosomal protein L41, mitochondrial	1.0	1.1	1.1	1.1	1.2	1.1	1.2	1.1	1.1	1.1	1.2	1.1	55.5	1	5	5	24	137	15.4	9.6
NP_001180432.1	301897479	ENO3	beta-enolase isoform 2	0.9	0.8	0.6	1.1	0.8	0.6	0.5	1.1	0.8	0.7	0.6	1.1	25.1	2	1	7	266	391	42.2	8.0
NP_057094.2	223555989	ZC2HC1A	zinc finger C2HC domain-containing protein 1A	0.9	1.1	0.9	1.1	1.0	1.0	1.0	1.1	1.0	1.1	1.0	1.1	2.2	1	1	1	1	325	35.1	9.8
NP_001273984.1	559098399	ZKSCAN1	zinc finger protein with KRAB and SCAN domains 1 isoform c	1.0	1.1	1.1	1.1	1.3	1.1	1.0	1.1	1.1	1.1	1.1	1.1	10.6	3	2	2	3	350	39.3	8.6
NP_996895.1	46370095;6912240	AP3M1	AP-3 complex subunit mu-1	1.0	1.1	1.1	1.1	1.0	1.0	1.1	1.1	1.0	1.0	1.1	1.1	24.9	2	7	7	15	418	46.9	6.9
NP_542391.2	259906440	ZC3HAV1L	zinc finger CCCH-type antiviral protein 1-like	1.1	1.1	1.1	1.2	0.9	0.9	1.1	0.9	1.0	1.0	1.1	1.1	6.7	1	1	1	1	300	32.9	8.1
NP_005723.2	19924129	RAD50	DNA repair protein RAD50	1.1	1.1	1.1	1.1	1.1	1.1	1.1	1.1	1.1	1.1	1.1	1.1	19.2	1	25	25	67	1312	153.8	6.9
NP_872578.1	33188463	MRPS9	28S ribosomal protein S9, mitochondrial	1.1	1.1	1.1	1.1	1.1	1.0	1.1	1.1	1.1	1.1	1.1	1.1	37.4	1	11	11	23	396	45.8	9.5
NP_077284.2	109715829	DHRS11	dehydrogenase/reductase SDR family member 11 precursor	1.0	1.0	1.0	1.0	1.2	1.0	1.2	1.1	1.1	1.0	1.1	1.1	4.6	1	1	1	1	260	28.3	6.6
NP_004696.2	19923268	PRKRIR	52 kDa repressor of the inhibitor of the protein kinase	1.1	1.0	1.1	1.1	1.0	1.1	1.1	1.1	1.0	1.0	1.1	1.1	5.3	1	3	3	3	761	87.6	5.9
NP_001076581.2	285002231;285002233	GPD2	glycerol-3-phosphate dehydrogenase, mitochondrial precursor	1.0	1.1	1.1	1.1	1.1	1.1	1.0	1.1	1.0	1.1	1.1	1.1	18.6	1	12	12	24	727	80.8	7.7
NP_775873.2	55749758	DIP2B	disco-interacting protein 2 homolog B	1.1	1.1	1.0	1.0	1.2	1.1	1.2	1.1	1.1	1.1	1.1	1.1	0.8	1	1	1	2	1576	171.4	8.1
NP_001139528.1	225703081	MBOAT7	lysophospholipid acyltransferase 7 isoform 2	1.2	1.1	1.2	1.1	1.0	1.0	1.1	1.1	1.1	1.1	1.2	1.1	11.8	3	4	4	10	399	44.7	8.9
NP_839943.2	39753961	IQGAP3	ras GTPase-activating-like protein IQGAP3	0.9	1.0	1.0	1.0	1.0	1.2	1.1	1.2	1.0	1.1	1.1	1.1	4.7	3	2	7	27	1631	184.5	7.6
NP_057558.3	108773808	CCDC174	coiled-coil domain-containing protein 174	1.4	1.2	1.3	1.2	0.9	1.0	0.9	1.0	1.1	1.1	1.1	1.1	12.0	1	3	3	3	467	53.9	6.3
NP_612352.1	19923891	KLC4	kinesin light chain 4 isoform c	1.1	1.0	1.0	1.1	1.1	1.1	1.1	1.1	1.1	1.1	1.1	1.1	8.3	4	1	2	2	315	35.1	5.5
NP_001180272.1	301069365	MRPS18A	28S ribosomal protein S18a, mitochondrial isoform 2 precursor	1.0	1.1	1.1	1.0	1.1	1.1	1.1	1.1	1.1	1.1	1.1	1.1	20.5	2	2	2	2	127	14.0	9.5
NP_932076.1	37574614	NME7	nucleoside diphosphate kinase 7 isoform b	1.0	1.0	1.0	1.1	1.0	1.1	1.0	1.1	1.0	1.1	1.0	1.1	3.2	2	1	1	2	340	38.2	6.8
NP_001213.2	26667191	CAMK2G	calcium/calmodulin-dependent protein kinase type II subunit gamma isoform 4	1.2	1.1	1.3	1.1	1.3	1.1	1.3	1.1	1.3	1.1	1.3	1.1	15.4	16	4	7	17	495	55.9	7.2
NP_001128527.1	205277463;4507521	TKT	transketolase isoform 1	1.1	1.1	1.1	1.1	1.1	1.1	1.1	1.1	1.1	1.1	1.1	1.1	69.7	2	30	30	338	623	67.8	7.7
NP_004206.1	4758230	EBAG9	receptor-binding cancer antigen expressed on SiSo cells	1.2	1.1	1.1	1.0	1.1	1.2	1.2	1.1	1.1	1.1	1.2	1.1	8.0	1	1	1	1	213	24.4	6.3
NP_001171835.1	296531375	FBXL20	F-box/LRR-repeat protein 20 isoform 2	1.2	1.1	1.0	1.0	1.0	1.1	1.0	1.2	1.1	1.1	1.0	1.1	7.2	3	2	2	3	404	45.0	7.1
NP_001268412.1	527498256	ARFGAP1	ADP-ribosylation factor GTPase-activating protein 1 isoform d	0.9	1.0	1.0	1.1	0.9	0.9	1.0	1.0	0.9	1.0	1.0	1.1	26.3	6	9	9	24	361	39.7	5.4
NP_002818.1	4506289	PTPN1	tyrosine-protein phosphatase non-receptor type 1 isoform 1	1.0	1.0	1.1	1.1	1.0	1.1	1.1	1.1	1.0	1.1	1.1	1.1	49.2	2	15	15	42	435	49.9	6.3
NP_061903.2	67782362	DHX29	ATP-dependent RNA helicase DHX29	1.2	1.1	1.0	1.1	1.1	1.1	1.2	1.1	1.1	1.1	1.1	1.1	13.6	1	14	15	27	1369	155.1	8.1
NP_005875.1	5031975;62422557	PAK4	serine/threonine-protein kinase PAK 4 isoform 1	0.9	0.9	0.9	1.0	1.2	1.2	1.1	1.2	1.1	1.1	1.0	1.1	10.5	3	5	5	14	591	64.0	9.7
NP_006827.1	54607053	GCN1L1	translational activator GCN1	1.0	1.0	1.0	1.1	1.0	1.0	1.0	1.1	1.0	1.0	1.0	1.1	30.7	1	60	60	208	2671	292.5	7.4
NP_116130.2	15100175	AGPAT1	1-acyl-sn-glycerol-3-phosphate acyltransferase alpha	1.0	0.9	0.9	0.9	1.2	1.1	1.1	1.2	1.1	1.0	1.0	1.1	13.1	1	3	3	4	283	31.7	9.4
NP_003666.1	4506123	PRPF18	pre-mRNA-splicing factor 18	0.9	1.0	1.0	1.0	1.0	1.2	1.1	1.1	0.9	1.1	1.0	1.1	12.9	1	3	3	4	342	39.8	8.1
NP_036277.2	42544174	DTYMK	thymidylate kinase isoform 1	1.1	1.0	1.2	1.1	1.0	1.0	1.1	1.1	1.1	1.0	1.1	1.1	27.4	2	6	6	13	212	23.8	8.3
NP_078943.1	13375926	VPS37B	vacuolar protein sorting-associated protein 37B	0.9	1.0	1.0	1.2	0.9	0.9	0.8	1.0	0.9	1.0	0.9	1.1	7.0	1	1	1	2	285	31.3	7.3
NP_057093.1	7705773	SH3GLB1	endophilin-B1 isoform 1	1.2	1.1	1.1	1.2	0.9	1.0	0.9	1.0	1.1	1.0	1.0	1.1	4.9	4	1	2	5	365	40.8	6.0
NP_060615.1	8922532	RMDN3	regulator of microtubule dynamics protein 3	1.1	1.1	1.1	1.1	1.0	1.2	1.0	1.1	1.0	1.2	1.1	1.1	13.0	1	4	4	5	470	52.1	5.1
NP_055961.1	149363661	Sep-08	septin-8 isoform b	1.0	1.0	1.0	1.1	1.0	1.0	0.9	1.0	1.0	1.0	1.0	1.1	22.8	6	4	7	11	429	49.8	6.1
NP_057191.2	34101286	ZFR	zinc finger RNA-binding protein	1.1	1.0	1.0	1.1	1.1	1.0	1.0	1.1	1.1	1.0	1.0	1.1	17.2	1	12	12	26	1074	116.9	9.0
NP_001743.1	4557014	CAT	catalase	1.0	1.0	1.0	1.1	1.1	1.0	0.9	1.1	1.0	1.0	1.0	1.1	40.0	1	15	15	39	527	59.7	7.4
NP_004362.2	148536853	COPA	coatomer subunit alpha isoform 2	1.2	1.1	1.2	1.1	1.2	1.1	1.2	1.1	1.2	1.1	1.2	1.1	44.4	2	38	38	148	1224	138.3	7.7
NP_002600.1	4505683	PDGFRB	platelet-derived growth factor receptor beta precursor	1.4	1.1	0.9	1.0	1.1	1.3	1.0	1.2	1.2	1.2	1.0	1.1	1.5	1	1	1	1	1106	123.9	5.0
NP_004365.1	4758040	COX6C	cytochrome c oxidase subunit 6C proprotein	0.9	1.0	1.0	1.1	1.0	1.1	1.1	1.1	0.9	1.1	1.0	1.1	10.7	1	1	1	2	75	8.8	10.4
NP_001248363.1	387528025	AARSD1	alanyl-tRNA editing protein Aarsd1	1.1	1.1	1.1	1.1	1.0	1.0	1.0	1.0	1.0	1.0	1.1	1.1	9.2	3	2	2	4	412	45.5	6.4
NP_116231.2	66348165	TMEM209	transmembrane protein 209 isoform 1	1.3	1.1	1.1	1.0	1.2	1.2	1.3	1.1	1.2	1.2	1.2	1.1	9.5	1	3	3	4	561	62.9	8.6
NP_065098.1	9966779	EIF2B3	translation initiation factor eIF-2B subunit gamma isoform 1	1.0	1.0	1.0	1.1	1.1	1.0	1.0	1.1	1.0	1.0	1.0	1.1	12.6	3	4	4	5	452	50.2	6.5
NP_001108585.2	264681556	WDR26	WD repeat-containing protein 26 isoform b	1.0	1.1	1.0	1.1	1.1	1.0	1.0	1.1	1.1	1.0	1.0	1.1	13.2	2	6	6	10	645	70.4	6.2
NP_057279.2	46049114	KIF20B	kinesin-like protein KIF20B isoform 2	1.0	1.1	1.1	1.1	1.0	1.1	1.2	1.1	1.0	1.1	1.1	1.1	4.6	2	5	5	9	1780	206.0	5.8
NP_060391.2	157426879	NPLOC4	nuclear protein localization protein 4 homolog	1.0	1.1	1.1	1.1	1.0	0.9	1.1	1.0	1.0	1.0	1.1	1.1	19.9	1	10	10	24	608	68.1	6.4
NP_064696.1	9945306	MGST1	microsomal glutathione S-transferase 1 isoform a	1.2	1.1	1.3	1.0	1.2	1.2	1.4	1.2	1.2	1.2	1.3	1.1	18.7	3	2	2	5	155	17.6	9.4
NP_001026866.1	72534684	PLD3	phospholipase D3	1.0	1.1	1.1	1.1	1.0	1.1	1.1	1.1	1.0	1.1	1.1	1.1	6.3	1	3	3	9	490	54.7	6.5
NP_006258.3	150418007	RANBP2	E3 SUMO-protein ligase RanBP2	1.1	1.1	1.1	1.1	1.1	1.0	1.1	1.1	1.1	1.0	1.1	1.1	17.1	9	39	40	94	3224	358.0	6.2
NP_009146.2	70887790	POLG2	DNA polymerase subunit gamma-2, mitochondrial	1.0	1.0	1.0	1.0	1.0	1.1	1.1	1.1	1.0	1.0	1.1	1.1	9.7	1	3	3	8	485	54.9	8.4
NP_005165.1	4885079	ATP5C1	ATP synthase subunit gamma, mitochondrial isoform H (heart) precursor	1.1	1.1	1.1	1.1	1.1	1.0	1.1	1.0	1.1	1.1	1.1	1.1	51.2	2	14	14	91	297	32.9	9.3
NP_942088.1	38683836	CD47	leukocyte surface antigen CD47 isoform 2 precursor	1.0	1.1	1.0	1.1	1.2	1.1	1.2	1.1	1.1	1.1	1.1	1.1	9.2	2	3	3	14	305	33.2	7.9
NP_004751.2	109255245	STK17A	serine/threonine-protein kinase 17A	1.3	1.2	1.3	1.0	1.2	1.2	1.1	1.1	1.3	1.2	1.2	1.1	4.1	1	1	1	1	414	46.5	5.1
NP_987100.1	45359846	G3BP2	ras GTPase-activating protein-binding protein 2 isoform b	1.1	1.0	1.0	1.1	1.0	1.0	1.1	1.1	1.1	1.0	1.1	1.1	22.1	3	7	8	20	449	50.8	5.4
NP_940916.2	42476013	NHLRC2	NHL repeat-containing protein 2	1.1	1.1	1.1	1.1	1.1	1.1	1.2	1.1	1.1	1.1	1.1	1.1	22.3	1	9	9	24	726	79.4	5.6
NP_003175.1	4507347	TAF2	transcription initiation factor TFIID subunit 2	1.2	1.1	1.1	1.1	1.1	1.2	1.1	1.1	1.1	1.1	1.1	1.1	5.6	1	5	5	10	1199	136.9	8.2
NP_078900.1	13375848	SMC6	structural maintenance of chromosomes protein 6	1.1	1.1	1.2	1.1	1.0	1.0	1.0	1.1	1.1	1.1	1.1	1.1	2.4	1	2	2	2	1091	126.2	7.0
NP_003944.1	4506357	MPZL1	myelin protein zero-like protein 1 isoform a precursor	1.1	1.4	1.4	1.1	1.1	1.2	1.2	1.1	1.1	1.3	1.3	1.1	13.0	2	4	4	6	269	29.1	8.7
NP_002386.1	4505143	ME1	NADP-dependent malic enzyme	1.3	1.3	1.1	1.1	1.2	1.1	1.1	1.1	1.2	1.2	1.1	1.1	25.7	1	11	11	50	572	64.1	6.1
NP_001257377.1	394025725	ACADVL	very long-chain specific acyl-CoA dehydrogenase, mitochondrial isoform 4	1.4	1.3	1.4	1.1	1.2	1.2	1.3	1.1	1.3	1.3	1.3	1.1	53.5	4	23	23	88	579	62.5	7.3
NP_061326.1	10092657	NDUFA12	NADH dehydrogenase [ubiquinone] 1 alpha subcomplex subunit 12 isoform a	1.1	1.1	1.1	1.1	1.2	1.0	1.1	1.1	1.1	1.1	1.1	1.1	18.6	2	3	3	4	145	17.1	9.6
NP_055975.1	24308013	PMPCA	mitochondrial-processing peptidase subunit alpha isoform 1 precursor	1.1	1.0	1.1	1.1	1.1	1.0	1.0	1.1	1.1	1.0	1.1	1.1	17.5	3	8	8	16	525	58.2	6.9
NP_057594.4	323668300	SCLY	selenocysteine lyase	0.9	1.1	1.1	1.1	1.1	1.0	0.9	1.1	1.0	1.0	1.0	1.1	8.4	1	2	2	4	453	48.8	7.1

Khan *et al.* , 2019. Multi-omics analysis to characterize cigarette smoke induced molecular alterations in esophageal cells
Supplementary Table 5. List of proteins quantified in untreated and chronically treated Het1A cells with cigarette smoke condensate for 8 months

NP_Accession	Protein group Accession	Gene ID	Description	Het-1A-Smoke - 2M/Parental	Het-1A-Smoke - 4M/Parental	Het-1A-Smoke - 6M/Parental	Het-1A-Smoke - 8M/Parental	Het-1A-Smoke - 2M/Parental	Het-1A-Smoke - 4M/Parental	Het-1A-Smoke - 6M/Parental	Het-1A-Smoke - 8M/Parental	Het-1A-Smoke - 2M/Parental	Het-1A-Smoke - 4M/Parental	Het-1A-Smoke - 6M/Parental	Het-1A-Smoke - 8M/Parental	Coverage (%)	Proteins	Unique Peptides	# Peptides	PSM	# AAs	MW [kDa]	calc. pI
				Replicate 1				Replicate 2				Average of replicates											
NP_001008660.1	56788368	PICALM	phosphatidylinositol-binding clathrin assembly protein isoform 2	1.3	1.3	1.3	1.1	1.3	1.1	1.2	1.0	1.3	1.2	1.3	1.1	13.1	4	5	5	17	610	66.4	8.8
NP_002471.1	4505317	PPP1R12A	protein phosphatase 1 regulatory subunit 12A isoform a	1.0	1.1	1.0	1.0	1.1	1.1	1.1	1.1	1.0	1.1	1.1	1.1	16.7	4	13	13	33	1030	115.2	5.4
NP_848934.1	30520329	AGPAT6	glycerol-3-phosphate acyltransferase 6	1.0	0.9	0.9	1.0	1.4	1.2	1.2	1.2	1.2	1.0	1.1	1.1	5.3	1	2	2	6	456	52.0	9.2
NP_001017992.1	63055057	ACTBL2	beta-actin-like protein 2	0.9	1.0	1.0	1.0	1.3	1.3	1.6	1.1	1.1	1.1	1.3	1.1	36.2	3	2	13	249	376	42.0	5.6
NP_001474.1	4503937	GBAS	protein NipSnap homolog 2 isoform 1	1.1	1.0	1.0	1.1	1.2	1.0	1.0	1.0	1.1	1.0	1.0	1.1	22.4	2	5	6	20	286	33.7	9.4
NP_001020376.1	68799814	AP2M1	AP-2 complex subunit mu isoform b	1.1	1.1	1.1	1.1	1.1	1.1	1.2	1.1	1.1	1.1	1.2	1.1	33.5	2	14	14	47	433	49.4	9.5
NP_065795.1	50838795	MTA3	metastasis-associated protein MTA3 isoform b	1.0	1.1	1.0	1.1	0.9	0.9	0.8	1.0	1.0	1.0	0.9	1.1	13.4	2	2	5	12	515	58.8	8.2
NP_060300.1	8923427	OCIAD1	OCIA domain-containing protein 1 isoform 1	1.0	1.1	1.1	1.1	1.0	1.1	1.1	1.1	1.0	1.1	1.1	1.1	14.7	4	3	3	5	245	27.6	7.5
NP_110400.1	13562114	TUBB1	tubulin beta-1 chain	1.1	1.1	0.9	1.0	0.9	1.2	0.9	1.1	1.0	1.2	0.9	1.1	22.6	1	2	7	82	451	50.3	5.2
NP_004137.2	10764847	NDUFB7	NADH dehydrogenase [ubiquinone] 1 beta subcomplex subunit 7	1.1	1.3	1.1	1.1	1.2	1.2	1.1	1.1	1.2	1.2	1.1	1.1	48.9	1	5	5	11	137	16.4	8.9
NP_001263347.1	449083351	SEC16A	protein transport protein Sec16A isoform 2	1.0	1.0	1.0	1.0	1.1	1.2	1.2	1.1	1.1	1.1	1.1	1.1	9.7	2	11	11	19	2334	249.3	5.8
NP_056262.3	186659512	CAMSAP1	calmodulin-regulated spectrin-associated protein 1	1.1	1.1	1.2	1.1	1.0	1.0	1.0	1.1	1.0	1.1	1.1	1.1	2.4	1	2	2	2	1602	177.9	6.7
NP_112182.1	13569879	ANP32E	acidic leucine-rich nuclear phosphoprotein 32 family member E isoform 1	0.9	1.1	1.1	1.0	0.8	1.1	1.0	1.1	0.9	1.1	1.1	1.1	57.1	5	10	10	119	268	30.7	3.9
NP_848927.2	223555917	MTDH	protein LYRIC	1.3	1.2	1.1	1.1	1.1	1.1	1.2	1.1	1.2	1.2	1.1	1.1	37.6	1	15	15	48	582	63.8	9.3
NP_036562.2	14591919	SEC22A	vesicle-trafficking protein SEC22a	1.1	1.1	1.2	1.2	0.8	0.9	0.9	1.0	1.0	1.0	1.1	1.1	3.6	1	1	1	1	307	34.9	8.2
NP_001032897.1	83700233;31243 3975	EIF3C	eukaryotic translation initiation factor 3 subunit C isoform a	1.1	1.0	1.1	1.1	1.0	1.0	1.1	1.1	1.0	1.0	1.1	1.1	33.0	3	27	27	92	913	105.3	5.7
NP_006635.2	42544159	HSPH1	heat shock protein 105 kDa isoform 1	1.0	1.1	1.0	1.1	1.0	1.1	1.1	1.1	1.0	1.1	1.1	1.1	65.4	8	39	42	187	858	96.8	5.4
NP_057562.3	66348045	ZC3HC1	nuclear-interacting partner of ALK isoform 1	1.1	1.1	1.1	1.2	1.0	0.9	1.0	1.0	1.1	1.0	1.0	1.1	16.5	3	5	5	10	502	55.2	5.6
NP_005796.1	5031981	PSMD14	26S proteasome non-ATPase regulatory subunit 14	0.9	0.8	0.9	0.9	1.1	1.1	1.1	1.2	1.0	1.0	1.0	1.1	30.7	1	6	6	7	310	34.6	6.5
NP_000812.2	21361163	GGCX	vitamin K-dependent gamma-carboxylase isoform 1	1.1	1.0	1.1	1.1	1.0	1.0	1.0	1.1	1.1	1.0	1.1	1.1	1.7	1	1	1	2	758	87.5	8.0
NP_001350.1	4503301	DECR1	2,4-dienoyl-CoA reductase, mitochondrial precursor	1.1	1.1	1.1	1.1	1.0	1.0	1.1	1.1	1.1	1.1	1.1	1.1	36.1	1	10	10	40	335	36.0	9.3
NP_005111.2	110347429	MED12	mediator of RNA polymerase II transcription subunit 12	1.3	0.9	1.0	1.1	1.0	1.1	1.1	1.1	1.2	1.0	1.1	1.1	2.4	2	2	2	3	2177	242.9	7.0
NP_055899.2	186928850	MRPS27	28S ribosomal protein S27, mitochondrial isoform 2	1.4	1.1	1.2	1.1	1.0	1.1	1.1	1.0	1.2	1.1	1.1	1.1	32.6	3	13	13	41	414	47.6	6.2
NP_001371.1	4503355	DOCK1	dedicator of cytokinesis protein 1 isoform 2	1.1	1.1	1.1	1.1	1.1	1.0	1.0	1.1	1.1	1.1	1.1	1.1	3.8	2	5	5	7	1865	215.2	7.6
NP_079224.1	13376431	FAM188A	protein FAM188A	1.3	1.2	1.2	1.1	1.4	1.0	1.0	1.1	1.3	1.1	1.1	1.1	8.5	1	2	2	5	445	49.7	4.8
NP_057107.3	157364937	OTUD6B	OTU domain-containing protein 6B isoform 1	1.1	1.2	1.2	1.1	1.0	1.2	1.1	1.1	1.1	1.2	1.1	1.1	26.9	2	7	7	22	323	37.3	6.7
NP_002851.2	21361368	ALDH18A1	delta-1-pyrroline-5-carboxylate synthase isoform 1	1.1	1.0	1.2	1.1	1.1	1.1	1.2	1.0	1.1	1.0	1.2	1.1	36.6	1	1	20	53	795	87.2	7.1
NP_060616.1	8922534	RNMTL1	rRNA methyltransferase 3, mitochondrial	0.9	1.0	1.0	1.1	1.0	1.1	1.1	1.1	1.0	1.0	1.1	1.1	7.9	1	2	2	3	420	47.0	8.7
NP_006694.1	5729804	NUDT3	diphosphoinositol polyphosphate phosphohydrolase 1	1.0	0.9	1.0	1.1	1.2	1.0	1.0	1.1	1.1	1.0	1.0	1.1	4.7	1	1	1	1	172	19.5	6.3
NP_006419.2	26051229	MRPL28	39S ribosomal protein L28, mitochondrial	1.0	1.1	1.2	1.1	1.1	1.0	1.1	1.0	1.1	1.1	1.2	1.1	40.2	1	8	8	15	256	30.1	8.3
NP_001177765.1	299890871	DCTN1	dynactin subunit 1 isoform 5	1.0	1.0	1.0	1.1	1.0	1.0	1.0	1.1	1.0	1.0	1.0	1.1	27.8	6	24	24	65	1236	136.7	5.4
NP_003894.3	118402580	CDC16	cell division cycle protein 16 homolog	1.1	1.2	1.1	1.0	1.1	1.2	1.3	1.1	1.1	1.2	1.2	1.1	10.7	1	5	5	5	620	71.6	5.8
NP_036344.2	226059133;2260 59159	PTGR1	prostaglandin reductase 1 isoform 1	1.0	0.9	0.8	1.0	0.9	0.9	0.8	1.1	0.9	0.9	0.8	1.1	45.3	2	10	10	28	329	35.8	8.3
NP_002844.1	4506385	RAD1	cell cycle checkpoint protein RAD1	1.2	1.1	1.1	1.1	1.1	1.0	1.0	1.0	1.2	1.0	1.0	1.1	11.0	1	2	2	4	282	31.8	4.8
NP_001032238.1	81230485	ACOT1	acyl-coenzyme A thioesterase 1	1.0	1.1	1.0	1.1	1.1	1.0	0.9	1.1	1.1	1.0	1.0	1.1	29.0	2	8	8	26	421	46.2	7.3
NP_001166299.1	290543591	YTHDF2	YTH domain-containing family protein 2 isoform 2	0.9	1.0	0.9	1.0	0.9	0.9	0.9	1.1	0.9	1.0	0.9	1.1	9.8	2	4	6	19	529	56.8	8.9
NP_057479.2	117168248	HACD3	very-long-chain (3R)-3-hydroxyacyl-CoA dehydratase 3	1.1	0.9	0.9	1.1	1.0	1.0	0.9	1.1	1.0	0.9	0.9	1.1	16.0	1	4	4	18	362	43.1	8.9
NP_009090.2	56550043	SLMAP	sarcolemmal membrane-associated protein isoform b	1.0	0.9	1.0	1.0	1.0	1.2	1.1	1.2	1.0	1.1	1.1	1.1	4.8	5	4	4	4	811	93.1	5.4
NP_001269965.1	545687684	RTFDC1	protein RTF2 homolog isoform c	0.7	0.8	0.7	1.1	0.8	0.8	0.9	1.1	0.7	0.8	0.8	1.1	8.9	4	2	2	6	305	33.7	8.7
NP_001002257.1	50659059	LCLAT1	lysocardiolipin acyltransferase 1 isoform 2	1.0	1.0	1.1	1.1	1.3	1.0	1.0	1.1	1.1	1.0	1.1	1.1	12.8	3	4	4	8	376	44.5	8.6
NP_064695.3	223468663	AKR1B10	aldo-keto reductase family 1 member B10	1.0	1.2	1.0	1.1	1.0	1.1	1.1	1.1	1.0	1.1	1.0	1.1	2.5	2	1	1	1	316	36.0	7.8
NP_001035526.1	94681057	YARS2	tyrosine--tRNA ligase, mitochondrial precursor	1.2	1.0	1.2	1.0	1.2	1.1	1.2	1.1	1.2	1.1	1.2	1.1	21.0	1	7	7	18	477	53.2	9.0
NP_006757.2	150378493	KAT6A	histone acetyltransferase KAT6A	1.1	1.1	1.0	1.1	1.1	1.1	1.0	1.1	1.1	1.1	1.0	1.1	2.7	1	2	2	3	2004	224.9	5.7
NP_002898.2	14591904	RECQL	ATP-dependent DNA helicase Q1	1.3	1.1	1.2	1.0	1.1	1.2	1.2	1.1	1.2	1.1	1.2	1.1	32.7	1	19	19	57	649	73.4	7.9
NP_001244290.1	383209660	AMPD2	AMP deaminase 2 isoform 4	1.1	1.0	1.0	1.0	1.2	1.2	1.1	1.1	1.1	1.1	1.0	1.1	14.3	5	10	10	17	761	88.2	6.2
NP_002038.2	116805340	GARS	glycine--tRNA ligase precursor	1.5	1.5	1.2	1.1	1.4	1.5	1.3	1.1	1.5	1.5	1.3	1.1	54.4	1	32	32	195	739	83.1	7.0
NP_075067.2	18105056	VPS33A	vacuolar protein sorting-associated protein 33A	1.0	1.0	1.0	1.0	0.8	1.0	1.0	1.1	0.9	1.0	1.0	1.1	14.4	1	4	4	9	596	67.6	7.0
NP_003751.2	10092601	EIF4G3	eukaryotic translation initiation factor 4 gamma 3 isoform 3	1.0	1.0	1.1	1.0	1.1	1.1	1.2	1.1	1.1	1.1	1.1	1.1	9.1	4	9	14	37	1585	176.5	5.4
NP_005038.1	4826952	PSMD5	26S proteasome non-ATPase regulatory subunit 5 isoform 1	1.1	1.1	1.1	1.1	1.1	1.0	1.1	1.0	1.1	1.0	1.1	1.1	30.8	2	10	10	26	504	56.2	5.5
NP_000258.1	4557793	NF1	neurofibromin isoform 2	1.0	1.0	1.0	1.1	1.0	0.9	1.0	1.1	1.0	1.0	1.0	1.1	1.3	3	3	3	5	2818	316.8	7.3
NP_065701.2	28626510	RCN3	reticulocalbin-3 precursor	1.1	1.0	1.6	1.0	1.1	1.1	1.2	1.1	1.1	1.0	1.4	1.1	17.4	1	3	3	7	328	37.5	4.9
NP_001071158.1	118026933	ALG9	alpha-1,2-mannosyltransferase ALG9 isoform b	1.0	1.0	1.0	1.1	1.0	1.0	1.0	1.0	1.0	1.0	1.0	1.1	5.1	4	2	2	5	611	69.8	8.7
NP_036329.3	164519084	RABGAP1	rab GTPase-activating protein 1	1.1	1.1	1.1	1.2	1.0	1.0	1.0	1.0	1.1	1.1	1.1	1.1	15.4	1	11	13	30	1069	121.7	5.2
NP_001596.2	109148542	AARS	alanine--tRNA ligase, cytoplasmic	1.2	1.1	1.0	1.1	1.2	1.1	1.0	1.1	1.2	1.1	1.0	1.1	53.0	1	38	38	163	968	106.7	5.5
NP_001092872.1	150417989	CCNK	cyclin-K	1.1	0.9	0.9	1.1	1.0	1.0	1.0	1.1	1.1	0.9	1.0	1.1	8.3	1	4	4	5	580	64.2	8.4
NP_071769.2	208610000	ATL2	atlastin-2 isoform 1	1.1	1.1	1.2	1.1	1.1	1.1	1.2	1.1	1.1	1.1	1.2	1.1	16.2	5	6	6	8	579	66.0	6.1
NP_001407.1	4503529	EIF4A1	eukaryotic initiation factor 4A-I isoform 1	1.0	1.0	1.0	1.1	1.0	1.1	1.1	1.1	1.0	1.1	1.1	1.1	73.2	2	12	22	331	406	46.1	5.5
NP_003452.1	4508047;585308 45	ZYX	zyxin	0.8	1.0	0.9	1.1	1.0	1.0	1.0	1.1	0.9	1.0	1.0	1.1	19.8	1	6	6	19	572	61.2	6.7
NP_056315.1	119226222	C2CD2	C2 domain-containing protein 2 isoform 1 precursor	1.0	1.0	1.0	1.1	0.9	1.1	0.9	1.1	1.0	1.0	1.0	1.1	4.7	2	2	2	4	696	75.5	6.9
NP_001128663.1	206597522	ASAP2	arf-GAP with SH3 domain, ANK repeat and PH domain-containing protein 2 isoform b	1.1	1.0	1.2	1.1	1.1	1.1	1.1	1.1	1.1	1.0	1.1	1.1	6.4	2	3	4	11	961	106.9	6.5
NP_005085.2	40807357	SLC27A4	long-chain fatty acid transport protein 4	1.0	1.0	0.9	1.1	1.0	1.0	1.0	1.0	1.0	1.0	1.0	1.1	13.8	2	7	7	9	643	72.0	8.5
NP_055580.1	7657257	TOMM20	mitochondrial import receptor subunit TOM20 homolog	1.0	1.0	1.1	1.1	1.1	1.0	1.1	1.1	1.0	1.0	1.1	1.1	18.6	1	2	2	29	145	16.3	8.6
NP_003153.2	51242945	STRN	striatin	1.0	0.9	1.0	1.0	1.0	1.0	1.0	1.1	1.0	1.0	1.0	1.1	18.9	3	8	9	16	780	86.1	5.3
NP_057445.4	523498731	ACP6	lysophosphatidic acid phosphatase type 6 precursor	1.5	1.2	1.3	1.1	1.1	1.1	0.9	1.1	1.3	1.1	1.1	1.1	4.9	1	1	1	2	428	48.9	6.5
NP_006003.1	5174419	CLPP	ATP-dependent Clp protease proteolytic subunit, mitochondrial precursor	0.9	0.9	0.9	1.0	1.1	1.0	1.0	1.1	1.0	0.9	0.9	1.1	6.5	1	1	1	4	277	30.2	8.1
NP_036516.1	6912568	GMEB2	glucocorticoid modulatory element-binding protein 2	0.9	1.0	1.0	1.2	0.8	1.0	0.9	1.0	0.9	1.0	0.9	1.1	9.4	1	2	3	5	530	56.4	5.6
NP_004498.1	4758576	HUS1	checkpoint protein HUS1	0.7	0.9	0.8	0.9	1.0	1.1	1.0	1.2	0.9	1.0	0.9	1.1	7.1	1	1	1	2	280	31.7	6.8
NP_001138521.1	223005903	GPN1	GPN-loop GTPase 1 isoform d	1.2	1.0	1.0	1.0	1.0	1.1	0.9	1.1	1.1	1.0	1.0	1.1	14.7	4	3	3	8	279	31.8	4.7
NP_570721.1	18677735	PRRC1	protein PRRC1 isoform 2	1.2	1.0	1.0	1.1	1.2	1.1	1.1	1.0	1.2	1.1	1.1	1.1	23.6	2	6	6	29	445	46.7	5.8
NP_057642.1	7706089	SCAND1	SCAN domain-containing protein 1 isoform 1	1.4	1.4	1.4	1.2	4.6	0.9	0.8	0.9	3.0	1.1	1.1	1.1	15.1	2	1	1	3	179	19.1	9.7
NP_056281.1	24308073	PTPN23	tyrosine-protein phosphatase non-receptor type 23 isoform 1	1.1	1.1	1.0	1.1	1.0	1.0	1.0	1.0	1.1	1.0	1.0	1.1	8.4	2	8	8	14	1636	178.9	6.9
NP_001619.1	4502049	AKR1B1	aldose reductase	1.0	1.0	1.0	1.1	1.1	1.0	1.0	1.1	1.0	1.0	1.0	1.1	51.9	1	11	12	42	316	35.8	7.0
NP_919424.1	37595752;37595 750	LBR	lamin-B receptor	1.0	1.1	1.2	1.1	1.1	1.1	1.3	1.1	1.1	1.1	1.2	1.1	11.5	1	8	8	10	615	70.7	9.4
NP_068593.2	16950603	MRPS35	28S ribosomal protein S35, mitochondrial isoform 1 precursor	1.0	1.0	1.0	1.1	1.1	1.1	1.2	1.1	1.1	1.0	1.1	1.1	24.2	2	6	6	11	323	36.8	8.2
NP_003105.2	27262636;27262 638	SPAG1	sperm-associated antigen 1	1.0	1.1	1.4	1.2	0.9	1.0	1.0	1.0	0.9	1.0	1.2	1.1	5.9	1	2	2	3	926	103.6	6.9
NP_001277151.1	589811532	NQO2	ribosyldihydronicotinamide dehydrogenase [quinone] isoform 2	1.0	1.0	1.0	1.0	1.1	1.0	1.0	1.1	1.0	1.0	1.0	1.1	10.4	2	2	2	3	193	21.5	6.5
NP_004530.1	4758762	NARS	asparagine--tRNA ligase, cytoplasmic	1.2	1.2	1.1	1.1	1.2	1.1	1.1	1.1	1.2	1.1	1.1	1.1	42.3	1	19	19	86	548	62.9	6.3
NP_036244.2	20127519	TPX2	targeting protein for Xklp2	0.8	1.0	1.0	1.1	1.0	1.0	1.0	1.1	0.9	1.0	1.0	1.1	25.2	1	14	14	40	747	85.6	9.2
NP_001161337.1	269846910	NSMCE4A	non-structural maintenance of chromosomes element 4 homolog A isoform 2	1.2	1.1	1.1	1.1	1.0	1.1	1.0	1.1	1.1	1.1	1.1	1.1	15.9	2	3	3	5	384	44.1	5.4
NP_055703.1	7661714	FAM3C	protein FAM3C precursor	1.0	1.0	0.9	1.1	1.1	1.0	1.0	1.1	1.1	1.0	0.9	1.1	14.5	1	2	2	13	227	24.7	8.3
NP_006588.1	5729877	HSPA8	heat shock cognate 71 kDa protein isoform 1	1.1	1.0	1.1	1.1	1.0	1.0	1.0	1.1	1.0	1.0	1.0	1.1	86.1	2	33	47	864	646	70.9	5.5
NP_001129704.1	210032390	SEC13	protein SEC13 homolog isoform 2	1.0	1.1	1.1	1.1	1.0	1.0	1.1	1.1	1.0	1.1	1.1	1.1	17.9	5	4	4	8	308	34.0	5.7
NP_076425.1	13027604	MRPS34	28S ribosomal protein S34, mitochondrial isoform 2	1.1	1.1	1.1	1.1	1.0	1.0	1.0	1.0	1.0	1.1	1.0	1.1	30.7	2	6	6	11	218	25.6	10.0
NP_055950.1	57634534	NUP205	nuclear pore complex protein Nup205	1.1	1.0	1.2	1.1	1.1	1.1	1.1	1.1	1.1	1.1	1.1	1.1	24.2	1	36	36	95	2012	227.8	6.2
NP_004485.1	4758516	HDGF	hepatoma-derived growth factor isoform a	0.9	1.1	1.0	1.1	0.9	1.1	1.1	1.1	0.9	1.1	1.1	1.1	81.3	3	20	21	159	240	26.8	4.7
NP_005024.2	77812670	EXOSC9	exosome complex component RRP45 isoform 2	1.0	1.0	1.1	1.1	1.0	1.0	1.0	1.1	1.0	1.0	1.0	1.1	12.8	2	5	5	22	439	48.9	5.3
NP_003029.2	21314632	SLC1A4	neutral amino acid transporter A isoform 1	1.3	1.4	1.0	1.1	1.3	1.2	1.0	1.0	1.3	1.3	1.0	1.1	10.3	2	4	4	8	532	55.7	6.3
NP_055426.1	24415404	MDN1	midasin	1.1	1.1	1.2	1.1	1.0	1.1	1.1	1.0	1.1	1.1	1.2	1.1	6.7	1	26	27	49	5596	632.4	5.7

Khan *et al.* , 2019. Multi-omics analysis to characterize cigarette smoke induced molecular alterations in esophageal cells
Supplementary Table 5. List of proteins quantified in untreated and chronically treated Het1A cells with cigarette smoke condensate for 8 months

NP_Accession	Protein group Accession	Gene ID	Description	Het-1A-Smoke - 2M/Parental	Het-1A-Smoke - 4M/Parental	Het-1A-Smoke - 6M/Parental	Het-1A-Smoke - 8M/Parental	Het-1A-Smoke - 2M/Parental	Het-1A-Smoke - 4M/Parental	Het-1A-Smoke - 6M/Parental	Het-1A-Smoke - 8M/Parental	Het-1A-Smoke - 2M/Parental	Het-1A-Smoke - 4M/Parental	Het-1A-Smoke - 6M/Parental	Het-1A-Smoke - 8M/Parental	Coverage (%)	Proteins	Unique Peptides	# Peptides	PSM	# AAs	MW [kDa]	calc. pI
				Replicate 1				Replicate 2				Average of replicates											
NP_004915.2	12025678	**ACTN4**	alpha-actinin-4	1.1	1.1	1.3	1.1	1.0	1.1	1.2	1.1	**1.0**	**1.1**	**1.3**	**1.1**	69.6	1	41	60	540	911	104.8	5.4
NP_001139685.2	299758464	**TBC1D15**	TBC1 domain family member 15 isoform 3	1.1	1.0	1.1	1.0	1.1	1.1	1.1	1.1	**1.1**	**1.1**	**1.1**	**1.1**	8.9	3	6	6	12	674	77.3	5.4
NP_005307.1	4885365	**GTF2H1**	general transcription factor IIH subunit 1	1.1	1.0	1.0	1.0	0.9	1.1	1.0	1.2	**1.0**	**1.1**	**1.0**	**1.1**	4.6	1	2	2	4	548	62.0	8.7
NP_001012745.1	69122971	**AGPAT2**	1-acyl-sn-glycerol-3-phosphate acyltransferase beta isoform b precursor	1.1	1.3	1.1	1.2	1.0	0.9	0.9	1.0	**1.0**	**1.1**	**1.0**	**1.1**	5.3	2	1	1	1	246	27.3	8.9
NP_004896.1	4758638	**PRDX6**	peroxiredoxin-6	1.0	1.0	1.1	1.1	0.9	0.9	1.0	1.1	**1.0**	**0.9**	**1.0**	**1.1**	79.5	1	17	17	192	224	25.0	6.4
NP_056273.2	19923424	**MTMR9**	myotubularin-related protein 9	1.5	1.4	1.4	1.2	1.0	1.0	1.0	0.9	**1.3**	**1.2**	**1.2**	**1.1**	3.1	1	1	1	2	549	63.4	6.4
NP_036453.1	6912486	**LSM4**	U6 snRNA-associated Sm-like protein LSm4 isoform 1	0.8	1.0	1.0	1.1	0.9	1.0	1.0	1.0	**0.9**	**1.0**	**1.0**	**1.1**	13.0	2	2	2	3	139	15.3	10.0
NP_001182068.1	304376294	**COG4**	conserved oligomeric Golgi complex subunit 4 isoform 2	1.0	1.2	1.1	1.1	1.2	1.1	1.1	1.0	**1.1**	**1.1**	**1.1**	**1.1**	4.2	2	3	3	5	768	87.1	5.2
NP_054872.2	27414497	**ZC3H7A**	zinc finger CCCH domain-containing protein 7A	1.0	1.0	1.0	1.0	0.9	1.0	1.0	1.2	**0.9**	**1.0**	**1.0**	**1.1**	8.3	1	7	7	9	971	110.5	7.3
NP_055768.3	190014623	**DIS3**	exosome complex exonuclease RRP44 isoform a	1.1	1.1	1.2	1.1	1.1	1.1	1.1	1.0	**1.1**	**1.1**	**1.1**	**1.1**	17.8	2	14	14	37	958	108.9	7.1
NP_073591.2	23618867	**SFXN1**	sideroflexin-1	1.1	1.0	1.0	1.1	1.1	1.1	1.1	1.1	**1.1**	**1.0**	**1.0**	**1.1**	55.3	1	11	12	73	322	35.6	9.1
NP_001002762.2	194306640;194306642	**DNAJB12**	dnaJ homolog subfamily B member 12	1.0	1.1	1.1	1.0	1.0	1.2	1.3	1.1	**1.0**	**1.1**	**1.2**	**1.1**	13.7	1	4	4	7	409	45.5	9.4
NP_005989.3	63162572	**CCT3**	T-complex protein 1 subunit gamma isoform a	1.0	1.0	1.0	1.1	1.0	1.0	1.0	1.1	**1.0**	**1.0**	**1.0**	**1.1**	58.2	3	29	30	236	545	60.5	6.5
NP_055045.2	24497620	**SRP68**	signal recognition particle subunit SRP68 isoform 1	1.0	1.0	1.0	1.1	1.1	1.1	1.1	1.1	**1.1**	**1.1**	**1.0**	**1.1**	21.9	3	12	13	46	627	70.7	8.6
NP_001239225.1	356582519	**CEBPG**	CCAAT/enhancer-binding protein gamma	1.1	1.1	1.0	1.1	1.0	1.0	0.9	1.1	**1.0**	**1.1**	**1.0**	**1.1**	20.7	1	1	1	4	150	16.4	9.8
NP_005104.3	526118265	**GOLGA5**	Golgin subfamily A member 5	1.2	1.1	1.0	1.1	1.2	1.1	1.1	1.1	**1.2**	**1.1**	**1.0**	**1.1**	8.8	1	4	4	6	731	82.9	5.8
NP_003128.3	47419936	**SRPK1**	SRSF protein kinase 1	1.1	0.9	1.1	1.1	1.0	1.0	1.1	1.0	**1.0**	**1.0**	**1.1**	**1.1**	17.1	4	7	9	17	655	74.3	6.2
NP_005959.2	14141152	**HNRNPM**	heterogeneous nuclear ribonucleoprotein M isoform a	0.9	1.0	1.0	1.1	0.9	0.9	0.9	1.1	**0.9**	**1.0**	**1.0**	**1.1**	45.1	3	28	28	175	730	77.5	8.7
NP_003357.2	50592988	**UQCRC2**	cytochrome b-c1 complex subunit 2, mitochondrial precursor	1.1	1.1	1.2	1.0	1.1	1.2	1.2	1.1	**1.1**	**1.2**	**1.2**	**1.1**	40.6	1	14	14	67	453	48.4	8.6
NP_056060.2	140161500	**ANKS1A**	ankyrin repeat and SAM domain-containing protein 1A	0.9	1.1	1.0	1.1	0.9	0.9	0.9	1.0	**0.9**	**1.0**	**1.0**	**1.1**	4.9	13	4	4	6	1134	123.0	6.4
NP_001268223.1	526253066	**ZNF281**	zinc finger protein 281 isoform 2	0.9	1.0	1.0	1.0	0.9	0.9	1.2	1.2	**0.9**	**0.9**	**1.1**	**1.1**	4.0	2	2	2	3	859	93.2	8.4
NP_075567.2	150417999	**UBE2Z**	ubiquitin-conjugating enzyme E2 Z	1.2	1.1	1.2	1.1	0.9	0.9	0.9	1.0	**1.1**	**1.0**	**1.0**	**1.1**	13.8	1	5	5	7	354	38.2	5.6
NP_001116106.1	170295797	**CPS1**	carbamoyl-phosphate synthase [ammonia], mitochondrial isoform c	1.0	1.1	1.0	1.1	1.1	0.9	1.0	1.0	**1.0**	**1.0**	**1.0**	**1.1**	2.7	3	1	3	6	1049	116.0	6.0
NP_060060.3	27881484	**ZC3H7B**	zinc finger CCCH domain-containing protein 7B	1.3	1.1	1.1	1.1	0.9	0.9	0.9	1.0	**1.1**	**1.0**	**1.0**	**1.1**	6.7	1	3	3	3	977	109.8	7.3
NP_996888.1	46877102	**USF1**	upstream stimulatory factor 1 isoform 2	0.9	1.0	1.0	1.1	1.0	0.9	0.9	1.1	**1.0**	**1.0**	**0.9**	**1.1**	14.7	2	3	3	5	251	27.4	6.3
NP_005717.3	171846268	**TSFM**	elongation factor Ts, mitochondrial isoform 2 precursor	1.0	1.0	1.0	1.1	1.1	1.1	1.0	1.1	**1.0**	**1.1**	**1.0**	**1.1**	26.8	4	5	5	11	325	35.4	8.4
NP_004060.2	70906430	**AP2S1**	AP-2 complex subunit sigma isoform AP17	1.1	1.1	1.1	1.1	1.0	1.0	1.0	1.0	**1.0**	**1.0**	**1.1**	**1.1**	39.4	5	6	6	29	142	17.0	6.2
NP_001258610.1	410991931	**PYCR2**	pyrroline-5-carboxylate reductase 2 isoform 2	1.2	1.1	1.1	1.1	1.1	1.1	1.1	1.0	**1.1**	**1.1**	**1.1**	**1.1**	37.4	2	6	7	22	246	25.9	9.3
NP_056155.1	7661872	**LARS2**	probable leucine--tRNA ligase, mitochondrial	1.1	1.0	1.1	1.0	1.3	1.1	1.2	1.2	**1.2**	**1.0**	**1.1**	**1.1**	11.6	1	8	8	10	903	101.9	8.2
NP_060336.3	34147498	**TMEM70**	transmembrane protein 70, mitochondrial isoform a	1.1	1.1	1.0	1.1	1.2	1.0	1.0	1.0	**1.1**	**1.0**	**1.0**	**1.1**	8.1	1	1	1	4	260	29.0	8.9
NP_002129.2	14110414	**HNRNPD**	heterogeneous nuclear ribonucleoprotein D0 isoform c	0.8	1.0	0.8	1.0	1.1	1.0	0.8	1.2	**1.0**	**1.0**	**0.8**	**1.1**	43.1	2	2	17	136	306	32.8	8.2
NP_060012.3	22027480	**POGK**	pogo transposable element with KRAB domain	0.9	1.0	1.0	1.1	1.0	1.0	0.9	1.0	**1.0**	**1.0**	**0.9**	**1.1**	3.5	1	1	1	2	609	69.4	5.1
NP_001139443.1	225579078	**RDH13**	retinol dehydrogenase 13 isoform 1 precursor	1.2	1.0	1.1	1.0	1.0	1.1	1.2	1.1	**1.1**	**1.1**	**1.1**	**1.1**	3.9	1	1	1	2	331	35.9	8.1
NP_003631.2	38569394	**IKBKAP**	elongator complex protein 1	1.1	1.0	1.1	1.1	1.1	1.1	1.1	1.1	**1.1**	**1.1**	**1.1**	**1.1**	16.5	1	16	16	33	1332	150.2	5.9
NP_000017.1	4557269	**ADSL**	adenylosuccinate lyase isoform a	1.1	1.1	1.2	1.1	1.0	0.9	1.0	1.0	**1.1**	**1.0**	**1.1**	**1.1**	34.9	2	12	12	40	484	54.9	7.1
NP_060377.1	8923579	**LAMTOR1**	ragulator complex protein LAMTOR1	1.2	1.2	1.0	1.1	0.9	0.8	0.9	1.0	**1.1**	**1.0**	**0.9**	**1.1**	29.2	1	3	3	5	161	17.7	5.1
NP_001137451.1	221316642;8923110	**NDE1**	nuclear distribution protein nudE homolog 1	1.0	1.1	1.1	1.1	1.1	1.0	1.0	1.1	**1.1**	**1.1**	**1.1**	**1.1**	16.4	1	3	3	6	335	37.7	5.1
NP_115958.2	40556376	**GLYR1**	putative oxidoreductase GLYR1	1.1	1.3	1.1	1.2	1.0	1.0	0.9	1.0	**1.0**	**1.1**	**1.0**	**1.1**	12.5	1	5	5	16	553	60.5	9.2
NP_055819.2	189083688	**EXOSC7**	exosome complex component RRP42	1.0	1.0	1.0	1.0	1.0	1.0	1.1	1.1	**1.0**	**1.0**	**1.1**	**1.1**	24.7	1	5	5	14	291	31.8	5.2
NP_060708.1	8922701	**AGK**	acylglycerol kinase, mitochondrial precursor	1.1	1.1	1.2	1.1	1.0	1.1	1.1	1.1	**1.1**	**1.1**	**1.1**	**1.1**	35.6	1	11	11	29	422	47.1	8.1
NP_859530.1	32455256	**CERS2**	ceramide synthase 2	1.0	1.0	1.2	1.1	1.0	1.0	1.0	1.1	**1.0**	**1.0**	**1.1**	**1.1**	7.4	1	2	2	3	380	44.8	9.0
NP_001180438.1	301898023	**TTC27**	tetratricopeptide repeat protein 27 isoform 2	1.0	0.9	0.9	1.1	1.1	1.0	1.0	1.1	**1.0**	**1.0**	**1.0**	**1.1**	4.4	2	3	3	6	793	90.9	5.8
NP_803882.1	29171730	**CYP20A1**	cytochrome P450 20A1	1.1	1.1	1.1	1.1	1.1	1.0	1.1	1.0	**1.1**	**1.0**	**1.1**	**1.1**	1.7	1	1	1	1	462	52.4	6.2
NP_001852.1	4502981	**COX4I1**	cytochrome c oxidase subunit 4 isoform 1, mitochondrial precursor	1.0	1.1	1.1	1.0	1.0	1.1	1.1	1.1	**1.0**	**1.1**	**1.1**	**1.1**	30.8	1	5	5	48	169	19.6	9.5
NP_570849.2	224831251	**OPA1**	dynamin-like 120 kDa protein, mitochondrial isoform 7	1.1	1.1	1.1	1.1	1.1	1.1	1.2	1.1	**1.1**	**1.1**	**1.2**	**1.1**	29.9	8	23	23	59	997	115.8	7.9
NP_005054.3	53759151	**SCD**	acyl-CoA desaturase	1.2	1.2	1.0	1.1	1.1	1.3	1.2	1.1	**1.1**	**1.3**	**1.1**	**1.1**	15.6	1	4	4	14	359	41.5	9.0
NP_060587.8	13324688	**WDR11**	WD repeat-containing protein 11	1.0	1.0	1.0	1.0	1.2	1.1	1.2	1.1	**1.1**	**1.1**	**1.1**	**1.1**	17.3	1	14	14	32	1224	136.6	6.9
NP_056414.1	7661568	**PGM3**	phosphoacetylglucosamine mutase isoform 2	1.2	1.1	1.0	1.1	1.2	1.0	1.0	1.0	**1.2**	**1.0**	**1.0**	**1.1**	25.5	4	12	12	26	542	59.8	6.3
NP_055907.3	62243658	**SMG1**	serine/threonine-protein kinase SMG1	1.1	1.1	1.1	1.1	1.1	1.0	1.0	1.1	**1.1**	**1.1**	**1.1**	**1.1**	3.5	2	8	9	14	3661	410.2	6.5
NP_071354.2	21361891	**SH2D4A**	SH2 domain-containing protein 4A isoform a	1.3	1.3	1.2	1.1	1.0	1.1	1.1	1.1	**1.1**	**1.2**	**1.2**	**1.1**	12.1	2	3	3	3	454	52.7	8.1
NP_689485.1	22748641	**CHMP7**	charged multivesicular body protein 7	1.1	1.1	1.2	1.1	0.9	1.1	1.1	1.0	**1.0**	**1.1**	**1.1**	**1.1**	3.5	1	1	1	2	453	50.9	5.4
NP_038464.1	7305303	**NCKAP1**	nck-associated protein 1 isoform 1	1.0	1.0	1.0	1.1	1.0	1.0	1.0	1.1	**1.0**	**1.0**	**1.0**	**1.1**	10.8	2	9	9	27	1128	128.7	6.6
NP_001264744.1	481026579;481026582	**YTHDF3**	YTH domain-containing family protein 3 isoform c	1.0	1.0	1.1	1.1	1.0	1.0	1.0	1.0	**1.0**	**1.0**	**1.0**	**1.1**	24.2	3	8	11	38	534	58.3	9.2
NP_001093879.1	156105703	**SENP6**	sentrin-specific protease 6 isoform 2	1.2	1.0	1.0	1.1	1.0	1.0	1.0	1.0	**1.1**	**1.0**	**1.0**	**1.1**	3.0	2	2	2	3	1105	125.3	6.7
NP_057187.1	7705827;7570920 4	**SAR1B**	GTP-binding protein SAR1b	1.2	1.2	1.1	1.1	1.1	1.2	1.2	1.1	**1.1**	**1.2**	**1.1**	**1.1**	37.4	1	4	7	51	198	22.4	6.1
NP_004152.1	4758988	**RAB1A**	ras-related protein Rab-1A isoform 1	1.1	1.1	1.1	1.1	1.0	1.0	0.9	1.1	**1.0**	**1.0**	**1.0**	**1.1**	78.1	11	7	15	237	205	22.7	6.2
NP_001185781.1	311893307	**FOXJ3**	forkhead box protein J3 isoform 2	0.9	1.0	0.9	0.9	1.1	1.1	1.1	1.2	**1.0**	**1.1**	**1.0**	**1.1**	7.8	2	3	3	4	588	65.5	7.2
NP_005637.3	110825988	**TARBP1**	probable methyltransferase TARBP1	1.2	1.0	1.2	1.1	1.2	1.2	1.2	1.1	**1.2**	**1.1**	**1.2**	**1.1**	1.6	1	2	2	3	1621	181.6	7.0
NP_003115.1	4507185	**SPR**	sepiapterin reductase	1.1	1.0	1.0	1.1	1.0	0.9	0.9	1.0	**1.0**	**0.9**	**1.0**	**1.1**	27.2	1	5	5	16	261	28.0	8.0
NP_037450.2	149192855	**PRRC2B**	protein PRRC2B	1.0	0.9	1.0	1.1	1.0	1.1	1.0	1.1	**1.0**	**1.0**	**1.0**	**1.1**	2.6	1	3	4	5	2229	242.8	8.3
NP_001230693.1	344217735	**RABGAP1L**	rab GTPase-activating protein 1-like isoform D	0.8	1.0	0.8	1.1	0.9	1.0	1.1	1.0	**0.9**	**1.0**	**0.9**	**1.1**	10.3	2	1	2	5	253	29.0	5.3
NP_115689.1	14150078	**NOA1**	nitric oxide-associated protein 1	1.1	1.0	1.0	1.2	1.0	1.0	1.0	1.0	**1.0**	**1.0**	**1.0**	**1.1**	5.4	1	3	3	6	698	78.4	8.7
NP_064530.1	9910352	**SH3GLB2**	endophilin-B2 isoform b	1.2	1.0	1.1	1.1	1.1	1.0	1.0	1.0	**1.1**	**1.0**	**1.0**	**1.1**	14.7	2	5	6	15	395	43.9	6.0
NP_001018080.1	65787364	**CORO1B**	coronin-1B	1.0	1.0	1.0	1.0	1.1	1.1	1.2	1.2	**1.0**	**1.1**	**1.1**	**1.1**	18.2	1	8	8	15	489	54.2	5.9
NP_056298.2	223468659	**KBTBD2**	kelch repeat and BTB domain-containing protein 2	0.9	1.0	1.0	1.0	1.1	1.2	1.2	1.1	**1.0**	**1.1**	**1.1**	**1.1**	4.2	1	1	1	1	623	71.3	5.6
NP_004535.1	4758768	**NDUFA10**	NADH dehydrogenase [ubiquinone] 1 alpha subcomplex subunit 10, mitochondrial precursor	1.1	1.1	1.1	1.1	1.0	1.0	1.1	1.0	**1.1**	**1.1**	**1.1**	**1.1**	27.9	1	8	8	27	355	40.7	8.5
NP_478126.1	17402904	**EXOSC6**	exosome complex component MTR3	1.0	1.1	1.0	1.1	1.1	1.0	1.0	1.1	**1.0**	**1.0**	**1.0**	**1.1**	19.5	1	3	3	12	272	28.2	6.3
NP_064560.2	239788462	**DUS3L**	tRNA-dihydrouridine(47) synthase [NAD(P)(+)]-like isoform 1	1.0	1.2	1.1	1.0	1.5	1.2	1.2	1.1	**1.3**	**1.2**	**1.2**	**1.1**	1.9	1	1	1	1	650	72.5	8.0
NP_671701.1	22219465;4502125	**NUDT2**	bis(5'-nucleosyl)-tetraphosphatase [asymmetrical]	1.2	1.1	1.0	1.1	1.0	1.1	1.1	1.1	**1.1**	**1.1**	**1.1**	**1.1**	33.3	1	3	3	6	147	16.8	5.4
NP_055472.1	24307961	**TTI1**	TELO2-interacting protein 1 homolog	1.1	1.1	1.1	1.1	1.1	1.0	1.1	1.1	**1.1**	**1.0**	**1.1**	**1.1**	15.9	1	10	10	28	1089	122.0	6.0
NP_001159590.1	260763926	**NT5C3A**	cytosolic 5'-nucleotidase 3A isoform 3	1.0	1.0	1.0	1.1	0.9	1.0	1.0	1.1	**0.9**	**1.0**	**1.0**	**1.1**	11.9	3	3	3	7	285	32.5	5.7
NP_001268705.1	528881282	**ZMYND8**	protein kinase C-binding protein 1 isoform j	1.2	1.0	1.2	1.1	1.1	1.1	1.1	1.1	**1.2**	**1.1**	**1.1**	**1.1**	5.3	17	4	4	4	1107	123.4	6.4
NP_001184222.1	308818195	**DPYSL2**	dihydropyrimidinase-related protein 2 isoform 1	1.1	1.2	1.2	1.1	1.0	1.3	1.2	1.1	**1.1**	**1.2**	**1.2**	**1.1**	46.2	3	17	19	90	677	73.5	6.4
NP_001002020.1	70166634;70166599	**PUS1**	tRNA pseudouridine synthase A, mitochondrial isoform 2	1.1	1.1	1.1	1.1	1.1	1.1	1.1	1.1	**1.1**	**1.1**	**1.1**	**1.1**	11.3	2	4	4	11	399	44.3	7.7
NP_110438.1	14269586	**MRPS26**	28S ribosomal protein S26, mitochondrial	1.1	1.0	1.0	1.1	1.1	1.0	0.9	1.0	**1.1**	**1.0**	**1.0**	**1.1**	13.7	1	2	2	7	205	24.2	10.4
NP_689835.3	94721313	**MIER3**	mesoderm induction early response protein 3 isoform 3	1.0	0.9	1.0	0.9	1.0	1.2	0.9	1.2	**1.0**	**1.0**	**0.9**	**1.1**	2.0	3	1	1	1	549	61.3	4.5
NP_001036024.3	289577114	**EIF4G2**	eukaryotic translation initiation factor 4 gamma 2 isoform 2	1.0	1.0	1.1	1.1	1.1	1.1	1.1	1.1	**1.1**	**1.1**	**1.1**	**1.1**	20.8	2	16	16	39	869	98.1	7.0
NP_001273703.1	557947988	**OCIAD2**	OCIA domain-containing protein 2 isoform 3	1.1	1.0	1.0	1.1	1.4	1.1	1.1	1.0	**1.2**	**1.0**	**1.0**	**1.1**	10.9	2	1	1	1	92	9.9	8.5
NP_001229812.1	338968901	**CARKD**	ATP-dependent (S)-NAD(P)H-hydrate dehydratase isoform d	1.1	1.0	1.0	1.1	1.0	1.0	1.1	1.1	**1.0**	**1.0**	**1.0**	**1.1**	5.1	4	1	1	2	237	25.2	6.9
NP_116093.1	14389309	**TUBA1C**	tubulin alpha-1C chain isoform c	1.0	1.0	1.1	1.0	1.0	1.2	1.1	1.1	**1.0**	**1.1**	**1.1**	**1.1**	80.6	3	4	26	1039	449	49.9	5.1
NP_001191011.1	323276668	**MINOS1**	MICOS complex subunit MIC10 isoform b	1.0	1.0	1.1	1.1	1.1	1.0	1.1	1.0	**1.1**	**1.0**	**1.1**	**1.1**	12.9	3	1	1	2	62	7.0	7.3
NP_001243307.1	373838727	**MCMBP**	mini-chromosome maintenance complex-binding protein isoform 2	1.1	1.0	1.1	1.1	1.1	1.0	1.0	1.1	**1.1**	**1.0**	**1.0**	**1.1**	30.2	3	14	14	39	640	72.7	5.8
NP_036336.2	156119605	**GTF3C4**	general transcription factor 3C polypeptide 4	1.1	1.0	1.1	1.1	1.0	1.0	1.1	1.0	**1.0**	**1.0**	**1.1**	**1.1**	18.6	1	11	11	28	822	91.9	6.7
NP_001186084.1	312434011	**KAT7**	histone acetyltransferase KAT7 isoform 2	1.5	1.5	1.5	1.2	0.9	0.9	0.9	0.9	**1.2**	**1.2**	**1.2**	**1.1**	18.6	5	8	8	17	581	66.9	8.7
NP_001229852.1	339275831	**EIF3L**	eukaryotic translation initiation factor 3 subunit L isoform 2	1.1	1.0	1.1	1.1	1.1	1.0	1.1	1.1	**1.1**	**1.0**	**1.1**	**1.1**	36.4	2	15	15	84	516	61.0	7.0
NP_006187.2	222352151	**PCBP1**	poly(rC)-binding protein 1	1.0	1.0	0.9	1.1	0.9	0.9	0.9	1.1	**0.9**	**0.9**	**0.9**	**1.1**	64.9	3	10	15	87	356	37.5	7.1
NP_036557.1	6912638	**RSU1**	ras suppressor protein 1 isoform 1	1.1	1.1	1.1	1.1	1.1	1.1	1.2	1.1	**1.1**	**1.1**	**1.1**	**1.1**	48.0	2	9	9	46	277	31.5	8.6
NP_060281.2	22212943	**UBE2R2**	ubiquitin-conjugating enzyme E2 R2	1.0	0.9	0.9	1.0	1.0	1.1	1.1	1.1	**1.0**	**1.0**	**1.0**	**1.1**	4.6	1	1	1	2	238	27.1	4.4

Khan *et al.* , 2019. Multi-omics analysis to characterize cigarette smoke induced molecular alterations in esophageal cells
Supplementary Table 5. List of proteins quantified in untreated and chronically treated Het1A cells with cigarette smoke condensate for 8 months

NP_Accession	Protein group Accession	Gene ID	Description	Het-1A-Smoke - 2M/Parental	Het-1A-Smoke - 4M/Parental	Het-1A-Smoke - 6M/Parental	Het-1A-Smoke - 8M/Parental	Het-1A-Smoke - 2M/Parental	Het-1A-Smoke - 4M/Parental	Het-1A-Smoke - 6M/Parental	Het-1A-Smoke - 8M/Parental	Het-1A-Smoke - 2M/Parental	Het-1A-Smoke - 4M/Parental	Het-1A-Smoke - 6M/Parental	Het-1A-Smoke - 8M/Parental	Coverage (%)	Proteins	Unique Peptides	# Peptides	PSM	# AAs	MW [kDa]	calc. pI
				Replicate 1				Replicate 2				Average of replicates											
NP_001269105.1	532164706	CLPTM1	cleft lip and palate transmembrane protein 1 isoform 4	1.0	1.1	1.2	1.1	1.0	1.1	1.2	1.1	1.0	1.1	1.2	1.1	27.7	3	8	8	37	567	65.6	6.0
NP_003737.1	4505813;83267866	DYNLL1	dynein light chain 1, cytoplasmic	1.5	1.2	1.1	1.2	1.0	1.0	1.0	1.0	1.3	1.1	1.1	1.1	37.1	1	1	2	10	89	10.4	7.4
NP_631908.1	21040257	TRUB1	probable tRNA pseudouridine synthase 1	1.2	1.0	1.0	1.0	1.2	1.1	1.1	1.1	1.2	1.0	1.1	1.1	12.0	1	4	4	6	349	37.2	8.3
NP_009133.2	21493033	AKAP10	A-kinase anchor protein 10, mitochondrial precursor	1.0	0.9	1.0	1.1	1.1	1.1	1.0	1.0	1.1	1.0	1.0	1.1	3.2	1	1	1	2	662	73.8	6.4
NP_076920.1	13273315	HOXB4	homeobox protein Hox-B4	1.0	1.1	1.0	1.2	0.9	1.0	0.9	0.9	1.0	1.0	1.0	1.1	3.2	4	1	1	2	251	27.6	9.8
NP_115867.2	169636418	MRPL38	39S ribosomal protein L38, mitochondrial	1.1	1.0	1.0	1.0	1.1	1.1	1.0	1.1	1.1	1.0	1.0	1.1	23.2	1	7	7	21	380	44.6	7.5
NP_003163.1	4507319	SURF1	surfeit locus protein 1 isoform 1	1.1	1.1	1.0	1.1	1.1	1.1	1.1	1.0	1.1	1.1	1.0	1.1	5.3	1	1	1	6	300	33.3	9.6
NP_056139.1	12758125	RRP8	ribosomal RNA-processing protein 8	1.0	1.1	1.2	1.1	1.1	1.1	1.1	1.0	1.1	1.1	1.2	1.1	3.7	1	2	2	2	456	50.7	9.4
NP_940953.2	122891870	MIA3	melanoma inhibitory activity protein 3 isoform 1 precursor	1.1	1.2	1.1	1.1	1.2	1.1	1.0	1.0	1.2	1.2	1.1	1.1	23.4	2	32	32	72	1907	213.6	4.8
NP_009057.1	6005942	VCP	transitional endoplasmic reticulum ATPase	1.0	1.0	1.1	1.1	1.0	1.0	1.1	1.1	1.0	1.0	1.1	1.1	69.7	2	40	40	237	806	89.3	5.3
NP_055640.2	194394141	URB1	nucleolar pre-ribosomal-associated protein 1	1.0	1.0	1.1	1.1	1.1	1.0	1.0	1.1	1.1	1.0	1.0	1.1	3.4	4	7	7	12	2271	254.2	6.5
NP_001157289.1	255308875;119943112	DHCR7	7-dehydrocholesterol reductase	1.4	1.4	1.3	1.1	1.5	1.5	1.3	1.1	1.5	1.5	1.3	1.1	16.4	1	7	7	35	475	54.5	8.7
NP_001248396.1	387849036	NSRP1	nuclear speckle splicing regulatory protein 1 isoform 2	0.9	1.1	1.0	1.1	1.0	1.2	1.1	1.1	1.0	1.1	1.0	1.1	15.9	2	6	6	16	504	60.4	8.9
NP_001257450.1	395455064	MED23	mediator of RNA polymerase II transcription subunit 23 isoform c	1.0	1.0	1.0	1.1	1.1	1.0	1.1	1.1	1.1	1.0	1.0	1.1	6.2	4	6	6	12	1359	155.5	7.4
NP_001244068.1	380420340	CLCNKA	chloride channel protein ClC-Ka isoform 3	1.1	1.2	1.1	1.0	1.0	1.2	1.1	1.1	1.1	1.2	1.1	1.1	2.3	3	1	1	1	644	70.4	7.7
NP_078880.2	225735591	RPAP3	RNA polymerase II-associated protein 3 isoform 1	1.0	1.0	1.0	1.0	0.9	1.1	0.9	1.1	1.0	1.1	1.0	1.1	19.3	3	12	12	29	665	75.7	6.8
NP_004478.3	148596984	GOLGB1	Golgin subfamily B member 1 isoform 2	1.6	1.3	1.3	1.1	1.1	1.1	1.1	1.0	1.3	1.2	1.2	1.1	24.1	3	1	64	129	3259	375.8	5.0
NP_060207.2	41581463	FNBP1L	formin-binding protein 1-like isoform 2	1.0	1.2	1.1	1.0	0.9	0.9	0.9	1.1	1.0	1.0	1.0	1.1	7.0	3	2	2	2	547	63.5	6.2
NP_003622.2	70610136	PARG	poly(ADP-ribose) glycohydrolase	2.0	1.1	1.1	1.0	1.1	1.1	1.2	1.1	1.6	1.1	1.2	1.1	3.1	3	1	2	2	976	111.0	6.4
NP_597728.1	145386517	PPP1R18	phostensin	1.2	1.2	1.2	1.1	0.9	1.0	1.0	1.0	1.1	1.1	1.1	1.1	5.9	1	3	3	3	613	67.9	5.4
NP_005264.2	20357529	GNB2	guanine nucleotide-binding protein G(I)/G(S)/G(T) subunit beta-2	1.0	1.0	1.1	1.1	1.0	1.0	1.1	1.0	1.0	1.0	1.1	1.1	36.2	1	3	8	44	340	37.3	6.0
NP_001160163.1	262399361	TTK	dual specificity protein kinase TTK isoform 2	1.0	1.0	1.0	1.1	0.9	1.0	1.0	1.1	0.9	1.0	1.0	1.1	13.3	2	6	6	12	856	96.9	8.2
NP_004949.1	4826730	MTOR	serine/threonine-protein kinase mTOR	1.0	1.1	1.0	1.0	1.0	1.0	1.0	1.1	1.0	1.1	1.0	1.1	8.4	2	12	13	25	2549	288.7	7.2
NP_036377.1	6912676	SNW1	SNW domain-containing protein 1	1.0	0.9	0.9	1.0	1.0	0.9	0.9	1.1	1.0	0.9	0.9	1.1	11.2	1	4	4	9	536	61.5	9.5
NP_072179.1	12545406	RASA1	ras GTPase-activating protein 1 isoform 2	1.1	1.1	1.2	1.1	1.2	1.1	1.1	1.0	1.1	1.1	1.1	1.1	2.5	2	2	2	8	870	100.3	7.6
NP_006318.1	5454122	TIMM23	mitochondrial import inner membrane translocase subunit Tim23	1.1	1.0	1.0	1.0	1.0	1.0	1.0	1.1	1.0	1.0	1.0	1.1	12.0	3	2	2	4	209	21.9	8.6
NP_001075028.1	125987603	CPSF4	cleavage and polyadenylation specificity factor subunit 4 isoform 2	1.0	1.0	1.0	1.1	1.0	1.0	1.0	1.0	1.0	1.0	1.0	1.1	9.0	2	1	1	6	244	27.5	8.2
NP_005543.2	33620730	KLC1	kinesin light chain 1 isoform 1	0.9	1.0	0.9	1.0	0.9	1.0	0.9	1.1	0.9	1.0	0.9	1.1	20.0	3	6	10	21	560	63.8	5.9
NP_001137160.1	219521928	DIS3L	DIS3-like exonuclease 1 isoform 1	0.8	0.9	0.9	1.1	1.1	0.9	1.1	1.0	1.0	0.9	1.0	1.1	2.6	2	2	2	3	1054	120.7	6.5
NP_001177258.1	298358756	ATP5G3	ATP synthase F(0) complex subunit C3, mitochondrial isoform B precursor	0.9	0.8	0.9	1.1	0.9	0.9	0.9	1.1	0.9	0.9	0.9	1.1	6.7	5	1	1	3	105	10.6	10.0
NP_036437.1	27477041	AP2A2	AP-2 complex subunit alpha-2 isoform 2	1.1	1.1	1.1	1.0	1.2	1.1	1.2	1.1	1.1	1.1	1.1	1.1	33.0	2	15	24	62	939	103.9	7.0
NP_060560.1	8922416	NECAP2	adaptin ear-binding coat-associated protein 2 isoform 1	1.0	1.0	1.0	1.0	0.9	1.1	1.0	1.1	0.9	1.1	1.0	1.1	22.1	3	4	5	10	263	28.3	8.4
NP_055444.2	62548864	ARHGEF10	rho guanine nucleotide exchange factor 10 isoform 1	0.9	0.9	0.9	0.9	1.2	1.0	1.3	1.2	1.0	1.0	1.1	1.1	8.6	3	8	8	14	1344	148.8	5.6
NP_055746.3	151101459	PPP6R1	serine/threonine-protein phosphatase 6 regulatory subunit 1	1.1	1.1	1.1	1.1	1.0	1.0	1.0	1.0	1.1	1.1	1.1	1.1	10.2	1	5	5	14	881	96.7	4.6
NP_004230.2	190194412	TRIP11	thyroid receptor-interacting protein 11	1.1	1.1	1.0	1.0	1.1	1.0	1.0	1.1	1.1	1.1	1.0	1.1	8.3	1	13	13	23	1979	227.4	5.3
NP_005338.1	16507237	HSPA5	78 kDa glucose-regulated protein precursor	1.4	1.4	1.2	1.1	1.3	1.3	1.1	1.1	1.3	1.3	1.2	1.1	68.8	3	44	47	781	654	72.3	5.2
NP_001012532.1	60302912	ITM2C	integral membrane protein 2C isoform 3	1.2	1.1	1.1	1.2	1.1	1.1	1.1	1.0	1.1	1.1	1.1	1.1	19.1	4	2	2	2	220	24.9	7.4
NP_055288.1	7657198	DIMT1	probable dimethyladenosine transferase	1.1	1.0	1.1	1.1	1.1	1.0	1.0	1.1	1.1	1.0	1.0	1.1	20.5	1	5	5	13	313	35.2	10.0
NP_005551.3	21264602	LAMA5	laminin subunit alpha-5 precursor	1.2	1.3	1.2	1.2	0.9	1.0	0.9	1.0	1.0	1.1	1.1	1.1	0.3	1	1	1	2	3695	399.5	7.0
NP_006414.2	222144309	RABAC1	prenylated Rab acceptor protein 1	1.3	1.2	1.1	1.0	1.2	1.2	1.1	1.1	1.2	1.2	1.1	1.1	11.4	1	2	2	6	185	20.6	7.3
NP_006810.1	5803181	STIP1	stress-induced-phosphoprotein 1 isoform b	1.1	1.2	1.1	1.1	1.1	1.1	1.0	1.0	1.1	1.2	1.0	1.1	51.2	3	29	29	136	543	62.6	6.8
NP_705717.1	41281798	TAB1	TGF-beta-activated kinase 1 and MAP3K7-binding protein 1 isoform beta	1.1	1.0	1.0	1.1	1.1	0.9	1.0	1.1	1.1	1.0	1.0	1.1	5.6	2	2	2	4	462	49.9	5.6
NP_996561.1	45643127	GNL3	guanine nucleotide-binding protein-like 3 isoform 2	1.0	1.1	1.0	1.1	1.0	1.0	1.0	1.0	1.0	1.0	1.0	1.1	26.8	2	13	13	42	537	60.5	8.8
NP_078816.2	22035588	MRPL24	39S ribosomal protein L24, mitochondrial	1.0	1.0	1.0	1.1	0.9	1.0	1.0	1.1	1.0	1.0	1.0	1.1	37.0	1	6	6	11	216	24.9	9.3
NP_005091.2	21493022	AKAP12	A-kinase anchor protein 12 isoform 1	0.9	1.0	1.2	1.1	0.9	1.1	1.2	1.1	0.9	1.1	1.2	1.1	68.6	2	77	77	414	1782	191.4	4.4
NP_057131.1	7706326	SF3B6	splicing factor 3B subunit 6	1.0	1.0	1.0	1.1	0.9	1.0	0.9	1.0	1.0	1.0	1.0	1.1	45.6	1	5	5	36	125	14.6	9.4
NP_036286.2	29171734	AGO2	protein argonaute-2 isoform 1	1.1	1.1	1.1	1.1	1.2	1.1	1.1	1.1	1.1	1.1	1.1	1.1	10.1	2	3	7	13	859	97.1	9.2
NP_037528.3	50312666	USP25	ubiquitin carboxyl-terminal hydrolase 25 isoform USP25a	1.1	1.0	1.0	1.0	1.1	1.1	1.1	1.1	1.1	1.0	1.0	1.1	2.2	3	2	2	4	1055	122.1	5.3
NP_076997.1	13129092	TMEM109	transmembrane protein 109 precursor	1.0	1.2	1.0	1.0	1.1	1.2	1.2	1.1	1.0	1.2	1.1	1.1	3.7	1	1	1	3	243	26.2	10.5
NP_071926.4	21264337	ARAP3	arf-GAP with Rho-GAP domain, ANK repeat and PH domain-containing protein 3	1.1	1.0	1.0	1.1	1.0	1.1	1.0	1.0	1.1	1.1	1.0	1.1	4.1	1	4	4	6	1544	169.7	7.1
NP_002258.2	34485722	KPNA3	importin subunit alpha-4	1.0	1.0	1.0	1.0	1.1	1.0	1.1	1.1	1.0	1.0	1.1	1.1	34.6	1	7	10	26	521	57.8	4.9
NP_005750.4	109689723	ABI2	abl interactor 2 isoform c	0.9	0.8	0.8	1.0	1.2	1.1	1.1	1.2	1.0	0.9	1.0	1.1	7.6	17	3	3	6	475	52.4	6.0
NP_064505.1	9910280	UGGT1	UDP-glucose:glycoprotein glucosyltransferase 1 precursor	1.2	1.1	1.1	1.0	1.2	1.2	1.2	1.1	1.2	1.2	1.1	1.1	27.2	1	30	30	92	1555	177.1	5.6
NP_542166.1	18375676;11693132	UPF2	regulator of nonsense transcripts 2	1.1	1.1	1.1	1.1	0.9	1.0	1.0	1.0	1.0	1.0	1.0	1.1	6.4	1	7	7	13	1272	147.7	5.7
NP_000704.1	4502419	BLVRB	flavin reductase (NADPH)	0.9	1.0	1.0	1.0	1.2	1.2	1.2	1.1	1.1	1.1	1.1	1.1	24.3	1	3	3	6	206	22.1	7.6
NP_001035236.1	94421448	CHTF8	chromosome transmission fidelity protein 8 homolog	1.0	1.1	1.1	1.1	1.0	1.0	1.0	1.0	1.0	1.0	1.0	1.1	6.6	1	1	1	1	121	13.3	8.2
NP_775885.1	27734709	NOMO2	nodal modulator 2 isoform 2 precursor	1.0	1.1	1.1	1.0	1.2	1.2	1.1	1.1	1.1	1.1	1.1	1.1	35.2	6	27	27	78	1222	134.1	5.8
NP_001258965.1	436408716	LRRC14	leucine-rich repeat-containing protein 14	1.0	1.0	1.1	1.0	1.1	1.2	1.2	1.1	1.1	1.1	1.1	1.1	10.6	1	3	3	4	493	54.5	6.9
NP_002682.2	156616275	POLD1	DNA polymerase delta catalytic subunit 1	1.1	1.0	1.1	1.1	1.0	1.0	1.0	1.0	1.0	1.0	1.1	1.1	25.3	2	18	19	44	1107	123.6	7.0
NP_005539.1	5031815	KARS	lysine--tRNA ligase isoform 2	1.1	1.1	1.1	1.0	1.0	1.1	1.1	1.1	1.1	1.1	1.1	1.1	47.1	2	22	22	123	597	68.0	6.4
NP_001673.2	48255945	ATP2B1	plasma membrane calcium-transporting ATPase 1 isoform 1b	1.0	1.1	1.1	1.1	1.3	1.0	1.1	1.1	1.2	1.1	1.1	1.1	26.1	6	14	20	44	1220	134.6	5.9
NP_006283.1	5454140	TSG101	tumor susceptibility gene 101 protein	1.0	1.0	1.0	1.0	1.0	1.1	1.1	1.1	1.0	1.1	1.0	1.1	13.6	1	5	5	10	390	43.9	6.5
NP_057023.2	19923475	TRMT6	tRNA (adenine(58)-N(1))-methyltransferase non-catalytic subunit TRM6 isoform 1	0.9	1.0	1.0	1.1	1.1	1.1	1.1	1.1	1.0	1.0	1.0	1.1	16.5	2	7	7	16	497	55.8	7.5
NP_036399.3	112363070;194294548	HSPBP1	hsp70-binding protein 1 isoform 2	1.0	0.9	1.0	1.1	1.0	1.0	1.0	1.1	1.0	1.0	1.0	1.1	29.3	2	6	6	24	359	39.3	5.2
NP_057733.2	18093112	ESF1	ESF1 homolog	1.1	1.1	1.0	1.1	1.0	1.1	1.0	1.0	1.0	1.1	1.0	1.1	11.1	1	8	8	14	851	98.7	5.1
NP_055416.2	21361462	EHD2	EH domain-containing protein 2	0.9	1.1	0.9	1.1	1.1	1.1	1.2	1.0	1.0	1.1	1.0	1.1	9.8	1	2	3	4	543	61.1	6.5
NP_001291476.1	751868077	MVB12A	multivesicular body subunit 12A isoform 2	1.1	1.0	1.1	1.1	1.1	1.1	1.0	1.0	1.1	1.1	1.0	1.1	8.3	2	1	1	3	181	19.4	9.7
NP_689759.2	103471995	SRFBP1	serum response factor-binding protein 1	0.9	1.0	0.9	1.0	0.9	1.1	1.0	1.1	0.9	1.0	1.0	1.1	7.2	1	3	3	6	429	48.6	9.6
NP_004472.1	4758412	GALNT2	polypeptide N-acetylgalactosaminyltransferase 2 precursor	1.1	1.1	1.1	1.0	1.1	1.0	1.1	1.1	1.1	1.0	1.1	1.1	30.1	3	16	16	36	571	64.7	8.4
NP_003469.2	40254446	CUL5	cullin-5	1.1	1.1	1.2	1.1	1.1	1.2	1.1	1.1	1.1	1.1	1.1	1.1	6.7	1	5	5	8	780	90.9	7.9
NP_004119.1	4758488	GTF2F2	general transcription factor IIF subunit 2	0.9	1.1	1.0	1.0	0.9	1.1	1.0	1.1	0.9	1.1	1.0	1.1	24.1	1	7	7	16	249	28.4	9.2
NP_059985.2	31542943	SLC35F2	solute carrier family 35 member F2	1.1	1.0	1.3	1.1	1.1	0.9	0.9	1.0	1.1	1.0	1.1	1.1	6.2	1	1	1	4	374	41.2	6.2
NP_039234.1	7330335	CLIC4	chloride intracellular channel protein 4	1.2	1.3	1.3	1.0	1.3	1.3	1.4	1.1	1.3	1.3	1.4	1.1	70.4	2	14	15	118	253	28.8	5.6
NP_036272.2	20149598	SLC25A10	mitochondrial dicarboxylate carrier isoform 2	1.0	1.0	1.0	1.1	1.1	1.1	1.1	1.1	1.0	1.1	1.1	1.1	22.3	3	6	6	12	287	31.3	9.5
NP_060668.2	217035105	DNAJC11	dnaJ homolog subfamily C member 11	1.1	1.1	1.1	1.1	1.0	1.1	1.1	1.1	1.1	1.1	1.1	1.1	22.7	1	10	10	18	559	63.2	8.4
NP_036552.1	6912630	IFIT5	interferon-induced protein with tetratricopeptide repeats 5	0.9	1.0	1.0	1.0	1.0	1.1	1.0	1.1	1.0	1.1	1.0	1.1	12.9	5	6	6	11	482	55.8	7.4
NP_061327.2	29568099	NSFL1C	NSFL1 cofactor p47 isoform b	1.2	1.1	1.4	1.1	1.1	1.2	1.5	1.1	1.2	1.1	1.4	1.1	6.8	3	2	2	4	339	37.3	5.1
NP_001032728.1	83641878	PARL	presenilins-associated rhomboid-like protein, mitochondrial isoform 2 preproprotein	1.3	1.2	1.1	1.1	1.4	1.2	1.3	1.1	1.3	1.2	1.2	1.1	6.7	2	2	2	8	329	36.6	9.9
NP_002255.3	222144293	KPNA1	importin subunit alpha-5	1.1	1.2	1.1	1.1	1.1	1.1	1.0	1.0	1.1	1.2	1.1	1.1	35.5	1	11	15	52	538	60.2	5.0
NP_056056.2	209862789	MICAL3	protein-methionine sulfoxide oxidase MICAL3 isoform 1	1.1	1.2	1.1	1.1	1.0	1.2	1.1	1.1	1.1	1.2	1.1	1.1	8.0	3	10	11	23	2002	224.2	5.6
NP_001096618.1	156766047	GIGYF2	PERQ amino acid-rich with GYF domain-containing protein 2 isoform c	1.1	1.1	1.2	1.1	0.9	0.9	1.0	1.0	1.0	1.0	1.1	1.1	2.8	3	3	3	4	1293	149.4	5.5
NP_005497.1	5031631	SCARB2	lysosome membrane protein 2 isoform 1 precursor	0.9	1.0	1.0	1.0	1.1	1.1	1.1	1.1	1.0	1.0	1.0	1.1	24.3	2	9	9	47	478	54.3	5.1
NP_005427.2	46852390	CCDC6	coiled-coil domain-containing protein 6	1.0	1.0	1.1	1.1	0.9	1.1	1.0	1.0	0.9	1.1	1.0	1.1	37.3	1	13	13	32	474	53.3	7.3
NP_001244923.1	384229064	TRMT2A	tRNA (uracil-5-)-methyltransferase homolog A isoform b	1.5	1.1	1.0	1.1	1.0	1.0	1.0	1.0	1.3	1.1	1.0	1.1	6.4	2	3	3	3	562	61.9	8.0
NP_060692.2	315583337	PARVA	alpha-parvin	1.0	1.1	1.2	1.0	1.0	1.1	1.2	1.1	1.0	1.1	1.2	1.1	25.5	1	7	7	16	412	46.6	8.4
NP_115649.1	14150017	TMEM126A	transmembrane protein 126A isoform 1	1.1	1.2	1.2	1.1	1.0	1.1	1.0	1.0	1.1	1.2	1.1	1.1	25.1	2	4	4	12	195	21.5	9.3
NP_001091.1	4501881	ACTA1	actin, alpha skeletal muscle	1.2	1.3	1.3	1.1	1.0	1.0	1.0	1.0	1.1	1.1	1.2	1.1	62.1	5	7	22	741	377	42.0	5.4

Khan *et al.*, 2019. Multi-omics analysis to characterize cigarette smoke induced molecular alterations in esophageal cells
Supplementary Table 5. List of proteins quantified in untreated and chronically treated Het1A cells with cigarette smoke condensate for 8 months

NP_Accession	Protein group Accession	Gene ID	Description	Het-1A-Smoke - 2M/Parental	Het-1A-Smoke - 4M/Parental	Het-1A-Smoke - 6M/Parental	Het-1A-Smoke - 8M/Parental	Het-1A-Smoke - 2M/Parental	Het-1A-Smoke - 4M/Parental	Het-1A-Smoke - 6M/Parental	Het-1A-Smoke - 8M/Parental	Het-1A-Smoke - 2M/Parental	Het-1A-Smoke - 4M/Parental	Het-1A-Smoke - 6M/Parental	Het-1A-Smoke - 8M/Parental	Coverage (%)	Proteins	Unique Peptides	# Peptides	PSM	# AAs	MW [kDa]	calc. pI
				Replicate 1				Replicate 2				Average of replicates											
NP_057582.2	51243055	MTFP1	mitochondrial fission process protein 1 isoform a	1.1	1.1	1.1	1.0	1.1	1.1	1.1	1.1	1.1	1.1	1.1	1.1	21.1	2	3	3	10	166	18.0	9.3
NP_055839.3	154448892	XPO7	exportin-7	1.2	1.1	1.2	1.1	1.2	1.1	1.1	1.0	1.2	1.1	1.2	1.1	11.1	1	9	9	25	1087	123.8	6.3
NP_001272807.1	551895085	CEBPB	CCAAT/enhancer-binding protein beta isoform b	1.0	1.0	0.9	1.1	1.2	1.1	0.9	1.1	1.1	1.1	0.9	1.1	12.7	3	3	3	8	322	33.6	8.2
NP_060337.2	157388914	C4orf27	UPF0609 protein C4orf27	1.0	1.0	1.0	1.1	1.0	1.0	1.0	1.1	1.0	1.0	1.0	1.1	14.7	1	4	4	5	346	39.4	6.8
NP_002052.1	4504011	GCLM	glutamate--cysteine ligase regulatory subunit isoform 1	1.2	1.2	1.1	1.1	1.1	1.1	1.0	1.1	1.2	1.2	1.1	1.1	36.5	2	6	6	20	274	30.7	6.0
NP_001269917.1	545478136	DHRS4	dehydrogenase/reductase SDR family member 4 isoform 3	1.1	1.0	1.0	1.1	1.1	1.0	1.0	1.0	1.1	1.0	1.0	1.1	7.0	3	2	2	2	244	25.9	9.2
NP_001186092.1	312596881	PSMC5	26S protease regulatory subunit 8 isoform 2	1.1	1.1	1.0	1.1	1.0	1.0	1.0	1.1	1.0	1.1	1.0	1.1	42.2	2	15	16	85	398	44.8	8.2
NP_001035863.1	109255230	CEP170	centrosomal protein of 170 kDa isoform beta	1.1	1.2	1.1	1.1	1.1	1.1	1.1	1.1	1.1	1.1	1.1	1.1	15.0	6	18	18	38	1486	164.4	7.2
NP_001123532.1	194239643	ATF7	cyclic AMP-dependent transcription factor ATF-7 isoform 3	0.9	1.0	0.9	1.1	1.0	0.9	0.9	1.0	1.0	1.0	0.9	1.1	7.6	3	2	2	3	462	49.6	8.3
NP_387506.1	16905526;315434212	DAP3	28S ribosomal protein S29, mitochondrial isoform 1	1.1	1.0	1.1	1.1	1.1	1.1	1.1	1.0	1.1	1.0	1.1	1.1	30.7	3	10	10	34	398	45.5	8.9
NP_006351.2	23397429	EIF3M	eukaryotic translation initiation factor 3 subunit M isoform 1	1.1	1.1	1.1	1.1	1.0	1.0	1.1	1.1	1.0	1.0	1.1	1.1	52.1	2	14	14	75	374	42.5	5.6
NP_001193963.1	333440442	RNF40	E3 ubiquitin-protein ligase BRE1B isoform 3	1.0	1.0	1.1	1.2	1.0	1.0	1.0	1.0	1.0	1.0	1.0	1.1	9.4	3	5	7	9	901	102.0	6.6
NP_001244031.1	380036038	NDUFB3	NADH dehydrogenase [ubiquinone] 1 beta subcomplex subunit 3	1.0	1.0	1.1	1.1	1.0	1.0	1.1	1.0	1.0	1.0	1.1	1.1	10.2	1	1	1	4	98	11.4	9.2
NP_006816.2	19920317	CKAP4	cytoskeleton-associated protein 4	1.2	1.2	1.2	1.1	1.2	1.2	1.2	1.1	1.2	1.2	1.2	1.1	45.5	5	24	24	89	602	66.0	5.9
NP_001123910.1	194595509	SPTAN1	spectrin alpha chain, non-erythrocytic 1 isoform 1	1.2	1.1	1.0	1.1	0.9	1.2	1.5	1.0	1.0	1.2	1.2	1.1	60.2	3	1	126	621	2477	284.9	5.4
NP_079236.3	24430167	PANK2	pantothenate kinase 2, mitochondrial isoform 2	1.3	1.0	1.0	1.1	1.1	1.0	0.9	1.0	1.2	1.0	0.9	1.1	3.2	5	1	1	1	279	30.7	6.1
NP_036347.1	11024698	MGEA5	protein O-GlcNAcase isoform a	1.1	1.1	1.1	1.1	1.1	1.1	1.1	1.1	1.1	1.1	1.1	1.1	30.1	2	17	18	64	916	102.8	4.9
NP_001136095.1	217330596	GHDC	GH3 domain-containing protein isoform 3 precursor	0.9	1.0	1.0	1.0	1.1	1.0	1.0	1.1	1.0	1.0	1.0	1.1	6.8	3	2	2	4	474	51.4	6.9
NP_001294906.1	808175962	WDR74	WD repeat-containing protein 74 isoform 2	0.9	1.0	1.0	1.0	1.2	1.2	1.3	1.1	1.0	1.1	1.1	1.1	7.7	2	2	2	2	366	40.2	8.4
NP_065095.2	21361837	PITHD1	PITH domain-containing protein 1	1.1	1.0	1.0	1.1	1.0	1.0	1.1	1.1	1.1	1.0	1.1	1.1	33.7	1	5	5	14	211	24.2	5.7
NP_361013.3	216548085	LUZP1	leucine zipper protein 1	1.0	1.0	1.1	1.1	1.1	1.0	1.1	1.0	1.1	1.0	1.1	1.1	17.8	1	14	14	23	1076	120.2	8.5
NP_658985.2	91984773	APOA1BP	NAD(P)H-hydrate epimerase precursor	1.0	0.9	1.0	1.1	1.2	1.1	1.1	1.1	1.1	1.0	1.1	1.1	37.9	1	8	8	43	288	31.7	7.7
NP_000844.2	167466164	GSTT1	glutathione S-transferase theta-1 isoform a	1.1	1.0	1.1	1.1	1.0	1.0	1.2	1.0	1.0	1.0	1.1	1.1	25.4	7	5	5	7	240	27.3	7.5
NP_005177.2	12408656;311893365	CAPN1	calpain-1 catalytic subunit	1.1	1.1	1.2	1.1	1.1	1.1	1.1	1.0	1.1	1.1	1.1	1.1	32.5	1	18	18	52	714	81.8	5.7
NP_001258795.1	428673522	GTF2H3	general transcription factor IIH subunit 3 isoform b	0.9	0.9	0.9	1.0	1.2	1.3	1.4	1.1	1.1	1.1	1.1	1.1	13.6	3	3	3	3	265	29.6	7.8
NP_060717.1	8922720	TMEM30A	cell cycle control protein 50A isoform 1	1.3	1.1	1.2	1.2	0.9	1.0	1.2	1.0	1.1	1.1	1.2	1.1	4.7	1	1	1	2	361	40.7	8.6
NP_001293009.1	807045912	LMO7	LIM domain only protein 7 isoform 3	1.3	1.4	1.8	1.2	1.1	1.1	1.2	1.0	1.2	1.3	1.5	1.1	5.0	1	1	8	13	1631	186.1	7.2
NP_001154485.1	238776801	ARMC10	armadillo repeat-containing protein 10 isoform f	1.0	1.0	1.1	1.1	1.1	1.1	1.1	1.1	1.0	1.0	1.1	1.1	7.1	6	1	1	2	225	24.7	7.5
NP_055316.2	112382377	UBE2S	ubiquitin-conjugating enzyme E2 S	1.0	1.2	1.2	1.1	1.0	1.1	1.1	1.0	1.0	1.1	1.2	1.1	63.1	1	9	9	38	222	23.8	8.4
NP_003472.2	148727247	USP5	ubiquitin carboxyl-terminal hydrolase 5 isoform 2	1.0	1.1	1.1	1.0	1.1	1.1	1.2	1.1	1.1	1.1	1.1	1.1	45.8	3	23	23	98	835	93.2	5.1
NP_002797.3	195539395	PSMC6	26S protease regulatory subunit 10B	1.0	1.0	0.9	1.1	1.0	1.0	1.0	1.1	1.0	1.0	0.9	1.1	52.1	1	17	17	75	403	45.8	7.8
NP_055230.2	166235167	ZBTB11	zinc finger and BTB domain-containing protein 11	1.1	1.1	1.1	1.0	1.2	1.2	1.1	1.1	1.1	1.1	1.1	1.1	5.4	1	4	4	7	1053	119.3	8.7
NP_004146.1	4758906	SERPINB9	serpin B9	1.0	0.9	0.8	1.1	1.0	0.9	1.0	1.0	1.0	0.9	0.9	1.1	7.7	1	3	3	4	376	42.4	5.9
NP_060648.2	21361716	GOLPH3L	Golgi phosphoprotein 3-like	1.0	1.1	1.0	1.0	1.3	1.2	0.9	1.1	1.2	1.1	0.9	1.1	19.7	1	2	4	10	285	32.7	5.8
NP_079023.2	31881785	HPS6	Hermansky-Pudlak syndrome 6 protein	1.1	1.1	1.1	1.1	0.8	0.7	0.9	1.0	0.9	0.9	1.0	1.1	8.3	1	3	3	4	775	82.9	6.3
NP_057122.2	28416940	SBDS	ribosome maturation protein SBDS	1.0	1.0	1.1	1.0	1.0	1.1	1.1	1.1	1.0	1.1	1.1	1.1	60.4	1	15	15	51	250	28.7	8.7
NP_005955.3	367460087	MYH10	myosin-10 isoform 2	0.9	0.9	0.9	1.1	1.0	0.9	1.0	1.1	1.0	0.9	0.9	1.1	40.4	3	46	72	364	1976	228.9	5.5
NP_057404.2	21264365	NUP98	nuclear pore complex protein Nup98-Nup96 isoform 1	1.1	1.1	1.1	1.1	1.1	1.0	1.1	1.0	1.1	1.0	1.1	1.1	9.9	4	13	13	32	1800	195.7	6.4
NP_001018119.1	66363697	PIR	pirin	1.8	1.5	1.5	1.1	1.5	1.4	1.4	1.0	1.6	1.4	1.5	1.1	24.1	1	5	5	18	290	32.1	6.9
NP_065908.1	17978485	VPS18	vacuolar protein sorting-associated protein 18 homolog	1.0	1.0	0.9	1.1	1.0	1.0	1.1	1.1	1.0	1.0	1.0	1.1	4.3	1	3	3	5	973	110.1	6.1
NP_116193.2	62177110	ADO	2-aminoethanethiol dioxygenase	1.0	1.1	1.1	1.1	1.0	1.1	1.2	1.0	1.0	1.1	1.2	1.1	9.3	1	2	2	3	270	29.7	6.0
NP_001922.2	31711992	DLAT	dihydrolipoyllysine-residue acetyltransferase component of pyruvate dehydrogenase complex, mitochondrial precursor	1.1	1.2	1.1	1.0	1.1	1.2	1.2	1.1	1.1	1.2	1.2	1.1	36.3	1	17	17	72	647	69.0	7.8
NP_001177645.1	299758394	DNM2	dynamin-2 isoform 5	1.0	1.0	1.0	1.0	1.0	1.0	1.0	1.1	1.0	1.0	1.0	1.1	28.3	5	13	19	54	869	97.9	7.4
NP_006108.2	45643119;260275230	ECI2	enoyl-CoA delta isomerase 2, mitochondrial isoform 1	1.1	1.0	1.1	1.1	1.1	1.1	1.0	1.0	1.1	1.1	1.0	1.1	35.2	2	6	6	21	364	40.2	8.8
NP_955399.1	40806173	SPG7	paraplegin isoform 2 precursor	1.1	1.1	1.1	1.0	1.3	1.0	0.9	1.1	1.2	1.0	1.0	1.1	2.9	2	2	2	2	489	53.9	9.6
NP_062559.2	122939167	WDR45B	WD repeat domain phosphoinositide-interacting protein 3	0.9	0.9	0.9	1.0	0.8	0.8	0.9	1.1	0.8	0.9	0.9	1.1	8.1	1	2	2	5	344	38.1	7.6
NP_002145.3	38327039	HSPA4	heat shock 70 kDa protein 4	1.0	1.0	1.0	1.1	1.1	1.0	1.0	1.0	1.0	1.0	1.0	1.1	71.6	3	49	53	454	840	94.3	5.2
NP_055871.1	57863269	RRP1B	ribosomal RNA processing protein 1 homolog B	1.0	1.0	1.0	1.1	0.9	0.9	0.9	1.0	0.9	1.0	0.9	1.1	24.8	1	13	13	31	758	84.4	9.8
NP_005330.1	4885417	UBE2K	ubiquitin-conjugating enzyme E2 K isoform 1	1.2	1.2	1.2	1.1	1.2	1.0	1.1	1.0	1.2	1.1	1.1	1.1	42.0	3	5	5	25	200	22.4	5.4
NP_085151.1	18139549	SETD7	histone-lysine N-methyltransferase SETD7 isoform 1	1.5	1.3	1.4	1.0	1.0	1.2	1.3	1.1	1.3	1.2	1.4	1.1	10.1	2	3	3	6	366	40.7	4.6
NP_006819.2	76880486	ASCC3	activating signal cointegrator 1 complex subunit 3 isoform a	1.0	1.0	1.1	1.1	1.0	1.0	1.0	1.0	1.0	1.0	1.0	1.1	8.3	2	13	13	26	2202	251.3	7.1
NP_008878.3	109638749	SRP72	signal recognition particle subunit SRP72 isoform 1	1.0	1.0	1.0	1.0	1.0	1.0	1.1	1.1	1.0	1.0	1.1	1.1	27.1	2	14	14	64	671	74.6	9.3
NP_060368.2	31542713	Mar-02	mitochondrial amidoxime reducing component 2 precursor	1.2	1.1	1.0	1.2	0.9	0.9	0.9	1.0	1.1	1.0	1.0	1.1	4.5	1	1	1	1	335	38.0	9.2
NP_060058.1	16554627;16554629	WDR5	WD repeat-containing protein 5	1.1	1.1	1.1	1.1	0.9	1.0	1.0	1.0	1.0	1.0	1.0	1.1	25.8	1	5	5	13	334	36.6	8.3
NP_005693.1	24307899	ERAL1	GTPase Era, mitochondrial	0.9	1.0	1.0	1.0	1.1	1.0	1.1	1.1	1.0	1.0	1.0	1.1	7.1	1	2	2	4	437	48.3	8.8
NP_055744.2	45267832	FASTKD2	FAST kinase domain-containing protein 2	0.9	0.9	0.9	1.1	1.1	1.1	1.3	1.0	1.0	1.0	1.1	1.1	2.8	1	2	2	3	710	81.4	8.0
NP_001275993.1	574274815	LIG1	DNA ligase 1 isoform 3	1.0	1.0	1.0	1.1	1.1	0.9	1.0	1.1	1.0	0.9	1.0	1.1	14.0	3	8	8	19	851	93.9	6.0
NP_001159584.1	260656039	PNPLA6	neuropathy target esterase isoform c	1.2	1.2	1.2	1.1	1.1	1.1	1.1	1.0	1.1	1.1	1.2	1.1	5.7	4	6	6	13	1300	143.3	7.7
NP_001276337.1	574957259;574957261	PMS1	PMS1 protein homolog 1 isoform d	1.1	1.0	1.1	1.0	1.1	0.9	1.0	1.1	1.1	1.0	1.0	1.1	9.9	4	7	7	12	756	86.5	6.5
NP_001273206.1	555943697	TUBGCP3	gamma-tubulin complex component 3 isoform 2	1.0	1.1	1.0	1.1	1.1	1.0	0.9	1.0	1.1	1.0	1.0	1.1	10.7	4	7	7	13	897	102.4	8.1
NP_001686.1	4502315	ATP6V1C1	V-type proton ATPase subunit C 1	1.0	0.9	1.1	1.1	1.0	1.0	1.0	1.1	1.0	1.0	1.1	1.1	36.9	3	10	10	40	382	43.9	7.5
NP_006331.1	5453565	BAIAP2	brain-specific angiogenesis inhibitor 1-associated protein 2 isoform 3	0.9	1.0	1.0	1.1	1.0	1.0	1.0	1.0	1.0	1.0	1.0	1.1	19.0	4	9	9	27	520	57.4	8.8
NP_872353.2	33457308	TMED4	transmembrane emp24 domain-containing protein 4 isoform 1 precursor	1.1	1.2	1.0	1.0	1.1	1.2	1.1	1.1	1.1	1.2	1.1	1.1	24.7	5	2	5	7	227	25.9	8.3
NP_001265660.1	523567531	HARS2	probable histidine--tRNA ligase, mitochondrial isoform 2	1.2	1.0	1.0	1.0	1.2	1.2	1.1	1.1	1.2	1.1	1.0	1.1	36.2	3	8	15	43	481	54.1	8.0
NP_056169.1	62955803	NUP188	nucleoporin NUP188 homolog	1.1	1.0	1.1	1.0	1.1	1.0	1.1	1.1	1.1	1.0	1.1	1.1	10.7	1	12	12	32	1749	195.9	6.7
NP_620601.1	20986499	MAPK7	mitogen-activated protein kinase 7 isoform 2	1.2	1.0	1.0	0.9	1.2	1.1	1.0	1.2	1.2	1.0	1.0	1.1	4.7	2	2	2	3	677	73.2	5.7
NP_003850.1	4503363	DPM1	dolichol-phosphate mannosyltransferase subunit 1	1.1	1.0	1.0	1.0	1.0	1.1	1.1	1.1	1.1	1.1	1.0	1.1	48.5	1	10	10	44	260	29.6	9.6
NP_003591.2	38327562;38327570	AURKA	aurora kinase A	1.0	1.0	1.0	1.1	0.9	1.0	1.1	1.1	1.0	1.0	1.0	1.1	24.1	1	8	8	22	403	45.8	9.4
NP_067075.1	10880989	RAB18	ras-related protein Rab-18 isoform 1	1.0	1.0	1.1	1.1	1.1	1.1	1.2	1.1	1.1	1.1	1.1	1.1	35.9	5	6	6	25	206	23.0	5.2
NP_057049.5	260763955	NDUFA13	NADH dehydrogenase [ubiquinone] 1 alpha subcomplex subunit 13	1.1	1.1	1.1	1.1	1.1	1.1	1.0	1.0	1.1	1.1	1.1	1.1	59.7	1	9	9	31	144	16.7	8.4
NP_001312.1	4503101	CSRP2	cysteine and glycine-rich protein 2	1.1	1.2	1.2	1.2	1.0	1.1	0.9	0.9	1.0	1.1	1.1	1.1	20.7	1	3	3	5	193	20.9	8.6
NP_001185792.1	311893345	CPNE1	copine-1 isoform c	1.0	0.8	0.8	1.1	1.0	0.9	0.9	1.0	1.0	0.9	0.8	1.1	25.6	3	10	10	52	536	58.9	5.8
NP_006355.2	38202214	SEC23A	protein transport protein Sec23A	1.1	1.1	1.1	1.0	1.2	1.1	1.1	1.1	1.1	1.1	1.1	1.1	32.9	1	16	18	52	765	86.1	7.1
NP_001375.2	21536476	DPH2	diphthamide biosynthesis protein 2 isoform a	0.9	0.9	0.9	1.0	1.0	1.1	1.1	1.1	1.0	1.0	1.0	1.1	8.2	2	3	3	4	489	52.0	5.5
NP_001019903.2	544346114	MECR	trans-2-enoyl-CoA reductase, mitochondrial isoform b	1.1	1.2	1.1	1.0	1.0	1.0	1.0	1.1	1.0	1.1	1.0	1.1	4.7	2	1	1	5	297	32.2	7.2
NP_071936.2	75677351	SUDS3	sin3 histone deacetylase corepressor complex component SDS3	0.9	0.9	1.0	1.1	0.9	1.0	0.8	1.0	0.9	0.9	0.9	1.1	3.1	1	1	1	2	328	38.1	5.7
NP_001627.2	156071462	SLC25A6	ADP/ATP translocase 3	1.2	1.1	1.2	1.1	1.0	1.1	1.1	1.0	1.1	1.1	1.2	1.1	66.1	2	4	17	219	298	32.8	9.7
NP_073753.3	30410805	CDCP1	CUB domain-containing protein 1 isoform 1 precursor	1.1	1.4	1.6	1.0	1.0	1.4	1.6	1.1	1.1	1.4	1.6	1.1	21.5	2	16	16	47	836	92.9	8.0
NP_031391.2	122939159	PADI2	protein-arginine deiminase type-2	1.0	1.3	1.3	1.0	1.1	1.4	1.3	1.1	1.1	1.3	1.3	1.1	20.2	1	9	9	19	665	75.5	5.6
NP_055599.1	7662086	ARHGEF11	rho guanine nucleotide exchange factor 11 isoform 1	1.2	1.0	0.9	1.0	1.1	1.2	1.0	1.1	1.1	1.1	1.0	1.1	4.1	2	3	3	5	1522	167.6	5.5
NP_001008491.1	56549636;4758158	Sep-02	septin-2 isoform a	1.0	1.0	1.0	1.0	1.0	1.0	1.0	1.1	1.0	1.0	1.0	1.1	44.9	3	11	11	34	361	41.5	6.6
NP_005436.1	4885399	SMC3	structural maintenance of chromosomes protein 3	1.0	1.0	1.1	1.1	1.0	1.0	1.1	1.1	1.0	1.0	1.1	1.1	31.8	1	31	32	83	1217	141.5	7.2
NP_006450.2	154800487;154800489	ERLIN1	erlin-1	1.0	1.0	1.1	1.0	0.9	0.9	0.9	1.1	1.0	0.9	1.0	1.1	28.7	2	6	9	39	348	39.1	7.9
NP_115646.2	19923729	LRRC8C	leucine-rich repeat-containing protein 8C	0.9	0.9	1.0	0.9	1.4	1.2	1.3	1.2	1.2	1.1	1.1	1.1	3.2	1	2	2	2	803	92.3	7.7
NP_065941.2	190194355	KIAA1524	protein CIP2A	1.0	1.1	1.1	1.1	1.1	1.1	1.1	1.0	1.0	1.1	1.1	1.1	9.4	1	7	7	12	905	102.1	6.2

Khan *et al.* , 2019. Multi-omics analysis to characterize cigarette smoke induced molecular alterations in esophageal cells
Supplementary Table 5. List of proteins quantified in untreated and chronically treated Het1A cells with cigarette smoke condensate for 8 months

NP_Accession	Protein group Accession	Gene ID	Description	Het-1A-Smoke - 2M/Parental	Het-1A-Smoke - 4M/Parental	Het-1A-Smoke - 6M/Parental	Het-1A-Smoke - 8M/Parental	Het-1A-Smoke - 2M/Parental	Het-1A-Smoke - 4M/Parental	Het-1A-Smoke - 6M/Parental	Het-1A-Smoke - 8M/Parental	Het-1A-Smoke - 2M/Parental	Het-1A-Smoke - 4M/Parental	Het-1A-Smoke - 6M/Parental	Het-1A-Smoke - 8M/Parental	Coverage (%)	Proteins	Unique Peptides	# Peptides	PSM	# AAs	MW [kDa]	calc. pI
				Replicate 1				Replicate 2				Average of replicates											
NP_001164666.1	283945529	**ELAVL2**	ELAV-like protein 2 isoform b	0.8	0.9	0.9	1.0	1.1	1.0	1.0	1.1	**1.0**	**0.9**	**1.0**	**1.1**	6.9	9	1	1	1	346	38.0	9.0
NP_055847.1	7657269	**PDS5B**	sister chromatid cohesion protein PDS5 homolog B	1.1	1.1	1.1	1.0	1.1	1.1	1.1	1.1	**1.1**	**1.1**	**1.1**	**1.1**	16.3	1	18	21	68	1447	164.6	8.5
NP_001259025.1	443287677	**STX4**	syntaxin-4 isoform 2	1.1	1.2	1.2	1.1	0.9	1.0	1.0	1.0	**1.0**	**1.1**	**1.1**	**1.1**	22.0	3	3	3	7	295	33.8	9.5
NP_056284.1	22267436	**NIPSNAP3A**	protein NipSnap homolog 3A	1.1	1.1	1.0	1.0	1.0	1.0	1.1	1.1	**1.1**	**1.1**	**1.0**	**1.1**	33.6	1	6	6	10	247	28.4	9.2
NP_003667.1	4505193	**DEGS1**	sphingolipid delta(4)-desaturase DES1	1.0	1.0	1.0	0.9	0.9	1.2	1.0	1.2	**1.0**	**1.1**	**1.0**	**1.1**	14.9	1	3	3	4	323	37.8	7.5
NP_006746.1	5803187	**TALDO1**	transaldolase	1.2	1.1	1.2	1.1	1.1	1.1	1.1	1.0	**1.1**	**1.1**	**1.2**	**1.1**	59.1	1	17	17	130	337	37.5	6.8
NP_001191786.1	325652033;5032161	**TCEB1**	transcription elongation factor B polypeptide 1 isoform a	1.1	0.9	1.2	1.0	1.4	1.0	1.6	1.1	**1.2**	**0.9**	**1.4**	**1.1**	38.4	2	3	3	6	112	12.5	4.8
NP_078844.2	373432617	**CHD1L**	chromodomain-helicase-DNA-binding protein 1-like isoform 3	1.2	1.2	1.2	1.1	1.2	1.1	1.2	1.0	**1.2**	**1.1**	**1.2**	**1.1**	17.9	5	9	9	21	784	88.4	6.9
NP_067585.2	82546845	**BCR**	breakpoint cluster region protein isoform 2	1.2	1.0	1.1	1.0	1.0	1.2	1.2	1.1	**1.1**	**1.1**	**1.1**	**1.1**	3.8	3	2	3	8	1227	137.6	7.2
NP_001273515.1	557440906	**CDIPT**	CDP-diacylglycerol--inositol 3-phosphatidyltransferase isoform 3	1.2	1.2	1.2	1.2	0.8	1.0	1.2	0.9	**1.0**	**1.1**	**1.2**	**1.1**	16.2	3	2	2	5	148	16.4	8.5
NP_659400.2	84000432	**FAM91A1**	protein FAM91A1	1.2	1.0	1.0	1.1	1.1	1.0	1.0	1.1	**1.1**	**1.0**	**1.0**	**1.1**	19.9	2	13	13	28	838	93.9	6.4
NP_057130.1	7705612	**EXOSC1**	exosome complex component CSL4	1.0	0.8	0.9	0.9	1.1	1.1	1.1	1.2	**1.0**	**0.9**	**1.0**	**1.1**	16.9	1	2	2	3	195	21.4	8.2
NP_057339.1	7705268	**FAM8A1**	protein FAM8A1	0.9	1.1	1.2	1.0	0.9	0.9	0.9	1.1	**0.9**	**1.0**	**1.0**	**1.1**	2.4	1	1	1	1	413	44.1	7.2
NP_063937.2	49574532	**GSK3A**	glycogen synthase kinase-3 alpha	1.3	1.0	1.2	1.1	1.0	1.0	1.0	1.0	**1.1**	**1.0**	**1.1**	**1.1**	26.3	1	6	6	16	483	50.9	8.7
NP_009135.4	256223453	**DDX20**	probable ATP-dependent RNA helicase DDX20	1.0	1.0	1.0	1.1	1.1	1.0	1.0	1.0	**1.1**	**1.0**	**1.0**	**1.1**	9.0	1	6	6	10	824	92.2	6.9
NP_001275589.1	568815692	**METTL9**	methyltransferase-like protein 9 isoform 4	1.0	1.2	1.0	1.2	1.1	1.0	1.3	0.9	**1.0**	**1.1**	**1.1**	**1.1**	5.8	4	1	1	1	277	32.3	5.7
NP_006160.1	5453790	**NNMT**	nicotinamide N-methyltransferase	1.0	0.8	0.8	1.0	0.9	0.9	0.8	1.1	**1.0**	**0.8**	**0.8**	**1.1**	62.5	1	10	10	114	264	29.6	5.7
NP_001139436.1	225579067	**SLC12A4**	solute carrier family 12 member 4 isoform e	1.0	1.0	1.1	1.1	1.0	1.1	1.1	1.0	**1.0**	**1.0**	**1.1**	**1.1**	1.9	10	2	2	2	1054	117.0	6.8
NP_055885.3	116008442	**ZC3H13**	zinc finger CCCH domain-containing protein 13	1.0	1.1	1.0	1.0	1.0	1.0	1.0	1.1	**1.0**	**1.1**	**1.0**	**1.1**	2.0	1	3	3	5	1564	184.8	9.5
NP_001229488.1	336020358	**MAP4K4**	mitogen-activated protein kinase kinase kinase kinase 4 isoform 4	1.1	1.1	1.1	1.1	1.1	1.1	1.0	1.0	**1.1**	**1.1**	**1.1**	**1.1**	6.9	13	4	7	10	1239	142.0	7.5
NP_060334.2	70608109	**INTS8**	integrator complex subunit 8	0.8	1.1	1.1	1.1	1.0	1.0	0.9	1.0	**0.9**	**1.0**	**1.0**	**1.1**	1.5	1	1	1	2	995	113.0	7.0
NP_001171812.1	296531339	**PLCB3**	1-phosphatidylinositol 4,5-bisphosphate phosphodiesterase beta-3 isoform 2	1.0	1.0	1.0	1.0	1.1	1.2	1.2	1.1	**1.1**	**1.1**	**1.1**	**1.1**	20.8	2	15	15	49	1167	131.1	5.9
NP_055921.2	168823443	**RAD54L2**	helicase ARIP4	0.9	0.9	1.0	1.0	1.1	1.1	1.3	1.2	**1.0**	**1.0**	**1.1**	**1.1**	2.1	1	3	3	5	1467	162.7	6.1
NP_036601.2	40807485	**PRPF6**	pre-mRNA-processing factor 6	1.0	1.0	1.0	1.1	1.0	1.0	1.1	1.1	**1.0**	**1.0**	**1.0**	**1.1**	24.2	1	18	18	56	941	106.9	8.3
NP_112200.2	20070349	**VMP1**	vacuole membrane protein 1	1.0	1.1	1.3	1.1	1.1	1.1	1.1	1.0	**1.0**	**1.1**	**1.2**	**1.1**	7.4	1	1	1	6	406	46.2	6.9
NP_001248342.1	387527974	**DCTN2**	dynactin subunit 2 isoform 3	1.0	0.9	0.9	1.1	1.0	0.9	1.0	1.0	**1.0**	**0.9**	**0.9**	**1.1**	38.2	3	12	12	51	401	44.2	5.2
NP_055238.1	7656879	**AFF4**	AF4/FMR2 family member 4	1.2	1.2	1.1	1.1	1.1	1.1	1.0	1.0	**1.1**	**1.1**	**1.0**	**1.1**	10.1	1	7	7	16	1163	127.4	9.3
NP_055567.2	162417971	**SPCS2**	signal peptidase complex subunit 2	1.1	1.1	1.0	1.1	1.0	1.0	1.0	1.0	**1.0**	**1.1**	**1.0**	**1.1**	42.0	1	8	8	46	226	25.0	8.5
NP_001265048.1	495529773	**ZKSCAN8**	zinc finger protein with KRAB and SCAN domains 8 isoform 1	1.1	1.0	1.0	1.2	0.9	0.9	0.8	0.9	**1.0**	**1.0**	**0.9**	**1.1**	9.0	2	3	3	4	578	65.8	7.4
NP_006491.2	71274107	**MCAM**	cell surface glycoprotein MUC18 precursor	1.1	1.3	1.4	1.0	1.2	1.3	1.4	1.1	**1.1**	**1.3**	**1.4**	**1.1**	22.0	1	9	9	38	646	71.6	5.8
NP_002759.2	103485496	**CHMP1A**	charged multivesicular body protein 1a isoform 2	1.2	1.0	1.3	1.2	1.0	0.9	1.1	0.9	**1.1**	**0.9**	**1.2**	**1.1**	4.1	1	1	1	2	196	21.7	8.1
NP_079291.2	119874213	**HSPA12A**	heat shock 70 kDa protein 12A	1.3	1.2	1.4	1.1	1.2	1.0	0.9	1.0	**1.2**	**1.1**	**1.2**	**1.1**	2.2	1	1	1	1	675	74.9	6.8
NP_001230881.1	345441817	**KIF2A**	kinesin-like protein KIF2A isoform 3	0.9	1.0	1.0	1.1	1.0	1.0	1.1	1.0	**1.0**	**1.0**	**1.0**	**1.1**	20.6	5	10	12	34	686	77.7	6.6
NP_067062.1	55741709	**RBM25**	RNA-binding protein 25	1.0	0.9	1.0	1.0	0.9	0.9	1.0	1.1	**1.0**	**0.9**	**1.0**	**1.1**	23.3	1	16	16	43	843	100.1	6.3
NP_001139647.1	226051634	**ZNF414**	zinc finger protein 414 isoform 1	1.3	1.2	1.1	1.3	0.9	0.9	0.7	0.9	**1.1**	**1.0**	**0.9**	**1.1**	4.1	1	1	1	2	390	41.0	7.6
NP_001012339.2	68077166	**DNAJC21**	dnaJ homolog subfamily C member 21 isoform 2	1.0	1.1	1.0	1.1	0.8	1.0	1.0	1.0	**0.9**	**1.1**	**1.0**	**1.1**	8.9	2	3	3	11	531	62.0	5.5
NP_006444.2	19913369	**TBL3**	transducin beta-like protein 3	1.0	1.0	1.0	1.1	1.0	1.0	1.1	1.0	**1.0**	**1.0**	**1.0**	**1.1**	30.1	1	16	16	36	808	89.0	6.9
NP_004625.2	51173722	**BRPF1**	peregrin isoform 2	0.9	0.9	1.0	1.0	0.9	1.0	1.1	1.1	**0.9**	**1.0**	**1.1**	**1.1**	2.5	2	2	2	4	1214	137.4	7.9
NP_001180529.1	302344762	**SCP2**	non-specific lipid-transfer protein isoform 6 proprotein	0.9	1.0	0.9	1.0	1.0	1.0	0.9	1.1	**1.0**	**1.0**	**0.9**	**1.1**	11.9	7	7	7	20	503	54.4	6.7
NP_001108089.1	167857780	**MGAT1**	alpha-1,3-mannosyl-glycoprotein 2-beta-N-acetylglucosaminyltransferase	1.2	1.1	1.0	1.1	1.1	1.1	1.1	1.0	**1.2**	**1.1**	**1.1**	**1.1**	2.5	1	1	1	4	445	50.8	9.2
NP_003672.1	4505701	**PDXK**	pyridoxal kinase	1.1	1.0	1.0	1.0	1.1	1.0	1.0	1.1	**1.1**	**1.0**	**1.0**	**1.1**	54.5	1	11	11	42	312	35.1	6.1
NP_002203.1	4504771;392494084	**EIF6**	eukaryotic translation initiation factor 6 isoform a	1.2	1.0	1.1	1.1	1.0	0.9	1.1	1.0	**1.1**	**0.9**	**1.1**	**1.1**	19.6	2	3	3	20	245	26.6	4.7
NP_004482.4	150417981	**ARHGAP35**	rho GTPase-activating protein 35	1.0	1.0	0.9	1.1	0.7	1.1	0.9	1.0	**0.8**	**1.0**	**0.9**	**1.1**	2.7	1	2	2	8	1499	170.4	6.6
NP_001007025.1	55770856	**GOSR1**	Golgi SNAP receptor complex member 1 isoform 3	1.0	1.0	1.0	1.1	0.9	1.0	0.9	1.1	**1.0**	**1.0**	**1.0**	**1.1**	15.1	3	2	2	4	185	21.2	9.6
NP_001119575.1	186910300	**RACGAP1**	rac GTPase-activating protein 1	1.0	1.1	1.2	1.1	1.0	1.0	1.1	1.0	**1.0**	**1.1**	**1.2**	**1.1**	25.6	1	12	12	24	632	71.0	8.9
NP_001229761.1	338827663	**ARFGAP2**	ADP-ribosylation factor GTPase-activating protein 2 isoform 2	1.0	1.1	1.1	1.0	1.0	1.1	1.0	1.1	**1.0**	**1.1**	**1.0**	**1.1**	11.6	2	5	5	10	493	53.4	8.5
NP_001248336.1	387157896	**MPDZ**	multiple PDZ domain protein isoform 3	0.9	1.0	1.0	1.0	1.1	1.3	1.2	1.1	**1.0**	**1.1**	**1.1**	**1.1**	2.4	3	3	3	3	2008	214.7	5.0
NP_001562.1	4504725	**IRF3**	interferon regulatory factor 3 isoform 1	1.0	1.1	1.1	1.1	1.0	1.0	1.0	1.0	**1.0**	**1.1**	**1.1**	**1.1**	27.2	6	7	7	27	427	47.2	5.3
NP_570603.2	19913416	**AP2A1**	AP-2 complex subunit alpha-1 isoform 2	1.1	1.1	1.1	1.1	1.1	1.0	1.1	1.0	**1.1**	**1.1**	**1.1**	**1.1**	40.7	2	17	26	66	955	105.3	7.7
NP_851999.2	194306543	**OXR1**	oxidation resistance protein 1 isoform 2	1.1	1.0	1.1	1.1	1.1	0.9	1.0	1.1	**1.1**	**1.0**	**1.0**	**1.1**	2.6	4	2	2	3	839	93.7	5.2
NP_057141.1	7706337	**COPZ1**	coatomer subunit zeta-1 isoform 1	1.1	1.1	1.3	1.0	1.4	1.1	1.2	1.1	**1.2**	**1.1**	**1.3**	**1.1**	35.0	4	3	3	21	177	20.2	4.8
NP_054760.3	193290128	**SPCS1**	signal peptidase complex subunit 1	1.1	1.1	1.0	1.0	1.1	1.1	1.1	1.1	**1.1**	**1.1**	**1.1**	**1.1**	7.1	1	1	1	8	169	18.3	8.7
NP_002204.2	20127446	**ITGB5**	integrin beta-5 precursor	1.1	1.2	1.3	1.0	1.0	1.0	1.1	1.1	**1.0**	**1.1**	**1.2**	**1.1**	3.9	1	2	2	5	799	88.0	6.1
NP_036364.2	42734430	**PTRF**	polymerase I and transcript release factor	0.9	0.9	1.1	1.0	0.9	1.0	1.2	1.1	**0.9**	**0.9**	**1.2**	**1.1**	30.8	1	11	11	57	390	43.4	5.6
NP_057142.1	7705590	**TPRKB**	EKC/KEOPS complex subunit TPRKB	1.0	0.8	0.9	1.1	1.0	0.9	0.9	1.0	**1.0**	**0.8**	**0.9**	**1.1**	33.1	1	3	3	12	175	19.6	6.8
NP_004697.2	15011972	**ARHGEF1**	rho guanine nucleotide exchange factor 1 isoform 2	1.0	1.0	1.1	1.0	1.1	1.0	1.1	1.1	**1.1**	**1.0**	**1.1**	**1.1**	18.2	3	13	13	35	912	102.4	5.7
NP_001273.1	4557469	**AP2B1**	AP-2 complex subunit beta isoform b	1.1	1.1	1.1	1.1	1.0	1.0	1.1	1.0	**1.0**	**1.0**	**1.1**	**1.1**	48.9	2	22	37	152	937	104.5	5.4
NP_848702.1	31083243	**PPP2R5C**	serine/threonine-protein phosphatase 2A 56 kDa regulatory subunit gamma isoform isoform c	1.2	1.1	1.2	1.1	1.1	1.0	1.1	1.0	**1.1**	**1.1**	**1.1**	**1.1**	8.5	5	2	3	4	449	52.6	7.2
NP_001099040.1	157739940	**NUDT19**	nucleoside diphosphate-linked moiety X motif 19, mitochondrial precursor	1.2	1.1	1.0	1.2	0.9	0.9	1.0	0.9	**1.1**	**1.0**	**1.0**	**1.1**	21.1	1	4	4	7	375	42.2	7.6
NP_110432.1	13540594	**LMAN2L**	VIP36-like protein isoform 2 precursor	1.1	1.1	1.1	1.0	1.1	1.0	1.0	1.1	**1.1**	**1.1**	**1.1**	**1.1**	2.3	2	1	1	1	348	39.7	8.4
NP_149079.2	56790935	**STRIP1**	striatin-interacting protein 1 isoform 1	1.0	1.0	0.9	1.0	1.2	1.1	1.0	1.1	**1.1**	**1.0**	**1.0**	**1.1**	5.4	2	3	3	4	837	95.5	6.3
NP_002865.1	4506387	**RAD23B**	UV excision repair protein RAD23 homolog B isoform 1	1.0	1.0	1.4	1.2	1.2	0.9	1.1	0.9	**1.1**	**0.9**	**1.3**	**1.1**	28.6	3	5	7	25	409	43.1	4.8
NP_006381.2	53759103	**IPO8**	importin-8 isoform 1	0.7	0.7	0.8	1.0	0.8	0.9	1.0	1.1	**0.7**	**0.8**	**0.9**	**1.1**	4.2	2	3	4	15	1037	119.9	5.2
NP_671692.1	312596924	**PSMG2**	proteasome assembly chaperone 2 isoform 2	1.1	1.0	1.1	1.0	1.1	1.0	1.0	1.1	**1.1**	**1.0**	**1.0**	**1.1**	26.2	2	6	6	29	233	26.3	8.0
NP_066553.3	48255924	**NDUFV3**	NADH dehydrogenase [ubiquinone] flavoprotein 3, mitochondrial isoform a precursor	1.0	1.1	1.0	1.0	0.9	1.2	1.0	1.1	**1.0**	**1.1**	**1.0**	**1.1**	16.3	1	5	5	9	473	51.0	9.2
NP_001017919.1	63054860	**RCCD1**	RCC1 domain-containing protein 1	0.8	0.8	0.8	0.9	1.2	1.2	1.2	1.2	**1.0**	**1.0**	**1.0**	**1.1**	7.2	1	1	1	2	376	40.1	5.3
NP_005887.2	28178825;538917488	**IDH1**	isocitrate dehydrogenase [NADP] cytoplasmic	1.2	1.1	1.0	1.0	1.2	1.2	1.1	1.1	**1.2**	**1.2**	**1.1**	**1.1**	48.1	1	17	18	64	414	46.6	7.0
NP_004740.2	40217812	**TBRG4**	protein TBRG4 isoform 1	0.9	1.0	1.0	1.1	0.9	0.9	0.9	1.0	**0.9**	**1.0**	**1.0**	**1.1**	7.0	3	3	3	8	631	70.7	7.4
NP_002476.2	33356172	**NBN**	nibrin	1.0	1.0	1.0	1.1	1.0	1.0	1.0	1.0	**1.0**	**1.0**	**1.0**	**1.1**	14.5	2	10	10	20	754	84.9	6.9
NP_003740.2	38683860	**IRS2**	insulin receptor substrate 2	1.1	1.0	0.9	1.1	1.0	1.0	1.0	1.0	**1.1**	**1.0**	**1.0**	**1.1**	4.4	1	5	5	6	1338	137.2	8.6
NP_005914.1	5174547	**MAP3K5**	mitogen-activated protein kinase kinase kinase 5	1.0	1.0	1.1	1.0	1.2	0.9	0.9	1.1	**1.1**	**1.0**	**1.0**	**1.1**	1.0	1	1	1	1	1374	154.4	5.8
NP_001257819.1	400153758	**HAUS6**	HAUS augmin-like complex subunit 6 isoform 2	1.1	0.9	1.1	1.0	1.2	1.1	1.2	1.1	**1.1**	**1.0**	**1.1**	**1.1**	1.5	2	1	1	2	920	104.2	6.3
NP_001165556.1	285026520	**TBP**	TATA-box-binding protein isoform 2	0.9	1.0	1.0	1.1	0.9	1.0	1.0	1.0	**0.9**	**1.0**	**1.0**	**1.1**	12.5	3	2	2	8	319	35.6	9.9
NP_002067.1	4504061	**GNS**	N-acetylglucosamine-6-sulfatase precursor	0.9	1.0	1.0	1.0	1.0	1.0	1.0	1.1	**1.0**	**1.0**	**1.0**	**1.1**	17.9	1	8	8	20	552	62.0	8.3
NP_001265481.1	514052659	**TUBA4A**	tubulin alpha-4A chain isoform 2	1.0	1.0	1.0	1.0	1.0	1.0	1.1	1.1	**1.0**	**1.0**	**1.1**	**1.1**	58.4	3	4	20	562	433	48.3	5.0
NP_001524.2	52632383	**HNRNPL**	heterogeneous nuclear ribonucleoprotein L isoform a	1.0	0.9	0.9	1.1	1.0	0.9	0.9	1.0	**1.0**	**0.9**	**0.9**	**1.1**	50.4	2	17	17	86	589	64.1	8.2
NP_055491.1	13491166	**PUM1**	pumilio homolog 1 isoform 2	1.1	1.1	1.1	1.2	0.9	1.0	1.0	1.0	**1.0**	**1.0**	**1.0**	**1.1**	4.9	2	2	5	10	1186	126.4	6.8
NP_001269109.1	532164718	**PPP2R5E**	serine/threonine-protein phosphatase 2A 56 kDa regulatory subunit epsilon isoform isoform b	1.1	1.0	1.0	1.1	0.9	0.9	1.0	1.0	**1.0**	**0.9**	**1.0**	**1.1**	17.5	3	6	6	11	462	54.0	6.6
NP_004172.2	21361091	**UCHL1**	ubiquitin carboxyl-terminal hydrolase isozyme L1	1.4	1.4	1.4	1.1	1.5	1.7	**2.3**	1.0	**1.5**	**1.6**	**1.8**	**1.1**	22.0	1	4	4	10	223	24.8	5.5
NP_116219.2	186928847	**CIRH1A**	cirhin	1.1	1.1	1.1	1.1	1.1	1.0	1.1	1.0	**1.1**	**1.0**	**1.1**	**1.1**	11.4	1	5	5	14	686	76.8	8.9
NP_005860.2	64276486	**CWC27**	peptidyl-prolyl cis-trans isomerase CWC27 homolog isoform 1	1.0	1.1	1.0	1.0	1.0	1.1	1.1	1.1	**1.0**	**1.1**	**1.0**	**1.1**	25.0	3	7	7	14	472	53.8	5.8
NP_003674.1	4503247	**RRP1**	ribosomal RNA processing protein 1 homolog A	1.0	1.0	1.0	1.1	0.9	1.0	0.9	1.0	**0.9**	**1.0**	**0.9**	**1.1**	21.9	1	9	9	23	461	52.8	9.3
NP_002777.1	4506179	**PSMA1**	proteasome subunit alpha type-1 isoform 2	1.0	1.0	1.0	1.0	1.1	1.0	1.0	1.1	**1.0**	**1.0**	**1.0**	**1.1**	81.8	3	17	17	78	263	29.5	6.6
NP_001269609.1	544161313	**GAPVD1**	GTPase-activating protein and VPS9 domain-containing protein 1 isoform 2	1.1	1.1	1.0	1.0	1.2	1.1	1.1	1.1	**1.2**	**1.1**	**1.1**	**1.1**	14.5	4	16	16	42	1460	162.8	5.2
NP_001229238.1	334085252	**PITRM1**	presequence protease, mitochondrial isoform 3 precursor	1.0	1.1	1.1	1.1	1.0	1.0	1.1	1.0	**1.0**	**1.1**	**1.1**	**1.1**	23.0	3	19	19	39	939	106.0	6.9
NP_001234925.1	351542238	**ASAP1**	arf-GAP with SH3 domain, ANK repeat and PH domain-containing protein 1 isoform 2	1.1	1.1	1.2	1.1	1.1	1.1	1.1	1.0	**1.1**	**1.1**	**1.1**	**1.1**	7.9	2	7	8	19	1122	124.7	7.3
NP_060691.2	148747437	**MOB1A**	MOB kinase activator 1A	0.9	1.0	0.9	1.0	0.9	0.9	0.9	1.1	**0.9**	**0.9**	**0.9**	**1.1**	22.2	4	4	4	17	216	25.1	6.9

Khan *et al.* , 2019. Multi-omics analysis to characterize cigarette smoke induced molecular alterations in esophageal cells

Supplementary Table 5. List of proteins quantified in untreated and chronically treated Het1A cells with cigarette smoke condensate for 8 months

NP_Accession	Protein group Accession	Gene ID	Description	Het-1A-Smoke - 2M/Parental	Het-1A-Smoke - 4M/Parental	Het-1A-Smoke - 6M/Parental	Het-1A-Smoke - 8M/Parental	Het-1A-Smoke - 2M/Parental	Het-1A-Smoke - 4M/Parental	Het-1A-Smoke - 6M/Parental	Het-1A-Smoke - 8M/Parental	Het-1A-Smoke - 2M/Parental	Het-1A-Smoke - 4M/Parental	Het-1A-Smoke - 6M/Parental	Het-1A-Smoke - 8M/Parental	Coverage (%)	Proteins	Unique Peptides	# Peptides	PSM	# AAs	MW [kDa]	calc. pI
				Replicate 1				Replicate 2				Average of replicates											
NP_001273294.1	556503401	MAP7D1	MAP7 domain-containing protein 1 isoform 2	0.8	0.9	0.9	0.8	1.2	1.6	1.3	1.3	1.0	1.2	1.1	1.1	8.2	3	5	5	17	803	88.7	10.1
NP_003701.1	4504329	SPINT1	kunitz-type protease inhibitor 1 isoform 2 precursor	1.1	1.1	1.1	1.1	1.1	1.1	1.1	1.0	1.1	1.1	1.1	1.1	2.0	2	1	1	1	513	56.8	6.2
NP_002701.1	4506007	PPP1CC	serine/threonine-protein phosphatase PP1-gamma catalytic subunit isoform 1	1.0	1.0	1.0	1.1	1.0	0.9	1.0	1.0	1.0	1.0	1.0	1.1	41.2	2	1	11	100	323	37.0	6.5
NP_005582.1	5031923	MRE11A	double-strand break repair protein MRE11A isoform 1	1.0	1.1	1.1	1.1	1.1	1.2	1.2	1.0	1.0	1.1	1.1	1.1	30.4	2	15	15	39	708	80.5	5.9
NP_003397.1	4507953;21735625	YWHAZ	14-3-3 protein zeta/delta	1.0	1.1	1.0	1.0	0.9	1.1	1.0	1.1	1.0	1.1	1.0	1.1	86.5	5	15	23	754	245	27.7	4.8
NP_003267.1	4507555	TMPO	thymopoietin isoform alpha	1.1	0.9	0.9	1.0	0.8	0.9	0.8	1.1	1.0	0.9	0.9	1.1	39.3	1	8	16	50	694	75.4	7.7
NP_085058.3	58331266	WDR59	WD repeat-containing protein 59	1.0	1.1	1.0	1.0	1.1	1.0	0.9	1.1	1.0	1.1	1.0	1.1	1.4	1	1	1	1	974	109.7	7.9
NP_056078.2	119120894	DMXL2	dmX-like protein 2 isoform 2	1.1	1.1	1.1	1.1	1.1	1.1	1.1	1.0	1.1	1.1	1.1	1.1	2.4	3	5	5	8	3036	339.4	6.4
NP_002486.1	4505369	NDUFS4	NADH dehydrogenase [ubiquinone] iron-sulfur protein 4, mitochondrial precursor	1.0	1.0	1.0	1.1	1.1	1.1	1.2	1.0	1.0	1.1	1.1	1.0	28.6	1	3	3	8	175	20.1	10.3
NP_057444.2	27545315	TACO1	translational activator of cytochrome c oxidase 1	1.1	1.1	1.0	1.1	0.9	1.0	0.9	1.0	1.0	1.0	1.0	1.0	41.1	1	7	7	22	297	32.5	8.1
NP_803188.1	29029591	FTSJ1	putative tRNA (cytidine(32)/guanosine(34)-2'-O)-methyltransferase isoform b	0.8	0.9	0.9	1.0	0.8	0.9	0.9	1.1	0.8	0.9	0.9	1.0	17.1	3	4	4	5	327	35.8	5.9
NP_001073279.1	119372312	GLB1	beta-galactosidase isoform b	1.1	1.2	1.1	1.1	1.2	1.2	1.1	1.0	1.2	1.2	1.1	1.0	15.6	3	8	8	26	647	72.7	6.3
NP_002853.2	21361370	PYGB	glycogen phosphorylase, brain form	1.2	1.2	1.3	1.0	1.1	1.1	1.3	1.1	1.1	1.2	1.3	1.0	34.4	3	23	27	87	843	96.6	6.9
NP_113611.2	117968353	NUF2	kinetochore protein Nuf2	1.0	1.0	1.0	1.0	0.9	1.1	1.1	1.1	0.9	1.0	1.1	1.0	16.4	1	6	6	13	464	54.3	8.3
NP_055683.3	34452681	RNF10	RING finger protein 10	1.1	1.4	1.3	1.1	1.1	1.3	1.2	1.0	1.1	1.3	1.2	1.0	1.0	1	1	1	1	811	89.9	6.9
NP_001070667.1	116089325	SREK1	splicing regulatory glutamine/lysine-rich protein 1 isoform a	1.1	1.0	1.0	1.1	1.0	1.0	0.9	1.0	1.0	1.0	1.0	1.0	5.0	4	3	3	9	624	71.6	10.2
NP_005496.4	132566684	SCARB1	scavenger receptor class B member 1 isoform 1	1.1	1.3	1.4	1.1	1.0	1.1	1.3	1.0	1.1	1.2	1.3	1.0	7.3	2	4	4	9	509	56.9	8.0
NP_003819.1	28872761	MTMR1	myotubularin-related protein 1	0.9	1.0	1.1	1.0	1.2	1.1	1.3	1.1	1.1	1.1	1.2	1.0	1.4	1	1	1	1	665	74.6	7.1
NP_001181926.1	303304987	CARS	cysteine--tRNA ligase, cytoplasmic isoform e	1.3	1.4	1.2	1.1	1.3	1.3	1.2	1.0	1.3	1.4	1.2	1.0	28.7	4	19	19	65	809	92.0	7.1
NP_001035700.1	94966752	EFTUD1	elongation factor Tu GTP-binding domain-containing protein 1 isoform 2	1.0	1.0	1.0	1.0	1.1	1.1	1.0	1.1	1.1	1.0	1.0	1.0	15.0	2	10	11	24	1069	119.8	5.8
NP_059133.1	34101288	CPSF2	cleavage and polyadenylation specificity factor subunit 2	1.0	0.9	0.9	1.1	1.0	0.9	1.0	1.0	1.0	0.9	1.0	1.0	20.3	1	9	9	17	782	88.4	5.1
NP_001789.2	16936528	CDK2	cyclin-dependent kinase 2 isoform 1	1.0	1.0	1.0	1.0	1.0	1.0	1.0	1.1	1.0	1.0	1.0	1.0	25.5	57	4	7	19	298	33.9	8.7
NP_006436.3	91208426	PRPF8	pre-mRNA-processing-splicing factor 8	1.0	1.0	1.1	1.1	1.0	1.0	1.1	1.0	1.0	1.0	1.1	1.0	35.8	1	64	64	222	2335	273.4	8.8
NP_002807.1	4506221	PSMD12	26S proteasome non-ATPase regulatory subunit 12 isoform 1	1.1	1.0	1.1	1.0	1.1	1.1	1.2	1.1	1.1	1.1	1.1	1.0	55.5	2	23	24	87	456	52.9	7.6
NP_036275.1	8393259;56788356	TFIP11	tuftelin-interacting protein 11	0.9	1.1	1.1	1.1	1.0	1.0	1.0	1.0	1.0	1.0	1.0	1.0	4.8	1	3	3	3	837	96.8	5.7
NP_004946.1	4826716;118582262	SLC29A1	equilibrative nucleoside transporter 1	1.0	0.9	1.1	1.0	1.0	0.9	1.0	1.1	1.0	0.9	1.1	1.0	7.0	5	3	3	5	456	50.2	8.3
NP_001883.4	68303572	CSNK1A1	casein kinase I isoform alpha isoform 2	0.8	0.9	0.9	0.9	1.2	1.1	1.2	1.2	1.0	1.0	1.0	1.0	19.0	5	5	5	8	337	38.9	9.6
NP_149975.1	15088795	PIGS	GPI transamidase component PIG-S	1.0	1.0	1.0	1.0	1.0	1.0	1.0	1.1	1.0	1.0	1.0	1.0	21.4	1	9	9	22	555	61.6	6.5
NP_115679.2	40254986	HSDL2	hydroxysteroid dehydrogenase-like protein 2 isoform 1	1.0	1.1	1.1	1.1	1.1	1.0	1.1	1.0	1.0	1.0	1.1	1.0	31.1	2	10	10	27	418	45.4	8.0
NP_065810.2	270265793	STARD9	stAR-related lipid transfer protein 9	1.1	1.1	1.0	1.1	1.0	1.0	1.0	1.0	1.0	1.0	1.0	1.0	0.4	7	1	2	4	4700	516.0	6.3
NP_006145.2	114520609	NEDD4	E3 ubiquitin-protein ligase NEDD4 isoform 1	1.1	1.1	1.1	1.1	1.1	1.0	1.1	1.0	1.1	1.1	1.1	1.0	18.2	5	9	12	48	900	104.2	6.0
NP_002705.2	25777671	PPP1R10	serine/threonine-protein phosphatase 1 regulatory subunit 10	1.0	1.0	1.2	1.1	1.0	0.9	1.0	1.0	1.0	1.0	1.1	1.0	4.4	1	4	4	8	940	99.0	9.2
NP_000042.3	71902540	ATM	serine-protein kinase ATM	1.0	1.1	1.2	1.0	1.1	1.1	1.1	1.1	1.1	1.1	1.1	1.0	4.3	1	9	9	18	3056	350.5	6.8
NP_055523.1	7661960	KNTC1	kinetochore-associated protein 1	1.0	1.0	1.2	1.0	1.1	1.1	1.2	1.1	1.1	1.0	1.2	1.0	6.9	1	10	10	21	2209	250.6	6.0
NP_065861.3	612339294	TRMT5	tRNA (guanine(37)-N1)-methyltransferase	1.0	1.0	1.0	1.1	1.0	1.0	1.1	1.0	1.0	1.0	1.0	1.0	10.0	1	5	5	9	509	58.2	8.6
NP_443100.1	16445029	IGSF8	immunoglobulin superfamily member 8 precursor	1.1	1.1	1.0	1.1	1.0	1.0	0.9	1.0	1.0	1.0	1.0	1.0	8.0	1	3	3	8	613	65.0	8.0
NP_060277.1	8923380	OSGEP	probable tRNA N6-adenosine threonylcarbamoyltransferase	1.0	0.9	1.0	1.0	1.1	1.0	1.0	1.1	1.1	1.0	1.0	1.0	28.7	1	5	5	13	335	36.4	6.4
NP_002784.1	4506193	PSMB1	proteasome subunit beta type-1	1.0	1.0	1.1	1.1	1.0	1.0	1.0	1.0	1.0	1.0	1.1	1.0	49.8	1	8	8	37	241	26.5	8.1
NP_055203.4	75677335	DIEXF	digestive organ expansion factor homolog	1.0	1.1	1.0	1.0	1.3	1.1	1.1	1.1	1.2	1.1	1.0	1.0	2.5	1	2	2	2	756	87.0	5.9
NP_001254746.1	392513684	OSTC	oligosaccharyltransferase complex subunit OSTC isoform 2	1.1	1.1	1.1	1.1	1.0	1.1	1.1	1.0	1.0	1.1	1.1	1.0	14.5	3	1	1	4	83	9.4	7.4
NP_037379.1	7019477	HTRA2	serine protease HTRA2, mitochondrial isoform 1 preproprotein	1.0	1.0	1.0	1.0	0.9	1.0	1.0	1.1	1.0	1.0	1.0	1.0	22.5	2	6	6	24	458	48.8	10.1
NP_001268370.1	527122094	WBSCR16	Williams-Beuren syndrome chromosomal region 16 protein isoform 3	1.2	1.1	1.2	1.2	0.9	0.9	0.9	0.9	1.0	1.0	1.1	1.0	11.2	3	2	2	3	358	38.3	8.6
NP_001026871.1	72534692	NT5DC3	5'-nucleotidase domain-containing protein 3	1.0	1.1	1.3	1.1	1.1	1.1	1.3	1.0	1.1	1.1	1.3	1.0	8.4	1	3	3	5	548	63.4	8.3
NP_006304.1	14149627	USP15	ubiquitin carboxyl-terminal hydrolase 15 isoform 2	1.1	1.1	1.2	1.0	1.2	1.0	1.2	1.1	1.1	1.1	1.2	1.0	14.0	3	8	9	20	952	109.2	5.1
NP_003423.1	84872173	ZNF131	zinc finger protein 131 isoform 2	0.9	1.0	1.0	1.1	0.9	1.0	1.1	0.9	0.9	1.0	1.1	1.0	3.7	2	1	1	2	589	67.3	5.1
NP_079094.1	13376212	UBA5	ubiquitin-like modifier-activating enzyme 5 isoform 1	1.3	1.4	1.4	1.1	1.2	1.1	1.2	1.0	1.2	1.2	1.3	1.0	23.5	2	6	6	13	404	44.8	4.8
NP_005144.2	119220605	USP10	ubiquitin carboxyl-terminal hydrolase 10 isoform 2	1.0	1.0	1.0	1.1	0.9	1.0	1.0	1.0	0.9	1.0	1.0	1.0	21.3	2	12	12	35	798	87.1	5.3
NP_001073312.1	119709834	CASZ1	zinc finger protein castor homolog 1 isoform a	1.0	1.1	1.2	1.0	0.8	1.0	0.8	1.1	0.9	1.0	1.0	1.0	2.2	1	1	1	1	1759	189.9	7.0
NP_001275583.1	568815653;191252797	DCAKD	dephospho-CoA kinase domain-containing protein	1.0	1.1	1.0	1.1	0.9	1.0	1.0	1.0	1.0	1.1	1.0	1.0	14.7	1	3	3	6	231	26.5	9.6
NP_001393.1	4503471	EEF1A1	elongation factor 1-alpha 1	1.0	0.9	1.0	1.0	1.0	1.0	1.0	1.1	1.0	1.0	1.0	1.0	72.7	1	15	26	995	462	50.1	9.0
NP_000312.2	108773787	RB1	retinoblastoma-associated protein	1.0	1.0	1.1	1.1	0.9	1.0	1.0	1.0	0.9	1.0	1.0	1.0	6.9	1	6	6	10	928	106.1	7.9
NP_060549.4	221136781	SRBD1	S1 RNA-binding domain-containing protein 1	1.1	1.1	1.1	1.1	1.1	1.0	1.0	1.0	1.1	1.1	1.0	1.0	19.7	1	14	15	44	995	111.7	8.7
NP_057535.1	7705369;221316630	COPB1	coatomer subunit beta	1.1	1.2	1.2	1.1	1.1	1.1	1.1	1.0	1.1	1.1	1.2	1.0	45.8	1	32	32	99	953	107.1	6.0
NP_060895.1	13559514	PI4K2A	phosphatidylinositol 4-kinase type 2-alpha	0.8	0.8	0.8	1.0	0.8	0.9	0.9	1.1	0.8	0.9	0.9	1.0	13.8	1	4	4	10	479	54.0	8.3
NP_001121167.1	189163485	CTSA	lysosomal protective protein isoform b precursor	1.1	1.1	1.0	1.1	1.0	1.0	1.0	1.0	1.0	1.0	1.0	1.0	5.8	3	3	3	6	480	54.4	6.6
NP_005406.3	31543630	SLC20A1	sodium-dependent phosphate transporter 1	1.1	1.1	1.2	1.1	1.2	1.2	1.3	1.0	1.2	1.1	1.2	1.0	6.5	1	4	4	5	679	73.7	7.1
NP_002215.2	153945846	ITPR3	inositol 1,4,5-trisphosphate receptor type 3	1.1	1.1	1.2	1.1	1.0	1.0	1.1	1.0	1.1	1.1	1.2	1.0	8.5	1	14	15	29	2671	303.9	6.5
NP_001120716.1	187829871	LRRC8A	leucine-rich repeat-containing protein 8A	1.0	1.0	1.1	1.0	1.1	1.0	1.1	1.0	1.0	1.0	1.1	1.0	6.4	1	3	3	6	810	94.1	7.9
NP_008974.5	117676403	KRR1	KRR1 small subunit processome component homolog	0.9	1.0	0.9	1.1	0.9	0.9	0.9	1.0	0.9	1.0	0.9	1.0	29.4	1	8	8	18	381	43.6	9.8
NP_060117.3	194097365	FTSJ3	pre-rRNA processing protein FTSJ3	1.0	1.1	1.0	1.1	0.9	1.0	0.9	1.0	0.9	1.0	0.9	1.0	35.2	1	19	19	72	847	96.5	8.4
NP_001106849.1	164607124	FANCI	Fanconi anemia group I protein isoform 1	1.0	1.0	1.0	1.1	1.0	1.0	1.0	1.0	1.0	1.0	1.0	1.0	19.0	2	20	21	53	1328	149.2	6.7
NP_057361.3	34485714;523425714	RAB23	ras-related protein Rab-23	1.0	1.0	1.0	1.1	1.0	0.9	0.9	1.0	1.0	0.9	0.9	1.0	30.0	1	5	5	14	237	26.6	6.6
NP_573566.2	31621305	LRPPRC	leucine-rich PPR motif-containing protein, mitochondrial precursor	1.1	1.0	1.1	1.0	1.1	1.0	1.1	1.0	1.1	1.0	1.1	1.0	60.3	1	70	70	365	1394	157.8	6.1
NP_001276977.1	586798196	ATL3	atlastin-3 isoform 2	1.0	1.1	1.1	1.0	1.0	1.1	1.1	1.1	1.0	1.1	1.1	1.0	44.4	2	17	17	45	523	58.7	5.6
NP_064535.1	9910542;217416369	SAR1A	GTP-binding protein SAR1a	1.0	1.0	1.1	1.0	1.1	1.0	1.2	1.1	1.1	1.0	1.2	1.0	49.0	1	5	8	63	198	22.4	6.7
NP_002708.1	4506019	PPP2R2A	serine/threonine-protein phosphatase 2A 55 kDa regulatory subunit B alpha isoform isoform 1	0.9	1.0	1.1	1.0	1.0	1.1	1.1	1.1	0.9	1.0	1.1	1.0	19.2	15	7	7	17	447	51.7	6.2
NP_001230106.1	342187211	ALDOA	fructose-bisphosphate aldolase A isoform 2	1.1	1.0	0.9	1.0	1.0	1.1	1.0	1.1	1.0	1.0	1.0	1.0	68.9	1	1	22	329	418	45.2	8.3
NP_079081.2	285394828	RBFA	putative ribosome-binding factor A, mitochondrial isoform 1 precursor	1.2	1.3	1.5	1.1	1.0	1.0	1.1	1.0	1.1	1.2	1.3	1.0	5.8	1	1	1	4	343	38.3	7.9
NP_003744.1	4503523	EIF3D	eukaryotic translation initiation factor 3 subunit D	1.1	1.1	1.1	1.1	1.0	1.0	1.0	1.0	1.0	1.0	1.1	1.0	47.5	1	15	15	46	548	63.9	6.0
NP_000104.1	4557541	TOR1A	torsin-1A precursor	0.9	0.8	0.9	1.1	1.1	1.0	1.0	1.0	1.0	0.9	0.9	1.0	7.8	1	1	1	4	332	37.8	7.0
NP_001003700.1	270132935	RREB1	ras-responsive element-binding protein 1 isoform 3	1.0	1.0	1.0	1.3	0.8	0.8	0.8	0.8	0.9	0.9	0.9	1.0	1.4	3	1	1	1	1476	159.2	8.1
NP_579866.2	194328736	PHF10	PHD finger protein 10 isoform b	0.8	0.9	0.9	1.0	0.9	0.9	0.9	1.1	0.8	0.9	0.9	1.0	19.2	2	5	5	18	496	55.8	6.8
NP_009200.2	118600975	STXBP3	syntaxin-binding protein 3	1.1	1.1	1.1	1.1	1.0	1.0	1.0	1.0	1.1	1.1	1.0	1.0	9.6	1	5	5	13	592	67.7	7.8
NP_085140.2	237757342	DDHD1	phospholipase DDHD1 isoform a	1.1	1.0	1.0	1.0	1.0	1.1	1.0	1.1	1.1	1.1	1.0	1.0	4.4	3	2	2	4	872	97.0	5.7
NP_001018169.1	66363686	NAE1	NEDD8-activating enzyme E1 regulatory subunit isoform b	1.2	1.1	1.1	1.1	1.2	1.1	1.1	1.0	1.2	1.1	1.1	1.0	36.4	4	11	11	40	528	59.4	5.2
NP_078914.1	13375872	QTRTD1	queuine tRNA-ribosyltransferase subunit QTRTD1 isoform 1	1.2	1.1	1.0	1.1	1.1	1.1	1.0	1.0	1.1	1.1	1.0	1.0	23.4	4	7	7	18	415	46.7	6.8
NP_002786.2	22538465	PSMB3	proteasome subunit beta type-3	1.1	1.0	1.1	1.0	1.1	1.0	1.1	1.1	1.1	1.0	1.0	1.0	38.5	1	6	6	42	205	22.9	6.6
NP_001026881.1	72534712	ERGIC1	endoplasmic reticulum-Golgi intermediate compartment protein 1	1.0	1.0	1.0	1.0	1.1	1.1	1.1	1.1	1.1	1.1	1.1	1.0	29.7	1	7	7	21	290	32.6	7.1
NP_001231855.1	349732206	PRPF4	U4/U6 small nuclear ribonucleoprotein Prp4 isoform 2	1.0	1.0	1.0	1.1	0.9	0.9	0.9	1.0	1.0	1.0	1.0	1.0	19.8	2	7	7	23	521	58.3	7.4
NP_057267.2	18490987	MRTO4	mRNA turnover protein 4 homolog	1.0	1.0	1.0	1.1	0.9	0.9	1.0	1.0	1.0	0.9	1.0	1.0	42.7	1	8	8	24	239	27.5	8.3
NP_085131.1	44771201	INTS5	integrator complex subunit 5	1.1	1.1	1.1	1.1	1.1	1.2	1.1	1.0	1.1	1.2	1.1	1.0	0.8	1	1	1	1	1019	107.9	7.0

Supplementary Table 5. List of proteins quantified in untreated and chronically treated Het1A cells with cigarette smoke condensate for 8 months

NP_Accession	Protein group Accession	Gene ID	Description	Het-1A-Smoke - 2M/Parental	Het-1A-Smoke - 4M/Parental	Het-1A-Smoke - 6M/Parental	Het-1A-Smoke - 8M/Parental	Het-1A-Smoke - 2M/Parental	Het-1A-Smoke - 4M/Parental	Het-1A-Smoke - 6M/Parental	Het-1A-Smoke - 8M/Parental	Het-1A-Smoke - 2M/Parental	Het-1A-Smoke - 4M/Parental	Het-1A-Smoke - 6M/Parental	Het-1A-Smoke - 8M/Parental	Coverage (%)	Proteins	Unique Peptides	# Peptides	PSM	# AAs	MW [kDa]	calc. pI
				Replicate 1				Replicate 2				Average of replicates											
NP_277035.2	188497750	HK1	hexokinase-1 isoform HKI-td	1.0	1.1	1.2	1.0	1.1	1.1	1.2	1.1	1.1	1.1	1.2	1.0	32.3	5	22	25	93	905	101.0	6.9
NP_002433.1	4505255	MSI1	RNA-binding protein Musashi homolog 1	0.9	0.6	1.0	0.9	1.2	0.9	0.9	1.2	1.1	0.8	1.0	1.0	14.9	1	1	3	5	362	39.1	7.9
NP_689601.2	113205077	TMEM237	transmembrane protein 237 isoform b	1.0	0.9	0.9	0.9	1.1	1.2	1.0	1.1	1.1	1.1	1.0	1.0	7.3	2	2	2	4	400	44.6	7.0
NP_001263223.1	443497970	NISCH	nischarin isoform 3	1.0	0.8	0.9	1.0	1.0	1.0	0.9	1.1	1.0	0.9	0.9	1.0	5.1	3	1	1	2	515	56.8	5.8
NP_079418.1	315139026	TAOK1	serine/threonine-protein kinase TAO1 isoform 2	1.0	1.0	1.0	1.0	1.9	1.1	1.0	1.1	1.4	1.1	1.0	1.0	10.7	5	5	7	15	853	97.5	6.9
NP_937845.1	38016922	POP5	ribonuclease P/MRP protein subunit POP5 isoform c	1.3	1.0	1.0	1.0	1.1	1.1	1.1	1.1	1.2	1.0	1.0	1.0	10.6	2	1	1	4	113	12.8	5.3
NP_056182.2	45243501	BCL2L13	bcl-2-like protein 13 isoform a	1.3	1.2	1.5	1.1	1.1	1.1	1.2	1.0	1.2	1.2	1.3	1.0	14.0	9	4	4	13	485	52.7	4.4
NP_079495.1	45504382	DNAJC5	dnaJ homolog subfamily C member 5	1.1	1.0	1.1	1.0	1.0	0.9	1.0	1.1	1.0	1.0	1.0	1.0	20.2	1	2	2	3	198	22.1	5.1
NP_055417.1	23943912	PIK3R4	phosphoinositide 3-kinase regulatory subunit 4	1.1	1.1	1.1	1.1	1.2	1.0	1.0	1.0	1.2	1.0	1.1	1.0	5.1	1	7	7	17	1358	153.0	7.2
NP_055423.1	24307969	CYFIP1	cytoplasmic FMR1-interacting protein 1 isoform a	1.0	0.9	1.0	1.0	1.0	1.0	1.0	1.0	1.0	1.0	1.0	1.0	15.8	5	14	14	31	1253	145.1	6.9
NP_001553.1	4504653	IL18	interleukin-18 isoform 1 proprotein	1.1	0.9	1.0	1.1	1.0	0.8	1.0	1.0	1.1	0.8	1.0	1.0	48.7	2	6	6	18	193	22.3	4.7
NP_079532.5	156142199	EHMT2	histone-lysine N-methyltransferase EHMT2 isoform b	1.1	1.2	1.1	1.2	0.9	0.8	0.9	0.9	1.0	1.0	1.0	1.0	4.2	3	2	2	3	1176	128.9	5.5
NP_001670.1	4502281	ATP1B3	sodium/potassium-transporting ATPase subunit beta-3	1.1	1.1	1.1	1.1	1.0	1.0	1.0	1.0	1.0	1.0	1.0	1.0	17.9	1	5	5	17	279	31.5	8.4
NP_001191763.1	325651952	ANO10	anoctamin-10 isoform 5	1.1	1.0	1.1	1.0	1.3	1.2	1.1	1.1	1.2	1.1	1.1	1.0	5.1	5	2	2	2	470	53.8	7.9
NP_060760.2	63055049	PGM2	phosphoglucomutase-2	1.1	1.1	1.1	1.0	1.0	1.2	1.2	1.0	1.1	1.1	1.2	1.0	29.4	1	13	13	34	612	68.2	6.7
NP_443089.3	145309298	ZNF830	zinc finger protein 830	1.1	0.8	0.9	1.2	0.7	0.7	0.8	0.9	0.9	0.8	0.8	1.0	10.0	1	2	2	4	372	42.0	5.3
NP_853530.2	148277064	CUX1	protein CASP isoform a	1.0	1.0	1.0	1.0	1.1	1.1	1.2	1.1	1.1	1.1	1.1	1.0	8.4	8	10	10	14	1505	164.1	5.9
NP_001610.2	148539876	ADRBK1	beta-adrenergic receptor kinase 1	0.9	1.0	1.1	1.1	0.9	1.0	0.9	1.0	0.9	1.0	1.0	1.0	1.3	1	1	1	1	689	79.5	7.3
NP_001264128.1	467094446	CNIH4	protein cornichon homolog 4 isoform 4	1.1	1.1	1.1	1.2	0.8	0.9	0.9	0.9	0.9	1.0	1.0	1.0	26.3	3	1	1	6	76	9.0	8.2
NP_061161.2	93102375	FAM114A2	protein FAM114A2	1.0	1.1	1.1	1.0	1.1	1.1	1.1	1.1	1.1	1.1	1.1	1.0	43.8	1	16	16	43	505	55.4	4.9
NP_001512.1	4504205	GTF3C2	general transcription factor 3C polypeptide 2	0.8	1.0	0.9	1.0	0.9	1.1	1.0	1.1	0.9	1.0	0.9	1.0	9.6	1	5	5	14	911	100.6	7.3
NP_056195.3	225543166	SAMM50	sorting and assembly machinery component 50 homolog	1.1	1.0	1.1	1.1	1.1	1.0	1.1	1.0	1.1	1.0	1.1	1.0	31.1	1	10	10	29	469	51.9	6.9
NP_055431.1	62632750	ATP11B	probable phospholipid-transporting ATPase IF	1.4	1.0	1.0	0.9	1.2	1.5	1.3	1.2	1.3	1.2	1.2	1.0	0.9	1	1	1	1	1177	134.1	7.0
NP_001230914.1	345842445	RELA	transcription factor p65 isoform 4	1.1	1.0	0.9	1.0	1.1	1.1	1.1	1.1	1.1	1.1	1.0	1.0	10.7	4	4	4	9	448	49.5	7.4
NP_055842.2	190341074	PDXDC1	pyridoxal-dependent decarboxylase domain-containing protein 1 isoform 1	1.2	1.2	1.1	1.1	1.1	1.1	1.0	1.0	1.1	1.1	1.1	1.0	32.9	11	14	14	41	788	86.7	5.4
NP_619649.1	20162572	NDNL2	melanoma-associated antigen G1	1.1	1.2	1.1	1.1	1.0	1.0	1.0	1.0	1.1	1.1	1.1	1.0	17.1	1	4	4	6	304	34.3	9.3
NP_001121164.1	189095269	PCCA	propionyl-CoA carboxylase alpha chain, mitochondrial isoform b	1.1	1.0	0.9	1.0	1.0	1.1	0.9	1.1	1.0	1.1	0.9	1.0	3.0	3	2	2	6	702	77.0	7.3
NP_075059.1	12597653	NT5DC2	5'-nucleotidase domain-containing protein 2 isoform 2	0.9	0.9	1.0	1.0	1.0	1.0	1.0	1.0	1.0	1.0	1.0	1.0	11.0	2	4	4	5	520	60.7	6.8
NP_001258692.1	419635906	DUS2	tRNA-dihydrouridine(20) synthase [NAD(P)+]-like isoform 2	1.1	1.1	1.1	1.1	1.0	1.0	1.0	1.0	1.1	1.1	1.0	1.0	12.5	2	4	4	5	458	51.4	7.3
NP_112493.2	61743969	SEH1L	nucleoporin SEH1 isoform 2	1.0	1.0	1.0	1.0	1.1	1.0	1.1	1.1	1.0	1.0	1.0	1.0	24.7	2	7	7	18	360	39.6	8.1
NP_001268667.1	528881083	ETFDH	electron transfer flavoprotein-ubiquinone oxidoreductase, mitochondrial isoform 3	1.0	1.1	1.2	1.1	1.1	1.1	1.1	1.0	1.1	1.1	1.1	1.0	5.6	3	3	3	5	556	61.3	6.8
NP_064507.3	46852178	KCMF1	E3 ubiquitin-protein ligase KCMF1	0.9	1.0	0.9	1.0	1.3	1.1	1.3	1.1	1.1	1.0	1.1	1.0	14.7	1	3	3	12	381	41.9	5.7
NP_110420.3	45505155	FBXO38	F-box only protein 38 isoform a	1.0	1.1	1.0	1.0	1.1	1.1	1.0	1.0	1.1	1.1	1.0	1.0	2.9	2	4	4	6	1113	125.9	6.2
NP_037397.2	8393009	VPS51	vacuolar protein sorting-associated protein 51 homolog	0.9	0.9	1.0	1.0	1.0	1.0	1.0	1.1	0.9	1.0	1.0	1.0	8.8	1	5	5	7	782	86.0	6.5
NP_071343.2	50658087	ABHD4	abhydrolase domain-containing protein 4	1.1	1.1	1.0	1.0	1.1	1.3	1.1	1.0	1.1	1.2	1.1	1.0	3.5	1	1	1	1	342	38.8	7.6
NP_666499.1	22547138;22547136	MRPL4	39S ribosomal protein L4, mitochondrial isoform a	1.0	1.0	1.0	1.0	1.1	1.0	1.1	1.1	1.1	1.0	1.1	1.0	32.2	2	7	7	17	311	34.9	9.7
NP_061895.3	38505222	TMX3	protein disulfide-isomerase TMX3 precursor	1.1	1.1	1.2	1.0	1.1	1.1	1.1	1.0	1.1	1.1	1.1	1.0	27.1	1	10	10	33	454	51.8	4.9
NP_004068.2	38327625	CS	citrate synthase, mitochondrial precursor	0.9	1.0	1.0	1.0	1.0	1.1	1.1	1.1	1.0	1.0	1.0	1.0	37.1	1	13	13	68	466	51.7	8.3
NP_001263205.1	442796460	MAZ	myc-associated zinc finger protein isoform 4	0.9	0.7	0.7	0.7	1.3	0.9	0.9	1.4	1.1	0.8	0.8	1.0	7.0	4	1	1	1	172	17.8	7.7
NP_001167575.1	291575163	CD14	monocyte differentiation antigen CD14 precursor	1.1	1.0	0.9	1.0	1.0	1.0	0.9	1.1	1.0	1.0	0.9	1.0	4.8	1	1	1	2	375	40.1	6.2
NP_005138.3	205360838	DNAJA3	dnaJ homolog subfamily A member 3, mitochondrial isoform 1	1.1	1.1	1.1	1.1	1.2	1.1	1.1	1.0	1.1	1.1	1.1	1.0	29.0	3	11	11	35	480	52.5	9.3
NP_001138574.1	223029440	SMAD3	mothers against decapentaplegic homolog 3 isoform 2	0.9	1.2	0.9	1.2	1.1	0.9	0.8	0.9	1.0	1.1	0.8	1.0	7.5	8	1	2	3	320	35.9	5.4
NP_056277.2	21361468	NOL11	nucleolar protein 11 isoform 1	1.0	1.0	1.1	1.0	1.0	1.0	1.1	1.1	1.0	1.0	1.1	1.0	20.6	2	12	12	26	719	81.1	6.1
NP_001177162.1	298231213	DNAJC19	mitochondrial import inner membrane translocase subunit TIM14 isoform 2	1.0	1.0	1.1	1.0	1.0	1.1	1.1	1.1	1.0	1.1	1.1	1.0	34.1	2	3	3	13	91	10.1	10.1
NP_056324.2	31542528	NECAP1	adaptin ear-binding coat-associated protein 1	1.0	1.0	0.9	1.0	1.1	1.1	1.0	1.1	1.0	1.0	0.9	1.0	17.1	1	2	3	6	275	29.7	6.8
NP_002072.2	167001141	GPC1	glypican-1 precursor	1.0	1.1	1.0	1.1	1.1	1.0	1.0	1.0	1.0	1.1	1.0	1.0	19.9	1	8	8	16	558	61.6	7.3
NP_006650.1	5729840	TUBGCP2	gamma-tubulin complex component 2 isoform 2	0.9	0.9	1.0	1.0	1.2	1.0	1.1	1.1	1.0	1.0	1.0	1.0	3.4	3	3	3	3	902	102.5	6.8
NP_001277144.1	589269185;4557499	CTBP2	C-terminal-binding protein 2 isoform 1	1.1	1.0	1.0	1.1	1.0	1.0	1.0	1.0	1.0	1.0	1.0	1.0	11.2	2	3	4	10	445	48.9	6.9
NP_071736.1	11641243	PDF	peptide deformylase, mitochondrial precursor	1.1	0.9	1.0	0.9	1.1	1.1	1.2	1.1	1.1	1.0	1.1	1.0	14.4	1	2	2	3	243	27.0	9.2
NP_659498.4	260763910	SKA3	spindle and kinetochore-associated protein 3 isoform 1	0.8	1.0	1.1	1.0	0.8	0.9	1.0	1.1	0.8	0.9	1.0	1.0	2.7	1	1	1	2	412	46.3	5.1
NP_005179.2	52426745	CBL	E3 ubiquitin-protein ligase CBL	1.0	1.0	1.0	1.0	1.0	1.1	1.1	1.1	1.0	1.0	1.0	1.0	2.8	1	2	2	2	906	99.6	6.5
NP_001230127.1	342307096	HIP1	huntingtin-interacting protein 1 isoform 2	1.1	1.1	1.1	1.1	0.9	0.9	1.0	1.0	1.0	1.0	1.0	1.0	3.5	2	3	3	3	986	110.6	5.3
NP_001530.1	4504511	DNAJA1	dnaJ homolog subfamily A member 1	0.9	1.1	1.1	1.0	0.9	1.0	1.0	1.0	0.9	1.1	1.0	1.0	50.4	1	14	15	94	397	44.8	7.1
NP_078846.2	186910286	RNASEH2B	ribonuclease H2 subunit B isoform 1	0.9	1.0	0.9	1.0	1.0	1.0	1.0	1.1	1.0	1.0	1.0	1.0	5.8	2	2	2	3	312	35.1	9.1
NP_001230710.1	344925834	FAM213A	redox-regulatory protein FAM213A isoform 2 precursor	1.0	1.0	1.1	1.1	1.1	1.0	1.2	1.0	1.1	1.0	1.1	1.0	29.4	3	6	6	33	218	24.4	9.0
NP_001013679.2	116642895	GDPGP1	GDP-D-glucose phosphorylase 1	1.0	1.1	0.9	1.1	0.9	0.9	0.9	0.9	1.0	1.0	0.9	1.0	3.9	1	1	1	1	385	42.3	6.5
NP_004044.3	51173724	BYSL	bystin	0.9	1.0	0.9	1.1	0.9	0.9	1.0	1.0	0.9	1.0	1.0	1.0	8.0	1	4	4	5	437	49.6	8.1
NP_078795.2	39995080	FAM65A	protein FAM65A isoform 1	1.1	1.2	1.1	1.1	1.2	1.0	1.0	1.0	1.1	1.1	1.1	1.0	1.4	4	1	1	6	1219	131.8	6.3
NP_001243631.1	375493565	USP3	ubiquitin carboxyl-terminal hydrolase 3 isoform 2	1.0	1.0	1.1	1.0	1.0	0.9	1.0	1.1	1.0	1.0	1.0	1.0	2.5	2	1	1	2	476	53.7	8.1
NP_001265434.1	511772975	GPATCH11	G patch domain-containing protein 11 isoform 2	0.9	0.9	0.9	1.1	1.0	0.9	0.9	1.0	0.9	0.9	0.9	1.0	5.8	2	1	1	2	156	18.6	5.0
NP_057128.2	156231349	FAHD2A	fumarylacetoacetate hydrolase domain-containing protein 2A	1.1	1.0	0.9	1.0	1.0	1.0	0.9	1.0	1.0	1.0	0.9	1.0	3.5	1	1	1	2	314	34.6	8.2
NP_077310.1	13236587	TMEM43	transmembrane protein 43	1.2	1.2	1.2	1.1	1.1	1.1	1.1	1.0	1.1	1.1	1.1	1.0	44.5	1	12	12	42	400	44.8	8.1
NP_001161820.1	270288818	NSUN5	putative methyltransferase NSUN5 isoform 4	0.9	1.0	0.9	1.0	1.1	1.1	1.1	1.1	1.0	1.1	1.0	1.0	7.7	4	3	3	5	391	42.5	7.7
NP_001269271.1	537361067	ARMC8	armadillo repeat-containing protein 8 isoform 5	1.0	1.0	1.0	1.0	1.0	1.1	1.1	1.0	1.0	1.0	1.1	1.0	17.0	5	8	8	12	600	67.1	6.7
NP_003008.1	4506901	SRSF3	serine/arginine-rich splicing factor 3	0.9	0.9	0.9	1.1	0.9	0.9	0.9	1.0	0.9	0.9	0.9	1.0	28.1	1	4	5	32	164	19.3	11.6
NP_945327.2	399788586	NELFCD	negative elongation factor C/D	0.9	0.9	0.9	1.0	1.1	1.1	1.1	1.1	1.0	1.0	1.0	1.0	17.5	1	8	8	17	599	67.3	5.2
NP_060445.3	110578655	ZWILCH	protein zwilch homolog isoform 1	1.1	1.0	1.1	1.0	1.1	1.1	1.2	1.1	1.1	1.0	1.1	1.0	19.5	2	9	9	16	591	67.2	6.3
NP_003786.1	4507143	SNX3	sorting nexin-3 isoform a	1.0	0.9	0.9	1.0	0.9	1.0	0.9	1.0	0.9	1.0	0.9	1.0	10.5	2	2	2	3	162	18.8	8.7
NP_001035830.1	108796653	PGP	phosphoglycolate phosphatase	1.1	1.1	1.1	1.0	1.2	1.2	1.2	1.1	1.2	1.1	1.2	1.0	27.4	1	6	6	27	321	34.0	6.1
NP_005435.1	4885579	RQCD1	cell differentiation protein RCD1 homolog isoform 2	1.0	1.0	1.0	1.1	1.1	1.0	1.1	1.0	1.1	1.0	1.1	1.0	20.7	3	6	6	10	299	33.6	8.0
NP_077268.1	13236510	UBL5	ubiquitin-like protein 5	0.9	0.9	0.9	1.0	0.9	0.9	0.9	1.1	0.9	0.9	0.9	1.0	28.8	1	1	1	1	73	8.5	8.4
NP_057050.1	7706278	ERGIC3	endoplasmic reticulum-Golgi intermediate compartment protein 3 isoform b	0.9	0.8	0.8	1.0	1.0	0.9	0.9	1.0	1.0	0.9	0.8	1.0	11.0	2	2	2	2	383	43.2	6.1
NP_071446.2	26667177	MRPL46	39S ribosomal protein L46, mitochondrial	1.0	1.0	1.0	1.0	1.0	1.0	1.0	1.0	1.0	1.0	1.0	1.0	21.9	1	4	4	9	279	31.7	7.0
NP_001129241.1	209413730	PVR	poliovirus receptor isoform gamma precursor	1.2	1.1	1.0	1.0	1.2	1.2	1.2	1.1	1.2	1.2	1.1	1.0	11.8	4	3	3	11	364	39.3	6.2
NP_060686.2	733216030	PANK4	pantothenate kinase 4	1.1	1.0	1.0	1.0	1.0	0.9	1.0	1.0	1.1	1.0	1.0	1.0	6.7	1	3	3	5	781	86.9	6.3
NP_056969.2	33620745	PCF11	pre-mRNA cleavage complex 2 protein Pcf11	0.9	1.0	1.1	1.0	1.0	1.0	1.1	1.1	1.0	1.0	1.1	1.0	2.3	1	3	3	5	1555	172.9	8.5
NP_056107.1	14149680	ESYT1	extended synaptotagmin-1 isoform 2	1.0	1.0	1.1	1.0	1.0	1.2	1.2	1.1	1.0	1.1	1.1	1.0	20.3	2	15	15	71	1104	122.8	5.8
NP_001254475.1	388890216	ARFRP1	ADP-ribosylation factor-related protein 1 isoform e	1.0	0.9	1.0	1.0	1.0	1.0	0.9	1.1	1.0	1.0	1.0	1.0	29.2	5	3	3	8	154	17.1	6.0
NP_004629.3	314122241;149158690	PRRC2A	protein PRRC2A	1.0	1.0	0.9	1.0	0.9	1.2	1.0	1.1	0.9	1.1	0.9	1.0	14.0	1	16	17	29	2157	228.7	9.5
NP_060612.2	83582809	INTS10	integrator complex subunit 10	1.0	1.0	1.0	1.0	1.0	1.1	1.1	1.0	1.0	1.1	1.0	1.0	2.4	1	2	2	2	710	82.2	7.4
NP_000779.1	4503269	DCK	deoxycytidine kinase	1.0	1.0	1.0	1.1	0.8	0.8	0.8	1.0	0.9	0.9	0.9	1.0	11.9	1	3	3	5	260	30.5	5.2
NP_002860.2	19923231	RAB6A	ras-related protein Rab-6A isoform a	1.0	1.1	0.9	1.0	0.9	1.1	1.1	1.1	1.0	1.1	1.0	1.0	46.6	12	2	8	45	208	23.5	5.5
NP_057085.2	118344456	UTP18	U3 small nucleolar RNA-associated protein 18 homolog	1.0	1.0	1.0	1.0	0.9	0.9	0.9	1.0	1.0	1.0	0.9	1.0	18.7	1	6	6	14	556	62.0	8.8
NP_000391.1	15834617	ERCC2	TFIIH basal transcription factor complex helicase XPD subunit isoform 1	1.0	1.0	1.0	1.0	1.0	1.1	1.1	1.1	1.0	1.1	1.1	1.0	4.0	2	3	3	5	760	86.9	7.2
NP_001119.3	71772942	AP1G1	AP-1 complex subunit gamma-1 isoform b	1.0	1.0	1.0	1.0	1.0	1.0	1.0	1.1	1.0	1.0	1.0	1.0	17.6	2	14	14	44	822	91.3	6.8
NP_001258698.1	419636284	AP3B1	AP-3 complex subunit beta-1 isoform 2	1.0	1.0	1.0	1.0	0.9	1.1	1.0	1.0	1.0	1.0	1.0	1.0	23.8	5	21	21	47	1045	116.1	6.3
NP_006582.1	38492356	POLD3	DNA polymerase delta subunit 3	1.1	1.1	1.0	1.0	1.1	1.0	1.1	1.1	1.1	1.1	1.1	1.0	17.4	1	8	8	26	466	51.4	9.3
NP_002857.1	4506367	RAB3A	ras-related protein Rab-3A	1.7	1.6	2.0	1.0	1.2	1.5	1.4	1.1	1.5	1.6	1.7	1.0	31.8	10	3	6	9	220	25.0	5.0
NP_001244067.1	380420338	ITCH	E3 ubiquitin-protein ligase Itchy homolog isoform 3	1.1	0.9	1.0	1.0	0.9	0.9	0.9	1.0	1.0	0.9	0.9	1.0	11.8	7	6	6	18	752	86.4	5.6
NP_115714.1	14150124	LLPH	protein LLP homolog	0.9	1.3	1.0	1.1	0.9	1.0	1.0	1.0	0.9	1.2	1.0	1.0	29.5	1	3	3	5	129	15.2	10.4
NP_001186550.1	313850978	NCOA7	nuclear receptor coactivator 7 isoform 3	1.1	1.1	1.1	1.0	1.0	1.0	1.1	1.0	1.0	1.1	1.1	1.0	5.0	4	3	3	5	827	92.4	5.2
NP_000604.1	11321561	HPX	hemopexin precursor	0.8	0.9	0.9	1.1	1.1	1.2	1.2	1.0	0.9	1.1	1.0	1.0	6.1	1	2	2	3	462	51.6	7.0

Khan *et al.* , 2019. Multi-omics analysis to characterize cigarette smoke induced molecular alterations in esophageal cells
Supplementary Table 5. List of proteins quantified in untreated and chronically treated Het1A cells with cigarette smoke condensate for 8 months

NP_Accession	Protein group Accession	Gene ID	Description	Het-1A-Smoke - 2M/Parental	Het-1A-Smoke - 4M/Parental	Het-1A-Smoke - 6M/Parental	Het-1A-Smoke - 8M/Parental	Het-1A-Smoke - 2M/Parental	Het-1A-Smoke - 4M/Parental	Het-1A-Smoke - 6M/Parental	Het-1A-Smoke - 8M/Parental	Het-1A-Smoke - 2M/Parental	Het-1A-Smoke - 4M/Parental	Het-1A-Smoke - 6M/Parental	Het-1A-Smoke - 8M/Parental	Coverage (%)	Proteins	Unique Peptides	# Peptides	PSM	# AAs	MW [kDa]	calc. pI
				Replicate 1				Replicate 2				Average of replicates											
NP_006089.1	5174447	GNB2L1	guanine nucleotide-binding protein subunit beta-2-like 1	1.0	1.0	1.0	1.0	1.1	1.1	1.0	1.1	1.0	1.0	1.0	1.0	69.7	1	18	18	129	317	35.1	7.7
NP_055152.1	7657473	PPIL2	peptidyl-prolyl cis-trans isomerase-like 2 isoform a	0.9	1.0	1.0	1.1	1.0	1.0	1.0	1.0	1.0	1.0	1.0	1.0	10.8	2	5	5	6	520	58.8	8.8
NP_001157285.1	255304948	C10orf2	twinkle protein, mitochondrial isoform C	0.9	1.0	1.2	0.9	1.1	1.3	1.4	1.2	1.0	1.2	1.3	1.0	6.1	2	1	1	2	230	26.0	7.5
NP_001275908.1	572876118	LIPA	lysosomal acid lipase/cholesteryl ester hydrolase isoform 2	1.0	1.0	1.2	1.0	1.0	1.1	1.2	1.1	1.0	1.1	1.2	1.0	6.4	2	2	2	5	283	32.5	7.8
NP_055970.1	57634536	LARP4B	la-related protein 4B	1.1	1.0	1.0	1.0	1.0	1.1	1.1	1.1	1.0	1.1	1.0	1.0	8.4	1	3	3	5	738	80.5	6.9
NP_001229806.1	338968890	ELP2	elongator complex protein 2 isoform 4	1.1	1.1	1.1	1.0	1.1	1.1	1.1	1.0	1.1	1.1	1.1	1.0	7.1	6	5	5	8	800	89.7	6.0
NP_001258753.1	425876770;41152086	SERPINB6	serpin B6 isoform a	1.1	1.0	1.1	1.0	1.1	1.0	1.0	1.1	1.1	1.0	1.1	1.0	27.9	4	8	8	28	376	42.6	5.3
NP_065905.2	109948292	KIAA1468	lisH domain and HEAT repeat-containing protein KIAA1468	1.3	1.2	1.1	1.1	1.0	1.0	1.0	1.0	1.2	1.1	1.1	1.0	1.6	1	1	1	1	1216	134.5	5.5
NP_061910.1	9506689	EXOSC4	exosome complex component RRP41	0.9	1.0	1.0	1.0	1.1	1.0	1.1	1.1	1.0	1.0	1.0	1.0	7.4	1	2	2	2	245	26.4	6.5
NP_078821.2	19923597	SAP130	histone deacetylase complex subunit SAP130 isoform b	0.8	0.9	1.0	1.0	0.9	1.1	1.0	1.1	0.9	1.0	1.0	1.0	1.1	2	1	1	1	1048	110.3	9.8
NP_001307.2	29029559	CSE1L	exportin-2 isoform 1	1.0	0.9	1.0	1.0	1.0	0.9	1.0	1.0	1.0	0.9	1.0	1.0	58.4	2	48	48	400	971	110.3	5.8
NP_001196.2	153946401	BNIP1	vesicle transport protein SEC20 isoform BNIP1	1.0	1.0	1.1	1.1	1.1	1.1	1.1	1.0	1.0	1.1	1.1	1.0	21.9	4	4	4	9	228	26.1	9.0
NP_075392.2	54607077	SOWAHC	ankyrin repeat domain-containing protein SOWAHC	1.0	1.0	1.0	1.0	0.9	0.9	0.9	1.0	1.0	1.0	1.0	1.0	7.4	1	3	3	5	525	55.6	7.0
NP_004250.4	148612816	RECQL5	ATP-dependent DNA helicase Q5 isoform 1	1.0	1.1	1.1	1.1	1.0	1.0	1.0	1.0	1.0	1.0	1.0	1.0	3.6	3	3	3	4	991	108.8	8.6
NP_001027005.1	73611906	PHKB	phosphorylase b kinase regulatory subunit beta isoform b	1.1	0.9	1.0	0.9	1.2	1.2	1.1	1.1	1.1	1.1	1.0	1.0	4.2	2	4	4	6	1086	123.7	6.6
NP_071353.4	109715835	HEATR6	HEAT repeat-containing protein 6	1.3	1.0	1.0	1.1	1.0	1.0	1.0	1.0	1.2	1.0	1.0	1.0	2.9	1	2	2	4	1181	128.7	7.0
NP_005473.1	23199983	PIGK	GPI-anchor transamidase precursor	1.0	1.0	0.9	1.0	1.0	0.9	0.9	1.0	1.0	1.0	0.9	1.0	18.5	1	6	6	11	395	45.2	6.2
NP_002428.1	4505241	MPV17	protein Mpv17	1.0	1.0	0.9	1.0	1.1	1.1	1.0	1.1	1.1	1.1	0.9	1.0	17.6	1	2	2	6	176	19.7	9.5
NP_055581.3	224465187	SCRN1	secernin-1 isoform a	1.2	1.1	1.3	1.0	1.2	1.1	1.2	1.1	1.2	1.1	1.3	1.0	32.4	3	9	9	43	414	46.4	4.8
NP_004715.1	4759344	ZW10	centromere/kinetochore protein zw10 homolog	1.2	1.1	1.2	1.1	1.1	1.0	1.1	1.0	1.1	1.1	1.2	1.0	21.7	1	12	12	28	779	88.8	6.3
NP_057531.2	21361598	MPP6	MAGUK p55 subfamily member 6	1.0	1.0	1.1	1.1	1.0	1.0	1.0	1.0	1.0	1.0	1.1	1.0	23.3	1	9	11	27	540	61.1	6.2
NP_005112.2	102468717	MED13	mediator of RNA polymerase II transcription subunit 13	1.0	0.8	0.8	0.9	1.0	1.0	0.9	1.1	1.0	0.9	0.9	1.0	2.6	1	2	2	2	2174	239.1	5.6
NP_001124297.1	195546902	SUPT5H	transcription elongation factor SPT5 isoform b	1.0	1.0	1.0	1.1	1.0	1.0	1.1	1.0	1.0	1.0	1.1	1.0	33.1	2	27	27	90	1083	120.4	5.1
NP_001247419.1	386781463	LSR	lipolysis-stimulated lipoprotein receptor isoform 5	1.0	0.9	0.9	1.0	1.0	1.0	1.0	1.1	1.0	1.0	0.9	1.0	8.9	5	3	3	4	541	59.7	9.4
NP_001166094.1	290656011	NEO1	neogenin isoform 2 precursor	0.9	1.0	0.9	0.9	1.1	1.1	0.9	1.1	1.0	1.1	0.9	1.0	2.3	3	2	2	8	1408	154.2	6.4
NP_078942.3	153251315	AAGAB	alpha- and gamma-adaptin-binding protein p34 isoform 1	1.0	1.1	1.0	1.1	0.9	0.9	0.9	1.0	0.9	1.0	1.0	1.0	36.2	2	6	6	22	315	34.6	4.6
NP_631898.3	194394146	DPP9	dipeptidyl peptidase 9	1.0	0.9	1.1	1.0	1.0	1.0	1.0	1.1	1.0	1.0	1.1	1.0	20.5	1	10	11	31	892	101.6	6.6
NP_115882.1	14210504	AP1M1	AP-1 complex subunit mu-1 isoform 2	0.9	0.9	0.9	1.0	1.0	0.9	1.0	1.1	1.0	0.9	1.0	1.0	47.8	4	16	16	33	423	48.6	7.3
NP_000867.2	119964726	IGF2R	cation-independent mannose-6-phosphate receptor precursor	0.9	1.0	1.0	1.0	0.9	1.1	1.0	1.0	0.9	1.0	1.0	1.0	19.9	1	35	35	81	2491	274.1	5.9
NP_000253.1	4557781	NAGA	alpha-N-acetylgalactosaminidase precursor	1.0	1.0	1.0	1.0	1.2	1.1	1.2	1.1	1.1	1.1	1.1	1.0	8.3	1	2	2	3	411	46.5	5.2
NP_002523.2	24497453	NUP88	nuclear pore complex protein Nup88	1.1	0.9	1.0	1.0	1.1	1.1	1.2	1.1	1.1	1.0	1.1	1.0	13.1	1	6	6	12	741	83.5	5.7
NP_001154180.1	238908505	POLR3B	DNA-directed RNA polymerase III subunit RPC2 isoform 2	1.0	1.0	1.0	1.1	1.0	1.1	1.0	1.0	1.0	1.0	1.0	1.0	0.9	2	1	1	2	1075	121.0	8.6
NP_005753.1	5032179	TRIM28	transcription intermediary factor 1-beta	1.0	1.0	1.0	1.0	0.9	0.9	1.0	1.0	1.0	1.0	1.0	1.0	47.0	1	26	26	174	835	88.5	5.8
NP_067051.2	226509740	SCAF1	splicing factor, arginine/serine-rich 19	1.0	1.0	1.0	1.0	1.0	1.1	1.3	1.1	1.0	1.1	1.1	1.0	3.8	1	3	3	4	1312	139.2	9.2
NP_001271297.1	548923784	NDUFB8	NADH dehydrogenase [ubiquinone] 1 beta subcomplex subunit 8, mitochondrial isoform 3	1.0	1.2	1.1	1.0	1.2	1.1	1.2	1.1	1.1	1.1	1.2	1.0	25.8	3	3	3	8	155	18.5	5.5
NP_001161400.1	268840214	IL1RAP	interleukin-1 receptor accessory protein isoform 1 precursor	0.8	1.0	0.9	1.0	1.0	1.0	1.0	1.1	0.9	1.0	1.0	1.0	1.8	2	1	1	2	570	65.4	8.1
NP_001243608.1	375493516	KIAA0391	mitochondrial ribonuclease P protein 3 isoform 3	1.1	0.9	1.1	1.0	1.1	1.1	1.0	1.0	1.1	1.0	1.0	1.0	11.9	4	4	4	5	488	56.5	7.8
NP_055192.1	94538370	DNAJC2	dnaJ homolog subfamily C member 2 isoform 1	1.0	1.0	1.1	1.0	1.0	1.0	1.0	1.1	1.0	1.0	1.0	1.0	23.7	2	12	12	32	621	72.0	8.7
NP_612638.3	189217863	HINT3	histidine triad nucleotide-binding protein 3	1.1	1.2	1.2	1.1	1.1	1.0	1.0	0.9	1.1	1.1	1.1	1.0	24.2	1	3	3	4	182	20.3	6.6
NP_001014445.1	62234438	NLE1	notchless protein homolog 1 isoform b	0.9	0.9	1.0	1.1	1.0	1.0	1.0	1.0	1.0	0.9	1.0	1.0	6.2	2	1	1	2	193	21.3	8.6
NP_001035247.1	93102389	CEP44	centrosomal protein of 44 kDa isoform a	1.1	1.0	1.1	1.2	1.0	1.0	0.9	0.9	1.1	1.0	1.0	1.0	3.6	2	1	1	1	390	44.1	5.2
NP_002618.1	11321601	PFKP	ATP-dependent 6-phosphofructokinase, platelet type isoform 1	1.1	1.1	1.3	1.0	1.1	1.2	1.3	1.1	1.1	1.1	1.3	1.0	31.8	2	15	19	86	784	85.5	7.5
NP_071918.1	11968023	ZNF106	zinc finger protein 106 isoform 1	1.0	1.0	1.1	1.1	1.4	1.0	1.0	1.0	1.2	1.0	1.0	1.0	0.7	1	1	1	1	1883	208.8	7.2
NP_055559.1	7661998	TBC1D5	TBC1 domain family member 5 isoform b	1.0	0.9	1.0	1.0	1.1	1.0	1.1	1.1	1.1	1.0	1.1	1.0	17.0	2	9	9	14	795	88.9	6.5
NP_077719.2	24041035	NOTCH2	neurogenic locus notch homolog protein 2 isoform 1 preproprotein	0.9	1.1	1.1	1.0	1.0	1.0	1.1	1.0	1.0	1.1	1.1	1.0	3.1	3	6	6	9	2471	265.2	5.1
NP_001258636.1	411147393	MKRN2	probable E3 ubiquitin-protein ligase makorin-2 isoform 2	1.0	0.9	1.0	1.1	1.0	0.9	0.9	1.0	1.0	0.9	1.0	1.0	12.9	2	4	4	7	373	42.0	7.1
NP_001124481.1	196114970	PTPN12	tyrosine-protein phosphatase non-receptor type 12 isoform 3	1.0	1.1	1.1	1.1	1.3	1.0	1.0	1.0	1.1	1.0	1.0	1.0	7.9	3	4	4	10	650	72.7	5.2
NP_055290.1	7657212	DHDH	trans-1,2-dihydrobenzene-1,2-diol dehydrogenase	1.6	1.3	1.1	1.0	1.2	1.2	1.2	1.0	1.4	1.3	1.1	1.0	3.3	1	1	1	1	334	36.4	7.2
NP_002660.1	4505895	PLRG1	pleiotropic regulator 1 isoform 1	1.0	1.0	1.0	1.1	1.0	1.0	1.0	1.0	1.0	1.0	1.0	1.0	20.6	2	7	7	14	514	57.2	9.2
NP_955475.1	40795667	USP4	ubiquitin carboxyl-terminal hydrolase 4 isoform b	1.1	1.0	1.0	1.1	1.0	0.9	0.8	1.0	1.1	1.0	0.9	1.0	12.3	3	5	7	14	916	103.9	5.5
NP_055642.3	114842410	ZC3H11A	zinc finger CCCH domain-containing protein 11A	1.0	1.1	1.0	1.0	1.0	1.1	1.0	1.1	1.0	1.1	1.0	1.0	21.0	2	14	15	28	810	89.1	8.4
NP_001124496.1	196259772	PEX5	peroxisomal biogenesis factor 5 isoform c	1.1	1.2	1.1	1.1	1.0	1.1	1.0	1.0	1.0	1.1	1.1	1.0	17.4	5	6	6	9	602	66.8	4.5
NP_001243763.1	378786658	AURKB	aurora kinase B isoform 2	1.0	1.0	1.1	1.0	1.0	1.0	1.0	1.0	1.0	1.0	1.1	1.0	45.5	3	9	9	18	303	34.7	9.1
NP_612428.2	39653315	MTFR2	mitochondrial fission regulator 2	0.9	0.9	1.0	1.0	1.0	1.0	1.0	1.0	1.0	1.0	1.0	1.0	3.1	1	1	1	2	385	43.4	7.0
NP_002856.1	4506365	RAB2A	ras-related protein Rab-2A isoform a	1.0	1.0	1.0	1.0	1.1	1.1	1.1	1.1	1.0	1.0	1.1	1.0	44.3	2	3	7	38	212	23.5	6.5
NP_054735.3	190014578	SACM1L	phosphatidylinositide phosphatase SAC1	1.0	1.0	1.0	1.1	1.0	1.0	1.0	1.0	1.0	1.0	1.0	1.0	13.8	1	7	7	8	587	66.9	7.1
NP_004701.1	4759202	SYNGR2	synaptogyrin-2	1.2	1.1	1.2	1.0	0.9	1.0	0.9	1.1	1.0	1.0	1.0	1.0	4.5	1	1	1	1	224	24.8	4.9
NP_076976.1	13129054	ZFYVE21	zinc finger FYVE domain-containing protein 21 isoform 2	1.1	1.2	1.2	1.2	1.2	1.0	1.1	0.9	1.2	1.1	1.1	1.0	8.6	2	1	1	1	234	26.5	8.4
NP_001186421.1	313760537	PDCD4	programmed cell death protein 4 isoform 3	1.0	1.0	0.9	1.0	1.0	0.9	0.9	1.0	1.0	0.9	0.9	1.0	25.1	3	9	9	29	455	50.2	5.5
NP_004514.2	13699824	KIF11	kinesin-like protein KIF11	1.0	1.0	1.1	1.0	1.0	1.0	1.1	1.0	1.0	1.0	1.1	1.0	20.5	1	18	18	49	1056	119.1	5.6
NP_079005.3	116284394	MYH14	myosin-14 isoform 2	1.0	0.9	1.0	1.1	1.1	1.1	1.1	1.0	1.0	1.0	1.1	1.0	13.4	3	10	22	97	1995	227.7	5.6
NP_001027392.1	73760415	STXBP1	syntaxin-binding protein 1 isoform b	1.1	1.2	1.1	1.0	1.1	1.2	1.2	1.1	1.1	1.2	1.1	1.0	27.1	2	10	10	21	594	67.5	7.0
NP_008879.3	48928056	HSPA13	heat shock 70 kDa protein 13 precursor	1.3	1.1	1.0	1.1	1.1	1.0	1.0	1.0	1.2	1.0	1.0	1.0	16.4	1	5	5	13	471	51.9	5.8
NP_001015054.1	62632771	MPG	DNA-3-methyladenine glycosylase isoform c	0.9	1.0	1.0	1.0	1.0	1.0	1.0	1.1	1.0	1.0	1.0	1.0	22.4	3	4	4	10	281	30.8	9.2
NP_115758.3	186928841	COG8	conserved oligomeric Golgi complex subunit 8	1.1	1.1	1.1	1.0	1.2	1.3	1.2	1.1	1.2	1.2	1.1	1.0	8.8	1	4	4	6	612	68.4	5.2
NP_060826.2	170763486	C5orf22	UPF0489 protein C5orf22	0.9	1.0	1.0	1.1	1.0	1.0	1.0	1.0	0.9	1.0	1.0	1.0	32.4	1	7	7	16	442	49.9	4.8
NP_057280.2	226497574	RBM19	probable RNA-binding protein 19	0.9	1.0	1.0	1.1	1.0	1.0	1.0	1.0	0.9	1.0	1.0	1.0	17.8	1	14	14	34	960	107.3	6.5
NP_064555.2	51873031	NCLN	nicalin precursor	1.2	1.1	1.2	1.1	1.0	1.0	1.0	1.0	1.1	1.1	1.1	1.0	19.9	1	7	7	18	563	62.9	6.9
NP_001645.1	4502193	ARAF	serine/threonine-protein kinase A-Raf isoform 1	1.1	1.1	1.2	1.1	1.0	1.0	1.0	1.0	1.0	1.0	1.1	1.0	5.0	2	1	2	3	606	67.5	9.0
NP_001258717.1	422398896;422398892	DYNC1I2	cytoplasmic dynein 1 intermediate chain 2 isoform 3	1.1	1.0	1.0	1.1	1.1	1.0	1.0	1.0	1.1	1.0	1.0	1.0	33.0	4	11	11	49	612	68.4	5.3
NP_060619.4	47777319	SMG8	protein SMG8	1.0	1.0	1.0	1.0	1.1	1.1	1.1	1.1	1.1	1.0	1.0	1.0	10.0	1	6	6	13	991	109.6	7.7
NP_001001485.1	48762687	ATP2C1	calcium-transporting ATPase type 2C member 1 isoform 1c	1.0	1.1	1.1	1.1	1.1	1.0	1.0	1.0	1.1	1.1	1.1	1.0	4.2	9	2	2	5	888	96.9	6.3
NP_001228.1	4502613	CCNA2	cyclin-A2	1.0	1.1	1.0	1.0	1.0	1.0	1.0	1.0	1.0	1.0	1.0	1.0	14.4	1	3	3	7	432	48.5	6.5
NP_004591.2	31377800;291084629	TROVE2	60 kDa SS-A/Ro ribonucleoprotein isoform 2	1.0	1.0	1.0	1.0	1.0	1.0	1.1	1.1	1.0	1.0	1.1	1.0	21.6	4	10	10	30	538	60.6	8.0
NP_002159.2	28178832	IDH2	isocitrate dehydrogenase [NADP], mitochondrial isoform 1 precursor	0.9	0.9	0.8	1.0	0.9	0.9	0.9	1.0	0.9	0.9	0.9	1.0	52.0	3	19	20	76	452	50.9	8.7
NP_001273200.1	555943926	HMOX2	heme oxygenase 2 isoform c	0.9	1.0	1.0	1.0	1.0	1.1	1.0	1.1	1.0	1.0	1.0	1.0	36.6	3	7	7	20	287	32.8	5.9
NP_987102.1	45359859	GRB2	growth factor receptor-bound protein 2 isoform 2	1.0	1.1	0.9	1.0	0.9	1.0	1.0	1.1	1.0	1.0	1.0	1.0	16.5	2	1	1	4	176	20.5	5.8
NP_064539.1	9910346	EMC7	ER membrane protein complex subunit 7 precursor	1.2	1.2	1.2	1.1	1.0	1.1	1.0	1.0	1.1	1.1	1.1	1.0	14.1	1	4	4	7	242	26.5	9.2
NP_848699.1	30795193	Sep-10	septin-10 isoform 2	1.0	0.9	1.0	1.1	0.9	0.9	0.9	1.0	1.0	0.9	1.0	1.0	14.9	2	3	4	7	431	50.0	7.0
NP_077297.2	21359951	RBM42	RNA-binding protein 42	1.0	1.0	1.0	1.0	1.0	1.0	1.0	1.1	1.0	1.0	1.0	1.0	17.7	1	5	5	19	480	50.4	9.6
NP_000692.2	21361181	ATP1A1	sodium/potassium-transporting ATPase subunit alpha-1 isoform a	1.0	1.0	1.0	1.0	1.0	1.0	1.0	1.0	1.0	1.0	1.0	1.0	40.8	10	37	39	239	1023	112.8	5.5
NP_001076048.1	133925805	RBFOX2	RNA binding protein fox-1 homolog 2 isoform 6	0.8	0.8	0.8	1.1	0.9	0.9	0.9	1.0	0.9	0.8	0.9	1.0	4.9	2	1	1	2	450	47.2	6.6
NP_003720.1	4506589	RTCA	RNA 3'-terminal phosphate cyclase isoform b	1.0	1.0	0.9	1.0	1.1	1.3	1.2	1.1	1.0	1.1	1.0	1.0	13.9	2	4	4	15	366	39.3	7.9
NP_550440.1	18426971	DGUOK	deoxyguanosine kinase, mitochondrial isoform b precursor	1.0	1.0	1.0	1.0	1.1	1.0	1.1	1.0	1.0	1.0	1.1	1.0	17.5	2	2	2	4	189	21.4	9.5
NP_001120697.1	187829070;187828910	CD59	CD59 glycoprotein preproprotein	1.1	1.1	1.1	1.1	0.8	1.0	1.2	1.0	1.0	1.1	1.1	1.0	17.2	1	2	2	4	128	14.2	6.5
NP_008830.2	28178821	IDH3B	isocitrate dehydrogenase [NAD] subunit beta, mitochondrial isoform a precursor	1.0	1.0	1.0	1.0	1.0	1.1	1.1	1.0	1.0	1.1	1.0	1.0	34.6	3	10	10	35	385	42.2	8.5
NP_008924.1	6857818	NPM3	nucleoplasmin-3	1.2	0.9	1.1	1.1	1.2	1.0	1.1	1.0	1.2	1.0	1.1	1.0	28.1	1	3	3	6	178	19.3	4.6
NP_055481.1	7661968	CLINT1	clathrin interactor 1 isoform 2	1.1	1.0	1.0	1.1	0.9	0.9	0.9	1.0	1.0	1.0	0.9	1.0	7.2	3	3	3	7	625	68.2	6.4

Khan *et al.* , 2019. Multi-omics analysis to characterize cigarette smoke induced molecular alterations in esophageal cells
Supplementary Table 5. List of proteins quantified in untreated and chronically treated Het1A cells with cigarette smoke condensate for 8 months

NP_Accession	Protein group Accession	Gene ID	Description	Het-1A-Smoke - 2M/Parental	Het-1A-Smoke - 4M/Parental	Het-1A-Smoke - 6M/Parental	Het-1A-Smoke - 8M/Parental	Het-1A-Smoke - 2M/Parental	Het-1A-Smoke - 4M/Parental	Het-1A-Smoke - 6M/Parental	Het-1A-Smoke - 8M/Parental	Het-1A-Smoke - 2M/Parental	Het-1A-Smoke - 4M/Parental	Het-1A-Smoke - 6M/Parental	Het-1A-Smoke - 8M/Parental	Coverage (%)	Proteins	Unique Peptides	# Peptides	PSM	# AAs	MW [kDa]	calc. pI
				Replicate 1				Replicate 2				Average of replicates											
NP_001239085.1	356461016	**GEMIN5**	gem-associated protein 5 isoform 2	1.0	1.1	1.0	1.0	1.1	1.1	1.1	1.0	1.1	1.1	1.1	1.0	28.0	2	35	35	116	1507	168.4	6.7
NP_115580.2	20270253	**ASCC2**	activating signal cointegrator 1 complex subunit 2 isoform 1	1.0	0.9	1.0	0.9	1.2	1.0	1.1	1.1	1.1	1.0	1.0	1.0	4.8	2	3	3	11	757	86.3	5.2
NP_002808.3	157502193	**PSMD13**	26S proteasome non-ATPase regulatory subunit 13 isoform 1	1.1	1.1	1.2	1.0	1.1	1.1	1.2	1.0	1.1	1.1	1.2	1.0	44.2	2	14	14	79	376	42.9	5.8
NP_001138999.1	224465235	**EHMT1**	histone-lysine N-methyltransferase EHMT1 isoform 2	1.0	1.0	1.1	1.0	1.0	1.0	1.0	1.0	1.0	1.0	1.0	1.0	7.7	2	3	3	4	808	86.6	6.3
NP_005711.1	5031601	**ARPC1B**	actin-related protein 2/3 complex subunit 1B	1.2	1.1	1.3	1.0	1.1	1.1	1.2	1.0	1.2	1.1	1.2	1.0	21.0	1	5	6	11	372	40.9	8.4
NP_055111.1	7656959	**CAPN7**	calpain-7	1.0	1.0	1.0	1.1	0.9	1.0	0.9	1.0	0.9	1.0	1.0	1.0	1.5	1	1	1	1	813	92.6	7.6
NP_003464.1	4507249	**STAM**	signal transducing adapter molecule 1	1.2	1.0	1.1	1.1	1.0	1.0	1.1	1.0	1.1	1.0	1.1	1.0	11.5	1	3	3	5	540	59.1	4.8
NP_078817.2	154350213	**C10orf76**	UPF0668 protein C10orf76	0.9	0.9	0.9	1.0	1.0	1.0	1.0	1.0	0.9	0.9	0.9	1.0	1.3	1	1	1	1	689	78.7	6.6
NP_002793.2	24430151	**PSMC1**	26S protease regulatory subunit 4	1.0	1.0	1.1	1.0	1.0	1.0	1.1	1.0	1.0	1.0	1.1	1.0	57.7	1	20	21	139	440	49.2	6.2
NP_001186883.1	316659409	**ACTG1**	actin, cytoplasmic 2	1.1	1.1	1.2	1.0	1.1	1.1	1.2	1.0	1.1	1.1	1.2	1.0	84.5	4	8	27	1564	375	41.8	5.5
NP_000378.1	4557403	**SLC25A20**	mitochondrial carnitine/acylcarnitine carrier protein	1.2	1.1	1.1	1.0	1.1	1.0	1.1	1.0	1.2	1.1	1.1	1.0	16.3	1	3	3	4	301	32.9	9.4
NP_002906.1	4506489	**RFC3**	replication factor C subunit 3 isoform 1	1.0	0.9	1.0	1.0	1.0	1.0	1.0	1.1	1.0	1.0	1.0	1.0	43.0	2	11	11	28	356	40.5	8.3
NP_005547.3	67782365	**KRT7**	keratin, type II cytoskeletal 7	1.0	1.0	0.9	1.0	1.0	1.0	0.9	1.0	1.0	1.0	0.9	1.0	56.1	10	21	28	209	469	51.4	5.5
NP_001243670.1	376319247	**CNOT10**	CCR4-NOT transcription complex subunit 10 isoform 2	1.1	1.0	1.1	1.1	1.1	1.0	1.1	1.0	1.1	1.0	1.1	1.0	3.4	3	2	2	2	717	79.3	7.8
NP_001092271.1	149408153	**FAM210A**	protein FAM210A	1.1	1.0	1.1	1.0	1.0	1.0	1.0	1.0	1.1	1.0	1.1	1.0	6.3	1	2	2	3	272	30.8	9.8
NP_060937.1	8923936	**USE1**	vesicle transport protein USE1	1.2	1.4	1.3	1.2	1.1	1.0	0.9	0.9	1.2	1.2	1.1	1.0	11.2	1	2	2	3	259	29.3	9.1
NP_001243657.1	376319206	**USP39**	U4/U6.U5 tri-snRNP-associated protein 2 isoform 4	0.9	1.0	1.0	1.1	1.0	1.0	1.0	1.0	1.0	1.0	1.0	1.0	34.9	4	11	11	47	462	53.5	8.6
NP_006136.1	5453690	**DNAJB1**	dnaJ homolog subfamily B member 1 isoform 1	0.8	0.9	0.9	1.0	1.0	1.0	1.0	1.0	0.9	1.0	1.0	1.0	36.8	2	12	13	38	340	38.0	8.6
NP_114428.1	14042970	**ITFG3**	protein ITFG3	1.1	1.1	1.0	1.1	1.1	1.1	1.0	1.0	1.1	1.1	1.0	1.0	12.5	1	4	4	6	552	59.6	6.3
NP_579899.1	19718759	**MYOF**	myoferlin isoform b	1.1	1.1	1.2	1.0	1.2	1.1	1.2	1.0	1.2	1.1	1.2	1.0	38.8	16	61	61	175	2048	233.3	6.2
NP_056461.1	7661678;58219792	**RAP1B**	ras-related protein Rap-1b isoform 1 precursor	1.0	1.2	1.1	1.1	1.0	1.1	1.2	1.0	1.0	1.1	1.2	1.0	42.9	4	2	5	37	184	20.8	5.8
NP_060496.2	30089916	**PACS1**	phosphofurin acidic cluster sorting protein 1	0.9	0.9	0.9	1.0	0.9	1.0	1.0	1.0	0.9	0.9	1.0	1.0	0.9	1	1	1	2	963	104.8	7.7
NP_001171723.1	296278206	**PARD3**	partitioning defective 3 homolog isoform 11	1.0	1.1	1.1	1.0	1.1	1.0	1.1	1.0	1.0	1.0	1.1	1.0	1.9	11	2	2	2	988	108.5	6.7
NP_057390.1	7706495	**DNAJB11**	dnaJ homolog subfamily B member 11 precursor	1.1	1.2	1.1	1.1	1.1	1.2	1.0	1.0	1.1	1.2	1.1	1.0	33.2	1	11	11	68	358	40.5	6.2
NP_057381.3	166795301	**PCYOX1**	prenylcysteine oxidase 1 precursor	1.0	1.0	1.0	1.0	1.0	1.0	1.0	1.0	1.0	1.0	1.0	1.0	20.0	1	8	8	24	505	56.6	6.2
NP_002262.3	24797086	**IPO5**	importin-5	1.0	1.0	1.1	1.0	1.0	1.0	1.1	1.0	1.0	1.0	1.1	1.0	40.5	3	29	29	139	1115	125.5	4.9
NP_055726.3	148277037	**AAK1**	AP2-associated protein kinase 1	1.1	1.1	1.1	1.1	1.2	1.0	1.1	1.0	1.1	1.1	1.1	1.0	10.7	1	7	7	10	961	103.8	6.5
NP_056955.2	154937327	**ZNF593**	zinc finger protein 593	1.3	1.1	0.9	1.0	1.0	1.0	0.9	1.0	1.1	1.0	0.9	1.0	30.6	1	3	3	10	134	15.2	9.8
NP_000421.1	4557741	**PAFAH1B1**	platelet-activating factor acetylhydrolase IB subunit alpha	1.1	1.1	1.2	1.0	1.1	1.1	1.1	1.0	1.1	1.1	1.1	1.0	44.9	1	14	15	49	410	46.6	7.4
NP_054753.1	7661592	**ASF1A**	histone chaperone ASF1A	1.0	0.9	1.1	1.1	0.9	1.0	1.0	1.0	1.0	1.0	1.0	1.0	21.1	1	2	2	4	204	23.0	4.4
NP_859526.1	32454748;32454746	**ORC4**	origin recognition complex subunit 4 isoform 1	1.0	0.9	1.0	1.0	1.0	1.0	1.0	1.1	1.0	1.0	1.0	1.0	22.5	7	8	8	15	436	50.3	8.0
NP_056106.1	56687498	**DNAJC16**	dnaJ homolog subfamily C member 16 isoform 1 precursor	1.2	1.1	1.1	1.1	0.9	1.1	1.0	1.0	1.1	1.1	1.0	1.0	3.5	2	2	2	8	782	90.5	7.1
NP_002445.2	169790958	**MTRR**	methionine synthase reductase isoform 1	1.0	1.0	1.1	1.1	1.1	1.1	1.2	1.0	1.1	1.1	1.1	1.0	3.9	2	2	2	2	698	77.6	6.5
NP_060850.2	256773275	**DDX28**	probable ATP-dependent RNA helicase DDX28	0.9	1.0	1.0	1.0	1.0	1.0	1.0	1.0	1.0	1.0	1.0	1.0	10.7	1	4	4	5	540	59.5	10.4
NP_055925.2	155722985	**SMC5**	structural maintenance of chromosomes protein 5	1.0	1.0	1.0	1.1	1.0	1.1	1.0	1.0	1.0	1.0	1.0	1.0	4.9	1	5	5	8	1101	128.7	8.4
NP_006149.2	105990539	**NEFL**	neurofilament light polypeptide	1.0	1.3	1.2	1.1	1.0	1.1	0.9	0.9	1.0	1.2	1.1	1.0	6.3	5	1	2	32	543	61.5	4.7
NP_066979.2	40254947	**TMX4**	thioredoxin-related transmembrane protein 4 precursor	1.1	1.0	1.1	1.2	0.9	1.0	0.9	0.9	1.0	1.0	1.0	1.0	11.5	1	3	3	5	349	38.9	4.4
NP_001166924.1	291219876	**TFCP2**	alpha-globin transcription factor CP2 isoform 3	1.0	1.1	1.1	1.1	1.0	1.0	1.0	1.0	1.0	1.0	1.1	1.0	5.6	4	1	2	3	450	51.3	5.4
NP_055866.1	52487191	**ERP44**	endoplasmic reticulum resident protein 44 precursor	1.1	1.0	1.1	1.0	1.1	1.1	1.1	1.0	1.1	1.1	1.1	1.0	24.4	1	7	8	16	406	46.9	5.3
NP_001014841.1	62526031	**NCDN**	neurochondrin isoform 2	1.0	0.9	1.0	1.0	1.0	1.0	1.1	1.0	1.0	0.9	1.1	1.0	6.3	2	4	4	11	712	77.2	5.5
NP_037365.2	115430252	**STK39**	STE20/SPS1-related proline-alanine-rich protein kinase	0.9	0.9	1.1	0.9	1.1	1.1	1.2	1.1	1.0	1.0	1.1	1.0	11.4	1	4	5	19	545	59.4	6.3
NP_075391.3	39995084	**INTS3**	integrator complex subunit 3	1.0	1.0	1.1	1.0	1.0	1.1	1.2	1.1	1.0	1.0	1.1	1.0	13.3	1	9	9	18	1042	117.9	5.8
NP_002787.2	22538467	**PSMB4**	proteasome subunit beta type-4	1.1	1.0	1.0	1.0	1.1	1.1	1.1	1.1	1.1	1.0	1.0	1.0	48.5	1	8	8	56	264	29.2	6.0
NP_291025.3	50086624	**INTS4**	integrator complex subunit 4	1.0	1.0	1.0	1.0	1.1	1.0	1.1	1.0	1.1	1.0	1.0	1.0	5.5	2	4	4	8	963	108.1	6.4
NP_116294.1	14249678	**RBM17**	splicing factor 45	1.0	0.9	0.9	1.0	0.9	1.0	1.0	1.1	0.9	1.0	0.9	1.0	8.5	1	2	3	7	401	44.9	6.0
NP_004539.1	4758774	**NDUFB10**	NADH dehydrogenase [ubiquinone] 1 beta subcomplex subunit 10	1.0	1.0	0.9	1.0	1.1	1.1	1.1	1.1	1.1	1.0	1.0	1.0	27.3	1	4	4	16	172	20.8	8.5
NP_001107869.1	167830436	**DHX36**	ATP-dependent RNA helicase DHX36 isoform 2	1.1	1.1	1.1	1.0	1.0	1.1	1.1	1.0	1.1	1.1	1.1	1.0	14.7	2	10	11	24	994	113.1	7.7
NP_620581.1	20986512	**MAPK14**	mitogen-activated protein kinase 14 isoform 2	1.0	1.0	1.0	1.0	1.0	1.0	1.1	1.1	1.0	1.0	1.0	1.0	32.2	4	8	8	21	360	41.3	5.8
NP_059990.2	41055989	**MPHOSPH8**	M-phase phosphoprotein 8	1.0	1.1	1.0	1.0	1.0	1.1	1.0	1.0	1.0	1.1	1.0	1.0	17.4	1	8	8	12	860	97.1	6.1
NP_002484.1	4505365	**NDUFB6**	NADH dehydrogenase [ubiquinone] 1 beta subcomplex subunit 6 isoform 1	1.0	1.1	1.1	1.0	1.1	1.0	1.2	1.0	1.1	1.0	1.1	1.0	31.3	3	4	4	15	128	15.5	9.6
NP_001269786.1	544583596	**XRN1**	5'-3' exoribonuclease 1 isoform c	1.1	1.1	1.0	1.0	1.1	1.1	1.1	1.0	1.1	1.1	1.0	1.0	5.2	3	5	5	7	1694	192.7	7.3
NP_001290041.1	733605691	**FAM45A**	protein FAM45A isoform 3	1.1	1.1	1.1	1.1	0.9	0.9	0.9	1.0	1.0	1.0	1.0	1.0	12.3	4	3	3	6	284	32.1	6.7
NP_066363.1	28626521	**ZNFX1**	NFX1-type zinc finger-containing protein 1	1.0	1.0	1.0	1.0	1.0	1.0	1.0	1.0	1.0	1.0	1.0	1.0	4.9	1	6	6	12	1918	220.1	7.3
NP_001073315.1	119943102	**CREBBP**	CREB-binding protein isoform b	0.8	0.9	0.9	1.0	0.9	1.0	1.0	1.0	0.9	1.0	0.9	1.0	2.1	2	2	4	8	2404	260.8	8.5
NP_001258824.1	429535836	**TARS2**	threonine--tRNA ligase, mitochondrial isoform b	1.0	1.0	0.9	1.1	0.8	0.9	0.8	1.0	0.9	0.9	0.9	1.0	8.2	3	4	4	5	636	71.4	7.2
NP_036361.1	6912598	**NT5C2**	cytosolic purine 5'-nucleotidase	0.9	0.9	1.0	1.0	1.0	1.1	1.1	1.1	0.9	1.0	1.0	1.0	14.4	1	7	7	17	561	64.9	6.1
NP_055535.2	41281453	**SLK**	STE20-like serine/threonine-protein kinase isoform 1	1.0	1.1	1.1	1.0	0.9	1.2	1.1	1.1	1.0	1.1	1.1	1.0	19.4	2	19	20	39	1235	142.6	5.1
NP_000695.2	51944966	**ATP4A**	potassium-transporting ATPase alpha chain 1	0.9	0.9	1.0	1.0	0.9	1.0	0.9	1.1	0.9	1.0	1.0	1.0	3.9	1	1	3	6	1035	114.0	5.8
NP_060947.1	8923712	**ACTR10**	actin-related protein 10	1.1	1.1	1.0	1.0	1.0	1.0	1.1	1.0	1.1	1.0	1.1	1.0	30.9	1	7	7	10	417	46.3	7.4
NP_002195.1	4504747	**ITGA3**	integrin alpha-3 isoform a precursor	0.9	0.9	1.0	1.0	0.9	1.0	1.1	1.0	0.9	0.9	1.0	1.0	5.9	2	5	5	14	1051	116.5	6.8
NP_036448.1	6912478	**KPNA6**	importin subunit alpha-7	1.0	1.0	1.0	1.0	1.0	1.0	1.0	1.1	1.0	1.0	1.0	1.0	41.8	1	12	16	37	536	60.0	5.0
NP_001120959.1	188595687	**FLNC**	filamin-C isoform b	1.1	1.0	1.0	1.0	1.1	1.1	1.0	1.0	1.1	1.0	1.0	1.0	7.8	2	7	18	56	2692	287.1	6.0
NP_002349.1	4505067	**MAD2L1**	mitotic spindle assembly checkpoint protein MAD2A	0.9	0.9	0.9	1.0	1.0	1.0	1.0	1.1	1.0	0.9	1.0	1.0	27.8	1	5	5	6	205	23.5	5.1
NP_004609.1	10835218	**TOP3A**	DNA topoisomerase 3-alpha	1.1	1.0	1.0	1.0	1.2	1.0	1.1	1.1	1.1	1.0	1.1	1.0	3.2	1	3	3	4	1001	112.3	8.3
NP_075559.2	148746220	**SPATS2**	spermatogenesis-associated serine-rich protein 2	1.2	0.8	1.1	1.0	0.8	0.9	0.9	1.0	1.0	0.8	1.0	1.0	7.3	1	2	2	7	545	59.5	8.9
NP_056046.1	54859722	**NUP160**	nuclear pore complex protein Nup160	1.1	1.1	1.2	1.1	1.1	1.0	1.1	1.0	1.1	1.0	1.1	1.0	17.1	1	17	17	57	1436	162.0	5.5
NP_115868.1	20806105	**MRPL36**	39S ribosomal protein L36, mitochondrial	1.0	0.9	1.0	1.0	1.0	1.0	1.0	1.0	1.0	1.0	1.0	1.0	5.8	1	1	1	1	103	11.8	11.3
NP_001191230.1	324021738	**APP**	amyloid beta A4 protein isoform h precursor	1.1	1.2	1.2	1.0	1.5	1.5	1.6	1.1	1.3	1.3	1.4	1.0	11.4	10	8	8	14	752	85.0	4.8
NP_078938.2	221316723	**NAT10**	N-acetyltransferase 10 isoform a	1.0	1.0	1.0	1.1	1.0	1.0	1.1	1.0	1.0	1.0	1.1	1.0	33.0	2	25	25	79	1025	115.6	8.3
NP_777576.1	28372497	**UBR1**	E3 ubiquitin-protein ligase UBR1	1.0	1.1	1.0	1.0	1.1	1.1	1.1	1.1	1.0	1.1	1.1	1.0	3.0	1	4	4	5	1749	200.1	6.0
NP_001269802.1	544583486	**UBR5**	E3 ubiquitin-protein ligase UBR5 isoform 2	1.1	1.0	1.1	1.1	1.0	0.9	1.0	1.0	1.0	1.0	1.0	1.0	10.0	2	20	20	39	2798	309.0	5.8
NP_005026.3	110618253	**POLRMT**	DNA-directed RNA polymerase, mitochondrial precursor	1.0	1.1	1.0	1.1	1.0	0.9	0.9	1.0	1.0	1.0	0.9	1.0	6.3	1	5	5	8	1230	138.5	9.0
NP_001165282.1	284807150	**GBA**	glucosylceramidase isoform 2	1.0	1.2	1.1	1.1	1.0	1.1	1.0	1.0	1.0	1.1	1.1	1.0	13.6	3	5	5	11	449	50.3	7.2
NP_114401.2	110681719	**TMEM245**	transmembrane protein 245	1.3	1.1	1.1	1.1	1.1	1.0	1.0	1.0	1.2	1.0	1.1	1.0	4.7	1	2	2	6	879	97.3	8.9
NP_036450.1	6912482	**LETM1**	LETM1 and EF-hand domain-containing protein 1, mitochondrial precursor	1.0	1.0	1.0	1.0	1.1	1.1	1.1	1.0	1.1	1.0	1.1	1.0	29.1	1	19	19	76	739	83.3	6.7
NP_002803.2	156631005	**PSMD8**	26S proteasome non-ATPase regulatory subunit 8	1.1	1.1	1.1	1.0	1.0	1.0	1.0	1.0	1.0	1.0	1.0	1.0	34.6	1	10	10	31	350	39.6	9.7
NP_004450.3	38788260	**BPTF**	nucleosome-remodeling factor subunit BPTF isoform 2	1.0	1.0	1.0	1.0	0.9	1.0	0.9	1.0	0.9	1.0	1.0	1.0	2.9	2	6	7	9	2903	322.0	6.4
NP_003892.2	31982936	**SGPL1**	sphingosine-1-phosphate lyase 1	1.0	1.1	1.1	1.0	1.1	1.0	1.0	1.0	1.1	1.0	1.1	1.0	19.4	1	10	10	24	568	63.5	9.2
NP_031389.3	34932414;224028246	**NONO**	non-POU domain-containing octamer-binding protein isoform 1	0.9	1.0	0.8	1.0	0.8	0.9	0.8	1.0	0.8	0.9	0.8	1.0	42.0	2	16	18	59	471	54.2	9.0
NP_996735.1	45827708	**QKI**	protein quaking isoform HQK-6	0.9	1.0	1.0	1.0	0.9	1.0	0.9	1.0	0.9	1.0	0.9	1.0	28.2	4	6	6	10	319	35.1	7.4
NP_775853.2	31377548	**PGM2L1**	glucose 1,6-bisphosphate synthase	1.1	0.9	0.9	1.1	1.0	1.1	1.0	0.9	1.0	1.0	1.0	1.0	6.4	1	3	3	3	622	70.4	7.2
NP_957717.1	41152107	**UBL7**	ubiquitin-like protein 7 isoform a	1.0	1.0	1.2	1.1	1.1	1.0	1.0	1.0	1.0	1.0	1.1	1.0	1.6	2	1	1	1	380	40.5	5.1
NP_660186.1	21735544	**MAP2K7**	dual specificity mitogen-activated protein kinase kinase 7	0.8	0.9	0.9	1.0	0.9	0.9	0.9	1.1	0.9	0.9	0.9	1.0	2.9	1	1	1	1	419	47.5	9.2
NP_077718.3	187281616	**NDUFS7**	NADH dehydrogenase [ubiquinone] iron-sulfur protein 7, mitochondrial	0.9	1.0	1.0	1.0	1.0	1.0	1.0	1.0	1.0	1.0	1.0	1.0	14.1	1	3	3	8	213	23.5	10.0
NP_006353.2	15487670	**NXF1**	nuclear RNA export factor 1 isoform 1	0.9	1.0	1.0	1.0	1.1	1.1	1.1	1.1	1.0	1.0	1.0	1.0	30.2	2	15	15	40	619	70.1	8.5
NP_004299.1	4757766	**ARHGAP1**	rho GTPase-activating protein 1	1.1	1.1	1.1	1.0	1.0	1.1	1.1	1.0	1.0	1.1	1.1	1.0	34.6	1	11	11	25	439	50.4	6.3
NP_067027.1	10864017	**ENOPH1**	enolase-phosphatase E1 isoform 1	1.0	0.9	1.0	1.0	1.1	1.0	1.1	1.0	1.0	1.0	1.0	1.0	17.6	2	3	3	9	261	28.9	4.8
NP_115687.2	29837655	**POLDIP3**	polymerase delta-interacting protein 3 isoform 1	0.9	0.9	0.9	1.0	0.9	0.9	0.9	1.0	0.9	0.9	0.9	1.0	17.3	3	5	5	13	421	46.1	10.0
NP_874384.1	33239443	**SYPL1**	synaptophysin-like protein 1 isoform b	0.7	0.8	0.9	1.0	1.1	0.9	1.0	1.1	0.9	0.9	0.9	1.0	19.5	2	3	3	3	241	26.4	7.2
NP_690869.1	23097254;206597468	**NEDD1**	protein NEDD1 isoform b	1.0	1.0	1.0	1.1	0.9	0.9	0.9	0.9	1.0	0.9	1.0	1.0	15.3	3	6	6	12	660	71.9	8.0
NP_054899.2	21265040	**MRPL22**	39S ribosomal protein L22, mitochondrial isoform a	1.1	1.1	1.0	1.0	1.1	1.0	1.0	1.0	1.1	1.0	1.0	1.0	39.8	2	7	7	42	206	23.6	9.9
NP_054765.1	7662645	**MRPS18B**	28S ribosomal protein S18b, mitochondrial	1.1	1.0	1.0	1.0	1.1	1.0	1.0	1.0	1.1	1.0	1.0	1.0	38.8	1	8	8	22	258	29.4	9.4
NP_057392.1	7706497	**CMPK1**	UMP-CMP kinase isoform a	1.0	0.9	0.8	1.0	1.0	0.9	0.9	1.1	1.0	0.9	0.9	1.0	36.4	2	6	6	22	228	25.8	8.0
NP_000066.1	4502735	**CDK4**	cyclin-dependent kinase 4	0.9	0.9	1.1	1.0	0.9	1.0	1.0	1.0	0.9	1.0	1.0	1.0	22.4	54	4	5	27	303	33.7	7.0

Khan *et al.* , 2019. Multi-omics analysis to characterize cigarette smoke induced molecular alterations in esophageal cells
Supplementary Table 5. List of proteins quantified in untreated and chronically treated Het1A cells with cigarette smoke condensate for 8 months

NP_Accession	Protein group Accession	Gene ID	Description	Het-1A-Smoke - 2M/Parental	Het-1A-Smoke - 4M/Parental	Het-1A-Smoke - 6M/Parental	Het-1A-Smoke - 8M/Parental	Het-1A-Smoke - 2M/Parental	Het-1A-Smoke - 4M/Parental	Het-1A-Smoke - 6M/Parental	Het-1A-Smoke - 8M/Parental	Het-1A-Smoke - 2M/Parental	Het-1A-Smoke - 4M/Parental	Het-1A-Smoke - 6M/Parental	Het-1A-Smoke - 8M/Parental	Coverage (%)	Proteins	Unique Peptides	# Peptides	PSM	# AAs	MW [kDa]	calc. pI
				Replicate 1				Replicate 2				Average of replicates											
NP_954592.1	40288288	CARM1	histone-arginine methyltransferase CARM1	1.0	0.9	1.0	1.1	1.0	0.9	1.0	0.9	1.0	0.9	1.0	1.0	9.7	1	6	6	8	608	65.8	6.7
NP_001136037.1	217035107	CPSF7	cleavage and polyadenylation specificity factor subunit 7 isoform 3	1.0	0.9	1.0	1.1	1.0	1.0	1.0	1.0	1.0	1.0	1.0	1.0	19.3	3	8	8	26	462	51.1	7.8
NP_036525.1	31657129	PFAS	phosphoribosylformylglycinamidine synthase	1.0	0.9	0.9	1.0	1.0	1.0	1.0	1.0	1.0	1.0	1.0	1.0	24.4	1	18	18	45	1338	144.6	5.8
NP_036585.1	7549793	TBL2	transducin beta-like protein 2 precursor	1.3	1.1	1.1	1.1	1.0	1.0	1.0	1.0	1.1	1.0	1.1	1.0	4.0	1	2	2	3	447	49.8	9.4
NP_008986.2	39725938	POLR3A	DNA-directed RNA polymerase III subunit RPC1	1.1	1.1	1.1	1.0	1.0	1.0	1.0	1.0	1.1	1.1	1.1	1.0	5.5	1	5	5	13	1390	155.5	8.5
NP_001245200.1	384871676	MLH1	DNA mismatch repair protein Mlh1 isoform 4	1.0	1.0	1.1	1.0	1.1	1.1	1.0	1.0	1.0	1.0	1.0	1.0	18.1	4	7	7	16	687	76.7	6.2
NP_036232.2	156416028	DNPEP	aspartyl aminopeptidase	1.1	0.9	1.1	1.0	1.1	1.1	1.1	1.1	1.1	1.0	1.1	1.0	25.6	1	7	7	17	485	53.4	7.6
NP_665813.1	22027554	CACNG6	voltage-dependent calcium channel gamma-6 subunit isoform a	1.3	1.2	1.2	1.1	1.0	1.0	1.1	1.0	1.1	1.1	1.1	1.0	2.7	1	1	1	1	260	28.1	9.2
NP_003913.3	126131099	HERC1	probable E3 ubiquitin-protein ligase HERC1	1.1	0.9	0.9	0.9	1.0	1.1	1.0	1.1	1.1	1.0	0.9	1.0	0.6	1	2	2	4	4861	531.9	6.0
NP_079330.2	36029914	VCPIP1	deubiquitinating protein VCIP135	1.1	0.9	0.9	0.8	1.4	1.6	1.4	1.3	1.2	1.2	1.2	1.0	1.3	1	1	1	1	1222	134.2	7.2
NP_001777.1	4502709	CDK1	cyclin-dependent kinase 1 isoform 1	0.9	0.9	1.0	1.0	1.0	1.0	1.1	1.0	0.9	1.0	1.0	1.0	71.7	56	14	17	103	297	34.1	8.4
NP_001007469.1	55956801	SMARCB1	SWI/SNF-related matrix-associated actin-dependent regulator of chromatin subfamily B member 1 isoform b	1.1	1.0	1.1	1.0	1.0	1.1	1.0	1.0	1.1	1.0	1.0	1.0	16.8	2	4	4	13	376	43.1	5.6
NP_000114.2	51988900	ERCC5	DNA repair protein complementing XP-G cells	1.1	0.9	1.0	0.9	1.1	1.1	1.0	1.1	1.1	1.0	1.0	1.0	5.4	2	4	5	5	1186	133.2	5.3
NP_001284637.1	663070987	CAMSAP2	calmodulin-regulated spectrin-associated protein 2 isoform 3	1.0	1.0	1.0	1.0	1.0	1.2	1.2	1.1	1.0	1.1	1.1	1.0	0.5	3	1	1	1	1462	165.0	6.7
NP_036365.1	55743118	RAB3GAP1	rab3 GTPase-activating protein catalytic subunit isoform 2	1.1	1.0	1.1	1.1	1.1	1.0	1.1	1.0	1.1	1.0	1.1	1.0	19.6	2	14	14	35	981	110.5	5.6
NP_066961.2	22027612	TRAF2	TNF receptor-associated factor 2	0.9	1.0	0.9	1.1	0.8	0.9	1.0	0.9	0.9	1.0	0.9	1.0	3.6	1	2	2	3	501	55.8	7.5
NP_060633.1	8922563	DNAJC17	dnaJ homolog subfamily C member 17	1.3	1.0	1.1	1.1	0.8	0.8	0.8	1.0	1.1	0.9	0.9	1.0	4.6	1	1	1	2	304	34.7	8.5
NP_001070957.1	117938765	GNAS	protein GNAS isoform g	1.0	1.0	1.0	1.0	1.0	0.9	1.0	1.0	1.0	0.9	1.0	1.0	36.7	11	9	10	64	379	44.2	6.3
NP_002788.1	4506201	PSMB5	proteasome subunit beta type-5 isoform 1	1.1	1.1	1.0	1.0	1.0	1.1	1.0	1.0	1.0	1.1	1.0	1.0	46.0	3	9	9	33	263	28.5	6.9
NP_631946.1	21327708	NAP1L1	nucleosome assembly protein 1-like 1 isoform 1	0.9	1.0	1.0	1.0	0.9	1.0	1.1	1.0	0.9	1.0	1.0	1.0	57.0	2	14	16	228	391	45.3	4.5
NP_057698.2	23510348	TDP2	tyrosyl-DNA phosphodiesterase 2	1.0	1.0	1.0	1.1	0.9	0.8	0.9	0.9	1.0	0.9	0.9	1.0	11.6	1	3	3	5	362	40.9	5.1
NP_001009814.1	57863273	VWA8	von Willebrand factor A domain-containing protein 8 isoform b precursor	1.1	1.2	1.2	1.2	1.1	0.8	0.9	0.9	1.1	1.0	1.0	1.0	5.5	2	4	4	6	1039	116.9	8.1
NP_004850.1	4758012	CLTC	clathrin heavy chain 1 isoform 1	1.1	1.1	1.1	1.0	1.1	1.0	1.1	1.0	1.1	1.1	1.1	1.0	62.5	4	77	77	485	1675	191.5	5.7
NP_079021.3	190358504	SHCBP1	SHC SH2 domain-binding protein 1	1.1	1.1	1.3	1.0	1.1	1.2	1.4	1.1	1.1	1.2	1.3	1.0	32.6	1	16	16	43	672	75.6	4.8
NP_006535.1	5730037	EXOC5	exocyst complex component 5	1.1	1.0	1.0	1.1	1.0	0.9	1.0	1.0	1.1	1.0	1.0	1.0	12.0	1	8	8	13	708	81.8	6.7
NP_001124164.1	194688149	PPP3CA	serine/threonine-protein phosphatase 2B catalytic subunit alpha isoform isoform 3	0.8	0.9	1.0	1.0	1.1	1.0	1.1	1.0	1.0	1.0	1.0	1.0	21.1	7	2	7	20	469	52.6	6.2
NP_060898.2	49574529	UTP6	U3 small nucleolar RNA-associated protein 6 homolog	1.0	1.0	1.0	1.0	1.0	1.0	1.0	1.0	1.0	1.0	1.0	1.0	13.2	1	6	6	14	597	70.1	7.3
NP_001157412.1	255653002	PYGL	glycogen phosphorylase, liver form isoform 2	1.1	1.0	1.1	1.0	1.1	1.0	1.1	1.0	1.1	1.0	1.1	1.0	45.6	2	28	32	188	813	93.1	7.3
NP_005768.1	5032033	RBM6	RNA-binding protein 6 isoform 1	1.0	1.1	1.0	1.1	0.9	1.0	1.0	1.0	1.0	1.1	1.0	1.0	4.8	2	4	4	5	1123	128.6	6.3
NP_056076.1	45356151	NCAPD3	condensin-2 complex subunit D3	1.0	1.0	1.0	1.0	0.9	1.0	1.2	1.1	1.0	1.0	1.1	1.0	8.5	1	7	7	19	1498	168.8	7.5
NP_003065.3	188536047	SMARCC1	SWI/SNF complex subunit SMARCC1	1.0	1.0	1.0	1.0	0.9	1.0	1.1	1.0	1.0	1.0	1.1	1.0	22.6	1	11	18	39	1105	122.8	5.8
NP_001075.1	4505891	PLOD3	procollagen-lysine,2-oxoglutarate 5-dioxygenase 3 precursor	1.0	1.1	1.1	1.1	1.0	1.0	1.1	1.0	1.0	1.0	1.1	1.0	23.3	1	12	12	29	738	84.7	6.0
NP_006342.2	33636719	TIMM44	mitochondrial import inner membrane translocase subunit TIM44	1.1	1.1	1.0	1.0	1.1	1.0	1.0	1.0	1.1	1.1	1.0	1.0	29.4	1	12	12	27	452	51.3	8.3
NP_078829.1	33438577	CCDC132	coiled-coil domain-containing protein 132 isoform b	1.0	1.0	1.0	1.1	0.8	0.9	0.9	1.0	0.9	1.0	0.9	1.0	9.8	3	2	2	3	327	37.2	6.9
NP_006053.2	154689858	SMYD5	SET and MYND domain-containing protein 5	1.1	1.1	1.0	1.0	1.0	1.0	1.0	1.1	1.0	1.0	1.0	1.0	15.3	1	4	4	9	418	47.3	5.0
NP_899236.1	34577047	DST	dystonin isoform 1	1.0	1.1	1.2	1.0	1.0	1.1	1.3	1.0	1.0	1.1	1.2	1.0	2.1	4	7	9	14	5497	629.4	5.7
NP_037373.2	118572599	FHOD1	FH1/FH2 domain-containing protein 1	0.9	1.0	0.9	1.0	1.0	1.0	0.9	1.0	0.9	1.0	0.9	1.0	13.1	1	11	11	33	1164	126.5	6.4
NP_000584.2	9665248	TAP1	antigen peptide transporter 1 isoform 1	1.0	1.1	1.0	1.1	1.0	1.0	1.0	1.0	1.0	1.0	1.0	1.0	8.8	2	4	4	8	808	87.2	8.0
NP_006380.1	5453832	HYOU1	hypoxia up-regulated protein 1 precursor	1.2	1.3	1.1	1.0	1.2	1.3	1.2	1.0	1.2	1.3	1.2	1.0	58.9	1	44	44	276	999	111.3	5.2
NP_443197.1	24308390	TSEN15	tRNA-splicing endonuclease subunit Sen15 isoform 1	1.3	1.1	1.2	1.1	1.2	1.1	1.2	1.0	1.3	1.1	1.2	1.0	9.4	1	1	1	1	171	18.6	4.6
NP_004721.1	4759034	ETF1	eukaryotic peptide chain release factor subunit 1 isoform 1	1.0	1.1	1.1	1.0	1.0	1.0	1.0	1.0	1.0	1.1	1.1	1.0	56.8	3	19	19	131	437	49.0	5.7
NP_001107803.1	167466160	CLCN7	H(+)/Cl(-) exchange transporter 7 isoform b	1.0	1.0	1.0	1.0	1.2	0.9	1.1	1.0	1.1	1.0	1.0	1.0	4.1	2	2	3	3	781	86.0	8.7
NP_003575.2	205277447	DUSP11	RNA/RNP complex-1-interacting phosphatase	0.9	0.8	0.8	1.0	1.3	1.0	0.9	1.0	1.1	0.9	0.9	1.0	5.0	1	1	1	2	377	43.7	9.3
NP_001288789.1	687170345	LPIN3	phosphatidate phosphatase LPIN3	1.1	1.1	1.2	1.1	1.0	0.9	1.0	1.0	1.1	1.0	1.1	1.0	4.2	1	1	1	4	852	93.6	5.5
NP_006741.1	5803177	SNTB2	beta-2-syntrophin	1.0	0.9	0.9	0.9	1.0	1.1	1.1	1.1	1.0	1.0	1.0	1.0	8.7	1	4	5	10	540	57.9	8.8
NP_031373.2	37675283	AP4E1	AP-4 complex subunit epsilon-1 isoform 1	0.9	0.9	1.0	0.9	1.0	1.1	1.0	1.1	1.0	1.0	1.0	1.0	1.7	2	2	2	3	1137	127.2	6.0
NP_057161.1	7706351	PTRH2	peptidyl-tRNA hydrolase 2, mitochondrial isoform b precursor	1.1	1.0	1.0	1.0	1.0	0.9	1.0	1.0	1.1	1.0	1.0	1.0	40.8	2	5	5	11	179	19.2	8.7
NP_001017408.1	62868213	GOPC	Golgi-associated PDZ and coiled-coil motif-containing protein isoform b	1.0	0.9	1.0	1.0	1.0	0.9	0.9	1.0	1.0	0.9	1.0	1.0	8.8	2	3	3	6	454	49.7	6.1
NP_006822.1	5803029	CLP1	polyribonucleotide 5'-hydroxyl-kinase Clp1 isoform 1	1.0	1.0	1.1	1.1	1.0	0.9	1.0	1.0	1.0	1.0	1.0	1.0	10.8	2	4	4	5	425	47.6	6.6
NP_001193928.1	332801082	CIT	citron Rho-interacting kinase isoform 1	1.0	1.1	1.1	1.1	1.2	1.0	1.0	1.0	1.1	1.0	1.1	1.0	8.2	2	16	16	25	2069	236.5	6.7
NP_003900.1	4503015	CPNE3	copine-3	1.1	1.0	1.1	1.0	1.1	1.1	1.1	1.0	1.1	1.0	1.1	1.0	35.6	10	14	15	47	537	60.1	5.8
NP_001180201.1	300796323	RBBP5	retinoblastoma-binding protein 5 isoform 2	1.1	1.1	1.2	1.1	1.0	1.0	1.1	0.9	1.1	1.1	1.1	1.0	14.2	3	5	5	12	500	55.0	5.0
NP_057118.1	7705805	MRPS2	28S ribosomal protein S2, mitochondrial	1.1	1.0	1.1	1.0	1.0	1.0	1.1	1.0	1.0	1.0	1.1	1.0	8.8	1	3	3	5	296	33.2	9.3
NP_620162.1	20270367	TAMM41	phosphatidate cytidylyltransferase, mitochondrial isoform b precursor	1.0	1.0	1.0	1.0	1.2	1.2	1.2	1.1	1.1	1.1	1.1	1.0	12.3	2	3	3	6	316	35.9	8.7
NP_004729.1	4759302	VAPB	vesicle-associated membrane protein-associated protein B/C isoform 1	1.0	0.9	1.0	1.0	1.0	1.0	1.1	1.1	1.0	1.0	1.0	1.0	56.4	2	11	12	71	243	27.2	7.3
NP_005643.2	429535832	TERF2	telomeric repeat-binding factor 2	0.9	1.0	1.0	1.0	1.0	1.0	1.0	1.0	1.0	1.0	1.0	1.0	27.1	1	10	10	24	542	59.6	9.3
NP_619634.1	20162550	PPP1R14B	protein phosphatase 1 regulatory subunit 14B	1.4	0.9	2.2	1.0	1.2	0.9	1.0	1.0	1.3	0.9	1.6	1.0	22.5	2	2	2	7	147	15.9	4.9
NP_115487.2	55769543	MRPL14	39S ribosomal protein L14, mitochondrial	1.1	1.1	1.1	1.1	0.9	1.0	1.1	1.0	1.0	1.0	1.1	1.0	16.6	1	2	2	5	145	15.9	10.2
NP_000143.2	119393891;119393895	GAA	lysosomal alpha-glucosidase preproprotein	0.9	1.0	1.0	1.0	1.2	1.0	0.9	1.0	1.1	1.0	0.9	1.0	21.0	1	9	9	20	952	105.3	6.0
NP_055144.3	45827771	EDC4	enhancer of mRNA-decapping protein 4	1.0	0.9	0.9	1.0	1.0	1.0	1.0	1.0	1.0	1.0	0.9	1.0	21.1	1	19	19	49	1401	151.6	5.9
NP_006238.1	5453958	PPP5C	serine/threonine-protein phosphatase 5 isoform 1	1.0	1.0	1.0	1.0	1.0	1.0	1.0	1.0	1.0	1.0	1.0	1.0	37.7	5	16	17	84	499	56.8	6.3
NP_057137.1	7705622	CCDC53	WASH complex subunit CCDC53 isoform 1	1.0	1.1	1.0	1.0	1.0	1.0	1.0	1.0	1.0	1.0	1.0	1.0	10.8	2	2	2	3	194	21.2	4.5
NP_002813.3	334724463	TWF1	twinfilin-1 isoform 2	0.9	1.0	1.0	1.0	0.9	1.0	1.0	1.0	0.9	1.0	1.0	1.0	20.6	2	6	6	17	350	40.3	7.0
NP_054886.2	156142178	CCDC59	thyroid transcription factor 1-associated protein 26	0.9	0.9	0.9	1.0	0.9	1.1	1.0	1.1	0.9	1.0	0.9	1.0	3.7	1	1	1	2	241	28.7	9.9
NP_060764.3	93352551	CWF19L1	CWF19-like protein 1 isoform 1	1.0	1.0	1.0	1.1	1.0	0.9	1.0	1.0	1.0	1.0	1.0	1.0	14.5	4	5	5	14	538	60.6	7.2
NP_065726.1	10190716	SPC25	kinetochore protein Spc25	1.0	1.1	1.0	1.0	1.1	1.0	0.9	1.0	1.0	1.0	1.0	1.0	12.5	1	2	2	3	224	26.1	8.0
NP_001028693.1	75709217;75709220	CSTF1	cleavage stimulation factor subunit 1	0.9	0.9	0.8	1.0	1.0	1.0	0.9	1.0	0.9	0.9	0.9	1.0	16.5	1	5	5	14	431	48.3	6.6
NP_001036189.1	112789532	PVRL2	nectin-2 isoform delta precursor	1.0	1.0	1.0	1.0	1.0	1.1	1.0	1.1	1.0	1.1	1.0	1.0	15.2	2	6	6	13	538	57.7	4.8
NP_620706.1	21237808	SMARCC2	SWI/SNF complex subunit SMARCC2 isoform b	1.1	1.1	1.1	1.0	1.1	1.1	1.0	1.0	1.1	1.1	1.0	1.0	20.4	3	10	17	41	1130	124.8	5.6
NP_004153.2	19923262	RAB5A	ras-related protein Rab-5A isoform 1	1.0	1.0	1.0	1.0	1.1	1.0	1.0	1.1	1.0	1.0	1.0	1.0	41.4	3	4	7	36	215	23.6	8.1
NP_001229714.1	338753400	HLCS	biotin--protein ligase	0.9	0.9	0.8	0.9	1.0	1.1	1.0	1.1	0.9	1.0	0.9	1.0	6.8	1	5	5	7	726	80.7	5.6
NP_009139.1	6005862	MRPL3	39S ribosomal protein L3, mitochondrial	1.2	1.1	1.2	1.0	1.2	1.0	1.0	1.0	1.2	1.1	1.1	1.0	37.1	1	11	11	20	348	38.6	9.5
NP_002403.2	197304670	MGMT	methylated-DNA--protein-cysteine methyltransferase	1.1	1.0	1.0	1.0	1.0	1.0	1.0	1.1	1.1	1.0	1.0	1.0	7.6	1	1	1	4	238	25.0	8.7
NP_068758.3	192448443	FKBP10	peptidyl-prolyl cis-trans isomerase FKBP10 precursor	1.0	1.0	1.0	1.0	0.9	1.0	1.0	1.0	1.0	1.0	1.0	1.0	32.5	1	14	14	37	582	64.2	5.6
NP_001231.2	17978466	CCNT1	cyclin-T1 isoform a	1.0	0.9	1.0	1.1	1.4	0.9	1.0	1.0	1.2	0.9	1.0	1.0	6.6	2	3	3	5	726	80.6	8.8
NP_001018062.1	65301135	POLR3H	DNA-directed RNA polymerase III subunit RPC8 isoform b	1.0	0.9	0.9	1.1	1.0	0.8	0.9	1.0	1.0	0.8	0.9	1.0	16.0	2	1	1	2	175	19.6	4.3
NP_003120.2	62865635	SQLE	squalene monooxygenase	1.2	1.3	1.1	1.0	1.3	1.4	1.1	1.1	1.3	1.4	1.1	1.0	25.3	1	11	11	40	574	63.9	8.6
NP_002323.2	126012562	LRP1	prolow-density lipoprotein receptor-related protein 1 precursor	1.1	1.3	1.2	1.2	0.9	1.0	0.8	0.8	1.0	1.1	1.0	1.0	0.2	1	1	1	1	4544	504.3	5.4
NP_002712.1	4506029	PPP6C	serine/threonine-protein phosphatase 6 catalytic subunit isoform b	1.1	1.1	1.0	1.1	1.0	1.1	1.1	1.0	1.1	1.1	1.1	1.0	27.2	3	6	6	12	305	35.1	5.7
NP_036457.1	6912494	MAPRE1	microtubule-associated protein RP/EB family member 1	1.0	0.8	0.9	1.0	1.0	0.9	1.0	1.0	1.0	0.9	0.9	1.0	72.4	1	13	14	101	268	30.0	5.1
NP_078944.2	46519154	ANKHD1	ankyrin repeat and KH domain-containing protein 1 isoform 3	1.1	1.1	1.2	1.0	1.1	1.1	1.0	1.0	1.1	1.1	1.1	1.0	11.0	3	1	5	16	627	64.9	4.5
NP_001255214.1	392996933	LRRC8E	leucine-rich repeat-containing protein 8E isoform 2	0.8	0.9	0.8	1.0	1.2	1.0	1.0	1.0	1.0	0.9	0.9	1.0	5.1	2	2	2	2	667	75.3	7.3
NP_006603.2	40254834	KIF1C	kinesin-like protein KIF1C	0.9	1.1	1.0	1.1	0.9	0.9	0.9	1.0	0.9	1.0	1.0	1.0	7.1	7	3	5	7	1103	122.9	6.9
NP_002558.1	4505621	PEBP1	phosphatidylethanolamine-binding protein 1 preproprotein	1.0	0.9	0.9	1.0	1.0	0.9	0.9	1.0	1.0	0.9	0.9	1.0	74.9	1	9	9	50	187	21.0	7.5
NP_057513.1	7705983	COPZ2	coatomer subunit zeta-2	1.3	1.1	1.1	1.1	1.2	1.1	1.1	0.9	1.3	1.1	1.1	1.0	9.1	1	1	1	2	210	23.5	5.2
NP_001138827.1	223941776	CBWD1	COBW domain-containing protein 1 isoform 2	1.0	0.9	1.1	1.0	1.0	0.9	0.9	1.0	1.0	0.9	1.0	1.0	12.3	11	3	3	4	359	40.4	5.6

Khan *et al.* , 2019. Multi-omics analysis to characterize cigarette smoke induced molecular alterations in esophageal cells
Supplementary Table 5. List of proteins quantified in untreated and chronically treated Het1A cells with cigarette smoke condensate for 8 months

NP_Accession	Protein group Accession	Gene ID	Description	Het-1A-Smoke - 2M/Parental	Het-1A-Smoke - 4M/Parental	Het-1A-Smoke - 6M/Parental	Het-1A-Smoke - 8M/Parental	Het-1A-Smoke - 2M/Parental	Het-1A-Smoke - 4M/Parental	Het-1A-Smoke - 6M/Parental	Het-1A-Smoke - 8M/Parental	Het-1A-Smoke - 2M/Parental	Het-1A-Smoke - 4M/Parental	Het-1A-Smoke - 6M/Parental	Het-1A-Smoke - 8M/Parental	Coverage (%)	Proteins	Unique Peptides	# Peptides	PSM	# AAs	MW [kDa]	calc. pI
				Replicate 1				Replicate 2				Average of replicates											
NP_112553.1	14165437	HNRNPK	heterogeneous nuclear ribonucleoprotein K isoform a	0.9	0.9	0.9	1.0	0.9	0.9	0.9	1.0	0.9	0.9	0.9	1.0	66.8	2	26	26	499	464	51.0	5.3
NP_945314.1	39777586	DHX57	putative ATP-dependent RNA helicase DHX57	1.0	1.1	1.0	1.1	1.0	1.0	1.0	1.0	1.0	1.1	1.0	1.0	5.3	1	5	6	7	1386	155.5	7.7
NP_787072.2	44921615	EXOC8	exocyst complex component 8	1.1	1.0	1.1	1.0	1.1	1.1	1.1	1.0	1.1	1.0	1.1	1.0	9.1	1	4	4	7	725	81.7	5.5
NP_000509.1	4504349	HBB	hemoglobin subunit beta	0.9	0.9	1.0	1.1	0.3	0.5	0.3	1.0	0.6	0.7	0.6	1.0	15.7	2	1	2	16	147	16.0	7.3
NP_057439.2	20149629	DDX47	probable ATP-dependent RNA helicase DDX47 isoform 1	1.0	1.0	1.1	1.0	1.1	1.1	1.2	1.0	1.1	1.1	1.2	1.0	29.5	2	10	10	29	455	50.6	9.1
NP_002065.1	11321585;54317 3169	GNB1	guanine nucleotide-binding protein G(I)/G(S)/G(T) subunit beta-1 isoform 1	1.0	1.1	1.0	1.1	0.9	0.9	0.9	1.0	1.0	1.0	1.0	1.0	36.8	4	5	9	50	340	37.4	6.0
NP_001231293.1	346986273	ARHGEF28	rho guanine nucleotide exchange factor 28 isoform 3	1.0	1.0	1.0	1.1	1.2	1.3	1.4	0.9	1.1	1.2	1.2	1.0	2.1	3	2	2	4	1392	157.2	6.4
NP_001263236.1	443929385	NOL3	nucleolar protein 3 isoform MYP	1.2	1.2	1.3	1.2	1.0	1.1	1.2	0.9	1.1	1.2	1.3	1.0	47.1	4	4	4	8	208	22.6	4.2
NP_002737.2	91718899	MAPK3	mitogen-activated protein kinase 3 isoform 1	1.0	1.0	0.9	1.0	1.0	1.1	1.1	1.0	1.0	1.0	1.0	1.0	40.9	7	7	12	33	379	43.1	6.7
NP_057195.2	225545550	TELO2	telomere length regulation protein TEL2 homolog	1.0	1.0	1.1	1.0	1.0	1.0	1.1	1.0	1.0	1.0	1.1	1.0	13.0	1	6	6	14	837	91.7	5.8
NP_000358.1	4507653	TPMT	thiopurine S-methyltransferase	1.0	0.9	1.0	0.9	1.0	0.8	0.9	1.1	1.0	0.9	0.9	1.0	9.4	1	2	2	2	245	28.2	6.2
NP_036546.2	19923790	RAB3GAP2	rab3 GTPase-activating protein non-catalytic subunit	1.0	1.0	1.0	1.0	1.1	1.0	1.0	1.0	1.0	1.0	1.0	1.0	15.4	1	15	15	29	1393	155.9	5.6
NP_059516.2	89276787	ADD2	beta-adducin isoform b	1.1	1.2	1.0	1.2	0.9	0.8	0.8	0.8	1.0	1.0	0.9	1.0	4.5	4	1	1	1	559	62.4	6.5
NP_001921.1	4503325	DHPS	deoxyhypusine synthase isoform a	1.0	1.0	0.8	0.8	1.1	1.1	1.1	1.2	1.1	1.0	1.0	1.0	8.4	3	2	2	4	369	40.9	5.4
NP_001243692.1	377520143	FAM49B	protein FAM49B	0.9	1.0	1.0	1.0	1.0	1.0	1.0	1.0	1.0	1.0	1.0	1.0	66.4	2	16	16	92	324	36.7	6.1
NP_076436.3	218083142;2180 83215	CHID1	chitinase domain-containing protein 1 isoform a precursor	1.1	1.2	1.1	1.0	1.0	1.0	1.0	1.0	1.0	1.1	1.0	1.0	14.5	3	4	4	4	393	44.9	8.6
NP_001264335.1	475808427	POTEI	POTE ankyrin domain family member I	1.2	1.1	1.0	1.0	1.0	1.0	1.1	1.0	1.1	1.1	1.0	1.0	12.9	11	3	11	276	1075	121.2	6.2
NP_004628.4	34147513	RAB7A	ras-related protein Rab-7a	1.0	1.0	1.1	1.0	1.0	1.1	1.0	1.0	1.0	1.0	1.0	1.0	69.6	1	12	12	89	207	23.5	6.7
NP_002697.1	4505995	PPM1B	protein phosphatase 1B isoform 1	0.9	0.9	0.9	1.0	1.0	0.9	1.1	1.0	0.9	0.9	1.0	1.0	23.6	5	7	10	28	479	52.6	5.0
NP_001019389.1	66932975	GPHN	gephyrin isoform 2	0.9	0.9	1.0	1.0	1.0	0.9	0.9	1.0	1.0	0.9	1.0	1.0	15.8	2	8	8	18	736	79.7	5.4
NP_061913.3	91208435	ELP4	elongator complex protein 4 isoform 1	1.1	1.2	1.1	1.1	0.9	0.9	0.9	0.9	1.0	1.0	1.0	1.0	11.8	3	3	3	7	424	46.6	8.5
NP_001041664.1	114796642	RCC1	regulator of chromosome condensation isoform c	1.0	1.1	1.1	1.0	1.0	0.9	1.0	1.0	1.0	1.0	1.0	1.0	39.4	3	11	11	61	421	44.9	7.5
NP_006747.1	5803191	TCEA1	transcription elongation factor A protein 1 isoform 1	1.0	1.0	0.9	1.0	1.0	1.0	0.9	1.0	1.0	1.0	0.9	1.0	22.3	4	7	7	23	301	33.9	8.4
NP_002783.1	4506189	PSMA7	proteasome subunit alpha type-7	1.0	1.0	1.0	1.0	1.0	1.0	1.0	1.0	1.0	1.0	1.0	1.0	64.9	4	13	13	84	248	27.9	8.5
NP_001032642.1	83267874	AGPAT3	1-acyl-sn-glycerol-3-phosphate acyltransferase gamma	0.9	1.0	1.0	1.0	1.0	1.0	1.0	1.1	1.0	1.0	1.0	1.0	5.6	1	2	2	2	376	43.4	8.7
NP_001265281.1	507834063;8659 555	ACO1	cytoplasmic aconitate hydratase	1.1	1.0	1.0	1.0	1.1	1.0	1.1	1.0	1.1	1.0	1.1	1.0	28.9	1	18	18	71	889	98.3	6.7
NP_002563.1	4505585	PAFAH1B2	platelet-activating factor acetylhydrolase IB subunit beta isoform a	1.3	1.0	1.3	1.0	1.3	1.0	1.2	1.0	1.3	1.0	1.3	1.0	48.0	4	5	5	13	229	25.6	5.9
NP_066283.1	10337595	PPM1A	protein phosphatase 1A isoform 1	0.8	0.8	0.8	1.0	1.0	0.9	1.0	1.1	0.9	0.9	0.9	1.0	16.2	3	3	5	8	382	42.4	5.4
NP_842565.2	112382252	SPTBN1	spectrin beta chain, non-erythrocytic 1 isoform 2	1.0	1.0	1.0	1.1	1.0	0.9	0.9	1.0	1.0	1.0	1.0	1.0	53.3	4	2	91	369	2155	251.2	5.5
NP_009228.2	237681121	BRCA1	breast cancer type 1 susceptibility protein isoform 3	1.1	1.1	1.0	1.0	1.0	1.1	0.9	1.0	1.0	1.1	1.0	1.0	4.0	5	3	3	5	1816	202.2	5.4
NP_001245237.1	385198059	NOP2	probable 28S rRNA (cytosine(4447)-C(5))-methyltransferase isoform 2	1.0	1.1	1.0	1.0	0.9	1.1	1.1	1.0	1.0	1.1	1.1	1.0	26.5	2	1	15	38	812	89.2	9.2
NP_001162023.1	271398379	NDC1	nucleoporin NDC1 isoform 2	1.0	0.9	1.0	1.0	1.0	1.0	1.0	1.0	1.0	0.9	1.0	1.0	8.5	2	4	4	7	634	71.9	9.1
NP_937887.1	38201627	EIF4G1	eukaryotic translation initiation factor 4 gamma 1 isoform 2	1.1	1.1	1.1	1.0	1.0	1.1	1.1	1.0	1.0	1.1	1.1	1.0	35.9	7	39	44	189	1512	166.5	5.2
NP_065994.1	55749769	CWC22	pre-mRNA-splicing factor CWC22 homolog	1.1	0.9	0.9	1.0	1.0	1.0	1.0	1.0	1.1	0.9	1.0	1.0	7.5	1	3	3	5	908	105.4	7.0
NP_009049.2	45439359	TRIO	triple functional domain protein	1.0	1.1	1.2	1.0	1.1	1.2	1.5	1.1	1.0	1.2	1.3	1.0	2.4	1	5	5	9	3097	346.7	6.4
NP_003788.2	24041020	EED	polycomb protein EED isoform a	0.9	0.9	1.0	1.0	1.0	1.0	1.1	1.0	1.0	1.0	1.0	1.0	19.5	3	8	8	16	441	50.2	7.0
NP_057231.1	7706645	PPME1	protein phosphatase methylesterase 1 isoform a	1.0	0.9	1.0	1.0	1.2	1.0	1.0	1.1	1.1	1.0	1.0	1.0	43.3	2	15	15	58	386	42.3	6.0
NP_001158884.1	259155302	NFKB1	nuclear factor NF-kappa-B p105 subunit isoform 2	1.1	1.1	1.1	1.1	0.9	0.8	1.0	0.9	1.0	1.0	1.1	1.0	4.1	2	2	2	3	968	105.3	5.4
NP_003889.1	26190608	SYNJ2	synaptojanin-2 isoform 1	1.0	1.1	1.1	0.9	1.1	1.2	1.1	1.1	1.1	1.2	1.1	1.0	3.7	2	4	4	9	1496	165.4	7.3
NP_001265392.1	510937022	DNM1L	dynamin-1-like protein isoform 4	1.1	1.0	1.1	1.0	1.1	1.0	1.2	1.0	1.1	1.0	1.1	1.0	33.0	7	17	17	45	725	80.5	6.7
NP_000681.2	25777732	ALDH2	aldehyde dehydrogenase, mitochondrial isoform 1 precursor	1.2	1.2	1.2	1.0	1.2	1.2	1.2	1.0	1.2	1.2	1.2	1.0	48.0	3	15	17	71	517	56.3	7.0
NP_775907.4	145580610	WDR62	WD repeat-containing protein 62 isoform 2	1.1	1.0	1.0	0.9	1.1	1.1	1.1	1.1	1.1	1.0	1.0	1.0	3.8	2	4	4	10	1518	165.8	5.9
NP_056202.2	41349449	MOB4	MOB-like protein phocein isoform 1	1.0	1.0	1.0	1.0	1.0	0.9	0.9	1.0	1.0	0.9	1.0	1.0	35.6	3	5	5	19	225	26.0	5.8
NP_001070863.1	117190324	DPH5	diphthine synthase isoform b	1.2	0.9	0.9	1.0	1.1	0.9	0.9	1.1	1.1	0.9	0.9	1.0	14.1	2	2	2	6	284	31.6	5.3
NP_054733.2	40217847	SNRNP200	U5 small nuclear ribonucleoprotein 200 kDa helicase	1.0	1.0	1.0	1.0	1.0	1.0	1.0	1.0	1.0	1.0	1.0	1.0	35.5	1	56	56	190	2136	244.4	6.1
NP_057487.2	116812573	CWC15	spliceosome-associated protein CWC15 homolog	1.0	1.0	0.9	1.0	1.1	1.0	1.0	1.0	1.0	1.0	1.0	1.0	8.3	1	3	3	3	229	26.6	5.7
NP_001188357.1	319803020	GORASP2	Golgi reassembly-stacking protein 2 isoform 2	1.0	1.0	1.0	1.0	1.1	1.0	1.0	1.0	1.1	1.0	1.0	1.0	19.0	2	5	5	13	384	39.7	4.6
NP_077005.2	56243583	WDR18	WD repeat-containing protein 18	1.1	1.0	1.0	1.0	1.1	1.0	1.0	1.0	1.1	1.0	1.0	1.0	6.7	1	3	3	8	432	47.4	6.7
NP_060656.2	40254931	C1orf112	uncharacterized protein C1orf112	0.8	1.1	1.0	1.0	0.7	0.9	0.7	1.0	0.8	1.0	0.9	1.0	2.2	1	1	1	2	853	96.5	6.0
NP_076869.1	13124770	VKORC1	vitamin K epoxide reductase complex subunit 1 isoform 1 precursor	0.9	1.0	1.0	1.0	1.0	1.1	1.2	1.0	1.0	1.0	1.1	1.0	12.3	2	2	2	2	163	18.2	9.4
NP_997636.1	47132581;51002 5468	PRKAR1A	cAMP-dependent protein kinase type I-alpha regulatory subunit isoform a	1.0	1.0	1.0	1.0	0.9	1.0	1.0	1.0	1.0	1.0	1.0	1.0	34.7	2	12	12	25	381	43.0	5.4
NP_060979.2	40254924	LRRC59	leucine-rich repeat-containing protein 59	0.9	0.9	0.9	1.0	0.9	0.9	0.9	1.0	0.9	0.9	0.9	1.0	52.1	1	13	13	104	307	34.9	9.6
NP_001273063.1	554790401	RIC8A	synembryn-A isoform 2	1.0	1.0	1.0	1.0	1.3	1.1	1.2	1.1	1.1	1.1	1.1	1.0	16.2	2	6	8	12	531	59.7	5.3
NP_005782.1	31317305	MPHOSPH10	U3 small nucleolar ribonucleoprotein protein MPP10	1.0	0.9	0.9	1.0	1.0	1.0	0.9	1.0	1.0	1.0	0.9	1.0	21.4	2	10	10	35	681	78.8	4.9
NP_001019979.1	67190736	BCL7A	B-cell CLL/lymphoma 7 protein family member A isoform b	0.9	0.8	1.0	1.3	0.9	0.8	0.8	0.8	0.9	0.8	0.9	1.0	18.1	3	1	2	3	210	22.8	5.1
NP_004278.2	16905522	GOSR2	Golgi SNAP receptor complex member 2 isoform A	1.0	1.3	1.1	1.0	1.0	1.0	1.0	1.0	1.0	1.2	1.0	1.0	17.0	3	2	2	6	212	24.8	8.1
NP_570710.1	18765731	SNAP23	synaptosomal-associated protein 23 isoform SNAP23B	0.9	0.8	0.9	1.0	1.0	0.9	0.9	1.0	0.9	0.8	0.9	1.0	8.9	2	1	1	4	158	17.8	4.7
NP_002202.2	19743813;19743 823	ITGB1	integrin beta-1 isoform 1A precursor	1.0	1.1	1.3	1.0	0.9	1.0	1.1	1.0	0.9	1.1	1.2	1.0	29.0	2	17	17	65	798	88.4	5.4
NP_002018.1	4503771	FNTA	protein farnesyltransferase/geranylgeranyltransferase type-1 subunit alpha	1.1	1.0	1.2	1.0	1.1	1.0	1.2	1.0	1.1	1.0	1.2	1.0	17.2	1	5	5	25	379	44.4	5.1
NP_065806.1	24308213	SERINC1	serine incorporator 1 precursor	1.0	1.0	0.9	1.0	1.0	0.9	1.0	1.0	1.0	0.9	1.0	1.0	3.8	1	1	1	4	453	50.5	5.8
NP_001252.1	4502747	CDK9	cyclin-dependent kinase 9	1.2	1.0	1.2	1.0	1.0	1.0	1.0	1.0	1.1	1.0	1.1	1.0	8.9	54	2	3	4	372	42.8	8.8
NP_004389.2	13514831	DDX10	probable ATP-dependent RNA helicase DDX10	1.1	1.2	1.1	1.0	1.2	1.1	1.1	1.0	1.1	1.1	1.1	1.0	16.7	1	9	9	22	875	100.8	8.6
NP_057632.2	29550838	GOLM1	Golgi membrane protein 1	0.9	1.1	1.0	1.0	1.1	1.2	1.1	1.0	1.0	1.1	1.1	1.0	19.5	1	7	7	14	401	45.3	5.0
NP_477352.3	348041302	PI4KA	phosphatidylinositol 4-kinase alpha	1.1	1.1	1.1	1.1	1.0	1.0	1.1	1.0	1.0	1.0	1.1	1.0	3.7	2	5	5	12	2102	236.7	7.1
NP_115597.3	29244924	CHD6	chromodomain-helicase-DNA-binding protein 6	0.9	0.9	0.9	0.9	1.0	1.0	1.0	1.1	1.0	1.0	1.0	1.0	3.7	1	7	8	15	2715	305.2	6.3
NP_000158.1	4504007	GK	glycerol kinase isoform b	1.0	1.2	0.9	1.2	1.0	0.8	0.8	0.8	1.0	1.0	0.8	1.0	2.5	4	1	1	2	524	57.5	6.3
NP_003742.2	33239445	EIF3B	eukaryotic translation initiation factor 3 subunit B	1.0	1.1	1.1	1.0	1.1	1.1	1.1	1.0	1.0	1.1	1.1	1.0	52.2	1	28	28	150	814	92.4	5.0
NP_001258798.1	428977040	SZRD1	SUZ domain-containing protein 1 isoform 3	1.0	1.1	0.9	1.0	1.0	1.0	0.9	1.0	1.0	1.0	0.9	1.0	8.0	2	1	1	3	151	16.9	9.0
NP_060962.2	18490991	PBK	lymphokine-activated killer T-cell-originated protein kinase isoform 1	0.9	1.0	1.0	1.0	0.9	1.0	1.0	1.0	0.9	1.0	1.0	1.0	21.4	2	6	6	15	322	36.1	5.1
NP_001317.1	4557495	CSTF3	cleavage stimulation factor subunit 3 isoform 1	1.0	0.9	0.9	1.0	1.0	1.0	0.9	1.0	1.0	1.0	0.9	1.0	21.6	3	12	12	29	717	82.9	8.1
NP_006504.2	16306548	SARS	serine--tRNA ligase, cytoplasmic	1.2	1.3	1.0	1.0	1.1	1.2	1.0	1.0	1.1	1.2	1.0	1.0	41.6	1	19	19	91	514	58.7	6.4
NP_005902.1	5174529	MAT2A	S-adenosylmethionine synthase isoform type-2	1.0	0.9	1.0	1.0	1.1	1.0	1.0	1.0	1.0	1.0	1.0	1.0	38.0	1	13	13	59	395	43.6	6.5
NP_001007226.1	56118219	IGF2BP2	insulin-like growth factor 2 mRNA-binding protein 2 isoform b	1.0	1.2	1.2	1.0	1.1	1.2	1.1	1.0	1.0	1.2	1.2	1.0	37.1	9	16	17	63	556	61.8	8.3
NP_055816.2	14790190	SPEN	msx2-interacting protein	1.1	1.0	0.9	1.0	1.1	1.1	1.0	1.1	1.1	1.1	1.0	1.0	3.9	1	9	10	15	3664	402.0	7.6
NP_001279.2	14251209	CLIC1	chloride intracellular channel protein 1	1.0	1.0	1.0	1.0	1.0	1.0	1.1	1.0	1.0	1.0	1.1	1.0	67.2	2	12	13	106	241	26.9	5.2
NP_065116.3	264681563	XPNPEP1	xaa-Pro aminopeptidase 1 isoform 1	1.0	1.0	1.1	1.0	1.0	1.1	1.1	1.0	1.0	1.0	1.1	1.0	20.6	2	10	10	26	666	74.8	5.9
NP_001240752.1	359465572;3358 9854	BLVRA	biliverdin reductase A precursor	1.1	1.0	1.0	1.0	1.0	1.0	1.0	1.0	1.0	1.0	1.0	1.0	31.8	1	7	7	34	296	33.4	6.4
NP_001026794.1	72255571	ZNF451	zinc finger protein 451 isoform 1	1.1	1.0	1.0	1.0	1.0	1.0	1.0	1.0	1.1	1.0	1.0	1.0	9.1	2	9	9	19	1061	121.4	6.8
NP_001265178.1	503775288	RNF214	RING finger protein 214 isoform 2	0.9	1.0	0.9	1.0	1.1	1.1	0.9	1.0	1.0	1.0	0.9	1.0	2.2	2	1	2	2	548	61.3	8.1
NP_054884.1	7661786	NDUFAF4	NADH dehydrogenase [ubiquinone] 1 alpha subcomplex assembly factor 4	1.0	0.9	1.0	1.0	1.0	0.9	0.9	1.0	1.0	0.9	1.0	1.0	20.0	1	4	4	10	175	20.3	8.8
NP_714916.3	153218487	KDELC2	KDEL motif-containing protein 2 precursor	1.0	1.1	1.1	1.0	1.2	1.2	1.1	1.1	1.1	1.1	1.1	1.0	13.8	1	5	5	9	507	58.5	8.2
NP_055693.4	109948283	KIAA0020	pumilio domain-containing protein KIAA0020	0.9	1.0	0.9	1.0	1.1	1.1	1.0	1.1	1.0	1.0	1.0	1.0	10.5	1	5	5	14	648	73.5	9.6
NP_001128715.1	207113160	TCOF1	treacle protein isoform d	0.9	1.0	1.0	1.0	0.8	1.0	0.9	1.0	0.9	1.0	1.0	1.0	28.2	3	3	41	136	1488	152.0	9.0
NP_006286.1	5454158	VARS	valine--tRNA ligase	1.0	1.0	0.9	1.0	1.0	1.0	0.9	1.0	1.0	1.0	0.9	1.0	21.8	1	19	19	71	1264	140.4	7.6
NP_001041675.1	115270970	CLCC1	chloride channel CLIC-like protein 1 isoform 1 precursor	1.1	1.1	1.1	1.1	1.0	1.0	0.9	0.9	1.1	1.1	1.0	1.0	19.1	4	9	9	15	551	62.0	5.6
NP_536724.1	17998700	PIGU	phosphatidylinositol glycan anchor biosynthesis class U protein precursor	0.9	0.9	0.9	0.9	1.0	1.0	0.9	1.1	0.9	0.9	0.9	1.0	2.3	1	1	1	2	435	50.0	7.7

Khan *et al.* , 2019. Multi-omics analysis to characterize cigarette smoke induced molecular alterations in esophageal cells
Supplementary Table 5. List of proteins quantified in untreated and chronically treated Het1A cells with cigarette smoke condensate for 8 months

NP_Accession	Protein group Accession	Gene ID	Description	Het-1A-Smoke - 2M/Parental	Het-1A-Smoke - 4M/Parental	Het-1A-Smoke - 6M/Parental	Het-1A-Smoke - 8M/Parental	Het-1A-Smoke - 2M/Parental	Het-1A-Smoke - 4M/Parental	Het-1A-Smoke - 6M/Parental	Het-1A-Smoke - 8M/Parental	Het-1A-Smoke - 2M/Parental	Het-1A-Smoke - 4M/Parental	Het-1A-Smoke - 6M/Parental	Het-1A-Smoke - 8M/Parental	Coverage (%)	Proteins	Unique Peptides	# Peptides	PSM	# AAs	MW [kDa]	calc. pI
				Replicate 1				Replicate 2				Average of replicates											
NP_073153.1	12056971	ANAPC1	anaphase-promoting complex subunit 1	1.0	1.2	1.2	1.0	1.0	0.9	1.0	1.0	1.0	1.1	1.1	1.0	2.4	3	4	4	5	1944	216.4	6.3
NP_001837.2	116256354	COL4A2	collagen alpha-2(IV) chain preproprotein	1.3	1.0	0.9	1.0	0.8	1.0	0.8	1.0	1.0	1.0	0.8	1.0	0.8	1	1	1	1	1712	167.4	8.7
NP_000150.1	4503943	GCDH	glutaryl-CoA dehydrogenase, mitochondrial isoform a precursor	1.0	1.0	1.0	1.0	1.1	1.1	1.1	1.0	1.1	1.1	1.1	1.0	30.6	2	8	8	21	438	48.1	8.1
NP_588611.2	94536805	YTHDC1	YTH domain-containing protein 1 isoform 2	1.0	1.0	1.0	1.0	1.0	1.0	1.0	1.0	1.0	1.0	1.0	1.0	8.6	2	4	4	7	709	82.6	6.0
NP_056004.1	149363656	EXOC6B	exocyst complex component 6B	1.0	0.9	0.9	1.0	1.0	0.9	1.0	1.0	1.0	0.9	0.9	1.0	3.7	1	2	3	3	811	94.1	6.5
NP_001253989.1	388596707	SNX17	sorting nexin-17 isoform 3	1.0	1.0	1.0	1.0	1.0	1.0	1.0	1.1	1.0	1.0	1.0	1.0	15.7	4	4	4	11	445	50.0	6.9
NP_057212.1	11559929	COPG1	coatomer subunit gamma-1	1.1	1.2	1.2	1.0	1.2	1.1	1.1	1.0	1.2	1.1	1.2	1.0	51.1	1	27	30	119	874	97.7	5.5
NP_001128567.1	205360934;205360932	FNDC3B	fibronectin type III domain-containing protein 3B	1.2	1.1	1.0	1.0	1.1	1.1	1.2	1.1	1.2	1.1	1.1	1.0	7.0	1	5	5	8	1204	132.8	5.9
NP_004026.2	30089972	ACOX1	peroxisomal acyl-coenzyme A oxidase 1 isoform a	1.0	1.0	0.9	1.0	1.0	1.0	1.0	1.0	1.0	1.0	0.9	1.0	15.5	3	5	5	15	660	74.6	7.6
NP_858058.1	32307148	OGT	UDP-N-acetylglucosamine--peptide N-acetylglucosaminyltransferase 110 kDa subunit isoform 1	1.0	1.1	1.1	1.0	1.1	1.1	1.1	1.0	1.1	1.1	1.1	1.0	8.4	2	7	7	11	1046	116.8	6.7
NP_001202.4	59814247	BUB1B	mitotic checkpoint serine/threonine-protein kinase BUB1 beta	1.2	1.1	1.1	1.0	0.9	0.9	0.9	1.0	1.1	1.0	1.0	1.0	2.1	1	2	2	5	1050	119.4	5.2
NP_001181988.1	303519091	PLEKHO2	pleckstrin homology domain-containing family O member 2 isoform 2	1.0	1.0	0.9	1.0	1.0	1.0	1.0	1.0	1.0	1.0	0.9	1.0	13.0	2	3	3	8	440	47.8	5.3
NP_004556.1	4758896	PEX14	peroxisomal membrane protein PEX14	0.8	0.7	0.7	0.8	1.5	1.2	1.5	1.2	1.2	1.0	1.1	1.0	19.1	1	5	5	8	377	41.2	4.9
NP_001275706.1	571026676	SLC39A7	zinc transporter SLC39A7 isoform 2	1.1	1.1	1.0	1.0	1.1	1.1	1.1	1.0	1.1	1.1	1.1	1.0	6.1	2	3	3	3	344	36.3	7.8
NP_001002926.1	50962817	TWISTNB	DNA-directed RNA polymerase I subunit RPA43	0.9	1.0	1.0	1.0	1.0	0.9	0.9	1.0	0.9	0.9	1.0	1.0	6.2	1	2	2	3	338	37.4	7.0
NP_001166175.1	289577111	GGA3	ADP-ribosylation factor-binding protein GGA3 isoform 2	0.9	1.1	1.0	1.0	1.2	1.0	0.9	1.0	1.1	1.0	1.0	1.0	13.3	5	3	3	5	592	62.9	5.9
NP_057365.3	148746218	TAOK3	serine/threonine-protein kinase TAO3	1.0	0.9	1.0	1.0	1.0	1.1	1.0	1.0	1.0	1.0	1.0	1.0	8.8	1	5	7	13	898	105.3	7.3
NP_055506.1	38788372	AQR	intron-binding protein aquarius	1.2	1.0	1.0	1.1	1.0	1.0	1.0	1.0	1.1	1.0	1.0	1.0	8.6	1	11	11	20	1485	171.2	6.4
NP_037520.1	7019503	PREB	prolactin regulatory element-binding protein	1.0	0.9	1.0	1.0	1.0	1.0	0.9	1.1	1.0	0.9	0.9	1.0	31.7	1	8	8	18	417	45.4	7.9
NP_114414.2	54873624	EIF2A	eukaryotic translation initiation factor 2A	1.1	1.1	1.1	1.0	1.1	1.1	1.1	1.0	1.1	1.1	1.1	1.0	42.7	1	20	20	71	585	64.9	8.9
NP_001185727.1	311771708;311771712	ASCC1	activating signal cointegrator 1 complex subunit 1 isoform b	1.1	0.9	1.0	1.0	1.1	0.8	0.9	1.0	1.1	0.9	1.0	1.0	17.9	2	4	4	6	357	41.2	5.5
NP_060102.1	8923040	CDKN2AIP	CDKN2A-interacting protein	1.2	1.0	1.1	1.1	1.0	1.0	1.0	0.9	1.1	1.0	1.0	1.0	10.5	1	4	4	8	580	61.1	9.0
NP_001243115.1	371874602	SNX12	sorting nexin-12 isoform 2	1.2	1.1	1.1	1.0	1.1	1.2	1.1	1.0	1.1	1.1	1.1	1.0	8.7	4	1	1	1	92	10.6	5.5
NP_057190.2	33469966	SCFD1	sec1 family domain-containing protein 1 isoform a	1.2	1.1	1.1	1.0	1.2	1.1	1.1	1.0	1.2	1.1	1.1	1.0	29.0	5	13	13	34	642	72.3	6.3
NP_079272.4	18390331	GFM1	elongation factor G, mitochondrial isoform 2	1.1	1.1	1.1	1.0	1.1	1.1	1.1	1.0	1.1	1.1	1.1	1.0	19.3	3	11	11	29	751	83.4	7.0
NP_001646.2	11863154	ARCN1	coatomer subunit delta isoform 1	1.2	1.0	1.2	1.0	1.2	1.1	1.1	1.0	1.2	1.0	1.2	1.0	37.0	2	19	19	65	511	57.2	6.2
NP_001258855.1	429484478	BCKDK	[3-methyl-2-oxobutanoate dehydrogenase [lipoamide]] kinase, mitochondrial isoform c	0.9	0.9	0.9	1.0	1.1	1.0	1.1	1.0	1.0	1.0	1.0	1.0	11.9	3	3	3	4	335	37.7	9.3
NP_002717.3	41349456	PREP	prolyl endopeptidase	1.0	0.9	1.0	1.0	1.0	0.9	1.0	1.0	1.0	0.9	1.0	1.0	47.2	1	26	26	113	710	80.6	5.9
NP_009038.1	6005884	SSR3	translocon-associated protein subunit gamma	1.1	0.9	0.9	1.0	1.1	1.0	1.0	1.0	1.1	1.0	1.0	1.0	7.6	1	1	1	7	185	21.1	9.6
NP_076983.1	13129068	NOC4L	nucleolar complex protein 4 homolog	1.2	1.1	1.2	1.1	0.9	0.9	0.9	0.9	1.0	1.0	1.0	1.0	16.1	1	4	4	12	516	58.4	7.5
NP_116262.2	24497612	UBASH3B	ubiquitin-associated and SH3 domain-containing protein B	1.1	1.1	1.2	1.0	1.1	1.1	1.1	1.0	1.1	1.1	1.2	1.0	1.5	1	1	1	1	649	72.6	6.9
NP_009160.2	148747351	PACSIN2	protein kinase C and casein kinase substrate in neurons protein 2 isoform A	1.0	1.0	1.0	1.0	1.0	1.0	1.0	1.0	1.0	1.0	1.0	1.0	17.7	3	9	9	24	486	55.7	5.2
NP_055859.1	9558751	GGA2	ADP-ribosylation factor-binding protein GGA2	1.0	0.9	0.9	1.0	1.0	1.0	1.0	1.0	1.0	0.9	1.0	1.0	3.4	1	2	2	4	613	67.1	6.6
NP_002941.1	4506675	RPN1	dolichyl-diphosphooligosaccharide--protein glycosyltransferase subunit 1 precursor	1.1	1.1	1.1	1.0	1.1	1.1	1.0	1.0	1.1	1.1	1.0	1.0	46.1	1	21	21	151	607	68.5	6.4
NP_037417.1	7019419	GNL2	nucleolar GTP-binding protein 2	0.9	1.0	0.9	1.0	0.9	1.0	0.9	1.1	0.9	1.0	0.9	1.0	17.2	1	12	12	25	731	83.6	9.2
NP_056329.1	111038133	NGDN	neuroguidin isoform 2	0.9	1.0	1.0	1.0	1.0	1.0	1.0	1.0	0.9	1.0	1.0	1.0	18.7	2	5	5	8	311	35.2	9.3
NP_955781.2	296923776	UQCC1	ubiquinol-cytochrome-c reductase complex assembly factor 1 isoform b	1.0	0.8	1.0	1.0	1.0	0.9	1.0	1.0	1.0	0.9	1.0	1.0	13.9	3	4	4	7	273	31.5	9.0
NP_060414.3	71774197	USP47	ubiquitin carboxyl-terminal hydrolase 47 isoform b	1.1	1.1	1.1	1.0	1.0	1.0	1.1	1.0	1.0	1.1	1.1	1.0	5.1	2	5	5	12	1287	147.1	5.1
NP_004757.1	4758032	COPB2	coatomer subunit beta'	1.2	1.2	1.2	1.0	1.2	1.1	1.1	1.0	1.2	1.2	1.1	1.0	43.6	1	32	32	115	906	102.4	5.3
NP_001349.2	68509926	DHX15	pre-mRNA-splicing factor ATP-dependent RNA helicase DHX15	1.0	1.0	1.1	1.0	1.1	1.0	1.1	1.0	1.0	1.0	1.1	1.0	47.9	1	29	30	117	795	90.9	7.5
NP_001273772.1	558472779	PHF19	PHD finger protein 19 isoform e	0.8	0.9	0.9	1.0	1.1	1.1	1.1	1.1	0.9	1.0	1.0	1.0	10.0	4	1	1	1	130	14.4	8.5
NP_076991.3	122114654	METTL16	methyltransferase-like protein 16	1.2	1.1	1.3	0.9	1.2	1.1	1.1	1.1	1.2	1.1	1.2	1.0	2.0	1	1	1	1	562	63.6	7.9
NP_003884.1	4504969	LDB1	LIM domain-binding protein 1 isoform 3	0.7	0.8	0.7	1.0	1.1	1.1	1.1	1.0	0.9	1.0	0.9	1.0	4.0	2	1	1	4	375	42.8	6.5
NP_064581.2	55770906	XAB2	pre-mRNA-splicing factor SYF1	0.9	0.9	0.9	1.0	1.0	1.0	1.0	1.0	0.9	1.0	0.9	1.0	12.2	1	8	8	15	855	99.9	6.2
NP_060842.3	55743124	LRIF1	ligand-dependent nuclear receptor-interacting factor 1 isoform 1	1.1	1.1	1.0	1.1	1.0	0.8	0.9	0.9	1.0	1.0	1.0	1.0	2.3	1	1	1	1	769	84.5	9.7
NP_057149.1	7705626	MRPS16	28S ribosomal protein S16, mitochondrial	1.0	1.0	1.0	1.0	0.9	1.0	1.0	1.0	1.0	1.0	1.0	1.0	34.3	1	4	4	7	137	15.3	9.5
NP_001186886.1	316660086	NDUFB5	NADH dehydrogenase [ubiquinone] 1 beta subcomplex subunit 5, mitochondrial isoform 2	0.9	1.1	1.1	1.0	1.0	1.0	1.1	1.0	1.0	1.1	1.1	1.0	29.2	2	4	4	6	137	15.7	7.6
NP_057406.2	19923483	RAB14	ras-related protein Rab-14	1.1	1.0	1.1	1.0	1.0	1.1	1.1	1.0	1.0	1.0	1.1	1.0	53.0	9	8	9	42	215	23.9	6.2
NP_852615.1	31652226	MRPL21	39S ribosomal protein L21, mitochondrial isoform d	1.0	1.0	1.0	0.9	1.0	1.0	1.0	1.1	1.0	1.0	1.0	1.0	33.7	2	6	6	16	205	22.8	9.9
NP_001278908.1	634743227	NOP14	nucleolar protein 14 isoform 2	1.0	1.0	1.0	1.1	0.9	1.0	0.9	1.0	0.9	1.0	1.0	1.0	6.1	2	4	4	10	806	91.3	6.6
NP_059116.3	142976638	SIX4	homeobox protein SIX4	1.1	1.2	1.2	1.0	0.9	1.1	0.9	1.0	1.0	1.1	1.0	1.0	3.7	1	1	1	2	781	82.9	5.7
NP_001244965.1	384475541	POLR3E	DNA-directed RNA polymerase III subunit RPC5 isoform 5	1.0	1.1	1.0	1.0	1.0	1.0	1.0	1.0	1.0	1.1	1.0	1.0	2.1	6	1	1	3	666	74.8	6.2
NP_001191006.1	323362983	UBE4A	ubiquitin conjugation factor E4 A isoform 2	1.3	1.2	1.0	1.0	1.1	1.1	1.1	1.1	1.2	1.2	1.1	1.0	3.7	2	2	2	4	1066	122.5	5.2
NP_056183.2	39995064	PANX1	pannexin-1	0.9	0.9	1.0	1.0	0.9	1.0	1.0	1.0	0.9	1.0	1.0	1.0	13.6	1	3	3	5	426	48.0	6.0
NP_002757.2	194018537	PRPSAP1	phosphoribosyl pyrophosphate synthase-associated protein 1	1.1	1.0	1.0	1.0	1.2	1.0	1.0	1.0	1.1	1.0	1.0	1.0	20.5	1	5	6	16	385	42.4	8.5
NP_005751.2	42542392	CEBPZ	CCAAT/enhancer-binding protein zeta	0.8	0.9	0.9	1.0	1.0	0.9	0.9	1.0	0.9	0.9	0.9	1.0	14.4	1	11	11	22	1054	120.9	5.9
NP_001186054.1	312284090	COPS3	COP9 signalosome complex subunit 3 isoform 2	1.0	1.1	1.0	1.0	1.0	1.0	0.9	1.0	1.0	1.0	1.0	1.0	29.3	2	8	8	17	403	45.7	6.7
NP_115553.2	66392146	PHAX	phosphorylated adapter RNA export protein	1.1	1.1	0.9	1.1	0.9	1.0	1.0	0.9	1.0	1.1	0.9	1.0	37.1	1	12	12	48	394	44.4	5.4
NP_036531.1	6912594	PITPNB	phosphatidylinositol transfer protein beta isoform isoform 1	1.1	1.0	1.1	1.0	1.1	1.1	1.1	1.1	1.1	1.1	1.1	1.0	52.4	3	12	13	53	271	31.5	6.9
NP_005393.2	33946329	RALA	ras-related protein Ral-A precursor	0.8	0.9	1.0	1.0	1.3	1.0	1.0	1.0	1.1	1.0	1.0	1.0	39.3	1	3	6	26	206	23.6	7.1
NP_002711.1	4506027	PPP4C	serine/threonine-protein phosphatase 4 catalytic subunit isoform 1	1.0	0.9	0.9	1.0	1.0	1.0	1.0	1.0	1.0	1.0	0.9	1.0	51.1	3	8	9	28	307	35.1	5.1
NP_057053.1	7705425	MRPS17	28S ribosomal protein S17, mitochondrial	1.0	0.9	0.9	1.0	1.0	1.0	1.0	1.0	1.0	1.0	1.0	1.0	51.5	1	4	4	10	130	14.5	9.8
NP_116558.1	14702171	POLR2C	DNA-directed RNA polymerase II subunit RPB3	1.0	0.9	1.0	1.0	1.0	0.9	0.9	1.0	1.0	0.9	1.0	1.0	12.7	1	2	2	5	275	31.4	4.9
NP_001107019.1	165905591	LIMA1	LIM domain and actin-binding protein 1 isoform 3	1.0	1.0	1.2	1.0	1.2	1.2	1.3	1.0	1.1	1.1	1.3	1.0	8.5	4	4	4	6	600	67.1	5.8
NP_002871.1	4506401	RAF1	RAF proto-oncogene serine/threonine-protein kinase	0.9	1.0	1.0	1.0	1.0	1.0	0.9	1.0	0.9	1.0	1.0	1.0	6.9	1	2	3	7	648	73.0	9.2
NP_001027563.1	74048545	LCMT1	leucine carboxyl methyltransferase 1 isoform b	1.1	1.0	1.0	1.0	1.0	0.9	0.9	1.1	1.1	0.9	0.9	1.0	25.5	2	6	6	10	279	32.2	6.3
NP_037499.2	41327749	ANAPC4	anaphase-promoting complex subunit 4 isoform 2	1.1	0.9	1.1	1.0	1.5	1.0	1.0	1.1	1.3	1.0	1.1	1.0	1.4	2	1	1	2	808	92.1	5.5
NP_076990.4	116089287	ATG9A	autophagy-related protein 9A	1.1	1.0	1.0	1.0	1.0	1.0	1.0	1.1	1.1	1.0	1.0	1.0	4.5	1	2	2	6	839	94.4	6.7
NP_076950.1	13129006	DDX50	ATP-dependent RNA helicase DDX50	1.0	1.0	1.0	1.0	1.0	1.0	1.0	1.0	1.0	1.0	1.0	1.0	24.2	3	12	14	34	737	82.5	9.2
NP_001269656.1	544346309	SLC25A17	peroxisomal membrane protein PMP34 isoform 3	1.2	1.1	1.1	1.0	1.4	1.2	1.1	1.0	1.3	1.1	1.1	1.0	7.7	3	1	1	4	234	26.3	10.5
NP_066012.1	24308265	METTL14	N6-adenosine-methyltransferase subunit METTL14	1.2	1.0	1.0	1.0	1.1	1.0	1.1	1.0	1.1	1.0	1.0	1.0	5.7	1	2	2	6	456	52.1	6.2
NP_001229529.1	336176066	RBM39	RNA-binding protein 39 isoform d	0.9	0.9	0.9	1.0	0.9	0.9	0.9	1.0	0.9	0.9	0.9	1.0	39.6	7	12	12	72	502	56.3	10.0
NP_061334.4	289063409	KLHL7	kelch-like protein 7 isoform 2	0.9	0.9	0.9	0.9	1.1	1.2	1.1	1.1	1.0	1.1	1.0	1.0	3.5	2	2	2	2	538	60.7	5.8
NP_001229753.1	338797808;209571584	FDPS	farnesyl pyrophosphate synthase isoform b	1.3	1.2	1.1	1.0	1.2	1.2	1.0	1.0	1.2	1.2	1.1	1.0	27.2	3	8	8	47	353	40.5	5.2
NP_003392.1	4507945;12408649	XRCC4	DNA repair protein XRCC4 isoform 1	1.0	0.9	0.9	1.0	0.9	0.9	0.9	1.0	0.9	0.9	0.9	1.0	31.7	2	9	9	23	334	38.0	5.0
NP_057715.2	22035565	PAXBP1	PAX3- and PAX7-binding protein 1 isoform 1	1.0	0.9	1.0	0.9	1.0	1.0	1.0	1.1	1.0	1.0	1.0	1.0	9.5	2	6	6	10	917	104.7	5.7
NP_620133.1	24308452	DPH7	diphthamide biosynthesis protein 7	1.2	1.0	1.1	1.1	1.1	1.1	1.0	0.9	1.1	1.0	1.1	1.0	7.5	1	2	2	2	452	50.5	6.6
NP_001182408.1	306774089	TFG	protein TFG isoform 2	1.1	1.0	0.9	1.0	1.0	0.9	0.9	1.0	1.1	1.0	0.9	1.0	30.1	2	7	7	34	396	43.0	5.2
NP_056072.2	195972879	TMEM194A	transmembrane protein 194A isoform b	0.9	1.0	1.0	1.0	1.1	1.1	1.1	1.0	1.0	1.1	1.1	1.0	2.2	2	1	1	1	371	42.0	6.2
NP_066018.1	15147335	NCOA5	nuclear receptor coactivator 5	0.9	0.8	0.9	1.0	0.9	0.9	0.9	1.0	0.9	0.9	0.9	1.0	10.9	1	4	4	7	579	65.5	9.6
NP_001121093.1	189083684;56237023	GALE	UDP-glucose 4-epimerase	1.0	1.0	1.1	1.0	1.1	1.1	1.2	1.0	1.1	1.0	1.2	1.0	25.3	1	7	7	24	348	38.3	6.7
NP_001073280.1	119395760	DIAPH1	protein diaphanous homolog 1 isoform 2	1.0	1.1	1.1	1.0	1.0	1.0	1.0	1.0	1.0	1.1	1.1	1.0	43.1	2	40	40	194	1263	140.2	5.4
NP_001070651.1	116256350	TSC2	tuberin isoform 4	1.1	1.0	1.0	0.9	1.0	1.0	1.0	1.1	1.1	1.0	1.0	1.0	1.3	3	2	2	4	1740	193.1	7.1
NP_006487.1	5729850	GNAI3	guanine nucleotide-binding protein G(k) subunit alpha	1.1	1.2	1.1	1.2	1.1	0.9	1.0	0.9	1.1	1.1	1.1	1.0	48.9	8	8	13	47	354	40.5	5.7

Khan *et al.* , 2019. Multi-omics analysis to characterize cigarette smoke induced molecular alterations in esophageal cells
Supplementary Table 5. List of proteins quantified in untreated and chronically treated Het1A cells with cigarette smoke condensate for 8 months

NP_Accession	Protein group Accession	Gene ID	Description	Het-1A-Smoke - 2M/Parental	Het-1A-Smoke - 4M/Parental	Het-1A-Smoke - 6M/Parental	Het-1A-Smoke - 8M/Parental	Het-1A-Smoke - 2M/Parental	Het-1A-Smoke - 4M/Parental	Het-1A-Smoke - 6M/Parental	Het-1A-Smoke - 8M/Parental	Het-1A-Smoke - 2M/Parental	Het-1A-Smoke - 4M/Parental	Het-1A-Smoke - 6M/Parental	Het-1A-Smoke - 8M/Parental	Coverage (%)	Proteins	Unique Peptides	# Peptides	PSM	# AAs	MW [kDa]	calc. pI
				Replicate 1				Replicate 2				Average of replicates											
NP_001155056.1	239787844	TEX10	testis-expressed sequence 10 protein isoform 2	1.0	0.9	1.0	1.0	1.1	1.0	1.0	1.0	**1.1**	**1.0**	**1.0**	**1.0**	15.4	2	12	12	28	913	103.8	9.4
NP_002728.1	4506067	PRKCA	protein kinase C alpha type	1.1	1.0	1.0	1.0	1.1	1.1	1.1	1.0	**1.1**	**1.0**	**1.1**	**1.0**	12.5	8	5	6	10	672	76.7	7.0
NP_001258972.1	440546397	RPS6KB1	ribosomal protein S6 kinase beta-1 isoform c	1.3	1.3	1.3	1.0	1.0	1.0	1.1	1.0	**1.1**	**1.2**	**1.2**	**1.0**	4.4	5	1	2	3	451	51.0	6.5
NP_001681.2	19913424	ATP6V1A	V-type proton ATPase catalytic subunit A	1.1	1.1	1.0	1.0	1.1	1.1	1.1	1.0	**1.1**	**1.1**	**1.1**	**1.0**	31.0	1	17	17	45	617	68.3	5.5
NP_689969.2	59891448	RICTOR	rapamycin-insensitive companion of mTOR isoform 1	1.3	1.1	1.1	1.0	1.0	1.1	1.1	1.0	**1.2**	**1.1**	**1.1**	**1.0**	0.5	2	1	1	1	1708	192.1	7.5
NP_001124433.1	195972873	PLCH1	1-phosphatidylinositol 4,5-bisphosphate phosphodiesterase eta-1 isoform c	1.1	1.3	1.1	1.0	1.0	1.3	1.1	1.0	**1.1**	**1.3**	**1.1**	**1.0**	0.8	3	1	1	22	1002	114.4	7.6
NP_003739.2	25777734	ALDH4A1	delta-1-pyrroline-5-carboxylate dehydrogenase, mitochondrial isoform a precursor	1.0	1.0	0.9	1.0	1.0	1.0	0.9	1.0	**1.0**	**1.0**	**0.9**	**1.0**	32.9	2	13	13	33	563	61.7	8.1
NP_054797.2	14165270	MRPL13	39S ribosomal protein L13, mitochondrial	1.0	1.0	1.0	1.0	0.9	0.9	1.0	1.0	**1.0**	**1.0**	**1.0**	**1.0**	28.7	1	5	5	10	178	20.7	9.2
NP_000088.3	41393599	CPOX	oxygen-dependent coproporphyrinogen-III oxidase, mitochondrial precursor	1.0	0.9	0.9	1.0	1.0	0.9	1.0	1.0	**1.0**	**0.9**	**0.9**	**1.0**	26.7	1	10	10	28	454	50.1	8.3
NP_001960.2	21361337;37537716	EIF5	eukaryotic translation initiation factor 5	1.1	1.0	0.9	1.0	1.1	1.0	0.9	1.0	**1.1**	**1.0**	**0.9**	**1.0**	36.9	1	12	12	64	431	49.2	5.6
NP_001782.1	4757952;89903012	CDC42	cell division control protein 42 homolog isoform 1 precursor	1.0	0.9	1.0	1.0	0.9	0.9	1.0	1.0	**0.9**	**0.9**	**1.0**	**1.0**	62.8	4	8	9	73	191	21.2	6.6
NP_002119.1	4504425	HMGB1	high mobility group protein B1	0.9	0.8	0.9	1.0	0.9	0.9	1.0	1.0	**0.9**	**0.9**	**1.0**	**1.0**	60.9	1	11	14	75	215	24.9	5.7
NP_060173.1	8923179	FBXL12	F-box/LRR-repeat protein 12	1.1	1.1	1.0	1.0	1.0	1.1	1.2	1.0	**1.0**	**1.1**	**1.1**	**1.0**	17.5	1	3	3	5	326	37.0	8.8
NP_001367.2	33350932	DYNC1H1	cytoplasmic dynein 1 heavy chain 1	1.1	1.0	1.1	1.0	1.1	1.0	1.1	1.0	**1.1**	**1.0**	**1.1**	**1.0**	44.2	1	154	155	718	4646	532.1	6.4
NP_115898.2	31543091	MAK16	protein MAK16 homolog	1.1	1.0	1.0	1.0	1.5	1.0	0.9	1.0	**1.3**	**1.0**	**1.0**	**1.0**	17.7	1	5	5	7	300	35.3	5.4
NP_005477.2	191252812	TOM1L1	TOM1-like protein 1	1.0	1.1	1.1	1.1	1.0	0.9	0.9	0.9	**1.0**	**1.0**	**1.0**	**1.0**	6.9	1	1	2	5	476	53.0	5.2
NP_003676.2	154355000	KHSRP	far upstream element-binding protein 2	0.9	0.9	0.9	1.0	0.9	1.0	0.9	1.0	**0.9**	**1.0**	**0.9**	**1.0**	34.7	1	17	20	101	711	73.1	7.3
NP_005078.2	61835148	FXR1	fragile X mental retardation syndrome-related protein 1 isoform a	1.0	1.0	1.1	1.0	1.0	1.1	1.1	1.0	**1.0**	**1.0**	**1.1**	**1.0**	51.2	3	21	22	109	621	69.7	6.2
NP_060822.2	153251297	TMA16	translation machinery-associated protein 16	0.9	1.1	1.1	1.0	0.9	1.1	1.0	1.0	**0.9**	**1.1**	**1.0**	**1.0**	28.1	1	5	5	15	203	23.8	9.3
NP_060082.2	38044290	ZCCHC8	zinc finger CCHC domain-containing protein 8	1.1	1.1	1.1	1.0	1.0	1.0	1.1	1.0	**1.1**	**1.1**	**1.1**	**1.0**	26.3	1	11	11	28	707	78.5	4.9
NP_004860.2	17865802	VPS4B	vacuolar protein sorting-associated protein 4B	1.0	1.0	1.1	1.0	1.0	0.9	1.0	1.0	**1.0**	**1.0**	**1.0**	**1.0**	16.7	1	4	7	14	444	49.3	7.2
NP_036252.1	11321634	CD2AP	CD2-associated protein	0.8	1.0	0.9	1.0	0.8	0.9	0.9	1.0	**0.8**	**1.0**	**0.9**	**1.0**	21.3	1	10	10	17	639	71.4	6.4
NP_001193730.1	332164786	RFC5	replication factor C subunit 5 isoform 4	1.0	0.9	0.9	1.0	0.9	1.0	0.9	1.0	**1.0**	**1.0**	**0.9**	**1.0**	38.0	4	10	10	32	337	38.1	7.8
NP_005882.2	148539872	ACAT2	acetyl-CoA acetyltransferase, cytosolic	1.2	1.2	1.0	1.0	1.2	1.2	1.0	1.0	**1.2**	**1.2**	**1.0**	**1.0**	57.2	2	14	14	96	397	41.3	6.9
NP_001121132.1	189083768	MFN2	mitofusin-2	1.0	1.0	1.0	1.0	1.2	1.0	1.1	1.0	**1.1**	**1.0**	**1.0**	**1.0**	1.7	1	1	1	2	757	86.3	7.0
NP_055982.2	125988389	JMJD6	bifunctional arginine demethylase and lysyl-hydroxylase JMJD6 isoform 2	1.0	1.0	1.0	1.0	1.0	1.0	1.0	1.0	**1.0**	**1.0**	**1.0**	**1.0**	17.1	2	5	5	9	403	46.4	8.7
NP_112534.1	13787215	SIRT5	NAD-dependent protein deacylase sirtuin-5, mitochondrial isoform 2	1.0	0.9	0.9	1.0	1.2	1.0	1.0	1.0	**1.1**	**0.9**	**0.9**	**1.0**	8.0	4	2	2	4	299	32.7	9.0
NP_001193775.1	332205965	ASPM	abnormal spindle-like microcephaly-associated protein isoform 2	1.0	0.9	1.0	0.9	1.0	1.2	1.1	1.1	**1.0**	**1.0**	**1.1**	**1.0**	2.1	2	3	3	3	1892	217.7	9.8
NP_064569.3	94681046	CNNM4	metal transporter CNNM4 precursor	1.1	1.0	1.0	0.9	1.3	1.1	1.0	1.1	**1.2**	**1.1**	**1.0**	**1.0**	7.4	4	5	5	8	775	86.6	6.1
NP_001167012.1	291084797	BANP	protein BANP isoform e	0.9	1.0	1.0	1.0	1.2	1.1	1.1	1.0	**1.0**	**1.0**	**1.0**	**1.0**	1.5	7	1	1	1	466	50.9	5.5
NP_001254738.1	392513662	ILF2	interleukin enhancer-binding factor 2 isoform 2	1.1	1.0	1.0	1.1	0.9	0.9	0.9	0.9	**1.0**	**1.0**	**0.9**	**1.0**	76.4	2	18	18	158	352	38.9	4.9
NP_705833.3	390979647	CRYBG3	beta/gamma crystallin domain-containing protein 3	1.2	1.2	1.1	1.1	1.1	1.1	1.0	0.9	**1.1**	**1.1**	**1.1**	**1.0**	0.3	1	1	1	1	2970	330.4	5.2
NP_001164191.1	282398125	BCAR1	breast cancer anti-estrogen resistance protein 1 isoform 8	0.8	0.8	0.9	1.0	1.0	1.0	1.0	1.1	**0.9**	**0.9**	**1.0**	**1.0**	5.8	9	2	2	3	722	77.7	5.4
NP_054745.1	7661734	DCPS	m7GpppX diphosphatase	1.1	0.9	1.0	1.0	1.1	1.0	1.0	1.0	**1.1**	**1.0**	**1.0**	**1.0**	23.4	1	6	6	20	337	38.6	6.4
NP_284941.2	45269137	MFN1	mitofusin-1	1.1	1.0	1.1	1.0	1.0	1.0	1.0	1.0	**1.1**	**1.0**	**1.0**	**1.0**	6.2	1	3	3	3	741	84.1	6.3
NP_001164015.1	281604138	PGAM5	serine/threonine-protein phosphatase PGAM5, mitochondrial isoform 2	1.1	1.0	1.1	1.0	1.1	1.0	1.0	1.0	**1.1**	**1.0**	**1.1**	**1.0**	27.1	3	1	6	18	288	31.9	8.7
NP_006818.3	98986464	TMED10	transmembrane emp24 domain-containing protein 10 precursor	1.0	1.0	0.9	1.0	1.0	1.1	1.0	1.1	**1.0**	**1.0**	**1.0**	**1.0**	42.9	1	7	7	41	219	25.0	7.4
NP_004710.2	117676384	SCAF11	protein SCAF11	0.9	1.1	1.0	1.0	0.9	1.0	0.9	1.0	**0.9**	**1.0**	**1.0**	**1.0**	7.9	1	9	9	13	1463	164.6	8.4
NP_060625.2	157388991	SLC25A36	solute carrier family 25 member 36 isoform b	1.1	1.2	1.1	1.1	1.1	0.9	1.0	0.9	**1.1**	**1.1**	**1.0**	**1.0**	7.7	3	2	2	3	310	34.1	8.7
NP_114172.1	14327896	CCNB1	G2/mitotic-specific cyclin-B1	0.8	1.1	1.2	1.0	0.9	1.1	1.1	1.0	**0.8**	**1.1**	**1.1**	**1.0**	22.6	1	5	5	20	433	48.3	7.5
NP_919415.2	94721252	VAPA	vesicle-associated membrane protein-associated protein A isoform 2	0.9	1.0	1.0	1.0	1.0	1.0	1.0	1.1	**0.9**	**1.0**	**1.0**	**1.0**	38.2	2	8	9	44	249	27.9	8.6
NP_060129.2	92110027	QPCTL	glutaminyl-peptide cyclotransferase-like protein isoform 1	1.1	1.0	1.2	1.0	1.1	1.1	1.2	1.0	**1.1**	**1.1**	**1.2**	**1.0**	13.4	2	3	3	5	382	42.9	9.8
NP_001073886.1	122937229	ZNF629	zinc finger protein 629	0.9	1.0	1.0	1.0	1.0	1.1	1.1	1.0	**1.0**	**1.1**	**1.0**	**1.0**	13.0	191	4	6	9	869	96.6	7.9
NP_954650.2	66773344	SOGA1	protein SOGA1 isoform 2	0.9	0.9	0.9	1.0	1.0	1.0	0.9	1.0	**0.9**	**1.0**	**0.9**	**1.0**	9.7	3	6	7	9	1016	115.7	5.5
NP_004166.1	4759160	SNRPD3	small nuclear ribonucleoprotein Sm D3	1.0	0.9	0.9	1.0	1.0	1.0	1.0	1.1	**1.0**	**0.9**	**0.9**	**1.0**	47.6	1	4	4	71	126	13.9	10.3
NP_003092.4	49533617	SOAT1	sterol O-acyltransferase 1 isoform 1	1.2	1.2	1.2	1.0	1.1	1.1	1.2	1.0	**1.1**	**1.2**	**1.2**	**1.0**	7.8	3	3	3	4	550	64.7	8.9
NP_006092.1	5174457	NDC80	kinetochore protein NDC80 homolog	0.8	0.9	0.9	1.0	1.0	1.0	1.1	1.0	**0.9**	**0.9**	**1.0**	**1.0**	13.2	1	8	8	14	642	73.9	5.6
NP_001136077.1	217272894	EFTUD2	116 kDa U5 small nuclear ribonucleoprotein component isoform b	1.0	1.1	1.0	1.0	1.0	1.0	1.0	1.0	**1.0**	**1.0**	**1.0**	**1.0**	58.5	4	33	34	179	937	105.3	5.2
NP_004118.3	47078240	GPS1	COP9 signalosome complex subunit 1 isoform 2	1.1	1.0	1.0	1.0	1.1	1.1	1.1	1.0	**1.1**	**1.0**	**1.1**	**1.0**	30.8	2	11	11	30	491	55.5	6.7
NP_037468.1	7019415	SEC61A1	protein transport protein Sec61 subunit alpha isoform 1	1.1	1.1	1.0	1.0	1.1	1.1	1.1	1.0	**1.1**	**1.1**	**1.0**	**1.0**	25.8	4	7	7	25	476	52.2	8.1
NP_065796.1	38569417	AARS2	alanine--tRNA ligase, mitochondrial	1.0	1.0	1.0	1.1	1.0	0.9	0.9	1.0	**1.0**	**1.0**	**0.9**	**1.0**	6.3	1	4	4	7	985	107.3	6.3
NP_001239053.1	355477269	PASK	PAS domain-containing serine/threonine-protein kinase isoform 4	0.9	0.9	0.9	1.0	1.0	1.0	0.9	1.0	**1.0**	**1.0**	**0.9**	**1.0**	3.7	4	3	3	4	1143	123.0	5.0
NP_001165775.1	288806590	MASTL	serine/threonine-protein kinase greatwall isoform 3	1.1	1.1	1.2	1.0	1.1	1.0	1.1	1.0	**1.1**	**1.1**	**1.1**	**1.0**	8.6	3	6	6	11	840	92.8	6.1
NP_003391.1	4507943	XPO1	exportin-1	1.1	1.0	1.0	1.0	1.0	1.0	1.0	1.0	**1.1**	**1.0**	**1.0**	**1.0**	44.5	1	37	37	279	1071	123.3	6.1
NP_001273897.1	558695424	JUND	transcription factor jun-D isoform deltaJunD	1.0	1.0	1.0	1.0	1.1	1.1	1.0	1.0	**1.0**	**1.0**	**1.0**	**1.0**	21.7	2	3	3	9	304	31.2	8.4
NP_073589.4	154744870	ZNF574	zinc finger protein 574	1.1	1.1	1.1	1.0	1.1	1.0	1.1	1.0	**1.1**	**1.0**	**1.1**	**1.0**	1.8	1	2	2	4	896	98.8	8.1
NP_001006947.1	55749480	SDC1	syndecan-1 precursor	1.0	1.0	0.9	1.0	0.9	1.1	1.0	1.0	**1.0**	**1.1**	**1.0**	**1.0**	5.5	1	1	1	4	310	32.5	4.6
NP_001295.2	22202611	CPD	carboxypeptidase D isoform 1 precursor	1.0	1.0	1.0	1.0	0.9	1.0	1.0	1.0	**0.9**	**1.0**	**1.0**	**1.0**	21.1	2	21	21	56	1380	152.8	6.0
NP_067642.1	11055998	GNB4	guanine nucleotide-binding protein subunit beta-4	1.1	1.0	1.1	1.1	1.2	0.9	0.9	0.9	**1.1**	**1.0**	**1.0**	**1.0**	28.8	1	3	7	42	340	37.5	6.0
NP_001034764.1	89179321	UNC45A	protein unc-45 homolog A isoform 3	0.9	0.9	0.9	1.0	0.9	0.9	0.9	1.0	**0.9**	**0.9**	**0.9**	**1.0**	40.3	2	30	30	142	929	101.6	6.1
NP_001243000.2	597709777	RNF213	E3 ubiquitin-protein ligase RNF213 isoform 3	1.1	1.0	1.0	1.0	1.1	1.0	1.1	1.0	**1.1**	**1.0**	**1.1**	**1.0**	6.1	2	21	21	35	5207	591.0	6.5
NP_001139248.1	224809329;4758384	FKBP5	peptidyl-prolyl cis-trans isomerase FKBP5 isoform 1	1.0	1.0	1.0	1.0	1.0	1.0	0.9	1.0	**1.0**	**1.0**	**0.9**	**1.0**	65.4	2	27	28	150	457	51.2	5.9
NP_003790.1	4506567	RNMT	mRNA cap guanine-N7 methyltransferase isoform 2	1.0	1.0	1.0	1.0	1.0	0.9	1.0	1.0	**1.0**	**1.0**	**1.0**	**1.0**	21.4	2	8	8	16	476	54.8	6.6
NP_004682.2	19913432	ATP6V0D1	V-type proton ATPase subunit d 1	1.0	1.0	1.0	1.0	1.0	1.0	1.0	1.0	**1.0**	**1.0**	**1.0**	**1.0**	24.8	1	8	8	20	351	40.3	5.0
NP_004386.2	18426915	DBN1	drebrin isoform a	1.0	1.2	1.1	1.0	1.1	1.1	1.1	1.0	**1.0**	**1.1**	**1.1**	**1.0**	51.0	2	20	20	201	649	71.4	4.4
NP_001096123.1	156630992	OTUD4	OTU domain-containing protein 4 isoform 3	0.9	0.9	0.9	1.0	1.1	1.1	1.1	1.1	**1.0**	**1.0**	**1.0**	**1.0**	6.6	2	4	4	7	1049	117.0	6.7
NP_112181.2	116235448	FAM83D	protein FAM83D	1.0	1.0	1.0	1.0	0.9	1.0	1.0	1.0	**0.9**	**1.0**	**1.0**	**1.0**	11.2	1	6	6	14	615	67.6	6.9
NP_056531.1	7657335	MINK1	misshapen-like kinase 1 isoform 1	1.1	1.1	1.0	1.0	1.0	1.0	1.0	1.0	**1.1**	**1.0**	**1.0**	**1.0**	4.0	12	2	5	10	1295	145.7	7.4
YP_003024029.1	251831110	COX2	cytochrome c oxidase subunit II	1.0	0.8	1.0	1.0	1.0	0.9	1.1	1.1	**1.0**	**0.9**	**1.0**	**1.0**	20.7	1	4	4	26	227	25.5	4.8
NP_001192051.1	326806958	ATG13	autophagy-related protein 13 isoform 4	1.0	1.0	0.9	1.0	1.1	1.0	0.9	1.0	**1.0**	**1.0**	**0.9**	**1.0**	3.2	4	1	1	2	401	44.1	4.8
NP_004809.2	41327771	DDX23	probable ATP-dependent RNA helicase DDX23	0.9	0.9	0.9	1.0	0.9	0.9	0.9	1.0	**0.9**	**0.9**	**0.9**	**1.0**	24.5	1	18	18	42	820	95.5	9.6
NP_203693.3	124494240	MYO1C	unconventional myosin-Ic isoform c	1.1	1.1	1.2	1.0	1.0	1.0	1.1	1.0	**1.1**	**1.1**	**1.2**	**1.0**	24.5	3	19	20	61	1028	117.8	9.4
NP_076422.1	13027600	FAM173A	protein FAM173A isoform 1	0.9	0.9	0.8	0.9	1.1	1.2	1.2	1.1	**1.0**	**1.0**	**1.0**	**1.0**	7.2	1	1	1	2	235	25.1	9.3
NP_000260.1	4557797	NME1	nucleoside diphosphate kinase A isoform b	1.3	0.9	1.0	1.0	1.3	0.9	0.9	1.0	**1.3**	**0.9**	**0.9**	**1.0**	85.5	2	10	10	90	152	17.1	6.2
NP_001177192.1	298231253	C1D	nuclear nucleic acid-binding protein C1D	1.1	1.0	1.1	1.0	0.9	0.9	0.9	1.0	**1.0**	**1.0**	**1.0**	**1.0**	9.9	2	1	2	2	141	16.0	9.0
NP_005712.1	5031573	ACTR3	actin-related protein 3 isoform 1	1.1	1.1	1.2	1.0	1.1	1.1	1.2	1.0	**1.1**	**1.1**	**1.2**	**1.0**	45.7	5	12	12	47	418	47.3	5.9
NP_079498.2	147904340	WDR82	WD repeat-containing protein 82	1.0	1.0	1.1	1.0	1.0	1.0	1.1	1.0	**1.0**	**1.0**	**1.1**	**1.0**	13.4	1	4	4	11	313	35.1	7.7
NP_079199.2	27477134	NUP210	nuclear pore membrane glycoprotein 210 precursor	1.0	1.0	1.1	1.0	1.2	1.1	1.1	1.0	**1.1**	**1.1**	**1.1**	**1.0**	20.5	1	22	22	42	1887	205.0	6.8
NP_001002836.2	152963637	ZNF787	zinc finger protein 787	0.9	1.0	1.0	1.0	0.8	1.1	0.9	1.0	**0.9**	**1.0**	**0.9**	**1.0**	9.2	1	2	2	3	382	40.4	8.0
NP_612398.2	288541299	FAM114A1	protein NOXP20	1.1	1.2	1.2	1.0	1.1	1.2	1.2	1.0	**1.1**	**1.2**	**1.2**	**1.0**	20.4	1	7	7	16	563	60.7	4.7
NP_056130.2	39725634	LARP1	la-related protein 1	0.9	1.0	1.0	1.0	1.0	1.1	1.0	1.0	**1.0**	**1.0**	**1.0**	**1.0**	38.5	1	26	30	102	1019	116.4	9.1
NP_055140.1	7656991;451172106	CORO1C	coronin-1C isoform b	1.0	1.0	1.2	1.0	1.0	1.0	1.1	1.0	**1.0**	**1.0**	**1.2**	**1.0**	30.8	2	15	15	63	474	53.2	7.1
NP_056163.1	150456424	TMEM131	transmembrane protein 131	1.0	1.1	1.1	1.1	1.0	0.9	1.0	1.0	**1.0**	**1.0**	**1.0**	**1.0**	1.1	1	1	1	1	1883	205.0	8.6
NP_057010.1	7706708	TEX264	testis-expressed sequence 264 protein isoform 1 precursor	1.0	0.9	0.9	1.0	1.1	1.0	1.0	1.0	**1.0**	**1.0**	**1.0**	**1.0**	24.3	2	4	4	9	313	34.2	4.9
NP_054767.3	38569480	MKL2	MKL/myocardin-like protein 2	0.9	1.0	1.0	1.0	0.9	1.0	0.9	1.0	**0.9**	**1.0**	**0.9**	**1.0**	4.6	2	3	3	3	1049	114.0	6.0
NP_055521.1	7661952	SART3	squamous cell carcinoma antigen recognized by T-cells 3	1.1	1.0	1.0	1.0	1.1	1.1	1.1	1.0	**1.1**	**1.0**	**1.0**	**1.0**	25.2	1	20	21	46	963	109.9	5.6
NP_001609.2	156523968	PARP1	poly [ADP-ribose] polymerase 1	1.0	1.0	1.0	1.0	1.0	1.0	1.0	1.0	**1.0**	**1.0**	**1.0**	**1.0**	60.0	1	51	51	281	1014	113.0	8.9

Khan *et al.* , 2019. Multi-omics analysis to characterize cigarette smoke induced molecular alterations in esophageal cells
Supplementary Table 5. List of proteins quantified in untreated and chronically treated Het1A cells with cigarette smoke condensate for 8 months

NP_Accession	Protein group Accession	Gene ID	Description	Het-1A-Smoke - 2M/Parental	Het-1A-Smoke - 4M/Parental	Het-1A-Smoke - 6M/Parental	Het-1A-Smoke - 8M/Parental	Het-1A-Smoke - 2M/Parental	Het-1A-Smoke - 4M/Parental	Het-1A-Smoke - 6M/Parental	Het-1A-Smoke - 8M/Parental	Het-1A-Smoke - 2M/Parental	Het-1A-Smoke - 4M/Parental	Het-1A-Smoke - 6M/Parental	Het-1A-Smoke - 8M/Parental	Coverage (%)	Proteins	Unique Peptides	# Peptides	PSM	# AAs	MW [kDa]	calc. pI
				Replicate 1				Replicate 2				Average of replicates											
NP_001243390.1	374253823	SLC12A2	solute carrier family 12 member 2 isoform 2	0.9	1.0	0.9	1.0	0.9	1.0	1.0	1.0	0.9	1.0	0.9	1.0	6.0	4	7	7	15	1196	129.6	6.5
NP_001271166.1	546232072	ZFYVE16	zinc finger FYVE domain-containing protein 16 isoform b	1.1	1.1	1.1	1.0	0.9	1.0	1.0	1.0	1.0	1.1	1.0	1.0	9.8	2	5	5	11	809	88.4	4.6
NP_001243364.1	374253762;532524975	PCYT2	ethanolamine-phosphate cytidylyltransferase isoform 4	1.3	1.3	1.1	1.0	1.1	1.2	1.1	1.0	1.2	1.2	1.1	1.0	22.8	6	6	6	13	311	35.2	6.5
NP_689608.2	285026434	NUDT16	U8 snoRNA-decapping enzyme isoform 2	1.1	1.0	1.1	0.9	1.2	1.1	1.2	1.1	1.1	1.0	1.2	1.0	30.8	3	3	4	6	195	21.3	6.9
NP_079366.3	122114651	USP36	ubiquitin carboxyl-terminal hydrolase 36	0.9	1.1	1.0	1.0	1.2	1.0	0.9	1.0	1.1	1.1	1.0	1.0	2.8	1	2	2	3	1123	122.8	9.7
NP_112196.3	163792194	TRMT1L	TRMT1-like protein isoform 1	0.9	0.9	0.9	1.0	1.0	1.0	1.0	1.0	1.0	0.9	1.0	1.0	16.5	2	8	8	17	733	81.7	7.9
NP_005086.2	44890068	ZMYM4	zinc finger MYM-type protein 4	1.1	1.0	1.0	1.0	0.9	1.1	1.0	1.0	1.0	1.0	1.0	1.0	4.4	1	5	5	5	1548	172.7	6.8
NP_058642.1	8393638	F11R	junctional adhesion molecule A precursor	0.8	0.9	0.8	0.8	1.1	1.1	1.1	1.2	1.0	1.0	0.9	1.0	14.1	1	2	2	4	299	32.6	7.9
NP_003983.2	153791372	CLK3	dual specificity protein kinase CLK3 isoform b	1.0	1.1	1.0	1.1	1.1	0.9	0.9	0.9	1.0	1.0	1.0	1.0	8.6	2	4	4	8	490	58.6	9.5
NP_004619.3	224809295	XPC	DNA repair protein complementing XP-C cells	1.1	1.0	1.1	1.0	1.2	1.1	1.1	1.0	1.1	1.1	1.1	1.0	9.0	2	4	4	10	940	105.9	8.9
NP_005981.3	126362971	STK10	serine/threonine-protein kinase 10	1.0	1.1	1.0	1.0	1.1	1.1	1.1	1.0	1.0	1.1	1.0	1.0	6.9	1	5	6	21	968	112.1	6.9
NP_003933.1	4506735	RPS6KA4	ribosomal protein S6 kinase alpha-4 isoform a	1.2	1.2	1.1	1.1	1.0	0.9	1.0	0.9	1.1	1.0	1.0	1.0	5.2	3	3	3	4	772	85.6	8.3
NP_001748.1	4502599	CBR1	carbonyl reductase [NADPH] 1 isoform 1	1.0	1.1	1.0	1.0	1.0	1.0	1.0	1.0	1.0	1.0	1.0	1.0	58.1	2	9	12	72	277	30.4	8.3
NP_001687.1	4502317	ATP6V1E1	V-type proton ATPase subunit E 1 isoform a	1.1	1.1	1.1	1.1	1.0	1.0	1.0	1.0	1.1	1.0	1.1	1.0	24.8	4	6	6	13	226	26.1	8.0
NP_006406.1	5454084	SPTLC1	serine palmitoyltransferase 1 isoform a	1.0	1.1	1.0	1.0	1.0	1.0	1.0	1.0	1.0	1.0	1.0	1.0	24.1	3	8	8	29	473	52.7	6.0
NP_951060.1	40068047	CNNM3	metal transporter CNNM3 isoform 2 precursor	0.8	0.9	0.9	0.9	1.1	1.1	1.0	1.1	1.0	1.0	0.9	1.0	6.8	2	4	4	6	659	70.6	7.1
NP_852128.1	31563362;31563358	MRPL55	39S ribosomal protein L55, mitochondrial isoform a	1.1	0.9	1.0	1.0	1.0	1.0	0.9	1.0	1.0	1.0	1.0	1.0	24.2	2	2	2	5	128	15.1	11.2
NP_006274.2	170763517	TACC1	transforming acidic coiled-coil-containing protein 1 isoform 1	1.1	1.0	1.1	1.0	0.9	1.0	0.9	1.0	1.0	1.0	1.0	1.0	22.1	3	10	12	25	805	87.7	4.9
NP_079359.2	19923613	EDC3	enhancer of mRNA-decapping protein 3	1.0	0.9	1.0	0.9	1.0	1.0	1.0	1.1	1.0	0.9	1.0	1.0	16.9	1	4	4	12	508	56.0	7.1
NP_001243094.1	371872799	TMCO1	transmembrane and coiled-coil domain-containing protein 1 isoform c	1.0	0.9	0.9	1.0	1.0	1.0	1.0	1.1	1.0	0.9	0.9	1.0	26.7	3	4	4	10	176	19.9	9.8
NP_056669.2	156105673	MBD1	methyl-CpG-binding domain protein 1 isoform 3	0.9	1.0	1.0	0.9	0.9	1.1	1.0	1.1	0.9	1.1	1.0	1.0	21.0	13	6	6	16	549	60.0	8.9
NP_001269892.1	545478902	HJURP	Holliday junction recognition protein isoform c	0.8	1.0	0.9	1.0	1.0	1.1	1.0	1.0	0.9	1.1	1.0	1.0	1.7	3	1	1	1	663	74.3	9.6
NP_004887.2	17978519	VPS26A	vacuolar protein sorting-associated protein 26A isoform 1	1.0	0.9	1.0	0.9	1.0	1.1	1.1	1.1	1.0	1.0	1.1	1.0	49.9	2	12	14	34	327	38.1	6.6
NP_005564.1	5031877	LMNB1	lamin-B1 isoform 1	0.9	0.9	0.8	1.0	0.9	0.9	0.9	1.0	0.9	0.9	0.9	1.0	61.1	2	27	32	206	586	66.4	5.2
NP_002858.2	19923750	RAB3B	ras-related protein Rab-3B	0.8	0.8	0.8	1.0	1.0	0.9	1.0	1.0	0.9	0.9	0.9	1.0	29.7	10	3	5	13	219	24.7	5.0
NP_039269.2	73747829	LIG3	DNA ligase 3 isoform alpha precursor	1.3	0.9	0.9	1.0	1.0	1.0	0.9	1.0	1.2	1.0	0.9	1.0	9.9	2	8	8	27	1009	112.8	9.0
NP_001427.2	12056465	FBL	rRNA 2'-O-methyltransferase fibrillarin	0.9	0.9	1.0	1.0	1.0	1.0	1.0	1.0	1.0	1.0	1.0	1.0	42.7	1	11	11	71	321	33.8	10.2
NP_003461.2	150378533	USP7	ubiquitin carboxyl-terminal hydrolase 7 isoform 1	1.0	1.0	1.0	1.0	1.0	1.0	1.0	1.0	1.0	1.0	1.0	1.0	35.7	3	27	27	96	1102	128.2	5.6
NP_000382.3	5729770	TPP1	tripeptidyl-peptidase 1 preproprotein	1.1	1.0	1.0	1.0	0.9	1.1	0.9	1.0	1.0	1.0	1.0	1.0	5.0	1	2	2	3	563	61.2	6.5
NP_689940.3	30181236	CPNE2	copine-2	1.0	0.9	1.0	1.0	1.0	1.1	1.1	1.0	1.0	1.0	1.0	1.0	4.2	10	1	2	5	548	61.2	6.1
NP_001138825.1	223941929	ELF1	ETS-related transcription factor Elf-1 isoform b	1.0	1.0	1.0	1.0	0.9	0.9	0.8	1.0	1.0	0.9	0.9	1.0	2.5	2	1	1	1	595	64.9	5.4
NP_036443.1	13124883	KIN	DNA/RNA-binding protein KIN17	0.9	1.0	1.0	1.1	1.0	0.9	1.0	0.9	1.0	1.0	1.0	1.0	3.1	1	1	1	2	393	45.3	9.0
NP_006311.2	291621647	PGRMC2	membrane-associated progesterone receptor component 2	1.0	1.0	1.0	1.0	1.1	1.1	1.1	1.0	1.0	1.0	1.0	1.0	27.5	1	4	5	12	247	26.2	5.3
NP_001257637.1	399124753	NRM	nurim isoform 3	1.0	1.0	1.0	1.0	1.1	0.9	1.0	1.0	1.0	0.9	1.0	1.0	9.0	4	1	1	1	111	11.9	9.0
NP_071334.1	13489073	EGLN1	egl nine homolog 1	0.8	1.0	1.0	0.9	1.0	1.3	1.0	1.1	0.9	1.1	1.0	1.0	4.5	1	2	2	2	426	46.0	8.5
NP_671717.1	22547149	FBXO22	F-box only protein 22 isoform a	1.0	1.0	1.0	1.1	0.9	0.8	0.8	0.9	1.0	0.9	0.9	1.0	18.9	2	5	5	11	403	44.5	7.0
NP_065700.1	10190682	CBX8	chromobox protein homolog 8	0.9	1.0	1.0	1.0	1.0	1.0	1.0	1.0	1.0	1.0	1.0	1.0	5.1	1	2	2	4	389	43.4	9.9
NP_001258029.1	402794208	PUF60	poly(U)-binding-splicing factor PUF60 isoform h	0.9	1.0	0.9	1.0	0.9	1.0	0.9	1.0	0.9	1.0	0.9	1.0	46.5	8	16	16	96	499	54.0	5.3
NP_065904.1	62821776	KIAA1467	uncharacterized protein KIAA1467	1.0	1.1	0.9	1.0	1.1	1.0	1.0	1.0	1.1	1.1	1.0	1.0	11.7	1	4	4	4	622	67.0	5.0
NP_835461.2	409264581	ZNF598	zinc finger protein 598	1.0	1.0	1.0	1.0	1.1	1.0	1.0	1.0	1.1	1.0	1.0	1.0	4.9	1	4	4	9	904	98.6	8.4
NP_005908.1	5174539	MDH1	malate dehydrogenase, cytoplasmic isoform 2	1.0	1.0	1.0	1.0	1.0	1.0	1.0	1.0	1.0	1.0	1.0	1.0	58.1	3	18	18	164	334	36.4	7.4
NP_057132.2	103471987	ISOC1	isochorismatase domain-containing protein 1	1.0	0.9	0.9	1.0	0.9	0.9	0.9	1.0	1.0	0.9	0.9	1.0	20.5	1	4	4	36	298	32.2	7.4
NP_005841.1	5032069	SF3B4	splicing factor 3B subunit 4	0.9	0.9	0.8	1.0	0.9	0.8	0.9	1.0	0.9	0.8	0.9	1.0	15.8	1	3	3	15	424	44.4	8.6
NP_001129225.1	209364550	ECD	protein SGT1 isoform 3	0.9	0.9	1.2	1.0	0.9	1.1	1.3	1.0	0.9	1.0	1.2	1.0	9.3	3	3	3	11	601	67.7	4.8
NP_003668.2	27501446	DENR	density-regulated protein	1.3	1.0	1.1	0.9	1.4	1.0	1.0	1.1	1.4	1.0	1.1	1.0	25.3	1	4	4	12	198	22.1	5.3
NP_005653.3	25188179	VDAC3	voltage-dependent anion-selective channel protein 3 isoform 1	0.9	0.9	0.9	1.0	1.0	1.0	1.0	1.0	1.0	1.0	1.0	1.0	34.3	2	7	8	86	283	30.6	8.7
NP_149098.2	111955139	GTPBP10	GTP-binding protein 10 isoform 2	0.9	1.0	1.1	1.1	0.9	0.9	1.0	0.9	0.9	0.9	1.0	1.0	3.9	2	2	2	3	387	42.9	9.0
NP_000991.1	4506647	RPL39	60S ribosomal protein L39	1.1	1.1	1.1	1.1	1.1	0.9	1.0	0.9	1.1	1.0	1.0	1.0	19.6	1	1	1	2	51	6.4	12.6
NP_057388.1	10047102	RSL24D1	probable ribosome biogenesis protein RLP24	1.0	1.0	0.9	1.0	0.9	1.0	1.0	1.0	0.9	1.0	1.0	1.0	16.6	1	2	2	8	163	19.6	10.0
NP_057308.1	7706706	SNX9	sorting nexin-9	1.0	1.0	1.0	1.0	1.1	1.0	1.1	1.0	1.0	1.0	1.0	1.0	44.4	1	16	16	55	595	66.5	5.6
NP_060695.2	109948304	SMU1	WD40 repeat-containing protein SMU1	1.0	1.0	1.0	1.0	1.0	1.0	1.0	1.0	1.0	1.0	1.0	1.0	34.5	1	14	14	43	513	57.5	7.2
NP_002809.2	30410792	PSME2	proteasome activator complex subunit 2	1.2	1.0	1.1	1.0	1.0	0.9	1.1	1.0	1.1	1.0	1.1	1.0	46.9	1	9	9	66	239	27.4	5.7
NP_008987.2	133922600	CLASRP	CLK4-associating serine/arginine rich protein isoform 1	1.0	1.0	0.9	1.0	1.0	1.0	0.9	1.0	1.0	1.0	0.9	1.0	10.1	3	5	6	7	674	77.1	10.4
NP_003874.2	13128862	HDAC3	histone deacetylase 3	1.0	1.0	1.1	1.1	0.8	0.9	0.9	0.9	0.9	1.0	1.0	1.0	9.8	1	2	3	5	428	48.8	5.2
NP_036382.2	21361416	RRAS2	ras-related protein R-Ras2 isoform a	1.3	1.2	1.3	1.0	1.3	1.1	1.2	1.0	1.3	1.2	1.3	1.0	48.0	3	7	8	14	204	23.4	6.0
NP_003869.1	4503987	GGH	gamma-glutamyl hydrolase precursor	1.1	1.0	1.0	1.1	1.0	0.9	1.0	0.9	1.1	1.0	1.0	1.0	14.8	1	4	4	7	318	35.9	7.1
NP_036335.1	6912396	GRHPR	glyoxylate reductase/hydroxypyruvate reductase	1.1	1.0	1.1	1.0	1.1	1.0	1.1	1.0	1.1	1.0	1.1	1.0	46.3	1	10	10	35	328	35.6	7.4
NP_004183.2	117553627	ASMTL	N-acetylserotonin O-methyltransferase-like protein isoform 1	1.1	1.0	1.1	1.0	1.1	1.0	1.1	1.0	1.1	1.0	1.1	1.0	35.6	3	15	15	39	621	68.8	6.1
NP_004035.2	20127454	ATIC	bifunctional purine biosynthesis protein PURH	1.1	1.0	1.0	1.0	1.1	1.0	1.0	1.0	1.1	1.0	1.0	1.0	61.5	1	27	27	132	592	64.6	6.7
NP_001123513.1	194239668	SHC1	SHC-transforming protein 1 isoform 4	1.0	1.0	1.0	1.0	0.9	1.0	1.0	1.0	1.0	1.0	1.0	1.0	13.7	6	4	4	7	473	51.6	7.2
NP_036330.1	6912388	GCA	grancalcin	1.2	1.0	1.1	1.1	1.2	1.0	0.9	0.9	1.2	1.0	1.0	1.0	10.1	1	1	1	2	217	24.0	5.2
NP_001138908.1	224177556	LSS	lanosterol synthase isoform 2	1.1	1.3	1.2	1.0	1.1	1.2	1.1	1.0	1.1	1.2	1.2	1.0	16.4	3	10	10	33	721	82.1	6.7
NP_065101.1	9966799	UTP3	something about silencing protein 10	0.8	1.0	0.9	1.0	0.9	0.9	0.9	1.0	0.8	0.9	0.9	1.0	17.1	1	7	7	11	479	54.5	5.6
NP_001153864.1	238550165	TRPT1	tRNA 2'-phosphotransferase 1 isoform 4	1.0	1.0	1.0	1.0	1.6	1.0	1.0	1.0	1.3	1.0	1.0	1.0	22.7	5	4	4	9	216	23.6	9.9
NP_001254792.1	392583865	SLIRP	SRA stem-loop-interacting RNA-binding protein, mitochondrial isoform 2 precursor	1.0	1.0	1.1	1.0	1.0	1.1	1.1	1.0	1.0	1.1	1.1	1.0	31.8	3	3	3	10	107	12.1	10.2
NP_008993.1	5902034	PWP1	periodic tryptophan protein 1 homolog	1.0	1.1	1.1	1.0	1.0	1.0	1.0	1.0	1.0	1.1	1.0	1.0	21.8	1	4	4	5	501	55.8	4.8
NP_690620.1	22902128	PTK7	inactive tyrosine-protein kinase 7 isoform c precursor	0.9	0.9	1.0	1.0	0.9	0.9	0.9	1.0	0.9	0.9	1.0	1.0	13.2	5	8	8	23	940	103.5	7.1
NP_001651.1	4502205	ARF4	ADP-ribosylation factor 4	1.2	1.1	1.1	1.0	1.2	1.1	1.2	1.0	1.2	1.1	1.2	1.0	83.3	9	7	11	137	180	20.5	7.1
NP_001275767.1	572153091	VRK2	serine/threonine-protein kinase VRK2 isoform 3	1.0	1.0	1.0	1.0	0.9	0.9	0.9	1.0	0.9	1.0	1.0	1.0	10.9	4	3	3	5	396	45.0	9.1
NP_060779.2	217035118	TBC1D23	TBC1 domain family member 23 isoform 2	1.1	1.1	1.1	1.0	1.1	1.0	1.0	1.0	1.1	1.1	1.1	1.0	3.1	2	2	2	2	684	76.5	5.4
NP_001275.1	4502861	AP3S1	AP-3 complex subunit sigma-1	1.0	1.0	1.0	1.0	1.1	1.1	1.0	1.0	1.1	1.0	1.0	1.0	7.8	1	1	1	9	193	21.7	5.4
NP_055850.1	28827782	ZHX3	zinc fingers and homeoboxes protein 3	0.9	1.1	1.0	1.0	0.9	1.0	0.9	1.0	0.9	1.0	1.0	1.0	2.9	1	2	2	3	956	104.6	6.1
NP_002676.1	4505917	EXOSC10	exosome component 10 isoform 2	1.0	1.0	1.0	1.0	1.0	0.9	1.0	1.0	1.0	1.0	1.0	1.0	23.6	2	16	16	40	860	98.0	8.4
NP_008937.1	5901926	NUDT21	cleavage and polyadenylation specificity factor subunit 5	1.0	0.9	0.9	1.0	0.9	1.0	1.0	1.0	1.0	0.9	0.9	1.0	32.2	1	7	7	15	227	26.2	8.8
NP_003550.1	4505819	PIP4K2B	phosphatidylinositol 5-phosphate 4-kinase type-2 beta	1.6	1.1	1.0	1.0	1.2	1.0	1.0	1.0	1.4	1.0	1.0	1.0	9.9	1	2	3	4	416	47.3	7.3
NP_001159434.1	260166619	ELAC2	zinc phosphodiesterase ELAC protein 2 isoform 3	1.0	1.0	0.9	1.0	1.0	1.0	1.1	1.0	1.0	1.0	1.0	1.0	25.8	3	15	15	39	786	87.5	8.0
NP_001240605.1	358439409	TBPL1	TATA box-binding protein-like protein 1	1.0	1.1	1.0	1.1	1.1	0.9	1.0	0.9	1.0	1.0	1.0	1.0	9.1	1	1	1	1	186	20.9	9.5
NP_998766.1	47106067	TMED8	protein TMED8	0.9	1.1	0.9	1.0	1.0	1.2	0.9	1.0	0.9	1.1	0.9	1.0	10.5	1	2	2	3	325	35.7	4.7
NP_001034581.1	87159836;87159832	FHL2	four and a half LIM domains protein 2	1.1	0.9	1.0	0.9	1.0	1.1	1.2	1.1	1.1	1.0	1.1	1.0	9.7	1	3	3	3	279	32.2	7.5
NP_001245142.1	384871583	PCID2	PCI domain-containing protein 2 isoform 3	1.0	0.9	1.0	1.0	1.1	0.8	0.9	1.0	1.1	0.9	0.9	1.0	8.3	3	3	3	7	397	45.7	8.5
NP_071760.2	21361912	DNAJC1	dnaJ homolog subfamily C member 1 precursor	1.3	1.3	1.2	1.0	1.2	1.1	1.0	1.0	1.2	1.2	1.1	1.0	3.8	1	2	2	3	554	63.8	8.6
NP_003972.1	4506039	PRC1	protein regulator of cytokinesis 1 isoform 1	1.0	1.0	0.9	1.0	0.9	1.0	0.9	1.0	0.9	1.0	0.9	1.0	46.8	2	1	23	87	620	71.6	6.6
NP_001505.1	4504193	GTF2B	transcription initiation factor IIB	1.1	1.0	1.0	1.0	0.9	1.0	0.9	1.0	1.0	1.0	1.0	1.0	33.9	1	7	7	10	316	34.8	8.4
NP_001254726.1	392583926;119220575	MED24	mediator of RNA polymerase II transcription subunit 24 isoform 2	1.0	1.0	1.0	1.0	1.1	0.9	1.0	1.0	1.1	1.0	1.0	1.0	5.6	2	4	4	9	976	108.9	6.9
NP_055928.3	73747881	ZZEF1	zinc finger ZZ-type and EF-hand domain-containing protein 1	1.0	1.0	1.0	1.2	0.9	0.9	0.9	0.8	1.0	0.9	1.0	1.0	0.7	1	2	2	3	2961	330.9	5.9
NP_001257411.1	394953908	PSMD11	26S proteasome non-ATPase regulatory subunit 11	1.0	1.0	1.1	1.0	1.0	1.0	1.1	1.0	1.0	1.0	1.1	1.0	34.8	1	12	12	64	422	47.4	6.5
NP_001035808.1	108773799	GDAP1	ganglioside-induced differentiation-associated protein 1 isoform b	0.9	0.9	0.8	1.1	1.1	0.8	0.9	0.9	1.0	0.9	0.8	1.0	7.2	2	2	2	3	290	33.5	9.0
NP_001093636.1	154354972	PHACTR2	phosphatase and actin regulator 2 isoform 4	1.1	1.0	1.0	1.0	1.0	1.0	1.0	1.0	1.1	1.0	1.0	1.0	2.4	4	1	1	1	554	61.4	6.0
NP_001106963.1	164698494	Sep-09	septin-9 isoform a	0.9	0.9	0.9	0.9	1.0	1.0	1.0	1.0	1.0	0.9	0.9	1.0	34.5	8	19	19	54	586	65.4	9.0
NP_001258898.1	431822401;20149594	HSP90AB1	heat shock protein HSP 90-beta isoform a	1.0	1.1	1.0	1.0	1.0	1.0	1.0	1.0	1.0	1.1	1.0	1.0	81.1	3	30	54	1197	724	83.2	5.0
NP_001021.1	4506711	RPS27	40S ribosomal protein S27	1.0	1.0	1.0	1.0	0.9	1.0	0.9	1.0	0.9	1.0	0.9	1.0	38.1	1	1	3	24	84	9.5	9.5

Khan *et al.* , 2019. Multi-omics analysis to characterize cigarette smoke induced molecular alterations in esophageal cells

Supplementary Table 5. List of proteins quantified in untreated and chronically treated Het1A cells with cigarette smoke condensate for 8 months

NP_Accession	Protein group Accession	Gene ID	Description	Het-1A-Smoke - 2M/Parental	Het-1A-Smoke - 4M/Parental	Het-1A-Smoke - 6M/Parental	Het-1A-Smoke - 8M/Parental	Het-1A-Smoke - 2M/Parental	Het-1A-Smoke - 4M/Parental	Het-1A-Smoke - 6M/Parental	Het-1A-Smoke - 8M/Parental	Het-1A-Smoke - 2M/Parental	Het-1A-Smoke - 4M/Parental	Het-1A-Smoke - 6M/Parental	Het-1A-Smoke - 8M/Parental	Coverage (%)	Proteins	Unique Peptides	# Peptides	PSM	# AAs	MW [kDa]	calc. pI
				Replicate 1				Replicate 2				Average of replicates											
NP_001138528.1	222831610	SLC44A2	choline transporter-like protein 2 isoform 2	0.9	1.0	0.9	1.0	1.1	1.1	0.9	1.0	1.0	1.1	0.9	1.0	3.1	2	2	2	5	704	79.8	8.5
NP_056295.1	11386199	PVRL3	poliovirus receptor-related protein 3 isoform 1 precursor	1.2	1.0	1.2	1.1	1.0	1.2	0.9	0.9	1.1	1.1	1.0	1.0	3.6	1	1	1	2	549	61.0	6.2
NP_005490.1	4885649	UBA2	SUMO-activating enzyme subunit 2	1.0	1.0	1.0	1.0	1.0	1.0	1.0	1.0	1.0	1.0	1.0	1.0	49.8	1	25	25	119	640	71.2	5.3
NP_932072.1	37574606	ZMYM2	zinc finger MYM-type protein 2	0.9	0.9	1.0	1.0	1.2	1.0	1.0	1.0	1.1	1.0	1.0	1.0	4.9	1	4	4	5	1377	154.8	6.3
NP_112574.3	31542509	ADPGK	ADP-dependent glucokinase precursor	1.1	1.0	1.0	0.9	1.0	0.9	1.0	1.1	1.0	1.0	1.0	1.0	16.1	1	5	5	8	496	53.9	6.2
NP_055781.2	20336290	DHX30	putative ATP-dependent RNA helicase DHX30 isoform 2	1.0	1.0	1.0	1.0	1.0	1.0	1.0	1.0	1.0	1.0	1.0	1.0	27.8	2	25	25	76	1155	129.4	8.5
NP_001138621.1	223468574	VAMP7	vesicle-associated membrane protein 7 isoform 2	0.9	1.1	0.9	1.2	0.9	0.8	0.7	0.8	0.9	0.9	0.8	1.0	11.7	3	2	2	3	179	20.1	9.3
NP_005125.1	4826934	PPP4R1	serine/threonine-protein phosphatase 4 regulatory subunit 1 isoform b	1.1	1.0	1.1	0.9	1.1	1.1	1.1	1.1	1.1	1.1	1.1	1.0	9.5	2	6	6	12	933	105.1	4.8
NP_005100.1	4826878	OXSR1	serine/threonine-protein kinase OSR1	1.2	1.2	1.3	1.0	1.3	1.1	1.3	1.0	1.3	1.2	1.3	1.0	38.0	1	14	15	46	527	58.0	6.4
NP_001139284.1	224926822;224926824	SH2B1	SH2B adapter protein 1 isoform 2	1.0	1.0	1.0	1.1	0.8	0.8	1.0	0.9	0.9	0.9	1.0	1.0	8.4	3	3	3	3	671	70.7	6.1
NP_008839.2	9845511	RAC1	ras-related C3 botulinum toxin substrate 1 isoform Rac1	1.0	1.0	1.0	1.0	0.9	1.0	1.1	1.0	0.9	1.0	1.1	1.0	34.9	6	6	7	22	192	21.4	8.5
NP_444505.1	16933546;4506667	RPLP0	60S acidic ribosomal protein P0	1.0	0.9	1.0	1.0	0.9	1.0	1.0	1.0	0.9	0.9	1.0	1.0	45.1	1	10	10	181	317	34.3	6.0
NP_115786.1	14165274	HOOK3	protein Hook homolog 3	1.0	1.1	1.2	1.1	1.0	1.0	1.1	0.9	1.0	1.0	1.1	1.0	5.6	1	3	3	7	718	83.1	5.2
NP_060524.4	54860105	ARHGAP17	rho GTPase-activating protein 17 isoform 2	1.0	0.9	1.0	1.0	1.0	1.0	1.0	1.0	1.0	1.0	1.0	1.0	11.8	3	6	6	12	803	87.6	7.6
NP_149072.2	301129165;301129170	DIDO1	death-inducer obliterator 1 isoform c	1.0	1.0	1.0	1.0	1.0	1.0	1.0	1.0	1.0	1.0	1.0	1.0	9.1	3	14	14	24	2240	243.7	7.9
NP_001129116.1	208431795	DCTN4	dynactin subunit 4 isoform c	1.0	0.9	0.9	1.0	0.9	1.0	1.0	1.0	0.9	1.0	1.0	1.0	35.5	3	8	8	31	403	45.7	7.6
NP_056464.1	24308115	IRF2BP1	interferon regulatory factor 2-binding protein 1	1.4	0.9	0.9	1.0	1.0	1.0	1.0	1.0	1.2	0.9	0.9	1.0	8.9	1	2	2	3	584	61.6	8.2
NP_060460.4	194306651	PDPR	pyruvate dehydrogenase phosphatase regulatory subunit, mitochondrial precursor	1.1	1.0	1.1	1.0	1.1	1.1	1.2	1.0	1.1	1.0	1.1	1.0	7.1	1	5	5	9	879	99.3	6.4
YP_003024030.1	251831111	ATP8	ATP synthase F0 subunit 8	1.1	0.9	1.1	1.0	1.0	1.0	1.0	1.0	1.0	1.0	1.0	1.0	29.4	1	2	2	2	68	8.0	9.9
NP_004274.1	4759000	RAB3D	ras-related protein Rab-3D	0.8	1.0	1.0	0.9	1.0	1.1	1.1	1.1	0.9	1.0	1.1	1.0	24.7	10	2	5	11	219	24.3	4.9
NP_612430.2	116235444	SAAL1	protein SAAL1	1.0	0.9	1.0	1.1	0.9	0.9	0.9	0.9	0.9	0.9	0.9	1.0	8.9	1	3	3	21	474	53.5	4.5
NP_056238.2	20357568	AASDHPPT	L-aminoadipate-semialdehyde dehydrogenase-phosphopantetheinyl transferase	0.9	0.9	1.0	1.0	1.0	1.0	1.0	1.0	0.9	0.9	1.0	1.0	28.8	1	7	7	22	309	35.8	6.8
NP_054907.1	7661832	SSU72	RNA polymerase II subunit A C-terminal domain phosphatase SSU72	1.0	1.0	1.0	1.1	1.0	0.9	1.0	0.9	1.0	0.9	1.0	1.0	13.9	1	2	2	4	194	22.6	5.3
NP_056133.2	41327769	ARHGEF18	rho guanine nucleotide exchange factor 18 isoform a	1.1	1.0	1.0	1.0	1.0	1.0	1.1	1.0	1.0	1.0	1.0	1.0	0.9	2	1	1	2	1015	114.0	6.2
NP_998776.1	47132595	SLC25A3	phosphate carrier protein, mitochondrial isoform b precursor	1.0	1.0	1.0	1.0	1.0	0.9	1.0	1.0	1.0	1.0	1.0	1.0	40.7	2	10	10	28	361	39.9	9.4
NP_002942.2	35493916	RPN2	dolichyl-diphosphooligosaccharide--protein glycosyltransferase subunit 2 isoform 1 precursor	1.1	1.0	1.1	1.0	1.1	1.0	1.1	1.0	1.1	1.0	1.1	1.0	56.6	2	24	24	134	631	69.2	5.7
NP_060697.4	150417996	UBA6	ubiquitin-like modifier-activating enzyme 6	1.0	1.0	1.1	1.0	1.0	1.0	1.0	1.0	1.0	1.0	1.1	1.0	20.0	1	18	18	41	1052	117.9	6.1
NP_037453.1	23943858	SNX8	sorting nexin-8	0.9	0.8	0.9	0.9	1.0	1.0	1.0	1.1	0.9	0.9	0.9	1.0	11.8	1	3	3	4	465	52.5	7.4
NP_004499.2	40018633	IDI1	isopentenyl-diphosphate Delta-isomerase 1	1.1	1.1	1.1	1.0	1.1	1.1	1.0	1.0	1.1	1.1	1.1	1.0	39.8	1	8	8	27	284	32.5	7.9
NP_006614.2	23308577	PHGDH	D-3-phosphoglycerate dehydrogenase	1.3	1.1	1.0	1.0	1.2	1.1	0.9	1.0	1.3	1.1	1.0	1.0	44.8	1	19	19	141	533	56.6	6.7
NP_005947.3	222136639	MTHFD1	C-1-tetrahydrofolate synthase, cytoplasmic	1.0	0.9	0.9	1.0	1.0	0.9	0.9	1.0	1.0	0.9	0.9	1.0	61.8	1	41	42	208	935	101.5	7.3
NP_005792.1	5032133	EIF1	eukaryotic translation initiation factor 1	1.2	1.0	1.1	1.1	1.5	1.0	1.1	0.9	1.3	1.0	1.1	1.0	38.9	2	4	4	13	113	12.7	7.4
NP_736606.1	28605137	PSMD10	26S proteasome non-ATPase regulatory subunit 10 isoform 2	0.8	1.0	0.8	0.9	1.0	1.0	1.0	1.1	0.9	1.0	0.9	1.0	6.6	2	1	1	2	151	16.1	5.9
NP_005042.1	4826960	QARS	glutamine--tRNA ligase isoform a	1.0	1.0	1.0	1.0	1.1	1.0	1.1	1.0	1.0	1.0	1.0	1.0	38.7	2	22	23	67	775	87.7	7.2
NP_005735.2	187761373	ARIH1	E3 ubiquitin-protein ligase ARIH1	1.0	1.0	1.0	1.0	0.9	0.9	0.8	1.0	1.0	1.0	0.9	1.0	11.5	1	4	4	9	557	64.1	5.1
NP_596869.4	291045225	TTN	titin isoform N2-A	0.9	1.1	1.1	1.0	0.9	1.0	1.0	1.0	0.9	1.0	1.1	1.0	0.1	8	3	6	8	33423	3711.3	6.5
NP_002802.2	25777615	PSMD7	26S proteasome non-ATPase regulatory subunit 7	1.1	1.1	1.1	1.0	0.9	1.0	1.0	1.0	1.0	1.0	1.1	1.0	33.3	1	8	8	35	324	37.0	6.8
NP_002843.2	167900484	PTX3	pentraxin-related protein PTX3 precursor	1.2	1.1	1.2	1.0	1.2	1.1	1.2	1.0	1.2	1.1	1.2	1.0	3.4	1	1	1	1	381	41.9	5.0
NP_060729.2	21361787	TTC17	tetratricopeptide repeat protein 17	0.9	0.9	0.9	0.9	0.9	1.1	1.1	1.1	0.9	1.0	1.0	1.0	1.5	1	1	1	1	1141	129.5	6.6
NP_872620.1	33356550	GPATCH4	G patch domain-containing protein 4 isoform 2	1.0	0.9	1.0	1.0	1.0	1.0	1.0	1.0	1.0	1.0	1.0	1.0	17.6	2	4	4	8	370	42.0	9.3
NP_031397.1	11067749	BRD3	bromodomain-containing protein 3	0.8	0.9	0.9	0.9	0.9	0.9	0.9	1.1	0.9	0.9	0.9	1.0	10.3	1	4	6	11	726	79.5	9.4
NP_004598.1	4759212	TBCA	tubulin-specific chaperone A	1.3	0.9	1.2	0.9	1.7	1.1	2.0	1.1	1.5	1.0	1.6	1.0	14.8	1	2	2	3	108	12.8	5.3
NP_064716.2	197100773	RARS2	probable arginine--tRNA ligase, mitochondrial precursor	1.0	1.0	1.1	1.1	1.0	0.8	1.0	0.9	1.0	0.9	1.0	1.0	11.1	1	6	6	10	578	65.5	8.2
NP_036333.2	54633312	GLG1	Golgi apparatus protein 1 isoform 1 precursor	1.1	0.9	1.0	1.0	1.0	1.0	1.0	1.0	1.0	1.0	1.0	1.0	17.0	3	17	17	39	1203	137.1	6.9
NP_001093894.1	154426312	SPATS2L	SPATS2-like protein isoform b	0.8	0.8	0.9	1.0	1.0	0.9	0.9	1.0	0.9	0.9	0.9	1.0	13.3	4	5	5	8	489	54.1	9.6
NP_001177738.1	299829255	DHX35	probable ATP-dependent RNA helicase DHX35 isoform 2	0.9	0.9	0.9	1.0	0.9	0.9	0.9	1.0	0.9	0.9	0.9	1.0	2.7	2	2	2	4	672	75.4	8.6
NP_055323.2	22907039	APOBEC3C	DNA dC->dU-editing enzyme APOBEC-3C	1.0	1.0	1.1	0.9	1.1	1.3	1.1	1.1	1.0	1.1	1.1	1.0	16.3	1	2	2	2	190	22.8	7.6
NP_054768.2	21361497	ACAD9	acyl-CoA dehydrogenase family member 9, mitochondrial	1.3	1.2	1.1	1.0	1.2	1.2	1.1	1.0	1.2	1.2	1.1	1.0	31.2	1	17	17	56	621	68.7	8.0
NP_061932.1	9506859	TOMM7	mitochondrial import receptor subunit TOM7 homolog	1.0	1.1	1.0	1.0	1.2	1.1	1.3	1.0	1.1	1.1	1.1	1.0	41.8	1	2	2	5	55	6.2	10.3
NP_000173.2	20127408	HADHA	trifunctional enzyme subunit alpha, mitochondrial precursor	1.0	1.2	1.1	1.0	1.0	1.1	1.1	1.0	1.0	1.1	1.1	1.0	46.9	1	30	30	169	763	82.9	9.0
NP_001162578.1	281182700	FAM21C	WASH complex subunit FAM21C isoform 3	1.0	1.1	1.0	0.9	1.1	1.2	1.0	1.1	1.1	1.2	1.0	1.0	17.4	3	1	13	48	1245	136.5	4.7
NP_059139.3	224586804	POLE3	DNA polymerase epsilon subunit 3	1.1	1.0	1.0	1.1	1.1	1.1	0.9	0.9	1.1	1.1	1.0	1.0	19.7	1	2	2	6	147	16.8	4.7
NP_003795.2	57242761	RIPK1	receptor-interacting serine/threonine-protein kinase 1	1.0	0.9	1.0	1.0	1.0	0.9	0.9	1.0	1.0	0.9	0.9	1.0	6.1	1	3	3	5	671	75.9	6.3
NP_060598.3	39780588	TSR1	pre-rRNA-processing protein TSR1 homolog	1.0	1.0	1.0	1.0	1.0	1.1	1.1	1.0	1.0	1.0	1.0	1.0	29.2	1	18	18	55	804	91.8	7.4
NP_073739.3	269847874	YTHDC2	probable ATP-dependent RNA helicase YTHDC2	1.1	1.1	1.1	1.0	0.9	1.0	0.9	1.0	1.0	1.0	1.0	1.0	3.9	1	4	4	6	1430	160.1	8.4
NP_598000.2	115529484	CD109	CD109 antigen isoform 1 preproprotein	0.8	0.8	1.0	1.0	0.8	0.8	0.9	1.0	0.8	0.8	0.9	1.0	19.8	3	19	19	60	1445	161.6	5.8
NP_001135974.1	215820635;63003907	PPP1R13L	relA-associated inhibitor	1.1	1.1	1.0	1.0	0.9	0.9	1.0	1.0	1.0	1.0	1.0	1.0	11.2	1	5	5	8	828	89.0	6.8
NP_003482.1	10835057	NAA10	N-alpha-acetyltransferase 10 isoform 1	1.1	1.1	1.0	1.0	0.9	1.0	1.0	0.9	1.0	1.0	1.0	1.0	45.5	4	8	8	32	235	26.4	5.6
NP_612450.2	115511030	MB21D1	cyclic GMP-AMP synthase	1.1	1.2	1.0	1.0	0.9	1.0	1.0	1.0	1.0	1.1	1.0	1.0	24.0	1	9	9	28	522	58.8	9.5
NP_059119.2	157502212	GALNT7	N-acetylgalactosaminyltransferase 7	1.4	1.1	1.2	1.0	1.0	1.1	1.2	1.0	1.2	1.1	1.2	1.0	2.1	1	1	1	2	657	75.3	7.1
NP_001131136.1	212549738	ANAPC7	anaphase-promoting complex subunit 7 isoform b	1.0	1.1	1.1	1.0	1.0	1.0	1.0	1.0	1.0	1.1	1.1	1.0	9.5	2	4	4	10	537	60.0	8.4
NP_001087.2	38569421	ACLY	ATP-citrate synthase isoform 1	1.2	1.2	1.1	1.0	0.9	1.0	0.9	1.0	1.0	1.1	1.0	1.0	53.5	4	49	49	320	1101	120.8	7.3
NP_001123654.1	194328760	DNAJA4	dnaJ homolog subfamily A member 4 isoform 2	1.1	1.1	1.1	1.0	1.0	1.0	1.0	1.0	1.0	1.0	1.1	1.0	19.1	3	5	6	16	397	44.8	7.6
NP_001273444.1	557357729	ATF7IP	activating transcription factor 7-interacting protein 1 isoform 4	1.0	0.9	1.0	1.0	1.0	1.0	1.2	1.0	1.0	1.0	1.1	1.0	4.2	4	3	3	4	1105	118.6	4.6
NP_076945.2	148596996	CUEDC2	CUE domain-containing protein 2	1.0	0.9	1.3	1.0	1.0	1.0	1.2	1.0	1.0	0.9	1.2	1.0	8.4	1	2	2	3	287	32.0	4.8
NP_002094.2	31542868	GYS1	glycogen [starch] synthase, muscle isoform 1	1.0	1.0	1.1	1.0	1.0	1.0	1.0	1.0	1.0	1.0	1.0	1.0	23.2	2	11	11	36	737	83.7	6.2
NP_001262.3	118421089	CHD2	chromodomain-helicase-DNA-binding protein 2 isoform 1	1.0	1.0	1.0	1.0	1.0	1.0	1.0	1.0	1.0	1.0	1.0	1.0	6.8	2	8	10	20	1828	211.2	8.1
NP_002256.2	19923142	KPNB1	importin subunit beta-1 isoform 1	1.0	0.9	1.0	1.0	1.0	1.0	1.0	1.0	1.0	1.0	1.0	1.0	61.6	2	32	32	285	876	97.1	4.8
NP_056417.2	39753957	TOR1AIP1	torsin-1A-interacting protein 1 isoform 2	1.0	1.0	1.1	1.1	1.0	0.9	1.0	0.9	1.0	1.0	1.1	1.0	37.7	2	15	15	48	583	66.2	8.2
NP_005031.1	4826940	PRCP	lysosomal Pro-X carboxypeptidase isoform 1 preproprotein	1.2	1.1	1.0	1.0	1.0	1.0	0.9	1.0	1.1	1.0	1.0	1.0	10.9	2	3	3	9	496	55.8	7.2
NP_005423.1	4885659	XRCC3	DNA repair protein XRCC3	1.0	1.0	0.9	1.1	0.9	0.9	0.9	0.9	0.9	0.9	0.9	1.0	4.1	1	1	1	1	346	37.8	8.5
NP_001193819.1	332688220	CTNND1	catenin delta-1 isoform 3A	1.0	1.0	1.0	1.0	1.0	1.0	1.0	1.0	1.0	1.0	1.0	1.0	26.8	15	16	16	33	832	92.6	7.2
NP_001340.2	45439306	DARS	aspartate--tRNA ligase, cytoplasmic isoform 1	1.0	1.0	1.0	1.0	1.0	1.0	1.0	1.0	1.0	1.0	1.0	1.0	54.1	2	25	25	103	501	57.1	6.6
NP_006242.5	94557301	PRKAA1	5'-AMP-activated protein kinase catalytic subunit alpha-1 isoform 1	1.0	0.9	1.0	1.0	1.0	1.1	1.0	1.0	1.0	1.0	1.0	1.0	21.8	3	9	9	23	559	64.0	8.1
NP_056289.2	38016914	SAMHD1	deoxynucleoside triphosphate triphosphohydrolase SAMHD1	0.9	0.9	0.9	1.0	0.9	0.8	0.9	1.0	0.9	0.8	0.9	1.0	34.4	1	18	18	48	626	72.2	7.1
NP_002286.2	9845502;59859885	RPSA	40S ribosomal protein SA isoform 1	1.0	1.0	1.0	1.0	1.0	1.0	1.0	1.0	1.0	1.0	1.0	1.0	59.0	2	13	13	146	295	32.8	4.9
NP_114108.1	13994259	MRPS5	28S ribosomal protein S5, mitochondrial	1.1	1.1	1.0	1.0	1.2	1.1	1.2	1.0	1.1	1.1	1.1	1.0	20.2	1	6	6	15	430	48.0	9.9
NP_001136122.1	217416379	HNRNPLL	heterogeneous nuclear ribonucleoprotein L-like isoform 2	1.0	0.9	1.0	1.0	1.0	0.9	1.0	1.0	1.0	0.9	1.0	1.0	11.0	2	4	4	6	537	59.6	7.7
NP_009167.1	6005731	CHP1	calcineurin B homologous protein 1	0.9	0.9	0.8	0.9	1.2	1.0	1.1	1.1	1.1	1.0	1.0	1.0	32.8	1	5	5	11	195	22.4	5.1
NP_001265114.1	499137509	HERC4	probable E3 ubiquitin-protein ligase HERC4 isoform c	1.0	1.0	1.1	1.0	1.0	1.0	1.1	1.0	1.0	1.0	1.1	1.0	10.6	5	7	7	11	979	109.5	6.5
NP_060855.2	223029424	LSG1	large subunit GTPase 1 homolog	1.0	1.0	1.0	1.0	1.0	1.0	1.1	1.0	1.0	1.0	1.1	1.0	14.3	1	7	7	10	658	75.2	6.4
NP_001103.1	4501919	ADARB1	double-stranded RNA-specific editase 1 isoform 1	1.0	1.0	1.1	0.9	1.0	1.0	1.0	1.0	1.0	1.0	1.0	1.0	9.3	4	4	4	4	701	76.6	9.0
NP_001265098.1	499591687	CDK5RAP1	CDK5 regulatory subunit-associated protein 1 isoform e	0.9	1.0	1.0	1.0	0.9	0.8	0.8	1.0	0.9	0.9	0.9	1.0	2.3	4	1	1	2	560	62.9	7.4
NP_112604.2	117189975;117190192	HNRNPC	heterogeneous nuclear ribonucleoproteins C1/C2 isoform a	1.0	1.0	1.0	1.0	1.1	1.1	1.0	1.0	1.0	1.1	1.0	1.0	59.8	3	1	21	305	306	33.6	5.1
NP_001655.1	10835049	RHOA	transforming protein RhoA precursor	1.0	1.0	1.1	0.9	0.9	0.9	0.9	1.1	0.9	0.9	1.0	1.0	61.1	1	3	8	54	193	21.8	6.1
NP_056518.2	25092725	RRP7A	ribosomal RNA-processing protein 7 homolog A	1.0	1.0	0.9	1.0	1.0	1.0	1.0	1.0	1.0	1.0	1.0	1.0	30.7	1	6	6	15	280	32.3	9.6

Khan *et al.* , 2019. Multi-omics analysis to characterize cigarette smoke induced molecular alterations in esophageal cells
Supplementary Table 5. List of proteins quantified in untreated and chronically treated Het1A cells with cigarette smoke condensate for 8 months

NP_Accession	Protein group Accession	Gene ID	Description	Het-1A-Smoke - 2M/Parental	Het-1A-Smoke - 4M/Parental	Het-1A-Smoke - 6M/Parental	Het-1A-Smoke - 8M/Parental	Het-1A-Smoke - 2M/Parental	Het-1A-Smoke - 4M/Parental	Het-1A-Smoke - 6M/Parental	Het-1A-Smoke - 8M/Parental	Het-1A-Smoke - 2M/Parental	Het-1A-Smoke - 4M/Parental	Het-1A-Smoke - 6M/Parental	Het-1A-Smoke - 8M/Parental	Coverage (%)	Proteins	Unique Peptides	# Peptides	PSM	# AAs	MW [kDa]	calc. pI
				Replicate 1				Replicate 2				Average of replicates											
NP_000131.2	60499021	FECH	ferrochelatase, mitochondrial isoform b precursor	1.0	1.0	1.0	1.0	1.1	1.0	1.0	1.0	1.1	1.0	1.0	1.0	18.0	2	5	5	9	423	47.8	8.7
NP_057291.1	7706427	CPSF3	cleavage and polyadenylation specificity factor subunit 3	0.8	0.9	0.9	1.0	1.0	1.0	1.0	1.0	0.9	1.0	0.9	1.0	15.8	1	7	7	15	684	77.4	5.6
NP_004260.2	28558979	MED27	mediator of RNA polymerase II transcription subunit 27 isoform 1	0.9	0.9	0.9	1.0	1.1	0.9	0.9	1.0	1.0	0.9	0.9	1.0	13.8	3	3	3	7	311	35.4	9.3
NP_060624.1	8922549	ASF1B	histone chaperone ASF1B	1.0	0.9	0.8	0.9	1.0	1.0	0.9	1.1	1.0	0.9	0.9	1.0	32.2	1	3	3	9	202	22.4	4.6
NP_036231.1	6912246	CD3EAP	DNA-directed RNA polymerase I subunit RPA34 isoform 2	1.0	1.1	1.0	1.0	0.9	1.0	1.0	1.0	0.9	1.0	1.0	1.0	31.8	2	9	9	45	510	55.0	8.5
NP_001226.2	32454741;333360851	SERPINH1	serpin H1 precursor	1.0	1.0	1.1	1.0	1.0	1.0	1.1	1.0	1.0	1.0	1.1	1.0	55.3	1	20	20	113	418	46.4	8.7
NP_078991.3	94421469	TXNDC15	thioredoxin domain-containing protein 15 precursor	1.0	1.0	1.0	1.0	1.0	0.9	0.9	1.0	1.0	1.0	1.0	1.0	3.9	1	1	1	1	360	39.9	4.8
NP_001091742.1	148298677;148298764	HMGCS1	hydroxymethylglutaryl-CoA synthase, cytoplasmic	1.3	1.2	0.9	1.0	1.4	1.1	1.0	1.0	1.3	1.2	0.9	1.0	23.7	3	12	12	40	520	57.3	5.4
NP_002800.2	25777612	PSMD3	26S proteasome non-ATPase regulatory subunit 3	1.0	1.1	1.1	1.0	1.0	1.0	1.0	1.0	1.0	1.0	1.0	1.0	44.8	1	21	21	71	534	60.9	8.4
NP_060140.2	109148508	OTUB1	ubiquitin thioesterase OTUB1	1.0	1.0	1.0	1.0	1.0	1.0	1.0	1.0	1.0	1.0	1.0	1.0	43.9	1	8	8	37	271	31.3	4.9
NP_060830.2	281182404	TXLNG	gamma-taxilin isoform 1	0.9	1.0	0.9	1.0	1.0	1.0	0.9	1.0	1.0	1.0	0.9	1.0	24.6	2	11	12	29	528	60.5	7.5
NP_056272.2	41152072	ZDHHC5	palmitoyltransferase ZDHHC5	1.1	1.0	1.0	0.9	1.0	1.0	1.0	1.1	1.1	1.0	1.0	1.0	2.5	1	2	2	3	715	77.5	9.0
NP_004347.1	4757944	CD81	CD81 antigen	1.1	1.1	1.1	1.0	1.0	1.1	1.2	1.0	1.0	1.1	1.1	1.0	8.5	1	1	1	4	236	25.8	5.3
NP_115707.2	153945707	ECE2	endothelin-converting enzyme 2 isoform C	1.1	1.0	1.2	1.0	0.9	0.9	0.9	1.0	1.0	1.0	1.1	1.0	5.9	2	1	1	2	255	28.3	6.0
NP_116249.2	21361875	LTV1	protein LTV1 homolog	0.8	1.1	0.9	1.0	0.9	1.1	0.9	1.0	0.9	1.1	0.9	1.0	9.5	1	4	4	8	475	54.8	4.9
NP_057026.3	34147676	MTERF3	transcription termination factor 3, mitochondrial isoform 1 precursor	1.0	1.1	1.0	1.0	1.1	1.1	0.9	1.0	1.0	1.1	1.0	1.0	9.8	2	4	4	5	417	47.9	8.5
NP_219484.1	15529982	IMP4	U3 small nucleolar ribonucleoprotein protein IMP4	0.9	0.9	0.9	1.0	1.0	1.0	0.9	1.0	1.0	1.0	0.9	1.0	22.3	1	5	5	10	291	33.7	9.5
NP_777573.1	28212272	NOP9	nucleolar protein 9 isoform 1	1.1	1.1	1.0	1.0	1.0	1.0	1.1	0.9	1.0	1.0	1.0	1.0	20.3	2	7	7	19	636	69.4	7.3
NP_005660.4	115430112	REEP5	receptor expression-enhancing protein 5	1.2	1.0	1.0	1.0	1.2	1.1	1.2	1.0	1.2	1.0	1.1	1.0	20.1	1	5	5	13	189	21.5	8.1
NP_056042.1	13273311	POFUT2	GDP-fucose protein O-fucosyltransferase 2 isoform A precursor	1.0	1.1	1.1	1.0	1.1	1.1	1.0	1.0	1.0	1.1	1.0	1.0	9.7	2	3	3	10	424	48.9	6.8
NP_001269637.1	544185998	EXOSC2	exosome complex component RRP4 isoform 2	1.0	0.9	1.1	1.0	1.0	0.9	1.0	1.0	1.0	0.9	1.0	1.0	20.2	3	4	4	11	267	30.0	7.1
NP_758862.1	27502409	CD46	membrane cofactor protein isoform 5 precursor	1.0	1.0	1.0	1.0	1.0	1.1	1.1	0.9	1.0	1.0	1.0	1.0	2.8	3	1	1	1	362	40.8	6.2
NP_056121.2	260064009	USP24	ubiquitin carboxyl-terminal hydrolase 24	1.0	1.0	1.0	1.0	1.1	1.0	1.0	1.0	1.0	1.0	1.0	1.0	7.1	1	14	16	30	2620	294.2	6.1
NP_863656.1	32528286	ACOT7	cytosolic acyl coenzyme A thioester hydrolase isoform hBACHd	1.0	1.0	1.0	1.0	1.0	1.0	1.0	1.0	1.0	1.0	1.0	1.0	43.2	4	10	10	66	329	36.5	7.8
NP_001273106.1	555290021	C2CD5	C2 domain-containing protein 5 isoform d	1.1	1.0	1.1	1.0	1.1	1.2	1.2	1.0	1.1	1.1	1.1	1.0	8.3	5	6	6	13	1042	114.9	6.1
NP_005070.1	4827038	TPD52	tumor protein D52 isoform 3	1.0	0.8	0.9	0.9	1.0	1.0	1.2	1.1	1.0	0.9	1.0	1.0	68.5	7	8	8	29	184	19.9	5.0
NP_079430.3	110227866	SUN1	SUN domain-containing protein 1 isoform b	1.0	1.1	1.1	1.0	1.2	1.1	1.1	1.0	1.1	1.1	1.1	1.0	13.0	5	5	6	14	702	77.9	7.1
NP_001009880.1	57863295	KIAA0930	uncharacterized protein KIAA0930 isoform b	1.0	0.9	1.0	1.0	1.2	1.1	1.1	1.0	1.1	1.0	1.0	1.0	3.2	2	1	1	2	404	45.8	7.9
NP_036419.3	187608516	ACAP2	arf-GAP with coiled-coil, ANK repeat and PH domain-containing protein 2	1.0	1.0	1.0	1.0	1.1	1.0	1.0	1.0	1.1	1.0	1.0	1.0	20.4	1	13	13	32	778	88.0	6.8
NP_060245.3	406601114	TTC19	tetratricopeptide repeat protein 19, mitochondrial isoform 1 precursor	1.2	1.1	1.1	1.0	1.1	1.1	1.0	1.0	1.1	1.1	1.0	1.0	15.5	2	6	6	9	380	42.4	5.8
NP_612565.1	20143967	KIF23	kinesin-like protein KIF23 isoform 1	1.1	1.1	1.1	1.1	0.9	0.9	0.9	0.9	1.0	1.0	1.0	1.0	34.4	2	2	27	90	960	110.0	8.5
NP_057541.2	19923468	PRKD2	serine/threonine-protein kinase D2 isoform A	1.1	0.9	0.9	1.0	1.1	1.1	1.1	1.0	1.1	1.0	1.0	1.0	9.0	12	6	6	8	878	96.7	6.8
NP_055791.1	70980549	PDCD11	protein RRP5 homolog	0.9	1.0	1.1	1.0	1.0	1.0	1.0	1.0	0.9	1.0	1.0	1.0	12.7	1	20	20	46	1871	208.6	8.9
NP_976060.1	44680133;17738292	BDH1	D-beta-hydroxybutyrate dehydrogenase, mitochondrial precursor	1.0	1.0	1.1	1.0	1.0	1.0	1.0	1.0	1.0	1.0	1.1	1.0	23.6	1	5	5	18	343	38.1	9.0
NP_006377.2	38201710	DDX17	probable ATP-dependent RNA helicase DDX17 isoform 1	1.0	1.0	1.0	1.0	1.0	1.0	1.0	1.0	1.0	1.0	1.0	1.0	39.9	2	18	26	116	729	80.2	8.3
NP_001121362.1	189217847	SPG21	maspardin isoform b	0.9	0.9	1.0	1.0	0.9	0.9	0.9	0.9	0.9	0.9	0.9	1.0	7.1	2	1	1	4	281	31.6	6.4
NP_001422.1	4503579	EPB41L2	band 4.1-like protein 2 isoform a	1.0	1.0	1.0	1.0	1.0	1.0	1.1	1.0	1.0	1.0	1.1	1.0	31.0	4	19	22	73	1005	112.5	5.4
NP_056203.2	49472828	YIPF3	protein YIPF3	1.1	1.1	1.0	1.2	1.0	1.0	0.9	0.8	1.0	1.0	0.9	1.0	10.3	1	2	2	2	350	38.2	5.8
NP_001190174.1	322303127	RPS10	40S ribosomal protein S10	0.9	0.9	0.9	1.0	0.9	1.0	0.9	1.0	0.9	0.9	0.9	1.0	63.6	2	10	11	102	165	18.9	10.2
NP_056044.3	87162455	CLUH	clustered mitochondria protein homolog	1.0	1.0	1.0	1.0	1.0	1.1	1.2	1.0	1.0	1.0	1.1	1.0	18.3	1	17	17	36	1309	146.6	6.1
NP_002622.2	40068518	PGD	6-phosphogluconate dehydrogenase, decarboxylating isoform 1	1.1	1.1	1.1	1.0	1.0	1.0	1.0	1.0	1.1	1.0	1.0	1.0	52.4	4	21	21	100	483	53.1	7.2
NP_114107.1	13994257	MRPS21	28S ribosomal protein S21, mitochondrial	1.1	0.8	0.9	0.9	1.1	1.0	1.0	1.0	1.1	0.9	1.0	1.0	16.1	1	1	1	3	87	10.7	10.2
NP_733934.1	25306272	MRPL11	39S ribosomal protein L11, mitochondrial isoform b	1.0	1.1	1.0	1.0	0.9	1.0	1.0	1.0	1.0	1.0	1.0	1.0	39.8	3	7	7	19	166	18.2	9.7
NP_001008727.1	56961676	ZNF121	zinc finger protein 121	0.9	0.9	0.8	1.0	1.1	1.1	1.0	1.0	1.0	1.0	0.9	1.0	5.4	1	1	1	2	390	44.7	8.0
NP_002769.1	11386147	PSAP	prosaposin isoform a preproprotein	1.5	1.2	1.8	1.0	1.4	1.1	1.5	1.0	1.4	1.2	1.7	1.0	20.0	3	10	10	41	524	58.1	5.2
NP_006000.2	17986283	TUBA1A	tubulin alpha-1A chain isoform 1	1.0	1.0	0.9	1.1	1.1	1.1	1.0	0.9	1.0	1.0	1.0	1.0	77.6	5	2	25	1134	451	50.1	5.1
NP_066930.1	11056056	MRPS12	28S ribosomal protein S12, mitochondrial precursor	1.0	1.1	1.1	1.0	1.0	1.1	1.1	1.0	1.0	1.1	1.1	1.0	38.4	1	4	4	8	138	15.2	10.3
NP_660298.2	22547114	MRPL10	39S ribosomal protein L10, mitochondrial isoform a precursor	1.2	1.0	1.1	1.0	1.0	0.9	1.0	1.0	1.1	1.0	1.0	1.0	22.6	2	4	4	14	261	29.3	9.6
NP_060294.1	8923415	Mar-05	E3 ubiquitin-protein ligase MARCH5	1.3	1.1	1.0	1.0	1.1	1.0	1.1	1.0	1.2	1.0	1.1	1.0	22.7	1	5	5	9	278	31.2	8.7
NP_055787.1	14149657	TCF25	transcription factor 25	1.0	1.0	1.0	1.0	1.2	1.0	1.0	1.0	1.1	1.0	1.0	1.0	6.5	1	2	2	2	676	76.6	6.4
NP_005708.1	5031593	ARPC5	actin-related protein 2/3 complex subunit 5 isoform 1	1.1	1.0	1.1	1.0	1.0	1.1	1.1	1.0	1.0	1.0	1.1	1.0	13.3	2	2	2	4	151	16.3	5.7
NP_624311.1	20911035	PPIL4	peptidyl-prolyl cis-trans isomerase-like 4	1.0	1.0	1.0	1.0	1.0	1.0	1.0	1.0	1.0	1.0	1.0	1.0	29.1	1	10	10	28	492	57.2	5.9
NP_808881.3	189027129	PDE12	2',5'-phosphodiesterase 12	1.0	0.9	0.9	1.0	1.0	1.0	1.0	1.0	1.0	1.0	1.0	1.0	5.6	1	3	3	6	609	67.3	6.6
NP_001159582.1	260656034	PALLD	palladin isoform 4	0.9	1.1	1.1	0.9	1.0	1.1	0.9	1.1	1.0	1.1	1.0	1.0	3.7	3	1	1	3	672	73.3	7.7
NP_001229829.1	339276044	PPP6R2	serine/threonine-protein phosphatase 6 regulatory subunit 2 isoform 4	1.2	1.2	1.3	1.1	1.0	0.8	1.0	0.9	1.1	1.0	1.1	1.0	3.3	4	2	3	3	927	100.9	4.9
NP_001011699.1	58761506	HAUS8	HAUS augmin-like complex subunit 8 isoform b	0.9	0.9	0.9	1.0	0.9	1.0	1.0	1.0	0.9	1.0	1.0	1.0	15.4	2	4	4	7	409	44.8	7.1
NP_001161302.1	282165817	TMED5	transmembrane emp24 domain-containing protein 5 isoform 2 precursor	1.2	1.2	1.3	1.0	1.1	1.0	1.0	0.9	1.1	1.1	1.1	1.0	29.0	2	4	4	9	193	21.7	4.7
NP_004724.1	4757708;300360496	SLC33A1	acetyl-coenzyme A transporter 1	1.1	1.2	1.1	1.0	1.1	1.1	1.0	0.9	1.1	1.1	1.0	1.0	4.0	1	2	2	10	549	60.9	7.3
NP_001035009.1	90193619	ZNF384	zinc finger protein 384 isoform c	1.1	1.2	1.1	1.0	1.0	1.1	1.0	1.0	1.1	1.1	1.1	1.0	13.0	4	3	3	8	461	50.5	8.5
NP_005745.1	5031703;38327552	G3BP1	ras GTPase-activating protein-binding protein 1	0.9	0.9	0.9	1.0	0.9	0.9	0.9	1.0	0.9	0.9	0.9	1.0	43.8	2	14	15	69	466	52.1	5.5
NP_001248323.1	386869309	NOL10	nucleolar protein 10 isoform 3	1.0	1.0	1.0	1.0	0.9	0.9	0.9	1.0	1.0	1.0	1.0	1.0	26.2	3	9	9	29	638	74.5	8.4
NP_817092.1	29826282	PPM1G	protein phosphatase 1G	0.9	1.0	0.9	1.0	0.9	1.0	0.9	1.0	0.9	1.0	0.9	1.0	52.6	1	20	20	109	546	59.2	4.4
NP_054747.2	30025026	OSTM1	osteopetrosis-associated transmembrane protein 1	1.2	1.0	1.0	1.0	1.2	1.2	1.1	1.0	1.2	1.1	1.1	1.0	10.5	1	3	3	7	334	37.2	6.0
NP_001005745.1	54144629	NUMB	protein numb homolog isoform 4	0.8	0.9	0.8	1.0	2.2	1.1	1.0	1.0	1.5	1.0	0.9	1.0	1.2	4	1	1	1	592	64.5	8.4
NP_006782.1	5803102	MORF4L1	mortality factor 4-like protein 1 isoform 1	1.4	0.9	0.9	1.0	0.8	0.8	0.8	1.0	1.1	0.9	0.8	1.0	14.9	3	3	4	21	323	37.2	9.2
NP_073210.1	13027630	DGCR14	protein DGCR14	1.0	1.0	0.9	1.0	1.1	1.1	0.9	1.0	1.0	1.1	0.9	1.0	21.6	1	6	6	12	476	52.5	7.6
NP_689939.1	22749443	MICU2	calcium uptake protein 2, mitochondrial	1.1	1.1	1.1	1.0	1.0	1.0	1.1	1.0	1.1	1.1	1.1	1.0	16.6	1	5	5	10	434	49.6	9.1
NP_001271245.1	546232251	ACSF3	acyl-CoA synthetase family member 3, mitochondrial isoform 2	0.8	0.9	0.8	0.9	1.0	1.1	1.2	1.1	0.9	1.0	1.0	1.0	2.6	2	1	1	1	311	35.1	7.1
NP_064601.3	40316915	RNPEP	aminopeptidase B	1.1	0.9	0.9	1.0	1.1	1.0	1.0	1.0	1.1	0.9	0.9	1.0	30.2	1	14	14	50	650	72.5	5.7
NP_077292.2	612407788	LENG1	leukocyte receptor cluster member 1	1.0	1.1	0.9	1.0	1.0	1.1	0.9	1.0	1.0	1.1	0.9	1.0	4.6	1	1	1	4	264	30.5	9.8
NP_001127894.1	197313672	CDV3	protein CDV3 homolog isoform a	1.0	1.1	1.2	1.0	0.9	1.0	1.1	1.0	1.0	1.0	1.1	1.0	56.3	6	6	6	19	213	22.1	5.8
NP_002624.2	21361621	PGM1	phosphoglucomutase-1 isoform 1	1.1	1.1	1.2	1.0	1.0	1.1	1.1	1.0	1.1	1.1	1.2	1.0	38.4	3	20	20	54	562	61.4	6.8
NP_056227.2	68299770	C3orf17	uncharacterized protein C3orf17	1.0	1.0	0.9	0.9	1.2	1.2	1.1	1.1	1.1	1.1	1.0	1.0	6.9	1	3	3	8	567	64.5	9.7
NP_055555.1	7661920	EIF4A3	eukaryotic initiation factor 4A-III	1.0	1.0	1.0	1.0	1.0	0.9	0.9	1.0	1.0	1.0	0.9	1.0	56.0	1	16	19	131	411	46.8	6.7
NP_061854.1	24308127	DNAJC10	dnaJ homolog subfamily C member 10 isoform 1 precursor	1.2	1.0	1.1	0.9	1.2	1.1	1.1	1.0	1.2	1.1	1.1	1.0	6.4	2	3	3	8	793	91.0	7.2
NP_001156896.1	254540132	MYEOV2	myeloma-overexpressed gene 2 protein isoform 2	1.2	1.0	1.2	1.0	1.1	0.8	1.2	1.0	1.1	0.9	1.2	1.0	35.1	1	1	1	8	57	6.2	3.8
NP_079434.3	94721336	RUFY1	RUN and FYVE domain-containing protein 1 isoform a	0.9	0.9	0.9	1.0	1.0	1.0	0.9	1.0	1.0	1.0	0.9	1.0	10.2	10	6	6	10	708	79.8	5.7
NP_001120868.1	188528901	STXBP2	syntaxin-binding protein 2 isoform b	1.0	0.9	0.9	1.0	1.0	1.0	1.0	1.0	1.0	1.0	0.9	1.0	24.9	3	10	10	22	590	66.1	6.6
NP_000138.2	119360348	FUCA1	tissue alpha-L-fucosidase precursor	0.9	0.9	0.8	1.1	0.7	0.7	0.7	0.9	0.8	0.8	0.7	1.0	4.7	1	2	2	6	466	53.7	6.8
NP_001093869.1	155030216	PDS5A	sister chromatid cohesion protein PDS5 homolog A isoform 1	1.0	1.0	1.0	1.0	1.0	1.0	1.1	1.0	1.0	1.0	1.0	1.0	22.9	2	21	24	58	1337	150.7	7.9
NP_006764.3	38327634	DDX18	ATP-dependent RNA helicase DDX18	0.9	1.0	0.9	1.0	1.0	1.0	0.9	1.0	0.9	1.0	0.9	1.0	27.3	1	16	16	73	670	75.4	9.5
NP_004418.2	37595530	PHC2	polyhomeotic-like protein 2 isoform b	0.9	1.0	1.0	1.0	1.0	1.0	1.0	1.0	0.9	1.0	1.0	1.0	21.1	3	5	6	16	323	35.7	8.6
NP_061856.1	9506713;15011916	GAR1	H/ACA ribonucleoprotein complex subunit 1	1.0	0.9	1.0	1.0	1.0	0.9	1.1	1.0	1.0	0.9	1.0	1.0	31.8	1	6	6	11	217	22.3	10.9

Khan *et al.*, 2019. Multi-omics analysis to characterize cigarette smoke induced molecular alterations in esophageal cells
Supplementary Table 5. List of proteins quantified in untreated and chronically treated Het1A cells with cigarette smoke condensate for 8 months

NP_Accession	Protein group Accession	Gene ID	Description	Het-1A-Smoke - 2M/Parental	Het-1A-Smoke - 4M/Parental	Het-1A-Smoke - 6M/Parental	Het-1A-Smoke - 8M/Parental	Het-1A-Smoke - 2M/Parental	Het-1A-Smoke - 4M/Parental	Het-1A-Smoke - 6M/Parental	Het-1A-Smoke - 8M/Parental	Het-1A-Smoke - 2M/Parental	Het-1A-Smoke - 4M/Parental	Het-1A-Smoke - 6M/Parental	Het-1A-Smoke - 8M/Parental	Coverage (%)	Proteins	Unique Peptides	# Peptides	PSM	# AAs	MW [kDa]	calc. pI
				Replicate 1				Replicate 2				Average of replicates											
NP_002785.1	4506195	PSMB2	proteasome subunit beta type-2 isoform 1	1.1	0.9	1.1	1.0	1.1	1.0	1.0	1.0	1.1	0.9	1.0	1.0	26.4	3	4	5	17	201	22.8	7.0
NP_071739.2	45505147	C17orf75	protein Njmu-R1	1.0	1.2	1.1	1.0	0.9	0.9	1.0	1.0	1.0	1.1	1.1	1.0	38.4	1	11	11	30	396	44.6	5.0
NP_001157855.1	256419001	STAU2	double-stranded RNA-binding protein Staufen homolog 2 isoform d	0.8	0.9	0.8	1.0	1.2	1.0	0.9	1.0	1.0	0.9	0.9	1.0	20.6	6	6	7	11	398	43.3	9.4
NP_060090.2	212549553	ILF3	interleukin enhancer-binding factor 3 isoform d	0.9	0.9	0.9	0.9	0.9	1.0	1.0	1.0	0.9	0.9	0.9	1.0	51.6	5	27	31	211	898	95.7	8.8
NP_932342.1	37620210	COX20	cytochrome c oxidase protein 20 homolog	1.1	1.1	1.1	1.1	1.1	1.1	1.2	0.9	1.1	1.1	1.1	1.0	10.2	1	1	1	2	118	13.3	8.8
NP_115570.1	39930469	RPF2	ribosome production factor 2 homolog isoform 1	0.9	1.0	0.9	1.0	1.0	1.0	0.9	1.0	0.9	1.0	0.9	1.0	16.0	2	5	5	8	306	35.6	10.0
NP_001268368.1	527122088	SMAP1	stromal membrane-associated protein 1 isoform C	0.9	0.9	0.9	1.0	0.9	0.9	0.9	1.0	0.9	0.9	0.9	1.0	7.6	4	3	3	6	436	47.8	8.4
NP_001258930.1	431822379	DOCK7	dedicator of cytokinesis protein 7 isoform 4	0.9	1.0	1.0	1.0	1.0	0.9	0.9	1.0	0.9	0.9	0.9	1.0	4.7	7	6	6	7	2098	238.1	6.8
NP_003290.1	4507677	HSP90B1	endoplasmin precursor	1.1	1.2	1.1	1.0	1.1	1.2	1.1	1.0	1.1	1.2	1.1	1.0	78.0	1	57	59	781	803	92.4	4.8
NP_000549.1	4504347	HBA1	hemoglobin subunit alpha	1.0	1.2	1.0	1.0	1.1	1.2	0.9	1.0	1.1	1.2	1.0	1.0	28.2	1	3	3	16	142	15.2	8.7
NP_002148.1	4504523	HSPE1	10 kDa heat shock protein, mitochondrial	1.1	1.1	1.1	1.0	0.9	1.0	1.0	1.0	1.0	1.0	1.1	1.0	50.0	1	5	5	7	102	10.9	8.9
NP_001011553.2	148352329	Sep-07	septin-7 isoform 2	1.1	1.1	1.0	1.0	1.0	1.0	1.0	1.0	1.0	1.0	1.0	1.0	35.6	10	13	13	35	436	50.5	8.6
NP_065761.1	24308207	LRRC47	leucine-rich repeat-containing protein 47	0.9	1.0	0.9	1.0	0.9	0.9	0.9	0.9	0.9	0.9	0.9	1.0	31.7	1	13	13	31	583	63.4	8.3
NP_001028739.2	570700820	RHOT1	mitochondrial Rho GTPase 1 isoform 4	1.1	1.0	1.0	1.0	1.0	0.9	0.9	0.9	1.1	1.0	0.9	1.0	9.6	7	2	3	5	491	56.4	6.9
NP_006661.1	5729718;262205665	TPBG	trophoblast glycoprotein precursor	1.1	1.1	1.1	1.0	1.0	1.1	1.1	1.0	1.1	1.1	1.1	1.0	15.5	1	5	5	18	420	46.0	6.8
NP_055956.1	24307999	GPD1L	glycerol-3-phosphate dehydrogenase 1-like protein	1.1	1.0	1.0	1.0	1.2	1.0	1.1	1.0	1.2	1.0	1.0	1.0	20.8	1	7	7	11	351	38.4	7.0
NP_002680.2	20127448	POLA2	DNA polymerase alpha subunit B	0.9	0.9	0.9	1.0	0.9	0.8	1.0	1.0	0.9	0.9	0.9	1.0	6.7	1	3	3	5	598	65.9	5.2
NP_064504.2	27477136	ZC3HAV1	zinc finger CCCH-type antiviral protein 1 isoform 1	1.0	0.9	1.0	1.0	1.0	1.0	1.0	1.0	1.0	1.0	1.0	1.0	22.8	2	15	15	41	902	101.4	8.4
NP_001961.1	4503545;219555710	EIF5A	eukaryotic translation initiation factor 5A-1 isoform B	1.3	1.1	1.7	1.0	1.3	1.1	1.7	1.0	1.3	1.1	1.7	1.0	61.0	4	8	8	96	154	16.8	5.2
NP_620634.1	20986519	MAPK8	mitogen-activated protein kinase 8 isoform beta1	1.0	1.2	1.1	1.1	1.0	0.9	1.2	0.9	1.0	1.0	1.2	1.0	18.0	6	4	5	8	384	44.0	7.5
NP_002444.2	53729337;53729339	MTIF2	translation initiation factor IF-2, mitochondrial precursor	1.0	1.0	1.0	1.0	0.9	1.0	1.0	1.0	1.0	1.0	1.0	1.0	16.5	1	9	9	18	727	81.3	7.2
NP_904325.2	41393559	KIF1B	kinesin-like protein KIF1B isoform alpha	0.9	1.1	0.9	1.0	1.0	1.0	0.9	0.9	0.9	1.0	0.9	1.0	6.9	4	4	6	8	1153	130.3	8.4
NP_115609.2	40068481	SETD3	histone-lysine N-methyltransferase setd3 isoform a	0.9	0.9	0.9	1.0	1.0	0.9	0.9	1.0	1.0	0.9	0.9	1.0	25.4	2	13	14	34	594	67.2	6.0
NP_036270.1	7657013	AATF	protein AATF	1.0	1.1	1.0	1.0	1.0	1.0	1.0	1.0	1.0	1.1	1.0	1.0	17.7	1	9	9	26	560	63.1	4.9
NP_060197.4	134152721	TMEM214	transmembrane protein 214 isoform 1	1.2	1.1	1.0	1.0	1.1	1.0	1.0	0.9	1.1	1.0	1.0	1.0	23.8	2	13	13	39	689	77.1	9.1
NP_612409.1	61097912	NOM1	nucleolar MIF4G domain-containing protein 1	0.9	0.9	0.9	1.0	1.0	0.9	0.9	1.0	0.9	0.9	0.9	1.0	16.9	1	12	12	27	860	96.2	8.1
NP_001136062.1	217272835	NFYC	nuclear transcription factor Y subunit gamma isoform 5	1.0	1.1	0.9	0.9	1.0	1.0	1.0	1.1	1.0	1.0	1.0	1.0	8.3	5	2	2	3	301	33.7	5.1
NP_004765.2	28559039	MED1	mediator of RNA polymerase II transcription subunit 1	1.0	1.0	0.9	1.0	0.9	1.1	0.9	1.0	0.9	1.0	0.9	1.0	7.7	1	8	8	17	1581	168.4	8.7
NP_689473.1	23308689	RPUSD2	RNA pseudouridylate synthase domain-containing protein 2 isoform 1	0.9	1.0	1.0	0.9	0.9	1.1	1.0	1.0	0.9	1.0	1.0	1.0	10.5	2	2	2	3	545	61.3	7.2
NP_733765.1	24638454	ATP2A2	sarcoplasmic/endoplasmic reticulum calcium ATPase 2 isoform b	1.1	1.0	1.0	1.0	1.0	1.0	1.0	1.0	1.0	1.0	1.0	1.0	36.3	8	21	33	165	1042	114.7	5.3
NP_612351.2	31543093	GLB1L2	beta-galactosidase-1-like protein 2 precursor	1.1	1.1	1.1	0.9	1.4	1.2	1.2	1.1	1.2	1.2	1.2	1.0	4.9	1	2	2	3	636	72.0	7.6
NP_001137325.1	219803946	WIBG	partner of Y14 and mago isoform 2	1.0	1.1	1.0	0.9	1.1	1.1	1.0	1.0	1.1	1.1	1.0	1.0	46.8	2	7	7	27	203	22.7	9.4
NP_001129123.1	208431827	EIF2AK2	interferon-induced, double-stranded RNA-activated protein kinase isoform a	0.9	0.8	0.9	0.9	0.9	0.9	0.9	1.0	0.9	0.9	0.9	1.0	31.0	2	14	14	64	551	62.1	8.4
NP_060392.3	156938331	PRPF39	pre-mRNA-processing factor 39	0.9	0.9	0.9	1.0	0.9	0.9	1.0	1.0	0.9	0.9	0.9	1.0	6.9	1	4	4	9	669	78.4	5.4
NP_036473.2	55953087	GTPBP4	nucleolar GTP-binding protein 1	1.0	1.0	0.9	1.0	0.9	0.9	1.0	1.0	0.9	1.0	0.9	1.0	31.9	2	18	18	45	634	73.9	9.5
NP_006215.1	5453908	PITPNA	phosphatidylinositol transfer protein alpha isoform	1.1	1.1	1.1	1.0	1.0	1.1	1.2	1.0	1.1	1.1	1.1	1.0	37.8	1	8	9	27	270	31.8	6.6
NP_000008.1	4557233	ACADS	short-chain specific acyl-CoA dehydrogenase, mitochondrial isoform 1 precursor	1.0	1.0	1.0	1.0	1.0	0.9	1.0	0.9	1.0	1.0	1.0	1.0	9.0	2	3	3	5	412	44.3	8.0
NP_005006.3	223718097	OXA1L	mitochondrial inner membrane protein OXA1L	1.0	1.0	0.8	1.0	1.1	1.0	0.9	0.9	1.1	1.0	0.9	1.0	4.0	1	2	2	3	495	55.2	9.7
NP_733821.1	27436946	LMNA	lamin isoform A	0.9	1.1	0.9	1.0	0.9	1.1	0.9	1.0	0.9	1.1	0.9	1.0	53.5	6	35	37	216	664	74.1	7.0
NP_068756.2	54607086	EEFSEC	selenocysteine-specific elongation factor	0.9	1.0	0.9	1.0	1.0	1.0	0.9	1.0	0.9	1.0	0.9	1.0	9.2	1	5	5	11	596	65.3	8.4
NP_073741.2	226371750	TUT1	speckle targeted PIP5K1A-regulated poly(A) polymerase	0.9	1.0	1.0	0.9	1.0	1.2	1.1	1.1	0.9	1.1	1.1	1.0	1.0	1	1	1	1	912	98.4	6.4
NP_000300.1	4506001	PPOX	protoporphyrinogen oxidase	1.0	1.0	0.9	0.9	1.2	1.1	1.1	1.0	1.1	1.0	1.0	1.0	10.9	1	3	3	3	477	50.7	8.2
NP_006796.1	5803036	HNRNPA0	heterogeneous nuclear ribonucleoprotein A0	0.8	0.9	0.8	1.0	0.8	0.9	0.8	1.0	0.8	0.9	0.8	1.0	49.5	1	9	11	89	305	30.8	9.3
NP_005709.1	5031595	ARPC4	actin-related protein 2/3 complex subunit 4 isoform a	1.1	1.0	1.1	1.0	1.1	1.0	1.0	1.0	1.1	1.0	1.1	1.0	51.8	4	6	6	23	168	19.7	8.4
NP_065118.2	76781492	REXO4	RNA exonuclease 4 isoform 1	1.0	1.0	1.0	1.0	0.9	0.9	0.9	0.9	0.9	1.0	0.9	1.0	16.8	4	5	5	7	422	46.6	9.8
NP_055969.1	74136552	MESDC2	LDLR chaperone MESD precursor	1.0	1.0	0.9	1.0	0.9	1.0	0.9	1.0	0.9	1.0	0.9	1.0	41.0	1	10	10	41	234	26.1	7.8
NP_001339.1	4557511	DAPK3	death-associated protein kinase 3	1.1	1.2	1.3	1.1	0.9	1.0	1.0	0.9	1.0	1.1	1.2	1.0	4.2	2	2	2	2	454	52.5	6.9
NP_001030590.1	78711824	SDHC	succinate dehydrogenase cytochrome b560 subunit, mitochondrial isoform 4 precursor	1.0	1.0	1.1	1.0	1.1	1.0	1.4	1.0	1.1	1.0	1.2	1.0	18.1	3	2	2	5	116	12.5	9.0
NP_004990.3	92859701	MYO6	unconventional myosin-VI isoform 1	0.9	1.0	0.9	1.0	0.9	0.9	0.9	0.9	0.9	0.9	0.9	1.0	10.3	2	9	9	23	1285	148.6	8.5
NP_005924.2	56550039	KMT2A	histone-lysine N-methyltransferase 2A isoform 2 precursor	1.2	1.0	0.9	1.0	1.0	1.1	1.0	1.0	1.1	1.0	1.0	1.0	4.6	2	13	13	21	3969	431.5	9.1
NP_005119.2	45827701	DOPEY2	protein dopey-2	0.9	1.0	1.0	1.0	1.0	1.0	0.9	0.9	0.9	1.0	1.0	1.0	0.6	1	1	1	2	2298	258.1	6.3
NP_001153576.1	231570186	ZC3H14	zinc finger CCCH domain-containing protein 14 isoform 6	0.9	0.9	0.8	1.0	0.9	0.9	0.8	1.0	0.9	0.9	0.8	1.0	14.8	5	7	7	13	730	82.3	7.3
NP_008896.2	190610012	ZNF24	zinc finger protein 24 isoform 1	0.8	0.9	0.8	0.8	1.1	1.3	1.0	1.1	1.0	1.1	0.9	1.0	10.9	2	4	4	6	368	42.1	6.2
NP_065797.2	83776598	MAVS	mitochondrial antiviral-signaling protein isoform 1	0.8	0.8	0.8	0.9	1.1	1.1	1.0	1.1	0.9	0.9	0.9	1.0	13.7	2	3	3	6	540	56.5	5.5
NP_055828.2	58761546	KDM1A	lysine-specific histone demethylase 1A isoform b	0.9	0.9	0.9	0.9	1.0	1.0	1.0	1.0	1.0	0.9	0.9	1.0	17.4	2	11	11	21	852	92.8	6.5
NP_037507.1	7019319	ABT1	activator of basal transcription 1	1.0	1.1	1.0	1.0	1.0	1.1	1.0	1.0	1.0	1.1	1.0	1.0	33.8	1	5	5	9	272	31.1	9.9
NP_006212.1	5453898	PIN1	peptidyl-prolyl cis-trans isomerase NIMA-interacting 1	0.7	0.9	0.9	1.0	0.8	0.9	0.9	1.0	0.7	0.9	0.9	1.0	9.2	1	1	1	1	163	18.2	8.8
NP_003508.1	24638446	HIST2H2AC	histone H2A type 2-C	1.5	1.3	1.5	1.2	0.9	0.9	0.9	0.8	1.2	1.1	1.2	1.0	65.1	2	2	8	994	129	14.0	10.9
NP_001186227.1	313151226;42476130	UIMC1	BRCA1-A complex subunit RAP80	0.8	0.9	0.9	1.0	1.0	0.9	0.8	0.9	0.9	0.9	0.9	1.0	8.5	1	4	4	9	719	79.7	5.5
NP_757364.1	27437023	CAMKK2	calcium/calmodulin-dependent protein kinase kinase 2 isoform 6	0.9	0.8	0.9	0.9	1.0	0.9	1.0	1.0	1.0	0.9	0.9	1.0	3.9	7	2	2	4	490	54.1	7.2
NP_006188.3	134142826	PCM1	pericentriolar material 1 protein	1.0	1.0	1.0	1.0	1.0	1.1	1.1	1.0	1.0	1.0	1.0	1.0	6.5	1	10	11	15	2024	228.4	5.0
NP_001191315.1	324120936	DLG1	disks large homolog 1 isoform 3	1.1	1.1	1.0	1.0	1.0	1.0	1.0	1.0	1.0	1.0	1.0	1.0	10.7	5	4	7	16	892	99.8	6.0
NP_077309.1	13236585	FSD1	fibronectin type III and SPRY domain-containing protein 1	1.0	1.0	1.0	1.0	0.9	0.7	0.8	1.0	0.9	0.9	0.9	1.0	13.7	1	4	4	4	496	55.8	7.0
NP_005688.2	5730031	SCAMP2	secretory carrier-associated membrane protein 2	1.0	0.9	0.9	0.9	1.1	1.0	1.0	1.0	1.0	1.0	0.9	1.0	13.7	1	3	3	26	329	36.6	6.1
NP_055445.2	108860697	ZNF592	zinc finger protein 592	1.0	1.0	1.0	1.0	1.0	1.0	0.9	1.0	1.0	1.0	1.0	1.0	5.3	1	4	4	8	1267	137.4	7.8
NP_002747.2	21618347	MAP2K3	dual specificity mitogen-activated protein kinase kinase 3 isoform A	1.0	1.0	1.0	1.0	1.1	1.1	1.2	1.0	1.1	1.1	1.1	1.0	27.4	5	6	6	23	318	36.1	6.3
NP_001801.1	21735415	CENPB	major centromere autoantigen B	0.9	1.0	0.9	0.9	0.9	1.1	1.0	1.0	0.9	1.0	1.0	1.0	7.7	1	4	4	9	599	65.1	4.6
NP_009172.2	21361380	SNF8	vacuolar-sorting protein SNF8	0.9	0.9	0.9	0.9	1.0	1.0	1.0	1.1	0.9	1.0	1.0	1.0	3.5	1	1	1	4	258	28.8	6.7
NP_001952.1	4503483	EEF2	elongation factor 2	0.9	0.9	0.9	1.0	1.0	0.9	1.0	1.0	1.0	0.9	1.0	1.0	71.8	1	48	49	659	858	95.3	6.8
NP_001007562.1	56090582	IRGQ	immunity-related GTPase family Q protein	0.9	0.9	0.9	0.9	1.1	1.0	1.0	1.0	1.0	1.0	1.0	1.0	7.5	1	3	3	6	623	62.7	4.9
NP_003943.2	55953117	RPS6KB2	ribosomal protein S6 kinase beta-2	0.9	0.9	0.9	0.9	1.1	1.2	1.1	1.1	1.0	1.1	1.0	1.0	4.4	1	1	2	5	482	53.4	7.3
NP_476528.1	17402912	VPS29	vacuolar protein sorting-associated protein 29 isoform 2	1.0	1.0	1.0	0.9	1.1	1.0	1.0	1.0	1.0	1.0	1.0	1.0	46.8	4	8	8	13	186	20.9	7.0
NP_006297.2	30581135	SMC1A	structural maintenance of chromosomes protein 1A isoform 1	0.9	1.0	0.9	1.0	1.0	0.9	1.0	1.0	1.0	1.0	1.0	1.0	30.3	2	32	32	93	1233	143.1	7.6
NP_060221.2	102467484	SMPD4	sphingomyelin phosphodiesterase 4 isoform 1	1.1	1.0	1.0	1.0	1.0	1.0	1.0	1.0	1.0	1.0	1.0	1.0	12.4	3	8	8	14	837	94.5	8.3
NP_006687.3	34452705	BRD8	bromodomain-containing protein 8 isoform 1	0.9	1.0	0.9	1.0	1.0	1.0	0.9	1.0	0.9	1.0	0.9	1.0	12.4	6	6	6	10	951	102.8	4.7
NP_004387.1	4758138	DDX5	probable ATP-dependent RNA helicase DDX5	0.9	0.8	0.8	1.0	0.9	0.8	0.8	1.0	0.9	0.8	0.8	1.0	46.4	1	19	27	140	614	69.1	8.9
NP_079353.3	156564398	TOE1	target of EGR1 protein 1	0.9	0.8	0.8	0.9	1.1	1.1	1.0	1.1	1.0	1.0	0.9	1.0	12.8	1	5	5	12	510	56.5	7.2
NP_036565.2	54112117	SF3B1	splicing factor 3B subunit 1 isoform 1	1.0	1.0	1.0	1.0	1.0	0.9	1.0	1.0	1.0	0.9	1.0	1.0	44.1	2	35	35	108	1304	145.7	7.1
NP_001858.1	4502993	COX7C	cytochrome c oxidase subunit 7C, mitochondrial precursor	0.9	0.9	0.9	1.0	0.9	1.0	1.0	1.0	0.9	1.0	0.9	1.0	28.6	1	2	2	5	63	7.2	10.3
NP_940915.1	39653325	PHF20L1	PHD finger protein 20-like protein 1 isoform 3	0.8	0.9	0.9	1.0	1.2	1.0	1.0	1.0	1.0	1.0	0.9	1.0	7.4	3	1	2	2	285	32.8	8.9
NP_663729.1	21735485	ATPAF2	ATP synthase mitochondrial F1 complex assembly factor 2	1.2	1.0	1.1	1.0	1.1	0.9	1.0	0.9	1.1	1.0	1.0	1.0	20.1	1	4	4	11	289	32.8	7.1
NP_065141.3	38570054	LYRM4	LYR motif-containing protein 4 isoform 1	1.1	1.0	1.0	1.0	1.0	0.9	1.0	0.9	1.0	0.9	1.0	1.0	19.8	3	2	2	2	91	10.8	10.7
NP_001269078.1	531034789	ABR	active breakpoint cluster region-related protein isoform e	1.0	0.9	1.0	1.2	0.8	0.8	0.8	0.8	0.9	0.9	0.9	1.0	4.4	4	1	2	6	641	73.5	7.7
NP_079428.2	157384956	NUBPL	iron-sulfur protein NUBPL isoform 1 precursor	1.1	1.0	1.0	0.9	1.0	1.0	1.0	1.0	1.0	1.0	1.0	1.0	19.1	3	4	4	6	319	34.1	9.0
NP_001265614.1	522838244	AMOTL2	angiomotin-like protein 2 isoform 3	0.9	0.9	1.0	1.0	0.6	0.9	0.7	1.0	0.8	0.9	0.8	1.0	5.0	3	3	3	4	777	85.4	7.2
NP_001091082.2	303304977	GPR89A	Golgi pH regulator A isoform 2	1.0	1.0	1.0	1.0	1.0	1.0	0.9	1.0	1.0	1.0	1.0	1.0	6.3	2	1	1	8	430	50.0	9.3
NP_057114.5	40548372	TRAPPC12	trafficking protein particle complex subunit 12	1.2	1.1	1.1	1.1	1.1	1.0	1.0	0.9	1.1	1.0	1.0	1.0	8.2	1	4	4	5	735	79.3	4.9

Khan *et al.* , 2019. Multi-omics analysis to characterize cigarette smoke induced molecular alterations in esophageal cells
Supplementary Table 5. List of proteins quantified in untreated and chronically treated Het1A cells with cigarette smoke condensate for 8 months

NP_Accession	Protein group Accession	Gene ID	Description	Het-1A-Smoke - 2M/Parental	Het-1A-Smoke - 4M/Parental	Het-1A-Smoke - 6M/Parental	Het-1A-Smoke - 8M/Parental	Het-1A-Smoke - 2M/Parental	Het-1A-Smoke - 4M/Parental	Het-1A-Smoke - 6M/Parental	Het-1A-Smoke - 8M/Parental	Het-1A-Smoke - 2M/Parental	Het-1A-Smoke - 4M/Parental	Het-1A-Smoke - 6M/Parental	Het-1A-Smoke - 8M/Parental	Coverage (%)	Proteins	Unique Peptides	# Peptides	PSM	# AAs	MW [kDa]	calc. pI
				Replicate 1				Replicate 2				Average of replicates											
NP_057665.2	20149633	ECSIT	evolutionarily conserved signaling intermediate in Toll pathway, mitochondrial isoform 1 precursor	1.0	0.9	0.9	0.9	1.3	0.9	1.0	1.0	1.2	0.9	1.0	1.0	26.2	4	6	6	11	431	49.1	6.3
NP_003362.2	40549448	VAV2	guanine nucleotide exchange factor VAV2 isoform 2	1.0	1.0	1.0	1.0	1.1	0.9	1.0	0.9	1.0	0.9	1.0	1.0	8.5	2	6	6	9	839	97.0	6.9
NP_851853.1	31044432	LEMD2	LEM domain-containing protein 2 isoform 1	0.9	1.0	1.0	1.0	0.9	0.9	0.9	1.0	0.9	1.0	1.0	1.0	8.0	2	3	3	14	503	56.9	9.0
NP_001035521.1	94536771	COA3	cytochrome c oxidase assembly factor 3 homolog, mitochondrial	1.2	1.1	1.1	1.0	1.2	1.0	1.0	1.0	1.2	1.1	1.1	1.0	27.4	1	3	3	8	106	11.7	9.6
NP_005691.2	18491024	DPP3	dipeptidyl peptidase 3 isoform 1	1.0	1.0	1.0	1.0	1.1	1.1	1.0	1.0	1.0	1.0	1.0	1.0	43.3	2	21	21	114	737	82.5	5.1
NP_001247.3	167466175	CDC27	cell division cycle protein 27 homolog isoform 2	1.0	1.1	1.0	1.0	1.1	1.1	1.0	1.0	1.0	1.1	1.0	1.0	9.8	4	6	6	13	824	91.8	7.0
NP_004313.1	10835069	BAD	bcl2 antagonist of cell death	1.1	1.0	1.1	0.9	1.1	1.1	1.2	1.0	1.1	1.1	1.1	1.0	14.3	1	2	2	2	168	18.4	7.2
NP_060116.2	31581534	TRIT1	tRNA dimethylallyltransferase, mitochondrial precursor	0.8	0.8	0.8	0.8	1.1	1.2	1.1	1.2	1.0	1.0	0.9	1.0	2.4	1	1	1	1	467	52.7	8.2
NP_036387.2	18860916	XRN2	5'-3' exoribonuclease 2	1.0	0.9	1.0	1.0	1.0	0.9	1.0	1.0	1.0	0.9	1.0	1.0	27.3	1	21	21	75	950	108.5	7.5
NP_076417.2	31982927	AACS	acetoacetyl-CoA synthetase	1.0	1.0	1.1	1.0	1.0	1.0	1.1	1.0	1.0	1.0	1.1	1.0	19.9	2	11	12	23	672	75.1	6.2
NP_060530.3	46852147	IARS2	isoleucine--tRNA ligase, mitochondrial precursor	1.0	0.9	1.0	1.0	1.0	0.9	1.0	1.0	1.0	0.9	1.0	1.0	27.7	1	23	23	56	1012	113.7	7.2
NP_001273278.1	556503362	KIRREL	kin of IRRE-like protein 1 isoform 2 precursor	1.4	1.0	1.1	1.0	1.1	1.0	1.1	0.9	1.3	1.0	1.1	1.0	5.6	2	3	3	3	657	72.7	5.8
NP_060810.2	153251270	CPPED1	serine/threonine-protein phosphatase CPPED1 isoform a	1.1	1.0	1.0	0.9	1.1	1.0	1.2	1.0	1.1	1.0	1.1	1.0	25.8	2	7	7	23	314	35.5	6.2
NP_001229526.1	336176058	TMEM147	transmembrane protein 147 isoform 2	1.0	1.0	1.0	0.8	1.1	1.2	1.1	1.1	1.0	1.1	1.0	1.0	9.7	2	1	1	2	175	19.5	6.9
NP_000928.1	4505939	POLR2A	DNA-directed RNA polymerase II subunit RPB1	1.0	0.9	0.9	1.0	1.0	1.0	1.0	1.0	1.0	0.9	0.9	1.0	8.9	1	15	15	28	1970	217.1	7.4
NP_002872.1	4506405	RALB	ras-related protein Ral-B	1.0	1.0	1.0	0.9	0.9	1.0	1.0	1.0	1.0	1.0	1.0	1.0	32.0	1	2	5	18	206	23.4	6.6
NP_055484.3	208609990	NUP93	nuclear pore complex protein Nup93 isoform 1	1.0	0.9	1.0	1.0	1.1	1.0	1.1	1.0	1.0	1.0	1.1	1.0	30.4	2	23	23	76	819	93.4	5.7
NP_958431.2	50980301	MPRIP	myosin phosphatase Rho-interacting protein isoform 2	1.1	1.0	1.1	1.0	1.0	0.9	0.9	0.9	1.0	1.0	1.0	1.0	9.2	2	7	7	15	1025	116.5	6.2
NP_002023.2	56682959	FTH1	ferritin heavy chain	0.9	0.8	0.8	1.0	0.9	0.7	0.7	0.9	0.9	0.7	0.8	1.0	17.5	1	3	3	5	183	21.2	5.6
NP_055259.2	38454194	TUBGCP4	gamma-tubulin complex component 4 isoform b	0.9	1.0	0.9	1.0	1.1	1.0	0.9	0.9	1.0	1.0	0.9	1.0	2.1	2	1	1	2	666	76.0	6.7
NP_003767.2	26638659	MRPL40	39S ribosomal protein L40, mitochondrial	0.9	1.0	1.0	1.0	1.0	1.2	1.0	1.0	1.0	1.1	1.0	1.0	21.8	1	3	3	6	206	24.5	9.6
NP_079422.1	13376735	NAA50	N-alpha-acetyltransferase 50 isoform 1	1.0	1.0	1.0	1.0	1.0	1.0	1.0	1.0	1.0	1.0	1.0	1.0	20.1	2	4	4	11	169	19.4	8.8
NP_001032726.1	83641885	BTF3	transcription factor BTF3 isoform A	0.9	1.0	1.0	1.0	0.9	1.0	1.0	1.0	0.9	1.0	1.0	1.0	67.0	2	11	13	88	206	22.2	9.4
NP_000908.2	63252886	P4HA1	prolyl 4-hydroxylase subunit alpha-1 isoform 1 precursor	0.9	1.0	1.2	1.0	0.9	1.0	1.1	0.9	0.9	1.0	1.2	1.0	34.5	3	12	12	41	534	60.9	6.0
NP_001118.3	260436862	AP1B1	AP-1 complex subunit beta-1 isoform a	1.0	1.0	1.1	1.0	1.1	1.1	1.1	1.0	1.1	1.0	1.1	1.0	46.8	3	16	31	102	949	104.5	5.1
NP_006806.1	5803149	TMED2	transmembrane emp24 domain-containing protein 2 precursor	1.1	1.1	1.0	1.0	1.1	1.0	1.0	1.0	1.1	1.0	1.0	1.0	30.9	1	6	6	29	201	22.7	5.2
NP_001246.2	118402582	CDC20	cell division cycle protein 20 homolog	1.0	1.0	1.0	1.1	0.8	0.8	0.8	0.9	0.9	0.9	0.9	1.0	14.0	1	4	4	9	499	54.7	9.2
NP_001348.2	100913206	DHX9	ATP-dependent RNA helicase A	0.9	0.9	1.0	1.0	1.0	1.0	1.0	1.0	1.0	0.9	1.0	1.0	42.1	1	41	41	270	1270	140.9	6.8
NP_001017926.1	63079683	ZHX1	zinc fingers and homeoboxes protein 1	1.3	1.0	0.8	0.9	1.1	1.1	1.0	1.0	1.2	1.0	0.9	1.0	3.7	1	2	2	3	873	98.0	6.0
NP_001446.1	4503739	FOXO3	forkhead box protein O3	0.8	0.9	0.9	0.9	0.8	1.0	0.9	1.1	0.8	1.0	0.9	1.0	6.7	1	3	3	6	673	71.2	5.2
NP_005207.2	20070197	DDOST	dolichyl-diphosphooligosaccharide--protein glycosyltransferase 48 kDa subunit precursor	1.0	1.0	1.0	1.0	1.0	1.0	1.1	1.0	1.0	1.0	1.0	1.0	48.7	1	16	16	78	456	50.7	6.4
NP_003807.1	4501915	ADAM9	disintegrin and metalloproteinase domain-containing protein 9 precursor	0.8	0.9	1.3	1.1	0.9	0.8	1.1	0.9	0.9	0.9	1.2	1.0	4.0	1	2	2	4	819	90.5	7.5
NP_071383.1	11559927	MRPS14	28S ribosomal protein S14, mitochondrial	1.2	1.0	1.1	0.9	1.1	1.1	1.1	1.0	1.1	1.0	1.1	1.0	11.7	1	1	1	2	128	15.1	11.4
NP_003147.2	21070997	STIM1	stromal interaction molecule 1 isoform 2 precursor	1.0	1.0	1.1	1.0	1.0	1.0	1.1	1.0	1.0	1.0	1.1	1.0	3.9	3	2	2	2	685	77.4	6.7
NP_060295.1	8923417	ADPRHL2	poly(ADP-ribose) glycohydrolase ARH3	1.0	0.7	0.8	1.0	0.9	0.9	0.9	1.0	1.0	0.8	0.8	1.0	9.4	1	3	3	8	363	38.9	5.1
NP_061848.2	52627149	TERF2IP	telomeric repeat-binding factor 2-interacting protein 1	1.0	1.0	0.9	1.0	1.0	1.0	1.0	1.0	1.0	1.0	1.0	1.0	17.8	1	5	5	11	399	44.2	4.7
NP_001035284.1	93588491	AGTRAP	type-1 angiotensin II receptor-associated protein isoform b	0.8	0.7	0.7	1.0	0.9	0.9	0.8	0.9	0.9	0.8	0.7	1.0	14.5	2	1	1	6	152	16.7	6.3
NP_057215.3	256222019	RAB10	ras-related protein Rab-10	0.9	1.0	1.0	0.9	1.0	1.0	1.1	1.0	0.9	1.0	1.0	1.0	71.5	10	9	12	31	200	22.5	8.4
NP_062535.2	146134388	YLPM1	YLP motif-containing protein 1	0.9	0.9	0.9	1.0	1.0	0.9	0.9	1.0	0.9	0.9	0.9	1.0	10.2	1	12	12	26	2146	241.5	6.6
NP_002547.1	4505531	OSBP	oxysterol-binding protein 1	1.1	0.9	1.0	1.0	1.0	1.0	1.0	1.0	1.1	1.0	1.0	1.0	21.7	1	10	10	23	807	89.4	7.3
NP_065143.2	170016077	ATP13A1	manganese-transporting ATPase 13A1	1.0	1.0	1.1	1.0	1.0	1.0	1.0	0.9	1.0	1.0	1.1	1.0	3.2	1	4	4	6	1204	132.9	8.1
NP_001273555.1	557636738	RNPS1	RNA-binding protein with serine-rich domain 1 isoform b	0.9	0.9	0.9	0.9	0.8	1.0	0.9	1.0	0.9	1.0	0.9	1.0	27.0	3	6	6	30	282	31.7	11.9
NP_001012780.1	61676108	CTU2	cytoplasmic tRNA 2-thiolation protein 2 isoform 2	0.9	0.9	0.9	1.1	1.0	1.0	1.0	0.9	0.9	1.0	0.9	1.0	1.9	2	1	1	1	485	52.4	6.9
NP_872282.1	32967305	COQ6	ubiquinone biosynthesis monooxygenase COQ6 isoform a	0.9	0.9	0.8	1.0	0.9	1.0	0.8	1.0	0.9	0.9	0.8	1.0	10.3	2	4	4	4	468	50.8	7.3
NP_001091896.1	148536864	SMARCD2	SWI/SNF-related matrix-associated actin-dependent regulator of chromatin subfamily D member 2	0.9	0.9	0.9	1.0	1.0	1.0	0.9	1.0	0.9	0.9	0.9	1.0	15.6	5	5	5	9	531	58.9	9.6
NP_002799.3	25777602	PSMD2	26S proteasome non-ATPase regulatory subunit 2 isoform 1	1.0	1.0	1.1	1.0	1.0	1.0	1.1	1.0	1.0	1.0	1.1	1.0	56.3	3	37	37	173	908	100.1	5.2
NP_038461.2	48675813	TNPO2	transportin-2 isoform 2	0.9	1.0	1.0	1.0	1.1	0.9	1.0	1.0	1.0	1.0	1.0	1.0	12.7	2	4	9	31	887	100.3	5.0
NP_000969.1	4506605	RPL23	60S ribosomal protein L23	1.0	1.0	1.1	1.0	1.0	1.0	1.0	1.0	1.0	1.0	1.1	1.0	67.9	1	8	8	61	140	14.9	10.5
NP_002328.1	4505021	LRPAP1	alpha-2-macroglobulin receptor-associated protein precursor	1.0	0.9	0.9	0.9	1.0	1.1	1.0	1.1	1.0	1.0	1.0	1.0	11.8	1	4	4	7	357	41.4	8.8
NP_006181.1	5453830	ORC2	origin recognition complex subunit 2	1.0	1.0	1.0	1.1	0.9	0.9	0.9	0.9	0.9	0.9	1.0	1.0	10.6	1	5	5	13	577	65.9	6.5
NP_001165783.1	288915536;223718250	PLS1	plastin-1	1.1	1.0	1.1	1.0	1.1	1.1	1.1	1.0	1.1	1.0	1.1	1.0	24.8	1	8	11	38	629	70.2	5.4
NP_116786.1	14790115	CASP3	caspase-3 preproprotein	1.1	0.9	0.9	0.9	1.0	1.0	1.0	1.1	1.0	1.0	1.0	1.0	18.1	1	4	4	9	277	31.6	6.5
NP_001137544.1	221316645	UBAC2	ubiquitin-associated domain-containing protein 2 isoform 1 precursor	1.1	0.9	0.9	1.0	1.2	1.0	1.0	1.0	1.1	0.9	0.9	1.0	8.7	2	2	2	3	344	38.9	9.0
NP_057225.2	166795297	DYNC1LI1	cytoplasmic dynein 1 light intermediate chain 1	1.0	1.0	1.0	1.0	1.1	0.9	1.0	1.0	1.0	1.0	1.0	1.0	21.4	1	8	8	17	523	56.5	6.4
NP_006169.2	156564401	NSF	vesicle-fusing ATPase	1.0	1.0	1.0	1.0	1.0	1.0	0.9	1.0	1.0	1.0	0.9	1.0	43.2	1	25	25	74	744	82.5	6.9
NP_062538.5	34878777	RNF20	E3 ubiquitin-protein ligase BRE1A	1.0	0.9	0.9	0.9	1.2	0.9	1.0	1.1	1.1	0.9	0.9	1.0	11.9	1	8	9	17	975	113.6	5.9
NP_000365.3	71051616	UROD	uroporphyrinogen decarboxylase	1.0	0.9	0.9	1.0	1.0	0.9	0.9	1.0	1.0	0.9	0.9	1.0	37.6	1	8	8	20	367	40.8	6.1
NP_079230.1	13376439	UBTD1	ubiquitin domain-containing protein 1	1.0	0.9	1.1	1.0	0.9	1.1	1.0	1.0	0.9	1.0	1.1	1.0	4.9	2	1	1	1	227	25.9	7.8
NP_065801.1	22748937	XPO5	exportin-5	1.0	0.9	0.9	0.9	1.0	1.0	1.0	1.0	1.0	0.9	1.0	1.0	30.7	1	27	27	83	1204	136.2	5.8
NP_001011713.2	190341107	NAA30	N-alpha-acetyltransferase 30	1.2	1.1	1.1	1.0	1.1	0.9	1.0	0.9	1.2	1.0	1.1	1.0	3.6	1	1	1	1	362	39.3	5.5
NP_001035951.1	109891938	DLGAP4	disks large-associated protein 4 isoform c	1.0	0.9	1.0	1.0	0.9	0.8	0.9	1.0	1.0	0.9	0.9	1.0	10.2	3	2	2	5	285	31.6	5.9
NP_055635.3	54607135	TOMM70A	mitochondrial import receptor subunit TOM70	1.1	1.1	1.0	1.0	1.1	1.0	1.1	1.0	1.1	1.1	1.1	1.0	30.6	1	16	16	57	608	67.4	7.1
NP_006452.3	73623035	SPAG5	sperm-associated antigen 5	0.8	0.9	0.9	0.9	1.1	1.1	1.1	1.1	1.0	1.0	1.0	1.0	9.5	1	8	8	12	1193	134.3	5.0
NP_778236.2	154759257	DDX51	ATP-dependent RNA helicase DDX51	1.0	0.9	1.0	1.0	1.0	1.0	0.9	1.0	1.0	0.9	0.9	1.0	8.9	1	4	4	6	666	72.4	8.2
NP_002071.2	73486658	GOT2	aspartate aminotransferase, mitochondrial isoform 1 precursor	1.1	1.0	1.1	0.9	1.1	1.0	1.0	1.0	1.1	1.0	1.1	1.0	49.1	2	20	20	91	430	47.5	9.0
NP_057143.1	7706339	PPIL1	peptidyl-prolyl cis-trans isomerase-like 1	1.0	0.9	0.9	1.0	1.0	1.1	1.0	1.0	1.0	1.0	1.0	1.0	35.5	1	5	5	7	166	18.2	8.0
NP_000933.1	4758950	PPIB	peptidyl-prolyl cis-trans isomerase B precursor	1.1	1.1	1.0	1.0	1.0	1.1	1.0	1.0	1.1	1.1	1.0	1.0	66.7	2	16	18	159	216	23.7	9.4
NP_109591.1	13489087	SERPINB1	leukocyte elastase inhibitor	1.0	1.1	1.1	1.1	1.0	0.9	0.9	0.9	1.0	1.0	1.0	1.0	20.3	1	5	5	10	379	42.7	6.3
NP_056235.4	229892270	DCAF13	DDB1- and CUL4-associated factor 13	0.9	1.0	1.0	1.0	1.0	1.0	0.9	1.0	0.9	1.0	1.0	1.0	14.2	1	6	6	17	597	67.5	9.3
NP_112485.1	13654274	FAHD1	acylpyruvase FAHD1, mitochondrial isoform 2	1.0	1.0	1.0	0.9	1.2	1.2	1.1	1.0	1.1	1.1	1.1	1.0	16.5	3	3	3	6	224	24.8	7.4
NP_695003.1	23397574	SNX33	sorting nexin-33	0.9	0.9	1.0	0.9	1.0	1.1	1.1	1.0	0.9	1.0	1.1	1.0	4.0	1	2	2	3	574	65.2	6.8
NP_001887.1	4503097	CSNK2A2	casein kinase II subunit alpha'	1.0	1.0	0.9	1.0	1.0	0.9	1.0	1.0	1.0	1.0	1.0	1.0	52.9	1	14	14	34	350	41.2	8.6
NP_055860.1	42734325	WAPAL	wings apart-like protein homolog	1.0	1.0	1.0	1.0	1.0	0.9	1.1	0.9	1.0	1.0	1.1	1.0	11.1	1	9	9	22	1190	132.9	5.4
NP_694550.1	23510358	RIOK1	serine/threonine-protein kinase RIO1 isoform 2	1.0	1.0	1.0	1.0	0.9	1.0	0.9	0.9	1.0	1.0	1.0	1.0	3.4	2	1	1	2	327	38.0	7.1
NP_002477.1	4505343	NCBP1	nuclear cap-binding protein subunit 1	1.2	1.0	1.0	1.0	1.0	1.0	1.1	1.0	1.1	1.0	1.0	1.0	31.3	1	16	16	45	790	91.8	6.4
NP_060755.1	8922794	IMP3	U3 small nucleolar ribonucleoprotein protein IMP3	1.0	0.9	0.9	1.0	0.9	0.9	0.9	1.0	0.9	0.9	0.9	1.0	24.5	1	4	4	8	184	21.8	9.5
NP_001268383.1	527498242	MBD3	methyl-CpG-binding domain protein 3 isoform 2	1.0	1.0	1.1	1.0	0.8	0.9	0.9	0.9	0.9	0.9	1.0	1.0	22.4	2	4	4	12	259	29.0	5.2
NP_001243205.1	372266169	RAB34	ras-related protein Rab-34 isoform 6	0.9	0.9	1.0	1.0	0.9	1.0	1.0	1.0	0.9	0.9	1.0	1.0	20.3	7	4	4	19	237	26.7	7.0
NP_001263991.1	457866836	MFF	mitochondrial fission factor isoform b	0.9	0.9	0.9	0.9	1.2	1.1	1.1	1.0	1.1	1.0	1.0	1.0	32.0	7	5	5	7	291	33.0	7.4
NP_008841.2	33620769	RBBP6	E3 ubiquitin-protein ligase RBBP6 isoform 1	0.9	0.9	0.9	1.0	0.9	1.0	0.9	1.0	0.9	1.0	0.9	1.0	7.1	3	14	14	23	1792	201.4	9.6
NP_078924.1	13375891	OGFOD3	2-oxoglutarate and iron-dependent oxygenase domain-containing protein 3 isoform 1	1.1	1.2	1.1	1.1	0.8	0.9	0.8	0.9	0.9	1.0	1.0	1.0	7.8	2	1	1	2	319	35.6	8.2
NP_001896.2	148491070	CTPS1	CTP synthase 1 isoform a	1.0	0.9	0.9	1.0	1.0	0.9	1.0	1.0	1.0	0.9	0.9	1.0	38.1	2	17	19	69	591	66.6	6.5
NP_056290.3	56699482	FAM98A	protein FAM98A isoform 1	1.0	1.0	1.0	1.0	0.9	1.0	1.0	1.0	1.0	1.0	1.0	1.0	16.6	2	6	6	19	518	55.2	9.0
NP_004614.3	156627581	TTC4	tetratricopeptide repeat protein 4 isoform 1	0.9	0.9	0.8	1.0	0.9	0.9	0.8	1.0	0.9	0.9	0.8	1.0	48.8	2	13	13	33	387	44.7	5.6
NP_079508.2	20149695	REEP4	receptor expression-enhancing protein 4 precursor	1.0	1.1	1.0	1.0	1.0	1.1	1.0	1.0	1.0	1.1	1.0	1.0	3.9	1	1	1	2	257	29.4	9.7
NP_037534.3	214831730	FADS1	fatty acid desaturase 1	1.0	1.0	1.0	0.9	1.0	1.1	1.2	1.0	1.0	1.1	1.1	1.0	7.6	1	2	3	4	501	57.8	9.5
NP_001171783.1	296434227	KLHDC4	kelch domain-containing protein 4 isoform 3	0.8	1.0	1.0	1.0	0.9	0.9	1.0	0.9	0.9	0.9	1.0	1.0	2.2	3	1	1	2	463	51.5	5.9
NP_036458.2	10800412	MAPRE3	microtubule-associated protein RP/EB family member 3	1.0	1.0	1.0	0.9	1.0	1.1	1.1	1.0	1.0	1.1	1.1	1.0	6.1	1	1	2	3	281	32.0	5.5
NP_061886.2	305632781	FAM64A	protein FAM64A isoform 1	1.0	1.0	1.0	1.0	1.1	1.0	1.0	1.0	1.1	1.0	1.0	1.0	34.0	2	5	5	9	238	26.2	9.9
NP_065104.1	9966841	AVEN	cell death regulator Aven	1.0	1.1	1.1	1.1	1.0	1.0	1.0	0.9	1.0	1.1	1.0	1.0	8.6	1	2	2	5	362	38.5	5.0
NP_060740.1	8922764	MRGBP	MRG/MORF4L-binding protein	1.2	0.7	0.8	0.8	1.0	1.0	1.0	1.1	1.1	0.9	0.9	1.0	6.4	1	1	1	3	204	22.4	5.8

Khan *et al.*, 2019. Multi-omics analysis to characterize cigarette smoke induced molecular alterations in esophageal cells
Supplementary Table 5. List of proteins quantified in untreated and chronically treated Het1A cells with cigarette smoke condensate for 8 months

NP_Accession	Protein group Accession	Gene ID	Description	Het-1A-Smoke - 2M/Parental	Het-1A-Smoke - 4M/Parental	Het-1A-Smoke - 6M/Parental	Het-1A-Smoke - 8M/Parental	Het-1A-Smoke - 2M/Parental	Het-1A-Smoke - 4M/Parental	Het-1A-Smoke - 6M/Parental	Het-1A-Smoke - 8M/Parental	Het-1A-Smoke - 2M/Parental	Het-1A-Smoke - 4M/Parental	Het-1A-Smoke - 6M/Parental	Het-1A-Smoke - 8M/Parental	Coverage (%)	Proteins	Unique Peptides	# Peptides	PSM	# AAs	MW [kDa]	calc. pI
				Replicate 1				Replicate 2				Average of replicates											
NP_001035548.1	94818891	ERAP1	endoplasmic reticulum aminopeptidase 1 isoform b precursor	1.0	1.0	1.1	1.0	1.0	1.0	1.0	1.0	1.0	1.0	1.0	1.0	8.6	2	7	7	14	941	107.2	6.5
NP_066997.3	24432106	CCAR2	cell cycle and apoptosis regulator protein 2	0.9	0.9	0.9	1.0	1.0	1.0	1.0	1.0	0.9	1.0	0.9	1.0	39.9	1	23	24	73	923	102.8	5.2
NP_001271446.1	548960904	PHF23	PHD finger protein 23 isoform 2	0.9	0.9	1.0	0.9	1.2	1.1	1.0	1.0	1.0	1.0	1.0	1.0	16.1	3	3	3	5	336	36.7	5.4
NP_002706.1	4506017	PPP2CA	serine/threonine-protein phosphatase 2A catalytic subunit alpha isoform	0.9	0.9	1.0	0.9	0.9	0.9	0.9	1.0	0.9	0.9	0.9	1.0	42.7	1	2	9	69	309	35.6	5.5
NP_056975.1	7706657	CDC40	pre-mRNA-processing factor 17	1.0	0.9	1.0	1.0	0.9	0.9	0.9	1.0	0.9	0.9	1.0	1.0	10.5	1	4	4	4	579	65.5	7.1
NP_056285.1	24308075	RAB11FIP5	rab11 family-interacting protein 5	1.0	0.9	1.0	1.1	0.9	0.8	0.8	0.9	0.9	0.9	0.9	1.0	6.7	1	3	3	5	653	70.4	9.2
NP_037524.1	7019333	NRBP1	nuclear receptor-binding protein	1.0	0.9	1.0	0.9	1.1	1.1	1.2	1.0	1.0	1.0	1.1	1.0	17.4	1	5	5	12	535	59.8	5.1
NP_001180308.1	301129207	UCKL1	uridine-cytidine kinase-like 1 isoform 2	1.0	1.0	0.9	1.0	1.0	1.0	1.1	1.0	1.0	1.0	1.0	1.0	9.0	2	4	4	4	533	59.4	8.0
NP_665906.1	22208967;22208971	HMGA1	high mobility group protein HMG-I/HMG-Y isoform a	1.0	1.1	1.1	0.9	1.0	1.4	1.2	1.0	1.0	1.2	1.2	1.0	30.8	2	4	4	101	107	11.7	10.3
NP_006022.3	81295809	PCNT	pericentrin	1.0	1.0	1.0	1.0	1.1	1.1	1.0	1.0	1.1	1.0	1.0	1.0	4.5	1	11	11	20	3336	377.8	5.6
NP_115881.3	190014601	JAGN1	protein jagunal homolog 1	1.0	1.0	1.1	1.0	0.8	0.9	1.0	0.9	0.9	0.9	1.0	1.0	13.1	1	2	2	5	183	21.1	9.7
NP_001166927.1	291084757	PDHA1	pyruvate dehydrogenase E1 component subunit alpha, somatic form, mitochondrial isoform 4 precursor	1.2	1.1	1.2	1.0	1.1	1.0	1.0	1.0	1.1	1.1	1.1	1.0	49.9	5	14	14	48	359	40.2	8.3
NP_003792.1	4504079	GPAA1	glycosylphosphatidylinositol anchor attachment 1 protein	0.9	0.9	1.0	0.9	1.0	1.1	1.0	1.0	1.0	1.0	1.0	1.0	4.2	1	3	3	4	621	67.6	8.1
NP_004588.1	4759158	SNRPD2	small nuclear ribonucleoprotein Sm D2 isoform 1	1.0	0.9	1.0	1.0	0.9	0.9	0.9	1.0	0.9	0.9	1.0	1.0	67.0	2	9	9	98	118	13.5	9.9
NP_001231902.1	350276245	NRP1	neuropilin-1 isoform e precursor	0.9	1.1	1.2	0.9	1.0	1.3	1.4	1.0	0.9	1.2	1.3	1.0	6.6	5	4	4	10	916	102.3	6.0
NP_116126.3	388240801	LMNB2	lamin-B2	0.9	0.9	0.8	1.0	0.9	0.9	0.9	1.0	0.9	0.9	0.9	1.0	40.0	1	21	26	90	620	69.9	5.6
NP_006113.2	51477716	MAN2A2	alpha-mannosidase 2x	1.0	0.9	1.0	1.0	1.2	1.0	1.1	0.9	1.1	1.0	1.0	1.0	3.7	1	3	3	6	1150	130.5	6.8
NP_001073901.1	122937263	FTO	alpha-ketoglutarate-dependent dioxygenase FTO	0.9	0.9	0.9	1.0	1.0	0.9	1.0	1.0	1.0	0.9	0.9	1.0	31.7	1	11	11	32	505	58.2	5.2
NP_057121.2	156415994	UTP11L	probable U3 small nucleolar RNA-associated protein 11	1.0	1.1	1.0	1.0	0.9	0.9	0.9	0.9	1.0	1.0	1.0	1.0	19.0	1	4	4	4	253	30.4	10.2
NP_001157751.1	256219564	SLC37A4	glucose-6-phosphate translocase isoform 3	1.0	1.0	1.0	1.1	0.9	0.7	0.8	0.8	0.9	0.8	0.9	1.0	2.8	3	1	1	5	356	38.2	8.8
NP_001273651.1	557878589	FAM120A	constitutive coactivator of PPAR-gamma-like protein 1 isoform b	1.0	1.0	1.0	1.0	1.0	1.0	1.1	1.0	1.0	1.0	1.1	1.0	22.8	6	21	21	55	1117	121.7	8.9
NP_064528.1	10047140	PNO1	RNA-binding protein PNO1	0.9	0.9	0.9	0.9	1.0	0.9	0.9	1.0	1.0	0.9	0.9	1.0	38.9	1	8	8	17	252	27.9	9.7
NP_001924.2	19923748	DLST	dihydrolipoyllysine-residue succinyltransferase component of 2-oxoglutarate dehydrogenase complex, mitochondrial isoform 1 precursor	1.0	0.9	0.9	1.0	1.0	0.9	0.9	1.0	1.0	0.9	0.9	1.0	28.9	2	11	11	68	453	48.7	9.0
NP_060362.3	151301228	PRPF40A	pre-mRNA-processing factor 40 homolog A	1.0	0.9	0.9	1.0	1.0	0.9	1.0	1.0	1.0	0.9	0.9	1.0	19.3	2	13	13	30	930	105.9	8.0
NP_065724.1	10190714	RAB22A	ras-related protein Rab-22A	1.2	0.9	0.8	1.0	0.9	0.8	0.8	1.0	1.1	0.9	0.8	1.0	16.5	2	2	2	6	194	21.8	8.1
NP_068749.3	62899047	RINT1	RAD50-interacting protein 1	0.9	0.9	1.0	1.0	1.0	1.0	1.0	1.0	0.9	1.0	1.0	1.0	2.9	1	1	1	2	792	90.6	5.5
NP_064708.1	40789233	COQ9	ubiquinone biosynthesis protein COQ9, mitochondrial precursor	1.0	0.7	1.0	0.9	1.1	1.1	1.0	1.1	1.0	0.9	1.0	1.0	24.5	1	4	4	11	318	35.5	5.9
NP_003907.3	22027655	AP1S2	AP-1 complex subunit sigma-2 isoform 2	0.9	1.0	0.9	0.9	1.1	1.0	1.0	1.0	1.0	1.0	1.0	1.0	5.7	2	1	1	2	157	18.6	5.5
NP_786886.1	28395033;111494251	RHOC	rho-related GTP-binding protein RhoC precursor	0.9	1.0	1.2	1.1	0.9	1.0	1.0	0.9	0.9	1.0	1.1	1.0	56.0	1	2	7	45	193	22.0	6.6
NP_061915.2	50727002	PUS7	pseudouridylate synthase 7 homolog	0.9	1.0	1.0	1.0	1.0	1.0	1.1	1.0	1.0	1.0	1.0	1.0	21.3	1	10	10	32	661	75.0	6.4
NP_001129064.1	208022622;294459921	RPS27A	ubiquitin-40S ribosomal protein S27a precursor	1.1	1.0	1.0	1.0	1.0	1.1	1.0	1.0	1.1	1.0	1.0	1.0	69.9	3	4	15	216	156	18.0	9.6
NP_001258358.1	406719590	EMC1	ER membrane protein complex subunit 1 isoform 4 precursor	1.1	1.0	1.0	1.0	1.0	1.0	1.0	1.0	1.1	1.0	1.0	1.0	17.8	4	14	14	51	971	109.4	7.9
NP_001073027.1	118601081	HNRNPUL2	heterogeneous nuclear ribonucleoprotein U-like protein 2	0.9	1.1	1.0	1.0	1.0	1.0	1.0	1.0	1.0	1.1	1.0	1.0	41.9	1	29	29	133	747	85.1	4.9
NP_653304.2	148839362	LSM14B	protein LSM14 homolog B	1.0	0.9	0.8	0.9	0.9	0.9	0.9	1.0	1.0	0.9	0.9	1.0	39.2	1	8	9	21	385	42.0	9.7
NP_003355.1	4507839;566006139	UPP1	uridine phosphorylase 1 isoform a	1.2	1.1	1.0	1.0	1.1	1.2	1.1	1.0	1.2	1.1	1.0	1.0	27.7	2	7	7	12	310	33.9	7.9
NP_008869.1	5902102	SNRPD1	small nuclear ribonucleoprotein Sm D1 isoform 1	1.0	0.9	1.0	1.0	1.0	1.0	1.0	0.9	1.0	1.0	1.0	1.0	37.0	2	4	4	132	119	13.3	11.6
NP_001139732.1	226371743	PIP4K2C	phosphatidylinositol 5-phosphate 4-kinase type-2 gamma isoform c	0.9	0.9	0.9	1.0	0.9	1.0	0.8	0.9	0.9	1.0	0.8	1.0	8.6	3	3	3	6	373	41.9	7.4
NP_060441.2	26638657	MRPL20	39S ribosomal protein L20, mitochondrial	1.0	0.9	1.0	1.0	1.0	1.0	1.0	1.0	1.0	1.0	1.0	1.0	12.8	1	2	2	8	149	17.4	10.9
NP_008985.3	46852174	KIF3A	kinesin-like protein KIF3A	1.0	0.9	0.9	1.0	1.0	0.9	0.9	0.9	1.0	0.9	0.9	1.0	16.3	3	8	9	17	699	80.0	6.5
NP_057321.2	20127553	ANAPC5	anaphase-promoting complex subunit 5 isoform a	0.9	0.9	1.0	0.9	1.3	1.1	1.1	1.1	1.1	1.0	1.1	1.0	2.4	1	1	1	2	755	85.0	6.9
NP_001958.2	83700235	EIF4A2	eukaryotic initiation factor 4A-II	1.0	1.0	1.1	1.0	0.9	1.0	1.0	0.9	0.9	1.0	1.1	1.0	54.8	1	7	17	224	407	46.4	5.5
NP_061138.3	119829191	VPS33B	vacuolar protein sorting-associated protein 33B isoform 1	1.0	0.9	0.9	1.0	0.9	0.8	0.8	1.0	1.0	0.9	0.8	1.0	9.7	3	5	5	11	617	70.5	6.7
NP_001509.3	169881252	GTF2I	general transcription factor II-I isoform 4	0.9	1.0	1.0	1.0	0.9	1.0	1.0	1.0	0.9	1.0	1.0	1.0	36.0	6	28	28	89	957	107.9	7.9
NP_006398.1	5453704	ARL6IP5	PRA1 family protein 3	1.0	1.0	1.1	1.0	1.0	1.0	1.1	1.0	1.0	1.0	1.1	1.0	13.8	1	2	2	6	188	21.6	9.8
NP_001009.1	4506687	RPS15	40S ribosomal protein S15 isoform 2	0.9	1.0	0.9	1.0	1.0	1.0	1.0	1.0	0.9	1.0	1.0	1.0	64.8	2	6	6	66	145	17.0	10.4
NP_004574.2	41393545;41393614	RAB5C	ras-related protein Rab-5C isoform a	0.9	0.9	0.9	0.9	1.0	0.9	0.9	1.0	1.0	0.9	0.9	1.0	66.7	2	7	9	70	216	23.5	8.4
NP_002061.1	4504041	GNAI2	guanine nucleotide-binding protein G(i) subunit alpha-2 isoform 1	1.1	1.1	1.1	1.0	0.9	1.0	1.0	0.9	1.0	1.0	1.1	1.0	36.9	13	6	12	60	355	40.4	5.5
NP_005920.2	134244281	MFI2	melanotransferrin isoform 1 precursor	1.0	0.9	1.2	1.0	1.0	1.0	1.2	0.9	1.0	0.9	1.2	1.0	12.7	2	6	6	14	738	80.2	5.9
NP_068806.1	11761621	GMPPB	mannose-1-phosphate guanyltransferase beta isoform 2	1.1	1.1	1.1	1.0	1.2	1.1	1.0	0.9	1.1	1.1	1.1	1.0	50.3	2	12	12	36	360	39.8	6.8
NP_078954.4	226491502	NARS2	probable asparagine--tRNA ligase, mitochondrial isoform 1 precursor	1.1	1.1	1.1	1.0	1.2	1.1	1.0	0.9	1.2	1.1	1.0	1.0	18.5	2	5	5	8	477	54.1	7.2
NP_001269530.1	543583723	PTGES3	prostaglandin E synthase 3 isoform b	1.0	0.8	1.0	0.9	1.0	0.9	1.1	1.0	1.0	0.9	1.0	1.0	48.2	6	6	6	27	139	16.5	4.8
NP_057385.3	256818742	GPN3	GPN-loop GTPase 3 isoform 1	1.1	1.0	1.0	1.0	0.9	0.8	0.9	0.9	1.0	0.9	0.9	1.0	20.8	3	4	4	6	284	32.7	4.5
NP_055965.1	41872577	RFTN1	raftlin	1.1	1.0	1.2	1.0	1.1	1.0	1.0	0.9	1.1	1.0	1.1	1.0	5.2	1	2	2	3	578	63.1	5.7
NP_061897.1	55749742	HEATR5B	HEAT repeat-containing protein 5B	1.1	1.1	1.0	1.0	0.9	0.9	0.9	0.9	1.0	1.0	1.0	1.0	1.5	1	2	2	2	2071	224.2	7.2
NP_597814.1	19743894	TADA3	transcriptional adapter 3 isoform b	0.9	1.0	0.9	0.9	0.9	1.0	1.0	1.0	0.9	1.0	0.9	1.0	19.0	2	4	4	7	369	41.4	5.5
NP_004583.2	23111062	SFSWAP	splicing factor, suppressor of white-apricot homolog isoform 2	1.0	1.1	1.0	1.1	1.0	1.0	1.1	0.9	1.0	1.1	1.0	1.0	2.1	2	2	2	2	951	104.8	8.0
NP_958928.1	41462412	SLC25A35	solute carrier family 25 member 35	1.3	1.2	1.1	1.0	1.1	0.9	1.1	0.9	1.2	1.1	1.1	1.0	2.7	1	1	1	1	295	31.2	9.3
NP_004508.1	4758606;62420873	ILK	integrin-linked protein kinase isoform 1	1.0	1.0	1.1	1.0	1.0	1.0	1.2	1.0	1.0	1.0	1.2	1.0	17.0	3	7	7	17	452	51.4	8.1
NP_653333.1	21536320	HNRNPUL1	heterogeneous nuclear ribonucleoprotein U-like protein 1 isoform d	1.0	1.0	1.1	1.0	1.0	1.0	1.1	1.0	1.0	1.0	1.1	1.0	34.3	2	20	21	63	756	84.7	8.8
NP_004879.1	4757818	ATP6V1G1	V-type proton ATPase subunit G 1	1.0	1.1	1.0	1.0	1.1	1.1	0.9	1.0	1.0	1.1	1.0	1.0	48.3	1	5	5	14	118	13.7	8.8
NP_002019.1	10835059	FNTB	protein farnesyltransferase subunit beta	1.0	1.0	1.0	1.0	0.9	0.9	0.9	0.9	1.0	0.9	1.0	1.0	3.7	1	1	1	2	437	48.7	5.8
NP_060522.3	39780552	VAC14	protein VAC14 homolog	1.0	1.0	1.1	1.0	1.0	0.9	1.1	0.9	1.0	1.0	1.1	1.0	13.2	1	7	7	15	782	87.9	6.1
NP_060550.2	66348091	VPS13C	vacuolar protein sorting-associated protein 13C isoform 1B	1.0	1.0	1.0	1.0	0.9	0.9	0.9	0.9	1.0	1.0	0.9	1.0	3.8	4	9	9	15	3585	402.8	6.4
NP_000980.1	4506631	RPL30	60S ribosomal protein L30	0.9	0.9	1.0	1.0	1.0	1.0	1.0	1.0	1.0	1.0	1.0	1.0	76.5	1	9	9	68	115	12.8	9.6
NP_067045.1	24308263	PRUNE	protein prune homolog isoform 1	1.0	1.0	1.0	0.9	1.0	0.9	1.0	1.0	1.0	0.9	1.0	1.0	5.3	2	2	2	2	453	50.2	5.5
NP_006558.1	5729820	FARS2	phenylalanine--tRNA ligase, mitochondrial precursor	1.0	0.9	0.9	0.9	1.2	1.0	1.0	1.0	1.1	1.0	1.0	1.0	1.8	1	1	1	2	451	52.3	7.5
NP_055488.1	7661910	EMC2	ER membrane protein complex subunit 2	1.1	0.9	1.0	1.0	1.0	1.0	1.0	1.0	1.0	0.9	0.9	1.0	18.9	1	4	4	12	297	34.8	6.6
NP_006668.1	57529246	USP19	ubiquitin carboxyl-terminal hydrolase 19 isoform 4	0.9	0.9	0.9	0.9	1.0	1.1	1.0	1.0	0.9	1.0	0.9	1.0	13.1	4	7	7	13	1318	145.6	6.3
NP_002192.2	6857800	ISG20	interferon-stimulated gene 20 kDa protein isoform a	0.9	1.2	1.8	1.0	1.0	1.0	1.5	1.0	1.0	1.1	1.6	1.0	4.4	1	1	1	2	181	20.4	8.9
NP_003929.4	117553580	AP3D1	AP-3 complex subunit delta-1 isoform 2	1.0	1.0	1.0	1.0	0.9	0.9	0.9	0.9	1.0	0.9	1.0	1.0	20.0	2	18	18	58	1153	130.1	8.5
NP_003923.2	19923193	ST13	hsc70-interacting protein isoform 1	1.0	1.0	1.1	1.0	1.0	1.0	1.0	1.0	1.0	1.0	1.1	1.0	43.6	2	14	14	120	369	41.3	5.3
NP_057055.2	186928852	MRPS7	28S ribosomal protein S7, mitochondrial	1.0	1.0	1.0	1.0	0.9	0.9	1.0	0.9	1.0	1.0	1.0	1.0	28.9	1	6	6	12	242	28.1	10.0
NP_060653.3	269846807	SBNO1	protein strawberry notch homolog 1 isoform 2	0.9	0.9	0.9	0.9	1.0	1.1	1.1	1.0	1.0	1.0	1.0	1.0	6.5	4	5	5	8	1392	154.1	7.9
NP_001013012.1	153791707	SNX30	sorting nexin-30	1.2	1.0	0.9	1.0	1.0	0.9	0.9	0.9	1.1	0.9	0.9	1.0	11.4	1	4	4	6	437	49.6	5.4
NP_005713.1	5031571	ACTR2	actin-related protein 2 isoform b	1.0	1.0	1.1	1.0	1.0	1.1	1.1	1.0	1.0	1.0	1.1	1.0	19.5	2	6	7	43	394	44.7	6.7
NP_001075019.1	125656165	THOC2	THO complex subunit 2	1.1	1.1	1.0	1.0	1.0	1.0	1.0	0.9	1.0	1.0	1.0	1.0	6.0	1	9	9	16	1593	182.7	8.4
NP_001122320.1	192807320	SMARCA4	transcription activator BRG1 isoform F	0.9	0.9	0.9	1.0	1.0	0.9	1.0	0.9	1.0	0.9	1.0	1.0	10.2	6	9	13	42	1613	181.1	8.0
NP_671713.1	22538446	TP53I3	quinone oxidoreductase PIG3 isoform 1	0.9	0.9	0.9	0.9	1.1	1.0	1.0	1.0	1.0	1.0	0.9	1.0	2.7	1	1	1	1	332	35.5	7.2
NP_065752.1	20149304	ISY1	pre-mRNA-splicing factor ISY1 homolog isoform 2	1.0	0.8	0.9	0.9	1.1	0.9	1.0	1.0	1.1	0.9	1.0	1.0	16.1	3	3	3	6	285	33.0	5.2
NP_085056.2	83921614	CYB5B	cytochrome b5 type B	1.0	1.0	1.0	1.0	1.0	1.0	1.0	1.0	1.0	1.0	1.0	1.0	58.0	1	6	6	44	150	16.7	5.0
NP_443107.1	16418381	VPS26B	vacuolar protein sorting-associated protein 26B	1.2	1.1	1.1	1.0	1.0	1.0	1.0	1.0	1.1	1.0	1.0	1.0	14.6	1	3	5	9	336	39.1	7.4
NP_001008528.1	56549131	MXRA7	matrix-remodeling-associated protein 7 isoform 1 precursor	1.0	0.9	1.0	0.9	0.9	1.0	1.0	1.0	1.0	0.9	1.0	1.0	22.1	3	4	4	11	204	21.5	4.3
NP_003760.1	4506903	SRSF9	serine/arginine-rich splicing factor 9	0.9	0.9	0.9	0.9	1.0	0.9	0.9	1.0	0.9	0.9	0.9	1.0	21.7	1	4	4	8	221	25.5	8.6

Khan *et al.* , 2019. Multi-omics analysis to characterize cigarette smoke induced molecular alterations in esophageal cells
Supplementary Table 5. List of proteins quantified in untreated and chronically treated Het1A cells with cigarette smoke condensate for 8 months

NP_Accession	Protein group Accession	Gene ID	Description	Het-1A-Smoke - 2M/Parental	Het-1A-Smoke - 4M/Parental	Het-1A-Smoke - 6M/Parental	Het-1A-Smoke - 8M/Parental	Het-1A-Smoke - 2M/Parental	Het-1A-Smoke - 4M/Parental	Het-1A-Smoke - 6M/Parental	Het-1A-Smoke - 8M/Parental	Het-1A-Smoke - 2M/Parental	Het-1A-Smoke - 4M/Parental	Het-1A-Smoke - 6M/Parental	Het-1A-Smoke - 8M/Parental	Coverage (%)	Proteins	Unique Peptides	# Peptides	PSM	# AAs	MW [kDa]	calc. pI
				Replicate 1				Replicate 2				Average of replicates											
NP_055629.1	7661914	PSMD6	26S proteasome non-ATPase regulatory subunit 6 isoform 2	1.0	1.0	1.0	1.0	1.1	1.0	1.1	1.0	1.0	1.0	1.0	1.0	49.6	4	18	18	61	389	45.5	5.6
NP_997005.1	46370069	EXT2	exostosin-2 isoform 2	1.1	1.1	0.8	1.0	1.1	1.1	0.9	1.0	1.1	1.1	0.9	1.0	2.8	3	2	2	2	718	82.2	6.6
NP_064627.1	9910266	KIF15	kinesin-like protein KIF15	0.9	0.9	0.9	1.0	1.0	0.9	0.9	1.0	0.9	0.9	0.9	1.0	5.3	1	6	6	15	1388	160.1	6.0
NP_476510.1	17149830	GIT2	ARF GTPase-activating protein GIT2 isoform 1	1.1	1.0	1.0	1.1	0.7	0.7	0.9	0.9	0.9	0.9	0.9	1.0	4.1	3	2	2	2	759	84.5	7.2
NP_001271292.1	556503457	TLK2	serine/threonine-protein kinase tousled-like 2 isoform B	1.0	0.9	1.0	1.0	0.9	1.0	0.9	1.0	1.0	0.9	1.0	1.0	3.5	3	1	1	2	718	82.3	8.3
NP_001243069.1	371502127	CAPG	macrophage-capping protein isoform 2	1.9	1.1	1.0	0.9	1.1	1.0	1.1	1.0	1.5	1.0	1.0	1.0	16.2	2	5	5	13	333	36.8	7.2
NP_057494.3	189409150	CHMP5	charged multivesicular body protein 5 isoform 1	1.1	1.0	1.1	1.0	1.2	1.1	1.1	1.0	1.2	1.1	1.1	1.0	43.8	2	7	7	47	219	24.6	4.8
NP_001159890.1	262073088	HDAC8	histone deacetylase 8 isoform 2	1.1	1.0	1.0	1.0	1.1	1.1	1.0	0.9	1.1	1.0	1.0	1.0	10.1	3	2	2	3	286	31.9	6.0
NP_055953.3	195976782	RTF1	RNA polymerase-associated protein RTF1 homolog	1.0	1.0	1.0	1.0	1.0	1.0	1.0	1.0	1.0	1.0	1.0	1.0	22.4	1	14	14	29	710	80.3	8.1
NP_002731.4	133908623	PRKCI	protein kinase C iota type	0.9	0.9	0.9	1.0	1.0	1.0	1.0	1.0	1.0	1.0	1.0	1.0	36.2	1	10	10	31	596	68.2	5.8
NP_001269952.1	545688070	GMPR2	GMP reductase 2 isoform 5	1.1	1.0	1.0	1.0	1.1	1.0	0.9	1.0	1.1	1.0	1.0	1.0	33.4	6	8	8	13	320	34.6	7.7
NP_001137484.1	221136993	TMX2	thioredoxin-related transmembrane protein 2 isoform 2	1.0	1.1	1.1	1.0	1.0	1.3	1.2	0.9	1.0	1.2	1.2	1.0	27.9	2	6	6	11	258	29.6	8.6
NP_001157739.1	256773178	WDR46	WD repeat-containing protein 46 isoform 2	0.9	0.9	1.0	1.0	1.0	1.0	1.0	1.0	1.0	1.0	1.0	1.0	20.5	2	5	5	25	556	61.7	9.3
NP_002086.1	4504195	GTF2E2	transcription initiation factor IIE subunit beta	0.9	1.0	0.9	0.9	0.9	1.0	0.9	1.0	0.9	1.0	0.9	1.0	37.5	1	10	10	29	291	33.0	9.7
NP_001132990.1	213417919	PRKRA	interferon-inducible double-stranded RNA-dependent protein kinase activator A isoform 3	0.9	0.9	0.9	1.0	1.0	0.9	0.9	1.0	1.0	0.9	0.9	1.0	17.0	3	3	3	12	288	31.6	8.4
NP_000113.1	4557563	ERCC3	TFIIH basal transcription factor complex helicase XPB subunit isoform a	1.1	1.1	1.1	1.1	0.9	1.0	1.0	0.8	1.0	1.1	1.1	1.0	7.3	2	3	3	14	782	89.2	7.2
NP_002097.1	4504255	H2AFZ	histone H2A.Z	0.8	1.0	0.9	1.0	0.9	1.0	1.0	1.0	0.8	1.0	0.9	1.0	53.9	6	3	5	327	128	13.5	10.6
NP_002904.3	32528306	RFC1	replication factor C subunit 1 isoform 1	1.0	1.0	0.9	0.9	0.9	1.1	1.0	1.0	1.0	1.0	1.0	1.0	21.4	2	18	18	42	1147	128.1	9.4
NP_001032242.1	82546824	FOXK1	forkhead box protein K1	0.9	1.0	1.0	0.9	1.1	1.0	1.0	1.0	1.0	1.0	1.0	1.0	15.6	1	8	8	21	733	75.4	9.3
NP_002087.2	156104891	GTF2F1	general transcription factor IIF subunit 1	1.0	1.1	1.1	1.0	1.0	1.0	1.0	0.9	1.0	1.1	1.1	1.0	12.6	1	6	6	13	517	58.2	7.5
NP_055851.1	148225659	ENDOD1	endonuclease domain-containing 1 protein precursor	1.1	1.1	1.1	1.0	1.0	1.1	1.0	0.9	1.1	1.1	1.1	1.0	19.0	1	5	5	15	500	55.0	5.7
NP_001188474.1	319996623	RBM15	putative RNA-binding protein 15 isoform 2	1.0	1.0	1.1	0.9	1.0	1.0	1.0	1.0	1.0	1.0	1.0	1.0	12.2	3	7	7	14	969	106.3	10.1
NP_005608.1	5032051	RPS14	40S ribosomal protein S14	0.9	0.9	0.9	0.9	0.9	0.9	0.9	1.0	0.9	0.9	0.9	1.0	62.9	2	6	6	78	151	16.3	10.1
NP_001278510.1	615276234	RABEP1	rab GTPase-binding effector protein 1 isoform 3	1.0	1.0	1.0	1.0	1.0	1.0	1.0	1.0	1.0	1.0	1.0	1.0	22.1	4	14	14	30	819	94.1	5.0
NP_006807.1	5803023	LMAN2	vesicular integral-membrane protein VIP36 precursor	1.1	1.1	1.0	1.0	1.0	1.1	1.0	1.0	1.0	1.1	1.0	1.0	34.0	1	8	8	34	356	40.2	6.9
NP_055156.1	7657345	MTCH1	mitochondrial carrier homolog 1 PSAP-LS	1.2	0.9	0.9	1.0	1.0	0.8	0.8	0.9	1.1	0.9	0.8	1.0	13.2	2	4	4	5	372	39.9	9.5
NP_690051.1	23510462;311893287	RBM12	RNA-binding protein 12	0.8	0.9	0.8	0.9	0.8	0.9	0.9	1.0	0.8	0.9	0.8	1.0	7.5	1	7	7	20	932	97.3	8.6
NP_003073.1	4507101	SNAPC1	snRNA-activating protein complex subunit 1	1.0	1.0	1.0	0.9	1.0	1.0	1.1	1.0	1.0	1.0	1.0	1.0	3.5	1	2	2	5	368	43.0	9.5
NP_004810.2	124028529	SYMPK	symplekin	0.9	0.9	1.0	0.9	0.9	0.9	0.9	1.0	0.9	0.9	1.0	1.0	17.5	1	16	16	38	1274	141.1	6.1
NP_003843.3	47419909	TRIM24	transcription intermediary factor 1-alpha isoform b	0.9	0.9	0.9	0.9	1.0	1.0	1.0	1.0	1.0	0.9	0.9	1.0	6.8	2	4	6	7	1016	112.9	6.8
NP_056268.2	166197706	THUMPD3	THUMP domain-containing protein 3	0.9	0.8	0.9	1.0	1.0	0.9	0.9	1.0	0.9	0.9	0.9	1.0	37.3	1	10	10	25	507	57.0	6.4
NP_004205.2	7262378	FIBP	acidic fibroblast growth factor intracellular-binding protein isoform b	0.9	0.9	1.0	0.9	1.1	1.1	1.2	1.0	1.0	1.0	1.1	1.0	10.1	2	3	3	6	357	41.2	6.8
NP_001122393.2	731441419	MARK3	MAP/microtubule affinity-regulating kinase 3 isoform e	0.9	0.8	0.8	0.9	1.0	1.0	1.0	1.0	0.9	0.9	0.9	1.0	3.0	5	1	2	2	659	74.0	9.8
NP_060641.2	24586663	APPL2	DCC-interacting protein 13-beta isoform 1	1.1	1.0	1.1	1.0	1.2	1.3	1.3	1.0	1.1	1.2	1.2	1.0	12.7	3	5	5	6	664	74.4	4.9
NP_149107.4	222136641	NEK9	serine/threonine-protein kinase Nek9	1.0	1.0	1.0	1.0	0.9	0.9	0.9	1.0	0.9	0.9	0.9	1.0	9.9	1	7	7	19	979	107.1	5.7
NP_490597.1	19718731	BRD4	bromodomain-containing protein 4 isoform long	0.9	0.9	1.0	1.0	0.9	0.9	0.9	0.9	0.9	0.9	0.9	1.0	10.0	2	8	10	15	1362	152.1	9.2
NP_689929.2	189217919	PATL1	protein PAT1 homolog 1	1.0	1.0	1.0	1.0	0.9	0.9	0.9	1.0	1.0	0.9	1.0	1.0	17.0	1	8	8	16	770	86.8	6.7
NP_001191297.1	324120898	MGST2	microsomal glutathione S-transferase 2 isoform 3 precursor	0.9	0.8	0.9	0.9	1.0	1.0	1.1	1.0	1.0	0.9	1.0	1.0	18.2	2	1	1	1	77	8.4	9.5
NP_006088.2	29568111	MYL9	myosin regulatory light polypeptide 9 isoform a	1.1	1.1	1.2	1.0	1.2	1.1	1.3	0.9	1.2	1.1	1.2	1.0	83.7	5	4	11	146	172	19.8	4.9
NP_003375.1	4507903	VRK1	serine/threonine-protein kinase VRK1	1.0	0.9	0.9	1.0	0.9	0.9	0.9	1.0	0.9	0.9	0.9	1.0	42.4	1	13	13	55	396	45.4	8.9
NP_001001890.1	49574546	RUNX1	runt-related transcription factor 1 isoform AML1b	1.3	0.8	1.2	0.9	1.0	1.1	1.0	1.0	1.2	1.0	1.1	1.0	11.0	3	3	3	4	453	48.7	9.3
NP_001012496.1	60279268	U2AF2	splicing factor U2AF 65 kDa subunit isoform b	0.9	0.9	0.9	1.0	1.0	1.0	1.0	1.0	0.9	0.9	0.9	1.0	50.7	2	14	14	108	471	53.1	9.1
NP_597709.2	123173757	RAVER1	ribonucleoprotein PTB-binding 1	0.9	1.0	0.9	1.0	0.9	1.0	1.0	1.0	0.9	1.0	0.9	1.0	32.4	1	12	12	30	756	79.5	8.9
NP_066964.1	10863945	XRCC5	X-ray repair cross-complementing protein 5	1.0	1.0	1.0	1.0	1.0	0.9	0.9	1.0	1.0	1.0	1.0	1.0	76.5	1	41	41	468	732	82.7	5.8
NP_835369.1	30410728	FUT8	alpha-(1,6)-fucosyltransferase isoform a	1.0	0.9	0.8	0.9	1.3	1.1	1.1	1.0	1.2	1.0	1.0	1.0	1.6	1	1	1	1	575	66.5	7.7
NP_001268662.1	528524505	SLC26A6	solute carrier family 26 member 6 isoform 6	1.0	0.9	1.0	1.0	0.9	0.9	0.9	1.0	0.9	0.9	0.9	1.0	1.4	6	1	1	2	651	71.5	8.5
NP_066918.2	256985183	SLC5A6	sodium-dependent multivitamin transporter	1.0	0.9	0.9	0.9	1.1	1.1	1.0	1.0	1.1	1.0	0.9	1.0	1.7	1	1	1	1	635	68.6	8.3
NP_076936.1	13128980	PRR14	proline-rich protein 14	1.0	1.0	1.0	1.0	0.8	0.8	0.9	0.9	0.9	0.9	0.9	1.0	3.8	1	2	2	4	585	64.3	10.3
NP_777596.2	31317307	PCSK9	proprotein convertase subtilisin/kexin type 9 preproprotein	1.1	1.1	1.1	1.1	1.1	1.2	1.0	0.9	1.1	1.1	1.1	1.0	19.9	1	6	6	12	692	74.2	6.6
NP_079120.1	13376259	NUP85	nuclear pore complex protein Nup85 isoform 1	1.0	0.9	1.0	1.0	1.1	0.9	1.0	1.0	1.0	0.9	1.0	1.0	18.6	2	9	9	29	656	75.0	5.6
NP_663731.1	22027648	LPIN1	phosphatidate phosphatase LPIN1 isoform 1	1.2	1.2	1.2	0.9	1.1	1.1	1.1	1.0	1.1	1.2	1.1	1.0	17.9	4	9	10	20	890	98.6	6.6
NP_003135.2	169404009	SSR1	translocon-associated protein subunit alpha isoform 1 precursor	1.1	1.0	0.9	1.0	1.1	1.1	1.0	1.0	1.1	1.1	1.0	1.0	21.0	2	4	4	11	286	32.2	4.5
NP_003895.1	4508021	ZPR1	zinc finger protein ZPR1	1.1	1.0	1.1	1.0	1.1	1.1	1.0	1.0	1.1	1.1	1.1	1.0	19.2	1	5	5	18	459	50.9	4.7
NP_001257558.1	397739073	MED4	mediator of RNA polymerase II transcription subunit 4 isoform 2	1.0	1.1	1.1	1.1	1.0	0.8	0.9	0.8	1.0	1.0	1.0	1.0	6.3	2	1	1	3	224	24.9	5.2
NP_001273005.1	554595586	RPA2	replication protein A 32 kDa subunit isoform 2	1.1	1.0	0.8	1.0	1.0	0.9	0.8	0.9	1.0	0.9	0.8	1.0	8.1	2	1	1	1	174	19.1	5.7
NP_001373.2	42794009	DPAGT1	UDP-N-acetylglucosamine--dolichyl-phosphate N-acetylglucosaminephosphotransferase	1.2	1.1	1.5	1.0	1.6	1.1	1.2	1.0	1.4	1.1	1.3	1.0	2.0	1	1	1	1	408	46.1	8.0
NP_006416.3	27477111	SLU7	pre-mRNA-splicing factor SLU7	1.0	0.9	1.0	1.0	0.9	0.9	1.0	0.9	1.0	0.9	1.0	1.0	10.2	1	4	4	6	586	68.3	7.1
NP_005654.3	194733742	NELFA	negative elongation factor A	1.1	0.9	1.0	0.9	1.0	1.0	1.0	1.0	1.1	1.0	1.0	1.0	26.0	1	7	7	17	539	58.5	9.3
NP_055664.3	216548223	SV2A	synaptic vesicle glycoprotein 2A isoform 1	1.0	1.0	0.9	0.9	1.0	1.0	1.0	1.0	1.0	1.0	0.9	1.0	5.4	1	3	3	13	742	82.6	5.6
NP_001230869.1	345478668	PRPSAP2	phosphoribosyl pyrophosphate synthase-associated protein 2 isoform 3	1.0	1.0	0.9	1.0	1.0	0.9	0.9	1.0	1.0	0.9	0.9	1.0	27.5	4	5	6	18	320	35.5	8.8
NP_006077.2	50592996	TUBB3	tubulin beta-3 chain isoform 1	0.9	0.8	1.0	1.0	1.0	0.9	1.0	1.0	0.9	0.9	1.0	1.0	66.9	2	6	21	546	450	50.4	4.9
NP_057654.2	50959176	ERGIC2	endoplasmic reticulum-Golgi intermediate compartment protein 2	0.7	0.8	0.8	1.0	1.0	0.9	0.9	0.9	0.9	0.8	0.8	1.0	5.8	1	1	1	2	377	42.5	6.8
NP_004302.1	4757774	ARL3	ADP-ribosylation factor-like protein 3	1.0	0.9	1.0	1.0	1.0	0.9	0.9	1.0	1.0	0.9	1.0	1.0	68.1	1	10	10	29	182	20.4	7.2
NP_954663.1	40353755;40353759	BRE	BRCA1-A complex subunit BRE isoform 3	1.0	1.1	1.0	1.1	1.0	0.9	1.0	0.9	1.0	1.0	1.0	1.0	13.6	4	3	3	23	376	42.7	5.4
NP_958782.1	41322916	PLEC	plectin isoform 1	0.9	0.9	1.0	1.0	0.9	0.9	0.9	1.0	0.9	0.9	0.9	1.0	40.3	8	151	154	520	4684	531.5	6.0
NP_009035.3	15431288	RPL10A	60S ribosomal protein L10a	0.9	1.0	1.0	1.0	0.9	0.9	0.9	1.0	0.9	0.9	1.0	1.0	47.9	1	11	11	66	217	24.8	9.9
NP_001269339.1	540344565	PPP1R7	protein phosphatase 1 regulatory subunit 7 isoform 3	1.0	1.0	1.0	1.0	1.0	0.9	0.9	1.0	1.0	0.9	1.0	1.0	25.7	7	6	6	15	280	32.0	4.7
NP_001191802.1	325652118	NPEPL1	probable aminopeptidase NPEPL1 isoform 3	1.0	0.8	0.9	0.8	1.0	1.0	0.8	1.1	1.0	0.9	0.9	1.0	1.7	3	1	1	2	475	50.5	6.5
NP_071344.1	11596859	MRPL17	39S ribosomal protein L17, mitochondrial	1.3	1.0	1.0	1.1	1.0	0.8	0.9	0.9	1.2	0.9	1.0	1.0	14.9	1	2	2	6	175	20.0	10.1
NP_060299.4	21914862	CECR5	cat eye syndrome critical region protein 5 isoform 1	1.0	1.0	1.0	1.0	1.0	0.9	1.0	0.9	1.0	0.9	1.0	1.0	41.5	2	8	8	18	393	43.6	6.9
NP_056536.2	122939157	GEMIN4	gem-associated protein 4	1.0	0.9	1.0	0.9	1.0	1.0	1.0	1.0	1.0	1.0	1.0	1.0	12.6	1	11	11	19	1058	120.0	6.0
NP_775738.3	51243059	MCAT	malonyl-CoA-acyl carrier protein transacylase, mitochondrial isoform a precursor	1.2	1.0	1.0	1.0	1.0	1.0	1.0	0.9	1.1	1.0	1.0	1.0	9.5	1	2	2	3	390	42.9	8.7
NP_078789.2	157738667	FYCO1	FYVE and coiled-coil domain-containing protein 1	1.1	1.0	1.0	1.0	0.9	0.9	0.9	0.9	1.0	0.9	1.0	1.0	7.8	3	10	11	16	1478	166.9	4.9
NP_056269.1	109809739;109809741	LARP7	la-related protein 7 isoform 1	0.9	1.0	0.9	1.0	0.9	0.9	0.9	0.9	0.9	0.9	0.9	1.0	27.8	2	16	16	40	582	66.9	9.6
NP_001229971.1	339882741	HLA-C	HLA class I histocompatibility antigen, Cw-1 alpha chain precursor	1.1	1.1	1.1	1.0	1.1	1.3	1.2	1.0	1.1	1.2	1.2	1.0	15.0	2	3	5	18	366	40.7	5.9
NP_003181.3	27436893	TAPBP	tapasin isoform 1 precursor	0.9	1.0	1.0	1.0	1.0	1.0	1.1	0.9	1.0	1.0	1.0	1.0	7.8	3	2	2	6	448	47.5	7.0
NP_006497.2	12545408	RASA2	ras GTPase-activating protein 2	1.1	1.0	1.1	1.0	1.0	1.0	1.0	0.9	1.1	1.0	1.1	1.0	3.4	1	2	2	3	849	96.5	7.2
NP_068596.2	56605983	NIF3L1	NIF3-like protein 1 isoform 2	1.0	1.0	1.0	0.9	1.0	0.9	1.0	1.0	1.0	1.0	1.0	1.0	16.3	3	5	5	11	350	39.0	6.4
NP_075068.2	18644728	NOL6	nucleolar protein 6 alpha isoform	1.0	1.0	1.0	1.0	1.0	0.9	0.9	0.9	1.0	0.9	0.9	1.0	6.8	2	6	6	13	1146	127.5	7.6
NP_703151.1	23592232	MED19	mediator of RNA polymerase II transcription subunit 19	0.9	0.9	0.8	0.9	1.0	1.1	1.2	1.0	0.9	1.0	1.0	1.0	25.3	1	2	2	2	194	20.4	9.4
NP_057070.3	156142186	CRLF3	cytokine receptor-like factor 3	1.0	1.0	1.1	1.0	1.0	0.8	0.9	0.9	1.0	0.9	1.0	1.0	2.3	1	1	1	2	442	49.7	5.1
NP_056393.2	166197710	LSM14A	protein LSM14 homolog A isoform b	1.0	0.9	0.8	1.0	0.8	0.9	0.8	1.0	0.9	0.9	0.8	1.0	8.4	2	3	4	18	463	50.5	9.5
NP_543012.1	18201880	OVCA2	ovarian cancer-associated gene 2 protein	1.0	1.0	0.9	0.9	1.1	1.0	1.0	1.0	1.1	1.0	0.9	1.0	22.5	1	3	3	7	227	24.4	6.9
NP_387449.2	48928048	FBXW11	F-box/WD repeat-containing protein 11 isoform A	1.0	0.9	0.9	1.0	1.0	1.0	1.0	0.9	1.0	1.0	0.9	1.0	8.3	6	3	3	4	508	58.4	7.0
NP_004131.3	62912476	LLGL1	lethal(2) giant larvae protein homolog 1	1.0	1.0	1.0	1.0	0.9	0.9	0.9	0.9	1.0	1.0	0.9	1.0	11.8	1	9	9	22	1064	115.3	6.3
NP_006026.3	115527097	CDC42BPB	serine/threonine-protein kinase MRCK beta	1.0	1.0	0.9	1.0	1.0	0.9	1.0	0.9	1.0	1.0	0.9	1.0	8.7	8	9	10	23	1711	194.2	6.4
NP_060671.3	190014603	TBC1D13	TBC1 domain family member 13 isoform a	1.1	1.0	1.0	0.9	1.2	1.1	1.1	1.1	1.2	1.1	1.0	1.0	12.8	2	4	4	4	400	46.5	5.2
NP_055217.2	83367083	UQCRQ	cytochrome b-c1 complex subunit 8	1.0	1.1	1.2	1.0	1.1	1.0	1.2	0.9	1.0	1.1	1.2	1.0	63.4	1	5	5	18	82	9.9	10.1
NP_006586.1	5729730	API5	apoptosis inhibitor 5 isoform b	1.0	0.9	1.0	1.0	1.0	0.9	1.0	1.0	1.0	0.9	1.0	1.0	48.0	4	20	20	57	504	56.7	5.9

Supplementary Table 5. List of proteins quantified in untreated and chronically treated Het1A cells with cigarette smoke condensate for 8 months

NP_Accession	Protein group Accession	Gene ID	Description	Het-1A-Smoke - 2M/Parental	Het-1A-Smoke - 4M/Parental	Het-1A-Smoke - 6M/Parental	Het-1A-Smoke - 8M/Parental	Het-1A-Smoke - 2M/Parental	Het-1A-Smoke - 4M/Parental	Het-1A-Smoke - 6M/Parental	Het-1A-Smoke - 8M/Parental	Het-1A-Smoke - 2M/Parental	Het-1A-Smoke - 4M/Parental	Het-1A-Smoke - 6M/Parental	Het-1A-Smoke - 8M/Parental	Coverage (%)	Proteins	Unique Peptides	# Peptides	PSM	# AAs	MW [kDa]	calc. pI
				Replicate 1				Replicate 2				Average of replicates											
NP_004789.1	4758646	**KIF3B**	kinesin-like protein KIF3B	0.9	0.9	0.9	1.0	0.9	0.9	0.9	0.9	**0.9**	**0.9**	**0.9**	**1.0**	13.7	2	8	8	12	747	85.1	7.7
NP_001035937.1	109689718	**ABHD12**	monoacylglycerol lipase ABHD12 isoform a	0.8	1.0	0.8	0.9	1.0	0.9	1.0	1.0	**0.9**	**1.0**	**0.9**	**1.0**	4.3	2	1	1	2	398	45.1	8.6
NP_001288139.1	667743700	**MYEF2**	myelin expression factor 2 isoform b	1.0	1.0	1.2	1.0	0.9	0.8	1.0	0.9	**1.0**	**0.9**	**1.1**	**1.0**	1.7	2	1	1	3	576	61.9	8.7
NP_004840.1	4757798	**ATG5**	autophagy protein 5 isoform a	1.1	1.1	1.0	0.9	1.3	1.0	1.0	1.0	**1.2**	**1.0**	**1.0**	**1.0**	14.9	3	4	4	7	275	32.4	5.8
NP_001019553.1	66933009	**HMBS**	porphobilinogen deaminase isoform 2	1.0	1.1	1.0	0.9	1.1	1.1	1.1	1.0	**1.0**	**1.1**	**1.1**	**1.0**	45.1	4	11	11	29	344	37.7	7.4
NP_109590.3	40807477	**RAI1**	retinoic acid-induced protein 1	0.9	0.9	1.0	0.9	1.0	0.8	0.9	1.0	**0.9**	**0.9**	**0.9**	**1.0**	7.0	1	6	7	13	1906	203.2	8.8
NP_005383.3	117190342	**PHF2**	lysine-specific demethylase PHF2	0.9	0.9	0.9	1.0	1.0	1.0	0.9	1.0	**0.9**	**1.0**	**0.9**	**1.0**	3.3	1	3	3	4	1096	120.7	9.2
NP_071381.1	11559925	**XPNPEP3**	probable Xaa-Pro aminopeptidase 3 isoform 1	1.1	1.1	1.1	1.0	0.9	0.9	0.9	0.9	**1.0**	**1.0**	**1.0**	**1.0**	15.4	1	4	5	74	507	57.0	6.8
NP_001157742.1	256017145	**RAD51**	DNA repair protein RAD51 homolog 1 isoform 3	0.8	1.0	0.9	0.9	1.0	1.0	1.0	1.0	**0.9**	**1.0**	**1.0**	**1.0**	2.5	3	2	2	2	280	31.0	5.7
NP_064580.2	116812630	**SDR39U1**	epimerase family protein SDR39U1 isoform 1	1.0	1.0	1.0	1.0	1.0	1.0	1.1	0.9	**1.0**	**1.0**	**1.0**	**1.0**	18.8	3	3	3	8	293	31.1	9.0
NP_001164108.1	282165721	**SRGAP2**	SLIT-ROBO Rho GTPase-activating protein 2 isoform c	1.0	1.0	1.0	1.0	1.0	0.9	1.0	0.9	**1.0**	**1.0**	**1.0**	**1.0**	9.0	7	7	7	16	1070	120.7	6.7
NP_001013009.2	111607441	**KNOP1**	lysine-rich nucleolar protein 1	1.0	0.9	0.9	0.9	1.5	1.0	0.9	1.0	**1.2**	**1.0**	**0.9**	**1.0**	17.5	1	5	5	9	458	51.6	9.9
NP_055037.1	7657369	**NDUFA8**	NADH dehydrogenase [ubiquinone] 1 alpha subcomplex subunit 8	1.1	1.1	1.1	0.9	1.1	1.0	0.9	1.0	**1.1**	**1.0**	**1.0**	**1.0**	20.4	1	2	2	2	172	20.1	7.6
NP_056444.3	221136939	**PRPF31**	U4/U6 small nuclear ribonucleoprotein Prp31	0.9	1.0	1.0	1.0	0.9	0.9	1.0	1.0	**0.9**	**0.9**	**1.0**	**1.0**	21.2	1	8	8	20	499	55.4	5.8
NP_005843.2	52630322	**CHD3**	chromodomain-helicase-DNA-binding protein 3 isoform 2	0.9	0.9	1.0	1.0	0.9	0.9	1.0	1.0	**0.9**	**0.9**	**1.0**	**1.0**	6.6	3	4	12	45	1966	222.7	7.5
NP_071930.2	117938274	**MTMR14**	myotubularin-related protein 14 isoform 3	1.0	1.0	0.9	1.0	1.1	1.0	1.1	0.9	**1.1**	**1.0**	**1.0**	**1.0**	3.4	3	1	1	1	538	60.0	6.2
NP_000678.1	9951915	**AHCY**	adenosylhomocysteinase isoform 1	0.9	0.9	0.9	1.0	0.9	0.9	0.8	1.0	**0.9**	**0.9**	**0.9**	**1.0**	57.9	2	24	25	212	432	47.7	6.3
NP_001018125.1	66528888	**FANCD2**	Fanconi anemia group D2 protein isoform b	0.8	0.8	0.8	1.0	0.9	0.9	0.9	0.9	**0.9**	**0.9**	**0.9**	**1.0**	9.1	3	11	11	22	1451	164.0	5.9
NP_005848.2	18379366	**ZMPSTE24**	CAAX prenyl protease 1 homolog	1.0	0.9	0.9	0.9	1.1	1.1	0.9	1.0	**1.0**	**1.0**	**0.9**	**1.0**	24.4	1	10	10	27	475	54.8	7.5
NP_057113.1	7706318	**DHRS7**	dehydrogenase/reductase SDR family member 7 precursor	1.1	0.9	1.0	0.9	1.1	1.0	1.0	1.0	**1.1**	**0.9**	**1.0**	**1.0**	3.5	1	1	1	2	339	38.3	8.3
NP_065799.1	24308211	**INTS2**	integrator complex subunit 2	0.9	0.9	0.9	0.9	1.1	1.1	1.1	1.1	**1.0**	**1.0**	**1.0**	**1.0**	3.0	1	2	2	4	1204	134.3	6.1
NP_056513.2	15811782	**GPKOW**	G patch domain and KOW motifs-containing protein	0.7	0.9	0.7	0.9	0.9	1.0	1.0	1.1	**0.8**	**1.0**	**0.8**	**1.0**	11.3	1	4	4	6	476	52.2	6.2
NP_001077415.1	145701028	**NDUFAF7**	NADH dehydrogenase [ubiquinone] complex I, assembly factor 7 isoform 3	1.0	0.9	0.9	0.9	1.0	1.1	1.0	1.0	**1.0**	**1.0**	**1.0**	**1.0**	18.7	2	5	5	11	343	38.0	8.6
NP_003353.1	6224979	**UNG**	uracil-DNA glycosylase isoform UNG1 precursor	1.2	1.1	1.1	1.0	1.0	0.9	0.9	0.9	**1.1**	**1.0**	**1.0**	**1.0**	11.5	2	2	2	4	304	33.9	9.5
NP_057427.3	55770834	**CENPF**	centromere protein F	0.9	1.0	1.1	1.0	0.9	1.0	1.0	1.0	**0.9**	**1.0**	**1.0**	**1.0**	10.3	4	25	27	44	3114	357.3	5.1
NP_004578.2	110611220;110611218	**RRBP1**	ribosome-binding protein 1	1.0	1.1	0.9	1.0	1.0	1.1	0.9	1.0	**1.0**	**1.1**	**0.9**	**1.0**	36.3	1	29	29	103	977	108.6	5.5
NP_060376.2	55769546	**PAK1IP1**	p21-activated protein kinase-interacting protein 1	0.9	0.9	0.9	1.0	1.0	1.0	1.0	1.0	**0.9**	**0.9**	**0.9**	**1.0**	20.2	1	7	7	18	392	43.9	8.9
NP_001171114.1	294997306;294997292	**DAG1**	dystroglycan preproprotein	1.0	1.1	1.1	0.9	1.1	1.1	1.1	1.0	**1.0**	**1.1**	**1.1**	**1.0**	11.6	1	7	7	22	895	97.5	8.6
NP_037392.1	9994179	**SAP30BP**	SAP30-binding protein isoform 2	0.9	0.8	0.9	0.9	0.9	0.9	1.0	1.0	**0.9**	**0.9**	**0.9**	**1.0**	19.2	3	5	5	11	308	33.8	4.8
NP_005794.1	5031699	**FLOT1**	flotillin-1	0.9	0.8	0.9	1.0	0.9	0.9	1.0	1.0	**0.9**	**0.9**	**0.9**	**1.0**	21.1	1	8	8	26	427	47.3	7.5
NP_003304.1	4507709	**TSTA3**	GDP-L-fucose synthase	1.1	1.0	1.0	1.0	1.0	1.1	1.1	1.0	**1.1**	**1.0**	**1.1**	**1.0**	20.6	1	5	5	13	321	35.9	6.6
NP_116253.2	24762236	**PRPF38A**	pre-mRNA-splicing factor 38A	1.0	0.9	0.9	1.0	0.9	0.9	0.9	0.9	**0.9**	**0.9**	**0.9**	**1.0**	9.0	1	3	3	12	312	37.5	10.0
NP_055701.1	7662677	**NSA2**	ribosome biogenesis protein NSA2 homolog isoform 1	0.9	1.1	1.0	1.0	0.9	1.0	0.9	0.9	**0.9**	**1.1**	**1.0**	**1.0**	20.0	2	3	3	7	260	30.0	10.3
NP_116045.2	29029601	**DHX37**	probable ATP-dependent RNA helicase DHX37	1.0	1.0	1.0	1.0	0.8	1.0	0.9	0.9	**0.9**	**1.0**	**0.9**	**1.0**	2.9	1	2	2	3	1157	129.5	8.1
NP_001263308.1	444909144	**LZTFL1**	leucine zipper transcription factor-like protein 1 isoform 3	1.2	0.9	1.0	0.9	1.0	1.0	1.1	1.0	**1.1**	**0.9**	**1.0**	**1.0**	10.2	3	2	2	3	256	29.3	5.5
NP_004452.1	4758340	**FARSA**	phenylalanine--tRNA ligase alpha subunit	1.0	1.0	1.0	1.0	1.1	1.0	0.9	1.0	**1.0**	**1.0**	**1.0**	**1.0**	27.4	1	11	11	71	508	57.5	7.8
NP_055912.2	333440449	**CLASP2**	CLIP-associating protein 2 isoform 1	1.0	0.9	1.0	1.0	1.1	1.0	0.9	0.9	**1.1**	**1.0**	**0.9**	**1.0**	14.6	2	11	14	26	1515	165.8	8.3
NP_001139628.1	225903437	**GSK3B**	glycogen synthase kinase-3 beta isoform 2	1.2	1.0	1.2	1.0	1.1	1.0	1.1	1.0	**1.1**	**1.0**	**1.1**	**1.0**	12.4	2	3	3	5	420	46.7	8.8
NP_005552.3	112380628	**LAMP1**	lysosome-associated membrane glycoprotein 1 precursor	0.9	0.9	0.9	0.9	0.8	0.9	0.9	1.0	**0.9**	**0.9**	**0.9**	**1.0**	8.4	1	3	3	14	417	44.9	8.7
NP_001128143.1	197927278	**DERL1**	derlin-1 isoform b	1.1	1.1	1.1	1.0	1.1	1.1	1.1	1.0	**1.1**	**1.1**	**1.1**	**1.0**	17.3	2	4	4	13	231	26.4	9.5
NP_112486.1	13654276	**QTRT1**	queuine tRNA-ribosyltransferase	1.1	1.1	1.0	0.9	1.0	0.9	0.9	1.0	**1.0**	**1.0**	**1.0**	**1.0**	17.9	1	4	4	9	403	44.0	7.2
NP_001098057.1	157388957	**SLFN11**	schlafen family member 11	1.0	1.0	0.9	0.9	1.1	1.0	1.0	1.0	**1.1**	**1.0**	**0.9**	**1.0**	34.7	2	17	21	77	901	102.8	7.8
NP_001007794.1	56550081	**BUB3**	mitotic checkpoint protein BUB3 isoform b	0.9	0.9	0.9	1.0	0.9	0.9	0.9	0.9	**0.9**	**0.9**	**0.9**	**1.0**	34.4	2	9	9	34	326	36.9	6.8
NP_001156769.1	254028240	**MARK2**	serine/threonine-protein kinase MARK2 isoform f	1.0	0.9	0.9	0.9	1.0	1.0	0.9	1.0	**1.0**	**1.0**	**0.9**	**1.0**	9.7	5	3	4	10	709	79.4	9.6
NP_001354.1	4503337	**DKC1**	H/ACA ribonucleoprotein complex subunit 4 isoform 1	0.9	0.9	1.0	0.9	1.0	1.0	1.0	1.0	**1.0**	**1.0**	**1.0**	**1.0**	33.3	3	13	13	43	514	57.6	9.4
NP_002063.2	40254462	**GNAQ**	guanine nucleotide-binding protein G(q) subunit alpha	0.9	1.0	1.0	1.0	1.1	1.0	0.9	0.9	**1.0**	**1.0**	**1.0**	**1.0**	17.6	1	3	5	22	359	42.1	5.7
NP_847884.2	195947382	**RILPL1**	RILP-like protein 1	1.0	1.0	0.9	0.9	1.0	1.0	1.0	1.0	**1.0**	**1.0**	**1.0**	**1.0**	13.2	1	4	4	15	403	47.1	5.2
NP_001136027.1	217416386	**ADCK4**	aarF domain-containing protein kinase 4 isoform b	0.9	0.8	0.8	1.1	0.7	0.8	0.6	0.8	**0.8**	**0.8**	**0.7**	**1.0**	1.2	2	1	1	4	503	55.8	7.4
NP_849193.1	30578410	**STT3B**	dolichyl-diphosphooligosaccharide--protein glycosyltransferase subunit STT3B	1.1	1.0	1.1	1.0	1.1	1.0	1.1	0.9	**1.1**	**1.0**	**1.1**	**1.0**	10.3	1	8	9	20	826	93.6	8.9
NP_775102.2	171543850	**RUNDC1**	RUN domain-containing protein 1	0.9	0.9	0.9	0.9	0.9	1.0	0.9	1.0	**0.9**	**0.9**	**0.9**	**1.0**	1.3	1	1	1	2	613	67.6	6.3
NP_001123544.1	194248095	**EPN1**	epsin-1 isoform b	1.0	1.0	1.1	0.9	1.0	1.0	1.0	1.0	**1.0**	**1.0**	**1.0**	**1.0**	9.6	3	5	5	7	576	60.3	4.8
NP_056342.3	167614488	**TBC1D10B**	TBC1 domain family member 10B	1.0	0.9	0.9	1.0	0.9	0.9	0.9	1.0	**1.0**	**0.9**	**0.9**	**1.0**	6.3	1	3	3	3	808	87.1	9.2
NP_982298.2	543583770	**VIMP**	selenoprotein S isoform 1	1.0	1.0	0.9	0.9	1.0	1.0	0.9	1.0	**1.0**	**1.0**	**0.9**	**1.0**	20.3	2	2	2	3	187	20.9	9.7
NP_644670.1	21264315	**EHD4**	EH domain-containing protein 4	0.9	1.1	1.1	1.0	0.9	1.0	1.1	0.9	**0.9**	**1.0**	**1.1**	**1.0**	34.0	1	11	13	31	541	61.1	6.8
NP_001124146.1	194578885	**CALU**	calumenin isoform b precursor	1.3	1.3	1.4	0.9	1.3	1.2	1.4	1.0	**1.3**	**1.3**	**1.4**	**1.0**	57.1	2	3	17	122	315	37.1	4.6
NP_065385.2	19923830	**PBXIP1**	pre-B-cell leukemia transcription factor-interacting protein 1	1.0	1.1	1.1	0.9	1.1	1.2	1.1	1.0	**1.0**	**1.1**	**1.1**	**1.0**	6.4	1	2	2	10	731	80.6	5.3
NP_002756.1	4506129	**PRPS2**	ribose-phosphate pyrophosphokinase 2 isoform 2	1.1	1.0	1.1	0.9	1.2	1.0	1.1	1.0	**1.1**	**1.0**	**1.1**	**1.0**	52.5	2	6	12	36	318	34.7	6.6
NP_061029.2	33354279	**GPALPP1**	GPALPP motifs-containing protein 1	1.0	1.1	1.0	1.0	0.9	1.0	0.9	0.9	**1.0**	**1.1**	**0.9**	**1.0**	20.6	1	6	6	19	340	38.1	5.4
NP_005853.2	62243696	**STAG1**	cohesin subunit SA-1	1.0	1.0	1.0	1.0	1.0	1.0	1.0	0.9	**1.0**	**1.0**	**1.0**	**1.0**	22.3	1	14	20	52	1258	144.3	5.6
NP_001185594.1	310832410	**ARHGEF12**	rho guanine nucleotide exchange factor 12 isoform 2	0.9	1.0	1.0	1.0	1.2	1.0	1.0	1.0	**1.0**	**1.0**	**1.0**	**1.0**	3.8	3	4	4	4	1525	171.0	5.7
NP_001098717.1	157426856	**ARMC5**	armadillo repeat-containing protein 5 isoform a precursor	0.8	0.9	0.7	0.9	0.9	1.0	1.1	1.0	**0.9**	**1.0**	**0.9**	**1.0**	1.7	3	1	1	2	935	97.6	7.1
NP_055204.3	503774453	**PELP1**	proline-, glutamic acid- and leucine-rich protein 1 isoform 1	1.0	1.0	1.0	0.9	1.0	1.1	1.1	1.0	**1.0**	**1.0**	**1.0**	**1.0**	22.7	2	16	16	48	1180	124.9	4.4
NP_076944.1	13128994	**MIS12**	protein MIS12 homolog	1.2	1.0	1.0	1.0	0.9	0.9	0.9	0.9	**1.0**	**1.0**	**1.0**	**1.0**	5.4	1	1	1	2	205	24.1	5.7
NP_006074.2	125988409	**IK**	protein Red	0.9	1.0	0.9	0.9	1.2	1.1	1.1	1.0	**1.0**	**1.1**	**1.0**	**1.0**	33.4	1	15	15	51	557	65.6	6.6
NP_001061.2	31543831	**TUBG1**	tubulin gamma-1 chain	1.0	1.0	1.0	1.0	1.0	0.9	1.0	0.9	**1.0**	**1.0**	**1.0**	**1.0**	39.0	2	9	9	23	451	51.1	6.1
NP_001271242.1	546231336	**DBNL**	drebrin-like protein isoform d	0.8	0.9	0.9	1.0	0.8	0.8	0.9	0.9	**0.8**	**0.8**	**0.9**	**1.0**	8.3	5	2	2	6	327	37.1	5.0
NP_001273061.1	554790364	**RPP40**	ribonuclease P protein subunit p40 isoform b	1.0	0.9	0.9	0.9	1.0	0.9	0.9	1.0	**1.0**	**0.9**	**0.9**	**1.0**	6.5	3	2	2	9	340	39.3	7.0
NP_009201.2	33469985	**FKBP9**	peptidyl-prolyl cis-trans isomerase FKBP9 isoform 1 precursor	0.9	0.9	0.9	1.0	0.7	0.7	0.8	0.9	**0.8**	**0.8**	**0.9**	**1.0**	5.8	3	3	3	4	570	63.0	5.1
NP_060899.2	109637791	**BDP1**	transcription factor TFIIIB component B" homolog	1.1	1.0	0.9	1.1	0.8	0.8	0.8	0.8	**0.9**	**0.9**	**0.8**	**1.0**	0.7	1	1	1	2	2624	293.7	5.1
NP_059980.2	39725636	**TMED9**	transmembrane emp24 domain-containing protein 9 precursor	1.0	1.1	1.0	1.0	1.1	1.0	1.0	0.9	**1.1**	**1.0**	**1.0**	**1.0**	30.6	1	4	7	24	235	27.3	8.0
NP_003696.2	21361103	**SLC25A12**	calcium-binding mitochondrial carrier protein Aralar1	0.9	0.9	1.0	1.0	1.0	0.9	1.0	0.9	**1.0**	**0.9**	**1.0**	**1.0**	14.6	1	6	9	28	678	74.7	8.4
NP_001783.2	14589889	**CDH2**	cadherin-2 preproprotein	1.1	1.1	0.8	0.9	0.9	1.0	1.1	1.0	**1.0**	**1.0**	**0.9**	**1.0**	10.8	1	4	4	5	906	99.7	4.8
NP_055448.1	7661950	**CTR9**	RNA polymerase-associated protein CTR9 homolog	1.0	1.0	1.0	1.0	1.0	1.0	1.0	1.0	**1.0**	**1.0**	**1.0**	**1.0**	18.6	6	20	20	28	1173	133.4	6.8
NP_004573.2	21359854	**RABGGTB**	geranylgeranyl transferase type-2 subunit beta	0.9	0.9	0.9	1.0	0.9	0.9	0.9	1.0	**0.9**	**0.9**	**0.9**	**1.0**	9.1	1	3	3	5	331	36.9	5.0
NP_001106819.1	164519138	**ECE1**	endothelin-converting enzyme 1 isoform 4	1.0	1.0	1.0	1.0	0.9	1.0	1.0	1.0	**1.0**	**1.0**	**1.0**	**1.0**	15.8	4	7	7	19	754	85.5	5.8
NP_005073.2	68160937	**TRIM25**	E3 ubiquitin/ISG15 ligase TRIM25	0.9	1.0	1.0	1.0	1.0	1.0	1.0	1.0	**1.0**	**1.0**	**1.0**	**1.0**	14.3	1	6	6	25	630	70.9	8.1
NP_001093640.1	154354966	**IMMT**	MICOS complex subunit MIC60 isoform 3	1.1	1.0	1.0	1.1	0.9	1.0	0.8	0.8	**1.0**	**1.0**	**0.9**	**1.0**	51.4	3	30	31	123	747	82.6	6.6
NP_937799.1	38045906	**VHL**	von Hippel-Lindau disease tumor suppressor isoform 2	1.0	0.9	1.0	0.9	1.1	1.0	0.9	1.0	**1.0**	**1.0**	**1.0**	**1.0**	14.5	2	1	1	2	172	19.6	4.7
NP_078922.1	55742693	**ZYG11B**	protein zyg-11 homolog B	1.1	1.0	1.1	1.0	1.0	0.9	0.9	0.9	**1.0**	**1.0**	**1.0**	**1.0**	1.9	1	1	1	1	744	83.9	6.9
NP_001034756.1	89264696	**ANGPTL4**	angiopoietin-related protein 4 isoform b precursor	0.8	1.3	1.3	1.0	0.8	1.2	1.2	0.9	**0.8**	**1.3**	**1.3**	**1.0**	3.8	2	1	1	2	368	40.9	9.2
NP_001015882.2	118498347	**DNAJC25**	dnaJ homolog subfamily C member 25 precursor	1.1	1.2	0.9	0.9	1.3	1.0	1.0	1.0	**1.2**	**1.1**	**0.9**	**1.0**	4.2	1	1	1	1	360	42.4	9.1
NP_004229.1	10863903	**TRIP12**	E3 ubiquitin-protein ligase TRIP12 isoform c	1.1	1.0	1.0	1.0	0.9	0.9	0.9	0.9	**1.0**	**0.9**	**1.0**	**1.0**	5.2	5	9	9	20	1992	220.3	8.5
NP_004308.2	50428938	**ASNA1**	ATPase ASNA1	0.9	0.8	0.9	1.0	1.0	0.8	0.9	1.0	**0.9**	**0.8**	**0.9**	**1.0**	34.5	1	9	9	35	348	38.8	4.9
NP_000929.1	4505941	**POLR2B**	DNA-directed RNA polymerase II subunit RPB2 isoform 1	0.9	0.9	0.9	1.0	1.0	0.9	1.0	0.9	**1.0**	**0.9**	**0.9**	**1.0**	14.7	3	14	14	24	1174	133.8	6.9
NP_000174.1	4504327	**HADHB**	trifunctional enzyme subunit beta, mitochondrial isoform 1 precursor	1.0	1.1	1.1	1.0	1.0	1.1	1.1	0.9	**1.0**	**1.1**	**1.1**	**1.0**	30.0	3	13	13	57	474	51.3	9.4
NP_005919.2	167830475	**MFGE8**	lactadherin isoform a preproprotein	1.0	1.1	1.1	0.9	1.1	1.1	1.1	1.0	**1.0**	**1.1**	**1.1**	**1.0**	27.9	2	9	9	21	387	43.1	8.1
NP_055904.1	24307991	**CUL9**	cullin-9	0.8	1.1	0.8	0.9	1.1	1.0	0.9	1.0	**1.0**	**1.1**	**0.8**	**1.0**	1.6	1	1	1	1	2517	281.0	5.5
NP_001241661.2	513788264	**SPECC1L**	cytospin-A isoform 2	1.0	1.1	1.1	1.0	1.0	1.0	1.0	0.9	**1.0**	**1.1**	**1.0**	**1.0**	12.9	2	9	10	23	1078	120.1	5.8
NP_004604.2	39777597	**TGM2**	protein-glutamine gamma-glutamyltransferase 2 isoform a	1.3	1.4	1.4	0.9	1.3	1.4	1.3	1.0	**1.3**	**1.4**	**1.4**	**1.0**	52.0	2	27	27	169	687	77.3	5.2
NP_055136.1	7657427	**ORC6**	origin recognition complex subunit 6	0.9	0.9	0.9	0.9	1.0	1.1	1.1	1.0	**1.0**	**1.0**	**1.0**	**1.0**	22.2	1	5	5	8	252	28.1	8.7
NP_003557.2	55770888	**EEA1**	early endosome antigen 1	0.9	0.9	0.9	1.0	0.9	0.9	0.9	0.9	**0.9**	**0.9**	**0.9**	**1.0**	10.7	1	13	13	30	1411	162.4	5.7

Supplementary Table 5. List of proteins quantified in untreated and chronically treated Het1A cells with cigarette smoke condensate for 8 months

NP_Accession	Protein group Accession	Gene ID	Description	Het-1A-Smoke - 2M/Parental	Het-1A-Smoke - 4M/Parental	Het-1A-Smoke - 6M/Parental	Het-1A-Smoke - 8M/Parental	Het-1A-Smoke - 2M/Parental	Het-1A-Smoke - 4M/Parental	Het-1A-Smoke - 6M/Parental	Het-1A-Smoke - 8M/Parental	Het-1A-Smoke - 2M/Parental	Het-1A-Smoke - 4M/Parental	Het-1A-Smoke - 6M/Parental	Het-1A-Smoke - 8M/Parental	Coverage (%)	Proteins	Unique Peptides	# Peptides	PSM	# AAs	MW [kDa]	calc. pI
				Replicate 1				Replicate 2				Average of replicates											
NP_954659.1	62750354;303227924	MATR3	matrin-3 isoform a	0.9	0.9	0.9	1.0	0.9	0.9	0.9	1.0	0.9	0.9	0.9	1.0	40.5	6	31	31	139	847	94.6	6.3
NP_005733.1	5031973	PDIA6	protein disulfide-isomerase A6 isoform d precursor	1.1	1.1	1.0	0.9	1.1	1.1	1.0	1.0	1.1	1.1	1.0	1.0	72.5	5	24	24	267	440	48.1	5.1
NP_060283.3	157388900	IMPAD1	inositol monophosphatase 3	0.9	0.8	1.0	1.0	0.8	0.8	0.8	0.9	0.8	0.8	0.9	1.0	8.1	1	1	1	1	359	38.7	6.9
NP_056229.2	16117794;16117796	RPL36	60S ribosomal protein L36	0.9	1.0	1.0	1.0	0.9	1.0	0.9	1.0	0.9	1.0	1.0	1.0	35.2	1	4	5	33	105	12.2	11.6
NP_067033.1	10864021;262263327	TRAPPC1	trafficking protein particle complex subunit 1	0.8	0.8	0.8	0.9	1.1	1.2	1.1	1.0	1.0	1.0	1.0	1.0	17.9	1	2	2	3	145	16.8	9.2
NP_001157791.1	256222415	FLNB	filamin-B isoform 4	1.0	1.4	1.3	0.9	1.2	1.2	1.2	1.0	1.1	1.3	1.2	1.0	64.2	3	1	114	739	2578	275.5	5.8
NP_066565.1	10835073	NMT1	glycylpeptide N-tetradecanoyltransferase 1	0.9	0.9	0.9	1.0	0.9	0.9	0.9	1.0	0.9	0.9	0.9	1.0	38.7	1	10	15	73	496	56.8	7.8
NP_001914.3	148529014	DDB1	DNA damage-binding protein 1	1.0	1.0	1.0	1.0	1.0	1.0	1.0	0.9	1.0	1.0	1.0	1.0	44.8	1	41	41	175	1140	126.9	5.3
NP_000302.1	4506113;122056628	PRNP	major prion protein preproprotein	1.1	1.2	1.0	1.0	0.9	0.9	0.8	0.9	1.0	1.1	0.9	1.0	9.1	1	2	2	5	253	27.6	9.0
NP_001129470.1	209862760	NDUFB11	NADH dehydrogenase [ubiquinone] 1 beta subcomplex subunit 11, mitochondrial isoform 2	1.0	1.0	1.0	1.0	1.0	0.9	0.9	0.9	1.0	0.9	1.0	1.0	22.2	2	2	2	14	153	17.3	5.2
NP_001135766.1	214830079;21703346	SPG20	spartin	0.8	0.9	0.9	1.0	0.8	0.8	0.8	0.9	0.8	0.9	0.8	1.0	6.8	1	4	4	7	666	72.8	5.9
NP_115564.2	226371636	KAT8	histone acetyltransferase KAT8 isoform 1	0.9	1.0	1.0	1.0	1.0	0.9	0.9	0.9	0.9	1.0	1.0	1.0	6.3	2	2	2	4	458	52.4	8.3
NP_001708.3	157276588	BNC1	zinc finger protein basonuclin-1	1.2	0.9	0.7	1.0	1.3	0.9	1.1	0.9	1.3	0.9	0.9	1.0	2.3	1	1	1	2	994	110.9	7.4
NP_064623.2	102467242	INCENP	inner centromere protein isoform 2	1.0	1.0	1.0	1.0	1.0	1.0	1.0	0.9	1.0	1.0	1.0	1.0	17.9	2	14	14	27	914	104.9	9.5
NP_588614.1	19115964	PLCD3	1-phosphatidylinositol 4,5-bisphosphate phosphodiesterase delta-3	0.9	1.0	1.0	1.0	0.9	1.0	0.9	1.0	0.9	1.0	1.0	1.0	2.9	1	1	1	4	789	89.2	7.0
NP_001276915.1	584613506	TIPIN	TIMELESS-interacting protein isoform 2	0.9	0.8	0.8	0.8	1.0	1.0	0.9	1.1	0.9	0.9	0.8	1.0	10.0	2	2	2	8	200	23.3	4.7
NP_055857.1	71725360	ZNF609	zinc finger protein 609	0.8	1.0	0.9	1.0	0.7	0.8	0.7	0.9	0.8	0.9	0.8	1.0	3.1	1	3	3	4	1411	151.1	8.0
NP_001269383.1	540344544	SNX5	sorting nexin-5 isoform b	0.9	1.0	0.9	1.0	0.9	1.0	0.9	0.9	0.9	1.0	0.9	1.0	55.5	2	14	16	41	299	34.9	8.4
NP_001243873.1	379643013	CLCN4	H(+)/Cl(-) exchange transporter 4 isoform 2	1.0	0.9	0.9	0.9	1.1	0.9	1.0	1.0	1.0	0.9	1.0	1.0	1.1	8	1	1	2	666	74.3	7.7
NP_060568.3	21735572	ECT2	protein ECT2 isoform b	1.0	1.0	1.0	1.0	0.9	0.9	1.0	0.9	1.0	1.0	1.0	1.0	10.7	2	7	7	13	883	100.0	7.5
NP_001164562.1	283806625	UBXN6	UBX domain-containing protein 6 isoform 2	1.0	1.0	1.0	1.0	1.1	0.9	0.9	0.9	1.1	0.9	1.0	1.0	8.5	2	2	2	3	388	43.8	5.7
NP_004198.1	4759112;332800979	SLC16A3	monocarboxylate transporter 4	0.8	1.0	1.0	1.0	0.8	0.9	0.9	0.9	0.8	0.9	0.9	1.0	13.1	1	6	6	28	465	49.4	8.0
NP_055456.2	132626688	MDC1	mediator of DNA damage checkpoint protein 1	1.0	1.0	1.0	1.0	0.9	1.0	0.9	1.0	0.9	1.0	1.0	1.0	25.2	1	28	28	84	2089	226.5	5.5
NP_079427.4	289547526	RAB11FIP1	rab11 family-interacting protein 1 isoform 1	0.9	0.9	1.0	0.9	1.0	1.0	0.9	1.0	0.9	1.0	0.9	1.0	2.5	2	1	1	2	649	70.9	9.3
NP_036359.3	604723316	GTPBP6	putative GTP-binding protein 6	1.0	1.0	1.1	0.9	1.0	1.1	1.1	1.0	1.0	1.0	1.1	1.0	25.0	1	10	10	24	516	56.9	9.4
NP_003303.2	17402865;395394071	TST	thiosulfate sulfurtransferase	0.9	1.1	1.1	1.0	1.0	1.0	1.1	0.9	0.9	1.0	1.1	1.0	10.8	1	3	3	8	297	33.4	7.3
NP_000103.2	100913030	SLC26A2	sulfate transporter	1.0	0.9	1.1	1.0	1.2	1.2	1.1	0.9	1.1	1.1	1.1	1.0	5.7	1	2	2	3	739	81.6	8.4
NP_757386.2	33469964	SUGP1	SURP and G-patch domain-containing protein 1	0.8	0.9	0.8	0.9	0.8	1.0	0.8	1.0	0.8	0.9	0.8	1.0	14.6	1	7	7	10	645	72.4	7.6
NP_859067.2	56118234	CENPV	centromere protein V	0.9	1.0	0.9	0.9	1.0	1.1	1.1	1.0	1.0	1.1	1.0	1.0	41.2	1	6	6	19	272	29.7	9.7
NP_078911.3	145275204	NAA35	N-alpha-acetyltransferase 35, NatC auxiliary subunit	1.2	0.8	1.0	0.9	1.0	0.9	0.9	1.0	1.1	0.9	0.9	1.0	1.7	1	1	1	2	725	83.6	7.0
NP_113638.2	208431769	C11orf68	UPF0696 protein C11orf68 isoform 2	1.0	1.0	1.0	1.0	1.1	1.0	1.0	0.9	1.0	1.0	1.0	1.0	29.1	2	6	6	16	292	31.4	6.3
NP_660213.1	21735604	MRPL30	39S ribosomal protein L30, mitochondrial precursor	1.3	1.0	1.0	1.0	1.0	0.9	1.0	0.9	1.2	1.0	1.0	1.0	17.4	1	2	2	6	161	18.5	10.0
NP_001263433.1	452405678	SDHD	succinate dehydrogenase [ubiquinone] cytochrome b small subunit, mitochondrial isoform c precursor	1.2	1.1	1.2	1.1	0.9	0.9	0.9	0.9	1.1	1.0	1.1	1.0	8.3	3	1	1	2	120	12.6	8.5
NP_001001992.1	50312664	USP16	ubiquitin carboxyl-terminal hydrolase 16 isoform b	1.0	0.8	0.9	0.9	0.9	0.9	1.0	1.0	0.9	0.9	0.9	1.0	7.1	2	3	3	5	822	93.4	6.9
NP_689821.3	148664216	SDE2	protein SDE2 homolog	1.0	1.1	1.0	0.9	1.0	0.9	1.0	1.0	1.0	1.0	1.0	1.0	1.8	1	1	1	1	451	49.7	6.0
NP_055278.1	7657315	LSM3	U6 snRNA-associated Sm-like protein LSm3	1.1	1.1	1.0	1.2	0.9	0.6	0.8	0.8	1.0	0.9	0.9	1.0	11.8	1	1	1	4	102	11.8	4.7
NP_001229856.1	339275847	ZNF410	zinc finger protein 410 isoform d	1.3	0.9	0.9	1.0	1.0	0.9	0.9	0.9	1.1	0.9	0.9	1.0	3.5	4	1	1	1	405	44.2	6.6
NP_055182.3	49355721	FAM162A	protein FAM162A	1.2	1.2	1.3	1.0	0.9	1.0	1.0	0.9	1.1	1.1	1.2	1.0	27.9	1	4	4	22	154	17.3	9.8
NP_002736.3	66932916;20986531	MAPK1	mitogen-activated protein kinase 1	0.9	0.9	1.0	1.0	1.0	1.0	1.0	1.0	1.0	0.9	1.0	1.0	46.1	2	8	13	42	360	41.4	7.0
NP_066928.1	10863877	PLSCR1	phospholipid scramblase 1	0.8	0.9	0.9	1.0	0.9	0.9	0.8	0.9	0.9	0.9	0.9	1.0	6.3	1	1	1	4	318	35.0	4.9
NP_006383.2	32483374	NOP56	nucleolar protein 56	1.0	1.0	1.0	1.0	1.0	1.0	1.0	0.9	1.0	1.0	1.0	1.0	37.2	1	17	17	84	594	66.0	9.2
NP_005379.3	88758594	PDCL	phosducin-like protein	1.1	1.0	1.0	1.0	1.0	0.9	1.0	1.0	1.0	0.9	1.0	1.0	12.6	1	3	3	4	301	34.3	4.7
NP_001186328.1	313661430	NEK1	serine/threonine-protein kinase Nek1 isoform 4	0.9	1.1	0.8	0.9	1.2	1.1	1.2	1.0	1.1	1.1	1.0	1.0	1.4	5	1	1	1	1189	135.2	5.6
NP_004484.1	4758504	HSD17B10	3-hydroxyacyl-CoA dehydrogenase type-2 isoform 1	1.1	1.3	1.0	1.0	1.1	1.1	0.9	0.9	1.1	1.2	1.0	1.0	41.8	2	6	6	21	261	26.9	7.8
NP_644671.1	21536301	STAT1	signal transducer and activator of transcription 1-alpha/beta isoform beta	1.1	1.0	0.9	0.9	1.1	1.0	1.0	1.0	1.1	1.0	1.0	1.0	18.3	2	10	10	24	712	83.0	6.4
NP_620305.2	520261969	SON	protein SON isoform F	0.9	1.0	0.9	1.0	0.9	1.0	0.9	0.9	0.9	1.0	0.9	1.0	18.0	4	24	24	87	2426	263.7	5.6
NP_981946.1	44890052	STMN1	stathmin isoform a	1.3	1.1	1.2	0.9	1.1	1.2	1.3	1.0	1.2	1.2	1.2	1.0	15.4	4	2	2	5	149	17.3	6.0
NP_001405.1	4503503	EIF2B1	translation initiation factor eIF-2B subunit alpha	1.0	1.0	1.0	1.0	1.0	1.0	1.0	0.9	1.0	1.0	1.0	1.0	36.7	1	11	11	31	305	33.7	7.3
NP_031375.3	93141033	PAXIP1	PAX-interacting protein 1	1.0	1.1	1.0	1.0	1.0	0.9	1.0	0.9	1.0	1.0	1.0	1.0	3.8	1	2	2	8	1069	121.3	6.7
NP_001269847.1	544711150	RALGAPB	ral GTPase-activating protein subunit beta isoform 2	1.0	0.8	0.8	0.9	1.0	0.9	1.0	1.0	1.0	0.9	0.9	1.0	1.3	2	1	1	2	1491	166.4	6.7
NP_001041683.1	115430243	SCYL1	N-terminal kinase-like protein isoform B	1.1	1.0	0.9	1.0	1.0	1.0	1.0	1.0	1.0	1.0	0.9	1.0	19.1	2	8	8	22	791	88.0	6.3
NP_001244118.1	380692328	DDX56	probable ATP-dependent RNA helicase DDX56 isoform 2	1.0	1.1	1.0	1.0	0.7	0.9	0.9	0.9	0.9	1.0	1.0	1.0	12.0	2	6	6	16	507	57.2	9.2
NP_056381.1	153792633	FAM169A	soluble lamin-associated protein of 75 kDa	0.9	0.9	0.9	0.9	1.0	1.0	0.9	1.0	0.9	1.0	0.9	1.0	16.0	1	5	5	12	670	74.9	4.6
NP_620119.2	242117893	BAX	apoptosis regulator BAX isoform sigma	0.9	0.8	0.8	0.9	1.0	0.9	1.0	1.0	1.0	0.9	0.9	1.0	27.4	8	4	4	22	179	19.7	5.4
NP_065691.2	36030883	DCUN1D1	DCN1-like protein 1 isoform 1	1.0	0.9	0.9	0.9	1.1	1.0	1.1	1.0	1.0	1.0	1.0	1.0	25.1	3	6	6	10	259	30.1	5.3
NP_004428.1	4758274	EPB41	protein 4.1 isoform 6	0.9	1.0	0.9	0.9	1.1	0.9	1.0	1.0	1.0	0.9	1.0	1.0	6.8	6	1	3	8	588	66.4	7.3
NP_112729.1	14165452	COL4A3BP	collagen type IV alpha-3-binding protein isoform 2	1.0	0.9	1.1	1.0	0.9	0.9	1.2	0.9	1.0	0.9	1.1	1.0	15.2	3	6	6	10	598	68.0	5.5
NP_937861.1	38201665	ING1	inhibitor of growth protein 1 isoform B	1.2	1.0	0.9	1.0	0.9	0.9	0.8	0.9	1.1	0.9	0.8	1.0	17.6	5	2	2	3	210	23.7	8.4
NP_065883.1	24308227	ZNF687	zinc finger protein 687	1.1	1.0	1.0	0.9	1.1	1.0	1.0	1.0	1.1	1.0	1.0	1.0	7.3	1	5	5	13	1237	129.4	8.2
NP_001273171.1	555943794	GTF3C1	general transcription factor 3C polypeptide 1 isoform 2	0.9	0.9	0.9	0.9	1.0	0.9	1.0	1.0	0.9	0.9	0.9	1.0	8.6	2	16	16	25	2084	236.1	7.5
NP_689775.2	44681484	CDCA2	cell division cycle-associated protein 2	0.9	0.9	1.0	1.0	0.9	0.9	1.0	0.9	0.9	0.9	1.0	1.0	21.3	1	11	12	29	1023	112.6	8.4
NP_004477.3	194097392	GOLGA2	Golgin subfamily A member 2	1.2	1.1	1.0	1.0	1.1	1.1	1.0	0.9	1.2	1.1	1.0	1.0	16.9	3	12	12	25	1002	113.0	5.0
NP_001288630.1	678246464	ERI3	ERI1 exoribonuclease 3 isoform 4	1.1	1.0	1.0	1.0	0.9	0.9	0.9	0.9	1.0	1.0	1.0	1.0	17.5	4	3	3	11	160	18.2	7.3
NP_006254.1	5453990	PSME1	proteasome activator complex subunit 1 isoform 1	1.2	1.0	1.1	0.9	1.1	1.0	1.1	1.0	1.2	1.0	1.1	1.0	61.9	4	16	16	67	249	28.7	6.0
NP_001062.1	4507751	TYMS	thymidylate synthase	0.9	1.0	0.9	1.0	0.8	0.8	0.8	0.9	0.9	0.9	0.8	1.0	16.3	1	4	4	5	313	35.7	7.0
NP_008975.1	5901990	KATNA1	katanin p60 ATPase-containing subunit A1 isoform 1	1.0	1.0	1.0	1.0	1.0	0.9	1.0	0.9	1.0	1.0	1.0	1.0	10.8	2	5	5	7	491	55.9	6.9
NP_003891.1	4505571	SQSTM1	sequestosome-1 isoform 1	1.0	1.1	1.0	1.0	1.0	1.1	1.0	1.0	1.0	1.1	1.0	1.0	37.5	2	8	8	23	440	47.7	5.2
NP_006619.1	5729732	ARPP19	cAMP-regulated phosphoprotein 19 isoform 2	1.1	0.8	1.2	0.9	1.1	0.9	1.1	1.0	1.1	0.9	1.1	1.0	17.9	9	2	2	8	112	12.3	9.1
NP_006411.2	150417986	ARFGEF2	brefeldin A-inhibited guanine nucleotide-exchange protein 2	1.0	0.9	1.0	1.0	0.9	0.9	0.9	0.9	0.9	0.9	0.9	1.0	9.1	1	10	12	22	1785	201.9	6.3
NP_001191445.1	325197191	KIFAP3	kinesin-associated protein 3 isoform 3	1.0	0.9	0.9	1.0	1.0	0.9	0.8	0.9	1.0	0.9	0.9	1.0	12.7	4	6	7	12	748	86.2	5.0
NP_061820.1	11128019	CYCS	cytochrome c	1.0	1.0	1.0	0.9	1.0	1.0	1.1	1.0	1.0	1.0	1.1	1.0	56.2	1	7	7	28	105	11.7	9.6
NP_115584.1	14149904	ANTXR1	anthrax toxin receptor 1 isoform 1 precursor	0.8	0.9	1.0	1.0	0.9	0.9	1.0	0.9	0.8	0.9	1.0	1.0	1.4	1	1	1	1	564	62.7	7.6
NP_060244.2	93277076	CDKAL1	threonylcarbamoyladenosine tRNA methylthiotransferase	0.9	0.9	0.9	0.9	1.1	0.9	1.0	1.0	1.0	0.9	0.9	1.0	4.5	1	3	3	4	579	65.1	7.5
NP_005868.1	5032087	SF3A1	splicing factor 3A subunit 1	0.9	0.9	0.8	0.9	0.9	0.9	0.9	1.0	0.9	0.9	0.9	1.0	29.4	2	18	18	71	793	88.8	5.2
NP_057297.2	32189376	TRIP4	activating signal cointegrator 1	0.9	0.9	0.9	1.0	0.9	0.9	0.9	0.9	0.9	0.9	0.9	1.0	17.7	1	7	7	20	581	66.1	7.9
NP_009202.1	6005814	STK38	serine/threonine-protein kinase 38	1.0	1.0	0.9	1.0	0.9	0.9	0.9	0.9	0.9	0.9	0.9	1.0	11.2	1	3	3	3	465	54.2	7.2
NP_056065.1	21735417	BICD2	protein bicaudal D homolog 2 isoform 2	0.9	1.0	0.9	1.0	1.0	1.0	1.0	0.9	1.0	1.0	1.0	1.0	16.6	2	10	10	22	824	93.5	5.4
NP_001252531.1	388240808;110347420	SMC2	structural maintenance of chromosomes protein 2	0.9	0.9	0.9	1.0	0.9	0.9	0.9	0.9	0.9	0.9	0.9	1.0	17.6	1	20	20	52	1197	135.6	8.4
NP_006825.1	5803133	RAB32	ras-related protein Rab-32	1.0	1.0	1.0	1.0	1.0	1.0	1.0	0.9	1.0	1.0	1.0	1.0	55.1	3	10	10	22	225	25.0	6.5
NP_004138.1	4758796	DRG1	developmentally-regulated GTP-binding protein 1	1.1	1.0	1.0	1.0	1.0	0.9	1.0	0.9	1.0	1.0	1.0	1.0	41.4	1	14	14	49	367	40.5	8.9
NP_443108.1	16418383	NACC1	nucleus accumbens-associated protein 1	1.0	0.9	0.9	1.0	1.1	0.9	0.8	0.9	1.0	0.9	0.9	1.0	13.5	1	6	6	15	527	57.2	5.7
NP_001257609.1	398303805	MCU	calcium uniporter protein, mitochondrial isoform 3	1.0	0.9	1.0	1.0	1.0	0.9	0.9	0.9	1.0	0.9	1.0	1.0	21.2	3	6	6	13	302	35.1	7.5
NP_996742.1	45827753	TACC2	transforming acidic coiled-coil-containing protein 2 isoform c	1.1	1.1	1.2	1.0	1.0	1.1	1.1	0.9	1.0	1.1	1.1	1.0	3.9	8	2	4	6	996	108.6	5.0
NP_071504.2	106049292	PC	pyruvate carboxylase, mitochondrial precursor	1.0	1.0	1.0	0.9	1.1	1.1	1.0	1.0	1.0	1.1	1.0	1.0	25.0	1	22	22	56	1178	129.6	6.8
NP_079467.3	163310725	EDEM3	ER degradation-enhancing alpha-mannosidase-like protein 3 precursor	1.4	1.0	1.1	1.1	0.9	0.9	0.9	0.8	1.2	1.0	1.0	1.0	4.9	1	3	3	5	932	104.6	4.9
NP_060700.2	26051235	NUP133	nuclear pore complex protein Nup133	1.1	1.0	1.2	1.0	1.0	0.9	1.0	0.9	1.0	1.0	1.1	1.0	32.9	1	25	25	81	1156	128.9	5.1
NP_001273615.1	557878731	CKAP2	cytoskeleton-associated protein 2 isoform 3	0.9	0.9	1.0	0.9	0.9	1.0	1.0	1.0	0.9	1.0	1.0	1.0	15.8	4	9	9	22	634	71.2	9.3

Khan *et al.* , 2019. Multi-omics analysis to characterize cigarette smoke induced molecular alterations in esophageal cells
Supplementary Table 5. List of proteins quantified in untreated and chronically treated Het1A cells with cigarette smoke condensate for 8 months

NP_Accession	Protein group Accession	Gene ID	Description	Het-1A-Smoke - 2M/Parental	Het-1A-Smoke - 4M/Parental	Het-1A-Smoke - 6M/Parental	Het-1A-Smoke - 8M/Parental	Het-1A-Smoke - 2M/Parental	Het-1A-Smoke - 4M/Parental	Het-1A-Smoke - 6M/Parental	Het-1A-Smoke - 8M/Parental	Het-1A-Smoke - 2M/Parental	Het-1A-Smoke - 4M/Parental	Het-1A-Smoke - 6M/Parental	Het-1A-Smoke - 8M/Parental	Coverage (%)	Proteins	Unique Peptides	# Peptides	PSM	# AAs	MW [kDa]	calc. pI
				Replicate 1				Replicate 2				Average of replicates											
NP_112243.1	13569962	RAB1B	ras-related protein Rab-1B	1.0	0.9	1.1	0.9	1.0	1.1	1.1	1.0	1.0	1.0	1.1	1.0	69.7	10	4	12	220	201	22.2	5.7
NP_647601.1	21536417	BIN1	myc box-dependent-interacting protein 1 isoform 10	1.1	1.1	1.0	0.9	1.0	1.1	1.0	1.0	1.1	1.1	1.0	1.0	40.3	10	11	11	44	409	45.5	5.5
NP_005726.1	11342680	ACTR1B	beta-centractin	1.0	0.9	1.0	1.0	0.9	0.8	0.8	0.9	1.0	0.9	0.9	1.0	20.5	2	1	7	15	376	42.3	6.4
NP_001230209.1	343168770	ALCAM	CD166 antigen isoform 2 precursor	1.0	1.0	1.1	1.0	1.0	1.0	1.1	0.9	1.0	1.0	1.1	1.0	34.2	4	12	12	22	570	63.6	7.3
NP_001157633.1	255918194	PPP6R3	serine/threonine-protein phosphatase 6 regulatory subunit 3 isoform 6	1.1	1.0	1.1	0.9	1.0	1.0	1.1	1.0	1.1	1.0	1.1	1.0	47.5	6	29	30	87	873	97.6	4.6
NP_115497.4	215983058	MAGT1	magnesium transporter protein 1	1.0	1.0	1.1	1.0	1.0	1.1	1.1	0.9	1.0	1.1	1.1	1.0	11.7	1	4	4	7	367	41.5	9.9
NP_036370.2	7657575	SIRT1	NAD-dependent protein deacetylase sirtuin-1 isoform a	1.0	0.9	1.0	1.0	0.9	1.0	1.0	0.9	1.0	1.0	1.0	1.0	14.1	2	5	5	12	747	81.6	4.7
NP_001158287.1	259906020	ACIN1	apoptotic chromatin condensation inducer in the nucleus isoform 3	0.9	0.9	0.8	0.9	1.7	1.2	1.0	1.0	1.3	1.1	0.9	1.0	31.5	5	33	33	102	1301	147.3	6.7
NP_976324.2	118722349	RBM12B	RNA-binding protein 12B	1.0	1.2	1.1	1.1	0.8	0.8	0.8	0.8	0.9	1.0	1.0	1.0	4.5	1	3	3	3	1001	118.0	6.8
NP_115744.2	21362062	LZIC	protein LZIC	1.1	0.8	1.1	0.9	1.1	0.9	1.1	1.0	1.1	0.9	1.1	1.0	27.9	1	4	4	6	190	21.5	4.9
NP_054779.1	7662502	MCTS1	malignant T-cell-amplified sequence 1 isoform 1	1.0	0.9	0.9	0.9	1.1	1.0	1.0	1.0	1.0	1.0	0.9	0.9	47.0	2	6	6	20	181	20.5	8.8
NP_036228.1	6912242	APPL1	DCC-interacting protein 13-alpha	0.9	0.8	0.9	0.9	1.1	1.1	1.1	1.0	1.0	1.0	1.0	0.9	4.0	1	2	2	3	709	79.6	5.4
NP_001188292.1	318067961	HMGN3	high mobility group nucleosome-binding domain-containing protein 3 isoform HMGN3d	0.9	0.8	0.7	0.8	0.8	1.0	0.9	1.1	0.9	0.9	0.8	0.9	28.5	4	5	5	13	130	13.9	9.9
NP_008941.3	612407837	DDX52	probable ATP-dependent RNA helicase DDX52 isoform 1	0.8	0.8	0.8	0.9	1.0	0.9	0.9	1.0	0.9	0.9	0.9	0.9	14.7	2	5	5	6	599	67.4	9.7
NP_115612.4	52630449	USP48	ubiquitin carboxyl-terminal hydrolase 48 isoform a	1.1	1.0	1.0	0.9	1.0	1.0	1.0	1.0	1.0	1.0	1.0	0.9	10.1	2	7	7	18	1035	119.0	6.0
NP_001139438.1	225543215	MKI67	antigen KI-67 isoform 2	0.9	1.0	1.0	1.0	0.8	0.9	0.9	0.9	0.9	1.0	1.0	0.9	49.9	1	1	107	435	2896	319.2	9.5
NP_003918.1	4505117	MBD2	methyl-CpG-binding domain protein 2 isoform 1	0.9	0.9	1.0	0.9	0.9	1.0	1.0	1.0	0.9	0.9	1.0	0.9	25.3	2	7	7	16	411	43.2	10.0
NP_003759.1	4505705	PEA15	astrocytic phosphoprotein PEA-15	1.8	1.1	2.0	1.0	1.4	0.9	1.4	0.9	1.6	1.0	1.7	0.9	14.6	1	1	1	2	130	15.0	5.0
NP_001171482.1	295821207	CDC45	cell division control protein 45 homolog isoform 3	1.0	1.0	0.9	0.9	1.1	1.0	1.1	0.9	1.0	1.0	1.0	0.9	1.7	3	1	1	2	520	60.2	5.8
NP_003560.2	170932494	STX7	syntaxin-7	1.0	1.0	0.9	0.9	1.0	0.9	0.9	1.0	1.0	0.9	0.9	0.9	35.6	1	5	6	12	261	29.8	5.6
NP_006485.2	156104872	ERF	ETS domain-containing transcription factor ERF	0.9	0.9	1.0	1.0	1.0	1.1	1.1	0.9	0.9	1.0	1.0	0.9	8.8	1	2	2	3	548	58.7	7.3
NP_005122.2	154448890	THOC1	THO complex subunit 1	1.0	1.0	1.0	1.0	1.0	0.9	0.9	0.9	1.0	1.0	1.0	0.9	17.2	1	8	8	17	657	75.6	5.0
NP_060289.2	117647226	TRMT10C	mitochondrial ribonuclease P protein 1 precursor	1.0	1.0	1.0	0.9	1.0	1.0	1.0	1.0	1.0	1.0	1.0	0.9	47.4	1	14	14	41	403	47.3	9.4
NP_001231514.1	347446678	LUC7L2	putative RNA-binding protein Luc7-like 2 isoform 3	0.9	0.9	0.9	0.9	0.9	1.1	0.9	1.0	0.9	1.0	0.9	0.9	26.0	4	6	10	48	389	46.2	10.1
NP_008933.2	28373192;28373194	ADRM1	proteasomal ubiquitin receptor ADRM1 isoform 1	0.9	0.8	1.0	0.9	1.0	0.9	0.9	1.0	0.9	0.8	1.0	0.9	14.3	2	5	5	24	407	42.1	5.1
NP_037407.4	56676397	ANKRD11	ankyrin repeat domain-containing protein 11	0.9	1.0	0.9	0.9	1.0	1.0	1.1	1.0	1.0	1.0	1.0	0.9	0.6	1	1	1	2	2663	297.7	7.1
NP_001077433.1	145312246	TDRKH	tudor and KH domain-containing protein isoform b	1.0	0.9	1.0	0.9	1.0	1.0	0.9	1.0	1.0	0.9	0.9	0.9	9.5	2	3	3	7	516	57.2	4.9
NP_056167.1	27436889	POFUT1	GDP-fucose protein O-fucosyltransferase 1 isoform 1 precursor	1.0	0.9	0.9	1.0	0.9	0.9	0.9	0.9	0.9	0.9	0.9	0.9	23.5	2	7	7	27	388	43.9	8.5
NP_009293.1	6382071	DIAPH2	protein diaphanous homolog 2 isoform 12C	1.2	1.0	1.1	0.9	1.0	1.0	1.0	0.9	1.1	1.0	1.1	0.9	4.3	2	3	3	4	1096	124.7	6.4
NP_036355.2	44889481	MYO1B	unconventional myosin-Ib isoform 2	1.0	0.9	1.0	0.9	1.0	1.0	1.0	1.0	1.0	1.0	1.0	0.9	26.2	3	22	22	69	1078	124.9	9.2
NP_061155.2	31657094	ANLN	actin-binding protein anillin isoform 1	1.0	1.0	1.1	0.9	1.0	1.0	1.0	1.0	1.0	1.0	1.1	0.9	25.9	3	25	25	61	1124	124.1	8.1
NP_003904.3	89276756	PRPF4B	serine/threonine-protein kinase PRP4 homolog	0.8	0.9	0.9	0.9	0.9	0.9	0.9	1.0	0.8	0.9	0.9	0.9	10.0	1	9	9	41	1007	116.9	10.3
NP_005505.2	17986001	HLA-B	major histocompatibility complex, class I, B precursor	0.8	0.8	0.8	0.9	1.0	1.1	1.0	1.0	0.9	1.0	0.9	0.9	17.1	3	2	5	19	362	40.4	5.8
NP_037471.2	38026892	ALG6	dolichyl pyrophosphate Man9GlcNAc2 alpha-1,3-glucosyltransferase precursor	1.0	1.0	0.9	1.0	0.8	0.8	0.8	0.9	0.9	0.9	0.8	0.9	4.9	1	2	2	4	507	58.1	8.5
NP_065987.1	41327779	DDX55	ATP-dependent RNA helicase DDX55	0.8	0.9	0.9	1.0	1.2	1.0	1.0	0.9	1.0	1.0	1.0	0.9	6.0	1	3	3	11	600	68.5	9.2
NP_112533.1	14043072	HNRNPA2B1	heterogeneous nuclear ribonucleoproteins A2/B1 isoform B1	0.9	1.0	0.9	1.0	0.9	1.0	0.9	0.9	0.9	1.0	0.9	0.9	73.9	2	17	20	263	353	37.4	9.0
NP_002944.2	20149547	RPS6KA1	ribosomal protein S6 kinase alpha-1 isoform a	0.9	0.8	0.8	0.8	1.1	1.1	0.9	1.1	1.0	0.9	0.9	0.9	14.4	4	4	8	12	735	82.7	7.8
NP_277071.2	148763345	CDK11A	cyclin-dependent kinase 11A isoform 4	1.1	0.9	1.0	1.0	0.9	0.9	0.9	0.9	1.0	0.9	0.9	0.9	9.2	2	1	7	17	770	90.0	5.3
NP_065196.1	39930397	SMEK2	serine/threonine-protein phosphatase 4 regulatory subunit 3B isoform 2	1.0	1.0	1.0	0.9	1.0	1.0	1.1	1.0	1.0	1.0	1.0	0.9	16.9	3	5	10	24	764	87.3	4.8
NP_001316.1	4557493	CSTF2	cleavage stimulation factor subunit 2	0.9	1.0	0.9	0.9	1.1	1.0	0.9	1.0	1.0	1.0	0.9	0.9	8.5	3	1	3	4	577	60.9	6.8
NP_006651.2	7242140	CLPX	ATP-dependent Clp protease ATP-binding subunit clpX-like, mitochondrial precursor	0.9	0.8	0.9	0.9	0.9	0.9	0.9	1.0	0.9	0.9	0.9	0.9	17.4	1	10	10	27	633	69.2	7.6
NP_003893.2	17402900	FUBP1	far upstream element-binding protein 1 isoform 2	1.0	1.0	0.9	0.9	1.0	1.0	0.9	0.9	1.0	1.0	0.9	0.9	39.3	2	16	20	72	644	67.5	7.6
NP_001011537.2	240849501	FYTTD1	UAP56-interacting factor isoform 2	1.0	0.9	1.0	0.9	1.0	0.9	0.9	1.0	1.0	0.9	0.9	0.9	8.2	2	2	2	3	292	33.0	11.8
NP_001244.1	11067747	CDC5L	cell division cycle 5-like protein	1.0	0.9	0.9	0.9	1.0	1.0	1.0	0.9	1.0	1.0	0.9	0.9	31.6	1	18	18	63	802	92.2	8.2
NP_005722.1	5031599;23238211	ARPC2	actin-related protein 2/3 complex subunit 2	1.1	1.0	1.2	0.9	1.0	1.0	1.1	1.0	1.1	1.0	1.1	0.9	40.0	1	9	9	40	300	34.3	7.4
NP_003494.1	4502715	CDC7	cell division cycle 7-related protein kinase	0.8	0.9	0.9	0.9	0.9	1.0	1.1	1.0	0.9	1.0	1.0	0.9	2.3	1	1	1	1	574	63.8	8.7
NP_057033.2	38570062	GET4	Golgi to ER traffic protein 4 homolog	0.9	0.9	0.9	0.9	1.0	0.8	0.9	1.0	0.9	0.8	0.9	0.9	14.4	1	4	4	10	327	36.5	5.4
NP_001003803.1	51558774	ATP5S	ATP synthase subunit s, mitochondrial isoform a precursor	1.1	0.9	1.0	1.0	1.2	0.9	0.9	0.9	1.2	0.9	1.0	0.9	5.1	1	1	1	2	215	24.8	7.6
NP_002943.2	15055539	RPS2	40S ribosomal protein S2	0.9	0.9	0.9	0.9	0.9	0.9	0.9	1.0	0.9	0.9	0.9	0.9	61.4	1	18	18	240	293	31.3	10.2
NP_001518.3	293336691	HDAC2	histone deacetylase 2	0.9	0.9	0.9	0.9	1.0	1.0	1.0	1.0	1.0	0.9	1.0	0.9	38.1	1	9	13	63	488	55.3	5.9
NP_001231775.1	349501061	CHEK1	serine/threonine-protein kinase Chk1 isoform 2	1.0	1.0	0.9	1.0	0.9	0.8	0.8	0.9	0.9	0.9	0.9	0.9	12.4	2	4	4	7	442	50.4	8.0
NP_001119590.1	371502119;454521557	TP53	cellular tumor antigen p53 isoform g	1.0	1.0	1.1	0.9	1.0	1.0	1.0	0.9	1.0	1.0	1.1	0.9	34.8	12	11	11	32	354	39.3	7.9
NP_001135763.2	334883182	MGRN1	E3 ubiquitin-protein ligase MGRN1 isoform 4	1.1	1.0	1.2	0.9	1.0	1.0	1.0	1.0	1.1	1.0	1.1	0.9	2.6	4	1	1	2	530	58.3	4.9
NP_003585.3	40807471	TTF2	transcription termination factor 2	0.9	1.0	1.0	0.9	1.0	1.1	1.0	1.0	1.0	1.1	1.0	0.9	1.6	1	1	2	3	1162	129.5	8.4
NP_001243222.1	372466577	KRT8	keratin, type II cytoskeletal 8 isoform 2	0.9	0.9	0.8	0.9	0.9	0.9	0.8	1.0	0.9	0.9	0.8	0.9	54.5	8	20	29	250	483	53.7	5.6
NP_006126.1	5453597	CAPZA1	F-actin-capping protein subunit alpha-1	1.0	0.9	1.0	1.0	1.0	0.9	1.0	0.9	1.0	0.9	1.0	0.9	53.5	2	10	10	70	286	32.9	5.7
NP_004449.1	4758332	ACSL4	long-chain-fatty-acid--CoA ligase 4 isoform 1	1.1	1.2	1.0	1.0	1.1	1.1	1.0	0.9	1.1	1.2	1.0	0.9	27.2	2	13	14	47	670	74.4	8.0
NP_068598.1	11141903	FASTKD5	FAST kinase domain-containing protein 5	1.0	0.9	1.0	1.0	0.9	0.9	1.0	0.9	1.0	0.9	1.0	0.9	2.2	1	2	2	3	764	86.5	8.1
NP_536348.1	18104982	PTPN2	tyrosine-protein phosphatase non-receptor type 2 isoform 3	1.1	1.0	1.0	1.0	0.9	0.9	0.9	0.9	1.0	1.0	1.0	0.9	10.5	5	2	2	6	353	41.0	6.4
NP_671714.1	22538393	AKAP9	A-kinase anchor protein 9 isoform 3	1.1	1.1	1.0	1.0	1.0	1.0	0.9	0.9	1.0	1.0	1.0	0.9	2.7	2	8	8	13	3899	451.8	5.0
NP_002874.1	4506411	RANGAP1	ran GTPase-activating protein 1	1.0	1.0	1.1	0.9	0.9	1.0	1.1	1.0	1.0	1.0	1.1	0.9	48.4	1	21	21	61	587	63.5	4.7
NP_000992.1	4506651	RPL36AL	60S ribosomal protein L36a-like	0.9	1.0	0.9	0.9	0.9	1.0	0.9	1.0	0.9	1.0	0.9	0.9	32.1	1	2	6	57	106	12.5	10.7
NP_443082.2	31543415	GADD45GIP1	growth arrest and DNA damage-inducible proteins-interacting protein 1	1.0	0.9	1.0	0.9	1.1	1.0	0.9	0.9	1.0	0.9	1.0	0.9	36.5	1	5	5	8	222	25.4	10.0
NP_004085.1	4758256	EIF2S1	eukaryotic translation initiation factor 2 subunit 1	1.0	0.9	0.9	0.9	0.9	0.9	0.8	0.9	1.0	0.9	0.9	0.9	68.3	1	19	19	91	315	36.1	5.1
NP_055805.1	51226124	RALGAPA1	ral GTPase-activating protein subunit alpha-1 isoform 1	1.0	0.9	0.9	0.9	1.0	1.0	1.0	1.0	1.0	0.9	1.0	0.9	1.3	4	2	2	3	2036	229.7	6.2
NP_004413.1	4758216	DVL2	segment polarity protein dishevelled homolog DVL-2	0.8	0.8	0.8	0.9	0.9	1.0	1.0	1.0	0.9	0.9	0.9	0.9	6.0	2	3	3	7	736	78.9	6.0
NP_001257869.1	401461771	XPO6	exportin-6 isoform 1	1.0	0.9	1.1	0.9	0.9	0.9	1.1	0.9	0.9	0.9	1.1	0.9	7.3	2	7	7	12	1111	127.3	6.5
NP_001277188.1	589908394	PHF3	PHD finger protein 3 isoform 2	0.9	0.9	0.9	0.9	0.9	0.9	0.9	1.0	0.9	0.9	0.9	0.9	14.5	3	18	18	33	1951	219.7	7.5
NP_005821.2	186928854	MRPS31	28S ribosomal protein S31, mitochondrial	1.0	1.1	1.0	0.9	1.0	1.2	1.0	1.0	1.0	1.1	1.0	0.9	32.7	1	12	12	41	395	45.3	9.3
NP_071435.2	50593008	TMBIM1	protein lifeguard 3	0.9	0.9	0.8	1.0	0.9	0.8	0.8	0.9	0.9	0.9	0.8	0.9	4.5	1	1	1	4	311	34.6	7.7
NP_001129669.1	209969678	FZR1	fizzy-related protein homolog isoform 3	1.0	0.9	0.9	1.0	1.0	1.0	1.0	0.9	1.0	0.9	1.0	0.9	4.0	3	1	1	1	404	44.8	9.3
NP_689472.3	118421085	TICRR	treslin	0.8	0.8	0.8	0.8	0.9	1.1	0.9	1.1	0.9	0.9	0.9	0.9	0.7	1	1	1	1	1910	210.7	8.8
NP_444271.2	151301053	RBM33	RNA-binding protein 33	1.0	0.8	0.8	1.0	1.0	0.9	0.9	0.9	1.0	0.9	0.9	0.9	5.6	1	5	5	10	1170	129.9	6.9
NP_001243731.1	378548190	RPS3	40S ribosomal protein S3 isoform 1	0.9	1.0	0.9	1.0	0.9	0.9	0.9	0.9	0.9	1.0	0.9	0.9	76.5	3	18	18	164	243	26.7	9.7
NP_003001.1	4506889	MAP2K4	dual specificity mitogen-activated protein kinase kinase 4 isoform 1	1.1	1.1	1.0	1.0	0.9	1.0	1.0	0.9	1.0	1.0	1.0	0.9	27.6	2	8	8	19	399	44.3	8.1
NP_001001521.1	48255968	UGP2	UTP--glucose-1-phosphate uridylyltransferase isoform b	0.9	0.9	1.0	1.0	0.9	0.9	1.0	0.9	0.9	0.9	1.0	0.9	33.8	2	13	13	41	497	55.6	7.9
NP_689596.4	134288890	DIS3L2	DIS3-like exonuclease 2 isoform 1	0.9	0.9	0.9	1.0	0.9	0.9	1.0	0.9	0.9	0.9	0.9	0.9	3.8	3	4	4	6	885	99.2	6.1
NP_543010.3	30795227	DTD1	D-tyrosyl-tRNA(Tyr) deacylase 1	1.3	1.2	1.2	1.0	1.1	1.0	1.0	0.9	1.2	1.1	1.1	0.9	23.9	1	3	3	6	209	23.4	8.2
NP_057673.2	59710109	TIMMDC1	complex I assembly factor TIMMDC1, mitochondrial precursor	1.1	1.1	1.1	1.0	1.0	0.9	0.8	0.9	1.0	1.0	0.9	0.9	7.7	1	2	2	3	285	32.2	8.5
NP_001138829.1	223941782;23397666	SIN3A	paired amphipathic helix protein Sin3a	1.0	0.9	0.9	0.9	1.0	1.0	1.0	1.0	1.0	0.9	0.9	0.9	17.8	1	18	18	40	1273	145.1	7.3
NP_004989.2	55956916	MYO1E	unconventional myosin-Ie	0.9	1.0	1.0	0.9	1.0	1.0	1.0	1.0	0.9	1.0	1.0	0.9	20.5	2	15	15	36	1108	127.0	8.9
NP_076977.3	51094101	DDX54	ATP-dependent RNA helicase DDX54 isoform 2	0.9	1.0	0.9	0.9	0.9	0.9	0.9	1.0	0.9	0.9	0.9	0.9	30.9	2	17	17	38	881	98.5	10.0
NP_060372.2	148596936	HIF1AN	hypoxia-inducible factor 1-alpha inhibitor	1.0	0.9	0.9	1.0	0.9	0.9	0.9	0.9	1.0	0.9	0.9	0.9	10.0	1	3	3	4	349	40.3	5.6
NP_001027566.1	74048422	ADGRG6	G-protein coupled receptor 126 isoform alpha 2 precursor	0.9	0.9	1.1	1.0	0.8	0.9	1.0	0.9	0.9	0.9	1.0	0.9	4.5	4	5	5	8	1193	133.6	7.8
NP_004515.2	62739161	LLGL2	lethal(2) giant larvae protein homolog 2 isoform a	0.8	0.8	0.8	1.0	0.7	0.7	0.7	0.9	0.8	0.8	0.8	0.9	2.8	2	1	1	2	1015	112.9	7.3

Khan *et al.*, 2019. Multi-omics analysis to characterize cigarette smoke induced molecular alterations in esophageal cells
Supplementary Table 5. List of proteins quantified in untreated and chronically treated Het1A cells with cigarette smoke condensate for 8 months

NP_Accession	Protein group Accession	Gene ID	Description	Het-1A-Smoke - 2M/Parental	Het-1A-Smoke - 4M/Parental	Het-1A-Smoke - 6M/Parental	Het-1A-Smoke - 8M/Parental	Het-1A-Smoke - 2M/Parental	Het-1A-Smoke - 4M/Parental	Het-1A-Smoke - 6M/Parental	Het-1A-Smoke - 8M/Parental	Het-1A-Smoke - 2M/Parental	Het-1A-Smoke - 4M/Parental	Het-1A-Smoke - 6M/Parental	Het-1A-Smoke - 8M/Parental	Coverage (%)	Proteins	Unique Peptides	# Peptides	PSM	# AAs	MW [kDa]	calc. pI
				Replicate 1				Replicate 2				Average of replicates											
NP_150644.1	15431306;4506663	RPL8	60S ribosomal protein L8	0.9	1.0	0.9	1.0	0.9	1.0	0.9	0.9	0.9	1.0	0.9	0.9	62.7	1	15	15	51	257	28.0	11.0
NP_114409.2	40068512	FUCA2	plasma alpha-L-fucosidase precursor	0.9	0.8	0.7	0.9	0.9	0.9	0.8	1.0	0.9	0.8	0.8	0.9	11.4	1	4	4	9	467	54.0	6.3
NP_003583.2	32307161	CUL1	cullin-1	1.1	1.0	1.1	1.0	1.0	1.1	1.1	0.9	1.1	1.0	1.1	0.9	14.6	1	9	9	19	776	89.6	8.0
NP_001268664.1	528524510	ZFYVE1	zinc finger FYVE domain-containing protein 1 isoform 2	1.3	1.2	1.0	1.1	0.8	0.8	0.7	0.8	1.0	1.0	0.9	0.9	4.1	3	1	1	1	362	40.2	7.6
NP_065784.1	153792110	HEG1	protein HEG homolog 1 precursor	0.9	0.9	0.9	0.9	0.9	0.9	0.9	1.0	0.9	0.9	0.9	0.9	1.9	1	2	2	3	1381	147.4	6.2
NP_078805.3	40018640	CDC73	parafibromin	0.9	0.9	1.0	0.9	1.0	1.0	1.0	1.0	1.0	1.0	1.0	0.9	33.7	1	15	15	36	531	60.5	9.6
NP_001265232.1	508083043	DIABLO	diablo homolog, mitochondrial isoform 5	1.0	0.9	0.9	0.9	1.1	0.9	0.9	0.9	1.0	0.9	0.9	0.9	53.0	5	7	7	27	166	18.9	4.8
NP_006546.1	5730120	YKT6	synaptobrevin homolog YKT6	1.0	0.9	0.9	0.9	1.1	0.9	1.0	1.0	1.0	0.9	0.9	0.9	32.8	1	6	6	21	198	22.4	6.9
NP_004883.3	380837121	SEC22B	vesicle-trafficking protein SEC22b precursor	1.0	0.9	0.9	0.9	1.1	1.0	1.1	0.9	1.1	1.0	1.0	0.9	38.6	1	7	7	25	215	24.7	8.5
NP_116259.2	154146193	PNISR	arginine/serine-rich protein PNISR	0.9	0.9	0.9	0.9	1.0	1.0	1.0	1.0	0.9	1.0	1.0	0.9	7.8	1	7	7	14	805	92.5	10.0
NP_067054.1	126723149	CACTIN	cactin	1.1	1.0	0.9	1.0	0.8	0.9	0.9	0.9	1.0	0.9	0.9	0.9	15.4	1	6	6	8	758	88.6	9.1
NP_694992.2	194394161	LRRC57	leucine-rich repeat-containing protein 57	0.9	0.8	0.9	0.9	0.9	0.8	0.9	1.0	0.9	0.8	0.9	0.9	4.6	1	1	1	4	239	26.7	8.4
NP_958357.1	41152093	ZNF644	zinc finger protein 644 isoform 1	1.0	1.0	1.0	1.0	0.8	0.8	0.9	0.9	0.9	0.9	1.0	0.9	4.5	2	4	4	6	1327	149.5	8.2
NP_005168.2	19913418	ATP6V0A1	V-type proton ATPase 116 kDa subunit a isoform 1 isoform c	1.0	0.9	0.9	0.9	1.0	1.0	0.9	1.0	1.0	1.0	0.9	0.9	12.5	4	7	7	16	831	95.7	6.7
NP_008996.1	5901922	CDC37	hsp90 co-chaperone Cdc37	0.9	0.9	0.9	0.9	0.9	0.9	1.0	1.0	0.9	0.9	1.0	0.9	31.5	1	12	12	92	378	44.4	5.2
NP_060670.1	8922633	HMG20A	high mobility group protein 20A isoform a	0.9	0.9	0.9	0.9	1.0	1.0	1.0	1.0	0.9	0.9	0.9	0.9	27.1	2	6	6	13	347	40.1	6.5
NP_055817.1	16306500	FBXO21	F-box only protein 21 isoform 2	0.9	0.9	0.9	0.9	0.9	1.0	0.9	1.0	0.9	0.9	0.9	0.9	8.5	2	3	3	6	621	71.4	6.0
NP_003578.2	255982614	DHX16	putative pre-mRNA-splicing factor ATP-dependent RNA helicase DHX16 isoform 1	1.0	1.0	0.9	1.0	1.0	1.0	0.9	0.9	1.0	1.0	0.9	0.9	15.1	2	10	11	34	1041	119.2	6.8
NP_001137545.1	221316570	CHORDC1	cysteine and histidine-rich domain-containing protein 1 isoform b	0.9	0.9	1.0	0.9	1.0	1.0	1.1	1.0	1.0	1.0	1.0	0.9	41.2	2	9	9	23	313	35.3	7.8
NP_001004304.1	51972182	ZNF740	zinc finger protein 740	0.8	1.0	0.9	0.9	1.0	1.0	0.9	1.0	0.9	1.0	0.9	0.9	6.7	1	1	1	1	193	21.8	9.2
NP_031385.2	15721939	CASC3	protein CASC3	0.8	1.0	0.9	0.9	0.9	1.0	1.0	1.0	0.9	1.0	0.9	0.9	5.0	1	3	3	6	703	76.2	6.5
NP_005460.2	34577075	ACOT8	acyl-coenzyme A thioesterase 8	1.1	1.0	1.0	0.9	1.0	0.9	0.9	0.9	1.0	0.9	1.0	0.9	21.0	1	5	5	8	319	35.9	7.6
NP_060252.4	296011010	FAM208B	protein FAM208B	1.0	0.9	0.9	0.9	1.1	0.9	1.0	1.0	1.0	0.9	0.9	0.9	2.3	1	4	4	4	2430	268.6	5.9
NP_001122326.1	192807336	SRRT	serrate RNA effector molecule homolog isoform e	0.9	0.9	1.0	0.9	0.9	1.0	0.9	0.9	0.9	1.0	0.9	0.9	25.8	5	17	17	70	871	100.1	6.0
NP_056418.1	7661690	CCDC9	coiled-coil domain-containing protein 9	1.0	1.1	1.0	0.9	1.0	1.0	0.9	0.9	1.0	1.1	0.9	0.9	10.0	1	5	5	10	531	59.7	5.4
NP_524558.1	17738287	SCAMP4	secretory carrier-associated membrane protein 4	0.9	1.0	0.9	0.9	1.1	1.0	1.1	1.0	1.0	1.0	1.0	0.9	21.0	1	3	3	7	229	25.7	8.8
NP_064536.2	151301035	STARD7	stAR-related lipid transfer protein 7, mitochondrial precursor	1.1	1.1	1.0	0.9	1.4	1.0	1.1	1.0	1.2	1.1	1.0	0.9	3.0	1	1	1	1	370	43.1	9.0
NP_982289.1	53832001;8051579	AK4	adenylate kinase 4, mitochondrial	1.0	1.0	0.9	1.0	0.9	0.9	0.9	0.9	0.9	1.0	0.9	0.9	40.8	1	6	6	19	223	25.3	8.4
NP_005021.2	21359873	PLK1	serine/threonine-protein kinase PLK1	0.8	1.0	1.1	1.0	0.8	0.9	1.0	0.9	0.8	0.9	1.1	0.9	18.9	1	8	8	19	603	68.2	8.9
NP_005850.1	5032007	PURA	transcriptional activator protein Pur-alpha	1.0	1.1	0.9	1.0	1.0	1.0	0.9	0.9	1.0	1.0	0.9	0.9	46.0	1	6	6	35	322	34.9	6.4
NP_001258761.1	425905337	PPP2R4	serine/threonine-protein phosphatase 2A activator isoform f	1.0	0.9	0.9	0.9	1.0	0.9	1.0	1.0	1.0	0.9	1.0	0.9	46.6	5	10	10	42	294	33.4	6.3
NP_057455.1	7705421	HSD17B7	3-keto-steroid reductase isoform 1	1.1	1.0	1.0	0.9	1.0	1.2	1.1	1.0	1.1	1.1	1.0	0.9	12.6	1	3	3	9	341	38.2	8.1
NP_001229790.1	338827761	PRKACB	cAMP-dependent protein kinase catalytic subunit beta isoform 8	0.9	1.0	0.9	1.0	0.8	1.0	0.9	0.9	0.8	1.0	0.9	0.9	34.3	13	3	7	21	321	37.0	8.2
NP_001277113.1	589269130	NCSTN	nicastrin isoform 2	1.0	1.0	0.9	1.0	0.9	0.9	0.9	0.9	0.9	1.0	0.9	0.9	13.1	3	7	7	14	689	76.7	5.7
NP_001073884.1	122937227	U2SURP	U2 snRNP-associated SURP motif-containing protein	1.0	1.0	1.0	1.0	0.9	0.9	0.9	0.9	0.9	0.9	0.9	0.9	17.5	1	14	14	32	1029	118.2	8.5
NP_001029196.1	78000213	DMAP1	DNA methyltransferase 1-associated protein 1	1.0	0.9	0.8	1.0	0.9	1.0	0.9	0.9	0.9	0.9	0.8	0.9	20.1	1	6	6	13	467	53.0	9.5
NP_005886.2	30089940	GOLGA3	Golgin subfamily A member 3 isoform 1	1.1	1.0	1.0	0.9	1.1	1.0	1.0	0.9	1.1	1.0	1.0	0.9	13.4	2	13	14	35	1498	167.3	5.4
NP_006378.3	119226260	CHERP	calcium homeostasis endoplasmic reticulum protein	0.9	0.9	0.9	0.9	0.8	0.9	0.9	0.9	0.9	0.9	0.9	0.9	14.9	1	9	9	30	916	103.6	9.0
NP_005900.2	153945728	MAP1B	microtubule-associated protein 1B	1.3	1.4	1.3	0.9	1.2	1.3	1.2	0.9	1.3	1.4	1.2	0.9	33.3	3	52	52	141	2468	270.5	4.8
NP_005517.1	5031767	HSF1	heat shock factor protein 1	0.8	0.9	0.9	0.9	1.0	1.2	1.2	1.0	0.9	1.0	1.1	0.9	6.8	1	2	2	5	529	57.2	5.2
NP_001002909.1	50962882	GPATCH8	G patch domain-containing protein 8 isoform 1	1.0	0.9	0.9	0.9	0.9	1.0	0.9	0.9	1.0	0.9	0.9	0.9	3.9	3	5	5	11	1502	164.1	8.7
NP_060590.1	8922479	ARMC1	armadillo repeat-containing protein 1 isoform 1	1.0	1.0	1.0	1.0	1.0	1.0	0.9	0.9	1.0	1.0	1.0	0.9	19.2	2	3	3	11	282	31.3	5.7
NP_056189.1	31742498;313760645	SUN2	SUN domain-containing protein 2 isoform b	1.1	1.2	1.3	0.9	1.1	1.2	1.3	1.0	1.1	1.2	1.3	0.9	21.8	2	9	10	34	717	80.3	6.7
NP_001171889.1	296785054	SH3KBP1	SH3 domain-containing kinase-binding protein 1 isoform c	1.0	1.0	1.0	0.9	1.1	1.2	1.1	1.0	1.0	1.1	1.0	0.9	7.0	3	2	2	7	427	46.6	8.8
NP_001277074.1	588480525	POLDIP2	polymerase delta-interacting protein 2 isoform 2	0.9	0.9	0.9	0.9	1.0	1.0	0.9	1.0	1.0	0.9	0.9	0.9	25.7	2	6	6	11	350	39.8	8.4
NP_001135746.1	214010175	CLASP1	CLIP-associating protein 1 isoform 3	0.9	0.9	0.9	0.9	1.0	1.0	1.0	1.0	0.9	1.0	0.9	0.9	11.8	4	10	13	26	1471	162.0	8.8
NP_073750.2	16554609	MRPS11	28S ribosomal protein S11, mitochondrial isoform a	1.0	1.0	1.0	1.0	0.9	0.9	0.9	0.9	1.0	1.0	0.9	0.9	21.7	2	3	3	7	194	20.6	10.8
NP_015556.1	6453818	KIF22	kinesin-like protein KIF22 isoform 1	0.9	0.9	0.8	0.9	0.9	1.0	0.9	0.9	0.9	0.9	0.9	0.9	21.8	2	11	11	29	665	73.2	9.5
NP_006507.2	166795299	SLC2A1	solute carrier family 2, facilitated glucose transporter member 1	0.8	1.0	1.0	0.8	0.9	1.1	1.1	1.1	0.8	1.0	1.0	0.9	13.4	6	8	8	21	492	54.0	8.7
NP_000166.2	18201905	GPI	glucose-6-phosphate isomerase isoform 2	0.9	0.9	1.0	0.9	1.0	1.0	1.0	1.0	1.0	0.9	1.0	0.9	54.7	5	25	25	227	558	63.1	8.3
NP_001229290.1	334358861	NRD1	nardilysin isoform c	1.0	1.0	1.0	0.9	1.0	1.0	1.0	1.0	1.0	1.0	1.0	0.9	16.7	3	15	15	48	1087	125.0	4.9
NP_054828.2	24497618	ATAD2	ATPase family AAA domain-containing protein 2	1.0	0.9	1.0	0.9	1.0	1.0	0.9	0.9	1.0	1.0	0.9	0.9	23.2	1	21	21	61	1390	158.5	6.3
NP_001193953.1	333440434	M6PR	cation-dependent mannose-6-phosphate receptor isoform 2 precursor	0.9	1.0	1.0	1.0	0.8	0.8	0.9	0.9	0.8	0.9	0.9	0.9	18.9	2	3	3	5	191	21.5	6.7
NP_694988.3	223633935	PROSER2	proline and serine-rich protein 2	0.7	0.9	0.9	1.0	0.8	0.8	0.9	0.9	0.8	0.9	0.9	0.9	3.5	1	1	1	2	435	45.8	7.2
NP_006222.2	62198237	POLE	DNA polymerase epsilon catalytic subunit A	1.0	1.0	1.0	1.0	0.9	0.9	0.9	0.9	1.0	0.9	0.9	0.9	8.1	1	11	11	20	2286	261.4	6.4
NP_892117.1	33859837;148238078	RPP38	ribonuclease P protein subunit p38	1.1	1.0	1.0	1.0	1.0	0.9	0.9	0.9	1.0	1.0	0.9	0.9	9.5	1	2	2	4	283	31.8	9.9
NP_001184058.1	308199460	SYS1	protein SYS1 homolog isoform a	0.9	1.0	0.9	0.9	1.1	0.9	1.0	0.9	1.0	1.0	1.0	0.9	5.8	1	1	1	1	156	17.6	7.7
NP_056988.3	84043963	EIF5B	eukaryotic translation initiation factor 5B	1.0	1.0	1.0	0.9	1.0	1.0	0.9	0.9	1.0	1.0	0.9	0.9	29.0	1	32	32	93	1220	138.7	5.5
NP_115501.2	148277052	TMEM222	transmembrane protein 222	1.1	1.1	0.8	0.9	0.9	0.9	0.8	1.0	1.0	1.0	0.8	0.9	12.5	1	1	1	2	208	23.2	6.5
NP_001243334.1	373938467	KDM2A	lysine-specific demethylase 2A isoform b	0.9	1.0	1.0	0.9	1.0	1.1	1.1	1.0	0.9	1.1	1.1	0.9	10.8	2	5	5	11	723	81.6	8.7
NP_003932.3	51702526	WASL	neural Wiskott-Aldrich syndrome protein	1.2	1.1	1.5	0.9	1.2	1.0	1.1	1.0	1.2	1.1	1.3	0.9	3.4	1	1	1	3	505	54.8	7.9
NP_940870.2	115583683	MMS22L	protein MMS22-like	1.0	1.0	0.9	0.9	1.0	0.9	1.1	1.0	1.0	1.0	1.0	0.9	1.5	1	1	1	1	1243	142.2	7.1
NP_000973.2	18104948	RPL21	60S ribosomal protein L21	1.0	1.0	1.0	0.9	0.9	1.0	0.9	0.9	0.9	1.0	1.0	0.9	35.0	1	7	7	51	160	18.6	10.5
NP_001137153.1	219842335	GSTK1	glutathione S-transferase kappa 1 isoform d	1.1	0.9	1.2	1.0	1.1	0.9	1.0	0.9	1.1	0.9	1.1	0.9	14.8	4	2	2	5	183	20.5	6.5
NP_078959.3	134053959	TEFM	transcription elongation factor, mitochondrial	1.0	1.0	0.9	0.9	1.5	1.0	0.9	1.0	1.3	1.0	0.9	0.9	19.2	1	5	5	11	360	41.6	9.3
NP_062552.2	47271406	MEPCE	7SK snRNA methylphosphate capping enzyme isoform A	0.9	0.9	0.9	0.9	0.8	0.9	0.9	1.0	0.9	0.9	0.9	0.9	8.6	2	5	5	9	689	74.3	9.6
NP_006808.1	5803013	ERP29	endoplasmic reticulum resident protein 29 isoform 1 precursor	1.3	1.1	1.0	0.9	1.4	1.1	1.1	1.0	1.4	1.1	1.1	0.9	44.8	2	8	8	38	261	29.0	7.3
NP_060146.2	116812606	GIN1	gypsy retrotransposon integrase-like protein 1	1.1	1.0	1.1	0.9	1.2	1.0	1.1	0.9	1.1	1.0	1.1	0.9	1.2	2	1	1	2	522	59.8	8.0
NP_065203.2	170932464	YIF1A	protein YIF1A	1.1	1.2	1.2	1.1	0.9	0.8	0.8	0.7	1.0	1.0	1.0	0.9	10.6	2	2	2	4	293	32.0	9.0
NP_006259.1	5454004	DPF2	zinc finger protein ubi-d4	0.9	1.0	0.9	0.9	0.9	0.9	0.9	0.9	0.9	0.9	0.9	0.9	35.6	1	9	9	25	391	44.1	6.3
NP_001275899.1	572878379	ACSF2	acyl-CoA synthetase family member 2, mitochondrial isoform 4	1.0	0.9	0.9	1.0	1.0	0.9	0.9	0.9	1.0	0.9	0.9	0.9	28.2	5	10	10	19	572	63.6	7.4
NP_387505.1	16445438	BRWD1	bromodomain and WD repeat-containing protein 1 isoform B	1.0	0.9	0.9	0.9	1.0	1.1	0.9	0.9	1.0	1.0	0.9	0.9	1.7	2	2	3	3	2269	257.1	8.4
NP_004772.1	4759300	VAMP3	vesicle-associated membrane protein 3	0.9	0.8	0.8	0.9	1.2	1.1	1.2	1.0	1.1	1.0	1.0	0.9	40.0	4	1	3	32	100	11.3	8.8
NP_001240795.1	359718987	YME1L1	ATP-dependent zinc metalloprotease YME1L1 isoform 4	1.0	0.9	0.9	0.9	0.9	0.9	0.9	0.9	0.9	0.9	0.9	0.9	16.8	3	8	8	20	683	75.9	8.6
NP_001028721.1	75709194;75709192	BABAM1	BRISC and BRCA1-A complex member 1 isoform 1	1.0	1.0	1.0	0.9	1.1	1.0	0.9	1.0	1.1	1.0	0.9	0.9	19.2	2	4	4	15	329	36.5	4.6
NP_542165.1	18375623;4758112	DDX39B	spliceosome RNA helicase DDX39B	0.9	0.9	0.9	0.9	1.0	0.9	1.0	1.0	1.0	0.9	0.9	0.9	58.9	1	10	20	141	428	49.0	5.7
NP_003240.1	4507491	THOP1	thimet oligopeptidase	1.0	0.9	1.0	1.0	1.0	0.8	0.9	0.9	1.0	0.9	1.0	0.9	23.2	1	15	15	32	689	78.8	6.0
NP_001278407.1	612407792	AKAP8L	A-kinase anchor protein 8-like isoform 2	1.2	1.0	1.0	0.9	1.0	1.0	1.0	1.0	1.1	1.0	1.0	0.9	20.2	2	9	9	30	585	65.2	5.1
NP_001122327.1	193082993	GTPBP3	tRNA modification GTPase GTPBP3, mitochondrial isoform III	1.0	1.1	1.2	1.1	1.0	1.1	0.8	0.8	1.0	1.1	1.0	0.9	4.5	4	1	1	2	471	49.8	6.8
NP_001269429.1	543173164;4507711	TTC1	tetratricopeptide repeat protein 1	0.9	1.0	0.9	0.9	0.9	1.0	0.9	0.9	0.9	1.0	0.9	0.9	47.6	1	11	11	44	292	33.5	4.8
NP_803172.1	29171753	PPFIA1	liprin-alpha-1 isoform a	1.0	1.0	0.9	1.0	1.0	0.9	0.9	0.9	1.0	0.9	0.9	0.9	10.8	11	9	10	17	1185	133.9	6.3
NP_005325.2	98986457	HCFC1	host cell factor 1	0.9	0.9	0.9	0.9	0.9	0.9	0.9	0.9	0.9	0.9	0.9	0.9	10.2	1	14	14	32	2035	208.6	7.5
NP_001161396.1	269847750	CMSS1	protein CMSS1 isoform 2	0.9	0.9	0.9	0.9	1.0	1.0	1.0	1.0	1.0	1.0	0.9	0.9	16.9	2	4	4	8	261	29.9	9.5
NP_005225.2	20070199	NR2F6	nuclear receptor subfamily 2 group F member 6	0.9	0.9	0.9	0.9	1.0	0.9	0.9	0.9	0.9	0.9	0.9	0.9	2.5	1	1	1	1	404	43.0	7.8
NP_001011.1	4506691	RPS16	40S ribosomal protein S16	0.9	0.9	0.9	0.9	0.9	1.0	0.9	0.9	0.9	1.0	0.9	0.9	52.7	1	9	9	110	146	16.4	10.2

Khan *et al.* , 2019. Multi-omics analysis to characterize cigarette smoke induced molecular alterations in esophageal cells
Supplementary Table 5. List of proteins quantified in untreated and chronically treated Het1A cells with cigarette smoke condensate for 8 months

NP_Accession	Protein group Accession	Gene ID	Description	Het-1A-Smoke - 2M/Parental	Het-1A-Smoke - 4M/Parental	Het-1A-Smoke - 6M/Parental	Het-1A-Smoke - 8M/Parental	Het-1A-Smoke - 2M/Parental	Het-1A-Smoke - 4M/Parental	Het-1A-Smoke - 6M/Parental	Het-1A-Smoke - 8M/Parental	Het-1A-Smoke - 2M/Parental	Het-1A-Smoke - 4M/Parental	Het-1A-Smoke - 6M/Parental	Het-1A-Smoke - 8M/Parental	Coverage (%)	Proteins	Unique Peptides	# Peptides	PSM	# AAs	MW [kDa]	calc. pI
				Replicate 1				Replicate 2				Average of replicates											
NP_001264003.1	459175660	CCDC12	coiled-coil domain-containing protein 12 2	1.0	0.9	1.1	0.9	0.9	0.9	0.9	0.9	1.0	0.9	1.0	0.9	42.2	2	5	5	7	166	19.2	7.3
NP_004799.1	4758816	NMT2	glycylpeptide N-tetradecanoyltransferase 2 isoform 1	1.0	1.1	1.1	0.9	0.8	0.9	1.0	0.9	0.9	1.0	1.0	0.9	14.1	2	2	6	24	498	56.9	7.6
NP_112570.2	16554611	MRPS15	28S ribosomal protein S15, mitochondrial	1.0	1.0	1.0	1.0	1.0	1.0	1.0	0.9	1.0	1.0	1.0	0.9	16.7	1	5	5	11	257	29.8	10.5
NP_060547.2	187960109	RBM28	RNA-binding protein 28 isoform 1	0.8	0.9	0.9	1.0	0.9	0.9	0.9	0.9	0.9	0.9	0.9	0.9	13.0	2	9	9	26	759	85.7	9.2
NP_001484.1	4503971	GDI1	rab GDP dissociation inhibitor alpha	1.2	0.9	1.0	1.0	1.1	0.9	0.9	0.9	1.1	0.9	0.9	0.9	51.0	1	10	16	88	447	50.6	5.1
NP_001230018.1	340545545	FOXM1	forkhead box protein M1 isoform 5	1.0	1.1	1.0	0.9	1.0	1.1	1.2	1.0	1.0	1.1	1.1	0.9	4.6	5	2	2	3	747	82.6	8.4
NP_002798.2	25777600	PSMD1	26S proteasome non-ATPase regulatory subunit 1 isoform 1	1.0	1.0	1.0	0.9	1.0	1.0	1.0	0.9	1.0	1.0	1.0	0.9	47.6	2	35	35	172	953	105.8	5.4
NP_071440.1	11995472	RRAGC	ras-related GTP-binding protein C isoform 1	1.0	0.9	0.9	1.0	1.2	0.9	0.9	0.9	1.1	0.9	0.9	0.9	18.1	3	6	6	9	399	44.2	5.1
NP_003286.1	4507669	TPT1	translationally-controlled tumor protein isoform 2	1.1	0.8	1.0	0.9	1.1	1.0	1.2	1.0	1.1	0.9	1.1	0.9	71.5	4	7	7	29	172	19.6	4.9
NP_002257.1	4504897	KPNA2	importin subunit alpha-1	0.9	0.9	1.0	0.9	0.9	0.9	1.0	0.9	0.9	0.9	1.0	0.9	58.0	1	21	21	171	529	57.8	5.4
NP_076994.2	176866369	KDELC1	KDEL motif-containing protein 1 precursor	0.8	1.0	1.0	1.0	0.9	0.8	1.0	0.9	0.9	0.9	1.0	0.9	1.6	1	1	1	1	502	58.0	7.7
NP_060783.3	93102371	PBRM1	protein polybromo-1	0.9	1.0	1.0	1.0	0.9	0.9	1.0	0.9	0.9	1.0	1.0	0.9	8.9	1	12	13	29	1582	181.0	6.9
NP_000970.1	4506607	RPL18	60S ribosomal protein L18 isoform 1	1.0	1.0	1.0	1.0	0.9	0.9	0.9	0.9	0.9	0.9	0.9	0.9	45.2	2	8	8	51	188	21.6	11.7
NP_004689.1	4758556	PRPF3	U4/U6 small nuclear ribonucleoprotein Prp3	1.0	0.9	1.0	1.0	0.9	0.9	0.9	0.9	0.9	0.9	0.9	0.9	21.8	1	11	11	27	683	77.5	9.5
NP_001157860.1	256600200	RAPGEF6	rap guanine nucleotide exchange factor 6 isoform 4	0.9	0.9	1.0	0.9	1.1	1.0	1.0	1.0	1.0	0.9	1.0	0.9	4.3	7	4	5	14	1504	168.8	6.4
NP_056261.4	262359929	AHCTF1	protein ELYS	1.0	1.0	1.0	0.9	1.0	1.0	1.0	0.9	1.0	1.0	1.0	0.9	18.4	1	31	31	87	2275	253.3	6.6
NP_001124160.1	194688133	HMGB2	high mobility group protein B2	0.9	1.0	1.0	0.9	0.9	1.0	1.0	1.0	0.9	1.0	1.0	0.9	56.0	1	13	17	115	209	24.0	7.8
NP_001653.1	4502209	ARF5	ADP-ribosylation factor 5	1.0	0.9	0.9	1.0	0.9	0.8	0.8	0.9	0.9	0.8	0.8	0.9	56.1	9	3	8	89	180	20.5	6.8
NP_001158730.1	259089438	TMEM14C	transmembrane protein 14C	0.9	1.0	0.9	1.0	0.9	1.0	1.2	0.8	0.9	1.0	1.0	0.9	15.2	1	1	1	7	112	11.6	9.9
NP_001269460.1	542133157;31563503	ADNP	activity-dependent neuroprotector homeobox protein	0.9	0.9	0.9	0.9	0.9	0.9	0.9	0.9	0.9	0.9	0.9	0.9	19.5	1	17	17	45	1102	123.5	7.3
NP_001275652.1	586597940	B3GAT3	galactosylgalactosylxylosylprotein 3-beta-glucuronosyltransferase 3 isoform 4	1.0	1.0	1.0	0.9	1.1	1.1	0.9	1.0	1.1	1.0	1.0	0.9	7.6	4	2	2	2	315	34.6	7.9
NP_056123.2	158534059	FNBP4	formin-binding protein 4	1.1	1.0	1.0	0.9	1.1	1.0	1.0	1.0	1.1	1.0	1.0	0.9	4.0	1	3	3	6	1017	110.2	4.7
NP_612382.1	19923903;66932923	MYADM	myeloid-associated differentiation marker	1.0	0.9	1.1	1.0	1.1	1.0	1.0	0.9	1.0	0.9	1.0	0.9	13.7	1	3	3	8	322	35.3	8.1
NP_001158088.1	257471001	FZD6	frizzled-6 isoform b	1.0	1.0	0.9	0.9	1.0	0.9	0.9	0.9	1.0	0.9	0.9	0.9	1.2	2	1	1	1	674	75.5	8.0
NP_003911.2	222136585	TIMELESS	protein timeless homolog	0.9	0.9	0.9	0.9	0.9	0.9	0.9	1.0	0.9	0.9	0.9	0.9	6.1	1	6	6	9	1208	138.6	5.4
NP_001014.1	4506697	RPS20	40S ribosomal protein S20 isoform 2	1.0	0.9	1.0	0.9	0.9	1.0	0.9	0.9	0.9	1.0	1.0	0.9	58.0	2	7	7	34	119	13.4	9.9
NP_001135890.1	215490021;215490041	MORF4L2	mortality factor 4-like protein 2	1.0	0.9	0.9	1.0	1.0	1.0	0.8	0.9	1.0	1.0	0.9	0.9	16.7	1	3	4	14	288	32.3	9.7
NP_006192.1	5453860	CDK16	cyclin-dependent kinase 16 isoform 1	1.0	0.9	0.9	0.9	1.0	0.8	0.9	0.9	1.0	0.9	0.9	0.9	3.8	56	1	2	3	496	55.7	7.6
NP_005960.1	5174613	NAP1L4	nucleosome assembly protein 1-like 4	1.0	1.1	1.1	0.9	1.0	1.1	1.1	0.9	1.0	1.1	1.1	0.9	50.9	1	13	15	111	375	42.8	4.7
NP_001164120.1	282403491	LAS1L	ribosomal biogenesis protein LAS1L isoform 2	1.0	1.0	0.9	1.0	0.9	0.9	0.9	0.9	0.9	0.9	0.9	0.9	17.2	3	8	8	18	717	81.2	4.7
NP_057474.2	38679912	C9orf114	uncharacterized protein C9orf114	1.0	0.9	0.9	0.9	1.0	0.9	0.8	1.0	1.0	0.9	0.9	0.9	3.2	1	1	1	2	376	42.0	7.4
NP_001107572.1	166235140	SEZ6L2	seizure 6-like protein 2 isoform 4 precursor	0.9	0.8	0.9	0.9	0.9	0.9	0.9	1.0	0.9	0.9	0.9	0.9	15.8	6	6	6	9	809	86.8	4.8
NP_002611.1	4505735	PF4V1	platelet factor 4 variant precursor	0.9	0.9	0.9	0.9	0.9	0.9	0.8	1.0	0.9	0.9	0.9	0.9	10.6	1	1	1	2	104	11.5	9.1
NP_005907.3	33469968	MCM7	DNA replication licensing factor MCM7 isoform 1	1.1	0.9	0.9	0.9	1.1	1.0	1.0	1.0	1.1	1.0	1.0	0.9	46.9	2	29	29	104	719	81.3	6.5
NP_000979.1	4506623	RPL27	60S ribosomal protein L27	0.9	0.9	0.9	0.9	0.9	1.0	1.0	0.9	0.9	1.0	0.9	0.9	52.2	1	6	7	91	136	15.8	10.6
NP_079480.2	33342278	TRABD	traB domain-containing protein	1.0	1.0	1.2	1.0	1.0	1.0	1.0	0.9	1.0	1.0	1.1	0.9	13.3	1	3	3	3	376	42.3	8.0
NP_001163886.1	282165804	TJP2	tight junction protein ZO-2 isoform 4	1.0	1.0	1.2	0.9	1.0	1.0	1.2	0.9	1.0	1.0	1.2	0.9	31.4	6	28	28	69	1157	130.4	7.6
NP_031372.2	33286446	OGFR	opioid growth factor receptor	0.8	1.0	0.9	1.0	0.8	1.0	0.9	0.9	0.8	1.0	0.9	0.9	22.9	1	9	9	22	677	73.3	4.8
NP_001182662.1	307691174	PIP5K1C	phosphatidylinositol 4-phosphate 5-kinase type-1 gamma isoform 1	0.9	1.2	0.9	1.0	1.4	0.8	0.8	0.8	1.2	1.0	0.8	0.9	10.0	5	2	5	10	640	70.2	5.4
NP_055721.3	30089948	PPM1E	protein phosphatase 1E	1.3	1.3	1.4	1.0	1.0	1.2	1.1	0.9	1.1	1.2	1.2	0.9	2.4	1	1	1	1	755	83.9	5.0
NP_060200.2	38570097	QRICH1	glutamine-rich protein 1	1.2	0.9	0.9	0.9	0.9	1.0	1.1	1.0	1.1	0.9	1.0	0.9	4.5	1	3	3	9	776	86.4	5.9
NP_001035810.1	108773793	G6PD	glucose-6-phosphate 1-dehydrogenase isoform b	1.2	1.1	0.9	0.9	1.2	1.0	0.9	0.9	1.2	1.1	0.9	0.9	59.0	2	26	26	111	515	59.2	6.8
NP_001070152.1	115529449	UBTF	nucleolar transcription factor 1 isoform b	0.8	0.9	0.9	0.9	0.8	0.9	0.8	0.9	0.8	0.9	0.9	0.9	21.2	2	14	14	26	727	84.9	5.6
NP_001291738.1	756141033;11321642	BRD1	bromodomain-containing protein 1 isoform 2	1.0	0.9	0.9	0.9	1.0	0.9	0.9	0.9	1.0	0.9	0.9	0.9	3.5	3	4	4	5	1058	119.4	8.6
NP_055118.1	7657455	PES1	pescadillo homolog isoform 1	0.9	1.0	1.0	0.9	0.9	1.0	1.0	1.0	0.9	1.0	1.0	0.9	24.7	3	14	14	46	588	68.0	7.3
NP_006100.2	20070220	PRMT5	protein arginine N-methyltransferase 5 isoform a	1.0	0.9	0.9	0.9	0.9	0.9	0.9	0.9	0.9	0.9	0.9	0.9	38.2	6	19	19	53	637	72.6	6.3
NP_060676.2	17999541	VPS35	vacuolar protein sorting-associated protein 35	1.3	1.0	1.1	0.9	1.0	1.0	1.1	1.0	1.2	1.0	1.1	0.9	40.5	1	24	24	107	796	91.6	5.5
NP_001274151.1	564473387	ARPC3	actin-related protein 2/3 complex subunit 3 isoform 2	0.9	1.0	1.0	1.0	1.0	1.0	1.0	0.9	1.0	1.0	1.0	0.9	11.3	2	2	2	16	177	20.4	8.8
NP_003669.4	50959085;50959115	THOC5	THO complex subunit 5 homolog	1.1	1.0	1.0	1.0	1.0	0.9	0.9	0.9	1.0	0.9	1.0	0.9	15.1	1	7	7	8	683	78.5	6.9
NP_004940.1	13435366	DSC2	desmocollin-2 isoform Dsc2b preproprotein	0.8	1.0	0.8	1.0	0.6	0.8	0.8	0.9	0.7	0.9	0.8	0.9	1.2	2	1	1	6	847	93.7	5.5
NP_061989.2	77917604	UBFD1	ubiquitin domain-containing protein UBFD1	0.9	0.9	1.1	0.9	1.1	1.0	1.3	0.9	1.0	0.9	1.2	0.9	9.1	1	3	3	7	309	33.4	5.8
NP_004374.1	4758078;187475373	CSK	tyrosine-protein kinase CSK	0.9	0.9	0.9	0.9	0.9	0.9	0.9	0.9	0.9	0.9	0.9	0.9	25.8	1	10	10	31	450	50.7	7.1
NP_001193996.1	333470717	BZW1	basic leucine zipper and W2 domain-containing protein 1 isoform 1	0.9	0.9	0.9	0.9	0.9	0.9	1.0	0.9	0.9	0.9	1.0	0.9	45.4	4	15	17	70	419	48.0	5.9
NP_060206.2	42476024	THUMPD1	THUMP domain-containing protein 1	0.8	0.9	0.8	0.9	0.9	0.9	1.0	1.0	0.9	0.9	0.9	0.9	32.9	1	9	9	33	353	39.3	7.9
NP_064516.2	40538799	UBQLN4	ubiquilin-4 isoform 1	0.9	0.8	0.9	1.0	1.0	0.9	0.9	0.9	0.9	0.8	0.9	0.9	4.8	2	1	2	10	601	63.8	5.2
NP_115914.1	14210536	TUBB6	tubulin beta-6 chain isoform 1	1.0	0.9	1.0	0.9	1.0	0.9	1.0	0.9	1.0	0.9	1.0	0.9	67.9	6	8	20	232	446	49.8	4.9
NP_009042.1	6005900	TFDP1	transcription factor Dp-1	0.8	0.7	0.8	0.8	0.9	1.0	0.9	1.0	0.8	0.9	0.9	0.9	3.7	1	1	1	2	410	45.0	6.0
NP_003809.1	20143480	CDS2	phosphatidate cytidylyltransferase 2	0.9	0.9	0.8	0.8	1.1	1.1	1.1	1.1	1.0	1.0	1.0	0.9	8.1	1	2	2	3	445	51.4	7.1
NP_976240.1	42734503	CAPRIN1	caprin-1 isoform 2	0.8	0.9	0.9	0.9	0.8	0.9	0.9	0.9	0.8	0.9	0.9	0.9	21.5	2	11	11	62	694	76.8	5.1
NP_005266.2	38788319	GNL1	guanine nucleotide-binding protein-like 1	1.2	0.9	0.9	0.9	0.9	0.9	0.9	0.9	1.1	0.9	0.9	0.9	35.4	1	17	17	46	607	68.6	5.8
NP_036555.1	6912634	RPL13A	60S ribosomal protein L13a isoform 1	0.9	0.9	0.9	0.9	0.9	1.0	0.9	1.0	0.9	0.9	0.9	0.9	45.3	2	13	13	91	203	23.6	10.9
NP_775823.1	27734895	C3orf58	deleted in autism protein 1 isoform a precursor	1.1	1.0	1.1	1.0	1.0	0.9	1.0	0.9	1.0	1.0	1.0	0.9	7.4	2	2	2	4	430	49.5	8.5
NP_005487.3	50658065;50658063	SMC4	structural maintenance of chromosomes protein 4 isoform 1	1.0	0.9	0.9	0.9	1.0	1.0	0.9	0.9	1.0	0.9	0.9	0.9	35.8	2	39	39	102	1288	147.1	6.8
NP_563615.3	54792129	DCBLD2	discoidin, CUB and LCCL domain-containing protein 2 precursor	0.9	0.9	1.2	0.9	0.9	1.0	1.1	0.9	0.9	1.0	1.1	0.9	1.0	1	1	1	1	775	85.0	7.2
NP_000967.1	4506597	RPL12	60S ribosomal protein L12	1.0	0.9	0.9	0.9	0.9	1.0	1.0	1.0	1.0	0.9	0.9	0.9	66.7	1	9	9	71	165	17.8	9.4
NP_741982.1	26051233	LACTB	serine beta-lactamase-like protein LACTB, mitochondrial isoform b precursor	0.9	1.0	1.0	1.0	1.1	1.0	1.0	0.9	1.0	1.0	1.0	0.9	14.5	3	3	3	9	373	41.2	8.2
NP_006836.2	166795250	KIF2C	kinesin-like protein KIF2C isoform 1	0.8	0.8	0.8	0.9	0.9	0.9	0.9	0.9	0.9	0.9	0.9	0.9	25.7	5	15	17	48	725	81.3	7.8
NP_001242943.1	365192539	PSMC3IP	homologous-pairing protein 2 homolog isoform 3	1.1	1.1	0.8	0.9	1.0	0.9	0.8	1.0	1.0	1.0	0.8	0.9	14.9	3	1	1	2	154	18.0	5.5
NP_001894.2	55770844	CTNNA1	catenin alpha-1 isoform 1	0.9	0.9	1.0	0.9	0.9	1.0	1.0	0.9	0.9	1.0	1.0	0.9	45.7	6	26	31	111	906	100.0	6.3
NP_003363.1	4507873	VBP1	prefoldin subunit 3	0.9	1.0	1.0	0.9	1.0	1.0	1.1	1.0	1.0	1.0	1.0	0.9	27.4	2	4	5	8	197	22.6	7.1
NP_783297.2	116875765	TJP1	tight junction protein ZO-1 isoform b	0.9	1.0	1.0	0.9	1.1	1.1	1.0	1.0	1.0	1.1	1.0	0.9	12.9	4	16	16	42	1668	186.9	6.8
NP_001186627.1	314122233	BAG6	large proline-rich protein BAG6 isoform d	1.0	0.9	0.9	0.9	0.9	1.0	0.9	1.0	1.0	0.9	0.9	0.9	21.0	4	10	10	26	1077	113.4	5.7
NP_982288.1	45007002	CERS6	ceramide synthase 6 isoform 2	1.1	0.8	1.0	1.0	1.0	0.8	1.0	0.9	1.0	0.8	1.0	0.9	5.2	2	1	1	5	384	44.9	7.6
NP_940993.1	38372923	BSG	basigin isoform 4	0.9	0.9	0.9	0.9	0.9	1.0	0.9	0.9	0.9	1.0	0.9	0.9	50.7	4	7	7	39	205	22.8	5.5
NP_004932.1	4826690	DHX8	ATP-dependent RNA helicase DHX8 isoform 1	0.9	1.1	1.0	1.0	1.0	0.9	1.0	0.8	1.0	1.0	1.0	0.9	8.0	2	6	7	16	1220	139.2	8.3
NP_071922.2	32130514	NDRG3	protein NDRG3 isoform b	0.8	0.8	0.8	0.9	0.8	0.9	0.8	1.0	0.8	0.9	0.8	0.9	15.2	2	3	3	8	363	40.0	5.4
NP_932332.1	37620194	GNPNAT1	glucosamine 6-phosphate N-acetyltransferase	1.2	1.0	1.1	1.0	1.0	0.8	0.9	0.9	1.1	0.9	1.0	0.9	50.5	1	5	5	14	184	20.7	8.0
NP_056451.3	78000156	EIF2B4	translation initiation factor eIF-2B subunit delta isoform 3	1.0	0.9	0.9	0.9	0.9	0.9	0.9	0.9	0.9	0.9	0.9	0.9	24.0	3	8	8	17	522	57.4	9.4
NP_003454.1	4506283	PTP4A1	protein tyrosine phosphatase type IVA 1	0.6	0.7	0.7	0.8	1.0	1.0	1.0	1.1	0.8	0.9	0.8	0.9	25.4	1	2	2	7	173	19.8	9.0
NP_001239268.1	356640224	CDH4	cadherin-4 isoform 3	0.9	0.8	0.8	0.9	1.1	1.0	1.1	1.0	1.0	0.9	0.9	0.9	3.3	3	2	2	4	842	92.3	4.8
NP_061945.1	24308141	SGTB	small glutamine-rich tetratricopeptide repeat-containing protein beta	1.1	1.0	1.1	1.0	1.1	1.0	1.2	0.9	1.1	1.0	1.1	0.9	28.0	1	5	5	15	304	33.4	4.9
NP_775956.1	27734761	NSMCE2	E3 SUMO-protein ligase NSE2	1.0	1.0	1.0	1.0	0.8	0.8	0.8	0.9	0.9	0.9	0.9	0.9	20.2	1	3	3	3	247	27.9	7.7
NP_002939.2	15431293;358356398	RPL15	60S ribosomal protein L15 isoform 1	0.9	0.9	0.9	0.9	0.9	0.9	0.9	0.9	0.9	0.9	0.9	0.9	43.1	2	9	9	56	204	24.1	11.6
NP_003225.2	189458817	TFRC	transferrin receptor protein 1	0.8	0.9	0.9	0.9	0.9	0.9	0.9	1.0	0.9	0.9	0.9	0.9	40.9	3	25	25	85	760	84.8	6.6
NP_000089.1	4503023	CPT2	carnitine O-palmitoyltransferase 2, mitochondrial precursor	0.9	0.9	0.9	0.8	1.1	1.0	1.0	1.0	1.0	1.0	1.0	0.9	24.2	1	11	11	23	658	73.7	8.2
NP_001180245.1	300934878	VIPAS39	spermatogenesis-defective protein 39 homolog isoform 2	0.9	0.8	0.8	0.9	1.0	0.9	0.8	0.9	1.0	0.8	0.8	0.9	16.7	2	5	5	6	444	51.5	6.9
NP_110445.1	13540614	CCDC130	coiled-coil domain-containing protein 130	0.9	0.8	0.8	1.0	1.1	0.9	1.0	0.9	1.0	0.9	0.9	0.9	7.8	1	2	2	2	396	44.8	8.2
NP_065998.3	205360930	TLDC1	TLD domain-containing protein 1	0.9	1.0	1.2	0.8	1.1	1.2	1.2	1.0	1.0	1.1	1.2	0.9	5.5	1	1	1	2	456	51.0	6.2

Khan *et al.* , 2019. Multi-omics analysis to characterize cigarette smoke induced molecular alterations in esophageal cells
Supplementary Table 5. List of proteins quantified in untreated and chronically treated Het1A cells with cigarette smoke condensate for 8 months

NP_Accession	Protein group Accession	Gene ID	Description	Het-1A-Smoke - 2M/Parental	Het-1A-Smoke - 4M/Parental	Het-1A-Smoke - 6M/Parental	Het-1A-Smoke - 8M/Parental	Het-1A-Smoke - 2M/Parental	Het-1A-Smoke - 4M/Parental	Het-1A-Smoke - 6M/Parental	Het-1A-Smoke - 8M/Parental	Het-1A-Smoke - 2M/Parental	Het-1A-Smoke - 4M/Parental	Het-1A-Smoke - 6M/Parental	Het-1A-Smoke - 8M/Parental	Coverage (%)	Proteins	Unique Peptides	# Peptides	PSM	# AAs	MW [kDa]	calc. pI
				Replicate 1				Replicate 2				Average of replicates											
NP_036243.1	6912280	AHSA1	activator of 90 kDa heat shock protein ATPase homolog 1	1.0	0.8	0.9	0.9	1.0	0.9	0.9	0.9	1.0	0.9	0.9	0.9	54.4	1	15	15	68	338	38.3	5.5
NP_001241.1	4507581	CD40	tumor necrosis factor receptor superfamily member 5 isoform 1 precursor	1.5	1.0	1.0	1.0	1.1	1.0	1.0	0.9	1.3	1.0	1.0	0.9	27.8	3	4	4	11	277	30.6	5.8
NP_000982.2	13904866	RPL28	60S ribosomal protein L28 isoform 2	0.9	0.9	0.9	0.9	0.9	0.9	0.9	0.9	0.9	0.9	0.9	0.9	36.5	5	6	6	21	137	15.7	12.0
NP_057475.2	148747209	NOP16	nucleolar protein 16 isoform 3	1.2	0.9	0.9	0.9	0.8	1.0	0.9	0.9	1.0	1.0	0.9	0.9	41.0	7	7	7	30	178	21.2	9.9
NP_002746.1	5579478	MAP2K1	dual specificity mitogen-activated protein kinase kinase 1	1.0	1.1	1.1	0.9	0.9	1.0	1.0	0.9	1.0	1.0	1.0	0.9	44.3	1	7	13	48	393	43.4	6.6
NP_004321.2	168480080	BNIP2	BCL2/adenovirus E1B 19 kDa protein-interacting protein 2	0.7	0.8	0.8	0.9	0.8	0.8	0.9	1.0	0.7	0.8	0.8	0.9	10.3	1	2	2	12	435	48.8	4.8
NP_115984.3	140972063	PPP1R9B	neurabin-2	1.0	1.2	1.0	1.0	1.1	0.9	0.9	0.9	1.1	1.0	0.9	0.9	5.6	6	4	4	8	817	89.3	5.0
NP_852669.1	31563514	NAA20	N-alpha-acetyltransferase 20 isoform c	1.0	1.1	1.1	1.0	0.9	0.9	0.9	0.9	1.0	1.0	1.0	0.9	8.1	2	1	1	1	111	12.7	4.7
NP_060024.2	154813199	PARP14	poly [ADP-ribose] polymerase 14	0.8	0.8	0.7	0.8	1.0	1.0	1.0	1.0	0.9	0.9	0.9	0.9	1.7	1	2	2	4	1801	202.7	7.2
NP_000468.1	4502027	ALB	serum albumin preproprotein	0.7	0.9	0.7	0.9	0.9	1.1	0.9	1.0	0.8	1.0	0.8	0.9	13.5	1	4	9	25	609	69.3	6.3
NP_009148.2	20127517;22538794	PDCD10	programmed cell death protein 10	1.0	0.9	0.9	0.9	1.0	1.0	1.0	1.0	1.0	0.9	1.0	0.9	19.3	1	4	4	15	212	24.7	8.2
NP_006046.1	5174445;212274337	LANCL1	lanC-like protein 1	1.1	1.0	0.9	0.9	1.1	1.0	1.0	0.9	1.1	1.0	1.0	0.9	27.8	1	9	9	22	399	45.3	7.8
NP_056326.2	18034690;428977337	AAR2	protein AAR2 homolog	0.9	0.8	0.9	0.9	0.8	0.8	0.8	0.9	0.8	0.8	0.9	0.9	15.1	1	4	4	11	384	43.4	6.0
NP_000961.2	16753227	RPL6	60S ribosomal protein L6	0.9	0.9	0.9	0.9	0.9	0.9	0.9	0.9	0.9	0.9	0.9	0.9	44.8	1	14	14	144	288	32.7	10.6
NP_060271.1	8923370	CMTM6	CKLF-like MARVEL transmembrane domain-containing protein 6	0.9	0.9	0.9	0.9	0.9	0.9	0.9	1.0	0.9	0.9	0.9	0.9	9.3	1	2	2	3	183	20.4	5.3
NP_001136026.1	216548445	TRMT1	tRNA (guanine(26)-N(2))-dimethyltransferase isoform 2	1.0	0.9	0.8	0.9	1.1	0.9	0.9	0.9	1.0	0.9	0.8	0.9	17.0	2	8	8	23	630	69.3	7.2
NP_061329.3	51036603	GNG12	guanine nucleotide-binding protein G(I)/G(S)/G(O) subunit gamma-12 precursor	2.1	1.0	1.1	1.0	1.2	0.9	1.0	0.8	1.7	0.9	1.0	0.9	51.4	1	3	3	22	72	8.0	9.0
NP_004680.2	115527080	MTA1	metastasis-associated protein MTA1 isoform MTA1	0.9	0.8	0.9	0.9	0.9	0.8	0.9	0.9	0.9	0.8	0.9	0.9	32.5	2	13	16	37	715	80.7	9.3
NP_001193988.1	333470760	VWA9	von Willebrand factor A domain-containing protein 9 isoform 5	0.9	0.9	1.0	0.9	1.1	1.0	1.1	0.9	1.0	1.0	1.0	0.9	7.6	2	2	2	4	461	50.7	5.1
NP_006568.2	9845238	B3GNT2	UDP-GlcNAc:betaGal beta-1,3-N-acetylglucosaminyltransferase 2	0.9	0.9	0.9	0.8	1.1	1.1	1.2	1.0	1.0	1.0	1.1	0.9	1.8	1	1	1	1	397	46.0	8.5
NP_001006942.1	55743086	ALG3	dol-P-Man:Man(5)GlcNAc(2)-PP-Dol alpha-1,3-mannosyltransferase isoform d	1.2	0.9	1.2	0.9	1.1	0.9	1.1	1.0	1.2	0.9	1.1	0.9	2.6	2	1	1	1	390	44.3	8.9
NP_001166969.1	291045220	MAN2B1	lysosomal alpha-mannosidase isoform 2 precursor	1.3	0.9	1.0	1.0	1.0	0.9	1.0	0.9	1.2	0.9	1.0	0.9	4.3	2	3	3	9	1010	113.5	7.3
NP_057154.2	16554604	MRPS23	28S ribosomal protein S23, mitochondrial	1.0	0.9	0.9	0.9	1.0	0.9	0.9	0.9	1.0	0.9	0.9	0.9	57.9	1	9	9	19	190	21.8	8.9
NP_004885.1	4758940	C14orf2	6.8 kDa mitochondrial proteolipid isoform 1	0.8	0.7	0.8	0.8	1.2	1.1	1.2	1.0	1.0	0.9	1.0	0.9	12.1	1	1	1	1	58	6.7	10.1
NP_001264.2	51599156	CHD4	chromodomain-helicase-DNA-binding protein 4 isoform 1	0.9	0.9	0.9	0.9	0.9	0.9	1.0	0.9	0.9	0.9	0.9	0.9	33.9	2	39	47	190	1912	217.9	5.9
NP_003127.1	4507215	SRP54	signal recognition particle 54 kDa protein isoform 1	1.0	1.0	1.1	0.9	1.0	1.0	1.0	1.0	1.0	1.0	1.0	0.9	20.0	2	8	8	19	504	55.7	8.7
NP_001186687.1	315113868	MTHFS	5-formyltetrahydrofolate cyclo-ligase isoform b	1.2	0.9	0.9	0.9	0.9	0.9	0.9	0.9	1.0	0.9	0.9	0.9	38.4	3	4	4	21	146	16.8	5.0
NP_006521.1	5729838	YEATS4	YEATS domain-containing protein 4 isoform 1	1.1	0.8	0.9	0.9	0.9	0.9	0.9	0.9	1.0	0.9	0.9	0.9	25.6	1	4	4	5	227	26.5	8.4
NP_002107.3	24797067	HLA-A	HLA class I histocompatibility antigen, A-1 alpha chain precursor	1.0	1.1	1.1	0.9	1.0	1.1	1.1	1.0	1.0	1.1	1.1	0.9	14.5	3	3	5	25	365	40.8	6.0
NP_003650.1	4501993	AGPS	alkyldihydroxyacetonephosphate synthase, peroxisomal precursor	1.0	1.1	1.0	0.9	1.1	1.0	1.0	0.9	1.1	1.0	1.0	0.9	31.9	1	15	15	30	658	72.9	7.3
NP_001005751.1	54124343	FAM21A	WASH complex subunit FAM21A isoform 1	1.0	1.3	1.0	1.0	1.0	1.0	0.9	0.9	1.0	1.2	1.0	0.9	16.8	2	2	14	49	1341	147.1	4.8
NP_620636.1	21071054;21071052	HLTF	helicase-like transcription factor	1.0	1.0	1.0	1.0	1.1	1.0	1.1	0.9	1.1	1.0	1.1	0.9	21.8	1	16	16	34	1009	113.9	8.6
NP_733827.2	31563383	SGK3	serine/threonine-protein kinase Sgk3 isoform 2	1.0	1.1	1.3	1.0	1.1	0.9	1.1	0.9	1.1	1.0	1.2	0.9	5.0	2	1	1	1	464	53.3	7.2
NP_148980.2	74027251	TRIM33	E3 ubiquitin-protein ligase TRIM33 isoform beta	1.0	1.0	0.9	1.0	0.9	0.9	0.9	0.9	1.0	0.9	0.9	0.9	9.8	2	6	7	10	1110	120.5	6.7
NP_055568.3	224589071	BMS1	ribosome biogenesis protein BMS1 homolog	1.0	1.0	1.0	0.9	1.0	1.0	1.0	0.9	1.0	1.0	1.0	0.9	24.0	5	19	19	54	1282	145.7	6.4
NP_056286.3	110349759	NSL1	kinetochore-associated protein NSL1 homolog isoform 1	1.0	1.0	0.9	0.9	0.9	0.9	0.9	0.9	1.0	0.9	0.9	0.9	10.3	2	2	2	3	281	32.1	6.8
NP_002815.3	46276863	PTMS	parathymosin	1.0	1.1	1.1	0.9	1.0	1.3	1.2	1.0	1.0	1.2	1.2	0.9	22.6	1	3	3	41	102	11.5	4.2
NP_056982.1	7705375	ZBTB7A	zinc finger and BTB domain-containing protein 7A	1.7	1.9	1.5	0.8	1.5	1.1	1.1	1.0	1.6	1.5	1.3	0.9	5.5	1	2	2	4	584	61.4	5.2
NP_055870.2	93102364	SWAP70	switch-associated protein 70	1.0	1.0	1.0	0.9	1.0	1.0	1.0	0.9	1.0	1.0	1.0	0.9	26.3	2	13	13	31	585	69.0	5.9
NP_000962.2	15431301	RPL7	60S ribosomal protein L7	0.9	0.9	0.9	0.9	0.9	0.9	0.9	0.9	0.9	0.9	0.9	0.9	53.2	3	16	16	137	248	29.2	10.7
NP_054749.2	41393573	GIT1	ARF GTPase-activating protein GIT1 isoform 2	0.9	0.9	0.9	0.9	0.9	0.9	0.9	0.9	0.9	0.9	0.9	0.9	6.3	2	3	3	6	761	84.3	6.8
NP_001243479.1	375065852	MPP5	MAGUK p55 subfamily member 5 isoform 2	0.9	0.9	0.8	0.9	0.9	0.9	0.9	0.9	0.9	0.9	0.9	0.9	5.0	2	3	3	8	641	73.4	6.3
NP_001012474.1	60279265	SEC61G	protein transport protein Sec61 subunit gamma	1.0	1.0	1.1	0.9	1.2	1.0	1.4	0.9	1.1	1.0	1.2	0.9	19.1	1	1	1	3	68	7.7	10.0
NP_003109.1	4507171	SPARC	SPARC precursor	1.1	1.1	1.2	0.9	1.1	1.0	1.1	0.9	1.1	1.1	1.1	0.9	30.0	1	6	6	20	303	34.6	4.8
NP_001180343.1	301171345	TUBA8	tubulin alpha-8 chain isoform 2	1.0	0.8	1.0	0.9	0.9	0.9	0.9	1.0	0.9	0.9	1.0	0.9	40.5	3	1	12	373	383	42.9	5.1
NP_001073922.2	160948599	INTS1	integrator complex subunit 1	1.0	1.0	0.9	0.9	1.1	1.0	1.0	0.9	1.0	1.0	1.0	0.9	4.3	1	6	6	11	2190	244.1	6.1
NP_002735.3	52486327	PRKCZ	protein kinase C zeta type isoform 1	1.2	1.2	1.1	1.1	0.9	0.7	0.8	0.8	1.0	0.9	0.9	0.9	4.4	1	1	1	1	592	67.6	5.8
NP_057301.1	7706435	HECA	headcase protein homolog	1.0	0.9	1.0	0.9	1.7	1.0	1.0	0.9	1.3	1.0	1.0	0.9	3.9	1	1	1	2	543	58.8	8.5
NP_001281269.1	661567300	ZC3H18	zinc finger CCCH domain-containing protein 18 isoform 1	0.8	1.0	0.9	1.0	0.8	0.9	0.8	0.9	0.8	0.9	0.8	0.9	8.3	1	1	6	13	977	108.9	8.4
NP_001003.1	4506743	RPS8	40S ribosomal protein S8	0.9	0.9	0.9	0.9	0.9	0.9	0.9	0.9	0.9	0.9	0.9	0.9	51.4	3	10	10	89	208	24.2	10.3
NP_115825.1	61742814	CC2D1B	coiled-coil and C2 domain-containing protein 1B	0.9	0.9	0.8	0.8	1.0	1.1	1.0	1.0	0.9	1.0	0.9	0.9	11.7	1	8	8	12	858	94.2	5.3
NP_057105.2	37577122	UBE2J1	ubiquitin-conjugating enzyme E2 J1	1.1	1.1	1.3	1.0	1.1	1.0	1.0	0.9	1.1	1.1	1.1	0.9	2.2	1	1	1	1	318	35.2	6.7
NP_005110.2	167234419	THRAP3	thyroid hormone receptor-associated protein 3	1.0	1.0	0.9	0.9	0.9	1.0	0.9	0.9	0.9	1.0	0.9	0.9	24.9	1	24	24	89	955	108.6	10.2
NP_056271.2	20070260	NELFB	negative elongation factor B	1.0	1.0	1.0	1.0	0.9	0.9	0.9	0.9	0.9	0.9	0.9	0.9	22.6	1	9	9	28	580	65.7	6.1
NP_005531.2	113722125	INPP5B	type II inositol 1,4,5-trisphosphate 5-phosphatase precursor	1.0	0.8	0.9	0.9	1.0	0.9	1.0	0.9	1.0	0.9	0.9	0.9	2.9	2	2	2	3	913	103.9	5.4
NP_689926.1	22749415	STT3A	dolichyl-diphosphooligosaccharide--protein glycosyltransferase subunit STT3A isoform a	1.0	1.0	1.0	0.9	1.1	0.9	1.0	0.9	1.1	1.0	1.0	0.9	21.6	2	15	16	36	705	80.5	8.1
NP_835227.1	30023820	FAM134C	protein FAM134C	0.9	0.9	0.9	1.0	1.0	1.0	0.9	0.9	0.9	0.9	0.9	0.9	9.9	1	2	2	4	466	51.4	5.0
NP_490647.1	17388799	DNAJB6	dnaJ homolog subfamily B member 6 isoform a	0.9	1.0	1.1	0.9	1.0	1.1	1.1	1.0	1.0	1.0	1.1	0.9	41.4	4	8	9	55	326	36.1	9.2
NP_115812.1	41281612;255918139	CHAMP1	chromosome alignment-maintaining phosphoprotein 1	0.9	0.9	1.0	0.9	1.0	1.0	1.1	1.0	1.0	1.0	1.0	0.9	8.5	1	6	6	10	812	89.0	8.4
NP_078932.2	31377697	COLGALT1	procollagen galactosyltransferase 1 precursor	0.9	1.0	1.0	1.0	0.9	1.0	1.0	0.9	0.9	1.0	1.0	0.9	24.4	4	14	14	48	622	71.6	7.3
NP_001018238.1	66363694	HAX1	HCLS1-associated protein X-1 isoform b	1.1	0.9	0.9	1.0	0.9	0.9	0.9	0.9	1.0	0.9	0.9	0.9	10.0	2	2	2	3	231	26.1	5.2
NP_078824.2	31377705	CEP97	centrosomal protein of 97 kDa isoform 1	0.9	1.1	0.9	0.9	1.0	1.0	1.0	1.0	1.0	1.0	1.0	0.9	7.3	2	5	5	12	865	96.9	5.0
NP_003592.3	325651836	SMARCA5	SWI/SNF-related matrix-associated actin-dependent regulator of chromatin subfamily A member 5	1.0	0.9	1.0	0.9	1.0	1.0	1.0	1.0	1.0	0.9	1.0	0.9	24.0	4	21	21	72	1052	121.8	8.1
NP_001274436.1	566559963	PPCS	phosphopantothenate--cysteine ligase isoform c	1.0	0.9	0.9	0.9	1.0	0.9	0.9	0.9	1.0	0.9	0.9	0.9	27.6	3	3	3	9	105	12.0	6.6
NP_942089.1	38570135	MAP4K5	mitogen-activated protein kinase kinase kinase kinase 5	0.9	0.8	0.8	0.9	1.3	1.0	0.9	1.0	1.1	0.9	0.8	0.9	1.7	1	1	1	1	846	95.0	7.7
NP_003898.2	83267879	EIF2B5	translation initiation factor eIF-2B subunit epsilon	1.0	1.0	0.9	0.9	1.1	1.0	1.0	1.0	1.0	1.0	1.0	0.9	24.6	1	10	10	31	721	80.3	5.1
NP_056651.1	7710154	WARS2	tryptophan--tRNA ligase, mitochondrial isoform 1 precursor	1.2	0.9	0.9	0.9	1.0	0.9	0.9	1.0	1.1	0.9	0.9	0.9	2.8	1	1	1	2	360	40.1	9.3
NP_085153.1	38176151	KIAA1715	protein lunapark isoform 4	1.0	0.9	1.0	0.9	1.1	1.0	1.0	1.0	1.1	1.0	1.0	0.9	19.4	5	4	4	6	428	47.7	5.1
NP_005678.3	124028525	FARSB	phenylalanine--tRNA ligase beta subunit	1.1	0.9	1.0	0.9	1.1	1.0	1.0	0.9	1.1	0.9	1.0	0.9	34.6	1	15	16	73	589	66.1	6.8
NP_015627.1	6594683	WHSC1	histone-lysine N-methyltransferase NSD2 isoform 4	0.8	0.8	0.9	0.9	0.9	0.9	1.0	0.9	0.8	0.9	0.9	0.9	1.3	3	1	1	1	629	69.4	9.3
NP_001093.1	4501891	ACTN1	alpha-actinin-1 isoform b	0.9	0.9	0.9	0.9	1.0	0.9	0.9	0.9	0.9	0.9	0.9	0.9	71.1	6	35	54	660	892	103.0	5.4
NP_064583.2	171906582	CCDC47	coiled-coil domain-containing protein 47 precursor	1.0	1.1	1.0	1.0	1.0	1.0	1.0	0.9	1.0	1.0	1.0	0.9	35.4	1	13	13	60	483	55.8	4.9
NP_002922.2	51479192	RING1	E3 ubiquitin-protein ligase RING1	1.1	1.1	1.1	1.0	0.9	0.9	0.8	0.9	1.0	1.0	0.9	0.9	16.5	2	4	4	6	406	42.4	5.6
NP_877429.2	38679907	TPRG1L	tumor protein p63-regulated gene 1-like protein	1.0	1.0	1.0	0.9	1.0	1.2	1.2	1.0	1.0	1.1	1.1	0.9	6.6	1	1	1	1	272	30.2	7.3
NP_002684.1	4505937	POLG	DNA polymerase subunit gamma-1	0.9	1.0	0.8	0.9	1.2	1.0	1.0	1.0	1.1	1.0	0.9	0.9	1.5	1	1	1	1	1239	139.5	6.9
NP_036617.2	217416398	HMMR	hyaluronan mediated motility receptor isoform c	1.0	1.2	1.1	0.9	1.0	1.1	1.0	0.9	1.0	1.1	1.0	0.9	32.0	4	19	20	59	709	82.3	5.9
NP_001271209.1	546231920	SMEK1	serine/threonine-protein phosphatase 4 regulatory subunit 3A isoform 1	0.9	0.9	0.9	1.0	0.8	0.8	0.8	0.9	0.9	0.8	0.8	0.9	27.9	2	11	16	66	820	93.8	4.9
NP_003396.1	4507951	YWHAH	14-3-3 protein eta	1.0	1.0	1.0	0.9	1.1	1.1	1.0	0.9	1.0	1.0	1.0	0.9	69.9	1	12	17	213	246	28.2	4.8
NP_001016.1	4506701	RPS23	40S ribosomal protein S23	1.0	1.0	1.0	0.9	0.9	0.9	1.0	0.9	1.0	1.0	1.0	0.9	57.3	3	9	9	183	143	15.8	10.5
NP_001018066.1	65301164	VLDLR	very low-density lipoprotein receptor isoform b precursor	1.0	1.0	0.8	0.9	0.9	0.9	0.8	0.9	0.9	0.9	0.8	0.9	2.5	2	2	2	3	845	93.3	4.8
NP_004921.1	4826659	CAPZB	F-actin-capping protein subunit beta isoform 1	1.0	0.9	0.9	0.9	1.1	1.0	1.0	1.0	1.0	0.9	1.0	0.9	54.8	4	13	13	101	272	30.6	6.0
NP_003104.2	19923236	SP100	nuclear autoantigen Sp-100 isoform 2	1.1	1.2	1.3	1.0	1.1	1.1	1.2	0.8	1.1	1.1	1.2	0.9	10.4	9	6	8	23	879	100.4	8.2
NP_060828.2	148612853	ABCF3	ATP-binding cassette sub-family F member 3	1.0	1.0	1.0	0.9	1.0	1.0	1.0	0.9	1.0	1.0	1.0	0.9	14.3	1	6	6	10	709	79.7	6.3
NP_001230367.1	343478201	SPECC1	cytospin-B isoform 5	1.1	1.0	0.9	1.0	0.9	0.9	0.9	0.8	1.0	1.0	0.9	0.9	2.4	5	2	2	3	703	79.0	5.3

Khan *et al.* , 2019. Multi-omics analysis to characterize cigarette smoke induced molecular alterations in esophageal cells

Supplementary Table 5. List of proteins quantified in untreated and chronically treated Het1A cells with cigarette smoke condensate for 8 months

NP_Accession	Protein group Accession	Gene ID	Description	Het-1A-Smoke - 2M/Parental	Het-1A-Smoke - 4M/Parental	Het-1A-Smoke - 6M/Parental	Het-1A-Smoke - 8M/Parental	Het-1A-Smoke - 2M/Parental	Het-1A-Smoke - 4M/Parental	Het-1A-Smoke - 6M/Parental	Het-1A-Smoke - 8M/Parental	Het-1A-Smoke - 2M/Parental	Het-1A-Smoke - 4M/Parental	Het-1A-Smoke - 6M/Parental	Het-1A-Smoke - 8M/Parental	Coverage (%)	Proteins	Unique Peptides	# Peptides	PSM	# AAs	MW [kDa]	calc. pI
				Replicate 1				Replicate 2				Average of replicates											
NP_076980.2	116875840	TSEN34	tRNA-splicing endonuclease subunit Sen34 isoform 1	0.8	0.7	0.8	0.7	1.2	1.1	1.1	1.1	1.0	0.9	1.0	0.9	6.5	2	1	1	2	310	33.6	8.4
NP_055070.1	7657176	CNPY2	protein canopy homolog 2 isoform 1 precursor	1.3	0.9	1.5	1.0	1.9	0.9	2.1	0.9	1.6	0.9	1.8	0.9	22.5	2	3	3	6	182	20.6	4.9
NP_001243194.1	372266129	FLII	protein flightless-1 homolog isoform 3	1.0	1.0	1.0	0.9	1.0	1.0	1.0	1.0	1.0	1.0	1.0	0.9	20.0	3	18	18	45	1214	138.4	6.1
NP_001070867.1	116642885	PNKD	probable hydrolase PNKD isoform 3 precursor	0.9	0.8	0.8	0.9	0.9	1.0	1.0	1.0	0.9	0.9	0.9	0.9	19.7	1	2	2	8	142	15.4	10.6
NP_055388.2	109948302	TMEM97	transmembrane protein 97	0.9	0.9	0.9	1.0	0.9	0.9	0.8	0.9	0.9	0.9	0.9	0.9	8.5	1	2	2	7	176	20.8	9.4
NP_057018.1	7706254	NOP58	nucleolar protein 58	0.9	0.9	1.0	0.9	1.0	0.9	1.0	0.9	0.9	0.9	1.0	0.9	41.0	1	16	16	57	529	59.5	8.9
NP_001164408.1	283135173	FUS	RNA-binding protein FUS isoform 3	0.8	0.7	0.8	0.9	0.9	0.8	0.8	1.0	0.9	0.8	0.8	0.9	26.6	3	9	11	71	522	53.2	9.4
NP_861968.1	32967264	NF2	merlin isoform 7	1.0	1.0	1.1	0.8	1.1	1.0	1.1	1.0	1.0	1.0	1.1	0.9	1.8	5	1	1	4	507	59.1	7.2
NP_001191187.1	324021671	NR3C1	glucocorticoid receptor isoform alpha-B	0.9	0.9	1.0	0.9	1.0	1.0	1.1	1.0	1.0	1.0	1.0	0.9	14.4	13	8	8	16	751	82.8	6.7
NP_001140160.1	226442779	KDM3A	lysine-specific demethylase 3A	1.1	1.1	1.1	1.0	0.9	0.8	0.8	0.8	1.0	1.0	0.9	0.9	1.4	1	1	1	1	1321	147.2	8.1
NP_004466.2	94538362	FLOT2	flotillin-2	0.8	0.8	0.8	0.9	0.8	0.8	0.8	1.0	0.8	0.8	0.8	0.9	23.8	1	10	10	24	428	47.0	5.2
NP_000986.2	16117787	RPL34	60S ribosomal protein L34	1.0	0.9	0.9	0.9	0.9	0.9	1.0	0.9	0.9	0.9	1.0	0.9	41.9	1	6	6	23	117	13.3	11.5
NP_001135750.1	214010185	APLP2	amyloid-like protein 2 isoform 4 precursor	1.1	1.1	1.1	0.9	1.3	1.3	1.2	0.9	1.2	1.2	1.2	0.9	9.2	6	5	5	7	522	59.1	5.6
NP_005975.1	21389315	SLC25A1	tricarboxylate transport protein, mitochondrial isoform a precursor	1.0	1.0	1.1	0.9	1.1	1.0	1.1	0.9	1.0	1.0	1.1	0.9	40.2	3	10	10	63	311	34.0	9.9
NP_001073341.1	121114302	CUL4B	cullin-4B isoform 2	1.0	1.0	1.0	0.9	1.0	1.1	1.1	0.9	1.0	1.0	1.1	0.9	33.1	2	19	27	107	895	102.2	7.9
NP_001123537.1	194272142	MYO9B	unconventional myosin-IXb isoform 2	0.9	1.0	0.9	0.9	0.9	0.9	0.9	0.9	0.9	0.9	0.9	0.9	8.1	2	12	12	24	2022	229.0	8.6
NP_004851.2	259013556	FXR2	fragile X mental retardation syndrome-related protein 2	1.0	1.0	0.9	0.9	1.0	1.1	1.0	0.9	1.0	1.1	1.0	0.9	37.0	1	15	17	35	673	74.2	6.2
NP_059988.3	66346717	HAUS7	HAUS augmin-like complex subunit 7	1.0	0.9	1.0	0.9	1.0	1.1	1.1	1.0	1.0	1.0	1.0	0.9	8.7	1	2	2	2	368	40.8	4.8
NP_005432.1	4885105	CHAF1B	chromatin assembly factor 1 subunit B	0.8	0.8	0.9	0.9	0.9	0.9	0.9	1.0	0.9	0.8	0.9	0.9	9.5	1	4	4	8	559	61.5	7.5
NP_694574.3	48928019	N4BP1	NEDD4-binding protein 1	1.1	1.1	1.1	0.9	1.0	1.1	1.0	1.0	1.1	1.1	1.0	0.9	6.0	1	5	5	8	896	100.3	5.4
NP_002069.2	6715600	GOLGA4	Golgin subfamily A member 4 isoform 2	1.0	0.9	1.0	0.9	1.0	1.0	0.9	0.9	1.0	1.0	1.0	0.9	7.5	2	14	15	32	2230	261.0	5.4
NP_005079.2	41152097	AKAP17A	A-kinase anchor protein 17A isoform 1	0.9	0.9	0.9	0.9	1.1	1.0	1.0	1.0	1.0	1.0	0.9	0.9	2.2	1	2	2	5	695	80.7	9.7
NP_116204.3	46447823	NFATC2IP	NFATC2-interacting protein	0.9	1.0	0.9	0.9	0.9	1.0	1.0	0.9	0.9	1.0	1.0	0.9	36.0	1	8	8	34	419	45.8	6.6
NP_001273187.1	555943914	PKNOX1	homeobox protein PKNOX1 isoform 2	0.8	0.9	0.8	0.9	0.8	1.0	0.9	0.9	0.8	0.9	0.9	0.9	10.3	3	2	2	3	319	34.5	6.3
NP_060418.4	46048234	NOL8	nucleolar protein 8 isoform a	1.1	1.0	1.0	0.9	1.1	1.0	1.0	0.9	1.1	1.0	1.0	0.9	12.7	2	11	11	17	1167	131.5	7.1
NP_998784.1	47717100	ATP6V1H	V-type proton ATPase subunit H isoform 2	1.1	1.1	1.2	0.9	1.1	1.1	1.1	0.9	1.1	1.1	1.1	0.9	11.2	2	4	4	6	465	54.1	6.5
NP_079222.1	13376429	FAM192A	protein FAM192A	1.2	0.9	1.0	1.0	1.0	0.8	0.9	0.9	1.1	0.8	1.0	0.9	16.1	1	3	3	4	254	28.9	5.5
NP_055160.2	120587019	ZNF318	zinc finger protein 318	1.0	1.0	0.9	0.9	1.0	1.0	0.9	0.9	1.0	1.0	0.9	0.9	1.3	1	3	3	4	2279	251.0	7.2
NP_054722.2	17999539	DHX38	pre-mRNA-splicing factor ATP-dependent RNA helicase PRP16	0.9	0.9	0.9	0.9	1.0	0.9	0.9	0.9	1.0	0.9	0.9	0.9	4.8	1	5	5	9	1227	140.4	6.5
NP_665878.2	194440732	GSTZ1	maleylacetoacetate isomerase isoform 2	0.9	0.9	0.8	1.0	0.8	0.8	0.7	0.9	0.9	0.8	0.8	0.9	28.2	3	4	4	12	174	19.4	7.2
NP_000960.2	14591909	RPL5	60S ribosomal protein L5	0.9	0.9	1.0	0.9	0.9	0.9	0.9	0.9	0.9	0.9	0.9	0.9	49.2	1	17	17	138	297	34.3	9.7
NP_006236.1	5453954	PPP2R5D	serine/threonine-protein phosphatase 2A 56 kDa regulatory subunit delta isoform isoform 1	0.9	0.9	0.9	0.9	0.9	0.9	0.9	0.9	0.9	0.9	0.9	0.9	20.4	4	8	10	15	602	69.9	8.1
NP_001018099.1	66346704	ICE2	little elongation complex subunit 2 isoform b	1.0	1.1	0.8	1.0	0.9	0.8	0.8	0.9	0.9	1.0	0.8	0.9	2.1	2	1	1	1	845	94.5	6.3
NP_000959.2	16579885	RPL4	60S ribosomal protein L4	0.9	0.9	0.9	0.9	0.9	0.9	0.9	0.9	0.9	0.9	0.9	0.9	53.9	1	23	24	229	427	47.7	11.1
NP_001070908.1	117938251	BCLAF1	bcl-2-associated transcription factor 1 isoform 2	0.9	0.9	0.9	0.9	0.9	0.9	0.9	0.9	0.9	0.9	0.9	0.9	29.3	2	23	23	77	869	100.2	9.9
NP_795352.2	112382257	INADL	inaD-like protein	0.8	0.5	0.5	0.6	1.0	1.1	1.0	1.2	0.9	0.8	0.8	0.9	0.8	1	2	2	2	1801	196.3	4.9
NP_569707.2	109633039	PTPRF	receptor-type tyrosine-protein phosphatase F isoform 2 precursor	0.9	0.9	0.9	0.9	0.9	0.9	0.9	0.9	0.9	0.9	0.9	0.9	11.0	2	13	15	30	1898	211.6	6.4
NP_001171649.1	296040505	GYG1	glycogenin-1 isoform 2	1.0	1.0	0.9	0.9	0.9	1.0	0.9	0.9	0.9	1.0	0.9	0.9	32.4	3	6	6	17	333	37.5	5.4
NP_071935.1	11968047	POLR1E	DNA-directed RNA polymerase I subunit RPA49 isoform 1	0.9	0.9	1.0	0.9	1.0	1.0	1.0	0.9	1.0	0.9	1.0	0.9	31.5	2	9	9	25	419	47.2	8.9
NP_003103.2	67010025	SP4	transcription factor Sp4	0.8	0.8	0.8	0.8	1.0	1.0	1.0	1.0	0.9	0.9	0.9	0.9	3.2	1	1	1	2	784	81.9	7.0
NP_001030335.1	78214522	RPL38	60S ribosomal protein L38	0.8	0.8	0.8	1.0	0.9	0.8	0.9	0.9	0.9	0.8	0.9	0.9	50.0	3	4	5	16	70	8.2	10.1
NP_067038.1	11034845	RPRD1B	regulation of nuclear pre-mRNA domain-containing protein 1B	0.9	0.9	0.9	1.0	0.9	0.9	0.9	0.9	0.9	0.9	0.9	0.9	36.8	1	7	8	35	326	36.9	6.0
NP_001278274.1	606215171	CDK11B	cyclin-dependent kinase 11B isoform 10	0.8	0.9	1.1	0.9	0.9	0.8	0.9	1.0	0.9	0.9	1.0	0.9	11.3	6	1	7	15	772	90.2	5.4
NP_001159968.1	262073007	SLC16A1	monocarboxylate transporter 1	0.8	0.9	0.9	0.9	1.0	1.0	1.0	1.0	0.9	0.9	0.9	0.9	10.0	1	4	4	12	500	53.9	8.7
NP_001171511.1	295842492	STX3	syntaxin-3 isoform 2	0.9	0.9	0.9	0.8	1.0	1.1	1.1	1.0	1.0	1.0	1.0	0.9	4.7	2	1	1	4	277	32.0	5.5
NP_001245259.1	385251401	EPB41L1	band 4.1-like protein 1 isoform d	1.1	1.0	1.1	1.0	0.9	0.9	0.9	0.9	1.0	1.0	1.0	0.9	8.4	4	2	4	13	701	78.8	7.1
NP_671492.1	22325356	PALM2-AKAP	PALM2-AKAP2 protein isoform 2	1.0	1.0	1.2	0.9	1.0	1.1	1.1	0.9	1.0	1.0	1.1	0.9	17.5	7	13	13	32	1090	120.6	5.0
NP_075046.1	12597631	C12orf43	uncharacterized protein C12orf43 isoform c	0.8	0.9	0.8	0.9	0.9	1.0	0.9	1.0	0.9	1.0	0.9	0.9	34.7	7	7	7	29	262	28.2	9.4
NP_060164.3	144953907	MFSD6	major facilitator superfamily domain-containing protein 6	1.0	1.0	0.9	0.9	0.8	0.7	0.8	0.9	0.9	0.9	0.8	0.9	3.3	1	1	1	1	791	88.0	5.5
NP_001243755.1	378744165	PAF1	RNA polymerase II-associated factor 1 homolog isoform 2	0.9	1.0	0.9	0.9	1.0	1.0	0.9	0.9	0.9	1.0	0.9	0.9	29.9	2	11	11	35	485	55.4	5.4
NP_003553.2	21361114	SLC25A11	mitochondrial 2-oxoglutarate/malate carrier protein isoform 1	1.2	1.0	1.1	0.9	1.3	1.0	1.1	0.9	1.3	1.0	1.1	0.9	35.7	3	9	9	28	314	34.0	9.9
NP_001161343.1	268832194	ATP5SL	ATP synthase subunit s-like protein isoform 5	1.1	1.0	0.9	0.9	1.2	1.0	1.0	0.9	1.1	1.0	1.0	0.9	7.8	3	1	1	2	230	26.3	5.9
NP_001278887.1	634743270	MAP3K4	mitogen-activated protein kinase kinase kinase 4 isoform c	0.9	0.8	0.9	0.9	0.9	0.9	1.0	0.9	0.9	0.9	1.0	0.9	5.8	4	5	5	12	1061	119.8	6.4
NP_004300.1	4757768	ARHGDIA	rho GDP-dissociation inhibitor 1 isoform a	1.1	0.9	1.1	0.9	1.0	0.9	1.1	0.9	1.0	0.9	1.1	0.9	57.8	5	8	8	52	204	23.2	5.1
NP_612478.2	375298729;140560994	R3HCC1L	coiled-coil domain-containing protein R3HCC1L isoform 2	0.9	0.9	0.9	0.9	1.1	1.0	0.9	1.0	1.0	1.0	0.9	0.9	3.2	2	2	2	3	778	86.3	5.1
NP_001167448.1	291190783	LRRC16A	leucine-rich repeat-containing protein 16A isoform 2	1.0	0.9	0.9	0.9	0.9	0.9	0.9	0.9	1.0	0.9	0.9	0.9	5.3	2	5	5	11	1365	150.8	7.8
NP_001129292.1	209447096	TMEM2	transmembrane protein 2 isoform b	0.9	1.0	0.9	0.9	1.3	0.9	0.8	0.9	1.1	0.9	0.8	0.9	0.9	2	1	1	1	1320	147.3	8.4
NP_001007258.1	55953110	PRDM2	PR domain zinc finger protein 2 isoform c	0.9	1.0	1.0	0.9	1.1	1.0	1.0	1.0	1.0	1.0	1.0	0.9	3.2	3	3	3	3	1481	162.0	6.8
NP_689512.2	148368978	NCAPH2	condensin-2 complex subunit H2 isoform 2	1.1	1.0	1.1	0.9	0.9	1.0	1.0	0.9	1.0	1.0	1.0	0.9	11.1	3	5	5	7	605	68.2	4.7
NP_061849.2	21361602	SLC38A2	sodium-coupled neutral amino acid transporter 2 isoform 1	0.9	0.9	1.1	0.9	1.0	1.0	1.2	0.9	1.0	1.0	1.1	0.9	7.3	2	2	2	14	506	56.0	8.0
NP_078905.2	38016935	CENPU	centromere protein U	0.9	0.9	0.9	0.9	0.9	0.9	0.9	1.0	0.9	0.9	0.9	0.9	4.8	1	2	2	3	418	47.5	9.1
NP_006556.1	5729790	CTCF	transcriptional repressor CTCF isoform 1	0.7	0.8	0.8	0.9	0.8	1.0	0.9	1.0	0.8	0.9	0.8	0.9	13.1	15	7	7	14	727	82.7	7.0
NP_115558.3	148529023	FAM175B	BRISC complex subunit Abro1	1.2	1.0	1.0	0.9	1.0	1.1	1.0	0.9	1.1	1.0	1.0	0.9	15.9	1	4	4	9	415	46.9	6.2
NP_036411.1	6912440	ZNF346	zinc finger protein 346 isoform a	1.0	0.9	0.9	1.0	0.9	0.9	0.8	0.9	1.0	0.9	0.9	0.9	15.7	8	4	4	4	294	32.9	9.1
NP_005129.2	153791313;281182727	SCO2	protein SCO2 homolog, mitochondrial precursor	0.8	0.7	0.8	0.8	1.0	0.9	0.9	1.1	0.9	0.8	0.9	0.9	10.5	1	2	2	4	266	29.8	8.9
NP_000160.1	4504009	GLA	alpha-galactosidase A precursor	1.0	0.9	0.9	0.8	1.1	1.1	1.0	1.0	1.0	1.0	0.9	0.9	7.0	1	2	2	3	429	48.7	5.6
NP_008958.2	194440660	TOPBP1	DNA topoisomerase 2-binding protein 1	1.0	1.0	1.0	1.0	0.8	0.9	0.9	0.9	0.9	0.9	0.9	0.9	6.9	1	5	6	9	1522	170.6	7.0
NP_003126.1	4507213	SRP19	signal recognition particle 19 kDa protein isoform 1	1.0	0.8	0.9	0.8	1.1	1.1	1.1	1.0	1.1	0.9	1.0	0.9	36.1	4	3	3	12	144	16.1	9.8
NP_056173.1	28872812	MORC3	MORC family CW-type zinc finger protein 3	1.0	0.9	0.9	0.8	1.0	1.0	0.9	1.0	1.0	0.9	0.9	0.9	11.5	1	8	8	14	939	107.0	5.6
NP_003757.1	4502395	BECN1	beclin-1	0.9	0.9	1.0	0.9	1.0	0.9	1.0	0.9	0.9	0.9	1.0	0.9	4.4	1	2	2	4	450	51.9	4.9
NP_775813.2	229093316	PLBD2	putative phospholipase B-like 2 isoform 1 precursor	0.9	0.9	0.9	0.9	0.9	0.9	0.9	0.9	0.9	0.9	0.9	0.9	8.7	2	4	4	7	589	65.4	6.8
NP_001229322.1	334724455	ACSS2	acetyl-coenzyme A synthetase, cytoplasmic isoform 3	1.3	1.2	1.0	0.9	1.3	1.2	1.0	0.9	1.3	1.2	1.0	0.9	22.0	3	10	10	25	606	67.6	6.2
NP_852136.1	31563534	RFC2	replication factor C subunit 2 isoform 1	1.0	0.9	0.9	0.9	1.0	0.9	0.9	0.9	1.0	0.9	0.9	0.9	42.9	5	12	12	32	354	39.1	6.4
NP_004554.3	614458221	PCK2	phosphoenolpyruvate carboxykinase [GTP], mitochondrial isoform 1 precursor	1.2	1.1	0.9	0.9	1.3	1.1	0.9	0.9	1.2	1.1	0.9	0.9	42.0	4	19	19	75	640	70.7	7.6
NP_073585.8	65288071	TNS3	tensin-3	0.9	0.9	1.0	0.9	0.9	0.8	0.9	0.9	0.9	0.9	1.0	0.9	5.3	1	5	5	8	1445	155.2	6.8
NP_006247.1	5453974	PKN2	serine/threonine-protein kinase N2	1.0	0.9	0.9	0.9	0.9	0.9	0.9	0.9	1.0	0.9	0.9	0.9	11.1	1	8	8	21	984	112.0	6.3
NP_054893.1	7661804;40806221	THYN1	thymocyte nuclear protein 1 isoform 1	1.0	1.0	1.0	0.9	0.9	1.0	0.9	0.9	0.9	1.0	0.9	0.9	51.6	2	11	11	65	225	25.7	9.2
NP_006793.1	5803167	SF3A3	splicing factor 3A subunit 3	0.9	0.9	0.8	0.9	1.0	1.0	1.0	1.0	0.9	0.9	0.9	0.9	38.3	1	14	14	72	501	58.8	5.4
NP_055135.1	7657603	HEBP2	heme-binding protein 2	0.9	0.8	1.0	1.0	1.0	0.9	1.0	0.8	1.0	0.8	1.0	0.9	20.5	1	3	3	7	205	22.9	4.6
NP_001253964.1	388452864	PWWP2A	PWWP domain-containing protein 2A isoform c	1.1	0.9	0.7	0.8	0.7	0.9	0.8	1.0	0.9	0.9	0.8	0.9	3.9	3	1	1	2	520	56.3	9.2
NP_002859.1	4506371	RAB5B	ras-related protein Rab-5B isoform 1	0.8	0.9	0.9	0.9	1.1	1.0	1.0	1.0	1.0	0.9	0.9	0.9	51.2	3	5	8	23	215	23.7	8.1
NP_001164006.1	283436222	ATAD3A	ATPase family AAA domain-containing protein 3A isoform 2	0.9	0.9	1.0	0.9	1.0	0.9	0.9	0.9	1.0	0.9	1.0	0.9	24.6	5	12	12	34	586	66.2	9.2
NP_001036783.1	112807226	ELMSAN1	ELM2 and SANT domain-containing protein 1	1.0	0.9	0.9	0.9	0.9	1.0	1.0	0.9	0.9	0.9	1.0	0.9	6.3	1	4	4	6	1045	114.9	9.2
NP_057525.1	10092639	CRIM1	cysteine-rich motor neuron 1 protein precursor	1.2	1.3	1.6	0.9	1.1	0.9	1.1	0.9	1.1	1.1	1.4	0.9	2.1	1	1	1	2	1036	113.7	5.2
NP_003277.1	11225260	TOP1	DNA topoisomerase 1	0.9	0.9	1.0	0.9	0.8	0.9	1.0	0.9	0.8	0.9	1.0	0.9	46.3	3	36	36	163	765	90.7	9.3
NP_001128714.1	207028748	NDRG1	protein NDRG1 isoform 1	0.8	1.0	1.1	0.9	0.9	0.9	1.1	0.9	0.9	1.0	1.1	0.9	21.3	3	5	5	7	394	42.8	5.8
NP_001276089.1	574584674	MICB	MHC class I polypeptide-related sequence B isoform 2	1.0	1.1	1.0	0.9	0.9	1.0	0.9	0.9	1.0	1.0	1.0	0.9	3.1	2	1	1	2	351	39.2	7.1
NP_005905.2	33469919;33469917	MCM4	DNA replication licensing factor MCM4	1.0	0.9	0.9	0.9	1.0	0.9	0.9	0.9	1.0	0.9	0.9	0.9	44.5	5	28	28	121	863	96.5	6.7
NP_569079.1	18426904	WRNIP1	ATPase WRNIP1 isoform 2	1.0	0.9	0.9	0.9	1.0	1.0	0.9	1.0	1.0	1.0	0.9	0.9	12.7	2	6	6	9	640	69.4	6.3

NP_Accession	Protein group Accession	Gene ID	Description	Het-1A-Smoke - 2M/Parental	Het-1A-Smoke - 4M/Parental	Het-1A-Smoke - 6M/Parental	Het-1A-Smoke - 8M/Parental	Het-1A-Smoke - 2M/Parental	Het-1A-Smoke - 4M/Parental	Het-1A-Smoke - 6M/Parental	Het-1A-Smoke - 8M/Parental	Het-1A-Smoke - 2M/Parental	Het-1A-Smoke - 4M/Parental	Het-1A-Smoke - 6M/Parental	Het-1A-Smoke - 8M/Parental	Coverage (%)	Proteins	Unique Peptides	# Peptides	PSM	# AAs	MW [kDa]	calc. pI
				Replicate 1				Replicate 2				Average of replicates											
NP_001263408.1	451327634	LONP1	lon protease homolog, mitochondrial isoform 2	1.1	1.0	1.0	0.9	1.1	1.0	1.0	0.9	1.1	1.0	1.0	0.9	54.2	3	34	34	159	895	100.3	6.7
NP_659471.1	21450775	TOR1AIP2	torsin-1A-interacting protein 2 isoform b	1.0	1.0	1.0	1.0	0.9	0.9	1.0	0.9	1.0	1.0	1.0	0.9	39.2	1	10	10	27	470	51.2	5.0
NP_115784.1	14670392	BAZ1B	tyrosine-protein kinase BAZ1B	0.9	0.9	1.0	0.9	0.9	0.9	0.9	0.9	0.9	0.9	1.0	0.9	10.3	1	12	12	30	1483	170.8	8.5
NP_003070.3	21264355	SMARCE1	SWI/SNF-related matrix-associated actin-dependent regulator of chromatin subfamily E member 1	0.9	0.9	0.9	0.9	1.0	1.0	1.0	0.9	0.9	1.0	1.0	0.9	35.0	1	12	12	32	411	46.6	4.9
NP_060917.1	8923857	EMC3	ER membrane protein complex subunit 3	0.9	1.0	1.0	0.9	1.0	1.0	0.9	0.9	0.9	1.0	0.9	0.9	34.1	1	7	7	17	261	29.9	6.8
NP_055082.1	7657234	C11orf58	small acidic protein	0.8	0.9	0.9	0.9	0.9	1.0	0.9	1.0	0.9	1.0	0.9	0.9	25.7	1	4	4	46	183	20.3	4.7
NP_001269026.1	530788244	YAP1	yorkie homolog isoform 6	0.8	0.7	1.0	0.8	1.0	0.7	0.8	1.0	0.9	0.7	0.9	0.9	33.9	9	8	8	22	466	50.0	5.2
NP_060644.4	50428935	MAP1S	microtubule-associated protein 1S isoform 1	0.8	0.9	0.8	0.9	0.9	0.9	0.9	0.9	0.9	0.9	0.9	0.9	18.1	2	12	12	32	1059	112.1	7.3
NP_079160.1	13376331	L2HGDH	L-2-hydroxyglutarate dehydrogenase, mitochondrial precursor	1.5	0.9	0.9	0.9	1.0	0.9	0.9	1.0	1.3	0.9	0.9	0.9	14.3	1	5	5	12	463	50.3	8.1
NP_115661.1	23943880	MRI1	methylthioribose-1-phosphate isomerase isoform 2	1.0	1.0	1.0	1.0	0.9	0.8	0.8	0.8	0.9	0.9	0.9	0.9	30.1	2	5	5	18	322	34.5	5.7
NP_006245.2	31377782;47157325	PRKCD	protein kinase C delta type	0.8	0.9	0.9	0.9	1.0	0.9	0.9	0.9	0.9	0.9	0.9	0.9	4.9	5	3	4	5	676	77.5	7.8
NP_000997.1	4506723	RPS3A	40S ribosomal protein S3a isoform 1	0.9	0.9	0.9	0.9	0.9	0.9	0.9	0.9	0.9	0.9	0.9	0.9	62.1	2	19	19	399	264	29.9	9.7
NP_002583.1	4505641;33239451	PCNA	proliferating cell nuclear antigen	0.9	0.9	0.9	0.9	0.9	0.8	0.9	0.9	0.9	0.9	0.9	0.9	90.4	3	18	18	109	261	28.8	4.7
NP_065971.2	114326455	CHD8	chromodomain-helicase-DNA-binding protein 8 isoform 2	0.9	0.9	0.9	0.9	0.9	0.9	0.8	0.9	0.9	0.9	0.9	0.9	4.4	5	7	8	12	2302	262.2	6.7
NP_060571.1	8922438;379056369	CDCA8	borealin	0.9	0.9	0.9	0.9	0.9	1.0	1.0	0.9	0.9	0.9	0.9	0.9	31.1	1	7	7	15	280	31.3	9.9
NP_005518.3	124256496	HSPA1L	heat shock 70 kDa protein 1-like	1.1	1.0	1.1	0.9	1.4	1.1	1.2	0.9	1.2	1.1	1.1	0.9	31.1	1	1	18	291	641	70.3	6.0
NP_078929.1	13375901	PRKRIP1	PRKR-interacting protein 1	0.9	0.9	0.9	1.0	0.9	0.9	0.8	0.9	0.9	0.9	0.8	0.9	21.7	1	4	4	5	184	21.0	9.8
NP_001378.1	4503379	DPYSL3	dihydropyrimidinase-related protein 3 isoform 2	0.9	1.0	0.9	0.9	0.9	1.0	1.0	0.9	0.9	1.0	1.0	0.9	36.5	3	13	15	65	570	61.9	6.5
NP_000149.3	189458812	GBE1	1,4-alpha-glucan-branching enzyme	0.9	0.9	1.1	0.9	1.0	1.0	1.1	0.9	1.0	1.0	1.1	0.9	31.3	1	17	17	40	702	80.4	6.3
NP_114368.1	14165466	PTBP1	polypyrimidine tract-binding protein 1 isoform c	0.9	0.9	0.9	0.9	0.9	0.9	0.9	0.9	0.9	0.9	0.9	0.9	66.1	3	17	20	185	531	57.2	9.2
NP_001017963.2	153792590	HSP90AA1	heat shock protein HSP 90-alpha isoform 1	0.8	0.9	0.8	0.9	0.9	0.8	0.8	0.9	0.8	0.9	0.8	0.9	61.7	1	1	52	1578	854	98.1	5.2
NP_055892.2	154090976	SARM1	sterile alpha and TIR motif-containing protein 1 precursor	1.0	0.9	0.9	0.9	1.0	0.9	0.9	0.9	1.0	0.9	0.9	0.9	9.1	1	5	5	9	724	79.3	6.6
NP_071761.1	11641289	TFB2M	dimethyladenosine transferase 2, mitochondrial	1.1	1.0	0.9	0.9	1.1	1.1	1.1	0.9	1.1	1.0	1.0	0.9	2.0	1	1	1	1	396	45.3	9.2
NP_055335.2	157694492	MYBBP1A	myb-binding protein 1A isoform 2	0.9	0.9	1.0	0.9	1.0	0.9	1.0	0.9	1.0	0.9	1.0	0.9	34.9	2	33	33	157	1328	148.8	9.3
NP_079007.2	31542246	KLHL36	kelch-like protein 36	1.1	0.9	1.0	0.9	1.0	0.9	0.9	0.9	1.0	0.9	1.0	0.9	2.1	1	1	1	1	616	69.9	6.2
NP_689942.2	38570156	NT5DC1	5'-nucleotidase domain-containing protein 1	0.8	0.8	0.8	0.9	0.9	0.8	0.8	0.9	0.8	0.8	0.8	0.9	28.8	1	9	9	25	455	51.8	6.4
NP_689735.1	22749091	ARL6IP6	ADP-ribosylation factor-like protein 6-interacting protein 6	0.9	1.0	0.9	0.9	0.9	0.8	0.8	0.9	0.9	0.9	0.9	0.9	5.3	1	1	1	2	226	24.7	6.3
NP_036580.2	21618344	STAT5B	signal transducer and activator of transcription 5B	0.8	0.6	0.7	0.8	1.5	0.8	0.8	1.0	1.1	0.7	0.8	0.9	6.7	1	2	4	9	787	89.8	6.0
NP_055478.2	98986459	KDM4A	lysine-specific demethylase 4A	0.9	1.0	0.8	0.8	1.1	1.1	1.0	1.0	1.0	1.0	0.9	0.9	3.9	1	2	2	2	1064	120.6	5.8
NP_003192.1	4507401	TFAM	transcription factor A, mitochondrial isoform 1 precursor	0.9	1.1	0.9	0.9	0.9	1.0	0.9	0.9	0.9	1.0	0.9	0.9	33.7	2	11	11	32	246	29.1	9.7
XP_005276554.1	530437751	LOC10193012	eukaryotic elongation factor 2 kinase	0.9	0.9	1.0	0.9	0.8	0.8	1.0	0.9	0.9	0.9	1.0	0.9	2.6	1	2	2	2	725	82.1	5.4
NP_004420.1	4758248	EFNB1	ephrin-B1 precursor	1.0	0.9	0.9	0.9	0.9	0.9	0.9	0.9	1.0	0.9	0.9	0.9	5.2	1	1	1	1	346	38.0	8.9
NP_003125.3	149999611	SRP14	signal recognition particle 14 kDa protein	0.9	0.9	0.9	0.9	0.9	0.9	0.9	0.9	0.9	0.9	0.9	0.9	33.8	1	5	5	18	136	14.6	10.0
NP_004453.3	67089147;567757574	FDFT1	squalene synthase isoform 1	1.1	1.1	1.0	0.9	1.0	1.0	0.9	0.9	1.0	1.1	1.0	0.9	19.9	5	6	6	20	417	48.1	6.5
NP_055412.2	54633315	DNTTIP2	deoxynucleotidyltransferase terminal-interacting protein 2	0.9	0.9	0.9	0.9	0.9	0.9	0.9	0.9	0.9	0.9	0.9	0.9	26.6	2	12	13	54	756	84.4	6.2
NP_009144.1	6005794	PRAF2	PRA1 family protein 2	1.0	0.9	0.9	0.9	0.9	0.9	0.9	0.9	1.0	0.9	0.9	0.9	20.2	1	3	3	32	178	19.2	9.2
NP_060506.5	118197272	ATG2B	autophagy-related protein 2 homolog B	1.0	0.9	1.0	1.0	1.0	1.1	0.9	0.9	1.0	1.0	0.9	0.9	3.9	1	5	5	9	2078	232.6	5.8
NP_000971.1	11415026	RPL18A	60S ribosomal protein L18a	0.9	0.9	0.9	0.9	1.0	0.9	0.9	0.9	0.9	0.9	0.9	0.9	51.7	1	10	10	58	176	20.7	10.7
NP_115754.3	304555581	EEF1D	elongation factor 1-delta isoform 1	0.8	0.7	0.7	0.9	0.8	0.7	0.8	0.9	0.8	0.7	0.7	0.9	24.6	4	13	16	84	647	71.4	6.4
NP_001269911.1	545688925	TLE3	transducin-like enhancer protein 3 isoform g	0.9	0.9	0.9	1.0	0.9	0.8	0.9	0.9	0.9	0.8	0.9	0.9	3.0	15	2	2	4	699	74.9	7.3
NP_542408.1	18087855	DYNLL2	dynein light chain 2, cytoplasmic	0.9	0.8	0.7	0.9	1.0	1.0	1.0	0.9	0.9	0.9	0.8	0.9	44.9	1	2	3	25	89	10.3	7.4
NP_001284.1	4502891	CLNS1A	methylosome subunit pICln	1.0	1.0	1.1	1.0	1.1	1.0	1.1	0.9	1.0	1.0	1.1	0.9	32.1	1	4	4	51	237	26.2	4.1
NP_003600.2	21396500	HIRIP3	HIRA-interacting protein 3 isoform 1	0.9	0.9	0.8	0.9	0.9	0.9	0.9	0.9	0.9	0.9	0.9	0.9	13.5	2	6	6	13	556	61.9	8.5
NP_001010.2	14165469	RPS15A	40S ribosomal protein S15a	1.0	0.9	1.0	0.9	1.0	0.9	0.9	0.9	1.0	0.9	0.9	0.9	56.9	1	7	7	42	130	14.8	10.1
NP_006178.2	45007007	OAS3	2'-5'-oligoadenylate synthase 3	1.0	1.0	0.9	1.0	0.9	0.9	0.9	0.9	0.9	0.9	0.9	0.9	3.2	1	3	3	8	1087	121.1	8.4
NP_006288.2	190684675	XRCC1	DNA repair protein XRCC1	1.0	0.9	0.9	0.9	0.9	0.9	0.9	0.9	0.9	0.9	0.9	0.9	7.3	1	5	5	6	633	69.5	6.3
NP_002617.3	48762920	PFKL	ATP-dependent 6-phosphofructokinase, liver type isoform b	0.9	0.9	0.9	0.9	0.9	0.9	0.9	0.9	0.9	0.9	0.9	0.9	21.5	2	11	15	52	780	85.0	7.5
NP_055680.3	178056552	NCAPD2	condensin complex subunit 1	0.9	0.9	0.9	0.9	0.9	0.9	0.9	0.9	0.9	0.9	0.9	0.9	31.6	1	34	34	83	1401	157.1	6.6
NP_001186308.1	313747584	GBF1	Golgi-specific brefeldin A-resistance guanine nucleotide exchange factor 1 isoform 3	0.9	0.9	1.0	0.9	1.0	1.0	1.0	0.9	0.9	1.0	1.0	0.9	22.0	3	26	27	65	1855	205.9	5.8
NP_444504.1	16933537	GLMN	glomulin	0.9	0.7	0.7	0.9	0.9	0.8	0.9	1.0	0.9	0.8	0.8	0.9	5.6	1	4	4	7	594	68.2	5.3
NP_055971.2	344925845	RCOR1	REST corepressor 1	1.0	1.0	1.0	0.9	0.8	1.0	0.8	0.9	0.9	1.0	0.9	0.9	22.3	2	4	7	22	485	53.3	7.0
NP_002902.2	18375673	UPF1	regulator of nonsense transcripts 1 isoform 2	0.9	0.9	0.9	0.9	0.9	0.9	0.9	0.9	0.9	0.9	0.9	0.9	31.4	2	25	25	88	1118	123.0	6.7
NP_003746.2	49472822	EIF3G	eukaryotic translation initiation factor 3 subunit G	0.9	1.0	0.9	0.9	0.9	1.0	1.0	0.9	0.9	1.0	0.9	0.9	53.4	1	15	15	126	320	35.6	6.1
NP_004243.1	4759140	SLC9A3R1	Na(+)/H(+) exchange regulatory cofactor NHE-RF1	1.0	1.0	1.0	1.0	1.0	0.8	0.9	0.8	1.0	0.9	0.9	0.9	22.6	1	6	6	12	358	38.8	5.8
NP_065937.1	16507200	USP28	ubiquitin carboxyl-terminal hydrolase 28	0.9	1.0	1.0	0.9	0.9	1.0	1.0	0.9	0.9	1.0	1.0	0.9	6.9	2	5	5	8	1077	122.4	5.2
NP_055312.2	21626468	ZNF638	zinc finger protein 638 isoform 1	0.9	1.0	0.9	0.9	1.0	1.0	0.9	0.9	0.9	1.0	0.9	0.9	12.9	2	19	19	51	1978	220.5	6.4
NP_004695.1	4759276	RRP9	U3 small nucleolar RNA-interacting protein 2	1.1	1.0	1.0	1.0	1.0	0.9	0.9	0.8	1.0	1.0	1.0	0.9	24.8	1	8	8	26	475	51.8	7.9
NP_003964.3	78000181;78000183	RPL14	60S ribosomal protein L14	0.9	0.9	0.9	0.9	0.9	0.9	0.9	0.9	0.9	0.9	0.9	0.9	32.6	1	7	7	60	215	23.4	10.9
NP_009045.2	110347443	TMF1	TATA element modulatory factor	1.1	1.2	1.1	0.9	1.2	1.1	1.0	0.9	1.2	1.1	1.1	0.9	19.9	1	17	17	39	1093	122.8	4.9
NP_001059.2	19913408	TOP2B	DNA topoisomerase 2-beta	1.0	1.0	1.0	0.9	1.0	1.0	0.9	0.9	1.0	1.0	1.0	0.9	36.0	2	36	53	212	1621	182.5	8.1
NP_009017.1	5901892	WDHD1	WD repeat and HMG-box DNA-binding protein 1 isoform 1	0.9	0.9	0.8	0.9	0.9	0.9	0.8	0.9	0.9	0.9	0.8	0.9	18.4	2	16	16	29	1129	125.9	5.6
NP_001278913.1	634743254	PTPRK	receptor-type tyrosine-protein phosphatase kappa isoform f precursor	0.9	1.0	0.9	1.0	0.9	1.0	0.9	0.8	0.9	1.0	0.9	0.9	2.3	4	2	2	3	1439	162.0	5.9
NP_001007074.1	55743128;4506635	RPL32	60S ribosomal protein L32	0.9	1.0	1.0	0.9	0.8	1.0	0.9	0.9	0.8	1.0	1.0	0.9	43.7	1	7	7	65	135	15.8	11.3
NP_060934.1	8923930	CISD1	CDGSH iron-sulfur domain-containing protein 1	0.9	0.9	0.9	0.9	0.9	0.9	0.9	0.9	0.9	0.9	0.9	0.9	36.1	1	3	3	14	108	12.2	9.1
NP_001013865.1	62244004	SLTM	SAFB-like transcription modulator isoform b	0.8	0.9	0.8	0.9	1.2	1.0	0.8	0.9	1.0	1.0	0.8	0.9	17.3	2	15	15	27	1016	115.3	8.1
NP_006577.2	33942072	CNPY3	protein canopy homolog 3 precursor	0.9	1.0	0.8	0.9	0.9	1.0	0.9	0.9	0.9	1.0	0.8	0.9	19.4	1	4	4	6	278	30.7	5.5
NP_001192191.1	327532738	RAD54B	DNA repair and recombination protein RAD54B isoform 2	0.9	0.9	0.9	0.9	0.9	0.9	0.9	0.9	0.9	0.9	0.9	0.9	11.1	2	1	1	1	117	13.3	9.3
NP_056973.2	21312134	MED15	mediator of RNA polymerase II transcription subunit 15 isoform b	1.0	0.7	0.8	0.9	1.2	1.2	1.2	0.9	1.1	1.0	1.0	0.9	6.4	5	2	2	3	748	82.5	9.3
NP_839955.1	30410716	EXOC1	exocyst complex component 1 isoform 2	1.2	1.0	1.1	1.1	0.9	0.9	0.8	0.7	1.1	0.9	0.9	0.9	6.0	2	5	5	7	879	100.2	6.6
NP_002755.1	4506127	PRPS1	ribose-phosphate pyrophosphokinase 1 isoform 1	1.1	0.9	0.9	0.9	1.3	1.0	1.1	0.9	1.2	1.0	1.0	0.9	35.5	2	3	8	29	318	34.8	7.0
NP_003371.2	62414289	VIM	vimentin	0.9	0.9	1.2	0.9	0.9	0.9	1.2	0.9	0.9	0.9	1.2	0.9	79.8	9	28	35	826	466	53.6	5.1
NP_060531.2	166235169	PRPF38B	pre-mRNA-splicing factor 38B	0.9	0.9	1.0	0.9	1.0	0.9	1.0	0.9	1.0	0.9	1.0	0.9	4.8	1	3	3	4	546	64.4	10.5
NP_078888.4	261878478	DHX40	probable ATP-dependent RNA helicase DHX40 isoform 1	1.0	0.9	0.8	0.9	0.9	0.9	0.9	0.9	0.9	0.9	0.9	0.9	10.3	2	6	6	11	779	88.5	8.6
NP_000958.1	4506649	RPL3	60S ribosomal protein L3 isoform a	0.9	0.9	1.0	0.9	0.9	0.9	0.9	0.9	0.9	0.9	0.9	0.9	51.9	3	18	18	238	403	46.1	10.2
NP_005795.2	21040371	DDX39A	ATP-dependent RNA helicase DDX39A	1.1	1.0	1.0	1.0	0.9	0.8	0.9	0.8	1.0	0.9	0.9	0.9	50.6	1	6	17	107	427	49.1	5.7
NP_055475.2	55769548	PHF14	PHD finger protein 14	1.1	1.0	1.1	0.9	0.9	0.9	1.0	0.9	1.0	1.0	1.0	0.9	10.0	1	7	7	8	888	100.0	5.3
NP_079133.3	26080431	ATAD5	ATPase family AAA domain-containing protein 5	0.9	0.8	0.8	0.9	1.0	0.9	1.0	1.0	1.0	0.9	0.9	0.9	2.0	1	3	3	4	1844	207.4	9.2
NP_057417.3	118572613	SRRM2	serine/arginine repetitive matrix protein 2	0.9	1.0	1.0	0.9	1.0	1.0	0.9	0.9	0.9	1.0	1.0	0.9	19.7	1	38	38	161	2752	299.4	12.1
NP_000407.1	4557880	IFNGR1	interferon gamma receptor 1 precursor	1.0	0.9	0.9	0.8	0.9	1.0	0.8	1.0	1.0	1.0	0.8	0.9	9.2	1	3	3	5	489	54.4	4.9
NP_003440.1	4508005	TRIM26	tripartite motif-containing protein 26	1.1	1.1	1.1	1.1	0.6	0.6	0.6	0.7	0.9	0.8	0.8	0.9	4.3	1	1	1	2	539	62.1	5.0
NP_149073.1	32129199	SARNP	SAP domain-containing ribonucleoprotein	0.9	0.9	0.9	0.9	0.9	1.0	0.9	0.9	0.9	1.0	0.9	0.9	32.9	1	6	6	22	210	23.7	6.4
NP_003861.1	4506787	IQGAP1	ras GTPase-activating-like protein IQGAP1	0.9	0.9	1.0	0.9	0.9	0.9	1.0	0.9	0.9	0.9	1.0	0.9	55.9	3	69	75	542	1657	189.1	6.5
NP_683692.2	87299628	BOD1L1	biorientation of chromosomes in cell division protein 1-like 1	1.0	1.0	0.9	0.9	1.0	1.0	1.0	0.9	1.0	1.0	1.0	0.9	12.4	3	26	26	62	3051	330.3	5.1
NP_115766.3	222352111	NIFK	MKI67 FHA domain-interacting nucleolar phosphoprotein	0.8	1.0	0.9	0.9	1.0	1.0	1.0	0.9	0.9	1.0	1.0	0.9	37.9	1	10	10	42	293	34.2	9.9
NP_000989.1	4506643	RPL37A	60S ribosomal protein L37a	0.8	0.8	0.9	0.9	0.9	0.9	1.0	0.9	0.9	0.9	0.9	0.9	58.7	1	6	6	16	92	10.3	10.4
NP_055894.6	194018476	TBC1D2B	TBC1 domain family member 2B isoform b	0.9	0.8	0.9	0.9	1.0	0.9	0.9	0.9	0.9	0.9	0.9	0.9	5.8	2	5	5	6	914	103.9	6.6

Khan *et al.* , 2019. Multi-omics analysis to characterize cigarette smoke induced molecular alterations in esophageal cells
Supplementary Table 5. List of proteins quantified in untreated and chronically treated Het1A cells with cigarette smoke condensate for 8 months

NP_Accession	Protein group Accession	Gene ID	Description	Het-1A-Smoke - 2M/Parental	Het-1A-Smoke - 4M/Parental	Het-1A-Smoke - 6M/Parental	Het-1A-Smoke - 8M/Parental	Het-1A-Smoke - 2M/Parental	Het-1A-Smoke - 4M/Parental	Het-1A-Smoke - 6M/Parental	Het-1A-Smoke - 8M/Parental	Het-1A-Smoke - 2M/Parental	Het-1A-Smoke - 4M/Parental	Het-1A-Smoke - 6M/Parental	Het-1A-Smoke - 8M/Parental	Coverage (%)	Proteins	Unique Peptides	# Peptides	PSM	# AAs	MW [kDa]	calc. pI
				Replicate 1				Replicate 2				Average of replicates											
NP_004266.2	30023851	MED20	mediator of RNA polymerase II transcription subunit 20	0.8	0.8	0.8	0.8	0.9	1.0	0.9	1.0	0.9	0.9	0.9	0.9	9.4	1	2	2	3	212	23.2	6.9
NP_009117.2	21735548	CEP250	centrosome-associated protein CEP250	1.1	0.9	0.9	0.9	1.0	0.9	0.9	0.9	1.1	0.9	0.9	0.9	3.0	1	5	5	7	2442	281.0	5.0
NP_005265.1	4885287	GNG5	guanine nucleotide-binding protein G(I)/G(S)/G(O) subunit gamma-5 precursor	1.1	0.8	0.8	0.9	0.8	0.7	0.8	0.9	1.0	0.8	0.8	0.9	33.8	1	3	3	11	68	7.3	9.8
NP_004730.2	14141170	MTA2	metastasis-associated protein MTA2	0.8	0.9	0.9	0.9	0.9	1.0	1.0	0.9	0.8	0.9	0.9	0.9	35.3	1	18	19	55	668	75.0	9.7
NP_006433.2	18426973	DRAP1	dr1-associated corepressor	0.9	0.9	0.9	0.9	0.9	1.0	0.9	0.9	0.9	0.9	0.9	0.9	31.2	1	5	5	19	205	22.3	5.2
NP_203754.2	110556636	TNKS1BP1	182 kDa tankyrase-1-binding protein	1.0	1.1	1.1	0.9	1.0	1.1	1.1	1.0	1.0	1.1	1.1	0.9	34.6	1	37	37	114	1729	181.7	4.9
NP_000978.1	4506621	RPL26	60S ribosomal protein L26	0.8	0.9	0.9	0.9	0.9	1.1	0.9	1.0	0.8	1.0	0.9	0.9	44.1	1	3	12	68	145	17.2	10.6
NP_036533.2	149363636	PLXNB2	plexin-B2 precursor	0.9	0.9	1.0	0.9	1.1	0.9	0.9	0.9	1.0	0.9	1.0	0.9	4.7	1	8	8	13	1838	205.0	6.2
NP_057662.3	38788333	RSF1	remodeling and spacing factor 1	1.0	1.0	1.1	0.9	1.1	1.1	1.1	0.9	1.1	1.1	1.1	0.9	16.8	1	18	18	55	1441	163.7	5.0
NP_038477.2	91176325	BAZ2A	bromodomain adjacent to zinc finger domain protein 2A isoform 1	0.9	0.9	1.0	0.9	0.9	0.9	1.0	0.9	0.9	0.9	1.0	0.9	3.8	2	4	4	5	1905	211.1	6.6
NP_006301.3	158937236	NPEPPS	puromycin-sensitive aminopeptidase	1.0	1.0	1.0	0.9	0.9	0.9	1.0	0.9	0.9	0.9	1.0	0.9	45.4	7	31	31	129	919	103.2	5.7
NP_116021.2	32490557	PAPOLA	poly(A) polymerase alpha isoform 1	0.9	0.7	0.8	0.9	0.8	0.7	0.8	0.9	0.8	0.7	0.8	0.9	23.0	7	13	13	30	745	82.8	7.4
NP_001273490.1	557440899	NUMA1	nuclear mitotic apparatus protein 1 isoform 2	0.9	0.9	0.9	0.9	0.9	1.0	1.0	0.9	0.9	0.9	0.9	0.9	41.7	2	68	69	193	2101	236.4	5.8
NP_006563.2	24111250	GNA13	guanine nucleotide-binding protein subunit alpha-13 isoform 1	0.9	0.9	0.8	0.9	0.9	0.9	0.9	0.9	0.9	0.9	0.8	0.9	12.2	8	3	4	33	377	44.0	8.0
NP_006833.2	55749531	SF3B2	splicing factor 3B subunit 2	0.8	0.8	0.8	0.9	0.9	0.9	0.9	0.9	0.9	0.8	0.8	0.9	44.5	1	32	32	122	895	100.2	5.7
NP_001014811.1	62420882;62420880	ME3	NADP-dependent malic enzyme, mitochondrial	1.0	1.0	1.0	0.9	0.9	0.9	1.0	0.9	0.9	1.0	1.0	0.9	6.5	1	2	2	4	604	67.0	8.0
NP_001268641.1	528281405	NCAPH	condensin complex subunit 2 isoform 4	1.0	1.0	1.0	0.9	1.0	0.9	0.9	0.9	1.0	0.9	0.9	0.9	22.3	4	8	8	21	605	67.5	4.9
NP_061119.1	8923920	H2AFY2	core histone macro-H2A.2	0.8	0.8	0.8	0.9	0.9	0.8	0.9	0.9	0.9	0.8	0.8	0.9	34.1	1	8	10	42	372	40.0	9.7
NP_056442.2	132626790	LDLRAP1	low density lipoprotein receptor adapter protein 1	1.0	0.9	0.9	0.9	1.0	0.9	0.9	0.9	1.0	0.9	0.9	0.9	19.5	1	4	4	7	308	33.9	6.7
NP_003091.2	23111038	SNX2	sorting nexin-2 isoform 1	1.0	0.9	0.9	0.9	1.0	1.0	1.0	0.9	1.0	1.0	0.9	0.9	36.2	2	11	14	56	519	58.4	5.1
NP_114032.2	74136883	HNRNPU	heterogeneous nuclear ribonucleoprotein U isoform a	0.7	1.0	0.8	0.9	0.9	0.9	0.9	1.0	0.8	0.9	0.8	0.9	47.0	1	3	37	425	825	90.5	6.0
NP_060308.1	8923444	NHP2	H/ACA ribonucleoprotein complex subunit 2 isoform a	1.2	1.0	0.9	0.9	1.0	1.0	1.0	0.9	1.1	1.0	0.9	0.9	75.2	2	6	6	33	153	17.2	8.2
NP_055898.1	157817073	CDK12	cyclin-dependent kinase 12 isoform 2	0.9	1.0	1.0	0.9	1.0	0.9	1.0	0.9	1.0	0.9	1.0	0.9	6.1	55	6	8	9	1481	163.1	9.5
NP_006561.1	5729999	RRAGA	ras-related GTP-binding protein A	1.0	0.9	0.9	0.9	0.9	0.9	1.0	0.9	0.9	0.9	1.0	0.9	5.1	1	1	1	8	313	36.5	7.7
NP_002076.2	75709200	GPX4	phospholipid hydroperoxide glutathione peroxidase, mitochondrial isoform A precursor	0.7	0.8	0.8	0.8	0.8	0.9	0.9	1.0	0.8	0.9	0.9	0.9	10.7	3	2	2	6	197	22.2	8.4
NP_002897.1	4506467	RDX	radixin isoform 2	1.0	1.0	1.0	0.9	1.0	1.1	1.1	0.9	1.0	1.1	1.0	0.9	51.8	5	17	36	208	583	68.5	6.4
NP_001379.1	4557537	DRG2	developmentally-regulated GTP-binding protein 2	1.1	0.9	0.9	0.9	1.0	0.9	1.0	0.9	1.1	0.9	0.9	0.9	29.1	1	9	9	34	364	40.7	8.9
NP_055040.2	21361399	PPP2R1A	serine/threonine-protein phosphatase 2A 65 kDa regulatory subunit A alpha isoform	0.9	0.9	0.9	0.9	0.9	0.9	0.9	0.9	0.9	0.9	0.9	0.9	49.6	1	18	24	206	589	65.3	5.1
NP_110395.1	13540531	ILKAP	integrin-linked kinase-associated serine/threonine phosphatase 2C	0.9	1.0	0.8	0.9	1.0	0.9	0.8	0.9	0.9	0.9	0.8	0.9	17.6	1	3	3	5	392	42.9	7.1
NP_057371.2	56676330	HP1BP3	heterochromatin protein 1-binding protein 3	0.9	1.0	1.1	0.9	0.9	1.0	1.1	0.9	0.9	1.0	1.1	0.9	50.6	1	21	21	123	553	61.2	9.7
NP_004703.1	4758528	HGS	hepatocyte growth factor-regulated tyrosine kinase substrate	1.3	1.0	1.1	0.8	1.0	1.0	1.0	1.0	1.1	1.0	1.0	0.9	1.7	1	1	1	2	777	86.1	6.2
NP_001229832.1	339275976;4502015	AKAP1	A-kinase anchor protein 1, mitochondrial precursor	1.1	0.9	0.9	1.0	0.9	0.8	0.9	0.9	1.0	0.9	0.9	0.9	7.9	1	5	5	8	903	97.3	4.9
NP_055389.3	142976675	STRN3	striatin-3 isoform 2	1.0	0.9	0.9	0.9	1.0	1.0	1.0	0.9	1.0	0.9	1.0	0.9	10.8	4	5	6	9	713	77.7	5.3
NP_002361.1	4505087	MAGOH	protein mago nashi homolog	0.9	0.8	0.9	0.9	1.0	0.9	0.9	0.9	1.0	0.9	0.9	0.9	67.1	3	8	8	34	146	17.2	6.1
NP_861974.1	32996709	TMED7	transmembrane emp24 domain-containing protein 7 precursor	1.1	1.1	1.2	1.0	1.2	1.1	1.4	0.8	1.2	1.1	1.3	0.9	43.3	3	7	7	24	224	25.2	6.9
NP_001276032.1	574280091	PRKCSH	glucosidase 2 subunit beta isoform 3 precursor	1.2	1.0	1.0	0.9	0.9	1.1	1.1	0.9	1.1	1.1	1.0	0.9	65.8	2	2	26	281	535	60.2	4.4
NP_002511.1	10835063	NPM1	nucleophosmin isoform 1	1.1	1.0	1.1	0.9	1.0	0.8	0.9	0.9	1.0	0.9	1.0	0.9	61.6	5	18	18	465	294	32.6	4.8
NP_115688.1	14150076	YIPF4	protein YIPF4	1.1	1.0	0.8	0.9	1.0	1.0	0.9	0.9	1.0	1.0	0.9	0.9	7.4	1	2	2	3	244	27.1	4.7
NP_002058.2	115511049	GNA11	guanine nucleotide-binding protein subunit alpha-11	0.9	0.9	0.9	0.8	0.9	1.0	1.0	1.0	0.9	0.9	0.9	0.9	28.4	2	6	8	27	359	42.1	5.7
NP_783313.1	28269681	NDUFA11	NADH dehydrogenase [ubiquinone] 1 alpha subcomplex subunit 11 isoform 1	0.9	1.0	0.9	0.8	0.9	0.9	0.9	1.0	0.9	1.0	0.9	0.9	10.6	2	1	1	2	141	14.8	8.7
NP_002825.3	33356177	PTPN11	tyrosine-protein phosphatase non-receptor type 11 isoform 1	1.1	0.9	0.9	0.9	0.9	0.9	0.9	0.9	1.0	0.9	0.9	0.9	34.9	3	18	18	38	593	68.0	7.3
NP_005817.1	5031755	HNRNPR	heterogeneous nuclear ribonucleoprotein R isoform 2	0.8	0.9	0.9	0.8	1.0	1.0	1.1	1.0	0.9	0.9	1.0	0.9	55.8	7	20	30	332	633	70.9	8.1
NP_076426.1	13027606	MRPL34	39S ribosomal protein L34, mitochondrial	0.9	1.0	1.0	0.9	1.1	1.0	1.0	0.9	1.0	1.0	1.0	0.9	10.9	1	1	1	4	92	10.2	12.2
NP_001002243.1	50409856	AFTPH	aftiphilin isoform c	1.0	1.1	1.0	0.9	1.1	1.0	1.1	0.9	1.1	1.0	1.1	0.9	5.7	3	3	3	3	908	99.3	4.6
NP_115635.1	14149987	WDR24	WD repeat-containing protein 24	1.3	1.1	1.0	1.0	0.9	0.8	0.8	0.8	1.1	1.0	0.9	0.9	2.8	1	2	2	2	790	88.1	6.5
NP_055865.1	24307983	CMTR1	cap-specific mRNA (nucleoside-2'-O-)-methyltransferase 1	0.9	0.8	0.8	0.8	1.0	0.9	0.9	1.0	0.9	0.9	0.8	0.9	17.7	1	10	10	17	835	95.3	7.0
NP_976063.1	42794779	MYO18A	unconventional myosin-XVIIIa isoform b	1.0	0.9	0.8	0.9	1.0	0.9	0.9	0.9	1.0	0.9	0.9	0.9	7.1	2	11	11	18	2039	231.1	6.2
NP_001186270.1	313569770;4506617	RPL17	60S ribosomal protein L17 isoform a	0.9	0.9	0.9	0.9	0.9	1.0	0.9	0.9	0.9	0.9	0.9	0.9	54.4	4	11	11	142	184	21.4	10.2
NP_112223.1	30794216	TRIM56	E3 ubiquitin-protein ligase TRIM56	0.9	0.9	0.8	0.9	0.9	0.9	0.9	0.9	0.9	0.9	0.9	0.9	8.7	1	5	5	10	755	81.4	7.7
NP_001139187.1	224589129	KPNA7	importin subunit alpha-8	1.0	1.0	1.1	0.9	0.9	0.8	1.1	0.9	0.9	0.9	1.1	0.9	2.1	1	1	1	1	516	56.9	6.0
NP_002196.2	56237029	ITGA5	integrin alpha-5 precursor	1.0	1.1	1.0	0.9	0.9	1.1	1.0	0.9	1.0	1.1	1.0	0.9	5.2	1	4	4	9	1049	114.5	5.8
NP_073624.2	20070321	NMNAT1	nicotinamide/nicotinic acid mononucleotide adenylyltransferase 1 isoform 1	1.0	0.9	0.9	0.9	0.9	0.9	0.8	0.9	1.0	0.9	0.9	0.9	25.5	1	6	6	20	279	31.9	8.9
NP_003841.1	11321583	SUCLA2	succinyl-CoA ligase [ADP-forming] subunit beta, mitochondrial precursor	1.0	0.9	0.9	0.9	1.0	0.9	1.0	0.9	1.0	0.9	1.0	0.9	25.9	1	8	9	22	463	50.3	7.4
NP_787048.1	28460688	TXLNA	alpha-taxilin	0.8	0.9	0.7	0.9	0.9	0.9	0.8	0.9	0.8	0.9	0.8	0.9	38.8	1	16	17	44	546	61.9	6.5
NP_115712.1	14150122	GINS4	DNA replication complex GINS protein SLD5	1.1	0.9	0.9	0.9	0.9	0.9	0.8	0.9	1.0	0.9	0.9	0.9	22.0	1	5	5	14	223	26.0	5.0
NP_056230.1	7661602	MPC2	mitochondrial pyruvate carrier 2	0.9	0.9	0.9	0.9	1.0	1.1	1.1	0.9	1.0	1.0	1.0	0.9	17.3	1	2	2	9	127	14.3	10.4
NP_000974.1	4506613	RPL22	60S ribosomal protein L22 proprotein	0.9	0.9	0.9	0.9	0.9	0.9	0.9	0.9	0.9	0.9	0.9	0.9	55.5	1	5	5	145	128	14.8	9.2
NP_077003.1	13129104	CCDC86	coiled-coil domain-containing protein 86	0.9	1.0	0.9	0.9	0.8	1.0	0.9	0.9	0.8	1.0	0.9	0.9	35.0	3	10	10	24	360	40.2	10.3
NP_852469.1	31652242	TCF20	transcription factor 20 isoform 2	0.9	0.9	0.9	0.9	1.4	1.0	1.2	0.9	1.2	0.9	1.0	0.9	5.6	2	6	7	9	1938	209.5	9.1
NP_001230575.1	343887347	CD2BP2	CD2 antigen cytoplasmic tail-binding protein 2	1.0	0.9	1.0	0.9	1.0	0.9	1.0	0.9	1.0	0.9	1.0	0.9	40.8	1	8	8	26	341	37.6	4.6
NP_001136070.1	217272861;63252893	P4HA2	prolyl 4-hydroxylase subunit alpha-2 isoform 2 precursor	0.8	0.8	0.9	0.9	0.9	0.9	1.0	0.9	0.8	0.9	0.9	0.9	17.3	2	7	7	15	533	60.6	5.7
NP_005511.1	5031753;381342476	HNRNPH1	heterogeneous nuclear ribonucleoprotein H	0.9	0.9	0.9	0.9	0.9	0.9	0.9	0.9	0.9	0.9	0.9	0.9	58.1	1	10	17	239	449	49.2	6.3
NP_055861.3	113722133	SETX	probable helicase senataxin	0.9	0.9	1.0	0.8	0.8	1.1	1.0	1.0	0.9	1.0	1.0	0.9	1.7	1	4	4	7	2677	302.7	7.2
NP_066545.3	126116594	GINS1	DNA replication complex GINS protein PSF1	0.8	0.8	0.8	0.9	0.9	0.8	0.8	0.9	0.8	0.8	0.8	0.9	16.3	1	2	2	5	196	23.0	7.4
NP_057059.2	20070280	TAF9B	transcription initiation factor TFIID subunit 9B	1.1	1.0	1.0	0.9	0.7	0.9	0.8	0.9	0.9	0.9	0.9	0.9	4.4	2	1	1	1	251	27.6	9.6
NP_060298.2	21361661	COMMD4	COMM domain-containing protein 4 isoform 1	0.9	0.8	0.9	0.9	0.9	0.9	0.9	0.9	0.9	0.9	0.9	0.9	29.7	4	4	4	13	199	21.8	7.3
NP_059145.2	111118978	EPHB2	ephrin type-B receptor 2 isoform 1 precursor	0.9	1.0	0.9	0.9	0.9	0.9	1.0	0.9	0.9	0.9	0.9	0.9	9.6	5	8	8	19	986	109.8	5.6
NP_689647.2	169234949	CWF19L2	CWF19-like protein 2	0.8	0.9	1.0	0.9	1.1	0.9	1.0	0.9	1.0	0.9	1.0	0.9	1.9	1	1	1	2	894	103.7	8.6
NP_006589.2	123701900	SLC12A7	solute carrier family 12 member 7	0.9	0.9	1.0	0.9	0.9	0.9	0.9	0.9	0.9	0.9	0.9	0.9	3.8	1	2	2	3	1083	119.0	6.7
NP_056474.2	118498359	RSL1D1	ribosomal L1 domain-containing protein 1	0.8	0.9	0.8	0.9	0.8	0.8	0.9	0.9	0.8	0.9	0.8	0.9	45.5	1	23	23	91	490	54.9	10.1
NP_071933.2	19526773	ATG3	ubiquitin-like-conjugating enzyme ATG3 isoform 1	1.0	1.0	0.9	0.9	1.0	0.9	0.9	0.9	1.0	0.9	0.9	0.9	24.8	2	8	8	25	314	35.8	4.7
NP_002480.1	4505357	NDUFA4	cytochrome c oxidase subunit NDUFA4	1.0	0.9	1.0	0.9	1.1	1.0	1.1	0.9	1.0	0.9	1.0	0.9	22.2	1	2	2	5	81	9.4	9.4
NP_072045.1	11968182	RPS18	40S ribosomal protein S18	1.0	0.9	1.0	0.9	0.9	0.9	0.9	0.9	1.0	0.9	0.9	0.9	43.4	1	8	8	60	152	17.7	11.0
NP_004490.2	55956921	HNRNPAB	heterogeneous nuclear ribonucleoprotein A/B isoform b	0.9	1.0	0.8	0.9	0.8	1.0	0.8	0.9	0.9	1.0	0.8	0.9	50.2	2	14	17	362	285	30.6	7.9
NP_064615.3	47174861	PPAN	suppressor of SWI4 1 homolog	0.9	0.9	0.9	0.9	0.9	0.9	0.9	0.9	0.9	0.9	0.9	0.9	25.8	3	9	9	23	473	53.2	10.1
NP_004791.1	4758874	TM9SF2	transmembrane 9 superfamily member 2 precursor	0.9	0.9	1.0	0.8	0.9	1.0	1.0	1.0	0.9	0.9	1.0	0.9	8.1	1	3	4	7	663	75.7	7.4
NP_078947.3	146198654	ZNF768	zinc finger protein 768	0.9	1.0	0.9	1.0	0.9	0.8	0.8	0.8	0.9	0.9	0.8	0.9	9.1	117	4	5	6	540	60.2	8.0
NP_689956.2	304555614	BRAT1	BRCA1-associated ATM activator 1	0.9	0.9	0.9	0.9	1.0	0.9	0.9	0.9	0.9	0.9	0.9	0.9	9.3	1	4	4	7	821	88.1	5.3
NP_001058.2	19913406	TOP2A	DNA topoisomerase 2-alpha	0.9	0.9	0.9	0.9	0.9	0.9	0.9	0.9	0.9	0.9	0.9	0.9	49.6	1	60	77	446	1531	174.3	8.7
NP_057136.2	126362983	RRP15	RRP15-like protein	0.8	0.9	0.9	0.9	1.1	1.1	1.2	0.9	0.9	1.0	1.1	0.9	11.0	1	4	4	7	282	31.5	5.5
NP_001183980.1	308081964	ZFP91	E3 ubiquitin-protein ligase ZFP91 isoform 2	1.1	1.0	0.9	0.9	0.9	1.1	1.0	0.9	1.0	1.1	0.9	0.9	10.9	2	5	5	15	569	63.3	7.4
NP_000377.1	4557367	BLMH	bleomycin hydrolase	0.8	0.8	0.8	0.9	0.9	0.8	0.8	0.9	0.9	0.8	0.8	0.9	27.5	1	9	9	30	455	52.5	6.3
NP_443149.2	145309313	GALNT13	polypeptide N-acetylgalactosaminyltransferase 13	0.7	1.0	0.8	0.9	0.7	1.1	0.8	0.9	0.7	1.1	0.8	0.9	1.1	1	1	1	2	556	64.0	6.8
NP_004366.1	4758034	COX11	cytochrome c oxidase assembly protein COX11, mitochondrial isoform 1	0.8	0.7	0.8	0.8	1.0	1.0	0.9	0.9	0.9	0.8	0.8	0.9	12.3	3	2	2	4	276	31.4	9.1
NP_037423.2	56676371	CPSF1	cleavage and polyadenylation specificity factor subunit 1	1.0	0.9	0.9	0.9	1.0	0.9	0.9	0.9	1.0	0.9	0.9	0.9	15.0	1	18	18	49	1443	160.8	6.4

Khan *et al.* , 2019. Multi-omics analysis to characterize cigarette smoke induced molecular alterations in esophageal cells
Supplementary Table 5. List of proteins quantified in untreated and chronically treated Het1A cells with cigarette smoke condensate for 8 months

NP_Accession	Protein group Accession	Gene ID	Description	Het-1A-Smoke - 2M/Parental	Het-1A-Smoke - 4M/Parental	Het-1A-Smoke - 6M/Parental	Het-1A-Smoke - 8M/Parental	Het-1A-Smoke - 2M/Parental	Het-1A-Smoke - 4M/Parental	Het-1A-Smoke - 6M/Parental	Het-1A-Smoke - 8M/Parental	Het-1A-Smoke - 2M/Parental	Het-1A-Smoke - 4M/Parental	Het-1A-Smoke - 6M/Parental	Het-1A-Smoke - 8M/Parental	Coverage (%)	Proteins	Unique Peptides	# Peptides	PSM	# AAs	MW [kDa]	calc. pI
				Replicate 1				Replicate 2				Average of replicates											
NP_001273001.1	554506491	MOV10	putative helicase MOV-10 isoform 2	0.9	0.8	0.8	0.8	1.0	0.9	0.9	1.0	1.0	0.9	0.9	0.9	11.8	2	9	9	20	947	107.1	8.8
NP_077295.1	13236559	C1orf35	multiple myeloma tumor-associated protein 2	0.9	0.8	0.8	0.9	0.8	0.8	0.8	0.9	0.8	0.8	0.8	0.9	17.5	1	4	4	13	263	29.4	10.0
NP_001139792.1	226443017	GRAMD3	GRAM domain-containing protein 3 isoform 3	1.2	1.2	1.1	0.9	1.3	1.2	1.1	0.9	1.2	1.2	1.1	0.9	7.6	5	1	1	1	328	36.6	7.8
NP_060423.3	282165823	ZNHIT6	box C/D snoRNA protein 1 isoform 1	1.0	1.1	1.1	0.9	0.8	0.9	0.9	0.9	0.9	1.0	1.0	0.9	5.7	2	3	3	4	470	53.9	5.8
NP_006752.1	5803225	YWHAE	14-3-3 protein epsilon	0.9	1.0	0.9	0.9	0.9	1.0	0.9	0.9	0.9	1.0	0.9	0.9	83.9	1	20	23	796	255	29.2	4.7
NP_001164040.1	283046676	CXorf56	UPF0428 protein CXorf56 isoform 2	0.8	0.8	0.8	0.8	0.9	0.9	0.9	1.0	0.9	0.9	0.8	0.9	28.9	3	5	5	13	173	20.0	9.5
NP_079131.3	151301041	ACTR5	actin-related protein 5	0.9	0.8	0.9	0.9	0.9	0.8	0.9	0.9	0.9	0.8	0.9	0.9	4.1	1	2	2	4	607	68.3	5.3
NP_004587.1	4759156	SNRPA	U1 small nuclear ribonucleoprotein A	1.0	0.9	0.9	0.9	0.9	0.9	0.9	0.9	1.0	0.9	0.9	0.9	42.9	1	8	10	49	282	31.3	9.8
NP_001244088.1	380692321	CEP41	centrosomal protein of 41 kDa isoform 3	0.8	0.8	0.9	0.9	1.1	1.0	1.1	0.9	1.0	0.9	1.0	0.9	11.2	3	2	2	3	285	31.6	7.5
NP_057460.3	110815813	ANKFY1	rabankyrin-5 isoform 1	1.0	1.0	1.0	0.9	1.0	1.0	0.9	0.9	1.0	1.0	0.9	0.9	17.5	2	14	14	27	1170	128.4	6.1
NP_620707.1	21237739	MAPK9	mitogen-activated protein kinase 9 isoform alpha1	1.0	0.9	0.9	0.9	1.1	1.0	1.0	0.9	1.0	1.0	1.0	0.9	15.2	9	4	5	13	382	44.0	6.4
NP_009120.1	27881506	ABCF2	ATP-binding cassette sub-family F member 2 isoform a	1.0	1.0	1.0	0.9	1.1	1.0	1.1	0.9	1.1	1.0	1.1	0.9	17.5	2	10	10	18	623	71.2	7.4
NP_006730.2	23510448	MCM5	DNA replication licensing factor MCM5	1.0	0.9	1.0	0.9	1.0	0.9	1.0	0.9	1.0	0.9	1.0	0.9	42.5	1	25	26	101	734	82.2	8.4
NP_001026884.3	149999378	INF2	inverted formin-2 isoform 2	1.0	0.9	0.9	0.9	1.0	0.9	1.0	0.9	1.0	0.9	0.9	0.9	19.8	3	12	12	39	1240	134.5	5.5
NP_113642.1	32880229	SELO	selenoprotein O	1.1	0.7	0.7	0.7	1.1	1.1	1.1	1.1	1.1	0.9	0.9	0.9	3.0	1	1	1	2	669	73.4	6.0
NP_056197.3	612339310	HECTD1	E3 ubiquitin-protein ligase HECTD1	0.9	0.8	0.9	1.0	0.9	0.8	0.9	0.8	0.9	0.8	0.9	0.9	1.2	1	3	3	6	2610	289.2	5.4
NP_003395.1	4507949	YWHAB	14-3-3 protein beta/alpha	0.8	0.8	0.7	0.9	0.8	0.8	0.7	0.9	0.8	0.8	0.7	0.9	83.3	1	9	19	443	246	28.1	4.8
NP_079104.3	262527223	CAAP1	caspase activity and apoptosis inhibitor 1 isoform 1	0.9	1.0	0.9	0.9	1.0	0.9	1.0	0.9	0.9	1.0	1.0	0.9	30.8	2	7	7	26	361	38.3	4.7
NP_001034680.2	145309311	USP9X	probable ubiquitin carboxyl-terminal hydrolase FAF-X isoform 4	1.0	0.9	0.9	0.9	1.0	1.0	1.0	0.9	1.0	0.9	0.9	0.9	13.9	3	26	26	66	2554	290.3	5.8
NP_061917.3	82775371	CCDC93	coiled-coil domain-containing protein 93	0.9	0.8	0.9	0.9	1.1	0.9	0.9	0.9	1.0	0.9	0.9	0.9	6.7	1	4	4	6	631	73.2	8.1
NP_079058.1	13376142	NHEJ1	non-homologous end-joining factor 1	0.8	0.7	0.9	0.8	0.9	0.7	0.9	1.0	0.8	0.7	0.9	0.9	7.0	1	1	1	2	299	33.3	6.0
NP_064572.2	54607104	HMCES	embryonic stem cell-specific 5-hydroxymethylcytosine-binding protein	0.9	0.9	0.9	0.9	0.9	0.9	0.9	0.9	0.9	0.9	0.9	0.9	9.0	1	2	2	3	354	40.5	8.1
NP_000963.1	4506661	RPL7A	60S ribosomal protein L7a	0.9	0.8	0.9	0.9	0.9	0.9	0.9	0.9	0.9	0.9	0.9	0.9	54.5	1	17	18	93	266	30.0	10.6
NP_150093.1	15147219	PURB	transcriptional activator protein Pur-beta	0.8	0.9	0.7	0.9	0.9	0.9	0.8	0.9	0.8	0.9	0.8	0.9	47.4	3	7	7	18	312	33.2	5.4
NP_060602.2	40068061	CENPQ	centromere protein Q	1.0	0.9	0.8	0.9	0.9	1.0	0.9	0.9	0.9	0.9	0.8	0.9	9.7	1	3	3	6	268	30.6	9.4
NP_001012.1	4506693	RPS17	40S ribosomal protein S17	0.9	1.0	1.0	0.9	0.9	1.0	0.9	0.9	0.9	1.0	1.0	0.9	59.3	1	8	8	72	135	15.5	9.8
NP_071441.1	11545906	FN3K	fructosamine-3-kinase	0.8	0.9	0.9	0.9	0.9	0.9	0.8	0.9	0.9	0.9	0.9	0.9	3.9	1	1	1	4	309	35.1	7.5
NP_004100.1	4758352	FDX1	adrenodoxin, mitochondrial precursor	1.1	1.0	1.2	0.9	1.4	1.0	1.5	0.9	1.3	1.0	1.3	0.9	4.9	1	1	1	1	184	19.4	5.8
NP_001112.2	62912453	ADD3	gamma-adducin isoform b	0.8	0.8	0.8	0.8	0.9	0.9	0.9	1.0	0.9	0.9	0.8	0.9	8.2	2	4	4	4	674	75.6	6.8
NP_001017405.1	62953131	MAEA	macrophage erythroblast attacher isoform 1	1.2	0.9	0.9	0.9	1.0	0.9	1.0	0.9	1.1	0.9	1.0	0.9	18.7	5	5	5	12	396	45.3	8.7
NP_001120654.1	187608545	CEP55	centrosomal protein of 55 kDa	0.9	0.9	1.0	0.9	1.0	0.9	0.9	0.9	0.9	0.9	0.9	0.9	1.7	1	1	1	1	464	54.1	7.0
NP_001139585.1	225735619	MIER1	mesoderm induction early response protein 1 isoform i	0.9	0.9	0.9	0.8	1.1	1.0	1.0	1.0	1.0	1.0	1.0	0.9	12.4	10	3	4	13	370	42.1	4.5
NP_056525.2	239787829	GLTSCR2	glioma tumor suppressor candidate region gene 2 protein	0.8	1.0	0.9	0.9	0.9	1.0	0.9	0.9	0.8	1.0	0.9	0.9	15.3	1	7	7	23	478	54.4	10.3
NP_061185.1	29789090;209969703	RCC2	protein RCC2	0.9	0.8	0.8	0.9	0.9	0.9	0.8	0.9	0.9	0.8	0.8	0.9	44.3	1	20	20	72	522	56.0	8.8
NP_078934.3	62460637	IPO4	importin-4	1.0	0.9	1.0	0.9	1.0	0.9	0.9	0.9	1.0	0.9	1.0	0.9	20.4	1	14	14	32	1081	118.6	5.0
NP_005605.1	5032041	RHEB	GTP-binding protein Rheb	1.1	1.1	1.2	0.9	0.9	0.9	1.0	0.8	1.0	1.0	1.1	0.9	42.4	1	6	6	22	184	20.5	5.9
NP_060366.1	8923557	GID8	glucose-induced degradation protein 8 homolog	1.0	0.8	0.9	0.9	1.1	0.9	1.0	0.9	1.0	0.9	1.0	0.9	42.5	1	6	6	29	228	26.7	5.0
NP_112420.1	14043070	HNRNPA1	heterogeneous nuclear ribonucleoprotein A1 isoform b	0.9	1.0	0.9	1.0	0.6	0.8	0.8	0.8	0.8	0.9	0.9	0.9	51.9	3	12	17	296	372	38.7	9.1
NP_001895.1	4503131;148233338	CTNNB1	catenin beta-1	0.9	0.8	0.9	0.9	1.0	0.9	1.0	0.9	0.9	0.9	0.9	0.9	34.7	1	19	21	46	781	85.4	5.9
NP_001231593.1	347658913	CHTOP	chromatin target of PRMT1 protein isoform 3	1.0	0.9	1.0	0.9	0.9	0.9	0.7	0.9	0.9	0.9	0.9	0.9	17.1	3	1	1	10	76	8.5	12.0
NP_446464.1	16950633;53759107	ASS1	argininosuccinate synthase	1.0	1.1	0.9	0.9	1.0	1.1	0.9	0.9	1.0	1.1	0.9	0.9	49.0	1	18	18	66	412	46.5	8.0
NP_003155.2	94400932	STX5	syntaxin-5 isoform 1	1.1	0.9	1.0	0.8	1.1	1.1	1.3	1.0	1.1	1.0	1.1	0.9	17.5	2	5	5	9	355	39.6	9.2
NP_005457.2	42544155	MED6	mediator of RNA polymerase II transcription subunit 6 isoform 2	1.0	0.7	0.9	0.9	1.0	0.7	0.8	0.9	1.0	0.7	0.9	0.9	3.7	3	1	1	2	246	28.4	8.6
NP_001006.1	4506681	RPS11	40S ribosomal protein S11	0.8	0.9	0.9	0.9	0.9	0.8	0.9	0.9	0.9	0.9	0.9	0.9	55.7	1	10	10	87	158	18.4	10.3
NP_963294.1	42544213;42544218	NDRG2	protein NDRG2 isoform b	0.9	0.8	0.8	0.8	1.0	0.9	1.0	0.9	1.0	0.8	0.9	0.9	12.6	6	3	3	5	357	39.3	5.4
NP_057508.2	19923485	LUC7L3	luc7-like protein 3	0.9	0.9	0.9	0.9	0.9	0.9	0.9	0.9	0.9	0.9	0.9	0.9	21.5	1	8	8	32	432	51.4	9.8
NP_112739.2	22035679	ARID4B	AT-rich interactive domain-containing protein 4B isoform 2	0.9	1.0	0.9	0.8	1.0	1.0	1.1	1.0	0.9	1.0	1.0	0.9	7.3	2	6	6	11	1226	137.5	5.5
NP_443080.1	16418351	CCDC97	coiled-coil domain-containing protein 97	1.1	0.9	0.8	0.9	1.0	0.9	0.9	0.9	1.1	0.9	0.8	0.9	17.5	1	4	4	8	343	38.9	4.6
NP_620175.1	20270389	HIGD2A	HIG1 domain family member 2A, mitochondrial	0.9	1.0	0.9	0.9	1.0	0.9	1.2	0.9	0.9	0.9	1.0	0.9	5.7	1	1	1	1	106	11.5	10.2
NP_149094.3	228008333	RHPN2	rhophilin-2	1.0	0.9	1.0	0.9	0.9	0.9	1.0	0.9	1.0	0.9	1.0	0.9	1.3	1	1	1	1	686	76.9	6.8
NP_004580.1	4759068	SCO1	protein SCO1 homolog, mitochondrial	0.9	0.8	0.8	0.9	1.0	0.8	0.9	0.9	0.9	0.8	0.9	0.9	18.3	1	3	3	5	301	33.8	8.9
NP_001161413.1	269847404	WDR76	WD repeat-containing protein 76 isoform 2	0.8	0.8	0.9	0.8	1.0	1.0	1.0	0.9	0.9	0.9	0.9	0.9	4.6	2	2	2	3	562	62.5	9.1
NP_002044.2	166706903	GBP1	interferon-induced guanylate-binding protein 1	1.0	1.0	0.9	0.9	1.0	1.0	0.9	0.9	1.0	1.0	0.9	0.9	21.5	3	11	11	26	592	67.9	6.3
NP_001420.2	50345997	EP300	histone acetyltransferase p300	1.0	0.9	0.9	0.9	0.9	0.9	1.0	0.9	0.9	0.9	0.9	0.9	4.4	1	3	4	8	2414	264.0	8.5
NP_036558.3	54112121	SF3B3	splicing factor 3B subunit 3	0.9	0.9	0.9	0.9	0.9	0.9	0.9	0.9	0.9	0.9	0.9	0.9	35.5	1	30	30	149	1217	135.5	5.3
NP_110382.3	151101292	TMX1	thioredoxin-related transmembrane protein 1 precursor	0.8	0.8	0.8	0.9	0.8	0.9	0.8	0.9	0.8	0.8	0.8	0.9	31.8	1	9	9	39	280	31.8	5.0
NP_001020092.1	67944630;15431303	RPL9	60S ribosomal protein L9	0.8	0.8	0.8	0.8	0.9	0.9	0.9	1.0	0.8	0.8	0.9	0.9	73.4	1	12	12	72	192	21.8	9.9
NP_149977.2	255918129	LMF2	lipase maturation factor 2	1.2	1.0	1.0	0.9	1.1	1.1	0.8	0.9	1.1	1.0	0.9	0.9	3.8	1	2	2	5	707	79.6	10.1
NP_612423.1	19923935	CCDC101	SAGA-associated factor 29 homolog	0.9	1.0	0.9	1.0	0.8	0.8	0.9	0.8	0.9	0.9	0.9	0.9	4.4	1	1	1	2	293	33.2	8.1
NP_443077.1	16418349	MMAB	cob(I)yrinic acid a,c-diamide adenosyltransferase, mitochondrial precursor	1.4	1.0	1.0	0.9	1.0	0.9	0.9	0.8	1.2	1.0	0.9	0.9	39.6	1	7	7	24	250	27.4	8.6
NP_150628.3	218505827	KNSTRN	small kinetochore-associated protein isoform a	0.8	1.0	1.0	1.0	0.7	0.8	0.9	0.8	0.7	0.9	0.9	0.9	24.7	3	4	4	7	316	35.4	6.3
NP_001121598.1	189409104	AP4S1	AP-4 complex subunit sigma-1 isoform 2	0.9	0.8	0.9	0.9	0.9	0.8	0.8	0.9	0.9	0.8	0.8	0.9	6.9	1	1	1	6	144	17.0	5.2
NP_064711.1	10092677	PDXP	pyridoxal phosphate phosphatase	1.1	1.2	1.1	0.9	1.0	1.0	1.0	0.9	1.1	1.1	1.1	0.9	14.9	1	3	3	7	296	31.7	6.6
NP_004955.2	13128860	HDAC1	histone deacetylase 1	0.9	0.9	0.8	0.8	1.0	1.0	1.0	0.9	1.0	0.9	0.9	0.9	32.8	1	9	13	30	482	55.1	5.5
NP_057066.2	156415990	YBX2	Y-box-binding protein 2	1.7	1.2	1.5	0.9	0.9	1.0	1.0	0.9	1.3	1.1	1.2	0.9	40.1	1	6	9	40	364	38.5	10.8
NP_001268424.1	527498276	CTNNBL1	beta-catenin-like protein 1 isoform 2	1.2	0.9	0.9	0.9	0.9	0.8	0.9	0.9	1.0	0.9	0.9	0.9	28.0	2	13	13	35	536	61.9	5.1
NP_001129125.1	208431833	PABPC4	polyadenylate-binding protein 4 isoform 1	0.9	0.9	0.8	0.9	0.9	0.9	0.8	0.9	0.9	0.9	0.8	0.9	43.8	5	16	24	174	660	72.3	9.3
NP_004259.3	28558975	MED17	mediator of RNA polymerase II transcription subunit 17	1.0	1.0	1.1	0.8	0.9	0.9	0.9	0.9	1.0	1.0	1.0	0.9	2.6	1	1	1	2	651	72.8	7.4
NP_057661.3	96975097	RAB6B	ras-related protein Rab-6B	0.9	0.9	0.8	0.9	1.0	0.9	0.8	0.9	1.0	0.9	0.8	0.9	28.4	9	1	6	27	208	23.4	5.5
NP_006550.1	5730027	KHDRBS1	KH domain-containing, RNA-binding, signal transduction-associated protein 1 isoform 1	0.8	0.8	0.8	0.9	0.9	0.9	0.8	0.9	0.8	0.8	0.8	0.9	35.0	5	11	13	85	443	48.2	8.7
NP_000349.1	4507467	TGFBI	transforming growth factor-beta-induced protein ig-h3 precursor	0.9	1.0	0.9	0.8	1.0	1.1	1.0	0.9	1.0	1.0	1.0	0.9	18.7	1	10	10	35	683	74.6	7.7
NP_006451.1	5453682	HEXIM1	protein HEXIM1	0.8	1.0	0.9	0.9	0.9	0.9	0.9	0.9	0.9	1.0	0.9	0.9	18.1	1	5	5	11	359	40.6	4.9
NP_006035.2	13128864	HDAC6	histone deacetylase 6	0.7	0.9	0.8	0.9	1.0	0.9	0.8	0.8	0.9	0.9	0.8	0.9	5.1	1	3	3	5	1215	131.3	5.3
NP_003161.2	27597090	SUPT6H	transcription elongation factor SPT6	0.9	0.9	0.9	0.9	0.9	1.0	1.0	0.9	0.9	0.9	0.9	0.9	26.9	1	31	31	83	1726	198.9	4.9
NP_001025.1	4557845	RRM2	ribonucleoside-diphosphate reductase subunit M2 isoform 2	0.9	0.8	0.9	0.9	0.9	0.9	0.9	0.9	0.9	0.8	0.9	0.9	24.2	2	5	6	18	389	44.8	5.4
NP_002443.3	40288274;40288276	NUDT1	7,8-dihydro-8-oxoguanine triphosphatase isoform p18	1.3	0.9	1.1	1.0	1.2	0.8	1.0	0.8	1.3	0.8	1.1	0.9	22.4	2	3	3	10	156	17.9	5.1
NP_005849.1	5031579	AKAP8	A-kinase anchor protein 8	0.9	1.0	1.0	0.9	1.0	1.0	1.0	0.9	1.0	1.0	1.0	0.9	30.9	1	10	10	32	692	76.1	5.1
NP_001264162.1	469663653	TM7SF2	delta(14)-sterol reductase isoform 2	0.8	0.8	0.9	0.9	1.0	1.0	1.0	0.9	0.9	0.9	0.9	0.9	6.4	2	2	2	9	391	43.3	8.2
NP_683705.1	22547171	OSBPL9	oxysterol-binding protein-related protein 9 isoform c	0.9	0.9	0.9	0.9	0.9	0.9	0.9	0.9	0.9	0.9	0.9	0.9	11.0	6	6	6	10	626	70.2	6.1
NP_006080.1	5174669	SCML2	sex comb on midleg-like protein 2	1.1	1.0	1.0	1.0	1.1	0.9	1.0	0.8	1.1	0.9	1.0	0.9	7.1	1	3	3	3	700	77.2	8.5
NP_004102.1	4758356	FEN1	flap endonuclease 1	1.0	0.9	0.9	0.9	0.9	0.9	0.8	0.9	1.0	0.9	0.9	0.9	49.7	1	15	15	93	380	42.6	8.6
NP_001116137.1	169881239;13514809	DDX3Y	ATP-dependent RNA helicase DDX3Y isoform 1	0.9	0.9	0.9	0.9	0.9	0.9	0.9	0.9	0.9	0.9	0.9	0.9	42.1	2	7	21	99	660	73.1	7.5
NP_037473.3	58761500	OLA1	obg-like ATPase 1 isoform 1	0.9	1.0	1.0	0.8	0.9	1.0	1.0	0.9	0.9	1.0	1.0	0.9	50.0	2	14	14	63	396	44.7	7.8
NP_003962.3	27436920	SPAG9	C-Jun-amino-terminal kinase-interacting protein 4 isoform 3	0.9	0.9	0.9	0.9	0.9	0.9	0.9	0.9	0.9	0.9	0.9	0.9	33.4	8	31	32	80	1307	144.6	5.2
NP_006316.1	5453555	RAN	GTP-binding nuclear protein Ran isoform 1	1.0	0.9	0.9	0.9	0.9	0.9	0.9	0.8	1.0	0.9	0.9	0.9	74.5	2	11	11	130	216	24.4	7.5
NP_612510.1	24475816	TECR	very-long-chain enoyl-CoA reductase	0.9	0.9	0.9	0.9	0.9	0.9	0.9	0.9	0.9	0.9	0.9	0.9	24.0	1	8	8	31	308	36.0	9.5
NP_079092.2	117956403	RABEP2	rab GTPase-binding effector protein 2	0.9	1.0	0.9	0.9	0.9	1.0	0.8	0.9	0.9	1.0	0.8	0.9	12.1	1	6	7	12	569	63.5	4.8
NP_689728.3	167466272	CKAP2L	cytoskeleton-associated protein 2-like isoform 1	0.9	1.0	0.9	0.9	0.9	1.0	0.9	0.9	0.9	1.0	0.9	0.9	5.8	1	3	3	4	745	83.5	9.8

Khan *et al.* , 2019. Multi-omics analysis to characterize cigarette smoke induced molecular alterations in esophageal cells
Supplementary Table 5. List of proteins quantified in untreated and chronically treated Het1A cells with cigarette smoke condensate for 8 months

NP_Accession	Protein group Accession	Gene ID	Description	Het-1A-Smoke - 2M/Parental	Het-1A-Smoke - 4M/Parental	Het-1A-Smoke - 6M/Parental	Het-1A-Smoke - 8M/Parental	Het-1A-Smoke - 2M/Parental	Het-1A-Smoke - 4M/Parental	Het-1A-Smoke - 6M/Parental	Het-1A-Smoke - 8M/Parental	Het-1A-Smoke - 2M/Parental	Het-1A-Smoke - 4M/Parental	Het-1A-Smoke - 6M/Parental	Het-1A-Smoke - 8M/Parental	Coverage (%)	Proteins	Unique Peptides	# Peptides	PSM	# AAs	MW [kDa]	calc. pI
				Replicate 1				Replicate 2				Average of replicates											
NP_004350.1	16357477	**CDC34**	ubiquitin-conjugating enzyme E2 R1	1.1	0.9	0.9	0.9	0.8	0.9	0.9	0.9	**1.0**	**0.9**	**0.9**	**0.9**	3.0	1	1	1	1	236	26.7	4.5
NP_057300.2	56549113	**DBR1**	lariat debranching enzyme	1.0	1.0	1.0	0.9	1.0	0.9	0.9	0.9	**1.0**	**1.0**	**0.9**	**0.9**	12.9	1	4	4	8	544	61.5	5.5
NP_000936.1	4506025	**PPP3R1**	calcineurin subunit B type 1	0.9	1.0	1.0	0.9	1.0	0.9	1.0	0.9	**1.0**	**0.9**	**1.0**	**0.9**	55.9	1	6	6	16	170	19.3	4.8
NP_001265528.1	520260399	**AXL**	tyrosine-protein kinase receptor UFO isoform 3	0.8	0.9	0.9	0.8	0.9	1.0	1.1	1.0	**0.9**	**1.0**	**1.0**	**0.9**	1.8	3	1	1	1	626	69.2	5.5
NP_001138471.1	223468595	**ITGAV**	integrin alpha-V isoform 2 precursor	0.9	1.0	1.0	0.9	0.9	1.0	1.1	0.9	**0.9**	**1.0**	**1.1**	**0.9**	14.3	3	9	10	16	1002	111.1	5.6
NP_078930.3	40217805	**NOL9**	polynucleotide 5'-hydroxyl-kinase NOL9	1.0	1.0	1.0	0.9	0.9	0.8	0.9	0.9	**1.0**	**0.9**	**0.9**	**0.9**	4.8	1	3	3	4	702	79.2	9.2
NP_004256.1	4758334	**FADS2**	fatty acid desaturase 2 isoform 1	1.0	1.1	1.2	0.9	0.9	1.1	1.1	0.9	**1.0**	**1.1**	**1.1**	**0.9**	17.6	3	7	8	12	444	52.2	8.8
NP_005769.1	5032031	**RBM5**	RNA-binding protein 5	0.9	1.0	0.9	0.9	0.9	0.9	0.9	0.9	**0.9**	**0.9**	**0.9**	**0.9**	9.7	1	6	7	15	815	92.1	6.3
NP_071751.3	186928835	**P3H1**	prolyl 3-hydroxylase 1 isoform 1 precursor	1.0	1.0	0.9	0.9	0.9	0.9	0.9	0.8	**0.9**	**0.9**	**0.9**	**0.9**	14.5	3	8	9	18	736	83.3	5.1
NP_006404.1	5454024	**RPP30**	ribonuclease P protein subunit p30 isoform b	0.9	0.9	0.8	0.9	0.8	0.7	0.8	0.9	**0.8**	**0.8**	**0.8**	**0.9**	38.8	2	8	8	23	268	29.3	8.9
NP_689476.2	114155140	**TPM3**	tropomyosin alpha-3 chain isoform Tpm3.12st	1.0	1.0	1.0	0.9	1.0	1.1	1.2	0.8	**1.0**	**1.1**	**1.1**	**0.9**	52.3	1	1	17	104	285	32.9	4.7
NP_001177938.1	300360554	**SRSF10**	serine/arginine-rich splicing factor 10 isoform 6	0.8	0.7	0.8	0.8	0.9	0.9	0.8	0.9	**0.8**	**0.8**	**0.8**	**0.9**	35.2	6	4	6	7	165	20.1	10.1
NP_000968.2	15431297;15431295	**RPL13**	60S ribosomal protein L13 isoform 1	0.9	0.9	0.9	0.9	0.9	0.9	0.9	0.9	**0.9**	**0.9**	**0.9**	**0.9**	41.7	3	10	10	80	211	24.2	11.6
NP_859056.2	91176333	**LYRM7**	complex III assembly factor LYRM7 isoform 1	1.3	0.9	1.2	0.9	1.1	0.8	1.1	0.9	**1.2**	**0.9**	**1.1**	**0.9**	46.2	1	4	4	8	104	11.9	9.7
NP_060851.2	154350198	**C19orf66**	UPF0515 protein C19orf66	0.9	1.0	0.9	0.9	1.0	1.0	1.0	0.9	**1.0**	**1.0**	**0.9**	**0.9**	22.3	1	4	4	8	291	33.1	7.3
NP_683718.1	22547189	**SHMT1**	serine hydroxymethyltransferase, cytosolic isoform 2	1.0	1.0	0.9	0.9	1.0	0.9	0.9	0.9	**1.0**	**0.9**	**0.9**	**0.9**	36.7	3	11	12	59	444	49.0	7.7
NP_005504.2	144922725	**GTF2E1**	general transcription factor IIE subunit 1	0.8	1.0	0.8	0.9	0.9	1.0	0.9	0.9	**0.9**	**1.0**	**0.9**	**0.9**	32.1	1	10	10	35	439	49.4	4.8
NP_660207.2	113374156	**VTI1A**	vesicle transport through interaction with t-SNAREs homolog 1A	0.9	0.9	0.9	1.0	0.8	0.8	1.0	0.8	**0.9**	**0.9**	**0.9**	**0.9**	2.8	3	1	1	1	217	25.2	6.4
NP_003237.2	40317626	**THBS1**	thrombospondin-1 precursor	1.1	1.4	1.3	0.9	0.9	1.1	1.2	0.9	**1.0**	**1.3**	**1.2**	**0.9**	9.7	1	8	8	16	1170	129.3	4.9
NP_001135441.1	215422388;4503257	**DAXX**	death domain-associated protein 6 isoform a	1.0	1.1	1.0	1.0	0.9	0.9	0.9	0.8	**1.0**	**1.0**	**1.0**	**0.9**	8.5	3	6	6	15	740	81.3	4.9
NP_000977.1	4506619	**RPL24**	60S ribosomal protein L24	0.9	0.9	0.9	0.9	0.9	0.8	0.9	0.9	**0.9**	**0.9**	**0.9**	**0.9**	36.3	1	8	8	52	157	17.8	11.3
NP_112179.2	40254978	**FIP1L1**	pre-mRNA 3'-end-processing factor FIP1 isoform 1	1.0	1.0	1.0	0.9	0.8	0.8	0.8	0.8	**0.9**	**0.9**	**0.9**	**0.9**	13.8	3	6	6	15	594	66.5	5.6
NP_653283.3	56119108	**SLFN13**	schlafen family member 13	1.0	0.9	1.0	0.9	1.0	0.9	1.0	0.8	**1.0**	**0.9**	**1.0**	**0.9**	7.3	1	1	5	15	897	102.0	7.0
NP_061981.2	145301567	**SMG9**	protein SMG9	0.9	0.8	0.8	0.8	1.0	1.0	1.0	1.0	**0.9**	**0.9**	**0.9**	**0.9**	2.5	1	1	1	2	520	57.6	7.0
NP_005834.4	188219625	**STAM2**	signal transducing adapter molecule 2	1.0	0.9	0.9	0.9	1.0	0.9	0.8	0.9	**1.0**	**0.9**	**0.9**	**0.9**	13.5	1	5	5	9	525	58.1	5.1
NP_002801.1	5292161	**PSMD4**	26S proteasome non-ATPase regulatory subunit 4	1.0	1.0	1.0	0.9	1.0	1.0	1.0	0.9	**1.0**	**1.0**	**1.0**	**0.9**	50.4	1	11	11	68	377	40.7	4.8
NP_036313.3	52630440	**FKBP8**	peptidyl-prolyl cis-trans isomerase FKBP8 isoform 1	0.9	1.0	1.0	0.9	0.8	0.8	0.8	0.9	**0.9**	**0.9**	**0.9**	**0.9**	26.9	2	7	7	32	413	44.6	4.8
NP_057306.2	21071032	**DDX41**	probable ATP-dependent RNA helicase DDX41	0.9	0.9	0.8	0.9	1.0	1.0	0.9	0.9	**0.9**	**1.0**	**0.9**	**0.9**	15.3	1	8	8	18	622	69.8	6.8
NP_620073.2	162461738	**GNAO1**	guanine nucleotide-binding protein G(o) subunit alpha isoform b	1.0	1.0	1.1	0.9	0.9	1.0	1.0	0.9	**1.0**	**1.0**	**1.1**	**0.9**	18.1	8	1	6	22	354	40.1	5.9
NP_001258218.1	404501455	**ZNF512**	zinc finger protein 512 isoform d	1.0	0.9	0.9	0.8	1.2	0.9	0.9	0.9	**1.1**	**0.9**	**0.9**	**0.9**	8.7	4	3	3	4	462	53.1	9.6
NP_002254.2	167555110	**KIFC1**	kinesin-like protein KIFC1	0.9	0.8	0.8	0.9	0.9	0.9	0.8	0.9	**0.9**	**0.8**	**0.8**	**0.9**	23.8	1	11	11	26	673	73.7	9.0
NP_075377.2	115334679	**ARID4A**	AT-rich interactive domain-containing protein 4A isoform III	0.9	0.9	0.9	0.9	1.0	1.0	0.9	0.9	**0.9**	**0.9**	**0.9**	**0.9**	6.1	3	5	5	9	1188	134.9	5.0
NP_998839.1	47519616	**TPM2**	tropomyosin beta chain isoform Tpm2.1sm/cy	1.1	1.1	1.2	0.9	1.0	0.9	0.9	0.9	**1.0**	**1.0**	**1.0**	**0.9**	64.1	2	6	22	150	284	33.0	4.7
NP_002678.2	33356174	**PNN**	pinin	0.8	0.8	0.8	0.9	0.9	0.9	0.8	0.9	**0.8**	**0.8**	**0.8**	**0.9**	33.2	1	16	16	53	717	81.6	7.1
NP_001116293.1	170763500	**SET**	protein SET isoform 1	0.9	1.0	0.9	0.9	1.1	1.0	0.9	0.9	**1.0**	**1.0**	**0.9**	**0.9**	57.9	4	2	12	278	290	33.5	4.3
NP_077289.1	13236553	**NOL12**	nucleolar protein 12	0.8	0.9	0.9	0.9	0.8	0.9	0.9	0.9	**0.8**	**0.9**	**0.9**	**0.9**	7.0	1	2	2	2	213	24.6	10.2
NP_055047.2	172072620	**VAMP2**	vesicle-associated membrane protein 2	0.9	0.7	0.7	0.7	1.1	1.1	1.1	1.0	**1.0**	**0.9**	**0.9**	**0.9**	34.5	4	1	3	20	116	12.7	8.1
NP_068809.1	11527390	**POLR2F**	DNA-directed RNA polymerases I, II, and III subunit RPABC2 isoform 1	0.9	0.8	1.0	0.7	1.2	0.9	1.1	1.1	**1.1**	**0.9**	**1.1**	**0.9**	5.5	2	1	1	2	127	14.5	4.2
NP_009169.3	34452734	**PXMP4**	peroxisomal membrane protein 4 isoform a	0.9	0.7	0.7	0.9	1.0	0.7	1.2	0.8	**0.9**	**0.7**	**1.0**	**0.9**	19.3	1	2	2	3	212	24.2	10.1
NP_612455.1	19923977	**MALSU1**	mitochondrial assembly of ribosomal large subunit protein 1	1.1	0.8	1.2	0.9	1.0	0.8	1.0	0.9	**1.0**	**0.8**	**1.1**	**0.9**	15.0	1	1	1	6	234	26.2	5.5
NP_778235.1	28195394	**HIST2H2AB**	histone H2A type 2-B	0.6	0.8	0.8	0.8	1.0	0.9	1.0	0.9	**0.8**	**0.9**	**0.9**	**0.9**	64.6	2	1	7	187	130	14.0	10.9
NP_001035242.1	94421473	**PEG10**	retrotransposon-derived protein PEG10 isoform 2	1.0	0.7	0.7	0.9	0.9	0.8	0.9	0.9	**1.0**	**0.8**	**0.8**	**0.9**	4.3	6	1	1	2	325	36.9	6.2
NP_001008240.1	56549656	**C18orf25**	uncharacterized protein C18orf25 isoform b	1.4	1.2	1.1	0.9	0.8	0.9	0.9	0.8	**1.1**	**1.0**	**1.0**	**0.9**	14.6	2	3	3	5	343	37.0	5.1
NP_001002857.1	50845386	**ANXA2**	annexin A2 isoform 2	0.9	0.8	0.9	0.9	0.9	0.8	0.9	0.9	**0.9**	**0.8**	**0.9**	**0.9**	75.5	2	27	27	722	339	38.6	7.8
NP_060189.3	156105705	**SNRK**	SNF-related serine/threonine-protein kinase	0.9	1.0	1.0	0.9	0.9	0.9	0.9	0.9	**0.9**	**0.9**	**1.0**	**0.9**	3.5	1	1	1	1	765	84.2	7.1
NP_060370.1	8923565	**AURKAIP1**	aurora kinase A-interacting protein	0.9	1.0	1.0	0.9	0.8	0.9	0.9	0.9	**0.9**	**1.0**	**0.9**	**0.9**	5.0	1	1	1	6	199	22.3	10.8
NP_001003684.1	51173876	**UQCR10**	cytochrome b-c1 complex subunit 9 isoform b	1.5	0.8	1.1	0.8	1.0	0.9	1.0	1.0	**1.2**	**0.9**	**1.0**	**0.9**	11.3	2	1	1	3	62	6.7	5.9
NP_001257824.1	401461824	**TRAPPC3**	trafficking protein particle complex subunit 3 isoform 3	0.9	0.9	0.9	0.9	0.8	0.8	0.8	0.8	**0.9**	**0.8**	**0.8**	**0.9**	20.2	4	3	3	5	134	15.0	4.9
NP_075384.3	145580615	**KRI1**	protein KRI1 homolog	0.8	0.9	0.9	0.9	0.8	0.9	0.9	0.9	**0.8**	**0.9**	**0.9**	**0.9**	11.3	1	6	6	18	709	83.2	5.2
NP_001184226.1	308818209;34452721	**GABPA**	GA-binding protein alpha chain	0.9	1.0	1.0	0.9	0.9	0.9	0.9	0.9	**0.9**	**1.0**	**0.9**	**0.9**	33.5	1	10	10	23	454	51.3	5.0
NP_851320.1	30725877	**GPR180**	integral membrane protein GPR180 precursor	0.9	0.9	0.9	0.9	0.9	0.8	0.7	0.9	**0.9**	**0.8**	**0.8**	**0.9**	2.3	1	1	1	1	440	49.4	7.4
NP_001136277.1	218563756	**SLC6A8**	sodium- and chloride-dependent creatine transporter 1 isoform 2	1.2	0.9	1.1	1.0	1.0	1.0	1.1	0.8	**1.1**	**0.9**	**1.1**	**0.9**	4.3	3	2	2	2	625	69.4	6.4
NP_057373.1	7706481;195927020	**CAB39**	calcium-binding protein 39	1.0	0.9	1.0	0.9	1.0	0.9	1.0	0.9	**1.0**	**0.9**	**1.0**	**0.9**	25.8	2	7	7	16	341	39.8	6.9
NP_004468.1	4758404	**FRG1**	protein FRG1	0.9	0.9	0.9	0.9	0.9	0.8	0.9	0.9	**0.9**	**0.9**	**0.9**	**0.9**	12.8	5	3	3	10	258	29.2	9.0
NP_001192108.1	326937484;326937486	**ALKBH2**	alpha-ketoglutarate-dependent dioxygenase alkB homolog 2 isoform 2	0.9	0.9	0.9	0.9	1.1	0.9	1.0	0.9	**1.0**	**0.9**	**1.0**	**0.9**	7.6	2	1	1	5	157	17.1	6.6
NP_003478.1	4507353	**TAF15**	TATA-binding protein-associated factor 2N isoform 2	0.8	0.8	0.8	0.8	0.8	0.8	0.8	0.9	**0.8**	**0.8**	**0.8**	**0.9**	15.5	2	5	7	48	589	61.5	8.0
NP_008855.1	5902076	**SRSF1**	serine/arginine-rich splicing factor 1 isoform 1	0.8	0.8	0.8	0.9	0.8	0.9	0.8	0.9	**0.8**	**0.9**	**0.8**	**0.9**	51.6	2	14	14	217	248	27.7	10.4
NP_056485.2	21361499	**SENP3**	sentrin-specific protease 3	1.0	0.8	0.9	0.8	1.0	0.9	0.9	0.9	**1.0**	**0.9**	**0.9**	**0.9**	11.7	1	5	5	17	574	65.0	8.6
NP_057230.1	7706667	**TRAPPC4**	trafficking protein particle complex subunit 4	0.9	0.8	0.9	0.9	0.9	0.9	0.9	0.8	**0.9**	**0.9**	**0.9**	**0.9**	20.1	1	4	4	12	219	24.3	6.2
NP_065970.2	40316935	**ALS2**	alsin isoform 1	1.0	1.1	1.2	1.0	0.9	0.7	0.9	0.8	**1.0**	**0.9**	**1.0**	**0.9**	1.1	1	1	1	2	1657	183.5	6.3
NP_001243239.1	373251164	**GLS**	glutaminase kidney isoform, mitochondrial isoform 2	1.1	0.9	1.0	0.8	1.0	1.0	1.1	1.0	**1.1**	**1.0**	**1.0**	**0.9**	17.2	2	9	9	28	598	65.4	7.9
NP_001000.2	13904870	**RPS5**	40S ribosomal protein S5	0.9	0.9	0.9	0.9	0.9	0.9	0.9	0.9	**0.9**	**0.9**	**0.9**	**0.9**	53.9	1	8	8	56	204	22.9	9.7
NP_055858.2	45597177	**TBC1D9B**	TBC1 domain family member 9B isoform b	0.9	0.9	0.9	0.9	1.0	0.9	0.8	0.9	**0.9**	**0.9**	**0.8**	**0.9**	13.9	3	13	13	27	1233	138.6	5.3
NP_002849.1	4506341	**ABCD3**	ATP-binding cassette sub-family D member 3 isoform a	1.0	0.9	1.0	0.9	1.0	0.9	0.9	0.9	**1.0**	**0.9**	**1.0**	**0.9**	14.3	2	8	8	20	659	75.4	9.4
NP_005830.2	42542379	**SRRM1**	serine/arginine repetitive matrix protein 1 isoform 2	0.9	0.9	0.9	0.9	0.8	0.9	0.9	0.9	**0.8**	**0.9**	**0.9**	**0.9**	12.9	3	9	9	37	904	102.3	11.8
NP_060439.2	217330641	**IWS1**	protein IWS1 homolog	1.0	1.1	1.0	0.9	1.0	1.0	1.0	0.9	**1.0**	**1.1**	**1.0**	**0.9**	11.8	1	9	9	24	819	91.9	4.7
NP_001185901.1	312176416	**KIAA1522**	uncharacterized protein KIAA1522 isoform 2	0.9	0.8	0.8	0.8	0.9	0.9	0.9	0.9	**0.9**	**0.9**	**0.9**	**0.9**	4.1	3	3	3	3	1035	107.0	9.7
NP_478070.1	17158023	**FAM207A**	protein FAM207A	0.9	1.0	0.9	0.9	0.9	0.8	0.8	0.8	**0.9**	**0.9**	**0.9**	**0.9**	26.1	1	4	4	16	230	25.4	11.1
NP_006004.2	223890243	**RPL10**	60S ribosomal protein L10 isoform a	0.9	0.9	0.9	0.9	0.9	0.9	0.9	0.9	**0.9**	**0.9**	**0.9**	**0.9**	37.4	8	7	7	49	214	24.6	10.1
NP_001191396.1	325120984	**RBM10**	RNA-binding protein 10 isoform 4	0.9	0.9	0.8	0.9	0.9	0.9	0.9	0.9	**0.9**	**0.9**	**0.9**	**0.9**	19.7	5	12	13	41	929	103.4	6.0
NP_001825.1	4502901	**CLTB**	clathrin light chain B isoform a	1.1	0.9	1.2	0.9	1.1	1.0	1.1	0.9	**1.1**	**1.0**	**1.2**	**0.9**	16.1	2	4	4	6	211	23.2	4.7
NP_001229822.1	338968934	**CSDE1**	cold shock domain-containing protein E1 isoform 2	0.8	0.9	0.8	0.9	0.8	0.9	0.8	0.8	**0.8**	**0.9**	**0.8**	**0.9**	34.4	4	25	25	68	767	85.7	6.2
NP_005557.1	5031857	**LDHA**	L-lactate dehydrogenase A chain isoform 1	0.8	0.9	1.0	0.9	0.8	0.9	1.0	0.9	**0.8**	**0.9**	**1.0**	**0.9**	84.9	8	25	28	393	332	36.7	8.3
NP_078889.1	13375827	**PLEKHF2**	pleckstrin homology domain-containing family F member 2	0.9	0.8	0.9	0.9	1.0	0.9	0.9	0.9	**0.9**	**0.8**	**0.9**	**0.9**	3.6	1	1	1	2	249	27.8	8.2
NP_037498.1	7019327	**ANAPC2**	anaphase-promoting complex subunit 2	1.0	1.1	1.1	1.0	0.9	0.9	0.9	0.8	**1.0**	**1.0**	**1.0**	**0.9**	3.8	1	2	2	3	822	93.8	5.2
NP_067017.2	52352803	**SLC30A1**	zinc transporter 1	0.9	0.8	0.8	0.7	1.1	1.0	1.0	1.0	**1.0**	**0.9**	**0.9**	**0.9**	2.2	1	1	1	2	507	55.3	6.5
NP_443183.1	16418441	**DNTTIP1**	deoxynucleotidyltransferase terminal-interacting protein 1	0.9	0.9	0.8	0.9	0.8	0.8	0.8	0.8	**0.8**	**0.9**	**0.8**	**0.9**	27.1	1	6	6	18	329	37.0	9.0
NP_068747.1	11345462	**SPCS3**	signal peptidase complex subunit 3	1.0	0.8	1.0	0.8	1.0	0.9	1.0	1.0	**1.0**	**0.9**	**1.0**	**0.9**	12.8	1	2	2	5	180	20.3	8.6
NP_006256.1	5453994	**RAD21**	double-strand-break repair protein rad21 homolog	1.0	1.0	1.0	0.9	0.9	0.9	0.9	0.9	**0.9**	**0.9**	**1.0**	**0.9**	34.2	1	16	16	48	631	71.6	4.7
NP_001170946.1	294489346	**MOSPD2**	motile sperm domain-containing protein 2 isoform 2	1.0	0.9	0.9	0.9	1.0	1.0	0.9	0.9	**1.0**	**0.9**	**0.9**	**0.9**	5.9	2	3	3	8	455	52.1	6.1
NP_065715.1	10190704	**DECR2**	peroxisomal 2,4-dienoyl-CoA reductase	1.0	0.9	1.0	0.9	1.3	0.9	1.0	0.9	**1.1**	**0.9**	**1.0**	**0.9**	5.5	1	1	1	2	292	30.8	9.2
NP_004692.1	4757930	**CCNB2**	G2/mitotic-specific cyclin-B2	0.9	0.8	0.9	0.9	0.9	0.9	0.9	0.9	**0.9**	**0.9**	**0.9**	**0.9**	15.6	1	5	5	8	398	45.3	8.9
NP_065874.1	153792042	**TMEM181**	transmembrane protein 181	0.9	0.8	0.8	0.9	0.9	0.8	1.0	0.9	**0.9**	**0.8**	**0.9**	**0.9**	2.6	1	1	1	2	612	69.3	8.9
NP_001273541.1	557636649	**REPS1**	ralBP1-associated Eps domain-containing protein 1 isoform d	1.0	0.9	1.0	0.9	0.8	0.8	0.8	0.9	**0.9**	**0.8**	**0.9**	**0.9**	5.8	6	3	3	7	705	76.8	5.8
NP_000265.1	4557809	**OAT**	ornithine aminotransferase, mitochondrial isoform 1 precursor	1.0	0.9	0.9	0.8	1.1	1.0	1.0	0.9	**1.0**	**1.0**	**0.9**	**0.9**	50.3	2	16	16	60	439	48.5	7.0
NP_078796.2	239582772	**C2orf47**	uncharacterized protein C2orf47, mitochondrial precursor	1.3	0.9	1.0	0.8	1.0	1.0	1.0	0.9	**1.1**	**1.0**	**1.0**	**0.9**	14.4	1	5	5	16	291	32.5	9.2
NP_937883.1	38044112	**CLIP1**	CAP-Gly domain-containing linker protein 1 isoform b	0.9	1.0	1.0	0.9	1.0	1.0	1.0	0.9	**1.0**	**1.0**	**1.0**	**0.9**	17.5	5	23	23	55	1392	156.7	5.4
NP_006517.1	5730124	**ZNF217**	zinc finger protein 217	0.8	0.8	0.8	0.9	0.8	0.9	0.8	0.9	**0.8**	**0.8**	**0.8**	**0.9**	4.6	54	2	3	4	1048	115.2	8.5
NP_001685.1	4502313	**ATP6V0C**	V-type proton ATPase 16 kDa proteolipid subunit	1.0	0.8	1.2	1.0	1.1	0.9	1.2	0.8	**1.0**	**0.9**	**1.2**	**0.9**	34.8	1	3	3	8	155	15.7	8.4

Khan *et al.* , 2019. Multi-omics analysis to characterize cigarette smoke induced molecular alterations in esophageal cells
Supplementary Table 5. List of proteins quantified in untreated and chronically treated Het1A cells with cigarette smoke condensate for 8 months

NP_Accession	Protein group Accession	Gene ID	Description	Het-1A-Smoke - 2M/Parental	Het-1A-Smoke - 4M/Parental	Het-1A-Smoke - 6M/Parental	Het-1A-Smoke - 8M/Parental	Het-1A-Smoke - 2M/Parental	Het-1A-Smoke - 4M/Parental	Het-1A-Smoke - 6M/Parental	Het-1A-Smoke - 8M/Parental	Het-1A-Smoke - 2M/Parental	Het-1A-Smoke - 4M/Parental	Het-1A-Smoke - 6M/Parental	Het-1A-Smoke - 8M/Parental	Coverage (%)	Proteins	Unique Peptides	# Peptides	PSM	# AAs	MW [kDa]	calc. pI
				Replicate 1				Replicate 2				Average of replicates											
NP_150091.2	19923653;190014588	PSIP1	PC4 and SFRS1-interacting protein isoform 2	0.8	0.9	0.8	0.9	0.8	0.9	0.8	0.9	0.8	0.9	0.8	0.9	42.8	2	22	23	119	530	60.1	9.1
NP_665696.1	21955172	PHLDB2	pleckstrin homology-like domain family B member 2 isoform c	1.0	1.0	1.0	1.0	0.9	0.8	0.9	0.8	1.0	0.9	0.9	0.9	5.6	4	5	6	10	1210	136.8	7.5
NP_004854.1	4758668	SPTLC2	serine palmitoyltransferase 2	1.0	0.9	0.9	0.9	0.9	0.8	0.8	0.9	0.9	0.8	0.9	0.9	24.7	1	10	10	19	562	62.9	7.8
NP_001081.1	10947135	ABCF1	ATP-binding cassette sub-family F member 1 isoform b	0.9	1.0	0.9	0.9	0.9	0.9	0.8	0.9	0.9	1.0	0.9	0.9	38.5	2	23	25	133	807	91.6	7.7
NP_073623.1	12232479	ARV1	protein ARV1	1.1	1.0	1.1	1.0	0.7	1.0	0.7	0.8	0.9	1.0	0.9	0.9	3.3	1	1	1	1	271	31.0	8.3
NP_006708.2	112293285	SPIN1	spindlin-1	0.8	0.8	0.8	0.9	0.8	0.8	0.8	0.9	0.8	0.8	0.8	0.9	16.4	2	3	3	6	262	29.6	7.0
NP_002467.1	4557038	MYL4	myosin light chain 4	0.9	0.5	0.2	0.5	29.4	5.2	1.8	1.3	15.2	2.9	1.0	0.9	4.1	1	1	1	1	197	21.6	5.0
NP_001263201.1	442796454	MBD4	methyl-CpG-binding domain protein 4 isoform 4	0.9	0.9	0.8	0.8	1.0	1.1	1.0	1.0	0.9	1.0	0.9	0.9	7.6	5	3	3	4	540	60.9	9.0
NP_079185.2	186910323	ATAT1	alpha-tubulin N-acetyltransferase 1 isoform 2	1.0	0.9	0.8	1.1	0.7	0.6	0.6	0.7	0.9	0.7	0.7	0.9	8.7	3	2	2	3	333	37.5	10.1
NP_057517.1	7705987	GLTP	glycolipid transfer protein	1.0	1.0	1.0	0.8	1.0	0.9	1.0	0.9	1.0	0.9	1.0	0.9	4.8	1	1	1	2	209	23.8	7.4
NP_002721.1	4506055	PRKACA	cAMP-dependent protein kinase catalytic subunit alpha isoform Calpha1	0.9	0.9	0.9	0.9	1.0	0.8	0.9	0.8	1.0	0.8	0.9	0.9	40.2	7	7	11	35	351	40.6	8.8
NP_001008.1	4506685	RPS13	40S ribosomal protein S13	0.9	0.9	0.9	0.9	0.8	0.9	0.9	0.9	0.8	0.9	0.9	0.9	57.0	1	10	10	115	151	17.2	10.5
NP_001137463.1	221136862;221136866	WRAP53	telomerase Cajal body protein 1	0.9	0.9	0.9	0.8	1.0	0.9	1.0	0.9	0.9	0.9	1.0	0.9	7.3	1	2	2	2	548	59.3	4.6
NP_919307.1	34996527	RPS19BP1	active regulator of SIRT1	0.9	0.9	0.9	0.9	0.9	0.9	0.9	0.9	0.9	0.9	0.9	0.9	24.3	1	3	3	7	136	15.4	10.7
NP_001254637.1	390979660;110227589	ZNF706	zinc finger protein 706	0.8	0.9	0.9	0.8	0.9	0.9	0.9	0.9	0.9	0.9	0.9	0.9	22.4	1	2	2	2	76	8.5	10.0
NP_001101.1	4557251	ADAM10	disintegrin and metalloproteinase domain-containing protein 10 precursor	1.0	1.0	1.2	1.0	0.8	0.8	0.8	0.7	0.9	0.9	1.0	0.9	9.5	1	4	4	9	748	84.1	7.8
NP_001010935.1	58331202	RAP1A	ras-related protein Rap-1A precursor	0.8	0.8	0.7	0.9	0.9	0.9	0.9	0.9	0.8	0.8	0.8	0.9	28.8	2	1	4	31	184	21.0	6.7
NP_006333.1	5454102	TACC3	transforming acidic coiled-coil-containing protein 3	0.8	1.0	0.8	0.8	1.0	1.0	0.9	0.9	0.9	1.0	0.9	0.9	24.6	1	14	14	34	838	90.3	5.0
NP_115715.3	42822891	MIEN1	migration and invasion enhancer 1	1.3	1.0	1.5	0.9	1.0	0.8	1.1	0.8	1.1	0.9	1.3	0.9	20.0	1	1	1	2	115	12.4	4.4
NP_008921.1	11386183	WASF2	wiskott-Aldrich syndrome protein family member 2 isoform 1	0.9	0.8	0.8	0.8	0.9	1.0	0.9	0.9	0.9	0.9	0.9	0.9	1.6	1	1	1	1	498	54.3	5.5
NP_071349.3	192449449	UBE2O	ubiquitin-conjugating enzyme E2 O	0.9	0.9	0.8	0.9	0.9	0.8	0.9	0.9	0.9	0.8	0.8	0.9	27.6	1	25	25	62	1292	141.2	5.1
NP_110390.1	13540523	HMGN5	high mobility group nucleosome-binding domain-containing protein 5	0.9	1.2	1.3	0.9	0.9	1.2	1.2	0.9	0.9	1.2	1.2	0.9	5.7	1	2	2	6	282	31.5	4.6
NP_003189.2	208022641	TCEB3	transcription elongation factor B polypeptide 3	1.0	0.9	0.9	0.9	1.0	1.0	0.9	0.9	1.0	0.9	0.9	0.9	13.5	1	8	8	15	798	89.9	9.6
NP_000293.2	32307144	PLOD1	procollagen-lysine,2-oxoglutarate 5-dioxygenase 1 precursor	0.9	0.9	1.0	0.8	0.9	1.0	1.1	1.0	0.9	0.9	1.0	0.9	34.5	1	17	17	46	727	83.5	6.9
NP_003283.2	114155142	TPR	nucleoprotein TPR	0.9	0.9	0.9	0.9	0.9	0.9	0.9	0.9	0.9	0.9	0.9	0.9	31.8	2	57	58	139	2363	267.1	5.0
NP_002559.2	46367787	PABPC1	polyadenylate-binding protein 1	0.9	0.9	0.9	0.9	0.9	0.9	0.9	0.9	0.9	0.9	0.9	0.9	52.7	4	19	27	273	636	70.6	9.5
NP_001135967.1	215982794	MYO5A	unconventional myosin-Va isoform 2	0.9	0.8	0.8	0.9	1.0	0.9	0.8	0.9	0.9	0.9	0.8	0.9	2.0	2	3	3	4	1828	212.1	8.6
NP_116114.1	14249338	BUD13	BUD13 homolog isoform 1	0.9	1.0	0.8	0.9	0.9	0.9	0.9	0.9	0.9	0.9	0.9	0.9	19.9	2	10	10	26	619	70.5	9.9
NP_079348.1	13376617	PTGES2	prostaglandin E synthase 2 isoform 1	1.2	1.0	1.0	0.9	1.0	0.9	0.9	0.9	1.1	1.0	0.9	0.9	13.3	2	4	4	10	377	41.9	9.2
NP_006744.2	19557702	SURF6	surfeit locus protein 6 isoform 1	0.9	0.9	0.8	0.9	0.8	0.8	0.8	0.9	0.8	0.9	0.8	0.9	27.4	2	7	7	14	361	41.4	10.6
NP_005906.2	7427519	MCM6	DNA replication licensing factor MCM6	1.0	0.9	0.9	0.8	1.1	0.9	0.9	0.9	1.1	0.9	0.9	0.9	46.0	1	34	34	136	821	92.8	5.4
NP_001460.1	4503841;573014815	XRCC6	X-ray repair cross-complementing protein 6 isoform 1	1.0	0.9	0.9	0.9	1.0	0.9	0.9	0.9	1.0	0.9	0.9	0.9	66.3	5	37	39	470	609	69.8	6.6
NP_079097.1	13376216	CCDC134	coiled-coil domain-containing protein 134 isoform 1 precursor	0.9	0.8	0.9	0.8	1.0	1.0	1.0	1.0	1.0	0.9	1.0	0.9	16.2	2	2	3	5	229	26.5	8.9
XP_005276741.1	530438702;15011936	LOC10192987	40S ribosomal protein S26	0.8	0.8	0.8	0.9	0.9	0.9	0.9	0.9	0.8	0.9	0.9	0.9	31.3	2	3	3	24	115	13.0	11.0
NP_001124508.1	196259805	ZCCHC9	zinc finger CCHC domain-containing protein 9	0.9	0.9	0.8	0.8	0.9	0.9	0.8	0.9	0.9	0.9	0.8	0.9	4.4	1	1	1	3	271	30.5	9.2
NP_060515.3	221316578	BSDC1	BSD domain-containing protein 1 isoform b	1.1	1.1	1.0	0.9	1.0	1.1	0.9	0.8	1.0	1.1	0.9	0.9	18.6	5	4	4	7	430	47.1	4.5
NP_004844.1	4759188	STX8	syntaxin-8	1.0	0.9	0.9	0.9	0.9	0.8	0.7	0.8	1.0	0.8	0.8	0.9	11.9	1	2	2	5	236	26.9	5.0
NP_005337.2	167466173;194248072	HSPA1B	heat shock 70 kDa protein 1A/1B	0.8	0.8	0.8	0.9	0.8	0.8	0.8	0.9	0.8	0.8	0.8	0.9	72.4	1	20	37	586	641	70.0	5.7
NP_689419.2	88703041	SNX6	sorting nexin-6 isoform b	0.9	0.8	0.8	0.9	0.9	0.8	0.8	0.9	0.9	0.8	0.8	0.9	24.6	5	11	12	26	418	47.8	6.4
NP_071908.2	33149331	NXN	nucleoredoxin isoform 1	0.9	0.8	0.8	0.8	1.0	0.9	0.9	0.9	0.9	0.8	0.8	0.9	24.1	2	6	6	15	435	48.4	5.0
NP_291028.3	41327715	TP53RK	TP53-regulating kinase	0.9	0.7	0.8	0.9	0.9	0.8	0.9	0.9	0.9	0.8	0.8	0.9	25.3	1	5	5	18	253	28.1	9.5
NP_001230153.1	342672046	RAD9A	cell cycle checkpoint control protein RAD9A isoform 2	1.0	0.9	0.8	0.9	1.0	1.0	0.9	0.9	1.0	0.9	0.8	0.9	7.0	2	1	1	2	315	34.2	5.2
NP_001265857.1	524557857	SURF2	surfeit locus protein 2 isoform 2	0.9	0.9	0.8	0.9	0.9	1.0	0.9	0.8	0.9	0.9	0.8	0.9	20.8	2	3	3	7	255	29.5	9.1
NP_001257906.1	401709935	IST1	IST1 homolog isoform d	0.9	0.9	0.9	0.9	1.0	0.8	0.8	0.8	1.0	0.8	0.9	0.9	23.9	5	5	5	13	335	36.6	5.4
NP_057164.3	217330598	GLOD4	glyoxalase domain-containing protein 4	1.0	0.9	0.8	0.9	0.9	0.8	0.8	0.9	0.9	0.8	0.8	0.9	49.7	1	12	12	49	298	33.2	5.6
NP_004647.1	4757836	BAP1	ubiquitin carboxyl-terminal hydrolase BAP1	0.5	0.7	0.7	0.8	0.8	0.9	0.9	1.0	0.7	0.8	0.8	0.9	1.0	1	1	1	2	729	80.3	6.8
NP_689497.1	22748643	CHMP4C	charged multivesicular body protein 4c	1.0	0.9	1.1	0.9	1.0	0.6	0.9	0.8	1.0	0.8	1.0	0.9	7.7	1	1	2	3	233	26.4	6.1
NP_076970.1	13129044	PDCL3	phosducin-like protein 3	0.9	0.7	0.9	0.9	0.9	0.8	0.9	0.9	0.9	0.8	0.9	0.9	20.9	1	3	3	6	239	27.6	4.8
NP_001094073.1	154736713	KDELR2	ER lumen protein-retaining receptor 2 isoform 2	1.0	0.9	1.0	0.8	1.1	0.9	1.0	1.0	1.0	0.9	1.0	0.9	4.3	3	1	1	1	186	21.2	9.1
NP_006079.1	5174735	TUBB4B	tubulin beta-4B chain	0.8	0.7	0.9	0.8	1.0	0.9	1.0	0.9	0.9	0.8	0.9	0.9	82.7	2	1	25	876	445	49.8	4.9
NP_003081.2	50593002	SNRPA1	U2 small nuclear ribonucleoprotein A'	0.9	1.0	1.0	0.9	0.9	0.9	0.9	0.9	0.9	1.0	0.9	0.9	62.4	1	13	13	122	255	28.4	8.6
NP_002957.1	4506761	S100A10	protein S100-A10	1.0	1.1	1.2	0.9	0.9	1.0	1.2	0.9	0.9	1.1	1.2	0.9	17.5	1	1	1	7	97	11.2	7.4
NP_665811.1	22202629	AIFM1	apoptosis-inducing factor 1, mitochondrial isoform 2 precursor	1.0	0.9	0.8	0.9	1.0	0.9	0.9	0.9	1.0	0.9	0.8	0.9	38.9	5	18	18	79	609	66.3	8.9
NP_004586.2	21264341	SMS	spermine synthase isoform 1	1.1	1.1	1.3	0.9	1.0	1.0	1.1	0.8	1.0	1.0	1.2	0.9	61.8	2	16	16	54	366	41.2	5.0
NP_055177.2	37594471	HIBCH	3-hydroxyisobutyryl-CoA hydrolase, mitochondrial isoform 1 precursor	1.1	1.0	1.0	0.9	1.5	1.1	0.9	0.8	1.3	1.0	0.9	0.9	15.5	2	5	5	18	386	43.5	8.2
NP_001335.1	4503253	DAD1	dolichyl-diphosphooligosaccharide--protein glycosyltransferase subunit DAD1	1.1	0.9	1.2	0.9	1.2	1.0	1.3	0.9	1.2	0.9	1.2	0.9	38.1	1	3	3	10	113	12.5	7.1
NP_001142.2	55749577	SLC25A4	ADP/ATP translocase 1	0.9	0.8	0.8	0.9	0.9	0.8	0.8	0.9	0.9	0.8	0.8	0.9	53.7	2	2	14	190	298	33.0	9.8
NP_001278918.1	635172861	RXRB	retinoic acid receptor RXR-beta isoform 3	1.0	1.0	1.0	0.9	1.0	0.9	0.7	0.8	1.0	0.9	0.8	0.9	23.1	3	4	4	10	347	38.5	8.1
NP_001139333.1	225007652;225007648	POP1	ribonucleases P/MRP protein subunit POP1	1.0	1.0	1.0	0.8	1.0	1.0	1.1	0.9	1.0	1.0	1.0	0.9	12.7	1	9	9	16	1024	114.6	9.2
NP_005137.1	10863889	SART1	U4/U6.U5 tri-snRNP-associated protein 1	0.8	0.9	0.8	0.9	0.9	0.9	0.9	0.9	0.9	0.9	0.9	0.9	48.0	1	31	31	130	800	90.2	6.1
NP_699163.2	31543184	ERI1	3'-5' exoribonuclease 1	0.9	0.9	0.9	0.9	1.0	0.9	1.0	0.9	0.9	0.9	0.9	0.9	2.0	1	1	1	1	349	40.0	6.7
NP_065190.2	40354197	THAP11	THAP domain-containing protein 11	0.9	0.8	0.8	0.9	0.8	0.8	0.7	0.8	0.8	0.8	0.8	0.9	2.6	1	1	1	2	314	34.4	9.0
NP_002958.2	21264343	SAFB	scaffold attachment factor B1 isoform 3	0.8	0.9	0.8	0.9	0.8	0.9	0.8	0.8	0.8	0.9	0.8	0.9	38.0	4	16	27	97	915	102.6	5.5
NP_002892.1	4506455	RCN1	reticulocalbin-1 precursor	1.5	1.1	1.5	0.9	1.4	1.1	1.4	0.9	1.5	1.1	1.4	0.9	52.9	1	13	13	76	331	38.9	5.0
NP_872589.1	32967605	BAZ1A	bromodomain adjacent to zinc finger domain protein 1A isoform b	1.0	1.0	1.0	0.9	1.0	0.8	1.0	0.8	1.0	0.9	1.0	0.9	4.2	2	5	5	7	1524	175.3	6.8
NP_055545.1	7661948	MLEC	malectin isoform1 precursor	0.9	0.8	0.8	0.9	0.9	0.8	0.8	0.9	0.9	0.8	0.8	0.9	20.2	3	4	4	21	292	32.2	5.4
NP_001036216.1	112789530;539846358	STAG2	cohesin subunit SA-2 isoform b	1.0	0.9	0.9	0.9	1.0	0.9	0.9	0.9	1.0	0.9	0.9	0.9	18.9	5	10	16	49	1231	141.2	5.4
NP_001158253.1	258614033	ATXN3	ataxin-3 isoform y	1.0	0.9	1.2	0.8	0.9	0.8	0.7	0.9	1.0	0.8	1.0	0.9	17.9	6	3	3	3	291	33.4	4.9
NP_001139487.1	226371669	DLGAP5	disks large-associated protein 5 isoform b	0.9	0.8	0.8	0.9	0.9	0.8	0.8	0.8	0.9	0.8	0.8	0.9	19.4	2	12	12	29	842	94.1	9.1
NP_055634.3	157412255	PJA2	E3 ubiquitin-protein ligase Praja-2	1.2	1.1	0.8	1.0	0.8	0.8	0.7	0.7	1.0	0.9	0.8	0.9	11.9	1	4	4	5	708	78.2	4.4
NP_005096.1	4826972	RBM8A	RNA-binding protein 8A	0.8	0.9	0.8	0.8	1.0	1.0	0.9	0.9	0.9	0.9	0.9	0.9	45.4	1	6	6	71	174	19.9	5.7
NP_001072989.1	118498356	KTN1	kinectin isoform a	0.8	0.8	0.8	0.9	0.8	0.8	0.8	0.9	0.8	0.8	0.8	0.9	44.9	4	51	52	184	1357	156.2	5.6
NP_036222.3	33188445	MACF1	microtubule-actin cross-linking factor 1	1.0	1.0	1.0	0.9	1.0	1.0	1.1	0.9	1.0	1.0	1.0	0.9	12.5	1	47	48	100	5430	620.0	5.4
NP_060774.2	88703045	PRR11	proline-rich protein 11	0.9	0.9	1.0	0.7	0.9	1.0	1.0	1.0	0.9	1.0	1.0	0.9	18.1	1	4	4	5	360	40.1	10.1
NP_060230.5	116812586	NCAPG2	condensin-2 complex subunit G2 isoform a	1.0	0.9	0.9	0.9	1.0	0.9	1.0	0.8	1.0	0.9	1.0	0.9	11.3	2	10	10	25	1143	130.9	6.9
NP_060834.2	38683851	RSBN1	round spermatid basic protein 1	0.9	0.9	1.0	0.9	0.7	0.7	0.8	0.8	0.8	0.8	0.9	0.9	0.9	1	1	1	2	802	90.0	8.6
NP_001139653.1	226052117	HNRNPCL3	heterogeneous nuclear ribonucleoprotein C-like 3	0.8	0.7	0.7	0.8	0.9	0.8	0.8	0.9	0.8	0.8	0.7	0.9	23.6	4	1	9	132	293	32.0	5.7
NP_001182729.1	307775416	LDLR	low-density lipoprotein receptor isoform 4 precursor	1.2	1.1	1.0	0.9	1.2	1.0	1.0	0.8	1.2	1.1	1.0	0.9	28.5	9	12	12	44	692	76.8	5.5
NP_000975.2	17105394	RPL23A	60S ribosomal protein L23a	0.8	1.0	0.9	0.9	0.8	0.9	0.8	0.8	0.8	1.0	0.9	0.9	51.9	1	13	13	189	156	17.7	10.4
NP_057078.1	7706757	ATP6V1D	V-type proton ATPase subunit D	1.0	1.0	0.9	0.9	0.9	0.9	0.9	0.8	0.9	0.9	0.9	0.9	17.8	1	3	3	13	247	28.2	9.4
NP_612451.1	19923969	CCDC124	coiled-coil domain-containing protein 124	0.8	0.9	0.8	0.8	0.8	0.9	0.8	0.9	0.8	0.9	0.8	0.9	52.5	1	13	13	136	223	25.8	9.5
NP_009096.2	21361376	SF3A2	splicing factor 3A subunit 2	0.9	0.8	0.8	0.9	0.8	0.7	0.7	0.8	0.8	0.8	0.8	0.9	11.0	1	5	5	18	464	49.2	9.6
NP_055984.1	14719402	RRS1	ribosome biogenesis regulatory protein homolog	0.8	0.9	0.8	0.9	0.8	0.9	0.9	0.9	0.8	0.9	0.8	0.9	35.3	1	11	11	43	365	41.2	10.7
NP_004326.1	4757876	BST2	bone marrow stromal antigen 2 precursor	0.7	0.8	0.9	0.9	0.8	0.7	0.9	0.8	0.8	0.8	0.9	0.9	13.9	1	2	2	6	180	19.8	5.6
NP_000180.2	15553127	HK2	hexokinase-2	0.8	0.9	0.9	0.9	0.9	0.9	0.9	0.9	0.8	0.9	0.9	0.9	16.1	2	9	12	32	917	102.3	6.0

Khan *et al.*, 2019. Multi-omics analysis to characterize cigarette smoke induced molecular alterations in esophageal cells
Supplementary Table 5. List of proteins quantified in untreated and chronically treated Het1A cells with cigarette smoke condensate for 8 months

NP_Accession	Protein group Accession	Gene ID	Description	Het-1A-Smoke - 2M/Parental	Het-1A-Smoke - 4M/Parental	Het-1A-Smoke - 6M/Parental	Het-1A-Smoke - 8M/Parental	Het-1A-Smoke - 2M/Parental	Het-1A-Smoke - 4M/Parental	Het-1A-Smoke - 6M/Parental	Het-1A-Smoke - 8M/Parental	Het-1A-Smoke - 2M/Parental	Het-1A-Smoke - 4M/Parental	Het-1A-Smoke - 6M/Parental	Het-1A-Smoke - 8M/Parental	Coverage (%)	Proteins	Unique Peptides	# Peptides	PSM	# AAs	MW [kDa]	calc. pI
				Replicate 1				Replicate 2				Average of replicates											
NP_055464.1	7661936	SAFB2	scaffold attachment factor B2	0.8	0.9	0.9	0.8	0.8	1.0	0.9	0.9	0.8	0.9	0.9	0.9	29.6	1	10	21	75	953	107.4	6.2
NP_004690.1	4758220	FAM50A	protein FAM50A	1.0	0.9	1.0	0.9	1.0	0.9	1.0	0.9	1.0	0.9	1.0	0.9	18.3	2	5	5	6	339	40.2	6.8
NP_060149.3	153218595	BCAS3	breast carcinoma-amplified sequence 3 isoform 2	1.0	0.8	0.8	0.8	1.0	1.0	1.0	0.9	1.0	0.9	0.9	0.9	2.4	2	2	2	4	913	99.6	6.6
NP_001276001.1	574275777	HELLS	lymphoid-specific helicase isoform 7	0.8	0.8	0.8	0.9	0.9	0.8	0.8	0.9	0.8	0.8	0.8	0.9	9.2	10	6	6	12	708	81.6	7.8
NP_001269591.1	544186098	MKL1	MKL/myocardin-like protein 1 isoform 3	0.9	0.9	1.0	0.9	0.7	0.8	0.8	0.8	0.8	0.9	0.9	0.9	3.6	3	1	1	4	798	85.0	7.8
NP_001171033.1	294774574	PPP2R1B	serine/threonine-protein phosphatase 2A 65 kDa regulatory subunit A beta isoform isoform d	0.9	0.8	0.8	0.8	1.0	0.9	1.0	0.9	0.9	0.8	0.9	0.9	28.6	5	6	12	60	556	61.3	4.9
NP_149103.1	24308350	RRP36	ribosomal RNA processing protein 36 homolog	0.9	0.9	0.9	0.9	0.9	0.9	0.8	0.8	0.9	0.9	0.9	0.9	17.0	1	3	3	5	259	29.8	10.2
NP_001137474.1	221316689	CTPS2	CTP synthase 2	1.0	0.9	0.9	0.8	1.0	1.0	1.0	0.9	1.0	0.9	0.9	0.9	24.4	1	10	12	24	586	65.6	6.9
NP_612144.1	19923717	DTX3L	E3 ubiquitin-protein ligase DTX3L	0.9	1.1	0.8	1.0	0.9	0.9	0.7	0.8	0.9	1.0	0.8	0.9	2.0	1	1	1	2	740	83.5	8.1
NP_940684.1	38257153	PTPLB	very-long-chain (3R)-3-hydroxyacyl-[acyl-carrier protein] dehydratase 2	0.8	0.9	0.7	0.8	1.0	1.0	0.9	1.0	0.9	0.9	0.8	0.9	3.9	1	1	1	1	254	28.4	9.6
NP_001019.1	4506707	RPS25	40S ribosomal protein S25	0.9	0.9	0.9	0.9	0.8	0.9	0.8	0.9	0.8	0.9	0.8	0.9	43.2	1	7	7	24	125	13.7	10.1
NP_060838.3	261878497	LMBRD1	probable lysosomal cobalamin transporter	0.8	0.8	0.7	0.8	0.9	0.8	0.8	1.0	0.8	0.8	0.8	0.9	3.3	1	1	1	3	540	61.3	7.8
NP_001182583.1	307548887	ZGPAT	zinc finger CCCH-type with G patch domain-containing protein isoform d	0.7	0.8	0.8	0.8	0.9	0.9	1.0	1.0	0.8	0.8	0.9	0.9	5.8	3	3	3	4	502	54.6	5.6
NP_006478.2	34734068	FBLN1	fibulin-1 isoform A precursor	1.1	1.2	1.4	0.9	1.1	0.9	1.2	0.8	1.1	1.0	1.3	0.9	1.9	4	1	1	1	566	61.5	5.0
NP_001071.1	4507229	ALDH5A1	succinate-semialdehyde dehydrogenase, mitochondrial isoform 2 precursor	0.9	0.8	0.8	0.9	0.9	0.8	0.8	0.8	0.9	0.8	0.8	0.9	30.1	2	8	8	21	535	57.2	8.3
NP_006043.1	5174425	DSCR3	Down syndrome critical region protein 3	1.0	0.7	0.8	0.8	0.9	0.9	0.8	0.9	1.0	0.8	0.8	0.9	8.1	1	2	2	2	297	33.0	7.7
NP_001015891.1	62865612	AK6	adenylate kinase isoenzyme 6 isoform c	1.0	0.8	1.0	0.9	0.9	0.8	0.9	0.8	0.9	0.8	0.9	0.9	24.9	2	3	3	7	169	19.8	4.7
NP_113651.4	226371731	HSDL1	inactive hydroxysteroid dehydrogenase-like protein 1 isoform a	0.9	0.7	0.7	0.9	0.8	0.9	0.8	0.8	0.9	0.8	0.8	0.9	9.1	2	3	3	4	330	37.0	8.7
NP_001002.1	4506741	RPS7	40S ribosomal protein S7	0.8	0.9	0.8	0.9	0.8	0.9	0.9	0.9	0.8	0.9	0.8	0.9	56.2	1	9	9	128	194	22.1	10.1
NP_004210.1	4758980;538260594	PTTG1	securin	0.8	0.9	0.8	0.9	0.8	0.8	0.8	0.9	0.8	0.8	0.8	0.9	29.7	2	3	3	7	202	22.0	6.6
NP_001545.2	31542331	CYR61	protein CYR61 precursor	1.2	1.1	1.2	0.9	1.1	1.1	1.1	0.9	1.2	1.1	1.2	0.9	37.5	1	13	13	54	381	42.0	8.2
NP_006362.1	5453601	CRTAP	cartilage-associated protein precursor	1.0	1.0	1.0	0.9	0.9	0.9	1.0	0.9	1.0	1.0	1.0	0.9	23.4	1	7	7	29	401	46.5	5.7
NP_653210.2	151101337	CCDC43	coiled-coil domain-containing protein 43 isoform 1	1.1	0.9	1.0	0.8	1.0	0.9	1.1	0.9	1.1	0.9	1.0	0.9	12.5	2	3	3	12	224	25.2	4.9
NP_078920.2	106879206	C14orf169	bifunctional lysine-specific demethylase and histidyl-hydroxylase NO66	0.9	0.8	0.8	0.9	0.8	0.7	0.7	0.8	0.8	0.8	0.8	0.9	8.4	1	4	4	8	641	71.0	6.5
NP_937863.1	38149981;4507123	SNRPB2	U2 small nuclear ribonucleoprotein B''	0.9	0.9	0.8	0.8	0.9	1.0	0.9	0.9	0.9	0.9	0.8	0.9	31.1	1	5	7	35	225	25.5	9.7
NP_060654.1	8922601	ARL8B	ADP-ribosylation factor-like protein 8B	0.8	0.7	0.8	0.8	0.8	0.7	0.8	0.9	0.8	0.7	0.8	0.9	30.1	2	4	4	9	186	21.5	8.4
NP_071387.1	18034767	PCIF1	phosphorylated CTD-interacting factor 1	0.8	0.8	0.8	0.8	1.2	0.9	0.9	1.0	1.0	0.9	0.8	0.9	9.8	1	5	5	9	704	80.6	7.4
NP_001165335.1	285395246	TUBAL3	tubulin alpha chain-like 3 isoform 2	0.9	0.8	0.8	0.9	0.9	0.9	1.0	0.9	0.9	0.8	0.9	0.9	10.3	2	1	4	49	406	45.5	6.3
NP_116235.2	21361884	RAB2B	ras-related protein Rab-2B isoform 1	0.8	1.0	1.0	0.9	0.9	0.8	0.8	0.9	0.9	0.9	0.9	0.9	26.9	2	1	5	32	216	24.2	7.8
NP_054909.2	29826323	ADD1	alpha-adducin isoform c	0.7	0.8	0.8	1.0	1.0	0.9	1.0	0.8	0.8	0.8	0.9	0.9	1.4	5	1	1	2	631	69.9	6.5
NP_001138629.1	223555953;223555951	NR2F2	COUP transcription factor 2 isoform c	0.8	0.8	0.9	0.8	0.8	0.9	1.0	0.9	0.8	0.8	0.9	0.9	11.5	4	3	3	6	261	29.1	6.4
NP_000988.1	4506641	RPL37	60S ribosomal protein L37	0.8	0.9	0.9	0.9	0.7	0.9	0.9	0.8	0.8	0.9	0.9	0.9	22.7	1	4	4	32	97	11.1	11.7
NP_000972.1	4506609	RPL19	60S ribosomal protein L19	0.9	0.9	0.9	0.9	0.8	0.9	0.9	0.8	0.9	0.9	0.9	0.9	34.2	3	8	9	54	196	23.5	11.5
NP_149062.1	23097308	SYNE1	nesprin-1 isoform 2	0.9	0.8	1.0	0.8	0.9	0.9	0.9	0.9	0.9	0.9	0.9	0.9	0.1	2	1	1	2	8749	1004.6	5.5
NP_001171737.1	296317276	CD99L2	CD99 antigen-like protein 2 isoform 4 precursor	0.9	0.9	0.9	0.9	0.8	0.9	0.7	0.8	0.8	0.9	0.8	0.9	21.2	6	3	3	9	189	20.0	5.0
NP_060502.1	8922297	LUC7L	putative RNA-binding protein Luc7-like 1 isoform a	0.8	0.9	0.9	0.9	0.9	0.9	0.8	0.9	0.8	0.9	0.8	0.9	24.3	2	3	7	18	325	38.4	10.0
NP_001013275.1	61742797	BCAM	basal cell adhesion molecule isoform 2 precursor	0.9	0.9	1.0	0.9	0.8	0.8	0.9	0.8	0.8	0.9	1.0	0.9	7.5	2	4	4	5	588	63.7	6.1
NP_689518.1	31982953	POGLUT1	protein O-glucosyltransferase 1 precursor	1.0	0.9	0.9	0.8	1.0	1.0	1.0	1.0	1.0	0.9	0.9	0.9	3.6	1	1	1	6	392	46.2	8.7
NP_001129202.1	209180450	TOM1	target of Myb protein 1 isoform 4	0.9	0.7	0.7	0.8	1.2	1.0	1.0	0.9	1.1	0.8	0.9	0.9	12.8	4	3	3	6	447	48.6	4.7
NP_004231.1	11342676	TRIP10	cdc42-interacting protein 4 isoform 2	1.0	0.9	0.9	0.9	0.9	0.8	0.8	0.8	1.0	0.8	0.9	0.9	26.2	3	9	9	24	545	62.6	5.3
NP_219488.1	15529970	TMEM259	membralin isoform 2	0.9	1.0	0.9	0.9	0.8	0.7	0.7	0.8	0.9	0.9	0.8	0.9	11.0	2	3	3	6	408	46.4	5.6
NP_001171134.1	295054210;295054220	RIF1	telomere-associated protein RIF1 isoform 2	0.9	0.9	0.9	0.8	1.0	1.0	0.9	0.9	1.0	0.9	0.9	0.9	22.9	2	36	36	82	2446	271.5	5.4
NP_974223.1	42544142;42544146	GIPC1	PDZ domain-containing protein GIPC1 isoform 2	0.8	0.8	0.9	0.8	0.8	0.8	0.9	0.9	0.8	0.8	0.9	0.9	13.6	3	2	2	5	236	26.1	5.6
NP_004392.1	4758148	DFFA	DNA fragmentation factor subunit alpha isoform 1	1.1	0.9	1.1	0.9	1.0	0.8	1.0	0.9	1.0	0.9	1.0	0.9	37.5	2	6	6	15	331	36.5	4.8
NP_006791.2	17975761	MSL3	male-specific lethal 3 homolog isoform c	1.0	0.9	1.0	0.9	0.8	0.9	0.7	0.9	0.9	0.9	0.8	0.9	7.6	5	2	2	8	355	40.6	8.9
NP_004994.1	4826852	NDUFAB1	acyl carrier protein, mitochondrial precursor	1.3	1.0	1.4	0.9	1.5	1.1	1.6	0.9	1.4	1.1	1.5	0.9	21.2	1	4	4	22	156	17.4	4.9
NP_940888.2	50053872	RPL7L1	60S ribosomal protein L7-like 1	0.9	0.9	0.9	0.9	0.8	0.9	0.9	0.9	0.9	0.9	0.9	0.9	31.7	1	7	7	19	246	28.6	10.5
NP_001276020.1	574272649	ZMYM1	zinc finger MYM-type protein 1 isoform b	1.2	0.9	0.8	0.8	0.9	0.9	0.9	0.9	1.0	0.9	0.8	0.9	2.9	3	2	2	3	782	89.5	7.2
NP_001273448.1	557357687	SLC35B2	adenosine 3'-phospho 5'-phosphosulfate transporter 1 isoform f	0.8	0.7	0.8	0.8	0.9	0.8	0.8	0.9	0.9	0.8	0.8	0.9	10.4	6	2	2	6	299	32.6	9.6
NP_114405.1	14042927	STARD3NL	MLN64 N-terminal domain homolog	0.9	0.9	0.9	0.9	0.8	0.8	0.9	0.8	0.8	0.8	0.9	0.9	4.7	1	1	1	1	234	26.6	4.8
NP_001278787.1	629266062	IGF1R	insulin-like growth factor 1 receptor isoform 2 precursor	0.9	0.9	0.9	1.0	0.9	0.7	0.8	0.8	0.9	0.8	0.9	0.9	4.5	2	5	6	9	1366	154.7	5.8
NP_055976.1	24308007	ARL6IP1	ADP-ribosylation factor-like protein 6-interacting protein 1	0.9	0.8	0.9	0.9	0.9	0.9	1.0	0.8	0.9	0.9	1.0	0.9	21.7	1	3	3	16	203	23.3	9.3
NP_071741.2	21359945	NCAPG	condensin complex subunit 3	0.9	0.9	0.9	0.9	1.0	0.8	0.8	0.8	1.0	0.9	0.9	0.9	27.5	1	21	21	60	1015	114.3	5.6
NP_055054.1	7657058	EIF2B2	translation initiation factor eIF-2B subunit beta	1.0	0.9	0.9	0.9	0.9	0.9	0.9	0.8	0.9	0.9	0.9	0.9	21.1	1	6	6	21	351	39.0	6.2
NP_000246.2	156105689	MUT	methylmalonyl-CoA mutase, mitochondrial precursor	1.0	0.9	1.0	0.9	1.0	0.9	0.9	0.9	1.0	0.9	0.9	0.9	19.5	1	10	10	26	750	83.1	6.9
NP_060365.7	224593278	DDX27	probable ATP-dependent RNA helicase DDX27	0.8	0.8	0.7	0.9	0.8	0.8	0.8	0.9	0.8	0.8	0.8	0.9	26.3	1	22	22	67	796	89.8	9.3
NP_001018065.1	64762484	BRCC3	lys-63-specific deubiquitinase BRCC36 isoform 2	0.9	0.9	0.9	0.8	0.9	1.0	1.0	0.9	0.9	0.9	0.9	0.9	11.0	3	2	2	7	291	33.1	5.9
NP_000981.1	4506625	RPL27A	60S ribosomal protein L27a	0.9	0.8	0.9	0.9	1.0	0.8	0.9	0.9	0.9	0.8	0.9	0.9	34.5	1	5	5	48	148	16.6	11.0
NP_660289.2	24432067	FRA10AC1	protein FRA10AC1	0.8	0.8	0.9	0.9	0.9	0.9	1.0	0.8	0.8	0.9	0.9	0.9	15.2	1	4	4	13	315	37.5	8.1
NP_004517.2	33356547	MCM2	DNA replication licensing factor MCM2	1.0	0.9	0.9	0.8	1.0	0.9	0.9	0.9	1.0	0.9	0.9	0.9	37.8	1	33	33	113	904	101.8	5.5
NP_004888.2	19923761	MINPP1	multiple inositol polyphosphate phosphatase 1 isoform 1 precursor	0.9	0.9	0.9	0.8	0.8	0.8	0.9	0.9	0.8	0.9	0.9	0.9	15.8	3	5	5	8	487	55.0	7.8
NP_003249.3	164698438	TK1	thymidine kinase, cytosolic	0.9	0.8	0.9	0.8	0.9	1.0	0.9	0.9	0.9	0.9	0.9	0.9	50.0	1	9	9	14	234	25.5	8.5
NP_057284.1	7706425	LSM8	LSM8 homolog, U6 small nuclear RNA associated	1.0	0.9	1.1	0.8	1.1	0.9	1.0	0.9	1.1	0.9	1.1	0.9	10.4	1	1	1	1	96	10.4	4.5
NP_852118.1	31563507	GCC2	GRIP and coiled-coil domain-containing protein 2	1.0	1.0	0.9	0.9	1.0	1.0	0.9	0.9	1.0	1.0	0.9	0.9	8.5	1	13	14	29	1684	195.8	5.1
NP_715638.1	24371268	NAP1L5	nucleosome assembly protein 1-like 5	0.9	1.0	1.0	0.9	0.8	0.8	0.9	0.8	0.9	0.9	0.9	0.9	7.1	1	1	1	1	182	19.6	4.2
NP_006849.1	5803040	TMED1	transmembrane emp24 domain-containing protein 1 precursor	0.9	0.9	0.8	0.8	0.9	0.9	0.9	0.9	0.9	0.9	0.9	0.9	17.2	1	3	3	6	227	25.2	4.5
NP_004127.1	41352693	IREB2	iron-responsive element-binding protein 2	0.9	0.8	0.8	0.8	0.9	0.9	0.8	0.9	0.9	0.9	0.8	0.9	4.5	1	2	2	3	963	105.0	7.0
NP_056073.1	150010552	FKBP15	FK506-binding protein 15	0.9	0.9	0.9	0.8	0.9	0.9	0.9	0.9	0.9	0.9	0.9	0.9	12.2	1	10	10	20	1219	133.5	5.2
NP_998816.1	47458041	SLC25A24	calcium-binding mitochondrial carrier protein SCaMC-1 isoform 2	1.5	0.9	0.9	0.8	0.9	0.9	1.0	0.9	1.2	0.9	1.0	0.9	15.3	2	4	4	6	458	51.3	5.9
NP_004361.3	93141047	COL12A1	collagen alpha-1(XII) chain long isoform precursor	0.9	0.8	0.8	0.8	0.9	0.9	0.8	0.8	0.9	0.8	0.8	0.9	3.3	2	8	8	14	3063	332.9	5.5
NP_004870.3	55956766	EI24	etoposide-induced protein 2.4 homolog isoform 1	0.9	0.8	0.9	0.9	0.9	0.8	0.9	0.8	0.9	0.8	0.9	0.9	3.2	1	1	1	6	340	38.9	9.7
NP_004756.2	20336477	BCL7C	B-cell CLL/lymphoma 7 protein family member C isoform 2	0.7	0.8	0.7	0.8	0.9	1.0	0.9	0.9	0.8	0.9	0.8	0.9	18.0	2	2	2	4	217	23.5	5.2
NP_008982.1	5901948	FAF1	FAS-associated factor 1	1.0	0.9	0.9	0.8	1.1	0.9	0.9	0.9	1.0	0.9	0.9	0.9	9.9	1	6	6	18	650	73.9	4.9
NP_005996.2	224994203;224994205	WFS1	wolframin	1.0	1.0	0.9	1.0	1.0	1.0	0.9	0.7	1.0	1.0	0.9	0.9	6.7	1	4	4	9	890	100.2	8.0
NP_003867.1	4505901	TMEM11	transmembrane protein 11, mitochondrial	1.0	1.0	1.0	0.9	0.9	0.8	0.8	0.8	0.9	0.9	0.9	0.9	4.7	1	1	1	2	192	21.5	7.4
NP_054762.2	40254866	CHMP2B	charged multivesicular body protein 2b isoform 1	1.0	0.9	0.9	0.8	1.0	0.9	1.0	0.9	1.0	0.9	1.0	0.9	28.2	3	7	7	15	213	23.9	8.8
NP_009055.2	110611228	UTRN	utrophin	0.9	0.9	0.9	0.8	1.0	1.0	1.0	0.9	1.0	0.9	1.0	0.9	14.4	11	38	38	89	3433	394.2	5.3
NP_570719.1	18677731	MOB3A	MOB kinase activator 3A	0.8	0.9	0.7	0.9	0.8	0.9	1.0	0.8	0.8	0.9	0.8	0.9	11.5	1	1	1	2	217	25.4	8.6
NP_001182375.1	306482694	SRSF7	serine/arginine-rich splicing factor 7 isoform 2	0.9	0.9	0.8	0.9	0.9	0.9	0.9	0.8	0.9	0.9	0.8	0.9	32.7	2	8	9	58	226	26.0	11.8
NP_001243349.1	374081840	MAPRE2	microtubule-associated protein RP/EB family member 2 isoform 4	0.9	0.8	0.7	0.9	0.7	0.7	0.6	0.8	0.8	0.7	0.7	0.9	11.3	4	2	2	8	274	30.7	5.8
NP_065881.1	18482373	WDFY1	WD repeat and FYVE domain-containing protein 1	1.0	1.0	1.0	0.9	0.9	0.8	0.8	0.8	0.9	0.9	0.9	0.9	6.8	1	2	2	2	410	46.3	7.3
NP_003855.1	4506783	SAP30	histone deacetylase complex subunit SAP30	0.9	0.9	1.0	0.9	0.7	0.9	1.3	0.9	0.8	0.9	1.2	0.9	9.1	1	2	2	2	220	23.3	9.2
NP_001010983.1	58331225	GLT8D1	glycosyltransferase 8 domain-containing protein 1	1.0	0.9	0.9	0.9	1.0	0.7	0.8	0.8	1.0	0.8	0.9	0.9	4.6	1	2	2	2	371	41.9	9.3
NP_055983.1	126723060	ZC3H4	zinc finger CCCH domain-containing protein 4	0.8	0.9	0.9	0.8	0.8	0.9	0.9	0.9	0.8	0.9	0.9	0.9	6.9	1	6	6	10	1303	140.2	6.3
NP_056299.1	7661636	SYF2	pre-mRNA-splicing factor SYF2 isoform 1	0.8	0.9	0.8	0.9	0.8	0.8	0.8	0.8	0.8	0.9	0.8	0.9	13.6	2	4	4	5	243	28.7	8.4

Khan *et al.* , 2019. Multi-omics analysis to characterize cigarette smoke induced molecular alterations in esophageal cells
Supplementary Table 5. List of proteins quantified in untreated and chronically treated Het1A cells with cigarette smoke condensate for 8 months

NP_Accession	Protein group Accession	Gene ID	Description	Het-1A-Smoke - 2M/Parental	Het-1A-Smoke - 4M/Parental	Het-1A-Smoke - 6M/Parental	Het-1A-Smoke - 8M/Parental	Het-1A-Smoke - 2M/Parental	Het-1A-Smoke - 4M/Parental	Het-1A-Smoke - 6M/Parental	Het-1A-Smoke - 8M/Parental	Het-1A-Smoke - 2M/Parental	Het-1A-Smoke - 4M/Parental	Het-1A-Smoke - 6M/Parental	Het-1A-Smoke - 8M/Parental	Coverage (%)	Proteins	Unique Peptides	# Peptides	PSM	# AAs	MW [kDa]	calc. pI
				Replicate 1				Replicate 2				Average of replicates											
NP_001229410.1	335334941;335334949	EIF1AD	probable RNA-binding protein EIF1AD	0.9	0.9	0.9	0.8	0.9	0.9	0.9	0.9	0.9	0.9	0.9	0.9	27.9	1	4	4	17	165	19.0	5.2
NP_001957.2	68989263	EHHADH	peroxisomal bifunctional enzyme isoform 1	0.9	0.7	0.8	0.8	1.0	0.8	0.8	0.9	1.0	0.8	0.8	0.9	3.7	2	2	2	3	723	79.4	9.1
NP_001154836.1	238624149	NPTN	neuroplastin isoform d precursor	0.7	0.9	0.8	0.8	0.8	0.9	1.0	0.9	0.8	0.9	0.9	0.9	19.4	4	4	4	5	278	30.8	7.8
NP_003899.2	29826335	EIF2S2	eukaryotic translation initiation factor 2 subunit 2	1.0	0.9	0.9	0.8	0.9	0.8	0.8	0.9	0.9	0.9	0.8	0.9	46.3	1	11	11	65	333	38.4	5.8
NP_006823.1	29789006	FERMT2	fermitin family homolog 2 isoform 1	0.8	0.8	0.8	0.8	0.9	0.8	0.9	0.9	0.8	0.8	0.8	0.9	41.2	4	21	21	56	680	77.8	6.7
NP_057538.1	7705997	EMC4	ER membrane protein complex subunit 4 isoform a	0.9	0.8	0.7	0.8	1.0	0.9	0.9	0.9	0.9	0.9	0.8	0.9	20.8	2	3	3	7	183	20.1	8.6
NP_001159367.1	259906403	CD320	CD320 antigen isoform 2 precursor	0.8	0.9	0.9	0.8	0.8	0.9	0.9	0.9	0.8	0.9	0.9	0.9	3.8	2	1	1	3	240	24.4	4.8
NP_612448.1	34147540	FLYWCH2	FLYWCH family member 2	0.9	0.9	1.0	0.8	1.0	0.9	0.9	0.9	0.9	0.9	0.9	0.9	47.1	1	4	4	14	140	14.6	8.5
NP_001190180.1	322303720	MAPT	microtubule-associated protein tau isoform 7	1.1	0.8	1.0	0.9	1.0	0.7	0.9	0.8	1.0	0.8	0.9	0.9	7.9	2	1	1	1	381	39.7	8.6
NP_054727.1	7661844	CCDC22	coiled-coil domain-containing protein 22	0.9	0.9	0.8	0.9	0.9	0.9	0.8	0.8	0.9	0.9	0.8	0.9	5.6	1	2	2	6	627	70.7	6.7
NP_006045.1	5174655	RTN3	reticulon-3 isoform a	0.9	0.9	0.8	0.8	1.0	1.0	0.9	0.9	0.9	0.9	0.9	0.9	7.2	5	2	2	4	236	25.6	8.5
NP_002344.2	166795236	TACSTD2	tumor-associated calcium signal transducer 2 precursor	0.9	0.8	0.8	0.8	0.8	0.8	0.8	0.9	0.8	0.8	0.8	0.9	18.0	1	4	4	9	323	35.7	8.9
NP_055601.2	21361458	ARHGEF17	rho guanine nucleotide exchange factor 17	1.1	0.9	0.9	0.9	1.0	1.0	0.7	0.8	1.0	1.0	0.8	0.9	2.3	1	2	2	3	2063	221.5	6.3
NP_055587.1	7662112	CTIF	CBP80/20-dependent translation initiation factor isoform 1	1.0	1.0	0.9	0.9	0.8	0.9	0.9	0.8	0.9	0.9	0.9	0.9	2.0	2	1	1	2	598	67.5	6.5
NP_060191.3	96974985	CC2D1A	coiled-coil and C2 domain-containing protein 1A	0.9	0.8	0.8	0.8	0.8	0.8	0.8	0.9	0.9	0.8	0.8	0.9	6.4	1	5	5	12	951	104.0	8.1
NP_055422.1	24307965	UBXN4	UBX domain-containing protein 4	1.6	1.0	0.9	0.9	1.0	0.9	0.9	0.8	1.3	1.0	0.9	0.9	10.0	1	3	3	10	508	56.7	6.4
NP_003281.1	4507651	TPM4	tropomyosin alpha-4 chain isoform Tpm4.2cy	1.0	1.0	1.1	0.9	1.0	1.0	1.1	0.8	1.0	1.0	1.1	0.9	63.7	2	10	20	193	248	28.5	4.7
NP_001093878.1	154350204	C17orf62	uncharacterized protein C17orf62 isoform b	0.7	0.8	0.7	0.8	0.9	0.9	0.9	0.9	0.8	0.8	0.8	0.9	4.6	2	1	1	2	173	19.4	6.8
NP_001431.1	4503617	EXTL3	exostosin-like 3	0.9	0.9	0.9	0.8	0.8	1.0	0.9	0.9	0.9	0.9	0.9	0.9	2.1	1	1	1	1	919	104.7	6.5
NP_055399.1	7657069	ERO1L	ERO1-like protein alpha precursor	0.9	0.8	0.9	0.8	0.9	0.9	1.0	0.9	0.9	0.8	0.9	0.9	45.9	1	15	16	67	468	54.4	5.7
NP_066357.2	316983124	RPL36A	60S ribosomal protein L36a isoform a	0.8	0.8	0.9	0.8	0.8	0.9	0.8	0.9	0.8	0.9	0.8	0.9	23.9	4	2	6	52	142	16.4	10.4
NP_004704.2	32130516	NEMF	nuclear export mediator factor NEMF isoform 1	0.9	0.9	0.8	0.8	0.9	0.8	0.8	0.9	0.9	0.9	0.8	0.9	7.5	2	7	7	16	1076	122.9	6.4
NP_068351.1	11496974	NFYA	nuclear transcription factor Y subunit alpha isoform 2	0.8	0.9	0.9	0.9	0.9	0.9	0.8	0.9	0.9	0.9	0.9	0.9	15.7	2	3	3	4	318	33.9	9.2
NP_065890.1	18874090	WDR48	WD repeat-containing protein 48 isoform 1	0.9	0.8	0.9	0.8	1.0	0.9	1.0	0.9	0.9	0.9	0.9	0.9	11.2	3	5	5	12	677	76.2	7.0
NP_001186731.1	315221152	RPL11	60S ribosomal protein L11 isoform 2	0.8	0.9	0.9	0.9	0.9	0.9	0.9	0.8	0.8	0.9	0.9	0.9	33.9	2	5	5	48	177	20.1	9.6
NP_004126.1	4758582	IDH3G	isocitrate dehydrogenase [NAD] subunit gamma, mitochondrial isoform a precursor	1.0	1.0	0.9	0.9	0.9	0.9	0.9	0.8	0.9	0.9	0.9	0.9	32.1	2	8	8	28	393	42.8	8.5
NP_002004.1	4503727	FKBP3	peptidyl-prolyl cis-trans isomerase FKBP3	0.8	0.8	0.8	0.8	0.8	0.8	0.8	0.9	0.8	0.8	0.8	0.9	62.5	1	12	12	77	224	25.2	9.3
NP_055438.1	7657326	MEA1	male-enhanced antigen 1	1.1	1.0	1.0	1.0	0.8	0.7	0.7	0.7	0.9	0.8	0.8	0.9	7.0	1	1	1	1	185	19.9	4.2
NP_055577.1	13375618	DHCR24	delta(24)-sterol reductase precursor	1.0	1.0	0.9	0.9	1.1	1.0	1.0	0.8	1.0	1.0	0.9	0.9	26.7	1	11	11	48	516	60.1	8.2
NP_055829.2	238859597	RBM34	RNA-binding protein 34 isoform 1	1.1	0.9	0.9	0.9	0.9	0.9	0.8	0.8	1.0	0.9	0.9	0.9	27.0	2	10	10	28	430	48.5	10.1
NP_005327.1	4885409	HDLBP	vigilin isoform a	0.9	0.9	0.9	0.8	1.0	0.9	0.9	0.9	0.9	0.9	0.9	0.8	45.7	4	46	47	185	1268	141.4	6.9
NP_909119.1	37577148	NCKIPSD	NCK-interacting protein with SH3 domain isoform 2	1.0	0.9	0.9	0.9	1.0	0.8	0.8	0.8	1.0	0.9	0.9	0.8	5.0	2	4	4	12	715	78.2	6.4
NP_037377.1	7019569	VPS4A	vacuolar protein sorting-associated protein 4A	0.9	0.8	0.8	0.8	0.9	0.8	0.8	0.9	0.9	0.8	0.8	0.8	17.4	1	5	8	14	437	48.9	7.8
NP_005522.2	112789562	IFI16	gamma-interferon-inducible protein 16 isoform 2	0.9	0.9	0.9	0.9	0.9	0.8	0.9	0.8	0.9	0.8	0.9	0.8	32.4	8	19	19	54	729	82.0	9.3
NP_001611.1	61743954	AHNAK	neuroblast differentiation-associated protein AHNAK isoform 1	0.8	1.0	1.0	0.8	0.8	1.0	1.0	0.9	0.8	1.0	1.0	0.8	61.6	12	187	190	846	5890	628.7	6.2
NP_005472.2	38146094	MED16	mediator of RNA polymerase II transcription subunit 16	0.8	0.8	0.8	0.9	0.7	0.9	0.9	0.8	0.8	0.8	0.9	0.8	1.3	1	1	1	4	877	96.7	7.4
NP_001265569.1	520975484	RANBP1	ran-specific GTPase-activating protein isoform 3	1.2	0.8	1.2	0.8	1.1	0.8	1.1	0.9	1.1	0.8	1.1	0.8	46.0	6	7	7	54	200	23.2	5.3
NP_071898.2	34878787	RNF25	E3 ubiquitin-protein ligase RNF25	0.8	0.8	0.8	0.8	0.9	0.9	0.9	0.9	0.9	0.9	0.8	0.8	9.6	1	3	3	6	459	51.2	6.5
NP_002130.2	56699409	RBMX	RNA-binding motif protein, X chromosome isoform 1	0.8	0.8	0.8	0.8	0.9	0.8	0.8	0.9	0.9	0.8	0.8	0.8	40.9	11	17	17	89	391	42.3	10.1
NP_000987.2	16117791	RPL35A	60S ribosomal protein L35a	0.8	0.8	0.9	0.8	0.8	0.8	0.9	0.9	0.8	0.8	0.9	0.8	44.6	1	6	6	26	110	12.5	11.1
NP_612568.1	20336239	PPP1R8	nuclear inhibitor of protein phosphatase 1 isoform beta/delta	0.9	0.9	0.8	0.9	0.7	0.7	0.7	0.8	0.8	0.8	0.7	0.8	18.7	3	2	2	4	209	22.7	5.9
NP_009118.1	6005948	WBP4	WW domain-binding protein 4	0.8	0.9	0.8	0.8	0.9	0.9	0.8	0.9	0.8	0.9	0.8	0.8	10.6	1	2	2	4	376	42.5	5.6
NP_055486.2	187960100	UBE3C	ubiquitin-protein ligase E3C	1.0	0.9	1.0	0.8	1.0	0.9	1.0	0.9	1.0	0.9	1.0	0.8	15.0	2	12	12	28	1083	123.8	6.7
NP_004593.2	82659083;82659089	STAU1	double-stranded RNA-binding protein Staufen homolog 1 isoform a	1.0	0.7	0.8	0.8	0.8	0.7	0.8	0.9	0.9	0.7	0.8	0.8	38.1	3	12	13	38	496	54.9	9.5
NP_005773.3	238776833	ALYREF	THO complex subunit 4	0.8	0.9	0.8	0.8	0.8	0.9	0.8	0.9	0.8	0.9	0.8	0.8	39.4	1	8	8	67	264	27.5	11.0
NP_001106673.1	163965364	NACA	nascent polypeptide-associated complex subunit alpha isoform b	0.9	0.9	1.0	0.8	1.0	0.9	1.0	0.8	1.0	0.9	1.0	0.8	35.8	2	6	6	127	215	23.4	4.6
NP_060228.3	148539642	ALKBH5	RNA demethylase ALKBH5	0.9	0.8	0.9	0.8	0.8	0.9	0.9	0.9	0.8	0.9	0.9	0.8	14.7	1	4	4	8	394	44.2	9.1
NP_000909.2	20070125	P4HB	protein disulfide-isomerase precursor	0.9	1.1	0.9	0.9	0.9	1.0	0.9	0.8	0.9	1.0	0.9	0.8	74.4	1	37	37	568	508	57.1	4.9
NP_478123.1	17402896	RAD51C	DNA repair protein RAD51 homolog 3 isoform 1	0.8	0.7	0.7	0.8	0.8	0.8	0.7	0.9	0.8	0.7	0.7	0.8	9.0	2	2	2	4	376	42.2	6.7
NP_079106.3	33946291	LPCAT1	lysophosphatidylcholine acyltransferase 1	0.8	0.8	0.8	0.8	0.8	0.7	0.9	0.8	0.8	0.8	0.8	0.8	9.0	1	4	4	7	534	59.1	6.0
NP_001001.2	17158044	RPS6	40S ribosomal protein S6	0.9	0.9	0.9	0.8	0.8	0.9	0.9	0.8	0.9	0.9	0.9	0.8	44.2	1	15	15	133	249	28.7	10.8
NP_056377.1	149944496	UBXN7	UBX domain-containing protein 7	1.1	0.9	1.0	0.8	1.0	0.9	1.0	0.8	1.1	0.9	1.0	0.8	32.9	1	12	12	35	489	54.8	5.2
NP_056515.2	50593025	NFU1	NFU1 iron-sulfur cluster scaffold homolog, mitochondrial isoform 1	1.1	0.8	1.1	0.8	1.1	1.0	1.2	0.9	1.1	0.9	1.2	0.8	19.1	3	3	3	7	230	25.9	4.7
NP_604391.1	19745184	CREB1	cyclic AMP-responsive element-binding protein 1 isoform B	1.7	0.8	0.8	0.8	0.9	0.9	0.9	0.9	1.3	0.9	0.8	0.8	27.3	9	4	6	18	341	36.7	5.6
NP_009123.1	6005757	SUPT16H	FACT complex subunit SPT16	1.0	0.8	0.8	0.8	0.9	0.8	0.8	0.8	0.9	0.8	0.8	0.8	49.4	1	45	45	184	1047	119.8	5.7
NP_940852.3	116812610	APOOL	MICOS complex subunit MIC27 precursor	0.9	0.9	1.0	0.9	0.9	1.0	1.1	0.8	0.9	1.0	1.0	0.8	10.8	1	3	3	4	268	29.1	9.5
XP_006724973.1	578839160	LOC10272401	spidroin-1-like isoform X2	0.7	0.9	0.7	0.8	0.7	0.9	0.8	0.9	0.7	0.9	0.7	0.8	4.8	6	2	2	4	270	27.9	12.0
NP_060083.1	8922149	DONSON	protein downstream neighbor of Son	1.0	0.9	1.0	1.0	0.7	0.7	0.8	0.7	0.9	0.8	0.9	0.8	4.1	1	1	1	1	566	62.7	8.7
NP_001135921.1	215490098	SPNS1	protein spinster homolog 1 isoform 4	0.9	0.7	0.9	0.9	0.9	0.9	1.0	0.8	0.9	0.8	0.9	0.8	9.7	4	2	2	4	454	49.0	7.8
NP_055109.1	7657655	TRAM1	translocating chain-associated membrane protein 1	1.0	0.9	0.9	0.8	0.9	0.9	0.9	0.9	0.9	0.9	0.9	0.8	4.8	1	2	2	6	374	43.0	9.6
NP_001263266.1	545309210	EIF4E2	eukaryotic translation initiation factor 4E type 2 isoform D	0.8	0.8	0.8	0.8	1.4	1.0	0.9	0.9	1.1	0.9	0.9	0.8	5.8	4	1	1	5	189	21.9	7.0
NP_003785.1	4507145	SNX4	sorting nexin-4	1.3	0.9	0.8	0.8	0.9	0.9	0.9	0.9	1.1	0.9	0.9	0.8	9.8	1	3	3	4	450	51.9	6.0
NP_057510.4	253970412	GTSE1	G2 and S phase-expressed protein 1	0.9	0.9	1.0	0.8	0.9	0.9	0.8	0.9	0.9	0.9	0.9	0.8	13.4	1	8	8	14	739	78.4	9.3
NP_004330.1	4757886	PTTG1IP	pituitary tumor-transforming gene 1 protein-interacting protein isoform 1 precursor	0.8	1.1	1.0	0.7	0.9	1.3	1.1	0.9	0.9	1.2	1.0	0.8	10.0	1	1	1	7	180	20.3	8.8
NP_079341.2	145275210	RPF1	ribosome production factor 1	0.7	0.7	0.8	0.7	0.9	1.0	0.9	0.9	0.8	0.9	0.9	0.8	16.6	1	3	3	4	349	40.1	10.0
NP_006308.3	30795231	BASP1	brain acid soluble protein 1	1.0	1.0	0.9	0.8	1.0	1.0	0.9	0.9	1.0	1.0	0.9	0.8	77.1	1	15	15	75	227	22.7	4.6
NP_079150.3	33359221	KIAA0319L	dyslexia-associated protein KIAA0319-like protein	0.9	1.0	0.8	0.9	0.7	0.7	0.7	0.8	0.8	0.8	0.7	0.8	4.8	1	5	5	7	1049	115.6	6.0
NP_002884.1	4506439	RBBP7	histone-binding protein RBBP7 isoform 2	0.9	0.8	0.9	0.8	0.9	0.9	0.9	0.9	0.9	0.8	0.9	0.8	33.9	2	7	12	48	425	47.8	5.0
NP_005917.2	50726968	MFAP1	microfibrillar-associated protein 1	0.8	0.8	0.7	0.8	0.9	1.0	0.8	0.9	0.8	0.9	0.8	0.8	15.7	1	8	8	16	439	51.9	5.0
NP_057151.1	7705630	MRPS18C	28S ribosomal protein S18c, mitochondrial	0.9	0.7	0.8	0.7	0.9	1.0	1.0	0.9	0.9	0.9	0.9	0.8	13.4	1	1	1	2	142	15.8	9.6
NP_060255.3	157388975	SPDL1	protein Spindly	1.0	0.9	0.8	0.9	0.7	0.7	0.7	0.8	0.9	0.8	0.7	0.8	5.5	1	3	3	3	605	70.1	5.5
NP_001034554.1	86991438	SRSF5	serine/arginine-rich splicing factor 5	0.8	0.9	0.8	0.8	0.8	0.9	0.8	0.8	0.8	0.9	0.8	0.8	27.2	1	7	8	79	272	31.2	11.6
NP_055699.2	224282117;224282119	SUGP2	SURP and G-patch domain-containing protein 2	0.9	0.8	0.8	0.9	0.8	0.7	0.7	0.8	0.8	0.8	0.7	0.8	21.3	1	14	14	23	1082	120.1	7.3
NP_005724.1	5032013	KIF20A	kinesin-like protein KIF20A	0.7	0.8	0.9	0.9	0.7	0.8	0.9	0.8	0.7	0.8	0.9	0.8	17.0	1	12	12	25	890	100.2	6.9
NP_002895.3	14670268	NELFE	negative elongation factor E	0.9	0.9	0.8	0.9	0.8	0.8	0.8	0.8	0.8	0.9	0.8	0.8	32.6	1	9	9	32	380	43.2	9.3
NP_009216.1	6005768	GABARAPL2	gamma-aminobutyric acid receptor-associated protein-like 2	0.8	0.8	0.9	0.8	0.9	0.9	1.0	0.8	0.9	0.8	0.9	0.8	25.6	1	2	3	9	117	13.7	8.1
NP_068814.2	13676857	HSPA2	heat shock-related 70 kDa protein 2	0.9	0.8	0.8	0.8	1.0	0.8	0.9	0.8	1.0	0.8	0.8	0.8	36.9	1	9	22	296	639	70.0	5.7
NP_004584.1	4759098	TRA2B	transformer-2 protein homolog beta isoform 1	0.8	0.9	0.8	0.8	0.9	0.9	0.8	0.9	0.8	0.9	0.8	0.8	26.7	2	6	6	78	288	33.6	11.3
NP_998827.1	47458820	STAT3	signal transducer and activator of transcription 3 isoform 3	0.9	0.8	0.8	0.9	0.8	0.8	0.8	0.8	0.8	0.8	0.8	0.8	22.3	3	11	11	22	722	83.1	7.1
NP_003007.2	47271443	SRSF2	serine/arginine-rich splicing factor 2	0.9	1.1	1.0	0.9	0.8	0.9	0.8	0.8	0.9	1.0	0.9	0.8	29.0	1	4	7	67	221	25.5	11.9
NP_001180346.1	301171475	DDX3X	ATP-dependent RNA helicase DDX3X isoform 3	0.8	0.8	0.8	0.8	0.9	0.8	0.9	0.9	0.9	0.8	0.8	0.8	41.5	3	7	21	90	646	71.3	6.6
NP_001269373.1	540344515	EHD1	EH domain-containing protein 1 isoform 1	0.9	1.0	0.9	0.8	0.9	0.9	0.9	0.8	0.9	0.9	0.9	0.8	42.5	2	12	15	41	534	60.6	6.8
NP_001177925.1	300360515	ARPC1A	actin-related protein 2/3 complex subunit 1A isoform 2	0.9	1.0	1.0	0.8	1.0	1.0	1.0	0.8	1.0	1.0	1.0	0.8	21.9	2	4	5	17	356	39.6	8.2
NP_001172011.1	297374791	FMR1	fragile X mental retardation protein 1 isoform ISO9	0.8	0.9	0.8	0.9	0.8	0.8	0.7	0.8	0.8	0.8	0.7	0.8	24.6	5	10	12	21	586	66.2	8.5
NP_002907.1	4506491;31881687	RFC4	replication factor C subunit 4	0.9	0.8	0.8	0.8	0.8	0.8	0.8	0.8	0.9	0.8	0.8	0.8	49.6	1	14	14	47	363	39.7	8.0
NP_001171122.1	294997330	SLC9A6	sodium/hydrogen exchanger 6 isoform c	1.0	0.9	0.9	0.8	0.9	0.9	0.8	0.8	1.0	0.9	0.9	0.8	4.8	3	2	2	6	649	72.2	5.7
NP_000937.1	4506051	PRIM1	DNA primase small subunit	0.9	0.8	0.9	0.9	0.9	0.7	0.8	0.8	0.9	0.8	0.9	0.8	22.4	1	7	7	12	420	49.9	8.2
NP_009131.2	21493029	AKAP13	A-kinase anchor protein 13 isoform 2	0.9	0.9	1.0	0.8	0.9	0.9	1.0	0.8	0.9	0.9	1.0	0.8	16.0	3	29	29	61	2813	307.4	5.2
NP_060248.2	13699813	WHSC1L1	histone-lysine N-methyltransferase NSD3 isoform short	0.9	0.8	0.8	0.9	0.9	0.8	1.0	0.8	0.9	0.8	0.9	0.8	7.4	2	2	2	6	645	72.6	9.1
NP_006518.1	5729862	SLBP	histone RNA hairpin-binding protein isoform 1	0.9	0.7	0.8	0.8	0.9	1.0	0.8	0.9	0.9	0.9	0.8	0.8	9.6	3	2	2	7	270	31.3	7.5

Khan *et al.* , 2019. Multi-omics analysis to characterize cigarette smoke induced molecular alterations in esophageal cells
Supplementary Table 5. List of proteins quantified in untreated and chronically treated Het1A cells with cigarette smoke condensate for 8 months

NP_Accession	Protein group Accession	Gene ID	Description	Het-1A-Smoke - 2M/Parental	Het-1A-Smoke - 4M/Parental	Het-1A-Smoke - 6M/Parental	Het-1A-Smoke - 8M/Parental	Het-1A-Smoke - 2M/Parental	Het-1A-Smoke - 4M/Parental	Het-1A-Smoke - 6M/Parental	Het-1A-Smoke - 8M/Parental	Het-1A-Smoke - 2M/Parental	Het-1A-Smoke - 4M/Parental	Het-1A-Smoke - 6M/Parental	Het-1A-Smoke - 8M/Parental	Coverage (%)	Proteins	Unique Peptides	# Peptides	PSM	# AAs	MW [kDa]	calc. pI
				Replicate 1				Replicate 2				Average of replicates											
NP_777567.1	28372531	**PPP4R2**	serine/threonine-protein phosphatase 4 regulatory subunit 2	0.8	0.8	0.8	0.8	0.9	0.9	0.9	0.9	**0.9**	**0.9**	**0.8**	**0.8**	28.5	1	8	8	25	417	46.9	4.5
NP_001193773.1	332205961	**TGOLN2**	trans-Golgi network integral membrane protein 2 isoform 4 precursor	0.9	0.9	0.9	0.8	1.0	0.9	0.9	0.9	**0.9**	**0.9**	**0.9**	**0.8**	13.5	4	4	4	9	379	39.5	5.6
NP_954981.1	40548332	**CCDC137**	coiled-coil domain-containing protein 137	0.9	0.8	0.9	0.8	0.7	0.9	0.8	0.9	**0.8**	**0.9**	**0.8**	**0.8**	6.6	1	2	2	2	289	33.2	10.9
NP_777569.1	28372533	**TMEM167A**	protein kish-A precursor	1.2	1.0	1.1	0.9	1.1	0.8	0.9	0.7	**1.2**	**0.9**	**1.0**	**0.8**	25.0	1	2	2	4	72	8.1	9.0
NP_542399.1	18087845	**CDCA5**	sororin	1.2	0.9	0.8	0.8	0.9	0.9	1.0	0.9	**1.0**	**0.9**	**0.9**	**0.8**	29.0	2	6	6	18	252	27.6	9.6
NP_004550.2	34098946	**YBX1**	nuclease-sensitive element-binding protein 1	0.8	0.9	0.8	0.8	0.9	1.0	0.8	0.9	**0.8**	**0.9**	**0.8**	**0.8**	53.4	1	10	13	158	324	35.9	9.9
NP_001123237.1	193211614;8393516	**NSDHL**	sterol-4-alpha-carboxylate 3-dehydrogenase, decarboxylating	1.1	1.0	0.9	0.9	1.1	0.9	0.8	0.7	**1.1**	**1.0**	**0.9**	**0.8**	30.0	1	10	10	28	373	41.9	8.1
NP_115726.1	14150149;197304708	**C7orf50**	uncharacterized protein C7orf50	0.8	1.0	0.8	0.8	0.9	1.0	0.8	0.8	**0.9**	**1.0**	**0.8**	**0.8**	50.0	1	8	8	27	194	22.1	9.6
NP_001950.1	4503477	**EEF1B2**	elongation factor 1-beta	1.6	0.9	1.6	0.8	1.5	0.9	1.4	0.8	**1.5**	**0.9**	**1.5**	**0.8**	76.0	1	12	15	80	225	24.7	4.7
NP_001230072.1	341865562	**NUSAP1**	nucleolar and spindle-associated protein 1 isoform 5	0.7	0.8	0.9	0.8	0.7	0.8	0.8	0.8	**0.7**	**0.8**	**0.9**	**0.8**	35.2	3	1	13	41	426	47.7	9.9
NP_001410.2	38201714	**ELAVL1**	ELAV-like protein 1	0.9	0.8	0.8	0.8	0.9	0.9	0.9	0.9	**0.9**	**0.9**	**0.8**	**0.8**	33.1	1	9	9	78	326	36.1	9.2
NP_056034.2	62241044	**EXOC7**	exocyst complex component 7 isoform 2	1.0	0.8	0.8	0.8	1.0	0.9	0.9	0.9	**1.0**	**0.9**	**0.8**	**0.8**	4.3	6	2	2	3	653	74.7	6.4
NP_000983.1	4506629	**RPL29**	60S ribosomal protein L29	0.7	0.9	0.8	0.8	0.8	0.9	0.9	0.9	**0.8**	**0.9**	**0.9**	**0.8**	22.0	1	4	4	33	159	17.7	11.7
NP_149124.3	94721261	**CNP**	2',3'-cyclic-nucleotide 3'-phosphodiesterase	0.8	0.8	0.8	0.9	0.8	0.7	0.7	0.8	**0.8**	**0.8**	**0.8**	**0.8**	20.0	1	7	7	31	421	47.5	9.1
NP_443167.4	294459996	**NT5C3B**	7-methylguanosine phosphate-specific 5'-nucleotidase	0.9	0.8	0.8	0.8	1.0	0.8	0.9	0.9	**1.0**	**0.8**	**0.8**	**0.8**	6.0	1	1	1	2	300	34.4	6.4
NP_543011.2	93277094	**FAM210B**	protein FAM210B	0.7	0.6	0.6	0.8	1.0	0.8	1.0	0.8	**0.8**	**0.7**	**0.8**	**0.8**	15.1	1	2	2	3	192	20.4	10.4
NP_612402.1	19923919	**REEP6**	receptor expression-enhancing protein 6	1.2	0.8	0.9	0.9	0.9	0.7	0.9	0.8	**1.1**	**0.8**	**0.9**	**0.8**	7.1	1	1	1	2	184	20.7	8.6
NP_001019637.1	67782309	**SOD2**	superoxide dismutase [Mn], mitochondrial isoform B precursor	0.8	0.8	0.8	0.8	0.9	0.9	0.8	0.9	**0.9**	**0.8**	**0.8**	**0.8**	3.8	2	1	1	2	183	20.7	8.3
NP_001171894.1	296841073	**WDR44**	WD repeat-containing protein 44 isoform 2	1.0	1.0	0.9	0.8	1.0	1.0	0.9	0.8	**1.0**	**1.0**	**0.9**	**0.8**	11.2	3	7	7	12	905	100.3	5.5
NP_001182085.1	304434704	**ZMYM6NB**	uncharacterized protein ZMYM6NB precursor	1.1	0.9	0.8	0.9	1.1	0.7	0.6	0.7	**1.1**	**0.8**	**0.7**	**0.8**	7.8	1	1	1	5	154	16.9	9.2
NP_057177.1	7705813	**RPL26L1**	60S ribosomal protein L26-like 1	0.7	1.0	0.8	0.9	0.8	0.9	0.7	0.8	**0.8**	**0.9**	**0.8**	**0.8**	37.9	1	1	10	51	145	17.2	10.6
NP_057251.2	13569843	**NOL7**	nucleolar protein 7	0.9	0.9	0.9	0.8	0.9	0.9	0.9	0.9	**0.9**	**0.9**	**0.9**	**0.8**	17.5	1	6	6	15	257	29.4	9.7
NP_055643.1	7662274	**TOX4**	TOX high mobility group box family member 4 isoform 1	0.9	0.9	0.9	0.9	0.8	0.9	0.9	0.8	**0.8**	**0.9**	**0.9**	**0.8**	12.7	8	6	6	15	621	66.2	5.1
NP_006817.1	5803227	**YWHAQ**	14-3-3 protein theta	0.9	1.0	0.9	0.8	1.0	1.0	1.0	0.8	**1.0**	**1.0**	**1.0**	**0.8**	71.0	1	13	19	249	245	27.7	4.8
NP_113584.3	61676188	**HUWE1**	E3 ubiquitin-protein ligase HUWE1	1.0	1.0	0.9	0.8	1.0	1.0	0.9	0.8	**1.0**	**1.0**	**0.9**	**0.8**	18.0	3	49	49	122	4374	481.6	5.2
NP_001257804.1	400153656	**MEAF6**	chromatin modification-related protein MEAF6 isoform 2	0.8	0.9	0.8	0.9	0.8	0.8	0.8	0.8	**0.8**	**0.8**	**0.8**	**0.8**	15.7	3	3	3	6	191	21.6	9.3
NP_060495.2	21361684	**GPATCH1**	G patch domain-containing protein 1	0.7	0.8	0.9	0.7	0.9	1.0	1.0	1.0	**0.8**	**0.9**	**0.9**	**0.8**	2.5	1	1	1	2	931	103.3	7.0
NP_001447.2	116063573	**FLNA**	filamin-A isoform 1	1.0	0.9	0.9	0.8	1.0	1.0	0.9	0.9	**1.0**	**0.9**	**0.9**	**0.8**	53.5	2	83	96	539	2639	279.8	6.0
NP_001177916.1	300244569	**SRSF11**	serine/arginine-rich splicing factor 11 isoform 2	1.0	0.8	0.8	0.9	0.8	0.8	0.8	0.8	**0.9**	**0.8**	**0.8**	**0.8**	9.5	2	3	3	4	483	53.4	10.5
NP_847896.1	30410798	**ATG4B**	cysteine protease ATG4B isoform b	0.9	0.8	0.8	0.8	0.9	0.7	0.8	0.8	**0.9**	**0.7**	**0.8**	**0.8**	22.4	2	5	5	17	380	42.6	5.5
NP_001166953.1	291045198	**CRBN**	protein cereblon isoform 2	0.9	0.8	0.9	0.8	1.0	0.8	0.9	0.9	**1.0**	**0.8**	**0.9**	**0.8**	8.6	2	3	3	5	441	50.4	5.6
NP_005633.2	14717407	**TAF7**	transcription initiation factor TFIID subunit 7	0.9	0.9	0.8	0.9	0.9	0.9	0.9	0.8	**0.9**	**0.9**	**0.8**	**0.8**	10.9	1	3	3	9	349	40.2	5.2
NP_001041648.1	115430211	**PHACTR4**	phosphatase and actin regulator 4 isoform 1	0.8	0.8	0.8	0.8	0.9	0.9	0.8	0.9	**0.9**	**0.9**	**0.8**	**0.8**	7.1	2	3	3	5	702	78.2	6.6
NP_109587.1	13489054	**MAP2K2**	dual specificity mitogen-activated protein kinase kinase 2	0.9	0.9	0.9	0.9	0.9	0.8	0.8	0.8	**0.9**	**0.9**	**0.9**	**0.8**	22.0	1	4	10	26	400	44.4	6.6
NP_001005849.1	54792071	**SUMO2**	small ubiquitin-related modifier 2 isoform b precursor	0.9	1.0	1.0	0.9	1.0	0.8	0.9	0.8	**0.9**	**0.9**	**0.9**	**0.8**	31.0	6	2	2	18	71	8.1	5.4
NP_005601.1	5032027	**RBBP4**	histone-binding protein RBBP4 isoform a	0.8	0.7	0.8	0.8	1.1	0.8	0.9	0.9	**1.0**	**0.8**	**0.9**	**0.8**	35.3	3	5	10	44	425	47.6	4.9
NP_065147.1	9966805	**DDX24**	ATP-dependent RNA helicase DDX24	0.8	0.8	0.8	0.8	0.9	0.9	0.8	0.9	**0.8**	**0.8**	**0.8**	**0.8**	33.9	1	21	22	61	859	96.3	9.1
NP_001008389.1	56605994	**CISD2**	CDGSH iron-sulfur domain-containing protein 2	0.8	0.7	0.8	0.7	0.9	0.9	0.9	0.9	**0.9**	**0.8**	**0.9**	**0.8**	28.9	1	4	4	8	135	15.3	9.6
NP_003300.1	62988322	**TSPYL1**	testis-specific Y-encoded-like protein 1	1.0	0.9	0.9	0.8	0.9	1.0	0.9	0.8	**0.9**	**0.9**	**0.9**	**0.8**	27.7	1	9	9	21	437	49.2	5.5
NP_001654.1	4502211	**ARF6**	ADP-ribosylation factor 6	0.8	0.7	0.8	0.8	0.8	0.8	0.8	0.8	**0.8**	**0.7**	**0.8**	**0.8**	62.9	12	6	7	23	175	20.1	9.0
NP_775918.2	284447287	**RNF149**	E3 ubiquitin-protein ligase RNF149 precursor	1.1	1.0	1.0	0.8	1.0	1.2	1.1	0.9	**1.1**	**1.1**	**1.1**	**0.8**	2.8	1	1	1	2	400	43.1	6.5
NP_653324.1	21389511	**ZMAT2**	zinc finger matrin-type protein 2	0.7	0.9	0.8	0.9	0.7	0.8	0.8	0.8	**0.7**	**0.9**	**0.8**	**0.8**	17.1	1	2	2	5	199	23.6	9.0
NP_060014.2	63003897;291084508	**NKRF**	NF-kappa-B-repressing factor isoform 2	0.9	0.9	0.8	0.9	0.9	0.8	0.7	0.8	**0.9**	**0.9**	**0.8**	**0.8**	15.1	2	10	10	15	690	77.6	8.8
NP_001491.1	4504031	**GMDS**	GDP-mannose 4,6 dehydratase isoform 1	0.8	0.8	0.8	0.8	0.8	0.8	0.7	0.8	**0.8**	**0.8**	**0.8**	**0.8**	32.3	2	8	8	20	372	41.9	7.3
NP_001265522.1	515870065	**ALDH6A1**	methylmalonate-semialdehyde dehydrogenase [acylating], mitochondrial isoform 2 precursor	0.9	0.8	0.7	0.8	1.0	0.8	0.8	0.8	**0.9**	**0.8**	**0.8**	**0.8**	17.8	3	8	8	19	522	56.2	8.4
NP_002936.1	4506583	**RPA1**	replication protein A 70 kDa DNA-binding subunit	0.9	0.7	0.7	0.8	0.9	0.8	0.8	0.9	**0.9**	**0.8**	**0.8**	**0.8**	25.2	1	12	12	41	616	68.1	7.2
NP_001273512.1	557440810	**PHRF1**	PHD and RING finger domain-containing protein 1 isoform 4	1.1	0.9	0.8	0.7	1.0	0.9	1.0	0.9	**1.0**	**0.9**	**0.9**	**0.8**	2.0	4	3	3	3	1645	178.1	9.0
NP_001265619.1	523498694	**FGFR1OP**	FGFR1 oncogene partner isoform c	0.8	0.8	0.8	0.8	0.9	1.0	0.9	0.9	**0.8**	**0.9**	**0.8**	**0.8**	33.9	3	8	8	23	351	38.1	4.7
NP_874356.1	33356154	**UBE2H**	ubiquitin-conjugating enzyme E2 H isoform 2	0.9	0.8	0.9	0.8	0.9	0.8	1.0	0.9	**0.9**	**0.8**	**1.0**	**0.8**	25.0	3	3	3	7	152	17.2	4.7
NP_001824.1	4502899	**CLTA**	clathrin light chain A isoform a	1.4	1.0	1.5	0.9	1.2	0.8	1.3	0.8	**1.3**	**0.9**	**1.4**	**0.8**	20.2	8	4	4	25	218	23.6	4.5
NP_005648.1	5032189	**TP53BP1**	tumor suppressor p53-binding protein 1 isoform 3	1.0	1.0	0.9	0.8	0.9	1.0	0.9	0.8	**1.0**	**1.0**	**0.9**	**0.8**	18.2	3	26	26	60	1972	213.4	4.7
NP_612480.1	39930523	**C11orf84**	uncharacterized protein C11orf84	1.0	0.9	0.9	0.9	0.7	0.7	0.8	0.8	**0.8**	**0.8**	**0.8**	**0.8**	17.1	1	4	4	10	381	41.0	5.0
NP_057737.2	82880648	**ZAK**	mitogen-activated protein kinase kinase kinase MLT isoform 1	1.0	0.9	0.9	0.9	0.8	0.8	0.8	0.7	**0.9**	**0.9**	**0.9**	**0.8**	5.1	2	3	3	9	800	91.1	7.9
NP_060544.2	31542652	**CCDC94**	coiled-coil domain-containing protein 94	0.9	0.8	0.9	0.8	0.9	0.9	0.8	0.9	**0.9**	**0.9**	**0.8**	**0.8**	17.0	1	5	5	10	323	37.1	5.9
NP_001370.1	4503351	**DNMT1**	DNA (cytosine-5)-methyltransferase 1 isoform b	0.9	0.8	0.8	0.8	0.9	0.8	0.8	0.8	**0.9**	**0.8**	**0.8**	**0.8**	23.0	2	30	30	83	1616	183.0	7.8
NP_001243695.1	377520157	**TMEM161A**	transmembrane protein 161A isoform 2 precursor	0.9	0.9	0.8	0.9	1.0	0.9	0.8	0.7	**1.0**	**0.9**	**0.8**	**0.8**	2.7	2	1	1	1	376	41.6	9.3
NP_001182257.1	305855090	**RAB9A**	ras-related protein Rab-9A	0.9	0.8	0.9	0.8	1.0	0.9	1.0	0.9	**1.0**	**0.8**	**0.9**	**0.8**	5.5	2	1	1	4	201	22.8	5.5
NP_148982.1	14916501	**RPS24**	40S ribosomal protein S24 isoform a	0.8	0.9	0.9	0.8	0.9	0.9	0.9	0.8	**0.9**	**0.9**	**0.9**	**0.8**	40.0	5	6	6	44	130	15.1	10.9
NP_006296.1	5453880	**ANP32A**	acidic leucine-rich nuclear phosphoprotein 32 family member A	0.8	0.8	0.7	0.7	1.0	1.0	0.8	0.9	**0.9**	**0.9**	**0.8**	**0.8**	49.8	3	8	13	229	249	28.6	4.1
NP_005408.1	4885609	**SRC**	proto-oncogene tyrosine-protein kinase Src	0.8	0.8	0.7	0.9	0.7	0.6	0.7	0.7	**0.7**	**0.7**	**0.7**	**0.8**	14.9	1	4	5	12	536	59.8	7.4
NP_689505.1	22748659	**TRMT10A**	tRNA methyltransferase 10 homolog A	0.7	0.7	0.7	0.7	0.9	0.9	0.9	0.9	**0.8**	**0.8**	**0.8**	**0.8**	4.1	1	1	1	1	339	39.7	7.6
NP_061167.1	8923911	**LANCL2**	lanC-like protein 2	0.8	0.8	0.8	0.7	0.9	0.9	0.9	0.9	**0.9**	**0.8**	**0.9**	**0.8**	15.1	1	5	6	12	450	50.8	7.4
NP_078813.1	13375695	**CARS2**	probable cysteine--tRNA ligase, mitochondrial	0.9	0.8	0.9	0.8	0.9	0.8	0.9	0.8	**0.9**	**0.8**	**0.9**	**0.8**	8.2	1	3	3	5	564	62.2	8.3
NP_001615.1	62988361	**AIM1**	absent in melanoma 1 protein	0.7	0.7	0.7	0.8	0.8	0.9	0.9	0.9	**0.8**	**0.8**	**0.8**	**0.8**	9.6	1	10	11	21	1723	188.5	5.9
NP_054878.5	197313748	**SETD2**	histone-lysine N-methyltransferase SETD2	0.9	0.9	0.9	0.9	0.8	0.8	0.9	0.8	**0.9**	**0.9**	**0.9**	**0.8**	2.6	1	5	5	10	2564	287.4	6.1
NP_115554.2	269315880	**SLC7A6OS**	probable RNA polymerase II nuclear localization protein SLC7A6OS	0.9	0.9	0.9	0.8	0.8	0.9	0.9	0.8	**0.9**	**0.9**	**0.9**	**0.8**	20.7	1	4	4	9	309	35.0	4.6
NP_036342.2	153792582;153791514	**TRIM32**	E3 ubiquitin-protein ligase TRIM32	0.9	0.9	0.9	0.9	1.0	0.7	0.9	0.8	**0.9**	**0.8**	**0.9**	**0.8**	4.0	1	2	2	6	653	71.9	7.0
NP_001972.1	4503593	**EPS15**	epidermal growth factor receptor substrate 15 isoform A	0.9	0.9	0.9	0.8	0.9	0.9	0.9	0.8	**0.9**	**0.9**	**0.9**	**0.8**	19.9	2	14	14	36	896	98.6	4.6
NP_690050.1	23238248	**SNX16**	sorting nexin-16 isoform b	0.9	0.8	0.8	0.7	0.9	1.0	0.9	0.9	**0.9**	**0.9**	**0.8**	**0.8**	4.8	2	1	1	1	315	35.5	4.5
NP_036420.1	6912450	**TRAM2**	translocating chain-associated membrane protein 2	1.0	0.9	0.9	1.0	0.8	0.7	0.8	0.7	**0.9**	**0.8**	**0.8**	**0.8**	4.3	1	1	1	3	370	43.3	9.2
NP_054901.1	7661820	**ORMDL2**	ORM1-like protein 2	1.0	1.0	1.0	0.8	0.9	0.9	0.8	0.8	**0.9**	**1.0**	**0.9**	**0.8**	11.8	1	1	1	1	153	17.4	9.6
NP_001020118.1	68226422	**YIPF5**	protein YIPF5 isoform a	1.1	0.9	0.9	0.8	1.0	0.9	0.9	0.8	**1.0**	**0.9**	**0.9**	**0.8**	4.7	1	1	1	6	257	28.0	4.4
NP_067064.2	151301215	**WIZ**	protein Wiz	0.8	0.9	0.9	0.8	0.8	0.8	0.8	0.8	**0.8**	**0.8**	**0.8**	**0.8**	2.9	1	2	2	3	794	85.3	9.1
NP_001070965.1	117320543	**PRR3**	proline-rich protein 3 isoform b	0.8	0.8	0.9	0.9	0.9	0.8	0.9	0.8	**0.9**	**0.8**	**0.9**	**0.8**	10.2	2	1	1	1	167	18.6	9.8
NP_005825.1	5032181	**TIMM17B**	mitochondrial import inner membrane translocase subunit Tim17-B isoform 2	1.0	0.9	0.9	0.8	1.0	0.9	0.9	0.8	**1.0**	**0.9**	**0.9**	**0.8**	34.9	2	4	4	6	172	18.3	9.0
NP_005862.1	5032113	**SMNDC1**	survival of motor neuron-related-splicing factor 30	0.8	0.9	0.8	0.8	0.8	0.8	0.8	0.8	**0.8**	**0.9**	**0.8**	**0.8**	37.0	1	6	6	32	238	26.7	7.2
NP_002379.3	394582093	**MCM3**	DNA replication licensing factor MCM3 isoform 1	1.0	0.9	0.8	0.8	0.9	0.9	0.8	0.8	**0.9**	**0.9**	**0.8**	**0.8**	42.4	2	30	30	204	853	95.8	5.8
NP_060909.1	8923819	**IMPACT**	protein IMPACT	0.9	0.9	0.9	0.9	0.8	0.6	0.8	0.7	**0.9**	**0.8**	**0.8**	**0.8**	6.3	1	1	1	2	320	36.4	5.0
NP_057110.3	166795268	**RDH11**	retinol dehydrogenase 11 isoform 1 precursor	0.9	0.9	0.8	0.8	1.0	0.8	0.8	0.8	**0.9**	**0.8**	**0.8**	**0.8**	15.1	3	4	4	13	318	35.4	8.8
NP_001008391.1	56550055	**CGGBP1**	CGG triplet repeat-binding protein 1	0.8	0.7	0.7	0.8	0.9	0.8	0.8	0.9	**0.8**	**0.8**	**0.8**	**0.8**	18.0	1	3	3	5	167	18.8	9.0
NP_001280108.1	643374324	**PRSS23**	serine protease 23 isoform c precursor	0.8	0.9	0.9	0.8	0.7	0.8	0.8	0.8	**0.8**	**0.8**	**0.9**	**0.8**	7.8	3	1	1	1	383	43.0	9.4
NP_001203.1	4502491	**C1QBP**	complement component 1 Q subcomponent-binding protein, mitochondrial precursor	1.0	1.0	1.0	0.8	1.0	1.0	1.0	0.8	**1.0**	**1.0**	**1.0**	**0.8**	52.1	1	9	9	207	282	31.3	4.8
NP_055097.2	157676331	**HABP4**	intracellular hyaluronan-binding protein 4	0.8	0.9	0.8	0.8	0.9	1.0	0.8	0.8	**0.8**	**1.0**	**0.8**	**0.8**	11.4	1	3	3	4	413	45.8	7.5
NP_786924.2	154426322	**UBR7**	putative E3 ubiquitin-protein ligase UBR7	0.9	0.8	0.8	0.8	0.7	0.6	0.7	0.8	**0.8**	**0.7**	**0.8**	**0.8**	13.9	1	4	4	9	425	48.0	4.8
NP_001131025.1	212276104	**LRRFIP1**	leucine-rich repeat flightless-interacting protein 1 isoform 5	0.9	0.9	0.9	0.8	1.0	1.0	0.9	0.8	**0.9**	**1.0**	**0.9**	**0.8**	47.3	8	17	23	80	752	82.6	4.6
NP_001135994.1	215982802	**FBXO5**	F-box only protein 5 isoform b	0.6	0.6	0.7	0.7	0.9	0.8	0.9	0.9	**0.8**	**0.7**	**0.8**	**0.8**	2.5	2	1	1	2	401	45.3	8.9
NP_057256.2	55770884	**UBAC1**	ubiquitin-associated domain-containing protein 1	1.0	0.8	0.9	0.8	1.0	0.9	0.9	0.9	**1.0**	**0.9**	**0.9**	**0.8**	4.4	1	2	2	3	405	45.3	4.9
NP_006271.1	5454090	**SSR4**	translocon-associated protein subunit delta isoform 2 precursor	1.0	0.8	0.8	0.8	1.0	1.0	1.1	0.8	**1.0**	**0.9**	**1.0**	**0.8**	47.4	3	5	5	13	173	19.0	6.2
NP_057359.2	42789380	**SELT**	selenoprotein T precursor	1.0	0.9	0.9	0.9	0.6	0.8	0.6	0.7	**0.8**	**0.8**	**0.8**	**0.8**	12.8	1	2	2	3	195	22.3	8.6

Khan *et al.* , 2019. Multi-omics analysis to characterize cigarette smoke induced molecular alterations in esophageal cells

Supplementary Table 5. List of proteins quantified in untreated and chronically treated Het1A cells with cigarette smoke condensate for 8 months

NP_Accession	Protein group Accession	Gene ID	Description	Het-1A-Smoke - 2M/Parental	Het-1A-Smoke - 4M/Parental	Het-1A-Smoke - 6M/Parental	Het-1A-Smoke - 8M/Parental	Het-1A-Smoke - 2M/Parental	Het-1A-Smoke - 4M/Parental	Het-1A-Smoke - 6M/Parental	Het-1A-Smoke - 8M/Parental	Het-1A-Smoke - 2M/Parental	Het-1A-Smoke - 4M/Parental	Het-1A-Smoke - 6M/Parental	Het-1A-Smoke - 8M/Parental	Coverage (%)	Proteins	Unique Peptides	# Peptides	PSM	# AAs	MW [kDa]	calc. pI
				Replicate 1				Replicate 2				Average of replicates											
NP_064519.2	19923821	DPYSL5	dihydropyrimidinase-related protein 5	0.7	0.8	0.7	0.7	0.8	0.9	0.8	0.9	0.7	0.9	0.8	0.8	1.2	1	1	1	16	564	61.4	7.2
NP_002366.2	47519639	MAP4	microtubule-associated protein 4 isoform 1	1.0	1.0	1.0	0.8	1.0	1.1	1.0	0.8	1.0	1.0	1.0	0.8	71.1	3	64	64	462	1152	120.9	5.4
NP_064522.3	46592991	GRIPAP1	GRIP1-associated protein 1	0.8	0.8	0.7	0.7	0.9	0.9	0.8	0.9	0.9	0.8	0.7	0.8	19.1	1	10	10	23	841	95.9	5.1
NP_060875.2	217416381	COPRS	coordinator of PRMT5 and differentiation stimulator	1.0	0.8	0.9	0.7	1.0	1.0	1.0	0.9	1.0	0.9	0.9	0.8	26.6	1	4	4	12	184	20.1	4.2
NP_001136040.1	217035121	BBX	HMG box transcription factor BBX isoform 1	0.9	1.0	0.9	0.8	0.9	0.8	0.8	0.8	0.9	0.9	0.9	0.8	7.9	3	5	5	5	941	105.1	8.8
NP_821133.1	29788785	TUBB	tubulin beta chain isoform b	0.8	0.7	0.8	0.8	0.8	0.8	0.8	0.8	0.8	0.8	0.8	0.8	83.1	5	5	26	1003	444	49.6	4.9
NP_003080.2	29568103	SNRNP70	U1 small nuclear ribonucleoprotein 70 kDa isoform 1	0.9	0.8	0.8	0.8	0.8	0.8	0.8	0.8	0.8	0.8	0.8	0.8	38.4	2	14	14	102	437	51.5	9.9
NP_000108.1	4557553	EMD	emerin	1.0	0.9	0.8	0.8	1.0	0.8	0.8	0.8	1.0	0.9	0.8	0.8	41.7	1	8	8	26	254	29.0	5.5
NP_064584.1	9910278	C5orf15	keratinocyte-associated transmembrane protein 2 precursor	0.9	0.9	0.9	0.8	0.9	0.9	0.8	0.8	0.9	0.9	0.9	0.8	4.9	1	1	1	5	265	29.2	5.1
NP_056005.1	27597059	DNAJC9	dnaJ homolog subfamily C member 9	0.9	0.9	0.8	0.8	0.9	1.0	0.9	0.9	0.9	0.9	0.9	0.8	65.8	1	16	16	94	260	29.9	5.7
NP_065779.1	45387945	ESYT2	extended synaptotagmin-2	0.9	0.9	1.0	0.8	0.9	1.0	1.1	0.8	0.9	0.9	1.0	0.8	19.2	4	12	12	36	893	98.8	8.7
NP_060388.2	194018570	CCDC109B	mitochondrial calcium uniporter regulatory subunit MCUb	0.9	0.8	1.0	0.8	0.9	0.8	1.0	0.8	0.9	0.8	1.0	0.8	3.6	1	1	1	2	336	39.1	9.2
NP_060669.1	8922631	EXD2	exonuclease 3'-5' domain-containing protein 2 isoform 2	0.8	0.7	0.8	0.8	0.8	0.7	0.7	0.9	0.8	0.7	0.7	0.8	14.1	2	5	5	12	496	56.3	8.3
NP_001078.2	55743075	AAMP	angio-associated migratory cell protein isoform 2	0.8	0.9	1.0	0.7	0.9	1.0	1.0	0.9	0.9	1.0	1.0	0.8	16.1	2	6	6	19	434	46.7	4.4
NP_444263.1	16950597	MRPS33	28S ribosomal protein S33, mitochondrial	1.0	0.8	0.9	0.8	1.0	0.9	1.0	0.8	1.0	0.8	1.0	0.8	8.5	1	1	1	4	106	12.6	10.1
NP_036238.1	6912268	ARL2BP	ADP-ribosylation factor-like protein 2-binding protein	1.1	0.7	0.8	0.7	1.2	0.8	0.9	0.9	1.2	0.8	0.9	0.8	20.3	1	3	3	7	163	18.8	4.3
NP_006852.1	5803135	RAB35	ras-related protein Rab-35 isoform 1	0.9	0.9	1.0	0.9	0.8	0.7	0.8	0.7	0.8	0.8	0.9	0.8	36.3	11	4	6	27	201	23.0	8.3
NP_734467.1	25014109	C11orf31	selenoprotein H	0.7	0.7	0.6	0.8	0.7	0.8	0.7	0.8	0.7	0.8	0.7	0.8	36.1	1	4	4	13	122	13.4	9.7
NP_004613.1	4759270	TSN	translin isoform 1	0.9	0.8	0.8	0.8	1.0	0.8	0.9	0.8	0.9	0.8	0.9	0.8	34.2	2	7	7	20	228	26.2	6.4
NP_001269688.1	544346329	TRA2A	transformer-2 protein homolog alpha isoform 3	0.8	0.8	0.8	0.8	0.8	0.9	0.9	0.8	0.8	0.8	0.9	0.8	32.2	3	5	5	17	180	20.6	10.2
NP_689478.1	56847620	BTF3L4	transcription factor BTF3 homolog 4 isoform 1	0.9	1.0	0.8	0.8	0.9	0.9	0.8	0.8	0.9	0.9	0.8	0.8	51.3	3	6	8	48	158	17.3	6.4
NP_001129970.1	211057411	CISD3	CDGSH iron-sulfur domain-containing protein 3, mitochondrial precursor	0.8	0.7	0.7	0.7	0.9	0.9	1.0	0.9	0.8	0.8	0.9	0.8	10.2	1	1	1	1	127	14.2	10.6
NP_006591.1	5729953	NUDC	nuclear migration protein nudC	0.9	0.7	0.8	0.8	0.9	0.8	0.8	0.8	0.9	0.7	0.8	0.8	32.0	1	10	10	32	331	38.2	5.4
NP_001013.1	4506695	RPS19	40S ribosomal protein S19	0.8	0.9	0.8	0.8	0.8	0.9	0.8	0.8	0.8	0.9	0.8	0.8	51.7	1	8	8	76	145	16.1	10.3
NP_001165462.1	285002259;169790849	HCCS	cytochrome c-type heme lyase	1.0	0.8	0.9	0.7	1.0	0.9	1.1	0.9	1.0	0.9	1.0	0.8	53.4	1	10	10	29	268	30.6	6.7
NP_064710.4	304766523	C16orf62	UPF0505 protein C16orf62	0.9	1.0	0.9	0.9	0.8	0.7	0.7	0.7	0.9	0.8	0.8	0.8	3.0	2	2	2	2	1052	118.5	7.8
XP_005275899.1	530356794	LOC10192920	zinc finger protein 254-like	0.9	0.9	0.9	0.9	0.8	0.8	0.9	0.7	0.8	0.9	0.9	0.8	12.9	118	1	2	2	171	19.7	9.5
NP_001243347.1	374081865	ATPAF1	ATP synthase mitochondrial F1 complex assembly factor 1 isoform 4	1.0	0.8	0.8	0.7	1.0	0.9	1.0	0.9	1.0	0.9	0.9	0.8	10.2	4	1	1	2	177	20.3	5.1
NP_004334.1	4757900	CALR	calreticulin precursor	0.9	1.1	0.9	0.8	0.9	1.1	0.9	0.8	0.9	1.1	0.9	0.8	76.7	1	27	27	475	417	48.1	4.4
NP_001269711.1	544346331	TRMU	mitochondrial tRNA-specific 2-thiouridylase 1 isoform d	1.0	1.0	0.9	0.9	0.9	0.8	0.7	0.7	1.0	0.9	0.8	0.8	8.8	3	2	2	2	307	34.6	9.0
NP_057250.1	7706637	PIAS1	E3 SUMO-protein ligase PIAS1	0.8	0.7	0.9	0.7	0.8	0.8	0.9	0.9	0.8	0.8	0.9	0.8	2.6	1	1	1	2	651	71.8	7.3
NP_695012.1	23510340;23510338	UBA1	ubiquitin-like modifier-activating enzyme 1	0.9	0.8	0.7	0.8	0.9	0.8	0.8	0.8	0.9	0.8	0.8	0.8	50.1	1	36	36	190	1058	117.8	5.8
NP_001406.1	4503507	EIF2S3	eukaryotic translation initiation factor 2 subunit 3	1.0	0.9	0.9	0.8	0.9	0.8	0.8	0.8	0.9	0.8	0.8	0.8	37.3	1	15	15	43	472	51.1	8.4
NP_002435.1	4505257	MSN	moesin	1.0	1.0	1.0	0.8	0.9	1.0	0.9	0.8	1.0	1.0	0.9	0.8	67.2	1	32	47	256	577	67.8	6.4
NP_001139578.1	226371718	PARP9	poly [ADP-ribose] polymerase 9 isoform c	0.9	0.7	0.7	0.9	0.8	0.7	0.7	0.8	0.9	0.7	0.7	0.8	1.7	3	1	1	2	710	80.2	8.1
NP_542193.3	19923665	BRI3BP	BRI3-binding protein precursor	0.9	0.8	0.9	0.8	1.0	0.8	0.8	0.8	1.0	0.8	0.9	0.8	9.6	1	2	2	5	251	27.8	9.4
NP_001174.2	17136148	ATP6AP1	V-type proton ATPase subunit S1 precursor	0.9	0.8	0.8	0.8	1.0	0.9	0.9	0.8	0.9	0.9	0.8	0.8	12.3	1	5	5	9	470	52.0	6.1
NP_001164608.1	283806705	STRBP	spermatid perinuclear RNA-binding protein isoform 2	1.7	0.8	0.8	0.8	0.8	0.7	0.7	0.8	1.2	0.8	0.7	0.8	11.9	2	3	7	44	658	71.9	8.6
NP_001153505.1	229892308	SGOL2	shugoshin-like 2 isoform 2	0.9	0.9	0.8	0.8	0.9	1.0	0.9	0.8	0.9	0.9	0.8	0.8	4.2	3	3	3	4	1260	143.9	7.7
NP_001185715.1	311771673	POU2F1	POU domain, class 2, transcription factor 1 isoform 3	0.9	0.8	0.9	0.8	0.9	0.8	0.9	0.8	0.9	0.8	0.9	0.8	6.1	9	3	3	5	703	72.0	5.8
NP_001028755.2	81295404	ACOT9	acyl-coenzyme A thioesterase 9, mitochondrial isoform b precursor	1.0	0.8	0.9	0.8	0.9	0.9	0.9	0.8	1.0	0.8	0.9	0.8	22.6	2	8	8	27	439	49.9	8.6
NP_068799.2	145386566	ZNF148	zinc finger protein 148	0.9	1.0	0.8	0.8	0.9	0.9	0.9	0.8	0.9	0.9	0.9	0.8	15.5	1	6	6	13	794	88.9	6.5
NP_062543.1	9624998	HNRNPH2	heterogeneous nuclear ribonucleoprotein H2	0.9	0.9	0.8	0.8	0.9	0.8	0.8	0.8	0.9	0.9	0.8	0.8	45.2	1	7	13	117	449	49.2	6.3
NP_733779.1	25777713	SKP1	S-phase kinase-associated protein 1 isoform b	1.0	0.8	1.1	0.7	1.1	0.9	1.2	0.9	1.1	0.9	1.1	0.8	69.3	2	10	11	34	163	18.6	4.5
NP_003016.1	4506929	SH3GL1	endophilin-A2 isoform 1	0.7	0.8	0.8	0.8	0.7	0.7	0.7	0.8	0.7	0.8	0.7	0.8	32.6	4	11	11	37	368	41.5	5.4
NP_005229.1	4885219	ETS1	protein C-ets-1 isoform 2	0.9	0.9	1.0	0.9	0.8	0.9	1.1	0.7	0.8	0.9	1.0	0.8	15.2	3	5	5	6	441	50.4	5.2
NP_060628.2	155722990	SLC4A1AP	kanadaptin	0.9	0.9	0.8	0.8	0.9	0.9	0.8	0.8	0.9	0.9	0.8	0.8	31.9	1	17	17	35	796	88.8	5.2
NP_056979.1	7705682;354681980	GMNN	geminin	0.8	0.7	0.7	0.8	0.9	0.8	0.7	0.8	0.9	0.8	0.7	0.8	32.1	1	5	5	10	209	23.6	5.0
NP_037438.2	22547191	SNX15	sorting nexin-15 isoform A	0.9	0.8	0.9	0.8	0.8	0.7	0.8	0.8	0.9	0.8	0.8	0.8	23.7	2	4	4	6	342	38.3	5.2
NP_689622.2	40255111	C5orf24	UPF0461 protein C5orf24	0.9	0.8	0.8	0.7	1.0	1.0	1.0	0.9	1.0	0.9	0.9	0.8	17.0	1	2	2	4	188	20.1	9.8
NP_002096.1	4504253	H2AFX	histone H2AX	0.8	0.9	0.9	0.8	0.9	0.9	0.9	0.8	0.8	0.9	0.9	0.8	69.2	2	5	10	231	143	15.1	10.7
NP_009188.1	11464969	PNMA2	paraneoplastic antigen Ma2	0.9	0.8	1.0	0.7	1.0	0.9	1.1	0.9	0.9	0.9	1.0	0.8	7.4	1	1	1	2	364	41.5	4.9
NP_006734.1	5803137	RBM3	RNA-binding protein 3	0.6	0.7	0.6	0.6	0.9	1.0	0.9	0.9	0.8	0.9	0.8	0.8	31.9	1	2	2	17	157	17.2	8.9
NP_001269913.1	545478189	FARP2	FERM, RhoGEF and pleckstrin domain-containing protein 2 isoform c	0.9	0.8	0.9	0.8	1.0	0.8	0.9	0.8	0.9	0.8	0.9	0.8	5.3	3	2	2	4	638	72.2	8.9
NP_065173.2	41152506	PTGFRN	prostaglandin F2 receptor negative regulator precursor	0.9	0.7	0.7	0.7	0.8	0.8	0.8	0.9	0.8	0.8	0.7	0.8	3.8	1	3	3	9	879	98.5	6.6
NP_001941.2	12669915	E2F4	transcription factor E2F4	0.8	0.8	1.0	0.8	0.9	0.9	1.0	0.8	0.9	0.8	1.0	0.8	7.0	1	2	2	4	413	43.9	4.8
NP_001988.1	4503659	FAU	ubiquitin-like protein fubi and ribosomal protein S30 precursor	0.9	0.8	0.9	0.8	0.9	0.8	0.9	0.8	0.9	0.8	0.9	0.8	7.5	1	1	1	67	133	14.4	10.2
NP_001258626.1	411147365	ABCB7	ATP-binding cassette sub-family B member 7, mitochondrial isoform 3	0.9	0.9	0.9	0.9	0.8	0.8	0.8	0.7	0.8	0.9	0.8	0.8	7.9	5	3	3	8	712	78.1	9.1
NP_005312.1	4885379	HIST1H1E	histone H1.4	0.7	0.8	0.7	0.7	0.7	0.9	0.7	0.8	0.7	0.9	0.7	0.8	52.1	2	6	17	407	219	21.9	11.0
NP_004209.2	190358517	RAB11B	ras-related protein Rab-11B	1.0	0.7	0.9	0.7	0.9	0.9	0.9	0.9	1.0	0.8	0.9	0.8	63.3	3	2	13	59	218	24.5	5.9
NP_003137.1	4507241	SSRP1	FACT complex subunit SSRP1	0.9	0.8	0.8	0.8	0.9	0.8	0.8	0.8	0.9	0.8	0.8	0.8	40.5	1	25	25	116	709	81.0	6.9
NP_005361.2	16933567	RAB8A	ras-related protein Rab-8A	0.8	0.8	0.8	0.8	0.8	0.8	0.8	0.8	0.8	0.8	0.8	0.8	50.2	11	5	10	33	207	23.7	9.1
NP_001177371.1	298919189	CAST	calpastatin isoform m	0.8	0.9	0.9	0.9	0.9	0.9	1.0	0.7	0.8	0.9	0.9	0.8	62.7	1	2	29	121	695	75.3	5.1
NP_001004.2	14141193	RPS9	40S ribosomal protein S9	0.8	0.8	0.8	0.8	0.8	0.9	0.8	0.8	0.8	0.9	0.8	0.8	52.6	3	13	14	112	194	22.6	10.7
NP_002289.2	167614506	LCP1	plastin-2	0.8	0.6	0.8	0.6	1.1	1.3	1.8	1.0	1.0	1.0	1.3	0.8	9.9	1	2	6	16	627	70.2	5.4
NP_001004456.1	52218828	OR1M1	olfactory receptor 1M1	0.9	1.3	1.0	0.8	0.9	1.0	0.9	0.8	0.9	1.2	1.0	0.8	2.2	1	1	1	2	313	34.8	8.9
NP_078976.2	21314720	SNIP1	smad nuclear-interacting protein 1	1.0	0.9	0.9	0.8	0.9	0.8	0.8	0.7	0.9	0.9	0.8	0.8	12.1	1	3	3	5	396	45.8	10.0
NP_000840.2	23065552	GSTM3	glutathione S-transferase Mu 3	1.0	0.8	0.9	0.8	1.0	0.8	0.9	0.8	1.0	0.8	0.9	0.8	47.1	1	10	10	47	225	26.5	5.5
NP_001041666.1	115430235;586798168	UHRF1	E3 ubiquitin-protein ligase UHRF1 isoform 1	0.9	0.8	0.8	0.8	0.8	0.8	0.9	0.8	0.8	0.8	0.8	0.8	26.4	2	16	17	44	793	89.8	7.6
NP_001271.1	4502847	CIRBP	cold-inducible RNA-binding protein isoform 1	0.7	0.8	0.7	0.8	0.8	0.8	0.8	0.8	0.7	0.8	0.7	0.8	41.3	3	6	6	80	172	18.6	9.5
NP_003370.2	21614499	EZR	ezrin	0.8	0.9	0.9	0.8	0.8	0.9	0.9	0.8	0.8	0.9	0.9	0.8	67.8	2	26	40	356	586	69.4	6.3
NP_000995.1	4506671	RPLP2	60S acidic ribosomal protein P2	1.2	0.7	1.1	0.8	1.2	0.7	1.1	0.8	1.2	0.7	1.1	0.8	91.3	2	6	7	57	115	11.7	4.5
NP_008964.3	116812591	RER1	protein RER1	0.7	0.7	0.7	0.7	0.8	0.8	0.8	0.8	0.8	0.8	0.8	0.8	18.9	1	3	3	5	196	22.9	9.5
NP_001119527.1	186700629	CASK	peripheral plasma membrane protein CASK isoform 3	0.8	0.8	0.7	0.8	0.8	0.8	0.8	0.8	0.8	0.8	0.7	0.8	4.6	3	3	4	7	897	102.0	6.4
NP_001009934.1	58430946	DNASE1L1	deoxyribonuclease-1-like 1 precursor	1.0	1.0	1.0	1.0	0.8	0.6	0.6	0.6	0.9	0.8	0.8	0.8	6.3	1	1	1	4	302	33.9	5.7
NP_060941.2	118150660	ZC3H15	zinc finger CCCH domain-containing protein 15	0.9	0.9	0.9	0.8	0.9	0.9	0.9	0.8	0.9	0.9	0.9	0.8	46.5	1	16	16	48	426	48.6	5.3
NP_001145.1	4502107	ANXA5	annexin A5	0.9	1.0	0.9	0.8	0.9	0.9	0.9	0.8	0.9	1.0	0.9	0.8	76.3	1	25	26	431	320	35.9	5.0
NP_054796.1	7661696	FAM32A	protein FAM32A	0.7	0.9	0.8	0.8	0.8	0.9	0.9	0.8	0.8	0.9	0.9	0.8	19.6	1	2	2	3	112	13.2	10.0
NP_054888.2	40548422	CHMP4A	charged multivesicular body protein 4a	1.0	0.9	0.9	0.7	1.0	0.9	0.9	0.8	1.0	0.9	0.9	0.8	15.9	1	3	4	11	265	29.8	5.1
NP_005756.2	15011918	ATP6AP2	renin receptor precursor	1.0	0.9	0.9	0.8	0.8	0.7	0.7	0.7	0.9	0.8	0.8	0.8	8.3	1	2	2	2	350	39.0	6.1
NP_620061.2	31543397	PGK2	phosphoglycerate kinase 2	0.7	0.9	0.7	0.8	0.8	0.9	0.8	0.8	0.8	0.9	0.8	0.8	17.5	1	1	8	58	417	44.8	8.5
NP_001018006.1	63252900	TPM1	tropomyosin alpha-1 chain isoform Tpm1.7cy	0.9	0.7	0.7	0.8	0.9	0.8	0.8	0.8	0.9	0.8	0.8	0.8	58.1	4	1	20	93	284	32.9	4.8
NP_066301.1	11321640	SNTB1	beta-1-syntrophin	0.8	0.8	0.8	0.9	0.8	0.7	0.7	0.7	0.8	0.8	0.8	0.8	6.5	1	2	3	3	538	58.0	8.6
NP_004577.1	4759050	RPS6KA3	ribosomal protein S6 kinase alpha-3	1.0	0.9	1.0	0.8	1.1	1.0	1.0	0.7	1.0	0.9	1.0	0.8	25.5	6	11	15	28	740	83.7	6.9
NP_620147.1	20270337	LEO1	RNA polymerase-associated protein LEO1 isoform 1	0.8	0.9	0.8	0.8	0.9	0.8	0.7	0.8	0.8	0.9	0.8	0.8	18.2	2	10	10	27	666	75.4	4.5
NP_057157.1	7705320	HDGFRP3	hepatoma-derived growth factor-related protein 3	0.7	0.7	0.6	0.8	0.7	0.7	0.7	0.8	0.7	0.7	0.7	0.8	32.0	1	4	6	50	203	22.6	8.0
NP_004806.3	134304853	ARHGAP29	rho GTPase-activating protein 29	0.9	0.9	0.9	0.8	0.8	0.9	0.9	0.8	0.8	0.9	0.9	0.8	5.5	1	4	5	11	1261	142.0	6.7
NP_001157882.1	256542306	CDK5	cyclin-dependent kinase 5 isoform 2	0.9	1.0	1.0	0.7	1.0	1.0	1.1	0.9	1.0	1.0	1.0	0.8	10.4	55	2	3	6	260	29.5	6.4
NP_077027.1	13129148	APOO	apolipoprotein O precursor	0.9	0.7	0.7	0.8	1.0	0.7	0.7	0.8	0.9	0.7	0.7	0.8	16.7	1	3	3	8	198	22.3	9.1
NP_000994.1	4506669	RPLP1	60S acidic ribosomal protein P1 isoform 1	1.4	0.7	1.2	0.8	1.3	0.7	1.3	0.8	1.3	0.7	1.3	0.8	71.9	2	3	4	49	114	11.5	4.3
NP_683758.1	23111032	SNX1	sorting nexin-1 isoform b	0.8	0.8	0.8	0.8	0.8	0.8	0.7	0.8	0.8	0.8	0.7	0.8	34.8	3	12	15	67	457	51.8	5.6
NP_005004.1	4826870	NUCB2	nucleobindin-2 precursor	1.1	1.0	1.1	0.8	1.1	1.0	1.0	0.8	1.1	1.0	1.0	0.8	41.0	1	12	12	42	420	50.2	5.1
NP_000422.1	4557769	MVK	mevalonate kinase isoform a	0.9	0.8	0.8	0.8	1.1	0.8	0.8	0.8	1.0	0.8	0.8	0.8	24.0	2	6	6	29	396	42.4	6.5

Khan *et al.* , 2019. Multi-omics analysis to characterize cigarette smoke induced molecular alterations in esophageal cells
Supplementary Table 5. List of proteins quantified in untreated and chronically treated Het1A cells with cigarette smoke condensate for 8 months

NP_Accession	Protein group Accession	Gene ID	Description	Het-1A-Smoke - 2M/Parental	Het-1A-Smoke - 4M/Parental	Het-1A-Smoke - 6M/Parental	Het-1A-Smoke - 8M/Parental	Het-1A-Smoke - 2M/Parental	Het-1A-Smoke - 4M/Parental	Het-1A-Smoke - 6M/Parental	Het-1A-Smoke - 8M/Parental	Het-1A-Smoke - 2M/Parental	Het-1A-Smoke - 4M/Parental	Het-1A-Smoke - 6M/Parental	Het-1A-Smoke - 8M/Parental	Coverage (%)	Proteins	Unique Peptides	# Peptides	PSM	# AAs	MW [kDa]	calc. pI
				Replicate 1				Replicate 2				Average of replicates											
NP_073568.2	56118310	**NUCKS1**	nuclear ubiquitous casein and cyclin-dependent kinase substrate 1	0.8	0.8	0.7	0.8	0.8	0.8	0.8	0.8	**0.8**	**0.8**	**0.7**	**0.8**	41.2	1	9	9	40	243	27.3	5.1
NP_002659.1	4505893	**PLP2**	proteolipid protein 2	1.1	0.8	0.8	0.6	0.9	0.9	0.7	0.9	**1.0**	**0.8**	**0.8**	**0.8**	8.6	1	1	1	2	152	16.7	7.2
NP_057163.1	7706353	**CHMP3**	charged multivesicular body protein 3 isoform 1	0.9	0.8	0.8	0.8	0.8	0.7	0.8	0.8	**0.8**	**0.8**	**0.8**	**0.8**	21.2	4	3	3	4	222	25.1	5.1
NP_001839.2	87196339	**COL6A1**	collagen alpha-1(VI) chain precursor	0.7	1.0	1.1	0.8	0.8	1.0	1.2	0.7	**0.7**	**1.0**	**1.1**	**0.8**	3.0	1	2	2	5	1028	108.5	5.4
NP_899058.1	34485709	**RAB27A**	ras-related protein Rab-27A	0.8	0.8	0.8	0.7	1.0	1.0	0.9	0.8	**0.9**	**0.9**	**0.8**	**0.8**	20.8	1	4	4	11	221	24.9	5.2
NP_001136175.1	218156273	**FAM111B**	protein FAM111B isoform b	0.8	0.8	0.8	0.8	0.8	0.8	0.7	0.8	**0.8**	**0.8**	**0.7**	**0.8**	7.2	2	5	5	7	704	81.1	8.7
NP_005474.2	50513245	**CHAF1A**	chromatin assembly factor 1 subunit A	0.7	0.8	0.7	0.7	0.8	0.9	0.8	0.8	**0.8**	**0.8**	**0.8**	**0.8**	19.3	1	14	14	24	956	106.8	5.9
NP_919223.1	34740329	**HNRNPA3**	heterogeneous nuclear ribonucleoprotein A3	0.8	0.8	0.8	0.8	0.8	0.7	0.7	0.8	**0.8**	**0.8**	**0.7**	**0.8**	36.8	1	12	15	175	378	39.6	9.0
NP_006266.2	20127499	**SRSF6**	serine/arginine-rich splicing factor 6	0.8	0.8	0.8	0.7	0.8	0.9	0.8	0.8	**0.8**	**0.8**	**0.8**	**0.8**	26.2	1	7	11	59	344	39.6	11.4
NP_803182.1	29171705;41350320	**MAGED2**	melanoma-associated antigen D2	1.0	1.0	0.8	0.8	1.1	1.1	0.8	0.7	**1.1**	**1.1**	**0.8**	**0.8**	44.2	6	18	19	59	606	64.9	9.3
NP_001143.2	156071459	**SLC25A5**	ADP/ATP translocase 2	0.9	0.9	0.8	0.8	0.9	0.9	0.8	0.8	**0.9**	**0.9**	**0.8**	**0.8**	60.7	2	6	18	257	298	32.8	9.7
NP_006799.1	5803165	**SEC61B**	protein transport protein Sec61 subunit beta	1.3	0.9	1.2	0.8	1.2	0.9	1.0	0.8	**1.2**	**0.9**	**1.1**	**0.8**	66.7	1	4	4	13	96	10.0	11.6
NP_001005413.1	53729320	**ZWINT**	ZW10 interactor isoform b	0.8	0.8	0.8	0.9	0.8	0.7	0.8	0.7	**0.8**	**0.8**	**0.8**	**0.8**	20.4	2	4	4	7	230	25.5	4.9
NP_001274433.1	566559898	**SSFA2**	sperm-specific antigen 2 isoform 4	1.0	0.8	0.8	0.8	0.8	0.8	1.0	0.8	**0.9**	**0.8**	**0.9**	**0.8**	4.3	4	4	4	5	1084	119.1	5.3
NP_001245304.1	385648257	**EPS15L1**	epidermal growth factor receptor substrate 15-like 1 isoform 3	1.1	0.9	0.9	0.8	1.1	0.9	1.0	0.8	**1.1**	**0.9**	**0.9**	**0.8**	22.0	4	13	13	42	754	83.2	5.1
NP_002507.1	5902724	**NOVA2**	RNA-binding protein Nova-2	1.3	0.8	0.7	0.8	0.6	0.7	0.7	0.8	**1.0**	**0.8**	**0.7**	**0.8**	1.6	1	1	1	1	492	49.0	8.2
NP_004642.2	24234683	**USP11**	ubiquitin carboxyl-terminal hydrolase 11	0.9	0.8	0.7	0.8	0.9	0.7	0.7	0.8	**0.9**	**0.7**	**0.7**	**0.8**	24.0	1	14	15	38	963	109.7	5.5
NP_001017369.1	62865628	**MSMO1**	methylsterol monooxygenase 1 isoform 2	1.5	1.1	1.1	0.8	1.5	1.1	1.0	0.8	**1.5**	**1.1**	**1.1**	**0.8**	13.0	2	2	2	5	162	19.5	7.7
NP_060037.3	295844834	**NAGK**	N-acetyl-D-glucosamine kinase	1.1	0.8	0.8	0.8	0.9	0.8	0.8	0.8	**1.0**	**0.8**	**0.8**	**0.8**	13.9	1	4	4	8	390	42.0	6.7
NP_892021.1	33946297	**ZNF326**	DBIRD complex subunit ZNF326 isoform 1	0.8	0.8	0.7	0.7	0.9	0.9	0.9	0.8	**0.8**	**0.8**	**0.8**	**0.8**	29.0	2	13	13	38	582	65.6	5.1
NP_009107.1	6005719	**C14orf1**	probable ergosterol biosynthetic protein 28	0.9	0.8	0.8	0.8	1.1	0.9	0.9	0.8	**1.0**	**0.8**	**0.9**	**0.8**	19.3	1	3	3	10	140	15.9	9.8
NP_001018077.1	66346679	**SERBP1**	plasminogen activator inhibitor 1 RNA-binding protein isoform 1	0.8	0.9	0.7	0.8	0.8	0.9	0.8	0.8	**0.8**	**0.9**	**0.7**	**0.8**	56.1	2	3	23	231	408	44.9	8.6
NP_001107590.1	166295179	**C17orf85**	uncharacterized protein C17orf85	0.9	0.9	0.9	0.8	0.9	0.9	0.8	0.7	**0.9**	**0.9**	**0.8**	**0.8**	26.1	5	13	13	33	620	70.5	5.7
NP_076943.1	13128992	**C19orf43**	uncharacterized protein C19orf43	0.7	0.8	0.8	0.8	0.7	0.8	0.8	0.8	**0.7**	**0.8**	**0.8**	**0.8**	26.1	1	4	4	13	176	18.4	9.4
NP_076423.2	24371248	**FUNDC2**	FUN14 domain-containing protein 2	0.7	0.7	0.7	0.7	0.9	0.9	0.8	0.9	**0.8**	**0.8**	**0.8**	**0.8**	11.6	1	2	2	3	189	20.7	9.7
NP_001155977.1	241982751	**TMEM189**	transmembrane protein 189 isoform 2	0.8	0.9	0.8	0.8	0.8	1.0	1.1	0.8	**0.8**	**0.9**	**1.0**	**0.8**	5.6	2	1	1	1	267	30.8	6.6
NP_005376.2	6631100	**NKTR**	NK-tumor recognition protein	0.8	0.8	0.9	0.7	0.9	0.9	0.9	0.8	**0.8**	**0.8**	**0.9**	**0.8**	9.5	1	11	11	28	1462	165.6	10.0
NP_001177748.1	299890795	**ORC1**	origin recognition complex subunit 1 isoform 2	0.8	0.7	0.7	0.8	0.9	0.8	0.8	0.8	**0.9**	**0.8**	**0.8**	**0.8**	5.8	2	3	3	4	856	96.7	9.2
NP_005446.2	42741682	**ZRANB2**	zinc finger Ran-binding domain-containing protein 2 isoform 2	0.9	0.9	0.9	0.7	0.9	0.9	0.8	0.8	**0.9**	**0.9**	**0.9**	**0.8**	26.9	2	9	9	46	320	36.3	9.8
NP_001028284.1	75677355	**PAIP2**	polyadenylate-binding protein-interacting protein 2	1.0	1.0	1.2	0.8	1.0	1.0	1.2	0.7	**1.0**	**1.0**	**1.2**	**0.8**	55.9	1	4	4	31	127	15.0	4.1
NP_006640.2	21361348	**UTP14A**	U3 small nucleolar RNA-associated protein 14 homolog A isoform 1	0.8	0.8	0.8	0.8	0.9	0.8	0.8	0.8	**0.9**	**0.8**	**0.8**	**0.8**	22.7	3	12	12	37	771	87.9	7.9
NP_078828.2	205360977	**CERS4**	ceramide synthase 4	0.8	0.6	0.6	0.8	0.9	0.7	0.7	0.8	**0.8**	**0.6**	**0.6**	**0.8**	2.0	1	1	1	1	394	46.4	9.1
NP_005154.2	62241011;62241013	**AKT1**	RAC-alpha serine/threonine-protein kinase	0.9	0.8	0.8	0.8	0.7	0.7	0.7	0.7	**0.8**	**0.7**	**0.8**	**0.8**	9.0	3	4	4	7	480	55.7	6.1
NP_009140.1	6005860	**RPL35**	60S ribosomal protein L35	0.7	0.8	0.8	0.8	0.8	0.9	0.8	0.8	**0.8**	**0.8**	**0.8**	**0.8**	44.7	1	7	8	63	123	14.5	11.0
NP_612437.3	258679461	**SMIM12**	small integral membrane protein 12	0.8	0.7	0.7	0.8	0.8	0.8	0.8	0.8	**0.8**	**0.8**	**0.8**	**0.8**	20.7	1	2	2	2	92	10.8	8.4
NP_002614.2	12408677	**PFDN4**	prefoldin subunit 4	1.2	0.9	1.4	0.7	1.2	0.8	1.5	0.8	**1.2**	**0.8**	**1.4**	**0.8**	20.2	1	2	2	6	134	15.3	4.5
NP_001403.1	4503499	**EIF1AX**	eukaryotic translation initiation factor 1A, X-chromosomal	0.8	0.9	0.9	0.7	0.9	1.0	1.1	0.8	**0.9**	**0.9**	**1.0**	**0.8**	70.1	1	1	10	114	144	16.5	5.2
NP_000998.1	4506725	**RPS4X**	40S ribosomal protein S4, X isoform X isoform	0.8	0.7	0.8	0.7	0.9	0.8	0.8	0.8	**0.8**	**0.7**	**0.8**	**0.8**	55.5	1	11	16	135	263	29.6	10.2
NP_006344.1	10835240	**HMGN4**	high mobility group nucleosome-binding domain-containing protein 4	0.8	1.0	0.8	0.8	1.0	1.0	0.8	0.8	**0.9**	**1.0**	**0.8**	**0.8**	26.7	1	2	3	8	90	9.5	10.5
NP_001275513.1	568384749	**FAM133B**	protein FAM133B isoform 2	0.7	0.7	0.7	0.8	0.8	0.8	0.7	0.8	**0.8**	**0.8**	**0.7**	**0.8**	3.4	3	1	1	2	237	27.2	10.0
NP_115883.2	217416362	**ZC3H8**	zinc finger CCCH domain-containing protein 8	0.8	1.1	0.9	0.9	0.9	0.8	0.8	0.7	**0.9**	**0.9**	**0.9**	**0.8**	7.9	1	1	1	2	291	33.6	8.3
NP_036366.3	112363092	**RYBP**	RING1 and YY1-binding protein	0.9	0.7	0.7	0.7	1.0	0.8	0.8	0.8	**0.9**	**0.8**	**0.7**	**0.8**	18.9	1	2	2	6	228	24.8	9.6
NP_001734.1	4502549;58218968	**CALM2**	calmodulin isoform 2	0.9	1.0	1.0	0.8	0.9	1.0	1.0	0.8	**0.9**	**1.0**	**1.0**	**0.8**	73.8	5	10	10	281	149	16.8	4.2
NP_057017.1	7705431	**TMA7**	translation machinery-associated protein 7	0.8	0.8	1.0	0.8	0.7	0.7	0.8	0.8	**0.7**	**0.8**	**0.9**	**0.8**	39.1	3	3	4	12	64	7.1	10.0
NP_001243331.1	373938452	**ELOVL1**	elongation of very long chain fatty acids protein 1 isoform 3	0.9	0.7	0.9	0.7	1.5	1.0	1.0	0.8	**1.2**	**0.9**	**1.0**	**0.8**	7.1	2	1	1	1	198	23.3	9.7
NP_001180573.1	302370946	**PEX19**	peroxisomal biogenesis factor 19 isoform c	0.9	0.9	0.9	0.8	1.0	0.8	0.9	0.7	**0.9**	**0.9**	**0.9**	**0.8**	41.9	2	8	8	18	279	30.8	4.4
NP_001164218.1	282847476	**PIN4**	peptidyl-prolyl cis-trans isomerase NIMA-interacting 4 isoform 2	0.9	0.8	0.8	0.8	0.8	0.7	0.7	0.7	**0.9**	**0.8**	**0.8**	**0.8**	9.0	2	1	1	2	133	14.2	10.2
NP_057260.2	18699732	**SDF4**	45 kDa calcium-binding protein isoform 2 precursor	0.9	0.8	0.8	0.8	0.7	0.7	0.7	0.7	**0.8**	**0.7**	**0.7**	**0.8**	31.5	2	5	5	12	362	41.8	4.9
NP_005219.2	29725609	**EGFR**	epidermal growth factor receptor isoform a precursor	0.9	0.8	0.9	0.8	0.9	0.8	0.9	0.8	**0.9**	**0.8**	**0.9**	**0.8**	20.4	4	19	19	41	1210	134.2	6.7
NP_004956.5	48255933	**HMGN1**	non-histone chromosomal protein HMG-14	0.8	0.9	0.8	0.7	0.8	0.9	0.8	0.8	**0.8**	**0.9**	**0.8**	**0.8**	52.0	1	8	8	73	100	10.7	9.6
NP_054890.1	7661798	**CRIPT**	cysteine-rich PDZ-binding protein	0.7	0.9	0.8	0.8	0.6	0.7	0.7	0.7	**0.7**	**0.8**	**0.7**	**0.8**	22.8	1	1	2	3	101	11.2	9.5
NP_004783.2	42560244	**PPIG**	peptidyl-prolyl cis-trans isomerase G	0.8	0.8	0.8	0.7	0.7	0.8	0.7	0.8	**0.8**	**0.8**	**0.8**	**0.8**	11.9	1	9	9	22	754	88.6	10.3
NP_778225.1	28173554	**HIST3H2BB**	histone H2B type 3-B	0.7	0.7	0.7	0.7	0.7	0.8	0.8	0.8	**0.7**	**0.7**	**0.7**	**0.8**	61.9	5	1	10	1039	126	13.9	10.3
NP_004902.1	4758304	**PDIA4**	protein disulfide-isomerase A4 precursor	1.0	1.1	1.0	0.7	1.0	1.1	1.0	0.8	**1.0**	**1.1**	**1.0**	**0.8**	55.8	1	34	34	272	645	72.9	5.1
NP_071357.2	38505209	**FAM111A**	protein FAM111A	0.9	0.9	0.9	0.9	0.7	0.7	0.8	0.7	**0.8**	**0.8**	**0.8**	**0.8**	13.8	1	6	6	16	611	70.2	8.5
NP_000984.1	4506633	**RPL31**	60S ribosomal protein L31 isoform 1	0.9	0.9	0.8	0.8	0.9	0.8	0.8	0.7	**0.9**	**0.8**	**0.8**	**0.8**	52.0	3	7	7	84	125	14.5	10.5
NP_612429.2	156766050	**AHNAK2**	protein AHNAK2	1.0	0.9	0.9	0.9	0.7	0.6	0.6	0.6	**0.8**	**0.7**	**0.8**	**0.8**	0.3	1	1	1	1	5795	616.2	5.4
NP_001132913.1	213511012	**BCAP31**	B-cell receptor-associated protein 31 isoform b	0.9	0.8	0.8	0.8	0.9	0.8	0.8	0.7	**0.9**	**0.8**	**0.8**	**0.8**	47.6	2	13	13	65	246	28.0	8.4
NP_005311.1	4885377	**HIST1H1D**	histone H1.3	0.6	0.8	0.7	0.7	0.7	0.8	0.8	0.8	**0.6**	**0.8**	**0.7**	**0.8**	45.3	2	7	16	375	221	22.3	11.0
NP_001077084.2	284172510	**MYO3B**	myosin-IIIb isoform 1	1.2	0.8	0.9	0.7	1.1	0.8	1.0	0.8	**1.2**	**0.8**	**1.0**	**0.8**	0.6	2	1	1	2	1314	148.5	7.8
NP_003535.1	4504313;4504301	**HIST1H4B**	histone H4	0.8	0.8	0.8	0.8	0.7	0.8	0.7	0.8	**0.7**	**0.8**	**0.8**	**0.8**	58.3	1	7	7	564	103	11.4	11.4
NP_001015877.1	62865858;28557677	**PHF6**	PHD finger protein 6 isoform 1	0.8	0.9	0.8	0.8	0.8	0.8	0.7	0.8	**0.8**	**0.8**	**0.8**	**0.8**	16.4	2	6	6	15	365	41.3	8.7
NP_001207.2	169636420	**CA9**	carbonic anhydrase 9 precursor	0.9	0.9	1.0	0.8	0.8	0.8	1.0	0.7	**0.9**	**0.9**	**1.0**	**0.8**	9.6	1	2	2	6	459	49.7	4.7
NP_001265571.1	522023554	**EDIL3**	EGF-like repeat and discoidin I-like domain-containing protein 3 isoform 2 precursor	0.9	1.0	1.1	0.8	1.0	1.0	1.1	0.8	**1.0**	**1.0**	**1.1**	**0.8**	15.5	2	5	5	7	470	52.7	7.8
NP_001186807.1	315506975	**SERF2**	small EDRK-rich factor 2 isoform d	0.8	0.8	0.8	0.8	0.7	0.7	0.7	0.8	**0.8**	**0.7**	**0.7**	**0.8**	55.6	4	3	3	5	45	5.2	10.1
NP_005313.1	4885381	**HIST1H1B**	histone H1.5	0.7	0.8	0.7	0.8	0.7	0.8	0.7	0.8	**0.7**	**0.8**	**0.7**	**0.8**	50.0	2	11	16	376	226	22.6	10.9
NP_620140.1	20302038	**GINM1**	glycoprotein integral membrane protein 1 precursor	0.8	0.8	0.9	0.7	1.2	0.9	1.1	0.8	**1.0**	**0.9**	**1.0**	**0.8**	3.9	1	1	1	1	330	36.8	4.9
NP_003133.1	10835067	**SSB**	lupus La protein	0.8	0.9	0.8	0.7	0.8	0.9	0.8	0.8	**0.8**	**0.9**	**0.8**	**0.8**	65.4	11	30	30	323	408	46.8	7.1
NP_001001520.1	48255931	**HDGFRP2**	hepatoma-derived growth factor-related protein 2 isoform 1	0.8	0.8	0.8	0.7	0.7	0.8	0.8	0.8	**0.8**	**0.8**	**0.8**	**0.8**	26.8	2	13	15	80	671	74.3	7.5
NP_002452.1	4505289	**MVD**	diphosphomevalonate decarboxylase	1.0	1.0	0.9	0.7	1.1	1.1	0.9	0.8	**1.1**	**1.0**	**0.9**	**0.8**	29.0	1	9	9	21	400	43.4	7.2
NP_057578.1	7706039	**RNF181**	E3 ubiquitin-protein ligase RNF181	0.9	0.7	0.9	0.6	1.0	0.9	0.9	0.9	**0.9**	**0.8**	**0.9**	**0.8**	24.2	1	3	3	4	153	17.9	5.1
NP_001268227.1	526253088	**EDF1**	endothelial differentiation-related factor 1 isoform 4	0.6	0.8	0.6	0.7	0.7	0.8	0.7	0.8	**0.7**	**0.8**	**0.7**	**0.8**	38.6	5	6	6	25	127	14.1	9.8
NP_004519.1	4758714	**MGST3**	microsomal glutathione S-transferase 3	0.9	0.8	1.1	0.7	1.1	0.9	1.2	0.8	**1.0**	**0.8**	**1.1**	**0.8**	34.2	1	2	2	3	152	16.5	9.4
NP_055995.4	118918403	**SYNE2**	nesprin-2 isoform 1	0.9	0.8	0.8	0.8	0.8	0.8	0.8	0.7	**0.9**	**0.8**	**0.8**	**0.8**	3.6	4	19	20	38	6885	795.9	5.4
NP_066403.2	31742503	**HIST2H3C**	histone H3.2	1.0	0.8	0.9	0.9	0.8	0.7	0.8	0.6	**0.9**	**0.7**	**0.9**	**0.8**	59.6	3	1	9	816	136	15.4	11.3
NP_057036.2	55953123	**RWDD1**	RWD domain-containing protein 1 isoform a	0.9	1.0	0.9	0.8	0.8	0.8	0.7	0.7	**0.9**	**0.9**	**0.8**	**0.7**	35.8	2	8	8	16	243	27.9	4.2
NP_001060.1	4507729	**TUBB2A**	tubulin beta-2A chain	1.0	0.8	0.9	0.7	1.1	0.9	1.0	0.8	**1.0**	**0.9**	**1.0**	**0.7**	71.0	1	1	22	819	445	49.9	4.9
NP_066299.2	17986258	**MYL6**	myosin light polypeptide 6 isoform 1	1.7	0.9	**2.0**	0.7	1.5	0.9	1.6	0.8	**1.6**	**0.9**	**1.8**	**0.7**	58.9	3	8	8	70	151	16.9	4.7
NP_008846.2	170016081	**RP2**	protein XRP2	0.9	0.9	0.9	0.8	1.1	0.7	0.8	0.7	**1.0**	**0.8**	**0.9**	**0.7**	8.6	1	2	2	6	350	39.6	5.1
NP_001019907.1	67188443	**CD276**	CD276 antigen isoform a precursor	0.8	0.8	0.7	0.8	0.9	0.8	0.8	0.7	**0.8**	**0.8**	**0.8**	**0.7**	25.5	2	5	5	19	534	57.2	4.9
NP_001164980.1	284005461;284005463	**MCFD2**	multiple coagulation factor deficiency protein 2 isoform B	1.4	0.9	1.4	0.7	1.4	1.1	1.6	0.8	**1.4**	**1.0**	**1.5**	**0.7**	69.2	3	4	4	15	94	10.7	4.4
NP_004356.2	46397403	**CETN3**	centrin-3 isoform 2	1.0	0.8	0.9	0.8	0.9	0.8	1.1	0.6	**1.0**	**0.8**	**1.0**	**0.7**	22.8	3	2	3	5	167	19.5	4.7
NP_001265672.1	523567561	**TSPAN6**	tetraspanin-6 isoform d precursor	1.1	1.0	0.9	0.9	0.6	0.5	**0.4**	0.5	**0.9**	**0.8**	**0.7**	**0.7**	6.2	4	1	1	1	129	14.9	7.2
NP_058633.2	106507301	**POLA1**	DNA polymerase alpha catalytic subunit	0.9	0.8	0.8	0.8	0.8	0.7	0.7	0.7	**0.8**	**0.8**	**0.8**	**0.7**	11.9	1	13	13	33	1462	165.8	5.8
NP_006185.1	7242167	**PAX9**	paired box protein Pax-9	0.6	0.6	0.6	0.7	0.8	0.7	0.8	0.8	**0.7**	**0.7**	**0.7**	**0.7**	2.9	1	1	1	1	341	36.3	9.3
NP_006392.1	5454088	**ANP32B**	acidic leucine-rich nuclear phosphoprotein 32 family member B	0.8	0.8	0.7	0.7	0.8	0.9	0.8	0.8	**0.8**	**0.8**	**0.8**	**0.7**	32.3	2	4	9	144	251	28.8	4.1
NP_005310.1	4885375	**HIST1H1C**	histone H1.2	0.6	0.8	0.7	0.7	0.6	0.8	0.7	0.8	**0.6**	**0.8**	**0.7**	**0.7**	54.0	2	6	16	372	213	21.4	10.9

Khan *et al.* , 2019. Multi-omics analysis to characterize cigarette smoke induced molecular alterations in esophageal cells
Supplementary Table 5. List of proteins quantified in untreated and chronically treated Het1A cells with cigarette smoke condensate for 8 months

NP_Accession	Protein group Accession	Gene ID	Description	Het-1A-Smoke - 2M/Parental	Het-1A-Smoke - 4M/Parental	Het-1A-Smoke - 6M/Parental	Het-1A-Smoke - 8M/Parental	Het-1A-Smoke - 2M/Parental	Het-1A-Smoke - 4M/Parental	Het-1A-Smoke - 6M/Parental	Het-1A-Smoke - 8M/Parental	Het-1A-Smoke - 2M/Parental	Het-1A-Smoke - 4M/Parental	Het-1A-Smoke - 6M/Parental	Het-1A-Smoke - 8M/Parental	Coverage (%)	Proteins	Unique Peptides	# Peptides	PSM	# AAs	MW [kDa]	calc. pI
				Replicate 1				Replicate 2				Average of replicates											
NP_003463.1	4503249	DEK	protein DEK isoform 1	0.8	0.8	0.8	0.7	0.8	0.8	0.8	0.8	0.8	0.8	0.8	0.7	35.5	2	18	18	118	375	42.6	8.6
NP_115753.1	14150203	ELOF1	transcription elongation factor 1 homolog	0.8	0.8	0.8	0.7	0.8	0.8	0.8	0.7	0.8	0.8	0.8	0.7	21.7	1	1	1	2	83	9.5	8.0
NP_003615.2	194097487	RANBP3	ran-binding protein 3 isoform RANBP3-a	0.8	0.8	0.8	0.7	0.9	0.8	0.8	0.8	0.9	0.8	0.8	0.7	35.6	4	16	16	48	562	59.7	4.8
NP_789782.1	28827795	CHMP4B	charged multivesicular body protein 4b	0.8	0.7	0.7	0.7	0.8	0.7	0.7	0.8	0.8	0.7	0.7	0.7	40.2	1	6	7	35	224	24.9	4.8
NP_001034288.1	85861243;85861250	TTPAL	alpha-tocopherol transfer protein-like isoform 1	0.8	0.8	0.7	0.7	0.9	0.8	0.8	0.7	0.8	0.8	0.7	0.7	13.5	2	3	3	8	342	38.5	6.4
NP_057951.1	8051631	RALY	RNA-binding protein Raly isoform 1	0.7	0.7	0.6	0.7	0.8	0.7	0.6	0.7	0.8	0.7	0.6	0.7	51.6	2	17	17	114	306	32.4	9.2
NP_987096.1	45269145	KEAP1	kelch-like ECH-associated protein 1	0.7	0.7	0.7	0.7	0.7	0.8	0.9	0.8	0.7	0.7	0.8	0.7	2.1	1	1	1	1	624	69.6	6.4
NP_005372.2	55956788	NCL	nucleolin	0.8	0.8	0.7	0.7	0.8	0.9	0.8	0.7	0.8	0.9	0.8	0.7	56.3	2	50	50	1197	710	76.6	4.7
NP_003752.2	14043026	VAMP8	vesicle-associated membrane protein 8	0.9	0.7	0.9	0.6	1.1	0.9	1.0	0.8	1.0	0.8	0.9	0.7	10.0	1	1	1	2	100	11.4	7.3
NP_004474.2	49574537	GCSH	glycine cleavage system H protein, mitochondrial precursor	1.3	0.9	1.2	0.8	1.2	0.7	0.9	0.7	1.3	0.8	1.0	0.7	17.3	2	2	2	15	173	18.9	4.9
NP_002098.1	4504279;4885385	H3F3A	histone H3.3	0.8	0.7	0.8	0.7	0.8	0.8	0.8	0.8	0.8	0.7	0.8	0.7	58.8	2	2	8	604	136	15.3	11.3
NP_821080.1	29788768	TUBB2B	tubulin beta-2B chain	1.5	1.3	1.0	0.8	1.6	1.3	1.0	0.7	1.5	1.3	1.0	0.7	79.8	1	1	23	827	445	49.9	4.9
NP_004090.4	38016911	STOM	erythrocyte band 7 integral membrane protein isoform a	0.7	0.6	0.8	0.7	0.7	0.7	0.8	0.7	0.7	0.7	0.8	0.7	28.8	4	7	7	20	288	31.7	7.9
NP_006658.1	5729875	PGRMC1	membrane-associated progesterone receptor component 1 isoform 1	0.9	0.9	0.7	0.7	0.8	0.9	0.8	0.8	0.8	0.9	0.8	0.7	34.9	2	3	4	23	195	21.7	4.7
NP_001091972.1	148612859	CHCHD4	mitochondrial intermembrane space import and assembly protein 40 isoform 1	2.0	1.0	1.5	0.7	1.9	1.1	1.7	0.7	1.9	1.0	1.6	0.7	25.4	2	3	3	10	142	16.0	4.3
NP_940938.1	63055043;224028279	TMEM205	transmembrane protein 205	0.8	0.7	0.8	0.7	0.8	0.7	0.7	0.7	0.8	0.7	0.7	0.7	20.6	1	3	3	12	189	21.2	8.6
NP_002151.2	153946395	TNC	tenascin precursor	0.8	0.8	0.7	0.8	0.6	0.6	0.6	0.6	0.7	0.7	0.7	0.7	4.8	1	6	6	11	2201	240.7	4.9
NP_036442.3	116686122	KIF4A	chromosome-associated kinesin KIF4A	0.8	0.8	0.8	0.8	0.7	0.7	0.7	0.7	0.7	0.8	0.7	0.7	25.4	2	21	21	54	1232	139.8	6.3
NP_001934.2	116534898	DSG2	desmoglein-2 preproprotein	0.8	0.8	0.7	0.8	0.8	0.6	0.6	0.7	0.8	0.7	0.7	0.7	7.9	1	7	7	11	1118	122.2	5.2
NP_001278434.1	613410239	PPP1R2	protein phosphatase inhibitor 2 isoform 3	1.1	0.8	1.0	0.7	1.2	0.8	1.3	0.7	1.2	0.8	1.1	0.7	19.0	3	3	3	12	179	20.3	5.0
NP_000480.3	530788276	ATRX	transcriptional regulator ATRX isoform 1	0.9	0.8	0.8	0.7	0.8	0.9	0.8	0.7	0.9	0.8	0.8	0.7	6.9	2	13	14	26	2492	282.4	6.6
NP_003012.1	4506921	SGTA	small glutamine-rich tetratricopeptide repeat-containing protein alpha	0.8	0.7	0.8	0.7	0.8	0.7	0.8	0.7	0.8	0.7	0.8	0.7	31.6	1	9	9	42	313	34.0	4.9
NP_872304.2	38788219	ZNF428	zinc finger protein 428	0.7	0.9	0.7	0.7	0.9	0.9	0.9	0.7	0.8	0.9	0.8	0.7	29.8	1	4	4	12	188	20.5	4.2
NP_542786.1	18152785	ROMO1	reactive oxygen species modulator 1	0.6	0.6	0.5	0.7	0.9	0.8	0.9	0.7	0.7	0.7	0.7	0.7	10.1	1	1	1	1	79	8.2	9.3
NP_001193831.1	332634744	TAX1BP1	tax1-binding protein 1 isoform 3	0.9	0.9	0.9	0.8	0.7	0.7	0.7	0.6	0.8	0.8	0.8	0.7	1.4	3	1	1	1	590	68.2	5.4
NP_002347.5	153070260	MARCKS	myristoylated alanine-rich C-kinase substrate	0.6	0.7	0.7	0.7	0.7	0.7	0.7	0.7	0.6	0.7	0.7	0.7	45.8	1	10	10	61	332	31.5	4.4
NP_803173.1	28933465	STX12	syntaxin-12	0.8	0.8	0.8	0.8	0.8	0.8	0.7	0.7	0.8	0.8	0.7	0.7	2.2	1	1	1	1	276	31.6	5.6
NP_001005782.1	54792067	SUMO1	small ubiquitin-related modifier 1 isoform b precursor	1.0	0.9	0.9	0.8	0.9	0.8	0.8	0.7	0.9	0.8	0.9	0.7	19.7	2	2	2	7	76	8.8	5.7
NP_002950.3	17149834	SORT1	sortilin isoform 1 preproprotein	0.8	0.8	0.8	0.8	0.6	0.6	0.7	0.6	0.7	0.7	0.7	0.7	8.5	2	4	5	10	831	92.0	5.7
NP_001164633.1	283837894	ZMYM3	zinc finger MYM-type protein 3 isoform 2	0.7	0.7	0.7	0.7	0.7	0.7	0.7	0.7	0.7	0.7	0.7	0.7	8.2	3	7	7	15	1358	151.0	6.4
NP_006278.1	5454114	TFPI	tissue factor pathway inhibitor isoform a precursor	1.0	1.0	1.1	0.8	0.9	0.9	1.0	0.7	1.0	1.0	1.1	0.7	9.9	2	2	2	3	304	35.0	8.3
NP_060045.4	115511020	SMG6	telomerase-binding protein EST1A isoform 1	0.7	0.7	0.7	0.7	0.9	0.8	0.8	0.8	0.8	0.8	0.8	0.7	2.0	3	2	2	4	1419	160.4	7.0
NP_006570.1	5729810	EBP	3-beta-hydroxysteroid-Delta(8), Delta(7)-isomerase	0.8	0.9	0.8	0.7	1.1	1.0	1.2	0.7	1.0	1.0	1.0	0.7	5.2	1	1	1	5	230	26.3	7.9
NP_001120700.1	187960037;5803076	CBX1	chromobox protein homolog 1	1.1	0.7	1.2	0.7	1.1	0.8	1.2	0.8	1.1	0.7	1.2	0.7	56.2	1	6	8	22	185	21.4	4.9
NP_060139.2	58331268	ERCC6L	DNA excision repair protein ERCC-6-like	0.8	0.7	0.7	0.7	0.8	0.8	0.7	0.7	0.8	0.7	0.7	0.7	13.7	1	11	11	23	1250	141.0	5.3
NP_891988.1	33636742	PLOD2	procollagen-lysine,2-oxoglutarate 5-dioxygenase 2 isoform 1 precursor	0.7	0.8	1.0	0.7	0.7	0.8	1.0	0.7	0.7	0.8	1.0	0.7	33.6	2	23	23	66	758	87.0	6.7
NP_000938.2	41349495	PRIM2	DNA primase large subunit isoform a	0.7	0.7	0.7	0.7	0.8	0.7	0.7	0.7	0.8	0.7	0.7	0.7	12.2	2	5	5	18	509	58.8	7.9
NP_006133.1	5454052	SFN	14-3-3 protein sigma	1.0	0.7	0.8	0.7	0.9	0.7	0.8	0.7	0.9	0.7	0.8	0.7	63.3	1	8	12	135	248	27.8	4.7
NP_006175.2	20070228	NUCB1	nucleobindin-1 precursor	1.0	0.8	1.0	0.7	1.0	0.8	0.9	0.7	1.0	0.8	0.9	0.7	34.5	1	12	12	36	461	53.8	5.2
NP_003394.1	4507955	YY1	transcriptional repressor protein YY1	0.8	0.8	0.7	0.7	0.8	0.8	0.7	0.7	0.8	0.8	0.7	0.7	26.6	3	6	6	24	414	44.7	6.3
NP_116185.2	19923855	SYAP1	synapse-associated protein 1	0.9	0.8	0.9	0.7	1.0	0.9	0.9	0.7	0.9	0.9	0.9	0.7	51.7	1	16	16	51	352	39.9	4.5
NP_000282.1	4505763	PGK1	phosphoglycerate kinase 1	0.8	0.8	0.7	0.7	0.8	0.8	0.7	0.7	0.8	0.8	0.7	0.7	79.4	1	23	30	337	417	44.6	8.1
NP_054766.1	7661742	C19orf53	leydig cell tumor 10 kDa protein homolog	0.6	0.7	0.6	0.7	0.6	0.7	0.6	0.7	0.6	0.7	0.6	0.7	32.3	1	5	5	9	99	10.6	11.5
NP_060136.1	24308171	ZNF280C	zinc finger protein 280C	0.8	0.7	0.7	0.7	0.8	0.7	0.5	0.7	0.8	0.7	0.6	0.7	8.3	1	4	4	5	737	83.0	9.1
NP_001121134.1	189083772;38044288	GSN	gelsolin isoform b	0.8	0.9	0.8	0.7	0.8	0.9	0.8	0.7	0.8	0.9	0.8	0.7	40.6	6	23	23	100	731	80.6	5.8
NP_002893.1	4506457	RCN2	reticulocalbin-2 isoform a precursor	0.8	0.8	0.8	0.7	0.8	0.8	0.8	0.7	0.8	0.8	0.8	0.7	78.9	2	16	16	165	317	36.9	4.4
NP_075386.1	12711674	UPF3B	regulator of nonsense transcripts 3B isoform 2	0.8	0.8	0.8	0.7	0.8	0.7	0.7	0.7	0.8	0.8	0.7	0.7	14.7	2	5	5	9	470	56.2	9.4
NP_003633.1	4504341	HAT1	histone acetyltransferase type B catalytic subunit	0.8	0.7	0.8	0.7	0.8	0.7	0.7	0.7	0.8	0.7	0.7	0.7	31.5	1	11	11	52	419	49.5	5.7
NP_001015048.1	62548854	BAG5	BAG family molecular chaperone regulator 5 isoform b	0.7	0.7	0.6	0.7	0.7	0.7	0.7	0.7	0.7	0.7	0.7	0.7	42.1	2	14	14	32	447	51.2	6.0
NP_001271246.1	546231794	CRELD2	cysteine-rich with EGF-like domain protein 2 isoform c precursor	1.3	0.9	1.1	0.7	1.3	1.0	1.1	0.7	1.3	0.9	1.1	0.7	13.2	4	4	4	11	325	35.3	4.7
NP_005333.2	71143137	HMGB3	high mobility group protein B3 isoform a	0.7	0.7	0.6	0.7	0.7	0.7	0.6	0.7	0.7	0.7	0.6	0.7	56.5	2	11	13	51	200	23.0	8.4
NP_001269267.1	537544626	PLS3	plastin-3 isoform 4	0.8	0.8	0.8	0.7	0.8	0.8	0.8	0.6	0.8	0.8	0.8	0.7	50.3	4	16	22	78	585	65.6	6.3
NP_002814.3	151101404	PTMA	prothymosin alpha isoform 2	0.7	0.8	0.7	0.7	0.8	0.8	0.7	0.7	0.8	0.8	0.7	0.7	22.7	3	2	4	77	110	12.1	3.8
NP_005309.1	4885371	H1F0	histone H1.0	0.9	0.8	0.8	0.6	0.9	0.9	0.9	0.7	0.9	0.9	0.8	0.7	33.5	1	7	7	81	194	20.9	10.8
NP_006315.1	5453567	CFDP1	craniofacial development protein 1	0.7	0.6	0.6	0.6	0.7	0.7	0.6	0.7	0.7	0.6	0.6	0.7	38.8	1	11	11	49	299	33.6	4.8
NP_071394.2	21735569	CLSPN	claspin isoform 1	0.8	0.8	0.6	0.7	0.8	0.7	0.6	0.6	0.8	0.7	0.6	0.7	12.1	2	10	10	33	1339	151.0	4.8
NP_002473.2	27262628	NASP	nuclear autoantigenic sperm protein isoform 2	0.8	0.7	0.6	0.6	0.8	0.8	0.6	0.7	0.8	0.8	0.6	0.7	75.3	3	41	43	566	788	85.2	4.3
NP_038472.2	16753207	UBQLN2	ubiquilin-2	0.6	0.5	0.6	0.6	0.9	0.7	0.9	0.7	0.7	0.6	0.7	0.7	3.7	1	1	2	3	624	65.7	5.2
NP_000413.1	4557701	KRT17	keratin, type I cytoskeletal 17	0.7	0.6	0.5	0.6	0.8	0.7	0.6	0.7	0.7	0.7	0.5	0.7	35.2	11	7	12	73	432	48.1	5.0
NP_005316.1	4885373	HIST1H1A	histone H1.1	0.5	0.5	0.5	0.6	0.6	0.6	0.6	0.7	0.5	0.6	0.6	0.7	41.9	3	6	10	163	215	21.8	11.0
NP_005382.1	4885545	PDK3	pyruvate dehydrogenase kinase, isozyme 3 isoform 2 precursor	0.9	0.7	0.8	0.6	0.8	0.8	0.8	0.7	0.9	0.7	0.8	0.7	7.9	2	2	2	6	406	46.9	8.4
NP_000185.1	4504483	HPRT1	hypoxanthine-guanine phosphoribosyltransferase	0.9	0.7	0.7	0.6	0.9	0.8	0.8	0.7	0.9	0.8	0.7	0.7	37.2	3	7	7	55	218	24.6	6.7
NP_005617.2	21361282	SRSF4	serine/arginine-rich splicing factor 4	0.7	0.7	0.7	0.6	0.7	0.8	0.7	0.7	0.7	0.8	0.7	0.7	22.7	1	8	12	51	494	56.6	11.5
NP_001165780.1	288856248	NEXN	nexilin isoform 2	0.8	0.8	1.1	0.7	0.8	0.8	1.2	0.6	0.8	0.8	1.2	0.7	1.6	2	1	1	2	611	72.7	5.1
NP_112594.1	13775228;223633884	DOHH	deoxyhypusine hydroxylase	0.7	0.8	1.0	0.7	0.8	0.7	0.9	0.6	0.8	0.7	1.0	0.6	15.2	1	2	2	9	302	32.9	4.8
NP_997239.2	224591399	C1orf174	UPF0688 protein C1orf174	0.7	0.7	0.6	0.7	0.7	0.7	0.6	0.6	0.7	0.7	0.6	0.6	28.0	1	3	3	5	243	26.0	6.9
NP_055095.2	112293277	DNAJC8	dnaJ homolog subfamily C member 8	0.7	0.6	0.6	0.6	0.7	0.7	0.6	0.7	0.7	0.7	0.6	0.6	46.3	1	10	10	29	253	29.8	9.1
NP_001245228.1	385198095	KIAA1598	shootin-1 isoform d	0.8	0.8	0.7	0.7	0.7	0.6	0.5	0.6	0.8	0.7	0.6	0.6	21.7	5	9	9	14	558	63.5	5.6
NP_057584.2	32306539	PBDC1	protein PBDC1 isoform 1	1.0	0.7	0.9	0.6	0.9	0.7	0.8	0.7	1.0	0.7	0.8	0.6	24.9	2	5	5	14	233	26.0	4.8
NP_006017.1	5174449	H1FX	histone H1x	0.7	0.7	0.7	0.6	0.7	0.7	0.6	0.6	0.7	0.7	0.7	0.6	28.6	1	6	6	31	213	22.5	10.8
NP_004983.1	4826830	MECP2	methyl-CpG-binding protein 2 isoform 1	0.7	0.7	0.6	0.6	0.8	0.8	0.6	0.7	0.8	0.7	0.6	0.6	20.0	2	8	8	30	486	52.4	9.9
NP_054701.1	7669503	LAMP2	lysosome-associated membrane glycoprotein 2 isoform B precursor	0.7	0.7	0.6	0.5	0.8	0.9	0.7	0.7	0.8	0.8	0.6	0.6	9.0	3	4	4	22	410	44.9	5.7
NP_066390.1	10645195	HIST1H2AE	histone H2A type 1-B/E	0.5	0.5	0.5	0.6	0.5	0.6	0.5	0.6	0.5	0.6	0.5	0.6	65.4	4	3	8	886	130	14.1	11.0
NP_001017371.3	289577125	SP3	transcription factor Sp3 isoform 2	0.8	0.9	0.8	0.7	0.6	0.6	0.6	0.5	0.7	0.8	0.7	0.6	2.2	3	1	1	4	713	75.3	5.3
NP_001193995.1	333470709	CXADR	coxsackievirus and adenovirus receptor isoform 5 precursor	0.7	0.7	0.8	0.6	0.6	0.6	0.7	0.6	0.7	0.6	0.7	0.6	8.8	5	2	2	3	352	38.9	7.5
NP_055551.1	7661906	KIAA0101	PCNA-associated factor isoform 1	0.7	0.7	0.7	0.7	0.7	0.6	0.6	0.6	0.7	0.7	0.6	0.6	60.4	2	6	6	16	111	12.0	9.8
NP_477511.1	24308388	TSR2	pre-rRNA-processing protein TSR2 homolog	1.0	0.8	0.8	0.6	0.9	0.7	0.7	0.6	1.0	0.7	0.8	0.6	6.3	1	1	1	9	191	20.9	4.4
NP_002696.3	45439327	PPL	periplakin	0.7	0.7	0.7	0.6	0.7	0.6	0.6	0.6	0.7	0.7	0.6	0.6	4.6	1	4	5	9	1756	204.6	5.6
NP_055315.2	21361437;253970456	HTATSF1	HIV Tat-specific factor 1	0.8	0.8	0.6	0.6	0.7	0.7	0.6	0.6	0.7	0.8	0.6	0.6	37.4	5	22	22	72	755	85.8	4.4
NP_066298.1	10440560;4504297	HIST1H3F	histone H3.1	0.8	0.6	0.8	0.6	0.7	0.6	0.8	0.6	0.7	0.6	0.8	0.6	59.6	3	1	9	632	136	15.4	11.1
NP_001035199.1	93141039	NRF1	nuclear respiratory factor 1	0.7	0.4	0.3	0.3	1.0	0.9	0.9	0.9	0.8	0.7	0.6	0.6	11.3	1	2	2	3	503	53.5	5.0
NP_001006934.1	55749431	TCEAL3	transcription elongation factor A protein-like 3	0.7	0.5	0.6	0.5	0.7	0.6	0.7	0.5	0.7	0.6	0.6	0.5	26.5	3	5	5	7	200	22.5	4.9
NP_005508.1	5031749	HMGN2	non-histone chromosomal protein HMG-17	0.6	0.6	0.5	0.5	0.6	0.6	0.4	0.5	0.6	0.6	0.5	0.5	44.4	1	4	5	36	90	9.4	10.0
NP_940874.2	148596951	FUOM	fucose mutarotase isoform 2	0.6	0.5	0.6	0.4	0.7	0.8	0.8	0.5	0.6	0.7	0.7	0.5	11.2	2	1	1	2	134	14.6	5.3
NP_036374.1	7110719	DKK1	dickkopf-related protein 1 precursor	0.8	0.6	0.8	0.5	0.7	0.6	0.6	0.4	0.7	0.6	0.7	0.4	16.9	1	3	3	5	266	28.7	8.4
NP_001006938.1	55749459;65505873	TCEAL4	transcription elongation factor A protein-like 4	0.6	0.6	0.5	0.4	0.5	0.5	0.4	0.4	0.6	0.5	0.4	0.4	23.7	2	4	4	8	215	24.6	5.2

Khan *et al.* , 2019. Multi-omics analysis to characterize cigarette smoke induced molecular alterations in esophageal cells

Supplementary Table 5. List of proteins quantified in untreated and chronically treated Het1A cells with cigarette smoke condensate for 8 months

NP_Accession	Protein group Accession	Gene ID	Description	Het-1A-Smoke - 2M/Parental	Het-1A-Smoke - 4M/Parental	Het-1A-Smoke - 6M/Parental	Het-1A-Smoke - 8M/Parental	Het-1A-Smoke - 2M/Parental	Het-1A-Smoke - 4M/Parental	Het-1A-Smoke - 6M/Parental	Het-1A-Smoke - 8M/Parental	Het-1A-Smoke - 2M/Parental	Het-1A-Smoke - 4M/Parental	Het-1A-Smoke - 6M/Parental	Het-1A-Smoke - 8M/Parental	Coverage (%)	Proteins	Unique Peptides	# Peptides	PSM	# AAs	MW [kDa]	calc. pI
				Replicate 1				Replicate 2				Average of replicates											
NP_003351.2	40254471	UGT8	2-hydroxyacylsphingosine 1-beta-galactosyltransferase precursor	1.3	2.0	2.3	2.6	-	-	-	-	1.3	2.0	2.3	2.6	2.8	1	1	1	2	541	61.4	9.5
NP_001229739.1	338797768	BRDT	bromodomain testis-specific protein isoform d	0.8	1.9	1.0	2.6	-	-	-	-	0.8	1.9	1.0	2.6	0.7	4	1	1	2	874	99.3	8.7
NP_690577.2	38045917	PIGO	GPI ethanolamine phosphate transferase 3 isoform 2	2.1	2.4	2.0	2.3	-	-	-	-	2.1	2.4	2.0	2.3	1.6	2	1	1	1	672	73.9	6.6
NP_060701.1	8922692	SLC38A7	putative sodium-coupled neutral amino acid transporter 7	0.0	1.5	1.0	2.3	-	-	-	-	0.0	1.5	1.0	2.3	10.2	1	1	1	1	462	49.9	5.1
NP_057238.3	82659107	RAB4B	ras-related protein Rab-4B	2.7	2.3	2.1	2.3	-	-	-	-	2.7	2.3	2.1	2.3	17.4	9	1	2	4	213	23.6	6.1
NP_056021.1	54606888	KIAA1024	UPF0258 protein KIAA1024	2.8	2.3	1.8	2.2	-	-	-	-	2.8	2.3	1.8	2.2	2.6	1	1	1	1	916	102.9	7.3
NP_001171753.1	296317343	PRMT7	protein arginine N-methyltransferase 7 isoform 2	1.6	1.5	1.5	1.9	-	-	-	-	1.6	1.5	1.5	1.9	2.7	2	1	1	1	642	73.1	5.5
NP_005720.1	5031987	PPIF	peptidyl-prolyl cis-trans isomerase F, mitochondrial precursor	1.3	1.8	1.3	1.8	-	-	-	-	1.3	1.8	1.3	1.8	14.0	2	2	4	6	207	22.0	9.4
NP_001344.2	5453543	AKR1C1	aldo-keto reductase family 1 member C1	1.4	1.3	1.5	1.8	-	-	-	-	1.4	1.3	1.5	1.8	11.2	4	2	2	4	323	36.8	7.9
NP_079197.3	193794853	POF1B	protein POF1B isoform 1	1.3	1.7	1.3	1.8	-	-	-	-	1.3	1.7	1.3	1.8	1.9	2	1	1	2	589	68.0	6.3
NP_001119595.1	186972148	ENOSF1	mitochondrial enolase superfamily member 1 isoform rTSalpha	1.4	1.3	1.5	1.7	-	-	-	-	1.4	1.3	1.5	1.7	4.7	3	1	1	2	361	41.0	6.5
NP_001269594.1	544063421	MICAL2	protein-methionine sulfoxide oxidase MICAL2 isoform c	1.4	1.8	2.0	1.7	-	-	-	-	1.4	1.8	2.0	1.7	3.6	5	1	2	6	934	106.1	8.1
NP_003245.1	4507509	TIMP1	metalloproteinase inhibitor 1 precursor	1.2	1.5	1.3	1.6	-	-	-	-	1.2	1.5	1.3	1.6	19.3	1	3	3	4	207	23.2	8.1
NP_443137.2	52485606	FMNL2	formin-like protein 2	1.3	1.5	1.4	1.6	-	-	-	-	1.3	1.5	1.4	1.6	4.6	3	4	4	6	1092	124.0	7.7
NP_775487.2	89357932	KRT78	keratin, type II cytoskeletal 78 isoform 1	1.3	1.6	1.0	1.6	-	-	-	-	1.3	1.6	1.0	1.6	5.2	6	2	3	17	520	56.8	6.0
NP_003638.1	4503313	DGKE	diacylglycerol kinase epsilon	1.3	1.3	1.4	1.6	-	-	-	-	1.3	1.3	1.4	1.6	2.3	1	1	1	2	567	63.9	7.7
NP_001273567.1	557786103	ZDHHC20	probable palmitoyltransferase ZDHHC20 isoform 2	1.6	1.4	1.4	1.6	-	-	-	-	1.6	1.4	1.4	1.6	8.6	2	2	2	2	292	33.7	8.0
NP_036605.2	21361403	TXN2	thioredoxin, mitochondrial precursor	1.9	2.0	2.0	1.6	-	-	-	-	1.9	2.0	2.0	1.6	9.0	1	1	1	1	166	18.4	8.3
NP_694951.1	23397479	ZNF524	zinc finger protein 524	0.9	2.1	1.5	1.5	-	-	-	-	0.9	2.1	1.5	1.5	19.3	1	1	1	2	264	28.7	8.7
NP_001912.2	61742819	DCTD	deoxycytidylate deaminase isoform b	1.2	1.3	1.2	1.5	-	-	-	-	1.2	1.3	1.2	1.5	10.1	2	1	1	2	178	20.0	7.6
NP_940857.2	134031945	SSPO	SCO-spondin precursor	1.2	1.5	1.3	1.5	-	-	-	-	1.2	1.5	1.3	1.5	0.3	1	1	1	1	5147	547.1	6.0
NP_001015.1	4506699	RPS21	40S ribosomal protein S21	1.1	1.6	1.3	1.5	-	-	-	-	1.1	1.6	1.3	1.5	25.3	1	2	2	4	83	9.1	8.5
NP_001248339.1	387157911	BCAR3	breast cancer anti-estrogen resistance protein 3 isoform 2	1.6	1.6	1.6	1.5	-	-	-	-	1.6	1.6	1.6	1.5	2.0	2	1	1	1	734	82.3	8.1
NP_659435.2	71559139	STRA13	centromere protein X isoform 2	1.6	1.6	1.2	1.5	-	-	-	-	1.6	1.6	1.2	1.5	14.3	2	1	1	2	63	7.0	6.5
NP_001244325.1	383872507	UBE2V1	ubiquitin-conjugating enzyme E2 variant 1 isoform f	1.3	1.5	1.1	1.5	-	-	-	-	1.3	1.5	1.1	1.5	58.1	9	1	5	25	105	11.8	5.5
NP_619646.1	20162566	MPLKIP	M-phase-specific PLK1-interacting protein	0.6	0.8	1.5	1.5	-	-	-	-	0.6	0.8	1.5	1.5	14.0	1	1	1	2	179	19.1	10.2
NP_006336.3	57164948	SLC30A9	zinc transporter 9	1.6	1.3	1.4	1.5	-	-	-	-	1.6	1.3	1.4	1.5	1.6	1	1	1	1	568	63.5	8.3
NP_000090.1	4503107	CST3	cystatin-C precursor	1.3	1.6	1.6	1.4	-	-	-	-	1.3	1.6	1.6	1.4	7.5	1	1	1	2	146	15.8	8.7
NP_689854.2	56549668	ARID2	AT-rich interactive domain-containing protein 2	1.3	1.5	1.6	1.4	-	-	-	-	1.3	1.5	1.6	1.4	0.5	1	1	1	1	1835	197.3	7.4
NP_001078897.1	146229327	ARSA	arylsulfatase A isoform b	1.1	1.3	1.3	1.4	-	-	-	-	1.1	1.3	1.3	1.4	4.0	2	1	1	2	423	44.9	5.9
NP_064625.1	9910378	CDC42SE2	CDC42 small effector protein 2	1.3	1.1	1.2	1.4	-	-	-	-	1.3	1.1	1.2	1.4	22.6	1	1	1	2	84	9.2	8.4
NP_057340.2	66346700	NAGPA	N-acetylglucosamine-1-phosphodiester alpha-N-acetylglucosaminidase precursor	1.2	1.3	1.1	1.4	-	-	-	-	1.2	1.3	1.1	1.4	2.5	1	1	1	1	515	56.0	6.6
NP_003857.2	156104895	INPP4B	type II inositol 3,4-bisphosphate 4-phosphatase	1.1	1.4	1.4	1.4	-	-	-	-	1.1	1.4	1.4	1.4	1.6	1	1	1	2	924	104.7	6.3
NP_115648.2	110349740	MAF1	repressor of RNA polymerase III transcription MAF1 homolog	1.2	1.1	1.3	1.4	-	-	-	-	1.2	1.1	1.3	1.4	5.1	1	1	1	1	256	28.8	4.6
NP_061865.1	9506651	KCTD5	BTB/POZ domain-containing protein KCTD5	1.1	1.2	1.2	1.4	-	-	-	-	1.1	1.2	1.2	1.4	15.8	1	2	2	3	234	26.1	6.2
NP_002791.1	4506205	PSMB9	proteasome subunit beta type-9 proprotein	1.1	1.0	1.0	1.4	-	-	-	-	1.1	1.0	1.0	1.4	8.7	1	2	2	4	219	23.2	5.0
NP_680092.1	22538435	CTSB	cathepsin B preproprotein	1.0	1.0	1.4	1.4	-	-	-	-	1.0	1.0	1.4	1.4	3.2	1	1	1	2	339	37.8	6.3
NP_056020.2	150421684	ATP11A	probable phospholipid-transporting ATPase IH isoform a	1.1	1.3	1.1	1.4	-	-	-	-	1.1	1.3	1.1	1.4	0.9	2	1	1	1	1134	129.7	6.6
NP_665857.2	90669511	CRYZL1	quinone oxidoreductase-like protein 1	1.2	1.3	1.1	1.4	-	-	-	-	1.2	1.3	1.1	1.4	6.0	1	1	1	2	349	38.7	5.8
NP_115602.2	110815844	ZCCHC7	zinc finger CCHC domain-containing protein 7	1.1	1.3	1.1	1.4	-	-	-	-	1.1	1.3	1.1	1.4	2.0	1	1	1	2	543	63.0	7.3
NP_002956.1	4506773	S100A9	protein S100-A9	1.0	1.4	1.1	1.4	-	-	-	-	1.0	1.4	1.1	1.4	11.4	1	1	1	1	114	13.2	6.1
NP_001180394.1	301500643	KANSL1	KAT8 regulatory NSL complex subunit 1 isoform 2	1.1	1.0	1.0	1.3	-	-	-	-	1.1	1.0	1.0	1.3	2.2	2	1	1	2	1104	120.8	8.7
NP_001257954.1	402478636	AKR1E2	1,5-anhydro-D-fructose reductase isoform 3	1.1	1.5	1.4	1.3	-	-	-	-	1.1	1.5	1.4	1.3	2.7	3	1	1	1	222	25.7	7.0
NP_079215.2	45935393	ESRP2	epithelial splicing regulatory protein 2	1.7	1.2	1.1	1.3	-	-	-	-	1.7	1.2	1.1	1.3	5.3	6	1	2	3	717	77.3	6.8
NP_110404.1	13540547	SLC2A10	solute carrier family 2, facilitated glucose transporter member 10	1.6	1.2	1.0	1.3	-	-	-	-	1.6	1.2	1.0	1.3	1.5	1	1	1	1	541	56.9	8.6
NP_001186715.1	315139020	FKBP1A	peptidyl-prolyl cis-trans isomerase FKBP1A isoform b	1.2	1.4	1.2	1.3	-	-	-	-	1.2	1.4	1.2	1.3	13.4	2	1	1	2	97	10.8	7.3
NP_997192.2	187936958	UAP1L1	UDP-N-acetylhexosamine pyrophosphorylase-like protein 1	1.1	1.2	1.2	1.3	-	-	-	-	1.1	1.2	1.2	1.3	5.7	1	1	2	5	507	57.0	6.3
NP_078916.3	58761510	YRDC	yrdC domain-containing protein, mitochondrial precursor	1.1	1.2	1.1	1.3	-	-	-	-	1.1	1.2	1.1	1.3	4.3	1	1	1	1	279	29.3	8.6
NP_001185625.1	311082415;14318424	AIFM2	apoptosis-inducing factor 2	1.4	1.6	1.3	1.3	-	-	-	-	1.4	1.6	1.3	1.3	8.0	1	3	3	4	373	40.5	9.1
NP_060769.4	402169569	UBE2W	ubiquitin-conjugating enzyme E2 W isoform 2	1.2	1.3	1.2	1.3	-	-	-	-	1.2	1.3	1.2	1.3	13.9	3	1	1	1	180	20.7	9.0
NP_057071.2	20336761	HEBP1	heme-binding protein 1	1.2	1.5	1.3	1.3	-	-	-	-	1.2	1.5	1.3	1.3	13.8	1	2	2	4	189	21.1	5.8
NP_955368.1	40786394	FAHD2B	fumarylacetoacetate hydrolase domain-containing protein 2B	1.3	1.0	1.1	1.3	-	-	-	-	1.3	1.0	1.1	1.3	8.0	1	1	1	1	314	34.6	7.8
NP_001106191.1	163310749	LIMCH1	LIM and calponin homology domains-containing protein 1 isoform e	1.2	1.1	1.3	1.3	-	-	-	-	1.2	1.1	1.3	1.3	1.5	7	1	1	2	890	100.7	6.3
NP_065109.1	32698724	PNPLA2	patatin-like phospholipase domain-containing protein 2	1.7	1.8	1.5	1.3	-	-	-	-	1.7	1.8	1.5	1.3	3.2	1	1	1	2	504	55.3	7.1
NP_077010.1	13129114	ALG12	dol-P-Man:Man(7)GlcNAc(2)-PP-Dol alpha-1,6-mannosyltransferase	1.2	1.4	1.4	1.3	-	-	-	-	1.2	1.4	1.4	1.3	3.3	1	1	1	2	488	54.6	9.6
NP_951008.1	39995078	TRAPPC11	trafficking protein particle complex subunit 11 isoform b	1.3	1.2	1.2	1.3	-	-	-	-	1.3	1.2	1.2	1.3	0.7	2	1	1	2	1086	123.3	7.0
NP_001268433.1	528078306	ATG4D	cysteine protease ATG4D isoform 2	1.0	1.3	1.0	1.3	-	-	-	-	1.0	1.3	1.0	1.3	3.7	2	1	1	1	411	46.4	8.3
NP_110402.2	17402919	WNT5B	protein Wnt-5b precursor	1.1	1.4	1.3	1.3	-	-	-	-	1.1	1.4	1.3	1.3	5.9	1	1	1	1	359	40.3	8.4
NP_003703.1	4505977	PPAP2C	lipid phosphate phosphohydrolase 2 isoform 1	1.2	1.2	1.2	1.3	-	-	-	-	1.2	1.2	1.2	1.3	3.5	2	1	1	2	288	32.6	8.4
NP_001093252.1	153266907	GGT5	gamma-glutamyltransferase 5 isoform c	0.9	1.0	1.1	1.3	-	-	-	-	0.9	1.0	1.1	1.3	2.7	3	1	1	1	554	59.0	7.8
NP_060604.2	229892326	IQCC	IQ domain-containing protein C isoform 2	1.1	1.3	1.4	1.3	-	-	-	-	1.1	1.3	1.4	1.3	1.5	2	1	1	1	466	53.0	8.0
NP_004999.1	4826860	NHP2L1	NHP2-like protein 1	1.4	1.0	1.1	1.3	-	-	-	-	1.4	1.0	1.1	1.3	9.4	1	1	1	2	128	14.2	8.5
NP_569119.1	18860859	PTPRE	receptor-type tyrosine-protein phosphatase epsilon isoform 2	0.9	1.3	1.2	1.3	-	-	-	-	0.9	1.3	1.2	1.3	1.3	2	1	1	1	642	74.5	7.5
NP_001017998.1	63055067	GNG10	guanine nucleotide-binding protein G(I)/G(S)/G(O) subunit gamma-10 precursor	1.1	1.0	1.1	1.3	-	-	-	-	1.1	1.0	1.1	1.3	22.1	2	1	1	6	68	7.2	7.9
NP_597998.1	19424126	SAT2	diamine acetyltransferase 2	1.2	1.1	1.2	1.3	-	-	-	-	1.2	1.1	1.2	1.3	4.1	1	1	1	1	170	19.1	6.0
NP_002748.1	4506101	MAP2K5	dual specificity mitogen-activated protein kinase kinase 5 isoform B	1.2	1.5	1.5	1.3	-	-	-	-	1.2	1.5	1.5	1.3	6.2	2	1	1	4	438	48.9	6.3
NP_116261.1	14249614	SYTL1	synaptotagmin-like protein 1 isoform 2	1.1	1.1	1.0	1.3	-	-	-	-	1.1	1.1	1.0	1.3	1.1	2	1	1	1	550	60.6	5.5
NP_003195.1	4505379	NFE2L1	nuclear factor erythroid 2-related factor 1	1.6	1.4	1.2	1.3	-	-	-	-	1.6	1.4	1.2	1.3	1.9	1	1	1	2	772	84.7	4.6
NP_002727.2	47132585	PRKAR2B	cAMP-dependent protein kinase type II-beta regulatory subunit	1.4	1.2	1.2	1.3	-	-	-	-	1.4	1.2	1.2	1.3	7.4	1	1	2	4	418	46.3	4.9
NP_001243462.1	374717332	MSTO1	protein misato homolog 1 isoform c	1.2	1.1	1.1	1.2	-	-	-	-	1.2	1.1	1.1	1.2	2.2	3	1	1	2	558	60.7	6.1
NP_057332.1	7706457	AKAP11	A-kinase anchor protein 11	1.5	1.1	1.0	1.2	-	-	-	-	1.5	1.1	1.0	1.2	0.7	1	1	1	1	1901	210.4	5.4
NP_001272390.1	550544226	IQGAP2	ras GTPase-activating-like protein IQGAP2 isoform 3	1.4	1.2	1.0	1.2	-	-	-	-	1.4	1.2	1.0	1.2	4.8	6	1	4	15	1071	122.7	6.7
NP_473365.3	46094009	MIA2	melanoma inhibitory activity protein 2 precursor	1.0	1.0	1.1	1.2	-	-	-	-	1.0	1.0	1.1	1.2	0.9	1	1	1	2	654	74.0	4.3
NP_112240.1	13569956	ARPC5L	actin-related protein 2/3 complex subunit 5-like protein	1.1	1.2	1.1	1.2	-	-	-	-	1.1	1.2	1.1	1.2	8.5	1	1	1	2	153	16.9	6.6
NP_258442.2	53692184	FBXO9	F-box only protein 9 isoform 3	1.2	1.3	1.4	1.2	-	-	-	-	1.2	1.3	1.4	1.2	5.5	3	1	1	1	403	47.3	8.4
NP_065727.4	189027141	ABHD6	monoacylglycerol lipase ABHD6	1.2	1.3	1.5	1.2	-	-	-	-	1.2	1.3	1.5	1.2	3.0	1	1	1	1	337	38.3	8.4
NP_001265488.1	514052677	ZNF316	zinc finger protein 316	0.9	1.2	0.9	1.2	-	-	-	-	0.9	1.2	0.9	1.2	4.0	1	2	2	2	1004	108.4	5.3
NP_001123498.2	293651621	FAM115C	protein FAM115C isoform B	1.2	1.4	1.1	1.2	-	-	-	-	1.2	1.4	1.1	1.2	10.2	2	2	6	9	815	90.6	7.7
NP_002761.1	4506147	PRSS2	trypsin-2 isoform 2 preproprotein	5.7	1.5	1.2	1.2	-	-	-	-	5.7	1.5	1.2	1.2	11.3	7	2	2	3	247	26.5	4.9
NP_055461.1	7662022	LPIN2	phosphatidate phosphatase LPIN2	1.0	1.0	1.0	1.2	-	-	-	-	1.0	1.0	1.0	1.2	2.3	1	1	2	3	896	99.3	5.3
NP_001254652.1	391353392	CCDC61	coiled-coil domain-containing protein 61	1.2	1.0	1.1	1.2	-	-	-	-	1.2	1.0	1.1	1.2	2.7	1	1	1	1	512	57.3	10.3
NP_002351.1	4505075	MAFK	transcription factor MafK	1.4	1.3	1.1	1.2	-	-	-	-	1.4	1.3	1.1	1.2	5.8	1	1	1	2	156	17.5	10.1
NP_001124400.1	195972805	MEF2A	myocyte-specific enhancer factor 2A isoform 4	1.1	1.1	1.1	1.2	-	-	-	-	1.1	1.1	1.1	1.2	2.3	3	1	1	1	429	45.5	9.3
NP_006338.1	5454154	PPIH	peptidyl-prolyl cis-trans isomerase H	1.2	1.3	1.1	1.2	-	-	-	-	1.2	1.3	1.1	1.2	22.0	2	3	5	8	177	19.2	8.1
NP_002607.2	194097341	PER1	period circadian protein homolog 1	1.1	1.2	1.1	1.2	-	-	-	-	1.1	1.2	1.1	1.2	1.2	1	1	1	2	1290	136.1	6.1
NP_001171434.1	295789085	MNAT1	CDK-activating kinase assembly factor MAT1 isoform 2	1.1	1.1	1.0	1.2	-	-	-	-	1.1	1.1	1.0	1.2	3.4	2	1	1	1	267	31.1	5.7
NP_065851.1	46409657	IFT80	intraflagellar transport protein 80 homolog isoform a	1.1	1.1	1.1	1.2	-	-	-	-	1.1	1.1	1.1	1.2	1.7	1	1	1	1	777	88.0	7.7
NP_001366.1	4503349	DNASE2	deoxyribonuclease-2-alpha precursor	1.2	1.4	1.2	1.2	-	-	-	-	1.2	1.4	1.2	1.2	7.8	1	3	3	4	360	39.6	8.0

Khan *et al.* , 2019. Multi-omics analysis to characterize cigarette smoke induced molecular alterations in esophageal cells
Supplementary Table 5. List of proteins quantified in untreated and chronically treated Het1A cells with cigarette smoke condensate for 8 months

NP_Accession	Protein group Accession	Gene ID	Description	Het-1A-Smoke - 2M/Parental	Het-1A-Smoke - 4M/Parental	Het-1A-Smoke - 6M/Parental	Het-1A-Smoke - 8M/Parental	Het-1A-Smoke - 2M/Parental	Het-1A-Smoke - 4M/Parental	Het-1A-Smoke - 6M/Parental	Het-1A-Smoke - 8M/Parental	Het-1A-Smoke - 2M/Parental	Het-1A-Smoke - 4M/Parental	Het-1A-Smoke - 6M/Parental	Het-1A-Smoke - 8M/Parental	Coverage (%)	Proteins	Unique Peptides	# Peptides	PSM	# AAs	MW [kDa]	calc. pI
				Replicate 1				Replicate 2				Average of replicates											
NP_963836.2	157837979	MED8	mediator of RNA polymerase II transcription subunit 8 isoform 4	1.1	1.3	1.1	1.2	-	-	-	-	1.1	1.3	1.1	1.2	13.4	3	2	2	5	268	29.1	7.4
NP_073607.2	21362088	GINS3	DNA replication complex GINS protein PSF3 isoform b	0.9	0.9	0.9	1.2	-	-	-	-	0.9	0.9	0.9	1.2	6.9	1	1	1	1	216	24.5	5.3
NP_038460.4	187608777	TONSL	tonsoku-like protein	1.0	1.2	1.1	1.2	-	-	-	-	1.0	1.2	1.1	1.2	2.5	1	2	2	2	1378	150.8	6.4
NP_056099.3	335353804	SZT2	protein SZT2	1.2	1.4	1.3	1.2	-	-	-	-	1.2	1.4	1.3	1.2	0.5	1	1	1	2	3375	371.6	6.3
XP_003846798.1	397138640	LOC10099678	mediator of RNA polymerase II transcription subunit 18-like	0.9	1.1	1.0	1.2	-	-	-	-	0.9	1.1	1.0	1.2	10.6	2	1	1	1	199	22.7	7.2
NP_065717.1	10190706	CLK4	dual specificity protein kinase CLK4	1.0	1.1	1.0	1.2	-	-	-	-	1.0	1.1	1.0	1.2	3.3	1	1	1	2	481	57.5	8.7
NP_001269348.1	538921154	NDUFA5	NADH dehydrogenase [ubiquinone] 1 alpha subcomplex subunit 5 isoform 2	1.1	1.1	1.4	1.2	-	-	-	-	1.1	1.1	1.4	1.2	9.9	5	1	1	3	101	12.0	6.1
YP_003024028.1	251831109	COX1	cytochrome c oxidase subunit I	1.1	1.1	1.1	1.2	-	-	-	-	1.1	1.1	1.1	1.2	6.0	1	1	1	4	513	57.0	6.7
NP_001139502.1	225637524	SEMA7A	semaphorin-7A isoform 3	1.3	1.6	1.7	1.2	-	-	-	-	1.3	1.6	1.7	1.2	5.6	3	2	2	4	501	57.0	6.8
NP_003087.1	4507133	SNRPG	small nuclear ribonucleoprotein G	1.2	1.3	1.4	1.2	-	-	-	-	1.2	1.3	1.4	1.2	15.8	1	1	1	1	76	8.5	8.9
NP_006692.1	5729802	TXNL4A	thioredoxin-like protein 4A	1.1	1.3	1.5	1.2	-	-	-	-	1.1	1.3	1.5	1.2	14.1	1	1	1	1	142	16.8	5.8
NP_002350.1	4505073	MAFG	transcription factor MafG	1.3	1.2	1.0	1.2	-	-	-	-	1.3	1.2	1.0	1.2	5.6	1	1	1	1	162	17.8	10.0
NP_036517.1	6912570	NUPR1	nuclear protein 1 isoform b	2.6	1.9	1.0	1.2	-	-	-	-	2.6	1.9	1.0	1.2	19.5	2	1	1	5	82	8.9	10.0
NP_116069.1	14249254	EFCAB4B	EF-hand calcium-binding domain-containing protein 4B isoform c	1.1	1.0	1.1	1.2	-	-	-	-	1.1	1.0	1.1	1.2	2.3	2	1	1	1	395	45.6	5.0
NP_001025062.1	71274132	PGAM4	phosphoglycerate mutase 4	1.4	1.5	1.7	1.2	-	-	-	-	1.4	1.5	1.7	1.2	40.6	1	1	7	69	254	28.8	6.7
NP_055020.1	7656936	ZNHIT2	zinc finger HIT domain-containing protein 2	1.1	1.2	1.0	1.2	-	-	-	-	1.1	1.2	1.0	1.2	10.7	1	2	2	4	403	42.9	6.0
NP_060793.2	224591401	PI4K2B	phosphatidylinositol 4-kinase type 2-beta	1.0	1.2	1.1	1.2	-	-	-	-	1.0	1.2	1.1	1.2	2.1	1	1	1	2	481	54.7	6.0
NP_057542.2	13124773	FAM203A	protein FAM203A	1.1	1.1	1.1	1.2	-	-	-	-	1.1	1.1	1.1	1.2	26.7	1	7	7	19	390	42.1	4.8
NP_060836.1	8922952	BLOC1S4	biogenesis of lysosome-related organelles complex 1 subunit 4	1.0	1.1	1.1	1.2	-	-	-	-	1.0	1.1	1.1	1.2	4.6	1	1	1	1	217	23.3	5.0
NP_001111.3	225703102	MFSD10	major facilitator superfamily domain-containing protein 10	1.1	1.2	1.2	1.2	-	-	-	-	1.1	1.2	1.2	1.2	2.0	1	1	1	1	455	48.3	9.6
NP_937826.1	38157986	UBE2L6	ubiquitin/ISG15-conjugating enzyme E2 L6 isoform 2	1.1	1.1	1.1	1.2	-	-	-	-	1.1	1.1	1.1	1.2	17.2	2	1	1	2	87	10.1	7.1
NP_064524.3	66933014	BDH2	3-hydroxybutyrate dehydrogenase type 2	1.3	1.1	1.1	1.2	-	-	-	-	1.3	1.1	1.1	1.2	4.1	1	1	1	2	245	26.7	7.6
NP_001136076.1	217272890	PPT1	palmitoyl-protein thioesterase 1 isoform 2 precursor	1.0	1.2	1.0	1.2	-	-	-	-	1.0	1.2	1.0	1.2	7.4	2	1	1	4	203	23.1	6.0
NP_001009571.2	148839284	CADPS2	calcium-dependent secretion activator 2 isoform b	1.5	1.9	1.8	1.2	-	-	-	-	1.5	1.9	1.8	1.2	0.6	6	1	1	1	1255	143.4	6.1
NP_612357.4	115334675	OTULIN	ubiquitin thioesterase otulin	1.2	1.2	1.2	1.2	-	-	-	-	1.2	1.2	1.2	1.2	8.0	1	1	1	1	352	40.2	5.5
YP_003024026.1	251831107	ND1	NADH dehydrogenase subunit 1	1.0	1.1	1.1	1.2	-	-	-	-	1.0	1.1	1.1	1.2	6.3	1	2	2	4	318	35.6	6.6
NP_063944.3	239582761	ERO1LB	ERO1-like protein beta precursor	1.2	1.2	1.1	1.2	-	-	-	-	1.2	1.2	1.1	1.2	8.6	1	3	4	11	467	53.5	8.0
NP_060315.1	8923458	COMMD8	COMM domain-containing protein 8	1.1	1.0	1.1	1.2	-	-	-	-	1.1	1.0	1.1	1.2	18.0	1	2	2	3	183	21.1	5.4
NP_001264232.1	471838917	POTEB2	POTE ankyrin domain family member B2	1.4	1.2	0.8	1.2	-	-	-	-	1.4	1.2	0.8	1.2	6.1	10	1	2	2	544	61.7	6.1
NP_001275959.1	573459784;573459786	GALK2	N-acetylgalactosamine kinase isoform 3	1.3	1.2	1.3	1.2	-	-	-	-	1.3	1.2	1.3	1.2	10.8	3	3	3	5	434	47.6	6.2
NP_001036010.1	110347437	LTBP4	latent-transforming growth factor beta-binding protein 4 isoform c precursor	1.4	1.2	1.2	1.2	-	-	-	-	1.4	1.2	1.2	1.2	1.0	3	1	1	1	1557	165.6	5.1
NP_003602.1	4503179	OFD1	oral-facial-digital syndrome 1 protein	1.0	1.1	1.0	1.2	-	-	-	-	1.0	1.1	1.0	1.2	1.0	1	1	1	1	1012	116.6	6.1
NP_005390.1	4885561	PRKAB2	5'-AMP-activated protein kinase subunit beta-2	1.0	1.1	1.0	1.2	-	-	-	-	1.0	1.1	1.0	1.2	9.6	1	1	2	3	272	30.3	6.5
NP_689474.1	22748615	TMEM263	transmembrane protein 263	1.1	1.1	1.2	1.2	-	-	-	-	1.1	1.1	1.2	1.2	13.8	2	1	1	2	116	11.7	9.3
NP_001007028.1	57165415	ALG8	probable dolichyl pyrophosphate Glc1Man9GlcNAc2 alpha-1,3-glucosyltransferase isoform b	0.9	1.0	1.0	1.2	-	-	-	-	0.9	1.0	1.0	1.2	3.0	2	1	1	1	467	53.1	9.2
NP_149988.1	15082242	C5orf30	UNC119-binding protein C5orf30	1.1	1.3	1.1	1.2	-	-	-	-	1.1	1.3	1.1	1.2	3.9	1	1	1	1	206	23.1	9.5
NP_660288.1	21687020	EVI5L	EVI5-like protein isoform 2	1.1	1.1	1.0	1.2	-	-	-	-	1.1	1.1	1.0	1.2	3.4	2	2	2	2	794	91.3	5.3
NP_689862.1	22749323	MLKL	mixed lineage kinase domain-like protein isoform 1	1.0	1.1	1.3	1.2	-	-	-	-	1.0	1.1	1.3	1.2	4.3	1	1	1	2	471	54.4	8.8
NP_004863.2	20070191	TMEM59	transmembrane protein 59 precursor	1.2	1.1	1.0	1.2	-	-	-	-	1.2	1.1	1.0	1.2	12.1	1	2	2	2	323	36.2	5.1
NP_002504.2	37693993	NME3	nucleoside diphosphate kinase 3 precursor	1.1	1.1	1.2	1.2	-	-	-	-	1.1	1.1	1.2	1.2	25.4	1	4	4	12	169	19.0	7.8
NP_620693.1	20544145	CSNK1D	casein kinase I isoform delta isoform 2	0.8	0.9	1.1	1.2	-	-	-	-	0.8	0.9	1.1	1.2	5.9	3	2	2	3	409	46.8	9.6
NP_060627.2	95113666	RIC8B	synembryn-B	1.1	1.1	1.0	1.2	-	-	-	-	1.1	1.1	1.0	1.2	5.6	1	1	2	2	520	58.8	5.7
NP_005237.2	119964721	FER	tyrosine-protein kinase Fer	1.3	1.2	1.2	1.2	-	-	-	-	1.3	1.2	1.2	1.2	1.0	1	1	1	1	822	94.6	7.1
NP_060342.2	89242148	THG1L	probable tRNA(His) guanylyltransferase	0.9	1.1	1.0	1.2	-	-	-	-	0.9	1.1	1.0	1.2	3.7	1	1	1	2	298	34.8	8.0
NP_001164433.1	283436133	IGSF1	immunoglobulin superfamily member 1 isoform 4	1.2	1.7	1.6	1.2	-	-	-	-	1.2	1.7	1.6	1.2	3.6	3	2	2	3	1327	147.9	5.8
NP_001017961.1	63003928	FAM78B	protein FAM78B	1.0	1.0	1.0	1.2	-	-	-	-	1.0	1.0	1.0	1.2	2.7	1	1	1	2	261	29.8	9.4
NP_079455.3	113722116	PLXNA2	plexin-A2 precursor	0.9	1.2	1.2	1.2	-	-	-	-	0.9	1.2	1.2	1.2	1.0	1	1	1	2	1894	211.0	6.5
NP_079402.2	37595537	RNF34	E3 ubiquitin-protein ligase RNF34 isoform 2	0.9	1.1	1.3	1.2	-	-	-	-	0.9	1.1	1.3	1.2	7.5	2	1	1	1	372	41.6	5.0
NP_001137313.1	219802797	TMEM106C	transmembrane protein 106C isoform b	1.0	1.1	1.0	1.2	-	-	-	-	1.0	1.1	1.0	1.2	7.4	2	1	1	3	231	25.8	6.8
NP_055056.3	82659105	HACD1	very-long-chain (3R)-3-hydroxyacyl-CoA dehydratase 1	1.2	1.1	1.0	1.2	-	-	-	-	1.2	1.1	1.0	1.2	4.2	1	1	1	1	288	32.4	9.0
NP_940927.2	203096856	KIF7	kinesin-like protein KIF7	1.1	1.0	1.0	1.2	-	-	-	-	1.1	1.0	1.0	1.2	2.0	1	2	2	3	1343	150.5	6.8
NP_060599.1	8922498	PNPO	pyridoxine-5'-phosphate oxidase	1.2	1.2	1.1	1.2	-	-	-	-	1.2	1.2	1.1	1.2	7.3	1	1	1	2	261	30.0	7.1
NP_689449.1	23238226	CDC42EP1	cdc42 effector protein 1	1.2	1.3	1.1	1.2	-	-	-	-	1.2	1.3	1.1	1.2	7.2	1	1	1	2	391	40.3	7.2
NP_001275540.1	568599844	HN1	hematological and neurological expressed 1 protein isoform 4	1.1	1.1	1.0	1.2	-	-	-	-	1.1	1.1	1.0	1.2	9.2	3	1	1	1	120	12.7	8.8
NP_001248767.1	387912557	PTER	phosphotriesterase-related protein isoform 3	1.0	1.1	1.0	1.2	-	-	-	-	1.0	1.1	1.0	1.2	4.5	3	1	1	1	200	22.6	7.0
NP_056369.1	51317380	GLCE	D-glucuronyl C5-epimerase	0.8	1.1	0.8	1.2	-	-	-	-	0.8	1.1	0.8	1.2	2.8	1	1	1	1	617	70.1	9.0
NP_002609.1	4505723	PEX13	peroxisome biogenesis factor 13	0.9	1.1	0.9	1.2	-	-	-	-	0.9	1.1	0.9	1.2	5.2	1	1	1	2	403	44.1	8.0
NP_001166102.1	289547517	TECPR2	tectonin beta-propeller repeat-containing protein 2 isoform 2	1.2	1.0	0.9	1.2	-	-	-	-	1.2	1.0	0.9	1.2	3.0	2	2	2	4	1267	137.9	5.5
NP_940890.3	157311635	FAM83H	protein FAM83H	1.0	1.1	1.2	1.2	-	-	-	-	1.0	1.1	1.2	1.2	1.8	1	1	1	2	1179	127.1	7.0
NP_004590.2	27477113	SREBF2	sterol regulatory element-binding protein 2	1.1	1.2	1.1	1.2	-	-	-	-	1.1	1.2	1.1	1.2	1.3	1	1	1	3	1141	123.6	8.4
NP_690865.1	23097246	PYDC1	pyrin domain-containing protein 1	1.4	1.4	1.3	1.2	-	-	-	-	1.4	1.4	1.3	1.2	12.4	1	1	1	1	89	10.1	6.8
NP_789784.2	39753953	ZNF800	zinc finger protein 800	1.1	1.1	1.1	1.2	-	-	-	-	1.1	1.1	1.1	1.2	4.1	1	2	2	3	664	75.2	9.5
NP_056506.2	38570149	WWC3	protein WWC3	1.4	1.4	1.3	1.2	-	-	-	-	1.4	1.4	1.3	1.2	1.0	1	1	1	1	1092	122.6	6.4
NP_006667.3	183397134;183396780	USP20	ubiquitin carboxyl-terminal hydrolase 20	1.1	1.1	1.0	1.2	-	-	-	-	1.1	1.1	1.0	1.2	1.3	1	1	1	2	914	101.9	6.1
NP_071745.1	11641261;194306629	ERAP2	endoplasmic reticulum aminopeptidase 2	0.9	1.1	1.3	1.2	-	-	-	-	0.9	1.1	1.3	1.2	1.6	1	2	2	2	960	110.4	6.7
NP_001265222.1	507834146	ARL6	ADP-ribosylation factor-like protein 6	1.2	1.2	1.1	1.2	-	-	-	-	1.2	1.2	1.1	1.2	10.2	1	1	1	2	186	21.1	8.5
NP_001124195.1	195539349	AHCYL2	putative adenosylhomocysteinase 3 isoform d	1.0	1.1	1.1	1.2	-	-	-	-	1.0	1.1	1.1	1.2	12.6	4	1	6	15	508	56.9	7.1
NP_001284635.1	663071061	ADGRL2	latrophilin-2 isoform 3 preproprotein	0.8	1.1	1.0	1.2	-	-	-	-	0.8	1.1	1.0	1.2	1.7	3	1	1	1	1123	126.1	6.9
NP_001230086.1	341926283	TAF1C	TATA box-binding protein-associated factor RNA polymerase I subunit C isoform 4	1.0	1.2	1.1	1.2	-	-	-	-	1.0	1.2	1.1	1.2	2.1	4	1	1	1	537	58.4	8.8
NP_001735.1	4502557	CAMK4	calcium/calmodulin-dependent protein kinase type IV	1.0	1.0	1.1	1.2	-	-	-	-	1.0	1.0	1.1	1.2	1.7	2	1	1	1	473	51.9	5.8
NP_001129522.1	209862901	DHRS1	dehydrogenase/reductase SDR family member 1	1.0	1.0	1.2	1.2	-	-	-	-	1.0	1.0	1.2	1.2	6.1	1	1	1	1	313	33.9	7.8
NP_073571.1	12232389	METTL17	methyltransferase-like protein 17, mitochondrial isoform 2 precursor	1.1	1.1	1.1	1.2	-	-	-	-	1.1	1.1	1.1	1.2	5.9	1	1	1	1	456	50.7	9.3
NP_683723.2	156671217	EPN2	epsin-2 isoform a	1.1	1.2	1.2	1.2	-	-	-	-	1.1	1.2	1.2	1.2	1.7	2	1	1	2	584	62.3	7.5
NP_942153.1	38570109	EFCAB6	EF-hand calcium-binding domain-containing protein 6 isoform b	1.0	1.2	1.0	1.2	-	-	-	-	1.0	1.2	1.0	1.2	1.1	2	1	1	1	1349	155.7	8.1
NP_001273740.1	558472816	LOC10099669	uncharacterized protein LOC100996693	1.0	1.1	1.1	1.1	-	-	-	-	1.0	1.1	1.1	1.1	2.5	2	1	1	1	238	26.1	5.6
NP_981951.1	42822880	MBLAC2	metallo-beta-lactamase domain-containing protein 2	1.3	1.1	1.1	1.1	-	-	-	-	1.3	1.1	1.1	1.1	7.5	1	1	1	1	279	31.3	6.9
NP_001073919.1	122937295	BEND3	BEN domain-containing protein 3	1.0	1.1	1.3	1.1	-	-	-	-	1.0	1.1	1.3	1.1	2.9	1	1	1	2	828	94.4	5.4
NP_001093859.1	154240704	TMEM192	transmembrane protein 192	1.0	1.1	1.0	1.1	-	-	-	-	1.0	1.1	1.0	1.1	8.9	1	2	2	4	271	30.9	8.0
NP_071386.3	268607677	ZNF667	zinc finger protein 667	1.0	1.2	1.2	1.1	-	-	-	-	1.0	1.2	1.2	1.1	1.3	1	1	1	1	610	70.1	9.7
NP_057726.4	485837026	SPTBN5	spectrin beta chain, non-erythrocytic 5	0.9	1.1	1.1	1.1	-	-	-	-	0.9	1.1	1.1	1.1	0.3	1	1	2	3	3674	416.5	6.7
NP_001258763.1	427197591	RSRC1	serine/Arginine-related protein 53 isoform 2	1.0	1.1	1.3	1.1	-	-	-	-	1.0	1.1	1.3	1.1	5.8	2	1	1	2	276	31.5	10.7
NP_066552.2	222080062	NDUFV2	NADH dehydrogenase [ubiquinone] flavoprotein 2, mitochondrial precursor	1.1	1.1	1.0	1.1	-	-	-	-	1.1	1.1	1.0	1.1	7.6	1	1	1	2	249	27.4	8.1
NP_001129611.1	209915594	TCF3	transcription factor E2-alpha isoform E47	0.8	1.0	0.8	1.1	-	-	-	-	0.8	1.0	0.8	1.1	2.2	2	1	1	2	651	67.2	6.7
NP_001124147.1	194578888;4758004	CLGN	calmegin precursor	0.9	1.1	1.2	1.1	-	-	-	-	0.9	1.1	1.2	1.1	5.9	1	3	4	4	610	70.0	4.7
NP_056504.3	154813195	DENND2A	DENN domain-containing protein 2A	1.0	1.0	1.1	1.1	-	-	-	-	1.0	1.0	1.1	1.1	1.7	3	1	2	3	1009	113.8	9.0
NP_001264984.1	491227401	SACS	sacsin isoform 2	1.1	1.2	1.1	1.1	-	-	-	-	1.1	1.2	1.1	1.1	0.2	2	1	1	1	4432	504.2	7.0
NP_001155899.1	241666464	TSC1	hamartin isoform 4	0.9	1.2	1.2	1.1	-	-	-	-	0.9	1.2	1.2	1.1	1.3	3	1	1	1	1113	123.9	6.2

Khan *et al.* , 2019. Multi-omics analysis to characterize cigarette smoke induced molecular alterations in esophageal cells
Supplementary Table 5. List of proteins quantified in untreated and chronically treated Het1A cells with cigarette smoke condensate for 8 months

NP_Accession	Protein group Accession	Gene ID	Description	Het-1A-Smoke - 2M/Parental	Het-1A-Smoke - 4M/Parental	Het-1A-Smoke - 6M/Parental	Het-1A-Smoke - 8M/Parental	Het-1A-Smoke - 2M/Parental	Het-1A-Smoke - 4M/Parental	Het-1A-Smoke - 6M/Parental	Het-1A-Smoke - 8M/Parental	Het-1A-Smoke - 2M/Parental	Het-1A-Smoke - 4M/Parental	Het-1A-Smoke - 6M/Parental	Het-1A-Smoke - 8M/Parental	Coverage (%)	Proteins	Unique Peptides	# Peptides	PSM	# AAs	MW [kDa]	calc. pI
				Replicate 1				Replicate 2				Average of replicates											
NP_001070148.1	116008154	LYRM9	LYR motif-containing protein 9	1.0	0.8	1.0	1.1	-	-	-	-	1.0	0.8	1.0	1.1	7.7	1	1	1	1	78	9.4	9.7
NP_001180404.1	301500682	PPP1R21	protein phosphatase 1 regulatory subunit 21 isoform 5	1.2	1.3	1.0	1.1	-	-	-	-	1.2	1.3	1.0	1.1	3.9	3	1	1	1	738	83.6	7.2
NP_005116.1	4826665	CCS	copper chaperone for superoxide dismutase	1.2	1.3	1.2	1.1	-	-	-	-	1.2	1.3	1.2	1.1	4.7	1	1	1	3	274	29.0	5.6
NP_060972.3	94536840	TMCO6	transmembrane and coiled-coil domain-containing protein 6	1.2	1.1	1.0	1.1	-	-	-	-	1.2	1.1	1.0	1.1	5.3	1	1	1	1	493	54.4	5.8
NP_065812.1	22094987	RPTOR	regulatory-associated protein of mTOR isoform 1	1.2	1.1	1.1	1.1	-	-	-	-	1.2	1.1	1.1	1.1	1.8	2	2	2	5	1335	148.9	6.9
NP_116195.2	31542693	POMGNT2	protein O-linked-mannose beta-1,4-N-acetylglucosaminyltransferase 2 precursor	1.2	1.1	1.2	1.1	-	-	-	-	1.2	1.1	1.2	1.1	7.6	1	2	2	6	580	66.6	8.6
NP_055299.1	7657339	MOCS3	adenylyltransferase and sulfurtransferase MOCS3	1.0	1.0	1.0	1.1	-	-	-	-	1.0	1.0	1.0	1.1	10.9	1	4	4	9	460	49.6	6.2
NP_003176.2	110832843	TAF4	transcription initiation factor TFIID subunit 4	1.2	1.0	1.1	1.1	-	-	-	-	1.2	1.0	1.1	1.1	3.0	2	2	2	3	1085	110.0	9.9
NP_059118.2	223278387	CALML5	calmodulin-like protein 5	1.4	1.3	1.1	1.1	-	-	-	-	1.4	1.3	1.1	1.1	5.5	1	1	1	1	146	15.9	4.4
NP_000194.2	110611239	IDUA	alpha-L-iduronidase precursor	0.9	1.1	1.0	1.1	-	-	-	-	0.9	1.1	1.0	1.1	3.7	1	1	1	1	653	72.6	9.1
NP_112494.3	148612831	KIF18A	kinesin-like protein KIF18A	1.0	1.2	1.2	1.1	-	-	-	-	1.0	1.2	1.2	1.1	2.3	1	2	2	2	898	102.2	8.9
NP_004098.1	4758346;209862839	FCGRT	IgG receptor FcRn large subunit p51 precursor	1.0	0.9	1.0	1.1	-	-	-	-	1.0	0.9	1.0	1.1	11.0	1	2	2	3	365	39.7	6.5
NP_149989.2	226495417	CCDC102A	coiled-coil domain-containing protein 102A	1.2	1.1	1.1	1.1	-	-	-	-	1.2	1.1	1.1	1.1	1.1	1	1	1	1	550	62.6	5.6
NP_853516.1	31559821	GJC3	gap junction gamma-3 protein	1.3	1.2	1.0	1.1	-	-	-	-	1.3	1.2	1.0	1.1	2.2	1	1	1	1	279	31.3	9.2
NP_055836.1	150170718	ZNF292	zinc finger protein 292	1.1	1.1	1.0	1.1	-	-	-	-	1.1	1.1	1.0	1.1	2.6	1	4	4	5	2723	304.6	7.4
NP_849144.2	38490688	IGSF10	immunoglobulin superfamily member 10 isoform 1 precursor	1.1	1.1	0.9	1.1	-	-	-	-	1.1	1.1	0.9	1.1	1.4	1	1	2	2	2623	290.7	9.1
NP_001257413.1	394953947	T	brachyury protein isoform 2	1.1	1.1	1.2	1.1	-	-	-	-	1.1	1.1	1.2	1.1	2.7	2	1	1	1	377	41.1	6.6
NP_733830.1	25777680	RASSF1	ras association domain-containing protein 1 isoform B	1.0	1.1	1.2	1.1	-	-	-	-	1.0	1.1	1.2	1.1	4.2	4	1	1	3	189	21.9	9.9
NP_005839.3	221307566	DENND4A	C-myc promoter-binding protein isoform 2	1.0	1.1	1.2	1.1	-	-	-	-	1.0	1.1	1.2	1.1	0.3	2	1	1	1	1863	209.1	7.3
NP_060620.2	46397375	RNF220	E3 ubiquitin-protein ligase RNF220	1.1	1.1	1.2	1.1	-	-	-	-	1.1	1.1	1.2	1.1	2.5	1	1	1	2	566	62.7	6.0
NP_055612.2	256600206	ZBTB24	zinc finger and BTB domain-containing protein 24 isoform 1	0.9	1.0	0.9	1.1	-	-	-	-	0.9	1.0	0.9	1.1	2.9	2	2	2	2	697	78.2	7.6
NP_055224.1	7657439	TAF5L	TAF5-like RNA polymerase II p300/CBP-associated factor-associated factor 65 kDa subunit 5L isoform a	1.1	1.0	1.1	1.1	-	-	-	-	1.1	1.0	1.1	1.1	1.7	1	1	1	1	589	66.1	5.9
NP_714923.1	24308516	TTL	tubulin--tyrosine ligase	1.2	1.0	1.1	1.1	-	-	-	-	1.2	1.0	1.1	1.1	7.4	1	1	1	2	377	43.2	6.7
NP_004536.2	38569473	NDUFB1	NADH dehydrogenase [ubiquinone] 1 beta subcomplex subunit 1	1.3	0.9	1.1	1.1	-	-	-	-	1.3	0.9	1.1	1.1	10.5	1	1	1	2	105	11.9	8.7
NP_001034806.1	89363030	C4orf29	uncharacterized protein C4orf29 precursor	1.0	1.0	1.1	1.1	-	-	-	-	1.0	1.0	1.1	1.1	1.5	1	1	1	1	414	46.9	9.3
NP_055903.2	148491080	TNRC6B	trinucleotide repeat-containing gene 6B protein isoform 2	1.0	1.3	1.2	1.1	-	-	-	-	1.0	1.3	1.2	1.1	1.1	2	1	1	2	1723	182.7	7.0
NP_060338.3	90669931	TTC12	tetratricopeptide repeat protein 12	1.0	0.9	1.0	1.1	-	-	-	-	1.0	0.9	1.0	1.1	2.3	1	2	2	2	705	78.7	5.7
NP_001268660.1	528881078	CERS5	ceramide synthase 5 isoform 2	1.1	1.2	1.0	1.1	-	-	-	-	1.1	1.2	1.0	1.1	2.1	2	1	1	1	334	39.2	8.6
NP_001258757.1	425905331	PRELID1	PRELI domain-containing protein 1, mitochondrial isoform 2 precursor	0.9	1.3	1.1	1.1	-	-	-	-	0.9	1.3	1.1	1.1	3.9	2	1	1	2	208	24.1	9.7
NP_598003.2	40255009	SLC30A7	zinc transporter 7	1.2	1.2	1.2	1.1	-	-	-	-	1.2	1.2	1.2	1.1	6.9	1	2	2	5	376	41.6	6.9
NP_004555.1	4758894	GATB	glutamyl-tRNA(Gln) amidotransferase subunit B, mitochondrial precursor	1.2	1.1	1.1	1.1	-	-	-	-	1.2	1.1	1.1	1.1	1.4	1	1	1	3	557	61.8	8.6
NP_001276972.1	586597929	TAP2	antigen peptide transporter 2 isoform 3	0.9	1.1	1.1	1.1	-	-	-	-	0.9	1.1	1.1	1.1	2.9	3	2	2	4	686	75.6	8.0
NP_065789.1	55741641	KIDINS220	kinase D-interacting substrate of 220 kDa	1.3	1.3	1.3	1.1	-	-	-	-	1.3	1.3	1.3	1.1	2.0	4	4	4	5	1771	196.4	6.6
NP_005789.2	47132521	TRIM13	E3 ubiquitin-protein ligase TRIM13 isoform 1	1.1	1.2	1.1	1.1	-	-	-	-	1.1	1.2	1.1	1.1	3.4	2	1	1	4	407	47.0	6.0
NP_065978.1	24308257	VAT1L	synaptic vesicle membrane protein VAT-1 homolog-like	1.2	1.3	1.3	1.1	-	-	-	-	1.2	1.3	1.3	1.1	6.4	1	2	2	3	419	45.9	5.1
NP_085133.1	29825823	HID1	protein HID1	1.3	1.2	1.3	1.1	-	-	-	-	1.3	1.2	1.3	1.1	1.4	1	1	1	2	788	88.7	6.1
NP_001128127.1	197383784	ZNF213	zinc finger protein 213	1.2	1.2	1.2	1.1	-	-	-	-	1.2	1.2	1.2	1.1	4.1	1	1	1	1	459	51.2	6.9
NP_612377.4	379317153	AP5B1	AP-5 complex subunit beta-1	1.1	1.0	1.0	1.1	-	-	-	-	1.1	1.0	1.0	1.1	1.4	1	1	1	2	878	93.9	5.9
NP_000850.1	4557643	HMGCR	3-hydroxy-3-methylglutaryl-Coenzyme A reductase isoform 1	1.2	1.1	1.1	1.1	-	-	-	-	1.2	1.1	1.1	1.1	4.3	2	3	3	5	888	97.4	6.7
NP_001257973.1	402534522	NFIX	nuclear factor 1 X-type isoform 3	1.0	1.1	1.1	1.1	-	-	-	-	1.0	1.1	1.1	1.1	6.0	3	2	2	3	433	47.9	8.5
NP_066575.2	188528626	ZNF8	zinc finger protein 8	1.1	1.2	1.0	1.1	-	-	-	-	1.1	1.2	1.0	1.1	1.7	1	1	1	2	575	64.9	7.4
NP_001193867.1	332635045	FAM115A	protein FAM115A isoform 2	1.0	1.1	1.1	1.1	-	-	-	-	1.0	1.1	1.1	1.1	8.7	3	6	6	11	919	101.8	6.5
NP_001020016.1	67782317	SLC6A9	sodium- and chloride-dependent glycine transporter 1 isoform 3	1.1	1.2	1.0	1.1	-	-	-	-	1.1	1.2	1.0	1.1	1.9	4	1	1	1	633	70.5	7.9
NP_777589.2	54607110	ZMIZ2	zinc finger MIZ domain-containing protein 2 isoform 2	1.1	1.0	1.1	1.1	-	-	-	-	1.1	1.0	1.1	1.1	1.6	4	2	2	2	894	93.9	7.2
NP_001159898.1	261878584	WDR13	WD repeat-containing protein 13 isoform 2	1.1	1.2	1.1	1.1	-	-	-	-	1.1	1.2	1.1	1.1	6.6	2	2	2	4	393	43.0	7.6
NP_001269335.1	538920129	KCTD14	BTB/POZ domain-containing protein KCTD14 isoform 2	1.1	1.2	1.2	1.1	-	-	-	-	1.1	1.2	1.2	1.1	16.9	2	3	3	3	225	26.2	7.4
NP_001265215.1	506949368	RPE	ribulose-phosphate 3-epimerase isoform 4	1.2	1.2	1.0	1.1	-	-	-	-	1.2	1.2	1.0	1.1	5.6	4	1	1	1	160	17.6	6.2
NP_001185909.1	312176435	SMAP2	stromal membrane-associated protein 2 isoform 4	1.2	1.1	1.0	1.1	-	-	-	-	1.2	1.1	1.0	1.1	4.9	4	1	1	2	349	37.7	9.1
NP_060867.2	217272839	CHDH	choline dehydrogenase, mitochondrial	1.3	1.1	1.0	1.1	-	-	-	-	1.3	1.1	1.0	1.1	2.0	1	1	1	1	594	65.3	8.3
NP_006334.2	66932918	MERTK	tyrosine-protein kinase Mer precursor	1.2	1.0	1.3	1.1	-	-	-	-	1.2	1.0	1.3	1.1	0.7	1	1	1	1	999	110.2	5.8
NP_112589.1	13876384	CDCA3	cell division cycle-associated protein 3 isoform 1	0.8	1.1	1.1	1.1	-	-	-	-	0.8	1.1	1.1	1.1	11.6	4	2	2	3	268	29.0	6.4
NP_742067.3	160948610	UBR3	E3 ubiquitin-protein ligase UBR3	1.1	1.2	1.3	1.1	-	-	-	-	1.1	1.2	1.3	1.1	1.1	1	1	1	1	1888	212.3	6.1
NP_683711.1	23200012	ABHD11	alpha/beta hydrolase domain-containing protein 11 isoform 2	1.2	1.2	1.2	1.1	-	-	-	-	1.2	1.2	1.2	1.1	3.3	2	1	1	2	308	33.8	9.2
NP_037469.2	56606061	TIMM22	mitochondrial import inner membrane translocase subunit Tim22	1.1	1.1	1.0	1.1	-	-	-	-	1.1	1.1	1.0	1.1	7.7	1	1	1	2	194	20.0	7.6
NP_001290024.1	734520325;13569960	ISG20L2	interferon-stimulated 20 kDa exonuclease-like 2	0.9	0.9	0.9	1.1	-	-	-	-	0.9	0.9	0.9	1.1	7.1	1	2	2	3	353	39.1	9.9
NP_006755.4	197333755	IFRD2	interferon-related developmental regulator 2	1.0	1.2	1.1	1.1	-	-	-	-	1.0	1.2	1.1	1.1	16.2	1	5	5	19	506	54.8	7.9
NP_005065.2	154800443	SLC17A1	sodium-dependent phosphate transport protein 1	1.0	1.2	1.1	1.1	-	-	-	-	1.0	1.2	1.1	1.1	1.3	1	1	1	1	467	51.1	8.6
NP_056391.1	34577114	ERC2	ERC protein 2	0.9	1.1	1.0	1.1	-	-	-	-	0.9	1.1	1.0	1.1	1.3	4	1	1	2	957	110.5	7.0
NP_776171.1	28603842	TTC9C	tetratricopeptide repeat protein 9C	1.0	0.9	1.0	1.1	-	-	-	-	1.0	0.9	1.0	1.1	4.7	1	1	1	2	171	20.0	8.9
NP_001278225.1	604723339	KPTN	kaptin isoform 2	0.9	1.1	1.0	1.1	-	-	-	-	0.9	1.1	1.0	1.1	6.6	2	2	2	2	380	42.0	5.4
NP_060272.3	157388904	DNAAF5	dynein assembly factor 5, axonemal	1.1	1.2	1.1	1.1	-	-	-	-	1.1	1.2	1.1	1.1	12.6	1	6	7	14	855	93.5	6.4
NP_076999.2	223555955	DSCC1	sister chromatid cohesion protein DCC1	1.0	1.1	1.0	1.1	-	-	-	-	1.0	1.1	1.0	1.1	11.2	1	3	3	7	393	44.8	5.2
NP_006253.2	21264345	PRPH	peripherin	0.9	1.0	0.9	1.1	-	-	-	-	0.9	1.0	0.9	1.1	8.3	1	1	5	42	470	53.6	5.5
NP_060091.1	8923019	ALKBH4	alpha-ketoglutarate-dependent dioxygenase alkB homolog 4	1.0	1.2	1.0	1.1	-	-	-	-	1.0	1.2	1.0	1.1	9.3	1	1	1	2	302	33.8	6.7
NP_680544.1	22325370	RPP25L	ribonuclease P protein subunit p25-like protein	1.1	1.1	1.1	1.1	-	-	-	-	1.1	1.1	1.1	1.1	19.6	1	2	2	4	163	17.6	10.3
NP_060844.2	40254893	TMEM106B	transmembrane protein 106B	0.9	0.9	1.0	1.1	-	-	-	-	0.9	0.9	1.0	1.1	3.7	1	1	1	2	274	31.1	7.0
NP_116072.2	116235468	MPV17L2	mpv17-like protein 2 precursor	1.3	1.2	1.1	1.1	-	-	-	-	1.3	1.2	1.1	1.1	7.8	1	1	1	3	206	23.2	9.2
NP_001070254.1	115647981	QSER1	glutamine and serine-rich protein 1	1.0	1.0	1.0	1.1	-	-	-	-	1.0	1.0	1.0	1.1	1.2	1	1	1	1	1735	189.9	7.1
NP_005698.1	22027541	PDCD7	programmed cell death protein 7	1.0	0.9	1.0	1.1	-	-	-	-	1.0	0.9	1.0	1.1	7.8	1	2	2	2	485	54.7	9.9
NP_689547.2	38570111	TARSL2	probable threonine--tRNA ligase 2, cytoplasmic	1.2	1.1	1.1	1.1	-	-	-	-	1.2	1.1	1.1	1.1	5.2	1	1	4	7	802	92.6	6.0
NP_996769.3	327315395	CCDC18	coiled-coil domain-containing protein 18 isoform b	0.7	0.8	0.8	1.1	-	-	-	-	0.7	0.8	0.8	1.1	0.5	2	1	1	1	1299	151.2	5.6
NP_006209.2	54792082	PIK3CA	phosphatidylinositol 4,5-bisphosphate 3-kinase catalytic subunit alpha isoform	1.2	1.1	1.1	1.1	-	-	-	-	1.2	1.1	1.1	1.1	0.8	1	1	1	2	1068	124.2	7.2
NP_000679.1	4502025;40316939	ALAS1	5-aminolevulinate synthase, nonspecific, mitochondrial precursor	1.1	1.3	1.1	1.1	-	-	-	-	1.1	1.3	1.1	1.1	11.3	2	4	4	5	640	70.5	8.5
NP_065901.1	40538736	RANBP10	ran-binding protein 10	1.1	1.2	1.2	1.1	-	-	-	-	1.1	1.2	1.2	1.1	1.6	1	1	1	1	620	67.2	6.8
NP_775837.2	209969819	PRR14L	protein PRR14L	1.2	1.1	1.1	1.1	-	-	-	-	1.2	1.1	1.1	1.1	0.5	1	1	1	1	2151	237.2	6.3
NP_055592.2	197333811	URB2	unhealthy ribosome biogenesis protein 2 homolog	1.0	1.0	1.2	1.1	-	-	-	-	1.0	1.0	1.2	1.1	1.8	1	2	2	5	1524	170.4	7.3
NP_997229.2	108389176	TSEN54	tRNA-splicing endonuclease subunit Sen54	1.1	1.3	1.0	1.1	-	-	-	-	1.1	1.3	1.0	1.1	1.9	1	1	1	1	526	58.8	7.9
NP_009208.2	31083351	EXOC3	exocyst complex component 3	1.1	1.1	1.1	1.1	-	-	-	-	1.1	1.1	1.1	1.1	9.4	1	5	5	9	745	85.5	6.3
NP_001275661.1	570359581;570359584	TEX2	testis-expressed sequence 2 protein isoform 2	1.1	0.9	1.1	1.1	-	-	-	-	1.1	0.9	1.1	1.1	2.7	2	2	2	3	1127	125.2	6.0
NP_006348.1	5454146	UBE2E3	ubiquitin-conjugating enzyme E2 E3	0.9	1.0	1.0	1.1	-	-	-	-	0.9	1.0	1.0	1.1	32.4	3	3	4	11	207	22.9	7.2
NP_001026997.1	73622130	BOLA2	BolA-like protein 2	1.1	1.0	1.1	1.1	-	-	-	-	1.1	1.0	1.1	1.1	5.9	1	1	1	1	152	16.9	8.2
NP_004676.3	134142348	MTMR6	myotubularin-related protein 6	1.0	1.3	1.1	1.1	-	-	-	-	1.0	1.3	1.1	1.1	5.5	1	3	3	3	621	71.9	7.7
NP_036514.1	7110679	TTC33	tetratricopeptide repeat protein 33	1.1	1.1	1.2	1.1	-	-	-	-	1.1	1.1	1.2	1.1	6.9	1	1	1	1	262	29.4	5.4
NP_056101.5	112382239	SYNM	synemin isoform B	1.1	1.0	1.1	1.1	-	-	-	-	1.1	1.0	1.1	1.1	1.4	2	1	1	2	1253	140.1	5.1
NP_005995.2	83627705	UQCRH	cytochrome b-c1 complex subunit 6, mitochondrial isoform 1 precursor	1.1	1.0	1.2	1.1	-	-	-	-	1.1	1.0	1.2	1.1	56.0	4	3	3	11	91	10.7	4.4

Supplementary Table 5. List of proteins quantified in untreated and chronically treated Het1A cells with cigarette smoke condensate for 8 months

NP_Accession	Protein group Accession	Gene ID	Description	Het-1A-Smoke - 2M/Parental	Het-1A-Smoke - 4M/Parental	Het-1A-Smoke - 6M/Parental	Het-1A-Smoke - 8M/Parental	Het-1A-Smoke - 2M/Parental	Het-1A-Smoke - 4M/Parental	Het-1A-Smoke - 6M/Parental	Het-1A-Smoke - 8M/Parental	Het-1A-Smoke - 2M/Parental	Het-1A-Smoke - 4M/Parental	Het-1A-Smoke - 6M/Parental	Het-1A-Smoke - 8M/Parental	Coverage (%)	Proteins	Unique Peptides	# Peptides	PSM	# AAs	MW [kDa]	calc. pI
				Replicate 1				Replicate 2				Average of replicates											
NP_055603.2	15208663	TRIM14	tripartite motif-containing protein 14	0.9	1.0	1.0	1.1	-	-	-	-	0.9	1.0	1.0	1.1	2.5	1	1	1	2	442	49.7	7.9
NP_068375.3	17978477	VPS11	vacuolar protein sorting-associated protein 11 homolog isoform 1	1.0	1.1	1.0	1.1	-	-	-	-	1.0	1.1	1.0	1.1	2.0	2	2	2	2	941	107.7	7.0
NP_001139150.1	224589077	KIAA0825	uncharacterized protein KIAA0825 isoform 1	1.1	1.1	1.3	1.1	-	-	-	-	1.1	1.1	1.3	1.1	1.1	1	1	1	1	1275	147.7	6.6
NP_056075.1	52138513	SIN3B	paired amphipathic helix protein Sin3b isoform 1	1.2	1.2	1.0	1.1	-	-	-	-	1.2	1.2	1.0	1.1	1.5	2	1	1	3	1162	133.0	6.9
NP_001189478.1	321400122	YNJ2BP-COX	SYNJ2BP-COX16 protein isoform 3	0.9	1.0	1.0	1.1	-	-	-	-	0.9	1.0	1.0	1.1	12.0	6	2	2	2	158	17.9	5.5
NP_663162.1	21735584	OSBPL3	oxysterol-binding protein-related protein 3 isoform d	1.0	0.9	1.2	1.1	-	-	-	-	1.0	0.9	1.2	1.1	4.8	4	3	3	3	820	93.7	6.5
NP_001291931.1	756761748	ITFG1	T-cell immunomodulatory protein isoform 2	1.1	1.2	1.0	1.1	-	-	-	-	1.1	1.2	1.0	1.1	2.6	2	1	1	2	464	52.1	5.4
NP_569712.2	110611233	COL18A1	collagen alpha-1(XVIII) chain isoform 2 precursor	1.0	1.4	1.1	1.1	-	-	-	-	1.0	1.4	1.1	1.1	2.3	2	1	1	2	1336	135.4	6.5
NP_001269720.1	544583552	PUM2	pumilio homolog 2 isoform 4	1.1	1.0	1.0	1.1	-	-	-	-	1.1	1.0	1.0	1.1	4.7	4	1	4	9	929	99.6	7.3
NP_001166.3	56676393	ARHGDIB	rho GDP-dissociation inhibitor 2	1.0	1.2	1.6	1.1	-	-	-	-	1.0	1.2	1.6	1.1	4.5	1	1	1	1	201	23.0	5.2
NP_005569.1	5031887	LPP	lipoma-preferred partner isoform a	1.0	1.1	1.1	1.1	-	-	-	-	1.0	1.1	1.1	1.1	6.5	2	2	2	2	612	65.7	7.4
NP_963920.1	42516561	USP33	ubiquitin carboxyl-terminal hydrolase 33 isoform 3	1.0	1.1	1.1	1.1	-	-	-	-	1.0	1.1	1.1	1.1	1.5	3	1	1	2	828	93.9	6.4
NP_612373.2	40538772	PRMT9	putative protein arginine N-methyltransferase 9	1.7	1.1	1.0	1.1	-	-	-	-	1.7	1.1	1.0	1.1	4.3	1	2	2	4	845	94.4	5.2
NP_000610.2	56786138	IFNG	interferon gamma precursor	0.9	1.0	1.0	1.1	-	-	-	-	0.9	1.0	1.0	1.1	3.6	1	1	1	3	166	19.3	9.5
NP_054868.3	222080092	WDR91	WD repeat-containing protein 91	1.0	1.0	0.9	1.1	-	-	-	-	1.0	1.0	0.9	1.1	2.3	1	1	1	2	747	83.3	6.6
NP_066362.2	148612842	IFITM3	interferon-induced transmembrane protein 3	0.9	1.0	0.9	1.1	-	-	-	-	0.9	1.0	0.9	1.1	30.8	3	2	2	3	133	14.6	7.0
NP_001139196.1	224809419	HOMER3	homer protein homolog 3 isoform 3	1.0	0.8	0.9	1.1	-	-	-	-	1.0	0.8	0.9	1.1	5.9	3	1	1	1	325	35.9	5.4
NP_005680.1	9955963	ABCB6	ATP-binding cassette sub-family B member 6, mitochondrial	1.1	1.1	1.0	1.1	-	-	-	-	1.1	1.1	1.0	1.1	3.3	1	2	2	3	842	93.8	8.5
NP_001165882.1	289547194	VANGL1	vang-like protein 1 isoform 2	1.1	1.3	1.3	1.1	-	-	-	-	1.1	1.3	1.3	1.1	2.1	2	1	1	2	522	59.7	8.8
NP_055918.2	157694524	PLXND1	plexin-D1 precursor	1.3	1.5	1.5	1.1	-	-	-	-	1.3	1.5	1.5	1.1	0.5	1	1	1	1	1925	211.9	7.2
NP_001120925.1	188536092	DFNA5	non-syndromic hearing impairment protein 5 isoform a	1.1	1.0	1.0	1.1	-	-	-	-	1.1	1.0	1.0	1.1	14.1	2	5	5	5	496	54.5	5.2
NP_006360.3	38569471	LRRC41	leucine-rich repeat-containing protein 41	1.1	1.2	1.1	1.1	-	-	-	-	1.1	1.2	1.1	1.1	8.1	1	5	5	7	812	88.6	8.4
NP_066926.1	10863895	TMSB10	thymosin beta-10	1.2	1.0	1.3	1.1	-	-	-	-	1.2	1.0	1.3	1.1	15.9	2	1	1	1	44	5.0	5.4
NP_002832.3	194097398	PTPRG	receptor-type tyrosine-protein phosphatase gamma	1.0	1.0	1.1	1.1	-	-	-	-	1.0	1.0	1.1	1.1	1.5	1	1	1	1	1445	161.9	6.4
NP_054900.2	156151366	LGALSL	galectin-related protein	1.1	0.9	0.9	1.1	-	-	-	-	1.1	0.9	0.9	1.1	6.4	1	1	1	2	172	19.0	5.4
NP_001027017.1	73623028	CPT1A	carnitine O-palmitoyltransferase 1, liver isoform isoform 2	1.0	1.0	1.0	1.1	-	-	-	-	1.0	1.0	1.0	1.1	2.3	2	1	1	2	756	86.2	8.4
NP_065130.1	9966875	CAMK1D	calcium/calmodulin-dependent protein kinase type 1D isoform 1	0.9	1.0	1.0	1.1	-	-	-	-	0.9	1.0	1.0	1.1	5.0	3	1	1	8	357	40.2	6.6
NP_001273719.1	557948059	CNOT6L	CCR4-NOT transcription complex subunit 6-like	1.0	1.0	1.0	1.1	-	-	-	-	1.0	1.0	1.0	1.1	4.1	2	2	2	3	555	63.0	6.7
NP_001284480.1	662033883	TMEM128	transmembrane protein 128 isoform 1	1.2	1.0	1.0	1.1	-	-	-	-	1.2	1.0	1.0	1.1	7.9	1	1	1	4	165	18.8	6.8
NP_001245.1	4502703	CDC6	cell division control protein 6 homolog	1.0	1.0	1.0	1.1	-	-	-	-	1.0	1.0	1.0	1.1	3.8	1	2	2	4	560	62.7	9.6
NP_057588.1	7706057	MRPL27	39S ribosomal protein L27, mitochondrial	0.9	1.1	1.0	1.1	-	-	-	-	0.9	1.1	1.0	1.1	17.6	1	2	2	3	148	16.1	10.4
NP_054742.2	41281564	WDR37	WD repeat-containing protein 37	1.2	1.2	1.1	1.1	-	-	-	-	1.2	1.2	1.1	1.1	2.6	1	1	1	4	494	54.6	7.2
NP_001159921.1	262050538	ITIH4	inter-alpha-trypsin inhibitor heavy chain H4 isoform 2 precursor	1.2	1.3	1.1	1.1	-	-	-	-	1.2	1.3	1.1	1.1	3.1	2	1	1	1	900	99.8	6.5
NP_061940.1	9506611	GNL3L	guanine nucleotide-binding protein-like 3-like protein	1.0	1.0	1.1	1.1	-	-	-	-	1.0	1.0	1.1	1.1	4.5	1	2	2	5	582	65.5	8.4
NP_001136017.1	217035081	ADCK1	uncharacterized aarF domain-containing protein kinase 1 isoform b	1.2	1.1	1.0	1.1	-	-	-	-	1.2	1.1	1.0	1.1	3.5	2	1	1	1	455	52.0	8.7
NP_002479.1	4505355	NDUFA2	NADH dehydrogenase [ubiquinone] 1 alpha subcomplex subunit 2 isoform 1	1.0	1.1	1.2	1.1	-	-	-	-	1.0	1.1	1.2	1.1	21.2	1	1	1	1	99	10.9	9.6
NP_001153245.1	229577444;20270339	CANT1	soluble calcium-activated nucleotidase 1	1.1	1.0	0.9	1.1	-	-	-	-	1.1	1.0	0.9	1.1	4.7	1	2	2	3	401	44.8	6.1
NP_002263.3	331999954	KRT4	keratin, type II cytoskeletal 4	1.5	1.8	0.9	1.1	-	-	-	-	1.5	1.8	0.9	1.1	9.2	4	1	5	21	520	56.1	6.6
NP_699204.1	24119277	PHYKPL	5-phosphohydroxy-L-lysine phospho-lyase isoform 2	1.1	1.1	1.0	1.1	-	-	-	-	1.1	1.1	1.0	1.1	2.0	1	1	1	1	450	49.7	6.8
NP_001036005.1	110349727	NCBP2	nuclear cap-binding protein subunit 2 isoform 2	1.3	1.1	1.1	1.1	-	-	-	-	1.3	1.1	1.1	1.1	10.7	2	1	1	1	103	11.9	8.9
NP_789790.2	33519450	NLRP9	NACHT, LRR and PYD domains-containing protein 9	1.2	1.2	1.1	1.1	-	-	-	-	1.2	1.2	1.1	1.1	1.2	1	1	1	1	991	113.2	6.5
NP_060395.5	408684407	DENND4C	DENN domain-containing protein 4C	1.0	1.1	1.0	1.1	-	-	-	-	1.0	1.1	1.0	1.1	1.6	1	2	2	2	1909	212.6	6.8
NP_001017920.2	217272872	DAPL1	death-associated protein-like 1	6.5	1.3	1.1	1.1	-	-	-	-	6.5	1.3	1.1	1.1	12.2	1	1	1	1	107	11.9	10.0
NP_055063.1	7657508	RBX1	E3 ubiquitin-protein ligase RBX1	0.9	1.1	1.0	1.1	-	-	-	-	0.9	1.1	1.0	1.1	5.6	1	1	1	1	108	12.3	7.0
NP_001035375.2	256818782	PAPD5	non-canonical poly(A) RNA polymerase PAPD5 isoform b	1.2	1.0	1.1	1.1	-	-	-	-	1.2	1.0	1.1	1.1	7.4	2	3	3	5	651	70.8	9.5
NP_078854.1	13375756	OCEL1	occludin/ELL domain-containing protein 1	1.1	1.1	1.2	1.1	-	-	-	-	1.1	1.1	1.2	1.1	3.0	1	1	1	1	264	29.4	10.0
NP_057410.1	7705933	CKLF	chemokine-like factor isoform c	1.0	1.1	1.2	1.1	-	-	-	-	1.0	1.1	1.2	1.1	16.4	4	1	1	2	67	7.6	9.8
NP_006867.1	5802984	B3GNT1	N-acetyllactosaminide beta-1,3-N-acetylglucosaminyltransferase	1.4	1.1	1.1	1.1	-	-	-	-	1.4	1.1	1.1	1.1	3.9	1	1	1	2	415	47.1	7.2
NP_001177255.1	298358598	DGCR8	microprocessor complex subunit DGCR8 isoform 2	1.1	1.4	1.2	1.1	-	-	-	-	1.1	1.4	1.2	1.1	6.8	2	2	2	2	740	82.7	5.9
NP_001248351.1	387527991	OS9	protein OS-9 isoform 7 precursor	1.2	1.0	1.0	1.1	-	-	-	-	1.2	1.0	1.0	1.1	12.0	8	4	4	5	560	63.1	4.7
NP_006312.1	5453557	ARIH2	E3 ubiquitin-protein ligase ARIH2	1.1	1.0	1.1	1.1	-	-	-	-	1.1	1.0	1.1	1.1	16.0	1	5	5	18	493	57.8	5.6
NP_115970.2	19923646	FAM126A	hyccin	1.0	1.0	1.2	1.1	-	-	-	-	1.0	1.0	1.2	1.1	3.8	1	2	2	4	521	57.6	8.2
NP_001186794.1	315434259	KIF16B	kinesin-like protein KIF16B isoform 3	0.9	1.1	0.9	1.0	-	-	-	-	0.9	1.1	0.9	1.0	1.6	7	1	2	4	1266	146.2	6.2
NP_068839.1	11527402	ITM2B	integral membrane protein 2B	1.1	1.1	1.1	1.0	-	-	-	-	1.1	1.1	1.1	1.0	8.3	1	2	2	2	266	30.3	5.1
NP_899064.1	39930541	C9orf142	protein PAXX	1.0	1.0	1.0	1.0	-	-	-	-	1.0	1.0	1.0	1.0	13.2	1	1	1	2	204	21.6	5.5
NP_932155.1	37594457	NUDT9	ADP-ribose pyrophosphatase, mitochondrial isoform b	0.9	0.9	1.1	1.0	-	-	-	-	0.9	0.9	1.1	1.0	3.7	3	1	1	1	300	33.8	6.8
NP_004066.1	4758072	CRY1	cryptochrome-1	0.9	1.0	1.0	1.0	-	-	-	-	0.9	1.0	1.0	1.0	3.8	3	2	2	2	586	66.4	8.0
NP_001650.1	4502203	ARF3	ADP-ribosylation factor 3	1.1	1.0	1.1	1.0	-	-	-	-	1.1	1.0	1.1	1.0	72.9	10	4	9	110	181	20.6	7.4
NP_001190984.1	323098337	NDUFC2	NADH dehydrogenase [ubiquinone] 1 subunit C2 isoform 3	1.2	1.0	1.2	1.0	-	-	-	-	1.2	1.0	1.2	1.0	8.3	2	1	1	1	96	11.4	8.1
NP_055620.1	7662294	EPM2AIP1	EPM2A-interacting protein 1	0.8	1.0	0.8	1.0	-	-	-	-	0.8	1.0	0.8	1.0	5.8	1	2	2	2	607	70.3	6.1
NP_710156.3	205360989	C2orf69	UPF0565 protein C2orf69 precursor	0.9	0.9	0.9	1.0	-	-	-	-	0.9	0.9	0.9	1.0	4.2	1	1	1	4	385	43.4	8.1
NP_006217.3	254939661	PLCL1	inactive phospholipase C-like protein 1	1.0	1.0	1.2	1.0	-	-	-	-	1.0	1.0	1.2	1.0	1.0	1	1	1	1	1095	122.6	5.6
NP_612635.3	226371708	EXOC3L2	exocyst complex component 3-like protein 2	1.0	0.9	1.0	1.0	-	-	-	-	1.0	0.9	1.0	1.0	2.7	1	1	1	1	409	45.8	7.7
NP_665862.1	22208951	ASB3	ankyrin repeat and SOCS box protein 3 isoform b	0.8	0.7	0.8	1.0	-	-	-	-	0.8	0.7	0.8	1.0	2.5	3	1	1	2	445	49.6	6.2
NP_115907.2	38570073	COL25A1	collagen alpha-1(XXV) chain isoform 2	1.0	1.0	1.0	1.0	-	-	-	-	1.0	1.0	1.0	1.0	2.0	2	1	1	1	642	63.6	8.4
NP_001276326.1	574957245	SMARCA2	probable global transcription activator SNF2L2 isoform c	1.0	1.2	1.1	1.0	-	-	-	-	1.0	1.2	1.1	1.0	4.2	6	3	7	13	1514	172.5	7.1
NP_078800.3	148839292	ATP13A3	probable cation-transporting ATPase 13A3	0.9	1.0	1.1	1.0	-	-	-	-	0.9	1.0	1.1	1.0	1.1	1	1	1	1	1226	138.0	6.6
NP_612401.2	223005950	SHKBP1	SH3KBP1-binding protein 1	0.9	1.1	1.0	1.0	-	-	-	-	0.9	1.1	1.0	1.0	3.3	1	1	1	2	707	76.3	8.3
NP_065690.2	41327754	RIPK4	receptor-interacting serine/threonine-protein kinase 4	0.9	0.9	0.9	1.0	-	-	-	-	0.9	0.9	0.9	1.0	1.2	1	1	1	1	784	86.4	7.1
NP_061174.1	24308179	CTTNBP2NL	CTTNBP2 N-terminal-like protein	1.0	1.1	1.1	1.0	-	-	-	-	1.0	1.1	1.1	1.0	1.4	1	1	1	1	639	70.1	8.1
NP_005025.1	4826924	POLR2K	DNA-directed RNA polymerases I, II, and III subunit RPABC4	0.9	0.8	0.9	1.0	-	-	-	-	0.9	0.8	0.9	1.0	12.1	1	1	1	2	58	7.0	9.1
NP_078799.2	22208850	GCC1	GRIP and coiled-coil domain-containing protein 1	1.0	0.9	0.9	1.0	-	-	-	-	1.0	0.9	0.9	1.0	1.2	1	1	1	1	775	87.8	5.5
NP_057623.2	300797577	SIRT6	NAD-dependent protein deacetylase sirtuin-6 isoform 1	1.0	1.0	1.0	1.0	-	-	-	-	1.0	1.0	1.0	1.0	10.1	2	3	3	3	355	39.1	9.2
NP_795361.1	28882043	NR2C2AP	nuclear receptor 2C2-associated protein	0.9	1.1	0.9	1.0	-	-	-	-	0.9	1.1	0.9	1.0	8.6	1	1	1	1	139	15.9	6.2
NP_115736.1	14150169	ACBD6	acyl-CoA-binding domain-containing protein 6	1.2	1.1	1.1	1.0	-	-	-	-	1.2	1.1	1.1	1.0	6.7	1	1	1	1	282	31.1	5.1
NP_065878.1	149944410	KIAA1430	UPF0501 protein KIAA1430 isoform 1	1.1	1.2	1.2	1.0	-	-	-	-	1.1	1.2	1.2	1.0	1.7	1	1	1	1	532	59.4	9.1
NP_000503.1	4503899	GALNS	N-acetylgalactosamine-6-sulfatase precursor	1.0	0.9	1.0	1.0	-	-	-	-	1.0	0.9	1.0	1.0	4.6	1	1	1	1	522	58.0	6.7
NP_001153172.1	228480207	FHL1	four and a half LIM domains protein 1 isoform 2	1.2	1.2	1.2	1.0	-	-	-	-	1.2	1.2	1.2	1.0	2.9	3	1	1	1	280	31.9	8.3
NP_612385.2	226498382	TTC5	tetratricopeptide repeat protein 5	1.1	1.0	1.0	1.0	-	-	-	-	1.1	1.0	1.0	1.0	4.6	1	2	2	3	440	48.9	6.5
NP_001138671.1	223633952	C12orf75	overexpressed in colon carcinoma 1 protein	1.0	1.2	1.0	1.0	-	-	-	-	1.0	1.2	1.0	1.0	34.9	1	1	1	1	63	6.4	6.6
NP_001271224.1	546231996	CHPF2	chondroitin sulfate glucuronyltransferase isoform 2	0.9	0.9	0.9	1.0	-	-	-	-	0.9	0.9	0.9	1.0	1.6	2	1	1	1	764	85.1	7.9
NP_001274324.1	565671697	MROH2A	maestro heat-like repeat-containing protein family member 2A	0.9	0.9	0.9	1.0	-	-	-	-	0.9	0.9	0.9	1.0	0.4	1	1	1	1	1688	191.1	6.9
NP_624361.1	21264575	ARID1A	AT-rich interactive domain-containing protein 1A isoform b	1.0	1.0	1.0	1.0	-	-	-	-	1.0	1.0	1.0	1.0	0.8	2	2	2	2	2068	218.2	6.5
NP_004709.2	18105037	COX7A2L	cytochrome c oxidase subunit 7A-related protein, mitochondrial	1.1	1.0	1.0	1.0	-	-	-	-	1.1	1.0	1.0	1.0	19.3	1	2	2	4	114	12.6	9.4
NP_001269881.1	545478937	COPS7B	COP9 signalosome complex subunit 7b isoform e	1.0	0.9	1.0	1.0	-	-	-	-	1.0	0.9	1.0	1.0	11.5	6	1	1	2	157	18.1	7.3
NP_115652.2	33300639	RHBDD1	rhomboid-related protein 4	1.0	1.1	1.0	1.0	-	-	-	-	1.0	1.1	1.0	1.0	2.9	1	1	1	1	315	35.8	8.2
NP_078849.1	13375746	C6orf211	UPF0364 protein C6orf211 isoform a	0.9	1.0	0.9	1.0	-	-	-	-	0.9	1.0	0.9	1.0	19.7	2	7	7	20	441	51.1	5.8
NP_660341.2	22907044	APOBEC3F	DNA dC->dU-editing enzyme APOBEC-3F isoform a	1.8	1.1	1.2	1.0	-	-	-	-	1.8	1.1	1.2	1.0	8.6	1	2	2	2	373	45.0	7.2
NP_001293066.1	807066289	TDRD15	tudor domain-containing protein 15	0.9	1.2	1.0	1.0	-	-	-	-	0.9	1.2	1.0	1.0	0.5	1	1	1	1	1934	221.6	7.0

Khan *et al.* , 2019. Multi-omics analysis to characterize cigarette smoke induced molecular alterations in esophageal cells
Supplementary Table 5. List of proteins quantified in untreated and chronically treated Het1A cells with cigarette smoke condensate for 8 months

NP_Accession	Protein group Accession	Gene ID	Description	Het-1A-Smoke - 2M/Parental	Het-1A-Smoke - 4M/Parental	Het-1A-Smoke - 6M/Parental	Het-1A-Smoke - 8M/Parental	Het-1A-Smoke - 2M/Parental	Het-1A-Smoke - 4M/Parental	Het-1A-Smoke - 6M/Parental	Het-1A-Smoke - 8M/Parental	Het-1A-Smoke - 2M/Parental	Het-1A-Smoke - 4M/Parental	Het-1A-Smoke - 6M/Parental	Het-1A-Smoke - 8M/Parental	Coverage (%)	Proteins	Unique Peptides	# Peptides	PSM	# AAs	MW [kDa]	calc. pI
				Replicate 1				Replicate 2				Average of replicates											
NP_001295098.1	815659680	**ACSM2A**	acyl-coenzyme A synthetase ACSM2A, mitochondrial isoform 2	1.0	1.1	0.9	1.0	-	-	-	-	1.0	1.1	0.9	1.0	2.6	4	1	1	1	498	55.0	7.4
NP_940869.2	158534055	**RSBN1L**	round spermatid basic protein 1-like protein	1.0	1.1	1.0	1.0	-	-	-	-	1.0	1.1	1.0	1.0	1.2	1	1	1	1	846	94.8	8.8
NP_004380.2	55770846	**CTNNA2**	catenin alpha-2 isoform 1	0.9	1.0	1.0	1.0	-	-	-	-	0.9	1.0	1.0	1.0	8.3	6	2	7	22	905	100.4	6.0
NP_878908.4	261337183	**WASH1**	WAS protein family homolog 1	1.2	1.1	1.0	1.0	-	-	-	-	1.2	1.1	1.0	1.0	8.8	1	2	3	10	465	50.3	5.7
NP_543150.1	18254476	**ASB5**	ankyrin repeat and SOCS box protein 5	1.1	1.0	1.1	1.0	-	-	-	-	1.1	1.0	1.1	1.0	3.0	1	1	1	1	329	36.3	6.8
NP_001139690.1	226246630	**WRB**	tail-anchored protein insertion receptor WRB isoform 2	1.0	1.1	1.0	1.0	-	-	-	-	1.0	1.1	1.0	1.0	5.0	2	1	1	1	140	16.0	9.8
NP_001013870.1	62243652	**EXOC6**	exocyst complex component 6 isoform b	1.1	1.0	1.0	1.0	-	-	-	-	1.1	1.0	1.0	1.0	6.3	2	3	4	5	799	93.3	6.0
NP_783859.1	148224884	**PTPMT1**	phosphatidylglycerophosphatase and protein-tyrosine phosphatase 1 isoform 1	1.1	1.1	1.0	1.0	-	-	-	-	1.1	1.1	1.0	1.0	16.9	2	3	3	13	201	22.8	9.8
NP_079114.3	153792148	**THNSL1**	threonine synthase-like 1	1.0	1.0	1.2	1.0	-	-	-	-	1.0	1.0	1.2	1.0	4.6	1	2	2	3	743	83.0	7.1
NP_008877.1	5902122	**SPTBN2**	spectrin beta chain, non-erythrocytic 2	1.2	1.0	1.1	1.0	-	-	-	-	1.2	1.0	1.1	1.0	4.7	3	2	11	31	2390	271.1	6.1
NP_079305.2	46094070	**MZT2B**	mitotic-spindle organizing protein 2B	0.9	1.0	1.0	1.0	-	-	-	-	0.9	1.0	1.0	1.0	13.3	2	2	2	2	158	16.2	10.2
NP_001136408.1	218931253	**DAGLB**	sn1-specific diacylglycerol lipase beta isoform 2	0.9	1.0	0.9	1.0	-	-	-	-	0.9	1.0	0.9	1.0	4.6	2	3	3	3	543	59.8	8.1
NP_005988.1	5174715	**VPS72**	vacuolar protein sorting-associated protein 72 homolog isoform 2	0.9	1.1	1.0	1.0	-	-	-	-	0.9	1.1	1.0	1.0	7.4	3	2	2	5	364	40.6	6.5
NP_060268.2	31377750	**YTHDF1**	YTH domain-containing family protein 1	1.1	1.0	1.1	1.0	-	-	-	-	1.1	1.0	1.1	1.0	8.2	1	1	4	18	559	60.8	8.8
NP_001289421.1	699964219	**ACP2**	lysosomal acid phosphatase isoform 6	1.0	1.1	1.0	1.0	-	-	-	-	1.0	1.1	1.0	1.0	4.7	5	1	1	2	236	26.6	5.4
NP_597719.1	33457330	**TTC14**	tetratricopeptide repeat protein 14 isoform a	0.9	0.9	1.0	1.0	-	-	-	-	0.9	0.9	1.0	1.0	2.3	1	1	1	1	770	88.3	8.6
NP_079060.1	13376146	**ZBTB3**	zinc finger and BTB domain-containing protein 3	1.2	1.1	1.2	1.0	-	-	-	-	1.2	1.1	1.2	1.0	1.7	1	1	1	2	574	61.8	5.6
NP_001093115.1	153791384	**RPL22L1**	60S ribosomal protein L22-like 1	1.3	1.2	1.2	1.0	-	-	-	-	1.3	1.2	1.2	1.0	19.7	1	1	1	1	122	14.6	9.4
NP_001013725.2	65287717	**EIF2AK4**	eukaryotic translation initiation factor 2-alpha kinase 4	1.0	1.0	1.0	1.0	-	-	-	-	1.0	1.0	1.0	1.0	1.6	1	2	2	2	1649	186.8	6.3
NP_003908.1	4503843	**AP1G2**	AP-1 complex subunit gamma-like 2 isoform 1	1.0	1.0	1.0	1.0	-	-	-	-	1.0	1.0	1.0	1.0	0.6	1	1	1	1	785	87.1	6.6
NP_038466.2	16753203	**UBQLN1**	ubiquilin-1 isoform 1	1.0	0.8	0.9	1.0	-	-	-	-	1.0	0.8	0.9	1.0	7.8	2	1	3	8	589	62.5	5.1
NP_001087225.1	148277004	**TRAPPC13**	trafficking protein particle complex subunit 13 isoform 3	1.0	0.9	1.0	1.0	-	-	-	-	1.0	0.9	1.0	1.0	1.7	4	1	1	1	411	45.9	5.7
NP_001012267.1	59709758	**CENPP**	centromere protein P isoform a	1.0	1.0	0.9	1.0	-	-	-	-	1.0	1.0	0.9	1.0	5.6	3	1	2	6	288	33.1	6.3
NP_065887.1	13124765	**BEGAIN**	brain-enriched guanylate kinase-associated protein	1.0	1.1	1.0	1.0	-	-	-	-	1.0	1.1	1.0	1.0	1.9	1	1	1	1	593	64.8	5.6
NP_149034.2	15431316	**KRT84**	keratin, type II cuticular Hb4	**2.6**	1.1	0.9	1.0	-	-	-	-	2.6	1.1	0.9	1.0	3.8	7	1	4	38	600	64.8	7.6
NP_694590.2	33356142	**C9orf91**	transmembrane protein C9orf91	1.4	1.3	1.2	1.0	-	-	-	-	1.4	1.3	1.2	1.0	9.4	1	1	1	1	342	37.5	5.3
NP_660215.1	21630277	**TRIM11**	E3 ubiquitin-protein ligase TRIM11	0.9	0.9	0.9	1.0	-	-	-	-	0.9	0.9	0.9	1.0	2.6	1	1	1	1	468	52.7	5.7
NP_001290423.1	745399100	**CBX6**	chromobox protein homolog 6 isoform 2	1.0	1.0	1.0	1.0	-	-	-	-	1.0	1.0	1.0	1.0	2.0	2	1	1	1	394	41.9	10.0
NP_003875.3	40805843	**KAT2B**	histone acetyltransferase KAT2B	1.0	1.0	1.0	1.0	-	-	-	-	1.0	1.0	1.0	1.0	1.7	2	2	2	2	832	93.0	9.0
NP_055213.2	38455385	**LAMP3**	lysosome-associated membrane glycoprotein 3 precursor	1.2	1.0	1.1	1.0	-	-	-	-	1.2	1.0	1.1	1.0	2.9	1	1	1	4	416	44.3	8.4
NP_835224.3	188528641	**SESTD1**	SEC14 domain and spectrin repeat-containing protein 1	0.9	0.9	0.9	1.0	-	-	-	-	0.9	0.9	0.9	1.0	6.0	1	2	2	2	696	79.3	5.1
NP_003232.2	156627577	**TGM4**	protein-glutamine gamma-glutamyltransferase 4	1.0	0.9	0.9	1.0	-	-	-	-	1.0	0.9	0.9	1.0	0.9	1	1	1	1	684	77.1	6.8
NP_001073918.2	320461728	**DNA2**	DNA replication ATP-dependent helicase/nuclease DNA2	**2.1**	1.8	**2.1**	1.0	-	-	-	-	2.1	1.8	2.1	1.0	1.1	1	1	1	1	1060	120.3	7.7
NP_004891.4	393715117	**APOBEC3B**	DNA dC->dU-editing enzyme APOBEC-3B isoform a	1.0	0.9	1.1	1.0	-	-	-	-	1.0	0.9	1.1	1.0	7.3	5	2	2	4	382	45.9	6.3
NP_115520.2	116875763	**RAB6C**	ras-related protein Rab-6C	0.9	0.9	0.8	1.0	-	-	-	-	0.9	0.9	0.8	1.0	15.0	2	1	3	6	254	28.3	7.7
NP_056162.4	140561070	**RIMBP2**	RIMS-binding protein 2	0.6	1.0	1.2	1.0	-	-	-	-	0.6	1.0	1.2	1.0	0.6	1	1	1	1	1052	116.0	5.3
NP_057169.2	18105012	**ATRAID**	all-trans retinoic acid-induced differentiation factor isoform a	1.3	0.9	1.2	1.0	-	-	-	-	1.3	0.9	1.2	1.0	22.8	3	1	1	1	171	18.6	7.3
XP_006726924.1	578847171	**LOC10193015**	WAS protein family homolog 6-like	1.2	1.1	1.0	1.0	-	-	-	-	1.2	1.1	1.0	1.0	6.0	1	1	2	6	451	48.3	6.0
NP_001036068.1	110618244	**KDM5A**	lysine-specific demethylase 5A	1.2	1.0	1.1	1.0	-	-	-	-	1.2	1.0	1.1	1.0	1.2	1	1	1	2	1690	192.0	6.5
NP_057174.1	9994185	**RBM7**	RNA-binding protein 7 isoform b	1.0	1.0	1.1	1.0	-	-	-	-	1.0	1.0	1.1	1.0	10.9	3	2	2	8	266	30.5	9.6
NP_037395.2	41350212	**BRD7**	bromodomain-containing protein 7 isoform 2	1.0	1.1	1.0	1.0	-	-	-	-	1.0	1.1	1.0	1.0	3.4	2	1	1	4	651	74.1	6.4
NP_076413.3	237649104	**BRD9**	bromodomain-containing protein 9 isoform 1	1.0	1.0	1.0	1.0	-	-	-	-	1.0	1.0	1.0	1.0	6.0	2	2	2	4	597	67.0	6.2
NP_001074961.1	125628632	**KRT80**	keratin, type II cytoskeletal 80 isoform K80.1	0.9	1.0	1.0	1.0	-	-	-	-	0.9	1.0	1.0	1.0	5.0	2	1	2	4	422	47.2	5.3
NP_001278947.1	635372927	**CYTH1**	cytohesin-1 isoform 3	0.9	1.0	1.0	1.0	-	-	-	-	0.9	1.0	1.0	1.0	3.2	6	1	1	1	339	39.4	7.0
NP_001287897.1	665821208	**CCDC181**	coiled-coil domain-containing protein 181 isoform a	0.9	0.9	1.0	1.0	-	-	-	-	0.9	0.9	1.0	1.0	1.2	2	1	1	1	508	59.9	5.9
NP_689502.2	206725458	**ZNF561**	zinc finger protein 561	1.1	1.0	1.0	1.0	-	-	-	-	1.1	1.0	1.0	1.0	2.1	1	1	1	1	486	55.2	8.8
NP_056103.4	40556370	**JADE2**	protein Jade-2 isoform 3	1.0	1.0	0.9	1.0	-	-	-	-	1.0	1.0	0.9	1.0	1.3	3	1	1	1	790	87.4	5.3
NP_001041714.1	115392144	**SMIM15**	small integral membrane protein 15	0.9	1.0	0.9	1.0	-	-	-	-	0.9	1.0	0.9	1.0	9.5	1	1	1	1	74	8.6	9.8
NP_078843.2	47059040	**HMBOX1**	homeobox-containing protein 1	1.1	1.0	0.9	1.0	-	-	-	-	1.1	1.0	0.9	1.0	3.8	1	1	1	1	420	47.2	6.1
NP_849150.3	155029550	**SPATA31E1**	spermatogenesis-associated protein 31E1	0.9	1.0	1.1	1.0	-	-	-	-	0.9	1.0	1.1	1.0	0.4	1	1	1	1	1445	157.0	9.1
NP_647476.1	21536359;5032147	**TAF6**	transcription initiation factor TFIID subunit 6 isoform alpha	1.1	1.0	1.1	1.0	-	-	-	-	1.1	1.0	1.1	1.0	5.6	2	3	3	3	677	72.6	8.6
NP_008966.1	5901992	**KERA**	keratocan precursor	0.5	0.8	1.2	1.0	-	-	-	-	0.5	0.8	1.2	1.0	10.5	1	1	1	1	352	40.5	7.5
NP_003604.3	192449445	**CILP**	cartilage intermediate layer protein 1 preproprotein	1.1	0.9	0.9	1.0	-	-	-	-	1.1	0.9	0.9	1.0	2.1	1	1	1	1	1184	132.5	8.3
NP_001181986.1	304282234	**DDIT3**	DNA damage-inducible transcript 3 protein isoform 2	1.0	1.1	0.9	1.0	-	-	-	-	1.0	1.1	0.9	1.0	7.1	2	1	1	1	169	19.2	4.6
NP_055679.1	7662150	**FAM20B**	glycosaminoglycan xylosylkinase	1.0	0.9	1.0	1.0	-	-	-	-	1.0	0.9	1.0	1.0	2.4	1	1	1	1	409	46.4	6.9
NP_001275882.1	572881594	**TTC7A**	tetratricopeptide repeat protein 7A isoform 3	1.2	1.0	1.1	1.0	-	-	-	-	1.2	1.0	1.1	1.0	7.0	4	2	2	3	824	92.7	6.6
NP_001273360.1	556695361	**REV3L**	DNA polymerase zeta catalytic subunit isoform b	1.0	1.0	1.1	1.0	-	-	-	-	1.0	1.0	1.1	1.0	0.2	2	1	1	1	3052	343.9	8.5
NP_060908.1	8922188	**FBXO6**	F-box only protein 6	1.1	0.9	0.8	1.0	-	-	-	-	1.1	0.9	0.8	1.0	8.9	1	1	1	1	293	33.9	6.1
NP_775105.1	27436873	**SHPRH**	E3 ubiquitin-protein ligase SHPRH isoform b	1.1	1.0	0.9	1.0	-	-	-	-	1.1	1.0	0.9	1.0	0.8	2	1	1	1	1659	190.4	7.6
NP_003174.3	73747889	**ADAM17**	disintegrin and metalloproteinase domain-containing protein 17 preproprotein	0.9	1.0	1.0	1.0	-	-	-	-	0.9	1.0	1.0	1.0	1.9	1	1	1	1	824	93.0	5.8
NP_057609.2	8394499	**UBAP1**	ubiquitin-associated protein 1 isoform 1	1.0	1.0	1.1	1.0	-	-	-	-	1.0	1.0	1.1	1.0	4.2	3	1	1	2	502	55.0	5.1
NP_079194.3	223972618;223890147	**DSN1**	kinetochore-associated protein DSN1 homolog isoform 1	0.9	0.9	0.9	1.0	-	-	-	-	0.9	0.9	0.9	1.0	6.2	3	3	3	4	356	40.0	7.0
NP_689494.2	225690529	**GORAB**	RAB6-interacting golgin isoform a precursor	1.1	1.1	1.1	1.0	-	-	-	-	1.1	1.1	1.1	1.0	4.6	1	1	1	1	394	45.0	7.1
NP_001294993.1	808688302	**INO80C**	INO80 complex subunit C isoform 3	0.9	1.0	1.0	1.0	-	-	-	-	0.9	1.0	1.0	1.0	12.4	3	1	1	1	137	15.2	9.6
NP_057407.2	110825982	**HERC5**	E3 ISG15--protein ligase HERC5	0.9	1.0	0.9	1.0	-	-	-	-	0.9	1.0	0.9	1.0	1.3	1	1	1	1	1024	116.8	7.6
NP_079461.2	157739945	**TANC2**	protein TANC2	1.0	1.0	1.1	1.0	-	-	-	-	1.0	1.0	1.1	1.0	0.4	1	1	1	1	1990	219.5	8.1
NP_008917.3	52632377	**MAGED1**	melanoma-associated antigen D1 isoform b	1.2	0.9	0.8	1.0	-	-	-	-	1.2	0.9	0.8	1.0	8.4	5	3	4	8	778	86.1	5.8
NP_612502.1	63003894	**CCDC167**	coiled-coil domain-containing protein 167	1.0	0.9	0.9	1.0	-	-	-	-	1.0	0.9	0.9	1.0	15.5	1	1	1	1	97	11.5	9.5
NP_001153844.1	238624124	**TRAPPC9**	trafficking protein particle complex subunit 9 isoform b	1.0	1.0	1.0	1.0	-	-	-	-	1.0	1.0	1.0	1.0	1.0	2	1	1	1	1148	128.4	6.6
NP_001135940.1	215599508	**C6orf203**	uncharacterized protein C6orf203 isoform a	1.1	1.0	1.1	1.0	-	-	-	-	1.1	1.0	1.1	1.0	8.3	2	2	2	3	240	27.9	9.3
NP_001129245.1	209413749	**SPATA2**	spermatogenesis-associated protein 2	1.1	1.1	1.0	1.0	-	-	-	-	1.1	1.1	1.0	1.0	2.3	1	1	1	1	520	58.4	8.7
NP_004496.2	109638751	**USP6**	ubiquitin carboxyl-terminal hydrolase 6	1.1	1.0	1.0	1.0	-	-	-	-	1.1	1.0	1.0	1.0	0.9	2	1	1	1	1406	158.6	7.7
NP_001275700.1	571026655	**C17orf80**	uncharacterized protein C17orf80 isoform d	0.9	1.0	0.8	1.0	-	-	-	-	0.9	1.0	0.8	1.0	3.3	4	1	1	1	547	60.8	9.2
NP_001166033.1	289191297	**INTS9**	integrator complex subunit 9 isoform 3	1.0	1.0	1.1	1.0	-	-	-	-	1.0	1.0	1.1	1.0	1.6	3	1	1	1	634	71.1	6.0
YP_003024027.1	251831108	**ND2**	NADH dehydrogenase subunit 2	1.0	1.0	1.0	1.0	-	-	-	-	1.0	1.0	1.0	1.0	2.6	1	1	1	2	347	38.9	9.8
NP_005397.1	4885583	**ROCK1**	rho-associated protein kinase 1	1.1	1.2	1.2	1.0	-	-	-	-	1.1	1.2	1.2	1.0	1.5	1	2	2	5	1354	158.1	5.9
NP_060464.1	8922229	**TMEM248**	transmembrane protein 248	1.2	1.1	1.0	1.0	-	-	-	-	1.2	1.1	1.0	1.0	3.2	1	1	1	2	314	35.0	6.4
NP_115788.1	14165278	**CYSTM1**	cysteine-rich and transmembrane domain-containing protein 1	1.1	1.2	1.0	1.0	-	-	-	-	1.1	1.2	1.0	1.0	10.3	1	1	1	2	97	10.6	4.3
NP_065756.1	89886453	**TBC1D24**	TBC1 domain family member 24 isoform 2	1.0	1.0	1.0	1.0	-	-	-	-	1.0	1.0	1.0	1.0	2.0	2	1	1	2	553	62.3	7.2
NP_055050.1	7657667	**UBL4A**	ubiquitin-like protein 4A	0.9	0.8	0.9	1.0	-	-	-	-	0.9	0.8	0.9	1.0	9.6	1	1	1	2	157	17.8	8.7
NP_000022.3	189083849	**ALAD**	delta-aminolevulinic acid dehydratase	1.1	1.2	1.1	1.0	-	-	-	-	1.1	1.2	1.1	1.0	5.8	1	1	1	2	330	36.3	6.8
NP_001278742.1	625180321	**SLC25A29**	mitochondrial basic amino acids transporter isoform 2	0.9	1.0	1.0	1.0	-	-	-	-	0.9	1.0	1.0	1.0	4.6	2	1	1	2	237	25.0	8.5
NP_078917.2	41393556	**MANEA**	glycoprotein endo-alpha-1,2-mannosidase	1.1	1.0	1.0	1.0	-	-	-	-	1.1	1.0	1.0	1.0	4.6	1	1	1	1	462	53.6	9.1
XP_006721060.2	767988043	**LOC400499**	putative uncharacterized protein LOC400499 isoform X2	0.8	1.0	0.9	1.0	-	-	-	-	0.8	1.0	0.9	1.0	0.2	1	1	1	1	3286	359.6	7.9
NP_005752.1	5032223	**PLXNC1**	plexin-C1 precursor	1.1	1.0	0.9	1.0	-	-	-	-	1.1	1.0	0.9	1.0	0.3	1	1	1	1	1568	175.6	7.6
NP_001129697.1	209977084	**RCOR3**	REST corepressor 3 isoform c	1.0	1.1	1.0	1.0	-	-	-	-	1.0	1.1	1.0	1.0	13.8	5	2	5	11	449	50.3	6.1
NP_001289968.1	733215354	**C7orf26**	uncharacterized protein C7orf26 isoform 2	0.9	1.1	1.1	1.0	-	-	-	-	0.9	1.1	1.1	1.0	6.3	2	2	2	5	352	39.0	6.5
NP_004832.1	4758808	**RASAL2**	ras GTPase-activating protein nGAP isoform 1	1.0	1.0	1.1	1.0	-	-	-	-	1.0	1.0	1.1	1.0	0.6	2	1	1	1	1139	128.5	8.2
NP_001274453.1	566559990	**CDC25B**	M-phase inducer phosphatase 2 isoform 9	1.3	1.0	0.9	1.0	-	-	-	-	1.3	1.0	0.9	1.0	2.3	9	1	1	1	388	44.9	5.9
NP_775935.1	27734809	**ARL10**	ADP-ribosylation factor-like protein 10	0.8	0.8	0.8	1.0	-	-	-	-	0.8	0.8	0.8	1.0	4.5	1	1	1	1	244	27.4	4.7
NP_001074002.1	122937500	**UNC119B**	protein unc-119 homolog B	1.0	1.0	1.0	1.0	-	-	-	-	1.0	1.0	1.0	1.0	22.7	3	4	4	4	251	28.1	5.7
NP_001258983.1	440546406	**MRFAP1**	MORF4 family-associated protein 1 isoform b	1.8	0.9	1.0	1.0	-	-	-	-	1.8	0.9	1.0	1.0	7.8	2	1	1	2	103	11.8	4.6
NP_078982.3	289547677	**ZNF668**	zinc finger protein 668 isoform a	0.9	1.0	0.9	1.0	-	-	-	-	0.9	1.0	0.9	1.0	2.1	2	1	1	1	619	67.9	8.9
NP_055751.1	7662358	**ENPP4**	bis(5'-adenosyl)-triphosphatase ENPP4 precursor	1.0	1.0	0.8	1.0	-	-	-	-	1.0	1.0	0.8	1.0	2.9	1	1	1	2	453	51.6	6.2
NP_067013.1	10863997	**PTBP2**	polypyrimidine tract-binding protein 2 isoform 6	1.0	0.8	0.9	1.0	-	-	-	-	1.0	0.8	0.9	1.0	6.6	7	3	4	23	531	57.5	8.7
NP_037448.2	56550057	**CNOT4**	CCR4-NOT transcription complex subunit 4 isoform a	1.0	1.0	0.9	1.0	-	-	-	-	1.0	1.0	0.9	1.0	2.0	4	1	1	1	639	70.3	7.1

Khan *et al.* , 2019. Multi-omics analysis to characterize cigarette smoke induced molecular alterations in esophageal cells
Supplementary Table 5. List of proteins quantified in untreated and chronically treated Het1A cells with cigarette smoke condensate for 8 months

NP_Accession	Protein group Accession	Gene ID	Description	Het-1A-Smoke - 2M/Parental	Het-1A-Smoke - 4M/Parental	Het-1A-Smoke - 6M/Parental	Het-1A-Smoke - 8M/Parental	Het-1A-Smoke - 2M/Parental	Het-1A-Smoke - 4M/Parental	Het-1A-Smoke - 6M/Parental	Het-1A-Smoke - 8M/Parental	Het-1A-Smoke - 2M/Parental	Het-1A-Smoke - 4M/Parental	Het-1A-Smoke - 6M/Parental	Het-1A-Smoke - 8M/Parental	Coverage (%)	Proteins	Unique Peptides	# Peptides	PSM	# AAs	MW [kDa]	calc. pI
				Replicate 1				Replicate 2				Average of replicates											
NP_001139805.1	226437577	SUMF2	sulfatase-modifying factor 2 isoform g	0.8	0.8	0.9	1.0	-	-	-	-	0.8	0.8	0.9	1.0	7.0	4	1	1	1	213	24.1	6.9
NP_054736.1	7661728	LAMTOR2	ragulator complex protein LAMTOR2 isoform 1	1.5	1.0	0.8	1.0	-	-	-	-	1.5	1.0	0.8	1.0	14.4	1	1	1	1	125	13.5	5.4
NP_005162.1	4885073	ATF1	cyclic AMP-dependent transcription factor ATF-1	0.8	0.9	0.9	1.0	-	-	-	-	0.8	0.9	0.9	1.0	8.1	1	1	3	6	271	29.2	8.4
NP_001177912.1	300244542	COQ7	ubiquinone biosynthesis protein COQ7 homolog isoform 2	1.5	1.0	0.9	1.0	-	-	-	-	1.5	1.0	0.9	1.0	14.5	2	2	2	2	179	20.1	6.0
NP_036466.2	154354979	MYO10	unconventional myosin-X	0.9	1.0	0.9	1.0	-	-	-	-	0.9	1.0	0.9	1.0	0.4	1	1	1	1	2058	237.2	6.2
NP_000775.1	4503211	CYP27A1	sterol 26-hydroxylase, mitochondrial precursor	0.9	0.7	0.6	1.0	-	-	-	-	0.9	0.7	0.6	1.0	4.5	1	1	1	2	531	60.2	8.9
NP_612426.1	19923939	KTI12	protein KTI12 homolog	1.1	1.2	1.1	1.0	-	-	-	-	1.1	1.2	1.1	1.0	8.8	1	2	2	3	354	38.6	6.9
NP_001268377.1	527122116	RBPJL	recombining binding protein suppressor of hairless-like protein isoform 2	1.0	1.2	1.1	1.0	-	-	-	-	1.0	1.2	1.1	1.0	2.8	6	1	1	1	471	51.2	8.6
NP_689676.2	260764007	EME1	crossover junction endonuclease EME1 isoform 2	1.3	0.9	1.0	1.0	-	-	-	-	1.3	0.9	1.0	1.0	4.9	2	1	1	2	570	63.2	7.0
NP_542377.1	18104950	TBX1	T-box transcription factor TBX1 isoform A	1.1	1.1	1.0	1.0	-	-	-	-	1.1	1.1	1.0	1.0	1.5	1	1	1	1	398	43.1	8.2
NP_056025.2	163644316	MTCL1	microtubule cross-linking factor 1	0.9	1.0	1.1	1.0	-	-	-	-	0.9	1.0	1.1	1.0	1.1	2	1	2	2	1586	177.8	5.9
NP_001171921.1	297139721	RNF135	E3 ubiquitin-protein ligase RNF135 isoform 3	0.9	0.9	0.9	1.0	-	-	-	-	0.9	0.9	0.9	1.0	3.5	2	1	1	1	286	31.0	6.6
NP_060242.2	40068063	TBC1D22B	TBC1 domain family member 22B	0.9	0.9	0.7	1.0	-	-	-	-	0.9	0.9	0.7	1.0	2.6	1	1	1	1	505	59.0	7.4
NP_060988.3	33383235	MCM10	protein MCM10 homolog isoform 2	1.1	1.0	0.9	1.0	-	-	-	-	1.1	1.0	0.9	1.0	1.6	2	1	1	2	874	98.0	8.7
NP_001264074.1	460417313	ZBTB10	zinc finger and BTB domain-containing protein 10 isoform c	1.3	0.9	0.8	1.0	-	-	-	-	1.3	0.9	0.8	1.0	2.1	2	1	1	1	579	64.9	5.5
NP_620278.1	20357588	TAF1	transcription initiation factor TFIID subunit 1 isoform 2	1.0	1.0	1.0	1.0	-	-	-	-	1.0	1.0	1.0	1.0	2.2	4	4	4	6	1872	212.5	5.1
NP_612355.1	25286703	KIAA2013	uncharacterized protein KIAA2013 precursor	1.0	1.0	1.1	1.0	-	-	-	-	1.0	1.0	1.1	1.0	8.0	1	3	3	7	634	69.1	8.2
NP_001184259.1	309747079	POLE2	DNA polymerase epsilon subunit 2 isoform 2	0.9	0.8	0.8	1.0	-	-	-	-	0.9	0.8	0.8	1.0	3.2	3	1	1	2	501	56.4	6.1
NP_060733.4	153792780	ASXL2	putative Polycomb group protein ASXL2	0.9	0.9	1.0	1.0	-	-	-	-	0.9	0.9	1.0	1.0	0.7	1	1	1	2	1435	153.7	8.8
NP_001138727.1	223671872	IKBKG	NF-kappa-B essential modulator isoform c	0.9	1.0	0.9	1.0	-	-	-	-	0.9	1.0	0.9	1.0	3.8	3	1	1	1	320	36.9	6.2
NP_001278203.1	602617451	HSH2D	hematopoietic SH2 domain-containing protein isoform 2	1.0	1.0	1.0	1.0	-	-	-	-	1.0	1.0	1.0	1.0	3.7	2	1	1	1	295	32.7	8.3
NP_060386.1	8923598	PIH1D1	PIH1 domain-containing protein 1	0.9	0.7	0.9	1.0	-	-	-	-	0.9	0.7	0.9	1.0	3.5	1	1	1	2	290	32.3	5.1
NP_001078868.1	146094506	RELL1	RELT-like protein 1 precursor	1.0	0.9	1.0	1.0	-	-	-	-	1.0	0.9	1.0	1.0	7.4	1	2	2	2	271	29.3	8.3
NP_000146.2	22165416	GALT	galactose-1-phosphate uridylyltransferase isoform 1	1.0	1.0	0.9	1.0	-	-	-	-	1.0	1.0	0.9	1.0	3.7	1	1	1	2	379	43.3	7.0
NP_061867.1	62955044	FBXO42	F-box only protein 42	1.1	0.9	0.9	1.0	-	-	-	-	1.1	0.9	0.9	1.0	2.8	1	2	2	3	717	77.8	7.4
NP_006446.1	5454038	P3H4	synaptonemal complex protein SC65 precursor	0.9	1.1	1.2	1.0	-	-	-	-	0.9	1.1	1.2	1.0	6.6	1	2	2	6	437	50.3	4.8
NP_001188405.1	319918864	C18orf21	UPF0711 protein C18orf21 isoform c	1.0	1.0	0.9	1.0	-	-	-	-	1.0	1.0	0.9	1.0	8.5	2	1	1	1	130	15.3	9.7
NP_001273569.1	557786105	MON1B	vacuolar fusion protein MON1 homolog B isoform 3	1.2	1.0	1.0	1.0	-	-	-	-	1.2	1.0	1.0	1.0	2.5	3	1	1	2	401	44.2	9.1
NP_542398.3	156564240	CCDC104	coiled-coil domain-containing protein 104 isoform b	1.0	1.1	1.0	1.0	-	-	-	-	1.0	1.1	1.0	1.0	26.0	2	5	5	17	342	39.4	5.0
NP_056481.1	24308117	MTG2	mitochondrial ribosome-associated GTPase 2	0.9	0.9	0.9	1.0	-	-	-	-	0.9	0.9	0.9	1.0	8.4	1	2	2	3	406	43.9	9.5
NP_037374.1	8392875	CFAP20	cilia- and flagella-associated protein 20	1.1	0.9	0.9	1.0	-	-	-	-	1.1	0.9	0.9	1.0	29.0	1	4	4	8	193	22.8	9.8
NP_001002913.1	50897284	PTRH1	probable peptidyl-tRNA hydrolase	1.2	1.0	0.9	1.0	-	-	-	-	1.2	1.0	0.9	1.0	4.7	1	1	1	1	214	22.9	10.6
NP_001017420.1	62899035	ESCO2	N-acetyltransferase ESCO2	1.0	1.0	0.8	1.0	-	-	-	-	1.0	1.0	0.8	1.0	5.7	1	2	2	3	601	68.3	9.4
NP_004873.3	40068059	CIR1	corepressor interacting with RBPJ 1	1.1	1.0	1.0	1.0	-	-	-	-	1.1	1.0	1.0	1.0	2.7	1	1	1	1	450	52.3	9.9
NP_001230332.1	343478149	CLEC16A	protein CLEC16A isoform 2	1.0	1.0	1.0	1.0	-	-	-	-	1.0	1.0	1.0	1.0	5.5	2	3	3	4	906	103.5	6.6
NP_078822.3	88759348	RNF219	RING finger protein 219	1.1	1.0	1.1	1.0	-	-	-	-	1.1	1.0	1.1	1.0	2.3	1	1	1	1	726	81.1	5.7
NP_690876.3	261878550;261878564	MTIF3	translation initiation factor IF-3, mitochondrial	1.1	1.1	1.1	1.0	-	-	-	-	1.1	1.1	1.1	1.0	8.6	1	3	3	3	278	31.7	9.7
NP_057181.1	7705819	IER3IP1	immediate early response 3-interacting protein 1 precursor	1.0	0.8	0.8	1.0	-	-	-	-	1.0	0.8	0.8	1.0	24.4	1	1	1	6	82	9.0	8.2
NP_079385.2	19923615	MYO19	unconventional myosin-XIX isoform 1	0.9	1.0	1.0	1.0	-	-	-	-	0.9	1.0	1.0	1.0	3.5	4	2	2	2	770	86.6	8.0
NP_001161414.1	269846912	TNFAIP8L1	tumor necrosis factor alpha-induced protein 8-like protein 1	1.0	1.1	1.1	1.0	-	-	-	-	1.0	1.1	1.1	1.0	10.8	1	1	1	2	186	20.8	9.5
NP_001008539.3	258614005	SLC7A2	cationic amino acid transporter 2 isoform 2	0.7	0.6	0.7	1.0	-	-	-	-	0.7	0.6	0.7	1.0	1.8	3	1	1	2	658	71.6	7.3
NP_056477.1	46358428	IFT172	intraflagellar transport protein 172 homolog	0.9	1.0	1.0	1.0	-	-	-	-	0.9	1.0	1.0	1.0	1.9	1	2	2	3	1749	197.5	6.1
NP_004725.1	4758128	DCLK1	serine/threonine-protein kinase DCLK1 isoform 1	1.0	0.9	0.9	1.0	-	-	-	-	1.0	0.9	0.9	1.0	1.5	1	1	1	1	729	81.0	8.7
NP_001229609.1	336455120	ZNF729	zinc finger protein 729	0.8	0.9	0.9	1.0	-	-	-	-	0.8	0.9	0.9	1.0	6.2	23	1	3	3	1252	144.9	9.5
NP_056120.2	224282153	ANGEL1	protein angel homolog 1 precursor	0.9	1.0	0.9	1.0	-	-	-	-	0.9	1.0	0.9	1.0	2.2	1	1	1	1	670	75.2	4.8
NP_001230574.1	343887344	CFL2	cofilin-2 isoform 2	1.0	0.9	1.0	1.0	-	-	-	-	1.0	0.9	1.0	1.0	36.9	2	1	5	33	149	16.9	8.6
NP_004533.1	4758772	NDUFA3	NADH dehydrogenase [ubiquinone] 1 alpha subcomplex subunit 3	1.4	1.3	1.3	1.0	-	-	-	-	1.4	1.3	1.3	1.0	28.6	1	1	1	1	84	9.3	8.5
NP_001273663.1	557878612	CENPE	centromere-associated protein E isoform 2	0.8	0.9	0.9	1.0	-	-	-	-	0.8	0.9	0.9	1.0	0.2	2	1	1	1	2580	301.6	5.6
NP_056161.2	38202205	ZFYVE26	zinc finger FYVE domain-containing protein 26	1.0	0.9	0.8	1.0	-	-	-	-	1.0	0.9	0.8	1.0	0.7	1	1	1	1	2539	284.4	6.4
NP_055103.3	27597075	ITGB3BP	centromere protein R isoform 2	1.0	1.1	1.0	1.0	-	-	-	-	1.0	1.1	1.0	1.0	12.4	2	1	1	1	177	20.2	9.1
NP_065145.2	31542306	CHMP1B	charged multivesicular body protein 1b	1.1	0.9	1.0	1.0	-	-	-	-	1.1	0.9	1.0	1.0	3.0	1	1	1	1	199	22.1	8.1
NP_001295166.1	815890985	ZZZ3	ZZ-type zinc finger-containing protein 3 isoform 2	0.9	1.0	1.0	1.0	-	-	-	-	0.9	1.0	1.0	1.0	2.0	2	1	1	1	409	47.7	7.7
NP_001190175.1	322506093	GPBP1	vasculin isoform 4	0.8	1.0	0.9	1.0	-	-	-	-	0.8	1.0	0.9	1.0	3.6	4	1	1	1	302	33.8	5.5
NP_002664.2	40254442	PLXNB1	plexin-B1 precursor	1.0	1.0	0.9	1.0	-	-	-	-	1.0	1.0	0.9	1.0	1.2	1	1	1	1	2135	232.2	5.5
NP_071735.2	70980547	RBSN	rabenosyn-5	1.1	1.0	1.1	1.0	-	-	-	-	1.1	1.0	1.1	1.0	1.5	1	1	1	2	784	88.8	5.5
NP_031390.1	6679189	TMED3	transmembrane emp24 domain-containing protein 3 precursor	1.0	1.0	0.9	1.0	-	-	-	-	1.0	1.0	0.9	1.0	26.3	1	4	4	7	217	24.8	5.6
NP_001240830.1	359806892	MEST	mesoderm-specific transcript homolog protein isoform d precursor	0.9	0.8	0.8	1.0	-	-	-	-	0.9	0.8	0.8	1.0	2.7	4	1	1	1	292	33.7	9.7
NP_001161870.1	270288773	SLC35A1	CMP-sialic acid transporter isoform b	0.8	1.0	0.9	1.0	-	-	-	-	0.8	1.0	0.9	1.0	2.9	2	1	1	2	278	29.9	9.1
NP_001273903.1	558695396	CTNNAL1	alpha-catulin isoform b	1.0	1.1	1.2	1.0	-	-	-	-	1.0	1.1	1.2	1.0	5.3	2	2	2	3	718	80.1	6.7
NP_055131.2	109715858	CARHSP1	calcium-regulated heat stable protein 1	0.9	0.9	0.9	1.0	-	-	-	-	0.9	0.9	0.9	1.0	5.4	1	1	1	1	147	15.9	8.2
NP_149074.3	38569495	EAF1	ELL-associated factor 1	0.9	0.9	0.9	1.0	-	-	-	-	0.9	0.9	0.9	1.0	3.0	1	1	1	1	268	29.0	5.4
NP_001244141.1	380748953	FGF1	fibroblast growth factor 1 isoform 4 precursor	1.2	1.0	1.0	1.0	-	-	-	-	1.2	1.0	1.0	1.0	7.8	2	1	1	1	154	17.3	7.0
NP_001295241.1	815930064	AP4B1	AP-4 complex subunit beta-1 isoform 3	1.0	0.8	0.9	1.0	-	-	-	-	1.0	0.8	0.9	1.0	3.7	3	1	1	4	571	64.5	5.5
NP_001184162.1	308522728	BTN2A1	butyrophilin subfamily 2 member A1 isoform 3	0.9	1.0	1.0	1.0	-	-	-	-	0.9	1.0	1.0	1.0	6.9	2	2	2	3	466	53.2	6.7
NP_079350.5	256000767	FRAS1	extracellular matrix protein FRAS1 isoform 1 precursor	1.0	0.9	0.9	0.9	-	-	-	-	1.0	0.9	0.9	0.9	0.3	1	2	2	2	4012	443.3	5.6
NP_002515.1	4505451	NRAS	GTPase NRas	1.0	1.0	1.0	0.9	-	-	-	-	1.0	1.0	1.0	0.9	52.4	5	6	6	17	189	21.2	5.2
NP_001276904.1	586798142	BAZ2B	bromodomain adjacent to zinc finger domain protein 2B isoform b	0.9	0.9	0.9	0.9	-	-	-	-	0.9	0.9	0.9	0.9	0.3	2	1	1	1	2132	236.3	6.5
NP_057485.2	21361535	C11orf73	protein Hikeshi	1.0	0.9	1.1	0.9	-	-	-	-	1.0	0.9	1.1	0.9	11.2	1	2	2	4	197	21.6	5.5
NP_001243095.1	390608639	BIRC2	baculoviral IAP repeat-containing protein 2 isoform 2	1.0	0.9	1.0	0.9	-	-	-	-	1.0	0.9	1.0	0.9	1.9	3	1	1	1	569	64.1	6.4
NP_001177720.1	299782608	ZNF317	zinc finger protein 317 isoform 2	0.9	1.0	0.9	0.9	-	-	-	-	0.9	1.0	0.9	0.9	1.2	2	1	1	1	563	64.4	9.1
NP_001036097.1	111118959	SNX21	sorting nexin-21 isoform c	0.9	1.0	0.9	0.9	-	-	-	-	0.9	1.0	0.9	0.9	4.6	4	1	1	1	152	16.3	4.3
NP_001165903.1	289063422	PHKG2	phosphorylase b kinase gamma catalytic chain, liver/testis isoform isoform 2	1.0	1.0	1.0	0.9	-	-	-	-	1.0	1.0	1.0	0.9	6.7	2	2	2	4	374	43.1	7.2
NP_671489.1	23065557	GSTM4	glutathione S-transferase Mu 4 isoform 2	1.2	1.1	1.0	0.9	-	-	-	-	1.2	1.1	1.0	0.9	26.7	4	3	4	9	195	22.8	5.1
NP_060536.3	88759337	GPN2	GPN-loop GTPase 2	1.0	0.9	0.8	0.9	-	-	-	-	1.0	0.9	0.8	0.9	2.3	1	1	1	1	310	34.5	5.2
NP_112189.2	190194420	TSPAN14	tetraspanin-14 isoform 1	1.0	1.1	1.4	0.9	-	-	-	-	1.0	1.1	1.4	0.9	10.4	2	2	2	6	270	30.7	6.8
NP_078832.1	13375721	FAM118B	protein FAM118B	1.0	1.0	0.9	0.9	-	-	-	-	1.0	1.0	0.9	0.9	4.6	1	1	1	4	351	39.5	6.0
NP_113678.2	31377667	LONP2	lon protease homolog 2, peroxisomal isoform 1	1.0	1.0	1.0	0.9	-	-	-	-	1.0	1.0	1.0	0.9	3.5	2	2	2	2	852	94.6	7.3
NP_001374.3	116686106	DPH1	diphthamide biosynthesis protein 1	0.8	1.0	1.0	0.9	-	-	-	-	0.8	1.0	1.0	0.9	1.6	1	1	1	2	443	48.8	8.2
NP_787103.1	28557745	ZADH2	zinc-binding alcohol dehydrogenase domain-containing protein 2 isoform 1	1.1	0.9	1.1	0.9	-	-	-	-	1.1	0.9	1.1	0.9	4.0	1	1	1	1	377	40.1	8.2
NP_001121190.1	189217428	LAMA3	laminin subunit alpha-3 isoform 4 precursor	1.1	1.4	1.4	0.9	-	-	-	-	1.1	1.4	1.4	0.9	0.4	4	1	1	1	1668	183.9	8.1
NP_001128626.1	205830426	SLC39A14	zinc transporter ZIP14 isoform c precursor	1.3	1.3	1.1	0.9	-	-	-	-	1.3	1.3	1.1	0.9	9.4	3	3	3	10	481	52.8	5.8
NP_001248379.1	387598085	UCK1	uridine-cytidine kinase 1 isoform c	0.9	0.9	0.8	0.9	-	-	-	-	0.9	0.9	0.8	0.9	6.7	4	1	2	3	268	30.7	7.0
NP_004661.2	34447231	PAPSS2	bifunctional 3'-phosphoadenosine 5'-phosphosulfate synthase 2 isoform a	1.1	1.0	0.8	0.9	-	-	-	-	1.1	1.0	0.8	0.9	4.6	2	2	2	3	614	69.5	8.0
NP_114432.2	301897118	BRIP1	Fanconi anemia group J protein	0.9	0.9	1.0	0.9	-	-	-	-	0.9	0.9	1.0	0.9	1.7	1	2	2	3	1249	140.8	6.9
NP_061157.3	291219911	C19orf80	betatrophin precursor	0.8	0.9	0.9	0.9	-	-	-	-	0.8	0.9	0.9	0.9	3.0	2	1	1	1	198	22.1	7.6
NP_940848.2	206725424	C1orf122	uncharacterized protein C1orf122 isoform 1	1.0	1.0	1.0	0.9	-	-	-	-	1.0	1.0	1.0	0.9	12.7	1	1	1	1	110	11.5	6.7
NP_068778.1	11386175	PPP1R11	protein phosphatase 1 regulatory subunit 11	0.9	0.9	0.9	0.9	-	-	-	-	0.9	0.9	0.9	0.9	20.6	1	1	1	1	126	13.9	7.0
NP_060380.3	222831587	TRMT61B	tRNA (adenine(58)-N(1))-methyltransferase, mitochondrial precursor	1.0	0.9	1.0	0.9	-	-	-	-	1.0	0.9	1.0	0.9	1.7	1	1	1	1	477	52.9	6.6
NP_001276324.1	574957240	TULP1	tubby-related protein 1 isoform 2	1.0	1.0	1.0	0.9	-	-	-	-	1.0	1.0	1.0	0.9	1.2	2	1	1	1	489	54.6	9.5
NP_055625.4	171184451	CEP350	centrosome-associated protein 350	1.0	1.0	1.0	0.9	-	-	-	-	1.0	1.0	1.0	0.9	1.1	1	2	2	4	3117	350.7	6.3
NP_653190.2	31542747	COMTD1	catechol O-methyltransferase domain-containing protein 1	1.1	1.0	1.0	0.9	-	-	-	-	1.1	1.0	1.0	0.9	23.7	1	2	2	2	262	28.8	8.4
NP_071327.2	56243533	SDF2L1	stromal cell-derived factor 2-like protein 1 precursor	1.1	1.0	1.2	0.9	-	-	-	-	1.1	1.0	1.2	0.9	5.9	1	1	1	2	221	23.6	7.0

Khan *et al.* , 2019. Multi-omics analysis to characterize cigarette smoke induced molecular alterations in esophageal cells
Supplementary Table 5. List of proteins quantified in untreated and chronically treated Het1A cells with cigarette smoke condensate for 8 months

NP_Accession	Protein group Accession	Gene ID	Description	Het-1A-Smoke - 2M/Parental	Het-1A-Smoke - 4M/Parental	Het-1A-Smoke - 6M/Parental	Het-1A-Smoke - 8M/Parental	Het-1A-Smoke - 2M/Parental	Het-1A-Smoke - 4M/Parental	Het-1A-Smoke - 6M/Parental	Het-1A-Smoke - 8M/Parental	Het-1A-Smoke - 2M/Parental	Het-1A-Smoke - 4M/Parental	Het-1A-Smoke - 6M/Parental	Het-1A-Smoke - 8M/Parental	Coverage (%)	Proteins	Unique Peptides	# Peptides	PSM	# AAs	MW [kDa]	calc. pI
				Replicate 1				Replicate 2				Average of replicates											
NP_001017995.1	63055059	**SH3PXD2B**	SH3 and PX domain-containing protein 2B	0.9	1.0	0.9	0.9	-	-	-	-	**0.9**	**1.0**	**0.9**	**0.9**	4.6	1	3	3	4	911	101.5	8.7
NP_068780.2	296434319	**TEAD1**	transcriptional enhancer factor TEF-1	0.9	0.9	1.0	0.9	-	-	-	-	**0.9**	**0.9**	**1.0**	**0.9**	7.8	9	4	4	10	426	47.9	8.1
NP_004188.1	4759180	**STK19**	serine/threonine-protein kinase 19 isoform 1	0.9	0.8	0.8	0.9	-	-	-	-	**0.9**	**0.8**	**0.8**	**0.9**	2.8	2	1	1	2	364	40.5	10.0
NP_056069.2	46852172	**KIF13B**	kinesin-like protein KIF13B	1.2	0.9	1.1	0.9	-	-	-	-	**1.2**	**0.9**	**1.1**	**0.9**	1.5	5	1	2	4	1826	202.7	5.9
NP_001153646.1	237649128	**NAT1**	arylamine N-acetyltransferase 1 isoform a	1.4	1.0	0.9	0.9	-	-	-	-	**1.4**	**1.0**	**0.9**	**0.9**	3.1	2	1	1	1	290	33.9	6.5
NP_006078.2	21361322	**TUBB4A**	tubulin beta-4A chain isoform 3	1.1	0.9	1.1	0.9	-	-	-	-	**1.1**	**0.9**	**1.1**	**0.9**	79.3	4	2	23	690	444	49.6	4.9
NP_000231.1	4557735	**MAOA**	amine oxidase [flavin-containing] A isoform 1	1.0	0.9	1.0	0.9	-	-	-	-	**1.0**	**0.9**	**1.0**	**0.9**	3.8	2	2	2	2	527	59.6	7.9
NP_001155048.2	341865550	**SAMD4A**	protein Smaug homolog 1 isoform 2	1.0	0.9	1.1	0.9	-	-	-	-	**1.0**	**0.9**	**1.1**	**0.9**	2.5	3	1	1	2	630	70.1	8.7
NP_000381.1	9966761	**CHM**	rab proteins geranylgeranyltransferase component A 1 isoform a	0.9	1.0	0.9	0.9	-	-	-	-	**0.9**	**1.0**	**0.9**	**0.9**	1.1	1	1	1	1	653	73.4	4.8
NP_653265.3	134288896	**FAM76B**	protein FAM76B	1.1	1.0	1.0	0.9	-	-	-	-	**1.1**	**1.0**	**1.0**	**0.9**	14.2	1	4	4	6	339	38.7	9.2
NP_001269618.1	544186119	**SECISBP2**	selenocysteine insertion sequence-binding protein 2 isoform 3	1.2	1.2	1.4	0.9	-	-	-	-	**1.2**	**1.2**	**1.4**	**0.9**	5.8	4	2	2	2	781	87.3	8.8
NP_000243.1	4557896	**MTM1**	myotubularin	1.0	1.1	0.9	0.9	-	-	-	-	**1.0**	**1.1**	**0.9**	**0.9**	3.2	1	1	1	2	603	69.9	8.2
NP_001021554.1	71051596	**CCDC7**	coiled-coil domain-containing protein 7 isoform a	0.7	0.8	0.8	0.9	-	-	-	-	**0.7**	**0.8**	**0.8**	**0.9**	1.4	2	1	1	1	486	55.7	7.8
NP_005764.2	37574603	**ZNF256**	zinc finger protein 256	0.9	0.7	0.7	0.9	-	-	-	-	**0.9**	**0.7**	**0.7**	**0.9**	2.4	1	1	1	1	627	71.8	8.8
NP_689541.1	22748717	**ADSSL1**	adenylosuccinate synthetase isozyme 1 isoform 2	0.8	0.8	0.8	0.9	-	-	-	-	**0.8**	**0.8**	**0.8**	**0.9**	5.5	2	2	2	6	457	50.2	8.6
XP_006726862.1	578846882;5803 207	**LOC10272459**	splicing factor U2AF 35 kDa subunit isoform X1	0.8	0.9	0.9	0.9	-	-	-	-	**0.8**	**0.9**	**0.9**	**0.9**	24.2	4	4	4	13	240	27.9	8.8
NP_071331.2	98986450	**CSNK1G1**	casein kinase I isoform gamma-1	0.9	0.9	0.9	0.9	-	-	-	-	**0.9**	**0.9**	**0.9**	**0.9**	4.0	8	2	2	4	422	48.5	9.0
NP_001139490.1	225690491	**TJAP1**	tight junction-associated protein 1 isoform b	0.7	0.8	0.6	0.9	-	-	-	-	**0.7**	**0.8**	**0.6**	**0.9**	2.2	2	1	1	2	547	60.7	5.9
NP_055132.2	50659086	**PDSS1**	decaprenyl-diphosphate synthase subunit 1	1.0	0.8	0.8	0.9	-	-	-	-	**1.0**	**0.8**	**0.8**	**0.9**	4.3	1	1	1	1	415	46.2	9.0
NP_001121632.1	189491632	**UBP1**	upstream-binding protein 1 isoform LBP-1a	1.0	0.9	1.0	0.9	-	-	-	-	**1.0**	**0.9**	**1.0**	**0.9**	13.3	2	3	3	4	504	56.4	6.3
NP_115548.1	79750944	**USP42**	ubiquitin carboxyl-terminal hydrolase 42	0.8	0.8	0.7	0.9	-	-	-	-	**0.8**	**0.8**	**0.7**	**0.9**	1.0	1	1	1	1	1316	144.2	8.5
NP_002822.2	18104989	**PTPN6**	tyrosine-protein phosphatase non-receptor type 6 isoform 1	1.0	0.7	0.7	0.9	-	-	-	-	**1.0**	**0.7**	**0.7**	**0.9**	2.0	3	1	1	1	595	67.5	7.8
NP_817123.1	29789445	**DSG4**	desmoglein-4 isoform 2 preproprotein	0.8	0.8	0.7	0.9	-	-	-	-	**0.8**	**0.8**	**0.7**	**0.9**	1.2	2	1	1	1	1040	113.8	4.6
NP_997174.1	46877105	**USF2**	upstream stimulatory factor 2 isoform 2	0.8	0.9	0.9	0.9	-	-	-	-	**0.8**	**0.9**	**0.9**	**0.9**	9.3	2	3	3	6	279	30.6	5.2
NP_112235.2	50428940	**MED25**	mediator of RNA polymerase II transcription subunit 25	1.0	0.9	0.8	0.9	-	-	-	-	**1.0**	**0.9**	**0.8**	**0.9**	2.8	1	1	1	1	747	78.1	8.3
NP_004043.3	734703979	**BNIP3**	BCL2/adenovirus E1B 19 kDa protein-interacting protein 3	1.1	0.9	1.0	0.9	-	-	-	-	**1.1**	**0.9**	**1.0**	**0.9**	3.1	1	1	1	1	259	27.8	7.2
NP_001121700.2	308193328	**TPRN**	taperin	0.9	1.0	0.9	0.9	-	-	-	-	**0.9**	**1.0**	**0.9**	**0.9**	2.3	1	1	1	1	711	75.5	7.3
XP_005275825.1	530356322	**LOC10106074**	3-phosphoinositide-dependent protein kinase 1-like isoform X2	1.7	1.0	1.1	0.9	-	-	-	-	**1.7**	**1.0**	**1.1**	**0.9**	7.3	7	1	1	4	218	24.6	8.8
NP_112598.3	525507390	**EPPK1**	epiplakin	0.9	0.9	1.0	0.9	-	-	-	-	**0.9**	**0.9**	**1.0**	**0.9**	3.8	1	2	3	6	5088	555.3	5.6
NP_079221.2	153791761	**RMI1**	recQ-mediated genome instability protein 1	1.0	1.0	1.0	0.9	-	-	-	-	**1.0**	**1.0**	**1.0**	**0.9**	4.5	1	2	2	3	625	70.1	5.0
XP_011546813.1	768054889	**LOC10537966**	uncharacterized protein LOC105379667	1.0	1.0	1.1	0.9	-	-	-	-	**1.0**	**1.0**	**1.1**	**0.9**	1.4	2	1	1	1	352	40.2	8.5
NP_694453.2	93277070	**RBM45**	RNA-binding protein 45	1.0	1.0	1.1	0.9	-	-	-	-	**1.0**	**1.0**	**1.1**	**0.9**	5.3	1	1	1	4	474	53.3	7.2
NP_002920.1	4506529	**GRK1**	rhodopsin kinase	1.4	1.0	0.9	0.9	-	-	-	-	**1.4**	**1.0**	**0.9**	**0.9**	2.1	1	1	1	1	563	63.5	5.9
NP_115710.2	223468687	**UTP23**	rRNA-processing protein UTP23 homolog	0.8	0.9	0.9	0.9	-	-	-	-	**0.8**	**0.9**	**0.9**	**0.9**	4.8	1	2	2	3	249	28.4	10.1
NP_660350.2	26190614	**RTKN2**	rhotekin-2 isoform 1	1.0	1.1	1.2	0.9	-	-	-	-	**1.0**	**1.1**	**1.2**	**0.9**	7.1	1	1	1	1	609	69.3	7.8
NP_003702.2	29171736	**PPAP2A**	lipid phosphate phosphohydrolase 1 isoform 1	1.0	0.9	0.9	0.9	-	-	-	-	**1.0**	**0.9**	**0.9**	**0.9**	3.5	1	1	1	1	284	32.1	8.0
NP_071905.3	68800430	**HS1BP3**	HCLS1-binding protein 3	1.0	1.2	1.2	0.9	-	-	-	-	**1.0**	**1.2**	**1.2**	**0.9**	8.9	1	1	1	1	392	42.8	5.0
NP_076933.3	226442763	**PCYOX1L**	prenylcysteine oxidase-like isoform 1 precursor	1.2	1.1	1.0	0.9	-	-	-	-	**1.2**	**1.1**	**1.0**	**0.9**	5.5	3	3	3	7	494	54.6	7.3
NP_056520.2	29648315	**SGSM3**	small G protein signaling modulator 3	1.0	0.9	1.0	0.9	-	-	-	-	**1.0**	**0.9**	**1.0**	**0.9**	0.8	1	1	1	1	749	85.3	6.0
NP_001164551.1	283806598	**RBM41**	RNA-binding protein 41 isoform 2	0.9	0.9	0.8	0.9	-	-	-	-	**0.9**	**0.9**	**0.8**	**0.9**	1.7	2	1	1	1	405	46.3	8.8
NP_000619.3	24430215	**IL10RB**	interleukin-10 receptor subunit beta precursor	0.9	0.8	0.8	0.9	-	-	-	-	**0.9**	**0.8**	**0.8**	**0.9**	4.0	1	1	1	1	325	37.0	5.2
NP_597732.1	126723390	**ANKRD24**	ankyrin repeat domain-containing protein 24	1.0	1.0	1.1	0.9	-	-	-	-	**1.0**	**1.0**	**1.1**	**0.9**	0.6	1	1	1	1	1146	124.1	5.0
NP_001171922.1	297139728	**RNF17**	RING finger protein 17 isoform 2	0.9	0.9	0.9	0.9	-	-	-	-	**0.9**	**0.9**	**0.9**	**0.9**	0.8	2	2	2	2	1619	184.1	5.4
NP_066288.2	38016945	**DEAF1**	deformed epidermal autoregulatory factor 1 homolog	0.8	0.8	1.0	0.9	-	-	-	-	**0.8**	**0.8**	**1.0**	**0.9**	3.2	1	1	1	2	565	59.3	5.3
NP_060039.1	8923735	**SUPT20H**	transcription factor SPT20 homolog isoform b	0.9	0.9	1.0	0.9	-	-	-	-	**0.9**	**0.9**	**1.0**	**0.9**	2.2	3	1	1	1	733	80.1	8.2
NP_001092102.1	148806906;4989 17186	**AKT1S1**	proline-rich AKT1 substrate 1 isoform b	0.8	0.8	1.0	0.9	-	-	-	-	**0.8**	**0.8**	**1.0**	**0.9**	7.8	2	2	2	5	256	27.4	4.8
NP_001291706.1	755571562	**DERL2**	derlin-2 isoform b	1.1	1.2	1.0	0.9	-	-	-	-	**1.1**	**1.2**	**1.0**	**0.9**	12.6	2	1	1	2	238	27.4	7.3
NP_057614.1	7706563	**RAB8B**	ras-related protein Rab-8B	0.8	0.8	0.8	0.9	-	-	-	-	**0.8**	**0.8**	**0.8**	**0.9**	42.5	11	4	9	25	207	23.6	9.1
NP_001010893.1	58219066	**SLC10A5**	sodium/bile acid cotransporter 5 precursor	1.0	1.0	1.1	0.9	-	-	-	-	**1.0**	**1.0**	**1.1**	**0.9**	1.4	1	1	1	1	438	48.8	8.8
NP_001034639.1	88501736	**DNAJB2**	dnaJ homolog subfamily B member 2 isoform a	0.8	0.8	0.7	0.9	-	-	-	-	**0.8**	**0.8**	**0.7**	**0.9**	7.9	3	1	2	16	277	30.5	5.1
NP_003712.1	4506499	**RFXANK**	DNA-binding protein RFXANK isoform a	0.9	1.0	1.0	0.9	-	-	-	-	**0.9**	**1.0**	**1.0**	**0.9**	11.2	1	1	1	1	260	28.1	4.6
NP_001161694.1	270265908	**TBC1D17**	TBC1 domain family member 17 isoform 2	0.9	0.9	0.9	0.9	-	-	-	-	**0.9**	**0.9**	**0.9**	**0.9**	9.9	2	4	4	7	615	68.9	5.1
NP_001287680.1	663429530	**LMBR1L**	protein LMBR1L isoform 3	1.2	0.8	1.2	0.9	-	-	-	-	**1.2**	**0.8**	**1.2**	**0.9**	1.9	4	1	1	2	469	53.2	7.9
NP_001269915.1	545479145	**MED30**	mediator of RNA polymerase II transcription subunit 30 isoform 2	0.9	1.1	1.0	0.9	-	-	-	-	**0.9**	**1.1**	**1.0**	**0.9**	6.3	2	1	1	2	143	16.3	9.2
NP_036477.2	166235163	**NUFIP1**	nuclear fragile X mental retardation-interacting protein 1	1.0	0.9	0.9	0.9	-	-	-	-	**1.0**	**0.9**	**0.9**	**0.9**	2.2	1	1	1	2	495	56.3	9.1
NP_002667.2	194097356	**PMM1**	phosphomannomutase 1	1.0	0.9	1.0	0.9	-	-	-	-	**1.0**	**0.9**	**1.0**	**0.9**	8.0	1	1	2	2	262	29.7	5.7
NP_079223.3	148612879	**PHC3**	polyhomeotic-like protein 3	1.0	1.0	1.1	0.9	-	-	-	-	**1.0**	**1.0**	**1.1**	**0.9**	6.4	2	2	3	7	995	107.4	6.6
NP_201576.1	58743363	**CENPL**	centromere protein L isoform 2	0.8	0.8	0.9	0.9	-	-	-	-	**0.8**	**0.8**	**0.9**	**0.9**	3.5	1	1	1	2	344	39.0	6.5
NP_064541.1	9910144	**C1GALT1**	glycoprotein-N-acetylgalactosamine 3-beta-galactosyltransferase 1	1.0	1.0	0.9	0.9	-	-	-	-	**1.0**	**1.0**	**0.9**	**0.9**	3.6	1	1	1	1	363	42.2	6.6
NP_001775.2	17978489	**CD97**	CD97 antigen isoform 2 preproprotein	0.8	0.9	0.8	0.9	-	-	-	-	**0.8**	**0.9**	**0.8**	**0.9**	2.7	3	1	1	2	742	81.7	7.0
NP_055979.2	221139728	**PLEKHM2**	pleckstrin homology domain-containing family M member 2	0.9	0.9	0.9	0.9	-	-	-	-	**0.9**	**0.9**	**0.9**	**0.9**	3.1	1	2	2	5	1019	112.7	4.9
NP_036331.1	6912352	**AGO1**	protein argonaute-1	0.9	0.9	1.0	0.9	-	-	-	-	**0.9**	**0.9**	**1.0**	**0.9**	9.5	4	3	7	11	857	97.2	9.2
NP_004654.1	4758984	**RAB11A**	ras-related protein Rab-11A isoform 1	1.1	1.2	1.1	0.9	-	-	-	-	**1.1**	**1.2**	**1.1**	**0.9**	61.6	3	1	12	50	216	24.4	6.6
NP_689592.2	63497679	**C1orf131**	uncharacterized protein C1orf131	0.8	0.8	0.8	0.9	-	-	-	-	**0.8**	**0.8**	**0.8**	**0.9**	3.8	1	1	1	2	293	32.6	9.9
NP_001245385.1	386642883	**TNIP1**	TNFAIP3-interacting protein 1 isoform 6	0.7	1.1	1.0	0.9	-	-	-	-	**0.7**	**1.1**	**1.0**	**0.9**	4.3	6	1	1	2	556	62.6	7.7
NP_001258734.1	424036676	**PFKFB1**	6-phosphofructo-2-kinase/fructose-2, 6-bisphosphatase 1 isoform 3	0.9	0.9	0.8	0.9	-	-	-	-	**0.9**	**0.9**	**0.8**	**0.9**	3.9	4	1	1	1	406	47.4	5.7
NP_689853.3	40548403	**DCP1B**	mRNA-decapping enzyme 1B	0.9	0.9	0.8	0.9	-	-	-	-	**0.9**	**0.9**	**0.8**	**0.9**	1.6	1	1	1	1	617	67.7	8.6
NP_001032252.1	81295416	**SUSD4**	sushi domain-containing protein 4 isoform b precursor	0.7	1.0	0.8	0.9	-	-	-	-	**0.7**	**1.0**	**0.8**	**0.9**	4.5	1	1	1	1	290	32.1	6.9
NP_000526.1	4505913	**PMS2**	mismatch repair endonuclease PMS2 isoform a	1.0	1.1	1.2	0.9	-	-	-	-	**1.0**	**1.1**	**1.2**	**0.9**	6.6	1	3	3	5	862	95.7	6.7
NP_058626.1	8394376	**STX18**	syntaxin-18	1.3	1.0	1.0	0.9	-	-	-	-	**1.3**	**1.0**	**1.0**	**0.9**	6.6	1	2	2	3	335	38.6	5.5
NP_004226.3	194248077	**KLF4**	Krueppel-like factor 4	1.3	1.3	1.4	0.9	-	-	-	-	**1.3**	**1.3**	**1.4**	**0.9**	4.2	1	1	1	1	479	51.0	8.4
NP_848657.1	30425534	**TSPAN33**	tetraspanin-33	0.8	0.8	0.8	0.9	-	-	-	-	**0.8**	**0.8**	**0.8**	**0.9**	5.0	1	1	1	1	283	31.5	7.2
NP_057413.2	53692193	**SFMBT1**	scm-like with four MBT domains protein 1	0.9	0.7	0.8	0.9	-	-	-	-	**0.9**	**0.7**	**0.8**	**0.9**	2.2	1	1	1	1	866	98.1	6.2
NP_001020414.1	68800350	**IRAK1**	interleukin-1 receptor-associated kinase 1 isoform 3	0.9	0.8	0.9	0.9	-	-	-	-	**0.9**	**0.8**	**0.9**	**0.9**	1.7	3	1	1	1	633	68.0	6.4
NP_002732.3	47132589	**PKN1**	serine/threonine-protein kinase N1 isoform 2	0.9	0.8	0.9	0.9	-	-	-	-	**0.9**	**0.8**	**0.9**	**0.9**	3.8	2	3	4	8	942	103.9	6.4
NP_001103444.1	158303333	**SYNPO**	synaptopodin isoform B	0.9	0.8	1.0	0.9	-	-	-	-	**0.9**	**0.8**	**1.0**	**0.9**	4.1	3	1	1	2	685	73.6	9.3
NP_001137250.1	221307473	**ASAP3**	arf-GAP with SH3 domain, ANK repeat and PH domain-containing protein 3 isoform b	1.1	1.0	1.1	0.9	-	-	-	-	**1.1**	**1.0**	**1.1**	**0.9**	0.8	2	1	1	1	894	97.8	6.4
NP_001578.2	13325070	**OCRL**	inositol polyphosphate 5-phosphatase OCRL-1 isoform b	1.2	0.9	0.9	0.9	-	-	-	-	**1.2**	**0.9**	**0.9**	**0.9**	3.6	2	3	3	5	893	103.2	6.8
NP_071347.2	37588869	**RNF123**	E3 ubiquitin-protein ligase RNF123	0.9	0.9	1.0	0.9	-	-	-	-	**0.9**	**0.9**	**1.0**	**0.9**	1.4	1	1	2	2	1314	148.4	6.7
NP_001274166.1	564730584	**COMMD5**	COMM domain-containing protein 5	0.9	0.9	0.8	0.9	-	-	-	-	**0.9**	**0.9**	**0.8**	**0.9**	6.7	1	2	2	2	224	24.7	7.0
NP_660275.2	222537719	**CTU1**	cytoplasmic tRNA 2-thiolation protein 1	1.2	0.9	1.0	0.9	-	-	-	-	**1.2**	**0.9**	**1.0**	**0.9**	4.3	1	1	1	2	348	36.4	9.2
NP_005080.1	4827046	**ZRSR2**	U2 small nuclear ribonucleoprotein auxiliary factor 35 kDa subunit-related protein 2	0.7	0.9	0.9	0.9	-	-	-	-	**0.7**	**0.9**	**0.9**	**0.9**	1.5	1	1	1	1	482	58.0	9.7
NP_008959.3	62865604	**TRIM31**	E3 ubiquitin-protein ligase TRIM31	0.8	0.9	0.8	0.9	-	-	-	-	**0.8**	**0.9**	**0.8**	**0.9**	1.4	1	1	1	1	425	48.2	7.6
NP_001191816.1	325910899	**RAB43**	ras-related protein Rab-43 isoform b	0.9	1.0	1.0	0.9	-	-	-	-	**0.9**	**1.0**	**1.0**	**0.9**	16.1	10	1	2	4	155	17.1	7.9
NP_001160006.1	262118318	**DDX4**	probable ATP-dependent RNA helicase DDX4 isoform 4	1.2	1.0	1.0	0.9	-	-	-	-	**1.2**	**1.0**	**1.0**	**0.9**	3.1	4	1	2	13	575	63.2	5.6
NP_776190.1	27883882	**SREK1IP1**	protein SREK1IP1	0.9	0.8	0.9	0.9	-	-	-	-	**0.9**	**0.8**	**0.9**	**0.9**	4.5	1	1	1	1	155	18.2	9.8
NP_689943.4	117414148	**TBC1D32**	protein broad-minded	0.9	1.0	0.9	0.9	-	-	-	-	**0.9**	**1.0**	**0.9**	**0.9**	0.5	1	1	1	1	1257	144.7	6.7
NP_660290.3	50593524	**SFR1**	swi5-dependent recombination DNA repair protein 1 homolog isoform b	0.9	0.9	0.9	0.9	-	-	-	-	**0.9**	**0.9**	**0.9**	**0.9**	12.1	2	2	2	3	232	26.7	6.8
NP_009292.1	6806898	**SNCA**	alpha-synuclein isoform NACP112	0.9	1.0	0.9	0.9	-	-	-	-	**0.9**	**1.0**	**0.9**	**0.9**	8.0	3	1	1	1	112	11.4	8.3
NP_778253.2	194018511	**KRT77**	keratin, type II cytoskeletal 1b	1.0	0.8	0.9	0.9	-	-	-	-	**1.0**	**0.8**	**0.9**	**0.9**	6.8	3	1	5	16	578	61.9	6.0

Khan *et al.* , 2019. Multi-omics analysis to characterize cigarette smoke induced molecular alterations in esophageal cells
Supplementary Table 5. List of proteins quantified in untreated and chronically treated Het1A cells with cigarette smoke condensate for 8 months

NP_Accession	Protein group Accession	Gene ID	Description	Het-1A-Smoke - 2M/Parental	Het-1A-Smoke - 4M/Parental	Het-1A-Smoke - 6M/Parental	Het-1A-Smoke - 8M/Parental	Het-1A-Smoke - 2M/Parental	Het-1A-Smoke - 4M/Parental	Het-1A-Smoke - 6M/Parental	Het-1A-Smoke - 8M/Parental	Het-1A-Smoke - 2M/Parental	Het-1A-Smoke - 4M/Parental	Het-1A-Smoke - 6M/Parental	Het-1A-Smoke - 8M/Parental	Coverage (%)	Proteins	Unique Peptides	# Peptides	PSM	# AAs	MW [kDa]	calc. pI
				Replicate 1				Replicate 2				Average of replicates											
NP_036219.2	170763506	GTF3C5	general transcription factor 3C polypeptide 5 isoform 2	0.8	0.9	0.9	0.9	-	-	-	-	0.8	0.9	0.9	0.9	13.3	3	5	5	8	519	59.5	6.9
NP_060069.3	155030244	VEZT	vezatin	0.9	1.0	1.0	0.9	-	-	-	-	0.9	1.0	1.0	0.9	1.9	1	1	1	4	779	88.6	5.2
NP_001171905.1	296923771	NCKAP1L	nck-associated protein 1-like isoform 2	0.9	1.0	1.0	0.9	-	-	-	-	0.9	1.0	1.0	0.9	1.5	2	1	1	1	1077	122.4	6.6
NP_004992.2	103472001	NDUFA7	NADH dehydrogenase [ubiquinone] 1 alpha subcomplex subunit 7	0.9	0.8	0.8	0.9	-	-	-	-	0.9	0.8	0.8	0.9	18.6	1	2	2	3	113	12.5	10.2
NP_005224.2	21361241	EPHA3	ephrin type-A receptor 3 isoform a precursor	1.1	0.9	1.0	0.9	-	-	-	-	1.1	0.9	1.0	0.9	0.6	1	1	1	1	983	110.1	6.8
NP_001036038.1	110431350	ENGASE	cytosolic endo-beta-N-acetylglucosaminidase	1.0	0.8	0.9	0.9	-	-	-	-	1.0	0.8	0.9	0.9	7.0	1	2	2	4	743	83.9	6.8
NP_001243412.1	374532802	EPHX2	bifunctional epoxide hydrolase 2 isoform c	1.1	0.8	0.7	0.9	-	-	-	-	1.1	0.8	0.7	0.9	1.4	3	1	1	1	489	55.6	6.4
NP_060401.2	116812608	TTC38	tetratricopeptide repeat protein 38	1.1	0.9	0.9	0.9	-	-	-	-	1.1	0.9	0.9	0.9	2.4	1	1	1	1	469	52.7	6.0
NP_008973.1	5902066;149192851	RPP14	ribonuclease P protein subunit p14	0.9	1.0	0.8	0.9	-	-	-	-	0.9	1.0	0.8	0.9	16.1	1	1	1	6	124	13.7	7.8
NP_001157019.1	254587994	SYNRG	synergin gamma isoform 7	1.2	0.9	1.0	0.9	-	-	-	-	1.2	0.9	1.0	0.9	2.0	7	1	1	2	1108	119.4	5.1
NP_005231.1	20270188	ETV3	ETS translocation variant 3 isoform 2	1.0	1.2	0.9	0.9	-	-	-	-	1.0	1.2	0.9	0.9	16.1	2	1	1	1	143	16.9	9.8
NP_001124535.1	197099958	SAP30L	histone deacetylase complex subunit SAP30L isoform 3	0.9	0.8	0.8	0.9	-	-	-	-	0.9	0.8	0.8	0.9	25.6	3	2	2	2	137	15.4	9.7
NP_001159750.1	261862237	DLG3	disks large homolog 3 isoform c	0.9	0.8	0.9	0.9	-	-	-	-	0.9	0.8	0.9	0.9	14.8	3	2	4	7	366	42.1	8.6
NP_004569.2	19923260	RAB4A	ras-related protein Rab-4A isoform 1	0.9	0.8	0.8	0.9	-	-	-	-	0.9	0.8	0.8	0.9	11.0	10	1	2	4	218	24.4	6.1
NP_001177910.1	300244535	IL6ST	interleukin-6 receptor subunit beta isoform 3 precursor	0.9	0.9	0.7	0.9	-	-	-	-	0.9	0.9	0.7	0.9	2.5	2	1	1	3	857	96.2	5.9
NP_001355.2	91199538	DLG2	disks large homolog 2 isoform 2	1.0	1.1	1.3	0.9	-	-	-	-	1.0	1.1	1.3	0.9	6.4	5	1	4	8	870	97.5	6.5
NP_001258152.2	404211881	OBSCN	obscurin isoform IC	1.0	0.8	0.9	0.9	-	-	-	-	1.0	0.8	0.9	0.9	0.1	1	1	1	1	8923	972.4	6.0
NP_004412.2	32479521	DVL1	segment polarity protein dishevelled homolog DVL-1	1.1	0.9	0.9	0.9	-	-	-	-	1.1	0.9	0.9	0.9	2.2	1	1	1	1	670	72.8	7.4
NP_001093858.1	154240677	C11orf88	UPF0722 protein C11orf88 isoform 2	1.6	1.1	0.7	0.9	-	-	-	-	1.6	1.1	0.7	0.9	14.8	2	1	1	1	169	19.3	8.9
NP_003784.2	6042196	CTSF	cathepsin F precursor	0.9	1.0	0.9	0.9	-	-	-	-	0.9	1.0	0.9	0.9	5.4	1	2	2	4	484	53.3	8.2
NP_997320.2	198442844	DNAH10	dynein heavy chain 10, axonemal	0.9	0.8	0.8	0.9	-	-	-	-	0.9	0.8	0.8	0.9	0.1	1	1	1	1	4471	514.5	5.9
NP_077733.3	84508631	PGS1	CDP-diacylglycerol--glycerol-3-phosphate 3-phosphatidyltransferase, mitochondrial precursor	0.9	0.9	0.8	0.9	-	-	-	-	0.9	0.9	0.8	0.9	2.5	1	1	1	1	556	62.7	8.9
NP_114171.2	56790919	GSG2	serine/threonine-protein kinase haspin	0.9	0.9	0.9	0.9	-	-	-	-	0.9	0.9	0.9	0.9	2.6	1	2	2	5	798	88.4	9.2
NP_001180486.1	302148485	ENY2	transcription and mRNA export factor ENY2 isoform 2	1.0	0.9	1.0	0.9	-	-	-	-	1.0	0.9	1.0	0.9	31.3	2	3	3	6	96	11.0	9.1
NP_001077377.1	145580586	DNAAF2	protein kintoun isoform 2	1.0	0.9	0.9	0.9	-	-	-	-	1.0	0.9	0.9	0.9	2.0	2	1	1	2	789	85.5	5.3
NP_115556.2	187936946	FAM161A	protein FAM161A isoform 2	0.9	0.9	0.9	0.9	-	-	-	-	0.9	0.9	0.9	0.9	2.4	2	1	1	2	660	76.7	8.0
NP_001138545.1	223005906	USP27X	ubiquitin carboxyl-terminal hydrolase 27	0.8	0.8	0.8	0.9	-	-	-	-	0.8	0.8	0.8	0.9	3.2	2	1	1	2	438	49.6	7.2
NP_689968.1	22749479	CNPY4	protein canopy homolog 4 precursor	1.0	1.0	1.0	0.9	-	-	-	-	1.0	1.0	1.0	0.9	5.2	1	1	1	4	248	28.3	4.6
NP_001275649.1	570359557	STAT5A	signal transducer and activator of transcription 5A isoform 3	0.8	0.8	0.8	0.9	-	-	-	-	0.8	0.8	0.8	0.9	5.2	3	1	3	5	763	87.3	6.6
NP_667339.1	23821044	ZNRF2	E3 ubiquitin-protein ligase ZNRF2	1.1	0.9	1.0	0.9	-	-	-	-	1.1	0.9	1.0	0.9	12.8	1	1	1	2	242	24.1	7.1
NP_006690.1	5729913	MAN1A2	mannosyl-oligosaccharide 1,2-alpha-mannosidase IB	1.0	1.0	1.0	0.9	-	-	-	-	1.0	1.0	1.0	0.9	3.6	1	1	1	1	641	73.0	7.6
NP_150636.1	15431332	CASP1	caspase-1 isoform delta	0.9	0.9	0.9	0.9	-	-	-	-	0.9	0.9	0.9	0.9	5.7	4	1	1	1	263	29.8	7.9
NP_115622.2	47716512	MEX3B	RNA-binding protein MEX3B	0.9	0.9	1.0	0.9	-	-	-	-	0.9	0.9	1.0	0.9	4.9	1	1	1	2	569	58.8	6.9
NP_001288579.1	675269448	R3HCC1	R3H and coiled-coil domain-containing protein 1 isoform b	1.3	1.0	0.9	0.9	-	-	-	-	1.3	1.0	0.9	0.9	3.0	2	1	1	1	398	44.3	5.0
NP_203747.2	37674210	SSH2	protein phosphatase Slingshot homolog 2 isoform 2	0.8	0.8	0.8	0.9	-	-	-	-	0.8	0.8	0.8	0.9	1.1	2	1	1	1	1423	158.1	5.4
NP_055764.2	55741675	KIAA0907	UPF0469 protein KIAA0907	1.1	0.9	1.0	0.9	-	-	-	-	1.1	0.9	1.0	0.9	7.8	1	1	1	2	614	64.8	8.7
NP_057727.2	214831336	ZNF771	zinc finger protein 771	1.0	1.0	0.9	0.9	-	-	-	-	1.0	1.0	0.9	0.9	3.8	1	1	1	2	317	35.7	8.8
NP_001129316.1	209529703	SIRPB1	signal-regulatory protein beta-1 isoform 3 precursor	0.9	1.0	1.0	0.9	-	-	-	-	0.9	1.0	1.0	0.9	3.5	2	1	1	1	398	43.3	7.8
NP_001154.2	22035548	APBA1	amyloid beta A4 precursor protein-binding family A member 1	1.0	1.0	1.0	0.9	-	-	-	-	1.0	1.0	1.0	0.9	1.0	1	1	1	1	837	92.8	4.9
NP_006691.1	5729828	TRAFD1	TRAF-type zinc finger domain-containing protein 1	1.0	0.8	0.8	0.9	-	-	-	-	1.0	0.8	0.8	0.9	6.7	1	2	2	3	582	64.8	5.3
NP_932068.2	188219595	C7orf55	UPF0562 protein C7orf55	0.9	1.0	0.9	0.9	-	-	-	-	0.9	1.0	0.9	0.9	10.6	1	1	1	5	113	12.7	10.1
NP_001284601.1	663070927	RGL1	ral guanine nucleotide dissociation stimulator-like 1 isoform 4	1.0	0.9	1.0	0.9	-	-	-	-	1.0	0.9	1.0	0.9	1.4	1	1	1	1	739	83.5	6.5
NP_443076.2	66267730	WDR34	WD repeat-containing protein 34	0.8	0.9	1.0	0.9	-	-	-	-	0.8	0.9	1.0	0.9	1.5	1	1	1	1	536	57.8	6.6
NP_000161.2	108773801	GLDC	glycine dehydrogenase (decarboxylating), mitochondrial precursor	0.9	0.8	0.9	0.9	-	-	-	-	0.9	0.8	0.9	0.9	0.8	1	1	1	1	1020	112.7	7.1
NP_001273015.1	554790405	LOC10228841	uncharacterized protein LOC102288414	0.9	1.0	1.0	0.9	-	-	-	-	0.9	1.0	1.0	0.9	26.8	1	3	4	10	123	14.2	11.5
NP_542781.3	148612890	SRSF12	serine/arginine-rich splicing factor 12	0.9	0.8	0.7	0.9	-	-	-	-	0.9	0.8	0.7	0.9	12.6	1	1	3	3	261	30.5	11.7
NP_055935.4	110349786	ALMS1	Alstrom syndrome protein 1	1.2	1.0	1.0	0.9	-	-	-	-	1.2	1.0	1.0	0.9	0.3	1	1	1	1	4169	460.9	6.3
NP_065768.2	118600967	SHROOM4	protein Shroom4	1.0	1.1	1.0	0.9	-	-	-	-	1.0	1.1	1.0	0.9	0.9	1	1	2	2	1493	164.8	6.7
NP_001269679.1	544346129	SUCO	SUN domain-containing ossification factor isoform 3 precursor	0.9	0.8	0.8	0.9	-	-	-	-	0.9	0.8	0.8	0.9	1.0	3	1	1	1	883	98.2	5.5
NP_057202.3	343403796	NUB1	NEDD8 ultimate buster 1 isoform 2	1.2	0.9	0.9	0.8	-	-	-	-	1.2	0.9	0.9	0.8	2.2	2	1	1	1	625	71.9	6.6
NP_689618.4	289547556	JMY	junction-mediating and -regulatory protein	1.1	1.0	1.2	0.8	-	-	-	-	1.1	1.0	1.2	0.8	2.4	1	1	1	2	988	111.4	6.2
NP_001179.1	4502363	BAK1	bcl-2 homologous antagonist/killer	0.9	0.8	0.9	0.8	-	-	-	-	0.9	0.8	0.9	0.8	5.2	1	1	1	2	211	23.4	6.0
NP_071938.1	11968051	NARFL	cytosolic Fe-S cluster assembly factor NARFL	0.9	0.9	0.9	0.8	-	-	-	-	0.9	0.9	0.9	0.8	6.9	1	1	1	3	476	53.0	7.2
NP_060532.2	49472819	FANCL	E3 ubiquitin-protein ligase FANCL isoform 2	0.7	0.8	0.8	0.8	-	-	-	-	0.7	0.8	0.8	0.8	2.7	2	1	1	1	375	42.9	6.5
NP_919288.2	155969701	ESPNL	espin-like protein isoform 1	0.8	0.9	0.9	0.8	-	-	-	-	0.8	0.9	0.9	0.8	0.6	2	1	1	1	1005	108.1	6.5
NP_005587.2	93004092	NFIB	nuclear factor 1 B-type isoform 3	0.8	0.9	0.8	0.8	-	-	-	-	0.8	0.9	0.8	0.8	3.3	3	1	1	1	420	47.4	8.9
NP_001191375.1	325053626	AP5S1	AP-5 complex subunit sigma-1	1.0	0.8	1.1	0.8	-	-	-	-	1.0	0.8	1.1	0.8	3.5	1	1	1	2	200	22.5	6.9
NP_001185761.2	525313622	PDE4DIP	myomegalin isoform 8	0.8	0.9	0.9	0.8	-	-	-	-	0.8	0.9	0.9	0.8	0.3	3	1	1	2	2240	253.9	5.4
NP_001185550.1	310750378	XIRP1	xin actin-binding repeat-containing protein 1 isoform 2	0.8	0.8	0.9	0.8	-	-	-	-	0.8	0.8	0.9	0.8	0.6	2	1	1	2	1121	122.1	5.3
NP_872434.2	217272874	CCDC37	coiled-coil domain-containing protein 37	0.9	0.8	0.8	0.8	-	-	-	-	0.9	0.8	0.8	0.8	0.8	13	1	1	2	611	71.1	7.1
NP_060556.2	64084766	FIGN	fidgetin	0.9	0.7	0.9	0.8	-	-	-	-	0.9	0.7	0.9	0.8	1.8	1	1	1	1	759	82.1	6.7
NP_009209.1	6005764	GABARAP	gamma-aminobutyric acid receptor-associated protein	1.0	0.9	1.1	0.8	-	-	-	-	1.0	0.9	1.1	0.8	29.9	2	2	3	8	117	13.9	8.8
NP_005388.2	33598950	PODXL	podocalyxin isoform 2 precursor	1.0	0.9	0.8	0.8	-	-	-	-	1.0	0.9	0.8	0.8	2.5	2	1	1	1	526	55.4	5.6
NP_848642.1	30425510	ZBTB8OS	protein archease	0.9	0.8	0.8	0.8	-	-	-	-	0.9	0.8	0.8	0.8	10.6	1	2	2	3	179	20.7	4.6
NP_001020949.1	71164888	VRK3	inactive serine/threonine-protein kinase VRK3 isoform 2	1.1	0.9	0.8	0.8	-	-	-	-	1.1	0.9	0.8	0.8	2.1	2	1	1	1	424	47.5	9.0
NP_001136118.1	217416360	TPRA1	transmembrane protein adipocyte-associated 1 isoform 2	1.1	1.0	1.0	0.8	-	-	-	-	1.1	1.0	1.0	0.8	4.4	2	1	1	1	293	32.1	8.9
NP_060662.2	27764882	P3H2	prolyl 3-hydroxylase 2 isoform a precursor	1.1	1.0	0.8	0.8	-	-	-	-	1.1	1.0	0.8	0.8	6.6	2	2	2	3	708	80.9	5.7
NP_004311.1	10835220	ATP2A1	sarcoplasmic/endoplasmic reticulum calcium ATPase 1 isoform a	1.1	0.9	0.9	0.8	-	-	-	-	1.1	0.9	0.9	0.8	15.2	3	1	13	58	994	109.2	5.2
NP_570122.1	18765707	INPP5K	inositol polyphosphate 5-phosphatase K isoform 2	1.0	1.0	1.0	0.8	-	-	-	-	1.0	1.0	1.0	0.8	4.0	2	1	1	2	372	42.8	6.5
NP_003100.1	218751903	SP1	transcription factor Sp1 isoform b	0.9	1.0	0.9	0.8	-	-	-	-	0.9	1.0	0.9	0.8	8.7	3	3	3	11	778	79.8	7.7
NP_001229468.1	335892792	NCOA6	nuclear receptor coactivator 6 isoform 2	1.1	0.7	1.0	0.8	-	-	-	-	1.1	0.7	1.0	0.8	0.6	2	1	1	1	1070	115.3	9.6
NP_542172.2	116268097	B3GALT6	beta-1,3-galactosyltransferase 6	1.5	0.7	0.9	0.8	-	-	-	-	1.5	0.7	0.9	0.8	2.7	1	1	1	2	329	37.1	9.7
NP_066974.2	31542325	CROT	peroxisomal carnitine O-octanoyltransferase isoform 2	0.7	0.8	0.7	0.8	-	-	-	-	0.7	0.8	0.7	0.8	2.8	2	1	1	2	612	70.1	7.1
NP_001230005.1	340523141	E2F3	transcription factor E2F3 isoform 2	0.9	0.9	0.8	0.8	-	-	-	-	0.9	0.9	0.8	0.8	3.0	2	1	1	2	334	37.0	5.2
NP_001182201.1	305632793	LOC10012952	uncharacterized protein LOC100129520	1.3	0.9	0.7	0.8	-	-	-	-	1.3	0.9	0.7	0.8	2.0	1	1	1	1	993	109.7	8.8
NP_150366.1	39930475	IRX2	iroquois-class homeodomain protein IRX-2	0.9	0.9	0.6	0.8	-	-	-	-	0.9	0.9	0.6	0.8	3.2	1	1	1	1	471	49.1	5.3
NP_787082.1	28557709	PRPS1L1	ribose-phosphate pyrophosphokinase 3	1.1	1.0	0.9	0.8	-	-	-	-	1.1	1.0	0.9	0.8	18.2	1	1	4	14	318	34.8	6.4
NP_443066.2	73747879	WDR7	WD repeat-containing protein 7 isoform 2	1.2	1.0	1.3	0.8	-	-	-	-	1.2	1.0	1.3	0.8	1.1	2	1	1	1	1457	160.4	7.0
NP_955459.2	162417982	ZFP64	zinc finger protein 64 isoform d	0.9	0.9	0.9	0.8	-	-	-	-	0.9	0.9	0.9	0.8	5.1	3	3	3	4	645	72.2	8.4
NP_001028746.1	75812976	AMZ2	archaemetzincin-2 isoform 2	0.9	0.8	0.9	0.8	-	-	-	-	0.9	0.8	0.9	0.8	14.6	2	1	1	1	302	34.6	7.7
NP_066361.1	10518344	RAP2A	ras-related protein Rap-2a precursor	0.8	0.9	0.8	0.8	-	-	-	-	0.8	0.9	0.8	0.8	11.5	3	2	2	3	183	20.6	4.8
NP_001273466.1	557440762	C2orf49	ashwin isoform 2	0.9	0.9	0.8	0.8	-	-	-	-	0.9	0.9	0.8	0.8	13.7	2	2	2	3	190	21.3	9.7
NP_001277486.1	594952027	ADIPOR1	adiponectin receptor protein 1	0.8	0.7	0.9	0.8	-	-	-	-	0.8	0.7	0.9	0.8	4.5	1	1	1	2	375	42.6	7.0
NP_919267.2	145580604	TMEM65	transmembrane protein 65	0.9	0.9	0.8	0.8	-	-	-	-	0.9	0.9	0.8	0.8	4.6	1	1	1	1	240	25.5	8.6
NP_001165420.1	285393969	KXD1	kxDL motif-containing protein 1	1.2	0.9	0.8	0.8	-	-	-	-	1.2	0.9	0.8	0.8	10.2	1	1	1	1	176	19.7	4.9
NP_001230037.1	340805823	PLD2	phospholipase D2 isoform PLD2B	0.9	0.9	0.9	0.8	-	-	-	-	0.9	0.9	0.9	0.8	2.3	2	1	1	1	922	104.6	7.3
NP_872607.1	33359691	UBE2E1	ubiquitin-conjugating enzyme E2 E1 isoform 2	0.8	0.9	1.1	0.8	-	-	-	-	0.8	0.9	1.1	0.8	15.9	3	1	2	8	176	19.5	8.9
NP_003233.4	67782324	TGFBR2	TGF-beta receptor type-2 isoform B precursor	0.8	0.9	1.0	0.8	-	-	-	-	0.8	0.9	1.0	0.8	7.8	2	4	4	7	567	64.5	5.9
NP_001269236.1	536288799;536288941	STK25	serine/threonine-protein kinase 25 isoform 3	0.9	0.8	0.8	0.8	-	-	-	-	0.9	0.8	0.8	0.8	8.7	4	1	3	8	332	37.3	7.7
NP_079157.3	164607128	SLC35E1	solute carrier family 35 member E1	0.9	0.8	0.8	0.8	-	-	-	-	0.9	0.8	0.8	0.8	2.7	1	1	1	2	410	44.7	9.8
NP_001274275.1	565324175	GPR107	protein GPR107 isoform 4	0.9	0.8	0.9	0.8	-	-	-	-	0.9	0.8	0.9	0.8	5.4	4	2	2	3	332	38.2	6.5
NP_001159934.1	262118263	MPP1	55 kDa erythrocyte membrane protein isoform 4	0.9	0.9	0.9	0.8	-	-	-	-	0.9	0.9	0.9	0.8	4.8	4	2	2	3	436	49.0	7.3
NP_077304.1	13236577	THTPA	thiamine-triphosphatase isoform 1	0.9	0.8	0.9	0.8	-	-	-	-	0.9	0.8	0.9	0.8	11.7	1	1	1	2	230	25.6	4.8
NP_054770.1	7662639	TMEM14A	transmembrane protein 14A	0.9	1.3	1.4	0.8	-	-	-	-	0.9	1.3	1.4	0.8	23.2	1	1	1	1	99	10.7	10.6

Khan *et al.* , 2019. Multi-omics analysis to characterize cigarette smoke induced molecular alterations in esophageal cells
Supplementary Table 5. List of proteins quantified in untreated and chronically treated Het1A cells with cigarette smoke condensate for 8 months

NP_Accession	Protein group Accession	Gene ID	Description	Het-1A-Smoke - 2M/Parental	Het-1A-Smoke - 4M/Parental	Het-1A-Smoke - 6M/Parental	Het-1A-Smoke - 8M/Parental	Het-1A-Smoke - 2M/Parental	Het-1A-Smoke - 4M/Parental	Het-1A-Smoke - 6M/Parental	Het-1A-Smoke - 8M/Parental	Het-1A-Smoke - 2M/Parental	Het-1A-Smoke - 4M/Parental	Het-1A-Smoke - 6M/Parental	Het-1A-Smoke - 8M/Parental	Coverage (%)	Proteins	Unique Peptides	# Peptides	PSM	# AAs	MW [kDa]	calc. pI
				Replicate 1				Replicate 2				Average of replicates											
NP_001029102.1	77539055;4507761	UBA52	ubiquitin-60S ribosomal protein L40 precursor	0.8	0.9	1.0	0.8	-	-	-	-	0.8	0.9	1.0	0.8	62.5	3	1	12	210	128	14.7	9.8
NP_002478.1	4505349	NDN	necdin	0.9	0.8	0.7	0.8	-	-	-	-	0.9	0.8	0.7	0.8	3.1	1	1	1	2	321	36.1	8.8
NP_775741.1	27735037	MMGT1	membrane magnesium transporter 1 precursor	1.0	1.0	0.8	0.8	-	-	-	-	1.0	1.0	0.8	0.8	18.3	1	1	1	2	131	14.7	9.2
NP_006723.2	166999613	FOSB	protein fosB isoform 1	0.9	0.9	0.7	0.8	-	-	-	-	0.9	0.9	0.7	0.8	7.1	1	1	1	2	338	35.9	4.9
NP_938146.1	291219924	STAT2	signal transducer and activator of transcription 2 isoform 2	1.1	0.8	0.9	0.8	-	-	-	-	1.1	0.8	0.9	0.8	3.5	2	2	2	4	847	97.4	5.5
NP_001265309.1	507834134	ARL6IP4	ADP-ribosylation factor-like protein 6-interacting protein 4 isoform 7	0.7	0.8	0.8	0.8	-	-	-	-	0.7	0.8	0.8	0.8	24.8	7	4	4	15	226	25.3	10.9
NP_055141.2	14670383	DAPK2	death-associated protein kinase 2	0.7	0.8	0.7	0.8	-	-	-	-	0.7	0.8	0.7	0.8	2.4	1	1	1	1	370	42.9	6.9
NP_659407.1	21450663	CXorf38	uncharacterized protein CXorf38	1.1	1.1	0.8	0.8	-	-	-	-	1.1	1.1	0.8	0.8	7.8	1	1	1	1	319	36.6	6.3
NP_443172.1	16506299	LRRC42	leucine-rich repeat-containing protein 42	0.7	0.7	0.6	0.8	-	-	-	-	0.7	0.7	0.6	0.8	4.4	1	1	1	1	428	48.5	7.5
NP_001274460.1	566559933	PQLC2	lysosomal amino acid transporter 1 homolog isoform 3	0.7	0.8	0.8	0.8	-	-	-	-	0.7	0.8	0.8	0.8	7.2	3	1	1	2	180	19.8	9.7
NP_963837.1	42544187;42544191	LGALS8	galectin-8 isoform b	0.7	0.7	0.7	0.8	-	-	-	-	0.7	0.7	0.7	0.8	11.7	2	4	4	7	317	35.8	8.3
NP_055796.1	150010558	MYH15	myosin-15 precursor	0.9	0.8	0.9	0.8	-	-	-	-	0.9	0.8	0.9	0.8	0.3	1	1	1	1	1946	224.5	5.8
NP_689500.2	40805102	ZNF276	zinc finger protein 276 isoform b	1.0	0.9	1.0	0.8	-	-	-	-	1.0	0.9	1.0	0.8	2.6	2	1	1	1	539	59.8	8.4
NP_001166040.1	289546653	MYD88	myeloid differentiation primary response protein MyD88 isoform 4	0.8	0.8	0.9	0.8	-	-	-	-	0.8	0.8	0.9	0.8	10.3	3	1	1	1	204	22.1	5.1
NP_005281.1	4885299	GPR15	G-protein coupled receptor 15	0.7	1.0	0.8	0.8	-	-	-	-	0.7	1.0	0.8	0.8	1.7	1	1	1	1	360	40.8	8.7
NP_776155.1	28603830	FUNDC1	FUN14 domain-containing protein 1	0.8	0.6	0.6	0.8	-	-	-	-	0.8	0.6	0.6	0.8	11.0	1	1	1	2	155	17.2	8.6
NP_003003.3	56117838	SFRP1	secreted frizzled-related protein 1 precursor	0.9	0.8	0.9	0.8	-	-	-	-	0.9	0.8	0.9	0.8	5.1	1	1	1	1	314	35.4	8.9
NP_569077.2	283484022	PTPRD	receptor-type tyrosine-protein phosphatase delta isoform 4 precursor	0.7	0.7	0.7	0.8	-	-	-	-	0.7	0.7	0.7	0.8	2.9	10	1	3	7	1496	168.6	6.7
NP_710160.1	24497544	HOXC6	homeobox protein Hox-C6 isoform 2	1.0	0.9	0.9	0.8	-	-	-	-	1.0	0.9	0.9	0.8	5.2	14	1	1	2	153	17.8	9.7
NP_001005179.2	94967016	OR56A4	olfactory receptor 56A4	0.8	0.7	0.7	0.8	-	-	-	-	0.8	0.7	0.7	0.8	8.8	1	1	1	1	365	41.2	9.3
NP_001265869.1	525313614	GTF2A1	transcription initiation factor IIA subunit 1 isoform 3	0.8	0.8	0.8	0.8	-	-	-	-	0.8	0.8	0.8	0.8	2.2	7	1	1	5	326	35.7	4.6
NP_073729.1	12383056	MAP1LC3B	microtubule-associated proteins 1A/1B light chain 3B	1.1	0.9	0.8	0.8	-	-	-	-	1.1	0.9	0.8	0.8	11.2	2	1	1	2	125	14.7	8.9
NP_036553.2	157671949	RLF	zinc finger protein Rlf	0.9	0.7	0.8	0.8	-	-	-	-	0.9	0.7	0.8	0.8	1.4	1	2	2	2	1914	217.8	6.8
NP_036471.1	6912530	TSPAN15	tetraspanin-15	0.7	0.7	0.8	0.8	-	-	-	-	0.7	0.7	0.8	0.8	4.1	1	1	1	2	294	33.1	5.5
NP_001137246.1	219555646	PHYHIPL	phytanoyl-CoA hydroxylase-interacting protein-like isoform 2	1.2	1.0	1.5	0.8	-	-	-	-	1.2	1.0	1.5	0.8	11.1	2	1	1	1	350	39.6	6.7
NP_004877.1	11342678	APBA3	amyloid beta A4 precursor protein-binding family A member 3	0.8	0.8	0.8	0.8	-	-	-	-	0.8	0.8	0.8	0.8	1.9	1	1	1	1	575	61.4	4.9
NP_001186793.1	315434255	CL2L2-PABP	BCL2L2-PABPN1 protein	0.9	0.8	0.8	0.8	-	-	-	-	0.9	0.8	0.8	0.8	28.8	5	8	8	34	333	37.2	8.4
NP_000337.1	4557853	SOX9	transcription factor SOX-9	0.9	0.8	0.7	0.7	-	-	-	-	0.9	0.8	0.7	0.7	2.4	1	1	1	1	509	56.1	6.8
NP_116191.2	21314755	SPPL2A	signal peptide peptidase-like 2A precursor	0.7	0.7	0.7	0.7	-	-	-	-	0.7	0.7	0.7	0.7	2.7	1	1	1	2	520	58.1	8.3
NP_001191765.1	325651958	ZNF568	zinc finger protein 568 isoform 3	0.7	0.7	0.8	0.7	-	-	-	-	0.7	0.7	0.8	0.7	2.9	121	1	2	2	580	67.0	8.6
NP_001245379.1	386642875	PKMYT1	membrane-associated tyrosine- and threonine-specific cdc2-inhibitory kinase isoform 3	0.8	0.7	0.8	0.7	-	-	-	-	0.8	0.7	0.8	0.7	3.0	3	1	1	4	430	47.3	6.1
NP_001073941.1	122937337	FITM2	fat storage-inducing transmembrane protein 2	0.7	0.7	0.7	0.7	-	-	-	-	0.7	0.7	0.7	0.7	5.0	1	1	1	2	262	29.8	8.6
NP_001243888.1	379698826	CLCN6	chloride transport protein 6 isoform 2	1.2	0.7	0.7	0.7	-	-	-	-	1.2	0.7	0.7	0.7	1.1	2	1	1	1	847	94.5	7.4
NP_003512.1	4504263	HIST1H2BM	histone H2B type 1-M	0.7	0.7	0.7	0.7	-	-	-	-	0.7	0.7	0.7	0.7	67.5	5	3	11	1489	126	14.0	10.3
NP_055260.1	7657552	SERP1	stress-associated endoplasmic reticulum protein 1	1.1	0.8	1.0	0.7	-	-	-	-	1.1	0.8	1.0	0.7	9.1	1	1	1	1	66	7.4	11.0
NP_114130.3	31543559	RSPH3	radial spoke head protein 3 homolog	0.8	0.8	0.7	0.7	-	-	-	-	0.8	0.8	0.7	0.7	2.1	1	1	1	1	560	63.6	5.7
NP_001158502.1	259013532	TMEM41B	transmembrane protein 41B isoform 2	0.9	0.8	0.8	0.7	-	-	-	-	0.9	0.8	0.8	0.7	15.0	2	1	1	1	127	14.1	9.4
NP_057529.1	7706643	PLEK2	pleckstrin-2	0.7	0.6	0.9	0.7	-	-	-	-	0.7	0.6	0.9	0.7	6.5	1	1	1	2	353	39.9	9.4
NP_061977.1	28144916	LIN37	protein lin-37 homolog	0.8	0.8	1.0	0.7	-	-	-	-	0.8	0.8	1.0	0.7	5.3	1	1	1	2	246	28.4	8.7
NP_001258596.1	410651504	DTNBP1	dysbindin isoform C	1.0	0.9	1.0	0.7	-	-	-	-	1.0	0.9	1.0	0.7	5.9	4	1	1	2	270	30.4	4.4
NP_001248324.1	386869312	CALCOCO2	calcium-binding and coiled-coil domain-containing protein 2 isoform 5	0.9	0.6	0.8	0.7	-	-	-	-	0.9	0.6	0.8	0.7	2.9	5	1	1	2	374	43.6	4.8
NP_001275572.1	568786303	ZNF286A	zinc finger protein 286A isoform 3	1.1	0.9	0.8	0.7	-	-	-	-	1.1	0.9	0.8	0.7	5.3	79	1	3	4	511	59.0	8.5
NP_004351.1	4757960	CDH1	cadherin-1 preproprotein	0.8	0.9	0.8	0.7	-	-	-	-	0.8	0.9	0.8	0.7	4.5	1	1	1	1	882	97.4	4.7
NP_000529.1	4506501	RFXAP	regulatory factor X-associated protein	0.7	0.9	0.8	0.7	-	-	-	-	0.7	0.9	0.8	0.7	12.5	1	2	2	4	272	28.2	5.5
NP_001135840.1	215277000	GSTM2	glutathione S-transferase Mu 2 isoform 2	0.8	0.7	0.6	0.7	-	-	-	-	0.8	0.7	0.6	0.7	35.6	2	4	5	14	191	22.6	5.3
NP_001161884.1	270483821	TSPAN3	tetraspanin-3 isoform 3	0.7	0.8	0.7	0.7	-	-	-	-	0.7	0.8	0.7	0.7	3.7	3	1	1	2	189	21.2	6.8
NP_061720.2	151301127	DNAH7	dynein heavy chain 7, axonemal	0.7	0.8	0.8	0.7	-	-	-	-	0.7	0.8	0.8	0.7	0.2	1	1	1	2	4024	460.9	6.0
NP_001192184.1	327478416	OCLN	occludin isoform b precursor	1.0	0.8	0.8	0.7	-	-	-	-	1.0	0.8	0.8	0.7	3.7	2	1	1	1	271	31.6	5.7
NP_003038.2	27777632	SLC9A1	sodium/hydrogen exchanger 1	0.7	0.7	0.7	0.7	-	-	-	-	0.7	0.7	0.7	0.7	3.3	1	1	1	4	815	90.7	7.2
NP_001287729.1	663855632	NAA40	N-alpha-acetyltransferase 40 isoform 2	0.6	0.7	0.7	0.7	-	-	-	-	0.6	0.7	0.7	0.7	5.6	2	1	1	2	216	24.7	6.2
NP_001542.1	4557663	IGBP1	immunoglobulin-binding protein 1	0.9	0.7	0.7	0.7	-	-	-	-	0.9	0.7	0.7	0.7	8.9	1	2	2	4	339	39.2	5.4
NP_001007532.1	56090620	NKAPL	NKAP-like protein	1.1	0.8	0.7	0.7	-	-	-	-	1.1	0.8	0.7	0.7	2.5	2	1	1	2	402	46.3	9.7
XP_006721691.1	578830221	LOC10192914	coiled-coil domain-containing protein 144B-like	0.8	0.8	0.7	0.7	-	-	-	-	0.8	0.8	0.7	0.7	5.2	2	1	1	1	496	55.6	4.9
NP_078792.1	13375654	PAGR1	PAXIP1-associated glutamate-rich protein 1	0.6	1.0	0.7	0.7	-	-	-	-	0.6	1.0	0.7	0.7	22.8	1	2	2	3	254	27.7	4.5
NP_001002862.1	50845411	DERL3	derlin-3 isoform 2	0.7	0.6	0.8	0.7	-	-	-	-	0.7	0.6	0.8	0.7	3.8	1	1	1	1	235	26.7	8.5
NP_004425.2	56790930	EML1	echinoderm microtubule-associated protein-like 1 isoform b	0.8	0.7	0.6	0.6	-	-	-	-	0.8	0.7	0.6	0.6	1.4	2	1	1	2	815	89.8	7.1
NP_001005366.1	54112380	KDM2B	lysine-specific demethylase 2B isoform b	1.0	0.6	0.9	0.6	-	-	-	-	1.0	0.6	0.9	0.6	1.0	2	1	1	1	1265	144.7	8.4
NP_001138502.1	222537754	TOPAZ1	testis- and ovary-specific PAZ domain-containing protein 1	0.9	0.6	0.8	0.6	-	-	-	-	0.9	0.6	0.8	0.6	0.5	1	1	1	1	1692	190.8	7.9
NP_036300.2	15812198	FBXO2	F-box only protein 2	0.7	0.8	0.8	0.6	-	-	-	-	0.7	0.8	0.8	0.6	4.1	1	1	1	4	296	33.3	4.4
NP_001003702.2	270265795	ARHGEF35	rho guanine nucleotide exchange factor 35	1.0	0.8	0.7	0.6	-	-	-	-	1.0	0.8	0.7	0.6	6.2	3	2	2	3	484	53.3	4.3
NP_001073285.1	119395738	INSR	insulin receptor isoform Short preproprotein	1.8	1.1	1.6	0.6	-	-	-	-	1.8	1.1	1.6	0.6	3.1	2	2	3	4	1370	155.0	6.2
NP_114033.2	87578392	MAP2	microtubule-associated protein 2 isoform 2	0.8	0.5	0.5	0.6	-	-	-	-	0.8	0.5	0.5	0.6	2.1	4	1	1	1	471	49.6	9.5
NP_065827.1	55749715	KIAA1328	uncharacterized protein KIAA1328	1.0	0.9	0.9	0.6	-	-	-	-	1.0	0.9	0.9	0.6	1.0	1	1	1	1	577	65.3	8.1
NP_001006640.1	55749406	TCEAL1	transcription elongation factor A protein-like 1	0.7	0.7	0.6	0.5	-	-	-	-	0.7	0.7	0.6	0.5	7.6	1	1	1	1	159	18.6	5.0
NP_001004135.1	51921273	OR2A12	olfactory receptor 2A12	1.1	0.8	0.8	0.5	-	-	-	-	1.1	0.8	0.8	0.5	8.7	1	1	1	1	310	35.2	9.0
NP_115285.1	15055543	SRSF8	serine/arginine-rich splicing factor 8	0.5	0.6	0.6	0.4	-	-	-	-	0.5	0.6	0.6	0.4	12.8	1	1	4	15	282	32.3	11.7
NP_689830.2	31377566	RNF168	E3 ubiquitin-protein ligase RNF168	0.8	0.9	0.7	0.4	-	-	-	-	0.8	0.9	0.7	0.4	1.9	1	1	1	2	571	65.0	8.0
NP_060688.1	41055953	USP40	ubiquitin carboxyl-terminal hydrolase 40	0.2	0.9	0.1	0.3	-	-	-	-	0.2	0.9	0.1	0.3	5.4	1	1	1	1	1247	141.5	5.7
NP_001193486.1	330864720	KIAA1432	protein RIC1 homolog isoform c	-	-	-	-	0.8	1.8	0.9	2.7	0.8	1.8	0.9	2.7	1.5	2	1	1	1	1386	155.1	6.2
NP_057421.1	7706687	EVL	ena/VASP-like protein	-	-	-	-	0.8	0.9	0.7	2.5	0.8	0.9	0.7	2.5	4.6	1	1	1	1	418	44.8	8.8
NP_059984.3	533112479	PLXNA3	plexin-A3 precursor	-	-	-	-	1.6	2.0	2.2	2.3	1.6	2.0	2.2	2.3	0.9	1	1	1	1	1871	207.6	7.3
NP_150377.1	15451844	ADAM19	disintegrin and metalloproteinase domain-containing protein 19 preproprotein	-	-	-	-	0.8	1.8	1.3	2.2	0.8	1.8	1.3	2.2	7.0	1	1	1	1	918	100.8	8.2
NP_000776.1	4503213	CYP27B1	25-hydroxyvitamin D-1 alpha hydroxylase, mitochondrial	-	-	-	-	1.6	1.9	1.9	2.0	1.6	1.9	1.9	2.0	3.9	1	1	1	2	508	56.5	9.2
NP_036199.2	41152114	AKR7A3	aflatoxin B1 aldehyde reductase member 3	-	-	-	-	1.9	2.5	2.2	1.8	1.9	2.5	2.2	1.8	9.1	1	1	2	4	331	37.2	7.2
NP_944489.2	119120861	FMNL3	formin-like protein 3 isoform 2	-	-	-	-	1.0	1.9	1.3	1.8	1.0	1.9	1.3	1.8	2.0	3	1	2	4	976	111.2	6.9
NP_004522.1	4758732	MOCS2	molybdopterin synthase catalytic subunit large subunit MOCS2B	-	-	-	-	1.3	1.5	1.2	1.8	1.3	1.5	1.2	1.8	17.0	1	2	2	3	188	20.9	5.4
NP_002955.2	21614544	S100A8	protein S100-A8	-	-	-	-	1.1	1.6	1.1	1.8	1.1	1.6	1.1	1.8	16.1	4	2	2	2	93	10.8	7.0
NP_001139698.1	226246665	KRT72	keratin, type II cytoskeletal 72 isoform 2	-	-	-	-	1.0	1.4	1.0	1.8	1.0	1.4	1.0	1.8	8.3	5	1	4	29	469	51.1	6.5
NP_000091.1	4503117	CSTB	cystatin-B	-	-	-	-	1.6	2.4	1.8	1.8	1.6	2.4	1.8	1.8	6.1	1	1	1	1	98	11.1	7.6
NP_003851.1	4502389;221316573	BANF1	barrier-to-autointegration factor	-	-	-	-	1.3	1.7	1.4	1.8	1.3	1.7	1.4	1.8	40.5	1	2	2	11	89	10.1	6.1
NP_001278797.1	630044883	NHS	Nance-Horan syndrome protein isoform 4	-	-	-	-	0.8	0.8	0.9	1.7	0.8	0.8	0.9	1.7	1.9	4	1	1	1	1453	158.3	7.0
NP_995317.1	45446745	AKR1C2	aldo-keto reductase family 1 member C2 isoform 1	-	-	-	-	1.4	1.3	1.5	1.7	1.4	1.3	1.5	1.7	14.2	5	3	3	4	323	36.7	7.5
NP_004555.1	4758894	PET112	glutamyl-tRNA(Gln) amidotransferase subunit B, mitochondrial precursor	-	-	-	-	1.5	1.4	1.3	1.7	1.5	1.4	1.3	1.7	4.5	1	1	1	4	557	61.8	8.6
NP_659495.1	21450828	RILPL2	RILP-like protein 2	-	-	-	-	1.4	1.3	1.2	1.6	1.4	1.3	1.2	1.6	9.0	1	1	1	2	211	24.0	5.0
NP_000013.2	47078295	ADA	adenosine deaminase	-	-	-	-	1.4	1.4	1.2	1.6	1.4	1.4	1.2	1.6	8.5	1	1	1	2	363	40.7	5.9
NP_003338.1	4507789	UBE2L3	ubiquitin-conjugating enzyme E2 L3 isoform 1	-	-	-	-	1.4	1.8	1.5	1.6	1.4	1.8	1.5	1.6	36.4	3	4	4	10	154	17.9	8.5
NP_958799.1	41406082	GPX1	glutathione peroxidase 1 isoform 2	-	-	-	-	0.9	1.1	1.1	1.6	0.9	1.1	1.1	1.6	10.2	2	1	1	2	98	10.3	10.2
NP_003705.1	4507267	STC2	stanniocalcin-2 precursor	-	-	-	-	1.9	2.0	1.8	1.6	1.9	2.0	1.8	1.6	7.6	1	1	1	2	302	33.2	7.3
NP_055688.1	7661996	LPGAT1	acyl-CoA:lysophosphatidylglycerol acyltransferase 1	-	-	-	-	1.3	1.5	1.7	1.6	1.3	1.5	1.7	1.6	4.3	1	1	1	1	370	43.1	8.9

Khan *et al.* , 2019. Multi-omics analysis to characterize cigarette smoke induced molecular alterations in esophageal cells
Supplementary Table 5. List of proteins quantified in untreated and chronically treated Het1A cells with cigarette smoke condensate for 8 months

NP_Accession	Protein group Accession	Gene ID	Description	Het-1A-Smoke - 2M/Parental	Het-1A-Smoke - 4M/Parental	Het-1A-Smoke - 6M/Parental	Het-1A-Smoke - 8M/Parental	Het-1A-Smoke - 2M/Parental	Het-1A-Smoke - 4M/Parental	Het-1A-Smoke - 6M/Parental	Het-1A-Smoke - 8M/Parental	Het-1A-Smoke - 2M/Parental	Het-1A-Smoke - 4M/Parental	Het-1A-Smoke - 6M/Parental	Het-1A-Smoke - 8M/Parental	Coverage (%)	Proteins	Unique Peptides	# Peptides	PSM	# AAs	MW [kDa]	calc. pI
				Replicate 1				Replicate 2				Average of replicates											
NP_001158255.1	258645172	BCKDHA	2-oxoisovalerate dehydrogenase subunit alpha, mitochondrial isoform 2 precursor	-	-	-	-	1.4	1.4	1.3	1.6	1.4	1.4	1.3	1.6	2.3	2	1	1	2	444	50.4	8.3
NP_001034842.2	223005862	EML6	echinoderm microtubule-associated protein-like 6	-	-	-	-	1.1	1.1	1.3	1.6	1.1	1.1	1.3	1.6	0.7	1	1	1	1	1958	217.8	7.4
NP_001240764.1	359465608	IGFBP7	insulin-like growth factor-binding protein 7 isoform 2 precursor	-	-	-	-	0.9	1.6	1.6	1.6	0.9	1.6	1.6	1.6	4.7	2	1	1	3	279	28.8	8.1
NP_775901.3	145046269	RTTN	rotatin	-	-	-	-	1.1	1.2	1.2	1.6	1.1	1.2	1.2	1.6	0.5	1	1	1	2	2226	248.5	6.7
NP_001120920.1	188536080	ABAT	4-aminobutyrate aminotransferase, mitochondrial precursor	-	-	-	-	1.5	1.8	1.8	1.6	1.5	1.8	1.8	1.6	3.0	1	1	1	1	500	56.4	8.0
NP_689633.1	22748889	C9orf41	UPF0586 protein C9orf41	-	-	-	-	1.2	0.9	1.3	1.5	1.2	0.9	1.3	1.5	2.7	1	1	1	2	409	47.2	6.3
NP_001274509.1	567316250	RBKS	ribokinase isoform 2	-	-	-	-	1.9	1.6	1.5	1.5	1.9	1.6	1.5	1.5	2.8	2	1	1	5	255	27.1	4.7
NP_001186803.1	315467844	B4GALT3	beta-1,4-galactosyltransferase 3	-	-	-	-	1.7	1.4	1.3	1.5	1.7	1.4	1.3	1.5	12.2	1	2	2	3	393	43.9	9.2
NP_057646.1	7706093	TLR7	toll-like receptor 7 precursor	-	-	-	-	0.9	1.4	1.0	1.5	0.9	1.4	1.0	1.5	0.6	1	1	1	1	1049	120.8	8.2
NP_005242.1	4885237	FOXC2	forkhead box protein C2	-	-	-	-	2.1	1.2	1.7	1.5	2.1	1.2	1.7	1.5	5.6	1	1	1	1	501	53.7	8.5
NP_473376.1	16936535	UNC119	protein unc-119 homolog A isoform b	-	-	-	-	1.2	1.2	1.2	1.5	1.2	1.2	1.2	1.5	6.4	2	1	1	3	220	24.1	7.3
NP_631909.2	59710085	C16orf71	uncharacterized protein C16orf71	-	-	-	-	1.3	1.3	1.4	1.5	1.3	1.3	1.4	1.5	2.7	1	1	1	1	520	55.6	4.9
NP_060458.3	47604944	SCYL2	SCY1-like protein 2	-	-	-	-	1.0	1.3	1.1	1.5	1.0	1.3	1.1	1.5	1.2	1	1	1	2	929	103.6	8.2
NP_000254.2	66346698	NAGLU	alpha-N-acetylglucosaminidase precursor	-	-	-	-	1.2	1.4	1.1	1.4	1.2	1.4	1.1	1.4	2.3	1	1	1	2	743	82.2	6.7
NP_079218.2	156139127	C10orf88	uncharacterized protein C10orf88	-	-	-	-	1.5	1.6	1.4	1.4	1.5	1.6	1.4	1.4	6.1	1	1	1	2	445	49.2	6.3
NP_115982.1	14211923	HINT2	histidine triad nucleotide-binding protein 2, mitochondrial precursor	-	-	-	-	1.1	1.3	1.3	1.4	1.1	1.3	1.3	1.4	22.1	1	3	3	7	163	17.2	9.2
NP_003815.1	4505229	FADD	FAS-associated death domain protein	-	-	-	-	1.9	1.7	1.5	1.4	1.9	1.7	1.5	1.4	12.5	1	1	1	1	208	23.3	5.7
NP_115521.3	34577055	FBXO30	F-box only protein 30	-	-	-	-	1.3	2.0	1.7	1.4	1.3	2.0	1.7	1.4	1.9	1	1	1	4	745	82.3	5.4
NP_001336.2	31542504;41872494	DGKA	diacylglycerol kinase alpha	-	-	-	-	1.1	1.2	1.2	1.4	1.1	1.2	1.2	1.4	7.2	1	3	3	7	735	82.6	6.7
NP_003754.2	34447229	STX16	syntaxin-16 isoform b	-	-	-	-	1.2	1.1	1.0	1.4	1.2	1.1	1.0	1.4	3.0	4	1	1	2	304	34.8	6.2
NP_005043.1	4826962	RAC3	ras-related C3 botulinum toxin substrate 3	-	-	-	-	1.0	1.2	1.0	1.4	1.0	1.2	1.0	1.4	35.9	1	1	5	21	192	21.4	8.1
NP_942590.1	38605733	NUP43	nucleoporin Nup43	-	-	-	-	1.1	1.1	1.1	1.4	1.1	1.1	1.1	1.4	4.2	1	1	1	2	380	42.1	5.6
NP_722517.3	109452597	CACUL1	CDK2-associated and cullin domain-containing protein 1	-	-	-	-	1.3	1.1	1.3	1.4	1.3	1.1	1.3	1.4	8.1	1	1	1	1	369	41.0	5.2
NP_060738.2	42716287	WDR41	WD repeat-containing protein 41	-	-	-	-	1.5	1.2	1.2	1.4	1.5	1.2	1.2	1.4	6.3	1	2	2	3	459	51.7	5.4
NP_001231742.1	348605229	FOXP1	forkhead box protein P1 isoform 6	-	-	-	-	1.4	1.2	1.2	1.4	1.4	1.2	1.2	1.4	3.3	9	1	1	1	577	64.7	6.6
NP_001230892.1	345478711	LRCH2	leucine-rich repeat and calponin homology domain-containing protein 2 isoform 2	-	-	-	-	1.0	1.1	1.1	1.4	1.0	1.1	1.1	1.4	2.1	2	1	1	1	748	82.8	6.8
NP_065885.2	158631177	HOMEZ	homeobox and leucine zipper protein Homez	-	-	-	-	1.4	1.0	1.0	1.4	1.4	1.0	1.0	1.4	9.5	1	1	1	1	550	61.2	4.9
NP_001944.1	4503445	TYMP	thymidine phosphorylase isoform 1 proprotein	-	-	-	-	1.3	1.3	1.2	1.4	1.3	1.3	1.2	1.4	12.0	2	3	3	5	482	49.9	5.5
NP_055752.1	7662414	INPP5F	phosphatidylinositide phosphatase SAC2 isoform 1	-	-	-	-	1.1	1.2	1.2	1.4	1.1	1.2	1.2	1.4	2.6	2	2	2	3	1132	128.3	7.0
NP_056084.1	57863246	ZCCHC11	terminal uridylyltransferase 4 isoform b	-	-	-	-	1.1	1.2	1.0	1.4	1.1	1.2	1.0	1.4	0.6	2	1	1	1	1644	185.0	8.0
NP_001127917.1	197313763	DDAH1	N(G),N(G)-dimethylarginine dimethylaminohydrolase 1 isoform 2	-	-	-	-	1.2	1.2	1.0	1.4	1.2	1.2	1.0	1.4	5.5	2	1	1	1	182	20.2	5.8
NP_001150.3	71773480	AOX1	aldehyde oxidase	-	-	-	-	1.0	0.8	1.1	1.4	1.0	0.8	1.1	1.4	6.0	1	5	5	9	1338	147.8	7.2
NP_001191807.1	325910869	BAG4	BAG family molecular chaperone regulator 4 isoform 2	-	-	-	-	1.3	1.1	1.4	1.3	1.3	1.1	1.4	1.3	5.0	2	1	2	4	421	45.4	5.0
NP_001287785.1	664806092	FOSL1	fos-related antigen 1 isoform 4	-	-	-	-	1.1	2.4	2.3	1.3	1.1	2.4	2.3	1.3	8.3	2	1	1	1	205	22.0	6.0
NP_001746.1	13124873	CBFB	core-binding factor subunit beta isoform 2	-	-	-	-	1.2	1.4	1.1	1.3	1.2	1.4	1.1	1.3	15.9	2	2	2	3	182	21.5	6.6
NP_536722.1	17998551	SERPINB12	serpin B12 isoform 2	-	-	-	-	1.0	1.2	1.1	1.3	1.0	1.2	1.1	1.3	6.4	2	1	1	2	405	46.2	5.5
NP_001435.1	4557581	FABP5	fatty acid-binding protein, epidermal	-	-	-	-	1.1	1.2	1.2	1.3	1.1	1.2	1.2	1.3	22.2	1	2	2	3	135	15.2	7.0
NP_859070.3	194239659	TMCO4	transmembrane and coiled-coil domain-containing protein 4	-	-	-	-	1.5	1.2	1.0	1.3	1.5	1.2	1.0	1.3	3.5	1	1	1	2	634	67.9	5.8
NP_001009894.2	61742164	C12orf29	uncharacterized protein C12orf29	-	-	-	-	1.0	1.1	1.4	1.3	1.0	1.1	1.4	1.3	6.5	1	1	1	2	325	37.5	7.1
NP_001273573.1	557786192	RFWD2	E3 ubiquitin-protein ligase RFWD2 isoform c precursor	-	-	-	-	1.0	1.0	1.1	1.3	1.0	1.0	1.1	1.3	2.0	2	1	1	1	491	55.6	6.4
NP_004283.2	68989256	RIN1	ras and Rab interactor 1	-	-	-	-	0.8	1.1	1.6	1.3	0.8	1.1	1.6	1.3	6.6	1	3	3	7	783	84.0	8.0
NP_079493.1	13376824	ULBP2	NKG2D ligand 2 preproprotein	-	-	-	-	1.2	1.3	1.3	1.3	1.2	1.3	1.3	1.3	4.1	1	1	1	1	246	27.3	7.3
NP_004167.3	22547195	SREBF1	sterol regulatory element-binding protein 1 isoform b	-	-	-	-	1.2	1.3	1.2	1.3	1.2	1.3	1.2	1.3	1.3	2	1	1	1	1147	121.6	8.1
NP_001257933.1	401871061	TFPI2	tissue factor pathway inhibitor 2 isoform 3 precursor	-	-	-	-	1.9	1.7	1.5	1.3	1.9	1.7	1.5	1.3	8.1	3	1	1	1	186	20.9	5.4
NP_001138890.1	224028289	TTC28	tetratricopeptide repeat protein 28	-	-	-	-	1.0	1.2	1.2	1.3	1.0	1.2	1.2	1.3	0.4	1	1	1	1	2481	270.7	6.9
NP_955400.1	41872443	TRPC4AP	short transient receptor potential channel 4-associated protein isoform b	-	-	-	-	1.1	1.1	1.2	1.3	1.1	1.1	1.2	1.3	3.0	2	1	1	2	789	90.0	7.6
NP_001240744.1	359465547	SLC52A2	solute carrier family 52, riboflavin transporter, member 2	-	-	-	-	1.2	1.4	1.4	1.3	1.2	1.4	1.4	1.3	2.0	1	1	1	1	445	45.7	7.2
NP_071748.2	116812636	OSGEPL1	probable tRNA N6-adenosine threonylcarbamoyltransferase, mitochondrial	-	-	-	-	1.0	1.2	0.9	1.3	1.0	1.2	0.9	1.3	2.9	1	1	1	1	414	45.1	8.6
NP_001103447.1	158341646	FAM160A1	protein FAM160A1	-	-	-	-	2.2	1.8	1.6	1.3	2.2	1.8	1.6	1.3	3.9	1	1	1	1	1040	116.5	4.9
NP_573571.1	18959210	WIPF2	WAS/WASL-interacting protein family member 2	-	-	-	-	0.8	1.0	0.9	1.3	0.8	1.0	0.9	1.3	3.6	1	1	1	1	440	46.3	10.9
NP_004626.1	4758700	MAPKAPK3	MAP kinase-activated protein kinase 3	-	-	-	-	1.6	1.1	1.2	1.3	1.6	1.1	1.2	1.3	3.1	1	1	1	2	382	43.0	7.3
NP_689814.1	22749235	ZNF709	zinc finger protein 709	-	-	-	-	1.3	1.5	1.3	1.3	1.3	1.5	1.3	1.3	4.5	76	1	2	2	641	74.6	9.0
NP_001266.1	4502805	CHGA	chromogranin-A preproprotein	-	-	-	-	1.7	1.5	1.3	1.3	1.7	1.5	1.3	1.3	5.0	1	1	1	1	457	50.7	4.6
NP_002989.2	40548378	SDC2	syndecan-2 precursor	-	-	-	-	1.1	1.2	1.1	1.3	1.1	1.2	1.1	1.3	7.5	1	1	1	1	201	22.1	4.9
NP_001180241.1	300863126	SUGCT	succinate--hydroxymethylglutarate CoA-transferase isoform 2	-	-	-	-	1.2	1.1	1.3	1.3	1.2	1.1	1.3	1.3	3.0	4	1	1	2	397	43.4	8.3
NP_001182670.1	307691194	FAM213B	prostamide/prostaglandin F synthase isoform f	-	-	-	-	1.0	1.3	1.1	1.3	1.0	1.3	1.1	1.3	5.8	6	1	1	1	191	20.5	5.9
NP_001158075.1	257195178	ASXL1	putative Polycomb group protein ASXL1 isoform 2	-	-	-	-	1.2	1.1	1.1	1.3	1.2	1.1	1.1	1.3	16.5	2	1	1	1	85	9.5	9.7
NP_659500.1	21450830	OARD1	O-acetyl-ADP-ribose deacetylase 1	-	-	-	-	1.3	1.2	1.2	1.3	1.3	1.2	1.2	1.3	19.1	1	3	3	4	152	17.0	8.3
NP_660308.1	21687127	CCDC127	coiled-coil domain-containing protein 127	-	-	-	-	1.2	1.2	1.4	1.3	1.2	1.2	1.4	1.3	4.2	1	1	1	2	260	30.8	9.2
NP_001138469.1	222418615	ITGA7	integrin alpha-7 isoform 3	-	-	-	-	1.0	1.3	1.3	1.3	1.0	1.3	1.3	1.3	0.7	3	1	1	1	1044	114.5	6.0
NP_055874.2	156938343	TLN2	talin-2	-	-	-	-	0.8	1.2	0.9	1.3	0.8	1.2	0.9	1.3	5.2	1	1	13	50	2542	271.4	5.6
#N/A	58331274	Sep-05	septin-5 isoform 2	-	-	-	-	1.1	1.0	1.0	1.3	1.1	1.0	1.0	1.3	2.6	2	1	1	1	346	39.3	7.2
NP_001153588.1	237858670	C15orf40	UPF0235 protein C15orf40 isoform e	-	-	-	-	1.1	1.1	1.2	1.3	1.1	1.1	1.2	1.3	11.4	5	1	1	1	149	15.9	8.6
NP_001160166.1	266454472	CHRNA3	neuronal acetylcholine receptor subunit alpha-3 isoform 2 precursor	-	-	-	-	0.7	2.0	0.8	1.3	0.7	2.0	0.8	1.3	3.3	2	1	1	1	489	55.6	6.6
NP_004776.3	71897229	SLC9A3R2	Na(+)/H(+) exchange regulatory cofactor NHE-RF2 isoform b	-	-	-	-	1.1	1.2	1.2	1.3	1.1	1.2	1.2	1.3	5.8	5	2	2	5	326	36.1	8.2
NP_004115.1	4758442	GMFB	glia maturation factor beta	-	-	-	-	1.3	1.1	1.2	1.3	1.3	1.1	1.2	1.3	22.5	2	2	2	5	142	16.7	5.3
NP_001245358.1	385731627	C20orf27	UPF0687 protein C20orf27 isoform 2	-	-	-	-	1.0	1.0	1.2	1.3	1.0	1.0	1.2	1.3	13.8	2	2	2	8	174	19.3	6.8
NP_001092264.1	149393138	FAM160A2	FTS and Hook-interacting protein isoform 2	-	-	-	-	4.6	1.4	1.2	1.3	4.6	1.4	1.2	1.3	2.1	2	1	1	1	972	105.5	6.8
NP_001127965.1	197927201	TOMM6	mitochondrial import receptor subunit TOM6 homolog	-	-	-	-	1.1	1.1	1.1	1.3	1.1	1.1	1.1	1.3	18.9	1	1	1	10	74	8.0	4.9
NP_001171928.1	297206754	KIAA0430	meiosis arrest female protein 1 isoform 3	-	-	-	-	1.1	1.2	1.1	1.3	1.1	1.2	1.1	1.3	0.6	3	1	1	2	1739	192.5	7.9
NP_612486.2	57222570	CDAN1	codanin-1	-	-	-	-	1.1	1.0	1.0	1.3	1.1	1.0	1.0	1.3	3.5	1	2	2	3	1227	134.0	6.8
NP_689481.2	34303926	PARS2	probable proline--tRNA ligase, mitochondrial precursor	-	-	-	-	1.4	1.4	1.2	1.3	1.4	1.4	1.2	1.3	5.5	1	1	1	1	475	53.2	8.1
NP_001243056.1	371123153	NSUN4	5-methylcytosine rRNA methyltransferase NSUN4 isoform b	-	-	-	-	1.4	1.2	1.2	1.3	1.4	1.2	1.2	1.3	8.7	2	3	3	3	335	37.3	6.5
NP_932343.1	37620206	C1orf52	UPF0690 protein C1orf52	-	-	-	-	1.0	1.4	1.1	1.3	1.0	1.4	1.1	1.3	7.7	1	1	1	1	182	20.6	5.2
NP_775746.1	27735047	DCUN1D3	DCN1-like protein 3	-	-	-	-	1.5	1.3	1.2	1.2	1.5	1.3	1.2	1.2	20.4	1	3	3	6	304	34.3	5.1
NP_001020410.1	68800032	TSPAN4	tetraspanin-4 isoform b	-	-	-	-	0.9	1.4	1.5	1.2	0.9	1.4	1.5	1.2	17.8	2	2	2	2	174	19.4	5.4
NP_056210.1	32698704	TECPR1	tectonin beta-propeller repeat-containing protein 1	-	-	-	-	1.1	1.2	1.3	1.2	1.1	1.2	1.3	1.2	0.5	1	1	1	1	1165	129.6	6.2
NP_036250.2	31083028	CCRN4L	nocturnin	-	-	-	-	1.1	1.3	1.2	1.2	1.1	1.3	1.2	1.2	3.5	1	1	1	1	431	48.2	7.2
NP_653188.2	50345833	BTBD16	BTB/POZ domain-containing protein 16	-	-	-	-	1.1	1.0	1.0	1.2	1.1	1.0	1.0	1.2	5.1	1	1	1	1	506	58.4	9.2
NP_036434.1	6912464	LPHN2	latrophilin-2 precursor	-	-	-	-	0.9	1.0	0.9	1.2	0.9	1.0	0.9	1.2	1.6	1	2	2	3	1403	157.1	6.2
NP_001186248.1	313482834	PEX26	peroxisome assembly protein 26 isoform b	-	-	-	-	1.2	1.4	1.1	1.2	1.2	1.4	1.1	1.2	9.8	2	1	1	4	256	28.4	5.5
NP_001073970.1	122937442	TMEM223	transmembrane protein 223	-	-	-	-	1.3	1.3	1.5	1.2	1.3	1.3	1.5	1.2	5.0	1	1	1	1	202	22.0	11.0
NP_001005377.1	53829381	PLAUR	urokinase plasminogen activator surface receptor isoform 3 precursor	-	-	-	-	1.0	1.3	1.2	1.2	1.0	1.3	1.2	1.2	14.1	4	3	3	6	290	32.0	6.3
NP_689979.1	22749499	TMEM256	transmembrane protein 256 precursor	-	-	-	-	1.0	1.1	1.0	1.2	1.0	1.1	1.0	1.2	38.9	1	2	2	4	113	11.7	8.9
NP_055134.2	7706607	LEMD3	inner nuclear membrane protein Man1 isoform 1	-	-	-	-	1.1	1.2	1.3	1.2	1.1	1.2	1.3	1.2	3.6	2	2	2	4	911	99.9	7.5
NP_057723.1	7706186	TNFRSF12A	tumor necrosis factor receptor superfamily member 12A precursor	-	-	-	-	1.2	1.5	1.5	1.2	1.2	1.5	1.5	1.2	23.3	1	2	2	8	129	13.9	9.0
NP_542395.1	18087837	DTD2	probable D-tyrosyl-tRNA(Tyr) deacylase 2	-	-	-	-	1.1	1.3	1.2	1.2	1.1	1.3	1.2	1.2	13.1	1	1	1	1	168	18.6	7.8
NP_001276908.1	586798132	NUMBL	numb-like protein isoform b	-	-	-	-	1.2	1.3	1.5	1.2	1.2	1.3	1.5	1.2	4.9	2	1	2	3	568	60.8	8.9

Khan *et al.* , 2019. Multi-omics analysis to characterize cigarette smoke induced molecular alterations in esophageal cells
Supplementary Table 5. List of proteins quantified in untreated and chronically treated Het1A cells with cigarette smoke condensate for 8 months

NP_Accession	Protein group Accession	Gene ID	Description	Het-1A-Smoke - 2M/Parental	Het-1A-Smoke - 4M/Parental	Het-1A-Smoke - 6M/Parental	Het-1A-Smoke - 8M/Parental	Het-1A-Smoke - 2M/Parental	Het-1A-Smoke - 4M/Parental	Het-1A-Smoke - 6M/Parental	Het-1A-Smoke - 8M/Parental	Het-1A-Smoke - 2M/Parental	Het-1A-Smoke - 4M/Parental	Het-1A-Smoke - 6M/Parental	Het-1A-Smoke - 8M/Parental	Coverage (%)	Proteins	Unique Peptides	# Peptides	PSM	# AAs	MW [kDa]	calc. pI
				Replicate 1				Replicate 2				Average of replicates											
NP_005092.1	4826774	ISG15	ubiquitin-like protein ISG15 precursor	-	-	-	-	1.0	0.9	0.9	1.2	1.0	0.9	0.9	1.2	12.1	1	1	1	2	165	17.9	7.4
NP_001702.1	4502403	BGN	biglycan preproprotein	-	-	-	-	1.1	1.3	1.2	1.2	1.1	1.3	1.2	1.2	8.4	1	2	2	3	368	41.6	7.5
NP_839952.1	30410790	TUSC3	tumor suppressor candidate 3 isoform b precursor	-	-	-	-	1.2	1.1	1.1	1.2	1.2	1.1	1.1	1.2	2.9	2	1	1	1	347	39.5	10.1
NP_001253987.1	388596702	CASP7	caspase-7 isoform f	-	-	-	-	1.4	1.3	1.1	1.2	1.4	1.3	1.1	1.2	8.3	4	1	1	2	278	31.6	7.6
NP_060684.4	95113664	LRRC1	leucine-rich repeat-containing protein 1	-	-	-	-	1.1	1.3	1.0	1.2	1.1	1.3	1.0	1.2	10.3	1	2	3	4	524	59.2	5.0
NP_002577.2	27436887	PBX2	pre-B-cell leukemia transcription factor 2	-	-	-	-	1.6	1.3	1.3	1.2	1.6	1.3	1.3	1.2	10.7	4	2	2	2	430	45.9	7.6
NP_006454.1	5453545	STAMBP	STAM-binding protein	-	-	-	-	1.0	1.0	0.8	1.2	1.0	1.0	0.8	1.2	2.1	1	1	1	4	424	48.0	6.3
NP_004220.2	28558973	MED14	mediator of RNA polymerase II transcription subunit 14	-	-	-	-	0.9	0.9	0.8	1.2	0.9	0.9	0.8	1.2	1.7	1	2	3	5	1454	160.5	8.7
NP_065178.1	9966913	ACTR3B	actin-related protein 3B isoform 1	-	-	-	-	1.3	1.0	1.0	1.2	1.3	1.0	1.0	1.2	15.1	3	1	5	28	418	47.6	5.9
NP_060383.2	66773038	CDC37L1	hsp90 co-chaperone Cdc37-like 1	-	-	-	-	1.1	0.9	0.9	1.2	1.1	0.9	0.9	1.2	2.7	1	1	1	1	337	38.8	5.3
NP_060694.2	90855771	COA1	cytochrome c oxidase assembly factor 1 homolog	-	-	-	-	1.1	1.2	1.1	1.2	1.1	1.2	1.1	1.2	24.7	1	2	2	3	146	16.7	8.8
NP_061331.2	46094065	SLC25A40	solute carrier family 25 member 40	-	-	-	-	1.4	1.4	1.3	1.2	1.4	1.4	1.3	1.2	8.3	1	2	2	3	338	38.1	9.3
NP_075566.2	198041662	PYCRL	pyrroline-5-carboxylate reductase 3	-	-	-	-	1.1	1.2	1.2	1.2	1.1	1.2	1.2	1.2	17.1	1	4	4	7	286	29.9	8.1
NP_001191809.1	325910882	UBE2D1	ubiquitin-conjugating enzyme E2 D1 isoform 2	-	-	-	-	1.2	1.3	1.2	1.2	1.2	1.3	1.2	1.2	34.9	3	2	2	6	109	12.4	7.9
NP_001243405.1	374429545	WDR92	WD repeat-containing protein 92 isoform 2	-	-	-	-	1.2	1.2	1.2	1.2	1.2	1.2	1.2	1.2	21.9	2	5	5	7	288	32.1	8.0
NP_001103132.3	359718912	HECTD4	probable E3 ubiquitin-protein ligase HECTD4	-	-	-	-	1.4	1.3	1.3	1.2	1.4	1.3	1.3	1.2	0.4	1	1	1	3	4284	469.7	6.2
NP_001597.2	45446740	ABCA2	ATP-binding cassette sub-family A member 2 isoform a	-	-	-	-	1.2	1.3	1.5	1.2	1.2	1.3	1.5	1.2	0.5	2	1	1	2	2436	269.8	6.8
NP_006323.2	29150261	IFI30	gamma-interferon-inducible lysosomal thiol reductase preproprotein	-	-	-	-	0.4	0.8	0.5	1.2	0.4	0.8	0.5	1.2	4.4	1	1	1	1	250	27.9	4.9
NP_116145.1	14249394	HPDL	4-hydroxyphenylpyruvate dioxygenase-like protein	-	-	-	-	1.2	1.1	1.1	1.2	1.2	1.1	1.1	1.2	10.5	1	2	2	3	371	39.4	7.0
NP_001189332.1	320202962	ASB6	ankyrin repeat and SOCS box protein 6 isoform 3	-	-	-	-	1.2	1.2	1.1	1.2	1.2	1.2	1.1	1.2	11.0	3	3	3	4	392	44.1	6.0
NP_001276037.1	574286889	FAM96A	MIP18 family protein FAM96A isoform b precursor	-	-	-	-	1.1	1.3	1.0	1.2	1.1	1.3	1.0	1.2	22.6	2	2	2	5	102	11.6	4.7
NP_115494.1	14149771	WDR54	WD repeat-containing protein 54	-	-	-	-	0.9	0.8	0.9	1.2	0.9	0.8	0.9	1.2	2.7	1	1	1	1	334	35.9	6.2
NP_002804.2	18543329	PSMD9	26S proteasome non-ATPase regulatory subunit 9 isoform 1	-	-	-	-	1.3	1.2	1.6	1.2	1.3	1.2	1.6	1.2	7.2	1	1	1	1	223	24.7	6.9
NP_005018.1	4826908	PIK3R2	phosphatidylinositol 3-kinase regulatory subunit beta	-	-	-	-	1.1	1.0	1.1	1.2	1.1	1.0	1.1	1.2	2.5	1	1	1	1	728	81.6	6.5
NP_071941.1	11968057	ACTR6	actin-related protein 6	-	-	-	-	1.1	1.3	1.2	1.2	1.1	1.3	1.2	1.2	3.0	1	1	1	1	396	45.8	5.0
NP_149123.2	118766337	ZIC5	zinc finger protein ZIC 5	-	-	-	-	1.1	1.1	1.0	1.2	1.1	1.1	1.0	1.2	2.6	1	1	1	1	663	68.4	8.8
NP_795364.2	116268093	TAS2R41	taste receptor type 2 member 41	-	-	-	-	1.3	1.1	1.4	1.2	1.3	1.1	1.4	1.2	4.2	1	1	1	1	307	35.9	10.0
NP_001258745.1	425871110	DCLRE1A	DNA cross-link repair 1A protein	-	-	-	-	1.3	1.2	1.0	1.2	1.3	1.2	1.0	1.2	5.0	1	2	2	3	1040	116.3	8.0
NP_001275717.1	571026641	TOM1L2	TOM1-like protein 2 isoform 6	-	-	-	-	0.9	1.1	0.9	1.2	0.9	1.1	0.9	1.2	11.6	6	3	3	8	389	42.9	4.8
NP_112571.2	157694526	FCRL5	Fc receptor-like protein 5 isoform 1 precursor	-	-	-	-	1.7	1.4	1.4	1.2	1.7	1.4	1.4	1.2	1.8	2	1	1	1	977	106.4	7.1
NP_115820.2	63252863	SLX4	structure-specific endonuclease subunit SLX4	-	-	-	-	1.2	1.1	1.1	1.2	1.2	1.1	1.1	1.2	1.1	1	1	1	2	1834	199.9	6.1
NP_004341.1	4757918	RUNX3	runt-related transcription factor 3 isoform 2	-	-	-	-	1.1	1.1	1.2	1.2	1.1	1.1	1.2	1.2	1.7	5	1	1	1	415	44.3	9.5
NP_060611.2	16554607	MRPS10	28S ribosomal protein S10, mitochondrial	-	-	-	-	1.1	1.2	1.2	1.2	1.1	1.2	1.2	1.2	14.4	1	2	2	7	201	23.0	8.0
NP_001140174.1	226442963	KDM5C	lysine-specific demethylase 5C isoform 2	-	-	-	-	1.0	1.0	1.0	1.2	1.0	1.0	1.0	1.2	1.4	3	1	1	1	1379	155.0	5.6
NP_004722.2	24431958	SLC16A7	monocarboxylate transporter 2	-	-	-	-	0.8	1.0	0.9	1.2	0.8	1.0	0.9	1.2	2.3	1	1	1	2	478	52.2	9.3
NP_055919.2	239047271	ATG2A	autophagy-related protein 2 homolog A	-	-	-	-	1.1	1.3	1.1	1.2	1.1	1.3	1.1	1.2	0.5	1	1	1	1	1938	212.7	5.9
NP_001091873.1	148491106	ZBTB21	zinc finger and BTB domain-containing protein 21 isoform S	-	-	-	-	1.2	1.1	1.1	1.2	1.2	1.1	1.1	1.2	11.2	2	5	5	7	865	95.7	6.5
NP_006224.1	5453930	POLR2I	DNA-directed RNA polymerase II subunit RPB9	-	-	-	-	1.2	1.1	1.3	1.2	1.2	1.1	1.3	1.2	18.4	1	1	1	2	125	14.5	5.1
NP_569830.2	209862987	MADD	MAP kinase-activating death domain protein isoform f	-	-	-	-	1.0	1.0	1.0	1.2	1.0	1.0	1.0	1.2	2.2	10	2	2	4	1479	163.9	6.3
NP_005524.2	306922358	IFI35	interferon-induced 35 kDa protein	-	-	-	-	1.0	1.1	0.9	1.2	1.0	1.1	0.9	1.2	5.6	1	1	1	1	288	31.8	6.1
NP_064533.3	191250771	SPIRE1	protein spire homolog 1 isoform b	-	-	-	-	1.1	1.2	1.1	1.2	1.1	1.2	1.1	1.2	5.1	3	2	3	3	742	83.9	8.6
NP_068594.1	13399304	APOBEC3G	DNA dC->dU-editing enzyme APOBEC-3G	-	-	-	-	1.3	1.3	1.2	1.2	1.3	1.3	1.2	1.2	3.7	1	1	1	1	384	46.4	8.0
NP_001013417.1	61676093	CCNC	cyclin-C isoform b	-	-	-	-	1.1	1.1	1.1	1.2	1.1	1.1	1.1	1.2	6.6	3	1	2	2	198	22.9	5.9
NP_001137469.1	221316669	OGDHL	2-oxoglutarate dehydrogenase-like, mitochondrial isoform c	-	-	-	-	1.4	1.1	0.9	1.2	1.4	1.1	0.9	1.2	12.5	3	2	9	33	801	91.2	6.8
NP_004861.2	116517313	MPDU1	mannose-P-dolichol utilization defect 1 protein	-	-	-	-	1.1	1.2	1.4	1.2	1.1	1.2	1.4	1.2	12.2	1	3	3	29	247	26.6	8.9
NP_001294870.1	808154199	SEC11C	signal peptidase complex catalytic subunit SEC11C isoform 2	-	-	-	-	1.1	1.1	1.2	1.2	1.1	1.1	1.2	1.2	12.4	2	1	1	2	161	18.1	8.9
NP_001138551.1	223468630	COG6	conserved oligomeric Golgi complex subunit 6 isoform 2	-	-	-	-	1.2	1.2	1.2	1.2	1.2	1.2	1.2	1.2	5.7	2	3	3	5	615	68.4	5.9
NP_055470.1	45237193	SLC25A44	solute carrier family 25 member 44 isoform 2	-	-	-	-	1.2	1.5	1.3	1.2	1.2	1.5	1.3	1.2	4.8	2	1	1	1	314	35.4	9.6
NP_660358.2	21918872	LACE1	lactation elevated protein 1	-	-	-	-	1.1	1.2	1.2	1.2	1.1	1.2	1.2	1.2	1.7	1	1	1	1	481	54.8	7.3
NP_001251.1	4502745	CDK8	cyclin-dependent kinase 8	-	-	-	-	1.0	1.1	1.0	1.2	1.0	1.1	1.0	1.2	1.5	1	1	1	1	464	53.2	8.6
NP_061332.2	56549091	BCAP29	B-cell receptor-associated protein 29 isoform b	-	-	-	-	1.0	1.0	1.1	1.2	1.0	1.0	1.1	1.2	5.8	2	2	2	2	241	28.3	9.5
NP_001269565.1	544063486	DBI	acyl-CoA-binding protein isoform 7	-	-	-	-	1.3	1.2	1.3	1.2	1.3	1.2	1.3	1.2	12.7	7	1	1	1	63	7.3	8.9
NP_001161205.1	268370295	VARS2	valine--tRNA ligase, mitochondrial isoform 3	-	-	-	-	1.1	1.3	0.8	1.2	1.1	1.3	0.8	1.2	1.6	3	1	1	1	923	102.4	6.6
NP_003922.1	4507913	WASF1	wiskott-Aldrich syndrome protein family member 1	-	-	-	-	1.0	1.0	1.1	1.2	1.0	1.0	1.1	1.2	9.3	1	2	2	2	559	61.6	6.5
NP_277055.1	41281603	C19orf33	immortalization up-regulated protein	-	-	-	-	1.3	1.2	1.8	1.2	1.3	1.2	1.8	1.2	29.3	1	2	2	4	106	10.9	9.7
NP_569709.1	18765702	ELMO1	engulfment and cell motility protein 1 isoform 2	-	-	-	-	1.0	1.1	1.2	1.2	1.0	1.1	1.2	1.2	6.9	2	1	2	3	247	28.7	6.4
NP_116260.2	21361873	RELT	tumor necrosis factor receptor superfamily member 19L precursor	-	-	-	-	1.2	1.5	1.2	1.2	1.2	1.5	1.2	1.2	4.2	1	1	1	1	430	46.1	8.4
NP_056355.2	24430139	RPAP1	RNA polymerase II-associated protein 1	-	-	-	-	0.9	1.0	0.9	1.2	0.9	1.0	0.9	1.2	0.8	1	1	1	2	1393	152.7	6.4
NP_689813.2	110681708	ZNF579	zinc finger protein 579	-	-	-	-	0.8	1.1	1.0	1.2	0.8	1.1	1.0	1.2	2.9	1	1	1	2	562	60.5	8.7
NP_065962.1	157738645	PLXNA4	plexin-A4 isoform 1 precursor	-	-	-	-	1.2	1.2	1.1	1.2	1.2	1.2	1.1	1.2	0.7	1	1	1	1	1894	212.3	6.9
NP_060480.1	8922256	IFT57	intraflagellar transport protein 57 homolog	-	-	-	-	1.1	1.0	1.1	1.2	1.1	1.0	1.1	1.2	2.3	1	1	1	2	429	49.1	5.0
NP_115759.2	19923642	HPS3	Hermansky-Pudlak syndrome 3 protein	-	-	-	-	0.9	1.2	0.9	1.2	0.9	1.2	0.9	1.2	4.2	2	3	3	5	1004	113.7	6.4
NP_000064.1	4557429	CD3G	T-cell surface glycoprotein CD3 gamma chain precursor	-	-	-	-	1.0	1.0	0.9	1.2	1.0	1.0	0.9	1.2	11.5	1	1	1	1	182	20.5	8.4
NP_001003398.1	51093830	BICD1	protein bicaudal D homolog 1 isoform 2	-	-	-	-	0.8	1.2	1.1	1.2	0.8	1.2	1.1	1.2	0.8	2	1	1	1	835	95.7	5.6
NP_114124.1	13994287	KLF16	Krueppel-like factor 16	-	-	-	-	1.2	1.2	1.2	1.2	1.2	1.2	1.2	1.2	10.3	1	1	1	2	252	25.4	9.9
NP_689822.2	213021160	CNST	consortin isoform 1	-	-	-	-	0.8	1.1	1.1	1.2	0.8	1.1	1.1	1.2	2.3	1	1	1	2	725	79.5	4.5
NP_005964.3	40807447	PRCC	proline-rich protein PRCC	-	-	-	-	1.1	1.0	1.0	1.2	1.1	1.0	1.0	1.2	27.5	1	6	6	13	491	52.4	5.1
NP_659451.1	21450731	HYLS1	hydrolethalus syndrome protein 1	-	-	-	-	1.0	1.2	0.9	1.2	1.0	1.2	0.9	1.2	7.0	1	1	1	1	299	34.3	7.4
NP_001017980.1	63025214	VMA21	vacuolar ATPase assembly integral membrane protein VMA21	-	-	-	-	0.8	1.2	0.7	1.2	0.8	1.2	0.7	1.2	11.9	1	1	1	3	101	11.3	7.2
NP_001273038.1	554790414	CLN3	battenin isoform d	-	-	-	-	0.9	0.9	0.9	1.2	0.9	0.9	0.9	1.2	4.2	4	1	1	3	360	39.1	6.4
NP_061924.1	21265096	MRPL50	39S ribosomal protein L50, mitochondrial	-	-	-	-	1.2	1.2	1.1	1.2	1.2	1.2	1.1	1.2	7.0	1	1	1	1	158	18.3	7.9
NP_057240.3	44680154	MTMR2	myotubularin-related protein 2 isoform 1	-	-	-	-	1.3	1.1	1.1	1.2	1.3	1.1	1.1	1.2	5.1	2	2	2	3	643	73.3	7.4
NP_001137307.1	219802034	NFRKB	nuclear factor related to kappa-B-binding protein isoform 1	-	-	-	-	2.5	1.2	1.1	1.2	2.5	1.2	1.1	1.2	1.9	2	1	1	1	1299	138.9	9.2
NP_004116.2	21361097	NAJC25-GNG	DNAJC25-GNG10 protein precursor	-	-	-	-	1.2	1.2	1.2	1.2	1.2	1.2	1.2	1.2	17.7	1	1	2	6	153	16.5	8.6
NP_060371.2	29725622	TPCN1	two pore calcium channel protein 1 isoform 2	-	-	-	-	1.2	1.3	1.4	1.2	1.2	1.3	1.4	1.2	4.4	3	3	3	3	816	94.1	8.3
NP_001287835.1	665505940	IBTK	inhibitor of Bruton tyrosine kinase isoform 2	-	-	-	-	1.0	1.2	1.1	1.2	1.0	1.2	1.1	1.2	1.4	2	2	2	2	1338	148.9	7.6
NP_001166936.1	291167764	KIF21A	kinesin-like protein KIF21A isoform 4	-	-	-	-	1.1	1.1	1.1	1.2	1.1	1.1	1.1	1.2	1.1	4	1	1	1	1621	180.6	6.3
NP_001288183.1	669173522	ACBD5	acyl-CoA-binding domain-containing protein 5 isoform 5	-	-	-	-	1.0	1.1	1.2	1.2	1.0	1.1	1.2	1.2	3.5	5	1	1	2	348	38.7	4.9
NP_057005.1	7706244	CUTA	protein CutA isoform 2 precursor	-	-	-	-	1.4	1.1	1.5	1.2	1.4	1.1	1.5	1.2	9.0	3	1	1	2	156	16.8	5.2
NP_955361.1	41056259;18252047	SLC43A3	solute carrier family 43 member 3 isoform 1	-	-	-	-	1.4	1.5	1.8	1.2	1.4	1.5	1.8	1.2	5.3	2	3	3	8	491	54.5	8.5
NP_872307.2	42740903	MTERF4	transcription termination factor 4, mitochondrial	-	-	-	-	1.7	1.1	1.0	1.2	1.7	1.1	1.0	1.2	2.9	1	1	1	1	381	43.9	4.8
NP_114129.1	151301171	TAF3	transcription initiation factor TFIID subunit 3	-	-	-	-	0.9	1.1	1.1	1.2	0.9	1.1	1.1	1.2	1.6	1	1	1	4	929	103.5	9.1
NP_001141.2	157266300	ANPEP	aminopeptidase N precursor	-	-	-	-	0.9	1.3	1.2	1.2	0.9	1.3	1.2	1.2	5.5	1	3	3	3	967	109.5	5.5
NP_000036.2	10947139	ARG1	arginase-1 isoform 2	-	-	-	-	1.0	1.2	1.2	1.2	1.0	1.2	1.2	1.2	2.2	2	1	1	1	322	34.7	7.2
NP_004273.1	4757834	BAG2	BAG family molecular chaperone regulator 2	-	-	-	-	1.1	1.2	1.1	1.2	1.1	1.2	1.1	1.2	35.1	1	6	6	12	211	23.8	6.7
NP_001273988.1	559098413	HECW1	E3 ubiquitin-protein ligase HECW1 isoform b	-	-	-	-	1.0	0.9	1.2	1.2	1.0	0.9	1.2	1.2	1.8	2	1	1	1	1572	175.6	5.5
NP_079216.4	117553586	DOCK5	dedicator of cytokinesis protein 5	-	-	-	-	1.3	1.0	1.0	1.2	1.3	1.0	1.0	1.2	0.5	1	1	1	1	1870	215.2	8.0
NP_009147.3	31657127;31657125	HPS5	Hermansky-Pudlak syndrome 5 protein isoform b	-	-	-	-	1.2	1.0	1.3	1.2	1.2	1.0	1.3	1.2	2.5	2	2	2	3	1015	114.8	5.4
NP_004081.1	4758208	DUSP3	dual specificity protein phosphatase 3	-	-	-	-	1.0	1.1	1.0	1.2	1.0	1.1	1.0	1.2	13.5	1	2	2	3	185	20.5	7.8
NP_001276863.1	584277014	LRCH4	leucine-rich repeat and calponin homology domain-containing protein 4 isoform 2	-	-	-	-	1.2	1.3	1.1	1.2	1.2	1.3	1.1	1.2	2.5	2	1	1	1	652	70.2	7.3
NP_001180444.1	301898545	SLC30A6	zinc transporter 6 isoform 4	-	-	-	-	1.1	1.1	1.2	1.2	1.1	1.1	1.2	1.2	2.8	3	1	1	2	432	47.6	8.3
NP_055731.2	38016937	LMTK2	serine/threonine-protein kinase LMTK2 precursor	-	-	-	-	1.3	1.2	1.0	1.2	1.3	1.2	1.0	1.2	1.7	1	2	2	2	1503	164.8	4.5
NP_001068566.1	116734668	MOCS1	molybdenum cofactor biosynthesis protein 1 isoform 4	-	-	-	-	1.4	1.1	1.0	1.2	1.4	1.1	1.0	1.2	5.7	2	1	1	2	385	43.1	9.3
NP_597713.2	289577096	SHROOM1	protein Shroom1 isoform 2	-	-	-	-	1.0	1.0	1.0	1.2	1.0	1.0	1.0	1.2	5.4	2	2	2	2	847	90.2	6.2

Khan *et al.* , 2019. Multi-omics analysis to characterize cigarette smoke induced molecular alterations in esophageal cells
Supplementary Table 5. List of proteins quantified in untreated and chronically treated Het1A cells with cigarette smoke condensate for 8 months

NP_Accession	Protein group Accession	Gene ID	Description	Het-1A-Smoke - 2M/Parental	Het-1A-Smoke - 4M/Parental	Het-1A-Smoke - 6M/Parental	Het-1A-Smoke - 8M/Parental	Het-1A-Smoke - 2M/Parental	Het-1A-Smoke - 4M/Parental	Het-1A-Smoke - 6M/Parental	Het-1A-Smoke - 8M/Parental	Het-1A-Smoke - 2M/Parental	Het-1A-Smoke - 4M/Parental	Het-1A-Smoke - 6M/Parental	Het-1A-Smoke - 8M/Parental	Coverage (%)	Proteins	Unique Peptides	# Peptides	PSM	# AAs	MW [kDa]	calc. pI
				Replicate 1				Replicate 2				Average of replicates											
NP_004272.2	14043024	BAG3	BAG family molecular chaperone regulator 3	-	-	-	-	0.9	1.2	1.0	1.2	0.9	1.2	1.0	1.2	6.8	1	3	3	5	575	61.6	6.9
NP_000683.3	25777730	ALDH1B1	aldehyde dehydrogenase X, mitochondrial precursor	-	-	-	-	1.2	1.2	1.1	1.2	1.2	1.2	1.1	1.2	10.3	1	3	4	7	517	57.2	7.0
NP_005150.1	4885049	ACTC1	actin, alpha cardiac muscle 1 proprotein	-	-	-	-	1.2	1.4	1.4	1.1	1.2	1.4	1.4	1.1	60.0	6	2	22	1153	377	42.0	5.4
NP_061907.2	38016957	RHOF	rho-related GTP-binding protein RhoF precursor	-	-	-	-	1.1	1.3	1.3	1.1	1.1	1.3	1.3	1.1	13.3	1	2	2	3	211	23.6	8.6
NP_001161827.1	270288820	OSMR	oncostatin-M-specific receptor subunit beta isoform 2 precursor	-	-	-	-	1.1	1.2	1.1	1.1	1.1	1.2	1.1	1.1	2.9	2	1	1	1	342	39.5	6.5
NP_060272.3	157388904	HEATR2	HEAT repeat-containing protein 2	-	-	-	-	1.1	1.2	1.2	1.1	1.1	1.2	1.2	1.1	7.3	1	5	5	11	855	93.5	6.4
NP_001129146.1	208610013	SMIM19	small integral membrane protein 19	-	-	-	-	0.9	1.0	1.0	1.1	0.9	1.0	1.0	1.1	20.6	1	1	1	1	107	12.4	5.5
NP_002760.1	4506145	PRSS1	trypsin-1 preproprotein	-	-	-	-	1.0	1.1	1.0	1.1	1.0	1.1	1.0	1.1	11.3	7	1	2	9	247	26.5	6.5
NP_009191.1	9966764	LYPLA2	acyl-protein thioesterase 2	-	-	-	-	0.8	1.0	0.9	1.1	0.8	1.0	0.9	1.1	7.8	1	1	1	4	231	24.7	7.2
NP_006282.2	26051240	TNFAIP2	tumor necrosis factor alpha-induced protein 2	-	-	-	-	1.1	1.0	1.0	1.1	1.1	1.0	1.0	1.1	2.8	1	1	1	2	654	72.6	6.5
NP_001265535.1	519666775	BFSP1	filensin isoform 3	-	-	-	-	1.1	1.1	1.2	1.1	1.1	1.1	1.2	1.1	1.9	4	1	1	1	526	58.5	5.0
NP_065963.1	62953136	FLYWCH1	FLYWCH-type zinc finger-containing protein 1 isoform b	-	-	-	-	1.0	1.1	1.0	1.1	1.0	1.1	1.0	1.1	4.7	2	1	2	3	703	78.6	8.5
NP_065194.2	56788364	TUBGCP6	gamma-tubulin complex component 6	-	-	-	-	1.0	1.2	1.0	1.1	1.0	1.2	1.0	1.1	3.0	1	3	3	4	1819	200.4	6.3
NP_938023.1	38093659	TMEM173	stimulator of interferon genes protein isoform 1	-	-	-	-	1.0	1.0	1.1	1.1	1.0	1.0	1.1	1.1	5.5	1	1	1	1	379	42.2	7.0
NP_115719.1	14150134	CHCHD6	coiled-coil-helix-coiled-coil-helix domain-containing protein 6, mitochondrial	-	-	-	-	1.1	1.1	1.0	1.1	1.1	1.1	1.0	1.1	6.8	1	1	1	2	235	26.4	8.9
NP_056224.3	331284125	EP400	E1A-binding protein p400	-	-	-	-	1.0	1.1	1.1	1.1	1.0	1.1	1.1	1.1	1.5	1	3	3	4	3123	339.6	9.2
NP_055945.2	139394668	TBC1D9	TBC1 domain family member 9	-	-	-	-	1.3	0.9	1.2	1.1	1.3	0.9	1.2	1.1	1.1	1	1	1	1	1266	143.1	5.3
NP_001273045.1	554790285	CCDC90B	coiled-coil domain-containing protein 90B, mitochondrial isoform b	-	-	-	-	1.2	1.1	1.2	1.1	1.2	1.1	1.2	1.1	16.7	5	3	3	11	245	28.0	5.3
NP_004036.1	4757804	ATOX1	copper transport protein ATOX1	-	-	-	-	1.1	1.2	1.1	1.1	1.1	1.2	1.1	1.1	11.8	1	1	1	2	68	7.4	7.2
NP_008919.3	50845384	ADAMTS1	A disintegrin and metalloproteinase with thrombospondin motifs 1 preproprotein	-	-	-	-	1.3	1.2	1.2	1.1	1.3	1.2	1.2	1.1	1.5	1	1	1	1	967	105.3	6.8
NP_001273696.1	557948000	USP46	ubiquitin carboxyl-terminal hydrolase 46 isoform 3	-	-	-	-	0.9	1.0	1.0	1.1	0.9	1.0	1.0	1.1	5.1	4	1	1	2	354	41.1	6.8
NP_001138844.1	223941891	PTPN3	tyrosine-protein phosphatase non-receptor type 3 isoform 6	-	-	-	-	1.2	1.0	1.1	1.1	1.2	1.0	1.1	1.1	1.9	6	1	1	1	581	65.8	6.2
NP_001135973.1	215820632	MON1A	vacuolar fusion protein MON1 homolog A isoform b	-	-	-	-	1.1	1.2	1.2	1.1	1.1	1.2	1.2	1.1	1.8	2	1	1	2	490	55.4	9.7
NP_001207423.1	333944039	GLYATL1	glycine N-acyltransferase-like protein 1 isoform 2	-	-	-	-	1.0	1.1	1.1	1.1	1.0	1.1	1.1	1.1	2.0	1	1	1	1	302	35.1	6.9
NP_002824.1	4506301	PTPN9	tyrosine-protein phosphatase non-receptor type 9	-	-	-	-	1.1	1.0	1.1	1.1	1.1	1.0	1.1	1.1	3.4	1	2	2	3	593	68.0	8.0
NP_001093096.1	153251766	MTFR1L	mitochondrial fission regulator 1-like isoform a	-	-	-	-	1.0	1.2	1.0	1.1	1.0	1.2	1.0	1.1	5.8	1	1	1	1	292	31.9	6.1
NP_004610.1	11321603	TRAF5	TNF receptor-associated factor 5	-	-	-	-	1.0	1.4	1.0	1.1	1.0	1.4	1.0	1.1	2.0	1	1	1	1	557	64.4	7.4
NP_690610.1	23510421	FAS	tumor necrosis factor receptor superfamily member 6 isoform 2 precursor	-	-	-	-	0.9	1.1	1.1	1.1	0.9	1.1	1.1	1.1	17.5	2	3	3	7	314	35.4	7.9
NP_775822.3	67906195	ANKS6	ankyrin repeat and SAM domain-containing protein 6	-	-	-	-	1.1	1.2	1.2	1.1	1.1	1.2	1.2	1.1	2.5	1	1	1	22	871	92.2	7.4
NP_001278931.1	635172817	ZNF330	zinc finger protein 330 isoform 2	-	-	-	-	1.1	1.0	1.2	1.1	1.1	1.0	1.2	1.1	12.3	2	2	2	4	260	29.2	5.0
NP_001175.2	157266317	ATR	serine/threonine-protein kinase ATR	-	-	-	-	1.2	1.2	1.1	1.1	1.2	1.2	1.1	1.1	0.8	1	1	1	1	2644	301.2	7.4
NP_006560.3	261278346	CGREF1	cell growth regulator with EF hand domain protein 1 isoform a precursor	-	-	-	-	1.2	1.2	1.2	1.1	1.2	1.2	1.2	1.1	13.8	2	2	2	2	318	33.4	4.4
NP_060211.3	98162802	DCAF16	DDB1- and CUL4-associated factor 16	-	-	-	-	1.0	1.0	1.1	1.1	1.0	1.0	1.1	1.1	6.9	1	1	1	2	216	24.2	6.0
NP_057041.2	166235186	APIP	methylthioribulose-1-phosphate dehydratase	-	-	-	-	1.1	1.1	1.1	1.1	1.1	1.1	1.1	1.1	5.8	1	1	1	1	242	27.1	7.1
NP_116167.3	23346418	MINA	bifunctional lysine-specific demethylase and histidyl-hydroxylase MINA isoform b	-	-	-	-	1.1	1.1	1.1	1.1	1.1	1.1	1.1	1.1	18.5	2	6	6	13	464	52.6	6.7
NP_620128.1	20270311	SLC25A46	solute carrier family 25 member 46	-	-	-	-	1.1	1.1	1.0	1.1	1.1	1.1	1.0	1.1	11.5	2	3	3	5	418	46.1	7.4
NP_543019.2	109689705	FAM65C	protein FAM65C isoform 1	-	-	-	-	1.0	1.2	1.1	1.1	1.0	1.2	1.1	1.1	1.1	2	1	1	1	946	105.2	7.4
NP_009129.1	6005842	PROSC	proline synthase co-transcribed bacterial homolog protein	-	-	-	-	1.1	1.0	1.0	1.1	1.1	1.0	1.0	1.1	16.0	1	4	4	12	275	30.3	7.5
NP_001273943.1	558757337	GLIPR2	Golgi-associated plant pathogenesis-related protein 1 isoform f	-	-	-	-	1.1	1.0	1.3	1.1	1.1	1.0	1.3	1.1	29.2	4	1	1	1	72	7.8	10.2
NP_001191407.1	325121006	VP23C-CDR1	uncharacterized protein LOC100533496	-	-	-	-	1.4	1.3	1.1	1.1	1.4	1.3	1.1	1.1	4.3	4	1	1	1	164	19.0	5.5
NP_008817.1	5901896	ATP5E	ATP synthase subunit epsilon, mitochondrial	-	-	-	-	1.1	0.9	1.2	1.1	1.1	0.9	1.2	1.1	29.4	1	2	2	4	51	5.8	9.9
NP_009206.1	6005760	TUSC2	tumor suppressor candidate 2	-	-	-	-	1.2	1.1	1.2	1.1	1.2	1.1	1.2	1.1	10.9	1	1	1	3	110	12.1	9.7
NP_001272988.1	554506503	RAB30	ras-related protein Rab-30	-	-	-	-	0.9	1.0	1.0	1.1	0.9	1.0	1.0	1.1	9.9	10	1	2	3	203	23.0	5.0
NP_001290170.1	735997432	CNOT6	CCR4-NOT transcription complex subunit 6	-	-	-	-	1.2	1.1	1.1	1.1	1.2	1.1	1.1	1.1	9.0	2	2	2	2	557	63.3	7.3
NP_005099.2	18860918	XYLB	xylulose kinase	-	-	-	-	1.1	1.0	0.9	1.1	1.1	1.0	0.9	1.1	2.8	1	1	1	2	536	58.3	5.9
NP_004897.2	21361159;395455090	WTAP	pre-mRNA-splicing regulator WTAP isoform 1	-	-	-	-	1.0	1.0	0.9	1.1	1.0	1.0	0.9	1.1	10.6	3	3	3	9	396	44.2	5.2
NP_055733.2	31542309	CHSY1	chondroitin sulfate synthase 1 precursor	-	-	-	-	1.1	1.1	1.0	1.1	1.1	1.1	1.0	1.1	2.6	1	1	1	2	802	91.7	9.2
NP_060312.2	209862796	SLC48A1	heme transporter HRG1	-	-	-	-	1.3	1.2	1.1	1.1	1.3	1.2	1.1	1.1	8.2	1	1	1	1	146	16.4	9.6
NP_001009924.1	58331122;42476068	TMEM230	transmembrane protein 230 isoform 2	-	-	-	-	1.1	1.1	1.1	1.1	1.1	1.1	1.1	1.1	21.7	2	2	2	3	120	13.2	9.3
NP_055067.1	7657585	SLC25A15	mitochondrial ornithine transporter 1	-	-	-	-	1.3	1.2	1.1	1.1	1.3	1.2	1.1	1.1	2.7	1	1	1	1	301	32.7	9.1
NP_005236.2	66346693	FAT1	protocadherin Fat 1 precursor	-	-	-	-	1.3	1.1	1.1	1.1	1.3	1.1	1.1	1.1	0.6	1	2	2	2	4588	506.0	5.0
NP_001006115.1	55769518	IP6K1	inositol hexakisphosphate kinase 1 isoform 2	-	-	-	-	1.0	1.0	1.0	1.1	1.0	1.0	1.0	1.1	6.5	2	2	2	3	276	31.5	8.6
NP_001274521.1	567316020	SLC50A1	sugar transporter SWEET1 isoform j	-	-	-	-	1.4	1.1	1.2	1.1	1.4	1.1	1.2	1.1	6.8	9	1	1	1	103	11.8	8.7
NP_055378.1	7657548	TRAPPC2	trafficking protein particle complex subunit 2 isoform 1	-	-	-	-	0.9	1.0	1.0	1.1	0.9	1.0	1.0	1.1	10.0	2	1	1	1	140	16.4	6.5
NP_001191343.1	324711034	TINAGL1	tubulointerstitial nephritis antigen-like isoform 2 precursor	-	-	-	-	1.0	1.2	1.1	1.1	1.0	1.2	1.1	1.1	2.1	2	1	1	1	436	48.8	7.3
NP_955389.1	40788018	ARHGAP11A	rho GTPase-activating protein 11A isoform 2	-	-	-	-	1.4	1.1	1.2	1.1	1.4	1.1	1.2	1.1	4.8	4	2	2	2	501	55.5	9.5
NP_001277194.1	594140567	CERS1	ceramide synthase 1 isoform 3	-	-	-	-	1.0	1.0	1.0	1.1	1.0	1.0	1.0	1.1	5.9	3	1	1	1	239	27.9	7.3
NP_113600.1	13899219	GABARAPL1	gamma-aminobutyric acid receptor-associated protein-like 1	-	-	-	-	1.2	1.0	1.1	1.1	1.2	1.0	1.1	1.1	14.5	2	1	1	4	117	14.0	8.7
NP_659402.1	21450645	TTC16	tetratricopeptide repeat protein 16	-	-	-	-	1.3	1.0	1.4	1.1	1.3	1.0	1.4	1.1	1.5	1	1	1	1	873	98.2	9.0
NP_055408.2	156142180	CXXC1	CXXC-type zinc finger protein 1 isoform 2	-	-	-	-	1.0	1.1	1.0	1.1	1.0	1.1	1.0	1.1	3.8	2	2	2	2	656	75.7	8.2
NP_115990.3	188035928	MCEE	methylmalonyl-CoA epimerase, mitochondrial precursor	-	-	-	-	1.1	1.2	1.0	1.1	1.1	1.2	1.0	1.1	7.4	1	1	1	2	176	18.7	9.1
NP_001278905.1	634743249;634743251	SPARCL1	SPARC-like protein 1 isoform 2	-	-	-	-	1.2	0.8	1.1	1.1	1.2	0.8	1.1	1.1	5.8	2	1	1	2	539	61.7	4.8
NP_001252511.1	387942392	URM1	ubiquitin-related modifier 1 isoform c	-	-	-	-	1.5	1.0	1.7	1.1	1.5	1.0	1.7	1.1	20.6	3	1	1	2	63	7.1	5.3
NP_640335.2	164663775	MTFMT	methionyl-tRNA formyltransferase, mitochondrial	-	-	-	-	1.0	1.0	0.9	1.1	1.0	1.0	0.9	1.1	9.0	1	3	3	4	389	43.8	9.7
NP_001182001.1	303519441	TMEM184B	transmembrane protein 184B isoform b	-	-	-	-	1.0	1.0	1.5	1.1	1.0	1.0	1.5	1.1	3.2	2	1	1	1	341	38.7	7.5
NP_647477.1	21536423	AMPH	amphiphysin isoform 2	-	-	-	-	1.1	1.0	0.9	1.1	1.1	1.0	0.9	1.1	3.7	6	1	3	7	653	71.9	4.7
NP_001007069.1	55749504	SDCBP	syntenin-1 isoform 2	-	-	-	-	1.8	1.2	1.2	1.1	1.8	1.2	1.2	1.1	6.5	3	1	1	2	292	31.7	7.5
NP_004285.2	34577120	MTRF1	peptide chain release factor 1, mitochondrial	-	-	-	-	1.1	1.2	1.0	1.1	1.1	1.2	1.0	1.1	4.3	1	1	1	2	445	52.3	8.5
NP_006773.2	33300635	ZFPL1	zinc finger protein-like 1	-	-	-	-	1.4	1.1	1.1	1.1	1.4	1.1	1.1	1.1	5.8	1	2	2	4	310	34.1	8.1
NP_001157787.1	256222280	ANKRD36	ankyrin repeat domain-containing protein 36A	-	-	-	-	1.0	0.9	1.0	1.1	1.0	0.9	1.0	1.1	1.1	2	2	2	4	1941	217.3	8.7
NP_001017390.1	62868225	SULT1A4	sulfotransferase 1A4	-	-	-	-	1.1	1.1	1.4	1.1	1.1	1.1	1.4	1.1	5.4	2	1	1	1	295	34.2	6.0
NP_003136.1	4507239	SSR2	translocon-associated protein subunit beta precursor	-	-	-	-	1.1	1.2	1.0	1.1	1.1	1.2	1.0	1.1	3.3	1	1	1	1	183	20.1	8.4
NP_057542.2	13124773	HGH1	protein HGH1 homolog	-	-	-	-	1.0	1.0	1.0	1.1	1.0	1.0	1.0	1.1	27.4	1	6	6	26	390	42.1	4.8
NP_060326.1	8923481	GEMIN8	gem-associated protein 8	-	-	-	-	1.0	1.0	1.0	1.1	1.0	1.0	1.0	1.1	6.6	1	1	1	1	242	28.6	6.8
NP_055309.2	116805348	TNRC6A	trinucleotide repeat-containing gene 6A protein	-	-	-	-	0.9	0.9	0.9	1.1	0.9	0.9	0.9	1.1	0.7	1	1	1	1	1962	210.2	7.0
NP_036520.1	6912574	BLOC1S6	biogenesis of lysosome-related organelles complex 1 subunit 6	-	-	-	-	1.1	1.1	1.1	1.1	1.1	1.1	1.1	1.1	24.4	1	2	2	6	172	19.7	6.4
NP_001242974.1	365777411	PIK3CB	phosphatidylinositol 4,5-bisphosphate 3-kinase catalytic subunit beta isoform isoform 2	-	-	-	-	1.0	1.3	1.0	1.1	1.0	1.3	1.0	1.1	5.3	3	2	2	6	582	66.8	8.1
NP_009143.1	6005747	RNF2	E3 ubiquitin-protein ligase RING2	-	-	-	-	0.9	0.9	0.9	1.1	0.9	0.9	0.9	1.1	14.3	1	3	4	7	336	37.6	6.8
NP_056511.2	15618997	GPX7	glutathione peroxidase 7 precursor	-	-	-	-	1.2	1.3	1.2	1.1	1.2	1.3	1.2	1.1	13.9	1	1	1	1	187	21.0	8.3
NP_001136002.1	216547715	BHLHB9	protein BHLHb9	-	-	-	-	1.1	1.0	1.0	1.1	1.1	1.0	1.0	1.1	1.1	1	1	1	1	547	60.3	7.6
NP_001240693.1	359338964	SRA1	steroid receptor RNA activator 1 isoform 2	-	-	-	-	1.0	1.1	1.0	1.1	1.0	1.1	1.0	1.1	5.8	2	1	1	1	208	23.4	10.2
NP_001230396.1	343478263	UBASH3A	ubiquitin-associated and SH3 domain-containing protein A isoform 3	-	-	-	-	1.2	1.2	1.3	1.1	1.2	1.2	1.3	1.1	1.9	3	1	1	1	526	59.1	8.0
NP_149017.2	25952122	BBS4	Bardet-Biedl syndrome 4 protein isoform 1	-	-	-	-	0.9	1.1	1.0	1.1	0.9	1.1	1.0	1.1	2.1	1	1	1	1	519	58.2	7.3
NP_001273694.1	557948004	KIAA0226L	uncharacterized protein KIAA0226-like isoform e	-	-	-	-	1.0	1.1	1.1	1.1	1.0	1.1	1.1	1.1	3.4	5	1	1	1	505	57.1	7.1
NP_001243620.1	375493542	MELK	maternal embryonic leucine zipper kinase isoform 7	-	-	-	-	1.0	1.1	1.1	1.1	1.0	1.1	1.1	1.1	4.6	9	2	3	4	571	65.6	9.0
NP_001909.3	392494079	DBT	lipoamide acyltransferase component of branched-chain alpha-keto acid dehydrogenase complex, mitochondrial precursor	-	-	-	-	1.0	1.0	1.0	1.1	1.0	1.0	1.0	1.1	6.4	1	2	2	3	482	53.5	8.5

Khan *et al.* , 2019. Multi-omics analysis to characterize cigarette smoke induced molecular alterations in esophageal cells
Supplementary Table 5. List of proteins quantified in untreated and chronically treated Het1A cells with cigarette smoke condensate for 8 months

NP_Accession	Protein group Accession	Gene ID	Description	Het-1A-Smoke - 2M/Parental	Het-1A-Smoke - 4M/Parental	Het-1A-Smoke - 6M/Parental	Het-1A-Smoke - 8M/Parental	Het-1A-Smoke - 2M/Parental	Het-1A-Smoke - 4M/Parental	Het-1A-Smoke - 6M/Parental	Het-1A-Smoke - 8M/Parental	Het-1A-Smoke - 2M/Parental	Het-1A-Smoke - 4M/Parental	Het-1A-Smoke - 6M/Parental	Het-1A-Smoke - 8M/Parental	Coverage (%)	Proteins	Unique Peptides	# Peptides	PSM	# AAs	MW [kDa]	calc. pI
				Replicate 1				Replicate 2				Average of replicates											
NP_005877.2	83779014	KATNB1	katanin p80 WD40 repeat-containing subunit B1	-	-	-	-	1.1	1.3	1.2	1.1	1.1	1.3	1.2	1.1	2.0	1	1	1	2	655	72.3	7.6
NP_004905.2	31795535	RAB36	ras-related protein Rab-36	-	-	-	-	1.0	1.1	1.0	1.1	1.0	1.1	1.0	1.1	3.6	1	1	1	1	333	36.3	7.9
NP_006522.2	28329439	IFT88	intraflagellar transport protein 88 homolog isoform 2	-	-	-	-	0.9	0.9	1.0	1.1	0.9	0.9	1.0	1.1	7.5	2	3	3	5	824	93.1	6.3
NP_003183.1	4507373	TBCC	tubulin-specific chaperone C	-	-	-	-	1.0	0.9	0.9	1.1	1.0	0.9	0.9	1.1	2.6	1	1	1	1	346	39.2	5.7
NP_001294976.1	808688285	PRUNE2	protein prune homolog 2 isoform 2	-	-	-	-	1.2	1.1	1.2	1.1	1.2	1.1	1.2	1.1	0.2	3	1	1	1	3062	337.4	4.4
NP_055106.1	7657118	GCAT	2-amino-3-ketobutyrate coenzyme A ligase, mitochondrial isoform 2 precursor	-	-	-	-	1.2	1.3	1.3	1.1	1.2	1.3	1.3	1.1	29.4	2	6	6	12	419	45.3	8.0
NP_775762.1	27735089	LSM11	U7 snRNA-associated Sm-like protein LSm11	-	-	-	-	1.2	0.9	1.2	1.1	1.2	0.9	1.2	1.1	5.0	1	1	1	5	360	39.5	11.0
NP_001019628.3	262118265	RGPD1	RANBP2-like and GRIP domain-containing protein 1	-	-	-	-	1.2	1.2	1.1	1.1	1.2	1.2	1.1	1.1	10.6	3	1	16	37	1748	196.5	6.1
NP_001171595.1	296011001	ABI1	abl interactor 1 isoform k	-	-	-	-	0.8	0.9	1.0	1.1	0.8	0.9	1.0	1.1	5.7	12	1	2	4	388	42.6	6.0
NP_001275927.1	573014822	GMIP	GEM-interacting protein isoform 2	-	-	-	-	1.3	1.0	1.2	1.1	1.3	1.0	1.2	1.1	1.9	3	1	1	1	941	103.6	5.9
NP_061878.3	109150419	MIOS	WD repeat-containing protein mio	-	-	-	-	1.0	1.1	1.0	1.1	1.0	1.1	1.0	1.1	3.0	1	2	2	3	875	98.5	6.7
NP_001338.2	40806175	DGKQ	diacylglycerol kinase theta	-	-	-	-	0.8	1.1	1.2	1.1	0.8	1.1	1.2	1.1	1.4	1	1	1	1	942	101.1	7.5
NP_001182156.1	304766677	TRPM4	transient receptor potential cation channel subfamily M member 4 isoform 2	-	-	-	-	1.1	1.2	1.2	1.1	1.1	1.2	1.2	1.1	2.1	2	1	1	1	1069	118.6	7.9
NP_077302.3	190194416	FBXL15	F-box/LRR-repeat protein 15	-	-	-	-	1.1	1.1	1.0	1.1	1.1	1.1	1.0	1.1	9.0	1	2	2	2	300	33.0	7.4
NP_112578.2	94536798	INO80B	INO80 complex subunit B	-	-	-	-	0.9	0.9	0.9	1.1	0.9	0.9	0.9	1.1	6.5	1	2	2	4	356	38.6	9.6
NP_001929.1	4503381	DR1	protein Dr1	-	-	-	-	1.5	1.0	1.7	1.1	1.5	1.0	1.7	1.1	7.4	1	1	1	2	176	19.4	4.8
NP_219486.1	15553097	METTL18	histidine protein methyltransferase 1 homolog	-	-	-	-	1.1	1.1	1.2	1.1	1.1	1.1	1.2	1.1	2.4	1	1	1	1	372	42.1	6.8
NP_001008398.2	192455698	GPX8	probable glutathione peroxidase 8 isoform a	-	-	-	-	1.1	1.1	1.2	1.1	1.1	1.1	1.2	1.1	17.2	3	4	4	5	209	23.9	9.3
NP_001026895.2	188035877	DDX59	probable ATP-dependent RNA helicase DDX59	-	-	-	-	1.0	1.1	1.0	1.1	1.0	1.1	1.0	1.1	5.0	2	2	3	4	619	68.8	7.4
NP_005754.2	13027640	AASS	alpha-aminoadipic semialdehyde synthase, mitochondrial	-	-	-	-	0.9	1.1	0.9	1.1	0.9	1.1	0.9	1.1	3.8	1	2	2	2	926	102.1	6.6
NP_001007103.1	55770872	L3MBTL3	lethal(3)malignant brain tumor-like protein 3 isoform b	-	-	-	-	1.1	1.1	1.1	1.1	1.1	1.1	1.1	1.1	3.7	2	2	2	2	755	85.7	6.4
NP_061947.1	9506545	DLL4	delta-like protein 4 precursor	-	-	-	-	0.7	1.0	0.6	1.1	0.7	1.0	0.6	1.1	5.8	1	1	1	1	685	74.6	6.9
NP_076415.3	226371704;224493912	ZSCAN18	zinc finger and SCAN domain-containing protein 18 isoform 3	-	-	-	-	1.1	1.2	1.0	1.1	1.1	1.2	1.0	1.1	15.9	3	3	3	9	510	54.8	4.8
NP_001185702.1	311771621	PI4KB	phosphatidylinositol 4-kinase beta isoform 2	-	-	-	-	0.9	1.0	1.0	1.1	0.9	1.0	1.0	1.1	4.0	3	3	3	3	801	89.8	6.4
NP_001172003.1	297374764	ZCCHC6	terminal uridylyltransferase 7 isoform 2	-	-	-	-	1.2	1.1	1.2	1.1	1.2	1.1	1.2	1.1	1.5	2	2	2	2	1259	144.6	6.9
NP_055684.3	50582989	IQSEC1	IQ motif and SEC7 domain-containing protein 1 isoform b	-	-	-	-	1.1	1.1	1.1	1.1	1.1	1.1	1.1	1.1	2.2	2	1	1	2	963	108.2	6.9
NP_004681.1	4758666	LATS1	serine/threonine-protein kinase LATS1 isoform 1	-	-	-	-	1.2	1.2	1.3	1.1	1.2	1.2	1.3	1.1	1.7	2	2	2	3	1130	126.8	8.7
NP_663306.1	21735566	MAP3K7	mitogen-activated protein kinase kinase kinase 7 isoform D	-	-	-	-	1.0	1.1	1.0	1.1	1.0	1.1	1.0	1.1	3.7	4	2	2	2	491	53.7	6.7
NP_000129.3	281485550	FBN1	fibrillin-1 precursor	-	-	-	-	0.9	1.1	0.9	1.1	0.9	1.1	0.9	1.1	0.5	1	1	1	2	2871	312.1	4.9
NP_055692.2	54792138	HELZ	probable helicase with zinc finger domain	-	-	-	-	1.0	0.9	1.0	1.1	1.0	0.9	1.0	1.1	0.9	1	1	1	2	1942	218.9	7.4
NP_000428.2	4758878	PAFAH2	platelet-activating factor acetylhydrolase 2, cytoplasmic	-	-	-	-	0.9	1.1	0.9	1.1	0.9	1.1	0.9	1.1	5.4	1	1	1	1	392	44.0	6.9
NP_004390.3	100913204	DDX11	probable ATP-dependent RNA helicase DDX11 isoform 2	-	-	-	-	0.8	1.2	1.1	1.1	0.8	1.2	1.1	1.1	2.6	4	1	1	1	856	96.1	7.6
NP_005883.2	33356148	FMNL1	formin-like protein 1	-	-	-	-	1.0	1.1	1.1	1.1	1.0	1.1	1.1	1.1	1.0	1	1	1	1	1100	121.8	5.7
NP_001264332.1	475808217	ZNF730	putative zinc finger protein 730	-	-	-	-	1.0	1.0	1.2	1.1	1.0	1.0	1.2	1.1	1.4	1	1	1	1	503	59.0	9.4
NP_653271.2	74271845	A2ML1	alpha-2-macroglobulin-like protein 1 isoform 1 precursor	-	-	-	-	1.0	1.1	1.0	1.1	1.0	1.1	1.0	1.1	0.5	1	1	1	1	1454	161.0	5.7
NP_001159465.1	260763878	GDPD1	glycerophosphodiester phosphodiesterase domain-containing protein 1 isoform 3	-	-	-	-	1.2	1.0	1.0	1.1	1.2	1.0	1.0	1.1	5.5	3	1	1	4	289	33.2	8.6
NP_002890.2	195976807	RBP1	retinol-binding protein 1 isoform a	-	-	-	-	1.1	1.1	1.1	1.1	1.1	1.1	1.1	1.1	8.1	1	1	1	1	197	22.3	6.1
NP_054887.2	92859575	METTL5	methyltransferase-like protein 5	-	-	-	-	0.9	1.0	1.1	1.1	0.9	1.0	1.1	1.1	5.3	1	1	1	1	209	23.7	6.7
NP_001274425.1	566559871	FIGNL1	fidgetin-like protein 1 isoform 2	-	-	-	-	1.0	1.0	1.1	1.1	1.0	1.0	1.1	1.1	5.3	2	1	2	7	563	61.6	7.8
NP_001161375.1	269847422	MFSD1	major facilitator superfamily domain-containing protein 1 isoform 2	-	-	-	-	1.0	1.1	1.0	1.1	1.0	1.1	1.0	1.1	2.5	2	1	1	2	475	51.7	7.4
NP_689637.3	124244056	AMER1	APC membrane recruitment protein 1	-	-	-	-	1.1	1.0	1.1	1.1	1.1	1.0	1.1	1.1	1.1	1	1	1	1	1135	124.0	4.8
NP_065897.1	149588928	SLAIN2	SLAIN motif-containing protein 2	-	-	-	-	1.0	1.0	0.9	1.1	1.0	1.0	0.9	1.1	2.8	1	1	1	1	581	62.5	9.5
NP_114160.1	13994353	KCTD10	BTB/POZ domain-containing adapter for CUL3-mediated RhoA degradation protein 3	-	-	-	-	1.0	1.1	0.9	1.1	1.0	1.1	0.9	1.1	2.6	1	1	1	1	313	35.4	6.3
NP_001257354.1	394025669	MAP4K3	mitogen-activated protein kinase kinase kinase kinase 3 isoform 2	-	-	-	-	0.9	1.0	1.0	1.1	0.9	1.0	1.0	1.1	3.0	2	2	2	4	873	98.9	7.9
NP_057211.4	42476193	SARAF	store-operated calcium entry-associated regulatory factor isoform 1 precursor	-	-	-	-	1.1	1.1	1.0	1.1	1.1	1.1	1.0	1.1	5.3	1	1	1	1	339	37.0	7.6
NP_056423.2	31742484	EDRF1	erythroid differentiation-related factor 1 isoform 2	-	-	-	-	1.0	1.0	1.0	1.1	1.0	1.0	1.0	1.1	2.6	2	3	3	5	1204	134.8	6.5
NP_001155000.1	238908508	LRRD1	leucine-rich repeat and death domain-containing protein 1	-	-	-	-	1.0	1.1	1.1	1.1	1.0	1.1	1.1	1.1	0.7	1	1	1	2	860	98.0	7.5
NP_663619.1	22035594	MRPL35	39S ribosomal protein L35, mitochondrial isoform b	-	-	-	-	1.0	1.2	1.2	1.1	1.0	1.2	1.2	1.1	4.1	2	1	1	1	170	19.2	11.8
NP_065733.2	109389364	AS3MT	arsenite methyltransferase	-	-	-	-	0.9	1.0	1.0	1.1	0.9	1.0	1.0	1.1	6.4	1	2	2	2	375	41.7	6.2
YP_003024036.1	251831117	ND5	NADH dehydrogenase subunit 5	-	-	-	-	1.1	1.1	1.1	1.1	1.1	1.1	1.1	1.1	7.3	1	2	2	5	603	67.0	9.0
NP_079379.2	151105229	IFT74	intraflagellar transport protein 74 homolog isoform a	-	-	-	-	1.0	1.1	1.0	1.1	1.0	1.1	1.0	1.1	6.8	2	3	3	3	600	69.2	6.0
NP_055685.3	139394556;139394579	ZBTB40	zinc finger and BTB domain-containing protein 40	-	-	-	-	1.2	1.1	1.1	1.1	1.2	1.1	1.1	1.1	2.3	1	2	2	3	1239	138.0	6.6
NP_001271332.1	548923939	PTCD2	pentatricopeptide repeat-containing protein 2, mitochondrial isoform 2	-	-	-	-	1.1	1.0	1.2	1.1	1.1	1.0	1.2	1.1	3.9	2	1	1	2	279	31.0	9.4
NP_116026.1	14249170	SKP2	S-phase kinase-associated protein 2 isoform 2	-	-	-	-	1.0	1.1	1.2	1.1	1.0	1.1	1.2	1.1	3.9	2	1	1	2	410	46.5	7.7
NP_060780.2	22035562	BRF2	transcription factor IIIB 50 kDa subunit	-	-	-	-	1.2	1.0	1.1	1.1	1.2	1.0	1.1	1.1	3.8	1	1	1	2	419	46.5	7.9
NP_005392.2	34328899	PTPN14	tyrosine-protein phosphatase non-receptor type 14	-	-	-	-	0.9	1.0	1.2	1.1	0.9	1.0	1.2	1.1	3.1	1	2	2	2	1187	135.2	8.3
NP_694978.2	55769539	C6orf223	uncharacterized protein C6orf223 isoform 1	-	-	-	-	1.1	1.0	0.9	1.1	1.1	1.0	0.9	1.1	11.6	1	1	1	1	242	26.1	12.2
NP_001191957.1	326381070	MDM1	nuclear protein MDM1 isoform 4	-	-	-	-	0.9	0.9	1.0	1.1	0.9	0.9	1.0	1.1	1.5	2	1	1	1	679	76.7	8.8
NP_001278675.1	619534182	REL	proto-oncogene c-Rel isoform 2	-	-	-	-	1.0	1.2	0.9	1.1	1.0	1.2	0.9	1.1	1.9	2	1	1	1	587	65.2	6.2
NP_001077086.1	134304846	RB1CC1	RB1-inducible coiled-coil protein 1 isoform 2	-	-	-	-	0.9	0.9	1.0	1.1	0.9	0.9	1.0	1.1	0.9	2	1	1	1	1591	182.7	5.4
NP_060264.4	614458185	FOCAD	focadhesin	-	-	-	-	1.0	1.0	1.0	1.1	1.0	1.0	1.0	1.1	0.8	1	1	1	2	1801	199.9	6.6
NP_776188.1	28395049	COX18	mitochondrial inner membrane protein COX18 isoform 2	-	-	-	-	1.7	1.2	1.1	1.1	1.7	1.2	1.1	1.1	2.7	2	1	1	1	333	37.0	9.8
NP_776195.2	304766239	YIPF6	protein YIPF6 isoform A	-	-	-	-	1.1	1.1	1.1	1.1	1.1	1.1	1.1	1.1	8.5	2	2	2	5	236	26.2	5.6
NP_001244905.1	384081602	CIZ1	cip1-interacting zinc finger protein isoform 6	-	-	-	-	0.9	1.1	0.9	1.1	0.9	1.1	0.9	1.1	2.1	6	1	1	1	797	88.5	6.0
NP_001273516.1	557440814	AIG1	androgen-induced gene 1 protein isoform b	-	-	-	-	1.0	1.4	1.2	1.1	1.0	1.4	1.2	1.1	4.0	2	1	1	1	228	26.3	6.9
NP_065894.2	225579092	SCAPER	S phase cyclin A-associated protein in the endoplasmic reticulum isoform a	-	-	-	-	0.8	0.9	0.8	1.1	0.8	0.9	0.8	1.1	0.8	1	1	1	2	1400	158.2	7.4
NP_006545.1	5729937	MTX2	metaxin-2	-	-	-	-	1.1	1.1	1.1	1.1	1.1	1.1	1.1	1.1	18.6	1	4	4	7	263	29.7	6.3
NP_001270164.1	545687403	TANGO2	transport and Golgi organization protein 2 homolog isoform g	-	-	-	-	1.4	1.8	1.2	1.1	1.4	1.8	1.2	1.1	20.8	4	1	1	1	178	19.5	5.3
NP_115493.1	14149769	MND1	meiotic nuclear division protein 1 homolog isoform 1	-	-	-	-	1.1	1.1	0.9	1.1	1.1	1.1	0.9	1.1	10.7	1	2	2	3	205	23.7	8.2
NP_689895.2	116256479	RWDD4	RWD domain-containing protein 4 isoform 1	-	-	-	-	1.2	0.9	1.2	1.1	1.2	0.9	1.2	1.1	5.3	1	1	1	1	188	21.2	5.3
NP_003556.1	4507831	ULK1	serine/threonine-protein kinase ULK1	-	-	-	-	0.9	1.0	0.8	1.1	0.9	1.0	0.8	1.1	1.6	1	1	1	1	1050	112.5	8.8
NP_598001.1	19424132	NEK7	serine/threonine-protein kinase Nek7	-	-	-	-	0.9	1.0	1.1	1.1	0.9	1.0	1.1	1.1	8.0	5	2	2	3	302	34.5	8.3
NP_001241872.1	363807222	CCDC88A	girdin isoform 3	-	-	-	-	1.1	1.0	1.0	1.1	1.1	1.0	1.0	1.1	1.5	3	2	2	2	1796	207.7	6.0
NP_001229813.1	338968907	ZBTB17	zinc finger and BTB domain-containing protein 17 isoform 1	-	-	-	-	1.1	1.2	1.0	1.1	1.1	1.2	1.0	1.1	2.2	4	1	1	2	721	79.2	7.0
NP_075050.3	39812115	ACTR8	actin-related protein 8	-	-	-	-	1.0	1.0	1.0	1.1	1.0	1.0	1.0	1.1	1.6	1	1	1	1	624	70.4	7.8
NP_005859.1	5031611	BET1	BET1 homolog	-	-	-	-	1.0	1.0	1.0	1.1	1.0	1.0	1.0	1.1	24.6	1	2	2	8	118	13.3	9.1
NP_640337.3	189217897	STXBP5	syntaxin-binding protein 5 isoform a	-	-	-	-	0.9	1.1	1.0	1.1	0.9	1.1	1.0	1.1	2.9	2	2	2	2	1115	123.4	7.1
NP_075045.2	16306568	PAPOLG	poly(A) polymerase gamma	-	-	-	-	1.0	1.0	1.0	1.1	1.0	1.0	1.0	1.1	12.1	1	5	7	8	736	82.8	9.1
NP_057461.2	330864783	AKAP7	A-kinase anchor protein 7 isoform gamma isoform gamma	-	-	-	-	1.1	1.0	1.1	1.1	1.1	1.0	1.1	1.1	4.3	1	1	1	1	348	39.5	6.2
NP_001020087.2	119120938	CBWD5	COBW domain-containing protein 5 isoform a	-	-	-	-	1.1	1.0	1.0	1.1	1.1	1.0	1.0	1.1	17.6	6	1	4	13	347	38.7	4.8
NP_037381.2	156546992	ZNF214	zinc finger protein 214	-	-	-	-	0.8	1.1	1.0	1.1	0.8	1.1	1.0	1.1	1.5	1	1	1	1	606	70.9	8.5
NP_005303.2	38373675	RAPGEF1	rap guanine nucleotide exchange factor 1 isoform a	-	-	-	-	1.1	1.1	1.0	1.1	1.1	1.1	1.0	1.1	2.2	3	2	2	2	1077	120.5	5.9
NP_002146.2	34419635	HSPA6	heat shock 70 kDa protein 6	-	-	-	-	1.0	1.0	1.0	1.1	1.0	1.0	1.0	1.1	20.7	1	1	11	174	643	71.0	6.1
NP_005350.1	4885457	SMAD4	mothers against decapentaplegic homolog 4	-	-	-	-	1.1	1.0	1.0	1.1	1.1	1.0	1.0	1.1	12.3	1	4	4	6	552	60.4	7.0
NP_001186346.1	313661470	ARHGAP23	rho GTPase-activating protein 23	-	-	-	-	1.2	1.0	1.0	1.1	1.2	1.0	1.0	1.1	3.2	1	2	2	3	1491	162.1	9.2
NP_001014402.1	62177112	KATNAL1	katanin p60 ATPase-containing subunit A-like 1	-	-	-	-	1.1	1.0	1.1	1.1	1.1	1.0	1.1	1.1	3.3	1	1	1	1	490	55.4	6.7
NP_001287694.1	663429614	LETMD1	LETM1 domain-containing protein 1 isoform 7	-	-	-	-	1.0	1.0	1.0	1.1	1.0	1.0	1.0	1.1	8.9	3	2	2	2	236	27.2	10.7
NP_056117.1	149944680	HAUS5	HAUS augmin-like complex subunit 5	-	-	-	-	1.0	1.1	0.9	1.1	1.0	1.1	0.9	1.1	12.3	1	4	4	4	633	71.6	8.5
NP_062826.2	21361827	METTL3	N6-adenosine-methyltransferase 70 kDa subunit	-	-	-	-	1.2	1.0	1.2	1.1	1.2	1.0	1.2	1.1	6.7	1	3	3	3	580	64.4	6.4

Khan *et al.* , 2019. Multi-omics analysis to characterize cigarette smoke induced molecular alterations in esophageal cells
Supplementary Table 5. List of proteins quantified in untreated and chronically treated Het1A cells with cigarette smoke condensate for 8 months

NP_Accession	Protein group Accession	Gene ID	Description	Het-1A-Smoke - 2M/Parental	Het-1A-Smoke - 4M/Parental	Het-1A-Smoke - 6M/Parental	Het-1A-Smoke - 8M/Parental	Het-1A-Smoke - 2M/Parental	Het-1A-Smoke - 4M/Parental	Het-1A-Smoke - 6M/Parental	Het-1A-Smoke - 8M/Parental	Het-1A-Smoke - 2M/Parental	Het-1A-Smoke - 4M/Parental	Het-1A-Smoke - 6M/Parental	Het-1A-Smoke - 8M/Parental	Coverage (%)	Proteins	Unique Peptides	# Peptides	PSM	# AAs	MW [kDa]	calc. pI
				Replicate 1				Replicate 2				Average of replicates											
NP_001138728.1	223671883	**IRAK4**	interleukin-1 receptor-associated kinase 4 isoform b	-	-	-	-	0.9	1.0	1.1	1.1	**0.9**	**1.0**	**1.1**	**1.1**	2.4	2	1	1	1	336	37.6	5.1
NP_001229848.1	339276081	**ZFAND6**	AN1-type zinc finger protein 6 isoform d	-	-	-	-	0.8	1.0	1.0	1.1	**0.8**	**1.0**	**1.0**	**1.1**	20.4	4	2	2	3	181	19.8	7.8
NP_001161354.1	268607595	**ANKRD50**	ankyrin repeat domain-containing protein 50 isoform 2	-	-	-	-	0.9	0.9	1.0	1.1	**0.9**	**0.9**	**1.0**	**1.1**	0.5	2	1	1	1	1250	136.4	6.4
NP_570923.2	104487295	**PTPRS**	receptor-type tyrosine-protein phosphatase S isoform 3 precursor	-	-	-	-	1.6	1.0	1.0	1.1	**1.6**	**1.0**	**1.0**	**1.1**	3.0	4	1	2	4	1501	168.3	6.5
NP_996734.1	45827776	**RTN1**	reticulon-1 isoform C	-	-	-	-	0.9	1.0	1.0	1.1	**0.9**	**1.0**	**1.0**	**1.1**	3.9	2	1	1	1	208	23.6	8.9
NP_001284700.1	663071119	**UEVLD**	ubiquitin-conjugating enzyme E2 variant 3 isoform h	-	-	-	-	1.0	1.1	1.1	1.1	**1.0**	**1.1**	**1.1**	**1.1**	5.1	7	1	1	2	215	24.2	7.9
NP_001265539.1	519666783	**MYBL2**	myb-related protein B isoform 2	-	-	-	-	1.0	0.9	0.9	1.1	**1.0**	**0.9**	**0.9**	**1.1**	2.2	2	1	1	1	676	75.8	6.8
NP_001123553.1	194272176	**PLD1**	phospholipase D1 isoform b	-	-	-	-	1.1	1.1	1.0	1.1	**1.1**	**1.1**	**1.0**	**1.1**	5.5	2	3	3	3	1036	119.6	8.7
NP_001289756.1	723586612	**CYB5R2**	NADH-cytochrome b5 reductase 2 isoform 2	-	-	-	-	1.1	1.0	1.0	1.1	**1.1**	**1.0**	**1.0**	**1.1**	4.8	2	1	1	1	230	25.8	9.8
NP_055199.1	7656849	**ACAD8**	isobutyryl-CoA dehydrogenase, mitochondrial	-	-	-	-	1.5	1.4	1.4	1.1	**1.5**	**1.4**	**1.4**	**1.1**	2.2	1	1	1	1	415	45.0	7.9
NP_996930.1	46389560	**ENSA**	alpha-endosulfine isoform 7	-	-	-	-	1.0	1.1	1.1	1.1	**1.0**	**1.1**	**1.1**	**1.1**	24.8	7	1	2	4	113	12.4	9.0
NP_001295018.1	809279635	**ANKS3**	ankyrin repeat and SAM domain-containing protein 3 isoform 3	-	-	-	-	1.0	0.9	1.0	1.1	**1.0**	**0.9**	**1.0**	**1.1**	4.0	3	1	1	2	527	58.0	6.2
NP_037474.1	7019371	**TFPT**	TCF3 fusion partner	-	-	-	-	0.9	1.1	1.0	1.1	**0.9**	**1.1**	**1.0**	**1.1**	5.9	1	1	1	4	253	28.3	5.2
NP_116147.1	14249398	**PHF5A**	PHD finger-like domain-containing protein 5A	-	-	-	-	1.0	1.0	1.1	1.1	**1.0**	**1.0**	**1.1**	**1.1**	12.7	1	2	2	2	110	12.4	8.4
NP_001161407.1	269308190	**CCM2**	cerebral cavernous malformations 2 protein isoform 4	-	-	-	-	1.1	1.1	1.1	1.1	**1.1**	**1.1**	**1.1**	**1.1**	7.1	4	2	2	3	353	39.4	6.0
NP_001093136.1	153792604	**PTAR1**	protein prenyltransferase alpha subunit repeat-containing protein 1	-	-	-	-	0.8	1.0	0.8	1.1	**0.8**	**1.0**	**0.8**	**1.1**	4.5	1	1	1	1	402	46.4	7.0
NP_001257502.1	396578108	**CSNK1G3**	casein kinase I isoform gamma-3 isoform 6	-	-	-	-	1.0	0.9	1.0	1.1	**1.0**	**0.9**	**1.0**	**1.1**	5.2	8	2	2	3	348	40.7	8.8
NP_009211.1	24307929	**OIP5**	protein Mis18-beta	-	-	-	-	1.1	1.0	1.1	1.1	**1.1**	**1.0**	**1.1**	**1.1**	16.6	1	2	2	6	229	24.7	7.4
NP_001254505.1	389616163	**USP43**	ubiquitin carboxyl-terminal hydrolase 43 isoform 2	-	-	-	-	1.0	1.1	1.1	1.1	**1.0**	**1.1**	**1.1**	**1.1**	1.3	2	1	1	1	1118	122.2	9.2
NP_443135.3	156616297	**TUBGCP5**	gamma-tubulin complex component 5 isoform a	-	-	-	-	1.1	1.0	1.0	1.1	**1.1**	**1.0**	**1.0**	**1.1**	1.2	2	1	1	1	1024	118.2	5.9
NP_001265659.1	523525241	**GAS2L1**	GAS2-like protein 1 isoform c	-	-	-	-	1.1	0.9	1.1	1.1	**1.1**	**0.9**	**1.1**	**1.1**	3.1	2	1	1	1	454	48.2	8.3
NP_005546.2	119703753	**KRT6B**	keratin, type II cytoskeletal 6B	-	-	-	-	1.0	1.3	1.2	1.1	**1.0**	**1.3**	**1.2**	**1.1**	28.7	6	1	18	187	564	60.0	8.0
NP_078868.1	13375785	**SRD5A3**	polyprenol reductase	-	-	-	-	1.2	1.0	0.9	1.1	**1.2**	**1.0**	**0.9**	**1.1**	2.2	1	1	1	1	318	36.5	9.4
NP_689763.3	222446607	**SH3RF2**	putative E3 ubiquitin-protein ligase SH3RF2	-	-	-	-	0.9	1.1	1.0	1.0	**0.9**	**1.1**	**1.0**	**1.0**	0.8	1	1	1	1	729	79.2	10.0
NP_037374.1	8392875	**C16orf80**	UPF0468 protein C16orf80	-	-	-	-	1.1	1.0	1.1	1.0	**1.1**	**1.0**	**1.1**	**1.0**	30.1	1	5	5	22	193	22.8	9.8
NP_079046.2	161377433	**METTL8**	methyltransferase-like protein 8	-	-	-	-	1.3	1.1	1.2	1.0	**1.3**	**1.1**	**1.2**	**1.0**	3.0	1	1	1	1	407	47.0	8.9
NP_001273596.1	557786190	**POSTN**	periostin isoform 7 precursor	-	-	-	-	1.1	1.4	1.3	1.0	**1.1**	**1.4**	**1.3**	**1.0**	3.5	9	1	2	3	721	80.3	8.1
NP_000283.1	4505781	**PHKA2**	phosphorylase b kinase regulatory subunit alpha, liver isoform	-	-	-	-	1.3	1.1	1.2	1.0	**1.3**	**1.1**	**1.2**	**1.0**	5.3	2	3	4	7	1235	138.3	6.4
NP_612393.2	93277113	**MTG1**	mitochondrial ribosome-associated GTPase 1	-	-	-	-	1.0	1.0	1.0	1.0	**1.0**	**1.0**	**1.0**	**1.0**	8.7	1	2	2	2	334	37.2	9.5
NP_057610.2	32261322	**BET1L**	BET1-like protein isoform 2	-	-	-	-	1.2	1.1	1.0	1.0	**1.2**	**1.1**	**1.0**	**1.0**	12.8	2	1	1	1	102	11.1	7.3
NP_002877.2	38201690	**RAP2B**	ras-related protein Rap-2b	-	-	-	-	1.0	1.1	1.1	1.0	**1.0**	**1.1**	**1.1**	**1.0**	23.0	3	4	4	9	183	20.5	4.8
NP_065764.1	34013528	**ZNF512B**	zinc finger protein 512B	-	-	-	-	1.1	1.0	1.0	1.0	**1.1**	**1.0**	**1.0**	**1.0**	5.6	1	4	4	5	892	97.2	9.8
NP_059989.2	297139703	**ARID1B**	AT-rich interactive domain-containing protein 1B isoform 1	-	-	-	-	1.1	1.1	1.1	1.0	**1.1**	**1.1**	**1.1**	**1.0**	2.9	4	4	4	7	2236	236.0	6.7
NP_071328.2	21630257	**MTBP**	mdm2-binding protein	-	-	-	-	1.0	1.0	0.9	1.0	**1.0**	**1.0**	**0.9**	**1.0**	2.1	1	1	1	1	904	102.1	7.8
NP_055770.1	42542405	**METTL13**	methyltransferase-like protein 13 isoform 2	-	-	-	-	1.0	0.9	1.1	1.0	**1.0**	**0.9**	**1.1**	**1.0**	7.5	3	3	3	6	613	68.6	6.7
NP_005863.1	5031653	**BCAS2**	pre-mRNA-splicing factor SPF27	-	-	-	-	1.2	1.1	1.1	1.0	**1.2**	**1.1**	**1.1**	**1.0**	20.0	1	3	3	4	225	26.1	5.7
NP_001123914.1	194363762;4885425	**HRAS**	GTPase HRas isoform 1	-	-	-	-	1.0	1.0	1.0	1.0	**1.0**	**1.0**	**1.0**	**1.0**	50.3	2	3	6	28	189	21.3	5.3
NP_001289706.1	720642346	**BPHL**	valacyclovir hydrolase isoform 2	-	-	-	-	1.2	1.1	1.2	1.0	**1.2**	**1.1**	**1.2**	**1.0**	9.1	2	2	2	3	274	31.1	8.5
NP_001234919.1	350994425	**CTAGE5**	cTAGE family member 5 isoform 7	-	-	-	-	1.1	1.0	1.0	1.0	**1.1**	**1.0**	**1.0**	**1.0**	7.0	8	4	4	5	729	82.4	5.1
NP_055467.3	224831257	**IPO13**	importin-13	-	-	-	-	1.1	1.1	1.1	1.0	**1.1**	**1.1**	**1.1**	**1.0**	2.7	1	2	2	3	963	108.1	5.3
NP_710155.2	119226229	**ZFP1**	zinc finger protein 1 homolog	-	-	-	-	1.0	1.0	0.9	1.0	**1.0**	**1.0**	**0.9**	**1.0**	3.0	1	1	1	2	407	47.5	8.6
NP_006014.2	221316620	**CDC123**	cell division cycle protein 123 homolog	-	-	-	-	1.4	1.5	1.6	1.0	**1.4**	**1.5**	**1.6**	**1.0**	1.8	1	1	1	1	336	39.1	4.8
NP_005998.1	5174755;156231049	**ZFAND5**	AN1-type zinc finger protein 5	-	-	-	-	1.2	1.2	1.1	1.0	**1.2**	**1.2**	**1.1**	**1.0**	20.7	1	2	2	6	213	23.1	8.5
NP_001130027.1	211971038	**TLK1**	serine/threonine-protein kinase tousled-like 1 isoform 3	-	-	-	-	1.0	1.0	0.9	1.0	**1.0**	**1.0**	**0.9**	**1.0**	6.9	3	2	3	4	670	77.1	9.0
NP_001182.1	4502381	**BCL2L1**	bcl-2-like protein 1 isoform 2	-	-	-	-	1.1	1.0	1.2	1.0	**1.1**	**1.0**	**1.2**	**1.0**	8.2	2	1	1	1	170	18.9	5.1
NP_055090.1	7710152	**MGAT4B**	alpha-1,3-mannosyl-glycoprotein 4-beta-N-acetylglucosaminyltransferase B isoform 1 precursor	-	-	-	-	1.0	1.2	1.1	1.0	**1.0**	**1.2**	**1.1**	**1.0**	2.4	2	1	1	1	548	63.2	7.9
NP_775925.1	39930531	**EOGT**	EGF domain-specific O-linked N-acetylglucosamine transferase isoform b precursor	-	-	-	-	0.9	1.0	1.0	1.0	**0.9**	**1.0**	**1.0**	**1.0**	5.6	2	2	2	3	443	52.3	6.8
NP_001123522.1	194239709	**DOCK9**	dedicator of cytokinesis protein 9 isoform d	-	-	-	-	0.9	1.1	1.1	1.0	**0.9**	**1.1**	**1.1**	**1.0**	0.8	6	1	1	1	1253	142.6	7.7
NP_001092198.1	148922852;109809737	**GTF2H2C**	general transcription factor IIH subunit 2-like protein	-	-	-	-	1.0	1.1	1.1	1.0	**1.0**	**1.1**	**1.1**	**1.0**	8.4	5	2	3	4	395	44.4	6.6
NP_001138363.1	222080055	**MBIP**	MAP3K12-binding inhibitory protein 1 isoform 2	-	-	-	-	0.9	1.0	1.0	1.0	**0.9**	**1.0**	**1.0**	**1.0**	5.8	2	1	1	2	343	39.1	7.2
XP_011542112.1	767901770	**LOC10272389**	LOW QUALITY PROTEIN: WAS protein family homolog 2-like isoform X7	-	-	-	-	1.1	1.2	1.1	1.0	**1.1**	**1.2**	**1.1**	**1.0**	6.3	2	3	3	11	478	51.5	6.4
NP_000386.1	4559408	**CSF2RB**	cytokine receptor common subunit beta precursor	-	-	-	-	1.1	1.1	1.0	1.0	**1.1**	**1.1**	**1.0**	**1.0**	3.8	1	1	1	1	897	97.3	5.5
NP_001258833.1	429535841	**GSKIP**	GSK3-beta interaction protein	-	-	-	-	1.1	0.9	1.0	1.0	**1.1**	**0.9**	**1.0**	**1.0**	23.7	1	1	1	1	139	15.6	4.5
NP_000055.2	115298678	**C3**	complement C3 precursor	-	-	-	-	1.1	1.1	1.0	1.0	**1.1**	**1.1**	**1.0**	**1.0**	0.7	1	1	1	3	1663	187.0	6.4
NP_115807.1	27436925	**SYVN1**	E3 ubiquitin-protein ligase synoviolin isoform a precursor	-	-	-	-	1.1	1.1	1.1	1.0	**1.1**	**1.1**	**1.1**	**1.0**	3.9	2	3	3	3	616	67.6	6.9
NP_954673.1	40806190	**IEM189-UBE2**	TMEM189-UBE2V1 fusion protein	-	-	-	-	1.0	1.1	1.0	1.0	**1.0**	**1.1**	**1.0**	**1.0**	28.7	9	4	9	54	370	42.2	6.6
NP_001276841.1	583966087;4503093	**CSNK1E**	casein kinase I isoform epsilon	-	-	-	-	0.9	1.0	1.0	1.0	**0.9**	**1.0**	**1.0**	**1.0**	20.9	3	8	8	15	416	47.3	9.7
NP_001138294.1	221316575	**CD58**	lymphocyte function-associated antigen 3 isoform 2	-	-	-	-	0.9	1.0	1.0	1.0	**0.9**	**1.0**	**1.0**	**1.0**	4.0	2	1	1	2	248	27.9	7.0
NP_056437.4	56699480	**CCZ1**	vacuolar fusion protein CCZ1 homolog	-	-	-	-	1.0	1.0	1.0	1.0	**1.0**	**1.0**	**1.0**	**1.0**	3.3	1	1	1	1	482	55.8	6.5
NP_694691.1	23510389	**MTMR3**	myotubularin-related protein 3 isoform b	-	-	-	-	1.2	1.2	1.1	1.0	**1.2**	**1.2**	**1.1**	**1.0**	1.5	3	1	1	4	1161	129.4	6.0
NP_689948.1	23308693	**ZBTB9**	zinc finger and BTB domain-containing protein 9	-	-	-	-	1.1	1.1	1.1	1.0	**1.1**	**1.1**	**1.1**	**1.0**	4.0	1	1	1	1	473	50.6	6.8
NP_004534.2	115527120	**NEB**	nebulin isoform 3	-	-	-	-	1.0	0.9	1.0	1.0	**1.0**	**0.9**	**1.0**	**1.0**	0.2	4	1	1	1	6669	772.4	9.1
NP_115670.1	14150045	**CAMKK1**	calcium/calmodulin-dependent protein kinase kinase 1 isoform a	-	-	-	-	1.0	1.1	1.1	1.0	**1.0**	**1.1**	**1.1**	**1.0**	1.8	2	1	1	1	505	55.7	5.8
NP_116076.1	14249268	**CYHR1**	cysteine and histidine-rich protein 1 isoform 2 precursor	-	-	-	-	0.9	1.0	1.1	1.0	**0.9**	**1.0**	**1.1**	**1.0**	3.1	1	1	1	1	192	19.9	6.5
NP_073586.5	89337270	**FAM160B2**	protein FAM160B2	-	-	-	-	1.1	1.1	1.2	1.0	**1.1**	**1.1**	**1.2**	**1.0**	1.1	1	1	1	1	743	82.3	5.5
NP_003236.3	189458821	**TGM3**	protein-glutamine gamma-glutamyltransferase E	-	-	-	-	1.0	1.1	1.1	1.0	**1.0**	**1.1**	**1.1**	**1.0**	2.6	1	1	1	2	693	76.6	5.9
NP_000934.1	4505991	**PPIC**	peptidyl-prolyl cis-trans isomerase C precursor	-	-	-	-	1.0	1.0	0.9	1.0	**1.0**	**1.0**	**0.9**	**1.0**	14.6	1	1	1	1	212	22.7	8.4
NP_001229875.2	345110633	**EPDR1**	mammalian ependymin-related protein 1 isoform 2 precursor	-	-	-	-	0.9	0.9	0.9	1.0	**0.9**	**0.9**	**0.9**	**1.0**	12.2	2	1	1	2	90	9.9	10.4
NP_001167531.1	291327480	**OSBPL10**	oxysterol-binding protein-related protein 10 isoform 2	-	-	-	-	1.3	1.0	1.4	1.0	**1.3**	**1.0**	**1.4**	**1.0**	3.9	2	2	2	4	700	77.2	7.7
NP_689509.1	22748667	**ATP1A3**	sodium/potassium-transporting ATPase subunit alpha-3 isoform 1	-	-	-	-	1.1	0.8	0.8	1.0	**1.1**	**0.8**	**0.8**	**1.0**	19.6	7	1	14	128	1013	111.7	5.4
NP_001153718.1	237858705	**RPAIN**	RPA-interacting protein isoform d	-	-	-	-	1.2	1.0	1.2	1.0	**1.2**	**1.0**	**1.2**	**1.0**	7.6	5	1	1	1	106	12.3	4.9
NP_201575.3	333609242	**SMDT1**	essential MCU regulator, mitochondrial precursor	-	-	-	-	1.2	1.1	1.0	1.0	**1.2**	**1.1**	**1.0**	**1.0**	19.6	1	1	1	4	107	11.4	7.3
NP_115678.1	14150060	**PSMG3**	proteasome assembly chaperone 3	-	-	-	-	1.0	1.0	1.1	1.0	**1.0**	**1.0**	**1.1**	**1.0**	42.6	1	3	3	5	122	13.1	7.9
NP_004976.2	15718761	**KRAS**	GTPase KRas isoform b	-	-	-	-	0.9	1.0	0.9	1.0	**0.9**	**1.0**	**0.9**	**1.0**	44.2	2	2	5	25	188	21.4	8.1
NP_942595.1	38787935	**BMP2K**	BMP-2-inducible protein kinase isoform a	-	-	-	-	1.1	1.0	1.0	1.0	**1.1**	**1.0**	**1.0**	**1.0**	5.4	2	4	4	9	1161	129.1	6.5
NP_001074318.1	124249392	**DNLZ**	DNL-type zinc finger protein	-	-	-	-	1.4	1.1	1.0	1.0	**1.4**	**1.1**	**1.0**	**1.0**	8.4	1	1	1	1	178	19.2	9.8
NP_002154.1	4504567	**IRF8**	interferon regulatory factor 8	-	-	-	-	1.3	1.1	1.1	1.0	**1.3**	**1.1**	**1.1**	**1.0**	1.9	1	1	1	2	426	48.3	6.8
NP_006794.1	5803000;197209857	**AP3M2**	AP-3 complex subunit mu-2	-	-	-	-	1.0	1.0	0.9	1.0	**1.0**	**1.0**	**0.9**	**1.0**	5.3	1	1	2	5	418	46.9	7.6
NP_066990.3	88759346	**GATAD1**	GATA zinc finger domain-containing protein 1	-	-	-	-	0.8	0.9	0.8	1.0	**0.8**	**0.9**	**0.8**	**1.0**	12.3	1	2	2	4	269	28.7	9.4
XP_011542159.1	767950464	**LOC10537924**	uncharacterized protein LOC105379247	-	-	-	-	1.0	1.1	1.1	1.0	**1.0**	**1.1**	**1.1**	**1.0**	2.4	1	1	1	1	254	27.9	9.7
NP_001155178.1	239787198	**FXN**	frataxin, mitochondrial isoform 3 preproprotein	-	-	-	-	1.1	1.0	1.1	1.0	**1.1**	**1.0**	**1.1**	**1.0**	9.9	3	1	1	2	171	19.1	8.8
NP_006340.1	5453617	**ZNHIT1**	zinc finger HIT domain-containing protein 1	-	-	-	-	1.0	0.9	1.0	1.0	**1.0**	**0.9**	**1.0**	**1.0**	7.8	1	1	1	2	154	17.5	9.4
NP_001269287.1	537544581	**NLRX1**	NLR family member X1 precursor	-	-	-	-	1.1	0.9	1.1	1.0	**1.1**	**0.9**	**1.1**	**1.0**	3.6	1	2	2	2	975	107.5	7.4
NP_116787.1	14916485	**CASP6**	caspase-6 isoform beta	-	-	-	-	0.8	1.0	1.0	1.0	**0.8**	**1.0**	**1.0**	**1.0**	9.8	2	1	1	2	204	22.6	7.2
NP_055991.1	7662158	**FBXO28**	F-box only protein 28 isoform a	-	-	-	-	1.0	0.9	1.0	1.0	**1.0**	**0.9**	**1.0**	**1.0**	13.6	2	3	3	6	368	41.1	9.6
NP_001288015.1	666637989	**CNOT8**	CCR4-NOT transcription complex subunit 8 isoform 4	-	-	-	-	0.9	1.0	1.0	1.0	**0.9**	**1.0**	**1.0**	**1.0**	20.3	4	2	2	3	128	14.6	5.3
NP_056093.3	45935385	**SASH1**	SAM and SH3 domain-containing protein 1	-	-	-	-	1.0	1.0	1.0	1.0	**1.0**	**1.0**	**1.0**	**1.0**	0.6	1	1	1	1	1247	136.6	6.1
NP_008832.2	156119615	**MYO9A**	unconventional myosin-IXa	-	-	-	-	1.0	1.0	1.0	1.0	**1.0**	**1.0**	**1.0**	**1.0**	0.9	1	2	2	3	2548	292.5	8.9

Khan *et al.* , 2019. Multi-omics analysis to characterize cigarette smoke induced molecular alterations in esophageal cells
Supplementary Table 5. List of proteins quantified in untreated and chronically treated Het1A cells with cigarette smoke condensate for 8 months

NP_Accession	Protein group Accession	Gene ID	Description	Het-1A-Smoke - 2M/Parental	Het-1A-Smoke - 4M/Parental	Het-1A-Smoke - 6M/Parental	Het-1A-Smoke - 8M/Parental	Het-1A-Smoke - 2M/Parental	Het-1A-Smoke - 4M/Parental	Het-1A-Smoke - 6M/Parental	Het-1A-Smoke - 8M/Parental	Het-1A-Smoke - 2M/Parental	Het-1A-Smoke - 4M/Parental	Het-1A-Smoke - 6M/Parental	Het-1A-Smoke - 8M/Parental	Coverage (%)	Proteins	Unique Peptides	# Peptides	PSM	# AAs	MW [kDa]	calc. pI
				Replicate 1				Replicate 2				Average of replicates											
NP_001191890.1	326320048	**PBX1**	pre-B-cell leukemia transcription factor 1 isoform 2	-	-	-	-	0.9	1.2	0.8	1.0	**0.9**	**1.2**	**0.8**	**1.0**	8.4	3	1	1	2	347	38.4	8.4
NP_065128.2	21361851	**INTS12**	integrator complex subunit 12	-	-	-	-	1.2	1.1	1.1	1.0	**1.2**	**1.1**	**1.1**	**1.0**	8.7	1	2	2	3	462	48.8	9.7
NP_001026883.1	72534716	**MCUR1**	mitochondrial calcium uniporter regulator 1	-	-	-	-	0.9	1.0	1.0	1.0	**0.9**	**1.0**	**1.0**	**1.0**	2.8	1	1	1	1	359	39.7	9.6
NP_579919.1	19718790	**RAD17**	cell cycle checkpoint protein RAD17 isoform 4	-	-	-	-	1.0	1.1	1.0	1.0	**1.0**	**1.1**	**1.0**	**1.0**	3.3	3	1	1	1	584	66.1	6.9
NP_057004.1	7705706	**RPS27L**	40S ribosomal protein S27-like	-	-	-	-	0.9	1.0	0.9	1.0	**0.9**	**1.0**	**0.9**	**1.0**	38.1	1	1	3	48	84	9.5	9.5
NP_078785.1	13375646	**LRFN3**	leucine-rich repeat and fibronectin type-III domain-containing protein 3 precursor	-	-	-	-	1.0	1.0	1.1	1.0	**1.0**	**1.0**	**1.1**	**1.0**	8.0	1	1	1	1	628	66.2	7.2
NP_004501.3	190343008	**SP110**	sp110 nuclear body protein isoform b	-	-	-	-	1.1	1.2	1.4	1.0	**1.1**	**1.2**	**1.4**	**1.0**	6.6	4	1	1	1	549	61.8	9.6
NP_055336.1	7657562	**SH3BP4**	SH3 domain-binding protein 4	-	-	-	-	0.9	1.0	0.9	1.0	**0.9**	**1.0**	**0.9**	**1.0**	3.4	1	2	2	6	963	107.4	7.7
NP_001243392.1	374253828	**CPSF3L**	integrator complex subunit 11 isoform 5	-	-	-	-	1.0	1.0	1.0	1.0	**1.0**	**1.0**	**1.0**	**1.0**	1.8	5	1	1	2	499	56.0	8.3
NP_036398.3	56549115	**DNAJB5**	dnaJ homolog subfamily B member 5 isoform 3	-	-	-	-	0.7	0.9	0.9	1.0	**0.7**	**0.9**	**0.9**	**1.0**	8.6	3	2	2	3	348	39.1	9.0
NP_005171.4	27883842	**BMI1**	polycomb complex protein BMI-1	-	-	-	-	0.9	0.9	1.0	1.0	**0.9**	**0.9**	**1.0**	**1.0**	2.5	3	1	1	2	326	36.9	8.6
NP_001879.1	4503065	**CRYM**	ketimine reductase mu-crystallin	-	-	-	-	1.2	1.3	1.4	1.0	**1.2**	**1.3**	**1.4**	**1.0**	3.2	1	1	1	2	314	33.8	5.1
NP_005676.3	312836811	**GTF2IRD1**	general transcription factor II-I repeat domain-containing protein 1 isoform 2	-	-	-	-	1.0	1.1	0.9	1.0	**1.0**	**1.1**	**0.9**	**1.0**	1.4	3	1	1	1	944	104.6	6.9
NP_002567.3	42794769	**PAK1**	serine/threonine-protein kinase PAK 1 isoform 2	-	-	-	-	0.9	1.0	0.9	1.0	**0.9**	**1.0**	**0.9**	**1.0**	12.3	2	2	4	9	545	60.6	5.8
NP_001036046.1	110611149	**SNUPN**	snurportin-1	-	-	-	-	1.0	0.9	1.0	1.0	**1.0**	**0.9**	**1.0**	**1.0**	7.8	1	3	3	4	360	41.1	6.6
NP_001138567.1	223029414	**HHLA1**	HERV-H LTR-associating protein 1 precursor	-	-	-	-	0.9	1.0	1.0	1.0	**0.9**	**1.0**	**1.0**	**1.0**	2.3	1	1	1	1	531	58.3	8.9
NP_937832.1	38027914	**COPS8**	COP9 signalosome complex subunit 8 isoform 2	-	-	-	-	0.9	1.0	0.9	1.0	**0.9**	**1.0**	**0.9**	**1.0**	19.4	2	2	2	6	160	17.9	7.3
NP_001184169.1	308522752	**BTN2A2**	butyrophilin subfamily 2 member A2 isoform e precursor	-	-	-	-	0.9	0.9	1.1	1.0	**0.9**	**0.9**	**1.1**	**1.0**	4.3	7	1	1	1	256	28.9	7.8
NP_001120983.2	306922386	**APC**	adenomatous polyposis coli protein isoform a	-	-	-	-	1.1	1.0	0.9	1.0	**1.1**	**1.0**	**0.9**	**1.0**	1.4	2	2	2	2	2825	308.5	8.0
NP_060040.1	48314820	**OPLAH**	5-oxoprolinase	-	-	-	-	1.0	1.0	1.1	1.0	**1.0**	**1.0**	**1.1**	**1.0**	3.6	1	3	3	3	1288	137.4	6.6
NP_062558.1	10257429	**IRGC**	interferon-inducible GTPase 5	-	-	-	-	1.0	0.9	1.0	1.0	**1.0**	**0.9**	**1.0**	**1.0**	1.3	2	1	1	1	463	50.3	5.4
NP_001177725.1	299829184	**NCK1**	cytoplasmic protein NCK1 isoform 2	-	-	-	-	1.2	1.0	1.1	1.0	**1.2**	**1.0**	**1.1**	**1.0**	7.7	2	2	2	3	313	35.5	5.7
NP_689482.1	22748627;303304971	**C12orf65**	probable peptide chain release factor C12orf65, mitochondrial	-	-	-	-	1.0	1.1	1.0	1.0	**1.0**	**1.1**	**1.0**	**1.0**	16.9	1	2	2	10	166	18.8	9.8
NP_001258968.1	440309853	**CDK5RAP2**	CDK5 regulatory subunit-associated protein 2 isoform c	-	-	-	-	1.1	1.2	1.2	1.0	**1.1**	**1.2**	**1.2**	**1.0**	0.9	3	1	1	1	1663	189.5	5.7
NP_573420.2	95147542	**APBB3**	amyloid beta A4 precursor protein-binding family B member 3 isoform a	-	-	-	-	0.9	1.1	1.0	1.0	**0.9**	**1.1**	**1.0**	**1.0**	3.5	4	1	1	1	484	52.3	6.4
NP_055875.1	57529131	**AVL9**	late secretory pathway protein AVL9 homolog	-	-	-	-	1.1	0.9	1.0	1.0	**1.1**	**0.9**	**1.0**	**1.0**	2.0	1	1	1	4	648	71.9	6.2
NP_001129663.1	209969812	**KANK2**	KN motif and ankyrin repeat domain-containing protein 2 isoform 2	-	-	-	-	1.0	1.0	1.0	1.0	**1.0**	**1.0**	**1.0**	**1.0**	2.2	2	2	2	3	851	91.1	5.6
NP_001265003.1	495528154	**COL5A1**	collagen alpha-1(V) chain isoform 2 preproprotein	-	-	-	-	1.0	1.0	0.9	1.0	**1.0**	**1.0**	**0.9**	**1.0**	1.1	2	1	1	2	1838	183.5	5.1
NP_001035515.1	94536809	**U2AF1L4**	splicing factor U2AF 26 kDa subunit isoform 1	-	-	-	-	0.9	0.8	0.8	1.0	**0.9**	**0.8**	**0.8**	**1.0**	17.7	2	1	2	2	181	21.3	8.1
NP_060699.3	224451116	**AP5M1**	AP-5 complex subunit mu-1	-	-	-	-	1.0	1.0	1.0	1.0	**1.0**	**1.0**	**1.0**	**1.0**	2.5	1	1	1	1	490	54.7	6.6
NP_078849.1	13375746	**ARMT1**	protein-glutamate O-methyltransferase isoform a	-	-	-	-	0.9	0.9	0.9	1.0	**0.9**	**0.9**	**0.9**	**1.0**	35.6	2	10	10	33	441	51.1	5.8
NP_001116835.1	182765478;83267877	**RGPD6**	RANBP2-like and GRIP domain-containing protein 5/6 isoform 1	-	-	-	-	1.1	1.0	1.0	1.0	**1.1**	**1.0**	**1.0**	**1.0**	12.1	4	1	18	44	1765	198.8	6.4
NP_008953.1	5901884	**CYB561D2**	cytochrome b561 domain-containing protein 2	-	-	-	-	1.1	1.1	1.0	1.0	**1.1**	**1.1**	**1.0**	**1.0**	5.4	1	1	1	1	222	24.0	9.9
NP_036221.2	171184400	**ABCB10**	ATP-binding cassette sub-family B member 10, mitochondrial	-	-	-	-	1.2	1.0	1.0	1.0	**1.2**	**1.0**	**1.0**	**1.0**	2.2	1	1	1	1	738	79.1	9.8
NP_001136336.2	257196142	**PIEZO1**	piezo-type mechanosensitive ion channel component 1	-	-	-	-	0.9	0.9	0.9	1.0	**0.9**	**0.9**	**0.9**	**1.0**	0.6	1	1	1	2	2521	286.6	7.5
NP_060375.4	157388921	**TMCO3**	transmembrane and coiled-coil domain-containing protein 3 precursor	-	-	-	-	1.2	1.1	1.0	1.0	**1.2**	**1.1**	**1.0**	**1.0**	1.9	1	1	1	1	677	75.5	6.9
NP_001201836.1	333805600	**ZNF48**	zinc finger protein 48 isoform 2	-	-	-	-	1.1	1.1	0.9	1.0	**1.1**	**1.1**	**0.9**	**1.0**	7.5	77	1	2	3	495	54.0	9.8
NP_001137443.1	221136756	**CDYL**	chromodomain Y-like protein isoform d	-	-	-	-	1.1	1.0	1.0	1.0	**1.1**	**1.0**	**1.0**	**1.0**	10.4	2	3	3	6	412	45.1	8.7
NP_612446.1	19923967	**GPRASP2**	G-protein coupled receptor-associated sorting protein 2	-	-	-	-	0.9	1.0	0.9	1.0	**0.9**	**1.0**	**0.9**	**1.0**	2.2	2	1	1	3	838	93.7	5.0
NP_659489.1	21450816	**UPRT**	uracil phosphoribosyltransferase homolog	-	-	-	-	0.9	0.9	1.0	1.0	**0.9**	**0.9**	**1.0**	**1.0**	8.7	1	1	1	2	309	33.8	6.0
NP_001123919.1	194394168	**HAUS2**	HAUS augmin-like complex subunit 2 isoform 2	-	-	-	-	0.8	0.8	0.9	1.0	**0.8**	**0.8**	**0.9**	**1.0**	8.3	2	1	1	4	204	23.3	7.6
NP_005501.2	20631980	**DXO**	decapping and exoribonuclease protein	-	-	-	-	1.0	1.0	1.0	1.0	**1.0**	**1.0**	**1.0**	**1.0**	6.6	1	1	1	1	396	44.9	7.7
NP_001028260.2	75677385	**NGRN**	neugrin precursor	-	-	-	-	0.9	1.1	1.0	1.0	**0.9**	**1.1**	**1.0**	**1.0**	12.0	1	3	3	5	291	32.4	9.1
NP_689866.1	22749327	**UBE2E2**	ubiquitin-conjugating enzyme E2 E2	-	-	-	-	0.8	0.9	1.0	1.0	**0.8**	**0.9**	**1.0**	**1.0**	18.4	3	1	3	8	201	22.2	7.7
NP_689520.2	123173772	**TRMT61A**	tRNA (adenine(58)-N(1))-methyltransferase catalytic subunit TRMT61A	-	-	-	-	1.0	1.1	1.2	1.0	**1.0**	**1.1**	**1.2**	**1.0**	22.2	1	3	3	5	289	31.4	7.4
NP_005783.2	118498352	**MPHOSPH6**	M-phase phosphoprotein 6	-	-	-	-	1.0	0.9	1.2	1.0	**1.0**	**0.9**	**1.2**	**1.0**	5.0	1	1	1	1	160	19.0	5.2
NP_653091.3	444299651	**CASC5**	protein CASC5 isoform 2	-	-	-	-	1.1	1.0	0.7	1.0	**1.1**	**1.0**	**0.7**	**1.0**	1.6	2	2	2	2	2316	262.5	5.4
NP_001273747.1	558472833	**KLF5**	Krueppel-like factor 5 isoform 2	-	-	-	-	1.0	1.0	1.0	1.0	**1.0**	**1.0**	**1.0**	**1.0**	8.7	77	1	2	2	366	41.2	8.7
NP_055666.2	55925608	**KLHL21**	kelch-like protein 21	-	-	-	-	1.0	1.3	1.2	1.0	**1.0**	**1.3**	**1.2**	**1.0**	4.5	1	1	1	1	597	66.6	5.4
NP_001230045.1	340805857	**NFKBIB**	NF-kappa-B inhibitor beta isoform 2	-	-	-	-	1.1	1.4	1.4	1.0	**1.1**	**1.4**	**1.4**	**1.0**	10.4	2	2	2	3	270	29.1	5.1
NP_037386.1	7019547	**TBK1**	serine/threonine-protein kinase TBK1	-	-	-	-	0.9	1.0	0.9	1.0	**0.9**	**1.0**	**0.9**	**1.0**	5.6	1	3	3	6	729	83.6	6.8
NP_056263.1	39930355	**DPCD**	protein DPCD	-	-	-	-	1.0	1.0	1.0	1.0	**1.0**	**1.0**	**1.0**	**1.0**	10.3	1	2	2	5	203	23.2	9.0
NP_001649.1	4502201;66879662;66879664	**ARF1**	ADP-ribosylation factor 1	-	-	-	-	1.1	1.0	1.0	1.0	**1.1**	**1.0**	**1.0**	**1.0**	72.9	8	4	10	176	181	20.7	6.8
NP_659474.1	21450781	**NXPE3**	NXPE family member 3 precursor	-	-	-	-	1.2	1.1	1.0	1.0	**1.2**	**1.1**	**1.0**	**1.0**	3.9	1	1	1	1	559	63.8	8.9
NP_001713.2	55769552	**POLR3D**	DNA-directed RNA polymerase III subunit RPC4	-	-	-	-	0.9	1.0	1.0	1.0	**0.9**	**1.0**	**1.0**	**1.0**	14.1	1	4	4	4	398	44.4	7.0
NP_006470.1	5453904	**RAD51AP1**	RAD51-associated protein 1 isoform b	-	-	-	-	0.9	0.8	0.8	1.0	**0.9**	**0.8**	**0.8**	**1.0**	8.4	2	2	2	2	335	36.7	9.2
NP_001240622.1	358679303	**MCOLN3**	mucolipin-3 isoform 2	-	-	-	-	0.9	0.9	1.0	1.0	**0.9**	**0.9**	**1.0**	**1.0**	1.4	2	1	1	2	497	57.7	6.5
NP_001186814.1	315468520	**HYPK**	huntingtin-interacting protein K isoform 2	-	-	-	-	1.3	1.1	1.5	1.0	**1.3**	**1.1**	**1.5**	**1.0**	9.9	2	1	1	1	81	9.1	4.7
NP_075568.1	12751497	**C8orf33**	UPF0488 protein C8orf33	-	-	-	-	0.8	1.0	0.8	1.0	**0.8**	**1.0**	**0.8**	**1.0**	10.5	1	2	2	6	229	25.0	9.9
NP_003360.2	21687212	**UVRAG**	UV radiation resistance-associated gene protein	-	-	-	-	1.1	1.1	1.1	1.0	**1.1**	**1.1**	**1.1**	**1.0**	1.4	1	1	1	2	699	78.1	8.3
NP_001796.2	28872800	**CEBPE**	CCAAT/enhancer-binding protein epsilon	-	-	-	-	1.0	1.0	0.9	1.0	**1.0**	**1.0**	**0.9**	**1.0**	4.3	1	1	1	1	281	30.6	9.0
NP_005375.2	52630429	**NFIL3**	nuclear factor interleukin-3-regulated protein	-	-	-	-	1.0	1.1	0.9	1.0	**1.0**	**1.1**	**0.9**	**1.0**	2.6	1	1	1	2	462	51.4	7.2
NP_064547.2	20336302	**DHX33**	putative ATP-dependent RNA helicase DHX33 isoform 1	-	-	-	-	1.0	1.0	1.0	1.0	**1.0**	**1.0**	**1.0**	**1.0**	2.1	1	1	1	2	707	78.8	8.9
NP_001689.1	4502327	**AUH**	methylglutaconyl-CoA hydratase, mitochondrial isoform 1 precursor	-	-	-	-	1.0	1.0	1.1	1.0	**1.0**	**1.0**	**1.1**	**1.0**	6.5	2	2	2	3	339	35.6	9.5
NP_056972.1	7705415	**HOOK1**	protein Hook homolog 1	-	-	-	-	1.0	0.8	0.7	1.0	**1.0**	**0.8**	**0.7**	**1.0**	4.8	1	2	2	2	728	84.6	5.1
NP_079358.3	126722969	**CENPT**	centromere protein T	-	-	-	-	1.1	1.0	0.9	1.0	**1.1**	**1.0**	**0.9**	**1.0**	4.5	1	2	2	2	561	60.4	6.6
NP_001014764.1	62339424	**EMC6**	ER membrane protein complex subunit 6	-	-	-	-	1.0	1.0	1.0	1.0	**1.0**	**1.0**	**1.0**	**1.0**	10.9	1	1	1	4	110	12.0	10.1
NP_443730.1	17402902	**GNB1L**	guanine nucleotide-binding protein subunit beta-like protein 1	-	-	-	-	0.9	0.9	1.0	1.0	**0.9**	**0.9**	**1.0**	**1.0**	3.1	1	1	1	2	327	35.6	8.0
XP_006710257.1	578795894	**LOC10050750**	uncharacterized protein LOC100507507	-	-	-	-	0.8	1.1	1.2	1.0	**0.8**	**1.1**	**1.2**	**1.0**	6.9	2	1	1	1	217	24.0	9.7
NP_004435.3	32528301	**EPHB4**	ephrin type-B receptor 4 precursor	-	-	-	-	0.9	1.0	0.9	1.0	**0.9**	**1.0**	**0.9**	**1.0**	4.6	9	4	5	7	987	108.2	6.9
NP_001268451.1	528078341	**MCM8**	DNA helicase MCM8 isoform 4	-	-	-	-	1.0	0.8	0.9	1.0	**1.0**	**0.8**	**0.9**	**1.0**	2.0	4	1	1	1	793	88.7	7.5
NP_060135.1	8923106	**ZCCHC10**	zinc finger CCHC domain-containing protein 10 isoform 4	-	-	-	-	0.9	0.9	1.0	1.0	**0.9**	**0.9**	**1.0**	**1.0**	8.2	4	1	1	6	170	18.4	8.8
NP_001291454.1	751557640	**MAD1L1**	mitotic spindle assembly checkpoint protein MAD1 isoform c	-	-	-	-	0.8	1.0	0.9	1.0	**0.8**	**1.0**	**0.9**	**1.0**	10.9	3	1	1	1	174	19.6	6.9
NP_001138349.1	221554543	**SCAI**	protein SCAI isoform 2	-	-	-	-	1.1	0.9	0.9	1.0	**1.1**	**0.9**	**0.9**	**1.0**	1.2	2	1	1	1	606	70.4	8.6
NP_003077.2	92859678	**SNAPC4**	snRNA-activating protein complex subunit 4	-	-	-	-	0.8	1.2	1.0	1.0	**0.8**	**1.2**	**1.0**	**1.0**	1.2	1	1	1	1	1469	159.3	8.3
NP_076931.1	13128970	**MRPL57**	ribosomal protein 63, mitochondrial	-	-	-	-	1.0	1.0	1.1	1.0	**1.0**	**1.0**	**1.1**	**1.0**	6.9	1	1	1	1	102	12.3	11.4
NP_060613.1	8922528	**KLHL11**	kelch-like protein 11 precursor	-	-	-	-	1.0	0.9	0.9	1.0	**1.0**	**0.9**	**0.9**	**1.0**	3.1	1	2	2	2	708	80.1	6.2
NP_002423.1	4505227	**MNDA**	myeloid cell nuclear differentiation antigen	-	-	-	-	0.9	0.9	1.0	1.0	**0.9**	**0.9**	**1.0**	**1.0**	1.5	1	1	1	1	407	45.8	9.8
NP_073616.6	17505907	**DDX31**	probable ATP-dependent RNA helicase DDX31 isoform 1	-	-	-	-	0.9	1.0	1.1	1.0	**0.9**	**1.0**	**1.1**	**1.0**	5.3	2	3	3	5	851	94.0	10.0
NP_001288031.1	666875839	**PPCDC**	phosphopantothenoylcysteine decarboxylase isoform c	-	-	-	-	0.9	0.8	0.9	1.0	**0.9**	**0.8**	**0.9**	**1.0**	21.5	5	2	2	5	172	18.7	7.7
NP_036537.1	6912430	**ICMT**	protein-S-isoprenylcysteine O-methyltransferase	-	-	-	-	1.0	0.9	0.9	1.0	**1.0**	**0.9**	**0.9**	**1.0**	10.6	1	2	2	2	284	31.9	8.0
NP_001803.2	68508961	**CENPC**	centromere protein C	-	-	-	-	1.0	0.9	1.0	1.0	**1.0**	**0.9**	**1.0**	**1.0**	2.2	1	1	1	1	943	106.8	9.4
NP_055446.2	55749544	**SH3PXD2A**	SH3 and PX domain-containing protein 2A	-	-	-	-	1.0	1.0	0.9	1.0	**1.0**	**1.0**	**0.9**	**1.0**	1.3	3	2	2	2	1105	121.8	8.4
NP_001158268.1	258645122	**SLC25A26**	S-adenosylmethionine mitochondrial carrier protein isoform b	-	-	-	-	1.0	1.1	1.1	1.0	**1.0**	**1.1**	**1.1**	**1.0**	23.1	2	3	3	7	186	20.1	9.6
NP_055524.3	110347427	**USP34**	ubiquitin carboxyl-terminal hydrolase 34	-	-	-	-	1.0	1.0	1.0	1.0	**1.0**	**1.0**	**1.0**	**1.0**	2.3	1	5	5	8	3546	404.0	5.8
NP_002889.1	4506449	**RBMS2**	RNA-binding motif, single-stranded-interacting protein 2	-	-	-	-	0.8	1.1	1.0	1.0	**0.8**	**1.1**	**1.0**	**1.0**	3.2	1	1	1	1	407	43.9	9.1
NP_149018.2	110556638	**LMLN**	leishmanolysin-like peptidase isoform 2	-	-	-	-	1.0	1.0	1.0	1.0	**1.0**	**1.0**	**1.0**	**1.0**	3.4	1	1	1	1	655	73.5	6.9
NP_004393.1	4758150	**DFFB**	DNA fragmentation factor subunit beta isoform 2	-	-	-	-	0.9	0.9	0.9	1.0	**0.9**	**0.9**	**0.9**	**1.0**	3.3	2	1	1	6	338	39.1	9.0
NP_001421.2	40254439	**EPAS1**	endothelial PAS domain-containing protein 1	-	-	-	-	1.1	1.1	1.0	1.0	**1.1**	**1.1**	**1.0**	**1.0**	0.7	1	1	1	1	870	96.4	6.3

Khan *et al.* , 2019. Multi-omics analysis to characterize cigarette smoke induced molecular alterations in esophageal cells
Supplementary Table 5. List of proteins quantified in untreated and chronically treated Het1A cells with cigarette smoke condensate for 8 months

NP_Accession	Protein group Accession	Gene ID	Description	Het-1A-Smoke - 2M/Parental	Het-1A-Smoke - 4M/Parental	Het-1A-Smoke - 6M/Parental	Het-1A-Smoke - 8M/Parental	Het-1A-Smoke - 2M/Parental	Het-1A-Smoke - 4M/Parental	Het-1A-Smoke - 6M/Parental	Het-1A-Smoke - 8M/Parental	Het-1A-Smoke - 2M/Parental	Het-1A-Smoke - 4M/Parental	Het-1A-Smoke - 6M/Parental	Het-1A-Smoke - 8M/Parental	Coverage (%)	Proteins	Unique Peptides	# Peptides	PSM	# AAs	MW [kDa]	calc. pI
				Replicate 1				Replicate 2				Average of replicates											
XP_005258156.1	530424835	**SOGA2**	protein SOGA2 isoform X2	-	-	-	-	1.0	0.9	1.0	1.0	**1.0**	**0.9**	**1.0**	**1.0**	0.6	2	1	1	1	1905	209.4	6.4
NP_001014842.1	62460635	**TM9SF1**	transmembrane 9 superfamily member 1 isoform b precursor	-	-	-	-	1.1	1.0	0.9	1.0	**1.1**	**1.0**	**0.9**	**1.0**	4.7	2	2	2	4	489	55.2	7.1
NP_056051.2	163792198	**ADGRL3**	latrophilin-3 precursor	-	-	-	-	1.1	0.9	0.9	1.0	**1.1**	**0.9**	**0.9**	**1.0**	2.0	1	1	1	1	1469	164.5	6.5
NP_055514.3	215820619	**ZNF646**	zinc finger protein 646	-	-	-	-	0.9	1.0	1.0	1.0	**0.9**	**1.0**	**1.0**	**1.0**	0.7	1	1	1	1	1832	200.7	7.2
NP_057146.1	7706343	**FAM96B**	mitotic spindle-associated MMXD complex subunit MIP18	-	-	-	-	1.1	0.9	1.0	1.0	**1.1**	**0.9**	**1.0**	**1.0**	26.4	1	2	2	3	163	17.7	5.2
NP_001850.1	4507015	**SLC31A1**	high affinity copper uptake protein 1	-	-	-	-	1.0	1.0	1.0	1.0	**1.0**	**1.0**	**1.0**	**1.0**	10.0	1	1	1	3	190	21.1	7.4
NP_689629.2	124517691	**NDUFAF6**	NADH dehydrogenase (ubiquinone) complex I, assembly factor 6 precursor	-	-	-	-	1.1	1.2	1.1	1.0	**1.1**	**1.2**	**1.1**	**1.0**	10.8	1	2	2	5	333	38.2	9.4
NP_001258371.1	407027851	**C21orf2**	protein C21orf2 isoform 4	-	-	-	-	1.1	0.9	0.9	1.0	**1.1**	**0.9**	**0.9**	**1.0**	6.5	4	1	1	1	214	23.6	5.7
NP_002863.1	4506381	**RAC2**	ras-related C3 botulinum toxin substrate 2	-	-	-	-	1.6	1.8	**2.0**	1.0	**1.6**	**1.8**	**2.0**	**1.0**	20.8	1	1	4	19	192	21.4	7.6
NP_006549.1	5730073	**KHDRBS3**	KH domain-containing, RNA-binding, signal transduction-associated protein 3	-	-	-	-	1.1	1.1	1.0	1.0	**1.1**	**1.1**	**1.0**	**1.0**	5.8	1	1	2	14	346	38.8	7.6
NP_001273145.1	555290135	**GTPBP2**	GTP-binding protein 2 isoform b	-	-	-	-	1.3	1.2	0.9	1.0	**1.3**	**1.2**	**0.9**	**1.0**	2.1	2	1	1	1	514	56.6	7.0
NP_003036.1	4507047	**SLC7A1**	high affinity cationic amino acid transporter 1	-	-	-	-	1.1	0.8	0.9	1.0	**1.1**	**0.8**	**0.9**	**1.0**	2.1	3	1	1	2	629	67.6	5.4
NP_057952.1	8051618	**LIMK2**	LIM domain kinase 2 isoform 2b	-	-	-	-	1.1	1.0	1.0	1.0	**1.1**	**1.0**	**1.0**	**1.0**	7.5	3	2	2	3	617	69.9	7.4
NP_036469.2	81295816	**CFAP45**	cilia- and flagella-associated protein 45	-	-	-	-	1.0	1.1	1.1	1.0	**1.0**	**1.1**	**1.1**	**1.0**	2.2	1	2	2	2	551	65.7	8.9
NP_689949.3	161353477	**ZSCAN26**	zinc finger and SCAN domain-containing protein 26 isoform c	-	-	-	-	1.0	1.0	0.9	1.0	**1.0**	**1.0**	**0.9**	**1.0**	7.6	4	2	2	2	344	39.7	8.5
NP_065199.1	10092689	**LYRM2**	LYR motif-containing protein 2	-	-	-	-	1.1	0.9	1.0	1.0	**1.1**	**0.9**	**1.0**	**1.0**	10.2	1	1	1	1	88	10.4	10.5
NP_001006621.1	56788399	**MAPKAP1**	target of rapamycin complex 2 subunit MAPKAP1 isoform 4	-	-	-	-	0.9	1.0	1.0	1.0	**0.9**	**1.0**	**1.0**	**1.0**	2.1	4	1	1	1	330	37.3	6.8
NP_001167595.1	291621680	**GOLGA7**	Golgin subfamily A member 7 isoform b	-	-	-	-	0.9	1.1	0.9	1.0	**0.9**	**1.1**	**0.9**	**1.0**	11.2	2	1	1	2	134	15.6	8.4
NP_060734.2	50726981	**TYW1**	S-adenosyl-L-methionine-dependent tRNA 4-demethylwyosine synthase	-	-	-	-	1.0	1.0	1.1	1.0	**1.0**	**1.0**	**1.1**	**1.0**	4.5	2	3	3	5	732	83.6	6.9
NP_031370.2	33356179	**TTF1**	transcription termination factor 1 isoform 1	-	-	-	-	0.9	1.0	0.9	1.0	**0.9**	**1.0**	**0.9**	**1.0**	4.9	2	3	3	5	905	103.0	9.4
NP_001229759.1	338827650	**ELOVL5**	elongation of very long chain fatty acids protein 5 isoform 3	-	-	-	-	1.0	1.0	1.0	1.0	**1.0**	**1.0**	**1.0**	**1.0**	6.5	3	2	2	4	262	30.7	7.5
NP_056170.2	197333809	**SUZ12**	polycomb protein SUZ12	-	-	-	-	1.0	1.0	1.0	1.0	**1.0**	**1.0**	**1.0**	**1.0**	11.8	1	4	4	6	739	83.0	8.8
NP_000552.2	23065544	**GSTM1**	glutathione S-transferase Mu 1 isoform 1	-	-	-	-	1.1	1.0	1.0	1.0	**1.1**	**1.0**	**1.0**	**1.0**	21.1	7	4	4	8	218	25.7	6.7
NP_001171507.1	295849272	**DPP10**	inactive dipeptidyl peptidase 10 isoform a	-	-	-	-	0.9	0.9	1.0	1.0	**0.9**	**0.9**	**1.0**	**1.0**	0.9	5	1	1	1	746	85.4	6.3
NP_068768.2	48255953	**ATP2B3**	plasma membrane calcium-transporting ATPase 3 isoform 3a	-	-	-	-	0.8	1.0	0.9	1.0	**0.8**	**1.0**	**0.9**	**1.0**	6.7	4	1	6	20	1173	128.5	5.9
NP_004882.1	4759048	**MRPL33**	39S ribosomal protein L33, mitochondrial isoform a	-	-	-	-	1.0	1.0	1.0	1.0	**1.0**	**1.0**	**1.0**	**1.0**	38.5	1	2	2	7	65	7.6	10.8
NP_001180209.1	300797197	**MID1**	E3 ubiquitin-protein ligase Midline-1 isoform 5	-	-	-	-	1.1	1.0	1.1	1.0	**1.1**	**1.0**	**1.1**	**1.0**	8.0	6	3	3	3	451	51.0	6.3
NP_001013863.1	62243769	**STAP2**	signal-transducing adaptor protein 2 isoform 2	-	-	-	-	1.3	1.2	1.2	1.0	**1.3**	**1.2**	**1.2**	**1.0**	9.4	2	2	2	2	403	44.9	8.2
NP_000287.3	205360962	**PKD1**	polycystin-1 isoform 2 precursor	-	-	-	-	1.1	1.0	1.1	1.0	**1.1**	**1.0**	**1.1**	**1.0**	0.2	2	1	1	8	4302	462.1	6.7
NP_015557.2	195947397	**PSEN1**	presenilin-1 isoform I-463	-	-	-	-	1.0	1.1	0.9	1.0	**1.0**	**1.1**	**0.9**	**1.0**	4.1	2	1	1	8	463	52.2	5.2
NP_001276988.1	586946394	**ALDH3B1**	aldehyde dehydrogenase family 3 member B1 isoform d	-	-	-	-	0.9	0.9	1.0	1.0	**0.9**	**0.9**	**1.0**	**1.0**	9.6	5	1	2	2	272	30.0	7.6
NP_001979.2	156104874	**EVPL**	envoplakin	-	-	-	-	1.0	1.0	0.9	1.0	**1.0**	**1.0**	**0.9**	**1.0**	0.5	1	1	1	1	2033	231.5	7.0
NP_006216.2	90265805	**PLCD1**	1-phosphatidylinositol 4,5-bisphosphate phosphodiesterase delta-1 isoform 2	-	-	-	-	1.1	1.0	1.0	1.0	**1.1**	**1.0**	**1.0**	**1.0**	2.3	2	2	2	2	756	85.6	6.7
NP_001294973.1	808688351	**RBM23**	probable RNA-binding protein 23 isoform 4	-	-	-	-	0.9	0.9	0.9	1.0	**0.9**	**0.9**	**0.9**	**1.0**	9.7	4	2	2	4	269	28.6	7.9
NP_001166366.1	290542359	**CAV1**	caveolin-1 isoform beta	-	-	-	-	1.0	1.1	0.9	1.0	**1.0**	**1.1**	**0.9**	**1.0**	14.3	2	1	1	1	147	17.0	5.5
NP_060896.1	55769589	**TMEM63B**	CSC1-like protein 2	-	-	-	-	0.9	0.9	0.9	1.0	**0.9**	**0.9**	**0.9**	**1.0**	1.1	1	1	1	1	832	94.9	7.4
NP_001277059.1	588480511	**POMGNT1**	protein O-linked-mannose beta-1,2-N-acetylglucosaminyltransferase 1 isoform 4	-	-	-	-	1.0	0.9	1.0	1.0	**1.0**	**0.9**	**1.0**	**1.0**	2.7	4	1	1	1	517	58.8	6.3
NP_001172056.1	297591831	**VAMP4**	vesicle-associated membrane protein 4 isoform 2	-	-	-	-	1.4	0.8	0.8	1.0	**1.4**	**0.8**	**0.8**	**1.0**	10.0	2	1	1	1	140	16.2	6.6
NP_005603.3	301897974	**REST**	RE1-silencing transcription factor	-	-	-	-	0.9	1.1	0.9	1.0	**0.9**	**1.1**	**0.9**	**1.0**	2.4	1	2	2	2	1097	121.8	6.7
NP_001092108.1	148839382	**RNF169**	E3 ubiquitin-protein ligase RNF169	-	-	-	-	1.0	1.0	0.9	1.0	**1.0**	**1.0**	**0.9**	**1.0**	9.0	1	4	4	5	708	77.1	9.1
NP_056142.2	187960090	**SMG5**	protein SMG5	-	-	-	-	1.0	0.9	1.1	1.0	**1.0**	**0.9**	**1.1**	**1.0**	1.6	1	1	1	2	1016	113.9	5.9
NP_002283.3	119703755	**LAMB2**	laminin subunit beta-2 precursor	-	-	-	-	1.0	1.1	1.0	1.0	**1.0**	**1.1**	**1.0**	**1.0**	0.6	1	1	1	1	1798	195.9	6.5
NP_694968.1	23397516	**GIMAP7**	GTPase IMAP family member 7	-	-	-	-	1.2	1.0	1.0	1.0	**1.2**	**1.0**	**1.0**	**1.0**	3.7	1	1	1	1	300	34.5	6.5
NP_071420.1	11545873	**SMOC1**	SPARC-related modular calcium-binding protein 1 isoform 2 precursor	-	-	-	-	0.8	0.8	0.8	1.0	**0.8**	**0.8**	**0.8**	**1.0**	1.8	2	1	1	2	434	48.1	8.2
NP_056109.1	15147333	**TRIM37**	E3 ubiquitin-protein ligase TRIM37	-	-	-	-	1.0	1.0	1.0	1.0	**1.0**	**1.0**	**1.0**	**1.0**	0.6	1	1	1	1	964	107.8	5.1
NP_036211.2	145864459	**DGAT1**	diacylglycerol O-acyltransferase 1	-	-	-	-	1.0	1.0	0.9	1.0	**1.0**	**1.0**	**0.9**	**1.0**	4.3	1	1	1	2	488	55.2	9.3
NP_055483.2	23397642	**GREB1**	protein GREB1 isoform a	-	-	-	-	1.1	1.1	1.0	1.0	**1.1**	**1.1**	**1.0**	**1.0**	0.8	1	1	1	1	1949	216.3	6.9
NP_149084.2	283046698	**TRIM5**	tripartite motif-containing protein 5 isoform delta	-	-	-	-	1.0	1.0	1.0	1.0	**1.0**	**1.0**	**1.0**	**1.0**	5.2	3	1	1	2	326	37.7	5.9
NP_055102.3	51944953	**NOMO1**	nodal modulator 1 precursor	-	-	-	-	1.0	1.1	1.0	1.0	**1.0**	**1.1**	**1.0**	**1.0**	30.8	3	2	26	110	1222	134.2	5.8
NP_055418.2	154759283	**CDR2L**	cerebellar degeneration-related protein 2-like	-	-	-	-	1.0	1.1	0.7	1.0	**1.0**	**1.1**	**0.7**	**1.0**	3.2	1	1	1	2	465	53.0	6.0
NP_689729.1	22749079	**COMMD1**	COMM domain-containing protein 1	-	-	-	-	1.0	0.8	1.0	1.0	**1.0**	**0.8**	**1.0**	**1.0**	3.2	1	1	1	1	190	21.2	6.2
NP_003250.3	189571583	**ICAM5**	intercellular adhesion molecule 5 precursor	-	-	-	-	1.3	1.3	1.1	1.0	**1.3**	**1.3**	**1.1**	**1.0**	1.4	1	1	1	2	924	97.1	5.9
NP_057520.2	18034775	**PHF20**	PHD finger protein 20	-	-	-	-	0.9	0.9	0.9	1.0	**0.9**	**0.9**	**0.9**	**1.0**	2.5	1	2	2	3	1012	115.3	7.0
NP_071379.3	38569426;38569428	**ANKEF1**	ankyrin repeat and EF-hand domain-containing protein 1 isoform 1	-	-	-	-	1.0	1.0	1.1	1.0	**1.0**	**1.0**	**1.1**	**1.0**	2.2	2	1	2	9	776	86.6	8.3
NP_077012.2	115527092;115527088	**TMUB2**	transmembrane and ubiquitin-like domain-containing protein 2 isoform a	-	-	-	-	1.0	1.0	1.1	1.0	**1.0**	**1.0**	**1.1**	**1.0**	14.0	2	2	2	3	301	31.6	4.9
NP_776152.1	29789403	**PDZD8**	PDZ domain-containing protein 8	-	-	-	-	0.8	1.0	1.0	1.0	**0.8**	**1.0**	**1.0**	**1.0**	1.2	1	1	1	1	1154	128.5	6.1
NP_055542.1	7662046	**KMT2B**	histone-lysine N-methyltransferase 2B	-	-	-	-	1.0	0.9	0.9	1.0	**1.0**	**0.9**	**0.9**	**1.0**	0.7	1	1	1	2	2715	293.3	8.2
NP_055490.4	612407805	**CROCC**	rootletin	-	-	-	-	1.0	0.8	1.0	1.0	**1.0**	**0.8**	**1.0**	**1.0**	0.8	1	1	2	4	2017	228.3	5.5
NP_001139283.1	224967050	**SOX6**	transcription factor SOX-6 isoform 3	-	-	-	-	1.0	0.9	1.1	1.0	**1.0**	**0.9**	**1.1**	**1.0**	0.8	4	1	1	1	801	88.9	7.2
NP_001265316.1	508083053;117168275	**SLC38A1**	sodium-coupled neutral amino acid transporter 1 isoform 1	-	-	-	-	1.3	0.8	1.4	1.0	**1.3**	**0.8**	**1.4**	**1.0**	8.8	2	2	2	2	487	54.0	7.0
NP_835471.1	30039706	**NMNAT3**	nicotinamide/nicotinic acid mononucleotide adenylyltransferase 3 isoform 1	-	-	-	-	1.0	0.9	0.9	1.0	**1.0**	**0.9**	**0.9**	**1.0**	4.7	1	1	1	1	215	24.1	9.1
NP_001138571.1	223029430	**SLC2A6**	solute carrier family 2, facilitated glucose transporter member 6 isoform 2	-	-	-	-	1.0	1.3	1.1	1.0	**1.0**	**1.3**	**1.1**	**1.0**	2.3	2	1	1	2	445	48.0	9.0
NP_476429.2	109148552	**KRT3**	keratin, type II cytoskeletal 3	-	-	-	-	1.3	1.1	0.9	1.0	**1.3**	**1.1**	**0.9**	**1.0**	10.4	6	1	7	71	628	64.4	6.5
NP_061975.2	24497517	**HOXA5**	homeobox protein Hox-A5	-	-	-	-	1.0	1.0	0.8	1.0	**1.0**	**1.0**	**0.8**	**1.0**	5.2	1	1	1	4	270	29.3	9.2
NP_997177.1	46411168	**MBNL1**	muscleblind-like protein 1 isoform d	-	-	-	-	0.9	0.9	1.0	1.0	**0.9**	**0.9**	**1.0**	**1.0**	10.6	14	2	2	2	302	33.0	8.8
NP_055759.3	57242755	**CLSTN1**	calsyntenin-1 isoform 2 precursor	-	-	-	-	1.0	1.1	1.0	1.0	**1.0**	**1.1**	**1.0**	**1.0**	2.8	3	2	2	2	971	108.6	4.9
NP_065968.1	55741659	**ZFP14**	zinc finger protein 14 homolog isoform 2	-	-	-	-	1.0	1.0	0.9	1.0	**1.0**	**1.0**	**0.9**	**1.0**	6.2	7	2	2	3	533	63.4	8.6
NP_006867.1	5802984	**B4GAT1**	beta-1,4-glucuronyltransferase 1	-	-	-	-	1.2	1.0	1.0	1.0	**1.2**	**1.0**	**1.0**	**1.0**	16.4	1	5	5	9	415	47.1	7.2
NP_001287896.1	665821271	**NPC1L1**	Niemann-Pick C1-like protein 1 isoform 3 precursor	-	-	-	-	1.0	1.1	1.1	1.0	**1.0**	**1.1**	**1.1**	**1.0**	1.1	3	1	1	2	724	79.1	5.7
NP_000109.1	4557555	**ENG**	endoglin isoform 2 precursor	-	-	-	-	1.0	1.1	1.0	1.0	**1.0**	**1.1**	**1.0**	**1.0**	2.9	2	1	1	1	625	67.5	6.6
NP_001248745.1	387849239	**PDPK1**	3-phosphoinositide-dependent protein kinase 1 isoform 3	-	-	-	-	1.0	1.0	1.0	1.0	**1.0**	**1.0**	**1.0**	**1.0**	9.7	3	4	4	7	454	50.8	6.1
NP_904320.1	34878753	**FBXO44**	F-box only protein 44 isoform 2	-	-	-	-	1.0	0.9	1.0	1.0	**1.0**	**0.9**	**1.0**	**1.0**	14.7	2	2	2	6	224	25.7	7.9
NP_060510.1	8922313	**GPATCH2**	G patch domain-containing protein 2	-	-	-	-	0.9	1.0	0.9	1.0	**0.9**	**1.0**	**0.9**	**1.0**	2.3	1	1	1	1	528	58.9	9.1
NP_001171556.1	296010880	**ZFX**	zinc finger X-chromosomal protein isoform 1	-	-	-	-	1.0	0.9	0.9	1.0	**1.0**	**0.9**	**0.9**	**1.0**	8.1	6	3	3	3	805	90.5	6.1
NP_068590.1	11141889	**SAV1**	protein salvador homolog 1	-	-	-	-	1.1	1.0	1.0	1.0	**1.1**	**1.0**	**1.0**	**1.0**	3.1	1	1	1	1	383	44.6	9.1
NP_009114.1	6005830	**PKP3**	plakophilin-3 isoform PKP3a	-	-	-	-	1.0	1.0	1.0	1.0	**1.0**	**1.0**	**1.0**	**1.0**	2.0	2	1	1	1	797	87.0	9.3
NP_061987.3	110624790	**EPB41L4B**	band 4.1-like protein 4B isoform 2	-	-	-	-	0.9	1.1	0.9	1.0	**0.9**	**1.1**	**0.9**	**1.0**	1.8	2	1	2	2	900	99.7	9.0
NP_006337.2	55769583	**PIBF1**	progesterone-induced-blocking factor 1	-	-	-	-	0.8	0.9	0.8	1.0	**0.8**	**0.9**	**0.8**	**1.0**	1.3	1	1	1	1	757	89.7	6.0
NP_775186.1	27886555	**NFATC3**	nuclear factor of activated T-cells, cytoplasmic 3 isoform 3	-	-	-	-	1.2	1.3	1.2	1.0	**1.2**	**1.3**	**1.2**	**1.0**	1.4	3	1	1	1	1065	115.0	6.4
NP_006749.1	5803207;578846882	**U2AF1**	splicing factor U2AF 35 kDa subunit isoform a	-	-	-	-	1.0	0.8	0.9	1.0	**1.0**	**0.8**	**0.9**	**1.0**	40.0	3	5	6	15	240	27.9	8.8
NP_001284501.1	662033917	**IL31RA**	interleukin-31 receptor subunit alpha isoform 7	-	-	-	-	1.1	1.0	1.1	1.0	**1.1**	**1.0**	**1.1**	**1.0**	1.4	8	1	1	1	440	50.5	7.6
NP_001253980.1	388490270	**GPR155**	integral membrane protein GPR155 isoform 2	-	-	-	-	0.9	0.9	0.8	1.0	**0.9**	**0.9**	**0.8**	**1.0**	1.5	2	1	1	1	842	93.6	6.5
NP_001151.1	4502123	**APAF1**	apoptotic protease-activating factor 1 isoform b	-	-	-	-	1.0	1.0	1.0	1.0	**1.0**	**1.0**	**1.0**	**1.0**	1.3	5	2	2	3	1194	135.9	6.4
NP_057619.1	7705495	**ZNF581**	zinc finger protein 581	-	-	-	-	**5.1**	1.2	1.1	1.0	**5.1**	**1.2**	**1.1**	**1.0**	5.6	1	1	1	1	197	22.0	8.8
NP_443160.2	156616308	**SMYD4**	SET and MYND domain-containing protein 4	-	-	-	-	1.0	1.0	1.0	1.0	**1.0**	**1.0**	**1.0**	**1.0**	3.5	1	2	2	2	804	89.2	6.8

Khan *et al.* , 2019. Multi-omics analysis to characterize cigarette smoke induced molecular alterations in esophageal cells
Supplementary Table 5. List of proteins quantified in untreated and chronically treated Het1A cells with cigarette smoke condensate for 8 months

NP_Accession	Protein group Accession	Gene ID	Description	Het-1A-Smoke - 2M/Parental	Het-1A-Smoke - 4M/Parental	Het-1A-Smoke - 6M/Parental	Het-1A-Smoke - 8M/Parental	Het-1A-Smoke - 2M/Parental	Het-1A-Smoke - 4M/Parental	Het-1A-Smoke - 6M/Parental	Het-1A-Smoke - 8M/Parental	Het-1A-Smoke - 2M/Parental	Het-1A-Smoke - 4M/Parental	Het-1A-Smoke - 6M/Parental	Het-1A-Smoke - 8M/Parental	Coverage (%)	Proteins	Unique Peptides	# Peptides	PSM	# AAs	MW [kDa]	calc. pI
				Replicate 1				Replicate 2				Average of replicates											
NP_060634.2	155030185	ASUN	protein asunder homolog	-	-	-	-	1.6	1.0	1.1	1.0	1.6	1.0	1.1	1.0	1.1	1	1	1	1	706	80.2	6.7
NP_060267.2	20127580	BTBD2	BTB/POZ domain-containing protein 2	-	-	-	-	1.0	0.9	0.9	1.0	1.0	0.9	0.9	1.0	1.5	1	1	1	2	525	55.9	5.9
NP_001171724.1	296317239	PEX11B	peroxisomal membrane protein 11B isoform 2	-	-	-	-	0.9	0.9	0.9	1.0	0.9	0.9	0.9	1.0	18.4	2	4	4	7	245	26.7	9.9
NP_001092270.1	149274614	MAGED4	melanoma-associated antigen D4 isoform 2	-	-	-	-	1.0	0.9	0.8	1.0	1.0	0.9	0.8	1.0	6.9	3	2	2	3	739	81.0	6.6
NP_001180417.1	301601630	LIMS1	LIM and senescent cell antigen-like-containing domain protein 1 isoform b	-	-	-	-	0.9	1.0	1.1	1.0	0.9	1.0	1.1	1.0	6.2	5	2	2	6	325	37.2	8.0
NP_001138866.1	223972636	TSEN2	tRNA-splicing endonuclease subunit Sen2 isoform 3	-	-	-	-	0.9	0.9	0.9	1.0	0.9	0.9	0.9	1.0	7.9	4	2	2	2	406	47.1	8.7
NP_037418.3	54607124	RBM15B	putative RNA-binding protein 15B	-	-	-	-	1.0	0.9	0.9	1.0	1.0	0.9	0.9	1.0	2.7	1	1	2	3	890	97.1	9.8
NP_001164626.1	283837866	PET100	protein PET100 homolog, mitochondrial	-	-	-	-	1.0	0.8	0.9	1.0	1.0	0.8	0.9	1.0	13.7	1	2	2	6	73	9.1	6.8
NP_689498.1	22748653	ARRDC1	arrestin domain-containing protein 1	-	-	-	-	1.0	1.1	1.1	1.0	1.0	1.1	1.1	1.0	9.5	1	2	2	2	433	46.0	7.0
NP_829884.1	38045898	ERC1	ELKS/Rab6-interacting/CAST family member 1 isoform epsilon	-	-	-	-	0.9	0.9	1.0	1.0	0.9	0.9	1.0	1.0	5.7	4	5	5	7	1116	128.0	6.0
NP_075565.2	282396088	COA7	cytochrome c oxidase assembly factor 7	-	-	-	-	0.9	1.1	1.1	1.0	0.9	1.1	1.1	1.0	6.1	1	1	1	1	231	25.7	6.0
NP_003315.2	21536436	TULP3	tubby-related protein 3 isoform 1	-	-	-	-	1.0	1.0	1.0	1.0	1.0	1.0	1.0	1.0	3.4	2	1	1	1	442	49.6	7.7
NP_898901.1	34304336	RNF8	E3 ubiquitin-protein ligase RNF8 isoform 2	-	-	-	-	0.9	1.0	0.9	1.0	0.9	1.0	0.9	1.0	3.1	2	1	1	1	448	50.8	5.9
NP_004046.2	37577157	CAPN5	calpain-5	-	-	-	-	1.2	1.1	0.8	1.0	1.2	1.1	0.8	1.0	4.4	1	2	2	2	640	73.1	7.6
NP_002399.1	4505163	MGAT2	alpha-1,6-mannosyl-glycoprotein 2-beta-N-acetylglucosaminyltransferase	-	-	-	-	1.1	1.0	1.0	1.0	1.1	1.0	1.0	1.0	4.9	1	2	2	8	447	51.5	8.8
NP_001161288.1	269847465	C20orf26	uncharacterized protein C20orf26 isoform 2	-	-	-	-	1.0	0.9	1.0	1.0	1.0	0.9	1.0	1.0	1.3	2	1	1	2	470	53.5	5.2
NP_001012398.1	61743933	AKTIP	AKT-interacting protein isoform 1	-	-	-	-	1.1	1.0	0.9	1.0	1.1	1.0	0.9	1.0	14.7	2	2	2	4	292	33.1	9.3
NP_006361.1	5454166	VTI1B	vesicle transport through interaction with t-SNAREs homolog 1B	-	-	-	-	0.9	0.8	0.8	1.0	0.9	0.8	0.8	1.0	18.1	1	3	3	7	232	26.7	9.0
NP_001278430.1	613410201	SMC1B	structural maintenance of chromosomes protein 1B isoform 2	-	-	-	-	0.8	0.9	0.9	1.0	0.8	0.9	0.9	1.0	2.8	2	3	5	7	1161	135.7	7.9
NP_001273204.1	555943827	KIAA1586	uncharacterized protein KIAA1586 isoform c	-	-	-	-	0.9	0.9	1.0	1.0	0.9	0.9	1.0	1.0	1.8	3	1	1	1	720	82.4	8.8
NP_001269670.1	544346317	OSBP2	oxysterol-binding protein 2 isoform f	-	-	-	-	1.0	1.1	0.9	1.0	1.0	1.1	0.9	1.0	6.5	6	2	3	5	659	74.6	6.5
NP_976313.1	42794758	ACSL5	long-chain-fatty-acid--CoA ligase 5 isoform b	-	-	-	-	1.0	1.0	0.9	1.0	1.0	1.0	0.9	1.0	3.5	2	1	2	6	683	75.9	6.9
NP_054769.1	7662637	MRPL42	39S ribosomal protein L42, mitochondrial precursor	-	-	-	-	1.0	0.9	1.0	1.0	1.0	0.9	1.0	1.0	10.6	1	1	1	4	142	16.7	8.4
NP_000889.3	38202207	MAOB	amine oxidase [flavin-containing] B	-	-	-	-	1.1	0.9	1.0	1.0	1.1	0.9	1.0	1.0	3.5	3	2	2	2	520	58.7	7.5
NP_001139745.1	226371762	TMEM64	transmembrane protein 64 isoform 2	-	-	-	-	0.9	0.9	1.0	1.0	0.9	0.9	1.0	1.0	2.7	2	1	1	1	328	33.9	8.9
NP_001623.3	94721246	ALPP	alkaline phosphatase, placental type preproprotein	-	-	-	-	0.9	0.9	0.9	1.0	0.9	0.9	0.9	1.0	1.9	1	1	1	1	535	57.9	6.3
NP_001032961.1	84043967	REV1	DNA repair protein REV1 isoform 2	-	-	-	-	0.9	0.9	1.0	1.0	0.9	0.9	1.0	1.0	0.6	2	1	1	2	1250	138.1	8.5
NP_059986.1	39930371	RAB39A	ras-related protein Rab-39A	-	-	-	-	0.9	0.8	0.9	1.0	0.9	0.8	0.9	1.0	8.3	10	1	2	3	217	25.0	7.6
NP_085148.1	24308303	SH3BP5L	SH3 domain-binding protein 5-like	-	-	-	-	0.9	0.9	0.9	1.0	0.9	0.9	0.9	1.0	14.5	1	4	4	12	393	43.5	5.8
NP_077277.1	13236528	FKRP	fukutin-related protein	-	-	-	-	1.1	1.0	1.0	1.0	1.1	1.0	1.0	1.0	2.2	1	1	1	2	495	54.5	7.4
NP_031379.2	42476111	GNA12	guanine nucleotide-binding protein subunit alpha-12 isoform 1	-	-	-	-	0.9	0.9	0.8	0.9	0.9	0.9	0.8	0.9	7.4	5	1	3	9	381	44.3	9.8
NP_004513.1	4758650	KIF5C	kinesin heavy chain isoform 5C	-	-	-	-	0.9	1.1	0.8	0.9	0.9	1.1	0.8	0.9	6.9	1	1	6	24	957	109.4	6.2
NP_004335.1	4757902	CETN2	centrin-2	-	-	-	-	1.0	0.7	1.0	0.9	1.0	0.7	1.0	0.9	7.0	1	1	1	1	172	19.7	5.0
NP_689848.1	22749297	OIT3	oncoprotein-induced transcript 3 protein precursor	-	-	-	-	0.9	0.8	1.0	0.9	0.9	0.8	1.0	0.9	1.1	1	1	1	1	545	60.0	5.6
NP_006036.1	65301139	ATP9A	probable phospholipid-transporting ATPase IIA	-	-	-	-	1.1	0.9	1.0	0.9	1.1	0.9	1.0	0.9	1.1	2	1	1	1	1047	118.5	7.8
NP_777571.1	28557798	FAM84B	protein FAM84B	-	-	-	-	1.1	0.8	1.1	0.9	1.1	0.8	1.1	0.9	4.8	1	1	1	2	310	34.5	5.5
NP_001099003.1	157671947	CPED1	cadherin-like and PC-esterase domain-containing protein 1 isoform 2 precursor	-	-	-	-	0.9	1.3	0.9	0.9	0.9	1.3	0.9	0.9	1.3	2	1	1	1	783	89.1	7.2
NP_060292.3	154426300	KANSL2	KAT8 regulatory NSL complex subunit 2	-	-	-	-	0.8	0.9	0.9	0.9	0.8	0.9	0.9	0.9	1.8	1	1	1	1	492	55.0	6.6
NP_620152.1	20270347	ANKRD54	ankyrin repeat domain-containing protein 54	-	-	-	-	1.0	1.0	1.2	0.9	1.0	1.0	1.2	0.9	9.7	1	1	1	1	300	32.5	6.3
NP_001284538.1	662236841	MAP3K6	mitogen-activated protein kinase kinase kinase 6 isoform 2	-	-	-	-	1.0	1.0	1.0	0.9	1.0	1.0	1.0	0.9	1.8	2	1	1	2	1280	141.6	7.2
NP_001171866.1	296531459	EPB41L5	band 4.1-like protein 5 isoform 2	-	-	-	-	1.0	1.0	0.9	0.9	1.0	1.0	0.9	0.9	7.7	4	3	4	5	687	77.5	6.8
NP_066289.3	601984520	UBC	polyubiquitin-C	-	-	-	-	1.1	1.3	1.3	0.9	1.1	1.3	1.3	0.9	96.2	5	1	11	351	685	77.0	7.7
NP_001760.1	4502693	CD9	CD9 antigen	-	-	-	-	1.0	1.2	1.3	0.9	1.0	1.2	1.3	0.9	10.5	1	3	3	12	228	25.4	7.2
NP_004679.2	188219599	NMI	N-myc-interactor	-	-	-	-	0.8	0.9	0.9	0.9	0.8	0.9	0.9	0.9	3.9	1	1	1	1	307	35.0	5.3
NP_001288758.1	685504974	ABCC4	multidrug resistance-associated protein 4 isoform 3	-	-	-	-	1.1	1.1	1.1	0.9	1.1	1.1	1.1	0.9	0.9	2	1	1	1	1278	144.1	8.2
NP_116272.1	14249636	TOX2	TOX high mobility group box family member 2 isoform c	-	-	-	-	0.9	1.2	1.1	0.9	0.9	1.2	1.1	0.9	4.3	3	1	2	3	464	49.1	7.3
NP_001229.1	17318559	CCNE1	G1/S-specific cyclin-E1	-	-	-	-	0.9	1.0	0.8	0.9	0.9	1.0	0.8	0.9	3.4	1	1	1	1	410	47.0	6.0
NP_004505.2	31563338	FOXK2	forkhead box protein K2	-	-	-	-	1.1	1.0	1.0	0.9	1.1	1.0	1.0	0.9	5.3	1	2	3	4	660	69.0	9.5
NP_001257808.1	399567832	MPC1	mitochondrial pyruvate carrier 1 isoform 2	-	-	-	-	1.0	1.1	1.1	0.9	1.0	1.1	1.1	0.9	13.6	2	1	1	1	66	7.5	9.7
NP_689424.2	297747325	SNX7	sorting nexin-7 isoform b	-	-	-	-	1.3	0.9	0.9	0.9	1.3	0.9	0.9	0.9	5.3	2	2	2	4	396	45.0	4.7
NP_001137499.1	221316707	NDOR1	NADPH-dependent diflavin oxidoreductase 1 isoform d	-	-	-	-	1.0	1.1	0.9	0.9	1.0	1.1	0.9	0.9	2.3	4	1	1	1	521	58.4	7.6
NP_001070729.2	331284176	NCOR2	nuclear receptor corepressor 2 isoform 2	-	-	-	-	1.0	0.9	0.9	0.9	1.0	0.9	0.9	0.9	0.9	4	1	2	3	2458	267.9	7.8
NP_060802.1	8922886	DDX19A	ATP-dependent RNA helicase DDX19A	-	-	-	-	1.2	0.9	1.0	0.9	1.2	0.9	1.0	0.9	39.8	6	2	15	53	478	53.9	6.6
NP_001083060.1	148229491	UQCRHL	ubiquinol-cytochrome c reductase hinge protein-like	-	-	-	-	1.7	1.1	1.4	0.9	1.7	1.1	1.4	0.9	37.4	4	2	2	8	91	10.7	4.4
NP_002415.1	4505221	MMP8	neutrophil collagenase preproprotein	-	-	-	-	1.1	1.2	1.0	0.9	1.1	1.2	1.0	0.9	3.4	1	1	1	1	467	53.4	6.9
NP_003205.2	42490753	TEAD3	transcriptional enhancer factor TEF-5	-	-	-	-	1.0	0.9	1.0	0.9	1.0	0.9	1.0	0.9	12.4	8	4	4	8	435	48.6	8.4
NP_001273159.1	555943901	DTL	denticleless protein homolog isoform 3	-	-	-	-	1.2	0.9	1.0	0.9	1.2	0.9	1.0	0.9	4.7	3	3	3	4	688	75.0	9.0
NP_005820.1	5031581	AP3S2	AP-3 complex subunit sigma-2	-	-	-	-	0.9	0.9	0.9	0.9	0.9	0.9	0.9	0.9	13.5	2	3	3	9	193	22.0	5.2
NP_001005290.1	52630338	PSRC1	proline/serine-rich coiled-coil protein 1 isoform b	-	-	-	-	1.0	0.9	0.8	0.9	1.0	0.9	0.8	0.9	8.7	2	2	2	2	310	33.6	9.7
NP_001176.1	4502337	AZGP1	zinc-alpha-2-glycoprotein precursor	-	-	-	-	0.8	0.9	0.9	0.9	0.8	0.9	0.9	0.9	7.1	1	1	1	1	298	34.2	6.0
NP_003359.3	31543910;63053524	USP1	ubiquitin carboxyl-terminal hydrolase 1	-	-	-	-	0.9	1.0	0.9	0.9	0.9	1.0	0.9	0.9	4.7	1	3	3	4	785	88.2	5.5
NP_001070865.1	116734706	IRF2BP2	interferon regulatory factor 2-binding protein 2 isoform B	-	-	-	-	1.0	1.1	1.0	0.9	1.0	1.1	1.0	0.9	2.8	2	1	1	1	571	59.4	8.6
NP_001139317.1	224994173	ROBO1	roundabout homolog 1 isoform d	-	-	-	-	0.8	0.9	0.9	0.9	0.8	0.9	0.9	0.9	0.7	3	1	1	1	1551	169.6	6.2
NP_001020951.1	71051602	ABHD17B	alpha/beta hydrolase domain-containing protein 17B isoform 2 precursor	-	-	-	-	1.1	1.1	1.1	0.9	1.1	1.1	1.1	0.9	9.0	2	1	1	1	288	32.2	6.3
NP_001284500.1	662033915	GNB3	guanine nucleotide-binding protein G(I)/G(S)/G(T) subunit beta-3 isoform 2	-	-	-	-	1.0	1.0	0.9	0.9	1.0	1.0	0.9	0.9	12.1	2	1	2	2	339	37.1	5.7
NP_001007795.1	56118223	CEPT1	choline/ethanolaminephosphotransferase 1	-	-	-	-	1.2	0.9	0.9	0.9	1.2	0.9	0.9	0.9	2.6	1	1	1	5	416	46.5	8.2
NP_072170.1	12408692	CSH2	chorionic somatomammotropin hormone 2 isoform 2 precursor	-	-	-	-	1.5	1.1	1.8	0.9	1.5	1.1	1.8	0.9	10.2	3	1	1	10	167	18.8	4.9
NP_078867.2	31542673	CHMP6	charged multivesicular body protein 6	-	-	-	-	0.9	0.9	0.8	0.9	0.9	0.9	0.8	0.9	10.5	1	2	2	3	201	23.5	5.3
NP_078907.2	189571647	MSANTD2	myb/SANT-like DNA-binding domain-containing protein 2 isoform 1	-	-	-	-	0.7	0.9	0.7	0.9	0.7	0.9	0.7	0.9	3.2	2	1	1	1	507	55.4	5.7
NP_000771.2	166295200	CYP7A1	cholesterol 7-alpha-monooxygenase	-	-	-	-	0.8	0.9	0.8	0.9	0.8	0.9	0.8	0.9	1.2	1	1	1	1	504	57.6	8.2
NP_061914.3	166795303	MTRF1L	peptide chain release factor 1-like, mitochondrial isoform 1	-	-	-	-	0.8	0.9	0.9	0.9	0.8	0.9	0.9	0.9	6.6	15	3	3	4	380	43.6	8.3
NP_001123619.1	194306660	CCDC77	coiled-coil domain-containing protein 77 isoform b	-	-	-	-	0.9	1.1	1.1	0.9	0.9	1.1	1.1	0.9	8.3	2	2	2	3	456	53.9	8.0
NP_060809.3	269973877	RFK	riboflavin kinase	-	-	-	-	1.0	1.0	0.9	0.9	1.0	1.0	0.9	0.9	5.2	1	1	1	1	155	17.6	8.1
YP_003024035.1	251831116	ND4	NADH dehydrogenase subunit 4	-	-	-	-	1.1	0.9	1.1	0.9	1.1	0.9	1.1	0.9	4.8	1	2	2	4	459	51.5	9.3
NP_004698.3	290560746	ATG12	ubiquitin-like protein ATG12 isoform 1	-	-	-	-	0.9	0.9	0.9	0.9	0.9	0.9	0.9	0.9	18.6	1	4	4	10	140	15.1	5.1
NP_001035546.1	94818790	RHBDD2	rhomboid domain-containing protein 2 isoform a	-	-	-	-	1.0	0.9	0.9	0.9	1.0	0.9	0.9	0.9	4.4	1	1	1	6	364	39.2	9.3
NP_001271234.1	546142142	TBC1D22A	TBC1 domain family member 22A isoform d	-	-	-	-	1.0	1.1	1.0	0.9	1.0	1.1	1.0	0.9	6.2	3	1	1	2	470	53.9	5.4
NP_004084.1	4758250	EFNB2	ephrin-B2 precursor	-	-	-	-	0.8	1.0	0.9	0.9	0.8	1.0	0.9	0.9	4.8	1	1	1	1	333	36.9	8.9
NP_001508.1	4504201	GTF2H4	general transcription factor IIH subunit 4	-	-	-	-	1.1	1.0	1.0	0.9	1.1	1.0	1.0	0.9	11.0	1	3	3	5	462	52.2	9.0
NP_940986.1	38348436	CA13	carbonic anhydrase 13	-	-	-	-	0.9	0.9	1.0	0.9	0.9	0.9	1.0	0.9	9.9	1	2	2	2	262	29.4	7.0
NP_061118.1	8923942	NOP10	H/ACA ribonucleoprotein complex subunit 3	-	-	-	-	1.0	0.9	0.9	0.9	1.0	0.9	0.9	0.9	20.3	1	1	1	4	64	7.7	10.0
NP_001136272.1	224451128	EYS	protein eyes shut homolog isoform 1 precursor	-	-	-	-	0.9	0.8	0.9	0.9	0.9	0.8	0.9	0.9	0.2	2	1	1	1	3144	348.2	5.7
NP_001698.2	20336473	BCL7B	B-cell CLL/lymphoma 7 protein family member B isoform 1	-	-	-	-	1.0	0.9	0.8	0.9	1.0	0.9	0.8	0.9	37.1	3	3	4	6	202	22.2	4.8
NP_004161.4	66773030	SLC1A1	excitatory amino acid transporter 3	-	-	-	-	0.6	0.7	0.6	0.9	0.6	0.7	0.6	0.9	5.3	1	1	1	1	524	57.1	5.7
NP_055496.2	38158022	DHX34	probable ATP-dependent RNA helicase DHX34	-	-	-	-	0.8	1.0	0.9	0.9	0.8	1.0	0.9	0.9	5.3	1	3	3	4	1143	128.0	7.6
NP_004062.2	67551261	CLK1	dual specificity protein kinase CLK1 isoform 1	-	-	-	-	0.9	1.0	1.1	0.9	0.9	1.0	1.1	0.9	1.7	2	1	1	2	484	57.3	8.9
NP_001265228.1	507144070	WIPI2	WD repeat domain phosphoinositide-interacting protein 2 isoform f	-	-	-	-	0.9	0.8	1.0	0.9	0.9	0.8	1.0	0.9	15.5	6	2	2	3	310	33.5	5.5
NP_066564.2	153791535	KAT2A	histone acetyltransferase KAT2A	-	-	-	-	1.0	0.9	0.9	0.9	1.0	0.9	0.9	0.9	2.6	1	2	2	3	837	93.9	9.0

Khan *et al.*, 2019. Multi-omics analysis to characterize cigarette smoke induced molecular alterations in esophageal cells
Supplementary Table 5. List of proteins quantified in untreated and chronically treated Het1A cells with cigarette smoke condensate for 8 months

NP_Accession	Protein group Accession	Gene ID	Description	Het-1A-Smoke - 2M/Parental	Het-1A-Smoke - 4M/Parental	Het-1A-Smoke - 6M/Parental	Het-1A-Smoke - 8M/Parental	Het-1A-Smoke - 2M/Parental	Het-1A-Smoke - 4M/Parental	Het-1A-Smoke - 6M/Parental	Het-1A-Smoke - 8M/Parental	Het-1A-Smoke - 2M/Parental	Het-1A-Smoke - 4M/Parental	Het-1A-Smoke - 6M/Parental	Het-1A-Smoke - 8M/Parental	Coverage (%)	Proteins	Unique Peptides	# Peptides	PSM	# AAs	MW [kDa]	calc. pI
				Replicate 1				Replicate 2				Average of replicates											
NP_835464.1	30039692	NEK8	serine/threonine-protein kinase Nek8	-	-	-	-	0.9	0.8	0.9	0.9	0.9	0.8	0.9	0.9	1.2	1	1	1	1	692	74.8	7.8
NP_057178.2	45545435	COMMD2	COMM domain-containing protein 2	-	-	-	-	0.9	0.9	0.9	0.9	0.9	0.9	0.9	0.9	3.5	1	1	1	1	199	22.7	6.7
NP_001185689.1	311771585	LY75-CD302	LY75-CD302 fusion protein isoform 2 precursor	-	-	-	-	1.0	0.9	0.9	0.9	1.0	0.9	0.9	0.9	2.1	5	2	2	3	1817	209.3	6.2
XP_006726657.1	578845912	LOC10272498	uncharacterized protein KIAA2013	-	-	-	-	0.9	1.0	0.9	0.9	0.9	1.0	0.9	0.9	1.3	2	1	1	2	633	68.8	7.6
NP_003322.3	187608615	TYK2	non-receptor tyrosine-protein kinase TYK2	-	-	-	-	0.8	0.8	0.9	0.9	0.8	0.8	0.9	0.9	1.3	1	1	1	1	1187	133.6	7.2
NP_001273015.1	554790405	C11orf98	uncharacterized protein C11orf98	-	-	-	-	0.8	1.0	0.9	0.9	0.8	1.0	0.9	0.9	39.0	1	5	5	21	123	14.2	11.5
NP_612361.1	39930517	SAMD1	atherin	-	-	-	-	1.0	0.8	0.9	0.9	1.0	0.8	0.9	0.9	1.9	1	1	1	1	538	56.0	7.6
NP_110418.1	13540569	SGPP1	sphingosine-1-phosphate phosphatase 1	-	-	-	-	0.7	0.9	0.8	0.9	0.7	0.9	0.8	0.9	3.4	1	1	1	1	441	49.1	8.8
NP_060843.2	157388993	SYNJ2BP	synaptojanin-2-binding protein	-	-	-	-	1.0	0.9	0.9	0.9	1.0	0.9	0.9	0.9	24.1	4	3	3	15	145	15.9	6.3
NP_056328.2	22095397	CRELD1	cysteine-rich with EGF-like domain protein 1 isoform 2 precursor	-	-	-	-	1.0	1.0	0.9	0.9	1.0	1.0	0.9	0.9	11.4	2	4	4	7	420	45.4	4.9
NP_001095123.1	156416007	COMMD9	COMM domain-containing protein 9 isoform 2	-	-	-	-	0.9	0.8	0.9	0.9	0.9	0.8	0.9	0.9	13.5	2	2	2	3	156	17.3	6.9
NP_001017987.1	63082035	C5orf45	UPF0544 protein C5orf45 isoform 2	-	-	-	-	0.9	0.9	0.9	0.9	0.9	0.9	0.9	0.9	5.2	2	1	1	1	288	31.8	9.1
NP_001093882.1	155030236	DROSHA	ribonuclease 3 isoform 2	-	-	-	-	0.8	0.9	0.9	0.9	0.8	0.9	0.9	0.9	1.2	2	1	1	2	1337	155.2	8.0
NP_001245217.1	385137128	SELENBP1	selenium-binding protein 1 isoform 2	-	-	-	-	0.8	1.0	0.9	0.9	0.8	1.0	0.9	0.9	4.4	3	1	1	1	410	45.4	5.5
NP_149112.1	38683799	ANKRD13A	ankyrin repeat domain-containing protein 13A	-	-	-	-	0.9	0.9	0.9	0.9	0.9	0.9	0.9	0.9	5.4	1	2	2	4	590	67.6	5.0
NP_660087.1	21704263	MAX	protein max isoform b	-	-	-	-	0.9	0.9	0.9	0.9	0.9	0.9	0.9	0.9	20.5	7	2	2	3	151	17.2	6.6
NP_005588.2	45505149	NFIC	nuclear factor 1 C-type isoform 5	-	-	-	-	0.8	0.9	0.9	0.9	0.8	0.9	0.9	0.9	21.7	5	6	6	12	428	47.6	8.2
NP_733759.1	24586679	HIST1H2BA	histone H2B type 1-A	-	-	-	-	1.0	1.0	1.0	0.9	1.0	1.0	1.0	0.9	44.9	1	1	6	953	127	14.2	10.3
NP_000762.2	13699818	CYP2C9	cytochrome P450 2C9 precursor	-	-	-	-	3.3	1.2	0.9	0.9	3.3	1.2	0.9	0.9	1.4	2	1	1	1	490	55.6	7.9
NP_036253.2	13786127	CDC42EP4	cdc42 effector protein 4	-	-	-	-	1.0	0.9	0.9	0.9	1.0	0.9	0.9	0.9	11.8	1	3	3	12	356	38.0	5.2
NP_001030177.1	78042607	C18orf32	UPF0729 protein C18orf32	-	-	-	-	1.0	1.1	0.9	0.9	1.0	1.1	0.9	0.9	13.2	1	1	1	2	76	8.7	9.1
NP_065838.2	172072608	ZNF624	zinc finger protein 624	-	-	-	-	0.9	0.8	1.0	0.9	0.9	0.8	1.0	0.9	3.0	150	1	3	3	865	99.9	8.9
NP_056254.1	27414491	CCDC28A	coiled-coil domain-containing protein 28A	-	-	-	-	1.5	1.0	0.9	0.9	1.5	1.0	0.9	0.9	5.5	1	1	1	4	274	30.3	9.2
NP_060498.2	55742695	SAMD4B	protein Smaug homolog 2	-	-	-	-	1.0	1.0	1.0	0.9	1.0	1.0	1.0	0.9	3.2	2	2	2	3	694	75.4	6.8
NP_001072987.1	118498366	FAM177A1	protein FAM177A1 isoform 2	-	-	-	-	1.2	1.1	1.1	0.9	1.2	1.1	1.1	0.9	4.7	2	1	1	1	213	23.7	4.4
NP_005973.1	5174681	SIX1	homeobox protein SIX1	-	-	-	-	0.8	0.9	0.8	0.9	0.8	0.9	0.8	0.9	6.3	2	1	1	1	284	32.2	9.1
NP_001265272.1	507588248	ACTN2	alpha-actinin-2 isoform 2	-	-	-	-	1.2	1.1	1.6	0.9	1.2	1.1	1.6	0.9	20.9	3	2	20	308	894	103.9	5.5
NP_004248.2	21359865	TGFBRAP1	transforming growth factor-beta receptor-associated protein 1	-	-	-	-	0.9	0.9	0.9	0.9	0.9	0.9	0.9	0.9	1.2	1	1	1	1	860	97.1	6.6
NP_004322.1	4757860	BNIP3L	BCL2/adenovirus E1B 19 kDa protein-interacting protein 3-like	-	-	-	-	1.0	0.9	1.0	0.9	1.0	0.9	1.0	0.9	6.9	1	1	1	1	219	23.9	5.8
NP_005624.2	15529996	SOS1	son of sevenless homolog 1	-	-	-	-	1.0	0.9	0.8	0.9	1.0	0.9	0.8	0.9	0.8	1	1	1	1	1333	152.4	6.8
NP_001273478.1	557440865	CAPS2	calcyphosin-2 isoform 4	-	-	-	-	1.0	0.9	0.9	0.9	1.0	0.9	0.9	0.9	3.4	4	1	1	2	204	23.4	5.5
NP_001736.1	4502559	CAMLG	calcium signal-modulating cyclophilin ligand	-	-	-	-	0.9	0.9	1.0	0.9	0.9	0.9	1.0	0.9	18.2	1	3	3	5	296	32.9	8.0
NP_001230131.1	342349354	RANBP6	ran-binding protein 6 isoform 2	-	-	-	-	1.1	1.0	1.0	0.9	1.1	1.0	1.0	0.9	9.6	2	2	5	30	753	85.2	5.1
NP_079068.1	13376162	FAM57A	protein FAM57A	-	-	-	-	1.0	0.9	1.0	0.9	1.0	0.9	1.0	0.9	3.1	1	1	1	1	257	29.4	9.4
NP_001008487.1	56549677	SLC41A3	solute carrier family 41 member 3 isoform 4	-	-	-	-	0.9	0.8	0.9	0.9	0.9	0.8	0.9	0.9	2.8	3	1	1	2	461	49.9	7.9
NP_006449.2	32454739	TRIM3	tripartite motif-containing protein 3 isoform 1	-	-	-	-	0.9	1.0	1.0	0.9	0.9	1.0	1.0	0.9	3.0	2	1	1	9	744	80.8	7.8
NP_001984.1	4503641	F3	tissue factor isoform 1 precursor	-	-	-	-	0.9	1.0	0.8	0.9	0.9	1.0	0.8	0.9	4.4	1	1	1	1	295	33.0	7.0
NP_001278501.1	615276331	NBR1	next to BRCA1 gene 1 protein isoform c	-	-	-	-	0.8	0.9	0.9	0.9	0.8	0.9	0.9	0.9	1.2	3	1	1	1	837	92.9	5.0
NP_001265154.1	501355147	RUFY2	RUN and FYVE domain-containing protein 2 isoform c	-	-	-	-	0.9	0.8	0.9	0.9	0.9	0.8	0.9	0.9	2.1	3	1	1	1	379	43.7	5.3
NP_001035814.1	109637774	CYLD	ubiquitin carboxyl-terminal hydrolase CYLD isoform 2	-	-	-	-	1.0	1.1	1.0	0.9	1.0	1.1	1.0	0.9	1.9	2	1	1	3	953	107.0	5.6
NP_005061.2	157671951	SLC4A3	anion exchange protein 3 isoform 1	-	-	-	-	0.9	0.9	1.0	0.9	0.9	0.9	1.0	0.9	0.7	2	1	1	4	1232	135.7	6.4
NP_002219.1	4758616	JUN	transcription factor AP-1	-	-	-	-	1.1	1.0	1.0	0.9	1.1	1.0	1.0	0.9	14.5	2	2	3	11	331	35.7	8.8
NP_068766.1	11345492	SRR	serine racemase	-	-	-	-	1.1	0.9	0.9	0.9	1.1	0.9	0.9	0.9	19.7	2	4	4	9	340	36.5	6.5
NP_002644.4	152963644	PITX1	pituitary homeobox 1	-	-	-	-	0.8	0.9	0.8	0.9	0.8	0.9	0.8	0.9	3.2	1	1	1	1	314	34.1	9.0
NP_000201.2	119395742	ITGA6	integrin alpha-6 isoform b precursor	-	-	-	-	0.9	0.9	0.9	0.9	0.9	0.9	0.9	0.9	2.2	2	2	2	2	1073	119.4	6.8
NP_001243234.1	372626421	HPGD	15-hydroxyprostaglandin dehydrogenase [NAD(+)] isoform 4	-	-	-	-	1.1	0.9	1.1	0.9	1.1	0.9	1.1	0.9	9.1	4	1	1	1	143	15.5	6.5
NP_659410.3	122891862	DENND5B	DENN domain-containing protein 5B isoform 1	-	-	-	-	0.9	0.8	0.9	0.9	0.9	0.8	0.9	0.9	0.6	2	1	1	1	1274	144.9	6.7
NP_001177906.1	300244514	YAF2	YY1-associated factor 2 isoform 3	-	-	-	-	0.7	0.8	0.7	0.9	0.7	0.8	0.7	0.9	18.1	3	1	1	2	138	15.1	9.9
NP_006010.2	19924145	TCIRG1	V-type proton ATPase 116 kDa subunit a isoform 3 isoform a	-	-	-	-	0.9	0.9	0.9	0.9	0.9	0.9	0.9	0.9	3.1	2	2	2	3	830	92.9	7.1
NP_056098.1	148839335	DPY19L1	probable C-mannosyltransferase DPY19L1	-	-	-	-	0.9	0.9	0.9	0.9	0.9	0.9	0.9	0.9	4.3	1	2	2	2	675	77.3	9.0
NP_001244330.1	383872547	CD63	CD63 antigen isoform D precursor	-	-	-	-	1.0	1.0	1.1	0.9	1.0	1.0	1.1	0.9	5.1	3	1	1	1	156	17.3	7.9
NP_002171.2	119392094	IGHMBP2	DNA-binding protein SMUBP-2	-	-	-	-	1.0	1.0	0.9	0.9	1.0	1.0	0.9	0.9	4.9	1	2	3	6	993	109.1	9.0
NP_060711.2	190358512	TMEM184C	transmembrane protein 184C	-	-	-	-	0.9	0.9	0.9	0.9	0.9	0.9	0.9	0.9	3.0	1	1	1	1	438	50.1	6.3
NP_003209.2	189409140	TERF1	telomeric repeat-binding factor 1 isoform 2	-	-	-	-	1.0	0.9	0.8	0.9	1.0	0.9	0.8	0.9	6.4	2	2	2	4	419	48.2	6.6
NP_001127841.1	197276647	LRRFIP2	leucine-rich repeat flightless-interacting protein 2 isoform 3	-	-	-	-	1.0	1.1	1.0	0.9	1.0	1.1	1.0	0.9	11.3	4	3	4	15	424	48.3	5.4
NP_004746.2	32528295	RPS6KA5	ribosomal protein S6 kinase alpha-5 isoform a	-	-	-	-	1.0	0.9	1.1	0.9	1.0	0.9	1.1	0.9	2.1	2	1	2	2	802	89.8	7.1
NP_001276027.1	574273242	PXK	PX domain-containing protein kinase-like protein isoform 4	-	-	-	-	1.0	1.1	1.0	0.9	1.0	1.1	1.0	0.9	12.2	7	3	3	5	515	58.7	9.2
NP_002745.1	4506085	MAPK13	mitogen-activated protein kinase 13	-	-	-	-	1.0	1.0	0.9	0.9	1.0	1.0	0.9	0.9	3.0	1	1	1	1	365	42.1	8.4
NP_001153773.1	237757300	DPYD	dihydropyrimidine dehydrogenase [NADP(+)] isoform 2	-	-	-	-	0.8	0.8	0.7	0.9	0.8	0.8	0.7	0.9	5.2	2	1	1	2	173	18.9	7.5
NP_478062.1	17158011	SMIM11	small integral membrane protein 11	-	-	-	-	0.9	0.9	1.4	0.9	0.9	0.9	1.4	0.9	25.9	1	1	1	3	58	6.9	9.9
NP_001004128.1	51873067	QSOX1	sulfhydryl oxidase 1 isoform b precursor	-	-	-	-	1.0	1.0	1.0	0.9	1.0	1.0	1.0	0.9	3.2	2	1	1	1	604	66.8	8.6
NP_055888.1	71143119	SIPA1L3	signal-induced proliferation-associated 1-like protein 3	-	-	-	-	1.0	0.8	0.9	0.9	1.0	0.8	0.9	0.9	0.8	1	1	1	1	1781	194.5	8.3
NP_006501.1	5730009	TRIM27	zinc finger protein RFP	-	-	-	-	0.9	0.9	0.9	0.9	0.9	0.9	0.9	0.9	2.7	1	1	1	1	513	58.5	6.2
NP_001254524.1	390131986	SENP1	sentrin-specific protease 1	-	-	-	-	2.0	1.0	0.8	0.9	2.0	1.0	0.8	0.9	1.6	1	1	1	1	644	73.4	8.5
NP_006330.2	83715964	HMG20B	SWI/SNF-related matrix-associated actin-dependent regulator of chromatin subfamily E member 1-related	-	-	-	-	0.8	0.9	0.8	0.9	0.8	0.9	0.8	0.9	10.4	1	2	2	3	317	35.8	9.3
NP_001040625.1	114145479	NET1	neuroepithelial cell-transforming gene 1 protein isoform 1	-	-	-	-	0.8	0.9	0.9	0.9	0.8	0.9	0.9	0.9	3.0	1	1	1	1	596	67.7	9.2
NP_061956.2	226493203	TRMT13	tRNA:m(4)X modification enzyme TRM13 homolog	-	-	-	-	0.8	0.8	0.8	0.9	0.8	0.8	0.8	0.9	1.7	1	1	1	2	481	54.2	8.1
NP_056981.2	24850133	PIAS4	E3 SUMO-protein ligase PIAS4	-	-	-	-	0.9	0.8	0.9	0.9	0.9	0.8	0.9	0.9	3.1	1	1	1	1	510	56.5	6.1
NP_071346.1	11545771	FAM204A	protein FAM204A	-	-	-	-	1.1	1.0	0.9	0.9	1.1	1.0	0.9	0.9	4.7	1	1	1	1	233	27.0	7.9
NP_055553.3	157502197	KIAA0195	uncharacterized protein KIAA0195	-	-	-	-	0.8	0.9	0.9	0.9	0.8	0.9	0.9	0.9	2.5	1	3	3	5	1356	151.1	6.4
NP_057480.2	100815975	CTDSPL2	CTD small phosphatase-like protein 2	-	-	-	-	0.9	0.9	0.9	0.9	0.9	0.9	0.9	0.9	10.1	1	4	4	6	466	53.0	6.4
NP_001905.1	4503183	CYB5A	cytochrome b5 isoform 2	-	-	-	-	1.0	0.9	0.9	0.9	1.0	0.9	0.9	0.9	49.0	3	3	3	4	98	11.3	5.1
NP_060886.1	8923842	FOXJ2	forkhead box protein J2	-	-	-	-	1.0	1.1	1.0	0.9	1.0	1.1	1.0	0.9	3.3	1	1	1	2	574	62.4	6.7
NP_065815.1	18079216	CASKIN1	caskin-1	-	-	-	-	0.9	1.0	1.0	0.9	0.9	1.0	1.0	0.9	0.5	1	1	1	1	1431	149.7	9.1
NP_064526.1	10047136	TMEM167B	protein kish-B precursor	-	-	-	-	1.1	1.0	0.9	0.9	1.1	1.0	0.9	0.9	9.5	1	1	1	1	74	8.3	9.6
NP_055815.1	24307971	STK38L	serine/threonine-protein kinase 38-like	-	-	-	-	0.8	0.9	0.8	0.9	0.8	0.9	0.8	0.9	2.2	1	1	1	1	464	54.0	6.8
NP_114403.1	15721937	MRPS24	28S ribosomal protein S24, mitochondrial precursor	-	-	-	-	1.1	0.9	0.9	0.9	1.1	0.9	0.9	0.9	10.2	1	1	1	6	167	19.0	9.4
NP_055754.2	42476076	TRAPPC8	trafficking protein particle complex subunit 8	-	-	-	-	1.2	1.0	1.2	0.9	1.2	1.0	1.2	0.9	1.5	1	2	2	2	1435	160.9	6.9
NP_758438.2	151301211	TFAP2D	transcription factor AP-2-delta	-	-	-	-	1.0	0.8	1.1	0.9	1.0	0.8	1.1	0.9	1.6	1	1	1	2	452	49.5	8.2
NP_008949.4	87298937	CNTRL	centriolin	-	-	-	-	0.8	0.8	0.8	0.9	0.8	0.8	0.8	0.9	0.3	1	1	1	1	2325	268.7	5.6
NP_002218.2	102469034	JAK1	tyrosine-protein kinase JAK1	-	-	-	-	0.9	0.9	0.9	0.9	0.9	0.9	0.9	0.9	3.6	1	2	2	4	1154	133.2	7.5
NP_003060.2	21071044	SMARCA1	probable global transcription activator SNF2L1 isoform a	-	-	-	-	0.8	0.8	0.9	0.9	0.8	0.8	0.9	0.9	7.0	3	5	9	29	1054	122.5	8.1
NP_149105.3	51243065	ZCRB1	zinc finger CCHC-type and RNA-binding motif-containing protein 1	-	-	-	-	0.7	1.0	0.9	0.9	0.7	1.0	0.9	0.9	6.9	1	1	1	2	217	24.6	8.5
NP_062556.2	21361809	RBMXL1	RNA binding motif protein, X-linked-like-1	-	-	-	-	1.1	0.8	0.9	0.9	1.1	0.8	0.9	0.9	34.6	2	3	14	84	390	42.1	9.9
NP_115677.2	124248558	FBXW9	F-box/WD repeat-containing protein 9	-	-	-	-	1.0	1.0	1.0	0.9	1.0	1.0	1.0	0.9	3.5	1	1	1	1	458	50.7	6.5
NP_004853.2	65787265	LITAF	lipopolysaccharide-induced tumor necrosis factor-alpha factor isoform 1	-	-	-	-	0.8	0.8	0.8	0.9	0.8	0.8	0.8	0.9	4.4	1	1	1	1	161	17.1	6.4
NP_689764.3	71143123	SNRNP48	U11/U12 small nuclear ribonucleoprotein 48 kDa protein	-	-	-	-	0.7	0.9	0.7	0.9	0.7	0.9	0.7	0.9	3.5	1	1	1	4	339	39.9	6.9
NP_062830.1	140560917	MYOM1	myomesin-1 isoform b	-	-	-	-	0.8	0.9	1.1	0.9	0.8	0.9	1.1	0.9	0.6	2	1	1	2	1589	177.6	6.8
NP_001229956.1	339895853	AKT2	RAC-beta serine/threonine-protein kinase isoform 2	-	-	-	-	0.7	0.8	0.9	0.9	0.7	0.8	0.9	0.9	4.3	2	2	2	4	419	48.5	6.2
NP_001073925.2	207028781	ZSCAN5B	zinc finger and SCAN domain-containing protein 5B	-	-	-	-	0.9	1.2	0.9	0.9	0.9	1.2	0.9	0.9	4.7	2	1	1	1	495	55.8	8.6
NP_001026904.2	685504978	FDX1L	adrenodoxin-like protein, mitochondrial precursor	-	-	-	-	0.9	1.0	1.0	0.9	0.9	1.0	1.0	0.9	5.9	1	1	1	1	186	19.9	5.5
NP_001244967.1	384475544	SPRY1	protein sprouty homolog 1	-	-	-	-	0.8	0.7	0.7	0.9	0.8	0.7	0.7	0.9	9.4	1	1	1	1	319	35.1	8.2
NP_005679.2	66529005	ABCC5	multidrug resistance-associated protein 5 isoform 1	-	-	-	-	1.2	1.0	1.0	0.9	1.2	1.0	1.0	0.9	0.6	1	1	1	1	1437	160.6	8.7
NP_689480.2	31542783	RNF185	E3 ubiquitin-protein ligase RNF185 isoform 1	-	-	-	-	1.1	1.0	1.0	0.9	1.1	1.0	1.0	0.9	7.8	1	1	1	1	192	20.4	6.5
NP_065397.3	258679433	CSRP2BP	cysteine-rich protein 2-binding protein	-	-	-	-	0.9	0.9	0.9	0.9	0.9	0.9	0.9	0.9	1.4	1	1	1	1	782	88.7	6.6
NP_116290.1	14249670	COX14	cytochrome c oxidase assembly protein COX14	-	-	-	-	1.0	1.1	0.9	0.9	1.0	1.1	0.9	0.9	12.3	1	1	1	1	57	6.6	9.6

Khan *et al.* , 2019. Multi-omics analysis to characterize cigarette smoke induced molecular alterations in esophageal cells

Supplementary Table 5. List of proteins quantified in untreated and chronically treated Het1A cells with cigarette smoke condensate for 8 months

NP_Accession	Protein group Accession	Gene ID	Description	Het-1A-Smoke - 2M/Parental	Het-1A-Smoke - 4M/Parental	Het-1A-Smoke - 6M/Parental	Het-1A-Smoke - 8M/Parental	Het-1A-Smoke - 2M/Parental	Het-1A-Smoke - 4M/Parental	Het-1A-Smoke - 6M/Parental	Het-1A-Smoke - 8M/Parental	Het-1A-Smoke - 2M/Parental	Het-1A-Smoke - 4M/Parental	Het-1A-Smoke - 6M/Parental	Het-1A-Smoke - 8M/Parental	Coverage (%)	Proteins	Unique Peptides	# Peptides	PSM	# AAs	MW [kDa]	calc. pI
				Replicate 1				Replicate 2				Average of replicates											
NP_001023.1	4506717	RPS29	40S ribosomal protein S29 isoform 1	-	-	-	-	0.8	0.9	0.9	0.9	0.8	0.9	0.9	0.9	30.4	2	2	2	5	56	6.7	10.1
NP_055189.2	153792539	REPIN1	replication initiator 1 isoform 1	-	-	-	-	1.0	0.9	1.0	0.9	1.0	0.9	1.0	0.9	2.7	2	1	1	2	567	63.5	10.0
NP_005093.2	110349754	FEZ2	fasciculation and elongation protein zeta-2 isoform 1	-	-	-	-	0.9	0.9	0.8	0.9	0.9	0.9	0.8	0.9	3.1	2	1	1	2	353	39.6	4.6
XP_005274558.1	530421074	KAL1	anosmin-1 isoform X1	-	-	-	-	1.2	1.3	1.7	0.9	1.2	1.3	1.7	0.9	7.4	2	1	1	1	420	46.6	8.6
NP_065821.1	16262452	CGN	cingulin	-	-	-	-	0.7	0.8	0.8	0.9	0.7	0.8	0.8	0.9	1.0	1	1	1	2	1203	137.0	5.5
NP_000156.1	4504001	GJA1	gap junction alpha-1 protein	-	-	-	-	1.2	0.9	0.9	0.9	1.2	0.9	0.9	0.9	3.7	1	1	1	1	382	43.0	8.8
NP_006157.1	5453780	NFYB	nuclear transcription factor Y subunit beta	-	-	-	-	1.1	1.1	1.1	0.9	1.1	1.1	1.1	0.9	16.4	1	2	2	5	207	22.8	4.6
NP_001276116.1	574584493	ZNF302	zinc finger protein 302 isoform c	-	-	-	-	1.0	0.9	0.9	0.9	1.0	0.9	0.9	0.9	2.8	3	1	1	1	399	45.9	8.5
NP_001136113.1	217416343	FBRSL1	fibrosin-1-like protein	-	-	-	-	0.9	0.8	0.9	0.9	0.9	0.8	0.9	0.9	1.0	1	1	1	1	1045	110.8	9.7
NP_115980.1	14211919	SLC9A7	sodium/hydrogen exchanger 7 isoform 2 precursor	-	-	-	-	0.8	0.9	0.9	0.9	0.8	0.9	0.9	0.9	2.3	2	2	2	3	725	80.1	6.4
NP_001257335.1	393715108	APOBEC3A	DNA dC->dU-editing enzyme APOBEC-3A isoform b	-	-	-	-	0.9	0.8	1.1	0.9	0.9	0.8	1.1	0.9	9.4	3	1	1	4	181	20.9	6.7
NP_001004450.1	52219202	OR1B1	olfactory receptor 1B1	-	-	-	-	2.2	1.0	0.9	0.9	2.2	1.0	0.9	0.9	2.2	1	1	1	2	318	35.3	8.2
NP_055321.1	14149653	TOR1B	torsin-1B precursor	-	-	-	-	1.6	0.9	0.9	0.9	1.6	0.9	0.9	0.9	7.7	1	3	3	4	336	38.0	8.5
NP_037455.2	23111026	SNX11	sorting nexin-11	-	-	-	-	0.7	0.7	0.7	0.9	0.7	0.7	0.7	0.9	7.8	1	1	1	2	270	30.4	7.0
NP_612367.1	24308410	C19orf52	uncharacterized protein C19orf52	-	-	-	-	0.9	0.9	0.9	0.9	0.9	0.9	0.9	0.9	11.5	1	2	2	3	260	29.2	8.1
NP_115879.2	110227846	C14orf142	uncharacterized protein C14orf142	-	-	-	-	1.1	0.8	1.1	0.9	1.1	0.8	1.1	0.9	41.0	1	2	2	7	100	10.9	4.3
NP_037487.2	40254851	PKN3	serine/threonine-protein kinase N3	-	-	-	-	0.9	0.9	0.9	0.9	0.9	0.9	0.9	0.9	2.4	1	2	2	2	889	99.4	8.5
NP_004483.1	4758486	GTF2A2	transcription initiation factor IIA subunit 2	-	-	-	-	0.8	0.8	0.8	0.9	0.8	0.8	0.8	0.9	6.4	1	1	1	1	109	12.4	6.6
NP_002886.2	34577079	RBL1	retinoblastoma-like protein 1 isoform a	-	-	-	-	1.0	0.9	0.8	0.9	1.0	0.9	0.8	0.9	1.2	1	1	1	2	1068	120.8	7.5
NP_060382.3	61563742	HERC6	probable E3 ubiquitin-protein ligase HERC6 isoform 1	-	-	-	-	1.1	1.0	1.3	0.9	1.1	1.0	1.3	0.9	0.9	1	1	1	1	1022	115.1	8.0
NP_116184.2	221316663	RPUSD4	RNA pseudouridylate synthase domain-containing protein 4 isoform a	-	-	-	-	1.2	1.0	1.0	0.9	1.2	1.0	1.0	0.9	11.9	2	2	2	6	377	42.2	9.9
NP_061902.2	83641876	CPVL	probable serine carboxypeptidase CPVL precursor	-	-	-	-	0.9	0.8	0.9	0.9	0.9	0.8	0.9	0.9	4.8	1	2	2	2	476	54.1	5.6
NP_005500.4	148528998	DMXL1	dmX-like protein 1 isoform 2	-	-	-	-	0.9	0.9	0.9	0.9	0.9	0.9	0.9	0.9	1.3	3	2	2	3	3027	337.6	6.3
NP_001186623.1	314122224	SLC4A2	anion exchange protein 2 isoform 3	-	-	-	-	0.9	1.2	1.1	0.9	0.9	1.2	1.1	0.9	1.6	3	1	1	1	1227	135.5	6.2
NP_068776.2	119372286	GYS2	glycogen [starch] synthase, liver	-	-	-	-	0.9	0.9	1.1	0.9	0.9	0.9	1.1	0.9	3.0	1	1	2	5	703	80.9	6.8
NP_001138500.1	222537747	PALM3	paralemmin-3	-	-	-	-	1.3	1.0	0.8	0.9	1.3	1.0	0.8	0.9	3.4	1	1	1	3	673	71.7	4.5
NP_057691.1	7705274;29540566	ARMCX3	armadillo repeat-containing X-linked protein 3	-	-	-	-	1.1	1.0	1.0	0.9	1.1	1.0	1.0	0.9	10.6	1	3	3	5	379	42.5	8.4
NP_060553.4	133922582	ZNF358	zinc finger protein 358	-	-	-	-	0.9	0.9	0.8	0.9	0.9	0.9	0.8	0.9	4.1	1	1	1	1	568	59.2	6.6
NP_060030.1	221139761	ZNF853	zinc finger protein 853	-	-	-	-	0.8	0.8	0.8	0.9	0.8	0.8	0.8	0.9	0.9	1	1	1	1	659	74.8	5.2
NP_689542.2	45439342	LRR1	leucine-rich repeat protein 1 isoform 1	-	-	-	-	1.0	0.9	1.0	0.9	1.0	0.9	1.0	0.9	6.3	1	2	2	3	414	46.7	9.1
NP_006846.1	6857802	KDELR3	ER lumen protein retaining receptor 3 isoform a	-	-	-	-	1.2	0.7	1.1	0.9	1.2	0.7	1.1	0.9	3.3	1	1	1	1	214	25.0	8.9
NP_001191118.1	323668276	TP73	tumor protein p73 isoform e	-	-	-	-	0.9	0.6	0.6	0.9	0.9	0.6	0.6	0.9	3.1	13	1	1	2	354	39.3	8.5
NP_115903.1	14210522	MAP1LC3A	microtubule-associated proteins 1A/1B light chain 3A isoform a	-	-	-	-	1.3	1.1	1.1	0.9	1.3	1.1	1.1	0.9	5.8	4	1	1	6	121	14.3	8.7
NP_919304.1	35493860	OTOF	otoferlin isoform d	-	-	-	-	0.8	0.7	0.8	0.9	0.8	0.7	0.8	0.9	1.0	5	1	1	1	1230	140.2	6.2
NP_001036096.1	111038124	SDHAF1	succinate dehydrogenase assembly factor 1, mitochondrial	-	-	-	-	1.0	1.0	1.2	0.9	1.0	1.0	1.2	0.9	13.9	1	1	1	1	115	12.8	11.5
NP_942582.1	38570103	TBC1D8B	TBC1 domain family member 8B isoform b	-	-	-	-	1.3	0.9	1.4	0.9	1.3	0.9	1.4	0.9	1.1	2	1	1	1	632	72.6	6.3
NP_006685.1	5729889	JTB	protein JTB precursor	-	-	-	-	0.9	0.8	1.0	0.9	0.9	0.8	1.0	0.9	8.9	1	1	1	2	146	16.3	8.3
NP_201572.1	15277321	RASL10B	ras-like protein family member 10B precursor	-	-	-	-	1.6	0.9	0.9	0.9	1.6	0.9	0.9	0.9	3.5	1	1	1	1	203	23.2	9.4
NP_001243398.1	374349207	KAT6B	histone acetyltransferase KAT6B isoform 3	-	-	-	-	0.9	0.9	0.9	0.9	0.9	0.9	0.9	0.9	3.1	3	2	2	2	1781	199.7	5.1
NP_006005.2	24430137	LAGE3	EKC/KEOPS complex subunit LAGE3	-	-	-	-	1.2	0.9	0.8	0.9	1.2	0.9	0.8	0.9	16.1	1	1	1	4	143	14.8	8.6
NP_000446.1	4507271	STK11	serine/threonine-protein kinase STK11	-	-	-	-	0.8	0.8	0.8	0.9	0.8	0.8	0.8	0.9	6.9	1	2	2	3	433	48.6	7.4
NP_004264.2	19923281	CHST3	carbohydrate sulfotransferase 3	-	-	-	-	1.1	1.1	0.8	0.9	1.1	1.1	0.8	0.9	3.3	1	1	1	1	479	54.7	8.6
NP_620832.1	20986495	YY1AP1	YY1-associated protein 1 isoform 5	-	-	-	-	0.8	0.7	0.8	0.9	0.8	0.7	0.8	0.9	2.3	10	1	1	2	684	75.3	8.9
NP_004251.3	284005309	RECQL4	ATP-dependent DNA helicase Q4	-	-	-	-	0.8	0.8	0.9	0.9	0.8	0.8	0.9	0.9	5.2	1	4	4	9	1208	133.0	8.1
NP_065206.3	299782546	PIGA	phosphatidylinositol N-acetylglucosaminyltransferase subunit A isoform 3	-	-	-	-	1.1	0.9	0.9	0.9	1.1	0.9	0.9	0.9	8.4	2	1	1	3	250	28.0	6.1
NP_919439.1	37577130;37577132	UBE2J2	ubiquitin-conjugating enzyme E2 J2 isoform 3	-	-	-	-	0.9	1.0	1.1	0.9	0.9	1.0	1.1	0.9	6.8	3	1	1	2	207	22.9	7.5
NP_001678.1	4502297	ATP5D	ATP synthase subunit delta, mitochondrial precursor	-	-	-	-	1.2	0.9	1.4	0.9	1.2	0.9	1.4	0.9	13.7	1	2	2	7	168	17.5	5.5
NP_112562.3	57863261	TEX14	inactive serine/threonine-protein kinase TEX14 isoform b	-	-	-	-	0.7	0.8	0.8	0.9	0.7	0.8	0.8	0.9	1.7	3	1	1	1	1451	162.6	5.2
NP_001253976.1	388490223	FRMD6	FERM domain-containing protein 6 isoform 3	-	-	-	-	0.9	0.8	0.9	0.8	0.9	0.8	0.9	0.8	5.7	3	1	1	2	264	29.2	5.5
NP_001269586.1	544063385	ZNF143	zinc finger protein 143 isoform 3	-	-	-	-	0.9	0.8	0.8	0.8	0.9	0.8	0.8	0.8	3.3	3	2	2	4	607	65.6	6.2
NP_002214.2	95147335	ITPR2	inositol 1,4,5-trisphosphate receptor type 2	-	-	-	-	0.9	0.8	1.0	0.8	0.9	0.8	1.0	0.8	2.2	3	1	8	19	2701	307.9	6.4
NP_899630.1	34452711	CADPS	calcium-dependent secretion activator 1 isoform 3	-	-	-	-	0.9	1.0	0.9	0.8	0.9	1.0	0.9	0.8	3.4	6	2	2	3	1274	144.0	5.7
NP_065386.1	10092625	IL22	interleukin-22 precursor	-	-	-	-	0.9	1.0	1.0	0.8	0.9	1.0	1.0	0.8	7.3	1	1	1	1	179	20.0	7.8
NP_079412.1	13376717	OPA3	optic atrophy 3 protein isoform b	-	-	-	-	1.0	1.1	1.1	0.8	1.0	1.1	1.1	0.8	16.8	1	1	1	2	179	20.0	8.9
NP_006792.1	5803048	KDELR1	ER lumen protein-retaining receptor 1	-	-	-	-	0.8	0.9	0.8	0.8	0.8	0.9	0.8	0.8	9.0	1	1	1	1	212	24.5	8.6
XP_011507330.1	767901670	LOC10537334	uncharacterized protein LOC105373347 isoform X1	-	-	-	-	0.7	0.9	0.4	0.8	0.7	0.9	0.4	0.8	3.6	1	1	1	1	169	18.6	6.0
NP_001287815.1	665505975	RHOD	rho-related GTP-binding protein RhoD isoform 2	-	-	-	-	1.1	0.9	1.0	0.8	1.1	0.9	1.0	0.8	7.6	2	1	1	2	144	15.7	8.8
NP_056995.1	7705692	ZNF691	zinc finger protein 691 isoform 2	-	-	-	-	0.7	0.9	0.7	0.8	0.7	0.9	0.7	0.8	4.6	2	1	1	1	284	32.5	8.3
NP_065107.1	9966847	C12orf4	uncharacterized protein C12orf4	-	-	-	-	0.9	0.9	0.8	0.8	0.9	0.9	0.8	0.8	4.4	1	1	1	1	552	63.8	6.4
NP_006724.2	41352697	CENPI	centromere protein I	-	-	-	-	0.9	1.0	0.9	0.8	0.9	1.0	0.9	0.8	1.5	1	1	1	2	756	86.7	8.8
NP_660339.1	21686977	CADM4	cell adhesion molecule 4 precursor	-	-	-	-	0.9	1.0	1.0	0.8	0.9	1.0	1.0	0.8	2.3	1	1	1	1	388	42.8	6.3
NP_001275659.1	570359577	DAPK1	death-associated protein kinase 1	-	-	-	-	0.8	0.9	1.0	0.8	0.8	0.9	1.0	0.8	0.6	1	1	1	2	1430	159.9	6.8
NP_001245302.1	385648255	SPATA20	spermatogenesis-associated protein 20 isoform 3	-	-	-	-	0.9	0.7	0.8	0.8	0.9	0.7	0.8	0.8	2.2	3	1	1	1	742	83.3	6.8
NP_001154948.1	241896933	WDR25	WD repeat-containing protein 25	-	-	-	-	0.9	0.8	1.0	0.8	0.9	0.8	1.0	0.8	4.0	1	1	1	1	544	60.1	9.0
NP_060017.1	8923708	FOXRED1	FAD-dependent oxidoreductase domain-containing protein 1	-	-	-	-	1.0	0.9	0.9	0.8	1.0	0.9	0.9	0.8	6.8	1	2	2	3	486	53.8	7.8
NP_001193870.1	332635057	TCAF1	protein FAM115A isoform 3	-	-	-	-	0.9	0.9	1.0	0.8	0.9	0.9	1.0	0.8	7.9	4	3	3	6	497	56.6	8.2
NP_003316.3	21536485	HIRA	protein HIRA	-	-	-	-	1.0	0.8	1.0	0.8	1.0	0.8	1.0	0.8	2.3	1	2	2	3	1017	111.8	8.1
NP_004804.1	4758898	PEX16	peroxisomal biogenesis factor 16 isoform 1	-	-	-	-	0.7	0.9	0.7	0.8	0.7	0.9	0.7	0.8	6.9	2	1	1	2	336	38.6	9.8
NP_004796.1	4758574	POLR2D	DNA-directed RNA polymerase II subunit RPB4	-	-	-	-	1.6	0.8	1.3	0.8	1.6	0.8	1.3	0.8	11.3	1	1	1	2	142	16.3	4.8
NP_001034738.1	89001102	ZMYM5	zinc finger MYM-type protein 5 isoform 2	-	-	-	-	0.7	0.8	0.6	0.8	0.7	0.8	0.6	0.8	2.9	1	1	1	1	208	22.7	4.9
NP_000205.1	4557679	JAG1	protein jagged-1 precursor	-	-	-	-	0.7	0.8	0.6	0.8	0.7	0.8	0.6	0.8	1.6	1	1	1	1	1218	133.7	6.1
NP_001035926.1	109698597;109698599	TRAPPC5	trafficking protein particle complex subunit 5	-	-	-	-	0.8	0.8	0.8	0.8	0.8	0.8	0.8	0.8	27.7	1	5	5	10	188	20.8	9.7
NP_001167566.1	291575190	ZEB1	zinc finger E-box-binding homeobox 1 isoform e	-	-	-	-	0.9	0.9	0.9	0.8	0.9	0.9	0.9	0.8	3.7	6	3	3	5	1057	116.5	5.1
NP_060108.2	188219520;188035867	MED18	mediator of RNA polymerase II transcription subunit 18	-	-	-	-	1.0	0.8	0.9	0.8	1.0	0.8	0.9	0.8	11.5	1	2	2	4	208	23.6	6.5
NP_078804.2	168229156	NKAP	NF-kappa-B-activating protein	-	-	-	-	0.8	0.8	0.7	0.8	0.8	0.8	0.7	0.8	5.8	2	2	2	3	415	47.1	10.1
NP_008867.2	48928058	SUMO3	small ubiquitin-related modifier 3 isoform 1 precursor	-	-	-	-	0.8	0.9	0.9	0.8	0.8	0.9	0.9	0.8	20.4	3	1	2	15	103	11.6	5.5
NP_542398.3	156564240	CFAP36	cilia- and flagella-associated protein 36 isoform b	-	-	-	-	0.9	0.9	0.9	0.8	0.9	0.9	0.9	0.8	29.0	2	7	7	22	342	39.4	5.0
NP_085048.1	13386480	NDFIP1	NEDD4 family-interacting protein 1	-	-	-	-	1.0	1.0	1.3	0.8	1.0	1.0	1.3	0.8	11.3	1	1	1	1	221	24.9	4.7
NP_004086.1	4758258	EIF4EBP1	eukaryotic translation initiation factor 4E-binding protein 1	-	-	-	-	0.9	0.9	0.8	0.8	0.9	0.9	0.8	0.8	32.2	1	2	2	2	118	12.6	5.5
NP_079231.4	156139139;156139141	FOXRED2	FAD-dependent oxidoreductase domain-containing protein 2 precursor	-	-	-	-	0.8	0.8	0.9	0.8	0.8	0.8	0.9	0.8	2.5	1	1	1	4	684	77.7	7.7
NP_056091.1	150010639	USP22	ubiquitin carboxyl-terminal hydrolase 22	-	-	-	-	0.9	0.9	1.0	0.8	0.9	0.9	1.0	0.8	5.3	2	3	3	5	525	59.9	8.0
NP_055021.1	7656934	TMEM258	transmembrane protein 258	-	-	-	-	2.4	2.6	3.7	0.8	2.4	2.6	3.7	0.8	39.2	1	1	1	1	79	9.1	5.8
NP_003621.1	4505727	PEX3	peroxisomal biogenesis factor 3	-	-	-	-	0.9	0.8	0.9	0.8	0.9	0.8	0.9	0.8	16.9	1	5	5	11	373	42.1	8.1
NP_055214.1	7657373	TSPAN13	tetraspanin-13	-	-	-	-	0.9	0.9	1.0	0.8	0.9	0.9	1.0	0.8	3.4	2	1	1	1	204	22.1	7.9
NP_001291435.1	751368086	ZKSCAN4	zinc finger protein with KRAB and SCAN domains 4 isoform 2	-	-	-	-	0.9	0.9	0.9	0.8	0.9	0.9	0.9	0.8	2.6	2	1	1	2	390	44.1	8.3
NP_078895.2	20149680	FN3KRP	ketosamine-3-kinase	-	-	-	-	0.9	0.9	0.8	0.8	0.9	0.9	0.8	0.8	15.9	1	4	4	10	309	34.4	7.3
NP_001245389.1	386642888	PHKG1	phosphorylase b kinase gamma catalytic chain, skeletal muscle/heart isoform isoform 3	-	-	-	-	0.9	1.0	1.0	0.8	0.9	1.0	1.0	0.8	1.9	3	1	1	1	378	44.0	6.8
NP_001012241.1	149944433	MSL1	male-specific lethal 1 homolog	-	-	-	-	0.7	0.7	0.7	0.8	0.7	0.7	0.7	0.8	5.1	1	1	1	1	351	40.8	9.1
NP_477097.1	17318565	CCNE2	G1/S-specific cyclin-E2	-	-	-	-	0.9	0.7	0.8	0.8	0.9	0.7	0.8	0.8	4.5	1	1	1	1	404	46.7	7.8
NP_004547.2	71274109	NFKBIE	NF-kappa-B inhibitor epsilon	-	-	-	-	0.8	0.6	0.6	0.8	0.8	0.6	0.6	0.8	4.8	1	1	1	2	500	52.8	6.7
NP_821138.1	29789451	CATSPER3	cation channel sperm-associated protein 3	-	-	-	-	0.9	0.9	0.9	0.8	0.9	0.9	0.9	0.8	6.3	1	1	1	1	398	46.4	6.3
NP_001295010.1	809281402	DTWD2	DTW domain-containing protein 2 isoform b	-	-	-	-	1.0	0.8	0.9	0.8	1.0	0.8	0.9	0.8	4.3	2	1	1	1	232	26.4	9.4
NP_776251.1	27777661	KRTCAP2	keratinocyte-associated protein 2	-	-	-	-	1.0	0.9	1.0	0.8	1.0	0.9	1.0	0.8	10.5	1	1	1	2	162	17.6	10.4
NP_001166988.1	291084575	MAP7D3	MAP7 domain-containing protein 3 isoform 3	-	-	-	-	0.7	0.9	0.7	0.8	0.7	0.9	0.7	0.8	4.2	3	3	3	4	841	94.6	9.2

Khan *et al.* , 2019. Multi-omics analysis to characterize cigarette smoke induced molecular alterations in esophageal cells
Supplementary Table 5. List of proteins quantified in untreated and chronically treated Het1A cells with cigarette smoke condensate for 8 months

NP_Accession	Protein group Accession	Gene ID	Description	Het-1A-Smoke - 2M/Parental	Het-1A-Smoke - 4M/Parental	Het-1A-Smoke - 6M/Parental	Het-1A-Smoke - 8M/Parental	Het-1A-Smoke - 2M/Parental	Het-1A-Smoke - 4M/Parental	Het-1A-Smoke - 6M/Parental	Het-1A-Smoke - 8M/Parental	Het-1A-Smoke - 2M/Parental	Het-1A-Smoke - 4M/Parental	Het-1A-Smoke - 6M/Parental	Het-1A-Smoke - 8M/Parental	Coverage (%)	Proteins	Unique Peptides	# Peptides	PSM	# AAs	MW [kDa]	calc. pI
				Replicate 1				Replicate 2				Average of replicates											
XP_006726868.1	578846900	LOC10272433	histone H2B type F-S-like	-	-	-	-	0.7	0.8	0.8	0.8	0.7	0.8	0.8	0.8	67.5	2	4	11	1920	126	13.9	10.3
NP_055798.2	241982802	HMGXB3	HMG domain-containing protein 3	-	-	-	-	0.8	0.8	0.7	0.8	0.8	0.8	0.7	0.8	0.7	1	1	1	1	1292	141.0	6.0
NP_115728.2	34147416	BRMS1L	breast cancer metastasis-suppressor 1-like protein	-	-	-	-	0.9	0.8	0.9	0.8	0.9	0.8	0.9	0.8	4.3	1	1	1	2	323	37.6	5.1
NP_116317.1	14249722	TMEM141	transmembrane protein 141	-	-	-	-	0.8	0.8	0.8	0.8	0.8	0.8	0.8	0.8	49.1	1	2	2	4	108	11.9	8.5
NP_057108.2	118600973	RBMX2	RNA-binding motif protein, X-linked 2	-	-	-	-	0.8	0.8	0.8	0.8	0.8	0.8	0.8	0.8	9.0	1	3	3	3	322	37.3	9.8
NP_001268.2	47078276	CHKA	choline kinase alpha isoform a	-	-	-	-	2.0	1.0	0.9	0.8	2.0	1.0	0.9	0.8	5.9	2	2	2	2	457	52.2	6.6
NP_057590.3	209693446	KBTBD4	kelch repeat and BTB domain-containing protein 4 isoform b	-	-	-	-	0.9	0.8	0.9	0.8	0.9	0.8	0.9	0.8	2.7	2	1	1	1	518	58.1	5.7
NP_001193856.1	332688227	DNAH8	dynein heavy chain 8, axonemal	-	-	-	-	1.2	0.9	1.3	0.8	1.2	0.9	1.3	0.8	0.1	1	1	1	1	4707	538.3	6.1
NP_892030.3	88999601	TRIM69	E3 ubiquitin-protein ligase TRIM69 isoform a	-	-	-	-	0.7	0.8	0.9	0.8	0.7	0.8	0.9	0.8	1.2	1	1	1	1	500	57.4	6.5
NP_061133.1	8923892	PXMP2	peroxisomal membrane protein 2	-	-	-	-	0.8	0.8	0.9	0.8	0.8	0.8	0.9	0.8	3.6	1	1	1	1	195	22.2	10.6
NP_057046.1	7705730	FCF1	rRNA-processing protein FCF1 homolog	-	-	-	-	0.9	0.8	0.9	0.8	0.9	0.8	0.9	0.8	10.1	1	2	2	4	198	23.4	9.7
NP_001010881.1	613410225	C1orf167	uncharacterized protein C1orf167	-	-	-	-	0.7	0.8	0.8	0.8	0.7	0.8	0.8	0.8	0.6	1	1	1	2	1449	160.4	10.9
NP_001528.1	4557647	HSBP1	heat shock factor-binding protein 1	-	-	-	-	1.4	0.9	1.5	0.8	1.4	0.9	1.5	0.8	14.5	1	1	1	1	76	8.5	4.4
NP_056445.3	194272186	EPC2	enhancer of polycomb homolog 2	-	-	-	-	0.7	0.6	0.7	0.8	0.7	0.6	0.7	0.8	1.4	1	1	1	1	807	91.0	8.7
NP_060626.2	54607141	VPS13D	vacuolar protein sorting-associated protein 13D isoform 2	-	-	-	-	0.9	0.7	0.7	0.8	0.9	0.7	0.7	0.8	0.3	2	1	1	1	4363	488.8	6.6
NP_001099038.1	157738629	KIF13A	kinesin-like protein KIF13A isoform d	-	-	-	-	1.0	1.1	0.8	0.8	1.0	1.1	0.8	0.8	1.1	4	1	1	2	1749	195.9	5.7
NP_003247.1	4507515	TIMP4	metalloproteinase inhibitor 4 precursor	-	-	-	-	0.9	1.0	0.9	0.8	0.9	1.0	0.9	0.8	5.8	1	1	1	1	224	25.5	7.8
NP_689426.1	23199978	ZMAT3	zinc finger matrin-type protein 3 isoform 2	-	-	-	-	1.1	0.9	1.3	0.8	1.1	0.9	1.3	0.8	10.1	2	1	1	1	288	32.0	8.9
NP_060147.2	21361759	MTMR8	myotubularin-related protein 8	-	-	-	-	0.8	0.7	0.7	0.8	0.8	0.7	0.7	0.8	3.0	1	1	1	1	704	78.9	6.7
NP_001123368.1	193804862	SYTL4	synaptotagmin-like protein 4	-	-	-	-	0.8	0.8	0.8	0.8	0.8	0.8	0.8	0.8	1.2	1	1	1	1	671	76.0	9.0
NP_002510.2	155969714	NPAT	protein NPAT	-	-	-	-	1.1	0.9	0.9	0.8	1.1	0.9	0.9	0.8	2.2	1	2	2	2	1427	154.2	5.9
NP_001180462.1	302058308	NEK4	serine/threonine-protein kinase Nek4 isoform 2	-	-	-	-	0.9	1.0	0.8	0.8	0.9	1.0	0.8	0.8	2.4	2	1	1	2	752	84.3	7.8
NP_001129599.1	209915561	DNM3	dynamin-3 isoform b	-	-	-	-	1.0	1.1	1.0	0.8	1.0	1.1	1.0	0.8	16.5	3	1	14	35	859	96.6	8.2
NP_056206.1	32698710	ANAPC13	anaphase-promoting complex subunit 13	-	-	-	-	1.8	0.9	1.8	0.8	1.8	0.9	1.8	0.8	13.5	1	1	1	1	74	8.5	4.2
NP_060101.3	222831595	DDX60	probable ATP-dependent RNA helicase DDX60	-	-	-	-	0.9	0.8	0.7	0.8	0.9	0.8	0.7	0.8	0.5	1	1	1	1	1712	197.7	7.6
NP_525021.3	153792144	ABCA10	ATP-binding cassette sub-family A member 10	-	-	-	-	0.9	0.7	0.9	0.8	0.9	0.7	0.9	0.8	0.4	1	1	1	1	1543	175.7	6.7
NP_001161831.1	270132304	TDRD6	tudor domain-containing protein 6 isoform 2	-	-	-	-	0.7	0.8	0.8	0.8	0.7	0.8	0.8	0.8	0.7	2	2	2	3	2066	233.1	5.3
NP_036451.3	153252201	SLC39A6	zinc transporter ZIP6 isoform 1 precursor	-	-	-	-	1.0	0.8	0.8	0.8	1.0	0.8	0.8	0.8	1.2	1	1	1	1	755	85.0	6.9
NP_006327.2	33589814	ZER1	protein zer-1 homolog	-	-	-	-	0.8	0.9	0.9	0.8	0.8	0.9	0.9	0.8	1.3	1	1	1	2	766	88.1	5.6
NP_056118.2	57863275	VPS8	vacuolar protein sorting-associated protein 8 homolog isoform b	-	-	-	-	0.8	0.9	0.9	0.8	0.8	0.9	0.9	0.8	1.1	2	1	1	2	1426	161.5	5.6
NP_001238892.1	354459394	ARHGEF26	rho guanine nucleotide exchange factor 26 isoform 2	-	-	-	-	1.1	0.9	1.1	0.8	1.1	0.9	1.1	0.8	2.3	2	1	1	1	791	88.1	9.2
NP_057077.1	7705755	PLLP	plasmolipin	-	-	-	-	1.2	1.0	0.8	0.8	1.2	1.0	0.8	0.8	12.6	1	1	1	1	182	20.0	9.4
NP_037408.2	74315356	SHPK	sedoheptulokinase	-	-	-	-	0.8	0.8	0.8	0.8	0.8	0.8	0.8	0.8	3.1	1	1	1	1	478	51.5	6.8
NP_001128165.1	197927408	OST4	dolichyl-diphosphooligosaccharide--protein glycosyltransferase subunit 4	-	-	-	-	1.3	1.3	1.9	0.8	1.3	1.3	1.9	0.8	91.9	1	1	1	1	37	4.2	7.2
NP_079243.1	13376460	ZNF556	zinc finger protein 556	-	-	-	-	0.8	0.8	0.9	0.8	0.8	0.8	0.9	0.8	3.1	1	1	1	1	456	51.5	9.9
NP_009077.2	56118214	VEZF1	vascular endothelial zinc finger 1	-	-	-	-	1.1	0.8	0.6	0.8	1.1	0.8	0.6	0.8	5.0	1	1	1	2	521	56.9	9.5
NP_078956.2	38505226	E2F8	transcription factor E2F8	-	-	-	-	0.9	0.8	1.0	0.8	0.9	0.8	1.0	0.8	1.9	1	1	1	1	867	94.1	9.0
NP_112557.2	145309300	CDK13	cyclin-dependent kinase 13 isoform 2	-	-	-	-	0.7	0.8	0.7	0.8	0.7	0.8	0.7	0.8	2.5	51	1	3	6	1452	158.3	9.7
NP_001269500.1	543173125	ZCCHC17	nucleolar protein of 40 kDa isoform f	-	-	-	-	0.8	0.8	0.8	0.8	0.8	0.8	0.8	0.8	22.4	7	3	3	3	179	20.4	9.9
NP_073208.1	13027642	SNRNP35	U11/U12 small nuclear ribonucleoprotein 35 kDa protein isoform a	-	-	-	-	1.0	1.1	1.0	0.8	1.0	1.1	1.0	0.8	11.4	2	2	2	3	246	29.4	9.9
NP_036295.1	16306591	LRRC29	leucine-rich repeat-containing protein 29	-	-	-	-	0.7	0.9	0.9	0.8	0.7	0.9	0.9	0.8	5.4	1	1	1	1	223	23.8	7.9
NP_001138600.2	237858799	AK9	adenylate kinase 9 isoform 1	-	-	-	-	0.8	0.8	0.8	0.8	0.8	0.8	0.8	0.8	0.6	1	1	1	2	1911	221.3	5.0
NP_004634.1	4758876	PABPN1	polyadenylate-binding protein 2	-	-	-	-	0.8	0.9	0.7	0.8	0.8	0.9	0.7	0.8	64.4	4	11	11	53	306	32.7	5.1
NP_001258886.1	430768587	SLC39A1	zinc transporter ZIP1 isoform a	-	-	-	-	1.1	0.7	0.8	0.8	1.1	0.7	0.8	0.8	3.7	1	1	1	1	324	34.2	5.9
NP_031368.1	6679074	NUPL2	nucleoporin-like protein 2	-	-	-	-	0.9	0.8	0.7	0.8	0.9	0.8	0.7	0.8	3.8	1	1	1	1	423	44.8	9.2
NP_001242970.1	365733629	MYO1A	unconventional myosin-Ia	-	-	-	-	0.8	0.9	0.9	0.8	0.8	0.9	0.9	0.8	2.6	1	1	3	8	1043	118.3	9.3
NP_056009.1	51100974	MYO1D	unconventional myosin-Id isoform 1	-	-	-	-	0.8	0.8	0.8	0.8	0.8	0.8	0.8	0.8	2.8	3	3	3	3	1006	116.1	9.4
NP_001251503.1	387942371	KIF18B	kinesin-like protein KIF18B isoform 2	-	-	-	-	0.8	0.8	0.8	0.8	0.8	0.8	0.8	0.8	2.0	2	1	1	1	833	91.0	8.3
NP_001035924.1	109659847	FILIP1L	filamin A-interacting protein 1-like isoform 3	-	-	-	-	0.9	0.9	0.9	0.8	0.9	0.9	0.9	0.8	0.6	2	1	1	2	1133	130.1	6.6
NP_001116370.1	171543879	CD99	CD99 antigen isoform b precursor	-	-	-	-	0.8	0.8	0.8	0.8	0.8	0.8	0.8	0.8	5.9	2	1	1	2	169	17.1	4.6
NP_005052.1	4826988	RPL3L	60S ribosomal protein L3-like	-	-	-	-	0.7	0.7	0.9	0.8	0.7	0.7	0.9	0.8	6.1	1	1	4	25	407	46.3	10.4
NP_115518.3	255003833	CEP192	centrosomal protein of 192 kDa	-	-	-	-	0.8	0.8	0.8	0.8	0.8	0.8	0.8	0.8	0.8	1	1	1	1	2537	278.9	5.5
NP_001267730.1	526253083	RNF130	E3 ubiquitin-protein ligase RNF130 isoform 2 precursor	-	-	-	-	0.8	0.9	0.7	0.8	0.8	0.9	0.7	0.8	4.4	2	1	1	1	384	42.4	9.1
NP_060218.1	8923271	CWC25	pre-mRNA-splicing factor CWC25 homolog	-	-	-	-	0.8	0.8	0.7	0.7	0.8	0.8	0.7	0.7	2.8	1	1	1	2	425	49.6	10.2
NP_722516.1	24429572	TAF1L	transcription initiation factor TFIID subunit 1-like	-	-	-	-	0.9	1.0	0.9	0.7	0.9	1.0	0.9	0.7	0.9	4	1	1	2	1826	207.2	5.4
NP_653280.1	21389577	ENTHD2	AP-4 complex accessory subunit tepsin	-	-	-	-	0.8	0.6	0.6	0.7	0.8	0.6	0.6	0.7	3.4	1	1	1	2	525	55.1	7.0
NP_006463.3	171184421	TXNIP	thioredoxin-interacting protein	-	-	-	-	0.7	0.8	0.8	0.7	0.7	0.8	0.8	0.7	5.1	1	2	2	3	391	43.6	7.5
NP_057697.2	32526911	FAM198B	protein FAM198B isoform 2	-	-	-	-	0.8	0.9	0.9	0.7	0.8	0.9	0.9	0.7	1.2	2	1	1	1	519	57.5	9.7
NP_001076439.1	131888281	C11orf57	uncharacterized protein C11orf57 isoform b	-	-	-	-	0.9	0.8	0.8	0.7	0.9	0.8	0.8	0.7	4.5	2	1	1	2	292	34.1	9.7
NP_001013424.1	61742817	KRIT1	krev interaction trapped protein 1 isoform 2	-	-	-	-	0.8	0.8	0.8	0.7	0.8	0.8	0.8	0.7	1.5	2	1	1	2	688	78.6	8.8
NP_054775.2	153085463	HIGD1A	HIG1 domain family member 1A, mitochondrial isoform b	-	-	-	-	1.3	1.0	1.7	0.7	1.3	1.0	1.7	0.7	28.0	2	2	2	5	93	10.1	9.8
NP_612383.1	210031219	ZNF845	zinc finger protein 845	-	-	-	-	0.7	0.8	0.7	0.7	0.7	0.8	0.7	0.7	2.5	76	1	3	3	970	113.1	9.3
NP_689718.1	22749049	LCA5L	Lebercilin-like protein	-	-	-	-	0.8	0.7	0.7	0.7	0.8	0.7	0.7	0.7	4.6	1	1	2	2	670	76.5	9.5
NP_001269491.1	543173157	EYA3	eyes absent homolog 3 isoform d	-	-	-	-	0.7	0.7	0.7	0.7	0.7	0.7	0.7	0.7	3.1	4	1	1	2	520	56.8	5.8
NP_071436.1	62339432	C10orf54	platelet receptor Gi24 precursor	-	-	-	-	0.8	0.7	0.7	0.7	0.8	0.7	0.7	0.7	11.3	1	1	1	1	311	33.9	6.9
NP_001414.1	4503559	EMP1	epithelial membrane protein 1	-	-	-	-	1.6	0.9	4.5	0.7	1.6	0.9	4.5	0.7	26.8	1	1	1	1	157	17.6	8.0
NP_001004704.1	52317237	OR4C6	olfactory receptor 4C6	-	-	-	-	0.8	0.7	0.7	0.7	0.8	0.7	0.7	0.7	17.5	1	1	1	1	309	34.5	8.1
NP_001182212.1	305632820	FLVCR2	feline leukemia virus subgroup C receptor-related protein 2 isoform 2	-	-	-	-	0.8	0.7	0.7	0.7	0.8	0.7	0.7	0.7	10.6	2	1	1	1	321	35.2	5.6
NP_001033707.1	84452151	NARF	nuclear prelamin A recognition factor isoform c	-	-	-	-	0.9	0.8	0.9	0.7	0.9	0.8	0.9	0.7	3.0	4	1	1	1	397	44.7	7.5
NP_054895.1	7661808	UBE2T	ubiquitin-conjugating enzyme E2 T	-	-	-	-	0.8	0.7	0.7	0.7	0.8	0.7	0.7	0.7	15.2	1	2	2	2	197	22.5	8.0
NP_001001431.1	48255881	TNNT2	troponin T, cardiac muscle isoform 3	-	-	-	-	0.8	0.9	0.7	0.7	0.8	0.9	0.7	0.7	14.7	2	1	1	1	285	34.2	5.1
NP_945352.3	558695408	CYSRT1	cysteine-rich tail protein 1	-	-	-	-	0.8	0.7	0.7	0.7	0.8	0.7	0.7	0.7	13.0	1	1	1	1	184	19.7	7.9
NP_056178.3	33354273	ZIM2	zinc finger imprinted 2	-	-	-	-	0.8	0.7	0.7	0.7	0.8	0.7	0.7	0.7	3.8	3	1	1	1	527	61.1	6.8
NP_005420.1	4885653	VEGFC	vascular endothelial growth factor C preproprotein	-	-	-	-	0.8	0.7	0.7	0.7	0.8	0.7	0.7	0.7	3.3	1	1	1	1	419	46.9	7.5
NP_060087.3	148833508	NOTCH1	neurogenic locus notch homolog protein 1 preproprotein	-	-	-	-	0.8	0.7	0.7	0.7	0.8	0.7	0.7	0.7	1.1	1	1	1	1	2555	272.3	5.1
NP_060492.1	8922277	TMEM51	transmembrane protein 51	-	-	-	-	0.6	0.7	0.7	0.7	0.6	0.7	0.7	0.7	5.1	1	1	1	2	253	27.7	5.5
NP_079089.2	226246608	RPAP2	putative RNA polymerase II subunit B1 CTD phosphatase RPAP2	-	-	-	-	1.5	0.7	0.6	0.7	1.5	0.7	0.6	0.7	2.3	1	1	1	1	612	69.5	7.8
NP_113659.3	28626504	FERMT3	fermitin family homolog 3 short form	-	-	-	-	0.9	0.6	0.8	0.7	0.9	0.6	0.8	0.7	1.5	2	1	1	1	663	75.4	6.8
NP_079544.1	13376888	TMEM121	transmembrane protein 121	-	-	-	-	1.3	0.9	0.7	0.7	1.3	0.9	0.7	0.7	12.9	1	1	1	1	319	35.8	9.3
NP_004767.1	4757828	B4GALT5	beta-1,4-galactosyltransferase 5	-	-	-	-	1.0	0.8	0.8	0.7	1.0	0.8	0.8	0.7	4.4	1	1	1	1	388	45.1	8.1
NP_003632.3	150010589	IFITM1	interferon-induced transmembrane protein 1	-	-	-	-	0.6	0.5	0.6	0.7	0.6	0.5	0.6	0.7	12.8	3	1	1	4	125	13.9	7.9
NP_001265440.1	511772994	AP3B2	AP-3 complex subunit beta-2 isoform 3	-	-	-	-	0.6	0.5	0.4	0.7	0.6	0.5	0.4	0.7	3.4	3	1	3	5	1050	115.2	5.5
NP_954984.1	40548328	NACA2	nascent polypeptide-associated complex subunit alpha-2	-	-	-	-	0.8	0.8	0.9	0.7	0.8	0.8	0.9	0.7	6.1	1	1	1	1	215	23.2	4.7
NP_001229718.1	338753412	BRF1	transcription factor IIIB 90 kDa subunit isoform 7	-	-	-	-	0.7	0.6	0.7	0.7	0.7	0.6	0.7	0.7	2.5	6	1	1	2	439	47.9	4.8
NP_115681.1	14150064	POLR3GL	DNA-directed RNA polymerase III subunit RPC7-like	-	-	-	-	0.8	1.0	0.9	0.7	0.8	1.0	0.9	0.7	17.4	1	1	1	1	218	25.3	4.5
NP_001153242.1	229577422	SLC39A11	zinc transporter ZIP11 isoform 1	-	-	-	-	0.7	0.7	0.7	0.7	0.7	0.7	0.7	0.7	2.1	1	1	1	1	342	35.4	5.6
NP_001138947.1	224458301	FAM186A	protein FAM186A	-	-	-	-	0.7	0.7	0.7	0.7	0.7	0.7	0.7	0.7	0.7	1	1	1	3	2351	262.6	8.6
NP_001001709.1	48717423	C9orf170	uncharacterized protein C9orf170 precursor	-	-	-	-	3.8	1.2	1.1	0.7	3.8	1.2	1.1	0.7	5.0	1	1	1	1	121	13.4	10.6
NP_002190.2	153082752	IRF2	interferon regulatory factor 2	-	-	-	-	0.9	0.8	0.8	0.7	0.9	0.8	0.8	0.7	2.3	1	1	1	1	349	39.3	7.5
NP_001123498.2	194097428	TCAF2	protein FAM115C isoform A	-	-	-	-	0.6	0.6	1.0	0.7	0.6	0.6	1.0	0.7	8.8	5	1	5	9	845	91.9	7.3
NP_112192.2	45580709	UNC93B1	protein unc-93 homolog B1	-	-	-	-	0.7	0.6	0.7	0.7	0.7	0.6	0.7	0.7	2.0	1	1	1	1	597	66.6	7.0
NP_057626.2	15011880	STK26	serine/threonine-protein kinase 26 isoform 1	-	-	-	-	0.9	0.7	0.8	0.7	0.9	0.7	0.8	0.7	15.9	7	2	6	20	416	46.5	5.3
NP_001027458.1	73808084	NR2C1	nuclear receptor subfamily 2 group C member 1 isoform b	-	-	-	-	0.7	0.6	0.7	0.7	0.7	0.6	0.7	0.7	3.6	3	1	1	1	467	51.2	6.2
NP_003484.1	4504299	HIST3H3	histone H3.1t	-	-	-	-	0.7	0.7	0.8	0.6	0.7	0.7	0.8	0.6	68.4	2	1	12	834	136	15.5	11.1
NP_001035869.1	109150435	ANKZF1	ankyrin repeat and zinc finger domain-containing protein 1 isoform 1	-	-	-	-	0.7	0.6	0.7	0.6	0.7	0.6	0.7	0.6	1.7	1	1	1	1	726	80.9	8.4
NP_653297.3	51093836	AXDND1	axonemal dynein light chain domain-containing protein 1	-	-	-	-	1.1	0.8	0.9	0.6	1.1	0.8	0.9	0.6	1.2	1	1	1	1	1012	118.0	5.7
NP_612411.4	134133279	SP140L	nuclear body protein SP140-like protein isoform 1	-	-	-	-	0.5	0.7	0.5	0.6	0.5	0.7	0.5	0.6	2.9	3	1	2	7	580	67.0	8.5
NP_940937.1	110626177	ZNF699	zinc finger protein 699	-	-	-	-	0.6	0.6	0.6	0.6	0.6	0.6	0.6	0.6	2.8	76	1	2	2	642	73.9	8.2

Khan *et al.*, 2019. Multi-omics analysis to characterize cigarette smoke induced molecular alterations in esophageal cells

Supplementary Table 5. List of proteins quantified in untreated and chronically treated Het1A cells with cigarette smoke condensate for 8 months

NP_Accession	Protein group Accession	Gene ID	Description	Het-1A-Smoke - 2M/Parental	Het-1A-Smoke - 4M/Parental	Het-1A-Smoke - 6M/Parental	Het-1A-Smoke - 8M/Parental	Het-1A-Smoke - 2M/Parental	Het-1A-Smoke - 4M/Parental	Het-1A-Smoke - 6M/Parental	Het-1A-Smoke - 8M/Parental	Het-1A-Smoke - 2M/Parental	Het-1A-Smoke - 4M/Parental	Het-1A-Smoke - 6M/Parental	Het-1A-Smoke - 8M/Parental	Coverage (%)	Proteins	Unique Peptides	# Peptides	PSM	# AAs	MW [kDa]	calc. pI
				Replicate 1				Replicate 2				Average of replicates											
NP_005426.2	50959191	ARHGEF5	rho guanine nucleotide exchange factor 5	-	-	-	-	0.8	0.7	0.7	0.6	0.8	0.7	0.7	0.6	3.1	4	3	3	8	1597	176.7	5.5
NP_001640.1	4502175	SHROOM2	protein Shroom2	-	-	-	-	0.7	0.7	0.6	0.6	0.7	0.7	0.6	0.6	1.1	1	1	2	2	1616	176.3	7.1
NP_055853.1	149773456	KIAA0754	uncharacterized protein KIAA0754	-	-	-	-	0.8	0.8	0.7	0.6	0.8	0.8	0.7	0.6	0.5	1	1	1	1	1427	150.4	4.3
NP_001008212.1	56549107	OPTN	optineurin	-	-	-	-	0.7	0.7	0.7	0.6	0.7	0.7	0.7	0.6	2.3	1	1	1	1	577	65.9	5.2
NP_695009.1	24497478	SLC22A6	solute carrier family 22 member 6 isoform c	-	-	-	-	0.6	0.7	0.5	0.6	0.6	0.7	0.5	0.6	11.7	4	1	1	1	506	55.8	8.7
NP_775820.2	282847455	ERICH5	glutamate-rich protein 5 isoform 1	-	-	-	-	0.8	0.7	0.6	0.6	0.8	0.7	0.6	0.6	3.5	1	1	1	2	374	39.9	4.5
NP_065192.1	148235699	PAIP2B	polyadenylate-binding protein-interacting protein 2B	-	-	-	-	0.9	0.7	0.7	0.6	0.9	0.7	0.7	0.6	6.5	1	1	1	2	123	14.2	4.2
NP_689706.2	38570115	ZNF362	zinc finger protein 362	-	-	-	-	0.6	0.5	0.5	0.6	0.6	0.5	0.5	0.6	12.9	1	2	3	4	420	45.8	9.6
NP_003460.2	68160947	SCG2	secretogranin-2 precursor	-	-	-	-	1.0	1.0	1.3	0.6	1.0	1.0	1.3	0.6	1.1	1	1	1	1	617	70.9	4.8
NP_005928.2	57222568	MLLT6	protein AF-17	-	-	-	-	0.6	0.8	0.7	0.5	0.6	0.8	0.7	0.5	2.1	1	1	1	1	1093	112.0	8.6
NP_002733.2	115529463	PRKD1	serine/threonine-protein kinase D1	-	-	-	-	0.6	0.4	0.5	0.5	0.6	0.4	0.5	0.5	5.0	9	1	3	8	912	101.6	6.6
NP_002345.2	218505670	EPCAM	epithelial cell adhesion molecule precursor	-	-	-	-	0.7	0.6	0.5	0.5	0.7	0.6	0.5	0.5	5.1	1	1	1	2	314	34.9	7.5
NP_059996.1	8923785	LEPROT	leptin receptor gene-related protein isoform 1	-	-	-	-	0.7	0.5	0.6	0.5	0.7	0.5	0.6	0.5	9.9	2	1	1	2	131	14.2	6.5
NP_003793.2	110624781	MYH13	myosin-13	-	-	-	-	1.2	0.7	0.6	0.5	1.2	0.7	0.6	0.5	1.2	1	1	1	1	1938	223.5	5.7
NP_006647.3	117190519	NEU3	sialidase-3	-	-	-	-	0.6	0.4	0.5	0.5	0.6	0.4	0.5	0.5	4.1	1	1	1	1	461	51.6	6.7
NP_005767.1	5031639	CNIH1	protein cornichon homolog 1	-	-	-	-	0.7	0.8	1.3	0.4	0.7	0.8	1.3	0.4	15.3	1	1	1	1	144	16.7	5.7
NP_008864.4	614458153	SLC5A3	sodium/myo-inositol cotransporter	-	-	-	-	0.6	0.6	0.4	0.4	0.6	0.6	0.4	0.4	4.6	1	1	1	2	718	79.6	7.5
NP_001278367.1	612149762	OR8G2	olfactory receptor 8G2	-	-	-	-	0.3	0.4	0.5	0.3	0.3	0.4	0.5	0.3	10.9	1	1	1	1	304	33.9	7.4
