# Supplementary material for: Multi-Omics Analysis to Characterize Cigarette Smoke Induced Molecular Alterations in Esophageal Cells
Source: Front Oncol. 2020 Nov 5;10:1666. doi: 10.3389/fonc.2020.01666 (PMC7675040; doi:10.3389/fonc.2020.01666)
Supplement: Supplementary Table 7 — List of differentially expressed proteins identified in smoke exposed Het-1A cells across both replicates. [file Table_7.pdf]

Khan *et al.* , 2019. Multi-omics analysis to characterize cigarette smoke induced molecular alterations in esophageal cells
Supplementary Table 7. List of differentially expressed proteins identified in smoke exposed Het-1A cells across both replicates.

NP_Accession	Protein group Accession	Gene ID	Description	Het-1A-Smoke - 2M/Parental	Het-1A-Smoke - 4M/Parental	Het-1A-Smoke - 6M/Parental	Het-1A-Smoke - 8M/Parental	Het-1A-Smoke - 2M/Parental	Het-1A-Smoke - 4M/Parental	Het-1A-Smoke - 6M/Parental	Het-1A-Smoke - 8M/Parental	Het-1A-Smoke - 2M/Parental	Het-1A-Smoke - 4M/Parental	Het-1A-Smoke - 6M/Parental	Het-1A-Smoke - 8M/Parental
				Replicate 1				Replicate 2				Average of replicates			
NP_002766.1	4506141	**HTRA1**	serine protease HTRA1 precursor	1.4	2.8	2.4	3.6	1.3	2.7	2.1	3.4	1.4	2.7	2.2	**3.5**
NP_000415.2	119395754	**KRT5**	keratin, type II cytoskeletal 5	1.1	2.1	1.0	2.6	1.1	2.0	1.1	2.5	1.1	2.0	1.0	**2.6**
NP_000414.2	47132620	**KRT2**	keratin, type II cytoskeletal 2 epidermal	1.1	2.1	1.0	2.5	1.0	2.0	1.0	2.5	1.1	2.0	1.0	**2.5**
NP_000412.3	195972866	**KRT10**	keratin, type I cytoskeletal 10	1.1	2.1	1.0	2.6	1.1	1.7	1.0	2.2	1.1	1.9	1.0	**2.4**
NP_006112.3	119395750	**KRT1**	keratin, type II cytoskeletal 1	1.1	1.8	1.0	2.2	1.0	1.8	1.0	2.4	1.0	1.8	1.0	**2.3**
NP_444513.1	16751921	**DCD**	dermcidin preproprotein	1.1	1.9	0.9	2.3	1.1	1.7	0.8	2.3	1.1	1.8	0.9	**2.3**
NP_000217.2	55956899	**KRT9**	keratin, type I cytoskeletal 9	1.0	1.7	1.0	2.2	1.0	1.8	1.0	2.3	1.0	1.7	1.0	**2.2**
NP_005508.1	5031749	**HMGN2**	non-histone chromosomal protein HMG-17	0.6	0.6	0.5	0.5	0.6	0.6	0.4	0.5	0.6	0.6	0.5	**0.5**
NP_001006938.1	55749459;6550587	**TCEAL4**	transcription elongation factor A protein-like 4	0.6	0.6	0.5	0.4	0.5	0.5	0.4	0.4	0.6	0.5	0.4	**0.4**
NP_000294.1	4557839	**PMM2**	phosphomannomutase 2	5.2	4.4	3.7	4.3	1.1	1.1	1.2	1.1	3.2	2.8	2.4	**2.7**
NP_056535.1	7657465	**PODXL2**	podocalyxin-like protein 2 precursor	1.6	3.2	2.0	3.4	0.9	1.0	1.0	1.2	1.3	2.1	1.5	**2.3**
NP_001009931.1	57864582	**HRNR**	hornerin	1.1	2.6	1.1	3.1	1.0	1.1	0.9	1.3	1.0	1.9	1.0	**2.2**
NP_851825.1	31317278	**WDR20**	WD repeat-containing protein 20 isoform 3	3.3	4.4	3.2	3.4	1.0	1.0	1.1	0.9	2.2	2.7	2.1	**2.1**
NP_061980.1	33457348	**MYDGF**	UPF0556 protein C19orf10 precursor	1.1	1.4	1.2	2.2	1.1	1.3	1.2	1.9	1.1	1.4	1.2	**2.1**
NP_001092315.2	524552373	**ANXA8L1**	annexin A8-like protein 1 isoform 1	1.0	0.9	1.0	1.9	1.2	1.2	1.3	2.2	1.1	1.1	1.2	**2.0**
NP_076968.2	229577436	**SPATA5L1**	spermatogenesis-associated protein 5-like protein 1	1.9	2.0	2.4	2.8	1.1	1.0	1.0	1.2	1.5	1.5	1.7	**2.0**
NP_001006934.1	55749431	**TCEAL3**	transcription elongation factor A protein-like 3	0.7	0.5	0.6	0.5	0.7	0.6	0.7	0.5	0.7	0.6	0.6	**0.5**
NP_940874.2	148596951	**FUOM**	fucose mutarotase isoform 2	0.6	0.5	0.6	0.4	0.7	0.8	0.8	0.5	0.6	0.7	0.7	**0.5**
NP_036374.1	7110719	**DKK1**	dickkopf-related protein 1 precursor	0.8	0.6	0.8	0.5	0.7	0.6	0.6	0.4	0.7	0.6	0.7	**0.4**
NP_003351.2	40254471	**UGT8**	2-hydroxyacylsphingosine 1-beta-galactosyltransferase precursor	1.3	2.0	2.3	2.6	-	-	-	-	1.3	2.0	2.3	**2.6**
NP_001229739.1	338797768	**BRDT**	bromodomain testis-specific protein isoform d	0.8	1.9	1.0	2.6	-	-	-	-	0.8	1.9	1.0	**2.6**
NP_690577.2	38045917	**PIGO**	GPI ethanolamine phosphate transferase 3 isoform 2	2.1	2.4	2.0	2.3	-	-	-	-	2.1	2.4	2.0	**2.3**
NP_060701.1	8922692	**SLC38A7**	putative sodium-coupled neutral amino acid transporter 7	0.0	1.5	1.0	2.3	-	-	-	-	0.0	1.5	1.0	**2.3**
NP_057238.3	82659107	**RAB4B**	ras-related protein Rab-4B	2.7	2.3	2.1	2.3	-	-	-	-	2.7	2.3	2.1	**2.3**
NP_056021.1	54606888	**KIAA1024**	UPF0258 protein KIAA1024	2.8	2.3	1.8	2.2	-	-	-	-	2.8	2.3	1.8	**2.2**
NP_001004135.1	51921273	**OR2A12**	olfactory receptor 2A12	1.1	0.8	0.8	0.5	-	-	-	-	1.1	0.8	0.8	**0.5**
NP_115285.1	15055543	**SRSF8**	serine/arginine-rich splicing factor 8	0.5	0.6	0.6	0.4	-	-	-	-	0.5	0.6	0.6	**0.4**
NP_689830.2	31377566	**RNF168**	E3 ubiquitin-protein ligase RNF168	0.8	0.9	0.7	0.4	-	-	-	-	0.8	0.9	0.7	**0.4**
NP_060688.1	41055953	**USP40**	ubiquitin carboxyl-terminal hydrolase 40	0.2	0.9	0.1	0.3	-	-	-	-	0.2	0.9	0.1	**0.3**
NP_001193486.1	330864720	**KIAA1432**	protein RIC1 homolog isoform c	-	-	-	-	0.8	1.8	0.9	2.7	0.8	1.8	0.9	**2.7**
NP_057421.1	7706687	**EVL**	ena/VASP-like protein	-	-	-	-	0.8	0.9	0.7	2.5	0.8	0.9	0.7	**2.5**
NP_059984.3	533112479	**PLXNA3**	plexin-A3 precursor	-	-	-	-	1.6	2.0	2.2	2.3	1.6	2.0	2.2	**2.3**
NP_150377.1	15451844	**ADAM19**	disintegrin and metalloproteinase domain-containing protein 19 preproprotein	-	-	-	-	0.8	1.8	1.3	2.2	0.8	1.8	1.3	**2.2**

Khan *et al.* , 2019. Multi-omics analysis to characterize cigarette smoke induced molecular alterations in esophageal cells
Supplementary Table 7. List of differentially expressed proteins identified in smoke exposed Het-1A cells across both replicates.

NP_Accession	Protein group Accession	Gene ID	Description	Het-1A-Smoke - 2M/Parental	Het-1A-Smoke - 4M/Parental	Het-1A-Smoke - 6M/Parental	Het-1A-Smoke - 8M/Parental	Het-1A-Smoke - 2M/Parental	Het-1A-Smoke - 4M/Parental	Het-1A-Smoke - 6M/Parental	Het-1A-Smoke - 8M/Parental	Het-1A-Smoke - 2M/Parental	Het-1A-Smoke - 4M/Parental	Het-1A-Smoke - 6M/Parental	Het-1A-Smoke - 8M/Parental
				Replicate 1				Replicate 2				Average of replicates			
NP_000776.1	4503213	**CYP27B1**	25-hydroxyvitamin D-1 alpha hydroxylase, mitochondrial	-	-	-	-	1.6	1.9	1.9	2.0	1.6	1.9	1.9	**2.0**
NP_006647.3	117190519	**NEU3**	sialidase-3	-	-	-	-	0.6	0.4	0.5	0.5	0.6	0.4	0.5	**0.5**
NP_005767.1	5031639	**CNIH1**	protein cornichon homolog 1	-	-	-	-	0.7	0.8	1.3	0.4	0.7	0.8	1.3	**0.4**
NP_008864.4	614458153	**SLC5A3**	sodium/myo-inositol cotransporter	-	-	-	-	0.6	0.6	0.4	0.4	0.6	0.6	0.4	**0.4**
NP_001278367.1	612149762	**OR8G2**	olfactory receptor 8G2	-	-	-	-	0.3	0.4	0.5	0.3	0.3	0.4	0.5	**0.3**
