# Supplementary material for: Multi-Omics Analysis to Characterize Cigarette Smoke Induced Molecular Alterations in Esophageal Cells
Source: Front Oncol. 2020 Nov 5;10:1666. doi: 10.3389/fonc.2020.01666 (PMC7675040; doi:10.3389/fonc.2020.01666)
Supplement: Supplementary Table 8 — List of phosphopeptides identified and quantified in untreated and chronically treated Het1A cells with cigarette smoke condensate for 8 months using SequestHT and Mascot search algorithms with a PhosphoRS probability score ≥75%. [file Table_8.pdf]

Khan et al., 2019. Multi-omics analysis to characterize cigarette smoke induced molecular alterations in esophageal cells

Supplementary Table 8. List of phosphopeptides identified and quantified in untreated and chronically treated Het1A cells with cigarette smoke condensate for 8 months using SequestHT and Mascot search algorithms with a PhosphoRS probability score ≥75%

Protein group Accession	NP_Accession	Gene ID	Gene Symbol	Protein Description	PhosphoSite (Protein)	Het-1A-Smoke - 2M/Parental	Het-1A-Smoke - 4M/Parental	Het-1A-Smoke - 6M/Parental	Het-1A-Smoke - 8M/Parental	Het-1A-Smoke - 2M/Parental	Het-1A-Smoke - 4M/Parental	Het-1A-Smoke - 6M/Parental	Het-1A-Smoke - 8M/Parental	Het-1A-Smoke - 2M/Parental	Het-1A-Smoke - 4M/Parental	Het-1A-Smoke - 6M/Parental	Het-1A-Smoke - 8M/Parental	Het-1A-Smoke - 2M/Parental	Het-1A-Smoke - 4M/Parental	Het-1A-Smoke - 6M/Parental	Het-1A-Smoke - 8M/Parental	Het-1A-Smoke - 2M/Parental	Het-1A-Smoke - 4M/Parental	Het-1A-Smoke - 6M/Parental	Het-1A-Smoke - 8M/Parental	Peptide Sequence	Phosphowindow	PhosphoSite (Peptide)	Phosphosite Plus Evidence	phosphoRS Site Probabilities	# PSMs	# Proteins	# Protein Groups	# Missed Cleavages	Modifications
						Replicate 1				Replicate 2				Normalized phosphoprotein ratios with respect to corresponding total protein ratios. Replicate 1				Replicate 2				Average of replicates													
47132620	NP_000414.2	3849	KRT2	keratin, type II cytoskeletal 2 epidermal	S62	0.6	4.2	0.5	5.8	0.8	4.0	1.0	4.9	0.6	2.1	0.5	2.3	0.7	2.0	0.9	2.0	0.7	2.0	0.7	2.1	sLVGLGGTK	GGGFGSRsLVGLGGT	S1;	Yes	S(1): 100.0; T(8): 0.0	6	1	1	0	N-Term(TMT6plex); S1(Phospho); K9(TMT6plex)
226437581	NP_001139807.1	55117	SLC6A15	sodium-dependent neutral amino acid transporter B(0)AT2 isoform3	S580	1.4	2.6	2.3	3.0	1.0	1.4	4.0	6.5	-	-	-	-	-	-	-	-	-	-	-	-	IPSEMPsPNFGK	KIPSEMPsPNFGKNI	S7;	No	S(3): 0.0; S(7): 100.0	3	2	1	0	N-Term(TMT6plex); M5(Oxidation); S7(Phospho); K12(TMT6plex)
38708299	NP_001523.2	3177	SLC29A2	equilibrative nucleoside transporter 2	S252	1.0	0.9	0.6	2.2	2.9	2.3	5.1	5.5	-	-	-	-	-	-	-	-	-	-	-	-	AELLQSDENGIPSsPQK	DENGIPSsPQKVALT	S14;	Yes	S(6): 0.0; S(13): 0.0; S(14): 100.0	1	1	1	0	N-Term(TMT6plex); S14(Phospho); K17(TMT6plex)
153945728	NP_005900.2	4131	MAP1B	microtubule-associated protein 1B	S1915	2.5	4.7	3.4	3.8	1.9	1.8	2.0	3.6	2.0	3.4	2.8	4.0	1.5	1.3	1.6	3.9	1.7	2.4	2.2	3.9	sPSDSGYSYETIGK	KIERTTKsPSDSGYS	S1;	Yes	S(1): 97.6; S(3): 1.2; S(5): 1.2; Y(7): 0.0; S(8): 0.0; Y(9): 0.0; T(11): 0.0	49	1	1	0	N-Term(TMT6plex); S1(Phospho); K14(TMT6plex)
33356148	NP_005883.2	752	FMNL1	formin-like protein 1	S624	1.0	1.1	1.6	1.3	2.8	3.8	3.0	5.6	0.9	1.0	1.4	1.2	2.8	3.6	2.7	5.2	1.9	2.3	2.1	3.2	RDsELGPGVK	DALGRRDsELGPGVK	S3;	Yes	S(3): 100.0	1	1	1	1	N-Term(TMT6plex); S3(Phospho); K10(TMT6plex)
70166599	NP_001002019.1	80324	PUS1	tRNA pseudouridine synthase A, mitochondrial	T398	2.4	1.8	2.1	2.4	5.2	2.8	5.1	3.9	2.2	1.6	1.9	2.3	4.7	2.6	4.6	3.6	3.4	2.1	3.2	2.9	VPSPLEGSEGDGDtD	GSEGDGDtD	T14;	No	S(3): 0.0; S(8): 0.0; T(14): 100.0	5	2	1	0	N-Term(TMT6plex); T14(Phospho)
148612879	NP_079223.3	80012	PHC3	polyhomeotic-like protein 3	T621; S628	0.8	1.7	1.7	0.9	3.9	4.0	4.9	4.7	0.8	1.8	1.5	1.0	3.9	4.1	4.3	5.1	2.4	2.9	2.9	3.1	MDRtPPPPTLsPAAITVGR	ECVRMDRtPPPPTLS;TPPPPTLsPAAITVG	T4; S11;	No;No	T(4): 100.0; T(9): 1.8; S(11): 98.2; T(16): 0.0	2	1	1	1	M1(Oxidation); N-Term(TMT6plex); T4(Phospho); S11(Phospho)
93141204	NP_060866.2	55798	METTL2B	methyltransferase-like protein 2B	T154	1.3	0.9	1.3	1.3	3.5	3.4	4.5	4.2	-	-	-	-	-	-	-	-	-	-	-	-	TQtPPVEENVTQK	SLEHKTQtPPVEENV	T3;	Yes	T(1): 0.7; T(3): 99.3; T(11): 0.0	1	1	1	0	N-Term(TMT6plex); T3(Phospho); K13(TMT6plex)
153945728	NP_005900.2	4131	MAP1B	microtubule-associated protein 1B	S2209; S2211	0.6	0.9	0.8	0.9	3.0	3.9	4.1	4.4	0.5	0.6	0.6	0.9	2.3	2.9	3.3	4.7	1.4	1.8	2.0	2.8	HMDPPPAPVQDRsPsR	PAPVQDRsPSPRHPD;PVQDRSPsPRHPDVS	S13; S15;	Yes;Yes	S(13): 100.0; S(15): 100.0	49	1	1	1	N-Term(TMT6plex); M2(Oxidation); S13(Phospho); S15(Phospho)
344313201	NP_001230704.1	51474	LIMA1	LIM domain and actin-binding protein 1 isoform4	S188	3.3	3.2	3.7	4.0	1.7	1.7	1.5	1.2	3.0	2.9	2.9	4.0	1.5	1.6	1.2	1.2	2.3	2.3	2.1	2.6	ETPHsPGVEDAPIAK	NARETPHsPGVEDAP	S5;	No	T(2): 0.0; S(5): 100.0	5	4	1	0	N-Term(TMT6plex); S5(Phospho); K15(TMT6plex)
109255232	NP_001035864.1	9859	CEP170	centrosomal protein of 170 kDa isoform gamma	T662	3.4	3.3	3.7	4.0	0.6	1.2	1.1	1.2	3.1	2.9	3.3	3.8	0.5	1.0	1.0	1.1	1.8	2.0	2.1	2.4	EEAQWtPTK	QREEAQWtPTKLSSK	T6;	No	T(6): 100.0; T(8): 0.0	11	3	1	0	N-Term(TMT6plex); T6(Phospho); K9(TMT6plex)
296011010	NP_060252.4	54906	FAM208B	protein FAM208B	S340	0.4	0.9	1.0	1.4	1.0	1.4	2.5	3.7	0.4	1.0	1.1	1.5	1.0	1.4	2.6	3.9	0.7	1.2	1.9	2.7	VFPLsPASNLR	KKRVFPLsPASNLRV	S5;	Yes	S(5): 100.0; S(8): 0.0	4	1	1	0	N-Term(TMT6plex); S5(Phospho)
57864582	NP_001009931.1	388697	HRNR	hornerin	S654	0.7	1.8	1.0	1.8	1.0	2.7	1.5	3.3	0.7	1.0	1.0	0.8	1.0	1.5	1.5	1.5	0.8	1.2	1.2	1.2	QGSGSGQSPsR	SGSGQSPsRGRHGSD	S3;	No	S(3): 1.8; S(5): 1.8; S(8): 1.8; S(10): 94.5	1	1	1	0	N-Term(TMT6plex); S5(Phospho)
57864582	NP_001009931.1	388697	HRNR	hornerin	S654	1.0	2.7	1.5	3.3	0.7	1.8	1.0	1.8	1.0	1.5	1.5	1.5	0.7	1.0	1.0	0.8	0.8	1.2	1.2	1.2	QGsGSGQSPSR	SRYGQQGsGSGQSPS	S3;	No	S(3): 98.4; S(5): 1.6; S(8): 0.0; S(10): 0.0	10	1	1	0	N-Term(TMT6plex); S5(Phospho)
157388923	NP_060417.2	55034	MOCOS	molybdenum cofactor sulfurase	S530	2.0	2.8	1.9	1.4	4.0	4.5	2.9	3.6	1.5	2.0	1.6	1.1	3.0	3.2	2.4	2.8	2.3	2.6	2.0	1.9	SLsPQEDALTGSR	VMGRRSLsPQEDALT	S3;	Yes	S(1): 0.0; S(3): 100.0; T(10): 0.0; S(12): 0.0	1	1	1	0	N-Term(TMT6plex); S3(Phospho)
150417989	NP_001092872.1	8812	CCNK	cyclin-K	S340	0.9	1.2	1.1	1.1	2.1	2.7	2.6	3.8	0.8	1.3	1.1	1.0	1.9	2.9	2.7	3.5	1.4	2.1	1.9	2.3	AVVVsPKEENK	VKRAVVVsPKEENKA	S5;	Yes	S(5): 100.0	11	1	1	1	N-Term(TMT6plex); S5(Phospho); K7(TMT6plex); K11(TMT6plex)
261278315	NP_001159693.1	10813	UTP14A	U3 small nucleolar RNA-associated protein 14 homolog A isoform2	S393	2.5	3.9	1.9	3.0	1.4	1.8	1.4	1.7	3.0	4.6	2.5	3.9	1.6	2.2	1.8	2.2	2.3	3.4	2.1	3.0	SELSQDAEPAGsQETK	QDAEPAGsQETKDSG	S12;	No	S(1): 0.0; S(4): 0.0; S(12): 100.0; T(15): 0.0	2	2	1	0	N-Term(TMT6plex); S12(Phospho); K16(TMT6plex)
41872631	NP_004095.4	2194	FASN	fatty acid synthase	S207	1.0	1.9	1.3	1.7	2.8	3.9	3.6	3.0	0.8	1.5	1.0	1.6	2.3	3.0	2.9	2.7	1.6	2.2	2.0	2.1	LGMLsPEGTCK	ELRLGMLsPEGTCKA	S5;	Yes	S(5): 100.0; T(9): 0.0	14	1	1	0	N-Term(TMT6plex); M3(Oxidation); S5(Phospho); C10(Carbamidomethyl); K11(TMT6plex)
19913396	NP_599031.1	6525	SMTN	smoothelin isoform b	S779	1.3	2.1	1.4	2.0	1.4	2.4	1.6	2.6	1.1	1.6	1.1	1.8	1.1	1.8	1.2	2.3	1.1	1.7	1.1	2.0	EGAAGsPGGPR	EKEGAAGsPGGPRAA	S6;	Yes	S(6): 100.0	7	5	1	0	N-Term(TMT6plex); S6(Phospho)
572881956	NP_001275884.1	57217	TTC7A	tetratricopeptide repeat protein 7A isoform4	S293	2.8	0.9	2.6	1.4	4.1	3.8	3.7	3.0	2.4	0.9	2.3	1.4	3.5	3.7	3.3	3.0	2.9	2.3	2.8	2.2	DGsEGEGLTMK	GGLEKDGsEGEGLTM	S3;	No	S(3): 100.0; T(9): 0.0	1	4	1	0	N-Term(TMT6plex); S3(Phospho); M10(Oxidation); K11(TMT6plex)
61743954	NP_001611.1	79026	AHNAK	neuroblast differentiation-associated protein AHNAK isoform1	S5110	2.1	3.4	2.8	3.9	0.9	1.2	1.2	0.4	2.5	3.5	2.9	4.5	1.1	1.3	1.3	0.5	1.8	2.4	2.1	2.5	FKAEAPLPsPK	KAEAPLPsPKLEGEL	S9;	Yes	S(9): 100.0	79	1	1	1	N-Term(TMT6plex); K2(TMT6plex); S9(Phospho); K11(TMT6plex)
41322916	NP_958782.1	5339	PLEC	plectin isoform1	S125	2.5	1.1	3.0	2.2	3.5	2.0	3.1	2.0	2.8	1.2	3.2	2.3	3.9	2.2	3.3	2.1	3.3	1.7	3.2	2.2	TPHVQAVQGPLGsPPK	AVQGPLGsPPKRGPL	S13;	Yes	T(1): 0.0; S(13): 100.0	17	1	1	0	N-Term(TMT6plex); S13(Phospho); K16(TMT6plex)
21704281	NP_065166.2	57158	JPH2	junctophilin-2 isoform1	T490	1.4	1.8	1.3	1.4	1.0	1.4	1.7	2.8	-	-	-	-	-	-	-	-	-	-	-	-	ETPRPEGGSPSPAGtPPQPK	GSPSPAGtPPQPKRP	T15;	Yes	T(2): 33.6; S(9): 33.6; S(11): 34.5; T(15): 98.2	1	1	1	0	N-Term(TMT6plex); T2(Phospho); T15(Phospho); K20(TMT6plex)
21704281	NP_065166.2	57158	JPH2	junctophilin-2 isoform1	T490	1.0	1.4	1.7	2.8	1.4	1.8	1.3	1.4	-	-	-	-	-	-	-	-	-	-	-	-	ETPRPEGGSPsPAGtPPQPK	RPEGGSPsPAGTPPQ;GSPSPAGtPPQPKRP	T15;	Yes;Yes	T(2): 2.9; S(9): 2.9; S(11): 94.5; T(15): 99.7	2	1	1	0	N-Term(TMT6plex); T2(Phospho); T15(Phospho); K20(TMT6plex)
41281453	NP_055535.2	9748	SLK	STE20-like serine/threonine-protein kinase	S779	1.7	1.8	1.5	2.4	1.7	1.7	1.8	1.7	1.8	1.6	1.4	2.4	1.8	1.5	1.6	1.7	1.8	1.5	1.5	2.0	DSGsISLQETR	SKTKDSGsISLQETR	S4;	Yes	S(2): 0.0; S(4): 99.1; S(6): 0.9; T(10): 0.0	6	1	1	0	N-Term(TMT6plex); S4(Phospho)
110611149	NP_001036046.1	10073	SNUPN	snurportin-1	S350	1.0	1.1	1.3	1.3	1.0	0.9	1.9	2.8	1.0	1.2	1.3	1.3	1.0	1.0	2.0	2.8	1.0	1.1	1.6	2.0	GSSHsPDHPGCLMEN	KLKGSSHsPDHPGCL	S5;	Yes	S(2): 0.0; S(3): 0.0; S(5): 100.0	1	1	1	0	N-Term(TMT6plex); S5(Phospho); C11(Carbamidomethyl); M13(Oxidation)
22208971	NP_665908.1	3159	HMGA1	high mobility group protein HMG-I/HMG-Y isoform a	S9	2.2	4.0	2.6	2.1	2.1	3.1	2.4	2.0	2.2	3.2	2.3	2.2	2.1	2.5	2.1	2.1	2.2	2.9	2.2	2.1	SsQPLASK	SESSSKSsQPLASKQ	S2;	Yes	S(1): 0.0; S(2): 100.0; S(7): 0.0	39	2	1	0	N-Term(TMT6plex); S2(Phospho); K8(TMT6plex)
47578101	NP_996809.1	57190	SEPN1	selenoprotein N isoform2 precursor	S13	2.6	2.5	1.8	3.1	1.1	1.0	0.9	1.0	-	-	-	-	-	-	-	-	-	-	-	-	GPPsPGPAAQPPAPPR	PGQRGPPsPGPAAQP	S4;	Yes	S(4): 100.0	1	2	1	0	N-Term(TMT6plex); S4(Phospho)
109255232	NP_001035864.1	9859	CEP170	centrosomal protein of 170 kDa isoform gamma	T546	1.0	0.9	1.1	2.0	0.7	0.8	1.9	2.1	1.0	0.8	1.0	1.9	0.6	0.7	1.7	2.0	0.8	0.7	1.4	1.9	tLPQLPNEEK	QGERRRRtLPQLPNE	T1;	No	T(1): 100.0	11	3	1	0	N-Term(TMT6plex); T1(Phospho); K10(TMT6plex)
118572613	NP_057417.3	23524	SRRM2	serine/arginine repetitive matrix protein 2	T2144	2.6	1.5	1.9	2.7	0.4	1.0	0.7	1.4	2.8	1.5	2.0	3.0	0.5	1.0	0.8	1.5	1.6	1.3	1.4	2.3	tPMSVLQQAGGSMMDGPGPR	EPLGSSRtPMSVLQQ	T1;	Yes	T(1): 100.0; S(4): 0.0; S(12): 0.0	436	1	1	0	N-Term(TMT6plex); T1(Phospho); M3(Oxidation); M13(Oxidation); M14(Oxidation)
7662340	NP_055756.1	22880	MORC2	MORC family CW-type zinc finger protein 2	S681	2.0	3.0	2.2	3.4	0.9	0.7	0.7	0.6	1.6	2.6	2.0	3.1	0.7	0.6	0.6	0.6	1.2	1.6	1.3	1.9	SVAVsDEEEVEEEAER	RKRSVAVsDEEEVEE	S5;	No	S(1): 0.0; S(5): 100.0	2	1	1	0	N-Term(TMT6plex); S5(Phospho)
34452681	NP_055683.3	9921	RNF10	RING finger protein 10	S128	0.4	0.7	0.7	0.9	1.6	2.3	1.9	3.1	0.4	0.5	0.6	0.9	1.4	1.7	1.5	3.0	0.9	1.1	1.1	1.9	AEEsPAQESGPK	EAQRAEEsPAQESGP	S4;	Yes	S(4): 100.0; S(9): 0.0	1	1	1	0	N-Term(TMT6plex); S4(Phospho); K12(TMT6plex)
296531339	NP_001171812.1	5331	PLCB3	1-phosphatidylinositol 4,5-bisphosphate phosphodiesterase beta-3 isoform2	S470	1.8	1.8	1.7	1.7	2.1	1.8	1.5	2.3	1.7	1.6	1.5	1.6	1.9	1.6	1.3	2.1	1.8	1.6	1.4	1.9	sLGDEGLNR	PSLEPQKsLGDEGLN	S1;	No	S(1): 100.0	4	2	1	0	N-Term(TMT6plex); S1(Phospho)
4503739	NP_001446.1	2309	FOXO3	forkhead box protein O3	S284	0.4	0.6	0.2	0.5	2.1	2.5	2.0	3.5	0.5	0.7	0.3	0.5	2.6	2.6	2.3	3.6	1.6	1.6	1.3	2.0	AALQTAPESADDsPSQLSK	APESADDsPSQLSKW	S13;	Yes	T(5): 0.0; S(9): 1.3; S(13): 98.7; S(15): 0.0; S(18): 0.0	4	1	1	0	N-Term(TMT6plex); S13(Phospho); K19(TMT6plex)
225007652	NP_001139333.1	10940	POP1	ribonucleases P/MRP protein subunit POP1	S367	1.0	0.8	0.6	0.9	2.3	2.4	2.4	3.1	1.0	0.8	0.5	1.0	2.2	2.4	2.3	3.5	1.6	1.6	1.4	2.3	SAVCIADPLPTPsQEK	ADPLPTPsQEKSQTE	S13;	Yes	S(1): 0.0; T(11): 0.0; S(13): 100.0	2	1	1	0	N-Term(TMT6plex); C4(Carbamidomethyl); S13(Phospho); K16(TMT6plex)
56788356	NP_001008697.1	24144	TFIP11	tuftelin-interacting protein 11	S210	1.3	1.6	2.1	0.8	2.8	0.9	2.6	3.1	1.3	1.5	2.1	0.8	2.9	0.9	2.6	3.0	2.1	1.2	2.3	1.9	TTQSMQDFPVVDsEEEAEEEFQK	QDFPVVDsEEEAEEE	S13;	Yes	T(1): 0.0; T(2): 0.0; S(4): 0.0; S(13): 100.0	1	1	1	0	N-Term(TMT6plex); M5(Oxidation); S13(Phospho); K23(TMT6plex)
5454002	NP_006434.1	10591	DNPH1	2'-deoxynucleoside 5'-phosphate N-hydrolase 1 isoform1	S169	1.0	1.3	2.2	2.5	1.6	1.0	1.3	1.4	0.8	1.2	2.1	2.1	1.3	1.0	1.3	1.2	1.1	1.1	1.7	1.7	YFEADPPGQVAAsPDPTT	PPGQVAAsPDPTT_	S13;	No	Y(1): 0.0; S(13): 98.4; T(17): 1.5; T(18): 0.0	4	1	1	0	N-Term(TMT6plex); S13(Phospho)
61743954	NP_001611.1	79026	AHNAK	neuroblast differentiation-associated protein AHNAK isoform1	T4430	2.0	2.2	1.7	1.9	2.5	3.8	3.9	2.0	2.4	2.2	1.7	2.2	3.0	3.9	4.0	2.4	2.7	3.0	2.8	2.3	GPSLDIDtPDVNIEGPEGK	GPSLDIDtPDVNIEG	T8;	Yes	S(3): 0.0; T(8): 100.0	79	1	1	0	N-Term(TMT6plex); T8(Phospho); K19(TMT6plex)
62739181	NP_001015056.1	6242	RTKN	rhotekin isoform c	S56	1.3	1.6	1.4	1.7	0.9	2.1	1.5	2.2	1.1	1.2	1.1	1.3	0.8	1.6	1.3	1.8	0.9	1.4	1.2	1.6	RPsDSGPPAER	GKTSRRPsDSGPPAE	S3;	No	S(3): 100.0; S(5): 0.0	4	3	1	0	N-Term(TMT6plex); S3(Phospho)
21493024	NP_653080.1	9590	AKAP12	A-kinase anchor protein 12 isoform2	S385	1.1	0.8	1.8	1.5	1.0	1.1	1.6	2.4	1.3	0.8	1.5	1.4	1.1	1.1	1.3	2.2	1.2	0.9	1.4	1.8	ETCVSGEDPTQGADLsPDEK	PTQGADLsPDEKVLS	S16;	No	T(2): 0.0; S(5): 0.0; T(10): 0.0; S(16): 100.0	103	2	1	0	N-Term(TMT6plex); C3(Carbamidomethyl); S16(Phospho); K20(TMT6plex)
24497453	NP_002523.2	4927	NUP88	nuclear pore complex protein Nup88	T525	1.2	1.4	1.5	2.1	1.1	1.4	1.1	1.8	1.1	1.4	1.4	2.0	1.1	1.3	1.0	1.7	1.1	1.4	1.2	1.8	VLAEtPDSFEK	PLRVLAEtPDSFEKH	T5;	Yes	T(5): 100.0; S(8): 0.0	3	1	1	0	N-Term(TMT6plex); T5(Phospho); K11(TMT6plex)
386643034	NP_001245362.1	10397	NDRG1	protein NDRG1 isoform3	S249	1.8	1.7	1.6	1.2	1.0	0.9	0.6	2.6	2.0	1.7	1.5	1.3	1.2	0.9	0.5	2.8	1.6	1.3	1.0	2.1	TAsGSSVTSLDGTR	LMRSRTAsGSSVTSL	S3;	No	T(1): 1.4; S(3): 98.6; S(5): 0.0; S(6): 0.0; T(8): 0.0; S(9): 0.0; T(13): 0.0	3	3	1	0	N-Term(TMT6plex); S3(Phospho)
155723000	NP_115811.2	84451	KIAA1804	mitogen-activated protein kinase kinase kinase MLK4	S528	0.9	0.7	1.1	1.0	1.5	2.3	1.5	2.7	-	-	-	-	-	-	-	-	-	-	-	-	ITVQAsPNLDK	HKITVQAsPNLDKRR	S6;	No	T(2): 0.0; S(6): 100.0	1	1	1	0	N-Term(TMT6plex); S6(Phospho); K11(TMT6plex)
634743290	NP_001278893.1	4301	MLLT4	afadin isoform4	S175	0.8	0.8	0.9	1.2	1.0	1.2	1.0	2.4	0.8	0.7	0.8	1.1	0.9	1.1	0.9	2.1	0.8	0.9	0.8	1.6	TIsNPEVVMK	TSFTRTIsNPEVVMK	S3;	No	T(1): 0.0; S(3): 100.0	16	3	1	0	N-Term(TMT6plex); S3(Phospho); M9(Oxidation); K10(TMT6plex)
573014817	NP_001275906.1	2547	XRCC6	X-ray repair cross-complementing protein 6 isoform2	T414	1.7	1.7	1.6	2.2	1.1	1.3	1.4	1.4	1.7	1.8	1.7	2.6	1.2	1.4	1.5	1.6	1.4	1.6	1.6	2.1	IMAtPEQVGK	ETEKIMAtPEQVGKM	T4;	No	T(4): 100.0	28	3	1	0	N-Term(TMT6plex); M2(Oxidation); T4(Phospho); K10(TMT6plex)
157739945	NP_079461.2	26115	TANC2	protein TANC2	S1530; S1534	1.4	2.4	1.7	2.7	1.1	1.1	1.1	1.0	1.5	2.3	1.6	2.7	1.2	1.0	1.0	1.0	1.3	1.7	1.3	1.8	EYPsPPPsPLR	GQGKEYPsPPPSPLR;EYPSPPPsPLRRGPQ	S4; S8;	Yes;Yes	Y(2): 0.0; S(4): 100.0; S(8): 100.0	6	1	1	0	N-Term(TMT6plex); S4(Phospho); S8(Phospho)
33188459	NP_004324.2	673	BRAF	serine/threonine-protein kinase B-raf	T401	1.6	1.5	1.5	1.4	1.2	1.6	1.8	2.2	-	-	-	-	-	-	-	-	-	-	-	-	GDGGSTTGLSAtPPASLPGSLTNVK	STTGLSAtPPASLPG	T12;	Yes	S(5): 0.0; T(6): 0.0; T(7): 0.0; S(10): 0.0; T(12): 100.0; S(16): 0.0; S(20): 0.0; T(22): 0.0	4	1	1	0	N-Term(TMT6plex); T12(Phospho); K25(TMT6plex)
7657339	NP_055299.1	27304	MOCS3	adenylyltransferase and sulfurtransferase MOCS3	S47	1.7	1.1	1.5	2.4	0.9	1.2	1.4	1.2	1.6	1.1	1.6	2.1	0.8	1.2	1.5	1.1	1.2	1.1	1.5	1.6	LVPVsPLPPK	PERLVPVsPLPPKAA	S5;	Yes	S(5): 100.0	4	1	1	0	N-Term(TMT6plex); S5(Phospho); K10(TMT6plex)
18426904	NP_569079.1	56897	WRNIP1	ATPase WRNIP1 isoform2	T116	1.1	0.6	0.6	1.0	1.0	1.1	1.4	2.6	1.1	0.6	0.7	1.1	1.0	1.2	1.5	2.9	1.1	0.9	1.1	2.0	ESYDAPPtPSGAR	ESYDAPPtPSGARLI	T8;	Yes	S(2): 0.0; Y(3): 0.0; T(8): 100.0; S(10): 0.0	5	2	1	0	N-Term(TMT6plex); T8(Phospho)
115298682	NP_055987.2	23215	PRRC2C	protein PRRC2C	S2105	1.1	1.3	1.0	1.5	1.7	1.8	1.8	2.1	1.2	1.3	1.0	1.3	1.8	1.7	1.8	1.9	1.5	1.5	1.4	1.6	LPDLsPVENK	AQKLPDLsPVENKEH	S5;	Yes	S(5): 100.0	18	1	1	0	N-Term(TMT6plex); S5(Phospho); K10(TMT6plex)
15082258	NP_009207.2	11335	CBX3	chromobox protein homolog 3	S95	1.9	1.7	2.4	2.1	1.6	1.4	2.3	1.4	1.6	1.6	1.4	1.9	1.3	1.2	1.4	1.3	1.5	1.4	1.4	1.6	SLsDSESDDSK	GTKRKSLsDSESDDS	S3;	Yes	S(1): 0.0; S(3): 100.0; S(5): 0.0; S(7): 0.0; S(10): 0.0	3	1	1	0	N-Term(TMT6plex); S3(Phospho); K11(TMT6plex)
254028240	NP_001156769.1	2011	MARK2	serine/threonine-protein kinase MARK2 isoform f	S455	1.2	2.2	2.4	2.5	1.0	1.5	1.3	1.1	1.2	2.3	2.5	2.6	1.0	1.5	1.4	1.1	1.1	1.9	2.0	1.9	VPAsPLPGLER	STAKVPAsPLPGLER	S4;	No	S(4): 100.0	2	5	1	0	N-Term(TMT6plex); S4(Phospho)
19923973	NP_612453.1	115207	KCTD12	BTB/POZ domain-containing protein KCTD12	S185	1.0	1.0	0.6	1.1	2.0	1.4	3.4	2.4	0.9	0.9	0.5	0.7	1.7	1.2	2.7	1.6	1.3	1.0	1.6	1.2	sPSGGAAGPLLTPSQSLDGSR	TLELASRsPSGGAAG	S1;	Yes	S(1): 98.3; S(3): 1.7; T(12): 0.0; S(14): 0.0; S(16): 0.0; S(20): 0.0	1	1	1	0	N-Term(TMT6plex); S1(Phospho)
190014588	NP_001121689.1	11168	PSIP1	PC4 and SFRS1-interacting protein isoform2	T272; S273	0.8	0.5	0.7	0.5	4.9	3.9	4.5	3.1	1.0	0.5	0.9	0.5	6.0	4.2	5.6	3.5	3.5	2.4	3.2	2.0	TGVTSTSDsEEEGDDQEGEK	AKTGVTStSDSEEEG;KTGVTSTsDSEEEGD	T6; S7;	Yes;Yes	T(1): 0.0; T(4): 0.0; S(5): 2.1; T(6): 97.9; S(7): 99.9; S(9): 0.0	27	2	1	0	N-Term(TMT6plex); T6(Phospho); S9(Phospho); K20(TMT6plex)
190014588	NP_001121689.1	11168	PSIP1	PC4 and SFRS1-interacting protein isoform2	T272; S273	4.9	3.9	4.5	3.1	0.8	0.5	0.7	0.5	6.0	4.2	5.6	3.5	1.0	0.5	0.9	0.5	3.5	2.4	3.2	2.0	TGVTStSDsEEEGDDQEGEK	GVTSTSDsEEEGDDQ	T6; S7;	Yes	T(1): 0.0; T(4): 0.0; S(5): 0.1; T(6): 2.8; S(7): 2.8; S(9): 94.3	1	2	1	0	N-Term(TMT6plex); S9(Phospho); K20(TMT6plex)
224591430	NP_001139197.1	55646	LYAR	cell growth-regulating nucleolar protein	S260	1.3	1.9	1.5	1.7	1.2	1.6	1.6	1.8	1.3	1.6	1.4	1.5	1.2	1.4	1.5	1.5	1.3	1.5	1.4	1.5	DSAsEEEAR	KQRKDSAsEEEARVG	S4;	No	S(2): 0.0; S(4): 100.0	10	1	1	0	N-Term(TMT6plex); S4(Phospho)
82659109	NP_065816.2	23352	UBR4	E3 ubiquitin-protein ligase UBR4	S178	1.3	1.3	1.3	1.6	1.4	1.4	1.4	2.0	1.2	1.2	1.1	1.4	1.2	1.2	1.2	1.8	1.2	1.2	1.2	1.6	ELAsPVsPELR	EDQKELAsPVSPELR	S4;	Yes	S(4): 100.0; S(7): 0.0	2	1	1	0	N-Term(TMT6plex); S4(Phospho)
82659109	NP_065816.2	23352	UBR4	E3 ubiquitin-protein ligase UBR4	S178	1.4	1.4	1.4	2.0	1.3	1.3	1.3	1.6	1.2	1.2	1.2	1.8	1.2	1.2	1.1	1.4	1.2	1.2	1.2	1.6	ELAsPVSPELR	EDQKELAsPVSPELR	S4;	Yes	S(4): 100.0; S(7): 0.0	28	1	1	0	N-Term(TMT6plex); S4(Phospho)
85815829	NP_061830.3	23616	SH3BP1	SH3 domain-binding protein 1	T626	0.7	1.0	1.0	1.2	1.0	1.4	2.3	2.3	0.6	0.8	0.8	0.9	0.9	1.2	1.9	1.8	0.7	1.0	1.4	1.3	APTVPPPLPPtPPQPAR	VPPPLPPtPPQPARR	T11;	Yes	T(3): 0.0; T(11): 100.0	5	1	1	0	N-Term(TMT6plex); T11(Phospho)
153945728	NP_005900.2	4131	MAP1B	microtubule-associated protein 1B	T527	1.1	1.0	1.4	2.1	0.6	1.4	1.2	1.5	0.9	0.7	1.1	2.2	0.4	1.0	1.0	1.5	0.7	0.9	1.1	1.9	DLTGQVPtPVVK	DLTGQVPtPVVKQTK	T8;	Yes	T(3): 0.0; T(8): 100.0	49	1	1	0	N-Term(TMT6plex); T8(Phospho); K12(TMT6plex)
291190787	NP_055872.4	23077	MYCBP2	E3 ubiquitin-protein ligase MYCBP2	S2911	1.4	1.1	1.2	0.9	4.9	3.9	4.3	2.6	1.4	1.0	1.2	0.7	5.1	3.7	4.3	2.1	3.3	2.3	2.8	1.4	DSTDsPGSENR	VPKDSTDsPGSENRA	S5;	No	S(2): 0.0; T(3): 0.0; S(5): 99.4; S(8):	6	1	1	0	N-Term(TMT6plex); S5(Phospho)
18105007	NP_004332.2	790	CAD	CAD protein	S1859	1.3	1.9	1.1	2.4	1.3	1.3	1.0	1.1	1.2	1.9	1.2	2.0	1.3	1.3	1.0	1.0	1.3	1.6	1.1	1.5	AsDPGLPAEEPK	PPRIHRAsDPGLPAE	S2;	Yes	S(2): 100.0	2	1	1	0	N-Term(TMT6plex); S2(Phospho); K12(TMT6plex)
37537716	NP_892116.2	1983	EIF5	eukaryotic translation initiation factor 5	S389; S390	2.5	2.5	1.2	2.6	1.0	1.6	1.5	0.8	2.3	2.5	1.3	2.6	0.9	1.6	1.6	0.8	1.6	2.1	1.4	1.7	EAEEEssGGEEEDEDENIEVVYSK	LKEAEEEsSGGEEED;KEAEEESsGGEEEDE	S6; S7;	Yes;Yes	S(6): 100.0; S(7): 100.0; Y(22): 0.0; S(23): 0.0	2	1	1	0	N-Term(TMT6plex); S6(Phospho); S7(Phospho); K24(TMT6plex)
5032189	NP_005648.1	7158	TP53BP1	tumor suppressor p53-binding protein 1 isoform3	S294	1.4	1.8	2.0	2.4	1.0	0.9	0.6	1.1	1.4	1.8	2.2	2.9	1.0	0.9	0.6	1.4	1.2	1.3	1.4	2.1	sPEPEVLSTQEDLFDQSNK	SGLQIQKsPEPEVLS	S1;	Yes	S(1): 100.0; S(8): 0.0; T(9): 0.0; S(17): 0.0	62	3	1	0	N-Term(TMT6plex); S1(Phospho); K19(TMT6plex)
29826335	NP_003899.2	8894	EIF2S2	eukaryotic translation initiation factor 2 subunit 2	T111	0.8	0.9	0.9	0.9	2.9	0.9	1.4	2.6	0.9	1.1	1.0	1.0	3.1	1.0	1.7	3.0	2.0	1.0	1.3	2.0	IESDVQEPtEPEDDLDIMLGNK	ESDVQEPtEPEDDLD	T9;	Yes	S(3): 0.0; T(9): 100.0	3	1	1	0	N-Term(TMT6plex); T9(Phospho); M18(Oxidation); K22(TMT6plex)
116256336	NP_001070674.1	22872	SEC31A	protein transport protein Sec31A isoform4	S799	1.4	1.7	1.6	1.8	1.0	1.9	1.5	1.6	1.3	1.6	1.5	1.7	0.9	1.7	1.3	1.5	1.1	1.6	1.4	1.6	AQGEPVAGHEsPK	EPVAGHEsPKIPYEK	S11;	Yes	S(11): 100.0	4	5	1	0	N-Term(TMT6plex); S11(Phospho); K13(TMT6plex)
58530845	NP_001010972.1	7791	ZYX	zyxin	S344	0.3	1.1	1.1	1.6	0.7	1.3	1.2	1.8	0.3	1.1	1.1	1.5	0.8	1.3	1.3	1.7	0.5	1.2	1.2	1.6	sPGAPGPLTLK	QNQNQVRsPGAPGPL	S1;	Yes	S(1): 100.0; T(9): 0.0	3	1	1	0	N-Term(TMT6plex); S1(Phospho); K11(TMT6plex)
41281583	NP_056031.2	23262	PPIP5K2	inositol hexakisphosphate and diphosphoinositol-pentakisphosphate kinase 2 isoform2	S1006	1.4	1.5	1.3	1.7	2.0	2.3	2.1	1.8	1.2	1.1	1.0	1.3	1.6	1.7	1.7	1.4	1.4	1.4	1.3	1.3	sGEQITSSPVSPK	TGRRRRRsGEQITSS	S1;	Yes	S(1): 100.0; T(6): 0.0; S(7): 0.0; S(8): 0.0; S(11): 0.0	3	3	1	0	N-Term(TMT6plex); S1(Phospho); K13(TMT6plex)
15082242	NP_149988.1	90355	C5orf30	UNC119-binding protein C5orf30	S25	1.2	0.9	1.5	2.3	3.1	1.2	1.6	1.1	1.1	0.7	1.4	1.9	3.0	1.0	1.5	1.0	2.0	0.8	1.4	1.5	STLTTLPFPGAEANsPGK	FPGAEANsPGKAEAE	S15;	Yes	S(1): 0.0; T(2): 0.0; T(4): 0.0; T(5): 0.0; S(15): 100.0	1	1	1	0	N-Term(TMT6plex); S15(Phospho); K18(TMT6plex)
443497966	NP_001263219.1	5573	PRKAR1A	cAMP-dependent protein kinase type I-alpha regulatory subunit isoform b	S83	1.2	0.8	1.4	2.0	1.1	1.7	1.2	1.4	1.2	0.8	1.4	2.0	1.1	1.7	1.2	1.4	1.2	1.2	1.3	1.7	EDEIsPPPPNPVVK	DSREDEIsPPPPNPV	S5;	Yes	S(5): 100.0	5	2	1	0	N-Term(TMT6plex); S5(Phospho); K14(TMT6plex)
42518070	NP_004808.2	9414	TJP2	tight junction protein ZO-2 isoform1	S130	2.7	2.2	3.4	2.3	1.0	3.0	3.6	1.1	2.8	2.2	2.9	2.4	1.0	2.9	3.0	1.2	1.9	2.6	2.9	1.8	VQVAALQAsPPLDQDDR	QVAALQAsPPLDQDD	S9;	Yes	S(9): 100.0	13	6	1	0	N-Term(TMT6plex); S9(Phospho)
22749367	NP_689893.1	201799	TMEM154	transmembrane protein 154 precursor	S115	1.4	1.4	1.3	1.5	1.6	3.6	2.5	1.9	-	-	-	-	-	-	-	-	-	-	-	-	QEPSSQGsQSALQTYELGSENVK	QEPSSQGsQSALQTY	S8;	Yes	S(4): 0.0; S(5): 0.0; S(8): 98.3; S(10): 1.7; T(14): 0.0; Y(15): 0.0; S(19): 0.0	5	1	1	0	N-Term(TMT6plex); S8(Phospho); K23(TMT6plex)
150010552	NP_056073.1	23307	FKBP15	FK506-binding protein 15	S956	2.2	3.6	2.5	2.5	1.0	0.6	1.1	1.0	2.3	3.9	2.9	2.9	1.1	0.6	1.3	1.1	1.7	2.3	2.1	2.0	RPsQEQSASASSGQPQAPLNR	EERPRRPsQEQSASA	S3;	Yes	S(3): 100.0; S(7): 0.0; S(9): 0.0; S(11): 0.0; S(12): 0.0	9	1	1	0	N-Term(TMT6plex); S3(Phospho)
103472005	NP_002408.3	4288	MKI67	antigen KI-67 isoform1	T1233	0.7	0.6	1.3	2.3	1.0	2.7	0.8	1.1	0.8	0.6	1.4	2.4	1.2	2.7	0.8	1.2	1.0	1.7	1.1	1.8	ELEQtPGHTEELVAAGK	GEKELEQtPGHTEEL	T5;	Yes	T(5): 100.0; T(9): 0.0	118	2	1	0	N-Term(TMT6plex); T5(Phospho); K17(TMT6plex)
385251399	NP_001245260.1	2036	EPB41L1	band 4.1-like protein 1 isoform b	S504	2.4	2.3	2.9	1.4	1.5	2.4	2.0	1.9	2.4	2.4	2.8	1.5	1.5	2.5	1.9	2.1	1.9	2.4	2.4	1.8	APEsDTGDEDQDQER	PRPRAPEsDTGDEDQ	S4;	No	S(4): 100.0; T(6): 0.0	14	3	1	0	N-Term(TMT6plex); S4(Phospho)
30795231	NP_006308.3	10409	BASP1	brain acid soluble protein 1	T36	1.4	1.8	1.0	1.5	2.2	2.5	2.1	1.9	1.4	1.7	1.1	1.8	2.2	2.5	2.3	2.2	1.8	2.1	1.7	2.0	AEGAATEEEGtPKESEPQAAAEPAEAK	AATEEEGtPKESEPQ	T11;	Yes	T(6): 2.3; T(11): 97.7; S(15): 0.1	16	1	1	1	N-Term(TMT6plex); T11(Phospho); K13(TMT6plex); K27(TMT6plex)
15082258	NP_009207.2	11335	CBX3	chromobox protein homolog 3	S176	0.6	1.1	1.0	0.8	1.2	3.2	4.1	2.6	0.5	1.0	0.6	0.7	1.0	2.9	2.4	2.3	0.8	2.0	1.5	1.5	LTWHsCPEDEAQ	EEELTWHsCPEDEAQ	S5;	Yes	T(2): 0.0; S(5): 100.0	3	1	1	0	N-Term(TMT6plex); S5(Phospho); C6(Carbamidomethyl)
190341107	NP_001011713.2	122830	NAA30	N-alpha-acetyltransferase 30	S152	1.3	0.9	1.3	2.2	1.0	0.9	0.6	1.1	1.1	0.9	1.2	2.2	0.9	0.9	0.5	1.2	1.0	0.9	0.9	1.7	TAVPsPVEAAAASDPAAAR	NARTAVPsPVEAAAA	S5;	Yes	T(1): 0.0; S(5): 100.0; S(13): 0.0	2	1	1	0	N-Term(TMT6plex); S5(Phospho)
321400140	NP_001189485.1	960	CD44	CD44 antigen isoform7 precursor	S304	1.3	1.7	2.3	1.4	1.4	1.4	1.9	1.9	1.0	1.0	1.4	1.2	1.0	0.9	1.2	1.7	1.0	1.0	1.3	1.5	sQEMVHLVNK	LNGEASKsQEMVHLV	S1;	No	S(1): 100.0	11	6	1	0	N-Term(TMT6plex); S1(Phospho); M4(Oxidation); K10(TMT6plex)
17865802	NP_004860.2	9525	VPS4B	vacuolar protein sorting-associated protein 4B	S102	1.0	1.0	1.1	1.4	1.1	1.6	1.5	1.9	1.0	1.0	1.0	1.4	1.1	1.6	1.5	1.9	1.1	1.3	1.3	1.7	EGQPSPADEKGNDsDGEGESDDPEK	ADEKGNDsDGEGESD	S14;	Yes	S(5): 0.0; S(14): 100.0; S(20): 0.0	4	1	1	1	N-Term(TMT6plex); K10(TMT6plex); S14(Phospho); K25(TMT6plex)
18093112	NP_057733.2	51575	ESF1	ESF1 homolog	S153	1.3	0.9	1.3	0.8	4.7	2.1	1.9	2.5	1.2	0.8	1.2	0.7	4.6	1.9	1.9	2.4	2.9	1.3	1.5	1.6	IDSNIsPK	EKIDSNIsPKKDSKE	S6;	Yes	S(3): 0.0; S(6): 100.0	1	1	1	0	N-Term(TMT6plex); S6(Phospho); K8(TMT6plex)
75677385	NP_001028260.2	51335	NGRN	neugrin precursor	S41	1.1	1.5	1.7	2.0	1.8	1.6	1.4	1.3	1.2	1.3	1.7	2.0	1.9	1.5	1.4	1.3	1.6	1.4	1.5	1.6	EPDPDsDWEPEER	GREPDPDsDWEPEER	S6;	Yes	S(6): 100.0	2	1	1	0	N-Term(TMT6plex); S6(Phospho)
8922563	NP_060633.1	55192	DNAJC17	dnaJ homolog subfamily C member 17	S112	1.6	1.5	1.7	2.1	1.1	1.0	1.3	1.2	1.5	1.7	1.8	2.0	1.0	1.1	1.4	1.2	1.3	1.4	1.6	1.6	QAQAQEsEEEEESR	RQAQAQEsEEEEESR	S7;	Yes	S(7): 100.0; S(13): 0.0	6	1	1	0	N-Term(TMT6plex); S7(Phospho)
50845386	NP_001002857.1	302	ANXA2	annexin A2 isoform2	S12	1.2	1.1	1.2	1.6	0.8	0.6	0.9	1.7	1.3	1.3	1.3	1.8	0.9	0.8	1.0	1.9	1.1	1.0	1.1	1.9	LsLEGDHSTPPSAYGSVK	HEILCKLsLEGDHST	S2;	Yes	S(2): 100.0; S(8): 0.0; T(9): 0.0; S(12): 0.0; Y(14): 0.0; S(16): 0.0	18	2	1	0	N-Term(TMT6plex); S2(Phospho); K18(TMT6plex)
50845386	NP_001002857.1	302	ANXA2	annexin A2 isoform2	S12	0.8	0.6	0.9	1.7	1.2	1.1	1.2	1.6	0.9	0.8	1.0	1.9	1.3	1.3	1.3	1.8	1.1	1.0	1.1	1.9	LSLEGDHsTPPSAYGSVK	LSLEGDHsTPPSAYG	S2;	Yes	S(2): 0.0; S(8): 98.9; T(9): 1.1; S(12): 0.0; Y(14): 0.0; S(16): 0.0	2	2	1	0	N-Term(TMT6plex); S8(Phospho); K18(TMT6plex)
31324577	NP_203744.1	85377	MICALL1	MICAL-like protein 1	S621	1.1	1.8	1.4	1.7	1.3	1.5	1.5	1.6	1.0	1.5	1.3	1.5	1.2	1.3	1.4	1.4	1.1	1.4	1.3	1.5	SPVPSPGSSsPQLQVK	VPSPGSSsPQLQVKS	S10;	Yes	S(1): 0.0; S(5): 0.0; S(8): 0.0; S(9): 1.0; S(10): 99.0	12	1	1	0	N-Term(TMT6plex); S10(Phospho); K16(TMT6plex)
21493024	NP_653080.1	9590	AKAP12	A-kinase anchor protein 12 isoform2	S1297	0.6	1.2	1.2	1.2	0.4	1.1	1.4	2.1	0.7	1.1	1.0	1.1	0.4	1.1	1.2	2.0	0.6	1.1	1.1	1.5	EVSSLEGsPPPCLGQEEAVCTK	EVSSLEGsPPPCLGQ	S8;	No	S(3): 1.8; S(4): 1.8; S(8): 96.3; T(21): 0.0	103	2	1	0	N-Term(TMT6plex); S8(Phospho); C12(Carbamidomethyl); C20(Carbamidomethyl); K22(TMT6plex)
61743954	NP_001611.1	79026	AHNAK	neuroblast differentiation-associated protein AHNAK isoform1	S5110	1.0	5.7	2.5	1.4	0.9	1.9	1.5	1.8	1.3	5.8	2.5	1.7	1.0	1.9	1.5	2.2	1.1	3.9	2.0	1.9	AEAPLPsPK	KAEAPLPsPKLEGEL	S7;	Yes	S(7): 100.0	79	1	1	0	N-Term(TMT6plex); S7(Phospho); K9(TMT6plex)
148596949	NP_004732.2	9221	NOLC1	nucleolar and coiled-body phosphoprotein 1 isoform2	T607; T610	0.8	1.1	1.4	1.6	0.9	0.9	1.7	1.6	0.7	0.9	1.1	1.2	0.8	0.7	1.3	1.2	0.8	0.8	1.2	1.2	LQtPNtFPK	AKKIKLQtPNTFPKR;IKLQTPNtFPKRKKG	T3; T6;	Yes;Yes	T(3): 100.0; T(6): 100.0	33	3	1	0	N-Term(TMT6plex); T3(Phospho); T6(Phospho); K9(TMT6plex)
148596949	NP_004732.2	9221	NOLC1	nucleolar and coiled-body phosphoprotein 1 isoform2	T607; T610	0.9	0.9	1.7	1.6	0.8	1.1	1.4	1.6	0.8	0.7	1.3	1.2	0.7	0.9	1.1	1.2	0.8	0.8	1.2	1.2	LQTPNtFPK	AKKIKLQtPNTFPKR;IKLQTPNtFPKRKKG	T3; T6;	Yes;Yes	T(3): 100.0; T(6): 100.0	3	3	1	0	N-Term(TMT6plex); T3(Phospho); T6(Phospho); K9(TMT6plex)
259155302	NP_001158884.1	4790	NFKB1	nuclear factor NF-kappa-B p105 subunit isoform2	S893	0.5	0.6	0.6	0.6	1.0	0.9	1.6	2.7	0.5	0.6	0.6	0.6	1.0	0.9	1.5	2.6	0.8	0.8	1.1	1.6	QMGYTEAIEVIQAASsPVK	EVIQAASsPVKTTSQ	S16;	Yes	Y(4): 0.0; T(5): 0.0; S(15): 0.0; S(16): 100.0	2	2	1	0	N-Term(TMT6plex); M2(Oxidation); S16(Phospho); K19(TMT6plex)
559098432	NP_001273991.1	27342	RABGEF1	rab5 GDP/GTP exchange factor isoform c	S124	0.6	0.7	0.8	1.1	1.1	0.9	1.3	2.2	0.6	0.6	0.7	0.8	1.0	0.7	1.1	1.6	0.8	0.7	0.9	1.2	APsPSINR	IQEAKAPsPSINRQT	S3;	No	S(3): 100.0; S(5): 0.0	2	2	1	0	N-Term(TMT6plex); S3(Phospho)
372622371	NP_001243242.1	6788	STK3	serine/threonine-protein kinase 3 isoform3	S205	2.4	2.9	1.7	2.4	0.7	2.8	2.0	0.8	2.2	2.7	1.5	2.0	0.7	2.6	1.7	0.7	1.4	2.6	1.6	1.3	ELEEEENsDEDELDSHTMVK	LEEEEENsDEDELDS	S9;	No	S(9): 100.0; S(16): 0.0; T(18): 0.0	2	3	1	0	N-Term(TMT6plex); S9(Phospho); M19(Oxidation); K21(TMT6plex)
15011974	NP_004714.2	9181	ARHGEF2	rho guanine nucleotide exchange factor 2 isoform3	S904	1.1	1.5	1.4	1.4	1.6	1.8	1.5	1.8	0.8	1.2	1.2	1.3	1.2	1.4	1.3	1.6	1.0	1.3	1.3	1.4	QELGsPEER	RERQELGsPEERLQD	S5;	No	S(5): 100.0	5	3	1	0	N-Term(TMT6plex); S5(Phospho)
118572613	NP_057417.3	23524	SRRM2	serine/arginine repetitive matrix protein 2	S1336	1.0	0.4	0.8	0.7	1.2	1.7	1.6	2.6	1.0	0.4	0.9	0.7	1.3	1.8	1.7	2.8	1.2	1.1	1.3	1.8	NsGPLGTEMNTGFSSEVK	SPLEFRNsGPLGTEM	S2;	Yes	S(2): 100.0; T(7): 0.0; T(11): 0.0; S(14): 0.0; S(15): 0.0	436	1	1	0	N-Term(TMT6plex); S2(Phospho); M9(Oxidation); K18(TMT6plex)
219842214	NP_001137358.1	4659	PPP1R12A	protein phosphatase 1 regulatory subunit 12A isoform	S420	1.6	1.8	1.4	1.0	3.1	3.1	2.4	2.3	1.6	1.6	1.3	0.9	3.0	2.8	2.2	2.1	2.3	2.2	1.8	1.5	LAsTSDIEEK	TIPRRLAsTSDIEEK	S3;	No	S(3): 100.0; T(4): 0.0; S(5): 0.0	10	4	1	0	N-Term(TMT6plex); S3(Phospho); K10(TMT6plex)
157502203	NP_001098985.1	10257	ABCC4	multidrug resistance-associated protein 4 isoform2	T646	1.0	1.2	1.8	1.9	0.9	0.7	0.9	1.3	1.0	1.1	1.7	2.0	0.8	0.6	0.8	1.4	0.9	0.9	1.3	1.7	KDNEESEQPPVPGtPTLR	EQPPVPGtPTLRNRT	T14;	Yes	S(6): 0.0; T(14): 97.4; T(16): 2.6	2	2	1	1	N-Term(TMT6plex); K1(TMT6plex); T14(Phospho)
110349740	NP_115648.2	84232	MAF1	repressor of RNA polymerase III transcription MAF1 homolog	S75	0.8	1.0	1.0	1.1	1.7	1.9	0.9	2.1	0.6	0.9	0.8	0.8	1.3	1.7	0.7	1.6	1.0	1.3	0.8	1.2	sQGGEEEGPLSDK	SPSRLSKsQGGEEEG	S1;	Yes	S(1): 100.0; S(11): 0.0	1	1	1	0	N-Term(TMT6plex); S1(Phospho); K13(TMT6plex)
148596949	NP_004732.2	9221	NOLC1	nucleolar and coiled-body phosphoprotein 1 isoform2	T188	0.4	0.9	1.0	1.6	0.5	0.8	1.0	1.6	0.4	0.7	0.8	1.2	0.5	0.7	0.8	1.2	0.4	0.7	0.8	1.2	ItPVTVK	KNQKPKItPVTVKAQ	T2;	Yes	T(2): 100.0; T(5): 0.0	33	3	1	0	N-Term(TMT6plex); T2(Phospho); K7(TMT6plex)
224809474	NP_001138994.1	26064	RAI14	ankycorbin isoform b	T268	1.3	1.9	1.8	1.7	1.4	1.7	1.5	1.5	1.1	1.7	1.4	1.3	1.2	1.5	1.2	1.2	1.2	1.6	1.3	1.3	SITStPLSGK	SPRSITStPLSGKES	T5;	No	S(1): 0.0; T(3): 0.0; S(4): 0.0; T(5): 100.0; S(8): 0.0	4	4	1	0	N-Term(TMT6plex); T5(Phospho); K10(TMT6plex)
40807477	NP_109590.3	10743	RAI1	retinoic acid-induced protein 1	T1068	0.8	1.5	1.8	2.3	2.1	1.9	1.6	0.9	0.8	1.7	1.9	2.4	2.3	2.2	1.8	0.9	1.6	1.9	1.9	1.7	tPGPPGLTTTPAPPDK	TALSEPRtPGPPGLT	T1;	Yes	T(1): 100.0; T(8): 0.0; T(9): 0.0; T(10): 0.0	6	1	1	0	N-Term(TMT6plex); T1(Phospho); K16(TMT6plex)
340545545	NP_001230018.1	2305	FOXM1	forkhead box protein M1 isoform5	S714	0.5	1.0	1.0	0.6	2.8	1.5	2.7	2.6	0.5	0.9	0.9	0.6	2.8	1.4	2.5	2.8	1.6	1.2	1.7	1.7	SLTEGLVLDTMNDsLSK	VLDTMNDsLSKILLD	S14;	No	S(1): 50.0; T(3): 50.0; T(10): 0.0; S(14): 100.0; S(16): 0.0	9	5	1	0	N-Term(TMT6plex); S1(Phospho); M11(Oxidation); S14(Phospho); K17(TMT6plex)
10716563	NP_001737.1	821	CANX	calnexin precursor	S554; S564	1.6	1.9	1.4	1.6	1.5	2.0	1.4	1.6	1.6	1.5	1.4	1.4	1.5	1.5	1.4	1.5	1.5	1.5	1.4	1.4	QKsDAEEDGGTVsQEEEDR	KLEEKQKsDAEEDGG;EEDGGTVsQEEEDRK	S3; S13;	Yes;Yes	S(3): 100.0; T(11): 0.0; S(13): 100.0	54	1	1	1	N-Term(TMT6plex); K2(TMT6plex); S3(Phospho); S13(Phospho)
39725634	NP_056130.2	23367	LARP1	la-related protein 1	T647	1.0	0.9	0.6	1.1	2.8	0.6	1.8	2.1	1.1	0.9	0.6	1.1	2.9	0.6	1.9	2.1	2.0	0.7	1.2	1.6	EQFDTLtPEPPVDPNQEVPPGPP	REQFDTLtPEPPVDP	T7;	Yes	T(5): 0.0; T(7): 100.0	28	1	1	0	N-Term(TMT6plex); T7(Phospho)
296011010	NP_060252.4	54906	FAM208B	protein FAM208B	S2009	1.8	2.0	2.3	2.4	0.7	1.0	1.2	0.8	1.7	2.2	2.5	2.5	0.7	1.1	1.3	0.8	1.2	1.6	1.9	1.7	EsPTQISIGAFPSTK	ANVFPKEsPTQISIG	S2;	Yes	S(2): 100.0; T(4): 0.0; S(7): 0.0; S(13): 0.0; T(14): 0.0	4	1	1	0	N-Term(TMT6plex); S2(Phospho); K15(TMT6plex)
4505255	NP_002433.1	4440	MSI1	RNA-binding protein Musashi homolog 1	S191	1.0	1.6	1.2	1.7	1.0	1.3	1.2	1.5	0.9	2.1	1.3	1.6	1.0	1.7	1.3	1.5	0.9	1.9	1.3	1.5	EVMsPTGSAR	AQPKEVMsPTGSARG	S4;	Yes	S(4): 100.0; T(6): 0.0; S(8): 0.0	1	1	1	0	N-Term(TMT6plex); M3(Oxidation); S4(Phospho)
4506667	NP_000993.1	6175	RPLP0	60S acidic ribosomal protein P0	S304; S307	1.2	2.2	1.9	1.9	1.1	1.0	1.3	1.3	1.2	2.3	2.0	1.9	1.2	1.1	1.3	1.3	1.2	1.7	1.6	1.6	VEAKEEsEEsDEDMGFGLFD	KVEAKEEsEESDEDM;AKEESEEsDEDMGFG	S7; S10;	Yes;Yes	S(7): 100.0; S(10): 100.0	3	1	1	1	N-Term(TMT6plex); K4(TMT6plex); S7(Phospho); S10(Phospho); M14(Oxidation)
11968023	NP_071918.1	64397	ZNF106	zinc finger protein 106 isoform1	S1025; S1026	0.9	1.5	1.1	1.5	1.3	1.0	0.9	1.7	0.8	1.5	1.1	1.5	1.1	1.0	0.9	1.6	0.9	1.2	1.0	1.5	ATGDGssPELPSLER	RRATGDGsSPELPSL;RATGDGSsPELPSLE	S6; S7;	No;No	T(2): 0.1; S(6): 99.9; S(7): 99.9; S(12): 0.0	6	3	1	0	N-Term(TMT6plex); S6(Phospho); S7(Phospho)
7706139	NP_057689.1	51307	FAM53C	protein FAM53C	S273	1.6	1.6	1.8	1.8	1.4	1.3	1.5	1.4	-	-	-	-	-	-	-	-	-	-	-	-	sQPCDLDAR	RNLPRSRsQPCDLDA	S1;	Yes	S(1): 100.0	1	1	1	0	N-Term(TMT6plex); S1(Phospho); C4(Carbamidomethyl)
66348045	NP_057562.3	51530	ZC3HC1	nuclear-interacting partner of ALK isoform1	S321	0.9	1.6	1.5	2.0	1.0	3.6	2.5	1.1	0.9	1.6	1.4	1.9	1.0	3.6	2.4	1.0	1.0	2.6	1.9	1.5	LPLVPEsPRR	RLPLVPEsPRRMMTR	S7;	Yes	S(7): 100.0	8	3	1	1	N-Term(TMT6plex); S7(Phospho)
4885487	NP_005430.1	8079	MLF2	myeloid leukemia factor 2	S238	1.0	1.4	1.2	1.4	1.8	2.4	1.9	1.7	-	-	-	-	-	-	-	-	-	-	-	-	LAIQGPEDsPSR	AIQGPEDsPSRQSRR	S9;	Yes	S(9): 99.2; S(11): 0.8	3	1	1	0	N-Term(TMT6plex); S9(Phospho)
223555917	NP_848927.2	92140	MTDH	protein LYRIC	S298	2.2	1.8	2.0	1.5	2.3	2.1	2.1	1.6	1.8	1.6	1.8	1.4	1.9	1.8	1.9	1.5	1.8	1.7	1.8	1.5	LSSQIsAGEEK	VKLSSQIsAGEEKWN	S6;	Yes	S(2): 0.0; S(3): 0.0; S(6): 100.0	16	1	1	0	N-Term(TMT6plex); S6(Phospho); K11(TMT6plex)
46852390	NP_005427.2	8030	CCDC6	coiled-coil domain-containing protein 6	S244	2.7	1.2	1.1	1.5	2.3	1.3	1.0	1.7	2.8	1.1	1.1	1.4	2.5	1.3	0.9	1.6	2.7	1.2	1.0	1.5	LDQPVSAPPsPR	QPVSAPPsPRDISMF	S10;	Yes	S(6): 0.0; S(10): 100.0	11	1	1	0	N-Term(TMT6plex); S10(Phospho)
528881309	NP_001268709.1	23613	ZMYND8	protein kinase C-binding protein 1 isoform n	S299	1.5	1.1	1.9	2.1	1.2	1.2	0.6	1.0	1.2	1.0	1.6	2.0	1.0	1.2	0.5	1.0	1.1	1.1	1.1	1.5	ELSESVQQQSTPVPLIsPK	STPVPLIsPKRQIRS	S17;	No	S(3): 0.0; S(5): 0.0; S(10): 50.0; T(11): 50.0; S(17): 100.0	8	17	1	0	N-Term(TMT6plex); S10(Phospho); S17(Phospho); K19(TMT6plex)
57864582	NP_001009931.1	388697	HRNR	hornerin	S903	0.7	1.8	0.9	1.3	1.0	1.8	1.1	1.8	0.6	1.0	0.9	0.6	1.0	1.0	1.0	0.8	0.8	1.0	1.0	0.7	GSGSGQsPSYGR	RGSGSGQsPSYGRHG	S7;	No	S(2): 0.0; S(4): 0.0; S(7): 100.0; S(9): 0.0; Y(10): 0.0	10	1	1	0	N-Term(TMT6plex); S7(Phospho)
190341065	NP_055526.3	9738	CCP110	centriolar coiled-coil protein of 110 kDa isoform2	S372	1.7	1.6	1.6	2.2	0.8	1.0	0.8	0.9	-	-	-	-	-	-	-	-	-	-	-	-	LPSPEPSMsPK	PSPEPSMsPKMHRRR	S9;	Yes	S(3): 0.0; S(7): 0.0; S(9): 100.0	8	2	1	0	N-Term(TMT6plex); M8(Oxidation); S9(Phospho); K11(TMT6plex)
6912398	NP_036218.1	9330	GTF3C3	general transcription factor 3C polypeptide 3 isoform1	S43	1.8	0.9	1.3	1.5	0.7	1.0	1.9	1.6	1.8	1.0	1.3	1.3	0.7	1.0	1.9	1.5	1.3	1.0	1.6	1.4	GKLsAEENPDDSEVPSSSGINSTK	LQEKGKLsAEENPDD	S4;	Yes	S(4): 100.0; S(12): 0.0; S(16): 0.0; S(17): 0.0; S(18): 0.0; S(22): 0.0; T(23): 0.0	8	2	1	1	N-Term(TMT6plex); K2(TMT6plex); S4(Phospho); K24(TMT6plex)
568214245	NP_001274744.1	9898	UBAP2L	ubiquitin-associated protein 2-like isoform c	S116	1.1	1.4	1.4	1.6	1.0	1.4	1.2	1.5	1.3	1.5	1.6	1.4	1.1	1.4	1.3	1.4	1.2	1.4	1.5	1.4	DGGQTEsNEEGKENR	KDGGQTEsNEEGKEN	S7;	Yes	T(5): 0.9; S(7): 99.1	23	4	1	1	N-Term(TMT6plex); S7(Phospho); K12(TMT6plex)
216548085	NP_361013.3	7798	LUZP1	leucine zipper protein 1	S394	0.8	1.5	1.3	1.7	1.0	1.4	1.3	1.4	0.8	1.4	1.2	1.6	0.9	1.3	1.2	1.3	0.8	1.3	1.2	1.5	ELsPQHK	KHTARELsPQHKRER	S3;	Yes	S(3): 100.0	7	1	1	0	N-Term(TMT6plex); S3(Phospho); K7(TMT6plex)
6912676	NP_036377.1	22938	SNW1	SNW domain-containing protein 1	S224	2.6	1.2	2.3	2.2	0.9	0.9	0.8	0.9	2.6	1.3	2.5	2.0	0.9	0.9	0.9	0.9	1.7	1.1	1.7	1.4	GPPsPPAPVMHSPSR	KIPRGPPsPPAPVMH	S4;	Yes	S(4): 100.0; S(12): 0.0; S(14): 0.0	3	1	1	0	N-Term(TMT6plex); S4(Phospho); M10(Oxidation)
15149465	NP_149131.1	800	CALD1	caldesmon isoform5	S196	1.7	2.1	2.8	1.6	1.9	1.4	2.3	1.5	1.7	1.5	2.1	1.4	1.9	1.1	1.8	1.3	1.8	1.3	1.9	1.3	RGsIGENQIK	EEKPKRGsIGENQIK	S3;	No	S(3): 100.0	4	2	1	1	N-Term(TMT6plex); S3(Phospho); K10(TMT6plex)
153791662	NP_075054.3	64925	CCDC71	coiled-coil domain-containing protein 71	S97	0.6	0.7	0.6	1.5	0.9	1.0	0.8	1.6	-	-	-	-	-	-	-	-	-	-	-	-	APNPTATsPPASAPR	APNPTATsPPASAPR	S8;	No	T(5): 0.0; T(7): 0.0; S(8): 100.0; S(12): 0.0	3	1	1	0	N-Term(TMT6plex); S8(Phospho)
281182836	NP_001162588.1	57620	STIM2	stromal interaction molecule 2 isoform3 precursor	S523	1.2	1.6	1.4	1.2	1.5	1.9	1.7	1.8	-	-	-	-	-	-	-	-	-	-	-	-	SIVPSsPQPQR	RRSIVPSsPQPQRAQ	S6;	Yes	S(1): 0.0; S(5): 1.2; S(6): 98.8	1	3	1	0	N-Term(TMT6plex); S5(Phospho)
281182836	NP_001162588.1	57620	STIM2	stromal interaction molecule 2 isoform3 precursor	S523	1.5	1.9	1.7	1.8	1.2	1.6	1.4	1.2	-	-	-	-	-	-	-	-	-	-	-	-	SIVPsSPQPQR	SRRSIVPsSPQPQRA	S6;	Yes	S(1): 0.0; S(5): 98.9; S(6): 1.1	2	3	1	0	N-Term(TMT6plex); S5(Phospho)
44955921	NP_005221.2	2004	ELK3	ETS domain-containing protein Elk-3	S396	0.3	0.7	0.6	0.4	0.8	3.2	4.1	2.7	-	-	-	-	-	-	-	-	-	-	-	-	AAsPVLLSSNSQK	PSLDRAAsPVLLSSN	S3;	Yes	S(3): 100.0; S(8): 0.0; S(9): 0.0; S(11): 0.0	2	1	1	0	N-Term(TMT6plex); S3(Phospho); K13(TMT6plex)
223633988	NP_001138678.1	85379	KIAA1671	uncharacterized protein KIAA1671	S1366	1.1	1.8	1.1	1.7	1.2	1.2	1.2	1.4	-	-	-	-	-	-	-	-	-	-	-	-	VSPKsPPTDQK	GVRVSPKsPPTDQKK	S5;	Yes	S(2): 1.4; S(5): 98.5; T(8): 0.0	2	1	1	1	N-Term(TMT6plex); K4(TMT6plex); S5(Phospho); K11(TMT6plex)
332164777	NP_001193726.1	5315	PKM	pyruvate kinase PKM isoform d	S37	0.9	1.3	1.2	1.3	1.9	1.8	1.5	1.7	0.8	0.9	0.9	1.1	1.6	1.3	1.2	1.4	1.2	1.1	1.0	1.3	LDIDsPPITAR	MCRLDIDsPPITARN	S5;	Yes	S(5): 100.0; T(9): 0.0	5	5	1	0	N-Term(TMT6plex); S5(Phospho)
151301228	NP_060362.3	55660	PRPF40A	pre-mRNA-processing factor 40 homolog A	T346	1.7	1.5	2.1	2.7	1.0	1.1	0.6	0.4	1.7	1.6	2.3	2.7	1.0	1.2	0.6	0.4	1.4	1.4	1.4	1.6	QETVADFtPK	QETVADFtPKKEEEE	T8;	No	T(3): 0.0; T(8): 100.0	7	1	1	0	N-Term(TMT6plex); T8(Phospho); K10(TMT6plex)
118572613	NP_057417.3	23524	SRRM2	serine/arginine repetitive matrix protein 2	S929	1.2	1.4	1.3	1.0	1.5	2.3	1.6	2.1	1.2	1.5	1.4	1.0	1.6	2.3	1.7	2.3	1.4	1.9	1.6	1.7	SHsGsSSPSPSR	IISPRQRsHSGSSSP	S1;	Yes	S(1): 100.0; S(3): 0.0; S(5): 0.0; S(6): 0.0; S(7): 0.0; S(9): 0.0; S(11): 0.0	436	1	1	0	N-Term(TMT6plex); S3(Phospho)

Khan et al., 2019. Multi-omics analysis to characterize cigarette smoke induced molecular alterations in esophageal cells

Supplementary Table 8. List of phosphopeptides identified and quantified in untreated and chronically treated Het1A cells with cigarette smoke condensate for 8 months using SequestHT and Mascot search algorithms with a PhosphoRS probability score ≥75%

														Normalized phosphoprotein ratios with respect to corresponding total protein ratios.								Average of replicates													
Protein group Accession	NP_Accession	Gene ID	Gene Symbol	Protein Description	PhosphoSite (Protein)	Het-1A-Smoke - 2M/Parental	Het-1A-Smoke - 4M/Parental	Het-1A-Smoke - 6M/Parental	Het-1A-Smoke - 8M/Parental	Het-1A-Smoke - 2M/Parental	Het-1A-Smoke - 4M/Parental	Het-1A-Smoke - 6M/Parental	Het-1A-Smoke - 8M/Parental	Het-1A-Smoke - 2M/Parental	Het-1A-Smoke - 4M/Parental	Het-1A-Smoke - 6M/Parental	Het-1A-Smoke - 8M/Parental	Het-1A-Smoke - 2M/Parental	Het-1A-Smoke - 4M/Parental	Het-1A-Smoke - 6M/Parental	Het-1A-Smoke - 8M/Parental	Het-1A-Smoke - 2M/Parental	Het-1A-Smoke - 4M/Parental	Het-1A-Smoke - 6M/Parental	Het-1A-Smoke - 8M/Parental	Peptide Sequence	Phosphowindow	PhosphoSite (Peptide)	Phosphosite Plus Evidence	phosphoRS Site Probabilities	# PSMs	# Proteins	# Protein Groups	# Missed Cleavages	Modifications
						Replicate 1				Replicate 2				Replicate 1				Replicate 2																	
5454102	NP_006333.1	10460	TACC3	transforming acidic coiled-coil-containing protein 3	S317	1.0	0.8	0.8	1.0	0.9	1.4	1.1	2.0	1.1	0.8	0.9	1.2	1.0	1.5	1.3	2.3	1.1	1.1	1.1	1.7	AMTLsPQEEVAAGQMASSSR	AGRAMTLsPQEEVAA	S5;	Yes	T(3): 1.2; S(5): 98.8; S(17): 0.0; S(18): 0.0; S(19): 0.0	12	1	1	0	N-Term(TMT6plex); M2(Oxidation); S5(Phospho); M15(Oxidation)
39930541	NP_899064.1	286257	C9orf142	uncharacterized protein C9orf142	S148	1.0	0.9	1.3	1.8	1.1	1.5	1.3	1.2	1.0	0.9	1.2	1.7	1.1	1.5	1.3	1.2	1.0	1.2	1.3	1.4	LAAAEETAVsPR	AAEETAVsPRKSPRP	S10;	Yes	T(7): 0.0; S(10): 100.0	4	1	1	0	N-Term(TMT6plex); S10(Phospho)
24308303	NP_085148.1	80851	SH3BP5L	SH3 domain-binding protein 5-like	S343	1.8	1.6	1.3	1.4	1.4	1.5	1.3	1.6	2.1	1.7	1.5	1.5	1.6	1.6	1.5	1.7	1.8	1.6	1.5	1.6	TVAsDLQK	LSLRTVAsDLQKCDS	S4;	Yes	T(1): 0.0; S(4): 100.0	2	1	1	0	N-Term(TMT6plex); S4(Phospho); K8(TMT6plex)
431822406	NP_001258900.1	3326	HSP90AB1	heat shock protein HSP 90-beta isoformb	S178	1.2	1.4	1.3	1.6	1.0	1.1	1.3	1.4	1.2	1.3	1.3	1.6	1.1	1.1	1.3	1.4	1.1	1.2	1.3	1.5	EIsDDEAEEEK	KEREKEIsDDEAEEE	S3;	No	S(3): 100.0	54	3	1	0	N-Term(TMT6plex); S3(Phospho); K11(TMT6plex)
18699734	NP_036606.2	7371	UCK2	uridine-cytidine kinase 2	S254	2.1	2.8	1.6	1.9	1.2	1.3	1.1	1.1	1.6	2.4	1.3	1.5	0.9	1.1	0.9	0.9	1.3	1.7	1.1	1.2	QAsESSSRPH	PSRKRQAsESSSRPH	S3;	Yes	S(3): 100.0; S(5): 0.0; S(6): 0.0; S(7): 0.0	3	1	1	0	N-Term(TMT6plex); S3(Phospho)
21237805	NP_003066.2	6601	SMARCC2	SWI/SNF complex subunit SMARCC2 isoforma	S302	1.1	1.2	1.2	1.1	1.2	1.7	1.9	1.9	1.0	1.1	1.2	1.0	1.1	1.6	1.8	1.9	1.1	1.4	1.5	1.5	SPsPSPTPEAK	NYKKRKRsPSPSPTP;KKRKRSPsPSPTPEA	S1;	Yes;Yes	S(1): 100.0; S(3): 100.0; S(5): 0.0; T(7): 0.0	1	3	1	0	N-Term(TMT6plex); S1(Phospho); S3(Phospho); K11(TMT6plex)
21237805	NP_003066.2	6601	SMARCC2	SWI/SNF complex subunit SMARCC2 isoforma	S302	1.2	1.7	1.9	1.9	1.1	1.2	1.2	1.1	1.1	1.6	1.8	1.9	1.0	1.1	1.2	1.0	1.1	1.4	1.5	1.5	sPsPSPTPEAK	NYKKRKRsPSPSPTP	S1;	Yes	S(1): 100.0; S(3): 0.0; S(5): 0.0; T(7): 0.0	12	3	1	0	N-Term(TMT6plex); S1(Phospho); K11(TMT6plex)
110347427	NP_055524.3	9736	USP34	ubiquitin carboxyl-terminal hydrolase 34	T3381	1.9	1.2	2.0	2.7	0.6	0.9	0.9	0.3	2.0	1.2	2.0	2.8	0.6	0.9	0.9	0.3	1.3	1.1	1.5	1.5	EVLtPTSTSDNETR	TETREVLtPTSTSDN	T4;	Yes	T(4): 99.9; T(6): 0.1; S(7): 0.1; T(8): 0.0; S(9): 0.0; T(13): 0.0	1	1	1	0	N-Term(TMT6plex); T4(Phospho)
148596949	NP_004732.2	9221	NOLC1	nucleolar and coiled-body phosphoprotein 1 isoform2	T597	0.7	0.9	1.1	1.4	0.7	1.0	1.2	1.6	0.6	0.7	0.9	1.1	0.6	0.8	0.9	1.2	0.6	0.8	0.9	1.1	EAEtPQAK	EAAKEAEtPQAKKIK	T4;	Yes	T(4): 100.0	33	3	1	0	N-Term(TMT6plex); T4(Phospho); K8(TMT6plex)
132626688	NP_055456.2	9656	MDC1	mediator of DNA damage checkpoint protein 1	S513	1.1	1.1	1.1	1.4	1.6	1.9	1.9	1.6	1.2	1.1	1.1	1.5	1.6	1.9	2.0	1.7	1.4	1.5	1.6	1.6	sQASTTVDINTQVEK	PGIHLERsQASTTVD	S1;	Yes	S(1): 95.6; S(4): 1.5; T(5): 1.5; T(6): 1.5; T(11): 0.0	26	1	1	0	N-Term(TMT6plex); S1(Phospho); K15(TMT6plex)
48255945	NP_001673.2	490	ATP2B1	plasma membrane calcium-transporting ATPase 1 isoform1b	S1178	1.3	1.4	1.4	1.4	1.9	1.5	1.3	1.6	1.1	1.3	1.3	1.3	1.6	1.4	1.3	1.5	1.4	1.3	1.3	1.4	NSsPPPSPNK	APTKRNSsPPPSPNK	S3;	No	S(2): 0.9; S(3): 99.1; S(7): 0.0	3	1	1	0	N-Term(TMT6plex); S3(Phospho); K10(TMT6plex)
23110939	NP_687033.1	5684	PSMA3	proteasome subunit alpha type-3 isoform2	S243	1.6	1.2	1.2	1.5	1.3	1.5	1.5	1.5	1.4	1.2	1.2	1.3	1.2	1.4	1.4	1.2	1.3	1.3	1.3	1.2	ESLKEEDEsDDDNM	SLKEEDEsDDDNM	S9;	No	S(2): 0.0; S(9): 100.0	10	2	1	1	N-Term(TMT6plex); K4(TMT6plex); S9(Phospho); M14(Oxidation)
48255945	NP_001673.2	490	ATP2B1	plasma membrane calcium-transporting ATPase 1 isoform1b	S1178	1.9	1.5	1.3	1.6	1.3	1.4	1.4	1.4	1.6	1.4	1.3	1.5	1.1	1.3	1.3	1.3	1.4	1.3	1.3	1.4	NSsPPPsPNK	APTKRNSsPPPSPNK	S3;	No	S(2): 1.0; S(3): 99.0; S(7): 0.0	2	1	1	0	N-Term(TMT6plex); S3(Phospho); K10(TMT6plex)
32967311	NP_004422.2	1969	EPHA2	ephrin type-A receptor 2 precursor	S901	1.4	2.0	1.2	1.8	1.4	1.9	1.7	1.1	1.3	1.8	1.0	1.7	1.3	1.7	1.5	1.0	1.3	1.7	1.2	1.4	LPSTSGsEGVPFR	RLPSTSGsEGVPFRT	S7;	Yes	S(3): 0.0; T(4): 0.0; S(5): 0.0; S(7): 100.0	4	1	1	0	N-Term(TMT6plex); S7(Phospho)
39777594	NP_057438.3	28231	SLCO4A1	solute carrier organic anion transporter family member 4A1	S40	1.6	1.9	1.8	2.1	1.6	1.0	1.0	0.9	-	-	-	-	-	-	-	-	-	-	-	-	ASPGTPLsPGSLR	ASPGTPLsPGSLRSA	S8;	Yes	S(2): 0.0; T(5): 0.0; S(8): 100.0; S(11): 0.0	5	1	1	0	N-Term(TMT6plex); S8(Phospho)
157694511	NP_056473.2	26155	NOC2L	nucleolar complex protein 2 homolog	S56	1.4	1.5	1.4	1.5	1.4	1.3	1.3	1.5	1.5	1.6	1.4	1.3	1.5	1.5	1.3	1.4	1.5	1.5	1.4	1.4	sPDKPGGsPSASR	SPDKPGGsPSASRRK	S8;	Yes	S(1): 0.0; S(8): 100.0; S(10): 0.0; S(12): 0.0	6	1	1	0	N-Term(TMT6plex); K4(TMT6plex); S8(Phospho)
157694511	NP_056473.2	26155	NOC2L	nucleolar complex protein 2 homolog	S56	1.4	1.3	1.3	1.5	1.4	1.5	1.4	1.5	1.5	1.5	1.3	1.4	1.5	1.6	1.4	1.3	1.5	1.5	1.4	1.4	sPDKPGGSPSASR	EAREAARsPDKPGGS	S8;	Yes	S(1): 100.0; S(8): 0.0; S(10): 0.0; S(12): 0.0	3	1	1	0	N-Term(TMT6plex); S1(Phospho); K4(TMT6plex)
157694511	NP_056473.2	26155	NOC2L	nucleolar complex protein 2 homolog	S49; S56	1.3	1.4	1.2	1.2	2.0	1.5	1.4	1.8	1.3	1.5	1.3	1.1	2.1	1.6	1.4	1.6	1.7	1.6	1.3	1.4	EAARsPDKPGGsPSASR	EAREAARsPDKPGGS;SPDKPGGsPSASRRK	S5; S12;	Yes;Yes	S(5): 100.0; S(12): 98.1; S(14): 1.9; S(16): 0.0	6	1	1	1	N-Term(TMT6plex); S5(Phospho); K8(TMT6plex); S12(Phospho)
325652026	NP_001191785.1	79005	SCNM1	sodium channel modifier 1 isoform2	S148	2.4	1.3	1.0	1.8	0.9	1.0	0.8	1.2	-	-	-	-	-	-	-	-	-	-	-	-	EPEPAAGPQAEESATVSAPAPMsPTR	VSAPAPMsPTRRRAL	S23;	No	S(13): 0.0; T(15): 0.0; S(17): 0.0; S(23): 98.5; T(25): 1.5	3	2	1	0	N-Term(TMT6plex); M22(Oxidation); S23(Phospho)
118572613	NP_057417.3	23524	SRRM2	serine/arginine repetitive matrix protein 2	S1727	1.7	3.0	2.8	1.3	1.2	1.4	1.4	1.6	1.8	3.1	2.9	1.5	1.2	1.5	1.5	1.8	1.5	2.3	2.2	1.6	sPsVSSPEPAEK	TPPRHRRsPSVSSPE	S1;	Yes	S(1): 100.0; S(3): 0.0; S(5): 0.0; S(6): 0.0	2	1	1	0	N-Term(TMT6plex); S1(Phospho); K12(TMT6plex)
118572613	NP_057417.3	23524	SRRM2	serine/arginine repetitive matrix protein 2	S1727	1.2	1.4	1.4	1.6	1.7	3.0	2.8	1.3	1.2	1.5	1.5	1.8	1.8	3.1	2.9	1.5	1.5	2.3	2.2	1.6	sPSVSSPEPAEK	TPPRHRRsPSVSSPE	S1;	Yes	S(1): 99.1; S(3): 33.1; S(5): 33.9; S(6): 33.9	436	1	1	0	N-Term(TMT6plex); S1(Phospho); S3(Phospho); K12(TMT6plex)
31652242	NP_852469.1	6942	TCF20	transcription factor 20 isoform2	S1322	1.1	1.0	0.9	1.1	1.2	1.0	0.7	1.8	1.0	1.1	0.9	1.3	1.1	1.1	0.7	2.0	1.0	1.1	0.8	1.6	DLPsPDSR	DSSKDLPsPDSRNCP	S4;	Yes	S(4): 100.0; S(7): 0.0	14	2	1	0	N-Term(TMT6plex); S4(Phospho)
605052052	NP_001278268.1	79848	CSPP1	centrosome and spindle pole-associated protein 1 isoform2	S540	0.6	1.1	0.9	1.7	1.0	1.4	0.9	1.3	-	-	-	-	-	-	-	-	-	-	-	-	AQsPPVPAR	SSMSRAQsPPVPARK	S3;	No	S(3): 100.0	2	2	1	0	N-Term(TMT6plex); S3(Phospho)
56549117	NP_001005336.1	1759	DNM1	dynamin-1 isoform2	S774; S778	1.1	1.7	1.0	1.4	1.3	1.8	1.1	1.6	0.9	1.4	0.7	1.0	1.1	1.5	0.8	1.2	1.0	1.4	0.8	1.1	sPTSsPTPQR	SVPAGRRsPTSSPTP;GRRSPTSsPTPQRRA	S1; S5;	Yes;Yes	S(1): 99.0; T(3): 1.0; S(4): 1.0; S(5): 99.0; T(7): 0.0	2	4	1	0	N-Term(TMT6plex); S1(Phospho); S4(Phospho)
608788342	NP_001278327.1	387680	FAM21A	WASH complex subunit FAM21A isoform2	S333	1.0	1.4	0.8	1.6	1.1	1.2	1.3	1.3	1.0	1.2	0.8	1.8	1.2	1.0	1.3	1.4	1.1	1.1	1.1	1.6	TPsDDEEDNLFAPPK	KKERRTPsDDEEDNL	S3;	Yes	T(1): 1.6; S(3): 98.4	7	4	1	0	N-Term(TMT6plex); S3(Phospho); K15(TMT6plex)
148596949	NP_004732.2	9221	NOLC1	nucleolar and coiled-body phosphoprotein 1 isoform2	S538	1.0	1.6	1.4	1.7	0.9	1.3	1.4	1.2	0.8	1.3	1.1	1.3	0.7	1.0	1.1	0.9	0.8	1.2	1.1	1.1	GsPRPQAPK	EKLKGKGsPRPQAPK	S2;	Yes	S(2): 100.0	33	3	1	0	N-Term(TMT6plex); S2(Phospho); K9(TMT6plex)
56549117	NP_001005336.1	1759	DNM1	dynamin-1 isoform2	S774	1.3	1.2	1.5	1.5	1.3	1.4	1.3	1.4	1.1	1.0	1.1	1.1	1.1	1.2	0.9	1.1	1.1	1.1	1.0	1.1	RsPTSSPTPQR	SVPAGRRsPTSSPTP	S2;	Yes	S(2): 98.9; T(4): 0.0; S(5): 0.0; S(6): 1.1; T(8): 0.0	2	4	1	1	N-Term(TMT6plex); S2(Phospho)
24308115	NP_056464.1	26145	IRF2BP1	interferon regulatory factor 2-binding protein 1	S384	0.7	1.2	0.7	1.6	0.7	0.8	0.9	1.4	0.6	1.2	0.8	1.6	0.6	0.9	0.9	1.4	0.6	1.1	0.9	1.5	KAsPEPEGEAAGK	TPRRRKAsPEPEGEA	S3;	Yes	S(3): 100.0	1	1	1	1	N-Term(TMT6plex); K1(TMT6plex); S3(Phospho); K13(TMT6plex)
148233338	NP_001091679.1	1499	CTNNB1	catenin beta-1	S552	1.7	2.2	2.2	2.2	1.2	0.9	0.8	0.8	1.8	2.5	2.3	2.4	1.3	1.0	0.9	0.8	1.6	1.8	1.6	1.6	TsMGGTQQQFVEGVR	QDTQRRTsMGGTQQQ	S2;	Yes	T(1): 1.1; S(2): 98.9; T(6): 0.0	4	1	1	0	N-Term(TMT6plex); S2(Phospho); M3(Oxidation)
555943672	NP_001273158.1	51514	DTL	denticleless protein homolog isoform2	S408	1.9	2.1	1.5	1.6	2.4	2.6	1.9	1.3	1.7	2.3	1.5	1.7	2.1	2.8	1.9	1.4	1.9	2.5	1.7	1.5	sPSSQTPNSR	AENPSPRsPSSQTPN	S1;	No	S(1): 100.0; S(3): 0.0; S(4): 0.0; T(6): 0.0; S(9): 0.0	5	3	1	0	N-Term(TMT6plex); S1(Phospho)
21493024	NP_653080.1	9590	AKAP12	A-kinase anchor protein 12 isoform2	T1662	1.2	1.8	1.7	1.5	1.2	1.4	1.7	1.4	1.4	1.7	1.4	1.4	1.3	1.3	1.4	1.3	1.4	1.5	1.4	1.4	AItPQAQEELQK	SESDKAItPQAQEEL	T3;	No	T(3): 100.0	103	2	1	0	N-Term(TMT6plex); T3(Phospho); K12(TMT6plex)
50659095	NP_004719.2	9188	DDX21	nucleolar RNA helicase 2 isoform1	S71	0.8	0.7	1.0	1.6	0.8	0.7	1.0	1.4	0.7	0.5	0.7	1.3	0.7	0.6	0.7	1.1	0.7	0.6	0.7	1.2	KAEPSEVDMNsPK	PSEVDMNsPKSKKAK	S11;	Yes	S(5): 0.0; S(11): 100.0	23	1	1	1	N-Term(TMT6plex); K1(TMT6plex); M9(Oxidation); S11(Phospho); K13(TMT6plex)
262073007	NP_001159968.1	6566	SLC16A1	monocarboxylate transporter 1	S498	1.4	1.7	1.6	2.0	0.9	1.1	0.9	0.9	1.5	1.8	1.7	2.2	1.0	1.1	1.0	1.0	1.3	1.5	1.4	1.6	DTDGGPKEEEsPV	GGPKEEEsPV	S11;	Yes	T(2): 0.0; S(11): 100.0	3	1	1	1	N-Term(TMT6plex); K7(TMT6plex); S11(Phospho)
223555989	NP_057094.2	51101	ZC2HC1A	zinc finger C2HC domain-containing protein 1A	S179	0.7	1.0	0.9	1.4	0.7	1.0	0.8	1.5	0.8	1.0	0.9	1.3	0.7	1.0	0.9	1.4	0.7	1.0	0.9	1.3	SNsPGTASSGSSR	PALKKSNsPGTASSG	S3;	No	S(1): 0.0; S(3): 100.0; T(6): 0.0; S(8): 0.0; S(9): 0.0; S(11): 0.0; S(12): 0.0	3	1	1	0	N-Term(TMT6plex); S3(Phospho)
19923497	NP_061936.2	27436	EML4	echinoderm microtubule-associated protein-like 4 isoforma	S146	1.0	1.1	1.2	1.3	1.1	1.2	1.5	1.6	0.9	1.0	1.2	1.1	1.1	1.2	1.4	1.4	1.0	1.1	1.3	1.3	ASPsPQPSSQPLQIHR	PQIRASPsPQPSSQP	S4;	Yes	S(2): 1.3; S(4): 98.7; S(8): 0.0; S(9): 0.0	10	1	1	0	N-Term(TMT6plex); S4(Phospho)
22035679	NP_112739.2	51742	ARID4B	AT-rich interactive domain-containing protein 4B isoform2	S704; T707	1.0	2.5	1.8	2.5	1.0	1.5	1.2	0.4	1.1	2.5	1.8	2.8	1.0	1.5	1.2	0.5	1.1	2.0	1.5	1.6	DIEVLsEDtDYEEDEVTK	RKDIEVLsEDTDYEE;IEVLSEDtDYEEDEV	S6; T9;	No;Yes	S(6): 100.0; T(9): 100.0; Y(11): 0.0; T(17): 0.0	4	2	1	0	N-Term(TMT6plex); S6(Phospho); T9(Phospho); K18(TMT6plex)
410444379	NP_001258537.1	3927	LASP1	LIM and SH3 domain protein 1 isoformb	T48	0.6	1.1	1.0	1.4	0.8	1.5	1.4	1.5	0.7	1.0	0.8	1.0	0.8	1.3	1.1	1.2	0.7	1.1	1.0	1.1	GFSVVADtPELQR	GFSVVADtPELQRIK	T8;	No	S(3): 0.0; T(8): 100.0	4	2	1	0	N-Term(TMT6plex); T8(Phospho)
195546902	NP_001124297.1	6829	SUPT5H	transcription elongation factor SPT5 isoformb	S662	1.0	0.9	0.6	1.1	0.7	0.7	0.6	1.8	1.1	0.9	0.6	1.1	0.8	0.6	0.6	1.7	0.9	0.8	0.6	1.4	DVTNFTVGGFAPMsPR	VGGFAPMsPRISSPM	S14;	No	T(3): 0.0; T(6): 0.0; S(14): 100.0	2	2	1	0	N-Term(TMT6plex); M13(Oxidation); S14(Phospho)
33598946	NP_877963.1	5335	PLCG1	1-phosphatidylinositol 4,5-bisphosphate phosphodiesterase gamma-1 isoformb	S1248	1.4	1.5	1.4	1.4	1.5	1.5	1.4	1.5	1.4	1.5	1.5	1.2	1.4	1.5	1.5	1.4	1.4	1.5	1.5	1.3	EGsFESR	HGRAREGsFESRYQQ	S3;	Yes	S(3): 100.0; S(6): 0.0	2	2	1	0	N-Term(TMT6plex); S3(Phospho)
38788193	NP_005217.2	1903	S1PR3	sphingosine 1-phosphate receptor 3	S326	0.9	1.3	0.9	1.5	1.1	1.2	0.9	1.4	-	-	-	-	-	-	-	-	-	-	-	-	AsPIQPALDPSR	RGRGARAsPIQPALD	S2;	Yes	S(2): 100.0; S(11): 0.0	2	1	1	0	N-Term(TMT6plex); S2(Phospho)
47519639	NP_002366.2	4134	MAP4	microtubule-associated protein 4 isoform1	S1151	1.3	1.5	1.9	2.0	0.9	0.9	1.2	0.9	1.4	1.4	2.0	2.4	1.0	0.9	1.2	1.1	1.2	1.2	1.6	1.8	EAQTLDSQIQETsI	DSQIQETsI_	S13;	Yes	T(4): 0.0; S(7): 0.0; T(12): 0.7; S(13): 99.3	47	1	1	0	N-Term(TMT6plex); S13(Phospho)
414144880	NP_001258671.1	1452	CSNK1A1	casein kinase I isoform alpha isoform4	T260	0.9	2.0	2.1	1.6	1.0	1.6	1.5	1.3	0.9	2.1	2.1	1.5	1.1	1.6	1.5	1.2	1.0	1.8	1.8	1.4	AAQQAASSSGQGQQAQtPTGK	GQGQQAQtPTGKQTD	T17;	No	S(7): 0.0; S(8): 0.0; S(9): 0.0; T(17): 100.0; T(19): 0.0	3	3	1	0	N-Term(TMT6plex); T17(Phospho); K21(TMT6plex)
38788333	NP_057662.3	51773	RSF1	remodeling and spacing factor 1	T1305	1.8	1.9	1.5	2.1	1.0	0.6	1.0	0.8	1.7	1.7	1.4	2.3	0.9	0.5	0.9	0.9	1.3	1.1	1.2	1.6	IEtDEESCDNAHGDANQPAR	KRLHRIEtDEEESCD	T3;	Yes	T(3): 100.0; S(8): 0.0	15	1	1	0	N-Term(TMT6plex); T3(Phospho); C9(Carbamidomethyl)
15529978	NP_219482.1	90441	ZNF622	zinc finger protein 622	S143	1.4	1.2	1.2	1.7	0.7	0.9	0.8	1.2	1.2	1.0	1.0	1.3	0.6	0.8	0.7	0.9	0.9	0.9	0.9	1.1	AQPSMsPK	IKAQPSMsPKKAPPA	S6;	Yes	S(4): 0.0; S(6): 100.0	1	1	1	0	N-Term(TMT6plex); M5(Oxidation); S6(Phospho); K8(TMT6plex)
4506597	NP_000967.1	6136	RPL12	60S ribosomal protein L12	S38	1.8	1.1	0.8	1.6	1.1	0.9	0.7	1.3	1.9	1.1	0.8	1.7	1.2	1.0	0.7	1.4	1.5	1.1	0.8	1.5	IGPLGLsPK	KIGPLGLsPKKVGDD	S7;	Yes	S(7): 100.0	2	1	1	0	N-Term(TMT6plex); S7(Phospho); K9(TMT6plex)
211971074	NP_001130034.1	11217	AKAP2	A-kinase anchor protein 2 isoform2	S152	1.0	0.9	0.6	1.1	1.6	2.1	2.3	1.8	-	-	-	-	-	-	-	-	-	-	-	-	DALGDSLQVPVsPSSTTSSR	DSLQVPVsPSSTTSS	S12;	Yes	S(6): 0.0; S(12): 97.5; S(14): 2.2; S(15): 0.1; T(16): 0.1; T(17): 0.1; S(18): 0.1; S(19): 0.1	6	5	1	0	N-Term(TMT6plex); S12(Phospho)
50658063	NP_001002800.1	10051	SMC4	structural maintenance of chromosomes protein 4 isoform1	S22	1.4	1.1	1.2	1.2	0.8	0.9	1.3	1.7	1.5	1.1	1.2	1.2	0.8	0.9	1.4	1.8	1.1	1.0	1.3	1.5	EEGPPPPsPDGASSDAEPEPPSGR	EEGPPPPsPDGASSD	S8;	Yes	S(8): 100.0; S(13): 0.0; S(14): 0.0; S(22): 0.0	18	2	1	0	N-Term(TMT6plex); S8(Phospho)
50658063	NP_001002800.1	10051	SMC4	structural maintenance of chromosomes protein 4 isoform1	S22	0.8	0.9	1.3	1.7	1.4	1.1	1.2	1.2	0.8	0.9	1.4	1.8	1.5	1.1	1.2	1.2	1.1	1.0	1.3	1.5	EEGPPPPSPDGASsDAEPEPPSGR	PSPDGASsDAEPEPP	S8;	Yes	S(8): 0.0; S(13): 1.1; S(14): 98.9; S(22): 0.0	7	2	1	0	N-Term(TMT6plex); S14(Phospho)
15011974	NP_004714.2	9181	ARHGEF2	rho guanine nucleotide exchange factor 2 isoform3	S147	1.5	1.2	1.3	1.2	1.7	1.9	1.5	1.7	1.2	0.9	1.1	1.0	1.4	1.5	1.3	1.5	1.3	1.2	1.2	1.3	ILSQsTDSLNMR	LRRILSQsTDSLNMR	S5;	No	S(3): 1.1; S(5): 97.7; T(6): 1.1; S(8): [illegible]	5	3	1	0	N-Term(TMT6plex); S5(Phospho); M11(Oxidation)
55749804	NP_079172.2	79956	ERMP1	endoplasmic reticulum metallopeptidase 1	S53	1.3	1.5	2.0	1.5	1.1	1.2	1.8	1.4	1.2	1.5	1.7	1.3	1.1	1.1	1.5	1.2	1.2	1.3	1.6	1.2	sPGGSGGASR	GGRTRKRsPGGSGGA	S1;	Yes	S(1): 100.0; S(5): 0.0; S(9): 0.0	1	1	1	0	N-Term(TMT6plex); S1(Phospho)
257196142	NP_001136336.2	9780	PIEZO1	piezo-type mechanosensitive ion channel component 1	S1391	1.4	1.3	1.0	1.1	1.4	1.5	1.3	1.7	1.5	1.4	1.1	1.1	1.5	1.6	1.4	1.7	1.5	1.5	1.3	1.4	DPGLEPGPDsPGGSSPPR	GLEPGPDsPGGSSPP	S10;	Yes	S(10): 97.1; S(14): 1.5; S(15): 1.5	3	1	1	0	N-Term(TMT6plex); S10(Phospho)
604723372	NP_878315.2	91179	SCARF2	scavenger receptor class F member 2 isoform2 precursor	T836	1.2	1.2	1.2	1.5	1.6	1.7	1.5	1.4	-	-	-	-	-	-	-	-	-	-	-	-	AATDLPAPEtPR	TDLPAPEtPRKKTPI	T10;	No	T(3): 0.0; T(10): 100.0	7	2	1	0	N-Term(TMT6plex); T10(Phospho)
42542379	NP_005830.2	10250	SRRM1	serine/arginine repetitive matrix protein 1	S389	1.3	1.1	1.3	1.7	1.0	0.9	0.6	1.1	1.5	1.2	1.4	2.0	1.2	1.0	0.6	1.3	1.4	1.1	1.0	1.6	RLsPSASPPR	PRKTRRLsPSASPPR	S3;	Yes	S(3): 100.0; S(5): 50.0; S(7): 50.0	229	1	1	1	N-Term(TMT6plex); S3(Phospho); S7(Phospho)
50659095	NP_004719.2	9188	DDX21	nucleolar RNA helicase 2 isoform1	S121	1.7	1.5	2.0	1.5	1.6	1.5	1.9	1.3	1.5	1.2	1.5	1.3	1.4	1.2	1.4	1.1	1.5	1.2	1.5	1.2	NEEPsEEEIDAPKPK	VTKNEEPsEEEIDAP	S5;	Yes	S(5): 100.0	23	2	1	0	N-Term(TMT6plex); S5(Phospho); K13(TMT6plex); K15(TMT6plex)
22208971	NP_665908.1	3159	HMGA1	high mobility group protein HMG-I/HMG-Y isoforma	S102; S103	0.8	0.7	0.7	0.8	1.7	1.7	2.1	2.1	0.8	0.6	0.6	0.8	1.8	1.4	1.9	2.1	1.3	1.0	1.2	1.5	KLEKEEEEGIsQESSEEEQ	KEEEEGIsQESSEEE	S14; S15;	Yes	S(11): 95.0; S(14): 2.5; S(15): 2.5	1	2	1	2	N-Term(TMT6plex); K1(TMT6plex); K4(TMT6plex); S14(Phospho)
22208971	NP_665908.1	3159	HMGA1	high mobility group protein HMG-I/HMG-Y isoforma	S102; S103	1.7	1.7	2.1	2.1	0.8	0.7	0.7	0.8	1.8	1.4	1.9	2.1	0.8	0.6	0.6	0.8	1.3	1.0	1.2	1.5	KLEKEEEEGISQEssEEEQ	EEGISQEsSEEEQ_;EGISQESsEEEQ	S14; S15;	Yes;Yes	S(11): 0.0; S(14): 100.0; S(15): 100.0	39	2	1	2	N-Term(TMT6plex); K1(TMT6plex); K4(TMT6plex); S14(Phospho); S15(Phospho)
19920317	NP_006816.2	10970	CKAP4	cytoskeleton-associated protein 4	S17	1.2	1.2	1.2	1.5	0.8	1.0	1.1	1.4	1.0	1.0	1.0	1.4	0.7	0.8	0.9	1.3	0.8	0.9	0.9	1.3	GGHGAAsPSEK	KGGHGAAsPSEKGAH	S7;	Yes	S(7): 100.0; S(9): 0.0	11	1	1	0	N-Term(TMT6plex); S7(Phospho); K11(TMT6plex)
206597509	NP_001128661.1	3267	AGFG1	arf-GAP domain and FG repeat-containing protein 1 isoform4	T177; S181	1.4	1.2	1.7	1.3	1.1	1.5	1.1	1.5	1.2	1.1	1.8	1.1	1.0	1.4	1.1	1.2	1.1	1.3	1.5	1.1	GtPSQsPVVGR	TLHLNKGtPSQSPVV;NKGTPSQsPVVGRSQ	T2; S6;	Yes;Yes	T(2): 100.0; S(4): 1.2; S(6): 98.8	3	4	1	0	N-Term(TMT6plex); T2(Phospho); S6(Phospho)
206597509	NP_001128661.1	3267	AGFG1	arf-GAP domain and FG repeat-containing protein 1 isoform4	T177; S181	1.1	1.5	1.1	1.5	1.4	1.2	1.7	1.3	1.0	1.4	1.1	1.2	1.2	1.1	1.8	1.1	1.1	1.3	1.5	1.1	GTPsQSPVVGR	HLNKGTPsQSPVVGR	T2; S6;	Yes	T(2): 0.0; S(4): 99.3; S(6): 0.7	6	4	1	0	N-Term(TMT6plex); S6(Phospho)
8400715	NP_058525.1	4137	MAPT	microtubule-associated protein tau isoform4	S144	0.5	1.1	0.9	0.9	1.0	1.5	1.2	2.0	0.4	1.4	0.9	1.0	0.9	1.9	1.3	2.3	0.7	1.6	1.1	1.7	SGYSSPGsPGTPGSR	SGYSSPGsPGTPGSR	S8;	Yes	S(1): 0.0; Y(3): 0.0; S(4): 0.0; S(5): 0.0; S(8): 100.0; T(11): 0.0; S(14): 0.0	2	8	1	0	N-Term(TMT6plex); S8(Phospho)
38146107	NP_075388.2	65117	RSRC2	arginine/serine-rich coiled-coil protein 2	S30	1.3	1.4	1.6	1.6	1.0	1.2	1.2	1.2	-	-	-	-	-	-	-	-	-	-	-	-	EQSEVsVSPR	KKEQSEVsVSPRASK	S6;	Yes	S(3): 0.0; S(6): 100.0; S(8): 0.0	3	1	1	0	N-Term(TMT6plex); S8(Phospho)
14249678	NP_116294.1	84991	RBM17	splicing factor 45	S155	1.1	1.1	1.2	1.2	0.9	1.5	1.2	1.6	1.2	1.2	1.2	1.2	1.0	1.5	1.3	1.6	1.1	1.4	1.3	1.4	RPDPDsDEDEDYER	ARRPDPDsDEDEDYE	S6;	Yes	S(6): 100.0; Y(12): 0.0	5	1	1	0	N-Term(TMT6plex); S6(Phospho)
18379332	NP_567823.1	51322	WAC	WW domain-containing adapter protein with coiled-coil isoform3	S408	1.2	1.2	1.1	1.3	2.1	1.3	1.4	1.5	1.1	1.1	1.0	1.1	1.9	1.2	1.3	1.3	1.5	1.2	1.1	1.2	QQGHEPVsPR	QQGHEPVsPRSLQRS	S8;	No	S(8): 100.0	5	2	1	0	N-Term(TMT6plex); S8(Phospho)
10092601	NP_003751.2	8672	EIF4G3	eukaryotic translation initiation factor 4 gamma 3	S1582	1.5	1.6	1.4	1.5	1.2	1.3	1.2	1.3	1.4	1.5	1.3	1.4	1.1	1.2	1.1	1.2	1.2	1.3	1.2	1.3	EAEEEsEDN	LREAEEEsEDN	S6;	Yes	S(6): 100.0	8	3	1	0	N-Term(TMT6plex); S6(Phospho)
29826282	NP_817092.1	5496	PPM1G	protein phosphatase 1G	S6	1.1	2.0	0.9	1.0	1.5	2.6	1.5	1.8	1.2	2.1	1.0	1.0	1.6	2.7	1.7	1.8	1.4	2.4	1.3	1.4	GAYLsQPNTVK	MGAYLsQPNTVKC	S5;	No	Y(3): 0.0; S(5): 100.0; T(9): 0.0	7	1	1	0	N-Term(TMT6plex); S5(Phospho); K11(TMT6plex)
503774357	NP_001265139.1	9972	NUP153	nuclear pore complex protein Nup153 isoform3	S522	2.3	1.8	1.3	1.7	1.4	1.1	1.1	1.1	2.0	1.7	1.1	1.5	1.2	1.1	1.0	1.0	1.6	1.4	1.0	1.2	VQMTSPSSTGsPMFK	TSPSSTGsPMFKFSS	S11;	Yes	T(4): 0.0; S(5): 0.0; S(7): 0.0; S(8): 0.0; T(9): 0.0; S(11): 100.0	8	3	1	0	N-Term(TMT6plex); M3(Oxidation); S11(Phospho); M13(Oxidation); K15(TMT6plex)
194440660	NP_008958.2	11073	TOPBP1	DNA topoisomerase 2-binding protein 1	S888	1.2	1.0	1.4	1.8	2.6	1.7	2.3	1.0	1.3	1.1	1.5	2.0	2.9	1.8	2.5	1.1	2.1	1.4	2.0	1.5	NAVALSAsPQLK	NAVALSAsPQLKEAQ	S8;	Yes	S(6): 0.0; S(8): 100.0	5	1	1	0	N-Term(TMT6plex); S8(Phospho); K12(TMT6plex)
28372563	NP_777602.1	283431	GAS2L3	GAS2-like protein 3	S307	0.8	1.1	1.0	1.0	1.0	1.2	1.0	1.8	-	-	-	-	-	-	-	-	-	-	-	-	GVSNFSVPDsPAR	SNFSVPDsPARTPQP	S10;	Yes	S(3): 0.0; S(6): 0.0; S(10): 100.0	6	1	1	0	N-Term(TMT6plex); S10(Phospho)
359843219	NP_001241646.1	1616	DAXX	death domain-associated protein 6 isoformc	S593	1.0	0.9	0.6	1.1	1.5	0.9	0.6	1.7	1.1	0.9	0.6	1.3	1.5	0.9	0.6	1.9	1.3	0.9	0.6	1.6	ICTLPsPPSPLASLAPVADSSTR	KKICTLPsPPSPLAS	S6;	No	T(3): 2.3; S(6): 95.4; S(9): 2.3; S(13): 0.0; S(20): 0.0; S(21): 0.0; T(22): 0.0	3	3	1	0	N-Term(TMT6plex); C2(Carbamidomethyl); S9(Phospho)
11067749	NP_031397.1	8019	BRD3	bromodomain-containing protein 3	S563	1.0	1.0	1.9	1.7	2.4	2.3	2.5	1.1	1.2	1.1	2.1	1.7	2.7	2.5	2.8	1.1	1.9	1.8	2.5	1.4	QASASYDsEEEEEGLPMSYDEK	QASASYDsEEEEEGL	S8;	Yes	S(3): 0.0; S(5): 0.0; Y(6): 1.6; S(8): 98.4; S(18): 0.0; Y(19): 0.0	2	1	1	0	N-Term(TMT6plex); S8(Phospho); M17(Oxidation); K22(TMT6plex)
386869506	NP_001247436.1	6188	RPS3	40S ribosomal protein S3 isoform3	T95	1.1	1.1	1.3	1.3	1.6	1.7	1.4	1.5	1.2	1.2	1.5	1.4	1.7	1.7	1.6	1.6	1.4	1.5	1.5	1.5	DEILPTtPISEQK	KDEILPTtPISEQKG	T7;	No	T(6): 0.0; T(7): 100.0; S(10): 0.0	10	3	1	0	N-Term(TMT6plex); T7(Phospho); K13(TMT6plex)
543583787	NP_001269472.1	580	BARD1	BRCA1-associated RING domain protein 1 isoform2	S167	1.5	0.9	1.2	1.4	1.7	1.6	1.3	1.4	-	-	-	-	-	-	-	-	-	-	-	-	DASAQQDSYEFVSPsPPADVSER	SYEFVSPsPPADVSE	S15;	No	S(3): 0.0; S(8): 0.0; Y(9): 0.0; S(13): 0.0; S(15): 100.0; S(21): 0.0	4	2	1	0	N-Term(TMT6plex); S15(Phospho)
153945728	NP_005900.2	4131	MAP1B	microtubule-associated protein 1B	T1949	1.0	1.3	1.2	1.0	1.6	1.5	1.8	1.8	0.8	1.0	1.0	1.0	1.3	1.1	1.5	1.9	1.0	1.0	1.2	1.5	tPEEGGYSYDISEK	IIEKTTRtPEEGGYS	T1;	Yes	T(1): 100.0; Y(7): 0.0; S(8): 0.0; Y(9): 0.0; S(12): 0.0	49	1	1	0	N-Term(TMT6plex); T1(Phospho); K14(TMT6plex)
37595750	NP_002287.2	3930	LBR	lamin-B receptor	S99	1.3	1.7	1.7	1.5	1.4	1.8	1.5	1.3	1.2	1.6	1.4	1.4	1.3	1.6	1.2	1.2	1.3	1.6	1.3	1.3	SAsASHQADIK	KSARRSAsASHQADI	S3;	Yes	S(1): 0.0; S(3): 100.0; S(5): 0.0	8	1	1	0	N-Term(TMT6plex); S3(Phospho); K11(TMT6plex)
23510356	NP_113668.2	83732	RIOK1	serine/threonine-protein kinase RIO1 isoform1	S22	0.8	1.3	1.1	1.0	1.0	1.2	1.0	1.7	0.8	1.3	1.2	1.1	1.0	1.2	1.1	1.8	0.9	1.2	1.1	1.4	VVPGQFDDADSsDSENR	QFDDADSsDSENRDL	S12;	Yes	S(11): 0.0; S(12): 100.0; S(14): 0.0	4	1	1	0	N-Term(TMT6plex); S12(Phospho)
103472005	NP_002408.3	4288	MKI67	antigen KI-67 isoform1	S648	0.5	0.8	1.0	1.3	0.5	0.9	1.0	1.5	0.5	0.8	1.0	1.4	0.5	0.9	1.0	1.6	0.5	0.9	1.0	1.5	sGASEANLIVAK	MICSKRRsGASEANL	S1;	Yes	S(1): 100.0; S(4): 0.0	118	2	1	0	N-Term(TMT6plex); S1(Phospho); K12(TMT6plex)
157649073	NP_001099000.1	10487	CAP1	adenylyl cyclase-associated protein 1	S34	0.8	1.0	0.8	1.4	0.8	1.0	0.9	1.4	0.8	0.9	0.8	1.3	0.8	1.0	0.8	1.3	0.8	0.9	0.8	1.3	GYADsPSK	MHRGYADsPSKAGAA	S5;	Yes	Y(2): 0.0; S(5): 100.0; S(7): 0.0	15	1	1	0	N-Term(TMT6plex); S5(Phospho); K8(TMT6plex)
539846358	NP_001269347.1	10735	STAG2	cohesin subunit SA-2 isoformb	S1061; S1064	1.2	1.6	0.9	1.6	1.2	2.0	1.4	1.2	1.2	1.8	1.0	1.9	1.2	2.3	1.6	1.3	1.2	2.0	1.3	1.6	NSLLAGGDDDTMSVIsGIsSR	DDTMSVIsGISSRGS;MSVISGIsSRGSTVR	S16; S19;	Yes;Yes	S(2): 0.0; T(11): 0.0; S(13): 0.0; S(16): 100.0; S(19): 97.9; S(20): 2.2	2	2	1	0	N-Term(TMT6plex); M12(Oxidation); S16(Phospho); S19(Phospho)
311893345	NP_001185792.1	8904	CPNE1	copine-1 isoformc	S55	1.0	0.6	0.9	1.5	0.8	0.6	0.9	1.3	1.0	0.7	1.1	1.4	0.8	0.7	1.1	1.2	0.9	0.7	1.1	1.3	NCSsPEFSK	ERVRNCSsPEFSKTL	S4;	Yes	S(3): 0.8; S(4): 99.2; S(8): 0.0	2	3	1	0	N-Term(TMT6plex); C2(Carbamidomethyl); S4(Phospho); K9(TMT6plex)
289577131	NP_001166183.1	6670	SP3	transcription factor Sp3 isoform3	S70	1.6	1.0	0.6	1.6	1.0	1.0	0.8	1.1	2.2	1.3	0.9	2.6	1.4	1.3	1.1	1.8	1.8	1.3	1.0	2.2	IGPPsPGDDEEEAAAAAGAPAAAGATGDLASAQLGGAPNR	CSKIGPPsPGDDEEE	S5;	No	S(5): 100.0; T(26): 0.0; S(31): 0.0	2	2	1	0	N-Term(TMT6plex); S5(Phospho)
41872339	NP_958846.1	51535	PPHLN1	periphilin-1 isoform5	S157	0.9	1.0	1.2	1.3	0.9	1.1	1.4	1.5	1.0	0.9	1.1	1.2	1.0	1.0	1.2	1.3	1.0	1.0	1.1	1.2	DTsPSSGSAVSSSK	LKTSRDTsPSSGSAV	S3;	No	T(2): 1.0; S(3): 99.0; S(5): 0.0; S(6): 0.0; S(8): 0.0; S(11): 0.0; S(12): 0.0; S(13): 0.0	8	8	1	0	N-Term(TMT6plex); S3(Phospho); K14(TMT6plex)
604723372	NP_878315.2	91179	SCARF2	scavenger receptor class F member 2 isoform2 precursor	S713	1.0	1.0	0.9	1.1	1.2	1.5	1.0	1.6	-	-	-	-	-	-	-	-	-	-	-	-	SAHTVEHGsPR	AHTVEHGsPRTRDPT	S9;	No	S(1): 0.0; T(4): 0.0; S(9): 100.0	7	2	1	0	N-Term(TMT6plex); S9(Phospho)
20143480	NP_003809.1	8760	CDS2	phosphatidate cytidylyltransferase 2	S33	1.3	1.3	1.3	1.2	1.4	1.7	1.6	1.6	1.3	1.3	1.3	1.2	1.3	1.7	1.6	1.7	1.3	1.5	1.5	1.5	VDGETAsDSESR	KVDGETAsDSESRAE	S7;	Yes	T(5): 0.0; S(7): 100.0; S(9): 0.0; S(11): 0.0	6	1	1	0	N-Term(TMT6plex); S7(Phospho)
19923796	NP_057022.2	51068	NMD3	60S ribosomal export protein NMD3	T433	1.4	1.3	1.8	1.3	1.8	1.3	2.8	1.5	1.2	1.2	1.6	1.2	1.6	1.2	2.5	1.3	1.4	1.2	2.1	1.3	ENMDtDDER	RERENMDtDDERQYQ	T5;	Yes	T(5): 100.0	3	1	1	0	N-Term(TMT6plex); M3(Oxidation); T5(Phospho)
118344456	NP_057085.2	51096	UTP18	U3 small nucleolar RNA-associated protein 18 homolog	S121; S124	1.2	1.3	1.3	1.2	1.3	1.2	1.3	1.5	1.2	1.3	1.4	1.2	1.4	1.3	1.4	1.5	1.3	1.3	1.4	1.3	VQEHEDsGDsEVENEAK	RVQEHEDsGDSEVEN;EHEDSGDsEVENEAK	S7; S10;	Yes;Yes	S(7): 100.0; S(10): 100.0	6	1	1	0	N-Term(TMT6plex); S7(Phospho); S10(Phospho); K17(TMT6plex)
4557515	NP_000098.1	1643	DDB2	DNA damage-binding protein 2	S24	1.0	1.2	1.0	1.1	1.2	1.9	1.4	1.7	-	-	-	-	-	-	-	-	-	-	-	-	sRSPLELEPEAK	LRPRNKRsRSPLELE	S1;	Yes	S(1): 99.3; S(3): 0.7	1	1	1	1	N-Term(TMT6plex); S1(Phospho); K12(TMT6plex)
23397666	NP_056292.1	25942	SIN3A	paired amphipathic helix protein Sin3a	S832	1.2	1.7	1.7	1.5	1.0	1.4	1.4	1.2	1.2	1.8	1.8	1.6	1.1	1.5	1.5	1.3	1.2	1.7	1.7	1.5	GDLsDVEEEEEEEMDVDEATGAVK	FAQRGDLsDVEEEEE	S4;	Yes	S(4): 100.0; T(20): 0.0	3	1	1	0	N-Term(TMT6plex); S4(Phospho); M14(Oxidation); K24(TMT6plex)
556503401	NP_001273294.1	55700	MAP7D1	MAP7 domain-containing protein 1 isoform2	S796	1.4	1.5	1.4	1.5	1.4	1.5	1.0	1.2	1.4	1.2	1.2	1.4	1.4	1.2	0.9	1.2	1.4	1.2	1.1	1.3	AVVQsPQVTEVL	LKKAVVQsPQVTEVL	S5;	No	S(5): 100.0; T(9): 0.0	27	3	1	0	N-Term(TMT6plex); S5(Phospho)
61743954	NP_001611.1	79026	AHNAK	neuroblast differentiation-associated protein AHNAK isoform1	T2845	0.8	1.3	1.8	1.6	1.0	0.9	0.6	1.1	0.9	1.3	1.8	1.9	1.2	0.9	0.6	1.3	1.1	1.1	1.2	1.6	ISMPDIDLNLtGPK	PDIDLNLtGPKIKGD	T11;	Yes	S(2): 0.0; T(11): 100.0	79	1	1	0	N-Term(TMT6plex); M3(Oxidation); T11(Phospho); K14(TMT6plex)
70610136	NP_003622.2	8505	PARG	poly(ADP-ribose) glycohydrolase	S291	1.8	2.0	2.5	2.1	0.6	0.9	2.0	0.6	1.1	1.9	2.1	2.0	0.4	0.8	1.7	0.6	0.8	1.4	1.9	1.3	QESCLGNsPPFEK	QESCLGNsPPFEKES	S8;	Yes	S(3): 0.0; S(8): 100.0	4	1	1	0	N-Term(TMT6plex); C4(Carbamidomethyl); S8(Phospho); K13(TMT6plex)
20270186	NP_004233.1	9324	HMGN3	high mobility group nucleosome-binding domain-containing protein 3 isoform HMGN3a	S93	1.2	1.6	1.3	1.3	1.2	1.4	1.4	1.5	1.4	1.8	1.6	1.4	1.4	1.5	1.7	1.5	1.4	1.6	1.7	1.4	AEEAQKTEsVDNEGE	EEAQKTEsVDNEGE_	S9;	Yes	T(7): 0.0; S(9): 100.0	13	1	1	1	N-Term(TMT6plex); K6(TMT6plex); S9(Phospho)
34485722	NP_002258.2	3839	KPNA3	importin subunit alpha-4	S60	1.6	1.3	2.0	1.7	0.9	0.8	0.9	1.1	1.6	1.3	1.8	1.6	0.9	0.8	0.8	1.0	1.2	1.1	1.3	1.3	NVPQEESLEDsDVDADFK	QEESLEDsDVDADFK	S11;	Yes	S(7): 0.0; S(11): 100.0	4	1	1	0	N-Term(TMT6plex); S11(Phospho); K18(TMT6plex)
429535832	NP_005643.2	7014	TERF2	telomeric repeat-binding factor 2	S365	1.1	1.1	0.8	1.2	1.0	0.9	0.8	1.5	1.2	1.1	0.8	1.2	1.0	0.9	0.8	1.5	1.1	1.0	0.8	1.3	DLVLPTQALPAsPALK	PTQALPAsPALKNKR	S12;	Yes	T(6): 0.0; S(12): 100.0	4	1	1	0	N-Term(TMT6plex); S12(Phospho); K16(TMT6plex)
13994322	NP_114143.1	83874	TBC1D10A	TBC1 domain family member 10A isoform2	S18	1.1	1.3	1.1	1.4	1.0	1.1	0.9	1.3	0.9	1.1	1.0	1.3	0.8	0.9	0.8	1.2	0.9	1.0	0.9	1.2	APAAGEsLSGTR	RAPAAGEsLSGTRES	S7;	Yes	S(7): 100.0; S(9): 0.0; T(11): 0.0	1	2	1	0	N-Term(TMT6plex); S7(Phospho)
19923359	NP_006244.2	5564	PRKAB1	5'-AMP-activated protein kinase subunit beta-1	S24	1.3	1.2	1.1	1.2	1.4	1.3	1.2	1.5	1.2	1.1	1.1	1.0	1.3	1.3	1.2	1.3	1.2	1.2	1.1	1.2	RDsSGGTK	HKTPRRDsSGGTKDG	S3;	Yes	S(3): 100.0; S(4): 0.0; T(7): 0.0	2	1	1	1	N-Term(TMT6plex); S3(Phospho); K8(TMT6plex)
150010552	NP_056073.1	23307	FKBP15	FK506-binding protein 15	S356	1.7	1.7	1.5	1.8	0.8	1.3	1.5	0.9	1.9	1.9	1.7	2.1	0.9	1.4	1.7	1.1	1.4	1.6	1.7	1.6	SNSLSEQLAINTsPDAVK	EQLAINTsPDAVKAK	S13;	Yes	S(1): 0.0; S(3): 0.0; S(5): 0.0; T(12): 1.4; S(13): 98.6	9	1	1	0	N-Term(TMT6plex); S13(Phospho); K18(TMT6plex)
21735566	NP_663306.1	6885	MAP3K7	mitogen-activated protein kinase kinase kinase 7 isoformD	S412	0.7	1.1	0.8	0.8	1.0	2.3	3.8	2.0	0.7	1.0	0.7	0.7	1.0	2.1	3.6	1.8	0.9	1.6	2.2	1.3	sIQDLTVTGTEPGQVSSR	NGQPRRRsIQDLTVT	S1;	No	S(1): 100.0; T(6): 0.0; T(8): 0.0; T(10): 0.0; S(16): 0.0; S(17): 0.0	3	4	1	0	N-Term(TMT6plex); S1(Phospho)
150378533	NP_003461.2	7874	USP7	ubiquitin carboxyl-terminal hydrolase 7 isoform1	S18	1.0	1.1	0.8	0.7	2.6	2.4	1.7	2.0	1.0	1.1	0.8	0.7	2.6	2.4	1.7	2.0	1.8	1.7	1.3	1.4	AGEQQLsEPEDMEMEAGDTDDPPR	KAGEQQLsEPEDMEM	S7;	Yes	S(7): 100.0; T(19): 0.0	10	1	1	0	N-Term(TMT6plex); S7(Phospho); M12(Oxidation); M14(Oxidation)
124494240	NP_203693.3	4641	MYO1C	unconventional myosin-Ic isoformc	S373	1.7	1.7	1.6	1.4	1.5	1.8	1.5	1.3	1.6	1.5	1.4	1.4	1.4	1.6	1.3	1.3	1.5	1.6	1.3	1.4	DVEsPSWR	LASKDVEsPSWRSTT	S4;	No	S(4): 99.3; S(6): 0.7	2	3	1	0	N-Term(TMT6plex); S4(Phospho)
32698730	NP_065823.1	57532	NUFIP2	nuclear fragile X mental retardation-interacting protein 2	S212	0.7	1.1	1.1	1.1	0.9	1.3	1.5	1.6	0.7	1.1	1.0	0.9	0.9	1.3	1.4	1.3	0.8	1.2	1.2	1.1	GADNDGsGSESGYTTPK	KGADNDGsGSESGYT	S7;	Yes	S(7): 100.0; S(9): 0.0; S(11): 0.0; Y(13): 0.0; T(14): 0.0; T(15): 0.0	2	1	1	0	N-Term(TMT6plex); S7(Phospho); K17(TMT6plex)
4507447	NP_003214.1	7023	TFAP4	transcription factor AP-4	S124	0.5	1.1	1.3	2.0	0.8	0.5	0.9	0.7	0.5	0.9	1.1	1.6	1.0	0.4	0.8	0.5	0.7	0.7	0.9	1.1	FIQELSGSsPK	IQELSGSsPKRRRAE	S9;	Yes	S(6): 0.0; S(8): 0.0; S(9): 100.0	1	1	1	0	N-Term(TMT6plex); S9(Phospho); K11(TMT6plex)
190358534	NP_064544.2	56916	SMARCAD1	SWI/SNF-related matrix-associated actin-dependent regulator of chromatin subfamily A containing DEAD/H box 1 isoformb	T54	1.2	1.4	1.4	1.2	1.7	1.3	1.6	1.5	1.1	1.3	1.3	1.1	1.5	1.2	1.5	1.4	1.3	1.2	1.4	1.2	ANtPDSDITEK	GEVSRANtPDSDITE	T3;	Yes	T(3): 100.0; S(6): 0.0; T(9): 0.0	4	2	1	0	N-Term(TMT6plex); T3(Phospho); K11(TMT6plex)
4759302	NP_004729.1	9217	VAPB	vesicle-associated membrane protein-associated protein B/C isoform1	S206	0.9	0.9	1.3	1.5	1.0	0.9	1.1	1.2	0.9	0.9	1.3	1.4	1.0	1.0	1.1	1.2	1.0	1.0	1.2	1.3	TVQSNsPISALAPTGK	RKTVQSNsPISALAP	S6;	Yes	T(1): 0.0; S(4): 1.1; S(6): 98.9; S(9): 0.0; T(14): 0.0	3	1	1	0	N-Term(TMT6plex); S6(Phospho); K16(TMT6plex)
226509740	NP_067051.2	58506	SCAF1	splicing factor, arginine/serine-rich 19	S965	1.1	1.2	1.3	1.4	1.2	1.4	1.3	1.3	1.1	1.2	1.1	1.3	1.2	1.4	1.1	1.3	1.1	1.3	1.1	1.3	GAEETSWsGEER	GAEETSWsGEERAAK	S8;	Yes	T(5): 0.0; S(6): 0.0; S(8): 100.0	29	1	1	0	N-Term(TMT6plex); S8(Phospho)
291045183	NP_001166945.1	8623	ASMTL	N-acetylserotonin O-methyltransferase-like protein isoform3	S21	1.2	1.0	1.0	1.4	1.3	1.4	1.2	1.3	1.1	1.0	1.0	1.4	1.1	1.3	1.2	1.3	1.1	1.1	1.1	1.4	VVLASAsPR	RVVLASAsPRRQEIL	S7;	Yes	S(5): 0.0; S(7): 100.0	2	2	1	0	N-Term(TMT6plex); S7(Phospho)
30061567	NP_060548.2	55132	LARP1B	la-related protein 1B isoform1	S90	1.1	1.4	1.0	1.3	1.3	1.6	1.2	1.4	-	-	-	-	-	-	-	-	-	-	-	-	SEsQERPGSR	LDVVRSEsQERPGSR	S3;	Yes	S(1): 0.0; S(3): 100.0; S(9): 0.0	6	4	1	0	N-Term(TMT6plex); S3(Phospho)
160948599	NP_001073922.2	26173	INTS1	integrator complex subunit 1	S1327	1.2	1.3	1.1	1.3	1.2	1.2	1.2	1.4	1.2	1.3	1.1	1.4	1.1	1.2	1.2	1.6	1.1	1.3	1.2	1.5	SsPEQPIGQGR	EAPKPKSsPEQPIGQ	S2;	Yes	S(1): 1.0; S(2): 99.0	3	1	1	0	N-Term(TMT6plex); S2(Phospho)
58530845	NP_001010972.1	7791	ZYX	zyxin	S259	0.5	1.3	1.0	1.4	0.6	1.4	1.0	1.3	0.6	1.3	1.0	1.3	0.7	1.4	1.1	1.2	0.6	1.4	1.0	1.3	GPPASsPAPAPK	PRGPPASsPAPAPKF	S6;	Yes	S(5): 0.0; S(6): 100.0	3	1	1	0	N-Term(TMT6plex); S6(Phospho); K12(TMT6plex)
50345873	NP_065778.3	49854	ZBTB21	zinc finger and BTB domain-containing protein 21 isoformL	S435	1.1	1.1	1.2	1.3	1.6	1.3	1.5	1.4	0.9	1.0	1.1	1.1	1.4	1.2	1.4	1.2	1.1	1.1	1.2	1.1	TEPSsPLSDPSDIIR	RIKTEPSsPLSDPSD	S5;	Yes	T(1): 0.0; S(4): 0.0; S(5): 100.0; S(8): 0.0; S(11): 0.0	2	2	1	0	N-Term(TMT6plex); S5(Phospho)
50345873	NP_065778.3	49854	ZBTB21	zinc finger and BTB domain-containing protein 21 isoformL	S435	1.6	1.3	1.5	1.4	1.1	1.1	1.2	1.3	1.4	1.2	1.4	1.2	0.9	1.0	1.1	1.1	1.1	1.1	1.2	1.1	TEPsSPLSDPSDIIR	VRIKTEPsSPLSDPS	S5;	Yes	T(1): 0.0; S(4): 98.6; S(5): 1.4; S(8): 0.0; S(11): 0.0	4	2	1	0	N-Term(TMT6plex); S5(Phospho)
219802034	NP_001137307.1	4798	NFRKB	nuclear factor related to kappa-B-binding protein isoform1	S228	0.8	1.1	0.9	1.4	1.0	1.2	1.1	1.3	0.3	0.9	0.8	1.2	0.4	1.0	1.0	1.1	0.4	0.9	0.9	1.2	SPsPAVPLR	SSPARSPsPAVPLRV	S3;	Yes	S(1): 0.0; S(3): 100.0	1	2	1	0	N-Term(TMT6plex); S3(Phospho)
301601658	NP_001180424.1	103	ADAR	double-stranded RNA-specific adenosine deaminase isoformd	S530	0.9	1.0	1.1	1.3	1.0	1.2	1.2	1.4	0.8	1.0	1.0	1.2	0.9	1.2	1.1	1.2	0.9	1.1	1.1	1.2	sPEAQPK	TMLLLSRsPEAQPKT	S1;	No	S(1): 100.0	3	2	1	0	N-Term(TMT6plex); S1(Phospho); K7(TMT6plex)
13786127	NP_036253.2	23580	CDC42EP4	cdc42 effector protein 4	S64	1.0	1.7	0.6	1.3	1.4	1.7	1.1	1.4	1.0	1.9	0.6	1.4	1.3	1.9	1.2	1.5	1.2	1.9	0.9	1.5	AGEPDGEsLDEQPSSSSSK	AGEPDGEsLDEQPSS	S8;	Yes	S(8): 100.0; S(14): 0.0; S(15): 0.0; S(16): 0.0; S(17): 0.0; S(18): 0.0	8	1	1	0	N-Term(TMT6plex); S8(Phospho); K19(TMT6plex)
371121601	NP_001243048.1	8260	NAA10	N-alpha-acetyltransferase 10 isoform2	S190	1.0	1.3	1.5	1.3	1.0	1.4	1.3	1.4	1.0	1.3	1.5	1.3	1.0	1.4	1.3	1.4	1.0	1.3	1.4	1.3	GLAAEDsGGDSK	KGLAAEDsGGDSKDL	S7;	No	S(7): 100.0; S(11): 0.0	20	3	1	0	N-Term(TMT6plex); S7(Phospho); K12(TMT6plex)
55749531	NP_006833.2	10992	SF3B2	splicing factor 3B subunit 2	S289	2.5	0.9	1.9	1.1	2.0	1.7	2.8	1.6	2.8	1.1	2.3	1.2	2.2	2.0	3.3	1.7	2.5	1.5	2.8	1.5	QEEMNsQQEEEMETDAR	SRQEEMNsQQEEEME	S6;	Yes	S(6): 99.9; T(15): 0.1	14	1	1	0	N-Term(TMT6plex); M4(Oxidation); S6(Phospho); M13(Oxidation)
321400140	NP_001189485.1	960	CD44	CD44 antigen isoform7 precursor	T318	0.9	1.6	1.3	1.7	1.0	1.5	1.3	1.0	0.7	1.0	0.8	1.5	0.7	0.9	0.8	0.8	0.7	0.9	0.8	1.2	ESSEtPDQFMTADETR	VNKESSEtPDQFMTA	T5;	No	S(2): 0.0; S(3): 0.0; T(5): 100.0; T(11): 0.0; T(15): 0.0	11	6	1	0	N-Term(TMT6plex); T5(Phospho); M10(Oxidation)
50962882	NP_001002909.1	23131	GPATCH8	G patch domain-containing protein 8	S1107	1.4	1.4	1.2	1.2	1.2	1.6	1.6	1.5	1.5	1.5	1.3	1.3	1.2	1.7	1.8	1.6	1.4	1.6	1.5	1.4	KPsVSEEVQATPNK	RKVERKPsVSEEVQA	S3;	Yes	S(3): 100.0; S(5): 0.0; T(11): 0.0	17	1	1	0	N-Term(TMT6plex); K1(TMT6plex); S3(Phospho); K14(TMT6plex)
7661708	NP_054772.1	28982	FLVCR1	feline leukemia virus subgroup C receptor-related protein 1	S536	1.0	1.3	1.2	1.4	1.1	1.4	1.1	1.2	-	-	-	-	-	-	-	-	-	-	-	-	AIPADsPTDQEPK	VKAIPADsPTDQEPK	S6;	Yes	S(6): 100.0; T(8): 0.0	2	1	1	0	N-Term(TMT6plex); S6(Phospho); K13(TMT6plex)
4505573	NP_003890.1	8874	ARHGEF7	rho guanine nucleotide exchange factor 7 isoforma	S340	0.9	1.1	1.0	0.9	0.9	1.6	1.4	1.8	0.9	1.0	1.0	0.7	0.9	1.5	1.4	1.4	0.9	1.2	1.2	1.1	MsGFIYQGK	LSASPRMsGFIYQGK	S2;	No	S(2): 100.0; Y(6): 0.0	2	5	1	0	N-Term(TMT6plex); M1(Oxidation); S2(Phospho); K9(TMT6plex)
20149695	NP_079508.2	80346	REEP4	receptor expression-enhancing protein 4 precursor	S253	0.9	1.0	0.7	1.3	1.0	1.6	1.3	1.4	0.9	1.0	0.8	1.3	1.0	1.5	1.3	1.4	0.9	1.2	1.1	1.4	TVPSDVDs	TVPSDVDs	S4;	Yes	T(1): 0.0; S(4): 0.0; S(8): 100.0	3	1	1	0	N-Term(TMT6plex); S8(Phospho)
20149695	NP_079508.2	80346	REEP4	receptor expression-enhancing protein 4 precursor	S253	1.0	1.6	1.3	1.4	0.9	1.0	0.7	1.3	1.0	1.5	1.3	1.4	0.9	1.0	0.8	1.3	0.9	1.2	1.1	1.4	TVPsDVDS	TRKKTVPsDVDS	S4;	Yes	T(1): 0.0; S(4): 100.0; S(8): 0.0	2	1	1	0	N-Term(TMT6plex); S4(Phospho)
149158690	NP_542417.2	7916	PRRC2A	protein PRRC2A	S380	0.9	1.2	1.0	1.5	0.9	1.2	0.9	1.2	0.9	1.1	1.1	1.4	0.9	1.1	1.0	1.1	0.9	1.1	1.0	1.3	GNsPNSEPPTPK	ADGKKGNsPNSEPPT	S3;	Yes	S(3): 100.0; S(6): 0.0; T(10): 0.0	23	1	1	0	N-Term(TMT6plex); S3(Phospho); K12(TMT6plex)
103472005	NP_002408.3	4288	MKI67	antigen KI-67 isoform1	T1557	1.0	0.9	1.2	1.1	1.2	0.9	1.8	1.5	1.2	0.9	1.2	1.2	1.4	0.9	1.9	1.6	1.3	0.9	1.6	1.4	NIYAEMGtPVQK	NIYAEMGtPVQKLDL	T8;	Yes	Y(3): 0.0; T(8): 100.0	118	2	1	0	N-Term(TMT6plex); M6(Oxidation); T8(Phospho); K12(TMT6plex)
41152072	NP_056272.2	25921	ZDHHC5	palmitoyltransferase ZDHHC5	T348	0.9	1.2	1.4	1.3	1.4	1.8	1.5	1.4	0.9	1.2	1.3	1.3	1.3	1.7	1.5	1.4	1.1	1.5	1.4	1.3	DSPPtPTMYK	LAKDSPPtPTMYKYR	T5;	Yes	S(2): 0.0; T(5): 100.0; T(7): 0.0; Y(9): 0.0	8	1	1	0	N-Term(TMT6plex); T5(Phospho); M8(Oxidation); K10(TMT6plex)

Khan et al., 2019. Multi-omics analysis to characterize cigarette smoke induced molecular alterations in esophageal cells

Supplementary Table 8. List of phosphopeptides identified and quantified in untreated and chronically treated Het1A cells with cigarette smoke condensate for 8 months using SequestHT and Mascot search algorithms with a PhosphoRS probability score ≥75%

Protein group Accession	NP_Accession	Gene ID	Gene Symbol	Protein Description	PhosphoSite (Protein)	Het-1A-Smoke - 2M/Parental (Replicate 1)	Het-1A-Smoke - 4M/Parental (Replicate 1)	Het-1A-Smoke - 6M/Parental (Replicate 1)	Het-1A-Smoke - 8M/Parental (Replicate 1)	Het-1A-Smoke - 2M/Parental (Replicate 2)	Het-1A-Smoke - 4M/Parental (Replicate 2)	Het-1A-Smoke - 6M/Parental (Replicate 2)	Het-1A-Smoke - 8M/Parental (Replicate 2)	Normalized phosphoprotein ratios with respect to corresponding total protein ratios: Het-1A-Smoke - 2M/Parental (Replicate 1)	Het-1A-Smoke - 4M/Parental (Replicate 1)	Het-1A-Smoke - 6M/Parental (Replicate 1)	Het-1A-Smoke - 8M/Parental (Replicate 1)	Het-1A-Smoke - 2M/Parental (Replicate 2)	Het-1A-Smoke - 4M/Parental (Replicate 2)	Het-1A-Smoke - 6M/Parental (Replicate 2)	Het-1A-Smoke - 8M/Parental (Replicate 2)	Average of replicates: Het-1A-Smoke - 2M/Parental	Het-1A-Smoke - 4M/Parental	Het-1A-Smoke - 6M/Parental	Het-1A-Smoke - 8M/Parental	Peptide Sequence	Phosphowindow	PhosphoSite (Peptide)	Phosphosite Plus Evidence	phosphoRS Site Probabilities	# PSMs	# Proteins	# Protein Groups	# Missed Cleavages	Modifications
30795231	NP_006308.3	10409	BASP1	brain acid soluble protein 1	T36	1.3	1.2	1.1	1.3	1.3	1.3	1.3	1.4	1.3	1.2	1.2	1.5	1.3	1.2	1.4	1.6	1.3	1.2	1.3	1.6	AEGAATEEEGtPK	AATEEEGtPKESEPQ	T11;	Yes	T(6): 0.0; T(11): 100.0	16	1	1	0	N-Term(TMT6plex); T11(Phospho); K13(TMT6plex)
118572613	NP_057417.3	23524	SRRM2	serine/arginine repetitive matrix protein 2	S377	2.1	1.9	1.4	1.5	1.0	0.9	0.9	1.1	2.2	1.9	1.5	1.7	1.1	0.9	0.9	1.2	1.6	1.4	1.2	1.5	HGGsPQPLATTPLSQEPVNPPSEASPTR	LAERHGGsPQPLATT	S4;	Yes	S(4): 100.0; T(10): 0.0; T(11): 0.0; S(14): 0.0; S(22): 33.3; S(25): 33.3; T(27): 33.3	436	1	1	0	N-Term(TMT6plex); S4(Phospho); T27(Phospho)
239582767	NP_060327.3	54961	SSH3	protein phosphatase Slingshot homolog 3	S9	1.2	2.7	1.5	1.1	1.6	1.3	1.9	1.5	-	-	-	-	-	-	-	-	-	-	-	-	sPPGSGASTPVGPWDQAVQR	ALVTVSRsPPGSGAS	S1;	Yes	S(1): 100.0; S(5): 0.0; S(8): 0.0; T(9): 0.0	5	1	1	0	N-Term(TMT6plex); S1(Phospho)
385648270	NP_001245309.1	9497	SLC4A7	sodium bicarbonate cotransporter 3 isoform c	S233	1.2	1.0	1.0	1.1	1.4	1.2	1.2	1.5	1.0	0.9	0.8	1.0	1.2	1.0	1.0	1.4	1.1	1.0	0.9	1.2	sFADIGK	SRIPLVRsFADIGKK	S1;	Yes	S(1): 100.0	6	6	1	0	N-Term(TMT6plex); S1(Phospho); K7(TMT6plex)
20357556	NP_612632.1	2017	CTTN	src substrate cortactin isoform b	S368	0.8	1.6	1.8	1.1	1.5	2.4	2.0	1.6	0.7	1.3	1.2	1.0	1.2	1.9	1.3	1.4	0.9	1.6	1.3	1.2	TQtPPVSPAPQPTEER	KTQTPPVsPAPQPTE	S7;	Yes	T(1): 50.0; T(3): 50.0; S(7): 100.0; T(13): 0.0	8	3	1	0	N-Term(TMT6plex); T1(Phospho); S7(Phospho)
19913408	NP_001059.2	7155	TOP2B	DNA topoisomerase 2-beta	S1547	1.5	1.2	1.3	1.6	1.0	1.2	1.4	1.1	1.5	1.2	1.3	1.8	1.0	1.2	1.4	1.2	1.3	1.2	1.3	1.5	ASGsENEGDYNPGR	KKRKASGsENEGDYN	S4;	No	S(2): 0.0; S(4): 100.0; Y(10): 0.0	5	1	1	0	N-Term(TMT6plex); S4(Phospho)
7657269	NP_055847.1	23047	PDS5B	sister chromatid cohesion protein PDS5 homolog B	S1358	1.3	1.3	1.7	1.5	1.7	1.3	1.3	1.1	1.2	1.1	1.6	1.4	1.5	1.2	1.2	1.1	1.4	1.1	1.4	1.3	AEsPESSAIESTQSTPQK	RAQQRAEsPESSAIE	S3;	Yes	S(3): 100.0; S(6): 0.0; S(7): 0.0; S(11): 0.0; T(12): 0.0; S(14): 0.0; T(15): 0.0	34	1	1	0	N-Term(TMT6plex); S3(Phospho); K18(TMT6plex)
166795297	NP_057225.2	51143	DYNC1LI1	cytoplasmic dynein 1 light intermediate chain 1	S207	1.0	0.8	1.0	1.1	2.0	1.9	1.5	1.5	0.9	0.8	1.0	1.1	1.9	2.0	1.4	1.6	1.4	1.4	1.2	1.4	DEQYVEPGEDEPAsPQR	PGEDEPAsPQRRNTA	S15;	Yes	Y(5): 0.0; S(15): 100.0	10	1	1	0	N-Term(TMT6plex); S15(Phospho)
27477138	NP_078901.3	56829	ZC3HAV1	zinc finger CCCH-type antiviral protein 1 isoform 2	S378	0.7	0.7	0.7	1.0	1.1	1.2	1.6	1.7	0.7	0.8	0.7	1.0	1.1	1.2	1.6	1.7	0.9	1.0	1.1	1.3	TVFsPTLPAAR	ARRKTVFsPTLPAAR	S4;	Yes	T(1): 0.0; S(4): 100.0; T(6): 0.0	9	2	1	0	N-Term(TMT6plex); S4(Phospho)
302699211	NP_665739.3	23126	POGZ	pogo transposable element with ZNF domain isoform 3	S1265; T1273	0.9	1.7	2.2	0.9	0.8	1.5	1.6	1.7	0.9	1.6	1.9	0.8	0.7	1.4	1.4	1.6	0.8	1.5	1.7	1.2	LsGEHSESStPRPR	LEEQLKLsGEHSESS;GEHSESStPRPSSP	S2; T10;	No;No	S(2): 98.4; S(6): 1.6; S(8): 0.0; S(9): 1.6; T(10): 98.3	8	5	1	0	N-Term(TMT6plex); S6(Phospho); S8(Phospho)
302699211	NP_665739.3	23126	POGZ	pogo transposable element with ZNF domain isoform 3	S1265; T1273	0.8	1.5	1.6	1.7	0.9	1.7	2.2	0.9	0.7	1.4	1.4	1.6	0.9	1.6	1.9	0.8	0.8	1.5	1.7	1.2	LSGEHsESSTPRPR	LKLSGEHsESSTPRP	S2; T10;	No	S(2): 0.0; S(6): 99.9; S(8): 0.9; S(9): 49.6; T(10): 49.6	2	5	1	0	N-Term(TMT6plex); S9(Phospho); T10(Phospho)
19913408	NP_001059.2	7155	TOP2B	DNA topoisomerase 2-beta	S1545; S1547	1.7	1.7	1.8	1.6	1.8	1.5	1.4	1.1	1.7	1.7	1.8	1.7	1.8	1.5	1.4	1.2	1.8	1.6	1.6	1.4	KAsGsENEGDYNPGR	GAKKRKAsGSENEGD;KKRKASGsENEGDYN	S3; S5;	Yes;No	S(3): 100.0; S(5): 100.0; Y(11): 0.0	5	1	1	1	K1(TMT6plex); N-Term(TMT6plex); S3(Phospho); S5(Phospho)
132626688	NP_055456.2	9656	MDC1	mediator of DNA damage checkpoint protein 1	S955	0.8	1.4	1.2	1.3	1.2	1.7	1.5	1.4	0.9	1.5	1.3	1.3	1.2	1.7	1.6	1.4	1.1	1.6	1.4	1.4	GEPEGGsQDQK	RGEPEGGsQDQKGQA	S7;	Yes	S(7): 100.0	26	1	1	0	N-Term(TMT6plex); S7(Phospho); K11(TMT6plex)
12667788	NP_002464.1	4627	MYH9	myosin-9	S1943	1.6	1.5	1.6	1.2	1.4	1.4	1.7	1.5	1.3	1.3	1.3	1.0	1.2	1.2	1.4	1.3	1.3	1.2	1.3	1.2	GAGDGsDEEVDGK	RKGAGDGsDEEVDGK	S6;	Yes	S(6): 100.0	75	1	1	0	N-Term(TMT6plex); S6(Phospho); K13(TMT6plex)
148596949	NP_004732.2	9221	NOLC1	nucleolar and coiled-body phosphoprotein 1 isoform 2	S563	1.0	1.3	1.3	1.4	1.1	1.4	1.3	1.3	0.8	1.1	1.0	1.0	0.9	1.1	1.0	1.0	0.9	1.1	1.0	1.0	AAKNsEEEEEK	NGKAAKNsEEEEEEK	S5;	Yes	S(5): 100.0	33	3	1	1	N-Term(TMT6plex); K3(TMT6plex); S5(Phospho); K12(TMT6plex)
114155142	NP_003283.2	7175	TPR	nucleoprotein TPR	T1677	0.5	1.6	0.7	1.3	0.7	2.4	1.2	1.3	0.5	1.7	0.8	1.5	0.8	2.5	1.4	1.5	0.7	2.1	1.1	1.5	GIASTSDPPTANIKPTPVVStPSK	KPTPVVStPSKVTAA	T21;	Yes	S(4): 0.0; T(5): 0.0; S(6): 0.0; T(10): 0.0; T(16): 0.1; S(20): 2.3; T(21): 97.7; S(23): 0.0	29	1	1	0	N-Term(TMT6plex); K14(TMT6plex); T21(Phospho); K24(TMT6plex)
157276588	NP_001708.3	646	BNC1	zinc finger protein basonuclin-1	S988	1.3	1.3	1.4	1.2	1.1	1.5	1.4	1.4	1.0	1.5	1.6	1.3	0.9	1.6	1.5	1.4	0.9	1.5	1.6	1.4	SLAsSPSHLQ	NLHKSLAsSPSHLQ_	S4;	No	S(1): 0.0; S(4): 100.0; S(5): 0.0; S(7): 0.0	3	1	1	0	N-Term(TMT6plex); S4(Phospho)
157276588	NP_001708.3	646	BNC1	zinc finger protein basonuclin-1	S988	1.1	1.5	1.4	1.4	1.3	1.3	1.4	1.2	0.9	1.6	1.5	1.4	1.0	1.5	1.6	1.3	0.9	1.5	1.6	1.4	sLASSPSHLQ	QNPNLHKsLASSPSH	S4;	No	S(1): 100.0; S(4): 0.0; S(5): 0.0; S(7): 0.0	2	1	1	0	N-Term(TMT6plex); S4(Phospho)
153070260	NP_002347.5	4082	MARCKS	myristoylated alanine-rich C-kinase substrate	S101	0.8	1.1	0.8	1.5	0.8	1.1	0.8	1.2	1.2	1.5	1.2	2.0	1.2	1.5	1.2	1.6	1.2	1.5	1.2	1.8	GEPAAAAPEAGAsPVEK	AAPEAGAsPVEKEAP	S14;	Yes	S(14): 100.0	69	1	1	0	N-Term(TMT6plex); S14(Phospho); K18(TMT6plex)
7656879	NP_055238.1	27125	AFF4	AF4/FMR2 family member 4	S1058	1.0	1.0	1.2	1.0	1.3	1.4	1.6	1.6	0.9	0.9	1.1	0.9	1.2	1.2	1.5	1.5	1.1	1.0	1.3	1.2	AVGMPsPVsPK	VGMPSPVsPKLSPGN	S9;	Yes	S(6): 0.0; S(9): 100.0	16	1	1	0	N-Term(TMT6plex); M4(Oxidation); S9(Phospho); K11(TMT6plex)
7656879	NP_055238.1	27125	AFF4	AF4/FMR2 family member 4	S1058	1.3	1.4	1.6	1.6	1.0	1.0	1.2	1.0	1.2	1.2	1.5	1.5	0.9	0.9	1.1	0.9	1.1	1.0	1.3	1.2	AVGMPsPVSPK	SKAVGMPsPVSPKLS;VGMPSPVsPKLSPGN	S9;	Yes;Yes	S(6): 100.0; S(9): 100.0	2	1	1	0	N-Term(TMT6plex); M4(Oxidation); S6(Phospho); S9(Phospho); K11(TMT6plex)
571026657	NP_001275686.1	29086	BABAM1	BRISC and BRCA1-A complex member 1 isoform 2	S29	3.1	2.5	2.2	1.2	2.9	2.5	1.9	1.5	2.9	2.5	2.3	1.2	2.7	2.6	2.0	1.6	2.8	2.6	2.2	1.4	TRsNPEGAEDR	EPRPRTRsNPEGAED	S3;	Yes	T(1): 0.0; S(3): 100.0	17	2	1	1	N-Term(TMT6plex); S3(Phospho)
186700623	NP_078994.3	55684	RABL6	rab-like protein 6 isoform 1	S492	0.8	1.3	1.1	1.2	1.3	1.3	1.2	1.5	0.9	1.5	1.2	1.0	1.3	1.4	1.3	1.3	1.1	1.4	1.2	1.2	GPAPAPQQCsEPETK	APAPQQCsEPETKWS	S10;	Yes	S(10): 100.0; T(14): 0.0	1	2	1	0	N-Term(TMT6plex); C9(Carbamidomethyl); S10(Phospho); K15(TMT6plex)
4758958	NP_004148.1	5576	PRKAR2A	cAMP-dependent protein kinase type II-alpha regulatory subunit	S58	1.7	1.3	1.1	1.4	1.5	1.0	1.0	1.2	1.5	1.2	1.0	1.2	1.4	1.0	0.9	1.1	1.5	1.1	1.0	1.2	QsLGHPPPEPGPDR	PAATPRQsLGHPPPE	S2;	Yes	S(2): 100.0	2	1	1	0	N-Term(TMT6plex); S2(Phospho)
224028248	NP_001138882.1	4841	NONO	non-POU domain-containing octamer-binding protein isoform 2	T321	1.0	0.9	0.6	1.1	2.3	3.9	1.7	1.5	1.2	1.0	0.7	1.1	2.7	4.2	2.1	1.5	2.0	2.6	1.4	1.3	GAMPPAPVPAGtPAPPGPATMMPDGTLGLTPPTTER	PAPVPAGtPAPPGPA	T12;	No	T(12): 86.0; T(20): 4.5; T(26): 4.5; T(30): 4.5; T(33): 0.3; T(34): 0.3	5	2	1	0	N-Term(TMT6plex); M3(Oxidation); M21(Oxidation); M22(Oxidation); T30(Phospho)
224028248	NP_001138882.1	4841	NONO	non-POU domain-containing octamer-binding protein isoform 2	T321	2.3	3.9	1.7	1.5	1.0	0.9	0.6	1.1	2.7	4.2	2.1	1.5	1.2	1.0	0.7	1.1	2.0	2.6	1.4	1.3	GAMPPAPVPAGTPAPPGPATMMPDGTLGLtPPTTER	PDGTLGLtPPTTERF	T12;	No	T(12): 0.1; T(20): 0.1; T(26): 0.0; T(30): 99.7; T(33): 0.0; T(34): 0.0	1	2	1	0	N-Term(TMT6plex); M3(Oxidation); M21(Oxidation); M22(Oxidation); T30(Phospho)
51173724	NP_004044.3	705	BYSL	bystin	S98	0.6	1.6	1.3	1.6	0.9	0.3	0.4	1.1	0.7	1.6	1.3	1.5	1.0	0.3	0.4	1.0	0.9	1.0	0.9	1.3	MPQDGsDDEDEEWPTLEK	PRMPQDGsDDEDEEW	S6;	Yes	S(6): 99.9; T(15): 0.1	1	1	1	0	N-Term(TMT6plex); M1(Oxidation); S6(Phospho); K18(TMT6plex)
341865562	NP_001230072.1	51203	NUSAP1	nucleolar and spindle-associated protein 1 isoform 5	T334	1.0	1.3	1.8	1.5	1.0	0.9	0.6	1.1	1.4	1.5	2.1	1.8	1.4	1.1	0.7	1.3	1.4	1.3	1.4	1.6	LTTEATQtPVSNK	LTTEATQtPVSNKKP	T8;	No	T(2): 0.0; T(3): 0.0; T(6): 1.2; T(8): 98.8; S(11): 0.0	14	4	1	0	N-Term(TMT6plex); T8(Phospho); K13(TMT6plex)
148536853	NP_004362.2	1314	COPA	coatomer subunit alpha isoform 2	S402	1.7	1.8	1.7	1.2	2.1	1.9	1.8	1.4	1.4	1.5	1.4	1.1	1.8	1.7	1.5	1.3	1.6	1.6	1.4	1.2	DADsQNPDAPEGK	TIPKDADsQNPDAPE	S4;	Yes	S(4): 100.0	2	2	1	0	N-Term(TMT6plex); S4(Phospho); K13(TMT6plex)
134142826	NP_006188.3	5108	PCM1	pericentriolar material 1 protein	S65	1.3	0.8	1.2	1.3	1.5	1.5	1.4	1.3	1.3	0.8	1.2	1.3	1.5	1.4	1.3	1.4	1.4	1.1	1.3	1.3	VTNDIsPESsPGVGR	KRVTNDIsPESSPGV	S6;	Yes	T(2): 0.0; S(6): 100.0; S(9): 0.0; S(10): 0.0	3	1	1	0	N-Term(TMT6plex); S6(Phospho)
134142826	NP_006188.3	5108	PCM1	pericentriolar material 1 protein	S65	1.5	1.5	1.4	1.3	1.3	0.8	1.2	1.3	1.5	1.4	1.3	1.4	1.3	0.8	1.2	1.3	1.4	1.1	1.3	1.3	VTNDIsPESSPGVGR	KRVTNDIsPESSPGV	S6;	Yes	T(2): 0.0; S(6): 100.0; S(9): 0.0; S(10): 0.0	13	1	1	0	N-Term(TMT6plex); S6(Phospho)
10947061	NP_066193.1	112	ADCY6	adenylate cyclase type 6 isoform b	S54	1.0	0.9	1.0	1.5	1.3	1.3	0.8	1.2	-	-	-	-	-	-	-	-	-	-	-	-	DAEPPsPTPAGPPR	LRDAEPPsPTPAGPP	S6;	Yes	S(6): 100.0; T(8): 0.0	4	2	1	0	N-Term(TMT6plex); S6(Phospho)
41872631	NP_004095.4	2194	FASN	fatty acid synthase	T2204	0.7	1.1	1.2	1.2	1.2	1.3	1.9	1.4	0.5	0.8	0.9	1.1	0.9	1.0	1.5	1.3	0.7	0.9	1.2	1.2	ADEAsELACPTPK	SSKADEAsELACPTP	T11;	Yes	S(5): 100.0; T(11): 0.0	7	1	1	0	N-Term(TMT6plex); S5(Phospho); C9(Carbamidomethyl); K13(TMT6plex)
41872631	NP_004095.4	2194	FASN	fatty acid synthase	T2204	1.2	1.3	1.9	1.4	0.7	1.1	1.2	1.2	0.9	1.0	1.5	1.3	0.5	0.8	0.9	1.1	0.7	0.9	1.2	1.2	ADEASELACPtPK	ASELACPtPKEDGLA	T11;	Yes	S(5): 0.0; T(11): 100.0	14	1	1	0	N-Term(TMT6plex); C9(Carbamidomethyl); T11(Phospho); K13(TMT6plex)
544063449	NP_001269582.1	10963	STIP1	stress-induced-phosphoprotein 1 isoform c	S457	1.4	1.2	1.3	1.4	1.3	1.2	1.2	1.2	1.3	1.1	1.3	1.3	1.2	1.0	1.1	1.1	1.3	1.0	1.2	1.2	HDsPEDVK	AQYNRHDsPEDVKRR	S3;	No	S(3): 100.0	3	3	1	0	N-Term(TMT6plex); S3(Phospho); K8(TMT6plex)
186928821	NP_001119523.1	3068	HDGF	hepatoma-derived growth factor isoform c	S125; S126	1.0	1.3	1.4	1.2	1.4	1.7	1.7	1.4	1.1	1.2	1.3	1.1	1.5	1.6	1.6	1.3	1.3	1.4	1.4	1.2	GNAEGssDEEGKLVIDEPAK	KKGNAEGsSDEEGKL;KGNAEGSsDEEGKLV	S6; S7;	No;No	S(6): 100.0; S(7): 100.0	78	3	1	1	N-Term(TMT6plex); S6(Phospho); S7(Phospho); K12(TMT6plex); K20(TMT6plex)
186928821	NP_001119523.1	3068	HDGF	hepatoma-derived growth factor isoform c	S125; S126	1.4	1.7	1.7	1.4	1.0	1.3	1.4	1.2	1.5	1.6	1.6	1.3	1.1	1.2	1.3	1.1	1.3	1.4	1.4	1.2	GNAEGSsDEEGKLVIDEPAK	KKGNAEGsSDEEGKL;KGNAEGSsDEEGKLV	S6; S7;	No;No	S(6): 100.0; S(7): 100.0	2	3	1	1	N-Term(TMT6plex); S6(Phospho); S7(Phospho); K12(TMT6plex); K20(TMT6plex)
103472005	NP_002408.3	4288	MKI67	antigen KI-67 isoform 1	S538; T543	1.0	2.3	2.1	1.1	1.3	0.5	1.3	1.5	1.2	2.4	2.2	1.2	1.5	0.5	1.3	1.6	1.4	1.5	1.8	1.4	sLVMHtPPVLK	EAPTKRKsLVMHTPP;RKSLVMHtPPVLKKI	S1; T6;	Yes;Yes	S(1): 100.0; T(6): 100.0	118	2	1	0	N-Term(TMT6plex); S1(Phospho); M4(Oxidation); T6(Phospho); K11(TMT6plex)
82659109	NP_065816.2	23352	UBR4	E3 ubiquitin-protein ligase UBR4	T2724	1.4	1.8	1.4	1.4	1.5	1.6	1.5	1.2	1.3	1.6	1.2	1.3	1.3	1.4	1.3	1.1	1.3	1.5	1.3	1.2	SNtPMGDKDDDDDDDADEK	PSSPRSNtPMGDKDD	T3;	Yes	S(1): 0.0; T(3): 100.0	28	1	1	1	N-Term(TMT6plex); T3(Phospho); M5(Oxidation); K8(TMT6plex); K19(TMT6plex)
410991931	NP_001258610.1	29920	PYCR2	pyrroline-5-carboxylate reductase 2 isoform 2	S230	1.0	0.9	1.3	1.5	1.4	0.9	0.7	1.1	0.9	0.8	1.3	1.4	1.2	0.8	0.7	1.1	1.1	0.8	1.0	1.2	LESPTVSTLTPSsPGK	VSTLTPSsPGKLLTR	S13;	No	S(3): 0.0; T(5): 0.0; S(7): 0.0; T(8): 0.0; T(10): 0.0; S(12): 0.2; S(13): 99.8	1	2	1	0	N-Term(TMT6plex); S13(Phospho); K16(TMT6plex)
33457336	NP_078772.1	64207	IRF2BPL	interferon regulatory factor 2-binding protein-like	S547	0.9	1.2	0.8	1.5	1.0	1.2	1.0	1.2	0.9	1.1	0.8	1.2	1.0	1.1	1.0	1.0	0.9	1.1	0.9	1.1	KAsPEPPDSAEGALK	SLRKRKAsPEPPDSA	S3;	Yes	S(3): 100.0; S(9): 0.0	2	1	1	1	N-Term(TMT6plex); K1(TMT6plex); S3(Phospho); K15(TMT6plex)
9966881	NP_065134.1	57122	NUP107	nuclear pore complex protein Nup107	S37	1.4	1.4	1.1	1.4	1.2	1.2	0.9	1.2	1.3	1.4	1.0	1.3	1.1	1.1	0.8	1.1	1.2	1.2	0.9	1.2	VLLQAsQDENFGNTTPR	KRVLLQAsQDENFGN	S6;	Yes	S(6): 100.0; T(14): 0.0; T(15): 0.0	1	1	1	0	N-Term(TMT6plex); S6(Phospho)
122114651	NP_079366.3	57602	USP36	ubiquitin carboxyl-terminal hydrolase 36	S952	0.7	0.6	0.8	1.1	3.1	0.9	2.1	1.5	0.6	0.6	0.9	1.1	2.9	0.8	2.1	1.5	1.8	0.7	1.5	1.3	HSCSPMGDGDPEAMEEsPR	DPEAMEEsPRKKKKK	S17;	No	S(2): 0.0; S(4): 0.0; S(17): 100.0	6	1	1	0	N-Term(TMT6plex); C3(Carbamidomethyl); M6(Oxidation); M14(Oxidation); S17(Phospho)
156713451	NP_001096139.1	22800	RRAS2	ras-related protein R-Ras2 isoform b	S109	1.3	1.0	1.5	1.5	1.0	0.9	1.4	1.1	1.0	0.9	1.2	1.5	0.8	0.8	1.1	1.1	0.9	0.8	1.2	1.3	EQEQECPPsPEPTR	QEQECPPsPEPTRKE	S9;	No	S(9): 99.1; T(13): 0.9	4	3	1	0	N-Term(TMT6plex); C6(Carbamidomethyl); S9(Phospho)
39777604	NP_945328.1	9138	ARHGEF1	rho guanine nucleotide exchange factor 1 isoform 3	S830	1.1	1.7	1.6	1.3	1.7	1.6	1.8	1.3	1.1	1.6	1.5	1.3	1.6	1.5	1.6	1.2	1.4	1.6	1.6	1.2	DGDGVPGGGPLsPAR	VPGGGPLsPARTQEI	S12;	No	S(12): 100.0	1	3	1	0	N-Term(TMT6plex); S12(Phospho)
422398894	NP_001258719.1	1781	DYNC1I2	cytoplasmic dynein 1 intermediate chain 2 isoform 4	T89	0.7	1.2	0.9	1.1	1.5	1.3	0.9	1.5	0.6	1.1	0.9	1.1	1.4	1.2	0.9	1.4	1.0	1.2	0.9	1.3	SVStPSEAGSQDSGDGAVGSR	PSSKSVStPSEAGSQ	T4;	Yes	S(1): 0.0; S(3): 1.5; T(4): 97.0; S(6): 1.5; S(10): 0.0; S(13): 0.0; S(20): 0.0	10	4	1	0	N-Term(TMT6plex); T4(Phospho)
256818821	NP_064550.3	56852	RAD18	E3 ubiquitin-protein ligase RAD18	S471	0.8	0.9	1.2	0.8	1.6	1.5	2.1	1.8	0.6	0.7	0.9	0.6	1.3	1.2	1.7	1.3	0.9	0.9	1.3	1.0	NDLQDTEIsPR	DLQDTEIsPRQNRRT	S9;	Yes	T(6): 0.0; S(9): 100.0	8	1	1	0	N-Term(TMT6plex); S9(Phospho)
156119605	NP_036336.2	9329	GTF3C4	general transcription factor 3C polypeptide 4	S611	1.5	1.5	1.4	1.0	1.5	1.7	2.0	1.6	1.4	1.5	1.3	0.9	1.5	1.7	1.9	1.5	1.4	1.6	1.6	1.2	ILLVDsPGMGNADDEQQEEGTSSK	SKILLVDsPGMGNAD	S6;	Yes	S(6): 100.0; T(21): 0.0; S(22): 0.0; S(23): 0.0	2	1	1	0	N-Term(TMT6plex); S6(Phospho); M9(Oxidation); K24(TMT6plex)
134152683	NP_001077059.1	54867	TMEM214	transmembrane protein 214 isoform 2	T97	1.2	1.7	1.5	1.4	0.9	1.2	1.1	1.2	1.0	1.7	1.5	1.5	0.8	1.1	1.1	1.2	0.9	1.4	1.3	1.3	KVAtPPNQNQK	KQPKKVAtPPNQNQK	T4;	Yes	T(4): 100.0	1	2	1	1	N-Term(TMT6plex); K1(TMT6plex); T4(Phospho); K11(TMT6plex)
556695389	NP_001273359.1	123169	LEO1	RNA polymerase-associated protein LEO1 isoform 2	S598	1.2	1.1	1.1	1.1	1.0	0.9	1.1	1.5	1.4	1.2	1.4	1.3	1.2	1.1	1.4	2.0	1.3	1.2	1.4	1.7	KYVIsDEEEEDDD	KHKKYVIsDEEEEDD	S5;	No	Y(2): 0.0; S(5): 100.0	23	2	1	1	N-Term(TMT6plex); K1(TMT6plex); S5(Phospho)
32189362	NP_003651.1	8541	PPFIA3	liprin-alpha-3	T714	1.0	1.2	1.0	1.5	1.1	1.0	1.0	1.1	-	-	-	-	-	-	-	-	-	-	-	-	GEGPAIPGDtPPPTPR	GPAIPGDtPPPTPRS	T10;	Yes	T(10): 100.0; T(14): 0.0	5	1	1	0	N-Term(TMT6plex); T10(Phospho)
47717125	NP_001001132.1	6453	ITSN1	intersectin-1 isoform ITSN-s	S904	1.3	1.3	1.2	1.0	1.6	1.4	1.5	1.6	1.1	1.1	1.1	0.9	1.4	1.2	1.4	1.4	1.2	1.2	1.3	1.2	SAFTPATATGSSPsPVLGQGEK	TATGSSPsPVLGQGE	S14;	Yes	S(1): 0.0; T(4): 0.0; T(7): 0.0; T(9): 0.0; S(11): 0.0; S(12): 0.0; S(14): [illegible]	8	2	1	0	N-Term(TMT6plex); S14(Phospho); K22(TMT6plex)
4507521	NP_001055.1	7086	TKT	transketolase isoform 1	S295	1.5	1.5	1.3	1.5	1.0	1.3	0.6	1.1	1.3	1.4	1.2	1.4	0.9	1.2	0.5	1.0	1.1	1.3	0.9	1.2	ILATPPQEDAPsVDIANIR	PPQEDAPsVDIANIR	S12;	Yes	T(4): 0.1; S(12): 99.9	1	2	1	0	N-Term(TMT6plex); S12(Phospho)
212276104	NP_001131025.1	9208	LRRFIP1	leucine-rich repeat flightless-interacting protein 1	S16	0.3	0.9	1.0	1.0	0.7	0.9	0.7	1.5	0.3	0.9	1.1	1.3	0.7	0.9	0.8	1.9	0.5	0.9	1.0	1.6	EIDCLsPEAQK	SREIDCLsPEAQKLA	S6;	Yes	S(6): 100.0	12	4	1	0	N-Term(TMT6plex); C4(Carbamidomethyl); S6(Phospho); K11(TMT6plex)
151301171	NP_114129.1	83860	TAF3	transcription initiation factor TFIID subunit 3	S183	1.5	1.1	1.0	1.2	1.6	1.1	1.8	1.4	1.6	0.9	0.9	1.0	1.7	1.0	1.7	1.2	1.6	1.0	1.3	1.1	RPLDsPEAEELPAMK	LGKRPLDsPEAEELP	S5;	Yes	S(5): 100.0	4	1	1	0	N-Term(TMT6plex); S5(Phospho); M14(Oxidation); K15(TMT6plex)
555943905	NP_001273176.1	55103	RALGPS2	ras-specific guanine nucleotide-releasing factor RalGPS2 isoform 2	T326; S329	0.8	0.5	0.4	0.4	1.5	1.9	1.4	2.2	-	-	-	-	-	-	-	-	-	-	-	-	SVAAEGALLPQtPPsPR	EGALLPQtPPSPRNL;LLPQTPPsPRNLIPH	T12; S15;	Yes;Yes	S(1): 0.0; T(12): 100.0; S(15): 100.0	2	2	1	0	N-Term(TMT6plex); T12(Phospho); S15(Phospho)
555943905	NP_001273176.1	55103	RALGPS2	ras-specific guanine nucleotide-releasing factor RalGPS2 isoform 2	T326; S329	1.5	1.9	1.4	2.2	0.8	0.5	0.4	0.4	-	-	-	-	-	-	-	-	-	-	-	-	SVAAEGALLPQTPPsPR	LLPQTPPsPRNLIPH	T12; S15;	Yes	S(1): 0.0; T(12): 0.0; S(15): 100.0	3	2	1	0	N-Term(TMT6plex); S15(Phospho)
4505939	NP_000928.1	5430	POLR2A	DNA-directed RNA polymerase II subunit RPB1	Y1909; S1917	0.8	1.8	1.5	1.6	1.1	1.2	1.0	1.0	0.8	2.0	1.6	1.6	1.1	1.3	1.1	1.0	1.0	1.6	1.4	1.3	ySPTSPTYsPTSPK	YTPTSPKySPTSPTY;SPTSPTYsPTSPKYS	Y1; S9;	Yes;Yes	Y(1): 98.5; S(2): 1.5; T(4): 0.0; S(5): 0.0; T(7): 0.0; Y(8): 0.0; S(9): 100.0; T(11): 0.0; S(12): 0.0	13	1	1	0	N-Term(TMT6plex); Y1(Phospho); T4(Phospho); K14(TMT6plex)
21493024	NP_653080.1	9590	AKAP12	A-kinase anchor protein 12 isoform 2	S185	0.7	1.2	1.1	1.1	1.0	1.8	1.9	1.5	0.8	1.2	0.9	1.1	1.1	1.7	1.6	1.4	1.0	1.5	1.2	1.2	SAESPTsPVTSETGSTFK	KSAESPTsPVTSETG	S4;	No	S(1): 0.0; S(4): 0.0; T(6): 1.1; S(7): 97.8; T(10): 0.0; S(11): 1.1; T(13): 0.0; S(15): 0.0; T(16): 0.0	8	2	1	0	N-Term(TMT6plex); S7(Phospho); K18(TMT6plex)
21493024	NP_653080.1	9590	AKAP12	A-kinase anchor protein 12 isoform 2	S185	1.0	1.8	1.9	1.5	0.7	1.2	1.1	1.1	1.1	1.7	1.6	1.4	0.8	1.2	0.9	1.1	1.0	1.5	1.2	1.2	SAEsPTSPVTSETGSTFK	EPSKSAEsPTSPVTS	S4;	No	S(1): 1.4; S(4): 95.7; T(6): 1.4; S(7): 1.4; T(10): 0.0; S(11): 0.0; T(13): 0.0; S(15): 0.0; T(16): 0.0	103	2	1	0	N-Term(TMT6plex); S4(Phospho); K18(TMT6plex)
31324577	NP_203744.1	85377	MICALL1	MICAL-like protein 1	S640	0.9	1.3	1.1	1.2	1.0	1.3	1.1	1.4	0.8	1.1	1.1	1.0	0.9	1.1	1.0	1.3	0.9	1.1	1.0	1.1	KPsPAASPATK	NPENRKPsPAASPAT	S3;	Yes	S(3): 100.0; S(7): 0.0; T(10): 0.0	12	1	1	0	N-Term(TMT6plex); K1(TMT6plex); S3(Phospho); K11(TMT6plex)
45439359	NP_009049.2	7204	TRIO	triple functional domain protein	S2455	1.5	2.0	2.1	1.4	1.7	2.4	3.1	1.1	1.4	1.7	1.5	1.4	1.6	2.1	2.3	1.1	1.5	1.9	1.9	1.3	AGAAsPLNSPLSSAVPSLGK	KPRAGAAsPLNSPLS	S5;	Yes	S(5): 100.0; S(9): 0.0; S(12): 0.0; S(13): 0.0; S(17): 0.0	3	1	1	0	N-Term(TMT6plex); S5(Phospho); K20(TMT6plex)
338753400	NP_001229714.1	3141	HLCS	biotin--protein ligase	S79	1.3	1.2	1.0	1.3	1.3	1.2	1.0	1.3	1.4	1.2	1.1	1.3	1.4	1.2	1.1	1.3	1.4	1.2	1.1	1.3	RGsASGSEPAGDSDR	EPKQRRGsASGSEPA	S3;	Yes	S(3): 100.0; S(5): 0.0; S(7): 0.0; S(13): 0.0	7	1	1	1	N-Term(TMT6plex); S3(Phospho)
109255232	NP_001035864.1	9859	CEP170	centrosomal protein of 170 kDa isoform gamma	S1067	1.1	1.3	1.2	1.3	1.0	1.4	1.3	1.3	1.0	1.2	1.1	1.2	0.9	1.2	1.1	1.2	1.0	1.2	1.1	1.2	sDSEATISR	LGSLSARsDSEATIS	S1;	No	S(1): 100.0; S(3): 0.0; T(6): 0.0; S(8): 0.0	11	3	1	0	N-Term(TMT6plex); S1(Phospho)
56550039	NP_005924.2	4297	KMT2A	histone-lysine N-methyltransferase 2A isoform 2 precursor	S2098	1.2	1.6	2.4	1.8	0.7	1.6	0.9	0.8	1.0	1.5	2.5	1.8	0.6	1.6	0.9	0.8	0.8	1.6	1.7	1.3	TIAHsPTSFTESSSK	ENRTIAHsPTSFTES	S5;	No	T(1): 0.0; S(5): 100.0; T(7): 0.0; S(8): 0.0; T(10): 0.0; S(12): 0.0; S(13): 0.0; S(14): 0.0	15	2	1	0	N-Term(TMT6plex); S5(Phospho); K15(TMT6plex)
33356172	NP_002476.2	4683	NBN	nibrin	S432	1.1	1.2	0.8	1.1	1.1	1.1	0.9	1.4	1.0	1.2	0.8	1.1	1.1	1.1	0.9	1.4	1.1	1.1	0.8	1.2	IPNYQLsPTK	RIPNYQLsPTKLPSI	S7;	Yes	Y(4): 0.0; S(7): 100.0; T(9): 0.0	8	1	1	0	N-Term(TMT6plex); S7(Phospho); K10(TMT6plex)
308818200	NP_001184223.1	1809	DPYSL3	dihydropyrimidinase-related protein 3 isoform 1	S102	0.8	1.2	0.9	1.1	1.1	1.7	1.2	1.4	0.9	1.1	1.0	1.2	1.2	1.7	1.3	1.6	1.0	1.4	1.1	1.4	EESREPAPAsPAPAGVEIR	SREPAPAsPAPAGVE	S10;	Yes	S(3): 0.0; S(10): 100.0	8	1	1	1	N-Term(TMT6plex); S10(Phospho)
531034764	NP_001269071.1	7030	TFE3	transcription factor E3 isoform 2	S443	1.3	1.2	0.8	1.2	0.8	1.2	0.8	1.3	-	-	-	-	-	-	-	-	-	-	-	-	AASDPLLSSVsPAVsK	LSPLRAAsDPLLSSV	S3;	No	S(3): 100.0; S(8): 0.0; S(9): 0.0; S(11): 0.0; S(15): 0.0	4	2	1	0	N-Term(TMT6plex); S3(Phospho); K16(TMT6plex)
531034764	NP_001269071.1	7030	TFE3	transcription factor E3 isoform 2	S443	0.8	1.2	0.8	1.3	1.3	1.2	0.8	1.2	-	-	-	-	-	-	-	-	-	-	-	-	AAsDPLLSSVSPAVSK	LSPLRAAsDPLLSSV	S3;	No	S(3): 100.0; S(8): 0.0; S(9): 0.0; S(11): 0.0; S(15): 0.0	2	2	1	0	N-Term(TMT6plex); S3(Phospho); K16(TMT6plex)
13435145	NP_079069.1	23059	CLUAP1	clusterin-associated protein 1 isoform 2	S243	1.0	1.0	1.3	1.5	0.9	0.9	0.9	1.0	-	-	-	-	-	-	-	-	-	-	-	-	SEPLDEsDNDE	KSEPLDEsDNDE	S7;	No	S(1): 0.0; S(7): 100.0	2	2	1	0	N-Term(TMT6plex); S7(Phospho)
21493024	NP_653080.1	9590	AKAP12	A-kinase anchor protein 12 isoform 2	S1233	0.7	1.0	1.3	1.2	0.9	1.3	1.3	1.4	0.8	0.9	1.1	1.1	1.1	1.2	1.0	1.3	0.9	1.1	1.0	1.2	SPPsPVER	SHAKSPPsPVEREMV	S4;	No	S(1): 0.0; S(4): 100.0	103	2	1	0	N-Term(TMT6plex); S4(Phospho)
291190787	NP_055872.4	23077	MYCBP2	E3 ubiquitin-protein ligase MYCBP2	S2812	0.9	1.9	1.5	1.7	1.0	0.7	0.8	0.8	0.9	1.8	1.5	1.4	1.0	0.7	0.8	0.7	1.0	1.2	1.1	1.0	AEsPGPGSR	PSSSRAEsPGPGSRL	S3;	No	S(3): 100.0; S(8): 0.0	6	1	1	0	N-Term(TMT6plex); S3(Phospho)
4502285	NP_001672.1	488	ATP2A2	sarcoplasmic/endoplasmic reticulum calcium ATPase 2 isoform a	S663	0.9	1.4	1.0	1.3	1.1	1.3	1.0	1.2	0.9	1.5	1.0	1.4	1.1	1.4	1.0	1.2	1.0	1.4	1.0	1.3	EFDELNPsAQR	EFDELNPsAQRDACL	S8;	Yes	S(8): 100.0	1	2	1	0	N-Term(TMT6plex); S8(Phospho)
503774456	NP_001265170.1	27043	PELP1	proline-, glutamic acid- and leucine-rich protein 1 isoform 2	T858	1.1	1.0	0.9	1.4	1.6	0.9	1.3	1.1	1.0	1.0	0.9	1.5	1.6	0.9	1.3	1.2	1.3	0.9	1.1	1.3	VQPPPEtPAEEEMETETEAEALQEK	KVQPPPEtPAEEEME	T7;	No	T(7): 94.8; T(15): 2.6; T(17): 2.6	19	2	1	0	N-Term(TMT6plex); T7(Phospho); M13(Oxidation); K25(TMT6plex)
606215169	NP_277024.2	984	CDK11B	cyclin-dependent kinase 11B isoform 5	S13	1.4	1.5	1.4	1.6	1.0	0.9	0.9	1.0	1.6	1.7	1.5	1.7	1.1	1.0	0.9	1.0	1.4	1.4	1.2	1.4	RDsLEEGELR	DRDSKRDsLEEGELR	S3;	No	S(3): 100.0	8	6	1	1	N-Term(TMT6plex); S3(Phospho)
216548223	NP_055664.3	9900	SV2A	synaptic vesicle glycoprotein 2A isoform 1	S127	1.2	1.3	1.1	1.1	1.3	1.4	1.3	1.4	1.3	1.3	1.2	1.2	1.3	1.4	1.4	1.5	1.3	1.3	1.3	1.3	GGLsDGEGPPGGR	AGVRGGLsDGEGPPG	S4;	Yes	S(4): 100.0	1	1	1	0	N-Term(TMT6plex); S4(Phospho)
189217847	NP_001121362.1	51324	SPG21	maspardin isoform b	S277	0.9	1.2	1.1	1.2	1.1	1.6	1.0	1.4	0.9	1.3	1.1	1.2	1.2	1.7	1.1	1.4	1.1	1.5	1.1	1.3	GSLGIsQEEQ	QKGSLGIsQEEQ	S6;	No	S(2): 0.0; S(6): 100.0	6	2	1	0	N-Term(TMT6plex); S6(Phospho)
7706457	NP_057332.1	11215	AKAP11	A-kinase anchor protein 11	S448	1.3	1.6	1.3	1.3	1.2	1.3	1.2	1.2	0.9	1.4	1.3	1.0	0.8	1.1	1.2	1.0	0.9	1.3	1.3	1.0	EDSGLEsPIR	REDSGLEsPIRSSAE	S7;	Yes	S(3): 0.0; S(7): 100.0	3	1	1	0	N-Term(TMT6plex); S7(Phospho)
574274721	NP_001275992.1	3978	LIG1	DNA ligase 1 isoform 2	T152	0.7	1.0	0.9	1.4	0.6	1.0	0.8	1.2	0.6	1.1	1.0	1.3	0.6	1.1	0.8	1.1	0.6	1.1	0.9	1.2	EGEDGDQPTtPPKPLK	EDGDQPTtPPKPLKT	T10;	No	T(9): 1.3; T(10): 98.7	13	2	1	0	N-Term(TMT6plex); T10(Phospho); K13(TMT6plex); K16(TMT6plex)
14249158	NP_116020.1	84717	HDGFRP2	hepatoma-derived growth factor-related protein 2 isoform 2	S663	1.0	1.2	0.9	1.2	1.5	1.6	1.0	1.4	1.3	1.5	1.2	1.6	2.0	2.0	1.3	1.8	1.6	1.8	1.3	1.7	GDsEALDEES	RERARGDsEALDEES	S3;	No	S(3): 100.0; S(10): 0.0	21	2	1	0	N-Term(TMT6plex); S3(Phospho)
4505117	NP_003918.1	8932	MBD2	methyl-CpG-binding domain protein 2 isoform 1	S407	1.2	1.1	1.3	1.4	2.8	1.6	2.6	1.1	1.3	1.2	1.3	1.5	3.1	1.7	2.7	1.2	2.2	1.4	2.0	1.3	AADTEEMDIEMDsGDEA	EMDIEMDsGDEA	S13;	Yes	T(4): 0.0; S(13): 100.0	4	1	1	0	N-Term(TMT6plex); M7(Oxidation); M11(Oxidation); S13(Phospho)
4885105	NP_005432.1	8208	CHAF1B	chromatin assembly factor 1 subunit B	S556	0.8	0.7	0.9	0.9	1.0	0.9	1.6	1.6	0.9	0.8	1.0	1.0	1.2	1.1	1.8	1.8	1.1	0.9	1.4	1.4	GGTEsLDP	ENKGGTEsLDP	S5;	Yes	T(3): 0.8; S(5): 99.2	10	1	1	0	N-Term(TMT6plex); S5(Phospho)
31543419	NP_009185.2	11284	PNKP	bifunctional polynucleotide phosphatase/kinase	T118	1.6	1.5	0.8	1.1	1.2	0.9	1.4	1.4	1.0	1.4	0.7	1.0	0.8	0.9	1.2	1.2	0.9	1.1	1.0	1.1	TPESQPDtPPGtPLVSQDEK	TPESQPDtPPGTPLV	T8;	Yes	T(1): 0.0; S(4): 1.5; T(8): 98.4; T(12): 0.0; S(16): 0.0	5	1	1	0	N-Term(TMT6plex); T8(Phospho); K20(TMT6plex)
157739945	NP_079461.2	26115	TANC2	protein TANC2	S1476	1.1	1.1	1.2	1.2	1.2	1.1	1.3	1.3	1.2	1.0	1.1	1.2	1.2	1.1	1.2	1.3	1.2	1.1	1.2	1.3	SSSSVGsPTR	RSSSSVGsPTRQTYQ	S7;	Yes	S(1): 0.0; S(2): 0.0; S(3): 0.0; S(4): 0.0; S(7): 100.0; T(9): 0.0	6	1	1	0	N-Term(TMT6plex); S7(Phospho)
14670268	NP_002895.3	7936	NELFE	negative elongation factor E	S115	1.2	1.3	1.1	1.3	1.2	1.6	1.2	1.2	1.4	1.5	1.4	1.6	1.4	1.8	1.5	1.5	1.4	1.7	1.5	1.5	SIsADDDLQESSR	QPFQRSIsADDDLQE	S3;	Yes	S(1): 0.0; S(3): 100.0; S(11): 0.0; S(12): 0.0	23	1	1	0	N-Term(TMT6plex); S3(Phospho)
31543419	NP_009185.2	11284	PNKP	bifunctional polynucleotide phosphatase/kinase	T118	1.2	0.9	1.4	1.4	1.6	1.5	0.8	1.1	0.8	0.9	1.2	1.2	1.0	1.4	0.7	1.0	0.9	1.1	1.0	1.1	TPESQPDtPPGTPLVSQDEK	TPESQPDtPPGTPLV	T8;	Yes	T(1): 0.0; S(4): 0.0; T(8): 98.7; T(12): 1.3; S(16): 0.0	3	1	1	0	N-Term(TMT6plex); T8(Phospho); K20(TMT6plex)
82546824	NP_001032242.1	221937	FOXK1	forkhead box protein K1	S420	1.1	1.2	1.0	1.1	1.1	1.2	1.1	1.4	1.1	1.2	1.0	1.2	1.1	1.2	1.2	1.5	1.1	1.2	1.1	1.3	SAPASPTHPGLMsPR	FGPLSSRsAPASPTH;SSRSAPAsPTHPGLM	S5;	Yes;Yes	S(1): 100.0; S(5): 98.2; T(7): 1.8; S(13): 0.0	4	1	1	0	N-Term(TMT6plex); S1(Phospho); S5(Phospho); M12(Oxidation)
82546824	NP_001032242.1	221937	FOXK1	forkhead box protein K1	S420	1.1	1.2	1.1	1.4	1.1	1.2	1.0	1.1	1.1	1.2	1.2	1.5	1.1	1.2	1.0	1.2	1.1	1.2	1.1	1.3	sAPAsPTHPGLMSPR	SSRSAPAsPTHPGLM	S5;	Yes	S(1): 1.5; S(5): 98.5; T(7): 0.0; S(13): 0.0	6	1	1	0	N-Term(TMT6plex); S5(Phospho); M12(Oxidation)
24234756	NP_703194.1	3609	ILF3	interleukin enhancer-binding factor 3 isoform c	S62	1.8	1.6	1.3	1.7	0.7	0.9	0.8	0.9	2.0	1.7	1.4	1.7	0.8	0.9	0.8	0.9	1.4	1.3	1.1	1.3	GSSEQAEsDNMDVPPEDDSK	GSSEQAEsDNMDVPP	S8;	Yes	S(2): 0.0; S(3): 0.0; S(8): 100.0; S(19): 0.0	26	5	1	0	N-Term(TMT6plex); S8(Phospho); M11(Oxidation); K20(TMT6plex)
115298682	NP_055987.2	23215	PRRC2C	protein PRRC2C	T2682	1.0	1.3	1.1	1.2	1.0	1.3	1.0	1.3	1.1	1.2	1.1	1.1	1.1	1.2	1.0	1.2	1.1	1.2	1.0	1.2	STtPtSSPFR	PIAGRSTtPTSSPFR	T3;	Yes	S(1): 0.0; T(2): 0.0; T(3): 97.7; T(5): 0.0; S(6): 1.1; S(7): 1.1	1	1	1	0	N-Term(TMT6plex); T3(Phospho)
115298682	NP_055987.2	23215	PRRC2C	protein PRRC2C	T2682	1.0	1.3	1.0	1.3	1.0	1.3	1.1	1.2	1.1	1.2	1.0	1.2	1.1	1.2	1.1	1.1	1.1	1.2	1.0	1.2	STtPTSSPFR	PIAGRSTtPTSSPFR	T3;	Yes	S(1): 0.0; T(2): 0.0; T(3): 100.0; T(5): 0.0; S(6): 0.0; S(7): 0.0	18	1	1	0	N-Term(TMT6plex); T3(Phospho)
545746435	NP_001269978.1	9101	USP8	ubiquitin carboxyl-terminal hydrolase 8 isoform b	S312	1.0	0.9	1.4	1.1	0.7	1.4	1.3	1.4	1.0	0.8	1.4	1.0	0.7	1.3	1.3	1.2	0.8	1.1	1.3	1.1	sDVSPIIQPVPSIK	EPVAASKsDVSPIIQ	S1;	No	S(1): 99.0; S(4): 1.0; S(12): 0.0	5	2	1	0	N-Term(TMT6plex); S4(Phospho); K14(TMT6plex)
305632781	NP_061886.2	54478	FAM64A	protein FAM64A isoform 1	S16	0.8	1.0	1.2	1.0	1.6	1.5	1.6	1.5	0.8	1.0	1.2	1.1	1.5	1.5	1.6	1.5	1.1	1.3	1.4	1.3	sLQHQEQLEDSK	GTSVRRRsLQHQEQL	S1;	Yes	S(1): 100.0; S(11): 0.0	6	2	1	0	N-Term(TMT6plex); S1(Phospho); K12(TMT6plex)
194097365	NP_060117.3	117246	FTSJ3	pre-rRNA processing protein FTSJ3	S584	0.8	0.9	1.0	1.2	0.8	0.9	0.9	1.4	0.8	0.9	1.1	1.1	0.8	0.9	1.0	1.3	0.8	0.9	1.0	1.2	TEIMsPLYQDEAPK	CLKTEIMsPLYQDEA	S5;	Yes	T(1): 0.0; S(5): 100.0; Y(8): 0.0	12	1	1	0	N-Term(TMT6plex); M4(Oxidation); S5(Phospho); K14(TMT6plex)
55749531	NP_006833.2	10992	SF3B2	splicing factor 3B subunit 2	S343	1.1	1.4	1.2	1.2	1.3	0.9	1.0	1.4	1.3	1.7	1.5	1.3	1.4	1.0	1.2	1.5	1.3	1.3	1.3	1.4	GVsSESSGDR	PQRVRGVsSESSGDR	S3;	Yes	S(3): 100.0; S(4): 0.0; S(6): 0.0; S(7): 0.0	14	1	1	0	N-Term(TMT6plex); S3(Phospho)
29826323	NP_054909.2	118	ADD1	alpha-adducin isoform c	S12	1.6	1.2	0.9	1.1	1.3	1.2	1.2	1.5	1.9	1.4	1.0	1.2	1.5	1.4	1.3	1.7	1.7	1.4	1.1	1.5	AAVVTsPPPTTAPHK	SRAAVVTsPPPTTAP	S6;	Yes	T(5): 0.8; S(6): 99.2; T(10): 0.0; T(11): 0.0	12	5	1	0	N-Term(TMT6plex); S6(Phospho); K15(TMT6plex)
7662354	NP_055755.1	22879	MON1B	vacuolar fusion protein MON1 homolog B isoform 1	S59	1.3	1.4	1.7	1.3	1.6	1.4	1.5	1.2	1.1	1.4	1.6	1.4	1.3	1.3	1.5	1.2	1.2	1.4	1.5	1.3	DKDQPPsPSPPPQSEALSSTSR	KDKDQPPsPSPPPQS	S7;	Yes	S(7): 97.4; S(9): 2.6; S(14): 0.0; S(18): 0.0; S(19): 0.0; T(20): 0.0; S(21): 0.0	1	1	1	1	N-Term(TMT6plex); K2(TMT6plex); S7(Phospho)
66348045	NP_057562.3	51530	ZC3HC1	nuclear-interacting partner of ALK isoform 1	S62	2.1	1.9	1.6	1.1	1.1	1.0	1.2	1.4	1.9	1.9	1.6	1.1	1.0	1.0	1.2	1.3	1.5	1.4	1.4	1.2	DTSATSQSVNGsPQAEQPSLESTSK	TSQSVNGsPQAEQPS	S12;	Yes	T(2): 0.1; S(3): 0.1; T(5): 0.0; S(6): 0.1; S(8): 2.9; S(12): 96.8; S(19): 0.0; S(22): 0.0; T(23): 0.0; S(24): 0.0	8	3	1	0	N-Term(TMT6plex); S12(Phospho); K25(TMT6plex)
608788342	NP_001278327.1	387680	FAM21A	WASH complex subunit FAM21A isoform 2	S498	0.5	1.4	1.2	1.3	0.9	1.7	1.3	1.2	0.5	1.2	1.2	1.4	0.9	1.4	1.4	1.3	0.7	1.3	1.3	1.4	AVAsPEATVSQTDENK	FKEKAVAsPEATVSQ	S4;	Yes	S(4): 100.0; T(8): 0.0; S(10): 0.0; T(12): 0.0	7	5	1	0	N-Term(TMT6plex); S4(Phospho); K16(TMT6plex)
33239445	NP_003742.2	8662	EIF3B	eukaryotic translation initiation factor 3 subunit B	S125	1.0	1.0	1.2	1.0	1.3	1.4	1.5	1.5	0.9	0.9	1.1	1.0	1.3	1.3	1.4	1.5	1.1	1.1	1.3	1.2	AQAVsEDAGGNEGR	DSRAQAVsEDAGGNE	S5;	Yes	S(5): 100.0	10	1	1	0	N-Term(TMT6plex); S5(Phospho)
66529203	NP_066005.2	57674	RNF213	E3 ubiquitin-protein ligase RNF213 isoform 2	S217	1.7	1.5	1.4	1.0	1.3	0.8	1.7	1.5	1.6	1.5	1.3	1.0	1.3	0.8	1.6	1.5	1.4	1.1	1.4	1.3	GLSQEGtGPPTSAGEGHSR	RGLSQEGtGPPTSAG	S3;	No	S(3): 2.1; T(7): 97.9; T(11): 0.0; S(12): 0.0; S(18): 0.0	1	2	1	0	N-Term(TMT6plex); S3(Phospho)
110681719	NP_114401.2	23731	TMEM245	transmembrane protein 245	S15	0.8	1.1	0.9	1.4	0.7	0.9	0.8	1.1	0.7	1.0	0.8	1.3	0.6	0.9	0.7	1.1	0.6	1.0	0.8	1.2	sSPGPAPR	KDAPSLRsSPGPAPR	S1;	Yes	S(1): 100.0; S(2): 0.0	4	1	1	0	N-Term(TMT6plex); S1(Phospho)
66529203	NP_066005.2	57674	RNF213	E3 ubiquitin-protein ligase RNF213 isoform 2	S217	1.3	0.8	1.7	1.5	1.7	1.5	1.4	1.0	1.3	0.8	1.6	1.5	1.6	1.5	1.3	1.0	1.4	1.1	1.4	1.3	GLsQEGTGPPTSAGEGHSR	PSGGRGLsQEGTGPP	S3;	Yes	S(3): 100.0; T(7): 0.0; T(11): 0.0; S(12): 0.0; S(18): 0.0	1	2	1	0	N-Term(TMT6plex); S3(Phospho)
156766047	NP_001096618.1	26058	GIGYF2	PERQ amino acid-rich with GYF domain-containing protein 2 isoform c	S26	0.6	0.6	0.8	1.1	3.0	2.2	1.7	1.4	0.6	0.6	0.7	1.1	3.0	2.1	1.6	1.3	1.8	1.3	1.1	1.2	ALSSGGSITSPPLsPALPK	SSGGSITsPPLSPAL	S10;	Yes	S(3): 0.0; S(4): 0.0; S(7): 0.0; T(9): 0.0; S(10): 100.0; S(14): 0.0	9	3	1	0	N-Term(TMT6plex); S10(Phospho); K19(TMT6plex)
385251399	NP_001245260.1	2036	EPB41L1	band 4.1-like protein 1 isoform b	S768	1.7	1.8	1.5	1.4	1.6	1.2	1.3	1.1	1.7	1.8	1.4	1.5	1.6	1.2	1.2	1.2	1.7	1.5	1.3	1.4	ETDPsPEER	VYRETDPsPEERDKK	S5;	No	T(2): 0.0; S(5): 100.0	14	4	1	0	N-Term(TMT6plex); S5(Phospho)
555943794	NP_001273171.1	2975	GTF3C1	general transcription factor 3C polypeptide 1 isoform 2	S1068	1.2	1.2	1.4	1.4	1.2	1.1	1.6	1.1	1.3	1.3	1.4	1.4	1.2	1.2	1.7	1.2	1.3	1.2	1.6	1.3	NSSTDQGsDEEGSLQK	NSSTDQGsDEEGSLQ	S8;	Yes	S(2): 0.0; S(3): 0.0; T(4): 0.0; S(8): 100.0; S(13): 0.0	9	2	1	0	N-Term(TMT6plex); S8(Phospho); K16(TMT6plex)
42542379	NP_005830.2	10250	SRRM1	serine/arginine repetitive matrix protein 1	S901	0.9	1.2	0.9	1.0	0.9	1.4	1.0	1.5	1.1	1.2	1.0	1.1	1.1	1.5	1.1	1.7	1.1	1.4	1.1	1.4	AQVsPQS	SMRKAQVsPQS	S4;	Yes	S(4): 100.0; S(7): 0.0	229	1	1	0	N-Term(TMT6plex); S4(Phospho)
71143119	NP_055888.1	23094	SIPA1L3	signal-induced proliferation-associated 1-like protein 3	S100	0.9	1.0	1.1	1.2	0.8	1.2	1.2	1.3	0.9	1.2	1.3	1.4	0.8	1.4	1.3	1.4	0.9	1.3	1.3	1.4	EHSNPsPSQDTDGTK	LREHSNPsPSQDTDG	S6;	Yes	S(3): 0.0; S(6): 100.0; S(8): 0.0; T(11): 0.0; T(14): 0.0	7	1	1	0	N-Term(TMT6plex); S6(Phospho); K15(TMT6plex)
5032189	NP_005648.1	7158	TP53BP1	tumor suppressor p53-binding protein 1 isoform 3	S380	1.4	1.1	1.4	1.4	1.1	1.7	0.8	1.1	1.4	1.1	1.6	1.7	1.1	1.7	0.8	1.4	1.3	1.4	1.2	1.5	STPFIVPSsPTEQEGR	TPFIVPSsPTEQEGR	S9;	Yes	S(1): 0.0; T(2): 0.0; S(8): 0.0; S(9): 100.0; T(11): 0.0	62	3	1	0	N-Term(TMT6plex); S9(Phospho)
68989256	NP_004283.2	9610	RIN1	ras and Rab interactor 1	S333; S337	2.0	0.9	1.1	1.0	1.2	1.2	1.4	1.5	2.4	0.8	0.7	0.7	1.5	1.1	0.9	1.2	2.0	0.9	0.8	0.9	GsPATsPHLGR	GVPGSRGsPATSPHL;SRGSPATsPHLGRRR	S2; S6;	Yes;Yes	S(2): 97.9; T(5): 4.2; S(6): 97.9	3	1	1	0	N-Term(TMT6plex); T5(Phospho); S6(Phospho)
547235537	NP_001271264.1	8669	EIF3J	eukaryotic translation initiation factor 3 subunit J	S127	0.9	1.3	1.4	1.2	0.9	1.0	1.2	1.3	0.9	1.1	1.3	1.1	0.9	0.9	1.1	1.1	0.9	1.0	1.2	1.1	LQEEsDLELAK	LKKLQEEsDLELAKE	S5;	Yes	S(5): 100.0	1	3	1	0	N-Term(TMT6plex); S5(Phospho); K11(TMT6plex)
30089937	NP_821174.1	54972	TMEM132A	transmembrane protein 132A isoform b precursor	S529	2.5	1.3	1.8	1.0	1.0	1.2	1.2	1.5	-	-	-	-	-	-	-	-	-	-	-	-	VPGPAEGPAEPAAEAsDEAER	AEPAAEAsDEAERRA	S16;	Yes	S(16): 100.0	1	2	1	0	N-Term(TMT6plex); S16(Phospho)
8923532	NP_060352.1	54982	CLN6	ceroid-lipofuscinosis neuronal protein 6	S31	1.2	1.2	1.2	1.3	1.2	1.2	1.5	1.2	-	-	-	-	-	-	-	-	-	-	-	-	HGsVSADEAAR	FLQARHGsVSADEAA	S3;	Yes	S(3): 100.0; S(5): 0.0	3	1	1	0	N-Term(TMT6plex); S3(Phospho)
215820635	NP_001135974.1	10848	PPP1R13L	relA-associated inhibitor	S358	1.0	1.1	1.2	1.3	1.1	1.2	1.1	1.2	1.1	1.1	1.2	1.3	1.1	1.2	1.1	1.2	1.1	1.2	1.2	1.3	IPMPPSsPQPR	RIPMPPSsPQPRGAP	S7;	Yes	S(6): 0.7; S(7): 99.3	4	1	1	0	N-Term(TMT6plex); M3(Oxidation); S7(Phospho)
18426915	NP_004386.2	1627	DBN1	drebrin isoform a	T331	1.0	1.9	1.6	1.5	0.8	1.6	1.3	1.0	0.9	1.6	1.4	1.5	0.8	1.4	1.2	1.0	0.8	1.5	1.3	1.2	MAPtPIPTRSPSDSSTASTPVAEQIER	SHRRMAPtPIPTRSP	T4;	Yes	T(4): 94.5; T(8): 1.8; S(10): 1.8; S(12): 1.8; S(14): 0.0; S(15): 0.0; T(16): 0.0; S(18): 0.0; T(19): 0.0	10	2	1	1	N-Term(TMT6plex); M1(Oxidation); T8(Phospho)
19923497	NP_061936.2	27436	EML4	echinoderm microtubule-associated protein-like 4 isoform a	S978	1.1	1.6	1.5	1.6	0.8	0.8	0.8	0.9	1.1	1.5	1.4	1.4	0.8	0.8	0.8	0.8	0.9	1.2	1.1	1.1	ATLLEDQQDPsPSS	LEDQQDPsPSS_	S11;	Yes	T(2): 0.0; S(11): 97.5; S(13): 1.2; S(14): 1.2	10	2	1	0	N-Term(TMT6plex); S11(Phospho)
4507311	NP_003159.1	6827	SUPT4H1	transcription elongation factor SPT4	T5	1.0	1.1	1.3	1.2	1.1	1.8	2.0	1.3	-	-	-	-	-	-	-	-	-	-	-	-	MALEtVPK	MALEtVPKDLRH	T5;	No	T(5): 100.0	1	1	1	0	N-Term(TMT6plex); M1(Oxidation); T5(Phospho); K8(TMT6plex)
348041385	NP_001231696.1	92597	MOB1B	MOB kinase activator 1B isoform 3	T35	0.9	1.5	1.2	1.0	0.8	1.0	1.1	1.5	-	-	-	-	-	-	-	-	-	-	-	-	HAEAtLGSGNLR	LLKHAEAtLGSGNLR	T5;	Yes	T(5): 100.0; S(8): 0.0	3	4	1	0	N-Term(TMT6plex); T5(Phospho)
118572613	NP_057417.3	23524	SRRM2	serine/arginine repetitive matrix protein 2	T1844	1.4	1.8	1.3	1.3	1.0	1.4	1.1	1.2	1.5	1.9	1.4	1.4	1.1	1.4	1.1	1.3	1.3	1.6	1.2	1.4	SRtPPTSR	RRRGRSRtPPTSRKR	T3;	Yes	S(1): 0.0; T(3): 100.0; T(6): 0.0; S(7): 0.0	436	1	1	1	N-Term(TMT6plex); T3(Phospho)

Khan et al., 2019. Multi-omics analysis to characterize cigarette smoke induced molecular alterations in esophageal cells

Supplementary Table 8. List of phosphopeptides identified and quantified in untreated and chronically treated Het1A cells with cigarette smoke condensate for 8 months using SequestHT and Mascot search algorithms with a PhosphoRS probability score ≥75%

														Normalized phosphoprotein ratios with respect to corresponding total protein ratios.								Average of replicates													
Protein group Accession	NP_Accession	Gene ID	Gene Symbol	Protein Description	PhosphoSite (Protein)	Het-1A-Smoke - 2M/Parental	Het-1A-Smoke - 4M/Parental	Het-1A-Smoke - 6M/Parental	Het-1A-Smoke - 8M/Parental	Het-1A-Smoke - 2M/Parental	Het-1A-Smoke - 4M/Parental	Het-1A-Smoke - 6M/Parental	Het-1A-Smoke - 8M/Parental	Het-1A-Smoke - 2M/Parental	Het-1A-Smoke - 4M/Parental	Het-1A-Smoke - 6M/Parental	Het-1A-Smoke - 8M/Parental	Het-1A-Smoke - 2M/Parental	Het-1A-Smoke - 4M/Parental	Het-1A-Smoke - 6M/Parental	Het-1A-Smoke - 8M/Parental	Het-1A-Smoke - 2M/Parental	Het-1A-Smoke - 4M/Parental	Het-1A-Smoke - 6M/Parental	Het-1A-Smoke - 8M/Parental	Peptide Sequence	Phosphowindow	PhosphoSite (Peptide)	Phosphosite Plus Evidence	phosphoRS Site Probabilities	# PSMs	# Proteins	# Protein Groups	# Missed Cleavages	Modifications
						Replicate 1				Replicate 2				Replicate 1				Replicate 2																	
115430211	NP_001041648.1	65979	PHACTR4	phosphatase and actin regulator 4 isoform 1	S116	1.2	1.4	1.2	1.2	1.0	1.3	1.0	1.3	1.5	1.6	1.5	1.4	1.1	1.5	1.3	1.6	1.3	1.5	1.4	1.5	sSSPVQVEEEPVR	TPIGNARsSSPVQVE	S1;	Yes	S(1): 98.9; S(2): 1.1; S(3): 0.0	3	2	1	0	N-Term(TMT6plex); S3(Phospho)
115430211	NP_001041648.1	65979	PHACTR4	phosphatase and actin regulator 4 isoform 1	S116	1.0	1.3	1.0	1.3	1.2	1.4	1.2	1.2	1.1	1.5	1.3	1.6	1.5	1.6	1.5	1.4	1.3	1.5	1.4	1.5	SSsPVQVEEEPVR	IGNARSSsPVQVEE	S1;	Yes	S(1): 0.0; S(2): 0.0; S(3): 100.0	7	2	1	0	N-Term(TMT6plex); S3(Phospho)
301897977	NP_001180437.1	5978	REST	RE1-silencing transcription factor	S864	1.6	2.3	2.0	1.6	0.7	0.7	0.8	0.9	1.7	2.1	2.3	1.6	0.8	0.7	0.9	0.9	1.3	1.4	1.6	1.3	ESVSTEDLSPPsPPLPK	TEDLSPPsPPLPKEN	S12;	Yes	S(2): 0.0; S(4): 0.0; T(5): 0.0; S(9): 0.0; S(12): 100.0	4	1	1	0	N-Term(TMT6plex); S12(Phospho); K17(TMT6plex)
76150625	NP_001028886.1	4839	NOP2	putative ribosomal RNA methyltransferase NOP2 isoform 1	S728	0.7	1.0	0.5	0.8	1.3	0.8	1.2	1.7	0.7	0.9	0.5	0.8	1.3	0.8	1.1	1.6	1.0	0.8	0.8	1.2	GTDTQTPAVLsPSK	TQTPAVLsPSKTQAT	S11;	No	T(2): 0.0; T(4): 0.0; T(6): 0.0; S(11): 100.0; S(13): 0.0	12	3	1	0	N-Term(TMT6plex); S11(Phospho); K14(TMT6plex)
24431933	NP_722550.1	57142	RTN4	reticulon-4 isoform B	S107	1.1	0.9	1.1	1.1	1.4	1.7	2.1	1.3	0.9	0.9	1.0	0.9	1.1	1.7	1.8	1.1	1.0	1.3	1.4	1.0	QPsWDPSPVSSTVPAPSPLSAAAVSPSK	VAPERQPsWDPSPVS	S3;	Yes	S(3): 100.0; S(7): 0.0; S(10): 0.0; S(11): 0.0; T(12): 0.0; S(17): 0.0; S(20): 0.0; S(25): 0.0; S(27): 0.0	6	3	1	0	N-Term(TMT6plex); S3(Phospho); K28(TMT6plex)
24431933	NP_722550.1	57142	RTN4	reticulon-4 isoform B	S107	1.4	1.7	2.1	1.3	1.1	0.9	1.1	1.1	1.1	1.7	1.8	1.1	0.9	0.9	1.0	0.9	1.0	1.3	1.4	1.0	QPSWDPsPVSSTVPAPSPLSAAAVSPSK	RQPSWDPsPVSSTVP	S3;	Yes	S(3): 0.0; S(7): 99.9; S(10): 0.0; S(11): 0.0; T(12): 0.0; S(17): 0.0; S(20): 0.0; S(25): 0.0; S(27): 0.0	6	3	1	0	N-Term(TMT6plex); S7(Phospho); K28(TMT6plex)
50659095	NP_004719.2	9188	DDX21	nucleolar RNA helicase 2 isoform 1	S89	0.9	1.3	1.6	1.2	1.1	1.8	2.0	1.3	0.8	1.0	1.2	1.0	1.0	1.4	1.5	1.1	0.9	1.2	1.3	1.0	KEEPSQNDIsPK	EPSQNDIsPKTKSLR	S10;	Yes	S(5): 0.0; S(10): 100.0	23	2	1	1	N-Term(TMT6plex); K1(TMT6plex); S10(Phospho); K12(TMT6plex)
13027630	NP_073210.1	8220	DGCR14	protein DGCR14	T386	1.5	1.4	1.5	0.8	1.5	1.1	2.0	1.7	1.5	1.3	1.7	0.8	1.5	1.0	2.2	1.7	1.5	1.1	1.9	1.3	VTENLASLtPK	TENLASLtPKGLSPA	T9;	Yes	T(2): 0.0; S(7): 0.0; T(9): 100.0	4	1	1	0	N-Term(TMT6plex); T9(Phospho); K11(TMT6plex)
50962882	NP_001002909.1	23131	GPATCH8	G patch domain-containing protein 8	S653	1.2	1.2	1.2	1.3	1.5	1.2	1.3	1.2	1.2	1.3	1.3	1.4	1.6	1.3	1.3	1.3	1.4	1.3	1.3	1.3	QEPGGSHGsETEDTGR	EPGGSHGsETEDTGR	S9;	Yes	S(6): 0.0; S(9): 100.0; T(11): 0.0; T(14): 0.0	17	1	1	0	N-Term(TMT6plex); S9(Phospho)
195972805	NP_001124400.1	4205	MEF2A	myocyte-specific enhancer factor 2A isoform 4	S330	1.3	1.1	1.2	1.4	0.8	1.0	1.0	1.1	1.2	1.1	1.1	1.1	0.7	1.0	0.9	0.9	1.0	1.0	1.0	1.0	SEPIsPPRDR	SIKSEPIsPPRDRMT	S5;	No	S(1): 0.0; S(5): 100.0	1	4	1	1	N-Term(TMT6plex); S5(Phospho)
47578107	NP_056199.2	25836	NIPBL	nipped-B-like protein isoform B	S2658	1.4	1.8	1.5	1.0	1.0	2.0	1.6	1.5	1.2	1.6	1.3	0.9	0.9	1.8	1.4	1.4	1.0	1.7	1.4	1.1	AITSLLGGGsPK	TSLLGGGsPKNNTAA	S10;	Yes	T(3): 0.0; S(4): 0.0; S(10): 100.0	8	2	1	0	N-Term(TMT6plex); S10(Phospho); K12(TMT6plex)
4758384	NP_004108.1	2289	FKBP5	peptidyl-prolyl cis-trans isomerase FKBP5 isoform 1	S13	1.7	1.2	1.3	1.0	1.0	1.0	1.8	1.5	1.7	1.2	1.4	1.0	1.0	1.0	1.9	1.5	1.4	1.1	1.6	1.2	NNEEsPTATVAEQGEDITSK	GAKNNEEsPTATVAE	S5;	Yes	S(5): 98.5; T(7): 1.5; T(9): 0.0; T(18): 0.0; S(19): 0.0	15	2	1	0	N-Term(TMT6plex); S5(Phospho); K20(TMT6plex)
212549782	NP_001131139.1	9994	CASP8AP2	CASP8-associated protein 2	S875	0.9	1.0	1.2	0.8	0.4	1.3	1.3	1.7	-	-	-	-	-	-	-	-	-	-	-	-	NEsPPQVPVYNNSHK	AKVLRNEsPPQVPVY	S3;	Yes	S(3): 100.0; Y(10): 0.0; S(13): 0.0	2	1	1	0	N-Term(TMT6plex); S3(Phospho); K15(TMT6plex)
267844828	NP_001161213.1	345778	MTX3	metaxin-3 isoform 1	S250	2.8	2.1	2.1	1.4	1.0	1.0	1.2	1.0	2.1	1.8	1.8	1.1	0.8	0.8	1.0	0.8	1.5	1.3	1.4	0.9	LTPAEEENNSEQRLsP	NNSEQRLsP	S15;	No	T(2): 0.0; S(10): 0.0; S(15): 100.0	1	1	1	1	N-Term(TMT6plex); S10(Phospho)
545746435	NP_001269978.1	9101	USP8	ubiquitin carboxyl-terminal hydrolase 8 isoform b	S357	0.8	0.8	0.9	1.1	0.8	0.9	0.9	1.3	0.7	0.7	0.8	1.0	0.8	0.8	0.9	1.2	0.8	0.8	0.8	1.1	SESTNHEQQsPQSGK	STNHEQQsPQSGKVI	S10;	No	S(1): 0.0; S(3): 0.0; T(4): 0.0; S(10): 99.2; S(13): 0.8	5	2	1	0	N-Term(TMT6plex); S10(Phospho); K15(TMT6plex)
545746335	NP_001271143.1	9320	TRIP12	E3 ubiquitin-protein ligase TRIP12 isoform a	S990	0.5	0.8	1.0	1.5	0.3	0.4	0.7	1.0	0.5	0.8	1.0	1.5	0.3	0.4	0.7	1.0	0.4	0.6	0.8	1.3	HLAESESLLTsPPK	ESESLLTsPPKACTN	S11;	No	S(5): 0.0; S(7): 0.0; T(10): 0.0; S(11): 100.0	23	4	1	0	N-Term(TMT6plex); S11(Phospho); K14(TMT6plex)
282847485	NP_001164228.1	159090	FAM122B	protein FAM122B isoform 5	S103	1.4	1.5	1.3	1.5	0.7	0.9	1.0	1.0	-	-	-	-	-	-	-	-	-	-	-	-	MFVSSSGLPPsPVPSPR	SSSGLPPsPVPSPRR	S11;	No	S(4): 0.0; S(5): 0.0; S(6): 0.0; S(11): 100.0; S(15): 0.0	3	5	1	0	N-Term(TMT6plex); M1(Oxidation); S11(Phospho)
325053642	NP_001191380.1	9236	CCPG1	cell cycle progression protein 1 isoform 3	S186	1.7	1.6	1.1	1.3	1.3	1.2	0.8	1.2	-	-	-	-	-	-	-	-	-	-	-	-	TVsASESEDR	RARKKTVsASESEDR	S3;	Yes	T(1): 0.0; S(3): 100.0; S(5): 0.0; S(7): 0.0	2	3	1	0	N-Term(TMT6plex); S3(Phospho)
394582099	NP_001257401.1	4172	MCM3	DNA replication licensing factor MCM3 isoform 2	S738	1.2	1.4	1.4	1.1	1.4	1.7	1.5	1.3	1.2	1.6	1.6	1.4	1.5	1.9	1.8	1.6	1.4	1.8	1.7	1.5	TADsQETK	HTPKTADsQETKESQ	S4;	No	T(1): 0.0; S(4): 100.0; T(7): 0.0	10	2	1	0	N-Term(TMT6plex); S4(Phospho); K8(TMT6plex)
544583552	NP_001269720.1	23369	PUM2	pumilio homolog 2 isoform 4	S126	1.5	1.8	1.7	1.6	1.0	1.0	1.0	0.9	1.4	1.8	1.6	1.4	0.9	0.9	1.0	0.8	1.1	1.4	1.3	1.1	QAsPTEVVER	TPGSRQAsPTEVVER	S3;	No	S(3): 100.0; T(5): 0.0	1	4	1	0	N-Term(TMT6plex); S3(Phospho)
47132589	NP_002732.3	5585	PKN1	serine/threonine-protein kinase N1 isoform 2	S562	0.5	0.8	1.0	1.0	0.8	0.9	1.2	1.5	0.6	1.1	1.1	1.1	0.9	1.1	1.3	1.6	0.8	1.1	1.2	1.4	LNLGTDSDSsPQK	LGTDSDSsPQKSSRD	S10;	Yes	T(5): 0.0; S(7): 0.0; S(9): 0.9; S(10): 99.1	8	2	1	0	N-Term(TMT6plex); S10(Phospho); K13(TMT6plex)
116008442	NP_055885.3	23091	ZC3H13	zinc finger CCCH domain-containing protein 13	S1014; S1017	0.7	0.6	0.7	1.0	1.1	1.1	1.1	1.4	0.6	0.6	0.7	1.0	1.1	1.0	1.1	1.4	0.9	0.8	0.9	1.2	SKGDsDIsDEEAAQQSK	RKKSKGDsDISDEEA;SKGDSDIsDEEAAQQ	S5; S8;	Yes;Yes	S(1): 2.4; S(5): 97.7; S(8): 99.9; S(16): 0.0	28	1	1	1	N-Term(TMT6plex); K2(TMT6plex); S5(Phospho); S8(Phospho); K17(TMT6plex)
197333809	NP_056170.2	23512	SUZ12	polycomb protein SUZ12	S546	1.3	1.6	1.6	1.5	0.9	0.9	1.2	1.0	1.3	1.7	1.6	1.5	0.9	0.9	1.2	1.0	1.1	1.3	1.4	1.2	ASMSEELEsEDGEVEQQR	SMSEELEsEDGEVEQ	S9;	Yes	S(2): 0.0; S(4): 0.0; S(9): 100.0	3	1	1	0	N-Term(TMT6plex); M3(Oxidation); S9(Phospho)
351542238	NP_001234925.1	50807	ASAP1	arf-GAP with SH3 domain, ANK repeat and PH domain-containing protein 1 isoform 2	S1001	1.3	1.2	1.8	1.2	1.6	1.6	2.0	1.3	1.2	1.1	1.6	1.1	1.4	1.4	1.8	1.2	1.3	1.3	1.7	1.2	SQTGDVsPK	KSQTGDVsPKAQQPS	S7;	No	S(1): 0.0; T(3): 0.0; S(7): 100.0	3	2	1	0	N-Term(TMT6plex); S7(Phospho); K9(TMT6plex)
38044290	NP_060082.2	55596	ZCCHC8	zinc finger CCHC domain-containing protein 8	S427	1.2	1.2	1.2	1.2	1.1	1.3	1.4	1.2	1.1	1.1	1.1	1.2	1.1	1.2	1.3	1.2	1.1	1.1	1.2	1.2	SSSHSSPGsPK	SSHSSPGsPKKQKNE	S9;	Yes	S(1): 0.0; S(2): 0.0; S(3): 0.0; S(5): 0.0; S(6): 0.0; S(9): 100.0	21	1	1	0	N-Term(TMT6plex); S9(Phospho); K11(TMT6plex)
33188445	NP_036222.3	23499	MACF1	microtubule-actin cross-linking factor 1	S5334	1.7	1.3	1.1	1.5	1.4	1.0	0.9	0.9	1.7	1.2	1.1	1.7	1.3	0.9	0.8	1.1	1.5	1.1	1.0	1.4	TSLAGDTSNSSsPASTGAK	GDTSNSSsPASTGAK	S12;	No	T(1): 0.0; S(2): 0.0; T(7): 0.0; S(8): 0.0; S(10): 0.0; S(11): 1.7; S(12): 98.2; S(15): 0.0; T(16): 0.0	4	1	1	0	N-Term(TMT6plex); S12(Phospho); K19(TMT6plex)
314122233	NP_001186627.1	7917	BAG6	large proline-rich protein BAG6 isoform d	S967	1.2	1.3	1.1	1.3	1.0	0.9	1.0	1.1	1.3	1.4	1.3	1.4	1.0	1.0	1.1	1.2	1.1	1.2	1.2	1.3	ENAsPAPGTTAEEAMSR	EPQRENAsPAPGTTA	S4;	No	S(4): 100.0; T(9): 0.0; T(10): 0.0; S(16): 0.0	15	3	1	0	N-Term(TMT6plex); S4(Phospho); M15(Oxidation)
61743954	NP_001611.1	79026	AHNAK	neuroblast differentiation-associated protein AHNAK isoform 1	T3716	1.2	1.9	2.0	1.1	1.6	1.4	2.1	1.3	1.5	1.9	2.0	1.4	1.9	1.4	2.2	1.5	1.7	1.7	2.1	1.4	VDIDtPDINIEGSEGK	GPKVDIDtPDINIEG	T5;	Yes	T(5): 100.0; S(13): 0.0	79	1	1	0	N-Term(TMT6plex); T5(Phospho); K16(TMT6plex)
7662394	NP_055719.1	22841	RAB11FIP2	rab11 family-interacting protein 2	S277	1.7	1.2	1.5	1.2	1.4	1.3	1.2	1.3	-	-	-	-	-	-	-	-	-	-	-	-	TLsFDTSK	SPHRRTLsFDTSKMN	S3;	Yes	T(1): 0.0; S(3): 100.0; T(6): 0.0; S(7): 0.0	2	1	1	0	N-Term(TMT6plex); S3(Phospho); K8(TMT6plex)
14165435	NP_112552.1	3190	HNRNPK	heterogeneous nuclear ribonucleoprotein K isoform b	S284	0.9	0.7	0.7	1.2	0.8	1.0	0.9	1.2	1.0	0.7	0.8	1.2	0.9	1.1	1.0	1.2	0.9	0.9	0.9	1.2	DYDDMsPR	RRDYDDMsPRRGPPP	S6;	Yes	Y(2): 0.0; S(6): 100.0	2	2	1	0	N-Term(TMT6plex); M5(Oxidation); S6(Phospho)
170763500	NP_001116293.1	6418	SET	protein SET isoform 1	S63	1.0	1.1	1.2	1.2	0.9	1.2	1.3	1.3	1.0	1.1	1.3	1.3	0.9	1.2	1.4	1.4	0.9	1.2	1.4	1.4	LNEQAsEEILK	DRLNEQAsEEILKVE	S6;	Yes	S(6): 100.0	7	4	1	0	N-Term(TMT6plex); S6(Phospho); K11(TMT6plex)
347582619	NP_001231573.1	25978	CHMP2B	charged multivesicular body protein 2b isoform 2	S158	1.2	1.1	1.3	1.4	1.1	1.1	1.3	1.0	1.1	1.2	1.3	1.6	1.1	1.2	1.3	1.2	1.1	1.2	1.3	1.4	ATIsDEEIER	STSKATIsDEEIERQ	S4;	No	T(2): 0.0; S(4): 100.0	2	2	1	0	N-Term(TMT6plex); S4(Phospho)
33356174	NP_002678.2	5411	PNN	pinin	S66	1.3	1.3	1.7	1.1	1.8	1.6	1.5	1.4	1.5	1.6	2.1	1.2	2.1	1.8	1.8	1.6	1.8	1.7	2.0	1.4	GEsDSGGGPPAK	LLLRRGEsDSGGGPP	S3;	Yes	S(3): 100.0; S(5): 0.0	7	1	1	0	N-Term(TMT6plex); S3(Phospho); K12(TMT6plex)
298358598	NP_001177255.1	54487	DGCR8	microprocessor complex subunit DGCR8 isoform 2	S377	0.8	0.9	0.9	0.8	2.1	1.7	2.0	1.6	0.7	0.6	0.8	0.8	1.9	1.2	1.7	1.5	1.3	0.9	1.2	1.2	EQSSDLTPSGDVsPVKPLSR	LTPSGDVsPVKPLSR	S13;	Yes	S(3): 0.0; S(4): 0.0; T(7): 0.0; S(9): 0.0; S(13): 99.9; S(19): 0.0	3	2	1	0	N-Term(TMT6plex); S13(Phospho); K16(TMT6plex)
39753957	NP_056417.2	26092	TOR1AIP1	torsin-1A-interacting protein 1 isoform 2	S242	1.3	1.4	1.6	1.2	1.2	1.4	1.5	1.2	1.2	1.5	1.5	1.2	1.2	1.5	1.4	1.2	1.2	1.5	1.5	1.2	SRDsDESGDK	VKARSRDsDESGDKT	S4;	No	S(1): 0.0; S(4): 100.0; S(7): 0.0	4	2	1	1	N-Term(TMT6plex); S4(Phospho); K10(TMT6plex)
341865562	NP_001230072.1	51203	NUSAP1	nucleolar and spindle-associated protein 1 isoform 5	S120	1.6	0.9	1.3	1.3	1.0	1.0	1.0	1.2	2.2	1.0	1.5	1.5	1.3	1.2	1.1	1.4	1.8	1.1	1.3	1.5	VPsPPDEHQEAENAVSSGNR	RATAKVPsPPDEHQE	S3;	No	S(3): 100.0; S(16): 0.0; S(17): 0.0	14	5	1	0	N-Term(TMT6plex); S3(Phospho)
123173757	NP_597709.2	125950	RAVER1	ribonucleoprotein PTB-binding 1	T480	1.1	0.9	0.6	1.1	0.9	1.3	0.9	1.3	1.3	0.9	0.6	1.2	1.0	1.3	1.0	1.3	1.1	1.1	0.8	1.3	EALGLGPPAAQLtPPPAPVGLR	GPPAAQLtPPPAPVG	T13;	No	T(13): 100.0	3	1	1	0	N-Term(TMT6plex); T13(Phospho)
4503739	NP_001446.1	2309	FOXO3	forkhead box protein O3	S413	1.4	1.4	1.5	1.2	1.2	1.3	1.5	1.2	1.7	1.5	1.7	1.2	1.4	1.3	1.6	1.3	1.6	1.4	1.6	1.2	SSsFPYTTK	GLMQRSSsFPYTTKG	S3;	Yes	S(1): 0.0; S(2): 0.7; S(3): 99.3; Y(6): 0.0; T(7): 0.0; T(8): 0.0	4	1	1	0	N-Term(TMT6plex); S3(Phospho); K9(TMT6plex)
4759098	NP_004584.1	6434	TRA2B	transformer-2 protein homolog beta isoform 1	S26	0.8	1.7	1.4	1.4	0.7	1.1	0.9	1.0	0.9	1.9	1.7	1.7	0.9	1.2	1.0	1.2	0.9	1.5	1.4	1.5	SGSAHGsGK	RSGSAHGsGKSARHT	S7;	Yes	S(1): 0.0; S(3): 0.0; S(7): 100.0	11	1	1	0	N-Term(TMT6plex); S7(Phospho); K9(TMT6plex)
499590378	NP_001265132.1	23155	CLCC1	chloride channel CLIC-like protein 1 isoform 4	T297	0.9	0.9	0.9	1.1	1.2	0.9	1.0	1.3	0.8	0.8	0.9	1.1	1.1	0.8	1.0	1.3	1.0	0.8	1.0	1.2	ETGGILGEGtPK	GGILGEGtPKESSTE	T10;	No	T(2): 0.0; T(10): 100.0	6	4	1	0	N-Term(TMT6plex); T10(Phospho); K12(TMT6plex)
21396500	NP_003600.2	8479	HIRIP3	HIRA-interacting protein 3 isoform 1	S555	1.0	1.4	1.0	1.2	1.0	1.1	0.9	1.2	1.1	1.5	1.2	1.3	1.1	1.2	1.0	1.3	1.1	1.3	1.1	1.3	GIISSDGEsN	IISSDGEsN	S9;	Yes	S(4): 0.0; S(5): 0.0; S(9): 100.0	26	1	1	0	N-Term(TMT6plex); S9(Phospho)
110735439	NP_000544.2	7486	WRN	Werner syndrome ATP-dependent helicase	S1133	0.6	0.9	0.8	0.8	1.1	1.4	1.5	1.7	0.6	0.8	0.7	0.6	0.9	1.2	1.3	1.4	0.8	1.0	1.0	1.0	SIMVQsPEK	KKSIMVQsPEKAYSS	S6;	Yes	S(1): 0.0; S(6): 100.0	2	1	1	0	N-Term(TMT6plex); M3(Oxidation); S6(Phospho); K9(TMT6plex)
259906020	NP_001158287.1	22985	ACIN1	apoptotic chromatin condensation inducer in the nucleus isoform 3	S200	1.2	1.3	1.1	1.1	1.3	1.6	1.4	1.3	1.0	1.2	1.2	1.1	1.0	1.5	1.5	1.4	1.0	1.3	1.4	1.3	LsEGSQPAEEEEDQETPSR	QARAAKLsEGSQPAE	S2;	No	S(2): 100.0; S(5): 0.0; T(16): 0.0; S(18): 0.0	38	3	1	0	N-Term(TMT6plex); S2(Phospho)
259906020	NP_001158287.1	22985	ACIN1	apoptotic chromatin condensation inducer in the nucleus isoform 3	S200	1.3	1.6	1.4	1.3	1.2	1.3	1.1	1.1	1.0	1.5	1.5	1.4	1.0	1.2	1.2	1.1	1.0	1.3	1.4	1.3	LSEGsQPAEEEEDQETPSR	AAKLSEGsQPAEEEE	S2;	No	S(2): 0.0; S(5): 100.0; T(16): 0.0; S(18): 0.0	4	3	1	0	N-Term(TMT6plex); S5(Phospho)
31563503	NP_852107.1	23394	ADNP	activity-dependent neuroprotector homeobox protein	S921	1.2	1.2	1.1	1.2	0.9	1.1	1.0	1.3	1.4	1.3	1.3	1.3	1.0	1.2	1.1	1.3	1.2	1.2	1.2	1.3	VIPEDAsESEEK	KVIPEDAsESEEKLD	S7;	Yes	S(7): 99.4; S(9): 0.6	7	1	1	0	N-Term(TMT6plex); S7(Phospho); K12(TMT6plex)
449083351	NP_001263347.1	9919	SEC16A	protein transport protein Sec16A isoform 2	S1069	0.6	1.4	1.1	0.7	1.8	2.0	1.8	1.7	0.6	1.2	1.0	0.6	1.7	1.8	1.6	1.6	1.1	1.5	1.3	1.1	AQQELVPPQQQQAsPPQLPK	VPPQQQQAsPPQLPKA	S13;	Yes	S(13): 100.0	10	2	1	0	N-Term(TMT6plex); S13(Phospho); K19(TMT6plex)
5031877	NP_005564.1	4001	LMNB1	lamin-B1 isoform 1	T20; S23	0.5	0.6	0.6	1.4	0.6	0.8	0.6	1.0	0.5	0.7	0.7	1.4	0.6	0.8	0.8	1.0	0.6	0.8	0.7	1.2	AGGPTtPLsPTR	SRAGGPTtPLSPTRL;GGPTTPLsPTRLSRL	T6; S9;	Yes;Yes	T(5): 1.5; T(6): 98.5; S(9): 98.5; T(11): 1.5	14	1	1	0	N-Term(TMT6plex); T5(Phospho); S9(Phospho)
546231404	NP_001271231.1	54443	ANLN	actin-binding protein anillin isoform 3	S754	1.3	1.7	1.7	1.2	1.5	1.4	1.8	1.3	1.3	1.7	1.6	1.2	1.6	1.4	1.7	1.3	1.5	1.5	1.7	1.3	NKAsPQSEFMPSK	PQRKNKAsPQSEFMP	S4;	No	S(4): 100.0; S(7): 0.0; S(12): 0.0	21	3	1	1	N-Term(TMT6plex); K2(TMT6plex); S4(Phospho); M10(Oxidation); K13(TMT6plex)
116256489	NP_006631.2	10801	40057	septin-9 isoform c	S12	0.9	0.9	1.0	1.0	1.3	1.3	1.4	1.5	1.0	0.9	1.1	1.0	1.3	1.4	1.5	1.5	1.2	1.2	1.3	1.2	sFEVEEVETPNSTPPR	RISALKRsFEVEEVE	S1;	No	S(1): 100.0; T(9): 0.0; S(12): 0.0; T(13): 0.0	9	3	1	0	N-Term(TMT6plex); S1(Phospho)
4504445	NP_002127.1	3178	HNRNPA1	heterogeneous nuclear ribonucleoprotein A1 isoform a	S6	1.3	1.3	1.2	1.2	1.1	1.2	1.2	1.2	1.6	1.4	1.4	1.3	1.4	1.3	1.3	1.4	1.5	1.4	1.4	1.4	SEsPKEPEQLR	MSKSEsPKEPEQL	S3;	Yes	S(1): 0.8; S(3): 99.2	4	2	1	1	N-Term(TMT6plex); S3(Phospho); K5(TMT6plex)
148596949	NP_004732.2	9221	NOLC1	nucleolar and coiled-body phosphoprotein 1 isoform 2	S643	0.6	1.0	1.3	1.2	0.7	1.0	1.4	1.2	0.5	0.8	1.0	0.9	0.6	0.8	1.1	0.9	0.5	0.8	1.0	0.9	VADNsEDAK	DSRVADNsEDAKRGA	S5;	Yes	S(5): 100.0	33	3	1	0	N-Term(TMT6plex); S5(Phospho); K9(TMT6plex)
347800726	NP_001231663.1	153562	MARVELD2	MARVEL domain-containing protein 2 isoform 2	S161	1.1	1.1	1.1	1.4	0.9	1.0	0.9	1.0	-	-	-	-	-	-	-	-	-	-	-	-	DPYGsLDR	EPRDPYGsLDRHTQT	S5;	Yes	Y(3): 0.0; S(5): 100.0	1	2	1	0	N-Term(TMT6plex); S5(Phospho)
109809741	NP_057732.2	51574	LARP7	la-related protein 7 isoform 1	S300	1.3	1.2	0.9	1.1	1.4	1.8	0.9	1.3	1.5	1.2	1.0	1.2	1.5	1.9	1.0	1.3	1.5	1.6	1.0	1.3	SSsEDAESLAPR	GKRKRSSsEDAESLA	S3;	Yes	S(1): 0.0; S(2): 0.0; S(3): 100.0; S(8): 0.0	6	2	1	0	N-Term(TMT6plex); S3(Phospho)
109809741	NP_057732.2	51574	LARP7	la-related protein 7 isoform 1	S300	1.4	1.8	0.9	1.3	1.3	1.2	0.9	1.1	1.5	1.9	1.0	1.3	1.5	1.2	1.0	1.2	1.5	1.6	1.0	1.3	sSSEDAESLAPR	RTGKRKRsSSEDAES	S3;	Yes	S(1): 98.9; S(2): 1.1; S(3): 0.0; S(8):	2	2	1	0	N-Term(TMT6plex); S1(Phospho)
367460087	NP_005955.3	4628	MYH10	myosin-10 isoform 2	S1975	1.2	0.9	1.2	1.2	1.6	1.2	1.9	1.2	1.2	1.0	1.3	1.1	1.7	1.3	2.0	1.2	1.5	1.1	1.6	1.1	TSDVNETQPPQsE	NETQPPQsE_	S12;	Yes	T(1): 0.0; S(2): 0.0; T(7): 0.0; S(12): 100.0	19	3	1	0	N-Term(TMT6plex); S12(Phospho)
148727247	NP_003472.2	8078	USP5	ubiquitin carboxyl-terminal hydrolase 5 isoform 2	T623	1.0	1.0	1.2	1.3	1.0	0.9	1.3	1.1	1.0	0.9	1.1	1.2	0.9	0.8	1.2	1.1	1.0	0.8	1.1	1.1	GTGLQPGEEELPDIAPPLVtPDE	DIAPPLVtPDEPKAP	T20;	No	T(2): 0.0; T(20): 100.0	5	2	1	0	N-Term(TMT6plex); T20(Phospho); K25(TMT6plex)
13129110	NP_077007.1	79084	WDR77	methylosome protein 50	T5	1.4	1.4	1.5	0.9	0.6	1.1	0.7	1.5	1.2	1.3	1.3	0.5	0.5	1.0	0.6	0.9	0.9	1.1	0.9	0.7	KEtPPPLVPPAAR	MRKEtPPPLVPP	T3;	Yes	T(3): 100.0	1	1	1	1	N-Term(TMT6plex); K1(TMT6plex); T3(Phospho)
139394604	NP_055767.3	22893	BAHD1	bromo adjacent homology domain-containing 1 protein	S184	1.1	1.4	1.3	1.3	1.1	1.4	1.2	1.2	-	-	-	-	-	-	-	-	-	-	-	-	DLsPEPAPDEGPR	GGGSRDLsPEPAPDE	S3;	Yes	S(3): 100.0	4	1	1	0	N-Term(TMT6plex); S3(Phospho)
132626688	NP_055456.2	9656	MDC1	mediator of DNA damage checkpoint protein 1	T1425	1.0	1.8	1.1	1.3	1.0	0.9	0.6	1.1	1.1	1.8	1.1	1.4	1.1	0.9	0.6	1.2	1.1	1.3	0.9	1.3	LEPSTSTDQPVtPEPTSQATR	TSTDQPVtPEPTSQA	T12;	Yes	S(4): 0.0; T(5): 0.0; S(6): 0.0; T(7): 0.0; T(12): 100.0; T(16): 0.0; S(17): 0.0; T(20): 0.0	26	1	1	0	N-Term(TMT6plex); T12(Phospho)
555290083	NP_001273086.1	1783	DYNC1LI2	cytoplasmic dynein 1 light intermediate chain 2 isoform 2	S330	0.7	0.8	0.9	1.1	0.8	0.8	0.9	1.3	0.7	0.8	0.9	1.0	0.7	0.8	0.8	1.2	0.7	0.8	0.9	1.1	GGPASVPSSsPGTSVK	PASVPSSsPGTSVKK	S10;	No	S(5): 0.0; S(8): 0.0; S(9): 1.2; S(10): 98.8; T(13): 0.0; S(14): 0.0	11	2	1	0	N-Term(TMT6plex); S10(Phospho); K16(TMT6plex)
313482820	NP_001186232.1	4848	CNOT2	CCR4-NOT transcription complex subunit 2	S165	1.4	0.6	0.9	1.0	1.3	1.1	1.0	1.4	1.3	0.6	0.8	0.8	1.3	1.0	1.0	1.1	1.3	0.8	0.9	1.0	TNSMSSSGLGsPNR	MSSSGLGsPNRSSPS	S11;	Yes	T(1): 0.0; S(3): 0.0; S(5): 0.0; S(6): 0.0; S(7): 0.0; S(11): 100.0	8	1	1	0	N-Term(TMT6plex); M4(Oxidation); S11(Phospho)
12667788	NP_002464.1	4627	MYH9	myosin-9	S1943	1.6	1.5	1.7	1.2	1.6	1.4	1.5	1.2	1.4	1.3	1.3	1.1	1.3	1.2	1.2	1.1	1.3	1.2	1.3	1.1	KGAGDGsDEEVDGK	RKGAGDGsDEEVDGK	S7;	Yes	S(7): 100.0	75	1	1	1	N-Term(TMT6plex); K1(TMT6plex); S7(Phospho); K14(TMT6plex)
146198654	NP_078947.3	79724	ZNF768	zinc finger protein 768	S90; S97	0.9	1.1	0.8	1.3	1.0	0.9	0.8	1.1	1.0	1.2	1.0	1.4	1.1	1.0	1.0	1.3	1.1	1.1	1.0	1.3	sPGLVPPsPEFAPR	SPGFESRsPGLVPPS;SPGLVPPsPEFAPRS	S1; S8;	Yes;Yes	S(1): 100.0; S(8): 100.0	18	1	1	0	N-Term(TMT6plex); S1(Phospho); S8(Phospho)
333440471	NP_001193980.1	23332	CLASP1	CLIP-associating protein 1 isoform 4	S662	1.7	1.6	1.7	1.6	1.4	1.8	1.4	0.8	1.8	1.7	1.9	1.7	1.5	1.8	1.5	0.9	1.6	1.8	1.7	1.3	RQsSGSATNVASTPDNR	RIRTRRQsSGSATNV	S3;	No	S(3): 98.9; S(4): 1.1; S(6): 0.0; T(8): 0.0; S(12): 0.0; T(13): 0.0	14	4	1	1	N-Term(TMT6plex); S3(Phospho)
13376852	NP_079516.1	80381	CD276	CD276 antigen isoform b precursor	S307	0.7	0.8	0.7	0.7	1.4	1.4	1.4	1.7	0.8	1.0	0.9	0.9	1.6	1.7	1.8	2.3	1.2	1.3	1.4	1.6	HSDsKEDDGQEIA	QPLKHSDsKEDDGQE	S4;	No	S(2): 0.0; S(4): 100.0	4	2	1	1	N-Term(TMT6plex); S4(Phospho); K5(TMT6plex)
7656879	NP_055238.1	27125	AFF4	AF4/FMR2 family member 4	S836	2.3	2.4	1.6	1.4	1.4	1.6	0.9	1.0	2.0	2.2	1.5	1.3	1.3	1.4	0.9	1.0	1.6	1.8	1.2	1.1	TIsQSSSLK	GSRKRTIsQSSSLKS	S3;	Yes	T(1): 0.0; S(3): 100.0; S(5): 0.0; S(6): 0.0; S(7): 0.0	16	1	1	0	N-Term(TMT6plex); S3(Phospho); K9(TMT6plex)
156071452	NP_001095132.1	4898	NRD1	nardilysin isoform b precursor	S94	1.4	2.0	1.5	1.5	1.0	1.2	1.3	0.9	1.5	2.1	1.5	1.6	1.1	1.2	1.3	0.9	1.3	1.6	1.4	1.3	GsLSNAGDPEIVK	EEEGRRGsLSNAGDP	S2;	Yes	S(2): 100.0; S(4): 0.0	2	2	1	0	N-Term(TMT6plex); S2(Phospho); K13(TMT6plex)
149192871	NP_056022.1	23252	OTUD3	OTU domain-containing protein 3	S326	1.1	1.0	1.0	1.2	1.2	1.2	1.0	1.2	-	-	-	-	-	-	-	-	-	-	-	-	AQAsPSEENK	ENNKAQAsPSEENKA	S4;	No	S(4): 100.0; S(6): 0.0	2	1	1	0	N-Term(TMT6plex); S4(Phospho); K10(TMT6plex)
542133150	NP_001269458.1	55833	UBAP2	ubiquitin-associated protein 2 isoform 4	S206	0.8	1.2	1.1	1.4	0.6	0.8	0.7	1.0	0.8	1.1	1.2	1.2	0.6	0.8	0.8	0.9	0.7	1.0	1.0	1.1	ESTPGDsPSTVNK	RESTPGDsPSTVNKL	S7;	No	S(2): 0.0; T(3): 0.0; S(7): 100.0; S(9): 0.0; T(10): 0.0	1	2	1	0	N-Term(TMT6plex); S7(Phospho); K13(TMT6plex)
355390315	NP_056171.3	23513	SCRIB	protein scribble homolog isoform b	S853	1.3	0.9	1.4	1.1	1.1	1.3	0.7	1.3	1.2	0.8	1.4	1.0	1.0	1.2	0.7	1.2	1.1	1.0	1.1	1.1	LPLLPPEsPGPLR	LPLLPPEsPGPLRQR	S8;	Yes	S(8): 100.0	16	2	1	0	N-Term(TMT6plex); S8(Phospho)
154354972	NP_001093636.1	9749	PHACTR2	phosphatase and actin regulator 2 isoform 4	S157	1.0	1.0	1.3	1.3	0.8	1.1	1.1	1.1	0.9	1.0	1.2	1.3	0.8	1.1	1.1	1.1	0.8	1.1	1.2	1.2	ASAsPSTSSTSSRPK	TGSKASAsPSTSSTS	S4;	No	S(2): 0.0; S(4): 93.8; S(6): 0.0; T(7): 0.0; S(8): 1.2; S(9): 1.2; T(10): 1.2; S(11): 1.2; S(12): 1.2	2	4	1	0	N-Term(TMT6plex); S4(Phospho); K15(TMT6plex)
154355000	NP_003676.2	8570	KHSRP	far upstream element-binding protein 2	S181	0.8	1.2	0.9	1.2	1.0	1.2	0.9	1.2	0.9	1.3	1.0	1.2	1.1	1.2	1.0	1.2	1.0	1.3	1.0	1.2	VQIsPDSGGLPER	SGCKVQIsPDSGGLP	S4;	Yes	S(4): 100.0; S(7): 0.0	16	1	1	0	N-Term(TMT6plex); S4(Phospho)
298228964	NP_942593.2	55054	ATG16L1	autophagy-related protein 16-1 isoform 3	S124	1.8	1.4	1.3	1.2	1.4	1.5	1.4	1.2	1.7	1.4	1.3	0.9	1.3	1.5	1.4	0.9	1.5	1.4	1.3	0.9	sVSSFPVPQDNVDTHPGSGK	SRAATRRsVSSFPVP	S1;	No	S(1): 94.7; S(3): 2.7; S(4): 2.7; T(14): 0.0; S(18): 0.0	1	5	1	0	N-Term(TMT6plex); S1(Phospho); K20(TMT6plex)
66348045	NP_057562.3	51530	ZC3HC1	nuclear-interacting partner of ALK isoform 1	S335	1.2	1.1	1.3	1.3	1.0	0.9	1.1	1.1	1.1	1.1	1.2	1.2	0.9	0.9	1.1	1.0	1.0	1.0	1.1	1.1	SQDATFsPGSEQAEK	RSQDATFsPGSEQAE	S7;	Yes	S(1): 0.0; T(5): 0.0; S(7): 100.0; S(10): 0.0	8	2	1	0	N-Term(TMT6plex); S7(Phospho); K15(TMT6plex)
39725634	NP_056130.2	23367	LARP1	la-related protein 1	S697	0.7	1.4	1.1	1.1	1.1	1.8	1.1	1.3	0.7	1.3	1.1	1.1	1.1	1.7	1.1	1.3	0.9	1.5	1.1	1.2	SLPTTVPEsPNYR	LPTTVPEsPNYRNTR	S9;	No	S(1): 0.0; T(4): 0.0; T(5): 0.0; S(9): 99.2; Y(12): 0.8	28	1	1	0	N-Term(TMT6plex); S9(Phospho)
571026657	NP_001275686.1	29086	BABAM1	BRISC and BRCA1-A complex member 1 isoform 2	S66	1.4	1.2	1.1	1.4	1.0	0.8	1.0	1.0	1.3	1.3	1.1	1.4	0.9	0.9	1.1	1.1	1.1	1.1	1.1	1.3	SEGEGEAASADDGSLNTsGAGPK	DDGSLNTsGAGPKSW	S18;	Yes	S(1): 0.0; S(9): 0.0; S(14): 0.0; T(17): 0.0; S(18): 100.0	17	2	1	0	N-Term(TMT6plex); S18(Phospho); K23(TMT6plex)
102467242	NP_064623.2	3619	INCENP	inner centromere protein isoform 2	S263	1.1	1.0	1.0	1.3	1.9	0.9	2.0	1.1	1.1	0.9	1.0	1.3	1.9	0.9	2.0	1.2	1.5	0.9	1.5	1.2	IAQVsPGPR	KLRIAQVsPGPRDSP	S5;	Yes	S(5): 100.0	23	2	1	0	N-Term(TMT6plex); S5(Phospho)
253970412	NP_057510.4	51512	GTSE1	G2 and S phase-expressed protein 1	T465	0.8	1.6	1.5	1.2	0.6	1.0	0.8	1.1	0.9	1.8	1.6	1.5	0.7	1.1	0.9	1.4	0.8	1.4	1.3	1.4	VMPtPTNQEK	SKTKVMPtPTNQEKL	T4;	No	T(4): 100.0; T(6): 0.0	19	1	1	0	N-Term(TMT6plex); M2(Oxidation); T4(Phospho); K10(TMT6plex)
41281453	NP_055535.2	9748	SLK	STE20-like serine/threonine-protein kinase	S565	1.2	1.1	1.1	1.4	1.0	1.2	1.0	1.0	1.3	1.0	1.0	1.3	1.0	1.0	0.9	1.0	1.2	1.0	1.0	1.2	VDEDsAEDTQSNDGK	AQKVDEDsAEDTQSN	S5;	Yes	S(5): 100.0; T(9): 0.0; S(11): 0.0	6	1	1	0	N-Term(TMT6plex); S5(Phospho); K15(TMT6plex)
103472005	NP_002408.3	4288	MKI67	antigen KI-67 isoform 1	S2344	1.1	1.0	1.1	1.3	0.8	1.0	0.9	1.1	1.2	1.1	1.1	1.4	0.9	1.1	0.9	1.2	1.2	1.1	1.0	1.1	sPQPDPVDTPASTK	TTKIACKsPQPDPVD	S1;	Yes	S(1): 100.0; T(9): 0.0; S(12): 0.0; T(13): 0.0	118	2	1	0	N-Term(TMT6plex); S1(Phospho); K14(TMT6plex)
154355000	NP_003676.2	8570	KHSRP	far upstream element-binding protein 2	S480	0.9	0.6	0.9	1.0	0.7	0.9	0.9	1.4	1.0	0.6	1.0	1.0	0.7	0.9	1.0	1.4	1.4	0.9	0.8	1.2	GsPQQIDHAK	KLEIIRGsPQQIDHA	S2;	Yes	S(2): 100.0	16	1	1	0	N-Term(TMT6plex); S2(Phospho); K10(TMT6plex)
50345873	NP_065778.3	49854	ZBTB21	zinc finger and BTB domain-containing protein 21 isoform L	S714	0.9	1.1	0.9	1.3	0.8	1.2	0.9	1.0	0.7	1.0	0.8	1.1	0.7	1.1	0.8	0.9	0.7	1.0	0.8	1.0	EHAPLAsPVENK	KEHAPLAsPVENKEV	S7;	Yes	S(7): 100.0	2	1	1	0	N-Term(TMT6plex); S7(Phospho); K12(TMT6plex)
528281423	NP_001268464.1	23136	EPB41L3	band 4.1-like protein 3 isoform 4	S369; T378	0.5	0.9	1.9	1.0	0.6	1.0	1.8	1.4	0.5	0.8	1.2	0.7	0.6	0.9	1.1	1.0	0.5	0.8	1.1	0.8	GIsQTNLITTVtPEKK	YATTKGIsQTNLITT;TNLITTVtPEKKAEE	S3; T12;	No;No	S(3): 100.0; T(5): 0.0; T(9): 0.0; T(10): 0.0; T(12): 100.0	10	4	1	1	N-Term(TMT6plex); T5(Phospho); T9(Phospho); K15(TMT6plex); K16(TMT6plex)
156105693	NP_001032412.2	85441	HELZ2	helicase with zinc finger domain 2 isoform 1	S338	1.2	1.0	1.1	1.2	0.9	1.1	1.0	1.2	1.1	0.9	1.0	1.1	0.8	0.9	0.9	1.1	0.9	0.9	1.0	1.1	SSVASGPIsPTNYR	SVASGPIsPTNYRQR	S9;	Yes	S(1): 0.0; S(2): 0.0; S(5): 0.0; S(9): 99.0; T(11): 0.0; Y(13): 1.0	1	1	1	0	N-Term(TMT6plex); S9(Phospho)
19913396	NP_599031.1	6525	SMTN	smoothelin isoform b	S341	0.9	0.9	0.9	1.2	0.9	0.8	1.0	1.2	0.8	0.7	0.7	1.0	0.7	0.6	0.7	1.1	0.7	0.6	0.7	1.1	FTSDsPMAAR	VHKFTSDsPMAARLQ	S5;	Yes	T(2): 1.3; S(3): 1.3; S(5): 97.5	7	5	1	0	N-Term(TMT6plex); S5(Phospho); M7(Oxidation)
259155302	NP_001158884.1	4790	NFKB1	nuclear factor NF-kappa-B p105 subunit isoform 2	S907	1.2	0.9	1.0	1.0	1.0	0.9	1.0	1.4	1.2	0.9	0.9	1.0	1.0	0.9	1.0	1.4	1.1	0.9	0.9	1.2	TTSQAHSLPLsPASTR	QAHSLPLsPASTRQQ	S11;	Yes	T(1): 0.0; T(2): 0.0; S(3): 0.0; S(7): 0.0; S(11): 100.0; S(14): 0.0; T(15):	2	2	1	0	N-Term(TMT6plex); S11(Phospho)
315139026	NP_079418.1	57551	TAOK1	serine/threonine-protein kinase TAO1 isoform 2	S421	0.9	1.2	0.9	0.9	0.9	1.5	1.3	1.5	0.6	1.2	1.0	0.8	0.6	1.4	1.3	1.4	0.6	1.3	1.1	1.1	ASDPQsPPQVSR	TRASDPQsPPQVSRH	S6;	Yes	S(2): 0.0; S(6): 100.0; S(11): 0.0	6	2	1	0	N-Term(TMT6plex); S6(Phospho)
56550039	NP_005924.2	4297	KMT2A	histone-lysine N-methyltransferase 2A isoform 2 precursor	S1837	2.0	0.9	0.6	1.1	2.0	1.9	1.9	1.3	1.8	0.9	0.6	1.1	1.8	1.8	1.9	1.3	1.8	1.3	1.3	1.2	GPGEPDsPTPLHPPTPPILSTDR	KGPGEPDsPTPLHPP	S7;	No	S(7): 97.7; T(9): 2.3; T(15): 0.0; S(20): 0.0; T(21): 0.0	15	2	1	0	N-Term(TMT6plex); S7(Phospho)
56550039	NP_005924.2	4297	KMT2A	histone-lysine N-methyltransferase 2A isoform 2 precursor	S1837	2.0	1.9	1.9	1.3	2.0	0.9	0.6	1.1	1.8	1.8	1.9	1.3	1.8	0.9	0.6	1.1	1.8	1.3	1.3	1.2	GPGEPDsPTPLHPPtPPILSTDR	KGPGEPDsPTPLHPP;PTPLHPPtPPILSTD	S7;	No;No	S(7): 97.0; T(9): 6.0; T(15): 97.0; S(20): 0.0; T(21): 0.0	1	2	1	0	N-Term(TMT6plex); T9(Phospho); T15(Phospho)
8922301	NP_060504.1	55100	WDR70	WD repeat-containing protein 70	S638	1.2	1.4	1.3	1.4	1.0	1.0	1.0	1.0	1.2	1.2	1.3	1.3	1.0	0.9	1.0	0.9	1.1	1.1	1.1	1.1	TMEAQVEsDDEEAK	TMEAQVEsDDEEAKN	S8;	Yes	T(1): 0.0; S(8): 100.0	2	1	1	0	N-Term(TMT6plex); M2(Oxidation); S8(Phospho); K14(TMT6plex)
568214245	NP_001274744.1	9898	UBAP2L	ubiquitin-associated protein 2-like isoform c	S602	0.9	1.0	0.9	1.0	1.2	1.2	1.3	1.4	1.0	1.0	1.0	0.9	1.4	1.3	1.4	1.3	1.2	1.1	1.2	1.1	RYPSSISSsPQK	YPSSISSsPQKDLTQ	S9;	No	Y(2): 0.0; S(4): 0.0; S(5): 0.0; S(7): 0.0; S(8): 1.2; S(9): 98.7	23	4	1	1	N-Term(TMT6plex); S9(Phospho); K12(TMT6plex)
311771621	NP_001185702.1	5298	PI4KB	phosphatidylinositol 4-kinase beta isoform 2	S413	0.8	1.0	0.9	1.3	1.1	1.0	1.2	1.1	0.9	1.0	0.9	1.2	1.2	1.0	1.2	1.0	1.0	1.0	1.1	1.1	sVENLPECGITHEQR	NRIRSTRsVENLPEC	S1;	No	S(1): 100.0; T(11): 0.0	3	3	1	0	N-Term(TMT6plex); S1(Phospho); C8(Carbamidomethyl)
69354671	NP_001020262.1	23	ABCF1	ATP-binding cassette sub-family F member 1 isoform a	S166	1.2	1.0	0.9	1.0	1.2	1.5	1.0	1.4	1.3	1.0	1.1	1.1	1.3	1.5	1.2	1.6	1.3	1.3	1.1	1.4	AVsEEQQPALK	NRINKAVsEEQQPAL	S3;	Yes	S(3): 100.0	44	2	1	0	N-Term(TMT6plex); S3(Phospho); K11(TMT6plex)
223555989	NP_057094.2	51101	ZC2HC1A	zinc finger C2HC domain-containing protein 1A	S292	0.4	1.2	0.8	1.1	0.6	1.0	0.7	1.2	0.4	1.1	0.8	1.0	0.7	0.9	0.7	1.1	0.6	1.0	0.7	1.1	GIEGHsPGNLPK	IKGIEGHsPGNLPKE	S6;	Yes	S(6): 100.0	3	1	1	0	N-Term(TMT6plex); S6(Phospho); K12(TMT6plex)
157311635	NP_940890.3	286077	FAM83H	protein FAM83H	S925	1.2	1.3	1.0	1.0	1.5	1.4	1.2	1.4	1.2	1.2	0.8	0.9	1.4	1.2	1.0	1.2	1.3	1.2	0.9	1.0	SsPVPPVPER	PVPERRSsPVPPVPE	S2;	Yes	S(1): 1.0; S(2): 99.0	3	1	1	0	N-Term(TMT6plex); S2(Phospho)
42542379	NP_005830.2	10250	SRRM1	serine/arginine repetitive matrix protein 1	S636; S638	0.9	1.3	1.0	0.9	1.1	1.3	1.1	1.4	1.1	1.4	1.2	1.1	1.4	1.4	1.2	1.6	1.3	1.4	1.2	1.3	RVsHsPPPK	PPPKRRVsHSPPPKQ;PKRRVSHsPPPKQRS	S3; S5;	Yes;Yes	S(3): 100.0; S(5): 100.0	229	1	1	1	N-Term(TMT6plex); S3(Phospho); S5(Phospho); K9(TMT6plex)
19913408	NP_001059.2	7155	TOP2B	DNA topoisomerase 2-beta	S1608	1.1	2.1	0.6	1.1	1.1	1.0	0.7	1.3	1.2	2.1	0.6	1.2	1.1	1.0	0.7	1.4	1.1	1.6	0.6	1.3	YFAEsDEEEDDVDFAMEN	EVKYFAEsDEEEDDV	S5;	No	Y(1): 0.0; S(5): 100.0	5	1	1	0	N-Term(TMT6plex); S5(Phospho); M16(Oxidation)
21493024	NP_653080.1	9590	AKAP12	A-kinase anchor protein 12 isoform 2	S500	0.9	1.1	1.1	1.2	1.0	1.4	1.5	1.2	1.1	1.1	0.9	1.1	1.2	1.4	1.3	1.1	1.1	1.2	1.1	1.1	GLAEVQQDGEAEEGATsDGEK	EAEEGATsDGEKKRE	S17;	No	T(16): 0.0; S(17): 100.0	103	2	1	0	N-Term(TMT6plex); S17(Phospho); K21(TMT6plex)
39930517	NP_612361.1	90378	SAMD1	atherin	S161	0.8	1.0	1.2	1.3	0.8	1.1	1.0	1.1	0.8	1.2	1.3	1.4	0.9	1.2	1.0	1.2	0.8	1.2	1.1	1.3	AAAAAATAPPsPGPAQPGPR	AAATAPPsPGPAQPG	S11;	Yes	T(7): 0.0; S(11): 100.0	4	1	1	0	N-Term(TMT6plex); S11(Phospho)
50409856	NP_001002243.1	54812	AFTPH	aftiphilin isoform c	S518	1.4	1.5	1.3	1.0	1.6	2.1	1.8	1.4	1.3	1.4	1.2	1.1	1.5	2.0	1.7	1.5	1.4	1.7	1.4	1.3	KSsGTGTEPVAK	COLARKSsGTGTEPV	S3;	Yes	S(2): 0.9; S(3): 99.1; T(5): 0.0; T(7):	2	3	1	1	N-Term(TMT6plex); K1(TMT6plex); S3(Phospho); K12(TMT6plex)
7656879	NP_055238.1	27125	AFF4	AF4/FMR2 family member 4	S549	1.6	1.3	1.2	1.3	1.2	1.3	1.2	1.1	1.4	1.2	1.2	1.2	1.0	1.1	1.1	1.0	1.2	1.2	1.2	1.1	QKsPAQSDSTTQR	SGRGRQKsPAQSDST	S3;	Yes	S(3): 100.0; S(7): 0.0; S(9): 0.0; T(10): 0.0; T(11): 0.0	16	1	1	1	N-Term(TMT6plex); K2(TMT6plex); S3(Phospho)
4506921	NP_003012.1	6449	SGTA	small glutamine-rich tetratricopeptide repeat-containing protein alpha	S305	1.3	1.4	1.3	1.1	1.2	1.7	1.6	1.2	1.6	2.0	1.6	1.6	1.4	2.4	2.1	1.7	1.5	2.2	1.9	1.6	TPsASNDDQQE	QIRSRTPsASNDDQQ	S3;	Yes	T(1): 0.5; S(3): 99.5; S(5): 0.0	28	1	1	0	N-Term(TMT6plex); S3(Phospho)
255918139	NP_001157616.1	283489	CHAMP1	chromosome alignment-maintaining phosphoprotein 1	S416	0.7	1.1	1.3	0.9	1.0	1.5	1.4	1.4	0.7	1.2	1.2	1.0	1.1	1.5	1.4	1.6	0.9	1.3	1.3	1.3	AVPPVsPELR	WKAVPPVsPELRKPG	S6;	Yes	S(6): 100.0	24	1	1	0	N-Term(TMT6plex); S6(Phospho)
153791372	NP_003983.2	1198	CLK3	dual specificity protein kinase CLK3 isoform b	S67	1.5	1.2	1.2	1.1	1.1	0.9	0.9	1.2	1.5	1.2	1.2	1.1	1.1	0.9	0.9	1.2	1.3	1.0	1.1	1.2	RDsDTYR	RYRERRDsDTYRCEE	S3;	No	S(3): 100.0; T(5): 0.0; Y(6): 0.0	1	2	1	1	N-Term(TMT6plex); S3(Phospho)
50428935	NP_060644.4	55201	MAP1S	microtubule-associated protein 1S	S759	0.6	1.7	0.6	1.2	1.0	1.1	1.0	1.1	0.6	1.8	0.7	1.4	1.2	1.2	1.2	1.2	0.9	1.5	0.9	1.3	AVPMAPAPAsPGSSNDSSAR	PMAPAPAsPGSSNDS	S10;	Yes	S(10): 100.0; S(13): 0.0; S(14): 0.0; S(17): 0.0; S(18): 0.0	8	1	1	0	N-Term(TMT6plex); M4(Oxidation); S10(Phospho)
20127519	NP_036244.2	22974	TPX2	targeting protein for Xklp2	S738	0.6	0.8	1.0	1.3	0.6	0.8	0.8	1.1	0.6	0.8	1.1	1.2	0.7	0.9	0.8	1.0	0.6	0.8	0.9	1.1	SSDQPLTVPVsPK	QPLTVPVsPKFSTRF	S11;	Yes	S(1): 0.0; S(2): 0.0; T(7): 0.0; S(11): 100.0	6	1	1	0	N-Term(TMT6plex); S11(Phospho); K13(TMT6plex)
24476013	NP_722560.1	5747	PTK2	focal adhesion kinase 1 isoform a	S910	1.1	1.0	1.0	1.1	0.8	1.6	1.4	1.2	1.0	0.9	0.9	1.0	0.8	1.5	1.4	1.1	0.9	1.2	1.1	1.1	LQPQEIsPPPTANLDR	KLQPQEIsPPPTANL	S7;	Yes	S(7): 100.0; T(11): 0.0	5	3	1	0	N-Term(TMT6plex); S7(Phospho)
109809741	NP_057732.2	51574	LARP7	la-related protein 7 isoform 1	S254	1.1	1.3	0.8	1.2	0.9	1.2	1.0	1.2	1.2	1.4	0.9	1.2	1.0	1.2	1.1	1.2	1.1	1.3	1.0	1.2	sRPTSEGSDIESTEPQK	SISKMKRsRPTSEGS	S1;	Yes	S(1): 96.3; T(4): 1.2; S(5): 1.2; S(8): 1.2; S(12): 0.0; T(13): 0.0	6	2	1	0	N-Term(TMT6plex); S8(Phospho); K17(TMT6plex)
109809741	NP_057732.2	51574	LARP7	la-related protein 7 isoform 1	S254	0.9	1.2	1.0	1.2	1.1	1.3	0.8	1.2	1.0	1.2	1.1	1.2	1.2	1.4	0.9	1.2	1.1	1.3	1.0	1.2	SRPTSEGsDIESTEPQK	SRPTSEGsDIESTEP	S1;	Yes	S(1): 33.3; T(4): 33.3; S(5): 33.3; S(8): 100.0; S(12): 0.0; T(13): 0.0	1	2	1	0	N-Term(TMT6plex); S5(Phospho); S8(Phospho); K17(TMT6plex)
114842410	NP_055642.3	9877	ZC3H11A	zinc finger CCCH domain-containing protein 11A	S108	1.6	1.5	1.2	1.2	1.2	1.4	1.3	1.2	1.6	1.3	1.2	1.2	1.2	1.3	1.3	1.1	1.4	1.3	1.3	1.1	TVLPTVPEsPEEEVK	VLPTVPEsPEEEVKA	S9;	Yes	T(1): 0.0; T(5): 0.0; S(9): 100.0	7	1	1	0	N-Term(TMT6plex); S9(Phospho); K15(TMT6plex)
115392150	NP_001035088.2	644815	FAM83G	protein FAM83G	S760	1.4	1.6	1.6	1.6	0.7	1.0	0.6	0.7	-	-	-	-	-	-	-	-	-	-	-	-	LLPDPGsPR	RLLPDPGsPRLAQNA	S7;	Yes	S(7): 100.0	2	1	1	0	N-Term(TMT6plex); S7(Phospho)
545746375	NP_001271175.1	26037	SIPA1L1	signal-induced proliferation-associated 1-like protein 1 isoform 3	S1181	1.4	1.3	1.2	1.3	0.9	0.9	0.9	1.1	1.4	1.1	1.2	1.2	0.9	0.8	0.9	1.0	1.1	1.0	1.0	1.1	RsPASIDR	GFGVSRRsPASIDRQ	S2;	Yes	S(2): 100.0; S(5): 0.0	7	4	1	1	N-Term(TMT6plex); S2(Phospho)
60302875	NP_001012409.1	151648	SGOL1	shugoshin-like 1 isoform A1	S256	1.0	2.4	2.5	1.5	1.1	1.4	2.9	0.9	0.8	1.4	1.3	1.1	0.9	0.8	1.5	0.7	0.9	1.1	1.4	0.9	DQVNLsPK	SKDQVNLsPKLIQPG	S6;	Yes	S(6): 100.0	1	2	1	0	N-Term(TMT6plex); S6(Phospho); K8(TMT6plex)
260064009	NP_056121.2	23358	USP24	ubiquitin carboxyl-terminal hydrolase 24	S2047	0.9	0.9	0.8	0.7	1.7	1.2	1.6	1.7	0.8	0.9	0.8	0.7	1.6	1.2	1.6	1.7	1.2	1.1	1.2	1.2	VSDQNsPVLPK	QRVSDQNsPVLPKKS	S6;	Yes	S(2): 0.0; S(6): 100.0	10	1	1	0	N-Term(TMT6plex); S6(Phospho); K11(TMT6plex)
118572613	NP_057417.3	23524	SRRM2	serine/arginine repetitive matrix protein 2	S1064	2.2	2.1	1.5	1.2	0.9	1.2	1.6	1.1	2.3	2.2	1.6	1.3	0.9	1.2	1.7	1.3	1.6	1.7	1.7	1.3	GQSQTsPDHR	LKGQSQTsPDHRSDT	S6;	Yes	S(3): 0.0; T(5): 0.9; S(6): 99.1	436	1	1	0	N-Term(TMT6plex); S6(Phospho)
94536805	NP_588611.2	91746	YTHDC1	YTH domain-containing protein 1 isoform 2	S146	1.0	1.3	1.0	1.2	0.9	0.9	0.8	1.2	1.0	1.3	1.0	1.1	0.9	0.9	0.9	1.2	1.0	1.1	0.9	1.2	AKsPTPDGSER	DPERRAKsPTPDGSE	S3;	Yes	S(3): 98.7; T(5): 1.3; S(9): 0.0	2	2	1	1	N-Term(TMT6plex); K2(TMT6plex); S3(Phospho)
32698688	NP_009105.1	11113	CIT	citron Rho-interacting kinase isoform 2	S440	1.4	1.3	1.2	1.2	1.0	0.9	1.0	1.2	1.3	1.3	1.1	1.1	0.9	0.8	0.9	1.2	1.1	1.1	1.0	1.2	SESVVSGLDsPAK	SVVSGLDsPAKTSSM	S10;	Yes	S(1): 0.0; S(3): 0.0; S(6): 0.0; S(10): 100.0	9	2	1	0	N-Term(TMT6plex); S10(Phospho); K13(TMT6plex)
344217752	NP_001230676.1	8539	API5	apoptosis inhibitor 5 isoform d	S291	1.0	1.1	1.0	1.4	0.9	1.0	0.8	0.9	1.1	1.2	1.1	1.5	0.9	1.1	0.8	0.9	1.0	1.2	0.9	1.2	ASEDTTSGsPPK	SEDTTSGsPPKKSSA	S9;	No	S(2): 0.0; T(5): 0.0; T(6): 0.0; S(7): 0.0; S(9): 100.0	8	4	1	0	N-Term(TMT6plex); S9(Phospho); K12(TMT6plex)
33946327	NP_005076.3	8021	NUP214	nuclear pore complex protein Nup214	S678	1.6	1.7	1.4	1.1	1.1	1.2	1.1	1.2	1.5	1.6	1.2	1.0	1.0	1.2	1.0	1.1	1.3	1.4	1.1	1.1	ITPPAAKPGsPQAK	PPAAKPGsPQAKSLQ	S10;	Yes	T(2): 0.0; S(10): 100.0	8	1	1	0	N-Term(TMT6plex); K7(TMT6plex); S10(Phospho); K14(TMT6plex)
154759257	NP_778236.2	317781	DDX51	ATP-dependent RNA helicase DDX51	S83	1.6	1.8	1.5	0.9	0.9	1.5	1.5	1.5	1.6	1.9	1.6	0.9	0.9	1.6	1.7	1.5	1.2	1.8	1.6	1.2	VNDAEPGsPEAPQGK	VNDAEPGsPEAPQGK	S8;	Yes	S(8): 100.0	3	1	1	0	N-Term(TMT6plex); S8(Phospho); K15(TMT6plex)
54607124	NP_037418.3	29890	RBM15B	putative RNA-binding protein 15B	S267	1.0	0.9	0.6	1.1	1.5	1.8	1.2	1.2	1.0	1.0	0.6	1.2	1.5	2.0	1.3	1.3	1.3	1.5	1.0	1.2	sLSPVAAPPLR	GYQYKQRsLSPVAAP	S3;	Yes	S(1): 98.8; S(3): 1.2	1	1	1	0	N-Term(TMT6plex); S3(Phospho)
54607124	NP_037418.3	29890	RBM15B	putative RNA-binding protein 15B	S267	1.5	1.8	1.2	1.2	1.0	0.9	0.6	1.1	1.5	2.0	1.3	1.3	1.0	1.0	0.6	1.2	1.3	1.5	1.0	1.2	SLsPVAAPPLR	QYKQRSLsPVAAPPL	S3;	Yes	S(1): 0.0; S(3): 100.0	4	1	1	0	N-Term(TMT6plex); S3(Phospho)
33598968	NP_005349.3	4008	LMO7	LIM domain only protein 7 isoform 1	S1259	1.1	1.5	1.7	1.2	1.1	1.2	1.1	1.2	0.9	1.2	1.1	1.1	0.9	0.9	0.8	1.1	0.9	1.1	1.0	1.1	SHsPSASQSGSQLR	SPTPRSHsPSASQSG	S3;	No	S(1): 0.0; S(3): 100.0; S(5): 0.0; S(7): 0.0; S(9): 0.0; S(11): 0.0	19	2	1	0	N-Term(TMT6plex); S3(Phospho)
196114970	NP_001124481.1	5782	PTPN12	tyrosine-protein phosphatase non-receptor type 12 isoform 3	S543	0.9	0.8	1.2	1.2	1.0	1.2	1.0	1.1	0.8	0.8	1.1	1.2	0.9	1.2	1.0	1.1	0.8	1.0	1.0	1.1	DVDVSEDsPPPLPER	DVDVSEDsPPPLPER	S8;	No	S(5): 0.0; S(8): 100.0	8	3	1	0	N-Term(TMT6plex); S8(Phospho)
27886526	NP_775322.1	10725	NFAT5	nuclear factor of activated T-cells 5 isoform a	S153	1.4	1.4	1.8	1.4	1.0	1.1	1.0	0.9	-	-	-	-	-	-	-	-	-	-	-	-	DCEESNMDIEDADsAK	MDIEDADsAKAPHYV	S14;	No	S(5): 0.0; S(14): 100.0	2	4	1	0	N-Term(TMT6plex); C2(Carbamidomethyl); M7(Oxidation); S14(Phospho); K16(TMT6plex)
5174669	NP_006080.1	10389	SCML2	sex comb on midleg-like protein 2	S499; S511	0.8	0.5	0.7	0.7	1.2	1.0	1.2	1.7	0.7	0.6	0.7	0.8	1.1	1.1	1.2	1.9	0.9	0.8	1.0	1.3	sPQQTVPYVVPLsPK	ERQSTKRsPQQTVPY;VPYVVPLsPKLPKTK	S1; S13;	Yes;Yes	S(1): 98.7; T(5): 1.3; Y(8): 0.0; S(13): 100.0	4	1	1	0	N-Term(TMT6plex); S1(Phospho); S13(Phospho); K15(TMT6plex)

Khan et al., 2019. Multi-omics analysis to characterize cigarette smoke induced molecular alterations in esophageal cells

Supplementary Table 8. List of phosphopeptides identified and quantified in untreated and chronically treated Het1A cells with cigarette smoke condensate for 8 months using SequestHT and Mascot search algorithms with a PhosphoRS probability score ≥75%

Protein group Accession	NP_Accession	Gene ID	Gene Symbol	Protein Description	PhosphoSite (Protein)	Replicate 1: Het-1A-Smoke - 2M/Parental	Replicate 1: Het-1A-Smoke - 4M/Parental	Replicate 1: Het-1A-Smoke - 6M/Parental	Replicate 1: Het-1A-Smoke - 8M/Parental	Replicate 2: Het-1A-Smoke - 2M/Parental	Replicate 2: Het-1A-Smoke - 4M/Parental	Replicate 2: Het-1A-Smoke - 6M/Parental	Replicate 2: Het-1A-Smoke - 8M/Parental	Normalized phosphoprotein ratios with respect to corresponding total protein ratios. Replicate 1: Het-1A-Smoke - 2M/Parental	Normalized, Replicate 1: Het-1A-Smoke - 4M/Parental	Normalized, Replicate 1: Het-1A-Smoke - 6M/Parental	Normalized, Replicate 1: Het-1A-Smoke - 8M/Parental	Normalized, Replicate 2: Het-1A-Smoke - 2M/Parental	Normalized, Replicate 2: Het-1A-Smoke - 4M/Parental	Normalized, Replicate 2: Het-1A-Smoke - 6M/Parental	Normalized, Replicate 2: Het-1A-Smoke - 8M/Parental	Average of replicates: Het-1A-Smoke - 2M/Parental	Average of replicates: Het-1A-Smoke - 4M/Parental	Average of replicates: Het-1A-Smoke - 6M/Parental	Average of replicates: Het-1A-Smoke - 8M/Parental	Peptide Sequence	Phosphowindow	PhosphoSite (Peptide)	Phosphosite Plus Evidence	phosphoRS Site Probabilities	# PSMs	# Proteins	# Protein Groups	# Missed Cleavages	Modifications
5174669	NP_006080.1	10389	SCML2	sex comb on midleg-like protein 2	S499; S511	1.2	1.0	1.2	1.7	0.8	0.5	0.7	0.7	1.1	1.1	1.2	1.9	0.7	0.6	0.7	0.8	0.9	0.8	1.0	1.3	SPQQTVPYVVPLsPK	VPYVVPLsPKLPKTK	S1; S13;	Yes	S(1): 50.0; T(5): 50.0; Y(8): 0.0; S(13): 100.0	2	1	1	0	N-Term(TMT6plex); S1(Phospho); S13(Phospho); K15(TMT6plex)
528281423	NP_001268464.1	23136	EPB41L3	band 4.1-like protein 3 isoform4	T378	0.6	0.4	1.3	0.6	1.1	1.3	2.2	1.7	0.6	0.4	0.8	0.4	1.1	1.2	1.4	1.2	0.8	0.8	1.1	0.8	GISQTNLITTVtPEK	TNLITTVtPEKKAEE	T12;	No	S(3): 0.0; T(5): 0.0; T(9): 1.3; T(10): 1.3; T(12): 97.5	10	4	1	0	N-Term(TMT6plex); T12(Phospho); K15(TMT6plex)
255918139	NP_001157616.1	283489	CHAMP1	chromosome alignment-maintaining phosphoprotein 1	S542	1.1	1.1	1.1	1.0	1.4	1.3	1.3	1.3	1.2	1.1	1.1	1.1	1.4	1.3	1.3	1.4	1.3	1.2	1.2	1.3	TAPPAsPEAR	AKTAPPAsPEARKRA	S6;	Yes	T(1): 0.0; S(6): 100.0	24	1	1	0	N-Term(TMT6plex); S6(Phospho)
33598968	NP_005349.3	4008	LMO7	LIM domain only protein 7 isoform1	S1089	1.3	1.4	1.9	1.1	1.0	1.9	2.1	1.2	1.1	1.1	1.3	1.0	0.9	1.5	1.4	1.2	1.0	1.3	1.3	1.1	sTTELDDYSTNK	GDRNKSRsTTELDDY	S1;	No	S(1): 97.7; T(2): 1.2; T(3): 1.2; Y(8): 0.0; S(9): 0.0; T(10): 0.0	19	2	1	0	N-Term(TMT6plex); S1(Phospho); K12(TMT6plex)
125625350	NP_001781.2	995	CDC25C	M-phase inducer phosphatase 3 isoforma	S168	1.2	1.1	1.0	1.3	0.9	0.9	1.0	1.0	1.4	1.0	1.0	1.0	1.1	0.8	1.0	0.8	1.2	0.9	1.0	0.9	YLGsPITTVPK	SEMKYLGsPITTVPK	S4;	Yes	Y(1): 0.8; S(4): 99.2; T(7): 0.0; T(8):	8	3	1	0	N-Term(TMT6plex); S4(Phospho); K11(TMT6plex)
214830451	NP_001135771.1	8878	SQSTM1	sequestosome-1 isoform2	S188	0.6	0.7	1.0	1.7	0.7	0.3	0.9	0.6	0.6	0.7	1.0	1.8	0.6	0.3	0.8	0.7	0.6	0.5	0.9	1.2	LTPVsPESSSTEEK	RSRLTPVsPESSSTE	S5;	No	T(2): 0.0; S(5): 100.0; S(8): 0.0; S(9): 0.0; S(10): 0.0; T(11): 0.0	6	2	1	0	N-Term(TMT6plex); S5(Phospho); K14(TMT6plex)
61743954	NP_001611.1	79026	AHNAK	neuroblast differentiation-associated protein AHNAK isoform1	S5752	2.1	2.0	1.9	1.1	1.6	1.6	1.5	1.2	2.5	2.0	1.9	1.3	2.0	1.7	1.5	1.4	2.2	1.8	1.7	1.4	ASLGsLEGEAEAEASSPK	SSKASLGsLEGEAEA	S5;	Yes	S(2): 0.9; S(5): 99.1; S(15): 0.0; S(16): 0.0	79	1	1	0	N-Term(TMT6plex); S5(Phospho); K18(TMT6plex)
557128994	NP_001273407.1	8663	EIF3C	eukaryotic translation initiation factor 3 subunit C isoformb	S39	1.1	1.4	1.4	1.3	1.1	1.1	1.1	1.0	1.0	1.3	1.3	1.2	1.0	1.1	1.0	1.0	1.0	1.2	1.2	1.1	QPLLLsEDEEDTKR	GKQPLLLsEDEEDTK	S6;	Yes	S(6): 100.0; T(12): 0.0	16	3	1	1	N-Term(TMT6plex); S6(Phospho); K13(TMT6plex)
65288071	NP_073585.8	64759	TNS3	tensin-3	S1149	0.8	0.9	1.2	1.1	1.1	1.4	1.9	1.2	0.9	1.0	1.2	1.2	1.2	1.6	2.0	1.3	1.1	1.3	1.6	1.3	ASEAAsPLPDSPGDK	SKASEAAsPLPDSPG	S6;	Yes	S(2): 0.0; S(6): 100.0; S(11): 0.0	3	1	1	0	N-Term(TMT6plex); S6(Phospho); K15(TMT6plex)
4759156	NP_004587.1	6626	SNRPA	U1 small nuclear ribonucleoprotein A	S115	1.1	1.1	1.3	1.4	0.9	1.2	1.2	1.0	1.2	1.2	1.4	1.5	1.0	1.4	1.3	1.1	1.1	1.3	1.4	1.3	sQETPATK	RKRKPKsQETPATK	S1;	No	S(1): 100.0; T(4): 0.0; T(7): 0.0	1	1	1	0	N-Term(TMT6plex); S1(Phospho); K8(TMT6plex)
527122034	NP_001268366.1	11047	ADRM1	proteasomal ubiquitin receptor ADRM1 isoform2	T178	0.3	0.7	0.9	1.2	0.7	0.9	1.0	1.2	0.3	0.8	0.9	1.2	0.7	1.0	1.0	1.2	0.5	0.9	1.0	1.2	SQSAAVtPSSTTSSTR	RSQSAAVtPSSTTSS	T7;	No	S(1): 0.0; S(3): 0.0; T(7): 100.0; S(9): 0.0; S(10): 0.0; T(11): 0.0; T(12): 0.0; S(13): 0.0; S(14): 0.0; T(15): 0.0	1	2	1	0	N-Term(TMT6plex); T7(Phospho)
333440471	NP_001193980.1	23332	CLASP1	CLIP-associating protein 1 isoform4	T718	1.2	1.3	1.4	1.2	1.2	1.6	1.4	1.1	1.2	1.4	1.5	1.3	1.3	1.6	1.6	1.2	1.3	1.5	1.5	1.2	GPPVtPSSEK	SSRGPPVtPSSEKRS	T5;	No	T(5): 100.0; S(7): 0.0; S(8): 0.0	14	4	1	0	N-Term(TMT6plex); T5(Phospho); K10(TMT6plex)
134304846	NP_001077086.1	9821	RB1CC1	RB1-inducible coiled-coil protein 1 isoform2	S266	0.7	1.1	0.7	1.1	1.4	1.2	1.5	1.3	0.8	1.2	0.7	1.0	1.5	1.3	1.5	1.2	1.2	1.2	1.1	1.1	sVEHVSPDTADAESGK	LLTSFPKsVEHVSPD	S6;	Yes	S(1): 98.5; S(6): 1.5; T(9): 0.0; S(14): 0.0	1	2	1	0	N-Term(TMT6plex); S1(Phospho); K16(TMT6plex)
568214245	NP_001274744.1	9898	UBAP2L	ubiquitin-associated protein 2-like isoformc	S602	0.9	0.8	0.9	1.2	1.4	1.4	1.4	1.1	1.0	0.8	1.0	1.1	1.6	1.4	1.6	1.0	1.3	1.1	1.3	1.1	YPSSISSsPQK	YPSSISSsPQKDLTQ	S8;	No	Y(1): 0.0; S(3): 0.0; S(4): 0.0; S(6): 0.0; S(7): 0.0; S(8): 100.0	23	4	1	0	N-Term(TMT6plex); S8(Phospho); K11(TMT6plex)
134304846	NP_001077086.1	9821	RB1CC1	RB1-inducible coiled-coil protein 1 isoform2	S266	1.4	1.2	1.5	1.3	0.7	1.1	0.7	1.1	1.5	1.3	1.5	1.2	0.8	1.2	0.7	1.0	1.2	1.2	1.1	1.1	SVEHVsPDTADAESGK	PKSVEHVsPDTADAE	S6;	Yes	S(1): 0.0; S(6): 100.0; T(9): 0.0; S(14): 0.0	3	2	1	0	N-Term(TMT6plex); S6(Phospho); K16(TMT6plex)
359807035	NP_001240841.1	23216	TBC1D1	TBC1 domain family member 1 isoform2	S614	1.0	1.1	1.0	1.1	1.1	1.1	1.0	1.2	-	-	-	-	-	-	-	-	-	-	-	-	GsPGVSQR	PPQPARGsPGVSQRK	S2;	Yes	S(2): 100.0; S(6): 0.0	1	2	1	0	N-Term(TMT6plex); S2(Phospho)
333440471	NP_001193980.1	23332	CLASP1	CLIP-associating protein 1 isoform4	S691	1.3	1.4	1.2	1.1	1.3	1.2	1.2	1.2	1.4	1.4	1.3	1.2	1.4	1.3	1.3	1.3	1.4	1.4	1.3	1.2	VVSQSQPGsR	VSQSQPGsRSSSPGK	S9;	Yes	S(3): 0.0; S(5): 0.0; S(9): 100.0	14	1	1	0	N-Term(TMT6plex); S9(Phospho)
42490753	NP_003205.2	7005	TEAD3	transcriptional enhancer factor TEF-5	S148	1.1	1.8	0.9	1.2	1.0	0.9	0.6	1.1	1.1	2.0	0.9	1.3	1.0	1.0	0.6	1.2	1.1	1.5	0.8	1.2	FSPPsPLPQAVFSTSSR	QNKFSPPsPLPQAVF	S5;	Yes	S(2): 1.5; S(5): 98.5; S(13): 0.0; T(14): 0.0; S(15): 0.0; S(16): 0.0	2	1	1	0	N-Term(TMT6plex); S5(Phospho)
546231404	NP_001271231.1	54443	ANLN	actin-binding protein anillin isoform3	S323	0.6	0.9	0.9	1.0	1.0	1.5	1.3	1.4	0.6	0.9	0.9	1.0	1.0	1.4	1.3	1.4	0.8	1.2	1.1	1.2	TPIsPLK	LLPKTPIsPLKTGVS	S4;	Yes	T(1): 0.0; S(4): 100.0	21	3	1	0	N-Term(TMT6plex); S4(Phospho); K7(TMT6plex)
120587019	NP_055160.2	24149	ZNF318	zinc finger protein 318	S527	1.1	1.8	1.5	1.3	0.7	1.0	1.0	1.0	1.1	1.9	1.6	1.5	0.7	1.0	1.0	1.1	0.9	1.4	1.3	1.3	sFPDIEDEEK	TQEKRRRsFPDIEDE	S1;	Yes	S(1): 100.0	9	1	1	0	N-Term(TMT6plex); S1(Phospho); K10(TMT6plex)
5032179	NP_005753.1	10155	TRIM28	transcription intermediary factor 1-beta	S594	0.4	0.3	0.2	0.4	1.6	1.0	1.9	1.9	0.4	0.4	0.2	0.4	1.7	1.1	1.9	1.8	1.0	0.7	1.1	1.1	LAsPSGSTSSGLEVVAPEGTSAPGGGPGTLDDSATICR	AEGPRLAsPSGSTSS	S3;	Yes	S(3): 99.8; S(5): 0.1; S(7): 0.1; T(8): 0.1; S(9): 0.1; S(10): 0.0; T(20): 0.0; S(21): 0.0; T(29): 0.0; S(33): 0.0; T(35): 0.0	10	1	1	0	N-Term(TMT6plex); S3(Phospho); C37(Carbamidomethyl)
5453565	NP_006331.1	10458	BAIAP2	brain-specific angiogenesis inhibitor 1-associated protein 2 isoform3	S325	0.8	2.7	2.1	0.9	1.1	1.5	1.4	1.5	0.8	2.7	2.1	0.8	1.1	1.5	1.4	1.4	1.0	2.1	1.8	1.1	sLSPPQSQSK	RKAAQPKsLSPPQSQ	S3;	Yes	S(1): 99.1; S(3): 0.9; S(7): 0.0; S(9): 0.0	2	4	1	0	N-Term(TMT6plex); S1(Phospho); K10(TMT6plex)
5453565	NP_006331.1	10458	BAIAP2	brain-specific angiogenesis inhibitor 1-associated protein 2 isoform3	S325	1.1	1.5	1.4	1.5	0.8	2.7	2.1	0.9	1.1	1.5	1.4	1.4	0.8	2.7	2.1	0.8	1.0	2.1	1.8	1.1	SLsPPQSQSK	AAQPKSLsPPQSQSK	S3;	Yes	S(1): 0.0; S(3): 100.0; S(7): 0.0; S(9): 0.0	2	4	1	0	N-Term(TMT6plex); S3(Phospho); K10(TMT6plex)
8923579	NP_060377.1	55004	LAMTOR1	ragulator complex protein LAMTOR1	S27	1.0	0.8	1.0	1.1	0.8	1.0	0.9	1.2	0.9	0.8	1.0	1.1	0.7	1.0	1.0	1.1	0.8	0.9	1.0	1.1	LLLDPSsPPTK	KLLLDPSsPPTKALN	S7;	Yes	S(6): 0.8; S(7): 99.2; T(10): 0.0	1	1	1	0	N-Term(TMT6plex); S7(Phospho); K11(TMT6plex)
302699239	NP_004944.3	1981	EIF4G1	eukaryotic translation initiation factor 4 gamma 1	S1401	1.1	1.3	1.1	1.2	1.2	1.3	1.3	1.2	1.1	1.1	1.0	1.1	1.1	1.2	1.1	1.1	1.1	1.2	1.0	1.1	EAEEEsDHN	LREAEEEsDHN	S6;	No	S(6): 100.0	17	7	1	0	N-Term(TMT6plex); S6(Phospho)
7019371	NP_037474.1	29844	TFPT	TCF3 fusion partner	S252	0.9	0.9	0.6	1.2	1.0	0.9	1.5	1.1	1.0	0.8	0.6	1.1	1.1	0.8	1.5	1.1	1.0	0.8	1.1	1.1	LLPYPTLASPAsD	PTLASPAsD_	S12;	Yes	Y(4): 0.0; T(6): 0.0; S(9): 0.0; S(12): 100.0	12	1	1	0	N-Term(TMT6plex); S12(Phospho)
25777602	NP_002799.3	5708	PSMD2	26S proteasome non-ATPase regulatory subunit 2 isoform1	S16	1.2	1.5	1.4	1.3	1.1	1.1	1.2	1.0	1.2	1.5	1.3	1.3	1.1	1.0	1.1	1.1	1.1	1.2	1.2	1.2	APVQPQQsPAAAPGGTDEKPSGK	APVQPQQsPAAAPGG	S8;	Yes	S(8): 100.0; T(16): 0.0; S(21): 0.0	8	1	1	0	N-Term(TMT6plex); S8(Phospho); K19(TMT6plex); K23(TMT6plex)
171543895	NP_002964.3	6311	ATXN2	ataxin-2	T741	1.4	1.7	1.4	1.3	1.0	1.3	1.0	1.0	1.2	1.4	1.3	1.1	0.9	1.1	0.9	0.8	1.0	1.3	1.1	1.0	AVtPSSEAK	PASNRAVtPSSEAKD	T3;	Yes	T(3): 100.0; S(5): 0.0; S(6): 0.0	3	1	1	0	N-Term(TMT6plex); T3(Phospho); K9(TMT6plex)
4503247	NP_003674.1	8568	RRP1	ribosomal RNA processing protein 1 homolog A	T412	0.9	1.2	1.0	1.1	0.9	1.1	1.1	1.2	0.9	1.2	1.1	1.1	1.0	1.1	1.1	1.2	1.0	1.1	1.1	1.1	AEAGEQPGtAER	EAGEQPGtAERALLR	T9;	Yes	T(9): 100.0	5	1	1	0	N-Term(TMT6plex); T9(Phospho)
22208971	NP_665908.1	3159	HMGA1	high mobility group protein HMG-I/HMG-Y isoforma	S36	0.7	0.9	1.0	1.3	0.6	0.8	0.9	1.1	0.7	0.8	0.9	1.3	0.6	0.6	0.8	1.1	0.7	0.7	0.8	1.2	KQPPVsPGTALVGSQK	PRKQPPVsPGTALVG	S6;	Yes	S(6): 100.0; T(9): 0.0; S(14): 0.0	39	1	1	1	N-Term(TMT6plex); K1(TMT6plex); S6(Phospho); K16(TMT6plex)
198041728	NP_001128246.1	64837	KLC2	kinesin light chain 2 isoform2	S534	1.0	1.9	1.5	1.4	1.2	1.4	1.0	0.9	0.8	1.6	1.2	1.2	1.0	1.1	0.9	0.7	0.9	1.4	1.0	0.9	TLSSSsMDLSR	SRTLSSSsMDLSRRS	S6;	No	T(1): 0.0; S(3): 0.0; S(4): 1.6; S(5): 1.6; S(6): 96.8; S(10): 0.0	1	2	1	0	N-Term(TMT6plex); S4(Phospho); M7(Oxidation)
226371669	NP_001139487.1	9787	DLGAP5	disks large-associated protein 5 isoformb	S725	0.6	0.8	0.8	1.2	1.4	3.4	4.6	1.1	0.7	1.0	1.1	1.4	1.6	4.4	5.9	1.3	1.1	2.7	3.5	1.3	MsLPLLAGGVADDINTNK	CLSSERMsLPLLAGG	S2;	Yes	S(2): 100.0; T(16): 0.0	9	2	1	0	N-Term(TMT6plex); M1(Oxidation); S2(Phospho); K18(TMT6plex)
29826323	NP_054909.2	118	ADD1	alpha-adducin isoformc	S358	1.2	1.2	1.1	1.3	1.1	1.0	1.1	1.1	1.4	1.4	1.3	1.5	1.3	1.2	1.2	1.2	1.3	1.3	1.2	1.3	SPGsPVGEGTGSPPK	AKSRSPGsPVGEGTG	S4;	Yes	S(1): 0.0; S(4): 100.0; T(10): 0.0; S(12): 0.0	12	5	1	0	N-Term(TMT6plex); S4(Phospho); K15(TMT6plex)
118572613	NP_057417.3	23524	SRRM2	serine/arginine repetitive matrix protein 2	S250	1.1	1.1	1.0	1.3	0.7	0.8	0.8	1.1	1.2	1.1	1.0	1.4	0.8	0.8	0.8	1.2	1.0	1.0	0.9	1.3	sTTPAPK	KKRKRSRsTTPAPKS	S1;	Yes	S(1): 100.0; T(2): 0.0; T(3): 0.0	436	1	1	0	N-Term(TMT6plex); S1(Phospho); K7(TMT6plex)
13375654	NP_078792.1	79447	PAGR1	PAXIP1-associated glutamate-rich protein 1	S237	0.7	1.0	0.9	1.2	1.0	0.9	1.5	1.1	1.2	0.9	1.3	1.7	1.7	0.9	2.1	1.7	1.5	0.9	1.7	1.7	DLFSLDSEDPSPAsPPLR	SEDPSPAsPPLRSSG	S14;	Yes	S(4): 0.0; S(7): 0.0; S(11): 0.0; S(14): 100.0	4	1	1	0	N-Term(TMT6plex); S14(Phospho)
95147555	NP_002364.5	4130	MAP1A	microtubule-associated protein 1A	S1818	1.0	1.5	0.6	1.1	1.0	1.1	1.1	1.2	-	-	-	-	-	-	-	-	-	-	-	-	VPSAPGQEsPIPDPK	PSAPGQEsPIPDPKL	S9;	Yes	S(3): 0.0; S(9): 100.0	7	1	1	0	N-Term(TMT6plex); S9(Phospho); K15(TMT6plex)
57864582	NP_001009931.1	388697	HRNR	hornerin	S1125	0.9	1.2	0.9	1.2	1.0	2.3	0.6	1.1	0.9	0.7	0.9	0.5	1.0	1.3	0.6	0.5	0.9	1.0	0.7	0.5	QGSGSGQsPGHGQR	QGSGSGQsPGHGQRG	S8;	No	S(3): 0.0; S(5): 0.0; S(8): 100.0	10	1	1	0	N-Term(TMT6plex); S8(Phospho)
574274721	NP_001275992.1	3978	LIG1	DNA ligase 1 isoform2	S111	1.0	1.1	1.3	1.1	1.3	1.1	1.3	1.2	1.0	1.2	1.3	1.0	1.3	1.1	1.3	1.2	1.2	1.2	1.3	1.1	TIQEVLEEQsEDEDR	QEVLEEQsEDEDREA	S10;	No	T(1): 0.0; S(10): 100.0	13	2	1	0	N-Term(TMT6plex); S10(Phospho)
156671217	NP_683723.2	22905	EPN2	epsin-2 isoforma	S192; S195	1.1	1.2	1.1	1.1	1.6	1.4	1.3	1.2	1.0	0.9	1.0	0.9	1.4	1.1	1.1	1.1	1.2	1.0	1.0	1.0	AGGsPAsYHGSTSPR	EYGKAGGsPASYHGS;KAGGSPAsYHGSTSP	S4; S7;	Yes;Yes	S(4): 100.0; S(7): 100.0; Y(8): 0.0; S(11): 0.0; T(12): 0.0; S(13): 0.0	4	1	1	0	N-Term(TMT6plex); S4(Phospho); S7(Phospho)
148539876	NP_001610.2	156	ADRBK1	beta-adrenergic receptor kinase 1	S670	1.1	1.1	1.3	1.4	1.3	1.2	1.4	1.0	1.3	1.1	1.3	1.3	1.4	1.1	1.4	0.9	1.3	1.1	1.4	1.1	sPVVELSK	KMKNKPRsPVVELSK	S1;	Yes	S(1): 100.0; S(7): 0.0	2	1	1	0	N-Term(TMT6plex); S1(Phospho); K8(TMT6plex)
7661950	NP_055448.1	9646	CTR9	RNA polymerase-associated protein CTR9 homolog	S941; S943	1.1	1.1	1.1	0.8	1.3	1.5	1.3	1.5	1.0	1.1	1.1	0.8	1.3	1.5	1.3	1.6	1.2	1.3	1.2	1.2	KGsGsEQEGEDEEGGER	KKKRRKGsGSEQEGE;KRRKGSGsEQEGEDE	S3; S5;	Yes;Yes	S(3): 100.0; S(5): 100.0	12	1	1	1	K1(TMT6plex); N-Term(TMT6plex); S3(Phospho); S5(Phospho)
41322916	NP_958782.1	5339	PLEC	plectin isoform1	S149	1.0	0.7	1.1	1.1	1.4	1.3	1.0	1.2	1.1	0.8	1.2	1.1	1.6	1.4	1.1	1.3	1.4	1.1	1.1	1.2	ELEEVsPETPVVPATTQR	RKELEEVsPETPVVP	S6;	Yes	S(6): 100.0; T(9): 0.0; T(15): 0.0; T(16): 0.0	17	1	1	0	N-Term(TMT6plex); S6(Phospho)
126722969	NP_079358.3	80152	CENPT	centromere protein T	S397	1.2	1.0	0.8	1.1	1.1	1.2	0.9	1.2	1.1	0.9	0.9	1.1	1.0	1.2	1.0	1.2	1.0	1.1	0.9	1.2	AAsPESASSTPESLQAR	DASGRAAsPESASST	S3;	Yes	S(3): 100.0; S(6): 0.0; S(8): 0.0; S(9): 0.0; T(10): 0.0; S(13): 0.0	2	1	1	0	N-Term(TMT6plex); S3(Phospho)
27437011	NP_757343.1	84254	CAMKK1	calcium/calmodulin-dependent protein kinase kinase 1 isoforma	S52	1.0	1.1	1.4	1.2	1.2	1.3	1.4	1.1	1.1	1.1	1.3	1.1	1.3	1.2	1.3	1.1	1.2	1.1	1.3	1.1	AAsVIPGSTSR	PPRARAAsVIPGSTS	S3;	Yes	S(3): 100.0; S(8): 0.0; T(9): 0.0; S(10): 0.0	1	2	1	0	N-Term(TMT6plex); S3(Phospho)
38788193	NP_005217.2	1903	S1PR3	sphingosine 1-phosphate receptor 3	S348	0.9	1.1	0.9	1.2	0.7	0.9	0.7	1.1	-	-	-	-	-	-	-	-	-	-	-	-	SSSSNNSSHsPK	SSNNSSHsPKVKEDL	S10;	No	S(1): 0.0; S(2): 0.0; S(3): 0.0; S(4): 0.0; S(7): 0.0; S(8): 0.0; S(10): 100.0	2	1	1	0	N-Term(TMT6plex); S10(Phospho); K12(TMT6plex)
14249678	NP_116294.1	84991	RBM17	splicing factor 45	S222	1.0	1.9	1.6	1.3	1.3	1.5	1.1	1.1	1.1	2.0	1.7	1.2	1.4	1.5	1.1	1.0	1.2	1.8	1.4	1.1	sPTGPSNSFLANMGGTVAHK	EEQDRPRsPTGPSNS	S1;	Yes	S(1): 100.0; T(3): 0.0; S(6): 0.0; S(8): 0.0; T(16): 0.0	5	1	1	0	N-Term(TMT6plex); S1(Phospho); M13(Oxidation); K20(TMT6plex)
56550051	NP_006581.2	10713	USP39	U4/U6.U5 tri-snRNP-associated protein 2 isoform1	S82	1.1	1.0	1.3	1.1	0.9	0.8	1.3	1.2	1.2	1.0	1.3	1.1	1.0	0.8	1.2	1.2	1.1	0.9	1.3	1.1	EVDEDsEPER	EREVDEDsEPEREVR	S6;	Yes	S(6): 100.0	8	2	1	0	N-Term(TMT6plex); S6(Phospho)
226509740	NP_067051.2	58506	SCAF1	splicing factor, arginine/serine-rich 19	S612; S614	0.7	0.9	1.0	0.7	1.3	2.1	1.8	1.6	0.7	0.9	0.9	0.7	1.3	2.0	1.5	1.5	1.0	1.4	1.2	1.1	sAsPPPATSSSSSSR	KRRRRRRsASPPPAT;RRRRRSAsPPPATSS	S1; S3;	Yes;Yes	S(1): 100.0; S(3): 100.0; T(8): 0.0; S(9): 0.0; S(10): 0.0; S(11): 0.0; S(12): 0.0; S(13): 0.0; S(14): 0.0	29	1	1	0	N-Term(TMT6plex); S1(Phospho); S3(Phospho)
13128980	NP_076936.1	78994	PRR14	proline-rich protein 14	S277	0.5	0.5	0.4	1.0	1.2	2.0	1.5	1.3	0.5	0.5	0.5	1.0	1.3	2.2	1.6	1.4	0.9	1.4	1.0	1.2	TPQPPPPsPPMK	TPQPPPPsPPMKLEL	S8;	Yes	T(1): 0.0; S(8): 100.0	1	1	1	0	N-Term(TMT6plex); S8(Phospho); M11(Oxidation); K12(TMT6plex)
226509740	NP_067051.2	58506	SCAF1	splicing factor, arginine/serine-rich 19	S612; S614	1.3	2.1	1.8	1.6	0.7	0.9	1.0	0.7	1.3	2.0	1.5	1.5	0.7	0.9	0.9	0.7	1.0	1.4	1.2	1.1	SAsPPPATSSSSSSR	RRRRRSAsPPPATSS	S1; S3;	Yes	S(1): 0.0; S(3): 100.0; T(8): 0.0; S(9): 0.0; S(10): 0.0; S(11): 0.0; S(12): 0.0; S(13): 0.0; S(14): 0.0	1	1	1	0	N-Term(TMT6plex); S3(Phospho)
4826972	NP_005096.1	9939	RBM8A	RNA-binding protein 8A	S42	1.1	1.1	0.9	1.2	1.1	1.1	1.2	1.2	1.2	1.1	1.1	1.3	1.3	1.1	1.4	1.3	1.2	1.1	1.2	1.3	GFGsEEGSR	RKGRGFGsEEGSRAR	S4;	Yes	S(4): 100.0; S(8): 0.0	11	1	1	0	N-Term(TMT6plex); S4(Phospho)
296531339	NP_001171812.1	5331	PLCB3	1-phosphatidylinositol 4,5-bisphosphate phosphodiesterase beta-3 isoform2	S1038	1.4	1.3	1.4	1.2	1.4	1.0	1.4	1.1	1.3	1.1	1.3	1.1	1.3	0.9	1.2	1.0	1.3	1.0	1.3	1.1	HNsISEAK	LDRKRHNsISEAKMR	S3;	No	S(3): 100.0; S(5): 0.0	4	2	1	0	N-Term(TMT6plex); S3(Phospho); K8(TMT6plex)
391353392	NP_001254652.1	729440	CCDC61	coiled-coil domain-containing protein 61	T285	0.9	1.0	1.2	1.4	0.7	0.8	0.7	0.9	0.7	0.9	1.1	1.1	0.6	0.8	0.6	0.8	0.7	0.9	0.9	0.9	tPPVQPPPTR	LYKRGRRtPPVQPPP	T1;	No	T(1): 100.0; T(9): 0.0	1	1	1	0	N-Term(TMT6plex); T1(Phospho)
223890259	NP_438112.2	11043	MID2	probable E3 ubiquitin-protein ligase MID2 isoform2	S116	0.9	0.9	0.8	0.8	1.2	1.2	1.1	1.5	-	-	-	-	-	-	-	-	-	-	-	-	ASVSGPNsPSESR	ASVSGPNsPSESRRE	S8;	Yes	S(2): 0.0; S(4): 0.0; S(8): 100.0; S(10): 0.0; S(12): 0.0	2	2	1	0	N-Term(TMT6plex); S8(Phospho)
55769564	NP_003430.1	7586	ZKSCAN1	zinc finger protein with KRAB and SCAN domains 1 isoforma	S13	0.7	1.0	1.0	0.9	1.0	0.9	0.4	1.4	0.6	0.9	0.9	0.9	0.9	0.8	0.4	1.3	0.7	0.9	0.6	1.1	EATGLsPQAAQEK	SREATGLsPQAAQEK	S6;	Yes	T(3): 0.0; S(6): 100.0	1	1	1	0	N-Term(TMT6plex); S6(Phospho); K13(TMT6plex)
40217847	NP_054733.2	23020	SNRNP200	U5 small nuclear ribonucleoprotein 200 kDa helicase	S225	1.1	0.7	1.0	1.2	1.2	1.2	1.0	1.1	1.1	0.7	1.0	1.2	1.2	1.3	1.0	1.1	1.2	1.0	1.0	1.1	EEAsDDDMEGDEAVVR	GEVREEAsDDDMEGD	S4;	Yes	S(4): 100.0	12	1	1	0	N-Term(TMT6plex); S4(Phospho); M8(Oxidation)
546231404	NP_001271231.1	54443	ANLN	actin-binding protein anillin isoform3	S754	1.5	2.0	2.4	1.2	1.5	1.4	1.7	1.1	1.5	1.9	2.2	1.3	1.6	1.4	1.6	1.1	1.6	1.6	1.9	1.2	AsPQSEEMPSK	PQRKNKAsPQSEEMP	S2;	No	S(2): 100.0; S(5): 0.0; S(10): 0.0	21	3	1	0	N-Term(TMT6plex); S2(Phospho); M8(Oxidation); K11(TMT6plex)
21396500	NP_003600.2	8479	HIRIP3	HIRA-interacting protein 3 isoform1	S196; S199	1.3	1.6	1.0	1.1	1.1	1.6	1.0	1.2	1.5	1.7	1.2	1.2	1.3	1.8	1.2	1.4	1.4	1.7	1.2	1.3	QAREEsEEsEAEPVQR	RKQAREEsEESEAEP;AREESEEsEAEPVQR	S6; S9;	Yes;Yes	S(6): 100.0; S(9): 100.0	26	1	1	1	N-Term(TMT6plex); S6(Phospho); S9(Phospho)
34996489	NP_060404.3	55023	PHIP	PH-interacting protein	S1783	1.4	1.4	1.2	1.2	1.0	1.1	1.9	1.1	1.3	1.3	1.1	1.1	1.0	1.0	1.7	1.0	1.2	1.1	1.4	1.0	TAEYNEDDsEEEQR	AEYNEDDsEEEQRQL	S9;	Yes	T(1): 0.0; Y(4): 0.0; S(9): 100.0	9	1	1	0	N-Term(TMT6plex); S9(Phospho)
385648259	NP_001245305.1	58513	EPS15L1	epidermal growth factor receptor substrate 15-like 1 isoform4	T366	2.1	1.3	1.0	0.8	5.6	5.6	3.4	1.4	2.0	1.4	1.0	1.1	5.1	6.3	3.6	1.9	3.5	3.9	2.3	1.5	GtPGPDSSGSLGSGEFTGVK	VPPSERGtPGPDSSG	T2;	Yes	T(2): 100.0; S(7): 0.0; S(8): 0.0; S(10): 0.0; S(13): 0.0; T(17): 0.0	6	4	1	0	N-Term(TMT6plex); T2(Phospho); K20(TMT6plex)
253970412	NP_057510.4	51512	GTSE1	G2 and S phase-expressed protein 1	S594; S599	1.1	1.1	1.0	1.0	1.3	1.4	1.1	1.2	1.2	1.2	1.1	1.2	1.5	1.5	1.2	1.5	1.3	1.3	1.1	1.4	LVDVsPDRGsPPSR	DSRLVDVsPDRGSPP;DVSPDRGsPPSRVPQ	S5; S10;	No;No	S(5): 100.0; S(10): 100.0; S(13): 0.0	19	1	1	1	N-Term(TMT6plex); S5(Phospho); S10(Phospho)
157676331	NP_055097.2	22927	HABP4	intracellular hyaluronan-binding protein 4	S108	1.3	1.7	1.6	1.1	1.2	2.1	1.3	1.2	1.5	1.8	2.1	1.4	1.5	2.2	1.7	1.4	1.5	2.0	1.9	1.4	SLPAPVAQRPDsPGGGLQAPGQ	PVAQRPDsPGGGLQA	S12;	Yes	S(1): 0.0; S(12): 100.0	4	1	1	0	N-Term(TMT6plex); S12(Phospho); K23(TMT6plex)
148596949	NP_004732.2	9221	NOLC1	nucleolar and coiled-body phosphoprotein 1 isoform2	S563	1.4	1.1	1.5	1.1	1.2	1.1	1.5	1.2	1.2	0.9	1.2	0.8	1.1	0.9	1.1	0.9	1.1	0.9	1.1	0.9	NsEEEEEEK	NGKAAKNsEEEEEEK	S2;	Yes	S(2): 100.0	33	3	1	0	N-Term(TMT6plex); S2(Phospho); K9(TMT6plex)
53829368	NP_001005408.1	84461	NEURL4	neuralized-like protein 4 isoform2	S502	1.1	1.4	1.1	1.3	0.9	1.2	0.9	1.0	-	-	-	-	-	-	-	-	-	-	-	-	ALsPEGALR	NAILRALsPEGALRR	S3;	Yes	S(3): 100.0	1	2	1	0	N-Term(TMT6plex); S3(Phospho)
61743954	NP_001611.1	79026	AHNAK	neuroblast differentiation-associated protein AHNAK isoform1	S5731	0.8	1.4	1.2	1.1	1.1	1.6	1.3	1.2	1.0	1.4	1.2	1.3	1.4	1.6	1.3	1.4	1.2	1.5	1.3	1.3	GGVTGsPEASISGSK	GKGGVTGsPEASISG	S6;	Yes	T(4): 0.0; S(6): 100.0; S(10): 0.0; S(12): 0.0; S(14): 0.0	79	1	1	0	N-Term(TMT6plex); S6(Phospho); K15(TMT6plex)
61743954	NP_001611.1	79026	AHNAK	neuroblast differentiation-associated protein AHNAK isoform1	S5731	1.1	1.6	1.3	1.2	0.8	1.4	1.2	1.1	1.4	1.6	1.3	1.4	1.0	1.4	1.2	1.3	1.2	1.5	1.3	1.3	GGVTGsPEAsISGSK	GKGGVTGsPEASISG	S6;	Yes	T(4): 0.0; S(6): 100.0; S(10): 0.0; S(12): 0.0; S(14): 0.0	7	1	1	0	N-Term(TMT6plex); S6(Phospho); K15(TMT6plex)
156630992	NP_001096123.1	54726	OTUD4	OTU domain-containing protein 4 isoform3	S941	0.9	0.9	0.6	0.9	1.3	1.2	1.2	1.4	0.9	0.9	0.6	0.9	1.3	1.1	1.1	1.3	1.1	1.0	0.9	1.1	TAADVVsPGANSVDSR	KTAADVVsPGANSVD	S7;	No	T(1): 0.0; S(7): 100.0; S(12): 0.0; S(15): 0.0	6	1	1	0	N-Term(TMT6plex); S7(Phospho)
604723372	NP_878315.2	91179	SCARF2	scavenger receptor class F member 2 isoform2 precursor	S682	1.2	1.3	1.2	1.1	0.8	1.4	1.4	1.2	-	-	-	-	-	-	-	-	-	-	-	-	APsPPPPGSEAAPsPSK	AAAGRAPsPPPPGSE	S3;	No	S(3): 100.0; S(9): 0.0; S(14): 0.0; S(16): 0.0	2	2	1	0	N-Term(TMT6plex); S3(Phospho); K17(TMT6plex)
604723372	NP_878315.2	91179	SCARF2	scavenger receptor class F member 2 isoform2 precursor	S682	0.8	1.4	1.4	1.2	1.2	1.3	1.2	1.1	-	-	-	-	-	-	-	-	-	-	-	-	APsPPPPGSEAAPSPSK	AAAGRAPsPPPPGSE	S3;	No	S(3): 100.0; S(9): 0.0; S(14): 0.0; S(16): 0.0	7	2	1	0	N-Term(TMT6plex); S3(Phospho); K17(TMT6plex)
217330641	NP_060439.2	55677	IWS1	protein IWS1 homolog	S250	1.2	1.0	1.3	0.9	1.3	1.4	1.3	1.4	1.3	1.0	1.3	1.0	1.4	1.3	1.3	1.6	1.3	1.1	1.3	1.3	ISDsESEDPPR	PKPRISDsESEDPPR	S4;	Yes	S(2): 1.2; S(4): 97.6; S(6): 1.2	25	1	1	0	N-Term(TMT6plex); S4(Phospho)
313661470	NP_001186346.1	57636	ARHGAP23	rho GTPase-activating protein 23	S372	1.3	0.9	0.8	1.1	1.0	1.2	1.3	1.1	1.1	0.9	0.8	1.1	0.9	1.3	1.3	1.1	1.0	1.1	1.0	1.1	SAEALGPGALVsPR	LGPGALVsPRFERCG	S12;	Yes	S(1): 0.0; S(12): 100.0	4	1	1	0	N-Term(TMT6plex); S12(Phospho)
42558250	NP_005889.3	4076	CAPRIN1	caprin-1 isoform1	S21	1.0	0.9	0.8	1.1	1.2	0.9	0.6	1.1	1.3	1.0	0.9	1.2	1.5	1.0	0.7	1.2	1.4	1.0	0.8	1.2	SSGPPPPsGSSGSEAAAGAGAAAPASQHPATGTGAVQTEAMK	SSGPPPPsGSSGSEA	S8;	No	S(1): 0.0; S(2): 0.0; S(8): 90.5; S(10): 3.1; S(11): 3.1; S(13): 3.1; S(26): 0.0; T(31): 0.0; T(33): 0.0; T(38): 0.0	6	2	1	0	N-Term(TMT6plex); S8(Phospho); M41(Oxidation); K42(TMT6plex)
15529996	NP_005624.2	6654	SOS1	son of sevenless homolog 1	S1082	1.0	1.1	1.5	1.1	1.0	1.8	0.6	1.1	1.0	1.3	1.9	1.2	1.0	2.0	0.7	1.2	1.0	1.6	1.3	1.2	IPESETESTASAPNsPR	STASAPNsPRTPLTP	S15;	Yes	S(4): 0.0; T(6): 0.0; S(8): 0.0; T(9): 0.0; S(11): 0.0; S(15): 100.0	1	1	1	0	N-Term(TMT6plex); S15(Phospho)
98986457	NP_005325.2	3054	HCFC1	host cell factor 1	S1507	1.0	1.1	0.7	1.1	1.0	1.5	0.6	1.1	1.1	1.2	0.8	1.2	1.1	1.7	0.6	1.2	1.1	1.4	0.7	1.2	AVTTVTQSTPVPGPSVPPPEELQVsPGPR	PPEELQVsPGPRQQL	S25;	Yes	T(3): 0.0; T(4): 0.0; T(6): 0.0; S(8): 0.0; T(9): 0.0; S(15): 0.0; S(25): 100.0	9	1	1	0	N-Term(TMT6plex); S25(Phospho)
28372563	NP_777602.1	283431	GAS2L3	GAS2-like protein 3	S570	1.0	1.0	0.9	1.1	1.0	2.0	2.3	1.1	-	-	-	-	-	-	-	-	-	-	-	-	SALNLNQPVSVSSVsPVK	PVSVSSVsPVKATQK	S15;	Yes	S(1): 0.0; S(10): 0.0; S(12): 0.0; S(13): 0.0; S(15): 100.0	6	1	1	0	N-Term(TMT6plex); S15(Phospho); K18(TMT6plex)
532525004	NP_001269147.1	9024	BRSK2	serine/threonine-protein kinase BRSK2 isoform5	S333	1.0	0.9	0.6	1.1	1.0	1.3	0.6	1.1	-	-	-	-	-	-	-	-	-	-	-	-	SMEVLSVTDGGsPVPAR	LSVTDGGsPVPARRA	S12;	No	S(1): 0.0; S(6): 0.0; T(8): 0.0; S(12): 100.0	1	5	1	0	N-Term(TMT6plex); M2(Oxidation); S12(Phospho)
325651836	NP_003592.3	8467	SMARCA5	SWI/SNF-related matrix-associated actin-dependent regulator of chromatin subfamily A member 5	S66	1.0	0.9	0.8	1.1	1.0	1.2	1.2	1.1	1.1	1.0	0.8	1.2	1.1	1.2	1.2	1.2	1.1	1.1	1.0	1.2	GGPEGVAAQAVASAASAGPADAEMEEIFDDAsPGK	EEIFDDAsPGKQKEI	S32;	Yes	S(13): 0.0; S(16): 0.0; S(32): 100.0	1	1	1	0	N-Term(TMT6plex); M24(Oxidation); S32(Phospho); K35(TMT6plex)
5902034	NP_008993.1	11137	PWP1	periodic tryptophan protein 1 homolog	S50	1.0	0.9	1.1	1.1	1.0	0.9	0.6	1.1	1.1	0.8	1.1	1.1	1.0	0.8	0.6	1.1	1.1	0.8	0.8	1.1	LQEEGGGsDEEETGSPSEDGMQSAR	LQEEGGGsDEEETGS	S8;	Yes	S(8): 100.0; T(13): 0.0; S(15): 0.0; S(17): 0.0; S(23): 0.0	4	1	1	0	N-Term(TMT6plex); S8(Phospho); M21(Oxidation)
103472005	NP_002408.3	4288	MKI67	antigen KI-67 isoform1	S584	1.0	0.9	0.7	1.1	1.0	0.9	0.6	1.1	1.2	0.9	0.8	1.2	1.2	0.9	0.7	1.2	1.2	0.9	0.7	1.2	AQSLVISPPAPsPR	VISPPAPsPRKTPVA	S12;	Yes	S(3): 0.0; S(7): 0.0; S(12): 100.0	118	2	1	0	N-Term(TMT6plex); S12(Phospho)
166295179	NP_001107590.1	55421	C17orf85	uncharacterized protein C17orf85	S25	1.0	0.9	0.6	1.1	1.0	0.9	0.6	1.1	1.2	1.0	0.7	1.5	1.2	1.0	0.7	1.5	1.2	1.0	0.7	1.5	AEAPAGPALGLPsPEAESGVDR	GPALGLPsPEAESGV	S13;	Yes	S(13): 100.0; S(18): 0.0	5	1	1	0	N-Term(TMT6plex); S13(Phospho)
6912246	NP_036231.1	10849	CD3EAP	DNA-directed RNA polymerase I subunit RPA34	S285	1.0	0.9	0.6	1.1	1.0	0.9	0.6	1.1	1.1	0.9	0.6	1.1	1.1	0.9	0.6	1.1	1.1	0.9	0.6	1.1	QEQINTEPLEDTVLsPTK	PLEDTVLsPTKKRKR	S15;	Yes	T(6): 0.0; T(12): 0.0; S(15): 98.8; T(17): 1.2	17	1	1	0	N-Term(TMT6plex); S15(Phospho); K18(TMT6plex)
42558250	NP_005889.3	4076	CAPRIN1	caprin-1 isoform1	S21	1.2	0.9	0.6	1.1	1.0	0.9	0.8	1.1	1.5	1.0	0.7	1.2	1.3	1.0	0.9	1.2	1.4	1.0	0.8	1.2	SSGPPPPSGSSGsEAAAGAGAAAPASQHPATGTGAVQTEAMK	PPSGSSGsEAAAGAG	S8;	No	S(1): 0.0; S(2): 0.0; S(8): 2.8; S(10): 2.8; S(11): 2.8; S(13): 91.5; S(26): 0.0; T(31): 0.0; T(33): 0.0; T(38): 0.0	2	2	1	0	N-Term(TMT6plex); S13(Phospho); M41(Oxidation); K42(TMT6plex)
532525004	NP_001269147.1	9024	BRSK2	serine/threonine-protein kinase BRSK2 isoform5	S333	1.0	1.3	0.6	1.1	1.0	0.9	0.6	1.1	-	-	-	-	-	-	-	-	-	-	-	-	sMEVLSVTDGGsPVPAR	LSVTDGGsPVPARRA	S12;	No	S(1): 0.0; S(6): 0.0; T(8): 0.0; S(12): 100.0	4	5	1	0	N-Term(TMT6plex); M2(Oxidation); S12(Phospho)
217330641	NP_060439.2	55677	IWS1	protein IWS1 homolog	S250	1.3	1.4	1.3	1.4	1.2	1.0	1.3	0.9	1.4	1.3	1.3	1.6	1.3	1.0	1.3	1.0	1.3	1.1	1.3	1.3	ISDsESEDPPR	PKPRISDsESEDPPR	S4;	Yes	S(2): 1.1; S(4): 97.8; S(6): 1.1	2	1	1	0	N-Term(TMT6plex); S4(Phospho)
42542379	NP_005830.2	10250	SRRM1	serine/arginine repetitive matrix protein 1	S234	0.7	1.2	1.3	1.0	1.0	1.5	1.5	1.2	0.9	1.2	1.4	1.2	1.2	1.6	1.7	1.4	1.0	1.4	1.5	1.3	VKEPsVQEATSTSDILK	SVKVKEPsVQEATST	S5;	Yes	S(5): 100.0; T(10): 0.0; S(11): 0.0; T(12): 0.0; S(13): 0.0	229	1	1	1	N-Term(TMT6plex); K2(TMT6plex); S5(Phospho); K17(TMT6plex)
108773787	NP_000312.2	5925	RB1	retinoblastoma-associated protein	T821	1.3	1.4	1.1	1.2	1.0	1.0	0.9	1.1	1.4	1.4	1.0	1.1	1.1	1.0	0.8	1.0	1.3	1.2	0.9	1.1	ISEGLPtPTK	KISEGLPtPTKMTPR	T7;	Yes	S(2): 0.0; T(7): 100.0; T(9): 0.0	4	1	1	0	N-Term(TMT6plex); T7(Phospho); K10(TMT6plex)
257196142	NP_001136336.2	9780	PIEZO1	piezo-type mechanosensitive ion channel component 1	S1621	0.9	1.3	1.0	0.9	1.6	1.4	1.4	1.3	1.0	1.3	1.1	0.9	1.8	1.5	1.5	1.3	1.4	1.4	1.3	1.1	SGsEEAVTDPGER	GYHTRSGsEEAVTDP	S3;	Yes	S(1): 0.0; S(3): 100.0; T(8): 0.0	3	1	1	0	N-Term(TMT6plex); S3(Phospho)
167614488	NP_056342.3	26000	TBC1D10B	TBC1 domain family member 10B	S22	1.4	1.5	1.0	1.1	0.8	0.9	1.1	1.2	1.5	1.6	1.2	1.1	0.9	1.0	1.2	1.3	1.2	1.3	1.2	1.2	HGAPAAPsPPPR	HGAPAAPsPPPRGSR	S8;	Yes	S(8): 100.0	13	1	1	0	N-Term(TMT6plex); S8(Phospho)
118572613	NP_057417.3	23524	SRRM2	serine/arginine repetitive matrix protein 2	S1083	0.8	1.4	1.1	1.2	0.8	1.1	0.9	1.0	0.9	1.4	1.1	1.4	0.9	1.1	1.0	1.1	0.9	1.3	1.0	1.3	QSHSEsPSLQSK	VRQSHSEsPSLQSKS	S6;	Yes	S(2): 0.0; S(4): 0.0; S(6): 100.0; S(8): 0.0; S(11): 0.0	436	1	1	0	N-Term(TMT6plex); S6(Phospho); K12(TMT6plex)
513788281	NP_001265477.1	5599	MAPK8	mitogen-activated protein kinase 8 isoform5	S301	0.9	1.1	1.1	1.0	1.1	1.0	0.9	1.2	0.9	1.1	0.9	1.0	1.1	1.0	0.8	1.3	1.0	1.1	0.9	1.2	GQPsPLAQVQQ	GVIRGQPsPLAQVQQ	S4;	No	S(4): 100.0	3	3	1	0	N-Term(TMT6plex); S4(Phospho)
153945728	NP_005900.2	4131	MAP1B	microtubule-associated protein 1B	S1265	2.0	1.5	1.2	1.0	1.2	2.2	1.7	1.3	1.6	1.1	1.0	1.0	1.0	1.6	1.4	1.4	1.3	1.4	1.2	1.2	SPSLSPSPPsPLEK	SLSPSPPsPLEKTPL	S10;	Yes	S(1): 0.0; S(3): 0.0; S(5): 0.0; S(7): 0.0; S(10): 100.0	49	1	1	0	N-Term(TMT6plex); S10(Phospho); K14(TMT6plex)
148664216	NP_689821.3	163859	SDE2	protein SDE2 homolog	S278	1.2	1.1	1.3	1.2	0.8	0.9	0.8	1.1	1.2	1.1	1.2	1.3	0.8	0.9	0.7	1.1	1.0	1.0	1.0	1.2	VVNTDHGsPEQLQIPVTDSGR	VVNTDHGsPEQLQIP	S8;	Yes	T(4): 0.0; S(8): 100.0; T(17): 0.0; S(19): 0.0	3	1	1	0	N-Term(TMT6plex); S8(Phospho)
31542498	NP_689837.2	167227	DCP2	m7GpppN-mRNA hydrolase isoform1	S329	0.9	0.7	0.7	1.0	0.8	0.8	0.9	1.3	-	-	-	-	-	-	-	-	-	-	-	-	QYQDsPNQK	GRKQYQDsPNQKKRT	S5;	Yes	Y(2): 0.0; S(5): 100.0	1	1	1	0	N-Term(TMT6plex); S5(Phospho); K9(TMT6plex)
56118310	NP_073568.2	64710	NUCKS1	nuclear ubiquitous casein and cyclin-dependent kinase substrate 1	S181	1.0	2.1	1.0	1.1	0.9	1.8	0.8	1.2	1.3	2.5	1.4	1.4	1.2	2.1	1.0	1.5	1.2	2.3	1.2	1.5	ATVTPsPVKGK	LKATVTPsPVKGKGK	S6;	Yes	T(2): 0.0; T(4): 0.0; S(6): 100.0	109	1	1	1	N-Term(TMT6plex); S6(Phospho); K9(TMT6plex); K11(TMT6plex)
431822406	NP_001258900.1	3326	HSP90AB1	heat shock protein HSP 90-beta isoformb	S207	1.1	1.2	1.1	1.1	1.3	1.5	1.2	1.2	1.1	1.1	1.1	1.1	1.4	1.4	1.2	1.2	1.2	1.2	1.2	1.1	IEDVGsDEEDDSGKDK	PKIEDVGsDEEDDSG	S6;	No	S(6): 100.0; S(12): 0.0	54	3	1	1	N-Term(TMT6plex); S6(Phospho); K14(TMT6plex); K16(TMT6plex)
47578107	NP_056199.2	25836	NIPBL	nipped-B-like protein isoformB	T599	1.1	1.0	0.8	1.0	1.3	1.2	1.0	1.3	1.0	0.9	0.7	0.9	1.2	1.1	0.9	1.2	1.1	1.0	0.8	1.0	STPENHPEtPK	TPENHPEtPKKKSDP	T9;	Yes	S(1): 0.0; T(2): 0.0; T(9): 100.0	8	2	1	0	N-Term(TMT6plex); T9(Phospho); K11(TMT6plex)
566006176	NP_001274366.1	79960	JADE1	protein Jade-1 isoform3	S591	0.5	0.8	0.7	1.0	0.8	1.4	1.0	1.2	-	-	-	-	-	-	-	-	-	-	-	-	DPLQNsPGSEGK	KRDPLQNsPGSEGKT	S6;	No	S(6): 100.0; S(9): 0.0	2	2	1	0	N-Term(TMT6plex); S6(Phospho); K12(TMT6plex)
295054220	NP_001171136.1	55183	RIF1	telomere-associated protein RIF1 isoform2	S2367	1.0	1.2	1.1	1.0	1.2	1.4	1.2	1.2	1.1	1.2	1.3	1.2	1.3	1.5	1.3	1.4	1.2	1.4	1.3	1.3	SLsPDEER	GIENKSLsPDEERLV	S3;	No	S(1): 0.7; S(3): 99.3	15	2	1	0	N-Term(TMT6plex); S3(Phospho)
4557793	NP_000258.1	4763	NF1	neurofibromin isoform2	S864	1.1	1.0	1.2	1.2	1.3	1.5	1.4	1.0	1.1	1.1	1.1	1.2	1.3	1.5	1.4	1.0	1.2	1.3	1.3	1.1	SNSGLATYsPPMGPVSER	NSGLATYsPPMGPVS	S9;	Yes	S(1): 0.0; S(3): 0.0; T(7): 1.6; Y(8): 1.6; S(9): 96.9; S(16): 0.0	2	2	1	0	N-Term(TMT6plex); S9(Phospho); M12(Oxidation)
118572613	NP_057417.3	23524	SRRM2	serine/arginine repetitive matrix protein 2	T2409	1.0	1.4	1.0	1.3	1.0	1.0	1.3	1.0	1.0	1.4	1.1	1.4	1.1	1.0	1.4	1.1	1.1	1.2	1.2	1.2	tPPSAPSQSR	LDRARSRtPPSAPSQ	T1;	Yes	T(1): 100.0; S(4): 0.0; S(7): 0.0; S(9): 0.0	436	1	1	0	N-Term(TMT6plex); T1(Phospho)
262399361	NP_001160163.1	7272	TTK	dual specificity protein kinase TTK isoform2	S435	1.0	0.9	1.0	1.3	0.8	0.7	0.7	1.0	1.0	0.9	1.1	1.2	0.8	0.7	0.8	0.9	0.9	0.8	0.9	1.1	QsPPISTSK	VFSVSKQsPPISTSK	S2;	No	S(2): 100.0; S(6): 0.0; T(7): 0.0; S(8): 0.0	1	2	1	0	N-Term(TMT6plex); S2(Phospho); K9(TMT6plex)
50659095	NP_004719.2	9188	DDX21	nucleolar RNA helicase 2 isoform1	S71	0.7	0.7	0.8	1.3	0.3	0.7	0.8	0.9	0.6	0.6	0.6	1.1	0.3	0.5	0.6	0.8	0.5	0.6	0.6	0.9	AEPSEVDMNsPK	PSEVDMNsPKSKKAK	S10;	Yes	S(4): 0.0; S(10): 100.0	23	1	1	0	N-Term(TMT6plex); M8(Oxidation); S10(Phospho); K12(TMT6plex)
50582989	NP_055684.3	9922	IQSEC1	IQ motif and SEC7 domain-containing protein 1	S417	1.0	1.0	1.0	1.3	1.0	0.9	0.7	1.0	0.9	0.9	1.0	1.2	0.9	0.8	0.7	0.9	0.9	0.9	0.8	1.0	SLGGQQGsPK	SLGGQQGsPKHGPHS	S8;	No	S(1): 0.0; S(8): 100.0	1	2	1	0	N-Term(TMT6plex); S8(Phospho); K10(TMT6plex)
51173873	NP_001003681.1	10042	HMGXB4	HMG domain-containing protein 4	S101	0.8	1.0	1.0	1.0	0.8	1.3	1.3	1.2	0.7	0.8	0.9	0.8	0.7	1.0	1.2	1.0	0.7	0.9	1.1	0.9	sSPQSTDTAMDLLK	SQKKKKsSPQSTDT	S1;	No	S(1): 98.9; S(2): 1.1; S(5): 0.0; T(6): 0.0; T(8): 0.0	5	1	1	0	N-Term(TMT6plex); S2(Phospho); M10(Oxidation); K14(TMT6plex)
154146193	NP_116259.2	25957	PNISR	arginine/serine-rich protein PNISR	S211	1.0	0.9	1.4	1.1	1.1	1.4	1.6	1.1	1.1	0.9	1.4	1.2	1.2	1.4	1.6	1.2	1.2	1.2	1.5	1.2	QRsPIALPVK	SFRDRQRsPIALPVK	S3;	Yes	S(3): 100.0	3	1	1	1	N-Term(TMT6plex); S3(Phospho); K10(TMT6plex)
166197710	NP_056393.2	26065	LSM14A	protein LSM14 homolog A isoformb	S216	0.9	0.8	0.9	0.9	1.2	1.5	1.0	1.3	1.0	0.9	1.1	1.0	1.4	1.7	1.1	1.4	1.2	1.3	1.1	1.2	sPVSTRPLPSASQK	PAAVGRRsPVSTRPL	S1;	Yes	S(1): 99.9; S(4): 0.0; T(5): 0.0; S(10): 0.0; S(12): 0.0	8	2	1	0	N-Term(TMT6plex); S1(Phospho); K14(TMT6plex)
39725634	NP_056130.2	23367	LARP1	la-related protein 1	T449	0.9	1.3	1.1	1.2	1.0	1.3	1.0	1.1	0.9	1.2	1.2	1.2	1.0	1.2	1.0	1.1	0.9	1.2	1.1	1.1	AVtPVPTK	PGSPRAVtPVPTKTE	T3;	No	T(3): 100.0; T(7): 0.0	28	1	1	0	N-Term(TMT6plex); T3(Phospho); K8(TMT6plex)
19923796	NP_057022.2	51068	NMD3	60S ribosomal export protein NMD3	S468	0.9	1.2	1.2	1.0	1.0	0.9	0.8	1.3	0.8	1.1	1.0	0.9	0.8	0.8	0.7	1.2	0.8	1.0	0.9	1.0	DSAIPVEsDTDDEGAPR	DSAIPVEsDTDDEGA	S8;	Yes	S(2): 0.1; S(8): 97.1; T(10): 2.8	3	1	1	0	N-Term(TMT6plex); S8(Phospho)
4505531	NP_002547.1	5007	OSBP	oxysterol-binding protein 1	S190; S193	1.1	1.0	1.1	1.1	1.5	1.0	1.5	1.2	1.0	1.0	1.2	1.1	1.4	1.1	1.5	1.2	1.2	1.1	1.4	1.2	MLAESDEsGDEESVSQTDK	AVKMLAEsDESGDEE;MLAESDEsGDEESVS	S5; S8;	Yes;Yes	S(5): 100.0; S(8): 100.0; S(13): 0.0; S(15): 0.0; T(17): 0.0	2	1	1	0	N-Term(TMT6plex); M1(Oxidation); S5(Phospho); S8(Phospho); K19(TMT6plex)
4505531	NP_002547.1	5007	OSBP	oxysterol-binding protein 1	S190; S193	1.5	1.0	1.5	1.2	1.1	1.0	1.1	1.1	1.4	1.1	1.5	1.2	1.0	1.0	1.2	1.1	1.2	1.1	1.4	1.2	MLAEsDEsGDEESVSQTDK	AVKMLAEsDESGDEE;MLAESDEsGDEESVS	S5; S8;	Yes;Yes	S(5): 100.0; S(8): 100.0; S(13): 0.0; S(15): 0.0; T(17): 0.0	8	1	1	0	N-Term(TMT6plex); M1(Oxidation); S5(Phospho); S8(Phospho); K19(TMT6plex)
19923796	NP_057022.2	51068	NMD3	60S ribosomal export protein NMD3	S468	1.0	0.9	0.8	1.3	0.9	1.2	1.2	1.0	0.8	0.8	0.7	1.2	0.8	1.1	1.0	0.9	0.8	1.0	0.9	1.0	DSAIPVESDtDDEGAPR	AIPVESDtDDEGAPR	S8;	Yes	S(2): 0.0; S(8): 0.0; T(10): 100.0	3	1	1	0	N-Term(TMT6plex); T10(Phospho)
39930523	NP_612480.1	144097	C11orf84	uncharacterized protein C11orf84	S308	1.0	0.9	0.9	1.1	1.0	1.0	0.8	1.1	1.2	1.1	1.1	1.4	1.2	1.2	1.0	1.4	1.2	1.2	1.0	1.4	AEsPSPAPPPGLR	GGLPRAEsPSPAPPP	S3;	Yes	S(3): 98.8; S(5): 1.2	3	1	1	0	N-Term(TMT6plex); S3(Phospho)
302699239	NP_004944.3	1981	EIF4G1	eukaryotic translation initiation factor 4 gamma 1	S1014	1.0	1.1	1.3	1.2	1.0	1.0	1.1	1.1	1.0	1.0	1.2	1.2	1.0	0.9	1.0	1.1	1.0	0.9	1.1	1.1	AAsLTEDR	EGLRKAAsLTEDRDR	S3;	No	S(3): 100.0; T(5): 0.0	17	7	1	0	N-Term(TMT6plex); S3(Phospho)
33188459	NP_004324.2	673	BRAF	serine/threonine-protein kinase B-raf	S729	1.4	1.4	1.2	1.2	1.2	1.1	1.1	1.1	-	-	-	-	-	-	-	-	-	-	-	-	SAsEPSLNR	PKIHRSAsEPSLNRA	S3;	Yes	S(1): 0.0; S(3): 100.0; S(6): 0.0	4	1	1	0	N-Term(TMT6plex); S3(Phospho)
42542379	NP_005830.2	10250	SRRM1	serine/arginine repetitive matrix protein 1	S450	1.0	1.1	0.9	1.1	1.0	1.4	1.0	1.2	1.2	1.2	1.0	1.2	1.2	1.4	1.1	1.3	1.2	1.3	1.1	1.3	REsPSPAPKPR	KGTEKREsPSPAPKP	S3;	Yes	S(3): 100.0; S(5): 0.0	229	1	1	1	N-Term(TMT6plex); S3(Phospho); K9(TMT6plex)
61743954	NP_001611.1	79026	AHNAK	neuroblast differentiation-associated protein AHNAK isoform1	S5400	1.0	0.9	1.0	1.1	3.6	2.6	4.7	1.1	1.2	0.9	1.0	1.3	4.3	2.7	4.7	1.3	2.8	1.8	2.8	1.3	VSVGAPDLSLEASEGsIK	SLEASEGsIKLPKMK	S16;	Yes	S(2): 0.0; S(9): 0.0; S(13): 1.6; S(16): 98.4	79	1	1	0	N-Term(TMT6plex); S16(Phospho); K18(TMT6plex)
422398894	NP_001258719.1	1781	DYNC1I2	cytoplasmic dynein 1 intermediate chain 2 isoform4	S81	2.2	0.9	1.7	1.1	1.0	0.9	1.3	1.1	2.0	0.9	1.6	1.1	0.9	0.9	1.3	1.1	1.5	0.9	1.5	1.1	EAEALLQSMGLTPESPIVPPPMsPSSK	PIVPPPMsPSSKSVS	S23;	Yes	S(8): 0.0; T(12): 0.0; S(15): 0.0; S(23): 94.2; S(25): 2.9; S(26): 2.9	10	2	1	0	N-Term(TMT6plex); M9(Oxidation); M22(Oxidation); S23(Phospho); K27(TMT6plex)
7661532	NP_054781.1	28987	NOB1	RNA-binding protein NOB1	S184	1.0	1.2	1.7	1.1	1.4	0.9	0.6	1.1	1.0	1.1	1.6	1.0	1.4	0.9	0.5	1.0	1.2	1.0	1.2	1.0	GEDVPsEEEEEEENGEEDR	DRGEDVPsEEEEEEE	S6;	Yes	S(6): 100.0	3	1	1	0	N-Term(TMT6plex); S6(Phospho)

Khan et al., 2019. Multi-omics analysis to characterize cigarette smoke induced molecular alterations in esophageal cells

Supplementary Table 8. List of phosphopeptides identified and quantified in untreated and chronically treated Het1A cells with cigarette smoke condensate for 8 months using SequestHT and Mascot search algorithms with a PhosphoRS probability score ≥75%

														Normalized phosphoprotein ratios with respect to corresponding total protein ratios.								Average of replicates													
Protein group Accession	NP_Accession	Gene ID	Gene Symbol	Protein Description	PhosphoSite (Protein)	Het-1A-Smoke - 2M/Parental	Het-1A-Smoke - 4M/Parental	Het-1A-Smoke - 6M/Parental	Het-1A-Smoke - 8M/Parental	Het-1A-Smoke - 2M/Parental	Het-1A-Smoke - 4M/Parental	Het-1A-Smoke - 6M/Parental	Het-1A-Smoke - 8M/Parental	Het-1A-Smoke - 2M/Parental	Het-1A-Smoke - 4M/Parental	Het-1A-Smoke - 6M/Parental	Het-1A-Smoke - 8M/Parental	Het-1A-Smoke - 2M/Parental	Het-1A-Smoke - 4M/Parental	Het-1A-Smoke - 6M/Parental	Het-1A-Smoke - 8M/Parental	Het-1A-Smoke - 2M/Parental	Het-1A-Smoke - 4M/Parental	Het-1A-Smoke - 6M/Parental	Het-1A-Smoke - 8M/Parental	Peptide Sequence	Phosphowindow	PhosphoSite (Peptide)	Phosphosite Plus Evidence	phosphoRS Site Probabilities	# PSMs	# Proteins	# Protein Groups	# Missed Cleavages	Modifications
						Replicate 1				Replicate 2				Replicate 1				Replicate 2																	
110556636	NP_203754.2	85456	TNKS1BP1	182 kDa tankyrase-1-binding protein	S601	1.3	0.9	0.6	1.1	1.0	0.9	1.2	1.1	1.2	0.8	0.5	1.2	1.0	0.8	1.1	1.3	1.1	0.8	0.8	1.2	YESQEPLAGQEsPLPLATR	EPLAGQEsPLPLATR	S12;	Yes	Y(1): 0.2; S(3): 0.2; S(12): 99.5; T(18): 0.2	41	1	1	0	N-Term(TMT6plex); S12(Phospho)
7662158	NP_055991.1	23219	FBXO28	F-box only protein 28 isoform a	S344	2.8	1.1	1.2	1.1	5.3	1.6	2.0	1.1	2.7	1.2	1.2	1.1	5.1	1.7	2.1	1.1	3.9	1.5	1.6	1.1	EVMESAVGNSSGSGQNEEsPR	GSGQNEEsPRKRKKA	S19;	Yes	S(5): 0.0; S(10): 0.0; S(11): 0.0; S(13): 0.0; S(19): 100.0	4	1	1	0	N-Term(TMT6plex); M3(Oxidation); S19(Phospho)
545478902	NP_001269892.1	55355	HJURP	Holliday junction recognition protein isoform c	S388	1.0	0.9	0.6	1.1	1.0	0.9	0.6	1.1	1.1	0.8	0.6	1.1	1.1	0.8	0.6	1.1	1.1	0.8	0.6	1.1	GGPAsPGGLQGLETR	MYRGGPAsPGGLQGL	S5;	No	S(5): 100.0; T(14): 0.0	5	3	1	0	N-Term(TMT6plex); S5(Phospho)
347543829	NP_001231533.1	1808	DPYSL2	dihydropyrimidinase-related protein 2 isoform 3	T473	1.0	0.9	0.6	1.1	1.0	0.9	0.6	1.1	1.0	0.7	0.5	1.1	1.0	0.7	0.5	1.0	1.0	0.7	0.5	1.1	GLYDGPVCEVSVtPK	PVCEVSVtPKTVTPA	T13;	No	Y(3): 0.0; S(11): 0.0; T(13): 100.0	5	3	1	0	N-Term(TMT6plex); C8(Carbamidomethyl); T13(Phospho); K15(TMT6plex)
82880645	NP_001032411.1	9097	USP14	ubiquitin carboxyl-terminal hydrolase 14 isoform b	S108	1.0	0.9	0.6	1.1	1.0	0.9	0.6	1.1	1.0	0.8	0.6	0.9	1.0	0.8	0.6	0.9	1.0	0.8	0.6	0.9	AsGEMASAQYITAALR	YAGALRAsGEMASAQ	S2;	No	S(2): 100.0; S(7): 0.0; Y(10): 0.0; T(12): 0.0	2	2	1	0	N-Term(TMT6plex); S2(Phospho); M5(Oxidation)
216548445	NP_001136026.1	55621	TRMT1	tRNA (guanine(26)-N(2))-dimethyltransferase isoform 2	S596	1.6	1.2	1.1	1.3	0.8	0.7	0.7	1.0	1.5	1.3	1.3	1.4	0.8	0.8	0.9	1.1	1.2	1.0	1.1	1.2	GDQCCYSHsPPTPR	DQCCYSHsPPTPRVS	S9;	No	Y(6): 0.0; S(7): 1.1; S(9): 97.7; T(12): 1.1	2	2	1	0	N-Term(TMT6plex); C4(Carbamidomethyl); C5(Carbamidomethyl); S9(Phospho)
157311635	NP_940890.3	286077	FAM83H	protein FAM83H	S870	1.2	1.2	1.2	1.2	0.8	0.9	1.1	1.0	1.1	1.1	1.1	1.1	0.8	0.8	0.9	0.9	0.9	0.9	1.0	1.0	GsPTSAYPER	LHNESKGsPTSAYPE	S2;	Yes	S(2): 100.0; T(4): 0.0; S(5): 0.0; Y(7): 0.0	3	1	1	0	N-Term(TMT6plex); S2(Phospho)
317574227	NP_001188264.1	6936	GCFC2	GC-rich sequence DNA-binding factor 2 isoform 3	S174; S180	1.4	1.6	1.2	1.3	1.5	1.2	0.7	1.0	1.3	1.5	1.0	1.1	1.3	1.1	0.6	0.8	1.3	1.3	0.8	1.0	REsEDDPEsEPDDHEK	ISGMKREsEDDPESE;ESEDDPEsEPDDHEK	S3; S9;	Yes;Yes	S(3): 100.0; S(9): 100.0	4	3	1	1	N-Term(TMT6plex); S3(Phospho); S9(Phospho); K16(TMT6plex)
21396500	NP_003600.2	8479	HIRIP3	HIRA-interacting protein 3 isoform 1	S142; S143	1.6	1.4	1.2	1.0	1.0	1.5	1.0	1.3	1.8	1.5	1.4	1.1	1.1	1.6	1.2	1.4	1.5	1.6	1.3	1.2	AVEEssDEERQR	ASKAVEEsSDEERQR;SKAVEESsDEERQRD	S5; S6;	Yes;Yes	S(5): 100.0; S(6): 100.0	26	1	1	1	N-Term(TMT6plex); S5(Phospho); S6(Phospho)
7661990	NP_055572.1	9794	MAML1	mastermind-like protein 1	S360	0.9	1.2	1.2	1.4	0.9	1.0	0.7	0.8	-	-	-	-	-	-	-	-	-	-	-	-	ADNPsPNLMPASAQAQNAQR	QPRADNPsPNLMPAS	S5;	Yes	S(5): 100.0; S(12): 0.0	1	1	1	0	N-Term(TMT6plex); S5(Phospho); M9(Oxidation)
145309300	NP_112557.2	8621	CDK13	cyclin-dependent kinase 13 isoform 2	S325	1.2	1.0	1.1	1.2	0.9	1.0	1.1	1.1	1.7	1.3	1.5	1.5	1.3	1.2	1.6	1.4	1.5	1.3	1.5	1.5	DDsPVSHR	PLGGRDDsPVSHRAS	S3;	Yes	S(3): 100.0; S(6): 0.0	8	2	1	0	N-Term(TMT6plex); S3(Phospho)
4885399	NP_005436.1	9126	SMC3	structural maintenance of chromosomes protein 3	S1067	1.0	1.2	1.2	1.0	0.9	1.2	2.0	1.3	1.0	1.2	1.1	0.9	0.8	1.2	1.7	1.2	0.9	1.2	1.4	1.1	KGDVEGsQSQDEGEGSGESER	KKGDVEGsQSQDEGE	S9;	Yes	S(7): 98.2; S(9): 1.8; S(16): 0.0; S(19): 0.0	2	1	1	1	N-Term(TMT6plex); K1(TMT6plex); S7(Phospho)
29725609	NP_005219.2	1956	EGFR	epidermal growth factor receptor isoform a precursor	Y1197	1.0	0.7	0.7	1.0	1.2	0.8	0.7	1.2	1.2	0.8	0.8	1.4	1.4	1.0	0.8	1.6	1.3	0.9	0.8	1.5	GSTAENAEyLR	STAENAEyLRVAPQS	Y9;	Yes	S(2): 0.0; T(3): 0.0; Y(9): 100.0	8	1	1	0	N-Term(TMT6plex); Y9(Phospho)
116008442	NP_055885.3	23091	ZC3H13	zinc finger CCCH domain-containing protein 13	S877	1.3	1.6	1.3	1.2	1.1	1.3	1.1	1.1	1.3	1.5	1.3	1.1	1.0	1.2	1.2	1.0	1.2	1.4	1.2	1.1	SLsPSHLTEDR	PQESRSLsPSHLTED	S3;	Yes	S(1): 0.0; S(3): 99.2; S(5): 0.8; T(8):	28	1	1	0	N-Term(TMT6plex); S3(Phospho)
4885399	NP_005436.1	9126	SMC3	structural maintenance of chromosomes protein 3	S1067	0.9	1.2	2.0	1.3	1.0	1.2	1.2	1.0	0.8	1.2	1.7	1.2	1.0	1.2	1.1	0.9	0.9	1.2	1.4	1.1	KGDVEGSQsQDEGEGSGESER	GDVEGSQsQDEGEGS	S9;	Yes	S(7): 0.0; S(9): 100.0; S(16): 0.0; S(19): 0.0	13	1	1	1	N-Term(TMT6plex); K1(TMT6plex); S9(Phospho)
312032467	NP_003613.3	8496	PPFIBP1	liprin-beta-1 isoform 1	S449	1.1	1.2	1.3	1.1	1.0	1.2	1.3	1.2	0.8	1.0	0.9	1.0	0.8	0.9	0.9	1.0	0.8	0.9	0.9	1.0	SSsLGNLK	SSLQKSSsLGNLKKE	S3;	No	S(1): 0.0; S(2): 0.0; S(3): 100.0	3	4	1	0	N-Term(TMT6plex); S3(Phospho); K8(TMT6plex)
31652264	NP_071401.3	64062	RBM26	RNA-binding protein 26 isoform 3	S127	1.2	1.3	1.3	1.2	1.0	1.1	1.0	1.1	1.0	1.1	1.2	1.0	0.9	1.0	0.9	0.9	1.0	1.1	1.0	1.0	LNHsPPQSSSR	FSRRLNHsPPQSSSR	S4;	Yes	S(4): 100.0; S(8): 0.0; S(9): 0.0; S(10): 0.0	2	3	1	0	N-Term(TMT6plex); S4(Phospho)
355390315	NP_056171.3	23513	SCRIB	protein scribble homolog isoform b	S1475	1.0	1.0	0.9	1.0	0.9	1.0	0.9	1.2	0.9	0.9	0.9	0.9	0.8	0.9	0.9	1.1	0.9	0.9	0.9	1.0	VQsPEPPAPER	QERLRVQsPEPPAPE	S3;	Yes	S(3): 100.0	16	2	1	0	N-Term(TMT6plex); S3(Phospho)
24308382	NP_443157.1	114823	LENG8	leukocyte receptor cluster member 8	S351	1.2	1.1	1.1	1.3	0.8	0.8	0.8	0.9	1.1	0.9	1.1	1.2	0.7	0.7	0.8	0.8	0.9	0.8	1.0	1.0	EPVAEsPK	TREPVAEsPKKKRWE	S6;	No	S(6): 100.0	2	1	1	0	N-Term(TMT6plex); S6(Phospho); K8(TMT6plex)
253970412	NP_057510.4	51512	GTSE1	G2 and S phase-expressed protein 1	T485	0.9	1.3	1.2	1.0	0.8	0.8	1.2	1.3	1.0	1.4	1.4	1.2	0.8	0.8	1.3	1.5	0.9	1.1	1.3	1.3	FSIGDsPDSSTPK	PKFSIGDsPDSSTPK	T11;	No	S(2): 0.0; S(6): 100.0; S(9): 0.0; S(10): 0.0; T(11): 0.0	4	1	1	0	N-Term(TMT6plex); S6(Phospho); K13(TMT6plex)
308818200	NP_001184223.1	1809	DPYSL3	dihydropyrimidinase-related protein 3 isoform 1	S102	0.9	1.2	1.1	1.0	1.5	1.7	1.2	1.2	1.0	1.2	1.1	1.1	1.7	1.6	1.2	1.3	1.3	1.4	1.2	1.2	EPAPAsPAPAGVEIR	SREPAPAsPAPAGVE	S6;	Yes	S(6): 100.0	8	1	1	0	N-Term(TMT6plex); S6(Phospho)
13129104	NP_077003.1	79080	CCDC86	coiled-coil domain-containing protein 86	S217	1.3	1.7	1.2	1.1	1.2	1.6	1.1	1.1	1.6	1.7	1.3	1.3	1.5	1.6	1.2	1.2	1.5	1.6	1.2	1.2	KGsSSQAPASK	GAKKRKGsSSQAPAS	S3;	Yes	S(3): 100.0; S(4): 0.0; S(5): 0.0; S(10): 0.0	26	1	1	1	N-Term(TMT6plex); K1(TMT6plex); S3(Phospho); K11(TMT6plex)
253970412	NP_057510.4	51512	GTSE1	G2 and S phase-expressed protein 1	T485	0.8	0.8	1.2	1.3	0.9	1.3	1.2	1.0	0.8	0.8	1.3	1.5	1.0	1.4	1.4	1.2	0.9	1.1	1.3	1.3	FSIGDSPDSStPK	GDSPDSStPKLSRAQ	T11;	No	S(2): 0.0; S(6): 0.0; S(9): 0.0; S(10): 0.0; T(11): 99.9	19	1	1	0	N-Term(TMT6plex); T11(Phospho); K13(TMT6plex)
19913406	NP_001058.2	7153	TOP2A	DNA topoisomerase 2-alpha	S1354	0.8	0.7	0.7	0.9	1.1	0.8	1.1	1.3	0.9	0.8	0.8	1.0	1.2	0.9	1.3	1.4	1.1	0.9	1.1	1.2	TDDEDFVPSDAsPPK	DFVPSDAsPPKTKTS	S12;	Yes	T(1): 0.0; S(9): 0.0; S(12): 100.0	64	1	1	0	N-Term(TMT6plex); S12(Phospho); K15(TMT6plex)
33946327	NP_005076.3	8021	NUP214	nuclear pore complex protein Nup214	S430	1.0	0.7	0.8	1.1	1.1	1.0	1.0	1.2	0.9	0.7	0.7	1.0	1.1	1.0	0.9	1.0	1.0	0.9	0.8	1.0	sPGSTPTTPTSSQAPQK	EGERQPKsPGSTPTT	S1;	Yes	S(1): 95.2; S(4): 2.3; T(5): 2.3; T(7): 0.0; T(8): 0.1; T(10): 0.1; S(11): 0.1; S(12): 0.1	8	1	1	0	N-Term(TMT6plex); S1(Phospho); K17(TMT6plex)
33946327	NP_005076.3	8021	NUP214	nuclear pore complex protein Nup214	S430	1.1	1.0	1.0	1.2	1.0	0.7	0.8	1.1	1.1	1.0	0.9	1.0	0.9	0.7	0.7	1.0	1.0	0.9	0.8	1.0	SPGsTPTTPTSSQAPQK	RQPKSPGsTPTTPTS	S1;	Yes	S(1): 1.3; S(4): 97.4; T(5): 1.3; T(7): 0.0; T(8): 0.0; T(10): 0.0; S(11): 0.0; S(12): 0.0	3	1	1	0	N-Term(TMT6plex); S4(Phospho); K17(TMT6plex)
399154157	NP_001257697.1	85369	STRIP1	striatin-interacting protein 1 isoform 2	S240	1.3	0.9	0.6	1.1	2.0	1.8	1.6	1.1	1.2	0.9	0.6	1.1	1.9	1.8	1.7	1.1	1.6	1.3	1.1	1.1	AAsPPASASDLIEQQQK	IRNMRAAsPPASASD	S3;	No	S(3): 100.0; S(7): 0.0; S(9): 0.0	1	2	1	0	N-Term(TMT6plex); S3(Phospho); K17(TMT6plex)
296278202	NP_001171721.1	56288	PARD3	partitioning defective 3 homolog isoform 9	S849	1.9	1.9	2.1	1.3	1.0	1.4	1.1	1.0	1.8	1.9	1.9	1.2	1.0	1.4	1.0	0.9	1.4	1.6	1.5	1.1	sMDLGIADETK	VKTRKSKsMDLGIAD	S1;	No	S(1): 100.0; T(10): 0.0	11	5	1	0	N-Term(TMT6plex); S1(Phospho); M2(Oxidation); K11(TMT6plex)
296179431	NP_068765.3	63035	BCORL1	BCL-6 corepressor-like protein 1	S599	1.0	1.4	1.2	1.0	0.8	1.6	1.7	1.2	-	-	-	-	-	-	-	-	-	-	-	-	MPSGTEQQTEGTSVTFsPLK	EGTSVTFsPLKSPPQ	S17;	Yes	S(3): 0.0; T(5): 0.0; T(9): 0.0; T(12): 0.0; S(13): 0.0; T(15): 0.0; S(17): [illegible]	2	1	1	0	N-Term(TMT6plex); M1(Oxidation); S17(Phospho); K20(TMT6plex)
69354671	NP_001020262.1	23	ABCF1	ATP-binding cassette sub-family F member 1 isoform a	S140	1.0	0.9	1.8	1.1	2.3	2.0	0.6	1.1	1.1	0.9	2.1	1.3	2.5	2.1	0.7	1.3	1.8	1.5	1.4	1.3	GGNVFAALIQDQsEEEEEEEK	AALIQDQsEEEEEEE	S13;	Yes	S(13): 100.0	44	2	1	0	N-Term(TMT6plex); S13(Phospho); K21(TMT6plex)
525313694	NP_001265719.1	64689	GORASP1	Golgi reassembly-stacking protein 1 isoform 3	T61	1.0	0.9	0.6	1.1	1.0	1.3	2.6	1.1	-	-	-	-	-	-	-	-	-	-	-	-	KPPGtPPPSALPLGAPPPDALPPGPTPEDSPSLETGSR	YHKKPPGtPPPSALP	T5;	No	T(5): 99.8; S(9): 0.2; T(26): 0.0; S(30): 0.0; S(32): 0.0; T(35): 0.0; S(37): 0.0	1	3	1	0	N-Term(TMT6plex); K1(TMT6plex); T5(Phospho)
45827753	NP_996742.1	10579	TACC2	transforming acidic coiled-coil-containing protein 2 isoform c	S334	1.0	0.9	0.6	1.1	1.0	0.9	0.8	1.1	1.0	0.8	0.5	1.2	1.0	0.8	0.7	1.2	1.0	0.8	0.6	1.2	TLPLTTAPEAGEVTPSDSGGQEDsPAK	DSGGQEDsPAKGLSV	S24;	No	T(1): 0.0; T(5): 0.0; T(6): 0.0; T(14): 0.0; S(16): 0.0; S(18): 0.0; S(24): [illegible]	11	7	1	0	N-Term(TMT6plex); S24(Phospho); K27(TMT6plex)
54292123	NP_000072.2	1130	LYST	lysosomal-trafficking regulator	S2105	1.0	0.9	1.2	1.1	1.0	0.9	1.5	1.1	-	-	-	-	-	-	-	-	-	-	-	-	sLPAFPTSSLLTQSQK	AHMLRSRsLPAFPTS	S1;	Yes	S(1): 100.0; T(7): 0.0; S(8): 0.0; S(9): 0.0; T(12): 0.0; S(14): 0.0	1	1	1	0	N-Term(TMT6plex); S1(Phospho); K16(TMT6plex)
393715095	NP_001257329.1	7846	TUBA1A	tubulin alpha-1A chain isoform 2	S404	1.0	0.9	0.6	1.1	1.0	0.9	0.6	1.1	1.0	0.9	0.6	1.1	1.0	0.9	0.6	1.1	1.0	0.9	0.6	1.1	DYEEVGVDsVEGEGEEEGEEY	YEEVGVDsVEGEGEE	S9;	No	Y(2): 0.0; S(9): 97.0; Y(21): 3.0	8	3	1	0	N-Term(TMT6plex); S9(Phospho)
555943905	NP_001273176.1	55103	RALGPS2	ras-specific guanine nucleotide-releasing factor RalGPS2 isoform 2	S308	1.0	0.9	0.6	1.1	1.0	0.9	0.8	1.1	-	-	-	-	-	-	-	-	-	-	-	-	EDLVGPEVGAsPQSGR	VGPEVGAsPQSGRKS	S11;	Yes	S(11): 98.2; S(14): 1.8	2	2	1	0	N-Term(TMT6plex); S11(Phospho)
47519639	NP_002366.2	4134	MAP4	microtubule-associated protein 4 isoform 1	T521	1.0	0.9	0.6	1.1	1.0	0.9	0.6	1.1	1.1	0.9	0.7	1.4	1.0	0.9	0.6	1.4	1.1	0.9	0.6	1.4	DVtPPPETEVVLIK	MALGKDVtPPPETEV	T3;	Yes	T(3): 100.0; T(8): 0.0	47	2	1	0	N-Term(TMT6plex); T3(Phospho); K14(TMT6plex)
23238226	NP_689449.1	11135	CDC42EP1	cdc42 effector protein 1	S121	1.0	0.9	1.7	1.1	1.0	0.9	0.6	1.1	0.9	0.7	1.6	1.0	0.9	0.7	0.5	1.0	0.9	0.7	1.0	1.0	NAIsLPQLNQAAYDSLVVGK	PIIKNAIsLPQLNQA	S4;	Yes	S(4): 99.8; Y(13): 0.1; S(15): 0.1	3	1	1	0	N-Term(TMT6plex); S4(Phospho); K20(TMT6plex)
154354976	NP_006598.2	10744	PTTG2	securin-2	S165	1.0	0.9	0.6	1.1	1.0	0.9	0.6	1.1	-	-	-	-	-	-	-	-	-	-	-	-	LEQLGPPsPVK	LEQLGPPsPVKMPSP	S8;	Yes	S(8): 100.0	1	2	1	0	N-Term(TMT6plex); S8(Phospho); K11(TMT6plex)
331999998	NP_001193633.1	6672	SP100	nuclear autoantigen Sp-100 isoform 6	S327	1.0	0.9	0.7	1.1	1.0	0.9	1.7	1.1	1.0	0.8	0.5	1.2	0.9	0.8	1.4	1.2	1.0	0.8	1.0	1.2	SEPVINNDNPLEsNDEK	NNDNPLEsNDEKEGQ	S13;	No	S(1): 0.0; S(13): 100.0	4	6	1	0	N-Term(TMT6plex); S13(Phospho); K17(TMT6plex)
49472822	NP_003746.2	8666	EIF3G	eukaryotic translation initiation factor 3 subunit G	T38	1.0	0.9	0.6	1.1	1.0	0.9	0.6	1.1	1.1	0.9	0.6	1.2	1.1	0.9	0.6	1.2	1.1	0.9	0.6	1.2	GIPLAtGDTSPEPELLPGAPLPPP	LKGIPLAtGDTSPEP	T6;	Yes	T(6): 96.1; T(9): 2.0; S(10): 2.0	8	1	1	0	N-Term(TMT6plex); T6(Phospho); K25(TMT6plex)
31652242	NP_852469.1	6942	TCF20	transcription factor 20 isoform 2	S1335	1.0	0.9	1.2	1.1	1.0	0.9	0.8	1.1	0.9	1.0	1.1	1.2	0.9	1.0	0.8	1.2	0.9	1.0	1.0	1.2	NCPAVTLTsPAK	CPAVTLTsPAKTKIL	S9;	Yes	T(6): 1.1; T(8): 1.1; S(9): 97.9	14	2	1	0	N-Term(TMT6plex); C2(Carbamidomethyl); S9(Phospho); K12(TMT6plex)
31652242	NP_852469.1	6942	TCF20	transcription factor 20 isoform 2	S1335	1.0	0.9	0.8	1.1	1.0	0.9	1.2	1.1	0.9	1.0	0.8	1.2	0.9	1.0	1.1	1.2	0.9	1.0	1.0	1.2	NCPAVtLTSPAK	SRNCPAVtLTSPAKT	S9;	Yes	T(6): 100.0; T(8): 0.0; S(9): 0.0	1	2	1	0	N-Term(TMT6plex); C2(Carbamidomethyl); T6(Phospho); K12(TMT6plex)
49472822	NP_003746.2	8666	EIF3G	eukaryotic translation initiation factor 3 subunit G	T38	1.0	0.9	0.6	1.1	1.0	0.9	0.6	1.1	1.1	0.9	0.6	1.2	1.1	0.9	0.6	1.2	1.1	0.9	0.6	1.2	GIPLATGDTsPEPELLPGAPLPP	PLATGDTsPEPELLP	T6;	Yes	T(6): 0.0; T(9): 2.0; S(10): 98.0	6	1	1	0	N-Term(TMT6plex); S10(Phospho); K25(TMT6plex)
33946282	NP_056311.2	25962	KIAA1429	protein virilizer homolog isoform 1	S1579	1.2	1.2	1.2	1.1	1.0	1.1	1.1	1.2	1.0	1.1	1.0	1.0	0.9	1.0	0.9	1.1	0.9	1.1	1.0	1.0	SFLSEPSsPGR	SFLSEPSsPGRTKTT	S8;	Yes	S(1): 0.0; S(4): 0.0; S(7): 1.0; S(8):	4	1	1	0	N-Term(TMT6plex); S8(Phospho)
10716563	NP_001737.1	821	CANX	calnexin precursor	S583	1.3	1.5	1.0	1.1	1.2	1.6	1.1	1.1	1.3	1.2	1.0	1.0	1.2	1.2	1.1	1.0	1.2	1.2	1.0	1.0	AEEDEILNRsPR	EDEILNRsPRNRKPR	S10;	Yes	S(10): 100.0	54	1	1	1	N-Term(TMT6plex); S10(Phospho)
33946282	NP_056311.2	25962	KIAA1429	protein virilizer homolog isoform 1	S1579	1.0	1.1	1.1	1.2	1.2	1.2	1.2	1.1	0.9	1.0	0.9	1.1	1.0	1.1	1.0	1.0	0.9	1.1	1.0	1.0	SFLSEPsSPGR	RSFLSEPsSPGRTKT	S8;	Yes	S(1): 0.0; S(4): 0.0; S(7): 99.0; S(8):	4	1	1	0	N-Term(TMT6plex); S7(Phospho)
156104891	NP_002087.2	2962	GTF2F1	general transcription factor IIF subunit 1	S433	1.3	1.1	1.2	1.2	0.9	1.0	1.0	1.0	1.3	1.1	1.1	1.3	0.9	0.9	0.9	1.1	1.1	1.0	1.0	1.2	LDTGPQSLsGK	DTGPQSLsGKSTPQP	S9;	Yes	T(3): 0.0; S(7): 0.0; S(9): 100.0	20	1	1	0	N-Term(TMT6plex); S9(Phospho); K11(TMT6plex)
116008442	NP_055885.3	23091	ZC3H13	zinc finger CCCH domain-containing protein 13	S198	1.3	1.2	1.1	1.3	1.3	1.2	1.3	0.9	1.2	1.1	1.1	1.2	1.3	1.2	1.3	0.9	1.2	1.1	1.2	1.1	EVsPEVVR	IIKKEVsPEVVRSK	S3;	Yes	S(3): 100.0	28	1	1	0	N-Term(TMT6plex); S3(Phospho)
13514831	NP_004389.2	1662	DDX10	probable ATP-dependent RNA helicase DDX10	S539	0.9	1.1	1.2	0.9	1.3	1.3	1.4	1.3	0.8	0.9	1.1	0.9	1.2	1.2	1.3	1.3	1.0	1.0	1.2	1.1	APsLTNDEVEEFR	VIEPRAPsLTNDEVE	S3;	Yes	S(3): 100.0; T(5): 0.0	4	1	1	0	N-Term(TMT6plex); S3(Phospho)
146198654	NP_078947.3	79724	ZNF768	zinc finger protein 768	S125	1.4	1.4	1.4	1.3	1.2	1.2	1.3	0.9	1.5	1.6	1.6	1.4	1.3	1.4	1.5	1.0	1.4	1.5	1.6	1.2	YEPQsPGYEPR	SPRYEPQsPGYEPRS	S5;	Yes	Y(1): 2.1; S(5): 97.9; Y(8): 0.1	18	1	1	0	N-Term(TMT6plex); S5(Phospho)
24308039	NP_056138.1	23376	UFL1	E3 UFM1-protein ligase 1	S458	1.5	1.4	1.1	1.3	0.9	1.6	1.3	0.9	1.4	1.3	1.1	1.2	0.8	1.5	1.3	0.8	1.1	1.4	1.2	1.0	DDDsDDESQSSHTGK	KGRKDDDsDDESQSS	S4;	Yes	S(4): 100.0; S(8): 0.0; S(10): 0.0; S(11): 0.0; T(13): 0.0	1	1	1	0	N-Term(TMT6plex); S4(Phospho); K15(TMT6plex)
17975761	NP_006791.2	10943	MSL3	male-specific lethal 3 homolog isoform c	S234; T239	0.8	0.5	0.5	0.6	1.0	1.2	1.2	1.6	0.9	0.5	0.5	0.7	1.2	1.4	1.5	1.9	1.0	1.0	1.0	1.3	SSsPIPLtPSK	PVHSRSSsPIPLTPS;SSSPIPLtPSKEGSA	S3; T8;	No;No	S(1): 0.0; S(2): 1.1; S(3): 98.8; T(8): 98.9; S(10): 1.1	13	4	1	0	N-Term(TMT6plex); S3(Phospho); T8(Phospho); K11(TMT6plex)
4758384	NP_004108.1	2289	FKBP5	peptidyl-prolyl cis-trans isomerase FKBP5 isoform 1	S445	1.0	1.4	1.1	1.0	0.9	1.3	1.5	1.2	1.0	1.4	1.2	1.0	0.9	1.4	1.6	1.2	1.0	1.4	1.4	1.1	GTDsQAMEEEKPEGHV	TNEKGTDsQAMEEEK	S4;	Yes	T(2): 1.9; S(4): 98.1	15	1	1	0	N-Term(TMT6plex); S4(Phospho); M7(Oxidation); K11(TMT6plex)
164519084	NP_036329.3	23637	RABGAP1	rab GTPase-activating protein 1	T996	1.1	1.3	1.3	1.1	0.8	1.2	1.2	1.1	1.1	1.3	1.3	1.1	0.8	1.1	1.1	1.0	0.9	1.2	1.2	1.0	EVLDEDtDEEKETLK	KEVLDEDtDEEKETL	T7;	Yes	T(7): 100.0; T(13): 0.0	1	1	1	1	N-Term(TMT6plex); T7(Phospho); K11(TMT6plex); K15(TMT6plex)
4501915	NP_003807.1	8754	ADAM9	disintegrin and metalloproteinase domain-containing protein 9 precursor	S758	0.9	0.8	1.2	1.2	0.8	0.8	0.9	1.1	1.0	0.9	1.0	1.2	0.9	1.0	0.8	1.1	0.9	0.9	0.9	1.1	HVsPVTPPR	GSVPRHVsPVTPPRE	S3;	Yes	S(3): 100.0; T(6): 0.0	3	1	1	0	N-Term(TMT6plex); S3(Phospho)
17975761	NP_006791.2	10943	MSL3	male-specific lethal 3 homolog isoform c	S234; T239	1.0	1.2	1.2	1.6	0.8	0.5	0.5	0.6	1.2	1.4	1.5	1.9	0.9	0.5	0.5	0.7	1.0	1.0	1.0	1.3	SSSPIPLtPSK	PVHSRSSsPIPLTPS	S3; T8;	No	S(1): 0.7; S(2): 0.7; S(3): 98.7; T(8): 0.0; S(10): 0.0	8	4	1	0	N-Term(TMT6plex); S3(Phospho); K11(TMT6plex)
222352111	NP_115766.3	84365	NIFK	MKI67 FHA domain-interacting nucleolar phosphoprotein	T279	0.9	1.2	1.0	1.3	0.8	1.0	0.8	0.9	1.0	1.2	1.0	1.4	0.9	1.0	0.8	1.0	1.0	1.1	0.9	1.2	EEIQETQtPTHSR	EEIQETQtPTHSRKK	T8;	No	T(6): 0.0; T(8): 100.0; T(10): 0.0; S(12): 0.0	5	1	1	0	N-Term(TMT6plex); T8(Phospho)
46447823	NP_116204.3	84901	NFATC2IP	NFATC2-interacting protein	S84	1.0	1.2	1.4	1.2	0.9	1.0	1.0	1.0	1.1	1.2	1.5	1.3	1.0	1.0	1.0	1.1	1.0	1.1	1.3	1.2	GAADEVEVEPPEPPGPVAsR	EPPGPVAsRDNSNSD	S19;	Yes	S(19): 100.0	7	1	1	0	N-Term(TMT6plex); S19(Phospho)
7657269	NP_055847.1	23047	PDS5B	sister chromatid cohesion protein PDS5 homolog B	S1166	1.3	1.9	1.2	1.1	0.8	1.0	1.0	1.1	1.2	1.6	1.1	1.1	0.8	0.9	0.9	1.0	1.0	1.3	1.0	1.1	METVSNASSSSNPSsPGR	SSSSNPSsPGRIKGR	S15;	Yes	T(3): 0.0; S(5): 0.0; S(8): 49.1; S(9): 0.9; S(10): 0.9; S(11): 49.1; S(14): 1.8; S(15): 98.2	34	1	1	0	M1(Oxidation); N-Term(TMT6plex); S11(Phospho); S14(Phospho)
194578923	NP_001124000.1	9043	SPAG9	C-Jun-amino-terminal kinase-interacting protein 4 isoform 1	S251	0.8	0.7	0.7	1.2	0.8	0.9	0.9	1.0	0.8	0.7	0.8	1.3	0.9	1.0	1.1	1.2	0.9	0.9	0.9	1.3	VSNsPEPQK	TSLKVSNsPEPQKAV	S4;	Yes	S(2): 0.0; S(4): 100.0	13	1	1	0	N-Term(TMT6plex); S4(Phospho); K9(TMT6plex)
224809302	NP_001139241.1	7508	XPC	DNA repair protein complementing XP-C cells isoform	S94	1.3	1.5	1.4	1.3	0.9	1.1	0.9	0.9	1.1	1.5	1.3	1.3	0.8	1.0	0.8	0.9	0.9	1.2	1.1	1.1	VIKDEALsDGDDLR	VIKDEALsDGDDLRD	S8;	Yes	S(8): 100.0	2	2	1	1	N-Term(TMT6plex); K3(TMT6plex); S8(Phospho)
4507137	NP_003089.1	6640	SNTA1	alpha-1-syntrophin	S201	1.0	0.9	1.0	1.3	0.8	0.8	0.8	0.9	-	-	-	-	-	-	-	-	-	-	-	-	QPSsPGPTPR	PLQRQPSsPGPTPRN	S4;	Yes	S(3): 1.0; S(4): 99.0; T(8): 0.0	1	1	1	0	N-Term(TMT6plex); S4(Phospho)
117190342	NP_005383.3	5253	PHF2	lysine-specific demethylase PHF2	S899	1.0	1.3	1.2	1.3	1.4	1.5	1.4	1.0	1.0	1.3	1.3	1.3	1.5	1.6	1.6	1.0	1.3	1.4	1.5	1.2	KGsDDAPYSPTAR	RSKKRKGsDDAPYSP	S3;	Yes	S(3): 100.0; Y(8): 50.0; S(9): 50.0; T(11): 0.0	9	1	1	1	K1(TMT6plex); N-Term(TMT6plex); S3(Phospho); S9(Phospho)
215599015	NP_001135935.1	1736	DKC1	H/ACA ribonucleoprotein complex subunit 4 isoform 2	S489	1.0	0.8	1.0	1.0	1.0	1.1	1.0	1.2	1.1	0.8	1.0	1.1	1.0	1.1	1.0	1.2	1.0	1.0	1.0	1.2	AGLESGAEPGDGDsDTTK	AEPGDGDsDTTKKKK	S14;	No	S(5): 0.0; S(14): 100.0; T(16): 0.0; T(17): 0.0	22	2	1	0	N-Term(TMT6plex); S14(Phospho); K18(TMT6plex)
355330276	NP_001239007.1	9958	USP15	ubiquitin carboxyl-terminal hydrolase 15 isoform 1	S229	0.5	0.4	1.1	1.1	0.9	1.2	1.2	1.1	0.4	0.4	1.0	1.0	0.8	1.1	1.0	1.1	0.6	0.8	1.0	1.1	sPGASNFSTLPK	RGPSTPKsPGASNFS	S1;	Yes	S(1): 100.0; S(5): 0.0; S(8): 0.0; T(9): 0.0	2	1	1	0	N-Term(TMT6plex); S1(Phospho); K12(TMT6plex)
21359969	NP_006459.3	10623	POLR3C	DNA-directed RNA polymerase III subunit RPC3	S204	1.3	1.1	1.3	1.0	1.0	1.3	1.5	1.2	1.3	1.1	1.3	1.0	1.0	1.3	1.5	1.1	1.1	1.2	1.4	1.0	sSDEDAAGEPK	GKGKRRRsSDEDAAG	S1;	Yes	S(1): 100.0; S(2): 0.0	3	1	1	0	N-Term(TMT6plex); S1(Phospho); K11(TMT6plex)
19913396	NP_599031.1	6525	SMTN	smoothelin isoform b	S357	0.9	1.2	1.2	1.1	1.0	0.9	0.6	1.1	0.7	0.9	0.9	1.0	0.8	0.7	0.4	1.0	0.8	0.8	0.6	1.0	LQDGTPQAALsPLtPAR	GTPQAALsPLTPARL;QAALSPLtPARLLGP	S11;	Yes;Yes	T(5): 0.0; S(11): 100.0; T(14): 100.0	2	5	1	0	N-Term(TMT6plex); T5(Phospho); T14(Phospho)
19913396	NP_599031.1	6525	SMTN	smoothelin isoform b	S357	1.0	0.9	0.6	1.1	0.9	1.2	1.2	1.1	0.8	0.7	0.4	1.0	0.7	0.9	0.9	1.0	0.8	0.8	0.6	1.0	LQDGTPQAALsPLTPAR	GTPQAALsPLTPARL	S11;	Yes	T(5): 0.0; S(11): 100.0; T(14): 0.0	7	5	1	0	N-Term(TMT6plex); S11(Phospho)
19913406	NP_001058.2	7153	TOP2A	DNA topoisomerase 2-alpha	S1106	1.2	0.9	0.9	1.1	1.0	0.8	1.0	1.1	1.4	1.0	1.0	1.3	1.2	0.9	1.1	1.2	1.3	1.0	1.1	1.2	VPDEEENEEsDNEK	DEEENEEsDNEKETE	S10;	Yes	S(10): 100.0	64	1	1	0	N-Term(TMT6plex); S10(Phospho); K14(TMT6plex)
13491174	NP_075385.1	65108	MARCKSL1	MARCKS-related protein	T148	0.9	1.1	1.0	1.0	0.8	1.3	1.3	1.2	1.0	1.1	1.0	0.8	0.9	1.3	1.4	1.1	1.0	1.2	1.2	0.9	AAATPEsQEPQAK	KAAATPEsQEPQAKG	T4;	Yes	T(4): 1.2; S(7): 98.8	2	1	1	0	N-Term(TMT6plex); S7(Phospho); K13(TMT6plex)
13491174	NP_075385.1	65108	MARCKSL1	MARCKS-related protein	T148	0.8	1.3	1.3	1.2	0.9	1.1	1.0	1.0	0.9	1.3	1.4	1.1	1.0	1.1	1.0	0.8	1.0	1.2	1.2	0.9	AAAtPESQEPQAK	QEGKAAAtPESQEPQ	T4;	Yes	T(4): 100.0; S(7): 0.0	18	1	1	0	N-Term(TMT6plex); T4(Phospho); K13(TMT6plex)
556503401	NP_001273294.1	55700	MAP7D1	MAP7 domain-containing protein 1 isoform 2	S507	1.4	1.3	0.9	1.1	1.5	0.9	0.8	1.1	1.4	1.0	0.8	1.0	1.5	0.7	0.7	1.1	1.4	0.9	0.8	1.1	ESAAPAsPAPSPAPSPTPAPPQK	KESAAPAsPAPSPAP	S7;	No	S(2): 0.0; S(7): 100.0; S(11): 0.0; S(15): 0.0; T(17): 0.0	27	3	1	0	N-Term(TMT6plex); S7(Phospho); K23(TMT6plex)
118498359	NP_056474.2	26156	RSL1D1	ribosomal L1 domain-containing protein 1	S361	0.9	1.2	1.0	1.1	0.8	1.1	0.9	1.1	1.2	1.4	1.2	1.2	1.0	1.3	1.1	1.2	1.1	1.3	1.1	1.2	ATNEsEDEIPQLVPIGK	QVKATNEsEDEIPQL	S5;	Yes	T(2): 0.0; S(5): 100.0	19	1	1	0	N-Term(TMT6plex); S5(Phospho); K17(TMT6plex)
42542379	NP_005830.2	10250	SRRM1	serine/arginine repetitive matrix protein 1	S756	0.8	1.1	1.1	1.1	0.9	1.3	1.0	1.1	1.0	1.2	1.2	1.2	1.1	1.4	1.1	1.3	1.1	1.3	1.1	1.3	SVSGsPEPAAK	SSRSVSGsPEPAAKK	S5;	Yes	S(1): 0.0; S(3): 0.0; S(5): 100.0	229	1	1	0	N-Term(TMT6plex); S5(Phospho); K11(TMT6plex)
42542379	NP_005830.2	10250	SRRM1	serine/arginine repetitive matrix protein 1	S756	0.9	1.3	1.0	1.1	0.8	1.1	1.1	1.1	1.1	1.4	1.1	1.3	1.0	1.2	1.2	1.2	1.1	1.3	1.1	1.3	SVsGsPEPAAK	SSRSVSGsPEPAAKK	S5;	Yes	S(1): 0.0; S(3): 0.0; S(5): 100.0	8	1	1	0	N-Term(TMT6plex); S5(Phospho); K11(TMT6plex)
54873624	NP_114414.2	83939	EIF2A	eukaryotic translation initiation factor 2A	S506	0.9	1.0	0.9	0.8	1.3	1.5	1.3	1.4	0.8	0.9	0.8	0.8	1.2	1.4	1.2	1.4	1.0	1.2	1.0	1.1	SDKsPDLAPTPAPQSTPR	QEARSDKsPDLAPTP	S4;	Yes	S(1): 1.1; S(4): 98.9; T(10): 0.0; S(15): 0.0; T(16): 0.0	3	1	1	1	N-Term(TMT6plex); K3(TMT6plex); S4(Phospho)
54873624	NP_114414.2	83939	EIF2A	eukaryotic translation initiation factor 2A	S506	1.3	1.5	1.3	1.4	0.9	1.0	0.9	0.8	1.2	1.4	1.2	1.4	0.8	0.9	0.8	0.8	1.0	1.2	1.0	1.1	sDKSPDLAPTPAPQSTPR	QEARSDKsPDLAPTP	S4;	Yes	S(1): 0.0; S(4): 100.0; T(10): 0.0; S(15): 0.0; T(16): 0.0	3	1	1	1	N-Term(TMT6plex); K3(TMT6plex); S4(Phospho)
145580615	NP_075384.3	65095	KRI1	protein KRI1 homolog	S645	0.9	1.0	1.6	1.0	1.7	1.6	2.1	1.2	1.1	1.1	1.8	1.2	2.1	1.8	2.4	1.3	1.6	1.4	2.1	1.3	QLPALDGSLMGPEsPPAQEEEAPVsPHK	GSLMGPEsPPAQEEE;QEEEAPVsPHKKPAP	S25;	No;No	S(8): 0.0; S(14): 100.0; S(25): 100.0	5	1	1	0	N-Term(TMT6plex); M10(Oxidation); S14(Phospho); S25(Phospho); K28(TMT6plex)
255918139	NP_001157616.1	283489	CHAMP1	chromosome alignment-maintaining phosphoprotein 1	S376	0.8	0.9	1.2	1.1	0.9	1.1	0.9	1.1	0.8	0.9	1.1	1.2	1.0	1.1	0.9	1.2	0.9	1.0	1.0	1.2	SSSVsPSSWK	SWKSSSVsPSSWKSP	S5;	Yes	S(1): 0.0; S(2): 0.0; S(3): 1.1; S(5): 98.9; S(7): 0.0; S(8): 0.0	24	1	1	0	N-Term(TMT6plex); S5(Phospho); K10(TMT6plex)
145580615	NP_075384.3	65095	KRI1	protein KRI1 homolog	S645	1.7	1.6	2.1	1.2	0.9	1.0	1.6	1.0	2.1	1.8	2.4	1.3	1.1	1.1	1.8	1.2	1.6	1.4	2.1	1.3	QLPALDGSLMGPESPPAQEEEAPVsPHK	QEEEAPVsPHKKPAP	S25;	No	S(8): 50.0; S(14): 50.0; S(25): 100.0	13	1	1	0	N-Term(TMT6plex); M10(Oxidation); S14(Phospho); S25(Phospho); K28(TMT6plex)
33286446	NP_031372.2	11054	OGFR	opioid growth factor receptor	S349	0.9	1.7	1.3	1.2	0.9	1.1	0.8	1.0	1.1	1.8	1.4	1.3	1.0	1.2	0.9	1.1	1.1	1.5	1.1	1.2	sVEPQDAGPLER	DEGPQPRsVEPQDAG	S1;	Yes	S(1): 100.0	8	1	1	0	N-Term(TMT6plex); S1(Phospho)
313760537	NP_001186421.1	27250	PDCD4	programmed cell death protein 4 isoform 3	S443	1.6	1.6	1.0	1.1	1.7	1.8	1.0	1.1	1.6	1.7	1.0	1.1	1.7	1.9	1.1	1.1	1.6	1.8	1.0	1.1	FVsEGDGGR	RGRKRFVsEGDGGRL	S3;	No	S(3): 100.0	13	3	1	0	N-Term(TMT6plex); S3(Phospho)
311771673	NP_001185715.1	5451	POU2F1	POU domain, class 2, transcription factor 1 isoform 3	S408	1.1	1.1	1.1	1.2	0.8	0.7	0.8	1.0	1.2	1.3	1.2	1.5	0.9	0.9	0.9	1.3	1.0	1.1	1.1	1.4	INPPSSGGTSSsPIK	SSGGTSSsPIKAIFP	S12;	No	S(5): 0.0; S(6): 0.0; T(9): 0.0; S(10): 0.0; S(11): 0.0; S(12): 100.0	5	3	1	0	N-Term(TMT6plex); S12(Phospho); K15(TMT6plex)
5032189	NP_005648.1	7158	TP53BP1	tumor suppressor p53-binding protein 1 isoform 3	S525	0.7	1.2	0.9	1.0	1.0	0.9	0.8	1.2	0.8	1.2	1.0	1.2	1.0	0.9	0.9	1.4	0.9	1.0	1.0	1.3	LMLSTSEYsQsPK	MLSTSEYsQSPKMES;STSEYSQsPKMESLS	S11;	Yes;Yes	S(4): 0.0; T(5): 0.0; S(6): 0.0; Y(8): 0.0; S(9): 100.0; S(11): 100.0	2	3	1	0	N-Term(TMT6plex); M2(Oxidation); S9(Phospho); S11(Phospho); K13(TMT6plex)
5032189	NP_005648.1	7158	TP53BP1	tumor suppressor p53-binding protein 1 isoform 3	S525	1.0	0.9	0.8	1.2	0.7	1.2	0.9	1.0	1.0	0.9	0.9	1.4	0.8	1.2	1.0	1.2	0.9	1.0	1.0	1.3	LMLSTSEYSQsPK	STSEYSQsPKMESLS	S11;	Yes	S(4): 0.0; T(5): 0.0; S(6): 0.0; Y(8): 50.3; S(9): 50.3; S(11): 99.3	62	3	1	0	N-Term(TMT6plex); M2(Oxidation); S9(Phospho); S11(Phospho); K13(TMT6plex)
201023341	NP_001128410.1	81608	FIP1L1	pre-mRNA 3'-end-processing factor FIP1 isoform 3	S418	0.9	1.0	0.8	1.3	0.9	1.1	1.0	0.9	1.0	1.1	0.9	1.4	1.0	1.3	1.1	1.1	1.0	1.2	1.0	1.2	DHsPTPSVFNSDEER	RERERDHsPTPSVFN	S3;	No	S(3): 100.0; T(5): 0.0; S(7): 0.0; S(11): 0.0	6	3	1	0	N-Term(TMT6plex); S3(Phospho)
187608516	NP_036419.3	23527	ACAP2	arf-GAP with coiled-coil, ANK repeat and PH domain-containing protein 2	S521	2.1	1.1	1.9	1.5	0.6	0.9	1.3	0.7	2.0	1.1	1.8	1.5	0.6	0.9	1.3	0.7	1.3	1.0	1.6	1.1	YSISLsPPEQQK	DKYSISLsPPEQQKK	S6;	Yes	Y(1): 0.0; S(2): 0.0; S(4): 0.9; S(6): 99.1	14	1	1	0	N-Term(TMT6plex); S6(Phospho); K12(TMT6plex)
11545835	NP_071400.1	64061	TSPYL2	testis-specific Y-encoded-like protein 2	S16	1.5	1.6	1.2	1.2	1.1	1.4	0.9	1.0	1.0	1.3	1.0	1.1	0.8	1.1	0.8	0.9	0.9	1.2	0.9	1.0	RLsSSESPQR	PAKTRRLsSSESPQR	S3;	Yes	S(3): 100.0; S(4): 0.0; S(5): 0.0; S(7): 0.0	3	1	1	1	N-Term(TMT6plex); S3(Phospho)
5032031	NP_005769.1	10181	RBM5	RNA-binding protein 5	S59	0.8	1.0	0.9	0.9	1.0	1.3	1.2	1.3	0.9	1.1	1.0	1.0	1.2	1.4	1.3	1.5	1.0	1.2	1.2	1.2	DYDsPERER	DDYRDYDsPERERER	S4;	Yes	Y(2): 0.0; S(4): 100.0	6	1	1	1	N-Term(TMT6plex); S4(Phospho)
19923559	NP_064578.2	56946	C11orf30	protein EMSY	S209	1.2	1.1	1.1	1.1	1.5	1.2	1.2	1.1	-	-	-	-	-	-	-	-	-	-	-	-	TNsSSSPVVLK	RKRRRTNsSSSPVVV	S3;	No	T(1): 0.0; S(3): 98.1; S(4): 0.9; S(5): 0.9; S(6): 0.0; S(7): 0.0	6	1	1	0	N-Term(TMT6plex); S3(Phospho); K12(TMT6plex)
161169017	NP_001104489.1	89797	NAV2	neuron navigator 2 isoform 4	S382	1.5	2.0	1.4	1.5	1.5	1.2	1.0	0.7	-	-	-	-	-	-	-	-	-	-	-	-	YPDVAsPTLR	AKYPDVAsPTLRRLF	S6;	No	Y(1): 0.0; S(6): 100.0; T(8): 0.0	1	5	1	0	N-Term(TMT6plex); S6(Phospho)
32483374	NP_006383.2	10528	NOP56	nucleolar protein 56	S520	1.2	1.0	1.1	1.1	2.3	0.9	0.7	1.1	1.2	1.1	1.1	1.1	2.3	0.9	0.8	1.2	1.8	1.0	0.9	1.2	EELMSsDLEETAGSTSIPK	SKEELMSsDLEETAG	S6;	Yes	S(5): 1.4; S(6): 98.6; T(11): 0.0; S(14): 0.0; T(15): 0.0; S(16): 0.0	3	1	1	0	N-Term(TMT6plex); M4(Oxidation); S6(Phospho); K19(TMT6plex)
118572613	NP_057417.3	23524	SRRM2	serine/arginine repetitive matrix protein 2	S1478; S1482; S1483	0.9	0.9	1.0	1.1	0.8	1.0	1.1	1.2	0.9	1.0	1.1	1.2	0.9	1.1	1.1	1.3	0.9	1.0	1.1	1.2	GRsECDssPEPK	RTPSRGRsECDSSPE;RGRSECDsSPEPKAL;GRSECDSsPEPKALP	S3; S7; S8;	Yes;Yes;Yes	S(3): 100.0; S(7): 100.0; S(8): 100.0	436	1	1	1	N-Term(TMT6plex); S3(Phospho); C5(Carbamidomethyl); S7(Phospho); S8(Phospho); K12(TMT6plex)
118572613	NP_057417.3	23524	SRRM2	serine/arginine repetitive matrix protein 2	S1478; S1482; S1483	0.8	1.0	1.1	1.2	0.9	0.9	1.0	1.1	0.9	1.1	1.1	1.3	0.9	1.0	1.1	1.2	0.9	1.0	1.1	1.2	GRsECDSSPEPK	RTPSRGRsECDSSPE;RGRSECDsSPEPKAL;GRSECDSsPEPKALP	S3; S7; S8;	Yes;Yes;Yes	S(3): 100.0; S(7): 100.0; S(8): 100.0	2	1	1	1	N-Term(TMT6plex); S3(Phospho); C5(Carbamidomethyl); S7(Phospho); S8(Phospho); K12(TMT6plex)
9558749	NP_037434.1	29904	EEF2K	eukaryotic elongation factor 2 kinase	S18	0.9	1.0	1.3	1.2	0.9	0.9	1.0	1.0	-	-	-	-	-	-	-	-	-	-	-	-	LEGVDGGQsPR	EGVDGGQsPRAGHDG	S9;	Yes	S(9): 100.0	4	1	1	0	N-Term(TMT6plex); S9(Phospho)
209364600	NP_001129179.1	79777	ACBD4	acyl-CoA-binding domain-containing protein 4	S166	0.5	0.4	1.4	1.1	1.0	0.9	0.8	1.1	-	-	-	-	-	-	-	-	-	-	-	-	EPAPPsPESHSPR	PKEPAPPsPESHSPR	S6;	Yes	S(6): 100.0; S(9): 0.0; S(11): 0.0	2	2	1	0	N-Term(TMT6plex); S6(Phospho)
507834119	NP_061164.3	51329	ARL6IP4	ADP-ribosylation factor-like protein 6-interacting protein 4 isoform 1	S239	0.8	0.9	0.9	1.1	0.7	0.8	0.7	1.1	1.1	1.1	1.2	1.4	1.0	1.1	0.9	1.4	1.0	1.1	1.0	1.4	ASTAPGAEAsPSPCITER	TAPGAEAsPSPCITE	S10;	No	S(2): 0.0; T(3): 0.0; S(10): 100.0; S(12): 0.0; T(16): 0.0	5	1	1	0	N-Term(TMT6plex); S10(Phospho); C14(Carbamidomethyl)
149158690	NP_542417.2	7916	PRRC2A	protein PRRC2A	T610	0.9	1.5	0.9	1.1	0.8	1.3	1.2	1.1	0.9	1.4	0.9	1.1	0.9	1.2	1.3	1.0	0.9	1.3	1.1	1.1	EGPEPPEEVPPPTtPPVPK	EEVPPPTtPPVPKVE	T14;	No	T(13): 0.0; T(14): 100.0	23	1	1	0	N-Term(TMT6plex); T14(Phospho); K19(TMT6plex)
4506357	NP_003944.1	9019	MPZL1	myelin protein zero-like protein 1 isoform a precursor	S210	0.5	0.8	1.0	1.2	0.6	0.7	0.8	1.0	0.5	0.6	0.7	1.1	0.5	0.5	0.6	0.9	0.5	0.6	0.7	1.0	DYTGCSTSESLsPVK	CSTSESLsPVKQAPR	S12;	Yes	Y(2): 0.0; T(3): 0.0; S(6): 0.0; T(7): 0.0; S(8): 0.0; S(10): 0.0; S(12): 100.0	1	1	1	0	N-Term(TMT6plex); C5(Carbamidomethyl); S12(Phospho); K15(TMT6plex)
170650672	NP_001116241.1	167691	LCA5	Lebercilin	S7	1.0	0.9	0.8	1.0	1.1	0.9	0.8	1.2	-	-	-	-	-	-	-	-	-	-	-	-	AGsPGTDQER	MGERAGsPGTDQER	S3;	No	S(3): 100.0; T(6): 0.0	1	1	1	0	N-Term(TMT6plex); S3(Phospho)
103472005	NP_002408.3	4288	MKI67	antigen KI-67 isoform 1	T1111	0.7	1.1	1.0	1.2	0.8	1.1	1.1	1.0	0.8	1.2	1.1	1.2	1.0	1.1	1.1	1.1	0.9	1.1	1.1	1.2	ELFQtPGPSEESMTDEK	GFKELFQtPGPSEES	T5;	Yes	T(5): 100.0; S(9): 0.0; S(12): 0.0; T(14): 0.0	118	2	1	0	N-Term(TMT6plex); T5(Phospho); M13(Oxidation); K17(TMT6plex)
30025026	NP_054747.2	28962	OSTM1	osteopetrosis-associated transmembrane protein 1	S325	0.8	1.0	0.8	1.0	1.0	1.1	1.1	1.2	0.7	0.9	0.7	1.0	0.8	1.0	1.0	1.2	0.8	1.0	0.9	1.1	SSTsFANIQENSN	KRLKSSTsFANIQEN	S4;	Yes	S(1): 0.0; S(2): 0.0; T(3): 1.0; S(4): 99.0; S(12): 0.0	1	1	1	0	N-Term(TMT6plex); S4(Phospho)
30025026	NP_054747.2	28962	OSTM1	osteopetrosis-associated transmembrane protein 1	S325	1.0	1.1	1.1	1.2	0.8	1.0	0.8	1.0	0.8	1.0	1.0	1.2	0.7	0.9	0.7	1.0	0.8	1.0	0.9	1.1	SSTSFANIQENsN	FANIQENsN_	S4;	Yes	S(1): 0.0; S(2): 0.0; T(3): 0.0; S(4): 0.0; S(12): 100.0	3	1	1	0	N-Term(TMT6plex); S12(Phospho)
5031633	NP_005757.1	10160	FARP1	FERM, RhoGEF and pleckstrin domain-containing protein 1 isoform 1	S427	0.8	1.0	0.6	1.0	0.9	0.9	1.0	1.2	-	-	-	-	-	-	-	-	-	-	-	-	VSAGEPGSHPsPAPR	GEPGSHPsPAPRRSP	S11;	Yes	S(2): 0.0; S(8): 0.0; S(11): 100.0	2	2	1	0	N-Term(TMT6plex); S11(Phospho)
544063461	NP_001269550.1	10857	PGRMC1	membrane-associated progesterone receptor component 1 isoform 2	S57	1.4	1.2	1.1	1.0	1.0	1.2	0.9	1.1	1.7	1.3	1.5	1.4	1.1	1.3	1.2	1.6	1.4	1.3	1.3	1.5	GDQPAASGDsDDDEPPPLPR	QPAASGDsDDDEPPP	S10;	Yes	S(7): 0.0; S(10): 100.0	22	2	1	0	N-Term(TMT6plex); S10(Phospho)
21070997	NP_003147.2	6786	STIM1	stromal interaction molecule 1 isoform 2 precursor	S575	1.0	1.1	1.0	1.0	1.3	1.3	1.4	1.2	1.0	1.1	0.9	1.0	1.3	1.2	1.3	1.3	1.1	1.2	1.1	1.1	LPDsPALAK	LVEKLPDsPALAKKA	S4;	Yes	S(4): 100.0	6	2	1	0	N-Term(TMT6plex); S4(Phospho); K9(TMT6plex)
20162572	NP_619649.1	56160	NDNL2	melanoma-associated antigen G1	S64	1.1	0.9	1.1	0.8	2.0	0.8	1.7	1.4	1.0	0.8	1.0	0.7	1.8	0.8	1.6	1.4	1.4	0.8	1.3	1.1	GPGGSQGSQGPsPQGAR	SQGSQGPsPQGARRA	S12;	Yes	S(5): 0.0; S(8): 0.0; S(12): 100.0	2	1	1	0	N-Term(TMT6plex); S12(Phospho)
5032189	NP_005648.1	7158	TP53BP1	tumor suppressor p53-binding protein 1 isoform 3	S222	0.9	1.3	1.1	0.8	0.6	1.5	1.1	1.3	1.0	1.3	1.2	1.0	0.6	1.5	1.2	1.6	0.8	1.4	1.2	1.3	HEEQsNEDIPIAEQSSK	AIKHEEQsNEDIPIA	S5;	Yes	S(5): 100.0; S(15): 0.0; S(16): 0.0	62	3	1	0	N-Term(TMT6plex); S5(Phospho); K17(TMT6plex)
157739945	NP_079461.2	26115	TANC2	protein TANC2	S404	0.7	0.9	0.8	0.9	0.9	1.0	1.1	1.3	0.7	0.9	0.8	0.9	0.9	1.0	1.0	1.3	0.8	0.9	0.9	1.1	QIASDsPHAsPK	MRQIASDsPHASPKH;ASDSPHAsPKHVDAN	S10;	Yes;Yes	S(4): 0.9; S(6): 99.1; S(10): 100.0	2	1	1	0	N-Term(TMT6plex); S6(Phospho); S10(Phospho); K12(TMT6plex)
50843837	NP_060541.3	55701	ARHGEF40	rho guanine nucleotide exchange factor 40 isoform 1	S262	1.0	1.1	0.9	1.1	1.0	1.0	0.9	1.1	-	-	-	-	-	-	-	-	-	-	-	-	GSPTDAEGsPGLSR	SPTDAEGsPGLSRVR	S9;	Yes	S(2): 0.0; T(4): 0.0; S(9): 100.0; S(13): 0.0	6	1	1	0	N-Term(TMT6plex); S9(Phospho)
388596707	NP_001253989.1	9784	SNX17	sorting nexin-17 isoform 3	S384	0.9	1.2	1.3	1.2	0.9	1.4	1.2	1.0	0.9	1.2	1.2	1.2	0.9	1.4	1.2	1.0	0.9	1.3	1.2	1.1	SDsQQAVK	GTLRRSDsQQAVKSP	S3;	No	S(1): 0.0; S(3): 100.0	2	4	1	0	N-Term(TMT6plex); S3(Phospho); K8(TMT6plex)
157739945	NP_079461.2	26115	TANC2	protein TANC2	S404	0.9	1.0	1.1	1.3	0.7	0.9	0.8	0.9	0.9	1.0	1.0	1.3	0.7	0.9	0.8	0.9	0.8	0.9	0.9	1.1	QIASDSPHAsPK	ASDSPHAsPKHVDAN	S10;	Yes	S(4): 50.0; S(6): 50.0; S(10): 100.0	6	1	1	0	N-Term(TMT6plex); S6(Phospho); S10(Phospho); K12(TMT6plex)
148762969	NP_003473.3	8085	KMT2D	histone-lysine N-methyltransferase 2D	S1671	0.8	1.1	1.2	1.0	1.2	1.7	1.2	1.2	-	-	-	-	-	-	-	-	-	-	-	-	LEGPVsPDVEPGKEETEESK	IKLEGPVsPDVEPGK	S6;	No	S(6): 100.0; T(16): 0.0; S(19): 0.0	1	1	1	1	N-Term(TMT6plex); S6(Phospho); K13(TMT6plex); K20(TMT6plex)
193788632	NP_001123359.1	27000	DNAJC2	dnaJ homolog subfamily C member 2 isoform 2	S47	1.0	1.2	1.2	1.0	1.1	0.8	1.1	1.2	1.0	1.2	1.2	1.0	1.1	0.8	1.1	1.1	1.0	1.0	1.1	1.1	NAsASFQELEDK	KRRNRNAsASFQELE	S3;	Yes	S(3): 100.0; S(5): 0.0	2	2	1	0	N-Term(TMT6plex); S3(Phospho); K12(TMT6plex)
39995059	NP_116764.2	835	CASP2	caspase-2 isoform 1 preproprotein	S340	1.1	0.9	1.0	0.8	1.0	1.1	1.1	1.4	-	-	-	-	-	-	-	-	-	-	-	-	NHAGsPGCEESDAGK	DGKNHAGsPGCEESD	S5;	Yes	S(5): 100.0; S(11): 0.0	1	1	1	0	N-Term(TMT6plex); S5(Phospho); C8(Carbamidomethyl); K15(TMT6plex)
38683860	NP_003740.2	8660	IRS2	insulin receptor substrate 2	S679	0.8	0.8	0.8	1.0	1.0	1.2	1.0	1.2	0.7	0.8	0.8	0.9	1.0	1.2	1.1	1.2	0.9	1.0	1.0	1.0	SDDYMPMsPASVSAPK	SDDYMPMsPASVSAP	S8;	Yes	S(1): 0.0; Y(4): 0.0; S(8): 99.9; S(11): 0.0; S(13): 0.0	9	1	1	0	N-Term(TMT6plex); M5(Oxidation); M7(Oxidation); S8(Phospho); K16(TMT6plex)
62244004	NP_001013865.1	79811	SLTM	SAFB-like transcription modulator isoform b	S271	0.8	0.7	0.8	1.1	0.8	0.8	0.9	1.1	0.8	0.7	0.9	1.2	0.8	0.9	1.1	1.2	0.8	0.8	1.0	1.2	DGQDAIAQsPEK	GQDAIAQsPEKESKD	S9;	No	S(9): 100.0	12	2	1	0	N-Term(TMT6plex); S9(Phospho); K12(TMT6plex)
21396500	NP_003600.2	8479	HIRIP3	HIRA-interacting protein 3 isoform 1	S196; S199	0.9	1.3	1.3	1.4	1.0	0.9	0.8	0.8	1.0	1.4	1.6	1.5	1.2	1.0	0.9	0.9	1.1	1.2	1.2	1.2	EEsEEsEAEPVQR	RKQAREEsEESEAEP;AREESEEsEAEPVQR	S3; S6;	Yes;Yes	S(3): 100.0; S(6): 100.0	26	1	1	0	N-Term(TMT6plex); S3(Phospho); S6(Phospho)
21396500	NP_003600.2	8479	HIRIP3	HIRA-interacting protein 3 isoform 1	S406	0.6	1.4	1.2	1.0	0.4	0.9	1.0	1.2	0.7	1.5	1.4	1.1	0.5	0.9	1.1	1.3	0.6	1.2	1.3	1.2	SSSSSSDGsPEAK	SSSSSDGsPEAKGGK	S9;	No	S(1): 0.0; S(2): 0.0; S(3): 0.0; S(4): 0.0; S(5): 0.0; S(6): 0.0; S(9): 100.0	26	1	1	0	N-Term(TMT6plex); S9(Phospho); K13(TMT6plex)
42518070	NP_004808.2	9414	TJP2	tight junction protein ZO-2 isoform 1	S130	0.9	1.3	1.6	1.0	1.0	0.9	1.4	1.1	1.0	1.3	1.4	1.1	1.0	0.9	1.2	1.2	1.0	1.1	1.3	1.2	KVQVAALQAsPPLDQDDR	QVAALQAsPPLDQDD	S10;	Yes	S(10): 100.0	13	6	1	1	N-Term(TMT6plex); K1(TMT6plex); S10(Phospho)
33667117	NP_891554.1	79778	MICALL2	MICAL-like protein 2	S153	1.1	1.2	1.1	1.2	1.0	1.1	1.0	0.9	-	-	-	-	-	-	-	-	-	-	-	-	KPPLsPAQTNPVVQR	PARKPPLsPAQTNPV	S5;	Yes	S(5): 100.0; T(9): 0.0	1	1	1	0	N-Term(TMT6plex); K1(TMT6plex); S5(Phospho)
73760403	NP_001027453.1	7071	KLF10	Krueppel-like factor 10 isoform b	S238	1.0	1.0	0.8	1.3	1.0	0.9	0.7	0.9	-	-	-	-	-	-	-	-	-	-	-	-	SQPAPVsPQQK	RSQPAPVsPQQKSVL	S7;	No	S(1): 0.0; S(7): 100.0	3	2	1	0	N-Term(TMT6plex); S7(Phospho); K11(TMT6plex)
54112403	NP_060250.2	55636	CHD7	chromodomain-helicase-DNA-binding protein 7	S2559	1.0	1.3	1.3	1.1	1.0	0.7	1.1	1.1	-	-	-	-	-	-	-	-	-	-	-	-	NIPsPGQLDPDTR	PPTRNIPsPGQLDPD	S4;	Yes	S(4): 100.0; T(12): 0.0	5	1	1	0	N-Term(TMT6plex); S4(Phospho)
38201714	NP_001410.2	1994	ELAVL1	ELAV-like protein 1	S202	0.6	0.9	1.2	1.1	2.4	2.0	1.1	1.0	0.7	1.1	1.4	1.4	2.8	2.4	1.3	1.2	1.7	1.7	1.3	1.3	NVALLSQLYHsPAR	LLSQLYHsPARRFGG	S11;	Yes	S(6): 0.0; Y(9): 0.0; S(11): 100.0	1	1	1	0	N-Term(TMT6plex); S11(Phospho)

Khan et al., 2019. Multi-omics analysis to characterize cigarette smoke induced molecular alterations in esophageal cells
Supplementary Table 8. List of phosphopeptides identified and quantified in untreated and chronically treated Het1A cells with cigarette smoke condensate for 8 months using SequestHT and Mascot search algorithms with a PhosphoRS probability score ≥75%

														Normalized phosphoprotein ratios with respect to corresponding total protein ratios.								Average of replicates													
Protein group Accession	NP_Accession	Gene ID	Gene Symbol	Protein Description	PhosphoSite (Protein)	Het-1A-Smoke - 2M/Parental	Het-1A-Smoke - 4M/Parental	Het-1A-Smoke - 6M/Parental	Het-1A-Smoke - 8M/Parental	Het-1A-Smoke - 2M/Parental	Het-1A-Smoke - 4M/Parental	Het-1A-Smoke - 6M/Parental	Het-1A-Smoke - 8M/Parental	Het-1A-Smoke - 2M/Parental	Het-1A-Smoke - 4M/Parental	Het-1A-Smoke - 6M/Parental	Het-1A-Smoke - 8M/Parental	Het-1A-Smoke - 2M/Parental	Het-1A-Smoke - 4M/Parental	Het-1A-Smoke - 6M/Parental	Het-1A-Smoke - 8M/Parental	Het-1A-Smoke - 2M/Parental	Het-1A-Smoke - 4M/Parental	Het-1A-Smoke - 6M/Parental	Het-1A-Smoke - 8M/Parental	Peptide Sequence	Phosphowindow	PhosphoSite (Peptide)	Phosphosite Plus Evidence	phosphoRS Site Probabilities	# PSMs	# Proteins	# Protein Groups	# Missed Cleavages	Modifications
						Replicate 1				Replicate 2				Replicate 1				Replicate 2																	
118572613	NP_057417.3	23524	**SRRM2**	serine/arginine repetitive matrix protein 2	**T367**	1.2	0.9	0.6	1.1	1.0	0.9	0.6	1.1	1.3	0.9	0.6	1.2	1.1	0.9	0.7	1.2	1.2	0.9	0.6	1.2	SSTGPEPPAPtPLLAER	GPEPPAPtPLLAERH	T11;	Yes	S(1): 0.0; S(2): 0.0; T(3): 0.0; T(11): 100.0	436	1	1	0	N-Term(TMT6plex); T11(Phospho)
153945728	NP_005900.2	4131	**MAP1B**	microtubule-associated protein 1B	**S1252**	1.5	2.3	1.6	1.2	1.2	1.8	1.7	1.0	1.2	1.7	1.3	1.3	1.0	1.4	1.4	1.1	1.1	1.5	1.4	1.2	DSISAVSSEKVsPSK	AVSSEKVsPSKSPSL	S12;	Yes	S(2): 0.0; S(4): 0.0; S(7): 0.0; S(8): 0.0; S(12): 100.0; S(14): 0.0	49	1	1	1	N-Term(TMT6plex); K10(TMT6plex); S12(Phospho); K15(TMT6plex)
14670392	NP_115784.1	9031	**BAZ1B**	tyrosine-protein kinase BAZ1B	**S1468**	0.8	1.1	1.0	1.0	0.9	0.9	1.1	1.2	0.9	1.2	1.1	1.0	1.0	1.0	1.1	1.3	0.9	1.1	1.1	1.2	LAEDEGDsEPEAVGQSR	LAEDEGDsEPEAVGQ	S8;	Yes	S(8): 100.0; S(16): 0.0	6	1	1	0	N-Term(TMT6plex); S8(Phospho)
57634536	NP_055970.1	23185	**LARP4B**	la-related protein 4B	**S731; S736**	1.1	1.1	1.2	1.0	1.0	1.2	1.3	1.2	1.0	1.0	1.1	0.9	1.0	1.1	1.2	1.2	1.0	1.1	1.2	1.0	EQsTPPKsPQ	KRLSREQsTPPKSPQ;EQSTPPKsPQ	S3; S8;	Yes;Yes	S(3): 100.0; T(4): 0.0; S(8): 100.0	7	1	1	1	N-Term(TMT6plex); S3(Phospho); K7(TMT6plex); S8(Phospho)
557128994	NP_001273407.1	8663	**EIF3C**	eukaryotic translation initiation factor 3 subunit C isoform b	**S156**	1.4	1.0	1.3	1.0	1.3	1.1	1.1	1.1	1.3	0.9	1.2	1.0	1.3	1.1	1.1	1.0	1.3	1.0	1.1	1.0	QNPEQsADEDAEK	YKQNPEQsADEDAEK	S6;	No	S(6): 100.0	16	3	1	0	N-Term(TMT6plex); S6(Phospho); K13(TMT6plex)
57634536	NP_055970.1	23185	**LARP4B**	la-related protein 4B	**S731; S736**	1.0	1.2	1.3	1.2	1.1	1.1	1.2	1.0	1.0	1.1	1.2	1.2	1.0	1.0	1.1	0.9	1.0	1.1	1.2	1.0	EQSTPPKsPQ	EQSTPPKsPQ	S3; S8;	Yes	S(3): 50.0; T(4): 50.0; S(8): 100.0	2	1	1	1	N-Term(TMT6plex); T4(Phospho); K7(TMT6plex); S8(Phospho)
217035107	NP_001136037.1	79869	**CPSF7**	cleavage and polyadenylation specificity factor subunit 7 isoform 3	**T194**	0.9	1.0	1.0	1.2	0.8	0.7	0.9	1.0	0.9	1.1	1.0	1.2	0.8	0.8	1.0	0.9	0.8	0.9	1.0	1.1	AtPSENLVPSSAR	DSADGRAtPSENLVP	T2;	No	T(2): 99.9; S(4): 0.0; S(10): 0.0; S(11): 0.0	3	3	1	0	N-Term(TMT6plex); T2(Phospho)
5803177	NP_006741.1	6645	**SNTB2**	beta-2-syntrophin	**S95**	0.9	1.5	1.1	0.9	1.1	0.8	1.0	1.3	0.9	1.6	1.0	0.9	1.1	0.8	0.9	1.2	1.0	1.2	1.0	1.1	GLGPPsPPAPPR	SRGLGPPsPPAPPRG	S6;	Yes	S(6): 100.0	3	1	1	0	N-Term(TMT6plex); S6(Phospho)
22749367	NP_689893.1	201799	**TMEM154**	transmembrane protein 154 precursor	**S179**	1.2	2.3	2.5	1.0	1.3	3.8	6.0	1.1	-	-	-	-	-	-	-	-	-	-	-	-	ESNHNPsDSES	KESNHNPsDSES_	S7;	Yes	S(2): 0.0; S(7): 100.0; S(9): 0.0; S(11): 0.0	5	1	1	0	N-Term(TMT6plex); S7(Phospho)
74959747	NP_065988.1	57697	**FANCM**	Fanconi anemia group M protein	**S1437**	1.3	1.0	0.9	1.0	1.4	1.3	1.4	1.1	-	-	-	-	-	-	-	-	-	-	-	-	GNVLNsPEDQK	AKGNVLNsPEDQKNS	S6;	No	S(6): 100.0	1	1	1	0	N-Term(TMT6plex); S6(Phospho); K11(TMT6plex)
4759140	NP_004243.1	9368	**SLC9A3R1**	Na(+)/H(+) exchange regulatory cofactor NHE-RF1	**S290**	0.9	1.1	0.9	1.0	1.6	1.0	1.0	1.2	0.9	1.2	1.0	1.1	1.6	1.1	1.0	1.3	1.3	1.1	1.0	1.2	SAsSDTSEELNSQDSPPK	PALVRSAsSDTSEEL	S3;	Yes	S(1): 1.1; S(3): 97.7; S(4): 1.1; T(6): 0.0; S(7): 0.0; S(12): 0.0; S(15): 0.0	7	1	1	0	N-Term(TMT6plex); S3(Phospho); K18(TMT6plex)
4759140	NP_004243.1	9368	**SLC9A3R1**	Na(+)/H(+) exchange regulatory cofactor NHE-RF1	**S290**	1.6	1.0	1.0	1.2	0.9	1.1	0.9	1.0	1.6	1.1	1.0	1.3	0.9	1.2	1.0	1.1	1.3	1.1	1.0	1.2	sASSDTSEELNSQDSPPK	PRPALVRsASSDTSE	S3;	Yes	S(1): 98.5; S(3): 1.4; S(4): 0.0; T(6): 0.0; S(7): 0.0; S(12): 0.0; S(15): 0.0	3	1	1	0	N-Term(TMT6plex); S3(Phospho); K18(TMT6plex)
45505147	NP_071739.2	64149	**C17orf75**	protein Njmu-R1	**S395**	0.9	0.7	1.2	1.1	2.3	0.9	3.8	1.1	0.9	0.6	1.1	1.1	2.4	0.9	3.6	1.1	1.7	0.7	2.4	1.1	EALDQsE	MKEALDQsE	S6;	Yes	S(6): 100.0	3	1	1	0	N-Term(TMT6plex); S6(Phospho)
78000156	NP_056451.3	8890	**EIF2B4**	translation initiation factor eIF-2B subunit delta	**T85**	0.8	1.0	0.8	1.0	1.2	1.3	0.6	1.1	0.8	1.1	0.9	1.1	1.3	1.4	0.7	1.2	1.1	1.2	0.8	1.2	ELPESGIQLGtPR	ESGIQLGtPREKVPA	T11;	No	S(5): 0.0; T(11): 100.0	5	3	1	0	N-Term(TMT6plex); T11(Phospho)
400153758	NP_001257819.1	54801	**HAUS6**	HAUS augmin-like complex subunit 6 isoform 2	**S517**	1.0	1.1	1.1	1.1	0.7	0.8	1.0	1.1	0.8	1.0	1.0	1.1	0.6	0.8	0.9	1.0	0.7	0.9	1.0	1.0	AVLSDsPQLSEGK	ARAVLSDsPQLSEGK	S6;	No	S(4): 0.8; S(6): 99.2; S(10): 0.0	4	2	1	0	N-Term(TMT6plex); S6(Phospho); K13(TMT6plex)
72255574	NP_056370.2	26036	**ZNF451**	zinc finger protein 451 isoform 2	**S979**	1.0	1.1	1.2	1.0	0.9	1.0	1.0	1.2	0.9	1.0	1.2	1.0	0.8	1.0	1.0	1.2	0.9	1.0	1.1	1.1	ECDsDDNMGAK	DTTKECDsDDNMGAK	S4;	No	S(4): 100.0	2	2	1	0	N-Term(TMT6plex); C2(Carbamidomethyl); S4(Phospho); M8(Oxidation); K11(TMT6plex)
31543148	NP_077299.3	79173	**C19orf57**	uncharacterized protein C19orf57	**S370**	1.1	1.2	0.7	1.1	1.7	1.1	1.2	1.0	-	-	-	-	-	-	-	-	-	-	-	-	ALEVAGPDGQASAISPAsPR	ASAISPAsPRRKAAD	S18;	Yes	S(12): 0.0; S(15): 0.0; S(18): 100.0	4	1	1	0	N-Term(TMT6plex); S18(Phospho)
15721939	NP_031385.2	22794	**CASC3**	protein CASC3	**S363**	1.3	2.0	0.6	1.1	1.5	1.1	1.2	1.0	1.5	2.0	0.6	1.2	1.6	1.1	1.3	1.1	1.6	1.5	1.0	1.2	DPsPEADAPVLGSPEK	QTSVRDPsPEADAPV	S3;	Yes	S(3): 100.0; S(13): 0.0	10	1	1	0	N-Term(TMT6plex); S3(Phospho); K16(TMT6plex)
156105703	NP_001093879.1	26054	**SENP6**	sentrin-specific protease 6 isoform 2	**S912**	0.8	1.5	1.0	0.8	0.7	2.0	2.3	1.3	0.7	1.4	1.0	0.8	0.7	2.0	2.3	1.2	0.7	1.7	1.6	1.0	LNYSDEsPEAGK	RLNYSDEsPEAGKML	S7;	No	Y(3): 0.0; S(4): 0.0; S(7): 100.0	3	2	1	0	N-Term(TMT6plex); S7(Phospho); K12(TMT6plex)
371121601	NP_001243048.1	8260	**NAA10**	N-alpha-acetyltransferase 10 isoform 2	**S218**	1.4	1.0	1.0	1.1	1.1	1.0	1.0	1.0	1.4	1.0	1.0	1.1	1.1	0.9	1.0	1.0	1.3	0.9	1.0	1.1	DSSEASDsAS	DSSEASDsAS_	S8;	No	S(2): 0.0; S(3): 0.0; S(6): 0.0; S(8): 100.0; S(10): 0.0	20	3	1	0	N-Term(TMT6plex); S8(Phospho)
119120894	NP_056078.2	23312	**DMXL2**	dmX-like protein 2 isoform 2	**S1400**	1.7	1.9	1.7	1.3	1.2	1.3	1.4	0.8	1.6	1.8	1.6	1.3	1.1	1.2	1.3	0.8	1.4	1.5	1.4	1.0	TIsVSGSTAK	RHLSRTIsVSGSTAK	S3;	Yes	T(1): 0.0; S(3): 100.0; S(5): 0.0; S(7): 0.0; T(8): 0.0	1	2	1	0	N-Term(TMT6plex); S3(Phospho); K10(TMT6plex)
262359929	NP_056261.4	25909	**AHCTF1**	protein ELYS	**S1953**	1.3	1.1	1.0	1.0	1.1	0.9	1.1	1.2	1.3	1.1	1.0	1.0	1.1	0.9	1.1	1.3	1.2	1.0	1.1	1.2	EVsPSDVR	RVRGREVsPSDVRED	S3;	No	S(3): 100.0; S(5): 0.0	13	1	1	0	N-Term(TMT6plex); S3(Phospho)
325120986	NP_001191397.1	8241	**RBM10**	RNA-binding protein 10 isoform 5	**S788**	1.2	1.2	1.2	1.1	1.0	1.0	1.0	1.0	1.3	1.4	1.4	1.3	1.1	1.1	1.2	1.2	1.2	1.2	1.3	1.2	LASDDRPsPPR	LASDDRPsPPRGLVA	S8;	No	S(3): 0.0; S(8): 100.0	6	5	1	0	N-Term(TMT6plex); S8(Phospho)
118572613	NP_057417.3	23524	**SRRM2**	serine/arginine repetitive matrix protein 2	**S297**	1.0	1.4	1.3	1.1	1.0	1.3	1.3	1.1	1.1	1.4	1.3	1.2	1.1	1.3	1.4	1.2	1.1	1.3	1.3	1.2	SPsPASGR	ALAGRSPsPASGRRG	S3;	Yes	S(1): 0.0; S(3): 100.0; S(6): 0.0	436	1	1	0	N-Term(TMT6plex); S3(Phospho)
19913406	NP_001058.2	7153	**TOP2A**	DNA topoisomerase 2-alpha	**S1525**	0.9	0.9	0.7	1.0	1.0	1.0	0.9	1.1	1.0	1.0	0.8	1.1	1.2	1.1	1.0	1.3	1.1	1.0	0.9	1.2	YLEEsDEDDLF	PIKYLEEsDEDDLF	S5;	Yes	Y(1): 0.0; S(5): 100.0	64	1	1	0	N-Term(TMT6plex); S5(Phospho)
634743290	NP_001278893.1	4301	**MLLT4**	afadin isoform 4	**T1293**	1.4	1.0	1.2	1.1	1.1	1.4	1.2	1.1	1.3	0.9	1.1	0.9	1.0	1.2	1.0	1.0	1.1	1.0	1.0	0.9	SVTPAStLTK	KSVTPAStLTKSGPG	T7;	No	S(1): 0.0; T(3): 1.0; S(6): 0.0; T(7): 97.9; T(9): 1.0	16	3	1	0	N-Term(TMT6plex); T3(Phospho); K10(TMT6plex)
333108224	NP_001193937.1	4301	**MLLT4**	afadin isoform 1	**T1293**	1.1	1.4	1.2	1.1	1.4	1.0	1.2	1.1	1.0	1.2	1.0	1.0	1.3	0.9	1.1	0.9	1.1	1.0	1.0	0.9	SVtPASTLTK	NHSSKSVtPASTLTK	T7;	No	S(1): 0.0; T(3): 99.0; S(6): 0.0; T(7): 0.9; T(9): 0.0	1	3	1	0	N-Term(TMT6plex); T3(Phospho); K10(TMT6plex)
19718759	NP_579899.1	26509	**MYOF**	myoferlin isoform b	**S174**	1.5	1.3	1.6	1.1	1.4	1.5	1.5	1.1	1.3	1.1	1.3	1.0	1.2	1.4	1.2	1.1	1.3	1.2	1.2	1.0	GPVGTVsEAQLAR	KGPVGTVsEAQLARR	S7;	Yes	T(5): 0.0; S(7): 100.0	4	2	1	0	N-Term(TMT6plex); S7(Phospho)
7662655	NP_054754.1	28966	**SNX24**	sorting nexin-24	**S116**	0.7	0.9	1.0	1.1	1.0	1.2	1.0	1.0	-	-	-	-	-	-	-	-	-	-	-	-	AESCGsFDETESEESSK	PKAESCGsFDETESE	S6;	Yes	S(3): 1.7; S(6): 98.2; T(10): 0.0; S(12): 0.0; S(15): 0.0; S(16): 0.0	1	1	1	0	N-Term(TMT6plex); C4(Carbamidomethyl); S6(Phospho); K17(TMT6plex)
39725634	NP_056130.2	23367	**LARP1**	la-related protein 1	**S440; S444**	1.0	1.2	1.1	0.9	1.1	1.0	0.9	1.2	1.0	1.1	1.1	0.9	1.1	1.0	0.9	1.2	1.1	1.1	1.0	1.1	ETEsAPGsPR	HYQKETEsAPGSPRA;ETESAPGsPRAVTPV	S4; S8;	No;No	T(2): 0.0; S(4): 100.0; S(8): 100.0	28	1	1	0	N-Term(TMT6plex); S4(Phospho); S8(Phospho)
186928821	NP_001119523.1	3068	**HDGF**	hepatoma-derived growth factor isoform c	**S125**	0.8	0.9	1.0	0.9	1.1	1.2	1.4	1.2	0.9	0.8	0.9	0.8	1.2	1.1	1.3	1.2	1.0	1.0	1.1	1.0	GNAEGsSDEEGK	KKGNAEGsSDEEGKL	S6;	No	S(6): 100.0; S(7): 0.0	78	3	1	0	N-Term(TMT6plex); S6(Phospho); K12(TMT6plex)
118601081	NP_001073027.1	221092	**HNRNPUL2**	heterogeneous nuclear ribonucleoprotein U-like	**S161**	1.0	1.1	1.0	1.0	0.8	1.2	1.0	1.1	1.0	1.0	0.9	1.1	0.8	1.2	1.0	1.1	0.9	1.1	1.0	1.0	SGDEtPGsEVPGDK	EDEPEERsGDETPGS	S1;	Yes	S(1): 100.0; T(5): 0.0; S(8): 0.0	33	1	1	0	N-Term(TMT6plex); S1(Phospho); K14(TMT6plex)
62422557	NP_001014832.1	10298	**PAK4**	serine/threonine-protein kinase PAK 4 isoform 1	**S104**	1.0	1.1	1.0	1.1	1.1	1.1	1.1	1.0	0.9	1.0	1.0	1.0	1.0	1.1	1.0	1.0	1.0	1.0	1.0	1.0	RDsPPPPAR	SNSLRRDsPPPPARA	S3;	Yes	S(3): 100.0	7	1	1	1	N-Term(TMT6plex); S3(Phospho)
118601081	NP_001073027.1	221092	**HNRNPUL2**	heterogeneous nuclear ribonucleoprotein U-like protein 2	**S161**	0.8	1.2	1.0	1.1	1.0	1.1	1.0	1.0	0.8	1.2	1.0	1.1	1.0	1.0	0.9	1.1	0.9	1.1	1.0	1.1	SGDEtPGSEVPGDK	EERSGDEtPGSEVPG;SGDETPGsEVPGDKA	S1;	Yes;Yes	S(1): 1.4; T(5): 98.6; S(8): 100.0	1	1	1	0	N-Term(TMT6plex); T5(Phospho); S8(Phospho); K14(TMT6plex)
119220605	NP_005144.2	9100	**USP10**	ubiquitin carboxyl-terminal hydrolase 10 isoform 2	**S547**	0.6	0.8	0.8	0.9	1.1	1.1	1.2	1.3	0.7	0.8	0.7	0.8	1.1	1.1	1.1	1.2	0.9	0.9	0.9	1.0	LLsPSNEK	LNLKKLLsPSNEKLT	S3;	Yes	S(3): 100.0; S(5): 0.0	5	2	1	0	N-Term(TMT6plex); S3(Phospho); K8(TMT6plex)
344217752	NP_001230676.1	8539	**API5**	apoptosis inhibitor 5 isoform d	**S291**	0.8	1.0	0.9	1.0	1.1	1.3	1.1	1.1	0.8	1.1	1.0	1.0	1.2	1.4	1.2	1.2	1.0	1.2	1.1	1.1	ASEDTTSGsPPKK	SEDTTSGsPPKKSSA	S9;	No	S(2): 0.0; T(5): 0.0; T(6): 0.0; S(7): 0.0; S(9): 100.0	8	4	1	1	N-Term(TMT6plex); S9(Phospho); K12(TMT6plex); K13(TMT6plex)
45359846	NP_987100.1	9908	**G3BP2**	ras GTPase-activating protein-binding protein 2 isoform b	**S225**	1.0	0.9	0.9	1.0	1.0	1.0	1.4	1.1	0.9	0.9	0.9	0.9	1.0	0.9	1.4	1.1	0.9	0.9	1.1	1.0	sTTPPPAEPVSLPQEPPKPR	LEELEEKsTTPPPAE	S1;	Yes	S(1): 97.7; T(2): 1.2; T(3): 1.2; S(11): 0.0	3	1	1	0	N-Term(TMT6plex); S1(Phospho); K18(TMT6plex)
55749769	NP_065994.1	57703	**CWC22**	pre-mRNA-splicing factor CWC22 homolog	**S61**	1.1	1.2	1.2	1.1	1.0	1.2	1.1	1.1	1.0	1.3	1.2	1.0	1.0	1.3	1.2	1.1	1.0	1.3	1.2	1.1	sYDSSMESR	EHSRRGRsYDSSMES	S1;	Yes	S(1): 100.0; Y(2): 0.0; S(4): 0.0; S(5): 0.0; S(8): 0.0	3	1	1	0	N-Term(TMT6plex); S1(Phospho); M6(Oxidation)
23821044	NP_667339.1	223082	**ZNRF2**	E3 ubiquitin-protein ligase ZNRF2	**S135**	1.0	1.1	1.1	1.0	0.9	1.2	1.0	1.2	0.9	1.2	1.1	1.1	0.8	1.3	0.9	1.3	0.9	1.3	1.0	1.2	DRPVGGsPGGPR	RDRPVGGsPGGPRLV	S7;	Yes	S(7): 100.0	1	1	1	0	N-Term(TMT6plex); S7(Phospho)
223633935	NP_694988.3	254427	**PROSER2**	proline and serine-rich protein 2	**S212**	1.2	1.1	1.2	1.0	1.0	0.9	0.7	1.1	1.5	1.3	1.3	1.1	1.3	1.0	0.7	1.2	1.4	1.1	1.0	1.1	MAGNEALsPTSPFR	MAGNEALsPTSPFRE	S8;	Yes	S(8): 100.0; T(10): 0.0; S(11): 0.0	4	1	1	0	N-Term(TMT6plex); M1(Oxidation); S8(Phospho)
223633935	NP_694988.3	254427	**PROSER2**	proline and serine-rich protein 2	**S212**	1.0	0.9	0.7	1.1	1.2	1.1	1.2	1.0	1.3	1.0	0.7	1.2	1.5	1.3	1.3	1.1	1.4	1.1	1.0	1.1	MAGNEALSPTsPFR	NEALSPTsPFREGRP	S8;	Yes	S(8): 0.0; T(10): 0.0; S(11): 100.0	4	1	1	0	N-Term(TMT6plex); M1(Oxidation); S11(Phospho)
13129104	NP_077003.1	79080	**CCDC86**	coiled-coil domain-containing protein 86	**S113**	0.9	1.3	0.9	0.9	1.1	1.1	1.0	1.2	1.1	1.3	1.0	1.0	1.3	1.1	1.1	1.4	1.2	1.2	1.1	1.2	QPEYSPEsPR	QPEYSPEsPRCQPKP	S8;	Yes	Y(4): 0.0; S(5): 0.0; S(8): 100.0	26	1	1	0	N-Term(TMT6plex); S8(Phospho)
13129104	NP_077003.1	79080	**CCDC86**	coiled-coil domain-containing protein 86	**S113**	1.1	1.1	1.0	1.2	0.9	1.3	0.9	0.9	1.3	1.1	1.1	1.4	1.1	1.3	1.0	1.0	1.2	1.2	1.1	1.2	QPEYsPEsPR	PQRQPEYsPESPRCQ;QPEYSPEsPRCQPKP	S8;	Yes;Yes	Y(4): 1.3; S(5): 98.7; S(8): 100.0	2	1	1	0	N-Term(TMT6plex); S5(Phospho); S8(Phospho)
149588928	NP_065897.1	57606	**SLAIN2**	SLAIN motif-containing protein 2	**S63**	0.8	1.1	1.0	0.9	1.1	1.3	1.1	1.2	0.8	1.0	1.0	0.9	1.1	1.3	1.2	1.1	0.9	1.1	1.1	1.0	AGASIPSSGAAsPR	IPSSGAAsPRGFPLG	S12;	Yes	S(4): 0.0; S(7): 0.0; S(8): 0.0; S(12): 100.0	9	1	1	0	N-Term(TMT6plex); S12(Phospho)
11321634	NP_036252.1	23607	**CD2AP**	CD2-associated protein	**S233**	1.0	0.9	0.8	1.1	1.3	1.0	0.8	1.0	1.3	1.0	0.8	1.1	1.5	1.1	0.9	1.0	1.4	1.0	0.8	1.1	TSsSETEEK	KLRTRTSsSETEEKK	S3;	Yes	T(1): 0.0; S(2): 1.1; S(3): 98.9; S(4): 0.0; T(6): 0.0	1	1	1	0	N-Term(TMT6plex); S3(Phospho); K9(TMT6plex)
5453904	NP_006470.1	10635	**RAD51AP1**	RAD51-associated protein 1 isoform b	**S103**	1.0	1.2	1.2	1.3	0.8	1.5	1.3	0.9	1.1	1.4	1.5	1.3	0.8	1.8	1.7	0.9	1.0	1.6	1.6	1.1	ELPTVTTNVQNsQDK	VTTNVQNsQDKSIEK	S12;	No	T(4): 0.0; T(6): 0.0; T(7): 0.0; S(12): 100.0	2	2	1	0	N-Term(TMT6plex); S12(Phospho); K15(TMT6plex)
16418351	NP_443080.1	90324	**CCDC97**	coiled-coil domain-containing protein 97	**S337**	1.0	0.9	2.4	1.4	0.6	0.7	0.8	0.8	1.0	1.0	2.9	1.6	0.5	0.8	0.9	0.9	0.8	0.9	1.9	1.2	YFDEEEPEDAPsPELDGD	EEPEDAPsPELDGD	S12;	Yes	Y(1): 0.0; S(12): 100.0	1	1	1	0	N-Term(TMT6plex); S12(Phospho)
11276067	NP_003059.1	6591	**SNAI2**	zinc finger protein SNAI2	**S87**	1.0	1.0	1.1	0.8	1.0	1.1	1.4	1.3	-	-	-	-	-	-	-	-	-	-	-	-	VsPPPPSDTSSK	SSSLGRVsPPPPSDT	S2;	Yes	S(2): 100.0; S(7): 0.0; T(9): 0.0; S(10): 0.0; S(11): 0.0	4	1	1	0	N-Term(TMT6plex); S2(Phospho); K12(TMT6plex)
156151371	NP_057615.3	51274	**KLF3**	Krueppel-like factor 3	**S92**	0.7	0.8	1.1	0.9	1.0	1.2	1.2	1.3	-	-	-	-	-	-	-	-	-	-	-	-	RAsPGLsMPSSSPPIK	PSSHRRAsPGLSMPS;RRASPGLsMPSSSPP	S3;	Yes;Yes	S(3): 100.0; S(7): 99.9; S(10): 0.1; S(11): 0.0; S(12): 0.0	1	1	1	1	N-Term(TMT6plex); S3(Phospho); S7(Phospho); M8(Oxidation); K16(TMT6plex)
38788260	NP_004450.3	2186	**BPTF**	nucleosome-remodeling factor subunit BPTF isoform 2	**S216**	1.0	1.2	1.3	1.0	1.0	1.1	1.1	1.1	1.0	1.2	1.4	1.0	1.1	1.1	1.2	1.1	1.1	1.1	1.3	1.0	sPILEEK	PRVHRPRsPILEEKD	S1;	Yes	S(1): 100.0	11	2	1	0	N-Term(TMT6plex); S1(Phospho); K7(TMT6plex)
92087055	NP_072096.2	64599	**GIGYF1**	PERQ amino acid-rich with GYF domain-containing protein 1	**S230**	1.2	1.1	1.0	1.2	1.0	1.3	1.0	1.0	-	-	-	-	-	-	-	-	-	-	-	-	SAsPDGGPR	GDRWRSAsPDGGPRS	S3;	No	S(1): 0.0; S(3): 100.0	3	1	1	0	N-Term(TMT6plex); S3(Phospho)
119874201	NP_055845.1	285527	**FRYL**	protein furry homolog-like	**S2454**	1.0	1.0	1.1	1.1	1.2	1.6	1.8	1.0	0.9	0.8	1.0	1.0	1.1	1.4	1.5	0.9	1.0	1.1	1.2	1.0	sLDSIDK	NWGVRRRsLDSIDKG	S1;	Yes	S(1): 100.0; S(4): 0.0	3	1	1	0	N-Term(TMT6plex); S1(Phospho); K7(TMT6plex)
386642883	NP_001245385.1	10318	**TNIP1**	TNFAIP3-interacting protein 1 isoform 6	**S266**	0.8	1.4	1.0	1.1	0.8	1.3	1.0	1.1	1.1	1.3	1.0	1.2	1.1	1.2	1.0	1.2	1.1	1.2	1.0	1.2	EGASGRPGsPK	GASGRPGsPKMEGTG	S9;	Yes	S(4): 0.0; S(9): 100.0	2	6	1	0	N-Term(TMT6plex); S9(Phospho); K11(TMT6plex)
589269226	NP_001277136.1	55802	**DCP1A**	mRNA-decapping enzyme 1A isoform e	**S366**	1.3	1.4	1.5	1.2	1.0	1.0	1.1	0.9	1.2	1.3	1.1	1.0	0.9	1.0	0.8	0.8	1.0	1.2	0.9	0.9	ASSPsPLTIGTPESQR	ERKASSPsPLTIGTP	S5;	No	S(2): 0.9; S(3): 0.9; S(5): 98.1; T(8): 0.0; T(11): 0.0; S(14): 0.0	3	5	1	0	N-Term(TMT6plex); S5(Phospho)
319996623	NP_001188474.1	64783	**RBM15**	putative RNA-binding protein 15 isoform 2	**S294**	1.1	1.2	1.1	1.1	1.1	1.3	1.1	1.1	1.1	1.2	1.0	1.1	1.1	1.3	1.0	1.1	1.1	1.3	1.0	1.1	SLsPGGAALGYR	GGGQRSLsPGGAALG	S3;	Yes	S(1): 0.0; S(3): 100.0; Y(11): 0.0	6	2	1	0	N-Term(TMT6plex); S3(Phospho)
253970412	NP_057510.4	51512	**GTSE1**	G2 and S phase-expressed protein 1	**T532**	0.5	1.1	1.8	1.4	0.7	0.9	0.8	0.8	0.6	1.2	2.0	1.6	0.8	0.9	0.9	0.9	0.7	1.0	1.5	1.3	VSALPtPASR	RRVSALPtPASRRCS	T6;	No	S(2): 0.0; T(6): 100.0; S(9): 0.0	19	1	1	0	N-Term(TMT6plex); T6(Phospho)
231570186	NP_001153576.1	79882	**ZC3H14**	zinc finger CCCH domain-containing protein 14	**S515**	0.6	0.7	0.5	0.9	0.7	0.9	0.6	1.3	0.7	0.7	0.7	0.9	0.8	1.0	0.8	1.3	0.8	0.9	0.7	1.1	DLVQPDKPAsPK	VQPDKPAsPKFIVTL	S10;	Yes	S(10): 100.0	2	3	1	0	N-Term(TMT6plex); K7(TMT6plex); S10(Phospho); K12(TMT6plex)
269315880	NP_115554.2	84138	**SLC7A6OS**	probable RNA polymerase II nuclear localization protein SLC7A6OS	**S302; S308**	0.9	1.4	1.1	1.0	1.0	1.1	1.0	1.1	1.0	1.6	1.2	1.2	1.2	1.3	1.1	1.4	1.1	1.4	1.1	1.3	EFGYDsPHDLDsD	QKEFGYDsPHDLDSD;DSPHDLDsD	S6; S12;	Yes;Yes	Y(4): 0.0; S(6): 100.0; S(12): 100.0	1	1	1	0	N-Term(TMT6plex); S6(Phospho); S12(Phospho)
16445438	NP_387505.1	54014	**BRWD1**	bromodomain and WD repeat-containing protein 1 isoform B	**S1793**	1.1	0.9	0.9	0.9	0.6	0.8	0.9	1.2	1.1	0.9	1.0	1.0	0.7	0.8	1.0	1.3	0.9	0.9	1.0	1.1	AESISEEADsEPGR	SISEEADsEPGRSGG	S10;	Yes	S(3): 0.0; S(5): 1.9; S(10): 98.1	1	2	1	0	N-Term(TMT6plex); S10(Phospho)
19913396	NP_599031.1	6525	**SMTN**	smoothelin isoform b	**T243**	1.4	1.3	1.4	0.9	1.9	1.9	2.4	1.2	1.1	1.0	1.0	0.8	1.5	1.5	1.7	1.1	1.3	1.2	1.4	0.9	tTSPEPQESPTLPSTEGQVVNK	PPPSPPKtTSPEPQE	T1;	Yes	T(1): 95.9; T(2): 2.0; S(3): 2.0; S(9): 0.0; T(11): 0.0; S(14): 0.0; T(15): 0.0	7	5	1	0	N-Term(TMT6plex); S3(Phospho); K22(TMT6plex)
76150625	NP_001028886.1	4839	**NOP2**	putative ribosomal RNA methyltransferase NOP2 isoform 1	**S782**	1.1	1.1	1.1	1.0	1.0	1.1	1.1	1.1	1.1	1.0	1.1	1.0	1.1	1.1	1.0	1.1	1.1	1.0	1.1	1.0	GPQPPTVsPIR	GPQPPTVsPIRSSRP	S8;	No	T(6): 0.0; S(8): 100.0	12	3	1	0	N-Term(TMT6plex); S8(Phospho)
50053795	NP_001408.2	1975	**EIF4B**	eukaryotic translation initiation factor 4B	**S504**	0.5	0.9	1.0	1.0	1.0	1.5	1.0	1.1	0.5	0.8	1.0	0.8	1.1	1.4	1.0	0.9	0.8	1.1	1.0	0.9	SQSSDTEQQsPTSGGGK	SSDTEQQsPTSGGGK	S10;	Yes	S(1): 0.7; S(3): 49.7; S(4): 49.7; T(6): 0.0; S(10): 100.0; T(12): 0.0; S(13):	39	1	1	0	N-Term(TMT6plex); S4(Phospho); S10(Phospho); K17(TMT6plex)
333440471	NP_001193980.1	23332	**CLASP1**	CLIP-associating protein 1 isoform 4	**S572**	1.1	1.2	1.1	1.0	1.1	1.1	1.1	1.1	1.2	1.3	1.2	1.1	1.1	1.2	1.2	1.2	1.2	1.2	1.2	1.1	RsPTGSTTSR	RPLSAKRsPTGSTTS	S2;	Yes	S(2): 100.0; T(4): 0.0; S(6): 0.0; T(7): 0.0; T(8): 0.0; S(9): 0.0	14	4	1	1	N-Term(TMT6plex); S2(Phospho)
55770834	NP_057427.3	1063	**CENPF**	centromere protein F	**T1541**	0.4	0.8	1.0	0.9	0.4	0.8	1.0	1.2	0.5	0.8	1.0	0.9	0.5	0.8	1.0	1.3	0.5	0.8	1.0	1.1	KEtPSAPAK	ENLTRKEtPSAPAKG	T3;	No	T(3): 100.0; S(5): 0.0	11	1	1	1	N-Term(TMT6plex); K1(TMT6plex); T3(Phospho); K9(TMT6plex)
56682953	NP_004438.3	2059	**EPS8**	epidermal growth factor receptor kinase substrate 8	**T223**	1.0	1.0	1.1	0.6	1.0	1.4	1.8	1.5	0.7	0.9	0.8	0.5	0.7	1.2	1.4	1.3	0.7	1.1	1.1	0.9	APAPAPPGtVTQVDVR	PAPAPPGtVTQVDVR	T9;	Yes	T(9): 100.0; T(11): 0.0	2	1	1	0	N-Term(TMT6plex); T9(Phospho)
41152072	NP_056272.2	25921	**ZDHHC5**	palmitoyltransferase ZDHHC5	**S380**	0.9	1.2	1.3	1.2	0.8	1.0	1.1	0.9	0.8	1.2	1.3	1.2	0.8	0.9	1.1	0.9	0.8	1.1	1.2	1.1	GDsLKEPTSIAESSR	AKLSRGDsLKEPTSI	S3;	Yes	S(3): 100.0; T(8): 0.0; S(9): 0.0; S(13): 0.0; S(14): 0.0	8	1	1	1	N-Term(TMT6plex); S3(Phospho); K5(TMT6plex)
132626688	NP_055456.2	9656	**MDC1**	mediator of DNA damage checkpoint protein 1	**S376**	1.3	1.0	1.1	0.7	1.2	1.9	1.7	1.4	1.3	1.0	1.2	0.7	1.2	1.9	1.8	1.5	1.3	1.4	1.5	1.1	GPGAPGLAHLQESQAGsDTDVEGK	LQESQAGsDTDVEEG	S17;	Yes	S(13): 0.0; S(17): 99.9; T(19): 0.0	26	1	1	0	N-Term(TMT6plex); S17(Phospho); K25(TMT6plex)
215490041	NP_001135900.1	9643	**MORF4L2**	mortality factor 4-like protein 2	**S71**	0.9	1.0	0.8	1.1	0.8	0.8	0.8	1.0	0.9	1.0	1.0	1.2	0.8	0.8	0.9	1.1	0.9	0.9	1.0	1.1	SAENPPSGsVR	AENPPSGsVRKTRKN	S9;	Yes	S(1): 0.0; S(7): 0.7; S(9): 99.3	6	1	1	0	N-Term(TMT6plex); S9(Phospho)
132626688	NP_055456.2	9656	**MDC1**	mediator of DNA damage checkpoint protein 1	**S376**	1.2	1.9	1.7	1.4	1.3	1.0	1.1	0.7	1.2	1.9	1.8	1.5	1.3	1.0	1.2	0.7	1.3	1.4	1.5	1.1	GPGAPGLAHLQESQAGSDtDVEGK	ESQAGSDtDVEEGKA	S17;	Yes	S(13): 2.2; S(17): 2.2; T(19): 95.6	2	1	1	0	N-Term(TMT6plex); S17(Phospho); K25(TMT6plex)
4557495	NP_001317.1	1479	**CSTF3**	cleavage stimulation factor subunit 3 isoform 1	**S691**	1.2	1.0	1.0	1.1	1.0	0.9	0.9	1.0	1.2	1.0	1.1	1.1	1.0	0.9	1.0	1.0	1.1	1.0	1.1	1.0	RPNEDsDEDEEK	VKRPNEDsDEDEEKG	S6;	Yes	S(6): 100.0	3	1	1	0	N-Term(TMT6plex); S6(Phospho); K12(TMT6plex)
186928821	NP_001119523.1	3068	**HDGF**	hepatoma-derived growth factor isoform c	**S96**	1.0	1.1	1.1	1.2	0.8	1.1	1.0	1.0	1.1	1.0	1.1	1.1	0.9	1.0	0.9	0.9	1.0	1.0	1.0	1.0	ASGYQSsQK	KASGYQSsQKKSCVE	S7;	No	S(2): 0.0; Y(4): 0.0; S(6): 0.0; S(7): 100.0	78	3	1	0	N-Term(TMT6plex); S7(Phospho); K9(TMT6plex)
4506723	NP_000997.1	6189	**RPS3A**	40S ribosomal protein S3a isoform 1	**S263**	0.9	1.1	0.9	1.3	0.9	1.0	1.0	0.8	1.1	1.2	1.0	1.4	1.0	1.1	1.1	0.9	1.0	1.1	1.1	1.2	ADGYEPPVQEsV	YEPPVQEsV	S11;	Yes	Y(4): 0.0; S(11): 100.0	7	1	1	0	N-Term(TMT6plex); S11(Phospho)
34996489	NP_060404.3	55023	**PHIP**	PH-interacting protein	**S911**	1.1	1.3	1.2	1.0	1.2	1.2	1.1	1.1	1.1	1.2	1.1	0.9	1.2	1.1	1.0	1.0	1.1	1.1	1.0	1.0	DGPIsPK	EEKDGPIsPKKKKPK	S5;	Yes	S(5): 100.0	9	1	1	0	N-Term(TMT6plex); S5(Phospho); K7(TMT6plex)
19923613	NP_079359.2	80153	**EDC3**	enhancer of mRNA-decapping protein 3	**S131**	1.2	1.2	1.1	1.2	0.8	1.2	1.0	0.9	1.2	1.3	1.1	1.2	0.8	1.3	1.0	0.9	1.0	1.3	1.0	1.1	SQDVAVsPQQQQCSK	KSQDVAVsPQQQQCS	S7;	Yes	S(1): 0.0; S(7): 100.0; S(14): 0.0	2	1	1	0	N-Term(TMT6plex); S7(Phospho); C13(Carbamidomethyl); K15(TMT6plex)
55741709	NP_067062.1	58517	**RBM25**	RNA-binding protein 25	**S677**	1.1	1.1	1.3	1.0	1.1	0.8	0.9	1.1	1.1	1.2	1.4	0.9	1.1	0.9	0.9	1.1	1.1	1.1	1.1	1.0	LGASNsPGQPNSVK	LKLGASNsPGQPNSV	S6;	Yes	S(4): 0.0; S(6): 100.0; S(12): 0.0	11	1	1	0	N-Term(TMT6plex); S6(Phospho); K14(TMT6plex)
509155837	NP_001265241.1	9631	**NUP155**	nuclear pore complex protein Nup155 isoform 3	**S928**	1.0	1.2	1.1	1.2	0.8	1.0	1.0	0.9	0.9	1.1	1.0	1.0	0.8	0.9	0.8	0.8	0.8	1.0	0.9	0.9	AAPQsPSVPK	QSKAAPQsPSVPKKP	S5;	No	S(5): 100.0; S(7): 0.0	8	3	1	0	N-Term(TMT6plex); S5(Phospho); K10(TMT6plex)
38569421	NP_001087.2	47	**ACLY**	ATP-citrate synthase isoform 1	**S481**	1.0	1.3	1.1	1.1	1.2	1.4	1.1	1.0	1.0	1.2	1.1	1.1	1.2	1.3	1.1	1.0	1.1	1.2	1.1	1.1	AKPAMPQDSVPsPR	MPQDSVPsPRSLQGK	S12;	Yes	S(9): 0.0; S(12): 100.0	3	1	1	0	N-Term(TMT6plex); K2(TMT6plex); M5(Oxidation); S12(Phospho)
5032179	NP_005753.1	10155	**TRIM28**	transcription intermediary factor 1-beta	**S473**	0.7	1.2	1.2	0.7	1.0	1.4	0.9	1.4	0.7	1.2	1.2	0.7	1.0	1.5	0.9	1.3	0.9	1.4	1.1	1.0	sGEGEVSGLMR	SGVKRSRsGEGEVSG	S1;	Yes	S(1): 100.0; S(7): 0.0	10	1	1	0	N-Term(TMT6plex); S1(Phospho); M10(Oxidation)
5031805	NP_005535.1	3667	**IRS1**	insulin receptor substrate 1	**T453**	0.8	0.8	0.7	1.0	1.1	1.1	1.0	1.2	-	-	-	-	-	-	-	-	-	-	-	-	SVTPDSLGHtPPAR	TPDSLGHtPPARGEE	T10;	Yes	S(1): 0.0; T(3): 0.0; S(6): 0.0; T(10): 100.0	3	1	1	0	N-Term(TMT6plex); T10(Phospho)
226509740	NP_067051.2	58506	**SCAF1**	splicing factor, arginine/serine-rich 19	**T976**	1.2	1.1	1.1	1.0	1.0	1.0	1.0	1.1	1.2	1.0	1.0	1.0	1.0	0.9	0.9	1.1	1.1	1.0	0.9	1.0	VPStPPPK	RAAKVPStPPPKAAP	T4;	Yes	S(3): 0.0; T(4): 100.0	29	1	1	0	N-Term(TMT6plex); T4(Phospho); K8(TMT6plex)
226509740	NP_067051.2	58506	**SCAF1**	splicing factor, arginine/serine-rich 19	**T976**	1.0	1.0	1.0	1.1	1.2	1.1	1.1	1.0	1.0	0.9	0.9	1.1	1.2	1.0	1.0	1.0	1.1	1.0	0.9	1.0	VPsTPPPK	ERAAKVPsTPPPKAA	T4;	Yes	S(3): 100.0; T(4): 0.0	4	1	1	0	N-Term(TMT6plex); S3(Phospho); K8(TMT6plex)
103472005	NP_002408.3	4288	**MKI67**	antigen KI-67 isoform 1	**S357**	0.9	0.9	0.9	1.0	1.0	1.1	1.0	1.1	1.0	0.9	1.0	1.1	1.2	1.1	1.0	1.1	1.1	1.0	1.0	1.1	TPVQYSQQQNsPQK	QYSQQQNsPQKHKNK	S11;	Yes	T(1): 0.0; Y(5): 0.0; S(6): 0.0; S(11): 100.0	118	1	1	0	N-Term(TMT6plex); S11(Phospho); K14(TMT6plex)
209969686	NP_001129671.1	57655	**GRAMD1A**	GRAM domain-containing protein 1A isoform 2	**S260**	0.9	1.4	1.2	1.1	1.1	1.1	1.1	1.0	-	-	-	-	-	-	-	-	-	-	-	-	SQEPsPVGSR	ADRSQEPsPVGSRRG	S5;	No	S(1): 0.0; S(5): 100.0; S(9): 0.0	1	2	1	0	N-Term(TMT6plex); S5(Phospho)
13491174	NP_075385.1	65108	**MARCKSL1**	MARCKS-related protein	**S22**	0.6	0.8	0.9	1.0	0.7	1.0	0.9	1.1	0.7	0.9	1.0	0.9	0.8	1.0	1.0	1.0	0.7	0.9	1.0	0.9	GDVTAEEAAGAsPAK	AEEAAGAsPAKANGQ	S12;	Yes	T(4): 0.0; S(12): 100.0	18	1	1	0	N-Term(TMT6plex); S12(Phospho); K15(TMT6plex)
296531351	NP_001171826.1	23133	**PHF8**	histone lysine demethylase PHF8 isoform 3	**S667**	1.2	1.1	1.2	1.2	0.3	0.9	0.7	0.9	-	-	-	-	-	-	-	-	-	-	-	-	SSGSSSSGLGTVSNsPASQR	GLGTVSNsPASQRTP	S15;	No	S(1): 0.0; S(2): 0.0; S(4): 0.0; S(5): 0.0; S(6): 0.0; S(7): 0.0; T(11): 0.0; S(13): 1.8; S(15): 98.2; S(18): 0.0	7	4	1	0	N-Term(TMT6plex); S15(Phospho)
47271406	NP_062552.2	56257	**MEPCE**	7SK snRNA methylphosphate capping enzyme isoform A	**S69**	0.9	1.0	1.0	1.0	1.2	1.3	1.1	1.1	1.1	1.2	1.1	1.1	1.4	1.5	1.2	1.2	1.2	1.3	1.1	1.1	EsPGAAATSSSGPQAQQHR	AAAVGREsPGAAATS	S2;	Yes	S(2): 100.0; T(8): 0.0; S(9): 0.0; S(10): 0.0; S(11): 0.0	5	1	1	0	N-Term(TMT6plex); S2(Phospho)
6912464	NP_036434.1	23266	**LPHN2**	latrophilin-2 precursor	**S1374**	0.6	0.8	0.9	1.2	0.6	0.6	0.7	0.9	0.6	0.8	1.1	0.9	0.7	0.6	0.8	0.8	0.7	0.7	0.9	0.9	GNsDGYIIPINK	YQISRGNsDGYIIPI	S3;	No	S(3): 98.8; Y(6): 1.2	1	1	1	0	N-Term(TMT6plex); S3(Phospho); K12(TMT6plex)
156415990	NP_057066.2	51087	**YBX2**	Y-box-binding protein 2	**T76**	0.6	0.9	0.8	1.0	0.6	1.2	0.9	1.1	0.5	0.8	0.7	1.2	0.4	1.1	0.8	1.2	0.5	1.0	0.7	1.2	tPGNPATAVSGtPAPPAR	PATAVSGtPAPPARS	T12;	Yes	T(1): 0.0; T(7): 0.0; S(10): 0.0; T(12): 100.0	7	1	1	0	N-Term(TMT6plex); T12(Phospho)
156415990	NP_057066.2	51087	**YBX2**	Y-box-binding protein 2	**T76**	0.6	1.2	0.9	1.1	0.6	0.9	0.8	1.0	0.4	1.1	0.8	1.2	0.5	0.8	0.7	1.2	0.5	1.0	0.7	1.2	tPGNPATAVSGTPAPPAR	PSAPGSRtPGNPATA	T12;	Yes	T(1): 100.0; T(7): 0.0; S(10): 0.0; T(12): 0.0	2	1	1	0	N-Term(TMT6plex); T1(Phospho)
24308179	NP_061174.1	55917	**CTTNBP2NL**	CTTNBP2 N-terminal-like protein	**S481**	0.7	0.8	0.7	0.5	1.2	1.0	1.2	1.6	0.7	0.7	0.6	0.5	1.2	0.9	1.1	1.5	0.9	0.8	0.9	1.0	FQSQADQDQQASGLQsPPSR	QQASGLQsPPSRDLS	S16;	Yes	S(3): 0.0; S(12): 0.0; S(16): 98.0; S(19): 1.9	1	1	1	0	N-Term(TMT6plex); S16(Phospho)
27262665	NP_757350.1	1435	**CSF1**	macrophage colony-stimulating factor 1 isoform c precursor	**S235**	1.0	1.5	1.1	1.0	1.0	0.9	0.6	1.1	-	-	-	-	-	-	-	-	-	-	-	-	ADsPLEQPEGSPLTQDDR	QEPQRADsPLEQPEG	S3;	No	S(3): 100.0; S(11): 0.0; T(14): 0.0	2	3	1	0	N-Term(TMT6plex); S3(Phospho)
12751477	NP_075555.1	668	**FOXL2**	forkhead box protein L2	**S33**	0.9	1.3	1.1	1.1	0.9	1.3	0.9	1.0	-	-	-	-	-	-	-	-	-	-	-	-	EPEGPPPsPGK	EPEGPPPsPGKGGGG	S8;	Yes	S(8): 100.0	12	1	1	0	N-Term(TMT6plex); S8(Phospho); K11(TMT6plex)
312434011	NP_001186084.1	11143	**KAT7**	histone acetyltransferase KAT7 isoform 2	**S124**	1.0	1.0	1.0	1.1	1.0	0.9	1.0	1.0	0.9	0.8	0.8	1.0	0.8	0.8	0.8	0.9	0.8	0.8	0.8	1.0	NTADHDEsPPR	NTADHDEsPPRTPTG	S8;	Yes	T(2): 0.0; S(8): 100.0	12	2	1	0	N-Term(TMT6plex); S8(Phospho)
22027646	NP_570899.1	154810	**AMOTL1**	angiomotin-like protein 1	**S809**	1.0	1.1	0.7	1.2	0.8	1.0	0.7	0.9	-	-	-	-	-	-	-	-	-	-	-	-	QTsLTSSQLAEEK	GTHSRQTsLTSSQLA	S3;	Yes	T(2): 0.9; S(3): 99.1; T(5): 0.0; S(6): 0.0; S(7): 0.0	1	1	1	0	N-Term(TMT6plex); S3(Phospho); K13(TMT6plex)
8393116	NP_059140.1	54108	**CHRAC1**	chromatin accessibility complex protein 1	**S124**	0.6	0.7	1.0	1.2	0.8	0.6	0.6	0.9	0.7	0.7	1.1	1.0	0.8	0.7	0.6	0.8	0.7	0.7	0.8	0.9	REEDEENDNDNEsDHDEADS	ENDNDNEsDHDEADS	S13;	Yes	S(13): 100.0; S(20): 0.0	1	1	1	1	N-Term(TMT6plex); S13(Phospho)
55770895	NP_001006600.1	55914	**ERBB2IP**	protein LAP2 isoform 7	**S857**	0.7	1.0	1.0	0.7	0.9	1.8	1.9	1.4	0.6	0.9	0.8	0.6	0.8	1.5	1.5	1.2	0.7	1.2	1.2	0.9	sTEDLSPQK	VDLGISKsTEDLSPQ	S6;	Yes	S(1): 99.3; T(2): 0.7; S(6): 0.0	2	6	1	0	N-Term(TMT6plex); S1(Phospho); K9(TMT6plex)
186659512	NP_056262.3	157922	**CAMSAP1**	calmodulin-regulated spectrin-associated protein 1	**S862**	1.3	1.2	1.1	1.3	0.8	1.0	0.8	0.8	1.2	1.1	1.0	1.2	0.8	0.9	0.7	0.7	1.0	1.0	0.9	1.0	EQsPSQHGK	WRQKREQsPSQHGKD	S3;	Yes	S(3): 100.0; S(5): 0.0	2	1	1	0	N-Term(TMT6plex); S3(Phospho); K9(TMT6plex)
55770895	NP_001006600.1	55914	**ERBB2IP**	protein LAP2 isoform 7	**S857**	0.9	1.8	1.9	1.4	0.7	1.0	1.0	0.7	0.8	1.5	1.5	1.2	0.6	0.9	0.8	0.6	0.7	1.2	1.2	0.9	STEDLsPQK	SKSTEDLsPQKSGPV	S6;	Yes	S(1): 0.0; T(2): 0.0; S(6): 100.0	1	6	1	0	N-Term(TMT6plex); S6(Phospho); K9(TMT6plex)
223890153	NP_001138790.1	79980	**DSN1**	kinetochore-associated protein DSN1 homolog isoform 3	**S65**	1.0	1.2	1.1	1.0	1.3	1.3	0.8	1.1	1.1	1.3	1.2	1.0	1.4	1.4	0.9	1.1	1.2	1.3	1.0	1.1	SLHLsPQEQSASYQDR	QSKSLHLsPQEQSAS	S5;	No	S(1): 0.0; S(5): 100.0; S(10): 0.0; S(12): 0.0; Y(13): 0.0	1	2	1	0	N-Term(TMT6plex); S5(Phospho)
78000213	NP_001029196.1	55929	**DMAP1**	DNA methyltransferase 1-associated protein 1	**S454**	1.3	1.2	1.1	1.0	1.3	1.3	1.0	1.1	1.4	1.4	1.3	1.1	1.4	1.4	1.2	1.2	1.4	1.4	1.2	1.1	REsASSSSSVK	NSRKRREsASSSSSV	S3;	Yes	S(3): 100.0; S(5): 0.0; S(6): 0.0; S(7): 0.0; S(8): 0.0; S(9): 0.0	7	1	1	1	N-Term(TMT6plex); S3(Phospho); K11(TMT6plex)
7019333	NP_037524.1	29959	**NRBP1**	nuclear receptor-binding protein	**T433**	1.0	1.4	1.1	1.0	1.0	1.1	0.9	1.1	1.0	1.4	1.0	1.1	0.9	1.0	0.8	1.1	1.0	1.2	0.9	1.1	TPtPEPAEVETR	PPSVKTPtPEPAEVE	T3;	Yes	T(1): 0.0; T(3): 100.0; T(11): 0.0	2	1	1	0	N-Term(TMT6plex); T3(Phospho)
17865802	NP_004860.2	9525	**VPS4B**	vacuolar protein sorting-associated protein 4B	**S102**	1.0	1.0	0.9	0.8	1.3	1.5	1.5	1.3	1.0	1.0	0.9	0.8	1.3	1.6	1.4	1.3	1.2	1.3	1.2	1.0	GNDsDGEGESDDPEK	ADEKGNDsDGEGESD	S4;	Yes	S(4): 100.0; S(10): 0.0	4	1	1	0	N-Term(TMT6plex); S4(Phospho); K15(TMT6plex)
544711150	NP_001269847.1	57148	**RALGAPB**	ral GTPase-activating protein subunit beta isoform 2	**T379**	0.9	1.1	0.7	0.9	0.8	0.9	0.8	1.2	0.9	1.2	0.8	1.0	0.8	1.0	1.0	1.2	0.8	1.1	0.9	1.1	LSMPQSAAVSTtPPHNR	QSAAVSTtPPHNRRH	T12;	Yes	S(2): 0.0; S(6): 0.0; S(10): 1.3; T(11): 1.3; T(12): 97.5	1	2	1	0	N-Term(TMT6plex); M3(Oxidation); T12(Phospho)
194328736	NP_579866.2	55274	**PHF10**	PHD finger protein 10 isoform b	**S36**	0.8	0.9	1.0	1.0	0.8	1.0	0.9	1.0	1.0	1.1	1.1	1.0	0.9	1.1	1.0	1.0	1.0	1.1	1.0	1.0	DDNEDNsNDGTQPSK	KDDNEDNsNDGTQPS	S7;	Yes	S(7): 100.0; T(11): 0.0; S(14): 0.0	6	2	1	0	N-Term(TMT6plex); S7(Phospho); K15(TMT6plex)
30795231	NP_006308.3	10409	**BASP1**	brain acid soluble protein 1	**T196**	1.1	0.8	0.9	1.1	0.8	0.9	0.8	1.0	1.2	0.8	1.0	1.3	0.8	0.9	0.8	1.2	1.0	0.8	0.9	1.3	ETPAATEAPSStPK	ATEAPSStPKAQGPA	T12;	Yes	T(2): 0.0; T(6): 0.0; S(10): 0.0; S(11): 0.0; T(12): 100.0	16	1	1	0	N-Term(TMT6plex); T12(Phospho); K14(TMT6plex)
350994425	NP_001234919.1	4253	**CTAGE5**	cTAGE family member 5 isoform 7	**S560**	1.3	1.4	1.4	1.2	1.1	1.2	1.1	0.9	1.2	1.4	1.4	1.1	1.0	1.2	1.1	0.9	1.1	1.3	1.2	1.0	LsGPAELR	CSNSGRLsGPAELRS	S2;	No	S(2): 100.0	1	8	1	0	N-Term(TMT6plex); S2(Phospho)
157168349	NP_001093254.2	54620	**FBXL19**	F-box/LRR-repeat protein 19 isoform 1	**T225**	0.9	0.8	0.8	1.0	1.0	1.0	0.9	1.1	-	-	-	-	-	-	-	-	-	-	-	-	EAGNEPPtPR	EAGNEPPtPRKKVKG	T8;	Yes	T(8): 100.0	2	1	1	0	N-Term(TMT6plex); T8(Phospho)
83641870	NP_001032827.1	4869	**NPM1**	nucleophosmin isoform 3	**S243**	0.9	0.8	1.0	1.1	0.8	0.7	1.0	1.0	0.9	0.9	0.9	1.2	0.8	0.8	1.0	1.2	0.8	0.9	1.0	1.2	GPSsVEDIK	KTPKGPSsVEDIKAK	S4;	Yes	S(3): 0.6; S(4): 99.4	15	3	1	0	N-Term(TMT6plex); S4(Phospho); K9(TMT6plex)
126362971	NP_005981.3	6793	**STK10**	serine/threonine-protein kinase 10	**T952**	0.8	1.3	1.1	1.3	1.1	1.4	1.0	0.8	0.8	1.2	1.0	1.3	1.1	1.3	0.9	0.8	1.0	1.2	1.0	1.0	LSEEAECPNPStPSK	AECPNPStPSKAAKF	T12;	Yes	S(2): 0.0; S(11): 0.0; T(12): 100.0; S(14): 0.0	11	1	1	0	N-Term(TMT6plex); C7(Carbamidomethyl); T12(Phospho); K15(TMT6plex)
546231336	NP_001271242.1	28988	**DBNL**	drebrin-like protein isoform d	**S129**	0.8	0.8	1.1	1.0	0.7	0.8	1.1	1.1	1.0	1.0	1.2	1.1	1.0	0.9	1.2	1.1	1.0	1.0	1.2	1.1	YQEQGGEAsPQR	QEQGGEAsPQRTWEQ	S9;	No	Y(1): 0.0; S(9): 100.0	4	2	1	0	N-Term(TMT6plex); S9(Phospho)
112382226	NP_001036147.1	473	**RERE**	arginine-glutamic acid dipeptide repeats protein	**S102**	1.0	1.1	0.9	1.1	1.0	0.9	1.0	1.0	-	-	-	-	-	-	-	-	-	-	-	-	VAsDTEEADR	RQREKVAsDTEEADR	S3;	No	S(3): 100.0; T(5): 0.0	9	2	1	0	N-Term(TMT6plex); S3(Phospho)
83641870	NP_001032827.1	4869	**NPM1**	nucleophosmin isoform 3	**S70**	1.0	0.9	0.7	1.1	1.7	0.8	1.2	1.0	1.0	1.0	0.7	1.3	1.6	0.8	1.1	1.1	1.3	0.9	0.9	1.2	DELHIVEAEAMNYEGsPIK	EAMNYEGsPIKVTLA	S16;	Yes	Y(13): 1.1; S(16): 98.9	15	3	1	0	N-Term(TMT6plex); M11(Oxidation); S16(Phospho); K19(TMT6plex)
8922764	NP_060740.1	55257	**MRGBP**	MRG/MORF4L-binding protein	**S195**	0.9	0.7	1.0	1.1	1.1	0.9	1.5	1.0	0.8	0.8	1.1	1.1	1.0	1.0	1.7	1.1	0.9	0.9	1.4	1.1	VLTANSNPSsPSAAK	TANSNPSsPSAAKRR	S10;	Yes	T(3): 0.0; S(6): 0.0; S(9): 0.0; S(10): 100.0; S(12): 0.0	4	1	1	0	N-Term(TMT6plex); S10(Phospho); K15(TMT6plex)
38327552	NP_938405.1	10146	**G3BP1**	ras GTPase-activating protein-binding protein 1	**S232**	0.7	0.7	0.5	1.0	1.0	1.0	0.6	1.1	0.8	0.7	0.5	1.0	1.1	1.1	0.6	1.1	1.0	0.9	0.6	1.1	SSsPAPADIAQTVQEDLR	EDAQKSSsPAPADIA	S3;	Yes	S(1): 0.0; S(2): 1.4; S(3): 98.6; T(12): 0.0	6	1	1	0	N-Term(TMT6plex); S3(Phospho)
115529449	NP_001070152.1	7343	**UBTF**	nucleolar transcription factor 1 isoform b	**S601**	0.9	1.1	0.9	1.1	0.8	1.0	0.9	1.0	1.1	1.2	1.1	1.2	0.9	1.1	1.1	1.1	1.0	1.2	1.1	1.1	SLsPQDR	DLWVKSLsPQDRAAY	S3;	No	S(1): 0.0; S(3): 100.0	2	2	1	0	N-Term(TMT6plex); S3(Phospho)
7706501	NP_057396.1	51729	**WBP11**	WW domain-binding protein 11	**S237**	0.7	0.9	1.6	1.3	0.7	1.1	0.7	0.8	0.7	0.8	1.6	1.2	0.8	0.9	0.8	0.7	0.8	0.9	1.2	0.9	DEDMLYsPELAQR	RDEDMLYsPELAQRG	S7;	Yes	Y(6): 0.0; S(7): 100.0	3	1	1	0	N-Term(TMT6plex); M4(Oxidation); S7(Phospho)
146219843	NP_006653.2	10847	**SRCAP**	helicase SRCAP	**S568**	1.5	0.8	1.0	1.0	1.0	0.9	1.0	1.1	1.5	0.7	1.0	0.9	1.0	0.8	1.0	1.0	1.2	0.7	1.0	1.0	DEEQSEADAGsGPPTPGPTTLGPK	QSEADAGsGPPTPGP	S11;	Yes	S(5): 2.3; S(11): 95.5; T(15): 2.3; T(19): 0.0; T(20): 0.0	18	1	1	0	N-Term(TMT6plex); S11(Phospho); K24(TMT6plex)
151301228	NP_060362.3	55660	**PRPF40A**	pre-mRNA-processing factor 40 homolog A	**S861**	1.0	1.0	1.0	1.0	0.9	1.0	0.9	1.1	1.0	1.0	1.0	1.0	0.9	1.0	1.0	1.1	0.9	1.0	1.0	1.1	SDSPEsDAER	HKSDSPEsDAEREKD	S6;	No	S(1): 0.0; S(3): 0.0; S(6): 100.0	7	1	1	0	N-Term(TMT6plex); S6(Phospho)
192807320	NP_001122320.1	6597	**SMARCA4**	transcription activator BRG1 isoform F	**S1349**	0.7	0.9	1.1	1.1	0.7	1.1	1.1	1.0	0.7	1.0	1.1	1.1	0.7	1.2	1.1	1.0	0.7	1.1	1.1	1.1	EVDYSDsLTEK	KEVDYSDsLTEKQWL	S7;	No	Y(4): 0.0; S(5): 0.7; S(7): 99.3; T(9): [illegible]	5	6	1	0	N-Term(TMT6plex); S7(Phospho); K11(TMT6plex)
61743954	NP_001611.1	79026	**AHNAK**	neuroblast differentiation-associated protein AHNAK isoform 1	**T3366**	1.1	1.4	1.0	1.1	0.9	1.1	0.9	1.0	1.3	1.4	1.0	1.3	1.1	1.1	1.0	1.2	1.2	1.3	1.0	1.2	VQtPEVDVK	FSGSKVQtPEVDVKG	T3;	Yes	T(3): 100.0	79	1	1	0	N-Term(TMT6plex); T3(Phospho); K9(TMT6plex)
146219843	NP_006653.2	10847	**SRCAP**	helicase SRCAP	**S568**	1.0	0.9	1.0	1.1	1.5	0.8	1.0	1.0	1.0	0.8	1.0	1.0	1.5	0.7	1.0	0.9	1.2	0.7	1.0	1.0	DEEQSEADAGSGPPtPGPTTLGPK	DAGSGPPtPGPTTLG	S11;	Yes	S(5): 0.0; S(11): 0.0; T(15): 100.0; T(19): 0.0; T(20): 0.0	3	1	1	0	N-Term(TMT6plex); T15(Phospho); K24(TMT6plex)
196162711	NP_001124499.1	8572	**PDLIM4**	PDZ and LIM domain protein 4 isoform 2	**S112**	0.6	0.6	0.9	1.2	0.8	0.9	0.8	0.9	0.6	0.5	0.8	0.8	0.7	0.7	0.7	0.6	0.7	0.6	0.8	0.7	IHIDPEIQDGsPTTSR	DPEIQDGsPTTSRRP	S11;	Yes	S(11): 97.8; T(13): 1.1; T(14): 1.1; S(15): 0.0	1	2	1	0	N-Term(TMT6plex); S11(Phospho)
151301053	NP_444271.2	155435	**RBM33**	RNA-binding protein 33	**S765**	0.8	1.0	0.9	1.0	0.8	1.2	1.0	1.1	0.8	1.1	1.1	1.0	0.8	1.4	1.2	1.2	0.8	1.2	1.1	1.1	VKPAsPVAQPK	EVKVKPAsPVAQPKE	S5;	Yes	S(5): 100.0	2	1	1	0	N-Term(TMT6plex); K2(TMT6plex); S5(Phospho); K11(TMT6plex)
7657441	NP_055706.1	11333	**PDAP1**	28 kDa heat- and acid-stable phosphoprotein	**S60; S63**	1.2	1.0	0.9	1.1	1.0	1.0	1.2	1.0	1.2	0.9	0.8	1.0	1.0	0.9	1.1	0.9	1.1	0.9	0.9	0.9	SLDsDEsEDEEDDYQQK	KEKKSLDsDESEDEE;KSLDSDEsEDEEDDY	S4; S7;	Yes;Yes	S(1): 0.0; S(4): 100.0; S(7): 100.0; Y(14): 0.0	2	1	1	0	N-Term(TMT6plex); S4(Phospho); S7(Phospho); K17(TMT6plex)
50428935	NP_060644.4	55201	**MAP1S**	microtubule-associated protein 1S	**T638; S640**	0.9	0.9	0.8	0.9	1.1	1.1	1.0	1.2	1.1	1.0	0.9	1.0	1.3	1.2	1.1	1.3	1.2	1.1	1.0	1.1	tPsPESHR	SSIPRPRtPSPESHR;IPRPRTPsPESHRSP	T1; S3;	Yes;Yes	T(1): 100.0; S(3): 100.0; S(6): 0.0	8	1	1	0	N-Term(TMT6plex); T1(Phospho); S3(Phospho)
50428935	NP_060644.4	55201	**MAP1S**	microtubule-associated protein 1S	**T638; S640**	1.1	1.1	1.0	1.2	0.9	0.9	0.8	0.9	1.3	1.2	1.1	1.3	1.1	1.0	0.9	1.0	1.2	1.1	1.0	1.1	TPsPESHR	IPRPRTPsPESHRSP	T1; S3;	Yes	T(1): 0.8; S(3): 99.2; S(6): 0.0	2	1	1	0	N-Term(TMT6plex); S3(Phospho)
307746921	NP_001182183.1	54840	**APTX**	aprataxin isoform b	**S64**	1.1	1.0	1.0	0.9	1.3	1.5	1.1	1.2	-	-	-	-	-	-	-	-	-	-	-	-	sGNSDSIER	THRKRKRsGNSDSIE	S1;	No	S(1): 100.0; S(4): 0.0; S(6): 0.0	1	6	1	0	N-Term(TMT6plex); S1(Phospho)
145386517	NP_597728.1	170954	**PPP1R18**	phostensin	**S125**	0.9	1.0	0.9	1.0	1.0	1.2	0.9	1.1	0.9	0.9	0.9	1.0	0.9	1.1	0.9	1.0	0.9	1.0	0.9	1.0	RPsPGEMR	EARERRPsPGEMRDQ	S3;	Yes	S(3): 100.0	3	1	1	0	N-Term(TMT6plex); S3(Phospho); M7(Oxidation)
34328899	NP_005392.2	5784	**PTPN14**	tyrosine-protein phosphatase non-receptor type 14	**S593**	1.1	1.3	1.3	1.0	1.1	1.1	1.3	1.1	1.2	1.3	1.1	1.0	1.2	1.1	1.1	1.0	1.2	1.2	1.1	1.0	YVSGsSPDLVTR	RHKYVSGsSPDLVTR	S5;	Yes	Y(1): 0.0; S(3): 0.0; S(5): 100.0; S(6): 0.0; T(11): 0.0	11	1	1	0	N-Term(TMT6plex); S5(Phospho)
298919179	NP_001177367.1	9611	**NCOR1**	nuclear receptor corepressor 1 isoform 2	**S728**	1.1	1.0	1.3	1.1	0.9	1.2	1.1	1.0	1.1	0.9	1.1	0.9	0.9	1.0	0.9	0.8	1.0	0.9	1.0	0.8	ADsVDVEVR	PPATKADsVDVEVRV	S3;	No	S(3): 100.0	2	3	1	0	N-Term(TMT6plex); S3(Phospho)

Khan et al., 2019. Multi-omics analysis to characterize cigarette smoke induced molecular alterations in esophageal cells

Supplementary Table 8. List of phosphopeptides identified and quantified in untreated and chronically treated Het1A cells with cigarette smoke condensate for 8 months using SequestHT and Mascot search algorithms with a PhosphoRS probability score ≥75%

Protein group Accession	NP_Accession	Gene ID	Gene Symbol	Protein Description	PhosphoSite (Protein)	Het-1A-Smoke - 2M/Parental (Replicate 1)	Het-1A-Smoke - 4M/Parental (Replicate 1)	Het-1A-Smoke - 6M/Parental (Replicate 1)	Het-1A-Smoke - 8M/Parental (Replicate 1)	Het-1A-Smoke - 2M/Parental (Replicate 2)	Het-1A-Smoke - 4M/Parental (Replicate 2)	Het-1A-Smoke - 6M/Parental (Replicate 2)	Het-1A-Smoke - 8M/Parental (Replicate 2)	Normalized phosphoprotein ratios with respect to corresponding total protein ratios. Het-1A-Smoke - 2M/Parental (Replicate 1)	Normalized Het-1A-Smoke - 4M/Parental (Replicate 1)	Normalized Het-1A-Smoke - 6M/Parental (Replicate 1)	Normalized Het-1A-Smoke - 8M/Parental (Replicate 1)	Normalized Het-1A-Smoke - 2M/Parental (Replicate 2)	Normalized Het-1A-Smoke - 4M/Parental (Replicate 2)	Normalized Het-1A-Smoke - 6M/Parental (Replicate 2)	Normalized Het-1A-Smoke - 8M/Parental (Replicate 2)	Average of replicates Het-1A-Smoke - 2M/Parental	Average Het-1A-Smoke - 4M/Parental	Average Het-1A-Smoke - 6M/Parental	Average Het-1A-Smoke - 8M/Parental	Peptide Sequence	Phosphowindow	PhosphoSite (Peptide)	Phosphosite Plus Evidence	phosphoRS Site Probabilities	# PSMs	# Proteins	# Protein Groups	# Missed Cleavages	Modifications
153791514	NP_001093149.1	22954	TRIM32	E3 ubiquitin-protein ligase TRIM32	S335	1.0	1.0	0.9	1.1	0.7	0.9	1.0	1.0	1.0	1.2	1.1	1.4	0.7	1.1	1.1	1.2	0.9	1.2	1.1	1.3	EMDMSPEEVVAsPR	SPEEVVAsPRASPAK	S12;	Yes	S(5): 0.0; S(12): 100.0	1	1	1	0	N-Term(TMT6plex); M2(Oxidation); M4(Oxidation); S12(Phospho)
42542379	NP_005830.2	10250	SRRM1	serine/arginine repetitive matrix protein 1	S478	0.6	0.9	1.0	1.1	0.6	0.8	0.9	1.0	0.8	1.0	1.1	1.2	0.7	0.8	1.0	1.2	0.8	0.9	1.1	1.2	MAAADsVQQR	GKMAAADsVQQRRQY	S6;	Yes	S(6): 100.0	229	1	1	0	N-Term(TMT6plex); M1(Oxidation); S6(Phospho)
120587019	NP_055160.2	24149	ZNF318	zinc finger protein 318	S40	0.9	1.1	0.8	1.0	0.9	1.0	0.9	1.1	0.9	1.1	0.9	1.1	0.9	1.0	1.0	1.2	0.9	1.1	0.9	1.1	RSsPPPPPSGSSSR	SGPARRSsPPPPPSG	S3;	Yes	S(2): 0.7; S(3): 99.3; S(9): 0.0; S(11): 0.0; S(12): 0.0; S(13): 0.0	9	1	1	1	N-Term(TMT6plex); S3(Phospho)
259906020	NP_001158287.1	22985	ACIN1	apoptotic chromatin condensation inducer in the nucleus isoform3	S798	1.1	1.0	1.2	0.9	2.1	0.9	0.9	1.2	0.9	1.0	1.2	0.9	1.7	0.8	1.0	1.3	1.3	0.9	1.1	1.1	GVPAGNsDTEGGQPGR	KGVPAGNsDTEGGQP	S7;	No	S(7): 100.0; T(9): 0.0	38	5	1	0	N-Term(TMT6plex); S7(Phospho)
22208971	NP_665908.1	3159	HMGA1	high mobility group protein HMG-I/HMG-Y isoforma	S36	0.6	1.0	1.1	0.9	0.8	1.0	1.0	1.2	0.6	0.8	0.9	1.0	0.9	0.8	0.9	1.2	0.7	0.8	0.9	1.1	QPPVsPGTALVGSQK	PRKQPPVsPGTALVG	S5;	Yes	S(5): 100.0; T(8): 0.0; S(13): 0.0	39	1	1	0	N-Term(TMT6plex); S5(Phospho); K15(TMT6plex)
150418007	NP_006258.3	5903	RANBP2	E3 SUMO-protein ligase RanBP2	S21	1.1	0.8	0.9	0.8	1.3	1.3	1.2	1.3	1.0	0.8	0.9	0.7	1.1	1.3	1.1	1.2	1.1	1.0	1.0	1.0	YIASVQGSTPsPR	SVQGSTPsPRQKSMK	S11;	Yes	Y(1): 0.0; S(4): 0.0; S(8): 0.0; T(9): 0.0; S(11): 100.0	17	1	1	0	N-Term(TMT6plex); S11(Phospho)
371121601	NP_001243048.1	8260	NAA10	N-alpha-acetyltransferase 10 isoform2	S171	0.8	0.7	0.9	1.1	0.7	0.7	0.9	1.0	0.8	0.7	0.9	1.1	0.7	0.7	0.9	1.0	0.7	0.7	0.9	1.0	GNsPPSSGEACR	KVESKGNsPPSSGEA	S3;	No	S(3): 100.0; S(6): 0.0; S(7): 0.0	20	3	1	0	N-Term(TMT6plex); S3(Phospho); C11(Carbamidomethyl)
331999998	NP_001193633.1	6672	SP100	nuclear autoantigen Sp-100 isoform6	S122	1.1	1.4	1.0	1.2	1.1	1.3	1.3	0.9	1.0	1.2	0.8	1.3	1.0	1.1	1.0	1.0	1.0	1.2	0.9	1.1	LPLQEsEEEEREER	DKLPLQEsEEEEREE	S6;	No	S(6): 100.0	4	6	1	1	N-Term(TMT6plex); S6(Phospho)
146219843	NP_006653.2	10847	SRCAP	helicase SRCAP	S2787	1.0	0.8	0.7	0.9	1.0	1.1	0.6	1.1	0.9	0.6	0.7	0.9	1.0	0.9	0.6	1.0	0.9	0.8	0.6	1.0	GAASTLVPGVSETSAsPGSPSVR	GVSETSAsPGSPSVR	S16;	Yes	S(4): 0.0; T(5): 0.0; S(11): 0.0; T(13): 0.0; S(14): 1.3; S(16): 98.6; S(19): 0.0; S(21): 0.0	18	1	1	0	N-Term(TMT6plex); S16(Phospho)
197313748	NP_054878.5	29072	SETD2	histone-lysine N-methyltransferase SETD2	T1872	1.0	0.7	0.8	1.0	1.0	1.1	1.0	1.1	1.1	0.8	0.9	1.2	1.2	1.2	1.1	1.4	1.1	1.0	1.0	1.3	LSTEADTDtPK	STEADTDtPKKLMFR	T9;	Yes	S(2): 0.0; T(3): 0.0; T(7): 0.0; T(9): 100.0	12	1	1	0	N-Term(TMT6plex); T9(Phospho); K11(TMT6plex)
146219843	NP_006653.2	10847	SRCAP	helicase SRCAP	S2787	1.0	1.1	0.6	1.1	1.0	0.8	0.7	0.9	1.0	0.9	0.6	1.0	0.9	0.6	0.7	0.9	0.9	0.8	0.6	1.0	GAASTLVPGVSETSASPGsPSVR	ETSASPGsPSVRSMS	S16;	Yes	S(4): 0.0; T(5): 0.0; S(11): 0.0; T(13): 0.0; S(14): 0.0; S(16): 0.0; S(19): 98.5; S(21): 1.5	4	1	1	0	N-Term(TMT6plex); S19(Phospho)
47717125	NP_001001132.1	6453	ITSN1	intersectin-1 isoformITSN-s	S986	1.1	1.1	0.9	0.9	0.9	1.1	1.1	1.2	0.9	0.9	0.8	0.8	0.8	1.0	1.0	1.0	0.8	1.0	0.9	0.9	STSMDSGSSEsPASLK	MDSGSSEsPASLKRV	S11;	Yes	S(1): 0.0; T(2): 0.0; S(3): 0.0; S(6): 0.0; S(8): 0.0; S(9): 0.0; S(11): 100.0; S(14): 0.0	8	2	1	0	N-Term(TMT6plex); M4(Oxidation); S11(Phospho); K16(TMT6plex)
31543385	NP_005008.2	5130	PCYT1A	choline-phosphate cytidylyltransferase A	S343; S347	1.0	1.0	1.0	0.8	0.9	0.9	1.0	1.2	0.8	0.8	0.8	0.8	0.8	0.7	0.9	1.1	0.8	0.8	0.9	0.9	TsPPCsPANLSR	WPFSGKTsPPCSPAN;GKTSPPCsPANLSRH	S2; S6;	Yes;Yes	T(1): 1.4; S(2): 98.6; S(6): 100.0; S(11): 0.0	2	1	1	0	N-Term(TMT6plex); S2(Phospho); C5(Carbamidomethyl); S6(Phospho)
118572613	NP_057417.3	23524	SRRM2	serine/arginine repetitive matrix protein 2	S1451	0.8	1.3	0.8	0.9	0.7	1.0	1.1	1.2	0.8	1.3	0.8	1.0	0.8	1.1	1.1	1.3	0.8	1.2	1.0	1.1	DGsGTPSRHSLSGSsPGMK	SPGLRDGsGTPSRHS;RHSLSGSsPGMKDIP	S3;	Yes;Yes	S(3): 97.2; T(5): 2.8; S(7): 0.0; S(10): 49.3; S(12): 49.3; S(14): 4.2; S(15): 97.2	1	1	1	1	N-Term(TMT6plex); S7(Phospho); S10(Phospho); S12(Phospho); M18(Oxidation); K19(TMT6plex)
118572613	NP_057417.3	23524	SRRM2	serine/arginine repetitive matrix protein 2	S1451	0.7	1.0	1.1	1.2	0.8	1.3	0.8	0.9	0.8	1.1	1.1	1.3	0.8	1.3	0.8	1.0	0.8	1.2	1.0	1.1	DGsGTPSRHSLSGSSPGMK	SPGLRDGsGTPSRHS	S3;	Yes	S(3): 97.7; T(5): 2.2; S(7): 0.1; S(10): 50.0; S(12): 50.0; S(14): 50.0; S(15): 50.0	436	1	1	1	N-Term(TMT6plex); S10(Phospho); S12(Phospho); S14(Phospho); M18(Oxidation); K19(TMT6plex)
394582099	NP_001257401.1	4172	MCM3	DNA replication licensing factor MCM3 isoform2	T684	1.1	0.9	0.9	1.0	1.0	0.8	0.9	1.1	1.1	1.1	1.1	1.2	1.1	1.0	1.1	1.3	1.1	1.0	1.1	1.3	SEDEsETEDEEEK	KKRSEDEsETEDEEE	T7;	No	S(1): 0.0; S(5): 100.0; T(7): 0.0	1	2	1	0	N-Term(TMT6plex); S5(Phospho); K13(TMT6plex)
313151226	NP_001186227.1	51720	UIMC1	BRCA1-A complex subunit RAP80	S677	0.9	1.1	1.0	1.1	0.7	1.0	0.8	1.0	1.0	1.2	1.2	1.1	0.8	1.1	0.9	1.0	0.9	1.1	1.1	1.1	DLNEsPVK	TRRDLNEsPVKSFVS	S5;	Yes	S(5): 100.0	7	1	1	0	N-Term(TMT6plex); S5(Phospho); K8(TMT6plex)
42542379	NP_005830.2	10250	SRRM1	serine/arginine repetitive matrix protein 1	S616	0.9	1.4	1.1	1.1	0.8	1.2	1.1	1.0	1.2	1.5	1.2	1.3	1.0	1.3	1.2	1.1	1.1	1.4	1.2	1.2	TAsPPPPPKR	PPKRRTAsPPPPPKR	S3;	Yes	T(1): 0.0; S(3): 100.0	229	1	1	1	N-Term(TMT6plex); S3(Phospho); K9(TMT6plex)
442796454	NP_001263201.1	8930	MBD4	methyl-CpG-binding domain protein 4 isoform4	S387	0.7	0.8	0.9	1.0	0.7	1.1	0.8	1.1	0.8	0.8	1.0	1.1	0.7	1.1	0.9	1.3	0.8	1.0	0.9	1.2	GSEMDNNCsPTR	SEMDNNCsPTRKDFT	S9;	No	S(2): 0.0; S(9): 98.5; T(11): 1.5	3	4	1	0	N-Term(TMT6plex); M4(Oxidation); C8(Carbamidomethyl); S9(Phospho)
27477138	NP_078901.3	56829	ZC3HAV1	zinc finger CCCH-type antiviral protein 1 isoform2	S284	1.7	1.6	1.8	1.1	1.2	1.1	1.2	1.0	1.7	1.6	1.7	1.1	1.2	1.1	1.2	1.0	1.4	1.4	1.5	1.1	AsLEDAPVDDLTR	DQISHRAsLEDAPVD	S2;	Yes	S(2): 100.0; T(12): 0.0	9	2	1	0	N-Term(TMT6plex); S2(Phospho)
113722133	NP_055861.3	23064	SETX	probable helicase senataxin	S2612	2.2	1.7	2.2	0.9	1.0	0.9	1.3	1.1	2.6	1.7	2.3	1.0	1.2	0.9	1.3	1.3	1.9	1.3	1.8	1.2	GEPPAAsPEASTCQSK	RGEPPAAsPEASTCQ	S7;	Yes	S(7): 100.0; S(11): 0.0; T(12): 0.0; S(15): 0.0	9	1	1	0	N-Term(TMT6plex); S7(Phospho); C13(Carbamidomethyl); K16(TMT6plex)
118498359	NP_056474.2	26156	RSL1D1	ribosomal L1 domain-containing protein 1	T415	0.5	0.5	0.5	1.0	0.6	0.6	0.9	1.1	0.6	0.6	0.6	1.1	0.7	0.7	1.0	1.2	0.7	0.7	0.8	1.2	ALPASEtPK	KALPASEtPKAAESE	T7;	Yes	S(5): 0.0; T(7): 100.0	19	1	1	0	N-Term(TMT6plex); T7(Phospho); K9(TMT6plex)
545746335	NP_001271143.1	9320	TRIP12	E3 ubiquitin-protein ligase TRIP12 isoforma	S1084	1.1	1.0	1.2	1.2	0.9	0.8	0.9	0.9	1.1	1.1	1.2	1.3	0.9	0.9	0.9	0.9	1.0	1.0	1.1	1.1	SPTTTQsPK	KSPTTTQsPKSSFLA	S7;	No	S(1): 0.0; T(3): 0.0; T(4): 0.0; T(5): 0.0; S(7): 100.0	23	4	1	0	N-Term(TMT6plex); S7(Phospho); K9(TMT6plex)
106507164	NP_001020471.2	201475	RAB12	ras-related protein Rab-12	S21	0.9	1.2	1.0	1.0	1.1	1.2	1.0	1.1	0.9	1.2	0.9	0.9	1.0	1.1	0.9	1.0	0.9	1.1	0.9	0.9	AGGGGGLGAGsPALSGGQGR	GGGLGAGsPALSGGQ	S11;	Yes	S(11): 100.0; S(15): 0.0	5	1	1	0	N-Term(TMT6plex); S11(Phospho)
21359969	NP_006459.3	10623	POLR3C	DNA-directed RNA polymerase III subunit RPC3	S205	1.0	1.1	1.1	1.0	0.8	1.1	0.9	1.0	1.0	1.1	1.1	0.9	0.8	1.0	0.9	1.0	0.9	1.1	1.0	0.9	RSsDEDAAGEPK	KGKRRRSsDEDAAGE	S3;	Yes	S(2): 0.0; S(3): 100.0	3	1	1	1	N-Term(TMT6plex); S3(Phospho); K12(TMT6plex)
13540569	NP_110418.1	81537	SGPP1	sphingosine-1-phosphate phosphatase 1	S112	1.2	0.8	1.1	1.0	1.3	1.1	1.0	1.0	1.7	0.9	1.3	1.1	1.8	1.1	1.3	1.1	1.8	1.0	1.3	1.1	NsLTGEEGQLAR	AGALRRNsLTGEEGQ	S2;	Yes	S(2): 100.0; T(4): 0.0	4	1	1	0	N-Term(TMT6plex); S2(Phospho)
63252863	NP_115820.2	84464	SLX4	structure-specific endonuclease subunit SLX4	S1469	0.7	1.0	0.9	0.8	1.7	1.2	1.0	1.2	0.5	0.9	0.8	0.7	1.4	1.1	0.9	1.0	1.0	1.0	0.8	0.9	sPGLLDTTPIR	ADSRDCRsPGLLDTT	S1;	Yes	S(1): 100.0; T(7): 0.0; T(8): 0.0	8	1	1	0	N-Term(TMT6plex); S1(Phospho)
39930523	NP_612480.1	144097	C11orf84	uncharacterized protein C11orf84	S148	1.0	0.9	0.8	0.9	1.0	0.9	1.7	1.1	1.2	1.1	1.0	1.1	1.2	1.1	2.1	1.4	1.2	1.1	1.5	1.3	AEQPsPPNsDSGQDAHPDPDANPDAAR	DVRAEQPsPPNSDSG;EQPSPPNsDSGQDAH	S5;	Yes;Yes	S(5): 100.0; S(9): 97.2; S(11): 2.8	4	1	1	0	N-Term(TMT6plex); S5(Phospho); S9(Phospho)
39930523	NP_612480.1	144097	C11orf84	uncharacterized protein C11orf84	S148	1.0	0.9	1.7	1.1	1.0	0.9	0.8	0.9	1.2	1.1	2.1	1.4	1.2	1.1	1.0	1.1	1.2	1.1	1.5	1.3	AEQPsPPNSDSGQDAHPDPDANPDAAR	DVRAEQPsPPNSDSG	S5;	Yes	S(5): 98.5; S(9): 50.7; S(11): 50.7	3	1	1	0	N-Term(TMT6plex); S5(Phospho); S9(Phospho)
208609982	NP_001129141.1	9213	XPR1	xenotropic and polytropic retrovirus receptor 1 isoform	T625	1.0	1.2	1.2	1.1	0.8	0.8	0.9	0.9	-	-	-	-	-	-	-	-	-	-	-	-	VLIEDtDDEANT	TKVLIEDtDDEANT	T6;	No	T(6): 100.0; T(12): 0.0	1	2	1	0	N-Term(TMT6plex); T6(Phospho)
117938765	NP_001070957.1	2778	GNAS	protein GNAS isoformg	S337	1.3	0.8	1.0	1.1	1.4	0.9	1.0	1.0	1.2	0.9	1.0	1.0	1.3	1.0	1.0	1.0	1.3	0.9	1.0	1.0	ISTAsGDGR	ELRISTAsGDGRHYC	S5;	No	S(2): 0.0; T(3): 0.6; S(5): 99.4	2	5	1	0	N-Term(TMT6plex); S5(Phospho)
4506921	NP_003012.1	6449	SGTA	small glutamine-rich tetratricopeptide repeat-containing protein alpha	T81	1.0	1.0	0.9	1.1	0.9	1.1	0.9	1.0	1.2	1.4	1.2	1.5	1.1	1.6	1.2	1.4	1.1	1.5	1.2	1.4	SPARtPPSEEDSAEAER	DLRSPARtPPSEEDS	T5;	Yes	S(1): 0.0; T(5): 100.0; S(8): 0.0; S(12): 0.0	28	1	1	1	N-Term(TMT6plex); T5(Phospho)
148368962	NP_079052.2	79834	PEAK1	pseudopodium-enriched atypical kinase 1	T1151	0.8	0.6	1.1	0.7	1.4	0.9	1.0	1.4	-	-	-	-	-	-	-	-	-	-	-	-	TDQEAPNASQPtPPPLPK	APNASQPtPPPLPKK	T12;	Yes	T(1): 0.0; S(9): 0.0; T(12): 100.0	6	1	1	0	N-Term(TMT6plex); T12(Phospho); K18(TMT6plex)
186928821	NP_001119523.1	3068	HDGF	hepatoma-derived growth factor isoformc	S158	1.0	1.0	1.2	1.0	1.0	1.0	1.3	1.1	1.1	0.9	1.1	0.9	1.1	1.0	1.2	1.0	1.1	0.9	1.1	1.0	AGDLLEDsPK	AGDLLEDsPKRPKEA	S8;	No	S(8): 100.0	78	3	1	0	N-Term(TMT6plex); S8(Phospho); K10(TMT6plex)
373838727	NP_001243307.1	79892	MCMBP	mini-chromosome maintenance complex-binding protein isoform2	S154	1.1	1.1	1.0	1.0	1.2	1.1	1.0	1.0	1.1	1.1	1.0	1.0	1.1	1.1	0.9	1.0	1.1	1.1	1.0	1.0	VsPSTSYTPSR	NANQARVsPSTSYTP	S2;	Yes	S(2): 100.0; S(4): 0.0; T(5): 0.0; S(6): 0.0; Y(7): 0.0; T(8): 0.0; S(10): 0.0	7	2	1	0	N-Term(TMT6plex); S2(Phospho)
55741719	NP_055621.1	9853	RUSC2	iporin	S536	2.0	1.9	3.1	1.1	0.7	0.6	1.0	1.0	-	-	-	-	-	-	-	-	-	-	-	-	LSEGPAAMAGPGsPPR	AAMAGPGsPPRRVTS	S13;	Yes	S(2): 3.6; S(13): 96.4	1	1	1	0	N-Term(TMT6plex); M8(Oxidation); S13(Phospho)
33598968	NP_005349.3	4008	LMO7	LIM domain only protein 7 isoform1	S1176	0.9	0.9	1.3	1.0	0.6	1.1	1.2	1.0	0.8	0.7	0.9	1.0	0.5	0.8	0.8	1.0	0.7	0.8	0.8	1.0	GEsLDNLDSPR	GIMRRGEsLDNLDSP	S3;	Yes	S(3): 100.0; S(9): 0.0	19	2	1	0	N-Term(TMT6plex); S3(Phospho)
33598968	NP_005349.3	4008	LMO7	LIM domain only protein 7 isoform1	S1176	0.6	1.1	1.2	1.0	0.9	0.9	1.3	1.0	0.5	0.8	0.8	1.0	0.8	0.7	0.9	1.0	0.7	0.8	0.8	1.0	GESLDNLDsPR	ESLDNLDsPRSNSWR	S3;	No	S(3): 0.0; S(9): 100.0	3	2	1	0	N-Term(TMT6plex); S9(Phospho)
557440810	NP_001273512.1	57661	PHRF1	PHD and RING finger domain-containing protein 1 isoform4	S1356	1.0	1.2	1.2	1.0	1.0	1.1	1.1	1.0	1.0	1.3	1.3	1.3	0.9	1.2	1.3	1.2	1.0	1.2	1.3	1.3	AEAPSsPDVAPAGK	EKAEAPSsPDVAPAG	S6;	No	S(5): 0.8; S(6): 99.2	15	4	1	0	N-Term(TMT6plex); S6(Phospho); K14(TMT6plex)
109715858	NP_055131.2	23589	CARHSP1	calcium-regulated heat stable protein 1	S41	1.1	1.2	0.9	1.0	1.2	1.2	1.1	1.1	1.2	1.2	0.9	1.0	1.4	1.2	1.2	1.1	1.3	1.2	1.1	1.1	GNVVPsPLPTR	LRGNVVPsPLPTRRT	S6;	Yes	S(6): 100.0; T(10): 0.0	3	1	1	0	N-Term(TMT6plex); S6(Phospho)
22212927	NP_667345.1	4799	NFX1	transcriptional repressor NF-X1 isoform3	S150	0.9	0.9	1.0	1.0	0.9	0.9	1.0	1.1	-	-	-	-	-	-	-	-	-	-	-	-	EHsPSESEK	GTDLREHsPSESEKE	S3;	Yes	S(3): 100.0; S(5): 0.0; S(7): 0.0	1	3	1	0	N-Term(TMT6plex); S3(Phospho); K9(TMT6plex)
226509740	NP_067051.2	58506	SCAF1	splicing factor, arginine/serine-rich 19	S719; S725	0.8	1.1	1.1	1.0	1.0	1.2	1.2	1.1	0.8	1.1	1.0	1.0	1.0	1.1	1.0	1.0	0.9	1.1	1.0	1.0	GPsPAPASsPKR	KSDPRGPsPAPASSP;PSPAPASsPKREVLY	S3; S9;	Yes;Yes	S(3): 100.0; S(8): 1.3; S(9): 98.7	29	1	1	1	N-Term(TMT6plex); S3(Phospho); S8(Phospho); K11(TMT6plex)
331284125	NP_056224.3	57634	EP400	E1A-binding protein p400	S700	0.9	1.3	0.9	0.9	0.9	1.4	1.0	1.2	0.9	1.1	0.9	0.8	0.9	1.2	0.9	1.0	0.9	1.2	0.9	0.9	ALsPVTSR	SATNKALsPVTSRTP	S3;	No	S(3): 100.0; T(6): 0.0; S(7): 0.0	1	1	1	0	N-Term(TMT6plex); S3(Phospho)
157041224	NP_001096630.1	400745	SH2D5	SH2 domain-containing protein 5 isoform2	S147	1.1	1.1	1.1	1.0	1.1	1.0	1.2	1.1	-	-	-	-	-	-	-	-	-	-	-	-	LGNPYCsPTLVR	RLGNPYCsPTLVRKK	S7;	No	Y(5): 1.2; S(7): 98.8; T(9): 0.0	1	2	1	0	N-Term(TMT6plex); C6(Carbamidomethyl); S7(Phospho)
20270347	NP_620152.1	129138	ANKRD54	ankyrin repeat domain-containing protein 54	S63	1.0	1.0	1.1	1.1	1.0	0.9	1.0	1.0	1.0	1.0	0.9	1.1	0.9	0.9	0.9	1.0	1.0	0.9	0.9	1.1	ASGGAQsPLR	RASGGAQsPLRYLHV	S7;	Yes	S(2): 1.0; S(7): 99.0	2	1	1	0	N-Term(TMT6plex); S7(Phospho)
117553586	NP_079216.4	80005	DOCK5	dedicator of cytokinesis protein 5	S1834	2.4	1.5	2.2	1.3	1.0	0.7	1.2	0.8	1.9	1.5	2.2	1.1	0.8	0.7	1.2	0.7	1.4	1.1	1.7	0.9	NsTELAPPLPVR	YEGSQRNsTELAPPL	S2;	Yes	S(2): 100.0; T(3): 0.0	3	1	1	0	N-Term(TMT6plex); S2(Phospho)
42542379	NP_005830.2	10250	SRRM1	serine/arginine repetitive matrix protein 1	S560; S562	0.8	1.0	1.1	1.1	0.8	1.0	1.0	1.0	0.9	1.1	1.2	1.2	0.9	1.0	1.1	1.1	0.9	1.0	1.1	1.2	RsPsPAPPPR	PPTRRRRsPSPAPPP;TRRRRSPsPAPPPRR	S2; S4;	Yes;Yes	S(2): 100.0; S(4): 100.0	229	1	1	1	N-Term(TMT6plex); S2(Phospho); S4(Phospho)
27436946	NP_733821.1	4000	LMNA	lamin isoformA	S628	1.1	1.4	0.9	1.1	0.7	1.3	1.2	0.9	1.2	1.3	1.0	1.1	0.8	1.3	1.3	1.0	1.0	1.3	1.2	1.0	sVGGSGGGSFGDNLVTR	TVTRSYRsVGGSGGG	S1;	Yes	S(1): 100.0; S(5): 0.0; S(9): 0.0; T(16): 0.0	36	3	1	0	N-Term(TMT6plex); S1(Phospho)
554790410	NP_001273033.1	1201	CLN3	battenin isoformb	S12	0.5	1.0	0.9	0.6	0.7	1.3	1.2	1.5	0.6	1.0	1.0	0.5	0.7	1.3	1.4	1.2	0.7	1.2	1.2	0.9	RFsDSEGEETVPEPR	AGSRRRFsDSEGEET	S3;	Yes	S(3): 100.0; S(5): 0.0; T(10): 0.0	1	2	1	1	N-Term(TMT6plex); S3(Phospho)
183396804	NP_056018.2	23248	RPRD2	regulation of nuclear pre-mRNA domain-containing protein 2	S758	1.1	1.2	1.2	1.0	1.1	1.2	1.2	1.1	1.1	1.2	1.1	0.9	1.1	1.1	1.2	0.9	1.1	1.2	1.1	0.9	IIsPGSSTPSSTR	SLLSKIIsPGSSTPS	S3;	Yes	S(3): 99.9; S(6): 0.0; S(7): 0.0; T(8): 0.0; S(10): 0.0; S(11): 0.0; T(12): 0.0	8	1	1	0	N-Term(TMT6plex); S3(Phospho)
18087845	NP_542399.1	113130	CDCA5	sororin	S75	1.2	0.5	0.8	1.3	0.7	0.9	0.8	0.7	1.2	0.5	0.9	1.6	0.6	1.0	0.9	0.9	0.9	0.8	0.9	1.2	IVAHAVEVPAVQsPR	VEVPAVQsPRRSPRI	S13;	Yes	S(13): 100.0	6	1	1	0	N-Term(TMT6plex); S13(Phospho)
56550039	NP_005924.2	4297	KMT2A	histone-lysine N-methyltransferase 2A isoform2 precursor	T337	1.1	1.1	1.1	1.1	0.9	1.2	1.0	0.9	1.0	1.0	1.1	1.1	0.8	1.1	1.0	0.9	0.9	1.1	1.1	1.0	DKEGtPPLTK	VRKDKEGtPPLTKED	T5;	No	T(5): 100.0; T(9): 0.0	15	2	1	1	N-Term(TMT6plex); K2(TMT6plex); T5(Phospho); K10(TMT6plex)
118572613	NP_057417.3	23524	SRRM2	serine/arginine repetitive matrix protein 2	S1404	1.1	1.0	0.9	1.1	0.8	1.1	0.8	0.9	1.2	1.0	0.9	1.2	0.8	1.2	0.8	1.0	1.0	1.1	0.9	1.1	AGMSSNQSISsPVLDAVPR	SSNQSISsPVLDAVP	S11;	Yes	S(4): 0.0; S(5): 0.0; S(8): 0.0; S(10): 1.1; S(11): 98.9	436	1	1	0	N-Term(TMT6plex); M3(Oxidation); S11(Phospho)
609876591	NP_001278340.1	6651	SON	protein SON isoformF	S1769	0.7	0.6	1.0	1.2	0.7	1.0	0.8	0.9	0.7	0.6	1.1	1.3	0.7	1.0	0.9	0.9	0.7	0.8	1.0	1.1	SAAsPVVSSMPER	GRDRSAAsPVVSSMP	S4;	Yes	S(1): 0.0; S(4): 99.9; S(8): 0.0; S(9):	17	3	1	0	N-Term(TMT6plex); S4(Phospho); M10(Oxidation)
48255933	NP_004956.5	3150	HMGN1	non-histone chromosomal protein HMG-14	S86	0.9	1.0	0.9	0.9	1.0	1.0	0.9	1.2	1.1	1.2	1.2	1.1	1.3	1.2	1.2	1.6	1.2	1.2	1.2	1.3	TEEsPASDEAGEK	GETKTEEsPASDEAG	S4;	Yes	T(1): 0.0; S(4): 100.0; S(7): 0.0	28	1	1	0	N-Term(TMT6plex); S4(Phospho); K13(TMT6plex)
50980301	NP_958431.2	23164	MPRIP	myosin phosphatase Rho-interacting protein isoform2	S326	2.8	1.6	1.4	1.2	1.7	1.3	1.1	0.9	2.7	1.6	1.4	1.2	1.7	1.3	1.1	0.9	2.2	1.5	1.2	1.0	AEHMETNAVGPsPSSDTR	ETNAVGPsPSSDTRQ	S12;	Yes	T(6): 0.0; S(12): 100.0; S(14): 0.0; S(15): 0.0; T(17): 0.0	7	2	1	0	N-Term(TMT6plex); M4(Oxidation); S12(Phospho)
48255933	NP_004956.5	3150	HMGN1	non-histone chromosomal protein HMG-14	S86	1.0	1.0	0.9	1.2	0.9	1.0	0.9	0.9	1.3	1.2	1.2	1.6	1.1	1.2	1.2	1.1	1.2	1.2	1.2	1.3	TEEsPAsDEAGEK	GETKTEEsPASDEAG;KTEESPAsDEAGEKE	S4;	Yes;Yes	T(1): 0.0; S(4): 100.0; S(7): 100.0	3	1	1	0	N-Term(TMT6plex); S4(Phospho); S7(Phospho); K13(TMT6plex)
39777586	NP_945314.1	90957	DHX57	putative ATP-dependent RNA helicase DHX57	S127	0.9	1.1	1.0	1.1	1.0	1.1	1.1	1.0	0.9	1.0	1.0	1.1	1.0	1.1	1.1	1.0	0.9	1.0	1.0	1.0	DLQEQDADAGsER	EQDADAGsERGLSGE	S11;	Yes	S(11): 100.0	10	1	1	0	N-Term(TMT6plex); S11(Phospho)
5032189	NP_005648.1	7158	TP53BP1	tumor suppressor p53-binding protein 1 isoform3	S727	1.4	1.5	1.4	0.9	1.0	1.1	1.1	1.1	1.5	1.5	1.6	1.1	1.1	1.1	1.2	1.4	1.3	1.3	1.4	1.2	ECSEAMEVETSVISIDsPQK	TSVISIDsPQKLAIL	S17;	Yes	S(3): 0.0; T(10): 0.0; S(11): 0.0; S(14): 0.0; S(17): 100.0	62	3	1	0	N-Term(TMT6plex); C2(Carbamidomethyl); M6(Oxidation); S17(Phospho); K20(TMT6plex)
126032350	NP_001075109.1	5591	PRKDC	DNA-dependent protein kinase catalytic subunit	S3205	1.7	3.0	1.6	0.5	2.3	1.7	1.3	1.5	1.5	2.8	1.4	0.5	2.1	1.6	1.1	1.4	1.8	2.2	1.2	0.9	LTPLPEDNsMNVDQDGDPSDR	TPLPEDNsMNVDQDG	S9;	Yes	T(2): 0.1; S(9): 99.9; S(19): 0.0	5	2	1	0	N-Term(TMT6plex); S9(Phospho); M10(Oxidation)
118572613	NP_057417.3	23524	SRRM2	serine/arginine repetitive matrix protein 2	S2581	1.1	1.2	1.2	1.1	1.1	1.2	1.2	1.0	1.2	1.2	1.2	1.2	1.1	1.2	1.2	1.1	1.2	1.2	1.2	1.1	RVPsPTPAPK	VALKRVPsPTPAPKE	S4;	Yes	S(4): 100.0; T(6): 0.0	436	1	1	1	N-Term(TMT6plex); S4(Phospho); K10(TMT6plex)
385862224	NP_001245350.1	84936	ZFYVE19	zinc finger FYVE domain-containing protein 19	S179	1.0	1.1	0.9	0.8	1.2	1.2	1.1	1.2	-	-	-	-	-	-	-	-	-	-	-	-	LPDsDDDEDEETAIQR	QDYRLPDsDDDEDEE	S4;	No	S(4): 100.0; T(12): 0.0	2	4	1	0	N-Term(TMT6plex); S4(Phospho)
118572613	NP_057417.3	23524	SRRM2	serine/arginine repetitive matrix protein 2	T1003	1.0	1.5	1.3	1.0	1.0	1.4	1.1	1.1	1.1	1.6	1.4	1.1	1.1	1.5	1.2	1.2	1.1	1.5	1.3	1.1	VKAQtPPGPSLSGSK	YPKVKAQtPPGPSLS	T5;	Yes	T(5): 100.0; S(10): 0.0; S(12): 0.0; S(14): 0.0	436	1	1	1	N-Term(TMT6plex); K2(TMT6plex); T5(Phospho); K15(TMT6plex)
13124765	NP_065887.1	57596	BEGAIN	brain-enriched guanylate kinase-associated protein	S502	1.1	1.1	1.0	1.1	1.2	1.2	1.0	1.0	1.1	1.0	1.0	1.1	1.2	1.1	1.0	0.9	1.1	1.1	1.0	1.0	AGGDLSLsPGR	AGGDLSLsPGRSADP	S8;	Yes	S(6): 1.1; S(8): 98.9	1	1	1	0	N-Term(TMT6plex); S8(Phospho)
32528306	NP_002904.3	5981	RFC1	replication factor C subunit 1 isoform1	S368	1.0	1.3	1.0	1.0	1.2	1.3	1.1	1.0	1.0	1.2	1.0	1.0	1.2	1.3	1.1	1.1	1.1	1.3	1.1	1.1	ESVsPEDSEK	PAKKESVsPEDSEKK	S4;	Yes	S(2): 0.0; S(4): 100.0; S(8): 0.0	12	2	1	0	N-Term(TMT6plex); S4(Phospho); K10(TMT6plex)
41350201	NP_037465.2	29924	EPN1	epsin-1 isoformc	T434	1.2	1.0	0.7	0.9	1.5	1.6	1.7	1.1	1.2	1.0	0.7	1.0	1.5	1.6	1.6	1.2	1.3	1.3	1.2	1.1	GSLAEAVGSPPPAAtPTPTPPTR	GSPPPAAtPTPTPPT	T15;	No	S(2): 0.0; S(9): 0.0; T(15): 100.0; T(17): 0.0; T(19): 0.0; T(22): 0.0	5	3	1	0	N-Term(TMT6plex); T15(Phospho)
124028529	NP_004810.2	8189	SYMPK	symplekin	S1243	0.7	0.9	0.7	0.8	1.5	1.3	1.7	1.2	0.7	1.0	0.7	0.8	1.6	1.4	1.8	1.3	1.2	1.2	1.2	1.1	sPQTLAPVGEDAMK	LTLKEERsPQTLAPV	S1;	Yes	S(1): 100.0; T(4): 0.0	4	1	1	0	N-Term(TMT6plex); S1(Phospho); M13(Oxidation); K14(TMT6plex)
555943672	NP_001273158.1	51514	DTL	denticleless protein homolog isoform2	T245	1.2	1.2	0.9	0.9	0.9	1.2	0.9	1.1	1.0	1.3	0.9	1.0	0.8	1.3	0.9	1.2	0.9	1.3	0.9	1.1	TPSSSPPItPPASETK	PSSSPPItPPASETK	T9;	No	T(1): 0.0; S(3): 0.7; S(4): 49.6; S(5): 49.6; T(9): 100.0; S(13): 0.0; T(15):	5	3	1	0	N-Term(TMT6plex); S5(Phospho); T9(Phospho); K16(TMT6plex)
545746375	NP_001271175.1	26037	SIPA1L1	signal-induced proliferation-associated 1-like protein 1 isoform3	S208	0.9	0.9	0.8	1.0	0.9	1.0	0.8	1.1	0.9	0.8	0.8	0.9	0.9	0.9	0.8	1.0	0.9	0.9	0.8	0.9	EYGsTSSIDK	SLHREYGsTSSIDKQ	S4;	Yes	Y(2): 0.0; S(4): 100.0; T(5): 0.0; S(6): 0.0; S(7): 0.0	7	4	1	0	N-Term(TMT6plex); S4(Phospho); K10(TMT6plex)
559098408	NP_001273961.1	30968	STOML2	stomatin-like protein 2, mitochondrial isoformc	T276	0.9	1.2	1.0	1.1	0.9	0.9	0.7	0.9	0.8	0.9	0.8	0.9	0.7	0.7	0.6	0.7	0.8	0.8	0.7	0.8	APVPGtPDSLSSGSSR	TKAPVPGtPDSLSSG	T6;	No	T(6): 100.0; S(9): 0.0; S(11): 0.0; S(12): 0.0; S(14): 0.0; S(15): 0.0	7	4	1	0	N-Term(TMT6plex); T6(Phospho)
16933567	NP_005361.2	4218	RAB8A	ras-related protein Rab-8A	S185	1.0	0.8	0.9	0.8	0.7	0.8	1.1	1.3	1.2	1.0	1.1	1.0	0.8	1.0	1.3	1.6	1.0	1.0	1.2	1.3	LEGNsPQGsNQGVK	DKKLEGNsPQGSNQG;EGNSPQGsNQGVKIT	S9;	Yes;Yes	S(5): 100.0; S(9): 100.0	1	1	1	0	N-Term(TMT6plex); S5(Phospho); S9(Phospho); K14(TMT6plex)
18375673	NP_002902.2	5976	UPF1	regulator of nonsense transcripts 1	S1096	1.1	1.4	1.3	1.2	0.9	1.3	1.0	0.8	1.2	1.6	1.5	1.3	1.1	1.5	1.2	0.9	1.1	1.6	1.4	1.1	SQIDVALsQDSTYQGER	SQIDVALsQDSTYQG	S8;	No	S(1): 0.0; S(8): 98.9; S(11): 50.0; T(12): 50.0; Y(13): 1.1	1	1	1	0	N-Term(TMT6plex); S8(Phospho); S11(Phospho)
16933567	NP_005361.2	4218	RAB8A	ras-related protein Rab-8A	S185	0.7	0.8	1.1	1.3	1.0	0.8	0.9	0.8	0.8	1.0	1.3	1.6	1.2	1.0	1.1	1.0	1.0	1.0	1.2	1.3	LEGNSPQGsNQGVK	EGNSPQGsNQGVKIT	S9;	Yes	S(5): 0.0; S(9): 100.0	1	1	1	0	N-Term(TMT6plex); S9(Phospho); K14(TMT6plex)
39725634	NP_056130.2	23367	LARP1	la-related protein 1	S151	1.0	1.1	1.1	1.0	1.1	1.1	1.1	1.1	1.1	1.0	1.1	1.0	1.1	1.1	1.2	1.1	1.1	1.1	1.2	1.0	SDEsGEEK	PKTKSDEsGEEKNGD	S4;	No	S(1): 0.0; S(4): 100.0	28	1	1	0	N-Term(TMT6plex); S4(Phospho); K8(TMT6plex)
116008442	NP_055885.3	23091	ZC3H13	zinc finger CCCH domain-containing protein 13	T263; S265	1.5	1.4	1.1	1.0	0.9	1.4	1.5	1.0	1.5	1.3	1.1	1.0	0.9	1.3	1.5	1.0	1.2	1.3	1.3	1.0	tPsPPPPIPEDIALGK	SKKKGPRtPSPPPPI;KKGPRTPsPPPPIPE	T1; S3;	Yes;Yes	T(1): 100.0; S(3): 100.0	28	1	1	0	N-Term(TMT6plex); T1(Phospho); S3(Phospho); K16(TMT6plex)
339639597	NP_001229885.1	989	39326	septin-7 isoform3	T192	1.5	0.9	1.4	1.1	1.3	1.2	1.9	0.9	1.4	0.9	1.4	1.1	1.3	1.2	1.8	0.9	1.4	1.0	1.6	1.0	IYEFPEtDDEEENK	KIYEFPEtDDEEENK	T7;	No	Y(2): 0.0; T(7): 100.0	1	3	1	0	N-Term(TMT6plex); T7(Phospho); K14(TMT6plex)
38348332	NP_940910.1	346689	KLRG2	killer cell lectin-like receptor subfamily G member 2	S158	1.1	0.9	0.9	0.9	1.1	0.9	1.1	1.1	-	-	-	-	-	-	-	-	-	-	-	-	VPVPEsPAFSR	LKVPVPEsPAFSRHA	S6;	Yes	S(6): 100.0; S(10): 0.0	1	1	1	0	N-Term(TMT6plex); S6(Phospho)
4504897	NP_002257.1	3838	KPNA2	importin subunit alpha-1	S62	0.8	0.7	1.1	1.2	0.2	0.3	0.7	0.8	0.9	0.8	1.2	1.3	0.2	0.3	0.7	0.9	0.6	0.6	0.9	1.1	NVSSFPDDATsPLQENR	SFPDDATsPLQENRN	S11;	Yes	S(3): 0.0; S(4): 0.0; T(10): 0.0; S(11): 100.0	8	1	1	0	N-Term(TMT6plex); S11(Phospho)
385648259	NP_001245305.1	58513	EPS15L1	epidermal growth factor receptor substrate 15-like 1 isoform4	S229	1.0	0.9	0.6	1.1	1.0	0.9	0.8	0.9	1.0	1.0	0.6	1.5	0.9	1.1	0.8	1.2	0.9	1.0	0.7	1.3	TVFPGAVPVLPAsPPPK	AVPVLPAsPPPKDSL	S13;	Yes	T(1): 0.0; S(13): 100.0	6	4	1	0	N-Term(TMT6plex); S13(Phospho); K17(TMT6plex)
429484478	NP_001258855.1	10295	BCKDK	[3-methyl-2-oxobutanoate dehydrogenase [lipoamide]] kinase, mitochondrial isoformc	S31	1.1	1.2	1.1	1.1	1.2	1.2	1.1	1.0	1.1	1.2	1.1	1.0	1.2	1.2	1.1	1.0	1.2	1.2	1.1	1.0	sTSATDTHHVEMAR	ALALRARsTSATDTH	S1;	Yes	S(1): 100.0; T(2): 0.0; S(3): 0.0; T(5): 0.0; T(7): 0.0	1	3	1	0	N-Term(TMT6plex); S1(Phospho); M12(Oxidation)
114796642	NP_001041664.1	1104	RCC1	regulator of chromosome condensation isoformc	S11	1.2	1.1	1.3	1.1	1.2	1.2	1.4	1.0	1.2	1.1	1.3	1.1	1.2	1.2	1.4	0.9	1.2	1.1	1.3	1.0	sPPADAIPK	KRIAKRRsPPADAIP	S1;	Yes	S(1): 100.0	9	3	1	0	N-Term(TMT6plex); S1(Phospho); K9(TMT6plex)
47777319	NP_060619.4	55181	SMG8	protein SMG8	S668	0.9	0.8	1.0	1.2	0.8	1.5	1.8	0.9	0.9	0.7	1.0	1.1	0.8	1.5	1.7	0.8	0.8	1.1	1.3	1.0	NESsPAPPDSDADK	APAKNESsPAPPDSD	S4;	Yes	S(3): 1.2; S(4): 98.8; S(10): 0.0	1	1	1	0	N-Term(TMT6plex); S4(Phospho); K14(TMT6plex)
21314720	NP_078976.2	79753	SNIP1	smad nuclear-interacting protein 1	S35	0.9	1.0	0.8	0.9	1.0	0.9	0.6	1.1	1.0	1.1	0.9	1.1	1.1	1.0	0.7	1.4	1.0	1.1	0.8	1.3	LsPEVAPPAHR	VVKQERLsPEVAPPA	S2;	Yes	S(2): 100.0	10	1	1	0	N-Term(TMT6plex); S2(Phospho)
19913406	NP_001058.2	7153	TOP2A	DNA topoisomerase 2-alpha	S1474	1.4	1.5	1.4	1.0	0.6	0.8	0.8	1.1	1.7	1.7	1.6	1.1	0.7	0.9	0.9	1.2	1.2	1.3	1.3	1.1	KPSTSDDsDSNFEK	KPSTSDDsDSNFEKI	S8;	Yes	S(3): 0.0; T(4): 0.0; S(5): 0.0; S(8): 100.0; S(10): 0.0	64	1	1	0	N-Term(TMT6plex); K1(TMT6plex); S8(Phospho); K14(TMT6plex)
154146191	NP_005339.3	3320	HSP90AA1	heat shock protein HSP 90-alpha isoform2	S231	0.7	0.8	0.7	1.0	0.8	1.0	0.8	1.0	0.9	0.9	0.9	1.1	0.9	1.2	1.0	1.1	0.9	1.0	1.0	1.1	EVsDDEAEEK	KERDKEVsDDEAEEK	S3;	Yes	S(3): 100.0	35	2	1	0	N-Term(TMT6plex); S3(Phospho); K10(TMT6plex)
19913406	NP_001058.2	7153	TOP2A	DNA topoisomerase 2-alpha	S1474	0.6	0.8	0.8	1.1	1.4	1.5	1.4	1.0	0.7	0.9	0.9	1.2	1.7	1.7	1.6	1.1	1.2	1.3	1.3	1.1	KPsTSDDSDSNFEK	NRRKRKPsTSDDSDS	S8;	Yes	S(3): 99.9; T(4): 0.0; S(5): 0.0; S(8): 0.0; S(10): 0.0	1	1	1	0	N-Term(TMT6plex); K1(TMT6plex); S5(Phospho); K14(TMT6plex)
21493029	NP_009131.2	11214	AKAP13	A-kinase anchor protein 13 isoform2	S983	0.5	1.2	1.3	0.9	1.0	2.3	1.8	1.1	0.5	1.3	1.4	1.1	1.1	2.5	1.9	1.4	0.8	1.9	1.6	1.2	ALQLSNsPGASSAFLK	KALQLSNsPGASSAF	S7;	Yes	S(5): 0.0; S(7): 100.0; S(11): 0.0; S(12): 0.0	12	2	1	0	N-Term(TMT6plex); S7(Phospho); K16(TMT6plex)
513788264	NP_001241661.2	23384	SPECC1L	cytospin-A isoform2	S220	0.9	1.2	1.0	1.0	1.0	1.2	1.3	1.0	0.9	1.1	0.9	1.1	1.0	1.1	1.2	1.1	1.0	1.1	1.1	1.1	AQLGINEDHsEGDEK	LGINEDHsEGDEKSE	S10;	Yes	S(10): 100.0	1	2	1	0	N-Term(TMT6plex); S10(Phospho); K15(TMT6plex)
18379332	NP_567823.1	51322	WAC	WW domain-containing adapter protein with coiled-coil isoform3	S53	1.0	1.1	1.2	1.2	0.7	0.8	1.0	0.8	0.9	1.0	1.1	1.0	0.7	0.7	0.9	0.7	0.8	0.9	1.0	0.9	DAGDPsPPNK	MRDAGDPsPPNKMLR	S6;	Yes	S(6): 100.0	5	2	1	0	N-Term(TMT6plex); S6(Phospho); K10(TMT6plex)
19923830	NP_065385.2	57326	PBXIP1	pre-B-cell leukemia transcription factor-interacting protein 1	S45	0.9	1.3	1.4	0.9	0.8	1.0	1.0	1.1	0.9	1.1	1.3	1.0	0.8	0.9	0.9	1.1	0.8	1.0	1.1	1.1	ALQAPHsPSK	RALQAPHsPSKTDGK	S9;	Yes	S(7): 99.3; S(9): 0.7	1	1	1	0	N-Term(TMT6plex); S7(Phospho); K10(TMT6plex)
307344673	NP_001182551.1	10210	TOPORS	E3 ubiquitin-protein ligase Topors isoform2	S33	1.0	1.0	1.2	1.0	1.0	1.0	1.2	1.1	-	-	-	-	-	-	-	-	-	-	-	-	LQQTVPADAsPDSK	QTVPADAsPDSKCPI	S10;	No	T(4): 0.0; S(10): 100.0; S(13): 0.0	11	2	1	0	N-Term(TMT6plex); S10(Phospho); K14(TMT6plex)
19923830	NP_065385.2	57326	PBXIP1	pre-B-cell leukemia transcription factor-interacting protein 1	S45	0.8	1.0	1.0	1.1	0.9	1.3	1.4	0.9	0.8	0.9	0.9	1.1	0.9	1.1	1.3	1.0	0.8	1.0	1.1	1.1	ALQAPHSPsK	LQAPHSPsKTDGKEL	S9;	Yes	S(7): 0.9; S(9): 99.1	1	1	1	0	N-Term(TMT6plex); S7(Phospho); K10(TMT6plex)
302699239	NP_004944.3	1981	EIF4G1	eukaryotic translation initiation factor 4 gamma 1	S1036	1.2	1.2	1.0	0.9	0.9	1.1	1.2	1.1	1.1	1.1	0.9	0.9	0.9	1.0	1.1	1.1	1.0	1.0	1.0	1.0	EAALPPVsPLK	EAALPPVsPLKAALS	S8;	No	S(8): 100.0	17	7	1	0	N-Term(TMT6plex); S8(Phospho); K11(TMT6plex)
151301053	NP_444271.2	155435	RBM33	RNA-binding protein 33	S973	0.8	1.2	0.9	1.0	1.0	1.3	1.1	1.0	0.8	1.4	1.0	1.1	1.0	1.5	1.3	1.1	0.9	1.4	1.2	1.1	TNsGGGDGPHISSK	TVTHRTNsGGGDGPH	S3;	Yes	T(1): 1.8; S(3): 98.2; S(12): 0.0; S(13): 0.0	2	1	1	0	N-Term(TMT6plex); S3(Phospho); K14(TMT6plex)
109715835	NP_071353.4	63897	HEATR6	HEAT repeat-containing protein 6	S643	0.9	0.9	0.8	1.0	1.0	0.9	0.8	1.0	0.7	0.9	0.9	1.0	0.8	0.9	0.8	0.9	0.8	0.9	0.8	1.0	APAGPSLEETSVSsPK	LEETSVSsPKGSSEP	S14;	Yes	S(6): 0.0; T(10): 0.0; S(11): 0.0; S(13): 1.1; S(14): 98.9	1	1	1	0	N-Term(TMT6plex); S14(Phospho); K16(TMT6plex)
7019371	NP_037474.1	29844	TFPT	TCF3 fusion partner	T207	0.5	0.8	0.6	0.9	2.0	1.9	0.6	1.1	0.5	0.7	0.7	0.9	2.1	1.7	0.6	1.1	1.3	1.2	0.6	1.0	AGNALtPELAPVQIK	RRAGNALtPELAPVQ	T6;	Yes	T(6): 100.0	12	1	1	0	N-Term(TMT6plex); T6(Phospho); K15(TMT6plex)
7661690	NP_056418.1	26093	CCDC9	coiled-coil domain-containing protein 9	S376; S386	1.2	1.0	0.7	0.9	1.0	1.2	0.9	1.2	1.2	1.0	0.8	0.9	1.0	1.1	1.0	1.2	1.1	1.0	0.9	1.1	EGAAsPAPETPQPTsPETSPK	ETKEGAAsPAPETPQ;PETPQPTsPETSPKE	S5; S15;	Yes;Yes	S(5): 100.0; T(10): 0.0; T(14): 0.0; S(15): 100.0; T(18): 0.0; S(19): 0.0	8	1	1	0	N-Term(TMT6plex); S5(Phospho); S15(Phospho); K21(TMT6plex)
257467639	NP_542194.2	140710	SOGA1	protein SOGA1 isoform1	S1541	0.9	0.8	1.1	0.9	2.0	0.9	0.6	1.1	1.0	0.9	1.2	0.9	2.2	0.9	0.6	1.1	1.6	0.9	0.9	1.0	APsPTSSAGEEGTK	NVCGRAPsPTSSAGE	S3;	No	S(3): 100.0; T(5): 0.0; S(6): 0.0; S(7): 0.0; T(13): 0.0	4	1	1	0	N-Term(TMT6plex); S3(Phospho); K14(TMT6plex)
144953907	NP_060164.3	54842	MFSD6	major facilitator superfamily domain-containing protein	T10	1.3	0.7	1.3	0.9	1.3	2.1	2.0	1.1	1.4	0.8	1.6	1.0	1.4	2.4	2.4	1.2	1.4	1.6	2.0	1.1	VAILtDDEEEQK	DDKVAILtDDEEEQK	T5;	Yes	T(5): 100.0	2	1	1	0	N-Term(TMT6plex); T5(Phospho); K12(TMT6plex)
149363685	NP_056140.1	23379	KIAA0947	uncharacterized protein KIAA0947	S1854	1.1	0.9	0.9	0.9	0.9	0.8	1.0	1.1	-	-	-	-	-	-	-	-	-	-	-	-	LDTGsPEPETR	RLRLDTGsPEPETRG	S5;	Yes	T(3): 1.1; S(5): 98.9; T(10): 0.0	3	1	1	0	N-Term(TMT6plex); S5(Phospho)
118572613	NP_057417.3	23524	SRRM2	serine/arginine repetitive matrix protein 2	T866	1.0	0.9	1.0	0.7	1.4	0.9	1.1	1.3	1.0	1.0	1.0	0.8	1.4	0.9	1.2	1.4	1.2	0.9	1.1	1.1	QGSITSPQANEQSVtPQRR	QANEQSVtPQRRSCF	T15;	Yes	S(3): 0.0; T(5): 50.0; S(6): 50.0; S(13): 0.0; T(15): 100.0	436	1	1	1	N-Term(TMT6plex); T5(Phospho); T15(Phospho)
42542379	NP_005830.2	10250	SRRM1	serine/arginine repetitive matrix protein 1	S696	0.9	1.1	0.9	1.0	0.9	0.9	0.9	1.1	1.1	1.1	1.0	1.1	1.1	1.0	1.0	1.2	1.1	1.1	1.0	1.1	APQTSSsPPPVR	RAPQTSSsPPPVRRG	S7;	Yes	T(4): 0.0; S(5): 0.0; S(6): 0.0; S(7): 100.0	229	1	1	0	N-Term(TMT6plex); S7(Phospho)
42542379	NP_005830.2	10250	SRRM1	serine/arginine repetitive matrix protein 1	S696	0.9	0.9	0.9	1.1	0.9	1.1	0.9	1.0	1.1	1.0	1.0	1.2	1.1	1.1	1.0	1.1	1.1	1.1	1.0	1.1	APQtSSsPPPVR	PRPRAPQtSSSPPPV;RAPQTSSsPPPVRRG	S7;	Yes;Yes	T(4): 97.9; S(5): 2.1; S(6): 4.1; S(7): 95.9	4	1	1	0	N-Term(TMT6plex); S6(Phospho); S7(Phospho)
118572613	NP_057417.3	23524	SRRM2	serine/arginine repetitive matrix protein 2	T866	1.4	0.9	1.1	1.3	1.0	0.9	1.0	0.7	1.4	0.9	1.2	1.4	1.0	1.0	1.0	0.8	1.2	0.9	1.1	1.1	QGSITSPQANEQsVtPQRR	SPQANEQsVTPQRRS;QANEQSVtPQRRSCF	T15;	Yes;Yes	S(3): 1.3; T(5): 49.4; S(6): 49.4; S(13): 100.0; T(15): 100.0	1	1	1	1	N-Term(TMT6plex); T5(Phospho); S13(Phospho); T15(Phospho)
20336746	NP_613075.1	9555	H2AFY	core histone macro-H2A.1 isoform1	T178	1.2	1.0	0.9	1.1	0.7	1.1	0.9	0.9	1.2	0.9	0.9	1.0	0.8	1.0	0.9	0.8	1.0	0.9	0.9	0.9	AASADSTTEGtPADGFTVLSTK	ADSTTEGtPADGFTV	T11;	Yes	S(3): 0.0; S(6): 0.0; T(7): 0.0; T(8): 0.0; T(11): 100.0; T(17): 0.0; S(20): 0.0; T(21): 0.0	4	3	1	0	N-Term(TMT6plex); T11(Phospho); K22(TMT6plex)
556503401	NP_001273294.1	55700	MAP7D1	MAP7 domain-containing protein 1 isoform2	S112; S116	0.7	0.6	0.7	0.8	1.4	1.8	1.7	1.2	0.7	0.5	0.6	0.8	1.4	1.5	1.6	1.1	1.0	1.0	1.1	1.0	RsSQPsPTAVPASDSPPTK	DARPPRRsSQPSPTA;PRRSSQPsPTAVPAS	S2; S6;	Yes;Yes	S(2): 98.4; S(3): 1.7; S(6): 98.3; T(8): 1.6; S(13): 0.0; S(15): 0.0; T(18): 0.0	27	3	1	1	N-Term(TMT6plex); S3(Phospho); S6(Phospho); K19(TMT6plex)
556503440	NP_001273295.1	55700	MAP7D1	MAP7 domain-containing protein 1 isoform3	S112; S116	1.4	1.8	1.7	1.2	0.7	0.6	0.7	0.8	1.4	1.5	1.6	1.1	0.7	0.5	0.6	0.8	1.0	1.0	1.1	1.0	RSsQPSPTAVPASDsPPTK	ARPPRRSsQPSPTAV	S2; S6;	Yes	S(2): 1.3; S(3): 98.7; S(6): 0.0; T(8): 0.0; S(13): 0.0; S(15): 0.0; T(18): 0.0	5	3	1	1	N-Term(TMT6plex); S2(Phospho); K19(TMT6plex)
91176325	NP_038477.2	11176	BAZ2A	bromodomain adjacent to zinc finger domain protein	S1397	1.2	1.0	0.9	1.0	0.8	0.8	1.0	1.0	1.3	1.1	0.9	1.1	0.9	0.9	1.1	1.1	1.1	1.0	1.0	1.1	AGDPGEMPQsPTGLGQPK	DPGEMPQsPTGLGQP	S10;	Yes	S(10): 100.0; T(12): 0.0	17	1	1	0	N-Term(TMT6plex); M7(Oxidation); S10(Phospho); K18(TMT6plex)
225545550	NP_057195.2	9894	TELO2	telomere length regulation protein TEL2 homolog	S836	0.8	1.0	0.7	0.9	0.5	0.4	0.9	1.1	0.8	1.0	0.6	0.9	0.5	0.4	0.8	1.1	0.6	0.7	0.7	1.0	LLPPAsP	NRLLPPAsP	S6;	Yes	S(6): 100.0	2	1	1	0	N-Term(TMT6plex); S6(Phospho)
57634536	NP_055970.1	23185	LARP4B	la-related protein 4B	S601	0.8	0.9	1.0	1.0	0.9	1.0	0.9	1.0	0.8	0.9	0.9	0.9	0.9	1.0	0.8	1.0	0.8	0.9	0.9	1.0	SPsPAHLPDDPK	ATYERSPsPAHLPDD	S3;	Yes	S(1): 0.6; S(3): 99.4	7	1	1	0	N-Term(TMT6plex); S3(Phospho); K12(TMT6plex)
19913406	NP_001058.2	7153	TOP2A	DNA topoisomerase 2-alpha	S1374	0.7	1.0	0.9	0.9	1.0	1.3	0.9	1.2	0.8	1.1	1.1	1.0	1.2	1.4	1.1	1.3	1.0	1.3	1.1	1.1	sVVSDLEADDVK	KELKPQKsVVSDLEA	S1;	Yes	S(1): 100.0; S(4): 0.0	64	1	1	0	N-Term(TMT6plex); S1(Phospho); K12(TMT6plex)
116008442	NP_055885.3	23091	ZC3H13	zinc finger CCCH domain-containing protein 13	S993	1.2	1.2	1.3	1.0	1.0	1.0	0.9	1.0	1.2	1.2	1.3	0.9	1.0	0.9	0.9	1.0	1.1	1.0	1.1	1.0	GNIETtSEDGQVFsPK	SEDGQVFsPKKGQKK	S14;	Yes	T(5): 0.0; T(6): 0.0; S(7): 0.0; S(14): 100.0	8	1	1	0	N-Term(TMT6plex); S14(Phospho); K16(TMT6plex)
431822406	NP_001258900.1	3326	HSP90AB1	heat shock protein HSP 90-beta isoformb	S207	1.0	1.0	1.1	1.0	0.9	0.9	1.0	1.0	1.1	1.0	1.1	1.0	0.9	0.9	0.9	1.0	1.0	0.9	1.0	1.0	IEDVGsDEEDDSGK	PKIEDVGsDEEDDSG	S6;	No	S(6): 100.0; S(12): 0.0	54	3	1	0	N-Term(TMT6plex); S6(Phospho); K14(TMT6plex)
116008442	NP_055885.3	23091	ZC3H13	zinc finger CCCH domain-containing protein 13	S993	1.0	1.0	0.9	1.0	1.2	1.2	1.3	1.0	1.0	0.9	0.9	1.0	1.2	1.2	1.3	0.9	1.1	1.0	1.1	1.0	GNIETTSEDGQVFsPK	SEDGQVFsPKKGQKK	S14;	Yes	T(5): 0.0; T(6): 0.0; S(7): 0.0; S(14): 100.0	28	1	1	0	N-Term(TMT6plex); S14(Phospho); K16(TMT6plex)
19913406	NP_001058.2	7153	TOP2A	DNA topoisomerase 2-alpha	S1374	1.0	1.3	0.9	1.2	0.7	1.0	0.9	0.9	1.2	1.4	1.1	1.3	0.8	1.1	1.1	1.0	1.0	1.3	1.1	1.1	SVVsDLEADDVK	KPQKSVVsDLEADDV	S1;	Yes	S(1): 0.0; S(4): 100.0	11	1	1	0	N-Term(TMT6plex); S4(Phospho); K12(TMT6plex)
13129104	NP_077003.1	79080	CCDC86	coiled-coil domain-containing protein 86	S58	1.2	1.1	1.1	1.0	0.4	1.1	0.8	1.0	1.5	1.1	1.2	1.1	0.4	1.1	0.8	1.1	1.0	1.1	1.0	1.1	AGLGsPERPPK	VQRAGLGsPERPPKT	S5;	Yes	S(5): 100.0	26	1	1	0	N-Term(TMT6plex); S5(Phospho); K11(TMT6plex)
186928821	NP_001119523.1	3068	HDGF	hepatoma-derived growth factor isoformc	S195	0.8	0.9	1.1	0.9	1.1	1.2	1.2	1.1	0.9	0.8	1.0	0.8	1.2	1.1	1.1	1.1	1.0	0.9	1.1	0.9	NSTPsEPGSGR	VEKNSTPsEPGSGRG	S5;	No	S(2): 0.0; T(3): 0.0; S(5): 100.0; S(9): 0.0	78	3	1	0	N-Term(TMT6plex); S5(Phospho)
116063573	NP_001447.2	2316	FLNA	filamin-A isoform1	S1084	1.2	1.0	0.9	0.9	0.8	0.9	0.8	1.1	1.2	1.0	1.1	1.1	0.8	0.9	0.9	1.3	1.0	1.0	1.0	1.2	AFGPGLQGGSAGsPAR	LQGGSAGsPARFTID	S13;	Yes	S(10): 0.0; S(13): 100.0	14	2	1	0	N-Term(TMT6plex); S13(Phospho)
186928821	NP_001119523.1	3068	HDGF	hepatoma-derived growth factor isoformc	S195	1.1	1.2	1.2	1.1	0.8	0.9	1.1	0.9	1.2	1.1	1.1	1.1	0.9	0.8	1.0	0.8	1.0	0.9	1.1	0.9	NSTPsEPGsGR	VEKNSTPsEPGSGRG;STPSEPGsGRGPPQE	S5;	No;No	S(2): 0.0; T(3): 0.0; S(5): 100.0; S(9): 100.0	1	3	1	0	N-Term(TMT6plex); S5(Phospho); S9(Phospho)
27735089	NP_775762.1	134353	LSM11	U7 snRNA-associated Sm-like protein LSm11	S21	1.1	0.9	0.9	0.9	1.1	0.9	1.0	1.1	0.9	1.0	0.8	0.8	0.9	1.0	0.8	1.0	0.9	1.0	0.8	0.9	SAGAGsPARPPsPR	ARSAGAGsPARPPSP;GSPARPPsPRLDVSS	S12;	Yes;Yes	S(1): 1.7; S(6): 98.3; S(12): 100.0	1	1	1	0	N-Term(TMT6plex); S6(Phospho); S12(Phospho)
153945728	NP_005900.2	4131	MAP1B	microtubule-associated protein 1B	S995	1.4	1.5	1.5	1.0	1.2	1.5	1.4	1.0	1.1	1.1	1.3	1.0	0.9	1.1	1.1	1.1	1.0	1.1	1.2	1.1	ESVAsGDDR	EKRESVAsGDDRAEE	S5;	Yes	S(2): 0.0; S(5): 100.0	49	1	1	0	N-Term(TMT6plex); S5(Phospho)
322303127	NP_001190174.1	6204	RPS10	40S ribosomal protein S10	S130	0.7	0.8	1.0	1.0	0.7	0.9	0.9	1.0	0.8	0.9	1.1	1.0	0.8	1.0	1.0	1.1	0.8	0.9	1.1	1.0	sAVPPGADK	DRDTYRRsAVPPGAD	S1;	Yes	S(1): 100.0	1	2	1	0	N-Term(TMT6plex); S1(Phospho); K9(TMT6plex)
45505147	NP_071739.2	64149	C17orf75	protein Nimu-R1	S18	3.5	1.2	1.3	1.0	2.7	0.7	1.0	1.0	3.6	1.2	1.3	1.0	2.8	0.7	1.0	1.0	3.2	0.9	1.1	1.0	ELEsSEEGGSAEER	GDEKELEsSEEGGSA	S4;	Yes	S(4): 98.8; S(5): 1.2; S(10): 0.0	3	1	1	0	N-Term(TMT6plex); S4(Phospho)
42542379	NP_005830.2	10250	SRRM1	serine/arginine repetitive matrix protein 1	S414	1.0	0.9	1.1	0.9	1.0	1.0	1.0	1.1	1.2	1.0	1.3	1.0	1.2	1.0	1.1	1.3	1.2	1.0	1.2	1.1	HsPTPQQSNR	PPPKTRHsPTPQQSN	S2;	Yes	S(2): 100.0; T(4): 0.0; S(8): 0.0	229	1	1	0	N-Term(TMT6plex); S2(Phospho)
41152072	NP_056272.2	25921	ZDHHC5	palmitoyltransferase ZDHHC5	S621	0.9	1.3	1.0	1.3	0.8	1.4	1.1	0.7	0.8	1.3	0.9	1.3	0.8	1.3	1.1	0.7	0.8	1.3	1.0	1.0	GVGsPEPGPTAPYLGR	LRGRGVGsPEPGPTA	S4;	Yes	S(4): 100.0; T(10): 0.0; Y(13): 0.0	8	1	1	0	N-Term(TMT6plex); S4(Phospho)

Khan et al., 2019. Multi-omics analysis to characterize cigarette smoke induced molecular alterations in esophageal cells
Supplementary Table 8. List of phosphopeptides identified and quantified in untreated and chronically treated Het1A cells with cigarette smoke condensate for 8 months using SequestHT and Mascot search algorithms with a PhosphoRS probability score ≥75%

														Normalized phosphoprotein ratios with respect to corresponding total protein ratios.								Average of replicates													
Protein group Accession	NP_Accession	Gene ID	Gene Symbol	Protein Description	PhosphoSite (Protein)	Het-1A-Smoke - 2M/Parental	Het-1A-Smoke - 4M/Parental	Het-1A-Smoke - 6M/Parental	Het-1A-Smoke - 8M/Parental	Het-1A-Smoke - 2M/Parental	Het-1A-Smoke - 4M/Parental	Het-1A-Smoke - 6M/Parental	Het-1A-Smoke - 8M/Parental	Het-1A-Smoke - 2M/Parental	Het-1A-Smoke - 4M/Parental	Het-1A-Smoke - 6M/Parental	Het-1A-Smoke - 8M/Parental	Het-1A-Smoke - 2M/Parental	Het-1A-Smoke - 4M/Parental	Het-1A-Smoke - 6M/Parental	Het-1A-Smoke - 8M/Parental	Het-1A-Smoke - 2M/Parental	Het-1A-Smoke - 4M/Parental	Het-1A-Smoke - 6M/Parental	Het-1A-Smoke - 8M/Parental	Peptide Sequence	Phosphowindow	PhosphoSite (Peptide)	Phosphosite Plus Evidence	phosphoRS Site Probabilities	# PSMs	# Proteins	# Protein Groups	# Missed Cleavages	Modifications
						Replicate 1				Replicate 2				Replicate 1				Replicate 2																	
167234419	NP_005110.2	9967	THRAP3	thyroid hormone receptor-associated protein 3	S55	0.8	1.0	0.8	0.8	1.0	1.2	1.1	1.2	0.8	1.0	0.9	0.9	1.1	1.3	1.2	1.3	1.0	1.1	1.0	1.1	SYsPAHNR	RSRSRSYsPAHNRER	S3;	Yes	S(1): 0.0; Y(2): 0.0; S(3): 100.0	33	1	1	0	N-Term(TMT6plex); S3(Phospho)
306922386	NP_001120983.2	324	APC	adenomatous polyposis coli protein isoform a	S2265	0.7	0.9	0.8	0.9	0.8	1.1	1.0	1.1	0.6	0.8	0.9	0.9	0.8	1.0	1.1	1.1	0.7	0.9	1.0	1.0	SELsPVAR	PSVKSELsPVARQTS	S4;	No	S(1): 0.0; S(4): 100.0	9	2	1	0	N-Term(TMT6plex); S4(Phospho)
91176325	NP_038477.2	11176	BAZ2A	bromodomain adjacent to zinc finger domain protein 2A	S1783	1.0	0.9	0.8	1.0	0.7	0.8	0.9	1.0	1.1	1.0	0.9	1.1	0.7	0.8	1.0	1.1	0.9	0.9	0.9	1.1	YSEEGLsPSK	RYSEEGLsPSKRRRL	S7;	Yes	Y(1): 0.0; S(2): 0.0; S(7): 100.0; S(9): 0.0	17	1	1	0	N-Term(TMT6plex); S7(Phospho); K10(TMT6plex)
17158023	NP_478070.1	85395	FAM207A	protein FAM207A	T34	1.0	1.0	0.9	1.0	1.0	1.5	1.1	1.0	1.2	1.2	1.1	1.1	1.1	1.7	1.3	1.2	1.1	1.4	1.2	1.1	GEAAPGPAPPAPEAtPPPASAA	APPAPEAtPPPASAA	T15;	Yes	T(15): 100.0; S(20): 0.0	4	1	1	0	N-Term(TMT6plex); T15(Phospho); K24(TMT6plex)
4505939	NP_000928.1	5430	POLR2A	DNA-directed RNA polymerase II subunit RPB1	S1882	1.8	1.0	0.8	0.9	1.2	1.0	1.0	1.1	1.9	1.1	0.9	0.9	1.2	1.0	1.0	1.1	1.5	1.1	1.0	1.0	YSPTSPTYsPTTPK	SPTSPTYsPTTPKYS	S9;	Yes	Y(1): 0.0; S(2): 0.0; T(4): 0.0; S(5): 0.0; T(7): 0.0; Y(8): 1.0; S(9): 99.0; T(11): 0.0; T(12): 0.0	13	1	1	0	N-Term(TMT6plex); S9(Phospho); K14(TMT6plex)
226509740	NP_067051.2	58506	SCAF1	splicing factor, arginine/serine-rich 19	S498; S500	0.9	1.1	1.0	1.0	1.1	1.2	1.1	1.0	0.9	1.0	0.9	1.0	1.1	1.1	1.0	1.0	1.0	1.1	0.9	1.0	QRsPsPAPAPAPAAAAGPPTR	RERYRQRsPSPAPAP;RYRQRSPsPAPAPAP	S3; S5;	Yes;Yes	S(3): 100.0; S(5): 100.0; T(20): 0.0	29	1	1	1	N-Term(TMT6plex); S3(Phospho); S5(Phospho)
4505531	NP_002547.1	5007	OSBP	oxysterol-binding protein 1	S382	1.3	1.1	1.1	1.3	1.0	0.8	0.7	0.7	1.3	1.1	1.2	1.3	1.0	0.9	0.8	0.7	1.1	1.0	1.0	1.0	TGSNIsGASSDISLDEQYK	KRTGSNIsGASSDIS	S6;	Yes	T(1): 0.0; S(3): 0.0; S(6): 100.0; S(9): 0.0; S(10): 0.0; S(13): 0.0; Y(18): 0.0	8	1	1	0	N-Term(TMT6plex); S6(Phospho); K19(TMT6plex)
45439355	NP_958845.1	6917	TCEA1	transcription elongation factor A protein 1 isoform 2	S79	0.7	0.9	0.6	0.8	0.8	1.0	1.1	1.2	0.7	0.9	0.7	0.7	0.8	1.0	1.2	1.2	0.8	0.9	0.9	1.0	EPAITSQNsPEAR	PAITSQNsPEAREES	S9;	No	T(5): 0.0; S(6): 0.0; S(9): 100.0	10	2	1	0	N-Term(TMT6plex); S9(Phospho)
4505913	NP_000526.1	5395	PMS2	mismatch repair endonuclease PMS2 isoform a	T573	1.4	0.5	1.4	0.9	1.0	0.9	2.0	1.1	1.5	0.5	1.2	0.9	1.0	0.8	1.7	1.3	1.3	0.6	1.4	1.1	VLPQPTNLAtPNTK	PQPTNLAtPNTKREK	T10;	Yes	T(6): 0.0; T(10): 100.0; T(13): 0.0	1	1	1	0	N-Term(TMT6plex); T10(Phospho); K14(TMT6plex)
546231336	NP_001271242.1	28988	DBNL	drebrin-like protein isoform d	S172	0.6	0.6	0.8	0.9	0.6	0.9	1.2	1.1	0.8	0.8	0.9	0.9	0.7	1.0	1.3	1.2	0.8	0.9	1.1	1.0	AMsTTSISSPQPGK	KQKERAMsTTSISSP	S9;	No	S(3): 98.7; T(4): 1.3; T(5): 0.0; S(6): 0.0; S(8): 0.0; S(9): 0.0	4	5	1	0	N-Term(TMT6plex); M2(Oxidation); S3(Phospho); K14(TMT6plex)
7657234	NP_055082.1	10944	C11orf58	small acidic protein	S17	1.2	1.2	1.3	1.1	0.9	1.1	0.9	0.9	1.3	1.2	1.5	1.2	1.1	1.1	1.1	1.0	1.2	1.2	1.3	1.1	SAsPDDDLGSSNWEAADLGNEER	HGVKRSAsPDDDLGS	S3;	No	S(1): 0.0; S(3): 100.0; S(10): 0.0; S(11): 0.0	5	1	1	0	N-Term(TMT6plex); S3(Phospho)
546231336	NP_001271242.1	28988	DBNL	drebrin-like protein isoform d	S172	0.6	0.9	1.2	1.1	0.6	0.6	0.8	0.9	0.7	1.0	1.3	1.2	0.8	0.8	0.9	0.9	0.8	0.9	1.1	1.0	AMSTTSISsPQPGK	MSTTSISsPQPGKLR	S9;	No	S(3): 1.7; T(4): 1.7; T(5): 1.7; S(6): 1.7; S(8): 1.7; S(9): 91.4	4	5	1	0	N-Term(TMT6plex); M2(Oxidation); S9(Phospho); K14(TMT6plex)
609876591	NP_001278340.1	6651	SON	protein SON isoform E	S283	0.7	1.0	1.0	0.9	1.0	1.1	1.0	1.1	0.8	1.0	1.1	0.9	1.0	1.2	1.1	1.2	0.9	1.1	1.1	1.0	SVESTsPEPSK	LKSVESTsPEPSKIM	S6;	Yes	S(1): 0.0; S(4): 0.0; T(5): 0.0; S(6): 100.0; S(10): 0.0	17	3	1	0	N-Term(TMT6plex); S6(Phospho); K11(TMT6plex)
380837121	NP_004883.3	9554	SEC22B	vesicle-trafficking protein SEC22b precursor	S137	0.8	0.8	0.9	1.2	0.6	0.8	0.4	0.8	0.7	0.8	0.9	1.3	0.6	0.8	0.4	0.8	0.7	0.8	0.6	1.1	NLGsINTELQDVQR	RARRNLGsINTELQD	S4;	No	S(4): 100.0; T(7): 0.0	4	1	1	0	N-Term(TMT6plex); S4(Phospho)
56550039	NP_005924.2	4297	KMT2A	histone-lysine N-methyltransferase 2A isoform 2 precursor	S2201	2.3	1.4	1.1	1.0	4.5	1.1	1.0	1.0	2.1	1.3	1.2	1.1	4.0	1.1	1.1	1.0	3.1	1.2	1.1	1.0	HSTSSLsPQR	RHSTSSLsPQRSKLR	S7;	No	S(2): 0.0; T(3): 0.0; S(4): 0.0; S(5): 0.9; S(7): 99.1	15	2	1	0	N-Term(TMT6plex); S7(Phospho)
557128994	NP_001273407.1	8663	EIF3C	eukaryotic translation initiation factor 3 subunit C isoform b	S39	0.9	1.2	1.0	1.1	1.0	1.3	1.1	0.9	0.9	1.1	0.9	1.0	1.0	1.3	1.0	0.8	0.9	1.2	1.0	0.9	QPLLLsEDEEDTK	GKQPLLLsEDEEDTK	S6;	Yes	S(6): 100.0; T(12): 0.0	16	3	1	0	N-Term(TMT6plex); S6(Phospho); K13(TMT6plex)
50659095	NP_004719.2	9188	DDX21	nucleolar RNA helicase 2 isoform 1	S89	1.1	1.1	1.6	1.0	0.7	1.1	1.3	1.0	0.9	0.9	1.2	0.9	0.6	0.9	1.0	0.8	0.8	0.9	1.1	0.8	EEPSQNDIsPK	EPSQNDIsPKTKSLR	S9;	Yes	S(4): 0.0; S(9): 100.0	23	2	1	0	N-Term(TMT6plex); S9(Phospho); K11(TMT6plex)
555290083	NP_001273086.1	1783	DYNC1LI2	cytoplasmic dynein 1 light intermediate chain 2	S117	1.4	1.1	1.7	1.2	0.9	0.9	1.2	0.8	1.4	1.1	1.5	1.1	0.9	0.9	1.1	0.8	1.1	1.0	1.3	0.9	DFQDYMEPEEGCQGsPQR	PEEGCQGsPQRRGPL	S15;	No	Y(5): 0.0; S(15): 100.0	11	2	1	0	N-Term(TMT6plex); M6(Oxidation); C12(Carbamidomethyl); S15(Phospho)
51173735	NP_001003698.1	6239	RREB1	ras-responsive element-binding protein 1 isoform 2	S1320	1.1	0.9	0.9	0.8	1.1	0.9	1.0	1.2	1.2	1.0	1.0	0.8	1.3	1.0	1.1	1.1	1.2	1.0	1.1	1.0	QVAGDAPVEQATAETAsPVHR	QATAETAsPVHREEH	S17;	Yes	T(12): 0.0; T(15): 0.0; S(17): 100.0	10	2	1	0	N-Term(TMT6plex); S17(Phospho)
118572613	NP_057417.3	23524	SRRM2	serine/arginine repetitive matrix protein 2	S2702; S2706	0.9	1.1	1.0	1.0	1.0	1.1	1.1	1.0	1.0	1.0	1.2	1.0	1.1	1.0	1.1	1.2	1.1	1.0	1.1	1.1	RPsPQPsPR	PVERRRPsPQPSPRD;RRPSPQPsPRDQQS	S3; S7;	Yes;Yes	S(3): 100.0; S(7): 100.0	436	1	1	0	N-Term(TMT6plex); S3(Phospho); S7(Phospho)
307078125	NP_001182485.1	9685	CLINT1	clathrin interactor 1 isoform 3	S209	1.2	1.0	1.1	0.9	1.1	1.3	1.2	1.1	1.2	1.1	1.1	0.9	1.1	1.3	1.3	1.1	1.1	1.2	1.2	1.0	DREDsPER	RRKDREDsPERCSDS	S5;	No	S(5): 100.0	3	3	1	1	N-Term(TMT6plex); S5(Phospho)
109948292	NP_065905.2	57614	KIAA1468	lisH domain and HEAT repeat-containing protein KIAA1468	S453	0.7	1.0	0.9	0.9	0.7	0.8	0.9	1.0	0.6	0.9	0.8	0.9	0.6	0.7	0.8	1.0	0.6	0.8	0.8	1.0	ENsPNSFPR	STIPKENsPNSFPRR	S3;	Yes	S(3): 100.0; S(6): 0.0	3	1	1	0	N-Term(TMT6plex); S3(Phospho)
7657387	NP_055331.1	4849	CNOT3	CCR4-NOT transcription complex subunit 3	S299	1.0	1.1	1.1	1.1	0.9	1.0	1.0	0.9	1.0	1.0	1.0	0.9	0.9	0.9	0.9	0.7	0.9	0.9	1.0	0.8	STDSEVSQsPAK	TDSEVSQsPAKNGSK	S9;	Yes	S(1): 0.0; T(2): 0.0; S(4): 0.0; S(7): 0.0; S(9): 100.0	4	1	1	0	N-Term(TMT6plex); S9(Phospho); K12(TMT6plex)
55956921	NP_004490.2	3182	HNRNPAB	heterogeneous nuclear ribonucleoprotein A/B isoform b	S255	0.6	0.9	0.8	1.0	0.6	1.0	0.9	1.0	0.7	0.9	1.0	1.1	0.7	1.0	1.1	1.1	0.7	1.0	1.0	1.1	GsGGGGGGGGQGSTNYGK	RNRGNRGsGGGGGGG	S2;	Yes	S(2): 100.0; S(13): 0.0; T(14): 0.0; Y(16): 0.0	10	1	1	0	N-Term(TMT6plex); S2(Phospho); K18(TMT6plex)
5032189	NP_005648.1	7158	TP53BP1	tumor suppressor p53-binding protein 1 isoform 3	S500	1.0	1.7	1.2	1.1	1.5	1.3	1.8	0.9	1.0	1.7	1.3	1.4	1.6	1.3	2.0	1.1	1.3	1.5	1.7	1.2	NsPEDLGLSLTGDSCK	SEIEPKNsPEDLGLS	S2;	Yes	S(2): 100.0; S(9): 0.0; T(11): 0.0; S(14): 0.0	62	3	1	0	N-Term(TMT6plex); S2(Phospho); C15(Carbamidomethyl); K16(TMT6plex)
19718751	NP_550433.1	7374	UNG	uracil-DNA glycosylase isoform UNG2	S64	0.9	1.0	0.9	0.7	2.0	1.5	1.4	1.3	0.9	1.0	0.9	0.7	1.9	1.5	1.4	1.3	1.4	1.2	1.1	1.0	KAPAGQEEPGTPPSsPLSAEQLDR	EPGTPPSsPLSAEQL	S15;	Yes	T(11): 50.7; S(14): 50.7; S(15): 98.7; S(18): 0.0	7	2	1	1	K1(TMT6plex); N-Term(TMT6plex); S14(Phospho); S15(Phospho)
54112117	NP_036565.2	23451	SF3B1	splicing factor 3B subunit 1 isoform 1	S242	0.8	0.9	0.9	0.9	0.9	0.9	0.9	1.1	0.8	0.9	0.9	0.9	0.9	1.0	1.0	1.1	0.9	1.0	0.9	1.0	GsETPGATPGSK	TPGRAKGsETPGATP	S2;	Yes	S(2): 99.5; T(4): 0.5; T(8): 0.0; S(11): 0.0	13	1	1	0	N-Term(TMT6plex); T4(Phospho); K12(TMT6plex)
54112117	NP_036565.2	23451	SF3B1	splicing factor 3B subunit 1 isoform 1	S242	0.9	0.9	0.9	1.1	0.8	0.9	0.9	0.9	0.9	1.0	1.0	1.1	0.8	0.9	0.9	0.9	0.9	1.0	0.9	1.0	GSEtPGAtPGSK	TPGRAKGsETPGATP	S2;	Yes	S(2): 99.5; T(4): 0.5; T(8): 0.0; S(11): 0.0	5	1	1	0	N-Term(TMT6plex); T4(Phospho); K12(TMT6plex)
341865562	NP_001230072.1	51203	NUSAP1	nucleolar and spindle-associated protein 1 isoform 5	T229	0.4	0.5	0.4	0.7	1.1	0.9	0.9	1.3	0.6	0.6	0.5	0.9	1.5	1.0	1.1	1.5	1.0	0.8	0.8	1.2	LSVAStPISQR	GRLSVAStPISQRRS	T6;	No	S(2): 0.0; S(5): 0.0; T(6): 100.0; S(9): 0.0	14	5	1	0	N-Term(TMT6plex); T6(Phospho)
118572613	NP_057417.3	23524	SRRM2	serine/arginine repetitive matrix protein 2	S1497; S1499	0.9	1.2	0.9	1.0	1.1	1.0	1.0	1.0	1.0	1.2	0.9	1.1	1.2	1.1	1.0	1.1	1.1	1.1	1.0	1.1	sRsPSSPELNNK	PQTPRPRsRSPSSPE;TPRPRSRsPSSPELN	S1; S3;	Yes;Yes	S(1): 100.0; S(3): 100.0; S(5): 50.0; S(6): 50.0	436	1	1	1	N-Term(TMT6plex); S1(Phospho); S3(Phospho); S5(Phospho); K12(TMT6plex)
30795231	NP_006308.3	10409	BASP1	brain acid soluble protein 1	T36	1.1	1.0	0.9	1.0	1.1	1.3	0.9	1.0	1.1	1.0	1.0	1.1	1.1	1.2	1.0	1.2	1.1	1.1	1.0	1.2	KAEGAATEEEGtPK	AATEEEGtPKESEPQ	T12;	Yes	T(7): 0.0; T(12): 100.0	16	1	1	1	N-Term(TMT6plex); K1(TMT6plex); T12(Phospho); K14(TMT6plex)
118572613	NP_057417.3	23524	SRRM2	serine/arginine repetitive matrix protein 2	S1497; S1499	1.1	1.0	1.0	1.0	0.9	1.2	0.9	1.0	1.2	1.1	1.0	1.1	1.0	1.2	0.9	1.1	1.1	1.1	1.0	1.1	sRSPSSPELNNK	PQTPRPRsRSPSSPE;TPRPRSRsPSSPELN	S1; S3;	Yes;Yes	S(1): 100.0; S(3): 100.0; S(5): 0.0; S(6): 0.0	3	1	1	1	N-Term(TMT6plex); S1(Phospho); S3(Phospho); K12(TMT6plex)
55956788	NP_005372.2	4691	NCL	nucleolin	S184; S206	1.2	1.7	1.2	1.2	0.9	0.9	0.8	0.8	1.5	2.0	1.5	1.6	1.2	1.1	1.1	1.1	1.3	1.6	1.3	1.4	AAAAAPAsEDEDDEDDEDDEDDDDDEEDDsEEEAMETTPAK	AAAAAPAsEDEDDED;DDDEEDDsEEEAMET	S8; S30;	Yes;Yes	S(8): 100.0; S(30): 94.5; T(37): 5.2; T(38): 0.3	65	1	1	0	N-Term(TMT6plex); S8(Phospho); S30(Phospho); M35(Oxidation); K41(TMT6plex)
259013556	NP_004851.2	9513	FXR2	fragile X mental retardation syndrome-related protein 2	S601	1.6	1.3	1.3	0.9	1.7	1.2	1.2	1.0	1.5	1.2	1.3	1.0	1.6	1.2	1.3	1.1	1.6	1.2	1.3	1.1	TDGsISGDR	RGNRTDGsISGDRQP	S4;	Yes	T(1): 0.0; S(4): 100.0; S(6): 0.0	6	1	1	0	N-Term(TMT6plex); S4(Phospho)
594140512	NP_001277241.1	1495	CTNNA1	catenin alpha-1 isoform 5	S271	0.8	0.7	0.4	0.8	1.0	1.1	0.6	1.1	0.9	0.7	0.5	0.9	1.1	1.1	0.6	1.2	1.0	0.9	0.5	1.1	TPEELDDsDFETEDFDVR	TPEELDDsDFETEDF	S8;	No	T(1): 0.0; S(8): 97.9; T(12): 2.1	2	5	1	0	N-Term(TMT6plex); S8(Phospho)
215599015	NP_001135935.1	1736	DKC1	H/ACA ribonucleoprotein complex subunit 4 isoform 2	S508	1.2	1.4	1.1	1.1	1.1	1.4	0.8	0.9	1.2	1.5	1.1	1.1	1.2	1.5	0.8	0.9	1.2	1.5	0.9	1.0	EVELVsE	AKEVELVsE	S6;	No	S(6): 100.0	22	2	1	0	N-Term(TMT6plex); S6(Phospho)
118601081	NP_001073027.1	221092	HNRNPUL2	heterogeneous nuclear ribonucleoprotein U-like	S161	1.2	1.8	2.0	1.2	0.8	1.0	1.1	0.8	1.3	1.7	2.0	1.2	0.8	0.9	1.1	0.8	1.0	1.3	1.6	1.0	EEDEPEERsGDEtPGSEVPGDK	EDEPEERsGDEtPGS	S9;	Yes	S(9): 99.9; T(13): 0.1; S(16): 0.0	33	1	1	1	N-Term(TMT6plex); S9(Phospho); K22(TMT6plex)
255918139	NP_001157616.1	283489	CHAMP1	chromosome alignment-maintaining phosphoprotein 1	S675	0.9	0.9	1.1	0.8	0.7	1.1	1.5	1.2	0.9	0.9	1.1	0.8	0.8	1.1	1.4	1.3	0.8	1.0	1.3	1.1	MDMTsPEQSR	ENKMDMTsPEQSRNV	S5;	Yes	T(4): 0.9; S(5): 99.1; S(9): 0.0	24	1	1	0	N-Term(TMT6plex); M1(Oxidation); M3(Oxidation); S5(Phospho)
75677351	NP_071936.2	64426	SUDS3	sin3 histone deacetylase corepressor complex component SDS3	S234	1.0	1.4	1.1	0.9	1.3	1.0	0.8	1.1	1.1	1.5	1.2	0.8	1.5	1.1	0.9	1.0	1.3	1.3	1.1	0.9	RPAsPSSPEHLPATPAESPAQR	KSPKRPAsPSSPEHL	S4;	Yes	S(4): 98.1; S(6): 51.0; S(7): 51.0; T(14): 0.0; S(18): 0.0	1	1	1	0	N-Term(TMT6plex); S4(Phospho); S6(Phospho)
153945728	NP_005900.2	4131	MAP1B	microtubule-associated protein 1B	S1378	1.0	1.3	1.4	1.1	0.9	1.1	1.1	0.9	0.8	1.0	1.1	1.2	0.7	0.8	0.9	0.9	0.7	0.9	1.0	1.1	ASVsPMDEPVPDSESPIEK	ENERASVsPMDEPVP	S4;	Yes	S(2): 0.9; S(4): 99.1; S(13): 0.0; S(15): 0.0	49	1	1	0	N-Term(TMT6plex); S4(Phospho); M6(Oxidation); K19(TMT6plex)
134304846	NP_001077086.1	9821	RB1CC1	RB1-inducible coiled-coil protein 1 isoform 2	S237	1.0	1.1	1.1	0.8	0.9	1.2	1.0	1.2	1.1	1.2	1.1	0.8	1.0	1.4	0.9	1.1	1.0	1.3	1.0	0.9	sTELVLSPDMPR	EKAEMKRsTELVLSP	S1;	Yes	S(1): 100.0; T(2): 0.0; S(7): 0.0	3	2	1	0	N-Term(TMT6plex); S1(Phospho); M10(Oxidation)
54607124	NP_037418.3	29890	RBM15B	putative RNA-binding protein 15B	S609	1.0	1.0	0.8	1.1	0.8	0.9	0.8	0.9	1.0	1.1	0.8	1.1	0.9	1.0	0.9	1.0	0.9	1.1	0.9	1.0	TTHsPYEER	DRGRTTHsPYEERSR	S4;	Yes	T(1): 0.0; T(2): 0.0; S(4): 100.0; Y(6): 0.0	4	1	1	0	N-Term(TMT6plex); S4(Phospho)
57863269	NP_055871.1	23076	RRP1B	ribosomal RNA processing protein 1 homolog B	S513	0.8	1.0	0.9	1.0	0.7	0.8	0.9	0.9	0.8	1.0	1.0	1.0	0.8	0.9	0.9	0.9	0.8	0.9	1.0	0.9	GsPTGGAQLLK	HPQGPRGsPTGGAQL	S2;	Yes	S(2): 100.0; T(4): 0.0	17	1	1	0	N-Term(TMT6plex); S2(Phospho); K11(TMT6plex)
454544715	NP_001263626.1	7157	TP53	cellular tumor antigen p53 isoform i	S156	1.2	1.2	1.0	1.0	1.2	1.2	1.0	1.0	1.2	1.1	0.9	1.0	1.2	1.2	0.9	1.1	1.2	1.2	0.9	1.0	ALPNNTSSsPQPK	LPNNTSSsPQPKKKP	S9;	No	T(6): 0.7; S(7): 0.7; S(8): 0.7; S(9): [illegible]	27	12	1	0	N-Term(TMT6plex); S9(Phospho); K13(TMT6plex)
56549656	NP_001008240.1	147339	C18orf25	uncharacterized protein C18orf25 isoform b	S145	1.4	2.1	1.0	1.0	1.4	1.8	0.9	1.0	1.3	2.0	1.0	1.1	1.2	1.7	0.9	1.1	1.3	1.9	0.9	1.1	SRsESETSTMAAK	RKSRRSRsESETSTM	S3;	Yes	S(1): 0.0; S(3): 100.0; S(5): 0.0; T(7): 0.0; S(8): 0.0; T(9): 0.0	1	2	1	1	N-Term(TMT6plex); S3(Phospho); M10(Oxidation); K13(TMT6plex)
27436946	NP_733821.1	4000	LMNA	lamin isoform A	S392	0.7	1.1	0.8	1.1	0.8	1.3	0.8	0.9	0.7	1.0	0.9	1.1	0.9	1.2	0.9	0.9	0.8	1.1	0.9	1.0	LSPsPTSQR	ERLRLSPsPTSQRSR	S4;	Yes	S(2): 0.0; S(4): 100.0; T(6): 0.0; S(7): 0.0	36	6	1	0	N-Term(TMT6plex); S4(Phospho)
157817073	NP_055898.1	51755	CDK12	cyclin-dependent kinase 12 isoform 2	T692	0.9	1.0	1.2	1.0	0.9	1.1	1.1	0.9	1.0	1.0	1.2	1.1	0.9	1.2	1.0	1.0	1.0	1.1	1.1	1.1	AItPPQQPYK	SPEPKAItPPQQPYK	T3;	Yes	T(3): 100.0; Y(9): 0.0	10	2	1	0	N-Term(TMT6plex); T3(Phospho); K10(TMT6plex)
31563338	NP_004505.2	3607	FOXK2	forkhead box protein K2	S170	0.9	1.1	0.9	0.6	0.9	1.0	0.8	1.4	0.8	1.1	0.9	0.6	0.8	1.0	0.8	1.5	0.8	1.1	0.8	1.0	EKQEASEsPVK	EKQEASEsPVKAVQP	S8;	Yes	S(6): 0.0; S(8): 100.0	6	1	1	1	N-Term(TMT6plex); K2(TMT6plex); S8(Phospho); K11(TMT6plex)
554790401	NP_001273063.1	60626	RIC8A	synembryn-A isoform 2	S502	1.0	1.0	1.2	1.0	0.9	1.0	1.0	1.0	0.9	1.0	1.1	1.0	0.7	0.9	0.9	1.0	0.8	1.0	1.0	1.0	VIQPMGMsPR	VIQPMGMsPRGHLTS	S8;	Yes	S(8): 100.0	1	2	1	0	N-Term(TMT6plex); M5(Oxidation); M7(Oxidation); S8(Phospho)
256222415	NP_001157791.1	2317	FLNB	filamin-B isoform 4	S2454	1.3	0.9	1.8	1.1	0.7	1.8	0.6	0.8	1.2	0.7	1.5	1.2	0.7	1.4	0.5	0.9	0.9	1.0	1.0	1.0	LVsPGSANETSSILVESVTR	VTGQRLVsPGSANET	S3;	No	S(3): 100.0; S(6): 0.0; T(10): 0.0; S(11): 0.0; S(12): 0.0; S(17): 0.0;	6	4	1	0	N-Term(TMT6plex); S3(Phospho)
255652953	NP_653205.3	124245	ZC3H18	zinc finger CCCH domain-containing protein 18	T796	1.4	1.3	1.2	1.2	1.2	0.7	1.0	0.8	1.7	1.4	1.5	1.3	1.5	0.7	1.1	0.8	1.6	1.0	1.3	1.1	SSQQPStPQQAPPGQPQQGTFVAHK	KSSQQPStPQQAPPG	T7;	Yes	S(1): 0.0; S(2): 0.0; S(6): 1.9; T(7): 98.1; T(20): 0.0	34	1	1	0	N-Term(TMT6plex); T7(Phospho); K25(TMT6plex)
7662340	NP_055756.1	22880	MORC2	MORC family CW-type zinc finger protein 2	S553	1.0	1.0	1.2	0.9	1.3	1.8	1.9	1.1	0.8	0.9	1.1	0.8	1.0	1.6	1.7	1.0	0.9	1.2	1.4	0.9	sPPLPAVIR	RRPQRPRsPPLPAVI	S1;	No	S(1): 100.0	2	1	1	0	N-Term(TMT6plex); S1(Phospho)
211971074	NP_001130034.1	11217	AKAP2	A-kinase anchor protein 2 isoform 2	S393	0.4	1.2	1.0	0.9	0.7	0.6	0.3	1.1	-	-	-	-	-	-	-	-	-	-	-	-	GQKsPGALETPSAAGSQGNTASQGK	SAAKGQKsPGALETP	S4;	Yes	S(4): 100.0; T(10): 0.0; S(12): 0.0; S(16): 0.0; T(20): 0.0; S(22): 0.0	6	5	1	1	N-Term(TMT6plex); K3(TMT6plex); S4(Phospho); K25(TMT6plex)
197313748	NP_054878.5	29072	SETD2	histone-lysine N-methyltransferase SETD2	S614	1.1	0.9	0.9	1.0	0.8	0.8	0.8	0.9	1.3	1.0	1.0	1.3	0.9	0.9	0.9	1.1	1.1	1.0	1.0	1.2	AGsPAPSNR	PEREKAGsPAPSNRL	S3;	Yes	S(3): 100.0; S(7): 0.0	12	1	1	0	N-Term(TMT6plex); S3(Phospho)
194239729	NP_001123528.1	1936	EEF1D	elongation factor 1-delta isoform 4	S109	0.6	0.6	0.7	1.1	0.7	0.7	0.7	0.9	0.8	0.9	0.9	1.2	0.8	0.9	0.9	0.9	0.8	0.9	0.9	1.1	ATAPQTQHVsPMR	APQTQHVsPMRQVEP	S10;	No	T(2): 0.0; T(6): 0.0; S(10): 100.0	18	4	1	0	N-Term(TMT6plex); S10(Phospho); M12(Oxidation)
33695090	NP_059128.2	53635	PTOV1	prostate tumor-overexpressed gene 1 protein	S53	0.9	1.0	0.9	1.0	0.8	1.1	0.9	1.0	-	-	-	-	-	-	-	-	-	-	-	-	sAPPMEGAR	PPRIRARsAPPMEGA	S1;	Yes	S(1): 100.0	1	1	1	0	N-Term(TMT6plex); S1(Phospho); M5(Oxidation)
118572613	NP_057417.3	23524	SRRM2	serine/arginine repetitive matrix protein 2	S778; S780	1.0	1.0	1.2	0.8	1.0	1.2	1.3	1.1	1.0	1.1	1.3	0.9	1.0	1.2	1.3	1.2	1.0	1.1	1.3	1.1	RsLsGSSPCPK	SRLSLRRsLSGSSPC;LSLRRSLsGSSPCPK	S2; S4;	Yes;Yes	S(2): 100.0; S(4): 100.0; S(6): 0.0; S(7): 0.0	436	1	1	1	N-Term(TMT6plex); S2(Phospho); S4(Phospho); C9(Carbamidomethyl); K11(TMT6plex)
167234419	NP_005110.2	9967	THRAP3	thyroid hormone receptor-associated protein 3	S315	1.2	1.0	0.9	0.9	1.4	1.1	0.9	1.0	1.2	1.1	1.0	1.0	1.5	1.1	1.0	1.1	1.4	1.1	1.0	1.1	SPVGKsPPSTGSTYGSSQK	KKSPVGKsPPSTGST	S1;	Yes	S(1): 1.2; S(6): 98.8; S(9): 0.0; T(10): 0.0; S(12): 0.0; T(13): 0.0; Y(14): 0.0; S(16): 0.0; S(17): 0.0	5	1	1	1	N-Term(TMT6plex); K5(TMT6plex); S6(Phospho); K19(TMT6plex)
118601081	NP_001073027.1	221092	HNRNPUL2	heterogeneous nuclear ribonucleoprotein U-like	S185	1.0	1.2	1.4	1.0	1.0	1.0	1.2	1.0	1.0	1.1	1.4	1.0	1.0	0.9	1.1	1.0	1.0	1.0	1.3	1.0	AAEEQGDDQDsEK	EQGDDQDsEKSKPAG	S11;	Yes	S(11): 100.0	33	1	1	0	N-Term(TMT6plex); S11(Phospho); K13(TMT6plex)
167234419	NP_005110.2	9967	THRAP3	thyroid hormone receptor-associated protein 3	S315	1.4	1.1	0.9	1.0	1.2	1.0	0.9	0.9	1.5	1.1	1.0	1.1	1.2	1.1	1.0	1.0	1.4	1.1	1.0	1.1	sPVGKSPPSTGSTYGSSQK	SLSPSKKsPVGKSPP	S1;	Yes	S(1): 98.5; S(6): 1.5; S(9): 0.0; T(10): 0.0; S(12): 0.0; T(13): 0.0; Y(14): 0.0; S(16): 0.0; S(17): 0.0	33	1	1	1	N-Term(TMT6plex); K5(TMT6plex); S6(Phospho); K19(TMT6plex)
167234419	NP_005110.2	9967	THRAP3	thyroid hormone receptor-associated protein 3	S939	1.1	1.3	1.2	1.1	1.2	0.8	0.8	0.9	1.2	1.4	1.3	1.2	1.2	0.9	0.9	1.0	1.2	1.1	1.1	1.1	FSGEEGEIEDDEsGTENR	GEIEDDEsGTENREE	S13;	Yes	S(2): 0.0; S(13): 99.0; T(15): 1.0	33	1	1	0	N-Term(TMT6plex); S13(Phospho)
156766047	NP_001096618.1	26058	GIGYF2	PERQ amino acid-rich with GYF domain-containing protein 2 isoform c	S269	1.4	1.0	0.9	0.9	1.8	1.1	1.1	1.1	1.4	0.9	0.8	0.8	1.8	1.1	1.0	1.0	1.6	1.0	0.9	0.9	SGsGSIDDDR	YRRVRSGsGSIDDDR	S3;	No	S(1): 0.0; S(3): 100.0; S(5): 0.0	9	3	1	0	N-Term(TMT6plex); S3(Phospho)
27436946	NP_733821.1	4000	LMNA	lamin isoform A	S414	0.8	1.1	0.7	0.9	0.8	0.9	0.8	1.1	0.9	1.1	0.8	0.9	0.9	0.8	0.9	1.1	0.9	0.9	0.9	1.0	ASSHSSQTQGGGsVTK	SQTQGGGsVTKKRKL	S13;	Yes	S(2): 0.0; S(3): 0.0; S(5): 0.0; S(6): 0.0; T(8): 0.0; S(13): 100.0; T(15): 0.0	36	6	1	0	N-Term(TMT6plex); S13(Phospho); K16(TMT6plex)
27436946	NP_733821.1	4000	LMNA	lamin isoform A	S414	0.8	0.9	0.8	1.1	0.8	1.1	0.7	0.9	0.9	0.8	0.9	1.1	0.9	1.1	0.8	0.9	0.9	0.9	0.9	1.0	ASSHSsQTQGGGSVTK	SQTQGGGsVTKKRKL	S13;	Yes	S(2): 0.0; S(3): 0.0; S(5): 0.0; S(6): 0.0; T(8): 0.0; S(13): 100.0; T(15): 0.0	12	6	1	0	N-Term(TMT6plex); S13(Phospho); K16(TMT6plex)
28559039	NP_004765.2	5469	MED1	mediator of RNA polymerase II transcription subunit 1	T805	0.7	0.7	0.5	0.8	1.4	0.9	1.3	1.1	0.8	0.7	0.6	0.8	1.5	0.9	1.5	1.2	1.1	0.8	1.0	1.0	LPSTSDDCPAIGtPLR	DDCPAIGtPLRDSSS	T13;	Yes	S(3): 0.0; T(4): 0.0; S(5): 0.0; T(13): 100.0	16	1	1	0	N-Term(TMT6plex); C8(Carbamidomethyl); T13(Phospho)
37574606	NP_932072.1	7750	ZMYM2	zinc finger MYM-type protein 2	S838	0.9	0.8	1.0	0.9	1.0	1.1	1.1	1.1	0.9	0.9	1.0	0.9	0.9	1.2	1.1	1.1	0.9	1.0	1.1	1.0	MTGSAPPPsPTPNK	TGSAPPPsPTPNKEM	S9;	Yes	T(2): 0.0; S(4): 0.0; S(9): 100.0; T(11): 0.0	4	1	1	0	N-Term(TMT6plex); M1(Oxidation); S9(Phospho); K14(TMT6plex)
190014588	NP_001121689.1	11168	PSIP1	PC4 and SFRS1-interacting protein isoform 2	S106	1.3	1.5	1.0	[illegible]	0.8	1.1	0.8	0.9	1.6	1.6	1.3	1.2	1.0	1.3	1.0	1.1	1.3	1.4	1.1	1.1	QSNASsDVEVEEKETSVSK	TKQSNASsDVEVEEK	S6;	Yes	S(2): 0.0; S(5): 1.4; S(6): 98.6; T(15): 0.0; S(16): 0.0; S(18): 0.0	27	2	1	1	N-Term(TMT6plex); S6(Phospho); K13(TMT6plex); K19(TMT6plex)
56118310	NP_073568.2	64710	NUCKS1	nuclear ubiquitous casein and cyclin-dependent kinase substrate 1	S214	1.7	1.3	1.1	1.1	0.8	0.9	1.0	0.9	2.1	1.5	1.5	1.4	1.1	1.1	1.4	1.1	1.6	1.3	1.4	1.3	EEDEEPEsPPEK	EEDEEPEsPPEKKTS	S8;	Yes	S(8): 100.0	109	1	1	0	N-Term(TMT6plex); S8(Phospho); K12(TMT6plex)
326319990	NP_055820.2	283638	CEP170B	centrosomal protein of 170 kDa protein B isoform 2	S641	0.7	0.8	0.6	1.0	0.7	0.8	0.6	0.9	-	-	-	-	-	-	-	-	-	-	-	-	ADsPAGPESSR	LPSERADsPAGPESS	S3;	No	S(3): 100.0; S(9): 0.0; S(10): 0.0	5	2	1	0	N-Term(TMT6plex); S3(Phospho)
557440899	NP_001273490.1	4926	NUMA1	nuclear mitotic apparatus protein 1 isoform 2	S1743	1.2	1.0	1.0	1.2	1.0	1.1	0.9	0.7	1.3	1.1	1.1	1.3	1.2	1.2	1.0	0.8	1.2	1.1	1.0	1.1	TQPDGTSVPGEPAsPISQR	SVPGEPAsPISQRLP	S14;	No	T(1): 0.0; T(6): 0.0; S(7): 0.0; S(14): 100.0; S(17): 0.0	34	2	1	0	N-Term(TMT6plex); S14(Phospho)
42542379	NP_005830.2	10250	SRRM1	serine/arginine repetitive matrix protein 1	T581; S583	1.0	1.9	1.2	1.1	0.9	1.5	1.1	0.9	1.2	2.1	1.3	1.2	1.0	1.6	1.2	1.0	1.1	1.8	1.3	1.1	RRtPsPPPR	TPPPRRRtPSPPPRR;PPRRRTPsPPPRRRS	T3; S5;	Yes;Yes	T(3): 100.0; S(5): 100.0	229	1	1	2	N-Term(TMT6plex); T3(Phospho); S5(Phospho)
302699211	NP_665739.3	23126	POGZ	pogo transposable element with ZNF domain isoform 3	S350	1.5	0.9	1.2	1.1	0.8	0.8	1.0	0.8	1.4	0.8	1.1	1.0	0.8	0.7	0.8	0.8	1.1	0.8	0.9	0.9	TAPVASTPSSTPIPALsPPTK	STPIPALsPPTKVPE	S17;	No	T(1): 0.0; S(6): 0.0; T(7): 0.0; S(9): 0.0; S(10): 0.0; T(11): 0.0; S(17): 100.0; T(20): 0.0	8	5	1	0	N-Term(TMT6plex); S17(Phospho); K21(TMT6plex)
115298682	NP_055987.2	23215	PRRC2C	protein PRRC2C	S187	0.4	0.7	0.7	0.7	0.9	0.8	0.9	1.3	0.4	0.6	0.7	0.6	1.0	0.7	0.9	1.2	0.7	0.7	0.8	0.9	AAGsPSSSDQDEK	DGGKAAGsPSSSDQD	S4;	Yes	S(4): 100.0; S(6): 0.0; S(7): 0.0; S(8): 0.0	18	1	1	0	N-Term(TMT6plex); S4(Phospho); K13(TMT6plex)
9966841	NP_065104.1	57099	AVEN	cell death regulator Aven	S261	0.8	1.0	0.8	0.9	0.8	1.1	0.8	1.0	0.8	1.0	0.8	0.9	0.8	1.0	0.8	1.1	0.8	1.0	0.8	1.0	DNPsPGPSR	LLGKDNPsPGPSRDS	S4;	Yes	S(4): 100.0; S(8): 0.0	3	1	1	0	N-Term(TMT6plex); S4(Phospho)
255918139	NP_001157616.1	283489	CHAMP1	chromosome alignment-maintaining phosphoprotein 1	S204; S214	0.9	1.4	1.4	0.9	1.1	1.4	1.5	1.1	0.9	1.4	1.4	1.0	1.2	1.4	1.5	1.1	1.1	1.4	1.4	1.1	LAPVPsPEPQKPAPVsPESVK	QKLAPVPsPEPQKPA;PQKPAPVsPESVKAT	S6; S16;	Yes;Yes	S(6): 100.0; S(16): 100.0; S(19): 0.0	24	1	1	0	N-Term(TMT6plex); S6(Phospho); K11(TMT6plex); S16(Phospho); K21(TMT6plex)
255918139	NP_001157616.1	283489	CHAMP1	chromosome alignment-maintaining phosphoprotein 1	S204; S214	1.1	1.4	1.5	1.1	0.9	1.4	1.4	0.9	1.2	1.4	1.5	1.1	0.9	1.4	1.4	1.0	1.1	1.4	1.4	1.1	LAPVPSPEPQKPAPVsPESVK	QKLAPVPsPEPQKPA;PQKPAPVsPESVKAT	S6; S16;	Yes;Yes	S(6): 100.0; S(16): 100.0; S(19): 0.0	5	1	1	0	N-Term(TMT6plex); S6(Phospho); K11(TMT6plex); S16(Phospho); K21(TMT6plex)
51988900	NP_000114.2	2073	ERCC5	DNA repair protein complementing XP-G cells	S384	0.7	1.1	0.9	0.9	0.7	0.8	0.7	1.0	0.7	1.1	0.9	0.9	0.7	0.8	0.7	1.0	0.7	0.9	0.8	1.0	NAPAAVDEGSIsPR	AVDEGSIsPRTLSAL	S12;	Yes	S(10): 0.0; S(12): 100.0	11	2	1	0	N-Term(TMT6plex); S12(Phospho)
47174861	NP_064615.3	56342	PPAN	suppressor of SWI4 1 homolog	S359	0.8	1.0	1.0	0.9	1.1	0.9	0.9	1.0	0.9	1.1	1.1	1.0	1.2	1.0	1.0	1.1	1.1	1.0	1.1	1.1	VGGsDEEASGIPSR	KKARVGGsDEEASGI	S4;	Yes	S(4): 100.0; S(9): 0.0; S(13): 0.0	5	3	1	0	N-Term(TMT6plex); S4(Phospho)
306922386	NP_001120983.2	324	APC	adenomatous polyposis coli protein isoform a	T1420	0.8	1.0	1.0	1.0	1.0	1.2	0.9	1.0	0.7	0.9	1.1	1.0	0.9	1.1	1.0	1.0	0.8	1.0	1.1	1.0	SKtPPPPPQTAQTK	MPPSRSKtPPPPPQT	T3;	No	S(1): 0.7; T(3): 99.3; T(10): 0.0; T(13): 0.0	9	2	1	1	N-Term(TMT6plex); K2(TMT6plex); T3(Phospho); K14(TMT6plex)
45827753	NP_996742.1	10579	TACC2	transforming acidic coiled-coil-containing protein 2 isoform c	S395	0.8	1.6	2.2	0.8	2.1	0.9	1.6	1.1	0.7	1.4	1.9	0.9	2.0	0.8	1.4	1.2	1.4	1.1	1.6	1.0	LDNTPAsPPRSPAEPNDIPIAK	KLDNTPAsPPRSPAE	S7;	No	T(4): 0.0; S(7): 99.9; S(11): 0.0	11	8	1	1	N-Term(TMT6plex); S7(Phospho); K22(TMT6plex)
45827753	NP_996742.1	10579	TACC2	transforming acidic coiled-coil-containing protein 2 isoform c	S395	2.1	0.9	1.6	1.1	0.8	1.6	2.2	0.8	2.0	0.8	1.4	1.2	0.7	1.4	1.9	0.9	1.4	1.1	1.6	1.0	LDNTPASPPRsPAEPNDIPIAK	TPASPPRsPAEPNDI	S7;	No	T(4): 50.5; S(7): 50.5; S(11): 99.0	1	8	1	1	N-Term(TMT6plex); T4(Phospho); S11(Phospho); K22(TMT6plex)
5454088	NP_006392.1	10541	ANP32B	acidic leucine-rich nuclear phosphoprotein 32 family member B	T244	0.9	1.2	0.7	0.9	0.7	1.2	0.6	1.1	1.1	1.5	0.9	1.2	0.9	1.4	0.8	1.5	1.0	1.4	0.8	1.3	KREtDDEGEDD	GEKRKREtDDEGEDD	T4;	Yes	T(4): 100.0	9	1	1	2	N-Term(TMT6plex); K1(TMT6plex); T4(Phospho)
148612890	NP_542781.3	135295	SRSF12	serine/arginine-rich splicing factor 12	S197	1.0	0.7	0.4	1.1	0.8	0.7	0.5	0.9	1.1	0.9	0.5	1.2	0.9	0.8	0.6	1.1	1.0	0.9	0.6	1.1	SQsSSPQK	KSIGKSQsSSPQKQT	S3;	No	S(1): 0.0; S(3): 100.0; S(4): 50.0; S(5): 50.0	1	1	1	0	N-Term(TMT6plex); S3(Phospho); S4(Phospho); K8(TMT6plex)
62244004	NP_001013865.1	79811	SLTM	SAFB-like transcription modulator isoform b	S356	0.9	1.0	0.9	0.8	1.0	1.2	1.0	1.2	0.9	1.1	1.1	0.8	1.0	1.3	1.2	1.3	1.0	1.2	1.2	1.1	GSTSsTSGSSGSSTK	DDKGSTSsTSGSSGS	S5;	No	S(2): 0.1; T(3): 0.1; S(4): 2.1; S(5): 91.3; T(6): 2.1; S(7): 2.1; S(9): 2.1; S(10): 0.1; S(12): 0.1; S(13): 0.1; T(14): 0.1	12	2	1	0	N-Term(TMT6plex); S5(Phospho); K15(TMT6plex)
357933604	NP_001239542.1	27332	ZNF638	zinc finger protein 638 isoform 2	S128	1.1	1.0	0.9	1.0	1.1	1.2	1.0	0.9	1.2	1.0	1.0	1.1	1.2	1.2	1.1	1.0	1.2	1.1	1.1	1.1	QSSVTQVTEQsPK	VTQVTEQsPKVQSRY	S11;	Yes	S(2): 0.0; S(3): 0.0; T(5): 0.0; T(8): 0.0; S(11): 100.0	2	2	1	0	N-Term(TMT6plex); S11(Phospho); K13(TMT6plex)
557440899	NP_001273490.1	4926	NUMA1	nuclear mitotic apparatus protein 1 isoform 2	S169	0.6	0.7	1.2	1.1	0.5	0.6	0.8	0.9	0.7	0.8	1.3	1.2	0.6	0.7	0.8	1.0	0.6	0.7	1.1	1.1	APVPSTCSSTFPEELsPPSHQAK	STFPEELsPPSHQAK	S16;	Yes	S(5): 0.0; T(6): 0.0; S(8): 0.0; S(9): 0.0; T(10): 0.0; S(16): 100.0; S(19): [illegible]	34	2	1	0	N-Term(TMT6plex); C7(Carbamidomethyl); S16(Phospho); K23(TMT6plex)
29826323	NP_054909.2	118	ADD1	alpha-adducin isoform c	S465	0.9	0.6	0.9	1.0	0.9	0.8	0.9	1.0	1.1	0.7	1.0	1.2	1.0	0.9	1.0	1.1	1.1	0.8	1.0	1.1	GDEASEEGQNGSsPK	EEGQNGSsPKSKTKW	S13;	Yes	S(5): 0.0; S(12): 0.0; S(13): 100.0	12	5	1	0	N-Term(TMT6plex); S13(Phospho); K15(TMT6plex)
38045896	NP_829883.1	23085	ERC1	ELKS/Rab6-interacting/CAST family member 1 isoform delta	S21	0.8	1.0	0.9	0.8	1.1	1.1	1.2	1.2	0.9	1.1	0.9	0.8	1.1	1.2	1.2	1.2	1.0	1.1	1.0	1.0	VEPSSQSPGRsPR	SSQSPGRsPRLPRSP	S11;	Yes	S(4): 0.0; S(5): 0.0; S(7): 0.0; S(11): 100.0	7	2	1	1	N-Term(TMT6plex); S11(Phospho)
634743227	NP_001278908.1	8602	NOP14	nucleolar protein 14 isoform 2	S96	1.2	1.2	1.2	1.0	0.9	0.8	0.9	0.9	1.3	1.2	1.2	1.0	0.9	0.8	0.9	0.9	1.1	1.0	1.1	1.0	EGEYNSNMsPEEK	GEYNSNMsPEEKMMK	S9;	Yes	Y(4): 0.0; S(6): 1.6; S(9): 98.4	1	2	1	0	N-Term(TMT6plex); M8(Oxidation); S9(Phospho); K13(TMT6plex)
38045896	NP_829883.1	23085	ERC1	ELKS/Rab6-interacting/CAST family member 1 isoform delta	S21	1.1	1.1	1.2	1.2	0.8	1.0	0.9	0.8	1.1	1.2	1.2	1.2	0.9	1.1	0.9	0.8	1.0	1.1	1.0	1.0	VEPSSQsPGRsPR	SSQSPGRsPRLPRSP	S11;	Yes	S(4): 0.0; S(5): 0.0; S(7): 0.0; S(11): 100.0	1	2	1	1	N-Term(TMT6plex); S11(Phospho)
87299628	NP_683692.2	259282	BOD1L1	biorientation of chromosomes in cell division protein 1-like 1	S3029	0.9	1.2	1.0	0.9	1.1	1.2	1.0	1.0	0.9	1.1	1.0	1.0	1.1	1.2	1.1	1.1	1.0	1.2	1.0	1.1	EVsPPGAR	IKRKREVsPPGARTR	S3;	Yes	S(3): 100.0	36	1	1	0	N-Term(TMT6plex); S3(Phospho)
589908394	NP_001277188.1	23469	PHF3	PHD finger protein 3 isoform 2	S1045	0.7	1.1	0.9	0.9	0.9	1.0	0.9	1.1	0.8	1.2	1.0	0.9	1.0	1.1	1.0	1.1	0.9	1.1	1.0	1.0	MAPPVDDLsPK	APPVDDLsPKKVKVV	S9;	No	S(9): 100.0	11	2	1	0	N-Term(TMT6plex); M1(Oxidation); S9(Phospho); K11(TMT6plex)
528281423	NP_001268464.1	23136	EPB41L3	band 4.1-like protein 3 isoform 4	S631	1.0	1.1	3.2	1.4	0.9	1.4	1.4	0.5	1.0	1.0	2.0	1.0	0.9	1.2	0.9	0.4	1.0	1.1	1.4	0.7	TETISFGSVsPGGVK	TISFGSVsPGGVKLE	S10;	No	T(1): 0.0; T(3): 0.0; S(5): 0.0; S(8): 0.0; S(10): 100.0	10	4	1	0	N-Term(TMT6plex); S10(Phospho); K15(TMT6plex)
39753953	NP_789784.2	168850	ZNF800	zinc finger protein 800	S647	1.0	1.2	1.1	0.9	0.9	1.2	1.1	1.0	0.9	1.1	1.0	0.8	0.9	1.1	0.9	0.9	0.9	1.1	1.0	0.8	ANASNsPEGNK	HKANASNsPEGNKTK	S6;	No	S(4): 0.0; S(6): 100.0	13	1	1	0	N-Term(TMT6plex); S6(Phospho); K11(TMT6plex)
259906020	NP_001158287.1	22985	ACIN1	apoptotic chromatin condensation inducer in the nucleus isoform 3	S450	0.6	0.9	0.6	1.1	0.8	0.9	0.8	0.9	0.5	0.9	0.6	1.1	0.6	0.8	0.9	0.9	0.6	0.8	0.8	1.0	AEsPAEKVPEESVLPLVQK	PSDTKAEsPAEKVPE	S3;	No	S(3): 100.0; S(12): 0.0	38	3	1	1	N-Term(TMT6plex); S3(Phospho); K7(TMT6plex); K19(TMT6plex)
55749769	NP_065994.1	57703	CWC22	pre-mRNA-splicing factor CWC22 homolog	S829	1.3	1.5	1.2	1.1	1.1	1.2	1.0	0.9	1.2	1.6	1.3	1.1	1.0	1.3	1.1	0.8	1.1	1.5	1.2	1.0	RNsFSENEK	KRGERRNsFSENEKH	S3;	Yes	S(3): 100.0; S(5): 0.0	3	1	1	1	N-Term(TMT6plex); S3(Phospho); K9(TMT6plex)
110431352	NP_001036039.1	64784	CRTC3	CREB-regulated transcription coactivator 3 isoform b	S329	0.7	0.8	0.8	1.0	0.8	1.0	1.1	1.0	-	-	-	-	-	-	-	-	-	-	-	-	sNPSIQATLNK	SGLQSSRsNPSIQAT	S1;	Yes	S(1): 100.0; S(4): 0.0; T(8): 0.0	2	2	1	0	N-Term(TMT6plex); S1(Phospho); K11(TMT6plex)
239582767	NP_060327.3	54961	SSH3	protein phosphatase Slingshot homolog 3	S649	0.4	0.4	0.9	1.1	0.9	0.8	0.9	0.9	-	-	-	-	-	-	-	-	-	-	-	-	QAsVHDSGEEGA	RKVVRQAsVHDSGEE	S3;	Yes	S(3): 100.0; S(7): 0.0	5	1	1	0	N-Term(TMT6plex); S3(Phospho)
41393554	NP_958815.1	55692	LUC7L	putative RNA-binding protein Luc7-like 1 isoform b	S336	1.0	1.1	1.0	1.1	1.2	1.2	1.0	0.9	1.2	1.2	1.2	1.2	1.5	1.4	1.2	1.0	1.3	1.3	1.2	1.1	EEsWESGR	ERASREEsWESGRSE	S3;	Yes	S(3): 100.0; S(6): 0.0	4	1	1	0	N-Term(TMT6plex); S3(Phospho)
226509740	NP_067051.2	58506	SCAF1	splicing factor, arginine/serine-rich 19	S725	0.7	1.2	1.2	1.0	0.7	1.1	1.1	1.0	0.7	1.2	1.0	0.9	0.7	1.1	0.9	0.9	0.7	1.1	1.0	0.9	GPsPAPASSPK	PSPAPASsPKREVLY	S9;	Yes	S(3): 0.0; S(8): 0.0; S(9): 100.0	2	1	1	0	N-Term(TMT6plex); S9(Phospho); K11(TMT6plex)
226509740	NP_067051.2	58506	SCAF1	splicing factor, arginine/serine-rich 19	S725	0.7	1.1	1.1	1.0	0.7	1.2	1.2	1.0	0.7	1.1	0.9	0.9	0.7	1.2	1.0	0.9	0.7	1.1	1.0	0.9	GPSPAPASsPK	PSPAPASsPKREVLY	S9;	Yes	S(3): 0.0; S(8): 0.0; S(9): 100.0	29	1	1	0	N-Term(TMT6plex); S9(Phospho); K11(TMT6plex)
365812504	NP_001242982.1	22852	ANKRD26	ankyrin repeat domain-containing protein 26 isoform 2	S630	0.9	0.9	1.3	1.0	1.1	0.9	1.0	1.0	-	-	-	-	-	-	-	-	-	-	-	-	ESVNsPVFGK	TSKESVNsPVFGKAS	S5;	Yes	S(2): 0.0; S(5): 100.0	1	2	1	0	N-Term(TMT6plex); S5(Phospho); K10(TMT6plex)
120587023	NP_055318.2	27340	UTP20	small subunit processome component 20 homolog	T1741	1.7	1.4	1.5	1.4	1.1	0.5	0.6	0.5	1.6	1.3	1.4	1.3	1.0	0.5	0.6	0.5	1.3	0.9	1.0	0.9	SLSDNGQPGtPDPADSGGTSAK	SDNGQPGtPDPADSG	T10;	Yes	S(1): 0.0; S(3): 0.0; T(10): 100.0; S(16): 0.0; T(19): 0.0; S(20): 0.0	4	1	1	0	N-Term(TMT6plex); T10(Phospho); K22(TMT6plex)
190014588	NP_001121689.1	11168	PSIP1	PC4 and SFRS1-interacting protein isoform 2	T141	0.4	0.7	0.6	0.9	0.5	0.8	0.7	1.0	0.5	0.8	0.7	1.1	0.6	0.8	0.8	1.1	0.5	0.8	0.8	1.1	AVDItPK	TKAVDItPKAARRG	T6;	Yes	T(5): 0.0; T(6): 100.0	27	2	1	0	N-Term(TMT6plex); T5(Phospho); K8(TMT6plex)
171184451	NP_055625.4	9857	CEP350	centrosome-associated protein 350	S1828	1.1	1.5	1.0	0.8	1.3	2.1	1.7	1.2	1.1	1.5	1.0	0.8	1.3	2.1	1.7	1.2	1.2	1.8	1.3	1.0	sPSPISISSSETSSIMQK	PTDLETRsPSPISIS	S1;	Yes	S(1): 100.0; S(3): 0.0; S(6): 0.0; S(8): 0.0; S(9): 0.0; S(10): 0.0; T(12): 0.0; S(13): 0.0; S(14): 0.0	3	1	1	0	N-Term(TMT6plex); S3(Phospho); M16(Oxidation); K18(TMT6plex)
41281453	NP_055535.2	9748	SLK	STE20-like serine/threonine-protein kinase	S655	0.7	0.9	1.1	1.1	1.0	0.9	1.0	0.9	0.8	0.8	1.0	1.1	1.0	0.7	0.9	0.8	0.9	0.8	1.0	0.9	sVVADTDQK	TEEMEVRsVVADTDQ	S1;	Yes	S(1): 100.0; T(6): 0.0	6	1	1	0	N-Term(TMT6plex); S1(Phospho); K9(TMT6plex)
116008442	NP_055885.3	23091	ZC3H13	zinc finger CCCH domain-containing protein 13	S325	0.7	1.1	0.9	0.7	1.1	1.1	1.1	1.2	0.7	1.0	0.9	0.7	1.0	1.0	1.1	1.2	0.9	1.0	1.0	0.9	STSPAGQHHsPISSR	SPAGQHHsPISSRHH	S10;	Yes	S(1): 0.5; T(2): 49.7; S(3): 49.7; S(10): 100.0; S(13): 0.0; S(14): 0.0	28	1	1	0	N-Term(TMT6plex); S3(Phospho); S10(Phospho)
7657269	NP_055847.1	23047	PDS5B	sister chromatid cohesion protein PDS5 homolog B	S1283	0.8	0.8	0.7	1.1	1.1	1.3	1.1	0.8	0.8	0.7	0.6	1.1	1.0	1.2	1.0	0.8	0.9	0.9	0.8	0.9	EDILENEDEQNsPPK	ENEDEQNsPPKKGKR	S12;	Yes	S(12): 100.0	34	1	1	0	N-Term(TMT6plex); S12(Phospho); K15(TMT6plex)
116008442	NP_055885.3	23091	ZC3H13	zinc finger CCCH domain-containing protein 13	S325	1.1	1.1	1.1	1.2	0.7	1.1	0.9	0.7	1.0	1.0	1.1	1.2	0.7	1.0	0.9	0.7	0.9	1.0	1.0	0.9	sTSPAGQHHsPISSR	EKRDKPRsTSPAGQH;SPAGQHHsPISSRHH	S10;	Yes;Yes	S(1): 100.0; T(2): 50.0; S(3): 50.0; S(10): 100.0; S(13): 0.0; S(14): 0.0	1	1	1	0	N-Term(TMT6plex); S1(Phospho); S3(Phospho); S10(Phospho)
54607141	NP_060626.2	55187	VPS13D	vacuolar protein sorting-associated protein 13D isoform 2	T1761	1.1	1.3	1.1	0.9	0.7	1.0	1.1	1.0	1.3	1.8	1.5	1.2	0.8	1.4	1.6	1.2	1.0	1.6	1.6	1.2	EVQDKDYPLtPPPsPTVDEPK	QDKDYPLtPPPSPTV;YPLTPPPsPTVDEPK	T10;	Yes;Yes	Y(7): 0.0; T(10): 100.0; S(14): 100.0; T(16): 0.0	3	2	1	1	N-Term(TMT6plex); K5(TMT6plex); T10(Phospho); S14(Phospho); K21(TMT6plex)
54607141	NP_060626.2	55187	VPS13D	vacuolar protein sorting-associated protein 13D isoform 2	T1761	0.7	1.0	1.1	1.0	1.1	1.3	1.1	0.9	0.8	1.4	1.6	1.2	1.3	1.8	1.5	1.2	1.0	1.6	1.6	1.2	EVQDKDYPLtPPPSPTVDEPK	QDKDYPLtPPPSPTV	T10;	Yes	Y(7): 2.0; T(10): 98.0; S(14): 50.0; T(16): 50.0	1	2	1	1	N-Term(TMT6plex); K5(TMT6plex); T10(Phospho); S14(Phospho); K21(TMT6plex)
54860079	NP_001006635.1	55114	ARHGAP17	rho GTPase-activating protein 17 isoform 1	S676	1.1	1.4	1.4	0.8	1.0	0.9	1.9	1.1	1.1	1.5	1.4	0.8	1.0	0.9	1.8	1.1	1.0	1.2	1.6	1.0	SPsPPTQHTGQPPGQPSAPSQLSAPR	KPPTRSPsPPTQHTG	S3;	Yes	S(1): 2.1; S(3): 93.7; T(6): 2.1; T(9): 2.1; S(17): 0.0; S(20): 0.0; S(23): 0.0	3	2	1	0	N-Term(TMT6plex); S3(Phospho)
90903231	NP_002102.4	3064	HTT	huntingtin	S1201	1.0	1.0	0.7	0.9	1.1	1.1	0.9	1.1	0.9	0.8	0.7	0.7	1.0	0.9	0.8	0.9	0.9	0.9	0.7	0.8	EKEPGEQASVPLsPK	EQASVPLsPKKGSEA	S13;	No	S(9): 0.0; S(13): 100.0	4	1	1	1	N-Term(TMT6plex); K2(TMT6plex); S13(Phospho); K15(TMT6plex)
13236585	NP_077309.1	79187	FSD1	fibronectin type III and SPRY domain-containing protein 1	S313; S317	0.8	0.7	0.9	0.9	0.9	0.9	0.9	1.0	0.9	0.9	1.0	0.9	0.9	1.0	1.0	1.1	0.9	0.9	1.0	1.0	TAsPINsPAR	DGKGRTAsPINSPAR;RTASPINsPARGTPS	S3; S7;	Yes;Yes	T(1): 0.0; S(3): 100.0; S(7): 100.0	1	1	1	0	N-Term(TMT6plex); S3(Phospho); S7(Phospho)
21396500	NP_003600.2	8479	HIRIP3	HIRA-interacting protein 3 isoform 1	S370	0.8	0.9	1.0	0.8	0.8	0.8	1.1	1.1	1.0	0.9	1.2	0.9	0.9	0.9	1.3	1.2	0.9	0.9	1.2	1.1	EVsDSEAGGGPQGER	SDLEREVsDSEAGGG	S3;	Yes	S(3): 100.0; S(5): 0.0	26	1	1	0	N-Term(TMT6plex); S3(Phospho)

Khan et al., 2019. Multi-omics analysis to characterize cigarette smoke induced molecular alterations in esophageal cells

Supplementary Table 8. List of phosphopeptides identified and quantified in untreated and chronically treated Het1A cells with cigarette smoke condensate for 8 months using SequestHT and Mascot search algorithms with a PhosphoRS probability score ≥75%

														Normalized phosphoprotein ratios with respect to corresponding total protein ratios.								Average of replicates													
Protein group Accession	NP_Accession	Gene ID	Gene Symbol	Protein Description	PhosphoSite (Protein)	Het-1A-Smoke - 2M/Parental	Het-1A-Smoke - 4M/Parental	Het-1A-Smoke - 6M/Parental	Het-1A-Smoke - 8M/Parental	Het-1A-Smoke - 2M/Parental	Het-1A-Smoke - 4M/Parental	Het-1A-Smoke - 6M/Parental	Het-1A-Smoke - 8M/Parental	Het-1A-Smoke - 2M/Parental	Het-1A-Smoke - 4M/Parental	Het-1A-Smoke - 6M/Parental	Het-1A-Smoke - 8M/Parental	Het-1A-Smoke - 2M/Parental	Het-1A-Smoke - 4M/Parental	Het-1A-Smoke - 6M/Parental	Het-1A-Smoke - 8M/Parental	Het-1A-Smoke - 2M/Parental	Het-1A-Smoke - 4M/Parental	Het-1A-Smoke - 6M/Parental	Het-1A-Smoke - 8M/Parental	Peptide Sequence	Phosphowindow	PhosphoSite (Peptide)	Phosphosite Plus Evidence	phosphoRS Site Probabilities	# PSMs	# Proteins	# Protein Groups	# Missed Cleavages	Modifications
						Replicate 1				Replicate 2				Replicate 1				Replicate 2																	
15721939	NP_031385.2	22794	CASC3	protein CASC3	S148	0.8	1.0	0.9	1.0	0.9	1.1	1.2	1.0	0.9	1.0	0.9	1.0	1.0	1.1	1.3	1.0	1.0	1.0	1.1	1.0	QsGDGQESTEPVENK	TVTGERQsGDGQEST	S2;	Yes	S(2): 100.0; S(8): 0.0; T(9): 0.0	10	1	1	0	N-Term(TMT6plex); S2(Phospho); K15(TMT6plex)
7661958	NP_055554.1	9774	BCLAF1	bcl-2-associated transcription factor 1 isoform1	S496	1.0	1.1	1.1	1.0	1.0	1.1	1.1	0.9	1.1	1.2	1.2	1.1	1.1	1.2	1.2	1.0	1.1	1.2	1.2	1.0	KETQsPEQVK	TVKKETQsPEQVKSE	S5;	Yes	T(3): 0.0; S(5): 100.0	47	2	1	1	N-Term(TMT6plex); K1(TMT6plex); S5(Phospho); K10(TMT6plex)
395455090	NP_001257460.1	9589	WTAP	pre-mRNA-splicing regulator WTAP isoform1	S306	1.2	0.8	0.9	1.0	1.0	0.9	0.8	0.9	1.2	0.8	1.0	0.9	1.0	0.9	0.9	0.8	1.1	0.9	0.9	0.9	EGNTTEDDFPSsPGNGNK	TEDDFPSsPGNGNKS	S12;	Yes	T(4): 0.0; T(5): 0.0; S(11): 0.0; S(12): 100.0	4	1	1	0	N-Term(TMT6plex); S12(Phospho); K18(TMT6plex)
132626688	NP_055456.2	9656	MDC1	mediator of DNA damage checkpoint protein 1	T1589	1.1	0.9	1.3	1.1	0.8	0.9	0.9	0.8	1.2	0.9	1.3	1.1	0.8	0.9	0.9	0.9	1.0	0.9	1.1	1.0	NQLVtPEPTSR	TSRNQLVtPEPTSRA	T5;	Yes	T(5): 100.0; T(9): 0.0; S(10): 0.0	26	1	1	0	N-Term(TMT6plex); T5(Phospho)
146198654	NP_078947.3	79724	ZNF768	zinc finger protein 768	S139	0.9	0.9	0.7	0.9	0.8	1.0	0.8	1.0	1.0	1.0	0.9	1.0	0.9	1.2	0.9	1.1	0.9	1.1	0.9	1.1	sPGYESESSR	SPGYEPRsPGYESES	S1;	Yes	S(1): 100.0; Y(4): 0.0; S(6): 0.0; S(8): 0.0; S(9): 0.0	18	1	1	0	N-Term(TMT6plex); S1(Phospho)
528881309	NP_001268709.1	23613	ZMYND8	protein kinase C-binding protein 1 isoformn	S508	1.1	1.1	1.1	1.0	1.2	1.1	1.0	0.9	0.9	1.0	1.0	1.0	1.0	1.1	0.9	0.8	0.9	1.0	0.9	0.9	TPPSTTVGSHsPPETPVLTR	STTVGSHsPPETPVL	S11;	No	T(1): 0.0; S(4): 0.0; T(5): 0.0; T(6): 0.0; S(9): 0.0; S(11): 100.0; T(15): 0.0; T(19): 0.0	8	15	1	0	N-Term(TMT6plex); S11(Phospho)
8923920	NP_061119.1	55506	H2AFY2	core histone macro-H2A.2	S129	0.7	0.4	0.5	1.4	0.3	0.3	0.5	0.5	0.8	0.6	0.6	1.6	0.4	0.4	0.5	0.6	0.6	0.5	0.6	1.1	SETILsPPPEK	GKSETILsPPPEKRG	S6;	No	S(1): 1.2; T(3): 1.2; S(6): 97.6	2	1	1	0	N-Term(TMT6plex); S6(Phospho); K11(TMT6plex)
528281403	NP_001268640.1	23397	NCAPH	condensin complex subunit 2 isoform3	S63	0.8	0.6	0.7	0.9	0.8	0.7	0.8	1.0	0.8	0.7	0.7	1.0	0.8	0.8	0.8	1.1	0.8	0.8	0.8	1.1	LLAsPSSR	DSPRLLAsPSSRSID	S4;	No	S(4): 100.0; S(6): 0.0; S(7): 0.0	1	3	1	0	N-Term(TMT6plex); S4(Phospho)
385251399	NP_001245260.1	2036	EPB41L1	band 4.1-like protein 1 isoformb	S472	0.9	1.1	0.9	1.0	1.3	1.1	1.0	0.9	0.9	1.2	0.8	1.1	1.3	1.1	1.0	1.0	1.1	1.1	0.9	1.0	RLPSSPASPsPK	PSSPASPsPKGTPEK	S10;	Yes	S(4): 0.0; S(5): 0.0; S(8): 0.0; S(10): 100.0	14	4	1	1	N-Term(TMT6plex); S10(Phospho); K12(TMT6plex)
146198654	NP_078947.3	79724	ZNF768	zinc finger protein 768	S125	0.9	1.3	0.9	1.1	0.7	1.0	0.8	0.9	0.9	1.4	1.1	1.2	0.8	1.1	0.9	0.9	0.9	1.3	1.0	1.1	sPGYEPR	SPRYEPQsPGYEPRS	S1;	Yes	S(1): 100.0; Y(4): 0.0	18	1	1	0	N-Term(TMT6plex); S1(Phospho)
108773787	NP_000312.2	5925	RB1	retinoblastoma-associated protein	S249	0.8	0.9	1.2	1.2	0.7	0.5	0.8	0.7	0.8	0.9	1.1	1.2	0.7	0.5	0.7	0.7	0.8	0.7	0.9	0.9	TAVIPINGsPR	AVIPINGsPRTPRRG	S9;	Yes	T(1): 0.0; S(9): 100.0	4	1	1	0	N-Term(TMT6plex); S9(Phospho)
212276104	NP_001131025.1	9208	LRRFIP1	leucine-rich repeat flightless-interacting protein 1	S658	1.0	0.8	0.7	0.9	1.5	1.1	0.9	1.0	1.0	0.8	0.8	1.1	1.6	1.1	1.0	1.3	1.3	0.9	0.9	1.2	IDGATQSsPAEPK	IDGATQSsPAEPKSE	S8;	No	T(5): 0.9; S(7): 0.9; S(8): 98.2	12	3	1	0	N-Term(TMT6plex); S8(Phospho); K13(TMT6plex)
163838629	NP_056039.2	23272	FAM208A	protein FAM208A isoformb	S666	1.0	0.8	0.9	1.0	3.2	2.5	1.9	0.9	1.0	0.8	0.8	0.9	3.2	2.3	1.6	0.8	2.1	1.5	1.2	0.8	GGNLPPVsPNDSGAK	GGNLPPVsPNDSGAK	S8;	No	S(8): 100.0; S(12): 0.0	4	2	1	0	N-Term(TMT6plex); S8(Phospho); K15(TMT6plex)
296531351	NP_001171826.1	23133	PHF8	histone lysine demethylase PHF8 isoform3	S884	1.1	1.0	0.9	1.0	1.1	1.0	1.1	0.9	-	-	-	-	-	-	-	-	-	-	-	-	RPsVGSQSNQAGQGK	ELTQRRPsVGSQSNQ	S3;	No	S(3): 96.6; S(6): 1.7; S(8): 1.7	7	3	1	0	N-Term(TMT6plex); S3(Phospho); K15(TMT6plex)
388240801	NP_116126.3	84823	LMNB2	lamin-B2	S37	1.1	0.9	0.9	1.1	0.6	0.6	0.6	0.8	1.2	1.0	1.1	1.1	0.6	0.6	0.6	0.8	0.9	0.8	0.9	1.0	AGGPATPLsPTR	GGPATPLsPTRLSRL	S9;	No	T(6): 1.2; S(9): 98.8; T(11): 0.0	9	1	1	0	N-Term(TMT6plex); S9(Phospho)
211971074	NP_001130034.1	11217	AKAP2	A-kinase anchor protein 2 isoform2	S393	1.0	1.3	1.5	1.1	1.0	1.4	1.6	0.8	-	-	-	-	-	-	-	-	-	-	-	-	sPGALETPSAAGSQGNTASQGK	SAAKGQKsPGALETP	S1;	Yes	S(1): 100.0; T(7): 0.0; S(9): 0.0; S(13): 0.0; T(17): 0.0; S(19): 0.0	6	5	1	0	N-Term(TMT6plex); S1(Phospho); K22(TMT6plex)
557440899	NP_001273490.1	4926	NUMA1	nuclear mitotic apparatus protein 1 isoform2	S271	0.9	1.0	0.9	0.9	0.9	1.1	0.9	1.0	1.0	1.1	0.9	1.0	1.0	1.2	0.9	1.1	1.0	1.1	0.9	1.1	QAAsPLEPK	LNEKQAAsPLEPKEL	S4;	Yes	S(4): 100.0	34	2	1	0	N-Term(TMT6plex); S4(Phospho); K9(TMT6plex)
118421085	NP_689472.3	90381	TICRR	treslin	S599	0.9	0.8	1.1	1.0	0.7	0.8	0.8	0.9	1.0	0.8	1.2	1.0	0.9	0.9	0.9	1.0	0.9	0.8	1.1	1.0	LHPDGsPDVAGEK	QKLHPDGsPDVAGEK	S6;	Yes	S(6): 100.0	11	1	1	0	N-Term(TMT6plex); S6(Phospho); K13(TMT6plex)
166795262	NP_694939.2	121536	AEBP2	zinc finger protein AEBP2 isoforma	S206	1.5	1.2	1.3	1.4	0.8	0.7	0.7	0.6	-	-	-	-	-	-	-	-	-	-	-	-	RGsLEMSSDGEPLSR	TSGGRRGsLEMSSDG	S3;	Yes	S(3): 100.0; S(7): 0.0; S(8): 0.0; S(14): 0.0	7	2	1	1	N-Term(TMT6plex); S3(Phospho); M6(Oxidation)
443497966	NP_001263219.1	5573	PRKAR1A	cAMP-dependent protein kinase type I-alpha regulatory subunit isoformb	S83	1.1	1.1	1.1	0.9	1.0	1.1	1.0	1.0	1.1	1.1	1.2	0.9	1.0	1.1	1.1	1.0	1.1	1.1	1.1	0.9	TDSREDEIsPPPPNPVVK	DSREDEIsPPPPNPV	S9;	Yes	T(1): 0.0; S(3): 0.9; S(9): 99.1	5	2	1	1	N-Term(TMT6plex); S9(Phospho); K18(TMT6plex)
156071452	NP_001095132.1	4898	NRD1	nardilysin isoformb precursor	S94	0.9	1.0	0.8	0.8	1.0	1.2	1.0	1.1	1.0	1.0	0.8	0.9	1.0	1.2	1.0	1.1	1.0	1.1	0.9	1.0	RGsLSNAGDPEIVK	EEEGRRGsLSNAGDP	S3;	Yes	S(3): 100.0; S(5): 0.0	2	2	1	1	N-Term(TMT6plex); S3(Phospho); K14(TMT6plex)
50959115	NP_001002879.1	8563	THOC5	THO complex subunit 5 homolog	S28	1.1	0.7	1.0	0.9	1.3	1.1	1.2	1.0	1.1	0.8	1.1	0.9	1.3	1.2	1.3	1.1	1.2	1.0	1.2	1.0	NRsDTEQEGK	AEGKRNRsDTEQEGK	S3;	Yes	S(3): 100.0; T(5): 0.0	4	1	1	1	N-Term(TMT6plex); S3(Phospho); K10(TMT6plex)
118572613	NP_057417.3	23524	SRRM2	serine/arginine repetitive matrix protein 2	S2694	0.9	1.1	1.1	0.9	0.8	1.0	1.1	1.0	0.9	1.1	1.2	1.0	0.9	1.1	1.2	1.1	0.9	1.1	1.2	1.1	SLSYsPVER	DSRSLSYsPVERRRP	S5;	Yes	S(1): 0.0; S(3): 0.0; Y(4): 0.5; S(5): [illegible]	436	1	1	0	N-Term(TMT6plex); S5(Phospho)
56119108	NP_653283.3	146857	SLFN13	schlafen family member 13	S170	2.1	0.8	1.1	1.0	0.9	1.1	0.9	0.9	2.1	0.8	1.1	1.1	0.9	1.1	0.9	1.1	1.5	1.0	1.0	1.1	YNLINEGsPPSK	YNLINEGsPPSKIMK	S8;	No	Y(1): 0.0; S(8): 100.0; S(11): 0.0	3	1	1	0	N-Term(TMT6plex); S8(Phospho); K12(TMT6plex)
118572613	NP_057417.3	23524	SRRM2	serine/arginine repetitive matrix protein 2	S2694	0.8	1.0	1.1	1.0	0.9	1.1	1.1	0.9	0.9	1.1	1.2	1.1	0.9	1.1	1.2	1.0	0.9	1.1	1.2	1.1	SLsYSPVER	LRDSRSLsYSPVERR	S5;	Yes	S(1): 0.0; S(3): 100.0; Y(4): 0.0; S(5): 0.0	4	1	1	0	N-Term(TMT6plex); S3(Phospho)
156938343	NP_055874.2	83660	TLN2	talin-2	T1843	1.3	1.2	0.9	1.0	0.9	0.9	0.8	0.9	1.6	1.0	1.0	0.8	1.2	0.8	0.9	0.7	1.4	0.9	0.9	0.8	LDEGtPPEPK	MSKLDEGtPPEPKGT	T5;	Yes	T(5): 100.0	9	1	1	0	N-Term(TMT6plex); T5(Phospho); K10(TMT6plex)
130502140	NP_065953.1	57662	CAMSAP3	calmodulin-regulated spectrin-associated protein 3 isoform2	S334	0.6	0.8	0.7	0.8	1.0	1.2	1.1	1.1	-	-	-	-	-	-	-	-	-	-	-	-	DLPDGHAAsPR	LPDGHAAsPRGTEAS	S9;	Yes	S(9): 100.0	2	2	1	0	N-Term(TMT6plex); S9(Phospho)
134276943	NP_036423.4	9700	ESPL1	separin	S1508	0.7	0.8	0.9	1.0	0.8	0.7	0.9	0.9	-	-	-	-	-	-	-	-	-	-	-	-	GsDGEDSASGGK	SEEILRGsDGEDSAS	S2;	Yes	S(2): 100.0; S(7): 0.0; S(9): 0.0	2	1	1	0	N-Term(TMT6plex); S2(Phospho); K12(TMT6plex)
308818200	NP_001184223.1	1809	DPYSL3	dihydropyrimidinase-related protein 3 isoform1	T85	0.9	1.2	0.9	1.0	0.8	0.9	0.8	0.9	1.0	1.1	1.0	1.1	0.9	0.8	0.8	0.9	0.9	1.0	0.9	1.0	GSGSRPGIEGDtPR	RPGIEGDtPRRGQGR	T12;	Yes	S(2): 0.0; S(4): 0.0; T(12): 99.9	8	1	1	0	N-Term(TMT6plex); T12(Phospho)
5032189	NP_005648.1	7158	TP53BP1	tumor suppressor p53-binding protein 1 isoform3	T1648	0.4	1.2	1.4	0.8	1.0	0.9	1.8	1.1	0.4	1.2	1.5	1.0	1.0	0.9	1.9	1.4	0.7	1.0	1.7	1.2	SNVSSPATPTASSSSSTtPTR	ASSSSSTtPTRKITE	T18;	Yes	S(1): 0.0; S(4): 0.0; S(5): 0.0; T(8): 0.0; T(10): 0.0; S(12): 0.0; S(13): 0.0; S(14): 0.0; S(15): 1.5; S(16): 1.5; T(17): 1.5; T(18): 94.0; T(20): 1.5	62	3	1	0	N-Term(TMT6plex); T18(Phospho)
259906020	NP_001158287.1	22985	ACIN1	apoptotic chromatin condensation inducer in the nucleus isoform3	S670	1.1	1.1	1.0	0.9	1.3	1.5	1.0	1.0	0.8	1.0	1.1	0.9	1.0	1.4	1.1	1.1	0.9	1.2	1.1	1.0	RLsQPESAEK	SVQARRLsQPESAEK	S3;	No	S(3): 100.0; S(7): 0.0	38	3	1	1	N-Term(TMT6plex); S3(Phospho); K10(TMT6plex)
7661950	NP_055448.1	9646	CTR9	RNA polymerase-associated protein CTR9 homolog	S1085	1.1	1.1	1.1	1.0	1.2	1.2	1.1	0.9	1.1	1.2	1.1	1.0	1.1	1.2	1.1	1.0	1.1	1.2	1.1	1.0	SDQDsDSDQPSR	RQRSDQDsDSDQPSR	S5;	Yes	S(1): 0.0; S(5): 100.0; S(7): 0.0; S(11): 0.0	12	1	1	0	N-Term(TMT6plex); S5(Phospho)
5032189	NP_005648.1	7158	TP53BP1	tumor suppressor p53-binding protein 1 isoform3	T1648	1.0	0.9	1.8	1.1	0.4	1.2	1.4	0.8	1.0	0.9	1.9	1.4	0.4	1.2	1.5	1.0	0.7	1.0	1.7	1.2	SNVSSPAtPTASSSSSTTPTR	SNVSSPAtPTASSSS	T18;	Yes	S(1): 0.1; S(4): 3.1; S(5): 3.1; T(8): 90.3; T(10): 3.5; S(12): 14.2; S(13): 14.2; S(14): 14.2; S(15): 14.2; S(16): 14.2; T(17): 14.2; T(18): 14.2; T(20): 0.5	1	3	1	0	N-Term(TMT6plex); T8(Phospho); S16(Phospho)
52630322	NP_005843.2	1107	CHD3	chromodomain-helicase-DNA-binding protein 3 isoform2	S1601; S1605	1.3	0.9	0.8	0.8	1.2	1.3	0.8	1.1	1.4	1.0	0.8	0.8	1.3	1.4	0.8	1.2	1.3	1.2	0.8	1.0	METEADAPsPAPsLGER	ETEADAPsPAPSLGE;DAPSPAPsLGERLEP	S9; S13;	Yes;Yes	T(3): 0.1; S(9): 99.9; S(13): 100.0	1	3	1	0	M1(Oxidation); N-Term(TMT6plex); S9(Phospho); S13(Phospho)
225735619	NP_001139585.1	57708	MIER1	mesoderm induction early response protein 1 isoformi	S306	0.9	0.8	0.8	0.8	0.8	0.9	0.9	1.1	0.9	0.8	0.8	0.9	0.8	0.9	0.9	1.2	0.8	0.8	0.9	1.1	LLDESESAAsSR	DESESAAsSRAPSPP	S7;	No	S(5): 0.9; S(7): 0.9; S(10): 97.2; S(11): 0.9	3	9	1	0	N-Term(TMT6plex); S10(Phospho)
42542379	NP_005830.2	10250	SRRM1	serine/arginine repetitive matrix protein 1	S675	1.9	2.9	2.2	1.0	1.4	2.0	1.8	0.9	2.3	3.0	2.4	1.1	1.7	2.1	1.9	1.1	2.0	2.6	2.2	1.1	EARsPQPNK	RSTREARsPQPNKRH	S4;	Yes	S(4): 100.0	229	1	1	1	N-Term(TMT6plex); S4(Phospho); K9(TMT6plex)
40556376	NP_115958.2	84656	GLYR1	putative oxidoreductase GLYR1	S151	1.3	1.1	1.0	1.1	1.0	0.9	0.9	0.8	1.2	0.9	1.0	1.0	1.0	0.8	0.8	0.7	1.1	0.9	0.9	0.9	VSSGsSER	KKRVSSGsSERGSKS	S5;	Yes	S(2): 0.0; S(3): 0.6; S(5): 99.4; S(6): [illegible]	1	1	1	0	N-Term(TMT6plex); S5(Phospho)
225735619	NP_001139585.1	57708	MIER1	mesoderm induction early response protein 1 isoformi	S306	0.8	0.9	0.9	1.1	0.9	0.8	0.8	0.8	0.8	0.9	0.9	1.2	0.9	0.8	0.8	0.9	0.8	0.8	0.9	1.1	LLDESEsAASSR	RLLDESEsAASSRAP	S7;	No	S(5): 0.7; S(7): 99.3; S(10): 0.0; S(11): 0.0	2	9	1	0	N-Term(TMT6plex); S7(Phospho)
52630322	NP_005843.2	1107	CHD3	chromodomain-helicase-DNA-binding protein 3 isoform2	S1601; S1605	1.2	1.3	0.8	1.1	1.3	0.9	0.8	0.8	1.3	1.4	0.8	1.2	1.4	1.0	0.8	0.8	1.3	1.2	0.8	1.0	METEADAPsPAPSLGER	ETEADAPsPAPSLGE;DAPSPAPsLGERLEP	S9; S13;	Yes;Yes	T(3): 0.0; S(9): 100.0; S(13): 100.0	1	3	1	0	M1(Oxidation); N-Term(TMT6plex); S9(Phospho); S13(Phospho)
94536786	NP_077014.2	79091	METTL22	methyltransferase-like protein 22	S132	1.6	1.4	1.5	1.1	1.2	0.7	0.9	0.8	-	-	-	-	-	-	-	-	-	-	-	-	AAsDSNPAGPLR	VRRPRAAsDSNPAGP	S3;	Yes	S(3): 100.0; S(5): 0.0	1	1	1	0	N-Term(TMT6plex); S3(Phospho)
166197710	NP_056393.2	26065	LSM14A	protein LSM14 homolog A isoformb	S192	0.8	0.8	1.1	0.8	1.4	1.1	1.0	1.1	0.9	0.9	1.4	0.9	1.5	1.2	1.2	1.1	1.2	1.1	1.3	1.0	sPTMEQAVQTASAHLPAPAAVGR	QLDPLRKsPTMEQAV	S1;	Yes	S(1): 100.0; T(3): 0.0; T(10): 0.0; S(12): 0.0	8	2	1	0	N-Term(TMT6plex); S1(Phospho); M4(Oxidation)
93141029	NP_001035224.1	5064	PALM	paralemmin-1 isoform2	S116	0.8	0.8	0.6	0.9	0.9	0.9	0.6	1.1	-	-	-	-	-	-	-	-	-	-	-	-	ENAAAPsPVR	KENAAAPsPVRAPAP	S7;	Yes	S(7): 100.0	6	2	1	0	N-Term(TMT6plex); S7(Phospho)
103472005	NP_002408.3	4288	MKI67	antigen KI-67 isoform1	S128	1.3	1.3	1.5	1.0	1.1	1.2	1.1	0.9	1.5	1.3	1.5	1.0	1.3	1.2	1.1	1.0	1.4	1.3	1.3	1.0	SsESSDPDEK	ARRVSRSsESSDPDE	S2;	Yes	S(1): 1.1; S(2): 98.9; S(4): 0.0; S(5): [illegible]	118	1	1	0	N-Term(TMT6plex); S2(Phospho); K10(TMT6plex)
311771621	NP_001185702.1	5298	PI4KB	phosphatidylinositol 4-kinase beta isoform2	S294	0.8	0.6	0.8	0.9	0.8	0.6	0.9	1.0	0.9	0.6	0.8	0.8	0.9	0.6	0.8	0.9	0.9	0.6	0.8	0.9	TAsNPKVENEDEPVR	SNLKRTAsNPKVENE	S3;	Yes	T(1): 0.0; S(3): 100.0	3	1	1	1	N-Term(TMT6plex); S3(Phospho); K6(TMT6plex)
20149560	NP_004595.2	6810	STX4	syntaxin-4 isoform3	S14; S15	1.2	1.3	1.3	1.0	1.3	0.8	1.1	0.9	1.1	1.2	1.2	1.0	1.3	0.7	1.0	0.8	1.2	1.0	1.1	0.9	QGDDssDEEDKER	ELRQGDDsSDEEDKE;LRQGDDSsDEEDKER	S5; S6;	Yes;Yes	S(5): 100.0; S(6): 100.0	1	1	1	1	N-Term(TMT6plex); S5(Phospho); S6(Phospho); K11(TMT6plex)
374253823	NP_001243390.1	6558	SLC12A2	solute carrier family 12 member 2 isoform2	T217	1.1	0.8	0.9	1.0	1.1	0.9	0.9	0.9	1.2	0.8	1.0	1.0	1.2	0.9	1.0	0.9	1.2	0.8	1.0	0.9	TEGHNtMDAVPR	LRTEGHNtMDAVPRI	T6;	Yes	T(1): 0.0; T(6): 100.0	6	2	1	0	N-Term(TMT6plex); T6(Phospho); M7(Oxidation)
28372531	NP_777567.1	151987	PPP4R2	serine/threonine-protein phosphatase 4 regulatory subunit 2	S226	0.7	1.0	0.8	0.9	0.4	0.4	0.7	1.0	0.8	1.1	0.9	1.0	0.5	0.4	0.8	1.2	0.6	0.8	0.9	1.1	NHSDSSTSESEVSSVsPLK	ESEVSSVsPLKNKHP	S16;	Yes	S(3): 0.0; S(5): 0.0; S(6): 0.0; T(7): 0.0; S(8): 0.0; S(10): 0.0; S(13): 0.0; S(14): 0.0; S(16): 100.0	8	1	1	0	N-Term(TMT6plex); S16(Phospho); K19(TMT6plex)
42542379	NP_005830.2	10250	SRRM1	serine/arginine repetitive matrix protein 1	S740	0.9	1.0	1.0	1.0	1.0	1.2	1.1	0.9	1.1	1.1	1.1	1.1	1.2	1.3	1.2	1.1	1.1	1.2	1.1	1.1	AAsPSQSVR	IKKAASPsQSVRRV	S5;	Yes	S(3): 0.8; S(5): 99.2; S(8): 0.0	229	1	1	0	N-Term(TMT6plex); S3(Phospho)
71725360	NP_055857.1	23060	ZNF609	zinc finger protein 609	T746	0.6	0.7	0.7	0.9	0.8	0.9	0.7	1.0	0.8	0.9	0.9	0.9	1.0	1.1	0.9	1.1	0.9	1.0	0.9	1.0	ELESPLtPGK	KELESPLtPGKVCRA	T7;	Yes	S(4): 0.0; T(7): 100.0	6	1	1	0	N-Term(TMT6plex); T7(Phospho); K10(TMT6plex)
39753953	NP_789784.2	168850	ZNF800	zinc finger protein 800	S426	1.0	1.2	1.2	1.1	1.1	0.9	0.8	0.8	0.9	1.1	1.1	0.9	1.0	0.8	0.7	0.7	1.0	1.0	0.9	0.8	VEPADSVESSPPSITHsPQNELK	SPPSITHsPQNELKG	S17;	Yes	S(6): 0.0; S(9): 0.0; S(10): 0.0; S(13): 0.0; T(15): 1.4; S(17): 98.6	13	1	1	0	N-Term(TMT6plex); S17(Phospho); K23(TMT6plex)
116235444	NP_612430.2	113174	SAAL1	protein SAAL1	S6	0.8	0.8	0.8	0.9	0.9	1.0	0.9	1.0	0.9	0.9	0.9	0.9	1.0	1.1	1.0	1.0	0.9	1.0	1.0	1.0	NPsPPPPGR	MDRNPsPPPPGRD	S3;	Yes	S(3): 100.0	3	1	1	0	N-Term(TMT6plex); S3(Phospho)
31340735	NP_852466.1	353274	ZNF445	zinc finger protein 445	S927	0.8	1.3	1.2	1.0	1.0	1.2	1.1	0.9	-	-	-	-	-	-	-	-	-	-	-	-	AAQAERsPPAR	RAAQAERsPPARSSS	S7;	No	S(7): 100.0	1	1	1	1	N-Term(TMT6plex); S7(Phospho)
528078296	NP_001268421.1	2956	MSH6	DNA mismatch repair protein Msh6 isoform2	S14	0.6	0.7	0.8	0.9	0.6	0.8	0.8	1.0	0.6	0.7	0.8	0.8	0.6	0.9	0.8	0.9	0.6	0.8	0.8	0.9	sPALSDANK	LYSFFPKsPALSDAN	S1;	Yes	S(1): 100.0; S(5): 0.0	6	2	1	0	N-Term(TMT6plex); S1(Phospho); K9(TMT6plex)
4504253	NP_002096.1	3014	H2AFX	histone H2AX	S140	0.7	0.9	1.0	0.8	0.9	1.3	1.1	1.1	0.8	1.0	1.1	1.0	1.1	1.5	1.2	1.4	0.9	1.3	1.1	1.2	ATQAsQEY	GKKATQAsQEY	S5;	Yes	T(2): 0.0; S(5): 100.0; Y(8): 0.0	35	1	1	0	N-Term(TMT6plex); S5(Phospho)
109637788	NP_150247.2	5371	PML	protein PML isoform5	S527	1.1	0.9	1.0	0.7	0.7	0.9	1.3	1.2	1.0	0.9	0.9	0.6	0.6	0.9	1.2	1.0	0.8	0.9	1.1	0.8	AVSPPHLDGPPsPR	PHLDGPPsPRSPVIG	S12;	Yes	S(3): 0.0; S(12): 100.0	7	7	1	0	N-Term(TMT6plex); S12(Phospho)
42542379	NP_005830.2	10250	SRRM1	serine/arginine repetitive matrix protein 1	S414	0.8	1.3	0.9	0.8	0.9	1.1	1.0	1.1	1.0	1.4	1.0	0.9	1.1	1.1	1.1	1.2	1.0	1.3	1.0	1.1	TRHsPTPQQSNR	PPPKTRHsPTPQQSN	S4;	Yes	T(1): 0.0; S(4): 100.0; T(6): 0.0; S(10): 0.0	229	1	1	1	N-Term(TMT6plex); S4(Phospho)
217330641	NP_060439.2	55677	IWS1	protein IWS1 homolog	S300	1.0	1.1	1.1	0.8	0.9	0.9	1.1	1.1	1.1	1.0	1.1	1.0	1.0	0.9	1.1	1.2	1.0	0.9	1.1	1.1	VsDSESEGPQK	ELPKPRVsDSESEGP	S2;	Yes	S(2): 100.0; S(4): 0.0; S(6): 0.0	25	1	1	0	N-Term(TMT6plex); S2(Phospho); K11(TMT6plex)
54112117	NP_036565.2	23451	SF3B1	splicing factor 3B subunit 1 isoform1	T326	0.7	0.7	0.5	0.9	0.6	0.8	1.0	1.0	0.7	0.7	0.5	0.9	0.6	0.8	1.0	1.1	0.7	0.8	0.8	1.0	GGDSIGETPtPGASK	DSIGETPtPGASKRK	T8;	Yes	S(4): 0.0; T(8): 0.0; T(10): 100.0; S(14): 0.0	6	1	1	0	N-Term(TMT6plex); T10(Phospho); K15(TMT6plex)
112382250	NP_003119.2	6711	SPTBN1	spectrin beta chain, non-erythrocytic 1 isoform1	S2164	1.1	0.9	0.9	0.9	0.6	1.0	0.8	1.0	1.1	0.9	1.0	0.9	0.6	1.0	0.8	0.9	0.8	1.0	0.9	0.9	ESsPIPsPTSDR	KESSPIPsPTSDRKA	S2;	Yes	S(2): 0.0; S(3): 0.0; S(7): 100.0; T(9): 0.0; S(10): 0.0	1	1	1	0	N-Term(TMT6plex); S7(Phospho)
112382250	NP_003119.2	6711	SPTBN1	spectrin beta chain, non-erythrocytic 1 isoform1	S2164	0.6	1.0	0.8	1.0	1.1	0.9	0.9	0.9	0.6	1.0	0.8	0.9	1.1	0.9	1.0	0.9	0.8	1.0	0.9	0.9	ESSPIPsPTSDR	QRTSSKEsSPIPSPT	S2;	Yes	S(2): 98.3; S(3): 1.7; S(7): 50.0; T(9): 0.0; S(10): 50.0	31	1	1	0	N-Term(TMT6plex); S2(Phospho); S10(Phospho)
54112117	NP_036565.2	23451	SF3B1	splicing factor 3B subunit 1 isoform1	T326	0.6	0.8	1.0	1.0	0.7	0.7	0.5	0.9	0.6	0.8	1.0	1.1	0.7	0.7	0.5	0.9	0.7	0.8	0.8	1.0	GGDSIGEtPTPGASK	GGDSIGEtPTPGASK	T8;	Yes	S(4): 0.0; T(8): 100.0; T(10): 0.0; S(14): 0.0	13	1	1	0	N-Term(TMT6plex); T8(Phospho); K15(TMT6plex)
545688925	NP_001269911.1	7090	TLE3	transducin-like enhancer protein 3 isoformg	S147	0.7	0.3	0.7	1.1	0.7	0.6	0.8	0.8	0.8	0.3	0.8	1.2	0.8	0.8	0.9	0.9	0.8	0.5	0.9	1.0	ESSANNSVsPSESLR	SSANNSVsPSESLRA	S9;	No	S(2): 0.0; S(3): 0.0; S(7): 1.6; S(9): 95.1; S(11): 1.6; S(13): 1.6	1	7	1	0	N-Term(TMT6plex); S9(Phospho)
255652953	NP_653205.3	124245	ZC3H18	zinc finger CCCH domain-containing protein 18	S746	0.8	0.8	0.6	0.8	0.7	0.9	0.9	1.1	0.9	0.8	0.8	0.9	0.9	1.0	1.1	1.2	0.9	0.9	0.9	1.0	sPAPAQTR	VSSASSRsPAPAQTR	S1;	Yes	S(1): 100.0; T(7): 0.0	34	1	1	0	N-Term(TMT6plex); S1(Phospho)
40807504	NP_940997.1	60312	AFAP1	actin filament-associated protein 1 isoformB	S711	0.7	0.7	0.8	0.9	0.9	0.7	0.9	1.0	-	-	-	-	-	-	-	-	-	-	-	-	SQAAPGSsPCR	SQAAPGSsPCRGHVL	S8;	No	S(1): 0.0; S(7): 0.0; S(8): 100.0	6	2	1	0	N-Term(TMT6plex); S8(Phospho); C10(Carbamidomethyl)
38683860	NP_003740.2	8660	IRS2	insulin receptor substrate 2	S577	1.0	1.1	1.0	1.0	1.1	1.1	0.9	0.9	1.0	1.1	1.1	0.9	1.0	1.1	1.0	0.9	1.0	1.1	1.0	0.9	TYsLTTPAR	GLRKRTYsLTTPARQ	S3;	Yes	T(1): 0.0; Y(2): 0.0; S(3): 100.0; T(5): 0.0; T(6): 0.0	9	1	1	0	N-Term(TMT6plex); S3(Phospho)
188536047	NP_003065.3	6599	SMARCC1	SWI/SNF complex subunit SMARCC1	T398	0.9	0.9	0.9	0.9	0.9	0.8	0.9	1.0	0.9	0.9	0.8	0.9	0.9	0.8	0.9	0.9	0.9	0.9	0.8	0.9	DSENtPVK	LKKDSENtPVKGGTV	T5;	Yes	S(2): 0.0; T(5): 100.0	6	1	1	0	N-Term(TMT6plex); T5(Phospho); K8(TMT6plex)
612407792	NP_001278407.1	26993	AKAP8L	A-kinase anchor protein 8-like isoform2	S222	1.0	0.9	0.9	0.7	0.9	1.0	1.2	1.2	0.9	0.9	0.9	0.8	0.8	1.0	1.2	1.2	0.9	0.9	1.1	1.0	QGGsPDEPDSK	KKRQGGsPDEPDSK	S4;	No	S(4): 100.0; S(10): 0.0	4	2	1	0	N-Term(TMT6plex); S4(Phospho); K11(TMT6plex)
21361462	NP_055416.2	30846	EHD2	EH domain-containing protein 2	S438	0.7	1.2	0.9	0.8	1.0	0.9	1.0	1.1	0.7	1.1	0.9	0.7	1.0	0.8	0.9	1.1	0.9	0.9	0.9	0.9	GPDEAMEDGEEGsDDEAEWVVTK	MEDGEEGsDDEAEWV	S13;	Yes	S(13): 100.0; T(22): 0.0	1	1	1	0	N-Term(TMT6plex); M6(Oxidation); S13(Phospho); K23(TMT6plex)
8922438	NP_060571.1	55143	CDCA8	borealin	T106	1.0	0.9	0.6	1.1	0.6	0.6	0.6	0.8	1.2	1.0	0.6	1.2	0.7	0.6	0.6	0.9	0.9	0.8	0.6	1.0	LTAEAIQtPLK	LTAEAIQtPLKSAKT	T8;	Yes	T(2): 0.0; T(8): 100.0	1	1	1	0	N-Term(TMT6plex); T8(Phospho); K11(TMT6plex)
4506619	NP_000977.1	6152	RPL24	60S ribosomal protein L24	T83	0.8	1.3	1.0	0.8	1.0	1.2	1.2	1.1	0.9	1.4	1.1	0.9	1.2	1.3	1.3	1.3	1.0	1.4	1.2	1.1	AITGAsLADIMAK	QRAITGAsLADIMAK	T3;	Yes	T(3): 0.0; S(6): 100.0	2	1	1	0	N-Term(TMT6plex); S6(Phospho); M11(Oxidation); K13(TMT6plex)
4506619	NP_000977.1	6152	RPL24	60S ribosomal protein L24	T83	1.0	1.2	1.2	1.1	0.8	1.3	1.0	0.8	1.2	1.3	1.3	1.3	0.9	1.4	1.1	0.9	1.0	1.4	1.2	1.1	AItGASLADIMAK	VKFQRAItGASLADI	T3;	Yes	T(3): 99.0; S(6): 1.0	1	1	1	0	N-Term(TMT6plex); S6(Phospho); M11(Oxidation); K13(TMT6plex)
34147540	NP_612448.1	114984	FLYWCH2	FLYWCH family member 2	S21	0.5	0.4	0.8	0.5	1.0	1.0	1.4	1.4	0.5	0.5	0.9	0.6	1.1	1.1	1.5	1.6	0.8	0.8	1.2	1.1	ASQEPsPKPGTEVIPAAPR	VKASQEPsPKPGTEV	S6;	Yes	S(2): 0.0; S(6): 100.0; T(11): 0.0	3	1	1	0	N-Term(TMT6plex); S6(Phospho); K8(TMT6plex)
118572613	NP_057417.3	23524	SRRM2	serine/arginine repetitive matrix protein 2	T1472	1.0	1.3	0.9	1.1	0.9	1.1	0.9	0.8	1.0	1.3	1.0	1.2	1.0	1.1	0.9	0.9	1.0	1.2	0.9	1.0	DIPRtPSR	GMKDIPRtPSRGRSE	T5;	Yes	T(5): 99.1; S(7): 0.9	436	1	1	1	N-Term(TMT6plex); T5(Phospho)
68508961	NP_001803.2	1060	CENPC	centromere protein C	S73	0.8	1.1	0.7	1.0	0.8	1.0	0.8	0.9	0.8	1.2	0.6	1.0	0.8	1.1	0.7	0.9	0.8	1.1	0.7	1.0	DTCIQsPSK	IKDTCIQsPSKECQK	S6;	No	T(2): 0.0; S(6): 100.0; S(8): 0.0	13	1	1	0	N-Term(TMT6plex); C3(Carbamidomethyl); S6(Phospho); K9(TMT6plex)
163310749	NP_001106191.1	22998	LIMCH1	LIM and calponin homology domains-containing protein 1 isoforme	S63	0.8	1.0	1.6	0.6	1.1	0.9	1.1	1.3	0.7	0.9	1.3	0.4	0.9	0.8	0.9	1.0	0.8	0.8	1.1	0.7	QTPsPDVVLR	SRSRQTPsPDVVLRG	S4;	No	T(2): 0.0; S(4): 100.0	5	7	1	0	N-Term(TMT6plex); S4(Phospho)
217035107	NP_001136037.1	79869	CPSF7	cleavage and polyadenylation specificity factor subunit 7 isoform3	S48	1.0	0.9	1.0	1.0	1.0	0.9	1.0	0.9	1.0	1.0	1.0	0.9	1.0	1.0	1.1	0.9	1.0	1.0	1.1	0.9	SSsTEPPPPVR	PSDDRSSsTEPPPPV	S3;	Yes	S(1): 0.0; S(2): 0.0; S(3): 100.0; T(4): 0.0	3	3	1	0	N-Term(TMT6plex); S3(Phospho)
118572613	NP_057417.3	23524	SRRM2	serine/arginine repetitive matrix protein 2	S2272	0.8	0.9	1.0	1.0	0.6	1.0	0.9	0.9	0.9	1.0	1.1	1.1	0.7	1.0	1.0	1.0	0.8	1.0	1.0	1.0	TPAAAAAMNLAsPR	AAAMNLAsPRTAVAP	S12;	Yes	T(1): 0.0; S(12): 100.0	436	1	1	0	N-Term(TMT6plex); M8(Oxidation); S12(Phospho)
51242945	NP_003153.2	6801	STRN	striatin	S245	1.0	0.9	0.6	1.1	1.8	1.9	1.3	0.8	1.0	0.9	0.6	1.0	1.8	2.0	1.3	0.7	1.4	1.5	0.9	0.9	FLESAAADFsDEDEDDDVDGR	ESAAADFsDEDEDDD	S10;	Yes	S(4): 0.0; S(10): 100.0	1	1	1	0	N-Term(TMT6plex); S10(Phospho)
156104891	NP_002087.2	2962	GTF2F1	general transcription factor IIF subunit 1	S433	1.0	1.1	1.2	0.9	1.1	1.4	1.5	1.0	1.0	1.0	1.1	0.9	1.1	1.3	1.4	1.1	1.0	1.1	1.3	1.0	LDTGPQSLsGKSTPQPPSGK	DTGPQSLsGKSTPQP	S9;	Yes	T(3): 0.0; S(7): 0.0; S(9): 100.0; S(12): 50.0; T(13): 50.0; S(18): 0.0	20	1	1	1	N-Term(TMT6plex); S9(Phospho); K11(TMT6plex); S12(Phospho); K20(TMT6plex)
373938427	NP_001243323.1	55035	NOL8	nucleolar protein 8 isoformb	S297	0.6	0.7	0.9	1.0	0.7	0.9	0.9	0.9	0.6	0.7	0.9	1.0	0.7	0.9	0.9	1.0	0.6	0.8	0.9	1.0	VSCHDsDDDIMR	NRVSCHDsDDDIMRN	S6;	No	S(2): 0.0; S(6): 100.0	5	2	1	0	N-Term(TMT6plex); C3(Carbamidomethyl); S6(Phospho); M11(Oxidation)
70610136	NP_003622.2	8505	PARG	poly(ADP-ribose) glycohydrolase	S302	0.8	1.1	1.1	0.9	1.0	1.3	1.1	1.0	0.5	1.0	0.9	0.9	0.7	1.2	0.9	0.9	0.6	1.1	0.9	0.9	ESEPEsPMDVDNSK	EKESEPEsPMDVDNS	S6;	Yes	S(2): 0.0; S(6): 100.0; S(13): 0.0	4	1	1	0	N-Term(TMT6plex); S6(Phospho); M8(Oxidation); K14(TMT6plex)
197304670	NP_002403.2	4255	MGMT	methylated-DNA--protein-cysteine methyltransferase	S232	0.8	0.7	0.6	0.9	0.9	0.7	0.7	1.0	0.7	0.7	0.6	0.9	0.8	0.7	0.7	1.0	0.8	0.7	0.7	0.9	GAGATSGsPPAGR	GAGATSGsPPAGRN	S8;	No	T(5): 0.0; S(6): 0.0; S(8): 100.0	7	1	1	0	N-Term(TMT6plex); S8(Phospho)
66932902	NP_036367.2	22937	SCAP	sterol regulatory element-binding protein cleavage-activating protein	S851	0.8	1.0	0.9	1.1	0.7	0.5	1.1	0.8	-	-	-	-	-	-	-	-	-	-	-	-	AGPEEPGDsPPLR	GPEEPGDsPPLRHRP	S9;	Yes	S(9): 100.0	1	1	1	0	N-Term(TMT6plex); S9(Phospho)
118572613	NP_057417.3	23524	SRRM2	serine/arginine repetitive matrix protein 2	S1762; S1764	1.0	1.3	1.2	0.9	0.9	1.2	1.1	1.0	1.1	1.3	1.3	1.0	1.0	1.2	1.2	1.1	1.2	1.1	1.0	1.3	1.2	1.0	GRsPsPKPR	TTSRRGRsPSPKPRG;SRRGRSPsPKPRGLQ	S3; S5;	Yes;Yes	S(3): 100.0; S(5): 100.0	436	1	1	1	N-Term(TMT6plex); S3(Phospho); S5(Phospho); K7(TMT6plex)
23312364	NP_690856.1	115426	UHRF2	E3 ubiquitin-protein ligase UHRF2	S667	1.0	0.9	0.9	0.9	1.1	1.2	1.1	1.0	0.9	0.8	0.7	0.8	1.0	1.1	0.8	0.8	0.9	1.0	0.8	0.8	RPIsDDDCPSASK	GTTKRPIsDDDCPSA	S4;	Yes	S(4): 100.0; S(10): 0.0; S(12): 0.0	1	1	1	0	N-Term(TMT6plex); S4(Phospho); C8(Carbamidomethyl); K13(TMT6plex)
55743138	NP_001006945.1	8986	RPS6KA4	ribosomal protein S6 kinase alpha-4 isoformb	S739	1.5	1.3	1.1	1.1	1.3	1.1	0.8	0.8	1.3	1.3	1.1	1.1	1.2	1.0	0.8	0.7	1.3	1.1	0.9	0.9	RGsPAPANPGR	ATASRRGsPAPANPG	S3;	No	S(3): 100.0	3	2	1	1	N-Term(TMT6plex); S3(Phospho)
24308227	NP_065883.1	57592	ZNF687	zinc finger protein 687	S433	0.9	1.1	0.8	1.2	0.8	0.8	0.9	0.7	0.8	1.1	0.8	1.2	0.7	0.8	0.9	0.7	0.8	1.0	0.8	1.0	AVVLPGGTATsPK	LPGGTATsPKMIAKN	S11;	Yes	T(8): 0.0; T(10): 0.0; S(11): 100.0	10	1	1	0	N-Term(TMT6plex); S11(Phospho); K13(TMT6plex)
546231404	NP_001271231.1	54443	ANLN	actin-binding protein anillin isoform3	S515	0.5	0.7	1.4	1.1	1.0	1.2	1.0	0.8	0.6	0.7	1.3	1.2	1.0	1.1	0.9	0.8	0.8	0.9	1.1	1.0	EIEMsVDDDDINSSK	VIREIEMsVDDDDIN	S5;	No	S(5): 100.0; S(13): 0.0; S(14): 0.0	21	3	1	0	N-Term(TMT6plex); M4(Oxidation); S5(Phospho); K15(TMT6plex)
19718751	NP_550433.1	7374	UNG	uracil-DNA glycosylase isoform UNG2	S23	1.1	1.1	1.0	1.0	0.9	0.9	0.8	0.9	1.1	1.1	1.0	1.0	0.8	0.9	0.8	0.9	0.9	1.0	0.9	1.0	HAPsPEPAVQGTGVAGVPEESGDAAAIPAK	ARKRHAPsPEPAVQG	S4;	Yes	S(4): 100.0; T(12): 0.0; S(21): 0.0	7	1	1	0	N-Term(TMT6plex); S4(Phospho); K30(TMT6plex)
20986495	NP_620832.1	55249	YY1AP1	YY1-associated protein 1 isoform5	S612	0.7	1.0	0.8	0.9	0.6	0.5	0.8	0.9	0.9	1.5	1.0	1.1	0.8	0.7	0.9	1.1	0.8	1.1	0.9	1.1	VEHsPGPPPVDK	VEPKVEHsPGPPPVD	S4;	No	S(4): 100.0	1	7	1	0	N-Term(TMT6plex); S4(Phospho); K12(TMT6plex)
503774456	NP_001265170.1	27043	PELP1	proline-, glutamic acid- and leucine-rich protein 1 isoform2	S334	1.0	1.2	1.0	0.8	1.1	1.2	1.2	1.0	1.0	1.2	1.0	0.9	1.1	1.2	1.1	1.1	1.0	1.2	1.1	1.0	GsPDGSLQTGKPSAPK	KLRSPRGsPDGSLQT	S2;	No	S(2): 100.0; S(6): 0.0; T(9): 0.0; S(13): 0.0	19	2	1	0	N-Term(TMT6plex); S2(Phospho); K11(TMT6plex); K16(TMT6plex)
126362971	NP_005981.3	6793	STK10	serine/threonine-protein kinase 10	S438	1.0	1.3	1.2	0.9	1.1	1.2	1.0	1.0	0.9	1.1	1.1	0.9	1.1	1.1	1.0	1.0	1.0	1.1	1.0	0.9	QVAEQGGDLsPAANR	AEQGGDLsPAANRSQ	S10;	Yes	S(10): 100.0	11	1	1	0	N-Term(TMT6plex); S10(Phospho)
117190342	NP_005383.3	5253	PHF2	lysine-specific demethylase PHF2	S905	0.7	0.8	0.6	0.8	0.8	1.1	0.8	1.0	0.8	0.8	0.7	0.9	0.8	1.1	0.9	1.1	0.8	1.0	0.8	1.0	GSDDAPYsPTAR	GSDDAPYsPTARVGP	S8;	Yes	S(2): 0.0; Y(7): 1.1; S(8): 98.9; T(10): 0.0	9	1	1	0	N-Term(TMT6plex); S8(Phospho)
83641870	NP_001032827.1	4869	NPM1	nucleophosmin isoform3	S254	0.4	0.6	0.7	0.9	0.4	0.5	0.7	1.0	0.4	0.7	0.7	1.0	0.3	0.6	0.7	1.1	0.4	0.6	0.7	1.0	MQAsIEK	IKAKMQAsIEKAH	S4;	Yes	S(4): 100.0	15	3	1	0	N-Term(TMT6plex); M1(Oxidation); S4(Phospho); K7(TMT6plex)
14249158	NP_116020.1	84717	HDGFRP2	hepatoma-derived growth factor-related protein 2 isoform2	S137	0.9	0.9	0.9	0.9	1.0	0.9	1.1	1.0	1.2	1.1	1.1	1.2	1.3	1.2	1.4	1.3	1.3	1.1	1.2	1.2	GVMAVTAVTATAAsDR	AVTATAAsDRMESDS	S14;	Yes	T(6): 0.0; T(9): 0.0; T(11): 0.7; S(14): 99.3	21	2	1	0	N-Term(TMT6plex); M3(Oxidation); S14(Phospho)
178056552	NP_055680.3	9918	NCAPD2	condensin complex subunit 1	T1339	1.0	1.3	1.0	0.8	1.0	0.8	0.5	1.1	1.1	1.5	1.1	0.9	1.1	0.9	0.6	1.2	1.1	1.2	0.9	1.0	YQPLAStASDNDFVTPEPR	ASDNDFVtPEPRRTT	T15;	Yes	Y(1): 0.0; S(6): 49.3; T(7): 49.3; S(9): 1.5; T(15): 100.0	1	1	1	0	N-Term(TMT6plex); T7(Phospho); T15(Phospho)
178056552	NP_055680.3	9918	NCAPD2	condensin complex subunit 1	T1339	1.0	0.8	0.5	1.1	1.0	1.3	1.0	0.8	1.1	0.9	0.6	1.2	1.1	1.5	1.1	0.9	1.1	1.2	0.9	1.0	YQPLASTASDNDFVtPEPR	ASDNDFVtPEPRRTT	T15;	Yes	Y(1): 0.0; S(6): 1.5; T(7): 1.5; S(9): 0.0; T(15): 97.0	3	1	1	0	N-Term(TMT6plex); T15(Phospho)
8923896	NP_061147.1	55902	ACSS2	acetyl-coenzyme A synthetase, cytoplasmic isoform1	S267	1.5	1.7	1.4	1.0	1.2	1.2	1.0	0.9	1.2	1.4	1.4	1.1	1.0	1.0	1.0	0.9	1.1	1.2	1.2	1.0	AELGMGDSTSQsPPIK	MGDSTSQsPPIKRSC	S12;	Yes	S(8): 0.0; T(9): 0.0; S(10): 0.0; S(12): 100.0	7	3	1	0	N-Term(TMT6plex); M5(Oxidation); S12(Phospho); K16(TMT6plex)
15721939	NP_031385.2	22794	CASC3	protein CASC3	S125	0.9	0.8	0.9	1.0	0.9	0.9	0.9	0.9	1.0	0.8	1.0	1.0	1.0	0.9	1.0	1.0	1.0	0.8	1.0	1.0	SEANDAVNsSTK	EANDAVNsSTKEEKG	S9;	Yes	S(1): 0.0; S(9): 98.2; S(10): 0.9; T(11): 0.9	10	1	1	0	N-Term(TMT6plex); S9(Phospho); K12(TMT6plex)
42542379	NP_005830.2	10250	SRRM1	serine/arginine repetitive matrix protein 1	S549; S551	0.9	1.0	1.1	0.8	1.0	1.2	0.9	1.1	1.1	1.1	1.2	0.9	1.2	1.2	1.0	1.2	1.1	1.1	1.1	1.1	sPsPPPTR	PRGRRRRsPSPPPTR;GRRRRSPsPPPTRRR	S1; S3;	Yes;Yes	S(1): 100.0; S(3): 100.0; T(7): 0.0	229	1	1	0	N-Term(TMT6plex); S1(Phospho); S3(Phospho)
62244004	NP_001013865.1	79811	SLTM	SAFB-like transcription modulator isoformb	S271	0.9	1.3	1.0	0.9	0.9	0.9	0.9	1.0	0.9	1.4	1.2	0.9	0.8	1.0	1.1	1.1	0.9	1.2	1.2	1.0	DGQDAIAQsPEKESK	GQDAIAQsPEKESKD	S9;	No	S(9): 100.0; S(14): 0.0	12	2	1	1	N-Term(TMT6plex); S9(Phospho); K12(TMT6plex); K15(TMT6plex)
355390315	NP_056171.3	23513	SCRIB	protein scribble homolog isoformb	S1448	1.0	0.9	0.9	0.9	1.0	0.9	0.8	1.0	1.0	0.8	0.9	0.8	0.9	0.8	0.8	0.9	0.9	0.8	0.8	0.9	QSPAsPPPLGGGAPVR	TSRQSPAsPPPLGGG	S5;	Yes	S(2): 0.0; S(5): 100.0	16	2	1	0	N-Term(TMT6plex); S5(Phospho)
339275993	NP_001229835.1	84164	ASCC2	activating signal cointegrator 1 complex subunit 2 isoform2	S637	0.8	0.9	0.8	0.9	0.9	0.9	0.8	1.0	0.7	0.9	0.8	0.9	0.8	1.0	0.8	0.9	0.8	0.9	0.8	0.9	HDSSTAVAGsPR	SSTAVAGsPRGHGQS	S10;	No	S(3): 0.0; S(4): 0.0; T(5): 0.0; S(10): 100.0	4	2	1	0	N-Term(TMT6plex); S10(Phospho)
574274721	NP_001275992.1	3978	LIG1	DNA ligase 1 isoform2	S36	1.3	1.4	1.5	0.6	2.0	1.6	1.6	1.3	1.3	1.5	1.5	0.6	2.0	1.7	1.6	1.2	1.6	1.6	1.6	0.9	VLGSEGEEEDEALsPAK	KAARVLGsEGEEEDE;EEEDEALsPAKGQKP	S4;	No;No	S(4): 100.0; S(14): 100.0	1	3	1	0	N-Term(TMT6plex); S4(Phospho); S14(Phospho); K17(TMT6plex)
18426915	NP_004386.2	1627	DBN1	drebrin isoforma	S337	1.2	1.0	1.1	0.9	0.9	0.9	1.3	1.0	1.2	0.8	1.0	0.9	0.9	0.8	1.1	1.0	1.0	0.8	1.1	0.9	SPSDSSTAStPVAEQIER	SDSSTAStPVAEQIE	S1;	Yes	S(1): 0.0; S(3): 0.0; S(5): 0.0; S(6): 0.0; T(7): 0.0; S(9): 0.0; T(10): 100.0	11	2	1	0	N-Term(TMT6plex); T10(Phospho)
7661690	NP_056418.1	26093	CCDC9	coiled-coil domain-containing protein 9	S202	0.7	0.7	0.8	0.9	0.7	0.6	0.8	0.9	0.7	0.7	0.9	1.0	0.7	0.6	0.9	1.0	0.7	0.6	0.9	1.0	sGPLEESER	LDDPRRRsGPLEESE	S1;	Yes	S(1): 100.0; S(7): 0.0	8	1	1	0	N-Term(TMT6plex); S1(Phospho)
18426915	NP_004386.2	1627	DBN1	drebrin isoforma	S337	0.9	0.9	1.3	1.0	1.2	1.0	1.1	0.9	0.9	0.8	1.1	1.0	1.2	0.8	1.0	0.9	1.0	0.8	1.1	0.9	sPSDSSTASTPVAEQIER	PTPIPTRsPSDSSTA	S1;	Yes	S(1): 100.0; S(3): 0.0; S(5): 0.0; S(6): 0.0; T(7): 0.0; S(9): 0.0; T(10): 0.0	10	2	1	0	N-Term(TMT6plex); S1(Phospho)
574274721	NP_001275992.1	3978	LIG1	DNA ligase 1 isoform2	S36	2.0	1.6	1.6	1.3	1.3	1.4	1.5	0.6	2.0	1.7	1.6	1.2	1.3	1.5	1.5	0.6	1.6	1.6	1.6	0.9	VLGsEGEEEDEALSPAK	KAARVLGsEGEEEDE	S4;	No	S(4): 100.0; S(14): 0.0	13	3	1	0	N-Term(TMT6plex); S4(Phospho); K17(TMT6plex)
47519639	NP_002366.2	4134	MAP4	microtubule-associated protein 4 isoform1	S696	0.9	1.2	1.4	1.1	1.0	1.3	1.2	0.8	1.0	1.1	1.4	1.3	1.0	1.2	1.3	1.0	1.0	1.2	1.3	1.2	ELPPsPEK	PNKELPPsPEKKTKP	S5;	Yes	S(5): 100.0	47	2	1	0	N-Term(TMT6plex); S5(Phospho); K8(TMT6plex)
110556636	NP_203754.2	85456	TNKS1BP1	182 kDa tankyrase-1-binding protein	S672	1.4	1.4	1.0	0.7	1.5	1.5	1.3	1.1	1.3	1.2	1.0	0.8	1.4	1.3	1.2	1.3	1.4	1.3	1.1	1.0	AsPEPPGPESSSR	AQDLCRAsPEPPGPE	S2;	Yes	S(2): 100.0; S(10): 0.0; S(11): 0.0; S(12): 0.0	41	1	1	0	N-Term(TMT6plex); S2(Phospho)
238859599	NP_001155005.1	23029	RBM34	RNA-binding protein 34 isoform2	S28	0.8	1.1	1.1	1.0	0.9	0.8	0.9	0.9	0.8	1.3	1.2	1.1	0.9	0.9	1.0	1.1	0.9	1.1	1.1	1.1	SVQEGENPDDGVRGsPPEDYR	PDDGVRGsPPEDYRL	S15;	Yes	S(1): 0.0; S(15): 100.0; Y(20): 0.0	3	2	1	1	N-Term(TMT6plex); S15(Phospho)
507144070	NP_001265228.1	26100	WIPI2	WD repeat domain phosphoinositide-interacting protein 2 isoformf	S269	0.8	1.1	1.0	0.9	0.8	0.9	1.1	0.9	0.9	1.4	1.1	1.0	0.9	1.1	1.1	1.0	0.9	1.3	1.1	1.0	GTYVPSsPTR	KGTYVPSsPTRLAYT	S7;	No	T(2): 0.0; Y(3): 0.0; S(6): 0.0; S(7): 100.0; T(9): 0.0	1	3	1	0	N-Term(TMT6plex); S7(Phospho)
116008442	NP_055885.3	23091	ZC3H13	zinc finger CCCH domain-containing protein 13	S242	1.0	0.9	0.9	0.9	0.9	0.9	1.0	1.0	1.0	0.9	0.9	0.8	0.8	0.9	1.0	1.0	0.9	0.9	1.0	0.9	TSAVSsPLLDQR	RKTSAVSsPLLDQRR	S6;	Yes	T(1): 0.0; S(2): 0.0; S(5): 0.6; S(6): [illegible]	28	1	1	0	N-Term(TMT6plex); S6(Phospho)
156104891	NP_002087.2	2962	GTF2F1	general transcription factor IIF subunit 1	T446	1.7	0.6	1.1	0.7	1.0	0.9	2.0	1.1	1.7	0.6	1.0	0.7	1.0	0.8	1.8	1.2	1.4	0.7	1.4	1.0	TtPNSGDVQVTEDAVR	QPPSGKTtPNSGDVQ	T2;	Yes	T(1): 1.2; T(2): 98.8; S(5): 0.0; T(11): 0.0	20	1	1	0	N-Term(TMT6plex); T2(Phospho)
188497705	NP_006759.3	8315	BRAP	BRCA1-associated protein	S117	0.8	0.9	0.9	0.8	0.8	0.8	0.7	1.1	-	-	-	-	-	-	-	-	-	-	-	-	ECINAAPDsPSK	CINAAPDsPSKQLPD	S9;	Yes	S(9): 100.0; S(11): 0.0	5	1	1	0	N-Term(TMT6plex); C2(Carbamidomethyl); S9(Phospho); K12(TMT6plex)
61743954	NP_001611.1	79026	AHNAK	neuroblast differentiation-associated protein AHNAK isoform1	S216	0.9	1.0	1.2	0.9	0.9	1.4	1.2	0.9	1.1	1.0	1.2	1.1	1.1	1.4	1.2	1.1	1.1	1.2	1.2	1.1	LPSGSGAAsPTGSAVDIR	PSGSGAAsPTGSAVD	S9;	Yes	S(3): 0.0; S(5): 0.0; S(9): 98.9; S(11): 1.1; S(13): 0.0	79	1	1	0	N-Term(TMT6plex); S9(Phospho)
61743954	NP_001611.1	79026	AHNAK	neuroblast differentiation-associated protein AHNAK isoform1	S216	0.9	1.4	1.2	0.9	0.9	1.0	1.2	0.9	1.1	1.4	1.2	1.1	1.1	1.0	1.2	1.1	1.1	1.2	1.2	1.1	LPsGSGAAsPTGSAVDIR	PSGSGAAsPTGSAVD	S9;	Yes	S(3): 0.0; S(5): 0.0; S(9): 100.0; S(11): 0.0; S(13): 0.0	9	1	1	0	N-Term(TMT6plex); S9(Phospho)
351721563	NP_001234935.1	10612	TRIM3	tripartite motif-containing protein 3 isoform1	S7	1.2	1.4	0.9	0.9	1.2	1.3	1.2	0.9	1.2	1.4	0.9	1.0	1.3	1.3	1.1	1.0	1.2	1.3	1.0	1.0	REDsPGPEVQPMDK	MAKREDsPGPEVQP	S4;	Yes	S(4): 100.0	1	1	1	1	N-Term(TMT6plex); S4(Phospho); M12(Oxidation); K14(TMT6plex)
110681708	NP_689813.2	163033	ZNF579	zinc finger protein 579	S483	1.0	0.9	0.6	1.1	1.6	0.6	1.1	0.7	1.4	0.8	0.6	1.0	2.1	0.6	1.1	0.6	1.7	0.7	0.9	0.8	AAALQALQAQAPTsPPPPPPL	LQAQAPTsPPPPPPP	S14;	Yes	T(13): 1.0; S(14): 99.0	5	1	1	0	N-Term(TMT6plex); S14(Phospho); K23(TMT6plex)
457867147	NP_001263988.1	1.01E+08	ERCC6-PGBD3	Cockayne syndrome B-piggyBac fusion protein	S158	1.2	0.9	1.3	1.1	0.7	0.6	1.2	0.7	1.1	0.7	1.0	1.0	0.6	0.5	0.9	0.7	0.9	0.6	1.0	0.9	IIEQLsPQAATSR	NKIIEQLsPQAATSR	S6;	No	S(6): 100.0; T(11): 0.0; S(12): 0.0	2	2	1	0	N-Term(TMT6plex); S6(Phospho)
42794779	NP_976063.1	399687	MYO18A	unconventional myosin-XVIIIa isoformb	S1983	1.1	1.0	0.9	1.0	0.8	0.7	0.7	0.8	1.2	1.1	1.0	1.2	0.9	0.8	0.8	0.9	1.0	0.9	0.9	1.0	AAsDDGSLK	KGPSKAAsDDGSLKS	S3;	No	S(3): 100.0; S(7): 0.0	3	2	1	0	N-Term(TMT6plex); S3(Phospho); K9(TMT6plex)
42518068	NP_006455.2	10618	TGOLN2	trans-Golgi network integral membrane protein 2 isoform1 precursor	S70	1.0	0.9	1.4	1.1	0.6	0.8	0.8	0.8	1.1	1.1	1.5	1.3	0.6	0.9	0.9	0.9	0.8	1.0	1.2	1.1	sSAEAQTPEDTPNK	PKDSPSKsSAEAQTP	S1;	Yes	S(1): 99.0; S(2): 1.0; T(7): 0.0; T(11): 0.0	8	4	1	0	N-Term(TMT6plex); S1(Phospho); K14(TMT6plex)
194578923	NP_001124000.1	9043	SPAG9	C-Jun-amino-terminal kinase-interacting protein 4 isoform1	S815	1.1	0.9	1.0	0.8	0.7	1.1	0.7	1.1	1.2	1.1	1.2	0.9	0.8	1.2	0.8	1.2	1.0	1.1	1.0	1.0	ETDYPAGEDLSEsGQVDK	AGEDLSEsGQVDKAS	S13;	No	T(2): 0.0; Y(4): 0.0; S(11): 0.0; S(13): 100.0	13	4	1	0	N-Term(TMT6plex); S13(Phospho); K18(TMT6plex)
4505117	NP_003918.1	8932	MBD2	methyl-CpG-binding domain protein 2 isoform1	S181	0.6	0.7	0.7	0.8	0.6	0.7	0.9	1.0	0.6	0.7	0.7	0.9	0.6	0.7	0.9	1.1	0.6	0.7	0.8	1.0	SDVYYFsPSGK	KSDVYYFsPSGKKFR	S7;	Yes	S(1): 0.0; Y(4): 0.0; Y(5): 0.0; S(7): 100.0; S(9): 0.0	4	2	1	0	N-Term(TMT6plex); S7(Phospho); K11(TMT6plex)
94681046	NP_064569.3	26504	CNNM4	metal transporter CNNM4 precursor	S664	1.2	1.2	0.9	0.8	1.2	1.2	1.1	1.0	1.0	1.1	0.9	0.8	1.0	1.1	1.1	1.0	1.0	1.1	1.0	0.9	SAsLSYPDR	TPLSRSAsLSYPDRT	S3;	Yes	S(1): 0.0; S(3): 100.0; S(5): 0.0; Y(6): 0.0	1	1	1	0	N-Term(TMT6plex); S3(Phospho)
19913406	NP_001058.2	7153	TOP2A	DNA topoisomerase 2-alpha	S1106	0.8	0.9	0.8	1.0	0.7	0.9	0.9	0.9	0.9	1.0	1.0	1.1	0.9	1.0	1.0	1.0	0.9	1.0	1.0	1.0	VPDEEENEEsDNEKETEK	DEEENEEsDNEKETE	S10;	Yes	S(10): 100.0; T(16): 0.0	64	1	1	1	N-Term(TMT6plex); S10(Phospho); K14(TMT6plex); K18(TMT6plex)
634743290	NP_001278893.1	4301	MLLT4	afadin isoform4	S1141	0.9	1.0	1.1	0.7	1.1	1.1	1.2	1.1	0.8	0.9	1.0	0.6	1.0	0.9	1.0	1.0	0.9	0.9	1.0	0.8	SSPNVANQPPsPGGK	NVANQPPsPGGKSAY	S11;	No	S(1): 50.0; S(2): 50.0; S(11): 100.0	16	3	1	0	N-Term(TMT6plex); S2(Phospho); S11(Phospho); K15(TMT6plex)

Khan et al., 2019. Multi-omics analysis to characterize cigarette smoke induced molecular alterations in esophageal cells

Supplementary Table 8. List of phosphopeptides identified and quantified in untreated and chronically treated Het1A cells with cigarette smoke condensate for 8 months using SequestHT and Mascot search algorithms with a PhosphoRS probability score ≥75%

Protein group Accession	NP_Accession	Gene ID	Gene Symbol	Protein Description	PhosphoSite (Protein)	Replicate 1: Het-1A-Smoke - 2M/Parental	Replicate 1: Het-1A-Smoke - 4M/Parental	Replicate 1: Het-1A-Smoke - 6M/Parental	Replicate 1: Het-1A-Smoke - 8M/Parental	Replicate 2: Het-1A-Smoke - 2M/Parental	Replicate 2: Het-1A-Smoke - 4M/Parental	Replicate 2: Het-1A-Smoke - 6M/Parental	Replicate 2: Het-1A-Smoke - 8M/Parental	Normalized phosphoprotein ratios with respect to corresponding total protein ratios. Replicate 1: Het-1A-Smoke - 2M/Parental	Normalized, Replicate 1: Het-1A-Smoke - 4M/Parental	Normalized, Replicate 1: Het-1A-Smoke - 6M/Parental	Normalized, Replicate 1: Het-1A-Smoke - 8M/Parental	Normalized, Replicate 2: Het-1A-Smoke - 2M/Parental	Normalized, Replicate 2: Het-1A-Smoke - 4M/Parental	Normalized, Replicate 2: Het-1A-Smoke - 6M/Parental	Normalized, Replicate 2: Het-1A-Smoke - 8M/Parental	Average of replicates: Het-1A-Smoke - 2M/Parental	Average of replicates: Het-1A-Smoke - 4M/Parental	Average of replicates: Het-1A-Smoke - 6M/Parental	Average of replicates: Het-1A-Smoke - 8M/Parental	Peptide Sequence	Phosphowindow	PhosphoSite (Peptide)	Phosphosite Plus Evidence	phosphoRS Site Probabilities	# PSMs	# Proteins	# Protein Groups	# Missed Cleavages	Modifications
22208971	NP_665908.1	3159	HMGA1	high mobility group protein HMG-I/HMG-Y isoform a	T53	0.6	0.8	0.7	0.9	0.6	0.9	0.9	1.0	0.6	0.7	0.6	0.9	0.7	0.7	0.8	1.0	0.6	0.7	0.7	1.0	EPSEVPtPK	KEPSEVPtPKRPRGR	T7;	Yes	S(3): 0.0; T(7): 100.0	39	2	1	0	N-Term(TMT6plex); T7(Phospho); K9(TMT6plex)
333108224	NP_001193937.1	4301	MLLT4	afadin isoform 1	S1141	1.1	1.1	1.2	1.1	0.9	1.0	1.1	0.7	1.0	0.9	1.0	1.0	0.8	0.9	1.0	0.6	0.9	0.9	1.0	0.8	sSPNVANQPPSPGGK	NVANQPPsPGGKSAY	S11;	No	S(1): 50.0; S(2): 50.0; S(11): 100.0	4	3	1	0	N-Term(TMT6plex); S2(Phospho); S11(Phospho); K15(TMT6plex)
574274721	NP_001275992.1	3978	LIG1	DNA ligase 1 isoform 2	S170	1.0	1.4	1.0	1.1	0.9	0.9	0.9	0.8	0.9	1.5	1.0	1.0	0.9	1.0	1.0	0.8	0.9	1.2	1.0	0.9	AETPTESVsEPEVATK	ETPTESVsEPEVATK	S9;	No	T(3): 0.0; T(5): 0.0; S(7): 1.2; S(9): 98.8; T(15): 0.0	13	2	1	0	N-Term(TMT6plex); S9(Phospho); K16(TMT6plex)
55770834	NP_057427.3	1063	CENPF	centromere protein F	S821	0.7	1.0	1.3	1.1	0.7	1.1	0.9	0.7	0.8	1.0	1.2	1.2	0.8	1.1	0.9	0.7	0.8	1.0	1.1	1.0	LEADQsPK	CRLEADQsPKNSAIL	S6;	Yes	S(6): 100.0	11	1	1	0	N-Term(TMT6plex); S6(Phospho); K8(TMT6plex)
190014588	NP_001121689.1	11168	PSIP1	PC4 and SFRS1-interacting protein isoform 2	S106	1.0	0.9	1.0	0.9	1.0	1.1	1.1	1.0	1.2	1.0	1.2	1.0	1.3	1.2	1.3	1.1	1.2	1.1	1.3	1.1	QSNASsDVEVEEK	TKQSNASsDVEVEEK	S6;	Yes	S(2): 0.0; S(5): 0.0; S(6): 100.0	27	2	1	0	N-Term(TMT6plex); S6(Phospho); K13(TMT6plex)
296278202	NP_001171721.1	56288	PARD3	partitioning defective 3 homolog isoform 9	S187	1.3	1.0	1.3	0.9	1.3	0.9	1.4	0.9	1.2	0.9	1.2	0.9	1.2	0.9	1.3	0.9	1.2	0.9	1.2	0.9	QNTAGsPK	LKQNTAGsPKTCDRK	S6;	No	T(3): 0.0; S(6): 100.0	11	11	1	0	N-Term(TMT6plex); S6(Phospho); K8(TMT6plex)
545746335	NP_001271143.1	9320	TRIP12	E3 ubiquitin-protein ligase TRIP12 isoform a	S354	0.7	0.6	0.8	0.7	1.1	1.0	0.8	1.2	0.7	0.6	0.8	0.7	1.1	1.1	0.8	1.2	0.9	0.9	0.8	1.0	SEsPPAELPSLR	STKKRSEsPPAELPS	S3;	No	S(1): 0.6; S(3): 99.4; S(10): 0.0	23	3	1	0	N-Term(TMT6plex); S3(Phospho)
122114651	NP_079366.3	57602	USP36	ubiquitin carboxyl-terminal hydrolase 36	S742	1.0	0.9	0.9	0.8	1.0	1.0	1.1	1.0	0.9	0.9	0.9	0.8	1.0	0.9	1.1	1.0	0.9	0.9	1.0	0.9	AVsPAPQSSSR	VHRARAVsPAPQSSS	S3;	Yes	S(3): 100.0; S(8): 0.0; S(9): 0.0; S(10): 0.0	6	1	1	0	N-Term(TMT6plex); S3(Phospho)
61743954	NP_001611.1	79026	AHNAK	neuroblast differentiation-associated protein AHNAK isoform 1	S5841	0.8	1.5	1.1	0.9	0.7	1.0	1.1	1.0	1.0	1.6	1.1	1.0	0.9	1.0	1.1	1.1	0.9	1.3	1.1	1.1	GHYEVTGsDDETGK	GHYEVTGsDDETGKL	S8;	Yes	Y(3): 0.0; T(6): 0.0; S(8): 100.0; T(12): 0.0	79	1	1	0	N-Term(TMT6plex); S8(Phospho); K14(TMT6plex)
42542379	NP_005830.2	10250	SRRM1	serine/arginine repetitive matrix protein 1	S607	0.8	1.0	1.0	0.9	0.8	1.0	0.9	0.9	1.0	1.1	1.1	1.0	1.0	1.1	1.0	1.1	1.0	1.1	1.0	1.1	RYSPsPPPK	IQRRYSPsPPPKRRT	S5;	Yes	Y(2): 50.0; S(3): 50.0; S(5): 100.0	229	1	1	1	N-Term(TMT6plex); S3(Phospho); S5(Phospho); K9(TMT6plex)
42542379	NP_005830.2	10250	SRRM1	serine/arginine repetitive matrix protein 1	S607	0.8	1.0	0.9	0.9	0.8	1.0	1.0	0.9	1.0	1.1	1.0	1.1	1.0	1.1	1.1	1.0	1.0	1.1	1.0	1.1	RYsPsPPPK	PPIQRRYsPSPPPKR;IQRRYSPsPPPKRRT	S5;	Yes;Yes	Y(2): 0.7; S(3): 99.3; S(5): 100.0	9	1	1	1	N-Term(TMT6plex); S3(Phospho); S5(Phospho); K9(TMT6plex)
166197710	NP_056393.2	26065	LSM14A	protein LSM14 homolog A isoform b	S178	1.3	0.8	1.1	1.1	0.8	0.7	0.8	0.8	1.5	0.9	1.3	1.1	0.9	0.8	1.0	0.8	1.2	0.9	1.1	1.0	TQLsQGR	RSLKTQLsQGRSSPQ	S4;	Yes	T(1): 0.0; S(4): 100.0	8	2	1	0	N-Term(TMT6plex); S4(Phospho)
21396500	NP_003600.2	8479	HIRIP3	HIRA-interacting protein 3 isoform 1	S227	0.8	1.4	1.1	1.1	0.8	0.9	0.8	0.8	0.9	1.5	1.3	1.2	1.0	0.9	0.9	0.8	1.0	1.2	1.1	1.0	ESEQEsEEEILAQK	LKESEQEsEEEILAQ	S6;	Yes	S(2): 0.0; S(6): 100.0	26	1	1	0	N-Term(TMT6plex); S6(Phospho); K14(TMT6plex)
93141029	NP_001035224.1	5064	PALM	paralemmin-1 isoform 2	S124	0.7	1.0	0.8	0.9	1.0	1.2	1.0	0.9	-	-	-	-	-	-	-	-	-	-	-	-	APAPsPAKEER	PVRAPAPsPAKEERK	S5;	Yes	S(5): 100.0	6	2	1	1	N-Term(TMT6plex); S5(Phospho); K8(TMT6plex)
7662086	NP_055599.1	9826	ARHGEF11	rho guanine nucleotide exchange factor 11 isoform 1	S251; S255	0.9	0.9	0.9	0.9	1.2	1.3	1.1	0.9	0.8	0.8	0.9	0.9	1.0	1.2	1.1	0.9	0.9	1.0	1.0	0.9	NSVLSDPGLDsPRTSPVIMAR	LSDPGLDsPRTSPVI	S11; S15;	Yes	S(2): 0.0; S(5): 0.0; S(11): 98.4; T(14): 50.8; S(15): 50.8	1	2	1	1	N-Term(TMT6plex); S11(Phospho); S15(Phospho); M19(Oxidation)
112382250	NP_003119.2	6711	SPTBN1	spectrin beta chain, non-erythrocytic 1 isoform 1	S2138	0.9	1.1	0.9	0.7	1.0	1.1	1.1	1.1	0.9	1.1	0.9	0.7	1.0	1.1	1.1	1.1	1.0	1.1	1.0	0.9	GEQVSQNGLPAEQGsPR	GLPAEQGsPRMAETV	S15;	Yes	S(5): 0.0; S(15): 100.0	31	2	1	0	N-Term(TMT6plex); S15(Phospho)
194578923	NP_001124000.1	9043	SPAG9	C-Jun-amino-terminal kinase-interacting protein 4 isoform 1	T226	1.2	1.1	0.8	0.8	1.8	1.7	0.9	1.0	1.3	1.2	0.9	0.9	1.9	1.9	1.1	1.2	1.6	1.5	1.0	1.0	GGEtPGSEQWK	DAQKGGEtPGSEQWK	T4;	Yes	T(4): 100.0; S(7): 0.0	13	4	1	0	N-Term(TMT6plex); T4(Phospho); K11(TMT6plex)
42542379	NP_005830.2	10250	SRRM1	serine/arginine repetitive matrix protein 1	S549; S551	0.9	1.0	0.9	0.9	1.2	1.1	1.1	1.0	1.1	1.0	0.9	1.0	1.4	1.2	1.2	1.1	1.2	1.1	1.1	1.0	RsPsPPPTR	PRGRRRRsPSPPPTR;GRRRRSPsPPPTRR	S2; S4;	Yes;Yes	S(2): 100.0; S(4): 100.0; T(8): 0.0	229	1	1	1	N-Term(TMT6plex); S2(Phospho); S4(Phospho)
7661958	NP_055554.1	9774	BCLAF1	bcl-2-associated transcription factor 1 isoform 1	S177	0.8	1.0	0.9	1.0	1.0	0.8	0.9	0.8	0.9	1.2	1.0	1.1	1.0	0.9	1.0	0.9	1.0	1.0	1.0	1.0	AEGEPQEEsPLK	EGEPQEEsPLKSKSQ	S9;	Yes	S(9): 100.0	47	3	1	0	N-Term(TMT6plex); S9(Phospho); K12(TMT6plex)
118572613	NP_057417.3	23524	SRRM2	serine/arginine repetitive matrix protein 2	S1727; S1729	0.9	0.9	1.0	0.8	1.1	0.9	0.9	1.0	0.9	0.9	1.1	0.9	1.1	1.0	0.9	1.1	1.0	0.9	1.0	1.0	RsPsVSSPEPAEK	TPPRHRRsPSVSSPE;PRHRRSPsVSSPEPA	S2; S4;	Yes;Yes	S(2): 100.0; S(4): 100.0; S(6): 0.0; S(7): 0.0	436	1	1	1	N-Term(TMT6plex); S2(Phospho); S4(Phospho); K13(TMT6plex)
113722133	NP_055861.3	23064	SETX	probable helicase senataxin	S947	0.8	1.1	0.9	0.9	1.0	1.3	1.3	1.0	0.9	1.1	0.9	1.0	1.1	1.3	1.4	1.1	1.0	1.2	1.1	1.0	EQAPDIsPK	REQAPDIsPKSDTLT	S7;	Yes	S(7): 100.0	9	1	1	0	N-Term(TMT6plex); S7(Phospho); K9(TMT6plex)
154146191	NP_005339.3	3320	HSP90AA1	heat shock protein HSP 90-alpha isoform 2	S231	0.8	0.7	0.6	0.9	1.0	0.7	0.8	0.9	1.0	0.8	0.8	1.0	1.2	0.7	1.0	1.0	1.1	0.8	0.9	1.0	DKEVsDDEAEEK	KERDKEVsDDEAEEK	S5;	Yes	S(5): 100.0	35	2	1	1	N-Term(TMT6plex); K2(TMT6plex); S5(Phospho); K12(TMT6plex)
12408677	NP_002614.2	5203	PFDN4	prefoldin subunit 4	S125	1.6	0.7	1.6	0.9	1.1	0.8	1.1	0.9	1.4	0.9	1.1	1.2	0.9	1.0	0.8	1.2	1.1	0.9	0.9	1.2	FGsNINLEADES	QLYAKFGsNINLEAD	S3;	Yes	S(3): 100.0; S(12): 0.0	2	1	1	0	N-Term(TMT6plex); S3(Phospho)
118572613	NP_057417.3	23524	SRRM2	serine/arginine repetitive matrix protein 2	S1727; S1729	1.1	0.9	0.9	1.0	0.9	0.9	1.0	0.8	1.1	1.0	0.9	1.1	0.9	0.9	1.1	0.9	1.0	0.9	1.0	1.0	RsPsVSsPEPAEK	TPPRHRRsPSVSSPE;PRHRRSPsVSSPEPA;RRSPSVSsPEPAEKS	S2; S4;	Yes;Yes;Yes	S(2): 100.0; S(4): 97.9; S(6): 4.2; S(7): 97.9	1	1	1	1	N-Term(TMT6plex); S4(Phospho); S6(Phospho); S7(Phospho); K13(TMT6plex)
195927037	NP_001124295.1	1786	DNMT1	DNA (cytosine-5)-methyltransferase 1 isoform a	S730	0.7	0.8	0.7	1.0	0.6	0.7	0.7	0.9	0.8	1.0	0.8	1.2	0.7	0.8	0.9	1.0	0.8	0.9	0.8	1.1	EADDDEEVDDNIPEMPsPK	DNIPEMPsPKKMHQG	S17;	No	S(17): 100.0	14	2	1	0	N-Term(TMT6plex); M15(Oxidation); S17(Phospho); K19(TMT6plex)
7657575	NP_036370.2	23411	SIRT1	NAD-dependent protein deacetylase sirtuin-1 isoform	S47	1.1	1.0	1.0	0.8	1.0	1.1	1.0	1.0	1.1	1.0	1.0	0.9	1.0	1.1	1.0	1.1	1.0	1.1	1.0	1.0	sPGEPGGAAPER	DGPGLERsPGEPGGA	S1;	Yes	S(1): 100.0	6	1	1	0	N-Term(TMT6plex); S1(Phospho)
167234419	NP_005110.2	9967	THRAP3	thyroid hormone receptor-associated protein 3	S243	0.9	0.8	0.8	0.9	0.9	0.8	0.7	0.9	1.0	0.8	0.8	1.0	1.0	0.8	0.8	1.0	1.0	0.8	0.8	1.0	ASAVSELsPR	ASAVSELsPRERSPA	S8;	Yes	S(2): 0.0; S(5): 0.0; S(8): 100.0	33	1	1	0	N-Term(TMT6plex); S8(Phospho)
5031767	NP_005517.1	3297	HSF1	heat shock factor protein 1	S303; S307	0.8	1.0	1.0	0.9	0.8	1.1	1.0	1.0	0.9	1.0	1.0	0.9	0.9	1.1	0.9	1.0	0.9	1.0	0.9	1.0	VKEEPPsPPQsPR	RVKEEPPsPPQSPRV;EPPSPPQsPRVEEAS	S7; S11;	Yes;Yes	S(7): 100.0; S(11): 100.0	4	1	1	1	N-Term(TMT6plex); K2(TMT6plex); S7(Phospho); S11(Phospho)
151301215	NP_067064.2	58525	WIZ	protein Wiz	S660	1.1	0.8	1.1	0.9	0.8	1.1	1.1	0.9	1.3	1.0	1.3	1.1	1.0	1.3	1.4	1.1	1.1	1.1	1.3	1.1	AADGGERPLAAsPPGTVK	GERPLAAsPPGTVKA	S12;	No	S(12): 100.0; T(16): 0.0	1	1	1	0	N-Term(TMT6plex); S12(Phospho); K18(TMT6plex)
262359929	NP_056261.4	25909	AHCTF1	protein ELYS	T1219; S1231	0.8	0.8	0.8	0.9	0.9	1.0	0.8	0.9	0.8	0.8	0.8	1.0	0.9	1.0	0.8	1.0	0.8	0.9	0.8	1.0	StPLASPSPSPGRsPQR	LRSTLRStPLASPSP;PSPSPGRsPQRLKET	T2; S14;	No;No	S(1): 1.9; T(2): 98.0; S(6): 0.0; S(8): 0.0; S(10): 1.9; S(14): 98.1	13	1	1	1	N-Term(TMT6plex); S10(Phospho); S14(Phospho)
262359929	NP_056261.4	25909	AHCTF1	protein ELYS	T1219; S1231	0.9	1.0	0.8	0.9	0.8	0.8	0.8	0.9	0.9	1.0	0.8	1.0	0.8	0.8	0.8	1.0	0.8	0.9	0.8	1.0	STPLASPSPsPGRsPQR	PLASPSPsPGRSPQR;PSPSPGRsPQRLKET	T2; S14;	No;No	S(1): 0.3; T(2): 6.4; S(6): 3.3; S(8): 3.3; S(10): 90.3; S(14): 96.4	2	1	1	1	N-Term(TMT6plex); S10(Phospho); S14(Phospho)
123701900	NP_006589.2	10723	SLC12A7	solute carrier family 12 member 7	T973	0.7	0.8	0.8	0.9	0.8	0.9	0.9	0.9	0.7	0.9	0.8	1.0	0.9	1.0	0.9	1.0	0.8	1.0	0.9	1.0	TQAPPtPDK	ARTQAPPtPDKVQMT	T6;	Yes	T(1): 0.0; T(6): 100.0	1	1	1	0	N-Term(TMT6plex); T6(Phospho); K9(TMT6plex)
33620755	NP_060493.3	55689	YEATS2	YEATS domain-containing protein 2	S124	0.7	0.6	0.7	0.9	0.7	0.9	1.0	1.0	-	-	-	-	-	-	-	-	-	-	-	-	SSsPANQR	ESPSRSSsPANQRAE	S3;	Yes	S(1): 0.0; S(2): 0.0; S(3): 100.0	6	1	1	0	N-Term(TMT6plex); S3(Phospho)
13129104	NP_077003.1	79080	CCDC86	coiled-coil domain-containing protein 86	S47	0.7	0.8	0.7	1.0	1.1	1.1	0.9	0.9	0.8	0.8	0.8	1.1	1.3	1.1	1.0	1.0	1.1	0.9	0.9	1.0	EPGsPPSVQR	EETREPGsPPSVQRA	S4;	Yes	S(4): 100.0; S(7): 0.0	26	1	1	0	N-Term(TMT6plex); S4(Phospho)
117190342	NP_005383.3	5253	PHF2	lysine-specific demethylase PHF2	S1059	0.7	0.7	0.7	0.8	1.0	0.8	0.9	1.1	0.8	0.7	0.7	0.8	1.0	0.9	1.0	1.1	0.9	0.8	0.9	1.0	RPSASsPNNNTAAK	QRRPSASsPNNNTAA	S6;	Yes	S(3): 0.0; S(5): 1.3; S(6): 98.7; T(11): 0.0	9	1	1	0	N-Term(TMT6plex); S6(Phospho); K14(TMT6plex)
10835073	NP_066565.1	4836	NMT1	glycylpeptide N-tetradecanoyltransferase 1	S47	0.8	0.8	0.9	0.8	0.9	0.9	0.8	1.0	1.0	0.9	1.0	0.9	1.0	1.0	1.0	1.0	1.0	1.0	1.0	1.0	GGLsPANDTGAK	SYNRGGLsPANDTGA	S4;	Yes	S(4): 100.0; T(9): 0.0	12	1	1	0	N-Term(TMT6plex); S4(Phospho); K12(TMT6plex)
116235448	NP_112181.2	81610	FAM83D	protein FAM83D	S492	0.6	0.9	0.9	0.8	0.7	1.0	1.1	1.1	0.7	0.9	0.9	0.7	0.8	1.0	1.1	1.1	0.7	1.0	1.0	0.9	GTQSTEGsPVSK	GTQSTEGsPVSKMSV	S8;	No	T(2): 0.0; S(4): 0.0; T(5): 0.0; S(8): 100.0; S(11): 0.0	3	1	1	0	N-Term(TMT6plex); S8(Phospho); K12(TMT6plex)
194272142	NP_001123537.1	4650	MYO9B	unconventional myosin-IXb isoform 2	T1346	1.0	0.9	0.7	0.9	1.0	0.7	0.5	0.9	1.1	1.0	0.7	1.0	1.1	0.7	0.6	1.0	1.1	0.9	0.7	1.0	ATGAALtPTEER	RATGAALtPTEERRT	T7;	Yes	T(2): 0.0; T(7): 100.0; T(9): 0.0	14	2	1	0	N-Term(TMT6plex); T7(Phospho)
5174607	NP_005958.1	4665	NAB2	NGFI-A-binding protein 2	S6	0.9	1.4	1.2	1.0	0.8	1.0	0.9	0.9	0.8	1.2	1.1	0.8	0.8	0.9	0.8	0.7	0.8	1.0	0.9	0.8	APsPTAEQPPGGGDSAR	MHRAPsPTAEQPP	S3;	Yes	S(3): 98.6; T(5): 1.4; S(15): 0.0	2	1	1	0	N-Term(TMT6plex); S3(Phospho)
347543829	NP_001231533.1	1808	DPYSL2	dihydropyrimidinase-related protein 2 isoform 3	T478	0.9	0.9	1.0	0.8	0.9	1.0	1.1	1.0	0.8	0.8	0.8	0.8	0.8	0.8	0.9	0.9	0.8	0.8	0.8	0.9	TVtPASSAK	SVTPKTVtPASSAKT	T3;	No	T(1): 0.0; T(3): 100.0; S(6): 0.0; S(7): 0.0	5	3	1	0	N-Term(TMT6plex); T3(Phospho); K9(TMT6plex)
21536411	NP_647598.1	274	BIN1	myc box-dependent-interacting protein 1 isoform 6	S265	0.9	1.4	1.3	1.0	0.8	1.1	1.0	0.9	0.8	1.3	1.3	1.0	0.8	1.1	1.0	0.9	0.8	1.2	1.2	1.0	GNKsPSPPDGSPAATPEIR	APAKGNKsPSPPDGS	S4;	No	S(4): 100.0; S(6): 0.0; S(11): 0.0; T(15): 0.0	18	10	1	1	N-Term(TMT6plex); K3(TMT6plex); S4(Phospho)
510937006	NP_001265390.1	22837	COBLL1	cordon-bleu protein-like 1 isoform 4	T260	0.8	0.6	0.7	0.7	0.9	1.0	0.8	1.1	-	-	-	-	-	-	-	-	-	-	-	-	DQTASAPAtPLVNK	QTASAPAtPLVNKHR	T9;	No	T(3): 0.0; S(5): 0.0; T(9): 100.0	3	4	1	0	N-Term(TMT6plex); T9(Phospho); K14(TMT6plex)
118572613	NP_057417.3	23524	SRRM2	serine/arginine repetitive matrix protein 2	S1219	0.9	1.0	1.0	0.8	0.9	1.0	1.2	1.0	0.9	1.0	1.0	0.9	1.0	1.1	1.2	1.1	1.0	1.0	1.1	1.0	SGAGSsPETK	ERSGAGSsPETKEQN	S6;	Yes	S(1): 0.0; S(5): 0.0; S(6): 100.0; T(9): 0.0	436	1	1	0	N-Term(TMT6plex); S6(Phospho); K10(TMT6plex)
153945728	NP_005900.2	4131	MAP1B	microtubule-associated protein 1B	S1427	1.2	1.8	1.4	0.9	1.2	2.5	1.7	0.9	0.9	1.3	1.1	1.0	0.9	1.8	1.4	1.0	0.9	1.6	1.3	1.0	GAEsPFEEK	ASGRGAEsPFEEKSG	S4;	Yes	S(4): 100.0	49	1	1	0	N-Term(TMT6plex); S4(Phospho); K9(TMT6plex)
289547536	NP_079437.5	80233	C17orf70	Fanconi anemia-associated protein of 100 kDa isoform	S667	0.9	0.7	0.8	0.9	1.0	1.0	0.9	1.0	-	-	-	-	-	-	-	-	-	-	-	-	APsPLGPTR	PPHTRAPsPLGPTRD	S3;	No	S(3): 100.0; T(8): 0.0	1	1	1	0	N-Term(TMT6plex); S3(Phospho)
153945728	NP_005900.2	4131	MAP1B	microtubule-associated protein 1B	S1785	1.2	1.4	1.1	1.0	1.1	1.2	1.0	0.8	0.9	1.0	0.9	1.1	0.9	0.9	0.8	0.9	0.9	0.9	0.9	1.0	SDIsPLTPR	LSPKSDIsPLTPRES	S4;	Yes	S(1): 0.0; S(4): 100.0; T(7): 0.0	49	1	1	0	N-Term(TMT6plex); S4(Phospho)
217330641	NP_060439.2	55677	IWS1	protein IWS1 homolog	S398; S400	0.8	1.0	0.9	0.8	1.2	1.3	1.0	1.0	0.8	0.9	0.9	0.9	1.2	1.2	1.0	1.1	1.0	1.1	0.9	1.0	AAVLsDsEDEEKASAK	KRKAAVLsDSEDEEK;KAAVLSDsEDEEKAS	S5; S7;	Yes;Yes	S(5): 100.0; S(7): 100.0; S(14): 0.0	25	1	1	1	N-Term(TMT6plex); S5(Phospho); S7(Phospho); K12(TMT6plex); K16(TMT6plex)
449083351	NP_001263347.1	9919	SEC16A	protein transport protein Sec16A isoform 2	S342	1.5	1.2	1.2	0.9	1.5	1.5	1.5	0.9	1.4	1.1	1.1	0.8	1.4	1.4	1.4	0.9	1.4	1.2	1.2	0.8	GDsPENR	NPLARGDsPENRTHH	S3;	Yes	S(3): 100.0	10	2	1	0	N-Term(TMT6plex); S3(Phospho)
194097365	NP_060117.3	117246	FTSJ3	pre-rRNA processing protein FTSJ3	S688	0.6	0.6	0.5	0.8	0.6	0.7	0.8	1.0	0.6	0.5	0.6	0.8	0.6	0.7	0.9	0.9	0.6	0.6	0.7	0.9	DLIDNsFNR	KRDLIDNsFNRYTFN	S6;	Yes	S(6): 100.0	12	1	1	0	N-Term(TMT6plex); S6(Phospho)
4507353	NP_003478.1	8148	TAF15	TATA-binding protein-associated factor 2N isoform 2	S435	0.8	0.8	0.8	0.8	0.9	0.9	0.9	1.0	1.0	0.9	0.9	1.0	1.1	1.1	1.0	1.1	1.0	1.0	1.0	1.0	SSGGGYsGDR	RSSGGGYsGDRSGGG	S7;	No	S(1): 0.0; S(2): 0.0; Y(6): 0.6; S(7):	7	2	1	0	N-Term(TMT6plex); S7(Phospho)
61743954	NP_001611.1	79026	AHNAK	neuroblast differentiation-associated protein AHNAK isoform 1	S4908	0.7	0.5	0.7	0.7	1.0	2.4	1.1	1.1	0.9	0.5	0.7	0.8	1.2	2.5	1.1	1.3	1.0	1.5	0.9	1.1	MPsLEISAPK	GPSLKMPsLEISAPK	S3;	Yes	S(3): 100.0; S(7): 0.0	79	1	1	0	N-Term(TMT6plex); M1(Oxidation); S3(Phospho); K10(TMT6plex)
544161311	NP_001269610.1	26130	GAPVD1	GTPase-activating protein and VPS9 domain-containing protein 1 isoform 3	S881	1.1	1.1	1.1	0.9	1.2	1.1	1.0	0.9	0.9	1.0	1.1	0.9	1.0	1.0	1.0	0.9	1.0	1.0	1.0	0.9	sSDIVSSVR	ERLVRSRsSDIVSSV	S1;	No	S(1): 100.0; S(2): 0.0; S(6): 0.0; S(7): 0.0	3	3	1	0	N-Term(TMT6plex); S1(Phospho)
255918139	NP_001157616.1	283489	CHAMP1	chromosome alignment-maintaining phosphoprotein 1	S507	0.8	1.0	0.9	0.9	0.2	1.0	0.8	0.9	0.9	1.0	0.9	1.0	0.2	1.0	0.8	1.0	0.5	1.0	0.8	1.0	KPGPSGPSEsPK	GPSGPSEsPKAASDI	S10;	Yes	S(5): 0.0; S(8): 0.8; S(10): 99.2	24	1	1	0	N-Term(TMT6plex); K1(TMT6plex); S10(Phospho); K12(TMT6plex)
41581463	NP_060207.2	54874	FNBP1L	formin-binding protein 1-like isoform 2	S295	0.7	0.6	0.6	0.9	0.8	0.7	0.7	0.9	0.8	0.6	0.6	0.8	0.9	0.7	0.7	0.9	0.8	0.6	0.7	0.9	TIsDGTISASK	QHIYRTIsDGTISAS	S3;	Yes	T(1): 0.0; S(3): 100.0; T(6): 0.0; S(8): 0.0; S(10): 0.0	4	3	1	0	N-Term(TMT6plex); S3(Phospho); K11(TMT6plex)
21493024	NP_653080.1	9590	AKAP12	A-kinase anchor protein 12 isoform 2	S598	1.1	1.4	1.5	1.0	0.8	1.1	1.1	0.8	1.3	1.3	1.2	0.9	1.0	1.0	0.9	0.8	1.1	1.2	1.1	0.9	GsSSDEEGGPK	KKRARRGsSSDEEGG	S2;	No	S(2): 97.7; S(3): 1.2; S(4): 1.2	103	2	1	0	N-Term(TMT6plex); S2(Phospho); K11(TMT6plex)
170763517	NP_006274.2	6867	TACC1	transforming acidic coiled-coil-containing protein 1 isoform 1	T504	1.0	0.9	1.0	1.0	0.8	1.1	1.0	0.8	1.0	0.9	1.0	1.0	0.8	1.1	0.9	0.8	0.9	1.0	1.0	0.9	DGHAtDEEK	SNRDGHAtDEEKLAS	T5;	Yes	T(5): 100.0	5	3	1	0	N-Term(TMT6plex); T5(Phospho); K9(TMT6plex)
90903231	NP_002102.4	3064	HTT	huntingtin	S1201	0.8	1.2	1.0	1.1	0.9	1.0	0.7	0.7	0.7	1.0	0.9	1.0	0.8	0.8	0.7	0.6	0.7	0.9	0.8	0.8	EPGEQASVPLsPK	EQASVPLsPKKGSEA	S11;	No	S(7): 0.0; S(11): 100.0	4	1	1	0	N-Term(TMT6plex); S11(Phospho); K13(TMT6plex)
12751477	NP_075555.1	668	FOXL2	forkhead box protein L2	S33	1.1	1.3	1.1	1.1	0.9	0.8	0.6	0.7	-	-	-	-	-	-	-	-	-	-	-	-	TVKEPEGPPPsPGK	EPEGPPPsPGKGGGG	S11;	Yes	T(1): 0.0; S(11): 100.0	12	1	1	1	N-Term(TMT6plex); K3(TMT6plex); S11(Phospho); K14(TMT6plex)
631226398	NP_001278804.1	10644	IGF2BP2	insulin-like growth factor 2 mRNA-binding protein 2 isoform g	S28	0.9	1.3	0.4	0.3	2.6	1.7	2.3	1.5	0.8	1.1	0.3	0.3	2.4	1.4	2.0	1.5	1.6	1.3	1.2	0.9	ISYIPDEEVSsPSPPQR	DEEVSSPsPPQRAQR	S13;	No	S(2): 0.0; Y(3): 0.0; S(10): 50.5; S(11): 50.5; S(13): 99.1	1	7	1	0	N-Term(TMT6plex); S11(Phospho); S13(Phospho)
631226398	NP_001278804.1	10644	IGF2BP2	insulin-like growth factor 2 mRNA-binding protein 2 isoform g	S28	2.6	1.7	2.3	1.5	0.9	1.3	0.4	0.3	2.4	1.4	2.0	1.5	0.8	1.1	0.3	0.3	1.6	1.3	1.2	0.9	ISYIPDEEVSSPsPPQR	DEEVSSPsPPQRAQR	S13;	No	S(2): 0.0; Y(3): 0.0; S(10): 50.0; S(11): 50.0; S(13): 100.0	2	7	1	0	N-Term(TMT6plex); S11(Phospho); S13(Phospho)
38788333	NP_057662.3	51773	RSF1	remodeling and spacing factor 1	S604	1.2	1.2	1.1	1.0	1.2	0.9	1.2	0.8	1.1	1.1	1.0	1.1	1.1	0.9	1.1	0.9	1.1	1.0	1.1	1.0	DAQRLsPIPEEVPK	DKDAQRLsPIPEEVP	S6;	Yes	S(6): 100.0	15	1	1	1	N-Term(TMT6plex); S6(Phospho); K14(TMT6plex)
20127519	NP_036244.2	22974	TPX2	targeting protein for Xklp2	T369	0.6	0.6	0.8	0.9	0.6	0.6	0.8	0.9	0.7	0.6	0.9	0.9	0.6	0.6	0.9	0.8	0.6	0.6	0.9	0.8	DPQtPVLQTK	KICRDPQtPVLQTKH	T4;	Yes	T(4): 100.0; T(9): 0.0	6	1	1	0	N-Term(TMT6plex); T4(Phospho); K10(TMT6plex)
156104891	NP_002087.2	2962	GTF2F1	general transcription factor IIF subunit 1	T389	1.0	1.0	1.0	0.8	1.1	0.9	1.0	1.0	1.0	0.9	0.9	0.8	1.1	0.9	0.9	1.1	1.1	0.9	0.9	0.9	GNSRPGtPSAEGGSTSSTLR	RGNSRPGtPSAEGGS	T7;	Yes	S(3): 0.0; T(7): 100.0; S(9): 0.0; S(14): 0.0; T(15): 0.0; S(16): 0.0; S(17): 0.0; T(18): 0.0	20	1	1	0	N-Term(TMT6plex); T7(Phospho)
301336155	NP_001180384.1	54888	NSUN2	tRNA (cytosine(34)-C(5))-methyltransferase isoform 2	S708; S716	1.3	2.0	1.7	0.8	1.8	1.0	1.6	1.0	1.2	1.8	1.4	0.7	1.6	0.9	1.4	0.9	1.4	1.3	1.4	0.8	AGEPNsPDAEEANsPDVTAGCDPAGVHPPR	QRAGEPNsPDAEEAN;PDAEEANsPDVTAGC	S6; S14;	No;No	S(6): 100.0; S(14): 97.0; T(18): 3.0	8	2	1	0	N-Term(TMT6plex); S6(Phospho); S14(Phospho); C21(Carbamidomethyl)
359843219	NP_001241646.1	1616	DAXX	death domain-associated protein 6 isoform c	S420	0.9	1.2	1.1	1.0	0.9	1.2	1.0	0.8	0.9	1.3	1.2	1.2	0.9	1.3	1.1	0.9	0.9	1.3	1.1	1.0	DGDKsPMSSLQISNEK	AGKDGDKsPMSSLQI	S5;	No	S(5): 100.0; S(8): 0.0; S(9): 0.0; S(13): 0.0	3	3	1	1	N-Term(TMT6plex); K4(TMT6plex); S5(Phospho); M7(Oxidation); K16(TMT6plex)
145309300	NP_112557.2	8621	CDK13	cyclin-dependent kinase 13 isoform 2	T494	1.0	0.8	1.0	0.9	0.9	1.1	0.8	0.9	1.5	1.0	1.3	1.2	1.2	1.3	1.1	1.2	1.3	1.2	1.2	1.2	ASNTStPTK	AKASNTStPTKGNTE	T6;	Yes	S(2): 0.0; T(4): 0.0; S(5): 0.0; T(6): 100.0; T(8): 0.0	8	2	1	0	N-Term(TMT6plex); T6(Phospho); K9(TMT6plex)
283046706	NP_001164274.1	113251	LARP4	la-related protein 4 isoform d	S455	1.0	0.8	1.2	1.0	1.5	0.6	1.2	0.8	1.0	0.7	1.0	0.9	1.6	0.6	1.0	0.7	1.3	0.6	1.0	0.8	ASTAsPCNNNINAATAVALQEPR	PSRASTAsPCNNNIN	S5;	No	S(2): 0.0; T(3): 0.0; S(5): 100.0; T(15): 0.0	4	5	1	0	N-Term(TMT6plex); S5(Phospho); C7(Carbamidomethyl)
47078240	NP_004118.3	2873	GPS1	COP9 signalosome complex subunit 1 isoform 2	T479	0.8	0.9	1.2	0.9	0.9	1.0	1.0	0.9	0.7	0.9	1.2	0.9	0.9	0.9	0.9	0.9	0.8	0.9	1.0	0.9	EGSQGELtPANSQSR	EGSQGELtPANSQSR	T8;	Yes	S(3): 0.0; T(8): 99.9; S(12): 0.1; S(14): 0.1	1	2	1	0	N-Term(TMT6plex); T8(Phospho)
14043024	NP_004272.2	9531	BAG3	BAG family molecular chaperone regulator 3	S289	0.8	1.0	1.0	0.8	0.7	1.2	1.0	1.0	0.9	0.8	0.9	0.7	0.8	1.0	1.0	0.9	0.8	0.9	1.0	0.8	SSTPLHsPSPIR	RSSTPLHsPSPIRVH	S7;	Yes	S(1): 33.3; S(2): 33.3; T(3): 33.3; S(7): 98.2; S(9): 1.8	2	1	1	0	N-Term(TMT6plex); S2(Phospho); S7(Phospho)
26051235	NP_060700.2	55746	NUP133	nuclear pore complex protein Nup133	T512	1.0	1.0	1.3	0.8	0.9	1.0	1.5	1.0	0.9	1.0	1.2	0.9	0.9	1.0	1.4	1.0	0.9	1.0	1.3	1.0	NEtIAQEDK	ETTTKNEtIAQEDKL	T3;	Yes	T(3): 100.0	2	1	1	0	N-Term(TMT6plex); T3(Phospho); K9(TMT6plex)
13129104	NP_077003.1	79080	CCDC86	coiled-coil domain-containing protein 86	S91	1.5	1.2	0.9	1.0	1.0	0.9	0.9	0.8	1.8	1.2	1.0	1.1	1.1	0.9	1.0	0.9	1.4	1.0	1.0	1.0	LQQGAGLESPQGQPEPGAAsPQ	QPEPGAAsPQRQQDL	S20;	Yes	S(9): 0.0; S(20): 100.0	26	1	1	0	N-Term(TMT6plex); S20(Phospho)
568214245	NP_001274744.1	9898	UBAP2L	ubiquitin-associated protein 2-like isoform c	S409	0.6	0.8	0.6	0.9	0.7	0.6	0.6	0.9	0.7	0.8	0.6	0.8	0.9	0.6	0.7	0.8	0.8	0.7	0.7	0.8	NPSDSAVHsPFTK	PSDSAVHsPFTKRQA	S9;	No	S(3): 0.0; S(5): 0.0; S(9): 100.0; T(12): 0.0	23	4	1	0	N-Term(TMT6plex); S9(Phospho); K13(TMT6plex)
543583707	NP_001269532.1	10728	PTGES3	prostaglandin E synthase 3 isoform d	S80	1.1	0.9	1.5	1.0	1.2	0.8	1.1	0.8	1.1	1.0	1.5	1.1	1.2	1.0	1.1	0.8	1.1	1.0	1.3	0.9	DWEDDsDEDMSNFDR	WKDWEDDsDEDMSNF	S6;	No	S(6): 100.0; S(11): 0.0	4	4	1	0	N-Term(TMT6plex); S6(Phospho); M10(Oxidation)
197333695	NP_001127949.1	5073	PARN	poly(A)-specific ribonuclease PARN isoform 2	S558	1.3	1.2	1.2	1.1	1.1	1.3	1.0	0.7	1.2	1.2	1.2	1.0	1.1	1.2	1.0	0.6	1.1	1.2	1.1	0.8	ELsPAGSISK	KRMKKELsPAGSISK	S3;	No	S(3): 99.1; S(7): 0.9; S(9): 0.0	1	3	1	0	N-Term(TMT6plex); S3(Phospho); K10(TMT6plex)
41327779	NP_065987.1	57696	DDX55	ATP-dependent RNA helicase DDX55	S594	1.1	1.3	1.1	1.1	1.0	0.8	0.7	0.7	1.1	1.4	1.1	1.2	1.0	0.8	0.7	0.7	1.1	1.1	0.9	1.0	TVDLGIsDLEDDC	KTVDLGIsDLEDDC	S7;	Yes	T(1): 0.0; S(7): 100.0	4	1	1	0	N-Term(TMT6plex); S7(Phospho); C13(Carbamidomethyl)
32698936	NP_872363.1	283149	BCL9L	B-cell CLL/lymphoma 9-like protein	S21; S25	0.6	0.9	0.8	0.9	1.2	0.8	0.9	1.0	-	-	-	-	-	-	-	-	-	-	-	-	EAPGsPPLsPR	RRREAPGsPPLSPRG;APGSPPLsPRGHCPP	S5; S9;	Yes;Yes	S(5): 100.0; S(9): 100.0	5	1	1	0	N-Term(TMT6plex); S5(Phospho); S9(Phospho)
112293285	NP_006708.2	10927	SPIN1	spindlin-1	S199	0.9	0.9	0.6	1.0	0.7	0.7	0.6	0.8	1.1	1.1	0.7	1.1	0.9	0.8	0.8	1.0	1.0	0.9	0.8	1.0	IMPDSNDsPPAER	IMPDSNDsPPAEREP	S8;	Yes	S(5): 0.0; S(8): 100.0	2	1	1	0	N-Term(TMT6plex); M2(Oxidation); S8(Phospho)
319996623	NP_001188474.1	64783	RBM15	putative RNA-binding protein 15 isoform 2	S127	1.2	1.1	0.9	0.8	0.8	0.7	0.8	1.0	1.2	1.1	0.9	0.8	0.8	0.7	0.8	1.0	1.0	0.9	0.8	0.9	LHSYsSPSTK	SSRLHSYsSPSTKNS	S5;	Yes	S(3): 0.6; Y(4): 0.6; S(5): 98.3; S(6): 0.6; S(8): 0.0; T(9): 0.0	6	2	1	0	N-Term(TMT6plex); S5(Phospho); K10(TMT6plex)
45505149	NP_005588.2	4782	NFIC	nuclear factor 1 C-type isoform 5	S339	1.0	1.1	1.1	0.8	1.1	1.1	1.1	1.0	1.2	1.2	1.2	0.9	1.3	1.2	1.2	1.1	1.3	1.2	1.2	1.0	SPFNSPsPQDSPR	KSPFNSPsPQDSPRL	S7;	Yes	S(1): 0.0; S(5): 0.0; S(7): 100.0; S(11): 0.0	13	5	1	0	N-Term(TMT6plex); S7(Phospho)
4759158	NP_004588.1	6633	SNRPD2	small nuclear ribonucleoprotein Sm D2 isoform 1	T12	1.4	1.0	1.0	0.9	0.8	0.8	0.7	0.9	1.5	1.1	1.1	0.9	0.8	0.9	0.7	0.9	1.1	1.0	0.9	0.9	SEMtPEELQK	NKPKSEMtPEELQKR	T4;	Yes	S(1): 0.0; T(4): 100.0	5	1	1	0	N-Term(TMT6plex); M3(Oxidation); T4(Phospho); K10(TMT6plex)
319996623	NP_001188474.1	64783	RBM15	putative RNA-binding protein 15 isoform 2	S127	0.8	0.7	0.8	1.0	1.2	1.1	0.9	0.8	0.8	0.7	0.8	1.0	1.2	1.1	0.9	0.8	1.0	0.9	0.8	0.9	LHSYSsPSTK	SRLHSYSsPSTKNSS	S5;	Yes	S(3): 0.0; Y(4): 0.0; S(5): 0.5; S(6): 99.5; S(8): 0.0; T(9): 0.0	2	2	1	0	N-Term(TMT6plex); S5(Phospho); K10(TMT6plex)
61743954	NP_001611.1	79026	AHNAK	neuroblast differentiation-associated protein AHNAK isoform 1	S5857	0.8	0.4	1.1	1.1	0.7	0.8	1.1	0.7	1.0	0.4	1.1	1.2	0.8	0.8	1.1	0.9	0.9	0.6	1.1	1.1	LQGSGVSLAsK	GSGVSLAsKKSRLSS	S10;	Yes	S(4): 0.0; S(7): 0.0; S(10): 100.0	79	1	1	0	N-Term(TMT6plex); S10(Phospho); K11(TMT6plex)
41393563	NP_055889.2	23095	KIF1B	kinesin-like protein KIF1B isoform b	S1408	0.8	0.6	1.0	0.9	0.9	0.8	1.0	0.9	0.9	0.6	1.1	0.9	1.0	0.8	1.1	0.9	0.9	0.7	1.1	0.9	MSDTGsPGMQR	CKMSDTGsPGMQRRR	S6;	No	S(2): 0.0; T(4): 0.9; S(6): 99.1	1	1	1	0	N-Term(TMT6plex); M1(Oxidation); S6(Phospho); M9(Oxidation)
216548085	NP_361013.3	7798	LUZP1	leucine zipper protein 1	S903	1.1	1.0	0.8	0.6	1.5	1.5	1.0	1.2	1.1	0.9	0.7	0.6	1.4	1.4	0.9	1.1	1.3	1.2	0.8	0.8	sHSAPSEVGFSDAR	AETIRWKsHSAPSEV	S1;	No	S(1): 98.8; S(3): 1.2; S(6): 0.0; S(11): 0.0	7	1	1	0	N-Term(TMT6plex); S1(Phospho)
38327039	NP_002145.3	3308	HSPA4	heat shock 70 kDa protein 4	S552	0.9	1.1	0.9	0.8	1.0	1.1	1.0	1.0	0.8	1.1	0.9	0.7	1.0	1.1	1.0	1.0	0.9	1.1	0.9	0.8	AESEEMETsQAGSK	ESEEMETsQAGSKDK	S9;	Yes	S(3): 0.0; T(8): 0.0; S(9): 100.0; S(13): 0.0	40	1	1	0	N-Term(TMT6plex); M6(Oxidation); S9(Phospho); K14(TMT6plex)
454544715	NP_001263626.1	7157	TP53	cellular tumor antigen p53 isoform i	S233	0.9	0.9	0.9	0.8	0.9	1.3	1.2	1.0	0.9	0.8	0.8	0.8	1.0	1.2	1.1	1.1	0.9	1.0	1.0	0.9	TEGPDsD	FKTEGPDsD	S6;	No	T(1): 0.0; S(6): 100.0	27	4	1	0	N-Term(TMT6plex); S6(Phospho)
216548085	NP_361013.3	7798	LUZP1	leucine zipper protein 1	S903	1.5	1.5	1.0	1.2	1.1	1.0	0.8	0.6	1.4	1.4	0.9	1.1	1.1	0.9	0.7	0.6	1.3	1.2	0.8	0.8	SHsAPSEVGFSDAR	TIRWKSHsAPSEVGF	S1;	Yes	S(1): 1.0; S(3): 99.0; S(6): 0.0; S(11): 0.0	1	1	1	0	N-Term(TMT6plex); S3(Phospho)
63252863	NP_115820.2	84464	SLX4	structure-specific endonuclease subunit SLX4	S601	0.7	0.8	0.9	0.9	0.8	1.0	1.0	0.9	0.6	0.7	0.8	0.7	0.6	0.9	0.9	0.8	0.6	0.8	0.8	0.8	GPsPSASQR	GCGSRGPsPSASQRE	S3;	Yes	S(3): 100.0; S(5): 0.0; S(7): 0.0	8	1	1	0	N-Term(TMT6plex); S3(Phospho)
55769546	NP_060376.2	55003	PAK1IP1	p21-activated protein kinase-interacting protein 1	S320	0.7	0.9	0.8	0.9	0.6	0.9	0.8	0.9	0.8	0.9	0.9	0.9	0.6	1.0	0.8	0.9	0.7	0.9	0.9	0.9	ESLPPAAEPsPVSK	LPPAAEPsPVSKEQS	S10;	Yes	S(2): 0.0; S(10): 100.0; S(13): 0.0	6	1	1	0	N-Term(TMT6plex); S10(Phospho); K14(TMT6plex)
126723149	NP_067054.1	58509	CACTIN	cactin	S489	1.2	0.9	0.9	0.9	1.0	0.8	0.9	0.9	1.3	0.9	1.0	1.0	1.1	0.8	1.0	0.9	1.2	0.9	1.0	0.9	QEPQsPSR	ILKQEPQsPSRSLEP	S5;	Yes	S(5): 100.0; S(7): 0.0	4	1	1	0	N-Term(TMT6plex); S5(Phospho)
31324577	NP_203744.1	85377	MICALL1	MICAL-like protein 1	S578	0.8	1.0	0.8	1.3	0.5	0.4	0.5	0.5	0.7	0.9	0.8	1.1	0.4	0.4	0.5	0.5	0.6	0.6	0.6	0.8	VEQMPQAsPGLAPR	VEQMPQAsPGLAPRT	S8;	Yes	S(8): 100.0	12	1	1	0	N-Term(TMT6plex); M4(Oxidation); S8(Phospho)
42542379	NP_005830.2	10250	SRRM1	serine/arginine repetitive matrix protein 1	S636; S638	1.0	1.2	1.0	0.9	0.8	0.8	0.8	0.9	1.2	1.2	1.1	1.0	1.0	0.9	0.8	1.0	1.1	1.1	1.0	1.0	VsHsPPPK	PPPKRRVsHSPPPKQ;PKRRVSHsPPPKQRS	S2; S4;	Yes;Yes	S(2): 100.0; S(4): 100.0	229	1	1	0	N-Term(TMT6plex); S2(Phospho); S4(Phospho); K8(TMT6plex)
31543630	NP_005406.3	6574	SLC20A1	sodium-dependent phosphate transporter 1	S265	0.9	1.0	1.1	0.8	1.0	1.3	1.2	0.9	0.8	0.9	0.9	0.8	0.9	1.1	1.0	0.9	0.9	1.0	0.9	0.9	CsPSESPLMEK	IERFIKCsPSESPLM	S2;	Yes	S(2): 99.2; S(4): 0.8; S(6): 0.0	2	1	1	0	N-Term(TMT6plex); C1(Carbamidomethyl); S2(Phospho); M9(Oxidation); K11(TMT6plex)
47419936	NP_003128.3	6732	SRPK1	SRSF protein kinase 1	S51	1.1	1.2	1.7	1.1	0.6	1.3	1.6	0.7	1.1	1.3	1.6	1.1	0.6	1.3	1.5	0.6	0.8	1.3	1.5	0.8	GSAPHSESDLPEQEEEILGsDDDEQEDPNDYCK	QEEEILGsDDDEQED	S20;	Yes	S(2): 0.0; S(6): 0.0; S(8): 0.0; S(20): 100.0; Y(31): 0.0	4	1	1	0	N-Term(TMT6plex); S20(Phospho); C32(Carbamidomethyl); K33(TMT6plex)
112382250	NP_003119.2	6711	SPTBN1	spectrin beta chain, non-erythrocytic 1 isoform 1	S2102	0.7	1.0	0.8	1.0	0.6	0.7	0.7	0.8	0.7	1.0	0.8	0.9	0.6	0.8	0.7	0.8	0.6	0.9	0.8	0.9	RPPsPEPSTK	ERKRRPPsPEPSTKV	S4;	Yes	S(4): 100.0; S(8): 0.0; T(9): 0.0	31	2	1	0	N-Term(TMT6plex); S4(Phospho); K10(TMT6plex)
313850978	NP_001186550.1	135112	NCOA7	nuclear receptor coactivator 7 isoform 3	S269	0.8	0.8	0.7	0.8	0.9	0.8	0.7	1.0	0.8	0.8	0.6	0.8	0.8	0.8	0.7	0.9	0.8	0.8	0.6	0.9	ETSHGsPTVTK	SRETSHGsPTVTKLS	S6;	No	T(2): 0.0; S(3): 0.0; S(6): 100.0; T(8): 0.0; T(10): 0.0	1	3	1	0	N-Term(TMT6plex); S6(Phospho); K11(TMT6plex)
4758808	NP_004832.1	9462	RASAL2	ras GTPase-activating protein nGAP isoform 1	S758	1.0	0.9	1.0	1.1	0.8	0.6	0.8	0.7	1.0	1.0	0.9	1.1	0.8	0.6	0.7	0.7	0.9	0.8	0.8	0.9	ETQSTPQsAPQVR	ETQSTPQsAPQVRRP	S8;	Yes	T(2): 0.0; S(4): 0.0; T(5): 0.0; S(8): 100.0	5	2	1	0	N-Term(TMT6plex); S8(Phospho)
111955162	NP_001009811.2	22994	CEP131	5-azacytidine-induced protein 1 isoform b	S78	0.9	0.9	0.9	0.8	1.1	1.1	1.1	1.0	-	-	-	-	-	-	-	-	-	-	-	-	SNsTTQVSQPR	NNLRRSNsTTQVSQP	S3;	No	S(1): 0.0; S(3): 100.0; T(4): 0.0; T(5): 0.0; S(8): 0.0	2	3	1	0	N-Term(TMT6plex); S3(Phospho)
281427280	NP_001163878.1	983	CDK1	cyclin-dependent kinase 1 isoform 4	T14	0.9	1.0	1.1	0.8	0.7	0.9	1.0	1.0	1.0	1.0	1.1	0.8	0.8	0.9	1.0	0.9	0.9	1.0	1.0	0.9	IGEGtYGVVYK	IEKIGEGtYGVVYKG	T5;	Yes	T(5): 100.0; Y(6): 0.0; Y(10): 0.0	8	7	1	0	N-Term(TMT6plex); T5(Phospho); K11(TMT6plex)
38044290	NP_060082.2	55596	ZCCHC8	zinc finger CCHC domain-containing protein 8	T479; T485	0.5	0.7	0.8	0.6	1.0	0.9	0.6	1.1	0.5	0.7	0.8	0.6	0.9	0.8	0.5	1.1	0.7	0.8	0.7	0.9	GtPPPVFtPPLPK	TPPLPRGtPPPVFTP;GTPPPVFtPPLPKGT	T2; T8;	Yes;Yes	T(2): 100.0; T(8): 100.0	21	1	1	0	N-Term(TMT6plex); T2(Phospho); T8(Phospho); K13(TMT6plex)
27436946	NP_733821.1	4000	LMNA	lamin isoform A	S22	0.7	0.9	1.0	0.9	0.7	0.9	0.7	0.9	0.7	0.8	1.0	0.9	0.8	0.8	0.7	0.9	0.8	0.8	0.9	0.9	SGAQASSTPLsPTR	QASSTPLsPTRITRL	S11;	Yes	S(1): 0.0; S(6): 0.0; S(7): 0.0; T(8): 0.0; S(11): 99.4; T(13): 0.6	36	4	1	0	N-Term(TMT6plex); S11(Phospho)
42542379	NP_005830.2	10250	SRRM1	serine/arginine repetitive matrix protein 1	S738; S740	0.9	1.1	1.0	0.9	0.8	1.1	1.0	0.9	1.1	1.1	1.1	1.1	1.0	1.1	1.1	1.0	1.1	1.1	1.1	1.0	KAAsPsPQSVR	KKIKKAAsPSPQSVR;IKKAASPsPQSVRRV	S4; S6;	Yes;Yes	S(4): 100.0; S(6): 100.0; S(9): 0.0	229	1	1	1	K1(TMT6plex); N-Term(TMT6plex); S4(Phospho); S6(Phospho)
572874814	NP_001275913.1	55153	SDAD1	protein SDA1 homolog isoform 3	S488	1.1	0.9	1.0	1.1	1.0	0.9	1.1	0.7	1.2	0.9	1.0	1.0	1.1	0.8	1.1	0.6	1.2	0.9	1.1	0.8	YIEIDsDEEPR	RKYIEIDsDEEPRGE	S6;	No	Y(1): 0.0; S(6): 100.0	2	3	1	0	N-Term(TMT6plex); S6(Phospho)
223941846	NP_001138837.1	22834	ZNF652	zinc finger protein 652	T203	0.9	0.9	0.9	0.8	1.0	0.9	0.7	1.0	-	-	-	-	-	-	-	-	-	-	-	-	AASVAAATTsPTPR	SVAAATTsPTPRTTR	T9;	Yes	S(3): 0.0; T(8): 0.0; T(9): 0.0; S(10): 99.2; T(12): 0.8	5	1	1	0	N-Term(TMT6plex); S10(Phospho)
223941846	NP_001138837.1	22834	ZNF652	zinc finger protein 652	T203	1.0	0.9	0.7	1.0	0.9	0.9	0.9	0.8	-	-	-	-	-	-	-	-	-	-	-	-	AASVAAATtSPTPR	ASVAAATtSPTPRTT	T9;	No	S(3): 0.0; T(8): 0.9; T(9): 98.1; S(10): 0.9; T(12): 0.0	3	1	1	0	N-Term(TMT6plex); T9(Phospho)
217330641	NP_060439.2	55677	IWS1	protein IWS1 homolog	S377	1.0	0.9	0.8	0.6	1.1	1.1	1.1	1.1	1.0	0.9	0.8	0.7	1.2	1.1	1.1	1.3	1.1	1.0	1.0	1.0	MDsDEDEKEGEEEK	HKKQKMDsDEDEKEG	S3;	Yes	S(3): 100.0	25	1	1	1	M1(Oxidation); N-Term(TMT6plex); S3(Phospho); K8(TMT6plex); K14(TMT6plex)
51094101	NP_076977.3	79039	DDX54	ATP-dependent RNA helicase DDX54 isoform 2	S782	1.0	1.0	0.9	0.9	0.9	0.9	0.9	0.9	1.1	1.0	1.0	1.0	1.0	1.0	1.0	0.9	1.0	1.0	1.0	0.9	IDDRDsDEEGASDR	QKIDDRDsDEEGASD	S6;	Yes	S(6): 100.0; S(12): 0.0	4	2	1	1	N-Term(TMT6plex); S6(Phospho)
67514036	NP_001019837.1	30011	SH3KBP1	SH3 domain-containing kinase-binding protein 1 isoform b	S146	1.1	1.1	0.9	0.9	1.0	1.1	1.1	0.8	1.1	1.0	0.9	1.0	1.0	1.0	1.0	0.9	1.0	1.0	0.9	0.9	ETTGSEsDGGDSSSTK	RETTGSEsDGGDSSS	S7;	No	T(2): 0.0; T(3): 0.0; S(5): 0.0; S(7): 100.0; S(12): 0.0; S(13): 0.0; S(14): 0.0; T(15): 0.0	4	2	1	0	N-Term(TMT6plex); S7(Phospho); K16(TMT6plex)
118572613	NP_057417.3	23524	SRRM2	serine/arginine repetitive matrix protein 2	S1621	0.8	0.9	0.8	0.8	1.3	1.3	1.1	1.0	0.8	0.9	0.9	0.9	1.4	1.4	1.2	1.1	1.1	1.1	1.2	1.0	AQSGSDSsPEPK	AQSGSDSsPEPKAPA	S8;	Yes	S(3): 0.0; S(5): 0.0; S(7): 0.7; S(8):	436	1	1	0	N-Term(TMT6plex); S8(Phospho); K12(TMT6plex)
166795297	NP_057225.2	51143	DYNC1LI1	cytoplasmic dynein 1 light intermediate chain 1	S516	1.8	2.1	1.1	0.8	1.2	1.0	1.1	1.0	1.7	2.2	1.1	0.8	1.1	1.0	1.0	1.0	1.4	1.6	1.1	0.9	KPVTVSPtTPTSPTEGEAS	KPVTVSPtTPTSPTE	S12;	Yes	T(4): 0.0; S(6): 0.1; T(8): 93.7; T(9): 2.1; T(11): 2.1; S(12): 2.1; T(14): 0.1; S(19): 0.0	1	1	1	0	N-Term(TMT6plex); K1(TMT6plex); T11(Phospho)
103472005	NP_002408.3	4288	MKI67	antigen KI-67 isoform 1	T1139	0.8	0.8	0.8	0.9	0.7	0.7	0.8	0.9	0.9	0.8	0.9	0.9	0.8	0.7	0.9	1.0	0.9	0.7	0.9	0.9	SPPPESVDtPTSTK	PPPESVDtPTSTKQW	T9;	Yes	S(1): 0.0; S(6): 0.0; T(9): 100.0; T(11): 0.0; S(12): 0.0; T(13): 0.0	118	2	1	0	N-Term(TMT6plex); T9(Phospho); K14(TMT6plex)
166795297	NP_057225.2	51143	DYNC1LI1	cytoplasmic dynein 1 light intermediate chain 1	S516	1.2	1.0	1.1	1.0	1.8	2.1	1.1	0.8	1.1	1.0	1.0	1.0	1.7	2.2	1.1	0.8	1.1	0.8	1.4	1.6	KPVTVSPTTPTsPTEGEAS	VSPTTPTsPTEGEAS	S12;	Yes	T(4): 0.0; S(6): 0.0; T(8): 1.8; T(9): 1.8; T(11): 1.8; S(12): 92.8; T(14): 1.8; S(19): 0.0	10	1	1	0	N-Term(TMT6plex); K1(TMT6plex); S12(Phospho)
9558749	NP_037434.1	29904	EEF2K	eukaryotic elongation factor 2 kinase	S445	0.9	1.0	0.7	0.8	0.9	1.0	0.8	1.0	-	-	-	-	-	-	-	-	-	-	-	-	ESENSGDSGYPsEK	SGDSGYPsEKRGELD	S12;	Yes	S(2): 0.0; S(5): 0.0; S(8): 0.0; Y(10): 0.0; S(12): 100.0	4	1	1	0	N-Term(TMT6plex); S12(Phospho); K14(TMT6plex)
546231404	NP_001271231.1	54443	ANLN	actin-binding protein anillin isoform 3	S623	1.0	1.0	1.4	1.0	0.7	0.9	1.1	0.8	1.1	1.0	1.3	1.0	0.7	0.9	1.0	0.9	0.9	0.9	1.2	0.9	AESGDsLGSEDR	PRAESGDsLGSEDRD	S6;	No	S(3): 0.0; S(6): 100.0; S(9): 0.0	21	3	1	0	N-Term(TMT6plex); S6(Phospho)
103472005	NP_002408.3	4288	MKI67	antigen KI-67 isoform 1	T2406	0.4	0.6	0.8	0.8	0.5	0.6	0.8	1.0	0.5	0.6	0.9	0.9	0.6	0.6	0.8	1.0	0.5	0.6	0.8	0.9	NINTEVEtPVQK	NINTEVEtPVQKLDL	T8;	Yes	T(4): 0.0; T(8): 100.0	118	2	1	0	N-Term(TMT6plex); T8(Phospho); K12(TMT6plex)
4506401	NP_002871.1	5894	RAF1	RAF proto-oncogene serine/threonine-protein kinase	T258	1.3	1.2	1.2	1.1	1.1	0.9	0.9	0.7	1.3	1.2	1.2	1.1	1.1	0.9	0.9	0.7	1.2	1.0	1.1	0.9	StSTPNVHMVSTTLPVDSR	LSQRQRStSTPNVHM	T2;	Yes	S(1): 0.9; T(2): 98.2; S(3): 0.9; T(4): 0.0; S(11): 0.0; T(12): 0.0; T(13): 0.0; S(18): 0.0	10	1	1	0	N-Term(TMT6plex); T2(Phospho); M9(Oxidation)
291190783	NP_001167448.1	55604	LRRC16A	leucine-rich repeat-containing protein 16A isoform 2	S1354	1.1	1.1	1.1	1.0	0.9	1.0	0.8	0.8	1.1	1.2	1.2	1.1	0.9	1.1	0.9	0.9	1.0	1.1	1.0	1.0	SNDsGEEAEK	QGSKSNDsGEEAEKE	S4;	No	S(1): 0.0; S(4): 100.0	13	2	1	0	N-Term(TMT6plex); S4(Phospho); K10(TMT6plex)
29789060	NP_055617.1	9847	C2CD5	C2 domain-containing protein 5 isoform e	S284; T285	1.1	1.1	1.0	0.5	0.5	1.4	1.8	1.3	1.0	1.0	0.9	0.5	0.5	1.2	1.6	1.3	0.7	1.1	1.3	0.9	EIPFNEDPNPNtHSSGPStPLK	NEDPNPNtHSSGPST;THSSGPStPLKNQTY	S18; T19;	Yes;Yes	T(12): 93.8; S(14): 2.1; S(15): 2.1; S(18): 2.1; T(19): 99.9	3	2	1	0	N-Term(TMT6plex); T12(Phospho); T19(Phospho); K22(TMT6plex)
29789060	NP_055617.1	9847	C2CD5	C2 domain-containing protein 5 isoform e	S284; T285	0.5	1.4	1.8	1.3	1.1	1.1	1.0	0.5	0.5	1.2	1.6	1.3	1.0	1.0	0.9	0.5	0.7	1.1	1.3	0.9	EIPFNEDPNPNTHSSGPsStPLK	NTHSSGPsTPLKNQT;THSSGPStPLKNQTY	S18; T19;	Yes;Yes	T(12): 0.0; S(14): 0.0; S(15): 0.0; S(18): 99.9; T(19): 100.0	3	2	1	0	N-Term(TMT6plex); S18(Phospho); T19(Phospho); K22(TMT6plex)
124286830	NP_588615.2	114991	ZNF618	zinc finger protein 618	S336	0.9	0.7	0.8	0.9	0.8	0.8	0.8	0.9	-	-	-	-	-	-	-	-	-	-	-	-	sPPAVVEEK	IASNQSRsPPAVVEE	S1;	No	S(1): 100.0	1	1	1	0	N-Term(TMT6plex); S1(Phospho); K9(TMT6plex)
503774357	NP_001265139.1	9972	NUP153	nuclear pore complex protein Nup153 isoform 3	T657	0.9	0.8	0.8	0.9	0.7	0.8	0.8	0.9	0.7	0.7	0.7	0.8	0.6	0.7	0.7	0.8	0.7	0.7	0.7	0.8	QTGIEtPNK	AKQTGIEtPNKSGKT	T6;	No	T(2): 0.0; T(6): 100.0	8	3	1	0	N-Term(TMT6plex); T6(Phospho); K9(TMT6plex)
312176416	NP_001185901.1	57648	KIAA1522	uncharacterized protein KIAA1522 isoform 2	S669; S673	5.4	0.9	0.5	0.6	1.5	0.9	0.6	1.1	6.1	1.1	0.6	0.7	1.7	1.0	0.7	1.3	3.9	1.0	0.7	1.0	DQsPPPsPPPSYHPPPPPTK	PVISKDQsPPPSPPP;KDQSPPPsPPPSYHP	S3; S7;	Yes;Yes	S(3): 100.0; S(7): 100.0; S(11): 0.0; Y(12): 0.0; T(19): 0.0	22	2	1	0	N-Term(TMT6plex); S3(Phospho); S7(Phospho); K20(TMT6plex)
5032189	NP_005648.1	7158	TP53BP1	tumor suppressor p53-binding protein 1 isoform 3	S552	1.7	1.5	1.4	1.1	0.8	1.1	1.0	0.7	1.8	1.4	1.5	1.3	0.8	1.1	1.1	0.8	1.3	1.3	1.3	1.1	IDEDGENTQIEDTEPMsPVLNSK	IEDTEPMsPVLNSKF	S17;	Yes	T(8): 0.0; T(13): 0.0; S(17): 100.0; S(22): 0.0	62	3	1	0	N-Term(TMT6plex); M16(Oxidation); S17(Phospho); K23(TMT6plex)
317574227	NP_001188264.1	6936	GCFC2	GC-rich sequence DNA-binding factor 2 isoform 3	S180	0.9	0.6	0.9	0.8	1.1	1.0	1.0	0.9	0.8	0.6	0.8	0.7	1.0	1.0	0.9	0.8	0.9	0.8	0.8	0.8	ESEDDPEsEPDDHEK	ESEDDPEsEPDDHEK	S8;	Yes	S(2): 0.0; S(8): 100.0	4	3	1	0	N-Term(TMT6plex); S8(Phospho); K15(TMT6plex)

Khan et al., 2019. Multi-omics analysis to characterize cigarette smoke induced molecular alterations in esophageal cells

Supplementary Table 8. List of phosphopeptides identified and quantified in untreated and chronically treated Het1A cells with cigarette smoke condensate for 8 months using SequestHT and Mascot search algorithms with a PhosphoRS probability score ≥75%

Protein group Accession	NP_Accession	Gene ID	Gene Symbol	Protein Description	PhosphoSite (Protein)	Het-1A-Smoke - 2M/Parental	Het-1A-Smoke - 4M/Parental	Het-1A-Smoke - 6M/Parental	Het-1A-Smoke - 8M/Parental	Het-1A-Smoke - 2M/Parental	Het-1A-Smoke - 4M/Parental	Het-1A-Smoke - 6M/Parental	Het-1A-Smoke - 8M/Parental	Het-1A-Smoke - 2M/Parental	Het-1A-Smoke - 4M/Parental	Het-1A-Smoke - 6M/Parental	Het-1A-Smoke - 8M/Parental	Het-1A-Smoke - 2M/Parental	Het-1A-Smoke - 4M/Parental	Het-1A-Smoke - 6M/Parental	Het-1A-Smoke - 8M/Parental
						Replicate 1				Replicate 2				Normalized phosphoprotein ratios with respect to corresponding total protein ratios. Replicate 1				Replicate 2			
556695367	NP_001273336.1	27079	RPUSD2	RNA pseudouridylate synthase domain-containing protein 2 isoform 2	S68	1.0	1.0	1.0	0.9	0.8	1.2	1.0	0.9	1.1	1.0	1.0	0.9	0.9	1.2	1.0	0.9
63053524	NP_001017416.1	7398	USP1	ubiquitin carboxyl-terminal hydrolase 1	S475	1.4	0.9	0.9	1.2	0.6	0.7	0.8	0.6	1.5	1.0	1.0	1.2	0.7	0.8	0.8	0.6
134133279	NP_612411.4	93349	SP140L	nuclear body protein SP140-like protein	S180	0.7	0.9	0.8	0.9	0.7	0.9	0.8	0.9	1.3	1.2	1.4	1.4	1.3	1.3	1.5	1.4
116008442	NP_055885.3	23091	ZC3H13	zinc finger CCCH domain-containing protein 13	S110	1.0	1.0	1.0	0.9	1.1	0.9	0.9	0.9	0.9	0.9	1.0	0.8	1.1	0.8	1.0	0.8
255918139	NP_001157616.1	283489	CHAMP1	chromosome alignment-maintaining phosphoprotein 1	S432; S436	0.8	0.9	0.7	0.8	1.0	0.9	0.9	1.0	0.8	0.9	0.7	0.9	1.0	0.9	0.9	1.0
120587019	NP_055160.2	24149	ZNF318	zinc finger protein 318	S136	0.7	0.7	0.7	0.8	0.8	0.8	0.8	0.9	0.8	0.7	0.8	0.9	0.8	0.8	0.8	1.0
42542379	NP_005830.2	10250	SRRM1	serine/arginine repetitive matrix protein 1	S209; S211	0.6	0.9	1.3	0.9	0.9	0.9	1.1	0.9	0.8	1.0	1.4	1.0	1.0	0.9	1.3	1.0
41322916	NP_958782.1	5339	PLEC	plectin isoform 1	S1435	0.5	0.8	0.9	0.8	0.5	0.8	0.9	1.0	0.6	0.9	0.9	0.8	0.6	0.9	1.0	1.0
193083131	NP_001122393.1	4140	MARK3	MAP/microtubule affinity-regulating kinase 3 isoform	S390	0.8	0.7	0.7	0.9	0.7	0.6	0.8	0.9	0.9	0.8	0.8	0.9	0.7	0.7	0.8	0.9
85838513	NP_705902.2	80025	PANK2	pantothenate kinase 2, mitochondrial isoform 1 preproprotein	S169	1.0	1.6	1.1	0.9	0.7	1.1	1.0	0.8	0.8	1.5	1.2	0.9	0.6	1.1	1.0	0.8
55749431	NP_001006934.1	85012	TCEAL3	transcription elongation factor A protein-like 3	S65	1.0	1.2	1.2	0.8	1.2	1.4	1.4	1.0	1.5	2.1	1.8	1.6	1.7	2.5	2.2	1.9
296011010	NP_060252.4	54906	FAM208B	protein FAM208B	S1172	0.8	1.1	0.9	0.8	0.8	0.7	0.7	1.0	0.8	1.1	1.0	0.9	0.7	0.8	0.8	1.0
167234419	NP_005110.2	9967	THRAP3	thyroid hormone receptor-associated protein 3	S320	0.9	0.9	1.0	1.0	0.8	0.9	0.8	0.8	0.9	1.0	1.1	1.1	0.8	1.0	0.9	0.8
347800665	NP_001231653.1	5887	RAD23B	UV excision repair protein RAD23 homolog B isoform 3	S88	1.4	0.9	2.2	1.1	0.5	0.4	0.9	0.7	1.2	0.9	1.7	1.1	0.4	0.4	0.7	0.6
256000749	NP_001157711.1	8449	DHX16	putative pre-mRNA-splicing factor ATP-dependent RNA helicase DHX16 isoform 2	S100	0.9	0.8	0.9	0.8	1.0	0.8	0.9	0.9	0.9	0.8	1.0	0.9	1.0	0.9	1.0	1.0
217330641	NP_060439.2	55677	IWS1	protein IWS1 homolog	S511; S513	1.0	1.0	0.9	0.9	0.9	0.9	0.9	0.9	1.0	0.9	0.9	1.0	0.9	0.9	0.9	1.0
118572613	NP_057417.3	23524	SRRM2	serine/arginine repetitive matrix protein 2	T1208	0.9	0.9	0.9	0.9	1.0	1.1	0.9	0.9	0.9	1.0	0.9	0.9	1.0	1.1	0.9	1.0
103472005	NP_002408.3	4288	MKI67	antigen KI-67 isoform 1	T1193	0.5	0.7	0.8	0.9	0.4	0.6	1.2	0.9	0.6	0.7	0.8	0.9	0.4	0.6	1.2	0.9
42542379	NP_005830.2	10250	SRRM1	serine/arginine repetitive matrix protein 1	S560; S562	1.0	1.0	1.1	0.9	0.8	0.8	0.8	0.9	1.2	1.1	1.2	1.0	1.0	0.8	0.9	1.0
39753957	NP_056417.2	26092	TOR1AIP1	torsin-1A-interacting protein 1 isoform 2	S156; S157	1.0	0.8	0.7	0.4	1.3	1.7	1.5	1.3	1.0	0.8	0.7	0.4	1.2	1.8	1.4	1.3
114155142	NP_003283.2	7175	TPR	nucleoprotein TPR	T2137	1.3	1.4	1.0	1.0	0.4	0.7	0.6	0.8	1.4	1.5	1.2	1.2	0.5	0.7	0.7	0.9
33620755	NP_060493.3	55689	YEATS2	YEATS domain-containing protein 2	S536	0.6	0.6	0.7	0.8	0.7	0.5	0.7	1.0	-	-	-	-	-	-	-	-
7661690	NP_056418.1	26093	CCDC9	coiled-coil domain-containing protein 9	T95	0.7	1.2	0.8	0.9	0.7	1.1	0.9	0.9	0.7	1.2	0.8	0.9	0.7	1.0	0.9	1.0
28558977	NP_004822.2	9441	MED26	mediator of RNA polymerase II transcription subunit	S447	0.9	0.9	0.7	1.0	0.8	0.9	0.9	0.8	-	-	-	-	-	-	-	-
42542379	NP_005830.2	10250	SRRM1	serine/arginine repetitive matrix protein 1	S402; T406	0.7	0.8	0.8	0.8	0.8	0.9	0.8	0.9	0.8	0.9	0.9	0.9	1.0	1.0	0.9	1.1
42741682	NP_005446.2	9406	ZRANB2	zinc finger Ran-binding domain-containing protein 2 isoform 2	S153	1.3	1.4	1.1	0.9	1.1	1.3	1.0	0.9	1.4	1.5	1.3	1.1	1.2	1.3	1.2	1.2
42542379	NP_005830.2	10250	SRRM1	serine/arginine repetitive matrix protein 1	S402; T406	0.8	0.9	0.8	0.9	0.7	0.8	0.8	0.8	1.0	1.0	0.9	1.1	0.8	0.9	0.9	0.9
14249338	NP_116114.1	84811	BUD13	BUD13 homolog isoform 1	T159; S163	0.9	0.8	0.8	0.9	0.8	1.1	0.8	0.9	1.0	0.8	0.9	1.0	0.9	1.1	0.9	1.0
21396500	NP_003600.2	8479	HIRIP3	HIRA-interacting protein 3 isoform 1	T84; S87	0.6	0.9	0.8	1.0	0.6	1.1	0.7	0.8	0.8	1.0	0.9	1.1	0.7	1.2	0.9	0.9
39995080	NP_078795.2	79567	FAM65A	protein FAM65A isoform 1	S347	1.1	1.0	1.2	0.7	0.9	1.2	0.9	1.0	0.9	1.0	1.1	0.7	0.8	1.2	0.8	1.0
546231404	NP_001271231.1	54443	ANLN	actin-binding protein anillin isoform 3	S54	1.0	0.9	0.9	0.8	0.9	1.2	1.2	1.0	1.0	0.9	0.9	0.8	0.9	1.2	1.1	1.0
4507741	NP_000465.1	7291	TWIST1	twist-related protein 1	S68	1.0	0.7	1.0	1.0	1.2	1.0	0.9	0.8	-	-	-	-	-	-	-	-
118572613	NP_057417.3	23524	SRRM2	serine/arginine repetitive matrix protein 2	S1542	1.0	1.0	1.1	0.8	1.2	1.2	1.1	1.0	1.1	1.1	1.2	0.9	1.3	1.2	1.1	1.1
7661958	NP_055554.1	9774	BCLAF1	bcl-2-associated transcription factor 1 isoform 1	S397	0.9	1.1	1.0	0.8	0.9	1.1	1.1	0.9	1.0	1.2	1.1	0.9	1.0	1.2	1.1	1.0
118572613	NP_057417.3	23524	SRRM2	serine/arginine repetitive matrix protein 2	S1542	1.2	1.2	1.1	1.0	1.0	1.0	1.1	0.8	1.3	1.2	1.1	1.1	1.1	1.1	1.2	0.9
7019569	NP_037377.1	27183	VPS4A	vacuolar protein sorting-associated protein 4A	S97	0.8	0.8	0.5	0.8	0.9	0.9	0.9	1.0	0.9	1.0	0.6	0.9	1.0	1.1	1.1	1.2
88759348	NP_078822.3	79596	RNF219	RING finger protein 219	S719	0.8	0.4	0.7	0.6	1.0	0.9	1.0	1.1	0.7	0.4	0.7	0.6	0.9	0.9	1.0	1.2
288806590	NP_001165775.1	84930	MASTL	serine/threonine-protein kinase greatwall isoform 3	S370	2.0	0.8	2.2	1.0	0.7	0.6	1.2	0.7	1.9	0.7	2.0	1.0	0.6	0.5	1.1	0.7
153945728	NP_005900.2	4131	MAP1B	microtubule-associated protein 1B	S1076	1.0	1.0	1.1	0.8	1.1	1.1	1.1	0.9	0.8	0.8	0.9	0.9	0.9	0.8	0.9	1.0
167234419	NP_005110.2	9967	THRAP3	thyroid hormone receptor-associated protein 3	T954	0.8	0.8	0.7	0.7	1.0	1.0	0.9	1.0	0.8	0.8	0.8	0.8	1.1	1.0	1.0	1.1
21493024	NP_653080.1	9590	AKAP12	A-kinase anchor protein 12 isoform 2	S598	0.7	0.9	1.1	0.8	0.7	0.9	1.2	0.9	0.8	0.8	0.9	0.7	0.8	0.9	1.0	0.9
51599156	NP_001264.2	1108	CHD4	chromodomain-helicase-DNA-binding protein 4	S1535	0.9	0.9	0.9	0.8	0.9	0.8	0.8	0.9	1.0	1.0	1.0	0.9	1.0	0.9	0.8	1.0
21493024	NP_653080.1	9590	AKAP12	A-kinase anchor protein 12 isoform 2	S598	0.7	0.9	1.2	0.9	0.7	0.9	1.1	0.8	0.8	0.9	1.0	0.9	0.8	0.8	0.9	0.7
53829376	NP_001005476.1	8502	PKP4	plakophilin-4 isoform b	S273; S281	0.8	0.6	0.7	0.7	1.2	1.1	0.8	1.0	-	-	-	-	-	-	-	-
544709647	NP_001269861.1	10152	ABI2	abl interactor 2 isoform e	S121	0.9	0.8	0.9	0.9	0.9	0.6	0.9	0.9	0.8	0.8	0.9	0.8	0.9	0.7	1.0	0.8
385251399	NP_001245260.1	2036	EPB41L1	band 4.1-like protein 1 isoform b	S682	1.0	1.2	1.1	1.0	1.0	1.3	1.0	0.8	1.0	1.3	1.1	1.1	1.0	1.4	1.0	0.8
53829376	NP_001005476.1	8502	PKP4	plakophilin-4 isoform b	S273; S281	1.2	1.1	0.8	1.0	0.8	0.6	0.7	0.7	-	-	-	-	-	-	-	-
149158690	NP_542417.2	7916	PRRC2A	protein PRRC2A	T782	1.3	1.4	1.2	1.3	0.6	0.4	0.4	0.5	1.4	1.3	1.3	1.2	0.7	0.4	0.4	0.4
545746316	NP_001271144.1	9320	TRIP12	E3 ubiquitin-protein ligase TRIP12 isoform b	S280	0.9	0.9	1.0	0.8	1.0	1.0	1.0	0.9	0.9	1.0	1.0	0.9	1.0	1.1	1.0	1.0
545746335	NP_001271143.1	9320	TRIP12	E3 ubiquitin-protein ligase TRIP12 isoform a	S280	1.0	1.0	1.0	0.9	0.9	0.9	1.0	0.8	1.0	1.1	1.0	1.0	0.9	1.0	1.0	0.9
114155142	NP_003283.2	7175	TPR	nucleoprotein TPR	S379	0.7	0.5	0.5	0.9	0.7	0.8	0.8	0.9	0.8	0.6	0.6	1.0	0.8	0.9	0.9	1.0
217416350	NP_001136115.1	57513	CASKIN2	caskin-2 isoform b	S314	0.8	1.0	1.0	0.8	0.8	0.9	0.7	0.9	-	-	-	-	-	-	-	-
385648270	NP_001245309.1	9497	SLC4A7	sodium bicarbonate cotransporter 3 isoform c	S1089	0.9	1.2	1.2	0.8	1.6	1.1	1.6	0.9	0.8	1.1	1.0	0.7	1.3	1.0	1.4	0.8
91176325	NP_038477.2	11176	BAZ2A	bromodomain adjacent to zinc finger domain protein 2A	S509	0.6	0.6	0.6	0.6	0.8	0.8	1.0	1.1	0.7	0.7	0.6	0.7	0.9	0.8	1.1	1.2
155722990	NP_060628.2	22950	SLC4A1AP	kanadaptin	S312	1.2	1.6	1.0	1.0	0.8	0.9	0.8	0.7	1.4	1.8	1.2	1.2	0.9	1.1	1.0	0.9
38788219	NP_872304.2	126299	ZNF428	zinc finger protein 428	T108	0.9	0.6	0.6	0.7	1.0	0.8	0.7	1.1	1.1	0.7	0.7	0.9	1.2	0.8	0.9	1.5
29568103	NP_003080.2	6625	SNRNP70	U1 small nuclear ribonucleoprotein 70 kDa	S226	0.6	0.8	0.8	0.7	1.0	0.9	1.0	1.1	0.7	0.9	1.0	0.8	1.2	1.1	1.2	1.3
324021685	NP_001191194.1	2908	NR3C1	glucocorticoid receptor isoform GR-P	S45	0.7	0.7	0.7	0.7	0.8	0.9	1.1	1.1	0.8	0.7	0.7	0.7	0.8	0.9	1.0	1.1
42542379	NP_005830.2	10250	SRRM1	serine/arginine repetitive matrix protein 1	T220	0.8	1.0	0.9	0.9	1.1	0.9	1.2	0.8	1.0	1.1	1.0	1.1	1.4	1.0	1.4	0.9
24041035	NP_077719.2	4853	NOTCH2	neurogenic locus notch homolog protein 2 isoform 1 preproprotein	S1778	0.9	1.5	1.3	0.7	0.8	1.1	0.7	1.0	1.0	1.4	1.3	0.7	0.8	1.1	0.7	1.0
31563338	NP_004505.2	3607	FOXK2	forkhead box protein K2	S170	0.9	0.9	0.8	0.8	0.7	0.8	0.9	0.9	0.8	0.9	0.8	0.9	0.7	0.9	0.9	0.9
222136585	NP_003911.2	8914	TIMELESS	protein timeless homolog	S1173	0.7	0.6	0.4	1.0	1.1	1.0	0.5	0.7	0.8	0.6	0.4	1.1	1.3	1.1	0.6	0.8
42741682	NP_005446.2	9406	ZRANB2	zinc finger Ran-binding domain-containing protein 2 isoform 2	S188	1.1	1.2	1.0	0.8	0.9	1.0	0.9	0.9	1.2	1.3	1.2	1.0	1.0	1.0	1.1	1.2
118572613	NP_057417.3	23524	SRRM2	serine/arginine repetitive matrix protein 2	S1925; T1927	0.9	0.9	0.9	0.8	1.0	1.1	1.0	1.0	1.0	1.0	1.0	0.9	1.0	1.1	1.1	1.0
33620716	NP_061173.1	5930	RBBP6	E3 ubiquitin-protein ligase RBBP6 isoform 2	S1243	0.5	0.5	0.7	0.6	1.0	0.9	1.4	1.1	0.6	0.5	0.8	0.6	1.1	0.9	1.6	1.2
223555917	NP_848927.2	92140	MTDH	protein LYRIC	T143	0.8	1.2	0.8	0.8	0.7	0.7	0.7	1.0	0.6	1.1	0.7	0.7	0.6	0.6	0.6	0.9
28559039	NP_004765.2	5469	MED1	mediator of RNA polymerase II transcription subunit 1	S1042	0.8	1.0	1.0	0.9	0.8	0.9	0.9	0.9	0.9	1.0	1.1	0.9	0.8	0.9	1.0	0.9
29568103	NP_003080.2	6625	SNRNP70	U1 small nuclear ribonucleoprotein 70 kDa	S410	1.4	1.1	1.4	0.9	1.2	0.5	1.0	0.8	1.7	1.3	1.7	1.2	1.4	0.5	1.2	1.0
118572613	NP_057417.3	23524	SRRM2	serine/arginine repetitive matrix protein 2	S1387	0.7	0.9	0.7	0.9	1.1	1.2	0.9	0.9	0.7	0.9	0.8	0.9	1.1	1.2	1.0	1.0
118572613	NP_057417.3	23524	SRRM2	serine/arginine repetitive matrix protein 2	S1387	1.1	1.2	0.9	0.9	0.7	0.9	0.7	0.9	1.1	1.2	1.0	1.0	0.7	0.9	0.8	0.9
10835067	NP_003133.1	6741	SSB	lupus La protein	S350	0.7	0.8	0.7	0.8	0.5	0.7	0.7	0.9	0.9	0.9	0.9	1.1	0.6	0.8	0.9	1.2
45597177	NP_055858.2	23061	TBC1D9B	TBC1 domain family member 9B isoform b	S435	2.1	1.9	1.2	0.8	0.9	0.9	0.8	0.9	2.3	2.1	1.4	0.9	0.9	1.0	0.9	1.1
5902022	NP_008955.1	11070	TMEM115	transmembrane protein 115	S320	0.8	1.1	0.8	0.8	1.0	1.0	0.9	0.9	0.8	1.0	0.8	0.7	0.9	0.9	0.8	0.8
314122233	NP_001186627.1	7917	BAG6	large proline-rich protein BAG6 isoform d	S1062	1.1	1.1	1.2	1.1	1.5	1.4	1.6	0.6	1.2	1.2	1.4	1.2	1.5	1.5	1.9	0.7
312434011	NP_001186084.1	11143	KAT7	histone acetyltransferase KAT7 isoform 2	T88	0.9	0.6	0.7	0.8	0.9	1.0	0.8	1.0	0.7	0.5	0.6	0.7	0.8	0.8	0.7	0.9
14717407	NP_005633.2	6879	TAF7	transcription initiation factor TFIID subunit 7	S201	1.1	1.1	1.0	0.9	0.8	0.8	0.9	0.9	1.2	1.2	1.3	1.1	0.9	0.8	1.1	1.0
21361499	NP_056485.2	26168	SENP3	sentrin-specific protease 3	S212	0.7	0.8	0.8	1.2	0.8	0.9	0.7	0.5	0.7	0.9	0.8	1.3	0.8	1.0	0.8	0.6
42542379	NP_005830.2	10250	SRRM1	serine/arginine repetitive matrix protein 1	S260	0.9	1.2	1.2	0.8	0.6	1.1	0.9	0.9	1.1	1.3	1.4	0.9	0.8	1.2	1.0	1.0
291621647	NP_006311.2	10424	PGRMC2	membrane-associated progesterone receptor component 2	S114	0.9	1.0	0.9	0.7	1.0	1.0	0.8	1.0	0.8	1.0	0.9	0.7	1.0	1.0	0.8	1.0
118572613	NP_057417.3	23524	SRRM2	serine/arginine repetitive matrix protein 2	S818; S820	0.9	1.1	1.0	0.9	1.0	1.1	1.0	0.8	1.0	1.1	1.0	1.0	1.0	1.1	1.0	0.9
51226124	NP_055805.1	253959	RALGAPA1	ral GTPase-activating protein subunit alpha-1 isoform 1	S740	0.9	0.8	0.8	0.9	0.8	0.7	0.8	0.8	0.9	0.9	0.8	1.0	0.8	0.8	0.8	0.9
321267473	NP_001188269.1	6294	SAFB	scaffold attachment factor B1 isoform 4	S532	0.9	1.4	1.3	0.8	1.8	3.0	2.3	1.0	1.2	1.5	1.6	0.9	2.3	3.2	2.8	1.1
42542379	NP_005830.2	10250	SRRM1	serine/arginine repetitive matrix protein 1	T220	0.8	0.9	0.9	0.8	0.8	1.0	1.0	0.9	0.9	1.0	1.0	1.0	1.0	1.0	1.1	1.0
56118310	NP_073568.2	64710	NUCKS1	nuclear ubiquitous casein and cyclin-dependent kinase substrate 1	S181	0.9	0.9	0.7	0.9	0.9	1.0	0.7	0.9	1.1	1.1	0.9	1.1	1.1	1.2	0.9	1.1
321267473	NP_001188269.1	6294	SAFB	scaffold attachment factor B1 isoform 4	S532	1.8	3.0	2.3	1.0	0.9	1.4	1.3	0.8	2.3	3.2	2.8	1.1	1.2	1.5	1.6	0.9
41152097	NP_005079.2	8227	AKAP17A	A-kinase anchor protein 17A isoform 1	S537	0.9	1.2	1.1	0.7	1.2	1.0	1.0	1.0	0.8	1.3	1.2	0.8	1.1	1.0	1.1	1.1
42542379	NP_005830.2	10250	SRRM1	serine/arginine repetitive matrix protein 1	S874	1.1	1.1	1.0	0.8	0.9	1.1	1.0	0.9	1.3	1.2	1.1	0.9	1.1	1.2	1.1	1.1
42542379	NP_005830.2	10250	SRRM1	serine/arginine repetitive matrix protein 1	S874	0.9	1.1	1.0	0.9	1.1	1.1	1.0	0.8	1.1	1.2	1.1	1.1	1.3	1.2	1.1	0.9
5031805	NP_005535.1	3667	IRS1	insulin receptor substrate 1	T525; S531	0.9	1.1	1.2	0.6	0.9	0.9	0.9	1.1	-	-	-	-	-	-	-	-
302699211	NP_665739.3	23126	POGZ	pogo transposable element with ZNF domain isoform 3	S330	0.8	1.2	1.5	0.8	0.8	0.7	1.2	0.9	0.8	1.1	1.4	0.8	0.7	0.7	1.1	0.8
4505195	NP_002410.1	4296	MAP3K11	mitogen-activated protein kinase kinase kinase 11	S507	0.4	0.9	0.8	0.8	1.3	1.4	1.1	0.9	-	-	-	-	-	-	-	-
148612816	NP_004250.4	9400	RECQL5	ATP-dependent DNA helicase Q5 isoform 1	T839	0.6	0.7	0.5	0.9	0.4	0.8	1.0	0.8	0.6	0.7	0.5	0.8	0.4	0.8	0.9	0.8
5031805	NP_005535.1	3667	IRS1	insulin receptor substrate 1	T525; S531	0.9	0.9	0.9	1.1	0.9	1.1	1.2	0.6	-	-	-	-	-	-	-	-
54607124	NP_037418.3	29890	RBM15B	putative RNA-binding protein 15B	T631	0.8	0.7	0.7	0.8	0.9	0.8	0.9	1.0	0.8	0.8	0.8	0.8	0.9	1.0	1.0	1.0
110556636	NP_203754.2	85456	TNKS1BP1	182 kDa tankyrase-1-binding protein	S1666	1.2	1.3	1.4	0.8	1.0	1.2	1.3	0.9	1.2	1.1	1.3	0.9	0.9	1.1	1.3	1.0
21237805	NP_003066.2	6601	SMARCC2	SWI/SNF complex subunit SMARCC2 isoform a	S347	0.5	0.4	0.3	0.5	1.3	1.6	1.4	1.2	0.4	0.4	0.3	0.5	1.2	1.5	1.4	1.2
32307124	NP_006525.2	8202	NCOA3	nuclear receptor coactivator 3 isoform b	S551	0.7	0.6	0.6	0.5	1.3	0.8	1.2	1.2	-	-	-	-	-	-	-	-
103472005	NP_002408.3	4288	MKI67	antigen KI-67 isoform 1	S1815	0.7	0.6	0.8	0.8	0.9	0.6	1.0	0.9	0.9	0.6	0.8	0.9	1.1	0.7	1.0	1.0
18426904	NP_569079.1	56897	WRNIP1	ATPase WRNIP1 isoform 2	S65	0.8	0.9	0.8	0.8	0.9	0.9	0.9	0.9	0.8	0.9	0.9	0.9	0.9	0.9	1.0	1.0
126722969	NP_079358.3	80152	CENPT	centromere protein T	S47	0.8	0.9	0.6	0.9	0.6	1.0	0.7	0.8	0.7	0.8	0.7	0.9	0.6	1.0	0.8	0.8
103472005	NP_002408.3	4288	MKI67	antigen KI-67 isoform 1	T328	0.7	0.7	0.8	0.9	0.7	0.6	0.5	0.8	0.9	0.7	0.8	1.0	0.8	0.7	0.5	0.8
72256200	NP_004317.2	607	BCL9	B-cell CLL/lymphoma 9 protein	T315	0.9	1.2	0.8	0.7	1.2	1.1	1.1	1.0	-	-	-	-	-	-	-	-
47271443	NP_003007.2	6427	SRSF2	serine/arginine-rich splicing factor 2	S191	0.7	1.0	0.7	0.8	0.7	0.8	0.9	0.9	0.8	1.0	0.8	1.0	0.8	0.8	1.0	1.1
40807504	NP_940997.1	60312	AFAP1	actin filament-associated protein 1 isoform B	S668	0.8	0.7	0.9	0.8	0.9	0.9	1.1	0.9	-	-	-	-	-	-	-	-
10716563	NP_001737.1	821	CANX	calnexin precursor	S564	1.0	1.2	0.9	0.8	1.1	1.1	0.9	0.9	1.0	0.9	0.9	0.7	1.1	0.8	0.9	0.8
313760537	NP_001186421.1	27250	PDCD4	programmed cell death protein 4 isoform 3	S80	1.0	1.0	1.0	0.9	0.9	0.7	0.7	0.8	1.0	1.1	1.0	0.9	0.9	0.8	0.7	0.8
7661958	NP_055554.1	9774	BCLAF1	bcl-2-associated transcription factor 1 isoform 1	S177	0.9	1.0	0.9	0.8	0.7	0.9	0.8	0.9	1.0	1.1	1.0	0.9	0.8	0.9	0.9	0.9
66528888	NP_001018125.1	2177	FANCD2	Fanconi anemia group D2 protein isoform b	T1408	0.5	0.4	0.9	0.3	0.4	0.4	1.6	1.4	0.6	0.5	1.0	0.3	0.5	0.4	1.8	1.5
197304708	NP_001127867.1	84310	C7orf50	uncharacterized protein C7orf50	S175	1.1	1.3	1.1	0.9	1.0	1.2	1.0	0.8	1.3	1.3	1.4	1.1	1.2	1.2	1.2	0.9
56118310	NP_073568.2	64710	NUCKS1	nuclear ubiquitous casein and cyclin-dependent kinase substrate 1	S214	0.9	1.1	0.9	1.0	0.9	0.9	0.7	0.7	1.2	1.3	1.2	1.3	1.2	1.1	1.0	0.9
66528888	NP_001018125.1	2177	FANCD2	Fanconi anemia group D2 protein isoform b	T1408	0.4	0.4	1.6	1.4	0.5	0.4	0.9	0.3	0.5	0.4	1.8	1.5	0.6	0.5	1.0	0.3
14110414	NP_002129.2	3184	HNRNPD	heterogeneous nuclear ribonucleoprotein D0 isoform c	S80; S83	1.0	0.7	1.2	0.8	0.9	0.9	1.0	0.9	1.0	0.7	1.4	0.8	1.0	0.9	1.3	0.8
14110414	NP_002129.2	3184	HNRNPD	heterogeneous nuclear ribonucleoprotein D0 isoform c	S80; S83	0.9	0.9	1.0	0.9	1.0	0.7	1.2	0.8	1.0	0.9	1.3	0.8	1.0	0.7	1.4	0.8
333440471	NP_001193980.1	23332	CLASP1	CLIP-associating protein 1 isoform 4	S1162	1.0	0.9	0.9	0.9	0.8	0.9	0.8	0.8	1.0	0.9	1.0	0.9	0.8	0.9	0.9	0.9
6594683	NP_015627.1	7468	WHSC1	histone-lysine N-methyltransferase NSD2 isoform 4	T544	0.9	1.2	0.9	0.8	1.0	0.9	0.7	0.9	1.0	1.3	1.0	0.9	1.2	1.1	0.8	1.0
112421108	NP_055940.3	23152	CIC	protein capicua homolog	S431	0.6	0.6	0.7	0.8	0.5	1.3	1.0	0.9	-	-	-	-	-	-	-	-
87299628	NP_683692.2	259282	BOD1L1	biorientation of chromosomes in cell division protein 1-like 1	S3019	0.9	0.9	0.9	0.8	1.1	1.1	0.9	0.9	0.9	0.9	0.9	0.9	1.1	1.0	0.9	1.0
33598968	NP_005349.3	4008	LMO7	LIM domain only protein 7 isoform 1	S1176	0.8	0.7	0.8	0.9	1.3	0.7	1.4	0.8	0.7	0.5	0.6	0.9	1.1	0.6	0.9	0.7
112421108	NP_055940.3	23152	CIC	protein capicua homolog	S431	0.5	1.3	1.0	0.9	0.6	0.6	0.7	0.8	-	-	-	-	-	-	-	-
183396804	NP_056018.2	23248	RPRD2	regulation of nuclear pre-mRNA domain-containing protein 2	S374	0.6	1.3	1.2	0.6	1.0	0.9	1.4	1.1	0.6	1.3	1.2	0.5	1.0	0.9	1.4	1.0
14249338	NP_116114.1	84811	BUD13	BUD13 homolog isoform 1	T187	0.2	0.5	0.7	0.6	0.9	1.2	0.9	1.1	0.2	0.6	0.8	0.6	0.9	1.3	1.0	1.3
13489054	NP_109587.1	5605	MAP2K2	dual specificity mitogen-activated protein kinase	T394	0.9	0.8	0.6	1.0	1.2	0.7	1.0	0.7	1.1	0.9	0.7	1.2	1.4	0.9	1.1	0.9
14249338	NP_116114.1	84811	BUD13	BUD13 homolog isoform 1	T187	0.9	1.2	0.9	1.1	0.2	0.5	0.7	0.6	0.9	1.3	1.0	1.3	0.2	0.6	0.8	0.6
124028529	NP_004810.2	8189	SYMPK	symplekin	S1259	0.7	0.6	0.6	0.8	2.0	1.6	1.7	0.9	0.8	0.6	0.7	0.8	2.2	1.7	1.8	0.9

Protein group Accession	Average of replicates: Het-1A-Smoke - 2M/Parental	Het-1A-Smoke - 4M/Parental	Het-1A-Smoke - 6M/Parental	Het-1A-Smoke - 8M/Parental	Peptide Sequence	Phosphowindow	PhosphoSite (Peptide)	Phosphosite Plus Evidence	phosphoRS Site Probabilities	# PSMs	# Proteins	# Protein Groups	# Missed Cleavages	Modifications
556695367	1.0	1.1	1.0	0.9	VELsPGPPKPAGR	GDAKVELsPGPPKPA	S4;	Yes	S(4): 100.0	1	2	1	0	N-Term(TMT6plex); S4(Phospho); K9(TMT6plex)
63053524	1.1	0.9	0.9	0.9	VEESSEIsPEPK	VEESSEIsPEPKTEM	S8;	Yes	S(4): 0.0; S(5): 0.0; S(8): 100.0	7	1	1	0	N-Term(TMT6plex); S8(Phospho); K12(TMT6plex)
134133279	1.3	1.2	1.5	1.4	QGEVPEsPEAR	KQGEVPEsPEARKES	S7;	Yes	S(7): 100.0	4	1	1	0	N-Term(TMT6plex); S7(Phospho)
116008442	1.0	0.9	1.0	0.8	NTEESSsPVR	RNTEESSsPVRKESS	S7;	Yes	T(2): 0.0; S(5): 0.0; S(6): 0.0; S(7): 100.0	28	1	1	0	N-Term(TMT6plex); S7(Phospho)
255918139	0.9	0.9	0.8	1.0	sPAGsPELR	PLSPEIRsPAGSPEL;EIRSPAGsPELRKPS	S1; S5;	Yes;Yes	S(1): 100.0; S(5): 100.0	24	1	1	0	N-Term(TMT6plex); S1(Phospho); S5(Phospho)
120587019	0.8	0.8	0.8	1.0	sPGLCSDSLEK	DSGSRRRsPGLCSDS	S1;	Yes	S(1): 100.0; S(6): 0.0; S(8): 0.0	9	1	1	0	N-Term(TMT6plex); S1(Phospho); C5(Carbamidomethyl); K11(TMT6plex)
42542379	0.9	1.0	1.3	1.0	sPsPAPEK	RHRTKSRsPSPAPEK;RTKSRSPsPAPEKKE	S1; S3;	Yes;Yes	S(1): 100.0; S(3): 100.0	229	1	1	0	N-Term(TMT6plex); S1(Phospho); S3(Phospho); K8(TMT6plex)
41322916	0.6	0.9	1.0	0.9	AQLEPVAsPAK	AQLEPVAsPAKKPKV	S8;	Yes	S(8): 100.0	17	8	1	0	N-Term(TMT6plex); S8(Phospho); K11(TMT6plex)
193083131	0.8	0.7	0.8	0.9	GIAPAsPMLGNASNPNK	GKGIAPAsPMLGNAS	S6;	No	S(6): 100.0; S(13): 0.0	15	5	1	0	N-Term(TMT6plex); S6(Phospho); M8(Oxidation); K17(TMT6plex)
85838513	0.7	1.3	1.1	0.8	ASsASVPAVGASAEGTR	PLRRRASsASVPAVG	S3;	Yes	S(2): 1.1; S(3): 98.9; S(5): 0.0; S(12): 0.0; T(16): 0.0	3	1	1	0	N-Term(TMT6plex); S3(Phospho)
55749431	1.6	2.3	2.0	1.8	EDEGEPGDEGQLEDEGsQEK	GQLEDEGsQEKQGRS	S17;	Yes	S(17): 100.0	10	1	1	0	N-Term(TMT6plex); S17(Phospho); K20(TMT6plex)
296011010	0.8	1.0	0.9	0.9	SSSHLsPSEEVR	AKSSSHLsPSEEVRC	S6;	Yes	S(1): 0.0; S(2): 0.0; S(3): 0.0; S(6): 100.0; S(8): 0.0	4	1	1	0	N-Term(TMT6plex); S6(Phospho)
167234419	0.9	1.0	1.0	1.0	sPPSTGSTYGSSQK	KKSPVGKsPPSTGST	S1;	Yes	S(1): 98.4; S(4): 1.6; T(5): 0.0; S(7): 0.0; T(8): 0.0; Y(9): 0.0; S(11): 0.0; S(12): 0.0	33	1	1	0	N-Term(TMT6plex); S1(Phospho); K14(TMT6plex)
347800665	0.8	0.7	1.2	0.8	QEKPAEKPAETPVATsPTATDSTSGDSSR	AETPVATsPTATDST	S16;	No	T(11): 0.0; T(15): 4.0; S(16): 91.8; T(18): 4.0; T(20): 0.2; S(22): 0.0; T(23): 0.0; S(24): 0.0; S(27): 0.0; [illegible]	1	3	1	0	N-Term(TMT6plex); K3(TMT6plex); K7(TMT6plex); S16(Phospho)
256000749	1.0	0.8	1.0	0.9	QQTEKPEsEDEWER	QQTEKPEsEDEWERT	S8;	No	T(3): 0.0; S(8): 100.0	8	2	1	0	N-Term(TMT6plex); K5(TMT6plex); S8(Phospho)
217330641	0.9	0.9	0.9	1.0	EAEDsDsDDNIKR	QVKEAEDsDSDDNIK;KEAEDSDsDDNIKRG	S5; S7;	Yes;Yes	S(5): 100.0; S(7): 100.0	25	1	1	1	N-Term(TMT6plex); S5(Phospho); S7(Phospho); K12(TMT6plex)
118572613	1.0	1.0	0.9	1.0	DTLRtPPR	VFKDTLRtPPRERSG	T5;	Yes	T(2): 0.0; T(5): 100.0	436	1	1	1	N-Term(TMT6plex); T5(Phospho)
103472005	0.5	0.6	1.0	0.9	AEMGtPVQK	DIKAEMGtPVQKLDL	T5;	Yes	T(5): 100.0	118	2	1	0	N-Term(TMT6plex); M3(Oxidation); T5(Phospho); K9(TMT6plex)
42542379	1.1	1.0	1.1	1.0	sPsPAPPPR	PPTRRRRsPSPAPPP;TRRRRSPsPAPPPRR	S1; S3;	Yes;Yes	S(1): 100.0; S(3): 100.0	229	1	1	0	N-Term(TMT6plex); S1(Phospho); S3(Phospho)
39753957	1.1	1.3	1.0	0.9	DSHssEEDEASSQTDLSQTISK	RGLRDSHsSEEDEAS;GLRDSHSsEEDEASS	S4; S5;	Yes;Yes	S(2): 5.6; S(4): 96.9; S(5): 96.9; S(11): 0.2; S(12): 0.2; T(14): 0.2; S(17): 0.0; T(19): 0.0; S(21): 0.0	4	2	1	0	N-Term(TMT6plex); S4(Phospho); S5(Phospho); K22(TMT6plex)
114155142	0.9	1.1	0.9	1.0	TVPStPTLVVPHR	EDRTVPStPTLVVPH	T5;	Yes	T(1): 0.0; S(4): 0.8; T(5): 99.2; T(7): [illegible]	29	1	1	0	N-Term(TMT6plex); T5(Phospho)
33620755	-	-	-	-	ISTASQVSQGTGsPVPK	QVSQGTGsPVPKIHG	S13;	Yes	S(2): 0.0; T(3): 0.0; S(5): 0.0; S(8): 0.0; T(11): 0.0; S(13): 100.0	6	1	1	0	N-Term(TMT6plex); S13(Phospho); K17(TMT6plex)
7661690	0.7	1.1	0.9	0.9	GGRtPPQQGGR	GASKGGRtPPQQGGR	T4;	Yes	T(4): 100.0	8	1	1	1	N-Term(TMT6plex); T4(Phospho)
28558977	-	-	-	-	ADsPVHMEQQSR	KEPVRADsPVHMEQQ	S3;	Yes	S(3): 100.0; S(11): 0.0	5	1	1	0	N-Term(TMT6plex); S3(Phospho); M7(Oxidation)
42542379	0.9	0.9	0.9	1.0	HRPsPPAtPPPK	PRRRHRPsPPATPPP;HRPSPPAtPPPKTRH	S4; T8;	Yes;Yes	S(4): 100.0; T(8): 100.0	229	1	1	0	N-Term(TMT6plex); S4(Phospho); T8(Phospho); K12(TMT6plex)
42741682	1.3	1.4	1.3	1.1	EVEDKEsEGEEEDEDEDLSK	KEVEDKEsEGEEEDE	S7;	Yes	S(7): 100.0; S(19): 0.0	17	2	1	1	N-Term(TMT6plex); K5(TMT6plex); S7(Phospho); K20(TMT6plex)
42542379	0.9	0.9	0.9	1.0	HRPsPPATPPPK	PRRRHRPsPPATPPP;HRPSPPAtPPPKTRH	S4; T8;	Yes;Yes	S(4): 100.0; T(8): 100.0	6	1	1	0	N-Term(TMT6plex); S4(Phospho); T8(Phospho); K12(TMT6plex)
14249338	0.9	1.0	0.9	1.0	HDtPDPsPLR	PRRARHDtPDPSPLR;RHDTPDPsPLRGARH	T3; S7;	Yes;Yes	T(3): 100.0; S(7): 100.0	52	2	1	0	N-Term(TMT6plex); T3(Phospho); S7(Phospho)
21396500	0.7	1.1	0.9	1.0	RPPtPCsDPER	KKGKRPPtPCSDPER;KRPPTPCsDPERKRF	T4; S7;	Yes;Yes	T(4): 100.0; S(7): 100.0	26	2	1	0	N-Term(TMT6plex); T4(Phospho); C6(Carbamidomethyl); S7(Phospho)
39995080	0.9	1.1	1.0	0.8	FSTYSQsPPDTPSLR	RFSTYSQsPPDTPSL	S7;	No	S(2): 0.0; T(3): 0.0; Y(4): 0.0; S(5): 1.7; S(7): 98.3; T(11): 0.0; S(13): 0.0	1	4	1	0	N-Term(TMT6plex); S5(Phospho)
546231404	1.0	1.0	1.0	0.9	QPLSEASNQQPLsGGEEK	ASNQQPLsGGEEKSC	S13;	Yes	S(4): 0.0; S(7): 0.0; S(13): 100.0	21	3	1	0	N-Term(TMT6plex); S13(Phospho); K18(TMT6plex)
4507741	-	-	-	-	SAGGGAGPGGAAGGGVGGGDEPGsPAQGK	GGGDEPGsPAQGKRG	S24;	Yes	S(1): 0.0; S(24): 100.0	2	1	1	0	N-Term(TMT6plex); S24(Phospho); K29(TMT6plex)
118572613	1.2	1.1	1.2	1.0	sGSSQELDVKPSASPQER	PLGQRSRsGSSQELD	S4;	Yes	S(1): 96.8; S(3): 1.6; S(4): 1.6; S(12): 0.0; S(14): 0.0	2	1	1	0	N-Term(TMT6plex); S1(Phospho); K10(TMT6plex)
7661958	1.0	1.2	1.1	0.9	FNDsEGDDTEETEDYR	GKQKFNDsEGDDTEE	S4;	Yes	S(4): 100.0; T(9): 0.0; T(12): 0.0; Y(15): 0.0	47	2	1	0	N-Term(TMT6plex); S4(Phospho)
118572613	1.2	1.1	1.2	1.0	SGSsQELDVKPSASPQER	QRSRSGSsQELDVKP	S4;	Yes	S(1): 0.0; S(3): 2.1; S(4): 97.8; S(12): 50.0; S(14): 50.0	436	1	1	0	N-Term(TMT6plex); S4(Phospho); K10(TMT6plex); S12(Phospho)
7019569	0.9	1.0	0.8	1.0	GSDsDSEGDNPEK	SEGKGSDsDSEGDNP	S4;	Yes	S(2): 1.1; S(4): 98.9; S(6): 0.0	2	1	1	0	N-Term(TMT6plex); S4(Phospho); K13(TMT6plex)
88759348	0.8	0.7	0.8	0.9	IQSSLSSAsPSK	QSSLSSAsPSKATKS	S9;	Yes	S(3): 0.0; S(4): 0.0; S(6): 0.0; S(7): 0.0; S(9): 100.0; S(11): 0.0	4	1	1	0	N-Term(TMT6plex); S9(Phospho); K12(TMT6plex)
288806590	1.2	0.6	1.5	0.9	DLELALsPIHNSSALPTTGR	KDLELALsPIHNSSA	S7;	Yes	S(7): 100.0; S(12): 0.0; S(13): 0.0; T(17): 0.0; T(18): 0.0	1	3	1	0	N-Term(TMT6plex); S7(Phospho)
153945728	0.8	0.8	0.9	0.9	QLGAQsPGR	TKQLGAQsPGREPAS	S6;	Yes	S(6): 100.0	49	1	1	0	N-Term(TMT6plex); S6(Phospho)
167234419	0.9	0.9	0.9	0.9	DNIQPTtE	KDNIQPTtE	T7;	Yes	T(6): 0.8; T(7): 99.2	33	1	1	0	N-Term(TMT6plex); T7(Phospho)
21493024	0.8	0.8	1.0	0.8	RGSSsDEEGGPK	KKRARRGsSSDEEGG	S3;	No	S(3): 99.5; S(4): 0.5; S(5): 0.0	6	2	1	1	N-Term(TMT6plex); S3(Phospho); K12(TMT6plex)
51599156	1.0	0.9	0.9	0.9	MSQPGsPSPK	KKMSQPGsPSPKTPT	S6;	Yes	S(2): 0.0; S(6): 100.0; S(8): 0.0	28	1	1	0	N-Term(TMT6plex); M1(Oxidation); S6(Phospho); K10(TMT6plex)
21493024	0.8	0.8	1.0	0.8	RGsSSDEEGGPK	KKRARRGsSSDEEGG	S3;	No	S(3): 100.0; S(4): 0.0; S(5): 0.0	103	2	1	1	N-Term(TMT6plex); S3(Phospho); K12(TMT6plex)
53829376	-	-	-	-	AAsPYSQRPAsPTAIR	LPAARAAsPYSQRPA;PYSQRPAsPTAIRRI	S3; S11;	Yes;Yes	S(3): 98.0; Y(5): 0.1; S(6): 2.0; S(11): 98.0; T(13): 1.9	5	2	1	0	N-Term(TMT6plex); S6(Phospho); S11(Phospho)
544709647	0.9	0.7	1.0	0.8	TTPPTQKPPsPPMSGK	PPTQKPPsPPMSGKG	S10;	No	T(1): 0.0; T(2): 0.0; T(5): 0.0; S(10): 100.0; S(14): 0.0	6	5	1	0	N-Term(TMT6plex); K7(TMT6plex); S10(Phospho); M13(Oxidation); K16(TMT6plex)
385251399	1.0	1.3	1.0	0.9	SLsPIIGK	ATEIRSLsPIIGKDV	S3;	No	S(1): 0.7; S(3): 99.3	14	4	1	0	N-Term(TMT6plex); S3(Phospho); K8(TMT6plex)
53829376	-	-	-	-	AASPYSQRPAsPTAIR	PYSQRPAsPTAIRRI	S3; S11;	Yes	S(3): 0.0; Y(5): 0.0; S(6): 0.0; S(11): 99.9; T(13): 0.0	1	2	1	0	N-Term(TMT6plex); S11(Phospho)
149158690	1.0	0.9	0.9	0.8	ERGtPPVDPK	AMLRERGtPPVDPKL	T4;	No	T(4): 100.0	23	1	1	1	N-Term(TMT6plex); T4(Phospho); K10(TMT6plex)
545746316	0.9	1.0	1.0	0.9	SASSPsPR	SRSASSPsPRRSSRE	S3;	Yes	S(1): 0.0; S(3): 0.0; S(4): 0.0; S(6): 100.0	3	3	1	0	N-Term(TMT6plex); S6(Phospho)
545746335	0.9	1.0	1.0	0.9	SAsSPSPR	ARRSRSAsSPSPRRS	S3;	No	S(1): 0.0; S(3): 98.3; S(4): 0.8; S(6): [illegible]	23	3	1	0	N-Term(TMT6plex); S4(Phospho)
114155142	0.8	0.7	0.7	1.0	GAILSEEELAAMsPTAAAVAK	EEELAAMsPTAAAVA	S13;	Yes	S(5): 0.0; S(13): 100.0; T(15): 0.0	29	1	1	0	N-Term(TMT6plex); M12(Oxidation); S13(Phospho); K21(TMT6plex)
217416350	-	-	-	-	VGLSPDsPAGDR	RVGLSPDsPAGDRNS	S7;	No	S(4): 0.0; S(7): 100.0	2	2	1	0	N-Term(TMT6plex); S7(Phospho)
385648270	1.0	1.0	1.2	0.8	YVDAETsL	KYVDAETsL	S7;	No	Y(1): 0.0; T(6): 0.9; S(7): 99.1	6	3	1	0	N-Term(TMT6plex); S7(Phospho)
91176325	0.8	0.7	0.9	1.0	ASPVTSPAAAFPTAsPANK	AAAFPTAsPANKDVS	S15;	Yes	S(2): 0.0; T(5): 0.0; S(6): 0.0; T(13): 0.0; S(15): 100.0	17	1	1	0	N-Term(TMT6plex); S15(Phospho); K19(TMT6plex)
155722990	1.2	1.4	1.1	1.1	MLGEDsDEEEEMDTSER	KKMLGEDsDEEEEMD	S6;	Yes	S(6): 100.0; T(14): 0.0; S(15): 0.0	9	1	1	0	N-Term(TMT6plex); M1(Oxidation); S6(Phospho); M12(Oxidation)
38788219	1.2	0.8	0.8	1.2	SPLGEAPPGtPPCR	LGEAPPGtPPCRLCC	T10;	Yes	S(1): 0.0; T(10): 100.0	3	1	1	0	N-Term(TMT6plex); T10(Phospho); C13(Carbamidomethyl)
29568103	1.0	1.0	1.1	1.1	YDERPGPsPLPHR	YDERPGPsPLPHRDR	S8;	Yes	Y(1): 0.0; S(8): 100.0	24	1	1	0	N-Term(TMT6plex); S8(Phospho)
324021685	0.8	0.8	0.8	0.9	VSASsPSLAVASQSDSK	TVKVSASsPSLAVAS	S5;	Yes	S(2): 1.3; S(4): 1.3; S(5): 96.2; S(7): 1.3; S(12): 0.0; S(14): 0.0; S(16): 0.0	2	5	1	0	N-Term(TMT6plex); S5(Phospho); K17(TMT6plex)
42542379	1.2	1.0	1.2	1.0	tPELPEPSVK	APEKKEKtPELPEPS	T1;	Yes	T(1): 100.0; S(8): 0.0	229	1	1	0	N-Term(TMT6plex); T1(Phospho); K10(TMT6plex)
24041035	0.9	1.2	1.0	0.8	AEDEALLsEEDDPIDR	AEDEALLsEEDDPID	S8;	Yes	S(8): 100.0	2	1	1	0	N-Term(TMT6plex); S8(Phospho)
31563338	0.7	0.9	0.8	0.9	QEASEsPVK	EKQEASEsPVKAVQP	S6;	Yes	S(4): 0.0; S(6): 100.0	6	1	1	0	N-Term(TMT6plex); S6(Phospho); K9(TMT6plex)
222136585	1.0	0.9	0.5	0.9	QLLDsDEEQEEDEGR	KKRQLLDsDEEQEED	S5;	Yes	S(5): 100.0	1	1	1	0	N-Term(TMT6plex); S5(Phospho)
42741682	1.1	1.2	1.1	1.1	YNLDAsEEEDSNK	SKYNLDAsEEEDSNK	S6;	Yes	Y(1): 0.0; S(6): 100.0; S(11): 0.0	17	2	1	0	N-Term(TMT6plex); S6(Phospho); K13(TMT6plex)
118572613	1.0	1.0	1.0	1.0	sRtPPVTR	VSRRRSRsRTPPVTR;RRRSRSRtPPVTRRR	S1; T3;	Yes;Yes	S(1): 100.0; T(3): 100.0; T(7): 0.0	436	1	1	1	N-Term(TMT6plex); S1(Phospho); T3(Phospho)
33620716	0.9	0.7	1.2	0.9	VTGTEGSSSTLVDYTSTSSTGGsPVR	STSSTGGsPVRKSEE	S23;	No	T(2): 0.0; T(4): 0.0; S(7): 0.0; S(8): 0.0; S(9): 0.0; T(10): 0.0; Y(14): 0.0; T(15): 0.0; S(16): 0.0; T(17): 0.0; S(18): 0.0; S(19): 0.0; T(20): 0.0; [illegible]	4	2	1	0	N-Term(TMT6plex); S23(Phospho)
223555917	0.6	0.8	0.7	0.8	TVEVAEGEAVRtPQSVTAK	AEGEAVRtPQSVTAK	T12;	Yes	T(1): 0.0; T(12): 100.0; S(15): 0.0; T(17): 0.0	16	1	1	1	N-Term(TMT6plex); T12(Phospho); K19(TMT6plex)
28559039	0.8	1.0	1.1	0.9	sPGSAGR	TSTGGSKsPGSAGRS	S1;	Yes	S(1): 100.0; S(4): 0.0	16	1	1	0	N-Term(TMT6plex); S1(Phospho)
29568103	1.6	0.9	1.5	1.1	GGGGGQDNGLEGLGNDsR	LEGLGNDsRDMYMES	S17;	Yes	S(17): 100.0	24	1	1	0	N-Term(TMT6plex); S17(Phospho)
118572613	0.9	1.1	0.9	1.0	SSGHSSSELsPDAVEK	GHSSSELsPDAVEKA	S10;	Yes	S(1): 0.0; S(2): 0.0; S(5): 0.0; S(6): 0.0; S(7): 0.0; S(10): 100.0	436	1	1	0	N-Term(TMT6plex); S10(Phospho); K16(TMT6plex)
118572613	0.9	1.1	0.9	1.0	SSGHssSELsPDAVEK	STRSSGHsSSELSPD;GHSSSELsPDAVEKA	S10;	Yes;Yes	S(1): 0.0; S(2): 0.0; S(5): 100.0; S(6): 0.0; S(7): 0.0; S(10): 100.0	4	1	1	0	N-Term(TMT6plex); S6(Phospho); S10(Phospho); K16(TMT6plex)
10835067	0.7	0.9	0.9	1.1	AAQPGsGK	NKAAQPGsGKGKVQF	S6;	No	S(6): 100.0	3	1	1	0	N-Term(TMT6plex); S6(Phospho); K8(TMT6plex)
45597177	1.6	1.6	1.2	1.0	ASVVDPSTESSPAPQEGSEQPASPAsPLSSR	EQPASPAsPLSSRQS	S26;	Yes	S(2): 0.0; S(7): 0.0; T(8): 0.0; S(10): 0.0; S(11): 0.0; S(18): 0.0; S(23): 0.0; S(26): 99.9; S(29): 0.1; S(30): 0.0	5	2	1	0	N-Term(TMT6plex); S26(Phospho)
5902022	0.8	0.9	0.8	0.8	VDsPLPSDK	ESGAKVDsPLPSDKA	S3;	Yes	S(3): 100.0; S(7): 0.0	1	1	1	0	N-Term(TMT6plex); S3(Phospho); K9(TMT6plex)
314122233	1.4	1.3	1.6	0.9	LQEDPNYsPQR	LQEDPNYsPQRFPNA	S8;	No	Y(7): 0.9; S(8): 99.1	15	4	1	0	N-Term(TMT6plex); S8(Phospho)
312434011	0.8	0.6	0.6	0.8	SQQQPTPVtPK	QQQPTPVtPKKYPLR	T9;	Yes	S(1): 0.0; T(6): 0.0; T(9): 100.0	12	3	1	0	N-Term(TMT6plex); T9(Phospho); K11(TMT6plex)
14717407	1.0	1.0	1.2	1.0	EAENQGLDISsPGMSGHR	NQGLDISsPGMSGHR	S11;	Yes	S(10): 0.0; S(11): 100.0; S(15): 0.0	2	1	1	0	N-Term(TMT6plex); S11(Phospho); M14(Oxidation)
21361499	0.8	0.9	0.8	1.0	GsPPVPSGPPMEEDGLR	AEDGVRGsPPVPSGP	S2;	Yes	S(2): 100.0; S(7): 0.0	3	1	1	0	N-Term(TMT6plex); S2(Phospho); M11(Oxidation)
42542379	1.0	1.2	1.2	1.0	VPKPEPIPEPKEPsPEK	IPEPKEPsPEKNSKK	S14;	Yes	S(14): 100.0	229	1	1	1	N-Term(TMT6plex); K3(TMT6plex); K11(TMT6plex); S14(Phospho); K17(TMT6plex)
291621647	0.9	1.0	0.9	0.9	GLGAGAGAGEEsPATSLPR	GAGAGEEsPATSLPR	S12;	No	S(12): 100.0; T(15): 0.0; S(16): 0.0	3	1	1	0	N-Term(TMT6plex); S12(Phospho)
118572613	1.0	1.1	1.0	1.0	SsSsPPPK	PPRRSRSsSSPPPKQ;RRSRSSSsPPPKQKS	S2; S4;	Yes;Yes	S(1): 0.0; S(2): 100.0; S(3): 0.7; S(4): 99.3	436	1	1	0	N-Term(TMT6plex); S2(Phospho); S4(Phospho); K8(TMT6plex)
51226124	0.8	0.8	0.8	0.9	SATTTGsPGTEK	RSATTTGsPGTEKAR	S7;	Yes	S(1): 0.0; T(3): 0.0; T(4): 0.0; T(5): 0.0; S(7): 100.0; T(10): 0.0	6	4	1	0	N-Term(TMT6plex); S7(Phospho); K12(TMT6plex)
321267473	1.7	2.4	2.2	1.0	sVVSFDK	SASREKRsVVSFDKV	S1;	No	S(1): 100.0; S(4): 0.0	23	4	1	0	N-Term(TMT6plex); S1(Phospho); K7(TMT6plex)
42542379	1.0	1.0	1.0	1.0	EKtPELPEPSVK	APEKKEKtPELPEPS	T3;	Yes	T(3): 100.0; S(10): 0.0	229	1	1	1	N-Term(TMT6plex); K2(TMT6plex); T3(Phospho); K12(TMT6plex)
56118310	1.1	1.1	0.9	1.1	ATVTPsPVK	LKATVTPsPVKGKGK	S6;	Yes	T(2): 0.0; T(4): 0.0; S(6): 100.0	109	1	1	0	N-Term(TMT6plex); S6(Phospho); K9(TMT6plex)
321267473	1.7	2.4	2.2	1.0	sVVsFDK	SASREKRsVVSFDKV;REKRSVVsFDKVEP	S1;	No;No	S(1): 100.0; S(4): 100.0	1	4	1	0	N-Term(TMT6plex); S1(Phospho); S4(Phospho); K7(TMT6plex)
41152097	1.0	1.1	1.1	0.9	VVPEDGsPEKR	RVVPEDGsPEKRCPG	S7;	Yes	S(7): 100.0	1	1	1	1	N-Term(TMT6plex); S7(Phospho); K10(TMT6plex)
42542379	1.2	1.2	1.1	1.0	KEtESEAEDNLDDLEK	APEPKKEtESEAEDN	S5;	Yes	T(3): 100.0; S(5): 0.0	6	1	1	1	N-Term(TMT6plex); K1(TMT6plex); T3(Phospho); K16(TMT6plex)
42542379	1.2	1.2	1.1	1.0	KETEsEAEDNLDDLEK	EPKKETEsEAEDNLD	S5;	Yes	T(3): 0.0; S(5): 100.0	229	1	1	1	N-Term(TMT6plex); K1(TMT6plex); S5(Phospho); K16(TMT6plex)
5031805	-	-	-	-	tHSAGTSPTITHQK	DNRFRKRtHSAGTSP	T1; S7;	No	T(1): 100.0; S(3): 33.1; T(6): 33.1; S(7): 33.1; T(9): 0.7; T(11): 0.0	1	1	1	0	N-Term(TMT6plex); T1(Phospho); S7(Phospho); K14(TMT6plex)
302699211	0.8	0.9	1.2	0.8	SLDSEPSVPSAAKPPsPEK	PSAAKPPsPEKTAPV	S16;	No	S(1): 0.0; S(4): 0.0; S(7): 0.0; S(10): 0.0; S(16): 100.0	8	5	1	0	N-Term(TMT6plex); K13(TMT6plex); S16(Phospho); K19(TMT6plex)
4505195	-	-	-	-	ITVQAsPGLDR	HRITVQAsPGLDRRR	S6;	Yes	T(2): 0.0; S(6): 100.0	1	1	1	0	N-Term(TMT6plex); S6(Phospho)
148612816	0.5	0.7	0.7	0.8	DQGtPEVQPTPAK	CPPRDQGtPEVQPTP	T4;	Yes	T(4): 100.0; T(10): 0.0	2	1	1	0	N-Term(TMT6plex); T4(Phospho); K13(TMT6plex)
5031805	-	-	-	-	tHSAGTsPTITHQK	DNRFRKRtHSAGTSP;RTHSAGTsPTITHQK	T1; S7;	No;Yes	T(1): 98.0; S(3): 2.0; T(6): 1.9; S(7): 96.2; T(9): 1.9; T(11): 0.0	3	1	1	0	N-Term(TMT6plex); T1(Phospho); S7(Phospho); K14(TMT6plex)
54607124	0.8	0.9	0.9	0.9	GSDRtPER	SERGSDRtPERSRKE	T5;	No	S(2): 0.0; T(5): 100.0	4	1	1	1	N-Term(TMT6plex); T5(Phospho)
110556636	1.1	1.1	1.3	0.9	sAEEGELAESK	KLRPRNRsAEEGELA	S1;	Yes	S(1): 100.0; S(10): 0.0	41	1	1	0	N-Term(TMT6plex); S1(Phospho); K11(TMT6plex)
21237805	0.8	0.9	0.8	0.8	DMDEPsPVPNVEEVTLPK	TKDMDEPsPVPNVEE	S6;	Yes	S(6): 100.0; T(15): 0.0	12	3	1	0	N-Term(TMT6plex); M2(Oxidation); S6(Phospho); K18(TMT6plex)
32307124	-	-	-	-	LDNsPNMNITQPSK	PGPKLDNsPNMNITQ	S4;	Yes	S(4): 100.0; T(10): 0.0; S(13): 0.0	6	4	1	0	N-Term(TMT6plex); S4(Phospho); M7(Oxidation); K14(TMT6plex)
103472005	1.0	0.7	0.9	0.9	LDQPGNLPGsNR	QPGNLPGsNRRLQTR	S10;	Yes	S(10): 100.0	118	2	1	0	N-Term(TMT6plex); S10(Phospho)
18426904	0.8	0.9	0.9	0.9	AKGPsPPGAK	GERAKGPsPPGAKRR	S5;	Yes	S(5): 100.0	5	2	1	1	N-Term(TMT6plex); K2(TMT6plex); S5(Phospho); K10(TMT6plex)
126722969	0.6	0.9	0.7	0.9	ALLETAsPR	RALLETAsPRKLSGQ	S7;	Yes	T(5): 0.8; S(7): 99.2	2	1	1	0	N-Term(TMT6plex); S7(Phospho)
103472005	0.8	0.7	0.7	0.9	DVESVQtPSK	RDVESVQtPSKAVGA	T7;	Yes	S(4): 0.0; T(7): 99.3; S(9): 0.7	118	1	1	0	N-Term(TMT6plex); T7(Phospho); K10(TMT6plex)
72256200	-	-	-	-	AVtPVSQGSNSSSADPK	TPNNRAVtPVSQGSN	T3;	Yes	T(3): 100.0; S(6): 0.0; S(9): 0.0; S(11): 0.0; S(12): 0.0; S(13): 0.0	2	1	1	0	N-Term(TMT6plex); T3(Phospho); K17(TMT6plex)
47271443	0.8	0.9	0.9	1.0	SRsPPPVSK	RSRSRSRsPPPVSKR	S3;	Yes	S(1): 0.5; S(3): 99.5; S(8): 0.0	29	1	1	1	N-Term(TMT6plex); S3(Phospho); K9(TMT6plex)
40807504	-	-	-	-	SGTSSPQsPVFR	SGTSSPQsPVFRHRT	S8;	Yes	S(1): 0.0; T(3): 0.0; S(4): 0.0; S(5): 0.0; S(8): 100.0	6	2	1	0	N-Term(TMT6plex); S8(Phospho)
10716563	1.0	0.9	0.9	0.8	SDAEEDGGTVsQEEEDR	EEDGGTVsQEEEDRK	S11;	Yes	S(1): 0.0; T(9): 0.0; S(11): 100.0	54	1	1	0	N-Term(TMT6plex); S11(Phospho)
313760537	0.9	0.9	0.9	0.8	SGLTVPTsPK	SGLTVPTsPKGRLLD	S8;	No	S(1): 0.0; T(4): 0.0; T(7): 0.0; S(8): 100.0	13	3	1	0	N-Term(TMT6plex); S8(Phospho); K10(TMT6plex)
7661958	0.9	1.0	1.0	0.9	KAEGEPQEEsPLK	EGEPQEEsPLKSKSQ	S10;	Yes	S(10): 100.0	47	3	1	1	N-Term(TMT6plex); K1(TMT6plex); S10(Phospho); K13(TMT6plex)
66528888	0.6	0.5	1.4	0.9	SQNSQEStADESEDDMSSQASK	SQNSQEStADESEDD	T8;	Yes	S(1): 0.0; S(4): 0.0; S(7): 1.9; T(8): 96.2; S(12): 1.9; S(17): 0.0; S(18): 0.0; S(21): 0.0	3	2	1	0	N-Term(TMT6plex); T8(Phospho); M16(Oxidation); K22(TMT6plex)
197304708	1.2	1.3	1.3	1.0	ELDEEGsDPPLPGR	RELDEEGsDPPLPGR	S7;	Yes	S(7): 100.0	12	1	1	0	N-Term(TMT6plex); S7(Phospho)
56118310	1.2	1.2	1.1	1.1	TPSPKEEDEEPEsPPEK	EEDEEPEsPPEKKTS	S13;	Yes	T(1): 0.0; S(3): 0.0; S(13): 100.0	109	1	1	1	N-Term(TMT6plex); K5(TMT6plex); S13(Phospho); K17(TMT6plex)
66528888	0.6	0.5	1.4	0.9	SQNSQESTADEsEDDMSSQASK	QESTADEsEDDMSSQ	T8;	Yes	S(1): 0.0; S(4): 0.0; S(7): 0.0; T(8): 1.4; S(12): 97.1; S(17): 1.4; S(18): 0.0; S(21): 0.0	2	2	1	0	N-Term(TMT6plex); S12(Phospho); M16(Oxidation); K22(TMT6plex)
14110414	1.0	0.8	1.4	0.8	NEEDEGHsNSsPR	NEEDEGHsNSSPRHS;DEGHSNSsPRHSEAA	S8; S11;	Yes;Yes	S(8): 98.9; S(10): 2.1; S(11): 98.9	31	2	1	0	N-Term(TMT6plex); S8(Phospho); S10(Phospho)
14110414	1.0	0.8	1.4	0.8	NEEDEGHSNSsPR	DEGHSNSsPRHSEAA	S8; S11;	Yes	S(8): 0.0; S(10): 0.0; S(11): 100.0	11	2	1	0	N-Term(TMT6plex); S11(Phospho)
333440471	0.9	0.9	0.9	0.9	DGGAAsPATEGR	SRDGGAAsPATEGRG	S6;	No	S(6): 100.0; T(9): 0.0	14	4	1	0	N-Term(TMT6plex); S6(Phospho)
6594683	1.1	1.2	0.9	0.9	IQDPTEDAEAEDtPR	EDAEAEDtPRKRLRT	T13;	Yes	T(5): 0.0; T(13): 100.0	3	3	1	0	N-Term(TMT6plex); T13(Phospho)
112421108	-	-	-	-	AASEDMTsDEER	AASEDMTsDEERMVI	S3;	No	S(3): 0.0; T(7): 0.6; S(8): 99.4	2	1	1	0	N-Term(TMT6plex); M6(Oxidation); S8(Phospho)
87299628	1.0	1.0	0.9	0.9	TQLsPSIK	QRSKTQLsPSIKRKR	S4;	Yes	T(1): 0.0; S(4): 100.0; S(6): 0.0	36	1	1	0	N-Term(TMT6plex); S4(Phospho); K8(TMT6plex)
33598968	0.9	0.6	0.7	0.8	RGEsLDNLDSPR	GIMRRGEsLDNLDSP	S4;	Yes	S(4): 100.0; S(10): 0.0	19	2	1	1	N-Term(TMT6plex); S4(Phospho)
112421108	-	-	-	-	AAsEDMTSDEER	SRSQRAAsEDMTSDE	S3;	Yes	S(3): 100.0; T(7): 50.0; S(8): 50.0	8	1	1	0	N-Term(TMT6plex); S3(Phospho); M6(Oxidation); S8(Phospho)
183396804	0.8	1.1	1.3	0.7	DVEDMELsDVEDDGSK	DVEDMELsDVEDDGS	S8;	Yes	S(8): 100.0; S(15): 0.0	8	1	1	0	N-Term(TMT6plex); M5(Oxidation); S8(Phospho); K16(TMT6plex)
14249338	0.6	0.9	0.9	1.0	HDssDTSPPR	PRRIRHDsSDTSPPR;RRIRHDSsDTSPPRR	T6;	Yes;Yes	S(3): 100.0; S(4): 100.0; T(6): 0.0; S(7): 0.0	1	2	1	0	N-Term(TMT6plex); S4(Phospho); S7(Phospho)
13489054	1.2	0.9	0.9	1.0	LNQPGtPTR	LRLNQPGtPTRTAV	T6;	Yes	T(6): 100.0; T(8): 0.0	1	1	1	0	N-Term(TMT6plex); T6(Phospho)
14249338	0.6	0.9	0.9	1.0	HDSSDtSPPR	IRHDSSDtSPPRRAR	T6;	Yes	S(3): 0.0; S(4): 0.0; T(6): 98.8; S(7): [illegible]	52	2	1	0	N-Term(TMT6plex); T6(Phospho)
124028529	1.5	1.2	1.2	0.9	TPsPAAEDAR	EDAMKTPsPAAEDAR	S3;	Yes	T(1): 0.0; S(3): 100.0	4	1	1	0	N-Term(TMT6plex); S3(Phospho)

Khan et al., 2019. Multi-omics analysis to characterize cigarette smoke induced molecular alterations in esophageal cells

Supplementary Table 8. List of phosphopeptides identified and quantified in untreated and chronically treated Het1A cells with cigarette smoke condensate for 8 months using SequestHT and Mascot search algorithms with a PhosphoRS probability score ≥75%

														Normalized phosphoprotein ratios with respect to corresponding total protein ratios.								Average of replicates														
Protein group Accession	NP_Accession	Gene ID	Gene Symbol	Protein Description	PhosphoSite (Protein)	Het-1A-Smoke - 2M/Parental	Het-1A-Smoke - 4M/Parental	Het-1A-Smoke - 6M/Parental	Het-1A-Smoke - 8M/Parental	Het-1A-Smoke - 2M/Parental	Het-1A-Smoke - 4M/Parental	Het-1A-Smoke - 6M/Parental	Het-1A-Smoke - 8M/Parental	Het-1A-Smoke - 2M/Parental	Het-1A-Smoke - 4M/Parental	Het-1A-Smoke - 6M/Parental	Het-1A-Smoke - 8M/Parental	Het-1A-Smoke - 2M/Parental	Het-1A-Smoke - 4M/Parental	Het-1A-Smoke - 6M/Parental	Het-1A-Smoke - 8M/Parental	Het-1A-Smoke - 2M/Parental	Het-1A-Smoke - 4M/Parental	Het-1A-Smoke - 6M/Parental	Het-1A-Smoke - 8M/Parental	Peptide Sequence	Phosphowindow	PhosphoSite (Peptide)	Phosphosite Plus Evidence	phosphoRS Site Probabilities	# PSMs	# Proteins	# Protein Groups	# Missed Cleavages	Modifications
						Replicate 1				Replicate 2				Replicate 1				Replicate 2																		
34098946	NP_004550.2	4904	YBX1	nuclease-sensitive element-binding protein 1	S174	1.4	1.9	1.3	1.1	1.1	1.2	1.3	0.6	1.7	2.1	1.7	1.4	1.4	1.3	1.6	0.7	1.5	1.7	1.6	1.0	NYQQNYQNSESGEKNEGsESAPEGQAQQR	SGEKNEGsESAPEGQ	S18;	Yes	Y(2): 0.0; Y(6): 0.0; S(9): 0.0; S(11): 1.4; S(18): 97.1; S(20): 1.4	66	1	1	1	N-Term(TMT6plex); K14(TMT6plex); S18(Phospho)
157694492	NP_055335.2	10514	MYBBP1A	myb-binding protein 1A isoform 2	S1163	0.6	0.9	0.8	0.8	0.7	0.8	0.9	0.9	0.7	0.9	0.8	0.8	0.7	0.9	0.9	1.0	0.7	0.9	0.9	0.9	EIPSATQsPISK	EIPSATQsPISKKRK	S8;	Yes	S(4): 0.0; T(6): 0.0; S(8): 100.0; S(11): 0.0	15	2	1	0	N-Term(TMT6plex); S8(Phospho); K12(TMT6plex)
157694492	NP_055335.2	10514	MYBBP1A	myb-binding protein 1A isoform 2	S1163	0.7	0.8	0.9	0.9	0.6	0.9	0.8	0.8	0.7	0.9	0.9	1.0	0.7	0.9	0.8	0.8	0.7	0.9	0.9	0.9	EIPsATQSPISK	EIPSATQsPISKKRK	S8;	Yes	S(4): 0.0; T(6): 0.0; S(8): 100.0; S(11): 0.0	14	2	1	0	N-Term(TMT6plex); S8(Phospho); K12(TMT6plex)
40018638	NP_950251.1	25966	C2CD2	C2 domain-containing protein 2 isoform 2	T290	0.9	1.1	0.8	0.8	0.7	1.0	0.8	0.9	0.9	1.0	0.8	0.7	0.7	1.0	0.8	0.8	0.8	1.0	0.8	0.8	ASPLSSDsPVKtPIK	ASPLSSDsPVKTPIK;SSDSPVKtPIKVKVI	T12;	No;No	S(2): 0.0; S(5): 0.1; S(6): 2.3; S(8): 97.7; T(12): 100.0	1	2	1	1	N-Term(TMT6plex); S8(Phospho); K11(TMT6plex); T12(Phospho); K15(TMT6plex)
24432026	NP_116164.2	84861	KLHL22	kelch-like protein 22	T605	0.5	0.8	0.4	0.9	0.3	1.3	1.4	0.8	-	-	-	-	-	-	-	-	-	-	-	-	SLLLEPPRGtPDR	LLEPPRGtPDRSQAD	T10;	Yes	S(1): 0.0; T(10): 100.0	1	1	1	1	N-Term(TMT6plex); T10(Phospho)
156151371	NP_057615.3	51274	KLF3	Krueppel-like factor 3	S78	1.5	1.5	1.0	1.1	0.7	0.7	0.7	0.6	-	-	-	-	-	-	-	-	-	-	-	-	SSPPSAGNsPSSLK	SPPSAGNsPSSLKFP	S9;	Yes	S(1): 0.0; S(2): 0.0; S(5): 0.0; S(9): 100.0; S(11): 0.0; S(12): 0.0	2	1	1	0	N-Term(TMT6plex); S9(Phospho); K14(TMT6plex)
312176416	NP_001185901.1	57648	KIAA1522	uncharacterized protein KIAA1522 isoform 2	S979	0.7	0.5	0.8	0.7	1.0	0.9	1.0	1.0	0.8	0.6	0.9	0.8	1.2	1.1	1.1	1.1	1.0	0.8	1.0	1.0	KPSVGVPPPAsPSYPR	VGVPPPAsPSYPRAE	S11;	Yes	S(3): 0.0; S(11): 98.4; S(13): 0.8; Y(14): 0.8	22	2	1	0	N-Term(TMT6plex); K1(TMT6plex); S11(Phospho)
84043963	NP_056988.3	9669	EIF5B	eukaryotic translation initiation factor 5B	S164	1.3	1.1	1.1	1.1	0.9	0.8	0.7	0.6	1.4	1.1	1.2	1.2	0.9	0.8	0.7	0.6	1.1	1.0	1.0	0.9	WDGsEEDEDNSK	SNKKWDGsEEDEDNS	S4;	Yes	S(4): 100.0; S(11): 0.0	13	1	1	0	N-Term(TMT6plex); S4(Phospho); K12(TMT6plex)
256017159	NP_001074010.2	23269	MGA	MAX gene-associated protein isoform 2	S607	1.0	0.6	0.6	0.8	1.1	0.7	0.8	0.9	0.8	0.5	0.6	0.7	1.0	0.6	0.8	0.7	0.9	0.5	0.7	0.7	VVNANQNAsPNVPGK	VNANQNAsPNVPGKR	S9;	Yes	S(9): 100.0	1	2	1	0	N-Term(TMT6plex); S9(Phospho); K15(TMT6plex)
118572613	NP_057417.3	23524	SRRM2	serine/arginine repetitive matrix protein 2	S2581	0.8	1.1	1.0	0.8	0.9	1.1	1.1	0.9	0.9	1.1	1.0	0.9	0.9	1.2	1.1	0.9	0.9	1.1	1.1	0.9	VPsPTPAPK	VALKRVPsPTPAPKE	S3;	Yes	S(3): 100.0; T(5): 0.0	436	1	1	0	N-Term(TMT6plex); S3(Phospho); K9(TMT6plex)
14150015	NP_115675.1	84259	DCUN1D5	DCN1-like protein 5	S9	1.1	0.8	0.5	1.0	1.0	0.6	1.1	0.7	0.9	0.7	0.4	0.9	0.9	0.5	0.9	0.6	0.9	0.6	0.7	0.7	sPGVAAAVAEDGGLK	PVKKKRKsPGVAAAV	S1;	Yes	S(1): 100.0	3	1	1	0	N-Term(TMT6plex); S1(Phospho); K15(TMT6plex)
145580615	NP_075384.3	65095	KRI1	protein KRI1 homolog	S142	1.2	1.1	1.1	0.9	0.8	1.0	1.0	0.8	1.4	1.1	1.2	1.0	1.0	1.1	1.1	0.9	1.2	1.1	1.2	1.0	YVDEENsDGETSNHR	KYVDEENsDGETSNH	S7;	No	Y(1): 0.0; S(7): 100.0; T(11): 0.0; S(12): 0.0	13	1	1	0	N-Term(TMT6plex); S7(Phospho)
30578410	NP_849193.1	201595	STT3B	dolichyl-diphosphooligosaccharide--protein glycosyltransferase subunit STT3B	S498	1.4	1.4	1.4	1.0	1.2	1.0	1.0	0.7	1.3	1.4	1.2	1.1	1.1	1.0	0.9	0.7	1.2	1.2	1.1	0.9	ENPPVEDssDEDDKR	ENPPVEDsSDEDDKR	S8;	Yes	S(8): 99.2; S(9): 0.8	8	1	1	1	N-Term(TMT6plex); S8(Phospho); K14(TMT6plex)
118572613	NP_057417.3	23524	SRRM2	serine/arginine repetitive matrix protein 2	S1866; S1869	1.2	1.3	1.3	1.0	0.7	0.9	0.8	0.7	1.3	1.4	1.3	1.1	0.8	0.9	0.8	0.7	1.0	1.1	1.1	0.9	sRAsPATHR	APWKRSRsRASPATH;KRSRSRAsPATHRRS	S1; S4;	Yes;Yes	S(1): 100.0; S(4): 100.0; T(7): 0.0	436	1	1	1	N-Term(TMT6plex); S1(Phospho); S4(Phospho)
118572613	NP_057417.3	23524	SRRM2	serine/arginine repetitive matrix protein 2	T1003	1.0	1.2	1.1	0.7	0.9	1.1	1.2	1.0	1.0	1.2	1.2	0.8	1.0	1.1	1.3	1.1	1.0	1.2	1.2	0.9	AQtPPGPSLSGSK	YPKVKAQtPPGPSLS	T3;	Yes	T(3): 100.0; S(8): 0.0; S(10): 0.0; S(12): 0.0	436	1	1	0	N-Term(TMT6plex); T3(Phospho); K13(TMT6plex)
545746344	NP_001271136.1	8816	DCAF5	DDB1- and CUL4-associated factor 5 isoform 3	S566	1.1	1.1	1.2	0.9	0.8	1.0	0.7	0.7	-	-	-	-	-	-	-	-	-	-	-	-	AsPTSDIESVER	EIQPSRAsPTSDIES	S2;	No	S(2): 100.0; T(4): 0.0; S(5): 0.0; S(9): 0.0	4	3	1	0	N-Term(TMT6plex); S2(Phospho)
36287110	NP_919410.1	11116	FGFR1OP	FGFR1 oncogene partner isoform b	S156; S160	0.7	1.3	1.0	1.1	0.6	0.9	0.7	0.6	0.8	1.5	1.3	1.3	0.8	1.0	0.9	0.7	0.8	1.2	1.1	1.0	GPTTGEGALDLSDVHsPPKsPEGK	LDLSDVHsPPKSPEG;DVHSPPKsPEGKTSA	S16; S20;	Yes;Yes	T(3): 0.0; T(4): 0.0; S(12): 0.0; S(16): 100.0; S(20): 100.0	11	2	1	1	N-Term(TMT6plex); S16(Phospho); K19(TMT6plex); S20(Phospho); K24(TMT6plex)
118572613	NP_057417.3	23524	SRRM2	serine/arginine repetitive matrix protein 2	S1517; S1519	1.1	0.9	1.0	0.8	1.2	1.1	1.1	0.9	1.2	0.9	1.1	0.9	1.2	1.1	1.1	0.9	1.2	1.0	1.1	0.9	ERsGsESSVDQK	LTPQRERsGSESSVD;PQRERSGsESSVDQK	S3; S5;	Yes;Yes	S(3): 100.0; S(5): 100.0; S(7): 0.0; S(8): 0.0	436	1	1	1	N-Term(TMT6plex); S3(Phospho); S5(Phospho); K12(TMT6plex)
151101292	NP_110382.3	81542	TMX1	thioredoxin-related transmembrane protein 1 precursor	S247	1.0	0.9	0.6	0.8	0.8	0.8	0.9	0.9	1.3	1.1	0.7	0.9	1.0	0.9	1.2	1.0	1.1	1.0	1.0	0.9	VEEEQEADEEDVsEEEAESK	EADEEDVsEEEAESK	S13;	Yes	S(13): 100.0; S(19): 0.0	9	1	1	0	N-Term(TMT6plex); S13(Phospho); K20(TMT6plex)
557440762	NP_001273466.1	79074	C2orf49	ashwin isoform 2	S151	0.7	0.7	0.6	0.9	0.7	0.6	0.6	0.8	0.7	0.9	0.8	1.1	0.7	0.7	0.7	1.0	0.7	0.8	0.7	1.0	SPPLsPVGTTPVK	PVKSPPLsPVGTTPV	S5;	No	S(1): 0.8; S(5): 99.2; T(9): 0.0; T(10): 0.0	3	2	1	0	N-Term(TMT6plex); S5(Phospho); K13(TMT6plex)
557357687	NP_001273448.1	347734	SLC35B2	adenosine 3'-phospho 5'-phosphosulfate transporter 1 isoform f	S294	0.5	0.6	0.6	0.5	0.5	0.9	0.7	1.2	0.6	0.8	0.7	0.5	0.6	1.1	0.9	1.4	0.6	1.0	0.8	1.0	AVPVEsPVQK	KKAVPVEsPVQKV_	S6;	No	S(6): 100.0	3	6	1	0	N-Term(TMT6plex); S6(Phospho); K10(TMT6plex)
38683860	NP_003740.2	8660	IRS2	insulin receptor substrate 2	S736	0.8	1.0	0.9	0.8	0.9	1.0	0.9	0.9	0.7	1.0	0.9	0.7	0.9	1.0	0.9	0.9	0.8	1.0	0.9	0.8	ASSPAESsPEDSGYMR	ASSPAESsPEDSGYM	S8;	Yes	S(2): 0.0; S(3): 0.0; S(7): 1.9; S(8): 98.1; S(12): 0.0; Y(14): 0.0	9	1	1	0	N-Term(TMT6plex); S8(Phospho); M15(Oxidation)
14790190	NP_055816.2	23013	SPEN	msx2-interacting protein	S736; S740	0.9	1.0	1.0	0.8	1.1	1.1	1.1	0.9	0.8	0.9	1.1	0.8	1.0	1.0	1.1	0.9	0.9	1.0	1.1	0.8	RPQsPGAsPSQAER	VHLRRPQsPGASPSQ;RPQSPGAsPSQAERL	S4; S8;	Yes;Yes	S(4): 100.0; S(8): 100.0; S(10): 0.0	16	1	1	0	N-Term(TMT6plex); S4(Phospho); S8(Phospho)
38683860	NP_003740.2	8660	IRS2	insulin receptor substrate 2	S736	0.9	1.0	0.9	0.9	0.8	1.0	0.9	0.8	0.9	1.0	0.9	0.9	0.7	1.0	0.9	0.7	0.8	1.0	0.9	0.8	ASSPAEsSPEDSGYMR	KASSPAEsSPEDSGY	S8;	Yes	S(2): 0.0; S(3): 0.0; S(7): 98.2; S(8): 1.8; S(12): 0.0; Y(14): 0.0	1	1	1	0	N-Term(TMT6plex); S8(Phospho); M15(Oxidation)
193083131	NP_001122393.1	4140	MARK3	MAP/microtubule affinity-regulating kinase 3 isoform e	T462	1.2	0.9	1.0	0.8	0.8	0.9	0.9	0.9	1.2	0.9	1.0	0.8	0.9	1.0	0.9	0.9	1.1	1.0	1.0	0.9	TPVASTHSISsAATPDR	ASTHSISsAATPDRI	T6;	No	T(1): 0.0; S(5): 0.0; T(6): 0.0; S(8): 0.0; S(10): 1.1; S(11): 97.8; T(14): 1.1	2	5	1	0	N-Term(TMT6plex); T14(Phospho)
57863269	NP_055871.1	23076	RRP1B	ribosomal RNA processing protein 1 homolog B	S732	1.4	1.2	0.7	0.8	0.8	0.8	0.7	0.8	1.5	1.2	0.8	0.8	0.8	0.8	0.8	0.8	1.2	1.0	0.8	0.8	TPTSsPASSPLVAK	VLKTPTSsPASSPLV	S5;	Yes	T(1): 0.0; T(3): 0.0; S(4): 1.5; S(5): 98.4; S(8): 50.0; S(9): 50.0	17	1	1	0	N-Term(TMT6plex); S5(Phospho); S9(Phospho); K14(TMT6plex)
57863269	NP_055871.1	23076	RRP1B	ribosomal RNA processing protein 1 homolog B	S732	0.8	0.8	0.7	0.8	1.4	1.2	0.7	0.8	0.8	0.8	0.8	0.8	1.5	1.2	0.8	0.8	1.2	1.0	0.8	0.8	TPtSSPASSPLVAK	GVLKTPTsSPASSPL	S5;	Yes	T(1): 0.0; T(3): 1.5; S(4): 97.5; S(5): 34.0; S(8): 33.5; S(9): 33.5	5	1	1	0	N-Term(TMT6plex); S5(Phospho); S8(Phospho); K14(TMT6plex)
193083131	NP_001122393.1	4140	MARK3	MAP/microtubule affinity-regulating kinase 3 isoform e	T462	0.8	0.9	0.9	0.9	1.2	0.9	1.0	0.8	0.9	1.0	0.9	0.9	1.2	0.9	1.0	0.8	1.1	1.0	1.0	0.9	TPVAStHSISSAATPDR	QRTPVAStHSISSAA	T6;	No	T(1): 0.0; S(5): 1.5; T(6): 98.5; S(8): 0.0; S(10): 0.0; S(11): 0.0; T(14): 0.0	15	5	1	0	N-Term(TMT6plex); T6(Phospho)
132626688	NP_055456.2	9656	MDC1	mediator of DNA damage checkpoint protein 1	S1820	0.8	1.3	1.0	0.8	0.5	0.9	0.7	0.9	0.9	1.3	1.1	0.8	0.6	0.9	0.8	1.0	0.7	1.1	0.9	0.9	SLATMDsPPHQK	RSLATMDsPPHQKQP	S7;	Yes	S(1): 0.0; T(4): 0.0; S(7): 100.0	26	1	1	0	N-Term(TMT6plex); M5(Oxidation); S7(Phospho); K12(TMT6plex)
110611228	NP_009055.2	7402	UTRN	utrophin	S1405	0.3	0.9	1.0	0.8	0.7	1.0	1.1	0.9	0.3	1.0	1.0	0.9	0.7	1.1	1.1	1.0	0.5	1.0	1.1	1.0	SQPLTsPESR	MRSQPLTsPESRTAR	S6;	Yes	S(1): 0.0; T(5): 1.1; S(6): 98.9; S(9):	3	1	1	0	N-Term(TMT6plex); S6(Phospho)
427197591	NP_001258763.1	51319	RSRC1	serine/Arginine-related protein 53 isoform 2	S6	1.0	1.0	1.3	0.9	0.9	1.2	1.2	0.7	1.0	0.9	1.0	0.8	0.9	1.1	0.9	0.6	0.9	1.0	1.0	0.7	SsDTEEESR	_MGRRSsDTEEESR	S2;	No	S(1): 0.0; S(2): 100.0; T(4): 0.0; S(8): 0.0	6	2	1	0	N-Term(TMT6plex); S2(Phospho)
5032147	NP_005632.1	6878	TAF6	transcription initiation factor TFIID subunit 6 isoform	S653	1.0	0.7	0.9	0.7	1.0	1.0	1.1	1.0	0.9	0.7	0.8	0.7	0.9	1.0	1.1	1.0	0.9	0.9	0.9	0.8	QEAGDSPPPAPGtPK	SPPPAPGtPKANGSQ	S6;	Yes	S(6): 0.0; T(13): 100.0	4	2	1	0	N-Term(TMT6plex); T13(Phospho); K15(TMT6plex)
283837894	NP_001164633.1	9203	ZMYM3	zinc finger MYM-type protein 3 isoform 2	T814	0.8	0.6	0.7	0.9	0.8	0.8	0.7	0.8	1.1	0.8	1.1	1.2	1.1	1.2	1.1	1.1	1.1	1.0	1.1	1.2	SAPTAPTPPPPPPPAtPR	PPPPPPAtPRKNKAA	T16;	No	S(1): 0.0; T(4): 0.0; T(7): 0.0; T(16): 100.0	3	2	1	0	N-Term(TMT6plex); T16(Phospho)
5032147	NP_005632.1	6878	TAF6	transcription initiation factor TFIID subunit 6 isoform	S653	1.0	1.0	1.1	1.0	1.0	0.7	0.9	0.7	0.9	1.0	1.1	1.0	0.9	0.7	0.8	0.7	0.9	0.9	0.9	0.8	QEAGDsPPPAPGtPK	GKQEAGDsPPPAPGT	S6;	Yes	S(6): 100.0; T(13): 0.0	7	2	1	0	N-Term(TMT6plex); S6(Phospho); K15(TMT6plex)
118572613	NP_057417.3	23524	SRRM2	serine/arginine repetitive matrix protein 2	T2409	1.3	1.6	1.4	1.0	1.4	0.9	1.3	0.7	1.4	1.6	1.5	1.1	1.5	0.9	1.3	0.8	1.4	1.2	1.4	0.9	SRtPPSAPSQSR	LDRARSRtPPSAPSQ	T3;	Yes	S(1): 0.0; T(3): 100.0; S(6): 0.0; S(9): 0.0; S(11): 0.0	436	1	1	1	N-Term(TMT6plex); T3(Phospho)
190358534	NP_064544.2	56916	SMARCAD1	SWI/SNF-related matrix-associated actin-dependent regulator of chromatin subfamily A containing DEAD/H box 1 isoform b	S132	0.3	1.0	1.0	0.7	0.9	0.6	0.7	1.0	0.3	1.0	0.9	0.6	0.8	0.6	0.7	0.9	0.5	0.8	0.8	0.8	DTVIIVsEPSEDEESQGLPTMAR	KDTVIIVsEPSEDEE	S15;	Yes	T(2): 0.0; S(7): 99.0; S(10): 50.5; S(15): 50.5; T(20): 0.0	4	2	1	0	N-Term(TMT6plex); S7(Phospho); S10(Phospho); M21(Oxidation)
118572613	NP_057417.3	23524	SRRM2	serine/arginine repetitive matrix protein 2	S952; S954	0.7	1.0	0.8	0.7	0.7	0.9	0.8	0.9	0.7	1.0	0.9	0.8	0.7	0.9	0.9	1.0	0.7	0.9	0.9	0.9	sVsPCSNVESR	TTPRRSRsVSPCSNV;PRRSRSVsPCSNVES	S1; S3;	Yes;Yes	S(1): 100.0; S(3): 100.0; S(6): 0.0; S(10): 0.0	436	1	1	0	N-Term(TMT6plex); S1(Phospho); S3(Phospho); C5(Carbamidomethyl)
118572613	NP_057417.3	23524	SRRM2	serine/arginine repetitive matrix protein 2	S351; S353	1.1	0.9	1.0	0.9	1.0	0.9	1.0	0.8	1.1	0.9	1.1	1.0	1.0	0.9	1.1	0.8	1.1	0.9	1.1	0.9	SATRPsPsPER	EKSATRPsPSPERSS;SATRPSPsPERSSTG	S6; S8;	Yes;Yes	S(1): 0.0; T(3): 0.0; S(6): 100.0; S(8): 100.0	436	1	1	0	N-Term(TMT6plex); S6(Phospho); S8(Phospho)
190358534	NP_064544.2	56916	SMARCAD1	SWI/SNF-related matrix-associated actin-dependent regulator of chromatin subfamily A containing DEAD/H box 1 isoform b	S132	0.9	0.6	0.7	1.0	0.3	1.0	1.0	0.7	0.8	0.6	0.7	0.9	0.3	1.0	0.9	0.6	0.5	0.8	0.8	0.8	DTVIIVSEPSEDEEsQGLPTMAR	EPSEDEEsQGLPTMA	S15;	Yes	T(2): 0.0; S(7): 0.0; S(10): 2.1; S(15): 97.8; T(20): 0.0	4	2	1	0	N-Term(TMT6plex); S10(Phospho); M21(Oxidation)
4507271	NP_000446.1	6794	STK11	serine/threonine-protein kinase STK11	S31	0.9	1.3	0.9	0.8	0.7	1.1	0.8	0.8	1.0	1.6	1.1	1.0	0.8	1.5	1.0	1.0	0.9	1.5	1.1	1.0	IDsTEVIYQPR	TEIHRIDsTEVIYQP	S3;	Yes	S(3): 100.0; T(4): 0.0; Y(8): 0.0	2	1	1	0	N-Term(TMT6plex); S3(Phospho)
556695389	NP_001273359.1	123169	LEO1	RNA polymerase-associated protein LEO1 isoform 2	S598	1.4	1.2	1.1	1.1	1.1	1.1	0.9	0.6	1.6	1.4	1.4	1.4	1.3	1.2	1.1	0.7	1.4	1.3	1.3	1.1	YVIsDEEEEDDD	KHKKYVIsDEEEEDD	S4;	No	Y(1): 0.0; S(4): 100.0	23	2	1	0	N-Term(TMT6plex); S4(Phospho)
260764007	NP_689676.2	146956	EME1	crossover junction endonuclease EME1 isoform 2	S111	1.0	0.9	0.9	0.9	0.9	0.9	0.9	0.8	0.8	1.0	0.8	0.9	0.7	1.1	0.8	0.8	0.7	1.0	0.8	0.9	QLsPEDSSSPVK	FLTHKQLsPEDSSSP	S3;	Yes	S(3): 100.0; S(7): 0.0; S(8): 0.0; S(9): 0.0	5	2	1	0	N-Term(TMT6plex); S3(Phospho); K12(TMT6plex)
114155142	NP_003283.2	7175	TPR	nucleoprotein TPR	S1185	1.0	0.9	1.2	1.1	0.5	0.4	0.4	0.5	1.2	1.0	1.3	1.3	0.5	0.5	0.5	0.6	0.8	0.7	0.9	1.0	EGVQGPLNVsLSEEGK	VQGPLNVsLSEEGKS	S10;	Yes	S(10): 98.4; S(12): 1.6	29	1	1	0	N-Term(TMT6plex); S10(Phospho); K16(TMT6plex)
10863889	NP_005137.1	9092	SART1	U4/U6.U5 tri-snRNP-associated protein 1	S448	1.1	1.4	1.1	0.8	1.1	1.7	1.3	0.9	1.3	1.5	1.3	0.9	1.3	1.7	1.5	1.1	1.3	1.6	1.4	1.0	RVsEVEEEKEPVPQPLPSDDTR	GRGRRRVsEVEEEKE	S3;	Yes	S(3): 100.0; S(18): 0.0; T(21): 0.0	1	1	1	2	N-Term(TMT6plex); S3(Phospho); K9(TMT6plex)
118572613	NP_057417.3	23524	SRRM2	serine/arginine repetitive matrix protein 2	S1458; S1460	0.8	1.0	0.9	0.8	1.0	0.9	1.0	0.9	0.8	1.1	1.0	0.9	1.1	0.9	1.0	1.0	1.0	1.0	1.0	0.9	HSLSGsSPGMK	SRHSLSGsSPGMKDI	S2; S4;	Yes	S(2): 0.0; S(4): 1.1; S(6): 97.8; S(7): 1.1	2	1	1	0	N-Term(TMT6plex); S7(Phospho); M10(Oxidation); K11(TMT6plex)
118572613	NP_057417.3	23524	SRRM2	serine/arginine repetitive matrix protein 2	S1458; S1460	1.0	0.9	1.0	0.9	0.8	1.0	0.9	0.8	1.1	0.9	1.0	1.0	0.8	1.1	1.0	0.9	1.0	1.0	1.0	0.9	HsLsGSSPGMK	SGTPSRHsLSGSSPG;TPSRHSLsGSSPGMK	S2; S4;	Yes;Yes	S(2): 100.0; S(4): 100.0; S(6): 0.0; S(7): 0.0	436	1	1	0	N-Term(TMT6plex); S2(Phospho); S6(Phospho); M10(Oxidation); K11(TMT6plex)
112421108	NP_055940.3	23152	CIC	protein capicua homolog	S173	1.4	1.4	1.2	0.9	0.9	1.3	1.1	0.7	-	-	-	-	-	-	-	-	-	-	-	-	TQsLSALPK	PGKRRTQsLSALPKE	S3;	Yes	T(1): 0.0; S(3): 100.0; S(5): 0.0	8	1	1	0	N-Term(TMT6plex); S3(Phospho); K9(TMT6plex)
291084575	NP_001166988.1	79649	MAP7D3	MAP7 domain-containing protein 3 isoform 3	S406	0.8	0.7	0.8	0.9	0.7	0.7	0.7	0.8	1.1	0.8	1.0	1.1	1.0	0.8	0.9	1.0	1.0	0.8	1.0	1.0	ESMEAsPEAMVK	PKESMEAsPEAMVKA	S6;	No	S(2): 0.0; S(6): 100.0	3	3	1	0	N-Term(TMT6plex); M3(Oxidation); S6(Phospho); M10(Oxidation); K12(TMT6plex)
7661958	NP_055554.1	9774	BCLAF1	bcl-2-associated transcription factor 1 isoform 1	S760	0.6	0.6	0.7	0.7	0.7	1.3	0.9	1.0	0.7	0.6	0.8	0.8	0.8	1.4	1.0	1.0	0.8	1.0	0.9	0.9	SSSSSASPSsPSSR	SSSASPSsPSSREEK	S10;	Yes	S(1): 0.0; S(2): 0.0; S(3): 0.0; S(4): 0.0; S(5): 0.0; S(7): 0.0; S(9): 0.0; S(10): 100.0; S(12): 0.0; S(13): 0.0	47	3	1	0	N-Term(TMT6plex); S10(Phospho)
118572613	NP_057417.3	23524	SRRM2	serine/arginine repetitive matrix protein 2	S1179	0.8	1.0	1.0	0.8	0.8	1.0	0.8	0.8	0.9	1.0	1.1	0.9	0.9	1.0	0.9	0.9	0.9	1.0	1.0	0.9	MALPPQEDATAsPPR	PQEDATAsPPRQKDK	S12;	Yes	T(10): 0.0; S(12): 100.0	436	1	1	0	N-Term(TMT6plex); M1(Oxidation); S12(Phospho)
7661958	NP_055554.1	9774	BCLAF1	bcl-2-associated transcription factor 1 isoform 1	S760	0.7	1.3	0.9	1.0	0.6	0.6	0.7	0.7	0.8	1.4	1.0	1.0	0.7	0.6	0.8	0.8	0.8	1.0	0.9	0.9	SSsSSASPSSPSSR	QDHSRSSsSSASPSS	S10;	No	S(1): 0.0; S(2): 1.7; S(3): 98.3; S(4): 0.6; S(5): 33.0; S(7): 33.0; S(9): 0.6; S(10): 33.0; S(12): 0.0; S(13): 0.0	1	3	1	0	N-Term(TMT6plex); S4(Phospho); S5(Phospho)
118918403	NP_055995.4	23224	SYNE2	nesprin-2 isoform 1	S2781	0.5	0.4	0.6	0.5	1.0	0.9	1.4	1.1	0.6	0.5	0.7	0.7	1.2	1.1	1.7	1.5	0.9	0.8	1.2	1.1	QQsVESLAEEVK	GILARQQsVESLAEE	S3;	Yes	S(3): 100.0; S(6): 0.0	1	2	1	0	N-Term(TMT6plex); S3(Phospho); K12(TMT6plex)
67782362	NP_061903.2	54505	DHX29	ATP-dependent RNA helicase DHX29	S200	0.8	1.0	1.0	0.5	1.0	0.9	1.5	1.1	0.7	0.9	0.9	0.5	0.9	0.8	1.3	1.0	0.8	0.8	1.1	0.8	EQSPQIQATIsPPLQPK	PQIQATIsPPLQPKT	S11;	Yes	S(3): 0.0; T(9): 0.0; S(11): 100.0	3	1	1	0	N-Term(TMT6plex); S11(Phospho); K17(TMT6plex)
157694492	NP_055335.2	10514	MYBBP1A	myb-binding protein 1A isoform 2	S1241	0.8	0.9	1.2	0.8	1.0	1.3	1.0	0.9	0.9	1.0	1.2	0.8	1.1	1.4	0.9	1.0	1.0	1.2	1.0	0.9	SPAPGAPtRSPSTPAK	SPAPGAPtRSPSTPA	S10;	Yes	S(1): 0.0; T(8): 98.1; S(10): 1.9; S(12): 0.0; T(13): 0.0	1	2	1	1	N-Term(TMT6plex); T8(Phospho); K16(TMT6plex)
157694492	NP_055335.2	10514	MYBBP1A	myb-binding protein 1A isoform 2	S1241	1.0	1.3	1.0	0.9	0.8	0.9	1.2	0.8	1.1	1.4	0.9	1.0	0.9	1.0	1.2	0.8	1.0	1.2	1.0	0.9	SPAPGAPTRsPSTPAK	APGAPTRsPSTPAKS	S10;	Yes	S(1): 0.0; T(8): 1.6; S(10): 98.4; S(12): 0.0; T(13): 0.0	15	2	1	1	N-Term(TMT6plex); S10(Phospho); K16(TMT6plex)
195927037	NP_001124295.1	1786	DNMT1	DNA (cytosine-5)-methyltransferase 1 isoform a	S170	0.7	0.9	0.7	0.9	0.5	0.8	0.6	0.7	0.8	1.1	0.8	1.1	0.6	0.9	0.7	0.9	0.7	1.0	0.8	1.0	AEPSPsPR	IRAEPSPsPRITRKS	S6;	No	S(4): 0.0; S(6): 100.0	14	1	1	0	N-Term(TMT6plex); S6(Phospho)
42542379	NP_005830.2	10250	SRRM1	serine/arginine repetitive matrix protein 1	S713; S715	1.0	1.2	1.2	1.0	0.7	0.8	0.9	0.7	1.2	1.3	1.3	1.1	0.9	0.9	1.0	0.8	1.1	1.1	1.1	0.9	QsPsPSTRPIR	SSPQRRQsPSPSTRP;PQRRQSPsPSTRPIR	S2; S4;	Yes;Yes	S(2): 100.0; S(4): 97.4; S(6): 1.3; T(7): 1.3	229	1	1	0	N-Term(TMT6plex); S2(Phospho); S4(Phospho)
556695389	NP_001273359.1	123169	LEO1	RNA polymerase-associated protein LEO1 isoform 2	S154	0.7	0.9	0.9	1.0	0.6	0.8	0.8	0.7	0.8	1.0	1.2	1.2	0.7	0.9	1.1	0.9	0.8	1.0	1.1	1.1	SDQsDDEK	REDKSDQsDDEKIQN	S4;	Yes	S(1): 0.0; S(4): 100.0	23	2	1	0	N-Term(TMT6plex); S4(Phospho); K8(TMT6plex)
13376115	NP_079045.1	79827	CLMP	CXADR-like membrane protein precursor	T372	0.9	0.8	1.7	0.5	1.0	4.4	2.7	1.2	-	-	-	-	-	-	-	-	-	-	-	-	AFQtV	SQSRAFQtV	T4;	No	T(4): 100.0	1	1	1	0	N-Term(TMT6plex); T4(Phospho)
47519639	NP_002366.2	4134	MAP4	microtubule-associated protein 4 isoform 1	S507	1.3	1.4	1.4	0.8	0.7	0.5	0.6	0.9	1.3	1.4	1.4	1.0	0.7	0.5	0.6	1.1	1.0	0.9	1.0	1.0	DMsPLSETEMALGK	VGLLKDMsPLSETEM	S3;	Yes	S(3): 100.0; S(6): 0.0; T(8): 0.0	47	2	1	0	N-Term(TMT6plex); M2(Oxidation); S3(Phospho); M10(Oxidation); K14(TMT6plex)
118572613	NP_057417.3	23524	SRRM2	serine/arginine repetitive matrix protein 2	T866	0.9	0.9	0.9	0.7	1.1	1.0	0.9	1.0	1.0	1.0	1.0	0.7	1.1	1.1	0.9	1.1	1.1	1.0	1.0	0.9	QGSITSPQANEQSVtPQR	QANEQSVtPQRRSCF	T15;	Yes	S(3): 0.0; T(5): 0.0; S(6): 0.0; S(13): 0.0; T(15): 100.0	436	1	1	0	N-Term(TMT6plex); T15(Phospho)
34098946	NP_004550.2	4904	YBX1	nuclease-sensitive element-binding protein 1	S165	0.9	0.9	1.0	0.7	1.1	0.7	0.9	1.0	1.1	0.9	1.2	0.8	1.3	0.8	1.2	1.2	1.2	0.9	1.2	1.0	NYQQNYQNsESGEK	YQQNYQNsESGEKNE	S9;	Yes	Y(2): 0.0; Y(6): 0.0; S(9): 100.0; S(11): 0.0	66	1	1	0	N-Term(TMT6plex); S9(Phospho); K14(TMT6plex)
118572613	NP_057417.3	23524	SRRM2	serine/arginine repetitive matrix protein 2	S1694	0.9	1.0	1.0	0.9	0.7	0.9	0.8	0.7	1.0	1.0	1.0	1.0	0.8	0.9	0.8	0.8	0.9	1.0	0.9	0.9	SsPELTR	RRRSSRSsPELTRKA	S2;	Yes	S(1): 0.0; S(2): 100.0; T(6): 0.0	436	1	1	0	N-Term(TMT6plex); S2(Phospho)
5454102	NP_006333.1	10460	TACC3	transforming acidic coiled-coil-containing protein 3	S434	1.0	0.9	0.7	0.5	1.2	1.1	1.0	1.2	1.1	0.9	0.9	0.6	1.4	1.1	1.2	1.3	1.2	1.0	1.0	0.9	SGCSEAQPPEsPETR	SEAQPPEsPETRLGQ	S11;	Yes	S(1): 0.0; S(4): 0.0; S(11): 99.1; T(14): 0.9	12	1	1	0	N-Term(TMT6plex); C3(Carbamidomethyl); S11(Phospho)
39753953	NP_789784.2	168850	ZNF800	zinc finger protein 800	S460	0.9	1.0	0.9	0.8	0.8	0.9	1.0	0.8	0.8	0.9	0.8	0.7	0.7	0.8	0.8	0.7	0.7	0.9	0.8	0.7	sTSPSAAGGQQK	QDSESPKsTSPSAAG	S1;	Yes	S(1): 98.1; T(2): 1.0; S(3): 1.0; S(5):	13	1	1	0	N-Term(TMT6plex); S3(Phospho); K12(TMT6plex)
155030216	NP_001093869.1	23244	PDS5A	sister chromatid cohesion protein PDS5 homolog A isoform 1	S1305	0.8	0.9	0.7	0.9	0.7	0.8	1.0	0.8	0.8	0.8	0.7	0.9	0.7	0.8	1.0	0.8	0.8	0.8	0.8	0.8	AAVGQEsPGGLEAGNAK	RAAVGQEsPGGLEAG	S7;	Yes	S(7): 100.0	9	1	1	0	N-Term(TMT6plex); S7(Phospho); K17(TMT6plex)
20127499	NP_006266.2	6431	SRSF6	serine/arginine-rich splicing factor 6	S303	0.8	0.8	0.8	0.8	0.8	0.9	0.8	0.8	1.0	1.0	1.0	1.0	1.0	1.1	1.0	1.1	1.0	1.0	1.0	1.1	sNsPLPVPPSK	RSQSRSNsPLPVPPS	S3;	Yes	S(1): 0.5; S(3): 99.5; S(10): 0.0	35	1	1	0	N-Term(TMT6plex); S3(Phospho); K11(TMT6plex)
55749659	NP_001006948.1	23074	UHRF1BP1L	UHRF1-binding protein 1-like isoform b	S414	1.1	0.8	1.0	0.8	1.3	0.8	0.6	0.8	-	-	-	-	-	-	-	-	-	-	-	-	DHNVGsPPK	VKDHNVGsPPKSPTH	S6;	Yes	S(6): 100.0	1	2	1	0	N-Term(TMT6plex); S6(Phospho); K9(TMT6plex)
20127499	NP_006266.2	6431	SRSF6	serine/arginine-rich splicing factor 6	S303	0.8	0.9	0.8	0.8	0.8	0.8	0.8	0.8	1.0	1.1	1.0	1.1	1.0	1.0	1.0	1.0	1.0	1.0	1.0	1.1	SNsPLPVPPSK	RSQSRSNsPLPVPPS	S3;	Yes	S(1): 0.0; S(3): 100.0; S(10): 0.0	43	1	1	0	N-Term(TMT6plex); S3(Phospho); K11(TMT6plex)
190341107	NP_001011713.2	122830	NAA30	N-alpha-acetyltransferase 30	S55	0.6	0.9	0.7	0.9	1.0	0.9	1.0	0.8	0.6	0.9	0.7	0.9	0.9	0.9	0.9	0.8	0.7	0.9	0.8	0.8	sPAGGESATVAAK	HEGGGSRsPAGGESA	S1;	Yes	S(1): 100.0; S(7): 0.0; T(9): 0.0	2	1	1	0	N-Term(TMT6plex); S1(Phospho); K13(TMT6plex)
56682959	NP_002023.2	2495	FTH1	ferritin heavy chain	S179	1.0	0.9	0.9	0.8	1.0	0.6	0.9	0.9	1.1	1.2	1.1	0.8	1.1	0.8	1.1	0.9	1.1	1.0	1.1	0.8	HTLGDsDNES	DKHTLGDsDNES	S6;	Yes	T(2): 0.0; S(6): 100.0; S(10): 0.0	6	1	1	0	N-Term(TMT6plex); S6(Phospho)
21955172	NP_665696.1	90102	PHLDB2	pleckstrin homology-like domain family B member 2 isoform c	S204	0.9	0.9	0.8	0.8	1.0	0.9	1.0	0.8	0.9	1.0	0.9	0.9	1.0	1.0	1.1	1.0	1.0	1.0	1.0	0.9	SGAASMPSsPK	GAASMPSsPKQARKM	S9;	Yes	S(1): 0.0; S(5): 0.0; S(8): 0.0; S(9): 100.0	9	3	1	0	N-Term(TMT6plex); M6(Oxidation); S9(Phospho); K11(TMT6plex)
153070260	NP_002347.5	4082	MARCKS	myristoylated alanine-rich C-kinase substrate	T150	0.7	0.8	0.7	0.7	0.6	0.9	0.7	0.9	1.1	1.0	1.0	1.0	1.0	1.2	1.0	1.3	1.0	1.1	1.0	1.1	AEDGATPSPSNEtPKK	TPSPSNEtPKKKKKR	T13;	Yes	T(6): 0.0; S(8): 0.0; S(10): 1.3; T(13): 98.7	69	1	1	1	N-Term(TMT6plex); T13(Phospho); K15(TMT6plex); K16(TMT6plex)
153070260	NP_002347.5	4082	MARCKS	myristoylated alanine-rich C-kinase substrate	T150	0.6	0.9	0.7	0.9	0.7	0.8	0.7	0.7	1.0	1.2	1.0	1.3	1.1	1.0	1.0	1.0	1.0	1.1	1.0	1.1	AEDGATPsPSNEtPKK	TPSPSNEtPKKKKKR	T13;	Yes	T(6): 0.0; S(8): 0.0; S(10): 0.0; T(13): 100.0	6	1	1	1	N-Term(TMT6plex); T13(Phospho); K15(TMT6plex); K16(TMT6plex)
4758958	NP_004148.1	5576	PRKAR2A	cAMP-dependent protein kinase type II-alpha regulatory subunit	S99	0.9	1.2	1.5	1.3	0.9	0.8	1.1	0.3	0.8	1.2	1.4	1.1	0.8	0.8	0.9	0.3	0.8	1.0	1.2	0.7	RVsVCAETYNPDEEEEDTDPR	SRFNRRVsVCAETYN	S3;	Yes	S(3): 100.0; T(8): 0.0; Y(9): 0.0; T(18): 0.0	2	1	1	1	N-Term(TMT6plex); S3(Phospho); C5(Carbamidomethyl)
110556636	NP_203754.2	85456	TNKS1BP1	182 kDa tankyrase-1-binding protein	S1620	0.7	1.0	1.0	1.0	0.9	0.9	0.9	0.7	0.7	0.9	1.0	1.1	0.9	0.9	0.8	0.8	0.8	0.9	0.9	0.9	VPssDEEVVEEPQSR	PRASRVPsSDEEVVE	S3;	Yes	S(3): 99.0; S(4): 1.0; S(14): 0.0	41	1	1	0	N-Term(TMT6plex); S3(Phospho)
71164886	NP_057524.3	51231	VRK3	inactive serine/threonine-protein kinase VRK3 isoform 1	S59	0.7	0.9	0.8	0.8	1.1	1.2	1.2	0.9	0.7	1.0	1.0	0.9	1.1	1.4	1.5	1.1	0.9	1.2	1.2	1.0	GLNSSFETsPK	LNSSFETsPKKVKWS	S9;	Yes	S(4): 0.0; S(5): 0.0; T(8): 0.0; S(9): 100.0	3	1	1	0	N-Term(TMT6plex); S9(Phospho); K11(TMT6plex)
21389407	NP_653209.1	124790	HEXIM2	protein HEXIM2	S76	0.7	0.7	0.8	0.9	0.6	0.5	0.6	0.7	-	-	-	-	-	-	-	-	-	-	-	-	TQsPGGCSAEAVLAR	SRSPRTQsPGGCSAE	S3;	Yes	T(1): 0.0; S(3): 100.0; S(8): 0.0	5	1	1	0	N-Term(TMT6plex); S3(Phospho); C7(Carbamidomethyl)
134288896	NP_653265.3	143684	FAM76B	protein FAM76B	T215	0.9	0.9	0.9	0.8	1.0	0.8	0.8	0.9	0.8	0.9	0.9	0.8	0.9	0.9	0.9	0.9	0.9	0.9	0.9	0.9	SSATIQNEtPK	SATIQNEtPKKKPKL	T9;	Yes	S(1): 0.0; S(2): 0.0; T(4): 0.0; T(9): 100.0	3	1	1	0	N-Term(TMT6plex); T9(Phospho); K11(TMT6plex)
24308350	NP_149103.1	88745	RRP36	ribosomal RNA processing protein 36 homolog	S73	0.8	0.7	0.6	0.7	0.8	0.9	0.7	0.9	0.9	0.8	0.7	0.8	0.9	1.0	0.8	1.1	0.9	0.9	0.7	1.0	QLVAGNsPK	KQLVAGNsPKKQASR	S7;	Yes	S(7): 100.0	6	1	1	0	N-Term(TMT6plex); S7(Phospho); K9(TMT6plex)
14249338	NP_116114.1	84811	BUD13	BUD13 homolog isoform 1	S358	1.0	1.0	0.9	0.8	1.0	0.8	0.7	0.8	1.1	1.0	1.0	0.9	1.1	0.8	0.9	1.0	1.1	0.9	0.9	0.9	ATDSDLSsPR	ATDSDLSsPRHKQSP	S8;	Yes	T(2): 0.0; S(4): 0.0; S(7): 0.0; S(8): 100.0	52	2	1	0	N-Term(TMT6plex); S8(Phospho)
122937229	NP_001073886.1	23361	ZNF629	zinc finger protein 629	S28	0.6	1.0	0.8	0.8	0.5	1.1	1.1	0.8	0.6	0.9	0.8	0.8	0.5	1.0	1.1	0.8	0.6	1.0	1.0	0.8	GAEsENEEESPR	DAHRGAEsENEEESP	S4;	No	S(4): 100.0; S(10): 0.0	4	1	1	0	N-Term(TMT6plex); S4(Phospho)
363807394	NP_001241881.1	79969	ATAT1	alpha-tubulin N-acetyltransferase 1 isoform 4	S292	0.6	0.7	0.4	0.7	0.7	0.5	0.5	1.0	0.8	0.9	0.6	0.8	0.8	0.7	0.6	1.1	0.8	0.8	0.6	0.9	LLLAADPGGsPAQR	LAADPGGsPAQRRRT	S10;	No	S(10): 100.0	6	3	1	0	N-Term(TMT6plex); S10(Phospho)
410651504	NP_001258596.1	84062	DTNBP1	dysbindin isoform C	S270	0.9	1.1	1.1	0.8	1.0	1.6	1.5	0.9	0.9	1.2	1.1	1.1	1.0	1.8	1.6	1.2	1.0	1.5	1.3	1.1	EATPDGGEDSDs	DGGEDSDs	S12;	No	T(3): 0.0; S(10): 0.0; S(12): 100.0	4	4	1	0	N-Term(TMT6plex); S12(Phospho)
153946395	NP_002151.2	3371	TNC	tenascin precursor	S72	0.8	1.0	0.9	0.9	0.7	1.1	0.9	0.7	1.1	1.4	1.3	1.2	1.0	1.6	1.4	1.0	1.0	1.5	1.4	1.1	LPVGSQCSVDLESAsGEK	SVDLESAsGEKDLAP	S15;	Yes	S(5): 0.0; S(8): 0.0; S(13): 0.0; S(15): 100.0	1	1	1	0	N-Term(TMT6plex); C7(Carbamidomethyl); S15(Phospho); K18(TMT6plex)
282398123	NP_001164190.1	9564	BCAR1	breast cancer anti-estrogen resistance protein 1 isoform	S137	2.3	1.5	0.7	1.0	0.6	0.5	1.3	0.7	2.6	1.6	0.7	1.0	0.6	0.5	1.3	0.7	1.6	1.1	1.0	0.8	AQQGLYQVPGPSPQFQsPPAK	GPSPQFQsPPAKQTS	S17;	No	Y(6): 0.0; S(12): 0.0; S(17): 100.0	1	7	1	0	N-Term(TMT6plex); S17(Phospho); K21(TMT6plex)
112382257	NP_795352.2	10207	INADL	inaD-like protein	S645	0.7	0.6	0.9	0.6	0.6	0.6	0.8	1.1	0.7	0.8	1.2	0.6	0.7	0.7	1.1	1.2	0.7	0.7	1.1	0.9	LEDDEAsVDEPR	RLEDDEAsVDEPRRT	S7;	Yes	S(7): 100.0	3	1	1	0	N-Term(TMT6plex); S7(Phospho)
88853059	NP_001034678.1	1802	DPH2	diphthamide biosynthesis protein 2 isoform b	S260	1.1	0.9	0.9	0.4	1.3	1.8	1.5	1.3	1.1	0.9	0.9	0.3	1.4	1.8	1.5	1.2	1.2	1.4	1.2	0.8	GIAIAYEDEGsG	IAYEDEGsG	S11;	No	Y(6): 0.0; S(11): 100.0	2	2	1	0	N-Term(TMT6plex); S11(Phospho)
34098946	NP_004550.2	4904	YBX1	nuclease-sensitive element-binding protein 1	S176	0.9	0.8	0.9	0.7	1.2	1.0	1.0	0.9	1.0	0.9	1.2	0.9	1.4	1.1	1.3	1.1	1.2	1.0	1.2	1.0	NEGsESAPEGQAQQR	SGEKNEGsESAPEGQ	S6;	Yes	S(4): 100.0; S(6): 0.0	3	1	1	0	N-Term(TMT6plex); S6(Phospho)
14249338	NP_116114.1	84811	BUD13	BUD13 homolog isoform 1	T147; S151	1.0	0.8	0.8	0.8	0.9	1.2	1.2	0.8	1.0	0.8	0.9	0.9	1.0	1.3	1.4	1.0	1.0	1.1	1.2	0.9	HDtPDPsPR	PRKDRHDtPDPSPRR;RHDTPDPsPRRARHD	T3; S7;	Yes;Yes	T(3): 100.0; S(7): 100.0	52	2	1	0	N-Term(TMT6plex); T3(Phospho); S7(Phospho)
34098946	NP_004550.2	4904	YBX1	nuclease-sensitive element-binding protein 1	S176	1.2	1.0	1.0	0.9	0.9	0.8	0.9	0.7	1.4	1.1	1.3	1.1	1.0	0.9	1.2	0.9	1.2	1.0	1.2	1.0	NEGSEsAPEGQAQQR	EKNEGSEsAPEGQAQ	S6;	Yes	S(4): 0.0; S(6): 100.0	66	1	1	0	N-Term(TMT6plex); S6(Phospho)
63053524	NP_001017416.1	7398	USP1	ubiquitin carboxyl-terminal hydrolase 1	S313	0.7	0.6	0.7	0.8	0.8	0.7	0.8	0.9	0.8	0.7	0.7	0.8	0.9	0.8	0.9	0.9	0.8	0.7	0.8	0.9	ATSDTLEsPPK	ATSDTLEsPPKIIPK	S8;	Yes	T(2): 0.0; S(3): 0.0; T(5): 0.0; S(8): 100.0	7	1	1	0	N-Term(TMT6plex); S8(Phospho); K11(TMT6plex)
194272142	NP_001123537.1	4650	MYO9B	unconventional myosin-IXb isoform 2	S1290	1.0	1.0	1.1	0.9	0.7	0.9	0.7	0.7	1.1	1.1	1.2	1.0	0.8	1.0	0.7	0.8	0.9	1.0	1.0	0.9	VQEKPDsPGGSTQIQR	RVQEKPDsPGGSTQI	S7;	Yes	S(7): 100.0; S(11): 0.0; T(12): 0.0	14	2	1	0	N-Term(TMT6plex); K4(TMT6plex); S7(Phospho)
18379349	NP_006364.2	10493	VAT1	synaptic vesicle membrane protein VAT-1 homolog	S18	0.1	0.1	0.2	0.1	1.0	0.7	0.9	1.5	0.1	0.1	0.1	0.1	0.9	0.6	0.6	1.3	0.5	0.3	0.4	0.7	EVAEAATGEDASsPPPK	ATGEDASsPPPKTEA	S13;	Yes	T(7): 1.4; S(12): 1.4; S(13): 97.2	1	1	1	0	N-Term(TMT6plex); S13(Phospho); K17(TMT6plex)
419636284	NP_001258698.1	8546	AP3B1	AP-3 complex subunit beta-1 isoform 2	S227	1.0	1.0	1.1	0.8	1.3	1.0	1.1	0.8	1.1	1.0	1.1	0.8	1.3	0.9	1.1	0.8	1.2	0.9	1.1	0.8	NEYEsDDDQK	NGKNEYEsDDDQKEK	S5;	No	Y(3): 0.0; S(5): 100.0	2	2	1	0	N-Term(TMT6plex); S5(Phospho); K10(TMT6plex)
253970456	NP_001156752.1	27336	HTATSF1	HIV Tat-specific factor 1	S445	1.0	0.9	0.8	0.9	0.5	0.6	0.6	0.7	1.4	1.2	1.2	1.6	0.7	0.8	0.9	1.1	1.0	1.0	1.1	1.4	TEDGGEEEEGAsENNAK	GEEEEGAsENNAKES	S12;	Yes	T(1): 0.0; S(12): 100.0	16	1	1	0	N-Term(TMT6plex); S12(Phospho); K17(TMT6plex)
42542379	NP_005830.2	10250	SRRM1	serine/arginine repetitive matrix protein 1	S769; S781	0.6	0.8	0.7	0.6	0.5	1.0	0.7	1.0	0.7	0.9	0.8	0.7	0.6	1.1	0.7	1.2	0.7	1.0	0.8	0.9	KPPAPPsPVQSQSPSTNWsPAVPVK	KKPPAPPsPVQSQSP;QSPSTNWsPAVPVKK	S7; S19;	Yes;Yes	S(7): 100.0; S(11): 0.0; S(13): 0.0; S(15): 0.0; T(16): 2.0; S(19): 98.0	229	1	1	0	N-Term(TMT6plex); K1(TMT6plex); S7(Phospho); S19(Phospho); K25(TMT6plex)
42542379	NP_005830.2	10250	SRRM1	serine/arginine repetitive matrix protein 1	S769; S781	0.5	1.0	0.7	1.0	0.6	0.8	0.7	0.6	0.6	1.1	0.7	1.2	0.7	0.9	0.8	0.7	0.7	1.0	0.8	0.9	KPPAPPsPVQSQsPSTNWSPAVPVK	KKPPAPPsPVQSQSP;PSPVQSQsPSTNWSP	S7; S19;	Yes;Yes	S(7): 100.0; S(11): 1.9; S(13): 92.5; S(15): 1.9; T(16): 1.9; S(19): 1.9	5	1	1	0	N-Term(TMT6plex); K1(TMT6plex); S7(Phospho); S11(Phospho); K25(TMT6plex)
88703045	NP_060774.2	55771	PRR11	proline-rich protein 11	S40	1.0	0.9	0.6	1.1	0.8	1.5	1.0	0.5	1.2	0.9	0.6	1.3	0.9	1.6	1.0	0.6	1.0	1.2	0.8	0.9	LITPPPPPPsPER	TPPPPPPsPERVGIS	S10;	Yes	T(3): 2.8; S(10): 97.2	1	1	1	0	N-Term(TMT6plex); S10(Phospho)
51479177	NP_077313.3	79192	IRX1	iroquois-class homeodomain protein IRX-1	S433	1.2	0.7	0.6	0.7	2.3	1.4	0.9	1.0	-	-	-	-	-	-	-	-	-	-	-	-	sSPTLPER	GDKASVRsSPTLPER	S2;	No	S(1): 99.0; S(2): 1.0; T(4): 0.0	1	1	1	0	N-Term(TMT6plex); S1(Phospho)
21396500	NP_003600.2	8479	HIRIP3	HIRA-interacting protein 3 isoform 1	S143	1.0	1.0	1.0	0.9	0.7	0.7	0.8	0.8	1.1	1.1	1.1	0.9	0.8	0.8	1.0	0.8	1.0	0.9	1.0	0.9	AVEEssDEER	SKAVEESsDEERQRD	S6;	Yes	S(5): 0.4; S(6): 99.6	26	1	1	0	N-Term(TMT6plex); S6(Phospho)
103472005	NP_002408.3	4288	MKI67	antigen KI-67 isoform 1	T2389	0.5	0.8	0.9	0.9	0.3	0.7	0.8	0.7	0.5	0.8	0.9	1.0	0.4	0.7	0.9	0.7	0.5	0.7	0.9	0.9	AMDtPKPAVSDEK	SAGKAMDtPKPAVSD	T4;	Yes	T(4): 100.0; S(10): 0.0	118	2	1	0	N-Term(TMT6plex); M2(Oxidation); T4(Phospho); K6(TMT6plex); K13(TMT6plex)
14790190	NP_055816.2	23013	SPEN	msx2-interacting protein	T1947	0.9	0.9	0.6	0.9	0.6	0.7	0.6	0.7	0.8	0.8	0.6	0.9	0.6	0.6	0.6	0.7	0.7	0.7	0.6	0.8	ELQEAAAVPTtPR	EAAAVPTtPRRGRPP	T11;	Yes	T(10): 0.9; T(11): 99.1	16	1	1	0	N-Term(TMT6plex); T11(Phospho)
167614488	NP_056342.3	26000	TBC1D10B	TBC1 domain family member 10B	S132	0.6	0.6	0.6	0.5	2.1	2.0	1.3	1.1	0.6	0.6	0.6	0.5	2.3	2.2	1.5	1.2	1.4	1.4	1.1	0.8	ALAAGADsPK	ALAAGADsPKTEEAR	S8;	Yes	S(8): 100.0	13	1	1	0	N-Term(TMT6plex); S8(Phospho); K10(TMT6plex)
118498368	NP_004977.2	3895	KTN1	kinectin isoform c	T153	0.7	0.8	0.8	0.8	0.8	0.9	0.8	0.8	0.8	1.0	1.0	0.9	0.9	1.1	1.0	0.9	0.9	1.0	1.0	0.9	QPtPPSEAAASK	VPVTKQPtPPSEAAA	T3;	Yes	T(3): 100.0; S(6): 0.0; S(11): 0.0	9	4	1	0	N-Term(TMT6plex); T3(Phospho); K12(TMT6plex)
118572613	NP_057417.3	23524	SRRM2	serine/arginine repetitive matrix protein 2	S2398	1.2	1.0	1.3	0.9	1.0	1.0	1.1	0.7	1.3	1.0	1.4	1.0	1.1	1.0	1.2	0.8	1.2	1.0	1.3	0.9	TsPPLLDR	ERVSGRTsPPLLDRA	S2;	Yes	T(1): 0.8; S(2): 99.2	436	1	1	0	N-Term(TMT6plex); S2(Phospho)
155969714	NP_002510.2	4863	NPAT	protein NPAT	S207	0.9	1.0	1.0	1.0	0.7	0.7	0.7	0.6	0.9	1.1	1.2	1.2	0.6	0.8	0.8	0.8	0.7	1.0	1.0	1.0	AHASLMsPGR	KAHASLMsPGRRKSE	S7;	Yes	S(4): 0.0; S(7): 100.0	4	1	1	0	N-Term(TMT6plex); M6(Oxidation); S7(Phospho)
130502140	NP_065953.1	57662	CAMSAP3	calmodulin-regulated spectrin-associated protein 3 isoform 2	S756	0.8	0.7	0.6	0.8	0.9	0.7	0.6	0.8	-	-	-	-	-	-	-	-	-	-	-	-	AAsPSPAR	TTGPKAAsPSPARRV	S3;	Yes	S(3): 99.2; S(5): 0.8	2	2	1	0	N-Term(TMT6plex); S3(Phospho)
13559514	NP_060895.1	55361	PI4K2A	phosphatidylinositol 4-kinase type 2-alpha	S51	0.9	0.9	1.0	0.4	1.1	1.3	1.2	1.2	1.1	1.1	1.1	0.4	1.3	1.4	1.4	1.1	1.2	1.3	1.3	0.8	VAAAAGSGPsPPGsPGHDR	AAAGSGPsPPGSPGH;SGPSPPGsPGHDRER	S14;	Yes;Yes	S(7): 1.9; S(10): 98.1; S(14): 100.0	4	1	1	0	N-Term(TMT6plex); S10(Phospho); S14(Phospho)
13559514	NP_060895.1	55361	PI4K2A	phosphatidylinositol 4-kinase type 2-alpha	S51	1.1	1.3	1.2	1.2	0.9	0.9	1.0	0.4	1.3	1.4	1.4	1.1	1.1	1.1	1.1	0.4	1.2	1.3	1.3	0.8	VAAAAGSGPSPPGsPGHDR	SGPSPPGsPGHDRER	S14;	Yes	S(7): 50.0; S(10): 50.0; S(14): 100.0	4	1	1	0	N-Term(TMT6plex); S10(Phospho); S14(Phospho)
4758024	NP_004636.1	8161	COIL	coilin	T122	0.6	0.5	0.9	0.5	2.3	0.9	2.6	1.1	0.6	0.4	0.8	0.4	2.3	0.9	2.5	1.0	1.5	0.6	1.7	0.7	AFQLEEGEEtEPDCK	QLEEGEEtEPDCKYS	T10;	Yes	T(10): 100.0	8	1	1	0	N-Term(TMT6plex); T10(Phospho); C14(Carbamidomethyl); K15(TMT6plex)
18860916	NP_036387.2	22803	XRN2	5'-3' exoribonuclease 2	S448	1.2	0.8	1.0	0.9	0.9	0.7	0.9	0.8	1.2	0.9	1.0	0.9	0.9	0.8	0.9	0.8	1.1	0.8	1.0	0.8	NsPGSQVASNPR	HALGSRNsPGSQVAS	S2;	Yes	S(2): 99.9; S(5): 0.0; S(9): 0.0	3	1	1	0	N-Term(TMT6plex); S2(Phospho)
118498359	NP_056474.2	26156	RSL1D1	ribosomal L1 domain-containing protein 1	S427	0.7	0.9	0.7	0.9	0.7	0.8	0.7	0.7	0.9	1.1	0.8	1.0	0.8	0.9	0.8	0.8	0.9	1.0	0.8	0.9	AAESETPGKsPEK	ESETPGKsPEKKPKI	S10;	Yes	S(4): 0.0; T(6): 0.0; S(10): 100.0	19	1	1	1	N-Term(TMT6plex); K9(TMT6plex); S10(Phospho); K13(TMT6plex)
37674210	NP_203747.2	85464	SSH2	protein phosphatase Slingshot homolog 2 isoform 2	S487	0.7	0.7	0.6	0.7	0.8	0.8	0.7	0.9	0.8	0.8	0.8	0.8	0.9	0.9	0.9	1.0	0.8	0.9	0.8	0.9	DITTsADQIAEVK	NKKDITTsADQIAEV	S5;	Yes	T(3): 0.0; T(4): 0.9; S(5): 99.1	5	2	1	0	N-Term(TMT6plex); S5(Phospho); K13(TMT6plex)
112821681	NP_443131.2	114787	GPRIN1	G protein-regulated inducer of neurite outgrowth 1	S850	0.6	0.8	0.6	0.9	0.7	0.6	0.6	0.7	-	-	-	-	-	-	-	-	-	-	-	-	APsPPSR	QAAPRAPsPPSRRDA	S3;	Yes	S(3): 100.0; S(6): 0.0	2	1	1	0	N-Term(TMT6plex); S3(Phospho)
50053795	NP_001408.2	1975	EIF4B	eukaryotic translation initiation factor 4B	S422	1.0	0.8	0.8	0.8	1.5	1.4	1.0	0.9	1.1	0.7	0.8	0.6	1.6	1.3	1.0	0.7	1.4	1.0	0.9	0.7	TGsESsQTGTSTTSSR	RERSRTGsESSQTGT	S3;	Yes	T(1): 0.9; S(3): 99.1; S(5): 0.0; S(6): 0.0; T(8): 0.0; T(10): 0.0; S(11): 0.0; T(12): 0.0; T(13): 0.0; S(14): 0.0; S(15): 0.0	39	1	1	0	N-Term(TMT6plex); S3(Phospho)
18426904	NP_569079.1	56897	WRNIP1	ATPase WRNIP1 isoform 2	S153	0.8	0.7	0.7	0.8	0.7	0.8	0.8	0.9	0.8	0.8	0.8	0.8	0.7	0.8	0.8	0.9	0.8	0.8	0.8	0.9	RPAAAAAAGSAsPR	AAAAGSAsPRSWDEA	S12;	Yes	S(10): 0.0; S(12): 100.0	5	2	1	0	N-Term(TMT6plex); S12(Phospho)
50053795	NP_001408.2	1975	EIF4B	eukaryotic translation initiation factor 4B	S422	1.5	1.4	1.0	0.9	1.0	0.8	0.8	0.8	1.6	1.3	1.0	0.7	1.1	0.7	0.8	0.6	1.4	1.0	0.9	0.7	tGSESSQTGTSTTSSR	RERSRTGsESSQTGT;SRTGSESsQTGTSTT	S3;	Yes;Yes	T(1): 0.0; S(3): 100.0; S(5): 1.3; S(6): 98.7; T(8): 0.0; T(10): 0.0; S(11): 0.0; T(12): 0.0; T(13): 0.0; S(14): 0.0; S(15): 0.0	2	1	1	0	N-Term(TMT6plex); S5(Phospho); S6(Phospho)
4826690	NP_004932.1	1659	DHX8	ATP-dependent RNA helicase DHX8	S460	0.9	1.0	1.2	0.9	0.8	0.8	0.9	0.7	1.0	1.0	1.2	0.9	0.8	0.8	0.9	0.8	0.9	0.9	1.0	0.9	QSMDMsPIK	TKQSMDMsPIKIVKN	S6;	Yes	S(2): 0.0; S(6): 100.0	1	1	1	0	N-Term(TMT6plex); M3(Oxidation); M5(Oxidation); S6(Phospho); K9(TMT6plex)
110556636	NP_203754.2	85456	TNKS1BP1	182 kDa tankyrase-1-binding protein	S893	0.8	0.6	1.1	0.5	1.6	1.3	2.1	1.1	0.8	0.5	1.0	0.6	1.6	1.2	2.0	1.2	1.2	0.9	1.5	0.9	DsLGAYASQDANEQGQDLGK	WEFGKRDsLGAYASQ	S2;	Yes	S(2): 100.0; Y(6): 0.0; S(8): 0.0	41	1	1	0	N-Term(TMT6plex); S2(Phospho); K20(TMT6plex)
557440810	NP_001273512.1	57661	PHRF1	PHD and RING finger domain-containing protein 1 isoform 4	S1198	1.4	1.6	1.2	0.9	0.7	0.6	0.6	0.7	1.3	1.7	1.3	1.1	0.6	0.6	0.7	0.9	1.0	1.2	1.0	1.0	EAsPAPLAQGEPGR	KGAVREAsPAPLAQG	S3;	No	S(3): 100.0	15	4	1	0	N-Term(TMT6plex); S3(Phospho)
110611218	NP_001036041.1	6238	RRBP1	ribosome-binding protein 1	S185	0.6	0.8	0.7	0.8	0.7	0.8	0.8	0.8	0.6	0.8	0.8	0.9	0.7	0.7	0.8	0.8	0.6	0.7	0.8	0.8	NTDVAQsPEAPKQEAPAK	RNTDVAQsPEAPKQE	S7;	No	T(2): 0.0; S(7): 100.0	8	1	1	1	N-Term(TMT6plex); S7(Phospho); K12(TMT6plex); K18(TMT6plex)
112807226	NP_001036783.1	91748	ELMSAN1	ELM2 and SANT domain-containing protein 1	T715	2.0	0.9	1.3	1.1	0.6	0.6	0.6	0.5	2.1	1.0	1.3	1.2	0.6	0.7	0.6	0.5	1.4	0.8	1.0	0.9	TNSAEVTPPVLSVMGEAtPVSIEPR	LSVMGEAtPVSIEPR	T18;	Yes	T(1): 0.0; S(3): 0.0; T(7): 0.0; S(12): 0.0; T(18): 100.0; S(21): 0.0	4	1	1	0	N-Term(TMT6plex); M14(Oxidation); T18(Phospho)

Khan et al., 2019. Multi-omics analysis to characterize cigarette smoke induced molecular alterations in esophageal cells
Supplementary Table 8. List of phosphopeptides identified and quantified in untreated and chronically treated Het1A cells with cigarette smoke condensate for 8 months using SequestHT and Mascot search algorithms with a PhosphoRS probability score ≥75%

Protein group Accession	NP_Accession	Gene ID	Gene Symbol	Protein Description	PhosphoSite (Protein)	Het-1A-Smoke - 2M/Parental	Het-1A-Smoke - 4M/Parental	Het-1A-Smoke - 6M/Parental	Het-1A-Smoke - 8M/Parental	Het-1A-Smoke - 2M/Parental	Het-1A-Smoke - 4M/Parental	Het-1A-Smoke - 6M/Parental	Het-1A-Smoke - 8M/Parental	Het-1A-Smoke - 2M/Parental	Het-1A-Smoke - 4M/Parental	Het-1A-Smoke - 6M/Parental	Het-1A-Smoke - 8M/Parental	Het-1A-Smoke - 2M/Parental	Het-1A-Smoke - 4M/Parental	Het-1A-Smoke - 6M/Parental	Het-1A-Smoke - 8M/Parental	Het-1A-Smoke - 2M/Parental	Het-1A-Smoke - 4M/Parental	Het-1A-Smoke - 6M/Parental	Het-1A-Smoke - 8M/Parental	Peptide Sequence	Phosphowindow	PhosphoSite (Peptide)	Phosphosite Plus Evidence	phosphoRS Site Probabilities	# PSMs	# Proteins	# Protein Groups	# Missed Cleavages	Modifications
						Replicate 1				Replicate 2				Normalized phosphoprotein ratios with respect to corresponding total protein ratios. Replicate 1				Replicate 2				Average of replicates													
102467242	NP_064623.2	3619	INCENP	inner centromere protein isoform2	S214	0.6	0.9	0.9	0.8	1.0	1.0	0.8	0.8	0.6	0.9	0.9	0.8	0.9	1.0	0.8	0.9	0.8	0.9	0.8	0.8	TLSPTPASATAPTSQGIPTsDEESTPKK	TSQGIPTsDEESTPK	S20;	Yes	T(1): 0.0; S(3): 0.0; T(5): 0.0; S(8): 0.0; T(10): 0.0; T(13): 0.0; S(14): 0.0; T(19): 2.3; S(20): 96.6; S(24): 50.6; T(25): 50.6	23	2	1	1	N-Term(TMT6plex); S20(Phospho); S24(Phospho); K27(TMT6plex); K28(TMT6plex)
38044112	NP_937883.1	6249	CLIP1	CAP-Gly domain-containing linker protein 1 isoformb	T182	0.7	0.8	0.8	0.8	0.6	0.9	0.7	0.8	0.7	0.8	0.8	0.9	0.6	0.8	0.7	0.9	0.7	0.8	0.7	0.9	EPSAtPPISNLTK	AAKEPSAtPPISNLT	T5;	Yes	S(3): 1.1; T(5): 98.9; S(9): 0.0; T(12): 0.0	9	3	1	0	N-Term(TMT6plex); T5(Phospho); K13(TMT6plex)
55743138	NP_001006945.1	8986	RPS6KA4	ribosomal protein S6 kinase alpha-4 isoformb	S347	1.0	0.9	0.6	1.1	0.6	0.5	0.7	0.5	0.9	0.9	0.6	1.1	0.5	0.4	0.7	0.5	0.7	0.6	0.6	0.8	LEPVYSPPGsPPPGDPR	PVYSPPGsPPPGDPR	S10;	Yes	Y(5): 0.0; S(6): 0.0; S(10): 100.0	3	2	1	0	N-Term(TMT6plex); S10(Phospho)
33620755	NP_060493.3	55689	YEATS2	YEATS domain-containing protein 2	S627	0.6	0.8	0.7	0.8	0.7	0.8	0.7	0.8	-	-	-	-	-	-	-	-	-	-	-	-	GGHMIAVsPQK	GGHMIAVsPQKQVIT	S8;	Yes	S(8): 100.0	6	1	1	0	N-Term(TMT6plex); M4(Oxidation); S8(Phospho); K11(TMT6plex)
27436887	NP_002577.2	5089	PBX2	pre-B-cell leukemia transcription factor 2	S330	1.0	0.8	0.9	0.8	0.8	0.8	0.9	0.8	0.6	0.6	0.7	0.7	0.5	0.6	0.7	0.6	0.6	0.6	0.7	0.7	TAVSVTQGGHsR	SVTQGGHsRTSSPTP	S11;	Yes	T(1): 0.0; S(4): 0.0; T(6): 0.0; S(11): 100.0	2	1	1	0	N-Term(TMT6plex); S11(Phospho)
109637779	NP_001035891.1	1058	CENPA	histone H3-like centromeric protein A isoformb	S17; S19	0.9	0.9	0.9	0.6	1.2	1.1	1.2	1.0	-	-	-	-	-	-	-	-	-	-	-	-	sPsPTPTPGPSR	PEAPRRRsPSPTPTP;APRRRSPsPTPTPGP	S1; S3;	Yes;Yes	S(1): 100.0; S(3): 100.0; T(5): 0.0; T(7): 0.0; S(11): 0.0	1	2	1	0	N-Term(TMT6plex); S1(Phospho); S3(Phospho)
109637779	NP_001035891.1	1058	CENPA	histone H3-like centromeric protein A isoformb	S17; S19	1.2	1.1	1.2	1.0	0.9	0.9	0.9	0.6	-	-	-	-	-	-	-	-	-	-	-	-	SPsPTPTPGPSR	APRRRSPsPTPTPGP	S1; S3;	Yes	S(1): 0.0; S(3): 96.8; T(5): 1.6; T(7): 1.6; S(11): 0.0	2	2	1	0	N-Term(TMT6plex); S3(Phospho)
42542379	NP_005830.2	10250	SRRM1	serine/arginine repetitive matrix protein 1	S607	1.1	1.1	1.0	0.5	0.8	1.6	1.1	1.1	1.3	1.1	1.1	0.6	1.0	1.7	1.2	1.3	1.2	1.4	1.2	0.9	YsPsPPPK	IQRRYSPsPPPKRRT	S4;	Yes	Y(1): 0.0; S(2): 0.0; S(4): 100.0	1	1	1	0	N-Term(TMT6plex); S4(Phospho); K8(TMT6plex)
42542379	NP_005830.2	10250	SRRM1	serine/arginine repetitive matrix protein 1	S607	0.8	1.6	1.1	1.1	1.1	1.1	1.0	0.5	1.0	1.7	1.2	1.3	1.3	1.1	1.1	0.6	1.2	1.4	1.2	0.9	YSPsPPPK	IQRRYSPsPPPKRRT	S4;	Yes	Y(1): 0.0; S(2): 0.9; S(4): 99.1	229	1	1	0	N-Term(TMT6plex); S4(Phospho); K8(TMT6plex)
103472005	NP_002408.3	4288	MKI67	antigen KI-67 isoform1	T2113	1.2	1.1	0.9	0.7	0.8	0.9	0.8	1.0	1.4	1.1	0.9	0.7	0.9	0.9	0.9	1.0	1.2	1.0	0.9	0.8	SPPPESMDtPTSTR	PPPESMDtPTSTRRR	T9;	Yes	S(1): 0.0; S(6): 0.0; T(9): 99.9; T(11): 0.0; S(12): 0.0; T(13): 0.0	118	2	1	0	N-Term(TMT6plex); M7(Oxidation); T9(Phospho)
114155142	NP_003283.2	7175	TPR	nucleoprotein TPR	S2155	1.4	1.3	1.3	0.8	1.3	1.1	1.4	0.8	1.6	1.4	1.5	1.0	1.4	1.1	1.6	0.9	1.5	1.2	1.6	0.9	TDGEAEAIHsPQVAGVPR	GEAEAIHsPQVAGVP	S10;	Yes	T(1): 0.0; S(10): 100.0	29	1	1	0	N-Term(TMT6plex); S10(Phospho)
15055543	NP_115285.1	10929	SRSF8	serine/arginine-rich splicing factor 8	S26	0.9	0.8	1.0	0.9	0.6	0.7	0.7	0.7	1.7	1.5	1.7	2.1	1.2	1.3	1.3	1.5	1.4	1.4	1.5	1.8	TsPDSLR	DNLTYRTsPDSLRRV	S2;	Yes	T(1): 0.7; S(2): 99.3; S(5): 0.0	13	1	1	0	N-Term(TMT6plex); S2(Phospho)
296841073	NP_001171894.1	54521	WDR44	WD repeat-containing protein 44 isoform2	S96	0.9	1.1	0.9	0.8	0.9	1.5	0.7	0.8	0.9	1.1	1.0	0.9	0.9	1.5	0.8	1.0	0.9	1.3	0.9	1.0	ELSDQATAsPIVAR	LSDQATAsPIVARTD	S9;	Yes	S(3): 0.0; T(7): 0.0; S(9): 100.0	12	3	1	0	N-Term(TMT6plex); S9(Phospho)
19913406	NP_001058.2	7153	TOP2A	DNA topoisomerase 2-alpha	S1247	1.0	0.7	0.8	0.8	1.2	0.8	1.0	0.8	1.1	0.8	0.9	0.9	1.4	0.9	1.2	0.9	1.2	0.8	1.0	0.9	NENTEGsPQEDGVELEGLK	KNENTEGsPQEDGVE	S7;	Yes	T(4): 0.0; S(7): 100.0	64	1	1	0	N-Term(TMT6plex); S7(Phospho); K19(TMT6plex)
56118310	NP_073568.2	64710	NUCKS1	nuclear ubiquitous casein and cyclin-dependent kinase substrate 1	S19	0.8	1.1	0.8	0.7	1.0	1.1	0.9	0.9	1.1	1.4	1.1	0.9	1.2	1.3	1.3	1.2	1.1	1.3	1.2	1.0	VVDYSQFQEsDDADEDYGR	DYSQFQEsDDADEDY	S10;	Yes	Y(4): 0.0; S(5): 0.0; S(10): 100.0; Y(17): 0.0	109	1	1	0	N-Term(TMT6plex); S10(Phospho)
28559039	NP_004765.2	5469	MED1	mediator of RNA polymerase II transcription subunit 1	S1447	0.7	0.8	0.7	0.7	0.7	0.8	0.7	0.9	0.7	0.8	0.8	0.8	0.7	0.8	0.8	0.9	0.7	0.8	0.8	0.8	GsPSHSK	TPKHERGsPSHSKSP	S2;	No	S(2): 100.0; S(4): 0.0; S(6): 0.0	16	1	1	0	N-Term(TMT6plex); S2(Phospho); K7(TMT6plex)
52627149	NP_061848.2	54386	TERF2IP	telomeric repeat-binding factor 2-interacting protein 1	S203	0.9	0.8	0.9	0.8	1.0	0.9	0.9	0.8	0.9	0.8	0.9	0.8	1.0	0.9	0.9	0.8	1.0	0.8	0.9	0.8	YLLGDAPVsPSSQK	LLGDAPVsPSSQKLK	S9;	Yes	Y(1): 0.0; S(9): 100.0; S(11): 0.0; S(12): 0.0	6	1	1	0	N-Term(TMT6plex); S9(Phospho); K14(TMT6plex)
5729734	NP_006707.1	10926	DBF4	protein DBF4 homolog A	S359	0.4	1.0	0.7	0.4	0.7	1.5	0.9	1.2	-	-	-	-	-	-	-	-	-	-	-	-	YSVGSLsPVSASVLK	KYSVGSLsPVSASVL	S7;	Yes	Y(1): 0.0; S(2): 0.0; S(5): 0.0; S(7): 100.0; S(10): 0.0; S(12): 0.0	2	1	1	0	N-Term(TMT6plex); S7(Phospho); K15(TMT6plex)
110347443	NP_009045.2	7110	TMF1	TATA element modulatory factor	S77	0.9	0.8	0.8	0.7	1.4	1.5	0.9	0.9	0.7	0.7	0.8	0.8	1.2	1.3	0.9	1.0	1.0	1.0	0.8	0.9	SNTEPQSPPIAsPK	PQSPPIAsPKAITKP	S12;	Yes	S(1): 0.0; T(3): 0.0; S(7): 0.0; S(12): 100.0	2	1	1	0	N-Term(TMT6plex); S12(Phospho); K14(TMT6plex)
110347443	NP_009045.2	7110	TMF1	TATA element modulatory factor	S77	1.4	1.5	0.9	0.9	0.9	0.8	0.8	0.7	1.2	1.3	0.9	1.0	0.7	0.7	0.8	0.8	1.0	1.0	0.8	0.9	SNTEPQsPPIAsPK	PQSPPIAsPKAITKP	S12;	Yes	S(1): 0.0; T(3): 0.0; S(7): 0.0; S(12): 100.0	1	1	1	0	N-Term(TMT6plex); S12(Phospho); K14(TMT6plex)
61676188	NP_113584.3	10075	HUWE1	E3 ubiquitin-protein ligase HUWE1	S2362; T2366	0.8	0.9	0.9	0.8	0.8	1.1	0.9	0.8	0.9	0.9	1.0	1.0	0.8	1.2	1.0	1.0	0.8	1.1	1.0	1.0	DGGsGNStIIVSR	LLERDGGsGNSTIIV;DGGSGNStIIVSRSG	S4; T8;	Yes;Yes	S(4): 100.0; S(7): 1.7; T(8): 96.7; S(12): 1.7	20	1	1	0	N-Term(TMT6plex); S4(Phospho); S7(Phospho)
349585060	NP_001231800.1	1601	DAB2	disabled homolog 2 isoform2	S380	0.6	0.5	0.4	0.7	0.8	0.6	0.9	0.9	-	-	-	-	-	-	-	-	-	-	-	-	SSPNPEVGsPPK	SPNPEVGsPPKGLSL	S9;	No	S(1): 0.0; S(2): 0.0; S(9): 100.0	2	2	1	0	N-Term(TMT6plex); S9(Phospho); K12(TMT6plex)
151101292	NP_110382.3	81542	TMX1	thioredoxin-related transmembrane protein 1 precursor	S247	0.8	0.7	0.6	0.8	0.7	0.7	0.7	0.8	1.0	0.8	0.8	0.9	0.8	0.8	0.9	0.9	0.9	0.8	0.9	0.9	KVEEEQEADEEDVsEEEAESK	EADEEDVsEEEAESK	S14;	Yes	S(14): 100.0; S(20): 0.0	9	1	1	1	N-Term(TMT6plex); K1(TMT6plex); S14(Phospho); K21(TMT6plex)
118572613	NP_057417.3	23524	SRRM2	serine/arginine repetitive matrix protein 2	S837	0.8	1.0	1.0	0.8	0.8	1.0	1.0	0.8	0.9	1.0	1.0	0.9	0.9	1.0	1.0	0.9	0.9	1.0	1.0	0.9	QSHSsSSPHPK	PSRQSHSsSSPHPKV	S5;	Yes	S(2): 0.0; S(4): 0.0; S(5): 98.5; S(6): 0.7; S(7): 0.7	436	1	1	0	N-Term(TMT6plex); S5(Phospho); K11(TMT6plex)
154146191	NP_005339.3	3320	HSP90AA1	heat shock protein HSP 90-alpha isoform2	S263	1.0	0.7	0.8	0.9	0.8	0.6	0.7	0.7	1.1	0.8	1.0	0.9	0.9	0.7	0.9	0.8	1.0	0.8	0.9	0.9	ESEDKPEIEDVGsDEEEEK	PEIEDVGsDEEEEKK	S13;	Yes	S(2): 0.0; S(13): 100.0	35	2	1	0	N-Term(TMT6plex); K5(TMT6plex); S13(Phospho); K19(TMT6plex)
95147555	NP_002364.5	4130	MAP1A	microtubule-associated protein 1A	T504	1.2	0.9	0.7	0.9	0.8	0.7	0.5	0.7	-	-	-	-	-	-	-	-	-	-	-	-	ELSSEPQtPPAQK	ELSSEPQtPPAQKGT	T8;	Yes	S(3): 0.0; S(4): 0.0; T(8): 100.0	7	1	1	0	N-Term(TMT6plex); T8(Phospho); K13(TMT6plex)
556503401	NP_001273294.1	55700	MAP7D1	MAP7 domain-containing protein 1 isoform2	S125	0.8	0.8	0.9	0.6	0.9	1.3	0.7	1.0	0.8	0.6	0.8	0.6	0.9	1.1	0.6	0.9	0.8	0.8	0.7	0.8	SSQPSPTAVPASDsPPTK	TAVPASDsPPTKQEV	S14;	Yes	S(1): 0.0; S(2): 0.0; S(5): 0.0; T(7): 0.0; S(12): 0.0; S(14): 100.0; T(17): [illegible]	27	3	1	0	N-Term(TMT6plex); S14(Phospho); K18(TMT6plex)
556503440	NP_001273295.1	55700	MAP7D1	MAP7 domain-containing protein 1 isoform3	S125	0.9	1.3	0.7	1.0	0.8	0.8	0.9	0.6	0.9	1.1	0.6	0.9	0.8	0.6	0.8	0.6	0.8	0.8	0.7	0.8	SSQPsPTAVPASDsPPTK	PRRSSQPsPTAVPAS;TAVPASDsPPTKQEV	S14;	Yes;Yes	S(1): 1.8; S(2): 1.8; S(5): 94.7; T(7): 1.8; S(12): 0.0; S(14): 100.0; T(17): [illegible]	2	3	1	0	N-Term(TMT6plex); T7(Phospho); S14(Phospho); K18(TMT6plex)
6631100	NP_005376.2	4820	NKTR	NK-tumor recognition protein	S243	1.0	1.1	1.1	0.7	0.9	0.9	0.9	0.9	1.2	1.2	1.2	0.9	1.1	1.0	1.0	1.2	1.1	1.1	1.1	1.0	EAsSSEEPR	KKRRKEAsSSEEPRN	S3;	Yes	S(3): 99.5; S(4): 50.3; S(5): 50.3	6	1	1	0	N-Term(TMT6plex); S3(Phospho); S4(Phospho)
343887347	NP_001230575.1	10421	CD2BP2	CD2 antigen cytoplasmic tail-binding protein 2	S49	1.0	0.7	1.0	0.7	1.1	1.0	1.1	0.9	1.0	0.8	1.0	0.8	1.1	1.0	1.1	0.9	1.1	0.9	1.0	0.9	HSLDsDEEEDDDDGGSSK	KGKHSLDsDEEEDDD	S5;	Yes	S(2): 0.0; S(5): 100.0; S(16): 0.0; S(17): 0.0	7	1	1	0	N-Term(TMT6plex); S5(Phospho); K18(TMT6plex)
7706457	NP_057332.1	11215	AKAP11	A-kinase anchor protein 11	T1100	1.2	0.6	1.0	0.7	1.1	0.9	1.2	0.9	0.9	0.6	1.1	0.6	0.8	0.8	1.2	0.7	0.8	0.7	1.1	0.6	NVIPDTPPstPLVPSR	DRNVIPDtPPSTPLV	T6;	Yes	T(6): 100.0; S(9): 0.0; T(10): 0.0; S(15): 0.0	2	1	1	0	N-Term(TMT6plex); T6(Phospho)
7706457	NP_057332.1	11215	AKAP11	A-kinase anchor protein 11	T1100	1.1	0.9	1.2	0.9	1.2	0.6	1.0	0.7	0.8	0.8	1.2	0.7	0.9	0.6	1.1	0.6	0.8	0.7	1.1	0.6	NVIPDtPPSTPLVPSR	DRNVIPDtPPSTPLV	T6;	Yes	T(6): 98.0; S(9): 0.0; T(10): 2.0; S(15): 0.0	3	1	1	0	N-Term(TMT6plex); T6(Phospho)
221136993	NP_001137484.1	51075	TMX2	thioredoxin-related transmembrane protein 2 isoform2	S250	3.0	1.5	1.6	0.5	1.0	0.9	0.8	1.1	3.0	1.3	1.3	0.5	1.0	0.7	0.7	1.1	2.0	1.0	1.0	0.8	AGDNIPEEQPVASTPTTVsDGENKK	ASTPTTVsDGENKKD	S19;	No	S(13): 0.0; T(14): 0.0; T(16): 0.0; T(17): 0.0; S(19): 100.0	7	2	1	1	N-Term(TMT6plex); S19(Phospho); K24(TMT6plex); K25(TMT6plex)
42716295	NP_115664.2	84248	FYTTD1	UAP56-interacting factor isoform1	S16	0.7	0.7	0.7	0.7	0.9	0.6	0.8	0.9	0.7	0.8	0.8	0.7	0.9	0.6	0.8	1.0	0.8	0.7	0.8	0.8	LVGATATSsPPPK	VGATATSsPPPKARS	S9;	Yes	T(5): 0.0; T(7): 0.0; S(8): 0.9; S(9): [illegible]	4	1	1	0	N-Term(TMT6plex); S9(Phospho); K13(TMT6plex)
42716295	NP_115664.2	84248	FYTTD1	UAP56-interacting factor isoform1	S16	0.9	0.6	0.8	0.9	0.7	0.7	0.7	0.7	0.9	0.6	0.8	1.0	0.7	0.8	0.8	0.7	0.8	0.7	0.8	0.8	LVGATATsSPPPK	LVGATATsSPPPKAR	S9;	Yes	T(5): 0.8; T(7): 0.8; S(8): 97.6; S(9): [illegible]	6	1	1	0	N-Term(TMT6plex); S8(Phospho); K13(TMT6plex)
116686122	NP_036442.3	24137	KIF4A	chromosome-associated kinesin KIF4A	T1181	0.2	0.4	0.6	0.5	1.0	0.9	0.9	1.1	0.3	0.5	0.8	0.7	1.4	1.2	1.2	1.5	0.8	0.8	1.0	1.1	tPPAPSPEDLPELK	EQVLSKKtPPAPSPE	T1;	Yes	T(1): 100.0; S(6): 0.0	3	1	1	0	N-Term(TMT6plex); T1(Phospho); K14(TMT6plex)
325120986	NP_001191397.1	8241	RBM10	RNA-binding protein 10 isoform5	S30	1.0	0.9	0.8	0.7	1.2	1.1	1.0	0.9	1.1	1.1	0.9	0.7	1.3	1.2	1.2	1.1	1.2	1.1	1.0	0.9	GGGESLQEAsPR	GESLQEAsPRLADHG	S10;	Yes	S(5): 0.0; S(10): 100.0	6	1	1	0	N-Term(TMT6plex); S10(Phospho)
41152093	NP_958357.1	84146	ZNF644	zinc finger protein 644 isoform1	T165	0.2	0.4	0.4	0.6	0.7	0.7	0.8	0.9	0.2	0.4	0.4	0.7	0.7	0.8	0.8	1.0	0.5	0.6	0.6	0.8	VAADLQLStPQK	AADLQLStPQKASQH	T9;	Yes	S(8): 0.0; T(9): 100.0	2	1	1	0	N-Term(TMT6plex); T9(Phospho); K12(TMT6plex)
156630992	NP_001096123.1	54726	OTUD4	OTU domain-containing protein 4 isoform3	S481	0.7	0.7	0.7	0.8	0.7	0.9	0.9	0.8	0.8	0.6	0.7	0.8	0.7	0.8	0.9	0.8	0.7	0.7	0.8	0.8	YATVSsPSK	DKYATVSsPSKSKKL	S6;	No	Y(1): 0.0; T(3): 0.0; S(5): 0.9; S(6): 99.1; S(8): 0.0	6	1	1	0	N-Term(TMT6plex); S6(Phospho); K9(TMT6plex)
21493024	NP_653080.1	9590	AKAP12	A-kinase anchor protein 12 isoform2	S789	0.7	1.1	0.8	0.8	1.5	2.6	2.6	0.8	0.8	1.0	0.7	0.8	1.7	2.5	2.1	0.7	1.2	1.8	1.4	0.7	ELSEsQVHMMAAAVADGTR	VSKELSEsQVHMMAA	S5;	No	S(3): 2.3; S(5): 97.7; T(18): 0.0	103	2	1	0	N-Term(TMT6plex); S5(Phospho); M9(Oxidation); M10(Oxidation)
4758816	NP_004799.1	9397	NMT2	glycylpeptide N-tetradecanoyltransferase 2	S38	0.9	0.9	1.3	0.9	0.8	0.9	1.3	0.7	1.0	1.0	1.2	1.0	0.9	0.9	1.2	0.7	1.0	0.9	1.2	0.8	GsPGGYLGAK	ETEHAKGsPGGYLGA	S2;	Yes	S(2): 100.0; Y(6): 0.0	1	1	1	0	N-Term(TMT6plex); S2(Phospho); K10(TMT6plex)
166295179	NP_001107590.1	55421	C17orf85	uncharacterized protein C17orf85	S444	0.9	1.4	1.1	0.9	1.1	1.3	1.0	0.7	1.1	1.6	1.4	1.1	1.3	1.5	1.2	0.9	1.2	1.5	1.3	1.0	ADsVSSSNIK	RNSMRADsVSSSNIK	S3;	No	S(3): 100.0; S(5): 0.0; S(6): 0.0; S(7): 0.0	5	1	1	0	N-Term(TMT6plex); S3(Phospho); K10(TMT6plex)
153945728	NP_005900.2	4131	MAP1B	microtubule-associated protein 1B	S614	0.9	1.3	1.4	0.7	0.9	1.4	1.5	0.9	0.7	1.0	1.2	0.7	0.7	1.0	1.3	1.0	0.7	1.0	1.2	0.8	EEPsPVKAEVAEK	VPSKEEPsPVKAEVA	S4;	Yes	S(4): 100.0	49	1	1	1	N-Term(TMT6plex); S4(Phospho); K7(TMT6plex); K13(TMT6plex)
148839382	NP_001092108.1	254225	RNF169	E3 ubiquitin-protein ligase RNF169	T554	0.9	0.8	0.8	0.9	0.9	0.6	0.8	0.7	0.9	0.7	0.8	0.9	0.8	0.6	0.9	0.7	0.9	0.7	0.9	0.8	EQEEGLGStPDAK	QEEGLGStPDAKLDK	T9;	Yes	S(8): 1.0; T(9): 99.0	1	1	1	0	N-Term(TMT6plex); T9(Phospho); K13(TMT6plex)
291190783	NP_001167448.1	55604	LRRC16A	leucine-rich repeat-containing protein 16A isoform2	T916	0.8	0.6	0.8	0.6	0.9	0.8	0.8	1.0	0.8	0.6	0.8	0.7	0.9	0.8	0.9	1.1	0.8	0.7	0.8	0.9	LEDLDTCMMtPK	DLDTCMMtPKSKRKS	T10;	Yes	T(6): 0.0; T(10): 100.0	13	2	1	0	N-Term(TMT6plex); C7(Carbamidomethyl); M8(Oxidation); M9(Oxidation); T10(Phospho); K12(TMT6plex)
47519639	NP_002366.2	4134	MAP4	microtubule-associated protein 4 isoform1	S787	0.7	1.0	0.8	0.8	0.8	1.0	1.0	0.8	0.7	1.0	0.8	1.0	0.8	1.0	1.1	1.0	0.8	1.0	0.9	1.0	AsPSKPASAPASR	KAPEKRAsPSKPASA	S2;	Yes	S(2): 100.0; S(4): 0.0; S(8): 0.0; S(12): 0.0	47	2	1	0	N-Term(TMT6plex); S2(Phospho); K5(TMT6plex)
6912246	NP_036231.1	10849	CD3EAP	DNA-directed RNA polymerase I subunit RPA34	S490	0.9	1.0	0.9	0.9	1.5	1.2	1.3	0.7	1.0	1.0	0.9	0.9	1.6	1.1	1.3	0.7	1.3	1.1	1.1	0.8	MPETVPQEEMPGPPLNSEsGEEAPTGR	GPPLNSEsGEEAPTG	S19;	Yes	T(4): 0.0; S(17): 0.0; S(19): 100.0; T(25): 0.0	17	1	1	0	N-Term(TMT6plex); M1(Oxidation); M10(Oxidation); S19(Phospho)
4506039	NP_003972.1	9055	PRC1	protein regulator of cytokinesis 1 isoform1	T481	0.4	0.6	0.6	0.7	0.4	0.7	0.8	0.9	0.5	0.6	0.7	0.7	0.5	0.7	0.8	0.9	0.5	0.6	0.8	0.8	GLAPNtPGK	RRGLAPNtPGKARKL	T6;	Yes	T(6): 100.0	8	3	1	0	N-Term(TMT6plex); T6(Phospho); K9(TMT6plex)
546231404	NP_001271231.1	54443	ANLN	actin-binding protein anillin isoform3	T401	0.9	0.8	0.9	0.8	0.5	0.9	0.7	0.8	0.9	0.8	0.8	0.8	0.5	0.9	0.6	0.9	0.7	0.8	0.7	0.8	TPIItPNTK	PHRTPIItPNTKAIQ	T5;	Yes	T(1): 0.0; T(5): 100.0; T(8): 0.0	21	3	1	0	N-Term(TMT6plex); T5(Phospho); K9(TMT6plex)
255652953	NP_653205.3	124245	ZC3H18	zinc finger CCCH domain-containing protein 18	S532	0.9	0.9	0.7	0.7	1.0	1.2	1.0	0.8	1.1	1.0	0.8	0.8	1.2	1.2	1.2	0.9	1.2	1.1	1.0	0.9	LGVsVSPSR	PKKKLGVsVSPSRAR	S4;	Yes	S(4): 100.0; S(6): 50.0; S(8): 50.0	34	1	1	0	N-Term(TMT6plex); S4(Phospho); S6(Phospho)
255652953	NP_653205.3	124245	ZC3H18	zinc finger CCCH domain-containing protein 18	S46	0.9	0.9	0.9	0.8	0.7	0.9	0.9	0.8	1.1	1.0	1.1	0.9	0.9	0.9	1.0	0.8	1.0	0.9	1.0	0.9	AsDLEDEESAAR	DGAGVRAsDLEDEES	S2;	Yes	S(2): 100.0; S(9): 0.0	34	1	1	0	N-Term(TMT6plex); S2(Phospho)
255652953	NP_653205.3	124245	ZC3H18	zinc finger CCCH domain-containing protein 18	S532	1.0	1.2	1.0	0.8	0.9	0.9	0.7	0.7	1.2	1.2	1.2	0.9	1.1	1.0	0.8	0.8	1.2	1.1	1.0	0.9	LGVSVsPSR	PKKKLGVsVSPSRAR	S4;	Yes	S(4): 99.1; S(6): 50.4; S(8): 50.4	1	1	1	0	N-Term(TMT6plex); S4(Phospho); S6(Phospho)
55953110	NP_001007258.1	7799	PRDM2	PR domain zinc finger protein 2 isoformc	S442	1.1	1.0	1.1	0.9	0.7	0.7	0.7	0.7	1.1	1.0	1.0	1.0	0.7	0.7	0.7	0.7	0.9	0.9	0.9	0.9	TAsPPALPK	AKKRRTAsPPALPKL	S3;	No	T(1): 0.0; S(3): 100.0	6	3	1	0	N-Term(TMT6plex); S3(Phospho); K9(TMT6plex)
84043963	NP_056988.3	9669	EIF5B	eukaryotic translation initiation factor 5B	S137	0.5	0.8	0.9	0.5	1.2	1.6	1.2	1.1	0.5	0.8	1.0	0.5	1.2	1.6	1.2	1.2	0.9	1.2	1.1	0.8	VEMYSGsDDDDDENK	KVEMYSGsDDDDDEN	S7;	Yes	Y(4): 0.0; S(5): 1.5; S(7): 98.4	13	1	1	0	N-Term(TMT6plex); M3(Oxidation); S7(Phospho); K15(TMT6plex)
9966805	NP_065147.1	57062	DDX24	ATP-dependent RNA helicase DDX24	S82	1.1	0.9	1.0	0.8	1.0	1.0	0.8	0.8	1.3	1.1	1.3	1.0	1.2	1.1	1.1	1.0	1.2	1.1	1.2	1.0	AQAVsEEEEEEEGK	KRKAQAVsEEEEEEE	S5;	Yes	S(5): 100.0	10	1	1	0	N-Term(TMT6plex); S5(Phospho); K14(TMT6plex)
47578107	NP_056199.2	25836	NIPBL	nipped-B-like protein isoformB	S2672	2.2	1.6	2.6	1.1	0.8	0.8	0.9	0.5	1.9	1.4	2.3	1.0	0.7	0.7	0.8	0.4	1.3	1.1	1.5	0.7	NNTAAETEDDEsDGEDR	AETEDDEsDGEDRGG	S12;	Yes	T(3): 0.0; T(7): 0.0; S(12): 100.0	8	2	1	0	N-Term(TMT6plex); S12(Phospho)
13540569	NP_110418.1	81537	SGPP1	sphingosine-1-phosphate phosphatase 1	S112	1.0	0.8	0.7	0.7	0.9	0.7	0.9	0.9	1.4	0.8	0.8	0.8	1.3	0.8	1.1	0.9	1.4	0.8	1.0	0.9	RNsLTGEEGQLAR	AGALRRNsLTGEEGQ	S3;	Yes	S(3): 100.0; T(5): 0.0	4	1	1	1	N-Term(TMT6plex); S3(Phospho)
386781463	NP_001247419.1	51599	LSR	lipolysis-stimulated lipoprotein receptor isoform5	S385	1.0	0.9	0.6	1.1	0.4	0.4	0.6	0.4	1.1	0.9	0.6	1.1	0.4	0.4	0.7	0.4	0.7	0.7	0.7	0.8	sVDALDDLTPPSTAESGSR	ARRPRARsVDALDDL	S1;	No	S(1): 100.0; T(9): 0.0; S(12): 0.0; T(13): 0.0; S(16): 0.0; S(18): 0.0	1	5	1	0	N-Term(TMT6plex); S1(Phospho)
118572613	NP_057417.3	23524	SRRM2	serine/arginine repetitive matrix protein 2	S992; S994	0.9	1.0	0.9	0.8	0.9	1.0	0.9	0.8	0.9	1.0	1.0	0.9	1.0	1.1	1.0	0.8	0.9	1.1	1.0	0.9	QSHSGsIsPYPK	PRQSHSGsISPYPKV;QSHSGSIsPYPKVKA	S6; S8;	Yes;Yes	S(2): 0.0; S(4): 0.0; S(6): 100.0; S(8): 100.0; Y(10): 0.0	436	1	1	0	N-Term(TMT6plex); S6(Phospho); S8(Phospho); K12(TMT6plex)
118572613	NP_057417.3	23524	SRRM2	serine/arginine repetitive matrix protein 2	S295; S297	1.0	0.8	0.9	0.7	1.1	1.1	1.1	0.9	1.0	0.9	1.0	0.7	1.1	1.1	1.2	1.0	1.1	1.0	1.1	0.9	THTTALAGRsPsPASGR	TTALAGRsPSPASGR;ALAGRSPsPASGRRG	S10; S12;	Yes;Yes	T(1): 0.0; T(3): 0.0; T(4): 0.0; S(10): 100.0; S(12): 100.0; S(15): 0.0	436	1	1	1	N-Term(TMT6plex); S10(Phospho); S12(Phospho)
5032189	NP_005648.1	7158	TP53BP1	tumor suppressor p53-binding protein 1 isoform3	S771	0.5	1.0	0.8	0.8	0.6	0.9	0.8	0.8	0.5	1.0	0.8	1.0	0.6	0.9	0.8	0.9	0.5	1.0	0.8	1.0	VDVsCEPLEGVEK	PSPRVDVsCEPLEGV	S4;	Yes	S(4): 100.0	62	3	1	0	N-Term(TMT6plex); S4(Phospho); C5(Carbamidomethyl); K13(TMT6plex)
149158690	NP_542417.2	7916	PRRC2A	protein PRRC2A	S1147	0.8	0.8	0.6	1.0	0.9	0.5	0.6	0.6	0.8	0.7	0.6	0.9	0.9	0.5	0.6	0.6	0.9	0.6	0.6	0.8	EGTLTQVPLAPPPPGAPPsPAPAR	PPPGAPPsPAPARFT	S19;	Yes	T(3): 0.0; T(5): 0.0; S(19): 100.0	23	1	1	0	N-Term(TMT6plex); S19(Phospho)
4505257	NP_002435.1	4478	MSN	moesin	S576	1.0	1.6	2.0	1.1	0.7	1.1	0.7	0.4	1.1	1.7	2.1	1.4	0.7	1.1	0.8	0.6	0.9	1.4	1.4	1.0	IDEEFsM	QRIDEEFsM	S6;	Yes	S(6): 100.0	10	1	1	0	N-Term(TMT6plex); S6(Phospho); M7(Oxidation)
42542379	NP_005830.2	10250	SRRM1	serine/arginine repetitive matrix protein 1	S626; S628	1.1	1.0	0.8	0.6	1.0	1.4	1.1	1.0	1.4	1.1	0.9	0.7	1.2	1.5	1.2	1.1	1.3	1.3	1.0	0.9	RAsPSPPPK	PPPKRRAsPSPPPKR;PKRRASPsPPPKRRV	S3; S5;	Yes;Yes	S(3): 100.0; S(5): 100.0	1	1	1	1	N-Term(TMT6plex); S3(Phospho); S5(Phospho); K9(TMT6plex)
42542379	NP_005830.2	10250	SRRM1	serine/arginine repetitive matrix protein 1	S626; S628	1.0	1.4	1.1	1.0	1.1	1.0	0.8	0.6	1.2	1.5	1.2	1.1	1.4	1.1	0.9	0.7	1.3	1.3	1.0	0.9	RAsPsPPPK	PPPKRRAsPSPPPKR;PKRRASPsPPPKRRV	S3; S5;	Yes;Yes	S(3): 100.0; S(5): 100.0	229	1	1	1	N-Term(TMT6plex); S3(Phospho); S5(Phospho); K9(TMT6plex)
565324115	NP_001274264.1	57179	KIAA1191	putative monooxygenase p33MONOX isoformc	S93	0.7	0.9	0.5	0.7	0.9	1.1	0.6	0.9	-	-	-	-	-	-	-	-	-	-	-	-	QPASAQsTPSTTPHSSPK	RQPASAQsTPSTTPH	S7;	No	S(4): 0.0; S(7): 99.9; T(8): 0.0; S(10): 0.0; T(11): 0.0; T(12): 0.0; S(15): 0.0; S(16): 0.0	1	3	1	0	N-Term(TMT6plex); S16(Phospho); K18(TMT6plex)
112382257	NP_795352.2	10207	INADL	inaD-like protein	S522	0.8	0.8	0.8	0.8	0.8	1.0	0.8	0.8	0.9	1.0	1.0	0.8	0.9	1.2	1.0	0.9	0.9	1.1	1.0	0.9	VPDsPENELK	KLEKVPDsPENELKS	S4;	Yes	S(4): 100.0	3	1	1	0	N-Term(TMT6plex); S4(Phospho); K10(TMT6plex)
21361282	NP_005617.2	6429	SRSF4	serine/arginine-rich splicing factor 4	S431	1.1	1.3	1.0	0.8	0.9	1.3	0.8	0.8	1.5	1.6	1.5	1.2	1.3	1.6	1.1	1.2	1.4	1.6	1.3	1.2	EGRGEsENAGTNQETR	QREGRGEsENAGTNQ	S6;	Yes	S(6): 100.0; T(11): 0.0; T(15): 0.0	17	1	1	1	N-Term(TMT6plex); S6(Phospho)
565324115	NP_001274264.1	57179	KIAA1191	putative monooxygenase p33MONOX isoformc	S93	0.9	1.1	0.6	0.9	0.7	0.9	0.5	0.7	-	-	-	-	-	-	-	-	-	-	-	-	QPASAQSTPSTTPHSsPK	PSTTPHSsPKQRPRG	S7;	No	S(4): 0.0; S(7): 0.0; T(8): 0.0; S(10): 0.0; T(11): 0.0; T(12): 0.0; S(15): 1.4; S(16): 98.6	2	3	1	0	N-Term(TMT6plex); S16(Phospho); K18(TMT6plex)
209969678	NP_001129669.1	51343	FZR1	fizzy-related protein homolog isoform3	S40	0.6	0.5	0.6	1.0	0.8	0.6	0.9	0.6	0.6	0.5	0.6	1.0	0.8	0.7	1.0	0.7	0.7	0.6	0.8	0.8	TLTPASSPVSsPSK	PASSPVSsPSKHGDR	S11;	Yes	T(1): 0.0; T(3): 0.0; S(6): 0.0; S(7): 0.0; S(10): 1.0; S(11): 99.0; S(13): 0.0	1	3	1	0	N-Term(TMT6plex); S11(Phospho); K14(TMT6plex)
50428935	NP_060644.4	55201	MAP1S	microtubule-associated protein 1S	S759	1.1	0.9	0.6	1.1	0.7	1.0	0.8	0.5	1.3	1.0	0.7	1.2	0.8	1.1	0.9	0.5	1.0	1.1	0.8	0.9	KAVPMAPAPAsPGSSNDSSAR	PMAPAPAsPGSSNDS	S11;	Yes	S(11): 92.0; S(14): 2.0; S(15): 2.0; S(18): 2.0; S(19): 2.0	8	1	1	1	K1(TMT6plex); N-Term(TMT6plex); M5(Oxidation); S11(Phospho)
284005309	NP_004251.3	9401	RECQL4	ATP-dependent DNA helicase Q4	S251	1.5	0.7	0.6	0.6	1.7	0.9	1.0	1.0	1.8	0.8	0.7	0.7	2.0	1.1	1.1	1.1	1.9	1.0	0.9	0.9	VGsPQPSSSGGEK	EVSIRVGsPQPSSSG	S3;	Yes	S(3): 100.0; S(7): 0.0; S(8): 0.0; S(9): 0.0	4	1	1	0	N-Term(TMT6plex); S3(Phospho); K13(TMT6plex)
326319990	NP_055820.2	283638	CEP170B	centrosomal protein of 170 kDa protein B isoform2	S1109	0.8	1.0	1.0	0.5	0.8	0.9	0.9	1.0	-	-	-	-	-	-	-	-	-	-	-	-	AGsFTGTSDPEAAPAR	MPRKRAGsFTGTSDP	S3;	No	S(3): 100.0; T(5): 0.0; T(7): 0.0; S(8): 0.0	5	2	1	0	N-Term(TMT6plex); S3(Phospho)
12758125	NP_056139.1	23378	RRP8	ribosomal RNA-processing protein 8	S223	2.8	0.9	1.2	0.8	2.2	0.8	1.0	0.8	2.7	0.8	1.0	0.8	2.1	0.7	0.9	0.7	2.4	0.8	0.9	0.7	TEVsPVPR	PTEKTEVsPVPRTDS	S4;	Yes	T(1): 0.0; S(4): 100.0	1	1	1	0	N-Term(TMT6plex); S4(Phospho)
50843837	NP_060541.3	55701	ARHGEF40	rho guanine nucleotide exchange factor 40 isoform1	T371	0.9	0.9	0.7	0.9	0.8	0.9	0.6	0.7	-	-	-	-	-	-	-	-	-	-	-	-	GGGGGGQGAEGPPGtPR	GAEGPPGtPRRTGKG	T15;	Yes	T(15): 100.0	6	1	1	0	N-Term(TMT6plex); T15(Phospho)
21314720	NP_078976.2	79753	SNIP1	smad nuclear-interacting protein 1	S52	0.5	0.5	0.5	0.7	0.5	0.4	0.4	0.9	0.5	0.6	0.5	0.9	0.5	0.5	0.5	1.1	0.5	0.5	0.5	1.0	RPDHSGGsPSPPTSEPAR	RPDHSGGsPSPPTSE	S8;	Yes	S(5): 0.0; S(8): 100.0; S(10): 0.0; T(13): 0.0; S(14): 0.0	10	1	1	0	N-Term(TMT6plex); S8(Phospho)
42542379	NP_005830.2	10250	SRRM1	serine/arginine repetitive matrix protein 1	S616	0.7	1.0	0.9	0.7	0.8	1.0	1.1	0.9	0.9	1.1	1.0	0.8	0.9	1.0	1.2	1.0	0.9	1.1	1.1	0.9	tAsPPPPPK	PPKRRTAsPPPPPKR	S3;	Yes	T(1): 0.6; S(3): 99.4	229	1	1	0	N-Term(TMT6plex); S3(Phospho); K9(TMT6plex)
5729862	NP_006518.1	7884	SLBP	histone RNA hairpin-binding protein	S20; S23	0.8	0.8	0.9	0.5	1.0	0.7	1.1	1.1	0.8	0.9	1.1	0.6	1.1	0.8	1.4	1.3	1.0	0.8	1.3	0.9	CDGDAsPPSPAR	SRCDGDAsPPSPARW	S6; S9;	Yes	S(6): 100.0; S(9): 0.0	1	1	1	0	N-Term(TMT6plex); C1(Carbamidomethyl); S6(Phospho)
5729862	NP_006518.1	7884	SLBP	histone RNA hairpin-binding protein	S20; S23	1.0	0.7	1.1	1.1	0.8	0.8	0.9	0.5	1.1	0.8	1.4	1.3	0.8	0.9	1.1	0.6	1.0	0.8	1.3	0.9	CDGDAsPPsPAR	SRCDGDAsPPSPARW;DGDASPPsPARWSLG	S6; S9;	Yes;Yes	S(6): 100.0; S(9): 100.0	2	1	1	0	N-Term(TMT6plex); C1(Carbamidomethyl); S6(Phospho); S9(Phospho)
545746344	NP_001271136.1	8816	DCAF5	DDB1- and CUL4-associated factor 5 isoform3	S712	0.7	0.5	0.7	0.4	1.1	1.3	1.3	1.1	-	-	-	-	-	-	-	-	-	-	-	-	AEEPPsPPVPK	SRAEEPPsPPVPKAS	S6;	No	S(6): 100.0	4	3	1	0	N-Term(TMT6plex); S6(Phospho); K11(TMT6plex)
5032189	NP_005648.1	7158	TP53BP1	tumor suppressor p53-binding protein 1 isoform3	S265	0.9	0.9	0.9	0.8	0.6	0.5	0.6	0.8	0.9	0.9	1.0	0.9	0.6	0.5	0.6	1.0	0.8	0.7	0.8	1.0	SEDMPFsPK	RSEDMPFsPKASVAA	S7;	Yes	S(1): 0.0; S(7): 100.0	62	3	1	0	N-Term(TMT6plex); M4(Oxidation); S7(Phospho); K9(TMT6plex)
27777632	NP_003038.2	6548	SLC9A1	sodium/hydrogen exchanger 1	S785	1.4	1.0	1.3	0.7	1.0	0.8	1.3	0.9	1.9	1.5	1.9	1.0	1.4	1.1	1.9	1.2	1.6	1.3	1.9	1.1	ETSSPGTDDVFTPAPSDsPSSQR	FTPAPSDsPSSQRIQ	S18;	Yes	T(2): 0.0; S(3): 0.0; S(4): 0.0; T(7): 0.0; T(12): 0.0; S(16): 0.0; S(18): 99.9; S(20): 0.0; S(21): 0.0	4	1	1	0	N-Term(TMT6plex); S18(Phospho)
372266129	NP_001243194.1	2314	FLII	protein flightless-1 homolog isoform3	S801	1.7	0.8	1.6	1.0	0.9	0.6	1.0	0.6	1.7	0.8	1.6	1.0	0.9	0.6	1.0	0.7	1.3	0.7	1.3	0.8	NAEAVLQsPGLSGK	NAEAVLQsPGLSGKV	S8;	No	S(8): 100.0; S(12): 0.0	3	3	1	0	N-Term(TMT6plex); S8(Phospho); K14(TMT6plex)
82880648	NP_057737.2	51776	ZAK	mitogen-activated protein kinase kinase kinase MLT isoform1	S633	0.9	0.9	0.9	0.7	1.3	2.1	1.3	0.9	1.0	1.1	1.0	0.8	1.4	2.4	1.5	1.1	1.2	1.7	1.3	1.0	YQQITPVNQsR	QITPVNQsRSSSPTQ	S10;	Yes	Y(1): 0.0; T(5): 0.9; S(10): 99.1	9	1	1	0	N-Term(TMT6plex); S10(Phospho)
4826830	NP_004983.1	4204	MECP2	methyl-CpG-binding protein 2 isoform1	S80	0.8	0.9	0.7	0.8	0.9	1.0	1.0	0.7	1.0	1.3	1.2	1.3	1.2	1.3	1.6	1.2	1.1	1.3	1.4	1.3	AETSEGSGSAPAVPEASAsPK	AVPEASAsPKQRRSI	S19;	Yes	T(3): 0.0; S(4): 0.0; S(7): 0.0; S(9): 0.0; S(17): 0.0; S(19): 100.0	14	2	1	0	N-Term(TMT6plex); S19(Phospho); K21(TMT6plex)
87299628	NP_683692.2	259282	BOD1L1	biorientation of chromosomes in cell division protein 1-like 1	S2203	0.9	0.5	0.6	0.4	1.0	0.9	0.6	1.1	0.9	0.5	0.6	0.5	1.0	0.9	0.6	1.2	1.0	0.7	0.6	0.9	ATGPVLISTADFEGPMPSAPPEAEsPLASTSK	SAPPEAEsPLASTSK	S25;	No	T(2): 0.0; S(8): 0.0; T(9): 0.0; S(18): 0.1; S(25): 99.8; S(29): 0.1; T(30): 0.0; S(31): 0.0	36	1	1	0	N-Term(TMT6plex); S8(Phospho); M16(Oxidation); K32(TMT6plex)
291190783	NP_001167448.1	55604	LRRC16A	leucine-rich repeat-containing protein 16A isoform2	S1282	0.8	1.0	0.8	0.7	0.8	0.8	0.7	0.8	0.9	1.1	0.8	0.8	0.8	0.9	0.8	0.9	0.9	1.0	0.8	0.9	TASRPDDIPDsPSSPK	RPDDIPDsPSSPKVA	S11;	No	T(1): 0.0; S(3): 0.0; S(11): 100.0; S(13): 0.0; S(14): 0.0	13	2	1	0	N-Term(TMT6plex); S11(Phospho); K16(TMT6plex)
33356547	NP_004517.2	4171	MCM2	DNA replication licensing factor MCM2	S27	1.1	1.0	0.8	0.7	1.1	0.8	0.8	0.8	1.1	1.0	0.9	0.8	1.1	0.8	1.0	1.0	1.1	0.9	0.9	0.9	GNDPLTSsPGR	GNDPLTSsPGRSSRR	S8;	Yes	T(6): 0.0; S(7): 0.0; S(8): 100.0	9	1	1	0	N-Term(TMT6plex); S8(Phospho)
9257222	NP_002006.2	2308	FOXO1	forkhead box protein O1	S287	0.6	0.8	0.5	0.8	0.8	0.7	0.6	0.7	-	-	-	-	-	-	-	-	-	-	-	-	ASLQSGQEGAGDsPGSQFSK	GQEGAGDsPGSQFSK	S13;	Yes	S(2): 0.0; S(5): 0.0; S(13): 100.0; S(16): 0.0; S(19): 0.0	9	1	1	0	N-Term(TMT6plex); S13(Phospho); K20(TMT6plex)
50980301	NP_958431.2	23164	MPRIP	myosin phosphatase Rho-interacting protein isoform2	T295	0.7	1.0	1.3	0.5	1.0	0.9	1.1	1.1	0.6	1.0	1.3	0.5	1.0	0.9	1.1	1.1	0.8	1.0	1.2	0.8	AEEQQLPPPLsPPSPSTPNHR	QQLPPPLsPPSPSTP	T17;	Yes	S(11): 97.5; S(14): 3.7; S(16): 49.4; T(17): 49.4	1	2	1	0	N-Term(TMT6plex); S11(Phospho); S14(Phospho)
75677357	NP_001028733.1	57649	PHF12	PHD finger protein 12 isoform1	T671	1.2	0.9	0.8	0.8	1.4	0.8	1.7	0.8	-	-	-	-	-	-	-	-	-	-	-	-	VLtPPQAAGDGILATTANQR	PNATRVLtPPQAAGD	T3;	Yes	T(3): 100.0; T(15): 0.0; T(16): 0.0	4	3	1	0	N-Term(TMT6plex); T3(Phospho)
14165466	NP_114368.1	5725	PTBP1	polypyrimidine tract-binding protein 1 isoformc	S141	0.8	0.6	0.9	0.7	0.7	0.5	0.8	0.9	0.8	0.6	1.0	0.8	0.8	0.6	0.9	0.9	0.8	0.6	0.9	0.9	TDSsPNQAR	KELKTDSsPNQARAQ	S4;	Yes	T(1): 0.0; S(3): 0.0; S(4): 100.0	9	3	1	0	N-Term(TMT6plex); S3(Phospho)
47271443	NP_003007.2	6427	SRSF2	serine/arginine-rich splicing factor 2	S26	0.8	0.8	0.8	0.7	0.9	1.0	1.0	0.8	1.0	0.8	0.9	0.9	1.0	1.0	1.1	1.0	1.0	0.9	1.0	0.9	TsPDTLR	DNLTYRTsPDTLRRV	S2;	Yes	T(1): 0.0; S(2): 100.0; T(5): 0.0	29	1	1	0	N-Term(TMT6plex); S2(Phospho)
149158690	NP_542417.2	7916	PRRC2A	protein PRRC2A	T878	0.5	0.8	0.4	0.8	0.6	0.6	0.5	0.8	0.5	0.7	0.4	0.7	0.6	0.6	0.5	0.8	0.5	0.7	0.5	0.8	IQtPPPK	DEVAKIQtPPPKKEP	T3;	No	T(3): 100.0	23	1	1	0	N-Term(TMT6plex); T3(Phospho); K7(TMT6plex)
255652953	NP_653205.3	124245	ZC3H18	zinc finger CCCH domain-containing protein 18	S842	1.0	1.0	0.9	0.8	1.2	0.9	0.8	0.8	1.2	1.0	1.1	0.9	1.4	1.0	1.0	0.8	1.3	1.0	1.0	0.8	DRQsPPPAK	PSDKDRQsPPPAKRP	S4;	Yes	S(4): 100.0	34	1	1	1	N-Term(TMT6plex); S4(Phospho); K9(TMT6plex)
118572613	NP_057417.3	23524	SRRM2	serine/arginine repetitive matrix protein 2	S1482; S1483	0.7	0.8	0.8	0.7	0.8	0.9	0.9	0.8	0.7	0.8	0.9	0.8	0.8	0.9	0.9	0.9	0.8	0.9	0.9	0.9	SECDssPEPK	RGRSECDsSPEPKAL;GRSECDSsPEPKALP	S5; S6;	Yes;Yes	S(1): 0.0; S(5): 100.0; S(6): 100.0	436	1	1	0	N-Term(TMT6plex); C3(Carbamidomethyl); S5(Phospho); S6(Phospho); K10(TMT6plex)
69354671	NP_001020262.1	23	ABCF1	ATP-binding cassette sub-family F member 1 isoforma	S22	0.7	1.0	0.9	0.7	1.0	1.2	1.0	0.8	0.7	1.0	1.0	0.9	1.0	1.3	1.2	0.9	0.9	1.1	1.1	0.9	QQPPEPEWIGDGESTsPSDK	IGDGESTsPSDKVVK	S16;	Yes	S(14): 0.0; T(15): 0.0; S(16): 100.0; S(18): 0.0	44	2	1	0	N-Term(TMT6plex); S16(Phospho); K20(TMT6plex)
56118310	NP_073568.2	64710	NUCKS1	nuclear ubiquitous casein and cyclin-dependent kinase substrate 1	S75; S79	0.7	0.9	0.8	0.7	0.8	0.8	0.9	0.8	0.9	1.1	1.1	1.0	1.0	1.0	1.2	1.0	1.0	1.0	1.1	1.0	DDSHsAEDsEDEKEDHK	TKKDDSHsAEDSEDE;DSHSAEDsEDEKEDH	S5; S9;	Yes;Yes	S(3): 0.0; S(5): 100.0; S(9): 100.0	109	1	1	1	N-Term(TMT6plex); S5(Phospho); S9(Phospho); K13(TMT6plex); K17(TMT6plex)
187607445	NP_077269.3	79137	FAM134A	protein FAM134A	S385	1.1	1.1	0.9	0.8	0.8	1.1	1.0	0.7	-	-	-	-	-	-	-	-	-	-	-	-	QALDsEEEEEDVAAK	LSRQALDsEEEEEDV	S5;	Yes	S(5): 100.0	4	1	1	0	N-Term(TMT6plex); S5(Phospho); K15(TMT6plex)
56118310	NP_073568.2	64710	NUCKS1	nuclear ubiquitous casein and cyclin-dependent kinase substrate 1	S75; S79	0.8	0.8	0.9	0.8	0.7	0.9	0.8	0.7	1.0	1.0	1.2	1.0	0.9	1.1	1.1	1.0	1.0	1.0	1.1	1.0	DDSHsAEDsEDEKEDHK	TKKDDSHsAEDSEDE;DSHSAEDsEDEKEDH	S5; S9;	Yes;Yes	S(3): 0.0; S(5): 100.0; S(9): 100.0	2	1	1	1	N-Term(TMT6plex); S5(Phospho); S9(Phospho); K13(TMT6plex); K17(TMT6plex)
21314720	NP_078976.2	79753	SNIP1	smad nuclear-interacting protein 1	S128	0.9	1.1	0.8	0.7	1.2	1.0	0.8	0.9	0.9	1.3	0.9	0.9	1.3	1.2	1.0	1.1	1.1	1.3	1.0	1.0	EPsEQEHR	DRQHREPsEQEHRRA	S3;	Yes	S(3): 100.0	10	1	1	0	N-Term(TMT6plex); S3(Phospho)
119874201	NP_055845.1	285527	FRYL	protein furry homolog-like	T1959	0.9	1.0	1.2	0.9	1.0	1.4	1.1	0.7	0.9	0.8	1.0	0.8	0.9	1.2	0.9	0.6	0.9	1.0	1.0	0.7	SNtLDIMDGR	GDRRRSNtLDIMDGR	T3;	Yes	S(1): 1.3; T(3): 98.7	3	1	1	0	N-Term(TMT6plex); T3(Phospho); M7(Oxidation)
209862789	NP_056056.2	57553	MICAL3	protein-methionine sulfoxide oxidase MICAL3 isoform	S1310	1.0	0.9	1.4	1.1	1.1	1.3	1.5	0.4	0.9	0.8	1.2	1.1	1.0	1.1	1.3	0.4	1.0	0.9	1.3	0.7	LGsPLAVDEALR	DTKDRLGsPLAVDEA	S3;	Yes	S(3): 100.0	3	1	1	0	N-Term(TMT6plex); S3(Phospho)
21389407	NP_653209.1	124790	HEXIM2	protein HEXIM2	S29	1.3	1.2	1.0	0.8	1.4	1.2	1.0	0.8	-	-	-	-	-	-	-	-	-	-	-	-	TSGAPGsPQTPPER	KTSGAPGsPQTPPER	S7;	Yes	T(1): 0.0; S(2): 0.0; S(7): 100.0; T(10): 0.0	5	1	1	0	N-Term(TMT6plex); S7(Phospho)
44890052	NP_981946.1	3925	STMN1	stathmin isoforma	S38	0.6	0.5	1.2	0.6	1.0	0.8	1.3	0.9	0.5	0.4	0.9	0.7	0.8	0.7	1.1	1.0	0.6	0.6	1.0	0.8	ESVPEFPLsPPK	SVPEFPLsPPKKKDL	S9;	Yes	S(2): 0.0; S(9): 100.0	9	2	1	0	N-Term(TMT6plex); S9(Phospho); K12(TMT6plex)
311893307	NP_001185781.1	22887	FOXJ3	forkhead box protein J3 isoform2	S189	0.7	0.8	0.9	0.8	0.9	0.9	0.8	0.8	0.7	0.7	0.9	0.7	0.9	0.8	0.8	0.7	0.8	0.8	0.9	0.7	VTLYNTDQDGSDsPR	TDQDGSDsPRSSLNN	S13;	No	T(2): 0.0; Y(4): 0.0; T(6): 0.0; S(11): 1.1; S(13): 98.9	3	2	1	0	N-Term(TMT6plex); S13(Phospho)
103472005	NP_002408.3	4288	MKI67	antigen KI-67 isoform1	S1131	0.4	0.8	1.4	0.7	0.7	1.0	1.0	0.8	0.5	0.8	1.5	0.8	0.8	1.0	1.0	0.9	0.6	0.9	1.2	0.8	IACKsPPPESVDTPTSTK	TTKIACKsPPPESVD	S5;	Yes	S(5): 100.0; S(10): 0.0; T(13): 0.0; T(15): 0.0; S(16): 0.0; T(17): 0.0	118	2	1	1	N-Term(TMT6plex); C3(Carbamidomethyl); K4(TMT6plex); S5(Phospho); K18(TMT6plex)
40548332	NP_954981.1	339230	CCDC137	coiled-coil domain-containing protein 137	S19	0.8	0.9	0.7	0.8	0.7	0.7	0.7	0.8	0.9	1.0	0.8	0.9	0.9	0.8	0.9	1.0	0.9	0.9	0.9	0.9	VQAGPGsPR	RVQAGPGsPRRARGR	S7;	Yes	S(7): 100.0	2	1	1	0	N-Term(TMT6plex); S7(Phospho)
259906020	NP_001158287.1	22985	ACIN1	apoptotic chromatin condensation inducer in the nucleus isoform3	S176	0.9	0.7	0.9	0.8	1.0	0.9	0.9	0.7	0.7	0.7	1.0	0.9	0.8	0.8	1.0	0.8	0.7	0.7	1.0	0.8	SSSISEEKGDsDDEKPR	ISEEKGDsDDEKPRK	S11;	No	S(1): 0.0; S(2): 0.0; S(3): 0.0; S(5): 0.0; S(11): 100.0	38	3	1	1	N-Term(TMT6plex); K8(TMT6plex); S11(Phospho); K15(TMT6plex)
21536411	NP_647598.1	274	BIN1	myc box-dependent-interacting protein 1 isoform6	S272	0.9	0.7	1.1	1.0	0.5	0.4	0.3	0.5	0.8	0.7	1.1	1.1	0.4	0.4	0.3	0.5	0.6	0.5	0.7	0.8	SPSPPDGsPAATPEIR	SPSPPDGsPAATPEI	S8;	No	S(1): 50.0; S(3): 50.0; S(8): 100.0; T(12): 0.0	18	10	1	0	N-Term(TMT6plex); S3(Phospho); S8(Phospho)
21389577	NP_653280.1	146705	ENTHD2	AP-4 complex accessory subunit tepsin	S455	0.8	0.5	0.7	0.9	0.7	0.7	0.6	0.7	1.0	0.9	1.1	1.2	1.0	1.2	0.9	0.9	1.0	1.0	1.0	1.0	IPGGTDsPK	RIPGGTDsPKRGPSS	S7;	Yes	T(5): 0.0; S(7): 100.0	1	1	1	0	N-Term(TMT6plex); S7(Phospho); K9(TMT6plex)
262073007	NP_001159968.1	6566	SLC16A1	monocarboxylate transporter 1	S483	0.7	0.8	1.0	0.9	0.6	0.7	0.8	0.7	0.8	0.9	1.2	1.0	0.6	0.7	0.9	0.7	0.7	0.8	1.0	0.8	AAEsPDQK	EVTKAAEsPDQKDTD	S4;	Yes	S(4): 100.0	3	1	1	0	N-Term(TMT6plex); S4(Phospho); K8(TMT6plex)
110556636	NP_203754.2	85456	TNKS1BP1	182 kDa tankyrase-1-binding protein	S178	1.4	1.4	1.3	0.9	1.2	1.5	1.2	0.7	1.4	1.2	1.2	1.0	1.1	1.3	1.2	0.7	1.3	1.3	1.2	0.9	EVLAsPDR	PRKEVLAsPDRLWGS	S5;	Yes	S(5): 100.0	41	1	1	0	N-Term(TMT6plex); S5(Phospho)
4504445	NP_002127.1	3178	HNRNPA1	heterogeneous nuclear ribonucleoprotein A1 isoforma	S199	0.8	0.7	0.5	0.8	1.1	0.9	0.6	0.8	1.0	0.8	0.6	0.9	1.4	1.0	0.7	0.9	1.2	0.9	0.7	0.9	SGsGNFGGGR	SQRGRSGsGNFGGGR	S3;	Yes	S(1): 0.0; S(3): 100.0	4	2	1	0	N-Term(TMT6plex); S3(Phospho)
87299628	NP_683692.2	259282	BOD1L1	biorientation of chromosomes in cell division protein 1-like 1	S2905	0.9	0.7	0.9	1.0	0.7	0.8	0.8	0.6	1.0	0.7	1.0	1.1	0.8	0.7	0.8	0.7	0.9	0.7	0.9	0.9	TDTGIVTVEQsPSSSK	GIVTVEQsPSSSKLK	S11;	Yes	T(1): 0.0; T(3): 0.0; T(7): 0.0; S(11): 100.0; S(13): 0.0; S(14): 0.0; S(15): [illegible]	36	1	1	0	N-Term(TMT6plex); S11(Phospho); K16(TMT6plex)
566559802	NP_001274432.1	6744	SSFA2	sperm-specific antigen 2 isoform3	S270	0.5	0.4	0.5	0.8	0.8	0.5	0.4	0.7	0.5	0.5	0.5	1.0	0.8	0.6	0.5	1.0	0.7	0.5	0.5	1.0	IGsMSSVTSNK	TPLQRIGsMSSVTSN	S3;	Yes	S(3): 99.9; S(5): 0.0; S(6): 0.0; T(8): 0.0; S(9): 0.0	6	4	1	0	N-Term(TMT6plex); S3(Phospho); M4(Oxidation); K11(TMT6plex)
556503401	NP_001273294.1	55700	MAP7D1	MAP7 domain-containing protein 1 isoform2	S276	1.1	1.1	0.8	0.8	0.9	1.0	0.8	0.7	1.1	0.9	0.8	0.8	0.9	0.8	0.8	0.7	1.0	0.9	0.8	0.7	sAVTLPR	SELARSRsAVTLPRN	S1;	No	S(1): 100.0; T(4): 0.0	27	3	1	0	N-Term(TMT6plex); S1(Phospho)
5453994	NP_006256.1	5885	RAD21	double-strand-break repair protein rad21 homolog	S545	1.2	2.8	1.7	1.1	0.4	0.7	0.9	0.4	1.3	3.0	1.8	1.3	0.4	0.7	1.0	0.5	0.9	1.9	1.4	0.9	EKEDDEEEEDEDAsGGDQDQEE	EEEDEDAsGGDQDQE	S14;	Yes	S(14): 100.0	1	1	1	1	N-Term(TMT6plex); K2(TMT6plex); S14(Phospho)
253970412	NP_057510.4	51512	GTSE1	G2 and S phase-expressed protein 1	S555	0.3	0.6	0.8	0.6	0.6	0.8	0.8	0.9	0.3	0.7	0.9	0.7	0.7	0.9	0.9	1.1	0.5	0.8	0.9	0.9	AVGsPLCVPAR	TMPRAVGsPLCVPAR	S4;	No	S(4): 100.0	19	1	1	0	N-Term(TMT6plex); S4(Phospho); C7(Carbamidomethyl)
15721939	NP_031385.2	22794	CASC3	protein CASC3	S148	1.2	1.1	0.9	0.6	0.9	1.0	0.9	0.9	1.3	1.1	1.0	0.6	1.0	1.0	1.0	1.0	1.2	1.1	1.0	0.8	STVTGERQsGDGQESTEPVENK	TVTGERQsGDGQEST	S9;	Yes	S(1): 0.0; T(2): 0.0; T(4): 0.0; S(9): 100.0; S(15): 0.0; T(16): 0.0	10	1	1	1	N-Term(TMT6plex); S9(Phospho); K22(TMT6plex)
28559039	NP_004765.2	5469	MED1	mediator of RNA polymerase II transcription subunit 1	S1401	0.7	0.7	0.7	0.8	0.7	0.7	0.7	0.8	0.8	0.7	0.7	0.8	0.7	0.7	0.8	0.8	0.7	0.7	0.8	0.8	HDGGsPSIK	ISKHDGGsPSIKAKV	S5;	Yes	S(5): 100.0; S(7): 0.0	16	1	1	0	N-Term(TMT6plex); S5(Phospho); K9(TMT6plex)
8923896	NP_061147.1	55902	ACSS2	acetyl-coenzyme A synthetase, cytoplasmic isoform1	S30	2.3	1.4	0.8	0.8	1.4	1.3	1.0	0.7	1.8	1.2	0.8	0.9	1.1	1.1	1.0	0.8	1.4	1.1	0.9	0.8	SWsPPPEVSR	GGRARSWsPPPEVSR	S3;	Yes	S(1): 0.0; S(3): 98.6; S(9): 1.4	7	2	1	0	N-Term(TMT6plex); S3(Phospho)
5032189	NP_005648.1	7158	TP53BP1	tumor suppressor p53-binding protein 1 isoform3	S831	1.3	1.4	1.4	1.1	1.2	1.0	1.2	0.4	1.3	1.3	1.5	1.4	1.2	0.9	1.3	0.5	1.2	1.1	1.4	0.9	SGTAETEPVEQDSsQPSLPLVR	EPVEQDSsQPSLPLV	S14;	Yes	S(1): 0.0; T(3): 0.0; T(6): 0.0; S(13): 1.8; S(14): 98.2; S(17): 0.0	62	3	1	0	N-Term(TMT6plex); S14(Phospho)
13128864	NP_006035.2	10013	HDAC6	histone deacetylase 6	S22	0.9	0.7	0.7	0.9	0.8	0.8	0.7	0.7	1.1	0.8	0.8	1.0	1.0	0.9	0.8	0.8	1.0	0.8	0.8	0.9	QNPQsPPQDSSVTSK	RSRQNPQsPPQDSSV	S5;	Yes	S(5): 100.0; S(10): 0.0; S(11): 0.0; T(13): 0.0; S(14): 0.0	5	1	1	0	N-Term(TMT6plex); S5(Phospho); K15(TMT6plex)
24431975	NP_056467.2	26148	C10orf12	uncharacterized protein C10orf12	S1096	1.0	0.9	1.1	0.7	1.1	0.9	1.0	0.8	-	-	-	-	-	-	-	-	-	-	-	-	TEGSsPPDSK	RKRTEGSsPPDSKNK	S5;	No	T(1): 0.0; S(4): 0.9; S(5): 99.0; S(9): [illegible]	10	1	1	0	N-Term(TMT6plex); S5(Phospho); K10(TMT6plex)
11225260	NP_003277.1	7150	TOP1	DNA topoisomerase 1	S394	0.5	0.6	0.6	0.7	0.7	0.8	0.8	0.8	0.6	0.7	0.6	0.8	0.8	0.8	0.8	0.9	0.7	0.8	0.7	0.8	VPsPPPGHK	SKDAKVPsPPPGHKW	S3;	Yes	S(3): 100.0	2	1	1	0	N-Term(TMT6plex); S3(Phospho); K9(TMT6plex)

Khan et al., 2019. Multi-omics analysis to characterize cigarette smoke induced molecular alterations in esophageal cells
Supplementary Table 8. List of phosphopeptides identified and quantified in untreated and chronically treated Het1A cells with cigarette smoke condensate for 8 months using SequestHT and Mascot search algorithms with a PhosphoRS probability score ≥75%

														Normalized phosphoprotein ratios with respect to corresponding total protein ratios.								Average of replicates													
Protein group Accession	NP_Accession	Gene ID	Gene Symbol	Protein Description	PhosphoSite (Protein)	Het-1A-Smoke - 2M/Parental	Het-1A-Smoke - 4M/Parental	Het-1A-Smoke - 6M/Parental	Het-1A-Smoke - 8M/Parental	Het-1A-Smoke - 2M/Parental	Het-1A-Smoke - 4M/Parental	Het-1A-Smoke - 6M/Parental	Het-1A-Smoke - 8M/Parental	Het-1A-Smoke - 2M/Parental	Het-1A-Smoke - 4M/Parental	Het-1A-Smoke - 6M/Parental	Het-1A-Smoke - 8M/Parental	Het-1A-Smoke - 2M/Parental	Het-1A-Smoke - 4M/Parental	Het-1A-Smoke - 6M/Parental	Het-1A-Smoke - 8M/Parental	Het-1A-Smoke - 2M/Parental	Het-1A-Smoke - 4M/Parental	Het-1A-Smoke - 6M/Parental	Het-1A-Smoke - 8M/Parental	Peptide Sequence	Phosphowindow	PhosphoSite (Peptide)	Phosphosite Plus Evidence	phosphoRS Site Probabilities	# PSMs	# Proteins	# Protein Groups	# Missed Cleavages	Modifications
						Replicate 1				Replicate 2				Replicate 1				Replicate 2																	
46852390	NP_005427.2	8030	CCDC6	coiled-coil domain-containing protein 6	S254	0.8	0.6	0.7	0.6	1.1	1.0	0.8	0.9	0.9	0.6	0.7	0.6	1.1	0.9	0.8	0.9	1.0	0.8	0.7	0.7	DISMEIDsPENMMR	DISMEIDsPENMMRH	S8;	Yes	S(3): 0.0; S(8): 100.0	11	1	1	0	N-Term(TMT6plex); M4(Oxidation); S8(Phospho); M12(Oxidation); M13(Oxidation)
4885237	NP_005242.1	2303	FOXC2	forkhead box protein C2	S288	0.7	0.9	0.9	0.7	0.9	0.8	0.9	0.8	0.3	0.8	0.5	0.5	0.4	0.7	0.6	0.6	0.4	0.7	0.5	0.5	TSPPGGELsPGAGR	SPPGGELsPGAGRAG	S9;	Yes	T(1): 0.0; S(2): 0.0; S(9): 100.0	8	1	1	0	N-Term(TMT6plex); S9(Phospho)
41327769	NP_056133.2	23370	ARHGEF18	rho guanine nucleotide exchange factor 18 isoforma	S602	0.9	1.2	1.1	0.9	0.3	1.0	0.9	0.7	0.9	1.1	1.1	0.9	0.3	1.0	0.9	0.7	0.6	1.1	1.0	0.8	GPPNsPDLK	DWRGPPNsPDLKLSD	S5;	No	S(5): 100.0	2	2	1	0	N-Term(TMT6plex); S5(Phospho); K9(TMT6plex)
566006176	NP_001274366.1	79960	JADE1	protein Jade-1 isoform3	S731	1.0	0.8	0.9	0.9	0.6	0.6	0.8	0.6	-	-	-	-	-	-	-	-	-	-	-	-	VPTTPAsPVK	KVPTTPAsPVKNWGG	S7;	No	T(3): 0.0; T(4): 0.0; S(7): 100.0	2	2	1	0	N-Term(TMT6plex); S7(Phospho); K10(TMT6plex)
155722990	NP_060628.2	22950	SLC4A1AP	kanadaptin	S82	0.5	0.9	0.5	0.7	0.9	1.0	0.9	0.9	0.6	1.0	0.7	0.8	1.0	1.1	1.2	1.1	0.8	1.1	0.9	1.0	KPALPVsPAAR	KKPALPVsPAARSKA	S7;	Yes	S(7): 100.0	9	1	1	0	N-Term(TMT6plex); K1(TMT6plex); S7(Phospho)
14670268	NP_002895.3	7936	NELFE	negative elongation factor E	S181	0.8	1.0	0.7	0.8	0.8	0.9	0.7	0.7	0.9	1.1	0.9	0.9	0.9	1.0	0.9	0.9	0.9	1.1	0.9	0.9	SGAHSSAsPPR	SGAHSSAsPPRSRSR	S8;	Yes	S(1): 0.0; S(5): 0.0; S(6): 0.0; S(8): 100.0	23	1	1	0	N-Term(TMT6plex); S8(Phospho)
11560152	NP_071378.1	63925	ZNF335	zinc finger protein 335	S976	0.7	0.7	0.7	0.7	0.6	0.8	0.7	0.8	-	-	-	-	-	-	-	-	-	-	-	-	DGPEPPsPAK	RDGPEPPsPAKTHCV	S7;	Yes	S(7): 100.0	8	1	1	0	N-Term(TMT6plex); S7(Phospho); K10(TMT6plex)
4503249	NP_003463.1	7913	DEK	protein DEK isoform1	S32	0.7	0.9	1.0	0.8	0.8	0.5	1.0	0.7	0.9	1.1	1.2	1.1	1.0	0.7	1.3	1.0	0.9	0.9	1.3	1.0	EEsEEEDEDDEEEEEEEKEK	MPGPREEsEEEDED	S3;	Yes	S(3): 100.0	3	1	1	1	N-Term(TMT6plex); S3(Phospho); K19(TMT6plex); K21(TMT6plex)
20336477	NP_004756.2	9274	BCL7C	B-cell CLL/lymphoma 7 protein family member C isoform2	S126	0.4	0.5	0.3	0.4	0.8	1.3	0.9	1.1	0.6	0.6	0.4	0.5	1.0	1.5	1.1	1.3	0.8	1.0	0.7	0.9	GTEPSPGGTPQPSRPVsPAGPPEGVPEEAQPPR	PQPSRPVsPAGPPEG	S17;	Yes	T(2): 0.0; S(5): 0.0; T(9): 0.0; S(13): 2.4; S(17): 97.6	3	2	1	0	N-Term(TMT6plex); S17(Phospho)
21361282	NP_005617.2	6429	SRSF4	serine/arginine-rich splicing factor 4	S431	0.9	1.0	0.7	0.7	1.0	1.0	0.8	0.8	1.2	1.2	1.0	1.1	1.3	1.3	1.1	1.2	1.3	1.2	1.1	1.2	GEsENAGTNQETR	QREGRGEsENAGTNQ	S3;	Yes	S(3): 100.0; T(8): 0.0; T(12): 0.0	17	1	1	0	N-Term(TMT6plex); S3(Phospho)
41872339	NP_958846.1	51535	PPHLN1	periphilin-1 isoform5	T156	0.6	0.5	0.7	0.7	0.7	0.6	0.6	0.8	0.6	0.5	0.6	0.6	0.8	0.5	0.6	0.7	0.7	0.5	0.6	0.7	TSRDtSPSSGSAVSSSK	SLKTSRDtSPSSGSA	T5;	No	T(1): 50.0; S(2): 50.0; T(5): 98.0; S(6): 2.0; S(8): 0.0; S(9): 0.0; S(11): 0.0; S(14): 0.0; S(15): 0.0; S(16): 0.0	8	8	1	1	N-Term(TMT6plex); T1(Phospho); T5(Phospho); K17(TMT6plex)
4504809	NP_002220.1	3726	JUNB	transcription factor jun-B	T255	1.0	1.0	1.2	0.9	0.2	0.4	0.6	0.6	-	-	-	-	-	-	-	-	-	-	-	-	DAtPPVsPINMEDQER	EARSRDAtPPVSPIN	T3;	Yes	T(3): 100.0; S(7): 0.0	2	1	1	0	N-Term(TMT6plex); T3(Phospho); M11(Oxidation)
5453974	NP_006247.1	5586	PKN2	serine/threonine-protein kinase N2	S583	0.8	0.8	0.7	0.8	0.8	0.8	0.8	0.7	0.8	0.9	0.8	0.9	0.8	0.8	0.9	0.8	0.8	0.9	0.9	0.8	ASsLGEIDESSELR	PAPPRASsLGEIDES	S3;	Yes	S(2): 0.0; S(3): 100.0; S(10): 0.0; S(11): 0.0	9	1	1	0	N-Term(TMT6plex); S3(Phospho)
19923969	NP_612451.1	115098	CCDC124	coiled-coil domain-containing protein 124	S141	0.4	0.7	0.6	0.6	0.9	1.1	0.8	0.9	0.5	0.8	0.8	0.7	1.0	1.2	1.0	1.1	0.8	1.0	0.9	0.9	VLEEGsVEAR	RRVLEEGsVEARTIE	S6;	Yes	S(6): 100.0	1	1	1	0	N-Term(TMT6plex); S6(Phospho)
556695389	NP_001273359.1	123169	LEO1	RNA polymerase-associated protein LEO1 isoform2	S162	0.9	0.8	0.9	0.7	0.8	0.9	1.0	0.8	1.1	0.9	1.1	0.9	1.0	1.0	1.2	1.1	1.0	0.9	1.2	1.0	IQNsDDEER	DDEKIQNsDDEERAQ	S4;	Yes	S(4): 100.0	23	2	1	0	N-Term(TMT6plex); S4(Phospho)
170763500	NP_001116293.1	6418	SET	protein SET isoform1	S7	0.9	1.1	1.0	0.7	0.8	1.0	1.0	0.8	0.9	1.1	1.0	0.8	0.8	1.0	1.1	1.0	0.8	1.0	1.1	0.9	RQsPLPPQK	MAPKRQsPLPPQKK	S3;	Yes	S(3): 100.0	7	1	1	1	N-Term(TMT6plex); S3(Phospho); K9(TMT6plex)
118572613	NP_057417.3	23524	SRRM2	serine/arginine repetitive matrix protein 2	S1581; S1582	0.7	0.9	0.9	0.7	0.7	1.0	0.8	0.8	0.7	0.9	1.0	0.8	0.7	1.0	0.8	0.9	0.7	1.0	0.9	0.8	SGssPEVDSK	RQRSRSGsSPEVDSK;QRSRSGSsPEVDSKS	S3; S4;	Yes;Yes	S(1): 0.0; S(3): 100.0; S(4): 99.0; S(9): 1.0	436	1	1	0	N-Term(TMT6plex); S3(Phospho); S4(Phospho); K10(TMT6plex)
150417989	NP_001092872.1	8812	CCNK	cyclin-K	S340	1.2	0.9	1.0	0.9	0.7	0.6	0.8	0.7	1.1	1.0	1.0	0.8	0.6	0.6	0.9	0.6	0.9	0.8	1.0	0.7	AVVVsPK	VKRAVVVsPKEENKA	S5;	Yes	S(5): 100.0	11	1	1	0	N-Term(TMT6plex); S5(Phospho); K7(TMT6plex)
312176416	NP_001185901.1	57648	KIAA1522	uncharacterized protein KIAA1522 isoform2	S545	0.5	0.7	0.6	0.8	0.5	0.7	0.7	0.7	0.6	0.8	0.6	0.9	0.6	0.8	0.8	0.8	0.6	0.8	0.7	0.9	GLAGPPAsPGK	GLAGPPAsPGKAQPP	S8;	Yes	S(8): 100.0	22	2	1	0	N-Term(TMT6plex); S8(Phospho); K11(TMT6plex)
103472005	NP_002408.3	4288	MKI67	antigen KI-67 isoform1	T1923	0.6	0.8	0.9	0.8	0.6	0.8	1.0	0.7	0.7	0.8	1.0	0.8	0.8	0.8	1.0	0.8	0.7	0.8	1.0	0.8	DINTFVGtPVEK	DINTFVGtPVEKLDL	T8;	Yes	T(4): 0.0; T(8): 100.0	118	2	1	0	N-Term(TMT6plex); T8(Phospho); K12(TMT6plex)
22035679	NP_112739.2	51742	ARID4B	AT-rich interactive domain-containing protein 4B isoform2	S1041	0.6	0.8	0.8	0.8	0.4	0.7	0.7	0.7	0.6	0.8	0.8	0.9	0.4	0.7	0.7	0.8	0.5	0.7	0.7	0.8	DAQGGGsSSK	KDAQGGGsSSKKQKR	S7;	No	S(7): 100.0; S(8): 0.0; S(9): 0.0	4	2	1	0	N-Term(TMT6plex); S7(Phospho); K10(TMT6plex)
47519639	NP_002366.2	4134	MAP4	microtubule-associated protein 4 isoform1	S280	0.9	1.4	1.1	0.7	1.1	1.4	1.2	0.8	0.9	1.3	1.1	0.9	1.1	1.3	1.3	1.0	1.0	1.3	1.2	0.9	DMEsPTKLDVTLAK	ALAKDMEsPTKLDVT	S4;	Yes	S(4): 100.0; T(6): 0.0; T(11): 0.0	47	2	1	1	N-Term(TMT6plex); M2(Oxidation); S4(Phospho); K7(TMT6plex); K14(TMT6plex)
188536047	NP_003065.3	6599	SMARCC1	SWI/SNF complex subunit SMARCC1	S328; S330	0.9	0.4	1.0	0.5	1.0	0.9	1.1	1.0	1.0	0.4	0.9	0.5	1.0	0.9	1.1	1.0	1.0	0.7	1.0	0.7	KHsPsPPPTPTESR	NARKRKHsPSPPPPT;RKRKHSPsPPPPTPT	S3; S5;	Yes;Yes	S(3): 100.0; S(5): 100.0; T(10): 0.0; T(12): 0.0; S(14): 0.0	6	1	1	1	K1(TMT6plex); N-Term(TMT6plex); S3(Phospho); S5(Phospho)
588480516	NP_001277064.1	9538	EI24	etoposide-induced protein 2.4 homolog isoform3	S312; S316	1.2	0.9	1.1	1.0	0.4	0.4	0.6	0.5	1.3	1.2	1.2	1.1	0.5	0.5	0.6	0.6	0.9	0.8	0.9	0.9	FPsPHPsPAK	TSAEKFPsPHPSPAK;KFPSPHPsPAKLKAT	S3; S7;	No;No	S(3): 100.0; S(7): 100.0	1	2	1	0	N-Term(TMT6plex); S3(Phospho); S7(Phospho); K10(TMT6plex)
118572613	NP_057417.3	23524	SRRM2	serine/arginine repetitive matrix protein 2	S1101	1.1	1.1	1.1	0.6	0.9	1.0	1.0	0.9	1.2	1.1	1.2	0.7	0.9	1.0	1.0	1.0	1.1	1.1	1.1	0.8	SSsPVTELASR	PKGGRSRsSSPVTEL	S1;	Yes	S(1): 100.0; S(2): 50.0; S(3): 50.0; T(6): 0.0; S(10): 0.0	436	1	1	0	N-Term(TMT6plex); S1(Phospho); S2(Phospho)
157276588	NP_001708.3	646	BNC1	zinc finger protein basonuclin-1	S338	0.8	0.8	0.7	0.8	0.8	0.9	0.8	0.8	0.7	0.9	0.8	0.8	0.7	1.0	0.9	0.8	0.7	0.9	0.8	0.8	TQLsPEAK	KFERTQLsPEAKVKP	S4;	No	T(1): 0.0; S(4): 100.0	3	1	1	0	N-Term(TMT6plex); S4(Phospho); K8(TMT6plex)
374253823	NP_001243390.1	6558	SLC12A2	solute carrier family 12 member 2 isoform2	T230	1.4	0.9	1.1	0.9	1.2	1.0	1.0	0.6	1.5	0.9	1.2	0.9	1.3	1.0	1.1	0.6	1.4	0.9	1.1	0.8	HtAAQLGEK	RIDHYRHtAAQLGEK	T2;	Yes	T(2): 100.0	6	2	1	0	N-Term(TMT6plex); T2(Phospho); K9(TMT6plex)
21361458	NP_055601.2	9828	ARHGEF17	rho guanine nucleotide exchange factor 17	S735	1.2	1.1	1.1	0.9	1.0	0.7	0.9	0.6	1.2	1.2	1.4	1.0	1.0	0.8	1.1	0.7	1.1	1.0	1.2	0.9	SLsDPIPQR	GEAYRSLsDPIPQRH	S3;	Yes	S(1): 0.0; S(3): 100.0	2	1	1	0	N-Term(TMT6plex); S3(Phospho)
55953110	NP_001007258.1	7799	PRDM2	PR domain zinc finger protein 2 isoformc	S1283	0.9	1.1	0.8	0.7	0.8	0.9	0.7	0.8	0.9	1.1	0.8	0.8	0.8	0.9	0.7	0.9	0.9	1.0	0.7	0.8	KPLsPPK	SCPKKPLsPPKKKVS	S4;	No	S(4): 100.0	6	3	1	0	N-Term(TMT6plex); K1(TMT6plex); S4(Phospho); K7(TMT6plex)
594140512	NP_001277241.1	1495	CTNNA1	catenin alpha-1 isoform5	S285	1.0	1.3	0.7	0.4	2.8	1.9	1.9	1.1	1.1	1.4	0.7	0.4	3.0	2.0	2.0	1.2	2.1	1.7	1.3	0.8	TsVQTEDDQLIAGQSAR	FDVRSRTsVQTEDDQ	S2;	No	T(1): 1.7; S(2): 98.3; T(5): 0.0; S(15): 0.0	2	11	1	0	N-Term(TMT6plex); S2(Phospho)
363807394	NP_001241881.1	79969	ATAT1	alpha-tubulin N-acetyltransferase 1 isoform4	S253	0.8	0.6	0.6	0.8	0.6	0.4	0.5	0.7	0.9	0.8	0.7	1.0	0.7	0.6	0.6	0.8	0.8	0.7	0.7	0.9	SSSLGNsPER	RSSSLGNsPERGPLR	S7;	No	S(1): 0.0; S(2): 0.0; S(3): 0.0; S(7): 100.0	6	3	1	0	N-Term(TMT6plex); S7(Phospho)
149588928	NP_065897.1	57606	SLAIN2	SLAIN motif-containing protein 2	S48	0.6	0.8	0.6	0.8	0.6	0.8	0.7	0.7	0.5	0.8	0.6	0.7	0.6	0.8	0.7	0.7	0.6	0.8	0.7	0.7	SGAVQGAGSLGPGsPVR	AGSLGPGsPVRAGAS	S14;	Yes	S(1): 0.0; S(9): 0.0; S(14): 100.0	9	1	1	0	N-Term(TMT6plex); S14(Phospho)
21493024	NP_653080.1	9590	AKAP12	A-kinase anchor protein 12 isoform2	S1530	1.3	1.0	1.0	0.5	1.2	1.2	1.2	1.0	1.5	1.0	0.8	0.4	1.3	1.2	1.0	1.0	1.4	1.1	0.9	0.7	EESESTAVGQAHsDISK	TAVGQAHsDISKDMS	S13;	No	S(3): 0.0; S(5): 0.0; T(6): 0.0; S(13): 100.0; S(16): 0.0	103	2	1	0	N-Term(TMT6plex); S13(Phospho); K17(TMT6plex)
72256200	NP_004317.2	607	BCL9	B-cell CLL/lymphoma 9 protein	S1039	1.1	1.0	0.9	0.9	0.6	0.6	0.5	0.6	-	-	-	-	-	-	-	-	-	-	-	-	TVASSDDDsPPAR	VASSDDDsPPARSPN	S9;	Yes	T(1): 0.0; S(4): 0.0; S(5): 0.0; S(9): [illegible]	2	1	1	0	N-Term(TMT6plex); S9(Phospho)
156105703	NP_001093879.1	26054	SENP6	sentrin-specific protease 6 isoform2	S214	0.4	0.8	0.7	0.7	0.7	0.8	0.8	0.8	0.4	0.8	0.7	0.6	0.6	0.8	0.8	0.8	0.5	0.8	0.7	0.7	HCSTYQPTPPLsPASK	YQPTPPLsPASKKCL	S12;	No	S(3): 0.0; T(4): 0.0; Y(5): 0.0; T(8): 0.0; S(12): 100.0; S(15): 0.0	3	2	1	0	N-Term(TMT6plex); C2(Carbamidomethyl); S12(Phospho); K16(TMT6plex)
372266146	NP_001243198.1	3835	KIF22	kinesin-like protein KIF22 isoform2	T395	1.0	0.7	1.2	0.8	0.7	0.6	0.6	0.7	1.2	0.8	1.4	0.8	0.8	0.6	0.8	0.8	1.0	0.7	1.1	0.8	LLASQGSQGAPLLStPK	QGAPLLStPKRERMV	T15;	No	S(4): 0.0; S(7): 0.0; S(14): 0.0; T(15): 100.0	9	2	1	0	N-Term(TMT6plex); T15(Phospho); K17(TMT6plex)
22035569	NP_037461.2	94104	PAXBP1	PAX3- and PAX7-binding protein 1 isoform2	S262	1.1	0.8	1.0	0.8	1.2	0.7	1.0	0.7	1.2	0.8	1.0	0.8	1.2	0.7	1.0	0.7	1.2	0.7	1.0	0.7	EDENDAsDDEDDDEKR	REDENDAsDDEDDDE	S7;	Yes	S(7): 100.0	1	2	1	1	N-Term(TMT6plex); S7(Phospho); K15(TMT6plex)
56699409	NP_002130.2	27316	RBMX	RNA-binding motif protein, X chromosome isoform1	S208	0.7	0.6	0.8	1.0	0.7	0.4	0.5	0.5	0.8	0.7	1.0	1.1	0.8	0.5	0.7	0.6	0.8	0.6	0.9	0.9	DVYLsPR	SRRDVYLsPRDDGYS	S5;	Yes	Y(3): 0.0; S(5): 100.0	5	2	1	0	N-Term(TMT6plex); S5(Phospho)
32171238	NP_061330.2	55971	BAIAP2L1	brain-specific angiogenesis inhibitor 1-associated protein 2-like protein 1	S261	0.9	0.9	0.7	0.7	0.8	0.9	0.9	0.8	0.8	1.0	0.6	0.6	0.7	1.0	0.8	0.7	0.7	1.0	0.7	0.7	TPASTPVSGTPQAsPMIER	VSGTPQAsPMIERSN	S14;	Yes	T(1): 0.0; S(4): 0.0; T(5): 0.0; S(8): 0.0; T(10): 0.0; S(14): 100.0	3	1	1	0	N-Term(TMT6plex); S14(Phospho); M16(Oxidation)
118572613	NP_057417.3	23524	SRRM2	serine/arginine repetitive matrix protein 2	S1598	0.8	0.9	0.9	0.7	0.9	1.0	0.9	0.8	0.8	0.9	0.9	0.8	0.9	1.0	1.0	0.9	0.9	0.9	0.9	0.8	SGSsPEVK	RRSRSGSsPEVKDKP	S1;	Yes	S(1): 0.0; S(3): 0.5; S(4): 99.5	1	1	1	0	N-Term(TMT6plex); S4(Phospho); K8(TMT6plex)
14249338	NP_116114.1	84811	BUD13	BUD13 homolog isoform1	T135; S139	1.1	1.0	0.9	0.8	0.9	0.8	0.7	0.7	1.2	1.1	1.0	0.9	1.0	0.8	0.8	0.8	1.1	0.9	0.9	0.9	HGtPDPsPR	PRRVRHGtPDPSPRK;RHGTPDPsPRKDRHD	T3; S7;	Yes;Yes	T(3): 100.0; S(7): 100.0	52	2	1	0	N-Term(TMT6plex); T3(Phospho); S7(Phospho)
126362983	NP_057136.2	51018	RRP15	RRP15-like protein	S226	0.4	0.6	0.6	0.7	0.4	0.7	0.7	0.8	0.5	0.6	0.6	0.8	0.4	0.7	0.6	0.9	0.4	0.7	0.6	0.8	GMDGSTNETAsSR	GSTNETAsSRKKPKA	S11;	Yes	S(5): 0.0; T(6): 0.0; T(9): 0.0; S(11): 100.0; S(12): 0.0	2	1	1	0	N-Term(TMT6plex); M2(Oxidation); S11(Phospho)
259906020	NP_001158287.1	22985	ACIN1	apoptotic chromatin condensation inducer in the nucleus isoform3	S450	0.7	0.7	0.7	0.7	0.8	0.7	0.7	0.8	0.6	0.7	0.8	0.7	0.6	0.7	0.8	0.8	0.6	0.7	0.8	0.8	AEsPAEK	PSDTKAEsPAEKVPE	S3;	No	S(3): 100.0	38	3	1	0	N-Term(TMT6plex); S3(Phospho); K7(TMT6plex)
223718207	NP_001138784.1	2117	ETV3	ETS translocation variant 3 isoform1	S139	2.0	0.9	2.5	1.1	1.0	0.7	0.7	0.4	2.0	0.8	2.9	1.3	1.0	0.6	0.8	0.4	1.5	0.7	1.9	0.8	SSGVVPQsAPPVPTASSR	SSGVVPQsAPPVPTA	S8;	Yes	S(1): 0.1; S(2): 0.1; S(8): 99.8; T(14): 0.0; S(16): 0.0; S(17): 0.0	2	1	1	0	N-Term(TMT6plex); S8(Phospho)
118572613	NP_057417.3	23524	SRRM2	serine/arginine repetitive matrix protein 2	S778; S780	0.5	0.7	0.6	0.7	0.7	1.1	1.0	0.8	0.5	0.7	0.7	0.8	0.8	1.1	1.0	0.9	0.6	0.9	0.8	0.8	sLSGSSPCPK	SRLSLRRsLSGSSPC	S1; S3;	Yes	S(1): 98.9; S(3): 33.7; S(5): 33.7; S(6): 33.7	6	1	1	0	N-Term(TMT6plex); S3(Phospho); S5(Phospho); C8(Carbamidomethyl); K10(TMT6plex)
118572613	NP_057417.3	23524	SRRM2	serine/arginine repetitive matrix protein 2	S778; S780	0.7	1.1	1.0	0.8	0.5	0.7	0.6	0.7	0.8	1.1	1.0	0.9	0.5	0.7	0.7	0.8	0.6	0.9	0.8	0.8	sLsGSSPCPK	SRLSLRRsLSGSSPC;LSLRRSLsGSSPCPK	S1; S3;	Yes;Yes	S(1): 100.0; S(3): 100.0; S(5): 0.0; S(6): 0.0	436	1	1	0	S1(Phospho); N-Term(TMT6plex); S3(Phospho); C8(Carbamidomethyl); K10(TMT6plex)
33188445	NP_036222.3	23499	MACF1	microtubule-actin cross-linking factor 1	S2429	1.6	1.4	1.5	1.0	0.7	0.6	0.8	0.5	1.5	1.4	1.4	1.2	0.6	0.6	0.8	0.6	1.1	1.0	1.1	0.9	SMDAMSsPTK	KSMDAMSsPTKTETV	S7;	No	S(1): 0.0; S(6): 0.0; S(7): 100.0; T(9): 0.0	4	1	1	0	N-Term(TMT6plex); M2(Oxidation); M5(Oxidation); S7(Phospho); K10(TMT6plex)
226509740	NP_067051.2	58506	SCAF1	splicing factor, arginine/serine-rich 19	T989	0.5	0.5	0.7	0.5	0.8	0.9	1.2	1.0	0.5	0.5	0.6	0.5	0.8	0.9	1.0	1.0	0.6	0.7	0.8	0.7	AAPPPPALtPDSQTVDSSCK	APPPPALtPDSQTVD	T9;	Yes	T(9): 100.0; S(12): 0.0; T(14): 0.0; S(17): 0.0; S(18): 0.0	29	1	1	0	N-Term(TMT6plex); T9(Phospho); C19(Carbamidomethyl); K20(TMT6plex)
75677357	NP_001028733.1	57649	PHF12	PHD finger protein 12 isoform1	S131	0.9	0.8	0.8	0.8	0.9	0.9	0.8	0.7	-	-	-	-	-	-	-	-	-	-	-	-	TTsPSSDTDLLDR	KSGKRTTsPSSDTDL	S3;	Yes	T(1): 0.0; T(2): 1.1; S(3): 98.9; S(5): 0.0; S(6): 0.0; T(8): 0.0	4	3	1	0	N-Term(TMT6plex); S3(Phospho)
117190174	NP_004491.2	3183	HNRNPC	heterogeneous nuclear ribonucleoproteins C1/C2 isoformb	S247	1.0	0.7	0.6	0.9	0.8	0.5	0.5	0.6	1.0	0.7	0.6	0.9	0.7	0.4	0.5	0.6	0.9	0.6	0.6	0.8	MESEGGADDsAEEGDLLDDDDNEDR	SEGGADDsAEEGDLL	S10;	No	S(3): 0.0; S(10): 100.0	41	2	1	0	N-Term(TMT6plex); M1(Oxidation); S10(Phospho)
340545545	NP_001230018.1	2305	FOXM1	forkhead box protein M1 isoform5	T595	0.6	1.0	0.9	0.7	0.7	0.9	1.1	0.8	0.6	0.9	0.8	0.7	0.7	0.8	1.0	0.8	0.7	0.9	0.9	0.8	ETLPISStPSK	ETLPISStPSKSVLP	T8;	No	T(2): 0.0; S(6): 0.0; S(7): 0.5; T(8): 99.5; S(10): 0.0	9	5	1	0	N-Term(TMT6plex); T8(Phospho); K11(TMT6plex)
253970412	NP_057510.4	51512	GTSE1	G2 and S phase-expressed protein 1	S594	0.7	0.9	0.9	0.7	0.7	0.8	0.9	0.8	0.8	0.9	1.0	0.8	0.8	0.9	1.0	0.9	0.8	0.9	1.0	0.9	LVDVsPDR	DSRLVDVsPDRGSPP	S5;	No	S(5): 100.0	19	1	1	0	N-Term(TMT6plex); S5(Phospho)
103472005	NP_002408.3	4288	MKI67	antigen KI-67 isoform1	S2505	0.5	0.6	0.7	0.7	0.6	0.7	0.9	0.8	0.6	0.6	0.8	0.7	0.7	0.7	0.9	0.8	0.6	0.7	0.8	0.8	TsGETTQTHTEPTGDSK	VSKLTRTsGETTQTH	S2;	Yes	T(1): 1.1; S(2): 98.9; T(5): 0.0; T(6): 0.0; T(8): 0.0; T(10): 0.0; T(13): 0.0; S(16): 0.0	118	2	1	0	N-Term(TMT6plex); S2(Phospho); K17(TMT6plex)
194578923	NP_001124000.1	9043	SPAG9	C-Jun-amino-terminal kinase-interacting protein 4 isoform1	S332	0.6	0.6	0.9	0.7	0.8	0.9	1.2	0.8	0.7	0.7	1.0	0.8	0.9	1.0	1.4	0.9	0.8	0.9	1.2	0.8	NVSTGsAENEEK	TRNVSTGsAENEEKS	S6;	Yes	S(3): 0.6; T(4): 0.6; S(6): 98.8	13	4	1	0	N-Term(TMT6plex); S6(Phospho); K12(TMT6plex)
118572613	NP_057417.3	23524	SRRM2	serine/arginine repetitive matrix protein 2	S455; S456	0.9	1.2	0.8	0.7	1.0	1.2	1.0	0.8	0.9	1.3	0.8	0.8	1.0	1.2	1.1	0.9	1.0	1.2	1.0	0.8	EIsSsPTSK	GSHREISsSPTSKNR	S4; S5;	Yes	S(3): 0.8; S(4): 99.2; S(5): 0.0; T(7): 0.0; S(8): 0.0	6	1	1	0	N-Term(TMT6plex); S5(Phospho); K9(TMT6plex)
118572613	NP_057417.3	23524	SRRM2	serine/arginine repetitive matrix protein 2	S455; S456	1.0	1.2	1.0	0.8	0.9	1.2	0.8	0.7	1.0	1.2	1.1	0.9	0.9	1.3	0.8	0.8	1.0	1.2	1.0	0.8	EISssPTSK	GSHREISsSPTSKNR;SHREISSsPTSKNRS	S4; S5;	Yes;Yes	S(3): 0.0; S(4): 100.0; S(5): 98.9; T(7): 1.1; S(8): 0.0	436	1	1	0	N-Term(TMT6plex); S4(Phospho); S5(Phospho); K9(TMT6plex)
13514831	NP_004389.2	1662	DDX10	probable ATP-dependent RNA helicase DDX10	S7	1.0	1.1	1.1	0.6	0.8	0.9	0.9	0.8	0.8	0.9	1.0	0.6	0.7	0.8	0.8	0.8	0.8	0.9	0.9	0.7	TANsPGSGARPDPVR	MGKTANsPGSGARP	S4;	Yes	T(1): 0.1; S(4): 99.9; S(7): 0.1	4	1	1	0	N-Term(TMT6plex); S4(Phospho)
606215169	NP_277024.2	984	CDK11B	cyclin-dependent kinase 11B isoform5	S81	0.9	0.9	0.9	0.7	1.0	1.0	1.0	0.8	1.0	1.0	0.9	0.8	1.1	1.1	1.0	0.8	1.1	1.1	1.0	0.8	SHsAEGGK	KRRHRSHsAEGGKHA	S3;	No	S(1): 0.0; S(3): 100.0	8	3	1	0	N-Term(TMT6plex); S3(Phospho); K8(TMT6plex)
56550039	NP_005924.2	4297	KMT2A	histone-lysine N-methyltransferase 2A isoform2 precursor	S2938	3.3	1.2	0.9	0.7	1.7	0.8	1.1	0.8	3.0	1.2	0.9	0.7	1.5	0.7	1.1	0.8	2.2	1.0	1.0	0.8	sPTVPSQNPSR	LSVLTTRsPTVPSQN	S1;	No	S(1): 100.0; T(3): 0.0; S(6): 0.0; S(10): 0.0	15	2	1	0	N-Term(TMT6plex); S1(Phospho)
27414491	NP_056254.1	25901	CCDC28A	coiled-coil domain-containing protein 28A	S239	0.8	0.8	0.9	0.9	0.7	0.7	0.6	0.6	0.5	0.8	1.0	0.9	0.5	0.7	0.7	0.7	0.5	0.7	0.9	0.8	TAsDSNLDR	EDKRKTAsDSNLDRL	S3;	No	T(1): 0.0; S(3): 100.0; S(5): 0.0	2	1	1	0	N-Term(TMT6plex); S3(Phospho)
545746335	NP_001271143.1	9320	TRIP12	E3 ubiquitin-protein ligase TRIP12 isoforma	S1039	0.6	0.8	0.7	0.6	0.7	0.6	0.7	0.9	0.6	0.8	0.7	0.6	0.7	0.7	0.7	1.0	0.7	0.8	0.7	0.8	DDSLDLsPQGR	RDDSLDLsPQGRLSD	S7;	No	S(3): 0.0; S(7): 100.0	23	4	1	0	N-Term(TMT6plex); S7(Phospho)
394581985	NP_001257384.1	11060	WWP2	NEDD4-like E3 ubiquitin-protein ligase WWP2 isoform WWP2-N	S211	0.9	0.9	1.0	0.8	0.7	0.8	0.9	0.7	-	-	-	-	-	-	-	-	-	-	-	-	TTPATGEQsPGAR	TPATGEQsPGARSRH	S9;	Yes	T(1): 0.0; T(2): 0.0; T(5): 0.0; S(9): 100.0	4	3	1	0	N-Term(TMT6plex); S9(Phospho)
50513245	NP_005474.2	10036	CHAF1A	chromatin assembly factor 1 subunit A	T865	0.5	0.7	0.6	0.5	0.4	0.5	0.9	1.0	0.6	0.8	0.7	0.7	0.5	0.6	1.2	1.2	0.6	0.7	1.0	0.9	EDSGSVPSTGPSQGtPISLK	STGPSQGtPISLKRK	T15;	Yes	S(3): 0.0; S(5): 0.0; S(8): 0.0; T(9): 0.0; S(12): 0.0; T(15): 100.0; S(18): [illegible]	22	1	1	0	N-Term(TMT6plex); S12(Phospho); K20(TMT6plex)
118572613	NP_057417.3	23524	SRRM2	serine/arginine repetitive matrix protein 2	S484; S486	0.8	0.9	1.0	0.7	1.0	1.1	1.0	0.7	0.9	1.0	1.1	0.8	1.0	1.1	1.0	0.8	1.0	1.0	1.0	0.8	sRsPATAK	PSRRMGRsRSPATAK;RRMGRSRsPATAKRG	S1; S3;	Yes;Yes	S(1): 100.0; S(3): 100.0; T(6): 0.0	436	1	1	1	N-Term(TMT6plex); S1(Phospho); S3(Phospho); K8(TMT6plex)
333470709	NP_001193995.1	1525	CXADR	coxsackievirus and adenovirus receptor isoform5 precursor	S332	0.8	0.8	1.0	0.8	0.9	0.6	0.6	0.7	1.2	1.3	1.4	1.3	1.3	1.0	0.8	1.1	1.2	1.2	1.1	1.2	TPQsPTLPPAK	DFERTPQsPTLPPAK	S4;	Yes	T(1): 0.0; S(4): 100.0; T(6): 0.0	6	2	1	0	N-Term(TMT6plex); S4(Phospho); K11(TMT6plex)
126723060	NP_055983.1	23211	ZC3H4	zinc finger CCCH domain-containing protein 4	S1110	1.2	0.8	0.8	0.7	2.2	1.4	0.9	0.8	1.5	0.9	0.9	0.8	2.7	1.6	1.0	0.9	2.1	1.2	1.0	0.9	AAKPGPAEAPSPTASPSGDAsPPATAPYDPR	ASPSGDAsPPATAPY	S17;	Yes	S(11): 0.0; T(13): 0.0; S(15): 0.1; S(17): 2.5; S(21): 97.3; T(25): 0.1;	3	1	1	0	N-Term(TMT6plex); K3(TMT6plex); S21(Phospho)
126723060	NP_055983.1	23211	ZC3H4	zinc finger CCCH domain-containing protein 4	S1110	2.2	1.4	0.9	0.8	1.2	0.8	0.8	0.7	2.7	1.6	1.0	0.9	1.5	0.9	0.9	0.8	2.1	1.2	1.0	0.9	AAKPGPAEAPSPTASPsGDASPPATAPYDPR	PSPTASPsGDASPPA	S17;	Yes	S(11): 0.0; T(13): 0.0; S(15): 3.0; S(17): 93.9; S(21): 3.0; T(25): 0.1;	3	1	1	0	N-Term(TMT6plex); K3(TMT6plex); S17(Phospho)
146219843	NP_006653.2	10847	SRCAP	helicase SRCAP	S2869	0.8	0.9	0.8	0.8	0.9	0.8	0.9	0.7	0.8	0.8	0.8	0.7	0.8	0.7	0.8	0.7	0.8	0.7	0.8	0.7	NRsPADAGR	RPPKKNRsPADAGRG	S3;	Yes	S(3): 100.0	18	1	1	1	N-Term(TMT6plex); S3(Phospho)
39753953	NP_789784.2	168850	ZNF800	zinc finger protein 800	S595	0.9	0.9	1.2	0.7	0.8	0.9	1.0	0.7	0.8	0.8	1.0	0.6	0.7	0.9	0.9	0.6	0.7	0.9	0.9	0.6	HDGTSNsPSK	KHDGTSNsPSKKYEV	S7;	No	T(4): 0.0; S(5): 0.0; S(7): 100.0; S(9): 0.0	13	1	1	0	N-Term(TMT6plex); S7(Phospho); K10(TMT6plex)
312284070	NP_060166.2	254394	MCM9	DNA helicase MCM9 isoform1	S915	0.6	0.9	0.5	0.6	0.7	1.0	0.7	0.9	-	-	-	-	-	-	-	-	-	-	-	-	SLAQVEEPAIENVKPPGsPVAK	ENVKPPGsPVAKLAK	S18;	Yes	S(1): 0.0; S(18): 100.0	1	1	1	0	N-Term(TMT6plex); K14(TMT6plex); S18(Phospho); K22(TMT6plex)
262205665	NP_001159864.1	7162	TPBG	trophoblast glycoprotein precursor	S418	1.1	1.1	1.1	1.1	0.4	0.7	0.2	0.3	1.0	1.0	1.1	1.2	0.4	0.6	0.2	0.3	0.7	0.8	0.6	0.7	LTNLSSNsDV	LTNLSSNsDV_	S8;	Yes	T(2): 0.0; S(5): 0.0; S(6): 0.0; S(8): 100.0	2	1	1	0	N-Term(TMT6plex); S8(Phospho)
126723149	NP_067054.1	58509	CACTIN	cactin	T162	0.6	0.6	0.7	0.8	0.7	0.5	0.8	0.7	0.6	0.7	0.8	0.9	0.7	0.5	0.9	0.7	0.7	0.6	0.8	0.8	AEEtPEEK	ELMKAEEtPEEKRAR	T4;	No	T(4): 100.0	4	1	1	0	N-Term(TMT6plex); T4(Phospho); K8(TMT6plex)
37577148	NP_909119.1	51517	NCKIPSD	NCK-interacting protein with SH3 domain isoform2	T174	1.0	0.8	0.7	0.7	1.0	0.8	0.8	0.7	1.0	0.9	0.8	0.9	1.0	0.9	0.9	0.9	1.0	0.9	0.9	0.9	AAPTtPPPPVK	PRRAAPTtPPPPVKR	T5;	Yes	T(4): 0.0; T(5): 100.0	2	2	1	0	N-Term(TMT6plex); T5(Phospho); K11(TMT6plex)
37577148	NP_909119.1	51517	NCKIPSD	NCK-interacting protein with SH3 domain isoform2	T174	1.0	0.8	0.8	0.7	1.0	0.8	0.7	0.7	1.0	0.9	0.9	0.9	1.0	0.9	0.8	0.9	1.0	0.9	0.9	0.9	AAPtTPPPPVK	QPRRAAPtTPPPPVK	T5;	No	T(4): 100.0; T(5): 0.0	4	2	1	0	N-Term(TMT6plex); T5(Phospho); K11(TMT6plex)
118572613	NP_057417.3	23524	SRRM2	serine/arginine repetitive matrix protein 2	T2599	0.6	0.7	0.9	0.7	0.6	0.8	0.9	0.8	0.7	0.7	0.9	0.8	0.7	0.8	1.0	0.9	0.7	0.7	0.9	0.8	EGRPPEPtPAK	EGRPPEPtPAKRKRR	T8;	Yes	T(8): 100.0	436	1	1	0	N-Term(TMT6plex); T8(Phospho); K11(TMT6plex)
217330641	NP_060439.2	55677	IWS1	protein IWS1 homolog	S400	1.0	1.0	1.0	0.5	0.6	1.6	1.3	1.0	1.1	1.0	1.0	0.6	0.6	1.5	1.4	1.1	0.9	1.3	1.2	0.8	AAVLSDsEDEEK	KAAVLSDsEDEEKAS	S7;	Yes	S(5): 1.1; S(7): 98.9	25	1	1	0	N-Term(TMT6plex); S7(Phospho); K12(TMT6plex)
217330641	NP_060439.2	55677	IWS1	protein IWS1 homolog	S400	0.6	1.6	1.3	1.0	1.0	1.0	1.0	0.5	0.6	1.5	1.4	1.1	1.1	1.0	1.0	0.6	0.9	1.3	1.2	0.8	AAVLsDSEDEEK	KRKAAVLsDSEDEEK	S7;	Yes	S(5): 100.0; S(7): 0.0	2	1	1	0	N-Term(TMT6plex); S5(Phospho); K12(TMT6plex)
586798168	NP_001276980.1	29128	UHRF1	E3 ubiquitin-protein ligase UHRF1 isoform1	S651	0.5	0.7	0.9	0.7	0.4	0.6	0.7	0.8	0.6	0.8	1.1	0.9	0.5	0.8	0.8	1.0	0.5	0.8	1.0	0.9	sAGGGPSR	KGKWKRKsAGGGPSR	S1;	Yes	S(1): 100.0; S(7): 0.0	5	2	1	0	N-Term(TMT6plex); S1(Phospho)
124028529	NP_004810.2	8189	SYMPK	symplekin	S1259	0.8	0.7	0.8	0.8	0.9	1.1	0.9	0.6	0.9	0.8	0.9	0.9	0.9	1.3	1.0	0.6	0.9	1.0	0.9	0.8	TPsPAAEDAREPEAK	EDAMKTPsPAAEDAR	S3;	Yes	T(1): 0.0; S(3): 100.0	4	1	1	1	N-Term(TMT6plex); S3(Phospho); K15(TMT6plex)
29789060	NP_055617.1	9847	C2CD5	C2 domain-containing protein 5 isoforme	S260	1.0	0.9	0.8	1.1	1.0	0.9	1.1	0.4	0.9	0.8	0.7	1.1	0.9	0.8	0.9	0.4	0.9	0.8	0.8	0.7	LSSPAAFLPACNsPSK	AFLPACNsPSKEMKE	S13;	Yes	S(2): 0.0; S(3): 0.0; S(13): 98.5; S(15): 1.5	3	5	1	0	N-Term(TMT6plex); C11(Carbamidomethyl); S13(Phospho); K16(TMT6plex)
255652953	NP_653205.3	124245	ZC3H18	zinc finger CCCH domain-containing protein 18	S842	0.7	0.6	0.7	0.6	0.9	0.9	0.9	0.9	0.9	0.7	0.9	0.7	1.1	0.9	1.0	0.9	1.0	0.8	0.9	0.8	QsPPPAK	PSDKDRQsPPPAKRP	S2;	Yes	S(2): 100.0	34	1	1	0	N-Term(TMT6plex); S2(Phospho); K7(TMT6plex)
146219843	NP_006653.2	10847	SRCAP	helicase SRCAP	S3148	0.7	0.7	0.5	0.6	0.8	0.8	0.7	0.8	0.6	0.6	0.5	0.6	0.7	0.6	0.7	0.8	0.7	0.6	0.6	0.7	AGAPVGGsPGLAK	AGAPVGGsPGLAKRG	S8;	Yes	S(8): 100.0	18	1	1	0	N-Term(TMT6plex); S8(Phospho); K13(TMT6plex)
89276756	NP_003904.3	8899	PRPF4B	serine/threonine-protein kinase PRP4 homolog	S431	0.9	0.8	0.9	0.7	0.8	0.7	0.9	0.7	1.0	0.9	1.0	0.8	1.0	0.7	1.0	0.8	1.0	0.8	1.0	0.8	DAsPINR	RERSKDAsPINRWSP	S3;	Yes	S(3): 100.0	4	1	1	0	N-Term(TMT6plex); S3(Phospho)
118572613	NP_057417.3	23524	SRRM2	serine/arginine repetitive matrix protein 2	S1132	0.8	0.7	0.8	0.7	0.5	0.3	0.4	0.8	0.8	0.7	0.8	0.8	0.5	0.3	0.4	0.8	0.7	0.5	0.6	0.8	SGMsPEQSR	PMLKSGMsPEQSRFQ	S4;	Yes	S(1): 0.0; S(4): 100.0; S(8): 0.0	436	1	1	0	N-Term(TMT6plex); M3(Oxidation); S4(Phospho)
166295179	NP_001107590.1	55421	C17orf85	uncharacterized protein C17orf85	S415	1.0	0.9	0.8	0.6	0.7	0.9	0.9	0.9	1.2	1.0	0.9	0.8	0.9	1.1	1.1	1.1	1.0	1.0	1.0	0.9	MISTPsPK	LKMISTPsPKKSMKM	S6;	Yes	S(3): 0.0; T(4): 0.0; S(6): 100.0	5	1	1	0	N-Term(TMT6plex); M1(Oxidation); S6(Phospho); K8(TMT6plex)
166295179	NP_001107590.1	55421	C17orf85	uncharacterized protein C17orf85	S415	0.7	0.9	0.9	0.9	1.0	0.9	0.8	0.6	0.9	1.1	1.1	1.1	1.2	1.0	0.9	0.8	1.0	1.0	1.0	0.9	MIsTPsPK	LKMISTPsPKKSMKM	S6;	Yes	S(3): 0.0; T(4): 0.0; S(6): 100.0	6	1	1	0	N-Term(TMT6plex); M1(Oxidation); S6(Phospho); K8(TMT6plex)
193083172	NP_001122403.1	92345	NAF1	H/ACA ribonucleoprotein complex non-core subunit NAF1 isoformb	S315	0.9	0.8	1.1	0.8	0.7	0.8	0.7	0.7	-	-	-	-	-	-	-	-	-	-	-	-	NDQEPPPEALDFsDDEKEK	PPEALDFsDDEKEKE	S13;	Yes	S(13): 100.0	4	2	1	1	N-Term(TMT6plex); S13(Phospho); K17(TMT6plex); K19(TMT6plex)
14249636	NP_116272.1	84969	TOX2	TOX high mobility group box family member 2 isoform c	S150	0.6	0.9	0.9	0.3	1.0	1.2	2.4	1.1	0.6	0.7	0.8	0.4	1.1	1.0	2.3	1.2	0.9	0.9	1.5	0.8	SSIAHSSPsPPGSK	SIAHSSPsPPGSKSA	S9;	No	S(1): 0.0; S(2): 0.0; S(6): 0.0; S(7): 0.0; S(9): 100.0; S(13): 0.0	1	3	1	0	N-Term(TMT6plex); S9(Phospho); K14(TMT6plex)
157426864	NP_055548.3	9765	ZFYVE16	zinc finger FYVE domain-containing protein 16	S939	0.6	0.9	0.8	0.9	0.4	1.1	1.4	0.6	0.6	0.9	0.7	0.9	0.4	1.1	1.3	0.6	0.5	1.0	1.0	0.7	NEIIQsPISQVPSVEK	TRNEIIQsPISQVPS	S6;	Yes	S(6): 100.0; S(9): 0.0; S(13): 0.0	1	1	1	0	N-Term(TMT6plex); S6(Phospho); K16(TMT6plex)
51173735	NP_001003698.1	6239	RREB1	ras-responsive element-binding protein 1 isoform2	S1122	0.8	0.8	0.8	0.6	0.8	1.1	1.1	0.8	0.9	0.9	0.9	0.6	0.9	1.3	1.2	0.8	0.9	1.1	1.0	0.7	AATTATPAATTsPK	ATPAATTsPKESSEP	S12;	Yes	T(3): 0.0; T(4): 0.0; T(6): 0.0; T(10): 0.0; T(11): 0.0; S(12): 100.0	10	3	1	0	N-Term(TMT6plex); S12(Phospho); K14(TMT6plex)
13540614	NP_110445.1	81576	CCDC130	coiled-coil domain-containing protein 130	S362	1.0	0.9	1.1	0.7	0.9	0.9	0.9	0.8	1.0	1.0	1.2	0.7	0.9	1.0	1.1	0.9	0.9	1.0	1.2	0.8	QDKPLsPAGSSQEAADTPDTR	SRQDKPLsPAGSSQE	S6;	Yes	S(6): 99.9; S(10): 0.1; S(11): 0.1; T(17): 0.0; T(20): 0.0	1	1	1	0	N-Term(TMT6plex); K3(TMT6plex); S6(Phospho)
12711674	NP_075386.1	65109	UPF3B	regulator of nonsense transcripts 3B isoform2	T169	0.3	0.8	0.5	0.6	0.4	0.9	0.5	0.8	0.3	1.1	0.6	0.9	0.5	1.2	0.7	1.2	0.4	1.2	0.6	1.1	ELESYAtDNEK	KELESYAtDNEKMTS	T7;	Yes	S(4): 0.0; Y(5): 0.0; T(7): 100.0	1	2	1	0	N-Term(TMT6plex); T7(Phospho); K11(TMT6plex)
110556636	NP_203754.2	85456	TNKS1BP1	182 kDa tankyrase-1-binding protein	S435	0.6	0.8	0.8	0.8	0.6	1.2	1.1	0.7	0.6	0.7	0.8	0.9	0.6	1.0	1.0	0.7	0.6	0.9	0.9	0.8	ESEGVLQsPSQDQEK	ESEGVLQsPSQDQEK	S8;	Yes	S(2): 0.0; S(8): 100.0; S(10): 0.0	41	1	1	0	N-Term(TMT6plex); S8(Phospho); K15(TMT6plex)
4758616	NP_002219.1	3725	JUN	transcription factor AP-1	S73	1.5	1.3	0.8	1.1	1.2	1.4	0.8	0.3	1.3	1.4	0.8	1.2	1.1	1.5	0.8	0.4	1.2	1.4	0.8	0.8	LAsPELER	VGLLKLAsPELERLL	S3;	Yes	S(3): 100.0	4	3	1	0	N-Term(TMT6plex); S3(Phospho)
27532984	NP_775298.1	9063	PIAS2	E3 SUMO-protein ligase PIAS2 isoformalpha	S499	0.5	0.7	0.5	0.5	1.0	1.2	1.3	0.9	-	-	-	-	-	-	-	-	-	-	-	-	CIFMSETQSsPTK	FMSETQSsPTKGVLM	S10;	Yes	S(5): 0.0; T(7): 0.0; S(9): 1.3; S(10): 97.4; T(12): 1.3	1	2	1	0	N-Term(TMT6plex); C1(Carbamidomethyl); M4(Oxidation); T12(Phospho); K13(TMT6plex)
118572613	NP_057417.3	23524	SRRM2	serine/arginine repetitive matrix protein 2	S2044; S2046	0.8	0.9	0.9	0.6	0.8	0.9	0.9	0.9	0.9	0.9	0.9	0.7	0.9	1.0	0.9	1.0	0.9	0.9	0.9	0.8	sRsPLAIR	LPRKRSRsRSPLAIR;RKRSRSRsPLAIRR	S1; S3;	Yes;Yes	S(1): 100.0; S(3): 100.0	436	1	1	1	N-Term(TMT6plex); S1(Phospho); S3(Phospho)
262359929	NP_056261.4	25909	AHCTF1	protein ELYS	S1169	0.8	0.8	0.9	0.7	0.8	0.8	1.1	0.8	0.8	0.8	0.9	0.8	0.8	0.8	1.1	0.8	0.8	0.8	1.0	0.8	GsPQAISR	SSSQLKGsPQAISRA	S2;	No	S(2): 100.0; S(7): 0.0	13	1	1	0	N-Term(TMT6plex); S2(Phospho)
153070260	NP_002347.5	4082	MARCKS	myristoylated alanine-rich C-kinase substrate	T150	0.6	0.7	0.7	0.7	0.6	0.7	0.6	0.7	0.9	1.0	1.0	1.0	0.9	1.0	0.9	1.0	0.9	1.0	0.9	1.0	AEDGATPSPSNEtPK	TPSPSNEtPKKKKKR	T13;	Yes	T(6): 0.0; S(8): 0.0; S(10): 0.0; T(13): 100.0	69	1	1	0	N-Term(TMT6plex); T13(Phospho); K15(TMT6plex)
14249338	NP_116114.1	84811	BUD13	BUD13 homolog isoform1	S235	1.1	1.1	0.9	0.8	1.0	1.0	0.8	0.7	1.2	1.1	1.0	0.9	1.1	1.0	0.9	0.8	1.1	1.1	0.9	0.8	HGsSDISSPR	PRRARHGsSDISSPR	S3;	Yes	S(3): 99.1; S(4): 0.9; S(7): 50.0; S(8): 50.0	52	1	1	0	N-Term(TMT6plex); S4(Phospho); S7(Phospho)
50409856	NP_001002243.1	54812	AFTPH	aftiphilin isoformc	S382	1.1	0.9	0.9	0.8	1.0	1.1	1.0	0.6	1.0	0.9	0.9	0.9	0.9	1.1	0.9	0.7	1.0	1.0	0.9	0.8	TSDDEVGsPKEESR	TSDDEVGsPKEESRK	S8;	Yes	T(1): 0.0; S(2): 0.0; S(8): 100.0; S(13): 0.0	2	3	1	1	N-Term(TMT6plex); S8(Phospho); K10(TMT6plex)
62988361	NP_001615.1	202	AIM1	absent in melanoma 1 protein	S103	0.9	0.8	0.9	0.9	0.6	0.8	0.7	0.5	1.1	0.9	1.1	1.1	0.7	0.9	0.8	0.6	0.9	0.9	0.9	0.9	SKQPPPASsPTK	KQPPPASsPTKRKGR	S9;	Yes	S(1): 0.0; S(8): 1.9; S(9): 96.3; T(11): 1.9	8	1	1	1	N-Term(TMT6plex); K2(TMT6plex); S9(Phospho); K12(TMT6plex)
253970412	NP_057510.4	51512	GTSE1	G2 and S phase-expressed protein 1	S611	1.1	1.3	1.4	0.6	0.9	0.9	1.1	0.9	1.2	1.4	1.6	0.7	1.0	0.9	1.3	1.1	1.1	1.2	1.4	0.9	VPQALNFsPEESDSTFSK	VPQALNFsPEESDST	S8;	No	S(8): 98.4; S(12): 1.6; S(14): 0.0; T(15): 0.0; S(17): 0.0	19	1	1	0	N-Term(TMT6plex); S8(Phospho); K18(TMT6plex)
28372497	NP_777576.1	197131	UBR1	E3 ubiquitin-protein ligase UBR1	T21	3.2	2.7	1.4	1.1	0.9	1.5	0.6	0.3	3.1	2.4	1.3	1.1	0.8	1.4	0.6	0.3	2.0	1.9	1.0	0.7	MEISAELPQtPQR	ISAELPQtPQRLASW	T10;	Yes	S(4): 0.0; T(10): 100.0	2	1	1	0	N-Term(TMT6plex); M1(Oxidation); T10(Phospho)
188536047	NP_003065.3	6599	SMARCC1	SWI/SNF complex subunit SMARCC1	S310	1.0	0.8	1.0	0.7	0.9	0.8	0.9	0.7	1.0	0.8	0.9	0.7	1.0	0.8	0.9	0.7	1.0	0.8	0.9	0.7	NEEPVRsPER	KNEEPVRsPERRDRK	S7;	Yes	S(7): 100.0	6	1	1	1	N-Term(TMT6plex); S7(Phospho)
54112117	NP_036565.2	23451	SF3B1	splicing factor 3B subunit 1 isoform1	T211	0.8	0.7	0.7	0.9	0.8	0.7	0.7	0.6	0.8	0.7	0.7	0.9	0.8	0.8	0.8	0.6	0.8	0.8	0.8	0.7	WDQTADQTPGAtPK	ADQTPGAtPKKLSSW	T12;	Yes	T(4): 0.0; T(8): 0.0; T(12): 100.0	13	1	1	0	N-Term(TMT6plex); T12(Phospho); K14(TMT6plex)
149158690	NP_542417.2	7916	PRRC2A	protein PRRC2A	S1004	0.9	0.7	0.8	0.9	0.9	0.8	0.8	0.6	1.0	0.6	0.8	0.8	0.9	0.7	0.8	0.6	0.9	0.7	0.8	0.7	ETPPNGNLsPAPR	TPPNGNLsPAPRLRR	S9;	No	T(2): 0.0; S(9): 100.0	23	1	1	0	N-Term(TMT6plex); S9(Phospho)
118572613	NP_057417.3	23524	SRRM2	serine/arginine repetitive matrix protein 2	S323	0.8	1.2	1.0	0.7	0.6	1.0	0.8	0.7	0.8	1.2	1.0	0.8	0.6	1.0	0.8	0.8	0.7	1.1	0.9	0.8	GEGDAPFSEPGTTSTQRPSsPETATK	TSTQRPSsPETATKQ	S20;	Yes	S(8): 0.0; T(12): 0.0; T(13): 0.0; S(14): 0.0; T(15): 0.0; S(19): 0.0; S(20): 99.8; T(23): 0.0; T(25): 0.0	436	1	1	0	N-Term(TMT6plex); S20(Phospho); K26(TMT6plex)
118572613	NP_057417.3	23524	SRRM2	serine/arginine repetitive matrix protein 2	S323	0.6	1.0	0.8	0.7	0.8	1.2	1.0	0.7	0.6	1.0	0.8	0.8	0.8	1.2	1.0	0.8	0.7	1.1	0.9	0.8	GEGDAPFsEPGTTSTQRPSSPETATK	GEGDAPFsEPGTTST	S20;	Yes	S(8): 99.6; T(12): 0.1; T(13): 0.1; S(14): 0.1; T(15): 0.1; S(19): 0.1; S(20): 0.1; T(23): 0.0; T(25): 0.0	1	1	1	0	N-Term(TMT6plex); S20(Phospho); K26(TMT6plex)
7019371	NP_037474.1	29844	TFPT	TCF3 fusion partner	S180	0.8	0.6	1.1	0.6	1.0	1.0	0.7	0.9	0.9	0.5	1.2	0.5	1.1	0.9	0.8	0.8	1.0	0.7	1.0	0.7	TPAPPEPGsPAPGEGPSGR	PAPPEPGsPAPGEGP	S9;	Yes	T(1): 0.0; S(9): 100.0; S(17): 0.0	12	1	1	0	N-Term(TMT6plex); S9(Phospho)
153945728	NP_005900.2	4131	MAP1B	microtubule-associated protein 1B	S1438	1.3	1.6	1.7	0.6	1.0	1.3	1.2	0.8	1.1	1.2	1.4	0.6	0.8	1.0	1.0	0.9	0.9	1.1	1.2	0.8	QGsPDQVsPVSEMTSTSLYQDK	EKSGKQGsPDQVSPV;QGSPDQVsPVSEMTS	S3;	Yes;Yes	S(3): 100.0; S(8): 100.0; S(11): 0.0; T(14): 0.0; S(15): 0.0; T(16): 0.0; S(17): 0.0; Y(19): 0.0	2	1	1	0	N-Term(TMT6plex); S3(Phospho); S8(Phospho); M13(Oxidation); K22(TMT6plex)
153945728	NP_005900.2	4131	MAP1B	microtubule-associated protein 1B	S1438	1.0	1.3	1.2	0.8	1.3	1.6	1.7	0.6	0.8	1.0	1.0	0.9	1.1	1.2	1.4	0.6	0.9	1.1	1.2	0.8	QGsPDQVSPVSEMTSTSLYQDK	EKSGKQGsPDQVSPV	S3;	Yes	S(3): 100.0; S(8): 0.0; S(11): 0.0; T(14): 0.0; S(15): 0.0; T(16): 0.0; S(17): 0.0; Y(19): 0.0	49	1	1	0	N-Term(TMT6plex); S3(Phospho); M13(Oxidation); K22(TMT6plex)
118150660	NP_060941.2	55854	ZC3H15	zinc finger CCCH domain-containing protein 15	S381	1.1	0.9	1.2	0.8	0.9	1.0	1.0	0.7	1.2	1.0	1.4	1.0	1.0	1.1	1.2	0.8	1.1	1.0	1.3	0.9	AENGERsDLEEDNER	RAENGERsDLEEDNE	S7;	Yes	S(7): 100.0	4	1	1	1	N-Term(TMT6plex); S7(Phospho)
4502847	NP_001271.1	1153	CIRBP	cold-inducible RNA-binding protein	S148	0.9	1.4	0.9	0.9	0.8	1.2	0.9	0.6	1.2	1.7	1.3	1.1	1.2	1.5	1.2	0.7	1.2	1.6	1.2	0.9	SQsGGYSDR	YYSSRSQsGGYSDRS	S3;	Yes	S(1): 0.0; S(3): 100.0; Y(6): 0.0; S(7): 0.0	3	1	1	0	N-Term(TMT6plex); S3(Phospho)
544709647	NP_001269861.1	10152	ABI2	abl interactor 2 isoforme	S165	0.8	0.5	0.6	0.6	0.9	0.7	0.8	0.8	0.8	0.6	0.6	0.6	0.9	0.8	0.8	0.8	0.8	0.7	0.7	0.7	NMAPSQQsPVR	NMAPSQQsPVRTASV	S8;	No	S(5): 0.0; S(8): 100.0	6	5	1	0	N-Term(TMT6plex); M2(Oxidation); S8(Phospho)
304434531	NP_001182122.1	4678	NASP	nuclear autoantigenic sperm protein isoform4	S182	0.5	0.8	0.4	0.6	0.5	0.4	0.5	0.8	0.7	1.0	0.6	0.9	0.7	0.6	0.7	1.2	0.7	0.8	0.7	1.1	SVsGTDVQEECR	AEEEKSVsGTDVQEE	S3;	No	S(1): 0.0; S(3): 98.9; T(5): 1.1	20	2	1	0	N-Term(TMT6plex); S3(Phospho); C11(Carbamidomethyl)
606215169	NP_277024.2	984	CDK11B	cyclin-dependent kinase 11B isoform5	S236	0.7	1.1	0.6	0.6	0.9	0.7	0.7	0.8	0.8	1.3	0.6	0.7	1.0	0.8	0.7	0.9	0.9	1.0	0.7	0.8	DLLSDLQDIsDSER	LSDLQDIsDSERKTS	S10;	No	S(4): 0.0; S(10): 99.1; S(12): 0.9	8	8	1	0	N-Term(TMT6plex); S10(Phospho)
112293285	NP_006708.2	10927	SPIN1	spindlin-1	S124	0.5	0.4	0.3	0.5	0.4	0.6	0.7	0.9	0.6	0.5	0.3	0.6	0.5	0.8	0.9	1.1	0.6	0.6	0.6	0.8	IsDAHLADTMIGK	RVATSRIsDAHLADT	S2;	Yes	S(2): 100.0; T(9): 0.0	2	1	1	0	N-Term(TMT6plex); S2(Phospho); M10(Oxidation); K13(TMT6plex)
331284176	NP_001070729.2	9612	NCOR2	nuclear receptor corepressor 2 isoform2	S1469	0.4	0.5	0.5	0.6	0.4	0.7	0.6	0.8	0.4	0.6	0.6	0.6	0.4	0.8	0.6	0.9	0.4	0.7	0.6	0.8	SLIGsPGR	DVRSLIGsPGRTFPP	S5;	No	S(1): 0.0; S(5): 100.0	1	3	1	0	N-Term(TMT6plex); S5(Phospho)
5453593	NP_006357.1	10486	CAP2	adenylyl cyclase-associated protein 2	S301	0.5	0.7	0.7	0.7	0.7	0.7	0.7	0.8	-	-	-	-	-	-	-	-	-	-	-	-	AQGGQTQsPTK	AQGGQTQsPTKSHTP	S8;	Yes	T(6): 0.0; S(8): 100.0; T(10): 0.0	1	1	1	0	N-Term(TMT6plex); S8(Phospho); K11(TMT6plex)
318067961	NP_001188292.1	9324	HMGN3	high mobility group nucleosome-binding domain-containing protein 3 isoform HMGN3d	T103	0.5	0.6	0.6	0.8	0.5	0.7	0.7	0.7	0.6	0.7	0.8	0.8	0.6	0.8	0.9	0.7	0.6	0.7	0.8	0.8	GtPPSTLSVK	NVSTSRGtPPSTLSV	T2;	No	T(2): 100.0; S(5): 0.0; T(6): 0.0; S(8): 0.0	13	1	1	0	N-Term(TMT6plex); T2(Phospho); K10(TMT6plex)
102467242	NP_064623.2	3619	INCENP	inner centromere protein isoform2	S275	0.7	0.7	0.7	0.7	0.5	0.6	0.6	0.7	0.7	0.6	0.7	0.8	0.5	0.6	0.6	0.7	0.6	0.6	0.6	0.8	DSPAEPDsPWR	DSPAEPDsPWRERVL	S8;	Yes	S(2): 0.0; S(8): 100.0	23	2	1	0	N-Term(TMT6plex); S8(Phospho)
168480080	NP_004321.2	663	BNIP2	BCL2/adenovirus E1B 19 kDa protein-interacting protein 2	S235	0.8	0.8	0.8	0.7	0.7	0.7	0.7	0.7	1.0	1.0	0.9	0.8	1.0	0.9	0.9	0.8	1.0	1.0	0.9	0.8	KGsITEYTAAEEK	TEVIRKGsITEYTAA	S3;	No	S(3): 100.0; T(5): 0.0; Y(7): 0.0; T(8): 0.0	1	1	1	1	N-Term(TMT6plex); K1(TMT6plex); S3(Phospho); K13(TMT6plex)
40217847	NP_054733.2	23020	SNRNP200	U5 small nuclear ribonucleoprotein 200 kDa helicase	S2135	0.7	0.9	0.9	0.7	1.1	0.8	0.8	0.7	0.7	0.9	0.8	0.7	1.1	0.8	0.8	0.7	0.9	0.9	0.8	0.7	EAETDSDsD	EAETDSDsD	S8;	Yes	T(4): 0.0; S(6): 0.0; S(8): 100.0	12	1	1	0	N-Term(TMT6plex); S8(Phospho)
118572613	NP_057417.3	23524	SRRM2	serine/arginine repetitive matrix protein 2	S1329	0.7	0.8	0.7	0.7	0.9	0.8	1.0	0.8	0.8	0.9	0.8	0.7	0.9	0.8	1.1	0.9	0.9	0.8	0.9	0.8	ENSEGsPLEER	LRENSEGsPLEERNS	S6;	Yes	S(3): 0.9; S(6): 99.1	436	1	1	0	N-Term(TMT6plex); S6(Phospho)
61743954	NP_001611.1	79026	AHNAK	neuroblast differentiation-associated protein AHNAK isoform1	S5031	0.4	0.7	0.7	0.6	0.6	0.8	0.9	0.8	0.4	0.7	0.7	0.7	1.0	0.8	0.9	1.0	0.6	0.8	0.8	0.8	IHMsGPK	KMPKIHMsGPKIKAK	S4;	Yes	S(4): 100.0	79	1	1	0	N-Term(TMT6plex); M3(Oxidation); S4(Phospho); K7(TMT6plex)

Khan et al., 2019. Multi-omics analysis to characterize cigarette smoke induced molecular alterations in esophageal cells
Supplementary Table 8. List of phosphopeptides identified and quantified in untreated and chronically treated Het1A cells with cigarette smoke condensate for 8 months using SequestHT and Mascot search algorithms with a PhosphoRS probability score ≥75%

														Normalized phosphoprotein ratios with respect to corresponding total protein ratios.								Average of replicates													
Protein group Accession	NP_Accession	Gene ID	Gene Symbol	Protein Description	PhosphoSite (Protein)	Het-1A-Smoke - 2M/Parental	Het-1A-Smoke - 4M/Parental	Het-1A-Smoke - 6M/Parental	Het-1A-Smoke - 8M/Parental	Het-1A-Smoke - 2M/Parental	Het-1A-Smoke - 4M/Parental	Het-1A-Smoke - 6M/Parental	Het-1A-Smoke - 8M/Parental	Het-1A-Smoke - 2M/Parental	Het-1A-Smoke - 4M/Parental	Het-1A-Smoke - 6M/Parental	Het-1A-Smoke - 8M/Parental	Het-1A-Smoke - 2M/Parental	Het-1A-Smoke - 4M/Parental	Het-1A-Smoke - 6M/Parental	Het-1A-Smoke - 8M/Parental	Het-1A-Smoke - 2M/Parental	Het-1A-Smoke - 4M/Parental	Het-1A-Smoke - 6M/Parental	Het-1A-Smoke - 8M/Parental	Peptide Sequence	Phosphowindow	PhosphoSite (Peptide)	Phosphosite Plus Evidence	phosphoRS Site Probabilities	# PSMs	# Proteins	# Protein Groups	# Missed Cleavages	Modifications
						Replicate 1				Replicate 2				Replicate 1				Replicate 2																	
23097240	NP_690852.1	222229	LRWD1	leucine-rich repeat and WD repeat-containing protein 1	S251	0.8	0.9	0.9	0.8	0.9	0.9	1.0	0.6	0.8	0.9	0.9	0.7	0.9	0.9	1.0	0.6	0.8	0.9	0.9	0.6	ACAsPSAQVEGSPVAGSDGSQPAVK	PSKRACAsPSAQVEG	S4;	Yes	S(4): 98.5; S(6): 1.5; S(12): 0.0; S(17): 0.0; S(20): 0.0	5	1	1	0	N-Term(TMT6plex); C2(Carbamidomethyl); S4(Phospho); K25(TMT6plex)
50513245	NP_005474.2	10036	CHAF1A	chromatin assembly factor 1 subunit A	S206	0.8	0.7	0.7	0.9	0.6	0.6	0.6	0.6	1.0	0.9	0.9	1.1	0.8	0.7	0.8	0.7	0.9	0.8	0.8	0.9	sCPELTSGPR	SQECSPRsCPELTSG	S1;	Yes	S(1): 100.0; T(6): 0.0; S(7): 0.0	22	1	1	0	N-Term(TMT6plex); S1(Phospho); C2(Carbamidomethyl)
55956788	NP_005372.2	4691	NCL	nucleolin	T84	0.5	0.5	0.5	0.7	0.4	0.5	0.5	0.7	0.6	0.6	0.7	0.9	0.4	0.6	0.7	1.0	0.5	0.6	0.7	1.0	AAVtPGK	PAKKAAVtPGKKAAA	T4;	Yes	T(4): 100.0	65	1	1	0	N-Term(TMT6plex); T4(Phospho); K7(TMT6plex)
197313748	NP_054878.5	29072	SETD2	histone-lysine N-methyltransferase SETD2	S754	0.8	1.2	1.0	0.7	1.0	1.0	0.9	0.7	1.0	1.4	1.1	0.9	1.1	1.1	1.0	0.8	1.0	1.2	1.0	0.9	ETEPLVsPHQDK	RETEPLVsPHQDKLM	S7;	Yes	T(2): 0.0; S(7): 100.0	12	1	1	0	N-Term(TMT6plex); S7(Phospho); K12(TMT6plex)
166795262	NP_694939.2	121536	AEBP2	zinc finger protein AEBP2 isoform a	S24	0.8	1.0	1.0	1.0	1.0	0.8	0.8	0.5	-	-	-	-	-	-	-	-	-	-	-	-	LSPLPPGsPGSAAR	LSPLPPGsPGSAARG	S8;	Yes	S(2): 0.0; S(8): 100.0; S(11): 0.0	7	2	1	0	N-Term(TMT6plex); S8(Phospho)
134304853	NP_004806.3	9411	ARHGAP29	rho GTPase-activating protein 29	S1029	0.9	1.0	1.0	1.0	1.0	1.1	1.5	0.4	1.1	1.1	1.1	1.2	1.2	1.2	1.6	0.6	1.2	1.2	1.3	0.9	LLLAsPPNER	VDRLLLAsPPNERNG	S5;	Yes	S(5): 100.0	2	1	1	0	N-Term(TMT6plex); S5(Phospho)
112382226	NP_001036147.1	473	RERE	arginine-glutamic acid dipeptide repeats protein	S46	0.8	0.7	1.1	0.7	0.9	0.5	1.0	0.7	-	-	-	-	-	-	-	-	-	-	-	-	TsPINEDIR	ASPDGRTsPINEDIR	S2;	No	T(1): 0.0; S(2): 100.0	9	2	1	0	N-Term(TMT6plex); S2(Phospho)
5174669	NP_006080.1	10389	SCML2	sex comb on midleg-like protein 2	S267	1.2	0.9	1.0	0.7	1.0	0.6	0.7	0.7	1.1	1.0	1.0	0.8	0.9	0.6	0.7	0.8	1.0	0.8	0.9	0.8	TESSPSEASQHSMQsPQK	ASQHSMQsPQKTTLI	S15;	Yes	T(1): 0.0; S(3): 0.0; S(4): 0.0; S(6): 0.0; S(9): 0.0; S(12): 1.1; S(15): 98.9	4	1	1	0	N-Term(TMT6plex); M13(Oxidation); S15(Phospho); K18(TMT6plex)
132626688	NP_055456.2	9656	MDC1	mediator of DNA damage checkpoint protein 1	T659	0.8	0.7	0.8	1.0	0.7	0.2	0.7	0.4	0.9	0.7	0.9	1.1	0.7	0.2	0.7	0.4	0.8	0.5	0.8	0.7	ENLTDLVVDTDTLGEStQPQR	TDTLGEStQPQREGA	T17;	Yes	T(4): 0.0; T(10): 0.0; T(12): 0.0; S(16): 1.6; T(17): 98.4	26	1	1	0	N-Term(TMT6plex); T17(Phospho)
61676106	NP_001012777.1	348180	CTU2	cytoplasmic tRNA 2-thiolation protein 2 isoform1	S515	1.3	0.8	1.3	0.5	1.5	1.5	1.2	0.9	1.4	0.9	1.4	0.5	1.6	1.6	1.3	0.9	1.5	1.2	1.4	0.7	DCLIEDSDDEAGQs	SDDEAGQs	S14;	Yes	S(7): 1.7; S(14): 98.3	1	1	1	0	N-Term(TMT6plex); C2(Carbamidomethyl); S14(Phospho)
372266146	NP_001243198.1	3835	KIF22	kinesin-like protein KIF22 isoform2	S344	0.8	0.7	0.6	0.7	0.8	0.8	0.7	0.7	0.8	0.7	0.7	0.8	0.9	0.9	0.9	0.8	0.9	0.8	0.8	0.8	GPEEEEIGsPEPMAAPASASQK	PEEEEIGsPEPMAAP	S9;	No	S(9): 100.0; S(18): 0.0; S(20): 0.0	9	2	1	0	N-Term(TMT6plex); S9(Phospho); M13(Oxidation); K22(TMT6plex)
118572613	NP_057417.3	23524	SRRM2	serine/arginine repetitive matrix protein 2	S974	0.7	0.8	0.7	0.6	1.0	0.9	0.6	0.8	0.8	0.8	0.8	0.7	1.1	0.9	0.7	0.8	0.9	0.9	0.7	0.8	YSHSGSSsPDTK	YSHSGSSsPDTKVKP	S8;	Yes	T(1): 0.0; S(2): 0.0; S(4): 0.0; S(6): 50.0; S(7): 50.0; S(8): 100.0; T(11):	436	1	1	0	N-Term(TMT6plex); S7(Phospho); S8(Phospho); K12(TMT6plex)
118572613	NP_057417.3	23524	SRRM2	serine/arginine repetitive matrix protein 2	S974	1.0	0.9	0.6	0.8	0.7	0.8	0.7	0.6	1.1	0.9	0.7	0.8	0.8	0.8	0.8	0.7	0.9	0.9	0.7	0.8	YSHsGSssPDTK	PRYSHSGsSSPDTKV;RYSHSGSsSPDTKVK	S8;	Yes;Yes	Y(1): 0.0; S(2): 0.0; S(4): 0.0; S(6): 99.0; S(7): 99.0; S(8): 1.9; T(11): 0.0	5	1	1	0	N-Term(TMT6plex); S6(Phospho); S8(Phospho); K12(TMT6plex)
56118310	NP_073568.2	64710	NUCKS1	nuclear ubiquitous casein and cyclin-dependent kinase substrate 1	S234	1.4	1.4	1.1	0.9	1.2	1.7	1.2	0.5	1.8	1.7	1.4	1.2	1.5	2.0	1.7	0.6	1.6	1.8	1.5	0.9	SGDEGsEDEAPSGED	EKSGDEGsEDEAPSG	S6;	Yes	S(1): 0.0; S(6): 100.0; S(12): 0.0	109	1	1	0	N-Term(TMT6plex); S6(Phospho)
55956788	NP_005372.2	4691	NCL	nucleolin	T76	0.4	0.5	0.5	0.8	0.4	0.6	0.6	0.6	0.5	0.6	0.7	1.0	0.5	0.7	0.9	0.9	0.5	0.6	0.8	1.0	VAVAtPAK	TKKVAVAtPAKKAAV	T5;	Yes	T(5): 100.0	65	1	1	0	N-Term(TMT6plex); T5(Phospho); K8(TMT6plex)
42794622	NP_976308.1	147808	ZNF784	zinc finger protein 784	S12	0.9	0.9	0.7	0.9	0.5	0.5	0.5	0.5	-	-	-	-	-	-	-	-	-	-	-	-	sSPTPESR	RPEAQSRsSPTPESR	S1;	No	S(1): 99.0; S(2): 1.0; T(4): 0.0; S(7):	2	1	1	0	N-Term(TMT6plex); S2(Phospho)
103472005	NP_002408.3	4288	MKI67	antigen KI-67 isoform1	S2466	0.7	0.8	1.0	0.7	0.5	0.7	0.7	0.7	0.8	0.8	1.0	0.7	0.6	0.8	0.7	0.8	0.7	0.8	0.9	0.7	sPQPESK	ITEVSCKsPQPESK	S1;	Yes	S(1): 100.0; S(6): 0.0	118	2	1	0	N-Term(TMT6plex); S1(Phospho); K8(TMT6plex)
253970412	NP_057510.4	51512	GTSE1	G2 and S phase-expressed protein 1	S262	0.5	0.9	0.8	0.7	0.7	1.0	0.9	0.7	0.5	1.0	0.9	0.8	0.7	1.1	1.0	0.9	0.6	1.0	1.0	0.8	KEIPAsPSR	PKKEIPAsPSRTKIP	S6;	No	S(6): 99.2; S(8): 0.8	19	1	1	1	N-Term(TMT6plex); K1(TMT6plex); S6(Phospho)
62899035	NP_001017420.1	157570	ESCO2	N-acetyltransferase ESCO2	S75	0.7	1.0	0.7	0.9	0.9	0.8	0.5	0.5	0.7	0.9	0.9	0.9	0.9	0.8	0.6	0.5	0.8	0.9	0.7	0.7	LPSANQGsPFK	LPSANQGsPFKSALS	S8;	Yes	S(3): 0.0; S(8): 100.0	7	1	1	0	N-Term(TMT6plex); S8(Phospho); K11(TMT6plex)
355390315	NP_056171.3	23513	SCRIB	protein scribble homolog isoformb	S1140	0.9	0.9	0.9	0.8	0.6	0.7	0.6	0.6	0.8	0.8	0.9	0.7	0.5	0.6	0.6	0.5	0.7	0.7	0.7	0.6	VsPTGAAGR	GIFISKVsPTGAAGR	S2;	Yes	S(2): 100.0; T(4): 0.0	16	2	1	0	N-Term(TMT6plex); T4(Phospho)
50980301	NP_958431.2	23164	MPRIP	myosin phosphatase Rho-interacting protein isoform2	S362	0.9	0.4	1.3	1.0	1.3	1.1	1.2	0.4	0.8	0.4	1.2	1.0	1.2	1.1	1.2	0.4	1.0	0.7	1.2	0.7	DFTNEAPPAPLPDASAsPLSPHR	PLPDASAsPLSPHRR	S17;	Yes	T(3): 0.0; S(15): 1.4; S(17): 98.6; S(20): 0.0	7	2	1	0	N-Term(TMT6plex); S17(Phospho)
68508961	NP_001803.2	1060	CENPC	centromere protein C	T734	0.7	0.4	0.3	0.5	0.5	0.6	0.6	0.9	0.7	0.4	0.3	0.5	0.5	0.7	0.6	0.9	0.6	0.5	0.4	0.7	LVLPSNtPNVR	KLVLPSNtPNVRRTK	T7;	No	S(5): 0.0; T(7): 100.0	13	1	1	0	N-Term(TMT6plex); T7(Phospho)
51173873	NP_001003681.1	10042	HMGXB4	HMG domain-containing protein 4	S497	1.0	0.9	0.7	0.7	0.9	0.9	0.8	0.7	1.0	0.7	0.7	0.5	0.8	0.7	0.8	0.6	0.9	0.7	0.7	0.6	ASSVGVLsPQK	ASSVGVLsPQKKSPP	S8;	Yes	S(2): 0.0; S(3): 0.0; S(8): 100.0	5	1	1	0	N-Term(TMT6plex); S8(Phospho); K11(TMT6plex)
47519639	NP_002366.2	4134	MAP4	microtubule-associated protein 4 isoform1	S1073	0.9	0.9	0.6	0.7	0.7	0.7	0.6	0.7	0.9	0.9	0.6	0.8	0.7	0.6	0.7	0.9	0.8	0.8	0.6	0.9	VGsLDNVGHLPAGGAVK	KAQAKVGsLDNVGHL	S3;	Yes	S(3): 100.0	47	2	1	0	N-Term(TMT6plex); S3(Phospho); K17(TMT6plex)
22748659	NP_689505.1	93587	TRMT10A	tRNA methyltransferase 10 homolog A	S318	0.8	0.8	0.7	0.7	0.7	0.8	0.9	0.7	1.0	1.0	0.9	0.8	0.9	1.0	1.1	0.9	1.0	1.0	1.0	0.9	NELDsPHEEK	YSRNELDsPHEEKQD	S5;	Yes	S(5): 100.0	2	1	1	0	N-Term(TMT6plex); S5(Phospho); K10(TMT6plex)
79750944	NP_115548.1	84132	USP42	ubiquitin carboxyl-terminal hydrolase 42	S856	0.7	0.6	0.7	0.6	0.6	0.6	0.7	0.8	0.9	0.7	1.0	0.6	0.8	0.8	0.9	0.9	0.8	0.7	1.0	0.8	DSALAEAPEGLsPAPPAR	AEAPEGLsPAPPARS	S12;	Yes	S(2): 0.0; S(12): 100.0	4	1	1	0	N-Term(TMT6plex); S12(Phospho)
4885245	NP_005244.1	2355	FOSL2	fos-related antigen 2	S200	0.7	0.9	0.9	0.5	0.8	0.9	0.9	0.9	0.7	0.7	0.7	0.5	0.7	0.7	0.7	0.8	0.7	0.7	0.7	0.6	RsPPAPGLQPMR	ISPEERRsPPAPGLQ	S2;	Yes	S(2): 100.0	2	1	1	1	N-Term(TMT6plex); S2(Phospho); M11(Oxidation)
118572613	NP_057417.3	23524	SRRM2	serine/arginine repetitive matrix protein 2	S876	0.5	0.7	0.7	0.6	0.6	0.8	0.7	0.8	0.6	0.7	0.8	0.7	0.7	0.8	0.8	0.9	0.6	0.7	0.8	0.8	SCFESsPDPELK	RRSCFESsPDPELKS	S6;	Yes	S(1): 0.0; S(5): 0.0; S(6): 100.0	436	1	1	0	N-Term(TMT6plex); C2(Carbamidomethyl); S6(Phospho); K12(TMT6plex)
295054220	NP_001171136.1	55183	RIF1	telomere-associated protein RIF1 isoform2	S2243	1.1	0.8	0.8	0.7	1.3	0.9	0.8	0.7	1.1	0.9	0.9	0.8	1.4	0.9	0.9	0.8	1.2	0.9	0.9	0.8	TsPTTQSK	IGKSVKTsPTTQSKI	S2;	No	T(1): 0.0; S(2): 100.0; T(4): 0.0; T(5): 0.0; S(7): 0.0	15	2	1	0	N-Term(TMT6plex); S2(Phospho); K8(TMT6plex)
110556636	NP_203754.2	85456	TNKS1BP1	182 kDa tankyrase-1-binding protein	S672	1.3	1.4	1.5	1.0	0.9	1.0	1.0	0.4	1.2	1.3	1.4	1.1	0.9	0.9	0.9	0.5	1.0	1.1	1.1	0.8	TEAQDLCRAsPEPPGPESSSR	AQDLCRAsPEPPGPE	S10;	Yes	T(1): 0.0; S(10): 100.0; S(18): 0.0; S(19): 0.0; S(20): 0.0	41	1	1	1	N-Term(TMT6plex); C7(Carbamidomethyl); S10(Phospho)
42560244	NP_004783.2	9360	PPIG	peptidyl-prolyl cis-trans isomerase G	S415	1.0	0.8	0.9	0.6	1.0	0.8	0.9	0.8	1.4	1.0	1.1	0.8	1.3	1.0	1.2	1.0	1.4	1.0	1.1	0.9	NVSEsPNR	DHRNVSEsPNRKNEK	S5;	Yes	S(3): 0.0; S(5): 100.0	4	1	1	0	N-Term(TMT6plex); S5(Phospho)
385648259	NP_001245305.1	58513	EPS15L1	epidermal growth factor receptor substrate 15-like 1 isoform4	S255	1.6	1.2	1.4	0.8	1.7	0.6	0.6	0.6	1.4	1.4	1.5	1.1	1.6	0.7	0.6	0.7	1.5	1.0	1.0	0.9	STPSHGSVSSLNSTGSLsPK	LNSTGSLsPKHSLKQ	S18;	Yes	S(1): 0.0; T(2): 0.0; S(4): 0.0; S(7): 0.0; S(9): 0.0; S(10): 0.0; S(13): 0.0; T(14): 0.1; S(16): 2.4; S(18): 97.6	6	4	1	0	N-Term(TMT6plex); S18(Phospho); K20(TMT6plex)
7662046	NP_055542.1	9757	KMT2B	histone-lysine N-methyltransferase 2B	S821	0.4	0.4	0.5	0.5	1.0	1.1	1.2	0.9	0.4	0.4	0.6	0.5	1.0	1.2	1.3	1.0	0.7	0.8	0.9	0.7	VAASMPLsPGGQMEEVAGAVK	VAASMPLsPGGQMEE	S8;	Yes	S(4): 0.0; S(8): 100.0	2	1	1	0	N-Term(TMT6plex); M5(Oxidation); S8(Phospho); M13(Oxidation); K21(TMT6plex)
7657269	NP_055847.1	23047	PDS5B	sister chromatid cohesion protein PDS5 homolog B	S1259	0.6	0.7	0.6	0.4	1.5	1.5	1.2	1.0	0.6	0.6	0.5	0.4	1.4	1.4	1.1	0.9	1.0	1.0	0.8	0.7	GHTASEsDEQQWPEEK	RGHTASEsDEQQWPE	S7;	Yes	T(3): 0.0; S(5): 1.4; S(7): 98.5	34	1	1	0	N-Term(TMT6plex); S7(Phospho); K16(TMT6plex)
7657269	NP_055847.1	23047	PDS5B	sister chromatid cohesion protein PDS5 homolog B	S1259	1.5	1.5	1.2	1.0	0.6	0.7	0.6	0.4	1.4	1.4	1.1	0.9	0.6	0.6	0.5	0.4	1.0	1.0	0.8	0.7	GHTAsESDEQQWPEEK	RKRGHTAsESDEQQW	S7;	Yes	T(3): 0.9; S(5): 99.1; S(7): 0.0	2	1	1	0	N-Term(TMT6plex); S5(Phospho); K16(TMT6plex)
56118310	NP_073568.2	64710	NUCKS1	nuclear ubiquitous casein and cyclin-dependent kinase substrate 1	S58; S61	0.9	0.6	0.8	0.6	1.0	0.6	0.8	0.7	1.1	0.8	1.0	0.8	1.2	0.8	1.1	1.0	1.2	0.8	1.1	0.9	NSQEDsEDSEDKDVK	GKNSQEDsEDSEDKD	S6; S9;	Yes	S(2): 0.0; S(6): 98.9; S(9): 1.1	3	1	1	1	N-Term(TMT6plex); S6(Phospho); K12(TMT6plex); K15(TMT6plex)
56118310	NP_073568.2	64710	NUCKS1	nuclear ubiquitous casein and cyclin-dependent kinase substrate 1	S214	0.8	0.9	0.7	0.7	0.6	0.7	0.7	0.7	1.0	1.1	1.0	0.9	0.8	0.8	1.0	0.8	0.9	0.9	1.0	0.9	EEDEEPEsPPEKK	EEDEEPEsPPEKKTS	S8;	Yes	S(8): 100.0	109	1	1	1	N-Term(TMT6plex); S8(Phospho); K12(TMT6plex); K13(TMT6plex)
56118310	NP_073568.2	64710	NUCKS1	nuclear ubiquitous casein and cyclin-dependent kinase substrate 1	S58; S61	1.0	0.6	0.8	0.7	0.9	0.6	0.8	0.6	1.2	0.8	1.1	1.0	1.1	0.8	1.0	0.8	1.2	0.8	1.1	0.9	NSQEDsEDsEDKDVK	GKNSQEDsEDSEDKD;SQEDSEDsEDKDVKT	S6; S9;	Yes;Yes	S(2): 0.0; S(6): 100.0; S(9): 100.0	109	1	1	1	N-Term(TMT6plex); S6(Phospho); S9(Phospho); K12(TMT6plex); K15(TMT6plex)
345199283	NP_001230829.1	3069	HDLBP	vigilin isoformb	S911	0.5	0.6	1.0	0.8	0.5	0.6	0.8	0.6	0.5	0.7	1.1	0.9	0.5	0.6	0.9	0.7	0.5	0.7	1.0	0.8	DCDPGsPR	AKDCDPGsPRRCDII	S6;	No	S(6): 100.0	10	2	1	0	N-Term(TMT6plex); C2(Carbamidomethyl); S6(Phospho)
255652953	NP_653205.3	124245	ZC3H18	zinc finger CCCH domain-containing protein 18	S67	0.7	1.0	0.9	0.7	0.7	0.8	0.7	0.7	0.8	1.1	1.0	0.7	0.9	0.8	0.9	0.8	0.9	1.0	0.9	0.7	GPSQEEEDNHsDEEDR	QEEEDNHsDEEDRAS	S11;	Yes	S(3): 0.0; S(11): 100.0	34	1	1	0	N-Term(TMT6plex); S11(Phospho)
55956788	NP_005372.2	4691	NCL	nucleolin	T105	0.3	0.4	0.4	0.7	0.3	0.5	0.4	0.7	0.4	0.5	0.6	0.9	0.4	0.6	0.6	1.0	0.4	0.6	0.6	0.9	AVtPGK	VTPAKAVtPGKKGA	T3;	Yes	T(3): 99.4; T(4): 0.6	65	1	1	0	N-Term(TMT6plex); T3(Phospho); K7(TMT6plex)
556503401	NP_001273294.1	55700	MAP7D1	MAP7 domain-containing protein 1 isoform2	S423	0.8	0.8	0.7	0.7	0.7	0.8	0.8	0.7	0.8	0.6	0.7	0.6	0.7	0.7	0.7	0.7	0.7	0.7	0.7	0.7	LSASTASELsPK	ASTASELsPKSKARP	S10;	No	S(2): 0.0; S(4): 0.0; T(5): 0.0; S(7): 0.0; S(10): 100.0	27	3	1	0	N-Term(TMT6plex); S10(Phospho); K12(TMT6plex)
556503440	NP_001273295.1	55700	MAP7D1	MAP7 domain-containing protein 1 isoform3	S423	0.7	0.8	0.8	0.7	0.8	0.8	0.7	0.7	0.7	0.7	0.7	0.7	0.8	0.6	0.7	0.6	0.7	0.7	0.7	0.7	LsASTASELsPK	ASTASELsPKSKARP	S10;	No	S(2): 0.0; S(4): 0.0; T(5): 0.0; S(7): 0.0; S(10): 100.0	9	3	1	0	N-Term(TMT6plex); S10(Phospho); K12(TMT6plex)
116256458	NP_689531.2	121053	C12orf45	uncharacterized protein C12orf45	S178	0.6	0.5	0.5	0.7	0.6	0.2	0.5	0.7	-	-	-	-	-	-	-	-	-	-	-	-	IEVLDsPASK	GKIEVLDsPASKKKK	S6;	Yes	S(6): 100.0; S(9): 0.0	3	1	1	0	N-Term(TMT6plex); S6(Phospho); K10(TMT6plex)
225690497	NP_001139492.1	93643	TJAP1	tight junction-associated protein 1 isoformb	S535	0.7	1.0	1.0	0.8	0.5	0.7	0.5	0.6	1.0	1.3	1.6	0.9	0.8	0.9	0.8	0.6	0.9	1.1	1.2	0.8	KDsLTQAQEQGNLLN	HHLHRKDsLTQAQEQ	S3;	No	S(3): 100.0; T(5): 0.0	4	2	1	1	N-Term(TMT6plex); K1(TMT6plex); S3(Phospho)
103472005	NP_002408.3	4288	MKI67	antigen KI-67 isoform1	T2268	0.3	0.3	0.6	0.7	0.5	0.7	1.0	0.7	0.4	0.3	0.6	0.7	0.6	0.7	1.0	0.7	0.5	0.5	0.8	0.7	AMDtPKPAGGDEK	SVGKAMDtPKPAGGD	T4;	Yes	T(4): 100.0	118	2	1	0	N-Term(TMT6plex); M2(Oxidation); T4(Phospho); K6(TMT6plex); K13(TMT6plex)
32306539	NP_057584.2	51260	PBDC1	protein PBDC1	S197	1.0	0.8	1.1	0.7	1.0	0.8	0.9	0.6	1.1	1.2	1.3	1.2	1.0	1.0	1.1	1.0	1.0	1.1	1.2	1.1	GADsGEEKEEGINR	GGEKGADsGEEKEEG	S4;	No	S(4): 100.0	4	1	1	1	N-Term(TMT6plex); S4(Phospho); K8(TMT6plex)
18087845	NP_542399.1	113130	CDCA5	sororin	T159	0.9	1.0	0.9	1.0	0.2	0.7	0.7	0.4	0.8	1.1	1.0	1.2	0.2	0.8	0.8	0.4	0.5	0.9	0.9	0.8	LETLGSASTStPGR	LGSASTStPGRRSCF	T11;	Yes	T(3): 0.0; S(6): 0.0; S(8): 0.0; T(9): 0.0; S(10): 1.2; T(11): 98.8	6	1	1	0	N-Term(TMT6plex); T11(Phospho)
556695389	NP_001273359.1	123169	LEO1	RNA polymerase-associated protein LEO1 isoform2	S171; S179	1.1	0.8	1.0	0.6	0.9	1.0	1.0	0.8	1.3	0.9	1.3	0.8	1.1	1.1	1.2	1.0	1.2	1.0	1.2	0.9	AQGsDEDKLQNsDDDEK	DEERAQGsDEDKLQN;DEDKLQNsDDDEKMQ	S4; S12;	Yes;Yes	S(4): 100.0; S(12): 100.0	23	2	1	1	N-Term(TMT6plex); S4(Phospho); K8(TMT6plex); S12(Phospho); K17(TMT6plex)
507834119	NP_061164.3	51329	ARL6IP4	ADP-ribosylation factor-like protein 6-interacting protein 4 isoform1	S332	0.3	0.5	0.7	0.7	0.9	0.7	0.7	0.6	0.4	0.6	0.9	0.9	1.2	0.9	0.9	0.8	0.8	0.8	0.9	0.9	sAGEEEDGPVLTDEQK	SLDQWHRsAGEEEDG	S1;	No	S(1): 100.0; T(12): 0.0	5	6	1	0	N-Term(TMT6plex); S1(Phospho); K16(TMT6plex)
282847485	NP_001164228.1	159090	FAM122B	protein FAM122B isoform5	S85	0.8	1.0	0.9	0.7	1.0	0.8	0.7	0.7	-	-	-	-	-	-	-	-	-	-	-	-	IDFTPVSPAPsPTR	TPVSPAPsPTRGFGK	S11;	No	T(4): 50.0; S(7): 50.0; S(11): 97.5; T(13): 2.5	3	5	1	0	N-Term(TMT6plex); S7(Phospho); S11(Phospho)
282847485	NP_001164228.1	159090	FAM122B	protein FAM122B isoform5	S85	1.0	0.8	0.7	0.7	0.8	1.0	0.9	0.7	-	-	-	-	-	-	-	-	-	-	-	-	IDFTPVsPAPsPTR	RIDFTPVsPAPSPTR;TPVSPAPsPTRGFGK	S11;	No;No	T(4): 2.1; S(7): 97.9; S(11): 100.0; T(13): 0.0	1	5	1	0	N-Term(TMT6plex); S7(Phospho); S11(Phospho)
291575190	NP_001167566.1	6935	ZEB1	zinc finger E-box-binding homeobox 1 isoforme	S579	0.9	0.9	1.3	0.8	1.2	1.3	1.1	0.6	1.1	1.0	1.5	1.0	1.3	1.4	1.2	0.7	1.2	1.2	1.4	0.8	MQAGQISVQSSEPSsPEPGK	VQSSEPSsPEPGKVN	S15;	No	S(7): 0.0; S(10): 0.0; S(11): 0.0; S(14): 0.0; S(15): 99.9	5	6	1	0	N-Term(TMT6plex); M1(Oxidation); S15(Phospho); K20(TMT6plex)
122937229	NP_001073886.1	23361	ZNF629	zinc finger protein 629	S52	0.8	1.1	0.8	0.6	0.6	0.7	0.7	0.8	0.8	1.0	0.8	0.6	0.6	0.7	0.6	0.7	0.7	0.8	0.7	0.7	QESSGEEIIMGDPAQsPESK	IMGDPAQsPESKDST	S16;	No	S(3): 0.0; S(4): 0.0; S(16): 98.9; S(19): 1.1	4	1	1	0	N-Term(TMT6plex); M10(Oxidation); S16(Phospho); K20(TMT6plex)
315139026	NP_079418.1	57551	TAOK1	serine/threonine-protein kinase TAO1 isoform2	S817	0.8	0.8	0.9	0.6	0.8	0.7	0.7	0.7	0.5	0.7	0.9	0.6	0.6	0.7	0.8	0.7	0.5	0.7	0.8	0.7	NsPQALR	SSMGVRNsPQALRRT	S2;	No	S(2): 100.0	6	2	1	0	N-Term(TMT6plex); S2(Phospho)
194272142	NP_001123537.1	4650	MYO9B	unconventional myosin-IXb isoform2	T1271	1.0	0.8	1.0	0.7	1.3	1.0	1.0	0.7	1.1	0.9	1.2	0.7	1.4	1.0	1.1	0.8	1.3	0.9	1.1	0.7	VSPPAPGsAPEtPEDK	APGSAPEtPEDKSKP	T12;	Yes	S(2): 50.0; S(8): 50.0; T(12): 100.0	14	2	1	0	N-Term(TMT6plex); S8(Phospho); T12(Phospho); K16(TMT6plex)
194272142	NP_001123537.1	4650	MYO9B	unconventional myosin-IXb isoform2	T1271	1.3	1.0	1.0	0.7	1.0	0.8	1.0	0.7	1.4	1.0	1.1	0.8	1.1	0.9	1.2	0.7	1.3	0.9	1.1	0.7	VSPPAPGsAPEtPEDK	VSPPAPGsAPETPED;APGSAPEtPEDKSKP	T12;	Yes;Yes	S(2): 0.0; S(8): 100.0; T(12): 100.0	2	2	1	0	N-Term(TMT6plex); S8(Phospho); T12(Phospho); K16(TMT6plex)
291190783	NP_001167448.1	55604	LRRC16A	leucine-rich repeat-containing protein 16A isoform2	S1151	0.9	0.7	0.7	0.7	0.8	0.7	0.7	0.7	0.9	0.7	0.8	0.7	0.9	0.7	0.8	0.8	0.9	0.7	0.8	0.8	SsPQAGR	ERSDSKSsPQAGRRY	S2;	No	S(1): 0.0; S(2): 100.0	13	2	1	0	N-Term(TMT6plex); S2(Phospho)
9966805	NP_065147.1	57062	DDX24	ATP-dependent RNA helicase DDX24	S82; S94	0.5	0.4	0.6	0.6	1.0	0.7	0.7	0.8	0.7	0.5	0.8	0.7	1.2	0.8	0.9	1.0	0.9	0.7	0.8	0.8	AQAVsEEEEEEEGKSSSPK	KRKAQAVsEEEEEEE	S5; S17;	Yes	S(5): 100.0; S(15): 33.3; S(16): 33.3; S(17): 33.3	1	1	1	1	N-Term(TMT6plex); S5(Phospho); K14(TMT6plex); S15(Phospho); K19(TMT6plex)
255652953	NP_653205.3	124245	ZC3H18	zinc finger CCCH domain-containing protein 18	S67; S74	0.6	0.8	0.7	0.6	0.9	1.1	0.9	0.8	0.7	0.9	0.9	0.7	1.1	1.1	1.1	0.8	0.9	1.0	1.0	0.7	GPSQEEEDNHsDEEDRAsEPK	QEEEDNHsDEEDRAS;SDEEDRAsEPKSQDQ	S11; S18;	Yes;Yes	S(3): 0.0; S(11): 100.0; S(18): 100.0	34	1	1	1	N-Term(TMT6plex); S11(Phospho); S18(Phospho); K21(TMT6plex)
9966805	NP_065147.1	57062	DDX24	ATP-dependent RNA helicase DDX24	S82; S94	1.0	0.7	0.7	0.8	0.5	0.4	0.6	0.6	1.2	0.8	0.9	1.0	0.7	0.5	0.8	0.7	0.9	0.7	0.8	0.8	AQAVsEEEEEEEGKSSsPK	KRKAQAVsEEEEEEE;EEEGKSSsPKKKIKL	S5; S17;	Yes;Yes	S(5): 100.0; S(15): 1.2; S(16): 1.2; S(17): 97.6	10	1	1	1	N-Term(TMT6plex); S5(Phospho); K14(TMT6plex); S17(Phospho); K19(TMT6plex)
32306539	NP_057584.2	51260	PBDC1	protein PBDC1	S181	1.2	0.9	1.2	0.7	1.2	0.9	1.2	0.7	1.3	1.3	1.5	1.1	1.2	1.3	1.4	1.1	1.3	1.3	1.4	1.1	ADsGEEENTK	GGEKRADsGEEENTK	S3;	No	S(3): 100.0; T(9): 0.0	4	1	1	0	N-Term(TMT6plex); S3(Phospho); K10(TMT6plex)
7242167	NP_006185.1	5083	PAX9	paired box protein Pax-9	T175	1.1	1.6	1.0	0.9	0.4	1.0	0.4	0.5	1.5	2.5	1.5	1.2	0.6	1.5	0.5	0.6	1.1	2.0	1.0	0.9	VPtPPGVPAIPGSVAMPR	AAAAKVPtPPGVPAI	T3;	No	T(3): 100.0; S(13): 0.0	1	1	1	0	N-Term(TMT6plex); T3(Phospho); M16(Oxidation)
300797215	NP_001180210.1	4281	MID1	E3 ubiquitin-protein ligase Midline-1 isoform6	S92; S96	0.8	0.9	0.7	0.2	1.2	1.0	1.4	1.1	0.7	0.9	0.6	0.3	1.1	1.0	1.2	1.1	0.9	0.9	0.9	0.7	ASVSGPNsPSETR	RFQKASVsGPNSPSE;ASVSGPNsPSETRRE	S4; S8;	Yes;Yes	S(2): 0.1; S(4): 99.9; S(8): 99.9; S(10): 0.1; T(12): 0.0	2	6	1	0	N-Term(TMT6plex); S4(Phospho); S8(Phospho)
121114302	NP_001073341.1	8450	CUL4B	cullin-4B isoform2	T31; S35	0.4	0.7	0.8	0.5	0.8	0.9	0.8	0.8	0.4	0.7	0.7	0.6	0.8	0.9	0.8	0.9	0.6	0.8	0.7	0.7	SATDGNTSTtPPTSAK	TDGNTSTtPPTSAKK;TSTTPPTsAKKRKLN	T10; S14;	Yes;Yes	S(1): 0.0; T(3): 0.0; T(7): 0.0; S(8): 0.0; T(9): 0.0; T(10): 100.0; T(13): 1.3; S(14): 98.7	8	2	1	0	N-Term(TMT6plex); T10(Phospho); T13(Phospho); K16(TMT6plex)
118572613	NP_057417.3	23524	SRRM2	serine/arginine repetitive matrix protein 2	S924	0.8	1.1	1.0	0.7	0.7	0.7	0.5	0.7	0.8	1.2	1.0	0.7	0.8	0.7	0.6	0.8	0.8	0.9	0.8	0.8	AIIsPR	PKVKAIIsPRQRSHS	S4;	Yes	S(4): 100.0	436	1	1	0	N-Term(TMT6plex); S4(Phospho)
121114302	NP_001073341.1	8450	CUL4B	cullin-4B isoform2	T31; S35	0.8	0.9	0.8	0.8	0.4	0.7	0.8	0.5	0.8	0.9	0.8	0.9	0.4	0.7	0.7	0.6	0.6	0.8	0.7	0.7	SATDGNTSTTPPTsAK	TDGNTSTtPPTSAKK	T10; S14;	Yes	S(1): 0.0; T(3): 0.0; T(7): 0.0; S(8): 0.0; T(9): 1.0; T(10): 99.0; T(13): 0.0; S(14): 0.0	4	2	1	0	N-Term(TMT6plex); T10(Phospho); K16(TMT6plex)
223633935	NP_694988.3	254427	PROSER2	proline and serine-rich protein 2	S179	0.3	0.4	0.4	0.6	0.7	0.7	0.7	0.7	0.4	0.5	0.5	0.7	0.9	0.9	0.7	0.8	0.6	0.7	0.6	0.7	APsPPVEHPR	RRELRAPsPPVEHPR	S3;	Yes	S(3): 100.0	4	1	1	0	N-Term(TMT6plex); S3(Phospho)
33356174	NP_002678.2	5411	PNN	pinin	S100	0.9	0.5	0.9	0.6	1.0	0.7	1.0	0.8	1.0	0.6	1.1	0.7	1.2	0.8	1.2	0.9	1.1	0.7	1.1	0.8	QEsDPEDDDVK	RRESRQEsDPEDDDV	S3;	Yes	S(3): 100.0	7	1	1	0	N-Term(TMT6plex); S3(Phospho); K11(TMT6plex)
148229134	NP_057710.3	51320	MEX3C	RNA-binding E3 ubiquitin-protein ligase MEX3C	S88	0.8	0.8	0.6	0.9	0.5	0.6	0.6	0.5	0.8	0.7	0.6	0.8	0.5	0.6	0.6	0.4	0.6	0.6	0.6	0.6	AAELsPEER	ARRAAELsPEERAPP	S5;	Yes	S(5): 100.0	2	1	1	0	N-Term(TMT6plex); S5(Phospho)
164519146	NP_060130.3	54815	GATAD2A	transcriptional repressor p66-alpha	S100; S107	0.7	0.9	1.0	0.9	1.1	0.4	0.9	0.5	0.7	0.9	1.1	0.8	1.2	0.4	0.9	0.4	0.9	0.7	1.0	0.6	RPPsPDVIVLsDNEQPSSPR	KSERRPPsPDVIVLS;SPDVIVLsDNEQPSS	S4; S11;	Yes;Yes	S(4): 100.0; S(11): 100.0; S(17): 50.0; S(18): 50.0	5	1	1	0	N-Term(TMT6plex); S4(Phospho); S11(Phospho); S17(Phospho)
126723060	NP_055983.1	23211	ZC3H4	zinc finger CCCH domain-containing protein 4	S1269	1.1	0.7	1.0	0.6	0.8	0.7	0.8	0.7	1.3	0.8	1.1	0.7	1.0	0.8	0.9	0.8	1.2	0.8	1.0	0.8	TGSGsPFAGNSPAR	TPKTGSGsPFAGNSP	S5;	Yes	T(1): 0.0; S(3): 0.0; S(5): 100.0; S(11): 0.0	3	1	1	0	N-Term(TMT6plex); S5(Phospho)
42560244	NP_004783.2	9360	PPIG	peptidyl-prolyl cis-trans isomerase G	S687	0.9	1.1	0.8	0.8	0.7	0.9	0.7	0.6	1.1	1.4	1.0	1.0	0.9	1.2	0.9	0.8	1.0	1.3	0.9	0.9	ADRDQsPFSK	KKADRDQsPFSKIKQ	S6;	Yes	S(6): 100.0; S(9): 0.0	4	1	1	1	N-Term(TMT6plex); S6(Phospho); K10(TMT6plex)
87162455	NP_056044.3	23277	CLUH	clustered mitochondria protein homolog	S664	0.3	0.4	0.6	0.5	0.9	0.8	0.7	0.9	0.3	0.4	0.5	0.5	0.9	0.8	0.6	0.9	0.6	0.6	0.6	0.7	SEDPPGQEAGsEEEGSSASGLAK	PPGQEAGsEEEGSSA	S11;	Yes	S(1): 0.0; S(11): 100.0; S(16): 0.0; S(17): 0.0; S(19): 0.0	2	1	1	0	N-Term(TMT6plex); S11(Phospho); K23(TMT6plex)
304434531	NP_001182122.1	4678	NASP	nuclear autoantigenic sperm protein isoform4	S357	1.1	1.1	0.5	0.7	0.8	0.9	0.4	0.6	1.4	1.4	0.8	1.1	1.1	1.2	0.6	1.0	1.2	1.3	0.7	1.0	LVPsQEETK	VRAKLVPsQEETKLS	S4;	No	S(4): 100.0; T(8): 0.0	20	2	1	0	N-Term(TMT6plex); S4(Phospho); K9(TMT6plex)
103472005	NP_002408.3	4288	MKI67	antigen KI-67 isoform1	S308	0.7	0.8	1.0	0.6	0.8	1.1	1.4	0.8	0.8	0.8	1.0	0.6	1.0	1.1	1.4	0.8	0.9	1.0	1.2	0.7	SGGSGHAVAEPAsPEQELDQNK	HAVAEPAsPEQELDQ	S13;	Yes	S(1): 0.0; S(4): 0.0; S(13): 100.0	118	1	1	0	N-Term(TMT6plex); S13(Phospho); K22(TMT6plex)
21955172	NP_665696.1	90102	PHLDB2	pleckstrin homology-like domain family B member 2 isoformc	S334	0.9	0.9	0.7	0.8	1.3	1.0	1.3	0.5	0.9	0.9	0.7	0.9	1.4	1.1	1.4	0.6	1.1	1.0	1.1	0.8	TSASEGNPYVSSTLSVPAsPR	STLSVPAsPRVARKM	S19;	Yes	T(1): 0.0; S(2): 0.0; S(4): 0.0; Y(9): 0.0; S(11): 0.0; S(12): 0.0; T(13): 0.0; S(15): 0.0; S(19): 100.0	9	3	1	0	N-Term(TMT6plex); S19(Phospho)
42415492	NP_060823.3	55320	MIS18BP1	mis18-binding protein 1	S1104	0.7	1.9	2.6	0.8	0.5	0.7	0.8	0.5	0.7	1.8	2.5	0.7	0.5	0.7	0.8	0.5	0.6	1.2	1.6	0.6	TPENTDLGENsGIGK	NTDLGENsGIGKLFT	S11;	Yes	T(1): 0.0; T(5): 0.0; S(11): 100.0	2	1	1	0	N-Term(TMT6plex); S11(Phospho); K15(TMT6plex)
257467639	NP_542194.2	140710	SOGA1	protein SOGA1 isoform1	S175	0.8	0.9	0.6	0.9	0.4	0.9	1.3	0.4	0.8	0.9	0.7	0.9	0.4	1.0	1.4	0.4	0.6	0.9	1.0	0.7	TGQPAQPAPSAQQPPRPPAsPDEPSVAASSVGSSR	QPPRPPAsPDEPSVA	S20;	Yes	T(1): 0.0; S(10): 0.0; S(20): 100.0; S(25): 0.0; S(29): 0.0; S(30): 0.0; S(33): 0.0; S(34): 0.0	4	1	1	0	N-Term(TMT6plex); S20(Phospho)
24497453	NP_002523.2	4927	NUP88	nuclear pore complex protein Nup88	S35; S50	0.5	0.4	0.8	0.5	1.2	0.9	0.5	0.9	0.4	0.4	0.7	0.4	1.1	0.9	0.5	0.9	0.8	0.6	0.6	0.7	NQsPTEAEKPASSSLPSsPPPQLLTR	REGLKNQsPTEAEKP;ASSSLPSsPPPQLLT	S3; S18;	Yes;Yes	S(3): 96.8; T(5): 3.2; S(12): 0.0; S(13): 0.0; S(14): 0.0; S(17): 3.2; S(18): 96.8; T(25): 0.0	3	1	1	0	N-Term(TMT6plex); T5(Phospho); K9(TMT6plex); S18(Phospho)
24497453	NP_002523.2	4927	NUP88	nuclear pore complex protein Nup88	S35; S50	1.2	0.9	0.5	0.9	0.5	0.4	0.8	0.5	1.1	0.9	0.5	0.9	0.4	0.4	0.7	0.4	0.8	0.6	0.6	0.7	NQsPTEAEKPASSSLPSSPPPQLLTR	REGLKNQsPTEAEKP	S3; S18;	Yes	S(3): 96.8; T(5): 4.2; S(12): 33.0; S(13): 33.0; S(14): 33.0; S(17): 0.0; S(18): 0.0; T(25): 0.0	1	1	1	0	N-Term(TMT6plex); T5(Phospho); K9(TMT6plex); S18(Phospho)
27501446	NP_003668.2	8562	DENR	density-regulated protein	S73	0.8	0.6	0.8	0.7	1.1	0.7	1.0	0.7	0.6	0.6	0.7	0.7	0.8	0.7	1.0	0.7	0.7	0.7	0.9	0.7	LTVENsPK	AKLTVENsPKQEAGI	S6;	Yes	T(2): 0.0; S(6): 100.0	6	1	1	0	N-Term(TMT6plex); S6(Phospho); K8(TMT6plex)
42518070	NP_004808.2	9414	TJP2	tight junction protein ZO-2 isoform1	S978	0.5	0.8	0.9	0.7	0.5	0.6	0.7	0.6	0.6	0.8	0.7	0.8	0.6	0.6	0.6	0.7	0.6	0.7	0.7	0.7	AAsSDQLR	AQMRRAAsSDQLRDN	S3;	Yes	S(3): 99.2; S(4): 0.8	13	2	1	0	N-Term(TMT6plex); S3(Phospho)
259906020	NP_001158287.1	22985	ACIN1	apoptotic chromatin condensation inducer in the nucleus isoform3	S964	0.6	0.7	0.6	0.6	0.9	0.8	0.8	0.7	0.5	0.7	0.7	0.7	0.7	0.8	0.9	0.7	0.6	0.7	0.8	0.7	TAQVPsPPR	VRTAQVPsPPRGKIS	S6;	No	T(1): 0.0; S(6): 100.0	38	5	1	0	N-Term(TMT6plex); S6(Phospho)
145309311	NP_001034680.2	8239	USP9X	probable ubiquitin carboxyl-terminal hydrolase FAF-X isoform4	S2547	0.9	1.6	1.1	0.9	0.5	0.5	0.4	0.4	0.9	1.7	1.2	1.0	0.5	0.6	0.4	0.5	0.7	1.1	0.8	0.7	AQENYEGSEEVsPPQTK	YEGSEEVsPPQTKDQ	S12;	No	Y(5): 0.1; S(8): 2.7; S(12): 97.1; T(16): 0.1	5	2	1	0	N-Term(TMT6plex); S12(Phospho); K17(TMT6plex)
42741682	NP_005446.2	9406	ZRANB2	zinc finger Ran-binding domain-containing protein 2 isoform2	S120	1.0	1.0	0.8	0.7	0.7	0.7	0.8	0.7	1.1	1.1	0.9	0.9	0.8	0.8	0.9	0.9	1.0	0.9	0.9	0.9	EEsDGEYDEFGR	EYIEREEsDGEYDEF	S3;	Yes	S(3): 100.0; Y(7): 0.0	17	2	1	0	N-Term(TMT6plex); S3(Phospho)
22027648	NP_663731.1	23175	LPIN1	phosphatidate phosphatase LPIN1 isoform1	S294	0.9	1.0	0.9	0.6	0.7	0.9	0.9	0.7	0.8	0.8	0.8	0.7	0.6	0.8	0.8	0.7	0.7	0.8	0.8	0.7	ESsPLSSR	PHKMKESsPLSSRKI	S3;	Yes	S(2): 0.6; S(3): 99.4; S(6): 0.0; S(7):	3	4	1	0	N-Term(TMT6plex); S3(Phospho)
157671949	NP_036553.2	6018	RLF	zinc finger protein Rlf	S632	0.9	0.7	0.7	0.6	1.2	0.9	0.9	0.7	1.1	1.0	0.9	0.8	1.4	1.3	1.0	1.0	1.3	1.2	1.0	0.9	sPSAIPEQNHSLNDQAK	QKGICPKsPSAIPEQ	S1;	Yes	S(1): 100.0; S(3): 0.0; S(11): 0.0	4	1	1	0	N-Term(TMT6plex); S1(Phospho); K17(TMT6plex)
299522984	NP_001177410.1	63967	CLSPN	claspin isoform2	S1252	0.6	0.7	0.5	0.8	0.5	0.4	0.6	0.6	0.8	1.0	0.9	1.2	0.6	0.5	0.9	0.8	0.7	0.8	0.9	1.0	GPSFMTSPsPK	PSFMTSPsPKHLKTD	S9;	No	S(3): 0.0; T(6): 0.0; S(7): 0.0; S(9): 100.0	4	2	1	0	N-Term(TMT6plex); M5(Oxidation); S9(Phospho); K11(TMT6plex)
117190174	NP_004491.2	3183	HNRNPC	heterogeneous nuclear ribonucleoproteins C1/C2 isoformb	S286	0.8	0.7	0.8	0.7	0.7	0.7	0.9	0.6	0.7	0.7	0.8	0.7	0.7	0.7	0.9	0.6	0.7	0.7	0.8	0.7	EAEEGEDDRDsANGEDDS	EGEDDRDsANGEDDS	S11;	No	S(11): 100.0; S(18): 0.0	41	2	1	1	N-Term(TMT6plex); S11(Phospho)
288856248	NP_001165780.1	91624	NEXN	nexilin isoform2	S16	0.8	0.8	1.1	0.7	0.7	0.9	1.2	0.6	1.0	0.9	0.9	1.0	0.8	1.1	1.0	1.0	0.9	1.0	1.0	1.0	EMLAsDDEEDVSSK	EIKEMLAsDDEEDVS	S5;	Yes	S(5): 100.0; S(12): 0.0; S(13): 0.0	4	2	1	0	N-Term(TMT6plex); M2(Oxidation); S5(Phospho); K14(TMT6plex)
4506633	NP_000984.1	6160	RPL31	60S ribosomal protein L31 isoform1	S98	0.8	0.6	0.7	0.7	0.7	0.7	0.7	0.7	0.8	0.7	0.8	0.9	0.8	0.8	0.9	0.9	0.8	0.7	0.9	0.9	NEDEDsPNK	KRNEDEDsPNKLYTL	S6;	Yes	S(6): 100.0	39	3	1	0	N-Term(TMT6plex); S6(Phospho); K9(TMT6plex)
8922633	NP_060670.1	10363	HMG20A	high mobility group protein 20A	S105	0.7	0.7	0.7	0.6	0.6	0.9	0.7	0.7	0.8	0.7	0.7	0.6	0.6	0.9	0.8	0.8	0.7	0.8	0.8	0.7	sPLTGYVR	RDSNAPKsPLTGYVR	S1;	Yes	S(1): 100.0; T(4): 0.0; Y(6): 0.0	2	1	1	0	N-Term(TMT6plex); S1(Phospho)
4507555	NP_003267.1	7112	TMPO	thymopoietin isoformalpha	S351	0.6	0.8	0.8	0.6	0.5	0.7	0.6	0.7	0.6	0.9	1.0	0.6	0.5	0.7	0.7	0.7	0.6	0.8	0.8	0.6	SHISDQsPLSSK	RSHISDQsPLSSKRK	S7;	Yes	S(1): 0.0; S(4): 0.0; S(7): 100.0; S(10): 0.0; S(11): 0.0	8	1	1	0	N-Term(TMT6plex); S7(Phospho); K12(TMT6plex)
260064009	NP_056121.2	23358	USP24	ubiquitin carboxyl-terminal hydrolase 24	S1943	0.5	0.5	0.5	0.7	0.5	0.6	0.5	0.7	0.5	0.5	0.5	0.7	0.5	0.6	0.5	0.7	0.5	0.5	0.5	0.7	SVDQGGGGsPR	VDQGGGGsPRKKVAL	S9;	Yes	S(1): 0.0; S(9): 100.0	10	1	1	0	N-Term(TMT6plex); S9(Phospho)
4507955	NP_003394.1	7528	YY1	transcriptional repressor protein YY1	S118	0.8	0.8	0.8	0.7	0.7	0.7	0.7	0.7	1.1	1.0	1.1	1.0	0.9	0.9	1.0	0.9	1.0	0.9	1.1	0.9	EEVVGGDDsDGLR	EVVGGDDsDGLRAED	S9;	Yes	S(9): 100.0	2	1	1	0	N-Term(TMT6plex); S9(Phospho)
347543829	NP_001231533.1	1808	DPYSL2	dihydropyrimidinase-related protein 2 isoform3	T485; S486	0.7	0.8	0.7	0.7	0.5	0.8	0.6	0.6	0.6	0.6	0.6	0.7	0.5	0.7	0.5	0.6	0.5	0.7	0.5	0.6	TVTPASSAKtsPAK	TPASSAKtSPAKQQA;PASSAKTsPAKQQAP	T10; S11;	No;No	T(1): 0.0; T(3): 0.0; S(6): 1.5; S(7): 1.5; T(10): 97.0; S(11): 99.9	5	3	1	1	N-Term(TMT6plex); S6(Phospho); S7(Phospho); K9(TMT6plex); K14(TMT6plex)
223555917	NP_848927.2	92140	MTDH	protein LYRIC	S568	1.0	1.2	1.0	0.7	1.1	1.2	1.0	0.7	0.9	1.1	0.9	0.6	1.0	1.1	0.8	0.6	0.9	1.1	0.9	0.6	SETSWEsPK	KSETSWEsPKQIKKK	S7;	Yes	S(1): 0.0; T(3): 0.0; S(4): 0.0; S(7): 100.0	16	1	1	0	N-Term(TMT6plex); S7(Phospho); K9(TMT6plex)
6005948	NP_009118.1	11193	WBP4	WW domain-binding protein 4	S262	0.8	0.6	1.1	0.8	1.0	0.8	0.9	0.5	1.0	0.7	1.3	0.9	1.3	0.9	1.1	0.6	1.1	0.8	1.2	0.8	NSDGGsDPETQK	NKNSDGGsDPETQKE	S6;	Yes	S(2): 0.0; S(6): 100.0; T(10): 0.0	3	1	1	0	N-Term(TMT6plex); S6(Phospho); K12(TMT6plex)
298358598	NP_001177255.1	54487	DGCR8	microprocessor complex subunit DGCR8 isoform2	S271	1.9	1.8	1.8	0.4	1.1	2.2	1.3	0.9	1.7	1.3	1.5	0.4	1.0	1.6	1.1	0.8	1.4	1.4	1.3	0.6	YGGDsDHPsDGETSVQPMMTK	EEKYGGDsDHPSDGE;GGDSDHPsDGETSVQ	S5;	Yes;Yes	Y(1): 2.6; S(5): 97.4; S(9): 100.0; T(13): 0.0; S(14): 0.0; T(20): 0.0	1	2	1	0	N-Term(TMT6plex); S5(Phospho); S9(Phospho); M18(Oxidation); M19(Oxidation); K21(TMT6plex)
82546879	NP_004277.2	9567	GTPBP1	GTP-binding protein 1	S25	1.2	0.7	0.8	0.5	1.0	1.0	1.2	0.9	1.1	0.6	0.6	0.4	0.9	0.8	0.9	0.7	1.0	0.7	0.8	0.6	SAMDSPVPASMFAPEPSsPGAAR	MFAPEPSsPGAARAA	S18;	Yes	S(1): 0.0; S(5): 0.0; S(10): 0.0; S(17): 1.4; S(18): 98.6	4	1	1	0	N-Term(TMT6plex); M3(Oxidation); M11(Oxidation); S18(Phospho)
388452864	NP_001253964.1	114825	PWWP2A	PWWP domain-containing protein 2A isoformc	S81	0.7	0.7	0.5	0.6	0.5	0.6	0.6	0.7	0.8	0.8	0.7	0.7	0.6	0.6	0.8	0.8	0.7	0.7	0.8	0.8	sPEAVGPELEAEEK	PPGELARsPEAVGPE	S1;	Yes	S(1): 100.0	2	3	1	0	N-Term(TMT6plex); S1(Phospho); K14(TMT6plex)
291190783	NP_001167448.1	55604	LRRC16A	leucine-rich repeat-containing protein 16A isoform2	S1309	1.0	0.7	0.7	0.7	1.2	0.9	0.8	0.6	1.0	0.8	0.8	0.8	1.3	0.9	0.8	0.6	1.1	0.9	0.8	0.7	DGQSsPQPSPR	KERDGQSsPQPSPRT	S5;	No	S(4): 0.0; S(5): 100.0; S(9): 0.0	13	2	1	0	N-Term(TMT6plex); S5(Phospho)
298358598	NP_001177255.1	54487	DGCR8	microprocessor complex subunit DGCR8 isoform2	S271	1.1	2.2	1.3	0.9	1.9	1.8	1.8	0.4	1.0	1.6	1.1	0.8	1.7	1.3	1.5	0.4	1.4	1.4	1.3	0.6	YGGDsDHPSDGETSVQPMMTK	EEKYGGDsDHPSDGE	S5;	Yes	Y(1): 3.1; S(5): 96.9; S(9): 33.3; T(13): 33.3; S(14): 33.3; T(20): 0.0	3	2	1	0	N-Term(TMT6plex); S5(Phospho); S9(Phospho); M18(Oxidation); M19(Oxidation); K21(TMT6plex)
168229156	NP_078804.2	79576	NKAP	NF-kappa-B-activating protein	S9	0.9	0.9	0.7	0.5	0.8	1.0	0.6	0.8	1.1	1.1	0.9	0.6	1.0	1.2	0.8	1.0	1.1	1.1	0.9	0.8	sPDREASGSGGR	APVSGSRsPDREASG	S1;	Yes	S(1): 100.0; S(7): 0.0; S(9): 0.0	2	1	1	1	N-Term(TMT6plex); S1(Phospho)
42475970	NP_149014.3	85360	SYDE1	rho GTPase-activating protein SYDE1	S645	0.8	0.9	0.9	0.8	0.9	0.7	0.8	0.5	-	-	-	-	-	-	-	-	-	-	-	-	GGPEsPPSNR	RGRGGPEsPPSNRYA	S5;	Yes	S(5): 100.0; S(8): 0.0	1	1	1	0	N-Term(TMT6plex); S5(Phospho)
118572613	NP_057417.3	23524	SRRM2	serine/arginine repetitive matrix protein 2	T1413	0.5	1.3	1.1	0.5	2.1	0.6	2.4	0.8	0.5	1.3	1.1	0.6	2.2	0.7	2.5	0.9	1.3	1.0	1.8	0.7	AGMSSNQSISSPVLDAVPRtPSR	VLDAVPRtPSRERSS	T20;	Yes	S(4): 0.0; S(5): 0.0; S(8): 33.3; S(10): 33.3; S(11): 33.3; T(20): 97.3; S(22): 2.7	436	1	1	1	N-Term(TMT6plex); M3(Oxidation); S11(Phospho); S22(Phospho)
4505705	NP_003759.1	8682	PEA15	astrocytic phosphoprotein PEA-15	S116	3.1	0.8	2.3	0.6	2.6	0.8	2.1	0.7	2.0	0.7	1.3	0.7	1.7	0.8	1.2	0.7	1.8	0.8	1.3	0.7	QPsEEIIK	KDIIRQPsEEIIKLA	S3;	Yes	S(3): 100.0	4	1	1	0	N-Term(TMT6plex); S3(Phospho); K9(TMT6plex)
118572613	NP_057417.3	23524	SRRM2	serine/arginine repetitive matrix protein 2	T1413	2.1	0.6	2.4	0.8	0.5	1.3	1.1	0.5	2.2	0.7	2.5	0.9	0.5	1.3	1.1	0.6	1.3	1.0	1.8	0.7	AGMSSNQsISSPVLDAVPRtPSR	AGMSSNQsISSPVLD;VLDAVPRtPSRERSS	T20;	Yes;Yes	S(4): 1.9; S(5): 1.9; S(8): 92.4; S(10): 1.9; S(11): 1.9; T(20): 98.0; S(22): 2.0	1	1	1	1	N-Term(TMT6plex); M3(Oxidation); S11(Phospho); S22(Phospho)
550544215	NP_001272369.1	253260	RICTOR	rapamycin-insensitive companion of mTOR isoform3	S945	0.9	0.9	0.7	0.5	1.3	0.5	1.1	0.8	0.8	0.8	0.6	0.5	1.1	0.4	1.0	0.8	0.9	0.6	0.8	0.6	SQSFNTDTTTSGIsSMSSSPSR	DTTTSGIsSMSSSPS	S14;	No	S(1): 0.0; S(3): 0.0; T(6): 0.0; T(8): 1.8; T(9): 1.8; T(10): 0.0; S(11): 0.0; S(14): 88.9; S(15): 1.8; S(17): 1.8; S(18): 1.8; S(19): 1.8; S(21): 0.0	1	3	1	0	N-Term(TMT6plex); S14(Phospho); M16(Oxidation)
59891448	NP_689969.2	253260	RICTOR	rapamycin-insensitive companion of mTOR isoform1	S945	1.3	0.5	1.1	0.8	0.9	0.9	0.7	0.5	1.1	0.4	1.0	0.8	0.8	0.8	0.6	0.5	0.9	0.6	0.8	0.6	SQSFNTDTtTSGISSMSSSPSR	QSFNTDTtTSGISSM	S14;	Yes	S(1): 0.0; S(3): 0.0; T(6): 0.0; T(8): 2.1; T(9): 95.6; T(10): 2.1; S(11): 0.0; S(14): 0.0; S(15): 0.0; S(17): 0.0; S(18): 0.0; S(19): 0.0; S(21): 0.0	1	3	1	0	N-Term(TMT6plex); T9(Phospho); M16(Oxidation)
62988361	NP_001615.1	202	AIM1	absent in melanoma 1 protein	S484	0.7	1.0	0.7	1.0	0.7	0.8	0.5	0.4	0.9	1.2	0.8	1.2	0.9	1.0	0.7	0.4	0.9	1.1	0.8	0.8	DTCVQsPISSFPCTDLK	PKDTCVQsPISSFPC	S6;	Yes	T(2): 0.0; S(6): 100.0; S(9): 0.0; S(10): 0.0; T(14): 0.0	8	1	1	0	N-Term(TMT6plex); C3(Carbamidomethyl); S6(Phospho); C13(Carbamidomethyl); K17(TMT6plex)
146198654	NP_078947.3	79724	ZNF768	zinc finger protein 768	S83	0.8	0.9	0.6	0.5	0.7	0.7	0.4	0.8	0.9	1.0	0.7	0.5	0.8	0.7	0.5	0.9	0.9	0.9	0.6	0.7	FEPEsPGFESR	SPRFEPEsPGFESRS	S5;	Yes	S(5): 100.0; S(10): 0.0	18	1	1	0	N-Term(TMT6plex); S5(Phospho)
21735548	NP_009117.2	11190	CEP250	centrosome-associated protein CEP250	S2332	0.6	0.5	0.5	0.6	0.6	0.6	0.5	0.7	0.6	0.6	0.6	0.7	0.5	0.7	0.5	0.7	0.5	0.7	0.5	0.7	ATASsPTQQDGR	ISKATASsPTQQDGR	S5;	No	T(2): 0.0; S(4): 0.7; S(5): 99.3; T(7):	4	1	1	0	N-Term(TMT6plex); S5(Phospho)
530788278	NP_612114.2	546	ATRX	transcriptional regulator ATRX isoform2	S1310	0.8	1.0	0.8	0.7	0.6	0.7	0.8	0.6	0.9	1.2	1.0	1.0	0.7	0.8	0.9	0.8	0.8	1.0	1.0	0.9	LTVsDGESGEEK	LRHKLTVsDGESGEE	S4;	No	T(2): 0.0; S(4): 100.0; S(8): 0.0	17	2	1	0	N-Term(TMT6plex); S4(Phospho); K12(TMT6plex)
385198097	NP_001245229.1	57698	KIAA1598	shootin-1 isoforme	S446	0.7	0.6	0.5	0.7	0.6	0.7	0.5	0.6	0.9	0.9	0.9	1.0	0.8	1.0	0.8	1.0	0.8	0.9	0.9	1.0	sMPVLGSVSSVTK	LATSESKsMPVLGSV	S1;	No	S(1): 100.0; S(7): 0.0; S(9): 0.0; S(10): 0.0; T(12): 0.0	2	4	1	0	N-Term(TMT6plex); S1(Phospho); M2(Oxidation); K13(TMT6plex)
13489073	NP_071334.1	54583	EGLN1	egl nine homolog 1	S125	0.6	0.6	0.7	0.6	0.5	0.6	0.7	0.7	0.7	0.5	0.7	0.6	0.6	0.5	0.7	0.7	0.6	0.5	0.7	0.7	AKPPADPAAAAsPCR	ADPAAAAsPCRAAAG	S12;	Yes	S(12): 100.0	1	1	1	0	N-Term(TMT6plex); K2(TMT6plex); S12(Phospho); C14(Carbamidomethyl)
7706637	NP_057250.1	8554	PIAS1	E3 SUMO-protein ligase PIAS1	S488	1.2	1.3	1.7	0.7	0.6	0.2	0.8	0.6	1.6	1.7	1.9	0.8	0.8	0.3	0.9	0.8	1.2	1.0	1.4	0.8	TCPSLSPTsPLNNK	CPSLSPTsPLNNKGI	S9;	Yes	T(1): 0.0; S(4): 0.0; S(6): 0.0; T(8): 1.7; S(9): 98.3	1	1	1	0	N-Term(TMT6plex); C2(Carbamidomethyl); S9(Phospho); K14(TMT6plex)
112382250	NP_003119.2	6711	SPTBN1	spectrin beta chain, non-erythrocytic 1 isoform1	S2316	0.6	0.6	0.8	0.6	0.7	0.7	1.0	0.7	0.6	0.7	0.8	0.6	0.7	0.7	1.0	0.7	0.7	0.7	0.9	0.6	HEVSAsTQSTPASSR	DKHEVSAsTQSTPAS	S6;	Yes	S(4): 0.0; S(6): 98.6; T(7): 0.7; S(9): 0.0; T(10): 0.7; S(13): 0.0; S(14): 0.0	31	1	1	0	N-Term(TMT6plex); T10(Phospho)
112382250	NP_003119.2	6711	SPTBN1	spectrin beta chain, non-erythrocytic 1 isoform1	S2316	0.7	0.7	1.0	0.7	0.6	0.6	0.8	0.6	0.7	0.7	1.0	0.7	0.6	0.7	0.8	0.6	0.7	0.7	0.9	0.6	HEVSAStQSTPASSR	KHEVSAStQSTPASS	S6;	Yes	S(4): 0.0; S(6): 0.8; T(7): 98.4; S(9): 0.0; T(10): 0.8; S(13): 0.0; S(14): 0.0	4	1	1	0	N-Term(TMT6plex); T10(Phospho)
217416381	NP_060875.2	55352	COPRS	coordinator of PRMT5 and differentiation stimulator	S66	1.2	1.5	2.2	1.1	0.9	0.5	0.8	0.2	1.2	1.7	2.4	1.4	0.9	0.6	0.8	0.2	1.1	1.1	1.6	0.8	GTQSIPNDsPAR	TQSIPNDsPARGEGT	S9;	Yes	T(2): 0.0; S(4): 0.0; S(9): 100.0	1	1	1	0	N-Term(TMT6plex); S9(Phospho)
55956788	NP_005372.2	4691	NCL	nucleolin	T121	0.4	0.4	0.4	0.6	0.4	0.4	0.5	0.7	0.5	0.5	0.6	0.8	0.5	0.5	0.6	0.9	0.5	0.5	0.6	0.9	ALVAtPGK	PGKALVAtPGKKGAA	T5;	Yes	T(5): 100.0	65	1	1	0	N-Term(TMT6plex); T5(Phospho); K8(TMT6plex)

Khan et al., 2019. Multi-omics analysis to characterize cigarette smoke induced molecular alterations in esophageal cells

Supplementary Table 8. List of phosphopeptides identified and quantified in untreated and chronically treated Het1A cells with cigarette smoke condensate for 8 months using SequestHT and Mascot search algorithms with a PhosphoRS probability score ≥75%

Protein group Accession	NP_Accession	Gene ID	Gene Symbol	Protein Description	PhosphoSite (Protein)	Het-1A-Smoke - 2M/Parental (Replicate 1)	Het-1A-Smoke - 4M/Parental (Replicate 1)	Het-1A-Smoke - 6M/Parental (Replicate 1)	Het-1A-Smoke - 8M/Parental (Replicate 1)	Het-1A-Smoke - 2M/Parental (Replicate 2)	Het-1A-Smoke - 4M/Parental (Replicate 2)	Het-1A-Smoke - 6M/Parental (Replicate 2)	Het-1A-Smoke - 8M/Parental (Replicate 2)	Normalized phosphoprotein ratios with respect to corresponding total protein ratios. Het-1A-Smoke - 2M/Parental (Replicate 1)	Het-1A-Smoke - 4M/Parental (Replicate 1)	Het-1A-Smoke - 6M/Parental (Replicate 1)	Het-1A-Smoke - 8M/Parental (Replicate 1)	Het-1A-Smoke - 2M/Parental (Replicate 2)	Het-1A-Smoke - 4M/Parental (Replicate 2)	Het-1A-Smoke - 6M/Parental (Replicate 2)	Het-1A-Smoke - 8M/Parental (Replicate 2)	Average of replicates Het-1A-Smoke - 2M/Parental	Het-1A-Smoke - 4M/Parental	Het-1A-Smoke - 6M/Parental	Het-1A-Smoke - 8M/Parental	Peptide Sequence	Phosphowindow	PhosphoSite (Peptide)	Phosphosite Plus Evidence	phosphoRS Site Probabilities	# PSMs	# Proteins	# Protein Groups	# Missed Cleavages	Modifications
118572613	NP_057417.3	23524	SRRM2	serine/arginine repetitive matrix protein 2	S895	0.5	0.6	0.7	0.6	0.6	0.6	0.7	0.7	0.6	0.6	0.8	0.7	0.6	0.6	0.8	0.7	0.6	0.6	0.8	0.7	HSCSGSsPPR	RHSCSGSsPPRVKSS	S7;	Yes	S(2): 0.0; S(4): 0.0; S(6): 0.5; S(7):	436	1	1	0	N-Term(TMT6plex); C3(Carbamidomethyl); S7(Phospho)
118572613	NP_057417.3	23524	SRRM2	serine/arginine repetitive matrix protein 2	S895	0.6	0.6	0.7	0.7	0.5	0.6	0.7	0.6	0.6	0.6	0.8	0.7	0.6	0.6	0.8	0.7	0.6	0.6	0.8	0.7	HsCSGSSPPR	RHSCSGSsPPRVKSS	S7;	Yes	S(2): 0.0; S(4): 0.0; S(6): 0.5; S(7):	10	1	1	0	N-Term(TMT6plex); C3(Carbamidomethyl); S7(Phospho)
21450663	NP_659407.1	159013	CXorf38	uncharacterized protein CXorf38	T314	1.2	0.8	0.7	0.7	1.0	0.9	0.7	0.6	1.1	0.8	0.9	0.9	0.9	0.8	0.8	0.7	1.0	0.8	0.8	0.8	LDSQEPGRQtPDR	SQEPGRQtPDRKA	T10;	Yes	S(3): 0.0; T(10): 100.0	1	1	1	1	N-Term(TMT6plex); T10(Phospho)
259013556	NP_004851.2	9513	FXR2	fragile X mental retardation syndrome-related protein 2	S453	1.0	1.0	0.9	0.7	1.2	1.0	1.0	0.6	0.9	0.9	0.9	0.8	1.2	0.9	1.0	0.7	1.1	0.9	1.0	0.7	TGGPAYGPSSDVSTAsETESEK	SSDVSTAsETESEKR	S16;	Yes	T(1): 0.0; Y(6): 0.0; S(9): 0.0; S(10): 0.0; S(13): 0.0; T(14): 0.0; S(16): 100.0; T(18): 0.0; S(20): 0.0	6	1	1	0	N-Term(TMT6plex); S16(Phospho); K22(TMT6plex)
63478060	NP_115711.2	84295	PHF6	PHD finger protein 6 isoform 2	S205	0.8	0.8	0.9	0.7	0.8	0.8	0.7	0.6	1.0	0.9	1.1	0.9	1.0	0.9	0.9	0.8	1.0	0.9	1.0	0.9	SsPSDTRPK	DRSPHRSsPSDTRPK	S2;	No	S(1): 0.9; S(2): 99.1; S(4): 0.0; T(6):	1	2	1	0	N-Term(TMT6plex); S2(Phospho); K9(TMT6plex)
145580615	NP_075384.3	65095	KRI1	protein KRI1 homolog	S315	0.6	0.7	0.8	0.7	0.7	0.8	0.7	0.6	0.7	0.8	1.0	0.8	0.8	0.8	0.8	0.7	0.8	0.8	0.9	0.7	FEEPDSAsVK	FEEPDSAsVKTYPRS	S8;	No	S(6): 1.1; S(8): 98.9	13	1	1	0	N-Term(TMT6plex); S8(Phospho); K10(TMT6plex)
9257222	NP_002006.2	2308	FOXO1	forkhead box protein O1	S319	0.8	0.6	0.7	0.6	0.8	0.6	0.7	0.6	-	-	-	-	-	-	-	-	-	-	-	-	TSsNASTISGR	TFRPRTSsNASTISG	S3;	Yes	T(1): 0.0; S(2): 0.0; S(3): 100.0; S(6): 0.0; T(7): 0.0; S(9): 0.0	9	1	1	0	N-Term(TMT6plex); S3(Phospho)
61676188	NP_113584.3	10075	HUWE1	E3 ubiquitin-protein ligase HUWE1	S3662	1.0	0.8	0.7	0.7	0.7	0.6	0.6	0.6	1.0	0.8	0.7	0.8	0.8	0.6	0.7	0.7	0.9	0.7	0.7	0.8	AQCETLsPDGLPEEQPQTTK	RAQCETLsPDGLPEE	S7;	Yes	T(5): 1.9; S(7): 98.1; T(18): 0.0; T(19): 0.0	20	1	1	0	N-Term(TMT6plex); C3(Carbamidomethyl); S7(Phospho); K20(TMT6plex)
117190174	NP_004491.2	3183	HNRNPC	heterogeneous nuclear ribonucleoproteins C1/C2 isoform b	S220	0.9	0.9	0.9	0.7	0.9	0.8	0.8	0.6	0.9	0.9	0.9	0.7	0.9	0.8	0.8	0.6	0.9	0.8	0.9	0.6	NDKsEEEQSSSSVK	VEMKNDKsEEEQSSS	S4;	No	S(4): 100.0; S(9): 0.0; S(10): 0.0; S(11): 0.0; S(12): 0.0	41	2	1	1	N-Term(TMT6plex); K3(TMT6plex); S4(Phospho); K14(TMT6plex)
28558977	NP_004822.2	9441	MED26	mediator of RNA polymerase II transcription subunit	S373	0.7	0.6	0.6	0.6	0.8	0.7	0.7	0.7	-	-	-	-	-	-	-	-	-	-	-	-	AGFsPDSSK	LLSRAGFsPDSSKAD	S4;	Yes	S(4): 100.0; S(7): 0.0; S(8): 0.0	5	1	1	0	N-Term(TMT6plex); S4(Phospho); K9(TMT6plex)
118572613	NP_057417.3	23524	SRRM2	serine/arginine repetitive matrix protein 2	S1424	0.7	0.9	0.7	0.7	0.7	0.6	0.8	0.6	0.8	0.9	0.7	0.8	0.7	0.6	0.8	0.7	0.7	0.8	0.7	0.7	SSSASsPEMK	ERSSSASsPEMKDGL	S6;	Yes	S(1): 0.0; S(2): 0.0; S(3): 0.0; S(5): 0.7; S(6): 99.3	436	1	1	0	N-Term(TMT6plex); S6(Phospho); M9(Oxidation); K10(TMT6plex)
4758876	NP_004634.1	8106	PABPN1	polyadenylate-binding protein 2	S150	1.2	1.1	1.0	0.6	0.8	0.8	0.8	0.6	1.5	1.3	1.4	0.8	1.0	1.0	1.1	0.8	1.2	1.1	1.2	0.8	QMNMsPPPGNAGPVIMSIEEK	VEKQMNMsPPPGNAG	S5;	Yes	S(5): 100.0; S(17): 0.0	4	2	1	0	N-Term(TMT6plex); M2(Oxidation); M4(Oxidation); S5(Phospho); M16(Oxidation);
56676330	NP_057371.2	50809	HP1BP3	heterochromatin protein 1-binding protein 3	T51	0.9	0.7	0.9	0.6	1.1	0.9	1.3	0.7	1.0	0.7	0.8	0.6	1.2	0.9	1.2	0.8	1.1	0.8	1.0	0.7	TVNSTREtPPK	TVNSTREtPPKSKLA	T8;	Yes	T(1): 0.0; S(4): 0.0; T(5): 0.0; T(8): 100.0	5	1	1	1	N-Term(TMT6plex); T8(Phospho); K11(TMT6plex)
41281429	NP_055485.2	9689	BZW1	basic leucine zipper and W2 domain-containing protein 1 isoform 4	S345; S347	1.1	1.1	1.3	0.7	1.0	0.5	0.2	0.6	1.3	1.3	1.3	0.7	1.1	0.6	0.2	0.6	1.2	0.9	0.8	0.7	NAEEEsESEAEEGD	LKNAEEEsESEAEEG;NAEEESEsEAEEGD	S6; S8;	No;No	S(6): 100.0; S(8): 100.0	5	4	1	0	N-Term(TMT6plex); S6(Phospho); S8(Phospho)
29725609	NP_005219.2	1956	EGFR	epidermal growth factor receptor isoform a precursor	T693	1.0	1.1	0.9	0.8	1.0	1.4	1.3	0.5	1.1	1.3	1.0	1.0	1.2	1.7	1.5	0.6	1.2	1.5	1.3	0.8	ELVEPLtPSGEAPNQALLR	RELVEPLtPSGEAPN	T7;	Yes	T(7): 100.0; S(9): 0.0	8	1	1	0	N-Term(TMT6plex); T7(Phospho)
103472005	NP_002408.3	4288	MKI67	antigen KI-67 isoform 1	S1679	0.5	0.8	0.6	0.6	0.4	0.4	0.4	0.6	0.5	0.8	0.6	0.7	0.4	0.4	0.4	0.7	0.5	0.6	0.5	0.7	AFMEsPK	SMKAFMEsPKQILDS	S5;	Yes	S(5): 100.0	118	2	1	0	N-Term(TMT6plex); M3(Oxidation); S5(Phospho); K7(TMT6plex)
14790190	NP_055816.2	23013	SPEN	msx2-interacting protein	T1619	0.9	0.9	0.6	0.4	1.0	0.8	1.1	0.9	0.9	0.8	0.6	0.4	1.0	0.7	1.1	0.9	0.9	0.8	0.8	0.6	QEDTENHPKtPESAPENK	DTENHPKtPESAPEN	T10;	Yes	T(4): 0.0; T(10): 98.5; S(13): 1.5	16	1	1	1	N-Term(TMT6plex); K9(TMT6plex); T10(Phospho); K18(TMT6plex)
118572613	NP_057417.3	23524	SRRM2	serine/arginine repetitive matrix protein 2	S1124	0.5	0.6	0.6	0.6	0.5	0.7	0.6	0.6	0.5	0.6	0.6	0.7	0.5	0.7	0.6	0.7	0.5	0.7	0.6	0.7	GEFSAsPMLK	DRGEFSAsPMLKSGM	S6;	Yes	S(4): 0.0; S(6): 100.0	436	1	1	0	N-Term(TMT6plex); S6(Phospho); M8(Oxidation); K10(TMT6plex)
14790190	NP_055816.2	23013	SPEN	msx2-interacting protein	T1619	1.0	0.8	1.1	0.9	0.9	0.9	0.6	0.4	1.0	0.7	1.1	0.9	0.9	0.8	0.6	0.4	0.9	0.8	0.8	0.6	QEDTENHPKTPEsAPENK	NHPKTPEsAPENKDS	T10;	Yes	T(4): 0.0; T(10): 0.0; S(13): 100.0	1	1	1	1	N-Term(TMT6plex); K9(TMT6plex); T10(Phospho); K18(TMT6plex)
118572613	NP_057417.3	23524	SRRM2	serine/arginine repetitive matrix protein 2	S908	0.8	1.0	0.8	0.6	1.1	1.0	0.9	0.7	0.8	1.0	0.8	0.7	1.2	1.0	1.0	0.7	1.0	1.0	0.9	0.7	SStPPRQSPSR	PPRVKSStPPRQSPS	S8;	Yes	S(1): 0.0; S(2): 0.9; T(3): 99.1; S(8): 50.0; S(10): 50.0	2	1	1	1	N-Term(TMT6plex); T3(Phospho); S10(Phospho)
167614488	NP_056342.3	26000	TBC1D10B	TBC1 domain family member 10B	S678	0.7	0.8	0.5	0.6	0.7	0.8	0.6	0.7	0.7	0.8	0.6	0.6	0.8	0.9	0.7	0.7	0.7	0.9	0.7	0.7	AAGGAPsPPPPVR	RAAGGAPsPPPPVRR	S7;	Yes	S(7): 100.0	13	1	1	0	N-Term(TMT6plex); S7(Phospho)
42542379	NP_005830.2	10250	SRRM1	serine/arginine repetitive matrix protein 1	S10	0.7	1.0	1.2	0.6	0.6	0.9	1.0	0.7	0.8	1.0	1.4	0.7	0.8	1.0	1.1	0.8	0.8	1.0	1.2	0.7	GTsAEQDNR	AGFFRGTsAEQDNRF	S3;	Yes	T(2): 0.0; S(3): 100.0	229	1	1	0	N-Term(TMT6plex); S3(Phospho)
56118310	NP_073568.2	64710	NUCKS1	nuclear ubiquitous casein and cyclin-dependent kinase substrate 1	S58	0.9	1.0	0.6	0.4	0.9	0.9	0.8	0.8	1.1	1.1	0.8	0.6	1.1	1.0	1.0	1.0	1.1	1.1	0.9	0.8	NSQEDSEDsEDK	SQEDSEDsEDKDVKT	S6;	Yes	S(2): 0.0; S(6): 1.1; S(9): 98.9	1	1	1	0	N-Term(TMT6plex); S6(Phospho); K12(TMT6plex)
545746335	NP_001271143.1	9320	TRIP12	E3 ubiquitin-protein ligase TRIP12 isoform a	S169	0.5	0.6	0.5	0.6	0.6	0.7	0.6	0.7	0.5	0.6	0.5	0.6	0.6	0.7	0.6	0.7	0.6	0.6	0.6	0.7	SAQsPSTSK	QQLKSAQsPSTSKAH	S4;	No	S(1): 0.0; S(4): 98.7; S(6): 0.7; T(7): 0.7; S(8): 0.0	23	3	1	0	N-Term(TMT6plex); S4(Phospho); K9(TMT6plex)
56118310	NP_073568.2	64710	NUCKS1	nuclear ubiquitous casein and cyclin-dependent kinase substrate 1	S58	0.9	0.9	0.8	0.8	0.9	1.0	0.6	0.4	1.1	1.0	1.0	1.0	1.1	1.1	0.8	0.6	1.1	1.1	0.9	0.8	NSQEDsEDSEDK	GKNSQEDsEDSEDKD	S6;	Yes	S(2): 0.0; S(6): 100.0; S(9): 0.0	109	1	1	0	N-Term(TMT6plex); S6(Phospho); K12(TMT6plex)
24432106	NP_066997.3	57805	CCAR2	cell cycle and apoptosis regulator protein 2	S678	0.7	0.8	0.6	0.4	0.8	0.9	0.8	0.8	0.7	0.8	0.6	0.4	0.9	0.9	0.9	0.9	0.8	0.9	0.8	0.6	SVAsNQSEMEFSSLQDMPK	SEVRSVAsNQSEMEF	S4;	Yes	S(1): 0.0; S(4): 100.0; S(7): 0.0; S(12): 0.0; S(13): 0.0	11	1	1	0	N-Term(TMT6plex); S4(Phospho); M9(Oxidation); M17(Oxidation); K19(TMT6plex)
24432106	NP_066997.3	57805	CCAR2	cell cycle and apoptosis regulator protein 2	S678	0.8	0.9	0.8	0.8	0.7	0.8	0.6	0.4	0.9	0.9	0.9	0.9	0.7	0.8	0.6	0.4	0.8	0.9	0.8	0.6	SVASNQsEMEFSSLQDMPK	RSVASNQsEMEFSSL	S4;	Yes	S(1): 0.0; S(4): 1.1; S(7): 98.9; S(12): 0.0; S(13): 0.0	4	1	1	0	N-Term(TMT6plex); S7(Phospho); M9(Oxidation); M17(Oxidation); K19(TMT6plex)
284005309	NP_004251.3	9401	RECQL4	ATP-dependent DNA helicase Q4	S89	0.7	0.6	0.7	0.7	0.6	0.7	0.6	0.6	0.9	0.7	0.8	0.8	0.7	0.9	0.7	0.7	0.8	0.8	0.7	0.7	AATKsPQPTPGR	LNRAATKsPQPTPGR	S5;	No	T(3): 0.0; S(5): 100.0; T(9): 0.0	4	1	1	1	N-Term(TMT6plex); K4(TMT6plex); S5(Phospho)
557357687	NP_001273448.1	347734	SLC35B2	adenosine 3'-phospho 5'-phosphosulfate transporter 1 isoform f	S294	0.6	0.5	0.5	0.7	0.5	0.4	0.3	0.5	0.7	0.7	0.6	0.9	0.5	0.5	0.3	0.6	0.6	0.6	0.5	0.7	AVPVEsPVQKV	KKAVPVEsPVQKV_	S6;	No	S(6): 100.0	3	6	1	1	N-Term(TMT6plex); S6(Phospho); K10(TMT6plex)
118572613	NP_057417.3	23524	SRRM2	serine/arginine repetitive matrix protein 2	S2382	0.7	0.6	0.5	0.7	0.5	0.5	0.6	0.6	0.7	0.7	0.5	0.7	0.5	0.5	0.6	0.7	0.6	0.6	0.6	0.7	MAPALSGANLTsPR	LSGANLTsPRVPLSA	S12;	Yes	S(6): 0.0; T(11): 0.7; S(12): 99.3	436	1	1	0	N-Term(TMT6plex); M1(Oxidation); S12(Phospho)
55749531	NP_006833.2	10992	SF3B2	splicing factor 3B subunit 2	S309	0.9	1.0	0.9	0.8	0.9	0.9	1.1	0.5	1.0	1.2	1.0	0.9	1.1	1.0	1.3	0.5	1.1	1.1	1.2	0.7	SSLGQSAsETEEDTVSVSK	SSLGQSAsETEEDTV	S8;	Yes	S(1): 0.0; S(2): 0.0; S(6): 0.0; S(8): 100.0; T(10): 0.0; T(14): 0.0; S(16): 0.0; S(18): 0.0	14	1	1	0	N-Term(TMT6plex); S8(Phospho); K19(TMT6plex)
5803177	NP_006741.1	6645	SNTB2	beta-2-syntrophin	S110	0.2	0.2	0.1	0.2	1.0	1.2	0.9	1.1	0.2	0.2	0.1	0.1	0.9	1.3	0.8	1.1	0.5	0.7	0.5	0.6	GPAGEAGAsPPVR	PAGEAGAsPPVRRVR	S9;	Yes	S(9): 100.0	3	1	1	0	N-Term(TMT6plex); S9(Phospho)
166197710	NP_056393.2	26065	LSM14A	protein LSM14 homolog A isoform b	S183	0.9	0.5	0.5	0.6	0.7	0.6	0.6	0.7	1.0	0.6	0.5	0.6	0.8	0.6	0.7	0.7	0.9	0.6	0.6	0.6	SsPQLDPLR	QLSQGRSsPQLDPLR	S2;	Yes	S(1): 0.9; S(2): 99.1	8	2	1	0	N-Term(TMT6plex); S2(Phospho)
37674210	NP_203747.2	85464	SSH2	protein phosphatase Slingshot homolog 2 isoform 2	T1422	0.5	0.9	0.6	0.6	0.6	0.6	0.6	0.6	0.6	1.1	0.8	0.7	0.7	0.7	0.8	0.7	0.7	0.9	0.8	0.7	TTNPFYNtM	TTNPFYNtM_	T8;	Yes	T(1): 0.0; T(2): 0.0; Y(6): 0.0; T(8): 100.0	5	2	1	0	N-Term(TMT6plex); T8(Phospho); M9(Oxidation)
4758148	NP_004392.1	1676	DFFA	DNA fragmentation factor subunit alpha isoform 1	S315	1.2	0.6	1.0	0.6	1.3	0.6	1.2	0.7	1.2	0.7	1.0	0.7	1.2	0.7	1.1	0.8	1.2	0.7	1.1	0.7	AsPPGDLQNPK	SISASKAsPPGDLQN	S2;	Yes	S(2): 100.0	8	1	1	0	N-Term(TMT6plex); S2(Phospho); K11(TMT6plex)
62988361	NP_001615.1	202	AIM1	absent in melanoma 1 protein	S103	0.5	0.5	0.3	0.5	0.7	0.6	0.5	0.7	0.6	0.6	0.4	0.6	0.9	0.7	0.7	0.9	0.7	0.7	0.5	0.8	QPPPASsPTK	KQPPPASsPTKRKGR	S7;	Yes	S(6): 0.0; S(7): 100.0; T(9): 0.0	8	1	1	0	N-Term(TMT6plex); S7(Phospho); K10(TMT6plex)
118572613	NP_057417.3	23524	SRRM2	serine/arginine repetitive matrix protein 2	S1648	1.0	1.3	0.8	0.4	0.7	1.0	0.8	0.8	1.1	1.3	0.9	0.5	0.8	1.1	0.9	0.9	0.9	1.2	0.9	0.7	GPsPEGSSSTESSPEHPPK	SSKGRGPsPEGSSST	S3;	Yes	S(3): 100.0; S(7): 0.0; S(8): 0.0; S(9): 0.0; T(10): 0.0; S(12): 0.0; S(13): 0.0	436	1	1	0	N-Term(TMT6plex); S3(Phospho); K19(TMT6plex)
61743954	NP_001611.1	79026	AHNAK	neuroblast differentiation-associated protein AHNAK isoform 1	S5731; S5739	0.9	0.8	0.5	1.0	0.6	0.2	0.4	0.3	1.1	0.8	0.5	1.1	0.7	0.2	0.4	0.3	0.9	0.5	0.5	0.7	GGVTGsPEASISGsKGDLK	GKGGVTGsPEASISG;PEASISGsKGDLKSS	S6; S14;	Yes;Yes	T(4): 1.6; S(6): 98.4; S(10): 0.0; S(12): 1.6; S(14): 98.4	79	1	1	1	N-Term(TMT6plex); T4(Phospho); S14(Phospho); K15(TMT6plex); K19(TMT6plex)
571026657	NP_001275686.1	29086	BABAM1	BRISC and BRCA1-A complex member 1 isoform 2	S29	1.1	0.9	1.0	0.6	1.3	0.8	0.9	0.6	1.1	0.9	1.1	0.6	1.3	0.8	1.0	0.7	1.2	0.9	1.0	0.7	sNPEGAEDR	EPRPRTRsNPEGAED	S1;	Yes	S(1): 100.0	17	2	1	0	N-Term(TMT6plex); S1(Phospho)
347582593	NP_001231567.1	84159	ARID5B	AT-rich interactive domain-containing protein 5B isoform 2	S789	0.8	0.9	0.8	0.7	0.6	0.7	0.7	0.5	-	-	-	-	-	-	-	-	-	-	-	-	AVsPLDPSK	GKKARAVsPLDPSKE	S3;	No	S(3): 100.0; S(8): 0.0	3	2	1	0	N-Term(TMT6plex); S3(Phospho); K9(TMT6plex)
63252863	NP_115820.2	84464	SLX4	structure-specific endonuclease subunit SLX4	S1044	0.7	0.6	0.8	0.8	0.4	0.4	0.5	0.5	0.6	0.6	0.7	0.6	0.3	0.3	0.4	0.4	0.5	0.4	0.5	0.5	ELLGPPQGGsPR	LGPPQGGsPRGSHHT	S10;	Yes	S(10): 100.0	8	1	1	0	N-Term(TMT6plex); S10(Phospho)
226509740	NP_067051.2	58506	SCAF1	splicing factor, arginine/serine-rich 19	S498; S500	0.9	0.8	0.5	1.0	0.5	0.4	0.4	0.3	0.9	0.7	0.4	0.9	0.5	0.4	0.3	0.2	0.7	0.6	0.4	0.6	sPsPAPAPAPAAAAGPPTR	RERYRQRsPSPAPAP;RYRQRSPsPAPAPAP	S1; S3;	Yes;Yes	S(1): 100.0; S(3): 100.0; T(18): 0.0	29	1	1	0	N-Term(TMT6plex); S1(Phospho); S3(Phospho)
4557797	NP_000260.1	4830	NME1	nucleoside diphosphate kinase A isoform b	S122	1.0	1.2	0.7	0.5	1.0	0.8	0.8	0.7	0.8	1.3	0.7	0.5	0.8	0.9	0.9	0.7	0.8	1.1	0.8	0.6	NIIHGSDsVESAEK	NIIHGSDsVESAEKE	S8;	Yes	S(6): 1.4; S(8): 98.6; S(11): 0.0	1	2	1	0	N-Term(TMT6plex); S8(Phospho); K14(TMT6plex)
4502175	NP_001640.1	357	SHROOM2	protein Shroom2	S974	0.5	0.7	0.7	0.6	0.7	0.7	0.9	0.6	0.7	1.1	1.1	1.0	0.9	1.1	1.4	1.0	0.8	1.1	1.2	1.0	EGsPGSQQHPPSQK	DAQCREGsPGSQQHP	S3;	Yes	S(3): 98.8; S(6): 1.2; S(12): 0.0	2	1	1	0	N-Term(TMT6plex); S3(Phospho); K14(TMT6plex)
154146193	NP_116259.2	25957	PNISR	arginine/serine-rich protein PNISR	S726	0.7	1.0	1.2	0.7	0.8	1.0	1.0	0.5	0.8	1.0	1.2	0.7	0.8	1.0	1.0	0.6	0.8	1.0	1.1	0.6	SGSIsVK	FSRSGSIsVKIIRHD	S3;	No	S(1): 0.0; S(3): 0.6; S(5): 99.4	2	1	1	0	N-Term(TMT6plex); S3(Phospho); K7(TMT6plex)
112382226	NP_001036147.1	473	RERE	arginine-glutamic acid dipeptide repeats protein isoform b	S59	0.7	0.5	0.8	0.5	0.8	0.7	0.8	0.7	-	-	-	-	-	-	-	-	-	-	-	-	NsPSAASTSSNDSK	IRSSGRNsPSAASTS	S2;	No	S(2): 100.0; S(4): 0.0; S(7): 0.0; T(8): 0.0; S(9): 0.0; S(10): 0.0; S(13): 0.0	9	2	1	0	N-Term(TMT6plex); S2(Phospho); K14(TMT6plex)
189217919	NP_689929.2	219988	PATL1	protein PAT1 homolog 1	T194	1.2	1.1	0.9	0.9	0.8	0.7	0.6	0.3	1.2	1.2	0.9	0.9	0.8	0.7	0.6	0.3	1.0	0.9	0.7	0.6	AVPIGtPPK	VRAVPIGtPPKQMAV	T6;	Yes	T(6): 100.0	5	1	1	0	N-Term(TMT6plex); T6(Phospho); K9(TMT6plex)
545746335	NP_001271143.1	9320	TRIP12	E3 ubiquitin-protein ligase TRIP12 isoform a	S127	0.9	0.7	0.8	0.6	0.9	0.7	0.7	0.6	0.9	0.7	0.8	0.6	0.9	0.7	0.7	0.6	0.9	0.7	0.8	0.6	TNsPSSAK	PDYNRTNsPSSAKKP	S3;	No	T(1): 0.0; S(3): 100.0; S(5): 0.0; S(6): 0.0	23	3	1	0	N-Term(TMT6plex); S3(Phospho); K8(TMT6plex)
4503739	NP_001446.1	2309	FOXO3	forkhead box protein O3	S253	0.7	1.0	0.7	0.6	0.7	1.0	0.8	0.6	0.9	1.0	0.8	0.6	0.8	1.0	0.9	0.6	0.8	1.0	0.8	0.6	AVsMDNSNK	APRRRAVsMDNSNKY	S3;	Yes	S(3): 100.0; S(7): 0.0	4	1	1	0	N-Term(TMT6plex); S3(Phospho); M4(Oxidation); K9(TMT6plex)
114326455	NP_065971.2	57680	CHD8	chromodomain-helicase-DNA-binding protein 8 isoform 2	S1767	1.1	0.7	0.7	0.7	0.8	0.7	0.6	0.5	1.2	0.8	0.8	0.8	0.9	0.8	0.7	0.6	1.0	0.8	0.7	0.7	VsPSDTTPLVSR	QDYEMRVsPSDTTPL	S2;	No	S(2): 100.0; S(4): 0.0; T(6): 0.0; T(7): 0.0; S(11): 0.0	3	2	1	0	N-Term(TMT6plex); S2(Phospho)
556695389	NP_001273359.1	123169	LEO1	RNA polymerase-associated protein LEO1 isoform 2	S179	0.7	0.8	0.8	0.7	0.8	0.8	0.9	0.5	0.9	0.9	1.1	0.9	0.9	0.9	1.1	0.7	0.9	0.9	1.1	0.8	LQNsDDDEK	DEDKLQNsDDDEKMQ	S4;	Yes	S(4): 100.0	23	2	1	0	N-Term(TMT6plex); S4(Phospho); K9(TMT6plex)
190343008	NP_004501.3	3431	SP110	sp110 nuclear body protein isoform b	S380	0.3	0.6	0.9	0.4	0.7	0.7	0.7	0.8	0.3	0.5	0.7	0.4	0.6	0.6	0.5	0.8	0.5	0.6	0.6	0.6	VTQGAAsPGHGIQEK	RVTQGAAsPGHGIQE	S7;	Yes	T(2): 0.0; S(7): 100.0	3	4	1	0	N-Term(TMT6plex); S7(Phospho); K15(TMT6plex)
217330641	NP_060439.2	55677	IWS1	protein IWS1 homolog	S313	0.8	1.0	1.0	0.6	0.9	0.9	1.0	0.6	0.8	1.0	1.0	0.7	0.9	0.8	1.0	0.7	0.9	0.9	1.0	0.7	GPAsDSETEDASR	GPQKGPAsDSETEDA	S4;	Yes	S(4): 100.0; S(6): 0.0; T(8): 0.0; S(12): 0.0	25	1	1	0	N-Term(TMT6plex); S4(Phospho)
[illegible]	[illegible]	[illegible]	[illegible]	[illegible]	[illegible]	[illegible]	[illegible]	[illegible]	[illegible]	[illegible]	[illegible]	[illegible]	[illegible]	[illegible]	[illegible]	[illegible]	[illegible]	[illegible]	[illegible]	[illegible]	[illegible]	[illegible]	[illegible]	[illegible]	[illegible]	[illegible]	[illegible]	[illegible]	[illegible]	[illegible]	[illegible]	[illegible]	[illegible]	[illegible]	[illegible]

Khan et al., 2019. Multi-omics analysis to characterize cigarette smoke induced molecular alterations in esophageal cells

Supplementary Table 8. List of phosphopeptides identified and quantified in untreated and chronically treated Het1A cells with cigarette smoke condensate for 8 months using SequestHT and Mascot search algorithms with a PhosphoRS probability score ≥75%

Protein group Accession	NP_Accession	Gene ID	Gene Symbol	Protein Description	PhosphoSite (Protein)	Replicate 1: Het-1A-Smoke - 2M/Parental	Replicate 1: Het-1A-Smoke - 4M/Parental	Replicate 1: Het-1A-Smoke - 6M/Parental	Replicate 1: Het-1A-Smoke - 8M/Parental	Replicate 2: Het-1A-Smoke - 2M/Parental	Replicate 2: Het-1A-Smoke - 4M/Parental	Replicate 2: Het-1A-Smoke - 6M/Parental	Replicate 2: Het-1A-Smoke - 8M/Parental	Normalized phosphoprotein ratios with respect to corresponding total protein ratios. Replicate 1: Het-1A-Smoke - 2M/Parental	Normalized Replicate 1: Het-1A-Smoke - 4M/Parental	Normalized Replicate 1: Het-1A-Smoke - 6M/Parental	Normalized Replicate 1: Het-1A-Smoke - 8M/Parental	Normalized Replicate 2: Het-1A-Smoke - 2M/Parental	Normalized Replicate 2: Het-1A-Smoke - 4M/Parental	Normalized Replicate 2: Het-1A-Smoke - 6M/Parental	Normalized Replicate 2: Het-1A-Smoke - 8M/Parental	Average of replicates: Het-1A-Smoke - 2M/Parental	Average of replicates: Het-1A-Smoke - 4M/Parental	Average of replicates: Het-1A-Smoke - 6M/Parental	Average of replicates: Het-1A-Smoke - 8M/Parental	Peptide Sequence	Phosphowindow	PhosphoSite (Peptide)	Phosphosite Plus Evidence	phosphoRS Site Probabilities	# PSMs	# Proteins	# Protein Groups	# Missed Cleavages	Modifications
109255232	NP_001035864.1	9859	CEP170	centrosomal protein of 170 kDa isoform gamma	S1014	0.7	1.1	1.1	0.8	0.2	0.3	0.3	0.2	0.6	1.0	1.0	0.8	0.1	0.2	0.3	0.2	0.4	0.6	0.6	0.5	LGEAsDSELADADK	RARLGEAsDSELADA	S5;	No	S(5): 100.0; S(7): 0.0	11	3	1	0	N-Term(TMT6plex); S5(Phospho); K14(TMT6plex)
4885377	NP_005311.1	3007	HIST1H1D	histone H1.3	T147	0.6	0.4	0.3	0.4	0.7	0.5	0.4	0.6	0.9	0.5	0.5	0.6	1.1	0.6	0.6	0.7	1.0	0.5	0.5	0.6	VAGAAtPK	KKVAGAAtPKKSIKK	T6;	Yes	T(6): 100.0	20	1	1	0	N-Term(TMT6plex); T6(Phospho); K8(TMT6plex)
62955803	NP_056169.1	23511	NUP188	nucleoporin NUP188 homolog	S1717	0.3	0.4	0.5	0.5	0.9	0.7	0.9	0.5	0.3	0.4	0.5	0.4	0.8	0.7	0.8	0.5	0.5	0.5	0.7	0.5	GAPSSPATGVLPsPQGK	PATGVLPsPQGKSTS	S13;	Yes	S(4): 49.6; S(5): 49.6; T(8): 0.7; S(13): 100.0	5	1	1	0	N-Term(TMT6plex); S5(Phospho); S13(Phospho); K17(TMT6plex)
5032013	NP_005724.1	10112	KIF20A	kinesin-like protein KIF20A	S825	0.4	0.5	0.8	0.7	0.1	0.2	0.4	0.3	0.5	0.6	0.9	0.8	0.2	0.2	0.5	0.3	0.3	0.4	0.7	0.6	LQGQVsAK	LKLQGQVsAKKRLGT	S6;	Yes	S(6): 100.0	9	1	1	0	N-Term(TMT6plex); S6(Phospho); K8(TMT6plex)
115527080	NP_004680.2	9112	MTA1	metastasis-associated protein MTA1 isoform MTA1	T564	0.4	0.3	0.5	0.6	0.2	0.2	0.5	0.4	0.5	0.3	0.5	0.6	0.2	0.3	0.5	0.4	0.3	0.3	0.5	0.5	SVSSVLSSLtPAK	SSVLSSLtPAKVAPV	T10;	Yes	S(1): 0.0; S(3): 0.0; S(4): 0.0; S(7): 0.0; S(8): 0.0; T(10): 100.0	7	1	1	0	N-Term(TMT6plex); T10(Phospho); K13(TMT6plex)
253970456	NP_001156752.1	27336	HTATSF1	HIV Tat-specific factor 1	S579	0.7	0.6	0.5	0.5	0.7	0.6	0.4	0.4	0.9	0.7	0.9	0.8	0.9	0.7	0.7	0.7	0.9	0.7	0.8	0.8	DLDEEGsEK	KDLDEEGsEKELHEN	S7;	Yes	S(7): 100.0	16	1	1	0	N-Term(TMT6plex); S7(Phospho); K9(TMT6plex)
58331268	NP_060139.2	54821	ERCC6L	DNA excision repair protein ERCC-6-like	S1069	0.6	0.6	0.7	0.4	0.7	0.5	0.7	0.5	0.7	0.8	0.9	0.6	0.9	0.7	0.9	0.7	0.8	0.7	0.9	0.7	NDIsPPGR	STPKNDIsPPGREES	S4;	Yes	S(4): 100.0	1	1	1	0	N-Term(TMT6plex); S4(Phospho)
55749431	NP_001006934.1	85012	TCEAL3	transcription elongation factor A protein-like 3	S121	0.8	0.6	0.6	0.5	0.8	0.6	0.5	0.4	1.1	1.1	1.0	1.0	1.1	1.1	0.8	0.8	1.1	1.1	0.9	0.9	GTDDsPKDSQEDLQER	TDRGTDDsPKDSQED	S5;	Yes	T(2): 1.6; S(5): 98.4; S(9): 0.0	10	3	1	1	N-Term(TMT6plex); S5(Phospho); K7(TMT6plex)
50513245	NP_005474.2	10036	CHAF1A	chromatin assembly factor 1 subunit A	S141	0.6	0.5	0.6	0.5	0.6	0.5	0.6	0.5	0.8	0.6	0.8	0.6	0.8	0.6	0.8	0.6	0.8	0.6	0.8	0.6	LNSEAsPSR	NKLNSEAsPSREAIN	S6;	Yes	S(3): 0.0; S(6): 100.0; S(8): 0.0	22	1	1	0	N-Term(TMT6plex); S6(Phospho)
41327769	NP_056133.2	23370	ARHGEF18	rho guanine nucleotide exchange factor 18 isoform a	S945	1.1	1.3	1.0	0.6	0.6	1.1	0.6	0.3	1.0	1.3	0.9	0.6	0.6	1.1	0.6	0.3	0.8	1.2	0.8	0.5	SLsPILPGR	SRNRRSLsPILPGRH	S3;	No	S(1): 0.0; S(3): 100.0	2	2	1	0	N-Term(TMT6plex); S3(Phospho)
444299622	NP_001263247.1	90673	PPP1R3E	protein phosphatase 1 regulatory subunit 3E	S66	0.7	0.6	0.5	0.6	0.6	0.7	0.5	0.3	-	-	-	-	-	-	-	-	-	-	-	-	sAPAGGGGAR	SRGRRARsAPAGGGG	S1;	No	S(1): 100.0	1	1	1	0	N-Term(TMT6plex); S1(Phospho)
296841073	NP_001171894.1	54521	WDR44	WD repeat-containing protein 44 isoform 2	S50	1.0	0.4	0.6	0.5	0.8	0.6	0.6	0.4	1.0	0.4	0.6	0.6	0.8	0.6	0.7	0.5	0.9	0.5	0.7	0.6	VGNEsPVQELK	AYKVGNEsPVQELKQ	S5;	Yes	S(5): 100.0	12	2	1	0	N-Term(TMT6plex); S5(Phospho); K11(TMT6plex)
117190342	NP_005383.3	5253	PHF2	lysine-specific demethylase PHF2	S681	0.4	0.2	0.5	0.2	0.4	0.6	0.6	0.7	0.5	0.2	0.5	0.2	0.4	0.7	0.7	0.7	0.4	0.4	0.6	0.5	DEYEYVsDDGELK	RDEYEYVsDDGELKI	S7;	Yes	Y(3): 0.0; Y(5): 1.3; S(7): 98.7	9	1	1	0	N-Term(TMT6plex); S7(Phospho); K13(TMT6plex)
4885377	NP_005311.1	3007	HIST1H1D	histone H1.3	T147	0.4	0.4	0.4	0.5	0.4	0.4	0.3	0.4	0.6	0.5	0.5	0.6	0.5	0.5	0.4	0.6	0.6	0.5	0.5	0.6	KVAGAAtPK	KKVAGAAtPKKSIKK	T7;	Yes	T(7): 100.0	20	1	1	1	N-Term(TMT6plex); K1(TMT6plex); T7(Phospho); K9(TMT6plex)
190684675	NP_006288.2	7515	XRCC1	DNA repair protein XRCC1	S241	0.5	0.2	0.2	0.3	0.9	0.5	0.5	0.6	0.5	0.3	0.2	0.4	0.9	0.5	0.5	0.6	0.7	0.4	0.4	0.5	AIGSTSKPQEsPK	STSKPQEsPKGKRKL	S11;	Yes	S(4): 0.0; T(5): 0.0; S(6): 0.0; S(11): 100.0	2	1	1	0	N-Term(TMT6plex); K7(TMT6plex); S11(Phospho); K13(TMT6plex)
36287110	NP_919410.1	11116	FGFR1OP	FGFR1 oncogene partner isoform b	T170	0.8	0.7	0.7	0.3	0.7	0.7	0.6	0.6	1.0	0.8	0.9	0.3	0.8	0.7	0.8	0.8	0.9	0.8	0.8	0.5	tSAQTTPSK	GKTSAQTtPSKKAND	T6;	Yes	T(1): 0.0; S(2): 0.0; T(5): 0.0; T(6): 100.0; S(8): 0.0	3	2	1	0	N-Term(TMT6plex); T6(Phospho); K9(TMT6plex)
36287110	NP_919410.1	11116	FGFR1OP	FGFR1 oncogene partner isoform b	T170	0.7	0.7	0.6	0.6	0.8	0.7	0.7	0.3	0.8	0.7	0.8	0.8	1.0	0.8	0.9	0.3	0.9	0.8	0.8	0.5	TSAQTtPSK	GKTSAQTtPSKKAND	T6;	Yes	T(1): 0.0; S(2): 0.0; T(5): 0.0; T(6): 100.0; S(8): 0.0	11	2	1	0	N-Term(TMT6plex); T6(Phospho); K9(TMT6plex)
4506671	NP_000995.1	6181	RPLP2	60S acidic ribosomal protein P2	S79	1.0	0.9	1.9	0.6	0.3	0.5	0.9	0.3	0.9	1.3	1.7	0.8	0.3	0.8	0.8	0.3	0.6	1.0	1.3	0.6	LASVPAGGAVAVSAAPGsAAPAAGSAPAAAEEK	AVSAAPGsAAPAAGS	S18;	Yes	S(3): 0.0; S(13): 0.0; S(18): 99.9; S(25): 0.0	4	1	1	0	N-Term(TMT6plex); S18(Phospho); K33(TMT6plex)
21396480	NP_031393.2	22913	RALY	RNA-binding protein Raly isoform 2	S119	0.9	0.7	0.6	0.7	0.7	0.5	0.4	0.2	1.1	1.0	1.0	1.0	0.9	0.7	0.7	0.2	1.0	0.9	0.8	0.6	LsPVPVPR	FDYRGRLsPVPVPRA	S2;	No	S(2): 100.0	6	2	1	0	N-Term(TMT6plex); S2(Phospho)
229577185	NP_001153441.1	2060	EPS15	epidermal growth factor receptor substrate 15 isoform B	S482	0.8	0.6	0.6	0.6	0.7	0.5	0.5	0.3	0.9	0.7	0.7	0.7	0.8	0.6	0.6	0.4	0.8	0.6	0.6	0.6	LDsPDPFK	RSINKLDsPDPFKLN	S3;	No	S(3): 100.0	2	2	1	0	N-Term(TMT6plex); S3(Phospho); K8(TMT6plex)
7656879	NP_055238.1	27125	AFF4	AF4/FMR2 family member 4	S703	0.9	1.1	1.0	0.7	0.7	1.1	0.7	0.2	0.8	1.0	1.0	0.6	0.6	1.0	0.7	0.2	0.7	1.0	0.8	0.4	ELLsPLSEPDDR	MEEKELLsPLSEPDD	S4;	Yes	S(4): 100.0; S(7): 0.0	16	1	1	0	N-Term(TMT6plex); S4(Phospho)
225543461	NP_203752.2	85461	TANC1	protein TANC1 isoform 1	S1564	0.5	0.6	0.4	0.4	0.6	0.2	0.3	0.5	-	-	-	-	-	-	-	-	-	-	-	-	VQISSQNPPPsPMPGR	SSQNPPPsPMPGRIA	S11;	Yes	S(4): 0.0; S(5): 0.0; S(11): 100.0	2	1	1	0	N-Term(TMT6plex); S11(Phospho); M13(Oxidation)
47519639	NP_002366.2	4134	MAP4	microtubule-associated protein 4 isoform 1	T927	0.6	0.4	0.5	0.4	0.7	0.7	0.5	0.5	0.6	0.4	0.6	0.5	0.7	0.6	0.5	0.6	0.7	0.5	0.5	0.5	LATNtSAPDLK	LSRLATNtSAPDLKN	T5;	Yes	T(3): 0.0; T(5): 99.1; S(6): 0.9	47	2	1	0	N-Term(TMT6plex); S6(Phospho); K11(TMT6plex)
17318565	NP_477097.1	9134	CCNE2	G1/S-specific cyclin-E2	S21	0.5	0.5	0.6	0.5	0.4	0.6	0.5	0.4	0.6	0.7	0.8	0.6	0.4	0.8	0.6	0.5	0.5	0.7	0.7	0.5	QQPQPSQTEsPQEAQIIQAK	PQPSQTEsPQEAQII	S10;	Yes	S(6): 0.0; T(8): 0.0; S(10): 100.0	5	1	1	0	N-Term(TMT6plex); S10(Phospho); K20(TMT6plex)
7706254	NP_057018.1	51602	NOP58	nucleolar protein 58	S502	0.7	0.9	0.9	0.2	0.8	0.7	0.9	0.6	0.8	1.0	0.9	0.3	0.9	0.8	0.9	0.7	0.8	0.9	0.9	0.5	EEPLsEEEPCTSTAIASPEK	HIKEEPLsEEEPCTS	S5;	Yes	S(5): 100.0; T(11): 0.0; S(12): 0.0; T(13): 0.0; S(17): 0.0	4	1	1	0	N-Term(TMT6plex); S5(Phospho); C10(Carbamidomethyl); K20(TMT6plex)
4826830	NP_004983.1	4204	MECP2	methyl-CpG-binding protein 2 isoform 1	S229	0.5	0.5	0.5	0.5	0.4	0.6	0.4	0.4	0.7	0.7	0.8	0.7	0.6	0.8	0.7	0.6	0.6	0.7	0.7	0.7	MPFQTsPGGK	VKMPFQTsPGGKAEG	S6;	Yes	T(5): 0.0; S(6): 100.0	14	2	1	0	N-Term(TMT6plex); M1(Oxidation); S6(Phospho); K10(TMT6plex)
7669481	NP_004043.2	664	BNIP3	BCL2/adenovirus E1B 19 kDa protein-interacting protein 3	S66	0.7	0.9	0.7	0.6	0.2	0.3	0.5	0.3	0.6	1.0	0.7	0.6	0.2	0.3	0.5	0.3	0.4	0.6	0.6	0.4	SSHCDsPPR	SKSSHCDsPPRSQTP	S6;	Yes	S(1): 0.0; S(2): 0.0; S(6): 100.0	2	1	1	0	N-Term(TMT6plex); C4(Carbamidomethyl); S6(Phospho)
47519639	NP_002366.2	4134	MAP4	microtubule-associated protein 4 isoform 1	T571	0.6	0.5	0.9	0.4	0.6	1.1	0.9	0.4	0.7	0.4	0.9	0.5	0.6	1.1	0.9	0.5	0.6	0.7	0.9	0.5	DGVLTLANNVtPAK	LTLANNVtPAKDVPP	T11;	Yes	T(5): 0.0; T(11): 100.0	47	2	1	0	N-Term(TMT6plex); T11(Phospho); K14(TMT6plex)
253970456	NP_001156752.1	27336	HTATSF1	HIV Tat-specific factor 1	S616	0.7	0.5	0.6	0.4	0.5	0.5	0.4	0.4	1.0	0.7	0.9	0.6	0.7	0.7	0.7	0.7	0.8	0.7	0.8	0.7	VLDEEGsER	KVLDEEGsEREEDED	S7;	Yes	S(7): 100.0	16	1	1	0	N-Term(TMT6plex); S7(Phospho)
498917186	NP_001265089.1	84335	AKT1S1	proline-rich AKT1 substrate 1 isoform b	T246	0.7	0.7	0.8	0.4	0.9	1.0	1.0	0.4	1.0	0.9	0.8	0.4	1.2	1.3	1.0	0.5	1.1	1.1	0.9	0.4	LNtSDFQK	LPRPRLNtSDFQKLK	T3;	Yes	T(3): 100.0; S(4): 0.0	4	2	1	0	N-Term(TMT6plex); T3(Phospho); K8(TMT6plex)
4885379	NP_005312.1	3008	HIST1H1E	histone H1.4	T146	0.3	0.4	0.3	0.4	0.3	0.3	0.3	0.4	0.5	0.4	0.4	0.5	0.5	0.4	0.4	0.5	0.5	0.4	0.4	0.5	KATGAAtPK	KKATGAAtPKKSAKK	T7;	Yes	T(3): 0.0; T(7): 100.0	12	1	1	1	N-Term(TMT6plex); K1(TMT6plex); T7(Phospho); K9(TMT6plex)
634743290	NP_001278893.1	4301	MLLT4	afadin isoform 4	S1756	0.7	0.9	0.4	0.3	0.5	0.8	0.6	0.4	0.6	0.8	0.3	0.3	0.4	0.7	0.5	0.4	0.5	0.7	0.4	0.3	LEsQGQDVSNK	KERQRLEsQGQDVSN	S3;	No	S(3): 100.0; S(9): 0.0	16	2	1	0	N-Term(TMT6plex); S3(Phospho); K11(TMT6plex)
40254834	NP_006603.2	10749	KIF1C	kinesin-like protein KIF1C	S494	0.4	0.4	0.7	0.4	0.2	0.2	0.7	0.4	0.4	0.4	0.7	0.4	0.3	0.2	0.8	0.4	0.3	0.3	0.7	0.4	EDGGTVGVFsPK	GGTVGVFsPKKTPHL	S10;	Yes	T(5): 0.0; S(10): 100.0	2	1	1	0	N-Term(TMT6plex); S10(Phospho); K12(TMT6plex)
14670388	NP_116784.1	572	BAD	bcl2 antagonist of cell death	S99	0.7	0.6	0.6	0.5	0.3	0.2	0.2	0.2	0.7	0.6	0.5	0.5	0.3	0.2	0.2	0.2	0.5	0.4	0.4	0.4	sAPPNLWAAQR	PFRGRSRsAPPNLWA	S1;	Yes	S(1): 100.0	4	1	1	0	N-Term(TMT6plex); S1(Phospho)
31742503	NP_066403.2	126961	HIST2H3C	histone H3.2	S29	0.3	0.3	0.3	0.4	0.4	0.3	0.3	0.4	0.3	0.4	0.4	0.5	0.4	0.4	0.3	0.5	0.3	0.4	0.3	0.5	sAPATGGVK	ATKAARKsAPATGGV	S1;	No	S(1): 100.0; T(5): 0.0	6	3	1	0	N-Term(TMT6plex); S1(Phospho); K9(TMT6plex)
4885381	NP_005313.1	3009	HIST1H1B	histone H1.5	S18	0.3	0.3	0.3	0.4	0.3	0.3	0.3	0.4	0.5	0.4	0.5	0.5	0.5	0.4	0.4	0.5	0.5	0.4	0.5	0.5	SETAPAETATPAPVEKsPAKK	TPAPVEKsPAKKKAT	S17;	Yes	S(1): 0.0; T(3): 0.0; T(8): 0.0; T(10): 0.0; S(17): 100.0	17	1	1	2	N-Term(TMT6plex); K16(TMT6plex); S17(Phospho); K20(TMT6plex); K21(TMT6plex)
13786127	NP_036253.2	23580	CDC42EP4	cdc42 effector protein 4	S118	0.6	0.8	0.8	0.5	0.4	0.2	0.5	0.2	0.6	0.9	0.9	0.5	0.4	0.3	0.5	0.3	0.5	0.6	0.7	0.4	NAMsLPQLNEK	LEVKNAMsLPQLNEK	S4;	Yes	S(4): 100.0	8	1	1	0	N-Term(TMT6plex); M3(Oxidation); S4(Phospho); K11(TMT6plex)
56790930	NP_004425.2	2009	EML1	echinoderm microtubule-associated protein-like 1 isoform b	S160	1.1	0.6	0.6	0.4	1.2	0.5	0.5	0.4	1.4	0.9	0.9	0.6	1.5	0.8	0.7	0.5	1.5	0.8	0.8	0.6	TGsTSSSSGK	GNRNRTGsTSSSSG	S3;	Yes	T(1): 0.0; S(3): 100.0; T(4): 0.0; S(5): 0.0; S(6): 0.0; S(7): 0.0; S(8): 0.0; S(9): 0.0	2	2	1	0	N-Term(TMT6plex); S3(Phospho); K11(TMT6plex)
431822379	NP_001258930.1	85440	DOCK7	dedicator of cytokinesis protein 7 isoform 4	S900	0.5	0.8	1.1	0.4	0.6	0.5	0.6	0.3	0.5	0.8	1.2	0.4	0.6	0.5	0.7	0.3	0.6	0.7	0.9	0.4	SLSNsNPDISGTPTSPDDEVR	RSRSLSNsNPDISGT	S5;	Yes	S(1): 0.0; S(3): 0.0; S(5): 100.0; S(10): 0.0; T(12): 0.0; T(14): 0.0; S(15): 0.0	5	4	1	0	N-Term(TMT6plex); S5(Phospho)
8923857	NP_060917.1	55831	EMC3	ER membrane protein complex subunit 3	T204	0.7	1.0	0.9	0.5	1.8	1.5	1.1	0.2	0.7	1.1	1.0	0.5	1.9	1.5	1.2	0.3	1.3	1.3	1.1	0.4	MMQEQMtGAAMAMPADTNK	RMMQEQMtGAAMAMP	T7;	No	T(7): 100.0; T(17): 0.0	1	1	1	0	N-Term(TMT6plex); T7(Phospho); K19(TMT6plex)
9966805	NP_065147.1	57062	DDX24	ATP-dependent RNA helicase DDX24	S82	0.8	1.1	0.6	0.3	0.8	0.8	0.8	0.4	1.0	1.3	0.8	0.3	1.0	1.0	1.0	0.5	1.0	1.2	0.9	0.4	KAQAVsEEEEEEEGK	KRKAQAVsEEEEEEE	S6;	Yes	S(6): 100.0	10	1	1	1	N-Term(TMT6plex); K1(TMT6plex); S6(Phospho); K15(TMT6plex)
47419905	NP_005987.3	6926	TBX3	T-box transcription factor TBX3 isoform 1	S687	0.8	0.5	0.6	0.3	0.3	0.5	1.2	0.4	-	-	-	-	-	-	-	-	-	-	-	-	SSTLSSSSMSLsPK	SSSSMSLsPKLCAEK	S12;	No	S(1): 0.0; S(2): 0.0; T(3): 0.0; S(5): 0.0; S(6): 0.0; S(7): 0.0; S(8): 0.0; S(10): 0.0; S(12): 100.0	2	2	1	0	N-Term(TMT6plex); M9(Oxidation); S12(Phospho); K14(TMT6plex)
4885381	NP_005313.1	3009	HIST1H1B	histone H1.5	T11; S18	0.3	0.3	0.3	0.3	0.4	0.4	0.3	0.3	0.4	0.3	0.4	0.5	0.6	0.5	0.4	0.4	0.5	0.4	0.4	0.4	SETAPAETATPAPVEKsPAK	TAPAETAtPAPVEKS;TPAPVEKsPAKKKAT	T10; S17;	Yes;Yes	S(1): 0.0; T(3): 0.0; T(8): 0.0; T(10): 100.0; S(17): 100.0	17	1	1	1	N-Term(TMT6plex); T10(Phospho); K16(TMT6plex); S17(Phospho); K20(TMT6plex)
46358076	NP_079038.2	79818	ZNF552	zinc finger protein 552	S86	0.4	0.4	0.4	0.3	0.8	0.6	0.8	0.3	-	-	-	-	-	-	-	-	-	-	-	-	TPMAGVsPK	RTPMAGVsPKKAHPC	S7;	Yes	T(1): 0.0; S(7): 100.0	2	2	1	0	N-Term(TMT6plex); M3(Oxidation); S7(Phospho); K9(TMT6plex)
4885379	NP_005312.1	3008	HIST1H1E	histone H1.4	T18	0.4	0.3	0.4	0.3	0.3	0.2	0.2	0.2	0.5	0.3	0.5	0.4	0.5	0.3	0.3	0.3	0.5	0.3	0.4	0.3	SETAPAAPAAPAPAEKtPVK	APAPAEKtPVKKKAR	T17;	Yes	S(1): 0.0; T(3): 0.0; T(17): 100.0	12	1	1	1	N-Term(TMT6plex); K16(TMT6plex); T17(Phospho); K20(TMT6plex)
61743954	NP_001611.1	79026	AHNAK	neuroblast differentiation-associated protein AHNAK isoform 1	S4903	0.5	0.4	0.4	0.2	0.4	0.4	0.3	0.3	0.6	0.4	0.4	0.3	0.5	0.4	0.3	0.3	0.5	0.4	0.4	0.3	LSGPsLK	DAKLSGPsLKMPSLE	S5;	Yes	S(2): 0.0; S(5): 100.0	79	1	1	0	N-Term(TMT6plex); S5(Phospho); K7(TMT6plex)
222352111	NP_115766.3	84365	NIFK	MKI67 FHA domain-interacting nucleolar phosphoprotein	T223	0.6	0.3	0.3	0.2	0.4	0.5	0.5	0.3	0.6	0.3	0.3	0.2	0.5	0.5	0.5	0.3	0.5	0.4	0.4	0.3	VSGTLDtPEK	KVSGTLDtPEKTVDS	T7;	No	S(2): 0.0; T(4): 0.0; T(7): 100.0	5	1	1	0	N-Term(TMT6plex); T7(Phospho); K10(TMT6plex)
4758808	NP_004832.1	9462	RASAL2	ras GTPase-activating protein nGAP isoform 1	T620	0.1	0.1	0.1	0.1	0.1	0.1	0.1	0.1	0.1	0.1	0.1	0.1	0.1	0.1	0.1	0.1	0.1	0.1	0.1	0.1	SLTNPtPIQQQLR	TKSLTNPtPIQQQLR	T6;	Yes	S(1): 0.0; T(3): 0.0; T(6): 100.0	5	2	1	0	N-Term(TMT6plex); T6(Phospho)
57864582	NP_001009931.1	388697	HRNR	hornerin	S542	2.9	6.1	2.9	8.5	-	-	-	-	2.8	3.3	2.8	3.9	-	-	-	-	2.8	3.3	2.8	3.9	HGSGSGQSPsPSR	SGSGQSPsPSRGRHE	S10;	No	S(3): 0.0; S(5): 0.0; S(8): 1.2; S(10): 98.7; S(12): 0.0	10	1	1	0	N-Term(TMT6plex); S10(Phospho)
5032153	NP_005635.1	6883	TAF12	transcription initiation factor TFIID subunit 12	S51	1.9	2.4	3.1	4.2	-	-	-	-	-	-	-	-	-	-	-	-	-	-	-	-	LsPENNQVLTK	PGAGGRLsPENNQVL	S2;	Yes	S(2): 100.0; T(10): 0.0	1	1	1	0	N-Term(TMT6plex); S2(Phospho); K11(TMT6plex)
217330574	NP_001136088.1	23301	EHBP1	EH domain-binding protein 1 isoform 3	S752	4.3	2.9	3.4	3.9	-	-	-	-	4.5	2.9	3.3	3.3	-	-	-	-	4.5	2.9	3.3	3.3	QTEsDPDADR	ASLRQTEsDPDADRT	S4;	No	T(2): 1.0; S(4): 99.0	5	3	1	0	N-Term(TMT6plex); S4(Phospho)
16950636	NP_001392.2	1902	LPAR1	lysophosphatidic acid receptor 1	S341	3.9	4.0	4.4	3.4	-	-	-	-	-	-	-	-	-	-	-	-	-	-	-	-	SENPTGPTEGsDR	PTGPTEGsDRSASSL	S11;	No	S(1): 0.0; T(5): 1.7; T(8): 1.7; S(11): 96.6	1	1	1	0	N-Term(TMT6plex); S11(Phospho)
160948599	NP_001073922.2	26173	INTS1	integrator complex subunit 1	S104	2.0	2.5	2.5	3.3	-	-	-	-	1.9	2.6	2.6	3.5	-	-	-	-	1.9	2.6	2.6	3.5	AIsPSIK	VAEKRAIsPSIKEPS	S3;	Yes	S(3): 100.0; S(5): 0.0	3	1	1	0	N-Term(TMT6plex); S3(Phospho); K7(TMT6plex)
13346498	NP_056287.1	25937	WWTR1	WW domain-containing transcription regulator protein 1	S65	1.9	3.1	3.8	2.8	-	-	-	-	-	-	-	-	-	-	-	-	-	-	-	-	QsSTDSSGGHPGPR	SGSHSRQsSTDSSGG	S2;	Yes	S(2): 98.0; S(3): 1.0; T(4): 1.0; S(6): 0.0; S(7): 0.0	2	1	1	0	N-Term(TMT6plex); S2(Phospho)
74316006	NP_775786.2	154043	CNKSR3	connector enhancer of kinase suppressor of ras 3	S383	2.0	3.0	2.6	2.8	-	-	-	-	-	-	-	-	-	-	-	-	-	-	-	-	GSEsPNSFLDQESR	PGPKGSEsPNSFLDQ	S4;	Yes	S(2): 0.0; S(4): 99.9; S(7): 0.0; S(13): 0.0	1	1	1	0	N-Term(TMT6plex); S4(Phospho)
56550039	NP_005924.2	4297	KMT2A	histone-lysine N-methyltransferase 2A isoform 2 precursor	S1056	2.0	2.5	2.2	2.8	-	-	-	-	1.8	2.4	2.3	2.8	-	-	-	-	1.8	2.4	2.3	2.8	AQGQEsDSSETSVR	PKAQGQEsDSSETSV	S6;	No	S(6): 99.8; S(8): 0.1; S(9): 0.1; T(11): 0.0; S(12): 0.0	15	2	1	0	N-Term(TMT6plex); S6(Phospho)
54633315	NP_055412.2	30836	DNTTIP2	deoxynucleotidyltransferase terminal-interacting protein 2	T232	1.0	0.9	1.1	2.8	-	-	-	-	1.1	1.0	1.2	3.1	-	-	-	-	1.1	1.0	1.2	3.1	QIVGtPVNSEDSDTR	NEKQIVGtPVNSEDS	T5;	Yes	T(5): 99.9; S(9): 0.0; S(12): 0.0; T(14): 0.0	2	1	1	0	N-Term(TMT6plex); T5(Phospho)
374093212	NP_001243357.1	10611	PDLIM5	PDZ and LIM domain protein 5 isoform h	S106	1.7	2.2	2.3	2.7	-	-	-	-	1.7	1.9	2.0	2.1	-	-	-	-	1.7	1.9	2.0	2.1	RGsQGDSK	AEGQRRGsQGDSKQQ	S3;	No	S(3): 100.0; S(7): 0.0	6	6	1	1	N-Term(TMT6plex); S3(Phospho); K8(TMT6plex)
196114970	NP_001124481.1	5782	PTPN12	tyrosine-protein phosphatase non-receptor type 12 isoform 3	T389	0.9	2.7	1.5	2.7	-	-	-	-	0.8	2.7	1.5	2.6	-	-	-	-	0.8	2.7	1.5	2.6	VSVTPPEESQNSDtPPRPDR	EESQNSDtPPRPDRL	T14;	No	S(2): 0.1; T(4): 0.1; S(9): 2.7; S(12): 2.7; T(14): 94.4	8	3	1	0	N-Term(TMT6plex); T14(Phospho)
217416362	NP_115883.2	84524	ZC3H8	zinc finger CCCH domain-containing protein 8	T32	1.0	0.9	2.1	2.7	-	-	-	-	1.2	1.0	2.3	3.5	-	-	-	-	1.2	1.0	2.3	3.5	IDDEIDtEVEETQEEK	RIDDEIDtEVEETQE	T7;	Yes	T(7): 97.2; T(12): 2.8	1	1	1	0	N-Term(TMT6plex); T12(Phospho); K16(TMT6plex)
82546824	NP_001032242.1	221937	FOXK1	forkhead box protein K1	S445	2.2	0.9	1.3	2.7	-	-	-	-	2.1	0.9	1.3	2.8	-	-	-	-	2.1	0.9	1.3	2.8	EGsPIPHDPEFGSK	EGLSREGsPIPHDPE	S3;	Yes	S(3): 100.0; S(13): 0.0	6	1	1	0	N-Term(TMT6plex); S3(Phospho); K14(TMT6plex)
557636741	NP_001273556.1	10921	RNPS1	RNA-binding protein with serine-rich domain 1 isoform c	S102	4.9	4.8	5.6	2.6	-	-	-	-	5.6	5.1	6.5	2.7	-	-	-	-	5.6	5.1	6.5	2.7	SSSSSSSSGSPsPSR	SSSSGSPsPSRRRHD	S12;	No	S(1): 0.0; S(2): 0.0; S(3): 0.0; S(4): 0.0; S(5): 0.0; S(6): 0.0; S(7): 0.0; S(8): 0.0; S(10): 0.0; S(12): 100.0; [illegible]	1	3	1	0	N-Term(TMT6plex); S12(Phospho)
163310749	NP_001106191.1	22998	LIMCH1	LIM and calponin homology domains-containing protein 1 isoform e	S211	2.1	2.0	2.7	2.6	-	-	-	-	1.7	1.8	2.1	2.0	-	-	-	-	1.7	1.8	2.1	2.0	sVSQDLIK	RWKSRRRsVSQDLIK	S1;	No	S(1): 100.0; S(3): 0.0	5	7	1	0	N-Term(TMT6plex); S1(Phospho); K8(TMT6plex)
5032189	NP_005648.1	7158	TP53BP1	tumor suppressor p53-binding protein 1 isoform 3	S580	1.0	3.8	1.8	2.6	-	-	-	-	1.0	3.7	2.0	3.1	-	-	-	-	1.0	3.7	2.0	3.1	FVPAENDSILMNPAQDGEVQLsQNDDKTK	QDGEVQLsQNDDKTK	S22;	Yes	S(8): 0.0; S(22): 96.8; T(28): 3.2	62	3	1	1	N-Term(TMT6plex); M11(Oxidation); S22(Phospho); K27(TMT6plex); K29(TMT6plex)
224586882	NP_003642.3	8531	YBX3	Y-box-binding protein 3 isoform a	S79	1.0	2.7	4.2	2.5	-	-	-	-	0.7	1.7	2.4	2.1	-	-	-	-	0.7	1.7	2.4	2.1	SPVGSGAPQAAAPAPAAHVAGNPGGDAAPAATGTAAAASLATAAGsEDAEKK	SLATAAGsEDAEKKV	S46;	No	S(1): 0.0; S(5): 0.0; T(32): 0.0; T(34): 0.0; S(39): 0.0; T(42): 0.0; S(46): 100.0	14	2	1	1	N-Term(TMT6plex); S46(Phospho); K51(TMT6plex); K52(TMT6plex)
50962882	NP_001002909.1	23131	GPATCH8	G patch domain-containing protein 8	S451; T457; S459; S462	1.9	2.0	1.7	2.5	-	-	-	-	2.0	2.1	1.8	2.6	-	-	-	-	2.0	2.1	1.8	2.6	AAAsQGAEKtVsEVsEQPK	SCIKAAAsQGAEKTV;ASQGAEKtVSEVSEQ;QGAEKTVsEVSEQPK;EKTVSEVsEQPKETS	[illegible]4; T10; S12; S1[illegible]	Yes;No;Yes;N[illegible]	S(4): 75.0; T(10): 75.0; S(12): 75.0; S(15): 75.0	17	1	1	1	N-Term(TMT6plex); K9(TMT6plex); T10(Phospho); S12(Phospho); S15(Phospho); K19(TMT6plex)
20357556	NP_612632.1	2017	CTTN	src substrate cortactin isoform b	S381	2.5	3.2	4.7	2.4	-	-	-	-	2.0	2.5	3.2	2.2	-	-	-	-	2.0	2.5	3.2	2.2	LPSsPVYEDAASFK	TEERLPSsPVYEDAA	S4;	Yes	S(3): 0.9; S(4): 99.1; Y(7): 0.0; S(12): 0.0	8	3	1	0	N-Term(TMT6plex); S4(Phospho); K14(TMT6plex)
31377679	NP_079056.2	79838	TMC5	transmembrane channel-like protein 5 isoform c	S569	2.6	2.4	1.8	2.4	-	-	-	-	-	-	-	-	-	-	-	-	-	-	-	-	NIsLMMNFQPPSK	MFYSKNIsLMMNFQP	S3;	No	S(3): 100.0; S(12): 0.0	1	3	1	0	N-Term(TMT6plex); S3(Phospho); M5(Oxidation); M6(Oxidation); K13(TMT6plex)
7705425	NP_057053.1	51373	MRPS17	28S ribosomal protein S17, mitochondrial	S128	1.4	1.7	1.7	2.4	-	-	-	-	1.4	1.7	1.7	2.4	-	-	-	-	1.4	1.7	1.7	2.4	NLEELNISsAQ	LEELNISsAQ	S9;	Yes	S(8): 1.2; S(9): 98.8	1	1	1	0	N-Term(TMT6plex); S9(Phospho)
46389560	NP_996930.1	2029	ENSA	alpha-endosulfine isoform 7	S105	3.0	1.2	4.8	2.4	-	-	-	-	3.0	1.2	4.5	2.3	-	-	-	-	3.0	1.2	4.5	2.3	SsLVTSK	DLPQRKSsLVTSKLA	S2;	No	S(1): 0.0; S(2): 100.0; T(5): 0.0; S(6): 0.0	1	7	1	0	N-Term(TMT6plex); S2(Phospho); K7(TMT6plex)
7706457	NP_057332.1	11215	AKAP11	A-kinase anchor protein 11	T981	1.1	1.1	2.5	2.4	-	-	-	-	0.8	1.0	2.5	1.9	-	-	-	-	0.8	1.0	2.5	1.9	ESLPVSGEESQLtPEK	SGEESQLtPEKSPKF	T13;	Yes	S(2): 0.0; S(6): 0.0; S(10): 0.0; T(13): 100.0	3	1	1	0	N-Term(TMT6plex); T13(Phospho); K16(TMT6plex)
66392157	NP_001013860.1	146206	RLTPR	leucine-rich repeat-containing protein 16C	S1423	1.5	1.9	1.1	2.4	-	-	-	-	1.2	1.3	0.7	1.7	-	-	-	-	1.2	1.3	0.7	1.7	SPPsPATDQR	SPERSPPsPATDQRG	S4;	No	S(1): 0.0; S(4): 100.0; T(7): 0.0	5	1	1	0	N-Term(TMT6plex); S4(Phospho)
203098098	NP_065910.3	57619	SHROOM3	protein Shroom3	S1441	0.7	2.0	1.7	2.3	-	-	-	-	-	-	-	-	-	-	-	-	-	-	-	-	EDsLPEESSAPDEANLK	AHAAREDsLPEESSA	S3;	Yes	S(3): 99.9; S(8): 0.1; S(9): 0.1	1	1	1	0	N-Term(TMT6plex); S3(Phospho); K17(TMT6plex)
57864582	NP_001009931.1	388697	HRNR	hornerin	S775	0.5	2.1	1.0	2.3	-	-	-	-	0.5	1.1	1.0	1.0	-	-	-	-	0.5	1.1	1.0	1.0	QGSGSGHsPSR	QGSGSGHsPSRVRHG	S8;	Yes	S(3): 0.0; S(5): 0.0; S(8): 98.8; S(10): 1.2	10	1	1	0	N-Term(TMT6plex); S8(Phospho)
215820630	NP_115731.2	84315	MON1A	vacuolar fusion protein MON1 homolog A isoform a	S153	2.6	2.5	1.9	2.2	-	-	-	-	2.4	2.1	1.6	2.0	-	-	-	-	2.4	2.1	1.6	2.0	sYEDLTESEDGAASGDSHK	AMFVHARsYEDLTES	S1;	No	S(1): 97.8; Y(2): 2.1; T(6): 0.0; S(8): 0.0; S(14): 0.0; S(17): 0.0	1	1	1	0	N-Term(TMT6plex); Y2(Phospho); K19(TMT6plex)
32130514	NP_071922.2	57446	NDRG3	protein NDRG3 isoform b	S319	1.7	1.5	1.3	2.2	-	-	-	-	2.0	1.7	1.7	2.3	-	-	-	-	2.0	1.7	1.7	2.3	THsTSSSLGSGESPFSR	LARSRTHsTSSSLGS	S3;	No	T(1): 0.0; S(3): 99.9; T(4): 0.0; S(5): 0.0; S(6): 0.0; S(7): 0.0; S(10): 0.0; S(13): 0.0; S(16): 0.0	2	2	1	0	N-Term(TMT6plex); S3(Phospho)
7656879	NP_055238.1	27125	AFF4	AF4/FMR2 family member 4	T528	1.9	1.8	2.0	2.2	-	-	-	-	1.7	1.6	1.9	2.1	-	-	-	-	1.7	1.6	1.9	2.1	ETSSAtPGR	PKETSSAtPGRDSKT	T6;	Yes	T(2): 0.0; S(3): 0.0; S(4): 0.0; T(6): 100.0	16	1	1	0	N-Term(TMT6plex); T6(Phospho)
38373675	NP_005303.2	2889	RAPGEF1	rap guanine nucleotide exchange factor 1 isoform a	S281	0.9	0.8	0.5	2.1	-	-	-	-	0.8	0.7	0.5	2.0	-	-	-	-	0.8	0.7	0.5	2.0	VVDNsPPPALPPK	GIRVVDNsPPPALPP	S5;	Yes	S(5): 100.0	1	2	1	0	N-Term(TMT6plex); S5(Phospho); K13(TMT6plex)
253683544	NP_258443.2	94026	POM121L2	POM121-like protein 2	S983; S984	1.0	1.1	1.4	2.1	-	-	-	-	-	-	-	-	-	-	-	-	-	-	-	-	AGssVGFGMPFPPAQGSVGR	PGQAKAGsSVGFGMP;GQAKAGSsVGFGMPF	S3; S4;	No;No	S(3): 90.4; S(4): 90.4; S(17): 19.3	1	1	1	0	N-Term(TMT6plex); S3(Phospho); S4(Phospho)
545746392	NP_001271170.1	54462	CCSER2	serine-rich coiled-coil domain-containing protein 2 isoform 3	S206	2.5	1.4	1.9	2.1	-	-	-	-	-	-	-	-	-	-	-	-	-	-	-	-	SSSGESLAQsPDSSK	SGESLAQsPDSSKSI	S10;	Yes	S(1): 0.0; S(2): 0.0; S(3): 0.0; S(6): 0.0; S(10): 100.0; S(13): 0.0; S(14): [illegible]	1	3	1	0	N-Term(TMT6plex); S10(Phospho); K15(TMT6plex)
22547195	NP_004167.3	6720	SREBF1	sterol regulatory element-binding protein 1 isoform b	S117	0.8	0.8	1.5	2.0	-	-	-	-	0.7	0.6	1.3	1.5	-	-	-	-	0.7	0.6	1.3	1.5	MYPSMPAFsPGPGIK	YPSMPAFsPGPGIKE	S9;	Yes	Y(2): 0.0; S(4): 0.0; S(9): 100.0	2	2	1	0	M1(Oxidation); N-Term(TMT6plex); M5(Oxidation); S9(Phospho); K15(TMT6plex)
45359849	NP_987101.1	9908	G3BP2	ras GTPase-activating protein-binding protein 2 isoform a	S225	1.0	1.1	1.7	2.0	-	-	-	-	1.0	1.1	1.6	1.9	-	-	-	-	1.0	1.1	1.6	1.9	sTTPPPAEPVSLPQEPPK	LEELEEKsTTPPPAE	S1;	Yes	S(1): 98.1; T(2): 1.0; T(3): 1.0; S(11): 0.0	3	1	1	0	N-Term(TMT6plex); S1(Phospho); K18(TMT6plex)
148727247	NP_003472.2	8078	USP5	ubiquitin carboxyl-terminal hydrolase 5 isoform 2	S760	1.0	2.3	1.9	2.0	-	-	-	-	1.0	2.1	1.7	1.9	-	-	-	-	1.0	2.1	1.7	1.9	SAADsISESVPVGPK	EGRSAADsISESVPV	S5;	No	S(1): 0.0; S(5): 100.0; S(7): 0.0; S(9): 0.0	5	2	1	0	N-Term(TMT6plex); S5(Phospho); K15(TMT6plex)
7706254	NP_057018.1	51602	NOP58	nucleolar protein 58	S502; S514	1.3	1.1	2.1	1.9	-	-	-	-	1.4	1.2	2.2	2.1	-	-	-	-	1.4	1.2	2.2	2.1	EEPLsEEEPCTSTAIAsPEKK	HIKEEPLsEEEPCTS;CTSTAIAsPEKKKKK	S5; S17;	Yes;Yes	S(5): 100.0; T(11): 0.0; S(12): 0.0; T(13): 0.0; S(17): 100.0	4	1	1	1	N-Term(TMT6plex); S5(Phospho); C10(Carbamidomethyl); S17(Phospho); K20(TMT6plex); K21(TMT6plex)
4507653	NP_000358.1	7172	TPMT	thiopurine S-methyltransferase	S14	1.0	1.4	1.5	1.9	-	-	-	-	1.0	1.7	1.6	1.9	-	-	-	-	1.0	1.7	1.6	1.9	TSLDIEEYsDTEVQK	SLDIEEYsDTEVQKN	S9;	Yes	T(1): 0.0; S(2): 0.0; Y(8): 0.0; S(9): 98.8; T(11): 1.2	2	1	1	0	N-Term(TMT6plex); T11(Phospho); K15(TMT6plex)
153792539	NP_055189.2	29803	REPIN1	replication initiator 1 isoform 1	S27	0.5	1.3	1.5	1.9	-	-	-	-	0.5	1.4	1.5	2.2	-	-	-	-	0.5	1.4	1.5	2.2	LLSGPSQEsPQTLGK	LSGPSQEsPQTLGKE	S9;	Yes	S(3): 0.0; S(6): 0.0; S(9): 98.4; T(12): 1.5	1	2	1	0	N-Term(TMT6plex); S9(Phospho); K15(TMT6plex)
5031635	NP_005498.1	1072	CFL1	cofilin-1	T25	1.1	1.6	1.3	1.9	-	-	-	-	1.0	1.4	1.1	1.7	-	-	-	-	1.0	1.4	1.1	1.7	SStPEEVK	MKVRKSStPEEVKKR	T3;	Yes	S(1): 0.0; S(2): 1.0; T(3): 99.0	1	1	1	0	N-Term(TMT6plex); T3(Phospho); K8(TMT6plex)
21361304	NP_006471.2	10636	RGS14	regulator of G-protein signaling 14	S203	1.0	1.4	1.4	1.9	-	-	-	-	-	-	-	-	-	-	-	-	-	-	-	-	LGsPDATR	PGSSRLGsPDATRKK	S3;	Yes	S(3): 100.0; T(7): 0.0	1	1	1	0	N-Term(TMT6plex); S3(Phospho)
121114287	NP_056131.2	23368	PPP1R13B	apoptosis-stimulating of p53 protein 1	S709	2.2	1.5	1.3	1.9	-	-	-	-	-	-	-	-	-	-	-	-	-	-	-	-	sSITEPEGPGGPNIQK	PRPLKKRsSITEPEG	S1;	No	S(1): 100.0; S(2): 0.0; T(4): 0.0	2	1	1	0	N-Term(TMT6plex); S1(Phospho); K16(TMT6plex)
355390315	NP_056171.3	23513	SCRIB	protein scribble homolog isoform b	S1285	1.8	2.1	1.9	1.9	-	-	-	-	1.6	1.9	1.9	1.7	-	-	-	-	1.6	1.9	1.9	1.7	VPsGAAGGK	GSVQRVPsGAAGGKM	S3;	Yes	S(3): 100.0	16	2	1	0	N-Term(TMT6plex); S3(Phospho); K9(TMT6plex)
4507241	NP_003137.1	6749	SSRP1	FACT complex subunit SSRP1	S667	0.8	1.3	1.1	1.9	-	-	-	-	0.9	1.6	1.3	2.3	-	-	-	-	0.9	1.6	1.3	2.3	EFVsSDESSSGENK	FKSKEFVsSDESSSG	S4;	Yes	S(4): 98.8; S(5): 1.2; S(8): 0.0; S(9): 0.0; S(10): 0.0	2	1	1	0	N-Term(TMT6plex); S4(Phospho); K14(TMT6plex)
56550039	NP_005924.2	4297	KMT2A	histone-lysine N-methyltransferase 2A isoform 2 precursor	T3038	1.6	1.5	1.4	1.9	-	-	-	-	1.4	1.5	1.5	1.9	-	-	-	-	1.4	1.5	1.5	1.9	NSSTPGLQVPVSPtVPIQNQK	LQVPVSPtVPIQNQK	T14;	No	S(2): 0.0; S(3): 0.0; T(4): 0.0; S(12): 1.6; T(14): 98.4	15	2	1	0	N-Term(TMT6plex); T14(Phospho); K21(TMT6plex)
51873067	NP_001004128.1	5768	QSOX1	sulfhydryl oxidase 1 isoform b precursor	S426	1.8	2.6	2.0	1.9	-	-	-	-	1.8	2.6	2.0	2.1	-	-	-	-	1.8	2.6	2.0	2.1	QNVDHsQEAAK	ARQNVDHsQEAAKAK	S6;	No	S(6): 100.0	2	2	1	0	N-Term(TMT6plex); S6(Phospho); K11(TMT6plex)
7661950	NP_055448.1	9646	CTR9	RNA polymerase-associated protein CTR9 homolog	S1072	1.7	2.4	2.3	1.8	-	-	-	-	1.6	2.5	2.3	1.9	-	-	-	-	1.6	2.5	2.3	1.9	SGSEAGsPR	KSGSEAGsPRRPRRQ	S7;	Yes	S(1): 0.0; S(3): 0.0; S(7): 100.0	12	1	1	0	N-Term(TMT6plex); S7(Phospho)
29826282	NP_817092.1	5496	PPM1G	protein phosphatase 1G	S183	1.6	1.8	1.4	1.8	-	-	-	-	1.7	1.8	1.5	1.8	-	-	-	-	1.7	1.8	1.5	1.8	SGGGTGEEPGsQGLNGEAGPEDSTR	GTGEEPGsQGLNGEA	S11;	Yes	S(1): 5.1; T(5): 0.4; S(11): 84.2; S(23): 5.1; T(24): 5.1	7	1	1	0	N-Term(TMT6plex); S11(Phospho)
224591430	NP_001139197.1	55646	LYAR	cell growth-regulating nucleolar protein	S260	1.5	1.9	1.6	1.8	-	-	-	-	1.5	1.7	1.5	1.5	-	-	-	-	1.5	1.7	1.5	1.5	KDSAsEEEAR	KQRKDSAsEEEARVG	S5;	No	S(3): 0.5; S(5): 99.5	10	1	1	1	N-Term(TMT6plex); K1(TMT6plex); S5(Phospho)
209180450	NP_001129202.1	10043	TOM1	target of Myb protein 1 isoform 4	S416	1.6	1.5	1.2	1.8	-	-	-	-	1.5	1.7	1.4	2.1	-	-	-	-	1.5	1.7	1.4	2.1	LPNLsSPSAEGPPGPPSGPAPR	ADRLPNLsSPSAEGP	S5;	No	S(5): 98.4; S(6): 1.6; S(8): 0.0; S(17): 0.0	2	4	1	0	N-Term(TMT6plex); S5(Phospho)
566559990	NP_001274453.1	994	CDC25B	M-phase inducer phosphatase 2 isoform 9	S161	1.6	2.1	2.4	1.8	-	-	-	-	1.2	2.0	2.6	1.8	-	-	-	-	1.2	2.0	2.6	1.8	sVTPPEEQQEAEEPK	VQNKRRRsVTPPEEQ	S1;	No	S(1): 100.0; T(3): 0.0	1	9	1	0	N-Term(TMT6plex); S1(Phospho); K15(TMT6plex)
50962882	NP_001002909.1	23131	GPATCH8	G patch domain-containing protein 8	S1014	0.9	1.2	0.9	1.8	-	-	-	-	0.9	1.3	1.0	1.9	-	-	-	-	0.9	1.3	1.0	1.9	SWGHEsPEER	KRSWGHEsPEERHSG	S6;	Yes	S(1): 0.0; S(6): 100.0	17	1	1	0	N-Term(TMT6plex); S6(Phospho)
118572613	NP_057417.3	23524	SRRM2	serine/arginine repetitive matrix protein 2	S890; S892	0.7	1.5	1.1	1.7	-	-	-	-	0.7	1.6	1.2	1.9	-	-	-	-	0.7	1.6	1.2	1.9	TPSRHsCsGSSPPR	SRTPSRHsCSGSSPP;TPSRHSCsGSSPPRV	S6; S8;	Yes;Yes	T(1): 0.0; S(3): 0.0; S(6): 100.0; S(8): 97.4; S(10): 1.3; S(11): 1.3	436	1	1	1	N-Term(TMT6plex); S6(Phospho); C7(Carbamidomethyl); S11(Phospho)
27477138	NP_078901.3	56829	ZC3HAV1	zinc finger CCCH-type antiviral protein 1 isoform 2	T393	1.1	1.9	1.5	1.7	-	-	-	-	1.1	2.0	1.5	1.7	-	-	-	-	1.1	2.0	1.5	1.7	SSLGSLQtPEAVTTR	SSLGSLQtPEAVTTR	T8;	Yes	S(1): 0.0; S(2): 0.0; S(5): 0.0; T(8): 100.0; T(13): 0.0; T(14): 0.0	9	2	1	0	N-Term(TMT6plex); T8(Phospho)
291190787	NP_055872.4	23077	MYCBP2	E3 ubiquitin-protein ligase MYCBP2	S2839	1.0	1.5	1.3	1.7	-	-	-	-	1.1	1.4	1.4	1.4	-	-	-	-	1.1	1.4	1.4	1.4	SSPSGASsPR	SSPSGASsPRSSSPH	S8;	No	S(1): 0.0; S(2): 0.0; S(4): 0.0; S(7): 1.0; S(8): 99.0	6	1	1	0	N-Term(TMT6plex); S8(Phospho)
83641870	NP_001032827.1	4869	NPM1	nucleophosmin isoform 3	S218; T219	0.7	0.8	0.9	1.7	-	-	-	-	0.7	0.9	0.8	1.9	-	-	-	-	0.7	0.9	0.8	1.9	DSKPSstPR	GKDSKPSsTPRSKGQ;KDSKPSStPRSKGQE	S6; T7;	Yes;Yes	S(2): 0.0; S(5): 0.0; S(6): 100.0; T(7): 100.0	15	2	1	0	N-Term(TMT6plex); K3(TMT6plex); S6(Phospho); T7(Phospho)
148368978	NP_689512.2	29781	NCAPH2	condensin-2 complex subunit H2 isoform 2	S284	1.6	1.3	1.6	1.7	-	-	-	-	1.5	1.3	1.5	1.8	-	-	-	-	1.5	1.3	1.5	1.8	sPQQSAALPR	LEPKESRsPQQSAAL	S1;	Yes	S(1): 100.0; S(5): 0.0	2	2	1	0	N-Term(TMT6plex); S1(Phospho)
31543385	NP_005008.2	5130	PCYT1A	choline-phosphate cytidylyltransferase A	S362	1.3	1.8	1.4	1.7	-	-	-	-	1.1	1.5	1.2	1.5	-	-	-	-	1.1	1.5	1.2	1.5	AAAYDIsEDEED	KAAAYDIsEDEED	S7;	Yes	Y(4): 0.0; S(7): 100.0	2	1	1	0	N-Term(TMT6plex); S7(Phospho)
145580615	NP_075384.3	65095	KRI1	protein KRI1 homolog	S158	0.7	1.6	1.2	1.7	-	-	-	-	0.8	1.8	1.4	1.9	-	-	-	-	0.8	1.8	1.4	1.9	LQETSSQsYVEEQK	LQETSSQsYVEEQKQ	S8;	No	T(4): 0.0; S(5): 0.0; S(6): 1.1; S(8): 97.9; Y(9): 1.1	13	1	1	0	N-Term(TMT6plex); S8(Phospho); K14(TMT6plex)
50428925	NP_001272.2	1155	TBCB	tubulin-folding cofactor B	S173	1.3	1.5	1.5	1.7	-	-	-	-	1.0	1.2	1.4	1.4	-	-	-	-	1.0	1.2	1.4	1.4	AAGQsPR	EVRAAGQsPRRGTVM	S5;	Yes	S(5): 100.0	1	1	1	0	N-Term(TMT6plex); S5(Phospho)
300388183	NP_001177966.1	5707	PSMD1	26S proteasome non-ATPase regulatory subunit 1 isoform 2	S315	1.7	2.1	1.8	1.7	-	-	-	-	1.7	2.1	1.8	1.8	-	-	-	-	1.7	2.1	1.8	1.8	TPEAsPEPK	VGKTPEAsPEPKDQT	S5;	Yes	T(1): 0.0; S(5): 100.0	8	2	1	0	N-Term(TMT6plex); S5(Phospho); K9(TMT6plex)
256818798	NP_001157974.1	56478	EIF4ENIF1	eukaryotic translation initiation factor 4E transporter isoform b	S777	1.0	1.2	1.1	1.6	-	-	-	-	1.0	1.1	1.1	1.5	-	-	-	-	1.0	1.1	1.1	1.5	SSsPVGLAK	RPSQRSSsPVGLAKW	S3;	No	S(1): 1.1; S(2): 1.1; S(3): 97.8	4	2	1	0	N-Term(TMT6plex); S3(Phospho); K9(TMT6plex)
564730691	NP_001274177.1	641	BLM	Bloom syndrome protein isoform 3	T391	1.1	1.2	1.1	1.6	-	-	-	-	1.1	1.2	1.3	1.5	-	-	-	-	1.1	1.2	1.3	1.5	LLYVtPEK	IIKLLYVtPEKICAS	T5;	No	Y(3): 0.8; T(5): 99.2	1	3	1	0	N-Term(TMT6plex); Y3(Phospho); K8(TMT6plex)
156546890	NP_079225.5	80014	WWC2	protein WWC2	S1022	2.0	2.0	1.9	1.6	-	-	-	-	-	-	-	-	-	-	-	-	-	-	-	-	SDsDSSTLAK	CRLNRSDsDSSTLAK	S3;	Yes	S(1): 0.0; S(3): 100.0; S(5): 0.0; S(6): 0.0; T(7): 0.0	1	1	1	0	N-Term(TMT6plex); S3(Phospho); K10(TMT6plex)
589811544	NP_001277154.1	9791	PTDSS1	phosphatidylserine synthase 1 isoform 2	S296	0.9	1.5	1.6	1.6	-	-	-	-	0.8	1.4	1.4	1.5	-	-	-	-	0.8	1.4	1.4	1.5	GSEDsPPKHAGNNESHSSR	GTKGSEDsPPKHAGN	S5;	No	S(2): 1.6; S(5): 98.4; S(15): 0.0; S(17): 0.0; S(18): 0.0	1	2	1	1	N-Term(TMT6plex); S5(Phospho); K8(TMT6plex)
21237805	NP_003066.2	6601	SMARCC2	SWI/SNF complex subunit SMARCC2 isoform a	S302; S304	1.9	2.4	1.8	1.6	-	-	-	-	1.7	2.3	1.7	1.6	-	-	-	-	1.7	2.3	1.7	1.6	KRsPsPSPTPEAK	NYKKRKRsPSPSPTP;KKRKRSPsPSPTPEA	S3; S5;	Yes;Yes	S(3): 100.0; S(5): 100.0; S(7): 0.0; T(9): 0.0	12	3	1	2	N-Term(TMT6plex); K1(TMT6plex); S3(Phospho); S5(Phospho); K13(TMT6plex)
58331179	NP_079527.1	80728	ARHGAP39	rho GTPase-activating protein 39	S122	0.9	1.4	1.0	1.6	-	-	-	-	-	-	-	-	-	-	-	-	-	-	-	-	ASAEsPGR	SPRASAEsPGRGSS	S5;	No	S(2): 0.0; S(5): 99.4; S(6): 0.6	1	1	1	0	N-Term(TMT6plex); S6(Phospho)
217330574	NP_001136088.1	23301	EHBP1	EH domain-binding protein 1 isoform 3	S401	0.9	1.0	1.3	1.6	-	-	-	-	1.0	1.0	1.2	1.4	-	-	-	-	1.0	1.0	1.2	1.4	DLSTSPKPSPIPsPVLGR	PKPSPIPsPVLGRKP	S13;	No	S(3): 33.3; T(4): 33.3; S(5): 33.3; S(9): 0.0; S(13): 100.0	5	3	1	0	N-Term(TMT6plex); T4(Phospho); K7(TMT6plex); S13(Phospho)
4507525	NP_003253.1	7095	SEC62	translocation protein SEC62	S335	1.5	1.9	1.9	1.6	-	-	-	-	1.2	1.3	1.4	1.5	-	-	-	-	1.2	1.3	1.4	1.5	VGPGNHGTEGsGGER	GNHGTEGsGGERHSD	S11;	No	T(8): 0.0; S(11): 100.0	2	1	1	0	N-Term(TMT6plex); S11(Phospho)
116063534	NP_115515.2	84079	ANKRD27	ankyrin repeat domain-containing protein 27	S634	1.1	1.2	1.2	1.6	-	-	-	-	-	-	-	-	-	-	-	-	-	-	-	-	SSEAPVQsPQR	SSEAPVQsPQRSVDS	S8;	Yes	S(1): 0.0; S(2): 0.0; S(8): 100.0	1	1	1	0	N-Term(TMT6plex); S8(Phospho)
27262651	NP_680781.1	11273	ATXN2L	ataxin-2-like protein isoform D	S684	0.7	1.3	1.0	1.6	-	-	-	-	0.7	1.1	1.0	1.2	-	-	-	-	0.7	1.1	1.0	1.2	STSTPTsPGPR	KSTSTPTsPGPRTHS	S7;	Yes	S(1): 0.0; T(2): 0.0; S(3): 0.0; T(4): 1.2; T(6): 1.2; S(7): 97.7	15	6	1	0	N-Term(TMT6plex); S7(Phospho)
225543461	NP_203752.2	85461	TANC1	protein TANC1 isoform 1	S444	1.7	1.1	1.3	1.6	-	-	-	-	-	-	-	-	-	-	-	-	-	-	-	-	QIASNsPGSSPK	MRQIASNsPGSSPKT	S6;	Yes	S(4): 0.0; S(6): 97.8; S(9): 1.1; S(10): 1.1	2	2	1	0	N-Term(TMT6plex); S6(Phospho); K12(TMT6plex)
7657269	NP_055847.1	23047	PDS5B	sister chromatid cohesion protein PDS5 homolog B	S1283	1.3	1.5	1.1	1.6	-	-	-	-	1.2	1.3	1.0	1.5	-	-	-	-	1.2	1.3	1.0	1.5	EDILENEDEQNsPPKK	ENEDEQNsPPKKGKR	S12;	Yes	S(12): 100.0	34	1	1	1	N-Term(TMT6plex); S12(Phospho); K15(TMT6plex); K16(TMT6plex)
24308075	NP_056285.1	26056	RAB11FIP5	rab11 family-interacting protein 5	S176	2.0	1.8	1.3	1.6	-	-	-	-	2.2	2.0	1.4	1.6	-	-	-	-	2.2	2.0	1.4	1.6	NNLSAsMEDLSMK	TRNNLSAsMEDLSMK	S6;	Yes	S(4): 1.4; S(6): 98.6; S(11): 0.0	5	1	1	0	N-Term(TMT6plex); S6(Phospho); M7(Oxidation); M12(Oxidation); K13(TMT6plex)
34328899	NP_005392.2	5784	PTPN14	tyrosine-protein phosphatase non-receptor type 14	S314	0.9	1.1	1.2	1.6	-	-	-	-	1.0	1.1	1.0	1.4	-	-	-	-	1.0	1.1	1.0	1.4	ICTEQSNsPPPIR	ICTEQSNsPPPIRRQ	S8;	Yes	T(3): 0.0; S(6): 0.0; S(8): 100.0	11	1	1	0	N-Term(TMT6plex); C2(Carbamidomethyl); S8(Phospho)
27262651	NP_680781.1	11273	ATXN2L	ataxin-2-like protein isoform D	S339	1.1	1.4	1.0	1.5	-	-	-	-	1.2	1.2	1.1	1.2	-	-	-	-	1.2	1.2	1.1	1.2	EsPSLASR	RQGSGREsPSLASRE	S2;	Yes	S(2): 100.0; S(4): 0.0; S(7): 0.0	15	6	1	0	N-Term(TMT6plex); S2(Phospho)
4504253	NP_002096.1	3014	H2AFX	histone H2AX	S140	1.1	2.3	1.8	1.5	-	-	-	-	1.3	2.5	2.0	1.9	-	-	-	-	1.3	2.5	2.0	1.9	KATQAsQEY	GKKATQAsQEY	S6;	Yes	T(3): 0.0; S(6): 100.0; Y(9): 0.0	35	1	1	1	N-Term(TMT6plex); K1(TMT6plex); S6(Phospho)
25777671	NP_002705.2	5514	PPP1R10	serine/threonine-protein phosphatase 1 regulatory subunit 10	T256	1.3	1.1	2.0	1.5	-	-	-	-	1.2	1.1	1.9	1.4	-	-	-	-	1.2	1.1	1.9	1.4	QSNVAAPGDAtPPAEK	VAAPGDAtPPAEKKY	T11;	Yes	S(2): 0.0; T(11): 100.0	1	1	1	0	N-Term(TMT6plex); T11(Phospho); K16(TMT6plex)

Khan et al., 2019. Multi-omics analysis to characterize cigarette smoke induced molecular alterations in esophageal cells
Supplementary Table 8. List of phosphopeptides identified and quantified in untreated and chronically treated Het1A cells with cigarette smoke condensate for 8 months using SequestHT and Mascot search algorithms with a PhosphoRS probability score ≥75%

Protein group Accession	NP_Accession	Gene ID	Gene Symbol	Protein Description	PhosphoSite (Protein)	Het-1A-Smoke - 2M/Parental	Het-1A-Smoke - 4M/Parental	Het-1A-Smoke - 6M/Parental	Het-1A-Smoke - 8M/Parental	Het-1A-Smoke - 2M/Parental	Het-1A-Smoke - 4M/Parental	Het-1A-Smoke - 6M/Parental	Het-1A-Smoke - 8M/Parental	Het-1A-Smoke - 2M/Parental	Het-1A-Smoke - 4M/Parental	Het-1A-Smoke - 6M/Parental	Het-1A-Smoke - 8M/Parental	Het-1A-Smoke - 2M/Parental	Het-1A-Smoke - 4M/Parental	Het-1A-Smoke - 6M/Parental	Het-1A-Smoke - 8M/Parental	Het-1A-Smoke - 2M/Parental	Het-1A-Smoke - 4M/Parental	Het-1A-Smoke - 6M/Parental	Het-1A-Smoke - 8M/Parental	Peptide Sequence	Phosphowindow	PhosphoSite (Peptide)	Phosphosite Plus Evidence	phosphoRS Site Probabilities	# PSMs	# Proteins	# Protein Groups	# Missed Cleavages	Modifications
						Replicate 1				Replicate 2				Normalized phosphoprotein ratios with respect to corresponding total protein ratios. Replicate 1				Replicate 2				Average of replicates													
94158609	NP_001035307.1	55793	FAM63A	protein FAM63A isoform2	S240	1.2	1.7	1.1	1.5	-	-	-	-	-	-	-	-	-	-	-	-	-	-	-	-	GPGAEGGSGsPEK	GAEGGSGsPEKQLQV	S10;	No	S(8): 0.0; S(10): 100.0	1	4	1	0	N-Term(TMT6plex); S10(Phospho); K13(TMT6plex)
4506921	NP_003012.1	6449	SGTA	small glutamine-rich tetratricopeptide repeat-containing protein alpha	T81	1.3	1.2	1.3	1.5	-	-	-	-	1.5	1.8	1.6	2.1	-	-	-	-	1.5	1.8	1.6	2.1	tPPSEEDSAEAER	DLRSPARtPPSEEDS	T1;	Yes	T(1): 100.0; S(4): 0.0; S(8): 0.0	28	1	1	0	N-Term(TMT6plex); T1(Phospho)
594190970	NP_001277403.1	9854	C2CD2L	C2 domain-containing protein 2-like isoform2	S662	1.7	2.0	1.7	1.5	-	-	-	-	-	-	-	-	-	-	-	-	-	-	-	-	EAGLSQsHDDLSNATATPSVR	KEAGLSQsHDDLSNA	S7;	Yes	S(5): 1.9; S(7): 98.1; S(12): 0.0; T(15): 0.0; T(17): 0.0; S(19): 0.0	1	2	1	0	N-Term(TMT6plex); S7(Phospho)
256818821	NP_064550.3	56852	RAD18	E3 ubiquitin-protein ligase RAD18	S483	2.4	1.1	1.1	1.5	-	-	-	-	1.8	0.9	0.9	1.1	-	-	-	-	1.8	0.9	0.9	1.1	AAEsAEIEPR	RRTRAAEsAEIEPRN	S4;	Yes	S(4): 100.0	8	1	1	0	N-Term(TMT6plex); S4(Phospho)
293336691	NP_001518.3	3066	HDAC2	histone deacetylase 2	S422; S424	1.2	1.1	1.2	1.5	-	-	-	-	1.3	1.2	1.3	1.5	-	-	-	-	1.3	1.2	1.3	1.5	IACDEEFsDsEDEGEGGRR	IACDEEFsDSEDEGE;CDEEFSDsEDEGEGG	S8; S10;	Yes;Yes	S(8): 100.0; S(10): 100.0	1	1	1	1	N-Term(TMT6plex); C3(Carbamidomethyl); S8(Phospho); S10(Phospho)
335334962	NP_001229416.1	23053	ZSWIM8	zinc finger SWIM domain-containing protein 8 isoform	S567	1.1	1.6	1.4	1.5	-	-	-	-	-	-	-	-	-	-	-	-	-	-	-	-	RLsAEGGDK	QRGPRRLsAEGGDKA	S3;	Yes	S(3): 100.0	2	3	1	1	N-Term(TMT6plex); S3(Phospho); K9(TMT6plex)
32967311	NP_004422.2	1969	EPHA2	ephrin type-A receptor 2 precursor	Y921; T922; Y930; T931	2.1	4.4	1.6	1.4	-	-	-	-	2.0	3.8	1.3	1.3	-	-	-	-	2.0	3.8	1.3	1.3	MQQytEHFMAAGytAIEK	ESIKMQQyTEHFMAA;SIKMQQYtEHFMAAG;EHFMAAGyTAIEKVV;HFMAAGYtAIEKVVQ	Y4; T5; Y13; T14	Yes;Yes;Yes;No	Y(4): 75.0; T(5): 75.0; Y(13): 75.0; T(14): 75.0	4	1	1	0	N-Term(TMT6plex); Y4(Phospho); T5(Phospho); M9(Oxidation); T14(Phospho); K18(TMT6plex)
62243658	NP_055907.3	23049	SMG1	serine/threonine-protein kinase SMG1	T3573	1.0	0.9	1.6	1.4	-	-	-	-	0.9	0.8	1.5	1.3	-	-	-	-	0.9	0.8	1.5	1.3	NLATSADtPPSTVPGTGK	NLATSADtPPSTVPG	T8;	Yes	T(4): 2.2; S(5): 2.2; T(8): 95.6; S(11): 0.0; T(12): 0.0; T(16): 0.0	1	1	1	0	N-Term(TMT6plex); S5(Phospho); K18(TMT6plex)
255003833	NP_115518.3	55125	CEP192	centrosomal protein of 192 kDa	S2110	1.8	2.1	1.6	1.4	-	-	-	-	2.2	2.7	1.9	1.9	-	-	-	-	2.2	2.7	1.9	1.9	GPQGsPLLSR	PVKGPQGsPLLSRAA	S5;	Yes	S(5): 100.0; S(9): 0.0	2	1	1	0	N-Term(TMT6plex); S5(Phospho)
223633988	NP_001138678.1	85379	KIAA1671	uncharacterized protein KIAA1671	S1701	1.4	1.5	1.3	1.4	-	-	-	-	-	-	-	-	-	-	-	-	-	-	-	-	KEEsDEEETASK	KSPRKEEsDEEETAS	S4;	Yes	S(4): 100.0; T(9): 0.0; S(11): 0.0	2	1	1	1	N-Term(TMT6plex); K1(TMT6plex); S4(Phospho); K12(TMT6plex)
11560152	NP_071378.1	63925	ZNF335	zinc finger protein 335	S1007	1.9	1.5	1.4	1.4	-	-	-	-	-	-	-	-	-	-	-	-	-	-	-	-	ALGLAVPPsPPSAATAASK	LGLAVPPsPPSAATA	S9;	Yes	S(9): 100.0; S(12): 0.0; T(15): 0.0; S(18): 0.0	8	1	1	0	N-Term(TMT6plex); S9(Phospho); K19(TMT6plex)
20149675	NP_077305.2	79180	EFHD2	EF-hand domain-containing protein D2	S74	1.4	0.8	1.6	1.4	-	-	-	-	1.0	0.6	1.0	0.9	-	-	-	-	1.0	0.6	1.0	0.9	ADLNQGIGEPQsPSR	QGIGEPQsPSRRVFN	S12;	Yes	S(12): 98.3; S(14): 1.7	1	1	1	0	N-Term(TMT6plex); S12(Phospho)
11321642	NP_055392.1	23774	BRD1	bromodomain-containing protein 1	S1055	1.5	1.6	1.2	1.4	-	-	-	-	1.5	1.8	1.3	1.5	-	-	-	-	1.5	1.8	1.3	1.5	VHGEPTSDLsDID	GEPTSDLsDID	S10;	Yes	T(6): 0.0; S(7): 0.0; S(10): 100.0	1	1	1	0	N-Term(TMT6plex); S10(Phospho)
113205077	NP_689601.2	65062	TMEM237	transmembrane protein 237 isoformb	S54	1.5	1.5	1.7	1.4	-	-	-	-	1.4	1.4	1.8	1.4	-	-	-	-	1.4	1.4	1.8	1.4	RPsEGNEPSTK	QTAGRRPsEGNEPST	S3;	No	S(3): 100.0; S(9): 0.0; T(10): 0.0	2	2	1	0	N-Term(TMT6plex); S3(Phospho); K11(TMT6plex)
37595750	NP_002287.2	3930	LBR	lamin-B receptor	S86	1.6	1.6	2.6	1.4	-	-	-	-	1.5	1.5	2.1	1.3	-	-	-	-	1.5	1.5	2.1	1.3	sPGRPK	RSRSRSRsPGRPPKS	S1;	Yes	S(1): 100.0	8	1	1	0	N-Term(TMT6plex); S1(Phospho); K7(TMT6plex)
395394049	NP_001257448.1	9113	LATS1	serine/threonine-protein kinase LATS1 isoform2	T246	1.2	1.5	1.2	1.4	-	-	-	-	1.0	1.2	0.9	1.3	-	-	-	-	1.0	1.2	0.9	1.3	SVtPPPPR	PPQVRSVtPPPPPRG	T3;	Yes	S(1): 0.0; T(3): 100.0	6	2	1	0	N-Term(TMT6plex); T3(Phospho)
163838629	NP_056039.2	23272	FAM208A	protein FAM208A isoformb	S303	6.7	1.5	1.8	1.4	-	-	-	-	6.7	1.3	1.5	1.2	-	-	-	-	6.7	1.3	1.5	1.2	SVGGDsDTEDMR	KKSVGGDsDTEDMRS	S6;	No	S(1): 0.0; S(6): 100.0; T(8): 0.0	4	2	1	0	N-Term(TMT6plex); S6(Phospho); M11(Oxidation)
110347443	NP_009045.2	7110	TMF1	TATA element modulatory factor	S344	0.9	2.0	0.6	1.4	-	-	-	-	0.8	1.8	0.6	1.6	-	-	-	-	0.8	1.8	0.6	1.6	SVSEINsDDELSGK	RSVSEINsDDELSGK	S7;	Yes	S(1): 0.1; S(3): 0.1; S(7): 97.7; S(12): 2.2	2	1	1	0	N-Term(TMT6plex); S7(Phospho); K14(TMT6plex)
170932514	NP_001074324.1	5829	PXN	paxillin isoform2	S302	0.9	1.2	0.9	1.4	-	-	-	-	0.9	1.0	0.9	1.1	-	-	-	-	0.9	1.0	0.9	1.1	sSPGGQDEGGFMAQGK	WPRDGGRsSPGGQDE	S1;	Yes	S(1): 100.0; S(2): 0.0	3	1	1	0	N-Term(TMT6plex); S1(Phospho); M12(Oxidation); K16(TMT6plex)
19913406	NP_001058.2	7153	TOP2A	DNA topoisomerase 2-alpha	S1247	1.0	1.2	1.1	1.4	-	-	-	-	1.1	1.4	1.3	1.6	-	-	-	-	1.1	1.4	1.3	1.6	IKNENTEGsPQEDGVELEGLK	KNENTEGsPQEDGVE	S9;	Yes	T(6): 0.0; S(9): 100.0	64	1	1	1	N-Term(TMT6plex); K2(TMT6plex); S9(Phospho); K21(TMT6plex)
7657257	NP_055580.1	9804	TOMM20	mitochondrial import receptor subunit TOM20	S138	1.3	1.4	1.0	1.4	-	-	-	-	1.2	1.4	0.9	1.3	-	-	-	-	1.2	1.4	0.9	1.3	IVSAQsLAEDDVE	QRIVSAQsLAEDDVE	S6;	Yes	S(3): 0.0; S(6): 100.0	4	1	1	0	N-Term(TMT6plex); S6(Phospho)
110556644	NP_722520.2	161882	ZFPM1	zinc finger protein ZFPM1	S909	0.5	1.0	1.1	1.4	-	-	-	-	-	-	-	-	-	-	-	-	-	-	-	-	GPSPAPAPAAsPQPGSR	PAPAPAAsPQPGSRG	S11;	Yes	S(3): 0.0; S(11): 100.0; S(16): 0.0	1	1	1	0	N-Term(TMT6plex); S11(Phospho)
601984520	NP_066289.3	7316	UBC	polyubiquitin-C	S57	1.1	1.3	1.7	1.4	-	-	-	-	1.0	1.0	1.3	1.5	-	-	-	-	1.0	1.0	1.3	1.5	TLsDYNIQK	LEDGRTLsDYNIQKE	S3;	Yes	T(1): 0.0; S(3): 100.0; Y(5): 0.0	7	4	1	0	N-Term(TMT6plex); S3(Phospho); K9(TMT6plex)
42542379	NP_005830.2	10250	SRRM1	serine/arginine repetitive matrix protein 1	T614; S616	2.8	0.9	2.1	1.4	-	-	-	-	3.4	1.0	2.3	1.6	-	-	-	-	3.4	1.0	2.3	1.6	RtAsPPPPK	SPPPKRRtASPPPPP;PPKRRTAsPPPPPKR	T2; S4;	Yes;Yes	T(2): 100.0; S(4): 100.0	229	1	1	1	N-Term(TMT6plex); T2(Phospho); S4(Phospho); K10(TMT6plex)
386781227	NP_001247404.1	10785	WDR4	tRNA (guanine-N(7)-)-methyltransferase non-catalytic subunit WDR4 isoform3	S245	1.2	1.4	1.6	1.4	-	-	-	-	1.1	1.3	1.5	1.3	-	-	-	-	1.1	1.3	1.5	1.3	sPPPGPDGHAK	EKKQRRRsPPPGPDG	S1;	No	S(1): 100.0	4	3	1	0	N-Term(TMT6plex); S1(Phospho); K11(TMT6plex)
15011974	NP_004714.2	9181	ARHGEF2	rho guanine nucleotide exchange factor 2 isoform3	S928	3.4	4.4	2.5	1.4	-	-	-	-	2.6	3.4	2.1	1.2	-	-	-	-	2.6	3.4	2.1	1.2	LsPPHSPR	EEGSSRLsPPHSPRD	S2;	No	S(2): 100.0; S(6): 0.0	5	3	1	0	N-Term(TMT6plex); S2(Phospho)
295844834	NP_060037.3	55577	NAGK	N-acetyl-D-glucosamine kinase	S122	1.2	1.2	1.0	1.4	-	-	-	-	1.2	1.5	1.3	1.8	-	-	-	-	1.2	1.5	1.3	1.8	SLGLSLsGGDQEDAGR	RSLGLSLsGGDQEDA	S7;	No	S(1): 0.0; S(5): 0.0; S(7): 100.0	6	1	1	0	N-Term(TMT6plex); S7(Phospho)
7661958	NP_055554.1	9774	BCLAF1	bcl-2-associated transcription factor 1 isoform1	S177	0.8	1.4	1.1	1.4	-	-	-	-	0.9	1.5	1.2	1.5	-	-	-	-	0.9	1.5	1.2	1.5	AEGEPQEEsPLKSK	EGEPQEEsPLKSKSQ	S9;	Yes	S(9): 100.0; S(13): 0.0	47	3	1	1	N-Term(TMT6plex); S9(Phospho); K12(TMT6plex); K14(TMT6plex)
301897118	NP_114432.2	83990	BRIP1	Fanconi anemia group J protein	S226	1.2	1.0	1.4	1.4	-	-	-	-	1.4	1.2	1.4	1.5	-	-	-	-	1.4	1.2	1.4	1.5	QGNsQESSNTIK	CSTKQGNsQESSNTI	S4;	Yes	S(4): 93.8; S(7): 2.1; S(8): 2.1; T(10): 2.1	2	1	1	0	N-Term(TMT6plex); S4(Phospho); K12(TMT6plex)
91176325	NP_038477.2	11176	BAZ2A	bromodomain adjacent to zinc finger domain protein	S1061	1.2	1.2	1.9	1.4	-	-	-	-	1.4	1.3	2.0	1.5	-	-	-	-	1.4	1.3	2.0	1.5	IMEETSGMEEEEEEEsIAAVPGR	EEEEEEEsIAAVPGR	S16;	No	T(5): 2.0; S(6): 2.0; S(16): 95.9	17	1	1	0	N-Term(TMT6plex); M2(Oxidation); S6(Phospho); M8(Oxidation)
380503862	NP_001244110.1	6575	SLC20A2	sodium-dependent phosphate transporter 2	S256	1.4	1.4	1.2	1.4	-	-	-	-	-	-	-	-	-	-	-	-	-	-	-	-	VsDESLSK	EGALSRVsDESLSKV	S2;	Yes	S(2): 100.0; S(5): 0.0; S(7): 0.0	1	1	1	0	N-Term(TMT6plex); S2(Phospho); K8(TMT6plex)
217416350	NP_001136115.1	57513	CASKIN2	caskin-2 isoformb	S615; S618	0.8	1.0	0.8	1.4	-	-	-	-	-	-	-	-	-	-	-	-	-	-	-	-	SPsQEsIGAR	LPPARSPsQESIGAR;ARSPSQEsIGARSRG	S3; S6;	No;No	S(1): 0.0; S(3): 100.0; S(6): 100.0	2	2	1	0	N-Term(TMT6plex); S3(Phospho); S6(Phospho)
21735572	NP_060568.3	1894	ECT2	protein ECT2 isoformb	T342	1.0	1.8	1.8	1.4	-	-	-	-	1.1	1.9	1.8	1.4	-	-	-	-	1.1	1.9	1.8	1.4	SVSMLSLNtPNSNR	VSMLSLNtPNSNRKR	T9;	No	S(1): 0.0; S(3): 0.0; S(6): 0.0; T(9): 100.0; S(12): 0.0	6	2	1	0	N-Term(TMT6plex); M4(Oxidation); T9(Phospho)
55925574	NP_981949.1	271	AMPD2	AMP deaminase 2 isoform3	S49	2.2	1.7	1.6	1.4	-	-	-	-	2.0	1.5	1.5	1.3	-	-	-	-	2.0	1.5	1.5	1.3	QIsQDVK	QRLERQIsQDVKLEP	S3;	No	S(3): 100.0	1	4	1	0	N-Term(TMT6plex); S3(Phospho); K7(TMT6plex)
7662330	NP_055760.1	22885	ABLIM3	actin-binding LIM protein 3	S373	1.5	1.6	2.1	1.4	-	-	-	-	-	-	-	-	-	-	-	-	-	-	-	-	ASsPGYIDSPTYSR	LRQRRASsPGYIDSP	S3;	Yes	S(2): 2.7; S(3): 94.2; Y(6): 2.7; S(9): 0.1; T(11): 0.1; Y(12): 0.1; S(13): 0.1	2	1	1	0	N-Term(TMT6plex); S3(Phospho)
221139926	NP_001137532.1	57224	NHSL1	NHS-like protein 1 isoform2	S466	1.1	1.4	0.9	1.4	-	-	-	-	1.1	1.4	0.9	1.3	-	-	-	-	1.1	1.4	0.9	1.3	GDPQsPGR	AVKGDPQsPGRHWNE	S5;	No	S(5): 100.0	2	2	1	0	N-Term(TMT6plex); S5(Phospho)
545746375	NP_001271175.1	26037	SIPA1L1	signal-induced proliferation-associated 1-like protein 1 isoform3	S1564	1.4	1.2	1.0	1.4	-	-	-	-	1.4	1.1	1.0	1.3	-	-	-	-	1.4	1.1	1.0	1.3	TLsDESIYNSQR	RALHRTLsDESIYNS	S3;	No	T(1): 0.0; S(3): 100.0; S(6): 0.0; Y(8): 0.0; S(10): 0.0	7	4	1	0	N-Term(TMT6plex); S3(Phospho)
39753957	NP_056417.2	26092	TOR1AIP1	torsin-1A-interacting protein 1 isoform2	S79	1.5	2.0	1.3	1.4	-	-	-	-	1.5	2.0	1.2	1.4	-	-	-	-	1.5	2.0	1.2	1.4	ERsPVGK	PLVAKERsPVGKRTR	S3;	No	S(3): 100.0	4	2	1	1	N-Term(TMT6plex); S3(Phospho); K7(TMT6plex)
510937029	NP_001265395.1	10059	DNM1L	dynamin-1-like protein isoform7	S413	0.9	1.5	1.3	1.4	-	-	-	-	0.9	1.5	1.1	1.3	-	-	-	-	0.9	1.5	1.1	1.3	SKPIPIMPAsPQK	PIPIMPAsPQKGHAV	S10;	No	S(1): 0.0; S(10): 100.0	2	7	1	0	N-Term(TMT6plex); K2(TMT6plex); M7(Oxidation); S10(Phospho); K13(TMT6plex)
13994287	NP_114124.1	83855	KLF16	Krueppel-like factor 16	S109	0.8	1.3	0.9	1.4	-	-	-	-	0.7	1.1	0.8	1.2	-	-	-	-	0.7	1.1	0.8	1.2	GGPGAAPGGASPASSSSAASsPSSGR	SSSSAASsPSSGRAP	S21;	Yes	S(11): 0.0; S(14): 0.0; S(15): 0.0; S(16): 0.0; S(17): 0.0; S(20): 1.7; S(21): 98.3; S(23): 0.0; S(24): 0.0	2	1	1	0	N-Term(TMT6plex); S21(Phospho)
30061567	NP_060548.2	55132	LARP1B	la-related protein 1B isoform1	S60	2.1	1.2	1.2	1.4	-	-	-	-	-	-	-	-	-	-	-	-	-	-	-	-	LNGPGENVsEDEAQSSNQR	NGPGENVsEDEAQSS	S9;	Yes	S(9): 99.9; S(15): 0.1; S(16): 0.0	6	4	1	0	N-Term(TMT6plex); S9(Phospho)
14165274	NP_115786.1	84376	HOOK3	protein Hook homolog 3	S238	1.0	0.9	0.8	1.4	-	-	-	-	1.0	0.8	0.7	1.4	-	-	-	-	1.0	0.8	0.7	1.4	LNQSDSIEDPNsPAGR	DSIEDPNsPAGRRHL	S12;	Yes	S(4): 0.0; S(6): 0.0; S(12): 100.0	2	1	1	0	N-Term(TMT6plex); S12(Phospho)
223468564	NP_001138616.1	6510	SLC1A5	neutral amino acid transporter B(0) isoform2	S307	2.0	1.7	1.3	1.4	-	-	-	-	1.4	1.1	1.0	1.0	-	-	-	-	1.4	1.1	1.0	1.0	GPAGDATVAsEKESVM	AGDATVAsEKESVM	S10;	No	T(7): 1.2; S(10): 98.8; S(14): 0.0	1	3	1	1	N-Term(TMT6plex); S10(Phospho); K12(TMT6plex); M16(Oxidation)
255918077	NP_001157569.1	1462	VCAN	versican core protein isoform3 precursor	T1128	1.2	1.3	1.4	1.4	-	-	-	-	-	-	-	-	-	-	-	-	-	-	-	-	QEIESETtSEEQIQEEK	QEIESETtSEEQIQE	T8;	No	S(5): 0.0; T(7): 0.0; T(8): 98.5; S(9):	1	2	1	0	N-Term(TMT6plex); T8(Phospho); K17(TMT6plex)
113951734	NP_001030312.2	10011	SRA1	steroid receptor RNA activator 1 isoform1	S60	0.8	1.1	1.1	1.4	-	-	-	-	0.7	1.0	1.1	1.2	-	-	-	-	0.7	1.0	1.1	1.2	VAAPQDGsPR	VAAPQDGsPRVPASE	S8;	Yes	S(8): 100.0	1	1	1	0	N-Term(TMT6plex); S8(Phospho)
38146107	NP_075388.2	65117	RSRC2	arginine/serine-rich coiled-coil protein 2	S104	1.3	1.8	1.6	1.3	-	-	-	-	-	-	-	-	-	-	-	-	-	-	-	-	LNsSENGEDR	KGRERLNsSENGEDR	S3;	Yes	S(3): 100.0; S(4): 0.0	3	1	1	0	N-Term(TMT6plex); S3(Phospho)
61743954	NP_001611.1	79026	AHNAK	neuroblast differentiation-associated protein AHNAK isoform1	S176	0.9	1.5	0.9	1.3	-	-	-	-	1.1	1.5	0.9	1.6	-	-	-	-	1.1	1.5	0.9	1.6	DIDIsSPEFK	GAKDIDIsSPEFKIK	S5;	Yes	S(5): 99.0; S(6): 1.0	79	1	1	0	N-Term(TMT6plex); S5(Phospho); K10(TMT6plex)
109637788	NP_150247.2	5371	PML	protein PML isoform5	S403	0.9	1.1	1.2	1.3	-	-	-	-	0.8	1.0	1.1	1.1	-	-	-	-	0.8	1.0	1.1	1.1	KAsPEAASTPR	AAVSKKAsPEAASTP	S3;	Yes	S(3): 100.0; S(8): 0.0; T(9): 0.0	7	9	1	1	N-Term(TMT6plex); K1(TMT6plex); S3(Phospho)
38016919	NP_054836.2	29035	C16orf72	UPF0472 protein C16orf72	S167	0.9	1.1	0.9	1.3	-	-	-	-	-	-	-	-	-	-	-	-	-	-	-	-	LTVVsPNR	PPRLTVVsPNRATST	S5;	Yes	T(2): 0.0; S(5): 100.0	2	1	1	0	N-Term(TMT6plex); S5(Phospho)
148613886	NP_073752.5	64864	RFX7	DNA-binding protein RFX7	T378	0.9	1.3	1.1	1.3	-	-	-	-	-	-	-	-	-	-	-	-	-	-	-	-	QLVtSPSPMSSSDGK	QRTRQLVtSPSPMSS	T4;	No	T(4): 98.7; S(5): 1.3; S(7): 0.0; S(10): 0.0; S(11): 0.0; S(12): 0.0	3	1	1	0	N-Term(TMT6plex); T4(Phospho); M9(Oxidation); K15(TMT6plex)
34328899	NP_005392.2	5784	PTPN14	tyrosine-protein phosphatase non-receptor type 14	S512	1.1	0.8	1.1	1.3	-	-	-	-	1.2	0.8	0.9	1.2	-	-	-	-	1.2	0.8	0.9	1.2	LVsPSDQR	VYSNKLVsPSDQRNP	S3;	Yes	S(3): 100.0; S(5): 0.0	11	1	1	0	N-Term(TMT6plex); S3(Phospho)
87116683	NP_659419.3	196441	ZFC3H1	zinc finger C3H1 domain-containing protein	S948	1.2	1.2	1.3	1.3	-	-	-	-	1.0	1.1	1.3	1.2	-	-	-	-	1.0	1.1	1.3	1.2	LDsSPVSSPR	NKMMRLDsSPVSSPR	S3;	Yes	S(3): 99.4; S(4): 0.6; S(7): 0.0; S(8):	1	1	1	0	N-Term(TMT6plex); S4(Phospho)
103472005	NP_002408.3	4288	MKI67	antigen KI-67 isoform1	S1983	1.2	1.5	1.1	1.3	-	-	-	-	1.4	1.5	1.2	1.4	-	-	-	-	1.4	1.5	1.2	1.4	sPQPDPVK	ITEVSCKsPQPDPVK	S1;	Yes	S(1): 100.0	118	2	1	0	N-Term(TMT6plex); S1(Phospho); K8(TMT6plex)
66932923	NP_001018654.1	91663	MYADM	myeloid-associated differentiation marker	T14	1.0	0.9	1.4	1.3	-	-	-	-	1.0	0.9	1.4	1.4	-	-	-	-	1.0	0.9	1.4	1.4	TITTTTtTSSSGLGSPMIVGSPR	RTTITTTtTSSSGLG	T7;	No	T(1): 1.9; T(2): 0.0; T(4): 1.9; T(5): 0.0; T(6): 1.9; T(7): 94.2; T(8): 0.0; S(9): 0.0; S(10): 0.0; S(11): 0.0; S(15): 0.0; S(21): 0.0	2	1	1	0	N-Term(TMT6plex); T7(Phospho); M17(Oxidation)
349501061	NP_001231775.1	1111	CHEK1	serine/threonine-protein kinase Chk1 isoform2	S317	1.5	1.3	1.1	1.3	-	-	-	-	1.6	1.4	1.3	1.4	-	-	-	-	1.6	1.4	1.3	1.4	YSSsQPEPR	ENVKYSSsQPEPRTG	S4;	Yes	Y(1): 0.0; S(2): 0.0; S(3): 0.7; S(4):	2	2	1	0	N-Term(TMT6plex); S4(Phospho)
21361625	NP_060773.3	55770	EXOC2	exocyst complex component 2	S432	1.8	2.2	1.8	1.3	-	-	-	-	1.5	1.8	1.4	1.1	-	-	-	-	1.5	1.8	1.4	1.1	GSsEQSGR	ASLKRGSsEQSGRDD	S3;	Yes	S(2): 0.5; S(3): 99.5; S(6): 0.0	1	1	1	0	N-Term(TMT6plex); S3(Phospho)
91176325	NP_038477.2	11176	BAZ2A	bromodomain adjacent to zinc finger domain protein 2A	S1184	0.7	0.9	0.4	1.3	-	-	-	-	0.8	0.9	0.4	1.5	-	-	-	-	0.8	0.9	0.4	1.5	MELAGSNTTASsPAR	GSNTTASsPARARGR	S12;	Yes	S(6): 0.0; T(8): 0.0; T(9): 0.0; S(11): 1.1; S(12): 98.9	17	1	1	0	N-Term(TMT6plex); M1(Oxidation); S12(Phospho)
38683816	NP_942592.1	26057	ANKRD17	ankyrin repeat domain-containing protein 17 isoformb	S1458	1.1	1.1	1.0	1.3	-	-	-	-	0.9	1.1	0.9	1.2	-	-	-	-	0.9	1.1	0.9	1.2	LTVAsPK	NGKLTVAsPKRGQKR	S5;	No	T(2): 0.0; S(5): 100.0	5	3	1	0	N-Term(TMT6plex); S5(Phospho); K7(TMT6plex)
116256464	NP_997245.2	343990	KIAA1211L	uncharacterized protein KIAA1211-like	S356	1.3	0.6	0.5	1.3	-	-	-	-	-	-	-	-	-	-	-	-	-	-	-	-	VEPPsPPEGPPNPGPDGGK	TLRVEPPsPPEGPPN	S5;	Yes	S(5): 100.0	1	1	1	0	N-Term(TMT6plex); S5(Phospho); K19(TMT6plex)
4507877	NP_003364.1	7414	VCL	vinculin isoformVCL	S346	1.2	1.3	1.4	1.3	-	-	-	-	1.0	1.1	1.0	1.2	-	-	-	-	1.0	1.1	1.0	1.2	GQGSsPVAMQK	RARGQGSsPVAMQKA	S5;	Yes	S(4): 1.1; S(5): 98.9	5	2	1	0	N-Term(TMT6plex); S5(Phospho); M9(Oxidation); K11(TMT6plex)
557440810	NP_001273512.1	57661	PHRF1	PHD and RING finger domain-containing protein 1 isoform4	S987	0.8	1.2	1.3	1.3	-	-	-	-	0.7	1.3	1.5	1.6	-	-	-	-	0.7	1.3	1.5	1.6	VVELRPPsR	VVELRPPsRSRSTSS	S8;	No	S(8): 100.0	15	4	1	0	N-Term(TMT6plex); S8(Phospho)
112421108	NP_055940.3	23152	CIC	protein capicua homolog	S721	1.0	0.9	1.2	1.3	-	-	-	-	-	-	-	-	-	-	-	-	-	-	-	-	VPGGsPLGVSLVYSDK	GSRVPGGsPLGVSLV	S5;	Yes	S(5): 100.0; S(10): 0.0; Y(13): 0.0; S(14): 0.0	8	1	1	0	N-Term(TMT6plex); S5(Phospho); K16(TMT6plex)
133922582	NP_060553.4	140467	ZNF358	zinc finger protein 358	T485; S487	1.3	1.3	1.1	1.3	-	-	-	-	1.4	1.5	1.4	1.5	-	-	-	-	1.4	1.5	1.4	1.5	StPsPTPVESSDPK	PLPGSRStPSPTPVE;PGSRSTPsPTPVESS	T2; S4;	Yes;Yes	S(1): 1.7; T(2): 98.3; S(4): 98.3; T(6): 1.7; S(10): 0.0; S(11): 0.0	3	1	1	0	N-Term(TMT6plex); S1(Phospho); S4(Phospho); K14(TMT6plex)
14589931	NP_002580.2	5099	PCDH7	protocadherin-7 isoforma precursor	S974	0.6	0.9	1.2	1.3	-	-	-	-	-	-	-	-	-	-	-	-	-	-	-	-	LSDsPSMGR	VNEKLSDsPSMGRYR	S4;	Yes	S(2): 0.9; S(4): 98.2; S(6): 0.9	1	4	1	0	N-Term(TMT6plex); S4(Phospho); M7(Oxidation)
22035606	NP_663720.1	9448	MAP4K4	mitogen-activated protein kinase kinase kinase kinase 4 isoform3	S625	1.6	1.1	1.2	1.3	-	-	-	-	1.4	1.0	1.1	1.2	-	-	-	-	1.4	1.0	1.1	1.2	NsTSSIEPR	SQAGQRNsTSSIEPR	S2;	No	S(2): 100.0; T(3): 0.0; S(4): 0.0; S(5): 0.0	2	3	1	0	N-Term(TMT6plex); S2(Phospho)
18379332	NP_567823.1	51322	WAC	WW domain-containing adapter protein with coiled-coil isoform3	S64	1.3	1.4	1.3	1.3	-	-	-	-	1.2	1.3	1.2	1.1	-	-	-	-	1.2	1.3	1.2	1.1	SDsPENK	KMLRRSDsPENKYSD	S3;	Yes	S(1): 0.0; S(3): 100.0	5	2	1	0	N-Term(TMT6plex); S3(Phospho); K7(TMT6plex)
4757926	NP_004893.1	9584	RBM39	RNA-binding protein 39 isoformb	S97	1.0	1.0	1.0	1.3	-	-	-	-	1.0	1.1	1.1	1.3	-	-	-	-	1.0	1.1	1.1	1.3	YRsPYSGPK	RFRGRYRsPYSGPKF	S3;	Yes	Y(1): 0.6; S(3): 99.4; Y(5): 0.0; S(6): 0.0	4	2	1	1	N-Term(TMT6plex); S3(Phospho); K9(TMT6plex)
14702162	NP_115797.1	7461	CLIP2	CAP-Gly domain-containing linker protein 2 isoform2	S294	0.9	1.3	0.9	1.3	-	-	-	-	-	-	-	-	-	-	-	-	-	-	-	-	IGEPSTsPAK	RIGEPSTsPAKAKKT	S7;	Yes	S(5): 0.0; T(6): 1.1; S(7): 98.9	1	2	1	0	N-Term(TMT6plex); T6(Phospho); K10(TMT6plex)
319996739	NP_001188411.1	114880	OSBPL6	oxysterol-binding protein-related protein 6 isoforme	S44	1.5	1.5	1.3	1.3	-	-	-	-	0.7	0.9	0.9	0.9	-	-	-	-	0.7	0.9	0.9	0.9	TAsSSTEPSVSR	HILERTAsSSTEPSV	S3;	Yes	T(1): 0.0; S(3): 100.0; S(4): 0.0; S(5): 0.0; T(6): 0.0; S(9): 0.0; S(11): 0.0	1	5	1	0	N-Term(TMT6plex); S3(Phospho)
355390315	NP_056171.3	23513	SCRIB	protein scribble homolog isoformb	S1348	1.9	1.7	1.2	1.3	-	-	-	-	1.7	1.6	1.2	1.2	-	-	-	-	1.7	1.6	1.2	1.2	AFAAVPTSHPPEDAPAQPPTPGPAAsPEQLSFR	PTPGPAAsPEQLSFR	S26;	Yes	T(7): 0.0; S(8): 0.0; T(20): 0.0; S(26): 99.9; S(31): 0.1	16	2	1	0	N-Term(TMT6plex); S26(Phospho)
118498359	NP_056474.2	26156	RSL1D1	ribosomal L1 domain-containing protein 1	T454	0.7	0.9	0.9	1.3	-	-	-	-	0.9	1.1	1.1	1.4	-	-	-	-	0.9	1.1	1.1	1.4	DARQtPK	GKKDARQtPKKPEAK	T5;	Yes	T(5): 100.0	19	1	1	1	N-Term(TMT6plex); T5(Phospho); K7(TMT6plex)
110611226	NP_006368.3	10497	UNC13B	protein unc-13 homolog B	S1251	1.0	1.2	1.1	1.3	-	-	-	-	-	-	-	-	-	-	-	-	-	-	-	-	GTGNAsPDAR	VRGTGNAsPDARASA	S6;	No	T(2): 0.0; S(6): 100.0	1	1	1	0	N-Term(TMT6plex); S6(Phospho)
115298682	NP_055987.2	23215	PRRC2C	protein PRRC2C	S500	1.0	1.3	1.3	1.3	-	-	-	-	1.1	1.2	1.3	1.2	-	-	-	-	1.1	1.2	1.3	1.2	QPsPEEIR	GILEKQPsPEEIRER	S3;	Yes	S(3): 100.0	18	1	1	0	N-Term(TMT6plex); S3(Phospho)
8923477	NP_060324.1	54958	TMEM160	transmembrane protein 160 precursor	S48	0.7	1.2	1.0	1.3	-	-	-	-	0.6	1.0	0.8	1.2	-	-	-	-	0.6	1.0	0.8	1.2	AGAsPPPVSELDR	HGPRAGAsPPPVSEL	S4;	Yes	S(4): 100.0; S(9): 0.0	1	1	1	0	N-Term(TMT6plex); S4(Phospho)
210032580	NP_055863.1	23067	SETD1B	histone-lysine N-methyltransferase SETD1B	S1240	1.4	1.4	1.5	1.3	-	-	-	-	-	-	-	-	-	-	-	-	-	-	-	-	EPPEEPGLSQEGAMLLsPEPPA	QEGAMLLsPEPPAKE	S17;	No	S(9): 1.9; S(17): 98.1	2	1	1	0	N-Term(TMT6plex); M14(Oxidation); S17(Phospho); K23(TMT6plex)
145309300	NP_112557.2	8621	CDK13	cyclin-dependent kinase 13 isoform2	T1058	0.9	0.9	1.1	1.3	-	-	-	-	1.2	1.1	1.5	1.6	-	-	-	-	1.2	1.1	1.5	1.6	TNtPQGVLPSSQLK	LDDSRTNtPQGVLPS	T3;	Yes	T(1): 0.8; T(3): 99.2; S(10): 0.0; S(11): 0.0	8	2	1	0	N-Term(TMT6plex); T3(Phospho); K14(TMT6plex)
24308207	NP_065761.1	57470	LRRC47	leucine-rich repeat-containing protein 47	S520	1.1	1.3	1.5	1.3	-	-	-	-	1.2	1.4	1.7	1.3	-	-	-	-	1.2	1.4	1.7	1.3	EEGSLsDTEADAVSGQLPDPTTNPSAGK	NKEEGSLsDTEADAV	S6;	Yes	S(4): 0.0; S(6): 99.9; T(8): 0.0; S(14): 0.0; T(21): 0.0; T(22): 0.0; S(25): 0.0	7	1	1	0	N-Term(TMT6plex); S6(Phospho); K28(TMT6plex)
550544173	NP_001272377.1	23042	PDXDC1	pyridoxal-dependent decarboxylase domain-containing protein 1 isoform5	T600	1.1	1.3	1.3	1.3	-	-	-	-	0.9	1.2	1.2	1.2	-	-	-	-	0.9	1.2	1.2	1.2	AQGAGVTLPPtPSGSR	AGVTLPPtPSGSRTK	T11;	No	T(7): 2.9; T(11): 94.0; S(13): 0.1; S(15): 2.9	1	8	1	0	N-Term(TMT6plex); T11(Phospho)
427197591	NP_001258763.1	51319	RSRC1	serine/Arginine-related protein 53 isoform2	S6	1.7	1.5	1.7	1.3	-	-	-	-	1.6	1.4	1.2	1.1	-	-	-	-	1.6	1.4	1.2	1.1	RSsDTEESR	_MGRRSsDTEESR	S3;	No	S(2): 0.0; S(3): 100.0; T(5): 0.0; S(9): 0.0	6	2	1	1	N-Term(TMT6plex); S3(Phospho)
109637791	NP_060899.2	55814	BDP1	transcription factor TFIIIB component B" homolog	S965	1.0	1.2	1.2	1.3	-	-	-	-	1.0	1.3	1.4	1.3	-	-	-	-	1.0	1.3	1.4	1.3	ATGNESsPR	KATGNESsPREKTPE	S7;	No	T(2): 0.0; S(6): 0.0; S(7): 100.0	1	1	1	0	N-Term(TMT6plex); S7(Phospho)
148368978	NP_689512.2	29781	NCAPH2	condensin-2 complex subunit H2 isoform2	S284	1.1	1.4	1.3	1.3	-	-	-	-	1.1	1.4	1.3	1.4	-	-	-	-	1.1	1.4	1.3	1.4	ESRsPQQSAALPR	LEPKESRsPQQSAAL	S4;	Yes	S(2): 1.7; S(4): 98.3; S(8): 0.0	2	2	1	1	N-Term(TMT6plex); S4(Phospho)
4505971	NP_003618.1	8501	SLC43A1	large neutral amino acids transporter small subunit 3	S267	1.5	1.8	1.7	1.3	-	-	-	-	-	-	-	-	-	-	-	-	-	-	-	-	APsLEDGSDAFMSPQDVR	RLSQKAPsLEDGSDA	S3;	Yes	S(3): 100.0; S(8): 0.0; S(13): 0.0	1	1	1	0	N-Term(TMT6plex); S3(Phospho); M12(Oxidation)
4504195	NP_002086.1	2961	GTF2E2	transcription initiation factor IIE subunit beta	S34	0.7	0.8	0.6	1.3	-	-	-	-	0.8	0.8	0.7	1.3	-	-	-	-	0.8	0.8	0.7	1.3	SASSESSSsSSK	ASSESSSsSSKKKKT	S9;	No	S(1): 0.0; S(3): 0.0; S(4): 0.0; S(6): 0.0; S(7): 0.0; S(8): 1.1; S(9): 97.8; S(10): 1.1; S(11): 0.0	1	1	1	0	N-Term(TMT6plex); S9(Phospho); K12(TMT6plex)
5454088	NP_006392.1	10541	ANP32B	acidic leucine-rich nuclear phosphoprotein 32 family member B	T244	0.6	2.0	1.1	1.3	-	-	-	-	0.8	2.4	1.4	1.7	-	-	-	-	0.8	2.4	1.4	1.7	REtDDEGEDD	GEKRKREtDDEGEDD	T3;	Yes	T(3): 100.0	9	1	1	1	N-Term(TMT6plex); T3(Phospho)
14150015	NP_115675.1	84259	DCUN1D5	DCN1-like protein 5	S9	1.6	1.5	1.5	1.3	-	-	-	-	1.4	1.3	1.3	1.1	-	-	-	-	1.4	1.3	1.3	1.1	sPGVAAAVAEDGGLKK	PVKKKRKsPGVAAAV	S1;	Yes	S(1): 100.0	3	1	1	1	N-Term(TMT6plex); S1(Phospho); K15(TMT6plex); K16(TMT6plex)
45935385	NP_056093.3	23328	SASH1	SAM and SH3 domain-containing protein 1	S248	1.1	0.7	1.0	1.3	-	-	-	-	1.2	0.7	1.0	1.2	-	-	-	-	1.2	0.7	1.0	1.2	EQsDDETEESVK	NCNSREQsDDETEES	S3;	Yes	S(3): 100.0; T(7): 0.0; S(10): 0.0	2	1	1	0	N-Term(TMT6plex); S3(Phospho); K12(TMT6plex)
150456436	NP_060591.3	55719	FAM178A	protein FAM178A isoform1	S315	1.2	1.5	1.5	1.3	-	-	-	-	-	-	-	-	-	-	-	-	-	-	-	-	sSSDSWEPTSAGSK	GHLSRKRsSSDSWEP	S1;	No	S(1): 95.7; S(2): 1.4; S(3): 1.4; S(5): 1.4; T(9): 0.0; S(10): 0.0; S(13): 0.0	1	2	1	0	N-Term(TMT6plex); S5(Phospho); K14(TMT6plex)
262359929	NP_056261.4	25909	AHCTF1	protein ELYS	T1817	1.1	0.9	1.2	1.3	-	-	-	-	1.1	0.9	1.2	1.3	-	-	-	-	1.1	0.9	1.2	1.3	GLSQNQQIPQNSVtPR	QIPQNSVtPRRGRRK	T14;	No	S(3): 0.0; S(12): 1.5; T(14): 98.5	13	1	1	0	N-Term(TMT6plex); T14(Phospho)
62953134	NP_115672.2	84256	FLYWCH1	FLYWCH-type zinc finger-containing protein 1	S502	0.9	1.3	0.8	1.2	-	-	-	-	0.9	1.2	0.8	1.1	-	-	-	-	0.9	1.2	0.8	1.1	GsPGGPEELK	PNTAQRGsPGGPEEL	S2;	No	S(2): 100.0	2	2	1	0	N-Term(TMT6plex); S2(Phospho); K10(TMT6plex)
7661690	NP_056418.1	26093	CCDC9	coiled-coil domain-containing protein 9	S60	1.1	1.3	1.2	1.2	-	-	-	-	1.1	1.3	1.3	1.3	-	-	-	-	1.1	1.3	1.3	1.3	sVEKENVAVESEK	TAPRKGRsVEKENVA	S1;	Yes	S(1): 100.0; S(11): 0.0	8	1	1	1	N-Term(TMT6plex); S1(Phospho); K4(TMT6plex); K13(TMT6plex)
46852172	NP_056069.2	23303	KIF13B	kinesin-like protein KIF13B	S1644	0.6	1.0	0.9	1.2	-	-	-	-	0.5	1.0	0.8	1.3	-	-	-	-	0.5	1.0	0.8	1.3	ERPDLEAPAPGsPFR	LEAPAPGsPFRVRRV	S12;	Yes	S(12): 100.0	1	1	1	0	N-Term(TMT6plex); S12(Phospho)
13236559	NP_077295.1	79169	C1orf35	multiple myeloma tumor-associated protein 2	S217; S220	1.4	1.2	1.4	1.2	-	-	-	-	1.6	1.5	1.7	1.4	-	-	-	-	1.6	1.5	1.7	1.4	RPAEATSsPTsPERPR	RPAEATSsPTSPERP;EATSSPTsPERPRHH	S8; S11;	No;No	T(6): 0.0; S(7): 1.4; S(8): 97.2; T(10): 2.8; S(11): 98.6	3	1	1	0	N-Term(TMT6plex); S8(Phospho); S11(Phospho)
156766047	NP_001096618.1	26058	GIGYF2	PERQ amino acid-rich with GYF domain-containing protein 2 isoformc	S160	1.2	1.6	1.3	1.2	-	-	-	-	1.2	1.5	1.2	1.2	-	-	-	-	1.2	1.5	1.2	1.2	SQsWEER	REMHRSQsWEERGDR	S3;	Yes	S(1): 0.0; S(3): 100.0	9	3	1	0	N-Term(TMT6plex); S3(Phospho)
21493024	NP_653080.1	9590	AKAP12	A-kinase anchor protein 12 isoform2	S249	0.7	0.6	0.7	1.2	-	-	-	-	0.8	0.6	0.6	1.2	-	-	-	-	0.8	0.6	0.6	1.2	AEVAsEK	DGKAEVAsEKLTASE	S5;	No	S(5): 100.0	103	2	1	0	N-Term(TMT6plex); S5(Phospho); K7(TMT6plex)
37655165	NP_115530.2	84108	PCGF6	polycomb group RING finger protein 6 isoformb	S115	1.0	1.0	1.0	1.2	-	-	-	-	-	-	-	-	-	-	-	-	-	-	-	-	LEGGRQDsEDEEER	LEGGRQDsEDEEERL	S8;	Yes	S(8): 100.0	2	2	1	1	N-Term(TMT6plex); S8(Phospho)
5453974	NP_006247.1	5586	PKN2	serine/threonine-protein kinase N2	T124	1.1	1.2	1.0	1.2	-	-	-	-	1.1	1.3	1.1	1.3	-	-	-	-	1.1	1.3	1.1	1.3	TPDtPNNDPR	DCPRTPDtPNNDPRC	T4;	Yes	T(1): 0.0; T(4): 100.0	9	1	1	0	N-Term(TMT6plex); T4(Phospho)
166235163	NP_036477.2	26747	NUFIP1	nuclear fragile X mental retardation-interacting protein	S403	1.1	1.2	1.2	1.2	-	-	-	-	1.1	1.3	1.3	1.3	-	-	-	-	1.1	1.3	1.3	1.3	sPSQDVK	LDSSAPKsPSQDVKA	S1;	Yes	S(1): 100.0; S(3): 0.0	1	1	1	0	N-Term(TMT6plex); S1(Phospho); K7(TMT6plex)
556503401	NP_001273294.1	55700	MAP7D1	MAP7 domain-containing protein 1 isoform2	T97	0.7	0.8	0.8	1.2	-	-	-	-	0.7	0.7	0.8	1.2	-	-	-	-	0.7	0.7	0.8	1.2	GPtPPAMGPR	EAKSRGPtPPAMGPR	T3;	Yes	T(3): 100.0	27	3	1	0	N-Term(TMT6plex); T3(Phospho); M7(Oxidation)
194578923	NP_001124000.1	9043	SPAG9	C-Jun-amino-terminal kinase-interacting protein 4 isoform1	T1264	1.3	2.0	1.1	1.2	-	-	-	-	1.4	2.2	1.3	1.4	-	-	-	-	1.4	2.2	1.3	1.4	AGPSAQEPGSQtPLK	AQEPGSQtPLKSMLV	T12;	Yes	S(4): 0.0; S(10): 1.3; T(12): 98.7	13	4	1	0	N-Term(TMT6plex); T12(Phospho); K15(TMT6plex)
30061567	NP_060548.2	55132	LARP1B	la-related protein 1B isoform1	S906	1.1	1.3	1.3	1.2	-	-	-	-	-	-	-	-	-	-	-	-	-	-	-	-	NIsPESSDNSH	NSPRRNIsPESSDNS	S3;	Yes	S(3): 100.0; S(6): 0.0; S(7): 0.0; S(10): 0.0	6	1	1	0	N-Term(TMT6plex); S3(Phospho)
431822406	NP_001258900.1	3326	HSP90AB1	heat shock protein HSP 90-beta isoformb	S178	0.9	1.3	1.1	1.2	-	-	-	-	1.0	1.2	1.1	1.2	-	-	-	-	1.0	1.2	1.1	1.2	EKEIsDDEAEEEK	KEREKEIsDDEAEEE	S5;	No	S(5): 100.0	54	3	1	1	N-Term(TMT6plex); K2(TMT6plex); S5(Phospho); K13(TMT6plex)
21218438	NP_065750.1	57459	GATAD2B	transcriptional repressor p66-beta	S486	1.0	1.0	0.6	1.2	-	-	-	-	1.0	1.0	0.6	1.1	-	-	-	-	1.0	1.0	0.6	1.1	LQQQAALsPTTAPAVSSVSK	LQQQAALsPTTAPAV	S8;	Yes	S(8): 95.8; T(10): 2.1; T(11): 2.1; S(16): 0.0; S(17): 0.0; S(19): 0.0	3	1	1	0	N-Term(TMT6plex); S8(Phospho); K20(TMT6plex)
169790837	NP_001116079.1	861	RUNX1	runt-related transcription factor 1 isoformAML1a	S21	1.0	1.0	1.3	1.2	-	-	-	-	0.9	1.0	1.2	1.3	-	-	-	-	0.9	1.0	1.2	1.3	FTPPSTALsPGK	TPPSTALsPGKMSEA	S9;	Yes	T(2): 0.0; S(5): 0.0; T(6): 0.0; S(9): 100.0	2	3	1	0	N-Term(TMT6plex); S9(Phospho); K12(TMT6plex)
132626688	NP_055456.2	9656	MDC1	mediator ofDNA damage checkpoint protein 1	T1403	0.5	0.9	0.9	1.2	-	-	-	-	0.5	0.9	1.0	1.3	-	-	-	-	0.5	0.9	1.0	1.3	SSGKtPETLVPTAPK	KNRSSGKtPETLVPT	T5;	Yes	S(1): 50.5; S(2): 50.5; T(5): 99.0; T(8): 0.0; T(12): 0.0	26	1	1	1	N-Term(TMT6plex); S1(Phospho); S2(Phospho); K4(TMT6plex); K15(TMT6plex)
21624607	NP_066972.1	23406	COTL1	coactosin-like protein	T141	1.2	1.1	1.0	1.2	-	-	-	-	0.9	0.9	0.7	1.0	-	-	-	-	0.9	0.9	0.7	1.0	AGGANYDAQtE	GANYDAQtE	T10;	Yes	Y(6): 0.0; T(10): 100.0	1	1	1	0	N-Term(TMT6plex); T10(Phospho)
604723372	NP_878315.2	91179	SCARF2	scavenger receptor class F member 2 isoform2 precursor	S737	2.5	2.0	3.0	1.2	-	-	-	-	-	-	-	-	-	-	-	-	-	-	-	-	DPTPRPPGLPEEATALAAPsPPR	ATALAAPsPPRARAR	S20;	No	T(3): 0.0; T(14): 0.0; S(20): 100.0	7	2	1	0	N-Term(TMT6plex); S20(Phospho)
215820619	NP_055514.3	9726	ZNF646	zinc finger protein 646	T609	1.2	1.2	1.1	1.2	-	-	-	-	1.4	1.2	1.1	1.2	-	-	-	-	1.4	1.2	1.1	1.2	TEtTMSPPR	KENSRTEtTMSPPRA	T3;	No	T(1): 1.2; T(3): 96.4; T(4): 1.2; S(6):	1	1	1	0	N-Term(TMT6plex); M5(Oxidation); S6(Phospho)
571026676	NP_001275706.1	7922	SLC39A7	zinc transporter SLC39A7 isoform2	S151	1.4	1.3	1.1	1.2	-	-	-	-	1.3	1.2	1.1	1.2	-	-	-	-	1.3	1.2	1.1	1.2	QSsEEEEKETR	STKEKQSsEEEEKET	S3;	No	S(2): 0.8; S(3): 99.2; T(10): 0.0	1	2	1	1	N-Term(TMT6plex); S2(Phospho); K8(TMT6plex)
194272142	NP_001123537.1	4650	MYO9B	unconventional myosin-IXb isoform2	T733	0.7	1.1	1.0	1.2	-	-	-	-	0.7	1.1	1.1	1.3	-	-	-	-	0.7	1.1	1.1	1.3	GAStPSEK	ELPRGAStPSEKLYR	T4;	No	S(3): 0.0; T(4): 100.0; S(6): 0.0	14	2	1	0	N-Term(TMT6plex); T4(Phospho); K8(TMT6plex)
153945728	NP_005900.2	4131	MAP1B	microtubule-associated protein 1B	T744	0.9	1.6	1.3	1.2	-	-	-	-	0.7	1.2	1.1	1.3	-	-	-	-	0.7	1.2	1.1	1.3	SStPLSEAK	KDAKKSStPLSEAKK	T3;	Yes	S(1): 0.0; S(2): 1.0; T(3): 99.0; S(6):	49	1	1	0	N-Term(TMT6plex); T3(Phospho); K9(TMT6plex)
557440899	NP_001273490.1	4926	NUMA1	nuclear mitotic apparatus protein 1 isoform2	S1842	1.2	1.5	1.3	1.2	-	-	-	-	1.3	1.6	1.4	1.3	-	-	-	-	1.3	1.6	1.4	1.3	ATSSTQsLAR	RATSSTQsLARLGSP	S7;	No	T(2): 0.0; S(3): 1.4; S(4): 1.4; T(5): 0.0; S(7): 97.2	34	2	1	0	N-Term(TMT6plex); S7(Phospho)
119874201	NP_055845.1	285527	FRYL	protein furry homolog-like	S1978	1.4	1.3	1.2	1.2	-	-	-	-	1.3	1.1	1.0	1.1	-	-	-	-	1.3	1.1	1.0	1.1	sLSSLR	SSLARTRsLSSLREK	S1;	Yes	S(1): 100.0; S(3): 0.0; S(4): 0.0	3	5	1	0	N-Term(TMT6plex); S1(Phospho)
262359929	NP_056261.4	25909	AHCTF1	protein ELYS	S1542	1.1	1.4	1.3	1.2	-	-	-	-	1.1	1.4	1.3	1.3	-	-	-	-	1.1	1.4	1.3	1.3	LEAQDsGEEAR	EKLEAQDsGEEARNL	S6;	No	S(6): 100.0	13	1	1	0	N-Term(TMT6plex); S6(Phospho)
116063573	NP_001447.2	2316	FLNA	filamin-A isoform1	S2276	1.1	1.1	1.2	1.2	-	-	-	-	1.1	1.1	1.3	1.4	-	-	-	-	1.1	1.1	1.3	1.4	AEIsFEDR	GPSKAEIsFEDRKDG	S4;	No	S(4): 100.0	14	2	1	0	N-Term(TMT6plex); S4(Phospho)
42542379	NP_005830.2	10250	SRRM1	serine/arginine repetitive matrix protein 1	S530	1.2	1.8	1.4	1.2	-	-	-	-	1.5	1.9	1.5	1.4	-	-	-	-	1.5	1.9	1.5	1.4	SAsPSPR	HSPSRSAsPSPRKRQ	S3;	Yes	S(1): 0.8; S(3): 98.4; S(5): 0.8	229	1	1	0	N-Term(TMT6plex); S3(Phospho)
21536485	NP_003316.3	7290	HIRA	protein HIRA	T555	1.7	1.5	1.2	1.2	-	-	-	-	1.7	1.8	1.2	1.4	-	-	-	-	1.7	1.8	1.2	1.4	DSMNATSTPAALSPSVLTtPSK	LSPSVLTtPSKIEPM	T19;	Yes	S(2): 0.0; T(6): 0.0; S(7): 0.0; T(8): 0.0; S(13): 0.0; S(15): 0.0; T(18): 1.9; T(19): 98.1; S(21): 0.0	3	1	1	0	N-Term(TMT6plex); M3(Oxidation); T19(Phospho); K22(TMT6plex)
61743954	NP_001611.1	79026	AHNAK	neuroblast differentiation-associated protein AHNAK isoform1	S3412	1.5	1.8	1.5	1.2	-	-	-	-	1.7	1.9	1.5	1.4	-	-	-	-	1.7	1.9	1.5	1.4	MPFLSISsPK	MPFLSISsPKVSMPD	S8;	Yes	S(5): 0.0; S(7): 1.1; S(8): 98.9	79	1	1	0	N-Term(TMT6plex); M1(Oxidation); S8(Phospho); K10(TMT6plex)
31542528	NP_056324.2	25977	NECAP1	adaptin ear-binding coat-associated protein 1	T211	1.1	1.8	1.4	1.2	-	-	-	-	1.0	1.7	1.5	1.1	-	-	-	-	1.0	1.7	1.5	1.1	VTIPPPSSSVAISNHVtPPPIPK	VAISNHVtPPPIPKS	T17;	Yes	T(2): 0.0; S(7): 0.0; S(8): 0.0; S(9): 0.0; S(13): 0.0; T(17): 100.0	3	1	1	0	N-Term(TMT6plex); T17(Phospho); K23(TMT6plex)
154813195	NP_056504.3	27147	DENND2A	DENN domain-containing protein 2A	S365	1.1	1.2	0.8	1.2	-	-	-	-	1.1	1.1	0.7	1.0	-	-	-	-	1.1	1.1	0.7	1.0	TLsEENVYEDILDPPMK	LGLTRTLsEENVYED	S3;	Yes	T(1): 1.5; S(3): 98.5; Y(8): 0.0	4	1	1	0	N-Term(TMT6plex); S3(Phospho); M16(Oxidation); K17(TMT6plex)
21493024	NP_653080.1	9590	AKAP12	A-kinase anchor protein 12 isoform2	T1018	0.5	0.9	1.0	1.2	-	-	-	-	0.6	0.9	0.8	1.1	-	-	-	-	0.6	0.9	0.8	1.1	VVGQTtPESFEK	GKVVGQTtPESFEKA	T6;	No	T(5): 0.0; T(6): 100.0; S(9): 0.0	103	2	1	0	N-Term(TMT6plex); T6(Phospho); K12(TMT6plex)
7705375	NP_056982.1	51341	ZBTB7A	zinc finger and BTB domain-containing protein 7A	S337	0.8	0.9	0.9	1.2	-	-	-	-	0.5	0.6	0.7	1.3	-	-	-	-	0.5	0.6	0.7	1.3	AGAAAGDsDEESR	AGAAAGDsDEESRAD	S8;	Yes	S(8): 100.0; S(12): 0.0	2	1	1	0	N-Term(TMT6plex); S8(Phospho)
18087845	NP_542399.1	113130	CDCA5	sororin	T111	1.2	1.2	1.1	1.2	-	-	-	-	1.2	1.3	1.2	1.4	-	-	-	-	1.2	1.3	1.2	1.4	THSVPAtPTSTPVPNPEAESSSK	KTHSVPAtPTSTPVP	T7;	Yes	T(1): 0.0; S(3): 1.6; T(7): 98.3; T(9): 0.0; S(10): 0.0; T(11): 0.0; S(20): 0.0; S(21): 0.0; S(22): 0.0	6	1	1	0	N-Term(TMT6plex); T7(Phospho); K23(TMT6plex)
153070260	NP_002347.5	4082	MARCKS	myristoylated alanine-rich C-kinase substrate	S81	0.9	0.9	1.2	1.2	-	-	-	-	1.4	1.3	1.7	1.6	-	-	-	-	1.4	1.3	1.7	1.6	EEPAAAGSGAAsPSAAEK	AAGSGAAsPSAAEKG	S12;	Yes	S(8): 2.4; S(12): 95.2; S(14): 2.4	69	1	1	0	N-Term(TMT6plex); S12(Phospho); K18(TMT6plex)
546231210	NP_001271210.1	55671	SMEK1	serine/threonine-protein phosphatase 4 regulatory subunit 3A isoform3	S532	1.1	1.4	1.3	1.2	-	-	-	-	1.3	1.7	1.5	1.3	-	-	-	-	1.3	1.7	1.5	1.3	TNLTSQSSTTNLPGSPGsPGSPGSPGSPGSVPK	NLPGSPGsPGSPGSP	S18;	No	T(1): 0.0; T(4): 0.0; S(5): 0.0; S(7): 0.0; S(8): 0.0; T(9): 0.1; T(10): 0.0; S(15): 2.5; S(18): 94.9; S(21): 2.5; S(24): 0.1; S(27): 0.0; S(30): 0.0	3	2	1	0	N-Term(TMT6plex); S21(Phospho); K33(TMT6plex)
115298682	NP_055987.2	23215	PRRC2C	protein PRRC2C	S2694	0.7	1.4	1.0	1.2	-	-	-	-	0.8	1.4	1.0	1.1	-	-	-	-	0.8	1.4	1.0	1.1	ATSTsPNSQSSK	PFRATSTsPNSQSSK	S5;	Yes	T(2): 0.0; S(3): 0.0; T(4): 1.1; S(5): 98.9; S(8): 0.0; S(10): 0.0; S(11): 0.0	18	1	1	0	N-Term(TMT6plex); S5(Phospho); K12(TMT6plex)
297747356	NP_001172048.1	55892	MYNN	myoneurin isoformB	S289	1.2	1.0	1.1	1.2	-	-	-	-	-	-	-	-	-	-	-	-	-	-	-	-	SPYEAENsGEELDQR	SPYEAENsGEELDQR	S8;	Yes	S(1): 0.0; Y(3): 0.0; S(8): 100.0	1	2	1	0	N-Term(TMT6plex); S8(Phospho)
256818798	NP_001157974.1	56478	EIF4ENIF1	eukaryotic translation initiation factor 4E transporter isoformb	S578	1.0	1.6	1.2	1.2	-	-	-	-	1.0	1.5	1.2	1.1	-	-	-	-	1.0	1.5	1.2	1.1	DSsPTTNSK	PSADRDSsPTTNSKL	S3;	No	S(2): 0.7; S(3): 99.3; T(5): 0.0; T(6): 0.0; S(8): 0.0	4	2	1	0	N-Term(TMT6plex); S3(Phospho); K9(TMT6plex)
50962882	NP_001002909.1	23131	GPATCH8	G patch domain-containing protein 8	S758	0.9	1.0	1.2	1.2	-	-	-	-	1.0	1.1	1.2	1.2	-	-	-	-	1.0	1.1	1.2	1.2	sLPAEEGSGK	QDDSQRRsLPAEEGS	S1;	Yes	S(1): 100.0; S(8): 0.0; S(9): 0.0	17	1	1	0	N-Term(TMT6plex); S1(Phospho); K11(TMT6plex)
30795227	NP_543010.3	92675	DTD1	D-tyrosyl-tRNA(Tyr) deacylase 1	S197	1.5	1.2	1.3	1.2	-	-	-	-	1.3	1.1	1.2	1.2	-	-	-	-	1.3	1.1	1.2	1.2	SASsGAEGDVSSER	KEDRSASsGAEGDVS	S4;	Yes	S(1): 0.0; S(3): 0.0; S(4): 100.0; S(11): 0.0; S(12): 0.0	4	1	1	0	N-Term(TMT6plex); S4(Phospho)
256419001	NP_001157855.1	27067	STAU2	double-stranded RNA-binding protein Staufen homolog 2 isoformd	S320	0.4	1.0	1.0	1.2	-	-	-	-	0.4	1.1	1.1	1.2	-	-	-	-	0.4	1.1	1.1	1.2	GSSPTPPCsPVQPSK	SSPTPPCsPVQPSKQ	S9;	No	S(2): 49.6; S(3): 0.9; T(5): 49.6; S(9): 100.0; S(14): 0.0	3	6	1	0	N-Term(TMT6plex); S3(Phospho); C8(Carbamidomethyl); S9(Phospho); K15(TMT6plex)

Khan et al., 2019. Multi-omics analysis to characterize cigarette smoke induced molecular alterations in esophageal cells

Supplementary Table 8. List of phosphopeptides identified and quantified in untreated and chronically treated Het1A cells with cigarette smoke condensate for 8 months using SequestHT and Mascot search algorithms with a PhosphoRS probability score ≥75%

Protein group Accession	NP_Accession	Gene ID	Gene Symbol	Protein Description	PhosphoSite (Protein)	Het-1A-Smoke - 2M/Parental (Replicate 1)	Het-1A-Smoke - 4M/Parental (Replicate 1)	Het-1A-Smoke - 6M/Parental (Replicate 1)	Het-1A-Smoke - 8M/Parental (Replicate 1)	Het-1A-Smoke - 2M/Parental (Replicate 2)	Het-1A-Smoke - 4M/Parental (Replicate 2)	Het-1A-Smoke - 6M/Parental (Replicate 2)	Het-1A-Smoke - 8M/Parental (Replicate 2)	Normalized phosphoprotein ratios with respect to corresponding total protein ratios. Het-1A-Smoke - 2M/Parental (Replicate 1)	Het-1A-Smoke - 4M/Parental (Replicate 1)	Het-1A-Smoke - 6M/Parental (Replicate 1)	Het-1A-Smoke - 8M/Parental (Replicate 1)	Het-1A-Smoke - 2M/Parental (Replicate 2)	Het-1A-Smoke - 4M/Parental (Replicate 2)	Het-1A-Smoke - 6M/Parental (Replicate 2)	Het-1A-Smoke - 8M/Parental (Replicate 2)	Average of replicates Het-1A-Smoke - 2M/Parental	Het-1A-Smoke - 4M/Parental	Het-1A-Smoke - 6M/Parental	Het-1A-Smoke - 8M/Parental	Peptide Sequence	Phosphowindow	PhosphoSite (Peptide)	Phosphosite Plus Evidence	phosphoRS Site Probabilities	# PSMs	# Proteins	# Protein Groups	# Missed Cleavages	Modifications
498917186	NP_001265089.1	84335	AKT1S1	proline-rich AKT1 substrate 1 isoform b	S212	1.0	1.1	0.9	1.2	-	-	-	-	1.3	1.4	0.9	1.3	-	-	-	-	1.3	1.4	0.9	1.3	SSDEENGPPSsPDLDR	EENGPPSsPDLDRIA	S11;	Yes	S(1): 0.0; S(2): 0.0; S(10): 0.0; S(11): 100.0	4	2	1	0	N-Term(TMT6plex); S11(Phospho)
118421085	NP_689472.3	90381	TICRR	treslin	S1115	1.0	1.4	1.1	1.2	-	-	-	-	1.1	1.5	1.2	1.2	-	-	-	-	1.1	1.5	1.2	1.2	SLsFSK	KSHQKSLsFSKTTPR	S3;	Yes	S(1): 0.0; S(3): 100.0; S(5): 0.0	11	1	1	0	N-Term(TMT6plex); S3(Phospho); K6(TMT6plex)
150418007	NP_006258.3	5903	RANBP2	E3 SUMO-protein ligase RanBP2	S2276; S2280	0.8	1.2	1.1	1.2	-	-	-	-	0.7	1.2	1.0	1.1	-	-	-	-	0.7	1.2	1.0	1.1	SALsPSKsPAK	FSFKSALsPSKSPAK;SALSPSKsPAKLNQS	S4; S8;	Yes;Yes	S(1): 1.2; S(4): 98.8; S(6): 0.0; S(8): 100.0	17	2	1	1	N-Term(TMT6plex); S6(Phospho); K7(TMT6plex); S8(Phospho); K11(TMT6plex)
102467242	NP_064623.2	3619	INCENP	inner centromere protein isoform 2	S312	1.5	1.4	1.2	1.2	-	-	-	-	1.5	1.3	1.2	1.2	-	-	-	-	1.5	1.3	1.2	1.2	HSPIAPSsPSPQVLAQK	HSPIAPSsPSPQVLA	S8;	Yes	S(2): 0.0; S(7): 1.3; S(8): 97.4; S(10): 1.3	23	2	1	0	N-Term(TMT6plex); S8(Phospho); K17(TMT6plex)
5803221	NP_006775.1	10885	WDR3	WD repeat-containing protein 3	T943	1.1	0.9	0.6	1.2	-	-	-	-	1.0	0.9	0.6	1.0	-	-	-	-	1.0	0.9	0.6	1.0	LILTLt	EKLILTLt	T6;	Yes	T(4): 0.0; T(6): 100.0	3	1	1	0	N-Term(TMT6plex); T6(Phospho)
305632781	NP_061886.2	54478	FAM64A	protein FAM64A isoform 1	S135	1.1	1.2	1.3	1.2	-	-	-	-	1.1	1.1	1.3	1.2	-	-	-	-	1.1	1.1	1.3	1.2	GSGSPTHsLSQK	GSGSPTHsLSQKSTR	S8;	Yes	S(2): 49.5; S(4): 49.5; T(6): 1.0; S(8): 100.0; S(10): 0.0	6	2	1	0	N-Term(TMT6plex); S4(Phospho); S8(Phospho); K12(TMT6plex)
57222563	NP_055571.2	9793	CKAP5	cytoskeleton-associated protein 5 isoform b	S816	1.2	1.0	1.0	1.2	-	-	-	-	1.2	0.9	0.8	1.0	-	-	-	-	1.2	0.9	0.8	1.0	MQGQsPPAPTR	EEKMQGQsPPAPTRG	S5;	Yes	S(5): 100.0; T(10): 0.0	1	2	1	0	N-Term(TMT6plex); M1(Oxidation); S5(Phospho)
224994162	NP_001139310.1	9650	MTFR1	mitochondrial fission regulator 1 isoform 2	S257	1.2	1.4	1.5	1.2	-	-	-	-	-	-	-	-	-	-	-	-	-	-	-	-	SDsQDEVEK	AYRYRSDsQDEVEKG	S3;	No	S(1): 0.0; S(3): 100.0	1	3	1	0	N-Term(TMT6plex); S3(Phospho); K9(TMT6plex)
153792694	NP_057336.3	57448	BIRC6	baculoviral IAP repeat-containing protein 6	S488	1.4	1.5	1.5	1.2	-	-	-	-	1.2	1.3	1.2	1.0	-	-	-	-	1.2	1.3	1.2	1.0	sDSVTGHTSQK	DSEEHSRsDSVTGHT	S1;	No	S(1): 96.5; S(3): 1.7; T(5): 1.7; T(8): 0.0; S(9): 0.0	1	1	1	0	N-Term(TMT6plex); S3(Phospho); K11(TMT6plex)
374349207	NP_001243398.1	23522	KAT6B	histone acetyltransferase KAT6B isoform 3	S371	0.8	0.9	1.1	1.2	-	-	-	-	0.9	1.1	1.3	1.3	-	-	-	-	0.9	1.1	1.3	1.3	GsPDTEIK	NAETGRGsPDTEIKI	S2;	No	S(2): 100.0; T(5): 0.0	1	1	1	0	N-Term(TMT6plex); S2(Phospho); K8(TMT6plex)
40018644	NP_060442.2	55668	GPATCH2L	G patch domain-containing protein 2-like isoform 2	S86	0.6	1.0	1.0	1.2	-	-	-	-	-	-	-	-	-	-	-	-	-	-	-	-	EVAPVTNFsDSDDTMVAK	VAPVTNFsDSDDTMV	S9;	Yes	T(6): 1.2; S(9): 97.5; S(11): 1.2; T(14): 0.0	5	2	1	0	N-Term(TMT6plex); S9(Phospho); M15(Oxidation); K18(TMT6plex)
41393547	NP_056993.2	51594	NBAS	neuroblastoma-amplified sequence	S386; Y388	0.7	1.2	1.0	1.2	-	-	-	-	0.6	1.0	0.8	1.1	-	-	-	-	0.6	1.0	0.8	1.1	EsFyPLIDVNWWADSAVTLAR	KKIKDKEsFYPLIDV;IKDKESFyPLIDVNW	S2; Y4;	No;No	S(2): 100.0; Y(4): 100.0; S(15): 0.0; T(18): 0.0	3	1	1	0	N-Term(TMT6plex); S2(Phospho); Y4(Phospho)
13129016	NP_076955.1	79016	DDA1	DET1- and DDB1-associated protein 1	S95	1.0	1.3	1.3	1.1	-	-	-	-	-	-	-	-	-	-	-	-	-	-	-	-	TDsPDMHEDT	RKVARTDsPDMHEDT	S3;	Yes	T(1): 0.9; S(3): 99.1; T(10): 0.0	1	1	1	0	N-Term(TMT6plex); S3(Phospho); M6(Oxidation)
167234419	NP_005110.2	9967	THRAP3	thyroid hormone receptor-associated protein 3	S682	1.3	1.4	1.3	1.1	-	-	-	-	1.4	1.4	1.4	1.2	-	-	-	-	1.4	1.4	1.4	1.2	IDIsPSTFR	IHRRIDIsPSTFRKH	S4;	Yes	S(4): 99.3; S(6): 0.7; T(7): 0.0	33	1	1	0	N-Term(TMT6plex); S4(Phospho)
635172853	NP_001278937.1	6745	SSR1	translocon-associated protein subunit alpha isoform 2 precursor	S213	1.4	1.5	1.1	1.1	-	-	-	-	1.3	1.4	1.1	1.2	-	-	-	-	1.3	1.4	1.1	1.2	sVGSDE	RKRAQKRsVGSDE_	S1;	No	S(1): 100.0; S(4): 0.0	1	2	1	0	N-Term(TMT6plex); S1(Phospho)
6594683	NP_015627.1	7468	WHSC1	histone-lysine N-methyltransferase NSD2 isoform 4	S616	0.5	0.9	1.0	1.1	-	-	-	-	0.6	1.0	1.2	1.2	-	-	-	-	0.6	1.0	1.2	1.2	sSSPSASLTENEVK	SALGFSKsSSPSASL	S1;	No	S(1): 96.6; S(2): 1.7; S(3): 1.7; S(5): 0.0; S(7): 0.0; T(9): 0.0	3	1	1	0	N-Term(TMT6plex); S1(Phospho); K14(TMT6plex)
296278202	NP_001171721.1	56288	PARD3	partitioning defective 3 homolog isoform 9	S223	1.4	1.2	1.1	1.1	-	-	-	-	1.3	1.2	1.0	1.1	-	-	-	-	1.3	1.2	1.0	1.1	SSLsASHPMVGK	DNARSSLsASHPMVG	S4;	No	S(1): 1.3; S(2): 1.3; S(4): 97.4; S(6): [illegible]	11	9	1	0	N-Term(TMT6plex); S2(Phospho); M9(Oxidation); K12(TMT6plex)
13775602	NP_085096.1	22933	SIRT2	NAD-dependent protein deacetylase sirtuin-2 isoform 2	S331	1.1	0.8	1.0	1.1	-	-	-	-	-	-	-	-	-	-	-	-	-	-	-	-	EHASIDAQSGAGVPNPSTSAsPK	PNPSTSAsPKKSPPP	S21;	No	S(4): 0.0; S(9): 0.0; S(17): 0.0; T(18): 0.0; S(19): 1.1; S(21): 98.9	3	2	1	0	N-Term(TMT6plex); S21(Phospho); K23(TMT6plex)
39777594	NP_057438.3	28231	SLCO4A1	solute carrier organic anion transporter family member 4A1	S50	0.7	1.2	1.1	1.1	-	-	-	-	-	-	-	-	-	-	-	-	-	-	-	-	SAAHsPLDTSK	SLRSAAHsPLDTSKQ	S5;	Yes	S(1): 0.0; S(5): 100.0; T(9): 0.0; S(10): 0.0	5	1	1	0	N-Term(TMT6plex); S5(Phospho); K11(TMT6plex)
119395760	NP_001073280.1	1729	DIAPH1	protein diaphanous homolog 1 isoform 2	S22	1.2	1.1	1.4	1.1	-	-	-	-	1.2	1.1	1.3	1.1	-	-	-	-	1.2	1.1	1.3	1.1	GRsPDELPSAGGDGGK	RDKKKGRsPDELPSA	S3;	Yes	S(3): 100.0; S(9): 0.0	7	2	1	1	N-Term(TMT6plex); S3(Phospho); K16(TMT6plex)
110681719	NP_114401.2	23731	TMEM245	transmembrane protein 245	T36	0.5	0.8	1.1	1.1	-	-	-	-	0.4	0.8	1.0	1.1	-	-	-	-	0.4	0.8	1.0	1.1	AVGPSGGGGEtPR	PSGGGGEtPRTAALA	T11;	Yes	S(5): 0.0; T(11): 100.0	4	1	1	0	N-Term(TMT6plex); T11(Phospho)
18034767	NP_071387.1	63935	PCIF1	phosphorylated CTD-interacting factor 1	S305	0.6	0.5	0.5	1.1	-	-	-	-	0.6	0.6	0.5	1.3	-	-	-	-	0.6	0.6	0.5	1.3	SAsPDSR	LIESRSAsPDSRKVV	S3;	No	S(1): 0.0; S(3): 100.0; S(6): 0.0	2	1	1	0	N-Term(TMT6plex); S3(Phospho)
23238226	NP_689449.1	11135	CDC42EP1	cdc42 effector protein 1	S303	1.6	1.8	1.8	1.1	-	-	-	-	1.4	1.3	1.7	1.0	-	-	-	-	1.4	1.3	1.7	1.0	SsPVGGGPR	PVAEVKSsPVGGGPR	S2;	Yes	S(1): 0.0; S(2): 100.0	3	1	1	0	N-Term(TMT6plex); S2(Phospho)
21493024	NP_653080.1	9590	AKAP12	A-kinase anchor protein 12 isoform 2	S1489	0.9	1.1	1.3	1.1	-	-	-	-	1.0	1.0	1.1	1.1	-	-	-	-	1.0	1.0	1.1	1.1	ADsQDAGQETEK	AHVIKADsQDAGQET	S3;	No	S(3): 100.0; T(10): 0.0	103	2	1	0	N-Term(TMT6plex); S3(Phospho); K12(TMT6plex)
91208437	NP_057657.2	51291	GMIP	GEM-interacting protein isoform 1	S231	0.6	0.8	1.0	1.1	-	-	-	-	0.5	0.8	0.9	1.0	-	-	-	-	0.5	0.8	0.9	1.0	sQGSPEDSAPQASPGPSK	SEDLRARsQGSPEDS	S1;	Yes	S(1): 98.5; S(4): 1.5; S(8): 0.0; S(13): 0.0; S(17): 0.0	2	3	1	0	N-Term(TMT6plex); S1(Phospho); K18(TMT6plex)
503774456	NP_001265170.1	27043	PELP1	proline-, glutamic acid- and leucine-rich protein 1 isoform 2	T598	1.0	1.0	0.8	1.1	-	-	-	-	1.0	1.0	0.8	1.2	-	-	-	-	1.0	1.0	0.8	1.2	AGSNEDPILAPSGtPPPTIPPDETFGGR	PILAPSGtPPPTIPP	T14;	No	S(3): 0.0; S(12): 1.5; T(14): 98.5; T(18): 0.0; T(24): 0.0	19	2	1	0	N-Term(TMT6plex); T14(Phospho)
112382250	NP_003119.2	6711	SPTBN1	spectrin beta chain, non-erythrocytic 1 isoform 1	S2341	1.0	1.0	0.7	1.1	-	-	-	-	1.0	1.0	0.8	1.1	-	-	-	-	1.0	1.0	0.8	1.1	AQTLPTSVVTITSESsPGK	VTITSESsPGKREKD	S16;	Yes	T(3): 0.0; T(6): 0.0; S(7): 0.0; T(10): 0.0; T(12): 0.0; S(13): 0.0; S(15): 0.0; S(16): 100.0	31	1	1	0	N-Term(TMT6plex); S16(Phospho); K19(TMT6plex)
117553586	NP_079216.4	80005	DOCK5	dedicator of cytokinesis protein 5	S1802	1.0	1.5	2.1	1.1	-	-	-	-	0.8	1.5	2.0	1.0	-	-	-	-	0.8	1.5	2.0	1.0	TLsSPSLQTDGIAATPVPPPPPPK	PKATRTLsSPSLQTD	S3;	No	T(1): 1.3; S(3): 97.5; S(4): 1.3; S(6): 0.0; T(9): 0.0; T(15): 0.0	3	1	1	0	N-Term(TMT6plex); S3(Phospho); K24(TMT6plex)
153792633	NP_056381.1	26049	FAM169A	soluble lamin-associated protein of 75 kDa	S635	2.4	2.2	0.7	1.1	-	-	-	-	2.5	2.3	0.8	1.2	-	-	-	-	2.5	2.3	0.8	1.2	AVDsSSEEIEVEVPVVDR	SAEKAVDsSSEEIEV	S4;	Yes	S(4): 99.0; S(5): 0.9; S(6): 0.0	10	1	1	0	N-Term(TMT6plex); S4(Phospho)
21070997	NP_003147.2	6786	STIM1	stromal interaction molecule 1 isoform 2 precursor	S618	1.2	0.9	2.2	1.1	-	-	-	-	1.2	0.9	2.0	1.2	-	-	-	-	1.2	0.9	2.0	1.2	SHsPSSPDPDTPSPVGDSR	LDSSRSHsPSSPDPD	S3;	Yes	S(1): 1.3; S(3): 98.7; S(5): 0.0; S(6): 0.0; T(11): 0.0; S(13): 0.0; S(18): 0.0	6	2	1	0	N-Term(TMT6plex); S3(Phospho)
519666804	NP_001265547.1	5770	PTPN1	tyrosine-protein phosphatase non-receptor type 1 isoform 2	S279	1.0	1.1	1.1	1.1	-	-	-	-	1.0	1.0	1.0	1.0	-	-	-	-	1.0	1.0	1.0	1.0	GsPLNAAPYGIESMSQDTEVR	PIKEEKGsPLNAAPY	S2;	No	S(2): 100.0; Y(9): 0.0; S(13): 0.0; S(15): 0.0; T(18): 0.0	2	2	1	0	N-Term(TMT6plex); S2(Phospho); M14(Oxidation)
259906020	NP_001158287.1	22985	ACIN1	apoptotic chromatin condensation inducer in the nucleus isoform 3	T642	1.0	1.7	0.7	1.1	-	-	-	-	0.8	1.6	0.8	1.2	-	-	-	-	0.8	1.6	0.8	1.2	DPSSGQEVAtPPVPQLQVCEPK	SSGQEVAtPPVPQLQ	T10;	No	S(3): 0.0; S(4): 0.0; T(10): 99.9	38	3	1	0	N-Term(TMT6plex); T10(Phospho); C19(Carbamidomethyl); K22(TMT6plex)
306774089	NP_001182408.1	10342	TFG	protein TFG isoform 2	S197	2.3	1.8	0.6	1.1	-	-	-	-	2.2	1.9	0.7	1.1	-	-	-	-	2.2	1.9	0.7	1.1	NVMSAFGLTDDQVSGPPsAPAEDR	DQVSGPPsAPAEDRS	S18;	Yes	S(4): 0.0; T(9): 0.0; S(14): 0.0; S(18): 100.0	3	2	1	0	N-Term(TMT6plex); M3(Oxidation); S18(Phospho)
21493029	NP_009131.2	11214	AKAP13	A-kinase anchor protein 13 isoform 2	S793	1.0	0.9	0.6	1.1	-	-	-	-	1.1	1.0	0.6	1.4	-	-	-	-	1.1	1.0	0.6	1.4	ELVPDQAVISDSTFSLANSPGsESVTK	SLANSPGsESVTKDD	S22;	No	S(10): 0.0; S(12): 0.0; T(13): 1.9; S(15): 1.9; S(19): 1.9; S(22): 90.4; S(24): 1.9; T(26): 1.9	12	2	1	0	N-Term(TMT6plex); S19(Phospho); K27(TMT6plex)
4503729	NP_002005.1	2288	FKBP4	peptidyl-prolyl cis-trans isomerase FKBP4	S451	1.4	1.8	1.9	1.1	-	-	-	-	1.2	1.5	1.6	1.0	-	-	-	-	1.2	1.5	1.6	1.0	SNTAGsQSQVETEA	QKSNTAGsQSQVETE	S6;	Yes	S(1): 0.0; T(3): 1.0; S(6): 99.0; S(8): 0.0; T(12): 0.0	12	1	1	0	N-Term(TMT6plex); S6(Phospho)
354459394	NP_001238892.1	26084	ARHGEF26	rho guanine nucleotide exchange factor 26 isoform 2	S392	1.5	3.6	2.2	1.1	-	-	-	-	1.3	3.8	2.1	1.5	-	-	-	-	1.3	3.8	2.1	1.5	ALDIDsDEESEPK	EKALDIDsDEESEPK	S6;	Yes	S(6): 100.0; S(10): 0.0	2	2	1	0	N-Term(TMT6plex); S6(Phospho); K13(TMT6plex)
168229156	NP_078804.2	79576	NKAP	NF-kappa-B-activating protein	S149	1.0	1.2	1.3	1.1	-	-	-	-	1.3	1.4	1.8	1.4	-	-	-	-	1.3	1.4	1.8	1.4	IGELGAPEVWGLsPK	APEVWGLsPKNPEPD	S13;	Yes	S(13): 100.0	2	1	1	0	N-Term(TMT6plex); S13(Phospho); K15(TMT6plex)
163310749	NP_001106191.1	22998	LIMCH1	LIM and calponin homology domains-containing protein 1 isoform e	S357	1.2	0.9	0.6	1.1	-	-	-	-	1.0	0.8	0.5	0.9	-	-	-	-	1.0	0.8	0.5	0.9	ASVLDTSMSAGSGsPSK	SMSAGSGsPSKTVTP	S14;	No	S(2): 0.0; T(6): 0.0; S(7): 0.0; S(9): 0.0; S(12): 0.0; S(14): 100.0; S(16): [illegible]	5	7	1	0	N-Term(TMT6plex); M8(Oxidation); S14(Phospho); K17(TMT6plex)
163965364	NP_001106673.1	4666	NACA	nascent polypeptide-associated complex subunit alpha isoform b	S166	1.2	0.9	0.6	1.1	-	-	-	-	1.2	1.0	0.6	1.3	-	-	-	-	1.2	1.0	0.6	1.3	VQGEAVSNIQENTQTPTVQEEsEEEVDETGVEVK	TPTVQEEsEEEEVDE	S22;	No	S(7): 0.0; T(13): 2.6; T(15): 2.6; T(17): 2.6; S(22): 92.2; T(30): 0.1	5	2	1	0	N-Term(TMT6plex); S22(Phospho); K35(TMT6plex)
30061567	NP_060548.2	55132	LARP1B	la-related protein 1B isoform 1	S862	1.0	0.9	0.6	1.1	-	-	-	-	-	-	-	-	-	-	-	-	-	-	-	-	HsSTSGEESNR	EFGRKRHsSTSGEES	S2;	No	S(2): 99.0; S(3): 1.0; T(4): 0.0; S(5): 0.0; S(9): 0.0	6	1	1	0	N-Term(TMT6plex); S3(Phospho)
4503771	NP_002018.1	2339	FNTA	protein farnesyltransferase/geranylgeranyltransferase type-1 subunit alpha	S373	1.0	0.9	1.1	1.1	-	-	-	-	0.9	0.9	0.9	1.1	-	-	-	-	0.9	0.9	0.9	1.1	HSTENDsPTNVQQ	KHSTENDsPTNVQQ_	S7;	Yes	S(2): 0.0; T(3): 0.0; S(7): 100.0; T(9): 0.0	6	1	1	0	N-Term(TMT6plex); S7(Phospho)
102468717	NP_005112.2	9969	MED13	mediator of RNA polymerase II transcription subunit 13	T336	1.0	0.9	0.6	1.1	-	-	-	-	1.0	1.0	0.7	1.1	-	-	-	-	1.0	1.0	0.7	1.1	DPAMSSVTLTPPTSPEEVQtVDPQSVQK	TSPEEVQtVDPQSVQ	T20;	Yes	S(5): 1.7; S(6): 1.7; T(8): 1.7; T(10): 1.7; T(13): 47.7; S(14): 47.7; T(20): 97.8; S(25): 0.0	2	1	1	0	N-Term(TMT6plex); M4(Oxidation); S14(Phospho); T20(Phospho); K28(TMT6plex)
34996489	NP_060404.3	55023	PHIP	PH-interacting protein	S1525	1.0	0.9	0.6	1.1	-	-	-	-	1.0	0.8	0.5	1.0	-	-	-	-	1.0	0.8	0.5	1.0	VVVDPVVTEQPSTSsAAK	TEQPSTSsAAKTFIT	S15;	Yes	T(8): 0.0; S(12): 2.0; T(13): 2.0; S(14): 2.0; S(15): 94.1	9	1	1	0	N-Term(TMT6plex); S15(Phospho); K18(TMT6plex)
4504035	NP_003866.1	8833	GMPS	GMP synthase [glutamine-hydrolyzing]	S332	1.0	1.1	1.0	1.1	-	-	-	-	0.9	0.9	0.8	0.9	-	-	-	-	0.9	0.9	0.8	0.9	TLNMTTsPEEK	KTLNMTTsPEEKRKI	S7;	Yes	T(1): 0.0; T(5): 0.0; T(6): 0.0; S(7): 100.0	2	1	1	0	N-Term(TMT6plex); M4(Oxidation); S7(Phospho); K11(TMT6plex)
164698438	NP_003249.3	7083	TK1	thymidine kinase, cytosolic	S231	1.0	0.9	0.6	1.1	-	-	-	-	1.2	0.9	0.6	1.3	-	-	-	-	1.2	0.9	0.6	1.3	LEAPQQILQCsPAN	PQQILQCsPAN	S11;	Yes	S(11): 100.0	2	1	1	0	N-Term(TMT6plex); C10(Carbamidomethyl); S11(Phospho)
608788342	NP_001278327.1	387680	FAM21A	WASH complex subunit FAM21A isoform 2	S158	2.3	1.9	0.7	1.1	-	-	-	-	2.4	1.6	0.7	1.2	-	-	-	-	2.4	1.6	0.7	1.2	AGNsDSEEDDANGR	LDIKAGNsDSEEDDA	S4;	Yes	S(4): 100.0; S(6): 0.0	7	5	1	0	N-Term(TMT6plex); S4(Phospho)
389565511	NP_001254501.1	55357	TBC1D2	TBC1 domain family member 2A isoform 3	S460	1.0	0.9	0.6	1.1	-	-	-	-	1.0	0.9	0.6	1.0	-	-	-	-	1.0	0.9	0.6	1.0	AVSEGCAsEDEVEGEA	AVSEGCAsEDEVEGE	S8;	No	S(3): 0.0; S(8): 100.0	2	3	1	0	N-Term(TMT6plex); C6(Carbamidomethyl); S8(Phospho)
31563374	NP_852131.1	3692	EIF6	eukaryotic translation initiation factor 6 isoform c	S224	1.0	0.9	0.6	1.1	-	-	-	-	1.0	0.9	0.5	1.1	-	-	-	-	1.0	0.9	0.5	1.1	DSLIDsLT	MRDSLIDsLT	S6;	No	S(2): 0.0; S(6): 100.0; T(8): 0.0	1	2	1	0	N-Term(TMT6plex); S6(Phospho)
116008442	NP_055885.3	23091	ZC3H13	zinc finger CCCH domain-containing protein 13	S1279	1.0	1.1	1.1	1.1	-	-	-	-	0.9	1.0	1.1	1.1	-	-	-	-	0.9	1.0	1.1	1.1	SsPESDR	SHSSRRSsPESDRQV	S2;	No	S(1): 0.0; S(2): 100.0; S(5): 0.0	28	1	1	0	N-Term(TMT6plex); S2(Phospho)
63053524	NP_001017416.1	7398	USP1	ubiquitin carboxyl-terminal hydrolase 1	S67	1.1	1.3	1.0	1.1	-	-	-	-	1.2	1.3	1.1	1.2	-	-	-	-	1.2	1.3	1.1	1.2	ASEIDQVVPAAQSsPINCEK	VVPAAQSsPINCEKR	S14;	Yes	S(2): 0.0; S(13): 1.2; S(14): 98.8	7	1	1	0	N-Term(TMT6plex); S14(Phospho); C18(Carbamidomethyl); K20(TMT6plex)
4885237	NP_005242.1	2303	FOXC2	forkhead box protein C2	S232	0.9	1.1	0.9	1.1	-	-	-	-	0.4	0.9	0.6	0.8	-	-	-	-	0.4	0.9	0.6	0.8	VETLsPESALQGSPR	ITKVETLsPESALQG	S5;	Yes	T(3): 1.1; S(5): 98.9; S(8): 0.0; S(13): 0.0	8	1	1	0	N-Term(TMT6plex); S5(Phospho)
76880486	NP_006819.2	10973	ASCC3	activating signal cointegrator 1 complex subunit 3 isoform a	S2195	0.6	0.8	0.8	1.1	-	-	-	-	0.6	0.8	0.8	1.1	-	-	-	-	0.6	0.8	0.8	1.1	VSDsLTDLALK	VNTKVSDsLTDLALK	S4;	Yes	S(2): 0.9; S(4): 99.1; T(6): 0.0	3	1	1	0	N-Term(TMT6plex); S4(Phospho); K11(TMT6plex)
94536805	NP_588611.2	91746	YTHDC1	YTH domain-containing protein 1 isoform 2	S287	1.2	1.1	1.1	1.1	-	-	-	-	1.2	1.0	1.1	1.1	-	-	-	-	1.2	1.0	1.1	1.1	sGSGTDGSDEK	FTDGSVRsGSGTDGS	S1;	No	S(1): 100.0; S(3): 0.0; T(5): 0.0; S(8): 0.0	2	2	1	0	N-Term(TMT6plex); S1(Phospho); K11(TMT6plex)
109255232	NP_001035864.1	9859	CEP170	centrosomal protein of 170 kDa isoform gamma	S1062	1.3	1.4	1.2	1.1	-	-	-	-	1.2	1.2	1.1	1.1	-	-	-	-	1.2	1.2	1.1	1.1	LGsLSAR	PRRTRLGsLSARSDS	S3;	No	S(3): 100.0; S(5): 0.0	11	3	1	0	N-Term(TMT6plex); S3(Phospho)
81295809	NP_006022.3	5116	PCNT	pericentrin	S2177	1.0	0.8	0.9	1.1	-	-	-	-	1.0	0.7	0.9	1.2	-	-	-	-	1.0	0.7	0.9	1.2	NWDSLIPDEMPDsPIQEK	IPDEMPDsPIQEKSE	S13;	Yes	S(4): 0.0; S(13): 100.0	2	1	1	0	N-Term(TMT6plex); M10(Oxidation); S13(Phospho); K18(TMT6plex)
166235167	NP_055230.2	27107	ZBTB11	zinc finger and BTB domain-containing protein 11	S1050	1.5	1.2	1.6	1.1	-	-	-	-	1.3	1.0	1.4	1.1	-	-	-	-	1.3	1.0	1.4	1.1	VEVAHIsGGE	KVEVAHIsGGE	S7;	Yes	S(7): 100.0	5	1	1	0	N-Term(TMT6plex); S7(Phospho)
66932990	NP_001018088.1	2356	FPGS	folylpolyglutamate synthase, mitochondrial isoform b	S489	0.6	0.5	1.5	1.1	-	-	-	-	-	-	-	-	-	-	-	-	-	-	-	-	DPIEQPPsPPK	DPIEQPPsPPKGLLT	S8;	No	S(8): 100.0	1	3	1	0	N-Term(TMT6plex); S8(Phospho); K11(TMT6plex)
110556636	NP_203754.2	85456	TNKS1BP1	182 kDa tankyrase-1-binding protein	S851	1.1	1.3	1.2	1.1	-	-	-	-	1.0	1.2	1.2	1.2	-	-	-	-	1.0	1.2	1.2	1.2	DsQGTYSSR	WEFRKRDsQGTYSSR	S2;	Yes	S(2): 100.0; T(5): 0.0; Y(6): 0.0; S(7): 0.0; S(8): 0.0	41	1	1	0	N-Term(TMT6plex); S2(Phospho)
32484977	NP_057285.3	51421	AMOTL2	angiomotin-like protein 2 isoform 2	S540	1.5	1.0	1.1	1.1	-	-	-	-	1.9	1.1	1.4	1.1	-	-	-	-	1.9	1.1	1.4	1.1	QAGAPGGSSGSGGsPELSALR	GSSGSGGsPELSALR	S14;	Yes	S(8): 0.0; S(9): 0.0; S(11): 0.1; S(14): 99.9; S(18): 0.0	2	2	1	0	N-Term(TMT6plex); S14(Phospho)
132626688	NP_055456.2	9656	MDC1	mediator of DNA damage checkpoint protein 1	T1384	2.5	1.1	1.2	1.1	-	-	-	-	2.7	1.1	1.3	1.2	-	-	-	-	2.7	1.1	1.3	1.2	NPESTVPIAPELPPSTSTEQPVtPEPTSR	TSTEQPVtPEPTSRA	T23;	Yes	S(4): 0.0; T(5): 0.0; S(15): 0.0; T(16): 0.0; S(17): 0.0; T(18): 0.0; T(23): 98.1; T(27): 0.0; S(28): 1.9	26	1	1	0	N-Term(TMT6plex); T23(Phospho)
158631177	NP_065885.2	57594	HOMEZ	homeobox and leucine zipper protein Homez	T451	1.0	0.9	0.6	1.1	-	-	-	-	0.7	0.9	0.6	0.8	-	-	-	-	0.7	0.9	0.6	0.8	AEtPPLPIPPPPPDIQPLER	SLNERAEtPPLPIPP	T3;	Yes	T(3): 100.0	1	1	1	0	N-Term(TMT6plex); T3(Phospho)
42794769	NP_002567.3	5058	PAK1	serine/threonine-protein kinase PAK 1 isoform 2	T225	1.0	1.7	1.7	1.1	-	-	-	-	1.1	1.7	1.9	1.1	-	-	-	-	1.1	1.7	1.9	1.1	DVATSPISPtENNTTPPDALTR	ATSPISPtENNTTPP	T10;	Yes	T(4): 0.0; S(5): 0.0; S(8): 1.9; T(10): 94.3; T(14): 1.9; T(15): 1.9; T(21): 0.0	1	2	1	0	N-Term(TMT6plex); T15(Phospho)
26051235	NP_060700.2	55746	NUP133	nuclear pore complex protein Nup133	S50	1.0	0.9	0.6	1.1	-	-	-	-	1.0	0.9	0.5	1.2	-	-	-	-	1.0	0.9	0.5	1.2	GLPLGSAVSSPVLFsPVGR	VSSPVLFsPVGRRSS	S15;	Yes	S(6): 0.0; S(9): 0.0; S(10): 0.0; S(15): 100.0	2	1	1	0	N-Term(TMT6plex); S15(Phospho)
224028248	NP_001138882.1	4841	NONO	non-POU domain-containing octamer-binding protein isoform 2	T361	1.0	1.3	1.3	1.1	-	-	-	-	1.2	1.4	1.6	1.1	-	-	-	-	1.2	1.4	1.6	1.1	FGQAATMEGIGAIGGtPPAFNR	GIGAIGGtPPAFNRA	T16;	No	T(6): 0.0; T(16): 100.0	5	2	1	0	N-Term(TMT6plex); M7(Oxidation); T16(Phospho)
156231049	NP_001095891.1	7763	ZFAND5	AN1-type zinc finger protein 5	S48	1.0	1.8	0.6	1.1	-	-	-	-	0.9	1.5	0.5	1.1	-	-	-	-	0.9	1.5	0.5	1.1	MsPMGTASGSNSPTSDSASVQR	QQNSGRMsPMGTASG	S2;	Yes	S(2): 99.9; T(6): 0.1; S(8): 0.0; S(10): 0.0; S(12): 0.0; T(14): 0.0; S(15): 0.0; S(17): 0.0; S(19): 0.0	1	1	1	0	N-Term(TMT6plex); M1(Oxidation); S2(Phospho); M4(Oxidation)
32307124	NP_006525.2	8202	NCOA3	nuclear receptor coactivator 3 isoform b	S867	1.2	0.9	0.6	1.1	-	-	-	-	-	-	-	-	-	-	-	-	-	-	-	-	AVSLDSPVSVGSsPPVK	SPVSVGSsPPVKNIS	S13;	Yes	S(3): 0.0; S(6): 0.0; S(9): 1.4; S(12): 1.4; S(13): 97.1	6	4	1	0	N-Term(TMT6plex); S13(Phospho); K17(TMT6plex)
194328736	NP_579866.2	55274	PHF10	PHD finger protein 10 isoform b	S325	1.0	0.9	0.6	1.1	-	-	-	-	1.2	1.0	0.6	1.1	-	-	-	-	1.2	1.0	0.6	1.1	GTSDSSSGNVsEGESPPDSQEDSFQGR	DSSSGNVsEGESPPD	S11;	No	T(2): 0.0; S(3): 0.0; S(5): 0.1; S(6): 2.6; S(7): 2.6; S(11): 92.0; S(15): 2.6; S(19): 0.0; S(23): 0.0	6	2	1	0	N-Term(TMT6plex); S11(Phospho)
368711277	NP_001243023.1	1386	ATF2	cyclic AMP-dependent transcription factor ATF-2 isoform 5	T71	1.0	1.1	0.6	1.1	-	-	-	-	-	-	-	-	-	-	-	-	-	-	-	-	NDSVIVADQTPtPTR	IVADQTPtPTRFLKN	T12;	Yes	S(3): 0.0; T(10): 0.0; T(12): 98.4; T(14): 1.6	1	3	1	0	N-Term(TMT6plex); T12(Phospho)
33598968	NP_005349.3	4008	LMO7	LIM domain only protein 7 isoform 1	S692	1.0	0.9	1.7	1.1	-	-	-	-	0.9	0.7	1.2	1.1	-	-	-	-	0.9	0.7	1.2	1.1	ATLSSTSGLDLMSESGEGEIsPQR	ESGEGEIsPQREVSR	S21;	No	T(2): 0.0; S(4): 0.0; S(5): 0.0; T(6): 0.0; S(7): 0.0; S(13): 0.0; S(15): 0.1; S(21): 99.9	19	2	1	0	N-Term(TMT6plex); M12(Oxidation); S21(Phospho)
221136939	NP_056444.3	26121	PRPF31	U4/U6 small nuclear ribonucleoprotein Prp31	T455	1.0	0.9	0.6	1.1	-	-	-	-	1.1	1.0	0.6	1.2	-	-	-	-	1.1	1.0	0.6	1.2	SSGTASSVAFtPLQGLEIVNPQAAEK	TASSVAFtPLQGLEI	T11;	Yes	S(1): 0.0; S(2): 0.0; T(4): 0.0; S(6): 0.0; S(7): 0.0; T(11): 100.0	2	1	1	0	N-Term(TMT6plex); T11(Phospho); K26(TMT6plex)
42542379	NP_005830.2	10250	SRRM1	serine/arginine repetitive matrix protein 1	T846	1.0	0.9	0.6	1.1	-	-	-	-	1.3	1.0	0.6	1.3	-	-	-	-	1.3	1.0	0.6	1.3	AVAAAAAAAVtPAAIAAATTTLAQEEPVAAPEPK	AAAAAAVtPAAIAAA	T11;	Yes	T(11): 88.6; T(19): 3.8; T(20): 3.8; T(21): 3.8	229	1	1	0	N-Term(TMT6plex); T11(Phospho); K34(TMT6plex)
270288818	NP_001161820.1	55695	NSUN5	putative methyltransferase NSUN5 isoform 4	S289	1.0	0.9	1.0	1.1	-	-	-	-	1.0	0.9	1.0	1.1	-	-	-	-	1.0	0.9	1.0	1.1	QLEEPGAGTPsPVR	EPGAGTPsPVRLHAL	S11;	No	T(9): 0.0; S(11): 100.0	1	4	1	0	N-Term(TMT6plex); S11(Phospho)
544186098	NP_001269591.1	57591	MKL1	MKL/myocardin-like protein 1 isoform 3	S151	1.0	0.9	0.6	1.1	-	-	-	-	1.2	1.0	0.7	1.3	-	-	-	-	1.2	1.0	0.7	1.3	VSEPLLsATSASPTQVVSQLPMGR	RVSEPLLsATSASPT	S7;	No	S(2): 0.0; S(7): 91.5; T(9): 2.1; S(10): 2.1; S(12): 2.1; T(14): 2.1; S(18): 0.0	2	2	1	0	N-Term(TMT6plex); S7(Phospho); M22(Oxidation)
31711992	NP_001922.2	1737	DLAT	dihydrolipoyllysine-residue acetyltransferase component of pyruvate dehydrogenase complex, mitochondrial precursor	S100	1.3	1.5	1.0	1.1	-	-	-	-	1.1	1.2	0.9	1.1	-	-	-	-	1.1	1.2	0.9	1.1	VPLPSLsPTMQAGTIAR	KVPLPSLsPTMQAGT	S7;	Yes	S(5): 0.0; S(7): 98.4; T(9): 1.6; T(14): 0.0	2	1	1	0	N-Term(TMT6plex); S7(Phospho); M10(Oxidation)
605052052	NP_001278268.1	79848	CSPP1	centrosome and spindle pole-associated protein 1 isoform 2	S130	0.9	1.2	1.1	1.1	-	-	-	-	-	-	-	-	-	-	-	-	-	-	-	-	VAASGAQDPEKsPDR	GAQDPEKsPDRLKQF	S12;	No	S(4): 0.0; S(12): 100.0	2	2	1	1	N-Term(TMT6plex); K11(TMT6plex); S12(Phospho)
163644283	NP_055429.2	23198	PSME4	proteasome activator complex subunit 4	S1121	1.0	0.9	1.3	1.1	-	-	-	-	0.9	0.8	1.2	1.0	-	-	-	-	0.9	0.8	1.2	1.0	NPSINQILLsPEK	SINQILLsPEKIKEG	S10;	Yes	S(3): 0.0; S(10): 100.0	1	1	1	0	N-Term(TMT6plex); S10(Phospho); K13(TMT6plex)
312176416	NP_001185901.1	57648	KIAA1522	uncharacterized protein KIAA1522 isoform 2	S620	1.0	0.9	0.6	1.1	-	-	-	-	1.2	1.0	0.7	1.3	-	-	-	-	1.2	1.0	0.7	1.3	sPNPAAPALAAPAVVPGPVSTTDASPQSPPTPQTTLTPLQESPVISK	PPPSKPRsPNPAAPA	S1;	No	S(1): 93.9; S(20): 0.7; T(21): 0.7; T(22): 0.7; S(25): 0.7; S(28): 0.7; T(31): 0.7; T(34): 0.7; T(35): 0.7; T(37): 0.7; S(42): 0.1; S(46): 0.0	22	2	1	0	N-Term(TMT6plex); S1(Phospho); K47(TMT6plex)
54112403	NP_060250.2	55636	CHD7	chromodomain-helicase-DNA-binding protein 7	S2956	1.0	0.9	0.7	1.1	-	-	-	-	-	-	-	-	-	-	-	-	-	-	-	-	DGETLEGsDAEESLDK	DGETLEGsDAEESLD	S8;	Yes	T(4): 0.1; S(8): 99.9; S(13): 0.1	5	1	1	0	N-Term(TMT6plex); S8(Phospho); K16(TMT6plex)
100913021	NP_001035758.1	83596	BCL2L12	bcl-2-like protein 12 isoform 3	S272	0.6	0.5	1.5	1.1	-	-	-	-	-	-	-	-	-	-	-	-	-	-	-	-	ACPGPPPPsPEPLAR	CPGPPPPsPEPLARL	S9;	No	S(9): 100.0	2	2	1	0	N-Term(TMT6plex); C2(Carbamidomethyl); S9(Phospho)
119220575	NP_001072986.1	9862	MED24	mediator of RNA polymerase II transcription subunit 24 isoform 2	S860	1.0	0.9	0.6	1.1	-	-	-	-	1.0	0.9	0.6	1.1	-	-	-	-	1.0	0.9	0.6	1.1	LLSSNEDDANILSsPTDR	DDANILSsPTDRSMS	S14;	No	S(3): 0.0; S(4): 0.0; S(13): 2.4; S(14): 95.2; T(16): 2.4	1	2	1	0	N-Term(TMT6plex); S14(Phospho)
41406089	NP_005677.2	9580	SOX13	transcription factor SOX-13	S312	1.3	1.0	0.8	1.1	-	-	-	-	-	-	-	-	-	-	-	-	-	-	-	-	APELPNTSSSPsLK	PNTSSSPsLKMSSCV	S12;	Yes	T(7): 2.3; S(8): 2.3; S(9): 2.3; S(10): 2.3; S(12): 90.9	3	1	1	0	N-Term(TMT6plex); S10(Phospho); K14(TMT6plex)
190358534	NP_064544.2	56916	SMARCAD1	SWI/SNF-related matrix-associated actin-dependent regulator of chromatin subfamily A containing DEAD/H box 1 isoform b	T71	1.0	1.7	1.9	1.1	-	-	-	-	1.0	1.6	1.8	1.0	-	-	-	-	1.0	1.6	1.8	1.0	TEDSSVPEtPDNER	EDSSVPEtPDNERKA	T9;	Yes	T(1): 0.0; S(4): 0.0; S(5): 0.0; T(9): 99.9	4	2	1	0	N-Term(TMT6plex); T9(Phospho)
302370946	NP_001180573.1	5824	PEX19	peroxisomal biogenesis factor 19 isoform c	S35	2.2	2.1	1.8	1.1	-	-	-	-	2.3	2.4	1.9	1.5	-	-	-	-	2.3	2.4	1.9	1.5	AKPsPAPPSTTTAPDASGPQK	DFDKAKPsPAPPSTT	S4;	Yes	S(4): 100.0; S(9): 0.0; T(10): 0.0; T(11): 0.0; T(12): 0.0; S(17): 0.0	8	2	1	0	N-Term(TMT6plex); K2(TMT6plex); S4(Phospho); K21(TMT6plex)
61676188	NP_113584.3	10075	HUWE1	E3 ubiquitin-protein ligase HUWE1	T1722	1.0	1.0	0.7	1.1	-	-	-	-	1.1	1.0	0.8	1.3	-	-	-	-	1.1	1.0	0.8	1.3	GNDtPLALESTNTEK	KENKGNDtPLALEST	T4;	Yes	T(4): 100.0; S(10): 0.0; T(11): 0.0; T(13): 0.0	20	1	1	0	N-Term(TMT6plex); T4(Phospho); K15(TMT6plex)
568815684	NP_001275585.1	29855	UBN1	ubinuclein-1 isoform b	S660	1.0	0.9	0.6	1.1	-	-	-	-	-	-	-	-	-	-	-	-	-	-	-	-	ELPSQASGGLANPPPVNLEDsLDEDLIR	PPVNLEDsLDEDLIR	S21;	Yes	S(4): 0.0; S(7): 0.0; S(21): 100.0	1	2	1	0	N-Term(TMT6plex); S21(Phospho)
530424835	XP_005258156.1	23255	SOGA2	protein SOGA2 isoform X2	T1417; S1421	1.0	0.9	1.3	1.1	-	-	-	-	1.1	1.0	1.3	1.1	-	-	-	-	1.1	1.0	1.3	1.1	EDVtPPLsPDDLK	TSSKEDVtPPLSPDD;EDVTPPLsPDDLKYI	T4; S8;	Yes;Yes	T(4): 100.0; S(8): 100.0	6	6	1	0	N-Term(TMT6plex); T4(Phospho); S8(Phospho); K13(TMT6plex)
385648270	NP_001245309.1	9497	SLC4A7	sodium bicarbonate cotransporter 3 isoform c	S258	1.0	1.1	0.6	1.1	-	-	-	-	0.9	1.0	0.5	1.0	-	-	-	-	0.9	1.0	0.5	1.0	NGILASPQsAPGNLDNSK	GILASPQsAPGNLDN	S9;	No	S(6): 2.2; S(9): 97.7; S(17): 0.1	6	2	1	0	N-Term(TMT6plex); S9(Phospho); K18(TMT6plex)
5032189	NP_005648.1	7158	TP53BP1	tumor suppressor p53-binding protein 1 isoform 3	S63	1.0	1.1	0.6	1.1	-	-	-	-	1.0	1.1	0.6	1.4	-	-	-	-	1.0	1.1	0.6	1.4	ENPVLDVVsNPEQTAGEER	NPVLDVVsNPEQTAG	S9;	Yes	S(9): 99.9; T(14): 0.1	62	3	1	0	N-Term(TMT6plex); S9(Phospho)
387157911	NP_001248339.1	8412	BCAR3	breast cancer anti-estrogen resistance protein 3 isoform	S284	1.3	3.6	3.9	1.1	-	-	-	-	0.8	2.3	2.4	0.7	-	-	-	-	0.8	2.3	2.4	0.7	TGSEPALsPAVVR	TGSEPALsPAVVRRV	S8;	No	T(1): 0.0; S(3): 0.1; S(8): 99.9	1	2	1	0	N-Term(TMT6plex); S8(Phospho)
4758024	NP_004636.1	8161	COIL	coilin	S566	1.0	1.2	0.9	1.1	-	-	-	-	1.0	1.1	0.9	1.0	-	-	-	-	1.0	1.1	0.9	1.0	LIIEsPSNTSSTEPA	DPRLIIEsPSNTSST	S5;	Yes	S(5): 98.3; S(7): 1.7; T(9): 0.0; S(10): 0.0; S(11): 0.0; T(12): 0.0	8	1	1	0	N-Term(TMT6plex); S5(Phospho)
27477070	NP_003963.1	9044	BTAF1	TATA-binding protein-associated factor 172	S95	2.4	0.9	2.6	1.1	-	-	-	-	2.2	0.8	2.3	1.0	-	-	-	-	2.2	0.8	2.3	1.0	QEPTSESSMEDsPTTER	SESSMEDsPTTERLN	S12;	Yes	T(4): 0.0; S(5): 0.0; S(7): 2.3; S(8): 2.3; S(12): 91.0; T(14): 2.3; T(15): 2.3	5	1	1	0	N-Term(TMT6plex); M9(Oxidation); S12(Phospho)
335334962	NP_001229416.1	23053	ZSWIM8	zinc finger SWIM domain-containing protein 8 isoform	S1836	1.0	1.1	0.6	1.1	-	-	-	-	-	-	-	-	-	-	-	-	-	-	-	-	VSLEMATEsP	SLEMATEsP	S9;	Yes	S(2): 0.0; T(7): 0.0; S(9): 100.0	2	2	1	0	N-Term(TMT6plex); M5(Oxidation); S9(Phospho)
149158690	NP_542417.2	7916	PRRC2A	protein PRRC2A	T1347	1.0	0.9	0.6	1.1	-	-	-	-	1.1	0.8	0.6	1.1	-	-	-	-	1.1	0.8	0.6	1.1	EAPPPVLLtPK	APPPVLLtPKAVGTP	T9;	No	T(9): 100.0	23	1	1	0	N-Term(TMT6plex); T9(Phospho); K11(TMT6plex)
73760401	NP_001027455.1	7112	TMPO	thymopoietin isoform gamma	S245	1.0	0.9	0.6	1.1	-	-	-	-	1.1	1.0	0.7	1.1	-	-	-	-	1.1	1.0	0.7	1.1	EMFPYEAsTPTGISASCR	EMFPYEAsTPTGISA	S8;	No	Y(5): 2.0; S(8): 95.9; T(9): 2.0; T(11): 0.0; S(14): 0.0; S(16): 0.0	8	2	1	0	N-Term(TMT6plex); M2(Oxidation); Y5(Phospho); C17(Carbamidomethyl)
308199458	NP_001184057.1	3661	IRF3	interferon regulatory factor 3 isoform 6	S27	1.0	1.6	1.5	1.1	-	-	-	-	1.0	1.5	1.4	1.1	-	-	-	-	1.0	1.5	1.4	1.1	sPSLDNPTPFPNLGPSENPLK	PCPQPLRsPSLDNPT	S1;	No	S(1): 97.5; S(3): 2.5; T(8): 0.0; S(16): 0.0	2	6	1	0	N-Term(TMT6plex); S1(Phospho); K21(TMT6plex)
153792780	NP_060733.4	55252	ASXL2	putative Polycomb group protein ASXL2	S562	1.0	1.1	1.1	1.1	-	-	-	-	1.1	1.2	1.1	1.1	-	-	-	-	1.1	1.2	1.1	1.1	EPLATLVDQsPESLK	LATLVDQsPESLKRK	S10;	Yes	T(5): 2.5; S(10): 97.4; S(13): 0.1	2	1	1	0	N-Term(TMT6plex); S10(Phospho); K15(TMT6plex)
222537747	NP_001138500.1	342979	PALM3	paralemmin-3	T151	1.0	0.9	0.6	1.1	-	-	-	-	0.8	0.9	0.7	1.3	-	-	-	-	0.8	0.9	0.7	1.3	ASLPAGLVGtPPESPSEPR	LPAGLVGtPPESPSE	T10;	Yes	S(2): 0.0; T(10): 99.9; S(14): 50.0; S(16): 50.0	1	1	1	0	N-Term(TMT6plex); T10(Phospho); S16(Phospho)
547234776	NP_001271266.1	23223	RRP12	RRP12-like protein isoform 3	T88	1.0	1.1	0.6	1.1	-	-	-	-	1.0	1.0	0.6	1.0	-	-	-	-	1.0	1.0	0.6	1.0	SEAPETPMEEEAELVLtEK	EEAELVLtEKSSGTE	T17;	Yes	S(1): 0.0; T(6): 0.0; T(17): 100.0	5	3	1	0	N-Term(TMT6plex); M8(Oxidation); T17(Phospho); K19(TMT6plex)
45504380	NP_055830.1	23030	KDM4B	lysine-specific demethylase 4B	S566	1.0	0.9	0.6	1.1	-	-	-	-	-	-	-	-	-	-	-	-	-	-	-	-	EPVsPMELTGPEDGAASSGAGR	PTWKEPVsPMELTGP	S4;	Yes	S(4): 100.0; T(9): 0.0; S(17): 0.0; S(18): 0.0	1	1	1	0	N-Term(TMT6plex); S4(Phospho); M6(Oxidation)
291190787	NP_055872.4	23077	MYCBP2	E3 ubiquitin-protein ligase MYCBP2	S3505	1.0	0.9	0.6	1.1	-	-	-	-	1.1	0.9	0.6	0.9	-	-	-	-	1.1	0.9	0.6	0.9	VNsGDTEVGSSLLR	IPRRRVNsGDTEVGS	S3;	No	S(3): 98.0; T(6): 2.0; S(10): 0.0; S(11): 0.0	6	1	1	0	N-Term(TMT6plex); S3(Phospho)
45439359	NP_009049.2	7204	TRIO	triple functional domain protein	S2477	1.0	0.9	0.8	1.1	-	-	-	-	1.0	0.8	0.6	1.1	-	-	-	-	1.0	0.8	0.6	1.1	EPEPPSsPLQK	KEPEPPSsPLQKGGS	S7;	Yes	S(6): 1.3; S(7): 98.7	3	1	1	0	N-Term(TMT6plex); S7(Phospho); K11(TMT6plex)
11995472	NP_071440.1	64121	RRAGC	ras-related GTP-binding protein C isoform 1	S95	1.0	1.1	0.6	1.1	-	-	-	-	0.9	1.2	0.7	1.2	-	-	-	-	0.9	1.2	0.7	1.2	MSPNETLFLEsTNK	NETLFLEsTNKIYKD	S11;	Yes	S(2): 0.0; T(6): 0.0; S(11): 98.4; T(12): 1.6	1	2	1	0	N-Term(TMT6plex); M1(Oxidation); S11(Phospho); K14(TMT6plex)
50980301	NP_958431.2	23164	MPRIP	myosin phosphatase Rho-interacting protein isoform 2	S220	1.0	0.9	0.6	1.1	-	-	-	-	1.0	0.9	0.6	1.1	-	-	-	-	1.0	0.9	0.6	1.1	DQPDGSSLsPAQSPSQSQPPAASSLR	QPDGSSLsPAQSPSQ	S9;	Yes	S(6): 6.1; S(7): 6.1; S(9): 82.6; S(13): 34.9; S(15): 34.9; S(17): 34.9; S(23): 0.3; S(24): 0.3	7	2	1	0	N-Term(TMT6plex); S9(Phospho); S13(Phospho)
21955172	NP_665696.1	90102	PHLDB2	pleckstrin homology-like domain family B member 2 isoform c	T550	1.0	0.9	0.6	1.1	-	-	-	-	1.1	1.0	0.7	1.3	-	-	-	-	1.1	1.0	0.7	1.3	tPPPPSSTFPK	LLSDLTRtPPPPSST	T1;	Yes	T(1): 100.0; S(6): 0.0; S(7): 0.0; T(8): 0.0	9	3	1	0	N-Term(TMT6plex); T1(Phospho); K11(TMT6plex)
122114651	NP_079366.3	57602	USP36	ubiquitin carboxyl-terminal hydrolase 36	S682	1.0	0.9	0.6	1.1	-	-	-	-	1.0	0.8	0.6	1.1	-	-	-	-	1.0	0.8	0.6	1.1	LKSPVLSNTTTEPASTMsPPPAK	TEPASTMsPPPAKKL	S18;	Yes	S(3): 20.0; S(7): 20.0; T(9): 20.0; T(10): 20.0; T(11): 20.0; S(15): 0.0; T(16): 0.0; S(18): 99.9	6	1	1	1	N-Term(TMT6plex); K2(TMT6plex); T9(Phospho); M17(Oxidation); S18(Phospho); K23(TMT6plex)
117676384	NP_004710.2	9169	SCAF11	protein SCAF11	S608	1.0	0.9	0.6	1.1	-	-	-	-	1.2	0.9	0.6	1.1	-	-	-	-	1.2	0.9	0.6	1.1	TEELIEsPK	KTEELIEsPKLESSE	S7;	Yes	T(1): 0.0; S(7): 100.0	4	1	1	0	N-Term(TMT6plex); S7(Phospho); K9(TMT6plex)
557786103	NP_001273567.1	253832	ZDHHC20	probable palmitoyltransferase ZDHHC20 isoform 2	S242	1.0	1.8	3.6	1.1	-	-	-	-	0.7	1.3	2.7	0.7	-	-	-	-	0.7	1.3	2.7	0.7	LVGMDPEQAsVTNQNEYAR	GMDPEQAsVTNQNEY	S10;	No	S(10): 96.0; T(12): 3.8; Y(17): 0.2	1	2	1	0	N-Term(TMT6plex); M4(Oxidation); S10(Phospho)
611435010	NP_001278350.1	7403	KDM6A	lysine-specific demethylase 6A isoform 6	S533	1.3	1.1	1.9	1.1	-	-	-	-	-	-	-	-	-	-	-	-	-	-	-	-	TDNSVASSPSSAISTATPsPK	AISTATPsPKSTEQT	S19;	No	T(1): 0.0; S(4): 0.0; S(7): 0.0; S(8): 0.0; S(10): 0.1; S(11): 0.1; S(14): 0.1; T(15): 3.0; T(17): 3.0; S(19): 93.6	1	6	1	0	N-Term(TMT6plex); S19(Phospho); K21(TMT6plex)

Khan et al., 2019. Multi-omics analysis to characterize cigarette smoke induced molecular alterations in esophageal cells

Supplementary Table 8. List of phosphopeptides identified and quantified in untreated and chronically treated Het1A cells with cigarette smoke condensate for 8 months using SequestHT and Mascot search algorithms with a PhosphoRS probability score ≥75%

Protein group Accession	NP_Accession	Gene ID	Gene Symbol	Protein Description	PhosphoSite (Protein)	Het-1A-Smoke - 2M/Parental (Replicate 1)	Het-1A-Smoke - 4M/Parental (Replicate 1)	Het-1A-Smoke - 6M/Parental (Replicate 1)	Het-1A-Smoke - 8M/Parental (Replicate 1)	Het-1A-Smoke - 2M/Parental (Replicate 2)	Het-1A-Smoke - 4M/Parental (Replicate 2)	Het-1A-Smoke - 6M/Parental (Replicate 2)	Het-1A-Smoke - 8M/Parental (Replicate 2)	Normalized phosphoprotein ratios with respect to corresponding total protein ratios. Het-1A-Smoke - 2M/Parental (Replicate 1)	Het-1A-Smoke - 4M/Parental (Replicate 1)	Het-1A-Smoke - 6M/Parental (Replicate 1)	Het-1A-Smoke - 8M/Parental (Replicate 1)	Het-1A-Smoke - 2M/Parental (Replicate 2)	Het-1A-Smoke - 4M/Parental (Replicate 2)	Het-1A-Smoke - 6M/Parental (Replicate 2)	Het-1A-Smoke - 8M/Parental (Replicate 2)	Average of replicates Het-1A-Smoke - 2M/Parental	Het-1A-Smoke - 4M/Parental	Het-1A-Smoke - 6M/Parental	Het-1A-Smoke - 8M/Parental	Peptide Sequence	Phosphowindow	PhosphoSite (Peptide)	Phosphosite Plus Evidence	phosphoRS Site Probabilities	# PSMs	# Proteins	# Protein Groups	# Missed Cleavages	Modifications
116008442	NP_055885.3	23091	ZC3H13	zinc finger CCCH domain-containing protein 13	S1208	1.2	2.2	0.7	1.1	-	-	-	-	1.2	2.1	0.7	1.1	-	-	-	-	1.2	2.1	0.7	1.1	sPSNDSAHR	HTSGRLRsPSNDSAH	S1;	Yes	S(1): 100.0; S(3): 0.0; S(6): 0.0	28	1	1	0	N-Term(TMT6plex); S1(Phospho)
63252910	NP_001017977.1	55827	DCAF6	DDB1- and CUL4-associated factor 6 isoformb	T654; S657	1.3	0.9	1.1	1.1	-	-	-	-	-	-	-	-	-	-	-	-	-	-	-	-	DSALQDtDDsDDDPVLIPGAR	RDSALQDtDDSDDDP;ALQDTDDsDDDPVLI	T7; S10;	Yes;Yes	S(2): 0.6; T(7): 99.7; S(10): 99.7	4	4	1	0	N-Term(TMT6plex); T7(Phospho); S10(Phospho)
58761546	NP_055828.2	23028	KDM1A	lysine-specific histone demethylase 1A isoformb	S137	2.2	2.2	2.1	1.1	-	-	-	-	2.3	2.4	2.3	1.1	-	-	-	-	2.3	2.4	2.3	1.1	EMDESLANLSEDEYYsEEER	LSEDEYYsEEERNAK	S16;	Yes	S(5): 0.0; S(10): 34.8; Y(14): 34.8; Y(15): 34.8; S(16): 95.5	4	2	1	0	N-Term(TMT6plex); M2(Oxidation); Y14(Phospho); S16(Phospho)
5032189	NP_005648.1	7158	TP53BP1	tumor suppressor p53-binding protein 1 isoform3	S1462	1.0	0.9	0.6	1.1	-	-	-	-	1.0	0.9	0.6	1.4	-	-	-	-	1.0	0.9	0.6	1.4	SDsPEIPFQAAAGPSDGLDASSPGNSFVGLR	GALRRSDsPEIPFQA	S3;	Yes	S(1): 4.4; S(3): 82.2; S(15): 4.4; S(21): 4.4; S(22): 4.4; S(26): 0.3	62	3	1	0	N-Term(TMT6plex); S1(Phospho)
254587994	NP_001157019.1	11276	SYNRG	synergin gamma isoform7	S737	1.3	1.6	1.7	1.1	-	-	-	-	1.1	1.7	1.7	1.3	-	-	-	-	1.1	1.7	1.7	1.3	ETSFGSSENITMTsLSK	SENITMTsLSKVTTF	S14;	No	T(2): 2.9; S(3): 2.9; S(6): 2.9; S(7): 2.9; T(11): 2.9; T(13): 2.9; S(14): 82.7; S(16): 0.1	3	7	1	0	N-Term(TMT6plex); S7(Phospho); M12(Oxidation); K17(TMT6plex)
15149465	NP_149131.1	800	CALD1	caldesmon isoform5	T469	2.0	1.6	1.6	1.1	-	-	-	-	1.9	1.2	1.2	1.0	-	-	-	-	1.9	1.2	1.2	1.0	GNVFSSPTAAGtPNK	SSPTAAGtPNKETAG	T12;	No	S(5): 49.4; S(6): 49.4; T(8): 1.3; T(12): 99.9	4	5	1	0	N-Term(TMT6plex); T8(Phospho); T12(Phospho); K15(TMT6plex)
291575190	NP_001167566.1	6935	ZEB1	zinc finger E-box-binding homeobox 1 isoforme	S612	1.0	0.9	1.4	1.1	-	-	-	-	1.2	1.0	1.5	1.3	-	-	-	-	1.2	1.0	1.5	1.3	NNDQPQSANANEPQDSTVNLQsPLK	DSTVNLQsPLKMTNS	S22;	No	S(7): 0.0; S(16): 0.0; T(17): 0.0; S(22): 100.0	5	6	1	0	N-Term(TMT6plex); S22(Phospho); K25(TMT6plex)
109240550	NP_001035879.1	55269	PSPC1	paraspeckle component 1	S409	1.0	0.9	0.6	1.1	-	-	-	-	1.0	0.6	0.5	0.7	-	-	-	-	1.0	0.6	0.5	0.7	GAINMGDAFsPAPAGNQGPPPMMGMNMNNR	INMGDAFsPAPAGNQ	S10;	Yes	S(10): 100.0	2	1	1	0	N-Term(TMT6plex); M5(Oxidation); S10(Phospho)
345498499	NP_001186211.2	987	LRBA	lipopolysaccharide-responsive and beige-like anchor protein isoform1	S10	1.0	1.1	1.1	1.1	-	-	-	-	0.9	0.9	0.9	1.0	-	-	-	-	0.9	0.9	0.9	1.0	VPsPPPTGDDGGGGGR	SEDNRVPsPPPTGDD	S3;	Yes	S(3): 96.8; T(7): 3.2	3	2	1	0	N-Term(TMT6plex); S3(Phospho)
38683860	NP_003740.2	8660	IRS2	insulin receptor substrate 2	S620	0.7	0.8	0.8	1.1	-	-	-	-	0.7	0.8	0.8	1.1	-	-	-	-	0.7	0.8	0.8	1.1	LCPSCPASsPK	CPSCPASsPKVAYHP	S9;	Yes	S(4): 0.0; S(8): 1.0; S(9): 99.0	9	1	1	0	N-Term(TMT6plex); C2(Carbamidomethyl); C5(Carbamidomethyl); S9(Phospho); K11(TMT6plex)
58218984	NP_001009998.1	170463	SSBP4	single-stranded DNA-binding protein 4 isoformb	S320	0.9	1.1	0.9	1.1	-	-	-	-	-	-	-	-	-	-	-	-	-	-	-	-	SsPGAVAGLSNAPGTPR	MDGLPKSsPGAVAGL	S2;	No	S(1): 2.5; S(2): 97.5; S(10): 0.0; T(15): 0.0	1	2	1	0	N-Term(TMT6plex); S2(Phospho)
92859678	NP_003077.2	6621	SNAPC4	snRNA-activating protein complex subunit 4	S1224	0.9	1.1	1.0	1.1	-	-	-	-	1.1	1.0	1.0	1.1	-	-	-	-	1.1	1.0	1.0	1.1	GTPGsPSGTQEPR	EPRGTPGsPSGTQEP	S5;	Yes	T(2): 0.0; S(5): 100.0; S(7): 0.0; T(9): 0.0	1	1	1	0	N-Term(TMT6plex); S5(Phospho)
199559779	NP_114429.2	83987	CCDC8	coiled-coil domain-containing protein 8	S261	0.9	0.7	0.7	1.1	-	-	-	-	-	-	-	-	-	-	-	-	-	-	-	-	LGNAGDVCVPQAsPR	DVCVPQAsPRRWRPK	S13;	Yes	S(13): 100.0	1	1	1	0	N-Term(TMT6plex); C8(Carbamidomethyl); S13(Phospho)
239047414	NP_061857.3	54434	SSH1	protein phosphatase Slingshot homolog 1 isoform1	S937	1.1	1.2	1.1	1.1	-	-	-	-	-	-	-	-	-	-	-	-	-	-	-	-	SSsSDSIHSVR	SNLTRSSsSDSIHSV	S3;	Yes	S(1): 0.0; S(2): 0.0; S(3): 100.0; S(4): 0.0; S(6): 0.0; S(9): 0.0	2	1	1	0	N-Term(TMT6plex); S3(Phospho)
69354671	NP_001020262.1	23	ABCF1	ATP-binding cassette sub-family F member 1 isoforma	T108	1.3	1.1	1.1	1.1	-	-	-	-	1.4	1.2	1.2	1.3	-	-	-	-	1.4	1.2	1.2	1.3	LSVPtSDEEDEVPAPKPR	LKKLSVPtSDEEDEV	T5;	Yes	S(2): 0.0; T(5): 99.1; S(6): 0.9	44	2	1	0	N-Term(TMT6plex); T5(Phospho); K16(TMT6plex)
56181387	NP_005852.2	10273	STUB1	E3 ubiquitin-protein ligase CHIP	S19	1.3	0.8	0.7	1.1	-	-	-	-	1.3	0.7	0.7	0.9	-	-	-	-	1.3	0.7	0.7	0.9	LGAGGGsPEK	RLGAGGGsPEKSPSA	S7;	Yes	S(7): 100.0	1	1	1	0	N-Term(TMT6plex); S7(Phospho); K10(TMT6plex)
13994263	NP_114110.1	83786	FRMD8	FERM domain-containing protein 8	S21	1.4	1.1	0.9	1.1	-	-	-	-	-	-	-	-	-	-	-	-	-	-	-	-	SsVSSVGAR	AERSHRSsVSSVGAR	S2;	Yes	S(1): 1.0; S(2): 98.9; S(4): 50.0; S(5): 50.0	1	1	1	0	N-Term(TMT6plex); S2(Phospho); S4(Phospho)
33620730	NP_005543.2	3831	KLC1	kinesin light chain 1 isoform1	S460	0.5	0.9	0.6	1.1	-	-	-	-	0.5	0.9	0.7	1.0	-	-	-	-	0.5	0.9	0.7	1.0	VDsPTVTTTLK	YKACKVDsPTVTTTL	S3;	Yes	S(3): 100.0; T(5): 0.0; T(7): 0.0; T(8): 0.0; T(9): 0.0	1	3	1	0	N-Term(TMT6plex); S3(Phospho); K11(TMT6plex)
66392157	NP_001013860.1	146206	RLTPR	leucine-rich repeat-containing protein 16C	S1226	1.5	1.9	2.2	1.1	-	-	-	-	1.2	1.3	1.3	0.8	-	-	-	-	1.2	1.3	1.3	0.8	GsGGAEGK	RIGVSRGsGGAEGKR	S2;	No	S(2): 100.0	5	1	1	0	N-Term(TMT6plex); S2(Phospho); K8(TMT6plex)
221139926	NP_001137532.1	57224	NHSL1	NHS-like protein 1 isoform2	S1468	0.8	0.4	0.5	1.1	-	-	-	-	0.8	0.4	0.5	1.0	-	-	-	-	0.8	0.4	0.5	1.0	SEPSPDAPESPSSCsPSK	PESPSSCsPSKNRRA	S15;	No	S(1): 0.0; S(4): 0.0; S(10): 49.3; S(12): 49.3; S(13): 4.3; S(15): 94.3; S(17):	2	2	1	0	N-Term(TMT6plex); S13(Phospho); C14(Carbamidomethyl); S15(Phospho); K18(TMT6plex)
94421473	NP_001035242.1	23089	PEG10	retrotransposon-derived protein PEG10 isoform2	S316	1.1	1.1	0.9	1.1	-	-	-	-	1.1	1.4	1.1	1.2	-	-	-	-	1.1	1.4	1.1	1.2	SsPAGNSPAPL	PAKASKSsPAGNSPA	S2;	Yes	S(1): 0.9; S(2): 99.1; S(7): 0.0	5	3	1	0	N-Term(TMT6plex); S2(Phospho)
334085240	NP_001207706.1	1026	CDKN1A	cyclin-dependent kinase inhibitor 1 isoform1	S130	2.0	2.2	1.1	1.1	-	-	-	-	-	-	-	-	-	-	-	-	-	-	-	-	SGEQAEGsPGGPGDSQGR	SGEQAEGsPGGPGDS	S8;	Yes	S(1): 0.0; S(8): 100.0; S(15): 0.0	1	2	1	0	N-Term(TMT6plex); S8(Phospho)
40807504	NP_940997.1	60312	AFAP1	actin filament-associated protein 1 isoformB	S548	1.3	1.4	1.7	1.1	-	-	-	-	-	-	-	-	-	-	-	-	-	-	-	-	TGsNAAQYK	AVVKRTGsNAAQYKY	S3;	Yes	T(1): 0.0; S(3): 100.0; Y(8): 0.0	6	2	1	0	N-Term(TMT6plex); S3(Phospho); K9(TMT6plex)
32528306	NP_002904.3	5981	RFC1	replication factor C subunit 1 isoform1	S368	1.2	1.2	1.1	1.1	-	-	-	-	1.2	1.2	1.1	1.1	-	-	-	-	1.2	1.2	1.1	1.1	KESVsPEDSEK	PAKKESVsPEDSEKK	S5;	Yes	S(3): 0.0; S(5): 100.0; S(9): 0.0	12	1	1	1	N-Term(TMT6plex); K1(TMT6plex); S5(Phospho); K11(TMT6plex)
51988900	NP_000114.2	2073	ERCC5	DNA repair protein complementing XP-G cells	T567	2.0	1.1	0.8	1.1	-	-	-	-	1.9	1.1	0.8	1.1	-	-	-	-	1.9	1.1	0.8	1.1	FDSSLLSSDDEtK	LLSSDDEtKCKPNSA	T12;	Yes	S(3): 0.0; S(4): 0.0; S(7): 1.3; S(8): 1.3; T(12): 97.3	11	2	1	0	N-Term(TMT6plex); S8(Phospho); K13(TMT6plex)
283046706	NP_001164274.1	113251	LARP4	la-related protein 4 isoformd	S580	1.0	1.4	1.2	1.1	-	-	-	-	1.1	1.3	1.0	1.0	-	-	-	-	1.1	1.3	1.0	1.0	EQYVPPRsPK	EQYVPPRsPK	S8;	No	Y(3): 0.1; S(8): 99.9	4	5	1	1	N-Term(TMT6plex); S8(Phospho); K10(TMT6plex)
520261838	NP_001265553.1	152518	NFXL1	NF-X1-type zinc finger protein NFXL1	S50	1.3	1.3	1.5	1.1	-	-	-	-	1.2	1.1	1.2	0.9	-	-	-	-	1.2	1.1	1.2	0.9	GSVGAVPSGTsPGGVATTAAAGSR	GAVPSGTsPGGVATT	S11;	Yes	S(2): 0.0; S(8): 0.1; T(10): 3.1; S(11): 96.7; T(17): 0.0; T(18): 0.0; S(23): 0.1	1	1	1	0	N-Term(TMT6plex); S11(Phospho)
148596949	NP_004732.2	9221	NOLC1	nucleolar and coiled-body phosphoprotein 1 isoform2	S508	0.5	1.0	0.9	1.1	-	-	-	-	0.4	0.8	0.7	0.8	-	-	-	-	0.4	0.8	0.7	0.8	VAGGAAPSKPAsAK	AAPSKPAsAKKGKAE	S12;	Yes	S(8): 0.0; S(12): 100.0	33	3	1	0	N-Term(TMT6plex); K9(TMT6plex); S12(Phospho); K14(TMT6plex)
47271406	NP_062552.2	56257	MEPCE	7SK snRNA methylphosphate capping enzyme isoform A	T213	1.0	1.2	1.1	1.1	-	-	-	-	1.2	1.4	1.2	1.2	-	-	-	-	1.2	1.4	1.2	1.2	TLNAEtPKSSPLPAK	SRTLNAEtPKSSPLP	T6;	Yes	T(1): 0.0; T(6): 100.0; S(9): 50.0; S(10): 50.0	5	1	1	1	N-Term(TMT6plex); T1(Phospho); K8(TMT6plex); S10(Phospho); K15(TMT6plex)
314122233	NP_001186627.1	7917	BAG6	large proline-rich protein BAG6 isoformd	S958	1.0	1.1	1.0	1.1	-	-	-	-	1.1	1.2	1.1	1.2	-	-	-	-	1.1	1.2	1.1	1.2	AsPEPQR	VQGAERAsPEPQREN	S2;	No	S(2): 100.0	15	3	1	0	N-Term(TMT6plex); S2(Phospho)
75750474	NP_001028727.1	92521	SPECC1	cytospin-B isoform2	T59	1.4	1.1	1.0	1.1	-	-	-	-	1.4	1.1	1.0	1.2	-	-	-	-	1.4	1.1	1.0	1.2	SVSSPTSSNtPTPTK	SSPTSSNtPTPTKHL	T10;	No	S(1): 0.0; S(3): 0.0; S(4): 0.0; T(6): 0.0; S(7): 0.0; S(8): 0.0; T(10): 100.0; T(12): 0.0; T(14): 0.0	3	5	1	0	N-Term(TMT6plex); T10(Phospho); K15(TMT6plex)
157502203	NP_001098985.1	10257	ABCC4	multidrug resistance-associated protein 4 isoform2	T646	0.5	0.4	0.4	1.1	-	-	-	-	0.5	0.4	0.4	1.1	-	-	-	-	0.5	0.4	0.4	1.1	DNEESEQPPVPGtPTLR	EQPPVPGtPTLRNRT	T13;	Yes	S(5): 0.0; T(13): 98.8; T(15): 1.2	2	2	1	0	N-Term(TMT6plex); T13(Phospho)
153945728	NP_005900.2	4131	MAP1B	microtubule-associated protein 1B	S1312	1.1	1.5	1.1	1.1	-	-	-	-	0.9	1.1	0.9	1.1	-	-	-	-	0.9	1.1	0.9	1.1	VSAEAEVAPVSPEVTQEVVEEHCAsPEDK	VVEEHCAsPEDKTLE	S25;	Yes	S(2): 0.0; S(11): 0.0; T(15): 0.0; S(25): 100.0	49	1	1	0	N-Term(TMT6plex); C23(Carbamidomethyl); S25(Phospho); K29(TMT6plex)
17865802	NP_004860.2	9525	VPS4B	vacuolar protein sorting-associated protein 4B	S102	1.3	1.2	1.0	1.1	-	-	-	-	1.3	1.2	1.0	1.1	-	-	-	-	1.3	1.2	1.0	1.1	GNDsDGEGESDDPEKK	ADEKGNDsDGEGESD	S4;	Yes	S(4): 100.0; S(10): 0.0	4	1	1	1	N-Term(TMT6plex); S4(Phospho); K15(TMT6plex); K16(TMT6plex)
149158690	NP_542417.2	7916	PRRC2A	protein PRRC2A	S380	1.0	1.1	0.9	1.1	-	-	-	-	1.1	1.0	1.0	1.0	-	-	-	-	1.1	1.0	1.0	1.0	KGNsPNSEPPTPK	ADGKKGNsPNSEPPT	S4;	Yes	S(4): 100.0; S(7): 0.0; T(11): 0.0	23	1	1	1	N-Term(TMT6plex); K1(TMT6plex); S4(Phospho); K13(TMT6plex)
117168248	NP_057479.2	51495	PTPLAD1	very-long-chain (3R)-3-hydroxyacyl-[acyl-carrier protein] dehydratase 3	S114	1.1	1.2	1.0	1.1	-	-	-	-	-	-	-	-	-	-	-	-	-	-	-	-	WLDEsDAEMELR	FDRWLDEsDAEMELR	S5;	Yes	S(5): 100.0	1	1	1	0	N-Term(TMT6plex); S5(Phospho); M9(Oxidation)
167466175	NP_001247.3	996	CDC27	cell division cycle protein 27 homolog isoform2	S426	1.7	1.8	1.0	1.1	-	-	-	-	1.6	1.7	0.9	1.1	-	-	-	-	1.6	1.7	0.9	1.1	GGITQPNINDsLEITK	TQPNINDsLEITKLD	S11;	Yes	T(4): 0.0; S(11): 100.0; T(15): 0.0	2	2	1	0	N-Term(TMT6plex); S11(Phospho); K16(TMT6plex)
114155142	NP_003283.2	7175	TPR	nucleoprotein TPR	T1650	0.7	0.7	0.9	1.1	-	-	-	-	0.8	0.8	1.0	1.2	-	-	-	-	0.8	0.8	1.0	1.2	TtPASGER	RQITLKTtPASGERG	T2;	Yes	T(1): 0.0; T(2): 100.0; S(5): 0.0	29	1	1	0	N-Term(TMT6plex); T2(Phospho)
7657261	NP_055062.1	9693	RAPGEF2	rap guanine nucleotide exchange factor 2	S1022	1.1	1.1	0.9	1.1	-	-	-	-	-	-	-	-	-	-	-	-	-	-	-	-	SETsPVAPR	KPVKSETsPVAPRAG	S4;	Yes	S(1): 0.0; T(3): 0.7; S(4): 99.3	1	1	1	0	N-Term(TMT6plex); S4(Phospho)
256223453	NP_009135.4	11218	DDX20	probable ATP-dependent RNA helicase DDX20	S714	1.2	1.2	1.3	1.1	-	-	-	-	1.1	1.2	1.3	1.0	-	-	-	-	1.1	1.2	1.3	1.0	YQEsPGIQMK	NPEKYQEsPGIQMKT	S4;	Yes	Y(1): 0.0; S(4): 100.0	3	1	1	0	N-Term(TMT6plex); S4(Phospho); M9(Oxidation); K10(TMT6plex)
149193319	NP_001091947.1	2926	GRSF1	G-rich sequence factor 1 isoform2	S82	1.2	1.2	1.0	1.1	-	-	-	-	1.4	1.4	1.0	0.9	-	-	-	-	1.4	1.4	1.0	0.9	SsPVVNDGVVR	KSLQVKSsPVVNDGV	S2;	No	S(1): 1.4; S(2): 98.6	3	2	1	0	N-Term(TMT6plex); S2(Phospho)
56550039	NP_005924.2	4297	KMT2A	histone-lysine N-methyltransferase 2A isoform2 precursor	S3511	1.1	0.9	0.8	1.1	-	-	-	-	1.0	0.8	0.8	1.1	-	-	-	-	1.0	0.8	0.8	1.1	ALSSAVQASPTsPGGSPSSPSSGQR	AVQASPTsPGGSPSS	S12;	No	S(3): 0.0; S(4): 0.0; S(9): 0.1; T(11): 3.3; S(12): 92.9; S(16): 3.3; S(18): 0.1; S(19): 0.1; S(21): 0.0; S(22): 0.0	15	2	1	0	N-Term(TMT6plex); S12(Phospho)
50962882	NP_001002909.1	23131	GPATCH8	G patch domain-containing protein 8	S1081	0.7	0.7	0.8	1.1	-	-	-	-	0.7	0.7	0.9	1.1	-	-	-	-	0.7	0.7	0.9	1.1	GSEGDCsPEDKNSVTAK	RGSEGDCsPEDKNSV	S7;	Yes	S(2): 0.0; S(7): 100.0; S(13): 0.0; T(15): 0.0	17	1	1	1	N-Term(TMT6plex); C6(Carbamidomethyl); S7(Phospho); K11(TMT6plex); K17(TMT6plex)
124244056	NP_689637.3	139285	AMER1	APC membrane recruitment protein 1	S246	0.6	1.0	0.9	1.1	-	-	-	-	0.5	1.0	0.8	1.0	-	-	-	-	0.5	1.0	0.8	1.0	VSPTPEPsPPATEK	VSPTPEPsPPATEKM	S8;	Yes	S(2): 50.0; T(4): 50.0; S(8): 100.0; T(12): 0.0	1	1	1	0	N-Term(TMT6plex); T4(Phospho); S8(Phospho); K14(TMT6plex)
42542379	NP_005830.2	10250	SRRM1	serine/arginine repetitive matrix protein 1	S713	0.9	1.3	1.1	1.1	-	-	-	-	1.1	1.4	1.2	1.2	-	-	-	-	1.1	1.4	1.2	1.2	RQsPSPSTRPIR	SSPQRRQsPSPSTRP	S3;	Yes	S(3): 99.9; S(5): 0.1; S(7): 0.0; T(8):	229	1	1	1	N-Term(TMT6plex); S3(Phospho)
116008442	NP_055885.3	23091	ZC3H13	zinc finger CCCH domain-containing protein 13	S1190	1.4	1.4	0.9	1.1	-	-	-	-	1.4	1.3	0.9	1.0	-	-	-	-	1.4	1.3	0.9	1.0	SsSLGSNR	PMDQKRSsSLGSNRS	S2;	Yes	S(1): 0.7; S(2): 98.6; S(3): 0.7; S(6):	28	1	1	0	N-Term(TMT6plex); S3(Phospho)
42542379	NP_005830.2	10250	SRRM1	serine/arginine repetitive matrix protein 1	S393	1.0	1.2	1.1	1.1	-	-	-	-	1.2	1.3	1.2	1.2	-	-	-	-	1.2	1.3	1.2	1.2	LSPSAsPPR	RRLSPSAsPPRRRHR	S6;	Yes	S(2): 50.3; S(4): 50.3; S(6): 99.4	229	1	1	0	N-Term(TMT6plex); S4(Phospho); S6(Phospho)
153792633	NP_056381.1	26049	FAM169A	soluble lamin-associated protein of 75 kDa	S447	1.0	1.0	0.8	1.1	-	-	-	-	1.0	1.0	0.9	1.1	-	-	-	-	1.0	1.0	0.9	1.1	TSLITEEEDsTSEVLDEELK	LITEEEDsTSEVLDE	S10;	Yes	T(1): 0.0; S(2): 0.0; T(5): 0.0; S(10): 97.2; T(11): 1.4; S(12): 1.4	10	1	1	0	N-Term(TMT6plex); S10(Phospho); K20(TMT6plex)
55956921	NP_004490.2	3182	HNRNPAB	heterogeneous nuclear ribonucleoprotein A/B isoform	S242	0.4	1.1	0.4	1.0	-	-	-	-	0.4	1.1	0.5	1.2	-	-	-	-	0.4	1.1	0.5	1.2	EVYQQQQYGsGGR	YQQQQYGsGGRGNRN	S10;	Yes	Y(3): 0.0; Y(8): 0.0; S(10): 100.0	10	2	1	0	N-Term(TMT6plex); S10(Phospho)
31652242	NP_852469.1	6942	TCF20	transcription factor 20 isoform2	S559	1.1	1.3	1.2	1.0	-	-	-	-	1.0	1.4	1.2	1.2	-	-	-	-	1.0	1.4	1.2	1.2	AGSsPAQGAQNEPPR	ASEKAGSsPAQGAQN	S4;	Yes	S(3): 1.5; S(4): 98.5	14	2	1	0	N-Term(TMT6plex); S4(Phospho)
301129172	NP_001180299.1	11083	DIDO1	death-inducer obliterator 1 isoformb	S138	1.0	1.3	1.0	1.0	-	-	-	-	1.0	1.3	1.0	1.1	-	-	-	-	1.0	1.3	1.0	1.1	ERPAsSEK	AVKERPAsSEKVKGG	S5;	No	S(5): 100.0; S(6): 0.0	7	3	1	0	N-Term(TMT6plex); S5(Phospho); K8(TMT6plex)
166795297	NP_057225.2	51143	DYNC1LI1	cytoplasmic dynein 1 light intermediate chain 1	S405; T408	0.9	0.9	0.9	1.0	-	-	-	-	0.9	1.0	0.9	1.1	-	-	-	-	0.9	1.0	0.9	1.1	VPGGsPRtPNR	SPRVPGGsPRTPNRS;VPGGSPRtPNRSVSS	S5; T8;	Yes;Yes	S(5): 100.0; T(8): 100.0	10	1	1	1	N-Term(TMT6plex); S5(Phospho); T8(Phospho)
55770834	NP_057427.3	1063	CENPF	centromere protein F	T144	1.1	1.0	1.3	1.0	-	-	-	-	1.2	1.0	1.2	1.1	-	-	-	-	1.2	1.0	1.2	1.1	SQQAAQSADVSLNPCNtPQK	VSLNPCNtPQKIFTT	T17;	Yes	S(1): 0.0; S(7): 2.8; S(11): 2.8; T(17): 94.4	11	1	1	0	N-Term(TMT6plex); C15(Carbamidomethyl); T17(Phospho); K20(TMT6plex)
8923819	NP_060909.1	55364	IMPACT	protein IMPACT	S297	0.8	0.9	0.8	1.0	-	-	-	-	1.0	1.1	0.9	1.3	-	-	-	-	1.0	1.1	0.9	1.3	NYTNsPEESSK	VEKNYTNsPEESSKA	S5;	No	Y(2): 0.0; T(3): 0.0; S(5): 100.0; S(9): 0.0; S(10): 0.0	1	1	1	0	N-Term(TMT6plex); S5(Phospho); K11(TMT6plex)
156119615	NP_008832.2	4649	MYO9A	unconventional myosin-IXa	S1299	1.4	1.4	1.2	1.0	-	-	-	-	1.4	1.4	1.2	1.0	-	-	-	-	1.4	1.4	1.2	1.0	SLGGIsPSEDR	VRSLGGIsPSEDRRW	S6;	Yes	S(1): 0.0; S(6): 100.0; S(8): 0.0	6	1	1	0	N-Term(TMT6plex); S6(Phospho)
7657335	NP_056531.1	50488	MINK1	misshapen-like kinase 1 isoform1	S601	1.1	1.2	1.2	1.0	-	-	-	-	1.0	1.1	1.1	1.0	-	-	-	-	1.0	1.1	1.1	1.0	SQsLQDQPTR	APVPRSQsLQDQPTR	S3;	Yes	S(1): 0.0; S(3): 100.0; T(9): 0.0	4	3	1	0	N-Term(TMT6plex); S3(Phospho)
388490223	NP_001253976.1	122786	FRMD6	FERM domain-containing protein 6 isoform3	S14	0.9	1.1	1.2	1.0	-	-	-	-	1.0	1.3	1.3	1.2	-	-	-	-	1.0	1.3	1.3	1.2	ASGSsAGSMK	RSRASGSsAGSMKHK	S5;	No	S(2): 0.0; S(4): 1.2; S(5): 97.5; S(8):	2	3	1	0	N-Term(TMT6plex); S8(Phospho); M9(Oxidation); K10(TMT6plex)
14249158	NP_116020.1	84717	HDGFRP2	hepatoma-derived growth factor-related protein 2 isoform2	S240	0.6	0.9	0.9	1.0	-	-	-	-	0.8	1.2	1.2	1.4	-	-	-	-	0.8	1.2	1.2	1.4	ADsDGAKPEPVAMAR	DSDSKADsDGAKPEP	S3;	Yes	S(3): 100.0	21	2	1	0	N-Term(TMT6plex); S3(Phospho); K7(TMT6plex); M13(Oxidation)
313851097	NP_001186499.1	25999	CLIP3	CAP-Gly domain-containing linker protein 3	S32; T34	1.0	0.8	0.8	1.0	-	-	-	-	-	-	-	-	-	-	-	-	-	-	-	-	GEEEEEEEEDEPVPEAPsPtQERR	EPVPEAPsPTQERRQ;VPEAPSPtQERRQKP	S18; T20;	No;No	S(18): 100.0; T(20): 100.0	1	1	1	1	N-Term(TMT6plex); S18(Phospho); T20(Phospho)
13129136	NP_077021.1	79101	TAF1D	TATA box-binding protein-associated factor RNA polymerase I subunit D	S40	1.4	0.9	1.1	1.0	-	-	-	-	-	-	-	-	-	-	-	-	-	-	-	-	TQCIPYsPK	KTQCIPYsPKGEKRN	S7;	Yes	T(1): 0.0; Y(6): 0.0; S(7): 100.0	1	1	1	0	N-Term(TMT6plex); C3(Carbamidomethyl); S7(Phospho); K9(TMT6plex)
533112484	NP_001269207.1	9782	MATR3	matrin-3 isoformc	S367	0.9	1.0	1.1	1.0	-	-	-	-	0.9	1.1	1.2	1.1	-	-	-	-	0.9	1.1	1.2	1.1	DGsASAAAK	KAVKKDGsASAAAKK	S3;	No	S(3): 100.0; S(5): 0.0	3	3	1	0	N-Term(TMT6plex); S3(Phospho); K9(TMT6plex)
4504447	NP_002128.1	3181	HNRNPA2B1	heterogeneous nuclear ribonucleoproteins A2/B1 isoform A2	S219	0.7	1.0	0.9	1.0	-	-	-	-	0.7	1.1	1.0	1.1	-	-	-	-	0.7	1.1	1.0	1.1	GGsDGYGSGR	GSNFRGGsDGYGSGR	S3;	No	S(3): 100.0; Y(6): 0.0; S(8): 0.0	11	2	1	0	N-Term(TMT6plex); S3(Phospho)
555290002	NP_001273127.1	64897	C12orf43	uncharacterized protein C12orf43 isoform e	S96	0.7	1.1	0.7	1.0	-	-	-	-	0.8	1.1	0.8	1.1	-	-	-	-	0.8	1.1	0.8	1.1	EKEEsPQPR	GGREKEEsPQPRRKR	S5;	No	S(5): 100.0	3	7	1	1	N-Term(TMT6plex); K2(TMT6plex); S5(Phospho)
46409304	NP_997215.1	157697	ERICH1	glutamate-rich protein 1	S238	1.0	1.2	1.3	1.0	-	-	-	-	-	-	-	-	-	-	-	-	-	-	-	-	EEDGADAsEEDLTR	EEDGADAsEEDLTRA	S8;	Yes	S(8): 97.8; T(13): 2.2	1	1	1	0	N-Term(TMT6plex); S8(Phospho)
13491174	NP_075385.1	65108	MARCKSL1	MARCKS-related protein	T174	1.0	0.8	0.5	1.0	-	-	-	-	1.1	0.9	0.6	0.9	-	-	-	-	1.1	0.9	0.6	0.9	GAEASAASEEEAGPQAtEPSTPSGPESGPTPASAEQNE	EEAGPQAtEPSTPSG	T17;	Yes	S(5): 0.0; S(8): 0.0; T(17): 85.3; S(20): 2.9; T(21): 2.9; S(23): 2.9; S(27): 2.9; T(30): 2.9; S(33): 0.1	18	1	1	0	N-Term(TMT6plex); T17(Phospho)
61966689	NP_001013633.1	541468	LURAP1	leucine rich adaptor protein 1	S213	1.2	1.5	1.0	1.0	-	-	-	-	-	-	-	-	-	-	-	-	-	-	-	-	LQGGPPEsPEDESAK	LQGGPPEsPEDESAK	S8;	Yes	S(8): 100.0; S(13): 0.0	1	1	1	0	N-Term(TMT6plex); S8(Phospho); K15(TMT6plex)
189409140	NP_003209.2	7013	TERF1	telomeric repeat-binding factor 1 isoform2	S414	1.2	1.2	0.6	1.0	-	-	-	-	1.2	1.3	0.8	1.1	-	-	-	-	1.2	1.3	0.8	1.1	LIsSDSED	MKKLKLIsSDSED	S3;	No	S(3): 99.4; S(4): 0.6; S(6): 0.0	2	2	1	0	N-Term(TMT6plex); S3(Phospho)
71143119	NP_055888.1	23094	SIPA1L3	signal-induced proliferation-associated 1-like protein	S1364	1.0	0.9	1.0	1.0	-	-	-	-	1.1	1.1	1.1	1.1	-	-	-	-	1.1	1.1	1.1	1.1	EVsPAPAVAGQSK	ADRRREVsPAPAVAG	S3;	Yes	S(3): 100.0; S(12): 0.0	7	1	1	0	N-Term(TMT6plex); S3(Phospho); K13(TMT6plex)
66528888	NP_001018125.1	2177	FANCD2	Fanconi anemia group D2 protein isoformb	S592	0.8	0.9	1.1	1.0	-	-	-	-	1.0	1.0	1.2	1.1	-	-	-	-	1.0	1.0	1.2	1.1	SEsPSLTQER	MAADRSEsPSLTQER	S3;	Yes	S(1): 0.0; S(3): 98.9; S(5): 0.0; T(7):	3	2	1	0	N-Term(TMT6plex); S3(Phospho)
6005948	NP_009118.1	11193	WBP4	WW domain-binding protein 4	S262	1.1	1.1	1.3	1.0	-	-	-	-	1.3	1.2	1.5	1.2	-	-	-	-	1.3	1.2	1.5	1.2	NSDGGsDPETQKEK	NKNSDGGsDPETQKE	S6;	Yes	S(2): 0.0; S(6): 100.0; T(10): 0.0	3	1	1	1	N-Term(TMT6plex); S6(Phospho); K12(TMT6plex); K14(TMT6plex)
71143119	NP_055888.1	23094	SIPA1L3	signal-induced proliferation-associated 1-like protein	S1534	0.7	1.2	0.9	1.0	-	-	-	-	0.8	1.4	1.1	1.1	-	-	-	-	0.8	1.4	1.1	1.1	DTGQsPQK	QERDTGQsPQKGLQR	S5;	Yes	T(2): 0.0; S(5): 100.0	7	1	1	0	N-Term(TMT6plex); S5(Phospho); K8(TMT6plex)
65301112	NP_001018003.1	10174	SORBS3	vinexin isoform2	S221	1.1	1.4	1.1	1.0	-	-	-	-	-	-	-	-	-	-	-	-	-	-	-	-	SPADPIDLGGQTsPR	IDLGGQTsPRRTGES	S13;	Yes	S(1): 0.0; T(12): 1.8; S(13): 98.2	2	2	1	0	N-Term(TMT6plex); S13(Phospho)
58331266	NP_085058.3	79726	WDR59	WD repeat-containing protein 59	S603	1.1	0.9	0.9	1.0	-	-	-	-	1.0	0.9	0.9	1.0	-	-	-	-	1.0	0.9	0.9	1.0	LYSGsPTR	NLRLYSGsPTRSEKE	S5;	Yes	Y(2): 0.0; S(3): 0.9; S(5): 99.1; T(7):	1	1	1	0	N-Term(TMT6plex); S5(Phospho)
21493033	NP_009133.2	11216	AKAP10	A-kinase anchor protein 10, mitochondrial precursor	S52	0.6	0.9	0.9	1.0	-	-	-	-	0.6	0.9	0.9	1.0	-	-	-	-	0.6	0.9	0.9	1.0	ASISVHsPQK	KASISVHsPQKSTKN	S7;	Yes	S(2): 0.0; S(4): 0.0; S(7): 100.0	1	1	1	0	N-Term(TMT6plex); S7(Phospho); K10(TMT6plex)
157694492	NP_055335.2	10514	MYBBP1A	myb-binding protein 1A isoform2	S1290	0.6	0.8	0.8	1.0	-	-	-	-	0.6	0.9	0.8	1.1	-	-	-	-	0.6	0.9	0.8	1.1	sPLSALAR	KKGVLGKsPLSALAR	S1;	Yes	S(1): 100.0; S(4): 0.0	15	2	1	0	N-Term(TMT6plex); S1(Phospho)
33469917	NP_877423.1	4173	MCM4	DNA replication licensing factor MCM4	S31; S32	0.7	0.8	0.5	1.0	-	-	-	-	0.7	0.9	0.6	1.1	-	-	-	-	0.7	0.9	0.6	1.1	SEDARssPSQR	PRSEDARsSPSQRRR;RSEDARSsPSQRRRG	S6; S7;	Yes;Yes	S(1): 0.0; S(6): 100.0; S(7): 100.0; S(9): 0.0	1	1	1	1	N-Term(TMT6plex); S6(Phospho); S7(Phospho)
431822379	NP_001258930.1	85440	DOCK7	dedicator of cytokinesis protein 7 isoform4	S1398	1.7	1.0	1.1	1.0	-	-	-	-	1.8	1.1	1.1	1.0	-	-	-	-	1.8	1.1	1.1	1.0	SPSGSAFGsQENLR	PSGSAFGsQENLRWR	S9;	Yes	S(1): 0.0; S(3): 1.7; S(5): 1.7; S(9):	5	4	1	0	N-Term(TMT6plex); S9(Phospho)
7657427	NP_055136.1	23594	ORC6	origin recognition complex subunit 6	T195	0.7	0.9	0.8	1.0	-	-	-	-	0.8	0.9	0.8	1.1	-	-	-	-	0.8	0.9	0.8	1.1	IGQQVDREPGDVAtPPR	REPGDVAtPPRKRKK	T14;	Yes	T(14): 100.0	3	1	1	1	N-Term(TMT6plex); T14(Phospho)
157412270	NP_112480.2	4670	HNRNPM	heterogeneous nuclear ribonucleoprotein M isoformb	S662	0.8	0.6	0.9	1.0	-	-	-	-	0.9	0.6	1.0	1.0	-	-	-	-	0.9	0.6	1.0	1.0	FEsPEVAER	CGVVKFEsPEVAERA	S3;	No	S(3): 100.0	3	2	1	0	N-Term(TMT6plex); S3(Phospho)
46049114	NP_057279.2	9585	KIF20B	kinesin-like protein KIF20B isoform2	S1716	0.7	1.1	1.0	1.0	-	-	-	-	0.7	1.1	0.9	0.9	-	-	-	-	0.7	1.1	0.9	0.9	IIETMsSSK	KKIIETMsSSKLSNV	S6;	No	T(4): 0.8; S(6): 99.2; S(7): 0.0; S(8):	1	2	1	0	N-Term(TMT6plex); T4(Phospho); M5(Oxidation); K9(TMT6plex)
32307124	NP_006525.2	8202	NCOA3	nuclear receptor coactivator 3 isoformb	S728	0.7	0.9	0.9	1.0	-	-	-	-	-	-	-	-	-	-	-	-	-	-	-	-	QEQLsPK	VVKQEQLsPKKKENN	S5;	Yes	S(5): 100.0	6	4	1	0	N-Term(TMT6plex); S5(Phospho); K7(TMT6plex)
259906020	NP_001158287.1	22985	ACIN1	apoptotic chromatin condensation inducer in the nucleus isoform3	S858	1.9	1.2	0.8	1.0	-	-	-	-	1.5	1.1	0.9	1.1	-	-	-	-	1.5	1.1	0.9	1.1	IsEDETER	HADDSRIsEDETERN	S2;	No	S(2): 100.0; T(6): 0.0	38	5	1	0	N-Term(TMT6plex); S2(Phospho)
226371725	NP_001139504.1	115548	FCHO2	FCH domain only protein 2 isoformb	S361	0.9	1.0	1.1	1.0	-	-	-	-	-	-	-	-	-	-	-	-	-	-	-	-	HsPVQMNR	SPAISRHsPVQMNRN	S2;	No	S(2): 100.0	1	2	1	0	N-Term(TMT6plex); S2(Phospho); M6(Oxidation)
23308505	NP_694580.1	127428	TCEANC2	transcription elongation factor A N-terminal and central domain-containing protein 2	S14	0.8	0.7	0.6	1.0	-	-	-	-	-	-	-	-	-	-	-	-	-	-	-	-	IQNsPQK	RTPRIQNsPQKKDSG	S4;	No	S(4): 100.0	2	1	1	0	N-Term(TMT6plex); S4(Phospho); K7(TMT6plex)
36029914	NP_079330.2	80124	VCPIP1	deubiquitinating protein VCIP135	S757	0.9	1.4	1.0	1.0	-	-	-	-	0.7	1.2	0.8	1.0	-	-	-	-	0.7	1.2	0.8	1.0	DGPSsAPATPTK	TIRDGPSsAPATPTK	S5;	Yes	S(4): 1.1; S(5): 98.9; T(9): 0.0; T(11): 0.0	4	1	1	0	N-Term(TMT6plex); S4(Phospho); K12(TMT6plex)
321267473	NP_001188269.1	6294	SAFB	scaffold attachment factor B1 isoform4	S314	1.0	1.2	1.3	1.0	-	-	-	-	1.3	1.3	1.5	1.2	-	-	-	-	1.3	1.3	1.5	1.2	MsSPEDDSDTKR	EGADQKMsSPEDDSD	S2;	No	S(2): 100.0; S(3): 0.0; S(8): 0.0; T(10): 0.0	23	4	1	1	N-Term(TMT6plex); M1(Oxidation); S2(Phospho); K11(TMT6plex)
344030221	NP_001230656.1	51368	TEX264	testis-expressed sequence 264 protein isoform2	S170	1.1	1.1	0.9	1.0	-	-	-	-	1.1	1.1	0.9	1.0	-	-	-	-	1.1	1.1	0.9	1.0	ETSAATLsPGASSR	ETSAATLsPGASSRG	S8;	No	T(2): 0.0; S(3): 0.0; T(6): 0.0; S(8): 100.0; S(12): 0.0; S(13): 0.0	4	2	1	0	N-Term(TMT6plex); S8(Phospho)
20127499	NP_006266.2	6431	SRSF6	serine/arginine-rich splicing factor 6	S314	0.7	1.3	1.2	1.0	-	-	-	-	0.9	1.6	1.4	1.3	-	-	-	-	0.9	1.6	1.4	1.3	sVSPPPK	VPPSKARsVSPPPKR	S1;	Yes	S(1): 100.0; S(3): 0.0	43	1	1	0	N-Term(TMT6plex); S1(Phospho); K7(TMT6plex)
11067747	NP_001244.1	988	CDC5L	cell division cycle 5-like protein	T355	1.0	1.2	1.1	1.0	-	-	-	-	1.0	1.2	1.1	1.1	-	-	-	-	1.0	1.2	1.1	1.1	tPASQDR	VALRTPRtPASQDRI	T1;	Yes	T(1): 100.0; S(4): 0.0	4	1	1	0	N-Term(TMT6plex); T1(Phospho)
118601081	NP_001073027.1	221092	HNRNPUL2	heterogeneous nuclear ribonucleoprotein U-like	S193	1.4	1.6	1.4	1.0	-	-	-	-	1.5	1.5	1.4	1.0	-	-	-	-	1.5	1.5	1.4	1.0	SKPAGsDGER	EKSKPAGsDGERRGV	S6;	Yes	S(1): 0.0; S(6): 100.0	33	1	1	0	N-Term(TMT6plex); K2(TMT6plex); S6(Phospho)
239049591	NP_001155059.1	2997	GYS1	glycogen [starch] synthase, muscle isoform2	S589; S593	1.4	1.1	0.9	1.0	-	-	-	-	1.5	1.1	0.8	1.0	-	-	-	-	1.5	1.1	0.8	1.0	HSsPHQsEDEEDPR	PSLSRHSsPHQSEDE;RHSSPHQsEDEEDPR	S3; S7;	No;No	S(2): 1.4; S(3): 98.7; S(7): 100.0	1	2	1	0	N-Term(TMT6plex); S3(Phospho); S7(Phospho)
51873067	NP_001004128.1	5768	QSOX1	sulfhydryl oxidase 1 isoformb precursor	S493	1.4	1.1	1.1	1.0	-	-	-	-	1.4	1.1	1.1	1.1	-	-	-	-	1.4	1.1	1.1	1.1	LAGAPsEDPQEPK	ARLAGAPsEDPQEPK	S6;	No	S(6): 100.0	2	2	1	0	N-Term(TMT6plex); S6(Phospho); K13(TMT6plex)
45827753	NP_996742.1	10579	TACC2	transforming acidic coiled-coil-containing protein 2 isoformc	S304	1.0	1.1	1.1	1.0	-	-	-	-	0.9	1.0	0.9	1.1	-	-	-	-	0.9	1.0	0.9	1.1	VQNsPPVGR	GGGRVQNsPPVGRKT	S4;	No	S(4): 100.0	11	7	1	0	N-Term(TMT6plex); S4(Phospho)
118572613	NP_057417.3	23524	SRRM2	serine/arginine repetitive matrix protein 2	S1441	0.9	0.9	0.8	1.0	-	-	-	-	1.0	1.0	0.8	1.1	-	-	-	-	1.0	1.0	0.8	1.1	sGSSPGLR	TPSRRSRsGSSPGLR	S1;	Yes	S(1): 100.0; S(3): 50.0; S(4): 50.0	436	1	1	0	N-Term(TMT6plex); S1(Phospho); S3(Phospho)
84043967	NP_001032961.1	51455	REV1	DNA repair protein REV1 isoform2	S1096	0.9	0.8	0.6	1.0	-	-	-	-	0.9	0.8	0.7	1.1	-	-	-	-	0.9	0.8	0.7	1.1	LLNsPAK	LNNKLLNsPAKTLPG	S4;	No	S(4): 100.0	1	2	1	0	N-Term(TMT6plex); S4(Phospho); K7(TMT6plex)
118572613	NP_057417.3	23524	SRRM2	serine/arginine repetitive matrix protein 2	T1434	1.4	1.8	1.6	1.0	-	-	-	-	1.4	1.9	1.7	1.1	-	-	-	-	1.4	1.9	1.7	1.1	DGLPRtPSR	MKDGLPRtPSRRSRS	T6;	Yes	T(6): 98.8; S(8): 1.2	436	1	1	1	N-Term(TMT6plex); T6(Phospho)
13027630	NP_073210.1	8220	DGCR14	protein DGCR14	S292	0.9	1.1	1.2	1.0	-	-	-	-	0.9	1.0	1.3	1.0	-	-	-	-	0.9	1.0	1.3	1.0	ELIPQEsPR	KELIPQEsPRVGGFG	S7;	Yes	S(7): 100.0	4	1	1	0	N-Term(TMT6plex); S7(Phospho)
217330574	NP_001136088.1	23301	EHBP1	EH domain-binding protein 1 isoform3	S724	0.8	1.1	1.0	1.0	-	-	-	-	0.9	1.1	1.0	0.8	-	-	-	-	0.9	1.1	1.0	0.8	TSLsPTSK	PIKKTSLsPTSKLGY	S4;	No	T(1): 0.0; S(2): 0.0; S(4): 100.0; T(6): 0.0; S(7): 0.0	5	3	1	0	N-Term(TMT6plex); S4(Phospho); K8(TMT6plex)
150418007	NP_006258.3	5903	RANBP2	E3 SUMO-protein ligase RanBP2	S2276	1.1	1.1	1.2	1.0	-	-	-	-	1.0	1.1	1.1	0.9	-	-	-	-	1.0	1.1	1.1	0.9	SALsPSK	FSFKSALsPSKSPAK	S4;	Yes	S(1): 0.0; S(4): 100.0; S(6): 0.0	17	2	1	0	N-Term(TMT6plex); S4(Phospho); K7(TMT6plex)
36029914	NP_079330.2	80124	VCPIP1	deubiquitinating protein VCIP135	T771	1.2	1.2	1.1	1.0	-	-	-	-	1.0	1.0	1.0	1.0	-	-	-	-	1.0	1.0	1.0	1.0	APYSPTtSK	KAPYSPTtSKEKKIR	T7;	Yes	Y(3): 0.0; S(4): 0.6; T(6): 0.6; T(7): 98.1; S(8): 0.6	4	1	1	0	N-Term(TMT6plex); S4(Phospho); K9(TMT6plex)
18765756	NP_569122.1	1859	DYRK1A	dual specificity tyrosine-phosphorylation-regulated kinase 1A isoform5	Y321	1.2	0.9	1.1	1.0	-	-	-	-	1.2	0.7	1.0	0.9	-	-	-	-	1.2	0.7	1.0	0.9	IYQyIQSR	LGQRIYQyIQSRFYR	Y4;	Yes	Y(2): 0.0; Y(4): 100.0; S(7): 0.0	1	7	1	0	N-Term(TMT6plex); Y4(Phospho)
7656879	NP_055238.1	27125	AFF4	AF4/FMR2 family member 4	S1062	2.2	2.9	2.6	1.0	-	-	-	-	1.9	2.6	2.4	0.9	-	-	-	-	1.9	2.6	2.4	0.9	LsPGNSGNYSSGASSASASGSSVTIPQK	SPVSPKLsPGNSGNY	S2;	Yes	S(2): 99.9; S(6): 0.1; Y(9): 0.0; S(10): 0.0; S(11): 0.0; S(14): 0.0; S(15): 0.0; S(17): 0.0; S(19): 0.0; S(21): 0.0; S(22): 0.0; T(24): 0.0	16	1	1	0	N-Term(TMT6plex); S2(Phospho); K28(TMT6plex)
4504747	NP_002195.1	3675	ITGA3	integrin alpha-3 isoforma precursor	S1042	0.6	0.9	1.0	1.0	-	-	-	-	0.7	0.9	1.0	1.0	-	-	-	-	0.7	0.9	1.0	1.0	SQPsETER	AEMKSQPsETERLTD	S4;	Yes	S(1): 0.0; S(4): 100.0; T(6): 0.0	1	1	1	0	N-Term(TMT6plex); S4(Phospho)
118572599	NP_037373.2	29109	FHOD1	FH1/FH2 domain-containing protein 1	S523	1.9	2.7	1.7	1.0	-	-	-	-	2.0	2.7	1.8	1.0	-	-	-	-	2.0	2.7	1.8	1.0	EPLIPAsPK	KEPLIPAsPKAEPIW	S7;	Yes	S(7): 100.0	1	1	1	0	N-Term(TMT6plex); S7(Phospho); K9(TMT6plex)
224809474	NP_001138994.1	26064	RAI14	ankycorbin isoformb	S261	1.0	0.9	1.1	1.0	-	-	-	-	0.9	0.8	0.8	0.8	-	-	-	-	0.9	0.8	0.8	0.8	QLSDVSsPR	KQLSDVSsPRSITST	S7;	No	S(3): 0.0; S(6): 0.8; S(7): 99.2	4	1	1	0	N-Term(TMT6plex); S7(Phospho)
103472005	NP_002408.3	4288	MKI67	antigen KI-67 isoform1	S108	0.5	0.8	1.0	1.0	-	-	-	-	0.5	0.9	1.0	1.0	-	-	-	-	0.5	0.9	1.0	1.0	sTEFPR	SLQNGRKsTEFPRKI	S1;	Yes	S(1): 99.5; T(2): 0.5	118	2	1	0	N-Term(TMT6plex); S1(Phospho)
100913021	NP_001035758.1	83596	BCL2L12	bcl-2-like protein 12 isoform3	S241	0.8	0.8	1.0	1.0	-	-	-	-	-	-	-	-	-	-	-	-	-	-	-	-	LsSDSFAR	RSKLVRLsSDSFARL	S2;	No	S(2): 99.0; S(3): 1.0; S(5): 0.0	2	2	1	0	N-Term(TMT6plex); S2(Phospho)
71725360	NP_055857.1	23060	ZNF609	zinc finger protein 609	S576	0.9	0.8	1.0	1.0	-	-	-	-	1.1	1.0	1.3	1.0	-	-	-	-	1.1	1.0	1.3	1.0	LVEPHsPSPSSK	VRLVEPHsPSPSSKF	S6;	Yes	S(6): 100.0; S(8): 0.0; S(10): 0.0; S(11): 0.0	6	1	1	0	N-Term(TMT6plex); S6(Phospho); K12(TMT6plex)
5902076	NP_008855.1	6426	SRSF1	serine/arginine-rich splicing factor 1 isoform1	S199	1.1	1.3	1.2	1.0	-	-	-	-	1.4	1.5	1.5	1.1	-	-	-	-	1.4	1.5	1.5	1.1	VDGPRsPSYGR	VKVDGPRsPSYGRSR	S6;	Yes	S(6): 100.0; S(8): 0.0; Y(9): 0.0	6	1	1	1	N-Term(TMT6plex); S6(Phospho)
150378498	NP_056127.2	84162	KIAA1109	uncharacterized protein KIAA1109	S3894	0.9	1.0	1.3	1.0	-	-	-	-	-	-	-	-	-	-	-	-	-	-	-	-	TEsPNASR	SKTLKTEsPNASRGS	S3;	No	T(1): 0.8; S(3): 99.2; S(7): 0.0	2	1	1	0	N-Term(TMT6plex); S3(Phospho)
555290018	NP_001273128.1	22828	SCAF8	protein SCAF8 isoformd	S617	0.5	0.9	1.1	1.0	-	-	-	-	0.5	0.8	1.0	0.9	-	-	-	-	0.5	0.8	1.0	0.9	SSEPVKETVQTTQsPTPVEK	ETVQTTQsPTPVEKE	S14;	Yes	S(1): 0.0; S(2): 0.0; T(8): 0.0; T(11): 1.7; T(12): 1.7; S(14): 94.8; T(16): 1.7	3	4	1	1	N-Term(TMT6plex); K6(TMT6plex); S14(Phospho); K20(TMT6plex)
4504205	NP_001512.1	2976	GTF3C2	general transcription factor 3C polypeptide 2	S893	1.0	1.2	0.8	1.0	-	-	-	-	1.1	1.2	0.9	0.9	-	-	-	-	1.1	1.2	0.9	0.9	AHFNAMFQPSsPTR	NAMFQPSsPTRRPGF	S11;	Yes	S(10): 1.0; S(11): 98.0; T(13): 1.0	2	1	1	0	N-Term(TMT6plex); M6(Oxidation); S11(Phospho)
116292168	NP_115923.2	84626	KRBA1	protein KRBA1 isoform1	S355	1.2	1.3	1.3	1.0	-	-	-	-	-	-	-	-	-	-	-	-	-	-	-	-	QPLsPSATGDTR	QPGRQPLsPSATGDT	S4;	Yes	S(4): 99.9; S(6): 0.0; T(8): 0.0; T(11): 0.0	1	2	1	0	N-Term(TMT6plex); S4(Phospho)
6912674	NP_036569.1	23557	SNAPIN	SNARE-associated protein Snapin	S133	1.1	1.3	1.2	1.0	-	-	-	-	-	-	-	-	-	-	-	-	-	-	-	-	AMLDSGIYPPGsPGK	SGIYPPGsPGK	S12;	Yes	S(5): 0.0; Y(8): 0.0; S(12): 100.0	1	1	1	0	N-Term(TMT6plex); M2(Oxidation); S12(Phospho); K15(TMT6plex)
545746375	NP_001271175.1	26037	SIPA1L1	signal-induced proliferation-associated 1-like protein 1 isoform3	S1712	1.1	0.9	0.9	1.0	-	-	-	-	1.0	0.8	0.9	0.9	-	-	-	-	1.0	0.8	0.9	0.9	DSsPTLASK	PYSSKDSsPTLASKV	S3;	No	S(2): 0.0; S(3): 100.0; T(5): 0.0; S(8): 0.0	7	4	1	0	N-Term(TMT6plex); S3(Phospho); K9(TMT6plex)
8922313	NP_060510.1	55105	GPATCH2	G patch domain-containing protein 2	S115	0.5	1.0	1.4	1.0	-	-	-	-	0.5	1.0	1.5	1.0	-	-	-	-	0.5	1.0	1.5	1.0	DHsDSDDQMLVAK	NNNKKDHsDSDDQML	S3;	Yes	S(3): 98.1; S(5): 1.9	1	1	1	0	N-Term(TMT6plex); S5(Phospho); M9(Oxidation); K13(TMT6plex)
289577096	NP_597713.2	134549	SHROOM1	protein Shroom1 isoform2	S190	1.0	1.2	0.9	1.0	-	-	-	-	1.0	1.1	0.9	0.9	-	-	-	-	1.0	1.1	0.9	0.9	SASLsHPGGEGEPAR	HPRSASLsHPGGEGE	S5;	Yes	S(1): 0.0; S(3): 1.1; S(5): 98.9	2	2	1	0	N-Term(TMT6plex); S3(Phospho)
10047086	NP_061821.1	54206	ERRFI1	ERBB receptor feedback inhibitor 1	S369	0.6	1.0	1.1	1.0	-	-	-	-	-	-	-	-	-	-	-	-	-	-	-	-	QNsEGSASK	KALQRQNsEGSASKV	S3;	Yes	S(3): 98.5; S(6): 0.0; S(8): 1.5	1	1	1	0	N-Term(TMT6plex); S3(Phospho); K9(TMT6plex)
146219843	NP_006653.2	10847	SRCAP	helicase SRCAP	S2725	2.2	1.4	1.1	1.0	-	-	-	-	2.1	1.2	1.0	0.9	-	-	-	-	2.1	1.2	1.0	0.9	TsADVEIR	RPPRRRTsADVEIRG	S2;	Yes	T(1): 0.0; S(2): 100.0	18	1	1	0	N-Term(TMT6plex); S2(Phospho)
554790379	NP_001273057.1	4139	MARK1	serine/threonine-protein kinase MARK1 isoform4	S566	0.4	0.7	0.9	1.0	-	-	-	-	-	-	-	-	-	-	-	-	-	-	-	-	VPAAsPSAHSISTATPDR	TQRVPAAsPSAHSIS	S5;	No	S(5): 98.2; S(7): 1.8; S(10): 0.0; S(12): 0.0; T(13): 0.0; T(15): 0.0	1	4	1	0	N-Term(TMT6plex); S5(Phospho)
355390315	NP_056171.3	23513	SCRIB	protein scribble homolog isoformb	S1232	0.8	1.1	1.5	1.0	-	-	-	-	0.7	1.1	1.4	0.9	-	-	-	-	0.7	1.1	1.4	0.9	ELsPEGPGK	SSIDRELsPEGPGKE	S3;	Yes	S(3): 100.0	16	2	1	0	N-Term(TMT6plex); S3(Phospho); K9(TMT6plex)
21359951	NP_077297.2	79171	RBM42	RNA-binding protein 42	S135	0.8	0.9	1.0	1.0	-	-	-	-	0.8	0.9	1.0	0.9	-	-	-	-	0.8	0.9	1.0	0.9	SHLDsPEAR	GDRSHLDsPEAREAM	S5;	Yes	S(1): 0.0; S(5): 100.0	1	1	1	0	N-Term(TMT6plex); S5(Phospho)
42542379	NP_005830.2	10250	SRRM1	serine/arginine repetitive matrix protein 1	T572; T574	1.1	1.2	1.0	1.0	-	-	-	-	1.3	1.2	1.2	1.1	-	-	-	-	1.3	1.2	1.2	1.1	RtPtPPPR	PPPRRRRtPTPPPRR;PRRRRTPtPPPRRRT	T2; T4;	Yes;Yes	T(2): 100.0; T(4): 100.0	229	1	1	1	N-Term(TMT6plex); T2(Phospho); T4(Phospho)
145386517	NP_597728.1	170954	PPP1R18	phostensin	S224	0.8	1.5	1.1	1.0	-	-	-	-	0.7	1.3	1.0	0.9	-	-	-	-	0.7	1.3	1.0	0.9	LsPGESAYQK	KEVESRLsPGESAYQ	S2;	Yes	S(2): 100.0; S(6): 0.0; Y(8): 0.0	3	1	1	0	N-Term(TMT6plex); S2(Phospho); K10(TMT6plex)
82830440	NP_060663.2	55215	FANCI	Fanconi anemia group I protein isoform2	S409	0.9	1.0	1.1	1.0	-	-	-	-	0.9	1.0	1.0	0.9	-	-	-	-	0.9	1.0	1.0	0.9	TIETSPsLSR	KTIETSPsLSRMPNQ	S7;	No	T(1): 0.0; T(4): 1.0; S(5): 1.0; S(7): 98.0; S(9): 0.0	7	2	1	0	N-Term(TMT6plex); T4(Phospho)
112807226	NP_001036783.1	91748	ELMSAN1	ELM2 and SANT domain-containing protein 1	S923	0.9	0.9	0.9	1.0	-	-	-	-	1.0	0.9	0.9	1.1	-	-	-	-	1.0	0.9	0.9	1.1	REsPSEER	VPLPRREsPSEERLE	S3;	Yes	S(3): 100.0; S(5): 0.0	4	1	1	1	N-Term(TMT6plex); S3(Phospho)
306922386	NP_001120983.2	324	APC	adenomatous polyposis coli protein isoforma	S2431	0.9	1.1	0.8	1.0	-	-	-	-	0.8	1.0	1.0	1.0	-	-	-	-	0.8	1.0	1.0	1.0	EAPsPTLR	TEIKEAPsPTLRRKL	S4;	No	S(4): 100.0; T(6): 0.0	9	2	1	0	N-Term(TMT6plex); S4(Phospho)
167466272	NP_689728.3	150468	CKAP2L	cytoskeleton-associated protein 2-like	T648	0.8	0.9	0.9	1.0	-	-	-	-	0.9	0.9	1.1	1.1	-	-	-	-	0.9	0.9	1.1	1.1	EQVTAtPR	EREQVTAtPRIAKAE	T6;	Yes	T(4): 0.0; T(6): 100.0	1	1	1	0	N-Term(TMT6plex); T6(Phospho)
296010889	NP_001171559.1	8871	SYNJ2	synaptojanin-2 isoform2	S1196	0.9	1.5	1.0	1.0	-	-	-	-	0.8	1.3	0.9	1.0	-	-	-	-	0.8	1.3	0.9	1.0	sSDPLDSGTR	NNTWLSKsSDPLDSG	S1;	No	S(1): 100.0; S(2): 0.0; S(7): 0.0; T(9): 0.0	1	2	1	0	N-Term(TMT6plex); S1(Phospho)
54607077	NP_075392.2	65124	SOWAHC	ankyrin repeat domain-containing protein SOWAHC	S213	1.1	0.8	0.8	1.0	-	-	-	-	1.2	0.8	0.8	0.9	-	-	-	-	1.2	0.8	0.8	0.9	DLVMGSsPQLK	RDLVMGSsPQLKRSV	S7;	Yes	S(6): 0.8; S(7): 99.2	1	1	1	0	N-Term(TMT6plex); M4(Oxidation); S7(Phospho); K11(TMT6plex)
108773787	NP_000312.2	5925	RB1	retinoblastoma-associated protein	S37	0.9	0.8	0.5	1.0	-	-	-	-	1.0	0.8	0.5	0.9	-	-	-	-	1.0	0.8	0.5	0.9	TAATAAAAAAEPPAPPPPPPPEEDPEQDsGPEDLPLVR	EEDPEQDsGPEDLPL	S29;	Yes	T(1): 0.0; T(4): 0.0; S(29): 100.0	4	1	1	0	N-Term(TMT6plex); S29(Phospho)

Khan et al., 2019. Multi-omics analysis to characterize cigarette smoke induced molecular alterations in esophageal cells
Supplementary Table 8. List of phosphopeptides identified and quantified in untreated and chronically treated Het1A cells with cigarette smoke condensate for 8 months using SequestHT and Mascot search algorithms with a PhosphoRS probability score ≥75%

Protein group Accession	NP_Accession	Gene ID	Gene Symbol	Protein Description	PhosphoSite (Protein)	Het-1A-Smoke - 2M/Parental	Het-1A-Smoke - 4M/Parental	Het-1A-Smoke - 6M/Parental	Het-1A-Smoke - 8M/Parental	Het-1A-Smoke - 2M/Parental	Het-1A-Smoke - 4M/Parental	Het-1A-Smoke - 6M/Parental	Het-1A-Smoke - 8M/Parental	Het-1A-Smoke - 2M/Parental	Het-1A-Smoke - 4M/Parental	Het-1A-Smoke - 6M/Parental	Het-1A-Smoke - 8M/Parental	Het-1A-Smoke - 2M/Parental	Het-1A-Smoke - 4M/Parental	Het-1A-Smoke - 6M/Parental	Het-1A-Smoke - 8M/Parental	Het-1A-Smoke - 2M/Parental	Het-1A-Smoke - 4M/Parental	Het-1A-Smoke - 6M/Parental	Het-1A-Smoke - 8M/Parental	Peptide Sequence	Phosphowindow	PhosphoSite (Peptide)	Phosphosite Plus Evidence	phosphoRS Site Probabilities	# PSMs	# Proteins	# Protein Groups	# Missed Cleavages	Modifications
														Normalized phosphoprotein ratios with respect to corresponding total protein ratios.								Average of replicates													
						Replicate 1				Replicate 2				Replicate 1				Replicate 2																	
148613886	NP_073752.5	64864	RFX7	DNA-binding protein RFX7	S418	2.4	1.4	1.1	1.0	-	-	-	-	-	-	-	-	-	-	-	-	-	-	-	-	TPQNVPAsPGGDR	TPQNVPAsPGGDRSA	S8;	No	T(1): 0.0; S(8): 100.0	3	1	1	0	N-Term(TMT6plex); S8(Phospho)
154744870	NP_073589.4	64763	ZNF574	zinc finger protein 574	S717; S728	1.2	1.2	1.0	1.0	-	-	-	-	1.1	1.1	0.9	1.0	-	-	-	-	1.1	1.1	0.9	1.0	APVAsPAALGSTATAsPAAPAR	ATRAPVAsPAALGST;LGSTATAsPAAPARR	S5; S16;	Yes;Yes	S(5): 100.0; S(11): 0.0; T(12): 0.0; T(14): 0.0; S(16): 100.0	6	1	1	0	N-Term(TMT6plex); S5(Phospho); S16(Phospho)
10800140	NP_066406.1	3018	HIST1H2BB	histone H2B type 1-B	S113	0.9	1.1	0.8	1.0	-	-	-	-	-	-	-	-	-	-	-	-	-	-	-	-	HAVsEGTK	ELAKHAVsEGTKAVT	S4;	Yes	S(4): 100.0; T(7): 0.0	17	17	1	0	N-Term(TMT6plex); S4(Phospho); K8(TMT6plex)
21361272	NP_005925.2	4298	MLLT1	protein ENL	S292	0.7	0.8	0.8	1.0	-	-	-	-	0.7	0.7	0.8	0.9	-	-	-	-	0.7	0.7	0.8	0.9	RPATADsPKPSAK	KRPATADsPKPSAKK	S7;	Yes	T(4): 0.0; S(7): 100.0; S(11): 0.0	1	1	1	0	N-Term(TMT6plex); S7(Phospho); K9(TMT6plex); K13(TMT6plex)
24308075	NP_056285.1	26056	RAB11FIP5	rab11 family-interacting protein 5	S307	1.1	0.8	0.8	1.0	-	-	-	-	1.2	0.9	0.9	1.0	-	-	-	-	1.2	0.9	0.9	1.0	TYsDEANQMR	FTHKRTYsDEANQMR	S3;	Yes	T(1): 0.0; Y(2): 0.6; S(3): 99.4	5	1	1	0	N-Term(TMT6plex); S3(Phospho); M9(Oxidation)
84043963	NP_056988.3	9669	EIF5B	eukaryotic translation initiation factor 5B	S113	0.9	0.7	0.6	1.0	-	-	-	-	0.9	0.7	0.7	1.0	-	-	-	-	0.9	0.7	0.7	1.0	QSFDDNDsEELEDK	QSFDDNDsEELEDKD	S8;	Yes	S(2): 0.0; S(8): 100.0	13	1	1	0	N-Term(TMT6plex); S8(Phospho); K14(TMT6plex)
21361851	NP_065128.2	57117	INTS12	integrator complex subunit 12	S378	0.8	1.0	1.1	1.0	-	-	-	-	0.7	0.9	0.9	0.9	-	-	-	-	0.7	0.9	0.9	0.9	SVsCDNVSK	TGLSRSVsCDNVSKV	S3;	Yes	S(1): 0.0; S(3): 100.0; S(8): 0.0	2	1	1	0	N-Term(TMT6plex); S3(Phospho); C4(Carbamidomethyl); K9(TMT6plex)
209862987	NP_569830.2	8567	MADD	MAP kinase-activating death domain protein isoform f	S996	1.0	1.3	1.1	1.0	-	-	-	-	1.0	1.3	1.1	0.8	-	-	-	-	1.0	1.3	1.1	0.8	sPTESVNTPVGK	EPDKRKRsPTESVNT	S1;	No	S(1): 99.7; T(3): 0.1; S(5): 0.1; T(8):	4	10	1	0	N-Term(TMT6plex); S1(Phospho); K12(TMT6plex)
289547526	NP_079427.4	80223	RAB11FIP1	rab11 family-interacting protein 1 isoform 1	S280	1.0	0.7	0.7	1.0	-	-	-	-	1.0	0.7	0.7	1.0	-	-	-	-	1.0	0.7	0.7	1.0	TAsTDLK	MSHKRTAsTDLKQLN	S3;	Yes	T(1): 0.0; S(3): 100.0; T(4): 0.0	2	2	1	0	N-Term(TMT6plex); S3(Phospho); K7(TMT6plex)
291045268	NP_001166984.1	114112	TXNRD3	thioredoxin reductase 3 isoform 2	S4	0.7	0.7	0.7	1.0	-	-	-	-	-	-	-	-	-	-	-	-	-	-	-	-	sPPQSPGPGK	MERsPPQSPGP	S1;	No	S(1): 100.0; S(5): 0.0	2	2	1	0	N-Term(TMT6plex); S1(Phospho); K10(TMT6plex)
260763912	NP_001159489.1	221150	SKA3	spindle and kinetochore-associated protein 3 isoform 2	S155	0.9	2.0	2.6	1.0	-	-	-	-	1.2	2.2	2.5	0.9	-	-	-	-	1.2	2.2	2.5	0.9	sPQLSDFGLER	ISEKSPRsPQLSDFG	S1;	Yes	S(1): 100.0; S(5): 0.0	1	2	1	0	N-Term(TMT6plex); S1(Phospho)
33286446	NP_031372.2	11054	OGFR	opioid growth factor receptor	S378	0.9	1.1	0.9	1.0	-	-	-	-	1.0	1.2	1.0	1.0	-	-	-	-	1.0	1.2	1.0	1.0	SQGDEAGGHGEDRPEPLsPK	EDRPEPLsPKESKKR	S18;	Yes	S(1): 0.0; S(18): 100.0	8	1	1	0	N-Term(TMT6plex); S18(Phospho); K20(TMT6plex)
557440810	NP_001273512.1	57661	PHRF1	PHD and RING finger domain-containing protein 1 isoform 4	S1198	1.9	1.9	2.0	1.0	-	-	-	-	1.9	2.0	2.2	1.2	-	-	-	-	1.9	2.0	2.2	1.2	EAsPAPLAQGEPGREDLPTR	KGAVREAsPAPLAQG	S3;	No	S(3): 100.0; T(19): 0.0	15	4	1	1	N-Term(TMT6plex); S3(Phospho)
51599156	NP_001264.2	1108	CHD4	chromodomain-helicase-DNA-binding protein 4	S1531; S1535	1.1	1.1	1.1	1.0	-	-	-	-	1.2	1.1	1.2	1.0	-	-	-	-	1.2	1.1	1.2	1.0	KMsQPGsPSPK	VEENKKMsQPGSPSP;KKMSQPGsPSPKTPT	S3; S7;	Yes;Yes	S(3): 100.0; S(7): 97.6; S(9): 2.4	28	1	1	1	K1(TMT6plex); N-Term(TMT6plex); M2(Oxidation); S3(Phospho); S7(Phospho); K11(TMT6plex)
51173735	NP_001003698.1	6239	RREB1	ras-responsive element-binding protein 1 isoform 2	S1475	0.9	0.9	0.7	1.0	-	-	-	-	1.0	1.0	0.8	0.9	-	-	-	-	1.0	1.0	0.8	0.9	LAEETEGPsDGESAAEK	AEETEGPsDGESAAE	S9;	Yes	T(5): 0.0; S(9): 98.5; S(13): 1.4	10	2	1	0	N-Term(TMT6plex); S9(Phospho); K17(TMT6plex)
54792098	NP_001005862.1	2064	ERBB2	receptor tyrosine-protein kinase erbB-2 isoform b	S1048; S1053	0.9	0.8	1.7	1.0	-	-	-	-	-	-	-	-	-	-	-	-	-	-	-	-	SPLAPsEGAGsDVFDGDLGMGAAK	PRSPLAPsEGAGSDV;APSEGAGsDVFDGDL	S6; S11;	No;No	S(1): 0.7; S(6): 99.7; S(11): 99.7	1	3	1	0	N-Term(TMT6plex); S6(Phospho); S11(Phospho); M20(Oxidation); K24(TMT6plex)
341604748	NP_001230056.1	23241	PACS2	phosphofurin acidic cluster sorting protein 2 isoform 3	S378	0.7	0.7	0.8	1.0	-	-	-	-	-	-	-	-	-	-	-	-	-	-	-	-	ANsLDNER	PQNERANsLDNERCP	S3;	No	S(3): 100.0	1	3	1	0	N-Term(TMT6plex); S3(Phospho)
61743954	NP_001611.1	79026	AHNAK	neuroblast differentiation-associated protein AHNAK isoform 1	S5782	0.9	1.1	1.4	0.9	-	-	-	-	1.1	1.1	1.4	1.1	-	-	-	-	1.1	1.1	1.4	1.1	SNsFSDER	KPRHRSNsFSDEREF	S3;	Yes	S(1): 0.0; S(3): 100.0; S(5): 0.0	79	1	1	0	N-Term(TMT6plex); S3(Phospho)
255652953	NP_653205.3	124245	ZC3H18	zinc finger CCCH domain-containing protein 18	S893	0.8	1.0	1.0	0.9	-	-	-	-	1.0	1.1	1.2	1.0	-	-	-	-	1.0	1.1	1.2	1.0	QLsPQSK	ADRKRQLsPQSKSSS	S3;	Yes	S(3): 100.0; S(6): 0.0	34	1	1	0	N-Term(TMT6plex); S3(Phospho); K7(TMT6plex)
4757926	NP_004893.1	9584	RBM39	RNA-binding protein 39 isoform b	S136	0.8	0.8	1.0	0.9	-	-	-	-	0.9	0.9	1.2	0.9	-	-	-	-	0.9	0.9	1.2	0.9	DKsPVREPIDNLTPEER	SPERKDKsPVREPID	S3;	Yes	S(3): 100.0; T(13): 0.0	4	4	1	2	N-Term(TMT6plex); K2(TMT6plex); S3(Phospho)
103472005	NP_002408.3	4288	MKI67	antigen KI-67 isoform 1	S1375; S1376	0.9	0.8	0.5	0.9	-	-	-	-	1.0	0.8	0.5	1.0	-	-	-	-	1.0	0.8	0.5	1.0	MPCEssPPESADTPTSTR	TTKMPCEsSPPESAD;TKMPCESsPPESADT	S5; S6;	Yes;Yes	S(5): 94.4; S(6): 94.4; S(10): 2.9; T(13): 7.7; T(15): 0.3; S(16): 0.3;	118	2	1	0	M1(Oxidation); N-Term(TMT6plex); C3(Carbamidomethyl); S5(Phospho); S6(Phospho)
192807336	NP_001122326.1	51593	SRRT	serrate RNA effector molecule homolog isoform e	S492	0.7	0.8	0.8	0.9	-	-	-	-	0.8	0.8	0.9	1.0	-	-	-	-	0.8	0.8	0.9	1.0	ECELsPGVNR	RLRECELsPGVNRDL	S5;	No	S(5): 100.0	11	4	1	0	N-Term(TMT6plex); C2(Carbamidomethyl); S5(Phospho)
42741682	NP_005446.2	9406	ZRANB2	zinc finger Ran-binding domain-containing protein 2 isoform 2	S305	0.8	1.1	1.1	0.9	-	-	-	-	0.9	1.1	1.3	1.2	-	-	-	-	0.9	1.1	1.3	1.2	sRSPESQVIGENTK	RKKRRTRsRSPESQV	S1;	Yes	S(1): 99.0; S(3): 1.0; S(6): 0.0; T(13): 0.0	17	1	1	1	N-Term(TMT6plex); S1(Phospho); K14(TMT6plex)
557440899	NP_001273490.1	4926	NUMA1	nuclear mitotic apparatus protein 1 isoform 2	T2092	1.1	1.2	1.0	0.9	-	-	-	-	1.3	1.4	1.1	1.0	-	-	-	-	1.3	1.4	1.1	1.0	IATTTASAATAAAIGAtPR	TAAAIGAtPRAKGKA	T17;	No	T(3): 0.0; T(4): 0.0; T(5): 0.0; S(7): 0.0; T(10): 0.0; T(17): 100.0	34	2	1	0	N-Term(TMT6plex); T17(Phospho)
5032179	NP_005753.1	10155	TRIM28	transcription intermediary factor 1-beta	S697	0.9	1.0	0.9	0.9	-	-	-	-	0.9	1.0	0.9	0.9	-	-	-	-	0.9	1.0	0.9	0.9	LsPANQR	TGVVAKLsPANQRKC	S2;	Yes	S(2): 100.0	10	1	1	0	N-Term(TMT6plex); S2(Phospho)
256818821	NP_064550.3	56852	RAD18	E3 ubiquitin-protein ligase RAD18	T118	0.7	0.7	1.0	0.9	-	-	-	-	0.5	0.6	0.7	0.7	-	-	-	-	0.5	0.6	0.7	0.7	VYtPVASR	NLAVKVYtPVASRQS	T3;	Yes	Y(2): 0.5; T(3): 99.5; S(7): 0.0	8	1	1	0	N-Term(TMT6plex); T3(Phospho)
148539642	NP_060228.3	54890	ALKBH5	RNA demethylase ALKBH5	S384	1.1	0.8	0.7	0.9	-	-	-	-	1.3	0.9	0.7	1.1	-	-	-	-	1.3	0.9	0.7	1.1	SYESSEDCSEAAGsPAR	DCSEAAGsPARKVKM	S14;	Yes	S(1): 0.0; Y(2): 0.0; S(4): 0.0; S(5): 0.0; S(9): 0.0; S(14): 100.0	2	1	1	0	N-Term(TMT6plex); C8(Carbamidomethyl); S14(Phospho)
118600973	NP_057108.2	51634	RBMX2	RNA-binding motif protein, X-linked 2	S187	0.9	0.9	1.0	0.9	-	-	-	-	1.1	1.2	1.3	1.2	-	-	-	-	1.1	1.2	1.3	1.2	EVQAEQPSSsSPR	QAEQPSSsSPRRKTV	S10;	No	S(8): 0.0; S(9): 0.7; S(10): 99.3; S(11): 0.0	5	1	1	0	N-Term(TMT6plex); S10(Phospho)
414144878	NP_001258670.1	1452	CSNK1A1	casein kinase I isoform alpha isoform 3	T321	0.8	1.0	0.8	0.9	-	-	-	-	0.9	1.1	0.7	0.9	-	-	-	-	0.9	1.1	0.7	0.9	AAQQAASSSGQGQQAQtPTGF	GQGQQAQtPTGF_	T17;	Yes	S(7): 0.0; S(8): 0.0; S(9): 0.0; T(17): 98.1; T(19): 1.9	3	1	1	0	N-Term(TMT6plex); T17(Phospho)
71774197	NP_060414.3	55031	USP47	ubiquitin carboxyl-terminal hydrolase 47 isoform b	S858	1.5	1.2	1.4	0.9	-	-	-	-	1.4	1.1	1.3	0.9	-	-	-	-	1.4	1.1	1.3	0.9	SDsDVNNDR	QSEERSDsDVNNDRS	S3;	No	S(1): 0.0; S(3): 100.0	6	2	1	0	N-Term(TMT6plex); S3(Phospho)
344313201	NP_001230704.1	51474	LIMA1	LIM domain and actin-binding protein 1 isoform 4	S396	1.2	1.3	1.6	0.9	-	-	-	-	1.1	1.2	1.3	0.9	-	-	-	-	1.1	1.2	1.3	0.9	QQsPQEPK	NSELKQQsPQEPKSL	S3;	No	S(3): 100.0	5	4	1	0	N-Term(TMT6plex); S3(Phospho); K8(TMT6plex)
24234690	NP_005581.2	4361	MRE11A	double-strand break repair protein MRE11A isoform 2	S660; S661	1.0	0.8	1.0	0.9	-	-	-	-	1.0	0.7	0.9	0.9	-	-	-	-	1.0	0.7	0.9	0.9	GVDFEssEDDDDDPFMNTSSLR	SKGVDFEsSEDDDDD;KGVDFESsEDDDDDP	S6; S7;	No;No	S(6): 99.9; S(7): 99.9; T(18): 0.1; S(19): 0.1; S(20): 0.1	3	2	1	0	N-Term(TMT6plex); S6(Phospho); S7(Phospho); M16(Oxidation)
148536864	NP_001091896.1	6603	SMARCD2	SWI/SNF-related matrix-associated actin-dependent regulator of chromatin subfamily D member 2	T221	1.1	0.9	1.0	0.9	-	-	-	-	1.2	1.0	1.1	1.0	-	-	-	-	1.2	1.0	1.1	1.0	AEGDSAGTAGTPGGtPAGDK	TAGTPGGtPAGDKVA	T15;	Yes	S(5): 0.0; T(8): 0.0; T(11): 1.8; T(15): 98.2	1	1	1	0	N-Term(TMT6plex); T15(Phospho); K20(TMT6plex)
118402582	NP_001246.2	991	CDC20	cell division cycle protein 20 homolog	T157	0.6	0.8	0.7	0.9	-	-	-	-	0.7	0.8	0.8	1.0	-	-	-	-	0.7	0.8	0.8	1.0	AtPGSSR	VLYSQKAtPGSSRKT	T2;	Yes	T(2): 100.0; S(5): 0.0; S(6): 0.0	11	1	1	0	N-Term(TMT6plex); T2(Phospho)
46877102	NP_996888.1	7391	USF1	upstream stimulatory factor 1 isoform 2	S127	0.5	0.4	0.7	0.9	-	-	-	-	0.5	0.4	0.8	0.9	-	-	-	-	0.5	0.4	0.8	0.9	THPYsPK	APRTHPYsPKSEAPR	S5;	No	T(1): 0.0; Y(4): 0.0; S(5): 100.0	1	4	1	0	N-Term(TMT6plex); S5(Phospho); K7(TMT6plex)
4503179	NP_003602.1	8481	OFD1	oral-facial-digital syndrome 1 protein	S774	1.0	0.9	1.3	0.9	-	-	-	-	1.0	0.9	1.2	0.8	-	-	-	-	1.0	0.9	1.2	0.8	MPLPsPTESR	PDRMPLPsPTESRHS	S5;	Yes	S(5): 100.0; T(7): 0.0; S(9): 0.0	1	1	1	0	N-Term(TMT6plex); M1(Oxidation); S5(Phospho)
545478902	NP_001269892.1	55355	HJURP	Holliday junction recognition protein isoform c	S363	0.7	0.7	0.7	0.9	-	-	-	-	0.7	0.7	0.7	0.9	-	-	-	-	0.7	0.7	0.7	0.9	EYCLsPR	LHREYCLsPRNQPRR	S5;	No	Y(2): 0.0; S(5): 100.0	5	3	1	0	N-Term(TMT6plex); C3(Carbamidomethyl); S5(Phospho)
192807336	NP_001122326.1	51593	SRRT	serrate RNA effector molecule homolog isoform e	S319	1.4	1.3	1.3	0.9	-	-	-	-	1.5	1.4	1.4	1.0	-	-	-	-	1.5	1.4	1.4	1.0	QAENDsSNDDKTK	GKQAENDsSNDDKTK	S6;	Yes	S(6): 100.0; S(7): 0.0; T(12): 0.0	11	4	1	1	N-Term(TMT6plex); S6(Phospho); K11(TMT6plex); K13(TMT6plex)
151301096	NP_079107.6	96764	TGS1	trimethylguanosine synthase	S89	0.4	0.8	0.7	0.9	-	-	-	-	0.4	0.8	0.7	0.8	-	-	-	-	0.4	0.8	0.7	0.8	GIGLDESELDsEAELMR	LDESELDsEAELMRS	S11;	Yes	S(7): 0.0; S(11): 100.0	1	1	1	0	N-Term(TMT6plex); S11(Phospho); M16(Oxidation)
66346704	NP_001018099.1	79664	NARG2	NMDA receptor-regulated protein 2 isoform b	S279	1.3	1.2	1.2	0.9	-	-	-	-	-	-	-	-	-	-	-	-	-	-	-	-	sPSPASTSTVPNMTDAPTAPK	TTTKVSKsPSPASTS	S1;	No	S(1): 97.7; S(3): 2.3; S(6): 0.0; T(7): 0.0; S(8): 0.0; T(9): 0.0; T(14): 0.0; T(18): 0.0	1	2	1	0	N-Term(TMT6plex); S1(Phospho); M13(Oxidation); K21(TMT6plex)
217330641	NP_060439.2	55677	IWS1	protein IWS1 homolog	S438	0.7	0.7	0.6	0.9	-	-	-	-	0.8	0.7	0.6	1.1	-	-	-	-	0.8	0.7	0.6	1.1	TIAsDSEEEAGKELSDK	KREKTIAsDSEEEAG	S4;	Yes	T(1): 50.6; S(4): 98.8; S(6): 50.6; S(15): 0.0	25	1	1	1	N-Term(TMT6plex); S4(Phospho); S6(Phospho); K12(TMT6plex); K17(TMT6plex)
7661958	NP_055554.1	9774	BCLAF1	bcl-2-associated transcription factor 1 isoform 1	S564	0.7	0.9	1.2	0.9	-	-	-	-	0.8	1.0	1.3	1.0	-	-	-	-	0.8	1.0	1.3	1.0	MAPVPLDDSNRPAsLTK	DDSNRPAsLTKDRLL	S14;	Yes	S(9): 1.4; S(14): 97.2; T(16): 1.4	47	3	1	0	N-Term(TMT6plex); M1(Oxidation); S14(Phospho); K17(TMT6plex)
31563507	NP_852118.1	9648	GCC2	GRIP and coiled-coil domain-containing protein 2	S1483	0.8	1.0	0.7	0.9	-	-	-	-	0.8	1.0	0.8	1.1	-	-	-	-	0.8	1.0	0.8	1.1	sPVSSQQSLK	KNEPTTRsPVSSQQS	S1;	Yes	S(1): 100.0; S(4): 0.0; S(5): 0.0; S(8): 0.0	2	1	1	0	N-Term(TMT6plex); S1(Phospho); K10(TMT6plex)
47578107	NP_056199.2	25836	NIPBL	nipped-B-like protein isoform B	T713	1.1	1.0	1.2	0.9	-	-	-	-	1.0	0.9	1.1	0.8	-	-	-	-	1.0	0.9	1.1	0.8	GESRPEtPK	KGESRPEtPKQKSDG	T7;	Yes	S(3): 0.0; T(7): 100.0	8	2	1	0	N-Term(TMT6plex); T7(Phospho); K9(TMT6plex)
256600202	NP_001157861.1	51735	RAPGEF6	rap guanine nucleotide exchange factor 6 isoform 5	S1157	1.2	1.2	1.1	0.9	-	-	-	-	1.2	1.3	1.1	1.0	-	-	-	-	1.2	1.3	1.1	1.0	SSEMsPVPMR	SAKSSEMsPVPMRSA	S5;	Yes	S(1): 0.0; S(2): 0.0; S(5): 100.0	3	5	1	0	N-Term(TMT6plex); M4(Oxidation); S5(Phospho); M9(Oxidation)
48255945	NP_001673.2	490	ATP2B1	plasma membrane calcium-transporting ATPase 1 isoform 1b	T1218	2.1	1.5	1.4	0.9	-	-	-	-	1.8	1.4	1.3	0.9	-	-	-	-	1.8	1.4	1.3	0.9	SATSSSPGSPLHSLEtSL	SPLHSLEtSL_	T16;	No	S(1): 0.0; T(3): 0.0; S(4): 0.0; S(5): 0.0; S(6): 0.0; S(9): 0.0; S(13): 1.8; T(16): 96.4; S(17): 1.8	3	1	1	0	N-Term(TMT6plex); S13(Phospho)
4758556	NP_004689.1	9129	PRPF3	U4/U6 small nuclear ribonucleoprotein Prp3	S619	0.8	0.9	1.0	0.9	-	-	-	-	0.8	1.0	1.1	1.0	-	-	-	-	0.8	1.0	1.1	1.0	GDDDEEsDEEAVKK	KGDDDEEsDEEAVKK	S7;	Yes	S(7): 100.0	1	1	1	1	N-Term(TMT6plex); S7(Phospho); K13(TMT6plex); K14(TMT6plex)
89276756	NP_003904.3	8899	PRPF4B	serine/threonine-protein kinase PRP4 homolog	S257	1.3	1.9	1.4	0.9	-	-	-	-	1.6	2.1	1.6	1.0	-	-	-	-	1.6	2.1	1.6	1.0	ARsPTDDK	EKIGKARsPTDDKVK	S3;	Yes	S(3): 100.0; T(5): 0.0	4	1	1	1	N-Term(TMT6plex); S3(Phospho); K8(TMT6plex)
103472005	NP_002408.3	4288	MKI67	antigen KI-67 isoform 1	T1176	0.8	1.0	1.0	0.9	-	-	-	-	1.0	1.0	1.1	1.0	-	-	-	-	1.0	1.0	1.1	1.0	AMLtPKPAGGDEK	SAGKAMLtPKPAGGD	T4;	Yes	T(4): 100.0	118	2	1	0	N-Term(TMT6plex); M2(Oxidation); T4(Phospho); K6(TMT6plex); K13(TMT6plex)
75677325	NP_055053.1	8844	KSR1	kinase suppressor of Ras 1	S269	1.2	1.2	1.1	0.9	-	-	-	-	-	-	-	-	-	-	-	-	-	-	-	-	RTEsVPSDINNPVDR	TRLRRTEsVPSDINN	S4;	No	T(2): 0.0; S(4): 100.0; S(7): 0.0	1	1	1	1	N-Term(TMT6plex); S4(Phospho)
88759348	NP_078822.3	79596	RNF219	RING finger protein 219	S210	0.9	0.8	0.8	0.9	-	-	-	-	0.8	0.7	0.7	0.9	-	-	-	-	0.8	0.7	0.7	0.9	AEVDNRsPQK	KAEVDNRsPQKFGRF	S7;	Yes	S(7): 100.0	4	1	1	1	N-Term(TMT6plex); S7(Phospho); K10(TMT6plex)
85838513	NP_705902.2	80025	PANK2	pantothenate kinase 2, mitochondrial isoform 1 preproprotein	S189	1.4	1.2	1.2	0.9	-	-	-	-	1.2	1.2	1.3	0.9	-	-	-	-	1.2	1.2	1.3	0.9	LGsYSGPTSVSR	TRRDRLGsYSGPTSV	S3;	Yes	S(3): 100.0; Y(4): 0.0; S(5): 0.0; T(8): 0.0; S(9): 0.0; S(11): 0.0	3	1	1	0	N-Term(TMT6plex); S3(Phospho)
112382226	NP_001036147.1	473	RERE	arginine-glutamic acid dipeptide repeats protein	S40	1.0	0.9	0.9	0.9	-	-	-	-	-	-	-	-	-	-	-	-	-	-	-	-	QPAsPDGRTSPINEDIR	GRKQPAsPDGRTSP	S4;	No	S(4): 100.0; T(9): 50.0; S(10): 50.0	9	2	1	1	N-Term(TMT6plex); S4(Phospho); S10(Phospho)
42542379	NP_005830.2	10250	SRRM1	serine/arginine repetitive matrix protein 1	T572; T574	1.2	1.1	0.9	0.9	-	-	-	-	1.4	1.2	1.0	1.0	-	-	-	-	1.4	1.2	1.0	1.0	tPtPPPR	PPPRRRRtPTPPPRR;PRRRRTPtPPPRRRT	T1; T3;	Yes;Yes	T(1): 100.0; T(3): 100.0	229	1	1	0	N-Term(TMT6plex); T1(Phospho); T3(Phospho)
44681484	NP_689775.2	157313	CDCA2	cell division cycle-associated protein 2	S756	0.5	0.9	1.1	0.9	-	-	-	-	0.6	1.0	1.1	1.0	-	-	-	-	0.6	1.0	1.1	1.0	IsPDLNIK	LCQFFKIsPDLNIKC	S2;	Yes	S(2): 100.0	5	1	1	0	N-Term(TMT6plex); S2(Phospho); K8(TMT6plex)
120587019	NP_055160.2	24149	ZNF318	zinc finger protein 318	S69; S71	1.1	1.0	0.8	0.9	-	-	-	-	1.1	1.0	0.9	1.0	-	-	-	-	1.1	1.0	0.9	1.0	RAsPsPPR	GHRGRRAsPSPPRGR;RGRRASPsPPRGRRV	S3; S5;	Yes;Yes	S(3): 100.0; S(5): 100.0	9	1	1	1	N-Term(TMT6plex); S3(Phospho); S5(Phospho)
257471001	NP_001158088.1	8323	FZD6	frizzled-6 isoform b	T575	0.7	0.8	0.7	0.9	-	-	-	-	0.7	0.8	0.8	1.0	-	-	-	-	0.7	0.8	0.8	1.0	ADGAStPR	VKADGAStPRLREQD	T6;	No	S(5): 0.0; T(6): 100.0	1	2	1	0	N-Term(TMT6plex); T6(Phospho)
110556636	NP_203754.2	85456	TNKS1BP1	182 kDa tankyrase-1-binding protein	S936	0.8	0.8	0.9	0.9	-	-	-	-	0.7	0.7	0.8	1.0	-	-	-	-	0.7	0.7	0.8	1.0	DVsLGTYGSR	EFQKRDVsLGTYGSR	S3;	Yes	S(3): 100.0; T(6): 0.0; Y(7): 0.0; S(9): 0.0	41	1	1	0	N-Term(TMT6plex); S3(Phospho)
52630429	NP_005375.2	4783	NFIL3	nuclear factor interleukin-3-regulated protein	S353	1.4	1.4	1.0	0.9	-	-	-	-	1.4	1.3	1.1	0.9	-	-	-	-	1.4	1.3	1.1	0.9	LSsPIDMTSK	EATQKLSsPIDMTSK	S3;	Yes	S(2): 1.4; S(3): 98.6; T(8): 0.0; S(9):	3	1	1	0	N-Term(TMT6plex); S3(Phospho); M7(Oxidation); K10(TMT6plex)
22027648	NP_663731.1	23175	LPIN1	phosphatidate phosphatase LPIN1 isoform 1	S449	0.8	0.7	1.2	0.9	-	-	-	-	0.7	0.6	1.1	0.9	-	-	-	-	0.7	0.6	1.1	0.9	SANQSPQSVGSSGVDsGVESTSDGLR	VGSSGVDsGVESTSD	S16;	Yes	S(1): 0.0; S(5): 1.8; S(8): 1.8; S(11): 1.8; S(12): 1.8; S(16): 89.2; S(20): 0.0; T(21): 1.8; S(22): 1.8	3	3	1	0	N-Term(TMT6plex); S16(Phospho)
30089916	NP_060496.2	55690	PACS1	phosphofurin acidic cluster sorting protein 1	T428	1.2	1.3	1.1	0.9	-	-	-	-	1.3	1.4	1.1	0.9	-	-	-	-	1.3	1.4	1.1	0.9	DtTSPMELAALEK	KGSLGKDtTSPMELA	T2;	No	T(2): 97.7; T(3): 1.2; S(4): 1.2	3	1	1	0	N-Term(TMT6plex); T2(Phospho); M6(Oxidation); K13(TMT6plex)
63252863	NP_115820.2	84464	SLX4	structure-specific endonuclease subunit SLX4	S467	0.8	2.7	1.1	0.9	-	-	-	-	0.7	2.4	1.0	0.8	-	-	-	-	0.7	2.4	1.0	0.8	KPPVsPPLLLVQDSETTGR	RKKKPPVsPPLLLVQ	S5;	Yes	S(5): 100.0; S(14): 0.0; T(16): 0.0; T(17): 0.0	8	1	1	0	N-Term(TMT6plex); K1(TMT6plex); S5(Phospho)
306922386	NP_001120983.2	324	APC	adenomatous polyposis coli protein isoform a	T2251	0.9	1.1	1.0	0.9	-	-	-	-	0.8	1.0	1.2	0.9	-	-	-	-	0.8	1.0	1.2	0.9	SPSEGQTATtSPR	SEGQTATtSPRGAKP	T10;	No	S(1): 0.0; S(3): 0.0; T(7): 0.0; T(9): 0.8; T(10): 98.4; S(11): 0.8	9	2	1	0	N-Term(TMT6plex); S11(Phospho)
91208437	NP_057657.2	51291	GMIP	GEM-interacting protein isoform 1	S437	1.1	0.8	0.9	0.9	-	-	-	-	0.8	0.8	0.8	0.8	-	-	-	-	0.8	0.8	0.8	0.8	SLDsPTSSPGAGTR	SESRSLDsPTSSPGA	S4;	Yes	S(1): 0.0; S(4): 100.0; T(6): 0.0; S(7): 0.0; S(8): 0.0; T(13): 0.0	2	1	1	0	N-Term(TMT6plex); S4(Phospho)
45439355	NP_958845.1	6917	TCEA1	transcription elongation factor A protein 1 isoform 2	S79	0.9	1.3	1.0	0.9	-	-	-	-	0.9	1.3	1.0	0.9	-	-	-	-	0.9	1.3	1.0	0.9	KKEPAITSQNsPEAR	PAITSQNsPEAREES	S11;	No	T(7): 0.0; S(8): 0.0; S(11): 100.0	10	2	1	2	N-Term(TMT6plex); K1(TMT6plex); K2(TMT6plex); S11(Phospho)
374081840	NP_001243349.1	10982	MAPRE2	microtubule-associated protein RP/EB family member 2 isoform 4	S176	0.8	0.7	1.0	0.9	-	-	-	-	1.1	1.0	1.4	1.0	-	-	-	-	1.1	1.0	1.4	1.0	ASsSGSASK	SSAKRASsSGSASKS	S3;	No	S(2): 0.0; S(3): 100.0; S(4): 0.0; S(6): 0.0; S(8): 0.0	1	4	1	0	N-Term(TMT6plex); S3(Phospho); K9(TMT6plex)
224451135	NP_001138917.1	57466	SCAF4	splicing factor, arginine/serine-rich 15 isoform 3	T236	0.4	0.6	0.9	0.9	-	-	-	-	0.4	0.7	0.9	0.8	-	-	-	-	0.4	0.7	0.9	0.8	tTPTQPSEQK	AITAQLKtTPTQPSE	T1;	No	T(1): 99.0; T(2): 1.0; T(4): 0.0; S(7):	4	3	1	0	N-Term(TMT6plex); T4(Phospho); K10(TMT6plex)
410442518	NP_001258547.1	54776	PPP1R12C	protein phosphatase 1 regulatory subunit 12C isoform b	S508	1.9	1.4	1.2	0.9	-	-	-	-	-	-	-	-	-	-	-	-	-	-	-	-	IPEPEsPAKPNVPTASTAPPADSR	SRIPEPEsPAKPNVP	S6;	No	S(6): 100.0; T(14): 0.0; S(16): 0.0; T(17): 0.0; S(23): 0.0	3	2	1	0	N-Term(TMT6plex); S6(Phospho); K9(TMT6plex)
77812670	NP_005024.2	5393	EXOSC9	exosome complex component RRP45 isoform 2	S306	1.1	0.9	0.9	0.9	-	-	-	-	1.1	0.9	0.9	0.8	-	-	-	-	1.1	0.9	0.9	0.8	APIDTsDVEEK	EKAPIDTsDVEEKAE	S6;	Yes	T(5): 0.9; S(6): 99.1	2	2	1	0	N-Term(TMT6plex); S6(Phospho); K11(TMT6plex)
5032189	NP_005648.1	7158	TP53BP1	tumor suppressor p53-binding protein 1 isoform 3	S1656	0.9	1.0	0.9	0.9	-	-	-	-	0.9	1.0	1.0	1.1	-	-	-	-	0.9	1.0	1.0	1.1	ITEsPR	PTRKITEsPRASMGV	S4;	Yes	T(2): 0.0; S(4): 100.0	62	3	1	0	N-Term(TMT6plex); S4(Phospho)
208609990	NP_055484.3	9688	NUP93	nuclear pore complex protein Nup93 isoform 1	S52	1.1	0.7	1.3	0.9	-	-	-	-	1.1	0.8	1.2	0.9	-	-	-	-	1.1	0.8	1.2	0.9	TsQETADVK	SRTLTRTsQETADVK	S2;	Yes	T(1): 0.0; S(2): 100.0; T(5): 0.0	6	1	1	0	N-Term(TMT6plex); S2(Phospho); K9(TMT6plex)
7657218	NP_055114.1	23476	BRD4	bromodomain-containing protein 4 isoform short	S601	0.6	0.8	1.0	0.9	-	-	-	-	0.6	0.9	1.1	0.9	-	-	-	-	0.6	0.9	1.1	0.9	SKPPPTYEsEEEDK	KPPPTYEsEEEDKCK	S9;	Yes	S(1): 0.0; T(6): 0.0; Y(7): 0.0; S(9): 100.0	7	2	1	0	N-Term(TMT6plex); K2(TMT6plex); S9(Phospho); K14(TMT6plex)
42415492	NP_060823.3	55320	MIS18BP1	mis18-binding protein 1	S914	0.7	0.9	0.9	0.9	-	-	-	-	0.7	0.9	0.9	0.8	-	-	-	-	0.7	0.9	0.9	0.8	sPEECQR	AAAVGSRsPEECQRK	S1;	No	S(1): 100.0	2	1	1	0	N-Term(TMT6plex); S1(Phospho); C5(Carbamidomethyl)
63252910	NP_001017977.1	55827	DCAF6	DDB1- and CUL4-associated factor 6 isoform b	S336	0.9	0.7	0.8	0.9	-	-	-	-	-	-	-	-	-	-	-	-	-	-	-	-	DGEQsPNVSLMQR	RERDGEQsPNVSLMQ	S5;	Yes	S(5): 97.9; S(9): 2.1	4	4	1	0	N-Term(TMT6plex); S5(Phospho); M11(Oxidation)
157817073	NP_055898.1	51755	CDK12	cyclin-dependent kinase 12 isoform 2	T269	0.9	1.1	0.9	0.9	-	-	-	-	0.9	1.2	0.9	1.0	-	-	-	-	0.9	1.2	0.9	1.0	SPGStSR	YKKSPGStSRRQSVS	T5;	No	S(1): 0.7; S(4): 0.0; T(5): 99.2; S(6):	10	2	1	0	N-Term(TMT6plex); S1(Phospho)
20270186	NP_004233.1	9324	HMGN3	high mobility group nucleosome-binding domain-containing protein 3 isoform HMGN3a	S78	0.9	1.1	0.9	0.9	-	-	-	-	1.1	1.2	1.2	0.9	-	-	-	-	1.1	1.2	1.2	0.9	EGTAPsENGETKAEEAQK	GKEGTAPsENGETKA	S6;	Yes	T(3): 0.0; S(6): 97.9; T(11): 2.1	13	1	1	1	N-Term(TMT6plex); S6(Phospho); K12(TMT6plex); K18(TMT6plex)
150010552	NP_056073.1	23307	FKBP15	FK506-binding protein 15	S1162	1.4	1.1	1.2	0.9	-	-	-	-	1.5	1.3	1.4	1.0	-	-	-	-	1.5	1.3	1.4	1.0	SsLSGDEEDELFK	SHHSQRSsLSGDEED	S2;	Yes	S(1): 2.0; S(2): 98.0; S(4): 0.0	9	1	1	0	N-Term(TMT6plex); S2(Phospho); K13(TMT6plex)
62244004	NP_001013865.1	79811	SLTM	SAFB-like transcription modulator isoform b	S981	0.9	0.9	0.7	0.9	-	-	-	-	0.8	0.9	0.8	1.0	-	-	-	-	0.8	0.9	0.8	1.0	AGAGMITQHSsNASPINR	GMITQHSsNASPINR	S11;	No	T(7): 0.0; S(10): 0.0; S(11): 98.7; S(14): 1.3	12	2	1	0	N-Term(TMT6plex); M5(Oxidation); S14(Phospho)
148613886	NP_073752.5	64864	RFX7	DNA-binding protein RFX7	S322	0.7	1.2	0.6	0.9	-	-	-	-	-	-	-	-	-	-	-	-	-	-	-	-	LQsPLPGESAAK	QQEQKLQsPLPGESA	S3;	No	S(3): 100.0; S(9): 0.0	3	1	1	0	N-Term(TMT6plex); S3(Phospho); K12(TMT6plex)
45505149	NP_005588.2	4782	NFIC	nuclear factor 1 C-type isoform 5	S333	0.8	1.0	1.0	0.9	-	-	-	-	1.0	1.1	1.1	1.0	-	-	-	-	1.0	1.1	1.1	1.0	TEMDKsPFNSPSPQDSPR	KKTEMDKsPFNSPSP	S6;	Yes	T(1): 0.0; S(6): 100.0; S(10): 0.0; S(12): 0.0; S(16): 0.0	13	5	1	1	N-Term(TMT6plex); M3(Oxidation); K5(TMT6plex); S6(Phospho)
95147555	NP_002364.5	4130	MAP1A	microtubule-associated protein 1A	S2022	0.9	0.8	0.6	0.9	-	-	-	-	-	-	-	-	-	-	-	-	-	-	-	-	ELSSPIsPK	KELSSPIsPKSLQSD	S7;	Yes	S(3): 50.0; S(4): 50.0; S(7): 100.0	7	1	1	0	N-Term(TMT6plex); S4(Phospho); S7(Phospho); K9(TMT6plex)
61743954	NP_001611.1	79026	AHNAK	neuroblast differentiation-associated protein AHNAK isoform 1	S5865	1.1	0.9	1.3	0.9	-	-	-	-	1.3	0.9	1.4	1.0	-	-	-	-	1.3	0.9	1.4	1.0	LSSsSSNDSGNK	KKSRLSSsSSNDSGN	S4;	Yes	S(2): 0.0; S(3): 1.5; S(4): 98.4; S(5): 0.0; S(6): 0.0; S(9): 0.0	79	1	1	0	N-Term(TMT6plex); S4(Phospho); K12(TMT6plex)
32483399	NP_002568.2	5062	PAK2	serine/threonine-protein kinase PAK 2	S141	1.5	0.9	1.1	0.9	-	-	-	-	1.6	0.9	1.1	0.8	-	-	-	-	1.6	0.9	1.1	0.8	YLsFTPPEK	TVKQKYLsFTPPEKD	S3;	Yes	Y(1): 0.0; S(3): 100.0; T(5): 0.0	2	1	1	0	N-Term(TMT6plex); S3(Phospho); K9(TMT6plex)
545746335	NP_001271143.1	9320	TRIP12	E3 ubiquitin-protein ligase TRIP12 isoform a	S45	0.6	0.8	0.7	0.9	-	-	-	-	0.6	0.9	0.7	0.9	-	-	-	-	0.6	0.9	0.7	0.9	GYsPPESR	QAKHKGYsPPESRKS	S3;	Yes	Y(2): 0.8; S(3): 99.2; S(7): 0.0	23	1	1	0	N-Term(TMT6plex); S3(Phospho)
33356174	NP_002678.2	5411	PNN	pinin	S441	0.6	1.0	1.0	0.9	-	-	-	-	0.7	1.2	1.2	1.0	-	-	-	-	0.7	1.2	1.2	1.0	sLSPGKENVSALDMEK	EPDKECKsLSPGKEN	S1;	Yes	S(1): 98.9; S(3): 1.1; S(10): 0.0	7	1	1	1	N-Term(TMT6plex); S3(Phospho); K6(TMT6plex); M14(Oxidation); K16(TMT6plex)
24308075	NP_056285.1	26056	RAB11FIP5	rab11 family-interacting protein 5	S296	0.7	0.8	0.7	0.9	-	-	-	-	0.8	0.8	0.8	0.9	-	-	-	-	0.8	0.8	0.8	0.9	DSAQsPK	GGRDSAQsPKLFTHK	S5;	Yes	S(2): 0.0; S(5): 100.0	5	1	1	0	N-Term(TMT6plex); S5(Phospho); K7(TMT6plex)
373938427	NP_001243323.1	55035	NOL8	nucleolar protein 8 isoform b	S199	1.2	1.3	1.2	0.9	-	-	-	-	1.1	1.3	1.3	0.9	-	-	-	-	1.1	1.3	1.3	0.9	sSPVPVSDTQK	DSITPSKsSPVPVSD	S1;	No	S(1): 98.7; S(2): 1.3; S(7): 0.0; T(9):	5	2	1	0	N-Term(TMT6plex); S2(Phospho); K11(TMT6plex)
5174669	NP_006080.1	10389	SCML2	sex comb on midleg-like protein 2	S590	0.8	0.7	0.5	0.9	-	-	-	-	0.7	0.7	0.5	1.0	-	-	-	-	0.7	0.7	0.5	1.0	SVPGTTSSPLVGDIsPK	SPLVGDIsPKSSPHE	S15;	Yes	S(1): 0.0; T(5): 0.0; T(6): 0.0; S(7): 0.0; S(8): 0.0; S(15): 100.0	4	1	1	0	N-Term(TMT6plex); S15(Phospho); K17(TMT6plex)
37655165	NP_115530.2	84108	PCGF6	polycomb group RING finger protein 6 isoform b	S115	1.1	0.6	1.1	0.9	-	-	-	-	-	-	-	-	-	-	-	-	-	-	-	-	QDsEDEEER	LEGGRQDsEDEEERL	S3;	Yes	S(3): 100.0	2	2	1	0	N-Term(TMT6plex); S3(Phospho)
153070260	NP_002347.5	4082	MARCKS	myristoylated alanine-rich C-kinase substrate	S81	0.4	0.6	0.7	0.9	-	-	-	-	0.7	0.8	1.0	1.2	-	-	-	-	0.7	0.8	1.0	1.2	EELQANGSAPAADKEEPAAAGSGAAsPSAAEK	AAGSGAAsPSAAEKG	S26;	Yes	S(8): 0.0; S(22): 0.0; S(26): 100.0; S(28): 0.0	69	1	1	1	N-Term(TMT6plex); K14(TMT6plex); S26(Phospho); K32(TMT6plex)
118421085	NP_689472.3	90381	TICRR	treslin	S1430; S1433	0.8	0.9	0.8	0.9	-	-	-	-	0.9	0.9	0.9	0.9	-	-	-	-	0.9	0.9	0.9	0.9	GLSLsPQsPPER	DQKGLSLsPQSPPER;GLSLSPQsPPERRGY	S5; S8;	Yes;Yes	S(3): 0.0; S(5): 100.0; S(8): 100.0	11	1	1	0	N-Term(TMT6plex); S5(Phospho); S8(Phospho)
255652953	NP_653205.3	124245	ZC3H18	zinc finger CCCH domain-containing protein 18	S94	0.8	0.9	1.2	0.9	-	-	-	-	1.0	0.9	1.5	0.9	-	-	-	-	1.0	0.9	1.5	0.9	GPTsSPCEEEGDEGEEDR	ELSRGPTsSPCEEEG	S4;	Yes	T(3): 2.2; S(4): 95.5; S(5): 2.2	34	1	1	0	N-Term(TMT6plex); S4(Phospho); C7(Carbamidomethyl)
55741677	NP_055527.1	9739	SETD1A	histone-lysine N-methyltransferase SETD1A	S464	1.3	1.2	1.2	0.9	-	-	-	-	1.3	1.1	1.1	0.7	-	-	-	-	1.3	1.1	1.1	0.7	TSPRPAsPAR	RTSPRPAsPARSGSP	S7;	Yes	T(1): 50.0; S(2): 50.0; S(7): 100.0	4	1	1	0	N-Term(TMT6plex); S2(Phospho); S7(Phospho)
103472005	NP_002408.3	4288	MKI67	antigen KI-67 isoform 1	S1291	0.5	0.7	0.9	0.9	-	-	-	-	0.6	0.7	1.0	0.9	-	-	-	-	0.6	0.7	1.0	0.9	NLMPsAGK	ACRNLMPsAGKAMHT	S5;	Yes	S(5): 100.0	118	2	1	0	N-Term(TMT6plex); M3(Oxidation); S5(Phospho); K8(TMT6plex)
29171753	NP_803172.1	8500	PPFIA1	liprin-alpha-1 isoform a	S668	1.6	0.6	0.9	0.9	-	-	-	-	1.6	0.7	1.0	0.9	-	-	-	-	1.6	0.7	1.0	0.9	VGSGsLDNLGR	ESRVGSGsLDNLGRE	S5;	Yes	S(3): 0.0; S(5): 100.0	9	2	1	0	N-Term(TMT6plex); S5(Phospho)
150417986	NP_006411.2	10564	ARFGEF2	brefeldin A-inhibited guanine nucleotide-exchange protein 2	S277	0.9	0.9	0.8	0.9	-	-	-	-	0.9	1.0	0.8	0.9	-	-	-	-	0.9	1.0	0.8	0.9	GSsLSGTDDGAQEVVK	APRERGSsLSGTDDG	S3;	Yes	S(2): 2.0; S(3): 97.9; S(5): 0.0; T(7): 0.0	2	1	1	0	N-Term(TMT6plex); S3(Phospho); K16(TMT6plex)
431822406	NP_001258900.1	3326	HSP90AB1	heat shock protein HSP 90-beta isoform b	S178	0.8	1.0	0.9	0.9	-	-	-	-	0.8	1.0	0.9	0.9	-	-	-	-	0.8	1.0	0.9	0.9	EKEIsDDEAEEEKGEK	KEREKEIsDDEAEEE	S5;	No	S(5): 100.0	54	3	1	2	N-Term(TMT6plex); K2(TMT6plex); S5(Phospho); K13(TMT6plex); K16(TMT6plex)
118572613	NP_057417.3	23524	SRRM2	serine/arginine repetitive matrix protein 2	S220	1.1	1.2	1.1	0.9	-	-	-	-	1.1	1.2	1.1	0.9	-	-	-	-	1.1	1.2	1.1	0.9	sESESK	SKKKKHRsESESKKR	S1;	Yes	S(1): 100.0; S(3): 0.0; S(5): 0.0	436	1	1	0	N-Term(TMT6plex); S1(Phospho); K6(TMT6plex)
634743290	NP_001278893.1	4301	MLLT4	afadin isoform 4	S1736	0.8	2.5	1.5	0.9	-	-	-	-	0.7	2.2	1.4	0.8	-	-	-	-	0.7	2.2	1.4	0.8	SQDADsPGSSGAPENLTFK	SKSQDADsPGSSGAP	S6;	No	S(1): 0.0; S(6): 96.9; S(9): 1.5; S(10): 1.5; T(17): 0.0	16	2	1	0	N-Term(TMT6plex); S6(Phospho); K19(TMT6plex)
5730124	NP_006517.1	7764	ZNF217	zinc finger protein 217	S848	0.7	0.8	1.0	0.9	-	-	-	-	0.8	0.9	1.2	1.0	-	-	-	-	0.8	0.9	1.2	1.0	TSVsPAPDK	MEPKTSVsPAPDKTK	S4;	Yes	T(1): 0.0; S(2): 0.0; S(4): 100.0	9	1	1	0	N-Term(TMT6plex); S4(Phospho); K9(TMT6plex)
18860916	NP_036387.2	22803	XRN2	5'-3' exoribonuclease 2	S499	0.9	0.9	0.9	0.9	-	-	-	-	0.9	1.0	0.9	0.9	-	-	-	-	0.9	1.0	0.9	0.9	KAEDsDSEPEPEDNVR	IKRKAEDsDSEPEPE	S5;	Yes	S(5): 100.0; S(7): 0.0	3	1	1	1	N-Term(TMT6plex); K1(TMT6plex); S5(Phospho)
42794779	NP_976063.1	399687	MYO18A	unconventional myosin-XVIIIa isoform b	S1640	0.9	0.9	1.1	0.9	-	-	-	-	0.9	0.9	1.2	0.9	-	-	-	-	0.9	0.9	1.2	0.9	LATLsDQVNR	EGKLATLsDQVNRRD	S5;	Yes	T(3): 0.7; S(5): 99.3	3	2	1	0	N-Term(TMT6plex); S5(Phospho)
373938427	NP_001243323.1	55035	NOL8	nucleolar protein 8 isoform b	S1031	0.7	1.0	1.3	0.9	-	-	-	-	0.7	1.0	1.3	0.9	-	-	-	-	0.7	1.0	1.3	0.9	NSsPGEASLLEK	ETDHRNSsPGEASLL	S3;	No	S(2): 1.0; S(3): 99.0; S(8): 0.0	5	2	1	0	N-Term(TMT6plex); S3(Phospho); K12(TMT6plex)
56118310	NP_073568.2	64710	NUCKS1	nuclear ubiquitous casein and cyclin-dependent kinase substrate 1	S113	1.0	1.4	0.9	0.9	-	-	-	-	1.2	1.7	1.3	1.1	-	-	-	-	1.2	1.7	1.3	1.1	EMLMEDVGsEEEQEEEDEAPFQEK	MLMEDVGsEEEQEEE	S9;	Yes	S(9): 100.0	109	1	1	0	N-Term(TMT6plex); M2(Oxidation); M4(Oxidation); S9(Phospho); K24(TMT6plex)
187608516	NP_036419.3	23527	ACAP2	arf-GAP with coiled-coil, ANK repeat and PH domain-containing protein 2	S384	1.3	1.1	1.0	0.9	-	-	-	-	1.2	1.1	1.0	0.9	-	-	-	-	1.2	1.1	1.0	0.9	SSPSTGsLDSGNESK	KSSPSTGsLDSGNES	S7;	Yes	S(1): 0.0; S(2): 0.0; S(4): 0.0; T(5): 0.0; S(7): 100.0; S(10): 0.0; S(14): 0.0	14	1	1	0	N-Term(TMT6plex); S7(Phospho); K15(TMT6plex)
56550051	NP_006581.2	10713	USP39	U4/U6.U5 tri-snRNP-associated protein 2 isoform 1	S82	1.0	1.5	1.3	0.9	-	-	-	-	1.0	1.4	1.2	0.8	-	-	-	-	1.0	1.4	1.2	0.8	EVDEDsEPEREVR	EREVDEDsEPEREVR	S6;	Yes	S(6): 100.0	8	2	1	1	N-Term(TMT6plex); S6(Phospho)
42560244	NP_004783.2	9360	PPIG	peptidyl-prolyl cis-trans isomerase G	S397	1.7	1.7	1.7	0.9	-	-	-	-	2.2	2.1	2.1	1.1	-	-	-	-	2.2	2.1	2.1	1.1	ENQRsPVR	EIKENQRsPVRVKER	S5;	Yes	S(5): 100.0	4	1	1	1	N-Term(TMT6plex); S5(Phospho)
73760415	NP_001027392.1	6812	STXBP1	syntaxin-binding protein 1 isoform b	S593	1.0	0.8	1.0	0.9	-	-	-	-	0.9	0.7	0.9	0.8	-	-	-	-	0.9	0.7	0.9	0.8	TDEEIsS	NKTDEEIsS	S6;	Yes	T(1): 0.0; S(6): 99.5; S(7): 0.5	2	1	1	0	N-Term(TMT6plex); S7(Phospho)
5730124	NP_006517.1	7764	ZNF217	zinc finger protein 217	S407	0.8	1.0	1.1	0.8	-	-	-	-	1.0	1.2	1.3	1.0	-	-	-	-	1.0	1.2	1.3	1.0	AGAEsPTMSVDGR	DRRAGAEsPTMSVDG	S5;	Yes	S(5): 100.0; T(7): 0.0; S(9): 0.0	9	1	1	0	N-Term(TMT6plex); S5(Phospho); M8(Oxidation)
38788260	NP_004450.3	2186	BPTF	nucleosome-remodeling factor subunit BPTF isoform 2	S2471	0.8	0.9	0.9	0.8	-	-	-	-	0.9	0.9	0.9	0.8	-	-	-	-	0.9	0.9	0.9	0.8	VQsPSQTR	QSPVRVQsPSQTRIR	S3;	Yes	S(3): 100.0; S(5): 0.0; T(7): 0.0	11	2	1	0	N-Term(TMT6plex); S3(Phospho)
71725360	NP_055857.1	23060	ZNF609	zinc finger protein 609	S560	0.7	0.9	0.8	0.8	-	-	-	-	0.8	1.0	1.0	0.9	-	-	-	-	0.8	1.0	1.0	0.9	GSLsPAR	VSQKGSLsPARSATP	S4;	Yes	S(2): 0.0; S(4): 100.0	6	1	1	0	N-Term(TMT6plex); S4(Phospho)
117676384	NP_004710.2	9169	SCAF11	protein SCAF11	S771	0.7	1.0	0.8	0.8	-	-	-	-	0.8	1.0	0.9	0.8	-	-	-	-	0.8	1.0	0.9	0.8	VETVsQPSESPKDTIDK	DEKVETVsQPSESPK	S5;	Yes	T(3): 0.1; S(5): 99.9; S(8): 0.1; S(10): 50.0; T(14): 50.0	4	1	1	1	N-Term(TMT6plex); S5(Phospho); S8(Phospho); K12(TMT6plex); K17(TMT6plex)
533112484	NP_001269207.1	9782	MATR3	matrin-3 isoform c	S266	0.7	0.7	0.8	0.8	-	-	-	-	0.8	0.8	0.9	0.9	-	-	-	-	0.8	0.8	0.9	0.9	SYSPDGKEsPSDKK	YSPDGKEsPSDKKSK	S9;	No	S(1): 0.0; Y(2): 50.0; S(3): 50.0; S(9): 100.0; S(11): 0.0	3	3	1	2	N-Term(TMT6plex); S3(Phospho); K7(TMT6plex); S9(Phospho); K13(TMT6plex); K14(TMT6plex)
153945728	NP_005900.2	4131	MAP1B	microtubule-associated protein 1B	T1633	1.0	1.2	0.9	0.8	-	-	-	-	0.8	0.9	0.7	0.9	-	-	-	-	0.8	0.9	0.7	0.9	SRtPVQDHR	PKTAKSRtPVQDHRS	T3;	Yes	S(1): 0.0; T(3): 100.0	49	1	1	1	N-Term(TMT6plex); T3(Phospho)
394582099	NP_001257401.1	4172	MCM3	DNA replication licensing factor MCM3 isoform 2	T732	1.6	1.0	1.1	0.8	-	-	-	-	1.7	1.1	1.3	1.0	-	-	-	-	1.7	1.1	1.3	1.0	DGDSYDPYDFSDTEEEMPQVHtPK	EEMPQVHtPKTADSQ	T22;	No	S(4): 0.0; Y(5): 0.0; Y(8): 33.3; S(11): 33.3; T(13): 33.3; T(22): 100.0	10	2	1	0	N-Term(TMT6plex); T13(Phospho); M17(Oxidation); T22(Phospho); K24(TMT6plex)
54112117	NP_036565.2	23451	SF3B1	splicing factor 3B subunit 1 isoform 1	T278	1.2	0.7	1.0	0.8	-	-	-	-	1.2	0.8	1.0	0.9	-	-	-	-	1.2	0.8	1.0	0.9	GDTPGHAtPGHGGATSSAR	GDTPGHAtPGHGGAT	T8;	Yes	T(3): 0.0; T(8): 100.0; T(15): 0.0; S(16): 0.0; S(17): 0.0	13	1	1	0	N-Term(TMT6plex); T8(Phospho)
19913406	NP_001058.2	7153	TOP2A	DNA topoisomerase 2-alpha	S1393	0.8	1.0	0.9	0.8	-	-	-	-	1.0	1.1	1.0	0.9	-	-	-	-	1.0	1.1	1.0	0.9	GSVPLSSsPPATHFPDETEITNPVPK	GSVPLSSsPPATHFP	S8;	Yes	S(2): 0.0; S(6): 0.0; S(7): 1.9; S(8): 96.1; T(12): 1.9; T(18): 0.0; T(21): 0.0	64	1	1	0	N-Term(TMT6plex); S8(Phospho); K26(TMT6plex)
132626688	NP_055456.2	9656	MDC1	mediator of DNA damage checkpoint protein 1	S964	0.8	0.7	0.4	0.8	-	-	-	-	0.8	0.7	0.5	0.9	-	-	-	-	0.8	0.7	0.5	0.9	GQASsPTPEPGVGAGDLPGPTSAPVPSGSQSGGR	DQKGQASsPTPEPGV	S5;	Yes	S(4): 0.1; S(5): 97.3; T(7): 2.6; T(21): 0.0; S(22): 0.0; S(27): 0.0; S(29): 0.0; S(31): 0.0	26	1	1	0	N-Term(TMT6plex); S5(Phospho)
296531459	NP_001171866.1	57669	EPB41L5	band 4.1-like protein 5 isoform 2	S517	1.1	1.0	0.7	0.8	-	-	-	-	1.0	1.0	0.8	0.9	-	-	-	-	1.0	1.0	0.8	0.9	QLEMENsPLLSPR	KQLEMENsPLLSPRS	S7;	Yes	S(7): 100.0; S(11): 0.0	2	2	1	0	N-Term(TMT6plex); M4(Oxidation); S7(Phospho)
118572613	NP_057417.3	23524	SRRM2	serine/arginine repetitive matrix protein 2	S801	0.8	0.9	0.9	0.8	-	-	-	-	0.9	1.0	0.9	0.9	-	-	-	-	0.9	1.0	0.9	0.9	SGsSQPK	PRRSRSGsSQPKAKS	S3;	No	S(1): 0.0; S(3): 99.4; S(4): 0.6	436	1	1	0	N-Term(TMT6plex); S3(Phospho); K7(TMT6plex)
112421013	NP_065859.3	57568	SIPA1L2	signal-induced proliferation-associated 1-like protein	S1652	0.9	0.8	0.7	0.8	-	-	-	-	-	-	-	-	-	-	-	-	-	-	-	-	SPsPLTGK	TTGERSPsPLTGKVN	S3;	Yes	S(1): 1.0; S(3): 99.0; T(6): 0.0	1	1	1	0	N-Term(TMT6plex); S3(Phospho); K8(TMT6plex)
578794667	XP_006710193.1	1.01E+08	LOC10050962[illegible]	aquaporin-7-like isoform X5	T6; T8	0.8	0.7	0.4	0.8	-	-	-	-	-	-	-	-	-	-	-	-	-	-	-	-	MNAAVtFtNCALGR	_MNAAVtFTNCALG;MNAAVTFtNCALGRV	T6; T8;	No;No	T(6): 100.0; T(8): 100.0	1	3	1	0	M1(Oxidation); N-Term(TMT6plex); T6(Phospho); T8(Phospho); C10(Carbamidomethyl)
148612816	NP_004250.4	9400	RECQL5	ATP-dependent DNA helicase Q5 isoform 1	S727	1.1	1.0	0.9	0.8	-	-	-	-	1.1	0.9	0.9	0.8	-	-	-	-	1.1	0.9	0.9	0.8	GEVPGGSAHYGGPsPEK	SAHYGGPsPEKKAKS	S14;	Yes	S(7): 0.0; Y(10): 0.0; S(14): 100.0	2	1	1	0	N-Term(TMT6plex); S14(Phospho); K17(TMT6plex)
4506711	NP_001021.1	6232	RPS27	40S ribosomal protein S27	S11	0.6	0.8	0.7	0.8	-	-	-	-	0.6	0.8	0.8	0.8	-	-	-	-	0.6	0.8	0.8	0.8	DLLHPsPEEEK	AKDLLHPsPEEEKRK	S6;	Yes	S(6): 100.0	3	1	1	0	N-Term(TMT6plex); S6(Phospho); K11(TMT6plex)
48255933	NP_004956.5	3150	HMGN1	non-histone chromosomal protein HMG-14	S89	0.8	1.2	1.0	0.8	-	-	-	-	1.1	1.4	1.3	1.1	-	-	-	-	1.1	1.4	1.3	1.1	EDLPAENGETKTEESPAsDEAGEK	KTEESPAsDEAGEKE	S18;	Yes	T(10): 0.0; T(12): 0.0; S(15): 2.1; S(18): 97.8	28	1	1	1	N-Term(TMT6plex); K11(TMT6plex); S18(Phospho); K24(TMT6plex)
116235474	NP_443134.2	114790	STK11IP	serine/threonine-protein kinase 11-interacting protein	S772	0.9	0.8	0.9	0.8	-	-	-	-	-	-	-	-	-	-	-	-	-	-	-	-	AKNsPPQAPSTR	HLDRAKNsPPQAPST	S4;	Yes	S(4): 100.0; S(10): 0.0; T(11): 0.0	1	1	1	1	N-Term(TMT6plex); K2(TMT6plex); S4(Phospho)
109240550	NP_001035879.1	55269	PSPC1	paraspeckle component 1	S477	1.2	1.4	1.2	0.8	-	-	-	-	1.2	1.0	1.0	0.5	-	-	-	-	1.2	1.0	1.0	0.5	FPQGPPSQMGsPMGSR	GPPSQMGsPMGSRTG	S11;	Yes	S(7): 0.0; S(11): 98.2; S(15): 1.8	2	1	1	0	N-Term(TMT6plex); M9(Oxidation); S11(Phospho); M13(Oxidation)
634743290	NP_001278893.1	4301	MLLT4	afadin isoform 4	S1655	1.1	1.4	0.9	0.8	-	-	-	-	1.0	1.2	0.8	0.7	-	-	-	-	1.0	1.2	0.8	0.7	DYEPPSPsPAPGAPPPPPQR	DYEPPSPsPAPGAPP	S8;	No	Y(2): 0.0; S(6): 1.7; S(8): 98.3	16	1	1	0	N-Term(TMT6plex); S6(Phospho)
150418007	NP_006258.3	5903	RANBP2	E3 SUMO-protein ligase RanBP2	S1400	0.9	0.9	1.1	0.8	-	-	-	-	0.8	0.9	1.0	0.8	-	-	-	-	0.8	0.9	1.0	0.8	TsPENVQDR	TVFTPKTsPENVQDR	S2;	Yes	T(1): 0.0; S(2): 100.0	17	1	1	0	N-Term(TMT6plex); S2(Phospho)
66348045	NP_057562.3	51530	ZC3HC1	nuclear-interacting partner of ALK isoform 1	S24	0.9	1.0	1.0	0.8	-	-	-	-	0.9	1.0	1.0	0.8	-	-	-	-	0.9	1.0	1.0	0.8	sPEGTPQK	NWGAVVRsPEGTPQK	S1;	Yes	S(1): 100.0; T(5): 0.0	8	2	1	0	N-Term(TMT6plex); S1(Phospho); K8(TMT6plex)
149588534	NP_001092303.1	56970	ATXN7L3	ataxin-7-like protein 3 isoform b	S281	0.8	0.9	1.0	0.8	-	-	-	-	-	-	-	-	-	-	-	-	-	-	-	-	LQWDGSSDLsPSDSGSSK	WDGSSDLsPSDSGSS	S10;	Yes	S(6): 0.0; S(7): 0.0; S(10): 98.8; S(12): 1.2; S(14): 0.0; S(16): 0.0; S(17): 0.0	2	2	1	0	N-Term(TMT6plex); S10(Phospho); K18(TMT6plex)
194018478	NP_653173.1	23102	TBC1D2B	TBC1 domain family member 2B isoform a	S957	0.7	0.7	0.7	0.8	-	-	-	-	0.8	0.8	0.8	0.9	-	-	-	-	0.8	0.8	0.8	0.9	GELVsDEEEDT	PDKGELVsDEEEDT	S5;	Yes	S(5): 100.0; T(11): 0.0	2	1	1	0	N-Term(TMT6plex); S5(Phospho)
102467242	NP_064623.2	3619	INCENP	inner centromere protein isoform 2	T292	0.8	1.0	1.0	0.8	-	-	-	-	0.8	0.9	1.0	0.9	-	-	-	-	0.8	0.9	1.0	0.9	VLAPILPDNFStPTGSR	ILPDNFStPTGSRTD	T12;	Yes	S(11): 1.0; T(12): 98.0; T(14): 1.0; S(16): 0.0	23	2	1	0	N-Term(TMT6plex); T12(Phospho)
63053524	NP_001017416.1	7398	USP1	ubiquitin carboxyl-terminal hydrolase 1	S42	1.2	0.8	0.7	0.8	-	-	-	-	1.3	0.9	0.8	0.9	-	-	-	-	1.3	0.9	0.8	0.9	ALDETDsQENEEK	RALDETDsQENEEKA	S7;	Yes	T(5): 1.8; S(7): 98.2	7	1	1	0	N-Term(TMT6plex); S7(Phospho); K13(TMT6plex)
68348723	NP_068838.3	7552	ZNF711	zinc finger protein 711	S459	1.0	0.9	0.7	0.8	-	-	-	-	-	-	-	-	-	-	-	-	-	-	-	-	EAsPLSSNK	TRRYREAsPLSSNKL	S3;	Yes	S(3): 100.0; S(6): 0.0; S(7): 0.0	1	1	1	0	N-Term(TMT6plex); S3(Phospho); K9(TMT6plex)
21735415	NP_001801.1	1059	CENPB	major centromere autoantigen B	S156	1.0	1.1	1.0	0.8	-	-	-	-	1.1	1.1	1.0	0.8	-	-	-	-	1.1	1.1	1.0	0.8	TPAAPAsPAAVPSEGSGGSTTGWR	RTPAAPAsPAAVPSE	S7;	Yes	T(1): 0.0; S(7): 100.0; S(13): 0.0; S(16): 0.0; S(19): 0.0; T(20): 0.0;	1	1	1	0	N-Term(TMT6plex); S7(Phospho)

Khan et al., 2019. Multi-omics analysis to characterize cigarette smoke induced molecular alterations in esophageal cells

Supplementary Table 8. List of phosphopeptides identified and quantified in untreated and chronically treated Het1A cells with cigarette smoke condensate for 8 months using SequestHT and Mascot search algorithms with a PhosphoRS probability score ≥75%

Protein group Accession	NP_Accession	Gene ID	Gene Symbol	Protein Description	PhosphoSite (Protein)	Het-1A-Smoke - 2M/Parental	Het-1A-Smoke - 4M/Parental	Het-1A-Smoke - 6M/Parental	Het-1A-Smoke - 8M/Parental	Het-1A-Smoke - 2M/Parental	Het-1A-Smoke - 4M/Parental	Het-1A-Smoke - 6M/Parental	Het-1A-Smoke - 8M/Parental	Het-1A-Smoke - 2M/Parental	Het-1A-Smoke - 4M/Parental	Het-1A-Smoke - 6M/Parental	Het-1A-Smoke - 8M/Parental	Het-1A-Smoke - 2M/Parental	Het-1A-Smoke - 4M/Parental	Het-1A-Smoke - 6M/Parental	Het-1A-Smoke - 8M/Parental	Het-1A-Smoke - 2M/Parental	Het-1A-Smoke - 4M/Parental	Het-1A-Smoke - 6M/Parental	Het-1A-Smoke - 8M/Parental	Peptide Sequence	Phosphowindow	PhosphoSite (Peptide)	Phosphosite Plus Evidence	phosphoRS Site Probabilities	# PSMs	# Proteins	# Protein Groups	# Missed Cleavages	Modifications
						Replicate 1				Replicate 2				Normalized phosphoprotein ratios with respect to corresponding total protein ratios. Replicate 1				Replicate 2				Average of replicates													
162951884	NP_055740.3	22864	R3HDM2	R3H domain-containing protein 2	S361	1.0	0.6	0.6	0.8	-	-	-	-	-	-	-	-	-	-	-	-	-	-	-	-	GDsIGSSK	SILTRGDsIGSSKGG	S3;	Yes	S(3): 100.0; S(6): 0.0; S(7): 0.0	1	1	1	0	N-Term(TMT6plex); S3(Phospho); K8(TMT6plex)
314122224	NP_001186623.1	6522	SLC4A2	anion exchange protein 2 isoform 3	T155	0.7	0.7	0.8	0.8	-	-	-	-	0.8	0.6	0.8	1.0	-	-	-	-	0.8	0.6	0.8	1.0	tSPSSPAPLPHQEATPR	ADRKAERtSPSSPAP	T1;	No	T(1): 94.9; S(2): 1.7; S(4): 1.7; S(5): 1.7; T(15): 0.0	1	3	1	0	N-Term(TMT6plex); T1(Phospho)
89274169	NP_064709.2	57019	CIAPIN1	anamorsin	S183	0.9	0.9	1.3	0.8	-	-	-	-	0.8	0.8	1.1	0.7	-	-	-	-	0.8	0.8	1.1	0.7	SsPSVKPAVDPAAAK	LSITKKSsPSVKPAV	S2;	Yes	S(1): 1.3; S(2): 98.7; S(4): 0.0	2	1	1	0	N-Term(TMT6plex); S2(Phospho); K6(TMT6plex); K15(TMT6plex)
217330574	NP_001136088.1	23301	EHBP1	EH domain-binding protein 1 isoform 3	S964	0.6	0.7	0.6	0.8	-	-	-	-	0.7	0.7	0.6	0.7	-	-	-	-	0.7	0.7	0.6	0.7	sIQEDTK	PEMKRQRsIQEDTKK	S1;	No	S(1): 100.0; T(6): 0.0	5	3	1	0	N-Term(TMT6plex); S1(Phospho); K7(TMT6plex)
11345454	NP_068741.1	2178	FANCE	Fanconi anemia group E protein	S210	0.4	0.6	0.5	0.8	-	-	-	-	-	-	-	-	-	-	-	-	-	-	-	-	DSEEEAAsPEGK	DSEEEAAsPEGKRVP	S8;	Yes	S(2): 0.0; S(8): 100.0	1	1	1	0	N-Term(TMT6plex); S8(Phospho); K12(TMT6plex)
14042970	NP_114428.1	83986	ITFG3	protein ITFG3	S21	0.9	0.9	0.7	0.8	-	-	-	-	0.8	0.8	0.7	0.8	-	-	-	-	0.8	0.8	0.7	0.8	KsQENLGNPSK	LKNEERKsQENLGNP	S2;	Yes	S(2): 100.0; S(10): 0.0	1	1	1	1	N-Term(TMT6plex); K1(TMT6plex); S2(Phospho); K11(TMT6plex)
291045268	NP_001166984.1	114112	TXNRD3	thioredoxin reductase 3 isoform 2	S42	1.1	1.1	0.8	0.8	-	-	-	-	-	-	-	-	-	-	-	-	-	-	-	-	LSsPGPSR	GRRARLSsPGPSRSS	S3;	Yes	S(2): 0.7; S(3): 99.3; S(7): 0.0	2	2	1	0	N-Term(TMT6plex); S3(Phospho)
289547556	NP_689618.4	133746	JMY	junction-mediating and -regulatory protein	S115	0.9	0.9	1.0	0.8	-	-	-	-	0.8	0.9	0.8	1.0	-	-	-	-	0.8	0.9	0.8	1.0	SSAWAEGGsPR	SAWAEGGsPRSTRSL	S9;	Yes	S(1): 0.0; S(2): 0.0; S(9): 100.0	1	1	1	0	N-Term(TMT6plex); S9(Phospho)
259906020	NP_001158287.1	22985	ACIN1	apoptotic chromatin condensation inducer in the nucleus isoform 3	S617	0.8	1.0	0.8	0.8	-	-	-	-	0.7	1.0	0.9	0.9	-	-	-	-	0.7	1.0	0.9	0.9	SLsPGVSR	SNSRKSLsPGVSRDS	S3;	No	S(1): 0.0; S(3): 100.0; S(7): 0.0	38	3	1	0	N-Term(TMT6plex); S3(Phospho)
31652242	NP_852469.1	6942	TCF20	transcription factor 20 isoform 2	S1522	0.7	1.0	0.7	0.8	-	-	-	-	0.6	1.1	0.7	0.9	-	-	-	-	0.6	1.1	0.7	0.9	ENDTVTIsPK	ENDTVTIsPKQEGEP	S8;	Yes	T(4): 0.0; T(6): 0.0; S(8): 100.0	14	2	1	0	N-Term(TMT6plex); S8(Phospho); K10(TMT6plex)
208022641	NP_003189.2	6924	TCEB3	transcription elongation factor B polypeptide 3	S410	0.5	0.5	0.5	0.8	-	-	-	-	0.5	0.5	0.6	0.9	-	-	-	-	0.5	0.5	0.6	0.9	sLGSLPK	KTNLDRKsLGSLPKV	S1;	Yes	S(1): 100.0; S(4): 0.0	2	1	1	0	N-Term(TMT6plex); S1(Phospho); K7(TMT6plex)
42734434	NP_078863.2	79639	TMEM53	transmembrane protein 53	S23	1.0	0.7	0.8	0.8	-	-	-	-	-	-	-	-	-	-	-	-	-	-	-	-	NsPSPGGK	PCWSQKNsPSPGGKE	S2;	No	S(2): 100.0; S(4): 0.0	2	1	1	0	N-Term(TMT6plex); S2(Phospho); K8(TMT6plex)
30410716	NP_839955.1	55763	EXOC1	exocyst complex component 1 isoform 2	S455	1.4	1.0	1.0	0.8	-	-	-	-	1.4	1.0	1.0	0.9	-	-	-	-	1.4	1.0	1.0	0.9	LTGsTSSLNK	SSGKLTGsTSSLNKL	S4;	Yes	T(2): 0.0; S(4): 98.0; T(5): 34.0; S(6): 34.0; S(7): 34.0	4	2	1	0	N-Term(TMT6plex); S4(Phospho); S6(Phospho); K10(TMT6plex)
29789072	NP_056349.1	26009	ZZZ3	ZZ-type zinc finger-containing protein 3	S391	0.7	0.8	0.9	0.8	-	-	-	-	0.7	0.8	0.9	0.9	-	-	-	-	0.7	0.8	0.9	0.9	AAPTRGsPTK	RAAPTRGsPTKNSSP	S7;	No	T(4): 0.0; S(7): 100.0; T(9): 0.0	1	1	1	1	N-Term(TMT6plex); S7(Phospho); K10(TMT6plex)
442654297	NP_001263189.1	5998	RGS3	regulator of G-protein signaling 3 isoform 2	S73	1.8	2.1	1.6	0.8	-	-	-	-	-	-	-	-	-	-	-	-	-	-	-	-	DSATSEGsPPGPDAPPSK	DSATSEGsPPGPDAP	S8;	No	S(2): 1.2; T(4): 1.2; S(5): 1.2; S(8): 96.3; S(17): 0.0	1	4	1	0	N-Term(TMT6plex); S8(Phospho); K18(TMT6plex)
8923110	NP_060138.1	54820	NDE1	nuclear distribution protein nudE homolog 1	S231	0.7	1.1	1.1	0.8	-	-	-	-	0.7	1.0	1.0	0.8	-	-	-	-	0.7	1.0	1.0	0.8	GPSSSLNTPGsFR	SSLNTPGsFRRGLDD	S11;	Yes	S(3): 0.0; S(4): 0.0; S(5): 0.0; T(8): 1.6; S(11): 98.4	1	1	1	0	N-Term(TMT6plex); T8(Phospho)
149158690	NP_542417.2	7916	PRRC2A	protein PRRC2A	S363	0.9	1.0	1.0	0.8	-	-	-	-	0.9	0.9	1.1	0.8	-	-	-	-	0.9	0.9	1.1	0.8	DSQsASGEERPPEADGK	EGHRDSQsASGEERP	S4;	Yes	S(2): 0.0; S(4): 98.5; S(6): 1.5	23	1	1	0	N-Term(TMT6plex); S6(Phospho); K17(TMT6plex)
164663775	NP_640335.2	123263	MTFMT	methionyl-tRNA formyltransferase, mitochondrial	T85	1.1	0.9	1.1	0.8	-	-	-	-	1.1	0.9	1.2	0.7	-	-	-	-	1.1	0.9	1.2	0.7	LEVVtMPSPSPK	IDKLEVVtMPSPSPK	T5;	No	T(5): 100.0; S(8): 0.0; S(10): 0.0	1	1	1	0	N-Term(TMT6plex); T5(Phospho); M6(Oxidation); K12(TMT6plex)
284813529	NP_001165375.1	9730	VPRBP	protein VPRBP isoform 2	T705	0.7	0.8	0.7	0.8	-	-	-	-	0.7	0.6	0.6	0.6	-	-	-	-	0.7	0.6	0.6	0.6	EISGtPR	IGKEISGtPRKLPQ	T5;	No	S(3): 0.0; T(5): 100.0	1	2	1	0	N-Term(TMT6plex); T5(Phospho)
24308227	NP_065883.1	57592	ZNF687	zinc finger protein 687	S266; S271	1.0	1.0	0.9	0.8	-	-	-	-	0.9	1.0	0.9	0.8	-	-	-	-	0.9	1.0	0.9	0.8	QsPGHQsPLASPK	GVPFFKQsPGHQSPL;KQSPGHQsPLASPKV	S2; S7;	Yes;Yes	S(2): 100.0; S(7): 100.0; S(11): 0.0	10	1	1	0	N-Term(TMT6plex); S2(Phospho); S7(Phospho); K13(TMT6plex)
139394579	NP_001077090.1	9923	ZBTB40	zinc finger and BTB domain-containing protein 40	S728	0.7	0.7	0.8	0.8	-	-	-	-	0.5	0.6	0.7	0.7	-	-	-	-	0.5	0.6	0.7	0.7	EASAsPDPAK	QEKEASAsPDPAKKS	S5;	Yes	S(3): 0.0; S(5): 100.0	3	1	1	0	N-Term(TMT6plex); S5(Phospho); K10(TMT6plex)
4757918	NP_004341.1	864	RUNX3	runt-related transcription factor 3 isoform 2	S214	0.8	0.7	0.8	0.8	-	-	-	-	0.7	0.6	0.7	0.7	-	-	-	-	0.7	0.6	0.7	0.7	VTPSTPsPR	RVTPSTPsPRGSLST	S7;	Yes	T(2): 0.0; S(4): 1.0; T(5): 1.0; S(7):	1	2	1	0	N-Term(TMT6plex); S7(Phospho)
41350320	NP_957516.1	10916	MAGED2	melanoma-associated antigen D2	S265	1.4	1.6	0.9	0.8	-	-	-	-	1.3	1.5	1.2	1.0	-	-	-	-	1.3	1.5	1.2	1.0	LQSsQEPEAPPPR	RAAKLQSsQEPEAPP	S4;	Yes	S(3): 1.0; S(4): 99.0	7	1	1	0	N-Term(TMT6plex); S4(Phospho)
32698936	NP_872363.1	283149	BCL9L	B-cell CLL/lymphoma 9-like protein	S118	1.0	1.5	1.2	0.8	-	-	-	-	-	-	-	-	-	-	-	-	-	-	-	-	SVsVDSGEQR	VKRDRSVsVDSGEQR	S3;	Yes	S(1): 0.0; S(3): 100.0; S(6): 0.0	5	1	1	0	N-Term(TMT6plex); S3(Phospho)
510025487	NP_001265368.1	11129	CLASRP	CLK4-associating serine/arginine rich protein isoform	S485	0.9	0.9	0.8	0.8	-	-	-	-	0.9	0.9	0.9	0.8	-	-	-	-	0.9	0.9	0.9	0.8	LTRPAAsPAVGEK	KLTRPAAsPAVGEKL	S7;	No	T(2): 0.0; S(7): 100.0	1	2	1	0	N-Term(TMT6plex); S7(Phospho); K13(TMT6plex)
255652953	NP_653205.3	124245	ZC3H18	zinc finger CCCH domain-containing protein 18	T851	0.9	1.2	0.9	0.8	-	-	-	-	1.1	1.2	1.1	0.9	-	-	-	-	1.1	1.2	1.1	0.9	RPNtSPDR	PPAKRPNtSPDRGSR	T4;	Yes	T(4): 99.4; S(5): 0.6	34	1	1	0	N-Term(TMT6plex); S5(Phospho)
93204865	NP_066015.2	57724	EPG5	ectopic P granules protein 5 homolog	Y1022; S1033	1.0	1.3	1.3	0.8	-	-	-	-	-	-	-	-	-	-	-	-	-	-	-	-	AGMPIGCyLALSMTAVGHsIEK	AGMPIGCyLALSMTA;SMTAVGHsIEKFCAE	Y8; S19;	No;No	Y(8): 99.8; S(12): 51.5; T(14): 51.5; S(19): 97.2	1	1	1	0	N-Term(TMT6plex); M3(Oxidation); C7(Carbamidomethyl); Y8(Phospho); T14(Phospho); S19(Phospho); K22(TMT6plex)
21493024	NP_653080.1	9590	AKAP12	A-kinase anchor protein 12 isoform 2	S273	0.6	0.7	1.3	0.8	-	-	-	-	0.7	0.6	1.1	0.7	-	-	-	-	0.7	0.6	1.1	0.7	LsAEYEK	SAHEPRLsAEYEKVE	S2;	No	S(2): 100.0; Y(5): 0.0	103	2	1	0	N-Term(TMT6plex); S2(Phospho); K7(TMT6plex)
221219026	NP_001138231.1	23187	PHLDB1	pleckstrin homology-like domain family B member 1 isoform b	S443	0.8	0.9	1.0	0.8	-	-	-	-	-	-	-	-	-	-	-	-	-	-	-	-	TLQPPEsPR	RTLQPPEsPRLGRRG	S7;	Yes	T(1): 0.0; S(7): 100.0	1	2	1	0	N-Term(TMT6plex); S7(Phospho)
226371669	NP_001139487.1	9787	DLGAP5	disks large-associated protein 5 isoform b	S662	1.4	0.9	1.4	0.8	-	-	-	-	1.7	1.1	1.7	0.9	-	-	-	-	1.7	1.1	1.7	0.9	NEMGIPQQTTsPENAGPQNTK	GIPQQTTsPENAGPQ	S11;	Yes	T(9): 2.0; T(10): 2.0; S(11): 96.1; T(20): 0.0	9	2	1	0	N-Term(TMT6plex); M3(Oxidation); S11(Phospho); K21(TMT6plex)
53829376	NP_001005476.1	8502	PKP4	plakophilin-4 isoform b	S337	0.8	0.8	0.9	0.8	-	-	-	-	-	-	-	-	-	-	-	-	-	-	-	-	VASPSQGQVGSSsPKR	QGQVGSSsPKRSGMT	S13;	Yes	S(3): 0.0; S(5): 0.0; S(11): 0.0; S(12): 1.2; S(13): 98.8	5	2	1	1	N-Term(TMT6plex); S13(Phospho); K15(TMT6plex)
304434531	NP_001182122.1	4678	NASP	nuclear autoantigenic sperm protein isoform 4	S662	0.6	0.7	0.4	0.8	-	-	-	-	0.7	1.0	0.7	1.2	-	-	-	-	0.7	1.0	0.7	1.2	KPEEEsPRKDDAK	KRKPEEEsPRKDDAK	S6;	No	S(6): 100.0	20	3	1	2	K1(TMT6plex); N-Term(TMT6plex); S6(Phospho); K9(TMT6plex); K13(TMT6plex)
4507877	NP_003364.1	7414	VCL	vinculin isoform VCL	S290	0.9	1.0	1.0	0.8	-	-	-	-	0.8	0.8	0.7	0.7	-	-	-	-	0.8	0.8	0.7	0.7	DPSAsPGDAGEQAIR	WLRDPSAsPGDAGEQ	S5;	Yes	S(3): 1.6; S(5): 98.4	5	2	1	0	N-Term(TMT6plex); S5(Phospho)
34013528	NP_065764.1	57473	ZNF512B	zinc finger protein 512B	S409	1.0	1.0	0.6	0.8	-	-	-	-	0.9	1.0	0.6	0.8	-	-	-	-	0.9	1.0	0.6	0.8	AAGPAsPPEEDPER	LKAAGPAsPPEEDPE	S6;	Yes	S(6): 100.0	3	1	1	0	N-Term(TMT6plex); S6(Phospho)
4506671	NP_000995.1	6181	RPLP2	60S acidic ribosomal protein P2	S102; S105	0.3	0.3	1.0	0.8	-	-	-	-	0.2	0.4	0.9	1.0	-	-	-	-	0.2	0.4	0.9	1.0	KEEsEEsDDDMGFGLFD	KDEKKEEsEESDDDM;KKEESEEsDDDMGFG	S4; S7;	Yes;Yes	S(4): 100.0; S(7): 100.0	4	3	1	1	K1(TMT6plex); N-Term(TMT6plex); S4(Phospho); S7(Phospho); M11(Oxidation)
9994179	NP_037392.1	29115	SAP30BP	SAP30-binding protein	S113	0.9	1.0	0.9	0.8	-	-	-	-	1.0	1.2	1.0	0.8	-	-	-	-	1.0	1.2	1.0	0.8	NMsPDEIKIPPEPPGR	SERVRNMsPDEIKIP	S3;	Yes	S(3): 100.0	1	1	1	1	N-Term(TMT6plex); M2(Oxidation); S3(Phospho); K8(TMT6plex)
6005757	NP_009123.1	11198	SUPT16H	FACT complex subunit SPT16	S1013	0.9	0.9	0.7	0.8	-	-	-	-	1.0	1.1	0.9	0.9	-	-	-	-	1.0	1.1	0.9	0.9	YEEEEEQsR	YEEEEEQsRSMSRKR	S8;	Yes	Y(1): 0.0; S(8): 100.0	3	1	1	0	N-Term(TMT6plex); S8(Phospho)
344925845	NP_055971.2	23186	RCOR1	REST corepressor 1	S460	0.9	1.0	0.8	0.8	-	-	-	-	1.0	1.0	0.8	0.9	-	-	-	-	1.0	1.0	0.8	0.9	sPDNSIK	SNQKPVKsPDNSIKM	S1;	No	S(1): 100.0; S(5): 0.0	1	1	1	0	N-Term(TMT6plex); S1(Phospho); K7(TMT6plex)
317108193	NP_001186873.1	6455	SH3GL1	endophilin-A2 isoform 3	S223	0.9	1.1	0.8	0.8	-	-	-	-	1.3	1.4	1.1	1.0	-	-	-	-	1.3	1.4	1.1	1.0	IAASsSFR	APKIAASsSFRSSDK	S5;	No	S(4): 0.7; S(5): 98.7; S(6): 0.7	1	3	1	0	N-Term(TMT6plex); S6(Phospho)
151301171	NP_114129.1	83860	TAF3	transcription initiation factor TFIID subunit 3	S297	0.6	0.8	0.9	0.8	-	-	-	-	0.6	0.7	0.8	0.7	-	-	-	-	0.6	0.7	0.8	0.7	TAQSPAMVGsPIR	QSPAMVGsPIRSPKT	S10;	Yes	T(1): 0.0; S(4): 0.0; S(10): 100.0	4	1	1	0	N-Term(TMT6plex); M7(Oxidation); S10(Phospho)
4506039	NP_003972.1	9055	PRC1	protein regulator of cytokinesis 1 isoform 1	S620	0.9	0.9	0.9	0.8	-	-	-	-	0.9	1.0	1.0	0.8	-	-	-	-	0.9	1.0	1.0	0.8	SDATSGILNSTNIQs	LNSTNIQs_	S15;	Yes	S(1): 0.0; T(4): 0.0; S(5): 0.0; S(10): 1.5; T(11): 1.5; S(15): 97.0	8	2	1	0	N-Term(TMT6plex); S15(Phospho)
338797736	NP_061994.1	284352	PPP1R37	protein phosphatase 1 regulatory subunit 37	S601	2.5	1.3	0.9	0.8	-	-	-	-	-	-	-	-	-	-	-	-	-	-	-	-	AEPPASPTPPSPPPPPSPPAsPSLPPAGAIDTR	PPPSPPAsPSLPPAG	S21;	No	S(6): 0.0; T(8): 0.1; S(11): 0.0; S(17): 2.3; S(21): 95.4; S(23): 2.3; T(32): 0.0	3	1	1	0	N-Term(TMT6plex); S17(Phospho)
398303814	NP_001257627.1	94134	ARHGAP12	rho GTPase-activating protein 12 isoform 5	S103	0.7	0.8	1.0	0.8	-	-	-	-	-	-	-	-	-	-	-	-	-	-	-	-	sTENVNK	QSLHLQRsTENVNKL	S1;	No	S(1): 100.0; T(2): 0.0	1	6	1	0	N-Term(TMT6plex); S1(Phospho); K7(TMT6plex)
7019569	NP_037377.1	27183	VPS4A	vacuolar protein sorting-associated protein 4A	S97	1.0	0.9	0.9	0.8	-	-	-	-	1.1	1.1	1.0	0.9	-	-	-	-	1.1	1.1	1.0	0.9	GSDsDSEGDNPEKK	SEGKGSDsDSEGDNP	S4;	Yes	S(2): 0.0; S(4): 100.0; S(6): 0.0	2	1	1	1	N-Term(TMT6plex); S4(Phospho); K13(TMT6plex); K14(TMT6plex)
112789562	NP_005522.2	3428	IFI16	gamma-interferon-inducible protein 16 isoform 2	T123	0.6	0.5	0.7	0.8	-	-	-	-	0.6	0.6	0.8	0.9	-	-	-	-	0.6	0.6	0.8	0.9	TEGAEAtPGAQK	KTEGAEAtPGAQKRK	T7;	Yes	T(1): 0.0; T(7): 100.0	5	1	1	0	N-Term(TMT6plex); T7(Phospho); K12(TMT6plex)
40538732	NP_060089.1	55599	RNPC3	RNA-binding protein 40	S108	0.8	0.7	0.9	0.8	-	-	-	-	-	-	-	-	-	-	-	-	-	-	-	-	VHsPCPTSGSEK	KEQDRVHsPCPTSGS	S3;	Yes	S(3): 100.0; T(7): 0.0; S(8): 0.0; S(10): 0.0	1	1	1	0	N-Term(TMT6plex); S3(Phospho); C5(Carbamidomethyl); K12(TMT6plex)
109134351	NP_054874.3	29068	ZBTB44	zinc finger and BTB domain-containing protein 44	S194	1.0	1.0	0.9	0.8	-	-	-	-	-	-	-	-	-	-	-	-	-	-	-	-	SYIVMSPEsPVK	YIVMSPEsPVKCGTQ	S9;	Yes	S(1): 0.0; Y(2): 0.0; S(6): 0.0; S(9): 100.0	4	1	1	0	N-Term(TMT6plex); M5(Oxidation); S9(Phospho); K12(TMT6plex)
87299628	NP_683692.2	259282	BOD1L1	biorientation of chromosomes in cell division protein 1-like 1	S1531	1.0	1.5	1.2	0.8	-	-	-	-	1.1	1.5	1.2	0.9	-	-	-	-	1.1	1.5	1.2	0.9	DKDVTLsPVK	KDKDVTLsPVKAGPA	S7;	Yes	T(5): 0.0; S(7): 100.0	36	1	1	1	N-Term(TMT6plex); K2(TMT6plex); S7(Phospho); K10(TMT6plex)
38683851	NP_060834.2	54665	RSBN1	round spermatid basic protein 1	S92	0.9	0.9	0.9	0.8	-	-	-	-	1.1	1.1	1.0	0.9	-	-	-	-	1.1	1.1	1.0	0.9	SsSGGSQEK	VKRQRRSsSGGSQEK	S2;	Yes	S(1): 0.0; S(2): 99.4; S(3): 0.6; S(6):	2	1	1	0	N-Term(TMT6plex); S2(Phospho); K9(TMT6plex)
29570782	NP_056153.2	171023	ASXL1	putative Polycomb group protein ASXL1 isoform 1	S503	0.3	0.4	0.8	0.8	-	-	-	-	0.3	0.3	0.7	0.6	-	-	-	-	0.3	0.3	0.7	0.6	ASAsPDRIPSLPQETVDQEPK	NLARASAsPDRIPSL	S4;	Yes	S(2): 3.1; S(4): 93.7; S(10): 0.1; T(15): 3.1	1	1	1	1	N-Term(TMT6plex); S4(Phospho); K21(TMT6plex)
190014603	NP_060671.3	54662	TBC1D13	TBC1 domain family member 13 isoform a	S184	0.8	1.0	0.9	0.8	-	-	-	-	0.7	1.0	0.9	0.8	-	-	-	-	0.7	1.0	0.9	0.8	SGVTNMSsPHK	SGVTNMSsPHKNSVP	S8;	Yes	S(1): 0.0; T(4): 0.0; S(7): 1.1; S(8):	1	1	1	0	N-Term(TMT6plex); M6(Oxidation); S8(Phospho); K11(TMT6plex)
557440810	NP_001273512.1	57661	PHRF1	PHD and RING finger domain-containing protein 1 isoform 4	S1225	0.6	0.9	0.9	0.8	-	-	-	-	0.6	1.0	1.0	0.9	-	-	-	-	0.6	1.0	1.0	0.9	LPALGEAHVsPEVATADK	ALGEAHVsPEVATAD	S10;	No	S(10): 100.0; T(15): 0.0	15	4	1	0	N-Term(TMT6plex); S10(Phospho); K18(TMT6plex)
192807320	NP_001122320.1	6597	SMARCA4	transcription activator BRG1 isoform F	T1392	0.7	0.7	0.6	0.8	-	-	-	-	0.7	0.7	0.7	0.8	-	-	-	-	0.7	0.7	0.7	0.8	DSDAGSSTPtTSTR	DAGSSTPtTSTRSRD	T10;	No	S(2): 0.0; S(6): 0.0; S(7): 0.0; T(8): 0.0; T(10): 98.3; T(11): 0.9; S(12): 0.9; T(13): 0.0	5	6	1	0	N-Term(TMT6plex); T8(Phospho)
333440451	NP_001193973.1	23122	CLASP2	CLIP-associating protein 2 isoform 2	S370	1.0	0.6	0.8	0.8	-	-	-	-	0.9	0.7	0.8	0.8	-	-	-	-	0.9	0.7	0.8	0.8	SRsDIDVNAAAGAK	GSLQRSRsDIDVNAA	S3;	Yes	S(1): 1.1; S(3): 98.9	4	2	1	1	N-Term(TMT6plex); S3(Phospho); K14(TMT6plex)
56699409	NP_002130.2	27316	RBMX	RNA-binding motif protein, X chromosome isoform 1	S189	0.7	0.8	0.9	0.8	-	-	-	-	0.9	1.0	1.1	0.9	-	-	-	-	0.9	1.0	1.1	0.9	DsYGGPPR	PVSRGRDsYGGPPRR	S2;	Yes	S(2): 100.0; Y(3): 0.0	5	2	1	0	N-Term(TMT6plex); S2(Phospho)
18643383	NP_079470.1	80271	ITPKC	inositol-trisphosphate 3-kinase C	T336	0.4	0.6	1.1	0.8	-	-	-	-	-	-	-	-	-	-	-	-	-	-	-	-	LIITPEtPEPEAQPVGPPSR	RLIITPEtPEPEAQP	T7;	Yes	T(4): 2.0; T(7): 98.0; S(19): 0.0	1	1	1	0	N-Term(TMT6plex); T7(Phospho)
195947382	NP_847884.2	353116	RILPL1	RILP-like protein 1	S346	0.8	0.8	0.8	0.8	-	-	-	-	0.8	0.8	0.8	0.8	-	-	-	-	0.8	0.8	0.8	0.8	TsPQPESGIK	PIAHPRTsPQPESGI	S2;	No	T(1): 0.6; S(2): 99.4; S(7): 0.0	3	1	1	0	N-Term(TMT6plex); S2(Phospho); K10(TMT6plex)
17158044	NP_001001.2	6194	RPS6	40S ribosomal protein S6	S236; S240	1.1	1.2	0.6	0.8	-	-	-	-	1.2	1.4	0.7	0.9	-	-	-	-	1.2	1.4	0.7	0.9	LSsLRAsTSK	AKRRRLSsLRASTSK;RLSSLRAsTSKSESS	S3; S7;	Yes;Yes	S(2): 0.0; S(3): 100.0; S(7): 100.0; T(8): 0.0; S(9): 0.0	2	1	1	1	N-Term(TMT6plex); S3(Phospho); S7(Phospho); K10(TMT6plex)
186928821	NP_001119523.1	3068	HDGF	hepatoma-derived growth factor isoform c	S158	0.8	1.2	1.1	0.8	-	-	-	-	0.9	1.0	1.0	0.7	-	-	-	-	0.9	1.0	1.0	0.7	AGDLLEDsPKRPK	AGDLLEDsPKRPKEA	S8;	No	S(8): 100.0	78	3	1	1	N-Term(TMT6plex); S8(Phospho); K10(TMT6plex); K13(TMT6plex)
57863269	NP_055871.1	23076	RRP1B	ribosomal RNA processing protein 1 homolog B	S245	1.0	0.7	1.0	0.8	-	-	-	-	1.1	0.8	1.1	0.7	-	-	-	-	1.1	0.8	1.1	0.7	VGDGDLsAEEIPENEVSLR	KVGDGDLsAEEIPEN	S7;	Yes	S(7): 99.8; S(17): 0.2	17	1	1	0	N-Term(TMT6plex); S7(Phospho)
119120915	NP_443156.2	114822	RHPN1	rhophilin-1	S102; T106	0.2	0.4	0.5	0.8	-	-	-	-	-	-	-	-	-	-	-	-	-	-	-	-	EELEELSGGVDPGRHGsEAVtVPMIPLGLK	VDPGRHGsEAVTVPM;RHGSEAVtVPMIPLG	S17; T21;	No;No	S(7): 6.1; S(17): 94.3; T(21): 99.6	1	1	1	1	N-Term(TMT6plex); S17(Phospho); T21(Phospho); M24(Oxidation); K30(TMT6plex)
148276990	NP_001073881.2	253461	ZBTB38	zinc finger and BTB domain-containing protein 38	S1021	0.5	0.7	0.8	0.8	-	-	-	-	0.5	0.6	0.6	0.7	-	-	-	-	0.5	0.6	0.6	0.7	QEQsPSTLK	LCAKQEQsPSTLKMH	S4;	Yes	S(4): 97.0; S(6): 1.5; T(7): 1.5	2	1	1	0	N-Term(TMT6plex); S4(Phospho); K9(TMT6plex)
38045896	NP_829883.1	23085	ERC1	ELKS/Rab6-interacting/CAST family member 1 isoform delta	T35	0.9	0.8	1.0	0.8	-	-	-	-	0.9	0.8	1.0	0.8	-	-	-	-	0.9	0.8	1.0	0.8	tNSTGGSSGSSVGGGSGK	PRLGHRRtNSTGGSS	T1;	Yes	T(1): 98.9; S(3): 1.1; T(4): 0.0; S(7): 0.0; S(8): 0.0; S(10): 0.0; S(11): 0.0; S(16): 0.0	7	2	1	0	N-Term(TMT6plex); T1(Phospho); K18(TMT6plex)
24497618	NP_054828.2	29028	ATAD2	ATPase family AAA domain-containing protein 2	S60	1.2	1.0	0.8	0.8	-	-	-	-	1.2	1.1	0.9	0.8	-	-	-	-	1.2	1.1	0.9	0.8	AGDGsSVK	TAKAGDGsSVKEVET	S5;	Yes	S(5): 99.4; S(6): 0.6	2	1	1	0	N-Term(TMT6plex); S6(Phospho); K8(TMT6plex)
259013556	NP_004851.2	9513	FXR2	fragile X mental retardation syndrome-related protein 2	S453	1.1	0.8	0.9	0.8	-	-	-	-	1.0	0.7	1.0	0.8	-	-	-	-	1.0	0.7	1.0	0.8	TGGPAYGPSSDVSTAsETESEKR	SSDVSTAsETESEKR	S16;	Yes	T(1): 0.0; Y(6): 0.0; S(9): 0.0; S(10): 0.0; S(13): 0.0; T(14): 1.5; S(16): 98.4; T(18): 0.0; S(20): 0.0	6	1	1	1	N-Term(TMT6plex); S16(Phospho); K22(TMT6plex)
103472005	NP_002408.3	4288	MKI67	antigen KI-67 isoform 1	T2085	0.8	1.2	1.0	0.8	-	-	-	-	0.9	1.2	1.0	0.8	-	-	-	-	0.9	1.2	1.0	0.8	ELFQtPDHTEESTTDDK	GFKELFQtPDHTEES	T5;	Yes	T(5): 100.0; T(9): 0.0; S(12): 0.0; T(13): 0.0; T(14): 0.0	118	2	1	0	N-Term(TMT6plex); T5(Phospho); K17(TMT6plex)
33598968	NP_005349.3	4008	LMO7	LIM domain only protein 7 isoform 1	S1252	0.5	0.9	0.8	0.8	-	-	-	-	0.5	0.7	0.5	0.7	-	-	-	-	0.5	0.7	0.5	0.7	TSTTGVATTQsPTPR	TGVATTQsPTPRSHS	S11;	No	T(1): 0.0; S(2): 0.0; T(3): 0.0; T(4): 0.0; T(8): 0.0; T(9): 1.1; S(11): 97.7; T(13): 1.1	19	2	1	0	N-Term(TMT6plex); S11(Phospho)
300244514	NP_001177906.1	10138	YAF2	YY1-associated factor 2 isoform 3	S94	0.6	0.9	0.7	0.7	-	-	-	-	0.8	1.1	0.9	0.8	-	-	-	-	0.8	1.1	0.9	0.8	TKsPPASSAASADQHSQSGSSSDNTER	DFKEKTKsPPASSAA	S3;	No	T(1): 1.9; S(3): 98.1; S(7): 0.0; S(8): 0.0; S(11): 0.0; S(16): 0.0; S(18): 0.0; S(20): 0.0; S(21): 0.0; S(22): 0.0; T(25): 0.0	4	3	1	1	N-Term(TMT6plex); K2(TMT6plex); S3(Phospho)
19923592	NP_073613.2	114885	OSBPL11	oxysterol-binding protein-related protein 11	T27	0.6	1.0	0.9	0.7	-	-	-	-	0.6	0.9	1.0	0.7	-	-	-	-	0.6	0.9	1.0	0.7	LEGQATAVtPNK	EGQATAVtPNKNSSC	T9;	Yes	T(6): 0.0; T(9): 100.0	2	1	1	0	N-Term(TMT6plex); T9(Phospho); K12(TMT6plex)
495529773	NP_001265048.1	7745	ZKSCAN8	zinc finger protein with KRAB and SCAN domains 8 isoform 1	S193	0.9	0.9	0.7	0.7	-	-	-	-	0.9	1.0	0.8	0.7	-	-	-	-	0.9	1.0	0.8	0.7	AMSTSQsPTR	RAMSTSQsPTRSQKG	S7;	No	S(3): 0.0; T(4): 0.0; S(5): 0.0; S(7): 100.0; T(9): 0.0	1	1	1	0	N-Term(TMT6plex); M2(Oxidation); S7(Phospho)
545687835	NP_001269941.1	64798	DEPTOR	DEP domain-containing mTOR-interacting protein isoform 2	S164	1.0	1.0	0.9	0.7	-	-	-	-	-	-	-	-	-	-	-	-	-	-	-	-	STSFMSVsPSK	STSFMSVsPSKEIKI	S8;	No	S(1): 0.0; T(2): 0.0; S(3): 0.0; S(6): 0.0; S(8): 100.0; S(10): 0.0	2	2	1	0	N-Term(TMT6plex); M5(Oxidation); S8(Phospho); K11(TMT6plex)
314122233	NP_001186627.1	7917	BAG6	large proline-rich protein BAG6 isoform d	S958	0.7	0.7	1.4	0.7	-	-	-	-	0.7	0.8	1.6	0.8	-	-	-	-	0.7	0.8	1.6	0.8	VGDPPQPLPEEPMEVQGAERAsPEPQR	VQGAERAsPEPQREN	S22;	No	S(22): 100.0	15	3	1	1	N-Term(TMT6plex); M13(Oxidation); S22(Phospho)
255652953	NP_653205.3	124245	ZC3H18	zinc finger CCCH domain-containing protein 18	S775	0.8	1.1	0.9	0.7	-	-	-	-	1.0	1.1	1.0	0.8	-	-	-	-	1.0	1.1	1.0	0.8	DSsTQPPK	EKRKRDSsTQPPKSA	S3;	Yes	S(2): 0.7; S(3): 98.7; T(4): 0.7	34	1	1	0	N-Term(TMT6plex); S3(Phospho); K8(TMT6plex)
190014588	NP_001121689.1	11168	PSIP1	PC4 and SFRS1-interacting protein isoform 2	S129	0.8	0.9	0.8	0.7	-	-	-	-	0.9	1.0	1.1	0.8	-	-	-	-	0.9	1.0	1.1	0.8	EDTDHEEKAsNEDVTK	TDHEEKAsNEDVTKA	S10;	Yes	T(3): 0.0; S(10): 100.0; T(15): 0.0	27	2	1	1	N-Term(TMT6plex); K8(TMT6plex); S10(Phospho); K16(TMT6plex)
24431975	NP_056467.2	26148	C10orf12	uncharacterized protein C10orf12	S273	0.6	0.6	0.6	0.7	-	-	-	-	-	-	-	-	-	-	-	-	-	-	-	-	EITSHEEGGGDVsPR	EEGGGDVsPRKEPQE	S13;	Yes	T(3): 0.0; S(4): 0.0; S(13): 100.0	10	1	1	0	N-Term(TMT6plex); S13(Phospho)
24497618	NP_054828.2	29028	ATAD2	ATPase family AAA domain-containing protein 2	S1233	0.6	0.5	0.8	0.7	-	-	-	-	0.6	0.5	0.8	0.8	-	-	-	-	0.6	0.5	0.8	0.8	QQNAsESK	NEKQQNAsESKLELR	S5;	Yes	S(5): 99.0; S(7): 1.0	2	1	1	0	N-Term(TMT6plex); S5(Phospho); K8(TMT6plex)
224994205	NP_001139325.1	7466	WFS1	wolframin	S235	1.1	0.7	0.7	0.7	-	-	-	-	1.1	0.7	0.7	0.9	-	-	-	-	1.1	0.7	0.7	0.9	LVsSESK	RMLERLVsSESKNYI	S3;	Yes	S(3): 100.0; S(4): 0.0; S(6): 0.0	2	1	1	0	N-Term(TMT6plex); S3(Phospho); K7(TMT6plex)
33563376	NP_061893.2	125058	TBC1D16	TBC1 domain family member 16 isoform a	T99	0.9	0.9	0.8	0.7	-	-	-	-	-	-	-	-	-	-	-	-	-	-	-	-	YItPESSPVR	EEALRYItPESSPVR	T3;	Yes	Y(1): 0.0; T(3): 100.0; S(6): 50.0; S(7): 50.0	1	1	1	0	N-Term(TMT6plex); T3(Phospho); S7(Phospho)
157671929	NP_002636.2	5286	PIK3C2A	phosphatidylinositol 4-phosphate 3-kinase C2 domain-containing subunit alpha	S1553	1.2	0.7	1.1	0.7	-	-	-	-	0.9	0.6	0.9	0.6	-	-	-	-	0.9	0.6	0.9	0.6	SADAGSFsPTPGQIGGAVK	SADAGSFsPTPGQIG	S8;	Yes	S(1): 0.0; S(6): 1.5; S(8): 96.9; T(10): 1.5	1	1	1	0	N-Term(TMT6plex); S8(Phospho); K19(TMT6plex)
55770884	NP_057256.2	10422	UBAC1	ubiquitin-associated domain-containing protein 1	S98	0.8	0.9	0.7	0.7	-	-	-	-	0.8	1.1	0.8	0.9	-	-	-	-	0.8	1.1	0.8	0.9	RAPsPLPK	LIKKRAPsPLPKMAD	S4;	Yes	S(4): 100.0	1	1	1	1	N-Term(TMT6plex); S4(Phospho); K8(TMT6plex)
29171753	NP_803172.1	8500	PPFIA1	liprin-alpha-1 isoform a	S150	0.7	1.3	1.1	0.7	-	-	-	-	0.7	1.4	1.2	0.8	-	-	-	-	0.7	1.4	1.2	0.8	QAQsPAGVSSEVEVLK	VVKRQAQsPAGVSSE	S4;	Yes	S(4): 100.0; S(9): 0.0; S(10): 0.0	9	2	1	0	N-Term(TMT6plex); S4(Phospho); K16(TMT6plex)
110556636	NP_203754.2	85456	TNKS1BP1	182 kDa tankyrase-1-binding protein	S1138	0.7	1.2	0.9	0.7	-	-	-	-	0.7	1.1	0.9	0.8	-	-	-	-	0.7	1.1	0.9	0.8	VSGAGFsPSSK	RVSGAGFsPSSKMEG	S7;	Yes	S(2): 0.0; S(7): 100.0; S(9): 0.0; S(10): 0.0	41	1	1	0	N-Term(TMT6plex); S7(Phospho); K11(TMT6plex)
45597177	NP_055858.2	23061	TBC1D9B	TBC1 domain family member 9B isoform b	S463	0.6	0.5	0.8	0.7	-	-	-	-	0.7	0.6	1.0	0.8	-	-	-	-	0.7	0.6	1.0	0.8	NsPMEDLGAK	LKLEQKNsPMEDLGA	S2;	Yes	S(2): 100.0	5	2	1	0	N-Term(TMT6plex); S2(Phospho); M4(Oxidation); K10(TMT6plex)
194097365	NP_060117.3	117246	FTSJ3	pre-rRNA processing protein FTSJ3	S599	1.0	0.7	0.8	0.7	-	-	-	-	1.0	0.7	0.9	0.7	-	-	-	-	1.0	0.7	0.9	0.7	GTEASsGTEAATGLEGEEK	PKGTEASsGTEAATG	S6;	Yes	T(2): 0.0; S(5): 1.2; S(6): 98.7; T(8): 0.0; T(12): 0.0	12	1	1	0	N-Term(TMT6plex); S6(Phospho); K19(TMT6plex)
118421085	NP_689472.3	90381	TICRR	treslin	T1105	0.7	0.9	0.7	0.7	-	-	-	-	0.9	0.9	0.8	0.8	-	-	-	-	0.9	0.9	0.8	0.8	NTLDSEVPAAYQtPKK	EVPAAYQtPKKSHQK	T13;	Yes	T(2): 0.0; S(5): 0.0; Y(11): 1.3; T(13): 98.7	11	1	1	1	N-Term(TMT6plex); T13(Phospho); K15(TMT6plex); K16(TMT6plex)
217330641	NP_060439.2	55677	IWS1	protein IWS1 homolog	S438	0.8	1.2	0.8	0.7	-	-	-	-	0.9	1.1	0.9	0.8	-	-	-	-	0.9	1.1	0.9	0.8	TIAsDSEEEAGK	KREKTIAsDSEEEAG	S4;	Yes	T(1): 0.0; S(4): 99.9; S(6): 0.0	25	1	1	0	N-Term(TMT6plex); S4(Phospho); K12(TMT6plex)
5032189	NP_005648.1	7158	TP53BP1	tumor suppressor p53-binding protein 1 isoform 3	S316	0.6	0.9	1.1	0.7	-	-	-	-	0.7	0.9	1.1	0.9	-	-	-	-	0.7	0.9	1.1	0.9	TVSsDGCSTPSR	QSNKTVSsDGCSTPS	S4;	Yes	T(1): 0.0; S(3): 0.9; S(4): 99.1; S(8): 0.0; T(9): 0.0; S(11): 0.0	62	3	1	0	N-Term(TMT6plex); S4(Phospho); C7(Carbamidomethyl)
150010552	NP_056073.1	23307	FKBP15	FK506-binding protein 15	S1195	0.6	0.7	0.6	0.7	-	-	-	-	0.6	0.8	0.7	0.9	-	-	-	-	0.6	0.8	0.7	0.9	AQPEEEDEDEVsMK	EEDEDEVsMKGRPPP	S12;	Yes	S(12): 100.0	9	1	1	0	N-Term(TMT6plex); S12(Phospho); M13(Oxidation); K14(TMT6plex)
190343008	NP_004501.3	3431	SP110	sp110 nuclear body protein isoform b	S256	0.7	0.7	0.8	0.7	-	-	-	-	0.7	0.6	0.6	0.7	-	-	-	-	0.7	0.6	0.6	0.7	DNsPEPNDPEEPQEVSSTPSDKK	MPEIRDNsPEPNDPE	S3;	Yes	S(3): 100.0; S(16): 0.0; S(17): 0.0; T(18): 0.0; S(20): 0.0	3	4	1	1	N-Term(TMT6plex); S3(Phospho); K22(TMT6plex); K23(TMT6plex)
21450761	NP_659467.1	221908	PPP1R35	protein phosphatase 1 regulatory subunit 35	S87	0.8	1.2	1.3	0.7	-	-	-	-	-	-	-	-	-	-	-	-	-	-	-	-	LTPPsPVR	RERLTPPsPVRSEPQ	S5;	Yes	T(2): 0.0; S(5): 100.0	1	1	1	0	N-Term(TMT6plex); S5(Phospho)
21389403	NP_653207.1	201163	FLCN	folliculin isoform 2	S73	0.8	0.6	0.8	0.7	-	-	-	-	-	-	-	-	-	-	-	-	-	-	-	-	AHSPAEGASVESSsPGPK	GASVESSsPGPKKSD	S14;	Yes	S(3): 0.0; S(9): 1.4; S(12): 0.0; S(13): 0.0; S(14): 98.6	2	2	1	0	N-Term(TMT6plex); S14(Phospho); K18(TMT6plex)
428673536	NP_001258807.1	10657	KHDRBS1	KH domain-containing, RNA-binding, signal transduction-associated protein 1 isoform 2	S18	0.7	1.0	0.8	0.7	-	-	-	-	0.8	1.2	1.0	0.8	-	-	-	-	0.8	1.2	1.0	0.8	sGSMDPSGAHPSVR	MSRSSGRsGSMDPSG	S1;	Yes	S(1): 99.0; S(3): 1.0; S(7): 0.0; S(12): 0.0	4	2	1	0	N-Term(TMT6plex); S1(Phospho); M4(Oxidation)
93141029	NP_001035224.1	5064	PALM	paralemmin-1 isoform 2	S162	0.8	0.8	1.0	0.7	-	-	-	-	-	-	-	-	-	-	-	-	-	-	-	-	TVDGsPMMK	PLRTVDGsPMMKAVV	S5;	No	T(1): 0.0; S(5): 100.0	6	2	1	0	N-Term(TMT6plex); S5(Phospho); M7(Oxidation); M8(Oxidation); K9(TMT6plex)
295054220	NP_001171136.1	55183	RIF1	telomere-associated protein RIF1 isoform 2	S1952	0.7	0.9	1.1	0.7	-	-	-	-	0.7	1.0	1.2	0.8	-	-	-	-	0.7	1.0	1.2	0.8	NDDsEADTAK	ENKRNDDsEADTAKL	S4;	Yes	S(4): 100.0; T(8): 0.0	15	2	1	0	N-Term(TMT6plex); S4(Phospho); K10(TMT6plex)
499590378	NP_001265132.1	23155	CLCC1	chloride channel CLIC-like protein 1 isoform 4 precursor	S347	0.7	0.6	1.1	0.7	-	-	-	-	0.7	0.6	1.2	0.7	-	-	-	-	0.7	0.6	1.2	0.7	SEAAGSPDQGSTYsPAR	PDQGSTYsPARGVAG	S14;	No	S(1): 0.0; S(6): 0.0; S(11): 0.0; T(12): 1.5; Y(13): 1.5; S(14): 97.0	6	4	1	0	N-Term(TMT6plex); S14(Phospho)
57863246	NP_056084.1	23318	ZCCHC11	terminal uridylyltransferase 4 isoform b	S156	0.8	0.7	0.7	0.7	-	-	-	-	0.7	0.6	0.7	0.5	-	-	-	-	0.7	0.6	0.7	0.5	VPSsPAEAEK	KSEKVPSsPAEAEKG	S4;	Yes	S(3): 0.9; S(4): 99.1	3	2	1	0	N-Term(TMT6plex); S3(Phospho); K10(TMT6plex)
5730124	NP_006517.1	7764	ZNF217	zinc finger protein 217	S570	0.6	0.6	0.5	0.7	-	-	-	-	0.8	0.7	0.6	0.8	-	-	-	-	0.8	0.7	0.6	0.8	DVTGsPPAK	GAKDVTGsPPAKQLK	S5;	Yes	T(3): 0.0; S(5): 100.0	9	1	1	0	N-Term(TMT6plex); S5(Phospho); K9(TMT6plex)
112421108	NP_055940.3	23152	CIC	protein capicua homolog	S1397; S1409	1.2	0.8	1.1	0.7	-	-	-	-	-	-	-	-	-	-	-	-	-	-	-	-	KNsTDLDSAPEDPTsPK	YRKKRKNsTDLDSAP;SAPEDPTsPKRKMRR	S3; S15;	Yes;Yes	S(3): 98.0; T(4): 2.0; S(8): 0.0; T(14): 0.0; S(15): 100.0	8	1	1	1	N-Term(TMT6plex); K1(TMT6plex); T4(Phospho); S15(Phospho); K17(TMT6plex)
38788260	NP_004450.3	2186	BPTF	nucleosome-remodeling factor subunit BPTF isoform 2	S1231	0.6	0.6	0.6	0.7	-	-	-	-	0.7	0.6	0.6	0.7	-	-	-	-	0.7	0.6	0.6	0.7	NLSEsPVITK	SSKNLSEsPVITKAK	S5;	Yes	S(3): 0.0; S(5): 100.0; T(9): 0.0	11	2	1	0	N-Term(TMT6plex); S5(Phospho); K10(TMT6plex)
56699409	NP_002130.2	27316	RBMX	RNA-binding motif protein, X chromosome isoform 1	S328	1.0	1.1	1.2	0.7	-	-	-	-	1.1	1.5	1.5	0.8	-	-	-	-	1.1	1.5	1.5	0.8	DSYsSSR	GGSRDSYsSSRSDLY	S4;	Yes	S(2): 0.0; Y(3): 0.4; S(4): 99.6; S(5): 0.0; S(6): 0.0	5	2	1	0	N-Term(TMT6plex); S4(Phospho)
125656165	NP_001075019.1	57187	THOC2	THO complex subunit 2	T1414	1.2	0.8	0.9	0.7	-	-	-	-	1.2	0.8	0.9	0.7	-	-	-	-	1.2	0.8	0.9	0.7	IDtHPSPSHSSTVK	QKRRKIDtHPSPSHS	T3;	Yes	T(3): 98.2; S(6): 1.8; S(8): 0.0; S(10): 0.0; S(11): 0.0; T(12): 0.0	7	1	1	0	N-Term(TMT6plex); S6(Phospho); K14(TMT6plex)
519666783	NP_001265539.1	4605	MYBL2	myb-related protein B isoform 2	S258	0.9	1.1	0.6	0.7	-	-	-	-	0.9	1.2	0.7	0.7	-	-	-	-	0.9	1.2	0.7	0.7	REDQEGsPPETSLPYK	KREDQEGsPPETSLP	S7;	No	S(7): 100.0; T(11): 0.0; S(12): 0.0; Y(15): 0.0	1	2	1	1	N-Term(TMT6plex); S7(Phospho); K16(TMT6plex)
4827046	NP_005080.1	8233	ZRSR2	U2 small nuclear ribonucleoprotein auxiliary factor 35 kDa subunit-related protein 2	S349	1.2	1.1	1.8	0.7	-	-	-	-	1.8	1.3	2.1	0.8	-	-	-	-	1.8	1.3	2.1	0.8	DIYLsPDR	ANRDIYLsPDRTGSS	S5;	Yes	Y(3): 0.8; S(5): 99.2	1	1	1	0	N-Term(TMT6plex); S5(Phospho)
568214245	NP_001274744.1	9898	UBAP2L	ubiquitin-associated protein 2-like isoform c	S432	0.7	1.3	1.4	0.7	-	-	-	-	0.8	1.3	1.6	0.6	-	-	-	-	0.8	1.3	1.6	0.6	sPAVATSTAAPPPPSSPLPSK	EVFLQEKsPAVATST	S1;	No	S(1): 99.5; T(6): 0.2; S(7): 0.2; T(8): 0.1; S(15): 49.9; S(16): 49.9; S(20):	23	4	1	0	N-Term(TMT6plex); S7(Phospho); S15(Phospho); K21(TMT6plex)
525459422	NP_001265867.1	9166	EBAG9	receptor-binding cancer antigen expressed on SiSo cells	S36	1.3	1.4	1.5	0.7	-	-	-	-	1.1	1.3	1.3	0.7	-	-	-	-	1.1	1.3	1.3	0.7	KLsGDQITLPTTVDYSSVPK	SGRGRKLsGDQITLP	S3;	Yes	S(3): 100.0; T(8): 0.0; T(11): 0.0; T(12): 0.0; Y(15): 0.0; S(16): 0.0;	1	1	1	1	N-Term(TMT6plex); K1(TMT6plex); S3(Phospho); K20(TMT6plex)
237757326	NP_001153778.1	8867	SYNJ1	synaptojanin-1 isoform d	S1518	1.0	1.0	1.1	0.7	-	-	-	-	0.9	0.8	0.9	0.6	-	-	-	-	0.9	0.8	0.9	0.6	AsPTLDFTER	TTLASKAsPTLDFTE	S2;	No	S(2): 98.6; T(4): 1.4; T(8): 0.0	1	2	1	0	N-Term(TMT6plex); S2(Phospho)
47519639	NP_002366.2	4134	MAP4	microtubule-associated protein 4 isoform 1	S60	0.5	0.5	0.8	0.7	-	-	-	-	0.5	0.5	0.8	0.9	-	-	-	-	0.5	0.5	0.8	0.9	TGNsESK	VDEKTGNsESKKKPC	S4;	Yes	T(1): 0.0; S(4): 100.0; S(6): 0.0	47	3	1	0	N-Term(TMT6plex); S4(Phospho); K7(TMT6plex)
7019371	NP_037474.1	29844	TFPT	TCF3 fusion partner	S167	0.8	0.9	0.9	0.7	-	-	-	-	0.9	0.8	0.9	0.7	-	-	-	-	0.9	0.8	0.9	0.7	ETLsPPR	PPEKETLsPPRRTPA	S4;	Yes	T(2): 0.6; S(4): 99.4	12	1	1	0	N-Term(TMT6plex); S4(Phospho)
308081964	NP_001183980.1	80829	ZFP91	E3 ubiquitin-protein ligase ZFP91 isoform 2	S83	0.7	0.8	0.7	0.7	-	-	-	-	0.7	0.8	0.7	0.8	-	-	-	-	0.7	0.8	0.7	0.8	SsPSARPPDVPGQQPQAAK	YPRRRRSsPSARPPD	S2;	Yes	S(1): 1.0; S(2): 99.0; S(4): 0.0	5	2	1	0	N-Term(TMT6plex); S2(Phospho); K19(TMT6plex)
545746335	NP_001271143.1	9320	TRIP12	E3 ubiquitin-protein ligase TRIP12 isoform a	S119	0.9	0.8	0.9	0.7	-	-	-	-	0.9	0.8	0.9	0.7	-	-	-	-	0.9	0.8	0.9	0.7	SAsPDYNR	RGVKRSAsPDYNRTN	S3;	No	S(1): 0.0; S(3): 100.0; Y(6): 0.0	23	3	1	0	N-Term(TMT6plex); S3(Phospho)
47519639	NP_002366.2	4134	MAP4	microtubule-associated protein 4 isoform 1	S280	0.9	1.1	1.1	0.7	-	-	-	-	0.9	1.1	1.1	0.9	-	-	-	-	0.9	1.1	1.1	0.9	DMEsPTK	ALAKDMEsPTKLDVT	S4;	Yes	S(4): 100.0; T(6): 0.0	47	2	1	0	N-Term(TMT6plex); M2(Oxidation); S4(Phospho); K7(TMT6plex)
5031633	NP_005757.1	10160	FARP1	FERM, RhoGEF and pleckstrin domain-containing protein 1 isoform 1	T24	0.9	0.7	0.6	0.7	-	-	-	-	-	-	-	-	-	-	-	-	-	-	-	-	LGAPENSGIStLER	PENSGIStLERGQKP	T11;	Yes	S(7): 0.0; S(10): 1.1; T(11): 98.9	2	3	1	0	N-Term(TMT6plex); T11(Phospho)
14790190	NP_055816.2	23013	SPEN	msx2-interacting protein	S2101	0.6	0.5	0.7	0.7	-	-	-	-	0.6	0.4	0.7	0.7	-	-	-	-	0.6	0.4	0.7	0.7	NVDAAVsPR	KNVDAAVsPRGAAAQ	S7;	Yes	S(7): 100.0	16	1	1	0	N-Term(TMT6plex); S7(Phospho)
4758340	NP_004452.1	2193	FARSA	phenylalanine--tRNA ligase alpha subunit	T299	0.7	0.8	0.8	0.7	-	-	-	-	0.7	0.8	0.9	0.7	-	-	-	-	0.7	0.8	0.9	0.7	tHSQGGYGSQGYK	YVQRVKRtHSQGGYG	T1;	No	T(1): 98.7; S(3): 1.3; Y(7): 0.0; S(9): 0.0; Y(12): 0.0	1	1	1	0	N-Term(TMT6plex); T1(Phospho); K13(TMT6plex)
56699409	NP_002130.2	27316	RBMX	RNA-binding motif protein, X chromosome isoform 1	S352	1.1	0.9	1.1	0.7	-	-	-	-	1.2	1.2	1.4	0.8	-	-	-	-	1.2	1.2	1.4	0.8	GLPPsMER	QERGLPPsMERGYPP	S5;	Yes	S(5): 100.0	5	1	1	0	N-Term(TMT6plex); S5(Phospho); M6(Oxidation)
41406089	NP_005677.2	9580	SOX13	transcription factor SOX-13	S386	0.7	0.6	0.3	0.7	-	-	-	-	-	-	-	-	-	-	-	-	-	-	-	-	DLISLDSsPAK	DLISLDSsPAKERLE	S8;	Yes	S(4): 0.0; S(7): 0.8; S(8): 99.2	3	1	1	0	N-Term(TMT6plex); S8(Phospho); K11(TMT6plex)
183396804	NP_056018.2	23248	RPRD2	regulation of nuclear pre-mRNA domain-containing protein 2	S593	0.7	0.6	0.7	0.7	-	-	-	-	0.7	0.6	0.7	0.6	-	-	-	-	0.7	0.6	0.7	0.6	SFNYsPNSSTSEVSSTSASK	IPKSFNYsPNSSTSE	S5;	Yes	S(1): 0.0; Y(4): 1.5; S(5): 98.5; S(8): 0.0; S(9): 0.0; T(10): 0.0; S(11): 0.0; S(14): 0.0; S(15): 0.0; T(16): 0.0; S(17): 0.0; S(19): 0.0	8	1	1	0	N-Term(TMT6plex); S5(Phospho); K20(TMT6plex)
289577114	NP_001036024.3	1982	EIF4G2	eukaryotic translation initiation factor 4 gamma 2	T470	1.2	0.6	0.9	0.7	-	-	-	-	1.2	0.6	0.8	0.7	-	-	-	-	1.2	0.6	0.8	0.7	TQtPPLGQTPQLGLK	AQPPRTQtPPLGQTP	T3;	No	T(1): 1.0; T(3): 99.0; T(9): 0.0	6	2	1	0	N-Term(TMT6plex); T3(Phospho); K15(TMT6plex)
395627636	NP_001257468.1	92960	PEX11G	peroxisomal membrane protein 11C isoform 2	T179	2.5	1.3	1.0	0.7	-	-	-	-	-	-	-	-	-	-	-	-	-	-	-	-	AGGQAEATtP	GGQAEATtP	T9;	No	T(8): 0.0; T(9): 100.0	1	2	1	0	N-Term(TMT6plex); T9(Phospho)
13129104	NP_077003.1	79080	CCDC86	coiled-coil domain-containing protein 86	S66	0.8	0.9	1.0	0.7	-	-	-	-	1.0	0.9	1.1	0.8	-	-	-	-	1.0	0.9	1.1	0.8	TsPGSPR	PERPPKTsPGSPRLQ	S2;	Yes	T(1): 0.0; S(2): 99.2; S(5): 0.8	26	1	1	0	N-Term(TMT6plex); S2(Phospho)
24307929	NP_009211.1	11339	OIP5	protein Mis18-beta	T221	0.9	0.7	1.0	0.7	-	-	-	-	0.9	0.7	0.9	0.7	-	-	-	-	0.9	0.7	0.9	0.7	ILSEVtPDQSKPEN	MKILSEVtPDQSKPE	T6;	Yes	S(3): 0.0; T(6): 100.0; S(10): 0.0	1	1	1	0	N-Term(TMT6plex); T6(Phospho); K11(TMT6plex)
23111062	NP_004583.2	6433	SFSWAP	splicing factor, suppressor of white-apricot homolog isoform 2	S844; S846	1.0	0.8	1.0	0.7	-	-	-	-	1.0	0.7	1.0	0.7	-	-	-	-	1.0	0.7	1.0	0.7	sRsPHEK	ASRKRTRsRSPHEKK;RKRTRSRsPHEKKKK	S1; S3;	No;No	S(1): 100.0; S(3): 100.0	1	2	1	1	N-Term(TMT6plex); S1(Phospho); S3(Phospho); K7(TMT6plex)
300244569	NP_001177916.1	9295	SRSF11	serine/arginine-rich splicing factor 11 isoform 2	S448	0.7	0.8	0.6	0.7	-	-	-	-	0.8	0.9	0.8	0.8	-	-	-	-	0.8	0.9	0.8	0.8	KPIETGsPK	KKPIETGsPKTKECS	S7;	No	T(5): 0.9; S(7): 99.1	4	2	1	0	N-Term(TMT6plex); K1(TMT6plex); S7(Phospho); K9(TMT6plex)
103472005	NP_002408.3	4288	MKI67	antigen KI-67 isoform 1	S3005	0.6	0.9	0.8	0.7	-	-	-	-	0.7	0.9	0.8	0.7	-	-	-	-	0.7	0.9	0.8	0.7	DGsVTGTK	RGGGKDGsVTGTKRL	S3;	No	S(3): 99.2; T(5): 0.0; T(7): 0.8	118	2	1	0	N-Term(TMT6plex); S3(Phospho); K8(TMT6plex)
118572613	NP_057417.3	23524	SRRM2	serine/arginine repetitive matrix protein 2	S2132	0.9	0.9	1.1	0.7	-	-	-	-	0.9	0.9	1.2	0.8	-	-	-	-	0.9	0.9	1.2	0.8	sPGMLEPLGSSR	TPLDRCRsPGMLEPL	S1;	Yes	S(1): 100.0; S(10): 0.0; S(11): 0.0	436	1	1	0	N-Term(TMT6plex); S1(Phospho); M4(Oxidation)
255918139	NP_001157616.1	283489	CHAMP1	chromosome alignment-maintaining phosphoprotein 1	S405	1.3	0.5	1.3	0.7	-	-	-	-	1.3	0.5	1.2	0.7	-	-	-	-	1.3	0.5	1.2	0.7	TAPTLsPEHWK	RKTAPTLsPEHWKAV	S6;	Yes	T(1): 0.0; T(4): 1.2; S(6): 98.8	24	1	1	0	N-Term(TMT6plex); S6(Phospho); K11(TMT6plex)
112821681	NP_443131.2	114787	GPRIN1	G protein-regulated inducer of neurite outgrowth 1	S64	1.0	1.0	0.8	0.7	-	-	-	-	-	-	-	-	-	-	-	-	-	-	-	-	HTPDQsPGMESR	PRHTPDQsPGMESRH	S6;	Yes	T(2): 0.0; S(6): 100.0; S(11): 0.0	2	1	1	0	N-Term(TMT6plex); S6(Phospho); M9(Oxidation)
42542379	NP_005830.2	10250	SRRM1	serine/arginine repetitive matrix protein 1	S626; S628	0.6	0.9	0.9	0.7	-	-	-	-	0.7	0.9	1.0	0.8	-	-	-	-	0.7	0.9	1.0	0.8	AsPsPPPK	PPPKRRAsPSPPPKR;PKRRASPsPPPKRRV	S2; S4;	Yes;Yes	S(2): 100.0; S(4): 100.0	229	1	1	0	N-Term(TMT6plex); S2(Phospho); S4(Phospho); K8(TMT6plex)
543583787	NP_001269472.1	580	BARD1	BRCA1-associated RING domain protein 1 isoform 2	T280	0.7	0.7	0.9	0.7	-	-	-	-	-	-	-	-	-	-	-	-	-	-	-	-	NEVVtPEK	KSRNEVVtPEKVCKN	T5;	No	T(5): 100.0	4	2	1	0	N-Term(TMT6plex); T5(Phospho); K8(TMT6plex)
55741709	NP_067062.1	58517	RBM25	RNA-binding protein 25	S703	1.1	0.8	1.0	0.7	-	-	-	-	1.2	0.9	1.0	0.7	-	-	-	-	1.2	0.9	1.0	0.7	EEDEDsDDVPR	NKEEDEDsDDVPRKR	S6;	Yes	S(6): 100.0	11	1	1	0	N-Term(TMT6plex); S6(Phospho)
224994205	NP_001139325.1	7466	WFS1	wolframin	S32	1.1	1.0	1.0	0.7	-	-	-	-	1.1	1.0	1.1	0.8	-	-	-	-	1.1	1.0	1.1	0.8	LNATAsLEQER	SRLNATAsLEQERSE	S6;	Yes	T(4): 0.0; S(6): 100.0	2	1	1	0	N-Term(TMT6plex); S6(Phospho)

Khan et al., 2019. Multi-omics analysis to characterize cigarette smoke induced molecular alterations in esophageal cells

Supplementary Table 8. List of phosphopeptides identified and quantified in untreated and chronically treated Het1A cells with cigarette smoke condensate for 8 months using SequestHT and Mascot search algorithms with a PhosphoRS probability score ≥75%

														Normalized phosphoprotein ratios with respect to corresponding total protein ratios.								Average of replicates													
Protein group Accession	NP_Accession	Gene ID	Gene Symbol	Protein Description	PhosphoSite (Protein)	Het-1A-Smoke - 2M/Parental	Het-1A-Smoke - 4M/Parental	Het-1A-Smoke - 6M/Parental	Het-1A-Smoke - 8M/Parental	Het-1A-Smoke - 2M/Parental	Het-1A-Smoke - 4M/Parental	Het-1A-Smoke - 6M/Parental	Het-1A-Smoke - 8M/Parental	Het-1A-Smoke - 2M/Parental	Het-1A-Smoke - 4M/Parental	Het-1A-Smoke - 6M/Parental	Het-1A-Smoke - 8M/Parental	Het-1A-Smoke - 2M/Parental	Het-1A-Smoke - 4M/Parental	Het-1A-Smoke - 6M/Parental	Het-1A-Smoke - 8M/Parental	Het-1A-Smoke - 2M/Parental	Het-1A-Smoke - 4M/Parental	Het-1A-Smoke - 6M/Parental	Het-1A-Smoke - 8M/Parental	Peptide Sequence	Phosphowindow	PhosphoSite (Peptide)	Phosphosite Plus Evidence	phosphoRS Site Probabilities	# PSMs	# Proteins	# Protein Groups	# Missed Cleavages	Modifications
						Replicate 1				Replicate 2				Replicate 1				Replicate 2																	
20986499	NP_620601.1	5598	MAPK7	mitogen-activated protein kinase 7 isoform2	T594	0.3	0.2	0.2	0.7	-	-	-	-	0.2	0.2	0.2	0.6	-	-	-	-	0.2	0.2	0.2	0.6	SQVEDPLPPVFSGtPK	LPPVFSGtPKGSGAG	T14;	No	S(1): 0.0; S(12): 1.1; T(14): 98.9	1	2	1	0	N-Term(TMT6plex); T14(Phospho); K16(TMT6plex)
566559802	NP_001274432.1	6744	SSFA2	sperm-specific antigen 2 isoform3	S1112	0.9	0.8	1.0	0.7	-	-	-	-	0.9	1.0	1.1	0.9	-	-	-	-	0.9	1.0	1.1	0.9	TGVPSTAsVGK	TGVPSTAsVGKSKTP	S8;	No	T(1): 0.0; S(5): 2.0; T(6): 2.0; S(8):	6	4	1	0	N-Term(TMT6plex); S8(Phospho); K11(TMT6plex)
116256458	NP_689531.2	121053	C12orf45	uncharacterized protein C12orf45	S15	0.4	0.4	0.5	0.7	-	-	-	-	-	-	-	-	-	-	-	-	-	-	-	-	ASPSCSsPTR	KASPSCSsPTRDSSG	S7;	Yes	S(2): 0.0; S(4): 0.0; S(6): 0.0; S(7): 99.2; T(9): 0.8	3	1	1	0	N-Term(TMT6plex); C5(Carbamidomethyl); S7(Phospho)
62241044	NP_056034.2	23265	EXOC7	exocyst complex component 7 isoform2	S250	1.2	0.9	0.7	0.7	-	-	-	-	1.2	1.1	0.8	0.8	-	-	-	-	1.2	1.1	0.8	0.8	SSSSSGVPYsPAIPNKR	SSSGVPYsPAIPNKR	S10;	Yes	S(1): 0.0; S(2): 0.0; S(3): 0.0; S(4): 0.0; S(5): 0.0; Y(9): 2.1; S(10): 97.9	4	6	1	1	N-Term(TMT6plex); S10(Phospho); K16(TMT6plex)
118572613	NP_057417.3	23524	SRRM2	serine/arginine repetitive matrix protein 2	T1453	0.8	0.8	0.7	0.7	-	-	-	-	0.9	0.8	0.8	0.7	-	-	-	-	0.9	0.8	0.8	0.7	DGSGtPSR	GLRDGSGtPSRHSLS	T5;	Yes	S(3): 0.0; T(5): 100.0; S(7): 0.0	436	1	1	0	N-Term(TMT6plex); T5(Phospho)
612407805	NP_055490.4	9696	CROCC	rootletin	S2009	1.5	0.9	0.7	0.7	-	-	-	-	1.5	1.1	0.8	0.7	-	-	-	-	1.5	1.1	0.8	0.7	SSAPFsPPSGPPEK	RRSSAPFsPPSGPPE	S6;	Yes	S(1): 1.5; S(2): 1.5; S(6): 97.0; S(9):	1	1	1	0	N-Term(TMT6plex); S6(Phospho); K14(TMT6plex)
22208971	NP_665908.1	3159	HMGA1	high mobility group protein HMG-I/HMG-Y isoforma	S102; S103	1.0	1.8	1.1	0.7	-	-	-	-	1.0	1.4	1.0	0.7	-	-	-	-	1.0	1.4	1.0	0.7	EEEEGISQEssEEEQ	EEGISQEsSEEEQ_;EGISQESsEEEQ	S10; S11;	Yes;Yes	S(7): 0.2; S(10): 99.9; S(11): 99.9	39	2	1	0	N-Term(TMT6plex); S10(Phospho); S11(Phospho)
109809741	NP_057732.2	51574	LARP7	la-related protein 7 isoform1	S247	0.8	0.7	0.6	0.7	-	-	-	-	0.9	0.7	0.6	0.7	-	-	-	-	0.9	0.7	0.6	0.7	EENMDTSNTsISK	NMDTSNTsISKMKRS	S10;	Yes	T(6): 0.0; S(7): 1.3; T(9): 1.3; S(10): 97.4; S(12): 0.0	6	2	1	0	N-Term(TMT6plex); M4(Oxidation); S10(Phospho); K13(TMT6plex)
544346207	NP_001269716.1	4781	NFIB	nuclear factor 1 B-type isoform4	S12	0.8	0.7	0.8	0.7	-	-	-	-	1.0	0.8	1.0	0.8	-	-	-	-	1.0	0.8	1.0	0.8	SLsSPPSSK	VNLQRSLsSPPSSKR	S3;	No	S(1): 0.0; S(3): 100.0; S(4): 0.0; S(7): 0.0; S(8): 0.0	3	4	1	0	N-Term(TMT6plex); S3(Phospho); K9(TMT6plex)
156104891	NP_002087.2	2962	GTF2F1	general transcription factor IIF subunit 1	T437	1.1	0.7	0.8	0.7	-	-	-	-	1.1	0.7	0.8	0.7	-	-	-	-	1.1	0.7	0.8	0.7	StPQPPSGK	QSLSGKStPQPPSGK	T2;	Yes	S(1): 0.8; T(2): 99.2; S(7): 0.0	20	1	1	0	N-Term(TMT6plex); T2(Phospho); K9(TMT6plex)
14249158	NP_116020.1	84717	HDGFRP2	hepatoma-derived growth factor-related protein 2 isoform2	S651	0.6	0.7	0.7	0.7	-	-	-	-	0.8	0.9	0.9	0.9	-	-	-	-	0.8	0.9	0.9	0.9	EGPDLDRPGsDR	PDLDRPGsDRQERER	S10;	No	S(10): 100.0	21	2	1	0	N-Term(TMT6plex); S10(Phospho)
71143123	NP_689764.3	154007	SNRNP48	U11/U12 small nuclear ribonucleoprotein 48 kDa	S277	0.6	0.6	0.6	0.7	-	-	-	-	0.8	0.7	0.9	0.8	-	-	-	-	0.8	0.7	0.9	0.8	SAsVDSR	NEERRSAsVDSRQSG	S3;	No	S(1): 0.0; S(3): 100.0; S(6): 0.0	1	1	1	0	N-Term(TMT6plex); S3(Phospho)
4506921	NP_003012.1	6449	SGTA	small glutamine-rich tetratricopeptide repeat-containing protein alpha	S77	0.8	0.7	0.7	0.7	-	-	-	-	1.0	1.1	0.9	0.9	-	-	-	-	1.0	1.1	0.9	0.9	EMPQDLRsPAR	EMPQDLRsPARTPPS	S8;	Yes	S(8): 100.0	28	1	1	1	N-Term(TMT6plex); M2(Oxidation); S8(Phospho)
18378731	NP_064620.2	56987	BBX	HMG box transcription factor BBX isoform2	S814	1.0	0.9	0.9	0.7	-	-	-	-	1.1	1.0	1.0	0.8	-	-	-	-	1.1	1.0	1.0	0.8	VsPAGGTLDDKPK	RTADGRVsPAGGTLD	S2;	No	S(2): 100.0; T(7): 0.0	1	2	1	0	N-Term(TMT6plex); S2(Phospho); K11(TMT6plex); K13(TMT6plex)
302370946	NP_001180573.1	5824	PEX19	peroxisomal biogenesis factor 19 isoformc	S54	0.8	0.8	0.8	0.7	-	-	-	-	0.8	0.9	0.8	0.9	-	-	-	-	0.8	0.9	0.8	0.9	sPGDTAK	ASGPQKRsPGDTAKD	S1;	Yes	S(1): 100.0; T(5): 0.0	8	2	1	0	N-Term(TMT6plex); S1(Phospho); K7(TMT6plex)
115298654	NP_001041639.1	4595	MUTYH	A/G-specific adenine DNA glycosylase isoform4	S487	0.5	0.6	0.6	0.7	-	-	-	-	-	-	-	-	-	-	-	-	-	-	-	-	SQVSsPCSR	SKRSQVSsPCSRKKP	S5;	No	S(1): 0.0; S(4): 1.4; S(5): 98.5; S(8):	1	5	1	0	N-Term(TMT6plex); S5(Phospho); C7(Carbamidomethyl)
291167764	NP_001166936.1	55605	KIF21A	kinesin-like protein KIF21A isoform4	S1176	0.7	1.6	1.6	0.7	-	-	-	-	0.6	1.4	1.5	0.6	-	-	-	-	0.6	1.4	1.5	0.6	EKELsPPPGLPSK	SAREKELsPPPGLPS	S5;	No	S(5): 100.0; S(12): 0.0	3	4	1	1	N-Term(TMT6plex); K2(TMT6plex); S5(Phospho); K13(TMT6plex)
118402582	NP_001246.2	991	CDC20	cell division cycle protein 20 homolog	T55; T59	0.4	0.7	0.7	0.7	-	-	-	-	0.5	0.7	0.8	0.7	-	-	-	-	0.5	0.7	0.8	0.7	tPGRtPGK	RSHSAGRtPGRTPGK;AGRTPGRtPGKSSSK	T1; T5;	No;Yes	T(1): 100.0; T(5): 100.0	11	1	1	1	N-Term(TMT6plex); T1(Phospho); T5(Phospho); K8(TMT6plex)
157364934	NP_001098549.1	64319	FBRS	probable fibrosin-1	S281	0.6	0.8	0.7	0.7	-	-	-	-	-	-	-	-	-	-	-	-	-	-	-	-	VsPATPK	SRPQLRVsPATPKAR	S2;	Yes	S(2): 100.0; T(5): 0.0	1	1	1	0	N-Term(TMT6plex); S2(Phospho); K7(TMT6plex)
73611906	NP_001027005.1	5257	PHKB	phosphorylase b kinase regulatory subunit beta	S20	1.2	1.2	1.6	0.7	-	-	-	-	1.1	1.2	1.5	0.6	-	-	-	-	1.1	1.2	1.5	0.6	SGsVYEPLK	SAFLRSGsVYEPLKS	S3;	No	S(1): 0.0; S(3): 100.0; Y(5): 0.0	1	2	1	0	N-Term(TMT6plex); S3(Phospho); K9(TMT6plex)
62244004	NP_001013865.1	79811	SLTM	SAFB-like transcription modulator isoformb	S996	1.0	0.9	0.9	0.7	-	-	-	-	1.0	1.0	1.1	0.7	-	-	-	-	1.0	1.0	1.1	0.7	IVQISGNsMPR	IVQISGNsMPRGSGS	S8;	No	S(5): 1.5; S(8): 98.5	12	2	1	0	N-Term(TMT6plex); S8(Phospho); M9(Oxidation)
206597522	NP_001128663.1	8853	ASAP2	arf-GAP with SH3 domain, ANK repeat and PH domain-containing protein 2 isoformb	S701	0.8	1.2	1.2	0.6	-	-	-	-	0.8	1.1	1.0	0.6	-	-	-	-	0.8	1.1	1.0	0.6	LLHEDLDEsDDDMDEK	LHEDLDEsDDDMDEK	S9;	Yes	S(9): 100.0	1	2	1	0	N-Term(TMT6plex); S9(Phospho); M13(Oxidation); K16(TMT6plex)
17975761	NP_006791.2	10943	MSL3	male-specific lethal 3 homolog isoformc	S201	0.9	0.8	0.6	0.6	-	-	-	-	1.0	0.9	0.8	0.7	-	-	-	-	1.0	0.9	0.8	0.7	LSESSAsPQPK	RLSESSAsPQPKRRQ	S7;	No	S(2): 0.0; S(4): 0.0; S(5): 0.0; S(7): 100.0	13	5	1	0	N-Term(TMT6plex); S7(Phospho); K11(TMT6plex)
5730124	NP_006517.1	7764	ZNF217	zinc finger protein 217	S795	0.7	0.6	0.9	0.6	-	-	-	-	0.8	0.7	1.1	0.7	-	-	-	-	0.8	0.7	1.1	0.7	GKQsPPGPGK	PSAKGKQsPPGPGKA	S4;	Yes	S(4): 100.0	9	1	1	1	N-Term(TMT6plex); K2(TMT6plex); S4(Phospho); K10(TMT6plex)
32306539	NP_057584.2	51260	PBDC1	protein PBDC1	S197	1.0	0.8	0.9	0.6	-	-	-	-	1.1	1.2	1.1	1.0	-	-	-	-	1.1	1.2	1.1	1.0	GADsGEEK	GGEKGADsGEEKEEG	S4;	No	S(4): 100.0	4	1	1	0	N-Term(TMT6plex); S4(Phospho); K8(TMT6plex)
27477111	NP_006416.3	10569	SLU7	pre-mRNA-splicing factor SLU7	S215	1.0	1.1	1.0	0.6	-	-	-	-	1.0	1.2	1.0	0.7	-	-	-	-	1.0	1.2	1.0	0.7	LVEQANsPK	KLVEQANsPKHQWGE	S7;	Yes	S(7): 100.0	4	1	1	0	N-Term(TMT6plex); S7(Phospho); K9(TMT6plex)
544709647	NP_001269861.1	10152	ABI2	abl interactor 2 isoforme	S171	0.7	0.7	0.6	0.6	-	-	-	-	0.7	0.7	0.7	0.6	-	-	-	-	0.7	0.7	0.7	0.6	TAsVNQR	QSPVRTAsVNQRNRT	S3;	No	T(1): 0.0; S(3): 100.0	6	5	1	0	N-Term(TMT6plex); S3(Phospho)
149944496	NP_056377.1	26043	UBXN7	UBX domain-containing protein 7	S278	0.5	0.6	0.5	0.6	-	-	-	-	0.4	0.6	0.4	0.7	-	-	-	-	0.4	0.6	0.4	0.7	sESLIDASEDSQLEAAIR	PPKKCARsESLIDAS	S1;	Yes	S(1): 98.3; S(3): 1.7; S(8): 0.0; S(11): 0.0	1	1	1	0	N-Term(TMT6plex); S1(Phospho)
186910300	NP_001119575.1	29127	RACGAP1	rac GTPase-activating protein 1	S214	1.2	0.9	0.7	0.6	-	-	-	-	1.2	0.8	0.6	0.6	-	-	-	-	1.2	0.8	0.6	0.6	SIGSAVDQGNEsIVAK	AVDQGNEsIVAKTTV	S12;	Yes	S(1): 0.0; S(4): 0.0; S(12): 99.9	2	1	1	0	N-Term(TMT6plex); S12(Phospho); K16(TMT6plex)
62422557	NP_001014832.1	10298	PAK4	serine/threonine-protein kinase PAK 4 isoform1	S167	0.8	0.8	0.7	0.6	-	-	-	-	0.7	0.8	0.6	0.6	-	-	-	-	0.7	0.8	0.6	0.6	EGsGGPQESSR	PKSSREGsGGPQESS	S3;	Yes	S(3): 100.0; S(9): 0.0; S(10): 0.0	7	1	1	0	N-Term(TMT6plex); S3(Phospho)
9257222	NP_002006.2	2308	FOXO1	forkhead box protein O1	S256	1.0	0.8	0.7	0.6	-	-	-	-	-	-	-	-	-	-	-	-	-	-	-	-	AAsMDNNSK	SPRRRAAsMDNNSKF	S3;	Yes	S(3): 100.0; S(8): 0.0	9	1	1	0	N-Term(TMT6plex); S3(Phospho); M4(Oxidation); K9(TMT6plex)
132626688	NP_055456.2	9656	MDC1	mediator of DNA damage checkpoint protein 1	S495; S498	0.7	1.0	1.0	0.6	-	-	-	-	0.8	1.0	1.1	0.7	-	-	-	-	0.8	1.0	1.1	0.7	DQPPFGDsDDsVEADK	DQPPFGDsDDSVEAD;PFGDSDDsVEADKSS	S8; S11;	Yes;Yes	S(8): 100.0; S(11): 100.0	26	1	1	0	N-Term(TMT6plex); S8(Phospho); S11(Phospho); K16(TMT6plex)
132626790	NP_056442.2	26119	LDLRAP1	low density lipoprotein receptor adapter protein 1	S14	1.0	0.8	0.7	0.6	-	-	-	-	0.9	0.9	0.8	0.7	-	-	-	-	0.9	0.9	0.8	0.7	ALIRsPSLAK	AGRALIRsPSLAKQS	S5;	Yes	S(5): 100.0; S(7): 0.0	1	1	1	1	N-Term(TMT6plex); S5(Phospho); K10(TMT6plex)
253970412	NP_057510.4	51512	GTSE1	G2 and S phase-expressed protein 1	S322	1.0	1.1	0.8	0.6	-	-	-	-	1.1	1.2	0.9	0.7	-	-	-	-	1.1	1.2	0.9	0.7	APGsTSNLAR	TLLKAPGsTSNLARK	S4;	No	S(4): 98.7; T(5): 1.3; S(6): 0.0	19	1	1	0	N-Term(TMT6plex); S4(Phospho)
149363685	NP_056140.1	23379	KIAA0947	uncharacterized protein KIAA0947	S516	0.7	0.6	0.8	0.6	-	-	-	-	-	-	-	-	-	-	-	-	-	-	-	-	LSAsPAQEK	CIERLSAsPAQEKEA	S4;	Yes	S(2): 0.0; S(4): 100.0	3	1	1	0	N-Term(TMT6plex); S4(Phospho); K9(TMT6plex)
295054220	NP_001171136.1	55183	RIF1	telomere-associated protein RIF1 isoform2	S1579	0.8	0.8	1.0	0.6	-	-	-	-	0.9	0.9	1.1	0.7	-	-	-	-	0.9	0.9	1.1	0.7	SNEsVDIQDQEEK	WKNKSNEsVDIQDQE	S4;	Yes	S(1): 0.0; S(4): 100.0	15	2	1	0	N-Term(TMT6plex); S4(Phospho); K13(TMT6plex)
61743954	NP_001611.1	79026	AHNAK	neuroblast differentiation-associated protein AHNAK isoform1	S3054	0.6	0.7	1.8	0.6	-	-	-	-	0.7	0.8	1.8	0.7	-	-	-	-	0.7	0.8	1.8	0.7	ADLDVsGPK	PKADLDVsGPKVDID	S6;	Yes	S(6): 100.0	79	1	1	0	N-Term(TMT6plex); S6(Phospho); K9(TMT6plex)
116686122	NP_036442.3	24137	KIF4A	chromosome-associated kinesin KIF4A	T1218	1.1	1.2	1.2	0.6	-	-	-	-	1.5	1.5	1.6	0.8	-	-	-	-	1.5	1.5	1.6	0.8	ALASNtSFFSGCSPIEEEAH	KRALASNtSFFSGCS	T6;	No	S(4): 0.1; T(6): 92.8; S(7): 2.4; S(10): 2.4; S(13): 2.4	3	2	1	0	N-Term(TMT6plex); C12(Carbamidomethyl); S13(Phospho)
9558733	NP_037425.1	29896	TRA2A	transformer-2 protein homolog alpha isoform1	S100	0.6	0.5	1.2	0.6	-	-	-	-	0.7	0.6	1.4	0.8	-	-	-	-	0.7	0.6	1.4	0.8	SHsPMSNR	RRRSRSHsPMSNRRR	S3;	Yes	S(1): 0.0; S(3): 100.0; S(6): 0.0	2	1	1	0	N-Term(TMT6plex); S3(Phospho); M5(Oxidation)
530788244	NP_001269026.1	10413	YAP1	yorkie homolog isoform6	S302	0.5	0.4	1.0	0.6	-	-	-	-	0.5	0.5	1.1	0.7	-	-	-	-	0.5	0.5	1.1	0.7	NINPSTANsPK	INPSTANsPKCQELA	S9;	No	S(5): 0.0; T(6): 0.0; S(9): 100.0	11	5	1	0	N-Term(TMT6plex); S9(Phospho); K11(TMT6plex)
343478277	NP_001230403.1	90627	STARD13	stAR-related lipid transfer protein 13 isoform6	T429	0.5	0.8	0.8	0.6	-	-	-	-	0.4	0.7	0.8	0.5	-	-	-	-	0.4	0.7	0.8	0.5	TtPSDVER	SVSEGRTtPSDVERD	T2;	No	T(1): 0.0; T(2): 100.0; S(4): 0.0	1	6	1	0	N-Term(TMT6plex); T2(Phospho)
19557702	NP_006744.2	6838	SURF6	surfeit locus protein 6 isoform1	T229	0.5	0.6	0.6	0.6	-	-	-	-	0.6	0.7	0.7	0.7	-	-	-	-	0.6	0.7	0.7	0.7	GNLtPLTGR	QRVKGNLtPLTGRNY	T4;	Yes	T(4): 100.0; T(7): 0.0	1	1	1	0	N-Term(TMT6plex); T4(Phospho)
255652953	NP_653205.3	124245	ZC3H18	zinc finger CCCH domain-containing protein 18	S34	0.9	0.9	0.7	0.6	-	-	-	-	1.1	0.9	0.9	0.7	-	-	-	-	1.1	0.9	0.9	0.7	DSGsDQDLDGAGVR	DILRDSGsDQDLDGA	S4;	Yes	S(2): 1.1; S(4): 98.9	34	1	1	0	N-Term(TMT6plex); S4(Phospho)
226509740	NP_067051.2	58506	SCAF1	splicing factor, arginine/serine-rich 19	T849	0.8	0.8	0.7	0.6	-	-	-	-	0.7	0.7	0.6	0.6	-	-	-	-	0.7	0.7	0.6	0.6	EGVSSTtPAK	REGVSSTtPAKDAAS	T7;	Yes	S(4): 0.8; S(5): 0.8; T(6): 0.8; T(7):	29	1	1	0	N-Term(TMT6plex); T6(Phospho); K10(TMT6plex)
392996951	NP_001257292.1	5886	RAD23A	UV excision repair protein RAD23 homolog A isoform 3	T132	0.9	0.8	0.8	0.6	-	-	-	-	0.8	0.7	0.7	0.5	-	-	-	-	0.8	0.7	0.7	0.5	SPSEESAPTtSPESVSGSVPSSGSSGR	SEESAPTtSPESVSG	T10;	Yes	S(1): 0.0; S(3): 0.0; S(6): 0.0; T(9): 2.9; T(10): 94.1; S(11): 2.9; S(14): 0.1; S(16): 0.0; S(18): 0.0; S(21): 0.0; S(22): 0.0; S(24): 0.0; S(25): 0.0	1	3	1	0	N-Term(TMT6plex); T10(Phospho)
82830436	NP_001032625.1	6322	SCML1	sex comb on midleg-like protein 1 isoformc	S117	0.6	1.1	0.6	0.6	-	-	-	-	-	-	-	-	-	-	-	-	-	-	-	-	sPVENDGYIEEGSITK	APPSVTRsPVENDGY	S1;	No	S(1): 100.0; Y(8): 0.0; S(13): 0.0; T(15): 0.0	1	3	1	0	N-Term(TMT6plex); S1(Phospho); K16(TMT6plex)
28559039	NP_004765.2	5469	MED1	mediator of RNA polymerase II transcription subunit 1	S1467	0.5	0.9	0.3	0.6	-	-	-	-	0.6	0.8	0.3	0.6	-	-	-	-	0.6	0.8	0.3	0.6	SPAYTPQNLDSESEsGSSIAEK	NLDSESEsGSSIAEK	S15;	No	S(1): 0.0; Y(4): 0.0; T(5): 0.1; S(11): 2.2; S(13): 2.2; S(15): 95.5; S(17): 0.1; S(18): 0.1	16	1	1	0	N-Term(TMT6plex); S15(Phospho); K22(TMT6plex)
153070260	NP_002347.5	4082	MARCKS	myristoylated alanine-rich C-kinase substrate	S131; S135	0.7	0.8	0.5	0.6	-	-	-	-	1.1	1.0	0.8	0.8	-	-	-	-	1.1	1.0	0.8	0.8	EAPAEGEAAEPGSPTAAEGEAASAAsSTSsPK	GEAASAAsSTSSPKA;SAASSTSsPKAEDGA	S26; S30;	Yes;Yes	S(13): 0.0; T(15): 0.0; S(23): 0.0; S(26): 97.7; S(27): 2.3; T(28): 0.0; S(29): 2.3; S(30): 97.7	69	1	1	0	N-Term(TMT6plex); T15(Phospho); S30(Phospho); K32(TMT6plex)
47132589	NP_002732.3	5585	PKN1	serine/threonine-protein kinase N1 isoform2	S374	0.5	0.5	0.7	0.6	-	-	-	-	0.6	0.6	0.7	0.7	-	-	-	-	0.6	0.6	0.7	0.7	SGsLSGR	GLYSRSGsLSGRSSL	S3;	Yes	S(1): 0.0; S(3): 100.0; S(5): 0.0	8	2	1	0	N-Term(TMT6plex); S3(Phospho)
148839292	NP_078800.3	79572	ATP13A3	probable cation-transporting ATPase 13A3	S817	0.8	0.7	0.7	0.6	-	-	-	-	0.8	0.7	0.7	0.6	-	-	-	-	0.8	0.7	0.7	0.6	LVHDsLEDLQMTR	PVKLVHDsLEDLQMT	S5;	Yes	S(5): 100.0; T(12): 0.0	1	1	1	0	N-Term(TMT6plex); S5(Phospho); M11(Oxidation)
37620163	NP_065741.3	404734	NKHD1-EIF4EE	ANKHD1-EIF4EBP3 protein	S740	0.7	0.5	1.0	0.6	-	-	-	-	-	-	-	-	-	-	-	-	-	-	-	-	TSQENsPALLGVQK	DRTSQENsPALLGVQ	S6;	Yes	T(1): 1.5; S(2): 1.5; S(6): 97.1	7	2	1	0	N-Term(TMT6plex); S6(Phospho); K14(TMT6plex)
237681121	NP_009228.2	672	BRCA1	breast cancer type 1 susceptibility protein isoform3	S348; S351	0.6	0.6	0.4	0.6	-	-	-	-	0.6	0.6	0.4	0.6	-	-	-	-	0.6	0.6	0.4	0.6	SDELLGsDDsHDGESESNAK	RSDELLGsDDSHDGE;ELLGSDDsHDGESES	S7; S10;	No;No	S(1): 0.2; S(7): 99.8; S(10): 99.8; S(15): 0.2; S(17): 0.0	1	3	1	0	N-Term(TMT6plex); S7(Phospho); S10(Phospho); K20(TMT6plex)
296841073	NP_001171894.1	54521	WDR44	WD repeat-containing protein 44 isoform2	S403	0.6	0.7	1.4	0.6	-	-	-	-	0.6	0.7	1.6	0.7	-	-	-	-	0.6	0.7	1.6	0.7	EYVSNDAAQsDDEEKLQSQPTDTDGGR	VSNDAAQsDDEEKLQ	S10;	Yes	Y(2): 0.0; S(4): 0.0; S(10): 100.0; S(18): 0.0; T(21): 0.0; T(23): 0.0	12	3	1	1	N-Term(TMT6plex); S10(Phospho); K15(TMT6plex)
38683860	NP_003740.2	8660	IRS2	insulin receptor substrate 2	S1100	0.7	0.8	0.8	0.6	-	-	-	-	0.7	0.8	0.8	0.6	-	-	-	-	0.7	0.8	0.8	0.6	VAsPTSGVK	PEAARVAsPTSGVKR	S3;	Yes	S(3): 98.3; T(5): 0.9; S(6): 0.9	9	1	1	0	N-Term(TMT6plex); S3(Phospho); K9(TMT6plex)
14790190	NP_055816.2	23013	SPEN	msx2-interacting protein	S2120	0.5	0.5	0.7	0.6	-	-	-	-	0.5	0.4	0.7	0.6	-	-	-	-	0.5	0.4	0.7	0.6	ESGVVAVsPEK	ESGVVAVsPEKSESP	S8;	Yes	S(2): 0.0; S(8): 100.0	16	1	1	0	N-Term(TMT6plex); S8(Phospho); K11(TMT6plex)
4757930	NP_004692.1	9133	CCNB2	G2/mitotic-specific cyclin-B2	S92	0.5	0.5	0.8	0.6	-	-	-	-	0.5	0.6	0.8	0.7	-	-	-	-	0.5	0.6	0.8	0.7	GPsPTPEDVSMK	KLAPKGPsPTPEDVS	S3;	Yes	S(3): 100.0; T(5): 0.0; S(10): 0.0	1	1	1	0	N-Term(TMT6plex); S3(Phospho); M11(Oxidation); K12(TMT6plex)
570359584	NP_001275662.1	55852	TEX2	testis-expressed sequence 2 protein isoform2	S196	1.0	0.5	0.6	0.6	-	-	-	-	0.9	0.6	0.6	0.5	-	-	-	-	0.9	0.6	0.6	0.5	SLsTEVEPK	MSLVKSLsTEVEPKE	S3;	Yes	S(1): 0.9; S(3): 99.1; T(4): 0.0	5	2	1	0	N-Term(TMT6plex); S3(Phospho); K9(TMT6plex)
21493024	NP_653080.1	9590	AKAP12	A-kinase anchor protein 12 isoform2	T1166	0.9	1.4	1.2	0.6	-	-	-	-	1.1	1.3	1.0	0.5	-	-	-	-	1.1	1.3	1.0	0.5	TEGtQEADQYADEK	ILSKTEGtQEADQYA	T4;	No	T(1): 1.5; T(4): 98.5; Y(10): 0.0	103	2	1	0	N-Term(TMT6plex); T4(Phospho); K14(TMT6plex)
102468717	NP_005112.2	9969	MED13	mediator of RNA polymerase II transcription subunit 13	S1436; S1447	0.7	0.5	0.7	0.6	-	-	-	-	0.7	0.5	0.8	0.6	-	-	-	-	0.7	0.5	0.8	0.6	LVAEWFsQAADGNNEAFsK	KLVAEWFsQAADGNN;DGNNEAFsKLKLYAQ	S7; S18;	No;No	S(7): 100.0; S(18): 100.0	2	1	1	0	N-Term(TMT6plex); S7(Phospho); S18(Phospho); K19(TMT6plex)
41350335	NP_003187.1	6920	TCEA3	transcription elongation factor A protein 3	S140	0.8	0.7	0.5	0.6	-	-	-	-	-	-	-	-	-	-	-	-	-	-	-	-	SSASSsPK	SKSSASSsPKRPSVE	S6;	No	S(1): 0.0; S(2): 0.0; S(4): 0.8; S(5): 0.8; S(6): 98.3	1	1	1	0	N-Term(TMT6plex); S6(Phospho); K8(TMT6plex)
4885385	NP_005315.1	3021	H3F3B	histone H3.3	S29	0.4	0.4	0.5	0.6	-	-	-	-	-	-	-	-	-	-	-	-	-	-	-	-	sAPSTGGVK	ATKAARKsAPSTGGV	S1;	No	S(1): 100.0; S(4): 0.0; T(5): 0.0	5	1	1	0	N-Term(TMT6plex); S1(Phospho); K9(TMT6plex)
356461016	NP_001239085.1	25929	GEMIN5	gem-associated protein 5 isoform2	S777	1.3	1.1	1.0	0.6	-	-	-	-	1.2	1.0	0.9	0.6	-	-	-	-	1.2	1.0	0.9	0.6	ENSGPVENGVsDQEGEEQAR	GPVENGVsDQEGEEQ	S11;	No	S(3): 0.0; S(11): 100.0	2	2	1	0	N-Term(TMT6plex); S11(Phospho)
7661950	NP_055448.1	9646	CTR9	RNA polymerase-associated protein CTR9 homolog	S1081; S1085	1.0	0.5	0.8	0.6	-	-	-	-	1.0	0.5	0.8	0.6	-	-	-	-	1.0	0.5	0.8	0.6	QRsDQDsDSDQPSR	RRPRRQRsDQDSDSD;RQRSDQDsDSDQPSR	S3; S7;	Yes;Yes	S(3): 100.0; S(7): 100.0; S(9): 0.0; S(13): 0.0	12	1	1	1	N-Term(TMT6plex); S3(Phospho); S7(Phospho)
29171753	NP_803172.1	8500	PPFIA1	liprin-alpha-1 isoforma	S1172	0.7	0.6	0.9	0.6	-	-	-	-	0.7	0.7	1.0	0.6	-	-	-	-	0.7	0.7	1.0	0.6	VTSSMSsPSMQPK	RVTSSMSsPSMQPKK	S7;	Yes	T(2): 0.0; S(3): 0.0; S(4): 1.2; S(6): 1.2; S(7): 97.6; S(9): 0.0	9	2	1	0	N-Term(TMT6plex); M5(Oxidation); S7(Phospho); M10(Oxidation); K13(TMT6plex)
6631100	NP_005376.2	4820	NKTR	NK-tumor recognition protein	S776	0.7	0.7	0.7	0.6	-	-	-	-	0.9	0.8	0.8	0.7	-	-	-	-	0.9	0.8	0.8	0.7	HSSsSEK	SHKKHSSsSEKTLHS	S4;	No	S(2): 0.0; S(3): 0.5; S(4): 99.5; S(5):	6	1	1	0	N-Term(TMT6plex); S4(Phospho); K7(TMT6plex)
4759098	NP_004584.1	6434	TRA2B	transformer-2 protein homolog beta isoform1	S22; S26; S29	0.6	0.6	0.8	0.6	-	-	-	-	0.7	0.7	0.9	0.7	-	-	-	-	0.7	0.7	0.9	0.7	SGsAHGsGKsAR	RSASRSGsAHGSGKS;RSGSAHGsGKSARHT;SAHGSGKsARHTPAR	S3; S7; S10;	No;Yes;Yes	S(1): 1.3; S(3): 98.7; S(7): 100.0; S(10): 100.0	11	1	1	1	N-Term(TMT6plex); S3(Phospho); S7(Phospho); K9(TMT6plex); S10(Phospho)
4506921	NP_003012.1	6449	SGTA	small glutamine-rich tetratricopeptide repeat-containing protein alpha	T303	0.9	0.8	0.7	0.6	-	-	-	-	1.0	1.1	0.9	0.8	-	-	-	-	1.0	1.1	0.9	0.8	SRtPSASNDDQQE	RSQIRSRtPSASNDD	T3;	Yes	S(1): 1.1; T(3): 98.9; S(5): 0.0; S(7): 0.0	28	1	1	1	N-Term(TMT6plex); T3(Phospho)
14249158	NP_116020.1	84717	HDGFRP2	hepatoma-derived growth factor-related protein 2 isoform2	S490	0.7	0.5	0.5	0.6	-	-	-	-	1.0	0.7	0.7	0.7	-	-	-	-	1.0	0.7	0.7	0.7	VDsPDVK	KFALKVDsPDVKRCL	S3;	Yes	S(3): 100.0	21	2	1	0	N-Term(TMT6plex); S3(Phospho); K7(TMT6plex)
194097365	NP_060117.3	117246	FTSJ3	pre-rRNA processing protein FTSJ3	T573	0.2	0.3	0.4	0.6	-	-	-	-	0.2	0.3	0.4	0.5	-	-	-	-	0.2	0.3	0.4	0.5	QQLPQtPPSCLK	QKQQLPQtPPSCLKT	T6;	Yes	T(6): 100.0; S(9): 0.0	12	1	1	0	N-Term(TMT6plex); T6(Phospho); C10(Carbamidomethyl); K12(TMT6plex)
282403493	NP_001164121.1	81887	LAS1L	ribosomal biogenesis protein LAS1L isoform3	S558	0.8	1.3	0.9	0.6	-	-	-	-	0.9	1.4	1.0	0.6	-	-	-	-	0.9	1.4	1.0	0.6	MEVGPFSTGQEsPTAENAR	PFSTGQEsPTAENAR	S12;	No	S(7): 0.0; T(8): 0.0; S(12): 100.0; T(14): 0.0	2	3	1	0	N-Term(TMT6plex); M1(Oxidation); S12(Phospho)
69354671	NP_001020262.1	23	ABCF1	ATP-binding cassette sub-family F member 1 isoforma	S228	0.8	1.1	0.8	0.6	-	-	-	-	0.9	1.2	0.9	0.6	-	-	-	-	0.9	1.2	0.9	0.6	KAEQGsEEEGEGEEEEEEGGES	AKKAEQGsEEEGEGE	S6;	Yes	S(6): 100.0; S(22): 0.0	44	1	1	1	N-Term(TMT6plex); K1(TMT6plex); S6(Phospho); K23(TMT6plex)
304434531	NP_001182122.1	4678	NASP	nuclear autoantigenic sperm protein isoform4	S433	0.7	0.7	0.7	0.6	-	-	-	-	0.9	0.9	1.0	0.8	-	-	-	-	0.9	0.9	1.0	0.8	TEEMPNDsVLENK	TEEMPNDsVLENKSL	S8;	No	T(1): 0.0; S(8): 100.0	20	3	1	0	N-Term(TMT6plex); M4(Oxidation); S8(Phospho); K13(TMT6plex)
149158690	NP_542417.2	7916	PRRC2A	protein PRRC2A	S1219	0.4	0.7	0.6	0.6	-	-	-	-	0.5	0.7	0.7	0.5	-	-	-	-	0.5	0.7	0.7	0.5	LIPGPLsPVAR	KLIPGPLsPVARGGS	S7;	No	S(7): 100.0	23	1	1	0	N-Term(TMT6plex); S7(Phospho)
153945755	NP_065798.2	57507	ZNF608	zinc finger protein 608	S627; T636	0.4	0.9	0.7	0.6	-	-	-	-	-	-	-	-	-	-	-	-	-	-	-	-	APAsPGAGNPPGtPK	DQLKAPAsPGAGNPP;GAGNPPGtPKGKREL	S4; T13;	Yes;Yes	S(4): 100.0; T(13): 100.0	2	1	1	0	N-Term(TMT6plex); S4(Phospho); T13(Phospho); K15(TMT6plex)
153792110	NP_065784.1	57493	HEG1	protein HEG homolog 1 precursor	S1293	0.3	0.6	0.5	0.6	-	-	-	-	0.4	0.6	0.6	0.6	-	-	-	-	0.4	0.6	0.6	0.6	SGDFQMsPYAEYPK	KSGDFQMsPYAEYPK	S7;	Yes	S(1): 0.0; S(7): 98.7; Y(9): 1.3; Y(12): 0.0	1	1	1	0	N-Term(TMT6plex); M6(Oxidation); S7(Phospho); K14(TMT6plex)
299522984	NP_001177410.1	63967	CLSPN	claspin isoform2	S746	0.2	0.8	0.3	0.5	-	-	-	-	0.3	1.1	0.5	0.8	-	-	-	-	0.3	1.1	0.5	0.8	SPsPGLFR	AGGFRSPsPGLFRAS	S3;	No	S(1): 0.0; S(3): 100.0	4	2	1	0	N-Term(TMT6plex); S3(Phospho)
33356174	NP_002678.2	5411	PNN	pinin	S100	0.8	0.8	0.8	0.5	-	-	-	-	0.9	0.9	0.9	0.6	-	-	-	-	0.9	0.9	0.9	0.6	QEsDPEDDDVKKPALQSSVVATSK	RRESRQEsDPEDDDV	S3;	Yes	S(3): 100.0; S(17): 0.0; S(18): 0.0; T(22): 0.0; S(23): 0.0	7	1	1	1	N-Term(TMT6plex); S3(Phospho); K11(TMT6plex); K12(TMT6plex); K24(TMT6plex)
224589071	NP_055568.3	9790	BMS1	ribosome biogenesis protein BMS1 homolog	S552	0.5	0.7	0.9	0.5	-	-	-	-	0.5	0.7	0.8	0.6	-	-	-	-	0.5	0.7	0.8	0.6	AGLsPANCQSDR	EGSKAGLsPANCQSD	S4;	Yes	S(4): 100.0; S(10): 0.0	3	1	1	0	N-Term(TMT6plex); S4(Phospho); C8(Carbamidomethyl)
103472005	NP_002408.3	4288	MKI67	antigen KI-67 isoform1	S2105	0.3	0.5	0.7	0.5	-	-	-	-	0.3	0.5	0.7	0.6	-	-	-	-	0.3	0.5	0.7	0.6	IACKsPPPESMDTPTSTR	TTKIACKsPPPESMD	S5;	Yes	S(5): 100.0; S(10): 0.0; T(13): 0.0; T(15): 0.0; S(16): 0.0; T(17): 0.0	118	2	1	1	N-Term(TMT6plex); C3(Carbamidomethyl); K4(TMT6plex); S5(Phospho); M11(Oxidation)
31542661	NP_060672.2	55219	TMEM57	macoilin isoforma	S332	0.8	0.8	0.9	0.5	-	-	-	-	-	-	-	-	-	-	-	-	-	-	-	-	NASGVVNSsPR	ASGVVNSsPRSHSAT	S9;	Yes	S(3): 0.0; S(8): 0.0; S(9): 100.0	4	1	1	0	N-Term(TMT6plex); S9(Phospho)
634743319	NP_001278915.1	6046	BRD2	bromodomain-containing protein 2 isoform4	S513	0.5	0.9	1.3	0.5	-	-	-	-	0.5	0.9	1.2	0.5	-	-	-	-	0.5	0.9	1.2	0.5	TAPPALPTGYDsEEEEESRPMSYDEK	ALPTGYDsEEEEESR	S12;	No	T(1): 0.0; T(8): 2.0; Y(10): 2.0; S(12): 96.0; S(18): 0.0; S(22): 0.0; Y(23): 0.0	1	4	1	0	N-Term(TMT6plex); S12(Phospho); M21(Oxidation); K26(TMT6plex)
55750041	NP_001931.2	1822	ATN1	atrophin-1	S34	0.7	0.9	0.9	0.5	-	-	-	-	-	-	-	-	-	-	-	-	-	-	-	-	AsPGGVSTSSSDGK	LRSRGRAsPGGVSTS	S2;	Yes	S(2): 100.0; S(7): 0.0; T(8): 0.0; S(9): 0.0; S(10): 0.0; S(11): 0.0	3	1	1	0	N-Term(TMT6plex); S2(Phospho); K14(TMT6plex)
5729862	NP_006518.1	7884	SLBP	histone RNA hairpin-binding protein	S270	0.9	0.8	1.3	0.5	-	-	-	-	1.0	1.0	1.7	0.6	-	-	-	-	1.0	1.0	1.7	0.6	DFSAMs	LRDFSAMs	S6;	No	S(3): 0.0; S(6): 100.0	2	1	1	0	N-Term(TMT6plex); M5(Oxidation); S6(Phospho)
56676330	NP_057371.2	50809	HP1BP3	heterochromatin protein 1-binding protein 3	S248	0.7	0.7	0.7	0.5	-	-	-	-	0.8	0.7	0.7	0.6	-	-	-	-	0.8	0.7	0.7	0.6	sSAVDPEPQVK	SRNRKNRsSAVDPEP	S1;	Yes	S(1): 99.2; S(2): 0.8	5	1	1	0	N-Term(TMT6plex); S1(Phospho); K11(TMT6plex)
61676188	NP_113584.3	10075	HUWE1	E3 ubiquitin-protein ligase HUWE1	S2887	0.8	1.0	0.9	0.5	-	-	-	-	0.8	1.0	0.9	0.6	-	-	-	-	0.8	1.0	0.9	0.6	AGsSTPGDAPPAVAEVQGR	SEQPRAGsSTPGDAP	S3;	Yes	S(3): 97.3; S(4): 1.3; T(5): 1.3	20	1	1	0	N-Term(TMT6plex); S3(Phospho)
40807477	NP_109590.3	10743	RAI1	retinoic acid-induced protein 1	S1492	1.4	0.8	0.9	0.5	-	-	-	-	1.5	1.0	1.0	0.5	-	-	-	-	1.5	1.0	1.0	0.5	ASGTQGASEDNsGGGGK	QGASEDNsGGGGKKP	S12;	No	S(2): 0.0; T(4): 0.0; S(8): 2.4; S(12): 97.6	6	1	1	0	N-Term(TMT6plex); S12(Phospho); K17(TMT6plex)
5031767	NP_005517.1	3297	HSF1	heat shock factor protein 1	S363	1.0	1.2	1.1	0.5	-	-	-	-	1.1	1.1	1.0	0.5	-	-	-	-	1.1	1.1	1.0	0.5	GHTDTEGRPPsPPPTSTPEK	DTEGRPPsPPPTSTP	S11;	Yes	T(3): 0.0; T(5): 1.9; S(11): 92.2; T(15): 1.9; S(16): 1.9; T(17): 1.9	4	1	1	0	N-Term(TMT6plex); S11(Phospho); K20(TMT6plex)
212276104	NP_001131025.1	9208	LRRFIP1	leucine-rich repeat flightless-interacting protein 1 isoform5	S677	1.3	0.9	1.2	0.5	-	-	-	-	1.4	0.9	1.3	0.6	-	-	-	-	1.4	0.9	1.3	0.6	CTLPEHEsPSQDISDACEAESTER	CTLPEHEsPSQDISD	S8;	No	T(2): 0.0; S(8): 98.2; S(10): 1.8; S(14): 0.0; S(21): 0.0; T(22): 0.0	12	3	1	0	N-Term(TMT6plex); C1(Carbamidomethyl); S8(Phospho); C17(Carbamidomethyl)
114842410	NP_055642.3	9877	ZC3H11A	zinc finger CCCH domain-containing protein 11A	S738	0.9	0.8	1.2	0.5	-	-	-	-	0.9	0.8	1.2	0.5	-	-	-	-	0.9	0.8	1.2	0.5	DSLVLPPTQSSSDSsPPEVSGPSSSQMSMK	TQSSSDSsPPEVSGP	S15;	Yes	S(2): 0.0; T(8): 2.0; S(10): 2.0; S(11): 2.0; S(12): 2.0; S(14): 2.0; S(15): 90.0; S(20): 0.0; S(23): 0.0; S(24): 0.0; S(25): 0.0; S(28): 0.0	7	1	1	0	N-Term(TMT6plex); S14(Phospho); M27(Oxidation); M29(Oxidation); K30(TMT6plex)
42542379	NP_005830.2	10250	SRRM1	serine/arginine repetitive matrix protein 1	S802	0.9	0.6	1.1	0.5	-	-	-	-	1.0	0.7	1.2	0.6	-	-	-	-	1.0	0.7	1.2	0.6	NsDQEGGGK	SPSPPRNsDQEGGGK	S2;	Yes	S(2): 100.0	229	1	1	0	N-Term(TMT6plex); S2(Phospho); K9(TMT6plex)
87299628	NP_683692.2	259282	BOD1L1	biorientation of chromosomes in cell division protein 1-like 1	S2964	1.1	1.1	0.8	0.5	-	-	-	-	1.2	1.0	0.9	0.6	-	-	-	-	1.2	1.0	0.9	0.6	SLTVSDDAESsEPER	VSDDAESsEPERKRQ	S11;	Yes	S(1): 0.0; T(3): 0.0; S(5): 0.0; S(10): 1.5; S(11): 98.5	36	1	1	0	N-Term(TMT6plex); S11(Phospho)
148839362	NP_653304.2	149986	LSM14B	protein LSM14 homolog B	S165	1.7	1.1	0.4	0.5	-	-	-	-	1.8	1.2	0.4	0.5	-	-	-	-	1.8	1.2	0.4	0.5	SPMVEQAVQTGsADNLNAK	EQAVQTGsADNLNAK	S12;	Yes	S(1): 0.0; T(10): 0.8; S(12): 99.2	8	1	1	0	N-Term(TMT6plex); M3(Oxidation); S12(Phospho); K19(TMT6plex)
112382226	NP_001036147.1	473	RERE	arginine-glutamic acid dipeptide repeats protein	S87	0.9	0.8	0.6	0.5	-	-	-	-	-	-	-	-	-	-	-	-	-	-	-	-	EEAsSPLK	KKVKEEAsSPLKSNK	S4;	No	S(4): 99.2; S(5): 0.8	9	2	1	0	N-Term(TMT6plex); S4(Phospho); K8(TMT6plex)
544186100	NP_001269590.1	57591	MKL1	MKL/myocardin-like protein 1 isoform2	S154	0.8	0.5	0.4	0.5	-	-	-	-	0.9	0.6	0.5	0.6	-	-	-	-	0.9	0.6	0.5	0.6	VSEPLLSATsASPTQQSQPK	EPLLSATsASPTQQS	S10;	No	S(2): 0.0; S(7): 2.0; T(9): 2.0; S(10): 92.0; S(12): 2.0; T(14): 2.0; S(17): 0.0	2	1	1	0	N-Term(TMT6plex); S10(Phospho); K20(TMT6plex)
45827690	NP_996749.1	571	BACH1	transcription regulator protein BACH1	S445	0.6	0.8	1.3	0.5	-	-	-	-	-	-	-	-	-	-	-	-	-	-	-	-	ISEsPEPGQR	LGIRISEsPEPGQRT	S4;	Yes	S(2): 1.1; S(4): 98.9	1	1	1	0	N-Term(TMT6plex); S4(Phospho)
588480509	NP_001277058.1	55624	POMGNT1	protein O-linked-mannose beta-1,2-N-acetylglucosaminyltransferase 1 isoform3	S44	2.4	1.8	1.2	0.5	-	-	-	-	2.3	2.0	1.2	0.5	-	-	-	-	2.3	2.0	1.2	0.5	AIsEANEDPEPEQDYDEALGR	LDTRRAIsEANEDPE	S3;	No	S(3): 99.9; Y(15): 0.1	1	3	1	0	N-Term(TMT6plex); Y15(Phospho)
4826830	NP_004983.1	4204	MECP2	methyl-CpG-binding protein 2 isoform1	S423	1.6	1.0	1.4	0.5	-	-	-	-	2.2	1.4	2.4	0.8	-	-	-	-	2.2	1.4	2.4	0.8	GGsLESDGCPK	EKMPRGGsLESDGCP	S3;	Yes	S(3): 100.0; S(6): 0.0	14	2	1	0	N-Term(TMT6plex); S3(Phospho); C9(Carbamidomethyl); K11(TMT6plex)
190341065	NP_055526.3	9738	CCP110	centriolar coiled-coil protein of 110 kDa isoform2	S170	1.0	0.4	0.7	0.5	-	-	-	-	-	-	-	-	-	-	-	-	-	-	-	-	DSEGFNsPK	RDSEGFNsPKQCDSS	S7;	Yes	S(2): 0.0; S(7): 100.0	8	2	1	0	N-Term(TMT6plex); S7(Phospho); K9(TMT6plex)
5032189	NP_005648.1	7158	TP53BP1	tumor suppressor p53-binding protein 1 isoform3	T1648	0.7	0.6	0.8	0.5	-	-	-	-	0.7	0.6	0.9	0.6	-	-	-	-	0.7	0.6	0.9	0.6	SNVSSPATPTASSSSSTtPTRK	ASSSSSTtPTRKITE	T18;	Yes	S(1): 0.0; S(4): 0.0; S(5): 0.0; T(8): 0.0; T(10): 0.1; S(12): 0.1; S(13): 0.1; S(14): 2.1; S(15): 2.1; S(16): 2.1; T(17): 2.1; T(18): 89.1; T(20): 2.1	62	3	1	1	N-Term(TMT6plex); T18(Phospho); K22(TMT6plex)
332688220	NP_001193819.1	1500	CTNND1	catenin delta-1 isoform3A	S813	0.7	0.8	1.2	0.5	-	-	-	-	0.7	0.9	1.3	0.5	-	-	-	-	0.7	0.9	1.3	0.5	sGDLGDMEPLK	HNRTLDRsGDLGDME	S1;	No	S(1): 100.0	13	15	1	0	N-Term(TMT6plex); S1(Phospho); M7(Oxidation); K11(TMT6plex)
83267879	NP_003898.2	8893	EIF2B5	translation initiation factor eIF-2B subunit epsilon	S717	0.9	1.2	0.9	0.5	-	-	-	-	0.8	1.2	1.0	0.5	-	-	-	-	0.8	1.2	1.0	0.5	EAEEEsSEDD	LKEAEEEsSEDD	S6;	Yes	S(6): 98.7; S(7): 1.3	2	1	1	0	N-Term(TMT6plex); S6(Phospho)
402534571	NP_001257951.1	84126	ATRIP	ATR-interacting protein isoform3	S97	0.8	1.0	1.0	0.5	-	-	-	-	-	-	-	-	-	-	-	-	-	-	-	-	LAAPSVSHVsPR	APSVSHVsPRKNPSV	S10;	No	S(5): 0.0; S(7): 0.0; S(10): 100.0	1	4	1	0	N-Term(TMT6plex); S10(Phospho)
6631100	NP_005376.2	4820	NKTR	NK-tumor recognition protein	S1138	1.2	0.8	0.7	0.5	-	-	-	-	1.4	1.0	0.9	0.6	-	-	-	-	1.4	1.0	0.9	0.6	SsPAKVEETSPLGNAR	ICTPDRSsPAKVEET	S2;	No	S(1): 2.2; S(2): 97.8; T(9): 50.0; S(10): 50.0	6	1	1	1	N-Term(TMT6plex); S2(Phospho); K5(TMT6plex); S10(Phospho)
148539591	NP_060229.3	54891	INO80D	INO80 complex subunit D	S359	1.3	1.6	1.2	0.5	-	-	-	-	-	-	-	-	-	-	-	-	-	-	-	-	HAsDDDDAESR	LRYQRHAsDDDDAES	S3;	No	S(3): 98.2; S(10): 1.8	1	1	1	0	N-Term(TMT6plex); S3(Phospho)
33620716	NP_061173.1	5930	RBBP6	E3 ubiquitin-protein ligase RBBP6 isoform2	S1671	1.3	0.8	0.9	0.5	-	-	-	-	1.5	0.8	1.0	0.5	-	-	-	-	1.5	0.8	1.0	0.5	SHsPSGSQTR	VSPSRSHsPSGSQTR	S3;	No	S(1): 0.0; S(3): 100.0; S(5): 0.0; S(7): 0.0; T(9): 0.0	4	2	1	0	N-Term(TMT6plex); S3(Phospho)
85815829	NP_061830.3	23616	SH3BP1	SH3 domain-binding protein 1	S550	0.9	0.5	0.4	0.5	-	-	-	-	0.8	0.4	0.4	0.4	-	-	-	-	0.8	0.4	0.4	0.4	sPPETAAPVEDMAR	ASPKVTRsPPETAAP	S1;	Yes	S(1): 97.2; T(5): 2.8	5	1	1	0	N-Term(TMT6plex); S1(Phospho); M12(Oxidation)
304434531	NP_001182122.1	4678	NASP	nuclear autoantigenic sperm protein isoform4	S662	0.6	0.6	0.4	0.5	-	-	-	-	0.8	0.7	0.6	0.7	-	-	-	-	0.8	0.7	0.6	0.7	KPEEEsPR	KRKPEEEsPRKDDAK	S6;	No	S(6): 100.0	20	3	1	0	N-Term(TMT6plex); K1(TMT6plex); S6(Phospho)
530424835	XP_005258156.1	23255	SOGA2	protein SOGA2 isoform X2	S87	0.4	0.4	0.4	0.5	-	-	-	-	0.4	0.5	0.4	0.5	-	-	-	-	0.4	0.5	0.4	0.5	APPsPGSLAAPGR	LAGKAPPsPGSLAAP	S4;	Yes	S(4): 100.0; S(7): 0.0	6	2	1	0	N-Term(TMT6plex); S4(Phospho)
51479192	NP_002922.2	6015	RING1	E3 ubiquitin-protein ligase RING1	S38	0.8	0.4	0.2	0.5	-	-	-	-	0.8	0.5	0.3	0.5	-	-	-	-	0.8	0.5	0.3	0.5	TPQEAIMDGTEIAVsPR	DGTEIAVsPRSLHSE	S15;	Yes	T(1): 0.0; T(10): 0.0; S(15): 100.0	1	1	1	0	N-Term(TMT6plex); M7(Oxidation); S15(Phospho)
285393969	NP_001165420.1	79036	KXD1	kxDL motif-containing protein 1	T168	1.4	1.3	1.4	0.5	-	-	-	-	1.2	1.4	1.7	0.6	-	-	-	-	1.2	1.4	1.7	0.6	SQtDDEEMTGE	AINGRSQtDDEEMTG	T3;	Yes	S(1): 0.0; T(3): 100.0; T(9): 0.0	4	1	1	0	N-Term(TMT6plex); T3(Phospho); M8(Oxidation)
110611218	NP_001036041.1	6238	RRBP1	ribosome-binding protein 1	S185	0.9	1.0	0.9	0.5	-	-	-	-	0.9	0.9	1.0	0.5	-	-	-	-	0.9	0.9	1.0	0.5	NTDVAQsPEAPK	RNTDVAQsPEAPKQE	S7;	No	T(2): 0.0; S(7): 100.0	8	1	1	0	N-Term(TMT6plex); S7(Phospho); K12(TMT6plex)
38524622	NP_055848.1	23048	FNBP1	formin-binding protein 1	S296; S299	0.7	0.6	0.4	0.5	-	-	-	-	-	-	-	-	-	-	-	-	-	-	-	-	TVsDNsLSNSR	QPMKRTVsDNSLSNS;KRTVSDNsLSNSRGE	S3; S6;	Yes;Yes	T(1): 0.0; S(3): 100.0; S(6): 100.0; S(8): 0.0; S(10): 0.0	2	1	1	0	N-Term(TMT6plex); S3(Phospho); S6(Phospho)
224809302	NP_001139241.1	7508	XPC	DNA repair protein complementing XP-C cells isoform	S94	1.2	0.8	1.3	0.5	-	-	-	-	1.1	0.7	1.3	0.5	-	-	-	-	1.1	0.7	1.3	0.5	DEALsDGDDLR	VIKDEALsDGDDLRD	S5;	Yes	S(5): 100.0	2	2	1	0	N-Term(TMT6plex); S5(Phospho)
112363092	NP_036366.3	23429	RYBP	RING1 and YY1-binding protein	T215	1.5	1.2	0.9	0.5	-	-	-	-	1.7	1.6	1.2	0.6	-	-	-	-	1.7	1.6	1.2	0.6	SStPKGDMSAVNDESF	KGSSRSStPKGDMSA	T3;	Yes	S(1): 50.6; S(2): 50.6; T(3): 98.8; S(9): 0.0; S(15): 0.0	1	1	1	1	N-Term(TMT6plex); S1(Phospho); S2(Phospho); K5(TMT6plex); M8(Oxidation)
586798132	NP_001276908.1	9253	NUMBL	numb-like protein isoformb	T210	1.0	1.4	0.7	0.5	-	-	-	-	0.8	1.0	0.4	0.4	-	-	-	-	0.8	1.0	0.4	0.4	AEAAAAPtVAPGPAQPGHVSPTPATTSPGEK	AEAAAAPtVAPGPAQ	T8;	No	T(8): 82.2; S(20): 3.6; T(22): 3.6; T(25): 3.6; T(26): 3.6; S(27): 3.6	1	2	1	0	N-Term(TMT6plex); T26(Phospho); K31(TMT6plex)
108860697	NP_055445.2	9640	ZNF592	zinc finger protein 592	S460	0.4	0.5	0.4	0.5	-	-	-	-	0.4	0.5	0.4	0.5	-	-	-	-	0.4	0.5	0.4	0.5	ASDSSsPSCSSGPR	TKASDSSsPSCSSGP	S6;	Yes	S(2): 0.0; S(4): 0.0; S(5): 1.0; S(6): 98.9; S(8): 0.0; S(10): 0.0; S(11): 0.0	5	1	1	0	N-Term(TMT6plex); S6(Phospho); C9(Carbamidomethyl)
299758464	NP_001139685.2	64786	TBC1D15	TBC1 domain family member 15 isoform3	S658	0.7	0.5	0.6	0.5	-	-	-	-	0.7	0.4	0.5	0.4	-	-	-	-	0.7	0.4	0.5	0.4	NDsPTQIPVSSDVCR	ASGARNDsPTQIPVS	S3;	No	S(3): 98.2; T(5): 1.8; S(10): 0.0; S(11): 0.0	1	3	1	0	N-Term(TMT6plex); S3(Phospho); C14(Carbamidomethyl)
4507321	NP_003164.1	6839	SUV39H1	histone-lysine N-methyltransferase SUV39H1 isoform	S391	0.3	0.6	0.3	0.5	-	-	-	-	-	-	-	-	-	-	-	-	-	-	-	-	MDSNFGLAGLPGsPK	GLAGLPGsPKKRVRI	S13;	Yes	S(3): 0.0; S(13): 100.0	1	2	1	0	N-Term(TMT6plex); M1(Oxidation); S13(Phospho); K15(TMT6plex)
63025206	NP_001010924.1	221061	FAM171A1	protein FAM171A1 precursor	S849	0.9	1.3	0.8	0.4	-	-	-	-	-	-	-	-	-	-	-	-	-	-	-	-	TADAPSEPAAsPHQR	APSEPAAsPHQRRSA	S11;	Yes	T(1): 0.0; S(6): 0.0; S(11): 100.0	1	1	1	0	N-Term(TMT6plex); S11(Phospho)
11560152	NP_071378.1	63925	ZNF335	zinc finger protein 335	S986	0.4	0.5	0.6	0.4	-	-	-	-	-	-	-	-	-	-	-	-	-	-	-	-	THCVGDsQSSASSPPATSK	KTHCVGDsQSSASSP	S7;	No	T(1): 0.0; S(7): 98.5; S(9): 1.5; S(10): 0.0; S(12): 0.0; S(13): 0.0; T(17): 0.0; S(18): 0.0	8	1	1	0	N-Term(TMT6plex); C3(Carbamidomethyl); S10(Phospho); K19(TMT6plex)
225735619	NP_001139585.1	57708	MIER1	mesoderm induction early response protein 1 isoformi	S314	1.1	0.4	0.7	0.4	-	-	-	-	1.1	0.4	0.7	0.5	-	-	-	-	1.1	0.4	0.7	0.5	APsPPPTASNSSNSQSEK	AASSRAPsPPPTASN	S3;	No	S(3): 100.0; T(7): 0.0; S(9): 0.0; S(11): 0.0; S(12): 0.0; S(14): 0.0; S(16): 0.0	2	9	1	0	N-Term(TMT6plex); S3(Phospho); K18(TMT6plex)
7706367	NP_057179.1	51659	GINS2	DNA replication complex GINS protein PSF2	S182	1.7	1.5	1.2	0.4	-	-	-	-	-	-	-	-	-	-	-	-	-	-	-	-	TNLQPLESTQsQDF	QPLESTQsQDF_	S11;	Yes	T(1): 0.0; S(8): 1.7; T(9): 1.7; S(11): 96.6	1	1	1	0	N-Term(TMT6plex); S11(Phospho)
188595687	NP_001120959.1	2318	FLNC	filamin-C isoformb	S2200	0.9	0.8	0.4	0.4	-	-	-	-	0.8	0.8	0.5	0.4	-	-	-	-	0.8	0.8	0.5	0.4	LGsFGSITR	LGRERLGsFGSITRQ	S3;	No	S(3): 100.0; S(6): 0.0; T(8): 0.0	3	2	1	0	N-Term(TMT6plex); S3(Phospho)
87578392	NP_114033.2	4133	MAP2	microtubule-associated protein 2 isoform2	T275	0.4	0.4	0.3	0.4	-	-	-	-	0.5	0.7	0.6	0.8	-	-	-	-	0.5	0.7	0.6	0.8	TPHTPGtPK	RTPHTPGtPKSAILV	T7;	No	T(1): 0.0; T(4): 0.0; T(7): 100.0	1	4	1	0	N-Term(TMT6plex); T7(Phospho); K9(TMT6plex)
28559039	NP_004765.2	5469	MED1	mediator of RNA polymerase II transcription subunit 1	S1156	0.7	0.6	0.5	0.4	-	-	-	-	0.7	0.6	0.6	0.4	-	-	-	-	0.7	0.6	0.6	0.4	NSSQSGGKPGSsPITK	SGGKPGSsPITKHGL	S12;	Yes	S(2): 0.0; S(3): 0.0; S(5): 0.0; S(11): 2.1; S(12): 97.9; T(15): 0.0	16	1	1	0	N-Term(TMT6plex); K8(TMT6plex); S12(Phospho); K16(TMT6plex)
166795297	NP_057225.2	51143	DYNC1LI1	cytoplasmic dynein 1 light intermediate chain 1	S421	0.4	0.3	0.2	0.4	-	-	-	-	0.4	0.3	0.2	0.4	-	-	-	-	0.4	0.3	0.2	0.4	SVSSNVASVsPIPAGSK	SSNVASVsPIPAGSK	S10;	Yes	S(1): 0.0; S(3): 0.0; S(4): 0.0; S(8): 1.5; S(10): 98.5; S(16): 0.0	10	1	1	0	N-Term(TMT6plex); S10(Phospho); K17(TMT6plex)

Khan et al., 2019. Multi-omics analysis to characterize cigarette smoke induced molecular alterations in esophageal cells
Supplementary Table 8. List of phosphopeptides identified and quantified in untreated and chronically treated Het1A cells with cigarette smoke condensate for 8 months using SequestHT and Mascot search algorithms with a PhosphoRS probability score ≥75%

														Normalized phosphoprotein ratios with respect to corresponding total protein ratios.								Average of replicates													
Protein group Accession	NP_Accession	Gene ID	Gene Symbol	Protein Description	PhosphoSite (Protein)	Het-1A-Smoke - 2M/Parental	Het-1A-Smoke - 4M/Parental	Het-1A-Smoke - 6M/Parental	Het-1A-Smoke - 8M/Parental	Het-1A-Smoke - 2M/Parental	Het-1A-Smoke - 4M/Parental	Het-1A-Smoke - 6M/Parental	Het-1A-Smoke - 8M/Parental	Het-1A-Smoke - 2M/Parental	Het-1A-Smoke - 4M/Parental	Het-1A-Smoke - 6M/Parental	Het-1A-Smoke - 8M/Parental	Het-1A-Smoke - 2M/Parental	Het-1A-Smoke - 4M/Parental	Het-1A-Smoke - 6M/Parental	Het-1A-Smoke - 8M/Parental	Het-1A-Smoke - 2M/Parental	Het-1A-Smoke - 4M/Parental	Het-1A-Smoke - 6M/Parental	Het-1A-Smoke - 8M/Parental	Peptide Sequence	Phosphowindow	PhosphoSite (Peptide)	Phosphosite Plus Evidence	phosphoRS Site Probabilities	# PSMs	# Proteins	# Protein Groups	# Missed Cleavages	Modifications
						Replicate 1				Replicate 2				Replicate 1				Replicate 2																	
54633315	NP_055412.2	30836	DNTTIP2	deoxynucleotidyltransferase terminal-interacting protein 2	S434	1.0	0.4	1.0	0.4	-	-	-	-	1.1	0.5	1.1	0.4	-	-	-	-	1.1	0.5	1.1	0.4	LYTSAPNTsQGK	YTSAPNTsQGKDNSV	S9;	Yes	Y(2): 0.0; T(3): 0.0; S(4): 0.0; T(8): 1.6; S(9): 98.4	2	1	1	0	N-Term(TMT6plex); S9(Phospho); K12(TMT6plex)
300244569	NP_001177916.1	9295	SRSF11	serine/arginine-rich splicing factor 11 isoform 2	S433	1.4	0.7	0.7	0.4	-	-	-	-	1.6	0.9	0.9	0.5	-	-	-	-	1.6	0.9	0.9	0.5	DYDEEEQGYDsEK	EEEQGYDsEKEKKEE	S11;	No	Y(2): 0.0; Y(9): 0.0; S(11): 100.0	4	2	1	0	N-Term(TMT6plex); S11(Phospho); K13(TMT6plex)
73858561	NP_001027462.1	84722	PSRC1	proline/serine-rich coiled-coil protein 1 isoform a	S212	0.8	0.7	0.8	0.4	-	-	-	-	0.9	0.8	1.0	0.4	-	-	-	-	0.9	0.8	1.0	0.4	AAAsPPTPIR	PSGRAAAsPPTPIRS	S4;	Yes	S(4): 99.9; T(7): 0.1	3	1	1	0	N-Term(TMT6plex); S4(Phospho)
354681980	NP_001238918.1	51053	GMNN	geminin	S64	0.7	0.8	0.6	0.4	-	-	-	-	0.8	1.0	0.9	0.5	-	-	-	-	0.8	1.0	0.9	0.5	NDHLTSTTSsPGVIVPESSENK	HLTSTTSsPGVIVPE	S10;	Yes	T(5): 0.0; S(6): 0.0; T(7): 0.0; T(8): 0.0; S(9): 1.7; S(10): 98.2; S(18): 0.0; S(19): 0.0	1	1	1	0	N-Term(TMT6plex); S10(Phospho); K22(TMT6plex)
156564398	NP_079353.3	114034	TOE1	target of EGR1 protein 1	S5	0.6	0.7	0.6	0.4	-	-	-	-	0.6	0.7	0.7	0.4	-	-	-	-	0.6	0.7	0.7	0.4	AADsDDGAVSAPAASDGGVSK	_MAADsDDGAVSA	S4;	Yes	S(4): 100.0; S(10): 0.0; S(15): 0.0; S(20): 0.0	3	1	1	0	N-Term(TMT6plex); S4(Phospho); K21(TMT6plex)
55742678	NP_612435.1	113263	GLCCI1	glucocorticoid-induced transcript 1 protein	S108; T110	0.5	0.4	0.8	0.4	-	-	-	-	-	-	-	-	-	-	-	-	-	-	-	-	GPSPSsPtPPAAAAPAEQAPR	ARGPSPSsPTPPAAA;GPSPSSPtPPAAAAP	S6; T8;	Yes;Yes	S(3): 9.9; S(5): 9.9; S(6): 90.1; T(8): 90.1	1	1	1	0	N-Term(TMT6plex); S3(Phospho); S5(Phospho)
5454102	NP_006333.1	10460	TACC3	transforming acidic coiled-coil-containing protein 3	S177	0.5	0.7	0.5	0.4	-	-	-	-	0.6	0.7	0.5	0.4	-	-	-	-	0.6	0.7	0.5	0.4	VSGsPEQAVEENLSSYSLDR	SPGKVSGsPEQAVEE	S4;	Yes	S(2): 0.9; S(4): 99.1; S(14): 0.0; S(15): 0.0; Y(16): 0.0; S(17): 0.0	12	1	1	0	N-Term(TMT6plex); S4(Phospho)
4508007	NP_003441.1	7727	ZNF174	zinc finger protein 174 isoform a	S287	0.5	0.5	0.5	0.4	-	-	-	-	-	-	-	-	-	-	-	-	-	-	-	-	VEYISsPLK	CRVEYISsPLKSHPL	S6;	Yes	Y(3): 0.0; S(5): 0.0; S(6): 100.0	2	1	1	0	N-Term(TMT6plex); S6(Phospho); K9(TMT6plex)
306922386	NP_001120983.2	324	APC	adenomatous polyposis coli protein isoform a	S2812	0.6	0.4	0.7	0.4	-	-	-	-	0.5	0.4	0.8	0.4	-	-	-	-	0.5	0.4	0.8	0.4	TDSTESSGTQsPK	TESSGTQsPKRHSGS	S11;	No	T(1): 0.0; S(3): 0.0; T(4): 0.0; S(6): 1.6; S(7): 1.6; T(9): 1.6; S(11): 95.3	9	2	1	0	N-Term(TMT6plex); S11(Phospho); K13(TMT6plex)
74229008	NP_001028171.1	9139	CBFA2T2	protein CBFA2T2 isoform MTGR1c	S24	0.3	0.3	0.2	0.4	-	-	-	-	-	-	-	-	-	-	-	-	-	-	-	-	VPAMPGsPVEVK	RVPAMPGsPVEVKIQ	S7;	No	S(7): 100.0	1	2	1	0	N-Term(TMT6plex); M4(Oxidation); S7(Phospho); K12(TMT6plex)
150378498	NP_056127.2	84162	KIAA1109	uncharacterized protein KIAA1109	S2603	0.6	0.3	0.2	0.3	-	-	-	-	-	-	-	-	-	-	-	-	-	-	-	-	YTAGSAsPTPTFK	RYTAGSAsPTPTFKT	S7;	Yes	Y(1): 0.0; T(2): 0.0; S(5): 1.1; S(7): 97.7; T(9): 1.1; T(11): 0.0	2	1	1	0	N-Term(TMT6plex); S7(Phospho); K13(TMT6plex)
555943794	NP_001273171.1	2975	GTF3C1	general transcription factor 3C polypeptide 1 isoform 2	S739	0.8	0.7	0.7	0.3	-	-	-	-	0.8	0.7	0.7	0.4	-	-	-	-	0.8	0.7	0.7	0.4	TSQPPVPQGEAEEDsQGK	QGEAEEDsQGKEGPS	S15;	Yes	T(1): 0.0; S(2): 0.0; S(15): 100.0	9	2	1	0	N-Term(TMT6plex); S15(Phospho); K18(TMT6plex)
37620163	NP_065741.3	404734	NKHD1-EIF4EE	ANKHD1-EIF4EBP3 protein	S2598	0.8	0.7	0.9	0.3	-	-	-	-	-	-	-	-	-	-	-	-	-	-	-	-	GHNSSNsPSLQAGGAEGAGDR	RGHNSSNsPSLQAGG	S7;	Yes	S(4): 0.0; S(5): 0.0; S(7): 100.0; S(9): 0.0	7	1	1	0	N-Term(TMT6plex); S7(Phospho)
57165366	NP_067007.3	57827	C6orf47	uncharacterized protein C6orf47	S112	0.3	0.2	0.2	0.3	-	-	-	-	-	-	-	-	-	-	-	-	-	-	-	-	LEAGGAsPK	RLEAGGAsPKLRWDH	S7;	Yes	S(7): 100.0	1	1	1	0	N-Term(TMT6plex); S7(Phospho); K9(TMT6plex)
333440471	NP_001193980.1	23332	CLASP1	CLIP-associating protein 1 isoform 4	S600	0.7	0.7	0.6	0.3	-	-	-	-	0.8	0.7	0.6	0.3	-	-	-	-	0.8	0.7	0.6	0.3	SRsDIDVNAAASAK	GSLQRSRsDIDVNAA	S3;	Yes	S(1): 0.6; S(3): 99.4; S(12): 0.0	14	4	1	1	N-Term(TMT6plex); S3(Phospho); K14(TMT6plex)
32171238	NP_061330.2	55971	BAIAP2L1	brain-specific angiogenesis inhibitor 1-associated protein 2-like protein 1	S329	0.4	0.7	0.4	0.3	-	-	-	-	0.3	0.8	0.3	0.3	-	-	-	-	0.3	0.8	0.3	0.3	sVSVATGLNMMK	EDPSLQRsVSVATGL	S1;	Yes	S(1): 99.1; S(3): 0.9; T(6): 0.0	3	1	1	0	N-Term(TMT6plex); S1(Phospho); M10(Oxidation); M11(Oxidation); K12(TMT6plex)
29469071	NP_055530.2	9743	ARHGAP32	rho GTPase-activating protein 32 isoform 2	S543	0.9	1.1	1.0	0.3	-	-	-	-	-	-	-	-	-	-	-	-	-	-	-	-	VVYAFsPK	EKVVYAFsPKIGRKL	S6;	No	Y(3): 0.0; S(6): 100.0	1	2	1	0	N-Term(TMT6plex); S6(Phospho); K8(TMT6plex)
62988361	NP_001615.1	202	AIM1	absent in melanoma 1 protein	S22	0.3	0.3	0.6	0.3	-	-	-	-	0.4	0.4	0.7	0.4	-	-	-	-	0.4	0.4	0.7	0.4	STDsPGADAELPESAAR	GSQKSTDsPGADAEL	S4;	No	S(1): 0.0; T(2): 1.3; S(4): 98.7; S(14): 0.0	8	1	1	0	N-Term(TMT6plex); S4(Phospho)
555290002	NP_001273127.1	64897	C12orf43	uncharacterized protein C12orf43 isoform g	T170	0.3	0.2	0.6	0.3	-	-	-	-	0.4	0.2	0.6	0.3	-	-	-	-	0.4	0.2	0.6	0.3	VASVDSAVAATtPTSMATVQK	DSAVAATtPTSMATV	T12;	No	S(3): 0.0; S(6): 0.0; T(11): 1.5; T(12): 98.5; T(14): 0.0; S(15): 0.0; T(18): 0.0	3	7	1	0	N-Term(TMT6plex); T12(Phospho); M16(Oxidation); K21(TMT6plex)
38327034	NP_006039.2	10277	UBE4B	ubiquitin conjugation factor E4 B isoform 2	S105	0.2	0.2	0.2	0.3	-	-	-	-	0.2	0.2	0.2	0.2	-	-	-	-	0.2	0.2	0.2	0.2	SQsMDIDGVSCEK	QSLSRSQsMDIDGVS	S3;	Yes	S(1): 0.9; S(3): 99.1; S(10): 0.0	2	2	1	0	N-Term(TMT6plex); S3(Phospho); M4(Oxidation); C11(Carbamidomethyl); K13(TMT6plex)
344313201	NP_001230704.1	51474	LIMA1	LIM domain and actin-binding protein 1 isoform 4	S72	0.6	0.3	0.6	0.3	-	-	-	-	0.5	0.3	0.5	0.2	-	-	-	-	0.5	0.3	0.5	0.2	ASSLSESsPPK	ASSLSESsPPKAMKK	S8;	No	S(2): 0.0; S(3): 0.0; S(5): 0.0; S(7): 0.9; S(8): 99.1	5	4	1	0	N-Term(TMT6plex); S8(Phospho); K11(TMT6plex)
197313748	NP_054878.5	29072	SETD2	histone-lysine N-methyltransferase SETD2	S131	0.5	0.5	0.4	0.2	-	-	-	-	0.6	0.6	0.4	0.3	-	-	-	-	0.6	0.6	0.4	0.3	MEIGDTLSTAEESsPPK	LSTAEESsPPKSRVE	S14;	Yes	T(6): 0.0; S(8): 1.7; T(9): 0.0; S(13): 1.7; S(14): 96.5	12	1	1	0	N-Term(TMT6plex); M1(Oxidation); S8(Phospho); K17(TMT6plex)
566559802	NP_001274432.1	6744	SSFA2	sperm-specific antigen 2 isoform 3	S92	0.4	0.2	0.3	0.2	-	-	-	-	0.4	0.2	0.3	0.3	-	-	-	-	0.4	0.2	0.3	0.3	TPLGAsLDEQSSSTLK	CRTPLGAsLDEQSSS	S6;	Yes	T(1): 0.0; S(6): 100.0; S(11): 0.0; S(12): 0.0; S(13): 0.0; T(14): 0.0	6	3	1	0	N-Term(TMT6plex); S6(Phospho); K16(TMT6plex)
62899035	NP_001017420.1	157570	ESCO2	N-acetyltransferase ESCO2	S244	0.6	0.3	0.6	0.2	-	-	-	-	0.6	0.3	0.7	0.2	-	-	-	-	0.6	0.3	0.7	0.2	SEVIEDsDVETVSEK	KSEVIEDsDVETVSE	S7;	Yes	S(1): 0.0; S(7): 100.0; T(11): 0.0; S(13): 0.0	7	1	1	0	N-Term(TMT6plex); S7(Phospho); K15(TMT6plex)
296939604	NP_055638.2	65125	SGsGGGSAK	serine/threonine-protein kinase WNK1 isoform 2	S185	1.1	1.4	1.4	1.2	-	-	-	-	-	-	-	-	-	-	-	-	-	-	-	-	WNK1	PPPARSGsGGGSAKE	S3;	Yes	S(1): 0.0; S(3): 100.0; S(7): 0.0	3	4	1	0	N-Term(TMT6plex); S3(Phospho); K9(TMT6plex)
544583471	NP_001269783.1	219771	CCNY	cyclin-Y isoform 5	S193	-	-	-	-	3.4	4.8	5.9	4.3	-	-	-	-	-	-	-	-	-	-	-	-	SAsADNLTLPR	SARKRSAsADNLTLP	S3;	No	S(1): 0.0; S(3): 100.0; T(8): 0.0	1	4	1	0	N-Term(TMT6plex); S3(Phospho)
209862987	NP_569830.2	8567	MADD	MAP kinase-activating death domain protein isoform f	S777	-	-	-	-	1.4	2.7	3.1	4.2	-	-	-	-	1.5	2.6	3.2	3.5	1.5	2.6	3.2	3.5	LASDsDAESDSR	SLRLASDsDAESDSR	S5;	No	S(3): 0.0; S(5): 98.6; S(9): 1.4; S(11): 0.0	1	10	1	0	N-Term(TMT6plex); S5(Phospho)
85815829	NP_061830.3	23616	SH3BP1	SH3 domain-binding protein 1	S586	-	-	-	-	1.5	2.4	3.2	4.1	-	-	-	-	1.3	2.0	2.6	3.1	1.3	2.0	2.6	3.1	SsPPAPPLPPGSGSPGTPQALPR	QVSGSRSsPPAPPLP	S2;	Yes	S(1): 1.4; S(2): 98.6; S(12): 0.0; S(14): 0.0; T(17): 0.0	3	1	1	0	N-Term(TMT6plex); S2(Phospho)
212276104	NP_001131025.1	9208	LRRFIP1	leucine-rich repeat flightless-interacting protein 1 isoform 5	T82	-	-	-	-	2.8	2.5	1.9	3.6	-	-	-	-	3.0	2.5	2.1	4.5	3.0	2.5	2.1	4.5	NMPGLSAATLASLGGtSSR	TLASLGGtSSRRGSG	T16;	No	S(6): 0.0; T(9): 0.0; S(12): 0.0; T(16): 97.9; S(17): 1.1; S(18): 1.1	3	5	1	0	N-Term(TMT6plex); M2(Oxidation); T16(Phospho)
16757970	NP_443198.1	116496	FAM129A	protein Niban	S646	-	-	-	-	5.2	5.7	6.8	3.3	-	-	-	-	3.3	3.1	3.9	2.1	3.3	3.1	3.9	2.1	GESLSLPGPsPPPDGTEQVIISR	SLSLPGPsPPPDGTE	S10;	Yes	S(3): 0.0; S(5): 0.0; S(10): 100.0; T(16): 0.0; S(22): 0.0	1	1	1	0	N-Term(TMT6plex); S10(Phospho)
53829376	NP_001005476.1	8502	PKP4	plakophilin-4 isoform b	S337	-	-	-	-	3.0	3.0	5.3	3.0	-	-	-	-	-	-	-	-	-	-	-	-	VASPSQGQVGSSsPK	QGQVGSSsPKRSGMT	S13;	Yes	S(3): 0.0; S(5): 0.0; S(11): 0.0; S(12): 1.2; S(13): 98.8	2	2	1	0	N-Term(TMT6plex); S13(Phospho); K15(TMT6plex)
13540594	NP_110432.1	81562	LMAN2L	VIP36-like protein isoform 2 precursor	T223	-	-	-	-	1.3	3.0	2.0	2.9	-	-	-	-	1.2	2.8	1.9	2.8	1.2	2.8	1.9	2.8	HLtIMMDIDGK	RYVKRHLtIMMDIDG	T3;	No	T(3): 100.0	1	2	1	0	N-Term(TMT6plex); T3(Phospho); M5(Oxidation); M6(Oxidation); K11(TMT6plex)
544709647	NP_001269861.1	10152	ABI2	abl interactor 2 isoform e	S245	-	-	-	-	1.0	0.9	2.0	2.9	-	-	-	-	1.0	0.9	2.1	2.7	1.0	0.9	2.1	2.7	HTPPTIGGsLPYR	TPPTIGGsLPYRRPP	S9;	No	T(2): 0.0; T(5): 0.0; S(9): 98.9; Y(12): 1.1	2	5	1	0	N-Term(TMT6plex); S9(Phospho)
55741677	NP_055527.1	9739	SETD1A	histone-lysine N-methyltransferase SETD1A	S468	-	-	-	-	1.4	0.9	1.9	2.8	-	-	-	-	1.4	0.8	1.8	2.4	1.4	0.8	1.8	2.4	sGSPAPETTNESVPFAQHSSLDSR	RPASPARsGSPAPET	S1;	Yes	S(1): 97.9; S(3): 2.1; T(8): 0.0; T(9): 0.0; S(12): 0.0; S(19): 0.0; S(20): 0.0; S(23): 0.0	2	1	1	0	N-Term(TMT6plex); S1(Phospho)
321267522	NP_001189433.1	9150	CTDP1	RNA polymerase II subunit A C-terminal domain phosphatase isoform 3	S621	-	-	-	-	1.4	0.9	2.1	2.8	-	-	-	-	1.3	0.9	2.1	2.5	1.3	0.9	2.1	2.5	ENsPAAFPDR	TKAQRENsPAAFPDR	S3;	No	S(3): 100.0	1	3	1	0	N-Term(TMT6plex); S3(Phospho)
527498258	NP_001268413.1	55738	ARFGAP1	ADP-ribosylation factor GTPase-activating protein 1 isoform e	T76	-	-	-	-	1.5	0.9	2.0	2.7	-	-	-	-	1.7	0.9	2.0	2.5	1.7	0.9	2.0	2.5	YVGFGNtPPPQK	RYVGFGNtPPPQKKE	T7;	No	Y(1): 0.0; T(7): 100.0	1	5	1	0	N-Term(TMT6plex); T7(Phospho); K12(TMT6plex)
313151226	NP_001186227.1	51720	UIMC1	BRCA1-A complex subunit RAP80	S101	-	-	-	-	1.0	3.5	2.1	2.7	-	-	-	-	1.1	3.8	2.5	2.8	1.1	3.8	2.5	2.8	EVNsQEEEEEELLR	QEAREVNsQEEEEEE	S4;	Yes	S(4): 100.0	1	1	1	0	N-Term(TMT6plex); S4(Phospho)
310832410	NP_001185594.1	23365	ARHGEF12	rho guanine nucleotide exchange factor 12 isoform 2	S618	-	-	-	-	3.3	1.7	4.2	2.7	-	-	-	-	3.2	1.7	4.2	2.8	3.2	1.7	4.2	2.8	HLSTPSSVsPEPQDSAK	LSTPSSVsPEPQDSA	S9;	No	S(3): 0.0; T(4): 0.0; S(6): 0.0; S(7): 1.6; S(9): 98.3; S(15): 0.0	1	2	1	0	N-Term(TMT6plex); S9(Phospho); K17(TMT6plex)
156105703	NP_001093879.1	26054	SENP6	sentrin-specific protease 6 isoform 2	S345	-	-	-	-	4.1	3.1	2.1	2.7	-	-	-	-	3.8	3.0	2.0	2.5	3.8	3.0	2.0	2.5	ESIsPQPADSACSSPAPSTGK	TNRRESIsPQPADSA	S4;	No	S(2): 1.9; S(4): 98.1; S(10): 0.0; S(13): 0.0; S(14): 0.0; S(18): 0.0; T(19): 0.0	2	2	1	0	N-Term(TMT6plex); S4(Phospho); C12(Carbamidomethyl); K21(TMT6plex)
296939604	NP_055638.2	65125	WNK1	serine/threonine-protein kinase WNK1 isoform 2	S382	-	-	-	-	2.5	3.4	4.5	2.7	-	-	-	-	2.3	3.1	4.1	2.4	2.3	3.1	4.1	2.4	sVIGTPEEMAPEMYEEK	KRASFAKsVIGTPEE	S1;	Yes	S(1): 100.0; T(5): 0.0; Y(14): 0.0	2	5	1	0	N-Term(TMT6plex); S1(Phospho); M9(Oxidation); M13(Oxidation); K17(TMT6plex)
326319990	NP_055820.2	283638	CEP170B	centrosomal protein of 170 kDa protein B isoform 2	S759	-	-	-	-	1.0	0.9	1.4	2.6	-	-	-	-	-	-	-	-	-	-	-	-	KPLAAPGDGEGLGQTAQPsPPA	LGQTAQPsPPARDGV	S19;	No	T(15): 2.9; S(19): 97.1	1	2	1	0	N-Term(TMT6plex); K1(TMT6plex); S19(Phospho)
54112403	NP_060250.2	55636	CHD7	chromodomain-helicase-DNA-binding protein 7	S637	-	-	-	-	2.0	1.7	2.3	2.6	-	-	-	-	-	-	-	-	-	-	-	-	NSLDGsQEEK	NRNSLDGsQEEKKKK	S6;	Yes	S(2): 0.0; S(6): 100.0	1	1	1	0	N-Term(TMT6plex); S6(Phospho); K10(TMT6plex)
81295809	NP_006022.3	5116	PCNT	pericentrin	S2355	-	-	-	-	1.0	2.0	1.5	2.5	-	-	-	-	1.0	1.9	1.5	2.6	1.0	1.9	1.5	2.6	LSPGsGGPEAQTAGPVTPASISGR	GARLSPGsGGPEAQT	S5;	Yes	S(2): 2.8; S(5): 97.1; T(12): 0.1; T(17): 0.0; S(20): 0.0; S(22): 0.0	1	1	1	0	N-Term(TMT6plex); S5(Phospho)
27501458	NP_071375.1	63922	CHTF18	chromosome transmission fidelity protein 18 homolog	S73	-	-	-	-	0.6	2.4	1.7	2.5	-	-	-	-	0.5	2.4	1.5	2.0	0.5	2.4	1.5	2.0	GDAASSPAPAASVGsSQGGAR	APAASVGsSQGGARK	S15;	Yes	S(5): 50.0; S(6): 50.0; S(12): 0.0; S(15): 97.8; S(16): 2.1	1	1	1	0	N-Term(TMT6plex); S6(Phospho); S12(Phospho)
24234750	NP_036350.2	3609	ILF3	interleukin enhancer-binding factor 3 isoform a	T592	-	-	-	-	1.0	0.9	0.6	2.4	-	-	-	-	1.1	1.0	0.6	2.5	1.1	1.0	0.6	2.5	LFPDtPLALDANK	LEKLFPDtPLALDAN	T5;	Yes	T(5): 100.0	1	5	1	0	N-Term(TMT6plex); T5(Phospho); K13(TMT6plex)
148613886	NP_073752.5	64864	RFX7	DNA-binding protein RFX7	S454; S455	-	-	-	-	2.7	2.8	2.0	2.4	-	-	-	-	-	-	-	-	-	-	-	-	SPTTVLFTssPIK	PTTVLFTsSPIKTAV;TTVLFTSsPIKTAVV	S9; S10;	No;No	S(1): 2.2; T(3): 2.2; T(4): 2.2; T(8): 2.2; S(9): 91.2; S(10): 100.0	1	1	1	0	N-Term(TMT6plex); T3(Phospho); S10(Phospho); K13(TMT6plex)
251823858	NP_001156506.1	57521	RPTOR	regulatory-associated protein of mTOR isoform 2	S701; S705	-	-	-	-	4.1	3.9	2.9	2.4	-	-	-	-	3.3	3.6	2.8	2.1	3.3	3.6	2.8	2.1	VLDTSSLTQsAPAsPTNK	DTSSLTQsAPASPTN;LTQSAPAsPTNKGVH	S10; S14;	No;No	T(4): 0.0; S(5): 0.0; S(6): 0.0; T(8): 1.5; S(10): 98.5; S(14): 100.0; T(16): [illegible]	2	2	1	0	N-Term(TMT6plex); S10(Phospho); S14(Phospho); K18(TMT6plex)
503774453	NP_055204.3	27043	PELP1	proline-, glutamic acid- and leucine-rich protein 1 isoform 1	S1083	-	-	-	-	2.6	3.4	2.9	2.3	-	-	-	-	2.6	3.3	2.8	2.4	2.6	3.3	2.8	2.4	GADTAPTLAPEALPsQGEVER	LAPEALPsQGEVERE	S15;	No	T(4): 0.0; T(7): 0.0; S(15): 100.0	1	1	1	0	N-Term(TMT6plex); S15(Phospho)
194097347	NP_002637.3	5287	PIK3C2B	phosphatidylinositol 4-phosphate 3-kinase C2 domain-containing subunit beta	Y1298	-	-	-	-	7.4	1.2	0.6	2.3	-	-	-	-	-	-	-	-	-	-	-	-	yVYDALRPQDTEANATTYFTR	SDLEDLKyVYDALRP	Y1;	No	Y(1): 96.8; Y(3): 1.6; T(11): 1.6; T(16): 0.0; T(17): 0.0; Y(18): 0.0; [illegible]	1	1	1	0	N-Term(TMT6plex); Y1(Phospho)
153792195	NP_056487.1	85376	RIMBP3	RIMS-binding protein 3A	Y1580	-	-	-	-	1.1	0.5	1.3	2.3	-	-	-	-	-	-	-	-	-	-	-	-	IMIAALDyDPGDGQMGGGQK	IMIAALDyDPGDGQM	Y8;	No	Y(8): 100.0	1	3	1	0	N-Term(TMT6plex); M2(Oxidation); Y8(Phospho); M15(Oxidation); K20(TMT6plex)
38327642	NP_060176.2	54853	WDR55	WD repeat-containing protein 55	S14	-	-	-	-	0.4	1.3	1.0	2.3	-	-	-	-	0.4	1.2	0.9	2.0	0.4	1.2	0.9	2.0	TCEERPAEDGsDEEDPDSMEAPTR	ERPAEDGsDEEDPDS	S11;	Yes	T(1): 3.9; S(11): 96.1; S(18): 0.0; T(23): 0.0	1	1	1	0	N-Term(TMT6plex); C2(Carbamidomethyl); S11(Phospho); M19(Oxidation)
157502197	NP_055553.3	9772	KIAA0195	uncharacterized protein KIAA0195	S1354	-	-	-	-	0.6	0.4	0.8	2.2	-	-	-	-	0.7	0.5	0.9	2.5	0.7	0.5	0.9	2.5	LGMNsPE	ETKLGMNsPE	S5;	No	S(5): 100.0	1	1	1	0	N-Term(TMT6plex); M3(Oxidation); S5(Phospho)
568214245	NP_001274744.1	9898	UBAP2L	ubiquitin-associated protein 2-like isoform c	S460	-	-	-	-	1.4	0.9	2.8	2.2	-	-	-	-	1.6	0.9	3.0	2.0	1.6	0.9	3.0	2.0	STSAPQMsPGSSDNQSSSPQPAQQK	STSAPQMsPGSSDNQ	S8;	No	S(1): 0.0; T(2): 0.0; S(3): 0.0; S(8): 97.6; S(11): 2.3; S(12): 1.2; S(16): 1.2; S(17): 48.8; S(18): 48.8	2	4	1	0	N-Term(TMT6plex); M7(Oxidation); S8(Phospho); S11(Phospho); K25(TMT6plex)
566006176	NP_001274366.1	79960	JADE1	protein Jade-1 isoform 3	S691	-	-	-	-	0.9	2.1	1.3	2.2	-	-	-	-	-	-	-	-	-	-	-	-	AATsPGVGQSAPGTR	DNTRAATsPGVGQSA	S4;	No	T(3): 2.2; S(4): 95.4; S(10): 2.2; T(14): 0.1	1	2	1	0	N-Term(TMT6plex); S4(Phospho)
21071074	NP_009180.3	11278	KLF12	Krueppel-like factor 12	S308	-	-	-	-	1.0	1.7	1.6	2.2	-	-	-	-	-	-	-	-	-	-	-	-	SEsPDSR	RRQRRSEsPDSRKRR	S3;	No	S(1): 0.7; S(3): 99.3; S(6): 0.0	1	1	1	0	N-Term(TMT6plex); S3(Phospho)
5032189	NP_005648.1	7158	TP53BP1	tumor suppressor p53-binding protein 1 isoform 3	T855	-	-	-	-	1.0	1.9	2.0	2.2	-	-	-	-	1.0	1.9	2.2	2.6	1.0	1.9	2.2	2.6	LDQELQQPQtQEK	QELQQPQtQEKTSNS	T10;	Yes	T(10): 100.0	2	3	1	0	N-Term(TMT6plex); T10(Phospho); K13(TMT6plex)
384475541	NP_001244965.1	55718	POLR3E	DNA-directed RNA polymerase III subunit RPC5 isoform 5	S161; S162	-	-	-	-	2.6	1.7	1.1	2.2	-	-	-	-	2.6	1.6	1.1	2.1	2.6	1.6	1.1	2.1	EAANEAGDssQDEAEDDVK	AANEAGDsSQDEAED;ANEAGDSsQDEAEDD	S9; S10;	Yes;Yes	S(9): 100.0; S(10): 100.0	2	5	1	0	N-Term(TMT6plex); S9(Phospho); S10(Phospho); K19(TMT6plex)
7662074	NP_055687.1	9925	ZBTB5	zinc finger and BTB domain-containing protein 5	S371	-	-	-	-	2.8	1.9	2.0	2.2	-	-	-	-	-	-	-	-	-	-	-	-	IDLsPESSDR	SAEKIDLsPESSDRS	S4;	Yes	S(4): 100.0; S(7): 0.0; S(8): 0.0	1	1	1	0	N-Term(TMT6plex); S4(Phospho)
298919181	NP_001177369.1	9611	NCOR1	nuclear receptor corepressor 1 isoform 3	S2017	-	-	-	-	1.1	2.0	2.1	2.1	-	-	-	-	1.1	1.7	1.7	1.7	1.1	1.7	1.7	1.7	VsPENLVDK	QRPGSRVsPENLVDK	S2;	No	S(2): 100.0	1	2	1	0	N-Term(TMT6plex); S2(Phospho); K9(TMT6plex)
296179392	NP_848659.2	340371	NRBP2	nuclear receptor-binding protein 2	T411	-	-	-	-	1.7	2.1	1.4	2.1	-	-	-	-	-	-	-	-	-	-	-	-	TPtPEPFDSETR	VQKAKTPtPEPFDSE	T3;	Yes	T(1): 0.0; T(3): 100.0; S(9): 0.0; T(11): 0.0	2	1	1	0	N-Term(TMT6plex); T3(Phospho)
36029914	NP_079330.2	80124	VCPIP1	deubiquitinating protein VCIP135	S998	-	-	-	-	1.4	2.1	1.8	2.1	-	-	-	-	1.1	1.7	1.6	2.1	1.1	1.7	1.6	2.1	ESsPSHGLLK	TTRSRESsPSHGLLK	S3;	Yes	S(2): 0.7; S(3): 98.5; S(5): 0.7	1	1	1	0	N-Term(TMT6plex); S3(Phospho); K10(TMT6plex)
256017159	NP_001074010.2	23269	MGA	MAX gene-associated protein isoform 2	S924	-	-	-	-	1.3	1.8	1.7	2.1	-	-	-	-	1.1	1.4	1.5	1.7	1.1	1.4	1.5	1.7	SILPYPVsPK	SILPYPVsPKQKYSH	S8;	Yes	S(1): 0.0; Y(5): 0.0; S(8): 100.0	2	2	1	0	N-Term(TMT6plex); S8(Phospho); K10(TMT6plex)
76150625	NP_001028886.1	4839	NOP2	putative ribosomal RNA methyltransferase NOP2 isoform 1	S808	-	-	-	-	1.1	2.6	2.0	2.1	-	-	-	-	1.2	2.4	1.9	2.1	1.2	2.4	1.9	2.1	GNSQLLLs	GNSQLLLs_	S8;	No	S(3): 0.0; S(8): 100.0	2	3	1	0	N-Term(TMT6plex); S8(Phospho)
612339330	NP_001278376.1	6297	SALL2	sal-like protein 2 isoform c	S669	-	-	-	-	2.7	2.6	2.1	2.0	-	-	-	-	-	-	-	-	-	-	-	-	GDSEEAsGAEEEVGTVAAAATAGK	RGDSEEAsGAEEEVG	S7;	No	S(3): 1.6; S(7): 98.4; T(15): 0.0; T(21): 0.0	3	3	1	0	N-Term(TMT6plex); S7(Phospho); K24(TMT6plex)
16262452	NP_065821.1	57530	CGN	cingulin	S258	-	-	-	-	0.7	0.6	1.5	2.0	-	-	-	-	1.0	0.8	1.9	2.3	1.0	0.8	1.9	2.3	QPAQSQNLsPLSGFSR	PAQSQNLsPLSGFSR	S9;	No	S(5): 0.0; S(9): 100.0; S(12): 0.0; S(15): 0.0	1	1	1	0	N-Term(TMT6plex); S9(Phospho)
21536371	NP_009041.2	7011	TEP1	telomerase protein component 1	S42	-	-	-	-	1.2	1.6	1.3	2.0	-	-	-	-	-	-	-	-	-	-	-	-	LHQHVSTHsDILSLK	HQHVSTHsDILSLKN	S9;	No	S(6): 50.0; T(7): 50.0; S(9): 95.8; S(13): 4.2	1	1	1	0	N-Term(TMT6plex); S6(Phospho); S9(Phospho); K15(TMT6plex)
222136585	NP_003911.2	8914	TIMELESS	protein timeless homolog	S1149	-	-	-	-	1.8	1.8	1.6	2.0	-	-	-	-	2.0	2.0	1.8	2.1	2.0	2.0	1.8	2.1	AGLAsPEEEDAVGK	KKKAGLAsPEEEDAV	S5;	Yes	S(5): 100.0	2	1	1	0	N-Term(TMT6plex); S5(Phospho); K14(TMT6plex)
40548403	NP_689853.3	196513	DCP1B	mRNA-decapping enzyme 1B	S448	-	-	-	-	3.2	1.9	2.5	2.0	-	-	-	-	3.6	2.0	3.0	2.2	3.6	2.0	3.0	2.2	QTLPISGSQTGSSGVIsPQELLK	TGSSGVIsPQELLKK	S17;	Yes	T(2): 0.0; S(6): 0.0; S(8): 0.0; T(10): 0.0; S(12): 0.0; S(13): 0.0; S(17): [illegible]	2	1	1	0	N-Term(TMT6plex); S17(Phospho); K23(TMT6plex)
17978519	NP_004887.2	9559	VPS26A	vacuolar protein sorting-associated protein 26A	S315	-	-	-	-	0.4	0.7	1.2	1.9	-	-	-	-	0.4	0.7	1.1	1.9	0.4	0.7	1.1	1.9	EEsPESQASAEQPEM	NEHQREEsPESQASA	S3;	Yes	S(3): 99.9; S(6): 0.1; S(9): 0.1	1	1	1	0	N-Term(TMT6plex); S3(Phospho); M15(Oxidation)
609876591	NP_001278340.1	6651	SON	protein SON isoform E	S1697	-	-	-	-	2.0	1.1	1.7	1.9	-	-	-	-	2.2	1.2	1.8	2.0	2.2	1.2	1.8	2.0	ESDQTLAALLsPK	QTLAALLsPKESSGG	S11;	Yes	S(2): 0.0; T(5): 0.0; S(11): 100.0	3	3	1	0	N-Term(TMT6plex); S11(Phospho); K13(TMT6plex)
528281423	NP_001268464.1	23136	EPB41L3	band 4.1-like protein 3 isoform 4	S472	-	-	-	-	0.9	1.3	2.6	1.9	-	-	-	-	0.9	1.1	1.6	1.3	0.9	1.1	1.6	1.3	LSTsPVR	WEKRLSTsPVRLAAR	S4;	No	S(2): 0.0; T(3): 0.6; S(4): 99.4	1	4	1	0	N-Term(TMT6plex); S4(Phospho)
5902034	NP_008993.1	11137	PWP1	periodic tryptophan protein 1 homolog	S50	-	-	-	-	1.1	0.7	2.4	1.9	-	-	-	-	1.1	0.6	2.3	1.9	1.1	0.6	2.3	1.9	EKLQEEGGGsDEEETGSPSEDGMQSAR	LQEEGGGsDEEETGS	S10;	Yes	S(10): 100.0; T(15): 0.0; S(17): 0.0; S(19): 0.0; S(25): 0.0	1	1	1	1	N-Term(TMT6plex); K2(TMT6plex); S10(Phospho); M23(Oxidation)
115511030	NP_612450.2	115004	MB21D1	cyclic GMP-AMP synthase	S37	-	-	-	-	1.0	1.4	1.2	1.9	-	-	-	-	1.0	1.3	1.2	1.9	1.0	1.3	1.2	1.9	GAPMDPTEsPAAPEAALPK	APMDPTEsPAAPEAA	S9;	No	T(7): 1.4; S(9): 98.6	2	1	1	0	N-Term(TMT6plex); M4(Oxidation); S9(Phospho); K19(TMT6plex)
12056473	NP_061819.2	54187	NANS	sialic acid synthase	S275	-	-	-	-	1.1	1.9	1.5	1.9	-	-	-	-	1.0	1.4	1.2	1.5	1.0	1.4	1.2	1.5	ALGsPTK	LVERALGsPTKQLLP	S4;	Yes	S(4): 99.4; T(6): 0.6	2	1	1	0	N-Term(TMT6plex); S4(Phospho); K7(TMT6plex)
13236559	NP_077295.1	79169	C1orf35	multiple myeloma tumor-associated protein 2	S177	-	-	-	-	1.4	1.6	1.6	1.9	-	-	-	-	1.6	2.0	1.9	2.1	1.6	2.0	1.9	2.1	AEDQTEsSCESHR	RAEDQTEsSCESHRK	S7;	No	T(5): 0.0; S(7): 99.4; S(8): 0.6; S(11): 0.0	2	1	1	0	N-Term(TMT6plex); S7(Phospho); C9(Carbamidomethyl)
540344565	NP_001269339.1	5510	PPP1R7	protein phosphatase 1 regulatory subunit 7 isoform 3	S12	-	-	-	-	1.0	1.1	1.2	1.9	-	-	-	-	1.0	1.2	1.2	1.9	1.0	1.2	1.2	1.9	GAGQQQsQEMMEVDR	RGAGQQQsQEMMEVD	S7;	Yes	S(7): 100.0	1	2	1	0	N-Term(TMT6plex); S7(Phospho); M10(Oxidation); M11(Oxidation)
6594683	NP_015627.1	7468	WHSC1	histone-lysine N-methyltransferase NSD2 isoform 4	T110	-	-	-	-	1.0	0.6	0.6	1.8	-	-	-	-	1.1	0.6	0.7	2.0	1.1	0.6	0.7	2.0	GIGtPPNTTPIK	QEMKGIGtPPNTTPI	T4;	Yes	T(4): 97.2; T(8): 1.4; T(9): 1.4	1	3	1	0	N-Term(TMT6plex); T4(Phospho); K12(TMT6plex)
20357556	NP_612632.1	2017	CTTN	src substrate cortactin isoform b	S368	-	-	-	-	1.0	1.9	1.8	1.8	-	-	-	-	0.8	1.6	1.2	1.7	0.8	1.6	1.2	1.7	AKTQTPPVsPAPQPTEER	KTQTPPVsPAPQPTE	S9;	Yes	T(3): 50.0; T(5): 50.0; S(9): 100.0; T(15): 0.0	1	3	1	1	N-Term(TMT6plex); K2(TMT6plex); T3(Phospho); S9(Phospho)
59891448	NP_689969.2	253260	RICTOR	rapamycin-insensitive companion of mTOR isoform 1	S21	-	-	-	-	1.9	1.7	1.8	1.8	-	-	-	-	1.6	1.5	1.6	1.8	1.6	1.5	1.6	1.8	NDsGEENVPLDLTR	RVRGRNDsGEENVPL	S3;	Yes	S(3): 100.0; T(13): 0.0	1	2	1	0	N-Term(TMT6plex); S3(Phospho)
519666804	NP_001265547.1	5770	PTPN1	tyrosine-protein phosphatase non-receptor type 1 isoform 2	S313	-	-	-	-	0.8	0.9	1.3	1.8	-	-	-	-	0.8	0.8	1.2	1.6	0.8	0.8	1.2	1.6	GAQAAsPAK	LRGAQAAsPAKGEPS	S6;	No	S(6): 100.0	1	2	1	0	N-Term(TMT6plex); S6(Phospho); K9(TMT6plex)
42544123	NP_973727.1	7536	SF1	splicing factor 1 isoform 3	S80; S82	-	-	-	-	1.6	1.1	1.0	1.8	-	-	-	-	1.6	1.0	1.1	1.6	1.6	1.0	1.1	1.6	TGDLGIPPNPEDRsPsPEPIYNSEGK	PPNPEDRsPSPEPIY;NPEDRSPsPEPIYNS	S14; S16;	Yes;Yes	T(1): 0.2; S(14): 96.7; S(16): 96.7; Y(21): 6.3; S(23): 0.0	1	6	1	1	N-Term(TMT6plex); S14(Phospho); S16(Phospho); K26(TMT6plex)
117168275	NP_109599.3	81539	SLC38A1	sodium-coupled neutral amino acid transporter 1 isoform 1	S52; S56	-	-	-	-	1.7	2.3	1.6	1.8	-	-	-	-	1.3	2.8	1.1	1.8	1.3	2.8	1.1	1.8	sLTNsHLEK	SDRESRRsLTNSHLE;SRRSLTNsHLEKKKC	S1; S5;	Yes;Yes	S(1): 100.0; T(3): 0.0; S(5): 100.0	1	2	1	0	N-Term(TMT6plex); S1(Phospho); S5(Phospho); K9(TMT6plex)
189027090	NP_085130.2	80315	CPEB4	cytoplasmic polyadenylation element-binding protein	S97	-	-	-	-	1.2	2.1	1.6	1.8	-	-	-	-	-	-	-	-	-	-	-	-	QQLsPSPGQEAGILPETEK	PLEKQQLsPSPGQEA	S4;	Yes	S(4): 98.0; S(6): 2.0; T(17): 0.0	1	1	1	0	N-Term(TMT6plex); S4(Phospho); K19(TMT6plex)
149192855	NP_037450.2	84726	PRRC2B	protein PRRC2B	S1139	-	-	-	-	1.8	2.2	1.7	1.7	-	-	-	-	1.7	2.2	1.8	1.6	1.7	2.2	1.8	1.6	VASETHSEGsEYEELPK	SETHSEGsEYEELPK	S10;	Yes	S(3): 2.1; T(5): 2.1; S(7): 2.1; S(10): 91.6; Y(12): 2.1	1	1	1	0	N-Term(TMT6plex); S7(Phospho); K17(TMT6plex)
557357729	NP_001273444.1	55729	ATF7IP	activating transcription factor 7-interacting protein 1 isoform 4	S113	-	-	-	-	0.7	1.8	1.8	1.7	-	-	-	-	0.7	1.8	1.7	1.7	0.7	1.8	1.7	1.7	QDDDLNCEPLsPHNITPEPVSK	DLNCEPLsPHNITPE	S11;	Yes	S(11): 97.3; T(16): 2.7; S(21): 0.0	1	4	1	0	N-Term(TMT6plex); C7(Carbamidomethyl); S11(Phospho); K22(TMT6plex)
19923485	NP_057508.2	51747	LUC7L3	luc7-like protein 3	S425	-	-	-	-	1.8	1.9	2.3	1.7	-	-	-	-	2.0	2.2	2.5	1.9	2.0	2.2	2.5	1.9	NEVNGTSEDIKsEGDTQSN	GTSEDIKsEGDTQSN	S12;	Yes	T(6): 0.0; S(7): 0.0; S(12): 99.9; T(16): 0.0; S(18): 0.0	1	1	1	1	N-Term(TMT6plex); K11(TMT6plex); S12(Phospho)
156071452	NP_001095132.1	4898	NRD1	nardilysin isoform b precursor	S86	-	-	-	-	1.2	1.5	1.4	1.7	-	-	-	-	1.3	1.5	1.4	1.8	1.3	1.5	1.4	1.8	LGADEsEEEGR	ARLGADEsEEEGRRG	S6;	Yes	S(6): 100.0	2	2	1	0	N-Term(TMT6plex); S6(Phospho)
40538736	NP_065901.1	57610	RANBP10	ran-binding protein 10	S365	-	-	-	-	1.1	1.3	1.6	1.7	-	-	-	-	1.0	1.1	1.3	1.6	1.0	1.1	1.3	1.6	SQDSYPGsPSLSPR	SQDSYPGsPSLSPRH	S8;	Yes	S(1): 0.0; S(4): 0.0; Y(5): 0.0; S(8): 97.3; S(10): 1.3; S(12): 1.3	1	1	1	0	N-Term(TMT6plex); S8(Phospho)
222537719	NP_660275.2	90353	CTU1	cytoplasmic tRNA 2-thiolation protein 1	T332	-	-	-	-	1.3	1.6	1.7	1.7	-	-	-	-	1.1	1.7	1.8	1.9	1.1	1.7	1.8	1.9	GLDEEATPGtPGDPARPPASK	DEEATPGtPGDPARP	T10;	Yes	T(7): 3.2; T(10): 96.8; S(20): 0.0	1	1	1	0	N-Term(TMT6plex); T10(Phospho); K21(TMT6plex)
28559039	NP_004765.2	5469	MED1	mediator of RNA polymerase II transcription subunit 1	T1215	-	-	-	-	1.6	2.1	1.6	1.7	-	-	-	-	1.7	2.0	1.8	1.7	1.7	2.0	1.8	1.7	LASPMKPVPGtPPSSK	PMKPVPGtPPSSKAK	T11;	Yes	S(3): 0.1; T(11): 99.9; S(14): 0.0; S(15): 0.0	1	1	1	0	N-Term(TMT6plex); M5(Oxidation); K6(TMT6plex); T11(Phospho); K16(TMT6plex)
15529996	NP_005624.2	6654	SOS1	son of sevenless homolog 1	S1134	-	-	-	-	1.8	2.5	1.9	1.7	-	-	-	-	1.8	2.9	2.3	1.9	1.8	2.9	2.3	1.9	SAsVSSISLTK	PHGPRSAsVSSISLT	S3;	Yes	S(1): 1.4; S(3): 98.6; S(5): 0.0; S(6): 0.0; S(8): 0.0; T(10): 0.0	1	1	1	0	N-Term(TMT6plex); S3(Phospho); K11(TMT6plex)
11067747	NP_001244.1	988	CDC5L	cell division cycle 5-like protein	T385	-	-	-	-	0.9	2.1	1.3	1.7	-	-	-	-	0.9	2.2	1.4	1.8	0.9	2.2	1.4	1.8	GGLNtPLHESDFSGVTPQR	PLKGGLNtPLHESDF	T5;	Yes	T(5): 100.0; S(10): 0.9; S(13): 49.5; T(16): 49.5	2	1	1	0	N-Term(TMT6plex); T5(Phospho); T16(Phospho)
543583760	NP_001269506.1	7335	UBE2V1	ubiquitin-conjugating enzyme E2 variant 1 isoform h	Y89	-	-	-	-	1.6	2.7	2.7	1.7	-	-	-	-	1.2	1.9	2.3	1.1	1.2	1.9	2.3	1.1	LPQPPEGQCySN	QPPEGQCySN	Y10;	No	Y(10): 98.1; S(11): 1.9	1	8	1	0	N-Term(TMT6plex); C9(Carbamidomethyl); Y10(Phospho)
29244924	NP_115597.3	84181	CHD6	chromodomain-helicase-DNA-binding protein 6	S36	-	-	-	-	0.8	0.9	1.5	1.6	-	-	-	-	0.8	1.0	1.6	1.6	0.8	1.0	1.6	1.6	SPsPEDCSTDQEEK	NEDYKSPsPEDCSTD	S3;	No	S(1): 0.9; S(3): 99.1; S(8): 0.0; T(9): [illegible]	2	1	1	0	N-Term(TMT6plex); S3(Phospho); C7(Carbamidomethyl); K14(TMT6plex)
42544123	NP_973727.1	7536	SF1	splicing factor 1 isoform 3	S80; S82	-	-	-	-	1.7	1.6	1.5	1.6	-	-	-	-	1.7	1.5	1.6	1.4	1.7	1.5	1.6	1.4	sPsPEPIYNSEGK	PPNPEDRsPSPEPIY;NPEDRSPsPEPIYNS	S1; S3;	Yes;Yes	S(1): 100.0; S(3): 100.0; Y(8): 0.0; S(10): 0.0	1	6	1	0	N-Term(TMT6plex); S1(Phospho); S3(Phospho); K13(TMT6plex)
209915594	NP_001129611.1	6929	TCF3	transcription factor E2-alpha isoform E47	S379	-	-	-	-	1.3	1.2	1.0	1.6	-	-	-	-	1.5	1.2	1.3	1.4	1.5	1.2	1.3	1.4	AGAPGALsPSYDGGLHGLQSK	AGAPGALsPSYDGGL	S8;	Yes	S(8): 96.1; S(10): 1.9; Y(11): 1.9; S(20): 0.0	1	2	1	0	N-Term(TMT6plex); S8(Phospho); K21(TMT6plex)
94557301	NP_006242.5	5562	PRKAA1	5'-AMP-activated protein kinase catalytic subunit alpha-1 isoform 1	S508	-	-	-	-	1.7	1.5	1.6	1.6	-	-	-	-	1.7	1.5	1.6	1.6	1.7	1.5	1.6	1.6	SDsDAEAQGK	RSCQRSDsDAEAQGK	S3;	Yes	S(1): 0.0; S(3): 100.0	2	2	1	0	N-Term(TMT6plex); S3(Phospho); K10(TMT6plex)
153945728	NP_005900.2	4131	MAP1B	microtubule-associated protein 1B	S1203	-	-	-	-	1.4	1.6	1.5	1.6	-	-	-	-	1.1	1.2	1.2	1.7	1.1	1.2	1.2	1.7	DYNAsASTISPPSSMEEDK	DGKDYNAsASTISPP	S5;	Yes	Y(2): 1.9; S(5): 97.9; S(7): 0.0; T(8): 0.0; S(10): 0.0; S(13): 0.0; S(14): 0.0	1	1	1	0	N-Term(TMT6plex); S5(Phospho); M15(Oxidation); K19(TMT6plex)
100913030	NP_000103.2	1836	SLC26A2	sulfate transporter	S12	-	-	-	-	1.0	1.3	1.1	1.6	-	-	-	-	0.9	1.2	1.0	1.7	0.9	1.2	1.0	1.7	EQHNVsPR	SKEQHNVsPRDSAEG	S6;	Yes	S(6): 100.0	3	1	1	0	N-Term(TMT6plex); S6(Phospho)
54792782	NP_056041.1	23274	CLEC16A	protein CLEC16A isoform 1	T976	-	-	-	-	1.0	0.9	1.9	1.6	-	-	-	-	1.0	0.9	1.8	1.6	1.0	0.9	1.8	1.6	SAAVEtASLSPSLVPAR	ARSAAVEtASLSPSL	T6;	No	S(1): 0.0; T(6): 95.9; S(8): 2.0; S(10): 2.0; S(12): 0.0	1	1	1	0	N-Term(TMT6plex); T6(Phospho)
92087055	NP_072096.2	64599	GIGYF1	PERQ amino acid-rich with GYF domain-containing protein 1	S24	-	-	-	-	2.2	0.9	0.6	1.6	-	-	-	-	-	-	-	-	-	-	-	-	ALSGGGSVAsPPPsPAMPK	SGGGSVAsPPPSPAM	S10;	Yes	S(3): 0.0; S(7): 1.5; S(10): 98.5; S(14): 0.0	2	1	1	0	N-Term(TMT6plex); S10(Phospho); M17(Oxidation); K19(TMT6plex)
4557471	NP_001274.1	1174	AP1S1	AP-1 complex subunit sigma-1A	S147	-	-	-	-	1.0	1.2	1.0	1.5	-	-	-	-	1.0	1.1	0.9	1.4	1.0	1.1	0.9	1.4	AIEQADLLQEEDEsPR	LLQEEDEsPRSVLEE	S14;	Yes	S(14): 100.0	2	1	1	0	N-Term(TMT6plex); S14(Phospho)
217416350	NP_001136115.1	57513	CASKIN2	caskin-2 isoform b	S760	-	-	-	-	1.2	1.7	1.3	1.5	-	-	-	-	-	-	-	-	-	-	-	-	TsPSVTPTPAR	RSALVRTsPSVTPTP	S2;	No	T(1): 1.2; S(2): 98.8; S(4): 0.0; T(6): 0.0; T(8): 0.0	1	2	1	0	N-Term(TMT6plex); S2(Phospho)
332688220	NP_001193819.1	1500	CTNND1	catenin delta-1 isoform 3A	T799	-	-	-	-	0.6	0.9	1.2	1.5	-	-	-	-	0.6	1.0	1.2	1.6	0.6	1.0	1.2	1.6	SLDNNYStPNER	SLDNNYStPNERGDH	T8;	No	S(1): 0.0; Y(6): 0.0; S(7): 1.2; T(8): [illegible]	2	12	1	0	N-Term(TMT6plex); T8(Phospho)
85815829	NP_061830.3	23616	SH3BP1	SH3 domain-binding protein 1	S544	-	-	-	-	1.6	1.5	1.6	1.5	-	-	-	-	1.4	1.3	1.3	1.2	1.4	1.3	1.3	1.2	TESEVPPRPAsPK	EVPPRPAsPKVTRSP	S11;	Yes	T(1): 0.0; S(3): 0.0; S(11): 100.0	1	1	1	0	N-Term(TMT6plex); S11(Phospho); K13(TMT6plex)
301171613	NP_001180356.1	79723	SUV39H2	histone-lysine N-methyltransferase SUV39H2 isoform 4	S148	-	-	-	-	1.0	1.4	1.2	1.5	-	-	-	-	-	-	-	-	-	-	-	-	GSGDISSDSIDHsPAK	SSDSIDHsPAKKRVR	S13;	No	S(2): 0.0; S(6): 0.0; S(7): 0.0; S(9): 0.1; S(13): 99.9	1	4	1	0	N-Term(TMT6plex); S13(Phospho); K16(TMT6plex)
255652953	NP_653205.3	124245	ZC3H18	zinc finger CCCH domain-containing protein 18	S83	-	-	-	-	1.2	1.3	1.3	1.5	-	-	-	-	1.4	1.3	1.5	1.7	1.4	1.3	1.5	1.7	SQDQDsEVNELSR	PKSQDQDsEVNELSR	S6;	Yes	S(1): 0.0; S(6): 100.0; S(12): 0.0	1	1	1	0	N-Term(TMT6plex); S6(Phospho)
132626688	NP_055456.2	9656	MDC1	mediator of DNA damage checkpoint protein 1	T1567	-	-	-	-	1.5	2.7	1.9	1.5	-	-	-	-	1.6	2.7	1.9	1.6	1.6	2.7	1.9	1.6	SSVKtPESIVPIAPELQPSTSR	TNRSSVKtPESIVPI	T5;	Yes	S(1): 1.8; S(2): 1.8; T(5): 96.3; S(8): 0.0; S(19): 0.0; T(20): 0.0; S(21): 0.0	1	1	1	1	N-Term(TMT6plex); K4(TMT6plex); T5(Phospho)
37595750	NP_002287.2	3930	LBR	lamin-B receptor	S71	-	-	-	-	1.2	1.3	1.4	1.5	-	-	-	-	1.1	1.2	1.2	1.4	1.1	1.2	1.2	1.4	KGGSTSSsPSR	KGGSTSSsPSRRRGS	S8;	Yes	S(4): 0.0; T(5): 0.0; S(6): 0.0; S(7): 0.0; S(8): 99.1; S(10): 0.9	1	1	1	1	N-Term(TMT6plex); K1(TMT6plex); S8(Phospho)
151101301	NP_689621.2	134359	POC5	centrosomal protein POC5 isoform 2	S84	-	-	-	-	1.7	1.4	1.3	1.5	-	-	-	-	-	-	-	-	-	-	-	-	TDESSPVLsPR	DESSPVLsPRKPSHP	S9;	No	T(1): 0.0; S(4): 50.0; S(5): 50.0; S(9): 100.0	1	2	1	0	N-Term(TMT6plex); S5(Phospho); S9(Phospho)
544224621	NP_001269689.1	7091	TLE4	transducin-like enhancer protein 4 isoform 5	S87	-	-	-	-	1.3	1.3	1.4	1.5	-	-	-	-	-	-	-	-	-	-	-	-	SSSVsPSASFR	SIKSSSVsPSASFRG	S5;	No	S(1): 1.9; S(2): 0.0; S(3): 0.0; S(5): 98.0; S(7): 0.0; S(9): 0.0	1	5	1	0	N-Term(TMT6plex); S5(Phospho)
391353390	NP_001254651.1	6731	SRP72	signal recognition particle subunit SRP72 isoform 2	S560; T563	-	-	-	-	0.9	1.4	1.2	1.5	-	-	-	-	0.9	1.4	1.1	1.4	0.9	1.4	1.1	1.4	TVSsPPtSPRPGSAATVSASTSNIIPPR	DASKTVSsPPTSPRP;KTVSSPPtSPRPGSA	S4; T7;	No;No	T(1): 0.0; S(3): 2.6; S(4): 97.4; T(7): 97.4; S(8): 2.6; S(13): 0.0; T(16): 0.0; S(18): 0.0; S(20): 0.0; T(21): 0.0; S(22): 0.0	4	2	1	0	N-Term(TMT6plex); S4(Phospho); S8(Phospho)
118918397	NP_055738.3	22862	FNDC3A	fibronectin type-III domain-containing protein 3A isoform 2	S151	-	-	-	-	0.6	1.4	1.3	1.5	-	-	-	-	0.6	1.2	1.1	1.2	0.6	1.2	1.1	1.2	MSSPPSsPQK	KMSSPPSsPQKCPSP	S7;	No	S(2): 33.7; S(3): 33.7; S(6): 33.2; S(7): 99.5	2	2	1	0	N-Term(TMT6plex); M1(Oxidation); S6(Phospho); S7(Phospho); K10(TMT6plex)

Khan et al., 2019. Multi-omics analysis to characterize cigarette smoke induced molecular alterations in esophageal cells

Supplementary Table 8. List of phosphopeptides identified and quantified in untreated and chronically treated Het1A cells with cigarette smoke condensate for 8 months using SequestHT and Mascot search algorithms with a PhosphoRS probability score ≥75%

Protein group Accession	NP_Accession	Gene ID	Gene Symbol	Protein Description	PhosphoSite (Protein)	Het-1A-Smoke - 2M/Parental (Replicate 1)	Het-1A-Smoke - 4M/Parental (Replicate 1)	Het-1A-Smoke - 6M/Parental (Replicate 1)	Het-1A-Smoke - 8M/Parental (Replicate 1)	Het-1A-Smoke - 2M/Parental (Replicate 2)	Het-1A-Smoke - 4M/Parental (Replicate 2)	Het-1A-Smoke - 6M/Parental (Replicate 2)	Het-1A-Smoke - 8M/Parental (Replicate 2)	Normalized phosphoprotein ratios with respect to corresponding total protein ratios. Het-1A-Smoke - 2M/Parental (Replicate 1)	Normalized Het-1A-Smoke - 4M/Parental (Replicate 1)	Normalized Het-1A-Smoke - 6M/Parental (Replicate 1)	Normalized Het-1A-Smoke - 8M/Parental (Replicate 1)	Normalized Het-1A-Smoke - 2M/Parental (Replicate 2)	Normalized Het-1A-Smoke - 4M/Parental (Replicate 2)	Normalized Het-1A-Smoke - 6M/Parental (Replicate 2)	Normalized Het-1A-Smoke - 8M/Parental (Replicate 2)	Average of replicates Het-1A-Smoke - 2M/Parental	Average of replicates Het-1A-Smoke - 4M/Parental	Average of replicates Het-1A-Smoke - 6M/Parental	Average of replicates Het-1A-Smoke - 8M/Parental	Peptide Sequence	Phosphowindow	PhosphoSite (Peptide)	Phosphosite Plus Evidence	phosphoRS Site Probabilities	# PSMs	# Proteins	# Protein Groups	# Missed Cleavages	Modifications
4505939	NP_000928.1	5430	POLR2A	DNA-directed RNA polymerase II subunit RPB1	S1857	-	-	-	-	1.0	1.1	2.6	1.5	-	-	-	-	1.0	1.1	2.7	1.5	1.0	1.1	2.7	1.5	YTPTSPSYSPSSPEYTPTsPK	SPEYTPTsPKYSPTS	S19;	Yes	Y(1): 0.0; T(2): 0.0; T(4): 0.0; S(5): 0.0; S(7): 0.1; Y(8): 0.1; S(9): 0.1; S(11): 1.6; S(12): 1.6; Y(15): 48.3; T(16): 48.3; T(18): 3.2; S(19): 96.9	1	1	1	0	N-Term(TMT6plex); T16(Phospho); T18(Phospho); K21(TMT6plex)
295054220	NP_001171136.1	55183	RIF1	telomere-associated protein RIF1 isoform 2	S1810	-	-	-	-	1.0	2.2	1.0	1.5	-	-	-	-	1.1	2.4	1.1	1.7	1.1	2.4	1.1	1.7	EDNDTINDsLIVSETK	DNDTINDsLIVSETK	S9;	Yes	T(5): 0.1; S(9): 99.9; S(13): 0.0; T(15): 0.0	1	2	1	0	N-Term(TMT6plex); S9(Phospho); K16(TMT6plex)
193083131	NP_001122393.1	4140	MARK3	MAP/microtubule affinity-regulating kinase 3 isoform e	S298	-	-	-	-	1.3	1.2	1.0	1.5	-	-	-	-	1.3	1.4	1.1	1.5	1.3	1.4	1.1	1.5	SSELDASDSsSSSNLSLAK	ELDASDSsSSSNLSL	S10;	No	S(1): 0.0; S(2): 0.0; S(7): 0.0; S(9): 1.1; S(10): 98.9; S(11): 0.0; S(12): 0.0; S(13): 0.0; S(16): 0.0	4	4	1	0	N-Term(TMT6plex); S10(Phospho); K19(TMT6plex)
4506357	NP_003944.1	9019	MPZL1	myelin protein zero-like protein 1 isoform a precursor	Y263	-	-	-	-	1.4	1.1	1.2	1.5	-	-	-	-	1.3	0.9	0.9	1.4	1.3	0.9	0.9	1.4	SESVVyADIR	NKSESVVyADIRKN	Y6;	Yes	S(1): 0.0; S(3): 0.0; Y(6): 100.0	2	2	1	0	N-Term(TMT6plex); Y6(Phospho)
87299628	NP_683692.2	259282	BOD1L1	biorientation of chromosomes in cell division protein 1-like 1	S537	-	-	-	-	1.1	1.7	1.2	1.5	-	-	-	-	1.2	1.6	1.3	1.6	1.2	1.6	1.3	1.6	sSVDLEESSTK	KTKGQGRsSVDLEES	S1;	No	S(1): 99.3; S(2): 0.7; S(8): 0.0; S(9): 0.0; T(10): 0.0	4	1	1	0	N-Term(TMT6plex); S1(Phospho); K11(TMT6plex)
223633988	NP_001138678.1	85379	KIAA1671	uncharacterized protein KIAA1671	S1312	-	-	-	-	0.9	1.5	1.2	1.5	-	-	-	-	-	-	-	-	-	-	-	-	YPGGsPIPADPR	SERYPGGsPIPADPR	S5;	Yes	Y(1): 1.5; S(5): 98.5	1	1	1	0	N-Term(TMT6plex); S5(Phospho)
27262651	NP_680781.1	11273	ATXN2L	ataxin-2-like protein isoform D	S594	-	-	-	-	1.0	0.9	0.6	1.5	-	-	-	-	1.0	0.8	0.6	1.2	1.0	0.8	0.6	1.2	EKEVDGLLTSEPMGsPVSSK	LTSEPMGsPVSSKTE	S15;	Yes	T(9): 0.0; S(10): 0.0; S(15): 99.8; S(18): 0.0; S(19): 0.0	1	6	1	1	N-Term(TMT6plex); K2(TMT6plex); M13(Oxidation); S15(Phospho); K20(TMT6plex)
33356172	NP_002476.2	4683	NBN	nibrin	S604	-	-	-	-	1.2	1.0	1.2	1.5	-	-	-	-	1.1	1.0	1.1	1.4	1.1	1.0	1.1	1.4	MDIETNDTFsDEAVPESSK	IETNDTFsDEAVPES	S10;	Yes	T(5): 0.0; T(8): 2.2; S(10): 97.7; S(17): 0.0; S(18): 0.0	1	1	1	0	N-Term(TMT6plex); M1(Oxidation); S10(Phospho); K19(TMT6plex)
25777612	NP_002800.2	5709	PSMD3	26S proteasome non-ATPase regulatory subunit 3	S47	-	-	-	-	1.5	1.7	1.6	1.5	-	-	-	-	1.5	1.6	1.5	1.5	1.5	1.6	1.5	1.5	EEAATGGGsTGEADGK	EAATGGGsTGEADGK	S9;	No	T(5): 0.0; S(9): 98.8; T(10): 1.2	4	1	1	0	N-Term(TMT6plex); S9(Phospho); K16(TMT6plex)
71774197	NP_060414.3	55031	USP47	ubiquitin carboxyl-terminal hydrolase 47 isoform b	S822	-	-	-	-	1.3	1.6	1.1	1.5	-	-	-	-	1.3	1.5	1.0	1.4	1.3	1.5	1.0	1.4	STETSDFENIEsPLNER	SDFENIEsPLNERDS	S12;	No	S(1): 0.0; T(2): 0.0; T(4): 0.0; S(5): 0.0; S(12): 100.0	3	2	1	0	N-Term(TMT6plex); S12(Phospho)
195927017	NP_001124320.1	9600	PITPNM1	membrane-associated phosphatidylinositol transfer protein 1 isoform b	S621	-	-	-	-	1.4	1.4	1.1	1.5	-	-	-	-	-	-	-	-	-	-	-	-	GsPEPSALPPQR	VEGLGRGsPEPSALP	S2;	Yes	S(2): 100.0; S(6): 0.0	1	2	1	0	N-Term(TMT6plex); S2(Phospho)
109255232	NP_001035864.1	9859	CEP170	centrosomal protein of 170 kDa isoform gamma	S981	-	-	-	-	0.7	1.5	1.2	1.5	-	-	-	-	0.6	1.3	1.1	1.4	0.6	1.3	1.1	1.4	SKTsPVVSGSSSK	KVTKSKTsPVVSGSS	S4;	No	S(1): 0.0; T(3): 1.1; S(4): 98.9; S(8): 0.0; S(10): 0.0; S(11): 0.0; S(12): 0.0	1	3	1	1	N-Term(TMT6plex); K2(TMT6plex); S4(Phospho); K13(TMT6plex)
51479145	NP_006412.2	10565	ARFGEF1	brefeldin A-inhibited guanine nucleotide-exchange protein 1	T1083	-	-	-	-	1.1	1.5	1.7	1.4	-	-	-	-	1.1	1.5	1.7	1.3	1.1	1.5	1.7	1.3	EGSLTGtK	REGSLTGtKDQAPDE	T7;	Yes	S(3): 1.0; T(5): 1.0; T(7): 98.0	1	1	1	0	N-Term(TMT6plex); S3(Phospho); K8(TMT6plex)
7657575	NP_036370.2	23411	SIRT1	NAD-dependent protein deacetylase sirtuin-1 isoform	S569	-	-	-	-	1.1	1.5	1.5	1.4	-	-	-	-	1.1	1.5	1.5	1.5	1.1	1.5	1.5	1.5	SNDDLDVsESK	SNDDLDVsESKGCME	S8;	No	S(1): 0.0; S(8): 100.0; S(10): 0.0	1	2	1	0	N-Term(TMT6plex); S8(Phospho); K11(TMT6plex)
299890795	NP_001177748.1	4998	ORC1	origin recognition complex subunit 1 isoform 2	T375	-	-	-	-	0.8	1.3	0.6	1.4	-	-	-	-	1.0	1.7	0.8	1.9	1.0	1.7	0.8	1.9	AQNEATStPHR	AQNEATStPHRIRRK	T8;	Yes	T(6): 0.0; S(7): 0.0; T(8): 100.0	2	2	1	0	N-Term(TMT6plex); T8(Phospho)
169658378	NP_001073964.2	84629	TNRC18	trinucleotide repeat-containing gene 18 protein	T2146	-	-	-	-	1.3	1.6	1.8	1.4	-	-	-	-	-	-	-	-	-	-	-	-	GGAVERPLtPAPR	GAVERPLtPAPRSCL	T9;	Yes	T(9): 100.0	1	1	1	0	N-Term(TMT6plex); T9(Phospho)
199559438	NP_001128302.1	54806	AHI1	jouberin isoform a	S1123	-	-	-	-	1.1	1.2	1.0	1.4	-	-	-	-	-	-	-	-	-	-	-	-	ERsPPLSPEEK	PPEIKERsPPLSPEE	S3;	Yes	S(3): 100.0; S(7): 0.0	1	1	1	1	N-Term(TMT6plex); S3(Phospho); K11(TMT6plex)
192449449	NP_071349.3	63893	UBE2O	ubiquitin-conjugating enzyme E2 O	S515	-	-	-	-	1.0	1.1	1.4	1.4	-	-	-	-	1.2	1.2	1.7	1.6	1.2	1.2	1.7	1.6	sIPLSIK	SGTSRKKsIPLSIKN	S1;	Yes	S(1): 100.0; S(5): 0.0	2	1	1	0	N-Term(TMT6plex); S1(Phospho); K7(TMT6plex)
9966799	NP_065101.1	57050	UTP3	something about silencing protein 10	S365; S368	-	-	-	-	0.8	2.9	1.6	1.4	-	-	-	-	0.9	3.1	1.8	1.4	0.9	3.1	1.8	1.4	TSAAACAVTDLsDDsDFDEK	ACAVTDLsDDSDFDE;VTDLSDDsDFDEKAK	S12; S15;	Yes;Yes	T(1): 0.0; S(2): 0.0; T(9): 4.3; S(12): 97.8; S(15): 97.8	2	1	1	0	N-Term(TMT6plex); C6(Carbamidomethyl); S12(Phospho); S15(Phospho); K20(TMT6plex)
457866842	NP_001263993.1	56947	MFF	mitochondrial fission factor isoform d	S146	-	-	-	-	1.6	1.3	1.2	1.4	-	-	-	-	1.5	1.3	1.2	1.5	1.5	1.3	1.2	1.5	NDsLPVLR	GQLVRNDsLPVLRGG	S3;	Yes	S(3): 100.0	1	1	1	0	N-Term(TMT6plex); S3(Phospho)
122114654	NP_076991.3	79066	METTL16	methyltransferase-like protein 16	S419	-	-	-	-	1.5	1.7	1.8	1.4	-	-	-	-	1.3	1.6	1.5	1.4	1.3	1.6	1.5	1.4	ESGNsQELAR	TPKESGNsQELARGP	S5;	Yes	S(2): 0.0; S(5): 100.0	2	1	1	0	N-Term(TMT6plex); S5(Phospho)
23110942	NP_002781.2	5686	PSMA5	proteasome subunit alpha type-5 isoform 1	T55	-	-	-	-	1.2	2.0	1.6	1.4	-	-	-	-	1.2	2.2	1.5	1.1	1.2	2.2	1.5	1.1	ItSPLMEPSSIEK	LAVEKRItSPLMEPS	T2;	Yes	T(2): 99.1; S(3): 0.9; S(9): 0.0; S(10): 0.0	2	1	1	0	N-Term(TMT6plex); T2(Phospho); M6(Oxidation); K13(TMT6plex)
21955172	NP_665696.1	90102	PHLDB2	pleckstrin homology-like domain family B member 2 isoform c	T404	-	-	-	-	1.0	1.2	1.2	1.4	-	-	-	-	1.0	1.4	1.3	1.6	1.0	1.4	1.3	1.6	QASGtPQPALR	SLRQASGtPQPALRE	T5;	Yes	S(3): 0.0; T(5): 100.0	2	3	1	0	N-Term(TMT6plex); T5(Phospho)
57863269	NP_055871.1	23076	RRP1B	ribosomal RNA processing protein 1 homolog B	S706	-	-	-	-	3.7	0.9	1.1	1.4	-	-	-	-	4.0	1.0	1.2	1.3	4.0	1.0	1.2	1.3	SILVsPTGPSR	TDKSILVsPTGPSRV	S5;	Yes	S(1): 0.0; S(5): 100.0; T(7): 0.0; S(10): 0.0	2	1	1	0	N-Term(TMT6plex); S5(Phospho)
148664230	NP_055929.1	23141	ANKLE2	ankyrin repeat and LEM domain-containing protein 2	S528	-	-	-	-	0.7	1.0	0.9	1.4	-	-	-	-	-	-	-	-	-	-	-	-	AFAGPLsPAK	RAFAGPLsPAKAEDE	S7;	Yes	S(7): 100.0	2	1	1	0	N-Term(TMT6plex); S7(Phospho); K10(TMT6plex)
132626688	NP_055456.2	9656	MDC1	mediator of DNA damage checkpoint protein 1	T1630	-	-	-	-	0.9	1.3	1.0	1.4	-	-	-	-	0.9	1.3	1.1	1.4	0.9	1.3	1.1	1.4	TPEPVVPTAPEPHPTTSTDQPVtPK	TSTDQPVtPKLTSRA	T23;	Yes	T(1): 0.0; T(8): 0.0; T(15): 0.0; T(16): 0.0; S(17): 0.0; T(18): 0.0; T(23): [illegible]	3	1	1	0	N-Term(TMT6plex); T23(Phospho); K25(TMT6plex)
30089954	NP_056541.2	50717	DCAF8	DDB1- and CUL4-associated factor 8	S130	-	-	-	-	1.6	1.6	1.6	1.4	-	-	-	-	1.4	1.3	1.3	1.1	1.4	1.3	1.3	1.1	DQDSsDDER	ANRDQDSsDDERALE	S5;	Yes	S(4): 0.5; S(5): 99.5	2	1	1	0	N-Term(TMT6plex); S5(Phospho)
21735548	NP_009117.2	11190	CEP250	centrosome-associated protein CEP250	S2229	-	-	-	-	1.1	1.9	1.1	1.4	-	-	-	-	1.1	2.1	1.2	1.5	1.1	2.1	1.2	1.5	LHsPGATSTAELGSR	LEKERLHsPGATSTA	S3;	Yes	S(3): 100.0; T(7): 0.0; S(8): 0.0; T(9): 0.0; S(14): 0.0	2	1	1	0	N-Term(TMT6plex); S3(Phospho)
545479072	NP_001269877.1	221037	JMJD1C	probable JmjC domain-containing histone demethylation protein 2C isoform c	S435	-	-	-	-	1.0	1.6	1.6	1.4	-	-	-	-	-	-	-	-	-	-	-	-	sPPPETIK	EDKLHKRsPPPETIK	S1;	No	S(1): 100.0; T(6): 0.0	1	2	1	0	N-Term(TMT6plex); S1(Phospho); K8(TMT6plex)
385648268	NP_001245308.1	9497	SLC4A7	sodium bicarbonate cotransporter 3 isoform b	S1105	-	-	-	-	0.8	1.6	1.7	1.4	-	-	-	-	0.6	1.4	1.5	1.2	0.6	1.4	1.5	1.2	SNMsPDKPVSVK	KVTRSNMsPDKPVSV	S4;	No	S(1): 0.0; S(4): 100.0; S(10): 0.0	1	1	1	0	N-Term(TMT6plex); M3(Oxidation); S4(Phospho); K7(TMT6plex); K12(TMT6plex)
15011974	NP_004714.2	9181	ARHGEF2	rho guanine nucleotide exchange factor 2 isoform 3	S116	-	-	-	-	1.0	1.2	1.1	1.4	-	-	-	-	0.8	0.9	1.0	1.2	0.8	0.9	1.0	1.2	SsLSLAK	GSRRGRSsLSLAKSV	S2;	No	S(1): 0.0; S(2): 100.0; S(4): 0.0	2	3	1	0	N-Term(TMT6plex); S2(Phospho); K7(TMT6plex)
183396804	NP_056018.2	23248	RPRD2	regulation of nuclear pre-mRNA domain-containing protein 2	T358	-	-	-	-	1.2	1.0	1.1	1.4	-	-	-	-	1.2	1.0	1.1	1.2	1.2	1.0	1.1	1.2	SAtPEPVTDNR	MESEKSAtPEPVTDN	T3;	Yes	S(1): 0.0; T(3): 99.0; T(8): 1.0	1	1	1	0	N-Term(TMT6plex); T3(Phospho)
547235537	NP_001271264.1	8669	EIF3J	eukaryotic translation initiation factor 3 subunit J	T109	-	-	-	-	1.3	1.3	1.0	1.3	-	-	-	-	1.3	1.2	0.9	1.2	1.3	1.2	0.9	1.2	VLtPEEQLADK	PEEPKVLtPEEQLAD	T3;	Yes	T(3): 100.0	4	3	1	0	N-Term(TMT6plex); T3(Phospho); K11(TMT6plex)
42518070	NP_004808.2	9414	TJP2	tight junction protein ZO-2 isoform 1	S415	-	-	-	-	2.3	1.9	1.5	1.3	-	-	-	-	2.3	1.8	1.2	1.4	2.3	1.8	1.2	1.4	SFsPEER	IESNRSFsPEERRHQ	S3;	Yes	S(1): 0.7; S(3): 99.3	1	6	1	0	N-Term(TMT6plex); S3(Phospho)
110556636	NP_203754.2	85456	TNKS1BP1	182 kDa tankyrase-1-binding protein	T131	-	-	-	-	1.0	1.2	1.2	1.3	-	-	-	-	0.9	1.1	1.1	1.5	0.9	1.1	1.1	1.5	EEPPPLtPPAR	KEEPPPLtPPARCAA	T7;	Yes	T(7): 100.0	1	1	1	0	N-Term(TMT6plex); T7(Phospho)
387157896	NP_001248336.1	8777	MPDZ	multiple PDZ domain protein isoform 3	T350	-	-	-	-	0.8	0.4	0.5	1.3	-	-	-	-	0.8	0.3	0.5	1.3	0.8	0.3	0.5	1.3	TAPTALGItLSSSPTSTPELR	APTALGItLSSSPTS	T9;	No	T(1): 0.0; T(4): 0.0; T(9): 96.1; S(11): 0.0; S(12): 1.9; S(13): 1.9; T(15): 0.0; S(16): 0.0; T(17): 0.0	1	3	1	0	N-Term(TMT6plex); S12(Phospho)
306482694	NP_001182375.1	6432	SRSF7	serine/arginine-rich splicing factor 7 isoform 2	S221	-	-	-	-	0.8	1.2	1.4	1.3	-	-	-	-	0.9	1.4	1.7	1.6	0.9	1.4	1.7	1.6	SAsPER	GSPRRSAsPERMD	S3;	No	S(1): 0.0; S(3): 100.0	2	2	1	0	N-Term(TMT6plex); S3(Phospho)
186659512	NP_056262.3	157922	CAMSAP1	calmodulin-regulated spectrin-associated protein 1	T1146	-	-	-	-	1.1	1.5	1.2	1.3	-	-	-	-	1.1	1.4	1.1	1.2	1.1	1.4	1.1	1.2	TPtDPGLDSALEPSGDPHGK	SSHPRTPtDPGLDSA	T3;	No	T(1): 2.6; T(3): 97.3; S(9): 0.1; S(14): 0.0	1	1	1	0	N-Term(TMT6plex); T3(Phospho); K20(TMT6plex)
33286446	NP_031372.2	11054	OGFR	opioid growth factor receptor	S315	-	-	-	-	1.6	1.0	1.1	1.3	-	-	-	-	2.0	1.0	1.2	1.4	2.0	1.0	1.2	1.4	KVEEEGsPGDPDHEASTQGR	RKVEEEGsPGDPDHE	S7;	Yes	S(7): 100.0; S(16): 0.0; T(17): 0.0	1	1	1	1	N-Term(TMT6plex); K1(TMT6plex); S7(Phospho)
559098432	NP_001273991.1	27342	RABGEF1	rab5 GDP/GTP exchange factor isoform c	S132	-	-	-	-	1.8	1.3	1.1	1.3	-	-	-	-	1.7	1.1	1.0	1.0	1.7	1.1	1.0	1.0	QTsIETDR	PSINRQTsIETDRVS	S3;	No	T(2): 0.0; S(3): 100.0; T(6): 0.0	1	2	1	0	N-Term(TMT6plex); S3(Phospho)
93141029	NP_001035224.1	5064	PALM	paralemmin-1 isoform 2	T141; T145	-	-	-	-	1.6	0.8	1.1	1.3	-	-	-	-	-	-	-	-	-	-	-	-	KTEVVMNSQQtPVGtPK	VVMNSQQtPVGTPKD;SQQTPVGtPKDKRVS	T11; T15;	Yes;Yes	T(2): 0.0; S(8): 0.1; T(11): 99.9; T(15): 100.0	1	2	1	1	K1(TMT6plex); N-Term(TMT6plex); M6(Oxidation); T11(Phospho); T15(Phospho); K17(TMT6plex)
116008442	NP_055885.3	23091	ZC3H13	zinc finger CCCH domain-containing protein 13	S1366	-	-	-	-	1.5	1.5	1.4	1.3	-	-	-	-	1.4	1.5	1.4	1.3	1.4	1.5	1.4	1.3	LISDsVER	RERLISDsVERDRDR	S5;	Yes	S(3): 0.0; S(5): 100.0	2	1	1	0	N-Term(TMT6plex); S5(Phospho)
528881363	NP_001268713.1	23613	ZMYND8	protein kinase C-binding protein 1 isoform r	S401	-	-	-	-	0.7	1.2	1.1	1.3	-	-	-	-	0.6	1.2	1.0	1.2	0.6	1.2	1.0	1.2	LNFDMTAsPK	LNFDMTAsPKILMSK	S8;	No	T(6): 1.3; S(8): 98.7	1	17	1	0	N-Term(TMT6plex); M5(Oxidation); S8(Phospho); K10(TMT6plex)
27262651	NP_680781.1	11273	ATXN2L	ataxin-2-like protein isoform D	S424	-	-	-	-	1.2	1.3	1.1	1.3	-	-	-	-	1.2	1.1	1.2	1.0	1.2	1.1	1.2	1.0	TLSsPSNRPSGETSVPPPPAVGR	RGAKTLSsPSNRPSG	S4;	Yes	T(1): 0.0; S(3): 2.7; S(4): 97.0; S(6): 0.1; S(10): 0.1; T(13): 0.1; S(14): 0.1	2	6	1	0	N-Term(TMT6plex); S4(Phospho)
221219026	NP_001138231.1	23187	PHLDB1	pleckstrin homology-like domain family B member 1 isoform b	S461	-	-	-	-	0.4	1.1	1.0	1.3	-	-	-	-	-	-	-	-	-	-	-	-	ELPPLsPSLSR	MRELPPLsPSLSRRA	S6;	Yes	S(6): 100.0; S(8): 0.0; S(10): 0.0	2	2	1	0	N-Term(TMT6plex); S6(Phospho)
118421089	NP_001262.3	1106	CHD2	chromodomain-helicase-DNA-binding protein 2 isoform 1	S136	-	-	-	-	1.2	1.3	1.4	1.3	-	-	-	-	1.2	1.3	1.4	1.3	1.2	1.3	1.4	1.3	EEASSGSESGsPK	SSGSESGsPKRRGQR	S11;	No	S(4): 0.0; S(5): 0.0; S(7): 0.0; S(9): 0.0; S(11): 100.0	3	2	1	0	N-Term(TMT6plex); S11(Phospho); K13(TMT6plex)
387157878	NP_001248332.1	4791	NFKB2	nuclear factor NF-kappa-B p100 subunit isoform b	S23	-	-	-	-	0.6	0.9	1.9	1.3	-	-	-	-	0.6	0.8	1.5	1.1	0.6	0.8	1.5	1.1	LNSsIVEPK	DDFKLNSsIVEPKEP	S4;	Yes	S(3): 0.0; S(4): 100.0	3	2	1	0	N-Term(TMT6plex); S4(Phospho); K9(TMT6plex)
40354195	NP_954657.1	3875	KRT18	keratin, type I cytoskeletal 18	S7	-	-	-	-	0.8	0.9	1.0	1.3	-	-	-	-	0.8	0.9	1.1	1.0	0.8	0.9	1.1	1.0	sTFSTNYR	_MSFTTRsTFSTNYR	S1;	Yes	S(1): 100.0; T(2): 0.0; S(4): 0.0; T(5): 0.0; Y(7): 0.0	2	1	1	0	N-Term(TMT6plex); S1(Phospho)
46409304	NP_997215.1	157697	ERICH1	glutamate-rich protein 1	S254	-	-	-	-	0.7	1.3	0.6	1.3	-	-	-	-	-	-	-	-	-	-	-	-	QEEGADAsEEDPTPAGEEDVK	QEEGADAsEEDPTPA	S8;	Yes	S(8): 98.1; T(13): 1.9	2	1	1	0	N-Term(TMT6plex); S8(Phospho); K21(TMT6plex)
339275976	NP_001229832.1	8165	AKAP1	A-kinase anchor protein 1, mitochondrial precursor	T70	-	-	-	-	0.9	1.2	1.3	1.3	-	-	-	-	0.9	1.3	1.4	1.4	0.9	1.3	1.4	1.4	VVStPPSVTEPPEK	VCPKVVStPPSVTEP	T4;	Yes	S(3): 1.3; T(4): 98.7; S(7): 0.0; T(9): [illegible]	1	1	1	0	N-Term(TMT6plex); T4(Phospho); K14(TMT6plex)
7706139	NP_057689.1	51307	FAM53C	protein FAM53C	S122	-	-	-	-	0.7	1.5	1.2	1.3	-	-	-	-	-	-	-	-	-	-	-	-	SLsVPVDLSR	KRHCRSLsVPVDLSR	S3;	Yes	S(1): 0.0; S(3): 100.0; S(9): 0.0	2	1	1	0	N-Term(TMT6plex); S3(Phospho)
313747584	NP_001186308.1	8729	GBF1	Golgi-specific brefeldin A-resistance guanine nucleotide exchange factor 1 isoform 3	S1780	-	-	-	-	1.3	0.9	1.0	1.3	-	-	-	-	1.4	0.9	1.0	1.4	1.4	0.9	1.0	1.4	AASSSSPGsPVASSPSR	ASSSSPGsPVASSPS	S9;	No	S(3): 0.1; S(4): 2.3; S(5): 2.3; S(6): 2.3; S(9): 88.5; S(13): 2.3; S(14): 2.3; S(16): 0.1	1	3	1	0	N-Term(TMT6plex); S9(Phospho)
42490760	NP_963861.1	993	CDC25A	M-phase inducer phosphatase 1 isoform b	S243	-	-	-	-	2.0	1.3	1.5	1.3	-	-	-	-	-	-	-	-	-	-	-	-	SQEEsPPGSTK	PERSQEEsPPGSTKR	S5;	No	S(1): 0.0; S(5): 100.0; S(9): 0.0; T(10): 0.0	1	2	1	0	N-Term(TMT6plex); S5(Phospho); K11(TMT6plex)
449083351	NP_001263347.1	9919	SEC16A	protein transport protein Sec16A isoform 2	S1347; S1350; S1353	-	-	-	-	1.0	1.1	1.3	1.3	-	-	-	-	0.9	1.0	1.2	1.2	0.9	1.0	1.2	1.2	sVHsEHsAR	GEEVDRRsVHSEHSA;VDRRSVHsEHSARSL;RSVHSEHsARSLHSA	S1; S4; S7;	Yes;Yes;Yes	S(1): 100.0; S(4): 100.0; S(7): 100.0	1	2	1	0	N-Term(TMT6plex); S1(Phospho); S4(Phospho); S7(Phospho)
42516561	NP_963920.1	23032	USP33	ubiquitin carboxyl-terminal hydrolase 33 isoform 3	S439	-	-	-	-	1.3	1.3	1.2	1.3	-	-	-	-	1.4	1.2	1.0	1.2	1.4	1.2	1.0	1.2	LSAsPPK	VNPRLSAsPPKSGNL	S4;	Yes	S(2): 0.0; S(4): 100.0	3	3	1	0	N-Term(TMT6plex); S4(Phospho); K7(TMT6plex)
166795262	NP_694939.2	121536	AEBP2	zinc finger protein AEBP2 isoform a	S139	-	-	-	-	0.9	1.5	1.3	1.3	-	-	-	-	-	-	-	-	-	-	-	-	sLSPGAASSSSGDGDGK	SSSDETRsLSPGAAS	S1;	Yes	S(1): 99.3; S(3): 0.7; S(8): 0.0; S(9): 0.0; S(10): 0.0; S(11): 0.0	2	2	1	0	N-Term(TMT6plex); S1(Phospho); K17(TMT6plex)
297206754	NP_001171928.1	9665	KIAA0430	meiosis arrest female protein 1 isoform 3	S713	-	-	-	-	1.2	0.9	1.0	1.3	-	-	-	-	1.0	0.8	0.9	1.0	1.0	0.8	0.9	1.0	SVTSsPVEK	SARSVTSsPVEKKDK	S5;	No	S(1): 0.0; T(3): 0.0; S(4): 0.0; S(5): 100.0	3	3	1	0	N-Term(TMT6plex); S5(Phospho); K9(TMT6plex)
306774089	NP_001182408.1	10342	TFG	protein TFG isoform 2	S150	-	-	-	-	1.3	1.7	1.0	1.3	-	-	-	-	1.2	1.8	1.1	1.3	1.2	1.8	1.1	1.3	SAsDSSGK	GREEKSAsDSSGKQS	S3;	Yes	S(1): 0.0; S(3): 98.5; S(5): 0.7; S(6): [illegible]	2	2	1	0	N-Term(TMT6plex); S3(Phospho); K8(TMT6plex)
634743270	NP_001278887.1	4216	MAP3K4	mitogen-activated protein kinase kinase kinase 4	S681	-	-	-	-	1.0	1.2	0.9	1.3	-	-	-	-	1.2	1.3	1.0	1.4	1.2	1.3	1.0	1.4	GSsVPENDR	AHDTRGSsVPENDRL	S3;	No	S(2): 0.7; S(3): 99.3	1	3	1	0	N-Term(TMT6plex); S3(Phospho)
222136641	NP_149107.4	91754	NEK9	serine/threonine-protein kinase Nek9	T333	-	-	-	-	1.6	0.9	1.3	1.3	-	-	-	-	1.7	0.9	1.4	1.3	1.7	0.9	1.4	1.3	SStVTEAPIAVVTSR	TKRPRSStVTEAPIA	T3;	Yes	S(1): 1.5; S(2): 1.5; T(3): 97.0; T(5): 0.0; T(13): 0.0; S(14): 0.0	1	1	1	0	N-Term(TMT6plex); T3(Phospho)
271397437	NP_060933.3	55846	ITFG2	integrin-alpha FG-GAP repeat-containing protein 2	S220	-	-	-	-	1.1	1.0	1.0	1.3	-	-	-	-	1.1	0.9	0.8	1.1	1.1	0.9	0.8	1.1	KDTGsPPASEGPTDGSR	TWKKDTGsPPASEGP	S5;	Yes	T(3): 1.3; S(5): 98.7; S(9): 0.0; T(13): 0.0; S(16): 0.0	1	1	1	1	N-Term(TMT6plex); K1(TMT6plex); S5(Phospho)
236459851	NP_775840.3	254048	UBN2	ubinuclein-2	S250	-	-	-	-	1.0	1.4	1.3	1.3	-	-	-	-	-	-	-	-	-	-	-	-	QAsDTEEDDITDNQK	TLQERQAsDTEEDDI	S3;	Yes	S(3): 100.0; T(5): 0.0; T(11): 0.0	2	1	1	0	N-Term(TMT6plex); S3(Phospho); K15(TMT6plex)
223633988	NP_001138678.1	85379	KIAA1671	uncharacterized protein KIAA1671	S404	-	-	-	-	0.8	1.2	0.7	1.3	-	-	-	-	-	-	-	-	-	-	-	-	AAESPsPR	EKAAESPsPRLGRGL	S6;	Yes	S(4): 0.0; S(6): 100.0	1	1	1	0	N-Term(TMT6plex); S6(Phospho)
217035107	NP_001136037.1	79869	CPSF7	cleavage and polyadenylation specificity factor subunit 7 isoform 3	T194	-	-	-	-	0.7	0.9	0.9	1.3	-	-	-	-	0.7	1.0	1.0	1.2	0.7	1.0	1.0	1.2	DSSDSADGRAtPSENLVPSSAR	DSADGRAtPSENLVP	T11;	No	S(2): 0.0; S(3): 0.0; S(5): 0.0; T(11): 99.9; S(13): 0.1; S(19): 0.0; S(20): 0.0	1	3	1	1	N-Term(TMT6plex); T11(Phospho)
21264616	NP_073609.2	64787	EPS8L2	epidermal growth factor receptor kinase substrate 8-like protein 2	S217	-	-	-	-	1.0	1.6	1.8	1.3	-	-	-	-	0.9	1.3	1.4	1.1	0.9	1.3	1.4	1.1	GGDsPEAK	FQHRGGDsPEAKNRV	S4;	Yes	S(4): 100.0	1	1	1	0	N-Term(TMT6plex); S4(Phospho); K8(TMT6plex)
56549656	NP_001008240.1	147339	C18orf25	uncharacterized protein C18orf25 isoform b	S145	-	-	-	-	1.8	1.9	0.9	1.3	-	-	-	-	1.6	1.8	0.9	1.4	1.6	1.8	0.9	1.4	sESETSTMAAK	RKSRRSRsESETSTM	S1;	Yes	S(1): 98.9; S(3): 1.1; T(5): 0.0; S(6): 0.0; T(7): 0.0	5	2	1	0	N-Term(TMT6plex); S1(Phospho); M8(Oxidation); K11(TMT6plex)
68160947	NP_003460.2	7857	SCG2	secretogranin-2 precursor	S556	-	-	-	-	1.1	1.4	1.9	1.3	-	-	-	-	1.2	1.4	1.4	2.3	1.2	1.4	1.4	2.3	EHLNQGSsQETDK	EHLNQGSsQETDKLA	S8;	No	S(7): 1.0; S(8): 99.0; T(11): 0.0	1	1	1	0	N-Term(TMT6plex); S8(Phospho); K13(TMT6plex)
4507401	NP_003192.1	7019	TFAM	transcription factor A, mitochondrial isoform 1	S177	-	-	-	-	0.8	1.2	1.0	1.3	-	-	-	-	0.9	1.2	1.1	1.4	0.9	1.2	1.1	1.4	GDsPQEK	FQEAKGDsPQEKLKT	S3;	No	S(3): 100.0	1	1	1	0	N-Term(TMT6plex); S3(Phospho); K7(TMT6plex)
33620716	NP_061173.1	5930	RBBP6	E3 ubiquitin-protein ligase RBBP6 isoform 2	S1591	-	-	-	-	1.4	1.8	1.2	1.3	-	-	-	-	1.6	1.8	1.4	1.3	1.6	1.8	1.4	1.3	LsSDLTR	LSHSSRLsSDLTRET	S2;	No	S(2): 100.0; S(3): 0.0; T(6): 0.0	2	2	1	0	N-Term(TMT6plex); S2(Phospho)
14110414	NP_002129.2	3184	HNRNPD	heterogeneous nuclear ribonucleoprotein D0 isoform c	S82	-	-	-	-	3.5	2.1	1.2	1.3	-	-	-	-	3.6	2.1	1.5	1.2	3.6	2.1	1.5	1.2	IDASKNEEDEGHSNsSPR	EDEGHSNsSPRHSEA	S15;	Yes	S(4): 0.0; S(13): 0.0; S(15): 98.6; S(16): 1.4	1	2	1	1	N-Term(TMT6plex); K5(TMT6plex); S15(Phospho)
374532823	NP_001243417.1	2804	GOLGB1	Golgin subfamily B member 1 isoform 4	S578	-	-	-	-	1.9	1.5	1.4	1.3	-	-	-	-	1.5	1.3	1.1	1.2	1.5	1.3	1.1	1.2	TSEEIsLNDAGVELK	SRTSEEIsLNDAGVE	S6;	No	T(1): 0.0; S(2): 0.0; S(6): 100.0	2	4	1	0	N-Term(TMT6plex); S6(Phospho); K15(TMT6plex)
109637788	NP_150247.2	5371	PML	protein PML isoform 5	S505	-	-	-	-	1.2	1.4	1.3	1.2	-	-	-	-	1.1	1.3	1.2	1.1	1.1	1.3	1.2	1.1	SsPEQPRPSTSK	EARLARSsPEQPRPS	S2;	Yes	S(1): 1.6; S(2): 98.4; S(9): 0.0; T(10): 0.0; S(11): 0.0	2	7	1	0	N-Term(TMT6plex); S2(Phospho); K12(TMT6plex)
21327667	NP_056016.1	23246	BOP1	ribosome biogenesis protein BOP1	S126; S127	-	-	-	-	2.0	1.6	1.0	1.2	-	-	-	-	1.8	1.6	0.9	1.1	1.8	1.6	0.9	1.1	IGDEYAEDssDEEDIR	GDEYAEDsSDEEDIR;DEYAEDSsDEEDIRN	S9; S10;	Yes;Yes	Y(5): 0.0; S(9): 100.0; S(10): 100.0	1	1	1	0	N-Term(TMT6plex); S9(Phospho); S10(Phospho)
51988900	NP_000114.2	2073	ERCC5	DNA repair protein complementing XP-G cells	S341	-	-	-	-	1.2	1.3	1.4	1.2	-	-	-	-	1.1	1.3	1.3	1.2	1.1	1.3	1.3	1.2	TEKEPDATPPsPR	EPDATPPsPRTLLAM	S11;	Yes	T(1): 0.0; T(8): 0.0; S(11): 100.0	1	2	1	1	N-Term(TMT6plex); K3(TMT6plex); S11(Phospho)
36029914	NP_079330.2	80124	VCPIP1	deubiquitinating protein VCIP135	S747	-	-	-	-	0.9	1.1	0.9	1.2	-	-	-	-	0.7	0.9	0.8	1.2	0.7	0.9	0.8	1.2	TVsPSTIR	KGQPRTVsPSTIRDG	S3;	Yes	T(1): 0.0; S(3): 100.0; S(5): 0.0; T(6): 0.0	2	1	1	0	N-Term(TMT6plex); S3(Phospho)
84043963	NP_056988.3	9669	EIF5B	eukaryotic translation initiation factor 5B	S1168	-	-	-	-	1.3	1.5	1.2	1.2	-	-	-	-	1.4	1.5	1.2	1.3	1.4	1.5	1.2	1.3	IEPIPGEsPK	IEPIPGEsPKMEGRH	S8;	Yes	S(8): 100.0	2	1	1	0	N-Term(TMT6plex); S8(Phospho); K10(TMT6plex)
545746375	NP_001271175.1	26037	SIPA1L1	signal-induced proliferation-associated 1-like protein 1 isoform 3	S1528	-	-	-	-	0.4	0.5	0.9	1.2	-	-	-	-	0.4	0.4	0.9	1.1	0.4	0.4	0.9	1.1	LIDLEsPTPESQK	KKLIDLEsPTPESQK	S6;	No	S(6): 100.0; T(8): 0.0; S(11): 0.0	1	4	1	0	N-Term(TMT6plex); S6(Phospho); K13(TMT6plex)
118421089	NP_001262.3	1106	CHD2	chromodomain-helicase-DNA-binding protein 2 isoform 1	S1085; S1087	-	-	-	-	1.3	1.3	1.1	1.2	-	-	-	-	1.2	1.3	1.1	1.2	1.2	1.3	1.1	1.2	AQTNDsDsDTESKR	KKAQTNDsDSDTESK;AQTNDSDsDTESKRQ	S6; S8;	Yes;No	T(3): 0.0; S(6): 100.0; S(8): 99.9; T(10): 0.0; S(12): 0.0	1	1	1	1	N-Term(TMT6plex); S6(Phospho); S8(Phospho); K13(TMT6plex)
557440899	NP_001273490.1	4926	NUMA1	nuclear mitotic apparatus protein 1 isoform 2	S1833	-	-	-	-	0.9	1.0	1.1	1.2	-	-	-	-	1.1	1.1	1.2	1.4	1.1	1.1	1.2	1.4	SAPASQAsLR	SAPASQAsLRATSST	S8;	No	S(1): 0.0; S(5): 1.2; S(8): 98.8	1	2	1	0	N-Term(TMT6plex); S8(Phospho)
31543385	NP_005008.2	5130	PCYT1A	choline-phosphate cytidylyltransferase A	S319; S321	-	-	-	-	1.7	1.1	1.3	1.2	-	-	-	-	1.5	1.0	1.1	1.1	1.5	1.0	1.1	1.1	QsPsSSPTR	QAISPKQsPSSSPTR;ISPKQSPsSSPTRER	S2; S4;	Yes;No	S(2): 100.0; S(4): 97.9; S(5): 1.1; S(6): 1.1; T(8): 0.0	1	1	1	0	N-Term(TMT6plex); S2(Phospho); S4(Phospho)
567316262	NP_001274514.1	129401	NUP35	nucleoporin NUP53 isoform c	T155	-	-	-	-	1.3	1.3	1.2	1.2	-	-	-	-	1.1	1.2	1.1	1.0	1.1	1.2	1.1	1.0	TLGtPTQPGSTPR	PPIKTLGtPTQPGST	T4;	No	T(1): 0.0; T(4): 98.5; T(6): 1.5; S(10): 0.0; T(11): 0.0	1	3	1	0	N-Term(TMT6plex); T4(Phospho)
4506067	NP_002728.1	5578	PRKCA	protein kinase C alpha type	S319	-	-	-	-	3.4	1.2	1.2	1.2	-	-	-	-	3.2	1.2	1.2	1.2	3.2	1.2	1.2	1.2	VIsPSEDR	PAGNKVIsPSEDRKQ	S3;	Yes	S(3): 100.0; S(5): 0.0	1	1	1	0	N-Term(TMT6plex); S3(Phospho)
53829376	NP_001005476.1	8502	PKP4	plakophilin-4 isoform b	S314	-	-	-	-	0.9	1.2	0.9	1.2	-	-	-	-	-	-	-	-	-	-	-	-	VGsPLTLTDAQTR	QTTARVGsPLTLTDA	S3;	Yes	S(3): 98.0; T(6): 0.0; T(8): 2.0; T(12): 0.0	1	2	1	0	N-Term(TMT6plex); S3(Phospho)
4501867	NP_001089.1	50	ACO2	aconitate hydratase, mitochondrial precursor	S559	-	-	-	-	1.3	1.1	1.1	1.2	-	-	-	-	1.0	0.9	0.8	1.0	1.0	0.9	0.8	1.0	DSSGQHVDVsPTSQR	SGQHVDVsPTSQRLQ	S10;	Yes	S(2): 0.0; S(3): 0.0; S(10): 100.0; T(12): 0.0; S(13): 0.0	1	1	1	0	N-Term(TMT6plex); S10(Phospho)
21493024	NP_653080.1	9590	AKAP12	A-kinase anchor protein 12 isoform 2	S547	-	-	-	-	1.1	1.6	1.3	1.2	-	-	-	-	1.2	1.5	1.1	1.1	1.2	1.5	1.1	1.1	SATLSsTESTASEMQEEMK	VKSATLSsTESTASE	S6;	No	S(1): 0.0; T(3): 0.0; S(5): 1.3; S(6): 97.5; T(7): 1.3; S(9): 0.0; T(10): 0.0; S(12): 0.0	4	2	1	0	N-Term(TMT6plex); S6(Phospho); M14(Oxidation); M18(Oxidation); K19(TMT6plex)
42542379	NP_005830.2	10250	SRRM1	serine/arginine repetitive matrix protein 1	S797	-	-	-	-	1.1	1.8	1.5	1.2	-	-	-	-	1.3	1.9	1.7	1.4	1.3	1.9	1.7	1.4	SPTPSPsPPR	KSPTPSPsPPRNSDQ	S7;	Yes	S(1): 0.0; T(3): 0.0; S(5): 0.0; S(7): 100.0	1	1	1	0	N-Term(TMT6plex); S7(Phospho)
194097489	NP_015559.2	8498	RANBP3	ran-binding protein 3 isoform RANBP3-b	S176	-	-	-	-	1.3	0.8	1.0	1.2	-	-	-	-	1.5	1.0	1.2	1.7	1.5	1.0	1.2	1.7	NESSNAsEEEACEK	KNESSNAsEEEACEK	S7;	No	S(3): 0.0; S(4): 0.0; S(7): 99.9	1	3	1	0	N-Term(TMT6plex); S7(Phospho); C12(Carbamidomethyl); K14(TMT6plex)
149588928	NP_065897.1	57606	SLAIN2	SLAIN motif-containing protein 2	S413	-	-	-	-	0.8	1.2	1.0	1.2	-	-	-	-	0.8	1.1	1.1	1.1	0.8	1.1	1.1	1.1	sLPNLSR	NEEKLRRsLPNLSRT	S1;	Yes	S(1): 100.0; S(6): 0.0	2	1	1	0	N-Term(TMT6plex); S1(Phospho)
171184451	NP_055625.4	9857	CEP350	centrosome-associated protein 350	S1818	-	-	-	-	1.1	1.4	1.0	1.2	-	-	-	-	1.1	1.4	1.0	1.3	1.1	1.4	1.0	1.3	ATsPGPTDLETR	TKDNKATsPGPTDLE	S3;	Yes	T(2): 0.0; S(3): 100.0; T(7): 0.0; T(11): 0.0	3	1	1	0	N-Term(TMT6plex); S3(Phospho)
157388941	NP_060468.2	55071	C9orf40	uncharacterized protein C9orf40	S76	-	-	-	-	1.9	1.2	1.7	1.2	-	-	-	-	-	-	-	-	-	-	-	-	RDsGDNSAPSGQER	SPSKRRDsGDNSAPS	S3;	Yes	S(3): 100.0; S(7): 0.0; S(10): 0.0	2	1	1	1	N-Term(TMT6plex); S3(Phospho)
449083351	NP_001263347.1	9919	SEC16A	protein transport protein Sec16A isoform 2	S2083	-	-	-	-	1.7	1.4	1.6	1.2	-	-	-	-	1.6	1.2	1.4	1.1	1.6	1.2	1.4	1.1	ADSGPTQPPLSLsPAPETK	TQPPLSLsPAPETKR	S13;	Yes	S(3): 0.0; T(6): 0.0; S(11): 0.0; S(13): 100.0; T(18): 0.0	4	2	1	0	N-Term(TMT6plex); S13(Phospho); K19(TMT6plex)
56118234	NP_859067.2	201161	CENPV	centromere protein V	S42	-	-	-	-	1.0	1.0	1.1	1.2	-	-	-	-	1.1	1.0	1.1	1.3	1.1	1.0	1.1	1.3	sASQAGSK	SATRTRRsASQAGSK	S1;	No	S(1): 100.0; S(3): 0.0; S(7): 0.0	1	1	1	0	N-Term(TMT6plex); S1(Phospho); K8(TMT6plex)
33946282	NP_056311.2	25962	KIAA1429	protein virilizer homolog isoform 1	T1708	-	-	-	-	0.8	1.1	1.3	1.2	-	-	-	-	0.7	1.0	1.1	1.1	0.7	1.0	1.1	1.1	FFtPPASK	HSQNRFFtPPASKGN	T3;	Yes	T(3): 100.0; S(7): 0.0	1	1	1	0	N-Term(TMT6plex); T3(Phospho); K8(TMT6plex)
25777602	NP_002799.3	5708	PSMD2	26S proteasome non-ATPase regulatory subunit 2 isoform 1	S361	-	-	-	-	1.2	1.3	1.3	1.2	-	-	-	-	1.2	1.3	1.2	1.2	1.2	1.3	1.2	1.2	FGGsGSQVDSAR	ENNRFGGsGSQVDSA	S4;	Yes	S(4): 100.0; S(6): 0.0; S(10): 0.0	1	3	1	0	N-Term(TMT6plex); S4(Phospho)
98986457	NP_005325.2	3054	HCFC1	host cell factor 1	S598	-	-	-	-	0.9	0.9	0.9	1.2	-	-	-	-	0.9	1.1	1.0	1.3	0.9	1.1	1.0	1.3	VASsPVMVSNPATR	ATVKVASsPVMVSNP	S4;	Yes	S(3): 1.6; S(4): 98.4; S(9): 0.0; T(13): 0.0	2	1	1	0	N-Term(TMT6plex); S4(Phospho); M7(Oxidation)
27262651	NP_680781.1	11273	ATXN2L	ataxin-2-like protein isoform D	S594	-	-	-	-	0.6	0.9	1.2	1.2	-	-	-	-	0.6	0.8	1.2	0.9	0.6	0.8	1.2	0.9	EVDGLLTSEPMGsPVSSK	LTSEPMGsPVSSKTE	S13;	Yes	T(7): 0.0; S(8): 0.0; S(13): 100.0; S(16): 0.0; S(17): 0.0	2	6	1	0	N-Term(TMT6plex); M11(Oxidation); S13(Phospho); K18(TMT6plex)
110624774	NP_006030.2	9902	MRC2	C-type mannose receptor 2 precursor	S1456	-	-	-	-	1.1	1.3	1.1	1.2	-	-	-	-	0.9	1.1	1.0	1.0	0.9	1.1	1.0	1.0	SsSSPTEATEK	GARYSRSsSSPTEAT	S2;	No	S(1): 1.0; S(2): 98.0; S(3): 1.0; S(4): 0.0; T(6): 0.0; T(9): 0.0	1	1	1	0	N-Term(TMT6plex); S2(Phospho); K11(TMT6plex)
38044290	NP_060082.2	55596	ZCCHC8	zinc finger CCHC domain-containing protein 8	T492	-	-	-	-	1.0	0.9	1.0	1.2	-	-	-	-	0.9	0.8	0.9	1.2	0.9	0.8	0.9	1.2	GtPPLTPSDSPQTR	TPPLPKGtPPLTPSD	T2;	Yes	T(2): 100.0; T(6): 0.0; S(8): 0.8; S(10): 49.6; T(13): 49.6	1	1	1	0	N-Term(TMT6plex); T2(Phospho); S10(Phospho)
40548372	NP_057114.5	51112	TRAPPC12	trafficking protein particle complex subunit 12	S182	-	-	-	-	0.7	1.1	1.2	1.2	-	-	-	-	0.6	1.1	1.1	1.2	0.6	1.1	1.1	1.2	sPSFGGASEASAR	FEPQMVKsPSFGGAS	S1;	Yes	S(1): 100.0; S(3): 0.0; S(8): 0.0; S(11): 0.0	1	1	1	0	N-Term(TMT6plex); S1(Phospho)
215599015	NP_001135935.1	1736	DKC1	H/ACA ribonucleoprotein complex subunit 4 isoform 2	S508	-	-	-	-	1.1	1.5	1.2	1.2	-	-	-	-	1.1	1.6	1.2	1.2	1.1	1.6	1.2	1.2	AKEVELVsE	AKEVELVsE	S8;	No	S(8): 100.0	1	2	1	1	N-Term(TMT6plex); K2(TMT6plex); S8(Phospho)
503774357	NP_001265139.1	9972	NUP153	nuclear pore complex protein Nup153 isoform 3	S320	-	-	-	-	0.8	0.9	0.9	1.2	-	-	-	-	0.7	0.8	0.8	1.1	0.7	0.8	0.8	1.1	MSsPLADAK	QSLEKMSsPLADAKR	S3;	Yes	S(2): 0.0; S(3): 100.0	4	3	1	0	N-Term(TMT6plex); M1(Oxidation); S3(Phospho); K9(TMT6plex)
212276104	NP_001131025.1	9208	LRRFIP1	leucine-rich repeat flightless-interacting protein 1	S712	-	-	-	-	1.8	1.9	1.9	1.2	-	-	-	-	2.0	2.0	2.1	1.5	2.0	2.0	2.1	1.5	ALDSNsLENDDLSAPGR	RKALDSNsLENDDLS	S6;	No	S(4): 0.0; S(6): 100.0; S(13): 0.0	2	3	1	0	N-Term(TMT6plex); S6(Phospho)
158534059	NP_056123.2	23360	FNBP4	formin-binding protein 4	S464	-	-	-	-	1.1	1.4	1.1	1.2	-	-	-	-	1.0	1.4	1.1	1.3	1.0	1.4	1.1	1.3	ATsPESTSR	KMFVRATsPESTSRS	S3;	Yes	T(2): 0.0; S(3): 100.0; S(6): 0.0; T(7): 0.0; S(8): 0.0	4	1	1	0	N-Term(TMT6plex); S3(Phospho)
61743954	NP_001611.1	79026	AHNAK	neuroblast differentiation-associated protein AHNAK isoform 1	S4960	-	-	-	-	1.3	1.1	0.9	1.2	-	-	-	-	1.5	1.2	0.9	1.4	1.5	1.2	0.9	1.4	VEAPSLDVHMDsPDINIEGPDVK	SLDVHMDsPDINIEG	S12;	Yes	S(5): 0.2; S(12): 99.8	1	1	1	0	N-Term(TMT6plex); M10(Oxidation); S12(Phospho); K23(TMT6plex)
440309857	NP_001258978.1	10131	TRAP1	heat shock protein 75 kDa, mitochondrial isoform 2	S348	-	-	-	-	1.0	1.5	1.2	1.2	-	-	-	-	0.9	1.3	1.1	1.0	0.9	1.3	1.1	1.0	GVVDSEDIPLNLsR	EDIPLNLsRELLQES	S13;	No	S(5): 0.0; S(13): 100.0	2	2	1	0	N-Term(TMT6plex); S13(Phospho)
194272161	NP_002212.3	3707	ITPKB	inositol-trisphosphate 3-kinase B	T33	-	-	-	-	1.1	1.4	1.1	1.2	-	-	-	-	-	-	-	-	-	-	-	-	SGGGPGPSGSEtPPPPR	PGPSGSEtPPPPRRA	T12;	Yes	S(1): 0.0; S(8): 0.0; S(10): 0.0; T(12): 100.0	2	1	1	0	N-Term(TMT6plex); T12(Phospho)
67190736	NP_001019979.1	605	BCL7A	B-cell CLL/lymphoma 7 protein family member A isoform b	S207	-	-	-	-	1.2	1.5	1.8	1.2	-	-	-	-	1.4	1.9	2.0	1.2	1.4	1.9	2.0	1.2	LEASQQNsEEM	LEASQQNsEEM_	S8;	Yes	S(4): 0.0; S(8): 100.0	1	2	1	0	N-Term(TMT6plex); S8(Phospho); M11(Oxidation)
148612879	NP_079223.3	80012	PHC3	polyhomeotic-like protein 3	S275	-	-	-	-	1.2	1.3	1.2	1.2	-	-	-	-	1.2	1.4	1.0	1.3	1.2	1.4	1.0	1.3	DPsPESNK	KISQRDPsPESNKKG	S3;	No	S(3): 100.0; S(6): 0.0	1	1	1	0	N-Term(TMT6plex); S3(Phospho); K8(TMT6plex)
14591904	NP_002898.2	5965	RECQL	ATP-dependent DNA helicase Q1	S636	-	-	-	-	2.4	1.3	1.0	1.2	-	-	-	-	2.0	1.2	0.8	1.1	2.0	1.2	0.8	1.1	AANMLQQSGsK	NMLQQSGsKNTGAKK	S10;	No	S(8): 1.1; S(10): 98.9	1	1	1	0	N-Term(TMT6plex); M4(Oxidation); S8(Phospho); K11(TMT6plex)
148596949	NP_004732.2	9221	NOLC1	nucleolar and coiled-body phosphoprotein 1 isoform 2	S643	-	-	-	-	0.6	1.1	1.2	1.2	-	-	-	-	0.5	0.9	0.9	0.9	0.5	0.9	0.9	0.9	VADNsFDAKR	DSRVADNsFDAKRGA	S5;	Yes	S(5): 100.0	1	3	1	1	N-Term(TMT6plex); S5(Phospho); K9(TMT6plex)
157311635	NP_940890.3	286077	FAM83H	protein FAM83H	S523	-	-	-	-	0.4	0.6	0.6	1.2	-	-	-	-	0.3	0.5	0.5	1.0	0.3	0.5	0.5	1.0	HGsDPAFAPGPR	SREVRHGsDPAFAPG	S3;	Yes	S(3): 100.0	2	1	1	0	N-Term(TMT6plex); S3(Phospho)
58331266	NP_085058.3	79726	WDR59	WD repeat-containing protein 59	S564	-	-	-	-	1.1	1.1	0.9	1.2	-	-	-	-	1.0	1.0	0.9	1.1	1.0	1.0	0.9	1.1	AVsPTEPTPR	MTMHRAVsPTEPTPR	S3;	Yes	S(3): 99.4; T(5): 0.6; T(8): 0.0	2	1	1	0	N-Term(TMT6plex); S3(Phospho)
118572613	NP_057417.3	23524	SRRM2	serine/arginine repetitive matrix protein 2	S2702	-	-	-	-	1.2	1.5	1.2	1.2	-	-	-	-	1.3	1.5	1.2	1.3	1.3	1.5	1.2	1.3	RRPsPQPSPR	PVERRRPsPQPSPRD	S4;	Yes	S(4): 100.0; S(8): 0.0	1	1	1	1	N-Term(TMT6plex); S4(Phospho)
209862789	NP_056056.2	57553	MICAL3	protein-methionine sulfoxide oxidase MICAL3 isoform	S1637	-	-	-	-	1.4	1.7	1.4	1.2	-	-	-	-	1.2	1.4	1.3	1.1	1.2	1.4	1.3	1.1	KASsAPSQGK	PRPRKASsAPSQGKE	S4;	No	S(3): 1.0; S(4): 99.0; S(7): 0.0	2	1	1	1	N-Term(TMT6plex); K1(TMT6plex); S3(Phospho); K10(TMT6plex)
16306580	NP_036440.1	22992	KDM2A	lysine-specific demethylase 2A isoform a	S692	-	-	-	-	1.1	1.0	1.1	1.2	-	-	-	-	1.1	0.9	1.0	1.3	1.1	0.9	1.0	1.3	MEEsDEEAVQAK	QKRKMEEsDEEAVQA	S4;	Yes	S(4): 100.0	2	2	1	0	N-Term(TMT6plex); M1(Oxidation); S4(Phospho); K12(TMT6plex)
14249158	NP_116020.1	84717	HDGFRP2	hepatoma-derived growth factor-related protein 2 isoform 2	S663	-	-	-	-	1.0	1.3	1.0	1.2	-	-	-	-	1.3	1.7	1.3	1.6	1.3	1.6	1.3	1.7	ARGDsEALDEES	RERARGDsEALDEES	S5;	No	S(5): 100.0; S(12): 0.0	1	2	1	1	N-Term(TMT6plex); S5(Phospho)
168229174	NP_061862.1	54439	RBM27	RNA-binding protein 27	T888	-	-	-	-	0.7	0.6	0.7	1.2	-	-	-	-	0.7	0.6	0.7	1.0	0.7	0.6	0.7	1.0	TSSAVStPSK	KTSSAVStPSKVKTK	T7;	Yes	T(1): 0.0; S(2): 0.0; S(3): 0.0; S(6): 0.0; T(7): 100.0; S(9): 0.0	2	1	1	0	N-Term(TMT6plex); T7(Phospho); K10(TMT6plex)
60302875	NP_001012409.1	151648	SGOL1	shugoshin-like 1 isoform A1	S468	-	-	-	-	0.7	0.5	1.0	1.2	-	-	-	-	0.5	0.3	0.5	0.9	0.5	0.3	0.5	0.9	AsPAVALPK	SPKKNKAsPAVALPK	S2;	Yes	S(2): 100.0	2	6	1	0	N-Term(TMT6plex); S2(Phospho); K9(TMT6plex)
117676384	NP_004710.2	9169	SCAF11	protein SCAF11	S902	-	-	-	-	1.0	1.1	1.0	1.2	-	-	-	-	1.1	1.1	1.0	1.2	1.1	1.1	1.0	1.2	VKDSsPGEK	QPRVKDSsPGEKSRS	S5;	Yes	S(4): 0.7; S(5): 99.3	2	1	1	1	N-Term(TMT6plex); K2(TMT6plex); S5(Phospho); K9(TMT6plex)
30061567	NP_060548.2	55132	LARP1B	la-related protein 1B isoform 1	S343	-	-	-	-	1.0	1.3	1.6	1.2	-	-	-	-	-	-	-	-	-	-	-	-	IGSPLsPK	PRIGSPLsPKKNSET	S6;	Yes	S(3): 0.0; S(6): 100.0	1	2	1	0	N-Term(TMT6plex); S6(Phospho); K8(TMT6plex)
148276990	NP_001073881.2	253461	ZBTB38	zinc finger and BTB domain-containing protein 38	S244	-	-	-	-	0.9	1.3	1.9	1.2	-	-	-	-	0.8	1.1	1.4	1.1	0.8	1.1	1.4	1.1	EHDGSsPGNTGK	VREHDGSsPGNTGKE	S6;	No	S(5): 1.0; S(6): 99.0; T(10): 0.0	4	1	1	0	N-Term(TMT6plex); S6(Phospho); K12(TMT6plex)

Khan et al., 2019. Multi-omics analysis to characterize cigarette smoke induced molecular alterations in esophageal cells
Supplementary Table 8. List of phosphopeptides identified and quantified in untreated and chronically treated Het1A cells with cigarette smoke condensate for 8 months using SequestHT and Mascot search algorithms with a PhosphoRS probability score ≥75%

														Normalized phosphoprotein ratios with respect to corresponding total protein ratios.								Average of replicates													
Protein group Accession	NP_Accession	Gene ID	Gene Symbol	Protein Description	PhosphoSite (Protein)	Het-1A-Smoke - 2M/Parental	Het-1A-Smoke - 4M/Parental	Het-1A-Smoke - 6M/Parental	Het-1A-Smoke - 8M/Parental	Het-1A-Smoke - 2M/Parental	Het-1A-Smoke - 4M/Parental	Het-1A-Smoke - 6M/Parental	Het-1A-Smoke - 8M/Parental	Het-1A-Smoke - 2M/Parental	Het-1A-Smoke - 4M/Parental	Het-1A-Smoke - 6M/Parental	Het-1A-Smoke - 8M/Parental	Het-1A-Smoke - 2M/Parental	Het-1A-Smoke - 4M/Parental	Het-1A-Smoke - 6M/Parental	Het-1A-Smoke - 8M/Parental	Het-1A-Smoke - 2M/Parental	Het-1A-Smoke - 4M/Parental	Het-1A-Smoke - 6M/Parental	Het-1A-Smoke - 8M/Parental	Peptide Sequence	Phosphowindow	PhosphoSite (Peptide)	Phosphosite Plus Evidence	phosphoRS Site Probabilities	# PSMs	# Proteins	# Protein Groups	# Missed Cleavages	Modifications
						Replicate 1				Replicate 2				Replicate 1				Replicate 2																	
13376115	NP_079045.1	79827	CLMP	CXADR-like membrane protein precursor	T359	-	-	-	-	1.3	1.1	1.2	1.2	-	-	-	-	-	-	-	-	-	-	-	-	AETtPSMIPSQSR	NLTKAETtPSMIPSQ	T4;	No	T(3): 1.8; T(4): 96.3; S(6): 0.0; S(10): 1.8; S(12): 0.0	1	1	1	0	N-Term(TMT6plex); T4(Phospho); M7(Oxidation)
24307983	NP_055865.1	23070	CMTR1	cap-specific mRNA (nucleoside-2'-O-)-methyltransferase 1	S53	-	-	-	-	0.8	0.7	0.9	1.2	-	-	-	-	0.9	0.8	1.1	1.3	0.9	0.8	1.1	1.3	ASTTSLSGsDSETEGK	STTSLSGsDSETEGK	S9;	No	S(2): 0.0; T(3): 0.0; T(4): 0.0; S(5): 0.0; S(7): 1.5; S(9): 97.1; S(11): 1.5; T(13): 0.0	2	1	1	0	N-Term(TMT6plex); S9(Phospho); K16(TMT6plex)
289577096	NP_597713.2	134549	SHROOM1	protein Shroom1 isoform2	T103	-	-	-	-	0.7	1.1	0.8	1.2	-	-	-	-	0.7	1.1	0.8	1.0	0.7	1.1	0.8	1.0	SGPQPTEVPGtPGPLNR	QPTEVPGtPGPLNRQ	T11;	Yes	S(1): 0.0; T(6): 0.0; T(11): 100.0	1	2	1	0	N-Term(TMT6plex); T11(Phospho)
116008442	NP_055885.3	23091	ZC3H13	zinc finger CCCH domain-containing protein 13	S198	-	-	-	-	1.0	1.3	1.0	1.2	-	-	-	-	1.0	1.2	1.0	1.1	1.0	1.2	1.0	1.1	KEVsPEVVR	IIKKEVsPEVVRSK	S4;	Yes	S(4): 100.0	1	1	1	1	N-Term(TMT6plex); K1(TMT6plex); S4(Phospho)
206725523	NP_006738.3	6494	SIPA1	signal-induced proliferation-associated protein 1	S67	-	-	-	-	1.0	1.0	1.0	1.2	-	-	-	-	-	-	-	-	-	-	-	-	SGSDAGEARPPTPAsPR	ARPPTPAsPRARAHS	S15;	Yes	S(1): 0.0; S(3): 0.0; T(12): 0.0; S(15): 100.0	1	1	1	0	N-Term(TMT6plex); S15(Phospho)
343780938	NP_115543.3	92017	SNX29	sorting nexin-29	S810	-	-	-	-	1.3	0.9	1.1	1.2	-	-	-	-	-	-	-	-	-	-	-	-	NVEPQsGDL	TRNVEPQsGDL	S6;	Yes	S(6): 100.0	2	1	1	0	N-Term(TMT6plex); S6(Phospho)
27477138	NP_078901.3	56829	ZC3HAV1	zinc finger CCCH-type antiviral protein 1 isoform2	S275	-	-	-	-	1.2	1.3	1.2	1.2	-	-	-	-	1.2	1.4	1.2	1.2	1.2	1.4	1.2	1.2	SCTPsPDQISHR	AERSCTPsPDQISHR	S5;	Yes	S(1): 0.0; T(3): 1.2; S(5): 98.7; S(10): 0.0	2	2	1	0	N-Term(TMT6plex); C2(Carbamidomethyl); S5(Phospho)
149158690	NP_542417.2	7916	PRRC2A	protein PRRC2A	S1671	-	-	-	-	0.8	0.9	0.8	1.2	-	-	-	-	0.8	0.8	0.9	1.1	0.8	0.8	0.9	1.1	SsPDGGLK	GPCSQRSsPDGGLKG	S2;	No	S(1): 0.8; S(2): 99.2	1	1	1	0	N-Term(TMT6plex); S2(Phospho); K8(TMT6plex)
74271888	NP_075047.1	64900	LPIN3	phosphatidate phosphatase LPIN3	S224	-	-	-	-	1.0	0.9	0.9	1.2	-	-	-	-	0.9	0.9	0.9	1.1	0.9	0.9	0.9	1.1	sDSELEVR	GELTSPKsDSELEVR	S1;	Yes	S(1): 100.0; S(3): 0.0	1	1	1	0	N-Term(TMT6plex); S1(Phospho)
528078296	NP_001268421.1	2956	MSH6	DNA mismatch repair protein Msh6 isoform2	S700	-	-	-	-	0.7	0.6	0.5	1.2	-	-	-	-	0.7	0.7	0.6	1.0	0.7	0.7	0.6	1.0	IHNVGsPLK	SKIHNVGsPLKSQNH	S6;	No	S(6): 100.0	2	3	1	0	N-Term(TMT6plex); S6(Phospho); K9(TMT6plex)
319996623	NP_001188474.1	64783	RBM15	putative RNA-binding protein 15 isoform2	S109	-	-	-	-	1.1	1.1	1.0	1.1	-	-	-	-	1.1	1.2	0.9	1.2	1.1	1.2	0.9	1.2	GGsREYDTGGGSSSSR	KSSSRGGsREYDTGG	S3;	Yes	S(3): 100.0; Y(6): 0.0; T(8): 0.0; S(12): 0.0; S(13): 0.0; S(14): 0.0;	2	2	1	1	N-Term(TMT6plex); S3(Phospho)
32698730	NP_065823.1	57532	NUFIP2	nuclear fragile X mental retardation-interacting protein	S692	-	-	-	-	1.2	0.6	1.2	1.1	-	-	-	-	1.2	0.6	1.1	0.9	1.2	0.6	1.1	0.9	IITYNEAMDsPDQ	TYNEAMDsPDQ	S10;	Yes	T(3): 0.0; Y(4): 0.0; S(10): 100.0	4	1	1	0	N-Term(TMT6plex); M8(Oxidation); S10(Phospho)
4506185	NP_002780.1	5685	PSMA4	proteasome subunit alpha type-4 isoform1	S13	-	-	-	-	0.3	0.4	1.0	1.1	-	-	-	-	0.2	0.4	0.9	1.0	0.2	0.4	0.9	1.0	TTIFsPEGR	DSRTTIFsPEGRLYQ	S5;	Yes	T(1): 0.0; T(2): 0.0; S(5): 100.0	2	1	1	0	N-Term(TMT6plex); S5(Phospho)
307078125	NP_001182485.1	9685	CLINT1	clathrin interactor 1 isoform3	T290	-	-	-	-	0.9	1.1	1.2	1.1	-	-	-	-	0.9	1.1	1.3	1.1	0.9	1.1	1.3	1.1	ASPDQNASTHtPQSSVK	DQNASTHtPQSSVKT	T11;	No	S(2): 0.0; S(8): 0.0; T(9): 0.0; T(11): 100.0; S(14): 0.0; S(15): 0.0	2	3	1	0	N-Term(TMT6plex); T11(Phospho); K17(TMT6plex)
24234750	NP_036350.2	3609	ILF3	interleukin enhancer-binding factor 3 isoforma	S812	-	-	-	-	0.6	0.7	0.8	1.1	-	-	-	-	0.7	0.8	0.8	1.2	0.7	0.8	0.8	1.2	FNYSGsGGR	SKFNYSGsGGRSGGN	S6;	Yes	Y(3): 0.0; S(4): 0.0; S(6): 100.0	3	2	1	0	N-Term(TMT6plex); S6(Phospho)
217416369	NP_001136120.1	56681	SAR1A	GTP-binding protein SAR1a	T139	-	-	-	-	1.4	1.2	1.4	1.1	-	-	-	-	1.3	1.2	1.2	1.1	1.3	1.2	1.2	1.1	IDRtDAISEEK	LGNKIDRtDAISEEK	T4;	Yes	T(4): 100.0; S(8): 0.0	2	1	1	1	N-Term(TMT6plex); T4(Phospho); K11(TMT6plex)
216548085	NP_361013.3	7798	LUZP1	leucine zipper protein 1	S423	-	-	-	-	0.7	1.0	0.9	1.1	-	-	-	-	0.6	1.0	0.9	1.1	0.6	1.0	0.9	1.1	QVSsPSFTNR	LSNRQVSsPSFTNRR	S4;	Yes	S(3): 0.0; S(4): 100.0; S(6): 0.0; T(8): 0.0	2	1	1	0	N-Term(TMT6plex); S4(Phospho)
55769548	NP_055475.2	9678	PHF14	PHD finger protein 14	S530	-	-	-	-	1.0	1.1	1.2	1.1	-	-	-	-	1.0	1.2	1.1	1.3	1.0	1.2	1.1	1.3	QLsPEAQAR	QEREKQLsPEAQARI	S3;	Yes	S(3): 100.0	1	1	1	0	N-Term(TMT6plex); S3(Phospho)
262359929	NP_056261.4	25909	AHCTF1	protein ELYS	T1773	-	-	-	-	1.3	1.6	1.1	1.1	-	-	-	-	1.3	1.5	1.1	1.2	1.3	1.5	1.1	1.2	SAQQEASADVAtPK	EASADVAtPKMPGQS	T12;	No	S(1): 0.0; S(7): 0.0; T(12): 100.0	2	1	1	0	N-Term(TMT6plex); T12(Phospho); K14(TMT6plex)
32130516	NP_004704.2	9147	NEMF	nuclear export mediator factor NEMF	S417	-	-	-	-	1.0	0.9	0.6	1.1	-	-	-	-	1.2	1.0	0.7	1.3	1.2	1.0	0.7	1.3	NPYLLsEEEDDDVDGDVNVEK	LRNPYLLsEEEDDDV	S6;	Yes	Y(3): 0.0; S(6): 100.0	2	1	1	0	N-Term(TMT6plex); S6(Phospho); K21(TMT6plex)
237681121	NP_009228.2	672	BRCA1	breast cancer type 1 susceptibility protein isoform3	S1477	-	-	-	-	2.9	2.5	2.8	1.1	-	-	-	-	2.8	2.3	2.9	1.1	2.8	2.3	2.9	1.1	NYPsQEELIK	LQNRNYPsQEELIKV	S4;	No	Y(2): 0.6; S(4): 99.4	2	5	1	0	N-Term(TMT6plex); S4(Phospho); K10(TMT6plex)
18426915	NP_004386.2	1627	DBN1	drebrin isoforma	S381	-	-	-	-	1.0	2.0	2.1	1.1	-	-	-	-	1.0	1.7	1.9	1.1	1.0	1.7	1.9	1.1	ALDEVTSSQPPPLPPPPPPAQETQEPsPILDSEETR	AQETQEPsPILDSEE	S27;	Yes	T(6): 0.1; S(7): 2.4; S(8): 0.0; T(23): 0.1; S(27): 97.5; S(32): 0.0; T(35): 0.0	2	2	1	0	N-Term(TMT6plex); S27(Phospho)
109255232	NP_001035864.1	9859	CEP170	centrosomal protein of 170 kDa isoformgamma	S379	-	-	-	-	1.0	0.9	0.6	1.1	-	-	-	-	0.9	0.8	0.5	1.1	0.9	0.8	0.5	1.1	HDDGTQsDSENAGAHR	KHDDGTQsDSENAGA	S7;	Yes	T(5): 1.3; S(7): 97.4; S(9): 1.3	2	4	1	0	N-Term(TMT6plex); S7(Phospho)
387942381	NP_001252509.1	10144	FAM13A	protein FAM13A isoforme	S502	-	-	-	-	2.4	2.6	2.3	1.1	-	-	-	-	-	-	-	-	-	-	-	-	ANTIPIIGsPSSK	NTIPIIGsPSSKRRS	S9;	No	T(3): 0.0; S(9): 100.0; S(11): 0.0; S(12): 0.0	2	4	1	0	N-Term(TMT6plex); S9(Phospho); K13(TMT6plex)
73747844	NP_002302.2	3980	LIG3	DNA ligase 3 isoformbeta precursor	S210	-	-	-	-	1.0	0.9	0.8	1.1	-	-	-	-	0.9	0.9	0.9	1.1	0.9	0.9	0.9	1.1	LTTTGQVTsPVK	TTTGQVTsPVKGASF	S9;	Yes	T(2): 0.0; T(3): 0.0; T(4): 0.0; T(8): 0.0; S(9): 100.0	2	2	1	0	N-Term(TMT6plex); S9(Phospho); K12(TMT6plex)
33356172	NP_002476.2	4683	NBN	nibrin	S343	-	-	-	-	3.5	4.2	3.2	1.1	-	-	-	-	3.4	4.2	3.1	1.1	3.4	4.2	3.1	1.1	TTTPGPSLsQGVSVDEK	TTPGPSLsQGVSVDE	S9;	Yes	T(1): 0.0; T(2): 0.0; T(3): 0.0; S(7): 1.5; S(9): 98.4; S(13): 0.0	2	1	1	0	N-Term(TMT6plex); S9(Phospho); K17(TMT6plex)
38044112	NP_937883.1	6249	CLIP1	CAP-Gly domain-containing linker protein 1 isoformb	S200; S204	-	-	-	-	1.0	0.9	0.6	1.1	-	-	-	-	1.1	0.9	0.6	1.3	1.1	0.9	0.6	1.3	TASESISNLsEAGsIK	SESISNLsEAGSIKK;SNLSEAGsIKKGERE	S10; S14;	Yes;Yes	T(1): 0.0; S(3): 0.0; S(5): 0.0; S(7): 0.0; S(10): 100.0; S(14): 100.0	2	3	1	0	N-Term(TMT6plex); S10(Phospho); S14(Phospho); K16(TMT6plex)
34577112	NP_908932.1	226	ALDOA	fructose-bisphosphate aldolase A isoform1	S39	-	-	-	-	1.4	2.5	1.5	1.1	-	-	-	-	1.3	2.4	1.5	1.1	1.3	2.4	1.5	1.1	GILAADESTGsIAK	AADESTGsIAKRLQS	S11;	Yes	S(8): 0.0; T(9): 0.0; S(11): 100.0	3	2	1	0	N-Term(TMT6plex); S11(Phospho); K14(TMT6plex)
4885399	NP_005436.1	9126	SMC3	structural maintenance of chromosomes protein 3	S1067	-	-	-	-	1.0	0.9	2.0	1.1	-	-	-	-	1.0	0.9	1.7	1.1	1.0	0.9	1.7	1.1	GDVEGSQsQDEGEGSGESER	GDVEGSQsQDEGEGS	S8;	Yes	S(6): 0.0; S(8): 100.0; S(15): 0.0; S(18): 0.0	2	1	1	0	N-Term(TMT6plex); S8(Phospho)
10801345	NP_037366.1	27335	EIF3K	eukaryotic translation initiation factor 3 subunit K	S217	-	-	-	-	1.0	0.9	0.6	1.1	-	-	-	-	0.9	0.8	0.5	1.0	0.9	0.8	0.5	1.0	IDFDSVSSIMASsQ	VSSIMASsQ_	S13;	Yes	S(5): 0.0; S(7): 0.0; S(8): 0.0; S(12): 1.1; S(13): 98.9	2	1	1	0	N-Term(TMT6plex); M10(Oxidation); S13(Phospho)
27734727	NP_775889.1	283899	INO80E	INO80 complex subunit E	S227	-	-	-	-	2.3	1.6	2.7	1.1	-	-	-	-	-	-	-	-	-	-	-	-	QMFSDAGsGDDALDGDDDLVIDIPE	QMFSDAGsGDDALDG	S8;	No	S(4): 2.0; S(8): 98.0	2	1	1	0	N-Term(TMT6plex); M2(Oxidation); S8(Phospho)
148613886	NP_073752.5	64864	RFX7	DNA-binding protein RFX7	S1178	-	-	-	-	1.0	1.4	0.6	1.1	-	-	-	-	-	-	-	-	-	-	-	-	NLsGSTLYPVSNIPR	VHRQRNLsGSTLYPV	S3;	No	S(3): 100.0; S(5): 0.0; T(6): 0.0; Y(8): 0.0; S(11): 0.0	2	1	1	0	N-Term(TMT6plex); S3(Phospho)
66392157	NP_001013860.1	146206	RLTPR	leucine-rich repeat-containing protein 16C	S1246	-	-	-	-	1.0	0.9	0.8	1.1	-	-	-	-	0.8	0.6	0.5	0.8	0.8	0.6	0.5	0.8	AGsDGDIMDSSTEAPPISIK	GEIKKAGsDGDIMDS	S3;	Yes	S(3): 100.0; S(10): 0.0; S(11): 0.0; T(12): 0.0; S(18): 0.0	2	1	1	0	N-Term(TMT6plex); S3(Phospho); M8(Oxidation); K20(TMT6plex)
398774359	NP_001257662.1	23786	BCL2L13	bcl-2-like protein 13 isoforme	S146	-	-	-	-	1.0	2.8	2.3	1.1	-	-	-	-	0.8	2.4	1.8	1.1	0.8	2.4	1.8	1.1	EESLVEELsPASEK	ESLVEELsPASEKKP	S9;	No	S(3): 0.0; S(9): 100.0; S(12): 0.0	2	5	1	0	N-Term(TMT6plex); S9(Phospho); K14(TMT6plex)
5803221	NP_006775.1	10885	WDR3	WD repeat-containing protein 3	S241	-	-	-	-	1.1	1.0	1.0	1.1	-	-	-	-	1.1	0.9	0.9	1.0	1.1	0.9	0.9	1.0	GSsPGIQDTLEAEDGAFETDEAPEDR	PKKIKGSsPGIQDTL	S3;	Yes	S(2): 1.2; S(3): 98.8; T(9): 0.0; T(19): 0.0	5	1	1	0	N-Term(TMT6plex); S3(Phospho)
61743954	NP_001611.1	79026	AHNAK	neuroblast differentiation-associated protein AHNAK isoform1	S511	-	-	-	-	1.0	3.9	1.9	1.1	-	-	-	-	1.2	4.0	2.0	1.3	1.2	4.0	2.0	1.3	ISMQDVDLSLGsPK	DVDLSLGsPKLKGDI	S12;	Yes	S(2): 0.0; S(9): 0.0; S(12): 100.0	2	1	1	0	N-Term(TMT6plex); M3(Oxidation); S12(Phospho); K14(TMT6plex)
118402596	NP_004652.2	8697	CDC23	cell division cycle protein 23 homolog	T596	-	-	-	-	1.0	0.9	1.4	1.1	-	-	-	-	1.0	0.8	1.3	1.0	1.0	0.8	1.3	1.0	VSPLNLSSVtP	PLNLSSVtP_	T10;	Yes	S(2): 0.0; S(7): 0.0; S(8): 0.0; T(10): 100.0	2	1	1	0	N-Term(TMT6plex); T10(Phospho)
299522984	NP_001177410.1	63967	CLSPN	claspin isoform2	Y779; S782	-	-	-	-	1.0	1.7	1.8	1.1	-	-	-	-	1.3	2.4	2.9	1.7	1.3	2.4	2.9	1.7	LSEPSLPIEDSQDLyNAsPEPK	IEDSQDLyNASPEPK;SQDLYNAsPEPKTLF	Y15; S18;	No;No	S(2): 0.0; S(5): 0.0; S(11): 2.2; Y(15): 97.8; S(18): 99.9	3	2	1	0	N-Term(TMT6plex); Y15(Phospho); S18(Phospho); K22(TMT6plex)
24497620	NP_055045.2	6730	SRP68	signal recognition particle subunit SRP68 isoform1	S22	-	-	-	-	1.0	1.8	2.1	1.1	-	-	-	-	1.0	1.7	2.0	1.1	1.0	1.7	2.0	1.1	QVPGGGGGGGSGGGGGsGGGGSGGGR	GSGGGGGsGGGGSGG	S17;	Yes	S(11): 0.0; S(17): 98.3; S(22): 1.7	2	2	1	0	N-Term(TMT6plex); S17(Phospho)
223555963	NP_001138630.1	10767	HBS1L	HBS1-like protein isoform2	T169	-	-	-	-	1.3	0.9	2.3	1.1	-	-	-	-	1.1	0.7	1.8	0.9	1.1	0.7	1.8	0.9	GPPIEDAIASSDVLEtASK	ASSDVLEtASKSANP	T16;	No	S(10): 0.0; S(11): 0.0; T(16): 100.0; S(18): 0.0	2	2	1	0	N-Term(TMT6plex); T16(Phospho); K19(TMT6plex)
222352092	NP_001138442.1	23327	NEDD4L	E3 ubiquitin-protein ligase NEDD4-like isoform6	S305	-	-	-	-	1.0	1.9	2.0	1.1	-	-	-	-	1.0	1.6	1.6	1.0	1.0	1.6	1.6	1.0	sLSSPTVTLSAPLEGAK	PQIRRPRsLSSPTVT	S1;	No	S(1): 98.9; S(3): 1.1; S(4): 0.0; T(6): 0.0; T(8): 0.0; S(10): 0.0	8	7	1	0	N-Term(TMT6plex); S1(Phospho); K17(TMT6plex)
55770832	NP_001793.1	1039	CDR2	cerebellar degeneration-related protein 2	S309	-	-	-	-	1.0	0.9	0.6	1.1	-	-	-	-	-	-	-	-	-	-	-	-	sSSETILSSLAGSDIVK	HRKPLKRsSSETILS	S1;	Yes	S(1): 94.3; S(2): 1.9; S(3): 1.9; T(5): 1.9; S(8): 0.0; S(9): 0.0; S(13): 0.0	2	1	1	0	N-Term(TMT6plex); T5(Phospho); K17(TMT6plex)
110556644	NP_722520.2	161882	ZFPM1	zinc finger protein ZFPM1	S786	-	-	-	-	1.0	0.9	1.2	1.1	-	-	-	-	-	-	-	-	-	-	-	-	sPGPAADGPIDLSK	PGLAPARsPGPAADG	S1;	Yes	S(1): 100.0; S(13): 0.0	3	1	1	0	N-Term(TMT6plex); S1(Phospho); K14(TMT6plex)
14589866	NP_004309.2	444	ASPH	aspartyl/asparaginyl beta-hydroxylase isoforma	S29	-	-	-	-	1.0	2.8	1.5	1.1	-	-	-	-	0.9	2.0	1.2	1.0	0.9	2.0	1.2	1.0	SSGNSSSSGSGSGSTSAGSSsPGAR	STSAGSSsPGARRET	S21;	Yes	S(1): 0.0; S(2): 0.0; S(5): 0.0; S(6): 0.0; S(7): 0.0; S(8): 0.0; S(10): 0.0; S(12): 0.0; S(14): 0.0; T(15): 0.0; S(16): 0.0; S(19): 0.0; S(20): 0.0;	2	5	1	0	N-Term(TMT6plex); S21(Phospho)
155722990	NP_060628.2	22950	SLC4A1AP	kanadaptin	S535	-	-	-	-	0.9	0.4	1.1	1.1	-	-	-	-	1.0	0.5	1.5	1.4	1.0	0.5	1.5	1.4	ASSQVLSESPSQDsLDAFMSEMK	SESPSQDsLDAFMSE	S14;	Yes	S(2): 0.0; S(3): 0.0; S(7): 0.1; S(9): 2.5; S(11): 2.5; S(14): 92.4; S(20): 2.5	1	1	1	0	N-Term(TMT6plex); S9(Phospho); M19(Oxidation); M22(Oxidation); K23(TMT6plex)
50658063	NP_001002800.1	10051	SMC4	structural maintenance of chromosomes protein 4 isoform1	S41	-	-	-	-	1.1	0.8	0.8	1.1	-	-	-	-	1.1	0.9	0.8	1.2	1.1	0.9	0.8	1.2	TEsPATAAETASEELDNR	PPSGRTEsPATAAET	S3;	Yes	T(1): 1.0; S(3): 99.0; T(6): 0.0; T(10): 0.0; S(12): 0.0	6	1	1	0	N-Term(TMT6plex); S3(Phospho)
336020353	NP_004825.3	9448	MAP4K4	mitogen-activated protein kinase kinase kinase kinase 4 isoform1	S630	-	-	-	-	1.0	1.0	1.1	1.1	-	-	-	-	0.9	0.9	1.1	1.1	0.9	0.9	1.1	1.1	SEGsPSQR	SSSKSEGsPSQRLEN	S4;	No	S(1): 0.0; S(4): 100.0; S(6): 0.0	1	5	1	0	N-Term(TMT6plex); S4(Phospho)
4506631	NP_000980.1	6156	RPL30	60S ribosomal protein L30	S10	-	-	-	-	0.7	0.6	1.1	1.1	-	-	-	-	0.7	0.7	1.1	1.2	0.7	0.7	1.1	1.2	sLESINSR	AAKKTKKsLESINSR	S1;	Yes	S(1): 100.0; S(4): 0.0; S(7): 0.0	1	1	1	0	N-Term(TMT6plex); S1(Phospho)
24308039	NP_056138.1	23376	UFL1	E3 UFM1-protein ligase 1	S458	-	-	-	-	1.0	1.1	1.0	1.1	-	-	-	-	1.0	1.1	1.0	1.0	1.0	1.1	1.0	1.0	KDDDsDDESQSSHTGK	KGRKDDDsDDESQSS	S5;	Yes	S(5): 100.0; S(9): 0.0; S(11): 0.0; S(12): 0.0; T(14): 0.0	2	1	1	1	N-Term(TMT6plex); K1(TMT6plex); S5(Phospho); K16(TMT6plex)
197333879	NP_001127945.1	23199	GSE1	genetic suppressor element 1 isoform2	S722; S724	-	-	-	-	0.9	1.1	1.0	1.1	-	-	-	-	-	-	-	-	-	-	-	-	ERsPsPPTIQSK	RRMLRERsPSPPTIQ;MLRERSPsPPTIQSK	S3; S5;	No;No	S(3): 100.0; S(5): 100.0; T(8): 0.0; S(11): 0.0	1	3	1	1	N-Term(TMT6plex); S3(Phospho); S5(Phospho); K12(TMT6plex)
163792194	NP_112196.3	81627	TRMT1L	TRMT1-like protein isoform1	S68	-	-	-	-	0.7	0.9	1.0	1.1	-	-	-	-	0.7	0.9	1.1	1.1	0.7	0.9	1.1	1.1	DSAGVPAPAPDSALDSAPTPASAPAPAPALAQAPALSPsLASAPEEAK	QAPALSPsLASAPEE	S39;	No	S(2): 0.0; S(12): 0.0; S(16): 0.0; T(19): 0.0; S(22): 0.0; S(37): 3.8; S(39): 92.5; S(42): 3.8	2	1	1	0	N-Term(TMT6plex); S39(Phospho); K48(TMT6plex)
151101292	NP_110382.3	81542	TMX1	thioredoxin-related transmembrane protein 1 precursor	S270	-	-	-	-	2.0	0.9	1.0	1.1	-	-	-	-	2.4	1.0	1.3	1.3	2.4	1.0	1.3	1.3	sLGPSLATDK	QNAIRQRsLGPSLAT	S1;	Yes	S(1): 100.0; S(5): 0.0; T(8): 0.0	1	1	1	0	N-Term(TMT6plex); S1(Phospho); K10(TMT6plex)
27262651	NP_680781.1	11273	ATXN2L	ataxin-2-like protein isoformD	S111	-	-	-	-	0.9	1.0	0.9	1.1	-	-	-	-	0.9	0.9	0.9	0.9	0.9	0.9	0.9	0.9	GPPQsPVFEGVYNNSR	TGKGPPQsPVFEGVY	S5;	Yes	S(5): 100.0; Y(12): 0.0; S(15): 0.0	1	6	1	0	N-Term(TMT6plex); S5(Phospho)
39930375	NP_060682.2	55740	ENAH	protein enabled homolog isoformb	T489	-	-	-	-	0.7	0.9	0.6	1.1	-	-	-	-	0.7	0.7	0.6	0.8	0.7	0.7	0.6	0.8	ASSTStPEPTR	SKASSTStPEPTRKP	T6;	Yes	S(2): 0.0; S(3): 0.0; T(4): 0.0; S(5): 0.8; T(6): 99.2; T(10): 0.0	4	2	1	0	N-Term(TMT6plex); T6(Phospho)
47578107	NP_056199.2	25836	NIPBL	nipped-B-like protein isoformB	S350	-	-	-	-	1.0	2.0	1.3	1.1	-	-	-	-	0.9	1.8	1.1	1.0	0.9	1.8	1.1	1.0	AAMYDIISsPSK	AMYDIISsPSKDSTK	S9;	Yes	Y(4): 0.0; S(8): 0.0; S(9): 100.0; S(11): 0.0	1	2	1	0	N-Term(TMT6plex); M3(Oxidation); S9(Phospho); K12(TMT6plex)
27597090	NP_003161.2	6830	SUPT6H	transcription elongation factor SPT6	T1523	-	-	-	-	1.4	3.6	3.0	1.1	-	-	-	-	1.5	3.8	3.2	1.3	1.5	3.8	3.2	1.3	DHYQDPVPGItPSSSSR	QDPVPGItPSSSSRT	T11;	Yes	Y(3): 5.4; T(11): 89.2; S(13): 26.4; S(14): 26.4; S(15): 26.4; S(16): 26.4	1	1	1	0	N-Term(TMT6plex); T11(Phospho); S16(Phospho)
45356743	NP_065115.3	387893	SETD8	N-lysine methyltransferase SETD8	S59	-	-	-	-	1.3	0.9	2.4	1.1	-	-	-	-	-	-	-	-	-	-	-	-	IYSYMsPNK	SKIYSYMsPNKCSGM	S6;	No	Y(2): 1.4; S(3): 1.4; Y(4): 1.4; S(6): 95.8	1	1	1	0	N-Term(TMT6plex); M5(Oxidation); S6(Phospho); K9(TMT6plex)
218563739	NP_001136271.1	124944	C17orf49	chromatin complexes subunit BAP18 isoform3	S62	-	-	-	-	1.0	0.9	0.6	1.1	-	-	-	-	0.9	0.8	0.6	1.0	0.9	0.8	0.6	1.0	VYEDSGIPLPAEsPK	GIPLPAEsPKKGPKK	S13;	No	Y(2): 0.2; S(5): 0.2; S(13): 99.7	1	3	1	0	N-Term(TMT6plex); S13(Phospho); K15(TMT6plex)
14389309	NP_116093.1	84790	TUBA1C	tubulin alpha-1C chain	S439	-	-	-	-	1.0	0.9	0.6	1.1	-	-	-	-	1.0	0.8	0.5	1.0	1.0	0.8	0.5	1.0	DYEEVGADsADGEDEGEEY	YEEVGADsADGEDEG	S9;	Yes	Y(2): 0.0; S(9): 97.6; Y(19): 2.4	1	1	1	0	N-Term(TMT6plex); Y19(Phospho)
24415404	NP_055426.1	23195	MDN1	midasin	S4538	-	-	-	-	1.0	0.9	1.3	1.1	-	-	-	-	1.0	0.8	1.1	1.0	1.0	0.8	1.1	1.0	AEENTDQAsPQEDYAGFER	EENTDQAsPQEDYAG	S9;	Yes	T(5): 1.9; S(9): 98.1; Y(14): 0.0	1	1	1	0	N-Term(TMT6plex); S9(Phospho)
118498368	NP_004977.2	3895	KTN1	kinectin isoformc	S1256	-	-	-	-	1.0	0.9	0.6	1.1	-	-	-	-	1.2	1.1	0.7	1.3	1.2	1.1	0.7	1.3	AAGDTTVIENSDVsPETESSEK	VIENSDVsPETESSE	S14;	No	T(5): 0.0; T(6): 0.0; S(11): 2.2; S(14): 97.8; T(17): 0.1; S(19): 0.0; S(20): 0.0	1	4	1	0	N-Term(TMT6plex); S14(Phospho); K22(TMT6plex)
389886545	NP_001254509.1	9055	PRC1	protein regulator of cytokinesis 1 isoform4	S224	-	-	-	-	1.0	0.9	0.6	1.1	-	-	-	-	1.1	0.9	0.6	1.1	1.1	0.9	0.6	1.1	EAVATIMsGSK	EAVATIMsGSKAKVR	S8;	No	T(5): 0.0; S(8): 98.8; S(10): 1.2	1	3	1	0	N-Term(TMT6plex); M7(Oxidation); S8(Phospho); K11(TMT6plex)
145386566	NP_068799.2	7707	ZNF148	zinc finger protein 148	S306	-	-	-	-	2.1	0.9	0.6	1.1	-	-	-	-	2.4	1.0	0.7	1.4	2.4	1.0	0.7	1.4	GGLLTSEEDSGFSTsPK	EDSGFSTsPKDNSLP	S15;	Yes	T(5): 0.0; S(6): 0.1; S(10): 0.1; S(13): 0.1; T(14): 0.1; S(15): 99.7	1	1	1	0	N-Term(TMT6plex); S15(Phospho); K17(TMT6plex)
7705949	NP_057437.1	51201	ZDHHC2	palmitoyltransferase ZDHHC2	T255; S261; S265	-	-	-	-	1.0	2.3	1.6	1.1	-	-	-	-	-	-	-	-	-	-	-	-	HGtDKNGFsLGFsK	SPVFRHGtDKNGFSL;GTDKNGFsLGFSKNM;NGFSLGFsKNMRQVF	T3; S9; S13;	No;No;No	T(3): 100.0; S(9): 100.0; S(13): 100.0	1	1	1	1	N-Term(TMT6plex); T3(Phospho); K5(TMT6plex); S9(Phospho); S13(Phospho); K14(TMT6plex)
313760645	NP_001186509.1	25777	SUN2	SUN domain-containing protein 2 isoformb	T107	-	-	-	-	1.0	0.9	0.6	1.1	-	-	-	-	0.9	0.8	0.4	1.2	0.9	0.8	0.4	1.2	RGtGGSESSR	RVRRRRGtGGSESSR	T3;	Yes	T(3): 100.0; S(6): 0.0; S(8): 0.0; S(9): 0.0	1	2	1	1	N-Term(TMT6plex); T3(Phospho)
194018478	NP_653173.1	23102	TBC1D2B	TBC1 domain family member 2B isoforma	S957	-	-	-	-	1.0	2.2	0.6	1.1	-	-	-	-	1.1	2.6	0.7	1.2	1.1	2.6	0.7	1.2	DTSPDKGELVsDEEEDT	PDKGELVsDEEEDT_	S11;	Yes	T(2): 0.0; S(3): 0.0; S(11): 100.0; T(17): 0.0	1	1	1	1	N-Term(TMT6plex); K6(TMT6plex); S11(Phospho)
157266317	NP_001175.2	545	ATR	serine/threonine-protein kinase ATR	T1989	-	-	-	-	1.0	0.9	0.6	1.1	-	-	-	-	0.9	0.8	0.5	1.0	0.9	0.8	0.5	1.0	GVELCEPENEtPPEGK	LCEPENEtPPEGKNM	T11;	Yes	T(11): 100.0	1	1	1	0	N-Term(TMT6plex); C5(Carbamidomethyl); T11(Phospho); K16(TMT6plex)
260064009	NP_056121.2	23358	USP24	ubiquitin carboxyl-terminal hydrolase 24	T2559	-	-	-	-	1.3	0.9	0.6	1.1	-	-	-	-	1.2	0.9	0.6	1.1	1.2	0.9	0.6	1.1	tISAQDTLAYATALLNEK	TGKTFQRtISAQDTL	T1;	Yes	T(1): 95.8; S(3): 2.1; T(7): 2.1; Y(10): 0.0; T(12): 0.0	1	1	1	0	N-Term(TMT6plex); T1(Phospho); K18(TMT6plex)
54144629	NP_001005745.1	8650	NUMB	protein numb homolog isoform4	S575	-	-	-	-	1.0	0.9	0.6	1.1	-	-	-	-	0.7	0.9	0.6	1.1	0.7	0.9	0.6	1.1	TNPsPTNPFSSDLQK	SKQRTNPsPTNPFSS	S4;	No	T(1): 1.9; S(4): 96.2; T(6): 1.9; S(10): 0.0; S(11): 0.0	1	4	1	0	N-Term(TMT6plex); S4(Phospho); K15(TMT6plex)
71725360	NP_055857.1	23060	ZNF609	zinc finger protein 609	S467	-	-	-	-	3.4	1.2	1.3	1.1	-	-	-	-	4.3	1.4	1.7	1.2	4.3	1.4	1.7	1.2	TNsMGSATGPLPGTK	SKRVRTNsMGSATGP	S3;	Yes	T(1): 1.5; S(3): 98.5; S(6): 0.0; T(8): 0.0; T(14): 0.0	1	1	1	0	N-Term(TMT6plex); S3(Phospho); M4(Oxidation); K15(TMT6plex)
195546902	NP_001124297.1	6829	SUPT5H	transcription elongation factor SPT5 isoformb	T787	-	-	-	-	1.0	0.9	0.6	1.1	-	-	-	-	1.1	0.9	0.6	1.1	1.1	0.9	0.6	1.1	TPMYGSQtPLQDGSR	TPMYGSQtPLQDGSR	T8;	No	T(1): 0.0; Y(4): 0.0; S(6): 0.0; T(8): 99.9; S(14): 0.0	2	2	1	0	N-Term(TMT6plex); M3(Oxidation); T8(Phospho)
132626688	NP_055456.2	9656	MDC1	mediator of DNA damage checkpoint protein 1	T1548	-	-	-	-	1.0	0.9	1.1	1.1	-	-	-	-	1.1	0.9	1.1	1.2	1.1	0.9	1.1	1.2	TPETVVPAAPELQPSTSTDQPVtPEPTSR	TSTDQPVtPEPTSRA	T23;	Yes	T(1): 0.0; T(4): 0.0; S(15): 0.1; T(16): 0.1; S(17): 0.1; T(18): 0.1; T(23): 99.3; T(27): 0.1; S(28): 0.1	1	1	1	0	N-Term(TMT6plex); T23(Phospho)
31542947	NP_002147.2	3329	HSPD1	60 kDa heat shock protein, mitochondrial	S70	-	-	-	-	1.0	0.9	0.6	1.1	-	-	-	-	1.0	0.9	0.6	1.0	1.0	0.9	0.6	1.0	TVIIEQSWGsPK	IIEQSWGsPKVTKDG	S10;	Yes	T(1): 0.0; S(7): 0.0; S(10): 100.0	1	1	1	0	N-Term(TMT6plex); S10(Phospho); K12(TMT6plex)
222352111	NP_115766.3	84365	NIFK	MKI67 FHA domain-interacting nucleolar phosphoprotein	T234; T238	-	-	-	-	1.0	1.3	0.6	1.1	-	-	-	-	1.1	1.3	0.6	1.2	1.1	1.3	0.6	1.2	TVDSQGPtPVCtPTFLER	TVDSQGPtPVCTPTF;QGPTPVCtPTFLERR	T8; T12;	No;No	T(1): 0.0; S(4): 0.0; T(8): 100.0; T(12): 96.9; T(14): 3.1	1	1	1	0	N-Term(TMT6plex); T8(Phospho); C11(Carbamidomethyl); T12(Phospho)
555943672	NP_001273158.1	51514	DTL	denticleless protein homolog isoform2	T431	-	-	-	-	1.0	2.7	0.8	1.1	-	-	-	-	0.9	3.0	0.8	1.2	0.9	3.0	0.8	1.2	TLPSPVTItPSSMR	LPSPVTItPSSMRKI	T9;	No	T(1): 0.0; S(4): 0.0; T(7): 1.5; T(9): 98.5; S(11): 0.0; S(12): 0.0	1	3	1	0	N-Term(TMT6plex); T9(Phospho); M13(Oxidation)
50845416	NP_001002838.1	65267	WNK3	serine/threonine-protein kinase WNK3 isoform2	S1538	-	-	-	-	1.0	0.9	0.6	1.1	-	-	-	-	-	-	-	-	-	-	-	-	TQSTEIPLPPAsPR	EIPLPPAsPRRPRSF	S12;	No	T(1): 0.0; S(3): 0.0; T(4): 0.0; S(12): 100.0	1	2	1	0	N-Term(TMT6plex); S12(Phospho)
42476206	NP_057690.2	51308	REEP2	receptor expression-enhancing protein 2 isoform2	S251	-	-	-	-	1.0	0.9	1.3	1.1	-	-	-	-	-	-	-	-	-	-	-	-	TSGGGDsA	KTSGGGDsA	S7;	No	T(1): 0.0; S(2): 0.0; S(7): 100.0	1	2	1	0	N-Term(TMT6plex); S7(Phospho)
38505220	NP_060358.2	54988	ACSM5	acyl-coenzyme A synthetase ACSM5, mitochondrial precursor	S489; S500	-	-	-	-	1.0	0.9	0.6	1.1	-	-	-	-	-	-	-	-	-	-	-	-	IGPVEVEsALAEHPAVLEsAVVSSPDPIR	IGPVEVEsALAEHPA;EHPAVLEsAVVSSPD	S8; S19;	No;No	S(8): 99.4; S(19): 95.7; S(23): 52.4; S(24): 52.4	1	1	1	0	N-Term(TMT6plex); S8(Phospho); S19(Phospho); S23(Phospho)
6005900	NP_009042.1	7027	TFDP1	transcription factor Dp-1	S23	-	-	-	-	2.2	0.9	0.6	1.1	-	-	-	-	2.7	1.0	0.7	1.2	2.7	1.0	0.7	1.2	VFIDQNLsPGK	VFIDQNLsPGKGVVS	S8;	Yes	S(8): 100.0	1	1	1	0	N-Term(TMT6plex); S8(Phospho); K11(TMT6plex)
317574227	NP_001188264.1	6936	GCFC2	GC-rich sequence DNA-binding factor 2 isoform3	S40	-	-	-	-	1.0	0.9	0.6	1.1	-	-	-	-	0.9	0.8	0.5	1.0	0.9	0.8	0.5	1.0	ELPVPGsAEEEPPSGGGR	RELPVPGsAEEEPPS	S7;	Yes	S(7): 99.9; S(14): 0.1	1	2	1	0	N-Term(TMT6plex); S7(Phospho)
332801090	NP_001193929.1	9987	HNRNPDL	heterogeneous nuclear ribonucleoprotein D-like	S241	-	-	-	-	1.0	1.2	1.3	1.1	-	-	-	-	1.1	1.2	1.2	0.9	1.1	1.2	1.2	0.9	VFVGGLsPDTSEEQIK	KVFVGGLsPDTSEEQ	S7;	No	S(7): 98.4; T(10): 1.5; S(11): 0.0	1	2	1	0	N-Term(TMT6plex); S7(Phospho); K16(TMT6plex)
47519639	NP_002366.2	4134	MAP4	microtubule-associated protein 4 isoform1	S580	-	-	-	-	1.0	1.3	1.7	1.1	-	-	-	-	1.0	1.3	1.8	1.4	1.0	1.3	1.8	1.4	DVPPLsETEATPVPIK	AKDVPPLsETEATPV	S6;	Yes	S(6): 96.0; T(8): 2.0; T(11): 2.0	1	2	1	0	N-Term(TMT6plex); T11(Phospho); K16(TMT6plex)
168229156	NP_078804.2	79576	NKAP	NF-kappa-B-activating protein	S157; T161	-	-	-	-	1.0	0.9	0.6	1.1	-	-	-	-	1.3	1.1	0.8	1.3	1.3	1.1	0.8	1.3	NPEPDsDEHtPVEDEEPK	PKNPEPDsDEHTPVE;EPDSDEHtPVEDEEP	S6; T10;	Yes;Yes	S(6): 100.0; T(10): 100.0	1	1	1	0	N-Term(TMT6plex); S6(Phospho); T10(Phospho); K18(TMT6plex)
386642875	NP_001245379.1	9088	PKMYT1	membrane-associated tyrosine- and threonine-specific cdc2-inhibitory kinase isoform3	S400	-	-	-	-	1.0	0.9	0.6	1.1	-	-	-	-	1.2	1.2	0.8	1.5	1.2	1.2	0.8	1.5	DALDLsDINSEPPR	PRDALDLsDINSEPP	S6;	No	S(6): 100.0; S(10): 0.0	1	3	1	0	N-Term(TMT6plex); S6(Phospho)
31317307	NP_777596.2	255738	PCSK9	proprotein convertase subtilisin/kexin type 9	S688	-	-	-	-	1.0	0.9	0.6	1.1	-	-	-	-	0.9	0.8	0.5	1.2	0.9	0.8	0.5	1.2	HLAQAsQELQ	SRHLAQAsQELQ	S6;	Yes	S(6): 100.0	1	1	1	0	N-Term(TMT6plex); S6(Phospho)
33598968	NP_005349.3	4008	LMO7	LIM domain only protein 7 isoform1	S276	-	-	-	-	2.8	5.4	5.7	1.1	-	-	-	-	2.3	4.3	3.9	1.1	2.3	4.3	3.9	1.1	EGFEsDTDSEFTFK	GGREGFEsDTDSEFT	S5;	Yes	S(5): 99.9; T(7): 0.0; S(9): 0.0; T(12): 0.0	1	1	1	0	N-Term(TMT6plex); S5(Phospho); K14(TMT6plex)
22749327	NP_689866.1	7325	UBE2E2	ubiquitin-conjugating enzyme E2 E2	S11	-	-	-	-	1.0	2.0	1.8	1.1	-	-	-	-	1.3	2.2	1.8	1.1	1.3	2.2	1.8	1.1	VDDsPSTSGGSSDGDQR	EAQRVDDsPSTSGGS	S4;	No	S(4): 99.8; S(6): 0.0; T(7): 0.0; S(8): 0.0; S(11): 0.0; S(12): 0.0	3	1	1	0	N-Term(TMT6plex); S4(Phospho)
449083351	NP_001263347.1	9919	SEC16A	protein transport protein Sec16A isoform2	S122	-	-	-	-	1.0	0.9	0.9	1.1	-	-	-	-	0.9	0.8	0.8	1.0	0.9	0.8	0.8	1.0	AHAsPFSGALTPSAPPGPEMNR	TQPRAHAsPFSGALT	S4;	Yes	S(4): 96.6; S(7): 3.4; T(11): 0.0; S(13): 0.0	1	2	1	0	N-Term(TMT6plex); S7(Phospho); M20(Oxidation)
546231404	NP_001271231.1	54443	ANLN	actin-binding protein anillin isoform3	S182	-	-	-	-	1.0	0.9	0.6	1.1	-	-	-	-	1.1	0.9	0.6	1.2	1.1	0.9	0.6	1.2	AAsPPRPLLSNASATPVGR	PSEEKAAsPPRPLLS	S3;	Yes	S(3): 99.9; S(10): 0.1; S(13): 0.0; T(15): 0.0	1	3	1	0	N-Term(TMT6plex); S3(Phospho)
153945846	NP_002215.2	3710	ITPR3	inositol 1,4,5-trisphosphate receptor type 3	S1832	-	-	-	-	4.1	3.3	3.9	1.1	-	-	-	-	3.9	3.1	3.3	1.1	3.9	3.1	3.3	1.1	VAsFSIPGSSSR	TTKGRVAsFSIPGSS	S3;	Yes	S(3): 99.0; S(5): 1.0; S(9): 0.0; S(10): 0.0; S(11): 0.0	1	1	1	0	N-Term(TMT6plex); S3(Phospho)
38683816	NP_942592.1	26057	ANKRD17	ankyrin repeat domain-containing protein 17 isoformb	S2150	-	-	-	-	2.7	3.4	0.6	1.1	-	-	-	-	2.4	3.2	0.6	1.0	2.4	3.2	0.6	1.0	APsPAPSSVPLGSEKPSNVSQDR	SSGVRAPsPAPSSVP	S3;	No	S(3): 86.6; S(7): 4.3; S(8): 4.3; S(13): 4.3; S(17): 0.3; S(20): 0.3	1	3	1	0	N-Term(TMT6plex); S3(Phospho); K15(TMT6plex)
150418005	NP_008970.2	11099	PTPN21	tyrosine-protein phosphatase non-receptor type 21	S673	-	-	-	-	3.5	3.4	3.8	1.1	-	-	-	-	-	-	-	-	-	-	-	-	AVSVGsQPSVFTER	PRAVSVGsQPSVFTE	S6;	No	S(3): 2.3; S(6): 95.3; S(9): 0.1; T(12): 2.3	1	1	1	0	N-Term(TMT6plex); S6(Phospho)
112421108	NP_055940.3	23152	CIC	protein capicua homolog	S904	-	-	-	-	1.0	0.9	0.6	1.1	-	-	-	-	-	-	-	-	-	-	-	-	AQSVsPVQAPPPGGSAQLLPGK	PPKAQSVsPVQAPPP	S5;	No	S(3): 2.2; S(5): 97.8; S(15): 0.0	1	1	1	0	N-Term(TMT6plex); S5(Phospho); K22(TMT6plex)
281427168	NP_001163935.1	5128	CDK17	cyclin-dependent kinase 17 isoform2	S180	-	-	-	-	1.0	1.7	1.6	1.1	-	-	-	-	0.8	1.4	1.2	0.9	0.8	1.4	1.2	0.9	RAsLSEIGFGK	SRRSRRAsLSEIGFG	S3;	Yes	S(3): 100.0; S(5): 0.0	1	2	1	1	N-Term(TMT6plex); S3(Phospho); K11(TMT6plex)
325120986	NP_001191397.1	8241	RBM10	RNA-binding protein 10 isoform5	S154	-	-	-	-	1.0	0.9	1.5	1.1	-	-	-	-	1.1	1.0	1.7	1.3	1.1	1.0	1.7	1.3	HsPTGPPGFPR	RRRRHRHsPTGPPGF	S2;	No	S(2): 98.4; T(4): 1.6	1	3	1	0	N-Term(TMT6plex); S2(Phospho)
66392157	NP_001013860.1	146206	RLTPR	leucine-rich repeat-containing protein 16C	S991	-	-	-	-	1.0	0.9	0.6	1.1	-	-	-	-	0.8	0.6	0.3	0.8	0.8	0.6	0.3	0.8	GsPSPAAPGPPAGPLPR	AGPSARGsPSPAAPG	S2;	Yes	S(2): 98.1; S(4): 1.9	1	1	1	0	N-Term(TMT6plex); S2(Phospho)
22748641	NP_689485.1	91782	CHMP7	charged multivesicular body protein 7	S417	-	-	-	-	1.0	0.9	0.8	1.1	-	-	-	-	1.0	0.8	0.7	1.0	1.0	0.8	0.7	1.0	IsDAELEAELEK	SVPNPRIsDAELEAE	S2;	Yes	S(2): 100.0	1	1	1	0	N-Term(TMT6plex); S2(Phospho); K12(TMT6plex)
259906020	NP_001158287.1	22985	ACIN1	apoptotic chromatin condensation inducer in the nucleus isoform3	S674	-	-	-	-	0.8	0.9	0.9	1.1	-	-	-	-	0.6	0.8	1.0	1.2	0.6	0.8	1.0	1.2	LSQPEsAEK	RRLSQPEsAEKHVTQ	S6;	No	S(2): 0.0; S(6): 100.0	2	3	1	0	N-Term(TMT6plex); S6(Phospho); K9(TMT6plex)
82775371	NP_061917.3	54520	CCDC93	coiled-coil domain-containing protein 93	S305	-	-	-	-	1.0	0.9	1.5	1.1	-	-	-	-	1.0	1.0	1.7	1.2	1.0	1.0	1.7	1.2	QSELSAEEsPEK	SELSAEEsPEKLGTS	S9;	Yes	S(2): 0.0; S(5): 1.2; S(9): 98.8	1	1	1	0	N-Term(TMT6plex); S9(Phospho); K12(TMT6plex)
91718902	NP_733751.2	58508	KMT2C	histone-lysine N-methyltransferase 2C	S4267	-	-	-	-	1.0	0.9	0.9	1.1	-	-	-	-	-	-	-	-	-	-	-	-	ESIPSLPQsPMR	SIPSLPQsPMRETPS	S9;	No	S(2): 0.0; S(5): 0.0; S(9): 100.0	1	1	1	0	N-Term(TMT6plex); S9(Phospho); M11(Oxidation)
40018638	NP_950251.1	25966	C2CD2	C2 domain-containing protein 2 isoform2	S286	-	-	-	-	1.5	3.8	2.3	1.1	-	-	-	-	1.5	3.7	2.4	1.0	1.5	3.7	2.4	1.0	ASPLSSDsPVK	ASPLSSDsPVKTPIK	S8;	No	S(2): 0.0; S(5): 0.0; S(6): 0.0; S(8): 100.0	1	2	1	0	N-Term(TMT6plex); S8(Phospho); K11(TMT6plex)
312032467	NP_003613.3	8496	PPFIBP1	liprin-beta-1 isoform1	S573	-	-	-	-	1.7	1.7	2.3	1.1	-	-	-	-	1.3	1.3	1.7	1.0	1.3	1.3	1.7	1.0	NSPFQIPPPsPDSK	PFQIPPPsPDSKKKS	S10;	No	S(2): 0.0; S(10): 100.0; S(13): 0.0	1	4	1	0	N-Term(TMT6plex); S10(Phospho); K14(TMT6plex)
166295179	NP_001107590.1	55421	C17orf85	uncharacterized protein C17orf85	S30	-	-	-	-	1.0	0.9	0.6	1.1	-	-	-	-	1.2	1.0	0.7	1.4	1.2	1.0	0.7	1.4	AEAPAGPALGLPSPEAEsGVDRGEPEPMEVEEGELEIVPVR	LPSPEAEsGVDRGEP	S18;	Yes	S(13): 4.1; S(18): 95.9	1	1	1	1	N-Term(TMT6plex); S18(Phospho); M28(Oxidation)
169881252	NP_001509.3	2969	GTF2I	general transcription factor II-I isoform4	S207	-	-	-	-	1.0	0.9	0.6	1.1	-	-	-	-	1.1	0.9	0.6	1.2	1.1	0.9	0.6	1.2	sILSPGGSCGPIK	MVTDADRsILSPGGS	S1;	Yes	S(1): 98.7; S(4): 1.3; S(8): 0.0	1	6	1	0	N-Term(TMT6plex); S1(Phospho); C9(Carbamidomethyl); K13(TMT6plex)
223468564	NP_001138616.1	6510	SLC1A5	neutral amino acid transporter B(0) isoform2	S265	-	-	-	-	1.0	1.1	1.1	1.1	-	-	-	-	0.7	0.7	0.8	0.9	0.7	0.7	0.8	0.9	sTEPELIQVK	VDRTESRsTEPELIQ	S1;	No	S(1): 100.0; T(2): 0.0	3	3	1	0	N-Term(TMT6plex); S1(Phospho); K10(TMT6plex)
212276104	NP_001131025.1	9208	LRRFIP1	leucine-rich repeat flightless-interacting protein 1	S562	-	-	-	-	1.0	0.9	1.3	1.1	-	-	-	-	1.1	0.9	1.4	1.4	1.1	0.9	1.4	1.4	sAVEAQNEVTENPK	VKSTDRKsAVEAQNE	S1;	No	S(1): 100.0; T(10): 0.0	1	3	1	0	N-Term(TMT6plex); S1(Phospho); K14(TMT6plex)
8923657	NP_060415.1	55032	SLC35A5	probable UDP-sugar transporter protein SLC35A5	S419	-	-	-	-	1.3	1.3	1.3	1.1	-	-	-	-	-	-	-	-	-	-	-	-	SDEsDEDTE	TKPKSDEsDEDTE	S4;	Yes	S(1): 0.9; S(4): 99.1; T(8): 0.0	1	1	1	0	N-Term(TMT6plex); S4(Phospho)
377652346	NP_001243716.1	54477	PLEKHA5	pleckstrin homology domain-containing family A member 5 isoform5	S497	-	-	-	-	1.0	0.9	0.6	1.1	-	-	-	-	1.0	0.8	0.5	1.0	1.0	0.8	0.5	1.0	SVPAGLTLQsVSPQSLQGK	PAGLTLQsVSPQSLQ	S10;	No	S(1): 0.1; T(7): 2.2; S(10): 95.4; S(12): 2.2; S(15): 0.1	1	4	1	0	N-Term(TMT6plex); S12(Phospho); K19(TMT6plex)
7662086	NP_055599.1	9826	ARHGEF11	rho guanine nucleotide exchange factor 11 isoform1	T668; T672	-	-	-	-	1.0	0.9	1.3	1.1	-	-	-	-	0.9	0.8	1.3	1.1	0.9	0.8	1.3	1.1	SLENPtPPFtPK	TRSLENPtPPFTPKM;ENPTPPFtPKMGRRS	T6; T10;	Yes;Yes	S(1): 0.0; T(6): 100.0; T(10): 100.0	1	2	1	0	N-Term(TMT6plex); T6(Phospho); T10(Phospho); K12(TMT6plex)
19923729	NP_115646.2	84230	LRRC8C	leucine-rich repeat-containing protein 8C	S212	-	-	-	-	1.0	0.9	1.2	1.1	-	-	-	-	0.9	0.8	1.0	1.1	0.9	0.8	1.0	1.1	SNTIQSGPEGSLVNSQsLK	GSLVNSQsLKSIPEK	S17;	No	S(1): 0.0; T(3): 0.0; S(6): 0.0; S(11): 0.0; S(15): 1.9; S(17): 98.1	1	1	1	0	N-Term(TMT6plex); S17(Phospho); K19(TMT6plex)
342672046	NP_001230153.1	5883	RAD9A	cell cycle checkpoint control protein RAD9A isoform	S311	-	-	-	-	1.0	2.0	1.7	1.1	-	-	-	-	1.1	2.1	2.0	1.3	1.1	2.1	2.0	1.3	SPQGPSPVLAEDsEGEG	SPVLAEDsEGEG	S13;	No	S(1): 0.0; S(6): 0.2; S(13): 99.8	1	2	1	0	N-Term(TMT6plex); S13(Phospho)
284795266	NP_067026.3	58477	SRPRB	signal recognition particle receptor subunit beta	T220	-	-	-	-	1.0	0.9	0.8	1.1	-	-	-	-	0.8	0.7	0.7	0.9	0.8	0.7	0.7	0.9	SAAPSTLDSSStAPAQLGK	STLDSSStAPAQLGK	T12;	No	S(1): 0.0; S(5): 0.1; T(6): 0.1; S(9): 2.4; S(10): 2.4; S(11): 2.4; T(12): 92.8	1	1	1	0	N-Term(TMT6plex); S9(Phospho); K19(TMT6plex)
111955162	NP_001009811.2	22994	CEP131	5-azacytidine-induced protein 1 isoformb	S150	-	-	-	-	1.3	0.9	2.8	1.1	-	-	-	-	-	-	-	-	-	-	-	-	SSSALDsPAGPR	RSSSALDsPAGPRRK	S7;	No	S(1): 0.0; S(2): 1.4; S(3): 1.4; S(7):	1	3	1	0	N-Term(TMT6plex); S7(Phospho)
6754472	NP_004847.2	9493	KIF23	kinesin-like protein KIF23 isoform2	T823	-	-	-	-	1.0	0.9	1.6	1.1	-	-	-	-	1.0	0.9	1.6	1.1	1.0	0.9	1.6	1.1	SSTVAPAQPDGAESEWtDVETR	DGAESEWtDVETRCS	T17;	Yes	S(1): 0.0; S(2): 0.0; T(3): 0.0; S(14): 2.7; T(17): 97.2; T(21): 0.1	1	3	1	0	N-Term(TMT6plex); T17(Phospho)
57634536	NP_055970.1	23185	LARP4B	la-related protein 4B	S664	-	-	-	-	0.9	1.0	1.1	1.1	-	-	-	-	0.9	0.9	1.0	1.1	0.9	0.9	1.0	1.1	EPPSsPLQPQK	TSKEPPSsPLQPQKE	S5;	Yes	S(4): 0.8; S(5): 99.2	6	1	1	0	N-Term(TMT6plex); S5(Phospho); K11(TMT6plex)
118572599	NP_037373.2	29109	FHOD1	FH1/FH2 domain-containing protein 1	S367	-	-	-	-	1.1	1.0	1.0	1.1	-	-	-	-	1.2	1.0	1.0	1.1	1.2	1.0	1.0	1.1	sLEGGGCPAR	EGKRSRRsLEGGGCP	S1;	Yes	S(1): 100.0	1	1	1	0	N-Term(TMT6plex); S1(Phospho); C7(Carbamidomethyl)

Khan et al., 2019. Multi-omics analysis to characterize cigarette smoke induced molecular alterations in esophageal cells
Supplementary Table 8. List of phosphopeptides identified and quantified in untreated and chronically treated Het1A cells with cigarette smoke condensate for 8 months using SequestHT and Mascot search algorithms with a PhosphoRS probability score ≥75%

														Normalized phosphoprotein ratios with respect to corresponding total protein ratios.								Average of replicates													
Protein group Accession	NP_Accession	Gene ID	Gene Symbol	Protein Description	PhosphoSite (Protein)	Het-1A-Smoke - 2M/Parental	Het-1A-Smoke - 4M/Parental	Het-1A-Smoke - 6M/Parental	Het-1A-Smoke - 8M/Parental	Het-1A-Smoke - 2M/Parental	Het-1A-Smoke - 4M/Parental	Het-1A-Smoke - 6M/Parental	Het-1A-Smoke - 8M/Parental	Het-1A-Smoke - 2M/Parental	Het-1A-Smoke - 4M/Parental	Het-1A-Smoke - 6M/Parental	Het-1A-Smoke - 8M/Parental	Het-1A-Smoke - 2M/Parental	Het-1A-Smoke - 4M/Parental	Het-1A-Smoke - 6M/Parental	Het-1A-Smoke - 8M/Parental	Het-1A-Smoke - 2M/Parental	Het-1A-Smoke - 4M/Parental	Het-1A-Smoke - 6M/Parental	Het-1A-Smoke - 8M/Parental	Peptide Sequence	Phosphowindow	PhosphoSite (Peptide)	Phosphosite Plus Evidence	phosphoRS Site Probabilities	# PSMs	# Proteins	# Protein Groups	# Missed Cleavages	Modifications
						Replicate 1				Replicate 2				Replicate 1				Replicate 2																	
42518070	NP_004808.2	9414	TJP2	tight junction protein ZO-2 isoform1	S702	-	-	-	-	1.1	1.2	1.1	1.1	-	-	-	-	1.1	1.2	0.9	1.2	1.1	1.2	0.9	1.2	sREDLTAVVSVSTK	VKKNLRKsREDLTAV	S1;	Yes	S(1): 99.1; T(6): 0.9; S(10): 0.0; S(12): 0.0; T(13): 0.0	4	6	1	1	N-Term(TMT6plex); S1(Phospho); K14(TMT6plex)
533112484	NP_001269207.1	9782	MATR3	matrin-3 isoform c	S268	-	-	-	-	1.0	1.2	1.4	1.1	-	-	-	-	1.1	1.4	1.5	1.2	1.1	1.4	1.5	1.2	SYsPDGKESPsDK	PDGKESPsDKKSKTD	S11;	No	S(1): 0.0; Y(2): 0.0; S(3): 0.0; S(9): 1.6; S(11): 98.4	1	3	1	1	N-Term(TMT6plex); K7(TMT6plex); S9(Phospho); K13(TMT6plex)
62362414	NP_005148.2	25	ABL1	tyrosine-protein kinase ABL1 isoform a	T852	-	-	-	-	1.1	0.4	1.2	1.1	-	-	-	-	-	-	-	-	-	-	-	-	GSALGTPAAAEPVtPTSK	PAAAEPVtPTSKAGS	T14;	Yes	S(2): 0.0; T(6): 0.0; T(14): 96.7; T(16): 1.7; S(17): 1.7	1	2	1	0	N-Term(TMT6plex); T14(Phospho); K18(TMT6plex)
38683860	NP_003740.2	8660	IRS2	insulin receptor substrate 2	T520	-	-	-	-	0.9	0.9	1.4	1.1	-	-	-	-	0.8	0.9	1.5	1.1	0.8	0.9	1.5	1.1	SNtPESIAETPPAR	FCSHRSNtPESIAET	T3;	Yes	S(1): 2.5; T(3): 97.3; S(6): 0.1; T(10): 0.1	1	1	1	0	N-Term(TMT6plex); T3(Phospho)
557357729	NP_001273444.1	55729	ATF7IP	activating transcription factor 7-interacting protein 1 isoform4	S898	-	-	-	-	0.4	0.8	1.3	1.1	-	-	-	-	0.4	0.8	1.2	1.1	0.4	0.8	1.2	1.1	TSLPTVGPSGLYsPSTNR	VGPSGLYsPSTNRGP	S13;	No	T(1): 0.0; S(2): 0.0; T(5): 0.0; S(9): 0.0; Y(12): 1.5; S(13): 95.5; S(15): 1.5; T(16): 1.5	2	4	1	0	N-Term(TMT6plex); S13(Phospho)
66346693	NP_005236.2	2195	FAT1	protocadherin Fat 1 precursor	S4473	-	-	-	-	1.0	1.3	1.1	1.1	-	-	-	-	0.8	1.1	1.0	1.0	0.8	1.1	1.0	1.0	DMPAAGsLGSSSR	RDMPAAGsLGSSSRN	S7;	Yes	S(7): 100.0; S(10): 0.0; S(11): 0.0; S(12): 0.0	1	1	1	0	N-Term(TMT6plex); M2(Oxidation); S7(Phospho)
148612816	NP_004250.4	9400	RECQL5	ATP-dependent DNA helicase Q5 isoform1	S858	-	-	-	-	1.2	1.3	1.0	1.1	-	-	-	-	1.2	1.3	1.0	1.1	1.2	1.3	1.0	1.1	sQQENPESQPQK	WKGKRPRsQQENPES	S1;	No	S(1): 100.0; S(8): 0.0	1	1	1	0	N-Term(TMT6plex); S1(Phospho); K12(TMT6plex)
116256489	NP_006631.2	10801	40057	septin-9 isoform c	S314	-	-	-	-	1.3	1.0	1.1	1.1	-	-	-	-	1.3	1.1	1.1	1.1	1.3	1.1	1.1	1.1	SVQPTsEER	RKSVQPTsEERIPKT	S6;	No	S(1): 0.0; T(5): 0.8; S(6): 99.2	1	6	1	0	N-Term(TMT6plex); S6(Phospho)
19923969	NP_612451.1	115098	CCDC124	coiled-coil domain-containing protein 124	T116	-	-	-	-	0.5	0.8	0.7	1.1	-	-	-	-	0.6	0.8	0.9	1.3	0.6	0.8	0.9	1.3	EAPDtAEK	QLREAPDtAEKAKSH	T5;	No	T(5): 100.0	2	1	1	0	N-Term(TMT6plex); T5(Phospho); K8(TMT6plex)
24308213	NP_065806.1	57515	SERINC1	serine incorporator 1 precursor	S364	-	-	-	-	3.6	2.5	1.9	1.1	-	-	-	-	3.5	2.7	1.9	1.1	3.5	2.7	1.9	1.1	SDGsLEDGDDVHR	GGARSDGsLEDGDDV	S4;	Yes	S(1): 1.0; S(4): 99.0	4	1	1	0	N-Term(TMT6plex); S4(Phospho)
4505939	NP_000928.1	5430	POLR2A	DNA-directed RNA polymerase II subunit RPB1	T1894	-	-	-	-	1.3	1.4	1.3	1.1	-	-	-	-	1.3	1.4	1.4	1.1	1.3	1.4	1.4	1.1	YSPTSPtYSPTSPVYTPTSPK	KYSPTSPtYSPTSPV	T7;	Yes	Y(1): 0.8; S(2): 33.7; T(4): 33.7; S(5): 33.7; T(7): 89.6; Y(8): 2.1; S(9): 2.1; T(11): 2.1; S(12): 2.1; Y(15): 0.1; T(16): 0.0; T(18): 0.0; S(19): 0.0	4	1	1	0	N-Term(TMT6plex); S5(Phospho); T7(Phospho); K21(TMT6plex)
19923777	NP_006469.2	10634	GAS2L1	GAS2-like protein 1 isoform a	T334	-	-	-	-	1.0	1.1	1.3	1.1	-	-	-	-	0.9	1.2	1.2	1.0	0.9	1.2	1.2	1.0	EGPEtPPRPR	STKEGPEtPPRPRDQ	T5;	Yes	T(5): 100.0	2	1	1	0	N-Term(TMT6plex); T5(Phospho)
211971038	NP_001130027.1	9874	TLK1	serine/threonine-protein kinase tousled-like 1 isoform	S38	-	-	-	-	1.8	1.4	1.3	1.1	-	-	-	-	1.9	1.4	1.4	1.1	1.9	1.4	1.4	1.1	KAENQNESsQGK	AENQNESsQGKSIGG	S9;	No	S(8): 1.1; S(9): 98.9	2	3	1	1	N-Term(TMT6plex); K1(TMT6plex); S9(Phospho); K12(TMT6plex)
109637779	NP_001035891.1	1058	CENPA	histone H3-like centromeric protein A isoform b	S17; S19	-	-	-	-	0.8	1.2	1.1	1.1	-	-	-	-	-	-	-	-	-	-	-	-	RsPsPTPTPGPSR	PEAPRRRsPSPTPTP;APRRRSPsPTPTPGP	S2; S4;	Yes;Yes	S(2): 100.0; S(4): 100.0; T(6): 0.0; T(8): 0.0; S(12): 0.0	1	2	1	1	N-Term(TMT6plex); S2(Phospho); S4(Phospho)
237681121	NP_009228.2	672	BRCA1	breast cancer type 1 susceptibility protein isoform 3	S1192	-	-	-	-	1.5	1.5	1.3	1.1	-	-	-	-	1.4	1.4	1.3	1.1	1.4	1.4	1.3	1.1	VNNIPsQSTR	GKVNNIPsQSTRHST	S6;	No	S(6): 98.0; S(8): 1.0; T(9): 1.0	1	3	1	0	N-Term(TMT6plex); S6(Phospho)
28274701	NP_665805.2	118472	ZNF511	zinc finger protein 511	S185	-	-	-	-	1.0	1.0	1.2	1.1	-	-	-	-	-	-	-	-	-	-	-	-	sPASAEAPGDSGER	DKPKKSRsPASAEAP	S1;	Yes	S(1): 100.0; S(4): 0.0; S(11): 0.0	1	1	1	0	N-Term(TMT6plex); S1(Phospho)
410442515	NP_001258558.1	4209	MEF2D	myocyte-specific enhancer factor 2D isoform 2	S437	-	-	-	-	0.8	1.1	1.1	1.1	-	-	-	-	-	-	-	-	-	-	-	-	SEPVsPSR	SIKSEPVsPSRERSP	S5;	No	S(1): 0.0; S(5): 100.0; S(7): 0.0	1	2	1	0	N-Term(TMT6plex); S5(Phospho)
338753358	NP_001229707.1	3551	IKBKB	inhibitor of nuclear factor kappa-B kinase subunit beta isoform5	S613	-	-	-	-	0.9	1.2	1.4	1.1	-	-	-	-	0.8	1.0	1.2	0.9	0.8	1.0	1.2	0.9	GPVSGsPDSMNASR	VRGPVSGsPDSMNAS	S6;	No	S(4): 1.7; S(6): 98.3; S(9): 0.0; S(13): 0.0	1	3	1	0	N-Term(TMT6plex); S6(Phospho); M10(Oxidation)
291621630	NP_001167579.1	1E+08	ZBED6	zinc finger BED domain-containing protein 6	S381	-	-	-	-	1.1	1.5	1.7	1.1	-	-	-	-	-	-	-	-	-	-	-	-	SEsPIPVAEQTLMR	EVENRSEsPIPVAEQ	S3;	No	S(1): 1.3; S(3): 98.7; T(12): 0.0	2	1	1	0	N-Term(TMT6plex); S3(Phospho); M14(Oxidation)
367460087	NP_005955.3	4628	MYH10	myosin-10 isoform2	S1952	-	-	-	-	0.9	0.9	1.1	1.1	-	-	-	-	0.9	0.9	1.1	1.0	0.9	0.9	1.1	1.0	QLHLEGAsLELSDDDTESK	QLHLEGAsLELSDDD	S8;	Yes	S(8): 100.0; S(12): 49.1; T(16): 49.1; S(18): 1.8	2	3	1	0	N-Term(TMT6plex); S8(Phospho); S12(Phospho); K19(TMT6plex)
115527092	NP_077012.2	79089	TMUB2	transmembrane and ubiquitin-like domain-containing protein 2 isoform a	S132	-	-	-	-	1.0	0.9	0.8	1.1	-	-	-	-	0.9	0.9	0.7	1.1	0.9	0.9	0.7	1.1	QAGAGsSSPEAPLR	KRQAGAGsSSPEAPL	S6;	No	S(6): 98.9; S(7): 1.1; S(8): 0.0	1	2	1	0	N-Term(TMT6plex); S7(Phospho)
61676188	NP_113584.3	10075	HUWE1	E3 ubiquitin-protein ligase HUWE1	S2918	-	-	-	-	1.6	1.2	1.2	1.1	-	-	-	-	1.7	1.2	1.3	1.3	1.7	1.2	1.3	1.3	SDGSGESAQPPEDSsPPASSESSSTR	AQPPEDSsPPASSES	S15;	Yes	S(1): 0.0; S(4): 0.0; S(7): 0.0; S(14): 1.9; S(15): 98.1; S(19): 0.0; S(20): 0.0; S(22): 0.0; S(23): 0.0; S(24): 0.0; T(25): 0.0	2	1	1	0	N-Term(TMT6plex); S15(Phospho)
7662010	NP_055458.1	9658	ZNF516	zinc finger protein 516	S116	-	-	-	-	0.9	0.9	1.0	1.1	-	-	-	-	-	-	-	-	-	-	-	-	ASEGLDACAsPTK	EGLDACAsPTKSASA	S10;	Yes	S(2): 0.0; S(10): 100.0; T(12): 0.0	2	1	1	0	N-Term(TMT6plex); C8(Carbamidomethyl); S10(Phospho); K13(TMT6plex)
7661742	NP_054766.1	28974	C19orf53	leydig cell tumor 10 kDa protein homolog	T97	-	-	-	-	0.7	0.9	0.7	1.1	-	-	-	-	1.2	1.3	1.2	1.6	1.2	1.3	1.2	1.6	GAAAATSSKtPS	AAATSSKtPS_	T10;	No	T(6): 0.0; S(7): 0.0; S(8): 0.0; T(10): 100.0; S(12): 0.0	2	1	1	1	N-Term(TMT6plex); K9(TMT6plex); T10(Phospho)
134948605	NP_001077094.1	23253	ANKRD12	ankyrin repeat domain-containing protein 12 isoform2	S126	-	-	-	-	0.7	1.8	1.5	1.1	-	-	-	-	-	-	-	-	-	-	-	-	DNsPDSTPNHPSQTTPAQK	QMTARDNsPDSTPNH	S3;	No	S(3): 99.9; S(6): 0.0; T(7): 0.0; S(12): 0.0; T(14): 0.0; T(15): 0.0	1	2	1	0	N-Term(TMT6plex); S3(Phospho); K19(TMT6plex)
296278202	NP_001171721.1	56288	PARD3	partitioning defective 3 homolog isoform9	S383	-	-	-	-	0.9	1.3	1.2	1.1	-	-	-	-	0.8	1.3	1.1	1.0	0.8	1.3	1.1	1.0	FsPDSQYIDNR	NYYSSRFsPDSQYID	S2;	Yes	S(2): 100.0; S(5): 0.0; Y(7): 0.0	1	11	1	0	N-Term(TMT6plex); S2(Phospho)
256818798	NP_001157974.1	56478	EIF4ENIF1	eukaryotic translation initiation factor 4E transporter isoform b	S389	-	-	-	-	1.0	1.0	0.9	1.1	-	-	-	-	1.0	0.9	1.0	1.0	1.0	0.9	1.0	1.0	APsPPLSQVFQTR	LLGQRAPsPPLSQVF	S3;	No	S(3): 100.0; S(7): 0.0; T(12): 0.0	2	2	1	0	N-Term(TMT6plex); S3(Phospho)
578846010	XP_006726690.1	1.01E+08	LOC10106027	nuclear pore complex-interacting protein family member B4-like isoform X4	S911	-	-	-	-	1.1	1.1	1.1	1.1	-	-	-	-	-	-	-	-	-	-	-	-	AADVEPSsPEPK	AADVEPSsPEPKRRR	S8;	No	S(7): 0.0; S(8): 100.0	1	8	1	0	N-Term(TMT6plex); S8(Phospho); K12(TMT6plex)
573459761	NP_001275964.1	89953	KLC4	kinesin light chain 4 isoform d	S383	-	-	-	-	0.9	1.1	0.8	1.1	-	-	-	-	0.8	1.1	0.7	1.0	0.8	1.1	0.7	1.0	VSsPTVNTTLR	YKACKVSsPTVNTTL	S3;	No	S(2): 1.2; S(3): 98.8; T(5): 0.0; T(8): 0.0; T(9): 0.0	1	3	1	0	N-Term(TMT6plex); S3(Phospho)
13491166	NP_055491.1	9698	PUM1	pumilio homolog 1 isoform 2	S247	-	-	-	-	1.3	1.4	1.3	1.1	-	-	-	-	1.2	1.4	1.3	1.0	1.2	1.4	1.3	1.0	DADsDENDKGEK	EGPRDADsDENDKGE	S4;	No	S(4): 100.0	1	2	1	1	N-Term(TMT6plex); S4(Phospho); K9(TMT6plex); K12(TMT6plex)
153945728	NP_005900.2	4131	MAP1B	microtubule-associated protein 1B	S970	-	-	-	-	1.2	1.5	1.7	1.1	-	-	-	-	0.9	1.1	1.4	1.1	0.9	1.1	1.4	1.1	HsPTEDEESAK	CVSASKHsPTEDEES	S2;	Yes	S(2): 98.9; T(4): 1.1; S(9): 0.0	4	1	1	0	N-Term(TMT6plex); S2(Phospho); K11(TMT6plex)
19923592	NP_073613.2	114885	OSBPL11	oxysterol-binding protein-related protein 11	S181	-	-	-	-	1.1	1.1	1.0	1.1	-	-	-	-	0.9	1.0	1.1	1.0	0.9	1.0	1.1	1.0	SFSLASSSNsPISQR	SLASSSNsPISQRRP	S10;	Yes	S(1): 0.0; S(3): 0.0; S(6): 0.0; S(7): 0.0; S(8): 0.0; S(10): 100.0; S(13): 0.0	2	1	1	0	N-Term(TMT6plex); S10(Phospho)
40807477	NP_109590.3	10743	RAI1	retinoic acid-induced protein 1	S1108	-	-	-	-	1.1	0.5	1.0	1.1	-	-	-	-	1.2	0.6	1.1	1.1	1.2	0.6	1.1	1.1	AASsPSNPAALPVASDSSPMGSK	PSPKAASsPSNPAAL	S4;	Yes	S(3): 2.3; S(4): 97.6; S(6): 0.1; S(15): 33.3; S(17): 33.3; S(18): 33.3; S(22): 0.0	2	1	1	0	N-Term(TMT6plex); S6(Phospho); S15(Phospho); M20(Oxidation); K23(TMT6plex)
19913396	NP_599031.1	6525	SMTN	smoothelin isoform b	S277	-	-	-	-	1.0	1.3	1.3	1.1	-	-	-	-	0.8	1.0	1.0	0.9	0.8	1.0	1.0	0.9	ETPAAQsPTR	KETPAAQsPTRGPSD	S7;	Yes	T(2): 0.0; S(7): 100.0; T(9): 0.0	3	5	1	0	N-Term(TMT6plex); S7(Phospho)
7661950	NP_055448.1	9646	CTR9	RNA polymerase-associated protein CTR9 homolog	S970	-	-	-	-	1.3	1.2	1.2	1.1	-	-	-	-	1.3	1.2	1.2	1.1	1.3	1.2	1.2	1.1	GEEGsDDDETENGPKPK	HPKGEEGsDDDETEN	S5;	Yes	S(5): 100.0; T(10): 0.0	5	1	1	0	N-Term(TMT6plex); S5(Phospho); K15(TMT6plex); K17(TMT6plex)
15811782	NP_056513.2	27238	GPKOW	G patch domain and KOW motifs-containing protein	S42	-	-	-	-	1.3	1.2	1.3	1.1	-	-	-	-	1.5	1.2	1.5	1.1	1.5	1.2	1.5	1.1	LADSGDGAGPsPEEK	SGDGAGPsPEEKDFL	S11;	Yes	S(4): 0.0; S(11): 100.0	1	1	1	0	N-Term(TMT6plex); S11(Phospho); K15(TMT6plex)
312434011	NP_001186084.1	11143	KAT7	histone acetyltransferase KAT7 isoform2	S52	-	-	-	-	0.8	0.9	0.8	1.1	-	-	-	-	0.7	0.8	0.7	1.0	0.7	0.8	0.7	1.0	LSQsSQDSSPVR	SSARLSQsSQDSSPV	S4;	Yes	S(2): 1.6; S(4): 95.2; S(5): 0.0; S(8): 1.6; S(9): 1.6	3	3	1	0	N-Term(TMT6plex); S9(Phospho)
103472005	NP_002408.3	4288	MKI67	antigen KI-67 isoform1	T694	-	-	-	-	0.6	0.7	0.7	1.1	-	-	-	-	0.7	0.7	0.7	1.1	0.7	0.7	0.7	1.1	RPAtPK	KRQRRPAtPKKPVGE	T4;	Yes	T(4): 100.0	1	2	1	0	N-Term(TMT6plex); T4(Phospho); K6(TMT6plex)
118572599	NP_037373.2	29109	FHOD1	FH1/FH2 domain-containing protein 1	S498	-	-	-	-	0.8	0.8	1.1	1.1	-	-	-	-	0.8	0.8	1.2	1.0	0.8	0.8	1.2	1.0	TPQsPAPCVLLR	PAARTPQsPAPCVLL	S4;	Yes	T(1): 0.0; S(4): 100.0	1	1	1	0	N-Term(TMT6plex); S4(Phospho); C8(Carbamidomethyl)
34996489	NP_060404.3	55023	PHIP	PH-interacting protein	S911	-	-	-	-	0.8	1.3	1.2	1.1	-	-	-	-	0.8	1.2	1.1	1.0	0.8	1.2	1.1	1.0	VNEEKDGPIsPK	EEKDGPIsPKKKKPK	S10;	Yes	S(10): 100.0	1	1	1	1	N-Term(TMT6plex); K5(TMT6plex); S10(Phospho); K12(TMT6plex)
116008442	NP_055885.3	23091	ZC3H13	zinc finger CCCH domain-containing protein 13	S643	-	-	-	-	1.0	1.0	0.9	1.1	-	-	-	-	1.0	0.9	0.9	1.0	1.0	0.9	0.9	1.0	DQRPSsPIR	ERDQRPSsPIRHQGR	S6;	Yes	S(5): 1.0; S(6): 99.0	1	1	1	0	N-Term(TMT6plex); S6(Phospho)
14790190	NP_055816.2	23013	SPEN	msx2-interacting protein	T1946	-	-	-	-	1.3	1.1	0.8	1.1	-	-	-	-	1.2	1.0	0.8	1.0	1.2	1.0	0.8	1.0	ELQEAAAVPtTPRR	QEAAAVPtTPRRGRP	T10;	No	T(10): 99.1; T(11): 0.9	1	1	1	1	N-Term(TMT6plex); T10(Phospho)
112382226	NP_001036147.1	473	RERE	arginine-glutamic acid dipeptide repeats protein	S40	-	-	-	-	1.0	1.0	1.3	1.1	-	-	-	-	-	-	-	-	-	-	-	-	QPAsPDGR	GRKKQPAsPDGRTSP	S4;	No	S(4): 100.0	1	2	1	0	N-Term(TMT6plex); S4(Phospho)
190343008	NP_004501.3	3431	SP110	sp110 nuclear body protein isoform b	S256	-	-	-	-	0.9	1.6	1.5	1.1	-	-	-	-	0.9	1.3	1.1	1.0	0.9	1.3	1.1	1.0	DNsPEPNDPEEPQEVSSTPSDK	MPEIRDNsPEPNDPE	S3;	Yes	S(3): 100.0; S(16): 0.0; S(17): 0.0; T(18): 0.0; S(20): 0.0	3	4	1	0	N-Term(TMT6plex); S3(Phospho); K22(TMT6plex)
5174457	NP_006092.1	10403	NDC80	kinetochore protein NDC80 homolog	S5	-	-	-	-	0.7	0.9	1.0	1.1	-	-	-	-	0.7	0.9	1.0	1.1	0.7	0.9	1.0	1.1	RSsVSSGGAGR	MKRSsVSSGGAG	S3;	Yes	S(2): 1.1; S(3): 98.9; S(5): 0.0; S(6): [illegible]	1	1	1	1	N-Term(TMT6plex); S3(Phospho)
50962882	NP_001002909.1	23131	GPATCH8	G patch domain-containing protein 8	S738	-	-	-	-	1.0	1.1	1.1	1.1	-	-	-	-	1.0	1.1	1.1	1.0	1.0	1.1	1.1	1.0	GPKPEPPGsGSPAPPR	PKPEPPGsGSPAPPR	S9;	Yes	S(9): 98.7; S(11): 1.3	2	1	1	0	N-Term(TMT6plex); K3(TMT6plex); S9(Phospho)
237681091	NP_001153702.1	104	ADARB1	double-stranded RNA-specific editase 1 isoform 7	S26	-	-	-	-	1.0	1.0	1.1	1.1	-	-	-	-	1.0	1.0	1.1	1.1	1.0	1.0	1.1	1.1	NLDNVsPK	NRNLDNVsPKDGSTP	S6;	Yes	S(6): 100.0	3	4	1	0	N-Term(TMT6plex); S6(Phospho); K8(TMT6plex)
115529449	NP_001070152.1	7343	UBTF	nucleolar transcription factor 1 isoform b	S447	-	-	-	-	0.7	0.9	0.7	1.1	-	-	-	-	0.9	1.0	0.9	1.1	0.9	1.0	0.9	1.1	GKLPEsPK	ERGKLPEsPKRAEEI	S6;	No	S(6): 100.0	1	2	1	1	N-Term(TMT6plex); K2(TMT6plex); S6(Phospho); K8(TMT6plex)
134152708	NP_060481.3	55082	ARGLU1	arginine and glutamate-rich protein 1	S60	-	-	-	-	1.0	1.0	1.1	1.1	-	-	-	-	1.1	1.0	1.1	1.0	1.1	1.0	1.1	1.0	sTNTAVSR	ESRSRSRsTNTAVSR	S1;	Yes	S(1): 100.0; T(2): 0.0; T(4): 0.0; S(7): 0.0	1	1	1	0	N-Term(TMT6plex); S1(Phospho)
21450761	NP_659467.1	221908	PPP1R35	protein phosphatase 1 regulatory subunit 35	S52	-	-	-	-	1.3	1.9	1.4	1.1	-	-	-	-	-	-	-	-	-	-	-	-	APVPEPGLDLSLSPRPDsPQPR	SLSPRPDsPQPRHGS	S18;	Yes	S(11): 0.0; S(13): 0.0; S(18): 100.0	2	1	1	0	N-Term(TMT6plex); S18(Phospho)
23199998	NP_683759.1	55695	NSUN5	putative methyltransferase NSUN5 isoform1	T462	-	-	-	-	2.7	1.0	0.8	1.1	-	-	-	-	2.7	0.9	0.8	1.0	2.7	0.9	0.8	1.0	AAAGACtPPCT	RAAAGACtPPCT	T7;	Yes	T(7): 100.0; T(11): 0.0	1	1	1	0	N-Term(TMT6plex); C6(Carbamidomethyl); T7(Phospho); C10(Carbamidomethyl)
188497632	NP_071768.3	64224	HERPUD2	homocysteine-responsive endoplasmic reticulum-resident ubiquitin-like domain member 2 protein	T90	-	-	-	-	1.1	1.0	1.1	1.1	-	-	-	-	-	-	-	-	-	-	-	-	tPPSSPK	HLVCTSRtPPSSPKS	T1;	Yes	T(1): 100.0; S(4): 0.0; S(5): 0.0	2	1	1	0	N-Term(TMT6plex); T1(Phospho); K7(TMT6plex)
149158690	NP_542417.2	7916	PRRC2A	protein PRRC2A	T1108	-	-	-	-	0.8	1.0	0.9	1.1	-	-	-	-	0.9	0.9	1.0	1.0	0.9	0.9	1.0	1.0	GSEtGSETHESDLAPSDK	RRQRGSEtGSETHES	T4;	Yes	S(2): 0.0; T(4): 98.7; S(6): 1.2; T(8): 0.0; S(11): 0.0; S(16): 0.0	4	1	1	0	N-Term(TMT6plex); T4(Phospho); K18(TMT6plex)
134152667	NP_060224.3	54887	UHRF1BP1	UHRF1-binding protein 1	S1106	-	-	-	-	1.1	1.2	1.3	1.1	-	-	-	-	0.9	1.1	1.4	0.9	0.9	1.1	1.4	0.9	TVSQQsFDGVSLDSSGPEDR	KRTVSQQsFDGVSLD	S6;	Yes	T(1): 0.0; S(3): 0.0; S(6): 100.0; S(11): 0.0; S(14): 0.0; S(15): 0.0	1	1	1	0	N-Term(TMT6plex); S6(Phospho)
15812226	NP_277037.1	55809	TRERF1	transcriptional-regulating factor 1	S491	-	-	-	-	0.6	1.0	0.7	1.1	-	-	-	-	-	-	-	-	-	-	-	-	AQPGsPESSGQPK	DGRAQPGsPESSGQP	S5;	Yes	S(5): 96.9; S(8): 1.5; S(9): 1.5	1	1	1	0	N-Term(TMT6plex); S5(Phospho); K13(TMT6plex)
188497705	NP_006759.3	8315	BRAP	BRCA1-associated protein	S97	-	-	-	-	0.7	0.7	1.2	1.1	-	-	-	-	-	-	-	-	-	-	-	-	SSEAsPTAQR	ERKSSEAsPTAQRSK	S5;	Yes	S(1): 0.0; S(2): 0.0; S(5): 100.0; T(7): 0.0	2	1	1	0	N-Term(TMT6plex); S5(Phospho)
44890068	NP_005086.2	9202	ZMYM4	zinc finger MYM-type protein 4	S122	-	-	-	-	0.9	0.8	1.0	1.1	-	-	-	-	0.9	0.8	1.0	1.0	0.9	0.8	1.0	1.0	VTQHEsDNENEIQIQNK	RRVTQHEsDNENEIQ	S6;	Yes	T(2): 0.0; S(6): 100.0	2	1	1	0	N-Term(TMT6plex); S6(Phospho); K17(TMT6plex)
556695389	NP_001273359.1	123169	LEO1	RNA polymerase-associated protein LEO1 isoform2	S229; S238	-	-	-	-	0.8	0.9	1.0	1.1	-	-	-	-	0.9	1.1	1.2	1.3	0.9	1.1	1.2	1.3	QNsDDEEQPQLsDEEK	NDDEKQNsDDEEQPQ;DEEQPQLsDEEKMQN	S3; S12;	Yes;Yes	S(3): 100.0; S(12): 100.0	2	2	1	0	N-Term(TMT6plex); S3(Phospho); S12(Phospho); K16(TMT6plex)
188497637	NP_001120843.1	55536	CDCA7L	cell division cycle-associated 7-like protein isoform3	S215	-	-	-	-	0.6	0.9	1.1	1.1	-	-	-	-	-	-	-	-	-	-	-	-	AEsEGQITR	KTVRRAEsEGQITRR	S3;	No	S(3): 100.0; T(8): 0.0	1	3	1	0	N-Term(TMT6plex); S3(Phospho)
46370071	NP_997006.1	2549	GAB1	GRB2-associated-binding protein 1 isoform a	S383	-	-	-	-	1.2	1.8	1.3	1.0	-	-	-	-	-	-	-	-	-	-	-	-	TASDTDSSYCIPTAGMSPsR	PTAGMSPsRSNTIST	S19;	Yes	T(1): 0.0; S(3): 0.0; T(5): 0.0; S(7): 0.0; S(8): 0.0; Y(9): 0.0; T(13): 0.1; S(17): 2.8; S(19): 97.2	1	2	1	0	N-Term(TMT6plex); C10(Carbamidomethyl); M16(Oxidation); S17(Phospho)
109948292	NP_065905.2	57614	KIAA1468	lisH domain and HEAT repeat-containing protein KIAA1468	S170	-	-	-	-	0.9	0.8	0.7	1.0	-	-	-	-	0.8	0.7	0.7	1.0	0.8	0.7	0.7	1.0	EPSTAsGGGQLNR	GREPSTAsGGGQLNR	S6;	Yes	S(3): 0.0; T(4): 0.0; S(6): 100.0	2	1	1	0	N-Term(TMT6plex); S6(Phospho)
12667788	NP_002464.1	4627	MYH9	myosin-9	S1714	-	-	-	-	0.9	1.4	1.4	1.0	-	-	-	-	0.7	1.2	1.1	1.0	0.7	1.2	1.1	1.0	DELADEIANSsGK	ADEIANSsGKGALAL	S11;	Yes	S(10): 0.7; S(11): 99.3	3	1	1	0	N-Term(TMT6plex); S11(Phospho); K13(TMT6plex)
13236585	NP_077309.1	79187	FSD1	fibronectin type III and SPRY domain-containing protein 1	S494	-	-	-	-	1.1	1.0	0.7	1.0	-	-	-	-	1.2	1.1	0.7	1.1	1.2	1.1	0.7	1.1	GSATSSSNTsLT	ATSSSNTsLT_	S10;	Yes	S(2): 0.0; T(4): 0.0; S(5): 0.0; S(6): 1.3; S(7): 1.3; T(9): 1.3; S(10): 94.9; T(12): 1.3	2	1	1	0	N-Term(TMT6plex); T12(Phospho)
194097489	NP_015559.2	8498	RANBP3	ran-binding protein 3 isoform RANBP3-b	S265	-	-	-	-	1.2	1.2	1.0	1.0	-	-	-	-	1.4	1.6	1.3	1.4	1.4	1.6	1.3	1.4	VLsPPKLNEVSSDANR	NMSERVLsPPKLNEV	S3;	No	S(3): 99.9; S(11): 0.1; S(12): 0.1	1	3	1	1	N-Term(TMT6plex); S3(Phospho); K6(TMT6plex)
51173873	NP_001003681.1	10042	HMGXB4	HMG domain-containing protein 4	S512	-	-	-	-	1.0	1.1	2.6	1.0	-	-	-	-	1.0	0.8	2.4	0.8	1.0	0.8	2.4	0.8	SPPTTMLLPAsPAK	TTMLLPAsPAKAPET	S11;	Yes	S(1): 0.0; T(4): 0.0; T(5): 0.0; S(11): 100.0	5	1	1	0	N-Term(TMT6plex); M6(Oxidation); S11(Phospho); K14(TMT6plex)
7661998	NP_055559.1	9779	TBC1D5	TBC1 domain family member 5 isoform b	S539	-	-	-	-	1.0	0.8	1.0	1.0	-	-	-	-	0.9	0.8	0.9	1.0	0.9	0.8	0.9	1.0	NISSsPSVESLPGGR	SSKNISSsPSVESLP	S5;	Yes	S(3): 0.0; S(4): 1.1; S(5): 98.9; S(7): 0.0; S(10): 0.0	3	2	1	0	N-Term(TMT6plex); S5(Phospho)
557878731	NP_001273615.1	26586	CKAP2	cytoskeleton-associated protein 2 isoform3	T634	-	-	-	-	1.1	1.2	1.3	1.0	-	-	-	-	1.1	1.2	1.4	1.1	1.1	1.2	1.4	1.1	VYYEADTt	VYYEADTt_	T8;	No	Y(2): 0.0; Y(3): 0.0; T(7): 0.0; T(8): 100.0	2	3	1	0	N-Term(TMT6plex); T8(Phospho)
332205965	NP_001193775.1	259266	ASPM	abnormal spindle-like microcephaly-associated protein isoform2	T563	-	-	-	-	0.6	0.9	0.9	1.0	-	-	-	-	0.6	0.9	0.8	1.0	0.6	0.9	0.8	1.0	NEVtPSSTTASVAR	KSYKNEVtPSSTTAS	T4;	Yes	T(4): 99.8; S(6): 0.0; S(7): 0.0; T(8): 0.1; T(9): 0.1; S(11): 0.1	1	2	1	0	N-Term(TMT6plex); T4(Phospho)
20127519	NP_036244.2	22974	TPX2	targeting protein for Xklp2	S292	-	-	-	-	0.7	0.8	0.9	1.0	-	-	-	-	0.8	0.8	0.9	1.0	0.8	0.8	0.9	1.0	KHPsSPAR	SELRKHPsSPARVTK	S4;	Yes	S(4): 99.3; S(5): 0.7	1	1	1	1	N-Term(TMT6plex); K1(TMT6plex); S4(Phospho)
154813195	NP_056504.3	27147	DENND2A	DENN domain-containing protein 2A	S504	-	-	-	-	1.3	2.1	1.6	1.0	-	-	-	-	1.3	2.0	1.5	0.9	1.3	2.0	1.5	0.9	RLsQSMESNSGK	KKRVKRLsQSMESNS	S3;	Yes	S(3): 100.0; S(5): 0.0; S(8): 0.0; S(10): 0.0	1	1	1	1	N-Term(TMT6plex); S3(Phospho); M6(Oxidation); K12(TMT6plex)
314122233	NP_001186627.1	7917	BAG6	large proline-rich protein BAG6 isoform d	S113	-	-	-	-	1.5	2.2	2.1	1.0	-	-	-	-	1.6	2.4	2.4	1.1	1.6	2.4	2.4	1.1	APPQTHLPSGASSGTGSASATHGGGsPPGTR	SATHGGGsPPGTRGP	S26;	Yes	T(5): 0.0; S(9): 0.1; S(12): 0.1; S(13): 0.1; T(15): 0.1; S(17): 0.1; S(19): 0.1; T(21): 0.1; S(26): 96.8; T(30): 2.6	2	4	1	0	N-Term(TMT6plex); T21(Phospho)
7656879	NP_055238.1	27125	AFF4	AF4/FMR2 family member 4	S222	-	-	-	-	1.1	1.2	0.9	1.0	-	-	-	-	1.0	1.0	0.9	1.0	1.0	1.0	0.9	1.0	DPDANWDsPSR	DPDANWDsPSRVPFS	S8;	Yes	S(8): 100.0; S(10): 0.0	3	1	1	0	N-Term(TMT6plex); S8(Phospho)
333440451	NP_001193973.1	23122	CLASP2	CLIP-associating protein 2 isoform 2	S459	-	-	-	-	1.3	0.8	1.0	1.0	-	-	-	-	1.2	0.8	1.1	1.1	1.2	0.8	1.1	1.1	MVSQSQPGsR	VSQSQPGsRSGSPGR	S9;	Yes	S(3): 0.0; S(5): 0.0; S(9): 100.0	2	2	1	0	N-Term(TMT6plex); M1(Oxidation); S9(Phospho)
153070822	NP_056321.2	25974	MMACHC	methylmalonic aciduria and homocystinuria type C protein	S275; S279	-	-	-	-	0.8	0.9	0.6	1.0	-	-	-	-	-	-	-	-	-	-	-	-	VsPPAsPGP	SWLSPRVsPPASPGP;PRVSPPAsPGP	S2; S6;	Yes;Yes	S(2): 100.0; S(6): 100.0	1	1	1	0	N-Term(TMT6plex); S2(Phospho); S6(Phospho)
325651970	NP_001188388.1	130557	ZNF513	zinc finger protein 513 isoform 2	S478	-	-	-	-	1.1	0.9	0.9	1.0	-	-	-	-	-	-	-	-	-	-	-	-	AVHTDsS	SRAVHTDsS	S6;	No	T(4): 0.0; S(6): 100.0; S(7): 0.0	1	2	1	0	N-Term(TMT6plex); S6(Phospho)
211971074	NP_001130034.1	11217	AKAP2	A-kinase anchor protein 2 isoform 2	S748	-	-	-	-	1.0	1.2	1.3	1.0	-	-	-	-	-	-	-	-	-	-	-	-	QVLQSTQsPR	QVLQSTQsPRTKNAP	S8;	Yes	S(5): 0.0; T(6): 0.7; S(8): 99.3	5	5	1	0	N-Term(TMT6plex); S8(Phospho)
283436133	NP_001164433.1	3547	IGSF1	immunoglobulin superfamily member 1 isoform4	S1285	-	-	-	-	1.3	1.8	2.0	1.0	-	-	-	-	1.1	1.1	1.3	0.9	1.1	1.1	1.3	0.9	TRGsETDGR	PRLRTRGsETDGRDQ	S4;	No	T(1): 0.0; S(4): 100.0; T(6): 0.0	1	3	1	1	N-Term(TMT6plex); S4(Phospho)
374532779	NP_001243425.1	4123	MAN2C1	alpha-mannosidase 2C1 isoform4	S381	-	-	-	-	0.9	1.0	0.9	1.0	-	-	-	-	0.8	0.9	0.8	0.8	0.8	0.9	0.8	0.8	LsNTDGLPR	LDRLKRLsNTDGLPR	S2;	No	S(2): 100.0; T(4): 0.0	1	4	1	0	N-Term(TMT6plex); S2(Phospho)
21361716	NP_060648.2	55204	GOLPH3L	Golgi phosphoprotein 3-like	S38	-	-	-	-	1.2	1.3	1.1	1.0	-	-	-	-	1.1	1.1	1.2	1.0	1.1	1.1	1.2	1.0	SPDNEDsGDSK	KSPDNEDsGDSKDIR	S7;	No	S(1): 0.0; S(7): 100.0; S(10): 0.0	2	1	1	0	N-Term(TMT6plex); S7(Phospho); K11(TMT6plex)
24497447	NP_705931.1	10762	NUP50	nuclear pore complex protein Nup50 isoform a	S193	-	-	-	-	1.2	1.1	0.8	1.0	-	-	-	-	1.2	1.1	0.8	0.9	1.2	1.1	0.8	0.9	VAAETQsPSLFGSTK	KVAAETQsPSLFGST	S7;	No	T(5): 0.0; S(7): 100.0; S(9): 0.0; S(13): 0.0; T(14): 0.0	2	2	1	0	N-Term(TMT6plex); S7(Phospho); K15(TMT6plex)
110815844	NP_115602.2	84186	ZCCHC7	zinc finger CCHC domain-containing protein 7	S482	-	-	-	-	1.0	0.9	0.9	1.0	-	-	-	-	0.9	0.7	0.8	0.8	0.9	0.7	0.8	0.8	TYSsPGSFK	RGPKTYSsPGSFKTQ	S4;	Yes	T(1): 0.0; Y(2): 0.0; S(3): 1.0; S(4): 99.0; S(7): 0.0	1	1	1	0	N-Term(TMT6plex); S4(Phospho); K9(TMT6plex)
301500643	NP_001180394.1	284058	KANSL1	KAT8 regulatory NSL complex subunit 1 isoform2	S249	-	-	-	-	1.1	1.1	1.0	1.0	-	-	-	-	1.0	1.1	1.0	0.8	1.0	1.1	1.0	0.8	LsPGTDSSSNLGGVK	LQGSSRLsPGTDSSS	S2;	Yes	S(2): 100.0; T(5): 0.0; S(7): 0.0; S(8): 0.0; S(9): 0.0	1	2	1	0	N-Term(TMT6plex); S2(Phospho); K15(TMT6plex)
151301053	NP_444271.2	155435	RBM33	RNA-binding protein 33	S205	-	-	-	-	1.2	1.3	1.0	1.0	-	-	-	-	1.2	1.5	1.1	1.1	1.2	1.5	1.1	1.1	DIKEEsDEEEEDDEESGR	QKDIKEEsDEEEEDD	S6;	Yes	S(6): 100.0; S(16): 0.0	1	1	1	1	N-Term(TMT6plex); K3(TMT6plex); S6(Phospho)
27477111	NP_006416.3	10569	SLU7	pre-mRNA-splicing factor SLU7	S515	-	-	-	-	0.7	0.8	0.9	1.0	-	-	-	-	0.8	0.8	0.9	1.0	0.8	0.8	0.9	1.0	SSSDsDDEEK	HRKSSSDsDDEEKKH	S5;	Yes	S(1): 0.0; S(2): 0.0; S(3): 0.7; S(5): [illegible]	1	1	1	0	N-Term(TMT6plex); S5(Phospho); K10(TMT6plex)
267844811	NP_065176.3	89796	NAV1	neuron navigator 1 isoform1	S391	-	-	-	-	1.0	1.4	1.4	1.0	-	-	-	-	-	-	-	-	-	-	-	-	SGYMsDSDLMGK	DLKSGYMsDSDLMGK	S5;	Yes	S(1): 0.0; Y(3): 1.4; S(5): 98.5; S(7): [illegible]	1	1	1	0	N-Term(TMT6plex); M4(Oxidation); S5(Phospho); M10(Oxidation); K12(TMT6plex)
11342676	NP_004231.1	9322	TRIP10	cdc42-interacting protein 4 isoform2	S296	-	-	-	-	0.8	0.7	0.7	1.0	-	-	-	-	0.8	0.9	0.8	1.2	0.8	0.9	0.8	1.2	APsDSSLGTPSDGRPELR	QPMNRAPsDSSLGTP	S3;	Yes	S(3): 100.0; S(5): 50.0; S(6): 50.0; T(9): 0.0; S(11): 0.0	1	3	1	0	N-Term(TMT6plex); S3(Phospho); S5(Phospho)
262399389	NP_001161054.1	10180	RBM6	RNA-binding protein 6 isoform 2	S503	-	-	-	-	1.1	0.9	1.0	1.0	-	-	-	-	1.2	0.9	1.0	1.0	1.2	0.9	1.0	1.0	LQSEDsPER	EKLQSEDsPERKRIK	S6;	No	S(3): 0.0; S(6): 100.0	1	2	1	0	N-Term(TMT6plex); S6(Phospho)
341865562	NP_001230072.1	51203	NUSAP1	nucleolar and spindle-associated protein 1 isoform5	T299	-	-	-	-	1.7	0.6	0.5	1.0	-	-	-	-	2.3	0.7	0.6	1.2	2.3	0.7	0.6	1.2	SAHVTVSGGtPK	HVTVSGGtPKGEAVL	T10;	No	S(1): 0.0; T(5): 0.0; S(7): 0.9; T(10): 99.1	1	5	1	0	N-Term(TMT6plex); T10(Phospho); K12(TMT6plex)
45505149	NP_005588.2	4782	NFIC	nuclear factor 1 C-type isoform5	S192	-	-	-	-	0.7	0.8	0.9	1.0	-	-	-	-	0.8	0.8	1.0	1.1	0.8	0.8	1.0	1.1	DAEQsGSPR	RERDAEQsGSPRTGM	S5;	No	S(5): 99.5; S(7): 0.5	2	5	1	0	N-Term(TMT6plex); S7(Phospho)
13540555	NP_110410.1	81490	PTDSS2	phosphatidylserine synthase 2	S16	-	-	-	-	0.9	0.9	1.0	1.0	-	-	-	-	-	-	-	-	-	-	-	-	DAGGPRPEsPVPAGR	AGGPRPEsPVPAGRA	S9;	Yes	S(9): 100.0	1	1	1	0	N-Term(TMT6plex); S9(Phospho)
429535832	NP_005643.2	7014	TERF2	telomeric repeat-binding factor 2	S40	-	-	-	-	1.0	1.3	1.0	1.0	-	-	-	-	1.0	1.2	1.0	1.0	1.0	1.2	1.0	1.0	RsDTMAGGGGSSDGSGR	GGEGARRsDTMAGGG	S2;	No	S(2): 100.0; T(4): 0.0; S(11): 0.0; S(12): 0.0; S(15): 0.0	2	1	1	1	N-Term(TMT6plex); S2(Phospho); M5(Oxidation)
530424835	XP_005258156.1	23255	SOGA2	protein SOGA2 isoform X2	S1661	-	-	-	-	0.8	1.1	0.9	1.0	-	-	-	-	0.8	1.2	1.0	1.0	0.8	1.2	1.0	1.0	GSGVTSsPHK	RGSGVTSsPHKCLTP	S7;	Yes	S(2): 0.0; T(5): 0.0; S(6): 0.0; S(7): 100.0	1	6	1	0	N-Term(TMT6plex); S7(Phospho); K10(TMT6plex)
39995080	NP_078795.2	79567	FAM65A	protein FAM65A isoform1	S728	-	-	-	-	0.8	1.0	0.8	1.0	-	-	-	-	0.7	1.0	0.8	1.0	0.7	1.0	0.8	1.0	TPHPsPAHSSR	APRTPHPsPAHSSRK	S5;	No	T(1): 0.0; S(5): 100.0; S(9): 0.0; S(10): 0.0	1	4	1	0	N-Term(TMT6plex); S5(Phospho)
82659097	NP_001032358.1	50855	PARD6A	partitioning defective 6 homolog alpha isoform2	S277	-	-	-	-	0.9	0.8	0.5	1.0	-	-	-	-	-	-	-	-	-	-	-	-	LTGPPSAGPGPAEPDsDDDSSDLVIENR	PGPAEPDsDDDSSDL	S16;	No	T(2): 0.2; S(6): 4.2; S(16): 87.2; S(20): 4.2; S(21): 4.2	2	2	1	0	N-Term(TMT6plex); S16(Phospho)
11321642	NP_055392.1	23774	BRD1	bromodomain-containing protein 1	S18	-	-	-	-	1.1	1.1	1.1	1.0	-	-	-	-	1.1	1.3	1.2	1.1	1.1	1.3	1.2	1.1	HPSsPCSVK	SAARHPSsPCSVKHS	S4;	Yes	S(3): 0.7; S(4): 99.3; S(7): 0.0	2	1	1	0	N-Term(TMT6plex); S4(Phospho); C6(Carbamidomethyl); K9(TMT6plex)
68508961	NP_001803.2	1060	CENPC	centromere protein C	S710	-	-	-	-	0.9	1.2	1.0	1.0	-	-	-	-	0.9	1.3	1.0	1.0	0.9	1.3	1.0	1.0	NDVDDEEVHGSsDDSK	DEEVHGSsDDSKQSK	S12;	No	S(11): 1.1; S(12): 98.8; S(15): 0.0	2	1	1	0	N-Term(TMT6plex); S12(Phospho); K16(TMT6plex)
125625324	NP_001074960.1	10482	NXF1	nuclear RNA export factor 1 isoform2	S111	-	-	-	-	1.8	1.9	1.5	1.0	-	-	-	-	1.8	1.8	1.5	1.0	1.8	1.8	1.5	1.0	GGAGTsQDGTSK	ERGGAGTsQDGTSKN	S6;	No	T(5): 0.0; S(6): 100.0; T(10): 0.0; S(11): 0.0	1	2	1	0	N-Term(TMT6plex); S6(Phospho); K12(TMT6plex)
40548322	NP_954976.1	201176	ARHGAP27	rho GTPase-activating protein 27 isoform a	S291	-	-	-	-	0.9	1.0	1.0	1.0	-	-	-	-	-	-	-	-	-	-	-	-	VDEGsSER	SSRVDEGsSERLGSW	S5;	No	S(5): 100.0; S(6): 0.0	2	2	1	0	N-Term(TMT6plex); S5(Phospho)
5032189	NP_005648.1	7158	TP53BP1	tumor suppressor p53-binding protein 1 isoform3	S1665	-	-	-	-	0.9	1.2	1.1	1.0	-	-	-	-	0.9	1.2	1.2	1.2	0.9	1.2	1.2	1.2	ASMGVLsGK	RASMGVLsGKRKLIT	S7;	Yes	S(2): 0.0; S(7): 100.0	1	3	1	0	N-Term(TMT6plex); M3(Oxidation); S7(Phospho); K9(TMT6plex)
17158044	NP_001001.2	6194	RPS6	40S ribosomal protein S6	S244	-	-	-	-	1.7	1.3	0.9	1.0	-	-	-	-	2.0	1.5	1.0	1.2	2.0	1.5	1.0	1.2	sESSQK	LRASTSKsESSQK	S1;	Yes	S(1): 100.0; S(3): 50.0; S(4): 50.0	1	1	1	0	N-Term(TMT6plex); S1(Phospho); S3(Phospho); K6(TMT6plex)
22748853	NP_689613.1	132720	C4orf32	uncharacterized protein C4orf32	S38	-	-	-	-	0.9	1.1	1.1	1.0	-	-	-	-	-	-	-	-	-	-	-	-	EAAGTEWDPGAsPR	TEWDPGAsPRRRGQR	S12;	No	T(5): 0.0; S(12): 100.0	2	1	1	0	N-Term(TMT6plex); S12(Phospho)
14670388	NP_116784.1	572	BAD	bcl2 antagonist of cell death	S134	-	-	-	-	1.6	1.3	1.0	1.0	-	-	-	-	1.5	1.2	0.9	1.0	1.5	1.2	0.9	1.0	sAGTATQMR	KGLPRPKsAGTATQM	S1;	Yes	S(1): 100.0; T(4): 0.0; T(6): 0.0	1	1	1	0	N-Term(TMT6plex); S1(Phospho); M8(Oxidation)
116875765	NP_783297.2	7082	TJP1	tight junction protein ZO-1 isoform b	S175	-	-	-	-	0.9	1.1	1.1	1.0	-	-	-	-	0.8	1.0	1.1	1.1	0.8	1.0	1.1	1.1	sVASSQPAKPTK	SPRSDRRsVASSQPA	S1;	Yes	S(1): 100.0; S(4): 0.0; S(5): 0.0; T(11): 0.0	1	2	1	0	N-Term(TMT6plex); S1(Phospho); K9(TMT6plex); K12(TMT6plex)
295842266	NP_001171500.1	1632	ECI1	enoyl-CoA delta isomerase 1, mitochondrial isoform2 precursor	S90	-	-	-	-	0.8	0.9	1.0	1.0	-	-	-	-	0.7	0.7	0.8	0.8	0.7	0.7	0.8	0.8	sFRGVILTSDRPGVFSAGLDLTEMCGR	EKLENDKsFRGVILT	S1;	No	S(1): 100.0; T(8): 0.0; S(9): 0.0; S(16): 0.0; T(22): 0.0	1	2	1	1	N-Term(TMT6plex); S1(Phospho); M24(Oxidation); C25(Carbamidomethyl)
19913396	NP_599031.1	6525	SMTN	smoothelin isoform b	S304	-	-	-	-	0.8	1.1	1.1	1.0	-	-	-	-	0.6	0.8	0.8	0.9	0.6	0.8	0.8	0.9	SLSVLsPR	QRSLSVLsPRQPAQN	S6;	Yes	S(1): 0.0; S(3): 0.0; S(6): 100.0	2	5	1	0	N-Term(TMT6plex); S6(Phospho)
219842214	NP_001137358.1	4659	PPP1R12A	protein phosphatase 1 regulatory subunit 12A isoform	S608	-	-	-	-	0.9	1.0	1.2	1.0	-	-	-	-	0.8	0.9	1.1	0.9	0.8	0.9	1.1	0.9	sTQGVTLTDLQEAEK	QARQSRRsTQGVTLT	S1;	No	S(1): 98.2; T(2): 1.8; T(6): 0.0; T(8): [illegible]	2	4	1	0	N-Term(TMT6plex); S1(Phospho); K15(TMT6plex)
32528301	NP_004435.3	2050	EPHB4	ephrin type-B receptor 4 precursor	T976	-	-	-	-	0.6	0.9	0.8	1.0	-	-	-	-	0.7	1.0	0.8	1.0	0.7	1.0	0.8	1.0	SQAKPGtPGGTGGPAPQY	KSQAKPGtPGGTGGP	T7;	Yes	S(1): 0.1; T(7): 99.9; T(11): 0.1; Y(18): 0.0	1	1	1	0	N-Term(TMT6plex); K4(TMT6plex); T7(Phospho)
164519084	NP_036329.3	23637	RABGAP1	rab GTPase-activating protein 1	S42	-	-	-	-	1.2	1.2	1.0	1.0	-	-	-	-	1.2	1.1	0.9	0.9	1.2	1.1	0.9	0.9	QGDETPSTNNGsDDEK	TPSTNNGsDDEKTGL	S12;	Yes	T(5): 0.0; S(7): 0.0; T(8): 0.0; S(12): 100.0	2	1	1	0	N-Term(TMT6plex); S12(Phospho); K16(TMT6plex)
5032113	NP_005862.1	10285	SMNDC1	survival of motor neuron-related-splicing factor 30	S201	-	-	-	-	0.3	0.3	0.5	1.0	-	-	-	-	0.4	0.4	0.7	1.2	0.4	0.4	0.7	1.2	SIFAsPESVTGK	VKRSIFAsPESVTGK	S5;	Yes	S(1): 0.0; S(5): 99.4; S(8): 0.6; T(10): 0.0	4	1	1	0	N-Term(TMT6plex); S5(Phospho); K12(TMT6plex)
7661958	NP_055554.1	9774	BCLAF1	bcl-2-associated transcription factor 1 isoform1	S660	-	-	-	-	1.0	0.9	1.1	1.0	-	-	-	-	1.1	1.0	1.2	1.1	1.1	1.0	1.2	1.1	IDISPsTLR	RRIDISPsTLRKHTR	S6;	Yes	S(4): 0.6; S(6): 99.4; T(7): 0.0	8	3	1	0	N-Term(TMT6plex); S4(Phospho)
4503641	NP_001984.1	2152	F3	tissue factor isoform1 precursor	S290	-	-	-	-	1.0	1.3	1.0	1.0	-	-	-	-	1.2	1.4	1.2	1.1	1.2	1.4	1.2	1.1	ENsPLNVS	GQSWKENsPLNVS	S3;	Yes	S(3): 100.0; S(8): 0.0	1	1	1	0	N-Term(TMT6plex); S3(Phospho)
335334962	NP_001229416.1	23053	ZSWIM8	zinc finger SWIM domain-containing protein 8 isoform 2	S53	-	-	-	-	1.2	1.7	1.4	1.0	-	-	-	-	-	-	-	-	-	-	-	-	KQSAGPNsPTGGGGGGGSGGTR	KQSAGPNsPTGGGGG	S8;	Yes	S(3): 0.0; S(8): 100.0; T(10): 0.0; S(18): 0.0; T(21): 0.0	1	3	1	1	N-Term(TMT6plex); K1(TMT6plex); S8(Phospho)
338797736	NP_061994.1	284352	PPP1R37	protein phosphatase 1 regulatory subunit 37	S561	-	-	-	-	0.6	0.8	0.7	1.0	-	-	-	-	-	-	-	-	-	-	-	-	ISVSsPGR	EQRISVSsPGRGHKV	S5;	Yes	S(2): 0.0; S(4): 0.0; S(5): 100.0	2	1	1	0	N-Term(TMT6plex); S5(Phospho)
255652953	NP_653205.3	124245	ZC3H18	zinc finger CCCH domain-containing protein 18	T162	-	-	-	-	0.8	1.0	0.9	1.0	-	-	-	-	1.0	1.1	1.1	1.0	1.0	1.1	1.1	1.0	AGAEDDEEKGEGtPR	DEEKGEGtPREEGKA	T13;	Yes	T(13): 100.0	2	1	1	1	N-Term(TMT6plex); K9(TMT6plex); T13(Phospho)
55953087	NP_036473.2	23560	GTPBP4	nucleolar GTP-binding protein 1	S563	-	-	-	-	0.9	1.2	1.3	1.0	-	-	-	-	1.0	1.3	1.4	1.0	1.0	1.3	1.4	1.0	EDSAPPSsVAR	EDSAPPSsVARSGSC	S8;	Yes	S(3): 0.0; S(7): 1.1; S(8): 98.9	1	1	1	0	N-Term(TMT6plex); S8(Phospho)
42542379	NP_005830.2	10250	SRRM1	serine/arginine repetitive matrix protein 1	T581; S583	-	-	-	-	1.1	1.3	0.9	1.0	-	-	-	-	1.3	1.4	1.0	1.1	1.3	1.4	1.0	1.1	tPsPPPR	TPPPRRRtPSPPPRR;PPRRRTPsPPPRRRS	T1; S3;	Yes;Yes	T(1): 100.0; S(3): 100.0	2	1	1	0	N-Term(TMT6plex); T1(Phospho); S3(Phospho)
386642875	NP_001245379.1	9088	PKMYT1	membrane-associated tyrosine- and threonine-specific cdc2-inhibitory kinase isoform3	T386	-	-	-	-	1.1	0.9	1.4	1.0	-	-	-	-	1.4	1.3	1.9	1.3	1.4	1.3	1.9	1.3	TVGSTStPR	RTVGSTStPRSRCTP	T7;	No	T(1): 0.0; S(4): 1.1; T(5): 1.1; S(6): 1.1; T(7): 96.7	1	3	1	0	N-Term(TMT6plex); T7(Phospho)
171543895	NP_002964.3	6311	ATXN2	ataxin-2	S758	-	-	-	-	0.8	0.9	0.9	1.0	-	-	-	-	0.7	0.8	0.8	0.8	0.7	0.8	0.8	0.8	QNsPAGNK	LQDQRQNsPAGNKEN	S3;	Yes	S(3): 100.0	1	1	1	0	N-Term(TMT6plex); S3(Phospho); K8(TMT6plex)
197927147	NP_001128210.1	604	BCL6	B-cell lymphoma 6 protein isoform2	S361	-	-	-	-	1.1	1.1	1.4	1.0	-	-	-	-	-	-	-	-	-	-	-	-	NACILQASGsPPAK	CILQASGsPPAKSPT	S10;	No	S(8): 1.7; S(10): 98.3	1	2	1	0	N-Term(TMT6plex); C3(Carbamidomethyl); S10(Phospho); K14(TMT6plex)
16306580	NP_036440.1	22992	KDM2A	lysine-specific demethylase 2A isoform a	S28	-	-	-	-	0.3	0.8	1.0	1.0	-	-	-	-	0.3	0.8	0.9	1.0	0.3	0.8	0.9	1.0	YEDDGIsDDEIEGK	RYEDDGIsDDEIEGK	S7;	Yes	Y(1): 0.0; S(7): 100.0	1	1	1	0	N-Term(TMT6plex); S7(Phospho); K14(TMT6plex)
32455269	NP_775846.2	282974	STK32C	serine/threonine-protein kinase 32C	S18	-	-	-	-	0.9	1.1	1.1	1.0	-	-	-	-	-	-	-	-	-	-	-	-	GSSAAASPGsPPPGR	SAAASPGsPPPGRAR	S10;	Yes	S(2): 0.0; S(3): 0.0; S(7): 0.0; S(10): 100.0	2	1	1	0	N-Term(TMT6plex); S10(Phospho)
528078296	NP_001268421.1	2956	MSH6	DNA mismatch repair protein Msh6 isoform2	S97	-	-	-	-	1.2	1.0	1.2	1.0	-	-	-	-	1.1	1.1	1.2	0.9	1.1	1.1	1.2	0.9	SEEDNEIEsEEEVQPK	EEDNEIEsEEEVQPK	S9;	No	S(1): 0.0; S(9): 100.0	3	2	1	0	N-Term(TMT6plex); S9(Phospho); K16(TMT6plex)
75677357	NP_001028733.1	57649	PHF12	PHD finger protein 12 isoform1	T880	-	-	-	-	1.4	0.9	0.9	1.0	-	-	-	-	-	-	-	-	-	-	-	-	TPPtPPSSIVAK	FSEKTPPtPPSSIVA	T4;	Yes	T(1): 1.0; T(4): 99.0; S(7): 0.0; S(8): [illegible]	2	1	1	0	N-Term(TMT6plex); T4(Phospho); K12(TMT6plex)
30795227	NP_543010.3	92675	DTD1	D-tyrosyl-tRNA(Tyr) deacylase 1	S197	-	-	-	-	2.4	1.6	1.7	1.0	-	-	-	-	2.1	1.5	1.5	1.0	2.1	1.5	1.5	1.0	SASsGAEGDVSSEREP	KEDRSASsGAEGDVS	S4;	Yes	S(1): 0.0; S(3): 1.5; S(4): 98.5; S(11): 0.0; S(12): 0.0	1	1	1	1	N-Term(TMT6plex); S4(Phospho)

Khan et al., 2019. Multi-omics analysis to characterize cigarette smoke induced molecular alterations in esophageal cells

Supplementary Table 8. List of phosphopeptides identified and quantified in untreated and chronically treated Het1A cells with cigarette smoke condensate for 8 months using SequestHT and Mascot search algorithms with a PhosphoRS probability score ≥75%

Protein group Accession	NP_Accession	Gene ID	Gene Symbol	Protein Description	PhosphoSite (Protein)	Het-1A-Smoke - 2M/Parental (Replicate 1)	Het-1A-Smoke - 4M/Parental (Replicate 1)	Het-1A-Smoke - 6M/Parental (Replicate 1)	Het-1A-Smoke - 8M/Parental (Replicate 1)	Het-1A-Smoke - 2M/Parental (Replicate 2)	Het-1A-Smoke - 4M/Parental (Replicate 2)	Het-1A-Smoke - 6M/Parental (Replicate 2)	Het-1A-Smoke - 8M/Parental (Replicate 2)	Normalized phosphoprotein ratios with respect to corresponding total protein ratios. Het-1A-Smoke - 2M/Parental (Replicate 1)	Het-1A-Smoke - 4M/Parental (Replicate 1)	Het-1A-Smoke - 6M/Parental (Replicate 1)	Het-1A-Smoke - 8M/Parental (Replicate 1)	Het-1A-Smoke - 2M/Parental (Replicate 2)	Het-1A-Smoke - 4M/Parental (Replicate 2)	Het-1A-Smoke - 6M/Parental (Replicate 2)	Het-1A-Smoke - 8M/Parental (Replicate 2)	Average of replicates Het-1A-Smoke - 2M/Parental	Het-1A-Smoke - 4M/Parental	Het-1A-Smoke - 6M/Parental	Het-1A-Smoke - 8M/Parental	Peptide Sequence	Phosphowindow	PhosphoSite (Peptide)	Phosphosite Plus Evidence	phosphoRS Site Probabilities	# PSMs	# Proteins	# Protein Groups	# Missed Cleavages	Modifications
45827753	NP_996742.1	10579	TACC2	transforming acidic coiled-coil-containing protein 2 isoform c	S590	-	-	-	-	1.1	1.3	1.3	1.0	-	-	-	-	1.1	1.1	1.1	1.0	1.1	1.1	1.1	1.0	FSsPTEELDYR	VNETKFSsPTEELDY	S3;	No	S(2): 1.3; S(3): 97.4; T(5): 1.3; Y(10): 0.0	1	8	1	0	N-Term(TMT6plex); S3(Phospho)
166235169	NP_060531.2	55119	PRPF38B	pre-mRNA-splicing factor 38B	S527; S529	-	-	-	-	0.7	0.8	0.9	1.0	-	-	-	-	0.7	0.8	0.9	1.1	0.7	0.8	0.9	1.1	sQsIEQESQEK	DKHDRRRsQSIEQES;HDRRRSQsIEQESQE	S1; S3;	Yes;Yes	S(1): 100.0; S(3): 100.0; S(8): 0.0	2	1	1	0	N-Term(TMT6plex); S1(Phospho); S3(Phospho); K11(TMT6plex)
401709950	NP_001257881.1	7347	UCHL3	ubiquitin carboxyl-terminal hydrolase isozyme L3 isoform 1	S94	-	-	-	-	0.5	0.6	1.0	1.0	-	-	-	-	0.5	0.6	0.9	0.9	0.5	0.6	0.9	0.9	FLEESVSMsPEER	LEESVSMsPEERARY	S9;	No	S(5): 0.0; S(7): 0.9; S(9): 99.1	3	2	1	0	N-Term(TMT6plex); M8(Oxidation); S9(Phospho)
31652242	NP_852469.1	6942	TCF20	transcription factor 20 isoform 2	S574	-	-	-	-	0.8	1.0	1.0	1.0	-	-	-	-	0.7	1.1	1.0	1.1	0.7	1.1	1.0	1.1	LNAsPAAR	EPPRLNAsPAAREEA	S4;	Yes	S(4): 100.0	1	2	1	0	N-Term(TMT6plex); S4(Phospho)
566559861	NP_001274430.1	23288	IQCE	IQ domain-containing protein E isoform 5	S257	-	-	-	-	0.4	1.0	0.9	1.0	-	-	-	-	-	-	-	-	-	-	-	-	VLSTsPTISK	LDRVLSTsPTISKTQ	S5;	No	S(3): 0.0; T(4): 1.5; S(5): 96.9; T(7): 0.0; S(9): 1.5	2	5	1	0	N-Term(TMT6plex); S5(Phospho); K10(TMT6plex)
4504205	NP_001512.1	2976	GTF3C2	general transcription factor 3C polypeptide 2	S167	-	-	-	-	0.7	1.0	0.8	1.0	-	-	-	-	0.8	0.9	0.9	0.9	0.8	0.9	0.9	0.9	DLDRPESQsPK	LDRPESQsPKRPPED	S9;	Yes	S(7): 1.2; S(9): 98.8	1	1	1	0	N-Term(TMT6plex); S9(Phospho); K11(TMT6plex)
38327599	NP_937870.1	6002	RGS12	regulator of G-protein signaling 12 isoform 3	S202	-	-	-	-	0.8	1.1	0.7	1.0	-	-	-	-	-	-	-	-	-	-	-	-	ALPDSQQVPSsPASK	DSQQVPSsPASKHSL	S11;	No	S(5): 0.0; S(10): 0.0; S(11): 100.0; S(14): 0.0	2	3	1	0	N-Term(TMT6plex); S11(Phospho); K15(TMT6plex)
44955929	NP_982284.1	23271	CAMSAP2	calmodulin-regulated spectrin-associated protein 2	S1308	-	-	-	-	1.0	1.2	1.2	1.0	-	-	-	-	1.1	1.1	1.1	0.9	1.1	1.1	1.1	0.9	SESVEGFLsPSR	ESVEGFLsPSRCGSR	S9;	No	S(1): 0.0; S(3): 0.0; S(9): 100.0; S(11): 0.0	2	1	1	0	N-Term(TMT6plex); S9(Phospho)
223890153	NP_001138790.1	79980	DSN1	kinetochore-associated protein DSN1 homolog isoform	S315	-	-	-	-	0.8	0.8	0.7	1.0	-	-	-	-	0.8	0.9	0.8	1.0	0.8	0.9	0.8	1.0	SMQQLDPsPAR	SMQQLDPsPARKLLK	S8;	No	S(1): 0.0; S(8): 100.0	1	3	1	0	N-Term(TMT6plex); M2(Oxidation); S8(Phospho)
556503440	NP_001273295.1	55700	MAP7D1	MAP7 domain-containing protein 1 isoform 3	S125	-	-	-	-	0.9	0.9	0.6	1.0	-	-	-	-	0.9	0.7	0.5	0.9	0.9	0.7	0.5	0.9	SSQPSPTAVPASDsPPTKQEVK	TAVPASDsPPTKQEV	S14;	Yes	S(1): 0.0; S(2): 0.0; S(5): 0.0; T(7): 0.0; S(12): 0.0; S(14): 100.0; T(17): [illegible]	2	3	1	1	N-Term(TMT6plex); S14(Phospho); K18(TMT6plex); K22(TMT6plex)
223005903	NP_001138521.1	11321	GPN1	GPN-loop GTPase 1 isoform d	S243	-	-	-	-	1.3	0.9	1.1	1.0	-	-	-	-	1.1	0.9	1.2	0.9	1.1	0.9	1.2	0.9	GTLDEEDEEADsDTDDIDHR	EEDEEADsDTDDIDH	S12;	No	T(2): 0.0; S(12): 98.8; T(14): 1.2	3	4	1	0	N-Term(TMT6plex); S12(Phospho)
124028529	NP_004810.2	8189	SYMPK	symplekin	S1243	-	-	-	-	1.2	1.2	1.1	1.0	-	-	-	-	1.3	1.3	1.2	1.0	1.3	1.3	1.2	1.0	EERsPQTLAPVGEDAMK	LTLKEERsPQTLAPV	S4;	Yes	S(4): 100.0; T(7): 0.0	1	1	1	1	N-Term(TMT6plex); S4(Phospho); M16(Oxidation); K17(TMT6plex)
21361282	NP_005617.2	6429	SRSF4	serine/arginine-rich splicing factor 4	S456	-	-	-	-	0.8	0.9	0.7	1.0	-	-	-	-	1.1	1.1	1.0	1.4	1.1	1.1	1.0	1.4	SKPNLPsESR	KSKPNLPsESRSRSK	S7;	Yes	S(1): 0.0; S(7): 100.0; S(9): 0.0	1	1	1	0	N-Term(TMT6plex); K2(TMT6plex); S7(Phospho)
52352803	NP_067017.2	7779	SLC30A1	zinc transporter 1	S468	-	-	-	-	0.9	0.9	1.5	1.0	-	-	-	-	0.9	1.0	1.7	1.1	0.9	1.0	1.7	1.1	TPAVSIsCLELSNNLEK	KTPAVSIsCLELSNN	S7;	No	T(1): 0.0; S(5): 1.6; S(7): 98.3; S(12): 0.0	2	1	1	0	N-Term(TMT6plex); S7(Phospho); C8(Carbamidomethyl); K17(TMT6plex)
375331936	NP_001243597.1	8603	FAM193A	protein FAM193A isoform 4	S1144	-	-	-	-	2.0	0.6	1.1	1.0	-	-	-	-	-	-	-	-	-	-	-	-	LVLAEsPQPK	SKLVLAEsPQPKGKN	S6;	Yes	S(6): 100.0	3	4	1	0	N-Term(TMT6plex); S6(Phospho); K10(TMT6plex)
44917604	NP_065813.1	57522	SRGAP1	SLIT-ROBO Rho GTPase-activating protein 1	S906	-	-	-	-	1.0	1.1	1.1	1.0	-	-	-	-	-	-	-	-	-	-	-	-	GLNNDsPER	NRGLNNDsPERRRRP	S6;	Yes	S(6): 100.0	1	1	1	0	N-Term(TMT6plex); S6(Phospho)
308818200	NP_001184223.1	1809	DPYSL3	dihydropyrimidinase-related protein 3 isoform 1	T628; S632	-	-	-	-	0.8	0.9	0.8	0.9	-	-	-	-	0.9	0.9	0.8	1.0	0.9	0.9	0.8	1.0	GGtPAGsAR	TTTPKGGtPAGSARG;KGGTPAGsARGSPTR	T3; S7;	No;No	T(3): 100.0; S(7): 100.0	3	2	1	0	N-Term(TMT6plex); T3(Phospho); S7(Phospho)
45827771	NP_055144.3	23644	EDC4	enhancer of mRNA-decapping protein 4	S875	-	-	-	-	1.2	1.0	1.2	0.9	-	-	-	-	1.2	1.0	1.3	0.9	1.2	1.0	1.3	0.9	DSQDAsAEQSDHDDEVASLASASGGFGTK	QRDSQDAsAEQSDHD	S6;	Yes	S(2): 2.6; S(6): 94.8; S(10): 2.6; S(18): 0.0; S(21): 0.0; S(23): 0.0; T(28): 0.0	2	1	1	0	N-Term(TMT6plex); S10(Phospho); K29(TMT6plex)
355390315	NP_056171.3	23513	SCRIB	protein scribble homolog isoform b	S1486	-	-	-	-	0.7	1.0	1.0	0.9	-	-	-	-	0.7	0.9	1.0	0.9	0.7	0.9	1.0	0.9	ALsPAELR	PAPERALsPAELRAL	S3;	Yes	S(3): 100.0	2	2	1	0	N-Term(TMT6plex); S3(Phospho)
167614488	NP_056342.3	26000	TBC1D10B	TBC1 domain family member 10B	T148	-	-	-	-	0.9	1.0	0.8	0.9	-	-	-	-	1.0	1.1	0.9	1.0	1.0	1.1	0.9	1.0	TEEARPSPAPGPGtPTGTPTR	SPAPGPGtPTGTPTR	T14;	Yes	T(1): 0.0; S(7): 0.0; T(14): 95.2; T(16): 2.4; T(18): 2.4; T(20): 0.1	1	1	1	0	N-Term(TMT6plex); T14(Phospho)
226371725	NP_001139504.1	115548	FCHO2	FCH domain only protein 2 isoform b	S370	-	-	-	-	1.1	0.9	0.8	0.9	-	-	-	-	-	-	-	-	-	-	-	-	NLsNEELTK	VQMNRNLsNEELTKS	S3;	No	S(3): 100.0; T(8): 0.0	2	2	1	0	N-Term(TMT6plex); S3(Phospho); K9(TMT6plex)
338753424	NP_001229723.1	9892	SNAP91	clathrin coat assembly protein AP180 isoform c	S284	-	-	-	-	1.2	1.1	0.9	0.9	-	-	-	-	-	-	-	-	-	-	-	-	SGAPsPLSK	GNKSGAPsPLSKSSP	S5;	No	S(1): 0.0; S(5): 98.8; S(8): 1.2	1	3	1	0	N-Term(TMT6plex); S5(Phospho); K9(TMT6plex)
55770834	NP_057427.3	1063	CENPF	centromere protein F	T2949	-	-	-	-	0.3	0.8	0.8	0.9	-	-	-	-	0.3	0.8	0.8	1.0	0.3	0.8	0.8	1.0	GPTPAtPESFSK	GRGPTPAtPESFSKK	T6;	No	T(3): 0.0; T(6): 100.0; S(9): 0.0; S(11): 0.0	3	1	1	0	N-Term(TMT6plex); T6(Phospho); K12(TMT6plex)
209862789	NP_056056.2	57553	MICAL3	protein-methionine sulfoxide oxidase MICAL3 isoform	S977	-	-	-	-	1.0	1.2	1.0	0.9	-	-	-	-	0.9	1.0	0.9	0.9	0.9	1.0	0.9	0.9	GKsEEELEASK	HALLKGKsEEELEAS	S3;	No	S(3): 100.0; S(10): 0.0	2	1	1	1	N-Term(TMT6plex); K2(TMT6plex); S3(Phospho); K11(TMT6plex)
91718902	NP_733751.2	58508	KMT2C	histone-lysine N-methyltransferase 2C	S2831	-	-	-	-	0.9	1.0	1.4	0.9	-	-	-	-	-	-	-	-	-	-	-	-	TEVLSPNsK	TEVLSPNsKVESKCE	S8;	No	T(1): 0.0; S(5): 0.9; S(8): 99.1	1	1	1	0	N-Term(TMT6plex); S5(Phospho); K9(TMT6plex)
155722994	NP_055932.2	23144	ZC3H3	zinc finger CCCH domain-containing protein 3	S383	-	-	-	-	0.8	1.1	1.1	0.9	-	-	-	-	-	-	-	-	-	-	-	-	ASsPSASSSSFR	KYKWKASsPSASSSS	S3;	No	S(2): 0.0; S(3): 100.0; S(5): 0.0; S(7): 0.0; S(8): 0.0; S(9): 0.0; S(10): 0.0; S(11): 0.0	1	1	1	0	N-Term(TMT6plex); S3(Phospho)
10092601	NP_003751.2	8672	EIF4G3	eukaryotic translation initiation factor 4 gamma 3	S232	-	-	-	-	1.4	1.5	1.2	0.9	-	-	-	-	1.3	1.4	1.1	0.9	1.3	1.4	1.1	0.9	SPsPVLR	DPVLKSPsPVLRLVL	S3;	Yes	S(1): 0.0; S(3): 100.0	1	4	1	0	N-Term(TMT6plex); S3(Phospho)
155722994	NP_055932.2	23144	ZC3H3	zinc finger CCCH domain-containing protein 3	S810	-	-	-	-	1.0	1.2	1.2	0.9	-	-	-	-	-	-	-	-	-	-	-	-	AATsPAPGPSDATAR	HSRRAATsPAPGPSD	S4;	Yes	T(3): 1.4; S(4): 98.6; S(10): 0.0; T(13): 0.0	1	1	1	0	N-Term(TMT6plex); T3(Phospho)
186659512	NP_056262.3	157922	CAMSAP1	calmodulin-regulated spectrin-associated protein 1	S563	-	-	-	-	0.8	0.7	1.0	0.9	-	-	-	-	0.7	0.7	0.9	0.9	0.7	0.7	0.9	0.9	ADVVPQQADPEEPRAsPR	DPEEPRAsPRALGLT	S16;	Yes	S(16): 100.0	1	1	1	1	N-Term(TMT6plex); S16(Phospho)
5729862	NP_006518.1	7884	SLBP	histone RNA hairpin-binding protein	S111	-	-	-	-	1.3	1.4	0.8	0.9	-	-	-	-	1.4	1.6	1.0	1.1	1.4	1.6	1.0	1.1	KSsSGSSDSK	FGRERKSsSGSSDSK	S3;	Yes	S(2): 1.2; S(3): 98.8; S(4): 0.0; S(6): 0.0; S(7): 0.0; S(9): 0.0	1	1	1	1	N-Term(TMT6plex); K1(TMT6plex); S3(Phospho); K10(TMT6plex)
169881252	NP_001509.3	2969	GTF2I	general transcription factor II-I isoform 4	S633	-	-	-	-	0.7	0.9	1.1	0.9	-	-	-	-	0.8	0.9	1.1	1.0	0.8	0.9	1.1	1.0	sPGSNSK	QSPKRPRsPGSNSKV	S1;	Yes	S(1): 100.0; S(4): 0.0; S(6): 0.0	1	5	1	0	N-Term(TMT6plex); S1(Phospho); K7(TMT6plex)
50659095	NP_004719.2	9188	DDX21	nucleolar RNA helicase 2 isoform 1	S13	-	-	-	-	1.1	1.6	1.5	0.9	-	-	-	-	1.0	1.2	1.1	0.8	1.0	1.2	1.1	0.8	SDAGLEsDTAMK	RSDAGLEsDTAMKKG	S7;	Yes	S(1): 0.0; S(7): 100.0; T(9): 0.0	2	1	1	0	N-Term(TMT6plex); S7(Phospho); M11(Oxidation); K12(TMT6plex)
50513245	NP_005474.2	10036	CHAF1A	chromatin assembly factor 1 subunit A	S951	-	-	-	-	0.3	0.8	0.8	0.9	-	-	-	-	0.4	1.0	1.1	1.2	0.4	1.0	1.1	1.2	ATLTAsPLGAS	GKATLTAsPLGAS_	S6;	No	T(2): 0.0; T(4): 0.0; S(6): 100.0; S(11): 0.0	2	1	1	0	N-Term(TMT6plex); S6(Phospho)
21493033	NP_009133.2	11216	AKAP10	A-kinase anchor protein 10, mitochondrial precursor	S187	-	-	-	-	1.4	0.8	0.6	0.9	-	-	-	-	1.3	0.9	0.6	0.9	1.3	0.9	0.6	0.9	QSSLAEPVsPSK	SSLAEPVsPSKKHET	S9;	Yes	S(2): 0.0; S(3): 0.0; S(9): 100.0; S(11): 0.0	3	1	1	0	N-Term(TMT6plex); S9(Phospho); K12(TMT6plex)
186928821	NP_001119523.1	3068	HDGF	hepatoma-derived growth factor isoform c	S125; S126	-	-	-	-	1.1	1.1	1.2	0.9	-	-	-	-	1.2	1.0	1.1	0.9	1.2	1.0	1.1	0.9	KGNAEGssDEEGK	KKGNAEGsSDEEGKL;KGNAEGSsDEEGKLV	S7; S8;	No;No	S(7): 100.0; S(8): 100.0	1	3	1	1	N-Term(TMT6plex); K1(TMT6plex); S7(Phospho); S8(Phospho); K13(TMT6plex)
574956994	NP_001276339.1	84923	FAM104A	protein FAM104A isoform 3	S21	-	-	-	-	1.1	1.0	0.9	0.9	-	-	-	-	-	-	-	-	-	-	-	-	GADAGSSGGTGPTEGYsPPAASTR	TGPTEGYsPPAASTR	S17;	No	S(6): 0.0; S(7): 0.0; T(10): 0.0; T(13): 0.0; Y(16): 1.2; S(17): 98.8; S(22): 0.0; T(23): 0.0	2	4	1	0	N-Term(TMT6plex); S17(Phospho)
221139926	NP_001137532.1	57224	NHSL1	NHS-like protein 1 isoform 2	S1491	-	-	-	-	0.8	1.0	0.9	0.9	-	-	-	-	0.8	1.0	0.9	0.9	0.8	1.0	0.9	0.9	SLsFSGPR	GLMPRSLsFSGPRYG	S3;	No	S(1): 0.0; S(3): 100.0; S(5): 0.0	1	2	1	0	N-Term(TMT6plex); S3(Phospho)
568384749	NP_001275513.1	257415	FAM133B	protein FAM133B isoform 2	S236	-	-	-	-	0.8	0.9	0.9	0.9	-	-	-	-	1.1	1.1	1.2	1.2	1.1	1.1	1.2	1.2	AASSSPDsP	AASSSPDsP_	S8;	No	S(3): 0.0; S(4): 0.0; S(5): 0.0; S(8): 100.0	3	2	1	0	N-Term(TMT6plex); S8(Phospho)
16262452	NP_065821.1	57530	CGN	cingulin	S137	-	-	-	-	2.3	1.6	0.9	0.9	-	-	-	-	3.3	1.9	1.1	1.1	3.3	1.9	1.1	1.1	sHsQASLAGPGPVDPSNR	GKLLRSHsQASLAGP	S3;	No	S(1): 2.0; S(3): 98.0; S(6): 0.0; S(16): 0.0	1	1	1	0	N-Term(TMT6plex); S3(Phospho)
259906020	NP_001158287.1	22985	ACIN1	apoptotic chromatin condensation inducer in the nucleus isoform 3	S658	-	-	-	-	0.8	0.8	0.9	0.9	-	-	-	-	0.6	0.7	0.9	1.0	0.6	0.7	0.9	1.0	TsTSSSSVQAR	CEPKERTsTSSSSVQ	S2;	No	T(1): 0.0; S(2): 100.0; T(3): 0.0; S(4): 0.0; S(5): 0.0; S(6): 0.0; S(7): 0.0	1	3	1	0	N-Term(TMT6plex); S2(Phospho)
333440451	NP_001193973.1	23122	CLASP2	CLIP-associating protein 2 isoform 2	S489	-	-	-	-	0.7	0.8	0.8	0.9	-	-	-	-	0.6	0.9	0.9	1.0	0.6	0.9	0.9	1.0	VLVNSAsAQK	RVLVNSAsAQKRSKI	S7;	Yes	S(5): 0.0; S(7): 100.0	2	2	1	0	N-Term(TMT6plex); S7(Phospho); K10(TMT6plex)
18104982	NP_536348.1	5771	PTPN2	tyrosine-protein phosphatase non-receptor type 2 isoform 3	S304	-	-	-	-	0.8	0.9	0.7	0.9	-	-	-	-	0.8	0.9	0.7	1.0	0.8	0.9	0.7	1.0	EDLSPAFDHsPNK	LSPAFDHsPNKIMTE	S10;	Yes	S(4): 0.0; S(10): 100.0	3	4	1	0	N-Term(TMT6plex); S10(Phospho); K13(TMT6plex)
431822406	NP_001258900.1	3326	HSP90AB1	heat shock protein HSP 90-beta isoform b	S207	-	-	-	-	0.9	1.3	1.0	0.9	-	-	-	-	1.0	1.2	1.0	0.9	1.0	1.2	1.0	0.9	IEDVGsDEEDDSGKDKK	PKIEDVGsDEEDDSG	S6;	No	S(6): 100.0; S(12): 0.0	3	3	1	2	N-Term(TMT6plex); S6(Phospho); K14(TMT6plex); K16(TMT6plex); K17(TMT6plex)
239735519	NP_002963.2	6305	SBF1	myotubularin-related protein 5	S1228	-	-	-	-	0.7	0.8	1.2	0.9	-	-	-	-	0.7	0.8	1.1	0.8	0.7	0.8	1.1	0.8	AQNAPsPGQSQADSSSLEQEK	FKAQNAPsPGQSQAD	S6;	Yes	S(6): 89.1; S(10): 2.7; S(14): 2.7; S(15): 2.7; S(16): 2.7	1	1	1	0	N-Term(TMT6plex); S6(Phospho); K21(TMT6plex)
41872583	NP_004841.2	9475	ROCK2	rho-associated protein kinase 2	S1374	-	-	-	-	0.8	0.8	0.8	0.9	-	-	-	-	0.6	0.7	0.7	0.8	0.6	0.7	0.7	0.8	IQQNQsIR	MKIQQNQsIRRPSRQ	S6;	Yes	S(6): 100.0	2	1	1	0	N-Term(TMT6plex); S6(Phospho)
419636284	NP_001258698.1	8546	AP3B1	AP-3 complex subunit beta-1 isoform 2	T612	-	-	-	-	0.5	0.7	0.8	0.9	-	-	-	-	0.5	0.7	0.8	0.9	0.5	0.7	0.8	0.9	EWtPAGK	IELAKEWtPAGKAKQ	T3;	No	T(3): 100.0	1	2	1	0	N-Term(TMT6plex); T3(Phospho); K7(TMT6plex)
11034845	NP_067038.1	58490	RPRD1B	regulation of nuclear pre-mRNA domain-containing protein 1B	S134	-	-	-	-	0.9	0.7	0.7	0.9	-	-	-	-	0.9	0.8	0.8	1.0	0.9	0.8	0.8	1.0	LSMEDSKsPPPK	LSMEDSKsPPPKATE	S8;	Yes	S(2): 0.0; S(6): 1.0; S(8): 99.0	1	1	1	1	N-Term(TMT6plex); M3(Oxidation); K7(TMT6plex); S8(Phospho); K12(TMT6plex)
545688925	NP_001269911.1	7090	TLE3	transducin-like enhancer protein 3 isoform g	T272	-	-	-	-	0.9	0.7	0.9	0.9	-	-	-	-	0.9	0.8	0.9	1.0	0.9	0.8	0.9	1.0	NDAPtPGTSTTPGLR	TPRNDAPtPGTSTTP	T5;	No	T(5): 99.9; T(8): 0.0; S(9): 0.0; T(10): 0.0; T(11): 0.0	2	7	1	0	N-Term(TMT6plex); T5(Phospho)
82546824	NP_001032242.1	221937	FOXK1	forkhead box protein K1	S101	-	-	-	-	0.8	1.0	0.9	0.9	-	-	-	-	0.8	1.0	0.9	0.9	0.8	1.0	0.9	0.9	QsPGPALAR	AAASVRQsPGPALAR	S2;	Yes	S(2): 100.0	1	1	1	0	N-Term(TMT6plex); S2(Phospho)
116235474	NP_443134.2	114790	STK11IP	serine/threonine-protein kinase 11-interacting protein	S493	-	-	-	-	1.5	0.7	0.7	0.9	-	-	-	-	-	-	-	-	-	-	-	-	GPQEsPQK	EARGPQEsPQKMSEE	S5;	Yes	S(5): 100.0	2	1	1	0	N-Term(TMT6plex); S5(Phospho); K8(TMT6plex)
557636741	NP_001273556.1	10921	RNPS1	RNA-binding protein with serine-rich domain 1 isoform c	S132; S134	-	-	-	-	0.8	1.1	1.0	0.9	-	-	-	-	1.0	1.1	1.1	0.9	1.0	1.1	1.1	0.9	sPsPKPTK	EKERKRRsPSPKPTK;ERKRRSPsPKPTKVH	S1; S3;	No;No	S(1): 100.0; S(3): 100.0; T(7): 0.0	1	3	1	0	N-Term(TMT6plex); S1(Phospho); S3(Phospho); K5(TMT6plex); K8(TMT6plex)
42518070	NP_004808.2	9414	TJP2	tight junction protein ZO-2 isoform 1	S174	-	-	-	-	1.0	0.9	1.3	0.9	-	-	-	-	1.0	0.9	1.1	1.0	1.0	0.9	1.1	1.0	SWEDsPER	RSRSWEDsPERGRPH	S5;	Yes	S(1): 0.0; S(5): 100.0	1	6	1	0	N-Term(TMT6plex); S5(Phospho)
66528888	NP_001018125.1	2177	FANCD2	Fanconi anemia group D2 protein isoform b	S319	-	-	-	-	1.5	1.2	1.1	0.9	-	-	-	-	1.7	1.5	1.3	0.9	1.7	1.5	1.3	0.9	LQAsQVK	LPSRLQAsQVKLKSK	S4;	No	S(4): 100.0	1	2	1	0	N-Term(TMT6plex); S4(Phospho); K7(TMT6plex)
194272142	NP_001123537.1	4650	MYO9B	unconventional myosin-IXb isoform 2	S1122	-	-	-	-	1.0	1.2	1.0	0.9	-	-	-	-	1.1	1.3	1.1	1.0	1.1	1.3	1.1	1.0	EAPsPEK	SPEKEAPsPEKTLPP	S4;	Yes	S(4): 100.0	1	2	1	0	N-Term(TMT6plex); S4(Phospho); K7(TMT6plex)
145699133	NP_079143.3	79925	SPEF2	sperm flagellar protein 2 isoform 1	T879; T880	-	-	-	-	5.5	1.3	0.9	0.9	-	-	-	-	-	-	-	-	-	-	-	-	EILttEIAK	EKVKEILtTEIAKKK;KVKEILTtEIAKKKN	T4; T5;	No;No	T(4): 100.0; T(5): 100.0	1	1	1	0	N-Term(TMT6plex); T4(Phospho); T5(Phospho); K9(TMT6plex)
385251399	NP_001245260.1	2036	EPB41L1	band 4.1-like protein 1 isoform b	T612	-	-	-	-	1.2	1.0	0.8	0.9	-	-	-	-	1.2	1.1	0.8	1.0	1.2	1.1	0.8	1.0	GACtPDMPQEEPVK	PDRGACtPDMPQEE	T5;	No	S(4): 1.2; T(5): 98.8	1	3	1	0	N-Term(TMT6plex); C3(Carbamidomethyl); T5(Phospho); M8(Oxidation); K15(TMT6plex)
118572613	NP_057417.3	23524	SRRM2	serine/arginine repetitive matrix protein 2	S1857	-	-	-	-	1.0	0.9	1.0	0.9	-	-	-	-	1.0	0.9	1.1	1.0	1.0	0.9	1.1	1.0	TsPAPWK	KRSRSRTsPAPWKRS	S2;	Yes	T(1): 0.0; S(2): 100.0	2	1	1	0	N-Term(TMT6plex); S2(Phospho); K7(TMT6plex)
94721263	NP_001035536.1	54545	MTMR12	myotubularin-related protein 12	S564	-	-	-	-	0.8	1.0	1.7	0.9	-	-	-	-	0.7	0.8	1.5	0.7	0.7	0.8	1.5	0.7	QLsLPLTQSK	EKHQRQLsLPLTQSK	S3;	Yes	S(3): 100.0; T(7): 0.0; S(9): 0.0	1	1	1	0	N-Term(TMT6plex); S3(Phospho); K10(TMT6plex)
153791514	NP_001093149.1	22954	TRIM32	E3 ubiquitin-protein ligase TRIM32	S335; S339	-	-	-	-	0.7	2.0	2.1	0.9	-	-	-	-	0.7	2.5	2.4	1.1	0.7	2.5	2.4	1.1	EMDMSPEEVVAsPRAsPAK	SPEEVVAsPRASPAK;VVASPRAsPAKQRGP	S12; S16;	Yes;Yes	S(5): 0.0; S(12): 100.0; S(16): 100.0	2	1	1	1	N-Term(TMT6plex); M2(Oxidation); M4(Oxidation); S12(Phospho); S16(Phospho); K19(TMT6plex)
4758216	NP_004413.1	1856	DVL2	segment polarity protein dishevelled homolog DVL-2	S618	-	-	-	-	0.8	0.9	1.1	0.9	-	-	-	-	1.0	1.0	1.2	0.9	1.0	1.0	1.2	0.9	SGsGSESEPSSR	APESKSGsGSESEPS	S3;	No	S(1): 0.0; S(3): 100.0; S(5): 0.0; S(7): 0.0; S(10): 0.0; S(11): 0.0	2	1	1	0	N-Term(TMT6plex); S3(Phospho)
612339330	NP_001278376.1	6297	SALL2	sal-like protein 2 isoform c	S653	-	-	-	-	0.9	0.9	0.8	0.9	-	-	-	-	-	-	-	-	-	-	-	-	GSEsGGEK	LAGRGSEsGGEKAIS	S4;	No	S(2): 0.0; S(4): 100.0	1	3	1	0	N-Term(TMT6plex); S4(Phospho); K8(TMT6plex)
313661470	NP_001186346.1	57636	ARHGAP23	rho GTPase-activating protein 23	S423	-	-	-	-	0.7	0.8	0.9	0.9	-	-	-	-	0.6	0.8	1.0	0.8	0.6	0.8	1.0	0.8	SYsPSFQR	YIGYRSYsPSFQRRT	S3;	Yes	S(1): 0.0; Y(2): 1.0; S(3): 99.0; S(5): [illegible]	1	1	1	0	N-Term(TMT6plex); S3(Phospho)
157743267	NP_001099046.1	347454	SOWAHD	ankyrin repeat domain-containing protein SOWAHD	S63; S67	-	-	-	-	1.1	1.3	1.2	0.9	-	-	-	-	-	-	-	-	-	-	-	-	AAAAASREHPFPGTLMHsAAGsGR	FPGTLMHsAAGSGRR;LMHSAAGsGRRRGAL	S18; S22;	No;No	S(6): 0.3; T(14): 4.6; S(18): 95.4; S(22): 99.7	1	1	1	1	N-Term(TMT6plex); S18(Phospho); S22(Phospho)
21070997	NP_003147.2	6786	STIM1	stromal interaction molecule 1 isoform 2 precursor	S257	-	-	-	-	0.9	0.6	0.7	0.9	-	-	-	-	0.9	0.6	0.6	0.9	0.9	0.6	0.6	0.9	AEQsLHDLQER	GLHRAEQsLHDLQER	S4;	Yes	S(4): 100.0	1	3	1	0	N-Term(TMT6plex); S4(Phospho)
31044432	NP_851853.1	221496	LEMD2	LEM domain-containing protein 2 isoform 1	S139	-	-	-	-	0.9	0.9	0.8	0.9	-	-	-	-	0.9	1.0	0.8	0.9	0.9	1.0	0.8	0.9	GSsEEDEDAR	RASVRGSsEEDEDAR	S3;	Yes	S(2): 0.8; S(3): 99.2	1	1	1	0	N-Term(TMT6plex); S3(Phospho)
302699211	NP_665739.3	23126	POGZ	pogo transposable element with ZNF domain isoform 3	S339	-	-	-	-	0.3	1.2	1.3	0.9	-	-	-	-	0.3	1.1	1.2	0.8	0.3	1.1	1.2	0.8	TAPVAsTPSSTPIPALSPPTKVPEPNENVGDAVQTK	EKTAPVAsTPSSTPI	S6;	No	T(1): 0.0; S(6): 88.0; T(7): 2.4; S(9): 2.4; S(10): 2.4; T(11): 2.4; S(17): 2.4; T(20): 0.1; T(35): 0.0	2	5	1	1	N-Term(TMT6plex); S17(Phospho); K21(TMT6plex); K36(TMT6plex)
55770834	NP_057427.3	1063	CENPF	centromere protein F	S3079	-	-	-	-	0.9	1.1	1.0	0.9	-	-	-	-	1.0	1.1	1.0	0.9	1.0	1.1	1.0	0.9	SVPVNNLPERsPTDsPR	VNNLPERsPTDSPRE	S11;	No	S(1): 0.0; S(11): 96.2; T(13): 1.9; S(15): 1.9	1	1	1	1	N-Term(TMT6plex); S11(Phospho)
52630322	NP_005843.2	1107	CHD3	chromodomain-helicase-DNA-binding protein 3 isoform 2	S713	-	-	-	-	0.9	0.9	1.0	0.9	-	-	-	-	1.0	0.9	1.0	0.9	1.0	0.9	1.0	0.9	ELQGDGPPSsPTNDPTVK	QGDGPPSsPTNDPTV	S10;	Yes	S(9): 0.0; S(10): 100.0; T(12): 0.0; T(16): 0.0	2	3	1	0	N-Term(TMT6plex); S10(Phospho); K18(TMT6plex)
76150625	NP_001028886.1	4839	NOP2	putative ribosomal RNA methyltransferase NOP2 isoform 1	T772	-	-	-	-	0.7	1.1	0.8	0.9	-	-	-	-	0.7	1.0	0.7	0.9	0.7	1.0	0.7	0.9	QNDtPKGPQPPTVSPIR	AFQKQNDtPKGPQPP	T4;	No	T(4): 100.0; T(12): 50.0; S(14): 50.0	1	3	1	1	N-Term(TMT6plex); T4(Phospho); K6(TMT6plex); T12(Phospho)
259906020	NP_001158287.1	22985	ACIN1	apoptotic chromatin condensation inducer in the nucleus isoform 3	S689	-	-	-	-	0.9	1.1	0.8	0.9	-	-	-	-	0.7	1.0	0.9	0.9	0.7	1.0	0.9	0.9	LQPERGsPK	RLQPERGsPKKCEAE	S7;	No	S(7): 100.0	1	3	1	1	N-Term(TMT6plex); S7(Phospho); K9(TMT6plex)
153945728	NP_005900.2	4131	MAP1B	microtubule-associated protein 1B	T1067	-	-	-	-	0.8	1.2	0.7	0.9	-	-	-	-	0.6	0.9	0.6	0.9	0.6	0.9	0.6	0.9	AAEAGGAEEQYGFLTtPTK	EQYGFLTtPTKQLGA	T16;	Yes	Y(11): 0.0; T(15): 1.4; T(16): 97.1; T(18): 1.4	2	1	1	0	N-Term(TMT6plex); T16(Phospho); K19(TMT6plex)
19913396	NP_599031.1	6525	SMTN	smoothelin isoform b	S514	-	-	-	-	1.1	1.0	1.2	0.9	-	-	-	-	0.9	0.7	0.9	0.8	0.9	0.7	0.9	0.8	VNsPGTLAR	STITRVNsPGTLARL	S3;	Yes	S(3): 99.1; T(6): 0.9	1	5	1	0	N-Term(TMT6plex); S3(Phospho)
326319990	NP_055820.2	283638	CEP170B	centrosomal protein of 170 kDa protein B isoform 2	S1065	-	-	-	-	0.7	1.0	1.0	0.9	-	-	-	-	-	-	-	-	-	-	-	-	LGDAsDTEAADGER	RARLGDAsDTEAADG	S5;	No	S(5): 100.0; T(7): 0.0	3	2	1	0	N-Term(TMT6plex); S5(Phospho)
296179386	NP_001171690.1	1211	CLTA	clathrin light chain A isoform e	S105	-	-	-	-	1.3	1.0	1.1	0.9	-	-	-	-	1.0	1.1	0.8	1.1	1.0	1.1	0.8	1.1	LQsEPESIR	SQVDRLQsEPESIRK	S3;	Yes	S(3): 100.0; S(7): 0.0	1	5	1	0	N-Term(TMT6plex); S3(Phospho)
255652953	NP_653205.3	124245	ZC3H18	zinc finger CCCH domain-containing protein 18	S118	-	-	-	-	0.9	1.0	0.9	0.9	-	-	-	-	1.1	1.0	1.1	0.9	1.1	1.0	1.1	0.9	DEASsVTR	DLRDEASsVTRELDE	S5;	Yes	S(4): 0.7; S(5): 99.3; T(7): 0.0	1	1	1	0	N-Term(TMT6plex); S5(Phospho)
467091962	NP_001264125.1	51105	PHF20L1	PHD finger protein 20-like protein 1 isoform 4	S407	-	-	-	-	0.8	1.1	0.8	0.9	-	-	-	-	0.8	1.1	0.9	0.9	0.8	1.1	0.9	0.9	VSsPSPATDGK	DSVEKVSsPSPATDG	S3;	No	S(2): 1.3; S(3): 98.7; S(5): 0.0; T(8): [illegible]	2	2	1	0	N-Term(TMT6plex); S3(Phospho); K11(TMT6plex)
19913406	NP_001058.2	7153	TOP2A	DNA topoisomerase 2-alpha	S1426	-	-	-	-	0.6	0.8	0.8	0.9	-	-	-	-	0.7	0.9	0.9	1.0	0.7	0.9	0.9	1.0	SQSsTSTTGAK	TAAKSQSsTSTTGAK	S4;	No	S(1): 0.0; S(3): 0.0; S(4): 100.0; T(5): 0.0; S(6): 0.0; T(7): 0.0; T(8): 0.0	2	1	1	0	N-Term(TMT6plex); S4(Phospho); K11(TMT6plex)
48255933	NP_004956.5	3150	HMGN1	non-histone chromosomal protein HMG-14	S86; S89	-	-	-	-	1.1	1.4	1.0	0.9	-	-	-	-	1.4	1.6	1.3	1.1	1.4	1.6	1.3	1.1	TEEsPAsDEAGEKEAK	GETKTEEsPASDEAG;KTEESPAsDEAGEKE	S4; S7;	Yes;Yes	T(1): 0.0; S(4): 100.0; S(7): 100.0	1	1	1	1	N-Term(TMT6plex); S4(Phospho); S7(Phospho); K13(TMT6plex); K16(TMT6plex)
150417981	NP_004482.4	2909	ARHGAP35	rho GTPase-activating protein 35	S1179	-	-	-	-	1.1	0.9	1.0	0.9	-	-	-	-	1.3	0.9	1.1	0.8	1.3	0.9	1.1	0.8	TSFSVGsDDELGPIR	RTSFSVGsDDELGPI	S7;	Yes	T(1): 0.0; S(2): 0.0; S(4): 0.0; S(7): 100.0	2	1	1	0	N-Term(TMT6plex); S7(Phospho)
21493024	NP_653080.1	9590	AKAP12	A-kinase anchor protein 12 isoform 2	S416	-	-	-	-	0.6	0.7	1.0	0.9	-	-	-	-	0.7	0.7	0.8	0.8	0.7	0.7	0.8	0.8	VQGsPLK	ERMKVQGsPLKKLFT	S4;	No	S(4): 100.0	2	2	1	0	N-Term(TMT6plex); S4(Phospho); K7(TMT6plex)
38569421	NP_001087.2	47	ACLY	ATP-citrate synthase isoform 1	S455	-	-	-	-	1.5	1.2	1.0	0.9	-	-	-	-	1.4	1.1	1.0	0.9	1.4	1.1	1.0	0.9	TAsFSESR	PAPSRTAsFSESRAD	S3;	Yes	T(1): 0.0; S(3): 100.0; S(5): 0.0; S(7): 0.0	3	2	1	0	N-Term(TMT6plex); S3(Phospho)
5453860	NP_006192.1	5127	CDK16	cyclin-dependent kinase 16 isoform 1	S95	-	-	-	-	1.7	0.7	1.1	0.9	-	-	-	-	1.7	0.8	1.2	0.9	1.7	0.8	1.2	0.9	MGSDGESDQASATSSDEVQsPVR	TSSDEVQsPVRVRMR	S20;	Yes	S(3): 0.0; S(7): 0.0; S(11): 0.1; T(13): 0.1; S(14): 0.1; S(15): 2.8; S(20): 96.9	1	3	1	0	N-Term(TMT6plex); M1(Oxidation); S20(Phospho)
38372921	NP_940992.1	682	BSG	basigin isoform 3	S153	-	-	-	-	0.5	0.8	0.8	0.9	-	-	-	-	0.5	0.8	0.9	0.9	0.5	0.8	0.9	0.9	KPEDVLDDDDAGsAPLK	LDDDDAGsAPLKSSG	S13;	No	S(13): 100.0	2	4	1	0	N-Term(TMT6plex); K1(TMT6plex); S13(Phospho); K17(TMT6plex)
167555110	NP_002254.2	3833	KIFC1	kinesin-like protein KIFC1	T359	-	-	-	-	1.0	0.8	1.0	0.9	-	-	-	-	1.2	1.0	1.1	1.0	1.2	1.0	1.1	1.0	RGtLSGAPAPTR	RSDERRGtLSGAPAP	T3;	Yes	T(3): 100.0; S(5): 0.0; T(12): 0.0	1	1	1	1	N-Term(TMT6plex); T3(Phospho)
21237805	NP_003066.2	6601	SMARCC2	SWI/SNF complex subunit SMARCC2 isoform a	S283	-	-	-	-	0.7	0.9	1.1	0.9	-	-	-	-	0.7	0.8	1.0	0.8	0.7	0.8	1.0	0.8	TLTDEVNsPDSDRR	TLTDEVNsPDSDRRD	S8;	Yes	T(1): 0.0; T(3): 0.0; S(8): 100.0; S(11): 0.0	2	3	1	1	N-Term(TMT6plex); S8(Phospho)
155722987	NP_036213.2	22936	ELL2	RNA polymerase II elongation factor ELL2	S503	-	-	-	-	0.8	0.8	0.8	0.9	-	-	-	-	-	-	-	-	-	-	-	-	LNNSsPNSSGGVK	IAKLNNSsPNSSGGV	S5;	Yes	S(4): 1.3; S(5): 98.7; S(8): 0.0; S(9): [illegible]	1	1	1	0	N-Term(TMT6plex); S4(Phospho); K13(TMT6plex)
410442518	NP_001258547.1	54776	PPP1R12C	protein phosphatase 1 regulatory subunit 12C isoform	S426	-	-	-	-	0.9	0.9	1.0	0.9	-	-	-	-	-	-	-	-	-	-	-	-	TGsSGALGPPER	EGLLKTGsSGALGPP	S3;	No	T(1): 0.0; S(3): 100.0; S(4): 0.0	2	2	1	0	N-Term(TMT6plex); S3(Phospho)
31324577	NP_203744.1	85377	MICALL1	MICAL-like protein 1	S486	-	-	-	-	1.0	1.7	1.4	0.9	-	-	-	-	0.9	1.5	1.3	0.8	0.9	1.5	1.3	0.8	APSAsPLALHASR	APRAPSAsPLALHAS	S5;	Yes	S(3): 0.0; S(5): 100.0; S(12): 0.0	2	1	1	0	N-Term(TMT6plex); S5(Phospho)
6631100	NP_005376.2	4820	NKTR	NK-tumor recognition protein	S463	-	-	-	-	0.8	1.0	1.6	0.9	-	-	-	-	0.9	1.2	1.9	1.1	0.9	1.2	1.9	1.1	ILIPsDIESSK	KRRILIPsDIESSKS	S5;	Yes	S(5): 97.0; S(9): 1.5; S(10): 1.5	1	1	1	0	N-Term(TMT6plex); S5(Phospho); K11(TMT6plex)
10835073	NP_066565.1	4836	NMT1	glycylpeptide N-tetradecanoyltransferase 1	S69	-	-	-	-	0.8	1.0	1.1	0.9	-	-	-	-	1.0	1.1	1.2	0.9	1.0	1.1	1.2	0.9	EKGsETDSAQDQPVK	KKKKEKGsETDSAQD	S4;	No	S(4): 100.0; T(6): 0.0; S(8): 0.0	1	1	1	1	N-Term(TMT6plex); K2(TMT6plex); S4(Phospho); K15(TMT6plex)
148664186	NP_001005242.2	5318	PKP2	plakophilin-2 isoform 2a	S132	-	-	-	-	1.4	1.4	1.9	0.9	-	-	-	-	1.0	1.2	1.5	0.8	1.0	1.2	1.5	0.8	GTAQYSsQK	RGTAQYSsQKSVEER	S7;	Yes	T(2): 0.0; Y(5): 0.0; S(6): 0.0; S(7): 100.0	2	2	1	0	N-Term(TMT6plex); S7(Phospho); K9(TMT6plex)
32455273	NP_006639.3	65268	WNK2	serine/threonine-protein kinase WNK2 isoform 2	S560	-	-	-	-	1.4	1.0	1.2	0.9	-	-	-	-	-	-	-	-	-	-	-	-	EQQDVGsPDK	KEQQDVGsPDKARGP	S7;	Yes	S(7): 100.0	1	2	1	0	N-Term(TMT6plex); S7(Phospho); K10(TMT6plex)
54607124	NP_037418.3	29890	RBM15B	putative RNA-binding protein 15B	S109	-	-	-	-	0.9	0.8	0.9	0.9	-	-	-	-	0.9	0.9	1.0	0.9	0.9	0.9	1.0	0.9	ASGDPGASGMsPR	DPGASGMsPRASPLP	S11;	Yes	S(2): 0.0; S(8): 0.0; S(11): 100.0	2	1	1	0	N-Term(TMT6plex); M10(Oxidation); S11(Phospho)
332800979	NP_001193879.1	9123	SLC16A3	monocarboxylate transporter 4	S464	-	-	-	-	1.1	1.3	1.4	0.9	-	-	-	-	1.4	1.4	1.5	0.9	1.4	1.4	1.5	0.9	NGEVVHTPETsV	VVHTPETsV	S11;	Yes	T(7): 0.0; T(10): 1.1; S(11): 98.9	2	1	1	0	N-Term(TMT6plex); S11(Phospho)
194733742	NP_005654.3	7469	NELFA	negative elongation factor A	S244	-	-	-	-	0.9	0.6	1.1	0.9	-	-	-	-	0.9	0.7	1.1	0.9	0.9	0.7	1.1	0.9	SPTAPSVFsPTGNR	PTAPSVFsPTGNRTP	S9;	No	S(1): 0.0; T(3): 0.0; S(6): 0.0; S(9): 99.0; T(11): 1.0	3	1	1	0	N-Term(TMT6plex); S9(Phospho)
21536485	NP_003316.3	7290	HIRA	protein HIRA	T586	-	-	-	-	0.8	0.9	0.8	0.9	-	-	-	-	0.8	1.1	0.8	1.0	0.8	1.1	0.8	1.0	ATPGAPALTSMtPTAVER	APALTSMtPTAVERL	T12;	Yes	T(2): 0.0; T(9): 0.0; S(10): 0.0; T(12): 98.5; T(14): 1.5	2	1	1	0	N-Term(TMT6plex); M11(Oxidation); T12(Phospho)
112821681	NP_443131.2	114787	GPRIN1	G protein-regulated inducer of neurite outgrowth 1	S615	-	-	-	-	0.6	0.7	0.6	0.9	-	-	-	-	-	-	-	-	-	-	-	-	GsPVTTTK	SLPLEKGsPVTTTKA	S2;	Yes	S(2): 100.0; T(5): 0.0; T(6): 0.0; T(7): 0.0	1	1	1	0	N-Term(TMT6plex); S2(Phospho); K8(TMT6plex)
38788333	NP_057662.3	51773	RSF1	remodeling and spacing factor 1	S748	-	-	-	-	0.9	1.2	1.2	0.9	-	-	-	-	0.9	1.1	1.1	0.9	0.9	1.1	1.1	0.9	KPDsPPK	SRKKKPDsPPKVLEP	S4;	Yes	S(4): 100.0	1	1	1	0	N-Term(TMT6plex); K1(TMT6plex); S4(Phospho); K7(TMT6plex)
47419905	NP_005987.3	6926	TBX3	T-box transcription factor TBX3 isoform 1	S418	-	-	-	-	1.1	0.7	0.9	0.9	-	-	-	-	-	-	-	-	-	-	-	-	HsPATISSSTR	ASPDSRHsPATISSS	S2;	No	S(2): 100.0; T(5): 0.0; S(7): 0.0; S(8): 0.0; S(9): 0.0; T(10): 0.0	1	2	1	0	N-Term(TMT6plex); S2(Phospho)
119220605	NP_005144.2	9100	USP10	ubiquitin carboxyl-terminal hydrolase 10 isoform 2	Y364	-	-	-	-	0.8	0.8	1.0	0.9	-	-	-	-	0.8	0.7	0.9	0.8	0.8	0.7	0.9	0.8	ySPPAISPLVSEK	VAYVETKySPPAISP	Y1;	Yes	Y(1): 99.3; S(2): 0.7; S(7): 0.0; S(11): 0.0	5	2	1	0	N-Term(TMT6plex); S2(Phospho); K13(TMT6plex)
256223315	NP_001157798.1	10902	BRD8	bromodomain-containing protein 8 isoform 4	T572	-	-	-	-	0.7	0.7	0.9	0.9	-	-	-	-	0.7	0.7	1.0	0.9	0.7	0.7	1.0	0.9	GDEtPLTNVK	AIGKGDEtPLTNVKT	T4;	No	T(4): 100.0; T(7): 0.0	1	3	1	0	N-Term(TMT6plex); T4(Phospho); K10(TMT6plex)
51173722	NP_004625.2	7862	BRPF1	peregrin isoform 2	S860	-	-	-	-	0.9	1.1	0.9	0.9	-	-	-	-	0.9	1.1	0.8	0.8	0.9	1.1	0.8	0.8	DGQTDsAAEESSSQETSK	DKDGQTDsAAEESSS	S6;	Yes	T(4): 1.0; S(6): 99.0; S(11): 0.0; S(12): 0.0; S(13): 0.0; T(16): 0.0; S(17): 0.0	2	2	1	0	N-Term(TMT6plex); S6(Phospho); K18(TMT6plex)
386781463	NP_001247419.1	51599	LSR	lipolysis-stimulated lipoprotein receptor isoform 5	S422	-	-	-	-	1.2	0.8	0.8	0.9	-	-	-	-	1.2	0.9	0.9	0.8	1.2	0.9	0.9	0.8	sRDDLYDQDDSR	YMPPRSRsDDLYDQ	S1;	No	S(1): 100.0; Y(6): 0.0; S(11): 0.0	1	5	1	1	N-Term(TMT6plex); S1(Phospho)
134152674	NP_001077054.1	9135	RABEP1	rab GTPase-binding effector protein 1 isoform 2	S410	-	-	-	-	0.6	0.7	0.8	0.9	-	-	-	-	0.6	0.6	0.8	0.9	0.6	0.6	0.8	0.9	AQSTDsLGTSGSLQSK	RRAQSTDsLGTSGSL	S6;	Yes	S(3): 50.0; T(4): 50.0; S(6): 100.0; T(9): 0.0; S(10): 0.0; S(12): 0.0; S(15): [illegible]	2	3	1	0	N-Term(TMT6plex); S3(Phospho); S6(Phospho); K16(TMT6plex)
31563524	NP_852667.1	10398	MYL9	myosin regulatory light polypeptide 9 isoform b	S20	-	-	-	-	0.9	1.5	1.5	0.8	-	-	-	-	0.8	1.4	1.2	0.9	0.8	1.4	1.2	0.9	ATsNVFAMFDQSQIQEFK	KRPQRATsNVFAMFD	S3;	Yes	T(2): 1.0; S(3): 99.0; S(12): 0.0	2	4	1	0	N-Term(TMT6plex); S3(Phospho); M8(Oxidation); K18(TMT6plex)
112382377	NP_055316.2	27338	UBE2S	ubiquitin-conjugating enzyme E2 S	S73	-	-	-	-	0.7	1.0	1.0	0.8	-	-	-	-	0.7	0.9	0.9	0.8	0.7	0.9	0.9	0.8	DEPAsPPK	LGKDEPAsPPKGYEL	S5;	Yes	S(5): 100.0	2	1	1	0	N-Term(TMT6plex); S5(Phospho); K8(TMT6plex)
23397574	NP_695003.1	257364	SNX33	sorting nexin-33	S169	-	-	-	-	0.8	0.8	0.7	0.8	-	-	-	-	0.9	0.8	0.7	0.9	0.9	0.8	0.7	0.9	QDsLASAK	PPLERQDsLASAKRG	S3;	No	S(3): 100.0; S(6): 0.0	1	1	1	0	N-Term(TMT6plex); S3(Phospho); K8(TMT6plex)
223633988	NP_001138678.1	85379	KIAA1671	uncharacterized protein KIAA1671	S1224	-	-	-	-	0.8	1.1	1.0	0.8	-	-	-	-	-	-	-	-	-	-	-	-	RsPTVEPSTLPR	GEAQERRsPTVEPST	S2;	Yes	S(2): 100.0; T(4): 0.0; S(8): 0.0; T(9): 0.0	1	1	1	1	N-Term(TMT6plex); S2(Phospho)
118722349	NP_976324.2	389677	RBM12B	RNA-binding protein 12B	S254	-	-	-	-	0.9	1.0	1.1	0.8	-	-	-	-	1.0	1.0	1.1	0.9	1.0	1.0	1.1	0.9	SFEHsPPR	LRRSFEHsPPRGIND	S5;	Yes	S(1): 0.0; S(5): 100.0	3	1	1	0	N-Term(TMT6plex); S5(Phospho)
38016945	NP_066288.2	10522	DEAF1	deformed epidermal autoregulatory factor 1 homolog	T312	-	-	-	-	0.6	0.8	0.6	0.8	-	-	-	-	0.7	0.9	0.6	0.9	0.7	0.9	0.6	0.9	ENELPTtPVKK	KENELPTtPVKKDSP	T7;	Yes	T(6): 1.4; T(7): 98.6	1	1	1	1	N-Term(TMT6plex); T7(Phospho); K10(TMT6plex); K11(TMT6plex)
16262452	NP_065821.1	57530	CGN	cingulin	S153	-	-	-	-	0.6	1.0	0.8	0.8	-	-	-	-	0.8	1.2	1.1	1.0	0.8	1.2	1.1	1.0	sNSMLELAPK	PVDPSNRsNSMLELA	S1;	No	S(1): 99.2; S(3): 0.8	1	1	1	0	N-Term(TMT6plex); S1(Phospho); M4(Oxidation); K10(TMT6plex)
192449449	NP_071349.3	63893	UBE2O	ubiquitin-conjugating enzyme E2 O	S896	-	-	-	-	0.7	0.7	0.7	0.8	-	-	-	-	0.8	0.8	0.8	1.0	0.8	0.8	0.8	1.0	EDKPEGQsPVK	EDKPEGQsPVKAEWP	S8;	Yes	S(8): 100.0	1	1	1	0	N-Term(TMT6plex); K3(TMT6plex); S8(Phospho); K11(TMT6plex)
589269226	NP_001277136.1	55802	DCP1A	mRNA-decapping enzyme 1A isoform e	S366	-	-	-	-	0.9	1.0	0.7	0.8	-	-	-	-	0.8	1.0	0.5	0.7	0.8	1.0	0.5	0.7	KASSPsPLTIGTPESQR	ERKASSPsPLTIGTP	S6;	No	S(3): 0.0; S(4): 0.0; S(6): 100.0; T(9): 0.0; T(12): 0.0; S(15): 0.0	2	5	1	1	N-Term(TMT6plex); K1(TMT6plex); S6(Phospho)
533112479	NP_059984.3	55558	PLXNA3	plexin-A3 precursor	S1610	-	-	-	-	1.2	1.1	1.0	0.8	-	-	-	-	0.8	0.5	0.5	0.4	0.8	0.5	0.5	0.4	TASSPDsLR	RTASSPDsLRSRAPM	S7;	Yes	T(1): 0.0; S(3): 0.0; S(4): 0.0; S(7): 100.0	1	2	1	0	N-Term(TMT6plex); S7(Phospho)
10190682	NP_065700.1	57332	CBX8	chromobox protein homolog 8	S190	-	-	-	-	0.7	0.7	0.8	0.8	-	-	-	-	0.7	0.7	0.8	0.8	0.7	0.7	0.8	0.8	VDDKPsSPGDSSK	SRVDDKPsSPGDSSK	S6;	No	S(6): 98.4; S(7): 1.6; S(11): 0.0; S(12): 0.0	2	1	1	0	N-Term(TMT6plex); K4(TMT6plex); S6(Phospho); K13(TMT6plex)
296011010	NP_060252.4	54906	FAM208B	protein FAM208B	S1087	-	-	-	-	0.8	0.9	0.9	0.8	-	-	-	-	0.8	0.9	0.9	0.9	0.8	0.9	0.9	0.9	QVsPAASLR	KELIKQVsPAASLRH	S3;	Yes	S(3): 100.0; S(7): 0.0	2	1	1	0	N-Term(TMT6plex); S3(Phospho)
33356547	NP_004517.2	4171	MCM2	DNA replication licensing factor MCM2	S41	-	-	-	-	1.0	0.5	0.7	0.8	-	-	-	-	0.9	0.5	0.8	1.0	0.9	0.5	0.8	1.0	TDALtSsPGR	RTDALTSsPGRDLPP	S7;	Yes	T(1): 0.0; T(5): 0.0; S(6): 0.0; S(7): 100.0	2	1	1	0	N-Term(TMT6plex); S7(Phospho)
55769548	NP_055475.2	9678	PHF14	PHD finger protein 14	S302	-	-	-	-	0.8	0.9	1.1	0.8	-	-	-	-	0.8	1.0	1.1	0.9	0.8	1.0	1.1	0.9	SNEDsLILEK	QSKSNEDsLILEKSQ	S5;	Yes	S(1): 0.0; S(5): 100.0	2	1	1	0	N-Term(TMT6plex); S5(Phospho); K10(TMT6plex)
4758356	NP_004102.1	2237	FEN1	flap endonuclease 1	S351	-	-	-	-	0.8	0.8	0.8	0.8	-	-	-	-	0.8	0.9	1.0	0.9	0.8	0.9	1.0	0.9	VTGSLsSAK	FKVTGSLsSAKRKEP	S6;	No	T(2): 0.0; S(4): 1.2; S(6): 97.6; S(7): [illegible]	1	1	1	0	N-Term(TMT6plex); S6(Phospho); K9(TMT6plex)
148596953	NP_061877.1	54467	ANKIB1	ankyrin repeat and IBR domain-containing protein 1	S737	-	-	-	-	0.7	0.8	0.8	0.8	-	-	-	-	-	-	-	-	-	-	-	-	GVAPADsPEAPR	RGVAPADsPEAPRRS	S7;	Yes	S(7): 100.0	1	1	1	0	N-Term(TMT6plex); S7(Phospho)
118421085	NP_689472.3	90381	TICRR	treslin	T1307	-	-	-	-	0.8	0.7	0.8	0.8	-	-	-	-	1.0	0.7	0.9	0.9	1.0	0.7	0.9	0.9	RPGNSTVTSSPPVtPK	VTSSPPVtPKKLFTS	T14;	Yes	S(5): 0.0; T(6): 0.0; T(8): 0.0; S(9): 50.0; S(10): 50.0; T(14): 100.0	1	1	1	0	N-Term(TMT6plex); T8(Phospho); T14(Phospho); K16(TMT6plex)
56682953	NP_004438.3	2059	EPS8	epidermal growth factor receptor kinase substrate 8	S659	-	-	-	-	0.9	0.8	1.0	0.8	-	-	-	-	0.7	0.7	0.8	0.7	0.7	0.7	0.8	0.7	QNsSSSDSGGSIVR	ANITRQNsSSSDSGG	S3;	Yes	S(3): 100.0; S(4): 0.0; S(5): 0.0; S(6): 0.0; S(8): 0.0; S(11): 0.0	1	1	1	0	N-Term(TMT6plex); S3(Phospho)
394582099	NP_001257401.1	4172	MCM3	DNA replication licensing factor MCM3 isoform 2	S682; T684	-	-	-	-	0.6	0.5	0.8	0.8	-	-	-	-	0.6	0.6	1.0	1.0	0.6	0.6	1.0	1.0	SEDEsEtEDEEEKSQEDQEQK	KKRSEDEsETEDEEE;RSEDESEtEDEEEKS	S5; T7;	No;No	S(1): 0.1; S(5): 99.9; T(7): 100.0; S(14): 0.0	1	2	1	1	N-Term(TMT6plex); S1(Phospho); S5(Phospho); K13(TMT6plex); K21(TMT6plex)
363807222	NP_001241872.1	55704	CCDC88A	girdin isoform 3	S1386	-	-	-	-	0.8	0.9	0.7	0.8	-	-	-	-	0.7	0.9	0.7	0.8	0.7	0.9	0.7	0.8	EYDPsPPR	QYKEYDPsPPRRRGN	S5;	No	Y(2): 0.0; S(5): 100.0	1	3	1	0	N-Term(TMT6plex); S5(Phospho)
56549645	NP_624358.2	4928	NUP98	nuclear pore complex protein Nup98-Nup96 isoform 4	S1011	-	-	-	-	1.8	0.9	0.9	0.8	-	-	-	-	1.6	0.9	0.8	0.8	1.6	0.9	0.8	0.8	ADTSQEICsPR	DTSQEICsPRLPISA	S9;	No	T(3): 0.0; S(4): 0.0; S(9): 100.0	1	2	1	0	N-Term(TMT6plex); C8(Carbamidomethyl); S9(Phospho)
259906020	NP_001158287.1	22985	ACIN1	apoptotic chromatin condensation inducer in the nucleus isoform 3	S176	-	-	-	-	1.2	1.0	0.9	0.8	-	-	-	-	0.9	0.9	1.0	0.9	0.9	0.9	1.0	0.9	GDsDDEKPR	ISEEKGDsDDEKPRK	S3;	No	S(3): 100.0	1	3	1	0	N-Term(TMT6plex); S3(Phospho); K7(TMT6plex)

Khan et al., 2019. Multi-omics analysis to characterize cigarette smoke induced molecular alterations in esophageal cells
Supplementary Table 8. List of phosphopeptides identified and quantified in untreated and chronically treated Het1A cells with cigarette smoke condensate for 8 months using SequestHT and Mascot search algorithms with a PhosphoRS probability score ≥75%

Normalized phosphoprotein ratios with respect to corresponding total protein ratios. | Average of replicates

Protein group Accession	NP_Accession	Gene ID	Gene Symbol	Protein Description	PhosphoSite (Protein)	Het-1A-Smoke - 2M/Parental (Replicate 1)	Het-1A-Smoke - 4M/Parental (Replicate 1)	Het-1A-Smoke - 6M/Parental (Replicate 1)	Het-1A-Smoke - 8M/Parental (Replicate 1)	Het-1A-Smoke - 2M/Parental (Replicate 2)	Het-1A-Smoke - 4M/Parental (Replicate 2)	Het-1A-Smoke - 6M/Parental (Replicate 2)	Het-1A-Smoke - 8M/Parental (Replicate 2)	Normalized Het-1A-Smoke - 2M/Parental (Replicate 1)	Normalized Het-1A-Smoke - 4M/Parental (Replicate 1)	Normalized Het-1A-Smoke - 6M/Parental (Replicate 1)	Normalized Het-1A-Smoke - 8M/Parental (Replicate 1)	Normalized Het-1A-Smoke - 2M/Parental (Replicate 2)	Normalized Het-1A-Smoke - 4M/Parental (Replicate 2)	Normalized Het-1A-Smoke - 6M/Parental (Replicate 2)	Normalized Het-1A-Smoke - 8M/Parental (Replicate 2)	Average Het-1A-Smoke - 2M/Parental	Average Het-1A-Smoke - 4M/Parental	Average Het-1A-Smoke - 6M/Parental	Average Het-1A-Smoke - 8M/Parental	Peptide Sequence	Phosphowindow	PhosphoSite (Peptide)	Phosphosite Plus Evidence	phosphoRS Site Probabilities	# PSMs	# Proteins	# Protein Groups	# Missed Cleavages	Modifications
296010995	NP_001171592.1	10006	ABI1	abl interactor 1 isoform h	S290	-	-	-	-	0.8	0.8	0.9	0.8	-	-	-	-	1.1	0.9	0.9	0.8	1.1	0.9	0.9	0.8	HNsTTSSTSSGGYR	RQISRHNsTTSSTSS	S3;	No	S(3): 100.0; T(4): 0.0; T(5): 0.0; S(6): 0.0; S(7): 0.0; T(8): 0.0; S(9): 0.0; S(10): 0.0; Y(13): 0.0	2	8	1	0	N-Term(TMT6plex); S3(Phospho)
34577087	NP_899237.1	11342	RNF13	E3 ubiquitin-protein ligase RNF13	T380	-	-	-	-	1.2	1.0	0.8	0.8	-	-	-	-	-	-	-	-	-	-	-	-	DYNIANtV	RDYNIANtV	T7;	No	Y(2): 0.0; T(7): 100.0	1	1	1	0	N-Term(TMT6plex); T7(Phospho)
29826323	NP_054909.2	118	ADD1	alpha-adducin isoform c	S586	-	-	-	-	0.5	0.8	0.7	0.8	-	-	-	-	0.6	0.9	0.8	1.0	0.6	0.9	0.8	1.0	GsEENLDEAR	VERKQKGsEENLDEA	S2;	Yes	S(2): 100.0	2	5	1	0	N-Term(TMT6plex); S2(Phospho)
304434531	NP_001182122.1	4678	NASP	nuclear autoantigenic sperm protein isoform 4	S320	-	-	-	-	1.6	1.5	2.1	0.8	-	-	-	-	2.1	2.0	3.3	1.2	2.1	2.0	3.3	1.2	DGAVNGPsVVGDQTPIEPQTSIER	DGAVNGPsVVGDQTP	S8;	No	S(8): 99.9; T(14): 0.1; T(20): 0.0; S(21): 0.0	1	2	1	0	N-Term(TMT6plex); S8(Phospho)
343887347	NP_001230575.1	10421	CD2BP2	CD2 antigen cytoplasmic tail-binding protein 2	S195	-	-	-	-	1.0	0.8	1.1	0.8	-	-	-	-	1.0	0.9	1.1	0.9	1.0	0.9	1.1	0.9	KGPGQPsPQR	KGPGQPsPQRLDRL	S8;	Yes	S(7): 0.0; S(8): 100.0	2	1	1	1	N-Term(TMT6plex); K1(TMT6plex); S8(Phospho)
21314720	NP_078976.2	79753	SNIP1	smad nuclear-interacting protein 1	S74; S76	-	-	-	-	0.7	1.0	0.7	0.8	-	-	-	-	0.7	1.2	0.8	1.0	0.7	1.2	0.8	1.0	GVsRsPPK	GNRARGVsRSPPKKK;RARGVSRsPPKKKNK	S3; S5;	Yes;Yes	S(3): 100.0; S(5): 100.0	1	1	1	1	N-Term(TMT6plex); S3(Phospho); S5(Phospho); K8(TMT6plex)
209977056	NP_001129690.1	55092	TMEM51	transmembrane protein 51	S182	-	-	-	-	0.9	0.8	0.8	0.8	-	-	-	-	1.5	1.2	1.3	1.2	1.5	1.2	1.3	1.2	ADVEAsPGNPPDR	TRADVEAsPGNPPDR	S6;	Yes	S(6): 100.0	1	1	1	0	N-Term(TMT6plex); S6(Phospho)
4502193	NP_001645.1	369	ARAF	serine/threonine-protein kinase A-Raf isoform 1	S257	-	-	-	-	1.0	1.0	0.9	0.8	-	-	-	-	0.9	1.0	0.8	0.8	0.9	1.0	0.8	0.8	GsPSPASVSSGR	SDGTPRGsPSPASVS	S2;	Yes	S(2): 100.0; S(4): 0.0; S(7): 0.0; S(9): 0.0; S(10): 0.0	1	2	1	0	N-Term(TMT6plex); S2(Phospho)
38327034	NP_006039.2	10277	UBE4B	ubiquitin conjugation factor E4 B isoform 2	S83	-	-	-	-	0.7	2.0	1.1	0.8	-	-	-	-	0.7	1.9	1.2	0.7	0.7	1.9	1.2	0.7	SQSSEGVsSLSSSPSNSLETQSQSLSR	SQSSEGVsSLSSSPS	S8;	No	S(1): 0.0; S(3): 0.0; S(4): 0.0; S(8): 86.9; S(9): 2.2; S(11): 2.2; S(12): 2.2; S(13): 2.2; S(15): 2.2; S(17): 2.2; T(20): 0.1; S(22): 0.0; S(24): 0.0; [illegible]	2	2	1	0	N-Term(TMT6plex); S8(Phospho)
188536047	NP_003065.3	6599	SMARCC1	SWI/SNF complex subunit SMARCC1	S310	-	-	-	-	1.0	0.9	1.0	0.8	-	-	-	-	1.1	0.9	1.0	0.8	1.1	0.9	1.0	0.8	NEEPVRsPERR	KNEEPVRsPERRDRK	S7;	Yes	S(7): 100.0	1	1	1	2	N-Term(TMT6plex); S7(Phospho)
52630338	NP_001005290.1	84722	PSRC1	proline/serine-rich coiled-coil protein 1 isoform b	S140	-	-	-	-	0.6	0.7	0.7	0.8	-	-	-	-	0.7	0.8	0.9	0.9	0.7	0.8	0.9	0.9	STPsPSSLTPR	SLTRSTPsPSSLTPR	S4;	Yes	S(1): 0.0; T(2): 0.0; S(4): 98.6; S(6): 0.0; S(7): 0.0; T(9): 1.3	1	2	1	0	N-Term(TMT6plex); S4(Phospho)
29789060	NP_055617.1	9847	C2CD5	C2 domain-containing protein 5 isoform e	S295	-	-	-	-	0.9	0.7	1.1	0.8	-	-	-	-	0.8	0.6	1.0	0.8	0.8	0.6	1.0	0.8	NQTYSFsPSK	KNQTYSFsPSKSYSR	S7;	Yes	T(3): 0.0; Y(4): 0.0; S(5): 0.7; S(7): 99.3; S(9): 0.0	2	2	1	0	N-Term(TMT6plex); S7(Phospho); K10(TMT6plex)
47578107	NP_056199.2	25836	NIPBL	nipped-B-like protein isoform B	S318	-	-	-	-	0.7	0.6	1.2	0.8	-	-	-	-	0.6	0.6	1.1	0.7	0.6	0.6	1.1	0.7	DVPPDILLDsPER	PPDILLDsPERKQKK	S10;	Yes	S(10): 100.0	1	2	1	0	N-Term(TMT6plex); S10(Phospho)
281182404	NP_060830.2	55787	TXLNG	gamma-taxilin isoform 1	S97	-	-	-	-	0.9	0.9	0.7	0.8	-	-	-	-	0.9	0.9	0.8	0.8	0.9	0.9	0.8	0.8	NLVsPAYCTQESR	TENRNLVsPAYCTQE	S4;	Yes	S(4): 100.0; Y(7): 0.0; T(9): 0.0; S(12): 0.0	1	1	1	0	N-Term(TMT6plex); S4(Phospho); C8(Carbamidomethyl)
298919181	NP_001177369.1	9611	NCOR1	nuclear receptor corepressor 1 isoform 3	S1008	-	-	-	-	0.7	0.8	0.9	0.8	-	-	-	-	0.7	0.6	0.7	0.6	0.7	0.6	0.7	0.6	SSTsPCGTSK	LECRSSTsPCGTSKS	S4;	No	S(1): 0.0; S(2): 0.0; T(3): 1.4; S(4): 98.5; T(8): 0.0; S(9): 0.0	1	3	1	0	N-Term(TMT6plex); S4(Phospho); C6(Carbamidomethyl); K10(TMT6plex)
7661958	NP_055554.1	9774	BCLAF1	bcl-2-associated transcription factor 1 isoform 1	S285	-	-	-	-	0.5	0.9	0.7	0.8	-	-	-	-	0.5	0.9	0.8	0.9	0.5	0.9	0.8	0.9	YsPSQNSPIHHIPSR	GNGSSRYsPSQNSPI	S2;	Yes	Y(1): 1.7; S(2): 98.3; S(4): 50.0; S(7): 50.0; S(14): 0.0	1	3	1	0	N-Term(TMT6plex); S2(Phospho); S4(Phospho)
63252863	NP_115820.2	84464	SLX4	structure-specific endonuclease subunit SLX4	S1185	-	-	-	-	0.7	0.9	0.6	0.8	-	-	-	-	0.6	0.8	0.6	0.7	0.6	0.8	0.6	0.7	ALEIsPR	EKKALEIsPRSCELF	S5;	Yes	S(5): 100.0	1	1	1	0	N-Term(TMT6plex); S5(Phospho)
62414289	NP_003371.2	7431	VIM	vimentin	S10	-	-	-	-	0.7	0.5	1.2	0.8	-	-	-	-	0.8	0.6	1.0	0.9	0.8	0.6	1.0	0.9	SVSSSsYR	TRSVSSSsYRRMFGG	S6;	Yes	S(1): 0.0; S(3): 0.0; S(4): 0.0; S(5): 0.6; S(6): 99.4; Y(7): 0.0	1	1	1	0	N-Term(TMT6plex); S6(Phospho)
61743954	NP_001611.1	79026	AHNAK	neuroblast differentiation-associated protein AHNAK isoform 1	S1286	-	-	-	-	0.5	0.8	1.0	0.8	-	-	-	-	0.6	0.8	1.0	0.9	0.6	0.8	1.0	0.9	ADIDVsGPK	PKADIDVsGPKVDVE	S6;	Yes	S(6): 100.0	1	1	1	0	N-Term(TMT6plex); S6(Phospho); K9(TMT6plex)
209862789	NP_056056.2	57553	MICAL3	protein-methionine sulfoxide oxidase MICAL3 isoform	S1221	-	-	-	-	1.0	1.0	0.9	0.8	-	-	-	-	0.9	0.8	0.8	0.8	0.9	0.8	0.8	0.8	AVHsPIR	SDLKAVHsPIRSQPV	S4;	Yes	S(4): 100.0	1	1	1	0	N-Term(TMT6plex); S4(Phospho)
14717407	NP_005633.2	6879	TAF7	transcription initiation factor TFIID subunit 7	S159	-	-	-	-	1.0	0.9	1.0	0.8	-	-	-	-	1.2	1.0	1.2	1.0	1.2	1.0	1.2	1.0	YIEsPDVEK	AKKKYIEsPDVEKEV	S4;	Yes	Y(1): 0.0; S(4): 100.0	2	1	1	0	N-Term(TMT6plex); S4(Phospho); K9(TMT6plex)
222136583	NP_001558.3	3636	INPPL1	phosphatidylinositol 3,4,5-trisphosphate 5-	S132	-	-	-	-	0.8	0.8	0.7	0.8	-	-	-	-	0.7	0.7	0.6	0.7	0.7	0.7	0.6	0.7	DAsDGEDEKPPLPPR	PPDDRDAsDGEDEKP	S3;	Yes	S(3): 100.0	1	1	1	0	N-Term(TMT6plex); S3(Phospho); K9(TMT6plex)
253970412	NP_057510.4	51512	GTSE1	G2 and S phase-expressed protein 1	S520	-	-	-	-	0.6	0.9	1.1	0.8	-	-	-	-	0.7	1.0	1.2	1.0	0.7	1.0	1.2	1.0	SSGPAPQsLLSAR	SSGPAPQsLLSARRV	S8;	No	S(1): 0.0; S(2): 0.0; S(8): 99.0; S(11): 1.0	1	1	1	0	N-Term(TMT6plex); S11(Phospho)
21396500	NP_003600.2	8479	HIRIP3	HIRA-interacting protein 3 isoform 1	S159; S160	-	-	-	-	0.7	0.8	0.8	0.8	-	-	-	-	0.9	0.9	1.0	0.9	0.9	0.9	1.0	0.9	GEEssEEEEK	PAQRGEEsSEEEEKG;AQRGEESsEEEEKGY	S4; S5;	Yes;Yes	S(4): 100.0; S(5): 100.0	4	1	1	0	N-Term(TMT6plex); S4(Phospho); S5(Phospho); K10(TMT6plex)
260763910	NP_659498.4	221150	SKA3	spindle and kinetochore-associated protein 3 isoform 1	T190	-	-	-	-	0.7	0.6	0.4	0.8	-	-	-	-	0.9	0.7	0.4	0.8	0.9	0.7	0.4	0.8	EEPVIVtPPTK	KEEPVIVtPPTKQSL	T7;	Yes	T(7): 100.0; T(10): 0.0	1	2	1	0	N-Term(TMT6plex); T7(Phospho); K11(TMT6plex)
295148163	NP_001171189.1	8462	KLF11	Krueppel-like factor 11 isoform b	S107	-	-	-	-	1.1	1.1	0.7	0.8	-	-	-	-	-	-	-	-	-	-	-	-	TPVsPQVTDSK	PSTRTPVsPQVTDSK	S4;	No	T(1): 0.0; S(4): 100.0; T(8): 0.0; S(10): 0.0	1	2	1	0	N-Term(TMT6plex); S4(Phospho); K11(TMT6plex)
98991763	NP_742101.2	2070	EYA4	eyes absent homolog 4 isoform b	S338	-	-	-	-	1.6	0.6	1.1	0.8	-	-	-	-	1.3	0.3	0.6	0.4	1.3	0.3	0.6	0.4	NNPsPPPDSDLER	RGRKNNPsPPPDSDL	S4;	No	S(4): 100.0; S(9): 0.0	1	3	1	0	N-Term(TMT6plex); S4(Phospho)
82830440	NP_060663.2	55215	FANCI	Fanconi anemia group I protein isoform 2	S1252	-	-	-	-	1.8	1.1	0.9	0.8	-	-	-	-	1.9	1.1	0.9	0.8	1.9	1.1	0.9	0.8	EDGEDENEEGTAsEHGGQNK	ENEEGTAsEHGGQNK	S13;	No	T(11): 0.0; S(13): 100.0	2	2	1	0	N-Term(TMT6plex); S13(Phospho); K20(TMT6plex)
157817073	NP_055898.1	51755	CDK12	cyclin-dependent kinase 12 isoform 2	S1082	-	-	-	-	0.9	0.8	1.1	0.8	-	-	-	-	0.9	0.8	1.1	0.9	0.9	0.8	1.1	0.9	NsPAPPQPAPGK	STEPVKNsPAPPQP	S2;	Yes	S(2): 99.3; S(3): 0.7	5	2	1	0	N-Term(TMT6plex); S2(Phospho); K13(TMT6plex)
336020353	NP_004825.3	9448	MAP4K4	mitogen-activated protein kinase kinase kinase kinase 4 isoform 1	S625	-	-	-	-	1.0	1.0	1.1	0.8	-	-	-	-	0.9	0.9	1.0	0.8	0.9	0.9	1.0	0.8	SSsKSEGSPSQR	RFRVRSSsKSEGSPS	S3;	No	S(1): 0.0; S(2): 0.0; S(3): 100.0; S(5): 0.0; S(8): 0.0; S(10): 0.0	1	5	1	1	N-Term(TMT6plex); S3(Phospho); K4(TMT6plex)
87299628	NP_683692.2	259282	BOD1L1	biorientation of chromosomes in cell division protein 1-like 1	S266	-	-	-	-	0.8	1.1	1.0	0.8	-	-	-	-	0.8	1.1	1.1	0.9	0.8	1.1	1.1	0.9	STADsGGEGLETAPK	KEKSTADsGGEGLET	S5;	Yes	S(1): 0.1; T(2): 2.1; S(5): 97.9; T(12): 0.0	2	1	1	0	N-Term(TMT6plex); S5(Phospho); K15(TMT6plex)
194578923	NP_001124000.1	9043	SPAG9	C-Jun-amino-terminal kinase-interacting protein 4 isoform 1	S733	-	-	-	-	0.7	0.8	0.8	0.8	-	-	-	-	0.8	0.9	0.9	0.9	0.8	0.9	0.9	0.9	SASQSsLDK	QRSASQSsLDKLDQE	S6;	Yes	S(1): 50.0; S(3): 50.0; S(5): 1.0; S(6): 99.0	1	4	1	0	N-Term(TMT6plex); S3(Phospho); S6(Phospho); K9(TMT6plex)
4758274	NP_004428.1	2035	EPB41	protein 4.1 isoform 6	S333	-	-	-	-	0.7	0.6	0.6	0.8	-	-	-	-	0.7	0.7	0.6	0.8	0.7	0.7	0.6	0.8	sLDGAAAVDSADR	ASKRASRsLDGAAAV	S1;	No	S(1): 100.0; S(10): 0.0	1	6	1	0	N-Term(TMT6plex); S1(Phospho)
4506903	NP_003760.1	8683	SRSF9	serine/arginine-rich splicing factor 9	S204	-	-	-	-	0.7	0.6	0.6	0.8	-	-	-	-	0.7	0.7	0.7	0.8	0.7	0.7	0.7	0.8	GRDsPYQSR	SGSRGRDsPYQSRGS	S4;	Yes	S(4): 100.0; Y(6): 0.0; S(8): 0.0	1	1	1	1	N-Term(TMT6plex); S4(Phospho)
149158690	NP_542417.2	7916	PRRC2A	protein PRRC2A	S2113	-	-	-	-	0.7	0.6	0.4	0.8	-	-	-	-	0.8	0.6	0.4	0.8	0.8	0.6	0.4	0.8	VDLYQQAsPPDALR	VDLYQQAsPPDALRW	S8;	No	Y(4): 1.8; S(8): 98.2	1	1	1	0	N-Term(TMT6plex); S8(Phospho)
5032031	NP_005769.1	10181	RBM5	RNA-binding protein 5	S59	-	-	-	-	0.7	0.7	0.9	0.8	-	-	-	-	0.8	0.7	1.0	0.9	0.8	0.7	1.0	0.9	DYDsPER	DDYRDYDsPERERER	S4;	Yes	Y(2): 0.0; S(4): 100.0	1	1	1	0	N-Term(TMT6plex); S4(Phospho)
110556636	NP_203754.2	85456	TNKS1BP1	182 kDa tankyrase-1-binding protein	S1297	-	-	-	-	0.7	0.7	1.1	0.8	-	-	-	-	0.7	0.7	1.1	0.9	0.7	0.7	1.1	0.9	NMAPGAVCsPGESK	MAPGAVCsPGESKEL	S9;	Yes	S(9): 100.0; S(13): 0.0	2	1	1	0	N-Term(TMT6plex); M2(Oxidation); C8(Carbamidomethyl); S9(Phospho); K14(TMT6plex)
100815975	NP_057480.2	51496	CTDSPL2	CTD small phosphatase-like protein 2	S134	-	-	-	-	0.7	0.8	0.8	0.8	-	-	-	-	0.7	0.9	0.8	0.9	0.7	0.9	0.8	0.9	LEDNPSSGsPPR	EDNPSSGsPPRTTLL	S9;	Yes	S(6): 0.0; S(7): 0.7; S(9): 99.3	2	1	1	0	N-Term(TMT6plex); S9(Phospho)
301129172	NP_001180299.1	11083	DIDO1	death-inducer obliterator 1 isoform b	S1040	-	-	-	-	1.6	0.6	1.1	0.8	-	-	-	-	1.6	0.6	1.1	0.8	1.6	0.6	1.1	0.8	sPPEGDTTLFLSR	ISTSESRsPPEGDTT	S1;	Yes	S(1): 100.0; T(7): 0.0; T(8): 0.0; S(12): 0.0	1	2	1	0	N-Term(TMT6plex); S1(Phospho)
282847506	NP_001164237.1	84458	LCOR	ligand-dependent corepressor isoform 2	S42	-	-	-	-	0.9	1.1	1.0	0.8	-	-	-	-	-	-	-	-	-	-	-	-	ASPVTTsPTAATTQNPVLSK	KASPVTTsPTAATTQ	S7;	Yes	S(2): 0.0; T(5): 0.0; T(6): 1.2; S(7): 97.6; T(9): 1.2; T(12): 0.0; T(13): 0.0; S(19): 0.0	2	2	1	0	N-Term(TMT6plex); T9(Phospho); K20(TMT6plex)
167614506	NP_002289.2	3936	LCP1	plastin-2	S5	-	-	-	-	0.8	0.7	0.7	0.8	-	-	-	-	0.8	0.8	0.6	1.0	0.8	0.8	0.6	1.0	GsVSDEEMMELR	MARGsVSDEEMM	S2;	Yes	S(2): 100.0; S(4): 0.0	1	1	1	0	N-Term(TMT6plex); S2(Phospho); M8(Oxidation); M9(Oxidation)
103472005	NP_002408.3	4288	MKI67	antigen KI-67 isoform 1	S1861	-	-	-	-	0.7	0.8	0.7	0.8	-	-	-	-	0.8	0.8	0.7	0.8	0.8	0.8	0.7	0.8	sPQSDPADTPTNTK	TKKILCKsPQSDPAD	S1;	Yes	S(1): 100.0; S(4): 0.0; T(9): 0.0; T(11): 0.0; T(13): 0.0	1	2	1	0	N-Term(TMT6plex); S1(Phospho); K14(TMT6plex)
38679974	NP_942135.1	31	ACACA	acetyl-CoA carboxylase 1 isoform 4	S2265	-	-	-	-	0.3	0.3	0.9	0.8	-	-	-	-	0.3	0.2	0.7	0.7	0.3	0.2	0.7	0.7	ILSTMDsPST	RILSTMDsPST_	S7;	No	S(3): 0.0; T(4): 0.0; S(7): 100.0; S(9): 0.0; T(10): 0.0	2	4	1	0	N-Term(TMT6plex); M5(Oxidation); S7(Phospho)
50345997	NP_001420.2	2033	EP300	histone acetyltransferase p300	T887	-	-	-	-	1.6	0.6	0.4	0.8	-	-	-	-	1.7	0.7	0.4	0.9	1.7	0.7	0.4	0.9	QTPtPPTTQLPQQVQPSLPAAPSADQPQQQPR	PPPRQTPtPPTTQLP	T4;	Yes	T(2): 0.1; T(4): 93.8; T(7): 3.1; T(8): 3.1; S(17): 0.0; S(23): 0.0	1	1	1	0	N-Term(TMT6plex); T4(Phospho)
9910266	NP_064627.1	56992	KIF15	kinesin-like protein KIF15	S568	-	-	-	-	0.8	0.7	0.8	0.8	-	-	-	-	0.8	0.8	0.9	0.8	0.8	0.8	0.9	0.8	NQQGEsPK	DKNQQGEsPKAQKEP	S6;	Yes	S(6): 100.0	3	1	1	0	N-Term(TMT6plex); S6(Phospho); K8(TMT6plex)
73760401	NP_001027455.1	7112	TMPO	thymopoietin isoform gamma	S190	-	-	-	-	1.2	0.7	1.1	0.8	-	-	-	-	1.3	0.7	1.3	0.7	1.3	0.7	1.3	0.7	QNGSNDSDRYSDNEEDsK	YSDNEEDsKIELKLE	S17;	Yes	S(4): 0.0; S(7): 0.0; Y(10): 0.0; S(11): 0.0; S(17): 99.9	1	2	1	1	N-Term(TMT6plex); S11(Phospho); K18(TMT6plex)
118421085	NP_689472.3	90381	TICRR	treslin	T1633	-	-	-	-	0.8	0.9	0.9	0.8	-	-	-	-	0.9	0.9	1.0	0.8	0.9	0.9	1.0	0.8	LSHStPGK	CPRLSHStPGKSRGQ	T5;	Yes	S(2): 0.0; S(4): 0.0; T(5): 100.0	1	1	1	0	N-Term(TMT6plex); T5(Phospho); K8(TMT6plex)
302058308	NP_001180462.1	6787	NEK4	serine/threonine-protein kinase Nek4 isoform 2	S372	-	-	-	-	0.8	0.8	1.2	0.8	-	-	-	-	0.9	0.8	1.4	1.0	0.9	0.8	1.4	1.0	DQSLALsPK	KDQSLALsPKLECSG	S7;	No	S(3): 0.0; S(7): 100.0	2	2	1	0	N-Term(TMT6plex); S7(Phospho); K9(TMT6plex)
62244004	NP_001013865.1	79811	SLTM	SAFB-like transcription modulator isoform b	S353; S355	-	-	-	-	1.2	1.1	1.1	0.8	-	-	-	-	1.2	1.2	1.3	0.9	1.2	1.2	1.3	0.9	DDKGsTsSTSGSSGSSTK	SSKDDKGsTSSTSGS;KDDKGSTsSTSGSSG	S5; S7;	No;No	S(5): 98.5; T(6): 3.0; S(7): 98.4; S(8): 0.0; T(9): 0.0; S(10): 0.0; S(12): 0.0; S(13): 0.0; S(15): 0.0; S(16): 0.0; T(17): 0.0	1	2	1	1	N-Term(TMT6plex); K3(TMT6plex); S5(Phospho); S7(Phospho); K18(TMT6plex)
110556636	NP_203754.2	85456	TNKS1BP1	182 kDa tankyrase-1-binding protein	S9	-	-	-	-	0.8	0.8	0.9	0.8	-	-	-	-	0.8	0.7	0.9	0.9	0.8	0.7	0.9	0.9	EsSAMASPLPR	KVSTLREsSAMASPL	S2;	Yes	S(2): 97.6; S(3): 1.2; S(7): 1.2	1	1	1	0	N-Term(TMT6plex); S2(Phospho); M5(Oxidation)
149588928	NP_065897.1	57606	SLAIN2	SLAIN motif-containing protein 2	S349; S353	-	-	-	-	0.9	1.0	1.2	0.8	-	-	-	-	0.9	1.0	1.3	0.7	0.9	1.0	1.3	0.7	NsPRPsPK	FSPSPRNsPRPSPKQ;PRNSPRPsPKQSPRN	S2; S6;	Yes;Yes	S(2): 100.0; S(6): 100.0	2	1	1	0	N-Term(TMT6plex); S2(Phospho); S6(Phospho); K8(TMT6plex)
115430211	NP_001041648.1	65979	PHACTR4	phosphatase and actin regulator 4 isoform 1	S176	-	-	-	-	1.1	1.1	1.0	0.8	-	-	-	-	1.3	1.2	1.3	0.9	1.3	1.2	1.3	0.9	RPLsSSHEASEGQAK	LPPKRPLsSSHEASE	S4;	Yes	S(4): 98.1; S(5): 1.0; S(6): 1.0; S(10): 0.0	1	2	1	0	N-Term(TMT6plex); S4(Phospho); K15(TMT6plex)
41393547	NP_056993.2	51594	NBAS	neuroblastoma-amplified sequence	S475	-	-	-	-	1.0	1.1	1.2	0.8	-	-	-	-	0.9	0.9	1.0	0.7	0.9	0.9	1.0	0.7	AGEEDEGEEDSDsDYEISAK	EGEEDSDsDYEISAK	S13;	Yes	S(11): 1.3; S(13): 98.7; Y(15): 0.0; S(18): 0.0	2	1	1	0	N-Term(TMT6plex); S13(Phospho); K20(TMT6plex)
150418007	NP_006258.3	5903	RANBP2	E3 SUMO-protein ligase RanBP2	T1144	-	-	-	-	0.8	0.7	0.7	0.8	-	-	-	-	0.7	0.7	0.7	0.7	0.7	0.7	0.7	0.7	SVFGtPTLETANK	PGKSVFGtPTLETAN	T5;	Yes	S(1): 2.0; T(5): 94.0; T(7): 2.0; T(10): 2.0	1	1	1	0	N-Term(TMT6plex); T5(Phospho); K13(TMT6plex)
4502175	NP_001640.1	357	SHROOM2	protein Shroom2	S1036; S1039	-	-	-	-	0.9	1.0	0.8	0.8	-	-	-	-	1.3	1.5	1.2	1.2	1.3	1.5	1.2	1.2	AQsPGsPLHAR	DLGPRAQsPGSPLHA;PRAQSPGsPLHARGQ	S3; S6;	Yes;Yes	S(3): 100.0; S(6): 100.0	1	1	1	0	N-Term(TMT6plex); S3(Phospho); S6(Phospho)
239582751	NP_001155049.1	23034	SAMD4A	protein Smaug homolog 1 isoform 3	S10	-	-	-	-	0.8	1.2	1.1	0.8	-	-	-	-	0.9	1.3	0.9	0.8	0.9	1.3	0.9	0.8	AYsSPSTIPEAR	LTPIKAYsSPSTIPE	S3;	No	Y(2): 2.1; S(3): 76.3; S(4): 21.1; S(6): 21.1; T(7): 39.7; T(8): 39.7	1	3	1	0	N-Term(TMT6plex); S6(Phospho); T7(Phospho)
27477095	NP_758859.1	64324	NSD1	histone-lysine N-methyltransferase, H3 lysine-36 and H4 lysine-20 specific isoform a	S2202	-	-	-	-	0.7	0.6	1.1	0.8	-	-	-	-	-	-	-	-	-	-	-	-	AAsPHQVTPQADEK	RQKERAAsPHQVTPQ	S3;	No	S(3): 100.0; T(8): 0.0	1	2	1	0	N-Term(TMT6plex); S3(Phospho); K14(TMT6plex)
4885237	NP_005242.1	2303	FOXC2	forkhead box protein C2	S219	-	-	-	-	0.6	0.9	0.8	0.8	-	-	-	-	0.3	0.8	0.4	0.5	0.3	0.8	0.4	0.5	SEAAsPALPVITK	VIKSEAAsPALPVIT	S5;	Yes	S(1): 0.0; S(5): 100.0; T(12): 0.0	2	1	1	0	N-Term(TMT6plex); S5(Phospho); K13(TMT6plex)
20127499	NP_006266.2	6431	SRSF6	serine/arginine-rich splicing factor 6	S86	-	-	-	-	0.7	0.9	1.1	0.8	-	-	-	-	0.9	1.0	1.3	1.0	0.9	1.0	1.3	1.0	sGGGGYSSR	GYSYGSRsGGGGYSS	S1;	Yes	S(1): 98.5; Y(6): 0.0; S(7): 0.0; S(8): [illegible]	1	1	1	0	N-Term(TMT6plex); S8(Phospho)
7661998	NP_055559.1	9779	TBC1D5	TBC1 domain family member 5 isoform b	S554	-	-	-	-	1.0	0.8	1.0	0.8	-	-	-	-	0.9	0.8	0.9	0.7	0.9	0.8	0.9	0.7	EFTGsPPSSATK	GGREFTGsPPSSATK	S5;	Yes	T(3): 0.0; S(5): 99.9; S(8): 0.0; S(9): 0.0; T(11): 0.0	3	2	1	0	N-Term(TMT6plex); S5(Phospho); K12(TMT6plex)
148277037	NP_055726.3	22848	AAK1	AP2-associated protein kinase 1	T606	-	-	-	-	0.6	0.5	0.6	0.8	-	-	-	-	0.5	0.4	0.6	0.7	0.5	0.4	0.6	0.7	VQTtPPPAVQGQK	QQPKVQTtPPPAVQG	T4;	Yes	T(3): 1.7; T(4): 98.3	1	1	1	0	N-Term(TMT6plex); T4(Phospho); K13(TMT6plex)
207113160	NP_001128715.1	6949	TCOF1	treacle protein isoform d	S1111	-	-	-	-	0.5	0.8	1.1	0.8	-	-	-	-	0.5	0.8	1.1	0.7	0.5	0.8	1.1	0.7	TQPSSGVDSAVGTLPATsPQSTSVQAK	VGTLPATsPQSTSVQ	S18;	Yes	T(1): 0.0; S(4): 0.0; S(5): 0.0; S(9): 0.0; T(13): 0.0; T(17): 1.6; S(18): 96.8; S(21): 1.6; T(22): 0.0; S(23): 0.0	3	3	1	0	N-Term(TMT6plex); S18(Phospho); K27(TMT6plex)
24308089	NP_056372.1	26038	CHD5	chromodomain-helicase-DNA-binding protein 5	S1631	-	-	-	-	0.6	0.6	0.8	0.7	-	-	-	-	-	-	-	-	-	-	-	-	APPsPEQLPR	ETEKAPPsPEQLPRE	S4;	No	S(4): 100.0	2	1	1	0	N-Term(TMT6plex); S4(Phospho)
42741682	NP_005446.2	9406	ZRANB2	zinc finger Ran-binding domain-containing protein 2 isoform 2	S120	-	-	-	-	0.8	0.8	1.0	0.7	-	-	-	-	0.9	0.8	1.2	1.0	0.9	0.8	1.2	1.0	ENVEYIEREEsDGEYDEFGR	EYIEREEsDGEYDEF	S11;	Yes	Y(5): 0.0; S(11): 100.0; Y(15): 0.0	1	2	1	1	N-Term(TMT6plex); S11(Phospho)
150417986	NP_006411.2	10564	ARFGEF2	brefeldin A-inhibited guanine nucleotide-exchange protein 2	S1528	-	-	-	-	0.6	0.7	1.0	0.7	-	-	-	-	0.6	0.8	1.1	0.8	0.6	0.8	1.1	0.8	GQSQLsNPTDDSWK	ERGQSQLsNPTDDSW	S6;	Yes	S(3): 0.0; S(6): 99.9; T(9): 0.0; S(12): 0.0	1	1	1	0	N-Term(TMT6plex); S6(Phospho); K14(TMT6plex)
7662112	NP_055587.1	9811	CTIF	CBP80/20-dependent translation initiation factor isoform 1	S18	-	-	-	-	0.8	1.2	1.1	0.7	-	-	-	-	1.0	1.2	1.2	0.9	1.0	1.2	1.2	0.9	sQEIEELER	SEAGSSRsQEIEELE	S1;	Yes	S(1): 100.0	1	2	1	0	N-Term(TMT6plex); S1(Phospho)
153251771	NP_001093097.1	56181	MTFR1L	mitochondrial fission regulator 1-like isoform b	S103	-	-	-	-	0.9	0.7	0.7	0.7	-	-	-	-	0.9	0.6	0.7	0.7	0.9	0.6	0.7	0.7	NAsVPNLR	IVMQRNAsVPNLRGS	S3;	No	S(3): 100.0	1	2	1	0	N-Term(TMT6plex); S3(Phospho)
42415492	NP_060823.3	55320	MIS18BP1	mis18-binding protein 1	S110	-	-	-	-	0.7	0.6	0.5	0.7	-	-	-	-	0.6	0.6	0.5	0.7	0.6	0.6	0.5	0.7	ANYEsPGK	KNKANYEsPGKIELR	S5;	Yes	Y(3): 0.0; S(5): 100.0	2	1	1	0	N-Term(TMT6plex); S5(Phospho); K8(TMT6plex)
13899325	NP_113660.1	83707	TRPT1	tRNA 2'-phosphotransferase 1 isoform 2	S191	-	-	-	-	0.7	1.1	0.7	0.7	-	-	-	-	0.5	1.1	0.7	0.7	0.5	1.1	0.7	0.7	KPLSLAGDEETECQSsPK	EETECQSsPKHSSRE	S16;	No	S(4): 0.0; T(11): 0.0; S(15): 1.9; S(16): 98.1	2	4	1	0	N-Term(TMT6plex); K1(TMT6plex); C13(Carbamidomethyl); S16(Phospho); K18(TMT6plex)
4507525	NP_003253.1	7095	SEC62	translocation protein SEC62	T158	-	-	-	-	1.3	1.3	1.0	0.7	-	-	-	-	1.1	0.9	0.8	0.7	1.1	0.9	0.8	0.7	EETPGtPK	KKEETPGtPKKKETK	T6;	Yes	T(3): 0.0; T(6): 100.0	2	1	1	0	N-Term(TMT6plex); T6(Phospho); K8(TMT6plex)
166235169	NP_060531.2	55119	PRPF38B	pre-mRNA-splicing factor 38B	S491	-	-	-	-	0.7	0.8	0.8	0.7	-	-	-	-	0.8	0.9	0.9	0.8	0.8	0.9	0.9	0.8	EHsPSKEK	KSKKREHsPSKEKSR	S3;	Yes	S(3): 100.0; S(5): 0.0	1	1	1	1	N-Term(TMT6plex); S3(Phospho); K6(TMT6plex); K8(TMT6plex)
319918862	NP_001188403.1	83608	C18orf21	UPF0711 protein C18orf21 isoform b	T42	-	-	-	-	0.6	0.6	0.5	0.7	-	-	-	-	0.6	0.6	0.5	0.8	0.6	0.6	0.5	0.8	SNPAtPTSK	TLKSNPAtPTSKLSL	T5;	No	S(1): 0.0; T(5): 100.0; T(7): 0.0; S(8): 0.0	1	2	1	0	N-Term(TMT6plex); T5(Phospho); K9(TMT6plex)
8923271	NP_060218.1	54883	CWC25	pre-mRNA-splicing factor CWC25 homolog	S218	-	-	-	-	0.7	0.6	0.6	0.7	-	-	-	-	0.8	0.8	0.9	1.0	0.8	0.8	0.9	1.0	MANSsPVLSK	QKKMANSsPVLSKVP	S5;	Yes	S(4): 1.5; S(5): 98.5; S(9): 0.0	1	1	1	0	N-Term(TMT6plex); M1(Oxidation); S5(Phospho); K10(TMT6plex)
30840980	NP_005586.1	4774	NFIA	nuclear factor 1 A-type isoform 2	S287	-	-	-	-	0.8	0.8	0.7	0.7	-	-	-	-	-	-	-	-	-	-	-	-	SVEDEMDsPGEEPFYTGQGR	SVEDEMDsPGEEPFY	S8;	Yes	S(1): 3.6; S(8): 92.7; Y(15): 3.6; T(16): 0.2	1	4	1	0	N-Term(TMT6plex); M6(Oxidation); S8(Phospho)
47519639	NP_002366.2	4134	MAP4	microtubule-associated protein 4 isoform 1	S899	-	-	-	-	0.9	1.0	1.2	0.7	-	-	-	-	0.9	1.0	1.3	0.9	0.9	1.0	1.3	0.9	ATPMPSRPsTTPFIDK	TPMPSRPsTTPFIDK	S9;	Yes	T(2): 0.0; S(6): 1.5; S(9): 95.5; T(10): 1.5; T(11): 1.5	1	2	1	0	N-Term(TMT6plex); M4(Oxidation); S9(Phospho); K16(TMT6plex)
118572613	NP_057417.3	23524	SRRM2	serine/arginine repetitive matrix protein 2	S1517	-	-	-	-	0.9	1.0	1.0	0.7	-	-	-	-	0.9	1.0	1.0	0.8	0.9	1.0	1.0	0.8	sGSESSVDQK	LTPQRERsGSESSVD	S1;	Yes	S(1): 100.0; S(3): 0.0; S(5): 0.0; S(6): 0.0	1	1	1	0	N-Term(TMT6plex); S1(Phospho); K10(TMT6plex)
154146193	NP_116259.2	25957	PNISR	arginine/serine-rich protein PNISR	S465	-	-	-	-	0.8	1.1	0.8	0.7	-	-	-	-	0.8	1.2	0.8	0.8	0.8	1.2	0.8	0.8	EQNsLSLLEAR	EIHKEQNsLSLLEAR	S4;	Yes	S(4): 98.5; S(6): 1.5	1	1	1	0	N-Term(TMT6plex); S4(Phospho)
256017159	NP_001074010.2	23269	MGA	MAX gene-associated protein isoform 2	S2847	-	-	-	-	0.8	0.7	0.8	0.7	-	-	-	-	0.6	0.6	0.7	0.6	0.6	0.6	0.7	0.6	LGNsGASPSSAGK	VVAKLGNsGASPSSA	S4;	No	S(4): 98.3; S(7): 1.7; S(9): 0.0; S(10): 0.0	1	2	1	0	N-Term(TMT6plex); S7(Phospho); K13(TMT6plex)
295054220	NP_001171136.1	55183	RIF1	telomere-associated protein RIF1 isoform 2	S2195	-	-	-	-	1.3	1.2	1.2	0.7	-	-	-	-	1.4	1.3	1.3	0.8	1.4	1.3	1.3	0.8	SQEDEIsPVNK	RSQEDEIsPVNKVR	S7;	Yes	S(1): 0.0; S(7): 98.7; S(8): 1.2	1	2	1	0	N-Term(TMT6plex); S7(Phospho); K12(TMT6plex)
19557702	NP_006744.2	6838	SURF6	surfeit locus protein 6 isoform 1	S138	-	-	-	-	0.6	0.7	0.7	0.7	-	-	-	-	0.7	0.8	0.8	0.8	0.7	0.8	0.8	0.8	ELsPAALEK	QGSAKELsPAALEKR	S3;	Yes	S(3): 100.0	2	1	1	0	N-Term(TMT6plex); S3(Phospho); K9(TMT6plex)
62988361	NP_001615.1	202	AIM1	absent in melanoma 1 protein	S533	-	-	-	-	0.7	0.9	0.8	0.7	-	-	-	-	0.9	1.0	0.9	0.9	0.9	1.0	0.9	0.9	sPEAVGSECPSR	PPLSTERsPEAVGSE	S1;	Yes	S(1): 100.0; S(7): 0.0; S(11): 0.0	1	1	1	0	N-Term(TMT6plex); S1(Phospho); C9(Carbamidomethyl)
217330646	NP_006704.3	10923	SUB1	activated RNA polymerase II transcriptional coactivator p15	S118	-	-	-	-	0.6	0.5	0.6	0.7	-	-	-	-	0.6	0.4	0.5	0.5	0.6	0.4	0.5	0.5	EQIsDIDDAVR	SQLKEQIsDIDDAVR	S4;	Yes	S(4): 100.0	1	1	1	0	N-Term(TMT6plex); S4(Phospho)
21735572	NP_060568.3	1894	ECT2	protein ECT2 isoform b	T328	-	-	-	-	0.8	0.9	1.0	0.7	-	-	-	-	0.8	0.9	1.0	0.8	0.8	0.9	1.0	0.8	ANtPELK	YLYEKANtPELKKSV	T3;	No	T(3): 100.0	4	2	1	0	N-Term(TMT6plex); T3(Phospho); K7(TMT6plex)
209862789	NP_056056.2	57553	MICAL3	protein-methionine sulfoxide oxidase MICAL3 isoform 1	T1763	-	-	-	-	0.7	1.0	0.9	0.7	-	-	-	-	0.7	0.8	0.8	0.7	0.7	0.8	0.8	0.7	SCPStPSSGATVDSGK	DDKSCPStPSSGATV	T5;	Yes	S(1): 1.8; S(4): 1.8; T(5): 92.9; S(7): 1.8; S(8): 1.8; T(11): 0.0; S(14): 0.0	1	1	1	0	N-Term(TMT6plex); C2(Carbamidomethyl); S4(Phospho); K16(TMT6plex)
5032051	NP_005608.1	6208	RPS14	40S ribosomal protein S14	S139	-	-	-	-	0.4	0.5	0.6	0.7	-	-	-	-	0.4	0.6	0.7	0.7	0.4	0.6	0.7	0.7	IEDVTPIPSDsTR	VTPIPSDsTRRKGGR	S11;	Yes	T(5): 0.0; S(9): 0.0; S(11): 98.5; T(12): 1.4	1	1	1	0	N-Term(TMT6plex); S11(Phospho)
282165721	NP_001164108.1	23380	SRGAP2	SLIT-ROBO Rho GTPase-activating protein 2 isoform	S915	-	-	-	-	1.0	1.1	0.9	0.7	-	-	-	-	1.0	1.1	0.9	0.7	1.0	1.1	0.9	0.7	LDsPQIR	SLKNRLDsPQIRKTA	S3;	No	S(3): 100.0	1	2	1	0	N-Term(TMT6plex); S3(Phospho)
118572613	NP_057417.3	23524	SRRM2	serine/arginine repetitive matrix protein 2	S1318; S1320	-	-	-	-	1.0	0.8	0.8	0.7	-	-	-	-	1.1	0.8	0.9	0.8	1.1	0.8	0.9	0.8	ELsNsPLR	SPEHKELsNSPLREN;EHKELSNsPLRENSF	S3; S5;	Yes;Yes	S(3): 100.0; S(5): 100.0	1	1	1	0	N-Term(TMT6plex); S3(Phospho); S5(Phospho)
503774159	NP_001265128.1	6643	SNX2	sorting nexin-2 isoform 2	S160	-	-	-	-	1.0	1.4	0.8	0.7	-	-	-	-	1.0	1.4	0.9	0.8	1.0	1.4	0.9	0.8	AVNTQALsGAGILR	AVNTQALsGAGILRM	S8;	No	T(4): 0.0; S(8): 100.0	2	2	1	0	N-Term(TMT6plex); S8(Phospho)
333440471	NP_001193980.1	23332	CLASP1	CLIP-associating protein 1 isoform 4	S1069	-	-	-	-	1.1	1.0	1.1	0.7	-	-	-	-	1.1	1.0	1.1	0.8	1.1	1.0	1.1	0.8	NSSNTSVGsPSNTIGR	SSNTSVGsPSNTIGR	S9;	No	S(2): 0.0; S(3): 0.0; T(5): 0.0; S(6): 0.0; S(9): 100.0; S(11): 0.0; T(13): 0.0	3	4	1	0	N-Term(TMT6plex); S9(Phospho)
14790190	NP_055816.2	23013	SPEN	msx2-interacting protein	S2481; S2493	-	-	-	-	0.8	0.9	0.7	0.7	-	-	-	-	0.7	0.8	0.7	0.7	0.7	0.8	0.7	0.7	LsPPVASGGIPHQsPPTK	SVTAAKLsPPVASGG;SGGIPHQsPPTKVTE	S2; S14;	Yes;Yes	S(2): 100.0; S(7): 0.0; S(14): 98.7; T(17): 1.3	3	1	1	0	N-Term(TMT6plex); S2(Phospho); S14(Phospho); K18(TMT6plex)
296011010	NP_060252.4	54906	FAM208B	protein FAM208B	S1541	-	-	-	-	0.6	0.4	0.7	0.7	-	-	-	-	0.6	0.5	0.8	0.8	0.6	0.5	0.8	0.8	CTGDESPsPEK	CTGDESPsPEKLVKS	S8;	Yes	T(2): 0.0; S(6): 0.0; S(8): 100.0	1	1	1	0	N-Term(TMT6plex); C1(Carbamidomethyl); S8(Phospho); K11(TMT6plex)
556695389	NP_001273359.1	123169	LEO1	RNA polymerase-associated protein LEO1 isoform 2	S205	-	-	-	-	0.8	0.8	1.0	0.7	-	-	-	-	0.9	0.9	1.2	0.9	0.9	0.9	1.2	0.9	QQLsEEEK	DDERQQLsEEEKANS	S4;	Yes	S(4): 100.0	2	2	1	0	N-Term(TMT6plex); S4(Phospho); K8(TMT6plex)
209969819	NP_775837.2	253143	PRR14L	protein PRR14L	S1029	-	-	-	-	0.5	1.2	0.8	0.7	-	-	-	-	0.4	1.1	0.7	0.6	0.4	1.1	0.7	0.6	DLLVSSGSNNSLPCGsPK	NNSLPCGsPKKCNLK	S16;	Yes	S(5): 0.0; S(6): 0.0; S(8): 0.0; S(11): 0.0; S(16): 100.0	2	1	1	0	N-Term(TMT6plex); C14(Carbamidomethyl); S16(Phospho); K18(TMT6plex)
55956788	NP_005372.2	4691	NCL	nucleolin	S563	-	-	-	-	0.8	0.8	0.6	0.7	-	-	-	-	0.9	0.9	0.8	1.0	0.9	0.9	0.8	1.0	GsPNAR	ELQGPRGsPNARSQP	S2;	Yes	S(2): 100.0	1	1	1	0	N-Term(TMT6plex); S2(Phospho)
33188445	NP_036222.3	23499	MACF1	microtubule-actin cross-linking factor 1	S2429	-	-	-	-	1.0	1.8	1.3	0.7	-	-	-	-	1.0	1.7	1.3	0.8	1.0	1.7	1.3	0.8	SMDAMSsPTKTETVK	KSMDAMSsPTKTETV	S7;	No	S(1): 0.0; S(6): 1.2; S(7): 98.8; T(9): 0.0; T(11): 0.0; T(13): 0.0	3	1	1	1	N-Term(TMT6plex); M2(Oxidation); M5(Oxidation); S7(Phospho); K10(TMT6plex); K15(TMT6plex)
269847793	NP_001161711.1	27	ABL2	Abelson tyrosine-protein kinase 2 isoform i	S584	-	-	-	-	0.8	0.7	0.8	0.7	-	-	-	-	-	-	-	-	-	-	-	-	GAQASSGsPALPR	GAQASSGsPALPRKQ	S8;	No	S(5): 0.0; S(6): 0.0; S(8): 100.0	2	7	1	0	N-Term(TMT6plex); S8(Phospho)
46370071	NP_997006.1	2549	GAB1	GRB2-associated-binding protein 1 isoform a	S547	-	-	-	-	0.7	1.0	0.7	0.7	-	-	-	-	-	-	-	-	-	-	-	-	TDsQTIGDFATR	HGLERTDsQTIGDFA	S3;	Yes	T(1): 0.0; S(3): 100.0; T(5): 0.0; T(11): 0.0	2	1	1	0	N-Term(TMT6plex); S3(Phospho)
21361639	NP_060053.2	54765	TRIM44	tripartite motif-containing protein 44	T319	-	-	-	-	0.9	0.9	0.9	0.7	-	-	-	-	-	-	-	-	-	-	-	-	EQLDtSNESAEPK	QAKEQLDtSNESAEP	T5;	Yes	T(5): 98.1; S(6): 1.8; S(9): 0.0	1	1	1	0	N-Term(TMT6plex); T5(Phospho); K13(TMT6plex)
7662046	NP_055542.1	9757	KMT2B	histone-lysine N-methyltransferase 2B	T278	-	-	-	-	0.7	0.7	0.6	0.7	-	-	-	-	0.6	0.8	0.7	0.7	0.6	0.8	0.7	0.7	EGPGPGPGtPR	GPGPGPGtPRRGGQS	T9;	Yes	T(9): 100.0	1	1	1	0	N-Term(TMT6plex); T9(Phospho)
15721939	NP_031385.2	22794	CASC3	protein CASC3	S125	-	-	-	-	0.8	0.9	0.9	0.7	-	-	-	-	0.9	0.9	1.0	0.7	0.9	0.9	1.0	0.7	SEANDAVNsSTKEEK	EANDAVNsSTKEEKG	S9;	Yes	S(1): 0.0; S(9): 97.5; S(10): 1.3; T(11): 1.3	2	1	1	1	N-Term(TMT6plex); S9(Phospho); K12(TMT6plex); K15(TMT6plex)
255652953	NP_653205.3	124245	ZC3H18	zinc finger CCCH domain-containing protein 18	S868	-	-	-	-	0.7	0.8	0.7	0.7	-	-	-	-	0.8	0.9	0.8	0.7	0.8	0.9	0.8	0.7	LGsPKPER	KSGGRLGsPKPERQR	S3;	Yes	S(3): 100.0	1	1	1	0	N-Term(TMT6plex); S3(Phospho); K5(TMT6plex)
22027646	NP_570899.1	154810	AMOTL1	angiomotin-like protein 1	S793	-	-	-	-	0.6	1.0	0.7	0.7	-	-	-	-	-	-	-	-	-	-	-	-	sVPSIAAATGTHSR	SSLRPARsVPSIAAA	S1;	Yes	S(1): 100.0; S(4): 0.0; T(9): 0.0; T(11): 50.0; S(13): 50.0	1	1	1	0	N-Term(TMT6plex); S4(Phospho); S13(Phospho)
41322916	NP_958782.1	5339	PLEC	plectin isoform 1	S4396	-	-	-	-	0.8	0.7	0.8	0.7	-	-	-	-	0.9	0.8	0.8	0.7	0.9	0.8	0.8	0.7	SSSVGSSSSYPIsPAVSR	SSSSYPIsPAVSRTQ	S13;	Yes	S(1): 0.0; S(2): 0.0; S(3): 0.0; S(6): 0.0; S(7): 0.0; S(8): 0.0; S(9): 0.0; Y(10): 0.0; S(13): 100.0; S(17): 0.0	7	8	1	0	N-Term(TMT6plex); S13(Phospho)
150418005	NP_008970.2	11099	PTPN21	tyrosine-protein phosphatase non-receptor type 21	S660	-	-	-	-	0.9	0.9	0.9	0.7	-	-	-	-	-	-	-	-	-	-	-	-	TLSAsAAEVAPR	KERTLSAsAAEVAPR	S5;	No	T(1): 0.0; S(3): 1.5; S(5): 98.5	1	1	1	0	N-Term(TMT6plex); S5(Phospho)
9994179	NP_037392.1	29115	SAP30BP	SAP30-binding protein	S113	-	-	-	-	0.6	0.7	0.9	0.7	-	-	-	-	0.7	0.7	1.0	0.7	0.7	0.7	1.0	0.7	NMsPDEIK	SERVRNMsPDEIKIP	S3;	Yes	S(3): 100.0	2	1	1	0	N-Term(TMT6plex); M2(Oxidation); S3(Phospho); K8(TMT6plex)
217330641	NP_060439.2	55677	IWS1	protein IWS1 homolog	S420	-	-	-	-	1.1	0.6	1.3	0.7	-	-	-	-	1.1	0.5	1.3	0.8	1.1	0.5	1.3	0.8	VVSDADDsDSDAVSDK	VVSDADDsDSDAVSD	S8;	Yes	S(3): 0.0; S(8): 100.0; S(10): 0.0; S(14): 0.0	2	1	1	0	N-Term(TMT6plex); S8(Phospho); K16(TMT6plex)
339275976	NP_001229832.1	8165	AKAP1	A-kinase anchor protein 1, mitochondrial precursor	S151	-	-	-	-	0.7	0.3	0.5	0.7	-	-	-	-	0.8	0.4	0.6	0.8	0.8	0.4	0.6	0.8	SIPLECPLSsPK	PLECPLSsPKGVLFS	S10;	Yes	S(1): 0.0; S(9): 0.8; S(10): 99.2	2	1	1	0	N-Term(TMT6plex); C6(Carbamidomethyl); S10(Phospho); K12(TMT6plex)
42794779	NP_976063.1	399687	MYO18A	unconventional myosin-XVIIIa isoform b	S1992	-	-	-	-	0.9	0.8	0.7	0.7	-	-	-	-	0.9	0.8	0.8	0.8	0.9	0.8	0.8	0.8	SSsPTSYWK	DGSLKSSsPTSYWKS	S3;	No	S(1): 0.0; S(2): 1.2; S(3): 98.8; T(5): 0.0; S(6): 0.0; Y(7): 0.0	1	2	1	0	N-Term(TMT6plex); S3(Phospho); K9(TMT6plex)
84043963	NP_056988.3	9669	EIF5B	eukaryotic translation initiation factor 5B	S214	-	-	-	-	0.9	0.9	0.8	0.7	-	-	-	-	0.9	0.9	0.9	0.7	0.9	0.9	0.9	0.7	NKPGPNIEsGNEDDDASFK	KPGPNIEsGNEDDDA	S9;	Yes	S(9): 100.0; S(17): 0.0	5	1	1	0	N-Term(TMT6plex); K2(TMT6plex); S9(Phospho); K19(TMT6plex)
103472005	NP_002408.3	4288	MKI67	antigen KI-67 isoform 1	S2223; T2231	-	-	-	-	0.2	0.2	0.8	0.7	-	-	-	-	0.3	0.2	0.8	0.7	0.3	0.2	0.8	0.7	sPQPDPVGtPTIFKPQSK	TTKIACRsPQPDPVG;PQPDPVGtPTIFKPQ	S1; T9;	Yes;Yes	S(1): 100.0; T(9): 98.0; T(11): 2.0; S(17): 0.0	1	2	1	0	N-Term(TMT6plex); S1(Phospho); T9(Phospho); K14(TMT6plex); K18(TMT6plex)
50428935	NP_060644.4	55201	MAP1S	microtubule-associated protein 1S	S547	-	-	-	-	1.7	1.1	0.7	0.7	-	-	-	-	1.9	1.2	0.8	0.7	1.9	1.2	0.8	0.7	AASsVPNLK	EVRRAASsVPNLKKT	S4;	Yes	S(3): 0.8; S(4): 99.2	1	1	1	0	N-Term(TMT6plex); S3(Phospho); K9(TMT6plex)
33620745	NP_056969.2	51585	PCF11	pre-mRNA cleavage complex 2 protein Pcf11	S1520	-	-	-	-	1.0	0.8	1.0	0.7	-	-	-	-	1.0	0.8	0.9	0.7	1.0	0.8	0.9	0.7	NELQEPCDsPK	ELQEPCDsPKVKEER	S9;	Yes	S(9): 100.0	2	1	1	0	N-Term(TMT6plex); C7(Carbamidomethyl); S9(Phospho); K11(TMT6plex)
570359584	NP_001275662.1	55852	TEX2	testis-expressed sequence 2 protein isoform 2	S162	-	-	-	-	1.1	0.8	0.8	0.7	-	-	-	-	1.0	0.9	0.8	0.6	1.0	0.9	0.8	0.6	TSSSsPLSSPSK	EQKTSSSsPLSSPSK	S5;	Yes	T(1): 0.0; S(2): 0.0; S(3): 0.0; S(4): 1.1; S(5): 98.9; S(8): 0.0; S(9): 0.0; S(11): 0.0	2	2	1	0	N-Term(TMT6plex); S5(Phospho); K12(TMT6plex)
304434531	NP_001182122.1	4678	NASP	nuclear autoantigenic sperm protein isoform 4	S614	-	-	-	-	1.3	1.5	1.0	0.7	-	-	-	-	1.7	2.0	1.6	1.0	1.7	2.0	1.6	1.0	ATLVESsTSGFTPGGGGSSVSMIASR	KATLVESsTSGFTPG	S7;	No	T(2): 0.0; S(6): 2.9; S(7): 91.1; T(8): 2.9; S(9): 2.9; T(12): 0.1; S(18): 0.1; S(19): 0.1; S(21): 0.0; S(25): 0.0	2	3	1	0	N-Term(TMT6plex); S7(Phospho); M22(Oxidation)
530788278	NP_612114.2	546	ATRX	transcriptional regulator ATRX isoform 2	S837	-	-	-	-	0.8	0.8	0.8	0.7	-	-	-	-	1.0	0.9	1.0	0.9	1.0	0.9	1.0	0.9	TSQEGsSDDAER	LKTSQEGsSDDAERK	S6;	No	T(1): 0.0; S(2): 0.0; S(6): 99.4; S(7): [illegible]	4	2	1	0	N-Term(TMT6plex); S7(Phospho)
156142199	NP_079532.5	10919	EHMT2	histone-lysine N-methyltransferase EHMT2 isoform b	S119	-	-	-	-	0.7	0.8	0.8	0.7	-	-	-	-	0.7	0.8	0.8	0.6	0.7	0.8	0.8	0.6	SEPSsPSK	ATKSEPSsPSKGGSC	S5;	Yes	S(1): 0.0; S(4): 0.8; S(5): 99.2; S(7): [illegible]	1	3	1	0	N-Term(TMT6plex); S5(Phospho); K8(TMT6plex)
42542379	NP_005830.2	10250	SRRM1	serine/arginine repetitive matrix protein 1	T693	-	-	-	-	0.7	0.6	0.6	0.7	-	-	-	-	0.9	0.6	0.6	0.8	0.9	0.6	0.6	0.8	APQtSSSPPPVRR	PRPRAPQtSSSPPPV	T4;	Yes	T(4): 94.3; S(5): 1.9; S(6): 1.9; S(7): [illegible]	1	1	1	1	N-Term(TMT6plex); S6(Phospho)
4759050	NP_004577.1	6197	RPS6KA3	ribosomal protein S6 kinase alpha-3	S715	-	-	-	-	0.9	0.6	0.7	0.7	-	-	-	-	0.9	0.7	0.7	0.9	0.9	0.7	0.7	0.9	NQsPVLEPVGR	SALNRNQsPVLEPVG	S3;	Yes	S(3): 100.0	1	1	1	0	N-Term(TMT6plex); S3(Phospho)
22538393	NP_671714.1	10142	AKAP9	A-kinase anchor protein 9 isoform 3	S31	-	-	-	-	1.0	0.9	0.7	0.7	-	-	-	-	0.9	0.8	0.7	0.7	0.9	0.8	0.7	0.7	AQSDGQsPSK	KAQSDGQsPSKKQKK	S7;	No	S(3): 0.0; S(7): 100.0; S(9): 0.0	1	2	1	0	N-Term(TMT6plex); S7(Phospho); K10(TMT6plex)

Khan et al., 2019. Multi-omics analysis to characterize cigarette smoke induced molecular alterations in esophageal cells

Supplementary Table 8. List of phosphopeptides identified and quantified in untreated and chronically treated Het1A cells with cigarette smoke condensate for 8 months using SequestHT and Mascot search algorithms with a PhosphoRS probability score ≥75%

Protein group Accession	NP_Accession	Gene ID	Gene Symbol	Protein Description	PhosphoSite (Protein)	Replicate 1: Het-1A-Smoke - 2M/Parental	Replicate 1: Het-1A-Smoke - 4M/Parental	Replicate 1: Het-1A-Smoke - 6M/Parental	Replicate 1: Het-1A-Smoke - 8M/Parental	Replicate 2: Het-1A-Smoke - 2M/Parental	Replicate 2: Het-1A-Smoke - 4M/Parental	Replicate 2: Het-1A-Smoke - 6M/Parental	Replicate 2: Het-1A-Smoke - 8M/Parental	Normalized phosphoprotein ratios with respect to corresponding total protein ratios. Replicate 1: Het-1A-Smoke - 2M/Parental	Normalized Replicate 1: Het-1A-Smoke - 4M/Parental	Normalized Replicate 1: Het-1A-Smoke - 6M/Parental	Normalized Replicate 1: Het-1A-Smoke - 8M/Parental	Normalized Replicate 2: Het-1A-Smoke - 2M/Parental	Normalized Replicate 2: Het-1A-Smoke - 4M/Parental	Normalized Replicate 2: Het-1A-Smoke - 6M/Parental	Normalized Replicate 2: Het-1A-Smoke - 8M/Parental
56118310	NP_073568.2	64710	NUCKS1	nuclear ubiquitous casein and cyclin-dependent kinase substrate 1	S223	-	-	-	-	0.7	0.8	0.8	0.7	-	-	-	-	0.9	1.0	1.0	0.9
291575181	NP_001167563.1	84548	TMEM185A	transmembrane protein 185A isoform2	S274	-	-	-	-	0.7	0.5	0.6	0.7	-	-	-	-	-	-	-	-
39777586	NP_945314.1	90957	DHX57	putative ATP-dependent RNA helicase DHX57	S132	-	-	-	-	0.8	1.0	0.9	0.7	-	-	-	-	0.8	0.9	0.9	0.7
154146193	NP_116259.2	25957	PNISR	arginine/serine-rich protein PNISR	S601	-	-	-	-	1.3	1.0	1.2	0.7	-	-	-	-	1.4	1.1	1.3	0.7
38044112	NP_937883.1	6249	CLIP1	CAP-Gly domain-containing linker protein 1 isoformb	T287	-	-	-	-	0.8	1.0	0.9	0.7	-	-	-	-	0.8	1.0	1.0	0.8
96974985	NP_060191.3	54862	CC2D1A	coiled-coil and C2 domain-containing protein 1A	S455	-	-	-	-	0.7	0.9	0.8	0.7	-	-	-	-	0.8	1.0	1.0	0.8
209862789	NP_056056.2	57553	MICAL3	protein-methionine sulfoxide oxidase MICAL3 isoform	T1155	-	-	-	-	0.9	0.9	1.1	0.7	-	-	-	-	0.8	0.7	1.0	0.6
157676331	NP_055097.2	22927	HABP4	intracellular hyaluronan-binding protein 4	S74	-	-	-	-	0.7	1.0	0.9	0.7	-	-	-	-	0.9	1.0	1.2	0.8
321267473	NP_001188269.1	6294	SAFB	scaffold attachment factor B1 isoform4	S314	-	-	-	-	0.6	0.9	0.9	0.7	-	-	-	-	0.7	1.0	1.1	0.8
150378493	NP_006757.2	7994	KAT6A	histone acetyltransferase KAT6A	T1144	-	-	-	-	0.8	0.9	0.7	0.7	-	-	-	-	0.8	0.8	0.7	0.6
387942371	NP_001251503.1	146909	KIF18B	kinesin-like protein KIF18B isoform2	S694; S698	-	-	-	-	0.4	0.5	0.5	0.7	-	-	-	-	0.5	0.6	0.6	0.9
55749431	NP_001006934.1	85012	TCEAL3	transcription elongation factor A protein-like 3	S65	-	-	-	-	1.8	1.2	1.4	0.7	-	-	-	-	2.6	2.1	2.1	1.3
63053524	NP_001017416.1	7398	USP1	ubiquitin carboxyl-terminal hydrolase 1	S313	-	-	-	-	0.6	0.5	0.5	0.7	-	-	-	-	0.6	0.6	0.5	0.7
302058308	NP_001180462.1	6787	NEK4	serine/threonine-protein kinase Nek4 isoform2	S395	-	-	-	-	0.7	0.5	0.9	0.7	-	-	-	-	0.8	0.5	1.0	0.8
18034775	NP_057520.2	51230	PHF20	PHD finger protein 20	S519	-	-	-	-	0.7	0.6	0.6	0.7	-	-	-	-	0.8	0.7	0.7	0.7
110556636	NP_203754.2	85456	TNKS1BP1	182 kDa tankyrase-1-binding protein	S1545	-	-	-	-	0.9	1.4	1.3	0.7	-	-	-	-	0.9	1.2	1.2	0.7
38016922	NP_937845.1	51367	POP5	ribonuclease P/MRP protein subunit POP5 isoformc	S104	-	-	-	-	1.9	0.7	1.1	0.7	-	-	-	-	1.6	0.7	1.1	0.6
5031579	NP_005849.1	10270	AKAP8	A-kinase anchor protein 8	S328	-	-	-	-	1.0	0.5	0.9	0.7	-	-	-	-	1.0	0.5	0.9	0.7
41152093	NP_958357.1	84146	ZNF644	zinc finger protein 644 isoform1	S1189	-	-	-	-	1.0	0.7	0.7	0.7	-	-	-	-	1.0	0.7	0.8	0.7
229892308	NP_001153505.1	151246	SGOL2	shugoshin-like 2 isoform2	S895	-	-	-	-	0.5	0.8	0.7	0.6	-	-	-	-	0.6	0.8	0.8	0.8
117190174	NP_004491.2	3183	HNRNPC	heterogeneous nuclear ribonucleoproteins C1/C2 isoformb	S228	-	-	-	-	0.8	0.7	0.6	0.6	-	-	-	-	0.8	0.6	0.6	0.6
191252785	NP_444279.2	116840	CNTROB	centrobin isoformalpha	S837	-	-	-	-	1.2	1.1	1.5	0.6	-	-	-	-	-	-	-	-
385862220	NP_001245349.1	84936	ZFYVE19	zinc finger FYVE domain-containing protein 19	S144	-	-	-	-	0.8	0.9	1.0	0.6	-	-	-	-	-	-	-	-
112789562	NP_005522.2	3428	IFI16	gamma-interferon-inducible protein 16 isoform2	T105	-	-	-	-	0.7	0.6	0.8	0.6	-	-	-	-	0.7	0.7	0.9	0.8
51479145	NP_006412.2	10565	ARFGEF1	brefeldin A-inhibited guanine nucleotide-exchange protein 1	S1569	-	-	-	-	1.1	1.2	0.8	0.6	-	-	-	-	1.1	1.2	0.8	0.6
103472005	NP_002408.3	4288	MKI67	antigen KI-67 isoform1	T1963	-	-	-	-	0.8	0.7	0.8	0.6	-	-	-	-	0.9	0.7	0.8	0.7
87116683	NP_659419.3	196441	ZFC3H1	zinc finger C3H1 domain-containing protein	S28	-	-	-	-	0.6	0.7	0.6	0.6	-	-	-	-	0.5	0.7	0.6	0.6
61676188	NP_113584.3	10075	HUWE1	E3 ubiquitin-protein ligase HUWE1	S1395	-	-	-	-	0.8	0.7	0.8	0.6	-	-	-	-	0.8	0.8	0.8	0.8
24041035	NP_077719.2	4853	NOTCH2	neurogenic locus notch homolog protein 2 isoform1 preproprotein	S2115	-	-	-	-	0.6	0.7	1.0	0.6	-	-	-	-	0.6	0.7	1.0	0.6
4504969	NP_003884.1	8861	LDB1	LIM domain-binding protein 1 isoform3	S266	-	-	-	-	0.4	0.6	0.6	0.6	-	-	-	-	0.5	0.6	0.7	0.6
194097392	NP_004477.3	2801	GOLGA2	Golgin subfamily A member 2	S37	-	-	-	-	0.6	0.7	0.8	0.6	-	-	-	-	0.5	0.6	0.8	0.7
118572613	NP_057417.3	23524	SRRM2	serine/arginine repetitive matrix protein 2	S1502	-	-	-	-	0.6	0.9	0.8	0.6	-	-	-	-	0.6	0.9	0.9	0.7
109637788	NP_150247.2	5371	PML	protein PML isoform5	S8	-	-	-	-	0.7	0.9	0.8	0.6	-	-	-	-	0.6	0.9	0.7	0.5
112421108	NP_055940.3	23152	CIC	protein capicua homolog	S700	-	-	-	-	0.8	0.9	0.8	0.6	-	-	-	-	-	-	-	-
15011974	NP_004714.2	9181	ARHGEF2	rho guanine nucleotide exchange factor 2 isoform3	S858	-	-	-	-	0.6	2.4	0.8	0.6	-	-	-	-	0.4	1.9	0.7	0.5
4885381	NP_005313.1	3009	HIST1H1B	histone H1.5	T11	-	-	-	-	0.5	0.6	0.7	0.6	-	-	-	-	0.8	0.8	1.1	0.8
148536853	NP_004362.2	1314	COPA	coatomer subunit alpha isoform2	S173	-	-	-	-	0.3	0.3	0.7	0.6	-	-	-	-	0.2	0.3	0.6	0.6
149363685	NP_056140.1	23379	KIAA0947	uncharacterized protein KIAA0947	S925	-	-	-	-	1.2	0.8	0.9	0.6	-	-	-	-	-	-	-	-
87299628	NP_683692.2	259282	BOD1L1	biorientation of chromosomes in cell division protein 1-like 1	S482	-	-	-	-	0.2	0.7	0.6	0.6	-	-	-	-	0.3	0.6	0.6	0.7
259906020	NP_001158287.1	22985	ACIN1	apoptotic chromatin condensation inducer in the nucleus isoform3	S325	-	-	-	-	1.1	1.4	1.3	0.6	-	-	-	-	0.8	1.3	1.4	0.7
544583541	NP_001269787.1	54856	GON4L	GON-4-like protein isoforma	S2106	-	-	-	-	0.9	0.8	0.7	0.6	-	-	-	-	-	-	-	-
7656879	NP_055238.1	27125	AFF4	AF4/FMR2 family member 4	S1043	-	-	-	-	0.8	0.7	0.7	0.6	-	-	-	-	0.7	0.6	0.7	0.6
4759098	NP_004584.1	6434	TRA2B	transformer-2 protein homolog beta isoform1	S264; S266	-	-	-	-	1.0	0.6	1.0	0.6	-	-	-	-	1.2	0.7	1.2	0.7
21735417	NP_056065.1	23299	BICD2	protein bicaudal D homolog 2 isoform2	S582	-	-	-	-	0.7	0.5	0.9	0.6	-	-	-	-	0.7	0.6	0.9	0.6
34577112	NP_908932.1	226	ALDOA	fructose-bisphosphate aldolase A isoform1	T9	-	-	-	-	0.9	0.9	1.0	0.6	-	-	-	-	0.8	0.9	1.0	0.6
41393573	NP_054749.2	28964	GIT1	ARF GTPase-activating protein GIT1 isoform2	S362	-	-	-	-	0.2	0.5	0.5	0.6	-	-	-	-	0.3	0.5	0.5	0.7
110431336	NP_872378.3	284312	ZSCAN1	zinc finger and SCAN domain-containing protein 1	S280	-	-	-	-	0.8	0.8	0.7	0.6	-	-	-	-	-	-	-	-
14249158	NP_116020.1	84717	HDGFRP2	hepatoma-derived growth factor-related protein 2 isoform2	S490	-	-	-	-	0.5	0.5	0.4	0.6	-	-	-	-	0.6	0.7	0.6	0.8
513788264	NP_001241661.2	23384	SPECC1L	cytospin-A isoform2	S870	-	-	-	-	0.6	0.9	1.2	0.6	-	-	-	-	0.6	0.9	1.1	0.6
122937472	NP_001073981.1	80114	BICC1	protein bicaudal C homolog 1	S633	-	-	-	-	0.7	0.7	0.8	0.6	-	-	-	-	-	-	-	-
112789532	NP_001036189.1	5819	PVRL2	poliovirus receptor-related protein 2 isoformdelta precursor	T410	-	-	-	-	1.1	1.0	1.3	0.6	-	-	-	-	1.1	0.9	1.3	0.6
5032189	NP_005648.1	7158	TP53BP1	tumor suppressor p53-binding protein 1 isoform3	S975	-	-	-	-	0.5	0.7	0.7	0.6	-	-	-	-	0.5	0.7	0.7	0.7
31652242	NP_852469.1	6942	TCF20	transcription factor 20 isoform2	S1522	-	-	-	-	0.8	0.8	0.8	0.6	-	-	-	-	0.7	0.9	0.7	0.7
134304853	NP_004806.3	9411	ARHGAP29	rho GTPase-activating protein 29	S949	-	-	-	-	0.6	0.6	0.6	0.6	-	-	-	-	0.8	0.6	0.7	0.8
20986514	NP_620582.1	1432	MAPK14	mitogen-activated protein kinase 14 isoform3	Y182	-	-	-	-	0.9	1.7	1.0	0.6	-	-	-	-	0.9	1.7	1.0	0.6
55956788	NP_005372.2	4691	NCL	nucleolin	S67	-	-	-	-	0.7	0.8	0.6	0.6	-	-	-	-	0.9	0.9	0.7	0.8
300388183	NP_001177966.1	5707	PSMD1	26S proteasome non-ATPase regulatory subunit 1 isoform2	T311; S315	-	-	-	-	1.0	0.6	0.6	0.6	-	-	-	-	1.0	0.7	0.6	0.6
110618244	NP_001036068.1	5927	KDM5A	lysine-specific demethylase 5A	S1331	-	-	-	-	0.8	0.8	0.8	0.6	-	-	-	-	0.7	0.8	0.8	0.6
118572613	NP_057417.3	23524	SRRM2	serine/arginine repetitive matrix protein 2	S1102	-	-	-	-	0.9	1.1	0.9	0.6	-	-	-	-	0.9	1.1	1.0	0.6
7669481	NP_004043.2	664	BNIP3	BCL2/adenovirus E1B 19 kDa protein-interacting protein 3	T72	-	-	-	-	0.6	0.8	0.7	0.6	-	-	-	-	0.6	0.9	0.8	0.6
95147555	NP_002364.5	4130	MAP1A	microtubule-associated protein 1A	S667	-	-	-	-	0.7	0.5	0.5	0.6	-	-	-	-	-	-	-	-
58331268	NP_060139.2	54821	ERCC6L	DNA excision repair protein ERCC-6-like	T1063	-	-	-	-	0.6	0.6	0.6	0.6	-	-	-	-	0.7	0.9	0.8	0.8
333360918	NP_001193972.1	554223	LOC554223	histocompatibility antigen-related	T140	-	-	-	-	0.8	0.7	0.7	0.6	-	-	-	-	-	-	-	-
56118310	NP_073568.2	64710	NUCKS1	nuclear ubiquitous casein and cyclin-dependent kinase substrate 1	S214	-	-	-	-	0.8	0.8	0.7	0.6	-	-	-	-	1.1	1.0	1.0	0.7
226509740	NP_067051.2	58506	SCAF1	splicing factor, arginine/serine-rich 19	S738	-	-	-	-	0.6	1.5	1.0	0.6	-	-	-	-	0.6	1.4	0.9	0.6
221139693	NP_001137387.1	199870	FAM76A	protein FAM76A isoform5	S226	-	-	-	-	0.5	0.5	0.4	0.6	-	-	-	-	-	-	-	-
116256350	NP_001070651.1	7249	TSC2	tuberin isoform4	S664	-	-	-	-	0.5	0.5	0.7	0.6	-	-	-	-	0.5	0.5	0.7	0.6
33620755	NP_060493.3	55689	YEATS2	YEATS domain-containing protein 2	S447	-	-	-	-	0.5	0.9	0.7	0.6	-	-	-	-	-	-	-	-
132626688	NP_055456.2	9656	MDC1	mediator of DNA damage checkpoint protein 1	S793	-	-	-	-	0.3	0.3	0.2	0.6	-	-	-	-	0.3	0.3	0.3	0.6
132626688	NP_055456.2	9656	MDC1	mediator of DNA damage checkpoint protein 1	S453	-	-	-	-	1.0	0.3	1.0	0.6	-	-	-	-	1.1	0.3	1.1	0.6
4758258	NP_004086.1	1978	EIF4EBP1	eukaryotic translation initiation factor 4E-binding protein 1	S65; T68	-	-	-	-	3.4	0.8	2.0	0.6	-	-	-	-	3.7	0.9	2.4	0.7
306922386	NP_001120983.2	324	APC	adenomatous polyposis coli protein isoforma	S2332	-	-	-	-	0.8	1.1	0.7	0.6	-	-	-	-	0.7	1.0	0.8	0.6
109637759	NP_001035905.1	831	CAST	calpastatin isoformf	S34	-	-	-	-	0.6	0.6	0.7	0.6	-	-	-	-	0.7	0.7	0.7	0.7
506949341	NP_001265202.1	6733	SRPK2	SRSF protein kinase 2 isoformb precursor	S497	-	-	-	-	1.0	0.7	0.7	0.6	-	-	-	-	1.0	0.7	0.7	0.5
116812600	NP_006636.2	10809	STARD10	PCTP-like protein	S284	-	-	-	-	2.0	0.4	0.8	0.5	-	-	-	-	-	-	-	-
192807320	NP_001122320.1	6597	SMARCA4	transcription activator BRG1 isoformF	S1419	-	-	-	-	0.8	0.9	1.0	0.5	-	-	-	-	0.8	1.0	1.1	0.6
313661430	NP_001186328.1	4750	NEK1	serine/threonine-protein kinase Nek1 isoform4	T592	-	-	-	-	1.2	1.3	1.1	0.5	-	-	-	-	1.1	1.1	1.1	0.6
190341065	NP_055526.3	9738	CCP110	centriolar coiled-coil protein of 110 kDa isoform2	S551	-	-	-	-	1.2	1.2	1.0	0.5	-	-	-	-	-	-	-	-
4885379	NP_005312.1	3008	HIST1H1E	histone H1.4	T146	-	-	-	-	0.5	0.4	0.4	0.5	-	-	-	-	0.7	0.5	0.6	0.7
14249158	NP_116020.1	84717	HDGFRP2	hepatoma-derived growth factor-related protein 2 isoform2	S142	-	-	-	-	0.5	0.5	0.6	0.5	-	-	-	-	0.6	0.7	0.8	0.7
260763910	NP_659498.4	221150	SKA3	spindle and kinetochore-associated protein 3 isoform1	T384	-	-	-	-	0.5	0.4	0.3	0.5	-	-	-	-	0.6	0.5	0.3	0.5
5803145	NP_006779.1	10928	RALBP1	ralA-binding protein 1	S29	-	-	-	-	0.5	1.0	1.5	0.5	-	-	-	-	0.5	0.9	1.4	0.5
21361458	NP_055601.2	9828	ARHGEF17	rho guanine nucleotide exchange factor 17	S420	-	-	-	-	0.7	0.8	0.7	0.5	-	-	-	-	0.6	0.9	0.8	0.6
5032031	NP_005769.1	10181	RBM5	RNA-binding protein 5	S624	-	-	-	-	0.6	0.6	0.9	0.5	-	-	-	-	0.6	0.6	1.0	0.6
457867147	NP_001263988.1	1.01E+08	ERCC6-PGBD	Cockayne syndrome B-piggyBac fusion protein	S554	-	-	-	-	0.7	0.9	0.6	0.5	-	-	-	-	0.7	0.8	0.5	0.5
18765741	NP_005976.2	6615	SNAI1	zinc finger protein SNAI1	S90	-	-	-	-	0.9	1.1	0.8	0.5	-	-	-	-	-	-	-	-
297374764	NP_001172003.1	79670	ZCCHC6	terminal uridylyltransferase 7 isoform2	S172	-	-	-	-	0.6	0.9	0.7	0.5	-	-	-	-	0.5	0.8	0.6	0.5
30089937	NP_821174.1	54972	TMEM132A	transmembrane protein 132A isoformb precursor	S529	-	-	-	-	0.5	0.4	0.3	0.5	-	-	-	-	-	-	-	-
5032189	NP_005648.1	7158	TP53BP1	tumor suppressor p53-binding protein 1 isoform3	S1114	-	-	-	-	1.1	1.4	0.9	0.5	-	-	-	-	1.1	1.4	0.9	0.6
307133775	NP_001182502.1	23405	DICER1	endoribonuclease Dicer isoform2	S1160	-	-	-	-	0.5	0.4	0.6	0.5	-	-	-	-	0.3	0.3	0.5	0.4
296841073	NP_001171894.1	54521	WDR44	WD repeat-containing protein 44 isoform2	S162	-	-	-	-	0.4	0.6	0.3	0.5	-	-	-	-	0.5	0.6	0.3	0.6
108860697	NP_055445.2	9640	ZNF592	zinc finger protein 592	S361	-	-	-	-	1.1	0.6	1.0	0.5	-	-	-	-	1.1	0.6	1.0	0.5
56676397	NP_037407.4	29123	ANKRD11	ankyrin repeat domain-containing protein 11	S276	-	-	-	-	0.7	0.9	0.9	0.5	-	-	-	-	0.7	0.9	0.9	0.5
57863269	NP_055871.1	23076	RRP1B	ribosomal RNA processing protein 1 homolog B	S392	-	-	-	-	1.0	1.4	0.8	0.5	-	-	-	-	1.1	1.4	0.9	0.5
5032189	NP_005648.1	7158	TP53BP1	tumor suppressor p53-binding protein 1 isoform3	S208	-	-	-	-	0.5	1.0	1.1	0.5	-	-	-	-	0.5	1.0	1.2	0.6
217330641	NP_060439.2	55677	IWS1	protein IWS1 homolog	S287; S289	-	-	-	-	0.8	0.6	0.8	0.5	-	-	-	-	0.8	0.6	0.8	0.6
55750041	NP_001931.2	1822	ATN1	atrophin-1	S77	-	-	-	-	0.6	0.8	0.7	0.5	-	-	-	-	-	-	-	-
556503440	NP_001273295.1	55700	MAP7D1	MAP7 domain-containing protein 1 isoform3	S254	-	-	-	-	0.4	0.6	0.6	0.5	-	-	-	-	0.4	0.5	0.5	0.5
170932526	NP_001116314.1	135112	NCOA7	nuclear receptor coactivator 7 isoform2	S89	-	-	-	-	0.6	0.9	0.5	0.5	-	-	-	-	0.6	0.8	0.5	0.5
103472005	NP_002408.3	4288	MKI67	antigen KI-67 isoform1	S1628	-	-	-	-	0.4	0.5	0.8	0.5	-	-	-	-	0.4	0.6	0.8	0.5
631226398	NP_001278804.1	10644	IGF2BP2	insulin-like growth factor 2 mRNA-binding protein 2 isoformg	T414	-	-	-	-	1.3	0.9	1.7	0.5	-	-	-	-	1.2	0.7	1.5	0.5
13540614	NP_110445.1	81576	CCDC130	coiled-coil domain-containing protein 130	S332	-	-	-	-	0.4	0.3	0.6	0.5	-	-	-	-	0.4	0.3	0.7	0.5
5032189	NP_005648.1	7158	TP53BP1	tumor suppressor p53-binding protein 1 isoform3	S1426	-	-	-	-	1.7	2.1	1.6	0.5	-	-	-	-	1.7	2.1	1.7	0.6
5454088	NP_006392.1	10541	ANP32B	acidic leucine-rich nuclear phosphoprotein 32 family member B	T244	-	-	-	-	5.9	0.6	1.0	0.4	-	-	-	-	7.3	0.7	1.3	0.6
254587994	NP_001157019.1	11276	SYNRG	synergin gamma isoform7	S746	-	-	-	-	1.2	1.4	1.4	0.4	-	-	-	-	1.0	1.5	1.5	0.5
4503483	NP_001952.1	1938	EEF2	elongation factor 2	T435	-	-	-	-	1.3	1.0	0.7	0.4	-	-	-	-	1.3	1.0	0.8	0.4
39753961	NP_839943.2	128239	IQGAP3	ras GTPase-activating-like protein IQGAP3	S931	-	-	-	-	0.6	1.3	1.2	0.4	-	-	-	-	0.6	1.1	1.1	0.4
109637788	NP_150247.2	5371	PML	protein PML isoform5	S36	-	-	-	-	0.4	0.5	0.6	0.4	-	-	-	-	0.4	0.5	0.5	0.4
6031189	NP_006482.1	4857	NOVA1	RNA-binding protein Nova-1 isoform3	T158	-	-	-	-	0.5	0.4	0.3	0.4	-	-	-	-	-	-	-	-
4885371	NP_005309.1	3005	H1F0	histone H1.0	T153	-	-	-	-	0.4	0.7	0.5	0.4	-	-	-	-	0.4	0.8	0.6	0.6
155722985	NP_055925.2	23137	SMC5	structural maintenance of chromosomes protein 5	S12	-	-	-	-	1.1	0.4	0.9	0.4	-	-	-	-	1.0	0.4	0.8	0.4
190684675	NP_006288.2	7515	XRCC1	DNA repair protein XRCC1	T453	-	-	-	-	1.2	0.8	1.0	0.4	-	-	-	-	1.2	0.8	1.1	0.5
5292161	NP_002801.1	5710	PSMD4	26S proteasome non-ATPase regulatory subunit 4	S256	-	-	-	-	1.7	0.8	1.1	0.4	-	-	-	-	1.7	0.8	1.1	0.5
381342476	NP_001244222.1	3187	HNRNPH1	heterogeneous nuclear ribonucleoprotein H	S104	-	-	-	-	0.6	0.3	0.8	0.4	-	-	-	-	0.7	0.4	0.9	0.5
4507525	NP_003253.1	7095	SEC62	translocation protein SEC62	S335	-	-	-	-	1.0	0.5	0.6	0.4	-	-	-	-	0.8	0.4	0.5	0.4
7657575	NP_036370.2	23411	SIRT1	NAD-dependent protein deacetylase sirtuin-1 isoform	S26	-	-	-	-	1.0	1.0	1.1	0.4	-	-	-	-	1.0	1.0	1.1	0.4
613410239	NP_001278434.1	5504	PPP1R2	protein phosphatase inhibitor 2 isoform3	S101	-	-	-	-	0.4	0.3	0.2	0.4	-	-	-	-	0.3	0.4	0.2	0.6
150378552	NP_056366.1	26032	SUSD5	sushi domain-containing protein 5 precursor	S240	-	-	-	-	0.9	0.6	0.9	0.4	-	-	-	-	-	-	-	-
167234419	NP_005110.2	9967	THRAP3	thyroid hormone receptor-associated protein 3	S379	-	-	-	-	1.2	2.0	1.2	0.4	-	-	-	-	1.2	2.0	1.3	0.4
58331268	NP_060139.2	54821	ERCC6L	DNA excision repair protein ERCC-6-like	S790	-	-	-	-	0.3	0.4	0.5	0.4	-	-	-	-	0.4	0.6	0.7	0.5
63252863	NP_115820.2	84464	SLX4	structure-specific endonuclease subunit SLX4	S1070	-	-	-	-	1.0	0.4	0.8	0.4	-	-	-	-	0.8	0.3	0.7	0.3
23510323	NP_055917.1	261734	NPHP4	nephrocystin-4 isoforma	S481	-	-	-	-	0.9	1.0	1.0	0.4	-	-	-	-	-	-	-	-
515869342	NP_001265518.1	6767	ST13	hsc70-interacting protein isoform2	S69	-	-	-	-	0.3	1.6	0.7	0.4	-	-	-	-	0.3	1.6	0.6	0.4
4885375	NP_005310.1	3006	HIST1H1C	histone H1.2	T146	-	-	-	-	0.4	0.3	0.3	0.4	-	-	-	-	0.6	0.4	0.5	0.5
118421085	NP_689472.3	90381	TICRR	treslin	S1413	-	-	-	-	1.1	0.5	0.5	0.4	-	-	-	-	1.3	0.6	0.6	0.4
327315343	NP_001192157.1	6272	SORT1	sortilin isoform2	S688	-	-	-	-	0.3	0.6	0.5	0.4	-	-	-	-	0.5	0.9	0.7	0.5
578798047	XP_006710293.1	1.01E+08	LOC10050878	inositol hexakisphosphate and diphosphoinositol-pentakisphosphate kinase 1-like	S612	-	-	-	-	0.2	0.2	0.1	0.4	-	-	-	-	-	-	-	-

Protein group Accession	Gene Symbol	Average of replicates: Het-1A-Smoke - 2M/Parental	Average of replicates: Het-1A-Smoke - 4M/Parental	Average of replicates: Het-1A-Smoke - 6M/Parental	Average of replicates: Het-1A-Smoke - 8M/Parental	Peptide Sequence	Phosphowindow	PhosphoSite (Peptide)	Phosphosite Plus Evidence	phosphoRS Site Probabilities	# PSMs	# Proteins	# Protein Groups	# Missed Cleavages	Modifications
56118310	NUCKS1	0.9	1.0	1.0	0.9	TSTsPPPEK	PEKKTSTsPPPEKSG	S4;	Yes	T(1): 0.0; S(2): 0.0; T(3): 0.0; S(4): 100.0	6	1	1	0	N-Term(TMT6plex); S4(Phospho); K9(TMT6plex)
291575181	TMEM185A	-	-	-	-	VVITQsPGK	ARVVITQsPGKYVLP	S6;	No	T(4): 0.9; S(6): 99.1	1	3	1	0	N-Term(TMT6plex); S6(Phospho); K9(TMT6plex)
39777586	DHX57	0.8	0.9	0.9	0.7	GLsGEEEDDEPDCCNDER	AGSERGLsGEEEDDE	S3;	Yes	S(3): 100.0	2	1	1	0	N-Term(TMT6plex); S3(Phospho); C13(Carbamidomethyl); C14(Carbamidomethyl)
154146193	PNISR	1.4	1.1	1.3	0.7	SNRNsIER	RRRSNRNsIERERRR	S5;	Yes	S(1): 0.0; S(5): 100.0	1	1	1	1	N-Term(TMT6plex); S5(Phospho)
38044112	CLIP1	0.8	1.0	1.0	0.8	IGFPSTtPAK	KIGFPSTtPAKAKAN	T7;	Yes	S(5): 0.0; T(6): 0.0; T(7): 100.0	1	3	1	0	N-Term(TMT6plex); T7(Phospho); K10(TMT6plex)
96974985	CC2D1A	0.8	1.0	1.0	0.8	KQNsPVAPTAQPK	EVPKKQNsPVAPTAQ	S4;	Yes	S(4): 100.0; T(9): 0.0	2	1	1	1	N-Term(TMT6plex); K1(TMT6plex); S4(Phospho); K13(TMT6plex)
209862789	MICAL3	0.8	0.7	1.0	0.6	GPSQAtSPIR	ERGPSQAtSPIRSPQ	T6;	No	S(3): 0.0; T(6): 98.6; S(7): 1.4	1	1	1	0	N-Term(TMT6plex); T6(Phospho)
157676331	HABP4	0.9	1.0	1.2	0.8	sPAGASGHR	AGPRGGRsPAGASGH	S1;	Yes	S(1): 100.0; S(6): 0.0	1	1	1	0	N-Term(TMT6plex); S1(Phospho)
321267473	SAFB	0.7	1.0	1.1	0.8	MsSPEDDSDTK	EGADQKMsSPEDDSD	S2;	No	S(2): 100.0; S(3): 0.0; S(8): 0.0; T(10): 0.0	1	4	1	0	N-Term(TMT6plex); M1(Oxidation); S2(Phospho); K11(TMT6plex)
150378493	KAT6A	0.8	0.8	0.7	0.6	NSPLEPDTStPLKK	PLEPDTStPLKKKKG	T10;	Yes	S(2): 0.0; T(8): 1.0; S(9): 1.0; T(10): 98.0	1	1	1	1	N-Term(TMT6plex); T10(Phospho); K13(TMT6plex); K14(TMT6plex)
387942371	KIF18B	0.5	0.6	0.6	0.9	ASsPCHsPR	PKGERASsPCHSPRV;RASSPCHsPRVCPAT	S3; S7;	No;Yes	S(2): 1.2; S(3): 98.8; S(7): 100.0	1	2	1	0	N-Term(TMT6plex); S3(Phospho); C5(Carbamidomethyl); S7(Phospho)
55749431	TCEAL3	2.6	2.1	2.1	1.3	REDEGEPGDEGQLEDEGsQEK	GQLEDEGsQEKQGRS	S18;	Yes	S(18): 100.0	2	1	1	1	N-Term(TMT6plex); S18(Phospho); K21(TMT6plex)
63053524	USP1	0.6	0.6	0.5	0.7	KATSDTLEsPPK	ATSDTLEsPPKIIPK	S9;	Yes	T(3): 0.0; S(4): 0.0; T(6): 0.0; S(9): 100.0	2	1	1	1	N-Term(TMT6plex); K1(TMT6plex); S9(Phospho); K12(TMT6plex)
302058308	NEK4	0.8	0.5	1.0	0.8	LLGSSDsPASASR	RLLGSSDsPASASRV	S7;	No	S(4): 0.0; S(5): 1.3; S(7): 98.7; S(10): 0.0; S(12): 0.0	1	2	1	0	N-Term(TMT6plex); S7(Phospho)
18034775	PHF20	0.8	0.7	0.7	0.7	VSASsPTTK	RKRVSASsPTTKDKE	S5;	No	S(2): 0.0; S(4): 0.9; S(5): 97.2; T(7): 0.9; T(8): 0.9	1	1	1	0	N-Term(TMT6plex); S5(Phospho); K9(TMT6plex)
110556636	TNKS1BP1	0.9	1.2	1.2	0.7	RPsQGPPAR	AQTSRRPsQGPPARS	S3;	Yes	S(3): 100.0	2	1	1	0	N-Term(TMT6plex); S3(Phospho)
38016922	POP5	1.6	0.7	1.1	0.6	SCLLEEEEEsGEEAAEAME	LLEEEEEsGEEAAEA	S10;	No	S(1): 0.0; S(10): 100.0	1	2	1	0	N-Term(TMT6plex); C2(Carbamidomethyl); S10(Phospho); M18(Oxidation)
5031579	AKAP8	1.0	0.5	0.9	0.7	VDSEGDEsENDDAAGDER	VDSEGDEsENDDAAG	S8;	Yes	S(3): 0.0; S(8): 100.0	1	1	1	0	N-Term(TMT6plex); S8(Phospho)
41152093	ZNF644	1.0	0.7	0.8	0.7	NSAIsPQK	EERNSAIsPQKIHNQ	S5;	Yes	S(2): 0.0; S(5): 100.0	1	1	1	0	N-Term(TMT6plex); S5(Phospho); K8(TMT6plex)
229892308	SGOL2	0.6	0.8	0.8	0.8	sAEQNESK	KYVTDRKsAEQNESK	S1;	No	S(1): 100.0; S(7): 0.0	1	3	1	0	N-Term(TMT6plex); S1(Phospho); K8(TMT6plex)
117190174	HNRNPC	0.8	0.6	0.6	0.6	SEEEQSSSsVK	EEEQSSSsVKKDETN	S9;	No	S(1): 0.0; S(6): 0.0; S(7): 0.0; S(8): 0.0; S(9): 100.0	4	2	1	0	N-Term(TMT6plex); S9(Phospho); K11(TMT6plex)
191252785	CNTROB	-	-	-	-	RLEHsGTDGR	YLKRLEHsGTDGRGD	S5;	No	S(5): 100.0; T(7): 0.0	1	1	1	1	N-Term(TMT6plex); S5(Phospho)
385862220	ZFYVE19	-	-	-	-	WsPPQNYK	SANASKWsPPQNYKK	S2;	Yes	S(2): 100.0; Y(7): 0.0	1	3	1	0	N-Term(TMT6plex); S2(Phospho); K8(TMT6plex)
112789562	IFI16	0.7	0.7	0.9	0.8	EVDAtSPAPSTSSTVK	RKKEVDAtSPAPSTS	T5;	No	T(5): 99.0; S(6): 1.0; S(10): 0.0; T(11): 0.0; S(12): 0.0; S(13): 0.0; T(14): 0.0	4	2	1	0	N-Term(TMT6plex); T5(Phospho); K16(TMT6plex)
51479145	ARFGEF1	1.1	1.2	0.8	0.6	sVDIHDSIQPR	LDTISQKsVDIHDSI	S1;	Yes	S(1): 100.0; S(7): 0.0	1	1	1	0	N-Term(TMT6plex); S1(Phospho)
103472005	MKI67	0.9	0.7	0.8	0.7	ELFQtPGHTEESMTDDK	GFKELFQtPGHTEES	T5;	Yes	T(5): 100.0; T(9): 0.0; S(12): 0.0; T(14): 0.0	2	2	1	0	N-Term(TMT6plex); T5(Phospho); M13(Oxidation); K17(TMT6plex)
87116683	ZFC3H1	0.5	0.7	0.6	0.6	EEGELEDGEIsDDDNNSQIR	ELEDGEIsDDDNNSQ	S11;	Yes	S(11): 100.0; S(17): 0.0	2	1	1	0	N-Term(TMT6plex); S11(Phospho)
61676188	HUWE1	0.8	0.8	0.8	0.8	AEsPEEVACR	PMDQRAEsPEEVACR	S3;	Yes	S(3): 100.0	1	1	1	0	N-Term(TMT6plex); S3(Phospho); C9(Carbamidomethyl)
24041035	NOTCH2	0.6	0.7	1.0	0.6	STMPTsLPNLAK	AKSTMPTsLPNLAKE	S6;	Yes	S(1): 0.0; T(2): 0.0; T(5): 1.2; S(6): 98.7	2	1	1	0	N-Term(TMT6plex); M3(Oxidation); S6(Phospho); K12(TMT6plex)
4504969	LDB1	0.5	0.6	0.7	0.6	KMsGGSTMSSGGGNTNNSNSK	KRRKRKMsGGSTMSS	S3;	No	S(3): 92.6; S(6): 1.8; T(7): 1.8; S(9): 1.8; S(10): 1.8; T(15): 0.0; S(18): 0.0; S(20): 0.0	1	2	1	1	K1(TMT6plex); N-Term(TMT6plex); M2(Oxidation); S3(Phospho); M8(Oxidation); K21(TMT6plex)
194097392	GOLGA2	0.5	0.6	0.8	0.7	NsPGVPTGAK	REYQQRNsPGVPTGA	S2;	Yes	S(2): 100.0; T(7): 0.0	2	1	1	0	N-Term(TMT6plex); S2(Phospho); K10(TMT6plex)
118572613	SRRM2	0.6	0.9	0.9	0.7	SPSsPELNNK	PRSRSPSsPELNNKC	S4;	Yes	S(1): 0.0; S(3): 1.3; S(4): 98.7	1	1	1	0	N-Term(TMT6plex); S3(Phospho); K10(TMT6plex)
109637788	PML	0.6	0.9	0.7	0.5	sPRPQQDPARPQEPTMPPPETPSEGR	MEPAPARsPRPQQDP	S1;	Yes	S(1): 100.0; T(15): 0.0; T(21): 0.0; S(23): 0.0	1	9	1	0	N-Term(TMT6plex); S1(Phospho); M16(Oxidation)
112421108	CIC	-	-	-	-	AEAsPNDTAGAR	TSGRAEAsPNDTAGA	S4;	No	S(4): 100.0; T(8): 0.0	1	1	1	0	N-Term(TMT6plex); S4(Phospho)
15011974	ARHGEF2	0.4	1.9	0.7	0.5	sLPAGDALYLSFNPPQPSR	PVDPRRRsLPAGDAL	S1;	No	S(1): 100.0; Y(9): 0.0; S(11): 0.0; S(18): 0.0	2	3	1	0	N-Term(TMT6plex); S1(Phospho)
4885381	HIST1H1B	0.8	0.8	1.1	0.8	SETAPAETAtPAPVEK	TAPAETAtPAPVEKS	T10;	Yes	S(1): 0.0; T(3): 0.0; T(8): 1.0; T(10): 99.0	1	1	1	0	N-Term(TMT6plex); T10(Phospho); K16(TMT6plex)
148536853	COPA	0.2	0.3	0.6	0.6	NLsPGAVESDVR	GLRKKNLsPGAVESD	S3;	Yes	S(3): 100.0; S(9): 0.0	1	2	1	0	N-Term(TMT6plex); S3(Phospho)
149363685	KIAA0947	-	-	-	-	SIsPEVSASR	VAAVKSIsPEVSASR	S3;	Yes	S(1): 1.1; S(3): 98.9; S(7): 0.0; S(9):	1	1	1	0	N-Term(TMT6plex); S3(Phospho)
87299628	BOD1L1	0.3	0.6	0.6	0.7	YYsDSDDELTVEQR	YLYSKYYsDSDDELT	S3;	Yes	Y(1): 0.0; Y(2): 1.0; S(3): 98.0; S(5): 1.0; T(10): 0.0	2	1	1	0	N-Term(TMT6plex); S3(Phospho)
259906020	ACIN1	0.8	1.3	1.4	0.7	TTsPLEEEER	EKEMKTTsPLEEEER	S3;	No	T(1): 0.0; T(2): 1.0; S(3): 99.0	3	3	1	0	N-Term(TMT6plex); S3(Phospho)
544583541	GON4L	-	-	-	-	TCPVHESPSGIDTSETsPK	GIDTSETsPKAPRGG	S17;	No	T(1): 0.0; S(7): 0.0; S(9): 0.0; T(13): 1.6; S(14): 0.0; T(16): 1.6; S(17): 96.7	1	2	1	0	N-Term(TMT6plex); C2(Carbamidomethyl); S17(Phospho); K19(TMT6plex)
7656879	AFF4	0.7	0.6	0.7	0.6	NSYNNSQAPsPGLGSK	YNNSQAPsPGLGSKA	S10;	Yes	S(2): 0.0; Y(3): 0.0; S(6): 0.0; S(10): 100.0; S(15): 0.0	2	1	1	0	N-Term(TMT6plex); S10(Phospho); K16(TMT6plex)
4759098	TRA2B	1.2	0.7	1.2	0.7	sPsPYYSR	DQIYRRRsPSPYYSR;IYRRRSPsPYYSRGG	S1; S3;	Yes;Yes	S(1): 100.0; S(3): 100.0; Y(5): 0.0; Y(6): 0.0; S(7): 0.0	1	5	1	0	N-Term(TMT6plex); S1(Phospho); S3(Phospho)
21735417	BICD2	0.7	0.6	0.9	0.6	sPILLPK	PEARGRRsPILLPKG	S1;	Yes	S(1): 100.0	1	2	1	0	N-Term(TMT6plex); S1(Phospho); K7(TMT6plex)
34577112	ALDOA	0.8	0.9	1.0	0.6	PYQYPALtPEQK	PYQYPALtPEQKKEL	T8;	Yes	Y(2): 0.0; Y(4): 0.0; T(8): 100.0	1	1	1	0	N-Term(TMT6plex); T8(Phospho); K12(TMT6plex)
41393573	GIT1	0.3	0.5	0.5	0.7	SLSsPTDNLELSLR	QQGKSLSsPTDNLEL	S4;	Yes	S(1): 0.0; S(3): 1.8; S(4): 96.4; T(6): 1.8; S(12): 0.0	1	2	1	0	N-Term(TMT6plex); S4(Phospho)
110431336	ZSCAN1	-	-	-	-	GGTQEAVAGIsVVPR	QEAVAGIsVVPRGPR	S11;	No	T(3): 0.0; S(11): 100.0	1	1	1	0	N-Term(TMT6plex); S11(Phospho)
14249158	HDGFRP2	0.6	0.7	0.6	0.8	VDsPDVKR	KFALKVDsPDVKRCL	S3;	Yes	S(3): 100.0	1	2	1	1	N-Term(TMT6plex); S3(Phospho); K7(TMT6plex)
513788264	SPECC1L	0.6	0.9	1.1	0.6	TPLSPsPMK	PRTPLSPsPMKTPPA	S6;	Yes	T(1): 0.0; S(4): 0.0; S(6): 100.0	1	2	1	0	N-Term(TMT6plex); S6(Phospho); M8(Oxidation); K9(TMT6plex)
122937472	BICC1	-	-	-	-	sPSHSGNAGDLK	VEVGMPRsPSHSGNA	S1;	Yes	S(1): 100.0; S(3): 0.0; S(5): 0.0	2	1	1	0	N-Term(TMT6plex); S1(Phospho); K12(TMT6plex)
112789532	PVRL2	1.1	0.9	1.3	0.6	EQTLQGAEEDEDLEGPPSYKPPtPK	PPSYKPPtPKAKLEA	T23;	Yes	T(3): 0.0; S(18): 0.0; Y(19): 0.0; T(23): 100.0	2	1	1	0	N-Term(TMT6plex); K20(TMT6plex); T23(Phospho); K25(TMT6plex)
5032189	TP53BP1	0.5	0.7	0.7	0.7	GDsGAAPDVDDK	LGSGKGDsGAAPDVD	S3;	Yes	S(3): 100.0	2	3	1	0	N-Term(TMT6plex); S3(Phospho); K12(TMT6plex)
31652242	TCF20	0.7	0.9	0.7	0.7	AEEKENDTVTIsPK	ENDTVTIsPKQEGFP	S12;	Yes	T(8): 0.0; T(10): 0.0; S(12): 100.0	1	2	1	1	N-Term(TMT6plex); K4(TMT6plex); S12(Phospho); K14(TMT6plex)
134304853	ARHGAP29	0.8	0.6	0.7	0.8	ATsEEESER	KIEERATsEEESERK	S3;	Yes	T(2): 0.0; S(3): 100.0; S(7): 0.0	1	1	1	0	N-Term(TMT6plex); S3(Phospho)
20986514	MAPK14	0.9	1.7	1.0	0.6	HTDDEMTGyVATR	TDDEMTGyVATRWYR	Y9;	Yes	T(2): 0.0; T(7): 1.6; Y(9): 98.4; T(12): 0.0	2	4	1	0	N-Term(TMT6plex); M6(Oxidation); Y9(Phospho)
55956788	NCL	0.9	0.9	0.7	0.8	VVVsPTK	SAKKVVVsPTKKVAV	S4;	Yes	S(4): 99.4; T(6): 0.6	3	1	1	0	N-Term(TMT6plex); S4(Phospho); K7(TMT6plex)
300388183	PSMD1	1.0	0.7	0.6	0.6	TSSAFVGKtPEAsPEPK	SSAFVGKtPEASPEP;VGKTPEAsPEPKDQT	T9; S13;	Yes;Yes	T(1): 0.0; S(2): 0.0; S(3): 0.0; T(9): 100.0; S(13): 100.0	2	2	1	1	N-Term(TMT6plex); K8(TMT6plex); T9(Phospho); S13(Phospho); K17(TMT6plex)
110618244	KDM5A	0.7	0.8	0.8	0.6	VVSSVSSsPR	VVSSVSSsPRQTMDY	S8;	Yes	S(3): 0.0; S(4): 0.0; S(6): 0.0; S(7): 1.2; S(8): 98.7	1	1	1	0	N-Term(TMT6plex); S8(Phospho)
118572613	SRRM2	0.9	1.1	1.0	0.6	SsSPVTELASRSPIR	KGGRSRSsSPVTELA	S2;	Yes	S(1): 1.2; S(2): 97.6; S(3): 1.2; T(6): 0.0; S(10): 50.0; S(12): 50.0	2	1	1	1	N-Term(TMT6plex); S3(Phospho); S10(Phospho)
7669481	BNIP3	0.6	0.9	0.8	0.6	SQtPQDTNR	DSPPRSQtPQDTNRA	T3;	No	S(1): 0.0; T(3): 100.0; T(7): 0.0	2	1	1	0	N-Term(TMT6plex); T3(Phospho)
95147555	MAP1A	-	-	-	-	AELEEMEEVHPsDEEEEDATK	EMEEVHPsDEEEEDA	S12;	Yes	S(12): 100.0; T(20): 0.0	1	1	1	0	N-Term(TMT6plex); M6(Oxidation); S12(Phospho); K21(TMT6plex)
58331268	ERCC6L	0.7	0.9	0.8	0.8	QEDAStPK	VKQEDAStPKNDISP	T6;	Yes	S(5): 0.8; T(6): 99.2	2	1	1	0	N-Term(TMT6plex); T6(Phospho); K8(TMT6plex)
333360918	LOC554223	-	-	-	-	LDSDAtSPR	VRLDSDAtSPRMEPR	T6;	No	S(3): 0.0; T(6): 99.2; S(7): 0.8	3	1	1	0	N-Term(TMT6plex); T6(Phospho)
56118310	NUCKS1	1.1	1.0	1.0	0.7	TPSPKEEDEEPEsPPEKK	EEDEEPEsPPEKKTS	S13;	Yes	T(1): 0.0; S(3): 0.0; S(13): 100.0	2	1	1	2	N-Term(TMT6plex); K5(TMT6plex); S13(Phospho); K17(TMT6plex); K18(TMT6plex)
226509740	SCAF1	0.6	1.4	0.9	0.6	EVLYDSEGLsGEER	LYDSEGLsGEERGGK	S10;	Yes	Y(4): 0.0; S(6): 1.7; S(10): 98.3	2	1	1	0	N-Term(TMT6plex); S10(Phospho)
221139693	FAM76A	-	-	-	-	SGAITsP	EKSGAITsP	S6;	No	S(1): 0.0; T(5): 0.0; S(6): 100.0	2	5	1	0	N-Term(TMT6plex); S6(Phospho)
116256350	TSC2	0.5	0.5	0.7	0.6	TSGPLsPPTGPPGPAPAGPAVR	KKTSGPLsPPTGPPG	S6;	Yes	T(1): 3.0; S(2): 3.0; S(6): 93.8; T(9):	1	3	1	0	N-Term(TMT6plex); S6(Phospho)
33620755	YEATS2	-	-	-	-	IVPQSQVPNPEsPGK	SQVPNPEsPGKSFQP	S12;	Yes	S(5): 0.0; S(12): 100.0	4	1	1	0	N-Term(TMT6plex); S12(Phospho); K15(TMT6plex)
132626688	MDC1	0.3	0.3	0.3	0.6	AIPGDQHPEsPVHTEPMGIQGR	PGDQHPEsPVHTEPM	S10;	Yes	S(10): 100.0; T(14): 0.0	2	1	1	0	N-Term(TMT6plex); S10(Phospho); M17(Oxidation)
132626688	MDC1	1.1	0.3	1.1	0.6	SQTTTERDsDTDVEEEELPVENR	QTTTERDsDTDVEEE	S9;	Yes	S(1): 0.0; T(3): 0.0; T(4): 0.0; T(5): 0.0; S(9): 99.9; T(11): 0.0	1	1	1	1	N-Term(TMT6plex); S9(Phospho)
4758258	EIF4EBP1	3.7	0.9	2.4	0.7	NsPVtKTPPR	FLMECRNsPVTKTPP;ECRNSPVtKTPPRDL	S2; T5;	Yes;Yes	S(2): 100.0; T(5): 100.0; T(7): 0.0	1	1	1	1	N-Term(TMT6plex); S2(Phospho); T5(Phospho); K6(TMT6plex)
306922386	APC	0.7	1.0	0.8	0.6	TSsPSTASTK	SQLPRTSsPSTASTK	S3;	No	T(1): 0.0; S(2): 0.9; S(3): 99.1; S(5): 0.0; T(6): 0.0; S(8): 0.0; T(9): 0.0	1	2	1	0	N-Term(TMT6plex); S3(Phospho); K10(TMT6plex)
109637759	CAST	0.7	0.7	0.7	0.7	TSEsPSKPGEK	VSEKTSEsPSKPGEK	S4;	No	T(1): 0.0; S(2): 0.0; S(4): 98.9; S(6):	2	1	1	0	N-Term(TMT6plex); S4(Phospho); K7(TMT6plex); K11(TMT6plex)
506949341	SRPK2	1.0	0.7	0.7	0.5	TVSASsTGDLPK	SRTVSASsTGDLPKA	S6;	Yes	T(1): 0.0; S(3): 0.7; S(5): 0.7; S(6): 98.6; T(7): 0.0	2	2	1	0	N-Term(TMT6plex); S6(Phospho); K12(TMT6plex)
116812600	STARD10	-	-	-	-	MGGAGGEGsDDDTSLT	GGAGGEGsDDDTSLT	S9;	Yes	S(9): 100.0; T(13): 0.0; S(14): 0.0; T(16): 0.0	2	1	1	0	N-Term(TMT6plex); M1(Oxidation); S9(Phospho)
192807320	SMARCA4	0.8	1.0	1.1	0.6	LsPNPPNLTK	RPPAEKLsPNPPNLT	S2;	No	S(2): 100.0; T(9): 0.0	3	6	1	0	N-Term(TMT6plex); S2(Phospho); K10(TMT6plex)
313661430	NEK1	1.1	1.1	1.1	0.6	SSDVSPPLGQHEtGGSPSK	PPLGQHEtGGSPSKQ	T13;	No	S(1): 0.0; S(2): 0.0; S(5): 0.0; T(13): 98.8; S(16): 1.2; S(18): 0.0	1	5	1	0	N-Term(TMT6plex); S16(Phospho); K19(TMT6plex)
190341065	CCP110	-	-	-	-	NPsPLLMQNQTR	SYDVKNPsPLLMQNQ	S3;	Yes	S(3): 100.0; T(12): 0.0	2	2	1	0	N-Term(TMT6plex); S3(Phospho); M7(Oxidation)
4885379	HIST1H1E	0.7	0.5	0.6	0.7	ATGAAtPK	KKATGAAtPKKSAKK	T6;	Yes	T(2): 0.0; T(6): 100.0	2	1	1	0	N-Term(TMT6plex); T6(Phospho); K8(TMT6plex)
14249158	HDGFRP2	0.6	0.7	0.8	0.7	MEsDSDSDK	AASDRMEsDSDSDKS	S3;	No	S(3): 100.0; S(5): 0.0; S(7): 0.0	2	2	1	0	N-Term(TMT6plex); M1(Oxidation); S3(Phospho); K9(TMT6plex)
260763910	SKA3	0.6	0.5	0.3	0.5	YNSNLAtPIAIK	KYNSNLAtPIAIKAV	T7;	Yes	Y(1): 0.0; S(3): 0.0; T(7): 100.0	1	1	1	0	N-Term(TMT6plex); T7(Phospho); K12(TMT6plex)
5803145	RALBP1	0.5	0.9	1.4	0.5	TPsSEEISPTK	SGLTRTPsSEEISPT	S3;	Yes	T(1): 0.6; S(3): 98.9; S(4): 0.6; S(8): 0.0; T(10): 0.0	2	1	1	0	N-Term(TMT6plex); S3(Phospho); K11(TMT6plex)
21361458	ARHGEF17	0.6	0.9	0.8	0.6	SPsFGAGEGLLR	WGVYRSPsFGAGEGL	S3;	Yes	S(1): 0.0; S(3): 100.0	1	1	1	0	N-Term(TMT6plex); S3(Phospho)
5032031	RBM5	0.6	0.6	1.0	0.6	GLVAAYSGDsDNEEELVER	VAAYSGDsDNEEELV	S10;	Yes	Y(6): 0.0; S(7): 0.0; S(10): 100.0	2	1	1	0	N-Term(TMT6plex); S10(Phospho)
457867147	ERCC6-PGBD	0.7	0.8	0.5	0.5	DDsPDEVPSTFTVQQPPPSR	SYAPKDDsPDEVPST	S3;	No	S(3): 100.0; S(9): 0.0; T(10): 0.0; T(12): 0.0; S(19): 0.0	2	2	1	0	N-Term(TMT6plex); S3(Phospho)
18765741	SNAI1	-	-	-	-	VAELTsLSDEDSGK	PRVAELTsLSDEDSG	S6;	No	T(5): 1.5; S(6): 97.0; S(8): 1.5; S(12): 0.0	1	1	1	0	N-Term(TMT6plex); S6(Phospho); K14(TMT6plex)
297374764	ZCCHC6	0.5	0.8	0.6	0.5	DLTSLETTSEMEAGsPENK	TSEMEAGsPENKKQR	S15;	Yes	T(3): 0.0; S(4): 0.0; T(7): 0.0; T(8): 0.0; S(9): 0.0; S(15): 100.0	2	2	1	0	N-Term(TMT6plex); M11(Oxidation); S15(Phospho); K19(TMT6plex)
30089937	TMEM132A	-	-	-	-	VPGPAEGPAEPAAEAsDEAERR	AEPAAEAsDEAERRA	S16;	Yes	S(16): 100.0	1	2	1	1	N-Term(TMT6plex); S16(Phospho)
5032189	TP53BP1	1.1	1.4	0.9	0.6	MVIQGPSsPQGEAMVTDVLEDQ	MVIQGPSsPQGEAMV	S8;	Yes	S(7): 1.2; S(8): 98.8; T(16): 0.0	4	3	1	0	M1(Oxidation); N-Term(TMT6plex); S8(Phospho); M14(Oxidation); K23(TMT6plex)
307133775	DICER1	0.3	0.3	0.5	0.4	TLLSEsPGK	CRTLLSEsPGKLHVE	S6;	Yes	T(1): 0.0; S(4): 1.2; S(6): 98.8	1	2	1	0	N-Term(TMT6plex); S6(Phospho); K9(TMT6plex)
296841073	WDR44	0.5	0.6	0.3	0.6	LTQTSsTEQLNVLETETEVLNK	TKLTQTSsTEQLNVL	S6;	Yes	T(2): 0.0; T(4): 0.0; S(5): 1.9; S(6): 96.1; T(7): 1.9; T(15): 0.0; T(17): 0.0	2	3	1	0	N-Term(TMT6plex); S6(Phospho); K22(TMT6plex)
108860697	ZNF592	1.1	0.6	1.0	0.5	GsPSVAASSPPAIPK	SDSSSKGsPSVAASS	S2;	No	S(2): 98.2; S(4): 2.7; S(8): 49.6; S(9): 49.6	3	1	1	0	N-Term(TMT6plex); S4(Phospho); S8(Phospho); K15(TMT6plex)
56676397	ANKRD11	0.7	0.9	0.9	0.5	VANsPTMVNLLLGK	TPLKVANsPTMVNLL	S4;	Yes	S(4): 100.0; T(6): 0.0	2	1	1	0	N-Term(TMT6plex); S4(Phospho); M7(Oxidation); K14(TMT6plex)
57863269	RRP1B	1.1	1.4	0.9	0.5	VFCVEEEDsESSLQK	FCVEEEDsESSLQKR	S9;	Yes	S(9): 98.9; S(11): 1.1; S(12): 0.0	2	1	1	0	N-Term(TMT6plex); C3(Carbamidomethyl); S9(Phospho); K15(TMT6plex)
5032189	TP53BP1	0.5	1.0	1.2	0.6	LsDVDANTAK	NSGYTRLsDVDANTA	S2;	Yes	S(2): 100.0; T(9): 0.0	1	3	1	0	N-Term(TMT6plex); S2(Phospho); K11(TMT6plex)
217330641	IWS1	0.8	0.6	0.8	0.6	NQAsDsENEELPKPR	DPPRNQAsDSENEEL;PRNQASDsENEELPK	S4; S6;	Yes;Yes	S(4): 100.0; S(6): 100.0	1	1	1	0	N-Term(TMT6plex); S4(Phospho); S6(Phospho); K13(TMT6plex)
55750041	ATN1	-	-	-	-	SEEIsESESEETNAPK	QGRSEEIsESESEET	S5;	Yes	S(1): 1.7; S(5): 98.3; S(7): 0.0; S(9): 0.0; T(12): 0.0	1	1	1	0	N-Term(TMT6plex); S5(Phospho); K16(TMT6plex)
556503440	MAP7D1	0.4	0.5	0.5	0.5	CSVsAVNLPK	SGSRCSVsAVNLPKH	S4;	Yes	S(2): 0.0; S(4): 100.0	2	2	1	0	N-Term(TMT6plex); C1(Carbamidomethyl); S4(Phospho); K10(TMT6plex)
170932526	NCOA7	0.6	0.8	0.5	0.5	YYsIDDNQNK	TELKRYYsIDDNQNK	S3;	Yes	Y(1): 0.0; Y(2): 1.2; S(3): 98.8	1	2	1	0	N-Term(TMT6plex); S3(Phospho); K10(TMT6plex)
103472005	MKI67	0.4	0.6	0.8	0.5	SSQPDPDKNPAsSK	DPDKNPAsSKRRLKT	S12;	Yes	S(1): 0.0; S(2): 0.0; S(12): 100.0; S(13): 0.0	1	2	1	1	N-Term(TMT6plex); K8(TMT6plex); S12(Phospho); K14(TMT6plex)
631226398	IGF2BP2	1.2	0.7	1.5	0.5	DQtPDENEEVIVR	VIVPRDQtPDENEEV	T3;	No	T(3): 100.0	1	7	1	0	N-Term(TMT6plex); T3(Phospho)
13540614	CCDC130	0.4	0.3	0.7	0.5	VPEEAAQDRPMsPGDCPPETTETPK	AAQDRPMsPGDCPPE	S12;	Yes	S(12): 100.0; T(20): 0.0; T(21): 0.0; T(23): 0.0	2	1	1	0	N-Term(TMT6plex); M11(Oxidation); S12(Phospho); C16(Carbamidomethyl); K25(TMT6plex)
5032189	TP53BP1	1.7	2.1	1.7	0.6	ETAVPGPLGIEDIsPNLSPDDK	PLGIEDIsPNLSPDD	S14;	Yes	T(2): 0.0; S(14): 100.0; S(18): 0.0	2	3	1	0	N-Term(TMT6plex); S14(Phospho); K22(TMT6plex)
5454088	ANP32B	7.3	0.7	1.3	0.6	EtDDEGEDD	GEKRKREtDDEGEDD	T2;	Yes	T(2): 100.0	1	1	1	0	N-Term(TMT6plex); T2(Phospho)
254587994	SYNRG	1.0	1.5	1.5	0.5	VTTFVsEDALPETTFPALASFK	SKVTTFVsEDALPET	S6;	No	T(2): 0.0; T(3): 0.0; S(6): 96.6; T(13): 34.5; T(14): 34.5; S(20): 34.5	1	7	1	0	N-Term(TMT6plex); S6(Phospho); S20(Phospho); K22(TMT6plex)
4503483	EEF2	1.3	1.0	0.8	0.4	IMGPNYtPGK	RIMGPNYtPGKKEDL	T7;	Yes	Y(6): 0.0; T(7): 100.0	2	1	1	0	N-Term(TMT6plex); M2(Oxidation); T7(Phospho); K10(TMT6plex)
39753961	IQGAP3	0.6	1.1	1.1	0.4	EQLsDMMVLDK	KRNKEQLsDMMVLDK	S4;	No	S(4): 100.0	1	1	1	0	N-Term(TMT6plex); S4(Phospho); K11(TMT6plex)
109637788	PML	0.4	0.5	0.5	0.4	QPsPSPSPTER	PSEGRQPsPSPSPTE	S3;	Yes	S(3): 98.7; S(5): 50.0; S(7): 50.0; T(9): 1.3	1	9	1	0	N-Term(TMT6plex); S3(Phospho); S5(Phospho)
6031189	NOVA1	-	-	-	-	QTLPSSPTTtK	LPSSPTTtKSSPSDP	T10;	No	T(2): 0.0; S(5): 1.3; S(6): 1.3; T(8): 0.0; T(9): 0.0; T(10): 97.4	2	2	1	0	N-Term(TMT6plex); S6(Phospho); K11(TMT6plex)
4885371	H1F0	0.4	0.8	0.6	0.6	KLAAtPK	AKKKLAAtPKKAKKP	T5;	No	T(5): 100.0	1	1	1	1	N-Term(TMT6plex); K1(TMT6plex); T5(Phospho); K7(TMT6plex)
155722985	SMC5	1.0	0.4	0.8	0.4	TSTPsPQPSK	SKKTSTPsPQPSKRA	S5;	Yes	T(1): 0.0; S(2): 0.0; T(3): 0.0; S(5): 100.0; S(9): 0.0	2	1	1	0	N-Term(TMT6plex); S5(Phospho); K10(TMT6plex)
190684675	XRCC1	1.2	0.8	1.1	0.5	TKPTQAAGPSSPQKPPtPEETK	SSPQKPPtPEETKAA	T17;	Yes	T(1): 0.0; T(4): 0.0; S(10): 50.0; S(11): 50.0; T(17): 97.8; T(21): 2.2	1	1	1	0	N-Term(TMT6plex); K2(TMT6plex); S10(Phospho); S11(Phospho); K14(TMT6plex); K22(TMT6plex)
5292161	PSMD4	1.7	0.8	1.1	0.5	AAAASAAEAGIATTGTEDsDDALLK	ATTGTEDsDDALLKM	S19;	Yes	S(5): 0.0; T(13): 0.0; T(14): 0.0; T(16): 0.0; S(19): 99.9	2	1	1	0	N-Term(TMT6plex); S19(Phospho); K25(TMT6plex)
381342476	HNRNPH1	0.7	0.4	0.9	0.5	HTGPNsPDTANDGFVR	LKHTGPNsPDTANDG	S6;	Yes	T(2): 0.0; S(6): 100.0; T(9): 0.0	2	2	1	0	N-Term(TMT6plex); S6(Phospho)
4507525	SEC62	0.8	0.4	0.5	0.4	VGPGNHGTEGsGGERHSDTDSDR	GNHGTEGsGGERHSD	S11;	No	T(8): 0.0; S(11): 96.5; S(17): 1.7; T(19): 1.7; S(21): 0.0	1	1	1	1	N-Term(TMT6plex); S17(Phospho)
7657575	SIRT1	1.0	1.0	1.1	0.4	EAAsSPAGEPLR	GADREAAsSPAGEPL	S4;	Yes	S(4): 98.6; S(5): 1.4	1	1	1	0	N-Term(TMT6plex); S5(Phospho)
613410239	PPP1R2	0.3	0.4	0.2	0.6	IQEQESSGEEDsDLSPEER	ESSGEEDsDLSPEER	S12;	No	S(6): 50.8; S(7): 50.8; S(12): 94.8; S(15): 3.5	1	3	1	0	N-Term(TMT6plex); S7(Phospho); S12(Phospho)
150378552	SUSD5	-	-	-	-	TEADEDRGQGDsSEEAPK	EDRGQGDsSEEAPKQ	S12;	Yes	T(1): 0.0; S(12): 98.4; S(13): 1.6	1	1	1	1	N-Term(TMT6plex); S12(Phospho); K18(TMT6plex)
167234419	THRAP3	1.2	2.0	1.3	0.4	GSEsDTGLGDGK	IKEKGSEsDTGLGDG	S4;	Yes	S(2): 0.0; S(4): 100.0; T(6): 0.0	2	1	1	0	N-Term(TMT6plex); S4(Phospho); K12(TMT6plex)
58331268	ERCC6L	0.4	0.6	0.7	0.5	QDLsSIK	EGEKQDLsSIKVNVT	S4;	Yes	S(4): 99.2; S(5): 0.8	2	1	1	0	N-Term(TMT6plex); S4(Phospho); K7(TMT6plex)
63252863	SLX4	0.8	0.3	0.7	0.3	GGTSQVGsPTLLSPAVPSK	GGTSQVGsPTLLSPA	S8;	Yes	T(3): 2.3; S(4): 2.3; S(8): 90.6; T(10): 2.3; S(13): 2.3; S(18): 0.0	1	1	1	0	N-Term(TMT6plex); S8(Phospho); K19(TMT6plex)
23510323	NPHP4	-	-	-	-	KPPTSPSsPPAPVPR	KPPTSPSsPPAPVPR	S8;	Yes	T(4): 0.0; S(5): 2.0; S(7): 2.0; S(8):	1	1	1	0	N-Term(TMT6plex); K1(TMT6plex); S5(Phospho)
515869342	ST13	0.3	1.6	0.6	0.4	ADEPSSEEsDLEIDK	DEPSSEEsDLEIDKE	S9;	No	S(5): 1.8; S(6): 0.0; S(9): 98.2	2	2	1	0	N-Term(TMT6plex); S9(Phospho); K15(TMT6plex)
4885375	HIST1H1C	0.6	0.4	0.5	0.5	KAAGGAtPK	KKAAGGAtPKKSAKK	T7;	Yes	T(7): 100.0	1	1	1	1	N-Term(TMT6plex); K1(TMT6plex); T7(Phospho); K9(TMT6plex)
118421085	TICRR	1.3	0.6	0.6	0.4	SIVECQPDASATPGVGTADsPAAPTDSR	PGVGTADsPAAPTDS	S20;	Yes	S(1): 0.0; S(10): 3.2; T(12): 3.2; T(17): 3.2; S(20): 87.0; T(25): 3.2; S(27): 0.1	1	1	1	0	N-Term(TMT6plex); C5(Carbamidomethyl); T17(Phospho)
327315343	SORT1	0.5	0.9	0.7	0.5	SGYHDDsDEDLLE	KSGYHDDsDEDLLE	S7;	No	S(1): 0.0; Y(3): 0.0; S(7): 100.0	1	2	1	0	N-Term(TMT6plex); S7(Phospho)
578798047	LOC10050878	-	-	-	-	ALQTsPQPPEGPGLPR	PDRALQTsPQPPEGP	S5;	No	T(4): 1.7; S(5): 98.3	1	4	1	0	N-Term(TMT6plex); S5(Phospho)

Khan et al., 2019. Multi-omics analysis to characterize cigarette smoke induced molecular alterations in esophageal cells
Supplementary Table 8. List of phosphopeptides identified and quantified in untreated and chronically treated Het1A cells with cigarette smoke condensate for 8 months using SequestHT and Mascot search algorithms with a PhosphoRS probability score ≥75%

														Normalized phosphoprotein ratios with respect to corresponding total protein ratios.								Average of replicates													
**Protein group Accession**	**NP_Accession**	**Gene ID**	**Gene Symbol**	**Protein Description**	**PhosphoSite (Protein)**	**Het-1A-Smoke - 2M/Parental**	**Het-1A-Smoke - 4M/Parental**	**Het-1A-Smoke - 6M/Parental**	**Het-1A-Smoke - 8M/Parental**	**Het-1A-Smoke - 2M/Parental**	**Het-1A-Smoke - 4M/Parental**	**Het-1A-Smoke - 6M/Parental**	**Het-1A-Smoke - 8M/Parental**	**Het-1A-Smoke - 2M/Parental**	**Het-1A-Smoke - 4M/Parental**	**Het-1A-Smoke - 6M/Parental**	**Het-1A-Smoke - 8M/Parental**	**Het-1A-Smoke - 2M/Parental**	**Het-1A-Smoke - 4M/Parental**	**Het-1A-Smoke - 6M/Parental**	**Het-1A-Smoke - 8M/Parental**	**Het-1A-Smoke - 2M/Parental**	**Het-1A-Smoke - 4M/Parental**	**Het-1A-Smoke - 6M/Parental**	**Het-1A-Smoke - 8M/Parental**	**Peptide Sequence**	**Phosphowindow**	**PhosphoSite (Peptide)**	**Phosphosite Plus Evidence**	**phosphoRS Site Probabilities**	**# PSMs**	**# Proteins**	**# Protein Groups**	**# Missed Cleavages**	**Modifications**
						**Replicate 1**				**Replicate 2**				**Replicate 1**				**Replicate 2**																	
73532778	NP_004375.2	1456	CSNK1G3	casein kinase I isoform gamma-3 isoform 1	**S413**	-	-	-	-	**1.3**	**0.6**	**0.7**	**0.3**	-	-	-	-	**1.3**	**0.7**	**0.8**	**0.3**	**1.3**	**0.7**	**0.8**	**0.3**	sNAPITAPTEVEVMDETK	DDPTAGRsNAPITAP	S1;	Yes	S(1): 97.0; T(6): 2.8; T(9): 0.1; T(17): 0.1	1	1	1	0	N-Term(TMT6plex); S1(Phospho); M14(Oxidation); K18(TMT6plex)
66346681	NP_001018078.1	26135	SERBP1	plasminogen activator inhibitor 1 RNA-binding protein isoform 2	**S228**	-	-	-	-	**0.2**	**0.7**	**0.7**	**0.3**	-	-	-	-	**0.3**	**0.8**	**0.9**	**0.4**	**0.3**	**0.8**	**0.9**	**0.4**	DELTEsPK	VKDELTEsPKYIQKQ	S6;	No	T(4): 0.7; S(6): 99.3	2	2	1	0	N-Term(TMT6plex); S6(Phospho); K8(TMT6plex)
27436910	NP_078951.2	79728	PALB2	partner and localizer of BRCA2	**S376**	-	-	-	-	**0.7**	**1.1**	**0.9**	**0.3**	-	-	-	-	-	-	-	-	-	-	-	-	NENLQESEILsQPK	LQESEILsQPKSLSL	S11;	Yes	S(7): 0.0; S(11): 100.0	2	1	1	0	N-Term(TMT6plex); S11(Phospho); K14(TMT6plex)
226509740	NP_067051.2	58506	SCAF1	splicing factor, arginine/serine-rich 19	**S548**	-	-	-	-	**0.8**	**0.8**	**0.9**	**0.3**	-	-	-	-	**0.8**	**0.7**	**0.8**	**0.3**	**0.8**	**0.7**	**0.8**	**0.3**	EAASSSSGTQPAPPAPAsPWDSK	PAPPAPAsPWDSKKH	S18;	Yes	S(4): 0.0; S(5): 0.0; S(6): 0.0; S(7): 0.0; T(9): 0.0; S(18): 100.0; S(22): 0.0	2	1	1	0	N-Term(TMT6plex); S18(Phospho); K23(TMT6plex)
51173735	NP_001003698.1	6239	RREB1	ras-responsive element-binding protein 1 isoform 2	**S1167**	-	-	-	-	**0.7**	**1.3**	**0.8**	**0.3**	-	-	-	-	**0.8**	**1.4**	**0.8**	**0.3**	**0.8**	**1.4**	**0.8**	**0.3**	ANsGGVDLDSSGEFASIEK	RSRPRANsGGVDLDS	S3;	Yes	S(3): 100.0; S(10): 0.0; S(11): 0.0; S(16): 0.0	2	3	1	0	N-Term(TMT6plex); S3(Phospho); K19(TMT6plex)
62912453	NP_001112.2	120	ADD3	gamma-adducin isoform b	**S645**	-	-	-	-	**0.7**	**0.2**	**0.6**	**0.3**	-	-	-	-	**0.8**	**0.3**	**0.8**	**0.3**	**0.8**	**0.3**	**0.8**	**0.3**	IEEVLSPEGsPSK	EVLSPEGsPSKSPSK	S10;	No	S(6): 0.0; S(10): 100.0; S(12): 0.0	2	2	1	0	N-Term(TMT6plex); S10(Phospho); K13(TMT6plex)
519666783	NP_001265539.1	4605	MYBL2	myb-related protein B isoform 2	**S217**	-	-	-	-	**0.3**	**0.5**	**0.9**	**0.3**	-	-	-	-	**0.3**	**0.6**	**0.9**	**0.3**	**0.3**	**0.6**	**0.9**	**0.3**	EEENsEEELAAATTSK	TIKEEENsEEELAAA	S5;	No	S(5): 100.0; T(13): 0.0; T(14): 0.0; S(15): 0.0	2	2	1	0	N-Term(TMT6plex); S5(Phospho); K16(TMT6plex)
42741682	NP_005446.2	9406	ZRANB2	zinc finger Ran-binding domain-containing protein 2 isoform 2	**S307**	-	-	-	-	**0.9**	**0.8**	**1.1**	**0.3**	-	-	-	-	**1.0**	**0.9**	**1.3**	**0.4**	**1.0**	**0.9**	**1.3**	**0.4**	sPESQVIGENTK	KRRTRSRsPESQVIG	S1;	Yes	S(1): 100.0; S(4): 0.0; T(11): 0.0	2	1	1	0	N-Term(TMT6plex); S1(Phospho); K12(TMT6plex)
256818782	NP_001035375.2	64282	PAPD5	non-canonical poly(A) RNA polymerase PAPD5 isoform b	**S112**	-	-	-	-	**1.0**	**1.1**	**1.0**	**0.3**	-	-	-	-	**0.9**	**1.0**	**0.9**	**0.3**	**0.9**	**1.0**	**0.9**	**0.3**	AGsSASSPPSASSSPHPSAAVPAADPADSASGSSNK	AWARRAGsSASSPPS	S3;	No	S(3): 91.4; S(4): 2.9; S(6): 2.9; S(7): 2.9; S(10): 0.0; S(12): 0.0; S(13): 0.0; S(14): 0.0; S(18): 0.0; S(29): 0.0; S(31): 0.0; S(33): 0.0; S(34): 0.0	1	2	1	0	N-Term(TMT6plex); S3(Phospho); K36(TMT6plex)
117190342	NP_005383.3	5253	PHF2	lysine-specific demethylase PHF2	**S458**	-	-	-	-	**0.9**	**0.8**	**0.8**	**0.3**	-	-	-	-	**0.9**	**0.9**	**0.9**	**0.3**	**0.9**	**0.9**	**0.9**	**0.3**	AVRPEVNTVAsSDEVCDGDR	PEVNTVAsSDEVCDG	S11;	No	T(8): 0.0; S(11): 97.8; S(12): 2.2	1	1	1	0	N-Term(TMT6plex); S11(Phospho); C16(Carbamidomethyl)
222418615	NP_001138469.1	3679	ITGA7	integrin alpha-7 isoform 3	**S838**	-	-	-	-	**0.6**	**0.9**	**1.1**	**0.2**	-	-	-	-	**0.5**	**0.7**	**0.9**	**0.2**	**0.5**	**0.7**	**0.9**	**0.2**	QEPsMSWWPVSSAEK	PGERQEPsMSWWPVS	S4;	No	S(4): 96.7; S(6): 49.2; S(11): 3.3; S(12): 50.8	1	3	1	0	N-Term(TMT6plex); S11(Phospho); S12(Phospho); K15(TMT6plex)
31377548	NP_775853.2	283209	PGM2L1	glucose 1,6-bisphosphate synthase	**S175**	-	-	-	-	**0.2**	**0.4**	**0.3**	**0.2**	-	-	-	-	**0.2**	**0.4**	**0.3**	**0.2**	**0.2**	**0.4**	**0.3**	**0.2**	AVAGVMITAsHNR	AGVMITAsHNRKEDN	S10;	Yes	T(8): 1.8; S(10): 98.2	1	1	1	0	N-Term(TMT6plex); M6(Oxidation); S10(Phospho)
31542943	NP_059985.2	54733	SLC35F2	solute carrier family 35 member F2	**S371**	-	-	-	-	**1.5**	**1.1**	**0.7**	**0.2**	-	-	-	-	**1.4**	**1.1**	**0.7**	**0.2**	**1.4**	**1.1**	**0.7**	**0.2**	LEENLQETHsAVL	ENLQETHsAVL	S10;	Yes	T(8): 0.0; S(10): 100.0	1	1	1	0	N-Term(TMT6plex); S10(Phospho)
38570115	NP_689706.2	149076	ZNF362	zinc finger protein 362	**S410**	-	-	-	-	**0.3**	**0.5**	**0.3**	**0.2**	-	-	-	-	**0.5**	**1.1**	**0.7**	**0.4**	**0.5**	**1.1**	**0.7**	**0.4**	TEsPGIPVR	HSPQRTEsPGIPVRL	S3;	Yes	T(1): 0.9; S(3): 99.1	1	1	1	0	N-Term(TMT6plex); T1(Phospho)
54792129	NP_563615.3	131566	DCBLD2	discoidin, CUB and LCCL domain-containing protein 2 precursor	**S724**	-	-	-	-	**0.2**	**0.2**	**1.0**	**0.2**	-	-	-	-	**0.2**	**0.2**	**0.9**	**0.2**	**0.2**	**0.2**	**0.9**	**0.2**	TDsCSSAQAQYDTPK	TLLSRTDsCSSAQAQ	S3;	Yes	T(1): 1.2; S(3): 97.7; S(5): 1.2; S(6): 0.0; Y(11): 0.0; T(13): 0.0	2	1	1	0	N-Term(TMT6plex); S3(Phospho); C4(Carbamidomethyl); K15(TMT6plex)
194097489	NP_015559.2	8498	RANBP3	ran-binding protein 3 isoform RANBP3-b	**S40**	-	-	-	-	**0.2**	**0.4**	**0.1**	**0.2**	-	-	-	-	**0.2**	**0.5**	**0.1**	**0.3**	**0.2**	**0.5**	**0.1**	**0.3**	SAGGSSPEGGEDsDREDGNYCPPVK	SPEGGEDsDREDGNY	S13;	No	S(1): 0.1; S(5): 0.1; S(6): 2.4; S(13): 95.1; Y(20): 2.4	1	3	1	1	N-Term(TMT6plex); S13(Phospho); C21(Carbamidomethyl); K25(TMT6plex)
14670388	NP_116784.1	572	BAD	bcl2 antagonist of cell death	**S25**	-	-	-	-	**0.4**	**0.4**	**0.6**	**0.2**	-	-	-	-	**0.4**	**0.4**	**0.5**	**0.2**	**0.4**	**0.4**	**0.5**	**0.2**	GLGPsPAGDGPSGSGK	AERGLGPsPAGDGPS	S5;	Yes	S(5): 100.0; S(12): 0.0; S(14): 0.0	1	1	1	0	N-Term(TMT6plex); S5(Phospho); K16(TMT6plex)
41152093	NP_958357.1	84146	ZNF644	zinc finger protein 644 isoform 1	**S1000**	-	-	-	-	**0.5**	**0.5**	**0.6**	**0.2**	-	-	-	-	**0.5**	**0.5**	**0.6**	**0.2**	**0.5**	**0.5**	**0.6**	**0.2**	HVVsPEQIATSDK	YEARHVVsPEQIATS	S4;	Yes	S(4): 100.0; T(10): 0.0; S(11): 0.0	2	1	1	0	N-Term(TMT6plex); S4(Phospho); K13(TMT6plex)
73747879	NP_443066.2	23335	WDR7	WD repeat-containing protein 7 isoform 2	**S935**	-	-	-	-	**0.3**	**0.1**	**0.1**	**0.1**	-	-	-	-	**0.3**	**0.1**	**0.1**	**0.2**	**0.3**	**0.1**	**0.1**	**0.2**	GsPPTSSNIVQGQIK	DLSKARGsPPTSSNI	S2;	Yes	S(2): 94.8; T(5): 1.7; S(6): 1.7; S(7):	1	2	1	0	N-Term(TMT6plex); S2(Phospho); K15(TMT6plex)
