# Supplementary material for: Multi-Omics Analysis to Characterize Cigarette Smoke Induced Molecular Alterations in Esophageal Cells
Source: Front Oncol. 2020 Nov 5;10:1666. doi: 10.3389/fonc.2020.01666 (PMC7675040; doi:10.3389/fonc.2020.01666)
Supplement: Supplementary Table 9 — List of differentially phosphorylated protein in smoke exposed Het-1A cells across both replicates. [file Table_9.pdf]

Khan et al., 2019. Multi-omics analysis to characterize cigarette smoke induced molecular alterations in esophageal cells
Supplementary Table 9. List of differentially phosphorylated protein in smoke exposed Het-1A cells across both replicates.

													Normalized phosphoprotein ratios with respect to corresponding total protein ratios.								Average of replicates			
Protein group Accession	NP_Accession	Gene Symbol	Protein Description	PhosphoSite (Protein)	Het-1A-Smoke - 2M/Parental	Het-1A-Smoke - 4M/Parental	Het-1A-Smoke - 6M/Parental	Het-1A-Smoke - 8M/Parental	Het-1A-Smoke - 2M/Parental	Het-1A-Smoke - 4M/Parental	Het-1A-Smoke - 6M/Parental	Het-1A-Smoke - 8M/Parental	Het-1A-Smoke - 2M/Parental	Het-1A-Smoke - 4M/Parental	Het-1A-Smoke - 6M/Parental	Het-1A-Smoke - 8M/Parental	Het-1A-Smoke - 2M/Parental	Het-1A-Smoke - 4M/Parental	Het-1A-Smoke - 6M/Parental	Het-1A-Smoke - 8M/Parental	Het-1A-Smoke - 2M/Parental	Het-1A-Smoke - 4M/Parental	Het-1A-Smoke - 6M/Parental	Het-1A-Smoke - 8M/Parental
					Replicate 1				Replicate 2				Replicate 1				Replicate 2							
47132620	NP_004414.2	**KRT2**	keratin, type II cytoskeletal 2 epidermal	S62	0.6	4.2	0.5	5.8	0.8	4.0	1.0	4.9	0.6	2.1	0.5	2.3	0.7	2.0	0.9	2.0	0.7	2.0	0.7	**2.1**
153945728	NP_005900.2	**MAP1B**	microtubule-associated protein 1B	S1915	2.5	4.7	3.4	3.8	1.9	1.8	2.0	3.6	2.0	3.4	2.8	4.0	1.5	1.3	1.6	3.9	1.7	2.4	2.2	**3.9**
70166599	NP_001002019.1	**PUS1**	tRNA pseudouridine synthase A, mitochondrial isoform 2	T398	2.4	1.8	2.1	2.4	5.2	2.8	5.1	3.9	2.2	1.6	1.9	2.3	4.7	2.6	4.6	3.6	3.4	2.1	3.2	**2.9**
19913396	NP_599031.1	**SMTN**	smoothelin isoform b	S779	1.3	2.1	1.4	2.0	1.4	2.4	1.6	2.6	1.1	1.6	1.1	1.8	1.1	1.8	1.2	2.3	1.1	1.7	1.1	**2.0**
61743954	NP_001611.1	**AHNAK**	neuroblast differentiation-associated protein AHNAK isoform 1	S5110	2.1	3.4	2.8	3.9	0.9	1.2	1.2	0.4	2.5	3.5	2.9	4.5	1.1	1.3	1.3	0.5	1.8	2.4	2.1	**2.5**
41322916	NP_958782.1	**PLEC**	plectin isoform 1	S125	2.5	1.1	3.0	2.2	3.5	2.0	3.1	2.0	2.8	1.2	3.2	2.3	3.9	2.2	3.3	2.1	3.3	1.7	3.2	**2.2**
22208971	NP_665908.1	**HMGA1**	high mobility group protein HMG-I/HMG-Y isoform a	S9	2.2	4.0	2.6	2.1	2.1	3.1	2.4	2.0	2.2	3.2	2.3	2.2	2.1	2.5	2.1	2.1	2.2	2.9	2.2	**2.1**
4503739	NP_001446.1	**FOXO3**	forkhead box protein O3	S284	0.4	0.6	0.2	0.5	2.1	2.5	2.0	3.5	0.5	0.7	0.3	0.5	2.6	2.6	2.3	3.6	1.6	1.6	1.3	**2.0**
190014588	NP_001121689.1	**PSIP1**	PC4 and SFRS1-interacting protein isoform 2	T272; S273	0.8	0.5	0.7	0.5	4.9	3.9	4.5	3.1	1.0	0.5	0.9	0.5	6.0	4.2	5.6	3.5	3.5	2.4	3.2	**2.0**
190014588	NP_001121689.1	**PSIP1**	PC4 and SFRS1-interacting protein isoform 2	T272; S273	4.9	3.9	4.5	3.1	0.8	0.5	0.7	0.5	6.0	4.2	5.6	3.5	1.0	0.5	0.9	0.5	3.5	2.4	3.2	**2.0**
498917186	NP_001265089.1	**AKT1S1**	proline-rich AKT1 substrate 1 isoform b	T246	0.7	0.7	0.8	0.4	0.9	1.0	1.0	0.4	1.0	0.9	0.8	0.4	1.2	1.3	1.0	0.5	1.1	1.1	0.9	**0.4**
4885379	NP_005312.1	**HIST1H1E**	histone H1.4	T146	0.3	0.4	0.3	0.4	0.3	0.3	0.3	0.4	0.5	0.4	0.4	0.5	0.5	0.4	0.4	0.5	0.5	0.4	0.4	**0.5**
634743290	NP_001278893.1	**MLLT4**	afadin isoform 4	S1756	0.7	0.9	0.4	0.3	0.5	0.8	0.6	0.4	0.6	0.8	0.3	0.3	0.4	0.7	0.5	0.4	0.5	0.7	0.4	**0.3**
40254834	NP_006603.2	**KIF1C**	kinesin-like protein KIF1C	S494	0.4	0.4	0.7	0.4	0.2	0.2	0.7	0.4	0.4	0.4	0.7	0.4	0.3	0.2	0.8	0.4	0.3	0.3	0.7	**0.4**
31742503	NP_066403.2	**HIST2H3C**	histone H3.2	S29	0.3	0.3	0.3	0.4	0.4	0.3	0.3	0.4	0.3	0.4	0.4	0.5	0.4	0.4	0.3	0.5	0.3	0.4	0.3	**0.5**
4885381	NP_005313.1	**HIST1H1B**	histone H1.5	S18	0.3	0.3	0.3	0.4	0.3	0.3	0.3	0.4	0.5	0.4	0.5	0.5	0.5	0.4	0.4	0.5	0.5	0.4	0.5	**0.5**
13786127	NP_036253.2	**CDC42EP4**	cdc42 effector protein 4	S118	0.6	0.8	0.8	0.5	0.4	0.2	0.5	0.2	0.6	0.9	0.9	0.5	0.4	0.3	0.5	0.3	0.5	0.6	0.7	**0.4**
431822379	NP_001258930.1	**DOCK7**	dedicator of cytokinesis protein 7 isoform 4	S900	0.5	0.8	1.1	0.4	0.6	0.5	0.6	0.3	0.5	0.8	1.2	0.4	0.6	0.5	0.7	0.3	0.6	0.7	0.9	**0.4**
8923857	NP_060917.1	**EMC3**	ER membrane protein complex subunit 3	T204	0.7	1.0	0.9	0.5	1.8	1.5	1.1	0.2	0.7	1.1	1.0	0.5	1.9	1.5	1.2	0.3	1.3	1.3	1.1	**0.4**
9966805	NP_065147.1	**DDX24**	ATP-dependent RNA helicase DDX24	S82	0.8	1.1	0.6	0.3	0.8	0.8	0.8	0.4	1.0	1.3	0.8	0.3	1.0	1.0	1.0	0.5	1.0	1.2	0.9	**0.4**
4885381	NP_005313.1	**HIST1H1B**	histone H1.5	T11; S18	0.3	0.3	0.3	0.3	0.4	0.4	0.3	0.3	0.4	0.3	0.4	0.5	0.6	0.5	0.4	0.4	0.5	0.4	0.4	**0.4**
4885379	NP_005312.1	**HIST1H1E**	histone H1.4	T18	0.4	0.3	0.4	0.3	0.3	0.2	0.2	0.2	0.5	0.3	0.5	0.4	0.5	0.3	0.3	0.3	0.5	0.3	0.4	**0.3**
61743954	NP_001611.1	**AHNAK**	neuroblast differentiation-associated protein AHNAK isoform 1	S4903	0.5	0.4	0.4	0.2	0.4	0.4	0.3	0.3	0.6	0.4	0.4	0.3	0.5	0.4	0.3	0.3	0.5	0.4	0.4	**0.3**
222352111	NP_115766.3	**NIFK**	MKI67 FHA domain-interacting nucleolar phosphoprotein	T223	0.6	0.3	0.3	0.2	0.4	0.5	0.5	0.3	0.6	0.3	0.3	0.2	0.5	0.5	0.5	0.3	0.5	0.4	0.4	**0.3**
4758808	NP_004832.1	**RASAL2**	ras GTPase-activating protein nGAP isoform 1	T620	0.1	0.1	0.1	0.1	0.1	0.1	0.1	0.1	0.1	0.1	0.1	0.1	0.1	0.1	0.1	0.1	0.1	0.1	0.1	**0.1**
33356148	NP_005883.2	**FMNL1**	formin-like protein 1	S624	1.0	1.1	1.6	1.3	2.8	3.8	3.0	5.6	0.9	1.0	1.4	1.2	2.8	3.6	2.7	5.2	1.9	2.3	2.1	**3.2**
148612879	NP_079223.3	**PHC3**	polyhomeotic-like protein 3	T621; S628	0.8	1.7	1.7	0.9	3.9	4.0	4.9	4.7	0.8	1.8	1.5	1.0	3.9	4.1	4.3	5.1	2.4	2.9	2.9	**3.1**
153945728	NP_005900.2	**MAP1B**	microtubule-associated protein 1B	S2209; S2211	0.6	0.9	0.8	0.9	3.0	3.9	4.1	4.4	0.5	0.6	0.6	0.9	2.3	2.9	3.3	4.7	1.4	1.8	2.0	**2.8**
344313201	NP_001230704.1	**LIMA1**	LIM domain and actin-binding protein 1 isoform 4	S188	3.3	3.2	3.7	4.0	1.7	1.7	1.5	1.2	3.0	2.9	2.9	4.0	1.5	1.6	1.2	1.2	2.3	2.3	2.1	**2.6**
109255232	NP_001035864.1	**CEP170**	centrosomal protein of 170 kDa isoform gamma	T662	3.4	3.3	3.7	4.0	0.6	1.2	1.1	1.2	3.1	2.9	3.3	3.8	0.5	1.0	1.0	1.1	1.8	2.0	2.1	**2.4**
296011010	NP_060252.4	**FAM208B**	protein FAM208B	S340	0.4	0.9	1.0	1.4	1.0	1.4	2.5	3.7	0.4	1.0	1.1	1.5	1.0	1.4	2.6	3.9	0.7	1.2	1.9	**2.7**
150417989	NP_001092872.1	**CCNK**	cyclin-K	S340	0.9	1.2	1.1	1.1	2.1	2.7	2.6	3.8	0.8	1.3	1.1	1.0	1.9	2.9	2.7	3.5	1.4	2.1	1.9	**2.3**
261278315	NP_001159693.1	**UTP14A**	U3 small nucleolar RNA-associated protein 14 homolog A isoform 2	S393	2.5	3.9	1.9	3.0	1.4	1.8	1.4	1.7	3.0	4.6	2.5	3.9	1.6	2.2	1.8	2.2	2.3	3.4	2.1	**3.0**
41872631	NP_004095.4	**FASN**	fatty acid synthase	S207	1.0	1.9	1.3	1.7	2.8	3.9	3.6	3.0	0.8	1.5	1.0	1.6	2.3	3.0	2.9	2.7	1.6	2.2	2.0	**2.1**
572881956	NP_001275884.1	**TTC7A**	tetratricopeptide repeat protein 7A isoform 4	S293	2.8	0.9	2.6	1.4	4.1	3.8	3.7	3.0	2.4	0.9	2.3	1.4	3.5	3.7	3.3	3.0	2.9	2.3	2.8	**2.2**
41281453	NP_055535.2	**SLK**	STE20-like serine/threonine-protein kinase	S779	1.7	1.8	1.5	2.4	1.7	1.7	1.8	1.7	1.8	1.6	1.4	2.4	1.8	1.5	1.6	1.7	1.8	1.5	1.5	**2.0**
110611149	NP_001036046.1	**SNUPN**	snurportin-1	S350	1.0	1.1	1.3	1.3	1.0	0.9	1.9	2.8	1.0	1.2	1.3	1.3	1.0	1.0	2.0	2.8	1.0	1.1	1.6	**2.0**
118572613	NP_057417.3	**SRRM2**	serine/arginine repetitive matrix protein 2	T2144	2.6	1.5	1.9	2.7	0.4	1.0	0.7	1.4	2.8	1.5	2.0	3.0	0.5	1.0	0.8	1.5	1.6	1.3	1.4	**2.3**
225007652	NP_001139333.1	**POP1**	ribonucleases P/MRP protein subunit POP1	S367	1.0	0.8	0.6	0.9	2.3	2.4	2.4	3.1	1.0	0.8	0.5	1.0	2.2	2.4	2.3	3.5	1.6	1.6	1.4	**2.3**
61743954	NP_001611.1	**AHNAK**	neuroblast differentiation-associated protein AHNAK isoform 1	T4430	2.0	2.2	1.7	1.9	2.5	3.8	3.9	2.0	2.4	2.2	1.7	2.2	3.0	3.9	4.0	2.4	2.7	3.0	2.8	**2.3**
386643034	NP_001245362.1	**NDRG1**	protein NDRG1 isoform 3	S249	1.8	1.7	1.6	1.2	1.0	0.9	0.6	2.6	2.0	1.7	1.5	1.3	1.2	0.9	0.5	2.8	1.6	1.3	1.0	**2.1**
573014817	NP_001275906.1	**XRCC6**	X-ray repair cross-complementing protein 6 isoform 2	T414	1.7	1.7	1.6	2.2	1.1	1.3	1.4	1.4	1.7	1.8	1.7	2.6	1.2	1.4	1.5	1.6	1.4	1.6	1.6	**2.1**
5032189	NP_005648.1	**TP53BP1**	tumor suppressor p53-binding protein 1 isoform 3	S294	1.4	1.8	2.0	2.4	1.0	0.9	0.6	1.1	1.4	1.8	2.2	2.9	1.0	0.9	0.6	1.4	1.2	1.3	1.4	**2.1**
29826335	NP_003899.2	**EIF2S2**	eukaryotic translation initiation factor 2 subunit 2	T111	0.8	0.9	0.9	0.9	2.9	0.9	1.4	2.6	0.9	1.1	1.0	1.0	3.1	1.0	1.7	3.0	2.0	1.0	1.3	**2.0**
289577131	NP_001166183.1	**SP3**	transcription factor Sp3 isoform 3	S70	1.6	1.0	0.6	1.6	1.0	1.0	0.8	1.1	2.2	1.3	0.9	2.6	1.4	1.3	1.1	1.8	1.8	1.3	1.0	**2.2**
226442963	NP_001140174.1	**KDM5C**	lysine-specific demethylase 5C isoform 2	S234	0.9	0.6	0.8	0.7	0.5	0.7	0.6	0.5	0.9	0.6	0.7	0.6	0.5	0.7	0.6	0.4	0.7	0.6	0.6	**0.5**
355390315	NP_056171.3	**SCRIB**	protein scribble homolog isoform b	S1220	0.8	0.5	0.7	0.5	0.8	0.7	0.7	0.5	0.7	0.5	0.7	0.4	0.7	0.6	0.7	0.5	0.7	0.5	0.7	**0.5**
109255232	NP_001035864.1	**CEP170**	centrosomal protein of 170 kDa isoform gamma	S1014	0.7	1.1	1.1	0.8	0.2	0.3	0.3	0.2	0.6	1.0	1.0	0.8	0.1	0.2	0.3	0.2	0.4	0.6	0.6	**0.5**
62955803	NP_056169.1	**NUP188**	nucleoporin NUP188 homolog	S1717	0.3	0.4	0.5	0.5	0.9	0.7	0.9	0.5	0.3	0.4	0.5	0.4	0.8	0.7	0.8	0.5	0.5	0.5	0.7	**0.5**
41327769	NP_056133.2	**ARHGEF18**	rho guanine nucleotide exchange factor 18 isoform a	S945	1.1	1.3	1.0	0.6	0.6	1.1	0.6	0.3	1.0	1.3	0.9	0.6	0.6	1.1	0.6	0.3	0.8	1.2	0.8	**0.5**
117190342	NP_005383.3	**PHF2**	lysine-specific demethylase PHF2	S681	0.4	0.2	0.5	0.2	0.4	0.6	0.6	0.7	0.5	0.2	0.5	0.2	0.4	0.7	0.7	0.7	0.4	0.4	0.6	**0.5**
190684675	NP_006288.2	**XRCC1**	DNA repair protein XRCC1	S241	0.5	0.2	0.2	0.3	0.9	0.5	0.5	0.6	0.5	0.3	0.2	0.4	0.9	0.5	0.5	0.6	0.7	0.4	0.4	**0.5**
7656879	NP_055238.1	**AFF4**	AF4/FMR2 family member 4	S703	0.9	1.1	1.0	0.7	0.7	1.1	0.7	0.2	0.8	1.0	1.0	0.6	0.6	1.0	0.7	0.2	0.7	1.0	0.8	**0.4**
7706254	NP_057018.1	**NOP58**	nucleolar protein 58	S502	0.7	0.9	0.9	0.2	0.8	0.7	0.9	0.6	0.8	1.0	0.9	0.3	0.9	0.8	0.9	0.7	0.8	0.9	0.9	**0.5**
7669481	NP_004043.2	**BNIP3**	BCL2/adenovirus E1B 19 kDa protein-interacting protein 3	S66	0.7	0.9	0.7	0.6	0.2	0.3	0.5	0.3	0.6	1.0	0.7	0.6	0.2	0.3	0.5	0.3	0.4	0.6	0.6	**0.4**
14670388	NP_116784.1	**BAD**	bcl2 antagonist of cell death	S99	0.7	0.6	0.6	0.5	0.3	0.2	0.2	0.2	0.7	0.6	0.5	0.5	0.3	0.2	0.2	0.2	0.5	0.4	0.4	**0.4**
57864582	NP_001009931.1	**HRNR**	hornerin	S542	2.9	6.1	2.9	8.5	-	-	-	-	2.8	3.3	2.8	3.9	-	-	-	-	2.8	3.3	2.8	**3.9**
217330574	NP_001136088.1	**EHBP1**	EH domain-binding protein 1 isoform 3	S752	4.3	2.9	3.4	3.9	-	-	-	-	4.5	2.9	3.3	3.3	-	-	-	-	4.5	2.9	3.3	**3.3**
160948599	NP_001073922.2	**INTS1**	integrator complex subunit 1	S104	2.0	2.5	2.5	3.3	-	-	-	-	1.9	2.6	2.6	3.5	-	-	-	-	1.9	2.6	2.6	**3.5**
56550039	NP_005924.2	**KMT2A**	histone-lysine N-methyltransferase 2A isoform 2 precursor	S1056	2.0	2.5	2.2	2.8	-	-	-	-	1.8	2.4	2.3	2.8	-	-	-	-	1.8	2.4	2.3	**2.8**
54633315	NP_055412.2	**DNTTIP2**	deoxynucleotidyltransferase terminal-interacting protein 2	T232	1.0	0.9	1.1	2.8	-	-	-	-	1.1	1.0	1.2	3.1	-	-	-	-	1.1	1.0	1.2	**3.1**
374093212	NP_001243357.1	**PDLIM5**	PDZ and LIM domain protein 5 isoform h	S106	1.7	2.2	2.3	2.7	-	-	-	-	1.7	1.9	2.0	2.1	-	-	-	-	1.7	1.9	2.0	**2.1**
196114970	NP_001124481.1	**PTPN12**	tyrosine-protein phosphatase non-receptor type 12 isoform 3	T389	0.9	2.7	1.5	2.7	-	-	-	-	0.8	2.7	1.5	2.6	-	-	-	-	0.8	2.7	1.5	**2.6**
217416362	NP_115883.2	**ZC3H8**	zinc finger CCCH domain-containing protein 8	T32	1.0	0.9	2.1	2.7	-	-	-	-	1.2	1.0	2.3	3.5	-	-	-	-	1.2	1.0	2.3	**3.5**
82546824	NP_001032242.1	**FOXK1**	forkhead box protein K1	S445	2.2	0.9	1.3	2.7	-	-	-	-	2.1	0.9	1.3	2.8	-	-	-	-	2.1	0.9	1.3	**2.8**
557636741	NP_001273556.1	**RNPS1**	RNA-binding protein with serine-rich domain 1 isoform c	S102	4.9	4.8	5.6	2.6	-	-	-	-	5.6	5.1	6.5	2.7	-	-	-	-	5.6	5.1	6.5	**2.7**
5032189	NP_005648.1	**TP53BP1**	tumor suppressor p53-binding protein 1 isoform 3	S580	1.0	3.8	1.8	2.6	-	-	-	-	1.0	3.7	2.0	3.1	-	-	-	-	1.0	3.7	2.0	**3.1**
224586882	NP_003642.3	**YBX3**	Y-box-binding protein 3 isoform a	S79	1.0	2.7	4.2	2.5	-	-	-	-	0.7	1.7	2.4	2.1	-	-	-	-	0.7	1.7	2.4	**2.1**
50962882	NP_001002909.1	**GPATCH8**	G patch domain-containing protein 8	1; T457; S459;	1.9	2.0	1.7	2.5	-	-	-	-	2.0	2.1	1.8	2.6	-	-	-	-	2.0	2.1	1.8	**2.6**
20357556	NP_612632.1	**CTTN**	src substrate cortactin isoform b	S381	2.5	3.2	4.7	2.4	-	-	-	-	2.0	2.5	3.2	2.2	-	-	-	-	2.0	2.5	3.2	**2.2**
7705425	NP_057053.1	**MRPS17**	28S ribosomal protein S17, mitochondrial	S128	1.4	1.7	1.7	2.4	-	-	-	-	1.4	1.7	1.7	2.4	-	-	-	-	1.4	1.7	1.7	**2.4**
46389560	NP_996930.1	**ENSA**	alpha-endosulfine isoform 7	S105	3.0	1.2	4.8	2.4	-	-	-	-	3.0	1.2	4.5	2.3	-	-	-	-	3.0	1.2	4.5	**2.3**
32130514	NP_071922.2	**NDRG3**	protein NDRG3 isoform b	S319	1.7	1.5	1.3	2.2	-	-	-	-	2.0	1.7	1.7	2.3	-	-	-	-	2.0	1.7	1.7	**2.3**
7656879	NP_055238.1	**AFF4**	AF4/FMR2 family member 4	T528	1.9	1.8	2.0	2.2	-	-	-	-	1.7	1.6	1.9	2.1	-	-	-	-	1.7	1.6	1.9	**2.1**
7706254	NP_057018.1	**NOP58**	nucleolar protein 58	S502; S514	1.3	1.1	2.1	1.9	-	-	-	-	1.4	1.2	2.2	2.1	-	-	-	-	1.4	1.2	2.2	**2.1**
153792539	NP_055189.2	**REPIN1**	replication initiator 1 isoform 1	S27	0.5	1.3	1.5	1.9	-	-	-	-	0.5	1.4	1.5	2.2	-	-	-	-	0.5	1.4	1.5	**2.2**
4507241	NP_003137.1	**SSRP1**	FACT complex subunit SSRP1	S667	0.8	1.3	1.1	1.9	-	-	-	-	0.9	1.6	1.3	2.3	-	-	-	-	0.9	1.6	1.3	**2.3**
51873067	NP_001004128.1	**QSOX1**	sulfhydryl oxidase 1 isoform b precursor	S426	1.8	2.6	2.0	1.9	-	-	-	-	1.8	2.6	2.0	2.1	-	-	-	-	1.8	2.6	2.0	**2.1**
209180450	NP_001129202.1	**TOM1**	target of Myb protein 1 isoform 4	S416	1.6	1.5	1.2	1.8	-	-	-	-	1.5	1.7	1.4	2.1	-	-	-	-	1.5	1.7	1.4	**2.1**
4506921	NP_003012.1	**SGTA**	small glutamine-rich tetratricopeptide repeat-containing protein alpha	T81	1.3	1.2	1.3	1.5	-	-	-	-	1.5	1.8	1.6	2.1	-	-	-	-	1.5	1.8	1.6	**2.1**
634743319	NP_001278915.1	**BRD2**	bromodomain-containing protein 2 isoform 4	S513	0.5	0.9	1.3	0.5	-	-	-	-	0.5	0.9	1.2	0.5	-	-	-	-	0.5	0.9	1.2	**0.5**
114842410	NP_055642.3	**ZC3H11A**	zinc finger CCCH domain-containing protein 11A	S738	0.9	0.8	1.2	0.5	-	-	-	-	0.9	0.8	1.2	0.5	-	-	-	-	0.9	0.8	1.2	**0.5**
332688220	NP_001193819.1	**CTNND1**	catenin delta-1 isoform 3A	S813	0.7	0.8	1.2	0.5	-	-	-	-	0.7	0.9	1.3	0.5	-	-	-	-	0.7	0.9	1.3	**0.5**
33620716	NP_061173.1	**RBBP6**	E3 ubiquitin-protein ligase RBBP6 isoform 2	S1671	1.3	0.8	0.9	0.5	-	-	-	-	1.5	0.8	1.0	0.5	-	-	-	-	1.5	0.8	1.0	**0.5**
85815829	NP_061830.3	**SH3BP1**	SH3 domain-binding protein 1	S550	0.9	0.5	0.4	0.5	-	-	-	-	0.8	0.4	0.4	0.4	-	-	-	-	0.8	0.4	0.4	**0.4**
530424835	XP_005258156.1	**SOGA2**	protein SOGA2 isoform X2	S87	0.4	0.4	0.4	0.5	-	-	-	-	0.4	0.5	0.4	0.5	-	-	-	-	0.4	0.5	0.4	**0.5**
110611218	NP_001036041.1	**RRBP1**	ribosome-binding protein 1	S185	0.9	1.0	0.9	0.5	-	-	-	-	0.9	0.9	1.0	0.5	-	-	-	-	0.9	0.9	1.0	**0.5**
224809302	NP_001139241.1	**XPC**	DNA repair protein complementing XP-C cells isoform 2	S94	1.2	0.8	1.3	0.5	-	-	-	-	1.1	0.7	1.3	0.5	-	-	-	-	1.1	0.7	1.3	**0.5**
586798132	NP_001276908.1	**NUMBL**	numb-like protein isoform b	T210	1.0	1.4	0.7	0.5	-	-	-	-	0.8	1.0	0.4	0.4	-	-	-	-	0.8	1.0	0.4	**0.4**
108860697	NP_055445.2	**ZNF592**	zinc finger protein 592	S460	0.4	0.5	0.4	0.5	-	-	-	-	0.4	0.5	0.4	0.5	-	-	-	-	0.4	0.5	0.4	**0.5**
299758464	NP_001139685.2	**TBC1D15**	TBC1 domain family member 15 isoform 3	S658	0.7	0.5	0.6	0.5	-	-	-	-	0.7	0.4	0.5	0.4	-	-	-	-	0.7	0.4	0.5	**0.4**
225735619	NP_001139585.1	**MIER1**	mesoderm induction early response protein 1 isoform i	S314	1.1	0.4	0.7	0.4	-	-	-	-	1.1	0.4	0.7	0.5	-	-	-	-	1.1	0.4	0.7	**0.5**
188595687	NP_001120959.1	**FLNC**	filamin-C isoform b	S2200	0.9	0.8	0.4	0.4	-	-	-	-	0.8	0.8	0.5	0.4	-	-	-	-	0.8	0.8	0.5	**0.4**
28559039	NP_004765.2	**MED1**	mediator of RNA polymerase II transcription subunit 1	S1156	0.7	0.6	0.5	0.4	-	-	-	-	0.7	0.6	0.6	0.4	-	-	-	-	0.7	0.6	0.6	**0.4**
166795297	NP_057225.2	**DYNC1LI1**	cytoplasmic dynein 1 light intermediate chain 1	S421	0.4	0.3	0.2	0.4	-	-	-	-	0.4	0.3	0.2	0.4	-	-	-	-	0.4	0.3	0.2	**0.4**
54633315	NP_055412.2	**DNTTIP2**	deoxynucleotidyltransferase terminal-interacting protein 2	S434	1.0	0.4	1.0	0.4	-	-	-	-	1.1	0.5	1.1	0.4	-	-	-	-	1.1	0.5	1.1	**0.4**
300244569	NP_001177916.1	**SRSF11**	serine/arginine-rich splicing factor 11 isoform 2	S433	1.4	0.7	0.7	0.4	-	-	-	-	1.6	0.9	0.9	0.5	-	-	-	-	1.6	0.9	0.9	**0.5**
73858561	NP_001027462.1	**PSRC1**	proline/serine-rich coiled-coil protein 1 isoform a	S212	0.8	0.7	0.8	0.4	-	-	-	-	0.9	0.8	1.0	0.4	-	-	-	-	0.9	0.8	1.0	**0.4**
354681980	NP_001238918.1	**GMNN**	geminin	S64	0.7	0.8	0.6	0.4	-	-	-	-	0.8	1.0	0.9	0.5	-	-	-	-	0.8	1.0	0.9	**0.5**
156564398	NP_079353.3	**TOE1**	target of EGR1 protein 1	S5	0.6	0.7	0.6	0.4	-	-	-	-	0.6	0.7	0.7	0.4	-	-	-	-	0.6	0.7	0.7	**0.4**
5454102	NP_006333.1	**TACC3**	transforming acidic coiled-coil-containing protein 3	S177	0.5	0.7	0.5	0.4	-	-	-	-	0.6	0.7	0.5	0.4	-	-	-	-	0.6	0.7	0.5	**0.4**
306922386	NP_001120983.2	**APC**	adenomatous polyposis coli protein isoform a	S2812	0.6	0.4	0.7	0.4	-	-	-	-	0.5	0.4	0.8	0.4	-	-	-	-	0.5	0.4	0.8	**0.4**
555943794	NP_001273171.1	**GTF3C1**	general transcription factor 3C polypeptide 1 isoform 2	S739	0.8	0.7	0.7	0.3	-	-	-	-	0.8	0.7	0.7	0.4	-	-	-	-	0.8	0.7	0.7	**0.4**
333440471	NP_001193980.1	**CLASP1**	CLIP-associating protein 1 isoform 4	S600	0.7	0.7	0.6	0.3	-	-	-	-	0.8	0.7	0.6	0.3	-	-	-	-	0.8	0.7	0.6	**0.3**
32171238	NP_061330.2	**BAIAP2L1**	brain-specific angiogenesis inhibitor 1-associated protein 2-like protein 1	S329	0.4	0.7	0.4	0.3	-	-	-	-	0.3	0.8	0.3	0.3	-	-	-	-	0.3	0.8	0.3	**0.3**

Khan et al., 2019. Multi-omics analysis to characterize cigarette smoke induced molecular alterations in esophageal cells

Supplementary Table 9. List of differentially phosphorylated protein in smoke exposed Het-1A cells across both replicates.

													Normalized phosphoprotein ratios with respect to corresponding total protein ratios.								Average of replicates			
**Protein group Accession**	**NP_Accession**	**Gene Symbol**	**Protein Description**	**PhosphoSite (Protein)**	**Het-1A-Smoke - 2M/Parental**	**Het-1A-Smoke - 4M/Parental**	**Het-1A-Smoke - 6M/Parental**	**Het-1A-Smoke - 8M/Parental**	**Het-1A-Smoke - 2M/Parental**	**Het-1A-Smoke - 4M/Parental**	**Het-1A-Smoke - 6M/Parental**	**Het-1A-Smoke - 8M/Parental**	**Het-1A-Smoke - 2M/Parental**	**Het-1A-Smoke - 4M/Parental**	**Het-1A-Smoke - 6M/Parental**	**Het-1A-Smoke - 8M/Parental**	**Het-1A-Smoke - 2M/Parental**	**Het-1A-Smoke - 4M/Parental**	**Het-1A-Smoke - 6M/Parental**	**Het-1A-Smoke - 8M/Parental**	**Het-1A-Smoke - 2M/Parental**	**Het-1A-Smoke - 4M/Parental**	**Het-1A-Smoke - 6M/Parental**	**Het-1A-Smoke - 8M/Parental**
					**Replicate 1**				**Replicate 2**				**Replicate 1**				**Replicate 2**							
62988361	NP_001615.1	**AIM1**	absent in melanoma 1 protein	S22	0.3	0.3	0.6	0.3	-	-	-	-	0.4	0.4	0.7	0.4	-	-	-	-	0.4	0.4	0.7	**0.4**
555290002	NP_001273127.1	**C12orf43**	uncharacterized protein C12orf43 isoform g	T170	0.3	0.2	0.6	0.3	-	-	-	-	0.4	0.2	0.6	0.3	-	-	-	-	0.4	0.2	0.6	**0.3**
38327034	NP_006039.2	**UBE4B**	ubiquitin conjugation factor E4 B isoform 2	S105	0.2	0.2	0.2	0.3	-	-	-	-	0.2	0.2	0.2	0.2	-	-	-	-	0.2	0.2	0.2	**0.2**
344313201	NP_001230704.1	**LIMA1**	LIM domain and actin-binding protein 1 isoform 4	S72	0.6	0.3	0.6	0.3	-	-	-	-	0.5	0.3	0.5	0.2	-	-	-	-	0.5	0.3	0.5	**0.2**
197313748	NP_054878.5	**SETD2**	histone-lysine N-methyltransferase SETD2	S131	0.5	0.5	0.4	0.2	-	-	-	-	0.6	0.6	0.4	0.3	-	-	-	-	0.6	0.6	0.4	**0.3**
566559802	NP_001274432.1	**SSFA2**	sperm-specific antigen 2 isoform 3	S92	0.4	0.2	0.3	0.2	-	-	-	-	0.4	0.2	0.3	0.3	-	-	-	-	0.4	0.2	0.3	**0.3**
62899035	NP_001017420.1	**ESCO2**	N-acetyltransferase ESCO2	S244	0.6	0.3	0.6	0.2	-	-	-	-	0.6	0.3	0.7	0.2	-	-	-	-	0.6	0.3	0.7	**0.2**
209862987	NP_569830.2	**MADD**	MAP kinase-activating death domain protein isoform f	S777	-	-	-	-	1.4	2.7	3.1	4.2	-	-	-	-	1.5	2.6	3.2	3.5	1.5	2.6	3.2	**3.5**
85815829	NP_061830.3	**SH3BP1**	SH3 domain-binding protein 1	S586	-	-	-	-	1.5	2.4	3.2	4.1	-	-	-	-	1.3	2.0	2.6	3.1	1.3	2.0	2.6	**3.1**
212276104	NP_001131025.1	**LRRFIP1**	leucine-rich repeat flightless-interacting protein 1 isoform 5	T82	-	-	-	-	2.8	2.5	1.9	3.6	-	-	-	-	3.0	2.5	2.1	4.5	3.0	2.5	2.1	**4.5**
16757970	NP_443198.1	**FAM129A**	protein Niban	S646	-	-	-	-	5.2	5.7	6.8	3.3	-	-	-	-	3.3	3.1	3.9	2.1	3.3	3.1	3.9	**2.1**
13540594	NP_110432.1	**LMAN2L**	VIP36-like protein isoform 2 precursor	T223	-	-	-	-	1.3	3.0	2.0	2.9	-	-	-	-	1.2	2.8	1.9	2.8	1.2	2.8	1.9	**2.8**
544709647	NP_001269861.1	**ABI2**	abl interactor 2 isoform e	S245	-	-	-	-	1.0	0.9	2.0	2.9	-	-	-	-	1.0	0.9	2.1	2.7	1.0	0.9	2.1	**2.7**
55741677	NP_055527.1	**SETD1A**	histone-lysine N-methyltransferase SETD1A	S468	-	-	-	-	1.4	0.9	1.9	2.8	-	-	-	-	1.4	0.8	1.8	2.4	1.4	0.8	1.8	**2.4**
321267522	NP_001189433.1	**CTDP1**	RNA polymerase II subunit A C-terminal domain phosphatase isoform 3	S621	-	-	-	-	1.4	0.9	2.1	2.8	-	-	-	-	1.3	0.9	2.1	2.5	1.3	0.9	2.1	**2.5**
527498258	NP_001268413.1	**ARFGAP1**	ADP-ribosylation factor GTPase-activating protein 1 isoform e	T76	-	-	-	-	1.5	0.9	2.0	2.7	-	-	-	-	1.7	0.9	2.0	2.5	1.7	0.9	2.0	**2.5**
313151226	NP_001186227.1	**UIMC1**	BRCA1-A complex subunit RAP80	S101	-	-	-	-	1.0	3.5	2.1	2.7	-	-	-	-	1.1	3.8	2.5	2.8	1.1	3.8	2.5	**2.8**
310832410	NP_001185594.1	**ARHGEF12**	rho guanine nucleotide exchange factor 12 isoform 2	S618	-	-	-	-	3.3	1.7	4.2	2.7	-	-	-	-	3.2	1.7	4.2	2.8	3.2	1.7	4.2	**2.8**
156105703	NP_001093879.1	**SENP6**	sentrin-specific protease 6 isoform 2	S345	-	-	-	-	4.1	3.1	2.1	2.7	-	-	-	-	3.8	3.0	2.0	2.5	3.8	3.0	2.0	**2.5**
296939604	NP_055638.2	**WNK1**	serine/threonine-protein kinase WNK1 isoform 2	S382	-	-	-	-	2.5	3.4	4.5	2.7	-	-	-	-	2.3	3.1	4.1	2.4	2.3	3.1	4.1	**2.4**
81295809	NP_006022.3	**PCNT**	pericentrin	S2355	-	-	-	-	1.0	2.0	1.5	2.5	-	-	-	-	1.0	1.9	1.5	2.6	1.0	1.9	1.5	**2.6**
24234750	NP_036350.2	**ILF3**	interleukin enhancer-binding factor 3 isoform a	T592	-	-	-	-	1.0	0.9	0.6	2.4	-	-	-	-	1.1	1.0	0.6	2.5	1.1	1.0	0.6	**2.5**
251823858	NP_001156506.1	**RPTOR**	regulatory-associated protein of mTOR isoform 2	S701; S705	-	-	-	-	4.1	3.9	2.9	2.4	-	-	-	-	3.3	3.6	2.8	2.1	3.3	3.6	2.8	**2.1**
503774453	NP_055204.3	**PELP1**	proline-, glutamic acid- and leucine-rich protein 1 isoform 1	S1083	-	-	-	-	2.6	3.4	2.9	2.3	-	-	-	-	2.6	3.3	2.8	2.4	2.6	3.3	2.8	**2.4**
38327642	NP_060176.2	**WDR55**	WD repeat-containing protein 55	S14	-	-	-	-	0.4	1.3	1.0	2.3	-	-	-	-	0.4	1.2	0.9	2.0	0.4	1.2	0.9	**2.0**
157502197	NP_055553.3	**KIAA0195**	uncharacterized protein KIAA0195	S1354	-	-	-	-	0.6	0.4	0.8	2.2	-	-	-	-	0.7	0.5	0.9	2.5	0.7	0.5	0.9	**2.5**
568214245	NP_001274744.1	**UBAP2L**	ubiquitin-associated protein 2-like isoform c	S460	-	-	-	-	1.4	0.9	2.8	2.2	-	-	-	-	1.6	0.9	3.0	2.0	1.6	0.9	3.0	**2.0**
5032189	NP_005648.1	**TP53BP1**	tumor suppressor p53-binding protein 1 isoform 3	T855	-	-	-	-	1.0	1.9	2.0	2.2	-	-	-	-	1.0	1.9	2.2	2.6	1.0	1.9	2.2	**2.6**
384475541	NP_001244965.1	**POLR3E**	DNA-directed RNA polymerase III subunit RPC5 isoform 5	S161; S162	-	-	-	-	2.6	1.7	1.1	2.2	-	-	-	-	2.6	1.6	1.1	2.1	2.6	1.6	1.1	**2.1**
36029914	NP_079330.2	**VCPIP1**	deubiquitinating protein VCIP135	S998	-	-	-	-	1.4	2.1	1.8	2.1	-	-	-	-	1.1	1.7	1.6	2.1	1.1	1.7	1.6	**2.1**
76150625	NP_001028886.1	**NOP2**	putative ribosomal RNA methyltransferase NOP2 isoform 1	S808	-	-	-	-	1.1	2.6	2.0	2.1	-	-	-	-	1.2	2.4	1.9	2.1	1.2	2.4	1.9	**2.1**
16262452	NP_065821.1	**CGN**	cingulin	S258	-	-	-	-	0.7	0.6	1.5	2.0	-	-	-	-	1.0	0.8	1.9	2.3	1.0	0.8	1.9	**2.3**
222136585	NP_003911.2	**TIMELESS**	protein timeless homolog	S1149	-	-	-	-	1.8	1.8	1.6	2.0	-	-	-	-	2.0	2.0	1.8	2.1	2.0	2.0	1.8	**2.1**
40548403	NP_689853.3	**DCP1B**	mRNA-decapping enzyme 1B	S448	-	-	-	-	3.2	1.9	2.5	2.0	-	-	-	-	3.6	2.0	3.0	2.2	3.6	2.0	3.0	**2.2**
609876591	NP_001278340.1	**SON**	protein SON isoform E	S1697	-	-	-	-	2.0	1.1	1.7	1.9	-	-	-	-	2.2	1.2	1.8	2.0	2.2	1.2	1.8	**2.0**
13236559	NP_077295.1	**C1orf35**	multiple myeloma tumor-associated protein 2	S177	-	-	-	-	1.4	1.6	1.6	1.9	-	-	-	-	1.6	2.0	1.9	2.1	1.6	2.0	1.9	**2.1**
68160947	NP_003460.2	**SCG2**	secretogranin-2 precursor	S556	-	-	-	-	1.1	1.4	1.9	1.3	-	-	-	-	1.2	1.4	1.4	2.3	1.2	1.4	1.4	**2.3**
533112479	NP_059984.3	**PLXNA3**	plexin-A3 precursor	S1610	-	-	-	-	1.2	1.1	1.0	0.8	-	-	-	-	0.8	0.5	0.5	0.4	0.8	0.5	0.5	**0.4**
98991763	NP_742101.2	**EYA4**	eyes absent homolog 4 isoform b	S338	-	-	-	-	1.6	0.6	1.1	0.8	-	-	-	-	1.3	0.3	0.6	0.4	1.3	0.3	0.6	**0.4**
217330646	NP_006704.3	**SUB1**	activated RNA polymerase II transcriptional coactivator p15	S118	-	-	-	-	0.6	0.5	0.6	0.7	-	-	-	-	0.6	0.4	0.5	0.5	0.6	0.4	0.5	**0.5**
506949341	NP_001265202.1	**SRPK2**	SRSF protein kinase 2 isoform b precursor	S497	-	-	-	-	1.0	0.7	0.7	0.6	-	-	-	-	1.0	0.7	0.7	0.5	1.0	0.7	0.7	**0.5**
5803145	NP_006779.1	**RALBP1**	ralA-binding protein 1	S29	-	-	-	-	0.5	1.0	1.5	0.5	-	-	-	-	0.5	0.9	1.4	0.5	0.5	0.9	1.4	**0.5**
457867147	NP_001263988.1	**ERCC6-PGBD3**	Cockayne syndrome B-piggyBac fusion protein	S554	-	-	-	-	0.7	0.9	0.6	0.5	-	-	-	-	0.7	0.8	0.5	0.5	0.7	0.8	0.5	**0.5**
297374764	NP_001172003.1	**ZCCHC6**	terminal uridylyltransferase 7 isoform 2	S172	-	-	-	-	0.6	0.9	0.7	0.5	-	-	-	-	0.5	0.8	0.6	0.5	0.5	0.8	0.6	**0.5**
307133775	NP_001182502.1	**DICER1**	endoribonuclease Dicer isoform 2	S1160	-	-	-	-	0.5	0.4	0.6	0.5	-	-	-	-	0.3	0.3	0.5	0.4	0.3	0.3	0.5	**0.4**
57863269	NP_055871.1	**RRP1B**	ribosomal RNA processing protein 1 homolog B	S392	-	-	-	-	1.0	1.4	0.8	0.5	-	-	-	-	1.1	1.4	0.9	0.5	1.1	1.4	0.9	**0.5**
556503440	NP_001273295.1	**MAP7D1**	MAP7 domain-containing protein 1 isoform 3	S254	-	-	-	-	0.4	0.6	0.6	0.5	-	-	-	-	0.4	0.5	0.5	0.5	0.4	0.5	0.5	**0.5**
170932526	NP_001116314.1	**NCOA7**	nuclear receptor coactivator 7 isoform 2	S89	-	-	-	-	0.6	0.9	0.5	0.5	-	-	-	-	0.6	0.8	0.5	0.5	0.6	0.8	0.5	**0.5**
103472005	NP_002408.3	**MKI67**	antigen KI-67 isoform 1	S1628	-	-	-	-	0.4	0.5	0.8	0.5	-	-	-	-	0.4	0.6	0.8	0.5	0.4	0.6	0.8	**0.5**
631226398	NP_001278804.1	**IGF2BP2**	insulin-like growth factor 2 mRNA-binding protein 2 isoform g	T414	-	-	-	-	1.3	0.9	1.7	0.5	-	-	-	-	1.2	0.7	1.5	0.5	1.2	0.7	1.5	**0.5**
13540614	NP_110445.1	**CCDC130**	coiled-coil domain-containing protein 130	S332	-	-	-	-	0.4	0.3	0.6	0.5	-	-	-	-	0.4	0.3	0.7	0.5	0.4	0.3	0.7	**0.5**
254587994	NP_001157019.1	**SYNRG**	synergin gamma isoform 7	S746	-	-	-	-	1.2	1.4	1.4	0.4	-	-	-	-	1.0	1.5	1.5	0.5	1.0	1.5	1.5	**0.5**
4503483	NP_001952.1	**EEF2**	elongation factor 2	T435	-	-	-	-	1.3	1.0	0.7	0.4	-	-	-	-	1.3	1.0	0.8	0.4	1.3	1.0	0.8	**0.4**
39753961	NP_839943.2	**IQGAP3**	ras GTPase-activating-like protein IQGAP3	S931	-	-	-	-	0.6	1.3	1.2	0.4	-	-	-	-	0.6	1.1	1.1	0.4	0.6	1.1	1.1	**0.4**
109637788	NP_150247.2	**PML**	protein PML isoform 5	S36	-	-	-	-	0.4	0.5	0.6	0.4	-	-	-	-	0.4	0.5	0.5	0.4	0.4	0.5	0.5	**0.4**
155722985	NP_055925.2	**SMC5**	structural maintenance of chromosomes protein 5	S12	-	-	-	-	1.1	0.4	0.9	0.4	-	-	-	-	1.0	0.4	0.8	0.4	1.0	0.4	0.8	**0.4**
190684675	NP_006288.2	**XRCC1**	DNA repair protein XRCC1	T453	-	-	-	-	1.2	0.8	1.0	0.4	-	-	-	-	1.2	0.8	1.1	0.5	1.2	0.8	1.1	**0.5**
5292161	NP_002801.1	**PSMD4**	26S proteasome non-ATPase regulatory subunit 4	S256	-	-	-	-	1.7	0.8	1.1	0.4	-	-	-	-	1.7	0.8	1.1	0.5	1.7	0.8	1.1	**0.5**
381342476	NP_001244222.1	**HNRNPH1**	heterogeneous nuclear ribonucleoprotein H	S104	-	-	-	-	0.6	0.3	0.8	0.4	-	-	-	-	0.7	0.4	0.9	0.5	0.7	0.4	0.9	**0.5**
4507525	NP_003253.1	**SEC62**	translocation protein SEC62	S335	-	-	-	-	1.0	0.5	0.6	0.4	-	-	-	-	0.8	0.4	0.5	0.4	0.8	0.4	0.5	**0.4**
7657575	NP_036370.2	**SIRT1**	NAD-dependent protein deacetylase sirtuin-1 isoform a	S26	-	-	-	-	1.0	1.0	1.1	0.4	-	-	-	-	1.0	1.0	1.1	0.4	1.0	1.0	1.1	**0.4**
167234419	NP_005110.2	**THRAP3**	thyroid hormone receptor-associated protein 3	S379	-	-	-	-	1.2	2.0	1.2	0.4	-	-	-	-	1.2	2.0	1.3	0.4	1.2	2.0	1.3	**0.4**
63252863	NP_115820.2	**SLX4**	structure-specific endonuclease subunit SLX4	S1070	-	-	-	-	1.0	0.4	0.8	0.4	-	-	-	-	0.8	0.3	0.7	0.3	0.8	0.3	0.7	**0.3**
515869342	NP_001265518.1	**ST13**	hsc70-interacting protein isoform 2	S69	-	-	-	-	0.3	1.6	0.7	0.4	-	-	-	-	0.3	1.6	0.6	0.4	0.3	1.6	0.6	**0.4**
118421085	NP_689472.3	**TICRR**	treslin	S1413	-	-	-	-	1.1	0.5	0.5	0.4	-	-	-	-	1.3	0.6	0.6	0.4	1.3	0.6	0.6	**0.4**
73532778	NP_004375.2	**CSNK1G3**	casein kinase I isoform gamma-3 isoform 1	S413	-	-	-	-	1.3	0.6	0.7	0.3	-	-	-	-	1.3	0.7	0.8	0.3	1.3	0.7	0.8	**0.3**
66346681	NP_001018078.1	**SERBP1**	plasminogen activator inhibitor 1 RNA-binding protein isoform 2	S228	-	-	-	-	0.2	0.7	0.7	0.3	-	-	-	-	0.3	0.8	0.9	0.4	0.3	0.8	0.9	**0.4**
226509740	NP_067051.2	**SCAF1**	splicing factor, arginine/serine-rich 19	S548	-	-	-	-	0.8	0.8	0.9	0.3	-	-	-	-	0.8	0.7	0.8	0.3	0.8	0.7	0.8	**0.3**
51173735	NP_001003698.1	**RREB1**	ras-responsive element-binding protein 1 isoform 2	S1167	-	-	-	-	0.7	1.3	0.8	0.3	-	-	-	-	0.8	1.4	0.8	0.3	0.8	1.4	0.8	**0.3**
62912453	NP_001112.2	**ADD3**	gamma-adducin isoform b	S645	-	-	-	-	0.7	0.2	0.6	0.3	-	-	-	-	0.8	0.3	0.8	0.3	0.8	0.3	0.8	**0.3**
519666783	NP_001265539.1	**MYBL2**	myb-related protein B isoform 2	S217	-	-	-	-	0.3	0.5	0.9	0.3	-	-	-	-	0.3	0.6	0.9	0.3	0.3	0.6	0.9	**0.3**
42741682	NP_005446.2	**ZRANB2**	zinc finger Ran-binding domain-containing protein 2 isoform 2	S307	-	-	-	-	0.9	0.8	1.1	0.3	-	-	-	-	1.0	0.9	1.3	0.4	1.0	0.9	1.3	**0.4**
256818782	NP_001035375.2	**PAPD5**	non-canonical poly(A) RNA polymerase PAPD5 isoform b	S112	-	-	-	-	1.0	1.1	1.0	0.3	-	-	-	-	0.9	1.0	0.9	0.3	0.9	1.0	0.9	**0.3**
117190342	NP_005383.3	**PHF2**	lysine-specific demethylase PHF2	S458	-	-	-	-	0.9	0.8	0.8	0.3	-	-	-	-	0.9	0.9	0.9	0.3	0.9	0.9	0.9	**0.3**
222418615	NP_001138469.1	**ITGA7**	integrin alpha-7 isoform 3	S838	-	-	-	-	0.6	0.9	1.1	0.2	-	-	-	-	0.5	0.7	0.9	0.2	0.5	0.7	0.9	**0.2**
31377548	NP_775853.2	**PGM2L1**	glucose 1,6-bisphosphate synthase	S175	-	-	-	-	0.2	0.4	0.3	0.2	-	-	-	-	0.2	0.4	0.3	0.2	0.2	0.4	0.3	**0.2**
31542943	NP_059985.2	**SLC35F2**	solute carrier family 35 member F2	S371	-	-	-	-	1.5	1.1	0.7	0.2	-	-	-	-	1.4	1.1	0.7	0.2	1.4	1.1	0.7	**0.2**
38570115	NP_689706.2	**ZNF362**	zinc finger protein 362	S410	-	-	-	-	0.3	0.5	0.3	0.2	-	-	-	-	0.5	1.1	0.7	0.4	0.5	1.1	0.7	**0.4**
54792129	NP_563615.3	**DCBLD2**	discoidin, CUB and LCCL domain-containing protein 2 precursor	S724	-	-	-	-	0.2	0.2	1.0	0.2	-	-	-	-	0.2	0.2	0.9	0.2	0.2	0.2	0.9	**0.2**
194097489	NP_015559.2	**RANBP3**	ran-binding protein 3 isoform RANBP3-b	S40	-	-	-	-	0.2	0.4	0.1	0.2	-	-	-	-	0.2	0.5	0.1	0.3	0.2	0.5	0.1	**0.3**
14670388	NP_116784.1	**BAD**	bcl2 antagonist of cell death	S25	-	-	-	-	0.4	0.4	0.6	0.2	-	-	-	-	0.4	0.4	0.5	0.2	0.4	0.4	0.5	**0.2**
41152093	NP_958357.1	**ZNF644**	zinc finger protein 644 isoform 1	S1000	-	-	-	-	0.5	0.5	0.6	0.2	-	-	-	-	0.5	0.5	0.6	0.2	0.5	0.5	0.6	**0.2**
73747879	NP_443066.2	**WDR7**	WD repeat-containing protein 7 isoform 2	S935	-	-	-	-	0.3	0.1	0.1	0.1	-	-	-	-	0.3	0.1	0.1	0.2	0.3	0.1	0.1	**0.2**
