# Supplementary material for: Multi-Omics Analysis to Characterize Cigarette Smoke Induced Molecular Alterations in Esophageal Cells
Source: Front Oncol. 2020 Nov 5;10:1666. doi: 10.3389/fonc.2020.01666 (PMC7675040; doi:10.3389/fonc.2020.01666)
Supplement: Supplementary Table 10 — Top six biological networks identified by IPA. [file Table_10.pdf]

Khan *et al.* , 2019. Multi-omics analysis to characterize cigarette smoke induced molecular alterations in esophageal cells
Supplementary Table 10. Top six biological networks identified by IPA

Top networks	Associated molecules	Score	Focus molecules
[Dermatological Diseases and Conditions, Immunological Disease, Organismal Injury and Abnormalities]	**AHNAK**,Akt,**C1orf35**,CDK4/6,Cytokeratin,**DNTTIP2,DYNC1LI1,FMNL1,GPATCH8**,H1-4,**HMGN2,HRNR,KRT1,KRT10,KRT2,KRT5,KRT9,MAP7D1,MKI67,NEU3,NIFK,NOP2,NOP58,PLEC,POLR3E,POP1,RRBP1,SCAF1,SCG2,SERBP1,SNUPN,SRPK2,TOE1,TOM1,ZC3H11A**	67	32
[Cancer, Dermatological Diseases and Conditions, Organismal Injury and Abnormalities]	**ARHGEF12,BAIAP2L1,CCDC130,CGN,CLASP1,CSNK1G3,DCBLD2,EHBP1**,Filamin,**FOXK1**,GTPase,H1-5,Histone H1,**IQGAP3,ITPRID2,KIF1C,LIMA1,LRRFIP1,MADD,MRPS17**,NFkB (complex),**NUMBL**,P-TEFb,**PDLIM5,PLXNA3,RALBP1,RASAL2**,RIC1,**SENP6,SH3BP1,SLX4,SMC5**,TASOR2,**TBC1D15**,TUT7	61	30
[Cell cycle, Cellular Assembly and Organization, Cellular Compromise, DNA Replication, Recombination, and Repair]	**AHDC1,APP,ARFGAP1,ARHGEF40,CENPI,CSNK1D,CSNK1G1**,DBNDD2,FRMD6,KIAA0513,KIAA0825,LMNA,LPAR3,METTL16,MIGA1,MINAR1,MTCL1,**NUP188**,NXPE3,**PHC3,PIGO,PODXL2**,RSC1A1,**SLC38A7,SLX4,SON,SRSF8**,TASP1,**TICRR**,TMED7,**UBAP2L**,VIRMA,**WDR7,ZC3H8,ZNF555, ADH6,ANXA8/ANXA8L1,C12orf43,CCND1,CDK1**,Ces2c,**CNIH1,DCD,DCP1B**,EPS8L3,**ESCO2,EYA4,GAS2L3,** GRHL1,HNF4A,**KRAS,MDM1**,mir497,MSMB,NDC80,P2RY14,PIGF,**SEC62,SPATA5L1**,SQSTM1,T**CEAL3,TCEAL4,TCN2,TGFB1**,TMEM94,TMIGD1,TP53,URGCP,XPNPEP2,**ZNF644**	48	28
[Connective Tissue Disorders, Developmental Disorder, Gene Expression]	**ADD3,BRD2,BRDT**,CRYBG1,Ctbp,**DKK1**,Dynein,ERK,Gcn5l,**GMNN**,H3C14,Hdac,HISTONE,Histone h3,Histoneh4,**HTRA1,KDM5C,KMT2A,MIER1**,NIBAN1,**PCNT,**PELP1,PHF2,**PSIP1,REPIN1**,RPA,**RREB1,SETD1A,SETD2,SLK**,TCF,**THRAP3,TIMELESS,TP53BP1,ZNF592**	47	25
[Gene Expression, Infectious Diseases, RNA Post-Transcriptional Modification]	**AFF4**,Cbp/p300,**CCNK**,Ck2,Creb,**CTDP1,**Cyclin A,Cyclin E,C**YP27B1,DDX24**,E2f,**EIF2S2,HNRNPH**1,Holo RNA polymerase II,Hsp70,IGF2BP2,**ILF3,INTS1**,Jnk,**MYBL2**,PP1 protein complex group,Rb,**RBBP6**,RNA polymerase II,**RNPS1**,Rnr,**RRP1B,SRRM2,SRSF11,SSRP1,SUB1,UTP14A,XRCC6,YBX3,ZRANB2**	39	22
[Cell Morphology, Cellular Assembly and Organization, Cellular Function and Maintenance]	14-3-3,**AKT1S1,AMPK,BNIP3**,cytochrome C,**EEF2**,ERK1/2,**FASN,FOXO3**,growth factor,Hif1,**ITGA7**,Keratin,**MED1**,N-cor,**NCOA7,NDRG1,NDRG3**,Nr1h,p70 S6k,**PARP1**,P**ML,PP2A,PRKAA,RAB4B**,Rar,**RNF168,RPTOR,**Rxr,**S6K1,TFIIH,UBE4B,WNK1,XPC,XRCC1**	30	18

Dysregulated molecules identified in our data are highlighted in bold.
