# Supplementary material for: Multi-Omics Analysis to Characterize Cigarette Smoke Induced Molecular Alterations in Esophageal Cells
Source: Front Oncol. 2020 Nov 5;10:1666. doi: 10.3389/fonc.2020.01666 (PMC7675040; doi:10.3389/fonc.2020.01666)
Supplement: Supplementary Table 11 — Summary of the immunohistochemical validation for HMGN2 in ESCC tissues and adjacent normal tissues from smokers. [file Table_11.pdf]

Khan *et al.* , 2019. Multi-omics analysis to characterize cigarette smoke induced molecular alterations in esophageal cells

Supplementary Table 11. Summary of the immunohistochemical validation for HMGN2 in ESCC tissues and adjacent normal tissues from smokers

Intensity	Tumor tissues	Normal tissues	% of positive stained cells
Strong (3+)	0	6	70%
Moderate (2+)	0	4	35%
Negative	10	0	-
