# Supplementary material for: Multi-Omics Analysis to Characterize Cigarette Smoke Induced Molecular Alterations in Esophageal Cells
Source: Front Oncol. 2020 Nov 5;10:1666. doi: 10.3389/fonc.2020.01666 (PMC7675040; doi:10.3389/fonc.2020.01666)
Supplement: Supplementary Table 6 — List of all proteins and peptides identified in untreated and chronically treated Het1A cells with cigarette smoke condensate treated for 8 months. [file Table_6.pdf]

Khan et al., 2019. Multi-omics analysis to characterize cigarette smoke induced molecular alterations in esophageal cells

Supplementary Table 6. List of all proteins and peptides identified in untreated and chronically treated Het1A cells with cigarette smoke condensate for 8 months

Accession	RefSeq Accession	Gene Symbol	Entrez Gene ID	Description	Σ Coverage	Σ# Proteins	Σ# Unique Peptides	Σ# Peptides	Σ# PSMs	Het-1A-Smoke - 2M/Parental	Het-1A-Smoke - 4M/Parental	Het-1A-Smoke - 6M/Parental	Het-1A-Smoke - 8M/Parental	# AAs	MW [kDa]	calc. pI	Sequence	# PSMs	# Proteins	# Protein Groups	Protein Group Accessions	Modifications	MH+ [Da]	# Missed Cleavages	Het-1A-Smoke - 2M/Parental	Het-1A-Smoke - 4M/Parental	Het-1A-Smoke - 6M/Parental	Het-1A-Smoke - 8M/Parental
601984520	NP_066289.3	**UBC**	7316	**polyubiquitin-C**	96.93	4	1	13	570	**1.4**	**1.7**	**1.8**	**1.3**	685	77.0	7.66												
																	TITLEVEPSDTIENVK	171	6	3	294459921;208022622;601984520;4507761;77539055	N-Term(TMT6plex); K16(TMT6plex)	2246.3	0				
																	GGMQIFVKTLTGK	1	2	1	601984520	N-Term(TMT6plex); K8(TMT6plex); K13(TMT6plex)	2067.3	1	**1.4**	**1.7**	**1.8**	**1.3**
																	EGIPPDQQR	118	5	3	294459921;208022622;601984520;4507761;77539055	N-Term(TMT6plex)	1268.7	0				
																	IQDKEGIPPDQQR	47	4	3	294459921;208022622;601984520;4507761;77539055	N-Term(TMT6plex); K4(TMT6plex)	1982.1	1				
																	LIFAGKQLEDGR	18	4	3	294459921;208022622;601984520;4507761;77539055	N-Term(TMT6plex); K6(TMT6plex)	1805.1	1				
																	ESTLHLVLR	90	5	3	294459921;208022622;601984520;4507761;77539055	N-Term(TMT6plex)	1296.8	0				
																	TLSDYNIQK	83	5	3	294459921;208022622;601984520;4507761;77539055	N-Term(TMT6plex); K9(TMT6plex)	1539.9	0				
																	TLSDYNIQKESTLHLVLR	7	4	3	294459921;208022622;601984520;4507761;77539055	N-Term(TMT6plex); K9(TMT6plex)	2588.5	1				
																	MQIFVKTLTGK	1	4	3	294459921;208022622;601984520;4507761;77539055	N-Term(TMT6plex); K6(TMT6plex); K11(TMT6plex)	1953.2	1				
																	MQIFVK	15	4	3	294459921;208022622;601984520;4507761;77539055	N-Term(TMT6plex); K6(TMT6plex)	1223.8	0				
																	LIFAGK	13	5	3	294459921;208022622;601984520;4507761;77539055	N-Term(TMT6plex); K6(TMT6plex)	1106.7	0				
																	AKIQDKEGIPPDQQR	1	4	3	294459921;208022622;601984520;4507761;77539055	N-Term(TMT6plex); K2(TMT6plex); K6(TMT6plex)	2410.4	2				
																	QLEDGR	3	4	3	294459921;208022622;601984520;4507761;77539055	N-Term(TMT6plex)	946.5	0				
																	MQIFVK	2	4	3	294459921;208022622;601984520;4507761;77539055	N-Term(TMT6plex); M1(Oxidation); K6(TMT6plex)	1239.8	0				
197927408	NP_001128165.1	**OST4**	1E+08	**dolichyl-diphosphooligosaccharide--protein glycosyltransferase subunit 4**	91.89	1	1	1	1	**1.8**	**1.7**	**3.0**	**0.9**	37	4.2	7.24												
																	MITDVQLAIFANMLGVSLFLLVVLYHYVAVNNPK	1	1	1	197927408	N-Term(TMT6plex); K34(TMT6plex)	4264.4	0	**1.8**	**1.7**	**3.0**	**0.9**
4506671	NP_000995.1	**RPLP2**	6181	**60S acidic ribosomal protein P2**	91.30	2	6	7	154	**1.5**	**0.9**	**1.6**	**1.1**	115	11.7	4.54												
																	ILDSVGIEADDDR	21	1	1	4506671	N-Term(TMT6plex)	1646.8	0	**1.4**	**1.1**	**1.5**	**1.2**
																	ILDSVGIEADDDRLNK	12	1	1	4506671	N-Term(TMT6plex); K16(TMT6plex)	2231.2	1	**1.7**	**1.0**	**1.7**	**1.0**
																	LASVPAGGAVAVSAAPGSAAPAAGSAPAAAEEK	37	1	1	4506671	N-Term(TMT6plex); K33(TMT6plex)	3232.8	0	**1.5**	**1.1**	**1.5**	**1.1**
																	VISELNGK	4	1	1	4506671	N-Term(TMT6plex); K8(TMT6plex)	1317.8	0	**1.6**	**0.9**	**1.6**	**1.0**
																	YVASYLLAALGGNSSPSAK	38	1	1	4506671	N-Term(TMT6plex); K19(TMT6plex)	2327.3	0	**1.5**	**0.8**	**1.6**	**1.0**
																	NIEDVIAQGIGK	40	1	1	4506671	N-Term(TMT6plex); K12(TMT6plex)	1715.0	0	**1.5**	**0.8**	**1.6**	**0.9**
																	KEESEESDDDMGFGLFD	2	3	2	4506669;4506671	N-Term(TMT6plex); K1(TMT6plex); M11(Oxidation)	2424.1	1				
33239451;4505641	NP_872590.1	**PCNA**	5111	**proliferating cell nuclear antigen**	90.42	3	18	18	339	**1.2**	**1.2**	**1.3**	**1.3**	261	28.8	4.69												
																	AEDNADTLALVFEAPNQEK	49	1	1	33239451;4505641	N-Term(TMT6plex); K19(TMT6plex)	2533.3	0	**1.3**	**1.2**	**1.2**	**1.3**
																	ATPLSSTVTLSMSADVPLVVEYK	42	1	1	33239451;4505641	N-Term(TMT6plex); M12(Oxidation); K23(TMT6plex)	2882.6	0	**1.1**	**1.1**	**1.2**	**1.3**
																	SEGFDTYR	18	1	1	33239451;4505641	N-Term(TMT6plex)	1203.6	0	**1.2**	**1.1**	**1.2**	**1.2**
																	YLNFFTK	17	1	1	33239451;4505641	N-Term(TMT6plex); K7(TMT6plex)	1390.8	0	**1.1**	**1.0**	**1.2**	**1.1**
																	LMDLDVEQLGIPEQEYSCVVK	40	1	1	33239451;4505641	N-Term(TMT6plex); M2(Oxidation); C18(Carbamidomethyl); K21(TMT6plex)	2939.5	0	**1.3**	**1.3**	**1.4**	**1.3**
																	CAGNEDIITLR	5	1	1	33239451;4505641	N-Term(TMT6plex); C1(Carbamidomethyl)	1490.8	0	**1.6**	**1.0**	**1.1**	**1.1**
																	LMDLDVEQLGIPEQEYSCVVK	4	1	1	33239451;4505641	N-Term(TMT6plex); C18(Carbamidomethyl); K21(TMT6plex)	2923.5	0	**1.7**	**1.7**	**2.1**	**1.3**
																	NLAMGVNLTSMSK	29	1	1	33239451;4505641	N-Term(TMT6plex); M4(Oxidation); M11(Oxidation); K13(TMT6plex)	1856.0	0	**1.3**	**1.1**	**1.2**	**1.2**
																	DLINEACWDISSSGVNLQSMDSSHVSLVQLTLR	5	1	1	33239451;4505641	N-Term(TMT6plex); C7(Carbamidomethyl); M20(Oxidation)	3919.9	0	**1.6**	**1.6**	**1.7**	**1.5**
																	FSASGELGNGNIK	4	1	1	33239451;4505641	N-Term(TMT6plex); K13(TMT6plex)	1751.9	0	**1.1**	**0.8**	**1.0**	**1.1**
																	IADMGHLK	14	1	1	33239451;4505641	N-Term(TMT6plex); M4(Oxidation); K8(TMT6plex)	1358.8	0	**1.1**	**1.2**	**1.3**	**1.3**
																	LSQTSNVDKEEEAVTIEMNEPVQLTFALR	12	1	1	33239451;4505641	N-Term(TMT6plex); K9(TMT6plex); M18(Oxidation)	3765.9	1	**1.2**	**1.3**	**1.3**	**1.6**
																	IADMGHLK	24	1	1	33239451;4505641	N-Term(TMT6plex); K8(TMT6plex)	1342.8	0	**1.2**	**1.3**	**1.5**	**1.3**
																	NLAMGVNLTSMSK	4	1	1	33239451;4505641	N-Term(TMT6plex); K13(TMT6plex)	1824.0	0	**1.6**	**1.4**	**2.0**	**1.4**
																	DLSHIGDAVVISCAK	18	1	1	33239451;4505641	N-Term(TMT6plex); C13(Carbamidomethyl); K15(TMT6plex)	2043.1	0	**1.2**	**1.2**	**1.3**	**1.3**
																	LVQGSILK	21	1	1	33239451;4505641	N-Term(TMT6plex); K8(TMT6plex)	1315.9	0	**1.1**	**1.1**	**1.2**	**1.2**
																	ATPLSSTVTLSMSADVPLVVEYK	2	1	1	33239451;4505641	N-Term(TMT6plex); K23(TMT6plex)	2866.6	0	**1.9**	**1.7**	**1.6**	**1.4**
																	NLAMGVNLTSMSK	2	1	1	33239451;4505641	N-Term(TMT6plex); M4(Oxidation); K13(TMT6plex)	1840.0	0	**1.2**	**1.3**	**1.3**	**1.4**
																	VSDYEMK	11	1	1	33239451;4505641	N-Term(TMT6plex); M6(Oxidation); K7(TMT6plex)	1345.7	0	**1.4**	**1.2**	**1.3**	**1.3**
																	YYLAPK	4	1	1	33239451;4505641	N-Term(TMT6plex); K6(TMT6plex)	1212.7	0	**1.1**	**1.1**	**1.2**	**1.2**
																	VSDYEMK	1	1	1	33239451;4505641	N-Term(TMT6plex); K7(TMT6plex)	1329.7	0				
																	KVLEALK	2	1	1	33239451;4505641	N-Term(TMT6plex); K1(TMT6plex); K7(TMT6plex)	1488.0	1	**1.1**	**1.2**	**1.3**	**1.3**
																	VLEALK	7	3	1	33239451;4505641	N-Term(TMT6plex); K6(TMT6plex)	1130.8	0	**1.1**	**1.1**	**1.1**	**1.5**
																	MPSGEFAR	4	1	1	33239451;4505641	N-Term(TMT6plex)	1123.6	0	**1.2**	**1.3**	**1.5**	**1.2**
392513662	NP_001254738.1	**ILF2**	3608	**interleukin enhancer-binding factor 2 isoform 2**	89.49	2	21	21	418	**1.3**	**1.3**	**1.3**	**1.4**	352	38.9	4.94												
																	INNVIDNLIVAPGTFEVQIEEVR	28	2	1	392513662	N-Term(TMT6plex)	2811.5	0	**1.9**	**2.4**	**2.0**	**2.4**
																	VHTVMTLEQQDMVCYTAQTLVR	17	2	1	392513662	N-Term(TMT6plex); M5(Oxidation); M12(Oxidation); C14(Carbamidomethyl)	2884.4	0	**1.4**	**1.4**	**1.4**	**1.4**
																	KILGQEGDASYLASEISTWDGVIVTPSEK	13	2	1	392513662	N-Term(TMT6plex); K1(TMT6plex); K29(TMT6plex)	3781.1	1	**1.0**	**1.4**	**1.3**	**1.5**
																	AQDPSEVLTMLTNETGFEISSSDATVK	52	2	1	392513662	N-Term(TMT6plex); M10(Oxidation); K27(TMT6plex)	3344.7	0	**1.2**	**1.2**	**1.2**	**1.3**
																	ILGQEGDASYLASEISTWDGVIVTPSEK	61	2	1	392513662	N-Term(TMT6plex); K28(TMT6plex)	3423.8	0	**1.3**	**1.2**	**1.2**	**1.3**
																	AQDPSEVLTMLTNETGFEISSSDATVK	8	2	1	392513662	N-Term(TMT6plex); K27(TMT6plex)	3328.7	0	**1.5**	**1.6**	**1.6**	**1.5**
																	LDPELHLDIK	7	2	1	392513662	N-Term(TMT6plex); K10(TMT6plex)	1651.0	0	**1.3**	**1.2**	**1.2**	**1.3**
																	GTMTTGHNVADLVVILK	2	2	1	392513662	N-Term(TMT6plex); K17(TMT6plex)	2227.3	0	**1.3**	**1.6**	**1.4**	**1.4**
																	CLQILAAGLFLPGSVGITDPCESGNFR	2	2	1	392513662	N-Term(TMT6plex); C1(Carbamidomethyl); C21(Carbamidomethyl)	3121.6	0	**1.5**	**1.4**	**1.5**	**1.4**
																	ILPTLEAVAALGNK	39	2	1	392513662	N-Term(TMT6plex); K14(TMT6plex)	1868.2	0	**1.1**	**1.1**	**1.1**	**1.2**
																	VHTVMTLEQQDMVCYTAQTLVR	2	2	1	392513662	N-Term(TMT6plex); M12(Oxidation); C14(Carbamidomethyl)	2868.4	0	**1.6**	**1.6**	**1.7**	**1.6**
																	WFEENASQSTVK	14	2	1	392513662	N-Term(TMT6plex); K12(TMT6plex)	1884.0	0	**1.1**	**1.2**	**1.2**	**1.3**
																	KGTMTTGHNVADLVVILK	1	2	1	392513662	N-Term(TMT6plex); K1(TMT6plex); K18(TMT6plex)	2584.5	1	**1.5**	**1.5**	**1.6**	**1.4**
																	AYEKPPEK	3	2	1	392513662	N-Term(TMT6plex); K4(TMT6plex); K8(TMT6plex)	1649.0	0	**1.0**	**1.1**	**1.1**	**1.3**
																	ILITTVPPNLR	50	2	1	392513662	N-Term(TMT6plex)	1465.9	0	**1.3**	**1.2**	**1.3**	**1.3**
																	VKPAPDETSFSEALLK	18	2	1	392513662	N-Term(TMT6plex); K2(TMT6plex); K16(TMT6plex)	2419.4	0	**1.2**	**1.4**	**1.4**	**1.4**
																	KLDPELHLDIK	29	2	1	392513662	N-Term(TMT6plex); K1(TMT6plex); K11(TMT6plex)	2008.2	1	**1.0**	**1.2**	**1.2**	**1.4**
																	NQDLAPNSAEQASILSLVTK	29	2	1	392513662	N-Term(TMT6plex); K20(TMT6plex)	2557.4	0	**1.2**	**1.0**	**1.2**	**1.0**
																	VLQSALAAIR	18	2	1	392513662	N-Term(TMT6plex)	1270.8	0	**1.3**	**1.0**	**1.3**	**1.3**
																	GTMTTGHNVADLVVILK	15	2	1	392513662	N-Term(TMT6plex); M3(Oxidation); K17(TMT6plex)	2243.3	0	**1.1**	**1.2**	**1.1**	**1.3**
																	QPLALNVAYR	4	2	1	392513662	N-Term(TMT6plex)	1373.8	0	**1.3**	**1.3**	**1.3**	**1.4**
																	FPGFEPLTPWILDLLGHYAVMNNPTR	2	2	1	392513662	N-Term(TMT6plex); M21(Oxidation)	3243.7	0	**1.7**	**1.1**	**1.4**	**1.4**
																	EGEEEEENTEEPPQGEEEESMETQE	3	2	1	392513662	N-Term(TMT6plex); M21(Oxidation)	3169.2	0	**1.0**	**1.0**	**1.0**	**1.0**
																	QVGSYK	1	2	1	392513662	N-Term(TMT6plex); K6(TMT6plex)	1139.7	0	**1.0**	**1.1**	**1.1**	**1.2**
316659409	NP_001186883.1	**ACTG1**	71	**actin, cytoplasmic 2**	88.80	4	10	30	3796	**1.4**	**1.5**	**1.6**	**1.4**	375	41.8	5.48												
																	KDLYANTVLSGGTTMYPGIADR	113	2	1	316659409	N-Term(TMT6plex); K1(TMT6plex); M15(Oxidation)	2817.5	1	**1.3**	**1.4**	**1.6**	**1.4**
																	SYELPDGQVITIGNER	184	10	4	4885049;63055057;316659409;4501881	N-Term(TMT6plex)	2020.0	0				
																	VAPEEHPVLLTEAPLNPK	333	2	1	316659409	N-Term(TMT6plex); K18(TMT6plex)	2412.4	0	**1.3**	**1.4**	**1.5**	**1.4**
																	TTGIVMDSGDGVTHTVPIYEGYALPHAILR	45	2	1	316659409	N-Term(TMT6plex); M6(Oxidation)	3428.8	0	**1.5**	**1.5**	**1.5**	**1.5**
																	DLYANTVLSGGTTMYPGIADR	284	2	1	316659409	N-Term(TMT6plex); M14(Oxidation)	2460.2	0	**1.4**	**1.4**	**1.5**	**1.4**
																	DSYVGDEAQSK	313	6	3	4885049;316659409;4501881	N-Term(TMT6plex); K11(TMT6plex)	1656.8	0				
																	GYSFTTTAER	168	2	1	316659409	N-Term(TMT6plex)	1361.7	0	**1.3**	**1.3**	**1.5**	**1.4**
																	EITALAPSTMK	216	7	3	4885049;316659409;4501881	N-Term(TMT6plex); M10(Oxidation); K11(TMT6plex)	1635.9	0				
																	DLTDYLMK	52	8	4	4885049;63055057;316659409;4501881	N-Term(TMT6plex); K8(TMT6plex)	1456.8	0				
																	DLTDYLMK	164	8	4	4885049;63055057;316659409;4501881	N-Term(TMT6plex); M7(Oxidation); K8(TMT6plex)	1472.8	0				
																	HQGVMVGMGQK	118	7	4	4885049;63055057;316659409;4501881	N-Term(TMT6plex); M5(Oxidation); M8(Oxidation); K11(TMT6plex)	1661.9	0				
																	GILTLK	148	10	4	4885049;475808427;316659409;4501881	N-Term(TMT6plex); K6(TMT6plex)	1102.8	0				
																	CPEALFQPSFLGMESCGIHETTFNSIMK	105	2	1	316659409	N-Term(TMT6plex); C1(Carbamidomethyl); M13(Oxidation); C16(Carbamidomethyl); M27(Oxidation); K28(TMT6plex)	3721.8	0	**1.3**	**1.3**	**1.6**	**1.3**
																	IIAPPERK	4	8	4	4885049;63055057;316659409;4501881	N-Term(TMT6plex); K8(TMT6plex)	1381.9	1				
																	MTQIMFETFNTPAMYVAIQAVLSLYASGR	23	2	1	316659409	N-Term(TMT6plex)	3482.8	0	**1.8**	**1.7**	**2.4**	**1.2**
																	LCYVALDFEQEMATAASSSSLEK	346	3	1	316659409	N-Term(TMT6plex); C2(Carbamidomethyl); M12(Oxidation); K23(TMT6plex)	3024.5	0	**1.4**	**1.4**	**1.6**	**1.4**
																	MTQIMFETFNTPAMYVAIQAVLSLYASGR	19	2	1	316659409	N-Term(TMT6plex); M1(Oxidation)	3498.8	0	**1.9**	**1.6**	**2.5**	**1.1**
																	MTQIMFETFNTPAMYVAIQAVLSLYASGR	32	2	1	316659409	N-Term(TMT6plex); M1(Oxidation); M5(Oxidation)	3514.8	0	**1.6**	**1.3**	**2.1**	**1.1**
																	EITALAPSTMK	42	7	3	4885049;316659409;4501881	N-Term(TMT6plex); K11(TMT6plex)	1619.9	0				
																	HQGVMVGMGQK	15	7	4	4885049;63055057;316659409;4501881	N-Term(TMT6plex); K11(TMT6plex)	1629.9	0				
																	HQGVMVGMGQK	56	7	4	4885049;63055057;316659409;4501881	N-Term(TMT6plex); M5(Oxidation); K11(TMT6plex)	1645.9	0				

Khan et al., 2019. Multi-omics analysis to characterize cigarette smoke induced molecular alterations in esophageal cells
Supplementary Table 6. List of all proteins and peptides identified in untreated and chronically treated Het1A cells with cigarette smoke condensate for 8 months

Accession	RefSeq Accession	Gene Symbol	Entrez Gene ID	Description	Σ Coverage	Σ# Proteins	Σ# Unique Peptides	Σ# Peptides	Σ# PSMs	Het-1A-Smoke - 2M/Parental	Het-1A-Smoke - 4M/Parental	Het-1A-Smoke - 6M/Parental	Het-1A-Smoke - 8M/Parental	# AAs	MW [kDa]	calc. pI	Sequence	# PSMs	# Proteins	# Protein Groups	Protein Group Accessions	Modifications	MH+ [Da]	# Missed Cleavages	Het-1A-Smoke - 2M/Parental	Het-1A-Smoke - 4M/Parental	Het-1A-Smoke - 6M/Parental	Het-1A-Smoke - 8M/Parental
																	IWHHTFYNELR	84	8	4	475808427;4885049;316659409;4501881	N-Term(TMT6plex)	1744.9	0				
																	YSVWIGGSILASLSTFQQMWISK	11	5	3	4885049;63055057;316659409	N-Term(TMT6plex); K23(TMT6plex)	3060.7	0				
																	KYSVWIGGSILASLSTFQQMWISK	11	5	3	4885049;63055057;316659409	N-Term(TMT6plex); K1(TMT6plex); M20(Oxidation); K24(TMT6plex)	3433.9	1				
																	YSVWIGGSILASLSTFQQMWISK	12	5	3	4885049;63055057;316659409	N-Term(TMT6plex); M19(Oxidation); K23(TMT6plex)	3076.7	0				
																	CPEALFQPSFLGMESCGIHETTFNSIMK	10	2	1	316659409	N-Term(TMT6plex); C1(Carbamidomethyl); C16(Carbamidomethyl); K28(TMT6plex)	3689.8	0	**2.3**	**2.1**	**3.3**	**1.6**
																	AGFAGDDAPR	215	11	4	4885049;475808427;316659409;4501881	N-Term(TMT6plex)	1205.6	0				
																	AVFPSIVGRPR	16	10	4	4885049;475808427;316659409;4501881	N-Term(TMT6plex)	1427.9	0				
																	LCYVALDFEQEMATAASSSSLEK	469	3	1	316659409	N-Term(TMT6plex); C2(Carbamidomethyl); K23(TMT6plex)	3008.5	0	**1.6**	**1.7**	**2.1**	**1.4**
																	DLYANTVLSGGTTMYPGIADR	12	2	1	316659409	N-Term(TMT6plex)	2444.2	0	**1.4**	**1.5**	**1.6**	**1.6**
																	DSYVGDEAQSKR	3	6	3	4885049;316659409;4501881	N-Term(TMT6plex); K11(TMT6plex)	1813.0	1				
																	KDLYANTVLSGGTTMYPGIADR	20	2	1	316659409	N-Term(TMT6plex); K1(TMT6plex)	2801.5	1	**1.4**	**1.6**	**1.8**	**1.4**
																	TTGIVMDSGDGVTHTVPIYEGYALPHAILR	8	2	1	316659409	N-Term(TMT6plex)	3412.8	0	**1.3**	**1.2**	**1.4**	**1.3**
																	QEYDESGPSIVHR	32	6	2	475808427;316659409	N-Term(TMT6plex)	1745.9	0				
																	HQGVMVGMGQKDSYVGDEAQSK	1	6	3	4885049;316659409;4501881	N-Term(TMT6plex); M5(Oxidation); M8(Oxidation); K11(TMT6plex); K22(TMT6plex)	3070.6	1				
																	KYSVWIGGSILASLSTFQQMWISK	4	5	3	4885049;63055057;316659409	N-Term(TMT6plex); K1(TMT6plex); K24(TMT6plex)	3417.9	1				
																	MTQIMFETFNTPAMYVAIQAVLSLYASGR	12	2	1	316659409	N-Term(TMT6plex); M1(Oxidation); M5(Oxidation); M14(Oxidation)	3530.8	0	**2.0**	**1.8**	**2.3**	**1.5**
																	CPEALFQPSFLGMESCGIHETTFNSIMK	48	2	1	316659409	N-Term(TMT6plex); C1(Carbamidomethyl); M13(Oxidation); C16(Carbamidomethyl); K28(TMT6plex)	3705.8	0	**1.6**	**1.8**	**2.4**	**1.5**
																	YPIEHGIVTNWDDMEK	2	2	1	316659409	N-Term(TMT6plex); M14(Oxidation); K16(TMT6plex)	2421.2	0	**1.4**	**1.7**	**2.1**	**1.6**
																	IIAPPERKYSVWIGGSILASLSTFQQMWISK	1	5	3	4885049;63055057;316659409	N-Term(TMT6plex); K8(TMT6plex); K31(TMT6plex)	4194.3	2				
																	YSVWIGGSILASLSTFQQMWISKQEYDESGPSIVHR	1	2	1	316659409	N-Term(TMT6plex); M19(Oxidation); K23(TMT6plex)	4574.3	1	**1.4**	**1.2**	**2.0**	**0.5**
																	IIAPPERKYSVWIGGSILASLSTFQQMWISK	1	5	3	4885049;63055057;316659409	N-Term(TMT6plex); K8(TMT6plex); M27(Oxidation); K31(TMT6plex)	4210.4	2				
																	IIAPPER	29	8	4	4885049;63055057;316659409;4501881	N-Term(TMT6plex)	1024.6	0				
																	LDLAGR	10	15	7	5031569;5031571;4885049;475808427;63055057;316659409;4501881	N-Term(TMT6plex)	873.5	0				
																	ILTER	1	13	6	4885049;475808427;63055057;316659409;4501881;24233517	N-Term(TMT6plex)	860.5	0				
																	CDVDIR	3	3	2	63055057;316659409	N-Term(TMT6plex); C1(Carbamidomethyl)	1006.5	0				
5803225	NP_006752.1	**YWHAE**	7531	**14-3-3 protein epsilon**	88.24	1	23	26	2310	**1.2**	**1.3**	**1.2**	**1.2**	255	29.2	4.74												
																	AAFDDAIAELDTLSEESYK	387	1	1	5803225	N-Term(TMT6plex); K19(TMT6plex)	2546.3	0	**1.2**	**1.3**	**1.3**	**1.2**
																	DSTLIMQLLR	298	7	7	21735625;208973244;4507951;5454052;5803227;21464101;4507949;5803225	N-Term(TMT6plex); M6(Oxidation)	1434.8	0				
																	LICCDILDVLDK	316	1	1	5803225	N-Term(TMT6plex); C3(Carbamidomethyl); C4(Carbamidomethyl); K12(TMT6plex)	1935.0	0	**1.3**	**1.5**	**1.3**	**1.2**
																	VAGMDVELTVEER	102	1	1	5803225	N-Term(TMT6plex); M4(Oxidation)	1692.9	0	**1.3**	**1.3**	**1.3**	**1.3**
																	NLLSVAYK	199	7	7	21735625;208973244;4507951;5454052;5803227;21464101;4507949;5803225	N-Term(TMT6plex); K8(TMT6plex)	1365.9	0				
																	IISSIEQK	83	1	1	5803225	N-Term(TMT6plex); K8(TMT6plex)	1375.9	0	**1.2**	**1.1**	**1.1**	**1.1**
																	KVAGMDVELTVEER	21	1	1	5803225	N-Term(TMT6plex); K1(TMT6plex); M5(Oxidation)	2050.1	1	**1.2**	**1.4**	**1.2**	**1.3**
																	AASDIAMTELPPTHPIR	117	1	1	5803225	N-Term(TMT6plex); M7(Oxidation)	2065.1	0	**1.3**	**1.4**	**1.4**	**1.3**
																	YDEMVESMK	89	1	1	5803225	N-Term(TMT6plex); M4(Oxidation); M8(Oxidation); K9(TMT6plex)	1621.8	0	**1.3**	**1.4**	**1.3**	**1.2**
																	LAEQAER	18	7	7	21735625;208973244;4507951;5454052;5803227;21464101;4507949;5803225	N-Term(TMT6plex)	1045.6	0				
																	EALQDVEDENQ	46	1	1	5803225	N-Term(TMT6plex)	1518.7	0	**1.3**	**1.3**	**1.4**	**1.4**
																	DSTLIMQLLR	41	7	7	21735625;208973244;4507951;5454052;5803227;21464101;4507949;5803225	N-Term(TMT6plex)	1418.8	0				
																	EAAENSLVAYK	91	1	1	5803225	N-Term(TMT6plex); K11(TMT6plex)	1652.9	0	**1.1**	**1.3**	**1.2**	**1.2**
																	HLIPAANTGESK	66	1	1	5803225	N-Term(TMT6plex); K12(TMT6plex)	1696.0	0	**1.1**	**1.3**	**1.2**	**1.3**
																	KEAAENSLVAYK	19	1	1	5803225	N-Term(TMT6plex); K1(TMT6plex); K12(TMT6plex)	2010.2	1	**1.0**	**1.2**	**1.2**	**1.2**
																	AASDIAMTELPPTHPIR	10	1	1	5803225	N-Term(TMT6plex)	2049.1	0	**1.3**	**1.4**	**1.5**	**1.3**
																	IISSIEQKEENK	2	1	1	5803225	N-Term(TMT6plex); K8(TMT6plex); K12(TMT6plex)	2105.2	1	**1.4**	**1.8**	**1.3**	**1.7**
																	DNLTLWTSDMQGDGEEQNK	92	1	1	5803225	N-Term(TMT6plex); M10(Oxidation); K19(TMT6plex)	2655.3	0	**1.3**	**1.4**	**1.3**	**1.4**
																	VAGMDVELTVEER	7	1	1	5803225	N-Term(TMT6plex)	1676.9	0	**1.4**	**1.4**	**1.5**	**1.5**
																	DNLTLWTSDMQGDGEEQNK	21	1	1	5803225	N-Term(TMT6plex); K19(TMT6plex)	2639.3	0	**1.7**	**1.8**	**1.9**	**1.5**
																	YLAEFATGNDR	89	1	1	5803225	N-Term(TMT6plex)	1485.8	0	**1.2**	**1.2**	**1.2**	**1.1**
																	DNLTLWTSDMQGDGEEQNKEALQDVEDENQ	25	1	1	5803225	N-Term(TMT6plex); M10(Oxidation); K19(TMT6plex)	3925.8	1	**1.2**	**1.5**	**1.2**	**1.3**
																	EENKGGEDK	9	1	1	5803225	N-Term(TMT6plex); K4(TMT6plex); K9(TMT6plex)	1692.9	1	**1.0**	**1.2**	**1.0**	**1.1**
																	DNLTLWTSDMQGDGEEQNKEALQDVEDENQ	7	1	1	5803225	N-Term(TMT6plex); K19(TMT6plex)	3909.8	1	**1.7**	**2.1**	**2.1**	**1.8**
																	AAFDDAIAELDTLSEESYKDSTLIMQLLR	36	1	1	5803225	N-Term(TMT6plex); K19(TMT6plex); M25(Oxidation)	3732.9	1	**1.4**	**2.0**	**1.2**	**2.0**
																	YDEMVESMK	13	1	1	5803225	N-Term(TMT6plex); M8(Oxidation); K9(TMT6plex)	1605.8	0	**1.5**	**1.8**	**1.8**	**1.3**
																	QMVETELK	29	1	1	5803225	N-Term(TMT6plex); M2(Oxidation); K8(TMT6plex)	1451.8	0	**1.3**	**1.4**	**1.3**	**1.2**
																	EDLVYQAK	22	1	1	5803225	N-Term(TMT6plex); K8(TMT6plex)	1423.8	0	**1.0**	**1.1**	**1.0**	**1.0**
																	QMVETELK	28	1	1	5803225	N-Term(TMT6plex); K8(TMT6plex)	1435.8	0	**1.6**	**1.9**	**1.8**	**1.3**
																	LGLALNFSVFYYEILNSPDR	15	1	1	5803225	N-Term(TMT6plex)	2560.3	0	**1.5**	**1.3**	**2.2**	**0.9**
																	YDEMVESMK	8	1	1	5803225	N-Term(TMT6plex); K9(TMT6plex)	1589.8	0	**1.7**	**1.9**	**2.1**	**1.4**
																	YLAEFATGNDRK	1	1	1	5803225	N-Term(TMT6plex); K12(TMT6plex)	1843.0	1	**1.1**	**1.6**	**1.2**	**1.4**
																	NVIGAR	2	1	1	5803225	N-Term(TMT6plex)	858.5	0	**1.2**	**1.0**	**1.1**	**0.9**
																	VFYYK	1	1	1	5803225	N-Term(TMT6plex); K5(TMT6plex)	1177.7	0	**1.0**	**1.2**	**1.1**	**1.1**
30795231;409971425	NP_006308.3	**BASP1**	10409	**brain acid soluble protein 1**	88.11	1	18	18	185	**1.3**	**1.4**	**1.3**	**1.2**	227	22.7	4.63												
																	ETPAATEAPSSTPK	20	1	1	30795231;409971425	N-Term(TMT6plex); K14(TMT6plex)	1845.0	0	**1.3**	**1.4**	**1.2**	**1.2**
																	AQGPAASAEEPKPVEAPAANSDQTVTVK	12	1	1	30795231;409971425	N-Term(TMT6plex); K12(TMT6plex); K28(TMT6plex)	3450.9	0	**1.2**	**1.3**	**1.2**	**1.1**
																	KAEGAATEEEGTPK	13	1	1	30795231;409971425	N-Term(TMT6plex); K1(TMT6plex); K14(TMT6plex)	2105.2	1	**1.4**	**1.4**	**1.3**	**1.2**
																	AAEAAAAPAESAAPAAGEEPSKEEGEPK	23	1	1	30795231;409971425	N-Term(TMT6plex); K22(TMT6plex); K28(TMT6plex)	3323.7	1	**1.5**	**1.6**	**1.5**	**1.4**
																	KTEAPAAPAAQETK	23	1	1	30795231;409971425	N-Term(TMT6plex); K1(TMT6plex); K14(TMT6plex)	2100.2	1	**1.3**	**1.4**	**1.4**	**1.2**
																	AEPEKTEGAAEAK	8	1	1	30795231;409971425	N-Term(TMT6plex); K5(TMT6plex); K13(TMT6plex)	2018.1	1	**1.3**	**1.5**	**1.3**	**1.2**
																	ESEPQAAAEPAEAK	12	1	1	30795231;409971425	N-Term(TMT6plex); K14(TMT6plex)	1886.0	0	**1.4**	**1.6**	**1.4**	**1.4**
																	AQGPAASAEEPKPVEAPAANSDQTVTVKE	14	1	1	30795231;409971425	N-Term(TMT6plex); K12(TMT6plex); K28(TMT6plex)	3579.9	1	**1.1**	**1.4**	**1.2**	**1.2**
																	TEGAAEAK	2	1	1	30795231;409971425	N-Term(TMT6plex); K8(TMT6plex)	1234.7	0				
																	SDGAPASDSKPGSSEAAPSSK	13	1	1	30795231;409971425	N-Term(TMT6plex); K10(TMT6plex); K21(TMT6plex)	2620.4	0	**1.2**	**1.2**	**1.3**	**1.1**
																	AEGAATEEEGTPK	9	1	1	30795231;409971425	N-Term(TMT6plex); K13(TMT6plex)	1747.9	0	**1.4**	**1.2**	**1.4**	**1.1**
																	GYNVNDEK	8	1	1	30795231;409971425	N-Term(TMT6plex); K8(TMT6plex)	1396.7	0	**1.1**	**1.3**	**1.2**	**1.0**
																	EKPDQDAEGK	6	1	1	30795231;409971425	N-Term(TMT6plex); K2(TMT6plex); K10(TMT6plex)	1804.0	0	**1.1**	**1.3**	**1.2**	**1.0**
																	EKPDQDAEGKAEEK	2	1	1	30795231;409971425	N-Term(TMT6plex); K2(TMT6plex); K10(TMT6plex); K14(TMT6plex)	2490.4	1	**1.3**	**1.8**	**1.4**	**1.4**
																	KGYNVNDEK	4	1	1	30795231;409971425	N-Term(TMT6plex); K1(TMT6plex); K9(TMT6plex)	1754.0	1	**1.2**	**1.4**	**1.3**	**1.2**
																	APEQEQAAPGPAAGGEAPK	4	1	1	30795231;409971425	N-Term(TMT6plex); K19(TMT6plex)	2234.2	0	**1.5**	**1.4**	**1.4**	**1.2**
																	AEPPKAPEQEQAAPGPAAGGEAPK	11	1	1	30795231;409971425	N-Term(TMT6plex); K5(TMT6plex); K24(TMT6plex)	2985.6	1	**1.4**	**1.4**	**1.3**	**1.3**
																	DAAAAK	1	1	1	30795231;409971425	N-Term(TMT6plex); K6(TMT6plex)	1004.6	0	**1.4**	**1.2**	**1.3**	**1.3**
4503477	NP_001950.1	**EEF1B2**	1933	**elongation factor 1-beta**	88.00	1	14	17	224	**2.0**	**1.3**	**2.1**	**1.1**	225	24.7	4.67												
																	YGPADVEDTTGSGATDSK	4	1	1	4503477	N-Term(TMT6plex); K18(TMT6plex)	2229.1	0	**2.1**	**0.9**	**2.2**	**0.7**
																	LVPVGYGIK	15	1	1	4503477	N-Term(TMT6plex); K9(TMT6plex)	1403.9	0	**2.0**	**1.2**	**2.0**	**1.1**
																	LQIQCVVEDDKVGTDMLEEQITAFEDYVQSMDVAAFNK	4	1	1	4503477	N-Term(TMT6plex); C5(Carbamidomethyl); K11(TMT6plex); M16(Oxidation); M31(Oxidation); K38(TMT6plex)	5098.5	1	**2.0**	**1.0**	**2.3**	**0.8**
																	KPALVAK	14	5	2	304555581;4503477	N-Term(TMT6plex); K1(TMT6plex); K7(TMT6plex)	1414.0	0				
																	SYIEGYVPSQADVAVFEAVSSPPPADLCHALR	27	1	1	4503477	N-Term(TMT6plex); C28(Carbamidomethyl)	3674.8	0	**1.5**	**1.4**	**1.5**	**1.3**
																	LAQYESK	15	1	1	4503477	N-Term(TMT6plex); K7(TMT6plex)	1296.8	0	**1.8**	**1.1**	**1.8**	**1.1**
																	SSILLDVKPWDDETDMAK	11	1	1	4503477	N-Term(TMT6plex); K8(TMT6plex); K18(TMT6plex)	2750.5	0	**3.7**	**1.8**	**3.5**	**1.5**
																	SSILLDVKPWDDETDMAK	16	1	1	4503477	N-Term(TMT6plex); K8(TMT6plex); M16(Oxidation); K18(TMT6plex)	2766.5	0	**2.2**	**1.4**	**2.2**	**1.3**
																	VGTDMLEEQITAFEDYVQSMDVAAFNK	15	1	1	4503477	N-Term(TMT6plex); M5(Oxidation); M20(Oxidation); K27(TMT6plex)	3541.7	0	**1.7**	**1.1**	**1.6**	**1.0**
																	LQIQCVVEDDK	4	5	2	304555581;4503477	N-Term(TMT6plex); C5(Carbamidomethyl); K11(TMT6plex)	1805.0	0				
																	VGTDMLEEQITAFEDYVQSMDVAAFNK	13	1	1	4503477	N-Term(TMT6plex); M5(Oxidation); K27(TMT6plex)	3525.7	0	**3.5**	**1.5**	**2.4**	**1.3**
																	LQIQCVVEDDKVGTDMLEEQITAFEDYVQSMDVAAFNK	4	1	1	4503477	N-Term(TMT6plex); C5(Carbamidomethyl); K11(TMT6plex); M16(Oxidation); K38(TMT6plex)	5082.5	1	**2.6**	**1.3**	**3.3**	**1.0**
																	SPAGLQVLNDYLADK	53	1	1	4503477	N-Term(TMT6plex); K15(TMT6plex)	2062.2	0	**1.8**	**1.2**	**1.9**	**1.1**

Khan et al., 2019. Multi-omics analysis to characterize cigarette smoke induced molecular alterations in esophageal cells
Supplementary Table 6. List of all proteins and peptides identified in untreated and chronically treated Het1A cells with cigarette smoke condensate for 8 months

Accession	RefSeq Accession	Gene Symbol	Entrez Gene ID	Description	Σ Coverage	Σ# Proteins	Σ# Unique Peptides	Σ# Peptides	Σ# PSMs	Het-1A-Smoke - 2M/Parental	Het-1A-Smoke - 4M/Parental	Het-1A-Smoke - 6M/Parental	Het-1A-Smoke - 8M/Parental	# AAs	MW [kDa]	calc. pI	Sequence	# PSMs	# Proteins	# Protein Groups	Protein Group Accessions	Modifications	MH+ [Da]	# Missed Cleavages	Het-1A-Smoke - 2M/Parental	Het-1A-Smoke - 4M/Parental	Het-1A-Smoke - 6M/Parental	Het-1A-Smoke - 8M/Parental
																	KLQIQCVVEDDK	6	5	2	304555581;4503477	N-Term(TMT6plex); K1(TMT6plex); C6(Carbamidomethyl); K12(TMT6plex)	2162.2	1				
																	SIQADGLVWGSSK	15	1	1	4503477	N-Term(TMT6plex); K13(TMT6plex)	1806.0	0	**2.1**	**1.3**	**1.8**	**1.2**
																	KLQIQCVVEDDKVGTDMLEEQITAFEDYVQSMDVAAFNK	2	1	1	4503477	N-Term(TMT6plex); K1(TMT6plex); C6(Carbamidomethyl); K12(TMT6plex); M17(Oxidation); M32(Oxidation); K39(TMT6plex)	5455.8	2	**1.6**	**1.3**	**1.9**	**1.4**
																	KLQIQCVVEDDKVGTDMLEEQITAFEDYVQSMDVAAFNK	1	1	1	4503477	N-Term(TMT6plex); K1(TMT6plex); C6(Carbamidomethyl); K12(TMT6plex); M32(Oxidation); K39(TMT6plex)	5439.8	2	**1.8**	**1.8**	**2.5**	**1.7**
																	WYNHIK	2	1	1	4503477	N-Term(TMT6plex); K6(TMT6plex)	1318.8	0	**2.3**	**1.0**	**2.4**	**0.9**
																	YGPADVEDTTGSGATDSKDDDDIDLFGSDDEEESEEAKR	1	1	1	4503477	N-Term(TMT6plex); K18(TMT6plex); K38(TMT6plex)	4868.2	2	**2.2**	**2.0**	**3.0**	**1.4**
																	LEECVR	1	1	1	4503477	N-Term(TMT6plex); C4(Carbamidomethyl)	1034.6	0	**2.1**	**1.1**	**2.1**	**1.0**
																	ASLPGVK	1	1	1	4503477	N-Term(TMT6plex); K7(TMT6plex)	1129.7	0	**1.5**	**1.3**	**1.4**	**1.4**
4503481	NP_001395.1	**EEF1G**	1937	**elongation factor 1-gamma**	87.19	1	33	33	837	**1.4**	**1.4**	**1.6**	**1.5**	437	50.1	6.67												
																	VPAFEGDDGFCVFESNAIAYYVSNEELR	215	1	1	4503481	N-Term(TMT6plex); C11(Carbamidomethyl)	3427.6	0	**1.6**	**1.6**	**1.7**	**1.6**
																	FPEELTQTFMSCNLITGMFQR	19	1	1	4503481	N-Term(TMT6plex); M10(Oxidation); C12(Carbamidomethyl); M18(Oxidation)	2811.3	0	**1.7**	**1.7**	**1.7**	**1.7**
																	VLSAPPHFHFGQTNR	13	1	1	4503481	N-Term(TMT6plex)	1937.0	0	**1.3**	**1.3**	**1.5**	**1.5**
																	LDPGSEETQTLVR	15	1	1	4503481	N-Term(TMT6plex)	1673.9	0	**1.3**	**1.3**	**1.4**	**1.5**
																	STFVLDEFKR	18	1	1	4503481	N-Term(TMT6plex); K9(TMT6plex)	1700.0	1	**1.4**	**1.4**	**1.6**	**1.5**
																	YSNEDTLSVALPYFWEHFDK	12	1	1	4503481	N-Term(TMT6plex); K20(TMT6plex)	2919.5	0	**1.3**	**1.2**	**1.5**	**1.4**
																	KAAAPAPEEEMDECEQALAAEPK	30	1	1	4503481	N-Term(TMT6plex); K1(TMT6plex); M11(Oxidation); C14(Carbamidomethyl); K23(TMT6plex)	3188.6	1	**1.4**	**1.6**	**1.5**	**1.7**
																	ILGLLDAYLK	50	1	1	4503481	N-Term(TMT6plex); K10(TMT6plex)	1577.0	0	**1.7**	**1.2**	**1.7**	**1.4**
																	STFVLDEFK	18	1	1	4503481	N-Term(TMT6plex); K9(TMT6plex)	1543.9	0	**1.5**	**1.5**	**1.7**	**1.6**
																	KAAAPAPEEEMDECEQALAAEPK	6	1	1	4503481	N-Term(TMT6plex); K1(TMT6plex); C14(Carbamidomethyl); K23(TMT6plex)	3172.6	1	**1.8**	**2.1**	**2.0**	**1.3**
																	ALIAAQYSGAQVR	41	1	1	4503481	N-Term(TMT6plex)	1576.9	0	**1.4**	**1.4**	**1.4**	**1.4**
																	AVLGEVK	21	1	1	4503481	N-Term(TMT6plex); K7(TMT6plex)	1173.8	0	**1.3**	**1.4**	**1.5**	**1.4**
																	VTLADITVVCTLLWLYK	13	1	1	4503481	N-Term(TMT6plex); C10(Carbamidomethyl); K17(TMT6plex)	2466.4	0	**1.8**	**1.7**	**2.3**	**1.7**
																	KFAETQPK	13	1	1	4503481	N-Term(TMT6plex); K1(TMT6plex); K8(TMT6plex)	1636.0	1	**1.3**	**1.4**	**1.5**	**1.5**
																	KLDPGSEETQTLVR	16	1	1	4503481	N-Term(TMT6plex); K1(TMT6plex)	2031.1	1	**1.5**	**1.6**	**1.7**	**1.7**
																	GQELAFPLSPDWQVDYESYTWR	60	1	1	4503481	N-Term(TMT6plex)	2916.4	0	**1.4**	**1.4**	**1.4**	**1.4**
																	AAAPAPEEEMDECEQALAAEPK	30	1	1	4503481	N-Term(TMT6plex); M10(Oxidation); C13(Carbamidomethyl); K22(TMT6plex)	2831.3	0	**1.5**	**1.7**	**1.9**	**1.5**
																	AAGTLYTYPENWR	1	1	1	4503481	N-Term(TMT6plex)	1770.9	0	**1.5**	**1.4**	**1.5**	**1.4**
																	KYSNEDTLSVALPYFWEHFDK	8	1	1	4503481	N-Term(TMT6plex); K1(TMT6plex); K21(TMT6plex)	3276.7	1	**1.1**	**1.2**	**1.7**	**1.4**
																	GSTPEAAAQVVQWVSFADSDIVPPASTWVFPTLGIMHHNK	14	1	1	4503481	N-Term(TMT6plex); M36(Oxidation); K40(TMT6plex)	4765.5	0	**1.4**	**1.7**	**1.7**	**1.6**
																	FPEELTQTFMSCNLITGMFQR	7	1	1	4503481	N-Term(TMT6plex); M10(Oxidation); C12(Carbamidomethyl)	2795.3	0	**1.3**	**1.4**	**1.4**	**1.4**
																	AKDPFAHLPK	10	1	1	4503481	N-Term(TMT6plex); K2(TMT6plex); K10(TMT6plex)	1811.1	1	**1.1**	**1.5**	**1.6**	**1.6**
																	EYFSWEGAFQHVGK	52	1	1	4503481	N-Term(TMT6plex); K14(TMT6plex)	2143.1	0	**1.3**	**1.4**	**1.6**	**1.4**
																	WFLTCINQPQFR	28	1	1	4503481	N-Term(TMT6plex); C5(Carbamidomethyl)	1839.0	0	**1.6**	**1.4**	**1.7**	**1.4**
																	DPFAHLPK	18	1	1	4503481	N-Term(TMT6plex); K8(TMT6plex)	1382.8	0	**1.3**	**1.3**	**1.5**	**1.5**
																	QVLEPSFR	38	1	1	4503481	N-Term(TMT6plex)	1204.7	0	**1.4**	**1.4**	**1.5**	**1.5**
																	DGWSLWYSEYR	20	1	1	4503481	N-Term(TMT6plex)	1690.8	0	**1.5**	**1.4**	**1.6**	**1.4**
																	AAAPAPEEEMDECEQALAAEPK	2	1	1	4503481	N-Term(TMT6plex); C13(Carbamidomethyl); K22(TMT6plex)	2815.4	0	**1.7**	**1.7**	**2.2**	**1.4**
																	FPEELTQTFMSCNLITGMFQR	2	1	1	4503481	N-Term(TMT6plex); C12(Carbamidomethyl)	2779.3	0	**1.7**	**2.2**	**1.7**	**2.0**
																	QATENAKEEVR	2	1	1	4503481	N-Term(TMT6plex); K7(TMT6plex)	1733.0	1	**1.9**	**2.1**	**2.0**	**2.3**
																	MAQFDAK	13	1	1	4503481	N-Term(TMT6plex); M1(Oxidation); K7(TMT6plex)	1284.7	0	**1.4**	**1.4**	**1.6**	**1.5**
																	TFLVGER	4	1	1	4503481	N-Term(TMT6plex)	1050.6	0	**1.7**	**1.3**	**1.7**	**1.4**
																	MAQFDAK	5	1	1	4503481	N-Term(TMT6plex); K7(TMT6plex)	1268.7	0	**1.7**	**1.8**	**2.3**	**1.7**
																	QAFPNTNR	7	1	1	4503481	N-Term(TMT6plex)	1176.6	0	**1.5**	**1.4**	**1.5**	**1.5**
																	KNAFASVILFGTNNSSSISGVWVFR	1	1	1	4503481	N-Term(TMT6plex); K1(TMT6plex)	3159.7	1	**1.4**	**1.3**	**1.7**	**1.0**
																	NAFASVILFGTNNSSSISGVWVFR	1	1	1	4503481	N-Term(TMT6plex)	2802.5	0	**2.5**	**2.5**	**3.8**	**1.7**
																	FAETQPK	10	1	1	4503481	N-Term(TMT6plex); K7(TMT6plex)	1278.7	0	**1.3**	**1.2**	**1.6**	**1.4**
																	AFNQGK	4	1	1	4503481	N-Term(TMT6plex); K6(TMT6plex)	1122.7	0	**1.3**	**1.4**	**1.5**	**1.4**
5031857	NP_005557.1	**LDHA**	3939	**L-lactate dehydrogenase A chain isoform 1**	87.05	8	28	31	980	**1.1**	**1.2**	**1.4**	**1.2**	332	36.7	8.27												
																	DLADELALVDVIEDK	235	5	1	5031857	N-Term(TMT6plex); K15(TMT6plex)	2116.2	0	**1.0**	**1.2**	**1.3**	**1.2**
																	QVVESAYEVIK	62	3	1	5031857	N-Term(TMT6plex); K11(TMT6plex)	1723.0	0	**1.1**	**1.2**	**1.3**	**1.2**
																	SADTLWGIQK	57	3	1	5031857	N-Term(TMT6plex); K10(TMT6plex)	1576.9	0	**1.1**	**1.2**	**1.3**	**1.2**
																	DDVFLSVPCILGQNGISDLVK	13	3	1	5031857	N-Term(TMT6plex); C9(Carbamidomethyl); K21(TMT6plex)	2747.5	0	**1.3**	**1.2**	**1.6**	**1.3**
																	FIIPNVVK	29	4	1	5031857	N-Term(TMT6plex); K8(TMT6plex)	1387.9	0	**1.1**	**1.2**	**1.3**	**1.2**
																	LVIITAGAR	46	4	1	5031857	N-Term(TMT6plex)	1142.7	0	**1.1**	**1.2**	**1.4**	**1.2**
																	DQLIYNLLK	98	5	1	5031857	N-Term(TMT6plex); K9(TMT6plex)	1578.0	0	**1.0**	**1.1**	**1.4**	**1.2**
																	DQLIYNLLKEEQTPQNK	4	5	1	5031857	N-Term(TMT6plex); K9(TMT6plex); K17(TMT6plex)	2761.6	1	**1.1**	**1.7**	**1.4**	**1.8**
																	LKGEMMDLQHGSLFLR	7	5	1	5031857	N-Term(TMT6plex); K2(TMT6plex); M5(Oxidation); M6(Oxidation)	2365.3	1	**1.0**	**1.2**	**1.2**	**1.3**
																	GEMMDLQHGSLFLR	15	5	1	5031857	N-Term(TMT6plex); M3(Oxidation); M4(Oxidation)	1894.9	0	**1.2**	**1.4**	**1.4**	**1.3**
																	DYNVTANSK	37	4	1	5031857	N-Term(TMT6plex); K9(TMT6plex)	1469.8	0	**1.1**	**1.2**	**1.3**	**1.3**
																	ITVVGVGAVGMACAISILMK	54	5	1	5031857	N-Term(TMT6plex); C13(Carbamidomethyl); K20(TMT6plex)	2448.4	0	**1.5**	**1.2**	**1.5**	**1.3**
																	GYTSWAIGLSVADLAESIMK	49	3	1	5031857	N-Term(TMT6plex); M19(Oxidation); K20(TMT6plex)	2586.4	0	**1.2**	**1.2**	**1.5**	**1.2**
																	EEQTPQNK	38	5	1	5031857	N-Term(TMT6plex); K8(TMT6plex)	1431.8	0	**1.0**	**1.1**	**1.3**	**1.2**
																	TLHPDLGTDK	4	5	1	5031857	N-Term(TMT6plex); K10(TMT6plex)	1554.9	0	**1.0**	**1.1**	**1.3**	**1.1**
																	GLYGIKDDVFLSVPCILGQNGISDLVK	2	3	1	5031857	N-Term(TMT6plex); K6(TMT6plex); C15(Carbamidomethyl); K27(TMT6plex)	3608.0	1	**1.1**	**1.4**	**1.5**	**1.5**
																	VHPVSTMIK	2	3	1	5031857	N-Term(TMT6plex); K9(TMT6plex)	1469.9	0	**1.3**	**1.5**	**2.0**	**1.3**
																	VHPVSTMIK	19	3	1	5031857	N-Term(TMT6plex); M7(Oxidation); K9(TMT6plex)	1485.9	0	**1.1**	**1.3**	**1.6**	**1.2**
																	DLADELALVDVIEDKLK	1	5	1	5031857	N-Term(TMT6plex); K15(TMT6plex); K17(TMT6plex)	2586.5	1	**1.4**	**1.9**	**1.5**	**1.8**
																	VTLTSEEEAR	42	3	1	5031857	N-Term(TMT6plex)	1363.7	0	**1.1**	**1.2**	**1.3**	**1.2**
																	GYTSWAIGLSVADLAESIMK	4	3	1	5031857	N-Term(TMT6plex); K20(TMT6plex)	2570.4	0	**1.3**	**1.6**	**2.0**	**1.5**
																	LGVHPLSCHGWVLGEHGDSSVPVWSGMNVAGVSLK	4	5	1	5031857	N-Term(TMT6plex); C8(Carbamidomethyl); M27(Oxidation); K35(TMT6plex)	4143.1	0	**1.0**	**1.3**	**1.5**	**1.3**
																	LLIVSNPVDILTYVAWK	10	5	1	5031857	N-Term(TMT6plex); K17(TMT6plex)	2402.4	0	**1.6**	**1.2**	**1.8**	**2.2**
																	VIGSGCNLDSAR	55	8	2	5031857;291575128;4557032	N-Term(TMT6plex); C6(Carbamidomethyl)	1477.8	0				
																	ITVVGVGAVGMACAISILMK	43	5	1	5031857	N-Term(TMT6plex); C13(Carbamidomethyl); M19(Oxidation); K20(TMT6plex)	2464.4	0	**1.3**	**1.4**	**1.7**	**1.3**
																	GEMMDLQHGSLFLR	12	5	1	5031857	N-Term(TMT6plex); M4(Oxidation)	1878.9	0	**1.4**	**1.7**	**1.7**	**1.5**
																	TLHPDLGTDKDK	4	5	1	5031857	N-Term(TMT6plex); K10(TMT6plex); K12(TMT6plex)	2027.2	1	**1.0**	**1.5**	**1.7**	**1.4**
																	LGVHPLSCHGWVLGEHGDSSVPVWSGMNVAGVSLK	4	5	1	5031857	N-Term(TMT6plex); C8(Carbamidomethyl); K35(TMT6plex)	4127.1	0	**1.4**	**1.9**	**2.3**	**1.5**
																	TLHPDLGTDKDKEQWK	2	5	1	5031857	N-Term(TMT6plex); K10(TMT6plex); K12(TMT6plex); K16(TMT6plex)	2827.6	2	**0.8**	**1.1**	**1.4**	**1.2**
																	ITVVGVGAVGMACAISILMK	4	5	1	5031857	N-Term(TMT6plex); M11(Oxidation); C13(Carbamidomethyl); M19(Oxidation); K20(TMT6plex)	2480.4	0	**1.4**	**1.3**	**2.1**	**1.0**
																	KSADTLWGIQK	1	3	1	5031857	N-Term(TMT6plex); K1(TMT6plex); K11(TMT6plex)	1934.2	1				
																	QQEGESR	4	5	2	5031857;291575128;4557032	N-Term(TMT6plex)	1062.5	0				
																	LLIVSNPVDILTYVAWKISGFPKNR	1	4	1	5031857	N-Term(TMT6plex); K17(TMT6plex); K23(TMT6plex)	3531.1	2	**1.9**	**1.3**	**3.1**	**1.0**
																	LKGEMMDLQHGSLFLR	1	5	1	5031857	N-Term(TMT6plex); K2(TMT6plex); M6(Oxidation)	2349.3	1	**1.0**	**1.6**	**1.6**	**1.5**
																	LNLVQR	3	7	2	5031857;291575128;4557032	N-Term(TMT6plex)	971.6	0				
																	ISGFPK	7	6	1	5031857	N-Term(TMT6plex); K6(TMT6plex)	1106.7	0	**1.1**	**1.0**	**1.4**	**1.1**
																	NVNIFK	6	4	1	5031857	N-Term(TMT6plex); K6(TMT6plex)	1192.7	0	**1.0**	**0.9**	**1.3**	**0.9**
																	GLYGIK	1	4	1	5031857	N-Term(TMT6plex); K6(TMT6plex)	1108.7	0	**1.2**	**1.1**	**1.4**	**1.2**
5729877	NP_006588.1	**HSPA8**	3312	**heat shock cognate 71 kDa protein isoform 1**	87.00	2	35	49	2373	**1.3**	**1.3**	**1.4**	**1.5**	646	70.9	5.52												
																	GPAVGIDLGTTYSCVGVFQHGK	58	2	1	5729877	N-Term(TMT6plex); C14(Carbamidomethyl); K22(TMT6plex)	2721.4	0	**1.3**	**1.5**	**1.6**	**1.5**
																	QTQTFTTYSDNQPGVLIQVYEGER	22	2	1	5729877	N-Term(TMT6plex)	3003.5	0	**1.4**	**1.3**	**1.4**	**1.5**
																	NQVAMNPTNTVFDAK	72	2	1	5729877	N-Term(TMT6plex); M5(Oxidation); K15(TMT6plex)	2124.1	0	**1.4**	**1.1**	**1.3**	**1.1**
																	SENVQDLLLLDVTPLSLGIETAGGVMTVLIK	134	2	1	5729877	N-Term(TMT6plex); M26(Oxidation); K31(TMT6plex)	3713.1	0	**1.3**	**1.2**	**1.3**	**1.6**
																	SINPDEAVAYGAAVQAAILSGDK	359	2	1	5729877	N-Term(TMT6plex); K23(TMT6plex)	2718.5	0	**1.4**	**1.4**	**1.5**	**1.5**
																	LLQDFFNGK	23	4	3	13676857;5729877;34419635	N-Term(TMT6plex); K9(TMT6plex)	1539.9	0				
																	IINEPTAAAIAYGLDKK	15	3	2	13676857;5729877	N-Term(TMT6plex); K16(TMT6plex); K17(TMT6plex)	2475.5	1				
																	ATVEDEK	29	1	1	5729877	N-Term(TMT6plex); K7(TMT6plex)	1249.7	0	**2.2**	**1.5**	**1.4**	**1.5**
																	GTLDPVEK	46	2	1	5729877	N-Term(TMT6plex); K8(TMT6plex)	1316.8	0	**1.2**	**1.3**	**1.4**	**1.5**
																	MVNHFIAEFK	55	2	1	5729877	N-Term(TMT6plex); M1(Oxidation); K10(TMT6plex)	1709.9	0	**1.4**	**1.4**	**1.6**	**1.5**
																	SFYPEEVSSMVLTK	108	2	1	5729877	N-Term(TMT6plex); M10(Oxidation); K14(TMT6plex)	2091.1	0	**1.3**	**1.4**	**1.5**	**1.5**

Accession	RefSeq Accession	Gene Symbol	Entrez Gene ID	Description	Σ Coverage	Σ# Proteins	Σ# Unique Peptides	Σ# Peptides	Σ# PSMs	Het-1A-Smoke - 2M/Parental	Het-1A-Smoke - 4M/Parental	Het-1A-Smoke - 6M/Parental	Het-1A-Smoke - 8M/Parental	# AAs	MW [kDa]	calc. pI	Sequence	# PSMs	# Proteins	# Protein Groups	Protein Group Accessions	Modifications	MH+ [Da]	# Missed Cleavages	Het-1A-Smoke - 2M/Parental	Het-1A-Smoke - 4M/Parental	Het-1A-Smoke - 6M/Parental	Het-1A-Smoke - 8M/Parental
																	LYQSAGGMPGGMPGGFPGGGAPPSGGASSGPTIEEVD	56	1	1	5729877	N-Term(TMT6plex); M8(Oxidation); M12(Oxidation)	3607.6	0	1.6	1.4	1.5	1.5
																	TTPSYVAFTDTER	87	6	5	13676857;5729877;194248072;167466173;124256496;34419635	N-Term(TMT6plex)	1716.9	0				
																	ATVEDEKLQGK	4	1	1	5729877	N-Term(TMT6plex); K7(TMT6plex); K11(TMT6plex)	1905.1	1	1.7	3.8	2.7	1.6
																	RFDDAVVQSDMK	38	2	1	5729877	N-Term(TMT6plex); M11(Oxidation); K12(TMT6plex)	1885.0	1	1.3	1.4	1.4	1.5
																	IINEPTAAAIAYGLDK	103	5	4	13676857;5729877;16507237;124256496	N-Term(TMT6plex); K16(TMT6plex)	2118.2	0				
																	DAGTIAGLNVLR	73	2	1	5729877	N-Term(TMT6plex)	1428.8	0	1.3	1.3	1.4	1.5
																	NSLESYAFNMK	13	1	1	5729877	N-Term(TMT6plex); K11(TMT6plex)	1761.9	0	1.8	1.7	2.1	1.5
																	VEIIANDQGNR	80	7	6	13676857;5729877;16507237;194248072;167466173;124256496;34419635	N-Term(TMT6plex)	1457.8	0				
																	SFYPEEVSSMVLTK	33	2	1	5729877	N-Term(TMT6plex); K14(TMT6plex)	2075.1	0	1.7	1.8	2.1	1.6
																	CNEIINWLDK	51	1	1	5729877	N-Term(TMT6plex); C1(Carbamidomethyl); K10(TMT6plex)	1763.0	0	1.4	1.1	1.5	1.1
																	NVLIFDLGGGTFDVSILTIEDGIFEVK	17	3	2	13676857;5729877	N-Term(TMT6plex); K27(TMT6plex)	3369.9	0				
																	TVTNAVVTVPAYFNDSQR	58	2	1	5729877	N-Term(TMT6plex)	2211.2	0	1.4	1.3	1.5	1.4
																	SQIHDIVLVGGSTR	62	2	1	5729877	N-Term(TMT6plex)	1711.0	0	1.3	1.4	1.4	1.5
																	EIAEAYLGK	41	2	1	5729877	N-Term(TMT6plex); K9(TMT6plex)	1451.9	0	1.2	1.4	1.5	1.6
																	FELTGIPPAPR	21	1	1	5729877	N-Term(TMT6plex)	1426.8	0	1.3	1.2	1.3	1.2
																	NSLESYAFNMK	13	1	1	5729877	N-Term(TMT6plex); M10(Oxidation); K11(TMT6plex)	1777.9	0	1.3	1.3	1.4	1.5
																	FDDAVVQSDMK	11	2	1	5729877	N-Term(TMT6plex); K11(TMT6plex)	1712.9	0	1.7	1.6	1.9	1.5
																	STAGDTHLGGEDFDNR	42	3	2	13676857;5729877	N-Term(TMT6plex)	1920.9	0				
																	FEELNADLFR	100	3	2	13676857;5729877	N-Term(TMT6plex)	1482.8	0				
																	VCNPIITK	32	1	1	5729877	N-Term(TMT6plex); C2(Carbamidomethyl); K8(TMT6plex)	1402.9	0	1.6	1.4	1.5	1.4
																	INDEDKQK	3	1	1	5729877	N-Term(TMT6plex); K6(TMT6plex); K8(TMT6plex)	1677.0	1	2.2	6.0	5.2	1.7
																	TLSSSTQASIEIDSLYEGIDFYTSITR	158	2	1	5729877	N-Term(TMT6plex)	3226.6	0	1.4	1.5	1.5	1.5
																	GVPQIEVTFDIDANGILNVSAVDK	17	1	1	5729877	N-Term(TMT6plex); K24(TMT6plex)	2972.6	0	1.2	1.3	1.4	1.5
																	LYQSAGGMPGGMPGGFPGGGAPPSGGASSGPTIEEVD	7	1	1	5729877	N-Term(TMT6plex)	3575.7	0	1.6	1.5	1.8	1.5
																	LYQSAGGMPGGMPGGFPGGGAPPSGGASSGPTIEEVD	29	1	1	5729877	N-Term(TMT6plex); M8(Oxidation)	3591.7	0	1.5	1.4	1.6	1.4
																	NQVAMNPTNTVFDAK	4	2	1	5729877	N-Term(TMT6plex); K15(TMT6plex)	2108.1	0	1.7	1.4	1.9	1.2
																	DNNLLGK	23	3	2	13676857;5729877	N-Term(TMT6plex); K7(TMT6plex)	1231.7	0				
																	HWPFMVVNDAGRPK	7	2	1	5729877	N-Term(TMT6plex); M5(Oxidation); K14(TMT6plex)	2128.2	0	1.1	1.3	1.3	1.5
																	NQTAEKEEFEHQQK	15	1	1	5729877	N-Term(TMT6plex); K6(TMT6plex); K14(TMT6plex)	2433.3	1	1.2	0.9	1.3	1.0
																	MKEIAEAYLGK	6	2	1	5729877	N-Term(TMT6plex); M1(Oxidation); K2(TMT6plex); K11(TMT6plex)	1956.1	1	1.2	1.5	1.4	1.7
																	MKEIAEAYLGK	1	2	1	5729877	N-Term(TMT6plex); K2(TMT6plex); K11(TMT6plex)	1940.1	1	1.2	1.8	1.6	1.6
																	FDDAVVQSDMK	49	2	1	5729877	N-Term(TMT6plex); M10(Oxidation); K11(TMT6plex)	1728.9	0	1.3	1.3	1.5	1.4
																	RFDDAVVQSDMK	10	2	1	5729877	N-Term(TMT6plex); K12(TMT6plex)	1869.0	1	1.8	1.9	2.1	1.5
																	LIGDAAK	87	6	5	13676857;5729877;16507237;194248072;167466173;124256496	N-Term(TMT6plex); K7(TMT6plex)	1145.7	0				
																	EEFEHQQK	5	1	1	5729877	N-Term(TMT6plex); K8(TMT6plex)	1532.8	0	1.3	1.3	1.4	1.6
																	MVNHFIAEFK	8	2	1	5729877	N-Term(TMT6plex); K10(TMT6plex)	1694.0	0	1.7	1.7	2.1	1.6
																	CNEIINWLDKNQTAEKEEFEHQQK	1	1	1	5729877	N-Term(TMT6plex); C1(Carbamidomethyl); K10(TMT6plex); K16(TMT6plex); K24(TMT6plex)	3948.1	2	1.6	2.1	1.9	2.3
																	HWPFMVVNDAGRPK	2	2	1	5729877	N-Term(TMT6plex); K14(TMT6plex)	2112.2	0	2.0	2.2	2.1	1.9
																	YKAEDEK	7	1	1	5729877	N-Term(TMT6plex); K2(TMT6plex); K7(TMT6plex)	1569.9	1	1.3	1.3	1.4	1.5
																	NTTIPTK	18	4	3	13676857;5729877;24234688	N-Term(TMT6plex); K7(TMT6plex)	1232.8	0				
																	ITITNDK	26	5	5	13676857;5729877;194248072;167466173;124256496;34419635	N-Term(TMT6plex); K7(TMT6plex)	1262.8	0				
																	KDISENK	1	2	1	5729877	N-Term(TMT6plex); K1(TMT6plex); K7(TMT6plex)	1520.9	1	1.3	1.1	1.3	1.4
																	MVQEAEK	4	2	2	5729877;194248072;167466173	N-Term(TMT6plex); K7(TMT6plex)	1292.7	0				
																	DKVSSK	1	1	1	5729877	N-Term(TMT6plex); K2(TMT6plex); K6(TMT6plex)	1350.9	1	1.0	1.9	0.5	1.7
																	DISENKR	2	2	1	5729877	N-Term(TMT6plex); K6(TMT6plex)	1319.8	1	1.1	1.4	1.4	1.7
																	MVQEAEK	9	2	2	5729877;194248072;167466173	N-Term(TMT6plex); M1(Oxidation); K7(TMT6plex)	1308.7	0				
																	VQVEYK	13	3	2	13676857;5729877	N-Term(TMT6plex); K6(TMT6plex)	1223.7	0				
																	DISENK	3	2	1	5729877	N-Term(TMT6plex); K6(TMT6plex)	1163.7	0	1.5	1.2	1.4	1.6
																	LSKEDIER	1	1	1	5729877	N-Term(TMT6plex); K3(TMT6plex)	1447.9	1	1.5	2.6	1.7	2.9
21735625;208973244	NP_663723.1	YWHAZ	7534	14-3-3 protein zeta/delta	86.53	5	15	24	1749	1.3	1.5	1.4	1.5	245	27.7	4.79												
																	GIVDQSQQAYQEAFEISK	157	1	1	21735625;208973244	N-Term(TMT6plex); K18(TMT6plex)	2499.3	0	1.2	1.5	1.4	1.5
																	TAFDEAIAELDTLSEESYK	336	1	1	21735625;208973244	N-Term(TMT6plex); K19(TMT6plex)	2590.3	0	1.3	1.4	1.5	1.4
																	DSTLIMQLLR	298	7	7	21735625;208973244;4507951;5454052;5803227;21464101;4507949;5803225	N-Term(TMT6plex); M6(Oxidation)	1434.8	0				
																	FLIPNASQAESK	60	1	1	21735625;208973244	N-Term(TMT6plex); K12(TMT6plex)	1763.0	0	1.3	1.5	1.4	1.5
																	KEMQPTHPIR	12	3	3	21735625;208973244;5803227;4507949	N-Term(TMT6plex); K1(TMT6plex); M3(Oxidation)	1711.0	1				
																	EMQPTHPIR	15	3	3	21735625;208973244;5803227;4507949	N-Term(TMT6plex); M2(Oxidation)	1353.7	0				
																	NLLSVAYK	199	7	7	21735625;208973244;4507951;5454052;5803227;21464101;4507949;5803225	N-Term(TMT6plex); K8(TMT6plex)	1365.9	0				
																	YLAEVAAGDDKK	30	1	1	21735625;208973244	N-Term(TMT6plex); K11(TMT6plex); K12(TMT6plex)	1967.1	1	1.2	1.4	1.3	1.4
																	LAEQAER	18	7	7	21735625;208973244;4507951;5454052;5803227;21464101;4507949;5803225	N-Term(TMT6plex)	1045.6	0				
																	YLAEVAAGDDK	25	1	1	21735625;208973244	N-Term(TMT6plex); K11(TMT6plex)	1609.9	0	1.2	1.3	1.3	1.4
																	SVTEQGAELSNEER	75	1	1	21735625;208973244	N-Term(TMT6plex)	1777.9	0	1.5	1.5	1.4	1.5
																	DSTLIMQLLR	41	7	7	21735625;208973244;4507951;5454052;5803227;21464101;4507949;5803225	N-Term(TMT6plex)	1418.8	0				
																	KGIVDQSQQAYQEAFEISK	23	1	1	21735625;208973244	N-Term(TMT6plex); K1(TMT6plex); K19(TMT6plex)	2856.6	1	1.1	1.5	1.4	1.5
																	DICNDVLSLLEK	110	1	1	21735625;208973244	N-Term(TMT6plex); C3(Carbamidomethyl); K12(TMT6plex)	1877.0	0	1.2	1.6	1.5	1.4
																	LGLALNFSVFYYEILNSPEK	11	2	2	21735625;208973244;4507949	N-Term(TMT6plex); K20(TMT6plex)	2775.5	0				
																	DNLTLWTSDTQGDEAEAGEGGEN	227	1	1	21735625;208973244	N-Term(TMT6plex)	2638.2	0	1.4	1.5	1.4	1.5
																	TAFDEAIAELDTLSEESYKDSTLIMQLLR	9	1	1	21735625;208973244	N-Term(TMT6plex); K19(TMT6plex); M25(Oxidation)	3777.0	1	1.5	2.0	1.5	1.8
																	YDDMAACMK	23	1	1	21735625;208973244	N-Term(TMT6plex); M4(Oxidation); C7(Carbamidomethyl); M8(Oxidation); K9(TMT6plex)	1594.7	0	1.4	1.7	1.7	1.4
																	VVSSIEQK	30	1	1	21735625;208973244	N-Term(TMT6plex); K8(TMT6plex)	1347.8	0	1.2	1.4	1.4	1.4
																	YDDMAACMK	15	1	1	21735625;208973244	N-Term(TMT6plex); C7(Carbamidomethyl); K9(TMT6plex)	1562.8	0	2.1	2.3	2.8	1.5
																	EMQPTHPIRLGLALNFSVFYYEILNSPEK	2	2	2	21735625;208973244;4507949	N-Term(TMT6plex); M2(Oxidation); K29(TMT6plex)	3881.1	1				
																	YDDMAACMK	13	1	1	21735625;208973244	N-Term(TMT6plex); C7(Carbamidomethyl); M8(Oxidation); K9(TMT6plex)	1578.7	0	2.0	2.4	2.7	1.5
																	EMQPTHPIR	2	3	3	21735625;208973244;5803227;4507949	N-Term(TMT6plex)	1337.7	0				
																	NVVGAR	7	4	4	21735625;208973244;4507951;21464101;4507949	N-Term(TMT6plex)	844.5	0				
																	NELVQK	5	1	1	21735625;208973244	N-Term(TMT6plex); K6(TMT6plex)	1188.7	0	1.2	1.0	1.3	1.0
																	TEGAEK	1	5	1	21735625;208973244	N-Term(TMT6plex); K6(TMT6plex)	1092.6	0	1.3	1.3	1.5	1.5
																	ACSLAK	3	2	2	21735625;208973244;4507949	N-Term(TMT6plex); C2(Carbamidomethyl); K6(TMT6plex)	1107.7	0				
																	IETELR	2	1	1	21735625;208973244	N-Term(TMT6plex)	989.6	0	1.2	1.3	1.3	1.3
4505357	NP_002480.1	NDUFA4	4697	NADH dehydrogenase [ubiquinone] 1 alpha subcomplex subunit 4	86.42	1	6	6	20	1.3	1.2	1.5	1.2	81	9.4	9.38												
																	FYSVNVDYSK	5	1	1	4505357	N-Term(TMT6plex); K10(TMT6plex)	1679.9	0	1.3	1.0	1.3	1.0
																	LALFNPDVCWDR	5	1	1	4505357	N-Term(TMT6plex); C9(Carbamidomethyl)	1734.9	0	1.3	1.3	1.4	1.4
																	NNPEPWNK	5	1	1	4505357	N-Term(TMT6plex); K8(TMT6plex)	1456.8	0	1.2	1.0	1.2	1.2
																	KHPSLIPLFVFIGTGATGATLYLLR	2	1	1	4505357	N-Term(TMT6plex); K1(TMT6plex)	3143.9	1	1.9	1.3	2.9	0.9
																	QIIGQAK	1	1	1	4505357	N-Term(TMT6plex); K7(TMT6plex)	1215.8	0	1.3	1.2	1.3	1.2
																	LGPNDQYK	2	1	1	4505357	N-Term(TMT6plex); K8(TMT6plex)	1392.8	0	1.2	1.3	1.4	1.4
4506457	NP_002893.1	RCN2	5955	reticulocalbin-2 isoform a precursor	86.12	2	19	20	392	1.0	1.1	1.1	0.9	317	36.9	4.40												
																	LSEEEILENPDLFLTSEATDYGR	47	2	1	4506457	N-Term(TMT6plex)	2870.4	0	1.2	1.2	1.3	1.2
																	WDPTANEDPEWILVEK	42	2	1	4506457	N-Term(TMT6plex); K16(TMT6plex)	2400.3	0	1.0	1.0	1.0	0.9
																	QLHDDYFYHDEL	10	2	1	4506457	N-Term(TMT6plex)	1823.8	0	1.2	1.3	1.3	1.2
																	FVNDYDKDNDGR	5	2	1	4506457	N-Term(TMT6plex); K7(TMT6plex)	1916.0	1	0.9	0.8	0.8	0.6
																	QQFVEYDK	19	2	1	4506457	N-Term(TMT6plex); K8(TMT6plex)	1514.8	0	1.0	0.9	1.0	0.8
																	HYAMQEAK	9	2	1	4506457	N-Term(TMT6plex); M4(Oxidation); K8(TMT6plex)	1451.8	0	0.9	1.0	1.0	1.0
																	LGHEEQQK	18	2	1	4506457	N-Term(TMT6plex); K8(TMT6plex)	1426.8	0	1.0	1.1	1.1	1.0
																	IDLDSDGFLTESELSSWIQMSFK	33	2	1	4506457	N-Term(TMT6plex); M20(Oxidation); K23(TMT6plex)	3122.6	0	1.1	1.1	1.1	1.0
																	NSDDTVTWDEYNIQMYDR	6	2	1	4506457	N-Term(TMT6plex)	2494.1	0	1.1	1.0	1.2	1.0
																	AEELHYPLGER	2	2	1	4506457	N-Term(TMT6plex)	1542.8	0				
																	EALLGVQEDVDEYVK	61	2	1	4506457	N-Term(TMT6plex); K15(TMT6plex)	2165.2	0	1.1	1.2	1.2	1.2
																	KIDLDSDGFLTESELSSWIQMSFK	20	2	1	4506457	N-Term(TMT6plex); K1(TMT6plex); M21(Oxidation); K24(TMT6plex)	3479.8	1	1.0	1.1	1.0	0.8
																	ANQDSGPGLSLEEFIAFEHPEEVDYMTEFVIQEALEEHDK	1	2	1	4506457	N-Term(TMT6plex); M26(Oxidation); K40(TMT6plex)	5067.4	0	1.3	1.2	1.1	1.1
																	KIDLDSDGFLTESELSSWIQMSFK	2	2	1	4506457	N-Term(TMT6plex); K1(TMT6plex); K24(TMT6plex)	3463.8	1	1.1	1.4	1.3	1.0
																	KLSEEEILENPDLFLTSEATDYGR	12	2	1	4506457	N-Term(TMT6plex); K1(TMT6plex)	3227.7	1	1.0	1.1	1.1	1.1

Khan et al., 2019. Multi-omics analysis to characterize cigarette smoke induced molecular alterations in esophageal cells
Supplementary Table 6. List of all proteins and peptides identified in untreated and chronically treated Het1A cells with cigarette smoke condensate for 8 months

Accession	RefSeq Accession	Gene Symbol	Entrez Gene ID	Description	Σ Coverage	Σ# Proteins	Σ# Unique Peptides	Σ# Peptides	Σ# PSMs	Het-1A-Smoke-2M/Parental	Het-1A-Smoke-4M/Parental	Het-1A-Smoke-6M/Parental	Het-1A-Smoke-8M/Parental	# AAs	MW [kDa]	calc. pI	Sequence	# PSMs	# Proteins	# Protein Groups	Protein Group Accessions	Modifications	MH+ [Da]	# Missed Cleavages	Het-1A-Smoke - 2M/Parental	Het-1A-Smoke - 4M/Parental	Het-1A-Smoke - 6M/Parental	Het-1A-Smoke - 8M/Parental
																	VIDFDENTALDDAEEESFR	71	2	1	4506457	N-Term(TMT6plex)	2444.1	0	**1.1**	**1.1**	**1.1**	**1.0**
																	IDLDSDGFLTESELSSWIQMSFK	2	2	1	4506457	N-Term(TMT6plex); K23(TMT6plex)	3106.6	0	**1.2**	**1.1**	**1.7**	**0.8**
																	NSDDTVTWDEYNIQMYDR	17	2	1	4506457	N-Term(TMT6plex); M15(Oxidation)	2510.1	0	**1.0**	**0.8**	**0.8**	**0.8**
																	LDPQELLPWVVPNNQGIAQEEALHL IDEMDLNGDK	2	2	1	4506457	N-Term(TMT6plex); K35(TMT6plex)	4412.3	0	**1.1**	**1.1**	**1.1**	**0.8**
																	LDPQELLPWVVPNNQGIAQEEALHL IDEMDLNGDKK	1	2	1	4506457	N-Term(TMT6plex); M29(Oxidation); K35(TMT6plex); K36(TMT6plex)	4785.5	1	**0.7**	**0.8**	**0.7**	**0.8**
																	NGDGFVSLEEFLGDYR	1	2	1	4506457	N-Term(TMT6plex)	2047.0	0	**1.1**	**1.1**	**1.2**	**1.0**
																	VIDFDENTALDDAEEESFRK	7	2	1	4506457	N-Term(TMT6plex); K20(TMT6plex)	2801.4	1	**1.0**	**1.2**	**1.1**	**1.0**
																	LDPQELLPWVVPNNQGIAQEEALHL IDEMDLNGDK	2	2	1	4506457	N-Term(TMT6plex); M29(Oxidation); K35(TMT6plex)	4428.3	0	**0.9**	**0.6**	**0.6**	**0.8**
																	LQAIIK	2	3	3	153792694;4506457;118918403	N-Term(TMT6plex); K6(TMT6plex)	1143.8	0				
4557797	NP_000260.1	**NME1**	4830	**nucleoside diphosphate kinase A isoform b**	85.53	2	2	10	240	**1.7**	**1.2**	**1.3**	**1.4**	152	17.1	6.19												
																	GLVGEIIK	23	5	2	66392203;4557797	N-Term(TMT6plex); K8(TMT6plex)	1286.8	0				
																	YMHSGPVVAMVWEGLNVVK	24	3	2	4557797;66392203	N-Term(TMT6plex); M2(Oxidation); M10(Oxidation); K19(TMT6plex)	2606.4	0				
																	GDFCIQVGR	27	4	2	66392203;4557797	N-Term(TMT6plex); C4(Carbamidomethyl)	1280.7	0				
																	NIIHGSDSVESAEK	31	2	1	4557797	N-Term(TMT6plex); K14(TMT6plex)	1944.0	0	**1.7**	**1.2**	**1.4**	**1.3**
																	FMQASEDLLK	4	3	2	4557797;66392203	N-Term(TMT6plex); K10(TMT6plex)	1639.9	0				
																	EHYVDLK	23	3	2	4557797;66392203	N-Term(TMT6plex); K7(TMT6plex)	1361.8	0				
																	FMQASEDLLK	27	3	2	4557797;66392203	N-Term(TMT6plex); M2(Oxidation); K10(TMT6plex)	1655.9	0				
																	VMLGETNPADSKPGTIR	36	4	2	66392203;4557797	N-Term(TMT6plex); M2(Oxidation); K12(TMT6plex)	2260.2	0				
																	TFIAIKPDGVQR	19	5	2	66392203;4557797	N-Term(TMT6plex); K6(TMT6plex)	1803.1	0				
																	VMLGETNPADSKPGTIR	11	4	2	66392203;4557797	N-Term(TMT6plex); K12(TMT6plex)	2244.2	0				
																	EIGLWFHPEELVDYTSCAQNWIYE	1	2	1	4557797	N-Term(TMT6plex); C17(Carbamidomethyl)	3228.5	0	**1.3**	**1.4**	**1.3**	**1.5**
																	DRPFFAGLVK	10	3	2	4557797;66392203	N-Term(TMT6plex); K10(TMT6plex)	1608.0	0				
																	YMHSGPVVAMVWEGLNVVK	4	3	2	4557797;66392203	N-Term(TMT6plex); K19(TMT6plex)	2574.4	0				
27262628	NP_002473.2	**NASP**	4678	**nuclear autoantigenic sperm protein isoform 2**	85.41	3	49	51	1242	**1.0**	**1.0**	**0.9**	**0.9**	788	85.2	4.30												
																	ATLVESSTSGFTPGGGGSSVSMIASR	71	3	1	27262628	N-Term(TMT6plex); M22(Oxidation)	2688.3	0	**1.2**	**1.3**	**1.2**	**1.3**
																	SLQENEEEEIGNLELAWDMLDLAK	125	3	1	27262628	N-Term(TMT6plex); M19(Oxidation); K24(TMT6plex)	3263.6	0	**1.0**	**1.1**	**0.9**	**1.0**
																	MAVLNEQVK	46	3	1	27262628	N-Term(TMT6plex); M1(Oxidation); K9(TMT6plex)	1505.9	0	**1.1**	**1.1**	**1.0**	**1.1**
																	EQVYDAMGEK	8	2	1	27262628	N-Term(TMT6plex); M7(Oxidation); K10(TMT6plex)	1643.8	0	**0.8**	**0.7**	**0.5**	**0.7**
																	QGEVIVSIEEKPK	12	2	1	27262628	N-Term(TMT6plex); K11(TMT6plex); K13(TMT6plex)	2143.3	0	**1.0**	**1.0**	**0.7**	**0.9**
																	AVLEQLVGQEVPPAEESPEVTTEAA EASAVEAGSEVSEKPGQEAPVLPK	11	2	1	27262628	N-Term(TMT6plex); K39(TMT6plex); K49(TMT6plex)	5671.0	0	**0.8**	**0.9**	**0.4**	**0.7**
																	VAQGATEK	8	2	1	27262628	N-Term(TMT6plex); K8(TMT6plex)	1261.8	0	**1.1**	**0.8**	**0.6**	**0.7**
																	QGTAVEVEAESLDPTVKPVDVGGD EPEEK	7	2	1	27262628	N-Term(TMT6plex); K17(TMT6plex); K29(TMT6plex)	3711.9	0	**1.1**	**0.8**	**0.7**	**0.8**
																	DGSGLEEK	9	2	1	27262628	N-Term(TMT6plex); K8(TMT6plex)	1292.7	0	**1.1**	**1.0**	**0.8**	**0.9**
																	HLVMGDIPAAVNAFQEAASLLGK	132	2	1	27262628	N-Term(TMT6plex); M4(Oxidation); K23(TMT6plex)	2826.6	0	**1.0**	**1.0**	**0.9**	**0.9**
																	EAEGSSAEYK	52	3	1	27262628	N-Term(TMT6plex); K10(TMT6plex)	1528.8	0	**0.9**	**0.9**	**0.8**	**0.8**
																	VVTSENEAGK	53	2	1	27262628	N-Term(TMT6plex); K10(TMT6plex)	1491.8	0	**1.3**	**1.0**	**0.8**	**0.9**
																	SAEEPQEK	14	2	1	27262628	N-Term(TMT6plex); K8(TMT6plex)	1375.7	0	**1.3**	**0.9**	**0.7**	**0.8**
																	LSVEESEAAGDGVDTK	35	2	1	27262628	N-Term(TMT6plex); K16(TMT6plex)	2065.1	0	**1.0**	**1.0**	**0.7**	**0.8**
																	EVSEEQPVVTLEK	37	2	1	27262628	N-Term(TMT6plex); K13(TMT6plex)	1945.1	0	**1.1**	**1.1**	**0.9**	**1.0**
																	YGETANECGEAFFFYGK	114	2	1	27262628	N-Term(TMT6plex); C8(Carbamidomethyl); K17(TMT6plex)	2448.2	0	**1.1**	**1.3**	**1.1**	**1.2**
																	VQIAANEETQER	15	2	1	27262628	N-Term(TMT6plex)	1616.8	0	**1.1**	**0.9**	**0.8**	**0.8**
																	VESLDVDSEAK	16	2	1	27262628	N-Term(TMT6plex); K11(TMT6plex)	1649.9	0	**0.9**	**0.8**	**0.7**	**0.7**
																	EAQLYAAQAHLK	22	3	1	27262628	N-Term(TMT6plex); K12(TMT6plex)	1801.0	0	**1.0**	**1.1**	**1.0**	**1.1**
																	GGAAPEGPNEAEVTSGKPEQEVPDA EEEK	18	2	1	27262628	N-Term(TMT6plex); K17(TMT6plex); K29(TMT6plex)	3638.8	0	**0.9**	**0.9**	**0.6**	**0.8**
																	EQVYDAMGEKEEAK	14	2	1	27262628	N-Term(TMT6plex); M7(Oxidation); K10(TMT6plex); K14(TMT6plex)	2330.2	1	**0.9**	**1.0**	**0.6**	**0.8**
																	LVPSQEETK	15	2	1	27262628	N-Term(TMT6plex); K9(TMT6plex)	1488.9	0	**0.9**	**0.8**	**0.7**	**0.9**
																	HLVMGDIPAAVNAFQEAASLLGK	33	2	1	27262628	N-Term(TMT6plex); K23(TMT6plex)	2810.6	0	**1.2**	**1.3**	**1.4**	**1.0**
																	LGEVSVESENYVQAVEEFQSCLNLQ EQYLEAHDR	82	3	1	27262628	N-Term(TMT6plex); C21(Carbamidomethyl)	4242.0	0	**1.3**	**1.2**	**1.1**	**1.1**
																	ATLVESSTSGFTPGGGGSSVSMIASR	4	3	1	27262628	N-Term(TMT6plex)	2672.3	0	**1.6**	**1.6**	**1.8**	**1.4**
																	SIEVIENR	19	3	1	27262628	N-Term(TMT6plex)	1188.7	0	**1.0**	**1.1**	**1.0**	**1.0**
																	IEDVPAPSTSADKVESLDVDSEAK	12	2	1	27262628	N-Term(TMT6plex); K13(TMT6plex); K24(TMT6plex)	3189.7	1	**0.8**	**0.8**	**0.7**	**0.8**
																	EIEELKELLPEIR	6	3	1	27262628	N-Term(TMT6plex); K6(TMT6plex)	2069.2	1	**1.2**	**1.5**	**1.0**	**1.0**
																	SLAKPETDKEQDSEMEK	8	2	1	27262628	N-Term(TMT6plex); K4(TMT6plex); K9(TMT6plex); M15(Oxidation); K17(TMT6plex)	2897.6	1	**0.8**	**0.9**	**0.5**	**0.7**
																	VDLTLDWLTETSEEAK	23	2	1	27262628	N-Term(TMT6plex); K16(TMT6plex)	2308.2	0	**1.0**	**1.0**	**0.8**	**0.8**
																	KYGETANECGEAFFFYGK	30	2	1	27262628	N-Term(TMT6plex); K1(TMT6plex); C9(Carbamidomethyl); K18(TMT6plex)	2805.4	1	**1.0**	**1.2**	**1.1**	**1.1**
																	SVSGTDVQEECR	8	2	1	27262628	N-Term(TMT6plex); C11(Carbamidomethyl)	1595.8	0	**1.0**	**0.9**	**0.6**	**0.7**
																	SLQENEEEEIGNLELAWDMLDLAK	19	3	1	27262628	N-Term(TMT6plex); K24(TMT6plex)	3247.6	0	**1.1**	**1.3**	**1.2**	**0.9**
																	LLAETHYQLGLAYGYNSQYDEAVA QFSK	13	3	1	27262628	N-Term(TMT6plex); K28(TMT6plex)	3637.9	0	**1.2**	**1.2**	**1.2**	**1.1**
																	KPTDGASSSNCVTDISHLVR	9	3	1	27262628	N-Term(TMT6plex); K1(TMT6plex); C11(Carbamidomethyl)	2602.4	0	**1.0**	**1.2**	**1.1**	**1.1**
																	MAVLNEQVK	7	3	1	27262628	N-Term(TMT6plex); K9(TMT6plex)	1489.9	0	**1.3**	**1.3**	**1.3**	**1.2**
																	DGAVNGPSVVGDQTPIEPQTSIER	5	2	1	27262628	N-Term(TMT6plex)	2695.4	0	**1.2**	**1.3**	**1.2**	**1.2**
																	SGNVAELALK	24	3	1	27262628	N-Term(TMT6plex); K10(TMT6plex)	1459.9	0	**1.0**	**0.9**	**0.9**	**0.8**
																	AAVEGTVEAGATVESTAC	6	3	1	27262628	N-Term(TMT6plex); C18(Carbamidomethyl)	1951.9	0	**1.1**	**1.2**	**1.3**	**1.1**
																	EAEGSSAEYKK	7	3	1	27262628	N-Term(TMT6plex); K10(TMT6plex); K11(TMT6plex)	1886.0	1	**0.8**	**1.4**	**0.9**	**1.1**
																	KPEEESPR	4	3	1	27262628	N-Term(TMT6plex); K1(TMT6plex)	1429.8	0	**1.0**	**1.2**	**1.2**	**1.0**
																	AKQEPEVNGGSGDAVPSGNEVSEN MEEEAENQAESR	2	3	1	27262628	N-Term(TMT6plex); K2(TMT6plex); M25(Oxidation)	4249.0	1	**1.0**	**1.0**	**1.0**	**0.8**
																	KLLGLGQK	2	2	1	27262628	N-Term(TMT6plex); K1(TMT6plex); K8(TMT6plex)	1544.0	1	**1.0**	**1.1**	**1.0**	**1.0**
																	VESLDVDSEAKK	1	2	1	27262628	N-Term(TMT6plex); K11(TMT6plex); K12(TMT6plex)	2007.2	1	**0.9**	**1.0**	**1.0**	**0.9**
																	VQIAANEETQEREEQMK	2	2	1	27262628	N-Term(TMT6plex); M16(Oxidation); K17(TMT6plex)	2507.3	1	**1.2**	**1.1**	**0.9**	**0.9**
																	IEDVPAPSTSADK	2	3	1	27262628	N-Term(TMT6plex); K13(TMT6plex)	1788.0	0	**0.9**	**0.8**	**0.8**	**0.8**
																	EGEETEGSEEDDKENDKTEEMPNDS VLENK	1	2	1	27262628	N-Term(TMT6plex); K13(TMT6plex); K17(TMT6plex); M21(Oxidation); K30(TMT6plex)	4359.1	2	**1.2**	**1.0**	**1.0**	**0.9**
																	TEEMPNDSVLENK	2	3	1	27262628	N-Term(TMT6plex); M4(Oxidation); K13(TMT6plex)	1980.0	0				
																	SLLELAR	28	2	1	27262628	N-Term(TMT6plex)	1030.6	0	**1.0**	**1.0**	**0.9**	**0.9**
																	LLGLGQK	13	2	1	27262628	N-Term(TMT6plex); K7(TMT6plex)	1186.8	0	**0.9**	**0.8**	**0.8**	**0.7**
																	KEIEELK	1	5	2	27262628;45006986	N-Term(TMT6plex); K1(TMT6plex); K7(TMT6plex)	1576.0	1				
																	EIEELK	13	7	8	27262628;46852174;117956403;33946327;45006986;32698866;10 9255234;513788264	N-Term(TMT6plex); K6(TMT6plex)	1218.7	0				
																	ELLPEIR	9	3	1	27262628	N-Term(TMT6plex)	1098.7	0	**1.0**	**1.0**	**1.0**	**0.9**
																	EKIEDAK	12	3	1	27262628	N-Term(TMT6plex); K2(TMT6plex); K7(TMT6plex)	1519.9	1	**0.7**	**0.9**	**0.7**	**0.7**
																	EDMDISK	1	2	1	27262628	N-Term(TMT6plex); M3(Oxidation); K7(TMT6plex)	1311.7	0	**0.9**	**0.8**	**0.7**	**0.8**
88999583	NP_524147.2	**MYL6**	4637	**myosin light polypeptide 6 isoform 2**	84.77	3	12	12	176	**2.1**	**1.2**	**2.5**	**1.0**	151	17.0	4.55												
																	VLDFEHFLPMLQTVAK	37	2	1	88999583	N-Term(TMT6plex); M10(Oxidation); K16(TMT6plex)	2362.3	0	**2.1**	**1.2**	**2.6**	**1.1**
																	HVLVTLGEK	17	2	1	88999583	N-Term(TMT6plex); K9(TMT6plex)	1453.9	0	**2.0**	**1.2**	**2.4**	**1.2**
																	ALGQNPTNAEVLK	29	3	1	88999583	N-Term(TMT6plex); K13(TMT6plex)	1813.1	0	**2.0**	**1.2**	**2.3**	**1.1**
																	EAFQLFDR	22	2	1	88999583	N-Term(TMT6plex)	1254.7	0	**2.1**	**1.3**	**2.5**	**1.1**
																	VLDFEHFLPMLQTVAK	16	2	1	88999583	N-Term(TMT6plex); K16(TMT6plex)	2346.3	0	**2.1**	**1.3**	**3.0**	**1.1**
																	DQGTYEDYVEGLR	17	2	1	88999583	N-Term(TMT6plex)	1773.8	0	**2.1**	**1.3**	**2.4**	**1.2**
																	ILYSQCGDVMR	10	3	1	88999583	N-Term(TMT6plex); C6(Carbamidomethyl); M10(Oxidation)	1586.8	0	**2.2**	**1.1**	**2.4**	**1.0**
																	NKDQGTYEDYVEGLR	6	2	1	88999583	N-Term(TMT6plex); K2(TMT6plex)	2245.1	1	**2.8**	**1.1**	**2.8**	**0.8**
																	ILYSQCGDVMR	2	3	1	88999583	N-Term(TMT6plex); C6(Carbamidomethyl)	1570.8	0	**2.5**	**1.5**	**3.2**	**1.2**
																	MTEEEVEMLVAGHEDSNGCINYEE LVR	3	1	1	88999583	N-Term(TMT6plex); M8(Oxidation); C19(Carbamidomethyl)	3398.6	0	**1.6**	**1.3**	**1.7**	**1.0**
																	MTEEEVEMLVAGHEDSNGCINYEE LVR	1	1	1	88999583	N-Term(TMT6plex); C19(Carbamidomethyl)	3382.5	0	**1.7**	**1.5**	**1.7**	**1.5**
																	SDEMNVK	11	2	1	88999583	N-Term(TMT6plex); M4(Oxidation); K7(TMT6plex)	1296.7	0	**2.6**	**1.3**	**3.2**	**0.9**
																	EGNGTVMGAEIR	2	2	1	88999583	N-Term(TMT6plex); M7(Oxidation)	1478.7	0	**1.9**	**1.3**	**1.6**	**1.1**
																	VFDKEGNGTVMGAEIR	1	2	1	88999583	N-Term(TMT6plex); K4(TMT6plex); M11(Oxidation)	2197.2	1	**1.4**	**1.3**	**1.3**	**1.1**
																	VLGNPK	2	3	1	88999583	N-Term(TMT6plex); K6(TMT6plex)	1085.7	0	**2.5**	**1.1**	**2.9**	**0.8**
45387955	NP_991330.1	**C19orf70**	125988	**protein QIL1 precursor**	84.75	1	7	7	27	**1.5**	**1.5**	**1.6**	**1.6**	118	13.1	9.42												
																	EGWEYVK	6	1	1	45387955	N-Term(TMT6plex); K7(TMT6plex)	1368.8	0	**1.2**	**1.4**	**1.4**	**1.5**
																	GSVAGGAVYLVYDQELLGPSDK	2	1	1	45387955	N-Term(TMT6plex); K22(TMT6plex)	2696.5	0	**1.3**	**1.5**	**1.5**	**1.8**
																	DSWNAGIMTVMSALSVAPSK	3	1	1	45387955	N-Term(TMT6plex); M8(Oxidation); M11(Oxidation); K20(TMT6plex)	2555.3	0	**1.7**	**1.7**	**1.5**	**1.6**

Accession	RefSeq Accession	Gene Symbol	Entrez Gene ID	Description	Σ Coverage	Σ# Proteins	Σ# Unique Peptides	Σ# Peptides	Σ# PSMs	Het-1A-Smoke - 2M/Parental	Het-1A-Smoke - 4M/Parental	Het-1A-Smoke - 6M/Parental	Het-1A-Smoke - 8M/Parental	# AAs	MW [kDa]	calc. pI	Sequence	# PSMs	# Proteins	# Protein Groups	Protein Group Accessions	Modifications	MH+ [Da]	# Missed Cleavages	Het-1A-Smoke - 2M/Parental	Het-1A-Smoke - 4M/Parental	Het-1A-Smoke - 6M/Parental	Het-1A-Smoke - 8M/Parental
																	SQAALQK	2	1	1	45387955	N-Term(TMT6plex); K7(TMT6plex)	1203.7	0	**1.8**	**1.2**	**1.3**	**1.3**
																	AGEVVPPAMYQFSQYVCQQTGLQIPQLPAPPK	8	1	1	45387955	N-Term(TMT6plex); C17(Carbamidomethyl); K32(TMT6plex)	4001.1	0	**1.7**	**1.8**	**2.0**	**1.8**
																	AGEVVPPAMYQFSQYVCQQTGLQIPQLPAPPK	1	1	1	45387955	N-Term(TMT6plex); M9(Oxidation); C17(Carbamidomethyl); K32(TMT6plex)	4017.1	0				
																	DSWNAGIMTVMSALSVAPSK	2	1	1	45387955	N-Term(TMT6plex); M8(Oxidation); K20(TMT6plex)	2539.3	0	**1.1**	**1.2**	**1.2**	**1.6**
																	IYFPIR	2	1	1	45387955	N-Term(TMT6plex)	1037.6	0	**1.6**	**1.5**	**1.5**	**1.6**
																	VWSLMR	1	1	1	45387955	N-Term(TMT6plex)	1020.6	0	**1.8**	**1.5**	**2.3**	**1.3**
21464101	NP_036611.2	**YWHAG**	7532	**14-3-3 protein gamma**	84.62	1	13	19	861	**1.4**	**1.6**	**1.6**	**1.6**	247	28.3	4.89												
																	DSTLIMQLLR	298	7	7	21735625;208973244;4507951;5454052;5803227;21464101;4507949;5803225	N-Term(TMT6plex); M6(Oxidation)	1434.8	0				
																	NLLSVAYK	199	7	7	21735625;208973244;4507951;5454052;5803227;21464101;4507949;5803225	N-Term(TMT6plex); K8(TMT6plex)	1365.9	0				
																	NCSETQYESK	18	1	1	21464101	N-Term(TMT6plex); C2(Carbamidomethyl); K10(TMT6plex)	1703.8	0	**1.5**	**1.4**	**1.7**	**1.3**
																	LAEQAER	18	7	7	21735625;208973244;4507951;5454052;5803227;21464101;4507949;5803225	N-Term(TMT6plex)	1045.6	0				
																	EHMQPTHPIR	2	1	1	21464101	N-Term(TMT6plex); M3(Oxidation)	1490.8	0	**1.2**	**2.0**	**1.6**	**1.8**
																	TAFDDAIAELDTLNEDSYK	39	1	1	21464101	N-Term(TMT6plex); K19(TMT6plex)	2589.3	0	**1.3**	**1.5**	**1.6**	**1.7**
																	DSTLIMQLLR	41	7	7	21735625;208973244;4507951;5454052;5803227;21464101;4507949;5803225	N-Term(TMT6plex)	1418.8	0				
																	VISSIEQK	57	5	5	4507951;5803227;21464101;4507949;5454052	N-Term(TMT6plex); K8(TMT6plex)	1361.8	0				
																	DNLTLWTSDQQDDDGGEGNN	23	1	1	21464101	N-Term(TMT6plex)	2423.0	0	**1.5**	**1.6**	**1.5**	**1.7**
																	TSADGNEK	4	1	1	21464101	N-Term(TMT6plex); K8(TMT6plex)	1279.7	0	**1.4**	**1.5**	**1.6**	**1.4**
																	ATVVESSEK	34	1	1	21464101	N-Term(TMT6plex); K9(TMT6plex)	1407.8	0	**1.5**	**1.6**	**1.5**	**1.7**
																	TAFDDAIAELDTLNEDSYKDSTLIMQLLR	5	1	1	21464101	N-Term(TMT6plex); K19(TMT6plex); M25(Oxidation)	3775.9	1	**1.5**	**2.0**	**1.5**	**2.1**
																	YLAEVATGEK	16	1	1	21464101	N-Term(TMT6plex); K10(TMT6plex)	1538.9	0	**1.2**	**1.6**	**1.6**	**1.6**
																	YDDMAAAMK	26	2	2	21464101;4507949	N-Term(TMT6plex); M4(Oxidation); M8(Oxidation); K9(TMT6plex)	1505.7	0				
																	LGLALNYSVFYYEIQNAPEQACHLAK	8	1	1	21464101	N-Term(TMT6plex); C22(Carbamidomethyl); K26(TMT6plex)	3470.8	0	**1.4**	**1.6**	**1.7**	**1.5**
																	NVTELNEPLSNEER	28	1	1	21464101	N-Term(TMT6plex)	1873.0	0	**1.5**	**1.7**	**1.7**	**1.7**
																	YDDMAAAMK	20	2	2	21464101;4507949	N-Term(TMT6plex); M8(Oxidation); K9(TMT6plex)	1489.7	0				
																	ELEAVCQDVLSLLDNYLIK	11	1	1	21464101	N-Term(TMT6plex); C6(Carbamidomethyl); K19(TMT6plex)	2693.5	0	**1.9**	**1.9**	**1.6**	**1.9**
																	AYSEAHEISK	4	1	1	21464101	N-Term(TMT6plex); K10(TMT6plex)	1592.9	0	**1.1**	**1.7**	**1.6**	**1.7**
																	YDDMAAAMK	2	2	2	21464101;4507949	N-Term(TMT6plex); K9(TMT6plex)	1473.7	0				
																	NVVGAR	7	4	4	21735625;208973244;4507951;21464101;4507949	N-Term(TMT6plex)	844.5	0				
																	EQLVQK	1	1	1	21464101	N-Term(TMT6plex); K6(TMT6plex)	1202.8	0	**1.1**	**1.8**	**1.7**	**1.9**
21264341	NP_004586.2	**SMS**	6611	**spermine synthase isoform 1**	84.43	2	23	23	200	**1.4**	**1.4**	**1.7**	**1.2**	366	41.2	5.02												
																	GDCYQVLIEDCIPVLK	14	2	1	21264341	N-Term(TMT6plex); C3(Carbamidomethyl); C11(Carbamidomethyl); K16(TMT6plex)	2380.3	0	**1.3**	**1.5**	**1.9**	**1.1**
																	MVTMVEIDQMVIDGCK	12	2	1	21264341	M1(Oxidation); N-Term(TMT6plex); M4(Oxidation); M10(Oxidation); C15(Carbamidomethyl); K16(TMT6plex)	2375.2	0	**1.5**	**1.4**	**1.5**	**1.4**
																	TCGDVLDNLK	8	2	1	21264341	N-Term(TMT6plex); C2(Carbamidomethyl); K10(TMT6plex)	1592.9	0	**1.4**	**1.1**	**1.8**	**0.9**
																	IYPHGLVLLDLQSYDGDAQGKEEIDSILNK	1	1	1	21264341	N-Term(TMT6plex); K21(TMT6plex); K30(TMT6plex)	4031.2	1	**1.6**	**1.8**	**1.5**	**1.9**
																	ADGETILK	14	2	1	21264341	N-Term(TMT6plex); K8(TMT6plex)	1304.8	0	**1.3**	**1.3**	**1.6**	**1.2**
																	GLQSIFQEQGMAESVHTWQDHGYLATYTNK	9	2	1	21264341	N-Term(TMT6plex); M11(Oxidation); K30(TMT6plex)	3913.9	0	**1.2**	**1.2**	**1.4**	**1.2**
																	MVTMVEIDQMVIDGCK	6	2	1	21264341	M1(Oxidation); N-Term(TMT6plex); M4(Oxidation); C15(Carbamidomethyl); K16(TMT6plex)	2359.2	0	**1.4**	**1.6**	**1.7**	**1.5**
																	LYCPVEFSK	8	2	1	21264341	N-Term(TMT6plex); C3(Carbamidomethyl); K9(TMT6plex)	1600.9	0	**1.3**	**1.4**	**1.6**	**1.2**
																	HSTLDFMLGAK	8	2	1	21264341	N-Term(TMT6plex); M7(Oxidation); K11(TMT6plex)	1693.9	0	**1.2**	**1.3**	**1.5**	**1.3**
																	LILDLSMK	4	2	1	21264341	N-Term(TMT6plex); K8(TMT6plex)	1390.9	0	**1.8**	**2.0**	**2.9**	**1.1**
																	EDYTGKDVLILGGGDGGILCEIVK	2	2	1	21264341	N-Term(TMT6plex); K6(TMT6plex); C20(Carbamidomethyl); K24(TMT6plex)	3208.8	1	**1.9**	**3.1**	**2.6**	**2.4**
																	LVEYDIDEVVYDEDSPYQNIK	28	2	1	21264341	N-Term(TMT6plex); K21(TMT6plex)	3004.5	0	**1.5**	**1.3**	**1.5**	**1.2**
																	EEIDSILNK	9	1	1	21264341	N-Term(TMT6plex); K9(TMT6plex)	1518.9	0	**1.2**	**1.2**	**1.5**	**1.1**
																	LILDLSMK	11	2	1	21264341	N-Term(TMT6plex); M7(Oxidation); K8(TMT6plex)	1406.9	0	**1.2**	**1.2**	**1.4**	**1.0**
																	EFDYVINDLTAVPISTSPEEDSTWEFLR	17	2	1	21264341	N-Term(TMT6plex)	3502.7	0	**1.4**	**1.3**	**1.4**	**1.3**
																	DVLILGGGDGGILCEIVK	22	2	1	21264341	N-Term(TMT6plex); C14(Carbamidomethyl); K18(TMT6plex)	2286.3	0	**1.5**	**1.4**	**1.8**	**1.1**
																	QFGNILILSGDVNLAESDLAYTR	8	2	1	21264341	N-Term(TMT6plex)	2738.4	0	**1.5**	**1.1**	**1.3**	**1.1**
																	GLQSIFQEQGMAESVHTWQDHGYLATYTNK	2	2	1	21264341	N-Term(TMT6plex); K30(TMT6plex)	3897.9	0	**1.6**	**1.8**	**2.0**	**1.4**
																	KTCGDVLDNLK	2	2	1	21264341	N-Term(TMT6plex); K1(TMT6plex); C3(Carbamidomethyl); K11(TMT6plex)	1950.1	1	**1.2**	**1.3**	**1.7**	**1.0**
																	EIVCVPSYLELWVFYTVWKKAKP	1	2	1	21264341	N-Term(TMT6plex); C4(Carbamidomethyl); K19(TMT6plex); K20(TMT6plex); K22(TMT6plex)	3772.1	2	**1.7**	**1.5**	**2.7**	**0.8**
																	YFTQGNCVNLTEALSLYEEQLGR	6	2	1	21264341	N-Term(TMT6plex); C7(Carbamidomethyl)	2934.4	0	**1.4**	**1.2**	**1.4**	**1.2**
																	ELSQDSTGR	2	1	1	21264341	N-Term(TMT6plex)	1221.6	0	**1.3**	**1.3**	**1.4**	**1.0**
																	YWPTADGR	2	2	1	21264341	N-Term(TMT6plex)	1194.6	0	**1.5**	**1.4**	**1.6**	**1.3**
																	EEIDSILNKVEER	1	1	1	21264341	N-Term(TMT6plex); K9(TMT6plex)	2032.1	1	**1.8**	**1.8**	**1.8**	**1.7**
																	EDYTGK	2	2	1	21264341	N-Term(TMT6plex); K6(TMT6plex)	1170.6	0	**1.3**	**1.3**	**1.6**	**1.0**
																	AIMGSGK	1	2	1	21264341	N-Term(TMT6plex); M3(Oxidation); K7(TMT6plex)	1137.7	0	**1.6**	**1.8**	**2.5**	**1.3**
5453740;740087125	NP_006462.1	**MYL12A**	10627	**myosin regulatory light chain 12A**	84.21	5	1	13	398	**1.9**	**2.4**	**2.4**	**1.8**	171	19.8	4.81												
																	ATSNVFAMFDQSQIQEFK	62	5	3	5453740;740087125;222144324;15809016;29568111	N-Term(TMT6plex); M8(Oxidation); K18(TMT6plex)	2565.3	0				
																	ATSNVFAMFDQSQIQEFK	40	5	3	5453740;740087125;222144324;15809016;29568111	N-Term(TMT6plex); K18(TMT6plex)	2549.3	0				
																	FTDEEVDELYR	46	2	2	5453740;740087125;222144324;15809016	N-Term(TMT6plex)	1644.8	0				
																	NPTDEYLDAMMNEAPGPINFTMFLTMFGEK	26	2	1	5453740;740087125	N-Term(TMT6plex); M10(Oxidation); M11(Oxidation); M22(Oxidation); M26(Oxidation); K30(TMT6plex)	3946.8	0	**2.0**	**2.2**	**2.3**	**1.7**
																	NPTDEYLDAMMNEAPGPINFTMFLTMFGEK	25	2	1	5453740;740087125	N-Term(TMT6plex); M10(Oxidation); M11(Oxidation); M26(Oxidation); K30(TMT6plex)	3930.8	0	**1.8**	**2.1**	**2.1**	**1.8**
																	ELLTTMGDR	23	5	3	5453740;740087125;222144324;15809016;29568111	N-Term(TMT6plex); M6(Oxidation)	1280.7	0				
																	EDLHDMLASLGK	2	3	3	5453740;740087125;222144324;15809016;29568111	N-Term(TMT6plex); M6(Oxidation); K12(TMT6plex)	1803.0	0				
																	EAFNMIDQNR	12	7	3	5453740;740087125;222144324;15809016;29568111	N-Term(TMT6plex)	1466.7	0				
																	EAFNMIDQNR	65	7	3	5453740;740087125;222144324;15809016;29568111	N-Term(TMT6plex); M5(Oxidation)	1482.7	0				
																	GNFNYIEFTR	21	2	2	5453740;740087125;222144324;15809016	N-Term(TMT6plex)	1489.8	0				
																	DGFIDKEDLHDMLASLGK	24	3	3	5453740;740087125;222144324;15809016;29568111	N-Term(TMT6plex); K6(TMT6plex); M12(Oxidation); K18(TMT6plex)	2707.4	1				
																	LNGTDPEDVIR	5	6	3	5453740;740087125;222144324;15809016;29568111	N-Term(TMT6plex)	1457.8	0				
																	NPTDEYLDAMMNEAPGPINFTMFLTMFGEK	12	2	1	5453740;740087125	N-Term(TMT6plex); M10(Oxidation); M11(Oxidation); K30(TMT6plex)	3914.8	0	**2.2**	**3.9**	**3.9**	**2.5**
																	DGFIDKEDLHDMLASLGK	19	3	3	5453740;740087125;222144324;15809016;29568111	N-Term(TMT6plex); K6(TMT6plex); K18(TMT6plex)	2691.5	1				
																	KGNFNYIEFTR	1	2	2	5453740;740087125;222144324;15809016	N-Term(TMT6plex); K1(TMT6plex)	1847.0	1				
																	ELLTTMGDR	4	5	3	5453740;740087125;222144324;15809016;29568111	N-Term(TMT6plex)	1264.7	0				
																	EDLHDMLASLGK	4	3	3	5453740;740087125;222144324;15809016;29568111	N-Term(TMT6plex); K12(TMT6plex)	1787.0	0				
																	NAFACFDEEATGTIQEDYLR	4	5	2	5453740;740087125;222144324;15809016	N-Term(TMT6plex); C5(Carbamidomethyl)	2579.2	0				
																	EAPIDKK	2	4	3	5453740;740087125;222144324;15809016;29568111	N-Term(TMT6plex); K6(TMT6plex); K7(TMT6plex)	1487.9	1				
																	DGFIDK	1	4	3	5453740;740087125;222144324;15809016;29568111	N-Term(TMT6plex); K6(TMT6plex)	1152.7	0				
4507949	NP_003395.1	**YWHAB**	7529	**14-3-3 protein beta/alpha**	84.15	1	10	21	1324	**1.0**	**1.0**	**1.0**	**1.2**	246	28.1	4.83												
																	YLSEVASGDNK	101	1	1	4507949	N-Term(TMT6plex); K11(TMT6plex)	1640.9	0	**1.1**	**1.1**	**1.1**	**1.2**
																	TAFDEAIAELDTLNEESYK	205	1	1	4507949	N-Term(TMT6plex); K19(TMT6plex)	2617.3	0	**1.1**	**1.1**	**1.1**	**1.2**
																	DSTLIMQLLR	298	7	7	21735625;208973244;4507951;5454052;5803227;21464101;4507949;5803225	N-Term(TMT6plex); M6(Oxidation)	1434.8	0				
																	KEMQPTHPIR	12	3	3	21735625;208973244;5803227;4507949	N-Term(TMT6plex); K1(TMT6plex); M3(Oxidation)	1711.0	1				
																	EMQPTHPIR	15	3	3	21735625;208973244;5803227;4507949	N-Term(TMT6plex); M2(Oxidation)	1353.7	0				
																	NLLSVAYK	199	7	7	21735625;208973244;4507951;5454052;5803227;21464101;4507949;5803225	N-Term(TMT6plex); K8(TMT6plex)	1365.9	0				
																	LAEQAER	18	7	7	21735625;208973244;4507951;5454052;5803227;21464101;4507949;5803225	N-Term(TMT6plex)	1045.6	0				
																	YLIPNATQPESK	60	1	1	4507949	N-Term(TMT6plex); K12(TMT6plex)	1819.0	0	**1.0**	**1.1**	**1.1**	**1.3**
																	AVTEQGHELSNEER	22	1	1	4507949	N-Term(TMT6plex)	1827.9	0	**1.1**	**1.0**	**1.0**	**1.3**
																	QTTVSNSQQAYQEAFEISK	57	1	1	4507949	N-Term(TMT6plex); K19(TMT6plex)	2617.4	0	**1.1**	**1.1**	**1.1**	**1.2**
																	DSTLIMQLLR	41	7	7	21735625;208973244;4507951;5454052;5803227;21464101;4507949;5803225	N-Term(TMT6plex)	1418.8	0				
																	VISSIEQK	57	5	5	4507951;5803227;21464101;4507949;5454052	N-Term(TMT6plex); K8(TMT6plex)	1361.8	0				
																	LGLALNFSVFYYEILNSPEK	11	2	2	21735625;208973244;4507949	N-Term(TMT6plex); K20(TMT6plex)	2775.5	0				
																	DNLTLWTSENQGDEGDAGEGEN	80	1	1	4507949	N-Term(TMT6plex)	2580.1	0	**1.2**	**1.2**	**1.2**	**1.4**
																	IEAELQDICNDVLELLDK	54	1	1	4507949	N-Term(TMT6plex); C9(Carbamidomethyl); K18(TMT6plex)	2588.4	0	**1.4**	**1.2**	**1.5**	**1.3**
																	TAFDEAIAELDTLNEESYKDSTLIMQLLR	24	1	1	4507949	N-Term(TMT6plex); K19(TMT6plex); M25(Oxidation)	3804.0	1	**1.3**	**1.6**	**1.3**	**1.9**
																	YDDMAAAMK	26	2	2	21464101;4507949	N-Term(TMT6plex); M4(Oxidation); M8(Oxidation); K9(TMT6plex)	1505.7	0				

Khan et al., 2019. Multi-omics analysis to characterize cigarette smoke induced molecular alterations in esophageal cells
Supplementary Table 6. List of all proteins and peptides identified in untreated and chronically treated Het1A cells with cigarette smoke condensate for 8 months

Accession	RefSeq Accession	Gene Symbol	Entrez Gene ID	Description	Σ Coverage	Σ# Proteins	Σ# Unique Peptides	Σ# Peptides	Σ# PSMs	Het-1A-Smoke - 2M/Parental	Het-1A-Smoke - 4M/Parental	Het-1A-Smoke - 6M/Parental	Het-1A-Smoke - 8M/Parental	# AAs	MW [kDa]	calc. pI	Sequence	# PSMs	# Proteins	# Protein Groups	Protein Group Accessions	Modifications	MH+ [Da]	# Missed Cleavages	Het-1A-Smoke - 2M/Parental	Het-1A-Smoke - 4M/Parental	Het-1A-Smoke - 6M/Parental	Het-1A-Smoke - 8M/Parental
																	EMQPTHPIRLGLALNFSVFYYEILNSPEK	2	2	2	21735625;208973244;4507949	N-Term(TMT6plex); M2(Oxidation); K29(TMT6plex)	3881.1	1				
																	YDDMAAAMK	20	2	2	21464101;4507949	N-Term(TMT6plex); M8(Oxidation); K9(TMT6plex)	1489.7	0				
																	YDDMAAAMK	2	2	2	21464101;4507949	N-Term(TMT6plex); K9(TMT6plex)	1473.7	0				
																	EMQPTHPIR	2	3	3	21735625;208973244;5803227;4507949	N-Term(TMT6plex)	1337.7	0				
																	EKIEAELQDICNDVLELLDK	2	1	1	4507949	N-Term(TMT6plex); K2(TMT6plex); C11(Carbamidomethyl); K20(TMT6plex)	3074.7	1	1.5	1.2	2.4	0.7
																	NVVGAR	7	4	4	21735625;208973244;4507951;21464101;4507949	N-Term(TMT6plex)	844.5	0				
																	ACSLAK	3	2	2	21735625;208973244;4507949	N-Term(TMT6plex); C2(Carbamidomethyl); K6(TMT6plex)	1107.7	0				
																	SELVQK	6	1	1	4507949	N-Term(TMT6plex); K6(TMT6plex)	1161.7	0	0.9	1.0	1.0	1.2
4758988	NP_004152.1	**RAB1A**	5861	**ras-related protein Rab-1A isoform 1**	83.90	12	7	17	358	1.4	1.4	1.4	1.5	205	22.7	6.21												
																	YASENVNK	22	2	2	4758988;13569962	N-Term(TMT6plex); K8(TMT6plex)	1382.8	0				
																	NATNVEQSFMTMAAEIK	20	2	1	4758988	N-Term(TMT6plex); M10(Oxidation); M12(Oxidation); K17(TMT6plex)	2375.2	0	1.4	1.3	1.3	1.4
																	MGPGATAGGAEK	12	2	1	4758988	N-Term(TMT6plex); M1(Oxidation); K12(TMT6plex)	1520.8	0	1.6	1.4	1.4	1.5
																	EFADSLGIPFLETSAK	77	2	2	4758988;13569962	N-Term(TMT6plex); K16(TMT6plex)	2183.2	0				
																	KVVDYTTAK	7	1	1	4758988	N-Term(TMT6plex); K1(TMT6plex); K9(TMT6plex)	1712.1	1	1.1	1.5	1.4	1.6
																	FADDTYTESYISTIGVDFK	142	3	2	4758988;13569962	N-Term(TMT6plex); K19(TMT6plex)	2630.3	0				
																	QWLQEIDR	17	2	2	4758988;13569962	N-Term(TMT6plex)	1316.7	0				
																	GAHGIIVVYDVTDQESFNNVK	7	1	1	4758988	N-Term(TMT6plex); K21(TMT6plex)	2763.5	0	1.4	1.5	1.5	1.5
																	MGPGATAGGAEK	2	2	1	4758988	N-Term(TMT6plex); K12(TMT6plex)	1504.8	0	2.0	2.2	2.3	1.8
																	TIELDGK	15	5	4	4758988;13569962;7706563;16933567	N-Term(TMT6plex); K7(TMT6plex)	1233.7	0				
																	LQIWDTAGQER	4	31	17	5803135;4758988;13569962;7706563;16933567;256222019;82659107;19923483;19923750;4759000;39930371;38679888;4506367;96975097;106507164;19923260;325910899	N-Term(TMT6plex)	1545.8	0				
																	LLLIGDSGVGK	14	10	7	5803135;4758988;13569962;7706563;16933567;256222019;4506363	N-Term(TMT6plex); K11(TMT6plex)	1530.0	0				
																	VVDYTTAK	6	1	1	4758988	N-Term(TMT6plex); K8(TMT6plex)	1354.8	0	1.3	1.7	1.3	2.1
																	NATNVEQSFMTMAAEIK	4	2	1	4758988	N-Term(TMT6plex); M12(Oxidation); K17(TMT6plex)	2359.2	0	1.3	1.4	1.5	1.5
																	TITSSYYR	2	2	2	4758988;13569962	N-Term(TMT6plex)	1219.7	0				
																	IQSTPVK	3	2	1	4758988	N-Term(TMT6plex); K7(TMT6plex)	1230.8	0	1.2	1.1	1.3	1.3
																	LLVGNK	1	5	3	4758988;13569962;5803135	N-Term(TMT6plex); K6(TMT6plex)	1101.7	0				
																	SCLLLR	1	3	2	4758988;13569962	N-Term(TMT6plex); C2(Carbamidomethyl)	990.6	0				
																	CDLTTK	2	1	1	4758988	N-Term(TMT6plex); C1(Carbamidomethyl); K6(TMT6plex)	1195.7	0	1.5	1.1	1.5	1.1
527498279	NP_001268425.1	**PHB**	5245	**prohibitin isoform 1**	83.82	2	18	18	245	1.6	1.7	1.8	1.7	272	29.8	5.76												
																	AAELIANSLATAGDGLIELR	54	1	1	527498279;528281407	N-Term(TMT6plex)	2227.3	0	1.6	1.5	1.6	1.6
																	NITYLPAGQSVLLQLPQ	27	1	1	527498279;528281407	N-Term(TMT6plex)	2084.2	0	1.4	1.4	1.4	1.4
																	NVPVITGSK	12	2	1	527498279;528281407	N-Term(TMT6plex); K9(TMT6plex)	1372.9	0	1.6	1.6	1.7	1.6
																	FDAGELITQR	11	1	1	527498279;528281407	N-Term(TMT6plex)	1378.8	0	1.6	1.5	1.6	1.6
																	GVQDIVVGEGTHFLIPWVQKPIIFDCR	6	2	1	527498279;528281407	N-Term(TMT6plex); K20(TMT6plex); C26(Carbamidomethyl)	3582.0	0	1.4	2.2	2.1	1.8
																	IFTSIGEDYDER	25	2	1	527498279;528281407	N-Term(TMT6plex)	1673.8	0	1.6	1.5	1.7	1.5
																	AAIISAEGDSK	22	1	1	527498279;528281407	N-Term(TMT6plex); K11(TMT6plex)	1519.9	0	1.9	1.7	1.6	1.6
																	DLQNVNITLR	9	2	1	527498279;528281407	N-Term(TMT6plex)	1414.8	0	1.6	1.6	1.5	1.5
																	VFESIGK	7	2	1	527498279;528281407	N-Term(TMT6plex); K7(TMT6plex)	1237.8	0	1.6	1.6	1.8	1.7
																	VLPSITTEILK	17	2	1	527498279;528281407	N-Term(TMT6plex); K11(TMT6plex)	1672.1	0	1.7	2.0	2.1	2.0
																	FGLALAVAGGVVNSALYNVDAGHR	12	2	1	527498279;528281407	N-Term(TMT6plex)	2600.4	0	1.6	1.5	2.0	1.4
																	QVAQQEAER	4	1	1	527498279;528281407	N-Term(TMT6plex)	1287.7	0	1.8	2.0	1.9	1.9
																	KLEAAEDIAYQLSR	23	1	1	527498279;528281407	N-Term(TMT6plex); K1(TMT6plex)	2065.2	1	1.7	2.0	2.0	1.9
																	ILFRPVASQLPR	2	2	1	527498279;528281407	N-Term(TMT6plex)	1626.0	0	1.8	2.0	2.1	2.2
																	AATFGLILDDVSLTHLTFGK	2	1	1	527498279;528281407	N-Term(TMT6plex); K20(TMT6plex)	2577.5	0	1.5	1.6	2.4	1.6
																	VFESIGKFGLALAVAGGVVNSALYNVDAGHR	2	2	1	527498279;528281407	N-Term(TMT6plex); K7(TMT6plex)	3590.0	1	1.8	1.3	2.6	0.6
																	EFTEAVEAK	8	1	1	527498279;528281407	N-Term(TMT6plex); K9(TMT6plex)	1481.8	0	1.6	1.8	1.9	1.7
																	AVIFDR	2	2	1	527498279;528281407	N-Term(TMT6plex)	949.6	0	1.7	2.0	2.0	2.0
4758516	NP_004485.1	**HDGF**	3068	**hepatoma-derived growth factor isoform a**	83.75	5	25	26	419	1.2	1.5	1.5	1.5	240	26.8	4.73												
																	ASGYQSSQK	22	3	1	4758516	N-Term(TMT6plex); K9(TMT6plex)	1413.8	0	1.1	1.5	1.6	1.4
																	EAENPEGEEK	24	3	1	4758516	N-Term(TMT6plex); K10(TMT6plex)	1589.8	0	1.1	1.6	1.6	1.5
																	SCVEEPEPEPEAAEGDGDK	7	3	1	4758516	N-Term(TMT6plex); C2(Carbamidomethyl); K19(TMT6plex)	2503.2	0	1.3	1.1	1.3	1.0
																	DLFPYEESK	34	3	1	4758516	N-Term(TMT6plex); K9(TMT6plex)	1585.9	0	1.5	1.6	1.6	1.5
																	GPPQEEEEEEDEEEEATKEDAEAPGIR	52	3	1	4758516	N-Term(TMT6plex); K18(TMT6plex)	3500.6	1	1.2	1.6	1.5	1.5
																	GPPQEEEEEEDEEEEATK	16	3	1	4758516	N-Term(TMT6plex); K18(TMT6plex)	2562.2	0	1.2	1.4	1.5	1.5
																	KGFSEGLWEIENNPTVK	19	3	1	4758516	N-Term(TMT6plex); K1(TMT6plex); K17(TMT6plex)	2635.5	1	1.1	1.5	1.5	1.4
																	EAATLEVERPLPMEVEK	14	3	1	4758516	N-Term(TMT6plex); M13(Oxidation); K17(TMT6plex)	2415.3	0	1.2	1.6	1.6	1.5
																	KSCVEEPEPEPEAAEGDGDKK	4	3	1	4758516	K1(TMT6plex); N-Term(TMT6plex); C3(Carbamidomethyl); K20(TMT6plex); K21(TMT6plex)	3217.7	2	1.4	1.5	1.6	1.3
																	GPPQEEEEEEDEEEEATKEDAEAPGIRDHESL	3	3	1	4758516	N-Term(TMT6plex); K18(TMT6plex)	4081.8	2	1.9	2.3	1.9	2.2
																	IDEMPEAAVK	56	1	1	4758516	N-Term(TMT6plex); M4(Oxidation); K10(TMT6plex)	1576.9	0	1.2	1.4	1.4	1.5
																	NSTPSEPGSGR	6	3	1	4758516	N-Term(TMT6plex)	1317.7	0	1.4	1.1	1.5	1.0
																	GYPHWPAR	32	6	5	48255931;7705320;190014586;4758516;190014588;19923653	N-Term(TMT6plex)	1212.6	0				
																	GNAEGSSDEEGKLVIDEPAK	1	3	1	4758516	N-Term(TMT6plex); K12(TMT6plex); K20(TMT6plex)	2732.5	1	1.0	1.5	1.3	1.5
																	YQVFFFGTHETAFLGPK	8	3	1	4758516	N-Term(TMT6plex); K17(TMT6plex)	2447.3	0	1.2	1.5	1.7	1.4
																	GFSEGLWEIENNPTVK	24	5	1	4758516	N-Term(TMT6plex); K16(TMT6plex)	2278.2	0	1.2	1.4	1.5	1.5
																	LVIDEPAK	8	3	1	4758516	N-Term(TMT6plex); K8(TMT6plex)	1342.8	0	1.0	1.3	1.4	1.3
																	LVIDEPAKEK	2	3	1	4758516	N-Term(TMT6plex); K8(TMT6plex); K10(TMT6plex)	1829.1	1	1.0	1.5	1.5	1.5
																	SCVEEPEPEPEAAEGDGDKK	47	5	1	4758516	N-Term(TMT6plex); C2(Carbamidomethyl); K19(TMT6plex); K20(TMT6plex)	2860.4	1	1.4	1.6	1.8	1.4
																	EDAEAPGIR	6	3	1	4758516	N-Term(TMT6plex)	1186.6	0	1.3	1.5	1.5	1.5
																	EAENPEGEEKEAATLEVERPLPMEVEK	1	3	1	4758516	N-Term(TMT6plex); K10(TMT6plex); K27(TMT6plex)	3740.9	1	1.6	2.3	2.6	1.6
																	CGDLVFAK	7	1	1	4758516	N-Term(TMT6plex); C1(Carbamidomethyl); K8(TMT6plex)	1367.8	0	1.3	1.2	1.5	1.1
																	AGDLLEDSPK	6	3	1	4758516	N-Term(TMT6plex); K10(TMT6plex)	1502.8	0	1.5	2.0	2.1	1.8
																	IDEMPEAAVK	4	1	1	4758516	N-Term(TMT6plex); K10(TMT6plex)	1560.9	0	1.5	1.9	2.2	1.6
																	KGNAEGSSDEEGK	6	3	1	4758516	N-Term(TMT6plex); K1(TMT6plex); K13(TMT6plex)	1995.1	1	0.7	1.0	1.2	1.1
																	GNAEGSSDEEGK	4	3	1	4758516	N-Term(TMT6plex); K12(TMT6plex)	1637.8	0	0.7	1.1	1.0	1.1
																	KSCVEEPEPEPEAAEGDGDK	6	3	1	4758516	N-Term(TMT6plex); K1(TMT6plex); C3(Carbamidomethyl); K20(TMT6plex)	2860.4	1	1.4	1.6	1.6	1.4
222144324;15809016	NP_001138416.1	**MYL12B**	103910	**myosin regulatory light chain 12B**	83.72	4	1	13	397	1.6	1.6	1.7	1.7	172	19.8	4.84												
																	ATSNVFAMFDQSQIQEFK	62	5	3	5453740;740087125;222144324;15809016;29568111	N-Term(TMT6plex); M8(Oxidation); K18(TMT6plex)	2565.3	0				
																	ATSNVFAMFDQSQIQEFK	40	5	3	5453740;740087125;222144324;15809016;29568111	N-Term(TMT6plex); K18(TMT6plex)	2549.3	0				
																	FTDEEVDELYR	46	2	2	5453740;740087125;222144324;15809016	N-Term(TMT6plex)	1644.8	0				
																	NPTDAYLDAMMNEAPGPINFTMFLTMFGEK	29	1	1	222144324;15809016	N-Term(TMT6plex); M10(Oxidation); M11(Oxidation); M22(Oxidation); M26(Oxidation); K30(TMT6plex)	3888.8	0	1.5	1.7	1.7	1.8
																	ELLTTMGDR	23	5	3	5453740;740087125;222144324;15809016;29568111	N-Term(TMT6plex); M6(Oxidation)	1280.7	0				
																	EDLHDMLASLGK	2	3	3	5453740;740087125;222144324;15809016;29568111	N-Term(TMT6plex); M6(Oxidation); K12(TMT6plex)	1803.0	0				
																	NPTDAYLDAMMNEAPGPINFTMFLTMFGEK	24	1	1	222144324;15809016	N-Term(TMT6plex); M11(Oxidation); M22(Oxidation); M26(Oxidation); K30(TMT6plex)	3872.8	0	1.7	1.6	1.6	1.6
																	EAFNMIDQNR	12	7	3	5453740;740087125;222144324;15809016;29568111	N-Term(TMT6plex)	1466.7	0				
																	EAFNMIDQNR	65	7	3	5453740;740087125;222144324;15809016;29568111	N-Term(TMT6plex); M5(Oxidation)	1482.7	0				
																	GNFNYIEFTR	21	2	2	5453740;740087125;222144324;15809016	N-Term(TMT6plex)	1489.8	0				
																	DGFIDKEDLHDMLASLGK	24	3	3	5453740;740087125;222144324;15809016;29568111	N-Term(TMT6plex); K6(TMT6plex); M12(Oxidation); K18(TMT6plex)	2707.4	1				
																	LNGTDPEDVIR	5	6	3	5453740;740087125;222144324;15809016;29568111	N-Term(TMT6plex)	1457.8	0				
																	DGFIDKEDLHDMLASLGK	19	3	3	5453740;740087125;222144324;15809016;29568111	N-Term(TMT6plex); K6(TMT6plex); K18(TMT6plex)	2691.5	1				
																	NPTDAYLDAMMNEAPGPINFTMFLTMFGEK	9	1	1	222144324;15809016	N-Term(TMT6plex); M11(Oxidation); M22(Oxidation); K30(TMT6plex)	3856.8	0	1.9	1.7	1.9	1.7
																	KGNFNYIEFTR	1	2	2	5453740;740087125;222144324;15809016	N-Term(TMT6plex); K1(TMT6plex)	1847.0	1				
																	ELLTTMGDR	4	5	3	5453740;740087125;222144324;15809016;29568111	N-Term(TMT6plex)	1264.7	0				
																	EDLHDMLASLGK	4	3	3	5453740;740087125;222144324;15809016;29568111	N-Term(TMT6plex); K12(TMT6plex)	1787.0	0				
																	NAFACFDEEATGTIQEDYLR	4	5	2	5453740;740087125;222144324;15809016	N-Term(TMT6plex); C5(Carbamidomethyl)	2579.2	0				
																	EAPIDKK	2	4	3	5453740;740087125;222144324;15809016;29568111	N-Term(TMT6plex); K6(TMT6plex); K7(TMT6plex)	1487.9	1				
																	DGFIDK	1	4	3	5453740;740087125;222144324;15809016;29568111	N-Term(TMT6plex); K6(TMT6plex)	1152.7	0				
29568111	NP_006088.2	**MYL9**	10398	**myosin regulatory light polypeptide 9 isoform a**	83.72	2	4	12	395	1.5	1.5	1.8	1.3	172	19.8	4.92												
																	NPTDEYLEGMMSEAPGPINFTMFLTMFGEK	23	1	1	29568111	N-Term(TMT6plex); M10(Oxidation); M11(Oxidation); M22(Oxidation); M26(Oxidation); K30(TMT6plex)	3919.8	0	1.5	1.4	1.7	1.4
																	ATSNVFAMFDQSQIQEFK	62	5	3	5453740;740087125;222144324;15809016;29568111	N-Term(TMT6plex); M8(Oxidation); K18(TMT6plex)	2565.3	0				
																	ATSNVFAMFDQSQIQEFK	40	5	3	5453740;740087125;222144324;15809016;29568111	N-Term(TMT6plex); K18(TMT6plex)	2549.3	0				

Supplementary Table 6. List of all proteins and peptides identified in untreated and chronically treated Het1A cells with cigarette smoke condensate for 8 months

Accession	RefSeq Accession	Gene Symbol	Entrez Gene ID	Description	Σ Coverage	Σ# Proteins	Σ# Unique Peptides	Σ# Peptides	Σ# PSMs	Het-1A-Smoke - 2M/Parental	Het-1A-Smoke - 4M/Parental	Het-1A-Smoke - 6M/Parental	Het-1A-Smoke - 8M/Parental	# AAs	MW [kDa]	calc. pI	Sequence	# PSMs	# Proteins	# Protein Groups	Protein Group Accessions	Modifications	MH+ [Da]	# Missed Cleavages	Het-1A-Smoke - 2M/Parental	Het-1A-Smoke - 4M/Parental	Het-1A-Smoke - 6M/Parental	Het-1A-Smoke - 8M/Parental
																	NPTDEYLEGMMSEAPGPINFTMFLTMFGEK	25	1	1	29568111	N-Term(TMT6plex); M10(Oxidation); M11(Oxidation); M22(Oxidation); K30(TMT6plex)	3903.8	0	1.5	1.6	1.7	1.4
																	ELLTTMGDR	23	5	3	5453740;740087125;222144324;15809016;29568111	N-Term(TMT6plex); M6(Oxidation)	1280.7	0				
																	EDLHDMLASLGK	2	3	3	5453740;740087125;222144324;15809016;29568111	N-Term(TMT6plex); M6(Oxidation); K12(TMT6plex)	1803.0	0				
																	EAFNMIDQNR	12	7	3	5453740;740087125;222144324;15809016;29568111	N-Term(TMT6plex)	1466.7	0				
																	EAFNMIDQNR	64	7	3	5453740;740087125;222144324;15809016;29568111	N-Term(TMT6plex); M5(Oxidation)	1482.7	0				
																	DGFIDKEDLHDMLASLGK	24	3	3	5453740;740087125;222144324;15809016;29568111	N-Term(TMT6plex); K6(TMT6plex); M12(Oxidation); K18(TMT6plex)	2707.4	1				
																	GNFNYVEFTR	10	2	1	29568111	N-Term(TMT6plex)	1475.7	0	1.4	1.5	1.6	1.3
																	LNGTDPEDVIR	5	6	3	5453740;740087125;222144324;15809016;29568111	N-Term(TMT6plex)	1457.8	0				
																	NPTDEYLEGMMSEAPGPINFTMFLTMFGEK	17	1	1	29568111	N-Term(TMT6plex); M10(Oxidation); M11(Oxidation); K30(TMT6plex)	3887.8	0	1.7	1.8	2.2	1.5
																	DGFIDKEDLHDMLASLGK	19	3	3	5453740;740087125;222144324;15809016;29568111	N-Term(TMT6plex); K6(TMT6plex); K18(TMT6plex)	2691.5	1				
																	NAFACFDEEASGFIHEDHLR	19	1	1	29568111	N-Term(TMT6plex); C5(Carbamidomethyl)	2594.2	0	1.4	1.3	1.4	1.2
																	FTDEEVDEMYR	35	2	1	29568111	N-Term(TMT6plex); M9(Oxidation)	1678.7	0	1.6	1.5	1.7	1.3
																	ELLTTMGDR	4	5	3	5453740;740087125;222144324;15809016;29568111	N-Term(TMT6plex)	1264.7	0				
																	FTDEEVDEMYR	4	2	1	29568111	N-Term(TMT6plex)	1662.8	0	2.0	1.9	2.5	1.3
																	EDLHDMLASLGK	4	3	3	5453740;740087125;222144324;15809016;29568111	N-Term(TMT6plex); K12(TMT6plex)	1787.0	0				
																	EAPIDKK	2	4	3	5453740;740087125;222144324;15809016;29568111	N-Term(TMT6plex); K6(TMT6plex); K7(TMT6plex)	1487.9	1				
																	DGFIDK	1	4	3	5453740;740087125;222144324;15809016;29568111	N-Term(TMT6plex); K6(TMT6plex)	1152.7	0				
6912494	NP_036457.1	**MAPRE1**	22919	**microtubule-associated protein RP/EB family member 1**	83.58	1	16	19	207	**1.3**	**1.2**	**1.3**	**1.4**	268	30.0	5.14												
																	QGQETAVAPSLVAPALNKPK	18	1	1	6912494	N-Term(TMT6plex); K18(TMT6plex); K20(TMT6plex)	2706.6	0	1.1	1.0	1.2	1.3
																	IVDILYATDEGFVIPDEGGPQEEQEEY	67	1	1	6912494	N-Term(TMT6plex)	3284.6	0	1.4	1.4	1.4	1.4
																	ILQAGFK	8	1	1	6912494	N-Term(TMT6plex); K7(TMT6plex)	1234.8	0	1.3	1.0	1.2	1.4
																	LTVEDLEKER	1	1	1	6912494	N-Term(TMT6plex); K8(TMT6plex)	1690.0	1	1.4	1.4	1.2	1.7
																	IEQLCSGAAYCQFMDMLFPGSIALK	6	1	1	6912494	N-Term(TMT6plex); C5(Carbamidomethyl); C11(Carbamidomethyl); M14(Oxidation); M16(Oxidation); K25(TMT6plex)	3340.6	0	1.2	0.9	1.0	1.3
																	IEQLCSGAAYCQFMDMLFPGSIALK	14	1	1	6912494	N-Term(TMT6plex); C5(Carbamidomethyl); C11(Carbamidomethyl); M16(Oxidation); K25(TMT6plex)	3324.7	0	1.4	1.1	1.2	1.3
																	NIELICQENEGENDPVLQR	12	1	1	6912494	N-Term(TMT6plex); C6(Carbamidomethyl)	2499.2	0	1.4	1.5	1.3	1.6
																	LTVEDLEK	17	1	1	6912494	N-Term(TMT6plex); K8(TMT6plex)	1404.8	0	1.3	1.1	1.2	1.5
																	KNPGVGNGDDEAAELMQQVNVLK	5	1	1	6912494	N-Term(TMT6plex); K1(TMT6plex); M16(Oxidation); K23(TMT6plex)	3129.7	1	1.1	1.1	1.1	1.4
																	IEQLCSGAAYCQFMDMLFPGSIALK	6	1	1	6912494	N-Term(TMT6plex); C5(Carbamidomethyl); C11(Carbamidomethyl); K25(TMT6plex)	3308.7	0	1.7	1.2	1.9	1.3
																	HDMLAWINESLQLNLTK	5	1	1	6912494	N-Term(TMT6plex); M3(Oxidation); K17(TMT6plex)	2500.4	0	1.2	1.0	1.1	1.2
																	HDMLAWINESLQLNLTK	2	1	1	6912494	N-Term(TMT6plex); K17(TMT6plex)	2484.4	0	1.2	1.1	1.2	1.1
																	LEHEYIQNFK	5	1	1	6912494	N-Term(TMT6plex); K10(TMT6plex)	1779.0	0	1.2	1.3	1.4	1.6
																	FQDNFEFVQWFK	12	1	1	6912494	N-Term(TMT6plex); K12(TMT6plex)	2093.1	0	1.4	1.1	1.3	1.5
																	DYDPVAAR	5	1	1	6912494	N-Term(TMT6plex)	1135.6	0	1.2	1.3	1.2	1.5
																	KFFDANYDGK	3	2	2	6912494;10800412	N-Term(TMT6plex); K1(TMT6plex); K10(TMT6plex)	1892.1	1				
																	KPLTSSSAAPQRPISTQR	8	1	1	6912494	N-Term(TMT6plex); K1(TMT6plex)	2383.4	0	1.1	1.1	1.2	1.5
																	FFDANYDGK	9	2	2	6912494;10800412	N-Term(TMT6plex); K9(TMT6plex)	1534.8	0				
																	FFDANYDGKDYDPVAAR	1	1	1	6912494	N-Term(TMT6plex); K9(TMT6plex)	2422.2	1	1.4	1.5	1.3	2.0
																	DFYFGK	1	5	2	374081840;6912494	N-Term(TMT6plex); K6(TMT6plex)	1234.7	0				
																	IIPVDK	1	1	1	6912494	N-Term(TMT6plex); K6(TMT6plex)	1142.8	0	1.1	1.0	1.1	1.5
																	TAAAPK	1	1	1	6912494	N-Term(TMT6plex); K6(TMT6plex)	1016.6	0	1.1	1.0	1.2	1.4
13569962	NP_112243.1	**RAB1B**	81876	**ras-related protein Rab-1B**	83.58	11	6	16	325	**1.3**	**1.4**	**1.5**	**1.3**	201	22.2	5.73												
																	VVDNTTAK	2	1	1	13569962	N-Term(TMT6plex); K8(TMT6plex)	1305.8	0	1.2	1.5	1.5	1.3
																	NATNVEQAFMTMAAEIK	12	1	1	13569962	N-Term(TMT6plex); M10(Oxidation); M12(Oxidation); K17(TMT6plex)	2359.2	0	1.3	1.4	1.4	1.3
																	YASENVNK	22	2	2	4758988;13569962	N-Term(TMT6plex); K8(TMT6plex)	1382.8	0				
																	EFADSLGIPFLETSAK	77	2	2	4758988;13569962	N-Term(TMT6plex); K16(TMT6plex)	2183.2	0				
																	FADDTYTESYISTIGVDFK	142	3	2	4758988;13569962	N-Term(TMT6plex); K19(TMT6plex)	2630.3	0				
																	QWLQEIDR	17	2	2	4758988;13569962	N-Term(TMT6plex)	1316.7	0				
																	GAHGIIVVYDVTDQESYANVK	2	1	1	13569962	N-Term(TMT6plex); K21(TMT6plex)	2736.5	0	1.3	1.3	1.4	1.4
																	TIELDGK	15	5	4	4758988;13569962;7706563;16933567	N-Term(TMT6plex); K7(TMT6plex)	1233.7	0				
																	LQIWDTAGQER	4	31	17	5803135;4758988;13569962;7706563;16933567;256222019;82659107;19923483;19923750;4759000;39930371;38679888;4506367;96975097;106507164;19923260;325910899	N-Term(TMT6plex)	1545.8	0				
																	LLLIGDSGVGK	14	10	7	5803135;4758988;13569962;7706563;16933567;256222019;4506363	N-Term(TMT6plex); K11(TMT6plex)	1530.0	0				
																	NATNVEQAFMTMAAEIK	2	1	1	13569962	N-Term(TMT6plex); K17(TMT6plex)	2327.2	0	1.5	1.4	2.1	1.1
																	KVVDNTTAK	6	1	1	13569962	N-Term(TMT6plex); K1(TMT6plex); K9(TMT6plex)	1663.0	1	1.2	1.6	1.7	1.5
																	NATNVEQAFMTMAAEIK	1	1	1	13569962	N-Term(TMT6plex); M12(Oxidation); K17(TMT6plex)	2343.2	0				
																	TITSSYYR	2	2	2	4758988;13569962	N-Term(TMT6plex)	1219.7	0				
																	MGPGAASGGERPNLK	1	1	1	13569962	N-Term(TMT6plex); M1(Oxidation); K15(TMT6plex)	1916.0	0	1.1	1.0	1.2	1.1
																	SDLTTK	4	1	1	13569962	N-Term(TMT6plex); K6(TMT6plex)	1122.7	0	1.3	1.3	1.5	1.3
																	LLVGNK	1	5	3	4758988;13569962;5803135	N-Term(TMT6plex); K6(TMT6plex)	1101.7	0				
																	SCLLLR	1	3	2	4758988;13569962	N-Term(TMT6plex); C2(Carbamidomethyl)	990.6	0				
4505763	NP_000282.1	**PGK1**	5230	**phosphoglycerate kinase 1**	83.45	1	27	34	723	**1.1**	**1.1**	**1.0**	**1.0**	417	44.6	8.10												
																	TGQATVASGIPAGWMGLDCGPESSK	19	1	1	4505763	N-Term(TMT6plex); M15(Oxidation); C19(Carbamidomethyl); K25(TMT6plex)	2951.5	0	1.3	1.0	1.2	1.2
																	VLNNMEIGTSLFDEEGAK	38	1	1	4505763	N-Term(TMT6plex); M5(Oxidation); K18(TMT6plex)	2441.3	0	1.1	1.0	1.1	0.8
																	VLPGVDALSNI	79	1	1	4505763	N-Term(TMT6plex)	1326.8	0	1.4	1.3	1.4	1.3
																	DCVGPEVEK	26	1	1	4505763	N-Term(TMT6plex); C2(Carbamidomethyl); K9(TMT6plex)	1490.8	0	1.2	1.1	1.0	1.0
																	ITLPVDFVTADKFDENAK	47	1	1	4505763	N-Term(TMT6plex); K12(TMT6plex); K18(TMT6plex)	2710.5	1	1.0	1.1	1.0	0.9
																	FHVEEEGK	39	2	2	4505763;31543397	N-Term(TMT6plex); K8(TMT6plex)	1432.8	0				
																	VDFNVPMK	28	2	2	4505763;31543397	N-Term(TMT6plex); M7(Oxidation); K8(TMT6plex)	1423.8	0				
																	ITLPVDFVTADK	44	1	1	4505763	N-Term(TMT6plex); K12(TMT6plex)	1777.1	0	1.0	1.0	1.1	0.9
																	ACANPAAGSVILLENLR	30	1	1	4505763	N-Term(TMT6plex); C2(Carbamidomethyl)	1998.1	0	1.3	1.3	1.4	1.1
																	AHSSMVGVNLPQK	6	1	1	4505763	N-Term(TMT6plex); K13(TMT6plex)	1826.0	0	1.3	1.4	1.4	1.1
																	ALMDEVVK	35	1	1	4505763	N-Term(TMT6plex); M3(Oxidation); K8(TMT6plex)	1378.8	0	1.2	1.2	1.1	1.0
																	NNQITNNQR	8	1	1	4505763	N-Term(TMT6plex)	1330.7	0	1.1	0.6	0.8	0.5
																	AHSSMVGVNLPQK	22	1	1	4505763	N-Term(TMT6plex); M5(Oxidation); K13(TMT6plex)	1842.0	0	1.1	1.2	1.1	1.1
																	IQLINNMLDK	42	1	1	4505763	N-Term(TMT6plex); M7(Oxidation); K10(TMT6plex)	1676.0	0	1.2	1.2	1.1	1.1
																	LGDVYVNDAFGTAHR	24	2	2	4505763;31543397	N-Term(TMT6plex)	1864.0	0				
																	VNEMIIGGGMAFTFLK	21	1	1	4505763	N-Term(TMT6plex); M4(Oxidation); M10(Oxidation); K16(TMT6plex)	2218.2	0	1.1	1.1	1.2	1.1
																	VSHVSTGGGASLELLEGK	22	2	2	4505763;31543397	N-Term(TMT6plex); K18(TMT6plex)	2199.2	0				
																	IQLINNMLDK	10	1	1	4505763	N-Term(TMT6plex); K10(TMT6plex)	1660.0	0	1.4	1.5	1.4	1.0
																	QIVWNGPVGVFEWEAFAR	29	1	1	4505763	N-Term(TMT6plex)	2334.2	0	1.2	1.1	1.3	1.2
																	VNEMIIGGGMAFTFLK	15	1	1	4505763	N-Term(TMT6plex); M10(Oxidation); K16(TMT6plex)	2202.2	0	1.1	1.1	1.3	1.0
																	VNEMIIGGGMAFTFLK	4	1	1	4505763	N-Term(TMT6plex); K16(TMT6plex)	2186.2	0	1.0	0.9	1.1	1.2
																	VLNNMEIGTSLFDEEGAK	2	1	1	4505763	N-Term(TMT6plex); K18(TMT6plex)	2425.3	0	1.5	1.3	1.7	1.0
																	YSLEPVAVELK	29	1	1	4505763	N-Term(TMT6plex); K11(TMT6plex)	1706.0	0	1.0	0.9	0.9	0.8
																	SVVLMSHLGRPDGVPMPDK	9	1	1	4505763	N-Term(TMT6plex); M5(Oxidation); M16(Oxidation); K19(TMT6plex)	2525.4	0	1.1	1.2	1.1	1.1
																	SVVLMSHLGRPDGVPMPDKYSLEPVAVELK	3	1	1	4505763	N-Term(TMT6plex); M5(Oxidation); M16(Oxidation); K19(TMT6plex); K30(TMT6plex)	3983.2	1	1.0	1.5	0.9	1.6
																	ALESPERPFLAILGGAK	27	1	1	4505763	N-Term(TMT6plex); K17(TMT6plex)	2227.3	0	1.0	1.1	1.0	0.9
																	ELNYFAK	12	1	1	4505763	N-Term(TMT6plex); K7(TMT6plex)	1342.8	0	1.0	1.0	1.0	0.9
																	GCITIIGGGDTATCCAK	8	1	1	4505763	N-Term(TMT6plex); C2(Carbamidomethyl); C14(Carbamidomethyl); C15(Carbamidomethyl); K17(TMT6plex)	2213.1	0	1.1	1.6	1.3	1.1
																	LTLDKLDVK	2	1	1	4505763	N-Term(TMT6plex); K5(TMT6plex); K9(TMT6plex)	1732.1	1	1.0	1.8	1.0	1.4
																	VDFNVPMK	4	2	2	4505763;31543397	N-Term(TMT6plex); K8(TMT6plex)	1407.8	0				
																	SVVLMSHLGRPDGVPMPDK	1	1	1	4505763	N-Term(TMT6plex); M5(Oxidation); K19(TMT6plex)	2509.4	0	1.4	1.3	1.3	1.1
																	KELNYFAK	1	1	1	4505763	N-Term(TMT6plex); K1(TMT6plex); K8(TMT6plex)	1700.0	1	1.1	2.0	1.1	2.4
																	DVLFLK	16	2	2	4505763;31543397	N-Term(TMT6plex); K6(TMT6plex)	1192.8	0				
																	KYAEAVTR	3	1	1	4505763	N-Term(TMT6plex); K1(TMT6plex)	1395.8	1	1.0	1.0	1.0	0.9
																	YAEAVTR	5	1	1	4505763	N-Term(TMT6plex)	1038.6	0	1.1	0.9	0.8	0.8
																	AGGFLMK	2	1	1	4505763	N-Term(TMT6plex); K7(TMT6plex)	1181.7	0	1.2	1.3	1.3	0.9
																	VKAEPAK	1	1	1	4505763	N-Term(TMT6plex); K2(TMT6plex); K7(TMT6plex)	1429.9	1	0.9	1.3	0.9	1.1
																	WNTEDK	2	2	2	4505763;31543397	N-Term(TMT6plex); K6(TMT6plex)	1250.7	0				
																	AAVPSIK	6	1	1	4505763	N-Term(TMT6plex); K7(TMT6plex)	1143.7	0	1.0	1.1	0.9	1.0
																	FDENAK	1	1	1	4505763	N-Term(TMT6plex); K6(TMT6plex)	1181.7	0	1.0	0.8	0.9	0.8
																	IEAFR	1	2	2	4505763;31543397	N-Term(TMT6plex)	864.5	0				
4502205	NP_001651.1	**ARF4**	378	**ADP-ribosylation factor 4**	83.33	7	7	12	302	**1.6**	**1.5**	**1.6**	**1.4**	180	20.5	7.14												
																	ILMVGLDAAGK	35	4	4	4502209;66879662;66879664;4502203;4502205	N-Term(TMT6plex); M3(Oxidation); K11(TMT6plex)	1561.9	0				
																	TWYVQATCATQGTGLYEGLDWLSNELSK	22	1	1	4502205	N-Term(TMT6plex); C8(Carbamidomethyl); K28(TMT6plex)	3649.8	0	1.7	1.4	1.7	1.3

Supplementary Table 6. List of all proteins and peptides identified in untreated and chronically treated Het1A cells with cigarette smoke condensate for 8 months

Accession	RefSeq Accession	Gene Symbol	Entrez Gene ID	Description	Σ Coverage	Σ# Proteins	Σ# Unique Peptides	Σ# Peptides	Σ# PSMs	Het-1A-Smoke - 2M/Parental	Het-1A-Smoke - 4M/Parental	Het-1A-Smoke - 6M/Parental	Het-1A-Smoke - 8M/Parental	# AAs	MW [kDa]	calc. pI	Sequence	# PSMs	# Proteins	# Protein Groups	Protein Group Accessions	Modifications	MH+ [Da]	# Missed Cleavages	Het-1A-Smoke - 2M/Parental	Het-1A-Smoke - 4M/Parental	Het-1A-Smoke - 6M/Parental	Het-1A-Smoke - 8M/Parental
																	MLLVDELR	10	1	1	4502205	N-Term(TMT6plex); M1(Oxidation)	1233.7	0	**1.5**	**1.3**	**1.3**	**1.4**
																	NICFTVWDVGGQDR	25	1	1	4502205	N-Term(TMT6plex); C3(Carbamidomethyl)	1895.9	0	**1.5**	**1.4**	**1.5**	**1.3**
																	DAVLLLFANK	48	1	1	4502205	N-Term(TMT6plex); K10(TMT6plex)	1562.0	0	**1.5**	**1.6**	**1.8**	**1.4**
																	QDLPNAMAISEMTDK	27	1	1	4502205	N-Term(TMT6plex); M7(Oxidation); M12(Oxidation); K15(TMT6plex)	2154.1	0	**1.5**	**1.5**	**1.5**	**1.5**
																	LGEIVTTIPTIGFNVETVEYK	34	4	4	4502209;66879662;66879664;4502203;4502205	N-Term(TMT6plex); K21(TMT6plex)	2781.6	0				
																	ILMVGLDAAGK	12	4	4	4502209;66879662;66879664;4502203;4502205	N-Term(TMT6plex); K11(TMT6plex)	1545.9	0				
																	IQEVADELQK	35	1	1	4502205	N-Term(TMT6plex); K10(TMT6plex)	1630.9	0	**1.6**	**1.5**	**1.6**	**1.5**
																	HYFQNTQGLIFVVDSNDRER	10	4	4	4502209;66879662;66879664;4502203;4502205	N-Term(TMT6plex)	2667.3	1				
																	QDLPNAMAISEMTDK	4	1	1	4502205	N-Term(TMT6plex); M12(Oxidation); K15(TMT6plex)	2138.1	0	**1.6**	**1.7**	**2.0**	**1.3**
																	HYFQNTQGLIFVVDSNDR	17	4	4	4502209;66879662;66879664;4502203;4502205	N-Term(TMT6plex)	2382.2	0				
																	LGLQSLR	14	1	1	4502205	N-Term(TMT6plex)	1015.6	0	**1.6**	**1.4**	**1.6**	**1.3**
																	TTILYK	8	11	5	4502209;66879662;66879664;4502203;4502211;4502205	N-Term(TMT6plex); K6(TMT6plex)	1196.8	0				
																	QDLPNAMAISEMTDK	1	1	1	4502205	N-Term(TMT6plex); K15(TMT6plex)	2122.1	0	**1.4**	**1.2**	**1.4**	**1.3**
20149594;431822403	NP_031381.2	**HSP90AB1**	3326	**heat shock protein HSP 90-beta isoform a**	83.29	4	34	62	3542	**1.3**	**1.4**	**1.4**	**1.4**	724	83.2	5.03												
																	YHTSQSGDEMTSLSEYVSR	115	3	1	20149594;431822403	N-Term(TMT6plex); M10(Oxidation)	2422.1	0	**1.5**	**1.5**	**1.4**	**1.5**
																	LGLGIDEDEVAAEEPNAAVPDEIPPLEGDEDASR	181	3	1	20149594;431822403	N-Term(TMT6plex)	3761.8	0	**1.4**	**1.5**	**1.4**	**1.4**
																	NPDDITQEEYGEFYK	160	3	1	20149594;431822403	N-Term(TMT6plex); K15(TMT6plex)	2306.1	0	**1.3**	**1.4**	**1.4**	**1.5**
																	ADLINNLGTIAK	105	4	2	154146191;20149594;431822403	N-Term(TMT6plex); K12(TMT6plex)	1701.0	0				
																	ELISNASDALDK	175	4	2	4507677;20149594;431822403	N-Term(TMT6plex); K12(TMT6plex)	1734.0	0				
																	EQVANSAFVER	61	3	1	20149594;431822403	N-Term(TMT6plex)	1478.8	0	**1.2**	**1.4**	**1.3**	**1.4**
																	GFEVVYMTEPIDEYCVQQLK	146	3	1	20149594;431822403	N-Term(TMT6plex); M7(Oxidation); C15(Carbamidomethyl); K20(TMT6plex)	2922.5	0	**1.3**	**1.6**	**1.6**	**1.4**
																	SLTNDWEDHLAVK	172	5	2	154146191;20149594;431822403	N-Term(TMT6plex); K13(TMT6plex)	1986.1	0				
																	EGLELPEDEEEK	71	5	2	154146191;20149594;431822403	N-Term(TMT6plex); K12(TMT6plex)	1875.0	0				
																	SIYYITGESK	63	3	1	20149594;431822403	N-Term(TMT6plex); K10(TMT6plex)	1618.9	0	**1.3**	**1.5**	**1.3**	**1.4**
																	HFSVEGQLEFR	373	5	2	154146191;20149594;431822403	N-Term(TMT6plex)	1577.8	0				
																	APFDLFENK	45	3	1	20149594;431822403	N-Term(TMT6plex); K9(TMT6plex)	1538.9	0	**1.2**	**1.5**	**1.4**	**1.5**
																	KHLEINPDHPIVETLR	21	3	1	20149594;431822403	N-Term(TMT6plex); K1(TMT6plex)	2369.4	1	**1.3**	**1.5**	**1.4**	**1.5**
																	VFIMDSCDELIPEYLNFIR	191	3	1	20149594;431822403	N-Term(TMT6plex); M4(Oxidation); C7(Carbamidomethyl)	2619.3	0	**1.4**	**1.5**	**1.5**	**1.4**
																	YESLTDPSK	181	5	2	154146191;20149594;431822403	N-Term(TMT6plex); K9(TMT6plex)	1497.8	0				
																	HSQFIGYPITLYLEK	75	3	1	20149594;431822403	N-Term(TMT6plex); K15(TMT6plex)	2267.3	0	**1.2**	**1.2**	**1.4**	**1.2**
																	HLEINPDHPIVETLR	81	3	1	20149594;431822403	N-Term(TMT6plex)	2012.1	0	**1.3**	**1.3**	**1.3**	**1.3**
																	IDIIPNPQER	86	2	1	20149594;431822403	N-Term(TMT6plex)	1423.8	0	**1.3**	**1.4**	**1.4**	**1.3**
																	EGLELPEDEEEKK	36	5	2	154146191;20149594;431822403	N-Term(TMT6plex); K12(TMT6plex); K13(TMT6plex)	2232.2	1				
																	EDQTEYLEER	70	5	2	154146191;20149594;431822403	N-Term(TMT6plex)	1540.7	0				
																	VFIMDSCDELIPEYLNFIR	66	3	1	20149594;431822403	N-Term(TMT6plex); C7(Carbamidomethyl)	2603.3	0	**1.4**	**1.6**	**1.7**	**1.2**
																	IEDVGSDEEDDSGKDK	12	3	1	20149594;431822403	N-Term(TMT6plex); K14(TMT6plex); K16(TMT6plex)	2425.2	1	**1.4**	**2.0**	**1.8**	**1.6**
																	YIDQEELNK	143	5	2	154146191;20149594;431822403	N-Term(TMT6plex); K9(TMT6plex)	1609.9	0				
																	RAPFDLFENK	4	3	1	20149594;431822403	N-Term(TMT6plex); K10(TMT6plex)	1695.0	1	**1.1**	**1.1**	**1.2**	**1.1**
																	HNDDEQYAWESSAGGSFTVR	222	5	2	154146191;20149594;431822403	N-Term(TMT6plex)	2485.1	0				
																	DNSTMGYMMAK	56	3	1	20149594;431822403	N-Term(TMT6plex); M5(Oxidation); M8(Oxidation); M9(Oxidation); K11(TMT6plex)	1754.8	0	**1.4**	**1.9**	**1.8**	**1.6**
																	TLTLVDTGIGMTK	67	4	2	154146191;20149594;431822403	N-Term(TMT6plex); M11(Oxidation); K13(TMT6plex)	1824.1	0				
																	VILHLKEDQTEYLEER	2	5	2	154146191;20149594;431822403	N-Term(TMT6plex); K6(TMT6plex)	2473.4	1				
																	GVVDSEDLPLNISR	47	7	3	154146191;20149594;431822403;440309857	N-Term(TMT6plex)	1742.9	0				
																	KHSQFIGYPITLYLEK	11	3	1	20149594;431822403	N-Term(TMT6plex); K1(TMT6plex); K16(TMT6plex)	2624.5	1	**1.3**	**1.3**	**1.4**	**1.4**
																	EKYIDQEELNK	1	5	2	154146191;20149594;431822403	N-Term(TMT6plex); K2(TMT6plex); K11(TMT6plex)	2096.2	1				
																	LVSSPCCIVTSTYGWTANMER	33	3	1	20149594;431822403	N-Term(TMT6plex); C6(Carbamidomethyl); C7(Carbamidomethyl); M19(Oxidation)	2677.3	0	**1.4**	**1.6**	**1.5**	**1.3**
																	DLVVLLFETALLSSGFSLEDPQTHSNR	22	3	1	20149594;431822403	N-Term(TMT6plex)	3217.7	0	**2.7**	**2.8**	**3.7**	**5.6**
																	LGIHEDSTNR	48	3	1	20149594;431822403	N-Term(TMT6plex)	1370.7	0	**1.2**	**1.3**	**1.3**	**1.4**
																	AFMEALQAGADISMIGQFGVGFYSAYLVAEK	27	3	2	154146191;20149594;431822403	N-Term(TMT6plex); M3(Oxidation); M14(Oxidation); K31(TMT6plex)	3774.9	0				
																	VVVITK	15	3	1	20149594;431822403	N-Term(TMT6plex); K6(TMT6plex)	1116.8	0	**1.1**	**1.2**	**1.2**	**1.2**
																	SLVSVTK	36	3	1	20149594;431822403	N-Term(TMT6plex); K7(TMT6plex)	1191.8	0	**1.1**	**1.4**	**1.3**	**1.3**
																	ALLFIPR	29	3	1	20149594;431822403	N-Term(TMT6plex)	1058.7	0	**1.2**	**1.2**	**1.1**	**1.2**
																	AEADKNDK	13	5	2	154146191;20149594;431822403	N-Term(TMT6plex); K5(TMT6plex); K8(TMT6plex)	1577.9	1				
																	AFMEALQAGADISMIGQFGVGFYSAYLVAEK	2	3	2	154146191;20149594;431822403	N-Term(TMT6plex); K31(TMT6plex)	3742.9	0				
																	IEDVGSDEEDDSGKDKK	1	3	1	20149594;431822403	N-Term(TMT6plex); K14(TMT6plex); K16(TMT6plex); K17(TMT6plex)	2782.5	2	**1.2**	**1.9**	**1.7**	**1.6**
																	GFEVVYMTEPIDEYCVQQLK	19	3	1	20149594;431822403	N-Term(TMT6plex); C15(Carbamidomethyl); K20(TMT6plex)	2906.5	0	**1.8**	**2.2**	**2.3**	**1.4**
																	PEEVHHGEEEVETFAFQAEIAQLMSLIINTFYSNK	1	3	1	20149594;431822403	N-Term(TMT6plex); M24(Oxidation); K35(TMT6plex)	4525.3	0	**1.0**	**1.1**	**0.9**	**1.5**
																	TLTLVDTGIGMTK	20	4	2	20149594;431822403;154146191	N-Term(TMT6plex); K13(TMT6plex)	1808.1	0				
																	YHTSQSGDEMTSLSEYVSR	11	3	1	20149594;431822403	N-Term(TMT6plex)	2406.1	0	**1.5**	**1.5**	**1.5**	**1.4**
																	ADHGEPIGR	2	3	1	20149594;431822403	N-Term(TMT6plex)	1180.6	0	**1.4**	**1.5**	**1.6**	**1.8**
																	SLTNDWEDHLAVKHFSVEGQLEFR	2	5	2	154146191;20149594;431822403	N-Term(TMT6plex); K13(TMT6plex)	3315.7	1				
																	EMLQQSK	2	5	2	154146191;20149594;431822403	N-Term(TMT6plex); K7(TMT6plex)	1321.8	0				
																	CLELFSELAEDK	4	3	1	20149594;431822403	N-Term(TMT6plex); C1(Carbamidomethyl); K12(TMT6plex)	1912.0	0	**1.3**	**1.1**	**1.2**	**1.2**
																	FYEAFSK	8	3	1	20149594;431822403	N-Term(TMT6plex); K7(TMT6plex)	1349.8	0	**1.2**	**1.3**	**1.3**	**1.2**
																	DNSTMGYMMAK	33	3	1	20149594;431822403	N-Term(TMT6plex); M5(Oxidation); M8(Oxidation); K11(TMT6plex)	1738.8	0	**1.5**	**2.3**	**2.1**	**1.6**
																	YESLTDPSKLDSGK	1	5	2	154146191;20149594;431822403	N-Term(TMT6plex); K9(TMT6plex); K14(TMT6plex)	2227.2	1				
																	PEEVHHGEEEVETFAFQAEIAQLMSLIINTFYSNK	3	3	1	20149594;431822403	N-Term(TMT6plex); K35(TMT6plex)	4509.3	0	**1.3**	**1.2**	**1.6**	**1.2**
																	AFMEALQAGADISMIGQFGVGFYSAYLVAEK	6	3	2	154146191;20149594;431822403	N-Term(TMT6plex); M14(Oxidation); K31(TMT6plex)	3758.9	0				
																	IEDVGSDEEDDSGK	9	3	1	20149594;431822403	N-Term(TMT6plex); K14(TMT6plex)	1952.9	0	**1.4**	**2.0**	**1.9**	**1.7**
																	CLELFSELAEDKENYK	21	3	1	20149594;431822403	N-Term(TMT6plex); C1(Carbamidomethyl); K12(TMT6plex); K16(TMT6plex)	2675.4	1	**1.1**	**0.9**	**0.9**	**0.8**
																	EGLELPEDEEEKKK	11	5	2	154146191;20149594;431822403	N-Term(TMT6plex); K12(TMT6plex); K13(TMT6plex); K14(TMT6plex)	2589.5	2				
																	APFDLFENKK	3	3	1	20149594;431822403	N-Term(TMT6plex); K9(TMT6plex); K10(TMT6plex)	1896.1	1	**1.1**	**1.7**	**1.5**	**1.6**
																	DNSTMGYMMAK	10	3	1	20149594;431822403	N-Term(TMT6plex); M5(Oxidation); K11(TMT6plex)	1722.8	0	**1.9**	**2.7**	**2.9**	**1.7**
																	LVSSPCCIVTSTYGWTANMER	14	3	1	20149594;431822403	N-Term(TMT6plex); C6(Carbamidomethyl); C7(Carbamidomethyl)	2661.3	0	**1.5**	**1.6**	**1.6**	**1.5**
																	CLELFSELAEDKENYKK	9	3	1	20149594;431822403	N-Term(TMT6plex); C1(Carbamidomethyl); K12(TMT6plex); K16(TMT6plex); K17(TMT6plex)	3032.7	2	**1.0**	**1.0**	**0.9**	**0.9**
																	QKAEADKNDK	2	5	2	154146191;20149594;431822403	N-Term(TMT6plex); K2(TMT6plex); K7(TMT6plex); K10(TMT6plex)	2063.2	2				
																	EMLQQSK	10	5	2	154146191;20149594;431822403	N-Term(TMT6plex); M2(Oxidation); K7(TMT6plex)	1337.7	0				
																	EEEDKDDEEKPK	1	3	1	20149594;431822403	N-Term(TMT6plex); K5(TMT6plex); K10(TMT6plex); K12(TMT6plex)	2407.3	1	**0.9**	**1.2**	**1.2**	**1.3**
																	LSELLR	43	8	3	313747584;154146191;20149594;431822403	N-Term(TMT6plex)	959.6	0				
																	QKAEADK	2	5	2	154146191;20149594;431822403	N-Term(TMT6plex); K2(TMT6plex); K7(TMT6plex)	1476.9	1				
																	VILHLK	7	5	2	154146191;20149594;431822403	N-Term(TMT6plex); K6(TMT6plex)	1180.8	0				
																	GEKEEEDKDDEEKPK	3	3	1	20149594;431822403	N-Term(TMT6plex); K3(TMT6plex); K8(TMT6plex); K13(TMT6plex); K15(TMT6plex)	2950.6	2	**1.2**	**1.6**	**1.4**	**1.6**
																	FENLCK	11	5	2	154146191;20149594;431822403	N-Term(TMT6plex); C5(Carbamidomethyl); K6(TMT6plex)	1268.7	0				
																	TKPIWTR	1	5	2	154146191;20149594;431822403	N-Term(TMT6plex); K2(TMT6plex)	1359.8	0				
																	VTISNR	5	3	1	20149594;431822403	N-Term(TMT6plex)	918.6	0	**1.2**	**1.3**	**1.3**	**1.3**
																	EIFLR	2	6	3	4507677;154146191;20149594;431822403	N-Term(TMT6plex)	906.6	0				
																	KNIVK	1	4	2	20149594;431822403;154146191	N-Term(TMT6plex); K1(TMT6plex); K5(TMT6plex)	1288.9	1				
154146191	NP_005339.3	**HSP90AA1**	3320	**heat shock protein HSP 90-alpha isoform 2**	83.20	2	40	67	4479	**1.2**	**1.1**	**1.2**	**1.3**	732	84.6	5.02												
																	LGLGIDEDDPTADDTSAAVTEEMPPLEGDDDTSR	140	2	1	154146191	N-Term(TMT6plex); M23(Oxidation)	3792.7	0	**1.4**	**1.3**	**1.3**	**1.4**
																	DQVANSAFVER	110	2	1	154146191	N-Term(TMT6plex)	1464.8	0	**1.2**	**1.2**	**1.1**	**1.3**
																	ADLINNLGTIAK	105	4	2	154146191;20149594;431822403	N-Term(TMT6plex); K12(TMT6plex)	1701.0	0				
																	LGLGIDEDDPTADDTSAAVTEEMPPLEGDDDTSR	28	2	1	154146191	N-Term(TMT6plex)	3776.7	0	**1.4**	**1.4**	**1.5**	**1.5**
																	SLTNDWEDHLAVK	172	5	2	154146191;20149594;431822403	N-Term(TMT6plex); K13(TMT6plex)	1986.1	0				
																	NPDDITNEEYGEFYK	237	2	1	154146191	N-Term(TMT6plex); K15(TMT6plex)	2292.1	0	**1.1**	**1.1**	**1.0**	**1.3**
																	EGLELPEDEEEK	71	5	2	154146191;20149594;431822403	N-Term(TMT6plex); K12(TMT6plex)	1875.0	0				
																	HFSVEGQLEFR	373	5	2	154146191;20149594;431822403	N-Term(TMT6plex)	1577.8	0				
																	TLTIVDTGIGMTK	189	4	2	154146191;20149594;431822403	N-Term(TMT6plex); M11(Oxidation); K13(TMT6plex)	1824.1	0				
																	LVTSPCCIVTSTYGWTANMER	82	2	1	154146191	N-Term(TMT6plex); C6(Carbamidomethyl); C7(Carbamidomethyl); M19(Oxidation)	2691.3	0	**1.3**	**1.3**	**1.3**	**1.3**
																	YESLTDPSK	181	5	2	154146191;20149594;431822403	N-Term(TMT6plex); K9(TMT6plex)	1497.8	0				

Supplementary Table 6. List of all proteins and peptides identified in untreated and chronically treated Het1A cells with cigarette smoke condensate for 8 months

Accession	RefSeq Accession	Gene Symbol	Entrez Gene ID	Description	Σ Coverage	Σ# Proteins	Σ# Unique Peptides	Σ# Peptides	Σ# PSMs	Het-1A-Smoke - 2M/Parental	Het-1A-Smoke - 4M/Parental	Het-1A-Smoke - 6M/Parental	Het-1A-Smoke - 8M/Parental	# AAs	MW [kDa]	calc. pI	Sequence	# PSMs	# Proteins	# Protein Groups	Protein Group Accessions	Modifications	MH+ [Da]	# Missed Cleavages	Het-1A-Smoke - 2M/Parental	Het-1A-Smoke - 4M/Parental	Het-1A-Smoke - 6M/Parental	Het-1A-Smoke - 8M/Parental
																	YYTSASGDEMVSLK	312	2	1	154146191	N-Term(TMT6plex); M10(Oxidation); K14(TMT6plex)	2025.0	0	**1.1**	**1.0**	**1.1**	**1.1**
																	ELHINLIPNK	63	2	1	154146191	N-Term(TMT6plex); K10(TMT6plex)	1649.0	0	**1.0**	**1.1**	**1.1**	**1.3**
																	EGLELPEDEEEKK	36	5	2	154146191;20149594;431822403	N-Term(TMT6plex); K12(TMT6plex); K13(TMT6plex)	2232.2	1				
																	FYEQFSK	63	2	1	154146191	N-Term(TMT6plex); K7(TMT6plex)	1406.8	0	**1.1**	**0.9**	**1.0**	**1.1**
																	EDQTEYLEER	70	5	2	154146191;20149594;431822403	N-Term(TMT6plex)	1540.7	0				
																	ELISNSSDALDK	116	2	1	154146191	N-Term(TMT6plex); K12(TMT6plex)	1750.0	0	**1.2**	**1.3**	**1.2**	**1.4**
																	HIYYITGETK	106	2	1	154146191	N-Term(TMT6plex); K10(TMT6plex)	1683.0	0	**1.1**	**1.2**	**1.2**	**1.3**
																	VFIMDNCEELIPEYLNFIR	320	2	1	154146191	N-Term(TMT6plex); M4(Oxidation); C7(Carbamidomethyl)	2660.3	0	**1.4**	**1.3**	**1.4**	**1.4**
																	YIDQEELNK	143	5	2	154146191;20149594;431822403	N-Term(TMT6plex); K9(TMT6plex)	1609.9	0				
																	TDTGEPMGR	19	2	1	154146191	N-Term(TMT6plex); M7(Oxidation)	1208.6	0	**1.2**	**0.8**	**1.1**	**0.9**
																	KHGLEVIYMIEPIDEYCVQQLK	16	2	1	154146191	N-Term(TMT6plex); K1(TMT6plex); C17(Carbamidomethyl); K22(TMT6plex)	3392.8	1	**1.3**	**1.5**	**1.5**	**1.4**
																	HNDDEQYAWESSAGGSFTVR	222	5	2	154146191;20149594;431822403	N-Term(TMT6plex)	2485.1	0				
																	ELHINLIPNKQDR	10	2	1	154146191	N-Term(TMT6plex); K10(TMT6plex)	2048.2	1	**1.1**	**1.5**	**1.2**	**1.9**
																	VILHLKEDQTEYLEER	2	5	2	154146191;20149594;431822403	N-Term(TMT6plex); K6(TMT6plex)	2473.4	1				
																	GVVDSEDLPLNISR	47	7	3	154146191;20149594;431822403;440309857	N-Term(TMT6plex)	1742.9	0				
																	EKYIDQEELNK	1	5	2	154146191;20149594;431822403	N-Term(TMT6plex); K2(TMT6plex); K11(TMT6plex)	2096.2	1				
																	ALLFVPR	36	3	2	11559925;154146191	N-Term(TMT6plex)	1044.7	0				
																	DNSTMGYMAAK	39	2	1	154146191	N-Term(TMT6plex); M5(Oxidation); K11(TMT6plex)	1662.8	0	**1.4**	**1.7**	**1.7**	**1.4**
																	LGIHEDSQNR	50	2	1	154146191	N-Term(TMT6plex)	1397.7	0	**1.2**	**1.2**	**1.1**	**1.3**
																	DLVILLYETALLSSGFSLEDPQTHAN R	53	2	1	154146191	N-Term(TMT6plex)	3231.7	0	**1.8**	**2.3**	**1.9**	**1.6**
																	HSQFIGYPITLFVEK	167	2	1	154146191	N-Term(TMT6plex); K15(TMT6plex)	2237.3	0	**1.1**	**1.0**	**1.2**	**1.2**
																	DNSTMGYMAAK	89	2	1	154146191	N-Term(TMT6plex); M5(Oxidation); M8(Oxidation); K11(TMT6plex)	1678.8	0	**1.2**	**1.3**	**1.4**	**1.4**
																	AFMEALQAGADISMIGQFGVGFYSA YLVAEK	27	3	2	154146191;20149594;431822403	N-Term(TMT6plex); M3(Oxidation); M14(Oxidation); K31(TMT6plex)	3774.9	0				
																	AEADKNDK	13	5	2	154146191;20149594;431822403	N-Term(TMT6plex); K5(TMT6plex); K8(TMT6plex)	1577.9	1				
																	AFMEALQAGADISMIGQFGVGFYSA YLVAEK	2	3	2	154146191;20149594;431822403	N-Term(TMT6plex); K31(TMT6plex)	3742.9	0				
																	HGLEVIYMIEPIDEYCVQQLK	80	2	1	154146191	N-Term(TMT6plex); M8(Oxidation); C16(Carbamidomethyl); K21(TMT6plex)	3051.6	0	**1.1**	**1.0**	**1.1**	**1.2**
																	ESEDKPEIEDVGSDEEEEK	3	2	1	154146191	N-Term(TMT6plex); K5(TMT6plex); K19(TMT6plex)	2880.4	0	**1.1**	**1.3**	**1.3**	**1.7**
																	TLTIVDTGIGMTK	50	4	2	20149594;431822403;154146191	N-Term(TMT6plex); K13(TMT6plex)	1808.1	0				
																	DNSTMGYMAAK	4	2	1	154146191	N-Term(TMT6plex); K11(TMT6plex)	1646.8	0	**1.7**	**1.6**	**1.9**	**1.3**
																	KHSQFIGYPITLFVEK	29	2	1	154146191	N-Term(TMT6plex); K1(TMT6plex); K16(TMT6plex)	2594.5	1	**1.0**	**1.0**	**1.1**	**1.2**
																	LVTSPCCIVTSTYGWTANMER	25	2	1	154146191	N-Term(TMT6plex); C6(Carbamidomethyl); C7(Carbamidomethyl)	2675.3	0	**1.7**	**1.5**	**1.7**	**1.5**
																	YYTSASGDEMVSLK	14	2	1	154146191	N-Term(TMT6plex); K14(TMT6plex)	2009.0	0	**1.2**	**1.1**	**1.3**	**1.1**
																	KCLELFTELAEDKENYK	4	2	1	154146191	K1(TMT6plex); N-Term(TMT6plex); C2(Carbamidomethyl); K13(TMT6plex); K17(TMT6plex)	3046.7	2	**1.0**	**1.2**	**1.0**	**1.2**
																	SLTNDWEDHLAVKHFSVEGQLEFR	2	5	2	154146191;20149594;431822403	N-Term(TMT6plex); K13(TMT6plex)	3315.7	1				
																	EMLQQSK	2	5	2	154146191;20149594;431822403	N-Term(TMT6plex); K7(TMT6plex)	1321.8	0				
																	CLELFTELAEDK	8	2	1	154146191	N-Term(TMT6plex); C1(Carbamidomethyl); K12(TMT6plex)	1926.0	0	**1.0**	**0.6**	**0.8**	**0.8**
																	VFIMDNCEELIPEYLNFIR	160	2	1	154146191	N-Term(TMT6plex); C7(Carbamidomethyl)	2644.3	0	**1.5**	**1.7**	**1.7**	**1.4**
																	TLVSVTK	31	2	1	154146191	N-Term(TMT6plex); K7(TMT6plex)	1205.8	0	**1.1**	**1.0**	**1.1**	**1.2**
																	HLEINPDHSIIETLR	92	2	1	154146191	N-Term(TMT6plex)	2016.1	0	**1.1**	**1.2**	**1.2**	**1.2**
																	YESLTDPSKLDSGK	1	5	2	154146191;20149594;431822403	N-Term(TMT6plex); K9(TMT6plex); K14(TMT6plex)	2227.2	1				
																	KHLEINPDHSIIETLR	31	2	1	154146191	N-Term(TMT6plex); K1(TMT6plex)	2373.4	1	**1.1**	**1.2**	**1.2**	**1.4**
																	KHGLEVIYMIEPIDEYCVQQLK	30	2	1	154146191	N-Term(TMT6plex); K1(TMT6plex); M9(Oxidation); C17(Carbamidomethyl); K22(TMT6plex)	3408.9	1	**1.0**	**1.1**	**1.1**	**1.3**
																	PEETQTQDQPMEEEEVETFAFQAEI AQLMSLIINTFYSNK	1	1	1	154146191	N-Term(TMT6plex); M29(Oxidation); K40(TMT6plex)	5153.5	0	**1.0**	**0.7**	**1.2**	**0.8**
																	AFMEALQAGADISMIGQFGVGFYSA YLVAEK	6	3	2	154146191;20149594;431822403	N-Term(TMT6plex); M14(Oxidation); K31(TMT6plex)	3758.9	0				
																	TKFENLCK	5	2	1	154146191	N-Term(TMT6plex); K2(TMT6plex); C7(Carbamidomethyl); K8(TMT6plex)	1727.0	1	**1.0**	**1.0**	**1.0**	**1.2**
																	CLELFTELAEDKENYK	39	2	1	154146191	N-Term(TMT6plex); C1(Carbamidomethyl); K12(TMT6plex); K16(TMT6plex)	2689.4	1	**1.0**	**0.7**	**0.8**	**0.8**
																	RAPFDLFENR	6	2	1	154146191	N-Term(TMT6plex)	1493.8	1	**1.1**	**1.2**	**1.0**	**1.3**
																	APFDLFENR	23	2	1	154146191	N-Term(TMT6plex)	1337.7	0	**1.3**	**1.2**	**1.2**	**1.3**
																	EGLELPEDEEEKKK	11	5	2	154146191;20149594;431822403	N-Term(TMT6plex); K12(TMT6plex); K13(TMT6plex); K14(TMT6plex)	2589.5	2				
																	CLELFTELAEDKENYKK	11	2	1	154146191	N-Term(TMT6plex); C1(Carbamidomethyl); K12(TMT6plex); K16(TMT6plex); K17(TMT6plex)	3046.7	2	**1.0**	**0.9**	**0.9**	**1.0**
																	KFYEQFSK	2	2	1	154146191	N-Term(TMT6plex); K1(TMT6plex); K8(TMT6plex)	1764.0	1	**1.1**	**1.1**	**1.1**	**1.5**
																	KCLELFTELAEDK	2	2	1	154146191	N-Term(TMT6plex); K1(TMT6plex); C2(Carbamidomethyl); K13(TMT6plex)	2283.3	1	**1.1**	**1.2**	**1.1**	**1.3**
																	LGLGIDEDDPTADDTSAAVTEEMPP LEGDDDTSRMEEVD	4	2	1	154146191	N-Term(TMT6plex); M23(Oxidation); M35(Oxidation)	4411.9	1	**1.0**	**1.5**	**0.8**	**1.6**
																	ESEDKPEIEDVGSDEEEEKKDGDK	1	2	1	154146191	N-Term(TMT6plex); K5(TMT6plex); K19(TMT6plex); K20(TMT6plex); K24(TMT6plex)	3882.0	2	**1.2**	**1.5**	**1.7**	**1.9**
																	QKAEADKNDK	2	5	2	154146191;20149594;431822403	N-Term(TMT6plex); K2(TMT6plex); K7(TMT6plex); K10(TMT6plex)	2063.2	2				
																	ESEDKPEIEDVGSDEEEEKK	1	2	1	154146191	N-Term(TMT6plex); K5(TMT6plex); K19(TMT6plex); K20(TMT6plex)	3237.7	1				
																	EMLQQSK	10	5	2	154146191;20149594;431822403	N-Term(TMT6plex); M2(Oxidation); K7(TMT6plex)	1337.7	0				
																	LSELLR	43	8	3	313747584;154146191;20149594;431822403	N-Term(TMT6plex)	959.6	0				
																	QKAEADK	2	5	2	154146191;20149594;431822403	N-Term(TMT6plex); K2(TMT6plex); K7(TMT6plex)	1476.9	1				
																	VILHLK	7	5	2	154146191;20149594;431822403	N-Term(TMT6plex); K6(TMT6plex)	1180.8	0				
																	KLSELLR	4	2	1	154146191	N-Term(TMT6plex); K1(TMT6plex)	1316.9	1	**1.0**	**0.9**	**0.9**	**1.1**
																	FENLCK	11	5	2	154146191;20149594;431822403	N-Term(TMT6plex); C5(Carbamidomethyl); K6(TMT6plex)	1268.7	0				
																	VTVITK	6	2	1	154146191	N-Term(TMT6plex); K6(TMT6plex)	1118.8	0	**0.9**	**0.9**	**0.9**	**1.2**
																	MKENQK	1	2	1	154146191	N-Term(TMT6plex); K2(TMT6plex); K6(TMT6plex)	1464.9	1	**1.3**	**1.6**	**1.8**	**1.3**
																	IKEIVK	2	2	1	154146191	N-Term(TMT6plex); K2(TMT6plex); K6(TMT6plex)	1417.0	1	**0.9**	**1.3**	**1.0**	**1.6**
																	TKPIWTR	1	5	2	154146191;20149594;431822403	N-Term(TMT6plex); K2(TMT6plex)	1359.8	0				
																	EIFLR	2	6	3	4507677;154146191;20149594;431822403	N-Term(TMT6plex)	906.6	0				
29788785	NP_821133.1	**TUBB**	203068	**tubulin beta chain**	83.11	5	4	26	2671	**1.1**	**1.1**	**1.2**	**1.1**	444	49.6	4.89												
																	AILVDLEPGTMDSVR	141	7	4	29788785;29788768;50592996;4507729	N-Term(TMT6plex); M11(Oxidation)	1861.0	0				
																	ALTVPELTQQVFDAK	332	1	2	29788785;5174735	N-Term(TMT6plex); K15(TMT6plex)	2118.2	0				
																	GHYTEGAELVDSVLDVVR	235	11	5	29788785;5174735;29788768;50592996;4507729	N-Term(TMT6plex)	2188.1	0				
																	MAVTFIGNSTAIQELFK	65	1	1	29788785	N-Term(TMT6plex); M1(Oxidation); K17(TMT6plex)	2344.3	0	**1.1**	**1.1**	**1.2**	**1.2**
																	NSSYFVEWIPNNVK	95	20	7	29788785;5174735;14210536;746815975;29788768;50592996;213 61322;574584816;4507729	N-Term(TMT6plex); K14(TMT6plex)	2155.2	0				
																	SGPFGQIFRPDNFVFGQSGAGNNWA K	124	8	5	29788785;5174735;29788768;21361322;574584816;4507729	N-Term(TMT6plex); K26(TMT6plex)	3256.7	0				
																	EVDEQMLNVQNK	195	9	4	29788785;5174735;29788768;4507729	N-Term(TMT6plex); M6(Oxidation); K12(TMT6plex)	1921.0	0				
																	FWEVISDEHGIDPTGTYHGDSDLQL DR	39	1	1	29788785	N-Term(TMT6plex)	3331.6	0	**1.3**	**1.1**	**1.2**	**1.1**
																	VSDTVVEPYNATLSVHQLVENTDET YCIDNEALYDICFR	50	8	5	29788785;5174735;14210536;746815975;29788768;21361322;574 584816	N-Term(TMT6plex); C27(Carbamidomethyl); C37(Carbamidomethyl)	4822.2	0				
																	IMNTFSVVPSPK	47	8	4	29788785;5174735;50592996;21361322;574584816	N-Term(TMT6plex); M2(Oxidation); K12(TMT6plex)	1794.0	0				
																	LTTPTYGDLNHLVSATMSGVTTCLR	171	12	5	29788785;5174735;29788768;21361322;574584816;4507729	N-Term(TMT6plex); M17(Oxidation); C23(Carbamidomethyl)	2953.5	0				
																	YLTVAAVFR	63	9	3	29788785;5174735;21361322;574584816	N-Term(TMT6plex)	1268.8	0				
																	EIVHIQAGQCGNQIGAK	36	8	6	29788785;29788768;50592996;4507729;5174735;21361322;57458 4816	N-Term(TMT6plex); C10(Carbamidomethyl); K17(TMT6plex)	2281.2	0				
																	LHFFMPGFAPLTSR	71	18	6	29788785;5174735;14210536;746815975;29788768;21361322;574 584816;4507729	N-Term(TMT6plex)	1850.0	0				
																	KLAVNMVPFPR	11	21	8	29788785;5174735;14210536;746815975;29788768;50592996;213 61322;574584816;4507729;13562114	N-Term(TMT6plex); K1(TMT6plex); M6(Oxidation)	1746.0	1				
																	TAVCDIPPR	171	14	5	29788785;5174735;29788768;21361322;574584816;4507729	N-Term(TMT6plex); C4(Carbamidomethyl)	1257.7	0				
																	ISEQFTAMFR	20	10	6	29788785;5174735;29788768;50592996;21361322;574584816;450 7729	N-Term(TMT6plex)	1458.8	0				
																	ISEQFTAMFR	244	10	6	29788785;5174735;29788768;50592996;21361322;574584816;450 7729	N-Term(TMT6plex); M8(Oxidation)	1474.8	0				
																	EAESCDCLQGFQLTHSLGGGTGSGM GTLLISK	32	7	3	29788785;5174735;21361322;574584816	N-Term(TMT6plex); C5(Carbamidomethyl); C7(Carbamidomethyl); M25(Oxidation); K32(TMT6plex)	3785.9	0				
																	MAVTFIGNSTAIQELFK	34	1	1	29788785	N-Term(TMT6plex); K17(TMT6plex)	2328.3	0	**1.4**	**1.4**	**1.7**	**1.2**
																	LTTPTYGDLNHLVSATMSGVTTCLR	35	12	5	29788785;5174735;29788768;21361322;574584816;4507729	N-Term(TMT6plex); C23(Carbamidomethyl)	2937.5	0				
																	FPGQLNADLR	98	13	8	29788785;5174735;14210536;746815975;29788768;50592996;213 61322;574584816;4507729;13562114	N-Term(TMT6plex)	1359.8	0				
																	AILVDLEPGTMDSVR	22	7	4	29788785;29788768;50592996;4507729	N-Term(TMT6plex)	1845.0	0				

Khan et al., 2019. Multi-omics analysis to characterize cigarette smoke induced molecular alterations in esophageal cells
Supplementary Table 6. List of all proteins and peptides identified in untreated and chronically treated Het1A cells with cigarette smoke condensate for 8 months

Accession	RefSeq Accession	Gene Symbol	Entrez Gene ID	Description	Σ Coverage	Σ# Proteins	Σ# Unique Peptides	Σ# Peptides	Σ# PSMs	Het-1A-Smoke - 2M/Parental	Het-1A-Smoke - 4M/Parental	Het-1A-Smoke - 6M/Parental	Het-1A-Smoke - 8M/Parental	# AAs	MW [kDa]	calc. pI	Sequence	# PSMs	# Proteins	# Protein Groups	Protein Group Accessions	Modifications	MH+ [Da]	# Missed Cleavages	Het-1A-Smoke - 2M/Parental	Het-1A-Smoke - 4M/Parental	Het-1A-Smoke - 6M/Parental	Het-1A-Smoke - 8M/Parental
																	LAVNMVPFPR	20	21	8	29788785;5174735;14210536;746815975;29788768;50592996;21361322;574584816;4507729;13562114	N-Term(TMT6plex)	1372.8	0				
																	EVDEQMLNVQNK	12	9	4	29788785;5174735;29788768;4507729	N-Term(TMT6plex); K12(TMT6plex)	1905.0	0				
																	LHFFMPGFAPLTSR	135	18	6	29788785;5174735;14210536;746815975;29788768;21361322;574584816;4507729	N-Term(TMT6plex); M5(Oxidation)	1866.0	0				
																	KEAESCDCLQGFQLTHSLGGGTGSGMGTLLISK	7	7	3	29788785;5174735;21361322;574584816	K1(TMT6plex); N-Term(TMT6plex); C6(Carbamidomethyl); C8(Carbamidomethyl); M26(Oxidation); K33(TMT6plex)	4143.1	1				
																	ISVYYNEATGGK	75	3	1	29788785	N-Term(TMT6plex); K12(TMT6plex)	1760.0	0	**1.0**	**1.0**	**1.1**	**1.1**
																	LAVNMVPFPR	54	21	8	29788785;5174735;14210536;746815975;29788768;50592996;21361322;574584816;4507729;13562114	N-Term(TMT6plex); M5(Oxidation)	1388.8	0				
																	IREEYPDR	9	6	5	29788785;5174735;29788768;4507729;13562114	N-Term(TMT6plex)	1306.7	1				
																	IMNTFSVVPSPK	8	8	4	29788785;5174735;50592996;21361322;574584816	N-Term(TMT6plex); K12(TMT6plex)	1778.0	0				
																	KEAESCDCLQGFQLTHSLGGGTGSGMGTLLISK	1	7	3	29788785;5174735;21361322;574584816	N-Term(TMT6plex); K1(TMT6plex); C6(Carbamidomethyl); C8(Carbamidomethyl); K33(TMT6plex)	4127.1	1				
																	EAESCDCLQGFQLTHSLGGGTGSGMGTLLISK	2	7	3	29788785;5174735;21361322;574584816	N-Term(TMT6plex); C5(Carbamidomethyl); C7(Carbamidomethyl); K32(TMT6plex)	3769.8	0				
																	NMMAACDPR	5	20	7	29788785;5174735;14210536;746815975;29788768;50592996;21361322;574584816;4507729	N-Term(TMT6plex); M2(Oxidation); M3(Oxidation); C6(Carbamidomethyl)	1326.6	0				
																	MAVTFIGNSTAIQELFKR	1	1	1	29788785	N-Term(TMT6plex); M1(Oxidation); K17(TMT6plex)	2500.4	1	**1.3**	**1.1**	**1.2**	**1.2**
																	GSQQYR	5	12	7	29788785;5174735;14210536;746815975;29788768;50592996;21361322;574584816;4507729	N-Term(TMT6plex)	967.5	0				
																	NMMAACDPR	4	20	7	29788785;5174735;14210536;746815975;29788768;50592996;21361322;574584816;4507729	N-Term(TMT6plex); M3(Oxidation); C6(Carbamidomethyl)	1310.6	0				
																	NMMAACDPR	2	20	7	29788785;5174735;14210536;746815975;29788768;50592996;21361322;574584816;4507729	N-Term(TMT6plex); C6(Carbamidomethyl)	1294.6	0				
34147513	NP_004628.4	**RAB7A**	7879	**ras-related protein Rab-7a**	83.09	1	17	17	213	**1.3**	**1.4**	**1.5**	**1.4**	207	23.5	6.70												
																	EAINVEQAFQTIAR	26	1	1	34147513	N-Term(TMT6plex)	1819.0	0	**1.4**	**1.4**	**1.4**	**1.3**
																	ATIGADFLTK	22	1	1	34147513	N-Term(TMT6plex); K10(TMT6plex)	1494.9	0	**1.3**	**1.4**	**1.5**	**1.2**
																	DPENFPFVVLGNK	25	1	1	34147513	N-Term(TMT6plex); K13(TMT6plex)	1934.1	0	**1.2**	**1.4**	**1.4**	**1.4**
																	QETEVELYNEFPEPIK	11	1	1	34147513	N-Term(TMT6plex); K16(TMT6plex)	2423.3	0	**1.4**	**1.4**	**1.5**	**1.5**
																	NALKQETEVELYNEFPEPIK	7	1	1	34147513	N-Term(TMT6plex); K4(TMT6plex); K20(TMT6plex)	3078.7	1	**1.2**	**1.3**	**1.3**	**1.3**
																	LVTMQIWDTAGQER	4	1	1	34147513	N-Term(TMT6plex)	1877.0	0	**1.7**	**1.6**	**1.7**	**1.5**
																	TSLMNQYVNK	2	1	1	34147513	N-Term(TMT6plex); K10(TMT6plex)	1655.9	0	**1.7**	**1.4**	**2.1**	**1.2**
																	TSLMNQYVNK	5	1	1	34147513	N-Term(TMT6plex); M4(Oxidation); K10(TMT6plex)	1671.9	0	**1.5**	**1.2**	**1.6**	**1.2**
																	NNIPYFETSAK	5	1	1	34147513	N-Term(TMT6plex); K11(TMT6plex)	1742.0	0	**1.3**	**1.0**	**1.3**	**1.1**
																	GADCCVLVFDVTAPNTFK	22	1	1	34147513	N-Term(TMT6plex); C4(Carbamidomethyl); C5(Carbamidomethyl); K18(TMT6plex)	2472.3	0	**1.3**	**1.6**	**1.6**	**1.4**
																	VIILGDSGVGK	29	1	1	34147513	N-Term(TMT6plex); K11(TMT6plex)	1516.0	0	**1.3**	**1.5**	**1.5**	**1.4**
																	LVTMQIWDTAGQER	20	1	1	34147513	N-Term(TMT6plex); M4(Oxidation)	1893.0	0	**1.3**	**1.6**	**1.4**	**1.4**
																	TLDSWRDEFLIQASPR	7	1	1	34147513	N-Term(TMT6plex)	2163.1	1	**1.3**	**1.3**	**1.4**	**1.4**
																	DEFLIQASPR	10	1	1	34147513	N-Term(TMT6plex)	1404.8	0	**1.3**	**1.4**	**1.5**	**1.3**
																	FQSLGVAFYR	4	1	1	34147513	N-Term(TMT6plex)	1416.8	0	**1.4**	**1.1**	**1.3**	**1.1**
																	AQAWCYSK	8	1	1	34147513	N-Term(TMT6plex); C5(Carbamidomethyl); K8(TMT6plex)	1471.8	0	**1.3**	**1.5**	**1.5**	**1.4**
																	KFSNQYK	1	1	1	34147513	N-Term(TMT6plex); K1(TMT6plex); K7(TMT6plex)	1602.0	1	**1.4**	**1.6**	**1.6**	**1.5**
																	QETEVELYNEFPEPIKLDK	3	1	1	34147513	N-Term(TMT6plex); K16(TMT6plex); K19(TMT6plex)	3008.7	1				
																	EVMVDDR	2	1	1	34147513	N-Term(TMT6plex); M3(Oxidation)	1108.5	0	**1.3**	**1.4**	**1.4**	**1.5**
10863945	NP_066964.1	**XRCC5**	7520	**X-ray repair cross-complementing protein 5**	83.06	1	50	50	924	**1.3**	**1.3**	**1.4**	**1.3**	732	82.7	5.81												
																	DQVTAQEIFQDNHEDGPTAK	27	1	1	10863945	N-Term(TMT6plex); K20(TMT6plex)	2701.3	0	**1.3**	**1.2**	**1.4**	**1.3**
																	LFQCLLHR	18	1	1	10863945	N-Term(TMT6plex); C4(Carbamidomethyl)	1315.7	0	**1.3**	**1.2**	**1.4**	**1.3**
																	TLFPLIEAK	39	1	1	10863945	N-Term(TMT6plex); K9(TMT6plex)	1489.9	0	**1.3**	**1.2**	**1.3**	**1.2**
																	IQPGSQQADFLDALIVSMDVIQHETIGK	27	1	1	10863945	N-Term(TMT6plex); M18(Oxidation); K28(TMT6plex)	3527.9	0	**1.3**	**1.4**	**1.5**	**1.4**
																	ASFEEASNQLINHIEQFLDTNETPYFMK	49	1	1	10863945	N-Term(TMT6plex); M27(Oxidation); K28(TMT6plex)	3790.9	0	**1.3**	**1.5**	**1.4**	**1.6**
																	ETVYCLNDDDETEVLKEDIIQGFR	21	1	1	10863945	N-Term(TMT6plex); C5(Carbamidomethyl); K16(TMT6plex)	3359.7	1	**1.4**	**1.4**	**1.4**	**1.3**
																	EGLEIVK	10	1	1	10863945	N-Term(TMT6plex); K7(TMT6plex)	1245.8	0	**1.3**	**1.3**	**1.4**	**1.3**
																	GITEQQK	12	1	1	10863945	N-Term(TMT6plex); K7(TMT6plex)	1261.8	0	**1.2**	**1.3**	**1.3**	**1.3**
																	EDGSGDRGDGPFR	6	1	1	10863945	N-Term(TMT6plex)	1593.7	1	**1.4**	**1.3**	**1.3**	**1.4**
																	LTIGSNLSIR	5	1	1	10863945	N-Term(TMT6plex)	1302.8	0	**1.2**	**1.3**	**1.3**	**1.3**
																	EPLPPIQQHIWNMLNPPAEVTTK	39	1	1	10863945	N-Term(TMT6plex); M13(Oxidation); K23(TMT6plex)	3127.7	0	**1.3**	**1.3**	**1.3**	**1.4**
																	TEQGGAHFSVSSLAEGSVTSVGSVNPAENFR	26	1	1	10863945	N-Term(TMT6plex)	3350.6	0	**1.6**	**1.5**	**1.5**	**1.5**
																	EEASGSSVTAEEAK	31	1	1	10863945	N-Term(TMT6plex); K14(TMT6plex)	1853.0	0	**1.2**	**1.2**	**1.2**	**1.2**
																	HLMLPDFDLLEDIESK	40	1	1	10863945	N-Term(TMT6plex); M3(Oxidation); K16(TMT6plex)	2389.3	0	**1.4**	**1.3**	**1.5**	**1.3**
																	FFMGNQVLK	12	1	1	10863945	N-Term(TMT6plex); M3(Oxidation); K9(TMT6plex)	1557.9	0	**1.2**	**1.1**	**1.3**	**1.1**
																	KFLAPK	6	1	1	10863945	N-Term(TMT6plex); K1(TMT6plex); K6(TMT6plex)	1390.9	1	**1.1**	**1.3**	**1.3**	**1.5**
																	DKPSGDTAAVFEEGGDVDDLLDMI	74	1	1	10863945	N-Term(TMT6plex); K2(TMT6plex); M23(Oxidation)	2983.4	0	**1.3**	**1.4**	**1.4**	**1.3**
																	YAPTEAQLNAVDALIDSMSLAK	40	1	1	10863945	N-Term(TMT6plex); M18(Oxidation); K22(TMT6plex)	2795.5	0	**1.4**	**1.4**	**1.4**	**1.3**
																	DKPSGDTAAVFEEGGDVDDLLDMI	12	1	1	10863945	N-Term(TMT6plex); K2(TMT6plex)	2967.4	0	**1.8**	**1.9**	**2.1**	**1.5**
																	ANPQVGVAFPHIK	14	1	1	10863945	N-Term(TMT6plex); K13(TMT6plex)	1836.1	0	**1.3**	**1.3**	**1.4**	**1.4**
																	HNYECLVYVQLPFMEDLR	13	1	1	10863945	N-Term(TMT6plex); C5(Carbamidomethyl); M14(Oxidation)	2571.3	0	**1.4**	**1.4**	**1.4**	**1.4**
																	EEASGSSVTAEEAKK	3	1	1	10863945	N-Term(TMT6plex); K14(TMT6plex); K15(TMT6plex)	2210.2	1	**1.4**	**1.7**	**1.5**	**1.7**
																	ASFEEASNQLINHIEQFLDTNETPYFMK	21	1	1	10863945	N-Term(TMT6plex); K28(TMT6plex)	3774.9	0	**1.6**	**1.8**	**1.9**	**1.4**
																	HLMLPDFDLLEDIESK	11	1	1	10863945	N-Term(TMT6plex); K16(TMT6plex)	2373.3	0	**1.8**	**1.8**	**1.9**	**1.5**
																	DDEAAAVALSSLIHALDDLDMVAIVR	54	1	1	10863945	N-Term(TMT6plex); M21(Oxidation)	2968.6	0	**1.6**	**1.5**	**1.7**	**1.4**
																	EPLPPIQQHIWNMLNPPAEVTTK	1	1	1	10863945	N-Term(TMT6plex); K23(TMT6plex)	3111.7	0	**1.5**	**1.8**	**1.4**	**1.7**
																	KASFEEASNQLINHIEQFLDTNETPYFMK	4	1	1	10863945	N-Term(TMT6plex); K1(TMT6plex); M28(Oxidation); K29(TMT6plex)	4148.1	1	**1.4**	**1.3**	**1.3**	**1.3**
																	QVFAENKDEIALVLFGTDGTDNPLSGGDQYQNITVHR	9	1	1	10863945	N-Term(TMT6plex); K7(TMT6plex)	4520.3	1	**1.3**	**1.4**	**1.5**	**1.4**
																	TDTLEDLFPTTK	22	1	1	10863945	N-Term(TMT6plex); K12(TMT6plex)	1839.0	0	**1.3**	**1.1**	**1.3**	**1.0**
																	YAPTEAQLNAVDALIDSMSLAK	57	1	1	10863945	N-Term(TMT6plex); K22(TMT6plex)	2779.5	0	**1.5**	**1.5**	**1.8**	**1.4**
																	ETVYCLNDDDETEVLK	13	1	1	10863945	N-Term(TMT6plex); C5(Carbamidomethyl); K16(TMT6plex)	2401.2	0	**1.4**	**1.3**	**1.3**	**1.2**
																	KYAPTEAQLNAVDALIDSMSLAK	10	1	1	10863945	N-Term(TMT6plex); K1(TMT6plex); M19(Oxidation); K23(TMT6plex)	3152.7	1	**1.2**	**1.3**	**1.2**	**1.5**
																	QLNHFWEIVVQDGITLITK	4	1	1	10863945	N-Term(TMT6plex); K19(TMT6plex)	2712.5	0	**1.7**	**1.3**	**1.9**	**1.1**
																	TWTVVDAK	13	1	1	10863945	N-Term(TMT6plex); K8(TMT6plex)	1377.8	0	**1.3**	**1.0**	**1.3**	**1.1**
																	HIEIFTDLSSR	11	1	1	10863945	N-Term(TMT6plex)	1546.8	0	**1.3**	**1.3**	**1.4**	**1.3**
																	EDIIQGFR	7	1	1	10863945	N-Term(TMT6plex)	1206.7	0	**1.4**	**1.4**	**1.5**	**1.4**
																	LGGHGPSFPLK	13	1	1	10863945	N-Term(TMT6plex); K11(TMT6plex)	1567.9	0	**1.3**	**1.2**	**1.3**	**1.2**
																	SQLDIIIHSLK	17	1	1	10863945	N-Term(TMT6plex); K11(TMT6plex)	1725.1	0	**1.4**	**1.4**	**1.4**	**1.3**
																	DDEAAAVALSSLIHALDDLDMVAIVR	21	1	1	10863945	N-Term(TMT6plex)	2952.6	0	**1.6**	**1.5**	**1.5**	**1.5**
																	MVMISLEGEDGLDEIYSFSESLR	30	1	1	10863945	N-Term(TMT6plex); M1(Oxidation); M3(Oxidation)	2881.4	0	**1.1**	**1.2**	**1.2**	**1.1**
																	AAVVLCMDVGFTMSNSIPGIESPFEQAK	8	1	1	10863945	N-Term(TMT6plex); C6(Carbamidomethyl); M7(Oxidation); M13(Oxidation); K28(TMT6plex)	3488.7	0	**1.4**	**1.3**	**1.3**	**1.5**
																	HNYECLVYVQLPFMEDLR	4	1	1	10863945	N-Term(TMT6plex); C5(Carbamidomethyl)	2555.3	0	**1.4**	**1.4**	**1.5**	**1.5**
																	KASFEEASNQLINHIEQFLDTNETPYFMK	4	1	1	10863945	N-Term(TMT6plex); K1(TMT6plex); K29(TMT6plex)	4132.1	1	**1.7**	**2.0**	**1.9**	**1.6**
																	KKDQVTAQEIFQDNHEDGPTAK	2	1	1	10863945	N-Term(TMT6plex); K1(TMT6plex); K2(TMT6plex); K22(TMT6plex)	3415.9	2	**1.1**	**1.4**	**1.2**	**1.4**
																	KTWTVVDAK	2	1	1	10863945	N-Term(TMT6plex); K1(TMT6plex); K9(TMT6plex)	1735.1	1	**1.2**	**1.3**	**1.3**	**1.4**
																	IQPGSQQADFLDALIVSMDVIQHETIGK	8	1	1	10863945	N-Term(TMT6plex); K28(TMT6plex)	3511.9	0	**1.4**	**1.5**	**1.7**	**1.5**
																	VITMFVQR	3	1	1	10863945	N-Term(TMT6plex)	1222.7	0	**1.4**	**1.5**	**1.6**	**1.4**
																	YGSDIVPFSK	5	1	1	10863945	N-Term(TMT6plex); K10(TMT6plex)	1570.9	0	**1.3**	**1.6**	**1.4**	**1.6**
																	CFSVLGFCK	2	1	1	10863945	N-Term(TMT6plex); C1(Carbamidomethyl); C8(Carbamidomethyl); K9(TMT6plex)	1575.8	0	**1.5**	**1.2**	**1.5**	**1.3**
																	QYMFSSLK	2	1	1	10863945	N-Term(TMT6plex); K8(TMT6plex)	1461.8	0	**1.6**	**1.5**	**1.9**	**1.4**
																	KVITMFVQR	2	1	1	10863945	N-Term(TMT6plex); K1(TMT6plex)	1580.0	1	**1.4**	**1.8**	**1.8**	**1.5**
																	MVMISLEGEDGLDEIYSFSESLR	3	1	1	10863945	N-Term(TMT6plex); M3(Oxidation)	2865.4	0	**1.3**	**1.3**	**1.4**	**1.4**
																	VDEEQMK	10	1	1	10863945	N-Term(TMT6plex); M6(Oxidation); K7(TMT6plex)	1352.7	0	**1.6**	**1.4**	**1.4**	**1.3**
																	AFREEAIK	1	1	1	10863945	N-Term(TMT6plex); K8(TMT6plex)	1421.9	1	**1.2**	**1.5**	**1.3**	**1.6**
																	SQIPLSK	17	1	1	10863945	N-Term(TMT6plex); K7(TMT6plex)	1230.8	0	**1.3**	**1.2**	**1.3**	**1.3**
																	VITMFVQR	2	1	1	10863945	N-Term(TMT6plex); M4(Oxidation)	1238.7	0	**1.5**	**1.2**	**1.3**	**1.4**
																	DEKTDTLEDLFPTTK	1	1	1	10863945	N-Term(TMT6plex); K3(TMT6plex); K15(TMT6plex)	2440.3	1	**1.4**	**1.7**	**1.8**	**1.7**
																	HSIHWPCR	1	1	1	10863945	N-Term(TMT6plex); C7(Carbamidomethyl)	1321.7	0	**1.2**	**1.2**	**1.3**	**1.3**
																	KLCVFK	1	1	1	10863945	N-Term(TMT6plex); K1(TMT6plex); C3(Carbamidomethyl); K6(TMT6plex)	1481.9	1	**1.2**	**1.3**	**1.4**	**1.3**
																	FSEEQR	1	1	1	10863945	N-Term(TMT6plex)	1024.5	0	**1.4**	**1.1**	**1.3**	**1.1**
																	QYMFSSLK	2	1	1	10863945	N-Term(TMT6plex); M3(Oxidation); K8(TMT6plex)	1477.8	0	**1.2**	**1.2**	**1.3**	**1.4**

Accession	RefSeq Accession	Gene Symbol	Entrez Gene ID	Description	Σ Coverage	Σ# Proteins	Σ# Unique Peptides	Σ# Peptides	Σ# PSMs	Het-1A-Smoke - 2M/Parental	Het-1A-Smoke - 4M/Parental	Het-1A-Smoke - 6M/Parental	Het-1A-Smoke - 8M/Parental	# AAs	MW [kDa]	calc. pI	Sequence	# PSMs	# Proteins	# Protein Groups	Protein Group Accessions	Modifications	MH+ [Da]	# Missed Cleavages	Het-1A-Smoke - 2M/Parental	Het-1A-Smoke - 4M/Parental	Het-1A-Smoke - 6M/Parental	Het-1A-Smoke - 8M/Parental
																	SILQER	1	1	1	10863945	N-Term(TMT6plex)	974.6	0	**1.5**	**1.3**	**1.3**	**1.4**
7669492	NP_002037.2	**GAPDH**	2597	**glyceraldehyde-3-phosphate dehydrogenase isoform 1**	82.99	3	20	20	1558	**1.3**	**1.5**	**1.7**	**1.5**	335	36.0	8.46												
																	VIHDNFGIVEGLMTTVHAITATQK	87	2	1	7669492	N-Term(TMT6plex); M13(Oxidation); K24(TMT6plex)	3069.7	0	**1.3**	**1.5**	**1.7**	**1.6**
																	LISWYDNEFGYSNR	170	2	1	7669492	N-Term(TMT6plex)	1993.0	0	**1.4**	**1.5**	**1.6**	**1.5**
																	WGDAGAEYVVESTGVFTTMEK	607	2	1	7669492	N-Term(TMT6plex); M19(Oxidation); K21(TMT6plex)	2751.4	0	**1.4**	**1.6**	**1.7**	**1.6**
																	VIISAPSADAPMFVMGVNHEK	123	2	1	7669492	N-Term(TMT6plex); M12(Oxidation); M15(Oxidation); K21(TMT6plex)	2703.4	0	**1.5**	**1.7**	**1.9**	**1.5**
																	GALQNIIPASTGAAK	105	2	1	7669492	N-Term(TMT6plex); K15(TMT6plex)	1870.1	0	**1.2**	**1.5**	**1.6**	**1.6**
																	VVDLMAHMASK	39	2	1	7669492	N-Term(TMT6plex); M5(Oxidation); M8(Oxidation); K11(TMT6plex)	1691.9	0	**1.4**	**1.5**	**1.6**	**1.6**
																	VIHDNFGIVEGLMTTVHAITATQK	25	2	1	7669492	N-Term(TMT6plex); K24(TMT6plex)	3053.7	0	**1.5**	**1.9**	**2.2**	**1.8**
																	VPTANVSVVDLTCR	41	2	1	7669492	N-Term(TMT6plex); C13(Carbamidomethyl)	1760.0	0	**1.4**	**1.6**	**1.6**	**1.5**
																	AGAHLQGGAK	24	2	1	7669492	N-Term(TMT6plex); K10(TMT6plex)	1367.8	0	**1.1**	**1.5**	**1.7**	**1.6**
																	VIISAPSADAPMFVMGVNHEK	63	2	1	7669492	N-Term(TMT6plex); M12(Oxidation); K21(TMT6plex)	2687.4	0	**1.5**	**1.8**	**2.1**	**1.6**
																	WGDAGAEYVVESTGVFTTMEK	44	2	1	7669492	N-Term(TMT6plex); K21(TMT6plex)	2735.4	0	**1.6**	**1.8**	**2.0**	**1.7**
																	IISNASCTTNCLAPLAK	27	2	1	7669492	N-Term(TMT6plex); C7(Carbamidomethyl); C11(Carbamidomethyl); K17(TMT6plex)	2292.2	0	**1.4**	**1.7**	**1.8**	**1.6**
																	VDIVAINDPFIDLNYMVYMFQYDSTHGK	4	1	1	7669492	N-Term(TMT6plex); M19(Oxidation); K28(TMT6plex)	3782.9	0	**1.5**	**2.4**	**1.9**	**2.1**
																	VDIVAINDPFIDLNYMVYMFQYDSTHGK	2	1	1	7669492	N-Term(TMT6plex); K28(TMT6plex)	3766.9	0	**2.7**	**3.0**	**2.7**	**2.6**
																	VIISAPSADAPMFVMGVNHEK	4	2	1	7669492	N-Term(TMT6plex); K21(TMT6plex)	2671.4	0	**1.5**	**1.4**	**2.1**	**1.2**
																	GILGYTEHQVVSSDFNSDTHSSTFDAGAGIALNDHFVK	30	2	1	7669492	N-Term(TMT6plex); K38(TMT6plex)	4495.2	0	**1.1**	**1.5**	**1.6**	**1.5**
																	VVDLMAHMASKE	1	2	1	7669492	N-Term(TMT6plex); M5(Oxidation); M8(Oxidation); K11(TMT6plex)	1821.0	1	**1.2**	**1.8**	**1.5**	**1.8**
																	VIPELNGK	7	2	1	7669492	N-Term(TMT6plex); K8(TMT6plex)	1327.8	0	**1.3**	**1.4**	**1.6**	**1.4**
																	AAFNSGK	21	1	1	7669492	N-Term(TMT6plex); K7(TMT6plex)	1152.7	0	**1.2**	**1.5**	**1.7**	**1.6**
																	QASEGPLK	41	2	1	7669492	N-Term(TMT6plex); K8(TMT6plex)	1287.8	0	**1.4**	**1.5**	**1.6**	**1.5**
																	VDIVAINDPFIDLNYMVYMFQYDSTHGK	6	1	1	7669492	N-Term(TMT6plex); M16(Oxidation); M19(Oxidation); K28(TMT6plex)	3798.9	0	**1.6**	**2.0**	**1.6**	**2.1**
																	VVDLMAHMASK	10	2	1	7669492	N-Term(TMT6plex); K11(TMT6plex)	1659.9	0	**1.5**	**2.0**	**2.5**	**1.7**
																	VVDLMAHMASK	6	2	1	7669492	N-Term(TMT6plex); M5(Oxidation); K11(TMT6plex)	1675.9	0	**1.3**	**1.8**	**1.9**	**1.7**
																	LVINGNPITIFQER	9	2	1	7669492	N-Term(TMT6plex)	1843.1	0	**1.6**	**1.5**	**1.8**	**1.7**
																	TVDGPSGK	37	2	1	7669492	N-Term(TMT6plex); K8(TMT6plex)	1218.7	0	**1.2**	**1.4**	**1.5**	**1.4**
																	LTGMAFR	4	3	1	7669492	N-Term(TMT6plex)	1024.6	0	**1.8**	**1.7**	**2.8**	**1.3**
																	YDNSLK	18	2	1	7669492	N-Term(TMT6plex); K6(TMT6plex)	1197.7	0	**1.2**	**1.2**	**1.6**	**1.2**
																	LTGMAFR	1	3	1	7669492	N-Term(TMT6plex); M4(Oxidation)	1040.6	0	**1.3**	**1.3**	**1.5**	**1.4**
																	LEKPAK	2	2	1	7669492	N-Term(TMT6plex); K3(TMT6plex); K6(TMT6plex)	1372.9	0	**0.7**	**1.6**	**1.6**	**1.1**
5174735	NP_006079.1	**TUBB4B**	10383	**tubulin beta-4B chain**	82.70	3	1	25	2287	**1.1**	**1.1**	**1.3**	**1.2**	445	49.8	4.89												
																	GHYTEGAELVDSVLDVVR	235	11	5	29788785;5174735;29788768;50592996;4507729	N-Term(TMT6plex)	2188.1	0				
																	NSSYFVEWIPNNVK	95	20	7	29788785;5174735;14210536;746815975;29788768;50592996;21361322;574584816;4507729	N-Term(TMT6plex); K14(TMT6plex)	2155.2	0				
																	ALTVPELTQQMFDAK	71	7	3	5174735;50592996;21361322;574584816	N-Term(TMT6plex); M11(Oxidation); K15(TMT6plex)	2166.2	0				
																	SGPFGQIFRPDNFVFGQSGAGNNWAK	124	8	5	29788785;5174735;29788768;21361322;574584816;4507729	N-Term(TMT6plex); K26(TMT6plex)	3256.7	0				
																	AVLVDLEPGTMDSVR	89	5	2	5174735;21361322;574584816	N-Term(TMT6plex); M11(Oxidation)	1847.0	0				
																	FWEVISDEHGIDPTGTYHGDSDLQLER	36	4	2	5174735;21361322;574584816	N-Term(TMT6plex)	3345.6	0				
																	EVDEQMLNVQNK	195	9	4	29788785;5174735;29788768;4507729	N-Term(TMT6plex); M6(Oxidation); K12(TMT6plex)	1921.0	0				
																	ALTVPELTQQMFDAK	47	7	3	5174735;50592996;21361322;574584816	N-Term(TMT6plex); K15(TMT6plex)	2150.2	0				
																	VSDTVVEPYNATLSVHQLVENTDETYCIDNEALYDICFR	50	8	5	29788785;5174735;14210536;746815975;29788768;21361322;574584816	N-Term(TMT6plex); C27(Carbamidomethyl); C37(Carbamidomethyl)	4822.2	0				
																	IMNTFSVVPSPK	47	8	4	29788785;5174735;50592996;21361322;574584816	N-Term(TMT6plex); M2(Oxidation); K12(TMT6plex)	1794.0	0				
																	LTTPTYGDLNHLVSATMSGVTTCLR	171	12	5	29788785;5174735;29788768;21361322;574584816;4507729	N-Term(TMT6plex); M17(Oxidation); C23(Carbamidomethyl)	2953.5	0				
																	YLTVAAVFR	63	9	3	29788785;5174735;21361322;574584816	N-Term(TMT6plex)	1268.8	0				
																	MSATFIGNSTAIQELFK	18	4	3	5174735;29788768;4507729	N-Term(TMT6plex); M1(Oxidation); K17(TMT6plex)	2332.3	0				
																	INVYYNEATGGK	66	1	1	5174735	N-Term(TMT6plex); K12(TMT6plex)	1787.0	0	**1.1**	**1.1**	**1.3**	**1.2**
																	LHFFMPGFAPLTSR	71	18	6	29788785;5174735;14210536;746815975;29788768;21361322;574584816;4507729	N-Term(TMT6plex)	1850.0	0				
																	KLAVNMVPFPR	11	21	8	29788785;5174735;14210536;746815975;29788768;50592996;21361322;574584816;4507729;13562114	N-Term(TMT6plex); K1(TMT6plex); M6(Oxidation)	1746.0	1				
																	TAVCDIPPR	171	14	5	29788785;5174735;29788768;21361322;574584816;4507729	N-Term(TMT6plex); C4(Carbamidomethyl)	1257.7	0				
																	ISEQFTAMFR	20	10	6	29788785;5174735;29788768;50592996;21361322;574584816;4507729	N-Term(TMT6plex)	1458.8	0				
																	ISEQFTAMFR	244	10	6	29788785;5174735;29788768;50592996;21361322;574584816;4507729	N-Term(TMT6plex); M8(Oxidation)	1474.8	0				
																	EAESCDCLQGFQLTHSLGGGTGSGMGTLLISK	32	7	3	29788785;5174735;21361322;574584816	N-Term(TMT6plex); C5(Carbamidomethyl); C7(Carbamidomethyl); M25(Oxidation); K32(TMT6plex)	3785.9	0				
																	EIVHLQAGQCGNQIGAK	14	8	6	29788785;29788768;50592996;4507729;5174735;21361322;574584816	N-Term(TMT6plex); C10(Carbamidomethyl); K17(TMT6plex)	2281.2	0				
																	LTTPTYGDLNHLVSATMSGVTTCLR	35	12	5	29788785;5174735;29788768;21361322;574584816;4507729	N-Term(TMT6plex); C23(Carbamidomethyl)	2937.5	0				
																	FPGQLNADLR	98	13	8	29788785;5174735;14210536;746815975;29788768;50592996;21361322;574584816;4507729;13562114	N-Term(TMT6plex)	1359.8	0				
																	LAVNMVPFPR	20	21	8	29788785;5174735;14210536;746815975;29788768;50592996;21361322;574584816;4507729;13562114	N-Term(TMT6plex)	1372.8	0				
																	EVDEQMLNVQNK	12	9	4	29788785;5174735;29788768;4507729	N-Term(TMT6plex); K12(TMT6plex)	1905.0	0				
																	LHFFMPGFAPLTSR	135	18	6	29788785;5174735;14210536;746815975;29788768;21361322;574584816;4507729	N-Term(TMT6plex); M5(Oxidation)	1866.0	0				
																	KEAESCDCLQGFQLTHSLGGGTGSGMGTLLISK	7	7	3	29788785;5174735;21361322;574584816	K1(TMT6plex); N-Term(TMT6plex); C6(Carbamidomethyl); C8(Carbamidomethyl); M26(Oxidation); K33(TMT6plex)	4143.1	1				
																	AVLVDLEPGTMDSVR	17	5	2	5174735;21361322;574584816	N-Term(TMT6plex)	1831.0	0				
																	LAVNMVPFPR	54	21	8	29788785;5174735;14210536;746815975;29788768;50592996;21361322;574584816;4507729;13562114	N-Term(TMT6plex); M5(Oxidation)	1388.8	0				
																	IREEYPDR	9	6	5	29788785;5174735;29788768;4507729;13562114	N-Term(TMT6plex)	1306.7	1				
																	MSATFIGNSTAIQELFK	3	4	3	5174735;29788768;4507729	N-Term(TMT6plex); K17(TMT6plex)	2316.3	0				
																	IMNTFSVVPSPK	8	8	4	29788785;5174735;50592996;21361322;574584816	N-Term(TMT6plex); K12(TMT6plex)	1778.0	0				
																	KEAESCDCLQGFQLTHSLGGGTGSGMGTLLISK	1	7	3	29788785;5174735;21361322;574584816	N-Term(TMT6plex); K1(TMT6plex); C6(Carbamidomethyl); C8(Carbamidomethyl); K33(TMT6plex)	4127.1	1				
																	EAESCDCLQGFQLTHSLGGGTGSGMGTLLISK	2	7	3	29788785;5174735;21361322;574584816	N-Term(TMT6plex); C5(Carbamidomethyl); C7(Carbamidomethyl); K32(TMT6plex)	3769.8	0				
																	NMMAACDPR	5	20	7	29788785;5174735;14210536;746815975;29788768;50592996;21361322;574584816;4507729	N-Term(TMT6plex); M2(Oxidation); M3(Oxidation); C6(Carbamidomethyl)	1326.6	0				
																	GSQQYR	5	12	7	29788785;5174735;14210536;746815975;29788768;50592996;21361322;574584816;4507729	N-Term(TMT6plex)	967.5	0				
																	NMMAACDPR	4	20	7	29788785;5174735;14210536;746815975;29788768;50592996;21361322;574584816;4507729	N-Term(TMT6plex); M3(Oxidation); C6(Carbamidomethyl)	1310.6	0				
																	NMMAACDPR	2	20	7	29788785;5174735;14210536;746815975;29788768;50592996;21361322;574584816;4507729	N-Term(TMT6plex); C6(Carbamidomethyl)	1294.6	0				
29788768	NP_821080.1	**TUBB2B**	347733	**tubulin beta-2B chain**	82.70	2	1	25	2050	**2.0**	**1.7**	**1.5**	**1.0**	445	49.9	4.89												
																	AILVDLEPGTMDSVR	141	7	4	29788785;29788768;50592996;4507729	N-Term(TMT6plex); M11(Oxidation)	1861.0	0				
																	GHYTEGAELVDSVLDVVR	235	11	5	29788785;5174735;29788768;50592996;4507729	N-Term(TMT6plex)	2188.1	0				
																	NSSYFVEWIPNNVK	95	20	7	29788785;5174735;14210536;746815975;29788768;50592996;21361322;574584816;4507729	N-Term(TMT6plex); K14(TMT6plex)	2155.2	0				
																	SGPFGQIFRPDNFVFGQSGAGNNWAK	124	8	5	29788785;5174735;29788768;21361322;574584816;4507729	N-Term(TMT6plex); K26(TMT6plex)	3256.7	0				
																	EVDEQMLNVQNK	195	9	4	29788785;5174735;29788768;4507729	N-Term(TMT6plex); M6(Oxidation); K12(TMT6plex)	1921.0	0				
																	ALTVPELTQQMFDSK	28	3	2	29788768;4507729	N-Term(TMT6plex); M11(Oxidation); K15(TMT6plex)	2182.2	0				
																	VSDTVVEPYNATLSVHQLVENTDETYCIDNEALYDICFR	50	8	5	29788785;5174735;14210536;746815975;29788768;21361322;574584816	N-Term(TMT6plex); C27(Carbamidomethyl); C37(Carbamidomethyl)	4822.2	0				
																	LTTPTYGDLNHLVSATMSGVTTCLR	171	12	5	29788785;5174735;29788768;21361322;574584816;4507729	N-Term(TMT6plex); M17(Oxidation); C23(Carbamidomethyl)	2953.5	0				
																	FWEVISDEHGIDPTGSYHGDSDLQLER	1	2	2	29788768;4507729	N-Term(TMT6plex)	3331.6	0				
																	MSATFIGNSTAIQELFK	18	4	3	5174735;29788768;4507729	N-Term(TMT6plex); M1(Oxidation); K17(TMT6plex)	2332.3	0				
																	EIVHIQAGQCGNQIGAK	36	8	6	29788785;29788768;50592996;4507729;5174735;21361322;574584816	N-Term(TMT6plex); C10(Carbamidomethyl); K17(TMT6plex)	2281.2	0				
																	INVYYNEATGNK	6	1	1	29788768	N-Term(TMT6plex); K12(TMT6plex)	1844.0	0	**2.0**	**1.7**	**1.5**	**1.0**
																	LHFFMPGFAPLTSR	71	18	6	29788785;5174735;14210536;746815975;29788768;21361322;574584816;4507729	N-Term(TMT6plex)	1850.0	0				
																	KESESCDCLQGFQLTHSLGGGTGSGMGTLLISK	4	2	2	29788768;4507729	K1(TMT6plex); N-Term(TMT6plex); C6(Carbamidomethyl); C8(Carbamidomethyl); M26(Oxidation); K33(TMT6plex)	4159.1	1				

Accession	RefSeq Accession	Gene Symbol	Entrez Gene ID	Description	Σ Coverage	Σ# Proteins	Σ# Unique Peptides	Σ# Peptides	Σ# PSMs	Het-1A-Smoke - 2M/Parental	Het-1A-Smoke - 4M/Parental	Het-1A-Smoke - 6M/Parental	Het-1A-Smoke - 8M/Parental	# AAs	MW [kDa]	calc. pI	Sequence	# PSMs	# Proteins	# Protein Groups	Protein Group Accessions	Modifications	MH+ [Da]	# Missed Cleavages	Het-1A-Smoke - 2M/Parental	Het-1A-Smoke - 4M/Parental	Het-1A-Smoke - 6M/Parental	Het-1A-Smoke - 8M/Parental
																	KLAVNMVPFPR	11	21	8	29788785;5174735;14210536;746815975;29788768;50592996;21361322;574584816;4507729;13562114	N-Term(TMT6plex); K1(TMT6plex); M6(Oxidation)	1746.0	1				
																	TAVCDIPPR	171	14	5	29788785;5174735;29788768;21361322;574584816;4507729	N-Term(TMT6plex); C4(Carbamidomethyl)	1257.7	0				
																	ISEQFTAMFR	20	10	6	29788785;5174735;29788768;50592996;21361322;574584816;4507729	N-Term(TMT6plex)	1458.8	0				
																	ISEQFTAMFR	244	10	6	29788785;5174735;29788768;50592996;21361322;574584816;4507729	N-Term(TMT6plex); M8(Oxidation)	1474.8	0				
																	ESESCDCLQGFQLTHSLGGGTGSGMGTLLISK	13	2	2	29788768;4507729	N-Term(TMT6plex); C5(Carbamidomethyl); C7(Carbamidomethyl); M25(Oxidation); K32(TMT6plex)	3801.9	0				
																	LTTPTYGDLNHLVSATMSGVTTCLR	35	12	5	29788785;5174735;29788768;21361322;574584816;4507729	N-Term(TMT6plex); C23(Carbamidomethyl)	2937.5	0				
																	FPGQLNADLR	98	13	8	29788785;5174735;14210536;746815975;29788768;50592996;21361322;574584816;4507729;13562114	N-Term(TMT6plex)	1359.8	0				
																	AILVDLEPGTMDSVR	22	7	4	29788785;29788768;50592996;4507729	N-Term(TMT6plex)	1845.0	0				
																	LAVNMVPFPR	20	21	8	29788785;5174735;14210536;746815975;29788768;50592996;21361322;574584816;4507729;13562114	N-Term(TMT6plex)	1372.8	0				
																	EVDEQMLNVQNK	12	9	4	29788785;5174735;29788768;4507729	N-Term(TMT6plex); K12(TMT6plex)	1905.0	0				
																	LHFFMPGFAPLTSR	135	18	6	29788785;5174735;14210536;746815975;29788768;21361322;574584816;4507729	N-Term(TMT6plex); M5(Oxidation)	1866.0	0				
																	LAVNMVPFPR	54	21	8	29788785;5174735;14210536;746815975;29788768;50592996;21361322;574584816;4507729;13562114	N-Term(TMT6plex); M5(Oxidation)	1388.8	0				
																	YLTVAAIFR	3	2	2	29788768;4507729	N-Term(TMT6plex)	1282.8	0				
																	IREEYPDR	9	6	5	29788785;5174735;29788768;4507729;13562114	N-Term(TMT6plex)	1306.7	1				
																	MSATFIGNSTAIQELFK	3	4	3	5174735;29788768;4507729	N-Term(TMT6plex); K17(TMT6plex)	2316.3	0				
																	IMNTFSVMPSPK	2	3	3	14210536;746815975;29788768;4507729	N-Term(TMT6plex); M2(Oxidation); M8(Oxidation); K12(TMT6plex)	1842.0	0				
																	ALTVPELTQQMFDSK	4	3	2	29788768;4507729	N-Term(TMT6plex); K15(TMT6plex)	2166.2	0				
																	KESESCDCLQGFQLTHSLGGGTGSGMGTLLISK	3	2	2	29788768;4507729	N-Term(TMT6plex); K1(TMT6plex); C6(Carbamidomethyl); C8(Carbamidomethyl); K33(TMT6plex)	4143.1	1				
																	NMMAACDPR	5	20	7	29788785;5174735;14210536;746815975;29788768;50592996;21361322;574584816;4507729	N-Term(TMT6plex); M2(Oxidation); M3(Oxidation); C6(Carbamidomethyl)	1326.6	0				
																	GSQQYR	5	12	7	29788785;5174735;14210536;746815975;29788768;50592996;21361322;574584816;4507729	N-Term(TMT6plex)	967.5	0				
																	NMMAACDPR	4	20	7	29788785;5174735;14210536;746815975;29788768;50592996;21361322;574584816;4507729	N-Term(TMT6plex); M3(Oxidation); C6(Carbamidomethyl)	1310.6	0				
																	NMMAACDPR	2	20	7	29788785;5174735;14210536;746815975;29788768;50592996;21361322;574584816;4507729	N-Term(TMT6plex); C6(Carbamidomethyl)	1294.6	0				
14251209	NP_001279.2	**CLIC1**	1192	**chloride intracellular channel protein 1**	82.57	1	15	16	317	**1.3**	**1.4**	**1.5**	**1.4**	241	26.9	5.17												
																	IEEFLEAVLCPPR	62	1	1	14251209	N-Term(TMT6plex); C10(Carbamidomethyl)	1802.0	0	**1.3**	**1.3**	**1.6**	**1.2**
																	LHIVQVVCK	10	1	1	14251209	N-Term(TMT6plex); C8(Carbamidomethyl); K9(TMT6plex)	1554.0	0	**1.4**	**1.6**	**1.6**	**1.5**
																	NSNPALNDNLEK	25	1	1	14251209	N-Term(TMT6plex); K12(TMT6plex)	1787.0	0	**1.3**	**1.2**	**1.4**	**1.3**
																	FLDGNELTLADCNLLPK	26	1	1	14251209	N-Term(TMT6plex); C12(Carbamidomethyl); K17(TMT6plex)	2391.3	0	**1.4**	**1.3**	**1.6**	**1.3**
																	LAALNPESNTAGLDIFAK	52	1	1	14251209	N-Term(TMT6plex); K18(TMT6plex)	2303.3	0	**1.3**	**1.5**	**1.6**	**1.4**
																	GVTFNVTTVDTK	26	1	1	14251209	N-Term(TMT6plex); K12(TMT6plex)	1740.0	0	**1.3**	**1.5**	**1.6**	**1.6**
																	VLDNYLTSPLPEEVDETSAEDEGVSQR	40	1	1	14251209	N-Term(TMT6plex)	3221.5	0	**1.3**	**1.4**	**1.3**	**1.3**
																	YLSNAYAR	4	1	1	14251209	N-Term(TMT6plex)	1186.6	0	**1.4**	**1.2**	**1.5**	**1.1**
																	LCPGGQLPFLLYGTEVHTDTNK	2	1	1	14251209	N-Term(TMT6plex); C2(Carbamidomethyl); K22(TMT6plex)	2918.6	0	**1.3**	**1.9**	**1.9**	**1.9**
																	KFLDGNELTLADCNLLPK	8	1	1	14251209	N-Term(TMT6plex); K1(TMT6plex); C13(Carbamidomethyl); K18(TMT6plex)	2748.6	1	**1.4**	**1.8**	**2.0**	**1.6**
																	IGNCPFSQR	4	1	1	14251209	N-Term(TMT6plex); C4(Carbamidomethyl)	1307.7	0	**1.4**	**1.3**	**1.5**	**1.3**
																	LFMVLWLK	6	1	1	14251209	N-Term(TMT6plex); K8(TMT6plex)	1507.9	0	**1.8**	**1.4**	**1.7**	**1.4**
																	EEFASTCPDDEEIELAYEQVAK	25	1	1	14251209	N-Term(TMT6plex); C7(Carbamidomethyl); K22(TMT6plex)	3031.4	0	**1.1**	**1.4**	**1.4**	**1.4**
																	GFTIPEAFR	15	1	1	14251209	N-Term(TMT6plex)	1266.7	0	**1.3**	**1.7**	**1.7**	**1.6**
																	LFMVLWLK	6	1	1	14251209	N-Term(TMT6plex); M3(Oxidation); K8(TMT6plex)	1523.9	0	**1.6**	**1.3**	**1.5**	**1.2**
																	FSAYIK	4	6	3	7330335;365733560;14251209	N-Term(TMT6plex); K6(TMT6plex)	1186.7	0				
																	TETVQK	2	1	1	14251209	N-Term(TMT6plex); K6(TMT6plex)	1163.7	0				
38455427	NP_006421.2	**CCT4**	10575	**T-complex protein 1 subunit delta isoform a**	82.56	2	35	36	474	**1.3**	**1.4**	**1.4**	**1.5**	539	57.9	7.83												
																	GDVTITNDGATILK	30	1	1	38455427	N-Term(TMT6plex); K14(TMT6plex)	1876.1	0	**1.2**	**1.4**	**1.5**	**1.5**
																	AFADAMEVIPSTLAENAGLNPISTVTELR	52	2	1	38455427	N-Term(TMT6plex); M6(Oxidation)	3275.7	0	**1.5**	**1.5**	**1.6**	**1.6**
																	GIHPTIISESFQK	5	2	1	38455427	N-Term(TMT6plex); K13(TMT6plex)	1915.1	0	**1.3**	**1.6**	**1.6**	**1.8**
																	IDDVVNTR	15	2	1	38455427	N-Term(TMT6plex)	1160.6	0	**1.5**	**1.7**	**1.6**	**1.7**
																	GIEILTDMSRPVELSDR	15	2	1	38455427	N-Term(TMT6plex); M8(Oxidation)	2176.1	0	**1.4**	**1.5**	**1.5**	**1.7**
																	TLSGMESYCVR	7	2	1	38455427	N-Term(TMT6plex); M5(Oxidation); C9(Carbamidomethyl)	1547.7	0	**1.5**	**1.3**	**1.4**	**1.3**
																	AFADAMEVIPSTLAENAGLNPISTVTELR	13	2	1	38455427	N-Term(TMT6plex)	3259.7	0	**1.4**	**1.6**	**1.8**	**1.7**
																	SIHDALCVIR	22	7	2	24307939;38455427	N-Term(TMT6plex); C7(Carbamidomethyl)	1412.8	0				
																	KLGGTIDDCELVEGLVLTQK	10	2	1	38455427	N-Term(TMT6plex); K1(TMT6plex); C9(Carbamidomethyl); K20(TMT6plex)	2875.6	1	**1.4**	**1.6**	**1.6**	**1.6**
																	TDMDNQIVVSDYAQMDR	12	2	1	38455427	N-Term(TMT6plex); M3(Oxidation)	2246.0	0	**1.9**	**1.9**	**2.4**	**1.9**
																	ITGCASPGK	12	2	1	38455427	N-Term(TMT6plex); C4(Carbamidomethyl); K9(TMT6plex)	1348.8	0	**1.6**	**1.3**	**1.5**	**1.3**
																	DALSDLALHFLNK	54	2	1	38455427	N-Term(TMT6plex); K13(TMT6plex)	1915.1	0	**1.4**	**1.5**	**1.5**	**1.7**
																	LGGTIDDCELVEGLVLTQK	29	2	1	38455427	N-Term(TMT6plex); C8(Carbamidomethyl); K19(TMT6plex)	2518.4	0	**1.3**	**1.4**	**1.4**	**1.3**
																	AQDIEAGDGTTSVVIIAGSLLDSCTK	30	2	1	38455427	N-Term(TMT6plex); C24(Carbamidomethyl); K26(TMT6plex)	3079.6	0	**1.4**	**1.5**	**1.5**	**1.6**
																	TGCNVLLIQK	6	2	1	38455427	N-Term(TMT6plex); C3(Carbamidomethyl); K10(TMT6plex)	1604.0	0	**1.3**	**1.2**	**1.4**	**1.3**
																	QMQVLHPAAR	2	1	1	38455427	N-Term(TMT6plex); M2(Oxidation)	1395.8	0	**1.3**	**1.0**	**1.1**	**1.4**
																	VVSQYSSLLSPMSVNAVMK	5	2	1	38455427	N-Term(TMT6plex); M12(Oxidation); M18(Oxidation); K19(TMT6plex)	2530.4	0	**1.4**	**1.4**	**1.3**	**1.5**
																	EDIEFICK	4	2	1	38455427	N-Term(TMT6plex); C7(Carbamidomethyl); K8(TMT6plex)	1511.8	0	**1.2**	**1.2**	**1.5**	**1.4**
																	ETLLNSATTSLNSK	26	2	1	38455427	N-Term(TMT6plex); K14(TMT6plex)	1937.1	0	**1.2**	**1.4**	**1.4**	**1.6**
																	AYILNLVK	17	2	1	38455427	N-Term(TMT6plex); K8(TMT6plex)	1391.9	0	**1.3**	**1.3**	**1.4**	**1.5**
																	KGGISNILEELVVQPLLVSVSALTLATETVR	7	2	1	38455427	N-Term(TMT6plex); K1(TMT6plex)	3708.2	1	**2.7**	**1.7**	**1.9**	**1.2**
																	VIDPATATSVDLR	24	2	1	38455427	N-Term(TMT6plex)	1586.9	0	**1.6**	**1.4**	**1.5**	**1.6**
																	KTGCNVLLIQK	3	2	1	38455427	N-Term(TMT6plex); K1(TMT6plex); C4(Carbamidomethyl); K11(TMT6plex)	1961.2	1	**1.2**	**1.5**	**1.5**	**1.3**
																	SGATAGAAGGR	8	2	1	38455427	N-Term(TMT6plex)	1104.6	0	**1.4**	**1.2**	**1.4**	**1.3**
																	TDMDNQIVVSDYAQMDR	7	2	1	38455427	N-Term(TMT6plex); M3(Oxidation); M15(Oxidation)	2262.0	0	**1.7**	**1.7**	**2.0**	**1.6**
																	ALIAGGGAPEIELALR	20	2	1	38455427	N-Term(TMT6plex)	1780.1	0	**1.4**	**1.3**	**1.5**	**1.4**
																	LVIEEAER	9	2	1	38455427	N-Term(TMT6plex)	1187.7	0	**1.3**	**1.3**	**1.3**	**1.4**
																	TLSGMESYCVR	2	2	1	38455427	N-Term(TMT6plex); C9(Carbamidomethyl)	1531.7	0	**1.7**	**1.5**	**1.9**	**1.2**
																	IGLIQFCLSAPK	2	2	1	38455427	N-Term(TMT6plex); C7(Carbamidomethyl); K12(TMT6plex)	1805.1	0	**1.2**	**1.4**	**1.3**	**1.4**
																	TVTIVVR	4	2	1	38455427	N-Term(TMT6plex)	1016.7	0	**1.3**	**1.3**	**1.4**	**1.3**
																	MLVELSK	13	2	1	38455427	N-Term(TMT6plex); M1(Oxidation); K7(TMT6plex)	1293.8	0	**1.2**	**1.4**	**1.4**	**1.6**
																	FSNISAAK	1	2	1	38455427	N-Term(TMT6plex); K8(TMT6plex)	1295.8	0	**1.3**	**1.2**	**1.3**	**1.2**
																	TIGTKPVAHIDQFTADMLGSAELAEEVNLNGSGK	1	2	1	38455427	N-Term(TMT6plex); K5(TMT6plex); M17(Oxidation); K34(TMT6plex)	4217.2	0	**1.1**	**1.6**	**1.2**	**1.5**
																	VSNSGITR	1	2	1	38455427	N-Term(TMT6plex)	1062.6	0	**1.3**	**1.2**	**1.3**	**1.3**
																	MIQDGKGDVTITNDGATILK	1	1	1	38455427	N-Term(TMT6plex); M1(Oxidation); K6(TMT6plex); K20(TMT6plex)	2793.6	1	**1.3**	**1.3**	**1.7**	**1.8**
																	TAGINVR	2	2	1	38455427	N-Term(TMT6plex)	959.6	0	**1.4**	**1.0**	**1.2**	**1.2**
																	AVADAIR	1	2	1	38455427	N-Term(TMT6plex)	944.6	0	**1.2**	**1.3**	**1.4**	**1.5**
																	GAYQDR	1	2	1	38455427	N-Term(TMT6plex)	938.5	0	**1.2**	**1.5**	**1.3**	**1.8**
																	DKPAQIR	1	2	1	38455427	N-Term(TMT6plex); K2(TMT6plex)	1285.8	0	**1.1**	**1.4**	**1.4**	**1.6**
20070125	NP_000909.2	**P4HB**	5034	**protein disulfide-isomerase precursor**	81.89	1	42	43	1634	**1.2**	**1.4**	**1.2**	**1.2**	508	57.1	4.87												
																	VDATEESDLAQQYGVR	106	1	1	20070125	N-Term(TMT6plex)	2010.0	0	**1.4**	**1.4**	**1.3**	**1.4**
																	ENLLDFIK	153	1	1	20070125	N-Term(TMT6plex); K8(TMT6plex)	1449.9	0	**1.1**	**1.5**	**1.2**	**1.2**
																	ILFIFIDSDHTDNQR	30	1	1	20070125	N-Term(TMT6plex)	2063.1	0	**1.4**	**1.5**	**1.4**	**1.3**
																	ALAPEYAK	27	1	1	20070125	N-Term(TMT6plex); K8(TMT6plex)	1320.8	0	**1.1**	**1.4**	**1.2**	**1.2**
																	SNFAEALAAHK	20	1	1	20070125	N-Term(TMT6plex); K11(TMT6plex)	1616.9	0	**1.3**	**1.4**	**1.3**	**1.2**
																	MDSTANEVEAVK	84	1	1	20070125	N-Term(TMT6plex); M1(Oxidation); K12(TMT6plex)	1767.9	0	**1.4**	**1.4**	**1.4**	**1.0**
																	YKPESEELTAER	40	1	1	20070125	N-Term(TMT6plex); K2(TMT6plex)	1910.0	0	**1.2**	**1.4**	**1.2**	**1.2**
																	QFLQAAEAIDDIPFGITSNSDVFSK	202	1	1	20070125	N-Term(TMT6plex); K25(TMT6plex)	3171.7	0	**1.2**	**1.3**	**1.2**	**1.1**
																	EADDIVNWLK	85	1	1	20070125	N-Term(TMT6plex); K10(TMT6plex)	1660.9	0	**1.3**	**1.6**	**1.2**	**1.2**
																	THILLFLPK	97	1	1	20070125	N-Term(TMT6plex); K9(TMT6plex)	1540.0	0	**1.2**	**1.4**	**1.2**	**1.0**
																	NFEDVAFDEK	35	1	1	20070125	N-Term(TMT6plex); K10(TMT6plex)	1671.9	0	**1.3**	**1.4**	**1.3**	**1.2**
																	DHENIVIAK	19	1	1	20070125	N-Term(TMT6plex); K9(TMT6plex)	1496.9	0	**1.0**	**1.3**	**1.2**	**1.1**
																	KSNFAEALAAHK	27	1	1	20070125	N-Term(TMT6plex); K1(TMT6plex); K12(TMT6plex)	1974.2	1	**1.2**	**1.4**	**1.3**	**1.2**
																	LGETYKDHENIVIAK	22	1	1	20070125	N-Term(TMT6plex); K6(TMT6plex); K15(TMT6plex)	2417.4	1	**1.2**	**1.4**	**1.3**	**1.3**

Supplementary Table 6. List of all proteins and peptides identified in untreated and chronically treated Het1A cells with cigarette smoke condensate for 8 months

Accession	RefSeq Accession	Gene Symbol	Entrez Gene ID	Description	Σ Coverage	Σ# Proteins	Σ# Unique Peptides	Σ# Peptides	Σ# PSMs	Het-1A-Smoke - 2M/Parental	Het-1A-Smoke - 4M/Parental	Het-1A-Smoke - 6M/Parental	Het-1A-Smoke - 8M/Parental	# AAs	MW [kDa]	calc. pI	Sequence	# PSMs	# Proteins	# Protein Groups	Protein Group Accessions	Modifications	MH+ [Da]	# Missed Cleavages	Het-1A-Smoke - 2M/Parental	Het-1A-Smoke - 4M/Parental	Het-1A-Smoke - 6M/Parental	Het-1A-Smoke - 8M/Parental
																	YQLDKDGVVLFK	19	1	1	20070125	N-Term(TMT6plex); K5(TMT6plex); K12(TMT6plex)	2112.3	1	1.1	1.7	1.2	1.3
																	KEECPAVR	18	1	1	20070125	N-Term(TMT6plex); K1(TMT6plex); C4(Carbamidomethyl)	1446.8	1	1.1	1.5	1.2	1.1
																	LITLEEEMTK	87	1	1	20070125	N-Term(TMT6plex); M8(Oxidation); K10(TMT6plex)	1680.9	0	1.3	1.4	1.3	1.3
																	TAAESFK	17	1	1	20070125	N-Term(TMT6plex); K7(TMT6plex)	1211.7	0	1.2	1.4	1.2	1.1
																	QLAPIWDK	26	1	1	20070125	N-Term(TMT6plex); K8(TMT6plex)	1428.9	0	1.2	1.4	1.2	1.1
																	SVSDYDGK	64	1	1	20070125	N-Term(TMT6plex); K8(TMT6plex)	1328.7	0	1.3	1.6	1.2	1.3
																	NFEDVAFDEKK	3	1	1	20070125	N-Term(TMT6plex); K10(TMT6plex); K11(TMT6plex)	2029.1	1	1.3	1.9	1.4	1.4
																	NNFEGEVTK	34	1	1	20070125	N-Term(TMT6plex); K9(TMT6plex)	1495.8	0	1.2	1.1	1.1	0.9
																	IKPHLMSQELPEDWDK	3	1	1	20070125	N-Term(TMT6plex); K2(TMT6plex); K16(TMT6plex)	2653.5	0	1.4	1.6	1.5	1.3
																	IKPHLMSQELPEDWDKQPVK	7	1	1	20070125	N-Term(TMT6plex); K2(TMT6plex); M6(Oxidation); K16(TMT6plex); K20(TMT6plex)	3350.9	1	1.0	1.7	1.2	1.3
																	TGPAATTLPDGAAAESLVESSEVAVIGFFK	89	1	1	20070125	N-Term(TMT6plex); K30(TMT6plex)	3393.8	0	1.3	1.2	1.3	1.0
																	ILEFFGLK	95	1	1	20070125	N-Term(TMT6plex); K8(TMT6plex)	1424.9	0	1.3	1.3	1.2	1.0
																	NVFVEFYAPWCGHCK	2	1	1	20070125	N-Term(TMT6plex); C11(Carbamidomethyl); C14(Carbamidomethyl); K15(TMT6plex)	2372.2	0	1.7	2.1	1.9	1.9
																	NNFEGEVTKENLLDFIK	1	1	1	20070125	N-Term(TMT6plex); K9(TMT6plex); K17(TMT6plex)	2697.5	1	1.5	2.4	1.4	1.7
																	FLESGGQDGAGDDDDLEDLEEAEEPDMEEDDDQK	33	1	1	20070125	N-Term(TMT6plex); M27(Oxidation); K34(TMT6plex)	4231.8	0	1.3	1.3	1.3	1.1
																	HNQLPLVIEFTEQTAPK	50	1	1	20070125	N-Term(TMT6plex); K17(TMT6plex)	2423.4	0	1.3	1.3	1.3	1.2
																	IKPHLMSQELPEDWDK	9	1	1	20070125	N-Term(TMT6plex); K2(TMT6plex); M6(Oxidation); K16(TMT6plex)	2669.5	0	1.0	1.4	1.2	1.2
																	FFPASADR	18	1	1	20070125	N-Term(TMT6plex)	1139.6	0	1.3	1.5	1.2	1.2
																	KFLESGGQDGAGDDDDLEDLEEAEEPDMEEDDDQK	2	1	1	20070125	N-Term(TMT6plex); K1(TMT6plex); M28(Oxidation); K35(TMT6plex)	4589.0	1	1.3	1.5	1.3	1.6
																	DVESDSAK	24	1	1	20070125	N-Term(TMT6plex); K8(TMT6plex)	1308.7	0	1.2	1.5	1.2	1.2
																	VHSFPTLK	24	1	1	20070125	N-Term(TMT6plex); K8(TMT6plex)	1386.8	0	1.2	1.3	1.2	1.1
																	LKAEGSEIR	6	1	1	20070125	N-Term(TMT6plex); K2(TMT6plex)	1460.9	1	1.2	1.7	1.2	1.2
																	FLESGGQDGAGDDDDLEDLEEAEEPDMEEDDDQK	5	1	1	20070125	N-Term(TMT6plex); K34(TMT6plex)	4215.8	0	1.8	1.9	1.8	1.5
																	TVIDYNGER	8	1	1	20070125	N-Term(TMT6plex)	1295.7	0	1.3	1.3	1.2	1.1
																	NGDTASPK	3	1	1	20070125	N-Term(TMT6plex); K8(TMT6plex)	1247.7	0	1.3	0.9	1.1	0.7
																	ITEFCHR	1	1	1	20070125	N-Term(TMT6plex); C5(Carbamidomethyl)	1191.6	0	1.4	1.7	1.5	1.4
																	IFGGEIK	12	1	1	20070125	N-Term(TMT6plex); K7(TMT6plex)	1221.8	0	1.2	1.2	1.2	1.0
																	DGVVLFK	15	1	1	20070125	N-Term(TMT6plex); K7(TMT6plex)	1235.8	0	1.2	1.3	1.5	1.1
																	EECPAVR	4	1	1	20070125	N-Term(TMT6plex); C3(Carbamidomethyl)	1089.6	0	1.3	1.7	1.3	1.3
																	EYTAGR	4	1	1	20070125	N-Term(TMT6plex)	925.5	0	1.2	1.5	1.2	1.2
																	GYPTIK	4	2	3	38505222;20070125;42794771	N-Term(TMT6plex); K6(TMT6plex)	1136.7	0				
4505753	NP_002620.1	PGAM1	5223	phosphoglycerate mutase 1	81.89	3	10	16	515	1.4	1.5	1.7	1.6	254	28.8	7.18												
																	DAGYEFDICFTSVQK	84	1	1	4505753	N-Term(TMT6plex); C9(Carbamidomethyl); K15(TMT6plex)	2238.1	0	1.4	1.6	1.7	1.6
																	YADLTEDQLPSCESLK	36	1	1	4505753	N-Term(TMT6plex); C12(Carbamidomethyl); K16(TMT6plex)	2327.2	0	1.4	1.5	1.7	1.4
																	HYGGLTGLNK	26	3	2	71274132;4505753	N-Term(TMT6plex); K10(TMT6plex)	1517.9	0				
																	SYDVPPPPMEPDHPFYSNISK	63	3	2	71274132;4505753	N-Term(TMT6plex); M9(Oxidation); K21(TMT6plex)	2891.4	0				
																	YADLTEDQLPSCESLKDTIAR	7	1	1	4505753	N-Term(TMT6plex); C12(Carbamidomethyl); K16(TMT6plex)	2883.5	1	1.7	2.3	2.0	2.1
																	KAMEAVAAQGK	11	3	1	4505753	N-Term(TMT6plex); K1(TMT6plex); M3(Oxidation); K11(TMT6plex)	1807.1	1	1.5	2.0	1.9	1.8
																	HLEGLSEEAIMELNLPTGIPIVYELDK	69	1	1	4505753	N-Term(TMT6plex); M11(Oxidation); K27(TMT6plex)	3497.9	0	1.4	1.5	1.7	1.5
																	AMEAVAAQGK	53	3	1	4505753	N-Term(TMT6plex); M2(Oxidation); K10(TMT6plex)	1449.8	0	1.2	1.5	1.6	1.4
																	HGESAWNLENR	11	1	1	4505753	N-Term(TMT6plex)	1541.8	0	1.7	1.3	1.4	1.7
																	TLWTVLDAIDQMWLPVVR	21	2	2	71274132;4505753	N-Term(TMT6plex); M12(Oxidation)	2401.3	0				
																	AMEAVAAQGK	4	3	1	4505753	N-Term(TMT6plex); K10(TMT6plex)	1433.8	0	1.7	1.9	2.4	1.5
																	HLEGLSEEAIMELNLPTGIPIVYELDK	16	1	1	4505753	N-Term(TMT6plex); K27(TMT6plex)	3481.9	0	1.5	1.7	2.0	1.5
																	FSGWYDADLSPAGHEEAK	22	1	1	4505753	N-Term(TMT6plex); K18(TMT6plex)	2438.2	0	1.4	1.4	1.7	1.4
																	ALPFWNEEIVPQIK	45	3	2	71274132;4505753	N-Term(TMT6plex); K14(TMT6plex)	2142.2	0				
																	VLIAAHGNSLR	6	2	1	4505753	N-Term(TMT6plex)	1379.8	0	1.2	1.3	1.3	1.3
																	SYDVPPPPMEPDHPFYSNISK	15	3	2	71274132;4505753	N-Term(TMT6plex); K21(TMT6plex)	2875.4	0				
																	HGEAQVK	6	2	2	71274132;4505753	N-Term(TMT6plex); K7(TMT6plex)	1226.7	0				
																	NLKPIKPMQFLGDEETVR	12	2	1	4505753	N-Term(TMT6plex); K3(TMT6plex); K6(TMT6plex); M8(Oxidation)	2818.6	0	1.3	1.4	1.6	1.4
																	NLKPIKPMQFLGDEETVR	3	2	1	4505753	N-Term(TMT6plex); K3(TMT6plex); K6(TMT6plex)	2802.6	0	1.9	2.1	2.9	1.6
																	TLWTVLDAIDQMWLPVVR	2	2	2	71274132;4505753	N-Term(TMT6plex)	2385.3	0				
																	AETAAK	3	3	2	4505753;71274132	N-Term(TMT6plex); K6(TMT6plex)	1048.6	0				
4506179	NP_002777.1	PSMA1	5682	proteasome subunit alpha type-1 isoform 2	81.75	3	19	19	142	1.4	1.4	1.5	1.5	263	29.5	6.61												
																	LVSLIGSK	4	2	1	4506179	N-Term(TMT6plex); K8(TMT6plex)	1274.8	0	1.2	1.2	1.3	1.2
																	AQSELAAHQK	14	3	1	4506179	N-Term(TMT6plex); K10(TMT6plex)	1540.9	0	1.2	1.3	1.3	1.4
																	THAVLVALK	6	3	1	4506179	N-Term(TMT6plex); K9(TMT6plex)	1409.9	0	1.2	1.4	1.5	1.3
																	KAQPAQPADEPAEKADEPMEH	1	2	1	4506179	N-Term(TMT6plex); K1(TMT6plex); K14(TMT6plex); M19(Oxidation)	2992.5	2	2.2	2.1	2.1	1.8
																	FVFDRPLPVSR	6	3	1	4506179	N-Term(TMT6plex)	1561.9	0	1.3	1.2	1.3	1.4
																	ETLPAEQDLTTK	19	2	1	4506179	N-Term(TMT6plex); K12(TMT6plex)	1804.0	0	1.4	1.4	1.5	1.6
																	NQYDNDVTVWSPQGR	19	3	1	4506179	N-Term(TMT6plex)	2008.0	0	1.5	1.2	1.4	1.4
																	DLEFTIYDDDDVSPFLEGLEERPQR	10	2	1	4506179	N-Term(TMT6plex)	3227.5	0	1.4	1.6	1.5	1.7
																	IHQIEYAMEAVK	4	3	1	4506179	N-Term(TMT6plex); M8(Oxidation); K12(TMT6plex)	1906.1	0	1.3	1.4	1.4	1.5
																	KAQPAQPADEPAEK	3	2	1	4506179	N-Term(TMT6plex); K1(TMT6plex); K14(TMT6plex)	2167.2	1	2.5	1.4	1.4	1.6
																	NVSIGIVGK	4	2	1	4506179	N-Term(TMT6plex); K9(TMT6plex)	1344.9	0	1.2	1.1	1.3	1.1
																	HMSEFMECNLNELVK	2	2	1	4506179	N-Term(TMT6plex); M2(Oxidation); M6(Oxidation); C8(Carbamidomethyl); K15(TMT6plex)	2371.2	0	1.2	1.3	1.4	1.5
																	HMSEFMECNLNELVK	5	2	1	4506179	N-Term(TMT6plex); M6(Oxidation); C8(Carbamidomethyl); K15(TMT6plex)	2355.2	0	1.2	1.2	1.4	1.3
																	QGSATVGLK	3	3	1	4506179	N-Term(TMT6plex); K9(TMT6plex)	1318.8	0	1.2	0.9	1.2	1.1
																	ILHVDNHIGISIAGLTADAR	2	3	1	4506179	N-Term(TMT6plex)	2315.3	0	1.4	1.4	1.7	1.3
																	AQPAQPADEPAEK	25	2	1	4506179	N-Term(TMT6plex); K13(TMT6plex)	1810.0	0	1.9	1.9	2.0	2.0
																	RPYGVGLLIAGYDDMGPHIFQTCPSANYFDCR	7	2	1	4506179	N-Term(TMT6plex); M15(Oxidation); C23(Carbamidomethyl); C31(Carbamidomethyl)	3935.8	0	1.4	1.5	1.6	1.6
																	IHQIEYAMEAVK	2	3	1	4506179	N-Term(TMT6plex); K12(TMT6plex)	1890.1	0	1.3	1.3	1.6	1.3
																	AQPAQPADEPAEKADEPMEH	1	2	1	4506179	N-Term(TMT6plex); K13(TMT6plex); M18(Oxidation)	2635.3	1	1.3	1.4	1.2	1.4
																	LLCNFMR	2	3	1	4506179	N-Term(TMT6plex); C3(Carbamidomethyl)	1182.6	0	1.9	1.8	2.3	1.5
																	HMSEFMECNLNELVK	1	2	1	4506179	N-Term(TMT6plex); C8(Carbamidomethyl); K15(TMT6plex)	2339.2	0	1.5	1.8	1.7	1.8
																	LLCNFMR	1	3	1	4506179	N-Term(TMT6plex); C3(Carbamidomethyl); M6(Oxidation)	1198.6	0	1.3	1.3	1.3	1.4
																	ADEPMEH	1	2	1	4506179	N-Term(TMT6plex); M5(Oxidation)	1073.5	0	1.4	1.3	1.5	1.6
221307584	NP_001138303.1	PHB2	11331	prohibitin-2 isoform 1	81.61	2	24	24	286	1.5	1.7	1.8	1.6	299	33.3	9.83												
																	EYTAAVEAK	14	2	1	221307584	N-Term(TMT6plex); K9(TMT6plex)	1439.8	0	1.7	2.0	1.9	1.7
																	DLQMVNISLR	11	2	1	221307584	N-Term(TMT6plex); M4(Oxidation)	1433.8	0	1.4	1.7	1.6	1.5
																	VLPSIVNEVLK	41	2	1	221307584	N-Term(TMT6plex); K11(TMT6plex)	1669.1	0	1.5	1.9	1.9	1.7
																	VLSRPNAQELPSMYQR	4	2	1	221307584	N-Term(TMT6plex); M13(Oxidation)	2134.1	0	1.8	1.7	1.9	1.6
																	LGLDYEER	10	2	1	221307584	N-Term(TMT6plex)	1223.6	0	1.5	1.6	1.8	1.6
																	IGGVQQDTILAEGLHFR	24	2	1	221307584	N-Term(TMT6plex)	2083.1	0	1.4	1.5	1.7	1.4
																	AQFLVEK	14	1	1	221307584	N-Term(TMT6plex); K7(TMT6plex)	1292.8	0	1.4	1.8	1.8	1.5
																	MLGEALSK	6	1	1	221307584	N-Term(TMT6plex); M1(Oxidation); K8(TMT6plex)	1322.8	0	1.4	1.4	1.5	1.4
																	LLLGAGAVAYGVR	17	2	1	221307584	N-Term(TMT6plex)	1488.9	0	1.6	1.8	1.8	1.8
																	IVQAEGEAEAAK	16	1	1	221307584	N-Term(TMT6plex); K12(TMT6plex)	1673.9	0	1.5	1.9	1.8	1.6
																	IYLTADNLVLNLQDESFTR	33	2	1	221307584	N-Term(TMT6plex)	2454.3	0	1.7	1.7	1.9	1.7
																	AKDFSLILDDVAITELSFSR	10	2	1	221307584	N-Term(TMT6plex); K2(TMT6plex)	2698.5	1	1.7	2.6	2.2	2.1
																	IPWFQYPIIYDIR	31	2	1	221307584	N-Term(TMT6plex)	1953.1	0	1.8	2.0	2.2	1.6
																	AQVSLLIR	9	2	1	221307584	N-Term(TMT6plex)	1128.7	0	1.5	1.9	1.9	1.8
																	KISSPTGSK	2	2	1	221307584	N-Term(TMT6plex); K1(TMT6plex); K9(TMT6plex)	1592.0	1	1.5	2.2	2.0	1.8
																	TIATSQNR	2	2	1	221307584	N-Term(TMT6plex)	1119.6	0	1.5	1.5	1.5	1.2
																	QVAQQEAQR	6	1	1	221307584	N-Term(TMT6plex)	1286.7	0	1.8	2.0	2.0	1.7
																	ESVFTVEGGHR	8	2	1	221307584	N-Term(TMT6plex)	1446.8	0	1.3	1.4	1.4	1.4
																	FNASQLITQR	6	2	1	221307584	N-Term(TMT6plex)	1406.8	0	1.4	1.4	1.4	1.3
																	ISSPTGSK	8	2	1	221307584	N-Term(TMT6plex); K8(TMT6plex)	1234.7	0	1.4	1.6	1.8	1.5
																	AAQNISK	6	2	1	221307584	N-Term(TMT6plex); K7(TMT6plex)	1189.7	0	1.3	1.6	1.6	1.5
																	AIFFNR	4	2	1	221307584	N-Term(TMT6plex)	996.6	0	1.7	2.2	2.1	1.8
																	GSDSLIK	2	2	1	221307584	N-Term(TMT6plex); K7(TMT6plex)	1177.7	0	1.5	2.0	2.0	1.6
																	NPGYIK	2	2	1	221307584	N-Term(TMT6plex); K6(TMT6plex)	1149.7	0	1.6	1.8	2.1	1.5
34577112;4557305	NP_908932.1	ALDOA	226	fructose-bisphosphate aldolase A isoform 1	81.59	1	1	26	1027	1.4	1.0	1.3	1.1	364	39.4	8.09												
																	ALQASALK	45	2	2	342187211;34577112;4557305	N-Term(TMT6plex); K8(TMT6plex)	1259.8	0				
																	IGEHTPSALAIMENANVLAR	51	2	2	342187211;34577112;4557305	N-Term(TMT6plex); M12(Oxidation)	2352.3	0				
																	PYQYPALTPEQK	5	1	1	34577112;4557305	N-Term(TMT6plex); K12(TMT6plex)	1893.0	0	1.4	1.0	1.3	1.1

Khan et al., 2019. Multi-omics analysis to characterize cigarette smoke induced molecular alterations in esophageal cells
Supplementary Table 6. List of all proteins and peptides identified in untreated and chronically treated Het1A cells with cigarette smoke condensate for 8 months

Accession	RefSeq Accession	Gene Symbol	Entrez Gene ID	Description	Σ Coverage	Σ# Proteins	Σ# Unique Peptides	Σ# Peptides	Σ# PSMs	Het-1A-Smoke - 2M/Parental	Het-1A-Smoke - 4M/Parental	Het-1A-Smoke - 6M/Parental	Het-1A-Smoke - 8M/Parental	# AAs	MW [kDa]	calc. pI	Sequence	# PSMs	# Proteins	# Protein Groups	Protein Group Accessions	Modifications	MH+ [Da]	# Missed Cleavages	Het-1A-Smoke - 2M/Parental	Het-1A-Smoke - 4M/Parental	Het-1A-Smoke - 6M/Parental	Het-1A-Smoke - 8M/Parental
																	CQYVTEK	14	3	3	342187211;34577112;4557305;4885063	N-Term(TMT6plex); C1(Carbamidomethyl); K7(TMT6plex)	1385.7	0				
																	VLAAVYK	40	4	3	342187211;34577112;4557305;4885063	N-Term(TMT6plex); K7(TMT6plex)	1221.8	0				
																	GGVVGIK	14	2	2	342187211;34577112;4557305	N-Term(TMT6plex); K7(TMT6plex)	1087.7	0				
																	TVPPAVTGITFLSGGQSEEEASINLNAINK	318	2	2	342187211;34577112;4557305	N-Term(TMT6plex); K30(TMT6plex)	3515.9	0				
																	YTPSGQAGAAASESLFVSNHAY	150	2	2	342187211;34577112;4557305	N-Term(TMT6plex)	2457.2	0				
																	GVVPLAGTNGETTTQGLDGLSER	32	2	2	342187211;34577112;4557305	N-Term(TMT6plex)	2501.3	0				
																	QLLLTADDR	31	2	2	342187211;34577112;4557305	N-Term(TMT6plex)	1273.7	0				
																	FSHEEIAMATVTALR	50	2	2	342187211;34577112;4557305	N-Term(TMT6plex); M8(Oxidation)	1921.0	0				
																	LQSIGTENTEENRR	33	2	2	342187211;34577112;4557305	N-Term(TMT6plex)	1876.0	1				
																	ELSDIAHR	4	2	2	342187211;34577112;4557305	N-Term(TMT6plex)	1169.6	0				
																	ALANSLACQGK	29	2	2	342187211;34577112;4557305	N-Term(TMT6plex); C8(Carbamidomethyl); K11(TMT6plex)	1590.9	0				
																	ADDGRPFPQVIK	16	2	2	342187211;34577112;4557305	N-Term(TMT6plex); K12(TMT6plex)	1801.0	0				
																	LQSIGTENTEENR	49	2	2	342187211;34577112;4557305	N-Term(TMT6plex)	1719.9	0				
																	DGADFAK	27	3	3	342187211;34577112;4557305;4885063	N-Term(TMT6plex); K7(TMT6plex)	1181.7	0				
																	GILAADESTGSIAK	36	2	2	342187211;34577112;4557305	N-Term(TMT6plex); K14(TMT6plex)	1791.0	0				
																	AAQEEYVK	32	2	2	342187211;34577112;4557305	N-Term(TMT6plex); K8(TMT6plex)	1395.8	0				
																	KELSDIAHR	2	2	2	342187211;34577112;4557305	N-Term(TMT6plex); K1(TMT6plex)	1526.9	1				
																	VNPCIGGVILFHETLYQK	8	2	2	342187211;34577112;4557305	N-Term(TMT6plex); C4(Carbamidomethyl); K18(TMT6plex)	2546.4	0				
																	IGEHTPSALAIMENANVLAR	15	2	2	342187211;34577112;4557305	N-Term(TMT6plex)	2336.3	0				
																	FSHEEIAMATVTALR	5	2	2	342187211;34577112;4557305	N-Term(TMT6plex)	1905.0	0				
																	KDGADFAK	7	3	3	342187211;34577112;4557305;4885063	N-Term(TMT6plex); K1(TMT6plex); K8(TMT6plex)	1538.9	1				
																	YASICQQNGIVPIVEPEILPDGDHDLKR	6	3	3	342187211;34577112;4557305;4885063	N-Term(TMT6plex); C5(Carbamidomethyl); K27(TMT6plex)	3634.9	1				
																	YASICQQNGIVPIVEPEILPDGDHDLK	2	3	3	342187211;34577112;4557305;4885063	N-Term(TMT6plex); C5(Carbamidomethyl); K27(TMT6plex)	3478.8	0				
																	CPLLKPWALTFSYGR	4	2	2	342187211;34577112;4557305	N-Term(TMT6plex); C1(Carbamidomethyl); K5(TMT6plex)	2267.3	0				
																	IGEHTPSALAIMENANVLARYASICQQNGIVPIVEPEILPDGDHDLK	1	2	2	342187211;34577112;4557305	N-Term(TMT6plex); C25(Carbamidomethyl); K47(TMT6plex)	5566.8	1				
																	IGEHTPSALAIMENANVLARYASICQQNGIVPIVEPEILPDGDHDLK	1	2	2	342187211;34577112;4557305	N-Term(TMT6plex); M12(Oxidation); C25(Carbamidomethyl); K47(TMT6plex)	5582.9	1				
256222019	NP_057215.3	**RAB10**	10890	**ras-related protein Rab-10**	81.50	9	12	16	83	**1.3**	**1.4**	**1.5**	**1.4**	200	22.5	8.38												
																	NIDEHANEDVER	9	1	1	256222019	N-Term(TMT6plex)	1669.8	0	**1.2**	**1.3**	**1.3**	**1.3**
																	AFLTLAEDILR	28	1	1	256222019	N-Term(TMT6plex)	1490.9	0	**1.4**	**1.5**	**1.6**	**1.5**
																	FSDDAFNTTFISTIGIDFK	4	1	1	256222019	N-Term(TMT6plex); K19(TMT6plex)	2597.3	0	**1.3**	**1.3**	**1.4**	**1.4**
																	FHTITTSYYR	1	1	1	256222019	N-Term(TMT6plex)	1517.8	0	**0.9**	**1.1**	**1.1**	**1.1**
																	KTPVKEPNSENVDISSGGGVTGWK	1	1	1	256222019	N-Term(TMT6plex); K1(TMT6plex); K5(TMT6plex); K24(TMT6plex)	3402.9	2	**1.1**	**1.3**	**1.3**	**1.2**
																	LQIWDTAGQER	4	31	17	5803135;4758988;13569962;7706563;16933567;256222019;82659107;19923483;19923750;4759000;39930371;38679888;4506367;96975097;106507164;19923260;325910899	N-Term(TMT6plex)	1545.8	0				
																	KTYDLLFK	5	1	1	256222019	N-Term(TMT6plex); K1(TMT6plex); K8(TMT6plex)	1715.1	1	**1.1**	**1.4**	**1.6**	**1.4**
																	LLLIGDSGVGK	14	10	7	5803135;4758988;13569962;7706563;16933567;256222019;4506363	N-Term(TMT6plex); K11(TMT6plex)	1530.0	0				
																	SFENISK	5	1	1	256222019	N-Term(TMT6plex); K7(TMT6plex)	1282.7	0	**1.2**	**1.4**	**1.5**	**1.3**
																	EPNSENVDISSGGGVTGWK	2	1	1	256222019	N-Term(TMT6plex); K19(TMT6plex)	2391.2	0	**1.3**	**1.2**	**1.7**	**1.4**
																	FFETSAK	4	3	2	4506363;256222019	N-Term(TMT6plex); K7(TMT6plex)	1287.7	0				
																	GAMGIMLVYDITNGK	1	1	1	256222019	N-Term(TMT6plex); M3(Oxidation); K15(TMT6plex)	2057.1	0	**1.3**	**1.3**	**1.4**	**1.1**
																	ANINIEK	2	1	1	256222019	N-Term(TMT6plex); K7(TMT6plex)	1259.8	0	**1.3**	**1.5**	**1.5**	**1.4**
																	TVELQGK	1	1	1	256222019	N-Term(TMT6plex); K7(TMT6plex)	1232.8	0	**1.3**	**1.4**	**1.4**	**1.3**
																	GKGEQIAR	1	1	1	256222019	N-Term(TMT6plex); K2(TMT6plex)	1316.8	1	**0.9**	**1.4**	**1.3**	**1.4**
																	TCVLFR	1	2	2	16933567;256222019	N-Term(TMT6plex); C2(Carbamidomethyl)	1024.6	0				
322303127	NP_001190174.1	**RPS10**	6204	**40S ribosomal protein S10**	81.21	1	15	16	293	**1.2**	**1.3**	**1.3**	**1.4**	165	18.9	10.15												
																	NVPNLHVMK	23	2	1	322303127	N-Term(TMT6plex); M8(Oxidation); K9(TMT6plex)	1525.9	0	**1.2**	**1.2**	**1.3**	**1.2**
																	SAVPPGADK	43	2	1	322303127	N-Term(TMT6plex); K9(TMT6plex)	1299.8	0	**1.1**	**1.2**	**1.2**	**1.2**
																	DYLHLPPEIVPATLR	56	2	1	322303127	N-Term(TMT6plex)	1963.1	0	**1.4**	**1.5**	**1.5**	**1.5**
																	IAIYELLFK	53	2	1	322303127	N-Term(TMT6plex); K9(TMT6plex)	1568.0	0	**1.2**	**1.2**	**1.2**	**1.3**
																	GLEGERPAR	10	2	1	322303127	N-Term(TMT6plex)	1213.7	0	**1.0**	**1.3**	**1.2**	**1.4**
																	AEAGAGSATEFQFR	24	1	1	322303127	N-Term(TMT6plex)	1670.8	0	**1.3**	**1.4**	**1.4**	**1.5**
																	KAEAGAGSATEFQFR	12	1	1	322303127	N-Term(TMT6plex); K1(TMT6plex)	2028.1	1	**1.1**	**1.3**	**1.2**	**1.5**
																	HFYWYLTNEGIQYLR	17	2	1	322303127	N-Term(TMT6plex)	2232.1	0	**1.2**	**1.3**	**1.4**	**1.3**
																	HPELADK	12	2	1	322303127	N-Term(TMT6plex); K7(TMT6plex)	1267.7	0	**1.0**	**1.2**	**1.2**	**1.5**
																	NVPNLHVMK	6	2	1	322303127	N-Term(TMT6plex); K9(TMT6plex)	1509.9	0	**1.6**	**1.5**	**1.8**	**1.4**
																	GEADRDTYR	11	2	1	322303127	N-Term(TMT6plex)	1311.6	1	**1.0**	**1.3**	**1.2**	**1.5**
																	EGVMVAK	14	3	2	322303127;41322916	N-Term(TMT6plex); M4(Oxidation); K7(TMT6plex)	1207.7	0				
																	SRPETGRPRPK	1	2	1	322303127	N-Term(TMT6plex); K11(TMT6plex)	1739.0	0	**1.2**	**3.1**	**1.3**	**3.2**
																	KDVHMPK	2	2	1	322303127	N-Term(TMT6plex); K1(TMT6plex); M5(Oxidation); K7(TMT6plex)	1557.9	1	**0.7**	**1.0**	**0.9**	**1.3**
																	DVHMPK	6	2	1	322303127	N-Term(TMT6plex); M4(Oxidation); K6(TMT6plex)	1200.7	0	**0.9**	**1.2**	**1.1**	**1.4**
																	EQFAWR	2	2	1	322303127	N-Term(TMT6plex)	1065.6	0	**1.2**	**1.2**	**1.2**	**1.3**
																	AMQSLK	1	2	1	322303127	N-Term(TMT6plex); K6(TMT6plex)	1135.7	0	**1.5**	**1.9**	**2.0**	**1.5**
62414289	NP_003371.2	**VIM**	7431	**vimentin**	81.12	9	30	38	1828	**1.2**	**1.3**	**1.7**	**1.3**	466	53.6	5.12												
																	QVQSLTCEVDALK	53	1	1	62414289	N-Term(TMT6plex); C7(Carbamidomethyl); K13(TMT6plex)	1949.1	0	**1.3**	**1.2**	**1.7**	**1.1**
																	ISLPLPNFSSLNLR	157	1	1	62414289	N-Term(TMT6plex)	1800.1	0	**1.2**	**1.3**	**1.6**	**1.3**
																	FADLSEAANR	36	1	1	62414289	N-Term(TMT6plex)	1322.7	0	**1.2**	**1.2**	**1.5**	**1.2**
																	SLYASSPGGVYATR	68	1	1	62414289	N-Term(TMT6plex)	1657.9	0	**1.4**	**1.2**	**1.6**	**1.3**
																	VELQELNDR	27	2	2	55749932;62414289	N-Term(TMT6plex)	1344.7	0				
																	LQDEIQNMK	46	1	1	62414289	N-Term(TMT6plex); M8(Oxidation); K9(TMT6plex)	1592.9	0	**1.2**	**1.2**	**1.6**	**1.2**
																	SVSSSSYR	10	1	1	62414289	N-Term(TMT6plex)	1101.6	0	**1.2**	**1.2**	**1.7**	**1.1**
																	DNLAEDIMR	18	1	1	62414289	N-Term(TMT6plex); M8(Oxidation)	1321.7	0	**1.2**	**1.3**	**1.5**	**1.2**
																	LQEEMLQR	24	1	1	62414289	N-Term(TMT6plex); M5(Oxidation)	1291.7	0	**1.5**	**1.3**	**1.6**	**1.3**
																	DGQVINETSQHHDDLE	49	1	1	62414289	N-Term(TMT6plex)	2066.0	0	**1.4**	**1.3**	**1.5**	**1.3**
																	MALDIEIATYR	30	2	2	62414289;21264345	N-Term(TMT6plex); M1(Oxidation)	1540.8	0				
																	KLLEGEESR	14	6	5	67782365;55749932;372466577;62414289;21264345	N-Term(TMT6plex); K1(TMT6plex)	1518.9	1				
																	ILLAELEQLK	129	1	1	62414289	N-Term(TMT6plex); K10(TMT6plex)	1628.0	0	**1.1**	**1.3**	**1.8**	**1.2**
																	ETNLDSLPLVDTHSK	57	1	1	62414289	N-Term(TMT6plex); K15(TMT6plex)	2127.1	0	**1.1**	**1.3**	**1.6**	**1.3**
																	EEAENTLQSFR	46	1	1	62414289	N-Term(TMT6plex)	1552.8	0	**1.1**	**1.2**	**1.6**	**1.2**
																	QQYESVAAK	34	1	1	62414289	N-Term(TMT6plex); K9(TMT6plex)	1481.8	0	**1.1**	**1.2**	**1.7**	**1.2**
																	QDVDNASLAR	16	1	1	62414289	N-Term(TMT6plex)	1317.7	0	**1.3**	**1.2**	**1.5**	**1.1**
																	QVDQLTNDK	34	1	1	62414289	N-Term(TMT6plex); K9(TMT6plex)	1518.9	0	**1.2**	**1.2**	**1.6**	**1.3**
																	EYQDLLNVK	51	7	3	55749932;62414289;105990539	N-Term(TMT6plex); K9(TMT6plex)	1579.9	0				
																	FLEQQNK	51	22	9	67782365;372466577;155969697;119395754;62414289;119703753;15431316;109148552;89357932	N-Term(TMT6plex); K7(TMT6plex)	1364.8	0				
																	EMEENFAVEAANYQDTIGR	123	1	1	62414289	N-Term(TMT6plex); M2(Oxidation)	2432.1	0	**1.3**	**1.4**	**1.5**	**1.3**
																	LLEGEESR	28	7	6	67782365;15431316;55749932;372466577;62414289;21264345	N-Term(TMT6plex)	1161.6	0				
																	LGDLYEEEMR	58	1	1	62414289	N-Term(TMT6plex); M9(Oxidation)	1499.7	0	**1.3**	**1.4**	**1.7**	**1.4**
																	FANYIDK	31	1	1	62414289	N-Term(TMT6plex); K7(TMT6plex)	1328.8	0	**1.2**	**1.1**	**1.6**	**1.1**
																	GTNESLER	7	4	1	62414289	N-Term(TMT6plex)	1134.6	0	**1.2**	**1.3**	**1.7**	**1.2**
																	LLQDSVDFSLADAINTEFK	394	1	1	62414289	N-Term(TMT6plex); K19(TMT6plex)	2584.4	0	**1.3**	**1.4**	**1.8**	**1.5**
																	VESLQEEIAFLK	18	1	1	62414289	N-Term(TMT6plex); K12(TMT6plex)	1864.1	0	**1.0**	**1.0**	**1.7**	**1.1**
																	KLHEEEIQELQAQIQEQHVQIDVDVSKPDLTAALR	4	1	1	62414289	N-Term(TMT6plex); K1(TMT6plex); K27(TMT6plex)	4738.6	1	**1.1**	**1.3**	**1.9**	**1.3**
																	LGDLYEEEMR	2	1	1	62414289	N-Term(TMT6plex)	1483.7	0	**1.4**	**1.8**	**2.2**	**1.6**
																	TYSLGSALRPSTSR	12	1	1	62414289	N-Term(TMT6plex)	1724.9	0	**1.3**	**1.2**	**1.5**	**1.2**
																	LQDEIQNMKEEMAR	1	1	1	62414289	N-Term(TMT6plex); K9(TMT6plex)	2193.1	1	**1.9**	**2.3**	**3.1**	**1.6**
																	LQDEIQNMKEEMAR	9	1	1	62414289	N-Term(TMT6plex); K9(TMT6plex); M12(Oxidation)	2209.1	1	**1.3**	**1.7**	**2.0**	**1.5**
																	NLQEAEEWYK	43	2	2	62414289;21264345	N-Term(TMT6plex); K10(TMT6plex)	1767.9	0				
																	DNLAEDIMR	8	1	1	62414289	N-Term(TMT6plex)	1305.7	0	**1.4**	**1.6**	**2.5**	**1.2**
																	KVESLQEEIAFLK	97	1	1	62414289	N-Term(TMT6plex); K1(TMT6plex); K13(TMT6plex)	2221.3	1	**1.1**	**1.3**	**1.7**	**1.3**
																	QESTEYR	11	1	1	62414289	N-Term(TMT6plex)	1141.6	0	**1.2**	**1.0**	**1.7**	**1.1**
																	SYVTTSTR	7	1	1	62414289	N-Term(TMT6plex)	1143.6	0	**1.4**	**1.1**	**1.5**	**1.1**
																	LQDEIQNMKEEMAR	3	1	1	62414289	N-Term(TMT6plex); M8(Oxidation); K9(TMT6plex); M12(Oxidation)	2225.1	1	**1.1**	**1.2**	**1.3**	**1.4**
																	EMEENFAVEAANYQDTIGR	6	1	1	62414289	N-Term(TMT6plex)	2416.1	0	**1.3**	**1.6**	**1.4**	**1.8**
																	LHEEEIQELQAQIQEQHVQIDVDVSKPDLTAALR	2	1	1	62414289	N-Term(TMT6plex); K26(TMT6plex)	4381.3	0	**1.8**	**1.6**	**1.7**	**1.3**
																	LQDEIQNMK	4	1	1	62414289	N-Term(TMT6plex); K9(TMT6plex)	1576.9	0	**1.3**	**1.4**	**1.6**	**1.3**
																	EKLQEEMLQR	1	1	1	62414289	N-Term(TMT6plex); K2(TMT6plex)	1762.0	1	**1.4**	**2.0**	**2.5**	**1.5**
																	TNEKVELQELNDR	4	2	2	55749932;62414289	N-Term(TMT6plex); K4(TMT6plex)	2046.1	1				
																	SSVPGVR	4	1	1	62414289	N-Term(TMT6plex)	930.6	0	**1.1**	**1.1**	**1.5**	**1.1**
																	EKLQEEMLQR	1	1	1	62414289	N-Term(TMT6plex); K2(TMT6plex); M7(Oxidation)	1778.0	1	**0.8**	**1.3**	**1.6**	**1.4**
4507645	NP_000356.1	**TPI1**	7167	**triosephosphate isomerase isoform 1**	81.12	3	17	17	376	**1.3**	**1.7**	**1.7**	**1.6**	249	26.7	6.90												
																	HVFGESDELIGQK	22	3	1	4507645	N-Term(TMT6plex); K13(TMT6plex)	1917.0	0	**1.3**	**1.6**	**1.6**	**1.5**
																	QSLGELIGTLNAAK	29	2	1	4507645	N-Term(TMT6plex); K14(TMT6plex)	1873.1	0	**1.3**	**1.5**	**1.6**	**1.5**

Accession	RefSeq Accession	Gene Symbol	Entrez Gene ID	Description	Σ Coverage	Σ# Proteins	Σ# Unique Peptides	Σ# Peptides	Σ# PSMs	Het-1A-Smoke - 2M/Parental	Het-1A-Smoke - 4M/Parental	Het-1A-Smoke - 6M/Parental	Het-1A-Smoke - 8M/Parental	# AAs	MW [kDa]	calc. pI	Sequence	# PSMs	# Proteins	# Protein Groups	Protein Group Accessions	Modifications	MH+ [Da]	# Missed Cleavages	Het-1A-Smoke - 2M/Parental	Het-1A-Smoke - 4M/Parental	Het-1A-Smoke - 6M/Parental	Het-1A-Smoke - 8M/Parental
																	VVLAYEPVWAIGTGK	60	3	1	4507645	N-Term(TMT6plex); K15(TMT6plex)	2061.2	0	1.4	1.9	1.7	1.8
																	VPADTEVVCAPPTAYIDFAR	39	2	1	4507645	N-Term(TMT6plex); C9(Carbamidomethyl)	2421.2	0	1.3	1.6	1.7	1.5
																	VAHALAEGLGVIACIGEK	22	3	1	4507645	N-Term(TMT6plex); C14(Carbamidomethyl); K18(TMT6plex)	2266.3	0	1.4	1.9	1.9	1.8
																	ELASQPDVDGFLVGGASLKPEFVDIINAK	45	3	1	4507645	N-Term(TMT6plex); K19(TMT6plex); K29(TMT6plex)	3717.1	0	1.1	2.1	2.0	2.0
																	SNVSDAVAQSTR	29	3	1	4507645	N-Term(TMT6plex)	1463.8	0	1.5	1.7	1.6	1.6
																	TATPQQAQEVHEK	16	3	1	4507645	N-Term(TMT6plex); K13(TMT6plex)	1925.0	0	1.4	1.6	1.7	1.6
																	IAVAAQNCYK	14	2	1	4507645	N-Term(TMT6plex); C8(Carbamidomethyl); K10(TMT6plex)	1595.9	0	1.4	1.7	1.6	1.7
																	IIYGGSVTGATCK	17	3	1	4507645	N-Term(TMT6plex); C12(Carbamidomethyl); K13(TMT6plex)	1785.0	0	1.4	1.6	1.6	1.5
																	KQSLGELIGTLNAAK	15	2	1	4507645	N-Term(TMT6plex); K1(TMT6plex); K15(TMT6plex)	2230.4	1	1.2	1.7	1.7	1.7
																	DCGATWVVLGHSER	29	3	1	4507645	N-Term(TMT6plex); C2(Carbamidomethyl)	1815.9	0	1.3	1.4	1.5	1.3
																	KFFVGGNWK	3	2	1	4507645	N-Term(TMT6plex); K1(TMT6plex); K9(TMT6plex)	1770.1	1	1.2	1.7	1.6	1.7
																	FFVGGNWK	15	2	1	4507645	N-Term(TMT6plex); K8(TMT6plex)	1412.8	0	1.4	1.7	1.7	1.6
																	VIADNVK	13	3	1	4507645	N-Term(TMT6plex); K7(TMT6plex)	1216.8	0	1.2	1.6	1.5	1.5
																	EAGITEK	7	3	1	4507645	N-Term(TMT6plex); K7(TMT6plex)	1205.7	0	1.6	1.6	1.5	1.6
																	VVFEQTK	1	3	1	4507645	N-Term(TMT6plex); K7(TMT6plex)	1308.8	0	1.1	1.5	1.3	1.3
4502049	NP_001619.1	**AKR1B1**	231	**aldose reductase**	81.01	1	17	18	131	**1.4**	**1.4**	**1.5**	**1.5**	316	35.8	6.98												
																	EFFPLDESGNVVPSDTNILDTWAAMEELVDEGLVK	18	1	1	4502049	N-Term(TMT6plex); M25(Oxidation); K35(TMT6plex)	4354.2	0	1.2	1.2	1.2	1.5
																	SPPGQVTEAVK	25	1	1	4502049	N-Term(TMT6plex); K11(TMT6plex)	1570.9	0	1.5	1.5	1.4	1.6
																	VCALLSCTSHK	4	1	1	4502049	N-Term(TMT6plex); C2(Carbamidomethyl); C7(Carbamidomethyl); K11(TMT6plex)	1733.9	0	1.5	1.4	1.6	1.5
																	VAIDVGYR	3	1	1	4502049	N-Term(TMT6plex)	1121.7	0	1.5	1.6	1.5	1.7
																	HIDCAHVYQNENEVGVAIQEK	3	1	1	4502049	N-Term(TMT6plex); C4(Carbamidomethyl); K21(TMT6plex)	2911.5	0	1.4	1.4	1.5	1.4
																	LIQYCQSK	2	1	1	4502049	N-Term(TMT6plex); C5(Carbamidomethyl); K8(TMT6plex)	1497.9	0	1.5	1.4	1.6	1.5
																	LDYLDLYLIHWPTGFKPGK	7	1	1	4502049	N-Term(TMT6plex); K16(TMT6plex); K19(TMT6plex)	2963.7	0	1.7	1.7	1.8	1.7
																	MPILGLGTWK	15	2	2	320202986;24497577;4502049	N-Term(TMT6plex); M1(Oxidation); K10(TMT6plex)	1589.9	0				
																	YKPAVNQIECHPYLTQEK	7	1	1	4502049	N-Term(TMT6plex); K2(TMT6plex); C10(Carbamidomethyl); K18(TMT6plex)	2905.6	0	1.3	1.6	1.6	1.5
																	REELFIVSK	6	1	1	4502049	N-Term(TMT6plex); K9(TMT6plex)	1579.0	1	1.3	1.4	1.3	1.4
																	TTAQVLIR	3	2	1	4502049	N-Term(TMT6plex)	1130.7	0	1.5	1.2	1.5	1.1
																	GIVVTAYSPLGSPDRPWAKPEDPSLLEDPR	5	1	1	4502049	N-Term(TMT6plex); K19(TMT6plex)	3721.0	0	1.4	1.7	1.5	1.8
																	VFDFELSSQDMTTLLSYNR	4	1	1	4502049	N-Term(TMT6plex)	2495.2	0	1.9	1.7	1.7	1.9
																	MPILGLGTWK	8	2	2	4502049;320202986;24497577	N-Term(TMT6plex); K10(TMT6plex)	1574.0	0				
																	AIGISNFNHLQVEMILNKPGLK	4	1	1	4502049	N-Term(TMT6plex); M14(Oxidation); K18(TMT6plex); K22(TMT6plex)	3139.8	0	1.1	1.3	1.2	1.5
																	NLVVIPK	7	1	1	4502049	N-Term(TMT6plex); K7(TMT6plex)	1240.8	0	1.4	1.2	1.3	1.3
																	EFFPLDESGNVVPSDTNILDTWAAMEELVDEGLVK	1	1	1	4502049	N-Term(TMT6plex); K35(TMT6plex)	4338.2	0	1.4	1.3	1.7	1.6
																	IAENFK	3	1	1	4502049	N-Term(TMT6plex); K6(TMT6plex)	1179.7	0	1.4	1.3	1.3	1.5
																	DYPFHEEF	4	1	1	4502049	N-Term(TMT6plex)	1312.6	0	1.1	1.4	1.3	1.3
																	TLSDLK	2	1	1	4502049	N-Term(TMT6plex); K6(TMT6plex)	1134.7	0	1.4	1.5	1.5	1.6
21624607	NP_066972.1	**COTL1**	23406	**coactosin-like protein**	80.99	1	13	13	105	**1.8**	**1.7**	**2.0**	**1.6**	142	15.9	5.67												
																	KELEEDFIK	7	1	1	21624607	N-Term(TMT6plex); K1(TMT6plex); K9(TMT6plex)	1838.1	1	1.7	1.7	1.9	1.7
																	DDGSAVIWVTFK	9	1	1	21624607	N-Term(TMT6plex); K12(TMT6plex)	1796.0	0	1.6	1.8	1.9	1.7
																	YDGSTIVPGEQGAEYQHFIQQCTDDVR	9	1	1	21624607	N-Term(TMT6plex); C22(Carbamidomethyl)	3342.6	0	1.8	1.3	1.7	1.4
																	ELEEDFIK	11	1	1	21624607	N-Term(TMT6plex); K8(TMT6plex)	1480.8	0	1.7	1.8	2.0	1.6
																	AGGANYDAQTE	6	1	1	21624607	N-Term(TMT6plex)	1325.6	0	1.6	1.4	1.5	1.4
																	FALITWIGENVSGLQR	27	1	1	21624607	N-Term(TMT6plex)	2033.1	0	1.7	1.3	1.8	1.3
																	EVVQNFAK	10	1	1	21624607	N-Term(TMT6plex); K8(TMT6plex)	1392.8	0	1.6	1.8	2.1	1.7
																	FTTGDAMSK	6	1	1	21624607	N-Term(TMT6plex); M7(Oxidation); K9(TMT6plex)	1431.8	0	1.8	1.4	1.8	1.5
																	EFVISDR	10	1	1	21624607	N-Term(TMT6plex)	1094.6	0	1.7	1.7	1.8	1.6
																	KAGGANYDAQTE	2	1	1	21624607	N-Term(TMT6plex); K1(TMT6plex)	1682.9	1	2.5	2.5	2.7	2.4
																	AAYNLVR	1	1	1	21624607	N-Term(TMT6plex)	1035.6	0				
																	LFAFVR	6	1	1	21624607	N-Term(TMT6plex)	981.6	0	2.2	1.7	2.2	1.7
																	SKFALITWIGENVSGLQR	1	1	1	21624607	N-Term(TMT6plex); K2(TMT6plex)	2477.4	1	1.7	1.4	2.2	1.2
17986283	NP_006000.2	**TUBA1A**	7846	**tubulin alpha-1A chain isoform 1**	80.93	5	3	27	2599	**1.5**	**1.4**	**1.3**	**1.3**	451	50.1	5.06												
																	AVFVDLEPTVIDEVR	460	6	2	14389309;17986283	N-Term(TMT6plex)	1931.1	0				
																	AYHEQLSVAEITNACFEPANQMVK	209	7	2	514052659;17986283	N-Term(TMT6plex); C15(Carbamidomethyl); M22(Oxidation); K24(TMT6plex)	3224.6	0				
																	FDGALNVDLTEFQTNLVPYPR	132	12	4	514052659;14389309;17986283;301171345	N-Term(TMT6plex)	2638.4	0				
																	AFVHWYVGEGMEEGEFSEAR	157	11	4	514052659;14389309;17986283;301171345	N-Term(TMT6plex); M11(Oxidation)	2575.2	0				
																	IHFPLATYAPVISAEK	266	10	3	514052659;14389309;17986283	N-Term(TMT6plex); K16(TMT6plex)	2215.3	0				
																	EDMAALEKDYEEVGVDSVEGEGEEEGEEY	35	3	1	17986283	N-Term(TMT6plex); M3(Oxidation); K8(TMT6plex)	3710.6	1	1.4	1.5	1.3	1.7
																	TIGGGDDSFNTFFSETGAGK	225	8	2	14389309;17986283	N-Term(TMT6plex); K20(TMT6plex)	2466.2	0				
																	VGINYQPPTVVPGGDLAK	212	12	4	514052659;14389309;17986283;301171345	N-Term(TMT6plex); K18(TMT6plex)	2283.3	0				
																	DVNAAIATIK	96	8	2	14389309;17986283	N-Term(TMT6plex); K10(TMT6plex)	1473.9	0				
																	QLFHPEQLITGK	67	12	4	514052659;14389309;17986283;301171345	N-Term(TMT6plex); K12(TMT6plex)	1869.1	0				
																	GHYTIGK	34	10	3	514052659;14389309;17986283	N-Term(TMT6plex); K7(TMT6plex)	1233.7	0				
																	EIIDLVLDR	208	4	2	14389309;17986283	N-Term(TMT6plex)	1314.8	0				
																	DYEEVGVDSVEGEGEEEGEEY	33	3	1	17986283	N-Term(TMT6plex)	2578.1	0	1.7	1.0	1.1	1.1
																	EDAANNYAR	39	13	5	514052659;14389309;285395246;17986283;301171345	N-Term(TMT6plex)	1252.6	0				
																	AVCMLSNTTAIAEAWAR	16	10	3	514052659;17986283;301171345	N-Term(TMT6plex); C3(Carbamidomethyl)	2094.1	0				
																	ECISIHVGQAGVQIGNACWELYCLEHGIQPDGQMPSDK	9	5	2	14389309;17986283	N-Term(TMT6plex); C2(Carbamidomethyl); C18(Carbamidomethyl); C23(Carbamidomethyl); M34(Oxidation); K38(TMT6plex)	4771.3	0				
																	ECISIHVGQAGVQIGNACWELYCLEHGIQPDGQMPSDK	4	5	2	14389309;17986283	N-Term(TMT6plex); C2(Carbamidomethyl); C18(Carbamidomethyl); C23(Carbamidomethyl); K38(TMT6plex)	4755.3	0				
																	FDLMYAK	16	15	5	514052659;14389309;285395246;17986283;301171345	N-Term(TMT6plex); K7(TMT6plex)	1345.8	0				
																	YMACCLLYR	2	8	4	514052659;14389309;285395246;17986283	N-Term(TMT6plex); C4(Carbamidomethyl); C5(Carbamidomethyl)	1478.7	0				
																	NLDIERPTYTNLNR	33	12	4	514052659;14389309;17986283;301171345	N-Term(TMT6plex)	1948.0	0				
																	YMACCLLYR	10	8	4	514052659;14389309;285395246;17986283	N-Term(TMT6plex); M2(Oxidation); C4(Carbamidomethyl); C5(Carbamidomethyl)	1494.7	0				
																	AVCMLSNTTAIAEAWAR	37	10	3	514052659;17986283;301171345	N-Term(TMT6plex); C3(Carbamidomethyl); M4(Oxidation)	2110.1	0				
																	TIQFVDWCPTGFK	50	7	3	14389309;17986283;301171345	N-Term(TMT6plex); C8(Carbamidomethyl); K13(TMT6plex)	2057.1	0				
																	LADQCTGLQGFLVFHSFGGGTGSGFTSLLMER	5	4	2	14389309;17986283	N-Term(TMT6plex); C5(Carbamidomethyl)	3619.8	0				
																	LADQCTGLQGFLVFHSFGGGTGSGFTSLLMER	32	4	2	14389309;17986283	N-Term(TMT6plex); C5(Carbamidomethyl); M30(Oxidation)	3635.8	0				
																	GHYTIGKEIIDLVLDR	4	4	2	14389309;17986283	N-Term(TMT6plex); K7(TMT6plex)	2300.3	1				
																	VGINYQPPTVVPGGDLAKVQR	3	10	4	514052659;14389309;17986283;301171345	N-Term(TMT6plex); K18(TMT6plex)	2666.5	1				
																	AFVHWYVGEGMEEGEFSEAR	55	11	4	514052659;14389309;17986283;301171345	N-Term(TMT6plex)	2559.2	0				
																	EDMAALEKDYEEVGVDSVEGEGEEEGEEY	3	3	1	17986283	N-Term(TMT6plex); K8(TMT6plex)	3694.6	1	1.5	1.9	1.7	1.6
																	LIGQIVSSITASLR	22	4	1	17986283	N-Term(TMT6plex)	1687.0	0	1.4	1.3	1.4	1.0
																	AYHEQLSVAEITNACFEPANQMVK	18	7	2	514052659;17986283	N-Term(TMT6plex); C15(Carbamidomethyl); K24(TMT6plex)	3208.6	0				
																	KLADQCTGLQGFLVFHSFGGGTGSGFTSLLMER	5	4	2	14389309;17986283	K1(TMT6plex); N-Term(TMT6plex); C6(Carbamidomethyl); M31(Oxidation)	3993.0	1				
																	KLADQCTGLQGFLVFHSFGGGTGSGFTSLLMER	1	4	2	14389309;17986283	N-Term(TMT6plex); K1(TMT6plex); C6(Carbamidomethyl)	3977.0	1				
																	FDLMYAK	44	15	5	514052659;14389309;285395246;17986283;301171345	N-Term(TMT6plex); M4(Oxidation); K7(TMT6plex)	1361.8	0				
																	LSVDYGK	44	9	3	514052659;14389309;17986283	N-Term(TMT6plex); K7(TMT6plex)	1239.7	0				
																	EDMAALEK	13	6	3	514052659;14389309;17986283	N-Term(TMT6plex); M3(Oxidation); K8(TMT6plex)	1380.7	0				
14389309	NP_116093.1	**TUBA1C**	84790	**tubulin alpha-1C chain**	80.85	3	4	27	2426	**1.3**	**1.5**	**1.5**	**1.5**	449	49.9	5.10												
																	AVFVDLEPTVIDEVR	460	6	2	14389309;17986283	N-Term(TMT6plex)	1931.1	0				
																	LISQIVSSITASLR	110	6	3	514052659;14389309;301171345	N-Term(TMT6plex)	1717.0	0				
																	FDGALNVDLTEFQTNLVPYPR	132	12	4	514052659;14389309;17986283;301171345	N-Term(TMT6plex)	2638.4	0				
																	AFVHWYVGEGMEEGEFSEAR	157	11	4	514052659;14389309;17986283;301171345	N-Term(TMT6plex); M11(Oxidation)	2575.2	0				
																	AYHEQLTVAEITNACFEPANQMVK	42	1	1	14389309	N-Term(TMT6plex); C15(Carbamidomethyl); M22(Oxidation); K24(TMT6plex)	3238.6	0	1.3	1.5	1.5	1.5
																	IHFPLATYAPVISAEK	266	10	3	514052659;14389309;17986283	N-Term(TMT6plex); K16(TMT6plex)	2215.3	0				
																	TIGGGDDSFNTFFSETGAGK	225	8	2	14389309;17986283	N-Term(TMT6plex); K20(TMT6plex)	2466.2	0				
																	VGINYQPPTVVPGGDLAK	212	12	4	514052659;14389309;17986283;301171345	N-Term(TMT6plex); K18(TMT6plex)	2283.3	0				
																	DVNAAIATIK	96	8	2	14389309;17986283	N-Term(TMT6plex); K10(TMT6plex)	1473.9	0				
																	QLFHPEQLITGK	67	12	4	514052659;14389309;17986283;301171345	N-Term(TMT6plex); K12(TMT6plex)	1869.1	0				
																	GHYTIGK	34	10	3	514052659;14389309;17986283	N-Term(TMT6plex); K7(TMT6plex)	1233.7	0				
																	EIIDLVLDR	208	4	2	14389309;17986283	N-Term(TMT6plex)	1314.8	0				

Accession	RefSeq Accession	Gene Symbol	Entrez Gene ID	Description	Σ Coverage	Σ# Proteins	Σ# Unique Peptides	Σ# Peptides	Σ# PSMs	Het-1A-Smoke - 2M/Parental	Het-1A-Smoke - 4M/Parental	Het-1A-Smoke - 6M/Parental	Het-1A-Smoke - 8M/Parental	# AAs	MW [kDa]	calc. pI	Sequence	# PSMs	# Proteins	# Protein Groups	Protein Group Accessions	Modifications	MH+ [Da]	# Missed Cleavages	Het-1A-Smoke - 2M/Parental	Het-1A-Smoke - 4M/Parental	Het-1A-Smoke - 6M/Parental	Het-1A-Smoke - 8M/Parental
																	EDMAALEKDYEEVGADSADGEDEGEEY	13	3	1	14389309	N-Term(TMT6plex); M3(Oxidation); K8(TMT6plex)	3440.5	1	1.4	1.7	1.4	1.8
																	EDAANNYAR	39	13	5	514052659;14389309;285395246;17986283;301171345	N-Term(TMT6plex)	1252.6	0				
																	AVCMLSNTTAVAEAWAR	15	1	1	14389309	N-Term(TMT6plex); C3(Carbamidomethyl); M4(Oxidation)	2096.0	0	1.4	1.4	1.4	1.4
																	DYEEVGADSADGEDEGEEY	11	1	1	14389309	N-Term(TMT6plex)	2307.9	0	1.1	1.2	1.6	1.3
																	ECISIHVGQAGVQIGNACWELYCLEHGIQPDGQMPSDK	9	5	2	14389309;17986283	N-Term(TMT6plex); C2(Carbamidomethyl); C18(Carbamidomethyl); C23(Carbamidomethyl); M34(Oxidation); K38(TMT6plex)	4771.3	0				
																	ECISIHVGQAGVQIGNACWELYCLEHGIQPDGQMPSDK	4	5	2	14389309;17986283	N-Term(TMT6plex); C2(Carbamidomethyl); C18(Carbamidomethyl); C23(Carbamidomethyl); K38(TMT6plex)	4755.3	0				
																	FDLMYAK	16	15	5	514052659;14389309;285395246;17986283;301171345	N-Term(TMT6plex); K7(TMT6plex)	1345.8	0				
																	YMACCLLYR	2	8	4	514052659;14389309;285395246;17986283	N-Term(TMT6plex); C4(Carbamidomethyl); C5(Carbamidomethyl)	1478.7	0				
																	NLDIERPTYTNLNR	33	12	4	514052659;14389309;17986283;301171345	N-Term(TMT6plex)	1948.0	0				
																	YMACCLLYR	10	8	4	514052659;14389309;285395246;17986283	N-Term(TMT6plex); M2(Oxidation); C4(Carbamidomethyl); C5(Carbamidomethyl)	1494.7	0				
																	TIQFVDWCPTGFK	50	7	3	14389309;17986283;301171345	N-Term(TMT6plex); C8(Carbamidomethyl); K13(TMT6plex)	2057.1	0				
																	LADQCTGLQGFLVFHSFGGGTGSGFTSLLMER	5	4	2	14389309;17986283	N-Term(TMT6plex); C5(Carbamidomethyl)	3619.8	0				
																	LADQCTGLQGFLVFHSFGGGTGSGFTSLLMER	32	4	2	14389309;17986283	N-Term(TMT6plex); C5(Carbamidomethyl); M30(Oxidation)	3635.8	0				
																	GHYTIGKEIIDLVLDR	4	4	2	14389309;17986283	N-Term(TMT6plex); K7(TMT6plex)	2300.3	1				
																	VGINYQPPTVVPGGDLAKVQR	3	10	4	514052659;14389309;17986283;301171345	N-Term(TMT6plex); K18(TMT6plex)	2666.5	1				
																	AFVHWYVGEGMEEGEFSEAR	55	11	4	514052659;14389309;17986283;301171345	N-Term(TMT6plex)	2559.2	0				
																	EDMAALEKDYEEVGADSADGEDEGEEY	1	3	1	14389309	N-Term(TMT6plex); K8(TMT6plex)	3424.5	1	1.3	1.7	1.5	1.3
																	AVCMLSNTTAVAEAWAR	6	1	1	14389309	N-Term(TMT6plex); C3(Carbamidomethyl)	2080.0	0	1.4	1.4	1.6	1.4
																	AYHEQLTVAEITNACFEPANQMVK	2	1	1	14389309	N-Term(TMT6plex); C15(Carbamidomethyl); K24(TMT6plex)	3222.6	0	1.4	1.5	1.6	1.7
																	KLADQCTGLQGFLVFHSFGGGTGSGFTSLLMER	5	4	2	14389309;17986283	K1(TMT6plex); N-Term(TMT6plex); C6(Carbamidomethyl); M31(Oxidation)	3993.0	1				
																	KLADQCTGLQGFLVFHSFGGGTGSGFTSLLMER	1	4	2	14389309;17986283	N-Term(TMT6plex); K1(TMT6plex); C6(Carbamidomethyl)	3977.0	1				
																	FDLMYAK	44	15	5	514052659;14389309;285395246;17986283;301171345	N-Term(TMT6plex); M4(Oxidation); K7(TMT6plex)	1361.8	0				
																	LSVDYGK	44	9	3	514052659;14389309;17986283	N-Term(TMT6plex); K7(TMT6plex)	1239.7	0				
																	EDMAALEK	13	6	3	514052659;14389309;17986283	N-Term(TMT6plex); M3(Oxidation); K8(TMT6plex)	1380.7	0				
5454052	NP_006133.1	**SFN**	2810	**14-3-3 protein sigma**	80.65	1	13	17	662	**1.2**	**1.0**	**1.2**	**1.0**	248	27.8	4.74												
																	DSTLIMQLLR	297	7	7	21735625;208973244;4507951;5454052;5803227;21464101;4507949;5803225	N-Term(TMT6plex); M6(Oxidation)	1434.8	0				
																	NLLSVAYK	199	7	7	21735625;208973244;4507951;5454052;5803227;21464101;4507949;5803225	N-Term(TMT6plex); K8(TMT6plex)	1365.9	0				
																	LAEQAER	18	7	7	21735625;208973244;4507951;5454052;5803227;21464101;4507949;5803225	N-Term(TMT6plex)	1045.6	0				
																	YLAEVATGDDK	18	1	1	5454052	N-Term(TMT6plex); K11(TMT6plex)	1639.9	0	1.1	1.1	1.2	1.1
																	VETELQGVCDTVLGLLDSHLIK	14	1	1	5454052	N-Term(TMT6plex); C9(Carbamidomethyl); K22(TMT6plex)	2897.6	0	1.2	1.1	1.2	1.1
																	DSTLIMQLLR	41	7	7	21735625;208973244;4507951;5454052;5803227;21464101;4507949;5803225	N-Term(TMT6plex)	1418.8	0				
																	VLSSIEQK	9	5	5	4507951;5803227;21464101;4507949;5454052	N-Term(TMT6plex); K8(TMT6plex)	1361.8	0				
																	DNLTLWTADNAGEEGGEAPQEPQS	12	1	1	5454052	N-Term(TMT6plex)	2758.3	0	1.2	1.2	1.2	1.1
																	TTFDEAMADLHTLSEDSYK	17	1	1	5454052	N-Term(TMT6plex); M7(Oxidation); K19(TMT6plex)	2648.3	0	1.2	1.0	1.1	1.0
																	SNEEGSEEKGPEVR	5	1	1	5454052	N-Term(TMT6plex); K9(TMT6plex)	2005.0	1	1.1	0.9	1.0	0.9
																	GAVEKGEELSCEER	1	1	1	5454052	N-Term(TMT6plex); K5(TMT6plex); C11(Carbamidomethyl)	2051.1	1	1.2	1.0	1.1	0.8
																	TTFDEAMADLHTLSEDSYK	2	1	1	5454052	N-Term(TMT6plex); K19(TMT6plex)	2632.3	0	1.4	0.9	1.3	0.8
																	SAYQEAMDISK	9	1	1	5454052	N-Term(TMT6plex); M7(Oxidation); K11(TMT6plex)	1716.9	0	1.3	0.9	1.1	1.0
																	YEDMAAFMK	2	1	1	5454052	N-Term(TMT6plex); K9(TMT6plex)	1563.8	0	1.7	1.2	1.6	0.9
																	EKVETELQGVCDTVLGLLDSHLIK	5	1	1	5454052	N-Term(TMT6plex); K2(TMT6plex); C11(Carbamidomethyl); K24(TMT6plex)	3383.9	1	1.0	1.1	1.3	1.1
																	SAYQEAMDISK	2	1	1	5454052	N-Term(TMT6plex); K11(TMT6plex)	1700.9	0	1.6	1.2	1.5	0.9
																	LGLALNFSVFHYEIANSPEEAISLAK	4	1	1	5454052	N-Term(TMT6plex); K26(TMT6plex)	3291.8	0	1.3	1.1	1.7	1.0
																	EAGDAESR	2	1	1	5454052	N-Term(TMT6plex)	1063.5	0	1.2	0.7	1.0	0.8
																	SNEEGSEEK	2	1	1	5454052	N-Term(TMT6plex); K9(TMT6plex)	1466.7	0	1.5	1.0	1.0	0.9
																	YEDMAAFMK	1	1	1	5454052	N-Term(TMT6plex); M4(Oxidation); M8(Oxidation); K9(TMT6plex)	1595.8	0				
																	YEDMAAFMK	1	1	1	5454052	N-Term(TMT6plex); M4(Oxidation); K9(TMT6plex)	1579.8	0	1.3	1.1	1.3	0.8
																	NVVGGQR	1	1	1	5454052	N-Term(TMT6plex)	958.6	0	1.1	1.3	1.3	1.2
6005942	NP_009057.1	**VCP**	7415	**transitional endoplasmic reticulum ATPase**	80.65	2	50	50	530	**1.3**	**1.4**	**1.5**	**1.5**	806	89.3	5.26												
																	LIVDEAINEDNSVVSLSQPK	30	1	1	6005942	N-Term(TMT6plex); K20(TMT6plex)	2628.4	0	1.2	1.4	1.4	1.5
																	LADDVDLEQVANETHGHVGADLAALCSEAALQAIR	10	1	1	6005942	N-Term(TMT6plex); C26(Carbamidomethyl)	3902.0	0	1.4	1.4	1.4	1.5
																	NAPAIIFIDELDAIAPK	47	1	1	6005942	N-Term(TMT6plex); K17(TMT6plex)	2269.3	0	1.4	1.3	1.4	1.3
																	GILLYGPPGTGK	17	2	1	6005942	N-Term(TMT6plex); K12(TMT6plex)	1631.0	0	1.2	1.4	1.5	1.4
																	GDDLSTAILK	9	1	1	6005942	N-Term(TMT6plex); K10(TMT6plex)	1490.9	0	1.2	1.4	1.4	1.6
																	VINQILTEMDGMSTK	14	1	1	6005942	N-Term(TMT6plex); M9(Oxidation); M12(Oxidation); K15(TMT6plex)	2170.2	0	1.4	1.4	1.5	1.5
																	LDQLIYIPLPDEK	31	1	1	6005942	N-Term(TMT6plex); K13(TMT6plex)	2015.2	0	1.3	1.3	1.6	1.4
																	MDLIDLEDETIDAEVMNSLAVTMDDFR	11	1	1	6005942	N-Term(TMT6plex); M1(Oxidation); M16(Oxidation); M23(Oxidation)	3378.5	0	1.9	1.8	2.0	2.2
																	GPELLTMWFGESEANVR	6	1	1	6005942	N-Term(TMT6plex); M7(Oxidation)	2181.1	0	1.2	1.3	1.4	1.4
																	QTNPSAMEVEEDDPVPEIR	27	1	1	6005942	N-Term(TMT6plex); M7(Oxidation)	2401.1	0	1.5	1.4	1.5	1.6
																	KYEMFAQTLQQSR	1	1	1	6005942	N-Term(TMT6plex); K1(TMT6plex); M4(Oxidation)	2104.1	1	1.1	1.2	1.1	1.3
																	AIANECQANFISIK	6	1	1	6005942	N-Term(TMT6plex); C6(Carbamidomethyl); K14(TMT6plex)	2037.1	0	1.4	1.6	1.5	1.8
																	ELQELVQYPVEHPDK	24	1	1	6005942	N-Term(TMT6plex); K15(TMT6plex)	2282.2	0	1.3	1.5	1.6	1.6
																	LGDVISIQPCPDVK	10	1	1	6005942	N-Term(TMT6plex); C10(Carbamidomethyl); K14(TMT6plex)	1999.1	0	1.2	1.3	1.4	1.5
																	LEILQIHTK	14	1	1	6005942	N-Term(TMT6plex); K9(TMT6plex)	1553.0	0	1.2	1.4	1.5	1.6
																	ETVVEVPQVTWEDIGGLEDVKR	16	1	1	6005942	N-Term(TMT6plex); K21(TMT6plex)	2956.6	1	1.2	1.4	1.5	1.6
																	VVETDPSPYCIVAPDTVIHCEGEPIKR	3	1	1	6005942	N-Term(TMT6plex); C10(Carbamidomethyl); C20(Carbamidomethyl); K26(TMT6plex)	3539.8	1	1.4	1.5	1.8	1.5
																	AVANETGAFFFLINGPEIMSK	9	1	1	6005942	N-Term(TMT6plex); M19(Oxidation); K21(TMT6plex)	2730.4	0	1.4	2.8	1.6	2.4
																	EVDIGIPDATGR	11	1	1	6005942	N-Term(TMT6plex)	1471.8	0	1.3	1.4	1.4	1.5
																	DVDLEFLAK	19	1	1	6005942	N-Term(TMT6plex); K9(TMT6plex)	1507.9	0	1.3	1.4	1.5	1.5
																	LAGESESNLR	8	1	1	6005942	N-Term(TMT6plex)	1304.7	0	1.2	1.3	1.4	1.4
																	ESIESEIR	7	1	1	6005942	N-Term(TMT6plex)	1191.6	0	1.3	1.5	1.5	1.7
																	EDEEESLNEVGYDDIGGCR	6	1	1	6005942	N-Term(TMT6plex); C18(Carbamidomethyl)	2415.0	0	1.0	1.1	1.3	1.2
																	IVSQLLTLMDGLK	23	1	1	6005942	N-Term(TMT6plex); M9(Oxidation); K13(TMT6plex)	1905.1	0	1.4	1.1	1.3	1.2
																	VINQILTEMDGMSTK	2	1	1	6005942	N-Term(TMT6plex); K15(TMT6plex)	2138.2	0	2.4	2.0	2.7	1.5
																	EAVCIVLSDDTCSDEK	10	1	1	6005942	N-Term(TMT6plex); C4(Carbamidomethyl); C12(Carbamidomethyl); K16(TMT6plex)	2299.1	0	1.4	1.5	1.5	1.5
																	FPSGNQGGAGPSQGSGGGTGGSVYTEDNDDDLYG	5	1	1	6005942	N-Term(TMT6plex)	3449.5	0	1.4	1.4	1.4	1.7
																	MDLIDLEDETIDAEVMNSLAVTMDDFR	22	1	1	6005942	N-Term(TMT6plex); M1(Oxidation); M16(Oxidation)	3362.6	0	1.5	1.7	1.6	1.5
																	AIGVKPPR	3	1	1	6005942	N-Term(TMT6plex); K5(TMT6plex)	1295.9	0	1.1	1.5	1.5	1.6
																	VINQILTEMDGMSTK	4	1	1	6005942	N-Term(TMT6plex); M12(Oxidation); K15(TMT6plex)	2154.1	0	1.5	1.5	1.6	1.4
																	EMVELPLR	2	1	1	6005942	N-Term(TMT6plex)	1215.7	0	1.6	1.7	1.9	1.6
																	NVFIIGATNRPDIIDPAILRPGR	6	1	1	6005942	N-Term(TMT6plex)	2747.6	0	1.7	1.6	1.7	1.5
																	GGNIGDGGGAADR	10	1	1	6005942	N-Term(TMT6plex)	1345.7	0	1.6	1.6	1.4	1.7
																	GVLFYGPPGCGK	20	1	1	6005942	N-Term(TMT6plex); C10(Carbamidomethyl); K12(TMT6plex)	1709.9	0	1.2	1.5	1.5	1.5
																	AHVIVMAATNRPNSIDPALR	7	1	1	6005942	N-Term(TMT6plex); M6(Oxidation)	2391.3	0	1.3	1.4	1.4	1.6
																	ETVVEVPQVTWEDIGGLEDVK	10	1	1	6005942	N-Term(TMT6plex); K21(TMT6plex)	2800.5	0	1.2	1.4	1.5	1.6
																	KMDLIDLEDETIDAEVMNSLAVTMDDFR	2	1	1	6005942	K1(TMT6plex); N-Term(TMT6plex); M2(Oxidation); M24(Oxidation)	3719.8	1	1.2	1.9	1.4	1.8
																	SVSDNDIR	6	1	1	6005942	N-Term(TMT6plex)	1134.6	0	1.2	1.3	1.3	1.4
																	VVETDPSPYCIVAPDTVIHCEGEPIK	2	1	1	6005942	N-Term(TMT6plex); C10(Carbamidomethyl); C20(Carbamidomethyl); K26(TMT6plex)	3383.7	0	1.5	1.4	1.5	1.5
																	AFEEAEK	10	1	1	6005942	N-Term(TMT6plex); K7(TMT6plex)	1281.7	0	1.3	1.6	1.6	1.6
																	KYEMFAQTLQQSR	1	1	1	6005942	N-Term(TMT6plex); K1(TMT6plex)	2088.1	1	1.6	1.6	2.0	1.5
																	WALSQSNPSALR	10	1	1	6005942	N-Term(TMT6plex)	1558.9	0	1.5	1.4	1.5	1.5
																	KGDIFLVR	4	1	1	6005942	N-Term(TMT6plex); K1(TMT6plex)	1405.9	1	1.2	1.3	1.3	1.4
																	IVSQLLTLMDGLK	6	1	1	6005942	N-Term(TMT6plex); K13(TMT6plex)	1889.2	0	1.7	1.4	1.8	1.3
																	KQLAQIK	1	1	1	6005942	N-Term(TMT6plex); K1(TMT6plex); K7(TMT6plex)	1516.0	1	1.2	1.8	1.5	2.0

Accession	RefSeq Accession	Gene Symbol	Entrez Gene ID	Description	Σ Coverage	Σ# Proteins	Σ# Unique Peptides	Σ# Peptides	Σ# PSMs	Het-1A-Smoke - 2M/Parental	Het-1A-Smoke - 4M/Parental	Het-1A-Smoke - 6M/Parental	Het-1A-Smoke - 8M/Parental	# AAs	MW [kDa]	calc. pI	Sequence	# PSMs	# Proteins	# Protein Groups	Protein Group Accessions	Modifications	MH+ [Da]	# Missed Cleavages	Het-1A-Smoke - 2M/Parental	Het-1A-Smoke - 4M/Parental	Het-1A-Smoke - 6M/Parental	Het-1A-Smoke - 8M/Parental
																	KMDLIDLEDETIDAEVMNSLAVTMDDFR	7	1	1	6005942	K1(TMT6plex); N-Term(TMT6plex); M2(Oxidation); M17(Oxidation); M24(Oxidation)	3735.8	1	1.3	1.3	1.3	1.4
																	KMDLIDLEDETIDAEVMNSLAVTMDDFR	2	1	1	6005942	N-Term(TMT6plex); K1(TMT6plex); M17(Oxidation)	3703.8	1	1.6	1.3	1.5	1.0
																	IHVLPIDDTVEGITGNLFEVYLKPYFLEAYRPIR	1	1	1	6005942	N-Term(TMT6plex); K23(TMT6plex)	4449.4	0	1.8	1.7	2.7	1.2
																	KAFEEAEK	1	1	1	6005942	N-Term(TMT6plex); K1(TMT6plex); K8(TMT6plex)	1639.0	1	1.2	1.5	1.5	1.5
																	GDIFLVR	5	1	1	6005942	N-Term(TMT6plex)	1048.6	0	1.2	1.4	1.4	1.5
																	EMVELPLR	3	1	1	6005942	N-Term(TMT6plex); M2(Oxidation)	1231.7	0	1.2	1.4	1.3	1.7
																	KSPVAK	2	1	1	6005942	N-Term(TMT6plex); K1(TMT6plex); K6(TMT6plex)	1316.9	1	1.1	1.5	1.5	1.6
																	QAAPCVLFFDELDSIAK	1	1	1	6005942	N-Term(TMT6plex); C5(Carbamidomethyl); K17(TMT6plex)	2382.3	0	2.7	1.2	2.7	3.1
																	RDHFEEAMR	2	1	1	6005942	N-Term(TMT6plex); M8(Oxidation)	1435.7	1	1.1	1.4	1.5	1.8
																	YEMFAQTLQQSR	1	1	1	6005942	N-Term(TMT6plex); M3(Oxidation)	1746.9	0	1.3	1.5	1.3	1.5
																	MDELQLFR	1	1	1	6005942	N-Term(TMT6plex); M1(Oxidation)	1296.7	0				
																	QLAQIK	1	1	1	6005942	N-Term(TMT6plex); K6(TMT6plex)	1158.8	0	1.1	1.4	1.4	1.5
																	GDTVLLK	1	1	1	6005942	N-Term(TMT6plex); K7(TMT6plex)	1203.8	0	1.2	1.4	1.4	1.5
4503529	NP_001407.1	**EIF4A1**	1973	**eukaryotic initiation factor 4A-I isoform 1**	80.54	2	14	26	772	**1.3**	**1.4**	**1.5**	**1.5**	406	46.1	5.48												
																	LQMEAPHIIVGTPGR	30	2	1	4503529	N-Term(TMT6plex); M3(Oxidation)	1864.0	0	1.3	1.4	1.4	1.4
																	MFVLDEADEMLSR	63	3	2	83700235;4503529	N-Term(TMT6plex); M1(Oxidation); M10(Oxidation)	1816.9	0				
																	EELTLEGIR	6	2	1	4503529	N-Term(TMT6plex)	1288.7	0	1.4	1.5	1.6	1.6
																	DIETFYNTSIEEMPLNVADLI	80	1	1	4503529	N-Term(TMT6plex); M13(Oxidation)	2672.3	0	1.5	1.6	1.6	1.6
																	DNGPDGMEPEGVIESNWNEIVDSFDDMNLSESLLR	16	2	1	4503529	N-Term(TMT6plex); M7(Oxidation); M27(Oxidation)	4184.9	0	1.5	1.6	1.6	1.3
																	DFTVSAMHGDMDQK	23	2	1	4503529	N-Term(TMT6plex); M7(Oxidation); M11(Oxidation); K14(TMT6plex)	2072.0	0	1.3	1.5	1.5	1.5
																	DIETFYNTSIEEMPLNVADLI	35	1	1	4503529	N-Term(TMT6plex)	2656.3	0	1.6	1.7	2.0	1.5
																	VDWLTEK	11	4	3	83700235;4503529;7661920	N-Term(TMT6plex); K7(TMT6plex)	1348.8	0				
																	KVDWLTEK	12	4	3	83700235;4503529;7661920	N-Term(TMT6plex); K1(TMT6plex); K8(TMT6plex)	1706.0	1				
																	LNSNTQVVLLSATMPSDVLEVTK	44	2	1	4503529	N-Term(TMT6plex); M14(Oxidation); K23(TMT6plex)	2933.6	0	1.4	1.3	1.5	1.4
																	TATFAISILQQIELDLK	19	2	1	4503529	N-Term(TMT6plex); K17(TMT6plex)	2362.4	0	1.4	1.2	1.6	1.4
																	ATQALVLAPTR	18	2	1	4503529	N-Term(TMT6plex)	1369.8	0	1.3	1.3	1.4	1.4
																	DFTVSAMHGDMDQK	6	2	1	4503529	N-Term(TMT6plex); M7(Oxidation); K14(TMT6plex)	2056.0	0	1.4	1.8	2.1	1.7
																	QFYINVER	14	3	2	83700235;4503529	N-Term(TMT6plex)	1297.7	0				
																	GIYAYGFEKPSAIQQR	11	4	3	83700235;4503529;7661920	N-Term(TMT6plex); K9(TMT6plex)	2286.3	0				
																	KGVAINMVTEEDKR	4	1	1	4503529	N-Term(TMT6plex); K1(TMT6plex); M7(Oxidation); K13(TMT6plex)	2293.3	2	1.4	1.6	1.6	1.5
																	GVAINMVTEEDKR	4	1	1	4503529	N-Term(TMT6plex); M6(Oxidation); K12(TMT6plex)	1936.1	1	0.9	1.1	1.1	1.1
																	LDTLCDLYETLTITQAVIFINTR	2	3	2	4503529;83700235	N-Term(TMT6plex); C5(Carbamidomethyl)	2942.6	0				
																	ELAQQIQK	37	3	2	83700235;4503529	N-Term(TMT6plex); K8(TMT6plex)	1415.9	0				
																	KEELTLEGIR	21	2	1	4503529	N-Term(TMT6plex); K1(TMT6plex)	1646.0	1	1.2	1.4	1.5	1.4
																	MFVLDEADEMLSR	4	3	2	83700235;4503529	N-Term(TMT6plex)	1784.9	0				
																	DNGPDGMEPEGVIESNWNEIVDSFDDMNLSESLLR	14	2	1	4503529	N-Term(TMT6plex); M7(Oxidation)	4168.9	0	1.2	1.4	1.9	1.5
																	GIDVQQVSLVINYDLPTNR	100	2	2	83700235;4503529	N-Term(TMT6plex)	2373.3	0				
																	MFVLDEADEMLSR	14	3	2	83700235;4503529	N-Term(TMT6plex); M10(Oxidation)	1800.9	0				
																	VVMALGDYMGASCHACIGGTNVR	6	2	1	4503529	N-Term(TMT6plex); M3(Oxidation); M9(Oxidation); C13(Carbamidomethyl); C16(Carbamidomethyl)	2700.3	0	1.2	1.3	1.2	1.4
																	GYDVIAQAQSGTGK	35	3	2	83700235;4503529	N-Term(TMT6plex); K14(TMT6plex)	1853.0	0				
																	VLITTDLLAR	18	2	2	83700235;4503529	N-Term(TMT6plex)	1343.8	0				
																	LNSNTQVVLLSATMPSDVLEVTK	20	2	1	4503529	N-Term(TMT6plex); K23(TMT6plex)	2917.6	0	1.9	1.6	2.4	1.4
																	GFKDQIYDIFQK	32	2	1	4503529	N-Term(TMT6plex); K3(TMT6plex); K12(TMT6plex)	2189.3	1	1.2	1.9	1.7	1.9
																	VVMALGDYMGASCHACIGGTNVR	8	2	1	4503529	N-Term(TMT6plex); M3(Oxidation); C13(Carbamidomethyl); C16(Carbamidomethyl)	2684.3	0	1.5	1.8	1.7	1.7
																	LQMEAPHIIVGTPGR	6	2	1	4503529	N-Term(TMT6plex)	1848.0	0	1.4	1.6	1.6	1.5
																	KGVAINMVTEEDKR	3	1	1	4503529	N-Term(TMT6plex); K1(TMT6plex); K13(TMT6plex)	2277.3	2	1.7	2.0	2.3	1.5
																	DQIYDIFQK	39	2	1	4503529	N-Term(TMT6plex); K9(TMT6plex)	1627.9	0	1.2	1.4	1.6	1.3
																	VFDMLNR	12	3	2	83700235;4503529	N-Term(TMT6plex); M4(Oxidation)	1139.6	0				
																	VFDMLNR	4	3	2	83700235;4503529	N-Term(TMT6plex)	1123.6	0				
																	ENYIHR	1	2	2	83700235;4503529	N-Term(TMT6plex)	1060.6	0				
66392203	NP_001018146.1	**NME1-NME2**	654364	**NME1-NME2 protein**	80.52	3	7	15	320	**1.7**	**1.3**	**1.6**	**1.5**	267	30.1	8.92												
																	YMNSGPVVAMVWEGLNVVK	34	2	1	66392203	N-Term(TMT6plex); M2(Oxidation); M10(Oxidation); K19(TMT6plex)	2583.4	0	1.8	1.3	1.4	1.5
																	GLVGEIIK	23	5	2	66392203;4557797	N-Term(TMT6plex); K8(TMT6plex)	1286.8	0				
																	YMHSGPVVAMVWEGLNVVK	24	3	2	4557797;66392203	N-Term(TMT6plex); M2(Oxidation); M10(Oxidation); K19(TMT6plex)	2606.4	0				
																	NIIHGSDSVK	19	2	1	66392203	N-Term(TMT6plex); K10(TMT6plex)	1527.9	0	1.4	1.2	1.4	1.4
																	GDFCIQVGR	27	4	2	66392203;4557797	N-Term(TMT6plex); C4(Carbamidomethyl)	1280.7	0				
																	FMQASEDLLK	4	3	2	4557797;66392203	N-Term(TMT6plex); K10(TMT6plex)	1639.9	0				
																	EHYVDLK	23	3	2	4557797;66392203	N-Term(TMT6plex); K7(TMT6plex)	1361.8	0				
																	FMQASEDLLK	27	3	2	4557797;66392203	N-Term(TMT6plex); M2(Oxidation); K10(TMT6plex)	1655.9	0				
																	VMLGETNPADSKPGTIR	36	4	2	66392203;4557797	N-Term(TMT6plex); M2(Oxidation); K12(TMT6plex)	2260.2	0				
																	TFIAIKPDGVQR	19	5	2	66392203;4557797	N-Term(TMT6plex); K6(TMT6plex)	1803.1	0				
																	VMLGETNPADSKPGTIR	11	4	2	66392203;4557797	N-Term(TMT6plex); K12(TMT6plex)	2244.2	0				
																	EISLWFKPEELVDYK	15	2	1	66392203	N-Term(TMT6plex); K7(TMT6plex); K15(TMT6plex)	2583.5	0	1.8	1.2	1.5	1.8
																	ASEEHLK	5	3	1	66392203	N-Term(TMT6plex); K7(TMT6plex)	1271.7	0	1.4	1.1	1.2	1.4
																	DRPFFPGLVK	18	3	1	66392203	N-Term(TMT6plex); K10(TMT6plex)	1634.0	0	1.7	1.2	1.6	1.6
																	QHYIDLK	8	3	1	66392203	N-Term(TMT6plex); K7(TMT6plex)	1374.8	0	1.4	1.2	1.4	1.4
																	DRPFFAGLVK	11	3	2	4557797;66392203	N-Term(TMT6plex); K10(TMT6plex)	1608.0	0				
																	SCAHDWVYE	4	2	1	66392203	N-Term(TMT6plex); C2(Carbamidomethyl)	1395.6	0	1.7	1.4	1.5	1.6
																	YMNSGPVVAMVWEGLNVVK	6	2	1	66392203	N-Term(TMT6plex); K19(TMT6plex)	2551.4	0	2.6	1.7	2.4	1.6
																	YMHSGPVVAMVWEGLNVVK	4	3	2	4557797;66392203	N-Term(TMT6plex); K19(TMT6plex)	2574.4	0				
																	YMNSGPVVAMVWEGLNVVK	2	2	1	66392203	N-Term(TMT6plex); M2(Oxidation); K19(TMT6plex)	2567.4	0	1.7	1.4	1.7	1.5
83367083	NP_055217.2	**UQCRQ**	27089	**cytochrome b-c1 complex subunit 8**	80.49	1	7	7	27	**1.4**	**1.5**	**1.7**	**1.3**	82	9.9	10.08												
																	AYPHVFTK	6	1	1	83367083	N-Term(TMT6plex); K8(TMT6plex)	1420.8	0	1.3	1.6	1.7	1.6
																	KNPAAYENDK	4	1	1	83367083	N-Term(TMT6plex); K1(TMT6plex); K10(TMT6plex)	1837.0	1	1.4	1.8	1.7	1.5
																	HVISYSLSPFEQR	3	1	1	83367083	N-Term(TMT6plex)	1792.0	0	1.2	1.4	1.4	1.4
																	VVPQFVVFYLIYTWGTEEFER	5	1	1	83367083	N-Term(TMT6plex)	2851.5	0	1.4	1.1	1.8	0.9
																	NPAAYENDK	6	1	1	83367083	N-Term(TMT6plex); K9(TMT6plex)	1479.8	0	1.5	1.5	1.6	1.3
																	GIPNVLR	2	1	1	83367083	N-Term(TMT6plex)	997.6	0	1.4	1.7	1.6	1.5
																	EFGNLTR	1	1	1	83367083	N-Term(TMT6plex)	1065.6	0	1.6	1.6	1.7	1.6
378548190;15718687	NP_001243731.1	**RPS3**	6188	**40S ribosomal protein S3 isoform 1**	80.25	3	19	19	503	**1.2**	**1.3**	**1.3**	**1.3**	243	26.7	9.66												
																	GCEVVVSGK	22	3	1	378548190;15718687	N-Term(TMT6plex); C2(Carbamidomethyl); K9(TMT6plex)	1392.8	0	1.2	1.4	1.4	1.3
																	GGKPEPPAMPQPVPTA	34	3	1	378548190;15718687	N-Term(TMT6plex); K3(TMT6plex); M9(Oxidation)	2048.1	0	1.3	1.4	1.5	1.4
																	KFVADGIFK	12	2	1	378548190;15718687	N-Term(TMT6plex); K1(TMT6plex); K9(TMT6plex)	1712.1	1	1.0	1.3	1.3	1.3
																	ACYGVLR	9	2	1	378548190;15718687	N-Term(TMT6plex); C2(Carbamidomethyl)	1067.6	0	1.3	1.4	1.3	1.4
																	IMLPWDPTGK	31	3	1	378548190;15718687	N-Term(TMT6plex); M2(Oxidation); K10(TMT6plex)	1631.9	0	1.3	1.4	1.4	1.3
																	KPLPDHVSIVEPK	14	3	1	378548190;15718687	N-Term(TMT6plex); K1(TMT6plex); K13(TMT6plex)	2146.3	0	1.1	1.3	1.3	1.3
																	FGFPEGSVELYAEK	41	1	1	378548190;15718687	N-Term(TMT6plex); K14(TMT6plex)	2031.1	0	1.3	1.3	1.5	1.3
																	FVDGLMIHSGDPVNYYVDTAVR	35	3	1	378548190;15718687	N-Term(TMT6plex); M6(Oxidation)	2713.3	0	1.4	1.5	1.5	1.4
																	TQNVLGEK	23	2	1	378548190;15718687	N-Term(TMT6plex); K8(TMT6plex)	1346.8	0	1.3	1.1	1.2	1.1
																	GLCAIAQAESLR	31	2	1	378548190;15718687	N-Term(TMT6plex); C3(Carbamidomethyl)	1517.8	0	1.3	1.4	1.4	1.4
																	DEILPTTPISEQK	62	3	1	378548190;15718687	N-Term(TMT6plex); K13(TMT6plex)	1929.1	0	1.2	1.4	1.4	1.3
																	ELTAVVQK	48	2	1	378548190;15718687	N-Term(TMT6plex); K8(TMT6plex)	1345.8	0	1.2	1.3	1.3	1.3
																	GGKPEPPAMPQPVPTA	4	3	1	378548190;15718687	N-Term(TMT6plex); K3(TMT6plex)	2032.1	0	1.4	1.6	1.6	1.2
																	ELAEDGYSGVEVR	29	2	1	378548190;15718687	N-Term(TMT6plex)	1652.8	0	1.3	1.4	1.3	1.4
																	TEIIILATR	22	2	1	378548190;15718687	N-Term(TMT6plex)	1258.8	0	1.3	1.2	1.3	1.2
																	LLGGLAVR	25	2	1	378548190;15718687	N-Term(TMT6plex)	1027.7	0	1.2	1.1	1.2	1.1
																	FVADGIFK	16	2	1	378548190;15718687	N-Term(TMT6plex); K8(TMT6plex)	1354.8	0	1.2	1.3	1.3	1.3
																	FVDGLMIHSGDPVNYYVDTAVR	5	3	1	378548190;15718687	N-Term(TMT6plex)	2697.4	0	1.4	1.5	1.6	1.4
																	AELNEFLTR	12	2	1	378548190;15718687	N-Term(TMT6plex)	1321.7	0	1.2	1.3	1.3	1.3
																	IMLPWDPTGK	11	3	1	378548190;15718687	N-Term(TMT6plex); K10(TMT6plex)	1615.9	0	1.6	1.8	2.0	1.6
																	FIMESGAK	10	2	1	378548190;15718687	N-Term(TMT6plex); M3(Oxidation); K8(TMT6plex)	1356.8	0	1.2	1.2	1.2	1.3
																	QGVLGIK	7	3	1	378548190;15718687	N-Term(TMT6plex); K7(TMT6plex)	1172.8	0	1.2	1.2	1.3	1.3
4502709	NP_001777.1	**CDK1**	983	**cyclin-dependent kinase 1 isoform 1**	80.13	48	16	19	189	**1.2**	**1.3**	**1.5**	**1.4**	297	34.1	8.40												
																	ALGTPNNEVWPEVESLQDYK	23	2	1	4502709	N-Term(TMT6plex); K20(TMT6plex)	2747.4	0	1.2	1.3	1.4	1.4
																	IGEGTYGVVYK	16	7	2	4502709;16936528	N-Term(TMT6plex); K11(TMT6plex)	1643.9	0				
																	MLIYDPAK	17	2	1	4502709	N-Term(TMT6plex); M1(Oxidation); K8(TMT6plex)	1424.8	0	1.2	1.2	1.3	1.3
																	NLDENGLDLLSK	4	2	1	4502709	N-Term(TMT6plex); K12(TMT6plex)	1789.0	0	1.2	1.1	1.5	1.3
																	YLDSIPPGQYMDSSLVK	11	3	1	4502709	N-Term(TMT6plex); M11(Oxidation); K17(TMT6plex)	2387.3	0	1.1	1.2	1.2	1.3
																	TTGQVVAMK	12	3	1	4502709	N-Term(TMT6plex); M8(Oxidation); K9(TMT6plex)	1408.8	0	1.1	1.0	1.3	1.1
																	VYTHEVVTLWYR	3	1	1	4502709	N-Term(TMT6plex)	1795.0	0	1.7	1.7	1.9	1.6

Khan et al., 2019. Multi-omics analysis to characterize cigarette smoke induced molecular alterations in esophageal cells
Supplementary Table 6. List of all proteins and peptides identified in untreated and chronically treated Het1A cells with cigarette smoke condensate for 8 months

Accession	RefSeq Accession	Gene Symbol	Entrez Gene ID	Description	Σ Coverage	Σ# Proteins	Σ# Unique Peptides	Σ# Peptides	Σ# PSMs	Het-1A-Smoke - 2M/Parental	Het-1A-Smoke - 4M/Parental	Het-1A-Smoke - 6M/Parental	Het-1A-Smoke - 8M/Parental	# AAs	MW [kDa]	calc. pI	Sequence	# PSMs	# Proteins	# Protein Groups	Protein Group Accessions	Modifications	MH+ [Da]	# Missed Cleavages	Het-1A-Smoke - 2M/Parental	Het-1A-Smoke - 4M/Parental	Het-1A-Smoke - 6M/Parental	Het-1A-Smoke - 8M/Parental
																	YSTPVDIWSIGTIFAELATK	46	2	1	4502709	N-Term(TMT6plex); K20(TMT6plex)	2670.5	0	**1.4**	**1.3**	**1.7**	**1.3**
																	WKPGSLASHVK	2	2	1	4502709	N-Term(TMT6plex); K2(TMT6plex); K11(TMT6plex)	1897.2	0	**1.0**	**1.2**	**1.4**	**1.3**
																	HPNIVSLQDVLMQDSR	5	3	1	4502709	N-Term(TMT6plex); M12(Oxidation)	2097.1	0	**1.1**	**1.2**	**1.5**	**1.5**
																	MALNHPYFNDLDNQIK	5	2	1	4502709	N-Term(TMT6plex); M1(Oxidation); K16(TMT6plex)	2407.2	0	**1.3**	**1.3**	**1.4**	**1.4**
																	SPEVLLGSAR	3	2	1	4502709	N-Term(TMT6plex)	1257.7	0	**1.1**	**1.3**	**1.4**	**1.4**
																	LESEEEGVPSTAIR	17	3	1	4502709	N-Term(TMT6plex)	1745.9	0	**1.2**	**1.3**	**1.4**	**1.3**
																	SYLYQILQGIVFCHSR	4	1	1	4502709	N-Term(TMT6plex); C13(Carbamidomethyl)	2213.2	0	**1.3**	**1.3**	**1.7**	**2.8**
																	LADFGLAR	4	74	10	16936528;4502709;157817073;4885661;4502735;145309300;4826675;281427168;5453860;4502747	N-Term(TMT6plex)	1091.6	0				
																	DLKPQNLLIDDK	5	1	1	4502709	N-Term(TMT6plex); K3(TMT6plex); K12(TMT6plex)	2099.3	0	**1.1**	**1.4**	**1.6**	**1.4**
																	MALNHPYFNDLDNQIK	2	2	1	4502709	N-Term(TMT6plex); K16(TMT6plex)	2391.3	0	**1.2**	**1.8**	**1.9**	**1.4**
																	LYLIFEFLSMDLK	2	3	1	4502709	N-Term(TMT6plex); K13(TMT6plex)	2090.2	0	**4.3**	**4.3**	**4.0**	**2.8**
																	AFGIPIR	5	1	1	4502709	N-Term(TMT6plex)	1002.6	0	**1.1**	**1.3**	**1.4**	**1.5**
																	YLDSIPPGQYMDSSLVK	1	3	1	4502709	N-Term(TMT6plex); K17(TMT6plex)	2371.3	0	**1.4**	**1.4**	**1.5**	**1.5**
																	EISLLK	2	6	2	4502709;16936528	N-Term(TMT6plex); K6(TMT6plex)	1160.8	0				
19923855	NP_116185.2	**SYAP1**	94056	**synapse-associated protein 1**	80.11	1	23	23	128	**1.2**	**1.2**	**1.3**	**1.0**	352	39.9	4.53												
																	DPPAGVQFNFDFDQMYPVALVMLQEDELLSK	10	1	1	19923855	N-Term(TMT6plex); M15(Oxidation); M22(Oxidation); K31(TMT6plex)	4047.0	0	**1.0**	**1.0**	**1.0**	**0.8**
																	KITESVAETAQTIK	4	1	1	19923855	N-Term(TMT6plex); K1(TMT6plex); K14(TMT6plex)	2206.3	1	**1.2**	**1.4**	**1.3**	**1.0**
																	DFGNYLFNFASAATK	19	1	1	19923855	N-Term(TMT6plex); K15(TMT6plex)	2124.1	0	**1.3**	**1.2**	**1.3**	**0.9**
																	FVEEQHTK	4	1	1	19923855	N-Term(TMT6plex); K8(TMT6plex)	1475.8	0	**1.2**	**1.3**	**1.2**	**1.0**
																	TIIGDFQK	9	1	1	19923855	N-Term(TMT6plex); K8(TMT6plex)	1379.8	0	**1.2**	**1.2**	**1.2**	**1.1**
																	SVEEGKIDGIIDK	2	1	1	19923855	N-Term(TMT6plex); K6(TMT6plex); K13(TMT6plex)	2090.2	1	**1.4**	**1.5**	**1.2**	**1.0**
																	SEAAVPPWVDTNDEETIQQQILALSADK	6	1	1	19923855	N-Term(TMT6plex); K28(TMT6plex)	3526.8	0	**1.1**	**1.0**	**1.4**	**0.9**
																	KSEAAVPPWVDTNDEETIQQQILALSADK	4	1	1	19923855	N-Term(TMT6plex); K1(TMT6plex); K29(TMT6plex)	3884.1	1	**1.3**	**1.4**	**1.3**	**1.0**
																	EMEQLVLDK	6	1	1	19923855	N-Term(TMT6plex); M2(Oxidation); K9(TMT6plex)	1578.9	0	**1.1**	**1.1**	**1.1**	**0.9**
																	ITESVAETAQTIK	7	1	1	19923855	N-Term(TMT6plex); K13(TMT6plex)	1849.1	0	**1.3**	**1.2**	**1.3**	**1.1**
																	DPPAGVQFNFDFDQMYPVALVMLQEDELLSK	2	1	1	19923855	N-Term(TMT6plex); M15(Oxidation); K31(TMT6plex)	4031.0	0	**1.3**	**1.4**	**1.4**	**0.9**
																	QSAQLTALAAQQQAAGK	8	1	1	19923855	N-Term(TMT6plex); K17(TMT6plex)	2143.2	0	**1.3**	**1.1**	**1.3**	**1.1**
																	QSAQLTALAAQQQAAGKEEK	2	1	1	19923855	N-Term(TMT6plex); K17(TMT6plex); K20(TMT6plex)	2758.6	1	**1.3**	**1.2**	**1.2**	**0.9**
																	KFVEEQHTK	4	1	1	19923855	N-Term(TMT6plex); K1(TMT6plex); K9(TMT6plex)	1833.1	1	**1.1**	**1.2**	**1.3**	**0.9**
																	TQEDEEEISTSPGVSEFVSDAFDACNLNQEDLR	6	1	1	19923855	N-Term(TMT6plex); C25(Carbamidomethyl)	3960.8	0	**1.7**	**1.4**	**1.6**	**1.3**
																	ELQQELQEYEVVTESEK	15	1	1	19923855	N-Term(TMT6plex); K17(TMT6plex)	2539.3	0	**1.1**	**1.1**	**1.1**	**0.9**
																	EQDLPLAEAVRPK	4	1	1	19923855	N-Term(TMT6plex); K13(TMT6plex)	1924.1	0	**1.2**	**1.3**	**1.3**	**1.1**
																	SEAAVPPWVDTNDEETIQQQILALSADKR	2	1	1	19923855	N-Term(TMT6plex); K28(TMT6plex)	3682.9	1	**1.4**	**1.5**	**1.4**	**1.1**
																	EMEQLVLDK	2	1	1	19923855	N-Term(TMT6plex); K9(TMT6plex)	1562.9	0	**2.0**	**1.9**	**2.2**	**1.3**
																	GLSSWLGLQQPVAGGGQPNGDAPPEQPSETVAESAEEELQQAGDQELLHQAK	2	1	1	19923855	N-Term(TMT6plex); K52(TMT6plex)	5836.8	0	**0.8**	**1.1**	**1.1**	**0.5**
																	DENWDKEIEK	1	1	1	19923855	N-Term(TMT6plex); K6(TMT6plex); K10(TMT6plex)	1993.1	1	**1.1**	**1.2**	**1.2**	**0.9**
																	IDGIIDK	6	1	1	19923855	N-Term(TMT6plex); K7(TMT6plex)	1231.8	0	**1.4**	**1.1**	**1.2**	**0.9**
																	RDENWDKEIEK	1	1	1	19923855	N-Term(TMT6plex); K7(TMT6plex); K11(TMT6plex)	2149.2	2	**1.2**	**1.3**	**1.3**	**1.0**
																	EMEQLVLDKK	1	1	1	19923855	N-Term(TMT6plex); M2(Oxidation); K9(TMT6plex); K10(TMT6plex)	1936.1	1	**1.1**	**1.3**	**1.1**	**1.1**
																	FALVPK	1	1	1	19923855	N-Term(TMT6plex); K6(TMT6plex)	1132.7	0	**1.4**	**1.3**	**1.3**	**0.9**
4501891	NP_001093.1	**ACTN1**	87	**alpha-actinin-1 isoform b**	80.04	3	35	62	1622	**1.3**	**1.2**	**1.3**	**1.3**	892	103.0	5.41												
																	ETADTDTADQVMASFK	49	3	1	4501891	N-Term(TMT6plex); M12(Oxidation); K16(TMT6plex)	2204.1	0	**1.2**	**1.2**	**1.3**	**1.3**
																	RDQALTEEHAR	9	3	1	4501891	N-Term(TMT6plex)	1554.8	1	**1.4**	**1.2**	**1.3**	**1.2**
																	ISIEMHGTLEDQLSHLR	36	3	1	4501891	N-Term(TMT6plex); M5(Oxidation)	2224.2	0	**1.3**	**1.4**	**1.4**	**1.4**
																	GISQEQMNEFR	37	3	1	4501891	N-Term(TMT6plex); M7(Oxidation)	1583.8	0	**1.3**	**1.3**	**1.3**	**1.3**
																	ELPPDQAEYCIAR	100	4	2	12025678;4501891	N-Term(TMT6plex); C10(Carbamidomethyl)	1790.9	0				
																	MVSDINNAWGCLEQVEK	27	3	1	4501891	N-Term(TMT6plex); M1(Oxidation); C11(Carbamidomethyl); K17(TMT6plex)	2467.2	0	**1.3**	**1.4**	**1.5**	**1.3**
																	TINEVENQILTR	44	4	2	12025678;4501891	N-Term(TMT6plex)	1658.9	0				
																	IDQLEGDHQLIQEALIFDNK	62	3	1	4501891	N-Term(TMT6plex); K20(TMT6plex)	2797.5	0	**1.3**	**1.1**	**1.4**	**1.2**
																	LLETIDQLYLEYAK	121	3	1	4501891	N-Term(TMT6plex); K14(TMT6plex)	2170.2	0	**1.2**	**1.1**	**1.3**	**1.3**
																	GYEEWLLNEIR	69	7	3	12025678;4501891;507588248	N-Term(TMT6plex)	1650.9	0				
																	EGLLLWCQR	22	8	3	12025678;4501891;507588248	N-Term(TMT6plex); C7(Carbamidomethyl)	1403.8	0				
																	TIPWLENR	18	6	2	4501891;507588248	N-Term(TMT6plex)	1257.7	0				
																	MAPYTGPDSVPGALDYMSFSTALYGESDL	22	3	1	4501891	N-Term(TMT6plex); M1(Oxidation); M17(Oxidation)	3316.5	0	**1.5**	**1.6**	**1.6**	**1.6**
																	DYETATLSEIK	10	3	1	4501891	N-Term(TMT6plex); K11(TMT6plex)	1727.9	0	**1.2**	**1.2**	**1.3**	**1.3**
																	HRPELIDYGK	18	3	1	4501891	N-Term(TMT6plex); K10(TMT6plex)	1686.0	0	**1.1**	**1.1**	**1.2**	**1.3**
																	LAILGIHNEVSK	27	3	1	4501891	N-Term(TMT6plex); K12(TMT6plex)	1752.1	0	**1.1**	**1.3**	**1.4**	**1.3**
																	LSNRPAFMPSEGR	7	3	1	4501891	N-Term(TMT6plex); M8(Oxidation)	1706.9	0	**1.3**	**1.2**	**1.4**	**1.2**
																	VPENTMHAMQQK	25	3	1	4501891	N-Term(TMT6plex); M6(Oxidation); M9(Oxidation); K12(TMT6plex)	1904.0	0	**1.2**	**1.5**	**1.5**	**1.3**
																	VLAVNQENEQLMEDYEK	38	3	1	4501891	N-Term(TMT6plex); M12(Oxidation); K17(TMT6plex)	2526.3	0	**1.3**	**1.3**	**1.4**	**1.4**
																	DDPLTNLNTAFDVAEK	36	3	1	4501891	N-Term(TMT6plex); K16(TMT6plex)	2221.2	0	**1.3**	**1.3**	**1.5**	**1.5**
																	VLAVNQENEQLMEDYEK	14	3	1	4501891	N-Term(TMT6plex); K17(TMT6plex)	2510.3	0	**1.6**	**1.7**	**1.9**	**1.5**
																	ICDQWDNLGALTQK	31	3	1	4501891	N-Term(TMT6plex); C2(Carbamidomethyl); K14(TMT6plex)	2120.1	0	**1.2**	**1.1**	**1.3**	**1.2**
																	HEAFESDLAAHQDR	4	9	3	12025678;4501891;507588248	N-Term(TMT6plex)	1854.9	0				
																	CQLEINFNTLQTK	27	9	3	12025678;4501891;507588248	N-Term(TMT6plex); C1(Carbamidomethyl); K13(TMT6plex)	2067.1	0				
																	VGWEQLLTTIAR	69	4	2	12025678;4501891	N-Term(TMT6plex)	1615.9	0				
																	NYITMDELR	13	3	1	4501891	N-Term(TMT6plex); M5(Oxidation)	1399.7	0	**1.3**	**1.2**	**1.3**	**1.2**
																	DHSGTLGPEEFK	22	2	1	4501891	N-Term(TMT6plex); K12(TMT6plex)	1774.9	0	**1.2**	**1.3**	**1.3**	**1.2**
																	ATLPDADKER	12	3	1	4501891	N-Term(TMT6plex); K8(TMT6plex)	1573.9	1	**1.2**	**1.2**	**1.3**	**1.3**
																	LASDLLEWIR	42	4	2	12025678;4501891	N-Term(TMT6plex)	1444.8	0				
																	MLDAEDIVGTARPDEK	3	3	1	4501891	N-Term(TMT6plex); K16(TMT6plex)	2218.2	0	**1.4**	**1.6**	**1.8**	**1.3**
																	MLDAEDIVGTARPDEK	20	3	1	4501891	N-Term(TMT6plex); M1(Oxidation); K16(TMT6plex)	2234.2	0	**1.4**	**1.3**	**1.4**	**1.3**
																	VEQIAAIAQELNELDYYDSPSVNAR	72	3	1	4501891	N-Term(TMT6plex)	3037.5	0	**1.5**	**1.3**	**1.5**	**1.3**
																	AETAANR	5	5	3	12025678;4501891;507588248	N-Term(TMT6plex)	961.5	0				
																	ASIHEAWTDGK	16	4	2	12025678;4501891	N-Term(TMT6plex); K11(TMT6plex)	1672.9	0				
																	QKDYETATLSEIK	6	3	1	4501891	N-Term(TMT6plex); K2(TMT6plex); K13(TMT6plex)	2213.3	1	**1.2**	**1.2**	**1.3**	**1.3**
																	AAPFNNWMEGAMEDLQDTFIVHTIEEIQGLTTAHEQFK	20	3	1	4501891	N-Term(TMT6plex); M8(Oxidation); M12(Oxidation); K38(TMT6plex)	4852.4	0	**1.0**	**1.0**	**1.1**	**1.2**
																	LMLLLEVISGER	65	8	3	12025678;4501891;507588248	N-Term(TMT6plex); M2(Oxidation)	1617.9	0				
																	AGTQIENIEEDFR	31	7	2	4501891;507588248	N-Term(TMT6plex)	1750.9	0				
																	KAGTQIENIEEDFRDGLK	1	3	1	4501891	N-Term(TMT6plex); K1(TMT6plex); K18(TMT6plex)	2750.5	2	**1.5**	**1.8**	**1.6**	**1.6**
																	FAIQDISVEETSAK	44	8	3	12025678;4501891;507588248	N-Term(TMT6plex); K14(TMT6plex)	1996.1	0				
																	HTNYTMEHIR	2	7	3	12025678;4501891;507588248	N-Term(TMT6plex); M6(Oxidation)	1546.8	0				
																	ALDFIASK	30	6	2	12025678;4501891	N-Term(TMT6plex); K8(TMT6plex)	1322.8	0				
																	KDDPLTNLNTAFDVAEK	25	3	1	4501891	N-Term(TMT6plex); K1(TMT6plex); K17(TMT6plex)	2578.4	1	**1.2**	**1.3**	**1.3**	**1.3**
																	AGTQIENIEEDFRDGLK	7	3	1	4501891	N-Term(TMT6plex); K17(TMT6plex)	2393.3	1	**1.3**	**1.4**	**1.4**	**1.4**
																	GISQEQMNEFR	3	3	1	4501891	N-Term(TMT6plex)	1567.8	0	**1.3**	**1.2**	**1.4**	**1.1**
																	LVSIGAEEIVDGNVK	19	5	2	4501891;507588248	N-Term(TMT6plex); K15(TMT6plex)	2001.2	0				
																	LSNRPAFMPSEGR	1	3	1	4501891	N-Term(TMT6plex)	1690.9	0	**1.4**	**1.5**	**1.7**	**1.3**
																	NYITMDELR	4	3	1	4501891	N-Term(TMT6plex)	1383.7	0	**1.6**	**1.6**	**1.8**	**1.3**
																	ASFNHFDR	11	8	2	4501891;507588248	N-Term(TMT6plex)	1222.6	0				
																	KAGTQIENIEEDFR	15	7	2	4501891;507588248	N-Term(TMT6plex); K1(TMT6plex)	2108.1	1				
																	KHEAFESDLAAHQDR	33	7	3	12025678;4501891;507588248	N-Term(TMT6plex); K1(TMT6plex)	2212.2	1				
																	DGLGFCALIHR	12	3	1	4501891	N-Term(TMT6plex); C6(Carbamidomethyl)	1487.8	0	**1.3**	**1.3**	**1.4**	**1.3**
																	MTLGMIWTIILR	10	8	3	12025678;4501891;507588248	N-Term(TMT6plex); M5(Oxidation)	1693.0	0				
																	SIVNYKPK	8	3	1	4501891	N-Term(TMT6plex); K6(TMT6plex); K8(TMT6plex)	1636.0	0	**1.1**	**1.2**	**1.3**	**1.2**
																	VPENTMHAMQQK	8	3	1	4501891	N-Term(TMT6plex); M6(Oxidation); K12(TMT6plex)	1888.0	0	**1.4**	**1.6**	**1.7**	**1.4**
																	MTLGMIWTIILR	11	8	3	12025678;4501891;507588248	N-Term(TMT6plex); M1(Oxidation); M5(Oxidation)	1709.0	0				
																	DLLLDPAWEK	25	7	3	12025678;4501891;507588248	N-Term(TMT6plex); K10(TMT6plex)	1657.9	0				
																	NVNIQNFHISWK	16	3	1	4501891	N-Term(TMT6plex); K12(TMT6plex)	1958.1	0	**1.1**	**1.0**	**1.3**	**1.1**
																	ACLISLGYDIGNDPQGEAEFAR	12	1	1	4501891	N-Term(TMT6plex); C2(Carbamidomethyl)	2625.3	0	**1.2**	**1.2**	**1.1**	**1.2**
																	TFTAWCNSHLR	4	7	3	12025678;4501891;507588248	N-Term(TMT6plex); C6(Carbamidomethyl)	1621.8	0				
																	MAPYTGPDSVPGALDYMSFSTALYGESDL	11	3	1	4501891	N-Term(TMT6plex); M1(Oxidation)	3300.5	0	**1.5**	**1.4**	**1.5**	**1.5**
																	DQALTEEHAR	2	3	1	4501891	N-Term(TMT6plex)	1398.7	0	**1.1**	**1.1**	**1.1**	**1.1**
																	IMSIVDPNR	2	3	1	4501891	N-Term(TMT6plex)	1273.7	0	**1.5**	**1.4**	**1.7**	**1.3**
																	MTLGMIWTIILR	6	8	3	12025678;4501891;507588248	N-Term(TMT6plex)	1677.0	0				
																	KTFTAWCNSHLR	1	7	3	12025678;4501891;507588248	N-Term(TMT6plex); K1(TMT6plex); C7(Carbamidomethyl)	1979.1	1				
																	LMLLLEVISGER	4	8	3	12025678;4501891;507588248	N-Term(TMT6plex)	1601.9	0				
																	QFGAQANVIGPWIQTK	1	3	1	4501891	N-Term(TMT6plex); K16(TMT6plex)	2216.2	0	**1.8**	**1.3**	**1.6**	**1.5**
																	AIMTYVSSFYHAFSGAQK	1	4	2	12025678;4501891	N-Term(TMT6plex); K18(TMT6plex)	2466.3	0				
																	LDHLAEK	21	4	2	12025678;4501891	N-Term(TMT6plex); K7(TMT6plex)	1283.8	0				

Supplementary Table 6. List of all proteins and peptides identified in untreated and chronically treated Het1A cells with cigarette smoke condensate for 8 months

Accession	RefSeq Accession	Gene Symbol	Entrez Gene ID	Description	Σ Coverage	Σ# Proteins	Σ# Unique Peptides	Σ# Peptides	Σ# PSMs	Het-1A-Smoke - 2M/Parental	Het-1A-Smoke - 4M/Parental	Het-1A-Smoke - 6M/Parental	Het-1A-Smoke - 8M/Parental	# AAs	MW [kDa]	calc. pI	Sequence	# PSMs	# Proteins	# Protein Groups	Protein Group Accessions	Modifications	MH+ [Da]	# Missed Cleavages	Het-1A-Smoke - 2M/Parental	Het-1A-Smoke - 4M/Parental	Het-1A-Smoke - 6M/Parental	Het-1A-Smoke - 8M/Parental
																	IMSIVDPNR	13	3	1	4501891	N-Term(TMT6plex); M2(Oxidation)	1289.7	0	**1.3**	**1.2**	**1.4**	**1.2**
																	LHKPPK	1	3	1	4501891	N-Term(TMT6plex); K3(TMT6plex); K6(TMT6plex)	1406.9	0	**1.0**	**1.2**	**1.3**	**1.3**
																	YLDIPK	7	6	2	12025678;4501891	N-Term(TMT6plex); K6(TMT6plex)	1206.7	0				
																	ISNVNK	2	3	1	4501891	N-Term(TMT6plex); K6(TMT6plex)	1132.7	0	**1.2**	**1.0**	**1.2**	**1.1**
380837121	NP_004883.3	**SEC22B**	9554	**vesicle-trafficking protein SEC22b precursor**	80.00	1	11	11	76	**1.4**	**1.4**	**1.4**	**1.3**	215	24.7	8.51												
																	DLQQYQSQAK	9	1	1	380837121	N-Term(TMT6plex); K10(TMT6plex)	1666.9	0	**1.2**	**1.2**	**1.1**	**1.2**
																	GEALSALDSK	9	1	1	380837121	N-Term(TMT6plex); K10(TMT6plex)	1448.8	0	**1.2**	**1.3**	**1.3**	**1.2**
																	NLGSINTELQDVQR	13	1	1	380837121	N-Term(TMT6plex)	1816.0	0	**1.3**	**1.3**	**1.3**	**1.3**
																	IMVANIEEVLQR	14	1	1	380837121	N-Term(TMT6plex); M2(Oxidation)	1659.9	0	**1.2**	**1.2**	**1.1**	**1.3**
																	VADGLPLAASMQEDEQSGR	10	1	1	380837121	N-Term(TMT6plex); M11(Oxidation)	2219.1	0	**1.7**	**1.5**	**1.6**	**1.5**
																	ANNLSSLSK	2	1	1	380837121	N-Term(TMT6plex); K9(TMT6plex)	1391.8	0	**1.2**	**1.2**	**1.2**	**1.2**
																	VADGLPLAASMQEDEQSGR	4	1	1	380837121	N-Term(TMT6plex)	2203.1	0	**1.6**	**1.6**	**1.5**	**1.5**
																	LAFAYLEDLHSEFDEQHGK	2	1	1	380837121	N-Term(TMT6plex); K19(TMT6plex)	2707.4	0	**0.9**	**0.9**	**0.8**	**1.0**
																	IMVANIEEVLQR	7	1	1	380837121	N-Term(TMT6plex)	1643.9	0	**1.5**	**1.5**	**1.7**	**1.4**
																	KVPTVSRPYSFIEFDTFIQK	1	1	1	380837121	N-Term(TMT6plex); K1(TMT6plex); K20(TMT6plex)	3089.8	1	**1.6**	**2.0**	**1.3**	**1.9**
																	LAAVAVFFIMLIVYVRFWWL	2	1	1	380837121	N-Term(TMT6plex)	2686.6	1	**2.3**	**1.5**	**2.8**	**0.7**
																	CTLEAGAMTFHYIIEQGVCYLVLCEAAFPK	2	1	1	380837121	N-Term(TMT6plex); C1(Carbamidomethyl); M8(Oxidation); C19(Carbamidomethyl); C24(Carbamidomethyl); K30(TMT6plex)	3965.9	0	**1.5**	**1.4**	**2.2**	**0.9**
																	KLNEQSPTR	1	1	1	380837121	N-Term(TMT6plex); K1(TMT6plex)	1530.9	1	**0.9**	**1.0**	**1.0**	**0.9**
50592996	NP_006077.2	**TUBB3**	10381	**tubulin beta-3 chain isoform 1**	80.00	2	8	23	1272	**1.3**	**1.2**	**1.4**	**1.3**	450	50.4	4.93												
																	AILVDLEPGTMDSVR	141	7	4	29788785;29788768;50592996;4507729	N-Term(TMT6plex); M11(Oxidation)	1861.0	0				
																	GHYTEGAELVDSVLDVVR	235	11	5	29788785;5174735;29788768;50592996;4507729	N-Term(TMT6plex)	2188.1	0				
																	NSSYFVEWIPNNVK	95	20	7	29788785;5174735;14210536;746815975;29788768;50592996;21361322;574584816;4507729	N-Term(TMT6plex); K14(TMT6plex)	2155.2	0				
																	ALTVPELTQQMFDAK	71	7	3	5174735;50592996;21361322;574584816	N-Term(TMT6plex); M11(Oxidation); K15(TMT6plex)	2166.2	0				
																	FWEVISDEHGIDPSGNYVGDSDLQLER	8	1	1	50592996	N-Term(TMT6plex)	3306.6	0	**1.2**	**1.2**	**1.4**	**1.2**
																	ALTVPELTQQMFDAK	47	7	3	5174735;50592996;21361322;574584816	N-Term(TMT6plex); K15(TMT6plex)	2150.2	0				
																	IMNTFSVVPSPK	47	8	4	29788785;5174735;50592996;21361322;574584816	N-Term(TMT6plex); M2(Oxidation); K12(TMT6plex)	1794.0	0				
																	EIVHIQAGQCGNQIGAK	36	8	6	29788785;29788768;50592996;4507729;5174735;21361322;574584816	N-Term(TMT6plex); C10(Carbamidomethyl); K17(TMT6plex)	2281.2	0				
																	ISVYYNEASSHK	5	1	1	50592996	N-Term(TMT6plex); K12(TMT6plex)	1856.0	0	**1.1**	**1.2**	**1.5**	**1.3**
																	KLAVNMVPFPR	11	21	8	29788785;5174735;14210536;746815975;29788768;50592996;21361322;574584816;4507729;13562114	N-Term(TMT6plex); K1(TMT6plex); M6(Oxidation)	1746.0	1				
																	KECENCDCLQGFQLTHSLGGGTGSGMGTLLISK	1	2	1	50592996	K1(TMT6plex); N-Term(TMT6plex); C3(Carbamidomethyl); C6(Carbamidomethyl); C8(Carbamidomethyl); M26(Oxidation); K33(TMT6plex)	4259.1	1	**1.1**	**1.4**	**1.0**	**1.6**
																	ISEQFTAMFR	20	10	6	29788785;5174735;29788768;50592996;21361322;574584816;4507729	N-Term(TMT6plex)	1458.8	0				
																	ISEQFTAMFR	244	10	6	29788785;5174735;29788768;50592996;21361322;574584816;4507729	N-Term(TMT6plex); M8(Oxidation)	1474.8	0				
																	EVDEQMLAIQSK	25	7	2	14210536;746815975;50592996	N-Term(TMT6plex); M6(Oxidation); K12(TMT6plex)	1865.0	0				
																	MSSTFIGNSTAIQELFK	16	2	1	50592996	N-Term(TMT6plex); M1(Oxidation); K17(TMT6plex)	2348.3	0	**1.2**	**1.0**	**1.2**	**1.2**
																	FPGQLNADLR	98	13	8	29788785;5174735;14210536;746815975;29788768;50592996;21361322;574584816;4507729;13562114	N-Term(TMT6plex)	1359.8	0				
																	AILVDLEPGTMDSVR	22	7	4	29788785;29788768;50592996;4507729	N-Term(TMT6plex)	1845.0	0				
																	LAVNMVPFPR	20	21	8	29788785;5174735;14210536;746815975;29788768;50592996;21361322;574584816;4507729;13562114	N-Term(TMT6plex)	1372.8	0				
																	VAVCDIPPR	5	8	3	14210536;746815975;50592996;13562114	N-Term(TMT6plex); C4(Carbamidomethyl)	1255.7	0				
																	LAVNMVPFPR	54	21	8	29788785;5174735;14210536;746815975;29788768;50592996;21361322;574584816;4507729;13562114	N-Term(TMT6plex); M5(Oxidation)	1388.8	0				
																	LHFFMPGFAPLTAR	6	2	1	50592996	N-Term(TMT6plex)	1834.0	0	**1.7**	**1.6**	**1.7**	**1.4**
																	LATPTYGDLNHLVSATMSGVTTSLR	8	2	1	50592996	N-Term(TMT6plex); M17(Oxidation)	2850.5	0	**1.3**	**1.3**	**1.5**	**1.4**
																	MSSTFIGNSTAIQELFK	14	2	1	50592996	N-Term(TMT6plex); K17(TMT6plex)	2332.3	0	**1.3**	**1.1**	**1.2**	**1.1**
																	YLTVATVFR	6	3	2	14210536;746815975;50592996	N-Term(TMT6plex)	1298.8	0				
																	EVDEQMLAIQSK	4	7	2	14210536;746815975;50592996	N-Term(TMT6plex); K12(TMT6plex)	1849.0	0				
																	LATPTYGDLNHLVSATMSGVTTSLR	3	2	1	50592996	N-Term(TMT6plex)	2834.5	0	**1.5**	**1.6**	**1.8**	**1.6**
																	IMNTFSVVPSPK	8	8	4	29788785;5174735;50592996;21361322;574584816	N-Term(TMT6plex); K12(TMT6plex)	1778.0	0				
																	LHFFMPGFAPLTAR	4	2	1	50592996	N-Term(TMT6plex); M5(Oxidation)	1850.0	0	**1.5**	**1.2**	**1.5**	**1.3**
																	NMMAACDPR	5	20	7	29788785;5174735;14210536;746815975;29788768;50592996;21361322;574584816;4507729	N-Term(TMT6plex); M2(Oxidation); M3(Oxidation); C6(Carbamidomethyl)	1326.6	0				
																	GSQQYR	5	12	7	29788785;5174735;14210536;746815975;29788768;50592996;21361322;574584816;4507729	N-Term(TMT6plex)	967.5	0				
																	VSDTVVEPYNATLSIHQLVENTDETYCIDNEALYDICFR	1	2	1	50592996	N-Term(TMT6plex); C27(Carbamidomethyl); C37(Carbamidomethyl)	4836.3	0	**1.4**	**1.3**	**1.8**	**1.6**
																	NMMAACDPR	4	20	7	29788785;5174735;14210536;746815975;29788768;50592996;21361322;574584816;4507729	N-Term(TMT6plex); M3(Oxidation); C6(Carbamidomethyl)	1310.6	0				
																	NMMAACDPR	2	20	7	29788785;5174735;14210536;746815975;29788768;50592996;21361322;574584816;4507729	N-Term(TMT6plex); C6(Carbamidomethyl)	1294.6	0				
																	SGAFGHLFRPDNFIFGQSGAGNNWAK	1	2	1	50592996	N-Term(TMT6plex); K26(TMT6plex)	3253.7	0	**1.7**	**2.1**	**2.2**	**1.6**
157502193	NP_002808.3	**PSMD13**	5719	**26S proteasome non-ATPase regulatory subunit 13 isoform 1**	79.79	2	25	25	200	**1.5**	**1.5**	**1.7**	**1.4**	376	42.9	5.81												
																	ETIEDVEEMLNNLPGVTSVHSR	10	2	1	157502193	N-Term(TMT6plex); M9(Oxidation)	2714.4	0	**1.8**	**1.4**	**1.4**	**1.5**
																	ETIEDVEEMLNNLPGVTSVHSR	7	2	1	157502193	N-Term(TMT6plex)	2698.4	0	**1.5**	**1.5**	**1.6**	**1.5**
																	ITVNEVELLVMK	28	2	1	157502193	N-Term(TMT6plex); M11(Oxidation); K12(TMT6plex)	1862.1	0	**1.5**	**1.5**	**1.7**	**1.6**
																	ALSVGLVK	8	2	1	157502193	N-Term(TMT6plex); K8(TMT6plex)	1244.8	0	**1.4**	**1.5**	**1.7**	**1.5**
																	QMTDPNVALTFLEK	11	2	1	157502193	N-Term(TMT6plex); M2(Oxidation); K14(TMT6plex)	2081.1	0	**1.5**	**1.3**	**1.5**	**1.4**
																	LNIGDLQVTK	12	2	1	157502193	N-Term(TMT6plex); K10(TMT6plex)	1559.0	0	**1.4**	**1.5**	**1.7**	**1.4**
																	LYENFISEFEHR	7	1	1	157502193	N-Term(TMT6plex)	1812.9	0	**1.6**	**1.4**	**1.4**	**1.3**
																	LEFWCTDVK	7	2	1	157502193	N-Term(TMT6plex); C5(Carbamidomethyl); K9(TMT6plex)	1655.9	0	**1.5**	**1.5**	**1.8**	**1.4**
																	VLDLQQIK	10	2	1	157502193	N-Term(TMT6plex); K8(TMT6plex)	1414.9	0	**1.5**	**1.5**	**1.7**	**1.4**
																	QMTDPNVALTFLEK	1	2	1	157502193	N-Term(TMT6plex); K14(TMT6plex)	2065.1	0	**1.4**	**1.7**	**1.9**	**1.5**
																	VNPLSLVEIILHVVR	9	2	1	157502193	N-Term(TMT6plex)	1930.2	0	**1.7**	**1.6**	**1.8**	**1.4**
																	TAWGQQPDLAANEAQLLR	16	2	1	157502193	N-Term(TMT6plex)	2211.2	0	**1.5**	**1.3**	**1.6**	**1.3**
																	ITVNEVELLVMK	4	2	1	157502193	N-Term(TMT6plex); K12(TMT6plex)	1846.1	0	**2.5**	**3.3**	**3.7**	**2.1**
																	QWLIDTLYAFNSGNVER	14	2	1	157502193	N-Term(TMT6plex)	2255.2	0	**1.7**	**1.8**	**1.9**	**1.6**
																	LEELYTK	8	2	1	157502193	N-Term(TMT6plex); K7(TMT6plex)	1353.8	0	**1.3**	**1.3**	**1.6**	**1.3**
																	DLPVSEQQER	9	2	1	157502193	N-Term(TMT6plex)	1429.8	0	**1.5**	**1.5**	**1.4**	**1.4**
																	SMEMLVEHQAHDILT	2	2	1	157502193	N-Term(TMT6plex); M2(Oxidation); M4(Oxidation)	2015.0	0	**1.2**	**1.2**	**1.3**	**1.2**
																	SSDEAVILCK	11	2	1	157502193	N-Term(TMT6plex); C9(Carbamidomethyl); K10(TMT6plex)	1579.9	0	**1.8**	**1.6**	**1.7**	**1.4**
																	YYQTIGNHASYYK	5	2	1	157502193	N-Term(TMT6plex); K13(TMT6plex)	2066.1	0	**1.5**	**1.4**	**1.7**	**1.4**
																	MKDVPGFLQQSQNSGPGQPAVWHR	3	2	1	157502193	N-Term(TMT6plex); M1(Oxidation); K2(TMT6plex)	3138.6	1	**1.5**	**1.6**	**1.6**	**1.6**
																	VHMTWVQPR	1	2	1	157502193	N-Term(TMT6plex)	1382.8	0				
																	QLTFEEIAK	4	2	1	157502193	N-Term(TMT6plex); K9(TMT6plex)	1536.9	0	**1.5**	**1.4**	**1.7**	**1.5**
																	FLGCVDIK	4	2	1	157502193	N-Term(TMT6plex); C4(Carbamidomethyl); K8(TMT6plex)	1409.8	0	**1.6**	**1.5**	**1.8**	**1.4**
																	GSIDEVDKR	3	2	1	157502193	N-Term(TMT6plex); K8(TMT6plex)	1476.8	1	**1.4**	**1.6**	**1.8**	**1.5**
																	FYDLSSK	2	2	1	157502193	N-Term(TMT6plex); K7(TMT6plex)	1317.7	0	**1.5**	**1.4**	**1.8**	**1.4**
																	TAIGALK	2	2	1	157502193	N-Term(TMT6plex); K7(TMT6plex)	1131.8	0	**1.4**	**1.3**	**1.6**	**1.2**
																	KLWHQLTLQVLDFVQDPCFAQGDGLIK	1	1	1	157502193	N-Term(TMT6plex); K1(TMT6plex); C18(Carbamidomethyl); K27(TMT6plex)	3857.1	1	**1.5**	**1.4**	**2.3**	**0.8**
																	DVPGFLQQSQNSGPGQPAVWHR	1	2	1	157502193	N-Term(TMT6plex)	2634.3	0	**1.2**	**1.3**	**1.4**	**1.3**
25777713	NP_733779.1	**SKP1**	6500	**S-phase kinase-associated protein 1 isoform b**	79.75	2	11	12	105	**1.4**	**1.2**	**1.6**	**1.1**	163	18.6	4.54												
																	RTDDIPVWDQEFLK	2	2	1	25777713	N-Term(TMT6plex); K14(TMT6plex)	2220.2	1	**1.4**	**1.3**	**1.5**	**1.2**
																	NDFTEEEEAQVR	16	1	1	25777713	N-Term(TMT6plex)	1695.8	0	**1.5**	**1.1**	**1.5**	**1.0**
																	TMLEDLGMDDEGDDDPVPLPNVNAAILK	20	2	1	25777713	N-Term(TMT6plex); M2(Oxidation); M8(Oxidation); K28(TMT6plex)	3487.7	0	**1.3**	**1.3**	**1.7**	**1.2**
																	TMLEDLGMDDEGDDDPVPLPNVNAAILK	13	2	1	25777713	N-Term(TMT6plex); M8(Oxidation); K28(TMT6plex)	3471.7	0	**1.6**	**1.4**	**2.1**	**1.3**
																	KENQWCEEK	3	1	1	25777713	N-Term(TMT6plex); K1(TMT6plex); C6(Carbamidomethyl); K9(TMT6plex)	1938.0	1	**1.7**	**1.1**	**1.6**	**0.9**
																	GLLDVTCK	4	2	1	25777713	N-Term(TMT6plex); C7(Carbamidomethyl); K8(TMT6plex)	1363.8	0	**1.2**	**1.2**	**1.5**	**1.1**
																	TDDIPVWDQEFLK	24	2	1	25777713	N-Term(TMT6plex); K13(TMT6plex)	2064.1	0	**1.5**	**0.9**	**1.5**	**0.9**
																	VIQWCTHHKDDPPPPEDDENKEK	2	2	1	25777713	N-Term(TMT6plex); C5(Carbamidomethyl); K9(TMT6plex); K21(TMT6plex); K23(TMT6plex)	3730.9	2	**1.1**	**1.2**	**1.6**	**1.0**
																	LQSSDGEIFEVDVEIAK	6	2	1	25777713	N-Term(TMT6plex); K17(TMT6plex)	2337.3	0	**1.4**	**1.1**	**1.5**	**1.1**
																	TMLEDLGMDDEGDDDPVPLPNVNAAILK	4	2	1	25777713	N-Term(TMT6plex); K28(TMT6plex)	3455.7	0	**1.9**	**1.8**	**2.3**	**1.5**
																	TVANMIK	1	2	1	25777713	N-Term(TMT6plex); K7(TMT6plex)	1234.8	0				

Khan et al., 2019. Multi-omics analysis to characterize cigarette smoke induced molecular alterations in esophageal cells

Supplementary Table 6. List of all proteins and peptides identified in untreated and chronically treated Het1A cells with cigarette smoke condensate for 8 months

Accession	RefSeq Accession	Gene Symbol	Entrez Gene ID	Description	Σ Coverage	Σ# Proteins	Σ# Unique Peptides	Σ# Peptides	Σ# PSMs	Het-1A-Smoke - 2M/Parental	Het-1A-Smoke - 4M/Parental	Het-1A-Smoke - 6M/Parental	Het-1A-Smoke - 8M/Parental	# AAs	MW [kDa]	calc. pI	Sequence	# PSMs	# Proteins	# Protein Groups	Protein Group Accessions	Modifications	MH+ [Da]	# Missed Cleavages	Het-1A-Smoke - 2M/Parental	Het-1A-Smoke - 4M/Parental	Het-1A-Smoke - 6M/Parental	Het-1A-Smoke - 8M/Parental
																	TVANMIK	4	2	1	25777713	N-Term(TMT6plex); M5(Oxidation); K7(TMT6plex)	1250.8	0	1.2	1.0	1.3	1.1
																	ENQWCEEK	2	1	1	25777713	N-Term(TMT6plex); C5(Carbamidomethyl); K8(TMT6plex)	1580.8	0	1.4	1.2	1.5	1.1
																	QSVTIK	2	2	1	25777713	N-Term(TMT6plex); K6(TMT6plex)	1133.7	0	1.2	0.8	1.3	0.9
																	TPEEIR	2	3	3	4502105;1002623478;25777713;4502107	N-Term(TMT6plex)	973.6	0				
31542947;41399285	NP_002147.2	**HSPD1**	3329	**60 kDa heat shock protein, mitochondrial**	79.58	1	38	38	1094	**1.3**	**1.4**	**1.4**	**1.5**	573	61.0	5.87												
																	AAVEEGIVLGGGCALLR	30	1	1	31542947;41399285	N-Term(TMT6plex); C13(Carbamidomethyl)	1914.1	0	1.3	1.5	1.4	1.5
																	KPLVIIAEDVDGEALSTLVLNR	78	1	1	31542947;41399285	N-Term(TMT6plex); K1(TMT6plex)	2823.7	0	1.4	1.5	1.4	1.5
																	ALMLQGVDLLADAVAVTMGPK	96	1	1	31542947;41399285	N-Term(TMT6plex); M3(Oxidation); M18(Oxidation); K21(TMT6plex)	2603.5	0	1.3	1.4	1.4	1.5
																	IQEIIEQLDVTTSEYEK	76	1	1	31542947;41399285	N-Term(TMT6plex); K17(TMT6plex)	2496.3	0	1.3	1.2	1.3	1.3
																	DMAIATGGAVFGEEGLTLNLEDVQPHDLGK	36	1	1	31542947;41399285	N-Term(TMT6plex); M2(Oxidation); K30(TMT6plex)	3571.8	0	1.4	1.6	1.5	1.6
																	IMQSSSEVGYDAMAGDFVNMVEK	20	1	1	31542947;41399285	N-Term(TMT6plex); M2(Oxidation); M13(Oxidation); M20(Oxidation); K23(TMT6plex)	3014.4	0	1.4	1.4	1.4	1.5
																	NAGVEGSLIVEK	24	1	1	31542947;41399285	N-Term(TMT6plex); K12(TMT6plex)	1674.0	0	1.6	1.2	1.3	1.4
																	TVIIEQSWGSPK	24	1	1	31542947;41399285	N-Term(TMT6plex); K12(TMT6plex)	1803.0	0	1.3	1.3	1.2	1.4
																	TLNDELEIIEGMK	45	1	1	31542947;41399285	N-Term(TMT6plex); M12(Oxidation); K13(TMT6plex)	1979.1	0	1.4	1.6	1.4	1.6
																	GVMLAVDAVIAELK	153	1	1	31542947;41399285	N-Term(TMT6plex); M3(Oxidation); K14(TMT6plex)	1903.1	0	1.2	1.2	1.2	1.5
																	KGVITVK	9	1	1	31542947;41399285	N-Term(TMT6plex); K1(TMT6plex); K7(TMT6plex)	1432.0	1	1.1	1.4	1.2	1.5
																	DGVTVAK	13	1	1	31542947;41399285	N-Term(TMT6plex); K7(TMT6plex)	1147.7	0	1.2	1.4	1.3	1.5
																	GVMLAVDAVIAELK	8	1	1	31542947;41399285	N-Term(TMT6plex); K14(TMT6plex)	1887.1	0	1.9	2.2	2.2	1.8
																	TLNDELEIIEGMK	14	1	1	31542947;41399285	N-Term(TMT6plex); K13(TMT6plex)	1963.1	0	1.7	2.1	1.9	1.7
																	VGGTSDVEVNEK	26	1	1	31542947;41399285	N-Term(TMT6plex); K12(TMT6plex)	1691.9	0	1.4	1.4	1.4	1.5
																	IMQSSSEVGYDAMAGDFVNMVEK	8	1	1	31542947;41399285	N-Term(TMT6plex); M13(Oxidation); M20(Oxidation); K23(TMT6plex)	2998.4	0	1.5	1.6	1.5	1.5
																	VGEVIVTK	22	1	1	31542947;41399285	N-Term(TMT6plex); K8(TMT6plex)	1302.8	0	1.2	1.3	1.3	1.4
																	APGFGDNR	5	1	1	31542947;41399285	N-Term(TMT6plex)	1062.6	0	1.2	1.4	1.3	1.8
																	EIGNIISDAMK	8	1	1	31542947;41399285	N-Term(TMT6plex); M10(Oxidation); K11(TMT6plex)	1664.9	0	1.1	1.2	1.1	1.4
																	CIPALDSLTPANEDQK	19	1	1	31542947;41399285	N-Term(TMT6plex); C1(Carbamidomethyl); K16(TMT6plex)	2230.2	0	1.4	1.3	1.4	1.3
																	CEFQDAYVLLSEK	19	1	1	31542947;41399285	N-Term(TMT6plex); C1(Carbamidomethyl); K13(TMT6plex)	2060.1	0	1.4	1.2	1.3	1.2
																	GANPVEIR	8	1	1	31542947;41399285	N-Term(TMT6plex)	1084.6	0	1.2	1.5	1.3	1.7
																	QSKPVTTPEEIAQVATISANGDKEIGNIISDAMK	4	1	1	31542947;41399285	N-Term(TMT6plex); K3(TMT6plex); K23(TMT6plex); M33(Oxidation); K34(TMT6plex)	4488.4	1	1.2	1.4	1.2	2.0
																	ALMLQGVDLLADAVAVTMGPK	57	1	1	31542947;41399285	N-Term(TMT6plex); M3(Oxidation); K21(TMT6plex)	2587.5	0	1.5	1.8	1.7	1.7
																	VGLQVVAVK	25	1	1	31542947;41399285	N-Term(TMT6plex); K9(TMT6plex)	1370.9	0	1.3	1.4	1.3	1.5
																	LVQDVANNTNEEAGDGTTTATVLAR	26	1	1	31542947;41399285	N-Term(TMT6plex)	2789.4	0	1.3	1.2	1.3	1.4
																	TALLDAAGVASLLTTAEVVVTEIPK	49	1	1	31542947;41399285	N-Term(TMT6plex); K25(TMT6plex)	2940.7	0	1.3	1.0	1.3	1.1
																	TALLDAAGVASLLTTAEVVVTEIPKEEK	13	1	1	31542947;41399285	N-Term(TMT6plex); K25(TMT6plex); K28(TMT6plex)	3556.1	1	1.4	1.1	1.4	1.2
																	QSKPVTTPEEIAQVATISANGDK	2	1	1	31542947;41399285	N-Term(TMT6plex); K3(TMT6plex); K23(TMT6plex)	3071.7	0	1.3	1.3	1.5	1.4
																	ISSIQSIVPALEIANAHR	20	1	1	31542947;41399285	N-Term(TMT6plex)	2148.2	0	1.3	1.3	1.5	1.3
																	ALMLQGVDLLADAVAVTMGPK	15	1	1	31542947;41399285	N-Term(TMT6plex); K21(TMT6plex)	2571.5	0	2.2	2.4	2.2	1.8
																	KISSIQSIVPALEIANAHR	19	1	1	31542947;41399285	N-Term(TMT6plex); K1(TMT6plex)	2505.5	1	1.3	1.4	1.4	1.4
																	VTDALNATR	27	1	1	31542947;41399285	N-Term(TMT6plex)	1189.7	0	1.4	1.3	1.3	1.4
																	LSDGVAVLK	22	1	1	31542947;41399285	N-Term(TMT6plex); K9(TMT6plex)	1359.9	0	1.3	1.4	1.3	1.4
																	IMQSSSEVGYDAMAGDFVNMVEK	3	1	1	31542947;41399285	N-Term(TMT6plex); M2(Oxidation); K23(TMT6plex)	2982.5	0	1.8	2.4	2.2	1.9
																	GYISPYFINTSK	10	1	1	31542947;41399285	N-Term(TMT6plex); K12(TMT6plex)	1848.0	0	1.4	2.1	1.4	2.2
																	EIGNIISDAMK	4	1	1	31542947;41399285	N-Term(TMT6plex); K11(TMT6plex)	1648.9	0	1.7	1.9	2.0	1.5
																	GVMLAVDAVIAELKK	5	1	1	31542947;41399285	N-Term(TMT6plex); M3(Oxidation); K14(TMT6plex); K15(TMT6plex)	2260.4	1	1.1	1.9	1.4	2.2
																	IQEIIEQLDVTTSEYEKEK	6	1	1	31542947;41399285	N-Term(TMT6plex); K17(TMT6plex); K19(TMT6plex)	2982.6	1	1.6	1.8	1.5	1.8
																	DMAIATGGAVFGEEGLTLNLEDVQPHDLGK	4	1	1	31542947;41399285	N-Term(TMT6plex); K30(TMT6plex)	3555.8	0	1.3	1.7	1.5	1.4
																	DPGMGAMGGMGGGMGGGMF	1	1	1	31542947;41399285	N-Term(TMT6plex); M4(Oxidation); M7(Oxidation); M10(Oxidation); M14(Oxidation); M18(Oxidation)	1983.8	0				
																	IGIEIIK	20	1	1	31542947;41399285	N-Term(TMT6plex); K7(TMT6plex)	1243.8	0	1.3	1.4	1.3	1.5
																	DDAMLLK	4	1	1	31542947;41399285	N-Term(TMT6plex); M4(Oxidation); K7(TMT6plex)	1279.7	0	1.1	1.4	1.1	1.7
																	GIIDPTK	9	1	1	31542947;41399285	N-Term(TMT6plex); K7(TMT6plex)	1201.8	0	1.2	1.5	1.4	1.5
																	IPAMTIAK	6	1	1	31542947;41399285	N-Term(TMT6plex); M4(Oxidation); K8(TMT6plex)	1318.8	0	1.3	1.5	1.5	1.6
																	IPAMTIAK	1	1	1	31542947;41399285	N-Term(TMT6plex); K8(TMT6plex)	1302.8	0	1.2	1.4	1.4	1.3
																	GVITVK	1	1	1	31542947;41399285	N-Term(TMT6plex); K6(TMT6plex)	1074.7	0	1.3	1.3	1.3	1.5
21536286	NP_001814.2	**CKB**	1152	**creatine kinase B-type**	79.53	2	20	20	288	**1.7**	**1.4**	**2.0**	**1.7**	381	42.6	5.59												
																	TDLNPDNLQGGDDLDPNYVLSSR	24	1	1	21536286	N-Term(TMT6plex)	2747.3	0	1.6	1.4	1.6	1.6
																	LAVEALSSLDGDLAGR	28	1	1	21536286	N-Term(TMT6plex)	1816.0	0	1.7	1.5	1.7	1.7
																	LGFSEVELVQMVVDGVK	45	1	1	21536286	N-Term(TMT6plex); M11(Oxidation); K17(TMT6plex)	2323.3	0	1.7	1.4	2.0	1.8
																	TFLVWVNEEDHLR	11	1	1	21536286	N-Term(TMT6plex)	1887.0	0	1.5	1.5	1.7	1.7
																	LGFSEVELVQMVVDGVK	27	1	1	21536286	N-Term(TMT6plex); K17(TMT6plex)	2307.3	0	1.6	1.6	2.1	1.8
																	FCTGLTQIETLFK	36	1	1	21536286	N-Term(TMT6plex); C2(Carbamidomethyl); K13(TMT6plex)	2016.1	0	1.9	1.1	2.3	1.5
																	FPAEDEFPDLSAHNNHMAK	10	1	1	21536286	N-Term(TMT6plex); M17(Oxidation); K19(TMT6plex)	2644.3	0	1.3	1.3	1.6	1.6
																	SMTEAEQQQLIDDHFLFDKPVSPLLLASGMAR	4	1	1	21536286	N-Term(TMT6plex); K19(TMT6plex); M30(Oxidation)	4062.1	0	2.9	2.7	3.4	3.2
																	HGGYKPSDEHK	3	1	1	21536286	N-Term(TMT6plex); K5(TMT6plex); K11(TMT6plex)	1942.1	0	1.7	1.3	2.1	1.7
																	LLIEMEQR	9	1	1	21536286	N-Term(TMT6plex); M5(Oxidation)	1276.7	0	1.4	1.4	1.6	1.5
																	SKDYEFMWNPHLGYILTCPSNLGTGLR	1	1	1	21536286	N-Term(TMT6plex); K2(TMT6plex); M7(Oxidation); C18(Carbamidomethyl)	3643.8	1	1.7	1.6	2.0	2.0
																	VLTPELYAELR	11	1	1	21536286	N-Term(TMT6plex)	1532.9	0	1.6	1.5	2.0	1.8
																	GTGGVDTAAVGGVFDVSNADR	13	1	1	21536286	N-Term(TMT6plex)	2194.1	0	1.6	1.5	2.2	1.9
																	LEQGQAIDDLMPAQK	15	1	1	21536286	N-Term(TMT6plex); K15(TMT6plex)	2115.2	0	1.6	1.5	2.0	1.5
																	GIWHNDNK	8	2	1	21536286	N-Term(TMT6plex); K8(TMT6plex)	1441.8	0	1.4	1.4	1.9	1.7
																	SMTEAEQQQLIDDHFLFDKPVSPLLLASGMAR	5	1	1	21536286	N-Term(TMT6plex); K19(TMT6plex)	4046.1	0	2.1	1.7	2.8	1.8
																	DLFDPIIEDR	10	1	1	21536286	N-Term(TMT6plex)	1461.8	0	1.5	1.5	1.8	1.7
																	LEQGQAIDDLMPAQK	10	1	1	21536286	N-Term(TMT6plex); M11(Oxidation); K15(TMT6plex)	2131.1	0	1.4	1.3	1.5	1.5
																	SMTEAEQQQLIDDHFLFDKPVSPLLLASGMAR	8	1	1	21536286	N-Term(TMT6plex); M2(Oxidation); K19(TMT6plex); M30(Oxidation)	4078.1	0	1.5	1.4	1.8	1.9
																	STPSGFTLDDVIQTGVDNPGHPYIMTVGCVAGDEESYEVFK	1	1	1	21536286	N-Term(TMT6plex); M25(Oxidation); C29(Carbamidomethyl); K41(TMT6plex)	4906.3	0	1.9	1.0	1.9	1.0
																	FPAEDEFPDLSAHNNHMAK	2	1	1	21536286	N-Term(TMT6plex); K19(TMT6plex)	2628.3	0	1.6	1.6	2.2	1.6
																	DYEFMWNPHLGYILTCPSNLGTGLR	1	1	1	21536286	N-Term(TMT6plex); M5(Oxidation); C16(Carbamidomethyl)	3199.6	0				
																	LLIEMEQR	3	1	1	21536286	N-Term(TMT6plex)	1260.7	0	1.7	1.7	2.0	1.6
																	FSEVLK	1	1	1	21536286	N-Term(TMT6plex); K6(TMT6plex)	1180.7	0	1.8	1.3	2.3	1.7
																	LPNLGK	1	1	1	21536286	N-Term(TMT6plex); K6(TMT6plex)	1099.7	0				
																	DWPDAR	1	1	1	21536286	N-Term(TMT6plex)	988.5	0	1.5	1.6	1.6	1.6
5031635	NP_005498.1	**CFL1**	1072	**cofilin-1**	79.52	1	15	19	317	**1.5**	**1.5**	**1.6**	**1.6**	166	18.5	8.09												
																	EILVGDVGQTVDDPYATFVK	113	1	1	5031635	N-Term(TMT6plex); K20(TMT6plex)	2624.4	0	1.4	1.6	1.7	1.6
																	AVLFCLSEDKK	23	1	1	5031635	N-Term(TMT6plex); C5(Carbamidomethyl); K10(TMT6plex); K11(TMT6plex)	1997.2	1	1.4	1.8	1.8	1.7
																	KEDLVFIFWAPESAPLK	40	3	2	5031635;343887344	N-Term(TMT6plex); K1(TMT6plex); K17(TMT6plex)	2677.6	1				
																	LGGSAVISLEGKPL	37	1	1	5031635	N-Term(TMT6plex); K12(TMT6plex)	1799.1	0	1.4	1.4	1.5	1.5
																	NIILEEGK	26	1	1	5031635	N-Term(TMT6plex); K8(TMT6plex)	1373.8	0	1.9	1.3	1.5	1.4
																	HELQANCYEEVK	10	1	1	5031635	N-Term(TMT6plex); C7(Carbamidomethyl); K12(TMT6plex)	1978.0	0	1.3	1.3	1.5	1.4
																	HELQANCYEEVKDR	8	1	1	5031635	N-Term(TMT6plex); C7(Carbamidomethyl); K12(TMT6plex)	2249.1	1	1.4	1.4	1.6	1.5
																	EDLVFIFWAPESAPLK	9	3	2	5031635;343887344	N-Term(TMT6plex); K16(TMT6plex)	2320.3	0				
																	KSSTPEEVK	5	1	1	5031635	N-Term(TMT6plex); K1(TMT6plex); K9(TMT6plex)	1692.0	1	1.3	1.5	1.5	1.5
																	YALYDATYETK	19	3	2	5031635;343887344	N-Term(TMT6plex); K11(TMT6plex)	1795.9	0				
																	NIILEEGKEILVGDVGQTVDDPYATFVK	8	1	1	5031635	N-Term(TMT6plex); K8(TMT6plex); K28(TMT6plex)	3750.1	1	2.9	1.5	1.5	1.4
																	AVLFCLSEDK	2	1	1	5031635	N-Term(TMT6plex); C5(Carbamidomethyl); K10(TMT6plex)	1639.9	0	1.4	1.5	1.6	1.5
																	MIYASSK	2	5	3	5031635;58530848;343887344	N-Term(TMT6plex); K7(TMT6plex)	1257.7	0				
																	SSTPEEVKK	1	1	1	5031635	N-Term(TMT6plex); K8(TMT6plex); K9(TMT6plex)	1692.0	1	1.3	1.5	1.6	1.5
																	SSTPEEVK	7	1	1	5031635	N-Term(TMT6plex); K8(TMT6plex)	1334.8	0	1.3	1.1	1.3	1.2
																	CTLAEK	1	1	1	5031635	N-Term(TMT6plex); C1(Carbamidomethyl); K6(TMT6plex)	1179.7	0	1.4	1.1	1.7	1.2
																	MIYASSK	3	5	3	5031635;58530848;343887344	N-Term(TMT6plex); M1(Oxidation); K7(TMT6plex)	1273.7	0				
																	KSSTPEEVKK	1	1	1	5031635	N-Term(TMT6plex); K1(TMT6plex); K9(TMT6plex); K10(TMT6plex)	2049.3	2	1.2	1.8	1.8	1.9
																	KLTGIK	1	1	1	5031635	N-Term(TMT6plex); K1(TMT6plex); K6(TMT6plex)	1346.9	1	1.2	1.4	1.6	1.4
																	MLPDKDCR	1	1	1	5031635	N-Term(TMT6plex); K5(TMT6plex); C7(Carbamidomethyl)	1492.8	1	2.1	2.6	3.1	1.9
4758638	NP_004896.1	**PRDX6**	9588	**peroxiredoxin-6**	79.46	1	17	17	423	**1.3**	**1.3**	**1.4**	**1.5**	224	25.0	6.38												
																	DGDSVMVLPTIPEEEAK	36	1	1	4758638	N-Term(TMT6plex); M6(Oxidation); K17(TMT6plex)	2304.2	0	1.3	1.3	1.5	1.5
																	ELAILLGMLDPAEK	18	1	1	4758638	N-Term(TMT6plex); M8(Oxidation); K14(TMT6plex)	1987.2	0	1.1	1.0	1.2	1.4
																	VVFVFGPDK	30	1	1	4758638	N-Term(TMT6plex); K9(TMT6plex)	1465.9	0	1.3	1.2	1.6	1.3

Supplementary Table 6. List of all proteins and peptides identified in untreated and chronically treated Het1A cells with cigarette smoke condensate for 8 months

Accession	RefSeq Accession	Gene Symbol	Entrez Gene ID	Description	Σ Coverage	Σ# Proteins	Σ# Unique Peptides	Σ# Peptides	Σ# PSMs	Het-1A-Smoke - 2M/Parental	Het-1A-Smoke - 4M/Parental	Het-1A-Smoke - 6M/Parental	Het-1A-Smoke - 8M/Parental	# AAs	MW [kDa]	calc. pI	Sequence	# PSMs	# Proteins	# Protein Groups	Protein Group Accessions	Modifications	MH+ [Da]	# Missed Cleavages	Het-1A-Smoke - 2M/Parental	Het-1A-Smoke - 4M/Parental	Het-1A-Smoke - 6M/Parental	Het-1A-Smoke - 8M/Parental
																	PGGLLLGDVAPNFEANTTVGR	8	1	1	4758638	N-Term(TMT6plex)	2327.3	0	**1.4**	**1.0**	**1.5**	**1.2**
																	LIALSIDSVEDHLAWSK	24	1	1	4758638	N-Term(TMT6plex); K17(TMT6plex)	2355.3	0	**1.2**	**1.3**	**1.5**	**1.5**
																	DINAYNCEEPTEK	38	1	1	4758638	N-Term(TMT6plex); C7(Carbamidomethyl); K13(TMT6plex)	2041.0	0	**1.3**	**1.3**	**1.5**	**1.4**
																	LPFPIIDDR	49	1	1	4758638	N-Term(TMT6plex)	1314.8	0	**1.3**	**1.3**	**1.5**	**1.5**
																	DGDSVMVLPTIPEEEAK	4	1	1	4758638	N-Term(TMT6plex); K17(TMT6plex)	2288.2	0	**1.5**	**1.8**	**2.1**	**1.7**
																	ELAILLGMLDPAEKDEK	59	1	1	4758638	N-Term(TMT6plex); M8(Oxidation); K14(TMT6plex); K17(TMT6plex)	2588.5	1	**1.1**	**1.2**	**1.3**	**1.5**
																	VVISLQLTAEK	10	1	1	4758638	N-Term(TMT6plex); K11(TMT6plex)	1659.0	0	**1.4**	**1.5**	**1.4**	**1.5**
																	FHDFLGDSWGILFSHPR	19	1	1	4758638	N-Term(TMT6plex)	2260.2	0	**1.3**	**1.2**	**1.7**	**1.5**
																	LAPEFAK	12	1	1	4758638	N-Term(TMT6plex); K7(TMT6plex)	1233.8	0	**1.2**	**1.3**	**1.4**	**1.5**
																	LSILYPATTGR	24	1	1	4758638	N-Term(TMT6plex)	1420.8	0	**1.3**	**1.3**	**1.5**	**1.4**
																	DFTPVCTTELGR	25	1	1	4758638	N-Term(TMT6plex); C6(Carbamidomethyl)	1624.8	0	**1.3**	**1.4**	**1.6**	**1.5**
																	ELAILLGMLDPAEK	26	1	1	4758638	N-Term(TMT6plex); K14(TMT6plex)	1971.2	0	**1.4**	**1.7**	**1.6**	**1.7**
																	VVFVFGPDKK	5	1	1	4758638	N-Term(TMT6plex); K9(TMT6plex); K10(TMT6plex)	1823.1	1	**1.2**	**1.5**	**1.5**	**1.8**
																	DGDSVMVLPTIPEEEAKK	6	1	1	4758638	N-Term(TMT6plex); M6(Oxidation); K17(TMT6plex); K18(TMT6plex)	2661.5	1	**1.1**	**1.2**	**1.3**	**1.3**
																	DGDSVMVLPTIPEEEAKK	2	1	1	4758638	N-Term(TMT6plex); K17(TMT6plex); K18(TMT6plex)	2645.5	1	**1.5**	**1.8**	**2.1**	**1.8**
																	NFDEILR	21	1	1	4758638	N-Term(TMT6plex)	1135.6	0	**1.4**	**1.3**	**1.4**	**1.4**
																	VATPVDWK	7	1	1	4758638	N-Term(TMT6plex); K8(TMT6plex)	1373.8	0	**1.1**	**0.9**	**1.3**	**1.1**
4502209	NP_001653.1	**ARF5**	381	**ADP-ribosylation factor 5**	79.44	7	5	11	228	**1.2**	**1.2**	**1.2**	**1.3**	180	20.5	6.79												
																	ILMVGLDAAGK	35	4	4	4502209;66879662;66879664;4502203;4502205	N-Term(TMT6plex); M3(Oxidation); K11(TMT6plex)	1561.9	0				
																	QDMPNAMPVSELTDK	20	1	1	4502209	N-Term(TMT6plex); M3(Oxidation); M7(Oxidation); K15(TMT6plex)	2166.1	0	**1.2**	**1.2**	**1.2**	**1.3**
																	LGEIVTTIPTIGFNVETVEYK	34	4	4	4502209;66879662;66879664;4502203;4502205	N-Term(TMT6plex); K21(TMT6plex)	2781.6	0				
																	VQESADELQK	14	1	1	4502209	N-Term(TMT6plex); K10(TMT6plex)	1604.9	0	**1.3**	**1.2**	**1.2**	**1.4**
																	ILMVGLDAAGK	12	4	4	4502209;66879662;66879664;4502203;4502205	N-Term(TMT6plex); K11(TMT6plex)	1545.9	0				
																	DAVLLVFANK	68	3	3	4502209;66879662;66879664;4502203	N-Term(TMT6plex); K10(TMT6plex)	1548.0	0				
																	HYFQNTQGLIFVVDSNDRER	10	4	4	4502209;66879662;66879664;4502203;4502205	N-Term(TMT6plex)	2667.3	1				
																	HYFQNTQGLIFVVDSNDR	17	4	4	4502209;66879662;66879664;4502203;4502205	N-Term(TMT6plex)	2382.2	0				
																	NICFTVWDVGGQDK	4	1	1	4502209	N-Term(TMT6plex); C3(Carbamidomethyl); K14(TMT6plex)	2097.1	0	**1.2**	**1.1**	**1.2**	**1.2**
																	TWYVQATCATQGTGLYDGLDWLSHELSK	4	1	1	4502209	N-Term(TMT6plex); C8(Carbamidomethyl); K28(TMT6plex)	3658.8	0	**1.2**	**1.1**	**1.2**	**1.1**
																	TTILYK	9	11	5	4502209;66879662;66879664;4502203;4502211;4502205	N-Term(TMT6plex); K6(TMT6plex)	1196.8	0				
																	MLQEDELR	1	1	1	4502209	N-Term(TMT6plex); M1(Oxidation)	1278.7	0	**1.4**	**1.3**	**1.4**	**1.4**
4503729	NP_002005.1	**FKBP4**	2288	**peptidyl-prolyl cis-trans isomerase FKBP4**	79.30	1	32	33	411	**1.5**	**1.6**	**1.7**	**1.5**	459	51.8	5.43												
																	FEIGEGENLDLPYGLER	35	1	1	4503729	N-Term(TMT6plex)	2180.1	0	**1.6**	**1.5**	**1.7**	**1.5**
																	FQIPPNAELK	27	1	1	4503729	N-Term(TMT6plex); K10(TMT6plex)	1615.0	0	**1.4**	**1.5**	**1.6**	**1.5**
																	EGTGTEMPMIGDR	17	1	1	4503729	N-Term(TMT6plex); M7(Oxidation); M9(Oxidation)	1654.8	0	**2.0**	**1.7**	**1.8**	**1.4**
																	GEGYAKPNEGAIVEVALEGYYK	15	1	1	4503729	N-Term(TMT6plex); K6(TMT6plex); K22(TMT6plex)	3044.7	0	**1.6**	**1.8**	**1.8**	**1.6**
																	SNTAGSQSQVETEA	14	1	1	4503729	N-Term(TMT6plex)	1637.8	0	**1.8**	**1.3**	**1.5**	**1.3**
																	AKESWEMNSEEK	4	1	1	4503729	N-Term(TMT6plex); K2(TMT6plex); M7(Oxidation); K12(TMT6plex)	2171.1	1	**1.7**	**1.6**	**1.5**	**1.5**
																	IVSWLEYESSFSNEEAQK	29	1	1	4503729	N-Term(TMT6plex); K18(TMT6plex)	2604.3	0	**1.6**	**1.6**	**1.7**	**1.5**
																	GEDLTEEEDGGIIR	18	1	1	4503729	N-Term(TMT6plex)	1761.9	0	**1.3**	**1.5**	**1.5**	**1.5**
																	VLQLYPNNK	23	1	1	4503729	N-Term(TMT6plex); K9(TMT6plex)	1546.9	0	**1.5**	**1.6**	**1.8**	**1.5**
																	YELHLK	13	1	1	4503729	N-Term(TMT6plex); K6(TMT6plex)	1260.8	0	**1.3**	**1.6**	**1.7**	**1.5**
																	TQLAVCQQR	7	1	1	4503729	N-Term(TMT6plex); C6(Carbamidomethyl)	1332.7	0	**1.7**	**1.3**	**1.7**	**1.2**
																	ALELDSNNEK	20	1	1	4503729	N-Term(TMT6plex); K10(TMT6plex)	1590.9	0	**1.5**	**1.8**	**1.7**	**1.8**
																	AEASSGDHPTDTEMKEEQK	16	1	1	4503729	N-Term(TMT6plex); M14(Oxidation); K15(TMT6plex); K19(TMT6plex)	2793.4	1	**1.9**	**2.1**	**2.2**	**1.8**
																	ATESGAQSAPLPMEGVDISPK	28	1	1	4503729	N-Term(TMT6plex); M13(Oxidation); K21(TMT6plex)	2559.3	0	**1.3**	**1.6**	**1.5**	**1.5**
																	AWDIAIATMK	22	1	1	4503729	N-Term(TMT6plex); M9(Oxidation); K10(TMT6plex)	1593.9	0	**1.5**	**1.6**	**1.7**	**1.5**
																	ESWEMNSEEK	35	1	1	4503729	N-Term(TMT6plex); M5(Oxidation); K10(TMT6plex)	1742.8	0	**1.8**	**1.9**	**2.1**	**1.6**
																	RGEAHLAVNDFELAR	6	1	1	4503729	N-Term(TMT6plex)	1927.0	1	**1.2**	**1.7**	**1.3**	**1.6**
																	GEAHLAVNDFELAR	2	1	1	4503729	N-Term(TMT6plex)	1770.9	0	**1.1**	**1.4**	**1.3**	**1.4**
																	ESWEMNSEEK	3	1	1	4503729	N-Term(TMT6plex); K10(TMT6plex)	1726.8	0	**2.2**	**2.0**	**2.3**	**1.5**
																	IPPNATLVFEVELFEFK	1	1	1	4503729	N-Term(TMT6plex); K17(TMT6plex)	2451.4	0	**1.3**	**1.4**	**1.5**	**1.4**
																	LEQSTIVK	4	1	1	4503729	N-Term(TMT6plex); K8(TMT6plex)	1375.9	0	**1.6**	**1.6**	**1.8**	**1.4**
																	QALLQYK	6	1	1	4503729	N-Term(TMT6plex); K7(TMT6plex)	1321.8	0	**1.4**	**1.3**	**1.5**	**1.3**
																	LASHLNLAMCHLK	2	1	1	4503729	N-Term(TMT6plex); C10(Carbamidomethyl); K13(TMT6plex)	1966.1	0	**1.4**	**1.5**	**2.0**	**1.4**
																	AWDIAIATMK	9	1	1	4503729	N-Term(TMT6plex); K10(TMT6plex)	1577.9	0	**1.8**	**2.1**	**2.3**	**1.7**
																	VGEVCHITCKPEYAYGSAGSPPK	3	1	1	4503729	N-Term(TMT6plex); C5(Carbamidomethyl); C9(Carbamidomethyl); K10(TMT6plex); K23(TMT6plex)	3194.7	0	**1.6**	**1.9**	**2.0**	**1.5**
																	GEHSIVYLKPSYAFGSVGK	7	1	1	4503729	N-Term(TMT6plex); K9(TMT6plex); K19(TMT6plex)	2726.5	0	**1.2**	**1.8**	**2.0**	**1.7**
																	ATESGAQSAPLPMEGVDISPK	6	1	1	4503729	N-Term(TMT6plex); K21(TMT6plex)	2543.3	0	**1.7**	**2.1**	**1.9**	**1.7**
																	LYANMFER	2	1	1	4503729	N-Term(TMT6plex)	1272.7	0	**1.9**	**1.7**	**2.3**	**1.4**
																	AEASSGDHPTDTEMKEEQK	2	1	1	4503729	N-Term(TMT6plex); K15(TMT6plex); K19(TMT6plex)	2777.4	1	**3.8**	**3.0**	**3.7**	**1.8**
																	VFVHYTGWLLDGTK	4	1	1	4503729	N-Term(TMT6plex); K14(TMT6plex)	2094.2	0	**1.6**	**1.6**	**1.9**	**1.4**
																	DKFSFDLGK	1	1	1	4503729	N-Term(TMT6plex); K2(TMT6plex); K9(TMT6plex)	1744.0	1	**1.2**	**1.9**	**1.8**	**1.6**
																	AEASSGDHPTDTEMK	3	1	1	4503729	N-Term(TMT6plex); M14(Oxidation); K15(TMT6plex)	2050.0	0	**1.4**	**1.9**	**2.0**	**1.5**
																	FSFDLGK	8	1	1	4503729	N-Term(TMT6plex); K7(TMT6plex)	1271.7	0	**1.5**	**1.6**	**1.7**	**1.4**
																	FDSSLDR	6	1	1	4503729	N-Term(TMT6plex)	1068.6	0	**1.6**	**1.3**	**1.7**	**1.2**
																	GTVYFK	4	2	2	4503729;4758384;224809329	N-Term(TMT6plex); K6(TMT6plex)	1172.7	0				
																	QDEGVLK	5	1	1	4503729	N-Term(TMT6plex); K7(TMT6plex)	1246.7	0	**1.6**	**1.6**	**1.7**	**1.6**
																	LAEEENK	2	1	1	4503729	N-Term(TMT6plex); K7(TMT6plex)	1290.7	0	**2.5**	**1.7**	**1.8**	**1.6**
																	LYANMFER	2	1	1	4503729	N-Term(TMT6plex); M5(Oxidation)	1288.7	0	**1.5**	**1.5**	**1.7**	**1.4**
21361322;574584816	NP_006078.2	**TUBB4A**	10382	**tubulin beta-4A chain isoform 3**	79.28	4	2	23	1796	**1.5**	**1.3**	**1.6**	**1.3**	444	49.6	4.88												
																	NSSYFVEWIPNNVK	95	20	7	29788785;5174735;14210536;746815975;29788768;50592996;21361322;574584816;4507729	N-Term(TMT6plex); K14(TMT6plex)	2155.2	0				
																	ALTVPELTQQMFDAK	71	7	3	5174735;50592996;21361322;574584816	N-Term(TMT6plex); M11(Oxidation); K15(TMT6plex)	2166.2	0				
																	SGPFGQIFRPDNFVFGQSGAGNNWAK	124	8	5	29788785;5174735;29788768;21361322;574584816;4507729	N-Term(TMT6plex); K26(TMT6plex)	3256.7	0				
																	AVLVDLEPGTMDSVR	89	5	2	5174735;21361322;574584816	N-Term(TMT6plex); M11(Oxidation)	1847.0	0				
																	FWEVISDEHGIDPTGTYHGDSDLQLER	36	4	2	5174735;21361322;574584816	N-Term(TMT6plex)	3345.6	0				
																	ALTVPELTQQMFDAK	47	7	3	5174735;50592996;21361322;574584816	N-Term(TMT6plex); K15(TMT6plex)	2150.2	0				
																	VSDTVVEPYNATLSVHQLVENTDETYCIDNEALYDICFR	50	8	5	29788785;5174735;14210536;746815975;29788768;21361322;574584816	N-Term(TMT6plex); C27(Carbamidomethyl); C37(Carbamidomethyl)	4822.2	0				
																	IMNTFSVVPSPK	47	8	4	29788785;5174735;50592996;21361322;574584816	N-Term(TMT6plex); M2(Oxidation); K12(TMT6plex)	1794.0	0				
																	LTTPTYGDLNHLVSATMSGVTTCLR	171	12	5	29788785;5174735;29788768;21361322;574584816;4507729	N-Term(TMT6plex); M17(Oxidation); C23(Carbamidomethyl)	2953.5	0				
																	YLTVAAVFR	63	9	3	29788785;5174735;21361322;574584816	N-Term(TMT6plex)	1268.8	0				
																	LHFFMPGFAPLTSR	71	18	6	29788785;5174735;14210536;746815975;29788768;21361322;574584816;4507729	N-Term(TMT6plex)	1850.0	0				
																	KLAVNMVPFPR	11	21	8	29788785;5174735;14210536;746815975;29788768;50592996;21361322;574584816;4507729;13562114	N-Term(TMT6plex); K1(TMT6plex); M6(Oxidation)	1746.0	1				
																	TAVCDIPPR	171	14	5	29788785;5174735;29788768;21361322;574584816;4507729	N-Term(TMT6plex); C4(Carbamidomethyl)	1257.7	0				
																	ISEQFTAMFR	20	10	6	29788785;5174735;29788768;50592996;21361322;574584816;4507729	N-Term(TMT6plex)	1458.8	0				
																	ISEQFTAMFR	244	10	6	29788785;5174735;29788768;50592996;21361322;574584816;4507729	N-Term(TMT6plex); M8(Oxidation)	1474.8	0				
																	EAESCDCLQGFQLTHSLGGGTGSGMGTLLISK	32	7	3	29788785;5174735;21361322;574584816	N-Term(TMT6plex); C5(Carbamidomethyl); C7(Carbamidomethyl); M25(Oxidation); K32(TMT6plex)	3785.9	0				
																	EIVHLQAGQCGNQIGAK	14	8	6	29788785;29788768;50592996;4507729;5174735;21361322;574584816	N-Term(TMT6plex); C10(Carbamidomethyl); K17(TMT6plex)	2281.2	0				
																	MAATFIGNSTAIQELFK	7	4	1	21361322;574584816	N-Term(TMT6plex); M1(Oxidation); K17(TMT6plex)	2316.3	0	**1.5**	**1.2**	**1.7**	**1.3**
																	LTTPTYGDLNHLVSATMSGVTTCLR	35	12	5	29788785;5174735;29788768;21361322;574584816;4507729	N-Term(TMT6plex); C23(Carbamidomethyl)	2937.5	0				
																	FPGQLNADLR	98	13	8	29788785;5174735;14210536;746815975;29788768;50592996;21361322;574584816;4507729;13562114	N-Term(TMT6plex)	1359.8	0				
																	LAVNMVPFPR	20	21	8	29788785;5174735;14210536;746815975;29788768;50592996;21361322;574584816;4507729;13562114	N-Term(TMT6plex)	1372.8	0				
																	LHFFMPGFAPLTSR	135	18	6	29788785;5174735;14210536;746815975;29788768;21361322;574584816;4507729	N-Term(TMT6plex); M5(Oxidation)	1866.0	0				
																	INVYYNEATGGNYVPR	2	3	1	21361322;574584816	N-Term(TMT6plex)	2059.0	0	**1.5**	**1.3**	**1.4**	**1.4**
																	KEAESCDCLQGFQLTHSLGGGTGSGMGTLLISK	7	7	3	29788785;5174735;21361322;574584816	K1(TMT6plex); N-Term(TMT6plex); C6(Carbamidomethyl); C8(Carbamidomethyl); M26(Oxidation); K33(TMT6plex)	4143.1	1				
																	AVLVDLEPGTMDSVR	17	5	2	5174735;21361322;574584816	N-Term(TMT6plex)	1831.0	0				
																	GHYTEGAELVDAVLDVVR	38	5	2	14210536;746815975;21361322;574584816	N-Term(TMT6plex)	2172.2	0				
																	LAVNMVPFPR	54	21	8	29788785;5174735;14210536;746815975;29788768;50592996;21361322;574584816;4507729;13562114	N-Term(TMT6plex); M5(Oxidation)	1388.8	0				
																	IMNTFSVVPSPK	8	8	4	29788785;5174735;50592996;21361322;574584816	N-Term(TMT6plex); K12(TMT6plex)	1778.0	0				

Accession	RefSeq Accession	Gene Symbol	Entrez Gene ID	Description	Σ Coverage	Σ# Proteins	Σ# Unique Peptides	Σ# Peptides	Σ# PSMs	Het-1A-Smoke - 2M/Parental	Het-1A-Smoke - 4M/Parental	Het-1A-Smoke - 6M/Parental	Het-1A-Smoke - 8M/Parental	# AAs	MW [kDa]	calc. pI	Sequence	# PSMs	# Proteins	# Protein Groups	Protein Group Accessions	Modifications	MH+ [Da]	# Missed Cleavages	Het-1A-Smoke - 2M/Parental	Het-1A-Smoke - 4M/Parental	Het-1A-Smoke - 6M/Parental	Het-1A-Smoke - 8M/Parental
																	KEAESCDCLQGFQLTHSLGGGTGSGMGTLLISK	1	7	3	29788785;5174735;21361322;574584816	N-Term(TMT6plex); K1(TMT6plex); C6(Carbamidomethyl); C8(Carbamidomethyl); K33(TMT6plex)	4127.1	1				
																	EAESCDCLQGFQLTHSLGGGTGSGMGTLLISK	2	7	3	29788785;5174735;21361322;574584816	N-Term(TMT6plex); C5(Carbamidomethyl); C7(Carbamidomethyl); K32(TMT6plex)	3769.8	0				
																	NMMAACDPR	5	20	7	29788785;5174735;14210536;746815975;29788768;50592996;21361322;574584816;4507729	N-Term(TMT6plex); M2(Oxidation); M3(Oxidation); C6(Carbamidomethyl)	1326.6	0				
																	GSQQYR	5	12	7	29788785;5174735;14210536;746815975;29788768;50592996;21361322;574584816;4507729	N-Term(TMT6plex)	967.5	0				
																	NMMAACDPR	4	20	7	29788785;5174735;14210536;746815975;29788768;50592996;21361322;574584816;4507729	N-Term(TMT6plex); M3(Oxidation); C6(Carbamidomethyl)	1310.6	0				
																	NMMAACDPR	2	20	7	29788785;5174735;14210536;746815975;29788768;50592996;21361322;574584816;4507729	N-Term(TMT6plex); C6(Carbamidomethyl)	1294.6	0				
4505621	NP_002558.1	**PEBP1**	5037	**phosphatidylethanolamine-binding protein 1 preproprotein**	79.14	1	10	10	99	**1.3**	**1.3**	**1.3**	**1.4**	187	21.0	7.53												
																	GNDISSGTVLSDYVGSGPPK	28	1	1	4505621	N-Term(TMT6plex); K20(TMT6plex)	2408.3	0	**1.3**	**1.4**	**1.4**	**1.5**
																	CDEPILSNR	5	1	1	4505621	N-Term(TMT6plex); C1(Carbamidomethyl)	1332.7	0	**1.4**	**1.2**	**1.3**	**1.3**
																	LYTLVLTDPDAPSR	12	1	1	4505621	N-Term(TMT6plex)	1790.0	0	**1.4**	**1.2**	**1.2**	**1.3**
																	NRPTSISWDGLDSGK	5	1	1	4505621	N-Term(TMT6plex); K15(TMT6plex)	2091.1	0	**1.3**	**1.1**	**1.2**	**1.3**
																	WSGPLSLQEVDEQPQHPLHVTYAGAAVDELGK	8	1	1	4505621	N-Term(TMT6plex); K32(TMT6plex)	3930.0	0	**1.2**	**1.3**	**1.2**	**1.5**
																	EWHHFLVVNMK	4	1	1	4505621	N-Term(TMT6plex); M10(Oxidation); K11(TMT6plex)	1914.0	0	**1.2**	**1.3**	**1.3**	**1.4**
																	APVAGTCYQAEWDDYVPK	17	1	1	4505621	N-Term(TMT6plex); C7(Carbamidomethyl); K18(TMT6plex)	2528.2	0	**1.4**	**1.4**	**1.3**	**1.4**
																	YVWLVYEQDRPLK	3	1	1	4505621	N-Term(TMT6plex); K13(TMT6plex)	2167.2	0	**1.2**	**1.2**	**1.3**	**1.5**
																	LYEQLSGK	4	1	1	4505621	N-Term(TMT6plex); K8(TMT6plex)	1395.8	0	**1.2**	**1.2**	**1.3**	**1.5**
																	VLTPTQVK	13	1	1	4505621	N-Term(TMT6plex); K8(TMT6plex)	1343.9	0	**1.3**	**1.2**	**1.2**	**1.4**
4507677	NP_003290.1	**HSP90B1**	7184	**endoplasmin precursor**	79.08	1	65	67	2085	**1.4**	**1.7**	**1.5**	**1.4**	803	92.4	4.84												
																	EEASDYLELDTIK	127	1	1	4507677	N-Term(TMT6plex); K13(TMT6plex)	1984.1	0	**1.4**	**1.8**	**1.5**	**1.4**
																	ELISNASDALDK	175	4	2	4507677;20149594;431822403	N-Term(TMT6plex); K12(TMT6plex)	1734.0	0				
																	LGVIEDHSNR	39	1	1	4507677	N-Term(TMT6plex)	1368.7	0	**1.3**	**1.5**	**1.4**	**1.4**
																	TETVEEPMEEEEAAK	8	1	1	4507677	N-Term(TMT6plex); M8(Oxidation); K15(TMT6plex)	2196.1	0	**1.4**	**1.3**	**1.4**	**1.1**
																	EEKEESDDEAAVEEEEEEK	8	1	1	4507677	N-Term(TMT6plex); K3(TMT6plex); K19(TMT6plex)	2940.4	1	**1.8**	**1.7**	**1.5**	**1.4**
																	ETLQQHK	25	1	1	4507677	N-Term(TMT6plex); K7(TMT6plex)	1341.8	0	**1.2**	**1.6**	**1.4**	**1.3**
																	TFEINPR	21	1	1	4507677	N-Term(TMT6plex)	1105.6	0	**1.5**	**1.6**	**1.5**	**1.3**
																	EFEPLLNWMK	62	1	1	4507677	N-Term(TMT6plex); M9(Oxidation); K10(TMT6plex)	1781.0	0	**1.3**	**1.6**	**1.5**	**1.3**
																	NLGTIAK	17	1	1	4507677	N-Term(TMT6plex); K7(TMT6plex)	1174.8	0	**1.3**	**1.3**	**1.3**	**1.1**
																	NLLHVTDTGVGMTR	27	1	1	4507677	N-Term(TMT6plex); M12(Oxidation)	1758.9	0	**1.4**	**1.3**	**1.4**	**1.4**
																	VFITDDFHDMMPK	28	1	1	4507677	N-Term(TMT6plex); M10(Oxidation); M11(Oxidation); K13(TMT6plex)	2086.0	0	**1.3**	**1.6**	**1.4**	**1.3**
																	LISLTDENALSGNEELTVK	275	1	1	4507677	N-Term(TMT6plex); K19(TMT6plex)	2504.4	0	**1.3**	**1.6**	**1.4**	**1.4**
																	KEAESSPFVER	17	1	1	4507677	N-Term(TMT6plex); K1(TMT6plex)	1737.0	1	**1.3**	**1.6**	**1.3**	**1.3**
																	EAESSPFVER	15	1	1	4507677	N-Term(TMT6plex)	1379.7	0	**1.4**	**1.6**	**1.5**	**1.4**
																	FAFQAEVNR	41	1	1	4507677	N-Term(TMT6plex)	1310.7	0	**1.3**	**1.4**	**1.3**	**1.2**
																	TDDEVVQREEEAIQLDGLNASQIR	68	1	1	4507677	N-Term(TMT6plex)	2957.5	1	**1.5**	**1.6**	**1.5**	**1.4**
																	SGYLLPDTK	46	1	1	4507677	N-Term(TMT6plex); K9(TMT6plex)	1451.9	0	**1.5**	**1.6**	**1.5**	**1.4**
																	TDDEVVQR	18	1	1	4507677	N-Term(TMT6plex)	1190.6	0	**1.3**	**1.4**	**1.4**	**1.2**
																	MTEAQEDGQSTSELIGQFGVGFYSAFLVADK	40	1	1	4507677	N-Term(TMT6plex); M1(Oxidation); K31(TMT6plex)	3799.9	0	**1.7**	**2.4**	**1.9**	**1.9**
																	FQSSHHPTDITSLDQYVER	43	1	1	4507677	N-Term(TMT6plex)	2489.2	0	**1.3**	**1.4**	**1.4**	**1.3**
																	IYFMAGSSR	4	1	1	4507677	N-Term(TMT6plex)	1260.7	0	**1.8**	**2.0**	**2.2**	**1.3**
																	GTTITLVLK	29	1	1	4507677	N-Term(TMT6plex); K9(TMT6plex)	1403.9	0	**1.3**	**1.8**	**1.5**	**1.4**
																	LIINSLYK	43	1	1	4507677	N-Term(TMT6plex); K8(TMT6plex)	1421.9	0	**1.4**	**1.8**	**1.6**	**1.4**
																	EVEEDEYK	32	1	1	4507677	N-Term(TMT6plex); K8(TMT6plex)	1498.8	0	**1.4**	**1.9**	**1.5**	**1.4**
																	EGVKFDESEK	5	1	1	4507677	N-Term(TMT6plex); K4(TMT6plex); K10(TMT6plex)	1855.0	1	**1.6**	**3.0**	**1.7**	**2.0**
																	LTESPCALVASQYGWSGNMER	54	1	1	4507677	N-Term(TMT6plex); C6(Carbamidomethyl); M19(Oxidation)	2601.2	0	**1.4**	**1.5**	**1.6**	**1.4**
																	AQAYQTGK	50	1	1	4507677	N-Term(TMT6plex); K8(TMT6plex)	1324.8	0	**1.3**	**1.8**	**1.5**	**1.3**
																	DISTNYYASQK	62	1	1	4507677	N-Term(TMT6plex); K11(TMT6plex)	1747.9	0	**1.4**	**1.7**	**1.5**	**1.4**
																	GVVDSDDLPLNVSR	53	1	1	4507677	N-Term(TMT6plex)	1714.9	0	**1.3**	**1.7**	**1.5**	**1.4**
																	EFEPLLNWMK	38	1	1	4507677	N-Term(TMT6plex); K10(TMT6plex)	1765.0	0	**1.7**	**2.6**	**2.2**	**1.6**
																	EESDDEAAVEEEEEEKKPK	1	1	1	4507677	N-Term(TMT6plex); K16(TMT6plex); K17(TMT6plex); K19(TMT6plex)	3136.6	1	**1.4**	**1.6**	**1.5**	**1.3**
																	SILFVPTSAPR	80	1	1	4507677	N-Term(TMT6plex)	1416.8	0	**1.4**	**1.6**	**1.4**	**1.3**
																	GLFDEYGSK	62	1	1	4507677	N-Term(TMT6plex); K9(TMT6plex)	1473.8	0	**1.2**	**1.8**	**1.5**	**1.3**
																	SGTSEFLNK	32	1	1	4507677	N-Term(TMT6plex); K9(TMT6plex)	1440.8	0	**1.4**	**1.5**	**1.5**	**1.2**
																	LTESPCALVASQYGWSGNMER	5	1	1	4507677	N-Term(TMT6plex); C6(Carbamidomethyl)	2585.2	0	**1.2**	**1.6**	**1.8**	**1.3**
																	KGYEVIYLTEPVDEYCIQALPEFDGK	4	1	1	4507677	N-Term(TMT6plex); K1(TMT6plex); C16(Carbamidomethyl); K26(TMT6plex)	3764.0	1	**1.4**	**1.9**	**1.7**	**1.3**
																	VEEEPEEEPEETAEDTTEDTEQDEDEEMDVGTDEEEETAK	2	1	1	4507677	N-Term(TMT6plex); M28(Oxidation); K40(TMT6plex)	5091.1	0	**1.0**	**2.2**	**1.0**	**1.0**
																	ESDDPMAYIHFTAEGEVTFK	4	1	1	4507677	N-Term(TMT6plex); M6(Oxidation); K20(TMT6plex)	2761.3	0	**1.6**	**1.8**	**1.4**	**1.8**
																	YSQFINFPIYVWSSK	47	1	1	4507677	N-Term(TMT6plex); K15(TMT6plex)	2337.3	0	**1.6**	**1.5**	**1.6**	**1.2**
																	GYEVIYLTEPVDEYCIQALPEFDGK	57	1	1	4507677	N-Term(TMT6plex); C15(Carbamidomethyl); K25(TMT6plex)	3406.7	0	**1.3**	**1.9**	**1.8**	**1.4**
																	GYEVIYLTEPVDEYCIQALPEFDGKR	8	1	1	4507677	N-Term(TMT6plex); C15(Carbamidomethyl); K25(TMT6plex)	3562.8	1	**1.5**	**3.1**	**1.9**	**1.9**
																	LSLNIDPDAK	48	1	1	4507677	N-Term(TMT6plex); K10(TMT6plex)	1543.9	0	**1.4**	**1.6**	**1.5**	**1.3**
																	KYSQFINFPIYVWSSK	30	1	1	4507677	N-Term(TMT6plex); K1(TMT6plex); K16(TMT6plex)	2694.5	1	**1.4**	**1.4**	**1.6**	**1.2**
																	EAVEKEFEPLLNWMK	4	1	1	4507677	N-Term(TMT6plex); K5(TMT6plex); K15(TMT6plex)	2550.4	1	**1.8**	**6.1**	**2.8**	**2.4**
																	VFITDDFHDMMPK	11	1	1	4507677	N-Term(TMT6plex); K13(TMT6plex)	2054.1	0	**2.1**	**2.7**	**2.7**	**1.5**
																	YNDTFWK	12	1	1	4507677	N-Term(TMT6plex); K7(TMT6plex)	1431.8	0	**1.5**	**1.6**	**1.6**	**1.2**
																	MTEAQEDGQSTSELIGQFGVGFYSAFLVADK	12	1	1	4507677	N-Term(TMT6plex); K31(TMT6plex)	3783.9	0	**2.0**	**2.5**	**2.3**	**1.8**
																	IADDKYNDTFWK	19	1	1	4507677	N-Term(TMT6plex); K5(TMT6plex); K12(TMT6plex)	2203.2	1	**1.3**	**1.9**	**1.5**	**1.4**
																	TVWDWELMNDIKPIWQRPSK	4	1	1	4507677	N-Term(TMT6plex); K12(TMT6plex); K20(TMT6plex)	3229.8	0	**1.4**	**3.1**	**1.9**	**1.8**
																	IYFMAGSSR	11	1	1	4507677	N-Term(TMT6plex); M4(Oxidation)	1276.7	0	**1.4**	**1.4**	**1.3**	**1.2**
																	IKEDEDDK	3	1	1	4507677	N-Term(TMT6plex); K2(TMT6plex); K8(TMT6plex)	1678.9	1	**1.3**	**1.6**	**1.5**	**1.2**
																	EEEAIQLDGLNASQIR	40	1	1	4507677	N-Term(TMT6plex)	2015.1	0	**1.3**	**1.4**	**1.4**	**1.3**
																	VFITDDFHDMMPK	26	1	1	4507677	N-Term(TMT6plex); M11(Oxidation); K13(TMT6plex)	2070.0	0	**1.6**	**2.2**	**1.9**	**1.5**
																	IKEDEDDKTVLDLAVVLFETATLR	12	1	1	4507677	N-Term(TMT6plex); K2(TMT6plex); K8(TMT6plex)	3420.9	2	**1.7**	**2.1**	**1.8**	**1.8**
																	NLLHVTDTGVGMTR	2	1	1	4507677	N-Term(TMT6plex)	1742.9	0	**1.3**	**1.4**	**1.5**	**1.3**
																	EAVEKEFEPLLNWMK	3	1	1	4507677	N-Term(TMT6plex); K5(TMT6plex); M14(Oxidation); K15(TMT6plex)	2566.4	1	**1.4**	**2.5**	**1.7**	**2.0**
																	EDEDDKTVLDLAVVLFETATLR	2	1	1	4507677	N-Term(TMT6plex); K6(TMT6plex)	2950.6	1	**1.6**	**1.6**	**1.8**	**1.3**
																	TETVEEPMEEEEAAKEEKEESDDEAAVEEEEEEK	1	1	1	4507677	N-Term(TMT6plex); K15(TMT6plex); K18(TMT6plex); K34(TMT6plex)	4872.3	2	**2.4**	**2.1**	**2.5**	**1.2**
																	TVLDLAVVLFETATLR	3	1	1	4507677	N-Term(TMT6plex)	1990.2	0	**2.0**	**1.8**	**1.8**	**1.7**
																	KGYEVIYLTEPVDEYCIQALPEFDGKR	2	1	1	4507677	N-Term(TMT6plex); K1(TMT6plex); C16(Carbamidomethyl); K26(TMT6plex)	3920.1	2	**1.4**	**3.0**	**1.9**	**2.0**
																	LSLNIDPDAKVEEEPEEEPEETAEDTTEDTEQDEDEEMDVGTDEEEETAK	2	1	1	4507677	N-Term(TMT6plex); K10(TMT6plex); M38(Oxidation); K50(TMT6plex)	6386.8	1	**1.6**	**2.3**	**1.5**	**1.4**
																	TETVEEPMEEEEAAKEEK	1	1	1	4507677	N-Term(TMT6plex); M8(Oxidation); K15(TMT6plex); K18(TMT6plex)	2811.4	1	**1.5**	**1.3**	**1.4**	**1.2**
																	KTFEINPR	2	1	1	4507677	N-Term(TMT6plex); K1(TMT6plex)	1462.9	1	**1.4**	**2.0**	**1.6**	**1.5**
																	EFGTNIK	20	1	1	4507677	N-Term(TMT6plex); K7(TMT6plex)	1266.7	0	**1.3**	**1.8**	**1.6**	**1.4**
																	HNNDTQHIWESDSNEFSVIADPR	2	1	1	4507677	N-Term(TMT6plex)	2940.4	0	**1.6**	**1.6**	**1.7**	**1.3**
																	YLNFVK	6	1	1	4507677	N-Term(TMT6plex); K6(TMT6plex)	1241.8	0	**1.4**	**1.4**	**1.5**	**1.1**
																	KTLDMIK	3	1	1	4507677	N-Term(TMT6plex); K1(TMT6plex); K7(TMT6plex)	1536.0	1	**1.6**	**2.9**	**2.3**	**1.5**
																	ESTAEKDEL	15	1	1	4507677	N-Term(TMT6plex); K6(TMT6plex)	1479.8	1	**1.3**	**1.7**	**1.4**	**1.3**
																	TETVEEPMEEEEAAKEEKEESDDEAAVEEEEEEK	1	1	1	4507677	N-Term(TMT6plex); M8(Oxidation); K15(TMT6plex); K18(TMT6plex); K34(TMT6plex)	4888.3	2	**1.7**	**0.9**	**1.4**	**0.9**
																	KSDYIK	4	1	1	4507677	N-Term(TMT6plex); K1(TMT6plex); K6(TMT6plex)	1440.9	1	**1.2**	**1.7**	**1.4**	**1.3**
																	GNTLGR	1	1	1	4507677	N-Term(TMT6plex)	846.5	0	**2.6**	**1.9**	**1.5**	**1.3**
																	VIVTSK	3	1	1	4507677	N-Term(TMT6plex); K6(TMT6plex)	1104.7	0	**1.3**	**1.6**	**1.4**	**1.3**
																	FDESEK	4	1	1	4507677	N-Term(TMT6plex); K6(TMT6plex)	1212.7	0	**1.5**	**1.6**	**1.5**	**1.2**
																	TLDMIK	1	1	1	4507677	N-Term(TMT6plex); K6(TMT6plex)	1178.7	0	**1.6**	**2.0**	**1.9**	**1.5**
																	FQNVAK	1	1	1	4507677	N-Term(TMT6plex); K6(TMT6plex)	1164.7	0	**1.3**	**1.2**	**1.3**	**0.9**
																	EIFLR	2	6	3	4507677;154146191;20149594;431822403	N-Term(TMT6plex)	906.6	0				
7330335	NP_039234.1	**CLIC4**	25932	**chloride intracellular channel protein 4**	79.05	2	17	19	327	**1.7**	**1.8**	**1.9**	**1.5**	253	28.8	5.59												
																	AGSDGESIGNCPFSQR	14	1	1	7330335	N-Term(TMT6plex); C11(Carbamidomethyl)	1910.9	0	**2.0**	**2.0**	**1.9**	**1.7**
																	IEEFLEEVLCPPK	81	1	1	7330335	N-Term(TMT6plex); C10(Carbamidomethyl); K13(TMT6plex)	2061.1	0	**1.8**	**1.7**	**2.1**	**1.4**
																	FLDGNEMTLADCNLLPK	16	1	1	7330335	N-Term(TMT6plex); M7(Oxidation); C12(Carbamidomethyl); K17(TMT6plex)	2425.2	0	**1.6**	**1.6**	**1.7**	**1.4**
																	NSRPEANEALER	7	1	1	7330335	N-Term(TMT6plex)	1614.8	0	**2.0**	**1.4**	**1.6**	**1.1**

Khan et al., 2019. Multi-omics analysis to characterize cigarette smoke induced molecular alterations in esophageal cells
Supplementary Table 6. List of all proteins and peptides identified in untreated and chronically treated Het1A cells with cigarette smoke condensate for 8 months

Accession	RefSeq Accession	Gene Symbol	Entrez Gene ID	Description	Σ Coverage	Σ# Proteins	Σ# Unique Peptides	Σ# Peptides	Σ# PSMs	Het-1A-Smoke - 2M/Parental	Het-1A-Smoke - 4M/Parental	Het-1A-Smoke - 6M/Parental	Het-1A-Smoke - 8M/Parental	# AAs	MW [kDa]	calc. pI	Sequence	# PSMs	# Proteins	# Protein Groups	Protein Group Accessions	Modifications	MH+ [Da]	# Missed Cleavages	Het-1A-Smoke - 2M/Parental	Het-1A-Smoke - 4M/Parental	Het-1A-Smoke - 6M/Parental	Het-1A-Smoke - 8M/Parental
																	TDVNKIEEFLEEVLCPPK	39	1	1	7330335	N-Term(TMT6plex); K5(TMT6plex); C15(Carbamidomethyl); K18(TMT6plex)	2847.6	1	**1.9**	**1.8**	**2.2**	**1.5**
																	DEFTNTCPSDK	14	1	1	7330335	N-Term(TMT6plex); C7(Carbamidomethyl); K11(TMT6plex)	1771.9	0	**1.7**	**1.6**	**1.9**	**1.3**
																	LDEYLNSPLPDEIDENSMEDIK	22	1	1	7330335	N-Term(TMT6plex); M18(Oxidation); K22(TMT6plex)	3053.5	0	**1.6**	**1.5**	**1.7**	**1.4**
																	KFLDGNEMTLADCNLLPK	17	1	1	7330335	N-Term(TMT6plex); K1(TMT6plex); M8(Oxidation); C13(Carbamidomethyl); K18(TMT6plex)	2782.5	1	**1.6**	**1.7**	**1.8**	**1.6**
																	HPESNTAGMDIFAK	31	1	1	7330335	N-Term(TMT6plex); M9(Oxidation); K14(TMT6plex)	1992.0	0	**1.5**	**1.7**	**1.6**	**1.5**
																	KFLDGNEMTLADCNLLPK	4	1	1	7330335	N-Term(TMT6plex); K1(TMT6plex); C13(Carbamidomethyl); K18(TMT6plex)	2766.5	1	**1.5**	**2.1**	**2.1**	**1.4**
																	HPESNTAGMDIFAK	2	1	1	7330335	N-Term(TMT6plex); K14(TMT6plex)	1976.0	0	**1.3**	**1.4**	**1.4**	**1.2**
																	DEFTNTCPSDKEVEIAYSDVAK	20	1	1	7330335	N-Term(TMT6plex); C7(Carbamidomethyl); K11(TMT6plex); K22(TMT6plex)	3205.6	1	**1.8**	**2.3**	**2.1**	**1.6**
																	EEDKEPLIELFVK	17	1	1	7330335	N-Term(TMT6plex); K4(TMT6plex); K13(TMT6plex)	2276.3	1	**1.7**	**1.8**	**2.1**	**1.5**
																	KPADLQNLAPGTHPPFITFNSEVK	8	1	1	7330335	N-Term(TMT6plex); K1(TMT6plex); K24(TMT6plex)	3308.9	0	**1.3**	**1.7**	**2.0**	**1.4**
																	RKPADLQNLAPGTHPPFITFNSEVK	1	1	1	7330335	N-Term(TMT6plex); K2(TMT6plex); K25(TMT6plex)	3465.0	1	**1.6**	**2.2**	**1.9**	**1.7**
																	GVVFSVTTVDLK	16	1	1	7330335	N-Term(TMT6plex); K12(TMT6plex)	1723.0	0	**1.6**	**1.9**	**1.9**	**1.4**
																	LDEYLNSPLPDEIDENSMEDIK	2	1	1	7330335	N-Term(TMT6plex); K22(TMT6plex)	3037.5	0	**1.4**	**1.5**	**1.6**	**1.2**
																	FLDGNEMTLADCNLLPK	4	1	1	7330335	N-Term(TMT6plex); C12(Carbamidomethyl); K17(TMT6plex)	2409.3	0	**2.4**	**2.5**	**3.1**	**1.6**
																	EVEIAYSDVAK	4	1	1	7330335	N-Term(TMT6plex); K11(TMT6plex)	1681.9	0	**1.3**	**1.5**	**1.6**	**1.4**
																	LFMILWLK	2	6	2	7330335;365733560	N-Term(TMT6plex); K8(TMT6plex)	1522.0	0				
																	FSAYIK	4	6	3	7330335;365733560;14251209	N-Term(TMT6plex); K6(TMT6plex)	1186.7	0				
																	YLTNAYSR	1	1	1	7330335	N-Term(TMT6plex)	1216.7	0	**1.4**	**1.3**	**1.5**	**1.3**
																	NFDIPK	1	1	1	7330335	N-Term(TMT6plex); K6(TMT6plex)	1191.7	0	**1.8**	**1.6**	**1.9**	**1.4**
545746425	NP_001271141.1	**CAST**	831	**calpastatin isoform n**	78.68	3	1	40	296	**1.0**	**1.1**	**1.4**	**0.8**	666	71.7	4.92												
																	SESELIDELSEDFDR	32	5	3	298919189;109637759;545746425	N-Term(TMT6plex)	2012.9	0				
																	GTVPDDAVEALADSLGK	26	5	3	298919189;109637759;545746425	N-Term(TMT6plex); K17(TMT6plex)	2116.1	0				
																	SAEQQPSEK	13	5	3	298919189;109637759;545746425	N-Term(TMT6plex); K9(TMT6plex)	1461.8	0				
																	AAAPAPVSEAVCR	13	5	3	298919189;109637759;545746425	N-Term(TMT6plex); C12(Carbamidomethyl)	1527.8	0				
																	QASDTGSNDAHNK	10	5	3	298919189;109637759;545746425	N-Term(TMT6plex); K13(TMT6plex)	1802.9	0				
																	QPDPDENKPMEDK	4	5	3	298919189;109637759;545746425	N-Term(TMT6plex); K8(TMT6plex); M10(Oxidation); K13(TMT6plex)	2246.2	0				
																	LGEKEETIPPDYR	4	5	3	298919189;109637759;545746425	N-Term(TMT6plex); K4(TMT6plex)	2005.1	1				
																	TSMCSIQSAPPEPATLK	9	5	3	298919189;109637759;545746425	N-Term(TMT6plex); M3(Oxidation); C4(Carbamidomethyl); K17(TMT6plex)	2292.2	0				
																	EQLPPMSEDFLLDALSEDFSGPQNASSLK	11	5	3	298919189;109637759;545746425	N-Term(TMT6plex); K29(TMT6plex)	3623.8	0				
																	EQLPPMSEDFLLDALSEDFSGPQNASSLK	27	5	3	298919189;109637759;545746425	N-Term(TMT6plex); M6(Oxidation); K29(TMT6plex)	3639.8	0				
																	TKPQDMISAGGESVAGITAISGKPGDK	6	4	3	298919189;109637759;545746425	N-Term(TMT6plex); K2(TMT6plex); M6(Oxidation); K23(TMT6plex); K27(TMT6plex)	3548.0	0				
																	CGEDDETIPSEYR	11	5	3	298919189;109637759;545746425	N-Term(TMT6plex); C1(Carbamidomethyl)	1799.8	0				
																	LAAAISEVVSQTPASTTQAGAPPR	20	5	3	298919189;109637759;545746425	N-Term(TMT6plex)	2552.4	0				
																	DDTIPPEYR	7	5	3	298919189;109637759;545746425	N-Term(TMT6plex)	1334.7	0				
																	DTSSDKDLDDALDK	2	1	1	545746425	N-Term(TMT6plex); K6(TMT6plex); K14(TMT6plex)	2225.2	1	**1.0**	**1.1**	**1.4**	**0.8**
																	SLTPAVPVESKPDKPSGK	2	4	3	298919189;109637759;545746425	N-Term(TMT6plex); K11(TMT6plex); K14(TMT6plex); K18(TMT6plex)	2753.7	0				
																	TTEETSKPK	2	5	3	298919189;109637759;545746425	N-Term(TMT6plex); K7(TMT6plex); K9(TMT6plex)	1708.0	0				
																	DLDDALDK	1	5	3	298919189;109637759;545746425	N-Term(TMT6plex); K8(TMT6plex)	1362.8	0				
																	KEGITGPPADSSKPIGPDDAIDALSSDFTCGSPTAAGK	3	4	2	109637759;545746425	K1(TMT6plex); N-Term(TMT6plex); K13(TMT6plex); C30(Carbamidomethyl); K38(TMT6plex)	4647.4	1				
																	EGITGPPADSSKPIGPDDAIDALSSDFTCGSPTAAGK	2	4	2	109637759;545746425	N-Term(TMT6plex); K12(TMT6plex); C29(Carbamidomethyl); K37(TMT6plex)	4290.2	0				
																	SGMDAALDDLIDTLGGPEETEEENTTYTGPEVSDPMSSTYIEELGK	4	5	3	298919189;109637759;545746425	N-Term(TMT6plex); M3(Oxidation); M36(Oxidation); K46(TMT6plex)	5397.5	0				
																	QASDTGSNDAHNKK	3	5	3	298919189;109637759;545746425	N-Term(TMT6plex); K13(TMT6plex); K14(TMT6plex)	2160.2	1				
																	HLLDDNGQDKPVKPPTK	3	5	3	298919189;109637759;545746425	N-Term(TMT6plex); K10(TMT6plex); K13(TMT6plex); K17(TMT6plex)	2818.6	0				
																	DTMSDQALEALSASLGTR	22	5	3	298919189;109637759;545746425	N-Term(TMT6plex); M3(Oxidation)	2111.0	0				
																	KPADDQDPIDALSGDLDSCPSTTETSQNTAK	11	5	3	298919189;109637759;545746425	N-Term(TMT6plex); K1(TMT6plex); C19(Carbamidomethyl); K31(TMT6plex)	3965.0	0				
																	SAAPPQEK	6	5	3	298919189;109637759;545746425	N-Term(TMT6plex); K8(TMT6plex)	1285.8	0				
																	TEKEESTEVLK	3	5	3	298919189;109637759;545746425	N-Term(TMT6plex); K3(TMT6plex); K11(TMT6plex)	1980.1	1				
																	DTMSDQALEALSASLGTR	6	5	3	298919189;109637759;545746425	N-Term(TMT6plex)	2095.1	0				
																	SGMDAALDDLIDTLGGPEETEEENTTYTGPEVSDPMSSTYIEELGK	4	5	3	298919189;109637759;545746425	N-Term(TMT6plex); M3(Oxidation); K46(TMT6plex)	5381.5	0				
																	TKPQDMISAGGESVAGITAISGKPGDK	2	4	3	298919189;109637759;545746425	N-Term(TMT6plex); K2(TMT6plex); K23(TMT6plex); K27(TMT6plex)	3532.0	0				
																	LSDSLGQR	4	5	3	298919189;109637759;545746425	N-Term(TMT6plex)	1104.6	0				
																	SESELIDELSEDFDRSECK	1	5	3	298919189;109637759;545746425	N-Term(TMT6plex); C18(Carbamidomethyl); K19(TMT6plex)	2746.3	1				
																	KTEKEESTEVLK	1	5	3	298919189;109637759;545746425	N-Term(TMT6plex); K1(TMT6plex); K4(TMT6plex); K12(TMT6plex)	2337.4	2				
																	EADPEDGKPVMDK	5	5	3	298919189;109637759;545746425	N-Term(TMT6plex); K8(TMT6plex); M11(Oxidation); K13(TMT6plex)	2134.1	0				
																	AQSAGTVR	3	5	3	298919189;109637759;545746425	N-Term(TMT6plex)	1018.6	0				
																	QPDPDENKPMEDK	1	5	3	298919189;109637759;545746425	N-Term(TMT6plex); K8(TMT6plex); K13(TMT6plex)	2230.2	0				
																	DKDGKPLLPK	1	5	3	298919189;109637759;545746425	N-Term(TMT6plex); K2(TMT6plex); K5(TMT6plex); K10(TMT6plex)	2027.3	1				
																	DGKPLLPEPEEKPKPR	1	5	3	298919189;109637759;545746425	N-Term(TMT6plex); K3(TMT6plex); K12(TMT6plex); K14(TMT6plex)	2746.7	0				
																	AASSSK	1	7	3	298919189;109637759;545746425	N-Term(TMT6plex); K6(TMT6plex)	1008.6	0				
																	EETIPPDYR	3	5	3	298919189;109637759;545746425	N-Term(TMT6plex)	1348.7	0				
																	TTEETSKPKDD	1	5	3	298919189;109637759;545746425	N-Term(TMT6plex); K7(TMT6plex); K9(TMT6plex)	1938.1	1				
																	QAEPELDLR	2	5	3	298919189;109637759;545746425	N-Term(TMT6plex)	1299.7	0				
																	EKPSKPTEK	1	5	3	298919189;109637759;545746425	N-Term(TMT6plex); K2(TMT6plex); K5(TMT6plex); K9(TMT6plex)	1960.2	0				
																	EVTIPPK	1	5	3	298919189;109637759;545746425	N-Term(TMT6plex); K7(TMT6plex)	1241.8	0				
																	AKEEDREK	1	5	3	298919189;109637759;545746425	N-Term(TMT6plex); K2(TMT6plex); K8(TMT6plex)	1692.0	2				
148539872	NP_005882.2	**ACAT2**	39	**acetyl-CoA acetyltransferase, cytosolic**	78.59	2	19	19	273	**1.6**	**1.6**	**1.4**	**1.4**	397	41.3	6.92												
																	IVSWSQVGVEPSIMGIGPIPAIK	69	2	1	148539872	N-Term(TMT6plex); M14(Oxidation); K23(TMT6plex)	2852.6	0	**1.7**	**1.6**	**1.4**	**1.4**
																	LKPYFLTDGTGTVTPANASGINDGAAAVVLMK	29	1	1	148539872	N-Term(TMT6plex); K2(TMT6plex); M31(Oxidation); K32(TMT6plex)	3896.2	0	**1.4**	**1.6**	**1.3**	**1.5**
																	IGEMPLTDSILCDGLTDAFHNCHMGITAENVAK	12	1	1	148539872	N-Term(TMT6plex); M4(Oxidation); C12(Carbamidomethyl); C22(Carbamidomethyl); M24(Oxidation); K33(TMT6plex)	4121.0	0	**1.4**	**1.4**	**1.2**	**1.4**
																	ELGLNPEK	21	2	1	148539872	N-Term(TMT6plex); K8(TMT6plex)	1357.8	0	**1.5**	**1.6**	**1.3**	**1.3**
																	AGHFDKEIVPVLVSTR	8	1	1	148539872	N-Term(TMT6plex); K6(TMT6plex)	2226.3	1	**1.7**	**1.9**	**1.5**	**1.7**
																	ATVAPEDVSEVIFGHVLAAGCGQNPVR	29	2	1	148539872	N-Term(TMT6plex); C21(Carbamidomethyl)	3022.6	0	**1.7**	**1.8**	**1.6**	**1.6**
																	AGWSLEDVDIFEINEAFAAVSAAIVK	4	1	1	148539872	N-Term(TMT6plex); K26(TMT6plex)	3223.7	0	**1.7**	**1.7**	**1.3**	**1.4**
																	ILVTLLHTLER	28	1	1	148539872	N-Term(TMT6plex)	1537.0	0	**1.5**	**1.2**	**1.3**	**1.1**
																	VNIEGGAIALGHPLGASGCR	14	1	1	148539872	N-Term(TMT6plex); C19(Carbamidomethyl)	2178.2	0	**1.5**	**1.6**	**1.6**	**1.5**
																	HGSNIEAMSK	4	1	1	148539872	N-Term(TMT6plex); K10(TMT6plex)	1531.8	0	**1.3**	**1.3**	**1.4**	**1.2**
																	VAVLSQNR	9	2	1	148539872	N-Term(TMT6plex)	1115.7	0	**1.5**	**1.6**	**1.4**	**1.4**
																	EIVPVLVSTR	12	1	1	148539872	N-Term(TMT6plex)	1341.8	0	**1.6**	**1.5**	**1.3**	**1.2**
																	HGSNIEAMSK	4	1	1	148539872	N-Term(TMT6plex); M8(Oxidation); K10(TMT6plex)	1547.8	0	**1.5**	**1.4**	**1.1**	**1.3**
																	KGLIEVK	1	1	1	148539872	N-Term(TMT6plex); K1(TMT6plex); K7(TMT6plex)	1474.0	1	**1.6**	**2.1**	**1.5**	**1.6**
																	IVSWSQVGVEPSIMGIGPIPAIK	10	2	1	148539872	N-Term(TMT6plex); K23(TMT6plex)	2836.6	0	**2.5**	**2.4**	**2.4**	**1.5**
																	AVCLAVQSIGIGDSSIVVAGGMENMSK	2	1	1	148539872	N-Term(TMT6plex); C3(Carbamidomethyl); M22(Oxidation); M25(Oxidation); K27(TMT6plex)	3183.6	0	**1.3**	**1.4**	**1.2**	**1.4**
																	TIIGSFNGALAAVPVQDLGSTVIK	6	1	1	148539872	N-Term(TMT6plex); K24(TMT6plex)	2829.6	0	**1.7**	**1.4**	**1.4**	**1.3**
																	IGEMPLTDSILCDGLTDAFHNCHMGITAENVAK	3	1	1	148539872	N-Term(TMT6plex); M4(Oxidation); C12(Carbamidomethyl); C22(Carbamidomethyl); K33(TMT6plex)	4105.0	0	**2.1**	**2.2**	**2.1**	**1.6**
																	QASVGAGIPYSVPAWSCQMICGSGLK	2	1	1	148539872	N-Term(TMT6plex); C17(Carbamidomethyl); M19(Oxidation); C21(Carbamidomethyl); K26(TMT6plex)	3198.6	0	**1.8**	**1.7**	**1.6**	**1.7**
																	GLTPLAR	2	1	1	148539872	N-Term(TMT6plex)	956.6	0	**1.5**	**1.7**	**1.4**	**1.4**
																	KSEADKR	1	1	1	148539872	N-Term(TMT6plex); K1(TMT6plex); K6(TMT6plex)	1520.9	2	**1.6**	**1.7**	**1.2**	**1.4**
																	GLIEVK	3	1	1	148539872	N-Term(TMT6plex); K6(TMT6plex)	1116.7	0	**1.4**	**1.7**	**1.3**	**1.5**
38327039	NP_002145.3	**HSPA4**	3308	**heat shock 70 kDa protein 4**	78.57	3	55	60	1106	**1.4**	**1.4**	**1.4**	**1.5**	840	94.3	5.19												
																	TSTVDLPIENQLLWQIDR	60	1	1	38327039	N-Term(TMT6plex)	2370.3	0	**1.5**	**1.3**	**1.5**	**1.3**
																	ELSTTLNADEAVTR	63	1	1	38327039	N-Term(TMT6plex)	1748.9	0	**1.5**	**1.4**	**1.4**	**1.6**
																	NFTTEQVTAMLLSK	25	1	1	38327039	N-Term(TMT6plex); M10(Oxidation); K14(TMT6plex)	2057.1	0	**1.4**	**1.3**	**1.4**	**1.4**
																	SVMDATQIAGLNCLR	22	1	1	38327039	N-Term(TMT6plex); M3(Oxidation); C13(Carbamidomethyl)	1894.0	0	**1.6**	**1.6**	**1.5**	**1.6**

Accession	RefSeq Accession	Gene Symbol	Entrez Gene ID	Description	Σ Coverage	Σ# Proteins	Σ# Unique Peptides	Σ# Peptides	Σ# PSMs	Het-1A-Smoke - 2M/Parental	Het-1A-Smoke - 4M/Parental	Het-1A-Smoke - 6M/Parental	Het-1A-Smoke - 8M/Parental	# AAs	MW [kDa]	calc. pI	Sequence	# PSMs	# Proteins	# Protein Groups	Protein Group Accessions	Modifications	MH+ [Da]	# Missed Cleavages	Het-1A-Smoke - 2M/Parental	Het-1A-Smoke - 4M/Parental	Het-1A-Smoke - 6M/Parental	Het-1A-Smoke - 8M/Parental
																	SEENEEPMETDQNAK	10	1	1	38327039	N-Term(TMT6plex); M8(Oxidation); K15(TMT6plex)	2225.0	0	**1.3**	**1.3**	**1.3**	**1.3**
																	AFSDPFVEAEK	42	1	1	38327039	N-Term(TMT6plex); K11(TMT6plex)	1697.9	0	**1.4**	**1.5**	**1.5**	**1.5**
																	LEDTENWLYEDGEDQPK	54	1	1	38327039	N-Term(TMT6plex); K17(TMT6plex)	2539.2	0	**1.5**	**1.6**	**1.5**	**1.5**
																	NAVEEYVYEMR	48	1	1	38327039	N-Term(TMT6plex); M10(Oxidation)	1647.8	0	**1.4**	**1.3**	**1.4**	**1.3**
																	LMNETTAVALAYGIYK	21	2	2	38327039;31541941	N-Term(TMT6plex); M2(Oxidation); K16(TMT6plex)	2232.2	0				
																	NAEQNGPVDGQGDNPGPQAAEQGTDTAVPSDSDK	6	1	1	38327039	N-Term(TMT6plex); K34(TMT6plex)	3824.8	0	**1.4**	**1.1**	**1.3**	**1.2**
																	QSLTMDPVVK	15	1	1	38327039	N-Term(TMT6plex); M5(Oxidation); K10(TMT6plex)	1591.9	0	**1.2**	**1.2**	**1.3**	**1.3**
																	VTPQSDGSSSK	23	1	1	38327039	N-Term(TMT6plex); K11(TMT6plex)	1550.8	0	**1.4**	**1.3**	**1.3**	**1.4**
																	SEENEEPMETDQNAKEEEK	5	1	1	38327039	N-Term(TMT6plex); M8(Oxidation); K15(TMT6plex); K19(TMT6plex)	2969.4	1	**1.3**	**1.3**	**1.4**	**1.6**
																	EMLNLYIENEGK	50	1	1	38327039	N-Term(TMT6plex); M2(Oxidation); K12(TMT6plex)	1927.0	0	**1.3**	**1.5**	**1.5**	**1.5**
																	NTVQGFK	13	1	1	38327039	N-Term(TMT6plex); K7(TMT6plex)	1251.7	0	**1.4**	**1.2**	**1.4**	**1.2**
																	QDLPALEEKPR	21	1	1	38327039	N-Term(TMT6plex); K9(TMT6plex)	1754.0	0	**1.3**	**1.4**	**1.4**	**1.4**
																	FQESEERPK	12	1	1	38327039	N-Term(TMT6plex); K9(TMT6plex)	1607.9	0	**1.3**	**1.3**	**1.3**	**1.4**
																	STNEAMEWMNNK	10	1	1	38327039	N-Term(TMT6plex); M6(Oxidation); K12(TMT6plex)	1928.9	0	**1.5**	**1.7**	**1.8**	**1.5**
																	MQVDQEEPHVEEQQQQTPAENKAESEEMETSQAGSK	5	1	1	38327039	N-Term(TMT6plex); M1(Oxidation); K22(TMT6plex); M28(Oxidation); K36(TMT6plex)	4806.3	1	**1.6**	**1.9**	**1.7**	**2.0**
																	AGGIETIANEYSDR	29	1	1	38327039	N-Term(TMT6plex)	1724.9	0	**1.5**	**1.6**	**1.5**	**1.6**
																	EDIYAVEIVGGATR	35	1	1	38327039	N-Term(TMT6plex)	1721.9	0	**1.4**	**1.4**	**1.4**	**1.4**
																	LMSANASDLPLSIECFMNDVDVSGTMNR	11	1	1	38327039	N-Term(TMT6plex); M2(Oxidation); C15(Carbamidomethyl); M17(Oxidation); M26(Oxidation)	3364.5	0	**1.3**	**1.4**	**1.3**	**1.4**
																	FLEMCNDLLAR	11	1	1	38327039	N-Term(TMT6plex); M4(Oxidation); C5(Carbamidomethyl)	1626.8	0	**1.4**	**1.3**	**1.3**	**1.5**
																	LSGEYEK	13	1	1	38327039	N-Term(TMT6plex); K7(TMT6plex)	1283.7	0	**1.4**	**1.3**	**1.4**	**1.4**
																	VTYMEEER	14	1	1	38327039	N-Term(TMT6plex); M4(Oxidation)	1301.6	0	**1.4**	**1.5**	**1.5**	**1.4**
																	MQVDQEEPHVEEQQQQTPAENK	20	1	1	38327039	N-Term(TMT6plex); M1(Oxidation); K22(TMT6plex)	3096.5	0	**1.6**	**1.7**	**1.7**	**1.7**
																	LKETAESVLK	5	1	1	38327039	N-Term(TMT6plex); K2(TMT6plex); K10(TMT6plex)	1805.1	1	**1.3**	**1.6**	**1.5**	**1.5**
																	STNEAMEWMNNK	11	1	1	38327039	N-Term(TMT6plex); M6(Oxidation); M9(Oxidation); K12(TMT6plex)	1944.9	0	**1.7**	**1.6**	**1.5**	**1.4**
																	VTYMEEER	2	1	1	38327039	N-Term(TMT6plex)	1285.6	0	**1.7**	**1.8**	**2.0**	**1.5**
																	LMSANASDLPLSIECFMNDVDVSGTMNR	2	1	1	38327039	N-Term(TMT6plex); M2(Oxidation); C15(Carbamidomethyl); M26(Oxidation)	3348.5	0	**1.5**	**1.5**	**1.5**	**1.4**
																	VLATAFDTTLGGR	26	1	1	38327039	N-Term(TMT6plex)	1550.9	0	**1.4**	**1.3**	**1.5**	**1.3**
																	STNEAMEWMNNK	2	1	1	38327039	N-Term(TMT6plex); K12(TMT6plex)	1912.9	0	**2.2**	**2.1**	**2.8**	**1.9**
																	SNLAYDIVQLPTGLTGIK	35	1	1	38327039	N-Term(TMT6plex); K18(TMT6plex)	2361.4	0	**1.5**	**1.4**	**1.8**	**1.4**
																	EPFTLEAYYSSPQDLPYPDPAIAQFSVQK	9	1	1	38327039	N-Term(TMT6plex); K29(TMT6plex)	3759.9	0	**1.2**	**1.6**	**1.5**	**1.7**
																	EMLNLYIENEGK	2	1	1	38327039	N-Term(TMT6plex); K12(TMT6plex)	1911.0	0	**1.5**	**1.7**	**1.5**	**1.6**
																	DKLSGEYEK	5	1	1	38327039	N-Term(TMT6plex); K2(TMT6plex); K9(TMT6plex)	1756.0	1	**1.2**	**1.8**	**1.5**	**1.8**
																	SVLEQTK	15	1	1	38327039	N-Term(TMT6plex); K7(TMT6plex)	1262.8	0	**1.3**	**1.5**	**1.4**	**1.6**
																	KPVVDCVVSVPCFYTDAER	16	1	1	38327039	K1(TMT6plex); N-Term(TMT6plex); C6(Carbamidomethyl); C12(Carbamidomethyl)	2699.4	0	**1.3**	**1.5**	**1.5**	**1.3**
																	KEPFTLEAYYSSPQDLPYPDPAIAQFSVQK	14	1	1	38327039	N-Term(TMT6plex); K1(TMT6plex); K30(TMT6plex)	4117.2	1	**1.3**	**1.8**	**1.6**	**1.9**
																	GCALQCAILSPAFK	21	6	3	38327039;42544159;31541941	N-Term(TMT6plex); C2(Carbamidomethyl); C6(Carbamidomethyl); K14(TMT6plex)	1994.1	0				
																	WNSPAEEGSSDCEVFSK	24	1	1	38327039	N-Term(TMT6plex); C12(Carbamidomethyl); K17(TMT6plex)	2387.1	0	**1.4**	**1.3**	**1.6**	**1.3**
																	CTPACISFGPK	13	1	1	38327039	N-Term(TMT6plex); C1(Carbamidomethyl); C5(Carbamidomethyl); K11(TMT6plex)	1695.9	0	**1.4**	**1.2**	**1.5**	**1.1**
																	AESEEMETSQAGSK	10	1	1	38327039	N-Term(TMT6plex); M6(Oxidation); K14(TMT6plex)	1957.9	0	**1.6**	**1.7**	**1.7**	**1.6**
																	LFEELGK	33	1	1	38327039	N-Term(TMT6plex); K7(TMT6plex)	1293.8	0	**1.4**	**1.4**	**1.4**	**1.5**
																	NLGQPIK	16	1	1	38327039	N-Term(TMT6plex); K7(TMT6plex)	1227.8	0	**1.3**	**1.1**	**1.4**	**1.2**
																	MQVDQEEPHVEEQQQQTPAENK	10	1	1	38327039	N-Term(TMT6plex); K22(TMT6plex)	3080.5	0	**2.2**	**2.4**	**2.2**	**1.7**
																	KFDEVLVNHFCEEFGK	16	1	1	38327039	N-Term(TMT6plex); K1(TMT6plex); C11(Carbamidomethyl); K16(TMT6plex)	2685.4	1	**1.3**	**1.4**	**1.5**	**1.4**
																	NFTTEQVTAMLLSK	11	1	1	38327039	N-Term(TMT6plex); K14(TMT6plex)	2041.1	0	**1.5**	**1.3**	**1.5**	**1.4**
																	NAEQNGPVDGQGDNPGPQAAEQGTDTAVPSDSDKK	1	1	1	38327039	N-Term(TMT6plex); K34(TMT6plex); K35(TMT6plex)	4182.0	1	**3.0**	**2.5**	**3.1**	**2.4**
																	LMNETTAVALAYGIYK	8	2	2	38327039;31541941	N-Term(TMT6plex); K16(TMT6plex)	2216.2	0				
																	LMSANASDLPLSIECFMNDVDVSGTMNR	2	1	1	38327039	N-Term(TMT6plex); C15(Carbamidomethyl); M26(Oxidation)	3332.5	0	**1.9**	**1.8**	**1.9**	**1.7**
																	SVMDATQIAGLNCLR	6	1	1	38327039	N-Term(TMT6plex); C13(Carbamidomethyl)	1878.0	0	**1.5**	**1.7**	**1.8**	**1.7**
																	SQVISNAK	10	1	1	38327039	N-Term(TMT6plex); K8(TMT6plex)	1304.8	0	**1.6**	**1.3**	**1.4**	**1.4**
																	ETAESVLK	10	1	1	38327039	N-Term(TMT6plex); K8(TMT6plex)	1334.8	0	**1.3**	**1.4**	**1.5**	**1.5**
																	AESEEMETSQAGSK	2	1	1	38327039	N-Term(TMT6plex); K14(TMT6plex)	1941.9	0	**1.3**	**1.4**	**1.7**	**1.3**
																	KMDQPPQAK	5	1	1	38327039	N-Term(TMT6plex); K1(TMT6plex); M2(Oxidation); K9(TMT6plex)	1746.0	1	**1.2**	**1.4**	**1.3**	**1.6**
																	NKEDQYDHLDAADMTK	13	1	1	38327039	N-Term(TMT6plex); K2(TMT6plex); M14(Oxidation); K16(TMT6plex)	2597.3	1	**1.3**	**1.4**	**1.4**	**1.5**
																	FVSEDDRNSFTLK	2	1	1	38327039	N-Term(TMT6plex); K13(TMT6plex)	2016.1	1	**1.4**	**1.5**	**1.5**	**1.7**
																	NKEDQYDHLDAADMTK	4	1	1	38327039	N-Term(TMT6plex); K2(TMT6plex); K16(TMT6plex)	2581.3	1	**1.8**	**2.0**	**2.2**	**1.6**
																	NHAAPFSK	4	5	2	38327039;42544159	N-Term(TMT6plex); K8(TMT6plex)	1329.8	0				
																	EFSITDVVPYPISLR	41	1	1	38327039	N-Term(TMT6plex)	1965.1	0	**1.4**	**1.3**	**1.4**	**1.4**
																	MDQPPQAK	2	1	1	38327039	N-Term(TMT6plex); K8(TMT6plex)	1372.8	0	**1.8**	**1.7**	**2.0**	**1.5**
																	LSQECEK	5	1	1	38327039	N-Term(TMT6plex); C5(Carbamidomethyl); K7(TMT6plex)	1351.7	0	**1.4**	**1.3**	**1.4**	**1.4**
																	NAVEEYVYEMR	4	1	1	38327039	N-Term(TMT6plex)	1631.8	0	**1.8**	**1.6**	**2.1**	**1.5**
																	FLEMCNDLLAR	4	1	1	38327039	N-Term(TMT6plex); C5(Carbamidomethyl)	1610.8	0	**1.9**	**1.9**	**2.2**	**1.5**
																	SKEIEAK	1	1	1	38327039	N-Term(TMT6plex); K2(TMT6plex); K7(TMT6plex)	1491.9	1	**1.2**	**1.7**	**1.6**	**1.8**
																	MDQPPQAK	20	1	1	38327039	N-Term(TMT6plex); M1(Oxidation); K8(TMT6plex)	1388.8	0	**2.1**	**1.4**	**1.5**	**1.3**
																	FVSEDDR	6	1	1	38327039	N-Term(TMT6plex)	1096.5	0	**1.2**	**1.3**	**1.3**	**1.3**
																	SIGAAAK	5	1	1	38327039	N-Term(TMT6plex); K7(TMT6plex)	1075.7	0	**1.2**	**1.3**	**1.3**	**1.4**
																	VEPPKEEQK	4	1	1	38327039	N-Term(TMT6plex); K5(TMT6plex); K9(TMT6plex)	1771.1	1	**1.3**	**1.6**	**1.6**	**1.7**
																	QVYVDK	5	4	2	38327039;31541941	N-Term(TMT6plex); K6(TMT6plex)	1209.7	0				
																	LNLQNK	1	1	1	38327039	N-Term(TMT6plex); K6(TMT6plex)	1187.8	0	**1.3**	**1.4**	**1.4**	**1.3**
																	IISSFK	2	2	2	38327039;55770834	N-Term(TMT6plex); K6(TMT6plex)	1152.7	0				
																	VLTFYR	2	1	1	38327039	N-Term(TMT6plex)	1027.6	0	**1.4**	**1.3**	**1.5**	**1.3**
																	VEPPLR	1	1	1	38327039	N-Term(TMT6plex)	939.6	0	**1.5**	**1.5**	**1.5**	**1.5**
4502107	NP_001145.1	**ANXA5**	308	**annexin A5**	78.44	1	27	28	1188	**1.2**	**1.3**	**1.2**	**1.1**	320	35.9	5.05												
																	DPDAGIDEAQVEQDAQALFQAGELK	202	1	1	4502107	N-Term(TMT6plex); K25(TMT6plex)	3116.6	0	**1.2**	**1.4**	**1.4**	**1.2**
																	ETSGNLEQLLLAVVK	164	1	1	4502107	N-Term(TMT6plex); K15(TMT6plex)	2072.2	0	**1.0**	**1.2**	**1.1**	**1.0**
																	GLGTDEESILTLLTSR	101	1	1	4502107	N-Term(TMT6plex)	1934.1	0	**1.4**	**1.5**	**1.4**	**1.3**
																	GTVTDFPGFDER	29	1	1	4502107	N-Term(TMT6plex)	1569.8	0	**1.2**	**1.4**	**1.3**	**1.2**
																	VLTEIIASR	51	1	1	4502107	N-Term(TMT6plex)	1230.8	0	**1.2**	**1.2**	**1.2**	**1.0**
																	GDTSGDYKK	4	1	1	4502107	N-Term(TMT6plex); K8(TMT6plex); K9(TMT6plex)	1657.9	1	**0.8**	**1.5**	**1.1**	**0.9**
																	DLLDDLKSELTGK	26	1	1	4502107	N-Term(TMT6plex); K7(TMT6plex); K13(TMT6plex)	2134.3	1	**1.2**	**1.7**	**1.3**	**1.2**
																	DLLDDLK	17	3	2	4502107;4502105;1002623478	N-Term(TMT6plex); K7(TMT6plex)	1289.8	0				
																	WGTDEEK	15	1	1	4502107	N-Term(TMT6plex); K7(TMT6plex)	1322.7	0	**1.2**	**1.3**	**1.2**	**1.0**
																	QVYEEEYGSSLEDDVVGDTSGYYQR	60	1	1	4502107	N-Term(TMT6plex)	3117.4	0	**1.4**	**1.4**	**1.4**	**1.4**
																	SIPAYLAETLYYAMK	133	1	1	4502107	N-Term(TMT6plex); M14(Oxidation); K15(TMT6plex)	2208.2	0	**1.1**	**1.4**	**1.3**	**1.1**
																	KNFATSLYSMIK	4	1	1	4502107	N-Term(TMT6plex); K1(TMT6plex); K12(TMT6plex)	2090.2	1	**1.1**	**1.7**	**1.6**	**1.1**
																	KNFATSLYSMIK	8	1	1	4502107	N-Term(TMT6plex); K1(TMT6plex); M10(Oxidation); K12(TMT6plex)	2106.2	1	**1.3**	**1.6**	**1.4**	**1.4**
																	SEIDLFNIR	41	1	1	4502107	N-Term(TMT6plex)	1335.7	0	**1.1**	**1.3**	**1.2**	**1.0**
																	NFATSLYSMIK	46	1	1	4502107	N-Term(TMT6plex); M9(Oxidation); K11(TMT6plex)	1749.0	0	**1.3**	**1.2**	**1.3**	**1.1**
																	GDTSGDYK	19	1	1	4502107	N-Term(TMT6plex); K8(TMT6plex)	1300.7	0	**1.1**	**1.3**	**1.1**	**1.0**
																	NFATSLYSMIK	14	1	1	4502107	N-Term(TMT6plex); K11(TMT6plex)	1733.0	0	**1.3**	**1.5**	**1.7**	**1.0**
																	QEISAAFK	26	1	1	4502107	N-Term(TMT6plex); K8(TMT6plex)	1351.8	0	**1.1**	**1.2**	**1.2**	**1.0**
																	LYDAYELK	29	1	1	4502107	N-Term(TMT6plex); K8(TMT6plex)	1472.8	0	**1.1**	**1.3**	**1.2**	**1.0**
																	FITIFGTR	35	1	1	4502107	N-Term(TMT6plex)	1183.7	0	**1.2**	**1.1**	**1.1**	**0.8**
																	MLVVLLQANRDPDAGIDEAQVEQDAQALFQAGELK	2	1	1	4502107	N-Term(TMT6plex); M1(Oxidation); K35(TMT6plex)	4270.3	1	**1.2**	**1.2**	**0.9**	**1.2**
																	YMTISGFQIEETIDR	44	1	1	4502107	N-Term(TMT6plex); M2(Oxidation)	2048.0	0	**1.3**	**1.3**	**1.4**	**1.1**
																	SIPAYLAETLYYAMK	23	1	1	4502107	N-Term(TMT6plex); K15(TMT6plex)	2192.2	0	**1.4**	**1.8**	**1.7**	**1.2**
																	ALLLLCGEDD	26	1	1	4502107	N-Term(TMT6plex); C6(Carbamidomethyl)	1347.7	0	**1.3**	**1.4**	**1.4**	**1.2**
																	MLVVLLQANR	13	1	1	4502107	N-Term(TMT6plex)	1385.9	0	**1.5**	**1.2**	**1.7**	**1.1**
																	GAGTDDHTLIR	22	1	1	4502107	N-Term(TMT6plex)	1384.7	0	**1.1**	**1.4**	**1.3**	**1.2**
																	YMTISGFQIEETIDRETSGNLEQLLLAVVK	4	1	1	4502107	N-Term(TMT6plex); M2(Oxidation); K30(TMT6plex)	3872.1	1	**1.4**	**1.4**	**1.3**	**1.0**
																	MLVVLLQANR	20	1	1	4502107	N-Term(TMT6plex); M1(Oxidation)	1401.8	0	**1.3**	**1.2**	**1.3**	**1.2**
																	SIPAYLAETLYYAMKGAGTDDHTLIR	2	1	1	4502107	N-Term(TMT6plex); K15(TMT6plex)	3328.8	1	**0.7**	**0.7**	**0.7**	**0.8**
																	ADAETLR	1	1	1	4502107	N-Term(TMT6plex)	1004.6	0	**1.4**	**1.2**	**1.2**	**1.2**
																	SELTGK	3	1	1	4502107	N-Term(TMT6plex); K6(TMT6plex)	1092.7	0	**1.1**	**1.2**	**1.2**	**1.1**

Accession	RefSeq Accession	Gene Symbol	Entrez Gene ID	Description	Σ Coverage	Σ# Proteins	Σ# Unique Peptides	Σ# Peptides	Σ# PSMs	Het-1A-Smoke - 2M/Parental	Het-1A-Smoke - 4M/Parental	Het-1A-Smoke - 6M/Parental	Het-1A-Smoke - 8M/Parental	# AAs	MW [kDa]	calc. pI	Sequence	# PSMs	# Proteins	# Protein Groups	Protein Group Accessions	Modifications	MH+ [Da]	# Missed Cleavages	Het-1A-Smoke - 2M/Parental	Het-1A-Smoke - 4M/Parental	Het-1A-Smoke - 6M/Parental	Het-1A-Smoke - 8M/Parental
																	GAGTNEK	4	1	1	4502107	N-Term(TMT6plex); K7(TMT6plex)	1134.7	0	**1.0**	**1.5**	**1.2**	**1.0**
256222415	NP_001157791.1	**FLNB**	2317	**filamin-B isoform 4**	78.28	3	1	139	1731	**1.3**	**1.8**	**1.8**	**1.3**	2578	275.5	5.78												
																	DAGYGGISLAVEGPSK	24	4	2	256222415;256222411	N-Term(TMT6plex); K16(TMT6plex)	1979.1	0				
																	VGEPGQAGNPALVSAYGTGLEGGTTGIQSEFFINTTR	23	4	2	256222415;256222411	N-Term(TMT6plex)	3926.0	0				
																	VDIQTEDLEDGTCK	24	4	2	256222415;256222411	N-Term(TMT6plex); C13(Carbamidomethyl); K14(TMT6plex)	2081.1	0				
																	DGTYAVTYIPDK	12	4	2	256222415;256222411	N-Term(TMT6plex); K12(TMT6plex)	1801.0	0				
																	GEAGVPAEFSIWTR	16	4	2	256222415;256222411	N-Term(TMT6plex)	1748.9	0				
																	VLSEDEEDVDFDIIHNANDTFTVK	16	4	2	256222415;256222411	N-Term(TMT6plex); K24(TMT6plex)	3223.6	0				
																	VHSPSGAVEECHVSELEPDK	8	4	2	256222415;256222411	N-Term(TMT6plex); C11(Carbamidomethyl); K20(TMT6plex)	2664.3	0				
																	IPYLPITNFNQNWQDGK	24	4	2	256222415;256222411	N-Term(TMT6plex); K17(TMT6plex)	2506.4	0				
																	DAGEGLLAVQITDQEGKPK	16	4	2	256222415;256222411	N-Term(TMT6plex); K17(TMT6plex); K19(TMT6plex)	2656.5	0				
																	VTASGPGLSSYGVPASLPVDFAIDAR	20	4	2	256222415;256222411	N-Term(TMT6plex)	2776.5	0				
																	VSYFPTVPGVYIVSTK	39	4	2	256222415;256222411	N-Term(TMT6plex); K16(TMT6plex)	2215.3	0				
																	LDVTILSPSR	9	4	2	256222415;256222411	N-Term(TMT6plex)	1329.8	0				
																	EAMQQADDWLGVPQVITPEEIIHPDVDEHSVMTYLSQFPK	26	4	2	256222415;256222411	N-Term(TMT6plex); M3(Oxidation); M32(Oxidation); K40(TMT6plex)	5083.5	0				
																	IQQNTFTR	7	8	4	116063573;256222415;256222411;188595687	N-Term(TMT6plex)	1236.7	0				
																	IPEINSSDMSAHVTSPSGR	18	4	2	256222415;256222411	N-Term(TMT6plex); M9(Oxidation)	2230.1	0				
																	FVPQEMGVHTVSVK	22	4	2	256222415;256222411	N-Term(TMT6plex); M6(Oxidation); K14(TMT6plex)	2032.1	0				
																	AEGPGLSK	6	4	2	256222415;256222411	N-Term(TMT6plex); K8(TMT6plex)	1216.7	0				
																	TATFTIVTEDAGEGGLDLAIEGPSK	9	4	2	256222415;256222411	N-Term(TMT6plex); K25(TMT6plex)	2950.6	0				
																	YAPTEVGLHEMHIK	11	4	2	256222415;256222411	N-Term(TMT6plex); M11(Oxidation); K14(TMT6plex)	2099.1	0				
																	ADIEMPFDPSK	18	4	2	256222415;256222411	N-Term(TMT6plex); M5(Oxidation); K11(TMT6plex)	1723.9	0				
																	LVSIDSK	7	8	4	116063573;256222415;256222411;188595687	N-Term(TMT6plex); K7(TMT6plex)	1219.8	0				
																	VTEAEIVPMGK	22	4	2	256222415;256222411	N-Term(TMT6plex); M9(Oxidation); K11(TMT6plex)	1647.9	0				
																	VPVKDVVDPSK	2	4	2	256222415;256222411	N-Term(TMT6plex); K4(TMT6plex); K11(TMT6plex)	1870.2	1				
																	VVPCLVTPVTGR	5	4	2	256222415;256222411	N-Term(TMT6plex); C4(Carbamidomethyl)	1526.9	0				
																	VGEAGLLSVDCSEAGPGALGLEAVSDSGTK	24	4	2	256222415;256222411	N-Term(TMT6plex); C11(Carbamidomethyl); K30(TMT6plex)	3304.7	0				
																	APSVATVGSICDLNLK	30	4	2	256222415;256222411	N-Term(TMT6plex); C11(Carbamidomethyl); K16(TMT6plex)	2103.2	0				
																	DGTCTVTYLPTLPGDYSILVK	30	4	2	256222415;256222411	N-Term(TMT6plex); C4(Carbamidomethyl); K21(TMT6plex)	2771.5	0				
																	YMIGVTYGGDDIPLSPYR	14	4	2	256222415;256222411	N-Term(TMT6plex)	2246.1	0				
																	NTVELLVEDK	25	4	2	256222415;256222411	N-Term(TMT6plex); K10(TMT6plex)	1617.9	0				
																	AHIANPSGASTECFVTDNADGTYQVEYTPFEK	10	4	2	256222415;256222411	N-Term(TMT6plex); C13(Carbamidomethyl); K32(TMT6plex)	3977.9	0				
																	GAGIGGLGITVEGPSESK	14	4	2	256222415;256222411	N-Term(TMT6plex); K18(TMT6plex)	2087.2	0				
																	QKDFLDGVYAFEYYPSTPGR	14	4	2	256222415;256222411	N-Term(TMT6plex); K2(TMT6plex)	2811.4	1				
																	AEITFDDHK	16	4	2	256222415;256222411	N-Term(TMT6plex); K9(TMT6plex)	1533.8	0				
																	DGSCSAEYIPFAPGDYDVNITYGGAHIPGSPFR	13	4	2	256222415;256222411	N-Term(TMT6plex); C4(Carbamidomethyl)	3759.8	0				
																	LLGWIQNK	32	8	4	116063573;256222415;256222411;188595687	N-Term(TMT6plex); K8(TMT6plex)	1429.9	0				
																	FNDEHIPESPYLVPVIAPSDDAR	16	4	2	256222415;256222411	N-Term(TMT6plex)	2810.4	0				
																	AEISCIDNK	6	4	2	256222415;256222411	N-Term(TMT6plex); C5(Carbamidomethyl); K9(TMT6plex)	1507.8	0				
																	TGEEVGFVVDAK	18	4	2	256222415;256222411	N-Term(TMT6plex); K12(TMT6plex)	1708.9	0				
																	WCNEHLK	8	8	4	116063573;256222415;256222411;188595687	N-Term(TMT6plex); C2(Carbamidomethyl); K7(TMT6plex)	1444.8	0				
																	VEVGKDQEFTVDTR	9	4	2	256222415;256222411	N-Term(TMT6plex); K5(TMT6plex)	2081.1	1				
																	AGVENGKPTHFTVYTK	2	4	2	256222415;256222411	N-Term(TMT6plex); K7(TMT6plex); K16(TMT6plex)	2436.4	0				
																	AGSNMLLIGVHGPTTPCEEVSMK	1	4	2	256222415;256222411	N-Term(TMT6plex); C17(Carbamidomethyl); K23(TMT6plex)	2886.5	0				
																	VAVTEGCQPSR	8	4	2	256222415;256222411	N-Term(TMT6plex); C7(Carbamidomethyl)	1432.7	0				
																	GDYVLAVK	9	4	2	256222415;256222411	N-Term(TMT6plex); K8(TMT6plex)	1322.8	0				
																	LVSPGSANETSSILVESVTR	22	4	2	256222415;256222411	N-Term(TMT6plex)	2275.2	0				
																	IEYNDQNDGSCDVK	18	4	2	256222415;256222411	N-Term(TMT6plex); C11(Carbamidomethyl); K14(TMT6plex)	2115.0	0				
																	AAGSGELGVTMK	26	4	2	256222415;256222411	N-Term(TMT6plex); M11(Oxidation); K12(TMT6plex)	1594.9	0				
																	QMQLENVSVALEFLDR	41	6	3	116063573;256222415;256222411	N-Term(TMT6plex); M2(Oxidation)	2137.1	0				
																	YMIGVTYGGDDIPLSPYR	23	4	2	256222415;256222411	N-Term(TMT6plex); M2(Oxidation)	2262.1	0				
																	YTPTQQGNMQVLVTYGGDPIPK	9	4	2	256222415;256222411	N-Term(TMT6plex); M9(Oxidation); K22(TMT6plex)	2881.5	0				
																	ANEPTHFTVDCTEAGEGDVSVGIK	15	4	2	256222415;256222411	N-Term(TMT6plex); C11(Carbamidomethyl); K24(TMT6plex)	2991.5	0				
																	SPFEVQVGPEAGMQK	18	4	2	256222415;256222411	N-Term(TMT6plex); M13(Oxidation); K15(TMT6plex)	2078.1	0				
																	AGLAPLEVR	10	4	2	256222415;256222411	N-Term(TMT6plex)	1154.7	0				
																	IFAQDGEGQR	5	4	2	256222415;256222411	N-Term(TMT6plex)	1349.7	0				
																	YGGELVPHFPAR	8	4	2	256222415;256222411	N-Term(TMT6plex)	1571.9	0				
																	TFEMSDFIVDTR	49	4	2	256222415;256222411	N-Term(TMT6plex); M4(Oxidation)	1705.8	0				
																	YGGPNHIVGSPFK	6	4	2	256222415;256222411	N-Term(TMT6plex); K13(TMT6plex)	1831.0	0				
																	AGSNMLLIGVHGPTTPCEEVSMK	9	4	2	256222415;256222411	N-Term(TMT6plex); M5(Oxidation); C17(Carbamidomethyl); M22(Oxidation); K23(TMT6plex)	2918.5	0				
																	WGEEHIPGSPFHVTVP	3	4	2	256222415;256222411	N-Term(TMT6plex)	2018.0	0				
																	EEGLYAVDVTYDGHPVPGSPYTVEASLPPDPSK	7	4	2	256222415;256222411	N-Term(TMT6plex); K33(TMT6plex)	3945.0	0				
																	EVGEHLVSIK	3	4	2	256222415;256222411	N-Term(TMT6plex); K10(TMT6plex)	1568.9	0				
																	LGSAADFLLDISETDLSSLTASIK	22	4	2	256222415;256222411	N-Term(TMT6plex); K24(TMT6plex)	2925.6	0				
																	IGNLQTDLSDGLR	14	4	2	256222415;256222411	N-Term(TMT6plex)	1630.9	0				
																	TFEMSDFIVDTR	6	4	2	256222415;256222411	N-Term(TMT6plex)	1689.8	0				
																	LTVMSLQESGLK	8	4	2	256222415;256222411	N-Term(TMT6plex); M4(Oxidation); K12(TMT6plex)	1780.0	0				
																	VTEAEIVPMGK	4	4	2	256222415;256222411	N-Term(TMT6plex); K11(TMT6plex)	1631.9	0				
																	VNQPASFAIR	4	4	2	256222415;256222411	N-Term(TMT6plex)	1331.8	0				
																	VLQSFTVDSSK	8	4	2	256222415;256222411	N-Term(TMT6plex); K11(TMT6plex)	1669.0	0				
																	VMYTPMAPGNYLISVK	10	4	2	256222415;256222411	N-Term(TMT6plex); M2(Oxidation); M6(Oxidation); K16(TMT6plex)	2274.2	0				
																	IECSDNGDGTCSVSYLPTKPGEYFVNILFEEVHIPGSPFK	3	4	2	256222415;256222411	N-Term(TMT6plex); C3(Carbamidomethyl); C11(Carbamidomethyl); K19(TMT6plex); K40(TMT6plex)	5190.6	0				
																	VKAEGPGLSK	2	4	2	256222415;256222411	N-Term(TMT6plex); K2(TMT6plex); K10(TMT6plex)	1673.1	1				
																	QMQLENVSVALEFLDR	5	6	3	116063573;256222415;256222411	N-Term(TMT6plex)	2121.1	0				
																	DGTYAVTYVPLTAGMYTLTMK	2	4	2	256222415;256222411	N-Term(TMT6plex); M20(Oxidation); K21(TMT6plex)	2770.4	0				
																	AAGSGELGVTMK	3	4	2	256222415;256222411	N-Term(TMT6plex); K12(TMT6plex)	1578.9	0				
																	GIEPTGNMVK	2	4	2	256222415;256222411	N-Term(TMT6plex); K10(TMT6plex)	1503.9	0				
																	DGTYAVTYVPLTAGMYTLTMK	13	4	2	256222415;256222411	N-Term(TMT6plex); M15(Oxidation); M20(Oxidation); K21(TMT6plex)	2786.4	0				
																	SADFVVESIGSEVGSLGFAIEGPSQAK	48	4	2	256222415;256222411	N-Term(TMT6plex); K27(TMT6plex)	3139.7	0				
																	DFLDGVYAFEYYPSTPGR	17	4	2	256222415;256222411	N-Term(TMT6plex)	2326.1	0				
																	SGCIVNNLAEFTVDPK	4	4	2	256222415;256222411	N-Term(TMT6plex); C3(Carbamidomethyl); K16(TMT6plex)	2222.2	0				
																	VTVLFAGQHISK	8	4	2	256222415;256222411	N-Term(TMT6plex); K12(TMT6plex)	1758.1	0				
																	AGPGTLSVTIEGPSK	11	4	2	256222415;256222411	N-Term(TMT6plex); K15(TMT6plex)	1872.1	0				
																	LPNNHIGISFIPR	2	4	2	256222415;256222411	N-Term(TMT6plex)	1707.0	0				
																	HIPGSPFTAK	14	6	3	256222415;256222411;188595687	N-Term(TMT6plex); K10(TMT6plex)	1512.9	0				
																	IAGPGLGSGVR	7	4	2	256222415;256222411	N-Term(TMT6plex)	1212.7	0				
																	SPFTVGVAAPLDLSK	10	4	2	256222415;256222411	N-Term(TMT6plex); K15(TMT6plex)	1960.1	0				
																	MDCQETPEGYK	2	4	2	256222415;256222411	N-Term(TMT6plex); C3(Carbamidomethyl); K11(TMT6plex)	1815.9	0				
																	AGSNMLLIGVHGPTTPCEEVSMK	4	4	2	256222415;256222411	N-Term(TMT6plex); C17(Carbamidomethyl); M22(Oxidation); K23(TMT6plex)	2902.5	0				
																	VVASGPGLEHGK	5	4	2	256222415;256222411	N-Term(TMT6plex); K12(TMT6plex)	1608.9	0				
																	DEPCLLK	5	4	2	256222415;256222411	N-Term(TMT6plex); C4(Carbamidomethyl); K7(TMT6plex)	1332.8	0				
																	LIALLEVLSQK	22	8	4	116063573;256222415;256222411;188595687	N-Term(TMT6plex); K11(TMT6plex)	1685.1	0				
																	GAGTGGLGLTVEGPCEAK	18	6	3	256222415;256222411;188595687	N-Term(TMT6plex); C15(Carbamidomethyl); K18(TMT6plex)	2132.1	0				
																	AWGPGLHGGIVGR	34	4	2	256222415;256222411	N-Term(TMT6plex)	1505.9	0				
																	SSTETCYSAIPK	16	4	2	256222415;256222411	N-Term(TMT6plex); C6(Carbamidomethyl); K12(TMT6plex)	1801.9	0				
																	APLNVQFNSPLPGDAVK	22	4	2	256222415;256222411	N-Term(TMT6plex); K17(TMT6plex)	2225.3	0				
																	DLAEDAPWK	24	8	4	116063573;256222415;256222411;188595687	N-Term(TMT6plex); K9(TMT6plex)	1502.8	0				
																	ADIEMPFDPSK	8	4	2	256222415;256222411	N-Term(TMT6plex); K11(TMT6plex)	1707.9	0				
																	CLATGPGIASTVK	6	4	2	256222415;256222411	N-Term(TMT6plex); C1(Carbamidomethyl); K13(TMT6plex)	1733.0	0				
																	EAMQQADDWLGVPQVITPEEIIHPDVDEHSVMTYLSQFPK	19	4	2	256222415;256222411	N-Term(TMT6plex); M3(Oxidation); K40(TMT6plex)	5067.5	0				
																	IPEINSSDMSAHVTSPSGR	5	4	2	256222415;256222411	N-Term(TMT6plex)	2214.1	0				
																	VLPTYDASK	12	4	2	256222415;256222411	N-Term(TMT6plex); K9(TMT6plex)	1451.8	0				
																	RLPNNHIGISFIPR	12	4	2	256222415;256222411	N-Term(TMT6plex)	1863.1	1				
																	IFFAGDTIPK	24	4	2	256222415;256222411	N-Term(TMT6plex); K10(TMT6plex)	1566.9	0				
																	AEVSIQNNK	11	4	2	256222415;256222411	N-Term(TMT6plex); K9(TMT6plex)	1460.8	0				

Accession	RefSeq Accession	Gene Symbol	Entrez Gene ID	Description	Σ Coverage	Σ# Proteins	Σ# Unique Peptides	Σ# Peptides	Σ# PSMs	Het-1A-Smoke - 2M/Parental	Het-1A-Smoke - 4M/Parental	Het-1A-Smoke - 6M/Parental	Het-1A-Smoke - 8M/Parental	# AAs	MW [kDa]	calc. pI	Sequence	# PSMs	# Proteins	# Protein Groups	Protein Group Accessions	Modifications	MH+ [Da]	# Missed Cleavages	Het-1A-Smoke - 2M/Parental	Het-1A-Smoke - 4M/Parental	Het-1A-Smoke - 6M/Parental	Het-1A-Smoke - 8M/Parental
																	TYSVEYLPK	14	4	2	256222415;256222411	N-Term(TMT6plex); K9(TMT6plex)	1557.9	0				
																	GLVEPVNVVDNGDGTHTVTYTPSQEGPYMVSVK	6	3	1	256222415	N-Term(TMT6plex); M29(Oxidation); K33(TMT6plex)	3964.0	0	**1.3**	**1.8**	**1.8**	**1.3**
																	VLFASQEIPASPFR	11	4	2	256222415;256222411	N-Term(TMT6plex)	1791.0	0				
																	YSIAITWGGHHIPK	4	4	2	256222415;256222411	N-Term(TMT6plex); K14(TMT6plex)	2038.2	0				
																	GAGGQGK	3	6	3	116063573;256222415;256222411	N-Term(TMT6plex); K7(TMT6plex)	1032.6	0				
																	SSFLVDCSK	12	4	2	256222415;256222411	N-Term(TMT6plex); C7(Carbamidomethyl); K9(TMT6plex)	1500.8	0				
																	AHGPGLEGGLVGKPAEFTIDTK	6	4	2	256222415;256222411	N-Term(TMT6plex); K13(TMT6plex); K22(TMT6plex)	2881.7	0				
																	GLHVVEVTYDDVPIPNSPFK	20	4	2	256222415;256222411	N-Term(TMT6plex); K20(TMT6plex)	2684.5	0				
																	VMYTPMAPGNYLISVK	3	4	2	256222415;256222411	N-Term(TMT6plex); M6(Oxidation); K16(TMT6plex)	2258.2	0				
																	TATPEIVDNK	15	4	2	256222415;256222411	N-Term(TMT6plex); K10(TMT6plex)	1545.9	0				
																	LTVMSLQESGLK	2	4	2	256222415;256222411	N-Term(TMT6plex); K12(TMT6plex)	1764.0	0				
																	VQAQGPGLK	17	4	2	256222415;256222411	N-Term(TMT6plex); K9(TMT6plex)	1355.8	0				
																	AIVDGNLK	6	8	4	116063573;256222415;256222411;188595687	N-Term(TMT6plex); K8(TMT6plex)	1287.8	0				
																	ATQTGDASK	7	4	2	256222415;256222411	N-Term(TMT6plex); K9(TMT6plex)	1336.7	0				
																	ASSDASK	2	4	2	256222415;256222411	N-Term(TMT6plex); K7(TMT6plex)	1123.6	0				
																	FADEHVPGSPFTVK	14	4	2	256222415;256222411	N-Term(TMT6plex); K14(TMT6plex)	1989.1	0				
																	HVGNQQYNVTYVVK	4	4	2	256222415;256222411	N-Term(TMT6plex); K14(TMT6plex)	2107.2	0				
																	AIVHDNKDGTYAVTYIPDK	1	4	2	256222415;256222411	N-Term(TMT6plex); K7(TMT6plex); K19(TMT6plex)	2807.6	1				
																	TATPEIVDNKDGTVTVR	2	4	2	256222415;256222411	N-Term(TMT6plex); K10(TMT6plex)	2274.3	1				
																	EAFTNKPNVFTVVTR	5	4	2	256222415;256222411	N-Term(TMT6plex); K6(TMT6plex)	2181.2	0				
																	VKVEPAVDTSR	3	4	2	256222415;256222411	N-Term(TMT6plex); K2(TMT6plex)	1659.0	1				
																	DLDIIDNYDYSHTVK	12	4	2	256222415;256222411	N-Term(TMT6plex); K15(TMT6plex)	2269.2	0				
																	VNIGQGSHPQK	6	4	2	256222415;256222411	N-Term(TMT6plex); K11(TMT6plex)	1622.9	0				
																	FIPHENGVHTIDVK	2	4	2	256222415;256222411	N-Term(TMT6plex); K14(TMT6plex)	2064.2	0				
																	YMGSHIPESPLQFYVNYPNSGSVSAYGPGLVYGVANK	6	4	2	256222415;256222411	N-Term(TMT6plex); M2(Oxidation); K37(TMT6plex)	4437.2	0				
																	SPFEVSVDK	8	4	2	256222415;256222411	N-Term(TMT6plex); K9(TMT6plex)	1465.8	0				
																	VMYTPMAPGNYLISVK	1	4	2	256222415;256222411	N-Term(TMT6plex); K16(TMT6plex)	2242.2	0				
																	GQHVTGSPFQFTVGPLGEGGAHK	10	4	2	256222415;256222411	N-Term(TMT6plex); K23(TMT6plex)	2766.5	0				
																	MDCQETPEGYK	8	4	2	256222415;256222411	N-Term(TMT6plex); M1(Oxidation); C3(Carbamidomethyl); K11(TMT6plex)	1831.9	0				
																	MDGTYACSYTPVK	10	4	2	256222415;256222411	N-Term(TMT6plex); M1(Oxidation); C7(Carbamidomethyl); K13(TMT6plex)	1967.0	0				
																	VEPAVDTSR	5	4	2	256222415;256222411	N-Term(TMT6plex)	1202.7	0				
																	EAGAGGLSIAVEGPSK	12	6	3	256222415;256222411;188595687	N-Term(TMT6plex); K16(TMT6plex)	1901.1	0				
																	GPGLEAVGNIANKPTYFDIYTAGAGVGDIGVEVEDPQGK	2	4	2	256222415;256222411	N-Term(TMT6plex); K13(TMT6plex); K39(TMT6plex)	4606.4	0				
																	VKVDPSHDASK	4	4	2	256222415;256222411	N-Term(TMT6plex); K2(TMT6plex); K11(TMT6plex)	1870.1	1				
																	HTIAVVWGGVNIPHSPYR	3	4	2	256222415;256222411	N-Term(TMT6plex)	2232.2	0				
																	AYGPGLEK	6	4	2	256222415;256222411	N-Term(TMT6plex); K8(TMT6plex)	1292.8	0				
																	YADEEIPR	1	4	2	256222415;256222411	N-Term(TMT6plex)	1221.6	0				
																	VDPSHDASK	4	6	3	256222415;256222411;188595687	N-Term(TMT6plex); K9(TMT6plex)	1413.8	0				
																	ALGALVDSCAPGLCPDWESWDPQKPVDNAR	8	4	2	256222415;256222411	N-Term(TMT6plex); C9(Carbamidomethyl); C14(Carbamidomethyl); K24(TMT6plex)	3782.9	0				
																	SPFVVQVGEACNPNACR	2	4	2	256222415;256222411	N-Term(TMT6plex); C11(Carbamidomethyl); C16(Carbamidomethyl)	2134.0	0				
																	EPGEYAVHIMCDDEDIKDSPYMAFIHPATGGYNPDLVR	1	4	2	256222415;256222411	N-Term(TMT6plex); M10(Oxidation); C11(Carbamidomethyl); K17(TMT6plex); M22(Oxidation)	4813.3	1				
																	KGEITGEVHMPSGK	1	4	2	256222415;256222411	N-Term(TMT6plex); K1(TMT6plex); M10(Oxidation); K14(TMT6plex)	2173.2	1				
																	GIEPTGNMVK	5	4	2	256222415;256222411	N-Term(TMT6plex); M8(Oxidation); K10(TMT6plex)	1519.9	0				
																	VFGPGIEGK	3	4	2	256222415;256222411	N-Term(TMT6plex); K9(TMT6plex)	1361.8	0				
																	AGGPGLER	5	6	3	116063573;256222415;256222411	N-Term(TMT6plex)	985.6	0				
																	GLEELVK	6	4	2	256222415;256222411	N-Term(TMT6plex); K7(TMT6plex)	1245.8	0				
																	AIVHDNK	2	4	2	256222415;256222411	N-Term(TMT6plex); K7(TMT6plex)	1254.8	0				
																	LKPGAPLKPK	3	4	2	256222415;256222411	N-Term(TMT6plex); K2(TMT6plex); K8(TMT6plex); K10(TMT6plex)	1965.3	0				
																	GAGLSK	2	4	2	256222415;256222411	N-Term(TMT6plex); K6(TMT6plex)	990.6	0				
																	DVVDPSK	1	4	2	256222415;256222411	N-Term(TMT6plex); K7(TMT6plex)	1217.7	0				
																	AQGDASK	3	4	2	256222415;256222411	N-Term(TMT6plex); K7(TMT6plex)	1134.7	0				
																	DGTVTVR	1	6	3	116063573;256222415;256222411	N-Term(TMT6plex)	976.6	0				
14141166	NP_114366.1	**PCBP2**	5094	**poly(rC)-binding protein 2 isoform b**	78.18	9	1	17	238	**1.5**	**1.8**	**2.0**	**1.7**	362	38.2	6.79												
																	YSTGSDSASFPHTTPSMCLNPDLEGPPLEAYTIQGQYAIPQPDLTK	2	4	2	193083110;14141166	N-Term(TMT6plex); M17(Oxidation); C18(Carbamidomethyl); K46(TMT6plex)	5469.7	0				
																	EVGSIIGK	22	12	3	193083110;222352151;14141166	N-Term(TMT6plex); K8(TMT6plex)	1260.8	0				
																	LSSETGGMGSS	10	7	2	193083110;14141166	N-Term(TMT6plex); M8(Oxidation)	1257.6	0				
																	LEEDISSSMTNSTAASRPPVTLR	9	7	2	193083110;14141166	N-Term(TMT6plex); M9(Oxidation)	2707.4	0				
																	INISEGNCPER	33	10	3	193083110;222352151;14141166	N-Term(TMT6plex); C8(Carbamidomethyl)	1517.8	0				
																	AFAMIIDKLEEDISSSMTNSTAASRPPVTLR	3	7	2	193083110;14141166	N-Term(TMT6plex); M4(Oxidation); K8(TMT6plex); M17(Oxidation)	3842.0	1				
																	IITLAGPTNAIFK	36	7	2	193083110;14141166	N-Term(TMT6plex); K13(TMT6plex)	1817.1	0				
																	ESTGAQVQVAGDMLPNSTER	14	10	3	193083110;222352151;14141166	N-Term(TMT6plex); M13(Oxidation)	2335.1	0				
																	AFAMIIDK	6	8	3	193083110;222352151;14141166	N-Term(TMT6plex); M4(Oxidation); K8(TMT6plex)	1382.8	0				
																	AITIAGIPQSIIECVK	29	7	2	193083110;14141166	N-Term(TMT6plex); C14(Carbamidomethyl); K16(TMT6plex)	2171.3	0				
																	LVVPASQCGSLIGK	16	9	2	193083110;14141166	N-Term(TMT6plex); C8(Carbamidomethyl); K14(TMT6plex)	1887.1	0				
																	GYWAGLDASAQTTSHELTIPNDLIGCIIGR	10	4	1	14141166	N-Term(TMT6plex); C26(Carbamidomethyl)	3458.8	0	**1.5**	**1.8**	**2.0**	**1.7**
																	QICVVMLESPPK	20	5	2	14141166;193083110	N-Term(TMT6plex); C3(Carbamidomethyl); M6(Oxidation); K12(TMT6plex)	1875.0	0				
																	AFAMIIDKLEEDISSSMTNSTAASRPPVTLR	1	7	2	193083110;14141166	N-Term(TMT6plex); K8(TMT6plex); M17(Oxidation)	3826.0	1				
																	QICVVMLESPPK	8	5	2	14141166;193083110	N-Term(TMT6plex); C3(Carbamidomethyl); K12(TMT6plex)	1859.1	0				
																	ESTGAQVQVAGDMLPNSTER	6	10	3	193083110;222352151;14141166	N-Term(TMT6plex)	2319.1	0				
																	QVTITGSAASISLAQYLINVR	2	7	2	193083110;14141166	N-Term(TMT6plex)	2434.4	0				
																	IANPVEGSTDR	9	7	2	193083110;14141166	N-Term(TMT6plex)	1387.7	0				
																	LHQLAMQQSHFPMTHGNTGFSGIESSPEVK	1	6	2	193083110;14141166	N-Term(TMT6plex); M6(Oxidation); M13(Oxidation); K31(TMT6plex)	3872.9	0				
																	QMSGAQIK	1	10	3	193083110;222352151;14141166	N-Term(TMT6plex); M2(Oxidation); K8(TMT6plex)	1336.8	0				
4757900	NP_004334.1	**CALR**	811	**calreticulin precursor**	78.18	1	29	29	1274	**1.2**	**1.5**	**1.3**	**1.1**	417	48.1	4.44												
																	FYALSASFEPFSNK	144	1	1	4757900	N-Term(TMT6plex); K14(TMT6plex)	2066.1	0	**1.2**	**1.3**	**1.3**	**1.0**
																	EQFLDGDGWTSR	61	1	1	4757900	N-Term(TMT6plex)	1639.8	0	**1.3**	**1.5**	**1.3**	**1.3**
																	KPEDWDEEMDGEWEPPVIQNPEYK	76	1	1	4757900	N-Term(TMT6plex); K1(TMT6plex); M9(Oxidation); K24(TMT6plex)	3663.8	0	**1.2**	**1.7**	**1.4**	**1.3**
																	IDDPTDSKPEDWDKPEHIPDPDAK	6	1	1	4757900	N-Term(TMT6plex); K8(TMT6plex); K14(TMT6plex); K24(TMT6plex)	3676.9	0	**1.2**	**1.6**	**1.4**	**1.1**
																	AKIDDPTDSKPEDWDKPEHIPDPDAK	1	1	1	4757900	N-Term(TMT6plex); K2(TMT6plex); K10(TMT6plex); K16(TMT6plex); K26(TMT6plex)	4105.2	1	**0.9**	**1.7**	**1.2**	**1.2**
																	DKGLQTSQDAR	2	1	1	4757900	N-Term(TMT6plex); K2(TMT6plex)	1676.9	1	**1.2**	**1.6**	**1.2**	**1.2**
																	LKEEEEDK	18	1	1	4757900	N-Term(TMT6plex); K2(TMT6plex); K8(TMT6plex)	1707.0	1	**1.1**	**1.4**	**1.3**	**1.1**
																	DKQDEEQR	17	1	1	4757900	N-Term(TMT6plex); K2(TMT6plex)	1505.8	1	**1.1**	**1.6**	**1.2**	**1.1**
																	SGTIFDNFLITNDEAYAEEFGNETWGVTK	276	1	1	4757900	N-Term(TMT6plex); K29(TMT6plex)	3726.8	0	**1.4**	**1.5**	**1.5**	**1.2**
																	IDNSQVESGSLEDDWDFLPPK	100	1	1	4757900	N-Term(TMT6plex); K21(TMT6plex)	2849.4	0	**1.2**	**1.5**	**1.3**	**1.2**
																	HEQNIDCGGGYVK	58	1	1	4757900	N-Term(TMT6plex); C7(Carbamidomethyl); K13(TMT6plex)	1935.0	0	**1.3**	**1.4**	**1.3**	**1.0**
																	GQTLVVQFTVK	72	1	1	4757900	N-Term(TMT6plex); K11(TMT6plex)	1678.0	0	**1.2**	**1.6**	**1.4**	**1.3**
																	CKDDEFTHLYTLIVRPDNTYEVK	17	1	1	4757900	N-Term(TMT6plex); C1(Carbamidomethyl); K2(TMT6plex); K23(TMT6plex)	3543.9	1	**1.2**	**1.5**	**1.4**	**1.1**
																	IDNSQVESGSLEDDWDFLPPKK	69	1	1	4757900	N-Term(TMT6plex); K21(TMT6plex); K22(TMT6plex)	3206.7	1	**1.1**	**1.6**	**1.4**	**1.2**
																	GLQTSQDAR	23	1	1	4757900	N-Term(TMT6plex)	1204.6	0	**1.2**	**1.5**	**1.2**	**1.1**
																	IKDPDASKPEDWDER	24	1	1	4757900	N-Term(TMT6plex); K2(TMT6plex); K8(TMT6plex)	2488.3	1	**1.2**	**1.5**	**1.3**	**1.1**
																	LFPNSLDQTDMHGDSEYNIMFGPDICGPGTK	18	1	1	4757900	N-Term(TMT6plex); M11(Oxidation); M20(Oxidation); C26(Carbamidomethyl); K31(TMT6plex)	3946.8	0	**1.6**	**2.0**	**1.8**	**1.2**
																	QIDNPDYK	71	1	1	4757900	N-Term(TMT6plex); K8(TMT6plex)	1450.8	0	**1.3**	**1.6**	**1.3**	**1.2**
																	VHVIFNYK	29	1	1	4757900	N-Term(TMT6plex); K8(TMT6plex)	1477.9	0	**1.1**	**1.4**	**1.3**	**1.1**
																	DDEFTHLYTLIVRPDNTYEVK	8	1	1	4757900	N-Term(TMT6plex); K21(TMT6plex)	3026.6	0	**1.2**	**1.4**	**1.4**	**1.2**
																	FYGDEEKDK	15	1	1	4757900	N-Term(TMT6plex); K7(TMT6plex); K9(TMT6plex)	1818.0	1	**1.2**	**1.4**	**1.2**	**1.0**
																	GTWIHPEIDNPEYSPDPSIYAYDNFGVLGLDLWQVK	12	1	1	4757900	N-Term(TMT6plex); K36(TMT6plex)	4607.3	0	**1.2**	**1.7**	**1.4**	**1.1**
																	KVHVIFNYK	32	1	1	4757900	N-Term(TMT6plex); K1(TMT6plex); K9(TMT6plex)	1835.1	1	**1.1**	**1.5**	**1.3**	**1.2**
																	DPDASKPEDWDER	5	1	1	4757900	N-Term(TMT6plex); K6(TMT6plex)	2018.0	0	**1.2**	**1.4**	**1.2**	**1.1**
																	KPEDWDEEMDGEWEPPVIQNPEYK	24	1	1	4757900	N-Term(TMT6plex); K1(TMT6plex); K24(TMT6plex)	3647.8	0	**1.7**	**2.5**	**2.1**	**1.4**

Accession	RefSeq Accession	Gene Symbol	Entrez Gene ID	Description	Σ Coverage	Σ# Proteins	Σ# Unique Peptides	Σ# Peptides	Σ# PSMs	Het-1A-Smoke - 2M/Parental	Het-1A-Smoke - 4M/Parental	Het-1A-Smoke - 6M/Parental	Het-1A-Smoke - 8M/Parental	# AAs	MW [kDa]	calc. pI	Sequence	# PSMs	# Proteins	# Protein Groups	Protein Group Accessions	Modifications	MH+ [Da]	# Missed Cleavages	Het-1A-Smoke - 2M/Parental	Het-1A-Smoke - 4M/Parental	Het-1A-Smoke - 6M/Parental	Het-1A-Smoke - 8M/Parental
																	LFPNSLDQTDMHGDSEYNIMFGPDICGPGTK	19	1	1	4757900	N-Term(TMT6plex); M11(Oxidation); C26(Carbamidomethyl); K31(TMT6plex)	3930.9	0	**1.5**	**2.3**	**2.1**	**1.3**
																	LFPNSLDQTDMHGDSEYNIMFGPDICGPGTK	8	1	1	4757900	N-Term(TMT6plex); C26(Carbamidomethyl); K31(TMT6plex)	3914.8	0	**2.3**	**3.5**	**3.2**	**1.5**
																	KIKDPDASKPEDWDER	2	1	1	4757900	N-Term(TMT6plex); K1(TMT6plex); K3(TMT6plex); K9(TMT6plex)	2845.6	2	**1.2**	**2.5**	**1.5**	**1.5**
																	FVLSSGK	46	1	1	4757900	N-Term(TMT6plex); K7(TMT6plex)	1195.7	0	**1.2**	**1.4**	**1.3**	**1.1**
																	FYGDEEK	11	1	1	4757900	N-Term(TMT6plex); K7(TMT6plex)	1345.7	0	**1.1**	**1.2**	**1.2**	**0.9**
																	NVLINK	9	1	1	4757900	N-Term(TMT6plex); K6(TMT6plex)	1158.8	0	**1.1**	**1.2**	**1.2**	**1.0**
																	GEWKPR	1	1	1	4757900	N-Term(TMT6plex); K4(TMT6plex)	1230.7	0	**1.0**	**1.7**	**1.4**	**1.2**
9506859	NP_061932.1	**TOMM7**	54543	**mitochondrial import receptor subunit TOM7 homolog**	78.18	1	4	4	11	**1.5**	**1.4**	**1.7**	**1.4**	55	6.2	10.29												
																	GADPGMPEPTVLSLLWG	7	1	1	9506859	N-Term(TMT6plex); M6(Oxidation)	1985.0	0	**1.3**	**1.4**	**1.4**	**1.4**
																	GSQFAIR	1	1	1	9506859	N-Term(TMT6plex)	1007.6	0	**1.4**	**1.4**	**1.4**	**1.4**
																	WGFIPLVIYLGFK	1	1	1	9506859	N-Term(TMT6plex); K13(TMT6plex)	2011.2	0	**2.0**	**1.7**	**2.7**	**1.3**
																	GADPGMPEPTVLSLLWG	1	1	1	9506859	N-Term(TMT6plex)	1969.0	0				
																	LQQLFK	1	1	1	9506859	N-Term(TMT6plex); K6(TMT6plex)	1234.8	0				
109637759	NP_001035905.1	**CAST**	831	**calpastatin isoform f**	78.13	3	6	46	317	**0.9**	**0.9**	**1.2**	**0.7**	750	80.2	5.15												
																	SESELIDELSEDFDR	32	5	3	298919189;109637759;545746425	N-Term(TMT6plex)	2012.9	0				
																	GTVPDDAVEALADSLGK	26	5	3	298919189;109637759;545746425	N-Term(TMT6plex); K17(TMT6plex)	2116.1	0				
																	SAEQQPSEK	13	5	3	298919189;109637759;545746425	N-Term(TMT6plex); K9(TMT6plex)	1461.8	0				
																	AAAPAPVSEAVCR	13	5	3	298919189;109637759;545746425	N-Term(TMT6plex); C12(Carbamidomethyl)	1527.8	0				
																	QASDTGSNDAHNK	10	5	3	298919189;109637759;545746425	N-Term(TMT6plex); K13(TMT6plex)	1802.9	0				
																	QPDPDENKPMEDK	4	5	3	298919189;109637759;545746425	N-Term(TMT6plex); K8(TMT6plex); M10(Oxidation); K13(TMT6plex)	2246.2	0				
																	LGEKEETIPPDYR	4	5	3	298919189;109637759;545746425	N-Term(TMT6plex); K4(TMT6plex)	2005.1	1				
																	TSESPSKPGEK	3	1	1	109637759	N-Term(TMT6plex); K7(TMT6plex); K11(TMT6plex)	1834.1	0	**1.0**	**0.9**	**1.3**	**0.7**
																	TSMCSIQSAPPEPATLK	9	5	3	298919189;109637759;545746425	N-Term(TMT6plex); M3(Oxidation); C4(Carbamidomethyl); K17(TMT6plex)	2292.2	0				
																	EQLPPMSEDFLLDALSEDFSGPQNASSLK	11	5	3	298919189;109637759;545746425	N-Term(TMT6plex); K29(TMT6plex)	3623.8	0				
																	EQLPPMSEDFLLDALSEDFSGPQNASSLK	27	5	3	298919189;109637759;545746425	N-Term(TMT6plex); M6(Oxidation); K29(TMT6plex)	3639.8	0				
																	TKPQDMISAGGESVAGITAISGKPGDK	6	4	3	298919189;109637759;545746425	N-Term(TMT6plex); K2(TMT6plex); M6(Oxidation); K23(TMT6plex); K27(TMT6plex)	3548.0	0				
																	CGEDDETIPSEYR	11	5	3	298919189;109637759;545746425	N-Term(TMT6plex); C1(Carbamidomethyl)	1799.8	0				
																	LAAAISEVVSQTPASTTQAGAPPR	20	5	3	298919189;109637759;545746425	N-Term(TMT6plex)	2552.4	0				
																	DDTIPPEYR	7	5	3	298919189;109637759;545746425	N-Term(TMT6plex)	1334.7	0				
																	DTSQSDKDLDDALDK	4	4	2	298919189;109637759	N-Term(TMT6plex); K7(TMT6plex); K15(TMT6plex)	2353.2	1				
																	VSASSGATSK	4	1	1	109637759	N-Term(TMT6plex); K10(TMT6plex)	1352.8	0	**0.9**	**1.0**	**1.2**	**0.7**
																	SLTPAVPVESKPDKPSGK	2	4	3	298919189;109637759;545746425	N-Term(TMT6plex); K11(TMT6plex); K14(TMT6plex); K18(TMT6plex)	2753.7	0				
																	TYAGGTASATK	5	1	1	109637759	N-Term(TMT6plex); K11(TMT6plex)	1485.8	0	**1.0**	**0.8**	**1.1**	**0.7**
																	TTEETSKPK	2	5	3	298919189;109637759;545746425	N-Term(TMT6plex); K7(TMT6plex); K9(TMT6plex)	1708.0	0				
																	DLDDALDK	1	5	3	298919189;109637759;545746425	N-Term(TMT6plex); K8(TMT6plex)	1362.8	0				
																	KEGITGPPADSSKPIGPDDAIDALSSDFTCGSPTAAGK	3	4	2	109637759;545746425	K1(TMT6plex); N-Term(TMT6plex); K13(TMT6plex); C30(Carbamidomethyl); K38(TMT6plex)	4647.4	1				
																	SSSMNPTETK	4	1	1	109637759	N-Term(TMT6plex); M4(Oxidation); K10(TMT6plex)	1555.8	0	**0.8**	**0.8**	**1.0**	**0.6**
																	EGITGPPADSSKPIGPDDAIDALSSDFTCGSPTAAGK	2	4	2	109637759;545746425	N-Term(TMT6plex); K12(TMT6plex); C29(Carbamidomethyl); K37(TMT6plex)	4290.2	0				
																	SGMDAALDDLIDTLGGPEETEEENTTYTGPEVSDPMSSTYIEELGK	4	5	3	298919189;109637759;545746425	N-Term(TMT6plex); M3(Oxidation); M36(Oxidation); K46(TMT6plex)	5397.5	0				
																	QASDTGSNDAHNKK	3	5	3	298919189;109637759;545746425	N-Term(TMT6plex); K13(TMT6plex); K14(TMT6plex)	2160.2	1				
																	HLLDDNGQDKPVKPPTK	3	5	3	298919189;109637759;545746425	N-Term(TMT6plex); K10(TMT6plex); K13(TMT6plex); K17(TMT6plex)	2818.6	0				
																	DTMSDQALEALSASLGTR	22	5	3	298919189;109637759;545746425	N-Term(TMT6plex); M3(Oxidation)	2111.0	0				
																	KPADDQDPIDALSGDLDSCPSTTETSQNTAK	11	5	3	298919189;109637759;545746425	N-Term(TMT6plex); K1(TMT6plex); C19(Carbamidomethyl); K31(TMT6plex)	3965.0	0				
																	SAAPPQEK	6	5	3	298919189;109637759;545746425	N-Term(TMT6plex); K8(TMT6plex)	1285.8	0				
																	TEKEESTEVLK	3	5	3	298919189;109637759;545746425	N-Term(TMT6plex); K3(TMT6plex); K11(TMT6plex)	1980.1	1				
																	DTMSDQALEALSASLGTR	6	5	3	298919189;109637759;545746425	N-Term(TMT6plex)	2095.1	0				
																	SGMDAALDDLIDTLGGPEETEEENTTYTGPEVSDPMSSTYIEELGK	4	5	3	298919189;109637759;545746425	N-Term(TMT6plex); M3(Oxidation); K46(TMT6plex)	5381.5	0				
																	TKPQDMISAGGESVAGITAISGKPGDK	2	4	3	298919189;109637759;545746425	N-Term(TMT6plex); K2(TMT6plex); K23(TMT6plex); K27(TMT6plex)	3532.0	0				
																	LSDSLGQR	4	5	3	298919189;109637759;545746425	N-Term(TMT6plex)	1104.6	0				
																	AASLGSSQSSR	2	1	1	109637759	N-Term(TMT6plex)	1279.7	0	**1.0**	**1.5**	**1.5**	**1.1**
																	SESELIDELSEDFDRSECK	1	5	3	298919189;109637759;545746425	N-Term(TMT6plex); C18(Carbamidomethyl); K19(TMT6plex)	2746.3	1				
																	KTEKEESTEVLK	1	5	3	298919189;109637759;545746425	N-Term(TMT6plex); K1(TMT6plex); K4(TMT6plex); K12(TMT6plex)	2337.4	2				
																	EADPEDGKPVMDK	5	5	3	298919189;109637759;545746425	N-Term(TMT6plex); K8(TMT6plex); M11(Oxidation); K13(TMT6plex)	2134.1	0				
																	AQSAGTVR	3	5	3	298919189;109637759;545746425	N-Term(TMT6plex)	1018.6	0				
																	QPDPDENKPMEDK	1	5	3	298919189;109637759;545746425	N-Term(TMT6plex); K8(TMT6plex); K13(TMT6plex)	2230.2	0				
																	DKDGKPLLPK	1	5	3	298919189;109637759;545746425	N-Term(TMT6plex); K2(TMT6plex); K5(TMT6plex); K10(TMT6plex)	2027.3	1				
																	DGKPLLPEPEEKPKPR	1	5	3	298919189;109637759;545746425	N-Term(TMT6plex); K3(TMT6plex); K12(TMT6plex); K14(TMT6plex)	2746.7	0				
																	AASSSK	1	7	3	298919189;109637759;545746425	N-Term(TMT6plex); K6(TMT6plex)	1008.6	0				
																	EETIPPDYR	3	5	3	298919189;109637759;545746425	N-Term(TMT6plex)	1348.7	0				
																	TTEETSKPKDD	1	5	3	298919189;109637759;545746425	N-Term(TMT6plex); K7(TMT6plex); K9(TMT6plex)	1938.1	1				
																	QAEPELDLR	2	5	3	298919189;109637759;545746425	N-Term(TMT6plex)	1299.7	0				
																	THEHVSEK	1	1	1	109637759	N-Term(TMT6plex); K8(TMT6plex)	1424.8	0	**0.8**	**0.8**	**0.8**	**1.0**
																	EKPSKPTEK	1	5	3	298919189;109637759;545746425	N-Term(TMT6plex); K2(TMT6plex); K5(TMT6plex); K9(TMT6plex)	1960.2	0				
																	EVTIPPK	1	5	3	298919189;109637759;545746425	N-Term(TMT6plex); K7(TMT6plex)	1241.8	0				
																	AKEEDREK	1	5	3	298919189;109637759;545746425	N-Term(TMT6plex); K2(TMT6plex); K8(TMT6plex)	1692.0	2				
193083110	NP_001122384.1	**PCBP2**	5094	**poly(rC)-binding protein 2 isoform e**	78.12	7	1	17	230	**1.5**	**1.7**	**1.7**	**1.8**	361	38.1	6.79												
																	YSTGSDSASFPHTTPSMCLNPDLEGPPLEAYTIQGQYAIPQPDLTK	2	4	2	193083110;14141166	N-Term(TMT6plex); M17(Oxidation); C18(Carbamidomethyl); K46(TMT6plex)	5469.7	0				
																	EVGSIIGK	22	12	3	193083110;222352151;14141166	N-Term(TMT6plex); K8(TMT6plex)	1260.8	0				
																	LSSETGGMGSS	10	7	2	193083110;14141166	N-Term(TMT6plex); M8(Oxidation)	1257.6	0				
																	LEEDISSSMTNSTAASRPPVTLR	9	7	2	193083110;14141166	N-Term(TMT6plex); M9(Oxidation)	2707.4	0				
																	INISEGNCPER	33	10	3	193083110;222352151;14141166	N-Term(TMT6plex); C8(Carbamidomethyl)	1517.8	0				
																	AFAMIIDKLEEDISSSMTNSTAASRPPVTLR	3	7	2	193083110;14141166	N-Term(TMT6plex); M4(Oxidation); K8(TMT6plex); M17(Oxidation)	3842.0	1				
																	IITLAGPTNAIFK	36	7	2	193083110;14141166	N-Term(TMT6plex); K13(TMT6plex)	1817.1	0				
																	ESTGAQVQVAGDMLPNSTER	14	10	3	193083110;222352151;14141166	N-Term(TMT6plex); M13(Oxidation)	2335.1	0				
																	AFAMIIDK	6	8	3	193083110;222352151;14141166	N-Term(TMT6plex); M4(Oxidation); K8(TMT6plex)	1382.8	0				
																	AITIAGIPQSIIECVK	29	7	2	193083110;14141166	N-Term(TMT6plex); C14(Carbamidomethyl); K16(TMT6plex)	2171.3	0				
																	LVVPASQCGSLIGK	16	9	2	193083110;14141166	N-Term(TMT6plex); C8(Carbamidomethyl); K14(TMT6plex)	1887.1	0				
																	QICVVMLESPPK	20	5	2	14141166;193083110	N-Term(TMT6plex); C3(Carbamidomethyl); M6(Oxidation); K12(TMT6plex)	1875.0	0				
																	AFAMIIDKLEEDISSSMTNSTAASRPPVTLR	1	7	2	193083110;14141166	N-Term(TMT6plex); K8(TMT6plex); M17(Oxidation)	3826.0	1				
																	GYWGLDASAQTTSHELTIPNDLIGCIIGR	2	2	1	193083110	N-Term(TMT6plex); C25(Carbamidomethyl)	3387.7	0	**1.5**	**1.7**	**1.7**	**1.8**
																	QICVVMLESPPK	8	5	2	14141166;193083110	N-Term(TMT6plex); C3(Carbamidomethyl); K12(TMT6plex)	1859.1	0				
																	ESTGAQVQVAGDMLPNSTER	6	10	3	193083110;222352151;14141166	N-Term(TMT6plex)	2319.1	0				
																	QVTITGSAASISLAQYLINVR	2	7	2	193083110;14141166	N-Term(TMT6plex)	2434.4	0				
																	IANPVEGSTDR	9	7	2	193083110;14141166	N-Term(TMT6plex)	1387.7	0				
																	LHQLAMQQSHFPMTHGNTGFSGIESSSPEVK	1	6	2	193083110;14141166	N-Term(TMT6plex); M6(Oxidation); M13(Oxidation); K31(TMT6plex)	3872.9	0				
																	QMSGAQIK	1	10	3	193083110;222352151;14141166	N-Term(TMT6plex); M2(Oxidation); K8(TMT6plex)	1336.8	0				
5453603	NP_006422.1	**CCT2**	10576	**T-complex protein 1 subunit beta isoform 1**	77.94	2	35	35	419	**1.5**	**1.6**	**1.6**	**1.9**	535	57.5	6.46												
																	LALVTGGEIASTFDHPELVK	22	2	1	5453603	N-Term(TMT6plex); K20(TMT6plex)	2555.4	0	**1.4**	**1.6**	**1.6**	**1.8**
																	ILIANTGMDTDK	14	2	1	5453603	N-Term(TMT6plex); M8(Oxidation); K12(TMT6plex)	1766.0	0	**1.5**	**1.6**	**1.6**	**1.9**
																	KLGGSLADSYLDEGFLLDK	4	2	1	5453603	N-Term(TMT6plex); K1(TMT6plex); K19(TMT6plex)	2728.5	1	**1.3**	**1.5**	**1.5**	**2.0**
																	VAEIEHAEK	4	2	1	5453603	N-Term(TMT6plex); K9(TMT6plex)	1483.9	0	**1.3**	**1.4**	**1.4**	**1.6**
																	VQDDEVGDGTTSVTVLAAELLR	31	2	1	5453603	N-Term(TMT6plex)	2517.3	0	**1.5**	**1.6**	**1.8**	**1.8**
																	GATQQILDEAER	21	2	1	5453603	N-Term(TMT6plex)	1559.8	0	**1.5**	**1.8**	**1.6**	**2.1**

Khan et al., 2019. Multi-omics analysis to characterize cigarette smoke induced molecular alterations in esophageal cells
Supplementary Table 6. List of all proteins and peptides identified in untreated and chronically treated Het1A cells with cigarette smoke condensate for 8 months

Accession	RefSeq Accession	Gene Symbol	Entrez Gene ID	Description	Σ Coverage	Σ# Proteins	Σ# Unique Peptides	Σ# Peptides	Σ# PSMs	Het-1A-Smoke - 2M/Parental	Het-1A-Smoke - 4M/Parental	Het-1A-Smoke - 6M/Parental	Het-1A-Smoke - 8M/Parental	# AAs	MW [kDa]	calc. pI	Sequence	# PSMs	# Proteins	# Protein Groups	Protein Group Accessions	Modifications	MH+ [Da]	# Missed Cleavages	Het-1A-Smoke - 2M/Parental	Het-1A-Smoke - 4M/Parental	Het-1A-Smoke - 6M/Parental	Het-1A-Smoke - 8M/Parental
																	TVYGGGCSEMLMAHAVTQLANR	10	2	1	5453603	N-Term(TMT6plex); C7(Carbamidomethyl); M10(Oxidation); M12(Oxidation)	2627.2	0	**1.6**	**1.5**	**1.5**	**1.7**
																	MLPTIIADNAGYDSADLVAQLR	38	2	1	5453603	N-Term(TMT6plex); M1(Oxidation)	2592.3	0	**1.3**	**1.5**	**1.4**	**1.5**
																	MLPTIIADNAGYDSADLVAQLR	6	2	1	5453603	N-Term(TMT6plex)	2576.3	0	**2.5**	**1.1**	**6.7**	**1.2**
																	QLIYNYPEQLFGAAGVMAIEHADFAGVER	14	2	1	5453603	N-Term(TMT6plex); M17(Oxidation)	3454.7	0	**1.7**	**1.5**	**1.4**	**2.1**
																	LIEEVMIGEDK	15	2	1	5453603	N-Term(TMT6plex); M6(Oxidation); K11(TMT6plex)	1750.0	0	**1.5**	**1.6**	**1.5**	**1.9**
																	LAVEAVLR	10	2	1	5453603	N-Term(TMT6plex)	1099.7	0	**1.7**	**1.7**	**1.7**	**2.3**
																	EAVAMESYAK	26	2	1	5453603	N-Term(TMT6plex); M5(Oxidation); K10(TMT6plex)	1572.8	0	**1.4**	**1.5**	**1.5**	**1.8**
																	EAVAMESYAK	6	2	1	5453603	N-Term(TMT6plex); K10(TMT6plex)	1556.8	0	**1.8**	**2.2**	**2.1**	**2.0**
																	EALLSSAVDHGSDEVK	9	2	1	5453603	N-Term(TMT6plex); K16(TMT6plex)	2115.1	0	**1.5**	**1.7**	**1.6**	**1.9**
																	NIGVDNPAAK	6	2	1	5453603	N-Term(TMT6plex); K10(TMT6plex)	1456.9	0	**1.5**	**1.4**	**1.5**	**1.8**
																	DASLMVTNDGATILK	2	2	1	5453603	N-Term(TMT6plex); K15(TMT6plex)	2007.1	0	**1.4**	**1.4**	**1.5**	**1.7**
																	EGTIGDMAILGITESFQVK	10	2	1	5453603	N-Term(TMT6plex); M7(Oxidation); K19(TMT6plex)	2483.3	0	**1.4**	**1.6**	**1.4**	**2.1**
																	SLHDALCVLAQTVK	14	2	1	5453603	N-Term(TMT6plex); C7(Carbamidomethyl); K14(TMT6plex)	2013.2	0	**1.4**	**1.6**	**1.8**	**1.8**
																	QVLLSAAEAAEVILR	29	2	1	5453603	N-Term(TMT6plex)	1812.1	0	**1.6**	**1.6**	**1.4**	**1.8**
																	HGINCFINR	2	2	1	5453603	N-Term(TMT6plex); C5(Carbamidomethyl)	1359.7	0	**1.3**	**1.3**	**1.4**	**1.4**
																	IHPQTIIAGWR	4	2	1	5453603	N-Term(TMT6plex)	1520.9	0	**1.2**	**1.3**	**1.2**	**1.4**
																	LGGSLADSYLDEGFLLDK	18	2	1	5453603	N-Term(TMT6plex); K18(TMT6plex)	2371.3	0	**1.5**	**1.3**	**1.6**	**1.9**
																	QLIYNYPEQLFGAAGVMAIEHADFAGVER	8	2	1	5453603	N-Term(TMT6plex)	3438.7	0	**1.6**	**2.0**	**1.9**	**1.7**
																	LIEEVMIGEDK	14	2	1	5453603	N-Term(TMT6plex); K11(TMT6plex)	1734.0	0	**2.2**	**2.7**	**2.7**	**2.3**
																	AAHSEGNTTAGLDMR	7	2	1	5453603	N-Term(TMT6plex); M14(Oxidation)	1775.9	0	**1.3**	**1.8**	**1.6**	**1.9**
																	LTSFIGAIAIGDLVK	24	1	1	5453603	N-Term(TMT6plex); K15(TMT6plex)	1976.2	0	**1.5**	**1.4**	**1.6**	**1.6**
																	DASLMVTNDGATILK	10	2	1	5453603	N-Term(TMT6plex); M5(Oxidation); K15(TMT6plex)	2023.1	0	**1.2**	**1.4**	**1.3**	**1.9**
																	LGGSLADSYLDEGFLLDKK	4	2	1	5453603	N-Term(TMT6plex); K18(TMT6plex); K19(TMT6plex)	2728.5	1	**1.7**	**2.1**	**1.8**	**2.6**
																	VAEIEHAEKEK	2	2	1	5453603	N-Term(TMT6plex); K9(TMT6plex); K11(TMT6plex)	1970.2	1	**1.4**	**1.9**	**1.6**	**2.1**
																	QDLMNIAGTTLSSK	7	2	1	5453603	N-Term(TMT6plex); M4(Oxidation); K14(TMT6plex)	1953.1	0	**1.4**	**1.3**	**1.4**	**1.8**
																	GSGNLEAIHIIK	5	2	1	5453603	N-Term(TMT6plex); K12(TMT6plex)	1710.0	0	**1.5**	**1.6**	**1.5**	**1.8**
																	TVYGGGCSEMLMAHAVTQLANR	2	2	1	5453603	N-Term(TMT6plex); C7(Carbamidomethyl); M12(Oxidation)	2611.3	0	**1.3**	**1.3**	**1.4**	**1.3**
																	EAESLIAK	8	2	1	5453603	N-Term(TMT6plex); K8(TMT6plex)	1318.8	0	**1.3**	**1.6**	**1.5**	**1.8**
																	ILIANTGMDTDK	1	2	1	5453603	N-Term(TMT6plex); K12(TMT6plex)	1750.0	0	**1.8**	**1.9**	**1.8**	**1.8**
																	IGVNQPK	3	2	1	5453603	N-Term(TMT6plex); K7(TMT6plex)	1213.8	0	**1.4**	**1.6**	**1.6**	**1.8**
																	VLVDMSR	3	2	1	5453603	N-Term(TMT6plex); M5(Oxidation)	1064.6	0	**1.4**	**1.4**	**1.4**	**1.8**
																	STLGPK	1	1	1	5453603	N-Term(TMT6plex); K6(TMT6plex)	1060.7	0	**1.5**	**1.4**	**1.4**	**1.6**
																	AGADEER	2	1	1	5453603	N-Term(TMT6plex)	976.5	0	**1.5**	**1.8**	**1.5**	**2.0**
																	VDNIIK	1	2	1	5453603	N-Term(TMT6plex); K6(TMT6plex)	1159.7	0				
																	ILLSSGR	1	2	1	5453603	N-Term(TMT6plex)	974.6	0	**1.3**	**1.2**	**1.3**	**1.3**
																	IFGSR	1	2	1	5453603	N-Term(TMT6plex)	808.5	0	**1.6**	**1.4**	**1.5**	**1.5**
302370946	NP_001180573.1	**PEX19**	5824	**peroxisomal biogenesis factor 19 isoform c**	77.78	2	14	14	63	**1.3**	**1.2**	**1.3**	**1.0**	279	30.8	4.37												
																	FEMVLDLMQQLQDLGHPPK	1	2	1	302370946	N-Term(TMT6plex); M3(Oxidation); M8(Oxidation); K19(TMT6plex)	2729.4	0				
																	NATDLQNSSMSEEELTK	4	2	1	302370946	N-Term(TMT6plex); M10(Oxidation); K17(TMT6plex)	2371.2	0	**1.1**	**0.9**	**0.9**	**0.9**
																	ELEELLESALDDFDK	5	2	1	302370946	N-Term(TMT6plex); K15(TMT6plex)	2224.2	0	**1.1**	**1.1**	**1.2**	**0.9**
																	FFQELFDSELASQATAEFEK	12	2	1	302370946	N-Term(TMT6plex); K20(TMT6plex)	2795.4	0	**1.5**	**1.1**	**1.5**	**0.9**
																	ICEQFEAETPTDSETTQK	4	2	1	302370946	N-Term(TMT6plex); C2(Carbamidomethyl); K18(TMT6plex)	2572.2	0	**1.2**	**1.2**	**1.1**	**1.0**
																	ELAEEEPHLVEQFQK	3	2	1	302370946	N-Term(TMT6plex); K15(TMT6plex)	2284.2	0	**1.1**	**1.2**	**1.3**	**1.1**
																	VGSDMTSQQEFTSCLK	10	2	1	302370946	N-Term(TMT6plex); M5(Oxidation); C14(Carbamidomethyl); K16(TMT6plex)	2292.1	0	**1.1**	**1.1**	**1.1**	**1.0**
																	DVLYPSLK	6	2	1	302370946	N-Term(TMT6plex); K8(TMT6plex)	1392.8	0	**1.0**	**1.2**	**1.2**	**1.0**
																	FEMVLDLMQQLQDLGHPPK	2	2	1	302370946	N-Term(TMT6plex); K19(TMT6plex)	2697.4	0	**1.5**	**1.5**	**1.4**	**1.2**
																	AKPSPAPPSTTTAPDASGPQK	6	2	1	302370946	N-Term(TMT6plex); K2(TMT6plex); K21(TMT6plex)	2693.5	0	**1.4**	**1.4**	**1.3**	**1.1**
																	YQEQHSVMCK	1	2	1	302370946	N-Term(TMT6plex); M8(Oxidation); C9(Carbamidomethyl); K10(TMT6plex)	1783.9	0	**1.1**	**1.1**	**1.1**	**1.1**
																	FEMVLDLMQQLQDLGHPPK	2	2	1	302370946	N-Term(TMT6plex); M3(Oxidation); K19(TMT6plex)	2713.5	0	**1.1**	**1.0**	**1.0**	**0.8**
																	VGSDMTSQQEFTSCLK	2	2	1	302370946	N-Term(TMT6plex); C14(Carbamidomethyl); K16(TMT6plex)	2276.1	0	**1.4**	**1.3**	**1.5**	**1.1**
																	AMEGLGMDEGDGEGNILPIMQSIMQNLLSK	2	2	1	302370946	N-Term(TMT6plex); M2(Oxidation); M7(Oxidation); M20(Oxidation); M24(Oxidation); K30(TMT6plex)	3713.8	0				
																	ESLPPEQFEK	1	2	1	302370946	N-Term(TMT6plex); K10(TMT6plex)	1661.9	0	**1.3**	**1.2**	**1.2**	**1.2**
																	DALFASQEK	1	2	1	302370946	N-Term(TMT6plex); K9(TMT6plex)	1466.8	0				
																	YPEWLQSHR	1	2	1	302370946	N-Term(TMT6plex)	1444.8	0	**1.9**	**2.2**	**2.1**	**1.4**
4503471	NP_001393.1	**EEF1A1**	1915	**elongation factor 1-alpha 1**	77.71	1	15	27	2152	**1.3**	**1.3**	**1.4**	**1.5**	462	50.1	9.01												
																	THINIVVIGHVDSGK	85	2	2	4503475;4503471	N-Term(TMT6plex); K15(TMT6plex)	2047.2	0				
																	NMITGTSQADCAVLIVAAGVGEFEAGISK	333	2	2	4503475;4503471	N-Term(TMT6plex); M2(Oxidation); C11(Carbamidomethyl); K29(TMT6plex)	3383.8	0				
																	IGYNPDTVAFVPISGWNGDNMLEPSANMPWFK	119	1	1	4503471	N-Term(TMT6plex); M21(Oxidation); M28(Oxidation); K32(TMT6plex)	4058.0	0	**1.3**	**1.4**	**1.5**	**1.4**
																	SGDAAIVDMVPGKPMCVESFSDYPPLGR	129	1	1	4503471	N-Term(TMT6plex); M9(Oxidation); K13(TMT6plex); M15(Oxidation); C16(Carbamidomethyl)	3485.7	0	**1.3**	**1.5**	**1.6**	**1.5**
																	VETGVLKPGMVVTFAPVNVTTEVK	161	1	1	4503471	N-Term(TMT6plex); K7(TMT6plex); M10(Oxidation); K24(TMT6plex)	3218.8	0	**1.2**	**1.2**	**1.4**	**1.4**
																	EHALLAYTLGVK	82	2	2	4503475;4503471	N-Term(TMT6plex); K12(TMT6plex)	1773.1	0				
																	DGNASGTTLLEALDCILPPTRPTDKPLR	173	1	1	4503471	N-Term(TMT6plex); C15(Carbamidomethyl); K25(TMT6plex)	3479.9	0	**1.3**	**1.4**	**1.4**	**1.4**
																	STTTGHLIYK	82	2	2	4503475;4503471	N-Term(TMT6plex); K10(TMT6plex)	1578.9	0				
																	QTVAVGVIK	75	2	2	4503475;4503471	N-Term(TMT6plex); K9(TMT6plex)	1372.9	0				
																	YEEIVK	29	1	1	4503471	N-Term(TMT6plex); K6(TMT6plex)	1238.7	0	**1.1**	**1.2**	**1.3**	**1.4**
																	QLIVGVNK	51	2	2	4503475;4503471	N-Term(TMT6plex); K8(TMT6plex)	1328.9	0				
																	EAAEMGK	21	2	2	4503475;4503471	N-Term(TMT6plex); M5(Oxidation); K7(TMT6plex)	1209.7	0				
																	IGYNPDTVAFVPISGWNGDNMLEPSANMPWFK	62	1	1	4503471	N-Term(TMT6plex); M21(Oxidation); K32(TMT6plex)	4042.0	0	**1.4**	**1.5**	**1.8**	**1.5**
																	KDGNASGTTLLEALDCILPPTRPTDKPLR	28	1	1	4503471	N-Term(TMT6plex); K1(TMT6plex); C16(Carbamidomethyl); K26(TMT6plex)	3837.1	1	**1.3**	**1.4**	**1.5**	**1.4**
																	IGGIGTVPVGR	98	2	2	4503475;4503471	N-Term(TMT6plex)	1254.8	0				
																	RYEEIVK	6	1	1	4503471	N-Term(TMT6plex); K7(TMT6plex)	1394.8	1	**1.4**	**1.0**	**0.9**	**1.3**
																	KLEDGPK	27	1	1	4503471	N-Term(TMT6plex); K1(TMT6plex); K7(TMT6plex)	1473.9	1	**1.3**	**1.5**	**1.5**	**1.6**
																	GNVAGDSK	37	1	1	4503471	N-Term(TMT6plex); K8(TMT6plex)	1205.7	0	**1.1**	**1.3**	**1.3**	**1.4**
																	EVSTYIK	70	1	1	4503471	N-Term(TMT6plex); K7(TMT6plex)	1297.8	0	**1.2**	**1.3**	**1.4**	**1.5**
																	MDSTEPPYSQK	7	1	1	4503471	N-Term(TMT6plex); K11(TMT6plex)	1740.9	0	**2.4**	**1.4**	**1.5**	**1.3**
																	SGDAAIVDMVPGKPMCVESFSDYPPLGR	8	1	1	4503471	N-Term(TMT6plex); K13(TMT6plex); C16(Carbamidomethyl)	3453.7	0	**1.7**	**1.7**	**2.0**	**1.6**
																	MDSTEPPYSQK	40	1	1	4503471	N-Term(TMT6plex); M1(Oxidation); K11(TMT6plex)	1756.9	0	**1.7**	**1.0**	**1.0**	**1.3**
																	NDPPMEAAGFTAQVIILNHPGQISAGYAPVLDCHTAHIACK	8	1	1	4503471	N-Term(TMT6plex); M5(Oxidation); C33(Carbamidomethyl); C40(Carbamidomethyl); K41(TMT6plex)	4859.5	0	**1.5**	**1.5**	**1.6**	**1.5**
																	LPLQDVYK	164	2	2	4503475;4503471	N-Term(TMT6plex); K8(TMT6plex)	1433.9	0				
																	YYVTIIDAPGHR	113	1	1	4503471	N-Term(TMT6plex)	1633.9	0	**1.4**	**1.4**	**1.5**	**1.6**
																	SGDAAIVDMVPGKPMCVESFSDYPPLGR	41	1	1	4503471	N-Term(TMT6plex); M9(Oxidation); K13(TMT6plex); C16(Carbamidomethyl)	3469.7	0	**1.7**	**1.7**	**2.1**	**1.7**
																	IGYNPDTVAFVPISGWNGDNMLEPSANMPWFK	7	1	1	4503471	N-Term(TMT6plex); K32(TMT6plex)	4026.0	0	**2.1**	**1.9**	**2.4**	**1.6**
																	NMITGTSQADCAVLIVAAGVGEFEAGISK	28	2	2	4503475;4503471	N-Term(TMT6plex); C11(Carbamidomethyl); K29(TMT6plex)	3367.8	0				
																	KIGYNPDTVAFVPISGWNGDNMLEPSANMPWFK	4	1	1	4503471	N-Term(TMT6plex); K1(TMT6plex); M22(Oxidation); M29(Oxidation); K33(TMT6plex)	4415.2	1	**1.2**	**1.7**	**1.2**	**2.0**
																	EAAEMGK	2	2	2	4503475;4503471	N-Term(TMT6plex); K7(TMT6plex)	1193.7	0				
																	VETGVLKPGMVVTFAPVNVTTEVK	33	1	1	4503471	N-Term(TMT6plex); K7(TMT6plex); K24(TMT6plex)	3202.9	0	**1.4**	**1.4**	**1.9**	**1.5**
																	YAWVLDK	18	2	2	4503475;4503471	N-Term(TMT6plex); K7(TMT6plex)	1352.8	0				
																	SVEMHHEALSEALPGDNVGFNVK	5	2	2	4503475;4503471	N-Term(TMT6plex); M4(Oxidation); K23(TMT6plex)	2954.5	0				
																	TIEKFEK	3	2	2	4503475;4503471	N-Term(TMT6plex); K4(TMT6plex); K7(TMT6plex)	1582.0	1				
																	AAGAGK	3	1	1	4503471	N-Term(TMT6plex); K6(TMT6plex)	932.6	0	**1.2**	**1.3**	**1.4**	**1.6**
55956899	NP_000217.2	**KRT9**	3857	**keratin, type I cytoskeletal 9**	77.69	2	31	33	511	**1.4**	**2.4**	**1.4**	**3.2**	623	62.0	5.24												
																	GGSGGSHGGGSGFGGESGGSYGGGEEASGSGGGYGGGSGK	15	1	1	55956899	N-Term(TMT6plex); K40(TMT6plex)	3681.6	0	**1.6**	**4.8**	**2.0**	**7.4**
																	SGGGGGGGLGSGGSIR	9	1	1	55956899	N-Term(TMT6plex)	1461.8	0	**1.5**	**1.6**	**1.3**	**1.6**
																	QEYEQLIAK	12	1	1	55956899	N-Term(TMT6plex); K9(TMT6plex)	1579.9	0	**1.2**	**2.5**	**1.3**	**3.8**
																	VQALEEANNDLENK	16	1	1	55956899	N-Term(TMT6plex); K14(TMT6plex)	2045.1	0	**1.4**	**2.5**	**1.4**	**3.7**
																	HGVQELEIELQSQLSK	58	1	1	55956899	N-Term(TMT6plex); K16(TMT6plex)	2296.3	0	**1.3**	**2.0**	**1.3**	**2.7**
																	NYSPYYNTIDDLKDQIVDLTVGNNK	18	1	1	55956899	N-Term(TMT6plex); K13(TMT6plex); K25(TMT6plex)	3589.9	1	**1.3**	**2.9**	**1.3**	**4.5**
																	DQIVDLTVGNNK	6	1	1	55956899	N-Term(TMT6plex); K12(TMT6plex)	1774.0	0	**1.6**	**1.8**	**1.4**	**2.2**
																	GGGGSFGYSYGGGSGGGFSASSLGGGFGGGSR	26	1	1	55956899	N-Term(TMT6plex)	2934.3	0	**1.4**	**1.7**	**1.5**	**1.9**
																	FSSSGGGGGGGR	6	1	1	55956899	N-Term(TMT6plex)	1211.6	0	**1.3**	**1.6**	**1.2**	**1.7**

Khan et al., 2019. Multi-omics analysis to characterize cigarette smoke induced molecular alterations in esophageal cells
Supplementary Table 6. List of all proteins and peptides identified in untreated and chronically treated Het1A cells with cigarette smoke condensate for 8 months

Accession	RefSeq Accession	Gene Symbol	Entrez Gene ID	Description	Σ Coverage	Σ# Proteins	Σ# Unique Peptides	Σ# Peptides	Σ# PSMs	Het-1A-Smoke - 2M/Parental	Het-1A-Smoke - 4M/Parental	Het-1A-Smoke - 6M/Parental	Het-1A-Smoke - 8M/Parental	# AAs	MW [kDa]	calc. pI	Sequence	# PSMs	# Proteins	# Protein Groups	Protein Group Accessions	Modifications	MH+ [Da]	# Missed Cleavages	Het-1A-Smoke - 2M/Parental	Het-1A-Smoke - 4M/Parental	Het-1A-Smoke - 6M/Parental	Het-1A-Smoke - 8M/Parental
																	SDLEMQYETLQEELMALK	65	1	1	55956899	N-Term(TMT6plex); M5(Oxidation); M15(Oxidation); K18(TMT6plex)	2661.3	0	1.8	3.0	1.9	4.6
																	EIETYHNLLEGGQEDFESSGAGK	41	1	1	55956899	N-Term(TMT6plex); K23(TMT6plex)	2968.5	0	1.4	2.2	1.6	2.8
																	GGSGGSYGGGGSGGGYGGGSGSR	27	1	1	55956899	N-Term(TMT6plex)	2020.9	0	1.1	2.3	1.2	2.8
																	TLLDIDNTR	14	1	1	55956899	N-Term(TMT6plex)	1289.7	0	1.3	2.0	1.5	2.3
																	GGSGGSYGGGSGSGGGSGGGYGGGSGGGHSGGSGGGHSGGSGGNYGGGSGSGGGSGGGYGGGSGSR	4	1	1	55956899	N-Term(TMT6plex)	5202.1	0	0.7	1.6	0.7	3.6
																	QFSSSYLSR	2	1	1	55956899	N-Term(TMT6plex)	1303.7	0	1.2	1.3	1.1	1.4
																	YCGQLQMIQEQISNLEAQITDVR	14	1	1	55956899	N-Term(TMT6plex); C2(Carbamidomethyl); M7(Oxidation)	2982.5	0	1.5	1.5	1.5	1.4
																	STMQELNSR	1	1	1	55956899	N-Term(TMT6plex)	1294.7	0	1.5	2.0	1.7	2.1
																	QVLDNLTMEK	12	1	1	55956899	N-Term(TMT6plex); M8(Oxidation); K10(TMT6plex)	1664.9	0	1.5	2.2	1.4	2.9
																	SDLEMQYETLQEELMALK	30	1	1	55956899	N-Term(TMT6plex); M5(Oxidation); K18(TMT6plex)	2645.3	0	1.6	4.8	2.0	6.0
																	DIENQYETQITQIEHEVSSSGQEVQSSAK	36	1	1	55956899	N-Term(TMT6plex); K29(TMT6plex)	3722.8	0	1.3	3.0	1.5	4.6
																	SDLEMQYETLQEELMALK	4	1	1	55956899	N-Term(TMT6plex); K18(TMT6plex)	2629.4	0	1.5	4.3	1.9	3.7
																	FSSSSGYGGGSSR	10	1	1	55956899	N-Term(TMT6plex)	1464.7	0	1.4	1.9	1.3	2.5
																	QGVDADINGLR	8	1	1	55956899	N-Term(TMT6plex)	1386.8	0	1.5	1.6	1.3	1.7
																	YCGQLQMIQEQISNLEAQITDVR	3	1	1	55956899	N-Term(TMT6plex); C2(Carbamidomethyl)	2966.5	0	1.5	1.7	1.6	1.6
																	QVLDNLTMEK	4	1	1	55956899	N-Term(TMT6plex); K10(TMT6plex)	1648.9	0	1.6	2.9	1.6	3.0
																	GGSGGSYGR	6	1	1	55956899	N-Term(TMT6plex)	1026.5	0	1.1	2.4	1.3	2.4
																	KDIENQYETQITQIEHEVSSSGQEVQSSAK	4	1	1	55956899	N-Term(TMT6plex); K1(TMT6plex); K30(TMT6plex)	4080.1	1	1.0	2.1	1.2	2.9
																	GGSGGSHGGGSGFGGESGGSYGGGEEASGSGGGYGGGSGKSSHS	4	1	1	55956899	N-Term(TMT6plex); K40(TMT6plex)	4079.8	1	1.0	0.4	0.4	0.4
																	NYSPYYNTIDDLK	3	1	1	55956899	N-Term(TMT6plex); K13(TMT6plex)	2064.1	0	1.3	2.0	1.3	2.6
																	NHKEEMSQLTGQNSGDVNVEINVAPGK	3	1	1	55956899	N-Term(TMT6plex); K3(TMT6plex); M6(Oxidation); K27(TMT6plex)	3598.9	1	1.3	2.8	1.4	4.9
																	SDLEMQYETLQEELMALKK	1	1	1	55956899	N-Term(TMT6plex); M5(Oxidation); M15(Oxidation); K18(TMT6plex); K19(TMT6plex)	3018.6	1	1.5	2.8	1.5	3.4
																	LASYLDK	32	8	5	4557701;55956899;15431310;195972866;24430192	N-Term(TMT6plex); K7(TMT6plex)	1267.8	0				
																	IQDWYDKK	1	1	1	55956899	N-Term(TMT6plex); K7(TMT6plex); K8(TMT6plex)	1783.0	1	1.2	2.9	1.4	4.1
																	GPAAIQK	2	1	1	55956899	N-Term(TMT6plex); K7(TMT6plex)	1142.7	0	1.2	2.5	1.5	3.4
																	MTLDDFR	4	1	1	55956899	N-Term(TMT6plex); M1(Oxidation)	1142.6	0	1.3	1.5	1.3	1.1
																	MTLDDFR	1	1	1	55956899	N-Term(TMT6plex)	1126.6	0	1.5	2.7	1.7	2.6
																	STMQELNSR	2	1	1	55956899	N-Term(TMT6plex); M3(Oxidation)	1310.7	0	1.3	3.0	1.2	5.1
																	KGPAAIQK	1	1	1	55956899	N-Term(TMT6plex); K1(TMT6plex); K8(TMT6plex)	1500.0	1	1.2	2.1	1.4	2.9
																	SLEDTK	6	5	2	34577047;55956899	N-Term(TMT6plex); K6(TMT6plex)	1150.7	0				
304571975	NP_001182147.1	DCXR	51181	L-xylulose reductase isoform 2	77.69	2	13	13	58	1.4	1.5	1.4	1.5	242	25.7	8.10												
																	ALGSVGPVDLLVNNAAVALLQPFLEVTK	10	2	1	304571975	N-Term(TMT6plex); K28(TMT6plex)	3306.9	0	1.3	1.6	1.3	1.6
																	VMALELGPHK	2	2	1	304571975	N-Term(TMT6plex); M2(Oxidation); K10(TMT6plex)	1568.9	0	1.0	1.2	1.0	1.4
																	GALDMLTK	2	2	1	304571975	N-Term(TMT6plex); K8(TMT6plex)	1306.8	0	1.5	1.6	1.6	1.4
																	GVPGAIVNVSSQCSQR	2	2	1	304571975	N-Term(TMT6plex); C13(Carbamidomethyl)	1888.0	0	2.1	1.2	1.1	1.3
																	VNAVNPTVVMTSMGQATWSDPHK	2	2	1	304571975	N-Term(TMT6plex); M10(Oxidation); M13(Oxidation); K23(TMT6plex)	2960.5	0	1.3	1.4	1.2	1.4
																	TQADLDSLVR	6	2	1	304571975	N-Term(TMT6plex)	1346.7	0	1.3	1.3	1.3	1.5
																	ECPGIEPVCVDLGDWEATER	10	2	1	304571975	N-Term(TMT6plex); C2(Carbamidomethyl); C9(Carbamidomethyl)	2561.2	0	1.5	1.6	1.5	1.6
																	GALDMLTK	3	2	1	304571975	N-Term(TMT6plex); M5(Oxidation); K8(TMT6plex)	1322.8	0	1.3	1.3	1.3	1.4
																	VLVTGAGK	4	2	1	304571975	N-Term(TMT6plex); K8(TMT6plex)	1202.8	0	1.2	1.2	1.2	1.6
																	VNAVNPTVVMTSMGQATWSDPHK	2	2	1	304571975	N-Term(TMT6plex); M13(Oxidation); K23(TMT6plex)	2944.5	0	1.2	1.3	1.3	1.3
																	AVIQVSQIVAR	6	2	1	304571975	N-Term(TMT6plex)	1412.9	0	1.4	1.4	1.3	1.6
																	GTVQALHATGAR	4	2	1	304571975	N-Term(TMT6plex)	1410.8	0	1.0	1.2	1.1	1.6
																	SGMTTGSTLPVEGGFWAC	1	2	1	304571975	N-Term(TMT6plex); M3(Oxidation); C18(Carbamidomethyl)	2103.0	0	1.3	1.6	1.4	1.4
																	FAEVEHVVNAILFLLSDR	2	2	1	304571975	N-Term(TMT6plex)	2301.3	0	2.3	2.0	2.8	1.7
																	SGMTTGSTLPVEGGFWAC	1	2	1	304571975	N-Term(TMT6plex); C18(Carbamidomethyl)	2087.0	0	1.5	1.5	1.6	1.5
																	VVAVSR	1	2	1	304571975	N-Term(TMT6plex)	859.6	0	1.4	1.3	1.2	1.6
50845386	NP_001002857.1	ANXA2	302	annexin A2 isoform 2	77.58	2	30	30	1627	1.2	1.1	1.3	1.2	339	38.6	7.75												
																	SLYYYIQQDTK	103	2	1	50845386	N-Term(TMT6plex); K11(TMT6plex)	1880.0	0	1.1	1.1	1.2	1.2
																	ALLYLCGGDD	51	2	1	50845386	N-Term(TMT6plex); C6(Carbamidomethyl)	1325.7	0	1.2	1.3	1.4	1.3
																	DALNIETAIK	86	2	1	50845386	N-Term(TMT6plex); K10(TMT6plex)	1545.9	0	1.2	1.3	1.3	1.4
																	LSLEGDHSTPPSAYGSVK	47	2	1	50845386	N-Term(TMT6plex); K18(TMT6plex)	2303.2	0	1.2	1.2	1.3	1.2
																	DLYDAGVK	59	2	1	50845386	N-Term(TMT6plex); K8(TMT6plex)	1338.8	0	1.1	1.1	1.2	1.2
																	TNQELQEINR	72	2	1	50845386	N-Term(TMT6plex)	1473.8	0	1.2	1.0	1.2	1.1
																	AYTNFDAERDALNIETAIK	2	2	1	50845386	N-Term(TMT6plex); K19(TMT6plex)	2613.4	1	0.9	1.1	0.9	0.9
																	TDLEKDIISDTSGDFR	45	2	1	50845386	N-Term(TMT6plex); K5(TMT6plex)	2270.2	1	1.3	1.2	1.3	1.3
																	GLGTDEDSLIEIICSR	131	2	1	50845386	N-Term(TMT6plex); C14(Carbamidomethyl)	2007.0	0	1.2	1.3	1.4	1.3
																	TPAQYDASELK	114	2	1	50845386	N-Term(TMT6plex); K11(TMT6plex)	1680.9	0	1.1	1.1	1.2	1.2
																	QDIAFAYQR	41	2	1	50845386	N-Term(TMT6plex)	1340.7	0	1.1	1.0	1.2	1.1
																	ELASALK	31	2	1	50845386	N-Term(TMT6plex); K7(TMT6plex)	1189.8	0	1.0	1.1	1.2	1.2
																	AYTNFDAER	65	2	1	50845386	N-Term(TMT6plex)	1315.6	0	1.2	1.1	1.2	1.2
																	AEDGSVIDYELIDQDAR	82	2	1	50845386	N-Term(TMT6plex)	2138.0	0	1.3	1.3	1.4	1.4
																	DIISDTSGDFR	89	2	1	50845386	N-Term(TMT6plex)	1454.7	0	1.2	1.1	1.2	1.2
																	GVDEVTIVNILTNR	266	2	1	50845386	N-Term(TMT6plex)	1772.0	0	1.2	1.3	1.3	1.3
																	SYSPYDMLESIR	37	2	1	50845386	N-Term(TMT6plex); M7(Oxidation)	1705.8	0	1.3	1.3	1.4	1.2
																	GDLENAFLNLVQCIQNKPLYFADR	45	2	1	50845386	N-Term(TMT6plex); C13(Carbamidomethyl); K17(TMT6plex)	3296.7	0	1.1	1.2	1.4	1.3
																	SYSPYDMLESIR	21	2	1	50845386	N-Term(TMT6plex)	1689.8	0	1.3	1.3	1.4	1.3
																	SALSGHLETVILGLLK	78	2	1	50845386	N-Term(TMT6plex); K16(TMT6plex)	2109.3	0	1.1	1.2	1.3	1.3
																	SEVDMLK	7	2	1	50845386	N-Term(TMT6plex); K7(TMT6plex)	1279.7	0	1.6	1.6	1.9	1.4
																	SVPHLQK	40	2	1	50845386	N-Term(TMT6plex); K7(TMT6plex)	1266.8	0	0.9	1.0	1.1	1.2
																	LMVALAK	13	2	1	50845386	N-Term(TMT6plex); K7(TMT6plex)	1203.8	0	1.3	1.4	1.8	1.3
																	KELASALK	7	2	1	50845386	N-Term(TMT6plex); K1(TMT6plex); K8(TMT6plex)	1547.0	1	1.1	1.1	1.3	1.2
																	KLMVALAK	3	2	1	50845386	N-Term(TMT6plex); K1(TMT6plex); K8(TMT6plex)	1561.0	1	1.1	1.3	1.4	1.4
																	RAEDGSVIDYELIDQDAR	9	2	1	50845386	N-Term(TMT6plex)	2294.1	1	1.2	1.4	1.4	1.5
																	SEVDMLK	20	2	1	50845386	N-Term(TMT6plex); M5(Oxidation); K7(TMT6plex)	1295.7	0	1.3	1.2	1.4	1.2
																	WISIMTER	21	2	1	50845386	N-Term(TMT6plex); M5(Oxidation)	1280.7	0	1.2	1.0	1.2	1.2
																	LMVALAK	26	2	1	50845386	N-Term(TMT6plex); M2(Oxidation); K7(TMT6plex)	1219.8	0	1.1	1.1	1.3	1.2
																	KGTDVPK	8	2	1	50845386	N-Term(TMT6plex); K1(TMT6plex); K7(TMT6plex)	1431.9	1	1.1	1.0	1.2	1.2
																	DIISDTSGDFRK	1	2	1	50845386	N-Term(TMT6plex); K12(TMT6plex)	1812.0	1	1.3	1.4	1.5	1.4
																	LYDSMK	4	2	1	50845386	N-Term(TMT6plex); K6(TMT6plex)	1214.7	0	1.2	1.3	1.6	1.0
																	GTDVPK	2	2	1	50845386	N-Term(TMT6plex); K6(TMT6plex)	1074.7	0	0.9	1.0	1.1	1.1
																	KLMVALAK	1	2	1	50845386	N-Term(TMT6plex); K1(TMT6plex); M3(Oxidation); K8(TMT6plex)	1577.0	1	0.9	1.1	1.1	1.4
4507669	NP_003286.1	TPT1	7178	translationally-controlled tumor protein isoform 2	77.33	4	8	8	88	1.5	1.2	1.5	1.3	172	19.6	4.93												
																	DLISHDEMFSDIYK	32	2	1	4507669	N-Term(TMT6plex); M8(Oxidation); K14(TMT6plex)	2187.1	0	1.5	1.3	1.7	1.3
																	DLISHDEMFSDIYK	5	2	1	4507669	N-Term(TMT6plex); K14(TMT6plex)	2171.1	0	1.9	1.6	2.1	1.5
																	TEGNIDDSLIGGNASAEGPEGEGTESTVITGVDIVMNHHLQETSFTK	6	3	1	4507669	N-Term(TMT6plex); M36(Oxidation); K47(TMT6plex)	5331.6	0	1.4	0.9	1.2	1.1
																	HILANFK	2	4	1	4507669	N-Term(TMT6plex); K7(TMT6plex)	1300.8	0	1.4	0.9	1.6	1.1
																	NYQFFIGENMNPDGMVALLDYR	13	3	1	4507669	N-Term(TMT6plex); M10(Oxidation); M15(Oxidation)	2868.4	0	1.6	1.3	1.6	1.4
																	VKPFMTGAAEQIK	6	4	1	4507669	N-Term(TMT6plex); K2(TMT6plex); M5(Oxidation); K13(TMT6plex)	2123.2	0	1.4	1.3	1.7	1.5
																	EIADGLCLEVEGK	17	2	1	4507669	N-Term(TMT6plex); C7(Carbamidomethyl); K13(TMT6plex)	1891.0	0	1.5	1.2	1.5	1.4
																	VKPFMTGAAEQIK	2	4	1	4507669	N-Term(TMT6plex); K2(TMT6plex); K13(TMT6plex)	2107.3	0	1.7	1.7	2.3	1.6
																	TEGNIDDSLIGGNASAEGPEGEGTESTVITGVDIVMNHHLQETSFTK	2	3	1	4507669	N-Term(TMT6plex); K47(TMT6plex)	5315.5	0	2.0	1.9	2.1	2.1
																	GKLEEQRPER	1	3	1	4507669	N-Term(TMT6plex); K2(TMT6plex)	1700.0	1	1.1	1.2	1.5	1.3
																	DGLEMEK	2	3	1	4507669	N-Term(TMT6plex); M5(Oxidation); K7(TMT6plex)	1295.7	0	1.3	1.0	1.4	1.3
4502599	NP_001748.1	CBR1	873	carbonyl reductase [NADPH] 1 isoform 1	77.26	2	14	18	166	1.3	1.4	1.4	1.4	277	30.4	8.32												
																	EYGGLDVLVNNAGIAFK	21	2	1	4502599	N-Term(TMT6plex); K17(TMT6plex)	2238.3	0	1.3	1.4	1.3	1.4
																	FHQLDIDDLQSIR	13	3	2	4502599;4502601	N-Term(TMT6plex)	1829.0	0				
																	GIGLAIVR	7	2	1	4502599	N-Term(TMT6plex)	1027.7	0	1.2	1.4	1.2	1.4
																	SETITEEELVGLMNK	25	1	1	4502599	N-Term(TMT6plex); M13(Oxidation); K15(TMT6plex)	2167.2	0	1.4	1.3	1.4	1.4
																	SETITEEELVGLMNK	10	1	1	4502599	N-Term(TMT6plex); K15(TMT6plex)	2151.2	0	1.5	1.5	1.6	1.4
																	VADPTPFHIQAEVTMK	6	2	1	4502599	N-Term(TMT6plex); M15(Oxidation); K16(TMT6plex)	2258.2	0	1.2	1.3	1.3	1.4
																	DVCTELLPLIKPQGR	2	1	1	4502599	N-Term(TMT6plex); C3(Carbamidomethyl); K11(TMT6plex)	2197.3	0	1.1	1.5	1.3	1.5
																	EGWPSSAYGVTK	3	1	1	4502599	N-Term(TMT6plex); K12(TMT6plex)	1739.9	0	1.3	1.4	1.3	1.5
																	ILLNACCPGWVR	12	1	1	4502599	N-Term(TMT6plex); C6(Carbamidomethyl); C7(Carbamidomethyl)	1687.9	0	1.4	1.5	1.4	1.4

Accession	RefSeq Accession	Gene Symbol	Entrez Gene ID	Description	Σ Coverage	Σ# Proteins	Σ# Unique Peptides	Σ# Peptides	Σ# PSMs	Het-1A-Smoke - 2M/Parental	Het-1A-Smoke - 4M/Parental	Het-1A-Smoke - 6M/Parental	Het-1A-Smoke - 8M/Parental	# AAs	MW [kDa]	calc. pI	Sequence	# PSMs	# Proteins	# Protein Groups	Protein Group Accessions	Modifications	MH+ [Da]	# Missed Cleavages	Het-1A-Smoke - 2M/Parental	Het-1A-Smoke - 4M/Parental	Het-1A-Smoke - 6M/Parental	Het-1A-Smoke - 8M/Parental
																	GQAAVQQLQAEGLSPR	12	3	2	4502599;4502601	N-Term(TMT6plex)	1882.0	0				
																	KEYGGLDVLVNNAGIAFK	12	2	1	4502599	N-Term(TMT6plex); K1(TMT6plex); K18(TMT6plex)	2595.5	1	**1.3**	**1.6**	**1.3**	**1.6**
																	SPEEGAETPVYLALLPPDAEGPHGQFVSEK	27	1	1	4502599	N-Term(TMT6plex); K30(TMT6plex)	3622.9	0	**1.2**	**1.4**	**1.2**	**1.4**
																	SCSPELQQK	4	1	1	4502599	N-Term(TMT6plex); C2(Carbamidomethyl); K9(TMT6plex)	1534.8	0	**1.3**	**1.3**	**1.2**	**1.3**
																	IGVTVLSR	2	2	2	4502601;4502599	N-Term(TMT6plex)	1073.7	0				
																	LFSGDVVLTAR	4	2	1	4502599	N-Term(TMT6plex)	1406.8	0	**1.4**	**1.0**	**1.1**	**1.1**
																	VVNVSSIMSVR	2	1	1	4502599	N-Term(TMT6plex); M8(Oxidation)	1435.8	0	**1.3**	**1.3**	**1.4**	**1.5**
																	TNFFGTR	2	2	1	4502599	N-Term(TMT6plex)	1071.6	0	**1.4**	**1.1**	**1.2**	**1.1**
																	FVEDTK	1	2	2	4502599;4502601	N-Term(TMT6plex); K6(TMT6plex)	1196.7	0				
																	TDMAGPK	1	1	1	4502599	N-Term(TMT6plex); M3(Oxidation); K7(TMT6plex)	1193.7	0	**1.6**	**1.2**	**1.2**	**1.2**
4503483	NP_001952.1	**EEF2**	1938	**elongation factor 2**	77.04	1	53	55	1702	**1.3**	**1.3**	**1.4**	**1.4**	858	95.3	6.83												
																	LMEPIYLVEIQCPEQVVGGIYGVLNR	79	1	1	4503483	N-Term(TMT6plex); M2(Oxidation); C12(Carbamidomethyl)	3234.7	0	**1.5**	**1.6**	**1.5**	**1.6**
																	STAISLFYELSENDLNFIK	152	1	1	4503483	N-Term(TMT6plex); K19(TMT6plex)	2662.4	0	**1.3**	**1.2**	**1.3**	**1.3**
																	ALLELQLEPEELYQTFQR	49	1	1	4503483	N-Term(TMT6plex)	2449.3	0	**1.3**	**1.3**	**1.4**	**1.4**
																	EGALCEENMR	24	1	1	4503483	N-Term(TMT6plex); C5(Carbamidomethyl); M9(Oxidation)	1453.7	0	**1.4**	**1.4**	**1.4**	**1.4**
																	VFDAIMNFK	31	1	1	4503483	N-Term(TMT6plex); K9(TMT6plex)	1542.9	0	**1.4**	**1.4**	**1.8**	**1.3**
																	DLEEDHACIPIKK	14	1	1	4503483	N-Term(TMT6plex); C8(Carbamidomethyl); K12(TMT6plex); K13(TMT6plex)	2255.3	1	**1.1**	**1.4**	**1.4**	**1.5**
																	VTDGALVVVDCVSGVCVQTETVLR	75	1	1	4503483	N-Term(TMT6plex); C11(Carbamidomethyl); C16(Carbamidomethyl)	2805.5	0	**1.4**	**1.4**	**1.3**	**1.4**
																	KIWCFGPDGTGPNILTDITK	26	1	1	4503483	N-Term(TMT6plex); K1(TMT6plex); C4(Carbamidomethyl); K20(TMT6plex)	2920.6	1	**1.7**	**1.7**	**1.9**	**2.2**
																	SDPVVSYR	22	1	1	4503483	N-Term(TMT6plex)	1151.6	0	**1.3**	**1.2**	**1.3**	**1.4**
																	VFDAIMNFK	42	1	1	4503483	N-Term(TMT6plex); M6(Oxidation); K9(TMT6plex)	1558.9	0	**1.3**	**1.2**	**1.4**	**1.3**
																	DSVVAGFQWATK	46	1	1	4503483	N-Term(TMT6plex); K12(TMT6plex)	1767.0	0	**1.2**	**1.3**	**1.4**	**1.4**
																	CLYASVLTAQPR	15	1	1	4503483	N-Term(TMT6plex); C1(Carbamidomethyl)	1607.9	0	**1.3**	**1.1**	**1.3**	**1.2**
																	AYLPVNESFGFTADLR	72	1	1	4503483	N-Term(TMT6plex)	2029.1	0	**1.3**	**1.4**	**1.5**	**1.4**
																	EGIPALDNFLDKL	128	1	1	4503483	N-Term(TMT6plex); K12(TMT6plex)	1903.1	1	**1.2**	**1.3**	**1.3**	**1.5**
																	STLTDSLVCK	25	1	1	4503483	N-Term(TMT6plex); C9(Carbamidomethyl); K10(TMT6plex)	1581.9	0	**1.2**	**1.2**	**1.3**	**1.3**
																	ETVSEESNVLCLSK	48	1	1	4503483	N-Term(TMT6plex); C11(Carbamidomethyl); K14(TMT6plex)	2053.1	0	**1.3**	**1.4**	**1.4**	**1.4**
																	WLPAGDALLQMITIHLPSPVTAQK	54	1	1	4503483	N-Term(TMT6plex); M11(Oxidation); K24(TMT6plex)	3074.7	0	**1.3**	**1.4**	**1.6**	**1.5**
																	GHVFEESQVAGTPMFVVK	13	1	1	4503483	N-Term(TMT6plex); K18(TMT6plex)	2420.3	0	**1.5**	**1.7**	**1.9**	**1.6**
																	GGGQIIPTAR	24	4	2	385298678;217272892;4503483	N-Term(TMT6plex)	1198.7	0				
																	YVEPIEDVPCGNIVGLVGVDQFLVK	55	1	1	4503483	N-Term(TMT6plex); C10(Carbamidomethyl); K25(TMT6plex)	3217.8	0	**1.4**	**1.6**	**1.7**	**1.6**
																	AGIIASAR	14	1	1	4503483	N-Term(TMT6plex)	987.6	0	**1.2**	**1.3**	**1.3**	**1.4**
																	IMGPNYTPGK	28	1	1	4503483	N-Term(TMT6plex); M2(Oxidation); K10(TMT6plex)	1551.9	0	**1.3**	**1.2**	**1.5**	**1.3**
																	TGTITTFEHAHNMR	18	1	1	4503483	N-Term(TMT6plex); M13(Oxidation)	1860.9	0	**1.2**	**1.2**	**1.3**	**1.3**
																	ARPFPDGLAEDIDKGEVSAR	7	1	1	4503483	N-Term(TMT6plex); K14(TMT6plex)	2601.4	1	**1.3**	**1.4**	**1.2**	**1.4**
																	VFSGLVSTGLK	31	1	1	4503483	N-Term(TMT6plex); K11(TMT6plex)	1566.0	0	**1.3**	**1.1**	**1.3**	**1.2**
																	YEWDVAEAR	26	1	1	4503483	N-Term(TMT6plex)	1367.7	0	**1.3**	**1.3**	**1.3**	**1.5**
																	SATSPEGK	22	1	1	4503483	N-Term(TMT6plex); K8(TMT6plex)	1234.7	0	**1.4**	**1.1**	**1.3**	**1.3**
																	DLEEDHACIPIK	18	1	1	4503483	N-Term(TMT6plex); C8(Carbamidomethyl); K12(TMT6plex)	1898.0	0	**1.2**	**1.3**	**1.3**	**1.2**
																	NPADLPK	20	1	1	4503483	N-Term(TMT6plex); K7(TMT6plex)	1212.7	0	**1.2**	**1.2**	**1.3**	**1.4**
																	LMEPIYLVEIQCPEQVVGGIYGVLNR	19	1	1	4503483	N-Term(TMT6plex); C12(Carbamidomethyl)	3218.7	0	**1.6**	**1.4**	**1.7**	**1.5**
																	IWCFGPDGTGPNILTDITK	66	1	1	4503483	N-Term(TMT6plex); C3(Carbamidomethyl); K19(TMT6plex)	2563.4	0	**1.3**	**1.3**	**1.4**	**1.4**
																	IMGPNYTPGK	5	1	1	4503483	N-Term(TMT6plex); K10(TMT6plex)	1535.9	0	**1.3**	**1.4**	**1.6**	**1.2**
																	GEGQLGPAER	12	1	1	4503483	N-Term(TMT6plex)	1242.7	0	**1.2**	**1.2**	**1.3**	**1.3**
																	SNTGGQAFPQCVFDHWQILPGDPFDNSSRPSQVVAETR	12	1	1	4503483	N-Term(TMT6plex); C11(Carbamidomethyl)	4474.1	0	**1.3**	**1.3**	**1.3**	**1.5**
																	DGAGFLINLIDSPGHVDFSSEVTAALR	22	1	1	4503483	N-Term(TMT6plex)	3030.6	0	**1.4**	**1.5**	**1.8**	**1.5**
																	TFCQLILDPIFK	108	1	1	4503483	N-Term(TMT6plex); C3(Carbamidomethyl); K12(TMT6plex)	1953.1	0	**1.3**	**1.1**	**1.3**	**1.1**
																	KEDLYLKPIQR	5	1	1	4503483	N-Term(TMT6plex); K1(TMT6plex); K7(TMT6plex)	2090.3	1	**1.1**	**1.2**	**1.3**	**1.4**
																	EGIPALDNFLDK	24	1	1	4503483	N-Term(TMT6plex); K12(TMT6plex)	1790.0	0	**1.1**	**1.0**	**1.2**	**1.2**
																	GHVFEESQVAGTPMFVVK	16	1	1	4503483	N-Term(TMT6plex); M14(Oxidation); K18(TMT6plex)	2436.3	0	**1.2**	**1.3**	**1.5**	**1.3**
																	VNFTVDQIR	7	1	1	4503483	N-Term(TMT6plex)	1320.7	0	**1.3**	**1.1**	**1.3**	**1.3**
																	WLPAGDALLQMITIHLPSPVTAQK	19	1	1	4503483	N-Term(TMT6plex); K24(TMT6plex)	3058.8	0	**1.3**	**1.5**	**1.9**	**1.4**
																	QFAEMYVAK	14	1	1	4503483	N-Term(TMT6plex); K9(TMT6plex)	1544.9	0	**1.4**	**1.4**	**1.7**	**1.4**
																	SDPMVQCIIEESGEHIIAGAGELHLEICLK	43	1	1	4503483	N-Term(TMT6plex); M4(Oxidation); C7(Carbamidomethyl); C28(Carbamidomethyl); K30(TMT6plex)	3822.9	0	**1.2**	**1.3**	**1.5**	**1.4**
																	NMSVIAHVDHGK	18	1	1	4503483	N-Term(TMT6plex); M2(Oxidation); K12(TMT6plex)	1782.0	0	**1.0**	**1.0**	**1.1**	**1.2**
																	GVQYLNEIK	42	1	1	4503483	N-Term(TMT6plex); K9(TMT6plex)	1521.9	0	**1.2**	**1.3**	**1.3**	**1.4**
																	QFAEMYVAK	13	1	1	4503483	N-Term(TMT6plex); M5(Oxidation); K9(TMT6plex)	1560.8	0	**1.3**	**1.3**	**1.4**	**1.4**
																	EDLYLKPIQR	7	1	1	4503483	N-Term(TMT6plex); K6(TMT6plex)	1733.0	0	**1.2**	**1.3**	**1.4**	**1.5**
																	YFDPANGK	6	1	1	4503483	N-Term(TMT6plex); K8(TMT6plex)	1369.8	0	**1.2**	**1.1**	**1.3**	**1.2**
																	IKPVLMMNK	5	1	1	4503483	N-Term(TMT6plex); K2(TMT6plex); M6(Oxidation); K9(TMT6plex)	1777.1	0	**1.3**	**1.6**	**1.8**	**1.5**
																	NMSVIAHVDHGK	1	1	1	4503483	N-Term(TMT6plex); K12(TMT6plex)	1766.0	0	**1.4**	**1.5**	**1.6**	**1.3**
																	TGTITTFEHAHNMR	2	1	1	4503483	N-Term(TMT6plex)	1844.9	0	**1.0**	**1.4**	**1.3**	**1.3**
																	IKPVLMMNK	4	1	1	4503483	N-Term(TMT6plex); K2(TMT6plex); K9(TMT6plex)	1761.1	0	**1.4**	**1.5**	**1.6**	**1.3**
																	CELLYEGPPDDEAAMGIK	3	1	1	4503483	N-Term(TMT6plex); C1(Carbamidomethyl); M15(Oxidation); K18(TMT6plex)	2482.2	0	**1.2**	**1.3**	**1.5**	**1.1**
																	LDSEDKDK	8	1	1	4503483	N-Term(TMT6plex); K6(TMT6plex); K8(TMT6plex)	1636.9	1	**1.3**	**1.0**	**1.2**	**1.2**
																	GPLMMYISK	2	1	1	4503483	N-Term(TMT6plex); M4(Oxidation); M5(Oxidation); K9(TMT6plex)	1529.8	0	**0.9**	**1.3**	**1.0**	**1.7**
																	GPLMMYISK	5	1	1	4503483	N-Term(TMT6plex); M5(Oxidation); K9(TMT6plex)	1513.9	0	**1.2**	**1.6**	**1.5**	**1.6**
																	FSVSPVVR	14	1	1	4503483	N-Term(TMT6plex)	1119.7	0	**1.4**	**1.1**	**1.3**	**1.3**
																	TILMMGR	1	1	1	4503483	N-Term(TMT6plex)	1050.6	0	**2.1**	**1.9**	**2.4**	**1.5**
																	KVEDMMK	1	1	1	4503483	N-Term(TMT6plex); K1(TMT6plex); K7(TMT6plex)	1567.9	1	**2.3**	**2.4**	**2.7**	**1.6**
																	IKPVLMMNK	7	1	1	4503483	N-Term(TMT6plex); K2(TMT6plex); M6(Oxidation); M7(Oxidation); K9(TMT6plex)	1793.1	0	**1.1**	**1.3**	**1.3**	**1.5**
																	EGKPLLK	1	1	1	4503483	N-Term(TMT6plex); K3(TMT6plex); K7(TMT6plex)	1472.0	0	**0.9**	**1.2**	**1.2**	**1.3**
																	VAVEAK	4	1	1	4503483	N-Term(TMT6plex); K6(TMT6plex)	1074.7	0	**1.1**	**1.2**	**1.3**	**1.3**
																	LDSEDKDKEGKPLLK	1	1	1	4503483	N-Term(TMT6plex); K6(TMT6plex); K8(TMT6plex); K11(TMT6plex); K15(TMT6plex)	2860.7	2	**1.2**	**1.7**	**1.3**	**1.8**
																	FYAFGR	5	1	1	4503483	N-Term(TMT6plex)	989.5	0	**1.4**	**1.0**	**1.2**	**1.2**
																	QAIAER	1	2	2	4503483;4506001	N-Term(TMT6plex)	916.5	0				
																	MVPTSDK	1	1	1	4503483	N-Term(TMT6plex); M1(Oxidation); K7(TMT6plex)	1251.7	0	**1.6**	**1.8**	**2.0**	**1.4**
57863257	NP_110379.2	**TCP1**	6950	**T-complex protein 1 subunit alpha isoform a**	76.98	2	34	34	643	**1.4**	**1.4**	**1.5**	**1.5**	556	60.3	6.11												
																	ASGATILSTLANLEGEETFEAAMLGQAEEVVQER	148	2	1	57863257	N-Term(TMT6plex); M23(Oxidation)	3809.9	0	**1.7**	**1.5**	**1.5**	**1.5**
																	ASGATILSTLANLEGEETFEAAMLGQAEEVVQER	15	2	1	57863257	N-Term(TMT6plex)	3793.9	0	**1.7**	**2.0**	**1.9**	**1.9**
																	SVVPGGGAVEAALSIYLENYATSMGSR	35	2	1	57863257	N-Term(TMT6plex); M24(Oxidation)	2944.5	0	**1.4**	**1.4**	**1.4**	**1.4**
																	SLHDALCVVK	8	2	1	57863257	N-Term(TMT6plex); C7(Carbamidomethyl); K10(TMT6plex)	1599.9	0	**1.2**	**1.4**	**1.5**	**1.5**
																	FATEAAITILR	17	2	1	57863257	N-Term(TMT6plex)	1434.9	0	**1.4**	**1.2**	**1.3**	**1.3**
																	EQLAIAEFAR	20	2	1	57863257	N-Term(TMT6plex)	1376.8	0	**1.3**	**1.4**	**1.4**	**1.5**
																	LGVQVVITDPEK	18	2	1	57863257	N-Term(TMT6plex); K12(TMT6plex)	1756.1	0	**1.3**	**1.5**	**1.5**	**1.5**
																	SQNVMAAASIANIVK	26	1	1	57863257	N-Term(TMT6plex); M5(Oxidation); K15(TMT6plex)	1991.1	0	**1.3**	**1.3**	**1.5**	**1.4**
																	YINENLIVNTDELGR	22	1	1	57863257	N-Term(TMT6plex)	1992.1	0	**1.4**	**1.5**	**1.5**	**1.3**
																	YFVEAGAMAVR	4	2	1	57863257	N-Term(TMT6plex)	1442.8	0	**1.7**	**1.7**	**2.0**	**1.4**
																	AFHNEAQVNPER	17	2	1	57863257	N-Term(TMT6plex)	1640.8	0	**1.2**	**1.2**	**1.2**	**1.3**
																	SQMESMLISGYALNCVVGSQGMPK	3	2	1	57863257	N-Term(TMT6plex); M3(Oxidation); M6(Oxidation); C15(Carbamidomethyl); M22(Oxidation); K24(TMT6plex)	3093.5	0	**1.2**	**1.4**	**1.4**	**1.6**
																	IHPTSVISGYR	16	1	1	57863257	N-Term(TMT6plex)	1458.8	0	**1.3**	**1.4**	**1.4**	**1.5**
																	MLVDDIGDVTITNDGATILK	46	1	1	57863257	N-Term(TMT6plex); M1(Oxidation); K20(TMT6plex)	2578.4	0	**1.5**	**1.6**	**1.7**	**1.6**
																	ICDDELILIK	22	2	1	57863257	N-Term(TMT6plex); C2(Carbamidomethyl); K10(TMT6plex)	1690.0	0	**1.4**	**1.5**	**1.6**	**1.5**
																	GANDFMCDEMER	8	2	1	57863257	N-Term(TMT6plex); M6(Oxidation); C7(Carbamidomethyl); M10(Oxidation)	1735.7	0	**1.4**	**1.6**	**1.7**	**1.7**
																	SQNVMAAASIANIVK	6	1	1	57863257	N-Term(TMT6plex); K15(TMT6plex)	1975.1	0	**1.4**	**1.4**	**1.7**	**1.5**
																	MLVDDIGDVTITNDGATILK	8	1	1	57863257	N-Term(TMT6plex); K20(TMT6plex)	2562.4	0	**1.8**	**2.2**	**2.3**	**1.7**
																	VLCELADLQDK	14	1	1	57863257	N-Term(TMT6plex); C3(Carbamidomethyl); K11(TMT6plex)	1762.0	0	**1.3**	**1.3**	**1.4**	**1.3**
																	SVVPGGGAVEAALSIYLENYATSMGSR	2	2	1	57863257	N-Term(TMT6plex)	2928.5	0	**1.6**	**1.8**	**1.5**	**1.5**
																	YPVNSVNILK	8	2	1	57863257	N-Term(TMT6plex); K10(TMT6plex)	1605.0	0	**1.3**	**1.4**	**1.4**	**1.5**
																	LLEVEHPAAK	21	1	1	57863257	N-Term(TMT6plex); K10(TMT6plex)	1564.9	0	**1.3**	**1.4**	**1.4**	**1.5**

Supplementary Table 6. List of all proteins and peptides identified in untreated and chronically treated Het1A cells with cigarette smoke condensate for 8 months

Accession	RefSeq Accession	Gene Symbol	Entrez Gene ID	Description	Σ Coverage	Σ# Proteins	Σ# Unique Peptides	Σ# Peptides	Σ# PSMs	Het-1A-Smoke - 2M/Parental	Het-1A-Smoke - 4M/Parental	Het-1A-Smoke - 6M/Parental	Het-1A-Smoke - 8M/Parental	# AAs	MW [kDa]	calc. pI	Sequence	# PSMs	# Proteins	# Protein Groups	Protein Group Accessions	Modifications	MH+ [Da]	# Missed Cleavages	Het-1A-Smoke - 2M/Parental	Het-1A-Smoke - 4M/Parental	Het-1A-Smoke - 6M/Parental	Het-1A-Smoke - 8M/Parental
																	VLCELADLQDKEVGDGTTSVVIIAAELLK	4	1	1	57863257	N-Term(TMT6plex); C3(Carbamidomethyl); K11(TMT6plex); K29(TMT6plex)	3787.1	1	**1.2**	**1.4**	**1.4**	**1.5**
																	LGVQVVITDPEKLDQIR	9	2	1	57863257	N-Term(TMT6plex); K12(TMT6plex)	2381.4	1	**1.4**	**1.6**	**1.5**	**1.6**
																	EVGDGTTSVVIIAAELLK	37	1	1	57863257	N-Term(TMT6plex); K18(TMT6plex)	2273.3	0	**1.3**	**1.3**	**1.7**	**1.5**
																	HGSYEDAVHSGALND	11	2	1	57863257	N-Term(TMT6plex)	1800.8	0	**1.3**	**1.3**	**1.3**	**1.5**
																	DDKHGSYEDAVHSGALND	5	2	1	57863257	N-Term(TMT6plex); K3(TMT6plex)	2388.1	1	**1.3**	**1.7**	**1.4**	**1.7**
																	IACLDFSLQK	17	2	1	57863257	N-Term(TMT6plex); C3(Carbamidomethyl); K10(TMT6plex)	1652.9	0	**1.3**	**1.4**	**1.5**	**1.5**
																	ILATGANVILTTGGIDDMCLK	6	2	1	57863257	N-Term(TMT6plex); C19(Carbamidomethyl); K21(TMT6plex)	2634.5	0	**1.7**	**1.6**	**2.1**	**1.5**
																	SSLGPVGLDK	22	1	1	57863257	N-Term(TMT6plex); K10(TMT6plex)	1430.9	0	**1.4**	**1.4**	**1.5**	**1.5**
																	NADELVK	14	1	1	57863257	N-Term(TMT6plex); K7(TMT6plex)	1246.7	0	**1.3**	**1.2**	**1.4**	**1.4**
																	DCLINAAK	4	1	1	57863257	N-Term(TMT6plex); C2(Carbamidomethyl); K8(TMT6plex)	1362.8	0	**1.2**	**1.3**	**1.4**	**1.4**
																	SQMESMLISGYALNCVVGSQGMPK	1	2	1	57863257	N-Term(TMT6plex); M6(Oxidation); C15(Carbamidomethyl); M22(Oxidation); K24(TMT6plex)	3077.5	0	**1.4**	**1.7**	**1.7**	**1.9**
																	GANDFMCDEMER	2	2	1	57863257	N-Term(TMT6plex); C7(Carbamidomethyl); M10(Oxidation)	1719.7	0	**1.5**	**2.2**	**2.3**	**1.7**
																	SLLVIPNTLAVNAAQDSTDLVAK	11	2	1	57863257	N-Term(TMT6plex); K23(TMT6plex)	2811.6	0	**1.5**	**1.7**	**1.5**	**1.8**
																	YFVEAGAMAVR	2	2	1	57863257	N-Term(TMT6plex); M8(Oxidation)	1458.8	0	**1.5**	**1.4**	**1.7**	**1.5**
																	ILATGANVILTTGGIDDMCLK	4	2	1	57863257	N-Term(TMT6plex); M18(Oxidation); C19(Carbamidomethyl); K21(TMT6plex)	2650.5	0	**1.6**	**1.8**	**1.9**	**1.5**
																	QAGVFEPTIVK	4	2	1	57863257	N-Term(TMT6plex); K11(TMT6plex)	1647.0	0	**1.2**	**1.2**	**1.2**	**1.3**
																	WIGLDLSNGKPR	1	2	1	57863257	N-Term(TMT6plex); K10(TMT6plex)	1814.0	0	**1.2**	**1.3**	**1.4**	**1.5**
																	TSASIILR	2	2	1	57863257	N-Term(TMT6plex)	1089.7	0	**1.6**	**1.2**	**1.5**	**1.3**
																	IDDLIK	2	2	1	57863257	N-Term(TMT6plex); K6(TMT6plex)	1174.7	0	**1.2**	**1.2**	**1.3**	**1.3**
																	ESDITK	1	2	1	57863257	N-Term(TMT6plex); K6(TMT6plex)	1150.7	0	**1.4**	**1.5**	**1.6**	**1.7**
4505591;32455266	NP_002565.1	**PRDX1**	5052	**peroxiredoxin-1**	76.88	1	11	14	211	**1.4**	**1.7**	**1.5**	**1.7**	199	22.1	8.13												
																	QITVNDLPVGR	15	2	2	32189392;4505591;32455266	N-Term(TMT6plex)	1440.8	0				
																	ATAVMPDGQFK	32	1	1	4505591;32455266	N-Term(TMT6plex); M5(Oxidation); K11(TMT6plex)	1638.9	0	**1.4**	**1.8**	**1.4**	**1.7**
																	IGHPAPNFK	15	1	1	4505591;32455266	N-Term(TMT6plex); K9(TMT6plex)	1438.9	0	**1.3**	**1.7**	**1.4**	**1.6**
																	LVQAFQFTDK	27	1	1	4505591;32455266	N-Term(TMT6plex); K10(TMT6plex)	1655.0	0	**1.5**	**1.9**	**1.5**	**1.7**
																	GLFIIDDK	33	2	2	5453549;4505591;32455266	N-Term(TMT6plex); K8(TMT6plex)	1378.8	0				
																	DISLSDYK	21	1	1	4505591;32455266	N-Term(TMT6plex); K8(TMT6plex)	1398.8	0	**1.5**	**1.9**	**1.4**	**1.8**
																	KQGGLGPMNIPLVSDPK	4	1	1	4505591;32455266	N-Term(TMT6plex); K1(TMT6plex); M8(Oxidation); K17(TMT6plex)	2454.4	1	**1.6**	**1.6**	**1.5**	**1.8**
																	QGGLGPMNIPLVSDPK	13	1	1	4505591;32455266	N-Term(TMT6plex); M7(Oxidation); K16(TMT6plex)	2097.2	0	**1.4**	**1.4**	**1.4**	**1.4**
																	YVVFFFYPLDFTFVCPTEIIAFSDR	4	1	1	4505591;32455266	N-Term(TMT6plex); C15(Carbamidomethyl)	3322.7	0	**1.9**	**1.5**	**2.5**	**1.1**
																	TIAQDYGVLK	21	1	1	4505591;32455266	N-Term(TMT6plex); K10(TMT6plex)	1565.9	0	**1.5**	**1.7**	**1.5**	**1.7**
																	HGEVCPAGWKPGSDTIKPDVQK	3	1	1	4505591;32455266	N-Term(TMT6plex); C5(Carbamidomethyl); K10(TMT6plex); K17(TMT6plex); K22(TMT6plex)	3322.8	0	**1.3**	**1.7**	**1.4**	**1.6**
																	ADEGISFR	9	1	1	4505591;32455266	N-Term(TMT6plex)	1123.6	0	**1.3**	**1.6**	**1.4**	**1.6**
																	SVDETLR	8	2	2	4505591;32455266;5453549	N-Term(TMT6plex)	1048.6	0				
																	SKEYFSK	2	1	1	4505591;32455266	N-Term(TMT6plex); K2(TMT6plex); K7(TMT6plex)	1575.9	1	**1.4**	**2.5**	**1.6**	**2.3**
																	ATAVMPDGQFK	2	1	1	4505591;32455266	N-Term(TMT6plex); K11(TMT6plex)	1622.9	0	**1.3**	**1.6**	**1.2**	**1.4**
																	QGGLGPMNIPLVSDPK	1	1	1	4505591;32455266	N-Term(TMT6plex); K16(TMT6plex)	2081.2	0	**1.6**	**1.5**	**1.8**	**1.6**
																	KQGGLGPMNIPLVSDPK	1	1	1	4505591;32455266	N-Term(TMT6plex); K1(TMT6plex); K17(TMT6plex)	2438.4	1	**1.3**	**1.6**	**1.4**	**1.8**
12025678	NP_004915.2	**ACTN4**	81	**alpha-actinin-4**	76.73	1	43	66	1343	**1.4**	**1.5**	**1.8**	**1.5**	911	104.8	5.44												
																	SFSTALYGESDL	28	1	1	12025678	N-Term(TMT6plex)	1518.8	0	**1.4**	**1.4**	**1.5**	**1.5**
																	LSGSNPYTTVTPQIINSK	18	1	1	12025678	N-Term(TMT6plex); K18(TMT6plex)	2378.3	0	**1.3**	**1.3**	**1.7**	**1.2**
																	QLEAIDQLHLEYAK	34	1	1	12025678	N-Term(TMT6plex); K14(TMT6plex)	2129.2	0	**1.4**	**1.5**	**1.8**	**1.5**
																	ELPPDQAEYCIAR	100	4	2	12025678;4501891	N-Term(TMT6plex); C10(Carbamidomethyl)	1790.9	0				
																	NFITAEELR	8	1	1	12025678	N-Term(TMT6plex)	1321.7	0	**1.5**	**1.4**	**1.9**	**1.3**
																	GISQEQMQEFR	48	1	1	12025678	N-Term(TMT6plex); M7(Oxidation)	1597.8	0	**1.4**	**1.8**	**2.0**	**1.7**
																	TINEVENQILTR	44	4	2	12025678;4501891	N-Term(TMT6plex)	1658.9	0				
																	DYETATLSDIK	14	1	1	12025678	N-Term(TMT6plex); K11(TMT6plex)	1713.9	0	**1.3**	**1.6**	**1.8**	**1.6**
																	EAILAIHK	15	1	1	12025678	N-Term(TMT6plex); K8(TMT6plex)	1352.9	0	**1.2**	**1.5**	**1.8**	**1.5**
																	KAGTQIENIDEDFR	7	1	1	12025678	N-Term(TMT6plex); K1(TMT6plex)	2094.1	1	**1.5**	**1.5**	**1.7**	**1.6**
																	GYEEWLLNEIR	69	7	3	12025678;4501891;507588248	N-Term(TMT6plex)	1650.9	0				
																	EGLLLWCQR	22	8	3	12025678;4501891;507588248	N-Term(TMT6plex); C7(Carbamidomethyl)	1403.8	0				
																	ISIEMNGTLEDQLSHLK	5	1	1	12025678	N-Term(TMT6plex); M5(Oxidation); K17(TMT6plex)	2402.3	0	**1.5**	**1.4**	**1.5**	**1.5**
																	HEAFESDLAAHQDR	4	9	3	12025678;4501891;507588248	N-Term(TMT6plex)	1854.9	0				
																	CQLEINFNTLQTK	27	9	3	12025678;4501891;507588248	N-Term(TMT6plex); C1(Carbamidomethyl); K13(TMT6plex)	2067.1	0				
																	VLAVNQENEHLMEDYEK	4	1	1	12025678	N-Term(TMT6plex); K17(TMT6plex)	2519.3	0	**1.8**	**2.4**	**3.3**	**2.1**
																	VGWEQLLTTIAR	69	4	2	12025678;4501891	N-Term(TMT6plex)	1615.9	0				
																	VLAVNQENEHLMEDYEK	25	1	1	12025678	N-Term(TMT6plex); M12(Oxidation); K17(TMT6plex)	2535.3	0	**1.3**	**1.6**	**1.8**	**1.6**
																	STLPDADRER	9	1	1	12025678	N-Term(TMT6plex)	1388.7	1	**1.4**	**1.6**	**1.8**	**1.5**
																	TIQEMQQK	27	1	1	12025678	N-Term(TMT6plex); M5(Oxidation); K8(TMT6plex)	1479.8	0	**1.4**	**1.5**	**1.8**	**1.5**
																	MLDAEDIVNTARPDEK	5	1	1	12025678	N-Term(TMT6plex); K16(TMT6plex)	2275.2	0	**1.7**	**2.0**	**2.6**	**1.5**
																	MLDAEDIVNTARPDEK	21	1	1	12025678	N-Term(TMT6plex); M1(Oxidation); K16(TMT6plex)	2291.2	0	**1.4**	**1.6**	**1.7**	**1.6**
																	LASDLLEWIR	42	4	2	12025678;4501891	N-Term(TMT6plex)	1444.8	0				
																	VEQIAAIAQELNELDYYDSHNVNTR	44	1	1	12025678	N-Term(TMT6plex)	3134.6	0	**1.4**	**1.4**	**1.5**	**1.4**
																	KDDPVTNLNNAFEVAEK	35	1	1	12025678	N-Term(TMT6plex); K1(TMT6plex); K17(TMT6plex)	2591.4	1	**1.3**	**1.5**	**1.7**	**1.5**
																	DHALLEEQSK	4	1	1	12025678	N-Term(TMT6plex); K10(TMT6plex)	1627.9	0	**1.4**	**1.5**	**1.7**	**1.3**
																	AETAANR	5	5	3	12025678;4501891;507588248	N-Term(TMT6plex)	961.5	0				
																	ASIHEAWTDGK	16	4	2	12025678;4501891	N-Term(TMT6plex); K11(TMT6plex)	1672.9	0				
																	KAGTQIENIDEDFRDGLK	3	1	1	12025678	N-Term(TMT6plex); K1(TMT6plex); K18(TMT6plex)	2736.5	2	**1.3**	**1.6**	**1.8**	**1.6**
																	IAESNHIK	7	1	1	12025678	N-Term(TMT6plex); K8(TMT6plex)	1369.8	0	**1.3**	**1.6**	**1.9**	**1.5**
																	ETTDTDTADQVIASFK	52	1	1	12025678	N-Term(TMT6plex); K16(TMT6plex)	2200.1	0	**1.4**	**1.6**	**1.8**	**1.5**
																	LMLLLEVISGER	65	8	3	12025678;4501891;507588248	N-Term(TMT6plex); M2(Oxidation)	1617.9	0				
																	SIVDYKPNLDLLEQQHQLIQEALIFDNK	12	1	1	12025678	N-Term(TMT6plex); K6(TMT6plex); K28(TMT6plex)	4012.2	0	**1.4**	**1.7**	**2.1**	**1.6**
																	QFASQANVVGPWIQTK	16	1	1	12025678	N-Term(TMT6plex); K16(TMT6plex)	2232.3	0	**1.5**	**1.5**	**1.8**	**1.4**
																	TIQEMQQK	2	1	1	12025678	N-Term(TMT6plex); K8(TMT6plex)	1463.8	0	**2.0**	**2.4**	**3.5**	**1.6**
																	QGEAEFNR	6	1	1	12025678	N-Term(TMT6plex)	1179.6	0	**1.6**	**1.4**	**1.6**	**1.4**
																	GISQEQMQEFR	5	1	1	12025678	N-Term(TMT6plex)	1581.8	0	**1.4**	**1.7**	**1.8**	**1.7**
																	LVSIGAEEIVDGNAK	43	1	1	12025678	N-Term(TMT6plex); K15(TMT6plex)	1973.1	0	**1.4**	**1.6**	**1.7**	**1.6**
																	FAIQDISVEETSAK	44	8	3	12025678;4501891;507588248	N-Term(TMT6plex); K14(TMT6plex)	1996.1	0				
																	HTNYTMEHIR	2	7	3	12025678;4501891;507588248	N-Term(TMT6plex); M6(Oxidation)	1546.8	0				
																	QQSNEHLR	6	1	1	12025678	N-Term(TMT6plex)	1240.7	0	**1.2**	**1.3**	**1.5**	**1.3**
																	ALDFIASK	30	6	2	12025678;4501891	N-Term(TMT6plex); K8(TMT6plex)	1322.8	0				
																	AGTQIENIDEDFRDGLK	9	1	1	12025678	N-Term(TMT6plex); K17(TMT6plex)	2379.3	1	**1.5**	**1.8**	**1.9**	**1.7**
																	MAPYQGPDAVPGALDYK	21	1	1	12025678	N-Term(TMT6plex); M1(Oxidation); K17(TMT6plex)	2267.2	0	**1.4**	**1.4**	**1.8**	**1.3**
																	DHGGALGPEEFK	6	1	1	12025678	N-Term(TMT6plex); K12(TMT6plex)	1714.9	0	**1.2**	**1.3**	**1.6**	**1.5**
																	RDHALLEEQSK	11	1	1	12025678	N-Term(TMT6plex); K11(TMT6plex)	1784.0	1	**1.3**	**1.6**	**1.8**	**1.5**
																	NVNVQNFHISWK	4	1	1	12025678	N-Term(TMT6plex); K12(TMT6plex)	1944.1	0	**1.2**	**1.4**	**1.8**	**1.3**
																	ASFNHFDKDHGGALGPEEFK	2	1	1	12025678	N-Term(TMT6plex); K8(TMT6plex); K20(TMT6plex)	2890.5	1	**1.2**	**1.8**	**2.1**	**1.7**
																	ASFNHFDK	3	1	1	12025678	N-Term(TMT6plex); K8(TMT6plex)	1423.8	0	**1.3**	**1.7**	**1.7**	**1.6**
																	KHEAFESDLAAHQDR	33	7	3	12025678;4501891;507588248	N-Term(TMT6plex); K1(TMT6plex)	2212.2	1				
																	ICDQWDALGSLTHSR	8	1	1	12025678	N-Term(TMT6plex); C2(Carbamidomethyl)	1988.0	0	**1.3**	**1.2**	**1.4**	**1.3**
																	ACLISLGYDVENDR	12	1	1	12025678	N-Term(TMT6plex); C2(Carbamidomethyl)	1853.9	0	**1.6**	**1.9**	**2.0**	**1.6**
																	MTLGMIWTIILR	10	8	3	12025678;4501891;507588248	N-Term(TMT6plex); M5(Oxidation)	1693.0	0				
																	VLAGDKNFITAEELR	2	1	1	12025678	N-Term(TMT6plex); K6(TMT6plex)	2134.2	1	**1.7**	**2.6**	**2.0**	**2.0**
																	MTLGMIWTIILR	11	8	3	12025678;4501891;507588248	N-Term(TMT6plex); M1(Oxidation); M5(Oxidation)	1709.0	0				
																	DGLAFNALIHR	27	2	1	12025678	N-Term(TMT6plex)	1455.8	0	**1.4**	**1.5**	**1.6**	**1.5**
																	AGTQIENIDEDFR	14	1	1	12025678	N-Term(TMT6plex)	1736.9	0	**1.3**	**1.4**	**1.7**	**1.5**
																	DDPVTNLNNAFEVAEK	16	1	1	12025678	N-Term(TMT6plex); K16(TMT6plex)	2234.2	0	**1.4**	**1.6**	**1.8**	**1.6**
																	DLLLDPAWEK	25	7	3	12025678;4501891;507588248	N-Term(TMT6plex); K10(TMT6plex)	1657.9	0				
																	HRPELIEYDK	10	1	1	12025678	N-Term(TMT6plex); K10(TMT6plex)	1758.0	0	**1.3**	**1.6**	**2.0**	**1.5**
																	TIPWLEDR	5	1	1	12025678	N-Term(TMT6plex)	1258.7	0	**1.4**	**1.5**	**1.8**	**1.5**
																	TFTAWCNSHLR	4	7	3	12025678;4501891;507588248	N-Term(TMT6plex); C6(Carbamidomethyl)	1621.8	0				
																	MVSDINNGWQHLEQAEK	2	1	1	12025678	N-Term(TMT6plex); M1(Oxidation); K17(TMT6plex)	2473.3	0	**1.4**	**1.3**	**1.5**	**1.4**
																	MTLGMIWTIILR	6	8	3	12025678;4501891;507588248	N-Term(TMT6plex)	1677.0	0				
																	KTFTAWCNSHLR	1	7	3	12025678;4501891;507588248	N-Term(TMT6plex); K1(TMT6plex); C7(Carbamidomethyl)	1979.1	1				
																	LMLLLEVISGER	4	8	3	12025678;4501891;507588248	N-Term(TMT6plex)	1601.9	0				
																	AIMTYVSSFYHAFSGAQK	1	4	2	12025678;4501891	N-Term(TMT6plex); K18(TMT6plex)	2466.3	0				
																	LDHLAEK	21	4	2	12025678;4501891	N-Term(TMT6plex); K7(TMT6plex)	1283.8	0				
																	VQQLVPK	18	1	1	12025678	N-Term(TMT6plex); K7(TMT6plex)	1269.8	0	**1.3**	**1.4**	**1.8**	**1.4**
																	STLPDADR	4	1	1	12025678	N-Term(TMT6plex)	1103.6	0	**1.2**	**1.4**	**1.7**	**1.3**
																	INNVNK	3	1	1	12025678	N-Term(TMT6plex); K6(TMT6plex)	1159.7	0	**1.5**	**1.3**	**1.9**	**1.4**
																	LSNRPAFMPSEGK	1	4	2	12025678;507588248	N-Term(TMT6plex); M8(Oxidation); K13(TMT6plex)	1908.0	0				
																	YLDIPK	7	6	2	12025678;4501891	N-Term(TMT6plex); K6(TMT6plex)	1206.7	0				
154354964	NP_006830.2	**IMMT**	10989	**mitochondrial inner membrane protein isoform 1**	76.65	2	1	45	307					758	83.6	6.48												
																	GIEQAVQSHAVAEEEAR	10	3	2	154354964;154354966	N-Term(TMT6plex)	2053.0	0				
																	LSQEQVDNFTLDINTAYAR	12	3	2	154354964;154354966	N-Term(TMT6plex)	2427.2	0				
																	AAMDNSEIAGEK	21	3	2	154354964;154354966	N-Term(TMT6plex); M3(Oxidation); K12(TMT6plex)	1709.9	0				

Accession	RefSeq Accession	Gene Symbol	Entrez Gene ID	Description	Σ Coverage	Σ# Proteins	Σ# Unique Peptides	Σ# Peptides	Σ# PSMs	Het-1A-Smoke - 2M/Parental	Het-1A-Smoke - 4M/Parental	Het-1A-Smoke - 6M/Parental	Het-1A-Smoke - 8M/Parental	# AAs	MW [kDa]	calc. pI	Sequence	# PSMs	# Proteins	# Protein Groups	Protein Group Accessions	Modifications	MH+ [Da]	# Missed Cleavages	Het-1A-Smoke - 2M/Parental	Het-1A-Smoke - 4M/Parental	Het-1A-Smoke - 6M/Parental	Het-1A-Smoke - 8M/Parental
																	TSSAETPTIPLGSAVEAIK	8	3	2	154354964;154354966	N-Term(TMT6plex); K19(TMT6plex)	2330.3	0				
																	GVYSEETLR	10	3	2	154354964;154354966	N-Term(TMT6plex)	1282.7	0				
																	AVDEAADALLK	13	3	2	154354964;154354966	N-Term(TMT6plex); K11(TMT6plex)	1573.9	0				
																	SIQSGPLK	12	3	2	154354964;154354966	N-Term(TMT6plex); K8(TMT6plex)	1287.8	0				
																	IAGAGLLFVGGGIGGTILYAK	1	3	2	154354964;154354966	N-Term(TMT6plex); K21(TMT6plex)	2406.5	0				
																	TDHPEIGEGKPTPALSEEASSSSIR	6	2	2	154354964;154354966	N-Term(TMT6plex); K10(TMT6plex)	3053.6	0				
																	QPASQLQK	6	3	2	154354964;154354966	N-Term(TMT6plex); K8(TMT6plex)	1357.8	0				
																	LHNMIVDLDNVVK	4	3	2	154354964;154354966	N-Term(TMT6plex); K13(TMT6plex)	1968.1	0				
																	VAMIDETR	3	3	2	154354964;154354966	N-Term(TMT6plex); M3(Oxidation)	1179.6	0				
																	GMSVSDLADK	7	3	2	154354964;154354966	N-Term(TMT6plex); M2(Oxidation); K10(TMT6plex)	1496.8	0				
																	GMSVSDLADK	2	3	2	154354964;154354966	N-Term(TMT6plex); K10(TMT6plex)	1480.8	0				
																	YSTSGSSGLTTGK	8	3	2	154354964;154354966	N-Term(TMT6plex); K13(TMT6plex)	1703.9	0				
																	QTASVTLQAIAAQNAAVQAVNAHSNILK	4	3	2	154354964;154354966	N-Term(TMT6plex); K28(TMT6plex)	3290.9	0				
																	SEFEQNLSEK	14	3	2	154354964;154354966	N-Term(TMT6plex); K10(TMT6plex)	1668.9	0				
																	VVSQYHELVVQAR	6	3	2	154354964;154354966	N-Term(TMT6plex)	1757.0	0				
																	QAAAHTDHLR	8	3	2	154354964;154354966	N-Term(TMT6plex)	1348.7	0				
																	ELDSITPEVLPGWK	10	3	2	154354964;154354966	N-Term(TMT6plex); K14(TMT6plex)	2042.2	0				
																	EVAGAKPHITAAEGK	2	3	2	154354964;154354966	N-Term(TMT6plex); K6(TMT6plex); K15(TMT6plex)	2166.3	0				
																	ISSVSEVMK	2	3	2	154354964;154354966	N-Term(TMT6plex); K9(TMT6plex)	1437.8	0				
																	LLSYASYCIEHGDLELAAK	6	3	2	154354964;154354966	N-Term(TMT6plex); C8(Carbamidomethyl); K19(TMT6plex)	2611.4	0				
																	KVQAAQSEAK	7	3	2	154354964;154354966	N-Term(TMT6plex); K1(TMT6plex); K10(TMT6plex)	1747.1	1				
																	ERPPEEVAAR	6	3	2	154354964;154354966	N-Term(TMT6plex)	1382.8	0				
																	LSTDDLNSLIAHAHR	8	3	2	154354964;154354966	N-Term(TMT6plex)	1892.0	0				
																	VQEQELK	5	3	2	154354964;154354966	N-Term(TMT6plex); K7(TMT6plex)	1331.8	0				
																	LHNMIVDLDNVVK	1	3	2	154354964;154354966	N-Term(TMT6plex); M4(Oxidation); K13(TMT6plex)	1984.1	0				
																	AHQLWLSVEALK	2	3	2	154354964;154354966	N-Term(TMT6plex); K12(TMT6plex)	1853.1	0				
																	LFEMVLGPAAYNVPLPK	4	3	2	154354964;154354966	N-Term(TMT6plex); K17(TMT6plex)	2317.3	0				
																	AFDSAVAK	4	3	2	154354964;154354966	N-Term(TMT6plex); K8(TMT6plex)	1266.7	0				
																	ANCSDNEFTQALTAAIPPESLTR	20	3	2	154354964;154354966	N-Term(TMT6plex); C3(Carbamidomethyl)	2735.4	0				
																	VQAAQSEAK	4	3	2	154354964;154354966	N-Term(TMT6plex); K9(TMT6plex)	1389.8	0				
																	LFEMVLGPAAYNVPLPK	25	3	2	154354964;154354966	N-Term(TMT6plex); M4(Oxidation); K17(TMT6plex)	2333.3	0				
																	QIVEILTAYASAVGIGTTQVQPE	5	3	2	154354964;154354966	N-Term(TMT6plex)	2617.4	0				
																	SEIQAEQDR	6	3	2	154354964;154354966	N-Term(TMT6plex)	1304.7	0				
																	SEIQAEQDRK	1	3	2	154354964;154354966	N-Term(TMT6plex); K10(TMT6plex)	1661.9	1				
																	QHITLALEK	4	3	2	154354964;154354966	N-Term(TMT6plex); K9(TMT6plex)	1510.9	0				
																	LSEQELQFR	4	3	2	154354964;154354966	N-Term(TMT6plex)	1378.8	0				
																	NSLYQYFLSYLQSLLLFPPQQLKPPPELCPEDINTFK	1	3	2	154354964;154354966	N-Term(TMT6plex); K23(TMT6plex); C29(Carbamidomethyl); K37(TMT6plex)	5128.7	0				
																	KAVDEAADALLK	2	3	2	154354964;154354966	N-Term(TMT6plex); K1(TMT6plex); K12(TMT6plex)	1931.2	1				
																	GDTPASATAPTEAAQIISAAGDTLSVPAPAVQPEESLK	3	2	1	154354964	N-Term(TMT6plex); K38(TMT6plex)	4120.2	0				
																	SVIENAK	2	3	2	154354964;154354966	N-Term(TMT6plex); K7(TMT6plex)	1218.7	0				
																	TVEGALK	4	3	2	154354964;154354966	N-Term(TMT6plex); K7(TMT6plex)	1175.7	0				
																	VAQDWLK	6	3	2	154354964;154354966	N-Term(TMT6plex); K7(TMT6plex)	1317.8	0				
																	ISSVSEVMK	2	3	2	154354964;154354966	N-Term(TMT6plex); M8(Oxidation); K9(TMT6plex)	1453.8	0				
																	SLEDALR	2	3	2	154354964;154354966	N-Term(TMT6plex)	1032.6	0				
																	AKEELEK	1	3	2	154354964;154354966	N-Term(TMT6plex); K2(TMT6plex); K7(TMT6plex)	1533.9	1				
																	LAQQEK	2	5	3	154354964;154354966;51988900	N-Term(TMT6plex); K6(TMT6plex)	1174.7	0				
																	VAMIDETR	1	3	2	154354964;154354966	N-Term(TMT6plex)	1163.6	0				
154354966	NP_001093640.1	**IMMT**	10989	**mitochondrial inner membrane protein isoform 3**	76.57	1	2	46	318	**1.4**	**1.4**	**1.4**	**1.5**	747	82.6	6.57												
																	GIEQAVQSHAVAEEEAR	10	3	2	154354964;154354966	N-Term(TMT6plex)	2053.0	0				
																	LSQEQVDNFTLDINTAYAR	12	3	2	154354964;154354966	N-Term(TMT6plex)	2427.2	0				
																	AAMDNSEIAGEK	21	3	2	154354964;154354966	N-Term(TMT6plex); M3(Oxidation); K12(TMT6plex)	1709.9	0				
																	TSSAETPTIPLGSAVEAIK	8	3	2	154354964;154354966	N-Term(TMT6plex); K19(TMT6plex)	2330.3	0				
																	QKGDTPASATAGDTLSVPAPAVQPEESLK	7	1	1	154354966	N-Term(TMT6plex); K2(TMT6plex); K29(TMT6plex)	3552.9	1	**1.3**	**1.4**	**1.3**	**1.4**
																	GVYSEETLR	10	3	2	154354964;154354966	N-Term(TMT6plex)	1282.7	0				
																	AVDEAADALLK	13	3	2	154354964;154354966	N-Term(TMT6plex); K11(TMT6plex)	1573.9	0				
																	SIQSGPLK	12	3	2	154354964;154354966	N-Term(TMT6plex); K8(TMT6plex)	1287.8	0				
																	IAGAGLLFVGGGIGGTILYAK	1	3	2	154354964;154354966	N-Term(TMT6plex); K21(TMT6plex)	2406.5	0				
																	GDTPASATAGDTLSVPAPAVQPEESLK	7	1	1	154354966	N-Term(TMT6plex); K27(TMT6plex)	3067.6	0	**1.7**	**1.5**	**1.5**	**1.6**
																	TDHPEIGEGKPTPALSEEASSSSIR	6	2	2	154354964;154354966	N-Term(TMT6plex); K10(TMT6plex)	3053.6	0				
																	QPASQLQK	6	3	2	154354964;154354966	N-Term(TMT6plex); K8(TMT6plex)	1357.8	0				
																	LHNMIVDLDNVVK	4	3	2	154354964;154354966	N-Term(TMT6plex); K13(TMT6plex)	1968.1	0				
																	VAMIDETR	3	3	2	154354964;154354966	N-Term(TMT6plex); M3(Oxidation)	1179.6	0				
																	GMSVSDLADK	7	3	2	154354964;154354966	N-Term(TMT6plex); M2(Oxidation); K10(TMT6plex)	1496.8	0				
																	GMSVSDLADK	2	3	2	154354964;154354966	N-Term(TMT6plex); K10(TMT6plex)	1480.8	0				
																	YSTSGSSGLTTGK	8	3	2	154354964;154354966	N-Term(TMT6plex); K13(TMT6plex)	1703.9	0				
																	QTASVTLQAIAAQNAAVQAVNAHSNILK	4	3	2	154354964;154354966	N-Term(TMT6plex); K28(TMT6plex)	3290.9	0				
																	SEFEQNLSEK	14	3	2	154354964;154354966	N-Term(TMT6plex); K10(TMT6plex)	1668.9	0				
																	VVSQYHELVVQAR	6	3	2	154354964;154354966	N-Term(TMT6plex)	1757.0	0				
																	QAAAHTDHLR	8	3	2	154354964;154354966	N-Term(TMT6plex)	1348.7	0				
																	ELDSITPEVLPGWK	10	3	2	154354964;154354966	N-Term(TMT6plex); K14(TMT6plex)	2042.2	0				
																	EVAGAKPHITAAEGK	2	3	2	154354964;154354966	N-Term(TMT6plex); K6(TMT6plex); K15(TMT6plex)	2166.3	0				
																	ISSVSEVMK	2	3	2	154354964;154354966	N-Term(TMT6plex); K9(TMT6plex)	1437.8	0				
																	LLSYASYCIEHGDLELAAK	6	3	2	154354964;154354966	N-Term(TMT6plex); C8(Carbamidomethyl); K19(TMT6plex)	2611.4	0				
																	KVQAAQSEAK	7	3	2	154354964;154354966	N-Term(TMT6plex); K1(TMT6plex); K10(TMT6plex)	1747.1	1				
																	ERPPEEVAAR	6	3	2	154354964;154354966	N-Term(TMT6plex)	1382.8	0				
																	LSTDDLNSLIAHAHR	8	3	2	154354964;154354966	N-Term(TMT6plex)	1892.0	0				
																	VQEQELK	5	3	2	154354964;154354966	N-Term(TMT6plex); K7(TMT6plex)	1331.8	0				
																	LHNMIVDLDNVVK	1	3	2	154354964;154354966	N-Term(TMT6plex); M4(Oxidation); K13(TMT6plex)	1984.1	0				
																	AHQLWLSVEALK	2	3	2	154354964;154354966	N-Term(TMT6plex); K12(TMT6plex)	1853.1	0				
																	LFEMVLGPAAYNVPLPK	4	3	2	154354964;154354966	N-Term(TMT6plex); K17(TMT6plex)	2317.3	0				
																	AFDSAVAK	4	3	2	154354964;154354966	N-Term(TMT6plex); K8(TMT6plex)	1266.7	0				
																	ANCSDNEFTQALTAAIPPESLTR	20	3	2	154354964;154354966	N-Term(TMT6plex); C3(Carbamidomethyl)	2735.4	0				
																	VQAAQSEAK	4	3	2	154354964;154354966	N-Term(TMT6plex); K9(TMT6plex)	1389.8	0				
																	LFEMVLGPAAYNVPLPK	25	3	2	154354964;154354966	N-Term(TMT6plex); M4(Oxidation); K17(TMT6plex)	2333.3	0				
																	QIVEILTAYASAVGIGTTQVQPE	5	3	2	154354964;154354966	N-Term(TMT6plex)	2617.4	0				
																	SEIQAEQDR	6	3	2	154354964;154354966	N-Term(TMT6plex)	1304.7	0				
																	SEIQAEQDRK	1	3	2	154354964;154354966	N-Term(TMT6plex); K10(TMT6plex)	1661.9	1				
																	QHITLALEK	4	3	2	154354964;154354966	N-Term(TMT6plex); K9(TMT6plex)	1510.9	0				
																	LSEQELQFR	4	3	2	154354964;154354966	N-Term(TMT6plex)	1378.8	0				
																	NSLYQYFLSYLQSLLLFPPQQLKPPPELCPEDINTFK	1	3	2	154354964;154354966	N-Term(TMT6plex); K23(TMT6plex); C29(Carbamidomethyl); K37(TMT6plex)	5128.7	0				
																	KAVDEAADALLK	2	3	2	154354964;154354966	N-Term(TMT6plex); K1(TMT6plex); K12(TMT6plex)	1931.2	1				
																	SVIENAK	2	3	2	154354964;154354966	N-Term(TMT6plex); K7(TMT6plex)	1218.7	0				
																	TVEGALK	4	3	2	154354964;154354966	N-Term(TMT6plex); K7(TMT6plex)	1175.7	0				
																	VAQDWLK	6	3	2	154354964;154354966	N-Term(TMT6plex); K7(TMT6plex)	1317.8	0				
																	ISSVSEVMK	2	3	2	154354964;154354966	N-Term(TMT6plex); M8(Oxidation); K9(TMT6plex)	1453.8	0				
																	SLEDALR	2	3	2	154354964;154354966	N-Term(TMT6plex)	1032.6	0				
																	AKEELEK	1	3	2	154354964;154354966	N-Term(TMT6plex); K2(TMT6plex); K7(TMT6plex)	1533.9	1				
																	LAQQEK	2	5	3	154354964;154354966;51988900	N-Term(TMT6plex); K6(TMT6plex)	1174.7	0				
																	VAMIDETR	1	3	2	154354964;154354966	N-Term(TMT6plex)	1163.6	0				
15431303	NP_000652.2	**RPL9**	6133	**60S ribosomal protein L9**	76.56	1	13	13	211	**1.1**	**1.2**	**1.2**	**1.3**	192	21.8	9.95												
																	FLDGIYVSEK	31	1	1	15431303	N-Term(TMT6plex); K10(TMT6plex)	1628.9	0	**1.2**	**1.2**	**1.3**	**1.2**
																	DFNHINVELSLLGK	43	1	1	15431303	N-Term(TMT6plex); K14(TMT6plex)	2057.2	0	**1.2**	**1.4**	**1.4**	**1.3**
																	DELILEGNDIELVSNSAALIQQATTVK	47	1	1	15431303	N-Term(TMT6plex); K27(TMT6plex)	3342.8	0	**1.3**	**1.3**	**1.3**	**1.4**
																	KFLDGIYVSEK	14	1	1	15431303	N-Term(TMT6plex); K1(TMT6plex); K11(TMT6plex)	1986.2	1	**1.1**	**1.3**	**1.3**	**1.3**
																	MRPGVACSVSQAQK	3	1	1	15431303	N-Term(TMT6plex); M1(Oxidation); C7(Carbamidomethyl); K14(TMT6plex)	1993.1	0	**1.1**	**1.1**	**1.0**	**1.1**
																	TILSNQTVDIPENVDITLK	30	1	1	15431303	N-Term(TMT6plex); K19(TMT6plex)	2571.5	0	**1.2**	**1.1**	**1.3**	**1.3**
																	SVYAHFPINVVIQENGSLVEIR	8	1	1	15431303	N-Term(TMT6plex)	2713.5	0	**1.4**	**1.8**	**1.8**	**1.6**
																	RDFNHINVELSLLGK	5	1	1	15431303	N-Term(TMT6plex); K15(TMT6plex)	2213.3	1	**1.1**	**1.5**	**1.4**	**1.5**
																	TICSHVQNMIK	5	1	1	15431303	N-Term(TMT6plex); C3(Carbamidomethyl); M9(Oxidation); K11(TMT6plex)	1805.0	0	**1.3**	**1.4**	**1.5**	**1.4**
																	TICSHVQNMIK	4	1	1	15431303	N-Term(TMT6plex); C3(Carbamidomethyl); K11(TMT6plex)	1789.0	0	**1.3**	**1.5**	**1.6**	**1.4**
																	GVTLGFR	14	1	1	15431303	N-Term(TMT6plex)	978.6	0	**1.1**	**1.2**	**1.2**	**1.3**
																	GTVQQADE	3	1	1	15431303	N-Term(TMT6plex)	1076.5	0	**1.0**	**1.1**	**0.8**	**1.0**
																	KELATVR	2	1	1	15431303	N-Term(TMT6plex); K1(TMT6plex)	1274.8	1	**1.0**	**1.3**	**1.2**	**1.3**
																	NFLGEK	2	1	1	15431303	N-Term(TMT6plex); K6(TMT6plex)	1165.7	0	**1.1**	**1.0**	**1.2**	**1.0**
4506631	NP_000980.1	**RPL30**	6156	**60S ribosomal protein L30**	76.52	1	8	9	160	**1.3**	**1.3**	**1.4**	**1.3**	115	12.8	9.63												

Khan et al., 2019. Multi-omics analysis to characterize cigarette smoke induced molecular alterations in esophageal cells
Supplementary Table 6. List of all proteins and peptides identified in untreated and chronically treated Het1A cells with cigarette smoke condensate for 8 months

Accession	RefSeq Accession	Gene Symbol	Entrez Gene ID	Description	Σ Coverage	Σ# Proteins	Σ# Unique Peptides	Σ# Peptides	Σ# PSMs	Het-1A-Smoke - 2M/Parental	Het-1A-Smoke - 4M/Parental	Het-1A-Smoke - 6M/Parental	Het-1A-Smoke - 8M/Parental	# AAs	MW [kDa]	calc. pI	Sequence	# PSMs	# Proteins	# Protein Groups	Protein Group Accessions	Modifications	MH+ [Da]	# Missed Cleavages	Het-1A-Smoke - 2M/Parental	Het-1A-Smoke - 4M/Parental	Het-1A-Smoke - 6M/Parental	Het-1A-Smoke - 8M/Parental
																	SMPEQTGEK	24	1	1	4506631	N-Term(TMT6plex); M2(Oxidation); K9(TMT6plex)	1480.8	0	1.2	1.2	1.3	1.4
																	KSEIEYYAMLAK	24	1	1	4506631	N-Term(TMT6plex); K1(TMT6plex); M9(Oxidation); K12(TMT6plex)	2149.2	1	1.1	1.2	1.2	1.3
																	VCTLAIIDPGDSDIIR	46	1	1	4506631	N-Term(TMT6plex); C2(Carbamidomethyl)	1987.1	0	1.3	1.3	1.4	1.3
																	KSEIEYYAMLAK	9	1	1	4506631	N-Term(TMT6plex); K1(TMT6plex); K12(TMT6plex)	2133.2	1	1.2	1.4	1.5	1.4
																	SEIEYYAMLAK	2	1	1	4506631	N-Term(TMT6plex); M8(Oxidation); K11(TMT6plex)	1792.0	0	1.1	1.3	1.2	1.3
																	TGVHHYSGNNIELGTACGK	5	1	1	4506631	N-Term(TMT6plex); C17(Carbamidomethyl); K19(TMT6plex)	2473.3	0	1.2	1.3	1.3	1.4
																	LVILANNCPALR	25	1	1	4506631	N-Term(TMT6plex); C8(Carbamidomethyl)	1582.9	0	1.3	1.3	1.4	1.3
																	SLESINSR	14	1	1	4506631	N-Term(TMT6plex)	1134.6	0	1.2	1.3	1.3	1.4
																	SMPEQTGEK	2	1	1	4506631	N-Term(TMT6plex); K9(TMT6plex)	1464.8	0	1.7	1.6	1.8	1.4
																	YVLGYK	8	1	1	4506631	N-Term(TMT6plex); K6(TMT6plex)	1200.7	0	1.1	1.3	1.3	1.3
																	LQLVMK	1	2	2	31541941;4506631	N-Term(TMT6plex); M5(Oxidation); K6(TMT6plex)	1205.8	0				
33286420;33286422	NP_872270.1	**PKM**	5315	**pyruvate kinase PKM isoform b**	76.46	9	5	38	2351	1.5	1.9	1.8	1.7	531	58.0	7.71												
																	IYVDDGLISLQVK	205	7	2	33286420;33286422;33286418	N-Term(TMT6plex); K13(TMT6plex)	1921.1	0				
																	DPVQEAWAEDVDLR	60	6	2	33286420;33286422;33286418	N-Term(TMT6plex)	1871.9	0				
																	AEGSDVANAVLDGADCIMLSGETAK	405	7	2	33286420;33286422;33286418	N-Term(TMT6plex); C16(Carbamidomethyl); M18(Oxidation); K25(TMT6plex)	2968.5	0				
																	GADFLVTEVENGGSLGSK	28	6	2	33286420;33286422;33286418	N-Term(TMT6plex); K18(TMT6plex)	2238.2	0				
																	GSGTAEVELK	70	6	2	33286420;33286422;33286418	N-Term(TMT6plex); K10(TMT6plex)	1448.8	0				
																	KGVNLPGAAVDLPAVSEK	67	7	2	33286420;33286422;33286418	N-Term(TMT6plex); K1(TMT6plex); K18(TMT6plex)	2452.5	1				
																	FDEILEASDGIMVAR	54	7	2	33286420;33286422;33286418	N-Term(TMT6plex); M12(Oxidation)	1911.0	0				
																	GDLGIEIPAEK	109	8	2	33286420;33286422;33286418	N-Term(TMT6plex); K11(TMT6plex)	1599.9	0				
																	GVNLPGAAVDLPAVSEK	74	7	2	33286420;33286422;33286418	N-Term(TMT6plex); K17(TMT6plex)	2095.2	0				
																	EAEAAMFHR	7	3	1	33286420;33286422	N-Term(TMT6plex); M6(Oxidation)	1306.6	0	1.2	1.5	1.4	1.4
																	FGVEQDVDMVFASFIR	255	7	2	33286420;33286422;33286418	N-Term(TMT6plex); M9(Oxidation)	2105.1	0				
																	GDYPLEAVR	52	7	2	33286420;33286422;33286418	N-Term(TMT6plex)	1248.7	0				
																	AGKPVICATQMLESMIK	60	6	2	33286420;33286422;33286418	N-Term(TMT6plex); K3(TMT6plex); C7(Carbamidomethyl); M11(Oxidation); M15(Oxidation); K17(TMT6plex)	2596.4	0				
																	VVEVGSK	31	7	2	33286420;33286422;33286418	N-Term(TMT6plex); K7(TMT6plex)	1175.7	0				
																	CDENILWLDYK	70	6	2	33286420;33286422;33286418	N-Term(TMT6plex); C1(Carbamidomethyl); K11(TMT6plex)	1927.0	0				
																	KASDVHEVR	11	6	2	33286420;33286422;33286418	N-Term(TMT6plex); K1(TMT6plex)	1498.9	1				
																	TATESFASDPILYRPVAVALDTK	48	5	2	33286420;33286422;33286418	N-Term(TMT6plex); K23(TMT6plex)	2923.6	0				
																	LDIDSPPITAR	40	6	2	33286420;33286422;33286418	N-Term(TMT6plex)	1426.8	0				
																	APIIAVTR	24	7	2	33286420;33286422;33286418	N-Term(TMT6plex)	1069.7	0				
																	GIFPVLCK	41	6	2	33286420;33286422;33286418	N-Term(TMT6plex); C7(Carbamidomethyl); K8(TMT6plex)	1391.8	0				
																	NTGIICTIGPASR	45	5	2	33286420;33286422;33286418	N-Term(TMT6plex); C6(Carbamidomethyl)	1588.9	0				
																	RFDEILEASDGIMVAR	193	7	2	33286420;33286422;33286418	N-Term(TMT6plex); M13(Oxidation)	2067.1	1				
																	VNFAMNVGK	39	7	2	33286420;33286422;33286418	N-Term(TMT6plex); M5(Oxidation); K9(TMT6plex)	1453.8	0				
																	GSGTAEVELKK	5	5	2	33286420;33286422;33286418	N-Term(TMT6plex); K10(TMT6plex); K11(TMT6plex)	1806.1	1				
																	ASSHSTDLMEAMAMGSVEASYK	8	4	1	33286420;33286422	N-Term(TMT6plex); M9(Oxidation); M12(Oxidation); M14(Oxidation); K22(TMT6plex)	2809.3	0	1.6	2.2	1.9	1.9
																	RFDEILEASDGIMVAR	17	7	2	33286420;33286422;33286418	N-Term(TMT6plex)	2051.1	1				
																	AGKPVICATQMLESMIK	14	6	2	33286420;33286422;33286418	N-Term(TMT6plex); K3(TMT6plex); C7(Carbamidomethyl); K17(TMT6plex)	2564.5	0				
																	FGVEQDVDMVFASFIR	101	7	2	33286420;33286422;33286418	N-Term(TMT6plex)	2089.1	0				
																	AGKPVICATQMLESMIK	11	6	2	33286420;33286422;33286418	N-Term(TMT6plex); K3(TMT6plex); C7(Carbamidomethyl); M11(Oxidation); K17(TMT6plex)	2580.5	0				
																	GIFPVLCKDPVQEAWAEDVDLR	15	6	2	33286420;33286422;33286418	N-Term(TMT6plex); C7(Carbamidomethyl); K8(TMT6plex)	3015.6	1				
																	KGDVVIVLTGWRPGSGFTNTMR	10	6	2	33286420;33286422;33286418	N-Term(TMT6plex); K1(TMT6plex); M21(Oxidation)	2865.6	1				
																	ITLDNAYMEK	36	7	2	33286420;33286422;33286418	N-Term(TMT6plex); M8(Oxidation); K10(TMT6plex)	1671.9	0				
																	VNFAMNVGK	8	7	2	33286420;33286422;33286418	N-Term(TMT6plex); K9(TMT6plex)	1437.8	0				
																	ITLDNAYMEK	12	7	2	33286420;33286422;33286418	N-Term(TMT6plex); K10(TMT6plex)	1655.9	0				
																	ASSHSTDLMEAMAMGSVEASYK	5	4	1	33286420;33286422	N-Term(TMT6plex); M14(Oxidation); K22(TMT6plex)	2777.3	0	1.7	2.0	1.8	1.7
																	LNFSHGTHEYHAETIK	26	5	2	33286420;33286422;33286418	N-Term(TMT6plex); K16(TMT6plex)	2342.2	0				
																	AEGSDVANAVLDGADCIMLSGETAK	28	7	2	33286420;33286422;33286418	N-Term(TMT6plex); C16(Carbamidomethyl); K25(TMT6plex)	2952.4	0				
																	LFEELVR	7	4	1	33286420;33286422	N-Term(TMT6plex)	1134.7	0	1.7	1.7	1.7	2.0
																	KLFEELVR	6	4	1	33286420;33286422	N-Term(TMT6plex); K1(TMT6plex)	1491.9	1	1.5	2.0	2.0	2.2
																	EAEAAMFHR	2	3	1	33286420;33286422	N-Term(TMT6plex)	1290.6	0	1.9	2.9	2.6	1.5
																	IENHEGVR	6	7	2	33286420;33286422;33286418	N-Term(TMT6plex)	1182.6	0				
																	FDEILEASDGIMVAR	7	7	2	33286420;33286422;33286418	N-Term(TMT6plex)	1895.0	0				
																	CLAAALIVLTESGR	2	3	1	33286420;33286422	N-Term(TMT6plex); C1(Carbamidomethyl)	1703.0	0	1.8	1.6	2.3	1.3
																	KGATLK	4	7	2	33286420;33286422;33286418	N-Term(TMT6plex); K1(TMT6plex); K6(TMT6plex)	1304.9	1				
																	VFLAQK	23	8	2	33286420;33286422;33286418	N-Term(TMT6plex); K6(TMT6plex)	1163.8	0				
																	KVLGEK	4	9	2	33286420;33286422;33286418	N-Term(TMT6plex); K1(TMT6plex); K6(TMT6plex)	1360.9	1				
																	SAHQVAR	4	7	2	33286420;33286422;33286418	N-Term(TMT6plex)	997.6	0				
																	DIQDLK	2	6	2	33286420;33286422;33286418	N-Term(TMT6plex); K6(TMT6plex)	1189.7	0				
47132620	NP_000414.2	**KRT2**	3849	**keratin, type II cytoskeletal 2 epidermal**	76.37	1	28	38	574	1.4	2.8	1.5	3.5	639	65.4	8.00												
																	GGSISGGGYGSGGGK	16	1	1	47132620	N-Term(TMT6plex); K15(TMT6plex)	1655.9	0	1.6	3.4	1.5	4.5
																	VLYDAEISQIHQSVTDTNVILSMDNSR	21	1	1	47132620	N-Term(TMT6plex); M23(Oxidation)	3293.7	0	1.6	1.9	1.5	2.3
																	TAAENDFVTLK	19	1	1	47132620	N-Term(TMT6plex); K11(TMT6plex)	1666.9	0	1.2	2.4	1.4	3.1
																	MSGDLSSNVTVSVTSSTISSNVASK	10	1	1	47132620	N-Term(TMT6plex); M1(Oxidation); K25(TMT6plex)	2932.5	0	1.4	1.7	1.3	2.1
																	GFSSGSAVVSGGSR	25	1	1	47132620	N-Term(TMT6plex)	1483.8	0	1.4	2.1	1.3	2.4
																	VDLLNQEIEFLK	56	1	1	47132620	N-Term(TMT6plex); K12(TMT6plex)	1919.1	0	1.5	3.7	1.5	5.2
																	GGGFGGGSSFGGGSGFSGGGFGGGGFGGGR	15	1	1	47132620	N-Term(TMT6plex)	2628.2	0	1.4	2.1	1.5	2.6
																	AQYEEIAQR	28	6	3	155969697;119703753;47132620	N-Term(TMT6plex)	1336.7	0				
																	DYQELMNVK	22	4	2	109148552;47132620	N-Term(TMT6plex); M6(Oxidation); K9(TMT6plex)	1613.9	0				
																	GGSGGGGSISGGGYGSGGGSGGR	15	1	1	47132620	N-Term(TMT6plex)	1970.9	0	1.4	3.9	1.4	5.4
																	FLEQQNQVLQTK	58	3	3	119395750;47132620;194018511	N-Term(TMT6plex); K12(TMT6plex)	1934.1	0				
																	DVDNAYMIK	13	1	1	47132620	N-Term(TMT6plex); M7(Oxidation); K9(TMT6plex)	1542.8	0	1.4	3.3	1.4	4.6
																	IEISELNR	25	2	2	119395750;47132620	N-Term(TMT6plex)	1202.7	0				
																	FGGFGGPGGVGGLGGPGGFGPGGYPGGIHEVSVNQSLLQPLNVK	13	1	1	47132620	N-Term(TMT6plex); K44(TMT6plex)	4550.4	0	1.3	3.5	1.4	5.4
																	VDPEIQNVK	19	1	1	47132620	N-Term(TMT6plex); K9(TMT6plex)	1499.9	0	1.4	3.0	1.5	4.0
																	YLDGLTAER	14	1	1	47132620	N-Term(TMT6plex)	1266.7	0	1.5	2.0	1.5	2.1
																	SKEEAEALYHSK	4	1	1	47132620	N-Term(TMT6plex); K2(TMT6plex); K12(TMT6plex)	2079.2	1	1.3	3.4	1.6	4.6
																	YEDEINK	3	13	8	372466577;119395750;109148552;155969697;119395754;119703753;47132620;194018511	N-Term(TMT6plex); K7(TMT6plex)	1368.7	0				
																	VLYDAEISQIHQSVTDTNVILSMDNSR	2	1	1	47132620	N-Term(TMT6plex)	3277.7	0	1.7	4.6	1.9	4.3
																	NLDLDSIIAEVK	57	7	4	155969697;119395754;119703753;47132620	N-Term(TMT6plex); K12(TMT6plex)	1788.1	0				
																	LNDLEEALQQAK	16	1	1	47132620	N-Term(TMT6plex); K12(TMT6plex)	1830.0	0	1.3	2.7	1.4	3.6
																	YEDEINKR	6	12	8	372466577;119395750;109148552;155969697;119395754;119703753;47132620;194018511	N-Term(TMT6plex); K7(TMT6plex)	1524.8	1				
																	DVDNAYMIK	6	1	1	47132620	N-Term(TMT6plex); K9(TMT6plex)	1526.8	0	1.4	5.0	1.8	4.9
																	YEELQVTVGR	9	1	1	47132620	N-Term(TMT6plex)	1422.8	0	1.4	2.1	1.4	2.6
																	TSQNSELNNMQDLVEDYK	3	1	1	47132620	N-Term(TMT6plex); M10(Oxidation); K18(TMT6plex)	2602.3	0	1.5	2.6	1.3	4.4
																	TSQNSELNNMQDLVEDYKK	10	1	1	47132620	N-Term(TMT6plex); M10(Oxidation); K18(TMT6plex); K19(TMT6plex)	2959.5	1	1.4	2.6	1.2	3.3
																	SLVGLGGTK	16	1	1	47132620	N-Term(TMT6plex); K9(TMT6plex)	1289.8	0	1.2	2.4	1.4	3.0
																	WELLQQMNVGTRPINLEPIFQGYIDSLK	6	1	1	47132620	N-Term(TMT6plex); M7(Oxidation); K28(TMT6plex)	3777.0	0	2.0	4.4	2.3	6.6
																	GGSSSGGGYGSGGGGSSSVK	5	1	1	47132620	N-Term(TMT6plex); K20(TMT6plex)	2047.0	0	1.5	4.4	1.7	6.4
																	HGGGGGGFGGGGFGSR	2	1	1	47132620	N-Term(TMT6plex)	1549.7	0	1.4	1.8	1.5	2.1
																	AAFGGSGGR	2	1	1	47132620	N-Term(TMT6plex)	1008.5	0	1.4	1.8	1.2	2.4
																	NVQDAIADAEQR	20	1	1	47132620	N-Term(TMT6plex)	1558.8	0	1.6	2.3	1.7	2.6
																	TSQNSELNNMQDLVEDYKK	2	1	1	47132620	N-Term(TMT6plex); K18(TMT6plex); K19(TMT6plex)	2943.5	1	1.4	4.3	1.7	3.8
																	KDVDNAYMIK	1	1	1	47132620	N-Term(TMT6plex); K1(TMT6plex); K10(TMT6plex)	1884.1	1	1.4	3.1	1.8	3.3
																	LALDVEIATYR	2	9	5	109148552;155969697;119395754;119703753;47132620	N-Term(TMT6plex)	1492.9	0				
																	GSSSGGGYSSGSSSYGSGGR	2	1	1	47132620	N-Term(TMT6plex)	1969.9	0	1.3	5.3	1.4	7.4
																	LQGEIAHVK	4	1	1	47132620	N-Term(TMT6plex); K9(TMT6plex)	1452.9	0	1.3	2.9	1.4	3.9
																	FASFIDK	22	20	11	67782365;15431316;226246665;372466577;28173564;109148552;155969697;119395754;119703753;47132620;194018511	N-Term(TMT6plex); K7(TMT6plex)	1285.8	0				
																	KDVDNAYMIK	1	1	1	47132620	N-Term(TMT6plex); K1(TMT6plex); M8(Oxidation); K10(TMT6plex)	1900.1	1				
																	KYEDEINK	1	3	2	109148552;47132620	N-Term(TMT6plex); K1(TMT6plex); K8(TMT6plex)	1726.0	1				
																	VELQSK	2	1	1	47132620	N-Term(TMT6plex); K6(TMT6plex)	1161.7	0	1.3	2.7	1.2	3.6
																	TAAENDFVTLKK	1	1	1	47132620	N-Term(TMT6plex); K11(TMT6plex); K12(TMT6plex)	2024.2	1	1.3	2.0	1.6	2.5

Accession	RefSeq Accession	Gene Symbol	Entrez Gene ID	Description	Σ Coverage	Σ# Proteins	Σ# Unique Peptides	Σ# Peptides	Σ# PSMs	Het-1A-Smoke - 2M/Parental	Het-1A-Smoke - 4M/Parental	Het-1A-Smoke - 6M/Parental	Het-1A-Smoke - 8M/Parental	# AAs	MW [kDa]	calc. pI	Sequence	# PSMs	# Proteins	# Protein Groups	Protein Group Accessions	Modifications	MH+ [Da]	# Missed Cleavages	Het-1A-Smoke - 2M/Parental	Het-1A-Smoke - 4M/Parental	Het-1A-Smoke - 6M/Parental	Het-1A-Smoke - 8M/Parental
4758256	NP_004085.1	**EIF2S1**	1965	**eukaryotic translation initiation factor 2 subunit 1**	76.19	1	22	22	210	**1.3**	**1.2**	**1.2**	**1.3**	315	36.1	5.08												
																	HAVSDPSILDSLDLNEDER	13	1	1	4758256	N-Term(TMT6plex)	2354.2	0	**1.6**	**1.6**	**1.6**	**1.7**
																	FPEVEDVVMVNVR	18	1	1	4758256	N-Term(TMT6plex); M9(Oxidation)	1777.9	0	**1.3**	**1.3**	**1.2**	**1.7**
																	AGLNCSTENMPIK	8	1	1	4758256	N-Term(TMT6plex); C5(Carbamidomethyl); M10(Oxidation); K13(TMT6plex)	1909.0	0	**1.3**	**1.2**	**1.3**	**1.2**
																	TEGLSVLSQAMAVIK	16	1	1	4758256	N-Term(TMT6plex); M11(Oxidation); K15(TMT6plex)	2021.2	0	**1.1**	**0.9**	**1.0**	**0.9**
																	VVTDTDETELAR	29	1	1	4758256	N-Term(TMT6plex)	1577.8	0	**1.3**	**1.3**	**1.3**	**1.5**
																	GVFNVQMEPK	20	1	1	4758256	N-Term(TMT6plex); M7(Oxidation); K10(TMT6plex)	1622.9	0	**1.1**	**1.1**	**1.1**	**1.2**
																	SIAEMGAYVSLLEYNNIEGMILLSELSR	2	1	1	4758256	N-Term(TMT6plex); M5(Oxidation); M20(Oxidation)	3376.7	0	**1.5**	**1.7**	**1.6**	**1.2**
																	FPEVEDVVMVNVR	3	1	1	4758256	N-Term(TMT6plex)	1761.9	0	**1.8**	**1.5**	**1.5**	**1.3**
																	HAVSDPSILDSLDLNEDEREVLINNINR	3	1	1	4758256	N-Term(TMT6plex)	3419.8	1	**1.3**	**1.3**	**1.2**	**1.5**
																	EVLINNINR	4	1	1	4758256	N-Term(TMT6plex)	1313.8	0	**1.2**	**1.1**	**1.2**	**1.2**
																	AGLNCSTENMPIK	2	1	1	4758256	N-Term(TMT6plex); C5(Carbamidomethyl); K13(TMT6plex)	1893.0	0	**1.5**	**1.5**	**1.8**	**1.2**
																	YVMTTTTLER	2	1	1	4758256	N-Term(TMT6plex)	1443.8	0	**2.0**	**2.0**	**2.1**	**1.5**
																	YVMTTTTLER	6	1	1	4758256	N-Term(TMT6plex); M3(Oxidation)	1459.8	0	**1.5**	**1.3**	**1.4**	**1.2**
																	HVAEVLEYTK	5	1	1	4758256	N-Term(TMT6plex); K10(TMT6plex)	1647.0	0	**1.3**	**1.3**	**1.3**	**1.3**
																	HVAEVLEYTKDEQLESLFQR	10	1	1	4758256	N-Term(TMT6plex); K10(TMT6plex)	2892.5	1	**1.1**	**1.2**	**1.2**	**1.3**
																	INLIAPPR	3	1	1	4758256	N-Term(TMT6plex)	1122.7	0	**1.3**	**1.1**	**1.2**	**1.2**
																	TAWVFDDK	10	1	1	4758256	N-Term(TMT6plex); K8(TMT6plex)	1439.8	0	**1.2**	**1.0**	**1.1**	**1.2**
																	GYIDLSK	10	1	1	4758256	N-Term(TMT6plex); K7(TMT6plex)	1253.7	0	**1.1**	**1.2**	**1.1**	**1.3**
																	ADIEVACYGYEGIDAVK	16	1	1	4758256	N-Term(TMT6plex); C7(Carbamidomethyl); K17(TMT6plex)	2331.2	0	**1.2**	**1.2**	**1.2**	**1.3**
																	RPGYGAYDAFK	5	1	1	4758256	N-Term(TMT6plex); K11(TMT6plex)	1702.9	0	**1.2**	**1.3**	**1.2**	**1.3**
																	TEGLSVLSQAMAVIK	4	1	1	4758256	N-Term(TMT6plex); K15(TMT6plex)	2005.2	0	**1.3**	**1.0**	**1.3**	**1.0**
																	VSPEEAIK	6	1	1	4758256	N-Term(TMT6plex); K8(TMT6plex)	1330.8	0	**1.2**	**1.1**	**1.1**	**1.3**
																	GVFNVQMEPK	2	1	1	4758256	N-Term(TMT6plex); K10(TMT6plex)	1606.9	0	**1.1**	**1.2**	**1.3**	**1.1**
																	SIAEMGAYVSLLEYNNIEGMILLSELSR	1	1	1	4758256	N-Term(TMT6plex); M5(Oxidation)	3360.7	0	**1.4**	**1.8**	**1.6**	**1.5**
																	ENAEVDGDDDAEEMEAK	2	1	1	4758256	N-Term(TMT6plex); M14(Oxidation); K17(TMT6plex)	2341.0	0	**1.6**	**1.4**	**1.3**	**1.3**
																	DEQLESLFQR	4	1	1	4758256	N-Term(TMT6plex)	1493.8	0	**1.2**	**1.2**	**1.2**	**1.3**
																	NECVVVIR	2	1	1	4758256	N-Term(TMT6plex); C3(Carbamidomethyl)	1217.7	0				
																	TVYSILR	4	1	1	4758256	N-Term(TMT6plex)	1080.7	0	**1.3**	**1.0**	**1.0**	**1.2**
50592988	NP_003357.2	**UQCRC2**	7385	**cytochrome b-c1 complex subunit 2, mitochondrial precursor**	76.16	1	23	23	127	**1.5**	**1.6**	**1.6**	**1.5**	453	48.4	8.63												
																	TIAQGNLSNTDVQAAK	10	1	1	50592988	N-Term(TMT6plex); K16(TMT6plex)	2089.2	0	**1.7**	**1.5**	**1.7**	**1.4**
																	ATAAPAGAPPQPQDLEFTK	23	1	1	50592988	N-Term(TMT6plex); K19(TMT6plex)	2368.3	0	**1.5**	**1.8**	**1.8**	**1.7**
																	ENMAYTVECLR	4	1	1	50592988	N-Term(TMT6plex); M3(Oxidation); C9(Carbamidomethyl)	1630.8	0	**1.7**	**2.2**	**1.8**	**1.9**
																	SMAASGNLGHTPFVDEL	12	1	1	50592988	N-Term(TMT6plex); M2(Oxidation)	1991.0	0	**1.4**	**1.5**	**1.5**	**1.4**
																	GDVDILMEFLLNVTTAPEFR	16	1	1	50592988	N-Term(TMT6plex); M7(Oxidation)	2525.3	0	**1.3**	**1.5**	**1.3**	**1.4**
																	YEDFSNLGTTHLLR	4	1	1	50592988	N-Term(TMT6plex)	1895.0	0	**1.5**	**1.5**	**1.6**	**1.5**
																	NALANPLYCPDYR	4	1	1	50592988	N-Term(TMT6plex); C9(Carbamidomethyl)	1795.9	0	**1.7**	**1.4**	**1.6**	**1.4**
																	QVAEQFLNMR	2	1	1	50592988	N-Term(TMT6plex); M9(Oxidation)	1480.8	0	**1.4**	**1.5**	**1.4**	**1.6**
																	GIEAVGGK	4	1	1	50592988	N-Term(TMT6plex); K8(TMT6plex)	1188.7	0	**1.2**	**1.6**	**1.7**	**1.5**
																	LTSSLTTK	4	1	1	50592988	N-Term(TMT6plex); K8(TMT6plex)	1308.8	0	**1.5**	**1.5**	**1.8**	**1.4**
																	MALIGLGVSHPVLK	2	1	1	50592988	N-Term(TMT6plex); K14(TMT6plex)	1893.2	0	**1.8**	**1.9**	**2.2**	**1.8**
																	SMAASGNLGHTPFVDEL	2	1	1	50592988	N-Term(TMT6plex)	1975.0	0	**1.4**	**1.7**	**1.8**	**1.7**
																	AVAFQNPQTHVIENLHAAAYR	2	1	1	50592988	N-Term(TMT6plex)	2579.4	0	**1.2**	**1.5**	**1.4**	**1.4**
																	WEVADLQPQLK	4	1	1	50592988	N-Term(TMT6plex); K11(TMT6plex)	1785.0	0	**1.3**	**1.4**	**1.4**	**1.2**
																	GGLGLSGAK	2	1	1	50592988	N-Term(TMT6plex); K9(TMT6plex)	1217.8	0	**1.5**	**1.9**	**1.9**	**1.5**
																	MALIGLGVSHPVLK	6	1	1	50592988	N-Term(TMT6plex); M1(Oxidation); K14(TMT6plex)	1909.2	0	**1.5**	**1.5**	**1.6**	**1.4**
																	GSNTTSHLHQAVAK	4	1	1	50592988	N-Term(TMT6plex); K14(TMT6plex)	1909.1	0	**1.5**	**2.0**	**2.0**	**1.7**
																	VTSEELHYFVQNHFTSAR	4	1	1	50592988	N-Term(TMT6plex)	2394.2	0	**1.5**	**1.6**	**1.8**	**1.6**
																	ATQQPFDVSAFNASYSDSGLFGIYTISQATAAGDVIK	7	1	1	50592988	N-Term(TMT6plex); K37(TMT6plex)	4299.2	0	**1.4**	**1.8**	**1.6**	**1.4**
																	LPNGLVIASLENYSPVSR	2	1	1	50592988	N-Term(TMT6plex)	2158.2	0	**1.1**	**1.1**	**2.0**	**1.3**
																	EQNGDSLVHAAFVAESAVAGSAEANAFSVLQHVLGAGPHVK	1	1	1	50592988	N-Term(TMT6plex); K41(TMT6plex)	4543.3	0				
																	LSVTATR	2	1	1	50592988	N-Term(TMT6plex)	976.6	0	**1.2**	**1.4**	**1.3**	**1.3**
																	AAYNQVK	1	1	1	50592988	N-Term(TMT6plex); K7(TMT6plex)	1251.7	0				
																	IGLFIK	4	1	1	50592988	N-Term(TMT6plex); K6(TMT6plex)	1148.8	0	**1.9**	**2.0**	**2.1**	**1.6**
																	FVSGQK	1	1	1	50592988	N-Term(TMT6plex); K6(TMT6plex)	1123.7	0	**1.4**	**1.5**	**1.7**	**1.5**
164698438	NP_003249.3	**TK1**	7083	**thymidine kinase, cytosolic**	76.07	1	15	15	66	**1.1**	**1.4**	**1.3**	**1.2**	234	25.5	8.51												
																	LTAVCMECFR	2	1	1	164698438	N-Term(TMT6plex); C5(Carbamidomethyl); C8(Carbamidomethyl)	1515.7	0	**1.2**	**1.4**	**1.5**	**1.3**
																	KPFGAILNLVPLAESVVK	23	1	1	164698438	N-Term(TMT6plex); K1(TMT6plex); K18(TMT6plex)	2582.6	0	**0.9**	**1.3**	**1.2**	**1.0**
																	LTAVCMECFR	2	1	1	164698438	N-Term(TMT6plex); C5(Carbamidomethyl); M6(Oxidation); C8(Carbamidomethyl)	1531.7	0	**1.2**	**1.1**	**1.2**	**1.0**
																	EVEVIGGADK	10	1	1	164698438	N-Term(TMT6plex); K10(TMT6plex)	1474.9	0	**1.1**	**1.1**	**1.2**	**1.2**
																	ASGQPAGPDNK	3	1	1	164698438	N-Term(TMT6plex); K11(TMT6plex)	1499.8	0	**1.0**	**1.0**	**1.1**	**0.9**
																	YSSSFCTHDR	2	1	1	164698438	N-Term(TMT6plex); C6(Carbamidomethyl)	1488.7	0	**1.0**	**1.1**	**1.2**	**1.0**
																	TVIVAALDGTFQR	4	1	1	164698438	N-Term(TMT6plex)	1619.9	0	**1.4**	**1.6**	**1.2**	**1.3**
																	ENCPVPGKPGEAVAAR	2	1	1	164698438	N-Term(TMT6plex); C3(Carbamidomethyl); K8(TMT6plex)	2110.1	0	**1.0**	**1.1**	**1.2**	**0.9**
																	GQIQVILGPMFSGK	7	1	1	164698438	N-Term(TMT6plex); M10(Oxidation); K14(TMT6plex)	1949.1	0	**1.1**	**1.2**	**1.2**	**1.2**
																	KASGQPAGPDNK	2	1	1	164698438	N-Term(TMT6plex); K1(TMT6plex); K12(TMT6plex)	1857.1	1	**0.9**	**0.9**	**1.0**	**1.0**
																	ASGQPAGPDNKENCPVPGKPGEAVAAR	1	1	1	164698438	N-Term(TMT6plex); K11(TMT6plex); C14(Carbamidomethyl); K19(TMT6plex)	3361.8	1	**1.4**	**2.3**	**1.7**	**1.5**
																	DVAQEALGVAVIGIDEGQFFPDIVEFCEAMANAGK	2	1	1	164698438	N-Term(TMT6plex); C27(Carbamidomethyl); M30(Oxidation); K35(TMT6plex)	4185.1	0	**1.4**	**1.2**	**1.4**	**1.2**
																	FQIAQYK	2	1	1	164698438	N-Term(TMT6plex); K7(TMT6plex)	1355.8	0	**1.3**	**1.2**	**1.5**	**1.2**
																	EAAYTK	2	1	1	164698438	N-Term(TMT6plex); K6(TMT6plex)	1140.7	0	**1.0**	**1.2**	**1.2**	**1.1**
																	KLFAPQQILQCSPAN	1	1	1	164698438	N-Term(TMT6plex); K1(TMT6plex); C11(Carbamidomethyl)	2173.2	1				
																	NTMEALPACLLR	1	1	1	164698438	N-Term(TMT6plex); M3(Oxidation); C9(Carbamidomethyl)	1633.9	0	**1.4**	**1.0**	**1.2**	**1.1**
5453607	NP_006420.1	**CCT7**	10574	**T-complex protein 1 subunit eta isoform a**	76.06	4	36	36	569	**1.3**	**1.4**	**1.4**	**1.5**	543	59.3	7.65												
																	INALTAASEAACLIVSVDETIK	29	4	1	5453607	N-Term(TMT6plex); C12(Carbamidomethyl); K22(TMT6plex)	2747.5	0	**1.4**	**1.3**	**1.5**	**1.4**
																	GGAEQFMEETER	17	4	1	5453607	N-Term(TMT6plex); M7(Oxidation)	1628.7	0	**1.4**	**1.6**	**1.6**	**1.8**
																	MVVDAVMMLDDLLQLK	95	3	1	5453607	N-Term(TMT6plex); M1(Oxidation); M7(Oxidation); M8(Oxidation); K16(TMT6plex)	2340.3	0	**1.3**	**1.4**	**1.4**	**1.6**
																	KVQGGALEDSQLVAGVAFK	8	3	1	5453607	N-Term(TMT6plex); K1(TMT6plex); K19(TMT6plex)	2604.5	1	**1.3**	**1.5**	**1.5**	**1.6**
																	EGTDSSQGIPQLVSNISACQVIAEAVR	46	1	1	5453607	N-Term(TMT6plex); C19(Carbamidomethyl)	3058.6	0	**1.5**	**1.4**	**1.5**	**1.6**
																	YNFFTGCPK	10	4	1	5453607	N-Term(TMT6plex); C7(Carbamidomethyl); K9(TMT6plex)	1591.8	0	**1.3**	**1.3**	**1.4**	**1.3**
																	STVDAPTAAGR	4	4	1	5453607	N-Term(TMT6plex)	1274.7	0	**1.1**	**1.3**	**1.5**	**1.6**
																	TFSYAGFEMQPK	21	4	1	5453607	N-Term(TMT6plex); M9(Oxidation); K12(TMT6plex)	1880.0	0	**1.3**	**1.2**	**1.3**	**1.4**
																	SLHDAIMIVR	8	4	1	5453607	N-Term(TMT6plex); M7(Oxidation)	1399.8	0	**1.3**	**1.4**	**1.4**	**1.4**
																	SLHDAIMIVR	4	4	1	5453607	N-Term(TMT6plex)	1383.8	0	**1.3**	**1.3**	**1.3**	**1.5**
																	KTFSYAGFEMQPK	4	4	1	5453607	N-Term(TMT6plex); K1(TMT6plex); K13(TMT6plex)	2221.2	1	**1.5**	**1.8**	**1.8**	**1.7**
																	QVKPYVEEGLHPQIIIR	11	3	1	5453607	N-Term(TMT6plex); K3(TMT6plex)	2477.5	0	**1.3**	**1.4**	**1.5**	**1.6**
																	SQDAEVGDGTTSVTLLAAEFLK	46	2	1	5453607	N-Term(TMT6plex); K22(TMT6plex)	2710.5	0	**1.4**	**1.5**	**1.5**	**1.6**
																	NDSVVAGGGAIEMELSK	16	4	1	5453607	N-Term(TMT6plex); M13(Oxidation); K17(TMT6plex)	2151.1	0	**1.3**	**1.0**	**1.2**	**1.1**
																	LLDVVHPAAK	12	2	1	5453607	N-Term(TMT6plex); K10(TMT6plex)	1521.0	0	**1.3**	**1.4**	**1.4**	**1.6**
																	TMMACGGSIQTSVNALSADVLGR	6	4	1	5453607	N-Term(TMT6plex); M3(Oxidation); C5(Carbamidomethyl)	2584.3	0	**1.4**	**1.5**	**1.6**	**1.4**
																	VHTVEDYQAIVDAEWNILYDK	28	4	1	5453607	N-Term(TMT6plex); K21(TMT6plex)	2979.6	0	**1.5**	**1.5**	**1.6**	**1.6**
																	CQVFEETQIGGER	2	4	1	5453607	N-Term(TMT6plex); C1(Carbamidomethyl)	1781.9	0	**1.2**	**1.0**	**1.2**	**1.0**
																	MVVDAVMMLDDLLQLK	24	3	1	5453607	N-Term(TMT6plex); M7(Oxidation); M8(Oxidation); K16(TMT6plex)	2324.3	0	**1.6**	**1.9**	**2.0**	**1.8**
																	ATISNDGATILK	18	2	1	5453607	N-Term(TMT6plex); K12(TMT6plex)	1662.0	0	**1.3**	**1.4**	**1.4**	**1.5**
																	LPIGDVATQYFADR	30	4	1	5453607	N-Term(TMT6plex)	1795.0	0	**1.4**	**1.4**	**1.4**	**1.7**
																	IKEIAVTVK	4	3	1	5453607	N-Term(TMT6plex); K2(TMT6plex); K9(TMT6plex)	1688.1	1	**1.3**	**1.8**	**1.5**	**2.0**
																	QQLLIGAYAK	17	4	1	5453607	N-Term(TMT6plex); K10(TMT6plex)	1563.0	0	**1.2**	**1.3**	**1.4**	**1.4**
																	QLCDNAGFDATNILNK	16	4	1	5453607	N-Term(TMT6plex); C3(Carbamidomethyl); K16(TMT6plex)	2252.2	0	**1.4**	**1.5**	**1.5**	**1.5**
																	TATQLAVNK	18	3	1	5453607	N-Term(TMT6plex); K9(TMT6plex)	1403.9	0	**1.5**	**1.1**	**1.3**	**1.3**
																	IHHSGAK	4	4	1	5453607	N-Term(TMT6plex); K7(TMT6plex)	1207.7	0	**1.2**	**1.3**	**1.4**	**1.6**
																	IALLNVELELK	4	4	1	5453607	N-Term(TMT6plex); K11(TMT6plex)	1713.1	0	**1.3**	**1.3**	**1.4**	**1.4**
																	TMMACGGSIQTSVNALSADVLGR	8	4	1	5453607	N-Term(TMT6plex); M2(Oxidation); M3(Oxidation); C5(Carbamidomethyl)	2600.3	0	**1.4**	**1.3**	**1.4**	**1.3**
																	TFSYAGFEMQPK	4	4	1	5453607	N-Term(TMT6plex); K12(TMT6plex)	1864.0	0	**1.4**	**1.3**	**1.7**	**1.3**
																	CAMTALSSK	2	3	1	5453607	N-Term(TMT6plex); C1(Carbamidomethyl); K9(TMT6plex)	1426.8	0	**1.9**	**1.4**	**2.0**	**1.4**
																	VQGGALEDSQLVAGVAFK	14	3	1	5453607	N-Term(TMT6plex); K18(TMT6plex)	2247.3	0	**1.3**	**1.4**	**1.5**	**1.5**
																	TLVDIAK	5	2	1	5453607	N-Term(TMT6plex); K7(TMT6plex)	1217.8	0	**1.2**	**1.3**	**1.3**	**1.6**
																	CAMTALSSK	4	3	1	5453607	N-Term(TMT6plex); C1(Carbamidomethyl); M3(Oxidation); K9(TMT6plex)	1442.8	0	**1.3**	**1.3**	**1.4**	**1.3**

Accession	RefSeq Accession	Gene Symbol	Entrez Gene ID	Description	Σ Coverage	Σ# Proteins	Σ# Unique Peptides	Σ# Peptides	Σ# PSMs	Het-1A-Smoke - 2M/Parental	Het-1A-Smoke - 4M/Parental	Het-1A-Smoke - 6M/Parental	Het-1A-Smoke - 8M/Parental	# AAs	MW [kDa]	calc. pI	Sequence	# PSMs	# Proteins	# Protein Groups	Protein Group Accessions	Modifications	MH+ [Da]	# Missed Cleavages	Het-1A-Smoke - 2M/Parental	Het-1A-Smoke - 4M/Parental	Het-1A-Smoke - 6M/Parental	Het-1A-Smoke - 8M/Parental
																	EIAVTVK	11	3	1	5453607	N-Term(TMT6plex); K7(TMT6plex)	1217.8	0	1.3	1.3	1.3	1.4
																	TCTFILR	2	4	1	5453607	N-Term(TMT6plex); C2(Carbamidomethyl)	1139.6	0	1.4	1.2	1.5	1.2
																	VPEEDLKR	1	4	1	5453607	N-Term(TMT6plex); K7(TMT6plex)	1443.9	1	1.2	1.6	1.4	1.9
																	DMFCAGR	1	4	1	5453607	N-Term(TMT6plex); M2(Oxidation); C4(Carbamidomethyl)	1101.5	0	1.2	1.5	1.4	1.7
																	ALEIIPR	8	4	1	5453607	N-Term(TMT6plex)	1040.7	0	1.3	1.5	1.5	1.6
																	LIVDGR	2	2	1	5453607	N-Term(TMT6plex)	901.6	0	1.3	1.5	1.3	1.8
																	LISQQK	3	3	1	5453607	N-Term(TMT6plex); K6(TMT6plex)	1174.8	0	1.4	1.2	1.3	1.3
																	KTFSYAGFEMQPK	1	4	1	5453607	N-Term(TMT6plex); K1(TMT6plex); M10(Oxidation); K13(TMT6plex)	2237.2	1	1.4	1.6	1.4	1.5
																	TTLGPR	1	1	1	5453607	N-Term(TMT6plex)	873.5	0	1.2	1.1	1.3	1.2
24307939	NP_036205.1	CCT5	22948	T-complex protein 1 subunit epsilon	75.97	5	37	38	304	1.4	1.4	1.5	1.7	541	59.6	5.66												
																	ETGANLAICQWGFDDEANHLLLQNNLPAVR	10	1	1	24307939	N-Term(TMT6plex); C9(Carbamidomethyl)	3608.8	0	1.4	1.6	1.5	1.8
																	DGDVTVTNDGATILSMMDVDHQIAK	12	1	1	24307939	N-Term(TMT6plex); M16(Oxidation); M17(Oxidation); K25(TMT6plex)	3136.5	0	1.4	1.3	1.4	1.5
																	LDVTSVEDYK	10	1	1	24307939	N-Term(TMT6plex); K10(TMT6plex)	1626.9	0	1.5	1.4	1.5	1.8
																	IADGYEQAAR	10	1	1	24307939	N-Term(TMT6plex)	1322.7	0	1.5	1.4	1.5	1.8
																	DVDFELIK	19	1	1	24307939	N-Term(TMT6plex); K8(TMT6plex)	1436.8	0	1.3	1.4	1.5	1.8
																	QQHVIETLIGK	4	1	1	24307939	N-Term(TMT6plex); K11(TMT6plex)	1724.0	0	1.5	1.6	1.7	2.0
																	LGFAGLVQEISFGTTK	37	1	1	24307939	N-Term(TMT6plex); K16(TMT6plex)	2126.2	0	1.5	1.4	1.5	1.6
																	DFSHPQMPK	4	1	1	24307939	N-Term(TMT6plex); M7(Oxidation); K9(TMT6plex)	1560.8	0	1.2	1.3	1.3	1.6
																	MMVDKDGDVTVTNDGATILSMMDVDHQIAK	1	1	1	24307939	N-Term(TMT6plex); M1(Oxidation); M2(Oxidation); K5(TMT6plex); M21(Oxidation); M22(Oxidation); K30(TMT6plex)	4001.9	1	1.6	2.1	2.3	2.4
																	GVIVDKDFSHPQMPK	3	1	1	24307939	N-Term(TMT6plex); K6(TMT6plex); M13(Oxidation); K15(TMT6plex)	2401.4	1	1.3	1.5	1.4	1.7
																	SQDDEIGDGTTGVVVLAGALLEEAEQLLDR	4	1	1	24307939	N-Term(TMT6plex)	3342.7	0	1.4	1.2	2.0	1.3
																	DTEPLIQTAK	11	5	1	24307939	N-Term(TMT6plex); K10(TMT6plex)	1573.9	0	1.4	1.3	1.5	1.7
																	AFADALEVIPMALSENSGMNPIQTMTEVR	6	1	1	24307939	N-Term(TMT6plex); M19(Oxidation)	3380.7	0	1.9	2.0	1.9	2.0
																	SLHDALCVIR	11	7	2	24307939;38455427	N-Term(TMT6plex); C7(Carbamidomethyl)	1412.8	0				
																	VVYGGGAAEISCALAVSQEADK	7	1	1	24307939	N-Term(TMT6plex); C12(Carbamidomethyl); K22(TMT6plex)	2653.4	0	1.5	1.3	1.4	1.5
																	EMNPALGIDCLHK	6	1	1	24307939	N-Term(TMT6plex); M2(Oxidation); C10(Carbamidomethyl); K13(TMT6plex)	1972.0	0	1.3	1.5	1.6	1.9
																	WVGGPEIELIAIATGGR	30	1	1	24307939	N-Term(TMT6plex)	1968.1	0	1.5	1.3	1.4	1.8
																	VVYGGGAAEISCALAVSQEADKCPTLEQYAMR	2	1	1	24307939	N-Term(TMT6plex); C12(Carbamidomethyl); K22(TMT6plex); C23(Carbamidomethyl); M31(Oxidation)	3919.0	1	1.4	1.8	1.9	1.8
																	AFADALEVIPMALSENSGMNPIQTMTEVR	16	1	1	24307939	N-Term(TMT6plex); M11(Oxidation); M19(Oxidation); M25(Oxidation)	3412.7	0	1.4	1.6	1.4	1.7
																	VAIEHLDK	4	1	1	24307939	N-Term(TMT6plex); K8(TMT6plex)	1382.8	0	1.4	1.3	1.4	1.8
																	MIIEEAK	2	1	1	24307939	N-Term(TMT6plex); K7(TMT6plex)	1291.8	0	1.7	1.9	2.1	2.0
																	IAILTCPFEPPKPK	6	1	1	24307939	N-Term(TMT6plex); C6(Carbamidomethyl); K12(TMT6plex); K14(TMT6plex)	2298.4	0	1.3	1.4	1.4	1.6
																	EKFEEMIQQIK	4	1	1	24307939	N-Term(TMT6plex); K2(TMT6plex); M6(Oxidation); K11(TMT6plex)	2126.2	1	1.2	1.4	1.4	2.0
																	FSELTAEK	10	1	1	24307939	N-Term(TMT6plex); K8(TMT6plex)	1382.8	0	1.4	1.3	1.4	1.6
																	ISDSVLVDIK	14	1	1	24307939	N-Term(TMT6plex); K10(TMT6plex)	1546.9	0	1.5	1.2	1.5	1.7
																	AFADALEVIPMALSENSGMNPIQTMTEVR	11	1	1	24307939	N-Term(TMT6plex); M11(Oxidation); M19(Oxidation)	3396.7	0	1.6	1.6	1.7	1.7
																	CPTLEQYAMR	3	1	1	24307939	N-Term(TMT6plex); C1(Carbamidomethyl); M9(Oxidation)	1513.7	0	1.4	1.1	1.3	1.6
																	ISDSVLVDIKDTEPLIQTAK	4	5	1	24307939	N-Term(TMT6plex); K10(TMT6plex); K20(TMT6plex)	2872.7	1	1.3	1.3	1.5	2.1
																	HKLDVTSVEDYK	4	5	1	24307939	N-Term(TMT6plex); K2(TMT6plex); K12(TMT6plex)	2121.2	1	1.1	1.3	1.4	1.7
																	MMVDKDGDVTVTNDGATILSMMDVDHQIAK	1	1	1	24307939	N-Term(TMT6plex); M1(Oxidation); M2(Oxidation); K5(TMT6plex); M21(Oxidation); K30(TMT6plex)	3986.0	1	1.3	1.3	1.4	1.7
																	QMAEIAVNAVLTVADMER	2	1	1	24307939	N-Term(TMT6plex); M16(Oxidation)	2206.1	0	1.4	1.6	1.3	1.2
																	SHIMAAK	2	1	1	24307939	N-Term(TMT6plex); K7(TMT6plex)	1215.7	0	1.5	1.6	1.7	1.5
																	QQISLATQMVR	6	1	1	24307939	N-Term(TMT6plex); M9(Oxidation)	1519.8	0	1.4	1.3	1.5	1.7
																	QMAEIAVNAVLTVADMER	1	1	1	24307939	N-Term(TMT6plex); M2(Oxidation); M16(Oxidation)	2222.1	0	1.4	1.4	1.8	1.7
																	LMGLEALK	1	1	1	24307939	N-Term(TMT6plex); K8(TMT6plex)	1332.8	0	1.4	1.3	1.4	1.5
																	LMGLEALK	2	1	1	24307939	N-Term(TMT6plex); M2(Oxidation); K8(TMT6plex)	1348.8	0	1.4	1.2	1.3	1.7
																	FEEMIQQIK	2	1	1	24307939	N-Term(TMT6plex); M4(Oxidation); K9(TMT6plex)	1639.9	0	1.4	1.2	1.4	1.4
																	LMVELSK	9	1	1	24307939	N-Term(TMT6plex); M2(Oxidation); K7(TMT6plex)	1293.8	0	1.4	1.3	1.5	1.7
																	SHIMAAK	2	1	1	24307939	N-Term(TMT6plex); M4(Oxidation); K7(TMT6plex)	1231.7	0	1.2	1.2	1.5	1.2
																	MLVIEQCK	4	1	1	24307939	N-Term(TMT6plex); M1(Oxidation); C7(Carbamidomethyl); K8(TMT6plex)	1494.8	0	1.4	1.4	1.4	1.5
																	TTLGSK	1	1	1	24307939	N-Term(TMT6plex); K6(TMT6plex)	1064.7	0	1.3	1.2	1.3	1.4
																	MIIEEAK	2	1	1	24307939	N-Term(TMT6plex); M1(Oxidation); K7(TMT6plex)	1307.8	0	1.4	1.3	1.3	1.9
																	AVTIFIR	2	1	1	24307939	N-Term(TMT6plex)	1048.7	0	1.4	1.6	1.6	1.9
																	KQQISLATQMVR	1	1	1	24307939	N-Term(TMT6plex); K1(TMT6plex); M10(Oxidation)	1877.1	1	1.3	1.3	1.5	1.5
																	AVANTMR	1	1	1	24307939	N-Term(TMT6plex); M6(Oxidation)	1007.6	0	1.3	1.3	1.3	2.0
c001	ALBU_BOVIN				75.95	1	44	49	562	1.2	1.6	1.4	4.1	607	69.2	6.18												
																	YICDNQDTISSK	44	1	1	c001	N-Term(TMT6plex); C3(Carbamidomethyl); K12(TMT6plex)	1902.0	0	1.4	1.6	1.5	5.8
																	DAFLGSFLYEYSR	30	1	1	c001	N-Term(TMT6plex)	1796.9	0	1.7	1.8	1.7	3.7
																	TCVADESHAGCEK	8	1	1	c001	N-Term(TMT6plex); C2(Carbamidomethyl); C11(Carbamidomethyl); K13(TMT6plex)	1921.9	0	1.4	1.4	1.5	4.4
																	KVPQVSTPTLVEVSR	26	2	2	4502027;c001	N-Term(TMT6plex); K1(TMT6plex)	2098.3	1				
																	AEFVEVTK	23	1	1	c001	N-Term(TMT6plex); K8(TMT6plex)	1380.8	0	1.1	1.5	1.3	3.8
																	MPCTEDYLSLILNR	4	1	1	c001	N-Term(TMT6plex); M1(Oxidation); C3(Carbamidomethyl)	1970.0	0	1.5	1.5	1.6	2.7
																	LVTDLTK	24	2	2	4502027;c001	N-Term(TMT6plex); K7(TMT6plex)	1247.8	0				
																	ECCHGDLLECADDR	4	1	1	c001	N-Term(TMT6plex); C2(Carbamidomethyl); C3(Carbamidomethyl); C10(Carbamidomethyl)	1978.8	0	1.7	1.7	1.7	3.7
																	LKPDPNTLCDEFK	20	1	1	c001	N-Term(TMT6plex); K2(TMT6plex); C9(Carbamidomethyl); K13(TMT6plex)	2264.3	0	1.3	1.6	1.5	5.8
																	DLGEEHFK	9	1	1	c001	N-Term(TMT6plex); K8(TMT6plex)	1432.8	0	1.1	1.5	1.3	3.0
																	NECFLSHKDDSPDLPK	1	1	1	c001	N-Term(TMT6plex); C3(Carbamidomethyl); K8(TMT6plex); K16(TMT6plex)	2589.4	1	1.2	1.4	1.4	4.2
																	FKDLGEEHFK	4	1	1	c001	N-Term(TMT6plex); K2(TMT6plex); K10(TMT6plex)	1937.1	1	1.1	2.0	1.4	7.2
																	TVMENFVAFVDK	19	1	1	c001	N-Term(TMT6plex); M3(Oxidation); K12(TMT6plex)	1874.0	0	1.3	1.5	1.4	4.1
																	CCTESLVNR	4	2	2	4502027;c001	N-Term(TMT6plex); C1(Carbamidomethyl); C2(Carbamidomethyl)	1367.7	0				
																	LVNELTEFAK	56	1	1	c001	N-Term(TMT6plex); K10(TMT6plex)	1622.0	0	1.2	1.6	1.4	4.9
																	EACFAVEGPK	6	1	1	c001	N-Term(TMT6plex); C3(Carbamidomethyl); K10(TMT6plex)	1565.8	0	1.2	1.3	1.3	5.4
																	DAIPENLPPLTADFAEDKDVCK	10	1	1	c001	N-Term(TMT6plex); K18(TMT6plex); C21(Carbamidomethyl); K22(TMT6plex)	3145.6	1	1.2	1.6	1.4	9.3
																	LVVSTQTALA	8	1	1	c001	N-Term(TMT6plex)	1231.7	0	1.2	1.4	1.5	1.8
																	ETYGDMADCCEK	4	1	1	c001	N-Term(TMT6plex); C9(Carbamidomethyl); C10(Carbamidomethyl); K12(TMT6plex)	1936.8	0	2.0	3.3	2.4	14.2
																	KQTALVELLK	14	1	1	c001	N-Term(TMT6plex); K1(TMT6plex); K10(TMT6plex)	1830.2	1	1.0	1.4	1.2	3.2
																	DDPHACYSTVFDK	14	1	1	c001	N-Term(TMT6plex); C6(Carbamidomethyl); K13(TMT6plex)	2013.0	0	1.1	1.5	1.3	3.8
																	CCAADDKEACFAVEGPK	4	1	1	c001	N-Term(TMT6plex); C1(Carbamidomethyl); C2(Carbamidomethyl); K7(TMT6plex); C10(Carbamidomethyl); K17(TMT6plex)	2615.3	1	1.4	1.4	1.5	5.2
																	QTALVELLK	15	1	1	c001	N-Term(TMT6plex); K9(TMT6plex)	1472.9	0	1.2	1.4	1.4	3.4
																	HLVDEPQNLIK	12	1	1	c001	N-Term(TMT6plex); K11(TMT6plex)	1764.0	0	1.2	1.5	1.4	2.8
																	SLHTLFGDELCK	13	1	1	c001	N-Term(TMT6plex); C11(Carbamidomethyl); K12(TMT6plex)	1878.0	0	1.5	1.6	1.6	3.3
																	LCVLHEK	10	1	1	c001	N-Term(TMT6plex); C2(Carbamidomethyl); K7(TMT6plex)	1356.8	0	1.2	1.5	1.5	4.5
																	RHPEYAVSVLLR	5	1	1	c001	N-Term(TMT6plex)	1669.0	1	1.4	1.5	1.5	1.8
																	TVMENFVAFVDK	2	1	1	c001	N-Term(TMT6plex); K12(TMT6plex)	1858.0	0	1.2	1.6	1.7	3.8
																	LFTFHADICTLPDTEK	9	1	1	c001	N-Term(TMT6plex); C9(Carbamidomethyl); K16(TMT6plex)	2366.2	0	1.3	1.6	1.7	4.0
																	VPQVSTPTLVEVSR	2	2	2	4502027;c001	N-Term(TMT6plex)	1741.0	0				
																	QNCDQFEK	10	1	1	c001	N-Term(TMT6plex); C3(Carbamidomethyl); K8(TMT6plex)	1526.8	0	1.3	1.5	1.5	6.4
																	NECFLSHK	2	1	1	c001	N-Term(TMT6plex); C3(Carbamidomethyl); K8(TMT6plex)	1492.8	0	1.2	1.2	1.5	4.0
																	LGEYGFQNALIVR	32	1	1	c001	N-Term(TMT6plex)	1709.0	0	1.3	1.5	1.4	3.0
																	ETYGDMADCCEK	13	1	1	c001	N-Term(TMT6plex); M6(Oxidation); C9(Carbamidomethyl); C10(Carbamidomethyl); K12(TMT6plex)	1952.8	0	1.3	1.6	1.5	9.2
																	YLYEIAR	9	2	2	4502027;c001	N-Term(TMT6plex)	1156.7	0				
																	HPEYAVSVLLR	4	1	1	c001	N-Term(TMT6plex)	1512.9	0	1.3	1.4	1.4	1.7
																	DTHKSEIAHR	8	1	1	c001	N-Term(TMT6plex); K4(TMT6plex)	1651.9	1	0.9	1.7	1.0	1.7
																	SHCIAEVEK	2	1	1	c001	N-Term(TMT6plex); C3(Carbamidomethyl); K9(TMT6plex)	1530.8	0	1.2	2.0	2.0	3.5
																	MPCTEDYLSLILNR	10	1	1	c001	N-Term(TMT6plex); C3(Carbamidomethyl)	1954.0	0	1.7	2.0	2.0	5.9
																	EYEATLEECCAK	17	1	1	c001	N-Term(TMT6plex); C9(Carbamidomethyl); C10(Carbamidomethyl); K12(TMT6plex)	1960.9	0	1.6	1.6	1.7	7.4

Accession	RefSeq Accession	Gene Symbol	Entrez Gene ID	Description	Σ Coverage	Σ# Proteins	Σ# Unique Peptides	Σ# Peptides	Σ# PSMs	Het-1A-Smoke - 2M/Parental	Het-1A-Smoke - 4M/Parental	Het-1A-Smoke - 6M/Parental	Het-1A-Smoke - 8M/Parental	# AAs	MW [kDa]	calc. pI	Sequence	# PSMs	# Proteins	# Protein Groups	Protein Group Accessions	Modifications	MH+ [Da]	# Missed Cleavages	Het-1A-Smoke - 2M/Parental	Het-1A-Smoke - 4M/Parental	Het-1A-Smoke - 6M/Parental	Het-1A-Smoke - 8M/Parental
																	GLVLIAFSQYLQQCPFDEHVK	7	1	1	c001	N-Term(TMT6plex); C14(Carbamidomethyl); K21(TMT6plex)	2950.6	0	**1.2**	**1.5**	**1.6**	**3.9**
																	SHCIAEVEKDAIPENLPPLTADFAEDKDVCK	2	1	1	c001	N-Term(TMT6plex); C3(Carbamidomethyl); K9(TMT6plex); K27(TMT6plex); C30(Carbamidomethyl); K31(TMT6plex)	4428.3	2	**1.3**	**1.6**	**1.4**	**6.8**
																	HPYFYAPELLYYANK	7	1	1	c001	N-Term(TMT6plex); K15(TMT6plex)	2347.2	0	**1.3**	**1.4**	**1.5**	**2.2**
																	RHPYFYAPELLYYANK	2	1	1	c001	N-Term(TMT6plex); K16(TMT6plex)	2503.4	1	**1.3**	**1.8**	**1.6**	**3.6**
																	RPCFSALTPDETYVPK	1	1	1	c001	N-Term(TMT6plex); C3(Carbamidomethyl); K16(TMT6plex)	2339.2	0	**1.8**	**1.9**	**1.8**	**2.8**
																	DAIPENLPPLTADFAEDK	2	1	1	c001	N-Term(TMT6plex); K18(TMT6plex)	2414.3	0	**1.1**	**1.4**	**1.4**	**3.0**
																	GACLLPK	3	1	1	c001	N-Term(TMT6plex); C3(Carbamidomethyl); K7(TMT6plex)	1216.7	0	**1.2**	**1.6**	**1.5**	**8.3**
																	ATEEQLK	6	1	1	c001	N-Term(TMT6plex); K7(TMT6plex)	1276.7	0	**1.2**	**1.6**	**1.4**	**3.7**
																	DDSPDLPK	16	1	1	c001	N-Term(TMT6plex); K8(TMT6plex)	1344.7	0	**1.6**	**1.5**	**1.4**	**4.9**
																	CCTKPESER	1	1	1	c001	N-Term(TMT6plex); C1(Carbamidomethyl); C2(Carbamidomethyl); K4(TMT6plex)	1624.8	0	**1.5**	**1.6**	**1.8**	**8.5**
																	NYQEAK	3	1	1	c001	N-Term(TMT6plex); K6(TMT6plex)	1210.7	0	**1.0**	**1.4**	**1.2**	**4.3**
																	LKECCDKPLLEK	1	1	1	c001	N-Term(TMT6plex); K2(TMT6plex); C4(Carbamidomethyl); C5(Carbamidomethyl); K7(TMT6plex); K12(TMT6plex)	2449.4	1	**1.3**	**1.9**	**1.7**	**9.6**
21614499	NP_003370.2	**EZR**	7430	**ezrin**	75.94	1	36	52	1054	**1.1**	**1.2**	**1.2**	**1.1**	586	69.4	6.27												
																	FYPEDVAEELIQDITQK	170	1	1	21614499	N-Term(TMT6plex); K17(TMT6plex)	2496.3	0	**1.1**	**1.3**	**1.3**	**1.1**
																	QLLTLSSELSQAR	41	1	1	21614499	N-Term(TMT6plex)	1675.0	0	**1.3**	**1.3**	**1.3**	**1.3**
																	IDEFEAL	40	1	1	21614499	N-Term(TMT6plex)	1065.6	0	**1.2**	**1.2**	**1.1**	**1.1**
																	VTTMDAELEFAIQPNTTGK	41	5	3	21614499;4505257;4506467	N-Term(TMT6plex); M4(Oxidation); K19(TMT6plex)	2540.3	0				
																	GTDLWLGVDALGLNIYEK	29	1	1	21614499	N-Term(TMT6plex); K18(TMT6plex)	2435.4	0	**1.0**	**1.2**	**1.2**	**1.1**
																	QLFDQVVK	26	4	3	21614499;4505257;4506467	N-Term(TMT6plex); K8(TMT6plex)	1434.9	0				
																	IGFPWSEIR	31	5	3	21614499;4505257;4506467	N-Term(TMT6plex)	1333.7	0				
																	SQEQLAAELAEYTAK	53	1	1	21614499	N-Term(TMT6plex); K15(TMT6plex)	2110.1	0	**1.1**	**1.2**	**1.2**	**1.1**
																	AKEAQDDLVK	3	1	1	21614499	N-Term(TMT6plex); K2(TMT6plex); K10(TMT6plex)	1804.1	1	**1.1**	**1.5**	**1.3**	**1.3**
																	IAQDLEMYGINYFEIK	20	1	1	21614499	N-Term(TMT6plex); K16(TMT6plex)	2405.3	0	**1.2**	**1.4**	**1.6**	**1.1**
																	IAQDLEMYGINYFEIK	72	1	1	21614499	N-Term(TMT6plex); M7(Oxidation); K16(TMT6plex)	2421.3	0	**1.2**	**1.3**	**1.3**	**1.1**
																	APDFVFYAPR	36	5	3	21614499;4505257;4506467	N-Term(TMT6plex)	1411.8	0				
																	THNDIIHNENMR	26	1	1	21614499	N-Term(TMT6plex); M11(Oxidation)	1738.8	0	**1.1**	**1.1**	**1.2**	**1.0**
																	DNAMLEYLK	35	1	1	21614499	N-Term(TMT6plex); M4(Oxidation); K9(TMT6plex)	1570.9	0	**1.1**	**1.3**	**1.3**	**1.1**
																	KPDTIEVQQMK	2	5	3	21614499;4505257;4506467	N-Term(TMT6plex); K1(TMT6plex); K11(TMT6plex)	2004.2	0				
																	KPDTIEVQQMK	8	5	3	21614499;4505257;4506467	N-Term(TMT6plex); K1(TMT6plex); M10(Oxidation); K11(TMT6plex)	2020.2	0				
																	RKPDTIEVQQMK	11	5	3	21614499;4505257;4506467	N-Term(TMT6plex); K2(TMT6plex); M11(Oxidation); K12(TMT6plex)	2176.3	1				
																	RKPDTIEVQQMK	2	5	3	21614499;4505257;4506467	N-Term(TMT6plex); K2(TMT6plex); K12(TMT6plex)	2160.3	1				
																	EGILSDEIYCPPETAVLLGSYAVQAK	55	1	1	21614499	N-Term(TMT6plex); C10(Carbamidomethyl); K26(TMT6plex)	3281.7	0	**1.3**	**1.3**	**1.5**	**1.3**
																	ALQLEEER	16	1	1	21614499	N-Term(TMT6plex)	1216.7	0	**1.1**	**1.2**	**1.2**	**1.1**
																	GFPTWLK	28	1	1	21614499	N-Term(TMT6plex); K7(TMT6plex)	1306.8	0	**0.9**	**1.2**	**1.1**	**1.0**
																	EVWYFGLHYVDNK	21	1	1	21614499	N-Term(TMT6plex); K13(TMT6plex)	2128.1	0	**1.1**	**1.3**	**1.3**	**1.1**
																	DNAMLEYLK	8	1	1	21614499	N-Term(TMT6plex); K9(TMT6plex)	1554.9	0	**1.3**	**1.8**	**1.8**	**1.1**
																	AQEEAER	6	1	1	21614499	N-Term(TMT6plex)	1061.5	0	**1.0**	**1.2**	**1.1**	**1.1**
																	TKEELHLVMTAPPPPPPPVYEPVSYHVQESLQDEGAEPTGYSAELSSEGIR	1	1	1	21614499	N-Term(TMT6plex); K2(TMT6plex); M9(Oxidation)	6033.0	1	**3.3**	**3.4**	**3.2**	**4.7**
																	KAPDFVFYAPR	9	5	3	21614499;4505257;4506467	N-Term(TMT6plex); K1(TMT6plex)	1769.0	1				
																	ILQLCMGNHELYMR	10	1	1	21614499	N-Term(TMT6plex); C5(Carbamidomethyl); M6(Oxidation); M13(Oxidation)	2039.0	0	**1.2**	**1.2**	**1.3**	**1.2**
																	KENPLQFK	9	4	2	21614499;4506467	N-Term(TMT6plex); K1(TMT6plex); K8(TMT6plex)	1691.0	1				
																	FVIKPIDK	10	5	3	21614499;4505257;4506467	N-Term(TMT6plex); K4(TMT6plex); K8(TMT6plex)	1647.1	0				
																	IALLEEAR	15	2	2	21614499;612407805	N-Term(TMT6plex)	1143.7	0				
																	LFFLQVK	45	5	3	21614499;4505257;4506467	N-Term(TMT6plex); K7(TMT6plex)	1352.9	0				
																	LQDYEEK	26	1	1	21614499	N-Term(TMT6plex); K7(TMT6plex)	1382.8	0	**1.1**	**1.2**	**1.2**	**1.1**
																	QAVDQIK	11	1	1	21614499	N-Term(TMT6plex); K7(TMT6plex)	1259.8	0	**0.9**	**1.1**	**1.1**	**1.0**
																	EAQDDLVK	22	1	1	21614499	N-Term(TMT6plex); K8(TMT6plex)	1375.8	0	**1.1**	**1.3**	**1.3**	**1.1**
																	ELSEQIQR	27	1	1	21614499	N-Term(TMT6plex)	1231.7	0	**1.1**	**1.3**	**1.2**	**1.1**
																	IQVWHAEHR	4	1	1	21614499	N-Term(TMT6plex)	1404.8	0	**1.1**	**1.3**	**1.2**	**1.2**
																	KGTDLWLGVDALGLNIYEK	20	1	1	21614499	N-Term(TMT6plex); K1(TMT6plex); K19(TMT6plex)	2792.6	1	**1.0**	**1.2**	**1.1**	**1.1**
																	ENPLQFK	8	4	2	21614499;4506467	N-Term(TMT6plex); K7(TMT6plex)	1333.8	0				
																	EDEVEEWQHR	2	1	1	21614499	N-Term(TMT6plex)	1585.7	0	**1.8**	**1.3**	**1.2**	**1.2**
																	GTDLWLGVDALGLNIYEKDDK	6	1	1	21614499	N-Term(TMT6plex); K18(TMT6plex); K21(TMT6plex)	3022.7	1	**1.0**	**1.4**	**1.2**	**1.1**
																	THNDIIHNENMR	4	1	1	21614499	N-Term(TMT6plex)	1722.9	0	**1.8**	**1.5**	**1.5**	**1.3**
																	ILQLCMGNHELYMR	2	1	1	21614499	N-Term(TMT6plex); C5(Carbamidomethyl); M6(Oxidation)	2023.0	0	**1.3**	**1.4**	**1.5**	**1.2**
																	VSAQEVR	2	1	1	21614499	N-Term(TMT6plex)	1017.6	0	**1.1**	**1.1**	**1.1**	**0.9**
																	VTTMDAELEFAIQPNTTGK	3	5	3	21614499;4505257;4506467	N-Term(TMT6plex); K19(TMT6plex)	2524.3	0				
																	KEDEVEEWQHR	2	1	1	21614499	N-Term(TMT6plex); K1(TMT6plex)	1943.0	1	**1.3**	**1.4**	**1.3**	**1.1**
																	QQLETEK	9	1	1	21614499	N-Term(TMT6plex); K7(TMT6plex)	1333.8	0	**1.2**	**1.3**	**1.3**	**1.1**
																	SGYLSSER	2	1	1	21614499	N-Term(TMT6plex)	1127.6	0	**1.2**	**1.1**	**1.2**	**1.0**
																	FVIKPIDKK	3	5	3	21614499;4505257;4506467	N-Term(TMT6plex); K4(TMT6plex); K8(TMT6plex); K9(TMT6plex)	2004.3	1				
																	QQLETEKK	1	1	1	21614499	N-Term(TMT6plex); K7(TMT6plex); K8(TMT6plex)	1691.0	1	**0.9**	**1.3**	**1.1**	**1.0**
																	ITEAEKNER	1	1	1	21614499	N-Term(TMT6plex); K6(TMT6plex)	1547.9	1	**1.2**	**1.6**	**1.3**	**1.2**
																	KFVIKPIDK	3	5	3	21614499;4505257;4506467	N-Term(TMT6plex); K1(TMT6plex); K5(TMT6plex); K9(TMT6plex)	2004.3	1				
																	NISFNDK	7	5	3	21614499;4505257;4506467	N-Term(TMT6plex); K7(TMT6plex)	1295.7	0				
																	EKEELMLR	2	1	1	21614499	N-Term(TMT6plex); K2(TMT6plex)	1505.9	1	**1.4**	**1.8**	**1.9**	**1.2**
																	DDRNEEKR	1	1	1	21614499	N-Term(TMT6plex); K7(TMT6plex)	1519.8	2	**0.8**	**1.3**	**1.0**	**1.0**
																	VMDQHK	3	1	1	21614499	N-Term(TMT6plex); M2(Oxidation); K6(TMT6plex)	1231.7	0	**0.8**	**1.1**	**1.0**	**1.0**
																	DDKLTPK	1	1	1	21614499	N-Term(TMT6plex); K3(TMT6plex); K7(TMT6plex)	1503.9	1	**0.9**	**1.3**	**1.2**	**1.1**
																	ITEAEK	1	1	1	21614499	N-Term(TMT6plex); K6(TMT6plex)	1148.7	0	**0.9**	**1.1**	**1.1**	**0.9**
																	NISFNDKK	1	5	3	21614499;4505257;4506467	N-Term(TMT6plex); K7(TMT6plex); K8(TMT6plex)	1653.0	1				
																	DDRNEEK	1	1	1	21614499	N-Term(TMT6plex); K7(TMT6plex)	1363.7	1	**1.0**	**1.1**	**1.2**	**1.1**
33286418	NP_002645.3	**PKM**	5315	**pyruvate kinase PKM isoform a**	75.89	8	4	37	2714	**1.2**	**1.2**	**1.3**	**1.4**	531	57.9	7.84												
																	IYVDDGLISLQVK	205	7	2	33286420;33286422;33286418	N-Term(TMT6plex); K13(TMT6plex)	1921.1	0				
																	DPVQEAWAEDVDLR	60	6	2	33286420;33286422;33286418	N-Term(TMT6plex)	1871.9	0				
																	AEGSDVANAVLDGADCIMLSGETAK	405	7	2	33286420;33286422;33286418	N-Term(TMT6plex); C16(Carbamidomethyl); M18(Oxidation); K25(TMT6plex)	2968.5	0				
																	GADFLVTEVENGGSLGSK	28	6	2	33286420;33286422;33286418	N-Term(TMT6plex); K18(TMT6plex)	2238.2	0				
																	LAPITSDPTEATAVGAVEASFK	269	3	1	33286418	N-Term(TMT6plex); K22(TMT6plex)	2633.4	0	**1.2**	**1.2**	**1.3**	**1.4**
																	GSGTAEVELK	70	6	2	33286420;33286422;33286418	N-Term(TMT6plex); K10(TMT6plex)	1448.8	0				
																	KGVNLPGAAVDLPAVSEK	67	7	2	33286420;33286422;33286418	N-Term(TMT6plex); K1(TMT6plex); K18(TMT6plex)	2452.5	1				
																	FDEILEASDGIMVAR	54	7	2	33286420;33286422;33286418	N-Term(TMT6plex); M12(Oxidation)	1911.0	0				
																	GDLGIEIPAEK	109	8	2	33286420;33286422;33286418	N-Term(TMT6plex); K11(TMT6plex)	1599.9	0				
																	GVNLPGAAVDLPAVSEK	74	7	2	33286420;33286422;33286418	N-Term(TMT6plex); K17(TMT6plex)	2095.2	0				
																	FGVEQDVDMVFASFIR	255	7	2	33286420;33286422;33286418	N-Term(TMT6plex); M9(Oxidation)	2105.1	0				
																	GDYPLEAVR	52	7	2	33286420;33286422;33286418	N-Term(TMT6plex)	1248.7	0				
																	AGKPVICATQMLESMIK	60	6	2	33286420;33286422;33286418	N-Term(TMT6plex); K3(TMT6plex); C7(Carbamidomethyl); M11(Oxidation); M15(Oxidation); K17(TMT6plex)	2596.4	0				
																	VVEVGSK	31	7	2	33286420;33286422;33286418	N-Term(TMT6plex); K7(TMT6plex)	1175.7	0				
																	CDENILWLDYK	70	6	2	33286420;33286422;33286418	N-Term(TMT6plex); C1(Carbamidomethyl); K11(TMT6plex)	1927.0	0				
																	KASDVHEVR	11	6	2	33286420;33286422;33286418	N-Term(TMT6plex); K1(TMT6plex)	1498.9	1				
																	TATESFASDPILYRPVAVALDTK	48	5	2	33286420;33286422;33286418	N-Term(TMT6plex); K23(TMT6plex)	2923.6	0				
																	LDIDSPPITAR	40	6	2	33286420;33286422;33286418	N-Term(TMT6plex)	1426.8	0				
																	APIIAVTR	24	7	2	33286420;33286422;33286418	N-Term(TMT6plex)	1069.7	0				
																	GIFPVLCK	41	6	2	33286420;33286422;33286418	N-Term(TMT6plex); C7(Carbamidomethyl); K8(TMT6plex)	1391.8	0				
																	NTGIICTIGPASR	45	5	2	33286420;33286422;33286418	N-Term(TMT6plex); C6(Carbamidomethyl)	1588.9	0				
																	RFDEILEASDGIMVAR	193	7	2	33286420;33286422;33286418	N-Term(TMT6plex); M13(Oxidation)	2067.1	1				
																	VNFAMNVGK	39	7	2	33286420;33286422;33286418	N-Term(TMT6plex); M5(Oxidation); K9(TMT6plex)	1453.8	0				
																	GSGTAEVELKK	5	5	2	33286420;33286422;33286418	N-Term(TMT6plex); K10(TMT6plex); K11(TMT6plex)	1806.1	1				
																	RFDEILEASDGIMVAR	17	7	2	33286420;33286422;33286418	N-Term(TMT6plex)	2051.1	1				
																	AGKPVICATQMLESMIK	14	6	2	33286420;33286422;33286418	N-Term(TMT6plex); K3(TMT6plex); C7(Carbamidomethyl); K17(TMT6plex)	2564.5	0				
																	FGVEQDVDMVFASFIR	101	7	2	33286420;33286422;33286418	N-Term(TMT6plex)	2089.1	0				
																	AGKPVICATQMLESMIK	11	6	2	33286420;33286422;33286418	N-Term(TMT6plex); K3(TMT6plex); C7(Carbamidomethyl); M11(Oxidation); K17(TMT6plex)	2580.5	0				
																	GIFPVLCKDPVQEAWAEDVDLR	15	6	2	33286420;33286422;33286418	N-Term(TMT6plex); C7(Carbamidomethyl); K8(TMT6plex)	3015.6	1				
																	KGDVVIVLTGWRPGSGFTNTMR	10	6	2	33286420;33286422;33286418	N-Term(TMT6plex); K1(TMT6plex); M21(Oxidation)	2865.6	1				
																	ITLDNAYMEK	36	7	2	33286420;33286422;33286418	N-Term(TMT6plex); M8(Oxidation); K10(TMT6plex)	1671.9	0				
																	VNFAMNVGK	8	7	2	33286420;33286422;33286418	N-Term(TMT6plex); K9(TMT6plex)	1437.8	0				
																	ITLDNAYMEK	12	7	2	33286420;33286422;33286418	N-Term(TMT6plex); K10(TMT6plex)	1655.9	0				
																	EAEAAIYHLQLFEELR	118	3	1	33286418	N-Term(TMT6plex)	2161.1	0	**1.3**	**1.3**	**1.5**	**1.4**
																	LNFSHGTHEYHAETIK	26	5	2	33286420;33286422;33286418	N-Term(TMT6plex); K16(TMT6plex)	2342.2	0				

Accession	RefSeq Accession	Gene Symbol	Entrez Gene ID	Description	Σ Coverage	Σ# Proteins	Σ# Unique Peptides	Σ# Peptides	Σ# PSMs	Het-1A-Smoke - 2M/Parental	Het-1A-Smoke - 4M/Parental	Het-1A-Smoke - 6M/Parental	Het-1A-Smoke - 8M/Parental	# AAs	MW [kDa]	calc. pI	Sequence	# PSMs	# Proteins	# Protein Groups	Protein Group Accessions	Modifications	MH+ [Da]	# Missed Cleavages	Het-1A-Smoke - 2M/Parental	Het-1A-Smoke - 4M/Parental	Het-1A-Smoke - 6M/Parental	Het-1A-Smoke - 8M/Parental
																	AEGSDVANAVLDGADCIMLSGETAK	28	7	2	33286420;33286422;33286418	N-Term(TMT6plex); C16(Carbamidomethyl); K25(TMT6plex)	2952.4	0				
																	CCSGAIIVLTK	12	3	1	33286418	N-Term(TMT6plex); C1(Carbamidomethyl); C2(Carbamidomethyl); K11(TMT6plex)	1680.0	0	1.2	1.1	1.4	1.1
																	IENHEGVR	6	7	2	33286420;33286422;33286418	N-Term(TMT6plex)	1182.6	0				
																	FDEILEASDGIMVAR	7	7	2	33286420;33286422;33286418	N-Term(TMT6plex)	1895.0	0				
																	EAEAAIYHLQLFEELRR	1	3	1	33286418	N-Term(TMT6plex)	2317.2	1	1.2	1.4	1.4	1.3
																	KGATLK	4	7	2	33286420;33286422;33286418	N-Term(TMT6plex); K1(TMT6plex); K6(TMT6plex)	1304.9	1				
																	VFLAQK	23	8	2	33286420;33286422;33286418	N-Term(TMT6plex); K6(TMT6plex)	1163.8	0				
																	KVLGEK	4	9	2	33286420;33286422;33286418	N-Term(TMT6plex); K1(TMT6plex); K6(TMT6plex)	1360.9	1				
																	SAHQVAR	4	7	2	33286420;33286422;33286418	N-Term(TMT6plex)	997.6	0				
																	DIQDLK	2	6	2	33286420;33286422;33286418	N-Term(TMT6plex); K6(TMT6plex)	1189.7	0				
4504897;1002341781	NP_002257.1	**KPNA2**	3838	**importin subunit alpha-1**	75.80	2	26	26	348	**1.1**	**1.2**	**1.4**	**1.3**	529	57.8	5.40												
																	NNQGTVNWSVDDIVK	8	1	1	4504897;1002341781	N-Term(TMT6plex); K15(TMT6plex)	2147.1	0	**0.9**	**0.8**	**1.2**	**0.9**
																	IEALQNHENESVYK	13	1	1	4504897;1002341781	N-Term(TMT6plex); K14(TMT6plex)	2132.1	0	**1.1**	**1.1**	**1.4**	**1.1**
																	NKNPAPPIDAVEQILPTLVR	38	1	1	4504897;1002341781	N-Term(TMT6plex); K2(TMT6plex)	2643.6	1	**1.2**	**1.3**	**1.6**	**1.3**
																	EATWTMSNITAGR	21	1	1	4504897;1002341781	N-Term(TMT6plex); M6(Oxidation)	1682.8	0	**1.2**	**1.4**	**1.5**	**1.4**
																	ASLSLIEK	17	1	1	4504897;1002341781	N-Term(TMT6plex); K8(TMT6plex)	1318.8	0	**1.1**	**1.3**	**1.4**	**1.3**
																	EKQPPIDNIIR	3	1	1	4504897;1002341781	N-Term(TMT6plex); K2(TMT6plex)	1781.1	1	**0.9**	**1.3**	**1.4**	**1.4**
																	LLGASELPIVTPALR	39	1	1	4504897;1002341781	N-Term(TMT6plex)	1779.1	0	**1.1**	**1.3**	**1.5**	**1.3**
																	AIGNIVTGTDEQTQVVIDAGALAVFPSLLTNPK	44	1	1	4504897;1002341781	N-Term(TMT6plex); K33(TMT6plex)	3811.1	0	**1.2**	**1.4**	**1.4**	**1.5**
																	NVSSFPDDATSPLQENR	20	1	1	4504897;1002341781	N-Term(TMT6plex)	2106.0	0	**1.2**	**1.0**	**1.4**	**1.2**
																	TGVVPQLVK	10	1	1	4504897;1002341781	N-Term(TMT6plex); K9(TMT6plex)	1398.9	0	**1.1**	**1.1**	**1.3**	**1.2**
																	AVVDGGAIPAFISLLASPHAHISEQAVWALGNIAGDGSVFR	4	1	1	4504897;1002341781	N-Term(TMT6plex)	4343.3	0	**1.3**	**1.5**	**1.7**	**1.4**
																	GINSSNVENQLQATQAAR	16	1	1	4504897;1002341781	N-Term(TMT6plex)	2130.1	0	**1.1**	**1.3**	**1.4**	**1.4**
																	LSIMIEECGGLDK	4	1	1	4504897;1002341781	N-Term(TMT6plex); M4(Oxidation); C8(Carbamidomethyl); K13(TMT6plex)	1939.0	0	**1.0**	**1.2**	**1.3**	**1.3**
																	NPAPPIDAVEQILPTLVR	24	1	1	4504897;1002341781	N-Term(TMT6plex)	2172.3	0	**1.5**	**1.5**	**1.6**	**1.4**
																	LSIMIEECGGLDKIEALQNHENESVYK	2	1	1	4504897;1002341781	N-Term(TMT6plex); M4(Oxidation); C8(Carbamidomethyl); K13(TMT6plex); K27(TMT6plex)	3823.0	1	**0.9**	**1.4**	**1.4**	**1.6**
																	YFSVEEEEDQNVVPETTSEGYTFQVQDGAPGTFNF	6	1	1	4504897;1002341781	N-Term(TMT6plex)	4189.9	0	**1.3**	**1.2**	**1.4**	**1.3**
																	YGAVDPLLALLAVPDMSSLACGYLR	26	1	1	4504897;1002341781	N-Term(TMT6plex); M16(Oxidation); C21(Carbamidomethyl)	2910.5	0	**1.5**	**1.4**	**1.7**	**1.4**
																	LSIMIEECGGLDKIEALQNHENESVYK	1	1	1	4504897;1002341781	N-Term(TMT6plex); C8(Carbamidomethyl); K13(TMT6plex); K27(TMT6plex)	3807.0	1	**1.3**	**1.6**	**2.0**	**1.5**
																	YGAVDPLLALLAVPDMSSLACGYLR	8	1	1	4504897;1002341781	N-Term(TMT6plex); C21(Carbamidomethyl)	2894.5	0	**1.5**	**1.5**	**1.5**	**1.4**
																	LLHHDDPEVLADTCWAISYLTDGPNER	4	1	1	4504897;1002341781	N-Term(TMT6plex); C14(Carbamidomethyl)	3366.6	0	**1.3**	**1.5**	**1.5**	**1.4**
																	EATWTMSNITAGR	6	1	1	4504897;1002341781	N-Term(TMT6plex)	1666.8	0	**1.3**	**1.5**	**1.8**	**1.4**
																	IEVNVELR	3	1	1	4504897;1002341781	N-Term(TMT6plex)	1200.7	0	**1.0**	**1.1**	**1.3**	**1.2**
																	QDQIQQVVNHGLVPFLVSVLSK	2	1	1	4504897;1002341781	N-Term(TMT6plex); K22(TMT6plex)	2906.7	0	**1.2**	**1.4**	**2.1**	**1.1**
																	TDCSPIQFESAWALTNIASGTSEQTK	2	1	1	4504897;1002341781	N-Term(TMT6plex); C3(Carbamidomethyl); K26(TMT6plex)	3300.6	0	**1.1**	**1.1**	**1.6**	**1.1**
																	NLTWTLSNLCR	1	2	1	4504897;1002341781	N-Term(TMT6plex); C10(Carbamidomethyl)	1606.9	0				
																	FVSFLGR	9	1	1	4504897;1002341781	N-Term(TMT6plex)	1054.6	0	**1.0**	**1.0**	**1.3**	**1.1**
																	QPPIDNIIR	15	1	1	4504897;1002341781	N-Term(TMT6plex)	1294.8	0	**1.1**	**1.3**	**1.3**	**1.4**
																	KDDQMLK	1	1	1	4504897;1002341781	N-Term(TMT6plex); K1(TMT6plex); K7(TMT6plex)	1564.9	1	**1.2**	**1.8**	**1.9**	**1.4**
																	IGMVVK	1	1	1	4504897;1002341781	N-Term(TMT6plex); K6(TMT6plex)	1104.7	0	**1.2**	**1.7**	**1.9**	**1.6**
13027378	NP_005462.1	**GNPDA1**	10007	**glucosamine-6-phosphate isomerase 1**	75.78	1	12	16	123	**1.4**	**1.4**	**1.4**	**1.5**	289	32.6	6.92												
																	TVFVCDEDATLELK	13	1	1	13027378	N-Term(TMT6plex); C5(Carbamidomethyl); K14(TMT6plex)	2098.1	0	**1.6**	**1.4**	**1.5**	**1.6**
																	TLAMDTILANAR	3	1	1	13027378	N-Term(TMT6plex); M4(Oxidation)	1534.8	0	**1.7**	**1.5**	**1.6**	**1.5**
																	LIILEHYSQASEWAAK	12	1	1	13027378	N-Term(TMT6plex); K16(TMT6plex)	2317.3	0	**1.3**	**1.4**	**1.3**	**1.6**
																	TFNMDEYVGLPR	9	2	2	13027378;19923881	N-Term(TMT6plex); M4(Oxidation)	1686.8	0				
																	IIQFNPGPEK	9	1	1	13027378	N-Term(TMT6plex); K10(TMT6plex)	1600.9	0	**1.5**	**1.4**	**1.3**	**1.5**
																	HIDIHPENTHILDGNAVDLQAECDAFEEK	2	1	1	13027378	N-Term(TMT6plex); C23(Carbamidomethyl); K29(TMT6plex)	3788.8	0	**1.3**	**1.4**	**1.5**	**1.6**
																	YFTLGLPTGSTPLGCYK	12	2	2	13027378;19923881	N-Term(TMT6plex); C15(Carbamidomethyl); K17(TMT6plex)	2333.3	0				
																	LVDPLYSIK	10	1	1	13027378	N-Term(TMT6plex); K9(TMT6plex)	1505.9	0	**1.6**	**1.6**	**1.6**	**1.6**
																	AIEEGVNHMWTVSAFQQHPR	6	4	2	13027378;19923881	N-Term(TMT6plex); M9(Oxidation)	2582.3	0				
																	FFDGELTK	12	1	1	13027378	N-Term(TMT6plex); K8(TMT6plex)	1414.8	0	**1.5**	**1.4**	**1.4**	**1.5**
																	KLIEYYK	5	1	1	13027378	N-Term(TMT6plex); K1(TMT6plex); K7(TMT6plex)	1644.0	1	**1.2**	**1.4**	**1.3**	**1.5**
																	AAGGIELFVGGIGPDGHIAFNEPGSSLVSR	4	1	1	13027378	N-Term(TMT6plex)	3153.6	0	**1.4**	**1.6**	**1.4**	**1.7**
																	TLAMDTILANAR	6	1	1	13027378	N-Term(TMT6plex)	1518.8	0	**1.5**	**1.4**	**1.7**	**1.4**
																	EVMILITGAHK	4	4	2	13027378;19923881	N-Term(TMT6plex); M3(Oxidation); K11(TMT6plex)	1686.0	0				
																	DHPESYHSFMWNNFFK	3	1	1	13027378	N-Term(TMT6plex); K16(TMT6plex)	2544.2	0	**1.5**	**1.5**	**1.5**	**1.5**
																	DHPESYHSFMWNNFFK	1	1	1	13027378	N-Term(TMT6plex); M10(Oxidation); K16(TMT6plex)	2560.2	0	**1.4**	**1.4**	**1.6**	**1.6**
																	GLMLVHNK	2	1	1	13027378	N-Term(TMT6plex); K8(TMT6plex)	1369.8	0	**1.4**	**1.7**	**1.7**	**1.7**
																	LIEYYK	5	1	1	13027378	N-Term(TMT6plex); K6(TMT6plex)	1286.8	0	**1.4**	**1.4**	**1.4**	**1.5**
																	GLMLVHNK	5	1	1	13027378	N-Term(TMT6plex); M3(Oxidation); K8(TMT6plex)	1385.8	0	**1.3**	**1.3**	**1.2**	**1.5**
33356163	NP_004672.2	**EIF1AY**	9086	**eukaryotic translation initiation factor 1A, Y-chromosomal isoform 1**	75.69	2	1	13	205	**1.4**	**1.6**	**1.6**	**1.5**	144	16.4	5.24												
																	LEALCFDGVK	20	2	1	33356163	N-Term(TMT6plex); C5(Carbamidomethyl); K10(TMT6plex)	1609.9	0	**1.4**	**1.6**	**1.6**	**1.5**
																	AYGELPEHAK	30	3	2	33356163;4503499	N-Term(TMT6plex); K10(TMT6plex)	1572.9	0				
																	EDGQEYAQVIK	42	3	2	33356163;4503499	N-Term(TMT6plex); K11(TMT6plex)	1737.9	0				
																	INETDTFGPGDDDEIQFDDIGDDDEDIDDI	32	3	2	33356163;4503499	N-Term(TMT6plex)	3587.5	0				
																	YNADEAR	6	3	2	33356163;4503499	N-Term(TMT6plex)	1067.5	0				
																	ELVFKEDGQEYAQVIK	3	3	2	33356163;4503499	N-Term(TMT6plex); K5(TMT6plex); K16(TMT6plex)	2583.5	1				
																	VWINTSDIILVGLR	50	2	2	33356163;4503499	N-Term(TMT6plex)	1828.1	0				
																	NENESEK	2	3	2	33356163;4503499	N-Term(TMT6plex); K7(TMT6plex)	1307.7	0				
																	GKNENESEK	3	3	2	33356163;4503499	N-Term(TMT6plex); K2(TMT6plex); K9(TMT6plex)	1722.0	1				
																	NENESEKR	6	3	2	33356163;4503499	N-Term(TMT6plex); K7(TMT6plex)	1463.8	1				
																	DYQDNK	7	2	2	33356163;4503499	N-Term(TMT6plex); K6(TMT6plex)	1240.7	0				
																	GKNENESEKR	1	3	2	33356163;4503499	N-Term(TMT6plex); K2(TMT6plex); K9(TMT6plex)	1878.1	2				
																	ADVILK	3	3	2	33356163;4503499	N-Term(TMT6plex); K6(TMT6plex)	1116.7	0				
4503499	NP_001403.1	**EIF1AX**	1964	**eukaryotic translation initiation factor 1A, X-chromosomal**	75.69	1	1	13	221	**1.2**	**1.2**	**1.4**	**1.1**	144	16.5	5.24												
																	LEAMCFDGVK	16	1	1	4503499	N-Term(TMT6plex); C5(Carbamidomethyl); K10(TMT6plex)	1627.9	0	**1.3**	**1.5**	**1.8**	**1.1**
																	AYGELPEHAK	30	3	2	33356163;4503499	N-Term(TMT6plex); K10(TMT6plex)	1572.9	0				
																	LEAMCFDGVK	20	1	1	4503499	N-Term(TMT6plex); M4(Oxidation); C5(Carbamidomethyl); K10(TMT6plex)	1643.9	0	**1.2**	**1.2**	**1.3**	**1.1**
																	EDGQEYAQVIK	42	3	2	33356163;4503499	N-Term(TMT6plex); K11(TMT6plex)	1737.9	0				
																	INETDTFGPGDDDEIQFDDIGDDDEDIDDI	32	3	2	33356163;4503499	N-Term(TMT6plex)	3587.5	0				
																	YNADEAR	6	3	2	33356163;4503499	N-Term(TMT6plex)	1067.5	0				
																	ELVFKEDGQEYAQVIK	3	3	2	33356163;4503499	N-Term(TMT6plex); K5(TMT6plex); K16(TMT6plex)	2583.5	1				
																	VWINTSDIILVGLR	50	2	2	33356163;4503499	N-Term(TMT6plex)	1828.1	0				
																	NENESEK	2	3	2	33356163;4503499	N-Term(TMT6plex); K7(TMT6plex)	1307.7	0				
																	GKNENESEK	3	3	2	33356163;4503499	N-Term(TMT6plex); K2(TMT6plex); K9(TMT6plex)	1722.0	1				
																	NENESEKR	6	3	2	33356163;4503499	N-Term(TMT6plex); K7(TMT6plex)	1463.8	1				
																	DYQDNK	7	2	2	33356163;4503499	N-Term(TMT6plex); K6(TMT6plex)	1240.7	0				
																	GKNENESEKR	1	3	2	33356163;4503499	N-Term(TMT6plex); K2(TMT6plex); K9(TMT6plex)	1878.1	2				
																	ADVILK	3	3	2	33356163;4503499	N-Term(TMT6plex); K6(TMT6plex)	1116.7	0				
194248072;167466173	NP_005336.3	**HSPA1A**	3303	**heat shock 70 kDa protein 1A/1B**	75.66	1	19	40	1440	**1.1**	**1.1**	**1.1**	**1.2**	641	70.0	5.66												
																	QTQIFTTYSDNQPGVLIQVYEGER	43	2	2	194248072;167466173;124256496	N-Term(TMT6plex)	3015.5	0				
																	AFYPEEISSMVLTK	82	2	2	194248072;167466173;124256496	N-Term(TMT6plex); M10(Oxidation); K14(TMT6plex)	2089.1	0				
																	SAVEDEGLK	49	1	1	194248072;167466173	N-Term(TMT6plex); K9(TMT6plex)	1405.8	0	**1.1**	**1.1**	**1.1**	**1.2**
																	IINEPTAAAIAYGLDR	102	2	2	194248072;167466173;34419635	N-Term(TMT6plex)	1917.0	0				
																	LVNHFVEEFK	34	1	1	194248072;167466173	N-Term(TMT6plex); K10(TMT6plex)	1720.0	0	**1.0**	**1.0**	**1.1**	**1.1**
																	GGSGSGPTIEEVD	24	1	1	194248072;167466173	N-Term(TMT6plex)	1433.7	0	**1.5**	**1.3**	**1.3**	**1.4**
																	AFYPEEISSMVLTK	31	2	2	194248072;167466173;124256496	N-Term(TMT6plex); K14(TMT6plex)	2073.1	0				
																	TTPSYVAFTDTER	87	6	5	13676857;5729877;194248072;167466173;124256496;34419635	N-Term(TMT6plex)	1716.9	0				
																	HWPFQVINDGDKPK	8	1	1	194248072;167466173	N-Term(TMT6plex); K12(TMT6plex); K14(TMT6plex)	2368.3	0	**1.0**	**1.0**	**1.1**	**1.1**
																	KDISQNK	3	2	2	194248072;167466173;124256496	N-Term(TMT6plex); K1(TMT6plex); K7(TMT6plex)	1519.9	1				

Supplementary Table 6. List of all proteins and peptides identified in untreated and chronically treated Het1A cells with cigarette smoke condensate for 8 months

Accession	RefSeq Accession	Gene Symbol	Entrez Gene ID	Description	Σ Coverage	Σ# Proteins	Σ# Unique Peptides	Σ# Peptides	Σ# PSMs	Het-1A-Smoke - 2M/Parental	Het-1A-Smoke - 4M/Parental	Het-1A-Smoke - 6M/Parental	Het-1A-Smoke - 8M/Parental	# AAs	MW [kDa]	calc. pI	Sequence	# PSMs	# Proteins	# Protein Groups	Protein Group Accessions	Modifications	MH+ [Da]	# Missed Cleavages	Het-1A-Smoke - 2M/Parental	Het-1A-Smoke - 4M/Parental	Het-1A-Smoke - 6M/Parental	Het-1A-Smoke - 8M/Parental
																	SINPDEAVAYGAAVQAAILMGDK	133	2	2	194248072;167466173;124256496	N-Term(TMT6plex); M20(Oxidation); K23(TMT6plex)	2778.5	0				
																	VEIIANDQGNR	80	7	6	13676857;5729877;16507237;194248072;167466173;124256496;34419635	N-Term(TMT6plex)	1457.8	0				
																	NQVALNPQNTVFDAK	32	1	1	194248072;167466173	N-Term(TMT6plex); K15(TMT6plex)	2117.2	0	**1.1**	**0.9**	**1.1**	**1.1**
																	EIAEAYLGYPVTNAVITVPAYFNDSQR	19	1	1	194248072;167466173	N-Term(TMT6plex)	3230.7	0	**1.5**	**1.7**	**1.8**	**1.8**
																	LLQDFFNGR	5	1	1	194248072;167466173	N-Term(TMT6plex)	1338.7	0	**1.1**	**1.0**	**1.1**	**1.1**
																	FEELCSDLFR	51	2	2	194248072;167466173;34419635	N-Term(TMT6plex); C5(Carbamidomethyl)	1544.8	0				
																	CQEVISWLDANTLAEKDEFEHK	9	1	1	194248072;167466173	N-Term(TMT6plex); C1(Carbamidomethyl); K16(TMT6plex); K22(TMT6plex)	3349.7	1	**1.2**	**0.9**	**1.0**	**0.9**
																	FGDPVVQSDMK	27	1	1	194248072;167466173	N-Term(TMT6plex); M10(Oxidation); K11(TMT6plex)	1696.9	0	**1.1**	**1.1**	**1.2**	**1.2**
																	DAGVIAGLNVLR	75	2	2	194248072;167466173;124256496	N-Term(TMT6plex)	1426.9	0				
																	ELEQVCNPIISGLYQGAGGPGPGGFGAQGPK	51	1	1	194248072;167466173	N-Term(TMT6plex); C6(Carbamidomethyl); K31(TMT6plex)	3513.8	0	**1.2**	**1.2**	**1.3**	**1.2**
																	NALESYAFNMK	20	2	2	194248072;167466173;124256496	N-Term(TMT6plex); M10(Oxidation); K11(TMT6plex)	1761.9	0				
																	AQIHDLVLVGGSTR	23	1	1	194248072;167466173	N-Term(TMT6plex)	1695.0	0	**1.2**	**1.3**	**1.3**	**1.3**
																	AAAIGIDLGTTYSCVGVFQHGK	3	1	1	194248072;167466173	N-Term(TMT6plex); C14(Carbamidomethyl); K22(TMT6plex)	2723.5	0	**1.2**	**1.7**	**1.4**	**1.6**
																	NSTIPTK	13	2	2	194248072;167466173;124256496	N-Term(TMT6plex); K7(TMT6plex)	1218.7	0				
																	ATAGDTHLGGEDFDNR	18	3	3	194248072;167466173;124256496;34419635	N-Term(TMT6plex)	1904.9	0				
																	SINPDEAVAYGAAVQAAILMGDK	95	2	2	194248072;167466173;124256496	N-Term(TMT6plex); K23(TMT6plex)	2762.5	0				
																	NALESYAFNMK	16	2	2	194248072;167466173;124256496	N-Term(TMT6plex); K11(TMT6plex)	1745.9	0				
																	SENVQDLLLLDVAPLSLGLETAGGVMTALIK	39	1	1	194248072;167466173	N-Term(TMT6plex); M26(Oxidation); K31(TMT6plex)	3655.1	0	**1.3**	**1.2**	**1.4**	**1.1**
																	YKAEDEVQR	14	2	2	194248072;167466173;124256496	N-Term(TMT6plex); K2(TMT6plex)	1595.9	1				
																	KFGDPVVQSDMK	30	1	1	194248072;167466173	N-Term(TMT6plex); K1(TMT6plex); M11(Oxidation); K12(TMT6plex)	2054.2	1	**0.9**	**1.0**	**1.0**	**1.3**
																	LIGDAAK	87	6	5	13676857;5729877;16507237;194248072;167466173;124256496	N-Term(TMT6plex); K7(TMT6plex)	1145.7	0				
																	MKEIAEAYLGYPVTNAVITVPAYFNDSQR	3	1	1	194248072;167466173	N-Term(TMT6plex); M1(Oxidation); K2(TMT6plex)	3735.0	1	**1.5**	**1.4**	**1.6**	**1.4**
																	STLEPVEK	23	2	2	194248072;167466173;34419635	N-Term(TMT6plex); K8(TMT6plex)	1360.8	0				
																	SENVQDLLLLDVAPLSLGLETAGGVMTALIK	11	1	1	194248072;167466173	N-Term(TMT6plex); K31(TMT6plex)	3639.1	0	**1.8**	**1.8**	**1.8**	**1.5**
																	TLSSSTQASLEIDSLFEGIDFYTSITR	22	1	1	194248072;167466173	N-Term(TMT6plex)	3210.6	0	**1.4**	**1.3**	**1.3**	**1.4**
																	FELSGIPPAPR	2	2	2	194248072;167466173;34419635	N-Term(TMT6plex)	1412.8	0				
																	LVNHFVEEFKR	11	1	1	194248072;167466173	N-Term(TMT6plex); K10(TMT6plex)	1876.1	1	**0.9**	**1.1**	**1.0**	**1.4**
																	KFGDPVVQSDMK	2	1	1	194248072;167466173	N-Term(TMT6plex); K1(TMT6plex); K12(TMT6plex)	2038.2	1	**1.6**	**1.7**	**1.8**	**1.3**
																	DEFEHK	1	1	1	194248072;167466173	N-Term(TMT6plex); K6(TMT6plex)	1262.7	0	**1.0**	**1.2**	**1.2**	**1.1**
																	AEDEVQR	7	2	2	194248072;167466173;124256496	N-Term(TMT6plex)	1075.6	0				
																	DNNLLGR	1	3	3	194248072;167466173;124256496;34419635	N-Term(TMT6plex)	1030.6	0				
																	ITITNDK	26	5	5	13676857;5729877;194248072;167466173;124256496;34419635	N-Term(TMT6plex); K7(TMT6plex)	1262.8	0				
																	MVQEAEK	4	2	2	5729877;194248072;167466173	N-Term(TMT6plex); K7(TMT6plex)	1292.7	0				
																	VQVSYK	8	1	1	194248072;167466173	N-Term(TMT6plex); K6(TMT6plex)	1181.7	0	**0.9**	**1.0**	**1.0**	**1.1**
																	DISQNK	7	2	2	194248072;167466173;124256496	N-Term(TMT6plex); K6(TMT6plex)	1162.7	0				
																	MVQEAEK	9	2	2	5729877;194248072;167466173	N-Term(TMT6plex); M1(Oxidation); K7(TMT6plex)	1308.7	0				
256222411	NP_001157789.1	**FLNB**	2317	**filamin-B isoform 1**	75.66	1	1	139	1727	**1.3**	**1.3**	**1.2**	**1.1**	2633	281.5	5.71												
																	DAGYGGISLAVEGPSK	24	4	2	256222415;256222411	N-Term(TMT6plex); K16(TMT6plex)	1979.1	0				
																	VGEPGQAGNPALVSAYGTGLEGGTTGIQSEFFINTTR	23	4	2	256222415;256222411	N-Term(TMT6plex)	3926.0	0				
																	VDIQTEDLEDGTCK	24	4	2	256222415;256222411	N-Term(TMT6plex); C13(Carbamidomethyl); K14(TMT6plex)	2081.1	0				
																	DGTYAVTYIPDK	12	4	2	256222415;256222411	N-Term(TMT6plex); K12(TMT6plex)	1801.0	0				
																	GEAGVPAEFSIWTR	16	4	2	256222415;256222411	N-Term(TMT6plex)	1748.9	0				
																	VLSEDEEDVDFDIIHNANDTFTVK	16	4	2	256222415;256222411	N-Term(TMT6plex); K24(TMT6plex)	3223.6	0				
																	VHSPSGAVEECHVSELEPDK	8	4	2	256222415;256222411	N-Term(TMT6plex); C11(Carbamidomethyl); K20(TMT6plex)	2664.3	0				
																	IPYLPITNFNQNWQDGK	24	4	2	256222415;256222411	N-Term(TMT6plex); K17(TMT6plex)	2506.4	0				
																	DAGEGLLAVQITDQEGKPK	16	4	2	256222415;256222411	N-Term(TMT6plex); K17(TMT6plex); K19(TMT6plex)	2656.5	0				
																	VTASGPGLSSYGVPASLPVDFAIDAR	20	4	2	256222415;256222411	N-Term(TMT6plex)	2776.5	0				
																	VSYFPTVPGVYIVSTK	39	4	2	256222415;256222411	N-Term(TMT6plex); K16(TMT6plex)	2215.3	0				
																	LDVTILSPSR	9	4	2	256222415;256222411	N-Term(TMT6plex)	1329.8	0				
																	EAMQQADDWLGVPQVITPEEIIHPDVDEHSVMTYLSQFPK	26	4	2	256222415;256222411	N-Term(TMT6plex); M3(Oxidation); M32(Oxidation); K40(TMT6plex)	5083.5	0				
																	IQQNTFTR	7	8	4	116063573;256222415;256222411;188595687	N-Term(TMT6plex)	1236.7	0				
																	IPEINSSDMSAHVTSPSGR	18	4	2	256222415;256222411	N-Term(TMT6plex); M9(Oxidation)	2230.1	0				
																	FVPQEMGVHTVSVK	22	4	2	256222415;256222411	N-Term(TMT6plex); M6(Oxidation); K14(TMT6plex)	2032.1	0				
																	AEGPGLSK	6	4	2	256222415;256222411	N-Term(TMT6plex); K8(TMT6plex)	1216.7	0				
																	TATFTIVTEDAGEGGLDLAIEGPSK	9	4	2	256222415;256222411	N-Term(TMT6plex); K25(TMT6plex)	2950.6	0				
																	YAPTEVGLHEMHIK	11	4	2	256222415;256222411	N-Term(TMT6plex); M11(Oxidation); K14(TMT6plex)	2099.1	0				
																	ADIEMPFDPSK	18	4	2	256222415;256222411	N-Term(TMT6plex); M5(Oxidation); K11(TMT6plex)	1723.9	0				
																	LVSIDSK	7	8	4	116063573;256222415;256222411;188595687	N-Term(TMT6plex); K7(TMT6plex)	1219.8	0				
																	VTEAEIVPMGK	22	4	2	256222415;256222411	N-Term(TMT6plex); M9(Oxidation); K11(TMT6plex)	1647.9	0				
																	VPVKDVVDPSK	2	4	2	256222415;256222411	N-Term(TMT6plex); K4(TMT6plex); K11(TMT6plex)	1870.2	1				
																	VVPCLVTPVTGR	5	4	2	256222415;256222411	N-Term(TMT6plex); C4(Carbamidomethyl)	1526.9	0				
																	VGEAGLLSVDCSEAGPGALGLEAVSDSGTK	24	4	2	256222415;256222411	N-Term(TMT6plex); C11(Carbamidomethyl); K30(TMT6plex)	3304.7	0				
																	APSVATVGSICDLNLK	30	4	2	256222415;256222411	N-Term(TMT6plex); C11(Carbamidomethyl); K16(TMT6plex)	2103.2	0				
																	DGTCTVTYLPTLPGDYSILVK	30	4	2	256222415;256222411	N-Term(TMT6plex); C4(Carbamidomethyl); K21(TMT6plex)	2771.5	0				
																	YMIGVTYGGDDIPLSPYR	14	4	2	256222415;256222411	N-Term(TMT6plex)	2246.1	0				
																	NTVELLVEDK	25	4	2	256222415;256222411	N-Term(TMT6plex); K10(TMT6plex)	1617.9	0				
																	AHIANPSGASTECFVTDNADGTYQVEYTPFEK	10	4	2	256222415;256222411	N-Term(TMT6plex); C13(Carbamidomethyl); K32(TMT6plex)	3977.9	0				
																	GAGIGGLGITVEGPSESK	14	4	2	256222415;256222411	N-Term(TMT6plex); K18(TMT6plex)	2087.2	0				
																	QKDFLDGVYAFEYYPSTPGR	14	4	2	256222415;256222411	N-Term(TMT6plex); K2(TMT6plex)	2811.4	1				
																	AEITFDDHK	16	4	2	256222415;256222411	N-Term(TMT6plex); K9(TMT6plex)	1533.8	0				
																	DGSCSAEYIPFAPGDYDVNITYGGAHIPGSPFR	13	4	2	256222415;256222411	N-Term(TMT6plex); C4(Carbamidomethyl)	3759.8	0				
																	LLGWIQNK	32	8	4	116063573;256222415;256222411;188595687	N-Term(TMT6plex); K8(TMT6plex)	1429.9	0				
																	FNDEHIPESPYLVPVIAPSDDAR	16	4	2	256222415;256222411	N-Term(TMT6plex)	2810.4	0				
																	AEISCIDNK	6	4	2	256222415;256222411	N-Term(TMT6plex); C5(Carbamidomethyl); K9(TMT6plex)	1507.8	0				
																	TGEEVGFVVDAK	18	4	2	256222415;256222411	N-Term(TMT6plex); K12(TMT6plex)	1708.9	0				
																	WCNEHLK	8	8	4	116063573;256222415;256222411;188595687	N-Term(TMT6plex); C2(Carbamidomethyl); K7(TMT6plex)	1444.8	0				
																	VEVGKDQEFTVDTR	9	4	2	256222415;256222411	N-Term(TMT6plex); K5(TMT6plex)	2081.1	1				
																	AGVENGKPTHFTVYTK	2	4	2	256222415;256222411	N-Term(TMT6plex); K7(TMT6plex); K16(TMT6plex)	2436.4	0				
																	AGSNMLLIGVHGPTTPCEEVSMK	1	4	2	256222415;256222411	N-Term(TMT6plex); C17(Carbamidomethyl); K23(TMT6plex)	2886.5	0				
																	VAVTEGCQPSR	8	4	2	256222415;256222411	N-Term(TMT6plex); C7(Carbamidomethyl)	1432.7	0				
																	GDYVLAVK	9	4	2	256222415;256222411	N-Term(TMT6plex); K8(TMT6plex)	1322.8	0				
																	LVSPGSANETSSILVESVTR	22	4	2	256222415;256222411	N-Term(TMT6plex)	2275.2	0				
																	IEYNDQNDGSCDVK	18	4	2	256222415;256222411	N-Term(TMT6plex); C11(Carbamidomethyl); K14(TMT6plex)	2115.0	0				
																	AAGSGELGVTMK	26	4	2	256222415;256222411	N-Term(TMT6plex); M11(Oxidation); K12(TMT6plex)	1594.9	0				
																	QMQLENVSVALEFLDR	41	6	3	116063573;256222415;256222411	N-Term(TMT6plex); M2(Oxidation)	2137.1	0				
																	YMIGVTYGGDDIPLSPYR	23	4	2	256222415;256222411	N-Term(TMT6plex); M2(Oxidation)	2262.1	0				
																	YTPTQQGNMQVLVTYGGDPIPK	9	4	2	256222415;256222411	N-Term(TMT6plex); M9(Oxidation); K22(TMT6plex)	2881.5	0				
																	ANEPTHFTVDCTEAGEGDVSVGIK	15	4	2	256222415;256222411	N-Term(TMT6plex); C11(Carbamidomethyl); K24(TMT6plex)	2991.5	0				
																	SPFEVQVGPEAGMQK	18	4	2	256222415;256222411	N-Term(TMT6plex); M13(Oxidation); K15(TMT6plex)	2078.1	0				
																	AGLAPLEVR	10	4	2	256222415;256222411	N-Term(TMT6plex)	1154.7	0				
																	IFAQDGEGQR	5	4	2	256222415;256222411	N-Term(TMT6plex)	1349.7	0				
																	YGGELVPHFPAR	8	4	2	256222415;256222411	N-Term(TMT6plex)	1571.9	0				
																	TFEMSDFIVDTR	49	4	2	256222415;256222411	N-Term(TMT6plex); M4(Oxidation)	1705.8	0				
																	YGGPNHIVGSPFK	6	4	2	256222415;256222411	N-Term(TMT6plex); K13(TMT6plex)	1831.0	0				
																	AGSNMLLIGVHGPTTPCEEVSMK	9	4	2	256222415;256222411	N-Term(TMT6plex); M5(Oxidation); C17(Carbamidomethyl); M22(Oxidation); K23(TMT6plex)	2918.5	0				
																	WGEEHIPGSPFHVTVP	3	4	2	256222415;256222411	N-Term(TMT6plex)	2018.0	0				
																	EEGLYAVDVTYDGHPVPGSPYTVEASLPPDPSK	7	4	2	256222415;256222411	N-Term(TMT6plex); K33(TMT6plex)	3945.0	0				
																	EVGEHLVSIK	3	4	2	256222415;256222411	N-Term(TMT6plex); K10(TMT6plex)	1568.9	0				
																	LGSAADFLLDISETDLSSLTASIK	22	4	2	256222415;256222411	N-Term(TMT6plex); K24(TMT6plex)	2925.6	0				
																	IGNLQTDLSDGLR	14	4	2	256222415;256222411	N-Term(TMT6plex)	1630.9	0				

Accession	RefSeq Accession	Gene Symbol	Entrez Gene ID	Description	Σ Coverage	Σ# Proteins	Σ# Unique Peptides	Σ# Peptides	Σ# PSMs	Het-1A-Smoke - 2M/Parental	Het-1A-Smoke - 4M/Parental	Het-1A-Smoke - 6M/Parental	Het-1A-Smoke - 8M/Parental	# AAs	MW [kDa]	calc. pI	Sequence	# PSMs	# Proteins	# Protein Groups	Protein Group Accessions	Modifications	MH+ [Da]	# Missed Cleavages	Het-1A-Smoke - 2M/Parental	Het-1A-Smoke - 4M/Parental	Het-1A-Smoke - 6M/Parental	Het-1A-Smoke - 8M/Parental
																	TFEMSDFIVDTR	6	4	2	256222415;256222411	N-Term(TMT6plex)	1689.8	0				
																	LTVMSLQESGLK	8	4	2	256222415;256222411	N-Term(TMT6plex); M4(Oxidation); K12(TMT6plex)	1780.0	0				
																	VTEAEIVPMGK	4	4	2	256222415;256222411	N-Term(TMT6plex); K11(TMT6plex)	1631.9	0				
																	VNQPASFAIR	4	4	2	256222415;256222411	N-Term(TMT6plex)	1331.8	0				
																	VLQSFTVDSSK	8	4	2	256222415;256222411	N-Term(TMT6plex); K11(TMT6plex)	1669.0	0				
																	VMYTPMAPGNYLISVK	10	4	2	256222415;256222411	N-Term(TMT6plex); M2(Oxidation); M6(Oxidation); K16(TMT6plex)	2274.2	0				
																	IECSDNGDGTCSVSYLPTKPGEYFVNILFEEVHIPGSPFK	3	4	2	256222415;256222411	N-Term(TMT6plex); C3(Carbamidomethyl); C11(Carbamidomethyl); K19(TMT6plex); K40(TMT6plex)	5190.6	0				
																	VKAEGPGLSK	2	4	2	256222415;256222411	N-Term(TMT6plex); K2(TMT6plex); K10(TMT6plex)	1673.1	1				
																	QMQLENVSVALEFLDR	5	6	3	116063573;256222415;256222411	N-Term(TMT6plex)	2121.1	0				
																	DGTYAVTYVPLTAGMYTLTMK	2	4	2	256222415;256222411	N-Term(TMT6plex); M20(Oxidation); K21(TMT6plex)	2770.4	0				
																	AAGSGELGVTMK	3	4	2	256222415;256222411	N-Term(TMT6plex); K12(TMT6plex)	1578.9	0				
																	GIEPTGNMVK	2	4	2	256222415;256222411	N-Term(TMT6plex); K10(TMT6plex)	1503.9	0				
																	DGTYAVTYVPLTAGMYTLTMK	13	4	2	256222415;256222411	N-Term(TMT6plex); M15(Oxidation); M20(Oxidation); K21(TMT6plex)	2786.4	0				
																	SADFVVESIGSEVGSLGFAIEGPSQAK	48	4	2	256222415;256222411	N-Term(TMT6plex); K27(TMT6plex)	3139.7	0				
																	DFLDGVYAFEYYPSTPGR	17	4	2	256222415;256222411	N-Term(TMT6plex)	2326.1	0				
																	SGCIVNNLAEFTVDPK	4	4	2	256222415;256222411	N-Term(TMT6plex); C3(Carbamidomethyl); K16(TMT6plex)	2222.2	0				
																	VTVLFAGQHISK	8	4	2	256222415;256222411	N-Term(TMT6plex); K12(TMT6plex)	1758.1	0				
																	AGPGTLSVTIEGPSK	11	4	2	256222415;256222411	N-Term(TMT6plex); K15(TMT6plex)	1872.1	0				
																	LPNNHIGISFIPR	2	4	2	256222415;256222411	N-Term(TMT6plex)	1707.0	0				
																	HIPGSPFTAK	14	6	3	256222415;256222411;188595687	N-Term(TMT6plex); K10(TMT6plex)	1512.9	0				
																	IAGPGLGSGVR	7	4	2	256222415;256222411	N-Term(TMT6plex)	1212.7	0				
																	SPFTVGVAAPLDLSK	10	4	2	256222415;256222411	N-Term(TMT6plex); K15(TMT6plex)	1960.1	0				
																	MDCQETPEGYK	2	4	2	256222415;256222411	N-Term(TMT6plex); C3(Carbamidomethyl); K11(TMT6plex)	1815.9	0				
																	AGSNMLLIGVHGPTTPCEEVSMK	4	4	2	256222415;256222411	N-Term(TMT6plex); C17(Carbamidomethyl); M22(Oxidation); K23(TMT6plex)	2902.5	0				
																	VVASGPGLEHGK	5	4	2	256222415;256222411	N-Term(TMT6plex); K12(TMT6plex)	1608.9	0				
																	DEPCLLK	5	4	2	256222415;256222411	N-Term(TMT6plex); C4(Carbamidomethyl); K7(TMT6plex)	1332.8	0				
																	LIALLEVLSQK	22	8	4	116063573;256222415;256222411;188595687	N-Term(TMT6plex); K11(TMT6plex)	1685.1	0				
																	GAGTGGLGLTVEGPCEAK	18	6	3	256222415;256222411;188595687	N-Term(TMT6plex); C15(Carbamidomethyl); K18(TMT6plex)	2132.1	0				
																	AWGPGLHGGIVGR	34	4	2	256222415;256222411	N-Term(TMT6plex)	1505.9	0				
																	SSTETCYSAIPK	16	4	2	256222415;256222411	N-Term(TMT6plex); C6(Carbamidomethyl); K12(TMT6plex)	1801.9	0				
																	APLNVQFNSPLPGDAVK	22	4	2	256222415;256222411	N-Term(TMT6plex); K17(TMT6plex)	2225.3	0				
																	DLAEDAPWK	24	8	4	116063573;256222415;256222411;188595687	N-Term(TMT6plex); K9(TMT6plex)	1502.8	0				
																	ADIEMPFDPSK	8	4	2	256222415;256222411	N-Term(TMT6plex); K11(TMT6plex)	1707.9	0				
																	CLATGPGIASTVK	6	4	2	256222415;256222411	N-Term(TMT6plex); C1(Carbamidomethyl); K13(TMT6plex)	1733.0	0				
																	EAMQQADDWLGVPQVITPEEIIHPDVDEHSVMTYLSQFPK	19	4	2	256222415;256222411	N-Term(TMT6plex); M3(Oxidation); K40(TMT6plex)	5067.5	0				
																	IPEINSSDMSAHVTSPSGR	5	4	2	256222415;256222411	N-Term(TMT6plex)	2214.1	0				
																	VLPTYDASK	12	4	2	256222415;256222411	N-Term(TMT6plex); K9(TMT6plex)	1451.8	0				
																	RLPNNHIGISFIPR	12	4	2	256222415;256222411	N-Term(TMT6plex)	1863.1	1				
																	IFFAGDTIPK	24	4	2	256222415;256222411	N-Term(TMT6plex); K10(TMT6plex)	1566.9	0				
																	AEVSIQNNK	11	4	2	256222415;256222411	N-Term(TMT6plex); K9(TMT6plex)	1460.8	0				
																	TYSVEYLPK	14	4	2	256222415;256222411	N-Term(TMT6plex); K9(TMT6plex)	1557.9	0				
																	VLFASQEIPASPFR	11	4	2	256222415;256222411	N-Term(TMT6plex)	1791.0	0				
																	YSIAITWGGHHIPK	4	4	2	256222415;256222411	N-Term(TMT6plex); K14(TMT6plex)	2038.2	0				
																	GAGGQGK	3	6	3	116063573;256222415;256222411	N-Term(TMT6plex); K7(TMT6plex)	1032.6	0				
																	SSFLVDCSK	12	4	2	256222415;256222411	N-Term(TMT6plex); C7(Carbamidomethyl); K9(TMT6plex)	1500.8	0				
																	AHGPGLEGGLVGKPAEFTIDTK	6	4	2	256222415;256222411	N-Term(TMT6plex); K13(TMT6plex); K22(TMT6plex)	2881.7	0				
																	GLHVVEVTYDDVPIPNSPFK	20	4	2	256222415;256222411	N-Term(TMT6plex); K20(TMT6plex)	2684.5	0				
																	VMYTPMAPGNYLISVK	3	4	2	256222415;256222411	N-Term(TMT6plex); M6(Oxidation); K16(TMT6plex)	2258.2	0				
																	TATPEIVDNK	15	4	2	256222415;256222411	N-Term(TMT6plex); K10(TMT6plex)	1545.9	0				
																	LTVMSLQESGLK	2	4	2	256222415;256222411	N-Term(TMT6plex); K12(TMT6plex)	1764.0	0				
																	VQAQGPGLK	17	4	2	256222415;256222411	N-Term(TMT6plex); K9(TMT6plex)	1355.8	0				
																	AIVDGNLK	6	8	4	116063573;256222415;256222411;188595687	N-Term(TMT6plex); K8(TMT6plex)	1287.8	0				
																	ATQTGDASK	7	4	2	256222415;256222411	N-Term(TMT6plex); K9(TMT6plex)	1336.7	0				
																	ASSDASK	2	4	2	256222415;256222411	N-Term(TMT6plex); K7(TMT6plex)	1123.6	0				
																	ALSEFFK	2	1	1	256222411	N-Term(TMT6plex); K7(TMT6plex)	1299.8	0	**1.3**	**1.3**	**1.2**	**1.1**
																	FADEHVPGSPFTVK	14	4	2	256222415;256222411	N-Term(TMT6plex); K14(TMT6plex)	1989.1	0				
																	HVGNQQYNVTYVVK	4	4	2	256222415;256222411	N-Term(TMT6plex); K14(TMT6plex)	2107.2	0				
																	AIVHDNKDGTYAVTYIPDK	1	4	2	256222415;256222411	N-Term(TMT6plex); K7(TMT6plex); K19(TMT6plex)	2807.6	1				
																	TATPEIVDNKDGTVTVR	2	4	2	256222415;256222411	N-Term(TMT6plex); K10(TMT6plex)	2274.3	1				
																	EAFTNKPNVFTVVTR	5	4	2	256222415;256222411	N-Term(TMT6plex); K6(TMT6plex)	2181.2	0				
																	VKVEPAVDTSR	3	4	2	256222415;256222411	N-Term(TMT6plex); K2(TMT6plex)	1659.0	1				
																	DLDIIDNYDYSHTVK	12	4	2	256222415;256222411	N-Term(TMT6plex); K15(TMT6plex)	2269.2	0				
																	VNIGQGSHPQK	6	4	2	256222415;256222411	N-Term(TMT6plex); K11(TMT6plex)	1622.9	0				
																	FIPHENGVHTIDVK	2	4	2	256222415;256222411	N-Term(TMT6plex); K14(TMT6plex)	2064.2	0				
																	YMGSHIPESPLQFYVNYPNSGSVSAYGPGLVYGVANK	6	4	2	256222415;256222411	N-Term(TMT6plex); M2(Oxidation); K37(TMT6plex)	4437.2	0				
																	SPFEVSVDK	8	4	2	256222415;256222411	N-Term(TMT6plex); K9(TMT6plex)	1465.8	0				
																	VMYTPMAPGNYLISVK	1	4	2	256222415;256222411	N-Term(TMT6plex); K16(TMT6plex)	2242.2	0				
																	GQHVTGSPFQFTVGPLGEGGAHK	10	4	2	256222415;256222411	N-Term(TMT6plex); K23(TMT6plex)	2766.5	0				
																	MDCQETPEGYK	8	4	2	256222415;256222411	N-Term(TMT6plex); M1(Oxidation); C3(Carbamidomethyl); K11(TMT6plex)	1831.9	0				
																	MDGTYACSYTPVK	10	4	2	256222415;256222411	N-Term(TMT6plex); M1(Oxidation); C7(Carbamidomethyl); K13(TMT6plex)	1967.0	0				
																	VEPAVDTSR	5	4	2	256222415;256222411	N-Term(TMT6plex)	1202.7	0				
																	EAGAGGLSIAVEGPSK	12	6	3	256222415;256222411;188595687	N-Term(TMT6plex); K16(TMT6plex)	1901.1	0				
																	GPGLEAVGNIANKPTYFDIYTAGAGVGDIGVEVEDPQGK	2	4	2	256222415;256222411	N-Term(TMT6plex); K13(TMT6plex); K39(TMT6plex)	4606.4	0				
																	VKVDPSHDASK	4	4	2	256222415;256222411	N-Term(TMT6plex); K2(TMT6plex); K11(TMT6plex)	1870.1	1				
																	HTIAVVWGGVNIPHSPYR	3	4	2	256222415;256222411	N-Term(TMT6plex)	2232.2	0				
																	AYGPGLEK	6	4	2	256222415;256222411	N-Term(TMT6plex); K8(TMT6plex)	1292.8	0				
																	YADEEIPR	1	4	2	256222415;256222411	N-Term(TMT6plex)	1221.6	0				
																	VDPSHDASK	4	6	3	256222415;256222411;188595687	N-Term(TMT6plex); K9(TMT6plex)	1413.8	0				
																	ALGALVDSCAPGLCPDWESWDPQKPVDNAR	8	4	2	256222415;256222411	N-Term(TMT6plex); C9(Carbamidomethyl); C14(Carbamidomethyl); K24(TMT6plex)	3782.9	0				
																	SPFVVQVGEACNPNACR	2	4	2	256222415;256222411	N-Term(TMT6plex); C11(Carbamidomethyl); C16(Carbamidomethyl)	2134.0	0				
																	EPGEYAVHIMCDDEDIKDSPYMAFIHPATGGYNPDLVR	1	4	2	256222415;256222411	N-Term(TMT6plex); M10(Oxidation); C11(Carbamidomethyl); K17(TMT6plex); M22(Oxidation)	4813.3	1				
																	KGEITGEVHMPSGK	1	4	2	256222415;256222411	N-Term(TMT6plex); K1(TMT6plex); M10(Oxidation); K14(TMT6plex)	2173.2	1				
																	GIEPTGNMVK	5	4	2	256222415;256222411	N-Term(TMT6plex); M8(Oxidation); K10(TMT6plex)	1519.9	0				
																	VFGPGIEGK	3	4	2	256222415;256222411	N-Term(TMT6plex); K9(TMT6plex)	1361.8	0				
																	AGGPGLER	5	6	3	116063573;256222415;256222411	N-Term(TMT6plex)	985.6	0				
																	GLEELVK	6	4	2	256222415;256222411	N-Term(TMT6plex); K7(TMT6plex)	1245.8	0				
																	AIVHDNK	2	4	2	256222415;256222411	N-Term(TMT6plex); K7(TMT6plex)	1254.8	0				
																	LKPGAPLKPK	3	4	2	256222415;256222411	N-Term(TMT6plex); K2(TMT6plex); K8(TMT6plex); K10(TMT6plex)	1965.3	0				
																	GAGLSK	2	4	2	256222415;256222411	N-Term(TMT6plex); K6(TMT6plex)	990.6	0				
																	DVVDPSK	1	4	2	256222415;256222411	N-Term(TMT6plex); K7(TMT6plex)	1217.7	0				
																	AQGDASK	3	4	2	256222415;256222411	N-Term(TMT6plex); K7(TMT6plex)	1134.7	0				
																	DGTVTVR	1	6	3	116063573;256222415;256222411	N-Term(TMT6plex)	976.6	0				
306966132	NP_001182461.1	**SPTAN1**	6709	**spectrin alpha chain, non-erythrocytic 1 isoform 3**	75.61	2	2	166	1445	**1.0**	**1.1**	**1.1**	**1.0**	2452	282.1	5.34												
																	ELPTAFDYVEFTR	12	3	2	194595509;306966132	N-Term(TMT6plex)	1816.9	0				
																	EAALTSEEVGADLEQVEVLQK	28	3	2	194595509;306966132	N-Term(TMT6plex); K21(TMT6plex)	2716.5	0				
																	QQVAPTDDETGK	11	3	2	194595509;306966132	N-Term(TMT6plex); K12(TMT6plex)	1746.9	0				
																	DLASVQALLR	27	3	2	194595509;306966132	N-Term(TMT6plex)	1314.8	0				
																	KFEEFQTDMAAHEER	10	3	2	194595509;306966132	N-Term(TMT6plex); K1(TMT6plex); M9(Oxidation)	2342.1	1				
																	DLNSQADSLMTSSAFDTSQVK	8	3	2	194595509;306966132	N-Term(TMT6plex); M10(Oxidation); K21(TMT6plex)	2719.4	0				
																	SQLLGSAHEVQR	4	3	2	194595509;306966132	N-Term(TMT6plex)	1553.9	0				
																	SSEEIESAFR	8	3	2	194595509;306966132	N-Term(TMT6plex)	1383.7	0				
																	NVEDIELWLYEVEGHLASDDYGK	9	3	2	194595509;306966132	N-Term(TMT6plex); K23(TMT6plex)	3152.6	0				
																	EAFLNTEDKGDSLDSVEALIK	23	3	2	194595509;306966132	N-Term(TMT6plex); K9(TMT6plex); K21(TMT6plex)	2981.6	1				
																	RQDLEDSLQAQQYFADANEAESWMR	11	3	2	194595509;306966132	N-Term(TMT6plex); M24(Oxidation)	3246.5	1				
																	GEIDAHEDSFK	12	3	2	194595509;306966132	N-Term(TMT6plex); K11(TMT6plex)	1705.9	0				
																	QWELLLEK	13	3	2	194595509;306966132	N-Term(TMT6plex); K8(TMT6plex)	1516.9	0				
																	DLASVNNLLK	7	3	2	194595509;306966132	N-Term(TMT6plex); K10(TMT6plex)	1544.9	0				
																	LSDDNTIGK	5	3	2	194595509;306966132	N-Term(TMT6plex); K9(TMT6plex)	1420.8	0				

Accession	RefSeq Accession	Gene Symbol	Entrez Gene ID	Description	Σ Coverage	Σ# Proteins	Σ# Unique Peptides	Σ# Peptides	Σ# PSMs	Het-1A-Smoke - 2M/Parental	Het-1A-Smoke - 4M/Parental	Het-1A-Smoke - 6M/Parental	Het-1A-Smoke - 8M/Parental	# AAs	MW [kDa]	calc. pI	Sequence	# PSMs	# Proteins	# Protein Groups	Protein Group Accessions	Modifications	MH+ [Da]	# Missed Cleavages	Het-1A-Smoke - 2M/Parental	Het-1A-Smoke - 4M/Parental	Het-1A-Smoke - 6M/Parental	Het-1A-Smoke - 8M/Parental
																	LKDLNSQADSLMTSSAFDTSQVK	4	3	2	194595509;306966132	N-Term(TMT6plex); K2(TMT6plex); M12(Oxidation); K23(TMT6plex)	3189.7	1				
																	LIQNNHYAMEDVATR	9	3	2	194595509;306966132	N-Term(TMT6plex); M9(Oxidation)	2020.0	0				
																	DVDETISWIK	13	3	2	194595509;306966132	N-Term(TMT6plex); K10(TMT6plex)	1663.9	0				
																	HQAFEAELHANADR	7	3	2	194595509;306966132	N-Term(TMT6plex)	1837.9	0				
																	LIQSHPESAEDLQEK	11	3	2	194595509;306966132	N-Term(TMT6plex); K15(TMT6plex)	2182.2	0				
																	QDEVNAAWQR	4	3	2	194595509;306966132	N-Term(TMT6plex)	1445.7	0				
																	LIDVNHYAK	9	3	2	194595509;306966132	N-Term(TMT6plex); K9(TMT6plex)	1530.9	0				
																	NNHHEENISSK	4	3	2	194595509;306966132	N-Term(TMT6plex); K11(TMT6plex)	1766.9	0				
																	MNEVISLWK	6	3	2	194595509;306966132	N-Term(TMT6plex); M1(Oxidation); K9(TMT6plex)	1593.9	0				
																	KVEDLFLTFAK	13	3	2	194595509;306966132	N-Term(TMT6plex); K1(TMT6plex); K11(TMT6plex)	1998.2	1				
																	QQVAPTDDETGKELVLALYDYQEK	4	3	2	194595509;306966132	N-Term(TMT6plex); K12(TMT6plex); K24(TMT6plex)	3440.8	1				
																	LGESQTLQQFSR	4	3	2	194595509;306966132	N-Term(TMT6plex)	1622.9	0				
																	ITALDEFATK	20	3	2	194595509;306966132	N-Term(TMT6plex); K10(TMT6plex)	1566.9	0				
																	DTEQVDNWMSK	6	3	2	194595509;306966132	N-Term(TMT6plex); K11(TMT6plex)	1810.9	0				
																	VASNPYTWFTMEALEETWR	19	3	2	194595509;306966132	N-Term(TMT6plex); M11(Oxidation)	2576.2	0				
																	GDILTLLNSTNK	4	3	2	194595509;306966132	N-Term(TMT6plex); K12(TMT6plex)	1747.0	0				
																	SLSAQEEK	10	3	2	194595509;306966132	N-Term(TMT6plex); K8(TMT6plex)	1349.8	0				
																	LQIASDENYKDPTNLQGK	9	3	2	194595509;306966132	N-Term(TMT6plex); K10(TMT6plex); K18(TMT6plex)	2721.5	1				
																	QFQDAGHFDAENIK	9	3	2	194595509;306966132	N-Term(TMT6plex); K14(TMT6plex)	2078.1	0				
																	LIQEQHPEEELIK	14	3	2	194595509;306966132	N-Term(TMT6plex); K13(TMT6plex)	2064.2	0				
																	YTEHSTVGLAQQWDQLDQLGMR	4	3	2	194595509;306966132	N-Term(TMT6plex); M21(Oxidation)	2821.4	0				
																	EELYQNLTR	9	3	2	194595509;306966132	N-Term(TMT6plex)	1394.7	0				
																	KLDPAQSASR	5	3	2	194595509;306966132	N-Term(TMT6plex); K1(TMT6plex)	1530.9	1				
																	LQTASDESYKDPTNIQSK	18	2	1	306966132	N-Term(TMT6plex); K10(TMT6plex); K18(TMT6plex)	2712.5	1	**1.0**	**1.1**	**1.1**	**1.0**
																	EKEPIAASTNR	5	3	2	194595509;306966132	N-Term(TMT6plex); K2(TMT6plex)	1674.0	1				
																	ENLLEEQGSIALR	13	3	2	194595509;306966132	N-Term(TMT6plex)	1700.9	0				
																	ASAFNSWFENAEEDLTDPVR	19	3	2	194595509;306966132	N-Term(TMT6plex)	2527.2	0				
																	EAIVTSEELGQDLEHVEVLQK	24	3	2	194595509;306966132	N-Term(TMT6plex); K21(TMT6plex)	2824.5	0				
																	VAEDLESEGLMAEEVQAVQQQEVYGMMPR	20	3	2	194595509;306966132	N-Term(TMT6plex); M11(Oxidation); M26(Oxidation); M27(Oxidation)	3543.7	0				
																	LTVLSEER	8	3	2	194595509;306966132	N-Term(TMT6plex)	1175.7	0				
																	GLVSSDELAK	8	3	2	194595509;306966132	N-Term(TMT6plex); K10(TMT6plex)	1476.9	0				
																	DVEDEETWIR	2	3	2	194595509;306966132	N-Term(TMT6plex)	1520.7	0				
																	SLGYDLPMVEEGEPDPEFEAILDTVDPNR	17	3	2	194595509;306966132	N-Term(TMT6plex); M8(Oxidation)	3492.6	0				
																	KIEDLGAAMEEALILDNK	6	3	2	194595509;306966132	N-Term(TMT6plex); K1(TMT6plex); M9(Oxidation); K18(TMT6plex)	2676.5	1				
																	AQLADSFHLQQFFR	12	3	2	194595509;306966132	N-Term(TMT6plex)	1937.0	0				
																	DTEQVDNWMSK	15	3	2	194595509;306966132	N-Term(TMT6plex); M9(Oxidation); K11(TMT6plex)	1826.9	0				
																	LEESLEYQQFVANVEEEEAWINEK	17	3	2	194595509;306966132	N-Term(TMT6plex); K24(TMT6plex)	3384.7	0				
																	GACAGSEDAVK	8	3	2	194595509;306966132	N-Term(TMT6plex); C3(Carbamidomethyl); K11(TMT6plex)	1522.8	0				
																	DMDDEESWIK	14	3	2	194595509;306966132	N-Term(TMT6plex); M2(Oxidation); K10(TMT6plex)	1741.8	0				
																	VNSLGETAER	5	3	2	194595509;306966132	N-Term(TMT6plex)	1304.7	0				
																	KHEDFEK	8	3	2	194595509;306966132	N-Term(TMT6plex); K1(TMT6plex); K7(TMT6plex)	1619.9	1				
																	ALCAEADR	4	3	2	194595509;306966132	N-Term(TMT6plex); C3(Carbamidomethyl)	1134.6	0				
																	LDENSAFLQFNWK	12	3	2	194595509;306966132	N-Term(TMT6plex); K13(TMT6plex)	2070.1	0				
																	DADETKEWIEEK	2	3	2	194595509;306966132	N-Term(TMT6plex); K6(TMT6plex); K12(TMT6plex)	2180.2	1				
																	ALSSEGKPYVTK	5	3	2	194595509;306966132	N-Term(TMT6plex); K7(TMT6plex); K12(TMT6plex)	1967.2	0				
																	DCEQAENWMAAR	5	3	2	194595509;306966132	N-Term(TMT6plex); C2(Carbamidomethyl); M9(Oxidation)	1725.8	0				
																	HQAFEAELSANQSR	9	3	2	194595509;306966132	N-Term(TMT6plex)	1816.9	0				
																	DLTSWVTEMK	2	3	2	194595509;306966132	N-Term(TMT6plex); K10(TMT6plex)	1667.9	0				
																	DVTGAEALLER	4	3	2	194595509;306966132	N-Term(TMT6plex)	1402.8	0				
																	DLIGVQNLLK	24	3	2	194595509;306966132	N-Term(TMT6plex); K10(TMT6plex)	1571.0	0				
																	QGFVPAAYVK	7	3	2	194595509;306966132	N-Term(TMT6plex); K10(TMT6plex)	1537.9	0				
																	TKQDEVNAAWQR	2	3	2	194595509;306966132	N-Term(TMT6plex); K2(TMT6plex)	1904.0	1				
																	DMDDEESWIK	1	3	2	194595509;306966132	N-Term(TMT6plex); K10(TMT6plex)	1725.8	0				
																	GVIDMGNSLIER	2	3	2	194595509;306966132	N-Term(TMT6plex)	1532.8	0				
																	ECEDVMDWINDKEAIVTSEELGQDLEHVEVLQK	6	3	2	194595509;306966132	N-Term(TMT6plex); C2(Carbamidomethyl); M6(Oxidation); K12(TMT6plex); K33(TMT6plex)	4604.3	1				
																	SLGYDLPMVEEGEPDPEFEAILDTVDPNR	2	3	2	194595509;306966132	N-Term(TMT6plex)	3476.7	0				
																	IEDLGAAMEEALILDNK	2	3	2	194595509;306966132	N-Term(TMT6plex); K17(TMT6plex)	2303.3	0				
																	MTLVASEDYGDTLAAIQGLLK	26	3	2	194595509;306966132	N-Term(TMT6plex); M1(Oxidation); K21(TMT6plex)	2683.4	0				
																	KGDILTLLNSTNK	6	3	2	194595509;306966132	N-Term(TMT6plex); K1(TMT6plex); K13(TMT6plex)	2104.3	1				
																	HQLLEADISAHEDR	6	3	2	194595509;306966132	N-Term(TMT6plex)	1863.0	0				
																	KHQLLEADISAHEDR	4	3	2	194595509;306966132	N-Term(TMT6plex); K1(TMT6plex)	2220.2	1				
																	MTLVASEDYGDTLAAIQGLLK	2	3	2	194595509;306966132	N-Term(TMT6plex); K21(TMT6plex)	2667.5	0				
																	WSQLLANSAAR	2	3	2	194595509;306966132	N-Term(TMT6plex)	1445.8	0				
																	EANQQQQFNR	5	3	2	194595509;306966132	N-Term(TMT6plex)	1491.7	0				
																	LAALADQWQFLVQK	12	3	2	194595509;306966132	N-Term(TMT6plex); K14(TMT6plex)	2089.2	0				
																	IDGITIQAR	6	3	2	194595509;306966132	N-Term(TMT6plex)	1215.7	0				
																	LEAELAAHEPAIQGVLDTGKK	2	3	2	194595509;306966132	N-Term(TMT6plex); K20(TMT6plex); K21(TMT6plex)	2877.7	1				
																	LGDSHDLQR	4	3	2	194595509;306966132	N-Term(TMT6plex)	1269.7	0				
																	NVEDIELWLYEVEGHLASDDYGKDLTNVQNLQK	1	3	2	194595509;306966132	N-Term(TMT6plex); K23(TMT6plex); K33(TMT6plex)	4535.3	1				
																	NQALNTDNYGHDLASVQALQR	6	3	2	194595509;306966132	N-Term(TMT6plex)	2557.3	0				
																	VNEVNQFAAK	8	3	2	194595509;306966132	N-Term(TMT6plex); K10(TMT6plex)	1577.9	0				
																	ADVVESWIGEK	10	3	2	194595509;306966132	N-Term(TMT6plex); K11(TMT6plex)	1690.9	0				
																	QQNFNTGIK	5	3	2	194595509;306966132	N-Term(TMT6plex); K9(TMT6plex)	1507.9	0				
																	LAQFVEHWK	17	3	2	194595509;306966132	N-Term(TMT6plex); K9(TMT6plex)	1615.9	0				
																	LHELNQK	12	3	2	194595509;306966132	N-Term(TMT6plex); K7(TMT6plex)	1339.8	0				
																	QEAFLLNEDLGDSLDSVEALLK	35	3	2	194595509;306966132	N-Term(TMT6plex); K22(TMT6plex)	2877.6	0				
																	SADESGQALLAAGHYASDEVR	20	3	2	194595509;306966132	N-Term(TMT6plex)	2376.2	0				
																	TYLLDGSCMVEESGTLESQLEATK	13	3	2	194595509;306966132	N-Term(TMT6plex); C8(Carbamidomethyl); M9(Oxidation); K24(TMT6plex)	3135.5	0				
																	ELVLALYDYQEK	10	3	2	194595509;306966132	N-Term(TMT6plex); K12(TMT6plex)	1942.1	0				
																	VLETAEDIQER	14	3	2	194595509;306966132	N-Term(TMT6plex)	1531.8	0				
																	LQQSHPLSATQIQVK	14	3	2	194595509;306966132	N-Term(TMT6plex); K15(TMT6plex)	2136.3	0				
																	IAALQAFADQLIAAGHYAK	21	3	2	194595509;306966132	N-Term(TMT6plex); K19(TMT6plex)	2430.4	0				
																	RLEAELAAHEPAIQGVLDTGK	14	3	2	194595509;306966132	N-Term(TMT6plex); K21(TMT6plex)	2676.5	1				
																	LDILDQER	9	3	2	194595509;306966132	N-Term(TMT6plex)	1230.7	0				
																	GNAMVEEGHFAAEDVK	11	3	2	194595509;306966132	N-Term(TMT6plex); M4(Oxidation); K16(TMT6plex)	2178.1	0				
																	ECEDVMDWINDK	12	3	2	194595509;306966132	N-Term(TMT6plex); C2(Carbamidomethyl); M6(Oxidation); K12(TMT6plex)	2027.9	0				
																	DLTGVQNLR	10	3	2	194595509;306966132	N-Term(TMT6plex)	1244.7	0				
																	TATDEAYKDPSNLQGK	12	3	2	194595509;306966132	N-Term(TMT6plex); K8(TMT6plex); K16(TMT6plex)	2425.3	1				
																	RQEENDK	4	3	2	194595509;306966132	N-Term(TMT6plex); K7(TMT6plex)	1376.8	1				
																	HEAFETDFTVHK	4	3	2	194595509;306966132	N-Term(TMT6plex); K12(TMT6plex)	1919.0	0				
																	AINVQEEK	13	3	2	194595509;306966132	N-Term(TMT6plex); K8(TMT6plex)	1388.8	0				
																	ALINADELASDVAGAEALLDR	76	3	2	194595509;306966132	N-Term(TMT6plex)	2356.3	0				
																	DLTNVQNLQK	9	3	2	194595509;306966132	N-Term(TMT6plex); K10(TMT6plex)	1631.0	0				
																	QETFDAGLQAFQQEGIANITALK	17	3	2	194595509;306966132	N-Term(TMT6plex); K23(TMT6plex)	2951.6	0				
																	DLSSVQTLLTK	18	3	2	194595509;306966132	N-Term(TMT6plex); K11(TMT6plex)	1663.0	0				
																	KIEDLGAAMEEALILDNK	6	3	2	194595509;306966132	N-Term(TMT6plex); K1(TMT6plex); K18(TMT6plex)	2660.5	1				
																	GVIDMGNSLIER	6	3	2	194595509;306966132	N-Term(TMT6plex); M5(Oxidation)	1548.8	0				
																	KHALLEADVAAHQDR	2	3	2	194595509;306966132	N-Term(TMT6plex); K1(TMT6plex)	2132.2	1				
																	QEFAQHANAFHQWIQETR	2	3	2	194595509;306966132	N-Term(TMT6plex)	2470.2	0				
																	REELITNWEQIR	4	3	2	194595509;306966132	N-Term(TMT6plex)	1816.0	1				
																	KHEALMSDLSAYGSSIQALR	2	3	2	194595509;306966132	N-Term(TMT6plex); K1(TMT6plex); M6(Oxidation)	2651.4	1				
																	LSDDNTIGKEEIQQR	8	3	2	194595509;306966132	N-Term(TMT6plex); K9(TMT6plex)	2204.2	1				
																	DVDEIEAWISEK	20	3	2	194595509;306966132	N-Term(TMT6plex); K12(TMT6plex)	1892.0	0				
																	AALLELWELR	11	3	2	194595509;306966132	N-Term(TMT6plex)	1442.9	0				
																	KHEAFETDFTVHK	4	3	2	194595509;306966132	N-Term(TMT6plex); K1(TMT6plex); K13(TMT6plex)	2276.3	1				
																	LLEATELK	10	3	2	194595509;306966132	N-Term(TMT6plex); K8(TMT6plex)	1374.9	0				
																	DAEELEK	6	3	2	194595509;306966132	N-Term(TMT6plex); K7(TMT6plex)	1291.7	0				
																	LDETGNLMISEGHFASETIR	2	3	2	194595509;306966132	N-Term(TMT6plex)	2449.2	0				
																	EKEQLMASDDFGR	1	3	2	194595509;306966132	N-Term(TMT6plex); K2(TMT6plex)	1984.0	1				
																	LMVHTVATFNSIK	1	3	2	194595509;306966132	N-Term(TMT6plex); M2(Oxidation); K13(TMT6plex)	1935.1	0				
																	CNSLEEIK	1	3	2	194595509;306966132	N-Term(TMT6plex); C1(Carbamidomethyl); K8(TMT6plex)	1450.8	0				
																	DLNSQADSLMTSSAFDTSQVK	2	3	2	194595509;306966132	N-Term(TMT6plex); K21(TMT6plex)	2703.4	0				
																	TYLLDGSCMVEESGTLESQLEATK	2	3	2	194595509;306966132	N-Term(TMT6plex); C8(Carbamidomethyl); K24(TMT6plex)	3119.6	0				
																	LQTASDESYK	2	3	2	194595509;306966132	N-Term(TMT6plex); K10(TMT6plex)	1599.9	0				
																	NTTGVTEEALK	9	3	2	194595509;306966132	N-Term(TMT6plex); K11(TMT6plex)	1620.9	0				

Khan et al., 2019. Multi-omics analysis to characterize cigarette smoke induced molecular alterations in esophageal cells
Supplementary Table 6. List of all proteins and peptides identified in untreated and chronically treated Het1A cells with cigarette smoke condensate for 8 months

Accession	RefSeq Accession	Gene Symbol	Entrez Gene ID	Description	Σ Coverage	Σ# Proteins	Σ# Unique Peptides	Σ# Peptides	Σ# PSMs	Het-1A-Smoke - 2M/Parental	Het-1A-Smoke - 4M/Parental	Het-1A-Smoke - 6M/Parental	Het-1A-Smoke - 8M/Parental	# AAs	MW [kDa]	calc. pI	Sequence	# PSMs	# Proteins	# Protein Groups	Protein Group Accessions	Modifications	MH+ [Da]	# Missed Cleavages	Het-1A-Smoke - 2M/Parental	Het-1A-Smoke - 4M/Parental	Het-1A-Smoke - 6M/Parental	Het-1A-Smoke - 8M/Parental
																	HALLEADVAAHQDR	5	3	2	194595509;306966132	N-Term(TMT6plex)	1774.9	0				
																	SSLSSAQADFNQLAELDR	7	3	2	194595509;306966132	N-Term(TMT6plex)	2181.1	0				
																	HQAFEAEVQANSGAIVK	8	3	2	194595509;306966132	N-Term(TMT6plex); K17(TMT6plex)	2257.2	0				
																	EANELQQWINEK	14	3	2	194595509;306966132	N-Term(TMT6plex); K12(TMT6plex)	1960.1	0				
																	DLTSWVTEMK	10	3	2	194595509;306966132	N-Term(TMT6plex); M9(Oxidation); K10(TMT6plex)	1683.9	0				
																	DLAALEDK	12	3	2	194595509;306966132	N-Term(TMT6plex); K8(TMT6plex)	1332.8	0				
																	HEALMSDLSAYGSSIQALR	3	3	2	194595509;306966132	N-Term(TMT6plex); M5(Oxidation)	2294.2	0				
																	CTELNQAWSSLGK	2	3	2	194595509;306966132	N-Term(TMT6plex); C1(Carbamidomethyl); K13(TMT6plex)	1952.0	0				
																	VASNPYTWFTMEALEETWR	2	3	2	194595509;306966132	N-Term(TMT6plex)	2560.2	0				
																	LIQNNHYAMEDVATR	2	3	2	194595509;306966132	N-Term(TMT6plex)	2004.0	0				
																	KHEGFER	2	3	2	194595509;306966132	N-Term(TMT6plex); K1(TMT6plex)	1360.8	1				
																	KFDDFQK	1	3	2	194595509;306966132	N-Term(TMT6plex); K1(TMT6plex); K7(TMT6plex)	1614.9	1				
																	MQHNLEQQIQAR	4	3	2	194595509;306966132	N-Term(TMT6plex); M1(Oxidation)	1740.9	0				
																	VNDVCTNGQDLIK	2	3	2	194595509;306966132	N-Term(TMT6plex); C5(Carbamidomethyl); K13(TMT6plex)	1934.0	0				
																	DCEQAENWMAAR	4	3	2	194595509;306966132	N-Term(TMT6plex); C2(Carbamidomethyl)	1709.8	0				
																	LFGAAEVQR	2	3	2	194595509;306966132	N-Term(TMT6plex)	1219.7	0				
																	GKDLIGVQNLLK	4	3	2	194595509;306966132	N-Term(TMT6plex); K2(TMT6plex); K12(TMT6plex)	1985.3	1				
																	KLSDDNTIGK	1	3	2	194595509;306966132	N-Term(TMT6plex); K1(TMT6plex); K10(TMT6plex)	1778.1	1				
																	YTEHSTVGLAQQWDQLDQLGMR	2	3	2	194595509;306966132	N-Term(TMT6plex)	2805.4	0				
																	QDLEDSLQAQQYFADANEAESWMR	2	3	2	194595509;306966132	N-Term(TMT6plex); M23(Oxidation)	3090.4	0				
																	EKEPIVGSTDYGKDEDSAEALLK	4	3	2	194595509;306966132	N-Term(TMT6plex); K2(TMT6plex); K13(TMT6plex); K23(TMT6plex)	3410.9	2				
																	EQADYCVSHMKPYVDGK	2	3	2	194595509;306966132	N-Term(TMT6plex); C6(Carbamidomethyl); M10(Oxidation); K11(TMT6plex); K17(TMT6plex)	2730.4	0				
																	AGTFQAFEQFGQQLLAHGHYASPEIK	4	3	2	194595509;306966132	N-Term(TMT6plex); K26(TMT6plex)	3333.7	0				
																	LDETGNLMISEGHFASETIR	7	3	2	194595509;306966132	N-Term(TMT6plex); M8(Oxidation)	2465.2	0				
																	LLVGSEDYGR	4	3	2	194595509;306966132	N-Term(TMT6plex)	1337.7	0				
																	DLAALGDK	2	3	2	194595509;306966132	N-Term(TMT6plex); K8(TMT6plex)	1260.8	0				
																	SLQQLAEER	4	3	2	194595509;306966132	N-Term(TMT6plex)	1302.7	0				
																	IEDLGAAMEEALILDNK	6	3	2	194595509;306966132	N-Term(TMT6plex); M8(Oxidation); K17(TMT6plex)	2319.2	0				
																	LLEAQSHFR	4	3	2	194595509;306966132	N-Term(TMT6plex)	1329.7	0				
																	KLLEATELK	2	3	2	194595509;306966132	N-Term(TMT6plex); K1(TMT6plex); K9(TMT6plex)	1732.1	1				
																	QEQIDNQYHSLLELGEK	4	1	1	306966132	N-Term(TMT6plex); K17(TMT6plex)	2502.3	0				
																	KHEDFDK	6	3	2	194595509;306966132	N-Term(TMT6plex); K1(TMT6plex); K7(TMT6plex)	1605.9	1				
																	DQLLAAK	3	3	2	194595509;306966132	N-Term(TMT6plex); K7(TMT6plex)	1216.8	0				
																	LNHQEFK	3	3	2	194595509;306966132	N-Term(TMT6plex); K7(TMT6plex)	1373.8	0				
																	QQYEQCMDLQLFYR	1	3	2	194595509;306966132	N-Term(TMT6plex); C6(Carbamidomethyl); M7(Oxidation)	2167.0	0				
																	QQVLDR	2	3	2	194595509;306966132	N-Term(TMT6plex)	987.6	0				
																	KASAFNSWFENAEEDLTDPVR	1	3	2	194595509;306966132	N-Term(TMT6plex); K1(TMT6plex)	2884.4	1				
																	EKEQLMASDDFGR	3	3	2	194595509;306966132	N-Term(TMT6plex); K2(TMT6plex); M6(Oxidation)	2000.0	1				
																	VEVNDR	2	3	2	194595509;306966132	N-Term(TMT6plex)	960.5	0				
																	EQLMASDDFGR	4	3	2	194595509;306966132	N-Term(TMT6plex); M4(Oxidation)	1513.7	0				
																	SWVNEK	1	3	2	194595509;306966132	N-Term(TMT6plex); K6(TMT6plex)	1220.7	0				
																	KQEALVAR	3	3	2	194595509;306966132	N-Term(TMT6plex); K1(TMT6plex)	1372.9	1				
																	DADETK	1	3	2	194595509;306966132	N-Term(TMT6plex); K6(TMT6plex)	1136.6	0				
																	KHEALMSDLSAYGSSIQALR	1	3	2	194595509;306966132	N-Term(TMT6plex); K1(TMT6plex)	2635.4	1				
																	FQFFQR	3	3	2	194595509;306966132	N-Term(TMT6plex)	1101.6	0				
																	EWIEEK	5	3	2	194595509;306966132	N-Term(TMT6plex); K6(TMT6plex)	1291.7	0				
																	QKLEDSYR	1	3	2	194595509;306966132	N-Term(TMT6plex); K2(TMT6plex)	1496.8	1				
																	FLADFR	1	3	2	194595509;306966132	N-Term(TMT6plex)	997.6	0				
																	DSDELK	1	4	2	194595509;306966132	N-Term(TMT6plex); K6(TMT6plex)	1164.7	0				
																	FLSDFR	2	3	2	194595509;306966132	N-Term(TMT6plex)	1013.6	0				
																	LVQYLR	3	3	2	194595509;306966132	N-Term(TMT6plex)	1020.6	0				
																	GLALQR	1	3	2	194595509;306966132	N-Term(TMT6plex)	886.6	0				
																	LQQLFR	1	3	2	194595509;306966132	N-Term(TMT6plex)	1033.6	0				
6677723	NP_031396.1	**RFC5**	5985	**replication factor C subunit 5 isoform 1**	75.59	4	23	23	81	**1.3**	**1.4**	**1.3**	**1.4**	340	38.5	7.20												
																	LVILDEADAMTQDAQNALR	19	4	1	6677723	N-Term(TMT6plex); M10(Oxidation)	2332.2	0	**1.4**	**1.5**	**1.4**	**1.6**
																	YRPQTLNDLISHQDILSTIQK	3	3	1	6677723	N-Term(TMT6plex); K21(TMT6plex)	2941.7	0	**1.3**	**1.3**	**1.2**	**1.3**
																	DKEFGSMVLELNASDDR	4	3	1	6677723	N-Term(TMT6plex); K2(TMT6plex); M7(Oxidation)	2400.2	1	**1.4**	**1.5**	**1.4**	**1.6**
																	FGPLTPELMVPR	7	4	1	6677723	N-Term(TMT6plex); M9(Oxidation)	1601.9	0	**1.3**	**1.3**	**1.3**	**1.3**
																	QQEQPAATK	4	2	1	6677723	N-Term(TMT6plex); K9(TMT6plex)	1458.8	0	**1.0**	**0.9**	**1.0**	**1.0**
																	GPILSFASTR	2	4	1	6677723	N-Term(TMT6plex)	1277.7	0	**1.2**	**1.4**	**1.3**	**1.5**
																	LEHVVEEEKVDISEDGMK	3	4	1	6677723	N-Term(TMT6plex); K9(TMT6plex); M17(Oxidation); K18(TMT6plex)	2789.5	1	**1.0**	**1.1**	**1.1**	**1.3**
																	LSVGTNEK	2	4	1	6677723	N-Term(TMT6plex); K8(TMT6plex)	1305.8	0				
																	ALVTLSSGDMR	2	4	1	6677723	N-Term(TMT6plex); M10(Oxidation)	1394.8	0	**1.5**	**1.3**	**1.3**	**1.3**
																	SDIANILDWMLNQDFTTAYR	4	4	1	6677723	N-Term(TMT6plex); M10(Oxidation)	2632.3	0	**1.4**	**1.5**	**1.7**	**1.4**
																	ALNILQSTNMAFGK	2	4	1	6677723	N-Term(TMT6plex); M10(Oxidation); K14(TMT6plex)	1982.1	0	**1.2**	**1.3**	**1.2**	**1.4**
																	TSTILACAK	2	3	1	6677723	N-Term(TMT6plex); C7(Carbamidomethyl); K9(TMT6plex)	1422.8	0	**1.3**	**1.1**	**1.3**	**1.1**
																	VTEETVYTCTGHPLK	1	4	1	6677723	N-Term(TMT6plex); C9(Carbamidomethyl); K15(TMT6plex)	2193.2	0	**1.3**	**1.5**	**1.4**	**1.4**
																	DKEFGSMVLELNASDDR	1	3	1	6677723	N-Term(TMT6plex); K2(TMT6plex)	2384.2	1	**1.4**	**1.7**	**1.5**	**1.6**
																	GLALHDILTEIHLFVHR	2	4	1	6677723	N-Term(TMT6plex)	2213.3	0	**1.0**	**1.8**	**1.0**	**1.0**
																	LPHLLLYGPPGTGK	4	3	1	6677723	N-Term(TMT6plex); K14(TMT6plex)	1921.1	0	**1.1**	**1.4**	**1.5**	**1.2**
																	LEHVVEEEK	4	4	1	6677723	N-Term(TMT6plex); K9(TMT6plex)	1569.9	0	**1.3**	**1.4**	**1.4**	**1.5**
																	VDISEDGMK	1	4	1	6677723	N-Term(TMT6plex); M8(Oxidation); K9(TMT6plex)	1467.8	0				
																	DLIVAEA	3	4	1	6677723	N-Term(TMT6plex)	959.6	0	**1.3**	**1.2**	**1.2**	**1.3**
																	NLPWVEK	2	2	1	6677723	N-Term(TMT6plex); K7(TMT6plex)	1343.8	0	**1.1**	**1.0**	**1.1**	**1.0**
																	IIPALQSR	2	4	1	6677723	N-Term(TMT6plex)	1126.7	0	**1.2**	**1.2**	**1.2**	**1.3**
																	MADIEYR	3	4	1	6677723	N-Term(TMT6plex); M1(Oxidation)	1142.6	0	**1.1**	**1.3**	**1.2**	**1.3**
																	VDFPSSVR	2	4	1	6677723	N-Term(TMT6plex)	1135.6	0	**1.3**	**1.2**	**1.3**	**1.2**
																	NITELK	2	3	1	6677723	N-Term(TMT6plex); K6(TMT6plex)	1175.7	0				
47519639	NP_002366.2	**MAP4**	4134	**microtubule-associated protein 4 isoform 1**	75.52	3	70	71	1135	**1.3**	**1.4**	**1.4**	**1.1**	1152	120.9	5.43												
																	NVVLPTETEVAPAK	36	2	1	47519639	N-Term(TMT6plex); K14(TMT6plex)	1926.1	0	**1.5**	**1.5**	**1.4**	**1.1**
																	NVCLPPEMEVALTEDQVPALK	28	2	1	47519639	N-Term(TMT6plex); C3(Carbamidomethyl); M8(Oxidation); K21(TMT6plex)	2827.5	0	**1.4**	**1.4**	**1.4**	**1.3**
																	TDYIPLLDVDEK	52	3	1	47519639	N-Term(TMT6plex); K12(TMT6plex)	1879.0	0	**1.4**	**1.2**	**1.4**	**0.9**
																	DMTLPPETNVILTK	41	2	1	47519639	N-Term(TMT6plex); M2(Oxidation); K14(TMT6plex)	2046.2	0	**1.2**	**1.5**	**1.4**	**1.2**
																	MYHDDDLADLVFPSSATADTSIFAGQNDPLK	39	2	1	47519639	N-Term(TMT6plex); M1(Oxidation); K31(TMT6plex)	3828.9	0	**1.4**	**1.6**	**1.5**	**1.0**
																	TEAPLAK	9	2	1	47519639	N-Term(TMT6plex); K7(TMT6plex)	1187.7	0	**1.3**	**1.2**	**1.3**	**1.0**
																	ALPLEAEVAPVKDMAQLPETEIAPAK	4	2	1	47519639	N-Term(TMT6plex); K12(TMT6plex); M14(Oxidation); K26(TMT6plex)	3434.9	1	**1.7**	**2.5**	**1.7**	**1.9**
																	STQTVAK	10	2	1	47519639	N-Term(TMT6plex); K7(TMT6plex)	1192.7	0	**1.2**	**1.3**	**1.3**	**1.0**
																	DMELPTEK	17	2	1	47519639	N-Term(TMT6plex); M2(Oxidation); K8(TMT6plex)	1436.8	0	**1.3**	**1.7**	**1.6**	**1.1**
																	DMQPSMESDMALVK	36	2	1	47519639	N-Term(TMT6plex); M2(Oxidation); M6(Oxidation); M10(Oxidation); K14(TMT6plex)	2088.0	0	**1.3**	**1.6**	**1.5**	**1.2**
																	EIEMASEERPPAQALEIMMGLK	10	2	1	47519639	N-Term(TMT6plex); M4(Oxidation); M18(Oxidation); M19(Oxidation); K22(TMT6plex)	2979.5	0	**1.5**	**1.6**	**1.4**	**1.4**
																	LATNTSAPDLK	11	2	1	47519639	N-Term(TMT6plex); K11(TMT6plex)	1588.9	0	**1.3**	**1.3**	**1.3**	**1.2**
																	VALSSETEVALAR	12	2	1	47519639	N-Term(TMT6plex)	1574.9	0	**1.4**	**1.5**	**1.5**	**1.3**
																	DVKPKPIADAK	2	2	1	47519639	N-Term(TMT6plex); K3(TMT6plex); K5(TMT6plex); K11(TMT6plex)	2098.3	0	**1.2**	**2.2**	**1.5**	**1.6**
																	GISEDSHLESLQDVGQSAAPTFMISPETVTGTGK	10	2	1	47519639	N-Term(TMT6plex); M23(Oxidation); K34(TMT6plex)	3964.0	0	**1.1**	**1.5**	**1.2**	**1.0**
																	ALPLEAEVAPVK	33	2	1	47519639	N-Term(TMT6plex); K12(TMT6plex)	1695.1	0	**1.2**	**1.5**	**1.4**	**1.1**
																	DVTPPPETEVVLIK	29	2	1	47519639	N-Term(TMT6plex); K14(TMT6plex)	1995.2	0	**1.2**	**1.5**	**1.4**	**1.2**
																	KPMSLASGLVPAAPPK	24	2	1	47519639	N-Term(TMT6plex); K1(TMT6plex); M3(Oxidation); K16(TMT6plex)	2267.4	0	**1.4**	**1.6**	**1.6**	**1.5**
																	TTTLSGTAPAAGVVPSR	28	2	1	47519639	N-Term(TMT6plex)	1815.0	0	**1.3**	**1.1**	**1.2**	**0.9**
																	EAQTLDSQIQETSI	10	1	1	47519639	N-Term(TMT6plex)	1791.9	0	**1.3**	**1.5**	**1.4**	**1.6**
																	TTTAAAVASTGPSSR	27	2	1	47519639	N-Term(TMT6plex)	1606.9	0	**1.3**	**1.1**	**1.2**	**0.9**
																	VSYSHIQSK	10	2	1	47519639	N-Term(TMT6plex); K9(TMT6plex)	1506.9	0	**1.3**	**1.4**	**1.3**	**1.2**
																	DGVLTLANNVTPAK	25	2	1	47519639	N-Term(TMT6plex); K14(TMT6plex)	1871.1	0	**1.3**	**1.4**	**1.4**	**1.2**
																	ATPMPSRPSTTPFIDK	1	2	1	47519639	N-Term(TMT6plex); K16(TMT6plex)	2204.2	0	**1.6**	**1.8**	**1.8**	**1.4**
																	DSYGMSPCNTAVVPQGWSVEALNSPHSESFVSPEAVAEPPQPTAVPLELAK	5	2	1	47519639	N-Term(TMT6plex); M5(Oxidation); C8(Carbamidomethyl); K51(TMT6plex)	5851.9	0	**1.1**	**1.5**	**1.5**	**1.1**
																	KPTAIK	7	2	1	47519639	N-Term(TMT6plex); K1(TMT6plex); K6(TMT6plex)	1344.9	0	**1.0**	**1.5**	**1.3**	**1.1**
																	CSLPAEEDSVLEK	10	2	1	47519639	N-Term(TMT6plex); C1(Carbamidomethyl); K13(TMT6plex)	1935.0	0	**1.2**	**1.1**	**1.3**	**0.8**
																	DMAQLPETEIAPAK	33	2	1	47519639	N-Term(TMT6plex); M2(Oxidation); K14(TMT6plex)	1988.1	0	**1.3**	**1.6**	**1.5**	**1.2**
																	HVPGGGNVQIQNK	13	2	1	47519639	N-Term(TMT6plex); K13(TMT6plex)	1806.0	0	**1.3**	**1.3**	**1.3**	**1.2**
																	KTEAAATTR	8	2	1	47519639	N-Term(TMT6plex); K1(TMT6plex)	1406.8	1	**1.2**	**1.4**	**1.3**	**1.1**

Accession	RefSeq Accession	Gene Symbol	Entrez Gene ID	Description	Σ Coverage	Σ# Proteins	Σ# Unique Peptides	Σ# Peptides	Σ# PSMs	Het-1A-Smoke - 2M/Parental	Het-1A-Smoke - 4M/Parental	Het-1A-Smoke - 6M/Parental	Het-1A-Smoke - 8M/Parental	# AAs	MW [kDa]	calc. pI	Sequence	# PSMs	# Proteins	# Protein Groups	Protein Group Accessions	Modifications	MH+ [Da]	# Missed Cleavages	Het-1A-Smoke - 2M/Parental	Het-1A-Smoke - 4M/Parental	Het-1A-Smoke - 6M/Parental	Het-1A-Smoke - 8M/Parental
																	DMEIAQTQK	11	2	1	47519639	N-Term(TMT6plex); M2(Oxidation); K9(TMT6plex)	1537.8	0	1.1	1.2	1.2	0.9
																	SPSTLLPK	9	2	1	47519639	N-Term(TMT6plex); K8(TMT6plex)	1300.8	0	1.3	1.5	1.4	1.2
																	KPCNSQPSELSSETSGIARPEEGRPVVSGTGNDITTPPNK	1	2	1	47519639	N-Term(TMT6plex); K1(TMT6plex); C3(Carbamidomethyl); K40(TMT6plex)	4881.5	0	1.5	1.6	1.3	1.3
																	VGSTENIK	7	2	1	47519639	N-Term(TMT6plex); K8(TMT6plex)	1305.8	0	1.4	1.5	1.5	1.2
																	ASPSKPASAPASR	10	2	1	47519639	N-Term(TMT6plex); K5(TMT6plex)	1685.0	0	1.2	1.5	1.3	1.2
																	LDVTLAK	6	2	1	47519639	N-Term(TMT6plex); K7(TMT6plex)	1217.8	0	1.4	1.4	1.4	1.3
																	DMQPSMESDMALVK	7	2	1	47519639	N-Term(TMT6plex); M2(Oxidation); M10(Oxidation); K14(TMT6plex)	2072.0	0	1.7	2.2	2.1	1.5
																	STSTSSMK	2	2	1	47519639	N-Term(TMT6plex); K8(TMT6plex)	1286.7	0	2.0	2.1	2.5	1.2
																	KCSLPAEEDSVLEK	2	2	1	47519639	N-Term(TMT6plex); K1(TMT6plex); C2(Carbamidomethyl); K14(TMT6plex)	2292.3	1	1.1	1.2	1.3	1.0
																	EIEMASEERPPAQALEIMMGLK	2	2	1	47519639	N-Term(TMT6plex); M18(Oxidation); K22(TMT6plex)	2947.5	0	1.8	2.4	2.2	1.5
																	TAGPIASAQK	14	2	1	47519639	N-Term(TMT6plex); K10(TMT6plex)	1401.9	0	1.3	1.2	1.3	1.1
																	WPTETDVSSAK	18	2	1	47519639	N-Term(TMT6plex); K11(TMT6plex)	1678.9	0	1.3	1.5	1.3	1.1
																	DMSPLSETEMALGK	23	2	1	47519639	N-Term(TMT6plex); M2(Oxidation); M10(Oxidation); K14(TMT6plex)	1999.0	0	1.2	1.4	1.3	1.2
																	KPMSLASGLVPAAPPK	4	2	1	47519639	N-Term(TMT6plex); K1(TMT6plex); K16(TMT6plex)	2251.4	0	1.4	1.9	1.7	1.4
																	DMSPLSETEMALGK	2	2	1	47519639	N-Term(TMT6plex); M10(Oxidation); K14(TMT6plex)	1983.0	0	1.3	1.6	1.8	1.5
																	TKPLATTQPAK	11	2	1	47519639	N-Term(TMT6plex); K2(TMT6plex); K11(TMT6plex)	1843.2	0	1.1	1.3	1.3	1.1
																	DVPPLSETEATPVPIKDMEIAQTQK	13	2	1	47519639	N-Term(TMT6plex); K16(TMT6plex); M18(Oxidation); K25(TMT6plex)	3440.9	1	1.4	1.9	1.6	1.5
																	DLVLLSEIEVAQANDIISSTEISSAEK	105	2	1	47519639	N-Term(TMT6plex); K27(TMT6plex)	3332.8	0	1.3	1.4	1.5	1.0
																	DFIATLEAEAFDDVVGETVGK	86	3	1	47519639	N-Term(TMT6plex); K21(TMT6plex)	2684.4	0	1.4	1.6	1.7	1.2
																	KTTTLSGTAPAAGVVPSR	8	2	1	47519639	N-Term(TMT6plex); K1(TMT6plex)	2172.3	1	1.2	1.3	1.3	1.2
																	RDFIATLEAEAFDDVVGETVGK	8	3	1	47519639	N-Term(TMT6plex); K22(TMT6plex)	2840.5	1	1.5	1.6	1.5	1.3
																	TTDMAPSK	13	2	1	47519639	N-Term(TMT6plex); M4(Oxidation); K8(TMT6plex)	1324.7	0	1.3	1.2	1.3	0.9
																	TTDMAPSK	4	2	1	47519639	N-Term(TMT6plex); K8(TMT6plex)	1308.7	0	1.6	1.6	2.0	1.1
																	DVPPLSETEATPVPIKDMEIAQTQK	9	2	1	47519639	N-Term(TMT6plex); K16(TMT6plex); K25(TMT6plex)	3424.9	1	1.4	2.4	1.9	1.3
																	ATPMPSRPSTTPFIDK	4	2	1	47519639	N-Term(TMT6plex); M4(Oxidation); K16(TMT6plex)	2220.2	0	1.3	1.7	1.4	1.4
																	DMAQLPETEIAPAK	6	2	1	47519639	N-Term(TMT6plex); K14(TMT6plex)	1972.1	0	1.6	1.9	2.0	1.2
																	VGSLDNVGHLPAGGAVK	14	2	1	47519639	N-Term(TMT6plex); K17(TMT6plex)	2049.2	0	1.3	1.4	1.4	1.3
																	DMELPTEK	2	2	1	47519639	N-Term(TMT6plex); K8(TMT6plex)	1420.8	0	1.5	2.1	2.2	1.5
																	TGNSESK	4	3	1	47519639	N-Term(TMT6plex); K7(TMT6plex)	1180.7	0	1.2	1.0	1.2	0.6
																	MAYQEYPNSQNWPEDTNFCFQPEQVVDPIQTDPFK	8	2	1	47519639	N-Term(TMT6plex); M1(Oxidation); C19(Carbamidomethyl); K35(TMT6plex)	4737.2	0	1.5	1.6	1.6	1.2
																	KVSYSHIQSK	2	2	1	47519639	N-Term(TMT6plex); K1(TMT6plex); K10(TMT6plex)	1864.1	1	1.0	1.5	1.4	1.2
																	DVPPLSETEATPVPIK	22	2	1	47519639	N-Term(TMT6plex); K16(TMT6plex)	2151.2	0	1.3	1.6	1.6	1.2
																	ETEMALAK	2	2	1	47519639	N-Term(TMT6plex); K8(TMT6plex)	1350.8	0	1.6	2.0	1.9	1.1
																	DMEIAQTQK	2	2	1	47519639	N-Term(TMT6plex); K9(TMT6plex)	1521.8	0	1.3	1.7	1.8	1.4
																	EIEMASEERPPAQALEIMMGLK	6	2	1	47519639	N-Term(TMT6plex); M4(Oxidation); M19(Oxidation); K22(TMT6plex)	2963.5	0	1.2	1.7	1.4	1.2
																	NVCLPPEMEVALTEDQVPALK	8	2	1	47519639	N-Term(TMT6plex); C3(Carbamidomethyl); K21(TMT6plex)	2811.5	0	1.7	1.8	2.1	1.2
																	QPAPTTIGGLNK	8	2	1	47519639	N-Term(TMT6plex); K12(TMT6plex)	1655.0	0	1.2	1.4	1.3	1.2
																	MYHDDDLADLVFPSSATADTSIFAGQNDPLK	7	2	1	47519639	N-Term(TMT6plex); K31(TMT6plex)	3812.9	0	1.7	2.0	1.8	1.3
																	GISEDSHLESLQDVGQSAAPTFMISPETVTGTGK	4	2	1	47519639	N-Term(TMT6plex); K34(TMT6plex)	3948.0	0	1.6	2.1	1.9	1.1
																	KPESNAVTK	5	2	1	47519639	N-Term(TMT6plex); K1(TMT6plex); K9(TMT6plex)	1661.0	0	0.9	1.3	1.1	0.9
																	DMSPLSETEMALGK	2	2	1	47519639	N-Term(TMT6plex); K14(TMT6plex)	1967.0	0	1.5	1.8	1.7	1.4
																	SVPADLSRPK	1	2	1	47519639	N-Term(TMT6plex); K10(TMT6plex)	1527.9	0	1.3	1.6	1.3	1.4
																	MDLAPSK	14	2	1	47519639	N-Term(TMT6plex); M1(Oxidation); K7(TMT6plex)	1235.7	0	1.4	1.5	1.5	1.1
																	ELPPSPEK	10	2	1	47519639	N-Term(TMT6plex); K8(TMT6plex)	1354.8	0	1.3	1.4	1.3	1.4
																	STSTSSMK	7	2	1	47519639	N-Term(TMT6plex); M7(Oxidation); K8(TMT6plex)	1302.7	0	1.1	1.1	1.1	0.9
																	TEGKPAEVK	2	2	1	47519639	N-Term(TMT6plex); K4(TMT6plex); K9(TMT6plex)	1646.0	0	1.1	1.1	1.2	0.9
																	TEVALAK	9	2	1	47519639	N-Term(TMT6plex); K7(TMT6plex)	1189.8	0	1.3	1.3	1.4	1.1
																	TQPTSLPK	6	2	1	47519639	N-Term(TMT6plex); K8(TMT6plex)	1329.8	0	1.3	1.3	1.4	1.1
																	DMALATK	4	2	1	47519639	N-Term(TMT6plex); M2(Oxidation); K7(TMT6plex)	1223.7	0	1.0	1.4	1.1	1.0
																	MDLAPSK	4	2	1	47519639	N-Term(TMT6plex); K7(TMT6plex)	1219.7	0	1.8	2.1	2.2	1.2
																	ETEMALAK	3	2	1	47519639	N-Term(TMT6plex); M4(Oxidation); K8(TMT6plex)	1366.8	0	1.3	1.4	1.3	1.1
																	TTDMAPSKETEMALAK	2	2	1	47519639	N-Term(TMT6plex); M4(Oxidation); K8(TMT6plex); M12(Oxidation); K16(TMT6plex)	2443.3	1	1.6	2.3	1.5	1.8
																	VQIVSK	2	2	1	47519639	N-Term(TMT6plex); K6(TMT6plex)	1131.8	0	1.2	1.4	1.3	1.2
																	KVDISK	2	2	2	47519639;118442828;4507375	N-Term(TMT6plex); K1(TMT6plex); K6(TMT6plex)	1376.9	1				
																	DVAPSTVK	2	2	1	47519639	N-Term(TMT6plex); K8(TMT6plex)	1274.8	0	1.2	1.6	1.3	1.2
																	DMESPTK	2	2	1	47519639	N-Term(TMT6plex); M2(Oxidation); K7(TMT6plex)	1281.7	0	1.2	1.4	1.2	1.2
																	KPTSAKPSSTTPR	1	2	1	47519639	N-Term(TMT6plex); K1(TMT6plex); K6(TMT6plex)	2045.2	0	1.3	1.7	1.4	1.4
																	DVTLLK	2	2	1	47519639	N-Term(TMT6plex); K6(TMT6plex)	1146.7	0	1.2	1.5	1.4	0.9
																	EVALVK	1	2	1	47519639	N-Term(TMT6plex); K6(TMT6plex)	1116.7	0	1.2	1.5	1.4	1.2
5453555	NP_006316.1	**RAN**	5901	**GTP-binding nuclear protein Ran**	75.46	2	13	13	360	**1.2**	**1.2**	**1.3**	**1.2**	216	24.4	7.49												
																	FNVWDTAGQEK	88	1	1	5453555	N-Term(TMT6plex); K11(TMT6plex)	1752.9	0	1.3	1.2	1.3	1.2
																	YVATLGVEVHPLVFHTNR	26	1	1	5453555	N-Term(TMT6plex)	2281.3	0	1.4	1.4	1.4	1.4
																	LIGDPNLEFVAMPALAPPEVVMDPALAAQYEHDLEVAQTTALPDEDDDL	28	1	1	5453555	N-Term(TMT6plex); M12(Oxidation); M22(Oxidation)	5520.7	0	1.2	1.2	1.0	1.3
																	NLQYYDISAK	43	2	1	5453555	N-Term(TMT6plex); K10(TMT6plex)	1672.9	0	1.3	1.0	1.3	1.0
																	KLIGDPNLEFVAMPALAPPEVVMDPALAAQYEHDLEVAQTTALPDEDDDL	7	2	1	5453555	N-Term(TMT6plex); K1(TMT6plex); M23(Oxidation)	5862.0	1	13.2	2.3	1.5	1.3
																	KLIGDPNLEFVAMPALAPPEVVMDPALAAQYEHDLEVAQTTALPDEDDDL	9	2	1	5453555	K1(TMT6plex); N-Term(TMT6plex); M13(Oxidation); M23(Oxidation)	5877.9	1	1.2	1.4	1.4	1.1
																	HLTGEFEK	22	1	1	5453555	N-Term(TMT6plex); K8(TMT6plex)	1418.8	0	1.2	1.2	1.3	1.2
																	LIGDPNLEFVAMPALAPPEVVMDPALAAQYEHDLEVAQTTALPDEDDDL	9	1	1	5453555	N-Term(TMT6plex); M22(Oxidation)	5504.7	0	1.6	1.9	1.6	1.4
																	LVLVGDGGTGK	24	1	1	5453555	N-Term(TMT6plex); K11(TMT6plex)	1473.9	0	1.2	1.2	1.2	1.2
																	LIGDPNLEFVAMPALAPPEVVMDPALAAQYEHDLEVAQTTALPDEDDDL	4	1	1	5453555	N-Term(TMT6plex)	5488.7	0	1.5	1.3	1.8	1.7
																	DGYYIQAQCAIIMFDVTSR	12	1	1	5453555	N-Term(TMT6plex); C9(Carbamidomethyl); M13(Oxidation)	2496.2	0	1.4	1.4	1.5	1.5
																	KLIGDPNLEFVAMPALAPPEVVMDPALAAQYEHDLEVAQTTALPDEDDDL	3	2	1	5453555	N-Term(TMT6plex); K1(TMT6plex)	5846.0	1	1.6	2.2	2.1	1.7
																	SNYNFEKPFLWLAR	29	1	1	5453555	N-Term(TMT6plex); K7(TMT6plex)	2243.2	0	1.2	1.2	1.4	1.2
																	VCENIPIVLCGNK	48	2	1	5453555	N-Term(TMT6plex); C2(Carbamidomethyl); C10(Carbamidomethyl); K13(TMT6plex)	1974.1	0	1.4	1.4	1.5	1.3
																	KYVATLGVEVHPLVFHTNR	2	1	1	5453555	N-Term(TMT6plex); K1(TMT6plex)	2638.5	1	1.4	1.4	1.7	1.2
																	KNLQYYDISAK	5	1	1	5453555	N-Term(TMT6plex); K1(TMT6plex); K11(TMT6plex)	2030.2	1	1.2	1.3	1.4	1.3
																	NVPNWHR	1	1	1	5453555	N-Term(TMT6plex)	1151.6	0	1.1	1.0	1.0	1.1
6912280	NP_036243.1	**AHSA1**	10598	**activator of 90 kDa heat shock protein ATPase homolog 1**	75.44	1	20	20	174	**1.3**	**1.2**	**1.3**	**1.3**	338	38.3	5.53												
																	FHMVDGNVSGEFTDLVPEK	15	1	1	6912280	N-Term(TMT6plex); M3(Oxidation); K19(TMT6plex)	2595.3	0	1.3	1.3	1.3	1.3
																	LDGEASINNR	13	1	1	6912280	N-Term(TMT6plex)	1317.7	0	1.5	1.2	1.2	1.2
																	YYFEGIK	11	1	1	6912280	N-Term(TMT6plex); K7(TMT6plex)	1377.8	0	1.2	1.2	1.3	1.3
																	VFTTQELVQAFTHAPATLEADR	22	1	1	6912280	N-Term(TMT6plex)	2674.4	0	1.4	1.2	1.4	1.2
																	ETFLTSPEELYR	19	1	1	6912280	N-Term(TMT6plex)	1713.9	0	1.4	1.3	1.4	1.3
																	ADATNVNNWHWTER	10	1	1	6912280	N-Term(TMT6plex)	1942.9	0	1.3	1.3	1.4	1.3
																	EAMGIYISTLK	18	1	1	6912280	N-Term(TMT6plex); M3(Oxidation); K11(TMT6plex)	1700.0	0	1.3	1.2	1.2	1.3
																	FHMVDGNVSGEFTDLVPEK	2	1	1	6912280	N-Term(TMT6plex); K19(TMT6plex)	2579.3	0	1.2	1.4	1.5	1.5
																	SWPEGHFATITLTFIDK	2	1	1	6912280	N-Term(TMT6plex); K17(TMT6plex)	2421.3	0	1.2	1.0	1.1	1.1
																	LIFFYEWSVK	5	1	1	6912280	N-Term(TMT6plex); K10(TMT6plex)	1790.0	0	1.3	1.0	1.3	1.2
																	DASNWSTDK	15	1	1	6912280	N-Term(TMT6plex); K9(TMT6plex)	1481.8	0	1.2	1.2	1.2	1.4
																	GIPAPEEER	8	1	1	6912280	N-Term(TMT6plex)	1226.7	0	1.3	1.3	1.3	1.4
																	GHVEIPNLSDENSVDEVEISVSLAK	7	1	1	6912280	N-Term(TMT6plex); K25(TMT6plex)	3138.7	0	1.6	1.6	1.6	1.6
																	QTFGYGAR	4	1	1	6912280	N-Term(TMT6plex)	1128.6	0	1.3	1.0	1.2	1.2
																	DEPDTNLVALMK	6	1	1	6912280	N-Term(TMT6plex); M11(Oxidation); K12(TMT6plex)	1820.0	0	1.3	1.3	1.3	1.4
																	DEPDTNLVALMK	6	1	1	6912280	N-Term(TMT6plex); K12(TMT6plex)	1804.0	0	1.3	1.2	1.5	1.2
																	NGETELCMEGR	2	1	1	6912280	N-Term(TMT6plex); C7(Carbamidomethyl); M8(Oxidation)	1540.7	0	1.4	0.7	1.0	0.8
																	TLFLAVQVQNEEGK	1	1	1	6912280	N-Term(TMT6plex); K14(TMT6plex)	2034.2	0	1.4	1.2	1.2	1.4
																	AKPAPSK	3	1	1	6912280	N-Term(TMT6plex); K2(TMT6plex); K7(TMT6plex)	1385.9	0	0.9	1.1	1.1	1.2

Accession	RefSeq Accession	Gene Symbol	Entrez Gene ID	Description	Σ Coverage	Σ# Proteins	Σ# Unique Peptides	Σ# Peptides	Σ# PSMs	Het-1A-Smoke - 2M/Parental	Het-1A-Smoke - 4M/Parental	Het-1A-Smoke - 6M/Parental	Het-1A-Smoke - 8M/Parental	# AAs	MW [kDa]	calc. pI	Sequence	# PSMs	# Proteins	# Protein Groups	Protein Group Accessions	Modifications	MH+ [Da]	# Missed Cleavages	Het-1A-Smoke - 2M/Parental	Het-1A-Smoke - 4M/Parental	Het-1A-Smoke - 6M/Parental	Het-1A-Smoke - 8M/Parental
																	WIVEER	3	1	1	6912280	N-Term(TMT6plex)	1060.6	0	**1.3**	**1.1**	**1.1**	**1.3**
																	TEFTQGMILPTMNGESVDPVGQPALK	1	1	1	6912280	N-Term(TMT6plex); M7(Oxidation); M12(Oxidation); K26(TMT6plex)	3250.6	0	**1.5**	**1.9**	**1.7**	**1.9**
																	SGVQYK	1	1	1	6912280	N-Term(TMT6plex); K6(TMT6plex)	1139.7	0	**1.0**	**1.1**	**1.1**	**1.1**
4507711	NP_003305.1	**TTC1**	7265	**tetratricopeptide repeat protein 1**	75.34	1	18	18	110	**1.2**	**1.4**	**1.3**	**1.3**	292	33.5	4.84												
																	VTDTQEAECAGPPVPDPK	19	1	1	4507711	N-Term(TMT6plex); C9(Carbamidomethyl); K18(TMT6plex)	2369.2	0	**1.3**	**1.4**	**1.3**	**1.3**
																	TDKLDEALEDYK	10	1	1	4507711	N-Term(TMT6plex); K3(TMT6plex); K12(TMT6plex)	2127.2	1	**1.2**	**1.1**	**1.1**	**1.0**
																	ALEMCPSCFQK	7	1	1	4507711	N-Term(TMT6plex); M4(Oxidation); C5(Carbamidomethyl); C8(Carbamidomethyl); K11(TMT6plex)	1844.9	0	**1.1**	**1.3**	**1.2**	**1.3**
																	EMAINDCSK	3	1	1	4507711	N-Term(TMT6plex); M2(Oxidation); C7(Carbamidomethyl); K9(TMT6plex)	1541.8	0	**1.2**	**1.4**	**1.2**	**1.3**
																	DDEAHLQEDQGEEECFHDCSASFEEEPGADKVENK	2	1	1	4507711	N-Term(TMT6plex); C15(Carbamidomethyl); C19(Carbamidomethyl); K31(TMT6plex); K35(TMT6plex)	4768.1	1	**1.3**	**1.5**	**1.3**	**1.4**
																	ALEMCPSCFQK	4	1	1	4507711	N-Term(TMT6plex); C5(Carbamidomethyl); C8(Carbamidomethyl); K11(TMT6plex)	1828.9	0	**1.5**	**1.7**	**1.7**	**1.4**
																	KGDYIEAESSYSR	14	1	1	4507711	N-Term(TMT6plex); K1(TMT6plex)	1963.0	1	**1.2**	**1.4**	**1.3**	**1.3**
																	AIQLNPSYIR	5	1	1	4507711	N-Term(TMT6plex)	1403.8	0	**1.3**	**1.5**	**1.4**	**1.4**
																	LKEEGNEQFK	3	1	1	4507711	N-Term(TMT6plex); K2(TMT6plex); K10(TMT6plex)	1909.1	1	**1.1**	**1.4**	**1.3**	**1.3**
																	SNEDVNSSELDEEYLIELEK	18	1	1	4507711	N-Term(TMT6plex); K20(TMT6plex)	2813.4	0	**1.3**	**1.7**	**1.3**	**1.7**
																	NQHSQSK	4	1	1	4507711	N-Term(TMT6plex); K7(TMT6plex)	1286.7	0	**1.1**	**1.3**	**1.1**	**1.2**
																	RAELYEK	3	1	1	4507711	N-Term(TMT6plex); K7(TMT6plex)	1366.8	1	**1.1**	**1.3**	**1.1**	**1.3**
																	SILEKDPSIHQAR	3	1	1	4507711	N-Term(TMT6plex); K5(TMT6plex)	1952.1	1	**1.2**	**1.5**	**1.5**	**1.5**
																	GDYIEAESSYSR	5	1	1	4507711	N-Term(TMT6plex)	1605.8	0	**1.5**	**1.5**	**1.6**	**1.3**
																	QDSSTGSYSINFVQNPNNNR	2	1	1	4507711	N-Term(TMT6plex)	2471.2	0	**1.2**	**1.2**	**1.2**	**1.3**
																	DLGNLVLRPFGLSTENFQIK	1	1	1	4507711	N-Term(TMT6plex); K20(TMT6plex)	2719.5	0	**1.2**	**2.2**	**1.6**	**2.1**
																	AELYEK	2	1	1	4507711	N-Term(TMT6plex); K6(TMT6plex)	1210.7	0	**1.1**	**1.7**	**1.3**	**1.5**
																	NMSDEEK	1	1	1	4507711	N-Term(TMT6plex); K7(TMT6plex)	1310.7	0	**5.5**	**2.1**	**2.0**	**1.2**
																	LKEEMLGK	1	1	1	4507711	N-Term(TMT6plex); K2(TMT6plex); K8(TMT6plex)	1635.0	1	**1.4**	**2.0**	**1.8**	**1.4**
																	NMSDEEK	3	1	1	4507711	N-Term(TMT6plex); M2(Oxidation); K7(TMT6plex)	1326.7	0	**1.1**	**1.0**	**1.0**	**1.0**
4758012	NP_004850.1	**CLTC**	1213	**clathrin heavy chain 1 isoform 1**	75.34	4	95	95	1228	**1.4**	**1.4**	**1.5**	**1.4**	1675	191.5	5.69												
																	SVNESLNNLFITEEDYQALR	30	2	1	4758012	N-Term(TMT6plex)	2584.3	0	**1.4**	**1.5**	**1.6**	**1.4**
																	FQEHLQLQNLGINPANIGFSTLTMESDK	22	2	1	4758012	N-Term(TMT6plex); M24(Oxidation); K28(TMT6plex)	3619.9	0	**1.5**	**1.5**	**1.4**	**1.4**
																	GQCDLELINVCNENSLFK	17	2	1	4758012	N-Term(TMT6plex); C3(Carbamidomethyl); C11(Carbamidomethyl); K18(TMT6plex)	2611.3	0	**1.4**	**1.5**	**1.6**	**1.4**
																	EVCFACVDGK	11	2	1	4758012	N-Term(TMT6plex); C3(Carbamidomethyl); C6(Carbamidomethyl); K10(TMT6plex)	1642.8	0	**1.4**	**1.4**	**1.5**	**1.2**
																	KFNALFAQGNYSEAAK	9	2	1	4758012	N-Term(TMT6plex); K1(TMT6plex); K16(TMT6plex)	2446.4	1	**1.4**	**1.4**	**1.5**	**1.4**
																	RPISADSAIMNPASK	22	2	1	4758012	N-Term(TMT6plex); M10(Oxidation); K15(TMT6plex)	2032.1	0	**1.4**	**1.5**	**1.4**	**1.5**
																	AFMTADLPNELIELLEK	86	4	1	4758012	N-Term(TMT6plex); M3(Oxidation); K17(TMT6plex)	2421.3	0	**1.5**	**1.6**	**1.8**	**1.5**
																	KAVDVFFPPEAQNDFPVAMQISEK	6	2	1	4758012	N-Term(TMT6plex); K1(TMT6plex); M19(Oxidation); K24(TMT6plex)	3410.8	1	**1.2**	**1.4**	**1.4**	**1.4**
																	EAIDSYIK	29	2	1	4758012	N-Term(TMT6plex); K8(TMT6plex)	1396.8	0	**1.3**	**1.5**	**1.5**	**1.4**
																	GQFSTDELVAEVEK	18	4	1	4758012	N-Term(TMT6plex); K14(TMT6plex)	2010.1	0	**1.4**	**1.5**	**1.7**	**1.5**
																	IYIDSNNNPER	24	2	1	4758012	N-Term(TMT6plex)	1563.8	0	**1.5**	**1.4**	**1.5**	**1.4**
																	QSVELCK	7	2	1	4758012	N-Term(TMT6plex); C6(Carbamidomethyl); K7(TMT6plex)	1321.8	0	**1.5**	**1.4**	**1.6**	**1.3**
																	KEEEQATETQPIVYGQPQLMLTAGPSVAVPPQAPFGYGYTAPPYGQPQPGFGYSM	3	2	1	4758012	K1(TMT6plex); N-Term(TMT6plex); M20(Oxidation); M55(Oxidation)	6361.1	1	**2.9**	**2.6**	**1.6**	**2.6**
																	ESYVETELIFALAK	21	2	1	4758012	N-Term(TMT6plex); K14(TMT6plex)	2071.2	0	**1.2**	**1.4**	**1.4**	**1.4**
																	SVDPTLALSVYLR	27	2	1	4758012	N-Term(TMT6plex)	1663.0	0	**1.9**	**1.5**	**1.6**	**1.5**
																	LPVVIGGLLDVDCSEDVIK	22	2	1	4758012	N-Term(TMT6plex); C13(Carbamidomethyl); K19(TMT6plex)	2499.4	0	**1.5**	**1.7**	**1.8**	**1.5**
																	LDNYDAPDIANIAISNELFEEAFAIFR	2	2	1	4758012	N-Term(TMT6plex)	3300.7	0	**1.9**	**1.8**	**2.3**	**1.6**
																	NNLAGAEELFAR	18	2	1	4758012	N-Term(TMT6plex)	1533.8	0	**1.4**	**1.1**	**1.4**	**1.2**
																	ADDPSSYMEVVQAANTSGNWEELVK	6	2	1	4758012	N-Term(TMT6plex); K25(TMT6plex)	3198.6	0	**1.6**	**2.0**	**2.2**	**1.3**
																	NLQNLLILTAIK	41	4	1	4758012	N-Term(TMT6plex); K12(TMT6plex)	1812.2	0	**1.5**	**1.3**	**1.6**	**1.3**
																	VGYTPDWIFLLR	30	4	1	4758012	N-Term(TMT6plex)	1709.0	0	**1.5**	**1.4**	**1.6**	**1.4**
																	VSQPIEGHAASFAQFK	8	2	1	4758012	N-Term(TMT6plex); K16(TMT6plex)	2175.2	0	**1.4**	**1.4**	**1.6**	**1.6**
																	TLQIFNIEMK	8	4	1	4758012	N-Term(TMT6plex); K10(TMT6plex)	1695.0	0	**1.8**	**1.7**	**2.1**	**1.4**
																	ADDPSSYMEVVQAANTSGNWEELVK	4	2	1	4758012	N-Term(TMT6plex); M8(Oxidation); K25(TMT6plex)	3214.6	0	**1.8**	**1.8**	**1.9**	**1.6**
																	VGEQAQVVIIDMNDPSNPIR	28	2	1	4758012	N-Term(TMT6plex); M12(Oxidation)	2440.3	0	**1.3**	**1.3**	**1.5**	**1.4**
																	IAAYLFK	26	2	1	4758012	N-Term(TMT6plex); K7(TMT6plex)	1283.8	0	**1.5**	**1.4**	**1.7**	**1.3**
																	LLEMNLMHAPQVADAILGNQMFTHYDR	7	2	1	4758012	N-Term(TMT6plex); M7(Oxidation); M21(Oxidation)	3389.7	0	**1.6**	**1.7**	**1.8**	**1.4**
																	ESNCYDPER	5	2	1	4758012	N-Term(TMT6plex); C4(Carbamidomethyl)	1398.6	0	**1.3**	**1.3**	**1.5**	**1.2**
																	NNRPSEGPLQTR	4	2	1	4758012	N-Term(TMT6plex)	1597.9	0	**1.3**	**1.2**	**1.2**	**1.2**
																	AVDVFFPPEAQNDFPVAMQISEK	27	2	1	4758012	N-Term(TMT6plex); M18(Oxidation); K23(TMT6plex)	3053.6	0	**1.4**	**1.4**	**1.6**	**1.5**
																	LLEMNLMHAPQVADAILGNQMFTHYDR	7	2	1	4758012	N-Term(TMT6plex); M4(Oxidation); M7(Oxidation); M21(Oxidation)	3405.6	0	**1.4**	**1.3**	**1.4**	**1.5**
																	KFDVNTSAVQVLIEHIGNLDR	11	2	1	4758012	N-Term(TMT6plex); K1(TMT6plex)	2826.6	1	**1.3**	**1.4**	**1.5**	**1.4**
																	AYEFAER	15	4	1	4758012	N-Term(TMT6plex)	1114.6	0	**1.4**	**1.5**	**1.6**	**1.5**
																	DAMQYASESK	39	2	1	4758012	N-Term(TMT6plex); M3(Oxidation); K10(TMT6plex)	1603.8	0	**1.6**	**1.5**	**1.5**	**1.4**
																	VANVELYYR	9	2	1	4758012	N-Term(TMT6plex)	1355.8	0	**1.4**	**1.4**	**1.4**	**1.4**
																	KGQVLSVCVEEENIIPYITNVLQNPDLALR	2	2	1	4758012	N-Term(TMT6plex); K1(TMT6plex); C8(Carbamidomethyl)	3883.1	1	**1.7**	**1.5**	**1.4**	**1.8**
																	VVGAMQLYSVDR	4	4	1	4758012	N-Term(TMT6plex); M5(Oxidation)	1582.8	0	**1.8**	**1.4**	**1.3**	**1.5**
																	KDPELWGSVLLESNPYR	3	2	1	4758012	N-Term(TMT6plex); K1(TMT6plex)	2461.3	1	**1.4**	**1.4**	**1.4**	**1.4**
																	YIEIYVQK	7	4	1	4758012	N-Term(TMT6plex); K8(TMT6plex)	1513.9	0	**1.5**	**1.5**	**1.6**	**1.3**
																	LAQMCGLHIVVHADELEELINYYQDR	3	2	1	4758012	N-Term(TMT6plex); M4(Oxidation); C5(Carbamidomethyl)	3374.7	0	**1.6**	**1.6**	**1.6**	**1.8**
																	KVGYTPDWIFLLR	8	4	1	4758012	N-Term(TMT6plex); K1(TMT6plex)	2066.2	1	**1.4**	**1.8**	**1.7**	**1.6**
																	LHIIEVGTPPTGNQPFPK	16	2	1	4758012	N-Term(TMT6plex); K18(TMT6plex)	2403.4	0	**1.3**	**1.5**	**1.5**	**1.6**
																	AHTMTDDVTFWK	4	2	1	4758012	N-Term(TMT6plex); M4(Oxidation); K12(TMT6plex)	1926.0	0	**1.4**	**1.6**	**1.5**	**1.6**
																	FDVNTSAVQVLIEHIGNLDR	6	2	1	4758012	N-Term(TMT6plex)	2469.3	0	**1.7**	**1.5**	**1.6**	**1.7**
																	AHMGMFTELAILYSK	14	4	1	4758012	N-Term(TMT6plex); M3(Oxidation); M5(Oxidation); K15(TMT6plex)	2202.2	0	**1.3**	**1.4**	**1.5**	**1.5**
																	YHEQLSTQSLIELFESFK	16	2	1	4758012	N-Term(TMT6plex); K18(TMT6plex)	2657.4	0	**1.6**	**1.5**	**1.8**	**1.4**
																	HDVVFLITK	15	2	1	4758012	N-Term(TMT6plex); K9(TMT6plex)	1529.9	0	**1.3**	**1.4**	**1.5**	**1.4**
																	KVSQPIEGHAASFAQFK	4	2	1	4758012	N-Term(TMT6plex); K1(TMT6plex); K17(TMT6plex)	2532.5	1	**1.3**	**1.9**	**1.8**	**1.8**
																	LLYNNVSNFGR	4	2	1	4758012	N-Term(TMT6plex)	1525.8	0	**1.3**	**1.4**	**1.5**	**1.4**
																	VDKLDASESLR	3	2	1	4758012	N-Term(TMT6plex); K3(TMT6plex)	1691.0	1	**1.6**	**1.5**	**1.6**	**1.5**
																	DAMQYASESK	4	2	1	4758012	N-Term(TMT6plex); K10(TMT6plex)	1587.8	0	**1.8**	**2.0**	**2.3**	**1.4**
																	VGEQAQVVIIDMNDPSNPIR	8	2	1	4758012	N-Term(TMT6plex)	2424.3	0	**1.6**	**1.8**	**1.9**	**1.4**
																	LASTLVHLGEYQAAVDGAR	34	2	1	4758012	N-Term(TMT6plex)	2200.2	0	**1.4**	**1.3**	**1.5**	**1.3**
																	VIQCFAETGQVQK	21	2	1	4758012	N-Term(TMT6plex); C4(Carbamidomethyl); K13(TMT6plex)	1966.1	0	**1.4**	**1.4**	**1.5**	**1.4**
																	FDFVHDLVLYLYR	15	2	1	4758012	N-Term(TMT6plex)	1929.0	0	**2.1**	**2.1**	**2.6**	**1.8**
																	AHMGMFTELAILYSK	6	4	1	4758012	N-Term(TMT6plex); K15(TMT6plex)	2170.2	0	**1.2**	**1.1**	**1.3**	**1.0**
																	IVLDNSVFSEHR	17	4	1	4758012	N-Term(TMT6plex)	1644.9	0	**1.2**	**1.3**	**1.3**	**1.3**
																	AAEQAHLWAELVFLYDK	4	4	1	4758012	N-Term(TMT6plex); K17(TMT6plex)	2462.3	0	**1.4**	**1.4**	**1.9**	**1.7**
																	ISGETIFVTAPHEATAGIIGVNR	20	2	1	4758012	N-Term(TMT6plex)	2582.4	0	**1.4**	**1.4**	**1.4**	**1.4**
																	LLLPWLEAR	24	2	1	4758012	N-Term(TMT6plex)	1339.8	0	**1.6**	**1.6**	**1.6**	**1.5**
																	RDPHLACVAYER	10	4	1	4758012	N-Term(TMT6plex); C7(Carbamidomethyl)	1715.9	1	**1.1**	**1.3**	**1.3**	**1.3**
																	YIQAACK	4	4	1	4758012	N-Term(TMT6plex); C6(Carbamidomethyl); K7(TMT6plex)	1311.7	0	**1.4**	**1.5**	**1.7**	**1.3**
																	WLLLTGISAQQNR	18	2	1	4758012	N-Term(TMT6plex)	1729.0	0	**1.5**	**1.3**	**1.7**	**1.3**
																	EHLELFWSR	24	2	1	4758012	N-Term(TMT6plex)	1445.8	0	**1.3**	**1.3**	**1.4**	**1.4**
																	SVQNHNNK	7	2	1	4758012	N-Term(TMT6plex); K8(TMT6plex)	1398.8	0	**1.3**	**1.4**	**1.4**	**1.3**
																	AHMGMFTELAILYSK	4	4	1	4758012	N-Term(TMT6plex); M3(Oxidation); K15(TMT6plex)	2186.2	0	**1.4**	**1.4**	**1.6**	**1.3**
																	TSIDAYDNFDNISLAQR	23	2	1	4758012	N-Term(TMT6plex)	2172.1	0	**1.5**	**1.4**	**1.4**	**1.3**
																	VAANAPK	7	2	1	4758012	N-Term(TMT6plex); K7(TMT6plex)	1128.7	0	**1.4**	**1.4**	**1.4**	**1.4**
																	YESLELCRPVLQQGR	3	2	1	4758012	N-Term(TMT6plex); C7(Carbamidomethyl)	2077.1	0	**1.4**	**1.5**	**1.5**	**1.5**
																	LAELEEFINGPNNAHIQQVGDR	8	2	1	4758012	N-Term(TMT6plex)	2693.4	0	**1.3**	**1.2**	**1.3**	**1.2**
																	DTELAEELLQWFLQEEK	16	2	1	4758012	N-Term(TMT6plex); K17(TMT6plex)	2579.4	0	**1.5**	**1.6**	**1.7**	**1.5**
																	LECSEELGDLVK	12	4	1	4758012	N-Term(TMT6plex); C3(Carbamidomethyl); K12(TMT6plex)	1850.0	0	**1.4**	**1.5**	**1.6**	**1.4**
																	RPLIDQVVQTALSETQDPEEVSVTVK	18	2	1	4758012	N-Term(TMT6plex); K26(TMT6plex)	3339.8	0	**1.3**	**1.5**	**1.6**	**1.5**
																	FNALFAQGNYSEAAK	8	2	1	4758012	N-Term(TMT6plex); K15(TMT6plex)	2089.1	0	**1.4**	**1.2**	**1.5**	**1.3**
																	IHEGCEEPATHNALAK	4	2	1	4758012	N-Term(TMT6plex); C5(Carbamidomethyl); K16(TMT6plex)	2235.2	0	**1.3**	**1.3**	**1.5**	**1.2**
																	ENPYYDSR	4	2	1	4758012	N-Term(TMT6plex)	1272.6	0	**1.5**	**1.6**	**1.7**	**1.5**
																	AHIAQLCEK	2	4	1	4758012	N-Term(TMT6plex); C7(Carbamidomethyl); K9(TMT6plex)	1527.9	0	**1.5**	**1.6**	**1.8**	**1.6**

Khan et al., 2019. Multi-omics analysis to characterize cigarette smoke induced molecular alterations in esophageal cells
Supplementary Table 6. List of all proteins and peptides identified in untreated and chronically treated Het1A cells with cigarette smoke condensate for 8 months

Accession	RefSeq Accession	Gene Symbol	Entrez Gene ID	Description	Σ Coverage	Σ# Proteins	Σ# Unique Peptides	Σ# Peptides	Σ# PSMs	Het-1A-Smoke - 2M/Parental	Het-1A-Smoke - 4M/Parental	Het-1A-Smoke - 6M/Parental	Het-1A-Smoke - 8M/Parental	# AAs	MW [kDa]	calc. pI	Sequence	# PSMs	# Proteins	# Protein Groups	Protein Group Accessions	Modifications	MH+ [Da]	# Missed Cleavages	Het-1A-Smoke - 2M/Parental	Het-1A-Smoke - 4M/Parental	Het-1A-Smoke - 6M/Parental	Het-1A-Smoke - 8M/Parental
																	YEEYDNAIITMMNHPTDAWK	2	2	1	4758012	N-Term(TMT6plex); M11(Oxidation); M12(Oxidation); K20(TMT6plex)	2932.4	0	**1.3**	**1.2**	**1.4**	**1.5**
																	DPELWGSVLLESNPYR	5	2	1	4758012	N-Term(TMT6plex)	2104.1	0	**1.6**	**1.7**	**1.7**	**1.7**
																	MEGNAEESTLFCFAVR	3	2	1	4758012	N-Term(TMT6plex); M1(Oxidation); C12(Carbamidomethyl)	2106.0	0	**1.4**	**1.4**	**1.4**	**1.5**
																	LEKHELIEFR	2	2	1	4758012	N-Term(TMT6plex); K3(TMT6plex)	1772.0	1	**1.3**	**2.2**	**1.6**	**2.1**
																	GQFSTDELVAEVEKR	4	4	1	4758012	N-Term(TMT6plex); K14(TMT6plex)	2166.2	1	**1.4**	**2.0**	**1.8**	**1.9**
																	LAQMCGLHIVVHADELEELINYYQDR	1	2	1	4758012	N-Term(TMT6plex); C5(Carbamidomethyl)	3358.7	0	**1.2**	**1.4**	**1.3**	**1.3**
																	TLQIFNIEMK	15	4	1	4758012	N-Term(TMT6plex); M9(Oxidation); K10(TMT6plex)	1711.0	0	**1.5**	**1.5**	**1.5**	**1.4**
																	EDKLECSEELGDLVK	6	4	1	4758012	N-Term(TMT6plex); K3(TMT6plex); C6(Carbamidomethyl); K15(TMT6plex)	2451.3	1	**1.3**	**1.5**	**1.6**	**1.4**
																	AFMTADLPNELIELLEK	8	4	1	4758012	N-Term(TMT6plex); K17(TMT6plex)	2405.3	0	**1.7**	**2.3**	**2.5**	**1.8**
																	HSSLAGCQIINYR	7	2	1	4758012	N-Term(TMT6plex); C7(Carbamidomethyl)	1747.9	0	**1.5**	**1.4**	**1.6**	**1.4**
																	HNIMDFAMPYFIQVMK	2	2	1	4758012	N-Term(TMT6plex); M4(Oxidation); M8(Oxidation); M15(Oxidation); K16(TMT6plex)	2491.3	0	**1.1**	**1.2**	**1.3**	**1.6**
																	LTDQLPLIIVCDR	22	4	1	4758012	N-Term(TMT6plex); C11(Carbamidomethyl)	1785.0	0	**1.3**	**1.3**	**1.4**	**1.2**
																	FQEHLQLQNLGINPANIGFSTLTMESDK	4	2	1	4758012	N-Term(TMT6plex); K28(TMT6plex)	3603.9	0	**1.8**	**1.7**	**2.1**	**1.4**
																	FQSVPAQPGQTSPLLQYFGILLDQGQLNK	19	2	1	4758012	N-Term(TMT6plex); K29(TMT6plex)	3646.0	0	**1.5**	**1.2**	**1.7**	**1.0**
																	RKDPELWGSVLLESNPYR	1	2	1	4758012	N-Term(TMT6plex); K2(TMT6plex)	2617.4	2	**1.3**	**1.3**	**1.4**	**1.4**
																	HELIEFR	1	2	1	4758012	N-Term(TMT6plex)	1172.7	0	**1.3**	**1.3**	**1.5**	**1.2**
																	AVVHTHLLNPEWLVNYFGSLSVEDSLECLR	2	2	1	4758012	N-Term(TMT6plex); C28(Carbamidomethyl)	3726.9	0	**1.6**	**1.4**	**2.2**	**1.0**
																	ALEHFTDLYDIK	12	2	1	4758012	N-Term(TMT6plex); K12(TMT6plex)	1923.1	0	**1.3**	**1.5**	**1.6**	**1.5**
																	YGYIHLYDLETGTCIYMNR	1	2	1	4758012	N-Term(TMT6plex); C14(Carbamidomethyl); M17(Oxidation)	2627.2	0	**1.4**	**1.3**	**1.4**	**1.5**
																	AVNYFSK	6	2	1	4758012	N-Term(TMT6plex); K7(TMT6plex)	1286.8	0	**1.4**	**1.6**	**1.7**	**1.6**
																	NLILVVR	11	2	1	4758012	N-Term(TMT6plex)	1055.7	0	**1.5**	**1.3**	**1.5**	**1.3**
																	VMEYINR	2	2	1	4758012	N-Term(TMT6plex); M2(Oxidation)	1169.6	0	**1.5**	**1.4**	**1.5**	**1.5**
																	QLPLVKPYLR	2	2	1	4758012	N-Term(TMT6plex); K6(TMT6plex)	1685.1	0	**1.4**	**1.8**	**1.7**	**1.9**
																	ANVPNK	1	2	1	4758012	N-Term(TMT6plex); K6(TMT6plex)	1100.7	0	**1.2**	**1.5**	**1.5**	**1.5**
																	KQLLEK	1	4	1	4758012	N-Term(TMT6plex); K1(TMT6plex); K6(TMT6plex)	1446.0	1	**1.3**	**1.7**	**1.7**	**1.6**
																	GQAGGK	1	2	1	4758012	N-Term(TMT6plex); K6(TMT6plex)	975.6	0	**1.1**	**1.4**	**1.6**	**1.5**
																	KDSLYK	1	2	1	4758012	N-Term(TMT6plex); K1(TMT6plex); K6(TMT6plex)	1440.9	1	**1.3**	**1.2**	**1.5**	**1.1**
																	IVLYAK	3	4	1	4758012	N-Term(TMT6plex); K6(TMT6plex)	1164.8	0	**1.4**	**1.5**	**1.5**	**1.5**
222352151	NP_006187.2	**PCBP1**	5093	**poly(rC)-binding protein 1**	75.28	3	11	16	266	**1.2**	**1.3**	**1.3**	**1.5**	356	37.5	7.09												
																	LEEDINSSMTNSTAASRPPVTLR	10	1	1	222352151	N-Term(TMT6plex); M9(Oxidation)	2734.4	0	**1.5**	**1.5**	**1.5**	**1.7**
																	VMTIPYQPMPASSPVICAGGQDR	10	1	1	222352151	N-Term(TMT6plex); M2(Oxidation); M9(Oxidation); C17(Carbamidomethyl)	2736.3	0	**1.1**	**1.1**	**1.2**	**1.4**
																	LVVPATQCGSLIGK	24	1	1	222352151	N-Term(TMT6plex); C8(Carbamidomethyl); K14(TMT6plex)	1901.1	0	**1.3**	**1.3**	**1.3**	**1.5**
																	EVGSIIGK	22	12	3	193083110;222352151;14141166	N-Term(TMT6plex); K8(TMT6plex)	1260.8	0				
																	IITLTGPTNAIFK	28	1	1	222352151	N-Term(TMT6plex); K13(TMT6plex)	1847.1	0	**1.0**	**1.1**	**1.2**	**1.4**
																	INISEGNCPER	33	10	3	193083110;222352151;14141166	N-Term(TMT6plex); C8(Carbamidomethyl)	1517.8	0				
																	GYWASLDASTQTTHELTIPNNLIGCIIGR	2	1	1	222352151	N-Term(TMT6plex); C25(Carbamidomethyl)	3430.8	0	**1.3**	**1.3**	**1.3**	**1.5**
																	VMTIPYQPMPASSPVICAGGQDR	4	1	1	222352151	N-Term(TMT6plex); M2(Oxidation); C17(Carbamidomethyl)	2720.3	0	**1.3**	**1.5**	**1.3**	**1.5**
																	QVTITGSAASISLAQYLINAR	16	1	1	222352151	N-Term(TMT6plex)	2406.4	0	**1.5**	**1.6**	**1.6**	**1.4**
																	ESTGAQVQVAGDMLPNSTER	14	10	3	193083110;222352151;14141166	N-Term(TMT6plex); M13(Oxidation)	2335.1	0				
																	AFAMIIDK	6	8	3	193083110;222352151;14141166	N-Term(TMT6plex); M4(Oxidation); K8(TMT6plex)	1382.8	0				
																	QICLVMLETLSQSPQGR	2	1	1	222352151	N-Term(TMT6plex); C3(Carbamidomethyl)	2189.2	0	**2.0**	**1.6**	**1.9**	**1.8**
																	QICLVMLETLSQSPQGR	25	1	1	222352151	N-Term(TMT6plex); C3(Carbamidomethyl); M6(Oxidation)	2205.1	0	**1.5**	**1.4**	**1.3**	**1.4**
																	AITIAGVPQSVTECVK	33	1	1	222352151	N-Term(TMT6plex); C14(Carbamidomethyl); K16(TMT6plex)	2131.2	0	**1.2**	**1.3**	**1.4**	**1.5**
																	QGANINEIR	11	1	1	222352151	N-Term(TMT6plex)	1243.7	0	**1.3**	**1.2**	**1.3**	**1.4**
																	IANPVEGSSGR	17	1	1	222352151	N-Term(TMT6plex)	1315.7	0	**1.3**	**1.2**	**1.1**	**1.4**
																	VMTIPYQPMPASSPVICAGGQDR	1	1	1	222352151	N-Term(TMT6plex); C17(Carbamidomethyl)	2704.4	0	**1.3**	**1.1**	**1.3**	**1.1**
																	CSDAAGYPHATHDLEGPPLDAYSIQGQHTISPLDLAK	1	1	1	222352151	N-Term(TMT6plex); C1(Carbamidomethyl); K37(TMT6plex)	4404.2	0	**1.4**	**1.3**	**1.4**	**1.4**
																	ESTGAQVQVAGDMLPNSTER	6	10	3	193083110;222352151;14141166	N-Term(TMT6plex)	2319.1	0				
																	QMSGAQIK	1	10	3	193083110;222352151;14141166	N-Term(TMT6plex); M2(Oxidation); K8(TMT6plex)	1336.8	0				
5031973	NP_005733.1	**PDIA6**	10130	**protein disulfide-isomerase A6 isoform d precursor**	75.23	5	28	29	520	**1.4**	**1.5**	**1.4**	**1.3**	440	48.1	5.08												
																	GSFSEQGINEFLR	33	5	1	5031973	N-Term(TMT6plex)	1712.9	0	**1.4**	**1.6**	**1.5**	**1.4**
																	LAAVDATVNQVLASR	68	5	1	5031973	N-Term(TMT6plex)	1757.0	0	**1.5**	**1.5**	**1.6**	**1.4**
																	DGELPVEDDIDLSDVELDDLGKDEL	19	5	1	5031973	N-Term(TMT6plex); K22(TMT6plex)	3216.6	1	**1.3**	**1.5**	**1.3**	**1.3**
																	SGGYSSGK	9	5	1	5031973	N-Term(TMT6plex); K8(TMT6plex)	1200.7	0	**1.3**	**1.2**	**1.3**	**1.1**
																	HHSLGGQYGVQGFPTIK	10	5	1	5031973	N-Term(TMT6plex); K17(TMT6plex)	2284.3	0	**1.3**	**1.4**	**1.4**	**1.3**
																	GSTAPVGGGAFPTIVER	46	5	1	5031973	N-Term(TMT6plex)	1845.0	0	**1.4**	**1.5**	**1.5**	**1.4**
																	NLEPEWAAAASEVK	36	5	1	5031973	N-Term(TMT6plex); K14(TMT6plex)	1973.1	0	**1.3**	**1.4**	**1.4**	**1.3**
																	ALDLFSDNAPPPELLEIINEDIAK	123	5	1	5031973	N-Term(TMT6plex); K24(TMT6plex)	3095.7	0	**1.4**	**1.5**	**1.5**	**1.3**
																	KDVIELTDDSFDK	8	5	1	5031973	N-Term(TMT6plex); K1(TMT6plex); K13(TMT6plex)	2212.2	1	**1.2**	**1.3**	**1.3**	**1.2**
																	GESPVDYDGGR	13	5	1	5031973	N-Term(TMT6plex)	1380.7	0	**1.3**	**1.4**	**1.3**	**1.3**
																	NSYLEVLLK	22	5	1	5031973	N-Term(TMT6plex); K9(TMT6plex)	1536.9	0	**1.4**	**1.1**	**1.4**	**1.1**
																	DVIELTDDSFDK	17	5	1	5031973	N-Term(TMT6plex); K12(TMT6plex)	1855.0	0	**1.3**	**1.4**	**1.4**	**1.2**
																	TCEEHQLCVVAVLPHILDTGAAGR	2	5	1	5031973	N-Term(TMT6plex); C2(Carbamidomethyl); C8(Carbamidomethyl)	2875.5	0	**1.3**	**1.7**	**1.5**	**1.5**
																	NRPEDYQGGR	8	5	1	5031973	N-Term(TMT6plex)	1420.7	0	**1.4**	**1.1**	**1.2**	**1.0**
																	GSTAPVGGGAFPTIVEREPWDGR	1	5	1	5031973	N-Term(TMT6plex)	2585.3	1	**1.3**	**1.8**	**1.5**	**1.7**
																	TGEAIVDAALSALR	68	5	1	5031973	N-Term(TMT6plex)	1615.9	0	**1.7**	**1.2**	**1.5**	**1.5**
																	VGAVDADK	14	5	1	5031973	N-Term(TMT6plex); K8(TMT6plex)	1232.7	0	**1.3**	**1.4**	**1.4**	**1.2**
																	DGELPVEDDIDLSDVELDDLGK	2	5	1	5031973	N-Term(TMT6plex); K22(TMT6plex)	2859.4	0	**1.4**	**1.3**	**1.3**	**1.3**
																	KAATALK	3	5	1	5031973	N-Term(TMT6plex); K1(TMT6plex); K7(TMT6plex)	1389.9	1	**1.1**	**1.6**	**1.3**	**1.4**
																	MALLVLGLVSCTFFLAVNGLYSSSDDVIELTPSNFNREVIQSDSLWLVEFYAPWCGHCQR	1	1	1	5031973	N-Term(TMT6plex); C11(Carbamidomethyl); C55(Carbamidomethyl); C58(Carbamidomethyl)	7153.5	1				
																	EPWDGRDGELPVEDDIDLSDVELDDLGKDEL	1	5	1	5031973	N-Term(TMT6plex); K28(TMT6plex)	3956.9	2	**2.0**	**2.2**	**1.8**	**2.6**
																	SDIVSR	5	5	1	5031973	N-Term(TMT6plex)	905.5	0	**1.3**	**1.3**	**1.3**	**1.2**
																	LTPEWK	1	5	1	5031973	N-Term(TMT6plex); K6(TMT6plex)	1231.7	0	**1.6**	**1.4**	**1.4**	**1.2**
																	ELSFGR	1	5	1	5031973	N-Term(TMT6plex)	937.5	0	**1.4**	**1.5**	**1.4**	**1.3**
																	AATALK	4	5	1	5031973	N-Term(TMT6plex); K6(TMT6plex)	1032.7	0	**1.3**	**1.5**	**1.4**	**1.3**
																	IFGSNK	2	5	2	5031973;24308007	N-Term(TMT6plex); K6(TMT6plex)	1123.7	0				
																	SDSSSK	1	5	1	5031973	N-Term(TMT6plex); K6(TMT6plex)	1068.6	0	**1.4**	**1.3**	**1.4**	**1.2**
																	GFPTIK	1	5	1	5031973	N-Term(TMT6plex); K6(TMT6plex)	1120.7	0	**1.2**	**1.5**	**1.3**	**1.3**
																	EPWDGR	1	5	1	5031973	N-Term(TMT6plex)	988.5	0	**1.7**	**1.6**	**1.5**	**1.4**
194595509	NP_001123910.1	**SPTAN1**	6709	**spectrin alpha chain, non-erythrocytic 1 isoform 1**	75.17	2	3	167	1437	**1.2**	**1.4**	**1.5**	**1.3**	2477	284.9	5.35												
																	ELPTAFDYVEFTR	12	3	2	194595509;306966132	N-Term(TMT6plex)	1816.9	0				
																	EAALTSEEVGADLEQVEVLQK	28	3	2	194595509;306966132	N-Term(TMT6plex); K21(TMT6plex)	2716.5	0				
																	QQVAPTDDETGK	11	3	2	194595509;306966132	N-Term(TMT6plex); K12(TMT6plex)	1746.9	0				
																	DLASVQALLR	27	3	2	194595509;306966132	N-Term(TMT6plex)	1314.8	0				
																	KFEEFQTDMAAHEER	10	3	2	194595509;306966132	N-Term(TMT6plex); K1(TMT6plex); M9(Oxidation)	2342.1	1				
																	DLNSQADSLMTSSAFDTSQVK	8	3	2	194595509;306966132	N-Term(TMT6plex); M10(Oxidation); K21(TMT6plex)	2719.4	0				
																	SQLLGSAHEVQR	4	3	2	194595509;306966132	N-Term(TMT6plex)	1553.9	0				
																	SSEEIESAFR	8	3	2	194595509;306966132	N-Term(TMT6plex)	1383.7	0				
																	NVEDIELWLYEVEGHLASDDYGK	9	3	2	194595509;306966132	N-Term(TMT6plex); K23(TMT6plex)	3152.6	0				
																	EAFLNTEDKGDSLDSVEALIK	23	3	2	194595509;306966132	N-Term(TMT6plex); K9(TMT6plex); K21(TMT6plex)	2981.6	1				
																	RQDLEDSLQAQQYFADANEAESWMR	11	3	2	194595509;306966132	N-Term(TMT6plex); M24(Oxidation)	3246.5	1				
																	GEIDAHEDSFK	12	3	2	194595509;306966132	N-Term(TMT6plex); K11(TMT6plex)	1705.9	0				
																	QWELLLEK	13	3	2	194595509;306966132	N-Term(TMT6plex); K8(TMT6plex)	1516.9	0				
																	DLASVNNLLK	7	3	2	194595509;306966132	N-Term(TMT6plex); K10(TMT6plex)	1544.9	0				
																	LSDDNTIGK	5	3	2	194595509;306966132	N-Term(TMT6plex); K9(TMT6plex)	1420.8	0				
																	LKDLNSQADSLMTSSAFDTSQVK	4	3	2	194595509;306966132	N-Term(TMT6plex); K2(TMT6plex); M12(Oxidation); K23(TMT6plex)	3189.7	1				
																	LIQNNHYAMEDVATR	9	3	2	194595509;306966132	N-Term(TMT6plex); M9(Oxidation)	2020.0	0				
																	DVDETISWIK	13	3	2	194595509;306966132	N-Term(TMT6plex); K10(TMT6plex)	1663.9	0				
																	HQAFEAELHANADR	7	3	2	194595509;306966132	N-Term(TMT6plex)	1837.9	0				
																	LIQSHPESAEDLQEK	11	3	2	194595509;306966132	N-Term(TMT6plex); K15(TMT6plex)	2182.2	0				
																	QDEVNAAWQR	4	3	2	194595509;306966132	N-Term(TMT6plex)	1445.7	0				
																	LIDVNHYAK	9	3	2	194595509;306966132	N-Term(TMT6plex); K9(TMT6plex)	1530.9	0				
																	NNHHEENISSK	4	3	2	194595509;306966132	N-Term(TMT6plex); K11(TMT6plex)	1766.9	0				
																	MNEVISLWK	6	3	2	194595509;306966132	N-Term(TMT6plex); M1(Oxidation); K9(TMT6plex)	1593.9	0				
																	KVEDLFLTFAK	13	3	2	194595509;306966132	N-Term(TMT6plex); K1(TMT6plex); K11(TMT6plex)	1998.2	1				

Accession	RefSeq Accession	Gene Symbol	Entrez Gene ID	Description	Σ Coverage	Σ# Proteins	Σ# Unique Peptides	Σ# Peptides	Σ# PSMs	Het-1A-Smoke - 2M/Parental	Het-1A-Smoke - 4M/Parental	Het-1A-Smoke - 6M/Parental	Het-1A-Smoke - 8M/Parental	# AAs	MW [kDa]	calc. pI	Sequence	# PSMs	# Proteins	# Protein Groups	Protein Group Accessions	Modifications	MH+ [Da]	# Missed Cleavages	Het-1A-Smoke - 2M/Parental	Het-1A-Smoke - 4M/Parental	Het-1A-Smoke - 6M/Parental	Het-1A-Smoke - 8M/Parental
																	QQVAPTDDETGKELVLALYDYQEK	4	3	2	194595509;306966132	N-Term(TMT6plex); K12(TMT6plex); K24(TMT6plex)	3440.8	1				
																	LGESQTLQQFSR	4	3	2	194595509;306966132	N-Term(TMT6plex)	1622.9	0				
																	ITALDEFATK	20	3	2	194595509;306966132	N-Term(TMT6plex); K10(TMT6plex)	1566.9	0				
																	DTEQVDNWMSK	6	3	2	194595509;306966132	N-Term(TMT6plex); K11(TMT6plex)	1810.9	0				
																	VASNPYTWFTMEALEETWR	19	3	2	194595509;306966132	N-Term(TMT6plex); M11(Oxidation)	2576.2	0				
																	GDILTLLNSTNK	4	3	2	194595509;306966132	N-Term(TMT6plex); K12(TMT6plex)	1747.0	0				
																	SLSAQEEK	10	3	2	194595509;306966132	N-Term(TMT6plex); K8(TMT6plex)	1349.8	0				
																	LQIASDENYKDPTNLQGK	9	3	2	194595509;306966132	N-Term(TMT6plex); K10(TMT6plex); K18(TMT6plex)	2721.5	1				
																	QFQDAGHFDAENIK	9	3	2	194595509;306966132	N-Term(TMT6plex); K14(TMT6plex)	2078.1	0				
																	LIQEQHPEEELIK	14	3	2	194595509;306966132	N-Term(TMT6plex); K13(TMT6plex)	2064.2	0				
																	YTEHSTVGLAQQWDQLDQLGMR	4	3	2	194595509;306966132	N-Term(TMT6plex); M21(Oxidation)	2821.4	0				
																	EELYQNLTR	9	3	2	194595509;306966132	N-Term(TMT6plex)	1394.7	0				
																	KLDPAQSASR	5	3	2	194595509;306966132	N-Term(TMT6plex); K1(TMT6plex)	1530.9	1				
																	EKEPIAASTNR	5	3	2	194595509;306966132	N-Term(TMT6plex); K2(TMT6plex)	1674.0	1				
																	ENLLEEQGSIALR	13	3	2	194595509;306966132	N-Term(TMT6plex)	1700.9	0				
																	ASAFNSWFENAEEDLTDPVR	19	3	2	194595509;306966132	N-Term(TMT6plex)	2527.2	0				
																	EAIVTSEELGQDLEHVEVLQK	24	3	2	194595509;306966132	N-Term(TMT6plex); K21(TMT6plex)	2824.5	0				
																	VAEDLESEGLMAEEVQAVQQQEVYGMMPR	20	3	2	194595509;306966132	N-Term(TMT6plex); M11(Oxidation); M26(Oxidation); M27(Oxidation)	3543.7	0				
																	LTVLSEER	8	3	2	194595509;306966132	N-Term(TMT6plex)	1175.7	0				
																	GLVSSDELAK	8	3	2	194595509;306966132	N-Term(TMT6plex); K10(TMT6plex)	1476.9	0				
																	DVEDEETWIR	2	3	2	194595509;306966132	N-Term(TMT6plex)	1520.7	0				
																	SLGYDLPMVEEGEPDPEFEAILDTVDPNR	17	3	2	194595509;306966132	N-Term(TMT6plex); M8(Oxidation)	3492.6	0				
																	QEQIDNQTR	5	2	1	194595509	N-Term(TMT6plex)	1360.7	0	**1.1**	**1.4**	**1.6**	**1.2**
																	KIEDLGAAMEEALILDNK	6	3	2	194595509;306966132	N-Term(TMT6plex); K1(TMT6plex); M9(Oxidation); K18(TMT6plex)	2676.5	1				
																	AQLADSFHLQQFFR	12	3	2	194595509;306966132	N-Term(TMT6plex)	1937.0	0				
																	DTEQVDNWMSK	15	3	2	194595509;306966132	N-Term(TMT6plex); M9(Oxidation); K11(TMT6plex)	1826.9	0				
																	LEESLEYQQFVANVEEEEAWINEK	17	3	2	194595509;306966132	N-Term(TMT6plex); K24(TMT6plex)	3384.7	0				
																	GACAGSEDAVK	8	3	2	194595509;306966132	N-Term(TMT6plex); C3(Carbamidomethyl); K11(TMT6plex)	1522.8	0				
																	DMDDEESWIK	14	3	2	194595509;306966132	N-Term(TMT6plex); M2(Oxidation); K10(TMT6plex)	1741.8	0				
																	VNSLGETAER	5	3	2	194595509;306966132	N-Term(TMT6plex)	1304.7	0				
																	KHEDFEK	8	3	2	194595509;306966132	N-Term(TMT6plex); K1(TMT6plex); K7(TMT6plex)	1619.9	1				
																	ALCAEADR	4	3	2	194595509;306966132	N-Term(TMT6plex); C3(Carbamidomethyl)	1134.6	0				
																	LDENSAFLQFNWK	12	3	2	194595509;306966132	N-Term(TMT6plex); K13(TMT6plex)	2070.1	0				
																	DADETKEWIEEK	2	3	2	194595509;306966132	N-Term(TMT6plex); K6(TMT6plex); K12(TMT6plex)	2180.2	1				
																	ALSSEGKPYVTK	5	3	2	194595509;306966132	N-Term(TMT6plex); K7(TMT6plex); K12(TMT6plex)	1967.2	0				
																	DCEQAENWMAAR	5	3	2	194595509;306966132	N-Term(TMT6plex); C2(Carbamidomethyl); M9(Oxidation)	1725.8	0				
																	HQAFEAELSANQSR	9	3	2	194595509;306966132	N-Term(TMT6plex)	1816.9	0				
																	DLTSWVTEMK	2	3	2	194595509;306966132	N-Term(TMT6plex); K10(TMT6plex)	1667.9	0				
																	DVTGAEALLER	4	3	2	194595509;306966132	N-Term(TMT6plex)	1402.8	0				
																	DLIGVQNLLK	24	3	2	194595509;306966132	N-Term(TMT6plex); K10(TMT6plex)	1571.0	0				
																	QGFVPAAYVK	7	3	2	194595509;306966132	N-Term(TMT6plex); K10(TMT6plex)	1537.9	0				
																	TKQDEVNAAWQR	2	3	2	194595509;306966132	N-Term(TMT6plex); K2(TMT6plex)	1904.0	1				
																	DMDDEESWIK	1	3	2	194595509;306966132	N-Term(TMT6plex); K10(TMT6plex)	1725.8	0				
																	GVIDMGNSLIER	2	3	2	194595509;306966132	N-Term(TMT6plex)	1532.8	0				
																	ECEDVMDWINDKEAIVTSEELGQDLEHVEVLQK	6	3	2	194595509;306966132	N-Term(TMT6plex); C2(Carbamidomethyl); M6(Oxidation); K12(TMT6plex); K33(TMT6plex)	4604.3	1				
																	SLGYDLPMVEEGEPDPEFEAILDTVDPNR	2	3	2	194595509;306966132	N-Term(TMT6plex)	3476.7	0				
																	IEDLGAAMEEALILDNK	2	3	2	194595509;306966132	N-Term(TMT6plex); K17(TMT6plex)	2303.3	0				
																	MTLVASEDYGDTLAAIQGLLK	26	3	2	194595509;306966132	N-Term(TMT6plex); M1(Oxidation); K21(TMT6plex)	2683.4	0				
																	KGDILTLLNSTNK	6	3	2	194595509;306966132	N-Term(TMT6plex); K1(TMT6plex); K13(TMT6plex)	2104.3	1				
																	HQLLEADISAHEDR	6	3	2	194595509;306966132	N-Term(TMT6plex)	1863.0	0				
																	KHQLLEADISAHEDR	4	3	2	194595509;306966132	N-Term(TMT6plex); K1(TMT6plex)	2220.2	1				
																	MTLVASEDYGDTLAAIQGLLK	2	3	2	194595509;306966132	N-Term(TMT6plex); K21(TMT6plex)	2667.5	0				
																	WSQLLANSAAR	2	3	2	194595509;306966132	N-Term(TMT6plex)	1445.8	0				
																	EANQQQQFNR	5	3	2	194595509;306966132	N-Term(TMT6plex)	1491.7	0				
																	LAALADQWQFLVQK	12	3	2	194595509;306966132	N-Term(TMT6plex); K14(TMT6plex)	2089.2	0				
																	IDGITIQAR	6	3	2	194595509;306966132	N-Term(TMT6plex)	1215.7	0				
																	LEAELAAHEPAIQGVLDTGKK	2	3	2	194595509;306966132	N-Term(TMT6plex); K20(TMT6plex); K21(TMT6plex)	2877.7	1				
																	LGDSHDLQR	4	3	2	194595509;306966132	N-Term(TMT6plex)	1269.7	0				
																	NVEDIELWLYEVEGHLASDDYGKDLTNVQNLQK	1	3	2	194595509;306966132	N-Term(TMT6plex); K23(TMT6plex); K33(TMT6plex)	4535.3	1				
																	NQALNTDNYGHDLASVQALQR	6	3	2	194595509;306966132	N-Term(TMT6plex)	2557.3	0				
																	VNEVNQFAAK	8	3	2	194595509;306966132	N-Term(TMT6plex); K10(TMT6plex)	1577.9	0				
																	ADVVESWIGEK	10	3	2	194595509;306966132	N-Term(TMT6plex); K11(TMT6plex)	1690.9	0				
																	QQNFNTGIK	5	3	2	194595509;306966132	N-Term(TMT6plex); K9(TMT6plex)	1507.9	0				
																	LAQFVEHWK	17	3	2	194595509;306966132	N-Term(TMT6plex); K9(TMT6plex)	1615.9	0				
																	LHELNQK	12	3	2	194595509;306966132	N-Term(TMT6plex); K7(TMT6plex)	1339.8	0				
																	QEAFLLNEDLGDSLDSVEALLK	35	3	2	194595509;306966132	N-Term(TMT6plex); K22(TMT6plex)	2877.6	0				
																	SADESGQALLAAGHYASDEVR	20	3	2	194595509;306966132	N-Term(TMT6plex)	2376.2	0				
																	TYLLDGSCMVEESGTLESQLEATK	13	3	2	194595509;306966132	N-Term(TMT6plex); C8(Carbamidomethyl); M9(Oxidation); K24(TMT6plex)	3135.5	0				
																	ELVLALYDYQEK	10	3	2	194595509;306966132	N-Term(TMT6plex); K12(TMT6plex)	1942.1	0				
																	VLETAEDIQER	14	3	2	194595509;306966132	N-Term(TMT6plex)	1531.8	0				
																	LQQSHPLSATQIQVK	14	3	2	194595509;306966132	N-Term(TMT6plex); K15(TMT6plex)	2136.3	0				
																	IAALQAFADQLIAAGHYAK	21	3	2	194595509;306966132	N-Term(TMT6plex); K19(TMT6plex)	2430.4	0				
																	RLEAELAAHEPAIQGVLDTGK	14	3	2	194595509;306966132	N-Term(TMT6plex); K21(TMT6plex)	2676.5	1				
																	LDILDQER	9	3	2	194595509;306966132	N-Term(TMT6plex)	1230.7	0				
																	GNAMVEEGHFAAEDVK	11	3	2	194595509;306966132	N-Term(TMT6plex); M4(Oxidation); K16(TMT6plex)	2178.1	0				
																	ECEDVMDWINDK	12	3	2	194595509;306966132	N-Term(TMT6plex); C2(Carbamidomethyl); M6(Oxidation); K12(TMT6plex)	2027.9	0				
																	DLTGVQNLR	10	3	2	194595509;306966132	N-Term(TMT6plex)	1244.7	0				
																	TATDEAYKDPSNLQGK	12	3	2	194595509;306966132	N-Term(TMT6plex); K8(TMT6plex); K16(TMT6plex)	2425.3	1				
																	RQEENDK	4	3	2	194595509;306966132	N-Term(TMT6plex); K7(TMT6plex)	1376.8	1				
																	HEAFETDFTVHK	4	3	2	194595509;306966132	N-Term(TMT6plex); K12(TMT6plex)	1919.0	0				
																	AINVQEEK	13	3	2	194595509;306966132	N-Term(TMT6plex); K8(TMT6plex)	1388.8	0				
																	ALINADELASDVAGAEALLDR	76	3	2	194595509;306966132	N-Term(TMT6plex)	2356.3	0				
																	DLTNVQNLQK	9	3	2	194595509;306966132	N-Term(TMT6plex); K10(TMT6plex)	1631.0	0				
																	QETFDAGLQAFQQEGIANITALK	17	3	2	194595509;306966132	N-Term(TMT6plex); K23(TMT6plex)	2951.6	0				
																	DLSSVQTLLTK	18	3	2	194595509;306966132	N-Term(TMT6plex); K11(TMT6plex)	1663.0	0				
																	KIEDLGAAMEEALILDNK	6	3	2	194595509;306966132	N-Term(TMT6plex); K1(TMT6plex); K18(TMT6plex)	2660.5	1				
																	GVIDMGNSLIER	6	3	2	194595509;306966132	N-Term(TMT6plex); M5(Oxidation)	1548.8	0				
																	KHALLEADVAAHQDR	2	3	2	194595509;306966132	N-Term(TMT6plex); K1(TMT6plex)	2132.2	1				
																	QEFAQHANAFHQWIQETR	2	3	2	194595509;306966132	N-Term(TMT6plex)	2470.2	0				
																	REELITNWEQIR	4	3	2	194595509;306966132	N-Term(TMT6plex)	1816.0	1				
																	KHEALMSDLSAYGSSIQALR	2	3	2	194595509;306966132	N-Term(TMT6plex); K1(TMT6plex); M6(Oxidation)	2651.4	1				
																	LSDDNTIGKEEIQQR	8	3	2	194595509;306966132	N-Term(TMT6plex); K9(TMT6plex)	2204.2	1				
																	LQTASDESYKDPTNIQLSK	2	1	1	194595509	N-Term(TMT6plex); K10(TMT6plex); K19(TMT6plex)	2825.6	1	**1.6**	**1.5**	**1.4**	**1.6**
																	DVDEIEAWISEK	20	3	2	194595509;306966132	N-Term(TMT6plex); K12(TMT6plex)	1892.0	0				
																	AALLELWELR	11	3	2	194595509;306966132	N-Term(TMT6plex)	1442.9	0				
																	KHEAFETDFTVHK	4	3	2	194595509;306966132	N-Term(TMT6plex); K1(TMT6plex); K13(TMT6plex)	2276.3	1				
																	LLEATELK	10	3	2	194595509;306966132	N-Term(TMT6plex); K8(TMT6plex)	1374.9	0				
																	QVEELYHSLLELGEK	7	2	1	194595509	N-Term(TMT6plex); K15(TMT6plex)	2245.3	0	**1.1**	**1.3**	**1.4**	**1.3**
																	DAEELEK	6	3	2	194595509;306966132	N-Term(TMT6plex); K7(TMT6plex)	1291.7	0				
																	LDETGNLMISEGHFASETIR	2	3	2	194595509;306966132	N-Term(TMT6plex)	2449.2	0				
																	EKEQLMASDDFGR	1	3	2	194595509;306966132	N-Term(TMT6plex); K2(TMT6plex)	1984.0	1				
																	LMVHTVATFNSIK	1	3	2	194595509;306966132	N-Term(TMT6plex); M2(Oxidation); K13(TMT6plex)	1935.1	0				
																	CNSLEEIK	1	3	2	194595509;306966132	N-Term(TMT6plex); C1(Carbamidomethyl); K8(TMT6plex)	1450.8	0				
																	DLNSQADSLMTSSAFDTSQVK	2	3	2	194595509;306966132	N-Term(TMT6plex); K21(TMT6plex)	2703.4	0				
																	TYLLDGSCMVEESGTLESQLEATK	2	3	2	194595509;306966132	N-Term(TMT6plex); C8(Carbamidomethyl); K24(TMT6plex)	3119.6	0				
																	LQTASDESYK	2	3	2	194595509;306966132	N-Term(TMT6plex); K10(TMT6plex)	1599.9	0				
																	NTTGVTEEALK	9	3	2	194595509;306966132	N-Term(TMT6plex); K11(TMT6plex)	1620.9	0				
																	HALLEADVAAHQDR	5	3	2	194595509;306966132	N-Term(TMT6plex)	1774.9	0				
																	SSLSSAQADFNQLAELDR	7	3	2	194595509;306966132	N-Term(TMT6plex)	2181.1	0				
																	HQAFEAEVQANSGAIVK	8	3	2	194595509;306966132	N-Term(TMT6plex); K17(TMT6plex)	2257.2	0				
																	EANELQQWINEK	14	3	2	194595509;306966132	N-Term(TMT6plex); K12(TMT6plex)	1960.1	0				
																	DLTSWVTEMK	10	3	2	194595509;306966132	N-Term(TMT6plex); M9(Oxidation); K10(TMT6plex)	1683.9	0				
																	DLAALEDK	12	3	2	194595509;306966132	N-Term(TMT6plex); K8(TMT6plex)	1332.8	0				
																	HEALMSDLSAYGSSIQALR	3	3	2	194595509;306966132	N-Term(TMT6plex); M5(Oxidation)	2294.2	0				
																	CTELNQAWSSLGK	2	3	2	194595509;306966132	N-Term(TMT6plex); C1(Carbamidomethyl); K13(TMT6plex)	1952.0	0				
																	VASNPYTWFTMEALEETWR	2	3	2	194595509;306966132	N-Term(TMT6plex)	2560.2	0				

Accession	RefSeq Accession	Gene Symbol	Entrez Gene ID	Description	Σ Coverage	Σ# Proteins	Σ# Unique Peptides	Σ# Peptides	Σ# PSMs	Het-1A-Smoke - 2M/Parental	Het-1A-Smoke - 4M/Parental	Het-1A-Smoke - 6M/Parental	Het-1A-Smoke - 8M/Parental	# AAs	MW [kDa]	calc. pI	Sequence	# PSMs	# Proteins	# Protein Groups	Protein Group Accessions	Modifications	MH+ [Da]	# Missed Cleavages	Het-1A-Smoke - 2M/Parental	Het-1A-Smoke - 4M/Parental	Het-1A-Smoke - 6M/Parental	Het-1A-Smoke - 8M/Parental
																	LIQNNHYAMEDVATR	2	3	2	194595509;306966132	N-Term(TMT6plex)	2004.0	0				
																	KHEGFER	2	3	2	194595509;306966132	N-Term(TMT6plex); K1(TMT6plex)	1360.8	1				
																	KFDDFQK	1	3	2	194595509;306966132	N-Term(TMT6plex); K1(TMT6plex); K7(TMT6plex)	1614.9	1				
																	MQHNLEQQIQAR	4	3	2	194595509;306966132	N-Term(TMT6plex); M1(Oxidation)	1740.9	0				
																	VNDVCTNGQDLIK	2	3	2	194595509;306966132	N-Term(TMT6plex); C5(Carbamidomethyl); K13(TMT6plex)	1934.0	0				
																	DCEQAENWMAAR	4	3	2	194595509;306966132	N-Term(TMT6plex); C2(Carbamidomethyl)	1709.8	0				
																	LFGAAEVQR	2	3	2	194595509;306966132	N-Term(TMT6plex)	1219.7	0				
																	GKDLIGVQNLLK	4	3	2	194595509;306966132	N-Term(TMT6plex); K2(TMT6plex); K12(TMT6plex)	1985.3	1				
																	KLSDDNTIGK	1	3	2	194595509;306966132	N-Term(TMT6plex); K1(TMT6plex); K10(TMT6plex)	1778.1	1				
																	YTEHSTVGLAQQWDQLDQLGMR	2	3	2	194595509;306966132	N-Term(TMT6plex)	2805.4	0				
																	QDLEDSLQAQQYFADANEAESWMR	2	3	2	194595509;306966132	N-Term(TMT6plex); M23(Oxidation)	3090.4	0				
																	EKEPIVGSTDYGKDEDSAEALLK	4	3	2	194595509;306966132	N-Term(TMT6plex); K2(TMT6plex); K13(TMT6plex); K23(TMT6plex)	3410.9	2				
																	EQADYCVSHMKPYVDGK	2	3	2	194595509;306966132	N-Term(TMT6plex); C6(Carbamidomethyl); M10(Oxidation); K11(TMT6plex); K17(TMT6plex)	2730.4	0				
																	AGTFQAFEQFGQQLLAHGHYASPEIK	4	3	2	194595509;306966132	N-Term(TMT6plex); K26(TMT6plex)	3333.7	0				
																	LDETGNLMISEGHFASETIR	7	3	2	194595509;306966132	N-Term(TMT6plex); M8(Oxidation)	2465.2	0				
																	LLVGSEDYGR	4	3	2	194595509;306966132	N-Term(TMT6plex)	1337.7	0				
																	DLAALGDK	2	3	2	194595509;306966132	N-Term(TMT6plex); K8(TMT6plex)	1260.8	0				
																	SLQQLAEER	4	3	2	194595509;306966132	N-Term(TMT6plex)	1302.7	0				
																	IEDLGAAMEEALILDNK	6	3	2	194595509;306966132	N-Term(TMT6plex); M8(Oxidation); K17(TMT6plex)	2319.2	0				
																	LLEAQSHFR	4	3	2	194595509;306966132	N-Term(TMT6plex)	1329.7	0				
																	KLLEATELK	2	3	2	194595509;306966132	N-Term(TMT6plex); K1(TMT6plex); K9(TMT6plex)	1732.1	1				
																	KHEDFDK	6	3	2	194595509;306966132	N-Term(TMT6plex); K1(TMT6plex); K7(TMT6plex)	1605.9	1				
																	DQLLAAK	3	3	2	194595509;306966132	N-Term(TMT6plex); K7(TMT6plex)	1216.8	0				
																	LNHQEFK	3	3	2	194595509;306966132	N-Term(TMT6plex); K7(TMT6plex)	1373.8	0				
																	QQYEQCMDLQLFYR	1	3	2	194595509;306966132	N-Term(TMT6plex); C6(Carbamidomethyl); M7(Oxidation)	2167.0	0				
																	QQVLDR	2	3	2	194595509;306966132	N-Term(TMT6plex)	987.6	0				
																	KASAFNSWFENAEEDLTDPVR	1	3	2	194595509;306966132	N-Term(TMT6plex); K1(TMT6plex)	2884.4	1				
																	EKEQLMASDDFGR	3	3	2	194595509;306966132	N-Term(TMT6plex); K2(TMT6plex); M6(Oxidation)	2000.0	1				
																	VEVNDR	2	3	2	194595509;306966132	N-Term(TMT6plex)	960.5	0				
																	EQLMASDDFGR	4	3	2	194595509;306966132	N-Term(TMT6plex); M4(Oxidation)	1513.7	0				
																	SWVNEK	1	3	2	194595509;306966132	N-Term(TMT6plex); K6(TMT6plex)	1220.7	0				
																	KQEALVAR	3	3	2	194595509;306966132	N-Term(TMT6plex); K1(TMT6plex)	1372.9	1				
																	DADETK	1	3	2	194595509;306966132	N-Term(TMT6plex); K6(TMT6plex)	1136.6	0				
																	KHEALMSDLSAYGSSIQALR	1	3	2	194595509;306966132	N-Term(TMT6plex); K1(TMT6plex)	2635.4	1				
																	FQFFQR	3	3	2	194595509;306966132	N-Term(TMT6plex)	1101.6	0				
																	EWIEEK	5	3	2	194595509;306966132	N-Term(TMT6plex); K6(TMT6plex)	1291.7	0				
																	QKLEDSYR	1	3	2	194595509;306966132	N-Term(TMT6plex); K2(TMT6plex)	1496.8	1				
																	FLADFR	1	3	2	194595509;306966132	N-Term(TMT6plex)	997.6	0				
																	DSDELK	1	4	2	194595509;306966132	N-Term(TMT6plex); K6(TMT6plex)	1164.7	0				
																	FLSDFR	2	3	2	194595509;306966132	N-Term(TMT6plex)	1013.6	0				
																	LVQYLR	3	3	2	194595509;306966132	N-Term(TMT6plex)	1020.6	0				
																	GLALQR	1	3	2	194595509;306966132	N-Term(TMT6plex)	886.6	0				
																	LQQLFR	1	3	2	194595509;306966132	N-Term(TMT6plex)	1033.6	0				
8923444	NP_060308.1	**NHP2**	55651	**H/ACA ribonucleoprotein complex subunit 2 isoform a**	75.16	2	7	7	67	**1.4**	**1.3**	**1.3**	**1.2**	153	17.2	8.22												
																	IKADPDGPEAQAEACSGER	8	2	1	8923444	N-Term(TMT6plex); K2(TMT6plex); C15(Carbamidomethyl)	2459.2	1	**1.5**	**1.6**	**1.4**	**1.3**
																	GIMVLAGDTLPIEVYCHLPVMCEDR	5	1	1	8923444	N-Term(TMT6plex); C16(Carbamidomethyl); M21(Oxidation); C22(Carbamidomethyl)	3133.5	0	**1.2**	**1.4**	**1.3**	**1.3**
																	TDLGAAAGSK	16	1	1	8923444	N-Term(TMT6plex); K10(TMT6plex)	1348.8	0	**1.3**	**1.2**	**1.3**	**1.1**
																	GIMVLAGDTLPIEVYCHLPVMCEDR	7	1	1	8923444	N-Term(TMT6plex); M3(Oxidation); C16(Carbamidomethyl); M21(Oxidation); C22(Carbamidomethyl)	3149.5	0	**1.4**	**1.6**	**1.3**	**1.4**
																	TYQELLVNQNPIAQPLASR	13	2	1	8923444	N-Term(TMT6plex)	2384.3	0	**1.3**	**1.3**	**1.4**	**1.3**
																	NLPYVYIPSK	14	1	1	8923444	N-Term(TMT6plex); K10(TMT6plex)	1652.0	0	**1.2**	**1.1**	**1.2**	**1.1**
																	GIMVLAGDTLPIEVYCHLPVMCEDR	2	1	1	8923444	N-Term(TMT6plex); C16(Carbamidomethyl); C22(Carbamidomethyl)	3117.5	0	**1.2**	**1.3**	**1.4**	**1.4**
																	RPTCVIMVKPHEEYQEAYDECLEEVQSLPLPL	1	1	1	8923444	N-Term(TMT6plex); C4(Carbamidomethyl); M7(Oxidation); K9(TMT6plex); C21(Carbamidomethyl)	4347.2	0	**4.1**	**1.7**	**1.2**	**1.8**
																	ADPDGPEAQAEACSGER	1	2	1	8923444	N-Term(TMT6plex); C13(Carbamidomethyl)	1988.9	0	**1.2**	**1.2**	**1.2**	**1.0**
19923142	NP_002256.2	**KPNB1**	3837	**importin subunit beta-1 isoform 1**	75.11	2	43	43	736	**1.3**	**1.3**	**1.4**	**1.4**	876	97.1	4.78												
																	AAVENLPTFLVELSR	66	1	1	19923142	N-Term(TMT6plex)	1888.1	0	**1.5**	**1.5**	**1.6**	**1.5**
																	LAATNALLNSLEFTK	42	2	1	19923142	N-Term(TMT6plex); K15(TMT6plex)	2064.2	0	**1.3**	**1.1**	**1.3**	**1.2**
																	ESTLEAIGYICQDIDPEQLQDK	21	2	1	19923142	N-Term(TMT6plex); C11(Carbamidomethyl); K22(TMT6plex)	3023.5	0	**1.6**	**1.6**	**1.7**	**1.5**
																	SNEILTAIIQGMR	33	2	1	19923142	N-Term(TMT6plex); M12(Oxidation)	1690.9	0	**1.4**	**1.4**	**1.4**	**1.4**
																	HFIMQVVCEATQCPDTR	13	2	1	19923142	N-Term(TMT6plex); M4(Oxidation); C8(Carbamidomethyl); C13(Carbamidomethyl)	2337.1	0	**1.5**	**1.3**	**1.3**	**1.4**
																	WLAIDANAR	7	1	1	19923142	N-Term(TMT6plex)	1258.7	0	**1.3**	**1.2**	**1.3**	**1.3**
																	SSAYESLMEIVK	30	2	1	19923142	N-Term(TMT6plex); M8(Oxidation); K12(TMT6plex)	1831.0	0	**1.3**	**1.3**	**1.4**	**1.4**
																	VQHQDALQISDVVMASLLR	11	2	1	19923142	N-Term(TMT6plex); M14(Oxidation)	2368.3	0	**1.2**	**1.4**	**1.4**	**1.3**
																	YLEVVLNTLQQASQAQVDK	50	2	1	19923142	N-Term(TMT6plex); K19(TMT6plex)	2605.4	0	**1.3**	**1.1**	**1.4**	**1.1**
																	IQFNDLQSLLCATLQNVLR	8	2	1	19923142	N-Term(TMT6plex); C11(Carbamidomethyl)	2475.4	0	**2.0**	**2.1**	**2.5**	**2.2**
																	TLATWATK	14	2	1	19923142	N-Term(TMT6plex); K8(TMT6plex)	1349.8	0	**1.3**	**1.2**	**1.4**	**1.2**
																	HFIMQVVCEATQCPDTR	4	2	1	19923142	N-Term(TMT6plex); C8(Carbamidomethyl); C13(Carbamidomethyl)	2321.1	0	**1.4**	**1.4**	**1.7**	**1.7**
																	ICELLPEAAINDVYLAPLLQCLIEGLSAEPR	2	2	1	19923142	N-Term(TMT6plex); C2(Carbamidomethyl); C21(Carbamidomethyl)	3710.0	0	**2.5**	**2.5**	**3.6**	**2.0**
																	SVKPQILSVFGDIALAIGGEFK	4	2	1	19923142	N-Term(TMT6plex); K3(TMT6plex); K22(TMT6plex)	2976.8	0	**1.5**	**1.2**	**1.7**	**1.5**
																	DTAAWTVGR	4	2	1	19923142	N-Term(TMT6plex)	1205.6	0	**1.2**	**1.2**	**1.2**	**1.4**
																	YMEAFKPFLGIGLK	5	2	1	19923142	N-Term(TMT6plex); K6(TMT6plex); K14(TMT6plex)	2301.4	0	**1.4**	**1.7**	**1.9**	**1.7**
																	YMEAFKPFLGIGLK	8	2	1	19923142	N-Term(TMT6plex); M2(Oxidation); K6(TMT6plex); K14(TMT6plex)	2317.4	0	**1.3**	**1.4**	**1.6**	**1.6**
																	LQQVLQMESHIQSTSDR	18	2	1	19923142	N-Term(TMT6plex); M7(Oxidation)	2245.1	0	**1.4**	**1.3**	**1.3**	**1.3**
																	VLANPGNSQVAR	19	1	1	19923142	N-Term(TMT6plex)	1454.8	0	**1.4**	**1.2**	**1.3**	**1.3**
																	LELEAAQK	8	1	1	19923142	N-Term(TMT6plex); K8(TMT6plex)	1359.8	0	**1.2**	**1.1**	**1.3**	**1.2**
																	KEEPSNNVK	13	2	1	19923142	N-Term(TMT6plex); K1(TMT6plex); K9(TMT6plex)	1732.0	1	**1.2**	**1.3**	**1.3**	**1.4**
																	GDQENVHPDVMLVQPR	18	2	1	19923142	N-Term(TMT6plex); M11(Oxidation)	2079.1	0	**1.5**	**1.6**	**1.5**	**1.5**
																	ESCLEAYTGIVQGLK	32	2	1	19923142	N-Term(TMT6plex); C3(Carbamidomethyl); K15(TMT6plex)	2126.2	0	**1.2**	**1.3**	**1.4**	**1.4**
																	MFQSTAGSGGVQEDALMAVSTLVEVLGGEFLK	9	2	1	19923142	N-Term(TMT6plex); M1(Oxidation); M17(Oxidation); K32(TMT6plex)	3761.9	0	**1.1**	**0.7**	**0.9**	**1.0**
																	SDIDEVALQGIEFWSNVCDEEMDLAIEASEAAEQGRPPEHTSK	14	2	1	19923142	N-Term(TMT6plex); C18(Carbamidomethyl); M22(Oxidation); K43(TMT6plex)	5277.5	0	**1.1**	**1.3**	**1.7**	**1.3**
																	ALQSNIIPFCDEVMQLLLENLGNENVHR	5	2	1	19923142	N-Term(TMT6plex); C10(Carbamidomethyl)	3495.8	0	**1.4**	**1.6**	**1.5**	**1.4**
																	DAAVMAFGCILEGPEPSQLKPLVIQAMPTLIELMK	6	2	1	19923142	N-Term(TMT6plex); M5(Oxidation); C9(Carbamidomethyl); K20(TMT6plex); M27(Oxidation); M34(Oxidation); K35(TMT6plex)	4546.5	0	**1.1**	**1.3**	**1.2**	**1.5**
																	ALQSNIIPFCDEVMQLLLENLGNENVHR	38	2	1	19923142	N-Term(TMT6plex); C10(Carbamidomethyl); M14(Oxidation)	3511.8	0	**1.7**	**1.6**	**1.5**	**1.7**
																	SDYDMVDYLNELR	24	2	1	19923142	N-Term(TMT6plex); M5(Oxidation)	1877.9	0	**1.3**	**1.2**	**1.3**	**1.3**
																	SDIDEVALQGIEFWSNVCDEEMDLAIEASEAAEQGRPPEHTSK	2	2	1	19923142	N-Term(TMT6plex); C18(Carbamidomethyl); K43(TMT6plex)	5261.5	0	**1.5**	**1.9**	**1.4**	**1.6**
																	VAAGLQIK	8	1	1	19923142	N-Term(TMT6plex); K8(TMT6plex)	1257.8	0	**1.2**	**1.2**	**1.3**	**1.2**
																	NYVLQTLGTETYRPSSASQCVAGIACAEIPVNQWPELIPQLVANVTNPNSTEHMK	2	1	1	19923142	N-Term(TMT6plex); C20(Carbamidomethyl); C26(Carbamidomethyl); M54(Oxidation); K55(TMT6plex)	6571.3	0	**1.5**	**0.7**	**1.7**	**1.0**
																	SDYDMVDYLNELR	4	2	1	19923142	N-Term(TMT6plex)	1861.9	0	**1.6**	**1.7**	**2.0**	**1.5**
																	DAAVMAFGCILEGPEPSQLKPLVIQAMPTLIELMK	3	2	1	19923142	N-Term(TMT6plex); M5(Oxidation); C9(Carbamidomethyl); K20(TMT6plex); M34(Oxidation); K35(TMT6plex)	4530.5	0	**1.2**	**1.7**	**1.7**	**1.6**
																	GALQYLVPILTQTLTK	60	2	1	19923142	N-Term(TMT6plex); K16(TMT6plex)	2217.4	0	**1.2**	**1.3**	**1.4**	**1.4**
																	LLETTDRPDGHQNNLR	10	2	1	19923142	N-Term(TMT6plex)	2108.1	0	**1.2**	**1.2**	**1.3**	**1.2**
																	VQHQDALQISDVVMASLLR	22	2	1	19923142	N-Term(TMT6plex)	2352.3	0	**1.5**	**1.6**	**1.7**	**1.5**
																	VAALQNLVK	17	2	1	19923142	N-Term(TMT6plex); K9(TMT6plex)	1413.9	0	**1.2**	**1.1**	**1.2**	**1.1**
																	GDQENVHPDVMLVQPR	11	2	1	19923142	N-Term(TMT6plex)	2063.1	0	**1.5**	**1.4**	**1.5**	**1.5**
																	EEPSNNVK	6	2	1	19923142	N-Term(TMT6plex); K8(TMT6plex)	1374.8	0	**1.3**	**1.3**	**1.3**	**1.4**
																	LQQVLQMESHIQSTSDR	2	2	1	19923142	N-Term(TMT6plex)	2229.1	0	**1.5**	**1.5**	**1.5**	**1.4**
																	SNEILTAIIQGMR	4	2	1	19923142	N-Term(TMT6plex)	1674.9	0	**2.2**	**2.1**	**2.3**	**2.2**
																	SSAYESLMEIVK	4	2	1	19923142	N-Term(TMT6plex); K12(TMT6plex)	1815.0	0	**1.5**	**1.7**	**1.9**	**1.4**
																	YLEVVLNTLQQASQAQVDKSDYDMVDYLNELR	2	2	1	19923142	N-Term(TMT6plex); K19(TMT6plex); M24(Oxidation)	4235.1	1	**1.5**	**1.5**	**1.5**	**1.6**
																	TVSPDRLELEAAQK	9	1	1	19923142	N-Term(TMT6plex); K14(TMT6plex)	2015.2	1	**1.3**	**1.3**	**1.4**	**1.4**
																	DCYPAVQK	15	2	1	19923142	N-Term(TMT6plex); C2(Carbamidomethyl); K8(TMT6plex)	1438.8	0	**1.3**	**1.6**	**1.6**	**1.4**

Accession	RefSeq Accession	Gene Symbol	Entrez Gene ID	Description	Σ Coverage	Σ# Proteins	Σ# Unique Peptides	Σ# Peptides	Σ# PSMs	Het-1A-Smoke - 2M/Parental	Het-1A-Smoke - 4M/Parental	Het-1A-Smoke - 6M/Parental	Het-1A-Smoke - 8M/Parental	# AAs	MW [kDa]	calc. pI	Sequence	# PSMs	# Proteins	# Protein Groups	Protein Group Accessions	Modifications	MH+ [Da]	# Missed Cleavages	Het-1A-Smoke - 2M/Parental	Het-1A-Smoke - 4M/Parental	Het-1A-Smoke - 6M/Parental	Het-1A-Smoke - 8M/Parental
																	KVQHQDALQISDVVMASLLR	1	2	1	19923142	N-Term(TMT6plex); K1(TMT6plex); M15(Oxidation)	2725.5	1	**1.4**	**1.3**	**1.6**	**1.5**
																	KYLEVVLNTLQQASQAQVDK	4	2	1	19923142	N-Term(TMT6plex); K1(TMT6plex); K20(TMT6plex)	2962.7	1	**1.2**	**1.4**	**1.5**	**1.4**
																	QDENDDDDDWNPCK	10	2	1	19923142	N-Term(TMT6plex); C13(Carbamidomethyl); K14(TMT6plex)	2224.0	0	**1.2**	**1.3**	**1.4**	**1.3**
																	NYAEYQVCLAAVGLVGDLCR	2	2	1	19923142	N-Term(TMT6plex); C8(Carbamidomethyl); C19(Carbamidomethyl)	2500.2	0	**1.3**	**1.4**	**1.4**	**1.3**
																	TTLVIMER	7	2	1	19923142	N-Term(TMT6plex); M6(Oxidation)	1207.7	0	**1.3**	**1.1**	**1.2**	**1.2**
																	EHIKNPDWR	1	2	1	19923142	N-Term(TMT6plex); K4(TMT6plex)	1652.9	1	**1.2**	**2.0**	**1.4**	**2.1**
																	ANFDKESER	2	2	1	19923142	N-Term(TMT6plex); K5(TMT6plex)	1553.8	1	**1.2**	**1.5**	**1.4**	**1.6**
																	AQYQQR	2	1	1	19923142	N-Term(TMT6plex)	1022.6	0	**1.3**	**1.4**	**1.3**	**1.4**
4506185	NP_002780.1	**PSMA4**	5685	**proteasome subunit alpha type-4 isoform 1**	75.10	2	15	15	205	**1.5**	**1.4**	**1.5**	**1.6**	261	29.5	7.72												
																	YLLQYQEPIPCEQLVTALCDIK	42	2	1	4506185	N-Term(TMT6plex); C11(Carbamidomethyl); C19(Carbamidomethyl); K22(TMT6plex)	3152.7	0	**1.7**	**1.6**	**1.7**	**1.5**
																	EVEQLIK	11	2	1	4506185	N-Term(TMT6plex); K7(TMT6plex)	1316.8	0	**1.3**	**1.4**	**1.4**	**1.6**
																	KHEEEEAK	10	2	1	4506185	N-Term(TMT6plex); K1(TMT6plex); K8(TMT6plex)	1687.0	1	**1.3**	**1.5**	**1.4**	**1.7**
																	VEIATLTR	8	2	1	4506185	N-Term(TMT6plex)	1131.7	0	**1.2**	**1.3**	**1.3**	**1.4**
																	LNEDMACSVAGITSDANVLTNELR	14	1	1	4506185	N-Term(TMT6plex); M5(Oxidation); C7(Carbamidomethyl)	2838.4	0	**1.4**	**1.4**	**1.4**	**1.5**
																	TTIFSPEGR	6	1	1	4506185	N-Term(TMT6plex)	1236.7	0	**1.5**	**1.4**	**1.5**	**1.6**
																	LYQVEYAMEAIGHAGTCLGILANDGVLLAAER	26	1	1	4506185	N-Term(TMT6plex); M8(Oxidation); C17(Carbamidomethyl)	3663.9	0	**1.3**	**1.2**	**1.4**	**1.3**
																	LLDEVFFSEK	48	1	1	4506185	N-Term(TMT6plex); K10(TMT6plex)	1685.0	0	**1.4**	**1.5**	**1.5**	**1.7**
																	RPFGVSLLYIGWDK	9	2	1	4506185	N-Term(TMT6plex); K14(TMT6plex)	2109.2	0	**1.4**	**1.3**	**1.6**	**1.4**
																	SALALAIK	11	2	1	4506185	N-Term(TMT6plex); K8(TMT6plex)	1244.8	0	**1.4**	**1.4**	**1.5**	**1.5**
																	LYQVEYAMEAIGHAGTCLGILANDGVLLAAER	4	1	1	4506185	N-Term(TMT6plex); C17(Carbamidomethyl)	3647.9	0	**1.7**	**1.5**	**1.8**	**1.7**
																	QAYTQFGGK	3	2	1	4506185	N-Term(TMT6plex); K9(TMT6plex)	1457.8	0	**1.6**	**1.5**	**1.6**	**1.6**
																	KHEEEEAKAER	1	2	1	4506185	N-Term(TMT6plex); K1(TMT6plex); K8(TMT6plex)	2043.1	2	**1.5**	**2.1**	**1.8**	**2.0**
																	HYGFQLYQSDPSGNYGGWK	2	2	1	4506185	N-Term(TMT6plex); K19(TMT6plex)	2662.3	0	**1.6**	**1.3**	**1.4**	**1.6**
																	LNEDMACSVAGITSDANVLTNELR	5	1	1	4506185	N-Term(TMT6plex); C7(Carbamidomethyl)	2822.4	0	**1.4**	**1.7**	**1.6**	**1.5**
																	ATCIGNNSAAAVSMLK	4	2	1	4506185	N-Term(TMT6plex); C3(Carbamidomethyl); K16(TMT6plex)	2066.1	0	**1.7**	**1.8**	**1.8**	**1.6**
																	EGEMTLK	1	2	1	4506185	N-Term(TMT6plex); K7(TMT6plex)	1265.7	0	**1.4**	**1.4**	**1.6**	**1.3**
4758384;224809329	NP_004108.1	**FKBP5**	2289	**peptidyl-prolyl cis-trans isomerase FKBP5 isoform 1**	75.05	2	34	35	369	**1.3**	**1.3**	**1.3**	**1.4**	457	51.2	5.90												
																	GEDLFEDGGIIR	16	2	1	4758384;224809329	N-Term(TMT6plex)	1549.8	0	**1.4**	**1.4**	**1.4**	**1.3**
																	NNEESPTATVAEQGEDITSK	12	2	1	4758384;224809329	N-Term(TMT6plex); K20(TMT6plex)	2578.3	0	**1.5**	**1.2**	**1.3**	**1.4**
																	IVSWLEMEYGLSEK	28	1	1	4758384;224809329	N-Term(TMT6plex); M7(Oxidation); K14(TMT6plex)	2158.2	0	**1.4**	**1.5**	**1.5**	**1.5**
																	AWDIGVATMK	21	2	1	4758384;224809329	N-Term(TMT6plex); M9(Oxidation); K10(TMT6plex)	1565.9	0	**1.4**	**1.4**	**1.3**	**1.4**
																	DVAFTVGEGEDHDIPIGIDK	26	2	1	4758384;224809329	N-Term(TMT6plex); K20(TMT6plex)	2585.3	0	**1.3**	**1.4**	**1.3**	**1.5**
																	FAEQDAKEEANK	5	1	1	4758384;224809329	N-Term(TMT6plex); K7(TMT6plex); K12(TMT6plex)	2067.1	1	**1.3**	**1.4**	**1.3**	**1.4**
																	KGEGYSNPNEGATVEIHLEGR	10	2	1	4758384;224809329	N-Term(TMT6plex); K1(TMT6plex)	2715.4	1	**1.2**	**1.2**	**1.4**	**1.4**
																	FGIEPNAELIYEVTLK	56	1	1	4758384;224809329	N-Term(TMT6plex); K16(TMT6plex)	2294.3	0	**1.3**	**1.4**	**1.4**	**1.3**
																	EKLEQAAIVK	8	1	1	4758384;224809329	N-Term(TMT6plex); K2(TMT6plex); K10(TMT6plex)	1816.2	1	**1.1**	**1.3**	**1.3**	**1.4**
																	KGEICHLLCKPEYAYGSAGSLPK	3	2	1	4758384;224809329	N-Term(TMT6plex); K1(TMT6plex); C5(Carbamidomethyl); C9(Carbamidomethyl); K10(TMT6plex); K23(TMT6plex)	3494.9	1	**1.1**	**1.4**	**1.3**	**1.4**
																	ALGLDSANEK	19	1	1	4758384;224809329	N-Term(TMT6plex); K10(TMT6plex)	1475.8	0	**1.3**	**1.3**	**1.3**	**1.4**
																	TSEGVTNEK	10	1	1	4758384;224809329	N-Term(TMT6plex); K9(TMT6plex)	1422.8	0	**1.2**	**1.0**	**1.2**	**1.1**
																	ESWEMDTK	10	1	1	4758384;224809329	N-Term(TMT6plex); M5(Oxidation); K8(TMT6plex)	1499.7	0	**1.3**	**1.4**	**1.3**	**1.4**
																	GTDSQAMEEEKPEGHV	12	1	1	4758384;224809329	N-Term(TMT6plex); M7(Oxidation); K11(TMT6plex)	2218.1	0	**1.3**	**1.3**	**1.3**	**1.4**
																	YGFGEAGKPK	2	1	1	4758384;224809329	N-Term(TMT6plex); K8(TMT6plex); K10(TMT6plex)	1741.0	0	**1.2**	**1.2**	**1.3**	**1.3**
																	IVSWLEMEYGLSEK	12	1	1	4758384;224809329	N-Term(TMT6plex); K14(TMT6plex)	2142.2	0	**1.7**	**1.7**	**1.9**	**1.5**
																	NEPFVFSLGK	10	2	1	4758384;224809329	N-Term(TMT6plex); K10(TMT6plex)	1595.9	0	**1.3**	**1.1**	**1.3**	**1.2**
																	AWDIGVATMK	4	2	1	4758384;224809329	N-Term(TMT6plex); K10(TMT6plex)	1549.9	0	**1.6**	**1.9**	**1.8**	**1.7**
																	KTSEGVTNEK	3	1	1	4758384;224809329	N-Term(TMT6plex); K1(TMT6plex); K10(TMT6plex)	1780.0	1	**1.3**	**1.6**	**1.4**	**1.5**
																	RGEAQLLMNEFESAK	11	1	1	4758384;224809329	N-Term(TMT6plex); K15(TMT6plex)	2181.2	1	**1.4**	**1.4**	**1.5**	**1.3**
																	FDSSHDRNEPFVFSLGK	1	2	1	4758384;224809329	N-Term(TMT6plex); K17(TMT6plex)	2440.3	1	**1.4**	**1.6**	**1.3**	**1.5**
																	RGEAQLLMNEFESAK	7	1	1	4758384;224809329	N-Term(TMT6plex); M8(Oxidation); K15(TMT6plex)	2197.2	1	**1.3**	**1.3**	**1.3**	**1.3**
																	GEGYSNPNEGATVEIHLEGR	2	2	1	4758384;224809329	N-Term(TMT6plex)	2358.2	0	**1.5**	**1.3**	**1.4**	**1.4**
																	VLEVNPQNK	11	1	1	4758384;224809329	N-Term(TMT6plex); K9(TMT6plex)	1498.9	0	**1.3**	**1.3**	**1.4**	**1.4**
																	EEQCILYLGPR	35	1	1	4758384;224809329	N-Term(TMT6plex); C4(Carbamidomethyl)	1606.8	0	**1.3**	**1.4**	**1.4**	**1.4**
																	YMQAVIQYGK	8	1	1	4758384;224809329	N-Term(TMT6plex); M2(Oxidation); K10(TMT6plex)	1674.9	0	**1.2**	**1.1**	**1.2**	**1.2**
																	LEQAAIVK	3	1	1	4758384;224809329	N-Term(TMT6plex); K8(TMT6plex)	1329.9	0	**1.3**	**1.3**	**1.4**	**1.3**
																	KFAEQDAKEEANK	1	1	1	4758384;224809329	N-Term(TMT6plex); K1(TMT6plex); K8(TMT6plex); K13(TMT6plex)	2424.4	2	**1.4**	**1.6**	**1.7**	**1.7**
																	LQISMCQK	4	1	1	4758384;224809329	N-Term(TMT6plex); M5(Oxidation); C6(Carbamidomethyl); K8(TMT6plex)	1481.8	0	**1.3**	**1.2**	**1.2**	**1.4**
																	KFAEQDAK	1	1	1	4758384;224809329	N-Term(TMT6plex); K1(TMT6plex); K8(TMT6plex)	1624.0	1	**1.1**	**1.2**	**1.2**	**1.4**
																	ASESFLLAAFLNLAMCYLK	1	1	1	4758384;224809329	N-Term(TMT6plex); C16(Carbamidomethyl); K19(TMT6plex)	2620.4	0	**1.6**	**1.1**	**2.0**	**0.9**
																	IYANMFK	2	1	1	4758384;224809329	N-Term(TMT6plex); K7(TMT6plex)	1344.8	0	**1.2**	**1.1**	**1.2**	**1.0**
																	FAEQDAK	4	1	1	4758384;224809329	N-Term(TMT6plex); K7(TMT6plex)	1266.7	0	**1.4**	**1.2**	**1.2**	**1.3**
																	TTDEGAK	1	2	1	4758384;224809329	N-Term(TMT6plex); K7(TMT6plex)	1179.7	0	**1.5**	**1.2**	**1.2**	**1.4**
																	GTVYFK	4	2	2	4503729;4758384;224809329	N-Term(TMT6plex); K6(TMT6plex)	1172.7	0				
																	VGNGEETPMIGDK	2	2	1	4758384;224809329	N-Term(TMT6plex); K13(TMT6plex)	1804.9	0	**2.4**	**2.0**	**2.2**	**1.6**
																	VYVHYK	2	2	1	4758384;224809329	N-Term(TMT6plex); K6(TMT6plex)	1266.8	0	**1.2**	**1.3**	**1.3**	**1.3**
																	VGNGEETPMIGDK	1	2	1	4758384;224809329	N-Term(TMT6plex); M9(Oxidation); K13(TMT6plex)	1820.9	0	**1.3**	**1.2**	**1.3**	**1.2**
																	AKESWEMDTK	1	1	1	4758384;224809329	N-Term(TMT6plex); K2(TMT6plex); M7(Oxidation); K10(TMT6plex)	1928.0	1	**1.3**	**1.4**	**1.2**	**1.6**
71773329	NP_001146.2	**ANXA6**	309	**annexin A6 isoform 1**	75.04	2	47	47	434	**1.5**	**1.6**	**1.8**	**1.6**	673	75.8	5.60												
																	LSGGDDDAAGQFFPEAAQVAYQMWELSAVAR	14	2	1	71773329	N-Term(TMT6plex); M23(Oxidation)	3545.7	0	**1.6**	**1.4**	**1.4**	**1.5**
																	SLHQAIEGDTSGDFLK	12	2	1	71773329	N-Term(TMT6plex); K16(TMT6plex)	2176.2	0	**1.3**	**1.5**	**1.6**	**1.4**
																	EDAQVAAEILEIADTPSGDK	28	2	1	71773329	N-Term(TMT6plex); K20(TMT6plex)	2530.3	0	**1.5**	**1.6**	**1.8**	**1.7**
																	GSIHDFPGFDPNQDAEALYTAMK	26	1	1	71773329	N-Term(TMT6plex); M22(Oxidation); K23(TMT6plex)	2998.5	0	**1.4**	**1.7**	**1.7**	**1.6**
																	LVFDEYLK	16	2	1	71773329	N-Term(TMT6plex); K8(TMT6plex)	1484.9	0	**1.6**	**1.7**	**2.0**	**1.7**
																	EAILDIITSR	8	2	1	71773329	N-Term(TMT6plex)	1359.8	0	**1.5**	**1.6**	**1.8**	**1.5**
																	TNEQMHQLVAAYK	8	2	1	71773329	N-Term(TMT6plex); M5(Oxidation); K13(TMT6plex)	2007.1	0	**1.3**	**1.3**	**1.6**	**1.5**
																	SEIDLLNIR	8	2	1	71773329	N-Term(TMT6plex)	1301.8	0	**1.6**	**1.8**	**1.9**	**1.7**
																	STPEYFAER	10	2	1	71773329	N-Term(TMT6plex)	1328.7	0	**1.4**	**1.5**	**1.7**	**1.5**
																	GLGTDEDTIIDIITHR	35	2	1	71773329	N-Term(TMT6plex)	1998.1	0	**1.6**	**1.5**	**2.0**	**1.6**
																	LSGGDDDAAGQFFPEAAQVAYQMWELSAVAR	6	2	1	71773329	N-Term(TMT6plex)	3529.7	0	**1.3**	**1.4**	**1.6**	**1.3**
																	AMEGAGTDEK	18	2	1	71773329	N-Term(TMT6plex); M2(Oxidation); K10(TMT6plex)	1482.8	0	**1.4**	**1.7**	**1.8**	**1.6**
																	QEVCQSYK	8	2	1	71773329	N-Term(TMT6plex); C4(Carbamidomethyl); K8(TMT6plex)	1499.8	0	**1.7**	**1.7**	**2.1**	**1.6**
																	ALIEILATR	10	2	1	71773329	N-Term(TMT6plex)	1228.8	0	**1.6**	**1.6**	**1.8**	**1.5**
																	NDTSGEYK	4	2	1	71773329	N-Term(TMT6plex); K8(TMT6plex)	1371.7	0	**1.7**	**1.1**	**2.0**	**1.1**
																	AINEAYK	4	2	1	71773329	N-Term(TMT6plex); K7(TMT6plex)	1266.7	0	**1.5**	**1.5**	**1.9**	**1.5**
																	AINEAYKEDYHK	3	2	1	71773329	N-Term(TMT6plex); K7(TMT6plex); K12(TMT6plex)	2168.2	1	**1.4**	**1.8**	**2.0**	**1.8**
																	EEGGENLDQAR	6	2	1	71773329	N-Term(TMT6plex)	1446.7	0	**1.5**	**1.8**	**1.8**	**1.6**
																	GSIHDFPGFDPNQDAEALYTAMK	2	1	1	71773329	N-Term(TMT6plex); K23(TMT6plex)	2982.5	0	**1.2**	**1.6**	**1.6**	**1.1**
																	DAISGIGTDEK	16	2	1	71773329	N-Term(TMT6plex); K11(TMT6plex)	1563.9	0	**1.4**	**1.6**	**1.7**	**1.5**
																	GFGSDKEAILDIITSR	2	2	1	71773329	N-Term(TMT6plex); K6(TMT6plex)	2180.2	1	**1.5**	**2.6**	**2.2**	**2.4**
																	EEDDVVSEDLVQQDVQDLYEAGELK	20	2	1	71773329	N-Term(TMT6plex); K25(TMT6plex)	3323.6	0	**1.5**	**1.7**	**1.8**	**1.6**
																	MLVVLLQGTREEDDVVSEDLVQQDVQDLYEAGELK	2	2	1	71773329	N-Term(TMT6plex); M1(Oxidation); K35(TMT6plex)	4450.3	1	**1.4**	**1.6**	**1.6**	**1.8**
																	VFQEFIK	6	2	1	71773329	N-Term(TMT6plex); K7(TMT6plex)	1368.8	0	**1.5**	**1.5**	**1.8**	**1.5**
																	DAFVAIVQSVK	22	2	1	71773329	N-Term(TMT6plex); K11(TMT6plex)	1635.0	0	**1.5**	**1.7**	**2.0**	**1.7**
																	MLVVLLQGTR	4	2	1	71773329	N-Term(TMT6plex)	1358.8	0	**1.7**	**1.8**	**2.5**	**1.5**
																	ILISLATGHR	11	2	1	71773329	N-Term(TMT6plex)	1309.8	0	**1.4**	**1.4**	**1.6**	**1.4**
																	LILGLMMPPAHYDAK	19	2	1	71773329	N-Term(TMT6plex); M6(Oxidation); M7(Oxidation); K15(TMT6plex)	2160.2	0	**1.3**	**1.4**	**1.3**	**1.5**
																	CLIEILASR	10	2	1	71773329	N-Term(TMT6plex); C1(Carbamidomethyl)	1303.8	0	**1.8**	**1.6**	**2.1**	**1.6**
																	SELDMLDIR	6	2	1	71773329	N-Term(TMT6plex); M5(Oxidation)	1336.7	0	**1.6**	**1.6**	**1.7**	**1.5**
																	NKPLFFADK	6	2	1	71773329	N-Term(TMT6plex); K2(TMT6plex); K9(TMT6plex)	1767.1	0	**1.4**	**1.4**	**1.9**	**1.5**
																	MLVVLLQGTR	2	2	1	71773329	N-Term(TMT6plex); M1(Oxidation)	1374.8	0	**1.3**	**1.3**	**1.4**	**1.4**
																	SLEDALSSDTSGHFR	6	2	1	71773329	N-Term(TMT6plex)	1850.9	0	**1.3**	**1.5**	**1.6**	**1.5**
																	GELSGDFEK	6	2	1	71773329	N-Term(TMT6plex); K9(TMT6plex)	1439.8	0	**1.5**	**1.9**	**2.1**	**1.9**
																	MTNYDVEHTIK	3	2	1	71773329	N-Term(TMT6plex); M1(Oxidation); K11(TMT6plex)	1825.0	0	**1.3**	**1.4**	**1.6**	**1.4**
																	DLEADIIGDTSGHFQK	14	2	1	71773329	N-Term(TMT6plex); K16(TMT6plex)	2204.1	0	**1.5**	**1.8**	**1.9**	**1.7**
																	TNEQMHQLVAAYK	1	2	1	71773329	N-Term(TMT6plex); K13(TMT6plex)	1991.1	0	**1.4**	**1.6**	**1.7**	**1.5**
																	WGTDEAQFIYILGNR	14	2	1	71773329	N-Term(TMT6plex)	2012.0	0	**1.3**	**1.3**	**1.4**	**1.3**
																	KAMEGAGTDEK	1	2	1	71773329	N-Term(TMT6plex); K1(TMT6plex); M3(Oxidation); K11(TMT6plex)	1840.0	1	**1.3**	**1.7**	**1.7**	**1.7**

Khan et al., 2019. Multi-omics analysis to characterize cigarette smoke induced molecular alterations in esophageal cells

Supplementary Table 6. List of all proteins and peptides identified in untreated and chronically treated Het1A cells with cigarette smoke condensate for 8 months

Accession	RefSeq Accession	Gene Symbol	Entrez Gene ID	Description	Σ Coverage	Σ# Proteins	Σ# Unique Peptides	Σ# Peptides	Σ# PSMs	Het-1A-Smoke - 2M/Parental	Het-1A-Smoke - 4M/Parental	Het-1A-Smoke - 6M/Parental	Het-1A-Smoke - 8M/Parental	# AAs	MW [kDa]	calc. pI	Sequence	# PSMs	# Proteins	# Protein Groups	Protein Group Accessions	Modifications	MH+ [Da]	# Missed Cleavages	Het-1A-Smoke - 2M/Parental	Het-1A-Smoke - 4M/Parental	Het-1A-Smoke - 6M/Parental	Het-1A-Smoke - 8M/Parental
																	ALLALCGGED	2	2	1	71773329	N-Term(TMT6plex); C6(Carbamidomethyl)	1247.6	0	**1.3**	**1.5**	**1.6**	**1.5**
																	SLYSMIK	2	2	1	71773329	N-Term(TMT6plex); K7(TMT6plex)	1299.8	0	**1.8**	**2.6**	**2.6**	**1.8**
																	GTVRPANDFNPDADAK	6	2	1	71773329	N-Term(TMT6plex); K16(TMT6plex)	2146.1	0	**1.7**	**1.6**	**1.6**	**1.7**
																	GAGTDEK	2	2	1	71773329	N-Term(TMT6plex); K7(TMT6plex)	1135.6	0	**1.4**	**1.5**	**1.3**	**1.2**
																	LIVGLMRPPAYCDAK	1	2	1	71773329	N-Term(TMT6plex); M6(Oxidation); C12(Carbamidomethyl); K15(TMT6plex)	2178.2	0	**1.6**	**2.2**	**1.8**	**2.5**
																	LMLAVVK	5	2	1	71773329	N-Term(TMT6plex); M2(Oxidation); K7(TMT6plex)	1247.8	0	**1.3**	**1.4**	**1.5**	**1.5**
																	DLIADLK	14	2	1	71773329	N-Term(TMT6plex); K7(TMT6plex)	1245.8	0	**1.5**	**1.9**	**2.0**	**1.7**
																	NDTSGEYKK	1	2	1	71773329	N-Term(TMT6plex); K8(TMT6plex); K9(TMT6plex)	1729.0	1	**1.4**	**1.4**	**2.1**	**1.3**
																	SEISGDLAR	1	2	1	71773329	N-Term(TMT6plex)	1176.6	0	**8.8**	**2.1**	**1.6**	**1.5**
																	EMSGDVR	1	2	1	71773329	N-Term(TMT6plex); M2(Oxidation)	1038.5	0	**2.2**	**2.6**	**3.2**	**1.9**
																	YELTGK	1	2	1	71773329	N-Term(TMT6plex); K6(TMT6plex)	1168.7	0	**1.7**	**1.6**	**1.8**	**1.5**
																	TTGKPIEASIR	1	2	1	71773329	N-Term(TMT6plex); K4(TMT6plex)	1631.0	0	**1.0**	**1.1**	**1.3**	**1.1**
48762932	NP_006576.2	**CCT8**	10694	**T-complex protein 1 subunit theta isoform 1**	74.82	4	38	38	651	**1.4**	**1.4**	**1.5**	**1.6**	548	59.6	5.60												
																	GSTDNLMDDIER	14	4	1	48762932	N-Term(TMT6plex); M7(Oxidation)	1610.8	0	**1.5**	**1.7**	**1.7**	**1.9**
																	NVGLDIEAEVPAVK	38	4	1	48762932	N-Term(TMT6plex); K14(TMT6plex)	1912.1	0	**1.4**	**1.3**	**1.5**	**1.4**
																	HEKEDGAISTIVLR	9	4	1	48762932	N-Term(TMT6plex); K3(TMT6plex)	2026.2	1	**1.4**	**1.5**	**1.5**	**1.6**
																	TAEELMNFSK	19	4	1	48762932	N-Term(TMT6plex); M6(Oxidation); K10(TMT6plex)	1643.9	0	**1.3**	**1.3**	**1.3**	**1.6**
																	ETEGDVTSVK	31	4	1	48762932	N-Term(TMT6plex); K10(TMT6plex)	1522.8	0	**1.4**	**1.5**	**1.5**	**1.7**
																	GEENLMDAQVK	54	4	1	48762932	N-Term(TMT6plex); M6(Oxidation); K11(TMT6plex)	1707.9	0	**1.4**	**1.6**	**1.7**	**1.6**
																	ANEVISK	8	4	1	48762932	N-Term(TMT6plex); K7(TMT6plex)	1218.7	0	**1.2**	**1.4**	**1.3**	**1.5**
																	KETEGDVTSVK	5	4	1	48762932	N-Term(TMT6plex); K1(TMT6plex); K11(TMT6plex)	1880.1	1	**1.2**	**1.3**	**1.4**	**1.6**
																	ELAQTTR	4	2	1	48762932	N-Term(TMT6plex)	1047.6	0	**1.4**	**1.6**	**1.5**	**1.9**
																	AVDDGVNTFK	32	4	1	48762932	N-Term(TMT6plex); K10(TMT6plex)	1523.8	0	**1.3**	**1.4**	**1.5**	**1.6**
																	AIADTGANVVVTGGK	34	4	1	48762932	N-Term(TMT6plex); K15(TMT6plex)	1831.1	0	**1.3**	**1.5**	**1.5**	**1.6**
																	DIDEVSSLLR	29	4	1	48762932	N-Term(TMT6plex)	1375.8	0	**1.4**	**1.5**	**1.5**	**1.6**
																	IAVYSCPFDGMITETK	30	4	1	48762932	N-Term(TMT6plex); C6(Carbamidomethyl); M11(Oxidation); K16(TMT6plex)	2306.2	0	**1.5**	**1.5**	**1.6**	**1.5**
																	LYAVHQEGNK	13	4	1	48762932	N-Term(TMT6plex); K10(TMT6plex)	1616.9	0	**1.5**	**1.5**	**1.5**	**1.6**
																	LFVTNDAATILR	11	3	1	48762932	N-Term(TMT6plex)	1562.9	0	**1.5**	**1.5**	**1.6**	**1.7**
																	LATNAAVTVLR	10	4	1	48762932	N-Term(TMT6plex)	1357.8	0	**1.5**	**1.3**	**1.5**	**1.5**
																	DMLEAGILDTYLGK	35	4	1	48762932	N-Term(TMT6plex); M2(Oxidation); K14(TMT6plex)	2013.1	0	**1.3**	**1.4**	**1.4**	**1.7**
																	ALAENSGVK	27	4	1	48762932	N-Term(TMT6plex); K9(TMT6plex)	1346.8	0	**1.3**	**1.4**	**1.4**	**1.5**
																	VADMALHYANK	10	4	1	48762932	N-Term(TMT6plex); M4(Oxidation); K11(TMT6plex)	1706.9	0	**1.4**	**1.5**	**1.4**	**1.6**
																	QITSYGETCPGLEQYAIK	12	4	1	48762932	N-Term(TMT6plex); C9(Carbamidomethyl); K18(TMT6plex)	2516.3	0	**1.4**	**1.5**	**1.6**	**1.6**
																	LTPPVLEEMGHCDSVYLSEVGDTQVVVFK	29	4	1	48762932	N-Term(TMT6plex); M9(Oxidation); C12(Carbamidomethyl); K29(TMT6plex)	3722.9	0	**1.5**	**1.6**	**1.7**	**1.7**
																	ILGSGISSSSVLHGMVFK	11	4	1	48762932	N-Term(TMT6plex); M15(Oxidation); K18(TMT6plex)	2293.3	0	**1.5**	**1.4**	**1.5**	**1.4**
																	LVPGGGATEIELAK	52	4	1	48762932	N-Term(TMT6plex); K14(TMT6plex)	1813.1	0	**1.4**	**1.5**	**1.5**	**1.5**
																	LIAQACVSIFPDSGHFNVDNIR	2	4	1	48762932	N-Term(TMT6plex); C6(Carbamidomethyl)	2702.4	0	**1.7**	**1.7**	**1.6**	**1.7**
																	AHEILPNLVCCSAK	2	4	1	48762932	N-Term(TMT6plex); C10(Carbamidomethyl); C11(Carbamidomethyl); K14(TMT6plex)	2070.1	0	**1.3**	**1.4**	**1.6**	**1.8**
																	GEENLMDAQVK	2	4	1	48762932	N-Term(TMT6plex); K11(TMT6plex)	1691.9	0	**1.8**	**2.2**	**2.3**	**1.7**
																	TVGATALPR	6	4	1	48762932	N-Term(TMT6plex)	1114.7	0	**1.5**	**1.3**	**1.4**	**1.4**
																	KFAEAFEAIPR	2	4	1	48762932	N-Term(TMT6plex); K1(TMT6plex)	1737.0	1	**1.3**	**1.5**	**1.5**	**1.7**
																	IAVYSCPFDGMITETK	13	4	1	48762932	N-Term(TMT6plex); C6(Carbamidomethyl); K16(TMT6plex)	2290.2	0	**2.1**	**2.0**	**2.5**	**1.7**
																	HFSGLEEAVYR	23	2	1	48762932	N-Term(TMT6plex)	1536.8	0	**1.3**	**1.3**	**1.4**	**1.3**
																	VADMALHYANK	4	4	1	48762932	N-Term(TMT6plex); K11(TMT6plex)	1690.9	0	**1.5**	**1.5**	**1.6**	**1.5**
																	FAEAFEAIPR	22	4	1	48762932	N-Term(TMT6plex)	1379.8	0	**1.6**	**1.4**	**1.6**	**1.6**
																	QYGNEVFLAK	10	4	1	48762932	N-Term(TMT6plex); K10(TMT6plex)	1626.9	0	**1.3**	**1.2**	**1.4**	**1.5**
																	ELEVQHPAAK	16	3	1	48762932	N-Term(TMT6plex); K10(TMT6plex)	1579.9	0	**1.1**	**1.4**	**1.3**	**1.4**
																	DMLEAGILDTYLGK	2	4	1	48762932	N-Term(TMT6plex); K14(TMT6plex)	1997.1	0	**1.9**	**2.2**	**2.7**	**2.0**
																	KAHEILPNLVCCSAK	2	4	1	48762932	N-Term(TMT6plex); K1(TMT6plex); C11(Carbamidomethyl); C12(Carbamidomethyl); K15(TMT6plex)	2427.4	1	**1.3**	**1.7**	**1.8**	**1.6**
																	MVINHLEK	5	3	1	48762932	N-Term(TMT6plex); M1(Oxidation); K8(TMT6plex)	1457.9	0	**1.4**	**1.4**	**1.3**	**1.5**
																	EDGAISTIVLR	5	4	1	48762932	N-Term(TMT6plex)	1402.8	0	**1.4**	**1.3**	**1.4**	**1.5**
																	ILGSGISSSSVLHGMVFK	2	4	1	48762932	N-Term(TMT6plex); K18(TMT6plex)	2277.3	0	**1.7**	**1.7**	**2.0**	**1.7**
																	APGFAQMLK	7	1	1	48762932	N-Term(TMT6plex); M7(Oxidation); K9(TMT6plex)	1436.8	0	**1.3**	**1.4**	**1.4**	**1.6**
																	GTVLIK	4	4	1	48762932	N-Term(TMT6plex); K6(TMT6plex)	1088.7	0	**1.3**	**1.5**	**1.5**	**1.6**
																	YNIMLVR	2	4	1	48762932	N-Term(TMT6plex)	1137.7	0	**1.7**	**1.8**	**2.3**	**1.7**
																	YNIMLVR	2	4	1	48762932	N-Term(TMT6plex); M4(Oxidation)	1153.7	0	**1.6**	**1.5**	**1.8**	**1.5**
																	KDWDDDQND	1	4	1	48762932	N-Term(TMT6plex); K1(TMT6plex)	1608.8	1	**1.3**	**1.2**	**1.2**	**1.4**
48255889	NP_002734.2	**PRKCSH**	5589	**glucosidase 2 subunit beta isoform 1 precursor**	74.62	2	1	27	534	**1.2**	**1.5**	**1.0**	**1.0**	528	59.4	4.41												
																	ESLQQMAEVTR	56	3	2	48255889;574280091	N-Term(TMT6plex); M6(Oxidation)	1536.8	0				
																	MPPYDEQTQAFIDAAQEAR	40	3	2	48255889;574280091	N-Term(TMT6plex); M1(Oxidation)	2426.1	0				
																	SLEDQVEMLR	39	3	2	48255889;574280091	N-Term(TMT6plex); M8(Oxidation)	1464.8	0				
																	CEYLMELMTPAACPEPPPEAPTEDDHDEL	10	3	2	48255889;574280091	N-Term(TMT6plex); C1(Carbamidomethyl); M5(Oxidation); M8(Oxidation); C13(Carbamidomethyl)	3618.5	0				
																	SEALPTDLPAPSAPDLTEPK	12	3	2	48255889;574280091	N-Term(TMT6plex); K20(TMT6plex)	2507.4	0				
																	TVKEEAEKPER	6	3	2	48255889;574280091	N-Term(TMT6plex); K3(TMT6plex); K8(TMT6plex)	2003.2	1				
																	KLIELQAGK	13	3	2	48255889;574280091	N-Term(TMT6plex); K1(TMT6plex); K9(TMT6plex)	1687.1	1				
																	VNDGVCDCCDGTDEYNSGVICENTCK	18	3	2	48255889;574280091	N-Term(TMT6plex); C6(Carbamidomethyl); C8(Carbamidomethyl); C9(Carbamidomethyl); C21(Carbamidomethyl); C25(Carbamidomethyl); K26(TMT6plex)	3499.5	0				
																	AQQEQELAADAFK	50	3	2	48255889;574280091	N-Term(TMT6plex); K13(TMT6plex)	1907.0	0				
																	KILIEDWK	6	3	2	48255889;574280091	N-Term(TMT6plex); K1(TMT6plex); K8(TMT6plex)	1732.1	1				
																	ELDDDMDGTVSVTELQTHPELDTDGDGALSEAEAQALLSGDTQTDATSFYDR	23	3	2	48255889;574280091	N-Term(TMT6plex); M6(Oxidation)	5790.6	0				
																	LGGSPTSLGTWGSWIGPDHDK	28	3	2	48255889;574280091	N-Term(TMT6plex); K21(TMT6plex)	2626.4	0				
																	ELDDDMDGTVSVTELQTHPELDTDGDGALSEAEAQALLSGDTQTDATSFYDR	6	3	2	48255889;574280091	N-Term(TMT6plex)	5774.6	0				
																	LIELQAGK	18	3	2	48255889;574280091	N-Term(TMT6plex); K8(TMT6plex)	1329.9	0				
																	YEQGTGCWQGPNR	33	3	2	48255889;574280091	N-Term(TMT6plex); C7(Carbamidomethyl)	1781.8	0				
																	ETMVTSTTEPSR	24	3	2	48255889;574280091	N-Term(TMT6plex); M3(Oxidation)	1583.8	0				
																	ILIEDWK	43	3	2	48255889;574280091	N-Term(TMT6plex); K7(TMT6plex)	1374.8	0				
																	GVSLTNHHFYDESKPFTCLDGSATIPFDQVNDDYCDCK	3	3	2	48255889;574280091	N-Term(TMT6plex); K14(TMT6plex); C18(Carbamidomethyl); C35(Carbamidomethyl); C37(Carbamidomethyl); K38(TMT6plex)	5140.4	0				
																	SLEDQVEMLR	2	3	2	48255889;574280091	N-Term(TMT6plex)	1448.8	0				
																	CEYLMELMTPAACPEPPPEAPTEDDHDEL	4	3	2	48255889;574280091	N-Term(TMT6plex); C1(Carbamidomethyl); M5(Oxidation); C13(Carbamidomethyl)	3602.6	0				
																	LWEEQLAAAK	54	3	2	48255889;574280091	N-Term(TMT6plex); K10(TMT6plex)	1616.9	0				
																	ETMVTSTTEPSR	9	3	2	48255889;574280091	N-Term(TMT6plex)	1567.8	0				
																	EEAEKPER	3	3	2	48255889;574280091	N-Term(TMT6plex); K5(TMT6plex)	1445.8	0				
																	MPPYDEQTQAFIDAAQEAR	6	3	2	48255889;574280091	N-Term(TMT6plex)	2410.1	0				
																	LGGSPTSLGTWGSWIGPDHDKFSAMK	4	3	2	48255889;574280091	N-Term(TMT6plex); K21(TMT6plex); M25(Oxidation); K26(TMT6plex)	3435.8	1				
																	AQQEQELAADAFKELDDDMDGTVSVTELQTHPELDTDGDGALSEAEAQALLSGDTQTDATSFYDR	2	3	2	48255889;574280091	N-Term(TMT6plex); K13(TMT6plex); M19(Oxidation)	7449.4	1				
																	LVSQKPK	5	3	2	48255889;574280091	N-Term(TMT6plex); K5(TMT6plex); K7(TMT6plex)	1487.0	0				
																	NKFEEAER	3	3	2	48255889;574280091	N-Term(TMT6plex); K2(TMT6plex)	1480.8	1				
																	DMEESIR	4	3	2	48255889;574280091	N-Term(TMT6plex); M2(Oxidation)	1124.5	0				
																	EEQPPVPSSPTEEEEEEEEEEEEEAEEEEEEEDSEEAPPPLSPPQPASPAEEDKMPPYDEQTQAFIDAAQEAR	1	1	1	48255889	N-Term(TMT6plex); K54(TMT6plex)	8708.8	1	**1.2**	**1.5**	**1.0**	**1.0**
																	VWAAIR	8	3	3	48255889;574280091;28212272	N-Term(TMT6plex)	944.6	0				
																	EQHQK	1	4	3	48255889;31563507;574280091	N-Term(TMT6plex); K5(TMT6plex)	1127.7	0				
29029559	NP_001307.2	**CSE1L**	1434	**exportin-2 isoform 1**	74.56	2	59	59	946	**1.3**	**1.3**	**1.4**	**1.4**	971	110.3	5.77												
																	EHDPVGQMVNNPK	30	2	1	29029559	N-Term(TMT6plex); M8(Oxidation); K13(TMT6plex)	1939.0	0	**1.4**	**1.4**	**1.4**	**1.5**
																	LLTECPPMMDTEYTK	25	2	1	29029559	N-Term(TMT6plex); C5(Carbamidomethyl); M8(Oxidation); M9(Oxidation); K15(TMT6plex)	2319.1	0	**1.4**	**1.4**	**1.6**	**1.5**
																	SQICDNAALYAQK	32	1	1	29029559	N-Term(TMT6plex); C4(Carbamidomethyl); K13(TMT6plex)	1940.0	0	**1.3**	**1.3**	**1.5**	**1.4**
																	NLFEDQNTLTSICEK	33	2	1	29029559	N-Term(TMT6plex); C13(Carbamidomethyl); K15(TMT6plex)	2270.2	0	**1.4**	**1.3**	**1.5**	**1.3**
																	ANIVHLMLSSPEQIQK	25	2	1	29029559	N-Term(TMT6plex); M7(Oxidation); K16(TMT6plex)	2282.3	0	**1.2**	**1.3**	**1.4**	**1.5**
																	FFEGPVTGIFSGYVNSMLQEYAK	28	2	1	29029559	N-Term(TMT6plex); M17(Oxidation); K23(TMT6plex)	3058.6	0	**1.4**	**1.3**	**1.5**	**1.5**
																	GSNTIASAAADK	35	2	1	29029559	N-Term(TMT6plex); K12(TMT6plex)	1563.9	0	**1.2**	**1.2**	**1.4**	**1.4**
																	QLSDAISIIGR	21	2	1	29029559	N-Term(TMT6plex)	1401.8	0	**1.3**	**1.3**	**1.4**	**1.4**
																	DAAIYLVTSLASK	45	2	1	29029559	N-Term(TMT6plex); K13(TMT6plex)	1810.1	0	**1.2**	**1.3**	**1.5**	**1.4**
																	WPDLLTEMVNR	27	2	1	29029559	N-Term(TMT6plex); M8(Oxidation)	1618.8	0	**1.4**	**1.3**	**1.4**	**1.5**

Khan et al., 2019. Multi-omics analysis to characterize cigarette smoke induced molecular alterations in esophageal cells
Supplementary Table 6. List of all proteins and peptides identified in untreated and chronically treated Het1A cells with cigarette smoke condensate for 8 months

Accession	RefSeq Accession	Gene Symbol	Entrez Gene ID	Description	Σ Coverage	Σ# Proteins	Σ# Unique Peptides	Σ# Peptides	Σ# PSMs	Het-1A-Smoke - 2M/Parental	Het-1A-Smoke - 4M/Parental	Het-1A-Smoke - 6M/Parental	Het-1A-Smoke - 8M/Parental	# AAs	MW [kDa]	calc. pI	Sequence	# PSMs	# Proteins	# Protein Groups	Protein Group Accessions	Modifications	MH+ [Da]	# Missed Cleavages	Het-1A-Smoke - 2M/Parental	Het-1A-Smoke - 4M/Parental	Het-1A-Smoke - 6M/Parental	Het-1A-Smoke - 8M/Parental
																	GSNTIASAAADKIPGLLGVFQK	22	2	1	29029559	N-Term(TMT6plex); K12(TMT6plex); K22(TMT6plex)	2845.7	1	1.3	1.5	1.7	1.8
																	IIIPEIQK	63	2	1	29029559	N-Term(TMT6plex); K8(TMT6plex)	1411.9	0	1.1	1.2	1.3	1.3
																	SQDNVIK	14	2	1	29029559	N-Term(TMT6plex); K7(TMT6plex)	1261.8	0	1.2	1.1	1.3	1.4
																	EDFPQKWPDLLTEMVNR	7	2	1	29029559	N-Term(TMT6plex); K6(TMT6plex)	2576.4	1	2.5	3.0	3.3	2.7
																	YDLLVSNAIQFLASVCERPHYK	11	1	1	29029559	N-Term(TMT6plex); C16(Carbamidomethyl); K22(TMT6plex)	3081.7	0	1.4	1.3	1.6	1.4
																	DLEGSDIDTR	18	2	1	29029559	N-Term(TMT6plex)	1349.7	0	1.5	1.4	1.4	1.5
																	SNELWTEIK	8	2	1	29029559	N-Term(TMT6plex); K9(TMT6plex)	1577.9	0	1.1	1.2	1.3	1.3
																	LLQTDDEEEAGLLELLK	49	1	1	29029559	N-Term(TMT6plex); K17(TMT6plex)	2387.3	0	1.2	1.1	1.4	1.4
																	ANDHQGFYLLNSIIEHMPPESVDQYR	7	2	1	29029559	N-Term(TMT6plex); M17(Oxidation)	3318.6	0	1.5	1.6	1.4	1.6
																	IVEDEPNKICEADR	9	2	1	29029559	N-Term(TMT6plex); K8(TMT6plex); C10(Carbamidomethyl)	2146.1	1	1.5	1.7	1.6	1.7
																	AADEEAFEDNSEEYIR	27	2	1	29029559	N-Term(TMT6plex)	2116.9	0	1.4	1.5	1.5	1.6
																	VPSMVSTSLNAEALQYLQGYLQAASVTLL	42	2	1	29029559	N-Term(TMT6plex); M4(Oxidation)	3312.8	0	2.3	1.5	1.5	1.5
																	TLDPDPAIR	5	2	1	29029559	N-Term(TMT6plex)	1226.7	0	1.4	1.3	1.4	1.5
																	FFEGPVTGIFSGYVNSMLQEYAK	8	2	1	29029559	N-Term(TMT6plex); K23(TMT6plex)	3042.6	0	1.4	1.3	1.6	1.3
																	WPDLLTEMVNR	10	2	1	29029559	N-Term(TMT6plex)	1602.9	0	1.7	1.7	2.2	1.6
																	VCASVTFK	4	2	1	29029559	N-Term(TMT6plex); C2(Carbamidomethyl); K8(TMT6plex)	1369.8	0	1.3	1.3	1.5	1.5
																	KEHDPVGQMVNNPK	2	2	1	29029559	N-Term(TMT6plex); K1(TMT6plex); M9(Oxidation); K14(TMT6plex)	2296.3	1	1.1	1.4	1.4	1.6
																	LLTECPPMMDTEYTK	4	2	1	29029559	N-Term(TMT6plex); C5(Carbamidomethyl); M9(Oxidation); K15(TMT6plex)	2303.1	0	1.7	1.8	2.3	1.3
																	NDIPSSYMALFPHLLQPVLWER	5	2	1	29029559	N-Term(TMT6plex); M8(Oxidation)	2871.5	0	1.5	1.4	1.5	1.5
																	TAHSLFK	6	2	1	29029559	N-Term(TMT6plex); K7(TMT6plex)	1261.8	0	1.1	1.2	1.4	1.4
																	EDFPQKWPDLLTEMVNR	3	2	1	29029559	N-Term(TMT6plex); K6(TMT6plex); M14(Oxidation)	2592.4	1	1.6	2.0	1.8	2.4
																	TGNIPALVR	2	2	1	29029559	N-Term(TMT6plex)	1169.7	0	1.4	1.0	1.2	1.2
																	FQSGDFHVINGVLR	4	2	1	29029559	N-Term(TMT6plex)	1818.0	0	1.3	1.3	1.3	1.4
																	LLQAFLER	21	2	1	29029559	N-Term(TMT6plex)	1218.7	0	1.3	1.1	1.4	1.3
																	ILFSSLILISK	23	2	1	29029559	N-Term(TMT6plex); K11(TMT6plex)	1692.1	0	1.6	1.0	1.7	1.2
																	QIFILLFQR	10	2	1	29029559	N-Term(TMT6plex)	1406.9	0	1.8	1.2	1.8	1.5
																	SANVNEFPVLK	20	2	1	29029559	N-Term(TMT6plex); K11(TMT6plex)	1676.0	0	1.3	1.3	1.4	1.5
																	IPGLLGVFQK	41	2	1	29029559	N-Term(TMT6plex); K10(TMT6plex)	1530.0	0	1.1	1.2	1.6	1.6
																	NPSVNWK	11	2	1	29029559	N-Term(TMT6plex); K7(TMT6plex)	1302.8	0	1.2	1.1	1.4	1.2
																	FLESVEGNQNYPLLLLTLLEK	5	2	1	29029559	N-Term(TMT6plex); K21(TMT6plex)	2891.6	0	1.7	1.5	1.9	1.6
																	ATIELCSTHANDASALR	12	2	1	29029559	N-Term(TMT6plex); C6(Carbamidomethyl)	2059.0	0	1.4	1.5	1.5	1.6
																	SFSLLQEAIIPYIPTLITQLTQK	10	2	1	29029559	N-Term(TMT6plex); K23(TMT6plex)	3075.8	0	1.2	1.0	1.6	1.1
																	ALTLPGSSENEYIMK	27	2	1	29029559	N-Term(TMT6plex); M14(Oxidation); K15(TMT6plex)	2127.1	0	1.1	1.2	1.3	1.3
																	EHDPVGQMVNNPK	2	2	1	29029559	N-Term(TMT6plex); K13(TMT6plex)	1923.0	0	1.7	1.7	1.9	1.3
																	YGALALQEIFDGIQPK	19	2	1	29029559	N-Term(TMT6plex); K16(TMT6plex)	2221.3	0	1.6	1.2	1.7	1.3
																	ALTLPGSSENEYIMK	9	2	1	29029559	N-Term(TMT6plex); K15(TMT6plex)	2111.1	0	1.5	1.5	1.8	1.5
																	ANIVHLMLSSPEQIQK	11	2	1	29029559	N-Term(TMT6plex); K16(TMT6plex)	2266.3	0	1.4	1.5	1.7	1.6
																	MFGMVLEK	3	2	1	29029559	N-Term(TMT6plex); K8(TMT6plex)	1412.8	0	1.7	1.6	2.2	1.4
																	VPSMVSTSLNAEALQYLQGYLQAASVTLL	8	2	1	29029559	N-Term(TMT6plex)	3296.8	0	1.7	2.0	1.8	1.9
																	LVLDAFALPLTNLFK	17	2	1	29029559	N-Term(TMT6plex); K15(TMT6plex)	2133.3	0	1.4	1.1	1.5	1.2
																	FVTAIWNLLVTTGQEVK	4	1	1	29029559	N-Term(TMT6plex); K17(TMT6plex)	2377.4	0	1.5	0.9	1.5	1.1
																	SFLVFINLYCIK	4	2	1	29029559	N-Term(TMT6plex); C10(Carbamidomethyl); K12(TMT6plex)	1975.1	0	1.7	1.5	2.2	1.2
																	GPNNATLFTAAEIAPFVEILLTNLFK	4	2	1	29029559	N-Term(TMT6plex); K26(TMT6plex)	3262.9	0	1.4	1.2	2.0	1.4
																	IVEDEPNK	12	2	1	29029559	N-Term(TMT6plex); K8(TMT6plex)	1401.8	0	1.2	1.2	1.4	1.4
																	HGITQANELVNLTEFFVNHILPDLK	3	2	1	29029559	N-Term(TMT6plex); K25(TMT6plex)	3320.8	0	1.6	1.3	2.2	1.3
																	ANPAAVVNFEEALFLVFTEILQNDVQEFIPYVFQVMSLLLETHK	1	2	1	29029559	N-Term(TMT6plex); K44(TMT6plex)	5525.0	0	0.9	1.1	2.6	0.9
																	HKDAAIYLVTSLASK	5	2	1	29029559	N-Term(TMT6plex); K2(TMT6plex); K15(TMT6plex)	2304.4	1	1.4	1.3	1.4	1.5
																	ICAVGITK	2	2	1	29029559	N-Term(TMT6plex); C2(Carbamidomethyl); K8(TMT6plex)	1319.8	0	1.2	1.2	1.5	1.3
																	VSGNVEK	6	2	1	29029559	N-Term(TMT6plex); K7(TMT6plex)	1190.7	0	1.2	1.1	1.3	1.3
																	YIMIFR	4	2	1	29029559	N-Term(TMT6plex)	1071.6	0	1.8	1.8	2.4	1.6
																	YDEEFQR	2	1	1	29029559	N-Term(TMT6plex)	1215.6	0	1.3	1.2	1.3	1.1
																	KQIFILLFQR	2	2	1	29029559	N-Term(TMT6plex); K1(TMT6plex)	1764.1	1	1.7	1.5	1.7	1.8
																	VIVPNMEFR	5	2	1	29029559	N-Term(TMT6plex); M6(Oxidation)	1349.7	0	1.3	1.4	1.5	1.5
																	AACDLVR	1	2	1	29029559	N-Term(TMT6plex); C3(Carbamidomethyl)	1033.6	0	1.5	1.5	1.7	1.6
																	MFGMVLEK	1	2	1	29029559	N-Term(TMT6plex); M1(Oxidation); M4(Oxidation); K8(TMT6plex)	1444.8	0	1.3	1.3	1.5	1.4
																	MFGMVLEK	1	2	1	29029559	N-Term(TMT6plex); M1(Oxidation); K8(TMT6plex)	1428.8	0	1.3	1.2	1.4	1.3
																	ICEADR	4	2	1	29029559	N-Term(TMT6plex); C2(Carbamidomethyl)	992.5	0	1.4	1.3	1.5	1.4
																	EDFPQK	1	2	1	29029559	N-Term(TMT6plex); K6(TMT6plex)	1221.7	0	1.1	1.1	1.3	1.3
																	LLAVSK	2	2	1	29029559	N-Term(TMT6plex); K6(TMT6plex)	1088.7	0	1.2	1.2	1.4	1.4
																	VIVPNMEFR	2	2	1	29029559	N-Term(TMT6plex)	1333.7	0	1.6	1.8	1.9	1.7
255958282	NP_005808.3	**PLIN3**	10226	**perilipin-3 isoform 1**	74.42	3	22	22	138	**1.4**	**1.3**	**1.5**	**1.6**	434	47.0	5.44												
																	GLDKLEENLPILQQPTEK	10	3	1	255958282	N-Term(TMT6plex); K4(TMT6plex); K18(TMT6plex)	2752.6	1	1.5	1.3	1.6	1.8
																	LEENLPILQQPTEK	2	3	1	255958282	N-Term(TMT6plex); K14(TMT6plex)	2110.2	0	1.2	1.1	1.2	1.4
																	DIAQQLQATCTSLGSSIQGLPTNVK	4	3	1	255958282	N-Term(TMT6plex); C10(Carbamidomethyl); K25(TMT6plex)	3088.7	0	1.6	1.3	1.8	1.9
																	QEQSYFVR	2	3	1	255958282	N-Term(TMT6plex)	1285.7	0	1.6	1.2	1.5	1.6
																	IATSLDGFDVASVQQQR	5	3	1	255958282	N-Term(TMT6plex)	2064.1	0	1.3	1.1	1.3	1.4
																	DTVATQLSEAVDATR	9	3	1	255958282	N-Term(TMT6plex)	1805.9	0	1.4	1.4	1.6	1.5
																	LGQMVLSGVDTVLGK	9	3	1	255958282	N-Term(TMT6plex); M4(Oxidation); K15(TMT6plex)	1991.2	0	1.6	1.4	1.6	1.6
																	VSGAQEMVSSAK	2	3	1	255958282	N-Term(TMT6plex); K12(TMT6plex)	1651.9	0	1.7	1.4	1.9	1.6
																	VSGAQEMVSSAK	10	3	1	255958282	N-Term(TMT6plex); M7(Oxidation); K12(TMT6plex)	1667.9	0	1.4	1.3	1.5	1.5
																	TLTAAAVSGAQPILSK	2	3	1	255958282	N-Term(TMT6plex); K16(TMT6plex)	1986.2	0	1.3	1.1	1.4	1.3
																	QVEDLQATFSSIHSFQDLSSSILAQSR	14	3	1	255958282	N-Term(TMT6plex)	3223.6	0	1.4	1.4	1.5	1.6
																	AQEALLQLSQVLSLMETVK	6	3	1	255958282	N-Term(TMT6plex); M15(Oxidation); K19(TMT6plex)	2575.5	0	1.2	1.1	1.3	1.3
																	EALDHMVEYVAQNTPVTWLVGPFAPGITEK	24	3	1	255958282	N-Term(TMT6plex); M6(Oxidation); K30(TMT6plex)	3787.0	0	1.4	1.4	1.4	1.7
																	SVVTGGVQSVMGSR	12	3	1	255958282	N-Term(TMT6plex); M11(Oxidation)	1608.9	0	1.6	1.4	1.5	1.6
																	AQEALLQLSQVLSLMETVK	2	3	1	255958282	N-Term(TMT6plex); K19(TMT6plex)	2559.5	0	1.5	1.0	1.6	1.4
																	TVCDAAEK	4	3	1	255958282	N-Term(TMT6plex); C3(Carbamidomethyl); K8(TMT6plex)	1351.7	0	1.4	1.1	1.6	1.4
																	LEPQIASASEYAHR	3	3	1	255958282	N-Term(TMT6plex)	1800.9	0	1.1	1.2	1.2	1.3
																	LGQMVLSGVDTVLGK	4	3	1	255958282	N-Term(TMT6plex); K15(TMT6plex)	1975.2	0	1.7	1.5	2.1	1.5
																	QHAYEHSLGK	2	3	1	255958282	N-Term(TMT6plex); K10(TMT6plex)	1627.9	0	1.3	1.1	1.4	1.5
																	LVEGQEK	2	3	1	255958282	N-Term(TMT6plex); K7(TMT6plex)	1260.8	0	1.1	1.0	1.2	1.4
																	VASMPLISSTCDMVSAAYASTK	4	3	1	255958282	N-Term(TMT6plex); M4(Oxidation); C11(Carbamidomethyl); K22(TMT6plex)	2764.4	0	1.6	1.6	1.7	1.7
																	SEEWADNHLPLTDAELAR	2	3	1	255958282	N-Term(TMT6plex)	2296.1	0	1.6	1.6	1.8	1.8
																	EALDHMVEYVAQNTPVTWLVGPFAPGITEK	1	3	1	255958282	N-Term(TMT6plex); K30(TMT6plex)	3771.0	0	1.8	2.1	3.1	2.0
																	LHQMWLSWNQK	1	3	1	255958282	N-Term(TMT6plex); M4(Oxidation); K11(TMT6plex)	1945.1	0	1.4	1.4	1.3	1.5
																	EPPKPEQVESR	1	2	1	255958282	N-Term(TMT6plex); K4(TMT6plex)	1754.0	0	1.2	1.1	1.5	1.3
																	VLADTK	1	2	1	255958282	N-Term(TMT6plex); K6(TMT6plex)	1104.7	0				
261862352;261862350	NP_001159831.1	**SHMT2**	6472	**serine hydroxymethyltransferase, mitochondrial isoform 3**	74.33	3	26	27	310	**1.6**	**1.8**	**1.7**	**1.7**	483	53.4	8.15												
																	VLELVSITANK	29	3	1	261862352;261862350	N-Term(TMT6plex); K11(TMT6plex)	1645.0	0	1.7	1.7	1.8	1.7
																	GYSLVSGGTDNHLVLVDLRPK	8	3	1	261862352;261862350	N-Term(TMT6plex); K21(TMT6plex)	2698.5	0	1.6	2.0	1.9	1.9
																	HADIVTTTTHK	8	3	1	261862352;261862350	N-Term(TMT6plex); K11(TMT6plex)	1682.0	0	1.4	1.5	1.7	1.6
																	SGLIFYR	6	3	1	261862352;261862350	N-Term(TMT6plex)	1084.6	0	1.9	1.8	1.7	1.8
																	YSEGYPGK	12	3	1	261862352;261862350	N-Term(TMT6plex); K8(TMT6plex)	1358.7	0	1.6	1.6	1.6	1.6
																	TGLIDYNQLALTAR	12	2	1	261862352;261862350	N-Term(TMT6plex)	1778.0	0	1.6	1.4	1.5	1.5
																	LIIAGTSAYAR	7	3	1	261862352;261862350	N-Term(TMT6plex)	1364.8	0	1.4	1.7	1.6	1.7
																	IMGLDLPDGGHLTHGYMSDVK	4	3	1	261862352;261862350	N-Term(TMT6plex); M17(Oxidation); K21(TMT6plex)	2730.4	0	1.3	1.7	1.5	1.6
																	AFPMPGFDEH	16	3	1	261862352;261862350	N-Term(TMT6plex); M4(Oxidation)	1392.6	0	1.5	1.7	1.6	1.7
																	VVDFIDEGVNIGLEVK	54	3	1	261862352;261862350	N-Term(TMT6plex); K16(TMT6plex)	2204.3	0	1.7	2.0	1.8	1.8
																	EIPYTFEDR	14	3	1	261862352;261862350	N-Term(TMT6plex)	1398.7	0	1.6	1.8	1.7	1.8
																	GLELIASENFCSR	6	3	1	261862352;261862350	N-Term(TMT6plex); C11(Carbamidomethyl)	1724.9	0	1.3	1.5	1.5	1.6
																	GWTGQESLSDSDPEMWELLQR	12	3	1	261862352;261862350	N-Term(TMT6plex); M15(Oxidation)	2709.3	0	1.5	1.7	1.6	1.7
																	ALEAFDLDPAQWGVNVQPYSGSPANLAVYTALLQPHDR	14	3	1	261862352;261862350	N-Term(TMT6plex)	4353.2	0	1.6	2.2	1.9	2.1
																	ISATSIFFESMPYK	17	6	2	22547189;261862352;261862350	N-Term(TMT6plex); K14(TMT6plex)	2079.1	0				
																	ISATSIFFESMPYK	28	6	2	22547189;261862352;261862350	N-Term(TMT6plex); M11(Oxidation); K14(TMT6plex)	2095.1	0				
																	EVCDEVK	7	3	1	261862352;261862350	N-Term(TMT6plex); C3(Carbamidomethyl); K7(TMT6plex)	1336.7	0	1.9	2.3	2.0	2.1
																	SAITPGGLR	4	3	1	261862352;261862350	N-Term(TMT6plex)	1100.7	0	1.3	1.3	1.2	1.2
																	GWTGQESLSDSDPEMWELLQR	9	3	1	261862352;261862350	N-Term(TMT6plex)	2693.3	0	1.6	2.1	1.9	1.8

Supplementary Table 6. List of all proteins and peptides identified in untreated and chronically treated Het1A cells with cigarette smoke condensate for 8 months

Accession	RefSeq Accession	Gene Symbol	Entrez Gene ID	Description	Σ Coverage	Σ# Proteins	Σ# Unique Peptides	Σ# Peptides	Σ# PSMs	Het-1A-Smoke - 2M/Parental	Het-1A-Smoke - 4M/Parental	Het-1A-Smoke - 6M/Parental	Het-1A-Smoke - 8M/Parental	# AAs	MW [kDa]	calc. pI	Sequence	# PSMs	# Proteins	# Protein Groups	Protein Group Accessions	Modifications	MH+ [Da]	# Missed Cleavages	Het-1A-Smoke - 2M/Parental	Het-1A-Smoke - 4M/Parental	Het-1A-Smoke - 6M/Parental	Het-1A-Smoke - 8M/Parental
																	AALEALGSCLNNK	10	3	1	261862352;261862350	N-Term(TMT6plex); C9(Carbamidomethyl); K13(TMT6plex)	1819.0	0	**1.4**	**1.6**	**1.6**	**1.6**
																	EYSLQVLK	6	3	1	261862352;261862350	N-Term(TMT6plex); K8(TMT6plex)	1437.9	0	**1.6**	**1.9**	**2.0**	**1.8**
																	AHLLADMAHISGLVAAK	4	3	1	261862352;261862350	N-Term(TMT6plex); K17(TMT6plex)	2176.3	0	**1.9**	**2.6**	**2.3**	**2.4**
																	AMADALLER	2	3	1	261862352;261862350	N-Term(TMT6plex)	1218.7	0	**1.8**	**2.4**	**2.3**	**1.8**
																	INFAVFPSLQGGPHNHAIAAVAVALK	5	3	1	261862352;261862350	N-Term(TMT6plex); K26(TMT6plex)	3100.8	0	**1.5**	**1.3**	**1.9**	**1.4**
																	AQHSNAAQTQTGEANR	2	3	1	261862352;261862350	N-Term(TMT6plex)	1912.9	0	**1.0**	**1.0**	**1.5**	**1.2**
																	VIPSPFK	5	3	1	261862352;261862350	N-Term(TMT6plex); K7(TMT6plex)	1245.8	0	**1.7**	**2.0**	**1.8**	**1.9**
																	AMADALLER	3	3	1	261862352;261862350	N-Term(TMT6plex); M2(Oxidation)	1234.7	0	**1.5**	**1.5**	**1.3**	**1.5**
																	DSETSQR	2	3	1	261862352;261862350	N-Term(TMT6plex)	1051.5	0	**1.6**	**1.8**	**1.8**	**2.1**
																	LGAPALTSR	2	3	1	261862352;261862350	N-Term(TMT6plex)	1114.7	0	**1.7**	**2.0**	**1.8**	**2.1**
																	VEQFAR	2	3	1	261862352;261862350	N-Term(TMT6plex)	978.6	0	**1.6**	**1.6**	**1.6**	**1.6**
4507521	NP_001055.1	**TKT**	7086	**transketolase isoform 1**	74.32	2	36	36	797	**1.5**	**1.5**	**1.5**	**1.5**	623	67.8	7.66												
																	QAFTDVATGSLGQGLGAACGMAYTGK	32	2	1	4507521	N-Term(TMT6plex); C19(Carbamidomethyl); M21(Oxidation); K26(TMT6plex)	3006.5	0	**1.7**	**1.7**	**1.7**	**1.5**
																	NMAEQIIQEIYSQIQSK	146	2	1	4507521	N-Term(TMT6plex); M2(Oxidation); K17(TMT6plex)	2497.3	0	**1.5**	**1.5**	**1.6**	**1.4**
																	ILATPPQEDAPSVDIANIR	61	2	1	4507521	N-Term(TMT6plex)	2249.2	0	**1.5**	**1.4**	**1.5**	**1.4**
																	SKDDQVTVIGAGVTLHEALAAAELLK	15	2	1	4507521	N-Term(TMT6plex); K2(TMT6plex); K26(TMT6plex)	3336.9	1	**1.5**	**1.7**	**1.7**	**1.7**
																	ISSDLDGHPVPK	23	2	1	4507521	N-Term(TMT6plex); K12(TMT6plex)	1723.0	0	**1.4**	**1.4**	**1.5**	**1.4**
																	LGQSDPAPLQHQMDIYQK	24	2	1	4507521	N-Term(TMT6plex); M13(Oxidation); K18(TMT6plex)	2543.3	0	**1.4**	**1.4**	**1.5**	**1.4**
																	NMAEQIIQEIYSQIQSK	45	2	1	4507521	N-Term(TMT6plex); K17(TMT6plex)	2481.3	0	**1.8**	**1.5**	**1.9**	**1.4**
																	GITGVEDK	10	2	1	4507521	N-Term(TMT6plex); K8(TMT6plex)	1276.8	0	**1.4**	**1.5**	**1.5**	**1.5**
																	ESWHGKPLPK	8	2	1	4507521	N-Term(TMT6plex); K6(TMT6plex); K10(TMT6plex)	1866.1	0	**1.3**	**1.5**	**1.5**	**1.5**
																	GITGVEDKESWHGKPLPK	2	2	1	4507521	N-Term(TMT6plex); K8(TMT6plex); K14(TMT6plex); K18(TMT6plex)	2894.7	1	**1.2**	**2.0**	**1.6**	**1.8**
																	SGKPAELLK	10	2	1	4507521	N-Term(TMT6plex); K3(TMT6plex); K9(TMT6plex)	1630.0	0	**1.3**	**1.4**	**1.5**	**1.4**
																	AYGQALAK	14	2	1	4507521	N-Term(TMT6plex); K8(TMT6plex)	1279.8	0	**1.4**	**1.4**	**1.6**	**1.5**
																	IIALDGDTK	36	2	1	4507521	N-Term(TMT6plex); K9(TMT6plex)	1403.8	0	**1.6**	**1.6**	**1.6**	**1.6**
																	AVELAANTK	24	2	1	4507521	N-Term(TMT6plex); K9(TMT6plex)	1374.8	0	**1.4**	**1.5**	**1.5**	**1.5**
																	KLILDSAR	3	2	1	4507521	N-Term(TMT6plex); K1(TMT6plex)	1373.9	1	**1.3**	**1.5**	**1.5**	**1.4**
																	SVPTSTVFYPSDGVATEK	56	2	1	4507521	N-Term(TMT6plex); K18(TMT6plex)	2343.2	0	**1.5**	**1.5**	**1.5**	**1.5**
																	ILTVEDHYYEGGIGEAVSSAVVGEPGITVTHLAVNR	14	2	1	4507521	N-Term(TMT6plex)	3982.1	0	**1.3**	**1.4**	**1.6**	**1.3**
																	DAIAQAVR	13	2	1	4507521	N-Term(TMT6plex)	1072.6	0	**1.3**	**1.3**	**1.3**	**1.3**
																	VLDPFTIKPLDR	26	2	1	4507521	N-Term(TMT6plex); K8(TMT6plex)	1872.1	0	**1.6**	**1.7**	**1.6**	**1.7**
																	LGQSDPAPLQHQMDIYQK	4	2	1	4507521	N-Term(TMT6plex); K18(TMT6plex)	2527.3	0	**2.0**	**1.9**	**2.3**	**1.6**
																	TVPFCSTFAAFFTR	4	2	1	4507521	N-Term(TMT6plex); C5(Carbamidomethyl)	1881.0	0	**1.7**	**1.8**	**1.8**	**1.7**
																	VYCLLGDGELSEGSVWEAMAFASIYK	6	2	1	4507521	N-Term(TMT6plex); C3(Carbamidomethyl); M19(Oxidation); K26(TMT6plex)	3369.7	0	**1.4**	**1.4**	**1.5**	**1.6**
																	VYCLLGDGELSEGSVWEAMAFASIYK	4	2	1	4507521	N-Term(TMT6plex); C3(Carbamidomethyl); K26(TMT6plex)	3353.7	0	**1.5**	**1.6**	**1.8**	**1.7**
																	LDNLVAILDINR	61	2	1	4507521	N-Term(TMT6plex)	1597.9	0	**1.7**	**1.3**	**1.6**	**1.3**
																	NSTFSEIFK	21	2	1	4507521	N-Term(TMT6plex); K9(TMT6plex)	1530.9	0	**1.6**	**1.2**	**1.6**	**1.2**
																	HQPTAIIAK	22	2	1	4507521	N-Term(TMT6plex); K9(TMT6plex)	1436.9	0	**1.3**	**1.3**	**1.4**	**1.4**
																	GHAAPILYAVWAEAGFLAEAELLNLR	3	2	1	4507521	N-Term(TMT6plex)	3024.6	0	**1.9**	**1.7**	**2.7**	**1.1**
																	MPSLPSYK	6	2	1	4507521	N-Term(TMT6plex); K8(TMT6plex)	1380.8	0	**1.8**	**2.0**	**2.1**	**1.7**
																	MAAISESNINLCGSHCGVSIGEDGPSQMALEDLAMFR	4	2	1	4507521	N-Term(TMT6plex); C12(Carbamidomethyl); C16(Carbamidomethyl); M28(Oxidation); M35(Oxidation)	4228.9	0	**1.4**	**1.6**	**1.8**	**1.6**
																	TSRPENAIIYNNNEDFQVGQAK	28	2	1	4507521	N-Term(TMT6plex); K22(TMT6plex)	2966.5	0	**1.6**	**1.2**	**1.5**	**1.3**
																	KAYGQALAK	14	2	1	4507521	N-Term(TMT6plex); K1(TMT6plex); K9(TMT6plex)	1637.0	1	**1.4**	**1.6**	**1.5**	**1.6**
																	QAFTDVATGSLGQGLGAACGMAYTGK	6	2	1	4507521	N-Term(TMT6plex); C19(Carbamidomethyl); K26(TMT6plex)	2990.5	0	**2.0**	**1.9**	**2.3**	**1.5**
																	KISSDLDGHPVPK	7	2	1	4507521	N-Term(TMT6plex); K1(TMT6plex); K13(TMT6plex)	2080.2	1	**1.4**	**1.8**	**1.7**	**1.7**
																	FIECYIAEQNMVSIAVGCATR	2	2	1	4507521	N-Term(TMT6plex); C4(Carbamidomethyl); C18(Carbamidomethyl)	2661.3	0	**1.4**	**1.8**	**1.5**	**1.5**
																	DDQVTVIGAGVTLHEALAAAELLK	6	2	1	4507521	N-Term(TMT6plex); K24(TMT6plex)	2892.6	0	**1.3**	**1.6**	**1.5**	**1.6**
																	MAAISESNINLCGSHCGVSIGEDGPSQMALEDLAMFR	2	2	1	4507521	N-Term(TMT6plex); M1(Oxidation); C12(Carbamidomethyl); C16(Carbamidomethyl); M28(Oxidation); M35(Oxidation)	4244.9	0	**1.3**	**1.5**	**1.5**	**1.6**
																	MPSLPSYK	11	2	1	4507521	N-Term(TMT6plex); M1(Oxidation); K8(TMT6plex)	1396.8	0	**1.5**	**1.6**	**1.5**	**1.6**
																	LILDSAR	13	2	1	4507521	N-Term(TMT6plex)	1016.6	0	**1.3**	**1.3**	**1.4**	**1.3**
																	GICFIR	6	2	1	4507521	N-Term(TMT6plex); C3(Carbamidomethyl)	994.6	0	**1.5**	**1.6**	**1.6**	**1.5**
																	AFDQIR	3	3	1	4507521	N-Term(TMT6plex)	978.6	0	**1.6**	**1.6**	**1.6**	**1.6**
																	AFGQAK	1	2	1	4507521	N-Term(TMT6plex); K6(TMT6plex)	1079.7	0	**1.3**	**1.4**	**1.4**	**1.3**
																	KEHPDR	1	2	1	4507521	N-Term(TMT6plex); K1(TMT6plex)	1239.7	1	**1.7**	**2.1**	**2.0**	**1.9**
298919189	NP_001177371.1	**CAST**	831	**calpastatin isoform m**	74.24	3	3	41	304	**1.1**	**1.3**	**1.3**	**1.1**	695	75.3	5.07												
																	SESELIDELSEDFDR	32	5	3	298919189;109637759;545746425	N-Term(TMT6plex)	2012.9	0				
																	GTVPDDAVEALADSLGK	26	5	3	298919189;109637759;545746425	N-Term(TMT6plex); K17(TMT6plex)	2116.1	0				
																	SAEQQPSEK	13	5	3	298919189;109637759;545746425	N-Term(TMT6plex); K9(TMT6plex)	1461.8	0				
																	AAAPAPVSEAVCR	13	5	3	298919189;109637759;545746425	N-Term(TMT6plex); C12(Carbamidomethyl)	1527.8	0				
																	QASDTGSNDAHNK	10	5	3	298919189;109637759;545746425	N-Term(TMT6plex); K13(TMT6plex)	1802.9	0				
																	QPDPDENKPMEDK	4	5	3	298919189;109637759;545746425	N-Term(TMT6plex); K8(TMT6plex); M10(Oxidation); K13(TMT6plex)	2246.2	0				
																	LGEKEETIPPDYR	4	5	3	298919189;109637759;545746425	N-Term(TMT6plex); K4(TMT6plex)	2005.1	1				
																	TSMCSIQSAPPEPATLK	9	5	3	298919189;109637759;545746425	N-Term(TMT6plex); M3(Oxidation); C4(Carbamidomethyl); K17(TMT6plex)	2292.2	0				
																	EQLPPMSEDFLLDALSEDFSGPQNASSLK	11	5	3	298919189;109637759;545746425	N-Term(TMT6plex); K29(TMT6plex)	3623.8	0				
																	EQLPPMSEDFLLDALSEDFSGPQNASSLK	27	5	3	298919189;109637759;545746425	N-Term(TMT6plex); M6(Oxidation); K29(TMT6plex)	3639.8	0				
																	TKPQDMISAGGESVAGITAISGKPGDK	6	4	3	298919189;109637759;545746425	N-Term(TMT6plex); K2(TMT6plex); M6(Oxidation); K23(TMT6plex); K27(TMT6plex)	3548.0	0				
																	CGEDDETIPSEYR	11	5	3	298919189;109637759;545746425	N-Term(TMT6plex); C1(Carbamidomethyl)	1799.8	0				
																	LAAAISEVVSQTPASTTQAGAPPR	20	5	3	298919189;109637759;545746425	N-Term(TMT6plex)	2552.4	0				
																	DDTIPPEYR	7	5	3	298919189;109637759;545746425	N-Term(TMT6plex)	1334.7	0				
																	DTSQSDKDLDDALDK	4	4	2	298919189;109637759	N-Term(TMT6plex); K7(TMT6plex); K15(TMT6plex)	2353.2	1				
																	SLTPAVPVESKPDKPSGK	2	4	3	298919189;109637759;545746425	N-Term(TMT6plex); K11(TMT6plex); K14(TMT6plex); K18(TMT6plex)	2753.7	0				
																	TTEETSKPK	2	5	3	298919189;109637759;545746425	N-Term(TMT6plex); K7(TMT6plex); K9(TMT6plex)	1708.0	0				
																	DLDDALDK	1	5	3	298919189;109637759;545746425	N-Term(TMT6plex); K8(TMT6plex)	1362.8	0				
																	AIPVSQQMEGPHLPNK	2	1	1	298919189	N-Term(TMT6plex); M8(Oxidation); K16(TMT6plex)	2220.2	0	**1.1**	**1.3**	**1.2**	**1.4**
																	SGMDAALDDLIDTLGGPEETEEENTTYTGPEVSDPMSSTYIEELGK	4	5	3	298919189;109637759;545746425	N-Term(TMT6plex); M3(Oxidation); M36(Oxidation); K46(TMT6plex)	5397.5	0				
																	LSVVHEK	8	3	1	298919189	N-Term(TMT6plex); K7(TMT6plex)	1269.8	0	**1.0**	**1.2**	**1.3**	**1.0**
																	QASDTGSNDAHNKK	3	5	3	298919189;109637759;545746425	N-Term(TMT6plex); K13(TMT6plex); K14(TMT6plex)	2160.2	1				
																	HLLDDNGQDKPVKPPTK	3	5	3	298919189;109637759;545746425	N-Term(TMT6plex); K10(TMT6plex); K13(TMT6plex); K17(TMT6plex)	2818.6	0				
																	DTMSDQALEALSASLGTR	22	5	3	298919189;109637759;545746425	N-Term(TMT6plex); M3(Oxidation)	2111.0	0				
																	KPADDQDPIDALSGDLDSCPSTTETSQNTAK	11	5	3	298919189;109637759;545746425	N-Term(TMT6plex); K1(TMT6plex); C19(Carbamidomethyl); K31(TMT6plex)	3965.0	0				
																	SAAPPQEK	6	5	3	298919189;109637759;545746425	N-Term(TMT6plex); K8(TMT6plex)	1285.8	0				
																	TEKEESTEVLK	3	5	3	298919189;109637759;545746425	N-Term(TMT6plex); K3(TMT6plex); K11(TMT6plex)	1980.1	1				
																	DTMSDQALEALSASLGTR	6	5	3	298919189;109637759;545746425	N-Term(TMT6plex)	2095.1	0				
																	SGMDAALDDLIDTLGGPEETEEENTTYTGPEVSDPMSSTYIEELGK	4	5	3	298919189;109637759;545746425	N-Term(TMT6plex); M3(Oxidation); K46(TMT6plex)	5381.5	0				
																	TKPQDMISAGGESVAGITAISGKPGDK	2	4	3	298919189;109637759;545746425	N-Term(TMT6plex); K2(TMT6plex); K23(TMT6plex); K27(TMT6plex)	3532.0	0				
																	LSDSLGQR	4	5	3	298919189;109637759;545746425	N-Term(TMT6plex)	1104.6	0				
																	SESELIDELSEDFDRSECK	1	5	3	298919189;109637759;545746425	N-Term(TMT6plex); C18(Carbamidomethyl); K19(TMT6plex)	2746.3	1				
																	KTEKEESTEVLK	1	5	3	298919189;109637759;545746425	N-Term(TMT6plex); K1(TMT6plex); K4(TMT6plex); K12(TMT6plex)	2337.4	2				
																	EADPEDGKPVMDK	5	5	3	298919189;109637759;545746425	N-Term(TMT6plex); K8(TMT6plex); M11(Oxidation); K13(TMT6plex)	2134.1	0				
																	AQSAGTVR	3	5	3	298919189;109637759;545746425	N-Term(TMT6plex)	1018.6	0				
																	QPDPDENKPMEDK	1	5	3	298919189;109637759;545746425	N-Term(TMT6plex); K8(TMT6plex); K13(TMT6plex)	2230.2	0				
																	DKDGKPLLPK	1	5	3	298919189;109637759;545746425	N-Term(TMT6plex); K2(TMT6plex); K5(TMT6plex); K10(TMT6plex)	2027.3	1				
																	DGKPLLPEPEEKPKPR	1	5	3	298919189;109637759;545746425	N-Term(TMT6plex); K3(TMT6plex); K12(TMT6plex); K14(TMT6plex)	2746.7	0				
																	AASSSK	1	7	3	298919189;109637759;545746425	N-Term(TMT6plex); K6(TMT6plex)	1008.6	0				
																	EETIPPDYR	3	5	3	298919189;109637759;545746425	N-Term(TMT6plex)	1348.7	0				
																	TTEETSKPKDD	1	5	3	298919189;109637759;545746425	N-Term(TMT6plex); K7(TMT6plex); K9(TMT6plex)	1938.1	1				
																	QAEPELDLR	2	5	3	298919189;109637759;545746425	N-Term(TMT6plex)	1299.7	0				

Supplementary Table 6. List of all proteins and peptides identified in untreated and chronically treated Het1A cells with cigarette smoke condensate for 8 months

Accession	RefSeq Accession	Gene Symbol	Entrez Gene ID	Description	Σ Coverage	Σ# Proteins	Σ# Unique Peptides	Σ# Peptides	Σ# PSMs	Het-1A-Smoke - 2M/Parental	Het-1A-Smoke - 4M/Parental	Het-1A-Smoke - 6M/Parental	Het-1A-Smoke - 8M/Parental	# AAs	MW [kDa]	calc. pI	Sequence	# PSMs	# Proteins	# Protein Groups	Protein Group Accessions	Modifications	MH+ [Da]	# Missed Cleavages	Het-1A-Smoke - 2M/Parental	Het-1A-Smoke - 4M/Parental	Het-1A-Smoke - 6M/Parental	Het-1A-Smoke - 8M/Parental
																	EKPSKPTEK	1	5	3	298919189;109637759;545746425	N-Term(TMT6plex); K2(TMT6plex); K5(TMT6plex); K9(TMT6plex)	1960.2	0				
																	EVTIPPK	1	5	3	298919189;109637759;545746425	N-Term(TMT6plex); K7(TMT6plex)	1241.8	0				
																	EHTEPK	1	3	1	298919189	N-Term(TMT6plex); K6(TMT6plex)	1198.7	0	1.4	1.3	1.4	1.0
																	AKEEDREK	1	5	3	298919189;109637759;545746425	N-Term(TMT6plex); K2(TMT6plex); K8(TMT6plex)	1692.0	2				
14043072	NP_112533.1	HNRNPA2B1	3181	heterogeneous nuclear ribonucleoproteins A2/B1 isoform B1	74.22	2	17	21	791	1.2	1.3	1.2	1.3	353	37.4	8.95												
																	KLFIGGLSFETTEESLR	14	2	1	14043072	N-Term(TMT6plex); K1(TMT6plex)	2385.3	1	1.2	1.7	1.6	1.5
																	LFIGGLSFETTEESLR	181	2	1	14043072	N-Term(TMT6plex)	2028.1	0	1.3	1.3	1.3	1.3
																	GGNFGFGDSR	49	2	1	14043072	N-Term(TMT6plex)	1242.6	0	1.2	1.3	1.2	1.3
																	EESGKPGAHVTVK	34	2	1	14043072	N-Term(TMT6plex); K5(TMT6plex); K13(TMT6plex)	2026.2	0	0.9	1.3	1.2	1.3
																	TLETVPLER	18	1	1	14043072	N-Term(TMT6plex)	1286.8	0	1.3	1.4	1.4	1.4
																	EDTEEHHLR	22	5	3	4504445;14043070;14043072	N-Term(TMT6plex)	1394.7	0				
																	QEMQEVQSSR	28	2	1	14043072	N-Term(TMT6plex); M3(Oxidation)	1466.7	0	1.3	1.4	1.4	1.2
																	GFGFVTFDDHDPVDK	58	2	1	14043072	N-Term(TMT6plex); K15(TMT6plex)	2154.1	0	1.1	1.4	1.3	1.4
																	NMGGPYGGGNYGPGGSGGSGGYGGR	6	2	1	14043072	N-Term(TMT6plex)	2419.1	0	1.4	1.4	1.6	1.3
																	NMGGPYGGGNYGPGGSGGSGGYGGR	82	2	1	14043072	N-Term(TMT6plex); M2(Oxidation)	2435.1	0	1.3	1.1	1.3	1.0
																	GGGGNFGPGPGSNFR	72	2	1	14043072	N-Term(TMT6plex)	1606.8	0	1.1	1.3	1.2	1.3
																	NYYEQWGK	42	2	1	14043072	N-Term(TMT6plex); K8(TMT6plex)	1545.8	0	1.2	1.1	1.2	1.1
																	LFVGGIKEDTEEHHLR	5	5	3	4504445;14043070;14043072	N-Term(TMT6plex); K7(TMT6plex)	2338.3	1				
																	QEMQEVQSSR	4	2	1	14043072	N-Term(TMT6plex)	1450.7	0	1.5	1.7	1.9	1.4
																	GGSDGYGSGR	10	2	1	14043072	N-Term(TMT6plex)	1141.5	0	1.1	1.3	1.2	1.3
																	DYFEEYGK	46	2	1	14043072	N-Term(TMT6plex); K8(TMT6plex)	1508.8	0	1.1	1.4	1.2	1.4
																	GGYGGGGPGYGNQGGGYGGGYDNYGGGNYGSGNYNDFGNYNQQPSNYGPMK	2	2	1	14043072	N-Term(TMT6plex); K51(TMT6plex)	5540.4	0	0.8	2.0	0.9	1.2
																	GFGDGYNGYGGGPGGGNFGGSPGYGGGR	14	2	1	14043072	N-Term(TMT6plex)	2724.2	0	1.4	1.4	1.4	1.4
																	IDTIEIITDR	37	2	1	14043072	N-Term(TMT6plex)	1417.8	0	1.3	1.2	1.3	1.1
																	GGYGGGGPGYGNQGGGYGGGYDNYGGGNYGSGNYNDFGNYNQQPSNYGPMK	7	2	1	14043072	N-Term(TMT6plex); M50(Oxidation); K51(TMT6plex)	5556.4	0	0.6	0.8	0.8	0.8
																	KLFVGGIK	14	7	5	14043072;34740329;4504445;14043070;5803036	N-Term(TMT6plex); K1(TMT6plex); K8(TMT6plex)	1549.0	1				
																	LTDCVVMR	3	2	1	14043072	N-Term(TMT6plex); C4(Carbamidomethyl)	1222.6	0	1.6	1.9	2.1	1.4
																	LTDCVVMR	22	2	1	14043072	N-Term(TMT6plex); C4(Carbamidomethyl); M7(Oxidation)	1238.6	0	1.3	1.4	1.3	1.3
																	SGNFGGSR	3	2	1	14043072	N-Term(TMT6plex)	1010.5	0	1.0	0.9	1.0	1.0
																	LFVGGIK	18	7	5	34740329;4504445;14043070;14043072;5803036	N-Term(TMT6plex); K7(TMT6plex)	1191.8	0				
4507879	NP_003365.1	VDAC1	7416	voltage-dependent anion-selective channel protein 1	74.20	1	16	17	552	1.5	1.6	1.6	1.7	283	30.8	8.54												
																	WTEYGLTFTEK	54	1	1	4507879	N-Term(TMT6plex); K11(TMT6plex)	1833.0	0	1.6	1.8	1.8	1.8
																	VTQSNFAVGYK	32	1	1	4507879	N-Term(TMT6plex); K11(TMT6plex)	1671.9	0	1.5	1.5	1.6	1.6
																	LTLSALLDGK	24	3	2	25188179;4507879	N-Term(TMT6plex); K10(TMT6plex)	1488.9	0				
																	EHINLGCDMDFDIAGPSIR	26	1	1	4507879	N-Term(TMT6plex); C7(Carbamidomethyl); M9(Oxidation)	2405.1	0	1.5	1.5	1.5	1.5
																	TDEFQLHTNVNDGTEFGGSIYQK	92	1	1	4507879	N-Term(TMT6plex); K23(TMT6plex)	3058.5	0	1.5	1.5	1.5	1.6
																	KLETAVNLAWTAGNSNTR	15	1	1	4507879	N-Term(TMT6plex); K1(TMT6plex)	2404.3	1	1.5	1.7	1.6	1.7
																	YQIDPDACFSAK	31	1	1	4507879	N-Term(TMT6plex); C8(Carbamidomethyl); K12(TMT6plex)	1873.0	0	1.5	1.8	1.7	1.7
																	SENGLEFTSSGSANTETTK	17	1	1	4507879	N-Term(TMT6plex); K19(TMT6plex)	2418.2	0	1.6	1.6	1.6	1.5
																	LTFDSSFSPNTGK	48	1	1	4507879	N-Term(TMT6plex); K13(TMT6plex)	1859.0	0	1.6	1.8	1.7	1.8
																	NVNAGGHK	6	1	1	4507879	N-Term(TMT6plex); K8(TMT6plex)	1254.7	0	1.3	1.5	1.5	1.5
																	TKSENGLEFTSSGSANTETTK	4	1	1	4507879	N-Term(TMT6plex); K2(TMT6plex); K21(TMT6plex)	2876.5	1	1.6	1.9	1.8	2.0
																	LETAVNLAWTAGNSNTR	5	1	1	4507879	N-Term(TMT6plex)	2047.1	0	1.6	1.5	1.4	1.5
																	WNTDNTLGTEITVEDQLAR	102	1	1	4507879	N-Term(TMT6plex)	2405.2	0	1.7	1.6	1.6	1.6
																	VTGSLETK	21	1	1	4507879	N-Term(TMT6plex); K8(TMT6plex)	1292.8	0	1.6	1.6	1.7	1.6
																	VNNSSLIGLGYTQTLKPGIK	37	1	1	4507879	N-Term(TMT6plex); K16(TMT6plex); K20(TMT6plex)	2790.7	0	1.4	1.6	1.7	1.6
																	LGLGLEFQA	20	1	1	4507879	N-Term(TMT6plex)	1176.7	0	1.5	1.5	1.6	1.7
																	GYGFGLIK	16	1	1	4507879	N-Term(TMT6plex); K8(TMT6plex)	1312.8	0	1.5	2.0	1.8	1.9
																	EHINLGCDMDFDIAGPSIR	2	1	1	4507879	N-Term(TMT6plex); C7(Carbamidomethyl)	2389.1	0	1.3	1.6	1.5	1.5
21493022	NP_005091.2	AKAP12	9590	A-kinase anchor protein 12 isoform 1	74.13	2	101	101	1098	1.1	1.4	1.7	1.5	1782	191.4	4.41												
																	VVGQTTPESFEK	24	2	1	21493022	N-Term(TMT6plex); K12(TMT6plex)	1780.0	0	1.2	1.5	1.7	1.5
																	EVIAEEEPPTVTEPLPENR	16	2	1	21493022	N-Term(TMT6plex)	2378.2	0	1.1	1.5	1.6	1.6
																	DVPFFEGLEGSIDTGITVSR	64	2	1	21493022	N-Term(TMT6plex)	2368.2	0	1.2	1.6	1.7	1.5
																	LTASEQAHPQEPAESAHEPR	10	2	1	21493022	N-Term(TMT6plex)	2414.2	0	1.2	1.4	1.7	1.5
																	EKEDAQEVELQEGK	28	2	1	21493022	N-Term(TMT6plex); K2(TMT6plex); K14(TMT6plex)	2319.3	1	1.1	1.5	1.7	1.5
																	LKETCVSGEDPTQGADLSPDEK	10	2	1	21493022	N-Term(TMT6plex); K2(TMT6plex); C5(Carbamidomethyl); K22(TMT6plex)	3063.6	1	1.3	1.6	1.7	1.6
																	EDEVEASEK	11	2	1	21493022	N-Term(TMT6plex); K9(TMT6plex)	1493.8	0	1.1	1.3	1.6	1.4
																	SAVVHDITDDGQEETPEIIEQIPSSESNLEELTQPTESQANDIGFK	10	2	1	21493022	N-Term(TMT6plex); K46(TMT6plex)	5498.7	0	1.2	1.5	1.8	1.6
																	SAEQPEQK	14	2	1	21493022	N-Term(TMT6plex); K8(TMT6plex)	1374.8	0	1.1	1.4	1.8	1.5
																	TEKPDTVQLLTVK	8	2	1	21493022	N-Term(TMT6plex); K3(TMT6plex); K13(TMT6plex)	2159.3	0	0.9	1.1	1.8	1.2
																	TEVEETAGSVPAEELVEMDAEPQEAEPAK	19	2	1	21493022	N-Term(TMT6plex); M18(Oxidation); K29(TMT6plex)	3559.7	0	1.2	1.4	1.7	1.4
																	SATLSSTESTASEMQEEMK	14	2	1	21493022	N-Term(TMT6plex); M14(Oxidation); M18(Oxidation); K19(TMT6plex)	2537.2	0	1.7	1.8	2.2	1.8
																	VTEVALKGEGTEEAECK	3	2	1	21493022	N-Term(TMT6plex); K7(TMT6plex); C16(Carbamidomethyl); K17(TMT6plex)	2537.4	1	1.2	1.7	1.8	1.7
																	ELSESQVHMMAAAVADGTR	8	2	1	21493022	N-Term(TMT6plex); M9(Oxidation); M10(Oxidation)	2264.1	0	1.3	1.2	1.3	1.4
																	VELPSEEQVSGSQGPSEEKPAPLATEVFDEK	12	2	1	21493022	N-Term(TMT6plex); K19(TMT6plex); K31(TMT6plex)	4001.1	0	1.3	1.7	2.0	1.7
																	LVQNIIQTAVDQFVR	20	2	1	21493022	N-Term(TMT6plex)	1973.1	0	1.2	1.4	2.0	1.3
																	GLAEVQQDGEAEEGATSDGEK	31	2	1	21493022	N-Term(TMT6plex); K21(TMT6plex)	2578.3	0	1.3	1.5	1.7	1.6
																	STPVIVSATTK	22	2	1	21493022	N-Term(TMT6plex); K11(TMT6plex)	1562.0	0	1.1	1.3	1.6	1.4
																	SPSWISASVTEPLEQVEAEAALLTEEVLER	3	2	1	21493022	N-Term(TMT6plex)	3512.8	0	1.1	2.1	2.2	1.5
																	AEAERPEEQAEASGLK	26	2	1	21493022	N-Term(TMT6plex); K16(TMT6plex)	2173.2	0	1.2	1.3	1.7	1.4
																	ERPPAPSSFVFQEETK	15	2	1	21493022	N-Term(TMT6plex); K16(TMT6plex)	2307.2	0	1.2	1.5	1.7	1.5
																	IEVHQEEVVAEVHVSTVEER	10	2	1	21493022	N-Term(TMT6plex)	2547.3	0	1.2	1.5	1.4	1.5
																	NEDFAAHPGEDAVPTGPDCQAK	15	2	1	21493022	N-Term(TMT6plex); C19(Carbamidomethyl); K22(TMT6plex)	2784.3	0	1.3	1.2	1.7	1.3
																	KLFTSTGLK	4	2	1	21493022	N-Term(TMT6plex); K1(TMT6plex); K9(TMT6plex)	1682.1	1	1.0	1.5	2.0	1.6
																	MEDTLEHTDKEVSVETVSILSK	4	2	1	21493022	N-Term(TMT6plex); M1(Oxidation); K10(TMT6plex); K22(TMT6plex)	3193.7	1	1.0	1.2	1.5	1.4
																	EQEPEKVDTEEDGKAEVASEK	1	2	1	21493022	N-Term(TMT6plex); K6(TMT6plex); K14(TMT6plex); K21(TMT6plex)	3263.7	2	1.1	1.5	1.6	1.4
																	LSAEYEKVELPSEEQVSGSQGPSEEKPAPLATEVFDEK	1	2	1	21493022	N-Term(TMT6plex); K7(TMT6plex); K26(TMT6plex); K38(TMT6plex)	5050.6	1	2.6	4.6	4.0	3.5
																	QSTEKPEETLK	5	2	1	21493022	N-Term(TMT6plex); K5(TMT6plex); K11(TMT6plex)	1977.1	0	1.0	1.1	1.8	1.3
																	TEPFTQGK	10	2	1	21493022	N-Term(TMT6plex); K8(TMT6plex)	1365.8	0	1.1	1.2	1.7	1.3
																	DEIVEIHEENEVASGTQSGGTEAEAVPAQK	14	2	1	21493022	N-Term(TMT6plex); K30(TMT6plex)	3582.8	0	1.1	1.4	1.8	1.6
																	EDAQEVELQEGK	5	2	1	21493022	N-Term(TMT6plex); K12(TMT6plex)	1833.0	0	1.3	1.6	1.9	1.4
																	EVSSLEGSPPPCLGQEEAVCTK	10	2	1	21493022	N-Term(TMT6plex); C12(Carbamidomethyl); C20(Carbamidomethyl); K22(TMT6plex)	2832.4	0	1.3	1.5	1.8	1.4
																	LEHETAVTVSEEVSK	8	2	1	21493022	N-Term(TMT6plex); K15(TMT6plex)	2116.2	0	1.2	2.1	1.8	1.6
																	AATIIEER	15	2	1	21493022	N-Term(TMT6plex)	1131.7	0	1.2	1.4	1.6	1.5
																	KDDALELQSHAK	10	2	1	21493022	N-Term(TMT6plex); K1(TMT6plex); K12(TMT6plex)	2042.2	1	1.1	1.4	1.5	1.5
																	LFTSTGLK	10	2	1	21493022	N-Term(TMT6plex); K8(TMT6plex)	1324.8	0	1.1	1.1	1.5	1.1
																	DMSEASEK	16	2	1	21493022	N-Term(TMT6plex); M2(Oxidation); K8(TMT6plex)	1370.7	0	1.1	1.6	2.1	1.5
																	EDEKGDDVDDPENQNSALADTDASGGLTK	19	2	1	21493022	N-Term(TMT6plex); K4(TMT6plex); K29(TMT6plex)	3693.8	1	1.4	1.7	1.9	1.6
																	SSEKNEDFAAHPGEDAVPTGPDCQAK	9	2	1	21493022	N-Term(TMT6plex); K4(TMT6plex); C23(Carbamidomethyl); K26(TMT6plex)	3444.7	1	1.0	1.4	1.7	1.4
																	EKTEAEPTHVNEEKLEHETAVTVSEEVSK	2	2	1	21493022	N-Term(TMT6plex); K2(TMT6plex); K14(TMT6plex); K29(TMT6plex)	4196.2	2	1.4	1.9	1.9	1.7
																	LSAEYEK	8	2	1	21493022	N-Term(TMT6plex); K7(TMT6plex)	1297.7	0	1.2	1.6	1.9	1.6
																	MEAQQAQK	6	2	1	21493022	N-Term(TMT6plex); M1(Oxidation); K8(TMT6plex)	1407.8	0	1.3	1.7	2.3	1.6
																	DDALELQSHAK	4	2	1	21493022	N-Term(TMT6plex); K11(TMT6plex)	1684.9	0	1.2	1.5	1.7	1.6
																	VDTEEDGKAEVASEK	2	2	1	21493022	N-Term(TMT6plex); K8(TMT6plex); K15(TMT6plex)	2294.2	1	1.2	1.2	1.6	1.4
																	EMVVQVER	3	2	1	21493022	N-Term(TMT6plex); M2(Oxidation)	1234.7	0	1.0	1.2	1.2	1.2
																	EKTEAEPTHVNEEK	6	2	1	21493022	N-Term(TMT6plex); K2(TMT6plex); K14(TMT6plex)	2328.3	1	1.0	1.4	1.5	1.4
																	SAESPTSPVTSETGSTFK	20	2	1	21493022	N-Term(TMT6plex); K18(TMT6plex)	2271.2	0	1.2	1.3	1.7	1.5
																	DMSEASEK	2	2	1	21493022	N-Term(TMT6plex); K8(TMT6plex)	1354.7	0	1.5	2.2	3.1	1.6

Khan et al., 2019. Multi-omics analysis to characterize cigarette smoke induced molecular alterations in esophageal cells
Supplementary Table 6. List of all proteins and peptides identified in untreated and chronically treated Het1A cells with cigarette smoke condensate for 8 months

Accession	RefSeq Accession	Gene Symbol	Entrez Gene ID	Description	Σ Coverage	Σ# Proteins	Σ# Unique Peptides	Σ# Peptides	Σ# PSMs	Het-1A-Smoke - 2M/Parental	Het-1A-Smoke - 4M/Parental	Het-1A-Smoke - 6M/Parental	Het-1A-Smoke - 8M/Parental	# AAs	MW [kDa]	calc. pI	Sequence	# PSMs	# Proteins	# Protein Groups	Protein Group Accessions	Modifications	MH+ [Da]	# Missed Cleavages	Het-1A-Smoke - 2M/Parental	Het-1A-Smoke - 4M/Parental	Het-1A-Smoke - 6M/Parental	Het-1A-Smoke - 8M/Parental
																	TQEVLQAVAEK	13	2	1	21493022	N-Term(TMT6plex); K11(TMT6plex)	1674.0	0	**1.1**	**1.2**	**1.6**	**1.2**
																	SDEVDEQVACQEVK	14	2	1	21493022	N-Term(TMT6plex); C10(Carbamidomethyl); K14(TMT6plex)	2094.1	0	**1.2**	**1.5**	**2.0**	**1.4**
																	TEETATEMLTSELQTQAHVIK	26	2	1	21493022	N-Term(TMT6plex); M8(Oxidation); K21(TMT6plex)	2834.5	0	**1.2**	**1.5**	**1.8**	**1.4**
																	QLLQTVNVPIIDGAK	20	2	1	21493022	N-Term(TMT6plex); K15(TMT6plex)	2067.3	0	**1.2**	**1.4**	**1.8**	**1.5**
																	EQSHAEISPPAESGQAVEECKEEGEEK	15	2	1	21493022	N-Term(TMT6plex); C20(Carbamidomethyl); K21(TMT6plex); K27(TMT6plex)	3671.8	1	**1.3**	**1.5**	**2.0**	**1.7**
																	TEGTQEADQYADEK	11	2	1	21493022	N-Term(TMT6plex); K14(TMT6plex)	2043.0	0	**1.3**	**1.3**	**1.8**	**1.3**
																	GEGTEEAECK	8	2	1	21493022	N-Term(TMT6plex); C9(Carbamidomethyl); K10(TMT6plex)	1567.8	0	**1.1**	**1.5**	**2.1**	**1.5**
																	ETCVSGEDPTQGADLSPDEK	4	2	1	21493022	N-Term(TMT6plex); C3(Carbamidomethyl); K20(TMT6plex)	2593.2	0	**1.9**	**1.2**	**2.4**	**2.1**
																	SPEQPPEGSSTPAEPEPSGGGPSAEAAPDTTADPAIAASDPATK	2	1	1	21493022	N-Term(TMT6plex); K44(TMT6plex)	4601.2	0	**0.6**	**0.6**	**1.1**	**0.6**
																	FFTQGWAGWR	4	2	1	21493022	N-Term(TMT6plex)	1484.8	0	**1.2**	**1.0**	**1.8**	**1.1**
																	GSVEEPKPEEPK	18	2	1	21493022	N-Term(TMT6plex); K7(TMT6plex); K12(TMT6plex)	2013.1	0	**1.3**	**1.6**	**1.8**	**1.5**
																	VKEESQLPGTGGPEDVLQPVQR	23	2	1	21493022	N-Term(TMT6plex); K2(TMT6plex)	2821.5	1	**1.2**	**1.4**	**1.6**	**1.4**
																	EESESTAVGQAHSDISK	22	2	1	21493022	N-Term(TMT6plex); K17(TMT6plex)	2233.1	0	**1.3**	**1.3**	**1.4**	**1.3**
																	VLGETANILETGETLEPAGAHLVLEEK	22	2	1	21493022	N-Term(TMT6plex); K27(TMT6plex)	3291.8	0	**1.1**	**1.5**	**1.9**	**1.5**
																	QEQAPVEDAGPTGANEDDSDVPAVVPLSEYDAVER	12	2	1	21493022	N-Term(TMT6plex)	3898.8	0	**1.3**	**1.5**	**1.8**	**1.5**
																	IQVQSSEASFTLTAAAEEEK	32	2	1	21493022	N-Term(TMT6plex); K20(TMT6plex)	2597.4	0	**1.4**	**1.5**	**1.8**	**1.4**
																	AITPQAQEELQK	46	2	1	21493022	N-Term(TMT6plex); K12(TMT6plex)	1814.0	0	**1.1**	**1.5**	**1.7**	**1.5**
																	GGGDEESGEHTQVPADSPDSQEEQK	10	2	1	21493022	N-Term(TMT6plex); K25(TMT6plex)	3071.4	0	**0.8**	**1.5**	**2.0**	**1.5**
																	TMTVEVEGSTVNDQQLEEVVLPSEEEGGGAGTK	18	2	1	21493022	N-Term(TMT6plex); M2(Oxidation); K33(TMT6plex)	3893.9	0	**1.2**	**1.3**	**1.5**	**1.3**
																	SVPEDDGHALLAER	22	2	1	21493022	N-Term(TMT6plex)	1737.9	0	**1.3**	**1.4**	**1.5**	**1.5**
																	APQVTESIESSELVTTCQAETLAGVK	20	2	1	21493022	N-Term(TMT6plex); C17(Carbamidomethyl); K26(TMT6plex)	3206.7	0	**1.1**	**1.6**	**1.9**	**1.5**
																	GLSSDLEGEK	12	2	1	21493022	N-Term(TMT6plex); K10(TMT6plex)	1492.8	0	**1.0**	**1.5**	**1.7**	**1.5**
																	KGLSSDLEGEK	6	2	1	21493022	N-Term(TMT6plex); K1(TMT6plex); K11(TMT6plex)	1850.1	1	**1.2**	**1.4**	**1.6**	**1.4**
																	TEETATEMLTSELQTQAHVIK	5	2	1	21493022	N-Term(TMT6plex); K21(TMT6plex)	2818.5	0	**1.5**	**1.8**	**2.1**	**1.6**
																	VHSESDK	4	2	1	21493022	N-Term(TMT6plex); K7(TMT6plex)	1259.7	0	**1.1**	**1.3**	**1.8**	**1.4**
																	VLSKPPEGVVSEVEMLSSQER	31	2	1	21493022	N-Term(TMT6plex); K4(TMT6plex); M15(Oxidation)	2774.5	0	**1.1**	**1.3**	**1.6**	**1.3**
																	SEDSIAGSGVEHSTPDTEPGKEESWVSIK	6	2	1	21493022	N-Term(TMT6plex); K21(TMT6plex); K29(TMT6plex)	3745.9	1	**1.1**	**1.7**	**2.0**	**1.7**
																	TKDVPFFEGLEGSIDTGITVSR	2	2	1	21493022	N-Term(TMT6plex); K2(TMT6plex)	2826.5	1	**1.3**	**1.7**	**1.7**	**1.6**
																	VLSKPPEGVVSEVEMLSSQER	5	2	1	21493022	N-Term(TMT6plex); K4(TMT6plex)	2758.5	0	**1.4**	**1.6**	**1.6**	**1.5**
																	VTEVALK	8	2	1	21493022	N-Term(TMT6plex); K7(TMT6plex)	1217.8	0	**1.1**	**1.4**	**1.6**	**1.4**
																	EKVTEVALK	3	2	1	21493022	N-Term(TMT6plex); K2(TMT6plex); K9(TMT6plex)	1704.1	1	**1.0**	**1.8**	**1.9**	**1.7**
																	AEAERPEEQAEASGLKK	4	2	1	21493022	N-Term(TMT6plex); K16(TMT6plex); K17(TMT6plex)	2530.4	1	**1.2**	**2.0**	**2.1**	**2.0**
																	ESPDTNGPK	2	2	1	21493022	N-Term(TMT6plex); K9(TMT6plex)	1402.8	0	**1.1**	**1.3**	**1.9**	**1.2**
																	SATLSSTESTASEMQEEMK	6	2	1	21493022	N-Term(TMT6plex); M14(Oxidation); K19(TMT6plex)	2521.2	0	**1.6**	**1.6**	**1.9**	**1.6**
																	GESSASSPEEPEEITCLEK	5	2	1	21493022	N-Term(TMT6plex); C16(Carbamidomethyl); K19(TMT6plex)	2537.2	0	**1.2**	**1.4**	**1.6**	**1.3**
																	AATEVSK	9	2	1	21493022	N-Term(TMT6plex); K7(TMT6plex)	1163.7	0	**1.1**	**1.4**	**1.6**	**1.5**
																	GSSSDEEGGPK	2	2	1	21493022	N-Term(TMT6plex); K11(TMT6plex)	1507.8	0	**2.0**	**3.1**	**3.6**	**1.7**
																	MEAQQAQK	2	2	1	21493022	N-Term(TMT6plex); K8(TMT6plex)	1391.8	0	**1.7**	**2.2**	**3.5**	**1.5**
																	ADSQDAGQETEK	2	2	1	21493022	N-Term(TMT6plex); K12(TMT6plex)	1736.9	0	**0.9**	**1.4**	**1.8**	**1.6**
																	GDDVDDPENQNSALADTDASGGLTK	2	2	1	21493022	N-Term(TMT6plex); K25(TMT6plex)	2963.4	0	**1.0**	**1.1**	**1.2**	**1.4**
																	TEAEPTHVNEEK	2	2	1	21493022	N-Term(TMT6plex); K12(TMT6plex)	1842.0	0	**0.9**	**1.1**	**1.5**	**1.3**
																	SEDSIAGSGVEHSTPDTEPGK	2	2	1	21493022	N-Term(TMT6plex); K21(TMT6plex)	2558.3	0	**1.3**	**1.4**	**1.7**	**1.4**
																	KPKEDEVEASEK	1	2	1	21493022	N-Term(TMT6plex); K1(TMT6plex); K3(TMT6plex); K12(TMT6plex)	2305.3	1	**1.1**	**1.4**	**1.6**	**1.6**
																	EQEPEKVDTEEDGK	1	2	1	21493022	N-Term(TMT6plex); K6(TMT6plex); K14(TMT6plex)	2320.2	1	**1.5**	**1.3**	**1.7**	**1.5**
																	QSTEKPEETLKR	1	2	1	21493022	N-Term(TMT6plex); K5(TMT6plex); K11(TMT6plex)	2133.2	1	**1.1**	**1.4**	**1.8**	**1.6**
																	GGGDEESGEHTQVPADSPDSQEEQKGESSASSPEEPEEITCLEK	1	2	1	21493022	N-Term(TMT6plex); K25(TMT6plex); C41(Carbamidomethyl); K44(TMT6plex)	5360.5	1	**0.9**	**1.0**	**2.9**	**1.5**
																	RPSESDKEDELDK	1	2	1	21493022	N-Term(TMT6plex); K7(TMT6plex); K13(TMT6plex)	2235.2	1	**1.9**	**2.1**	**2.4**	**1.6**
																	ETDVVLK	11	2	1	21493022	N-Term(TMT6plex); K7(TMT6plex)	1261.8	0	**1.3**	**1.6**	**1.9**	**1.6**
																	VDAQEAK	1	2	1	21493022	N-Term(TMT6plex); K7(TMT6plex)	1218.7	0	**1.4**	**1.5**	**1.8**	**1.5**
																	EKMEAQQAQK	1	2	1	21493022	N-Term(TMT6plex); K2(TMT6plex); M3(Oxidation); K10(TMT6plex)	1894.1	1	**1.0**	**1.5**	**1.7**	**1.7**
																	AMGGDHQK	2	2	1	21493022	N-Term(TMT6plex); M2(Oxidation); K8(TMT6plex)	1317.7	0	**1.0**	**1.6**	**1.7**	**1.6**
																	DSDKEMATK	1	2	1	21493022	N-Term(TMT6plex); K4(TMT6plex); M6(Oxidation); K9(TMT6plex)	1727.9	1	**1.0**	**1.3**	**1.8**	**1.6**
																	RPSESDKEDELDKVK	1	2	1	21493022	N-Term(TMT6plex); K7(TMT6plex); K13(TMT6plex); K15(TMT6plex)	2691.5	2	**1.5**	**2.2**	**2.3**	**1.6**
																	EGVTPWASFK	1	2	1	21493022	N-Term(TMT6plex); K10(TMT6plex)	1579.9	0	**1.1**	**1.5**	**1.6**	**1.4**
																	QEKEPSK	2	2	1	21493022	N-Term(TMT6plex); K3(TMT6plex); K7(TMT6plex)	1532.9	1	**0.9**	**1.4**	**1.8**	**1.4**
																	SLVEPK	2	2	1	21493022	N-Term(TMT6plex); K6(TMT6plex)	1130.7	0	**1.0**	**1.4**	**1.8**	**1.5**
																	ADEAGK	1	2	1	21493022	N-Term(TMT6plex); K6(TMT6plex)	1048.6	0	**1.0**	**1.2**	**1.7**	**1.3**
																	VQGSPLKK	1	2	1	21493022	N-Term(TMT6plex); K7(TMT6plex); K8(TMT6plex)	1544.0	1	**1.1**	**1.8**	**1.9**	**1.7**
4507947	NP_003671.1	**YARS**	8565	**tyrosine--tRNA ligase, cytoplasmic**	74.05	1	38	38	249	**1.8**	**1.8**	**1.5**	**1.7**	528	59.1	7.05												
																	APWELLELR	12	1	1	4507947	N-Term(TMT6plex)	1355.8	0	**2.2**	**2.3**	**1.6**	**2.1**
																	NLQEVLGEEK	4	1	1	4507947	N-Term(TMT6plex); K10(TMT6plex)	1616.9	0	**1.9**	**1.6**	**1.4**	**1.6**
																	VHLMNPMVPGLTGSK	1	1	1	4507947	N-Term(TMT6plex); M4(Oxidation); M7(Oxidation); K15(TMT6plex)	2071.1	0	**1.5**	**1.3**	**1.3**	**1.4**
																	HPDADSLYVEK	7	1	1	4507947	N-Term(TMT6plex); K11(TMT6plex)	1731.9	0	**1.4**	**1.6**	**1.5**	**1.6**
																	GTDYQLSK	6	1	1	4507947	N-Term(TMT6plex); K8(TMT6plex)	1369.8	0	**1.8**	**2.0**	**1.6**	**1.9**
																	QVEPLDPPAGSAPGEHVFVK	12	1	1	4507947	N-Term(TMT6plex); K20(TMT6plex)	2532.4	0	**1.7**	**1.7**	**1.4**	**1.6**
																	GVESQGMLLCASIEGINR	10	1	1	4507947	N-Term(TMT6plex); M7(Oxidation); C10(Carbamidomethyl)	2179.1	0	**1.5**	**1.7**	**1.5**	**1.7**
																	MSSSEEESK	8	1	1	4507947	N-Term(TMT6plex); M1(Oxidation); K9(TMT6plex)	1487.7	0	**2.1**	**1.9**	**1.4**	**1.7**
																	TYTAYVDLEK	6	1	1	4507947	N-Term(TMT6plex); K10(TMT6plex)	1660.9	0	**1.9**	**1.5**	**1.4**	**1.4**
																	TVVSGLVQFVPK	12	1	1	4507947	N-Term(TMT6plex); K12(TMT6plex)	1732.1	0	**2.7**	**1.9**	**1.6**	**1.9**
																	NSEPEEVIPSR	4	1	1	4507947	N-Term(TMT6plex)	1485.8	0	**1.9**	**1.7**	**1.5**	**1.6**
																	NSVEVALNK	7	1	1	4507947	N-Term(TMT6plex); K9(TMT6plex)	1431.9	0	**1.7**	**1.5**	**1.4**	**1.5**
																	AGCEVTILFADLHAYLDNMK	4	1	1	4507947	N-Term(TMT6plex); C3(Carbamidomethyl); K20(TMT6plex)	2739.4	0	**2.6**	**2.6**	**2.4**	**1.9**
																	HVLFPLK	8	1	1	4507947	N-Term(TMT6plex); K7(TMT6plex)	1311.9	0	**1.8**	**1.9**	**1.4**	**1.7**
																	QVEHPLLSGLLYPGLQALDEEYLK	23	1	1	4507947	N-Term(TMT6plex); K24(TMT6plex)	3183.7	0	**2.0**	**1.6**	**1.5**	**1.5**
																	AGCEVTILFADLHAYLDNMK	5	1	1	4507947	N-Term(TMT6plex); C3(Carbamidomethyl); M19(Oxidation); K20(TMT6plex)	2755.4	0	**1.9**	**1.6**	**1.5**	**1.6**
																	LVVVLCNLKPQK	13	1	1	4507947	N-Term(TMT6plex); C6(Carbamidomethyl); K9(TMT6plex); K12(TMT6plex)	2098.3	0	**2.0**	**2.5**	**2.0**	**2.0**
																	AFCEPGNVENNGVLSFIK	6	1	1	4507947	N-Term(TMT6plex); C3(Carbamidomethyl); K18(TMT6plex)	2453.3	0	**1.6**	**1.8**	**1.6**	**1.6**
																	KAGAEVVK	2	1	1	4507947	N-Term(TMT6plex); K1(TMT6plex); K8(TMT6plex)	1489.0	1	**1.3**	**1.6**	**1.4**	**1.4**
																	ISEECIAQWK	6	1	1	4507947	N-Term(TMT6plex); C5(Carbamidomethyl); K10(TMT6plex)	1721.9	0	**1.8**	**1.9**	**1.6**	**1.7**
																	LASAAYPDPSK	14	1	1	4507947	N-Term(TMT6plex); K11(TMT6plex)	1577.9	0	**1.5**	**1.6**	**1.5**	**1.7**
																	VSYYENVIK	25	1	1	4507947	N-Term(TMT6plex); K9(TMT6plex)	1572.9	0	**1.9**	**1.9**	**1.6**	**1.9**
																	VDAQFGGIDQR	4	1	1	4507947	N-Term(TMT6plex)	1434.8	0	**1.2**	**1.4**	**1.4**	**1.4**
																	LSSVVTQHDSK	4	1	1	4507947	N-Term(TMT6plex); K11(TMT6plex)	1658.9	0	**1.6**	**1.5**	**1.4**	**1.4**
																	IDVGEAEPR	4	1	1	4507947	N-Term(TMT6plex)	1214.7	0	**1.6**	**1.6**	**1.5**	**1.6**
																	IFTFAEK	2	1	1	4507947	N-Term(TMT6plex); K7(TMT6plex)	1313.8	0	**2.1**	**1.6**	**1.5**	**1.6**
																	DFAAEVVHPGDLK	10	1	1	4507947	N-Term(TMT6plex); K13(TMT6plex)	1856.0	0	**1.6**	**1.8**	**1.5**	**1.7**
																	YLPALGYSK	6	1	1	4507947	N-Term(TMT6plex); K9(TMT6plex)	1469.9	0	**2.0**	**2.2**	**1.5**	**1.9**
																	GQPDEELKPK	3	1	1	4507947	N-Term(TMT6plex); K8(TMT6plex); K10(TMT6plex)	1828.1	0	**1.7**	**2.1**	**1.5**	**1.8**
																	SEFVILR	4	1	1	4507947	N-Term(TMT6plex)	1092.7	0	**2.2**	**2.0**	**1.6**	**1.8**
																	AMLESIGVPLEK	3	1	1	4507947	N-Term(TMT6plex); M2(Oxidation); K12(TMT6plex)	1761.0	0	**1.5**	**1.7**	**1.4**	**1.6**
																	QTNFMTK	2	1	1	4507947	N-Term(TMT6plex); M5(Oxidation); K7(TMT6plex)	1343.7	0	**1.8**	**1.6**	**1.4**	**1.6**
																	EYTLDVYR	4	1	1	4507947	N-Term(TMT6plex)	1287.7	0	**1.6**	**1.6**	**1.4**	**1.5**
																	KIFTFAEK	1	1	1	4507947	N-Term(TMT6plex); K1(TMT6plex); K8(TMT6plex)	1671.0	1	**1.4**	**1.9**	**1.4**	**1.7**
																	IDLLDR	2	1	1	4507947	N-Term(TMT6plex)	973.6	0	**1.8**	**1.6**	**1.4**	**1.5**
																	LGSISCK	1	1	1	4507947	N-Term(TMT6plex); C6(Carbamidomethyl); K7(TMT6plex)	1222.7	0	**2.0**	**1.7**	**1.5**	**1.6**
																	IADFLK	2	1	1	4507947	N-Term(TMT6plex); K6(TMT6plex)	1164.7	0	**1.6**	**1.8**	**1.5**	**1.6**
																	IITVEK	1	1	1	4507947	N-Term(TMT6plex); K6(TMT6plex)	1160.8	0	**2.0**	**1.9**	**1.5**	**2.0**
																	AGAEVVK	1	1	1	4507947	N-Term(TMT6plex); K7(TMT6plex)	1131.7	0	**1.6**	**1.4**	**1.4**	**1.4**
61743954	NP_001611.1	**AHNAK**	79026	**neuroblast differentiation-associated protein AHNAK isoform 1**	74.04	10	254	256	2170	**1.1**	**1.3**	**1.4**	**1.2**	5890	628.7	6.15												
																	VDVEGPDVNIEGPEGK	14	1	1	61743954	N-Term(TMT6plex); K16(TMT6plex)	2112.1	0	**1.1**	**1.3**	**1.2**	**1.0**
																	VSVGAPDLSLEASEGSIK	6	1	1	61743954	N-Term(TMT6plex); K18(TMT6plex)	2217.2	0	**1.0**	**1.4**	**1.4**	**1.2**
																	VQANLGAPDINIEGLDAK	15	1	1	61743954	N-Term(TMT6plex); K18(TMT6plex)	2296.3	0	**1.3**	**1.4**	**1.4**	**1.4**
																	VNVEAPDVNLEGLGGK	11	1	1	61743954	N-Term(TMT6plex); K16(TMT6plex)	2069.2	0	**1.1**	**1.4**	**1.5**	**1.3**
																	AGAISASGPELQGAGHSK	6	1	1	61743954	N-Term(TMT6plex); K18(TMT6plex)	2096.1	0	**1.1**	**1.3**	**1.3**	**1.3**

Accession	RefSeq Accession	Gene Symbol	Entrez Gene ID	Description	Σ Coverage	Σ# Proteins	Σ# Unique Peptides	Σ# Peptides	Σ# PSMs	Het-1A-Smoke - 2M/Parental	Het-1A-Smoke - 4M/Parental	Het-1A-Smoke - 6M/Parental	Het-1A-Smoke - 8M/Parental	# AAs	MW [kDa]	calc. pI	Sequence	# PSMs	# Proteins	# Protein Groups	Protein Group Accessions	Modifications	MH+ [Da]	# Missed Cleavages	Het-1A-Smoke - 2M/Parental	Het-1A-Smoke - 4M/Parental	Het-1A-Smoke - 6M/Parental	Het-1A-Smoke - 8M/Parental
																	VDIDVPDVNVQGPDWHLK	14	1	1	61743954	N-Term(TMT6plex); K18(TMT6plex)	2504.4	0	**1.1**	**1.2**	**1.3**	**1.1**
																	VDVECPDVNIEGPEGK	6	1	1	61743954	N-Term(TMT6plex); C5(Carbamidomethyl); K16(TMT6plex)	2215.1	0	**1.0**	**1.2**	**1.4**	**0.9**
																	MDVNVGDIDIEGPEGK	14	1	1	61743954	N-Term(TMT6plex); M1(Oxidation); K16(TMT6plex)	2162.1	0	**1.2**	**1.3**	**1.5**	**1.1**
																	VDINAPDVDVQGPDWHLK	23	1	1	61743954	N-Term(TMT6plex); K18(TMT6plex)	2476.3	0	**1.1**	**1.3**	**1.3**	**1.1**
																	VDIDVPDVNIEGPEGK	15	1	1	61743954	N-Term(TMT6plex); K16(TMT6plex)	2154.2	0	**1.1**	**1.2**	**1.3**	**1.1**
																	VDVNAPDVQAPDWHLK	8	1	1	61743954	N-Term(TMT6plex); K16(TMT6plex)	2262.2	0	**1.1**	**1.3**	**1.4**	**1.2**
																	TPSFGISAPQVSIPDVNVNLK	15	1	1	61743954	N-Term(TMT6plex); K21(TMT6plex)	2641.5	0	**1.2**	**1.3**	**1.4**	**1.3**
																	IEGEMQVPDVDIR	4	1	1	61743954	N-Term(TMT6plex); M5(Oxidation)	1745.9	0	**1.3**	**1.3**	**1.4**	**1.1**
																	GDVDISLPK	14	1	1	61743954	N-Term(TMT6plex); K9(TMT6plex)	1401.8	0	**1.0**	**1.2**	**1.4**	**1.1**
																	GPSLQGDLAVSGDIK	10	1	1	61743954	N-Term(TMT6plex); K15(TMT6plex)	1915.1	0	**1.1**	**1.4**	**1.3**	**1.2**
																	GSEVGFHGAAPDISVK	6	1	1	61743954	N-Term(TMT6plex); K16(TMT6plex)	2029.1	0	**1.0**	**1.3**	**1.3**	**1.3**
																	ISMSEVDLNVAAPK	8	1	1	61743954	N-Term(TMT6plex); M3(Oxidation); K14(TMT6plex)	1948.1	0	**1.0**	**1.2**	**1.4**	**1.2**
																	ISMPDIDLNLK	54	1	1	61743954	N-Term(TMT6plex); M3(Oxidation); K11(TMT6plex)	1733.0	0	**1.1**	**1.4**	**1.4**	**1.2**
																	VDINAPDVDVHGPDWHLK	10	1	1	61743954	N-Term(TMT6plex); K18(TMT6plex)	2485.3	0	**1.0**	**1.3**	**1.4**	**1.1**
																	VDINAPDVEVHGPDWHLK	5	1	1	61743954	N-Term(TMT6plex); K18(TMT6plex)	2499.3	0	**1.1**	**1.3**	**1.2**	**1.3**
																	GHYEVTGSDDETGK	4	1	1	61743954	N-Term(TMT6plex); K14(TMT6plex)	1953.0	0	**1.3**	**1.6**	**1.6**	**1.5**
																	QGFDLNVPGGEIDASLK	10	1	1	61743954	N-Term(TMT6plex); K17(TMT6plex)	2218.2	0	**1.1**	**1.2**	**1.3**	**1.2**
																	VDISAPDVDVHGPDWHLK	7	1	1	61743954	N-Term(TMT6plex); K18(TMT6plex)	2458.3	0	**1.0**	**1.3**	**1.3**	**1.2**
																	GDFDVSVPK	8	1	1	61743954	N-Term(TMT6plex); K9(TMT6plex)	1421.8	0	**0.9**	**1.3**	**1.4**	**1.2**
																	IKGDVDVSAPK	7	1	1	61743954	N-Term(TMT6plex); K2(TMT6plex); K11(TMT6plex)	1816.1	1	**1.0**	**1.2**	**1.4**	**1.0**
																	LDFEGPDAK	7	1	1	61743954	N-Term(TMT6plex); K9(TMT6plex)	1449.8	0	**1.1**	**1.3**	**1.3**	**1.2**
																	GNVDISAPK	8	1	1	61743954	N-Term(TMT6plex); K9(TMT6plex)	1358.8	0	**1.2**	**1.4**	**1.5**	**1.3**
																	APDVEGQGLDWSLK	12	1	1	61743954	N-Term(TMT6plex); K14(TMT6plex)	1973.1	0	**1.2**	**1.5**	**1.6**	**1.3**
																	SPQISMSDIDLNLK	13	1	1	61743954	N-Term(TMT6plex); M6(Oxidation); K14(TMT6plex)	2035.1	0	**1.1**	**1.3**	**1.3**	**1.2**
																	GDVDVSLPEVEGEMK	2	1	1	61743954	N-Term(TMT6plex); M14(Oxidation); K15(TMT6plex)	2078.1	0	**1.2**	**1.2**	**1.4**	**1.1**
																	TDVDVSLPK	8	1	1	61743954	N-Term(TMT6plex); K9(TMT6plex)	1431.8	0	**1.2**	**1.2**	**1.4**	**1.2**
																	ISMPEIDLNLK	15	1	1	61743954	N-Term(TMT6plex); M3(Oxidation); K11(TMT6plex)	1747.0	0	**1.2**	**1.4**	**1.5**	**1.2**
																	LEGDLTGPSVDVEVPDVELECPDAK	8	1	1	61743954	N-Term(TMT6plex); C21(Carbamidomethyl); K25(TMT6plex)	3141.6	0	**1.0**	**1.1**	**1.4**	**0.9**
																	VKGDVDISLPK	4	1	1	61743954	N-Term(TMT6plex); K2(TMT6plex); K11(TMT6plex)	1858.2	1	**0.8**	**1.3**	**1.3**	**1.0**
																	GEGPEVDVK	7	1	1	61743954	N-Term(TMT6plex); K9(TMT6plex)	1387.8	0	**1.0**	**1.3**	**1.4**	**1.1**
																	SSGCDVNLPGVNVK	7	1	1	61743954	N-Term(TMT6plex); C4(Carbamidomethyl); K14(TMT6plex)	1904.0	0	**1.1**	**1.3**	**1.4**	**1.2**
																	AEAPLPSPK	6	1	1	61743954	N-Term(TMT6plex); K9(TMT6plex)	1367.8	0	**1.0**	**1.3**	**1.3**	**1.3**
																	GPTVGGGLPGIGVQGLEGNLQMPGIK	14	1	1	61743954	N-Term(TMT6plex); M22(Oxidation); K26(TMT6plex)	2920.6	0	**0.9**	**1.3**	**1.3**	**1.3**
																	VKGDVDVSLPK	18	1	1	61743954	N-Term(TMT6plex); K2(TMT6plex); K11(TMT6plex)	1844.2	1	**0.9**	**1.3**	**1.3**	**1.0**
																	GPQVSGELK	6	1	1	61743954	N-Term(TMT6plex); K9(TMT6plex)	1372.8	0	**1.0**	**1.5**	**1.4**	**1.3**
																	IKGDVDVTGPK	4	1	1	61743954	N-Term(TMT6plex); K2(TMT6plex); K11(TMT6plex)	1816.1	1	**1.1**	**1.4**	**1.4**	**1.2**
																	ISMPDLDLHLK	4	1	1	61743954	N-Term(TMT6plex); M3(Oxidation); K11(TMT6plex)	1756.0	0	**1.0**	**1.1**	**1.3**	**1.0**
																	FGTFGGLGSK	7	1	1	61743954	N-Term(TMT6plex); K10(TMT6plex)	1428.8	0	**1.2**	**1.0**	**1.4**	**1.0**
																	VKGDVDVTLPK	6	1	1	61743954	N-Term(TMT6plex); K2(TMT6plex); K11(TMT6plex)	1858.2	1	**1.0**	**1.4**	**1.4**	**1.3**
																	LSSSSSNDSGNK	5	1	1	61743954	N-Term(TMT6plex); K12(TMT6plex)	1640.9	0	**1.1**	**1.2**	**1.9**	**1.0**
																	VEGDLKGPEVDIK	7	1	1	61743954	N-Term(TMT6plex); K6(TMT6plex); K13(TMT6plex)	2086.2	1	**1.2**	**1.5**	**1.4**	**1.1**
																	VESEIKVPDVELK	3	1	1	61743954	N-Term(TMT6plex); K6(TMT6plex); K13(TMT6plex)	2172.3	1	**1.1**	**1.4**	**1.5**	**1.2**
																	VDIEGPDVNIEGPEGK	32	1	1	61743954	N-Term(TMT6plex); K16(TMT6plex)	2126.1	0	**1.2**	**1.3**	**1.3**	**1.1**
																	IPMPDFDLHLK	7	1	1	61743954	N-Term(TMT6plex); M3(Oxidation); K11(TMT6plex)	1800.0	0	**0.9**	**1.0**	**1.1**	**1.0**
																	APDVDVNIAGPDAALK	11	1	1	61743954	N-Term(TMT6plex); K16(TMT6plex)	2024.1	0	**0.9**	**1.4**	**1.3**	**1.2**
																	MDAEVPDVNIEGPDAK	12	1	1	61743954	N-Term(TMT6plex); M1(Oxidation); K16(TMT6plex)	2174.1	0	**1.1**	**1.2**	**1.5**	**1.0**
																	GEGPEVDVNLPK	37	1	1	61743954	N-Term(TMT6plex); K12(TMT6plex)	1712.0	0	**1.1**	**1.4**	**1.4**	**1.2**
																	ADLDVSGPK	46	1	1	61743954	N-Term(TMT6plex); K9(TMT6plex)	1359.8	0	**1.0**	**1.3**	**1.3**	**1.1**
																	VGVEVPDVNIEGPEGK	16	1	1	61743954	N-Term(TMT6plex); K16(TMT6plex)	2096.2	0	**1.1**	**1.2**	**1.4**	**1.4**
																	ISMPDVNLNLK	20	1	1	61743954	N-Term(TMT6plex); M3(Oxidation); K11(TMT6plex)	1718.0	0	**1.1**	**1.3**	**1.4**	**1.2**
																	VDIDAPDVDVHGPDWHLK	10	1	1	61743954	N-Term(TMT6plex); K18(TMT6plex)	2486.3	0	**1.0**	**1.2**	**1.4**	**1.1**
																	ISMPDVDLHVK	2	1	1	61743954	N-Term(TMT6plex); K11(TMT6plex)	1712.0	0	**1.3**	**1.7**	**1.9**	**1.4**
																	VDVDVPDVNIEGPDAK	29	1	1	61743954	N-Term(TMT6plex); K16(TMT6plex)	2140.2	0	**1.2**	**1.3**	**1.3**	**1.1**
																	VDVSAPDVEMQGPDWNLK	12	1	1	61743954	N-Term(TMT6plex); M10(Oxidation); K18(TMT6plex)	2474.3	0	**1.2**	**1.5**	**1.3**	**1.3**
																	ISMPDVGLNLK	14	1	1	61743954	N-Term(TMT6plex); M3(Oxidation); K11(TMT6plex)	1661.0	0	**1.1**	**1.3**	**1.3**	**1.2**
																	GDYDVTVPK	4	1	1	61743954	N-Term(TMT6plex); K9(TMT6plex)	1451.8	0	**1.2**	**1.3**	**1.5**	**1.1**
																	VKGDADVSVPK	11	1	1	61743954	N-Term(TMT6plex); K2(TMT6plex); K11(TMT6plex)	1802.1	1	**1.0**	**1.2**	**1.4**	**1.0**
																	LEGGEVDLK	7	1	1	61743954	N-Term(TMT6plex); K9(TMT6plex)	1417.8	0	**1.2**	**1.3**	**1.5**	**1.3**
																	GDMDISLPK	6	1	1	61743954	N-Term(TMT6plex); M3(Oxidation); K9(TMT6plex)	1449.8	0	**1.0**	**1.2**	**1.3**	**1.1**
																	ISMPEVDLNLK	12	1	1	61743954	N-Term(TMT6plex); M3(Oxidation); K11(TMT6plex)	1733.0	0	**1.1**	**1.3**	**1.4**	**1.2**
																	LPTGQISGPEIK	14	1	1	61743954	N-Term(TMT6plex); K12(TMT6plex)	1698.0	0	**1.2**	**1.5**	**1.5**	**1.4**
																	MEGGGAEVHAQLPSLEGDLR	10	1	1	61743954	N-Term(TMT6plex); M1(Oxidation)	2311.2	0	**1.1**	**1.2**	**1.1**	**1.3**
																	GEVDVDVPKLEGDLK	4	1	1	61743954	N-Term(TMT6plex); K9(TMT6plex); K15(TMT6plex)	2300.3	1	**1.1**	**1.3**	**1.4**	**1.1**
																	GKGGVTGSPEASISGSK	1	1	1	61743954	N-Term(TMT6plex); K2(TMT6plex); K17(TMT6plex)	2206.3	1	**1.3**	**1.6**	**1.5**	**1.5**
																	GEYDVTMPK	2	1	1	61743954	N-Term(TMT6plex); K9(TMT6plex)	1497.8	0	**1.4**	**1.7**	**1.9**	**1.6**
																	FAGGLHFSGPK	3	1	1	61743954	N-Term(TMT6plex); K11(TMT6plex)	1575.9	0	**1.1**	**1.3**	**1.4**	**1.4**
																	VDINAPDVDVR	4	1	1	61743954	N-Term(TMT6plex)	1441.8	0	**1.2**	**1.4**	**1.4**	**1.1**
																	GDVPSVGLEGPDVDLQGPEAK	10	1	1	61743954	N-Term(TMT6plex); K21(TMT6plex)	2537.3	0	**1.0**	**1.3**	**1.4**	**1.2**
																	GDINIEGPSMNIEGPDLNVEGPEGGLK	10	1	1	61743954	N-Term(TMT6plex); M10(Oxidation); K27(TMT6plex)	3225.6	0	**1.0**	**1.3**	**1.3**	**1.2**
																	VEAPSLDVHMDSPDINIEGPDVK	5	1	1	61743954	N-Term(TMT6plex); M10(Oxidation); K23(TMT6plex)	2951.5	0	**1.9**	**1.4**	**1.3**	**1.4**
																	LEGDLTGPSVGVEVPDVELECPDAK	25	1	1	61743954	N-Term(TMT6plex); C21(Carbamidomethyl); K25(TMT6plex)	3083.6	0	**1.3**	**1.4**	**1.5**	**1.1**
																	VDINAPDVGVQGPDWHLK	8	1	1	61743954	N-Term(TMT6plex); K18(TMT6plex)	2418.3	0	**1.4**	**1.2**	**1.3**	**1.0**
																	GDVDVSVPEVEGK	6	1	1	61743954	N-Term(TMT6plex); K13(TMT6plex)	1788.0	0	**0.9**	**1.1**	**1.2**	**0.9**
																	LKGEIDASVPELEGDLR	14	1	1	61743954	N-Term(TMT6plex); K2(TMT6plex)	2299.3	1	**1.3**	**1.3**	**1.5**	**1.4**
																	GDISISGPK	4	1	1	61743954	N-Term(TMT6plex); K9(TMT6plex)	1331.8	0	**1.0**	**1.3**	**1.5**	**1.5**
																	GPQITGPSLEGDLGLK	7	1	1	61743954	N-Term(TMT6plex); K16(TMT6plex)	2040.2	0	**1.1**	**1.3**	**1.5**	**1.4**
																	ADIDISGPNVDVDVPDVNIEGPDAK	9	1	1	61743954	N-Term(TMT6plex); K25(TMT6plex)	3022.6	0	**1.1**	**1.5**	**1.3**	**1.1**
																	ADVVVSGPK	13	1	1	61743954	N-Term(TMT6plex); K9(TMT6plex)	1329.8	0	**1.0**	**1.2**	**1.5**	**1.1**
																	VDIDAPDVSIEGPDAK	9	1	1	61743954	N-Term(TMT6plex); K16(TMT6plex)	2099.1	0	**1.1**	**1.3**	**1.4**	**1.1**
																	GEVDVDVPK	6	1	1	61743954	N-Term(TMT6plex); K9(TMT6plex)	1415.8	0	**1.6**	**1.4**	**1.5**	**1.1**
																	GPHVDVSGPDIDIEGPEGK	4	1	1	61743954	N-Term(TMT6plex); K19(TMT6plex)	2376.2	0	**1.0**	**1.4**	**1.2**	**1.2**
																	LEGELQAPDLELSLPAIHVEGLDIK	11	1	1	61743954	N-Term(TMT6plex); K25(TMT6plex)	3157.8	0	**1.1**	**1.4**	**1.5**	**1.5**
																	ANVDISAPK	11	1	1	61743954	N-Term(TMT6plex); K9(TMT6plex)	1372.8	0	**1.2**	**1.4**	**1.4**	**1.2**
																	ISMQDVDLSLGSPK	6	1	1	61743954	N-Term(TMT6plex); M3(Oxidation); K14(TMT6plex)	1964.1	0	**1.2**	**1.3**	**1.4**	**1.3**
																	EMGVDVHFPK	6	1	1	61743954	N-Term(TMT6plex); M2(Oxidation); K10(TMT6plex)	1632.9	0	**1.1**	**1.4**	**1.6**	**1.3**
																	ISMPDFDLHLK	15	1	1	61743954	N-Term(TMT6plex); M3(Oxidation); K11(TMT6plex)	1790.0	0	**1.1**	**1.3**	**1.4**	**1.2**
																	VSAPEVSVGHK	1	1	1	61743954	N-Term(TMT6plex); K11(TMT6plex)	1567.9	0	**1.3**	**1.2**	**1.1**	**1.1**
																	APNISMPDVDLNLK	3	1	1	61743954	N-Term(TMT6plex); M6(Oxidation); K14(TMT6plex)	2001.1	0	**1.1**	**1.2**	**1.2**	**1.1**
																	ISMPDLDLNLK	18	1	1	61743954	N-Term(TMT6plex); K11(TMT6plex)	1717.0	0	**1.3**	**1.7**	**2.1**	**1.0**
																	VSAPGVQGDVK	4	1	1	61743954	N-Term(TMT6plex); K11(TMT6plex)	1514.9	0	**1.1**	**1.3**	**1.4**	**1.3**
																	VSMPDVDLNLK	5	1	1	61743954	N-Term(TMT6plex); M3(Oxidation); K11(TMT6plex)	1705.0	0	**1.0**	**1.2**	**1.2**	**1.0**
																	VSMPDVELNLK	9	1	1	61743954	N-Term(TMT6plex); M3(Oxidation); K11(TMT6plex)	1719.0	0	**1.0**	**1.2**	**1.1**	**1.1**
																	ADVDVSGPK	24	1	1	61743954	N-Term(TMT6plex); K9(TMT6plex)	1345.8	0	**1.2**	**1.3**	**1.4**	**1.1**
																	GGVQVPAVDISSSLGGR	6	1	1	61743954	N-Term(TMT6plex)	1828.0	0	**1.3**	**1.3**	**1.4**	**1.4**
																	MKGDVVVSLPK	2	1	1	61743954	N-Term(TMT6plex); M1(Oxidation); K2(TMT6plex); K11(TMT6plex)	1876.2	1	**1.1**	**1.3**	**1.2**	**1.4**
																	AKGEVDVDVPK	1	1	1	61743954	N-Term(TMT6plex); K2(TMT6plex); K11(TMT6plex)	1844.1	1	**1.1**	**1.3**	**1.4**	**1.1**
																	ISMPDIDFNLK	13	1	1	61743954	N-Term(TMT6plex); M3(Oxidation); K11(TMT6plex)	1767.0	0	**1.0**	**1.2**	**1.2**	**1.1**
																	ISMPDFDLNLK	2	1	1	61743954	N-Term(TMT6plex); K11(TMT6plex)	1751.0	0	**1.2**	**1.7**	**2.0**	**1.0**
																	GSLGATGEIK	2	1	1	61743954	N-Term(TMT6plex); K10(TMT6plex)	1390.8	0	**1.1**	**1.6**	**1.6**	**1.5**
																	ISMPDFDLHLK	2	1	1	61743954	N-Term(TMT6plex); K11(TMT6plex)	1774.0	0	**1.1**	**1.5**	**1.9**	**0.9**
																	MPSLEISAPK	10	1	1	61743954	N-Term(TMT6plex); M1(Oxidation); K10(TMT6plex)	1546.9	0	**1.3**	**1.3**	**1.3**	**1.3**
																	GGQIGLQAPGLSVSGPQGHLESGSGK	8	1	1	61743954	N-Term(TMT6plex); K26(TMT6plex)	2876.6	0	**0.9**	**1.4**	**1.3**	**1.3**
																	VDIEAPDVSLEGPEGK	11	1	1	61743954	N-Term(TMT6plex); K16(TMT6plex)	2113.1	0	**1.1**	**1.3**	**1.4**	**1.1**
																	GPSLDIDTPDVNIEGPEGK	10	1	1	61743954	N-Term(TMT6plex); K19(TMT6plex)	2411.3	0	**0.9**	**1.4**	**1.4**	**1.0**
																	VDIDTPDINIEGSEGK	12	1	1	61743954	N-Term(TMT6plex); K16(TMT6plex)	2160.1	0	**0.9**	**1.2**	**1.2**	**0.9**
																	GEGPDVDVNLPK	27	1	1	61743954	N-Term(TMT6plex); K12(TMT6plex)	1697.9	0	**1.1**	**1.4**	**1.4**	**1.2**
																	HELTEISNVDVETQSGK	4	1	1	61743954	N-Term(TMT6plex); K17(TMT6plex)	2344.2	0	**1.1**	**1.4**	**1.5**	**1.3**
																	MYFPDVEFDIK	12	1	1	61743954	N-Term(TMT6plex); M1(Oxidation); K11(TMT6plex)	1878.0	0	**1.0**	**1.2**	**1.2**	**1.3**
																	GEVDVSLANVEGDLK	12	1	1	61743954	N-Term(TMT6plex); K15(TMT6plex)	2003.1	0	**1.0**	**1.3**	**1.4**	**1.0**
																	VEGDMQVPDLDIK	17	1	1	61743954	N-Term(TMT6plex); M5(Oxidation); K13(TMT6plex)	1933.0	0	**1.2**	**1.4**	**1.3**	**1.3**
																	ISMPDVDLNLK	10	1	1	61743954	N-Term(TMT6plex); M3(Oxidation); K11(TMT6plex)	1719.0	0	**1.0**	**1.1**	**1.2**	**1.1**
																	VDIDTPDIDIHGPEGK	7	1	1	61743954	N-Term(TMT6plex); K16(TMT6plex)	2179.2	0	**1.2**	**1.2**	**1.3**	**1.1**
																	GDMDVSVPK	11	1	1	61743954	N-Term(TMT6plex); M3(Oxidation); K9(TMT6plex)	1421.8	0	**1.5**	**1.3**	**1.4**	**1.1**
																	GPGVDVNLK	6	1	1	61743954	N-Term(TMT6plex); K9(TMT6plex)	1356.8	0	**0.7**	**1.1**	**1.2**	**1.0**
																	LNVGAPDVTLR	4	1	1	61743954	N-Term(TMT6plex)	1383.8	0	**1.1**	**1.3**	**1.3**	**1.3**
																	ADLGVSGPK	4	1	1	61743954	N-Term(TMT6plex); K9(TMT6plex)	1301.8	0	**1.1**	**1.3**	**1.4**	**1.1**
																	AEGPEVDVNLPK	24	1	1	61743954	N-Term(TMT6plex); K12(TMT6plex)	1726.0	0	**1.2**	**1.4**	**1.4**	**1.2**
																	VTAPDVDLHLK	6	1	1	61743954	N-Term(TMT6plex); K11(TMT6plex)	1666.0	0	**0.9**	**1.3**	**1.3**	**1.2**
																	ISMPDVDLHLK	24	1	1	61743954	N-Term(TMT6plex); M3(Oxidation); K11(TMT6plex)	1742.0	0	**1.2**	**1.4**	**1.7**	**1.2**

Accession	RefSeq Accession	Gene Symbol	Entrez Gene ID	Description	Σ Coverage	Σ# Proteins	Σ# Unique Peptides	Σ# Peptides	Σ# PSMs	Het-1A-Smoke - 2M/Parental	Het-1A-Smoke - 4M/Parental	Het-1A-Smoke - 6M/Parental	Het-1A-Smoke - 8M/Parental	# AAs	MW [kDa]	calc. pI	Sequence	# PSMs	# Proteins	# Protein Groups	Protein Group Accessions	Modifications	MH+ [Da]	# Missed Cleavages	Het-1A-Smoke - 2M/Parental	Het-1A-Smoke - 4M/Parental	Het-1A-Smoke - 6M/Parental	Het-1A-Smoke - 8M/Parental
																	VDVEVPDVSLEGPEGK	23	1	1	61743954	N-Term(TMT6plex); K16(TMT6plex)	2127.2	0	**1.2**	**1.4**	**1.5**	**1.1**
																	GPQVSSALNLDTSK	6	1	1	61743954	N-Term(TMT6plex); K14(TMT6plex)	1875.1	0	**1.0**	**1.4**	**1.4**	**1.3**
																	AEASIQAGAGDGEWEESEVK	10	1	1	61743954	N-Term(TMT6plex); K20(TMT6plex)	2521.2	0	**1.5**	**1.6**	**1.6**	**1.5**
																	VDVDIPDVNIEGPDAK	16	1	1	61743954	N-Term(TMT6plex); K16(TMT6plex)	2154.2	0	**1.0**	**1.2**	**1.3**	**1.1**
																	IKGDVPSVGLEGPDVDLQGPEAK	6	1	1	61743954	N-Term(TMT6plex); K2(TMT6plex); K23(TMT6plex)	3007.7	1	**1.4**	**1.8**	**1.6**	**1.5**
																	VPGIDATTK	5	1	1	61743954	N-Term(TMT6plex); K9(TMT6plex)	1359.8	0	**1.0**	**1.3**	**1.3**	**1.3**
																	MDIDAPDVEVQGPDWHLK	2	1	1	61743954	N-Term(TMT6plex); K18(TMT6plex)	2523.3	0	**1.2**	**1.4**	**1.5**	**1.2**
																	GDMDVSVPK	2	1	1	61743954	N-Term(TMT6plex); K9(TMT6plex)	1405.8	0	**1.5**	**1.8**	**2.3**	**1.1**
																	SPSLDVTVPEAELNLETPEISVGGK	9	1	1	61743954	N-Term(TMT6plex); K25(TMT6plex)	3039.6	0	**1.0**	**1.3**	**1.2**	**1.1**
																	IDVTAPDVSIEEPEGK	10	1	1	61743954	N-Term(TMT6plex); K16(TMT6plex)	2157.2	0	**1.3**	**1.4**	**1.6**	**1.4**
																	GKKPDIDITGPK	1	1	1	61743954	N-Term(TMT6plex); K2(TMT6plex); K3(TMT6plex); K12(TMT6plex)	2185.4	1	**1.0**	**1.6**	**1.5**	**1.3**
																	ADVDISGPK	1	1	1	61743954	N-Term(TMT6plex); K9(TMT6plex)	1359.8	0				
																	VDINAPDVEVQGK	10	1	1	61743954	N-Term(TMT6plex); K13(TMT6plex)	1842.0	0	**1.1**	**1.3**	**1.3**	**1.2**
																	GEGPEVDVTLPK	13	1	1	61743954	N-Term(TMT6plex); K12(TMT6plex)	1699.0	0	**1.1**	**1.4**	**1.4**	**1.1**
																	APEVNLNAPDVDVHGPDWNLK	16	1	1	61743954	N-Term(TMT6plex); K21(TMT6plex)	2758.5	0	**0.9**	**1.3**	**1.3**	**1.1**
																	MSLPDVDLDLK	9	1	1	61743954	N-Term(TMT6plex); M1(Oxidation); K11(TMT6plex)	1720.0	0	**1.0**	**1.2**	**1.3**	**1.1**
																	ISMPEVDLNLK	12	1	1	61743954	N-Term(TMT6plex); K11(TMT6plex)	1717.0	0	**1.5**	**1.8**	**2.1**	**1.1**
																	ISMPDVDLHLK	16	1	1	61743954	N-Term(TMT6plex); K11(TMT6plex)	1726.0	0	**1.3**	**1.7**	**2.2**	**1.1**
																	LPSGSGAASPTGSAVDIR	10	1	1	61743954	N-Term(TMT6plex)	1872.0	0	**1.3**	**1.5**	**1.5**	**1.5**
																	VDIDVPDVDVQGPDWHLK	12	1	1	61743954	N-Term(TMT6plex); K18(TMT6plex)	2505.3	0	**1.0**	**1.4**	**1.3**	**1.0**
																	ISMPDVDLNLK	8	1	1	61743954	N-Term(TMT6plex); K11(TMT6plex)	1703.0	0	**1.4**	**1.8**	**2.2**	**1.1**
																	GGVTGSPEASISGSK	10	1	1	61743954	N-Term(TMT6plex); K15(TMT6plex)	1792.0	0	**1.4**	**2.0**	**2.2**	**1.6**
																	ADIEISGPK	12	1	1	61743954	N-Term(TMT6plex); K9(TMT6plex)	1387.8	0	**1.2**	**1.5**	**1.4**	**1.2**
																	VDINTPDVDVHGPDWHLK	14	1	1	61743954	N-Term(TMT6plex); K18(TMT6plex)	2515.3	0	**1.0**	**1.3**	**1.4**	**1.2**
																	GDVDVTLPK	8	1	1	61743954	N-Term(TMT6plex); K9(TMT6plex)	1401.8	0	**1.0**	**1.3**	**1.4**	**1.2**
																	LKSEDGVEGDLGETQSR	4	1	1	61743954	N-Term(TMT6plex); K2(TMT6plex)	2278.2	1	**1.0**	**1.1**	**1.3**	**1.2**
																	DDGVFVQEVTQNSPAAR	7	2	1	61743954	N-Term(TMT6plex)	2062.0	0	**1.2**	**1.3**	**1.4**	**1.3**
																	MDIDVPDVEVQGPDWHLK	2	1	1	61743954	N-Term(TMT6plex); K18(TMT6plex)	2551.3	0	**1.3**	**1.7**	**2.0**	**1.3**
																	KPDIDITGPK	8	1	1	61743954	N-Term(TMT6plex); K1(TMT6plex); K10(TMT6plex)	1771.1	0	**0.9**	**1.2**	**1.3**	**1.1**
																	MPDMHVNMPK	3	1	1	61743954	N-Term(TMT6plex); M1(Oxidation); M4(Oxidation); M8(Oxidation); K10(TMT6plex)	1705.8	0	**1.3**	**1.5**	**1.5**	**1.4**
																	VDIDVPDVNLEAPEGK	6	1	1	61743954	N-Term(TMT6plex); K16(TMT6plex)	2168.2	0	**1.0**	**1.1**	**1.4**	**0.9**
																	ISAPNVDFNLEGPK	18	1	1	61743954	N-Term(TMT6plex); K14(TMT6plex)	1959.1	0	**1.1**	**1.3**	**1.5**	**1.1**
																	ISIPDVDLDLK	17	1	1	61743954	N-Term(TMT6plex); K11(TMT6plex)	1686.0	0	**1.3**	**1.5**	**1.5**	**1.2**
																	APNISMPDVDLNLK	8	1	1	61743954	N-Term(TMT6plex); K14(TMT6plex)	1985.1	0	**1.4**	**1.8**	**1.9**	**1.2**
																	GPEVDVSGPK	8	1	1	61743954	N-Term(TMT6plex); K10(TMT6plex)	1442.8	0	**0.9**	**1.4**	**1.4**	**1.1**
																	VDIETPNLEGTLTGPR	9	1	1	61743954	N-Term(TMT6plex)	1941.0	0	**1.2**	**1.4**	**1.5**	**1.4**
																	GEGPEFDVNLSK	20	1	1	61743954	N-Term(TMT6plex); K12(TMT6plex)	1749.9	0	**1.0**	**1.3**	**1.4**	**1.1**
																	ISMSEVDLNVAAPK	2	1	1	61743954	N-Term(TMT6plex); K14(TMT6plex)	1932.1	0	**1.7**	**1.8**	**2.3**	**1.3**
																	GDLDASVPSMK	7	1	1	61743954	N-Term(TMT6plex); M10(Oxidation); K11(TMT6plex)	1593.9	0	**0.9**	**1.3**	**1.2**	**1.2**
																	GDVVVSLPK	12	1	1	61743954	N-Term(TMT6plex); K9(TMT6plex)	1371.9	0	**1.1**	**1.3**	**1.4**	**1.2**
																	GDMDVSLPK	3	1	1	61743954	N-Term(TMT6plex); M3(Oxidation); K9(TMT6plex)	1435.8	0	**1.1**	**1.2**	**1.2**	**1.1**
																	GDVDVTGPK	6	1	1	61743954	N-Term(TMT6plex); K9(TMT6plex)	1345.8	0	**1.2**	**1.2**	**1.3**	**1.0**
																	ISIPDVGLHLK	13	1	1	61743954	N-Term(TMT6plex); K11(TMT6plex)	1650.0	0	**1.0**	**1.2**	**1.3**	**0.9**
																	GEETGIDVTLPTGEVTVPGVSGDVSLPEIATGGLEGK	10	1	1	61743954	N-Term(TMT6plex); K37(TMT6plex)	4039.1	0	**1.1**	**1.5**	**1.5**	**1.4**
																	GEYDVTMPK	2	1	1	61743954	N-Term(TMT6plex); M7(Oxidation); K9(TMT6plex)	1513.8	0	**0.8**	**1.3**	**1.3**	**1.2**
																	IHMSGPK	2	1	1	61743954	N-Term(TMT6plex); M3(Oxidation); K7(TMT6plex)	1243.7	0	**1.0**	**1.3**	**1.2**	**1.2**
																	GPAFNMASPESDFGINLK	9	1	1	61743954	N-Term(TMT6plex); M6(Oxidation); K18(TMT6plex)	2369.2	0	**1.3**	**1.5**	**1.4**	**1.5**
																	MDIDVPDVEVQGPDWHLK	6	1	1	61743954	N-Term(TMT6plex); M1(Oxidation); K18(TMT6plex)	2567.3	0	**1.0**	**1.4**	**1.3**	**1.3**
																	GGVDVTLPR	4	1	1	61743954	N-Term(TMT6plex)	1142.7	0	**1.2**	**1.4**	**1.5**	**1.4**
																	FKAEAPLPSPK	4	1	1	61743954	N-Term(TMT6plex); K2(TMT6plex); K11(TMT6plex)	1872.2	1	**1.1**	**1.3**	**1.4**	**1.3**
																	GDLDIAGPNLEGDFK	12	1	1	61743954	N-Term(TMT6plex); K15(TMT6plex)	2019.1	0	**1.0**	**1.3**	**1.3**	**1.1**
																	ISMPDFDLNLK	16	1	1	61743954	N-Term(TMT6plex); M3(Oxidation); K11(TMT6plex)	1767.0	0	**1.1**	**1.4**	**1.3**	**1.2**
																	VSMPDVDLNLK	6	1	1	61743954	N-Term(TMT6plex); K11(TMT6plex)	1689.0	0	**1.2**	**1.5**	**2.0**	**1.2**
																	VGIDTPDIDIHGPEGK	4	1	1	61743954	N-Term(TMT6plex); K16(TMT6plex)	2121.2	0	**1.1**	**1.3**	**1.2**	**1.2**
																	MPFLSISSPK	4	1	1	61743954	N-Term(TMT6plex); M1(Oxidation); K10(TMT6plex)	1580.9	0	**0.9**	**1.1**	**1.1**	**1.1**
																	VKGDLDIAGPNLEGDFK	6	1	1	61743954	N-Term(TMT6plex); K2(TMT6plex); K17(TMT6plex)	2475.4	1	**1.1**	**1.5**	**1.4**	**1.3**
																	GDVDVSVPK	4	1	1	61743954	N-Term(TMT6plex); K9(TMT6plex)	1373.8	0	**1.1**	**1.2**	**1.4**	**1.0**
																	ISMPDIDLNLTGPK	8	1	1	61743954	N-Term(TMT6plex); K14(TMT6plex)	1972.1	0	**1.3**	**1.6**	**1.9**	**1.1**
																	LQGSGVSLASK	6	1	1	61743954	N-Term(TMT6plex); K11(TMT6plex)	1504.9	0	**1.2**	**1.3**	**1.4**	**1.2**
																	VTAYTVDVTGR	4	1	1	61743954	N-Term(TMT6plex)	1410.8	0	**1.2**	**1.5**	**1.4**	**1.6**
																	ISMPDVDFNLK	4	1	1	61743954	N-Term(TMT6plex); K11(TMT6plex)	1737.0	0	**1.4**	**1.5**	**2.0**	**1.2**
																	GPEVDIEGPEGK	2	1	1	61743954	N-Term(TMT6plex); K12(TMT6plex)	1684.9	0	**1.2**	**1.7**	**1.7**	**1.4**
																	VDIDVPDVNIEGPDAK	7	1	1	61743954	N-Term(TMT6plex); K16(TMT6plex)	2154.2	0	**1.1**	**1.2**	**1.3**	**1.0**
																	MKGEVDVSLANVEGDLK	4	1	1	61743954	N-Term(TMT6plex); M1(Oxidation); K2(TMT6plex); K17(TMT6plex)	2507.4	1	**1.1**	**1.4**	**1.3**	**1.3**
																	LKGDVDVSGPK	4	1	1	61743954	N-Term(TMT6plex); K2(TMT6plex); K11(TMT6plex)	1802.1	1	**1.1**	**1.4**	**1.5**	**1.1**
																	ISMPDVDLHMK	1	1	1	61743954	N-Term(TMT6plex); M3(Oxidation); M10(Oxidation); K11(TMT6plex)	1775.9	0	**1.2**	**1.6**	**1.5**	**1.6**
																	MPSMNIQTHK	3	1	1	61743954	N-Term(TMT6plex); M1(Oxidation); M4(Oxidation); K10(TMT6plex)	1676.9	0	**1.0**	**1.1**	**1.1**	**1.1**
																	GGADVSGGVSAPDISLGEGHLSVK	10	1	1	61743954	N-Term(TMT6plex); K24(TMT6plex)	2667.4	0	**1.1**	**1.2**	**1.4**	**1.2**
																	ADIDVSGPSVDTDAPDLDIEGPEGK	9	1	1	61743954	N-Term(TMT6plex); K25(TMT6plex)	2970.5	0	**1.0**	**1.3**	**1.6**	**0.9**
																	VGIQLPEVELSVSTK	6	1	1	61743954	N-Term(TMT6plex); K15(TMT6plex)	2057.2	0	**1.3**	**1.5**	**1.7**	**1.4**
																	GEGPDGDVK	4	1	1	61743954	N-Term(TMT6plex); K9(TMT6plex)	1331.7	0	**1.0**	**1.2**	**1.3**	**0.9**
																	DIDISSPEFK	6	1	1	61743954	N-Term(TMT6plex); K10(TMT6plex)	1608.9	0	**1.1**	**1.6**	**1.5**	**1.4**
																	GDVDVSLPK	12	1	1	61743954	N-Term(TMT6plex); K9(TMT6plex)	1387.8	0	**1.0**	**1.1**	**1.3**	**0.9**
																	MPDVDISVPK	11	1	1	61743954	N-Term(TMT6plex); M1(Oxidation); K10(TMT6plex)	1574.9	0	**1.3**	**1.4**	**1.4**	**1.4**
																	EVDVNLPK	8	1	1	61743954	N-Term(TMT6plex); K8(TMT6plex)	1371.8	0	**1.2**	**1.3**	**1.4**	**1.2**
																	VKGDVDVSVPK	5	1	1	61743954	N-Term(TMT6plex); K2(TMT6plex); K11(TMT6plex)	1830.1	1	**1.0**	**1.3**	**1.4**	**1.1**
																	GGKPGLTIQAPQLEVSVPSANIEGLEGK	3	1	1	61743954	N-Term(TMT6plex); K3(TMT6plex); K28(TMT6plex)	3477.0	0	**1.0**	**1.4**	**1.3**	**1.3**
																	VKGEYDVTVPK	2	1	1	61743954	N-Term(TMT6plex); K2(TMT6plex); K11(TMT6plex)	1922.2	1	**1.1**	**1.4**	**1.6**	**1.3**
																	ELLLPNWQGSGSHGLTIAQR	2	2	1	61743954	N-Term(TMT6plex)	2406.3	0	**1.2**	**1.4**	**1.4**	**1.4**
																	GPTVGGGLPGIGVQGLEGNLQMPGIK	4	1	1	61743954	N-Term(TMT6plex); K26(TMT6plex)	2904.6	0	**1.2**	**1.8**	**1.7**	**1.3**
																	ISMPEIDLNLK	8	1	1	61743954	N-Term(TMT6plex); K11(TMT6plex)	1731.0	0	**1.2**	**1.7**	**1.9**	**1.2**
																	ISMPDLDLHLK	3	1	1	61743954	N-Term(TMT6plex); K11(TMT6plex)	1740.0	0	**1.3**	**1.4**	**1.7**	**1.1**
																	MPEMHFK	4	1	1	61743954	N-Term(TMT6plex); K7(TMT6plex)	1377.7	0	**1.6**	**2.0**	**2.8**	**1.1**
																	MPSLEISAPK	2	1	1	61743954	N-Term(TMT6plex); K10(TMT6plex)	1530.9	0	**1.3**	**1.8**	**1.9**	**1.4**
																	IPEMHLK	2	1	1	61743954	N-Term(TMT6plex); K7(TMT6plex)	1325.8	0	**1.1**	**1.5**	**1.7**	**1.0**
																	MPDMHFK	2	1	1	61743954	N-Term(TMT6plex); K7(TMT6plex)	1363.7	0	**1.6**	**2.1**	**3.0**	**1.2**
																	LPQFGISTPGSDLHVNAK	4	1	1	61743954	N-Term(TMT6plex); K18(TMT6plex)	2339.3	0	**1.2**	**1.4**	**1.4**	**1.3**
																	GPGVDLPSVNLSMPK	4	1	1	61743954	N-Term(TMT6plex); K15(TMT6plex)	1969.1	0	**1.5**	**1.9**	**2.3**	**1.3**
																	VDVSAPDVEAHGPEWNLK	4	1	1	61743954	N-Term(TMT6plex); K18(TMT6plex)	2421.3	0	**0.9**	**1.3**	**1.4**	**1.3**
																	MYFPDVEFDIK	8	1	1	61743954	N-Term(TMT6plex); K11(TMT6plex)	1862.0	0	**1.3**	**1.7**	**2.1**	**1.4**
																	VEGDLKGPEADIK	1	1	1	61743954	N-Term(TMT6plex); K6(TMT6plex); K13(TMT6plex)	2058.2	1	**1.0**	**1.7**	**1.4**	**1.3**
																	GDLDASVPSMK	2	1	1	61743954	N-Term(TMT6plex); K11(TMT6plex)	1577.9	0	**1.3**	**1.6**	**1.7**	**1.2**
																	IDVDAPDIDIHGPDAK	6	1	1	61743954	N-Term(TMT6plex); K16(TMT6plex)	2149.2	0	**1.0**	**1.4**	**1.3**	**1.0**
																	VDTNAPDLSLEGPEGK	5	1	1	61743954	N-Term(TMT6plex); K16(TMT6plex)	2100.1	0	**1.1**	**1.2**	**1.4**	**1.1**
																	AEGPDVAVDLPK	8	1	1	61743954	N-Term(TMT6plex); K12(TMT6plex)	1669.0	0	**0.9**	**1.3**	**1.3**	**1.2**
																	GEGPDVDVSLPK	6	1	1	61743954	N-Term(TMT6plex); K12(TMT6plex)	1670.9	0	**0.9**	**1.4**	**1.4**	**1.1**
																	GEGPDVDVTLPK	9	1	1	61743954	N-Term(TMT6plex); K12(TMT6plex)	1685.0	0	**0.9**	**1.3**	**1.4**	**1.0**
																	VSGPDLDLNLK	6	1	1	61743954	N-Term(TMT6plex); K11(TMT6plex)	1629.0	0	**1.0**	**1.3**	**1.4**	**1.2**
																	EFSGPSTPTGTLEFEGGEVSLEGGK	2	1	1	61743954	N-Term(TMT6plex); K25(TMT6plex)	2970.5	0	**1.1**	**1.6**	**1.9**	**1.2**
																	VDIDAPDVEVHDPDWHLK	2	1	1	61743954	N-Term(TMT6plex); K18(TMT6plex)	2558.3	0	**1.1**	**1.1**	**1.3**	**1.1**
																	LDADMPEVAVEGPNGK	2	1	1	61743954	N-Term(TMT6plex); M5(Oxidation); K16(TMT6plex)	2116.1	0	**1.3**	**1.4**	**1.6**	**1.0**
																	VGGSGVNVNAK	1	1	1	61743954	N-Term(TMT6plex); K11(TMT6plex)	1459.9	0	**2.5**	**1.5**	**1.3**	**1.5**
																	GEIDASVPELEGDLR	6	1	1	61743954	N-Term(TMT6plex)	1828.9	0	**1.2**	**1.3**	**1.3**	**1.4**
																	ISMPDIDLNLTGPK	6	1	1	61743954	N-Term(TMT6plex); M3(Oxidation); K14(TMT6plex)	1988.1	0	**0.9**	**1.2**	**1.2**	**1.1**
																	GPFVEAEVPDVDLECPDAK	6	1	1	61743954	N-Term(TMT6plex); C15(Carbamidomethyl); K19(TMT6plex)	2545.3	0	**0.9**	**1.1**	**1.5**	**0.8**
																	LKGPQITGPSLEGDLGLK	2	1	1	61743954	N-Term(TMT6plex); K2(TMT6plex); K18(TMT6plex)	2510.5	1	**1.0**	**1.6**	**1.6**	**1.4**
																	AVEVQGPSLESGDHGK	4	1	1	61743954	N-Term(TMT6plex); K16(TMT6plex)	2068.1	0	**1.2**	**1.3**	**1.3**	**1.2**
																	GPGVDLPSVNLSMPK	4	1	1	61743954	N-Term(TMT6plex); M13(Oxidation); K15(TMT6plex)	1985.1	0	**1.0**	**1.5**	**1.3**	**1.3**
																	IKGDVDVSVPEVEGK	2	1	1	61743954	N-Term(TMT6plex); K2(TMT6plex); K15(TMT6plex)	2258.3	1	**1.0**	**1.2**	**1.3**	**0.9**
																	ISMPDVDFNLK	2	1	1	61743954	N-Term(TMT6plex); M3(Oxidation); K11(TMT6plex)	1753.0	0	**1.1**	**1.4**	**1.2**	**1.0**
																	GEGPDVHMTLPK	1	1	1	61743954	N-Term(TMT6plex); M8(Oxidation); K12(TMT6plex)	1754.9	0	**1.2**	**1.4**	**1.4**	**1.4**
																	GEGPEVDMNLPK	7	1	1	61743954	N-Term(TMT6plex); M8(Oxidation); K12(TMT6plex)	1759.9	0	**1.0**	**1.1**	**1.2**	**1.0**
																	GPEIDVK	4	1	1	61743954	N-Term(TMT6plex); K7(TMT6plex)	1215.7	0	**1.0**	**1.2**	**1.4**	**1.1**
																	LEGPDVSLK	11	1	1	61743954	N-Term(TMT6plex); K9(TMT6plex)	1415.9	0	**1.2**	**1.4**	**1.4**	**1.3**

Accession	RefSeq Accession	Gene Symbol	Entrez Gene ID	Description	Σ Coverage	Σ# Proteins	Σ# Unique Peptides	Σ# Peptides	Σ# PSMs	Het-1A-Smoke - 2M/Parental	Het-1A-Smoke - 4M/Parental	Het-1A-Smoke - 6M/Parental	Het-1A-Smoke - 8M/Parental	# AAs	MW [kDa]	calc. pI	Sequence	# PSMs	# Proteins	# Protein Groups	Protein Group Accessions	Modifications	MH+ [Da]	# Missed Cleavages	Het-1A-Smoke - 2M/Parental	Het-1A-Smoke - 4M/Parental	Het-1A-Smoke - 6M/Parental	Het-1A-Smoke - 8M/Parental
																	VPDVEIK	7	1	1	61743954	N-Term(TMT6plex); K7(TMT6plex)	1257.8	0	1.2	1.3	1.5	1.1
																	SPEPGQTWTR	3	2	1	61743954	N-Term(TMT6plex)	1387.7	0	1.4	1.2	1.3	1.3
																	GSGGEWK	2	1	1	61743954	N-Term(TMT6plex); K7(TMT6plex)	1178.7	0	0.9	1.4	1.4	1.3
																	FSMPGFK	9	1	1	61743954	N-Term(TMT6plex); K7(TMT6plex)	1271.7	0	1.1	1.4	1.8	1.0
																	APDVDIK	4	1	1	61743954	N-Term(TMT6plex); K7(TMT6plex)	1215.7	0	1.0	1.2	1.4	1.0
																	GPEVDIK	9	1	1	61743954	N-Term(TMT6plex); K7(TMT6plex)	1215.7	0	0.9	1.3	1.4	1.0
																	VKGDMDVSLPK	1	1	1	61743954	N-Term(TMT6plex); K2(TMT6plex); M5(Oxidation); K11(TMT6plex)	1892.1	1	0.8	1.3	1.4	1.1
																	VKGEYDMTVPK	4	1	1	61743954	N-Term(TMT6plex); K2(TMT6plex); M7(Oxidation); K11(TMT6plex)	1970.1	1	1.1	1.2	1.3	1.2
																	MPEMNIK	2	1	1	61743954	N-Term(TMT6plex); K7(TMT6plex)	1320.7	0	1.6	2.3	2.7	1.3
																	IGFSGPK	2	1	1	61743954	N-Term(TMT6plex); K7(TMT6plex)	1163.7	0	1.0	1.3	1.4	1.2
																	MSLPDVDLDLK	2	1	1	61743954	N-Term(TMT6plex); K11(TMT6plex)	1704.0	0	1.4	1.5	1.7	1.2
																	VEGDLKGPEVDIR	1	1	1	61743954	N-Term(TMT6plex); K6(TMT6plex)	1885.1	1	1.5	1.7	1.7	1.3
																	APSLDIK	2	1	1	61743954	N-Term(TMT6plex); K7(TMT6plex)	1201.8	0	1.0	1.2	1.2	1.0
																	MQVGGDGVK	3	1	1	61743954	N-Term(TMT6plex); M1(Oxidation); K9(TMT6plex)	1364.8	0	1.1	1.4	1.3	1.3
																	APEVDIK	4	1	1	61743954	N-Term(TMT6plex); K7(TMT6plex)	1229.8	0	1.1	1.2	1.3	1.0
																	FSVSGLK	6	1	1	61743954	N-Term(TMT6plex); K7(TMT6plex)	1195.7	0	1.1	1.3	1.4	1.2
																	IGIPGVK	5	1	1	61743954	N-Term(TMT6plex); K7(TMT6plex)	1141.8	0	0.9	1.4	1.3	1.2
																	MPDMHVNMPK	2	1	1	61743954	N-Term(TMT6plex); M1(Oxidation); M4(Oxidation); K10(TMT6plex)	1689.9	0	1.3	1.4	1.5	1.4
																	GPQVALK	1	1	1	61743954	N-Term(TMT6plex); K7(TMT6plex)	1170.8	0	0.9	1.2	1.3	1.2
																	VEGDLKGPEIDIK	1	1	1	61743954	N-Term(TMT6plex); K6(TMT6plex); K13(TMT6plex)	2100.2	1	1.2	1.6	1.5	1.3
																	VPDVDIK	6	2	1	61743954	N-Term(TMT6plex); K7(TMT6plex)	1243.8	0	1.0	1.3	1.4	1.0
																	MPDVHFK	2	1	1	61743954	N-Term(TMT6plex); M1(Oxidation); K7(TMT6plex)	1347.7	0	1.0	1.2	1.3	1.2
																	SEDGVEGDLGETQSR	1	1	1	61743954	N-Term(TMT6plex)	1807.8	0	1.1	1.2	1.4	1.8
																	MPEMHFK	8	1	1	61743954	N-Term(TMT6plex); M1(Oxidation); M4(Oxidation); K7(TMT6plex)	1409.7	0	1.0	1.2	1.3	1.0
																	LEVPDMNIR	6	1	1	61743954	N-Term(TMT6plex); M6(Oxidation)	1331.7	0	1.1	1.3	1.3	1.1
																	GPALDIK	3	1	1	61743954	N-Term(TMT6plex); K7(TMT6plex)	1171.7	0	0.9	1.3	1.3	1.1
																	MPTFSTPGAK	2	1	1	61743954	N-Term(TMT6plex); M1(Oxidation); K10(TMT6plex)	1510.8	0	1.0	1.2	1.3	1.2
																	VKGDMDVSVPK	2	1	1	61743954	N-Term(TMT6plex); K2(TMT6plex); M5(Oxidation); K11(TMT6plex)	1878.1	1	1.1	1.4	1.3	1.2
																	FKIPEMHLK	2	1	1	61743954	N-Term(TMT6plex); K2(TMT6plex); M6(Oxidation); K9(TMT6plex)	1846.1	1	0.9	1.3	1.2	1.1
																	GPEVDIRDPK	1	1	1	61743954	N-Term(TMT6plex); K10(TMT6plex)	1583.9	1	1.0	1.4	1.3	1.3
																	LQVTMPGIK	1	1	1	61743954	N-Term(TMT6plex); M5(Oxidation); K9(TMT6plex)	1460.9	0	1.2	1.4	1.4	1.3
																	GPEADIK	2	1	1	61743954	N-Term(TMT6plex); K7(TMT6plex)	1187.7	0	0.8	1.3	1.3	1.1
																	FNFSKPK	1	1	1	61743954	N-Term(TMT6plex); K5(TMT6plex); K7(TMT6plex)	1555.0	0	1.0	1.3	1.5	1.2
																	FSVPGFK	3	1	1	61743954	N-Term(TMT6plex); K7(TMT6plex)	1239.8	0	1.1	1.3	1.6	1.1
																	MPEMNIK	1	1	1	61743954	N-Term(TMT6plex); M1(Oxidation); M4(Oxidation); K7(TMT6plex)	1352.7	0	1.0	1.2	1.2	1.2
																	LEVPDMNIR	1	1	1	61743954	N-Term(TMT6plex)	1315.7	0	1.4	1.6	1.8	1.3
																	VEGDLK	2	1	1	61743954	N-Term(TMT6plex); K6(TMT6plex)	1118.7	0	1.1	1.3	1.3	1.1
																	IEGDLK	6	14	3	61743954;13124875;110624781	N-Term(TMT6plex); K6(TMT6plex)	1132.7	0				
																	GPDWHLK	3	1	1	61743954	N-Term(TMT6plex); K7(TMT6plex)	1310.8	0	0.8	1.3	1.3	1.0
																	MPDMHFK	2	1	1	61743954	N-Term(TMT6plex); M1(Oxidation); M4(Oxidation); K7(TMT6plex)	1395.7	0	1.0	1.2	1.3	1.1
																	IKGDMDISVPK	2	1	1	61743954	N-Term(TMT6plex); K2(TMT6plex); M5(Oxidation); K11(TMT6plex)	1906.1	1	1.1	1.5	1.3	1.3
																	VKGDMDISLPK	1	1	1	61743954	N-Term(TMT6plex); K2(TMT6plex); M5(Oxidation); K11(TMT6plex)	1906.1	1	1.0	1.2	1.1	1.2
																	MPEMHFK	1	1	1	61743954	N-Term(TMT6plex); M1(Oxidation); K7(TMT6plex)	1393.7	0	0.9	1.0	1.1	0.9
																	LNIEGK	2	1	1	61743954	N-Term(TMT6plex); K6(TMT6plex)	1131.7	0	1.0	1.3	1.3	1.1
																	FGFGAK	2	1	1	61743954	N-Term(TMT6plex); K6(TMT6plex)	1084.7	0	1.0	1.2	1.3	1.1
																	FSMPGFK	1	1	1	61743954	N-Term(TMT6plex); M3(Oxidation); K7(TMT6plex)	1287.7	0	1.2	1.5	2.0	1.0
																	FSVSGAK	1	1	1	61743954	N-Term(TMT6plex); K7(TMT6plex)	1153.7	0	1.0	1.3	1.4	1.3
4507877	NP_003364.1	VCL	7414	vinculin isoform VCL	74.02	2	71	71	601	1.6	1.7	1.9	1.5	1066	116.6	6.09												
																	SLGEISALTSK	16	2	1	4507877	N-Term(TMT6plex); K11(TMT6plex)	1563.9	0	1.6	1.8	2.1	1.5
																	VDQLTAQLADLAAR	16	2	1	4507877	N-Term(TMT6plex)	1714.0	0	1.5	1.6	1.7	1.2
																	VLQLTSWDEDAWASK	21	2	1	4507877	N-Term(TMT6plex); K15(TMT6plex)	2207.2	0	1.7	1.8	2.2	1.6
																	EAFQPQEPDFPPPPPDLEQLR	16	2	1	4507877	N-Term(TMT6plex)	2676.4	0	1.6	1.6	1.7	1.5
																	SLLDASEEAIK	4	2	1	4507877	N-Term(TMT6plex); K11(TMT6plex)	1633.9	0	1.5	1.6	1.8	1.4
																	QILDEAGK	9	2	1	4507877	N-Term(TMT6plex); K8(TMT6plex)	1331.8	0	1.6	1.8	2.0	2.0
																	AANFENHSGK	4	2	1	4507877	N-Term(TMT6plex); K10(TMT6plex)	1532.8	0	1.5	1.7	2.0	1.5
																	TISPMVMDAK	7	2	1	4507877	N-Term(TMT6plex); M5(Oxidation); M7(Oxidation); K10(TMT6plex)	1582.9	0	1.5	1.5	1.5	1.5
																	ILGAVAK	7	2	1	4507877	N-Term(TMT6plex); K7(TMT6plex)	1129.8	0	1.4	1.4	1.7	1.3
																	GQGSSPVAMQK	10	2	1	4507877	N-Term(TMT6plex); M9(Oxidation); K11(TMT6plex)	1563.9	0	2.0	2.4	3.1	1.9
																	WIDNPTVDDR	9	2	1	4507877	N-Term(TMT6plex)	1459.7	0	1.6	1.9	2.1	1.7
																	ELLPVLISAMK	23	2	1	4507877	N-Term(TMT6plex); M10(Oxidation); K11(TMT6plex)	1688.0	0	1.4	1.9	2.0	1.6
																	IPTISTQLK	12	2	1	4507877	N-Term(TMT6plex); K9(TMT6plex)	1458.9	0	1.6	1.8	2.1	1.7
																	TNISDEESEQATEMLVHNAQNLMQSVK	4	2	1	4507877	N-Term(TMT6plex); M23(Oxidation); K27(TMT6plex)	3520.7	0	1.4	1.6	1.8	1.5
																	DMPPAFIK	4	2	1	4507877	N-Term(TMT6plex); K8(TMT6plex)	1376.8	0	1.7	2.0	2.3	1.7
																	NPGNQAAYEHFETMK	8	2	1	4507877	N-Term(TMT6plex); M14(Oxidation); K15(TMT6plex)	2211.1	0	1.7	1.7	1.7	1.6
																	NPGNQAAYEHFETMK	3	2	1	4507877	N-Term(TMT6plex); K15(TMT6plex)	2195.1	0	1.7	1.8	2.1	1.4
																	ETVQTTEDQILK	10	2	1	4507877	N-Term(TMT6plex); K12(TMT6plex)	1863.0	0	1.8	2.0	2.2	1.7
																	AVAGNISDPGLQK	17	2	1	4507877	N-Term(TMT6plex); K13(TMT6plex)	1728.0	0	1.6	1.6	1.9	1.5
																	MQEAMTQEVSDVFSDTTTPIK	13	2	1	4507877	N-Term(TMT6plex); M1(Oxidation); M5(Oxidation); K21(TMT6plex)	2848.4	0	1.5	1.7	1.8	1.6
																	GNDIIAAAK	14	2	1	4507877	N-Term(TMT6plex); K9(TMT6plex)	1330.8	0	1.6	1.7	1.9	1.6
																	MLGQMTDQVADLR	11	2	1	4507877	N-Term(TMT6plex); M1(Oxidation); M5(Oxidation)	1738.9	0	1.6	1.5	1.8	1.4
																	DPSASPGDAGEQAIR	16	2	1	4507877	N-Term(TMT6plex)	1699.8	0	1.7	1.9	2.0	1.5
																	STVEGIQASVK	15	2	1	4507877	N-Term(TMT6plex); K11(TMT6plex)	1576.9	0	1.5	1.5	1.7	1.4
																	MSAEINEIIR	3	2	1	4507877	N-Term(TMT6plex); M1(Oxidation)	1420.8	0	1.6	1.6	1.8	1.5
																	NQWIDNVEK	5	2	1	4507877	N-Term(TMT6plex); K9(TMT6plex)	1603.9	0	1.7	1.4	1.7	1.3
																	QVATALQNLQTK	11	2	1	4507877	N-Term(TMT6plex); K12(TMT6plex)	1773.1	0	1.5	1.5	1.8	1.5
																	AIPDLTAPVAAVQAAVSNLVR	42	2	1	4507877	N-Term(TMT6plex)	2305.3	0	1.7	1.8	2.1	1.6
																	GQGSSPVAMQK	2	2	1	4507877	N-Term(TMT6plex); K11(TMT6plex)	1547.9	0	1.9	2.1	3.0	1.5
																	TIESILEPVAQQISHLVIMHEEGEVDGK	9	2	1	4507877	N-Term(TMT6plex); M19(Oxidation); K28(TMT6plex)	3575.9	0	1.7	2.0	2.3	1.7
																	LVQAAQMLQSDPYSVPAR	2	2	1	4507877	N-Term(TMT6plex)	2203.2	0	1.7	1.5	1.5	1.4
																	GILEYLTVAEVVETMEDLVTYTK	13	2	1	4507877	N-Term(TMT6plex); M15(Oxidation); K23(TMT6plex)	3090.6	0	1.5	1.9	2.5	1.7
																	VAMANIQPQMLVAGATSIAR	6	2	1	4507877	N-Term(TMT6plex); M3(Oxidation); M10(Oxidation)	2303.2	0	1.5	1.4	1.4	1.4
																	TNLLQVCER	4	2	1	4507877	N-Term(TMT6plex); C7(Carbamidomethyl)	1361.7	0	1.8	1.6	2.1	1.7
																	DMPPAFIK	5	2	1	4507877	N-Term(TMT6plex); M2(Oxidation); K8(TMT6plex)	1392.8	0	1.3	1.7	1.9	1.6
																	EAEAASIK	4	2	1	4507877	N-Term(TMT6plex); K8(TMT6plex)	1276.8	0	1.5	1.7	1.9	1.6
																	AAAVGTANK	8	2	1	4507877	N-Term(TMT6plex); K9(TMT6plex)	1260.8	0	1.4	1.7	1.8	1.5
																	AAVHLEGK	6	2	1	4507877	N-Term(TMT6plex); K8(TMT6plex)	1282.8	0	1.1	1.3	1.4	1.4
																	IAELCDDPK	7	2	1	4507877	N-Term(TMT6plex); C5(Carbamidomethyl); K9(TMT6plex)	1518.8	0	1.3	1.4	1.6	1.5
																	ALASQLQDSLK	12	2	1	4507877	N-Term(TMT6plex); K11(TMT6plex)	1632.0	0	1.6	1.8	2.1	1.5
																	MLGQMTDQVADLR	4	2	1	4507877	N-Term(TMT6plex); M5(Oxidation)	1722.9	0	2.6	1.7	2.2	1.6
																	EVENSEDPK	8	2	1	4507877	N-Term(TMT6plex); K9(TMT6plex)	1504.8	0	1.5	1.6	1.9	1.4
																	AQQVSQGLDVLTAK	30	2	1	4507877	N-Term(TMT6plex); K14(TMT6plex)	1916.1	0	1.4	1.8	1.9	1.5
																	GILSGTSDLLLTFDEAEVR	23	2	1	4507877	N-Term(TMT6plex)	2265.2	0	1.3	1.8	1.6	1.6
																	ELTPQVVSAAR	6	2	1	4507877	N-Term(TMT6plex)	1399.8	0	1.6	1.9	2.0	1.6
																	NQGIEEALK	6	2	1	4507877	N-Term(TMT6plex); K9(TMT6plex)	1459.9	0	1.6	1.4	1.8	1.4
																	LVQAAQMLQSDPYSVPAR	11	2	1	4507877	N-Term(TMT6plex); M7(Oxidation)	2219.2	0	1.7	1.5	1.5	1.4
																	MQEAMTQEVSDVFSDTTTPIK	2	2	1	4507877	N-Term(TMT6plex); M5(Oxidation); K21(TMT6plex)	2832.4	0	1.8	1.8	2.3	1.6
																	SLLDASEEAIKK	6	2	1	4507877	N-Term(TMT6plex); K11(TMT6plex); K12(TMT6plex)	1991.2	1	1.5	1.9	2.2	1.6
																	LLAVAATAPPDAPNREEVFDER	8	2	1	4507877	N-Term(TMT6plex)	2610.4	1	1.3	1.5	1.6	1.4
																	AASDELSK	7	2	1	4507877	N-Term(TMT6plex); K8(TMT6plex)	1278.7	0	1.7	1.7	1.9	1.7
																	TNISDEESEQATEMLVHNAQNLMQSVK	12	2	1	4507877	N-Term(TMT6plex); M14(Oxidation); M23(Oxidation); K27(TMT6plex)	3536.7	0	1.4	1.4	1.5	1.5
																	ALASIDSK	2	2	1	4507877	N-Term(TMT6plex); K8(TMT6plex)	1262.8	0	1.4	1.8	2.2	1.6
																	EILGTCK	2	2	1	4507877	N-Term(TMT6plex); C6(Carbamidomethyl); K7(TMT6plex)	1278.7	0	1.5	1.9	2.2	1.5
																	VMLVNSMNTVK	3	2	1	4507877	N-Term(TMT6plex); M2(Oxidation); M7(Oxidation); K11(TMT6plex)	1726.0	0	1.4	1.7	1.8	1.7
																	VENACTK	4	2	1	4507877	N-Term(TMT6plex); C5(Carbamidomethyl); K7(TMT6plex)	1279.7	0	1.7	1.7	2.4	1.3
																	KLEAMTNSK	3	2	1	4507877	N-Term(TMT6plex); K1(TMT6plex); M5(Oxidation); K9(TMT6plex)	1725.0	1	1.3	1.4	1.5	1.3
																	AGEVINQPMMMAAR	4	2	1	4507877	N-Term(TMT6plex); M9(Oxidation); M10(Oxidation); M11(Oxidation)	1795.9	0	1.9	2.6	2.1	2.2
																	IDAAQNWLADPNGGPEGEEQIR	2	2	1	4507877	N-Term(TMT6plex)	2609.3	0	1.4	1.5	1.5	1.6
																	MQEAMTQEVSDVFSDTTTPIK	2	2	1	4507877	N-Term(TMT6plex); K21(TMT6plex)	2816.4	0	1.6	1.8	1.9	1.5

Supplementary Table 6. List of all proteins and peptides identified in untreated and chronically treated Het1A cells with cigarette smoke condensate for 8 months

Accession	RefSeq Accession	Gene Symbol	Entrez Gene ID	Description	Σ Coverage	Σ# Proteins	Σ# Unique Peptides	Σ# Peptides	Σ# PSMs	Het-1A-Smoke - 2M/Parental	Het-1A-Smoke - 4M/Parental	Het-1A-Smoke - 6M/Parental	Het-1A-Smoke - 8M/Parental	# AAs	MW [kDa]	calc. pI	Sequence	# PSMs	# Proteins	# Protein Groups	Protein Group Accessions	Modifications	MH+ [Da]	# Missed Cleavages	Het-1A-Smoke - 2M/Parental	Het-1A-Smoke - 4M/Parental	Het-1A-Smoke - 6M/Parental	Het-1A-Smoke - 8M/Parental
																	GEGESPQAR	4	2	1	4507877	N-Term(TMT6plex)	1159.6	0	**1.6**	**2.1**	**2.4**	**2.0**
																	QQELTHQEHR	2	2	1	4507877	N-Term(TMT6plex)	1534.8	0	**1.2**	**1.5**	**1.4**	**1.3**
																	GVGQAAIR	2	2	1	4507877	N-Term(TMT6plex)	1000.6	0	**1.6**	**1.8**	**2.0**	**1.5**
																	DYLIDGSR	2	2	1	4507877	N-Term(TMT6plex)	1167.6	0	**1.3**	**1.6**	**1.6**	**1.5**
																	MTGLVDEAIDTK	2	2	1	4507877	N-Term(TMT6plex); M1(Oxidation); K12(TMT6plex)	1767.0	0	**1.6**	**1.5**	**1.5**	**1.4**
																	KIDAAQNWLADPNGGPEGEEQIR	4	2	1	4507877	N-Term(TMT6plex); K1(TMT6plex)	2966.5	1	**1.5**	**1.7**	**1.5**	**1.5**
																	VGELCAGK	2	2	1	4507877	N-Term(TMT6plex); C5(Carbamidomethyl); K8(TMT6plex)	1291.7	0	**1.6**	**1.8**	**2.2**	**1.5**
																	DEEFPEQK	1	2	1	4507877	N-Term(TMT6plex); K8(TMT6plex)	1479.8	0	**1.4**	**1.5**	**1.7**	**1.2**
																	ASDEVTR	4	2	1	4507877	N-Term(TMT6plex)	1006.5	0	**1.6**	**1.9**	**2.2**	**1.5**
																	TDAGFTLR	4	2	1	4507877	N-Term(TMT6plex)	1109.6	0	**1.8**	**1.4**	**1.9**	**1.2**
																	LANVMMGPYR	1	2	1	4507877	N-Term(TMT6plex); M5(Oxidation); M6(Oxidation)	1412.7	0	**1.6**	**1.4**	**1.8**	**1.4**
																	TIESILEPVAQQISHLVIMHEEGEVDGK	1	2	1	4507877	N-Term(TMT6plex); K28(TMT6plex)	3559.9	0	**4.9**	**6.4**	**7.6**	**1.0**
																	REVENSEDPK	1	2	1	4507877	N-Term(TMT6plex); K10(TMT6plex)	1660.9	1	**1.4**	**1.5**	**1.5**	**1.6**
																	LGATAEK	1	2	1	4507877	N-Term(TMT6plex); K7(TMT6plex)	1147.7	0	**1.5**	**1.6**	**1.8**	**1.5**
																	IAELCDDPKER	1	2	1	4507877	N-Term(TMT6plex); C5(Carbamidomethyl); K9(TMT6plex)	1804.0	1	**1.5**	**1.7**	**1.8**	**1.6**
																	SFLDSGYR	2	2	1	4507877	N-Term(TMT6plex)	1173.6	0	**1.6**	**1.5**	**1.7**	**1.4**
																	LANVMMGPYR	1	2	1	4507877	N-Term(TMT6plex); M6(Oxidation)	1396.7	0	**1.5**	**1.7**	**2.0**	**1.5**
																	ILLVAK	7	2	1	4507877	N-Term(TMT6plex); K6(TMT6plex)	1114.8	0	**1.8**	**2.0**	**2.4**	**1.6**
																	NFTVEK	4	2	1	4507877	N-Term(TMT6plex); K6(TMT6plex)	1195.7	0	**1.6**	**1.5**	**2.1**	**1.3**
																	QDLLAK	1	2	1	4507877	N-Term(TMT6plex); K6(TMT6plex)	1145.7	0	**1.6**	**1.5**	**2.0**	**1.4**
																	IFVTTK	1	2	1	4507877	N-Term(TMT6plex); K6(TMT6plex)	1166.8	0	**1.5**	**1.6**	**2.0**	**1.4**
4507729	NP_001060.1	**TUBB2A**	7280	**tubulin beta-2A chain**	73.93	2	1	24	2008	**1.3**	**1.2**	**1.4**	**1.1**	445	49.9	4.89												
																	AILVDLEPGTMDSVR	141	7	4	29788785;29788768;50592996;4507729	N-Term(TMT6plex); M11(Oxidation)	1861.0	0				
																	GHYTEGAELVDSVLDVVR	235	11	5	29788785;5174735;29788768;50592996;4507729	N-Term(TMT6plex)	2188.1	0				
																	NSSYFVEWIPNNVK	95	20	7	29788785;5174735;14210536;746815975;29788768;50592996;21361322;574584816;4507729	N-Term(TMT6plex); K14(TMT6plex)	2155.2	0				
																	SGPFGQIFRPDNFVFGQSGAGNNWAK	124	8	5	29788785;5174735;29788768;21361322;574584816;4507729	N-Term(TMT6plex); K26(TMT6plex)	3256.7	0				
																	EVDEQMLNVQNK	195	9	4	29788785;5174735;29788768;4507729	N-Term(TMT6plex); M6(Oxidation); K12(TMT6plex)	1921.0	0				
																	ALTVPELTQQMFDSK	28	3	2	29788768;4507729	N-Term(TMT6plex); M11(Oxidation); K15(TMT6plex)	2182.2	0				
																	LTTPTYGDLNHLVSATMSGVTTCLR	171	12	5	29788785;5174735;29788768;21361322;574584816;4507729	N-Term(TMT6plex); M17(Oxidation); C23(Carbamidomethyl)	2953.5	0				
																	FWEVISDEHGIDPTGSYHGDSDLQLER	1	2	2	29788768;4507729	N-Term(TMT6plex)	3331.6	0				
																	MSATFIGNSTAIQELFK	18	4	3	5174735;29788768;4507729	N-Term(TMT6plex); M1(Oxidation); K17(TMT6plex)	2332.3	0				
																	INVYYNEAAGNK	14	1	1	4507729	N-Term(TMT6plex); K12(TMT6plex)	1814.0	0	**1.3**	**1.2**	**1.4**	**1.1**
																	EIVHIQAGQCGNQIGAK	36	8	6	29788785;29788768;50592996;4507729;5174735;21361322;574584816	N-Term(TMT6plex); C10(Carbamidomethyl); K17(TMT6plex)	2281.2	0				
																	LHFFMPGFAPLTSR	71	18	6	29788785;5174735;14210536;746815975;29788768;21361322;574584816;4507729	N-Term(TMT6plex)	1850.0	0				
																	KESESCDCLQGFQLTHSLGGGTGSGMGTLLISK	4	2	2	29788768;4507729	K1(TMT6plex); N-Term(TMT6plex); C6(Carbamidomethyl); C8(Carbamidomethyl); M26(Oxidation); K33(TMT6plex)	4159.1	1				
																	KLAVNMVPFPR	11	21	8	29788785;5174735;14210536;746815975;29788768;50592996;21361322;574584816;4507729;13562114	N-Term(TMT6plex); K1(TMT6plex); M6(Oxidation)	1746.0	1				
																	TAVCDIPPR	171	14	5	29788785;5174735;29788768;21361322;574584816;4507729	N-Term(TMT6plex); C4(Carbamidomethyl)	1257.7	0				
																	ISEQFTAMFR	20	10	6	29788785;5174735;29788768;50592996;21361322;574584816;4507729	N-Term(TMT6plex)	1458.8	0				
																	ISEQFTAMFR	244	10	6	29788785;5174735;29788768;50592996;21361322;574584816;4507729	N-Term(TMT6plex); M8(Oxidation)	1474.8	0				
																	ESESCDCLQGFQLTHSLGGGTGSGMGTLLISK	13	2	2	29788768;4507729	N-Term(TMT6plex); C5(Carbamidomethyl); C7(Carbamidomethyl); M25(Oxidation); K32(TMT6plex)	3801.9	0				
																	LTTPTYGDLNHLVSATMSGVTTCLR	35	12	5	29788785;5174735;29788768;21361322;574584816;4507729	N-Term(TMT6plex); C23(Carbamidomethyl)	2937.5	0				
																	FPGQLNADLR	98	13	8	29788785;5174735;14210536;746815975;29788768;50592996;21361322;574584816;4507729;13562114	N-Term(TMT6plex)	1359.8	0				
																	AILVDLEPGTMDSVR	22	7	4	29788785;29788768;50592996;4507729	N-Term(TMT6plex)	1845.0	0				
																	LAVNMVPFPR	20	21	8	29788785;5174735;14210536;746815975;29788768;50592996;21361322;574584816;4507729;13562114	N-Term(TMT6plex)	1372.8	0				
																	EVDEQMLNVQNK	12	9	4	29788785;5174735;29788768;4507729	N-Term(TMT6plex); K12(TMT6plex)	1905.0	0				
																	LHFFMPGFAPLTSR	135	18	6	29788785;5174735;14210536;746815975;29788768;21361322;574584816;4507729	N-Term(TMT6plex); M5(Oxidation)	1866.0	0				
																	LAVNMVPFPR	54	21	8	29788785;5174735;14210536;746815975;29788768;50592996;21361322;574584816;4507729;13562114	N-Term(TMT6plex); M5(Oxidation)	1388.8	0				
																	YLTVAAIFR	3	2	2	29788768;4507729	N-Term(TMT6plex)	1282.8	0				
																	IREEYPDR	9	6	5	29788785;5174735;29788768;4507729;13562114	N-Term(TMT6plex)	1306.7	1				
																	MSATFIGNSTAIQELFK	3	4	3	5174735;29788768;4507729	N-Term(TMT6plex); K17(TMT6plex)	2316.3	0				
																	IMNTFSVMPSPK	2	3	3	14210536;746815975;29788768;4507729	N-Term(TMT6plex); M2(Oxidation); M8(Oxidation); K12(TMT6plex)	1842.0	0				
																	ALTVPELTQQMFDSK	4	3	2	29788768;4507729	N-Term(TMT6plex); K15(TMT6plex)	2166.2	0				
																	KESESCDCLQGFQLTHSLGGGTGSGMGTLLISK	3	2	2	29788768;4507729	N-Term(TMT6plex); K1(TMT6plex); C6(Carbamidomethyl); C8(Carbamidomethyl); K33(TMT6plex)	4143.1	1				
																	NMMAACDPR	5	20	7	29788785;5174735;14210536;746815975;29788768;50592996;21361322;574584816;4507729	N-Term(TMT6plex); M2(Oxidation); M3(Oxidation); C6(Carbamidomethyl)	1326.6	0				
																	GSQQYR	5	12	7	29788785;5174735;14210536;746815975;29788768;50592996;21361322;574584816;4507729	N-Term(TMT6plex)	967.5	0				
																	NMMAACDPR	4	20	7	29788785;5174735;14210536;746815975;29788768;50592996;21361322;574584816;4507729	N-Term(TMT6plex); M3(Oxidation); C6(Carbamidomethyl)	1310.6	0				
																	NMMAACDPR	2	20	7	29788785;5174735;14210536;746815975;29788768;50592996;21361322;574584816;4507729	N-Term(TMT6plex); C6(Carbamidomethyl)	1294.6	0				
58218968;4502549	NP_005175.2	**CALM3**	808	**calmodulin**	73.83	4	10	10	755	**1.2**	**1.3**	**1.3**	**1.1**	149	16.8	4.22												
																	EADIDGDGQVNYEEFVQMMTAK	212	3	1	58218968;4502549	N-Term(TMT6plex); M18(Oxidation); M19(Oxidation); K22(TMT6plex)	2980.4	0	**1.2**	**1.3**	**1.3**	**1.2**
																	EADIDGDGQVNYEEFVQMMTAK	123	3	1	58218968;4502549	N-Term(TMT6plex); K22(TMT6plex)	2948.4	0	**1.5**	**1.6**	**1.7**	**1.3**
																	EADIDGDGQVNYEEFVQMMTAK	138	3	1	58218968;4502549	N-Term(TMT6plex); M18(Oxidation); K22(TMT6plex)	2964.4	0	**1.5**	**1.5**	**1.5**	**1.2**
																	DGNGYISAAELR	6	1	1	58218968;4502549	N-Term(TMT6plex)	1494.8	0	**1.2**	**0.9**	**1.2**	**0.8**
																	DTDSEEEIR	20	1	1	58218968;4502549	N-Term(TMT6plex)	1322.6	0	**1.3**	**1.2**	**1.2**	**0.9**
																	EAFSLFDK	48	2	1	58218968;4502549	N-Term(TMT6plex); K8(TMT6plex)	1414.8	0	**1.1**	**1.0**	**1.2**	**0.8**
																	EAFSLFDKDGDGTITTK	105	2	1	58218968;4502549	N-Term(TMT6plex); K8(TMT6plex); K17(TMT6plex)	2532.4	1	**1.2**	**1.3**	**1.3**	**1.0**
																	SLGQNPTEAELQDMINEVDADGNGTIDFPEFLTMMAR	12	3	1	58218968;4502549	N-Term(TMT6plex); M14(Oxidation); M34(Oxidation); M35(Oxidation)	4347.0	0	**1.6**	**1.6**	**1.4**	**1.4**
																	DGDGTITTK	44	1	1	58218968;4502549	N-Term(TMT6plex); K9(TMT6plex)	1365.8	0	**1.1**	**1.0**	**1.2**	**0.9**
																	VFDKDGNGYISAAELR	10	1	1	58218968;4502549	N-Term(TMT6plex); K4(TMT6plex)	2213.2	1	**1.2**	**1.0**	**1.3**	**0.8**
																	MKDTDSEEEIR	7	3	1	58218968;4502549	N-Term(TMT6plex); K2(TMT6plex)	1810.9	1	**1.6**	**1.9**	**2.0**	**1.2**
																	SLGQNPTEAELQDMINEVDADGNGTIDFPEFLTMMAR	11	3	1	58218968;4502549	N-Term(TMT6plex); M34(Oxidation); M35(Oxidation)	4331.0	0	**1.9**	**2.4**	**2.1**	**2.3**
																	SLGQNPTEAELQDMINEVDADGNGTIDFPEFLTMMAR	8	3	1	58218968;4502549	N-Term(TMT6plex); M34(Oxidation)	4315.0	0	**1.8**	**2.0**	**2.0**	**1.1**
																	MKDTDSEEEIR	10	3	1	58218968;4502549	N-Term(TMT6plex); M1(Oxidation); K2(TMT6plex)	1826.9	1	**1.2**	**1.1**	**1.3**	**0.9**
																	ELGTVMR	1	1	1	58218968;4502549	N-Term(TMT6plex)	1034.6	0	**1.9**	**1.7**	**2.2**	**1.2**
42544159	NP_006635.2	**HSPH1**	10808	**heat shock protein 105 kDa isoform 1**	73.66	8	47	51	374	**1.3**	**1.4**	**1.5**	**1.5**	858	96.8	5.39												
																	AGGIETIANEFSDR	10	2	1	42544159	N-Term(TMT6plex)	1708.9	0	**1.3**	**1.4**	**1.4**	**1.4**
																	SLDQDPVVR	8	4	1	42544159	N-Term(TMT6plex)	1257.7	0	**1.3**	**1.3**	**1.4**	**1.5**
																	LQHYAK	3	4	1	42544159	N-Term(TMT6plex); K6(TMT6plex)	1217.7	0	**1.0**	**1.3**	**1.4**	**1.4**
																	KPVTDCVISVPSFFTDAER	8	3	1	42544159	N-Term(TMT6plex); K1(TMT6plex); C6(Carbamidomethyl)	2626.4	0	**1.5**	**1.5**	**1.7**	**1.5**
																	NAVEEYVYEFR	10	4	1	42544159	N-Term(TMT6plex)	1647.8	0	**1.3**	**1.3**	**1.3**	**1.4**
																	LKETAENSLK	1	4	1	42544159	N-Term(TMT6plex); K2(TMT6plex); K10(TMT6plex)	1820.1	1	**1.2**	**1.5**	**1.4**	**1.7**
																	LVEHFCAEFK	9	4	1	42544159	N-Term(TMT6plex); C6(Carbamidomethyl); K10(TMT6plex)	1737.9	0	**1.2**	**1.5**	**1.5**	**1.5**
																	EFSVTDAVPFPISLIWNHDSEDTEGVHEVFSR	6	4	1	42544159	N-Term(TMT6plex)	3888.9	0	**2.0**	**3.1**	**2.9**	**2.6**
																	VEDVSAVEIVGGATR	15	4	1	42544159	N-Term(TMT6plex)	1730.9	0	**1.4**	**1.5**	**1.5**	**1.5**
																	SVLDAAQIVGLNCLR	8	3	1	42544159	N-Term(TMT6plex); C13(Carbamidomethyl)	1858.0	0	**1.4**	**1.5**	**1.4**	**1.4**
																	DKLCGPYEK	2	4	1	42544159	N-Term(TMT6plex); K2(TMT6plex); C4(Carbamidomethyl); K9(TMT6plex)	1797.0	1	**1.1**	**1.7**	**1.5**	**1.7**
																	QDLPSLDEKPR	5	3	1	42544159	N-Term(TMT6plex); K9(TMT6plex)	1756.0	0	**1.2**	**1.3**	**1.3**	**1.4**
																	QAYVDKLEELMK	2	4	1	42544159	N-Term(TMT6plex); K6(TMT6plex); M11(Oxidation); K12(TMT6plex)	2170.2	1	**1.1**	**1.2**	**1.2**	**1.5**
																	FQEAEERPK	3	4	1	42544159	N-Term(TMT6plex); K9(TMT6plex)	1591.9	0	**1.3**	**1.3**	**1.4**	**1.3**
																	DISTTLNADEAVAR	25	5	2	42544159;31541941	N-Term(TMT6plex)	1704.9	0				
																	FICEQDHQNFLR	5	4	1	42544159	N-Term(TMT6plex); C3(Carbamidomethyl)	1835.9	0	**1.3**	**1.4**	**1.5**	**1.6**
																	YNHIDESEMK	7	4	1	42544159	N-Term(TMT6plex); M9(Oxidation); K10(TMT6plex)	1739.9	0	**1.2**	**1.4**	**1.4**	**1.4**
																	LMSSNSTDLPLNIECFMNDK	4	4	1	42544159	N-Term(TMT6plex); M2(Oxidation); C15(Carbamidomethyl); M17(Oxidation); K20(TMT6plex)	2819.4	0	**1.3**	**1.4**	**1.3**	**1.5**

Accession	RefSeq Accession	Gene Symbol	Entrez Gene ID	Description	Σ Coverage	Σ# Proteins	Σ# Unique Peptides	Σ# Peptides	Σ# PSMs	Het-1A-Smoke - 2M/Parental	Het-1A-Smoke - 4M/Parental	Het-1A-Smoke - 6M/Parental	Het-1A-Smoke - 8M/Parental	# AAs	MW [kDa]	calc. pI	Sequence	# PSMs	# Proteins	# Protein Groups	Protein Group Accessions	Modifications	MH+ [Da]	# Missed Cleavages	Het-1A-Smoke - 2M/Parental	Het-1A-Smoke - 4M/Parental	Het-1A-Smoke - 6M/Parental	Het-1A-Smoke - 8M/Parental
																	GPFELEAFYSDPQGVPYPEAK	6	4	1	42544159	N-Term(TMT6plex); K21(TMT6plex)	2799.4	0	1.1	1.5	1.5	1.5
																	SVNEVMEWMNNVMNAQAK	6	4	1	42544159	N-Term(TMT6plex); M6(Oxidation); M13(Oxidation); K18(TMT6plex)	2585.3	0	1.3	1.5	1.5	1.7
																	NQQITHANNTVSNFK	6	4	1	42544159	N-Term(TMT6plex); K15(TMT6plex)	2174.2	0	1.2	1.2	1.4	1.3
																	LMNDMTAVALNYGIYK	2	3	1	42544159	N-Term(TMT6plex); M2(Oxidation); K16(TMT6plex)	2291.2	0	1.4	1.5	1.8	1.4
																	LLTETEDWLYEEGEDQAK	28	4	1	42544159	N-Term(TMT6plex); K18(TMT6plex)	2627.3	0	1.3	1.4	1.5	1.5
																	LYQECEK	5	5	2	42544159;31541941	N-Term(TMT6plex); C5(Carbamidomethyl); K7(TMT6plex)	1427.8	0				
																	QAYVDKLEELMK	1	4	1	42544159	N-Term(TMT6plex); K6(TMT6plex); K12(TMT6plex)	2154.2	1	1.5	2.0	2.2	1.5
																	SQFEELCAELLQK	10	4	1	42544159	N-Term(TMT6plex); C7(Carbamidomethyl); K13(TMT6plex)	2053.1	0	1.2	1.4	1.5	1.6
																	VMYMGEEHLFSVEQITAMLLTK	4	4	1	42544159	N-Term(TMT6plex); M2(Oxidation); M4(Oxidation); K22(TMT6plex)	3060.6	0	0.9	1.1	1.1	1.4
																	VVNVELPIEANLVWQLGK	10	4	1	42544159	N-Term(TMT6plex); K18(TMT6plex)	2479.5	0	1.2	1.4	1.5	1.5
																	VNTHGIFTISTASMVEK	2	4	1	42544159	N-Term(TMT6plex); K17(TMT6plex)	2293.3	0	1.7	1.7	1.5	1.5
																	VMYMGEEHLFSVEQITAMLLTK	4	4	1	42544159	N-Term(TMT6plex); M2(Oxidation); M4(Oxidation); M18(Oxidation); K22(TMT6plex)	3076.6	0	1.3	1.2	1.3	1.5
																	RGPFELEAFYSDPQGVPYPEAK	3	4	1	42544159	N-Term(TMT6plex); K22(TMT6plex)	2955.5	1	1.4	1.7	1.5	1.8
																	LMSSNSTDLPLNIECFMNDKDVSGK	2	4	1	42544159	N-Term(TMT6plex); M2(Oxidation); C15(Carbamidomethyl); M17(Oxidation); K20(TMT6plex); K25(TMT6plex)	3534.8	1	1.2	1.2	1.3	1.5
																	LMNDMTAVALNYGIYK	5	3	1	42544159	N-Term(TMT6plex); M2(Oxidation); M5(Oxidation); K16(TMT6plex)	2307.2	0	1.3	1.4	1.5	1.8
																	KVDQPPEAK	6	4	1	42544159	N-Term(TMT6plex); K1(TMT6plex); K9(TMT6plex)	1699.0	1	1.2	1.3	1.3	1.5
																	VLGTAFDPFLGGK	24	3	1	42544159	N-Term(TMT6plex); K13(TMT6plex)	1780.0	0	1.3	1.2	1.5	1.4
																	GCALQCAILSPAFK	21	6	3	38327039;42544159;31541941	N-Term(TMT6plex); C2(Carbamidomethyl); C6(Carbamidomethyl); K14(TMT6plex)	1994.1	0				
																	AFNDPFIQK	12	4	1	42544159	N-Term(TMT6plex); K9(TMT6plex)	1537.9	0	1.1	1.5	1.5	1.6
																	NVQQDNSEAGTQPQVQTDAQQTSQ SPPSPELTSEENKIPDADK	3	3	1	42544159	N-Term(TMT6plex); K37(TMT6plex); K43(TMT6plex)	5324.6	1	0.9	0.7	0.8	0.7
																	IEVPLYSLLEQTHLK	23	4	1	42544159	N-Term(TMT6plex); K15(TMT6plex)	2241.3	0	1.2	1.5	1.5	1.6
																	EKENLSYDLVPLK	14	4	1	42544159	N-Term(TMT6plex); K2(TMT6plex); K13(TMT6plex)	2235.3	1	1.4	1.9	1.6	1.7
																	VMYMGEEHLFSVEQITAMLLTK	2	4	1	42544159	N-Term(TMT6plex); M4(Oxidation); K22(TMT6plex)	3044.6	0	1.5	1.6	1.7	1.4
																	NHAAPFSK	4	5	2	38327039;42544159	N-Term(TMT6plex); K8(TMT6plex)	1329.8	0				
																	CTPSVISFGSK	2	2	1	42544159	N-Term(TMT6plex); C1(Carbamidomethyl); K11(TMT6plex)	1640.9	0	1.2	1.1	1.4	1.0
																	FVVQNVSAQK	4	4	1	42544159	N-Term(TMT6plex); K10(TMT6plex)	1577.9	0	1.2	1.3	1.3	1.4
																	DLLNMYIETEGK	8	4	1	42544159	N-Term(TMT6plex); M5(Oxidation); K12(TMT6plex)	1900.0	0	1.1	1.3	1.3	1.5
																	ETAENSLK	2	4	1	42544159	N-Term(TMT6plex); K8(TMT6plex)	1349.8	0	1.3	1.3	1.5	1.4
																	ENLSYDLVPLK	4	4	1	42544159	N-Term(TMT6plex); K11(TMT6plex)	1749.0	0	1.2	1.4	1.4	1.5
																	TIGVAAK	5	4	1	42544159	N-Term(TMT6plex); K7(TMT6plex)	1117.7	0	1.3	1.3	1.4	1.3
																	EEDLEDK	1	4	1	42544159	N-Term(TMT6plex); K7(TMT6plex)	1335.7	0	1.1	1.2	1.4	1.2
																	MFEELGQR	6	4	1	42544159	N-Term(TMT6plex); M1(Oxidation)	1254.6	0	1.3	1.5	1.5	1.4
																	KSLDQDPVVR	2	4	1	42544159	N-Term(TMT6plex); K1(TMT6plex)	1615.0	1	1.3	1.5	1.4	1.5
																	VLTFLR	3	8	1	42544159	N-Term(TMT6plex)	977.6	0	1.3	1.2	1.4	1.3
																	NSVNMDLD	1	4	1	42544159	N-Term(TMT6plex); M5(Oxidation)	1152.5	0	1.3	1.4	1.5	1.4
																	LCGPYEK	1	4	1	42544159	N-Term(TMT6plex); C2(Carbamidomethyl); K7(TMT6plex)	1324.7	0	1.1	1.2	1.4	1.2
																	VPTEENEMSSEADMECLNQRPPENP DTDK	1	3	1	42544159	N-Term(TMT6plex); M8(Oxidation); M14(Oxidation); C16(Carbamidomethyl); K29(TMT6plex)	3852.7	0	1.5	1.3	1.6	1.7
4885287	NP_005265.1	**GNG5**	2787	**guanine nucleotide-binding protein G(I)/G(S)/G(O) subunit gamma-5 precursor**	73.53	1	5	5	14	**1.4**	**1.1**	**1.1**	**1.3**	68	7.3	9.85												
																	VSQAAADLK	8	1	1	4885287	N-Term(TMT6plex); K9(TMT6plex)	1360.8	0	1.4	1.1	1.1	1.2
																	QFCLQNAQHDPLLTGVSSSTNPFRPQ K	2	1	1	4885287	N-Term(TMT6plex); C3(Carbamidomethyl); K27(TMT6plex)	3528.8	0	1.1	1.0	1.1	1.3
																	LEAGLNR	2	1	1	4885287	N-Term(TMT6plex)	1001.6	0				
																	KVVQQLR	1	1	1	4885287	N-Term(TMT6plex); K1(TMT6plex)	1328.9	1	1.3	1.3	1.1	1.4
																	VVQQLR	1	1	1	4885287	N-Term(TMT6plex)	971.6	0	1.1	1.1	1.1	1.3
4504253	NP_002096.1	**H2AFX**	3014	**histone H2AX**	73.43	2	6	11	1007	**1.1**	**1.2**	**1.3**	**1.1**	143	15.1	10.74												
																	LLGGVTIAQGGVLPNIQAVLLPK	153	3	2	28195394;4504253	N-Term(TMT6plex); K23(TMT6plex)	2729.7	0				
																	NDEELNK	248	13	4	10645195;28195394;24638446;4504253	N-Term(TMT6plex); K7(TMT6plex)	1319.7	0				
																	AGLQFPVGR	448	17	5	10645195;28195394;24638446;4504253;4504255	N-Term(TMT6plex)	1173.7	0				
																	HLQLAIR	25	17	4	10645195;4504255;24638446;4504253	N-Term(TMT6plex)	1079.7	0				
																	VGAGAPVYLAAVLEYLTAEILELAG NAAR	66	10	3	10645195;28195394;4504253	N-Term(TMT6plex)	3144.8	0				
																	TSATVGPK	16	1	1	4504253	N-Term(TMT6plex); K8(TMT6plex)	1218.7	0	1.1	1.0	1.2	0.9
																	KTSATVGPK	21	1	1	4504253	N-Term(TMT6plex); K1(TMT6plex); K9(TMT6plex)	1576.0	1	1.0	1.2	1.3	1.1
																	KATQASQEY	20	1	1	4504253	N-Term(TMT6plex); K1(TMT6plex)	1483.8	1	1.1	1.2	1.2	1.1
																	ATQASQEY	7	1	1	4504253	N-Term(TMT6plex)	1126.6	0	1.2	1.3	1.4	1.3
																	APSGGK	2	1	1	4504253	N-Term(TMT6plex); K6(TMT6plex)	974.6	0	0.9	1.3	1.2	1.0
																	GHYAER	1	1	1	4504253	N-Term(TMT6plex)	961.5	0	1.5	1.6	1.6	1.1
35494003;35493996	NP_919237.1	**UBE2I**	7329	**SUMO-conjugating enzyme UBC9**	73.42	1	8	8	59	**1.4**	**1.5**	**1.5**	**1.8**	158	18.0	8.66												
																	DHPFGFVAVPTK	2	1	1	35494003;35493996	N-Term(TMT6plex); K12(TMT6plex)	1773.0	0	1.2	1.4	1.5	1.8
																	GTPWEGGLFK	16	1	1	35494003;35493996	N-Term(TMT6plex); K10(TMT6plex)	1549.9	0	1.3	1.5	1.5	1.8
																	KDHPFGFVAVPTK	5	1	1	35494003;35493996	N-Term(TMT6plex); K1(TMT6plex); K13(TMT6plex)	2130.3	1	1.2	1.5	1.6	2.0
																	NPDGTMNLMNWECAIPGK	1	1	1	35494003;35493996	N-Term(TMT6plex); C13(Carbamidomethyl); K18(TMT6plex)	2506.2	0	1.5	1.5	1.6	1.6
																	DDYPSSPPK	11	1	1	35494003;35493996	N-Term(TMT6plex); K9(TMT6plex)	1463.8	0	1.3	1.3	1.3	1.6
																	NPDGTMNLMNWECAIPGK	8	1	1	35494003;35493996	N-Term(TMT6plex); M6(Oxidation); M9(Oxidation); C13(Carbamidomethyl); K18(TMT6plex)	2538.2	0	1.3	1.4	1.3	1.7
																	FEPPLFHPNVYPSGTVCLSILEEDKD WRPAITIK	1	1	1	35494003;35493996	N-Term(TMT6plex); C17(Carbamidomethyl); K25(TMT6plex); K34(TMT6plex)	4656.5	1	1.4	1.5	1.6	2.1
																	KGTPWEGGLFK	3	1	1	35494003;35493996	N-Term(TMT6plex); K1(TMT6plex); K11(TMT6plex)	1907.1	1	1.2	1.4	1.4	2.0
																	QILLGIQELLNEPNIQDPAQAEAYTIY CQNR	12	1	1	35494003;35493996	N-Term(TMT6plex); C28(Carbamidomethyl)	3845.0	0	1.5	1.7	1.6	1.9
4885585	NP_005491.1	**SAE1**	10055	**SUMO-activating enzyme subunit 1 isoform a**	73.41	3	19	19	136	**1.4**	**1.3**	**1.4**	**1.6**	346	38.4	5.30												
																	YCFSEMAPVCAVVGGILAQEIVK	4	1	1	4885585	N-Term(TMT6plex); C2(Carbamidomethyl); M6(Oxidation); C10(Carbamidomethyl); K23(TMT6plex)	3015.6	0	1.4	1.4	1.5	1.4
																	AQNLNPMVDVK	14	3	1	4885585	N-Term(TMT6plex); M7(Oxidation); K11(TMT6plex)	1703.0	0	1.3	1.4	1.3	1.8
																	LDSSETTMVK	11	3	1	4885585	N-Term(TMT6plex); M8(Oxidation); K10(TMT6plex)	1584.9	0	1.6	1.4	1.7	1.6
																	GLTMLDHEQVTPEDPGAQFLIR	7	3	1	4885585	N-Term(TMT6plex); M4(Oxidation)	2712.4	0	1.6	1.3	1.3	1.6
																	VDTEDIEK	6	3	1	4885585	N-Term(TMT6plex); K8(TMT6plex)	1406.8	0	1.6	1.5	1.4	1.6
																	VEKEEAGGGISEEEAAQYDR	3	3	1	4885585	N-Term(TMT6plex); K3(TMT6plex)	2625.3	1	1.2	1.3	1.4	1.9
																	NDVLDSLGISPDLLPEDFVR	15	1	1	4885585	N-Term(TMT6plex)	2443.3	0	1.3	1.0	1.3	1.3
																	VSQGVEDGPDTK	16	3	1	4885585	N-Term(TMT6plex); K12(TMT6plex)	1689.9	0	1.4	1.2	1.3	1.5
																	FFTGDVFGYHGYTFANLGEHEFVEE K	2	3	1	4885585	N-Term(TMT6plex); K26(TMT6plex)	3498.7	0	1.5	1.3	1.6	1.5
																	KPESFFTQFDAVCLTCCSR	2	3	1	4885585	K1(TMT6plex); N-Term(TMT6plex); C13(Carbamidomethyl); C16(Carbamidomethyl); C17(Carbamidomethyl)	2811.4	0	1.7	1.8	1.9	1.9
																	VDQICHK	2	3	1	4885585	N-Term(TMT6plex); C5(Carbamidomethyl); K7(TMT6plex)	1357.8	0	1.2	1.1	1.4	1.5
																	TTSDYFLLQVLLK	19	1	1	4885585	N-Term(TMT6plex); K13(TMT6plex)	1999.2	0	1.5	1.3	1.5	1.7
																	VVFCPVK	6	3	1	4885585	N-Term(TMT6plex); C4(Carbamidomethyl); K7(TMT6plex)	1306.8	0	1.2	1.3	1.4	1.5
																	EALEVDWSSEK	10	3	1	4885585	N-Term(TMT6plex); K11(TMT6plex)	1750.9	0	1.3	1.3	1.4	1.5
																	LWGLEAQK	6	3	1	4885585	N-Term(TMT6plex); K8(TMT6plex)	1402.8	0	1.4	1.5	1.5	1.6
																	DPPHNNFFFFDGMK	2	1	1	4885585	N-Term(TMT6plex); M13(Oxidation); K14(TMT6plex)	2187.1	0	1.0	1.3	1.2	1.6
																	GLGAEIAK	3	3	1	4885585	N-Term(TMT6plex); K8(TMT6plex)	1216.8	0	1.1	1.2	1.3	1.3
																	VLLVGLK	6	3	1	4885585	N-Term(TMT6plex); K7(TMT6plex)	1199.9	0	1.5	1.4	1.5	1.7
																	NLILAGVK	2	3	1	4885585	N-Term(TMT6plex); K8(TMT6plex)	1285.9	0	1.4	1.2	1.3	1.4
385298678;217272892	NP_001245282.1	**EFTUD2**	9343	**116 kDa U5 small nuclear ribonucleoprotein component isoform a**	73.35	5	48	49	460	**1.3**	**1.4**	**1.4**	**1.4**	972	109.4	5.00												
																	VLGENYTLEDEEDSQICTVGR	10	3	1	385298678;217272892	N-Term(TMT6plex); C17(Carbamidomethyl)	2656.3	0	1.6	1.5	1.5	1.3
																	AFIPAIDSFGFETDLR	26	3	1	385298678;217272892	N-Term(TMT6plex)	2028.1	0	1.4	1.4	1.4	1.4
																	YYPTAEEVYGPEVETIVQEEDTQPL TEPIIKPVK	8	3	1	385298678;217272892	N-Term(TMT6plex); K31(TMT6plex); K34(TMT6plex)	4592.4	0	1.1	1.5	1.5	1.4
																	FTLMEQTLPVTVYEMDFLADLMDN SELIR	12	3	1	385298678;217272892	N-Term(TMT6plex); M4(Oxidation); M15(Oxidation); M22(Oxidation)	3711.8	0	1.4	1.9	1.4	1.7
																	GLSEDVSISK	5	3	1	385298678;217272892	N-Term(TMT6plex); K10(TMT6plex)	1492.9	0	1.2	1.4	1.5	1.7
																	ITMIAEPLEK	7	3	1	385298678;217272892	N-Term(TMT6plex); M3(Oxidation); K10(TMT6plex)	1619.0	0	1.4	1.4	1.5	1.4
																	TLDELGIHLTK	4	3	1	385298678;217272892	N-Term(TMT6plex); K11(TMT6plex)	1698.0	0	1.2	1.4	1.5	1.4
																	SYLFNIMDTPGHVNFSDEVTAGLR	8	3	1	385298678;217272892	N-Term(TMT6plex); M7(Oxidation)	2928.4	0	1.3	1.5	1.6	1.5
																	LPPTDAYYK	14	3	1	385298678;217272892	N-Term(TMT6plex); K9(TMT6plex)	1525.9	0	1.2	1.4	1.4	1.4
																	GGGQIIPTAR	24	4	2	385298678;217272892;4503483	N-Term(TMT6plex)	1198.7	0				
																	FFDDPMLLELAK	31	3	1	385298678;217272892	N-Term(TMT6plex); M6(Oxidation); K12(TMT6plex)	1913.1	0	1.5	1.5	1.6	1.5
																	MYSEIDIK	6	3	1	385298678;217272892	N-Term(TMT6plex); M1(Oxidation); K8(TMT6plex)	1472.8	0	1.2	1.2	1.3	1.2
																	SYPSLTTK	3	3	1	385298678;217272892	N-Term(TMT6plex); K8(TMT6plex)	1354.8	0	1.1	1.2	1.2	1.1
																	SIVIRPLEPQPAPHLAR	5	3	1	385298678;217272892	N-Term(TMT6plex)	2123.3	0	1.1	1.3	1.4	1.3

Supplementary Table 6. List of all proteins and peptides identified in untreated and chronically treated Het1A cells with cigarette smoke condensate for 8 months

Accession	RefSeq Accession	Gene Symbol	Entrez Gene ID	Description	Σ Coverage	Σ# Proteins	Σ# Unique Peptides	Σ# Peptides	Σ# PSMs	Het-1A-Smoke - 2M/Parental	Het-1A-Smoke - 4M/Parental	Het-1A-Smoke - 6M/Parental	Het-1A-Smoke - 8M/Parental	# AAs	MW [kDa]	calc. pI	Sequence	# PSMs	# Proteins	# Protein Groups	Protein Group Accessions	Modifications	MH+ [Da]	# Missed Cleavages	Het-1A-Smoke - 2M/Parental	Het-1A-Smoke - 4M/Parental	Het-1A-Smoke - 6M/Parental	Het-1A-Smoke - 8M/Parental
																	IAVEPVNPSELPK	14	3	1	385298678;217272892	N-Term(TMT6plex); K13(TMT6plex)	1851.1	0	**1.3**	**1.4**	**1.5**	**1.4**
																	VEESGEHVILGTGELYLDCVMHDLR	4	3	1	385298678;217272892	N-Term(TMT6plex); C19(Carbamidomethyl); M21(Oxidation)	3116.5	0	**1.7**	**1.5**	**1.4**	**1.5**
																	IEHTYTGGVDSDLGEAMSDCDPDGPLMCHTTK	2	3	1	385298678;217272892	N-Term(TMT6plex); M17(Oxidation); C20(Carbamidomethyl); M27(Oxidation); C28(Carbamidomethyl); K32(TMT6plex)	3999.8	0	**2.0**	**1.8**	**1.6**	**1.5**
																	SFVEFILEPLYK	32	3	1	385298678;217272892	N-Term(TMT6plex); K12(TMT6plex)	1943.1	0	**1.2**	**1.5**	**1.5**	**1.4**
																	TATITEPR	4	3	1	385298678;217272892	N-Term(TMT6plex)	1117.6	0	**1.5**	**1.2**	**1.4**	**1.2**
																	GLAEDIENEVVQITWNR	24	3	1	385298678;217272892	N-Term(TMT6plex)	2215.2	0	**1.3**	**1.5**	**1.5**	**1.6**
																	VLSGTIHAGQPVK	4	3	1	385298678;217272892	N-Term(TMT6plex); K13(TMT6plex)	1765.1	0	**1.1**	**1.6**	**1.2**	**1.8**
																	ISDGVVLFIDAAEGVMLNTER	17	3	1	385298678;217272892	N-Term(TMT6plex); M16(Oxidation)	2494.3	0	**1.5**	**1.3**	**1.3**	**1.6**
																	VADPVVTFCETVVETSSLK	10	3	1	385298678;217272892	N-Term(TMT6plex); C9(Carbamidomethyl); K19(TMT6plex)	2539.4	0	**1.2**	**1.5**	**1.5**	**1.3**
																	TCFVDCLIEQTHPEIR	4	2	1	385298678;217272892	N-Term(TMT6plex); C2(Carbamidomethyl); C6(Carbamidomethyl)	2247.1	0	**1.5**	**1.4**	**1.4**	**1.5**
																	YDQDLCYTDILFTEQER	2	3	1	385298678;217272892	N-Term(TMT6plex); C6(Carbamidomethyl)	2438.1	0	**1.4**	**1.4**	**1.3**	**1.6**
																	ILAQVVGDVDTSLPR	34	3	1	385298678;217272892	N-Term(TMT6plex)	1812.0	0	**1.3**	**1.3**	**1.5**	**1.3**
																	VPAGNWVLIEGVDQPIVK	30	3	1	385298678;217272892	N-Term(TMT6plex); K18(TMT6plex)	2392.4	0	**1.2**	**1.5**	**1.6**	**1.4**
																	FFDDPMLLELAK	12	3	1	385298678;217272892	N-Term(TMT6plex); K12(TMT6plex)	1897.0	0	**1.9**	**2.0**	**2.3**	**1.4**
																	LMEPYYFVEVQAPADCVSAVYTVLAR	4	3	1	385298678;217272892	N-Term(TMT6plex); C16(Carbamidomethyl)	3220.6	0	**1.7**	**1.8**	**2.2**	**1.6**
																	SIWAFGPDATGPNILVDDTLPSEVDK	13	3	1	385298678;217272892	N-Term(TMT6plex); K26(TMT6plex)	3215.7	0	**1.3**	**1.5**	**1.5**	**1.4**
																	FTLMEQTLPVTVYEMDFLADLMDNSELIR	4	3	1	385298678;217272892	N-Term(TMT6plex); M15(Oxidation); M22(Oxidation)	3695.8	0	**1.4**	**1.9**	**1.7**	**1.6**
																	IYADTFGDINYQEFAK	6	3	1	385298678;217272892	N-Term(TMT6plex); K16(TMT6plex)	2353.2	0	**1.5**	**1.5**	**1.5**	**1.4**
																	GHVTQDAPIPGSPLYTIK	6	3	1	385298678;217272892	N-Term(TMT6plex); K18(TMT6plex)	2352.3	0	**1.2**	**1.3**	**1.5**	**1.2**
																	GNEEAQIFRPLK	3	3	1	385298678;217272892	N-Term(TMT6plex); K12(TMT6plex)	1860.1	0	**1.0**	**1.5**	**1.4**	**1.4**
																	LMEPYYFVEVQAPADCVSAVYTVLAR	3	3	1	385298678;217272892	N-Term(TMT6plex); M2(Oxidation); C16(Carbamidomethyl)	3236.6	0	**1.3**	**1.4**	**1.5**	**1.3**
																	DSIVQGFQWGTR	6	3	1	385298678;217272892	N-Term(TMT6plex)	1622.8	0	**1.2**	**1.4**	**1.4**	**1.4**
																	MYSTDDGVQFHAFGR	4	3	1	385298678;217272892	N-Term(TMT6plex); M1(Oxidation)	1975.9	0	**1.4**	**1.5**	**1.4**	**1.5**
																	ISDGVVLFIDAAEGVMLNTER	1	3	1	385298678;217272892	N-Term(TMT6plex)	2478.3	0	**1.6**	**1.5**	**1.4**	**1.6**
																	ITMIAEPLEK	2	3	1	385298678;217272892	N-Term(TMT6plex); K10(TMT6plex)	1603.0	0	**1.4**	**1.8**	**2.1**	**1.4**
																	LWGDIYFNPK	8	3	1	385298678;217272892	N-Term(TMT6plex); K10(TMT6plex)	1711.0	0	**1.2**	**1.2**	**1.3**	**1.2**
																	EGPLCDELIR	6	3	1	385298678;217272892	N-Term(TMT6plex); C5(Carbamidomethyl)	1430.8	0	**1.4**	**1.5**	**1.3**	**1.7**
																	KFTLMEQTLPVTVYEMDFLADLMDNSELIR	1	3	1	385298678;217272892	K1(TMT6plex); N-Term(TMT6plex); M5(Oxidation); M16(Oxidation); M23(Oxidation)	4069.1	1	**2.4**	**2.4**	**2.8**	**2.9**
																	KGLSEDVSISK	2	3	1	385298678;217272892	N-Term(TMT6plex); K1(TMT6plex); K11(TMT6plex)	1850.1	1	**1.0**	**1.3**	**1.3**	**1.3**
																	ILDAVVAQEPLHR	8	3	1	385298678;217272892	N-Term(TMT6plex)	1690.0	0	**1.4**	**1.3**	**1.5**	**1.3**
																	STPVTVVLPDTK	7	3	1	385298678;217272892	N-Term(TMT6plex); K12(TMT6plex)	1715.0	0	**1.2**	**1.2**	**1.3**	**1.2**
																	YDWDLLAAR	1	3	1	385298678;217272892	N-Term(TMT6plex)	1351.7	0	**1.4**	**1.6**	**1.5**	**1.5**
																	QDVVLNYPM	2	3	1	385298678;217272892	N-Term(TMT6plex); M9(Oxidation)	1323.7	0	**1.0**	**1.3**	**1.2**	**1.3**
																	DLDEMDDDDDDDVGDHDDDHPGMEVVLHEDKK	2	2	1	385298678;217272892	N-Term(TMT6plex); M5(Oxidation); M24(Oxidation); K32(TMT6plex); K33(TMT6plex)	4515.0	1	**1.0**	**1.3**	**1.3**	**1.3**
																	ALLGSVK	5	3	1	385298678;217272892	N-Term(TMT6plex); K7(TMT6plex)	1145.8	0	**1.4**	**1.6**	**1.5**	**1.5**
																	LWISVAR	2	3	1	385298678;217272892	N-Term(TMT6plex)	1073.7	0	**1.3**	**1.3**	**1.4**	**1.3**
																	LNIRPLLR	1	3	1	385298678;217272892	N-Term(TMT6plex)	1223.8	0	**1.1**	**1.4**	**1.4**	**1.4**
																	KAPTSSSQR	2	3	1	385298678;217272892	N-Term(TMT6plex); K1(TMT6plex)	1419.8	1	**1.0**	**1.4**	**1.5**	**1.4**
																	KFTLMEQTLPVTVYEMDFLADLMDNSELIR	1	3	1	385298678;217272892	K1(TMT6plex); N-Term(TMT6plex); M5(Oxidation); M16(Oxidation)	4053.1	1	**1.4**	**1.7**	**1.4**	**1.4**
																	LILELK	3	5	1	385298678;217272892	N-Term(TMT6plex); K6(TMT6plex)	1186.8	0				
																	LGEFFQTK	1	3	1	385298678;217272892	N-Term(TMT6plex); K8(TMT6plex)	1427.8	0	**1.3**	**1.2**	**1.5**	**1.0**
4506597	NP_000967.1	**RPL12**	6136	**60S ribosomal protein L12**	73.33	1	12	12	222	**1.2**	**1.3**	**1.3**	**1.3**	165	17.8	9.42												
																	CTGGEVGATSALAPK	13	1	1	4506597	N-Term(TMT6plex); C1(Carbamidomethyl); K15(TMT6plex)	1877.0	0	**1.3**	**1.0**	**1.2**	**1.0**
																	EILGTAQSVGCNVDGR	38	1	1	4506597	N-Term(TMT6plex); C11(Carbamidomethyl)	1905.0	0	**1.4**	**1.2**	**1.4**	**1.3**
																	IGPLGLSPK	40	1	1	4506597	N-Term(TMT6plex); K9(TMT6plex)	1339.9	0	**1.2**	**1.3**	**1.4**	**1.3**
																	HPHDIIDDINSGAVECPAS	46	1	1	4506597	N-Term(TMT6plex); C16(Carbamidomethyl)	2276.1	0	**1.4**	**1.4**	**1.3**	**1.4**
																	QAQIEVVPSASALIIK	35	1	1	4506597	N-Term(TMT6plex); K16(TMT6plex)	2125.3	0	**1.3**	**1.3**	**1.4**	**1.3**
																	HSGNITFDEIVNIAR	22	1	1	4506597	N-Term(TMT6plex)	1915.0	0	**1.3**	**1.3**	**1.4**	**1.3**
																	KVGDDIAK	10	1	1	4506597	N-Term(TMT6plex); K1(TMT6plex); K8(TMT6plex)	1533.0	1	**1.1**	**1.3**	**1.3**	**1.5**
																	VGDDIAK	5	1	1	4506597	N-Term(TMT6plex); K7(TMT6plex)	1175.7	0	**1.2**	**1.2**	**1.3**	**1.3**
																	ELSGTIK	9	1	1	4506597	N-Term(TMT6plex); K7(TMT6plex)	1205.7	0	**1.1**	**1.2**	**1.3**	**1.3**
																	ATGDWKGLR	1	1	1	4506597	N-Term(TMT6plex); K6(TMT6plex)	1461.9	1	**1.1**	**1.1**	**1.2**	**1.2**
																	ATGDWK	2	1	1	4506597	N-Term(TMT6plex); K6(TMT6plex)	1135.7	0	**1.1**	**1.2**	**1.3**	**1.4**
																	ALKEPPR	1	1	1	4506597	N-Term(TMT6plex); K3(TMT6plex)	1268.8	1	**1.0**	**1.5**	**1.3**	**1.5**
14210536;746815975	NP_115914.1	**TUBB6**	84617	**tubulin beta-6 chain**	73.32	6	9	22	794	**1.3**	**1.2**	**1.4**	**1.3**	446	49.8	4.88												
																	NSSYFVEWIPNNVK	95	20	7	29788785;5174735;14210536;746815975;29788768;50592996;21361322;574584816;4507729	N-Term(TMT6plex); K14(TMT6plex)	2155.2	0				
																	FWEVISDEHGIDPAGGYVGDSALQLER	15	1	1	14210536;746815975	N-Term(TMT6plex)	3189.6	0	**1.3**	**1.2**	**1.5**	**1.3**
																	MASTFIGNSTAIQELFK	67	5	1	14210536;746815975	N-Term(TMT6plex); M1(Oxidation); K17(TMT6plex)	2332.3	0	**1.3**	**1.3**	**1.6**	**1.3**
																	VSDTVVEPYNATLSVHQLVENTDETYCIDNEALYDICFR	50	8	5	29788785;5174735;14210536;746815975;29788768;21361322;574584816	N-Term(TMT6plex); C27(Carbamidomethyl); C37(Carbamidomethyl)	4822.2	0				
																	ALTVPELTQQMFDAR	17	1	1	14210536;746815975	N-Term(TMT6plex); M11(Oxidation)	1965.0	0	**1.6**	**1.4**	**1.5**	**1.5**
																	ALTVPELTQQMFDAR	4	1	1	14210536;746815975	N-Term(TMT6plex)	1949.0	0	**1.3**	**1.3**	**1.4**	**1.5**
																	LHFFMPGFAPLTSR	71	18	6	29788785;5174735;14210536;746815975;29788768;21361322;574584816;4507729	N-Term(TMT6plex)	1850.0	0				
																	KLAVNMVPFPR	11	21	8	29788785;5174735;14210536;746815975;29788768;50592996;21361322;574584816;4507729;13562114	N-Term(TMT6plex); K1(TMT6plex); M6(Oxidation)	1746.0	1				
																	EIVHIQAGQCGNQIGTK	5	1	1	14210536;746815975	N-Term(TMT6plex); C10(Carbamidomethyl); K17(TMT6plex)	2311.3	0	**1.2**	**1.2**	**1.3**	**1.3**
																	EVDEQMLAIQSK	25	7	2	14210536;746815975;50592996	N-Term(TMT6plex); M6(Oxidation); K12(TMT6plex)	1865.0	0				
																	FPGQLNADLR	98	13	8	29788785;5174735;14210536;746815975;29788768;50592996;21361322;574584816;4507729;13562114	N-Term(TMT6plex)	1359.8	0				
																	LAVNMVPFPR	20	21	8	29788785;5174735;14210536;746815975;29788768;50592996;21361322;574584816;4507729;13562114	N-Term(TMT6plex)	1372.8	0				
																	ISEQFSAMFR	3	1	1	14210536;746815975	N-Term(TMT6plex)	1444.7	0	**1.3**	**1.2**	**1.7**	**1.2**
																	SGPFGQLFRPDNFIFGQTGAGNNWAK	4	1	1	14210536;746815975	N-Term(TMT6plex); K26(TMT6plex)	3284.7	0	**1.1**	**1.0**	**1.4**	**1.0**
																	LHFFMPGFAPLTSR	135	18	6	29788785;5174735;14210536;746815975;29788768;21361322;574584816;4507729	N-Term(TMT6plex); M5(Oxidation)	1866.0	0				
																	AALVDLEPGTMDSVR	2	2	1	14210536;746815975	N-Term(TMT6plex)	1803.0	0	**1.5**	**1.5**	**1.5**	**1.5**
																	VAVCDIPPR	5	8	3	14210536;746815975;50592996;13562114	N-Term(TMT6plex); C4(Carbamidomethyl)	1255.7	0				
																	GHYTEGAELVDAVLDVVR	38	5	2	14210536;746815975;21361322;574584816	N-Term(TMT6plex)	2172.2	0				
																	LTTPTYGDLNHLVSATMSGVTTSLR	21	1	1	14210536;746815975	N-Term(TMT6plex); M17(Oxidation)	2880.5	0	**1.4**	**1.3**	**1.4**	**1.3**
																	LAVNMVPFPR	54	21	8	29788785;5174735;14210536;746815975;29788768;50592996;21361322;574584816;4507729;13562114	N-Term(TMT6plex); M5(Oxidation)	1388.8	0				
																	AALVDLEPGTMDSVR	13	2	1	14210536;746815975	N-Term(TMT6plex); M11(Oxidation)	1818.9	0	**1.1**	**1.5**	**1.5**	**1.5**
																	YLTVATVFR	6	3	2	14210536;746815975;50592996	N-Term(TMT6plex)	1298.8	0				
																	EVDEQMLAIQSK	4	7	2	14210536;746815975;50592996	N-Term(TMT6plex); K12(TMT6plex)	1849.0	0				
																	INVYYNESSSQK	10	2	1	14210536;746815975	N-Term(TMT6plex); K12(TMT6plex)	1890.0	0	**1.1**	**1.2**	**1.4**	**1.2**
																	LTTPTYGDLNHLVSATMSGVTTSLR	1	1	1	14210536;746815975	N-Term(TMT6plex)	2864.5	0	**0.9**	**1.0**	**1.0**	**1.2**
																	IMNTFSVMPSPK	2	3	3	14210536;746815975;29788768;4507729	N-Term(TMT6plex); M2(Oxidation); M8(Oxidation); K12(TMT6plex)	1842.0	0				
																	MASTFIGNSTAIQELFK	1	5	1	14210536;746815975	N-Term(TMT6plex); K17(TMT6plex)	2316.3	0	**1.3**	**1.4**	**1.5**	**1.1**
																	NMMAACDPR	5	20	7	29788785;5174735;14210536;746815975;29788768;50592996;21361322;574584816;4507729	N-Term(TMT6plex); M2(Oxidation); M3(Oxidation); C6(Carbamidomethyl)	1326.6	0				
																	GSQQYR	5	12	7	29788785;5174735;14210536;746815975;29788768;50592996;21361322;574584816;4507729	N-Term(TMT6plex)	967.5	0				
																	NMMAACDPR	4	20	7	29788785;5174735;14210536;746815975;29788768;50592996;21361322;574584816;4507729	N-Term(TMT6plex); M3(Oxidation); C6(Carbamidomethyl)	1310.6	0				
																	ISEQFSAMFR	1	1	1	14210536;746815975	N-Term(TMT6plex); M8(Oxidation)	1460.7	0	**1.4**	**1.3**	**1.4**	**1.3**
																	NMMAACDPR	2	20	7	29788785;5174735;14210536;746815975;29788768;50592996;21361322;574584816;4507729	N-Term(TMT6plex); C6(Carbamidomethyl)	1294.6	0				
16507237	NP_005338.1	**HSPA5**	3309	**78 kDa glucose-regulated protein precursor**	73.09	3	48	51	2240	**1.8**	**1.8**	**1.6**	**1.5**	654	72.3	5.16												
																	TFAPEEISAMVLTK	117	1	1	16507237	N-Term(TMT6plex); M10(Oxidation); K14(TMT6plex)	2011.1	0	**1.6**	**1.7**	**1.5**	**1.4**
																	IEWLESHQDADIEDFK	98	1	1	16507237	N-Term(TMT6plex); K16(TMT6plex)	2433.2	0	**1.8**	**1.9**	**1.6**	**1.4**
																	NQLTSNPENTVFDAK	47	1	1	16507237	N-Term(TMT6plex); K15(TMT6plex)	2136.1	0	**1.8**	**1.5**	**1.5**	**1.2**
																	IINEPTAAAIAYGLDKR	21	1	1	16507237	N-Term(TMT6plex); K16(TMT6plex)	2274.3	1	**1.9**	**2.2**	**1.7**	**1.6**
																	LYGSAGPPPTGEEDTAEKDEL	49	1	1	16507237	N-Term(TMT6plex); K18(TMT6plex)	2634.3	1	**1.8**	**1.6**	**1.6**	**1.4**
																	DNHLLGTFDLTGIPPAPR	91	1	1	16507237	N-Term(TMT6plex)	2163.2	0	**1.4**	**1.5**	**1.5**	**1.4**
																	ALSSQHQAR	8	1	1	16507237	N-Term(TMT6plex)	1226.7	0	**1.7**	**1.9**	**1.6**	**1.4**

Accession	RefSeq Accession	Gene Symbol	Entrez Gene ID	Description	Σ Coverage	Σ# Proteins	Σ# Unique Peptides	Σ# Peptides	Σ# PSMs	Het-1A-Smoke - 2M/Parental	Het-1A-Smoke - 4M/Parental	Het-1A-Smoke - 6M/Parental	Het-1A-Smoke - 8M/Parental	# AAs	MW [kDa]	calc. pI	Sequence	# PSMs	# Proteins	# Protein Groups	Protein Group Accessions	Modifications	MH+ [Da]	# Missed Cleavages	Het-1A-Smoke - 2M/Parental	Het-1A-Smoke - 4M/Parental	Het-1A-Smoke - 6M/Parental	Het-1A-Smoke - 8M/Parental
																	TWNDPSVQQDIK	59	1	1	16507237	N-Term(TMT6plex); K12(TMT6plex)	1889.0	0	**1.8**	**1.6**	**1.6**	**1.4**
																	TFAPEEISAMVLTK	30	1	1	16507237	N-Term(TMT6plex); K14(TMT6plex)	1995.1	0	**1.9**	**2.0**	**1.9**	**1.5**
																	FEELNMDLFR	65	1	1	16507237	N-Term(TMT6plex); M6(Oxidation)	1558.8	0	**1.8**	**1.6**	**1.6**	**1.3**
																	ITITNDQNR	49	1	1	16507237	N-Term(TMT6plex)	1303.7	0	**1.7**	**1.7**	**1.5**	**1.6**
																	ELEEIVQPIISK	136	1	1	16507237	N-Term(TMT6plex); K12(TMT6plex)	1856.1	0	**1.7**	**2.0**	**1.7**	**1.6**
																	AKFEELNMDLFR	10	1	1	16507237	N-Term(TMT6plex); K2(TMT6plex)	1971.1	1	**2.1**	**2.8**	**2.2**	**1.7**
																	LSSEDKETMEK	8	1	1	16507237	N-Term(TMT6plex); K6(TMT6plex); M9(Oxidation); K11(TMT6plex)	2000.1	1	**5.7**	**7.9**	**5.0**	**3.8**
																	KELEEIVQPIISK	8	1	1	16507237	N-Term(TMT6plex); K1(TMT6plex); K13(TMT6plex)	2213.4	1	**1.5**	**1.8**	**1.5**	**1.5**
																	VMEHFIK	19	1	1	16507237	N-Term(TMT6plex); M2(Oxidation); K7(TMT6plex)	1377.8	0	**1.7**	**1.9**	**1.7**	**1.5**
																	VYEGERPLTK	25	1	1	16507237	N-Term(TMT6plex); K10(TMT6plex)	1650.0	0	**1.9**	**1.8**	**1.5**	**1.4**
																	SQIFSTASDNQPTVTIK	71	1	1	16507237	N-Term(TMT6plex); K17(TMT6plex)	2295.3	0	**1.6**	**1.7**	**1.6**	**1.4**
																	AKFEELNMDLFR	12	1	1	16507237	N-Term(TMT6plex); K2(TMT6plex); M8(Oxidation)	1987.1	1	**1.7**	**2.2**	**1.6**	**1.6**
																	IINEPTAAAIAYGLDK	103	5	4	13676857;5729877;16507237;124256496	N-Term(TMT6plex); K16(TMT6plex)	2118.2	0				
																	DAGTIAGLNVMR	68	1	1	16507237	N-Term(TMT6plex); M11(Oxidation)	1462.8	0	**1.6**	**1.7**	**1.6**	**1.5**
																	VEIIANDQGNR	80	7	6	13676857;5729877;16507237;194248072;167466173;124256496;34419635	N-Term(TMT6plex)	1457.8	0				
																	ETAEAYLGK	54	1	1	16507237	N-Term(TMT6plex); K9(TMT6plex)	1439.8	0	**1.7**	**1.9**	**1.6**	**1.5**
																	TKPYIQVDIGGGQTK	35	1	1	16507237	N-Term(TMT6plex); K2(TMT6plex); K15(TMT6plex)	2292.4	0	**1.6**	**1.8**	**1.6**	**1.4**
																	VLEDSDLK	36	1	1	16507237	N-Term(TMT6plex); K8(TMT6plex)	1376.8	0	**1.9**	**2.0**	**1.7**	**1.6**
																	KSDIDEIVLVGGSTR	41	1	1	16507237	N-Term(TMT6plex); K1(TMT6plex)	2047.2	1	**1.7**	**1.9**	**1.7**	**1.5**
																	ITPSYVAFTPEGER	118	1	1	16507237	N-Term(TMT6plex)	1795.9	0	**1.7**	**1.7**	**1.6**	**1.5**
																	VTHAVVTVPAYFNDAQR	41	1	1	16507237	N-Term(TMT6plex)	2117.1	0	**1.4**	**1.4**	**1.4**	**1.3**
																	NELESYAYSLK	61	1	1	16507237	N-Term(TMT6plex); K11(TMT6plex)	1775.0	0	**1.6**	**1.6**	**1.5**	**1.4**
																	GINPDEAVAYGAAVQAGVLSGDQDTGDLVLLDVCPLTLGIETVGGVMTK	22	1	1	16507237	N-Term(TMT6plex); C34(Carbamidomethyl); M47(Oxidation); K49(TMT6plex)	5373.8	0	**1.9**	**2.3**	**2.0**	**1.4**
																	DAGTIAGLNVMR	10	1	1	16507237	N-Term(TMT6plex)	1446.8	0	**1.9**	**1.7**	**1.7**	**1.5**
																	MKETAEAYLGK	6	1	1	16507237	N-Term(TMT6plex); M1(Oxidation); K2(TMT6plex); K11(TMT6plex)	1944.1	1	**1.7**	**2.2**	**1.6**	**1.6**
																	VMEHFIK	12	1	1	16507237	N-Term(TMT6plex); K7(TMT6plex)	1361.8	0	**2.1**	**2.5**	**2.3**	**1.5**
																	FEELNMDLFR	23	1	1	16507237	N-Term(TMT6plex)	1542.8	0	**2.1**	**1.9**	**2.0**	**1.4**
																	MKETAEAYLGK	3	1	1	16507237	N-Term(TMT6plex); K2(TMT6plex); K11(TMT6plex)	1928.1	1	**2.0**	**2.5**	**2.1**	**1.7**
																	LSSEDKETMEK	2	1	1	16507237	N-Term(TMT6plex); K6(TMT6plex); K11(TMT6plex)	1984.1	1	**5.0**	**7.6**	**5.5**	**2.8**
																	SDIDEIVLVGGSTR	43	1	1	16507237	N-Term(TMT6plex)	1689.9	0	**1.5**	**1.6**	**1.5**	**1.3**
																	IEIESFYEGEDFSETLTR	347	1	1	16507237	N-Term(TMT6plex)	2394.2	0	**1.6**	**1.7**	**1.5**	**1.5**
																	GINPDEAVAYGAAVQAGVLSGDQDTGDLVLLDVCPLTLGIETVGGVMTK	13	1	1	16507237	N-Term(TMT6plex); C34(Carbamidomethyl); K49(TMT6plex)	5357.8	0	**3.2**	**3.2**	**3.1**	**2.5**
																	NQIGDKEK	6	1	1	16507237	N-Term(TMT6plex); K6(TMT6plex); K8(TMT6plex)	1619.0	1	**1.9**	**1.7**	**1.6**	**1.2**
																	KVTHAVVTVPAYFNDAQR	4	1	1	16507237	N-Term(TMT6plex); K1(TMT6plex)	2474.4	1	**1.4**	**1.5**	**1.6**	**1.5**
																	LIGDAAK	87	6	5	13676857;5729877;16507237;194248072;167466173;124256496	N-Term(TMT6plex); K7(TMT6plex)	1145.7	0				
																	NTVVPTK	20	1	1	16507237	N-Term(TMT6plex); K7(TMT6plex)	1216.8	0	**1.6**	**1.4**	**1.5**	**1.2**
																	KTKPYIQVDIGGGQTK	6	1	1	16507237	N-Term(TMT6plex); K1(TMT6plex); K3(TMT6plex); K16(TMT6plex)	2649.6	1	**1.5**	**2.4**	**1.7**	**1.8**
																	KSQIFSTASDNQPTVTIK	8	1	1	16507237	N-Term(TMT6plex); K1(TMT6plex); K18(TMT6plex)	2652.5	1	**1.6**	**1.8**	**1.6**	**1.5**
																	KKELEEIVQPIISK	6	1	1	16507237	N-Term(TMT6plex); K1(TMT6plex); K2(TMT6plex); K14(TMT6plex)	2570.6	2	**1.6**	**3.1**	**1.8**	**2.3**
																	LYGSAGPPPTGEEDTAEK	2	1	1	16507237	N-Term(TMT6plex); K18(TMT6plex)	2277.2	0	**1.3**	**1.2**	**1.3**	**1.3**
																	EDVGTVVGIDLGTTYSCVGVFK	2	1	1	16507237	N-Term(TMT6plex); C17(Carbamidomethyl); K22(TMT6plex)	2774.5	0	**2.6**	**2.2**	**1.5**	**2.5**
																	LTPEEIER	21	3	1	16507237	N-Term(TMT6plex)	1215.7	0	**1.8**	**1.9**	**1.6**	**1.5**
																	VLEDSDLKK	3	1	1	16507237	N-Term(TMT6plex); K8(TMT6plex); K9(TMT6plex)	1734.1	1	**1.8**	**2.2**	**1.7**	**1.5**
																	MVNDAEK	7	1	1	16507237	N-Term(TMT6plex); K7(TMT6plex)	1264.7	0	**3.1**	**3.6**	**3.4**	**1.7**
																	STMKPVQK	2	1	1	16507237	N-Term(TMT6plex); M3(Oxidation); K4(TMT6plex); K8(TMT6plex)	1622.0	0	**1.6**	**1.9**	**1.6**	**1.4**
																	VTAEDK	7	1	1	16507237	N-Term(TMT6plex); K6(TMT6plex)	1120.7	0	**1.6**	**1.8**	**1.6**	**1.4**
																	FAEEDKK	3	1	1	16507237	N-Term(TMT6plex); K6(TMT6plex); K7(TMT6plex)	1553.9	1	**2.1**	**2.2**	**1.8**	**1.4**
																	NTVVPTKK	2	1	1	16507237	N-Term(TMT6plex); K7(TMT6plex); K8(TMT6plex)	1574.0	1	**1.7**	**2.3**	**1.8**	**1.9**
																	IQQLVK	5	1	1	16507237	N-Term(TMT6plex); K6(TMT6plex)	1186.8	0	**1.8**	**1.8**	**1.6**	**1.5**
																	EFFNGK	2	1	1	16507237	N-Term(TMT6plex); K6(TMT6plex)	1199.7	0	**1.8**	**1.9**	**1.7**	**1.5**
																	NQIGDK	2	1	1	16507237	N-Term(TMT6plex); K6(TMT6plex)	1132.7	0	**1.9**	**1.3**	**1.6**	**1.0**
																	FAEEDK	2	1	1	16507237	N-Term(TMT6plex); K6(TMT6plex)	1196.7	0	**2.1**	**2.2**	**1.9**	**1.6**
																	STMKPVQK	1	1	1	16507237	N-Term(TMT6plex); K4(TMT6plex); K8(TMT6plex)	1606.0	0	**2.4**	**2.5**	**2.6**	**1.6**
																	MVNDAEK	1	1	1	16507237	N-Term(TMT6plex); M1(Oxidation); K7(TMT6plex)	1280.7	0	**2.9**	**3.4**	**3.1**	**1.6**
291575128;4557032	NP_001167568.1	**LDHB**	3945	**L-lactate dehydrogenase B chain**	73.05	2	20	23	978	**1.5**	**1.6**	**1.8**	**1.7**	334	36.6	6.05												
																	IVADKDYSVTANSK	20	1	1	291575128;4557032	N-Term(TMT6plex); K5(TMT6plex); K14(TMT6plex)	2198.3	1	**1.6**	**1.9**	**2.0**	**2.0**
																	ITVVGVGQVGMACAISILGK	41	2	1	291575128;4557032	N-Term(TMT6plex); M11(Oxidation); C13(Carbamidomethyl); K20(TMT6plex)	2447.4	0	**1.3**	**1.3**	**1.6**	**1.4**
																	MVVESAYEVIK	50	2	1	291575128;4557032	N-Term(TMT6plex); M1(Oxidation); K11(TMT6plex)	1742.0	0	**1.4**	**1.6**	**1.6**	**1.5**
																	SADTLWDIQK	27	1	1	291575128;4557032	N-Term(TMT6plex); K10(TMT6plex)	1634.9	0	**1.5**	**1.6**	**1.7**	**1.7**
																	IHPVSTMVK	18	1	1	291575128;4557032	N-Term(TMT6plex); M7(Oxidation); K9(TMT6plex)	1485.9	0	**1.2**	**1.4**	**1.5**	**1.3**
																	ITVVGVGQVGMACAISILGK	61	2	1	291575128;4557032	N-Term(TMT6plex); C13(Carbamidomethyl); K20(TMT6plex)	2431.4	0	**1.4**	**1.5**	**1.8**	**1.6**
																	GEMMDLQHGSLFLQTPK	9	2	1	291575128;4557032	N-Term(TMT6plex); K17(TMT6plex)	2390.3	0	**1.9**	**1.9**	**2.3**	**1.6**
																	IVVVTAGVR	25	2	1	291575128;4557032	N-Term(TMT6plex)	1142.7	0	**1.6**	**1.6**	**1.8**	**1.6**
																	GEMMDLQHGSLFLQTPK	27	2	1	291575128;4557032	N-Term(TMT6plex); M3(Oxidation); M4(Oxidation); K17(TMT6plex)	2422.3	0	**1.3**	**1.5**	**1.5**	**1.5**
																	LIAPVAEEEATVPNNK	71	1	1	291575128;4557032	N-Term(TMT6plex); K16(TMT6plex)	2153.2	0	**1.5**	**1.6**	**1.8**	**1.6**
																	GLTSVINQK	47	1	1	291575128;4557032	N-Term(TMT6plex); K9(TMT6plex)	1417.9	0	**1.4**	**1.6**	**1.7**	**1.6**
																	SLADELALVDVLEDKLK	16	1	1	291575128;4557032	N-Term(TMT6plex); K15(TMT6plex); K17(TMT6plex)	2558.5	1	**2.1**	**3.1**	**2.2**	**3.0**
																	GMYGIENEVFLSLPCILNAR	6	1	1	291575128;4557032	N-Term(TMT6plex); C15(Carbamidomethyl)	2525.3	0	**1.6**	**2.0**	**1.9**	**1.8**
																	GMYGIENEVFLSLPCILNAR	52	1	1	291575128;4557032	N-Term(TMT6plex); M2(Oxidation); C15(Carbamidomethyl)	2541.3	0	**1.5**	**1.7**	**1.7**	**1.7**
																	GYTNWAIGLSVADLIESMLK	10	1	1	291575128;4557032	N-Term(TMT6plex); M18(Oxidation); K20(TMT6plex)	2655.4	0	**2.1**	**1.7**	**3.8**	**3.1**
																	SLADELALVDVLEDK	287	2	1	291575128;4557032	N-Term(TMT6plex); K15(TMT6plex)	2088.2	0	**1.5**	**1.7**	**1.8**	**1.7**
																	GYTNWAIGLSVADLIESMLK	6	1	1	291575128;4557032	N-Term(TMT6plex); K20(TMT6plex)	2639.5	0	**1.9**	**2.1**	**2.6**	**2.0**
																	LKDDEVAQLK	51	2	1	291575128;4557032	N-Term(TMT6plex); K2(TMT6plex); K10(TMT6plex)	1846.1	1	**1.4**	**1.6**	**1.8**	**1.7**
																	LKGEMMDLQHGSLFLQTPK	9	1	1	291575128;4557032	N-Term(TMT6plex); K2(TMT6plex); M5(Oxidation); M6(Oxidation); K19(TMT6plex)	2892.6	1	**1.3**	**1.4**	**1.6**	**1.6**
																	VIGSGCNLDSAR	55	8	2	5031857;291575128;4557032	N-Term(TMT6plex); C6(Carbamidomethyl)	1477.8	0				
																	LKGEMMDLQHGSLFLQTPK	4	1	1	291575128;4557032	N-Term(TMT6plex); K2(TMT6plex); K19(TMT6plex)	2860.6	1	**2.2**	**2.5**	**3.1**	**2.0**
																	DYSVTANSK	14	1	1	291575128;4557032	N-Term(TMT6plex); K9(TMT6plex)	1442.8	0	**1.7**	**1.6**	**1.9**	**1.7**
																	YSPDCIIIVVSNPVDILTYVTWK	6	1	1	291575128;4557032	N-Term(TMT6plex); C5(Carbamidomethyl); K23(TMT6plex)	3153.7	0	**1.8**	**1.7**	**2.6**	**1.3**
																	MVVESAYEVIK	14	2	1	291575128;4557032	N-Term(TMT6plex); K11(TMT6plex)	1726.0	0	**2.0**	**2.1**	**2.6**	**1.9**
																	FIIPQIVK	35	2	1	291575128;4557032	N-Term(TMT6plex); K8(TMT6plex)	1415.9	0	**1.5**	**1.6**	**1.8**	**1.6**
																	QQEGESR	4	5	2	5031857;291575128;4557032	N-Term(TMT6plex)	1062.5	0				
																	LNLVQR	3	7	2	5031857;291575128;4557032	N-Term(TMT6plex)	971.6	0				
																	NVNVFK	9	1	1	291575128;4557032	N-Term(TMT6plex); K6(TMT6plex)	1178.7	0	**1.4**	**1.4**	**1.6**	**1.4**
																	DDEVAQLK	1	1	1	291575128;4557032	N-Term(TMT6plex); K8(TMT6plex)	1375.8	0	**1.3**	**1.3**	**1.5**	**1.3**
342187211	NP_001230106.1	**ALDOA**	226	**fructose-bisphosphate aldolase A isoform 2**	72.97	1	1	26	1025	**1.4**	**1.3**	**1.3**	**1.4**	418	45.2	8.25												
																	ALQASALK	45	2	2	342187211;34577112;4557305	N-Term(TMT6plex); K8(TMT6plex)	1259.8	0				
																	IGEHTPSALAIMENANVLAR	51	2	2	342187211;34577112;4557305	N-Term(TMT6plex); M12(Oxidation)	2352.3	0				
																	CQYVTEK	14	3	3	342187211;34577112;4557305;4885063	N-Term(TMT6plex); C1(Carbamidomethyl); K7(TMT6plex)	1385.7	0				
																	VLAAVYK	40	4	3	342187211;34577112;4557305;4885063	N-Term(TMT6plex); K7(TMT6plex)	1221.8	0				
																	GGVVGIK	14	2	2	342187211;34577112;4557305	N-Term(TMT6plex); K7(TMT6plex)	1087.7	0				
																	ELATTSTMPYQYPALTPEQK	3	1	1	342187211	N-Term(TMT6plex); M8(Oxidation); K20(TMT6plex)	2743.4	0	**1.4**	**1.3**	**1.3**	**1.4**
																	TVPPAVTGITFLSGGQSEEEASINLNAINK	318	2	2	342187211;34577112;4557305	N-Term(TMT6plex); K30(TMT6plex)	3515.9	0				
																	YTPSGQAGAAASESLFVSNHAY	150	2	2	342187211;34577112;4557305	N-Term(TMT6plex)	2457.2	0				
																	GVVPLAGTNGETTTQGLDGLSER	32	2	2	342187211;34577112;4557305	N-Term(TMT6plex)	2501.3	0				
																	QLLLTADDR	31	2	2	342187211;34577112;4557305	N-Term(TMT6plex)	1273.7	0				
																	FSHEEIAMATVTALR	50	2	2	342187211;34577112;4557305	N-Term(TMT6plex); M8(Oxidation)	1921.0	0				
																	LQSIGTENTEENRR	33	2	2	342187211;34577112;4557305	N-Term(TMT6plex)	1876.0	1				
																	ELSDIAHR	4	2	2	342187211;34577112;4557305	N-Term(TMT6plex)	1169.6	0				
																	ALANSLACQGK	29	2	2	342187211;34577112;4557305	N-Term(TMT6plex); C8(Carbamidomethyl); K11(TMT6plex)	1590.9	0				
																	ADDGRPFPQVIK	16	2	2	342187211;34577112;4557305	N-Term(TMT6plex); K12(TMT6plex)	1801.0	0				
																	LQSIGTENTEENR	49	2	2	342187211;34577112;4557305	N-Term(TMT6plex)	1719.9	0				
																	DGADFAK	27	3	3	342187211;34577112;4557305;4885063	N-Term(TMT6plex); K7(TMT6plex)	1181.7	0				
																	GILAADESTGSIAK	36	2	2	342187211;34577112;4557305	N-Term(TMT6plex); K14(TMT6plex)	1791.0	0				
																	AAQEEYVK	32	2	2	342187211;34577112;4557305	N-Term(TMT6plex); K8(TMT6plex)	1395.8	0				
																	KELSDIAHR	2	2	2	342187211;34577112;4557305	N-Term(TMT6plex); K1(TMT6plex)	1526.9	1				
																	VNPCIGGVILFHETLYQK	8	2	2	342187211;34577112;4557305	N-Term(TMT6plex); C4(Carbamidomethyl); K18(TMT6plex)	2546.4	0				
																	IGEHTPSALAIMENANVLAR	15	2	2	342187211;34577112;4557305	N-Term(TMT6plex)	2336.3	0				

Accession	RefSeq Accession	Gene Symbol	Entrez Gene ID	Description	Σ Coverage	Σ# Proteins	Σ# Unique Peptides	Σ# Peptides	Σ# PSMs	Het-1A-Smoke - 2M/Parental	Het-1A-Smoke - 4M/Parental	Het-1A-Smoke - 6M/Parental	Het-1A-Smoke - 8M/Parental	# AAs	MW [kDa]	calc. pI	Sequence	# PSMs	# Proteins	# Protein Groups	Protein Group Accessions	Modifications	MH+ [Da]	# Missed Cleavages	Het-1A-Smoke - 2M/Parental	Het-1A-Smoke - 4M/Parental	Het-1A-Smoke - 6M/Parental	Het-1A-Smoke - 8M/Parental
																	FSHEEIAMATVTALR	5	2	2	342187211;34577112;4557305	N-Term(TMT6plex)	1905.0	0				
																	KDGADFAK	7	3	3	342187211;34577112;4557305;4885063	N-Term(TMT6plex); K1(TMT6plex); K8(TMT6plex)	1538.9	1				
																	YASICQQNGIVPIVEPEILPDGDHDLKR	6	3	3	342187211;34577112;4557305;4885063	N-Term(TMT6plex); C5(Carbamidomethyl); K27(TMT6plex)	3634.9	1				
																	YASICQQNGIVPIVEPEILPDGDHDLK	2	3	3	342187211;34577112;4557305;4885063	N-Term(TMT6plex); C5(Carbamidomethyl); K27(TMT6plex)	3478.8	0				
																	CPLLKPWALTFSYGR	4	2	2	342187211;34577112;4557305	N-Term(TMT6plex); C1(Carbamidomethyl); K5(TMT6plex)	2267.3	0				
																	IGEHTPSALAIMENANVLARYASICQQNGIVPIVEPEILPDGDHDLK	1	2	2	342187211;34577112;4557305	N-Term(TMT6plex); C25(Carbamidomethyl); K47(TMT6plex)	5566.8	1				
																	IGEHTPSALAIMENANVLARYASICQQNGIVPIVEPEILPDGDHDLK	1	2	2	342187211;34577112;4557305	N-Term(TMT6plex); M12(Oxidation); C25(Carbamidomethyl); K47(TMT6plex)	5582.9	1				
7661906	NP_055551.1	**KIAA0101**	9768	**PCNA-associated factor isoform 1**	72.97	2	8	8	32	**0.9**	**0.9**	**0.9**	**0.9**	111	12.0	9.83												
																	ADSVPGTYR	4	2	1	7661906	N-Term(TMT6plex)	1194.6	0	**0.8**	**0.8**	**0.7**	**0.8**
																	ACPLQPDHTNDEKE	5	1	1	7661906	N-Term(TMT6plex); C2(Carbamidomethyl); K13(TMT6plex)	2112.0	1	**1.0**	**1.0**	**1.0**	**0.9**
																	ENQIPEEAGSSGLGK	4	1	1	7661906	N-Term(TMT6plex); K15(TMT6plex)	1974.1	0	**0.7**	**0.7**	**0.7**	**0.8**
																	KVLGSSTSATNSTSVSSR	2	2	1	7661906	N-Term(TMT6plex); K1(TMT6plex)	2227.2	1	**0.8**	**1.0**	**0.9**	**0.9**
																	DSEKENQIPEEAGSSGLGK	8	1	1	7661906	N-Term(TMT6plex); K4(TMT6plex); K19(TMT6plex)	2662.4	1	**0.9**	**1.0**	**1.0**	**0.9**
																	VLGSSTSATNSTSVSSR	5	2	1	7661906	N-Term(TMT6plex)	1870.0	0	**0.9**	**0.7**	**0.8**	**0.7**
																	YAGGNPVCVRPTPK	1	1	1	7661906	N-Term(TMT6plex); C8(Carbamidomethyl); K14(TMT6plex)	1974.1	0	**1.0**	**1.1**	**1.0**	**1.0**
																	GIGEFFR	3	1	1	7661906	N-Term(TMT6plex)	1054.6	0	**0.8**	**0.9**	**0.7**	**0.9**
4504483	NP_000185.1	**HPRT1**	3251	**hypoxanthine-guanine phosphoribosyltransferase**	72.94	3	12	12	133	**1.2**	**1.0**	**1.1**	**0.9**	218	24.6	6.68												
																	DLNHVCVISETGK	11	1	1	4504483	N-Term(TMT6plex); C6(Carbamidomethyl); K13(TMT6plex)	1930.0	0	**1.4**	**1.3**	**1.2**	**1.2**
																	SVGYKPDFVGFEIPDK	17	1	1	4504483	N-Term(TMT6plex); K5(TMT6plex); K16(TMT6plex)	2485.4	0	**1.1**	**1.1**	**1.0**	**0.9**
																	FFADLLDYIK	30	1	1	4504483	N-Term(TMT6plex); K10(TMT6plex)	1703.0	0	**1.2**	**1.0**	**1.2**	**0.9**
																	NVLIVEDIIDTGK	20	1	1	4504483	N-Term(TMT6plex); K13(TMT6plex)	1887.1	0	**1.2**	**1.0**	**1.0**	**0.8**
																	SYCNDQSTGDIK	9	1	1	4504483	N-Term(TMT6plex); C3(Carbamidomethyl); K12(TMT6plex)	1845.9	0	**1.2**	**1.0**	**1.0**	**0.9**
																	VIGGDDLSTLTGK	17	1	1	4504483	N-Term(TMT6plex); K13(TMT6plex)	1734.0	0	**1.1**	**1.2**	**1.1**	**1.1**
																	VFIPHGLIMDR	2	1	1	4504483	N-Term(TMT6plex)	1526.9	0	**1.5**	**1.7**	**1.6**	**1.4**
																	EMGGHHIVALCVLK	4	1	1	4504483	N-Term(TMT6plex); M2(Oxidation); C11(Carbamidomethyl); K14(TMT6plex)	2038.1	0	**0.9**	**0.9**	**0.9**	**0.9**
																	TMQTLLSLVR	2	1	1	4504483	N-Term(TMT6plex)	1390.8	0	**1.5**	**1.0**	**1.3**	**0.8**
																	TMQTLLSLVR	2	1	1	4504483	N-Term(TMT6plex); M2(Oxidation)	1406.8	0				
																	VFIPHGLIMDR	4	1	1	4504483	N-Term(TMT6plex); M9(Oxidation)	1542.9	0	**1.3**	**0.5**	**0.8**	**1.0**
																	SPGVVISDDEPGYDLDLFCIPNHYAEDLER	2	1	1	4504483	N-Term(TMT6plex); C19(Carbamidomethyl)	3664.7	0	**1.4**	**1.4**	**1.3**	**1.5**
																	VASLLVK	7	3	1	4504483	N-Term(TMT6plex); K7(TMT6plex)	1187.8	0	**1.2**	**1.0**	**1.1**	**1.0**
																	SIPMTVDFIR	4	1	1	4504483	N-Term(TMT6plex); M4(Oxidation)	1423.8	0	**1.1**	**1.0**	**0.9**	**0.9**
																	SIPMTVDFIR	2	1	1	4504483	N-Term(TMT6plex)	1407.8	0	**1.4**	**1.5**	**1.6**	**1.0**
66879662;66879664	NP_001019398.1	**ARF1**	375	**ADP-ribosylation factor 1**	72.93	7	1	10	279	**1.3**	**1.3**	**1.3**	**1.2**	181	20.7	6.80												
																	ILMVGLDAAGK	35	4	4	4502209;66879662;66879664;4502203;4502205	N-Term(TMT6plex); M3(Oxidation); K11(TMT6plex)	1561.9	0				
																	NISFTVWDVGGQDK	24	2	2	66879662;66879664;4502203	N-Term(TMT6plex); K14(TMT6plex)	2024.1	0				
																	QDLPNAMNAAEITDK	37	2	2	66879662;66879664;4502203	N-Term(TMT6plex); M7(Oxidation); K15(TMT6plex)	2105.1	0				
																	LGEIVTTIPTIGFNVETVEYK	34	4	4	4502209;66879662;66879664;4502203;4502205	N-Term(TMT6plex); K21(TMT6plex)	2781.6	0				
																	MLAEDELR	15	2	2	66879662;66879664;4502203	N-Term(TMT6plex); M1(Oxidation)	1221.6	0				
																	ILMVGLDAAGK	12	4	4	4502209;66879662;66879664;4502203;4502205	N-Term(TMT6plex); K11(TMT6plex)	1545.9	0				
																	DAVLLVFANK	68	3	3	4502209;66879662;66879664;4502203	N-Term(TMT6plex); K10(TMT6plex)	1548.0	0				
																	QDLPNAMNAAEITDK	10	2	2	66879662;66879664;4502203	N-Term(TMT6plex); K15(TMT6plex)	2089.1	0				
																	HYFQNTQGLIFVVDSNDRER	10	4	4	4502209;66879662;66879664;4502203;4502205	N-Term(TMT6plex)	2667.3	1				
																	HYFQNTQGLIFVVDSNDR	17	4	4	4502209;66879662;66879664;4502203;4502205	N-Term(TMT6plex)	2382.2	0				
																	NWYIQATCATSGDGLYEGLDWLSNQLR	8	1	1	66879662;66879664	N-Term(TMT6plex); C8(Carbamidomethyl)	3360.6	0	**1.3**	**1.3**	**1.3**	**1.2**
																	TTILYK	9	11	5	4502209;66879662;66879664;4502203;4502211;4502205	N-Term(TMT6plex); K6(TMT6plex)	1196.8	0				
4502203	NP_001650.1	**ARF3**	377	**ADP-ribosylation factor 3**	72.93	7	1	10	277	**1.3**	**1.1**	**1.4**	**1.1**	181	20.6	7.43												
																	ILMVGLDAAGK	35	4	4	4502209;66879662;66879664;4502203;4502205	N-Term(TMT6plex); M3(Oxidation); K11(TMT6plex)	1561.9	0				
																	NISFTVWDVGGQDK	24	2	2	66879662;66879664;4502203	N-Term(TMT6plex); K14(TMT6plex)	2024.1	0				
																	QDLPNAMNAAEITDK	37	2	2	66879662;66879664;4502203	N-Term(TMT6plex); M7(Oxidation); K15(TMT6plex)	2105.1	0				
																	LGEIVTTIPTIGFNVETVEYK	34	4	4	4502209;66879662;66879664;4502203;4502205	N-Term(TMT6plex); K21(TMT6plex)	2781.6	0				
																	MLAEDELR	15	2	2	66879662;66879664;4502203	N-Term(TMT6plex); M1(Oxidation)	1221.6	0				
																	ILMVGLDAAGK	12	4	4	4502209;66879662;66879664;4502203;4502205	N-Term(TMT6plex); K11(TMT6plex)	1545.9	0				
																	NWYIQATCATSGDGLYEGLDWLANQLK	6	1	1	4502203	N-Term(TMT6plex); C8(Carbamidomethyl); K27(TMT6plex)	3545.8	0	**1.3**	**1.1**	**1.4**	**1.1**
																	DAVLLVFANK	68	3	3	4502209;66879662;66879664;4502203	N-Term(TMT6plex); K10(TMT6plex)	1548.0	0				
																	QDLPNAMNAAEITDK	10	2	2	66879662;66879664;4502203	N-Term(TMT6plex); K15(TMT6plex)	2089.1	0				
																	HYFQNTQGLIFVVDSNDRER	10	4	4	4502209;66879662;66879664;4502203;4502205	N-Term(TMT6plex)	2667.3	1				
																	HYFQNTQGLIFVVDSNDR	17	4	4	4502209;66879662;66879664;4502203;4502205	N-Term(TMT6plex)	2382.2	0				
																	TTILYK	9	11	5	4502209;66879662;66879664;4502203;4502211;4502205	N-Term(TMT6plex); K6(TMT6plex)	1196.8	0				
4505257	NP_002435.1	**MSN**	4478	**moesin**	72.79	1	35	51	728	**1.3**	**1.3**	**1.3**	**1.1**	577	67.8	6.40												
																	FYPEDVSEELIQDITQR	76	1	1	4505257	N-Term(TMT6plex)	2311.2	0	**1.4**	**1.4**	**1.4**	**1.4**
																	VTTMDAELEFAIQPNTTGK	41	5	3	21614499;4505257;4506467	N-Term(TMT6plex); M4(Oxidation); K19(TMT6plex)	2540.3	0				
																	IAQDLEMYGVNYFSIK	38	1	1	4505257	N-Term(TMT6plex); M7(Oxidation); K16(TMT6plex)	2365.2	0	**1.3**	**1.4**	**1.4**	**1.1**
																	QLFDQVVK	26	4	3	21614499;4505257;4506467	N-Term(TMT6plex); K8(TMT6plex)	1434.9	0				
																	KTQEQLALEMAELTAR	12	1	1	4505257	N-Term(TMT6plex); K1(TMT6plex); M10(Oxidation)	2306.3	1	**1.4**	**1.2**	**1.2**	**1.2**
																	AQQELEEQTR	15	1	1	4505257	N-Term(TMT6plex)	1460.8	0	**1.3**	**1.3**	**1.2**	**1.1**
																	AQMVQEDLEK	37	1	1	4505257	N-Term(TMT6plex); M3(Oxidation); K10(TMT6plex)	1664.9	0	**1.3**	**1.4**	**1.4**	**1.1**
																	EALLQASR	13	1	1	4505257	N-Term(TMT6plex)	1116.7	0	**1.3**	**1.4**	**1.3**	**1.2**
																	ALELEQER	14	1	1	4505257	N-Term(TMT6plex)	1216.7	0	**1.3**	**1.3**	**1.3**	**1.1**
																	IGFPWSEIR	31	5	3	21614499;4505257;4506467	N-Term(TMT6plex)	1333.7	0				
																	GFSTWLK	13	1	1	4505257	N-Term(TMT6plex); K7(TMT6plex)	1296.8	0	**1.2**	**1.3**	**1.3**	**1.1**
																	KTANDMIHAENMR	4	1	1	4505257	K1(TMT6plex); N-Term(TMT6plex); M6(Oxidation); M12(Oxidation)	2021.0	1	**1.0**	**1.1**	**1.1**	**1.0**
																	IDEFESM	4	1	1	4505257	N-Term(TMT6plex); M7(Oxidation)	1115.5	0	**1.3**	**1.3**	**1.4**	**1.3**
																	APDFVFYAPR	36	5	3	21614499;4505257;4506467	N-Term(TMT6plex)	1411.8	0				
																	KPDTIEVQQMK	2	5	3	21614499;4505257;4506467	N-Term(TMT6plex); K1(TMT6plex); K11(TMT6plex)	2004.2	0				
																	KPDTIEVQQMK	8	5	3	21614499;4505257;4506467	N-Term(TMT6plex); K1(TMT6plex); M10(Oxidation); K11(TMT6plex)	2020.2	0				
																	KESPLLFK	15	1	1	4505257	N-Term(TMT6plex); K1(TMT6plex); K8(TMT6plex)	1649.1	1	**1.1**	**1.3**	**1.3**	**1.0**
																	RKPDTIEVQQMK	11	5	3	21614499;4505257;4506467	N-Term(TMT6plex); K2(TMT6plex); M11(Oxidation); K12(TMT6plex)	2176.3	1				
																	RKPDTIEVQQMK	2	5	3	21614499;4505257;4506467	N-Term(TMT6plex); K2(TMT6plex); K12(TMT6plex)	2160.3	1				
																	ESEAVEWQQK	22	1	1	4505257	N-Term(TMT6plex); K10(TMT6plex)	1691.9	0	**1.3**	**1.4**	**1.4**	**1.2**
																	KTQEQLALEMAELTAR	8	1	1	4505257	N-Term(TMT6plex); K1(TMT6plex)	2290.3	1	**1.5**	**1.7**	**1.6**	**1.2**
																	AQSEAEK	8	1	1	4505257	N-Term(TMT6plex); K7(TMT6plex)	1220.7	0	**1.2**	**1.3**	**1.2**	**1.0**
																	TANDMIHAENMR	11	1	1	4505257	N-Term(TMT6plex); M5(Oxidation); M11(Oxidation)	1663.8	0	**1.5**	**1.3**	**1.5**	**1.0**
																	IAQDLEMYGVNYFSIK	6	1	1	4505257	N-Term(TMT6plex); K16(TMT6plex)	2349.3	0	**1.5**	**1.9**	**2.0**	**1.1**
																	KESEAVEWQQK	2	1	1	4505257	N-Term(TMT6plex); K1(TMT6plex); K11(TMT6plex)	2049.2	1	**1.4**	**1.6**	**1.5**	**1.3**
																	ISQLEMAR	3	1	1	4505257	N-Term(TMT6plex)	1176.7	0	**1.6**	**1.6**	**1.8**	**1.3**
																	ILALCMGNHELYMR	5	4	2	4505257;4506467	N-Term(TMT6plex); C5(Carbamidomethyl); M6(Oxidation); M13(Oxidation)	1982.0	0				
																	KGSELWLGVDALGLNIYEQNDR	1	1	1	4505257	N-Term(TMT6plex); K1(TMT6plex)	2948.6	1	**1.4**	**1.8**	**1.6**	**1.4**
																	EGILNDDIYCPPETAVLLASYAVQSK	6	1	1	4505257	N-Term(TMT6plex); C10(Carbamidomethyl); K26(TMT6plex)	3324.8	0	**1.5**	**1.5**	**1.6**	**1.2**
																	KAPDFVFYAPR	9	5	3	21614499;4505257;4506467	N-Term(TMT6plex); K1(TMT6plex)	1769.0	1				
																	SGYLAGDK	15	1	1	4505257	N-Term(TMT6plex); K8(TMT6plex)	1268.7	0	**1.2**	**1.2**	**1.3**	**1.1**
																	FVIKPIDK	10	5	3	21614499;4505257;4506467	N-Term(TMT6plex); K4(TMT6plex); K8(TMT6plex)	1647.1	0				
																	EVWFFGLQYQDTK	7	1	1	4505257	N-Term(TMT6plex); K13(TMT6plex)	2119.1	0	**1.1**	**1.1**	**1.2**	**1.0**
																	LFFLQVK	45	5	3	21614499;4505257;4506467	N-Term(TMT6plex); K7(TMT6plex)	1352.9	0				
																	ILALCMGNHELYMR	2	4	2	4505257;4506467	N-Term(TMT6plex); C5(Carbamidomethyl); M13(Oxidation)	1966.0	0				
																	ALTSELANAR	8	1	1	4505257	N-Term(TMT6plex)	1274.7	0	**1.2**	**1.3**	**1.3**	**1.1**
																	KAQQELEEQTR	19	1	1	4505257	N-Term(TMT6plex); K1(TMT6plex)	1818.0	1	**1.2**	**1.3**	**1.3**	**1.2**
																	ILALCMGNHELYMR	10	4	2	4505257;4506467	N-Term(TMT6plex); C5(Carbamidomethyl)	1950.0	0				
																	EDAVLEYLK	32	1	1	4505257	N-Term(TMT6plex); K9(TMT6plex)	1537.9	0	**1.2**	**1.3**	**1.3**	**1.1**
																	GSELWLGVDALGLNIYEQNDR	12	1	1	4505257	N-Term(TMT6plex)	2591.3	0	**1.3**	**1.3**	**1.4**	**1.2**
																	QEAEEAK	8	1	1	4505257	N-Term(TMT6plex); K7(TMT6plex)	1262.7	0	**1.3**	**1.2**	**1.3**	**0.9**
																	AQMVQEDLEK	4	1	1	4505257	N-Term(TMT6plex); K10(TMT6plex)	1648.9	0	**1.6**	**1.9**	**2.0**	**1.3**
																	KVTAQDVR	2	1	1	4505257	N-Term(TMT6plex); K1(TMT6plex)	1374.8	1	**1.2**	**1.4**	**1.3**	**1.2**
																	TAMSTPHVAEPAENEQDEQDENGAEASADLR	8	1	1	4505257	N-Term(TMT6plex); M3(Oxidation)	3557.6	0	**1.4**	**1.0**	**1.2**	**0.8**
																	TQEQLALEMAELTAR	16	1	1	4505257	N-Term(TMT6plex); M9(Oxidation)	1949.0	0	**1.2**	**1.2**	**1.2**	**1.2**
																	VTTMDAELEFAIQPNTTGK	3	5	3	21614499;4505257;4506467	N-Term(TMT6plex); K19(TMT6plex)	2524.3	0				
																	ISQLEMAR	9	1	1	4505257	N-Term(TMT6plex); M6(Oxidation)	1192.7	0	**1.4**	**1.2**	**1.2**	**1.1**
																	FVIKPIDKK	3	5	3	21614499;4505257;4506467	N-Term(TMT6plex); K4(TMT6plex); K8(TMT6plex); K9(TMT6plex)	2004.3	1				
																	VTAQDVR	2	1	1	4505257	N-Term(TMT6plex)	1017.6	0	**1.2**	**1.1**	**1.2**	**0.9**

Accession	RefSeq Accession	Gene Symbol	Entrez Gene ID	Description	Σ Coverage	Σ# Proteins	Σ# Unique Peptides	Σ# Peptides	Σ# PSMs	Het-1A-Smoke - 2M/Parental	Het-1A-Smoke - 4M/Parental	Het-1A-Smoke - 6M/Parental	Het-1A-Smoke - 8M/Parental	# AAs	MW [kDa]	calc. pI	Sequence	# PSMs	# Proteins	# Protein Groups	Protein Group Accessions	Modifications	MH+ [Da]	# Missed Cleavages	Het-1A-Smoke - 2M/Parental	Het-1A-Smoke - 4M/Parental	Het-1A-Smoke - 6M/Parental	Het-1A-Smoke - 8M/Parental
																	QIEEQTK	4	1	1	4505257	N-Term(TMT6plex); K7(TMT6plex)	1333.8	0	1.2	1.2	1.2	0.9
																	KFVIKPIDK	3	5	3	21614499;4505257;4506467	N-Term(TMT6plex); K1(TMT6plex); K5(TMT6plex); K9(TMT6plex)	2004.3	1				
																	VLEQHK	4	4	2	4505257;4506467	N-Term(TMT6plex); K6(TMT6plex)	1211.8	0				
																	ESPLLFK	5	1	1	4505257	N-Term(TMT6plex); K7(TMT6plex)	1291.8	0	1.1	1.3	1.3	1.0
																	AMLENEK	2	1	1	4505257	N-Term(TMT6plex); M2(Oxidation); K7(TMT6plex)	1308.7	0	1.0	1.1	1.0	0.9
																	NISFNDK	7	5	3	21614499;4505257;4506467	N-Term(TMT6plex); K7(TMT6plex)	1295.7	0				
																	TTEAEK	2	1	1	4505257	N-Term(TMT6plex); K6(TMT6plex)	1136.7	0	1.1	0.9	1.1	0.7
																	TTEAEKNER	1	1	1	4505257	N-Term(TMT6plex); K6(TMT6plex)	1535.8	1	1.4	1.3	1.3	1.0
																	TANDMIHAENMR	1	1	1	4505257	N-Term(TMT6plex); M11(Oxidation)	1647.8	0	1.3	1.1	1.1	1.3
																	ADAMAK	2	1	1	4505257	N-Term(TMT6plex); K6(TMT6plex)	1064.6	0	1.6	1.9	2.1	1.1
																	EKEELMER	1	4	2	4505257;4506467	N-Term(TMT6plex); K2(TMT6plex); M6(Oxidation)	1537.8	1				
																	EKEELMER	1	4	2	4505257;4506467	N-Term(TMT6plex); K2(TMT6plex)	1521.8	1				
																	NISFNDKK	1	5	3	21614499;4505257;4506467	N-Term(TMT6plex); K7(TMT6plex); K8(TMT6plex)	1653.0	1				
4502171	NP_000476.1	APRT	353	adenine phosphoribosyltransferase isoform a	72.78	2	10	10	145	1.5	1.4	1.5	1.7	180	19.6	6.02												
																	SFPDFPTPGVVFR	23	2	1	4502171	N-Term(TMT6plex)	1694.9	0	1.6	1.5	1.5	1.8
																	VVVVDDLLATGGTMNAACELLGR	6	1	1	4502171	N-Term(TMT6plex); M14(Oxidation); C18(Carbamidomethyl)	2619.4	0	1.2	1.2	1.2	1.3
																	LAPVPFFSLLQYE	12	1	1	4502171	N-Term(TMT6plex)	1753.0	0	1.9	1.7	1.6	2.0
																	AAIGLLAR	9	2	1	4502171	N-Term(TMT6plex)	1013.7	0	1.5	1.3	1.4	1.8
																	VVVVDDLLATGGTMNAACELLGR	2	1	1	4502171	N-Term(TMT6plex); C18(Carbamidomethyl)	2603.4	0	1.3	1.5	1.7	1.3
																	LQAEVLECVSLVELTSLK	66	1	1	4502171	N-Term(TMT6plex); C8(Carbamidomethyl); K18(TMT6plex)	2489.4	0	1.5	1.3	1.5	1.7
																	AELEIQK	14	2	1	4502171	N-Term(TMT6plex); K7(TMT6plex)	1288.8	0	1.3	1.3	1.4	1.6
																	DISPVLKDPASFR	2	2	1	4502171	N-Term(TMT6plex); K7(TMT6plex)	1903.1	1	1.4	1.6	1.6	2.3
																	LPGPTLWASYSLEYGK	4	2	1	4502171	N-Term(TMT6plex); K16(TMT6plex)	2240.2	0	1.1	0.9	1.1	1.3
																	GFLFGPSLAQELGLGCVLIR	3	2	1	4502171	N-Term(TMT6plex); C16(Carbamidomethyl)	2376.3	0	1.6	1.5	2.4	1.3
																	DISPVLK	4	2	1	4502171	N-Term(TMT6plex); K7(TMT6plex)	1229.8	0	1.6	1.4	1.5	1.7
24431933	NP_722550.1	RTN4	57142	reticulon-4 isoform B	72.65	2	1	13	212	1.4	1.4	1.4	1.4	373	40.3	4.78												
																	EPEDEEEEEEEEEEDEDEDLEELEVLER	12	3	3	47519490;24431935;24431933	N-Term(TMT6plex)	3722.5	0				
																	KPAAGLSAAPVPTAPAAGAPLMDFGNDFVPPAPR	28	3	3	47519490;24431935;24431933	N-Term(TMT6plex); K1(TMT6plex); M22(Oxidation)	3746.0	0				
																	GPLPAAPPVAPER	34	3	3	47519490;24431935;24431933	N-Term(TMT6plex)	1500.9	0				
																	AYLESEVAISEELVQK	78	5	3	47519490;24431935;24431933	N-Term(TMT6plex); K16(TMT6plex)	2266.3	0				
																	LFLVDDLVDSLKFAVLMWVFTYVGALFNGLTLLILALISLFSVPVIYER	1	5	3	47519490;24431935;24431933	N-Term(TMT6plex); K12(TMT6plex)	6005.4	1				
																	GSSGSVVVDLLYWR	9	1	1	24431933	N-Term(TMT6plex)	1767.0	0	1.4	1.4	1.4	1.4
																	GVIQAIQK	2	5	3	47519490;24431935;24431933	N-Term(TMT6plex); K8(TMT6plex)	1314.9	0				
																	QPSWDPSPVSSTVPAPSPLSAAAVSPSK	12	3	3	47519490;24431935;24431933	N-Term(TMT6plex); K28(TMT6plex)	3205.7	0				
																	HQAQIDHYLGLANK	12	5	3	47519490;24431935;24431933	N-Term(TMT6plex); K14(TMT6plex)	2066.2	0				
																	LFLVDDLVDSLK	6	5	3	47519490;24431935;24431933	N-Term(TMT6plex); K12(TMT6plex)	1835.1	0				
																	LPEDDEPPARPPPPPPASVSPQAEPVWTPPAPAPAAPPSTPAAPK	6	3	3	47519490;24431935;24431933	N-Term(TMT6plex); K45(TMT6plex)	4950.6	0				
																	YSNSALGHVNCTIK	3	5	3	47519490;24431935;24431933	N-Term(TMT6plex); C11(Carbamidomethyl); K14(TMT6plex)	2022.1	0				
																	KPAAGLSAAPVPTAPAAGAPLMDFGNDFVPPAPR	7	3	3	47519490;24431935;24431933	N-Term(TMT6plex); K1(TMT6plex)	3730.0	0				
																	SDEGHPFR	2	5	3	47519490;24431935;24431933	N-Term(TMT6plex)	1173.6	0				
4504067	NP_002070.1	GOT1	2805	aspartate aminotransferase, cytoplasmic	72.64	1	19	19	167	1.8	1.8	1.8	1.8	413	46.2	7.01												
																	IVASTLSNPELFEEWTGNVK	22	1	1	4504067	N-Term(TMT6plex); K20(TMT6plex)	2692.5	0	2.0	1.9	1.9	2.0
																	EPESILQVLSQMEK	14	1	1	4504067	N-Term(TMT6plex); K14(TMT6plex)	2089.2	0	2.1	2.4	2.3	2.1
																	EPESILQVLSQMEK	22	1	1	4504067	N-Term(TMT6plex); M12(Oxidation); K14(TMT6plex)	2105.1	0	1.5	1.6	1.6	1.8
																	HIYLLPSGR	6	1	1	4504067	N-Term(TMT6plex)	1284.8	0	1.3	1.3	1.3	1.4
																	APPSVFAEVPQAQPVLVFK	12	1	1	4504067	N-Term(TMT6plex); K19(TMT6plex)	2482.4	0	1.4	1.7	1.6	1.7
																	IGADFLAR	6	1	1	4504067	N-Term(TMT6plex)	1091.6	0	1.8	1.6	1.8	1.8
																	ITWSNPPAQGAR	9	1	1	4504067	N-Term(TMT6plex)	1526.8	0	1.7	1.7	1.6	1.6
																	VGGVQSLGGTGALR	4	1	1	4504067	N-Term(TMT6plex)	1500.9	0	2.1	1.7	1.9	1.9
																	TDDCHPWVLPVVK	5	1	1	4504067	N-Term(TMT6plex); C4(Carbamidomethyl); K13(TMT6plex)	2024.1	0	1.5	1.5	1.4	1.4
																	VGNLTVVGK	1	1	1	4504067	N-Term(TMT6plex); K9(TMT6plex)	1344.9	0	1.6	1.6	1.7	1.7
																	NTPVYVSSPTWENHNAVFSAAGFK	8	1	1	4504067	N-Term(TMT6plex); K24(TMT6plex)	3081.6	0	1.9	1.5	1.8	1.7
																	NLDYVATSIHEAVTK	12	1	1	4504067	N-Term(TMT6plex); K15(TMT6plex)	2119.2	0	1.6	1.6	1.6	1.6
																	FLFPFFDSAYQGFASGNLER	12	1	1	4504067	N-Term(TMT6plex)	2542.3	0	1.6	1.5	1.9	1.3
																	LALGDDSPALK	13	1	1	4504067	N-Term(TMT6plex); K11(TMT6plex)	1557.9	0	1.7	1.8	1.6	1.8
																	KVNLGVGAYR	2	1	1	4504067	N-Term(TMT6plex); K1(TMT6plex)	1534.9	1	1.9	2.2	2.0	2.0
																	TPGTWNHITDQIGMFSFTGLNPK	2	1	1	4504067	N-Term(TMT6plex); M14(Oxidation); K23(TMT6plex)	3036.5	0	1.5	1.7	1.5	1.8
																	IANDNSLNHEYLPILGLAEFR	9	1	1	4504067	N-Term(TMT6plex)	2628.4	0	2.0	1.7	1.5	1.8
																	QVEYLVNEK	2	1	1	4504067	N-Term(TMT6plex); K9(TMT6plex)	1579.9	0	2.3	2.4	2.2	2.3
																	GLDLQGFLNDLENAPEFSIVVLHACAHNPTGIDPTPEQWK	4	1	1	4504067	N-Term(TMT6plex); C25(Carbamidomethyl); K40(TMT6plex)	4901.5	0	1.9	2.5	2.0	2.4
																	INVSGLTTK	2	1	1	4504067	N-Term(TMT6plex); K9(TMT6plex)	1390.9	0	2.1	2.0	1.9	2.0
5174447	NP_006089.1	GNB2L1	10399	guanine nucleotide-binding protein subunit beta-2-like 1	72.56	1	20	20	362	1.4	1.4	1.4	1.4	317	35.1	7.69												
																	AEPPQCTSLAWSADGQTLFAGYTDNLVR	57	1	1	5174447	N-Term(TMT6plex); C6(Carbamidomethyl)	3297.6	0	1.4	1.5	1.6	1.5
																	HLYTLDGGDIINALCFSPNR	24	1	1	5174447	N-Term(TMT6plex); C15(Carbamidomethyl)	2505.3	0	1.3	1.4	1.5	1.4
																	FSPNSSNPIIVSCGWDK	18	1	1	5174447	N-Term(TMT6plex); C13(Carbamidomethyl); K17(TMT6plex)	2366.2	0	1.5	1.6	1.7	1.5
																	QEVISTSSK	23	1	1	5174447	N-Term(TMT6plex); K9(TMT6plex)	1436.8	0	1.5	1.3	1.4	1.4
																	DGQAMLWDLNEGK	36	1	1	5174447	N-Term(TMT6plex); M5(Oxidation); K13(TMT6plex)	1951.0	0	1.3	1.4	1.5	1.5
																	IWDLEGK	20	1	1	5174447	N-Term(TMT6plex); K7(TMT6plex)	1318.8	0	1.4	1.3	1.4	1.3
																	IIVDELK	18	1	1	5174447	N-Term(TMT6plex); K7(TMT6plex)	1287.8	0	1.4	1.4	1.5	1.4
																	TNHIGHTGYLNTVTVSPDGSLCASGGK	7	1	1	5174447	N-Term(TMT6plex); C22(Carbamidomethyl); K27(TMT6plex)	3201.6	0	1.1	1.2	1.3	1.3
																	YTVQDESHSEWVSCVR	21	1	1	5174447	N-Term(TMT6plex); C14(Carbamidomethyl)	2211.0	0	1.4	1.4	1.4	1.4
																	YWLCAATGPSIK	22	1	1	5174447	N-Term(TMT6plex); C4(Carbamidomethyl); K12(TMT6plex)	1825.0	0	1.4	1.3	1.5	1.3
																	LWDLTTGTTTR	13	1	1	5174447	N-Term(TMT6plex)	1493.8	0	1.3	1.3	1.3	1.4
																	LWNTLGVCK	8	1	1	5174447	N-Term(TMT6plex); C8(Carbamidomethyl); K9(TMT6plex)	1548.9	0	1.5	1.1	1.5	1.0
																	VWQVTIGTR	9	1	1	5174447	N-Term(TMT6plex)	1288.8	0	1.4	1.3	1.4	1.3
																	IIVDELKQEVISTSSK	19	1	1	5174447	N-Term(TMT6plex); K7(TMT6plex); K16(TMT6plex)	2476.5	1	1.2	1.6	1.6	1.5
																	VWNLANCK	13	1	1	5174447	N-Term(TMT6plex); C7(Carbamidomethyl); K8(TMT6plex)	1462.8	0	1.4	1.3	1.5	1.3
																	DETNYGIPQR	17	1	1	5174447	N-Term(TMT6plex)	1421.7	0	1.4	1.3	1.4	1.4
																	DVLSVAFSSDNR	24	1	1	5174447	N-Term(TMT6plex)	1538.8	0	1.3	1.4	1.4	1.5
																	DGQAMLWDLNEGK	8	1	1	5174447	N-Term(TMT6plex); K13(TMT6plex)	1935.0	0	1.8	1.9	2.2	1.6
																	DKTIIMWK	1	1	1	5174447	N-Term(TMT6plex); K2(TMT6plex); K8(TMT6plex)	1722.1	1	1.1	2.7	1.5	2.2
																	TIIMWK	2	1	1	5174447	N-Term(TMT6plex); K6(TMT6plex)	1249.8	0	1.4	1.5	1.6	1.4
																	QIVSGSR	2	1	1	5174447	N-Term(TMT6plex)	975.6	0	1.4	1.6	1.4	1.6
4757774	NP_004302.1	ARL3	403	ADP-ribosylation factor-like protein 3	72.53	1	11	11	43	1.3	1.2	1.3	1.3	182	20.4	7.24												
																	QDLLTAAPASEIAEGLNLHTIR	4	1	1	4757774	N-Term(TMT6plex)	2562.4	0	1.8	1.8	1.8	1.7
																	FEETGQELAELLEEEK	7	1	1	4757774	N-Term(TMT6plex); K16(TMT6plex)	2352.2	0	1.7	1.3	1.3	1.4
																	RFEETGQELAELLEEEK	5	1	1	4757774	N-Term(TMT6plex); K17(TMT6plex)	2508.3	1	1.5	1.4	1.4	1.5
																	LNVWDIGGQR	2	1	1	4757774	N-Term(TMT6plex)	1386.8	0	1.4	1.3	1.4	1.4
																	NYFENTDILIYVIDSADR	3	1	1	4757774	N-Term(TMT6plex)	2390.2	0	1.1	1.0	1.1	1.2
																	ILLLGLDNAGK	7	1	1	4757774	N-Term(TMT6plex); K11(TMT6plex)	1585.0	0	1.4	1.5	1.6	1.6
																	LSCVPVLIFANK	9	1	1	4757774	N-Term(TMT6plex); C3(Carbamidomethyl); K12(TMT6plex)	1819.1	0	1.1	1.1	1.3	1.2
																	GLLSILR	2	1	1	4757774	N-Term(TMT6plex)	1000.7	0	1.1	1.1	1.1	1.3
																	SAPDQEVR	2	1	1	4757774	N-Term(TMT6plex)	1130.6	0	1.3	1.2	1.3	1.6
																	SVQSQGFK	1	1	1	4757774	N-Term(TMT6plex); K8(TMT6plex)	1338.8	0	1.3	1.2	1.2	1.3
																	QLASEDISHITPTQGFNIK	1	1	1	4757774	N-Term(TMT6plex); K19(TMT6plex)	2557.4	0	1.2	1.4	1.4	1.2
4502551	NP_001210.1	CALU	813	calumenin isoform a precursor	72.38	3	5	24	290	1.5	1.6	1.7	1.1	315	37.1	4.64												
																	DIVVQETMEDIDK	32	5	2	4502551;194578885	N-Term(TMT6plex); M8(Oxidation); K13(TMT6plex)	2009.1	0				
																	HLVYESDQNK	9	4	2	4502551;194578885	N-Term(TMT6plex); K10(TMT6plex)	1690.9	0				
																	MADKDGDLIATK	8	5	2	4502551;194578885	N-Term(TMT6plex); M1(Oxidation); K4(TMT6plex); K12(TMT6plex)	1981.1	1				
																	EEFTAFLHPEEYDYMK	32	5	2	4502551;194578885	N-Term(TMT6plex); M15(Oxidation); K16(TMT6plex)	2523.2	0				
																	IDGDKDGFVTVDELK	6	3	1	4502551	N-Term(TMT6plex); K5(TMT6plex); K15(TMT6plex)	2338.3	1	1.6	1.5	1.6	1.1
																	DWILPSDYDHAEAEAR	18	4	2	4502551;194578885	N-Term(TMT6plex)	2117.0	0				
																	EEIVDKYDLFVGSQATDFGEALVR	31	4	2	4502551;194578885	N-Term(TMT6plex); K6(TMT6plex)	3159.7	1				

Accession	RefSeq Accession	Gene Symbol	Entrez Gene ID	Description	Σ Coverage	Σ# Proteins	Σ# Unique Peptides	Σ# Peptides	Σ# PSMs	Het-1A-Smoke - 2M/Parental	Het-1A-Smoke - 4M/Parental	Het-1A-Smoke - 6M/Parental	Het-1A-Smoke - 8M/Parental	# AAs	MW [kDa]	calc. pI	Sequence	# PSMs	# Proteins	# Protein Groups	Protein Group Accessions	Modifications	MH+ [Da]	# Missed Cleavages	Het-1A-Smoke - 2M/Parental	Het-1A-Smoke - 4M/Parental	Het-1A-Smoke - 6M/Parental	Het-1A-Smoke - 8M/Parental
																	MDKEETKDWILPSDYDHAEAEAR	1	4	2	4502551;194578885	N-Term(TMT6plex); M1(Oxidation); K3(TMT6plex); K7(TMT6plex)	3452.7	2				
																	TFDQLTPEESKER	2	5	2	4502551;194578885	N-Term(TMT6plex); K11(TMT6plex)	2038.1	1				
																	NADGFIDLEEYIGDMYSHDGNTDEPEWVK	32	4	2	4502551;194578885	N-Term(TMT6plex); M15(Oxidation); K29(TMT6plex)	3833.8	0				
																	VHNDAQSFDYDHDAFLGAEEAK	11	5	2	4502551;194578885	N-Term(TMT6plex); K22(TMT6plex)	2937.4	0				
																	DIVVQETMEDIDK	5	5	2	4502551;194578885	N-Term(TMT6plex); K13(TMT6plex)	1993.1	0				
																	DIVVQETMEDIDKNADGFIDLEEYIGDMYSHDGNTDEPEWVK	2	4	2	4502551;194578885	N-Term(TMT6plex); M8(Oxidation); K13(TMT6plex); M28(Oxidation); K42(TMT6plex)	5594.6	1				
																	HLVYESDQNKDGK	10	4	2	4502551;194578885	N-Term(TMT6plex); K10(TMT6plex); K13(TMT6plex)	2220.2	1				
																	TFDQLTPEESK	29	5	2	4502551;194578885	N-Term(TMT6plex); K11(TMT6plex)	1752.9	0				
																	EEFTAFLHPEEYDYMK	10	5	2	4502551;194578885	N-Term(TMT6plex); K16(TMT6plex)	2507.2	0				
																	DGFVTVDELKDWIK	3	3	1	4502551	N-Term(TMT6plex); K10(TMT6plex); K14(TMT6plex)	2352.3	1	**1.5**	**1.6**	**1.9**	**1.0**
																	YDLFVGSQATDFGEALVR	12	4	2	4502551;194578885	N-Term(TMT6plex)	2217.1	0				
																	MADKDGDLIATK	1	5	2	4502551;194578885	N-Term(TMT6plex); K4(TMT6plex); K12(TMT6plex)	1965.1	1				
																	VHHEPQLSDK	10	5	2	4502551;194578885	N-Term(TMT6plex); K10(TMT6plex)	1647.9	0				
																	WIYEDVER	7	3	1	4502551	N-Term(TMT6plex)	1338.7	0	**1.8**	**1.6**	**1.7**	**1.1**
																	IDGDKDGFVTVDELKDWIK	3	3	1	4502551	N-Term(TMT6plex); K5(TMT6plex); K15(TMT6plex); K19(TMT6plex)	3109.8	2	**1.7**	**1.9**	**2.0**	**1.2**
																	GHDLNEDGLVSWEEYK	7	3	1	4502551	N-Term(TMT6plex); K16(TMT6plex)	2349.2	0	**1.3**	**1.4**	**1.4**	**1.3**
																	LTKEEIVDKYDLFVGSQATDFGEALVR	1	4	2	4502551;194578885	N-Term(TMT6plex); K3(TMT6plex); K9(TMT6plex)	3731.1	2				
																	EQFVEFR	5	4	2	4502551;194578885	N-Term(TMT6plex)	1183.6	0				
																	EEIVDK	2	5	3	4502551;194578885;45439327	N-Term(TMT6plex); K6(TMT6plex)	1190.7	0				
																	DGDLIATK	1	5	2	4502551;194578885	N-Term(TMT6plex); K8(TMT6plex)	1290.8	0				
4502013	NP_001616.1	**AK2**	204	**adenylate kinase 2, mitochondrial isoform a**	72.38	8	13	13	120	**1.5**	**1.5**	**1.4**	**1.5**	239	26.5	7.81												
																	AMVASGSELGK	9	3	1	4502013	N-Term(TMT6plex); M2(Oxidation); K11(TMT6plex)	1523.8	0	**1.5**	**1.5**	**1.3**	**1.6**
																	LVSDEMVVELIEK	29	3	1	4502013	N-Term(TMT6plex); M6(Oxidation); K13(TMT6plex)	1978.1	0	**1.3**	**1.5**	**1.3**	**1.6**
																	LDSVIEFSIPDSLLIR	12	2	1	4502013	N-Term(TMT6plex)	2046.2	0	**1.6**	**1.1**	**1.3**	**1.3**
																	QAEMLDDLMEK	13	7	1	4502013	N-Term(TMT6plex); M4(Oxidation); M9(Oxidation); K11(TMT6plex)	1812.9	0	**1.5**	**1.5**	**1.3**	**1.6**
																	LVSDEMVVELIEK	4	3	1	4502013	N-Term(TMT6plex); K13(TMT6plex)	1962.1	0	**2.0**	**2.3**	**2.0**	**1.7**
																	GIHSAIDASQTPDVVFASILAAFSK	4	3	1	4502013	N-Term(TMT6plex); K25(TMT6plex)	3003.7	0	**1.3**	**1.9**	**1.4**	**2.1**
																	LAENFCVCHLATGDMLR	4	3	1	4502013	N-Term(TMT6plex); C6(Carbamidomethyl); C8(Carbamidomethyl); M15(Oxidation)	2252.1	0	**1.2**	**1.3**	**1.2**	**1.3**
																	EKLDSVIEFSIPDSLLIR	17	2	1	4502013	N-Term(TMT6plex); K2(TMT6plex)	2532.5	1	**1.4**	**1.7**	**1.5**	**1.6**
																	AMVASGSELGK	2	3	1	4502013	N-Term(TMT6plex); K11(TMT6plex)	1507.9	0	**1.8**	**2.1**	**2.0**	**1.4**
																	LQAYHTQTTPLIEYYR	9	3	1	4502013	N-Term(TMT6plex)	2226.2	0	**1.3**	**1.4**	**1.6**	**1.4**
																	APSVPAAEPEYPK	3	6	1	4502013	N-Term(TMT6plex); K13(TMT6plex)	1814.0	0	**1.3**	**1.4**	**1.2**	**1.5**
																	SYHEEFNPPKEPMKDDITGEPLIR	2	3	1	4502013	N-Term(TMT6plex); K10(TMT6plex); M13(Oxidation); K14(TMT6plex)	3545.8	2	**1.1**	**1.4**	**1.2**	**1.5**
																	AVLLGPPGAGK	8	3	1	4502013	N-Term(TMT6plex); K11(TMT6plex)	1437.9	0	**1.3**	**1.5**	**1.4**	**1.3**
																	DLVMFI	1	1	1	4502013	N-Term(TMT6plex); M4(Oxidation)	982.5	0	**1.3**	**1.4**	**1.1**	**1.5**
																	NLETPLCK	3	3	1	4502013	N-Term(TMT6plex); C7(Carbamidomethyl); K8(TMT6plex)	1432.8	0	**1.8**	**1.5**	**1.6**	**1.3**
157168362	NP_000261.2	**PNP**	4860	**purine nucleoside phosphorylase**	72.32	1	15	15	157	**1.5**	**1.4**	**1.6**	**1.7**	289	32.1	6.95												
																	LGADAVGMSTVPEVIVAR	26	1	1	157168362	N-Term(TMT6plex); M8(Oxidation)	2030.1	0	**1.4**	**1.5**	**1.5**	**1.8**
																	LEQFVSILMASIPLPDK	6	1	1	157168362	N-Term(TMT6plex); M9(Oxidation); K17(TMT6plex)	2375.4	0	**1.8**	**1.1**	**1.5**	**1.8**
																	HRPQVAIICGSGLGGLTDK	5	1	1	157168362	N-Term(TMT6plex); C9(Carbamidomethyl); K19(TMT6plex)	2437.4	0	**1.3**	**1.3**	**1.3**	**1.6**
																	DHINLPGFSGQNPLR	12	1	1	157168362	N-Term(TMT6plex)	1894.0	0	**1.4**	**1.4**	**1.5**	**1.5**
																	ELQEGTYVMVAGPSFETVAECR	9	1	1	157168362	N-Term(TMT6plex); M9(Oxidation); C21(Carbamidomethyl)	2718.3	0	**1.4**	**1.2**	**1.4**	**1.4**
																	LTQAQIFDYGEIPNFPR	27	1	1	157168362	N-Term(TMT6plex)	2238.2	0	**1.6**	**1.5**	**1.7**	**1.9**
																	NTAEWLLSHTK	3	1	1	157168362	N-Term(TMT6plex); K11(TMT6plex)	1758.0	0	**1.3**	**1.3**	**1.4**	**1.7**
																	LGADAVGMSTVPEVIVAR	2	1	1	157168362	N-Term(TMT6plex)	2014.1	0	**2.2**	**2.1**	**2.5**	**2.4**
																	VIMDYESLEK	2	1	1	157168362	N-Term(TMT6plex); K10(TMT6plex)	1684.9	0	**1.8**	**2.2**	**2.4**	**2.5**
																	VIMDYESLEK	18	1	1	157168362	N-Term(TMT6plex); M3(Oxidation); K10(TMT6plex)	1700.9	0	**1.4**	**1.6**	**1.6**	**2.2**
																	FHMYEGYPLWK	1	1	1	157168362	N-Term(TMT6plex); M3(Oxidation); K11(TMT6plex)	1945.0	0	**1.3**	**1.5**	**1.7**	**1.6**
																	VFHLLGVDTLVVTNAAGGLNPK	12	1	1	157168362	N-Term(TMT6plex); K22(TMT6plex)	2693.5	0	**1.4**	**1.4**	**1.7**	**1.6**
																	FPAMSDAYDR	5	1	1	157168362	N-Term(TMT6plex); M4(Oxidation)	1417.7	0	**1.7**	**1.5**	**1.6**	**1.8**
																	ANHEEVLAAGK	4	1	1	157168362	N-Term(TMT6plex); K11(TMT6plex)	1596.9	0	**1.5**	**1.6**	**2.1**	**2.1**
																	FEVGDIMLIR	5	1	1	157168362	N-Term(TMT6plex); M7(Oxidation)	1437.8	0	**1.5**	**1.5**	**1.5**	**1.8**
																	VFGFSLITNK	10	1	1	157168362	N-Term(TMT6plex); K10(TMT6plex)	1584.0	0	**1.7**	**1.5**	**1.9**	**2.0**
																	FEVGDIMLIR	4	1	1	157168362	N-Term(TMT6plex)	1421.8	0	**1.7**	**1.7**	**1.9**	**2.0**
																	LEQFVSILMASIPLPDK	5	1	1	157168362	N-Term(TMT6plex); K17(TMT6plex)	2359.4	0	**1.7**	**1.5**	**1.7**	**1.7**
																	ALSTWK	1	1	1	157168362	N-Term(TMT6plex); K6(TMT6plex)	1163.7	0	**1.2**	**1.3**	**1.5**	**1.5**
27597059	NP_056005.1	**DNAJC9**	23234	**dnaJ homolog subfamily C member 9**	72.31	1	19	19	198	**1.2**	**1.3**	**1.2**	**1.1**	260	29.9	5.73												
																	NIIQQAIDAGEVPSYNAFVK	16	1	1	27597059	N-Term(TMT6plex); K20(TMT6plex)	2635.5	0	**1.2**	**1.2**	**1.3**	**1.2**
																	VSLQVHPDR	8	1	1	27597059	N-Term(TMT6plex)	1279.7	0	**1.1**	**1.1**	**1.1**	**1.1**
																	ELGLDEGVDSLK	16	1	1	27597059	N-Term(TMT6plex); K12(TMT6plex)	1733.0	0	**1.1**	**1.3**	**1.2**	**1.2**
																	EMDNFLAQMEAK	16	1	1	27597059	N-Term(TMT6plex); M2(Oxidation); M9(Oxidation); K12(TMT6plex)	1916.9	0	**1.2**	**1.2**	**1.3**	**1.3**
																	VGEGDKEDATR	4	1	1	27597059	N-Term(TMT6plex); K6(TMT6plex)	1634.9	1	**1.1**	**1.1**	**1.1**	**0.9**
																	GDMDQIMESVLCVQYTEEPR	4	1	1	27597059	N-Term(TMT6plex); M3(Oxidation); M7(Oxidation); C12(Carbamidomethyl)	2661.2	0	**0.9**	**1.1**	**1.0**	**1.1**
																	GLLDLCEEVFGTADLYR	42	1	1	27597059	N-Term(TMT6plex); C6(Carbamidomethyl)	2200.1	0	**1.3**	**1.4**	**1.4**	**1.3**
																	EMDNFLAQMEAK	2	1	1	27597059	N-Term(TMT6plex); K12(TMT6plex)	1885.0	0	**1.7**	**2.1**	**2.2**	**1.3**
																	KISLEDIQAFEK	4	1	1	27597059	N-Term(TMT6plex); K1(TMT6plex); K12(TMT6plex)	2108.3	1	**1.0**	**1.2**	**1.1**	**1.0**
																	EMDNFLAQMEAK	3	1	1	27597059	N-Term(TMT6plex); M9(Oxidation); K12(TMT6plex)	1901.0	0	**1.7**	**1.8**	**2.0**	**1.7**
																	AVYDEQGTVDEDSPVLTQDR	12	1	1	27597059	N-Term(TMT6plex)	2466.2	0	**1.5**	**1.1**	**1.3**	**1.2**
																	KELGLDEGVDSLK	2	1	1	27597059	N-Term(TMT6plex); K1(TMT6plex); K13(TMT6plex)	2090.2	1	**1.1**	**1.3**	**1.2**	**1.1**
																	VYSVLSDR	4	1	1	27597059	N-Term(TMT6plex)	1167.7	0	**1.6**	**1.1**	**1.3**	**1.0**
																	GSEEELADIK	17	1	1	27597059	N-Term(TMT6plex); K10(TMT6plex)	1548.9	0	**1.0**	**1.3**	**1.2**	**1.1**
																	ISLEDIQAFEK	31	1	1	27597059	N-Term(TMT6plex); K11(TMT6plex)	1751.0	0	**1.2**	**1.3**	**1.3**	**1.1**
																	AVYDEQGTVDEDSPVLTQDRDWEAYWR	2	1	1	27597059	N-Term(TMT6plex)	3472.6	1	**1.4**	**1.6**	**1.6**	**1.4**
																	GDMDQIMESVLCVQYTEEPR	2	1	1	27597059	N-Term(TMT6plex); C12(Carbamidomethyl)	2629.2	0	**1.4**	**1.5**	**1.4**	**1.6**
																	QKEMDNFLAQMEAK	1	1	1	27597059	N-Term(TMT6plex); K2(TMT6plex); M4(Oxidation); M11(Oxidation); K14(TMT6plex)	2402.3	1	**1.5**	**1.5**	**1.2**	**1.5**
																	AQEEAK	2	1	1	27597059	N-Term(TMT6plex); K6(TMT6plex)	1133.7	0	**1.2**	**1.2**	**1.1**	**1.0**
																	QAYLDFK	8	1	1	27597059	N-Term(TMT6plex); K7(TMT6plex)	1342.8	0	**1.1**	**1.2**	**1.2**	**1.0**
																	EASDGEVR	1	1	1	27597059	N-Term(TMT6plex)	1091.6	0				
																	FQILGK	1	1	1	27597059	N-Term(TMT6plex); K6(TMT6plex)	1163.8	0	**1.0**	**1.0**	**1.1**	**0.9**
5453990	NP_006254.1	**PSME1**	5720	**proteasome activator complex subunit 1 isoform 1**	72.29	4	19	19	175	**1.5**	**1.4**	**1.5**	**1.3**	249	28.7	6.02												
																	QLVHELDEAEYR	12	4	1	5453990	N-Term(TMT6plex)	1730.9	0	**1.6**	**1.4**	**1.4**	**1.4**
																	APLDIPVPDPVK	23	4	1	5453990	N-Term(TMT6plex); K12(TMT6plex)	1719.0	0	**1.4**	**1.3**	**1.5**	**1.3**
																	LEGFHTQISK	8	3	1	5453990	N-Term(TMT6plex); K10(TMT6plex)	1617.9	0	**1.4**	**1.4**	**1.6**	**1.4**
																	DVIEQLNLVTTWLQLQIPR	4	4	1	5453990	N-Term(TMT6plex)	2508.4	0	**1.7**	**1.6**	**1.7**	**1.8**
																	IEDGNNFGVAVQEK	18	3	1	5453990	N-Term(TMT6plex); K14(TMT6plex)	1978.1	0	**1.4**	**1.3**	**1.5**	**1.2**
																	NAYAVLYDIILK	20	2	1	5453990	N-Term(TMT6plex); K12(TMT6plex)	1854.1	0	**1.5**	**1.2**	**1.4**	**1.0**
																	TENLLGSYFPK	8	4	1	5453990	N-Term(TMT6plex); K11(TMT6plex)	1727.0	0	**1.6**	**1.0**	**1.4**	**1.0**
																	IVVLLQR	14	4	1	5453990	N-Term(TMT6plex)	1069.7	0	**1.7**	**1.4**	**1.6**	**1.3**
																	VFELMTSLHTK	1	3	1	5453990	N-Term(TMT6plex); M5(Oxidation); K11(TMT6plex)	1780.0	0				
																	KISELDAFLK	5	4	1	5453990	N-Term(TMT6plex); K1(TMT6plex); K10(TMT6plex)	1851.2	1	**1.4**	**1.3**	**1.6**	**1.3**
																	ISELDAFLK	4	4	1	5453990	N-Term(TMT6plex); K9(TMT6plex)	1493.9	0	**1.5**	**1.1**	**1.5**	**1.1**
																	ISELDAFLKEPALNEANLSNLK	12	4	1	5453990	N-Term(TMT6plex); K9(TMT6plex); K22(TMT6plex)	3116.8	1	**1.5**	**1.5**	**1.7**	**1.5**
																	EPALNEANLSNLK	17	4	1	5453990	N-Term(TMT6plex); K13(TMT6plex)	1871.1	0	**1.5**	**1.6**	**1.7**	**1.5**
																	VQPEAQAK	11	4	1	5453990	N-Term(TMT6plex); K8(TMT6plex)	1328.8	0	**1.5**	**1.3**	**1.4**	**1.2**
																	APLDIPVPDPVKEK	7	4	1	5453990	N-Term(TMT6plex); K12(TMT6plex); K14(TMT6plex)	2205.3	1	**1.4**	**1.7**	**1.5**	**1.7**
																	GEDEDKGPPCGPVNCNEK	2	4	1	5453990	N-Term(TMT6plex); K6(TMT6plex); C10(Carbamidomethyl); C15(Carbamidomethyl); K18(TMT6plex)	2689.3	1	**1.8**	**1.6**	**2.0**	**1.1**
																	QPHVGDYR	5	4	1	5453990	N-Term(TMT6plex)	1200.6	0	**1.0**	**1.4**	**1.0**	**1.2**
																	LKPEIK	2	4	1	5453990	N-Term(TMT6plex); K2(TMT6plex); K6(TMT6plex)	1415.0	0	**1.4**	**1.4**	**1.6**	**1.3**
																	LMVMEIR	1	4	1	5453990	N-Term(TMT6plex)	1120.6	0	**2.1**	**2.0**	**2.1**	**1.6**
																	LMVMEIR	1	4	1	5453990	N-Term(TMT6plex); M4(Oxidation)	1136.6	0	**1.8**	**1.7**	**1.9**	**1.4**
40354195	NP_954657.1	**KRT18**	3875	**keratin, type I cytoskeletal 18**	72.09	12	28	29	325	**1.3**	**1.3**	**1.3**	**1.7**	430	48.0	5.45												
																	GGMGSGGLATGIAGGLAGMGGIQNEK	18	1	1	40354195	N-Term(TMT6plex); M3(Oxidation); M19(Oxidation); K26(TMT6plex)	2751.4	0	**1.3**	**1.3**	**1.3**	**1.5**
																	LLEDGEDFNLGDALDSSNSMQTIQK	6	1	1	40354195	N-Term(TMT6plex); K25(TMT6plex)	3198.6	0	**1.5**	**1.7**	**1.8**	**1.8**
																	QSVENDIHGLR	13	1	1	40354195	N-Term(TMT6plex)	1496.8	0	**1.2**	**1.2**	**1.3**	**1.4**
																	YWSQQIEESTTVVTTQSAEVGAAETTLTELR	18	1	1	40354195	N-Term(TMT6plex)	3657.8	0	**1.4**	**1.3**	**1.7**	**1.6**

Khan et al., 2019. Multi-omics analysis to characterize cigarette smoke induced molecular alterations in esophageal cells
Supplementary Table 6. List of all proteins and peptides identified in untreated and chronically treated Het1A cells with cigarette smoke condensate for 8 months

Accession	RefSeq Accession	Gene Symbol	Entrez Gene ID	Description	Σ Coverage	Σ# Proteins	Σ# Unique Peptides	Σ# Peptides	Σ# PSMs	Het-1A-Smoke - 2M/Parental	Het-1A-Smoke - 4M/Parental	Het-1A-Smoke - 6M/Parental	Het-1A-Smoke - 8M/Parental	# AAs	MW [kDa]	calc. pI	Sequence	# PSMs	# Proteins	# Protein Groups	Protein Group Accessions	Modifications	MH+ [Da]	# Missed Cleavages	Het-1A-Smoke - 2M/Parental	Het-1A-Smoke - 4M/Parental	Het-1A-Smoke - 6M/Parental	Het-1A-Smoke - 8M/Parental
																	VVSETNDTK	21	1	1	40354195	N-Term(TMT6plex); K9(TMT6plex)	1450.8	0	**1.2**	**1.2**	**1.2**	**1.7**
																	AQYDELAR	15	1	1	40354195	N-Term(TMT6plex)	1194.6	0	**1.6**	**1.4**	**1.3**	**1.6**
																	YALQMEQLNGILLHLESELAQTR	1	1	1	40354195	N-Term(TMT6plex); M5(Oxidation)	2915.6	0	**1.3**	**1.2**	**1.6**	**1.5**
																	LLEDGEDFNLGDALDSSNSMQTIQK	40	1	1	40354195	N-Term(TMT6plex); M20(Oxidation); K25(TMT6plex)	3214.6	0	**1.4**	**1.4**	**1.4**	**1.7**
																	TVQSLEIDLDSMR	14	1	1	40354195	N-Term(TMT6plex); M12(Oxidation)	1751.9	0	**1.4**	**1.4**	**1.4**	**1.5**
																	QAQEYEALLNIK	22	1	1	40354195	N-Term(TMT6plex); K12(TMT6plex)	1878.1	0	**1.3**	**1.3**	**1.4**	**1.7**
																	VIDDTNITR	8	1	1	40354195	N-Term(TMT6plex)	1275.7	0	**1.3**	**1.4**	**1.1**	**1.7**
																	LAADDFR	30	18	5	40354195;4557701;15431310;195972866;24430192	N-Term(TMT6plex)	1036.6	0				
																	LQLETEIEALKEELLFMK	20	1	1	40354195	N-Term(TMT6plex); K11(TMT6plex); M17(Oxidation); K18(TMT6plex)	2880.7	1	**1.3**	**1.7**	**1.4**	**2.0**
																	LQLETEIEALKEELLFMK	9	1	1	40354195	N-Term(TMT6plex); K11(TMT6plex); K18(TMT6plex)	2864.7	1	**1.5**	**2.2**	**1.8**	**2.0**
																	YALQMEQLNGILLHLESELAQTR	2	1	1	40354195	N-Term(TMT6plex)	2899.6	0	**1.4**	**1.2**	**1.5**	**1.5**
																	GLQAQIASSGLTVEVDAPK	2	1	1	40354195	N-Term(TMT6plex); K19(TMT6plex)	2342.3	0	**1.1**	**1.8**	**1.3**	**2.1**
																	DWSHYFK	13	1	1	40354195	N-Term(TMT6plex); K7(TMT6plex)	1440.8	0	**1.2**	**1.5**	**1.2**	**1.8**
																	DWSHYFKIIEDLR	5	1	1	40354195	N-Term(TMT6plex); K7(TMT6plex)	2180.2	1	**1.2**	**0.9**	**1.4**	**2.2**
																	TVQSLEIDLDSMR	3	1	1	40354195	N-Term(TMT6plex)	1735.9	0	**1.5**	**1.5**	**1.6**	**1.5**
																	AQIFANTVDNAR	8	1	1	40354195	N-Term(TMT6plex)	1548.8	0	**1.4**	**1.5**	**1.3**	**1.8**
																	ASLENSLR	10	1	1	40354195	N-Term(TMT6plex)	1118.6	0	**1.3**	**1.3**	**1.3**	**1.6**
																	NHEEEVK	4	1	1	40354195	N-Term(TMT6plex); K7(TMT6plex)	1342.7	0	**1.0**	**1.1**	**1.0**	**1.5**
																	KNHEEEVK	2	1	1	40354195	N-Term(TMT6plex); K1(TMT6plex); K8(TMT6plex)	1700.0	1	**1.0**	**1.3**	**1.1**	**1.7**
																	IVLQIDNAR	9	2	1	40354195	N-Term(TMT6plex)	1270.8	0	**1.4**	**1.3**	**1.4**	**1.7**
																	STFSTNYR	3	1	1	40354195	N-Term(TMT6plex)	1204.6	0	**1.1**	**1.0**	**1.0**	**1.3**
																	GGMGSGGLATGIAGGLAGMGGIQNEK	2	1	1	40354195	N-Term(TMT6plex); K26(TMT6plex)	2719.4	0	**1.5**	**1.3**	**1.4**	**1.5**
																	LEAEIATYR	6	1	1	40354195	N-Term(TMT6plex)	1294.7	0	**1.3**	**1.1**	**1.3**	**1.6**
																	KVIDDTNITR	2	1	1	40354195	N-Term(TMT6plex); K1(TMT6plex)	1633.0	1	**1.2**	**1.2**	**1.3**	**1.5**
																	ETMQSLNDR	6	2	1	40354195	N-Term(TMT6plex); M3(Oxidation)	1338.7	0	**1.3**	**1.3**	**1.4**	**1.6**
																	LASYLDR	2	1	1	40354195	N-Term(TMT6plex)	1066.6	0	**1.7**	**1.0**	**1.2**	**1.5**
																	SLETENR	4	1	1	40354195	N-Term(TMT6plex)	1077.6	0	**1.3**	**1.3**	**1.3**	**1.9**
																	IIEDLR	5	5	1	40354195	N-Term(TMT6plex)	987.6	0	**1.1**	**1.4**	**1.2**	**1.6**
																	VKLEAEIATYR	1	1	1	40354195	N-Term(TMT6plex); K2(TMT6plex)	1751.0	1	**1.2**	**1.2**	**1.4**	**1.6**
																	IMADIR	1	1	1	40354195	N-Term(TMT6plex)	947.6	0	**1.4**	**1.3**	**1.3**	**1.4**
4506669	NP_000994.1	**RPLP1**	6176	**60S acidic ribosomal protein P1 isoform 1**	71.93	2	3	4	102	**1.7**	**1.0**	**1.8**	**1.1**	114	11.5	4.32												
																	AAGVNVEPFWPGLFAK	73	1	1	4506669	N-Term(TMT6plex); K16(TMT6plex)	2161.2	0	**1.7**	**1.1**	**1.8**	**1.2**
																	ALANVNIGSLICNVGAGGPAPAAGAAPAGGPAPSTAAAPAEEK	24	1	1	4506669	N-Term(TMT6plex); C12(Carbamidomethyl); K43(TMT6plex)	4267.3	0	**1.8**	**1.0**	**1.7**	**1.0**
																	KEESEESDDDMGFGLFD	2	3	2	4506669;4506671	N-Term(TMT6plex); K1(TMT6plex); M11(Oxidation)	2424.1	1				
																	INALIK	3	1	1	4506669	N-Term(TMT6plex); K6(TMT6plex)	1129.8	0	**1.8**	**0.7**	**1.9**	**0.8**
6996014	NP_002100.2	**HARS**	3035	**histidine--tRNA ligase, cytoplasmic isoform 1**	71.91	7	24	31	269	**1.4**	**1.4**	**1.6**	**1.5**	509	57.4	5.88												
																	ASAELIEEEVAK	12	6	1	6996014	N-Term(TMT6plex); K12(TMT6plex)	1747.0	0	**1.3**	**1.5**	**1.5**	**1.6**
																	HGAEVIDTPVFELK	5	4	1	6996014	N-Term(TMT6plex); K14(TMT6plex)	2013.1	0	**1.4**	**1.4**	**1.4**	**1.7**
																	EFYQCDFDIAGNFDPMIPDAECLK	10	3	1	6996014	N-Term(TMT6plex); C5(Carbamidomethyl); M16(Oxidation); C22(Carbamidomethyl); K24(TMT6plex)	3369.5	0	**1.5**	**1.4**	**1.6**	**1.5**
																	TGQPLCIC	3	7	1	6996014	N-Term(TMT6plex); C6(Carbamidomethyl); C8(Carbamidomethyl)	1177.6	0	**1.3**	**1.3**	**1.4**	**1.3**
																	GLAPEVADR	11	10	2	6996014;523567531	N-Term(TMT6plex)	1156.7	0				
																	TTETQVLVASAQK	8	7	1	6996014	N-Term(TMT6plex); K13(TMT6plex)	1834.1	0	**1.7**	**1.3**	**1.5**	**1.4**
																	LVSELWDAGIK	16	7	1	6996014	N-Term(TMT6plex); K11(TMT6plex)	1689.0	0	**1.4**	**1.5**	**1.7**	**1.7**
																	VFDVIIR	11	4	1	6996014	N-Term(TMT6plex)	1090.7	0	**1.5**	**1.3**	**1.5**	**1.5**
																	ISFDLSLAR	4	10	2	6996014;523567531	N-Term(TMT6plex)	1250.7	0				
																	TICSSVDK	4	7	1	6996014	N-Term(TMT6plex); C3(Carbamidomethyl); K8(TMT6plex)	1367.8	0	**1.4**	**1.3**	**1.7**	**1.4**
																	IFSIVEQR	4	7	1	6996014	N-Term(TMT6plex)	1220.7	0	**1.5**	**1.1**	**1.5**	**1.3**
																	AALEELVK	5	6	1	6996014	N-Term(TMT6plex); K8(TMT6plex)	1330.8	0	**1.3**	**1.4**	**1.5**	**1.6**
																	KVPCVGLSIGVER	2	7	1	6996014	N-Term(TMT6plex); K1(TMT6plex); C4(Carbamidomethyl)	1872.1	1	**1.5**	**1.6**	**1.6**	**1.5**
																	DQGGELLSLR	6	7	2	6996014;523567531	N-Term(TMT6plex)	1316.7	0				
																	YDGLVGMFDPK	5	10	2	6996014;523567531	N-Term(TMT6plex); M7(Oxidation); K11(TMT6plex)	1715.9	0				
																	QALEGLGDLK	10	10	2	6996014;523567531	N-Term(TMT6plex); K10(TMT6plex)	1501.9	0				
																	LLNQLQYCEEAGIPLVAIIGEQELK	27	7	1	6996014	N-Term(TMT6plex); C8(Carbamidomethyl); K25(TMT6plex)	3299.8	0	**1.4**	**1.1**	**1.5**	**1.3**
																	GLDYYTGVIYEAVLLQTPAQAGEEPLGVGSVAAGGR	38	7	1	6996014	N-Term(TMT6plex)	3851.0	0	**1.7**	**1.4**	**1.4**	**1.6**
																	ILDGMFAICGVSDSK	6	7	1	6996014	N-Term(TMT6plex); M5(Oxidation); C9(Carbamidomethyl); K15(TMT6plex)	2087.1	0	**1.5**	**1.5**	**1.4**	**1.6**
																	AQLGPDESK	14	7	1	6996014	N-Term(TMT6plex); K9(TMT6plex)	1402.8	0	**1.4**	**1.4**	**1.5**	**1.6**
																	YDLTVPFAR	7	7	2	6996014;523567531	N-Term(TMT6plex)	1310.7	0				
																	IMCEILSSLQIGDFLVK	25	7	1	6996014	N-Term(TMT6plex); M2(Oxidation); C3(Carbamidomethyl); K17(TMT6plex)	2440.4	0	**1.4**	**1.3**	**1.4**	**1.5**
																	LLFEYLTLFGIDDK	4	7	1	6996014	N-Term(TMT6plex); K14(TMT6plex)	2145.2	0	**1.7**	**1.8**	**2.3**	**1.7**
																	YDGLVGMFDPK	6	10	2	6996014;523567531	N-Term(TMT6plex); K11(TMT6plex)	1699.9	0				
																	IGDYVQQHGGVSLVEQLLQDPK	15	7	1	6996014	N-Term(TMT6plex); K22(TMT6plex)	2881.6	0	**1.4**	**1.3**	**1.5**	**1.7**
																	EFYQCDFDIAGNFDPMIPDAECLK	2	3	1	6996014	N-Term(TMT6plex); C5(Carbamidomethyl); C22(Carbamidomethyl); K24(TMT6plex)	3353.6	0	**2.6**	**2.5**	**3.2**	**2.2**
																	LEALEEK	2	7	1	6996014	N-Term(TMT6plex); K7(TMT6plex)	1289.8	0	**1.4**	**1.4**	**1.5**	**1.7**
																	VSWEEVK	1	7	1	6996014	N-Term(TMT6plex); K7(TMT6plex)	1334.8	0	**1.3**	**1.4**	**1.5**	**1.5**
																	TICSSVDKLDK	1	7	1	6996014	N-Term(TMT6plex); C3(Carbamidomethyl); K8(TMT6plex); K11(TMT6plex)	1953.1	1	**1.3**	**1.6**	**1.8**	**1.7**
																	LIYDLK	2	5	1	6996014	N-Term(TMT6plex); K6(TMT6plex)	1222.8	0	**1.4**	**1.5**	**1.6**	**1.7**
																	YLAMNK	1	7	2	6996014;523567531	N-Term(TMT6plex); K6(TMT6plex)	1197.7	0				
																	AELLYK	1	6	1	6996014	N-Term(TMT6plex); K6(TMT6plex)	1194.7	0	**1.3**	**1.5**	**1.6**	**1.7**
																	REDLVEEIK	1	7	1	6996014	N-Term(TMT6plex); K9(TMT6plex)	1588.9	1	**1.4**	**1.7**	**1.5**	**1.8**
5031877	NP_005564.1	**LMNB1**	4001	**lamin-B1 isoform 1**	71.84	2	38	43	406	**1.2**	**1.3**	**1.2**	**1.4**	586	66.4	5.16												
																	TTIPEEEEEEEEAAGVVVEEELFHQQGTPR	29	2	1	5031877	N-Term(TMT6plex)	3640.7	0	**1.3**	**1.2**	**1.1**	**1.3**
																	QLADETLLK	7	1	1	5031877	N-Term(TMT6plex); K9(TMT6plex)	1488.9	0	**1.1**	**1.3**	**1.2**	**1.4**
																	AEHDQLLLNYAK	12	1	1	5031877	N-Term(TMT6plex); K12(TMT6plex)	1873.1	0	**1.2**	**1.3**	**1.2**	**1.4**
																	LLEGEEER	12	9	3	5031877;27436946;388240801	N-Term(TMT6plex)	1203.6	0				
																	LALDMEISAYR	4	2	1	5031877	N-Term(TMT6plex); M5(Oxidation)	1526.8	0	**1.0**	**1.1**	**1.0**	**1.5**
																	NSQGEEVAQR	13	2	1	5031877	N-Term(TMT6plex)	1346.7	0	**1.2**	**1.0**	**1.1**	**1.1**
																	EQHDAQVR	8	2	1	5031877	N-Term(TMT6plex)	1211.6	0	**1.1**	**1.3**	**1.3**	**1.6**
																	DAALATALGDK	16	1	1	5031877	N-Term(TMT6plex); K11(TMT6plex)	1503.9	0	**1.2**	**1.2**	**1.2**	**1.4**
																	SLETENSALQLQVTER	23	1	1	5031877	N-Term(TMT6plex)	2047.1	0	**1.3**	**1.3**	**1.2**	**1.4**
																	ALYETELADAR	16	1	1	5031877	N-Term(TMT6plex)	1480.8	0	**1.3**	**1.4**	**1.2**	**1.5**
																	DQMQQQLNDYEQLLDVK	26	2	1	5031877	N-Term(TMT6plex); M3(Oxidation); K17(TMT6plex)	2582.3	0	**1.2**	**1.4**	**1.3**	**1.5**
																	LSSEMNTSTVNSAR	20	2	1	5031877	N-Term(TMT6plex); M5(Oxidation)	1741.9	0	**1.2**	**1.2**	**1.1**	**1.3**
																	KLLEGEEER	7	9	3	5031877;27436946;388240801	N-Term(TMT6plex); K1(TMT6plex)	1560.9	1				
																	NQNSWGTGEDVK	16	2	1	5031877	N-Term(TMT6plex); K12(TMT6plex)	1792.9	0	**1.2**	**1.0**	**1.1**	**1.1**
																	CQSLTEDLEFR	2	1	1	5031877	N-Term(TMT6plex); C1(Carbamidomethyl)	1626.8	0	**1.2**	**1.0**	**1.1**	**1.1**
																	AGQTVTIWAANAGVTASPPTDLIWK	2	2	1	5031877	N-Term(TMT6plex); K25(TMT6plex)	3026.7	0	**1.2**	**1.3**	**1.3**	**1.6**
																	IESLSSQLSNLQK	10	2	1	5031877	N-Term(TMT6plex); K13(TMT6plex)	1905.1	0	**1.2**	**1.1**	**1.1**	**1.3**
																	KIGDTSVSYK	2	2	1	5031877	N-Term(TMT6plex); K1(TMT6plex); K10(TMT6plex)	1785.1	1	**0.9**	**1.4**	**1.1**	**1.5**
																	IQELEDLLAK	36	2	1	5031877	N-Term(TMT6plex); K10(TMT6plex)	1630.0	0	**1.2**	**1.3**	**1.3**	**1.4**
																	KQLADETLLK	5	1	1	5031877	N-Term(TMT6plex); K1(TMT6plex); K10(TMT6plex)	1846.2	1	**1.0**	**1.4**	**1.3**	**1.4**
																	QIEYEYK	17	2	1	5031877	N-Term(TMT6plex); K7(TMT6plex)	1430.8	0	**1.2**	**1.3**	**1.2**	**1.5**
																	SLEGDLEDLKDQIAQLEASLAAAK	17	1	1	5031877	N-Term(TMT6plex); K10(TMT6plex); K24(TMT6plex)	3215.8	1	**1.1**	**1.3**	**1.2**	**1.5**
																	LYKEELEQTYHAK	10	2	1	5031877	N-Term(TMT6plex); K3(TMT6plex); K13(TMT6plex)	2339.3	1	**1.3**	**1.4**	**1.3**	**1.6**
																	IGDTSVSYK	8	2	1	5031877	N-Term(TMT6plex); K9(TMT6plex)	1427.8	0	**1.1**	**1.2**	**1.1**	**1.4**
																	NTSEQDQPMGGWEMIR	4	2	1	5031877	N-Term(TMT6plex); M9(Oxidation)	2124.0	0	**1.5**	**1.4**	**1.5**	**1.3**
																	SMYEEEINETR	17	1	1	5031877	N-Term(TMT6plex); M2(Oxidation)	1645.8	0	**1.3**	**1.4**	**1.2**	**1.5**
																	DQMQQQLNDYEQLLDVK	6	2	1	5031877	N-Term(TMT6plex); K17(TMT6plex)	2566.3	0	**1.5**	**2.4**	**1.8**	**1.9**
																	AGGPTTPLSPTR	6	1	1	5031877	N-Term(TMT6plex)	1383.8	0	**1.2**	**1.3**	**1.2**	**1.5**
																	NTSEQDQPMGGWEMIR	10	2	1	5031877	N-Term(TMT6plex); M9(Oxidation); M14(Oxidation)	2140.0	0	**1.2**	**1.0**	**1.0**	**1.3**
																	EYEAALNSK	7	1	1	5031877	N-Term(TMT6plex); K9(TMT6plex)	1482.8	0	**1.3**	**1.4**	**1.3**	**1.6**
																	LSSEMNTSTVNSAR	2	2	1	5031877	N-Term(TMT6plex)	1725.9	0	**2.4**	**1.9**	**2.2**	**1.4**
																	LVEVDSGR	8	2	1	5031877	N-Term(TMT6plex)	1103.6	0	**1.1**	**1.3**	**1.1**	**1.3**
																	LAQALHEMR	2	2	1	5031877	N-Term(TMT6plex); M8(Oxidation)	1313.7	0	**1.1**	**1.2**	**1.3**	**1.3**
																	CKAEHDQLLLNYAK	1	1	1	5031877	N-Term(TMT6plex); C1(Carbamidomethyl); K2(TMT6plex); K14(TMT6plex)	2390.3	1	**1.3**	**1.5**	**1.4**	**1.6**
																	LAVYIDK	6	1	1	5031877	N-Term(TMT6plex); K7(TMT6plex)	1279.8	0	**1.3**	**1.3**	**1.1**	**1.4**
																	ESDLNGAQIK	1	1	1	5031877	N-Term(TMT6plex); K10(TMT6plex)	1532.9	0	**1.2**	**1.5**	**1.3**	**1.6**
																	SLEGDLEDLK	2	1	1	5031877	N-Term(TMT6plex); K10(TMT6plex)	1576.9	0	**1.3**	**1.3**	**1.2**	**1.4**
																	LQIELGK	3	2	2	388240801;5031877	N-Term(TMT6plex); K7(TMT6plex)	1258.8	0				

Supplementary Table 6. List of all proteins and peptides identified in untreated and chronically treated Het1A cells with cigarette smoke condensate for 8 months

Accession	RefSeq Accession	Gene Symbol	Entrez Gene ID	Description	Σ Coverage	Σ# Proteins	Σ# Unique Peptides	Σ# Peptides	Σ# PSMs	Het-1A-Smoke - 2M/Parental	Het-1A-Smoke - 4M/Parental	Het-1A-Smoke - 6M/Parental	Het-1A-Smoke - 8M/Parental	# AAs	MW [kDa]	calc. pI	Sequence	# PSMs	# Proteins	# Protein Groups	Protein Group Accessions	Modifications	MH+ [Da]	# Missed Cleavages	Het-1A-Smoke - 2M/Parental	Het-1A-Smoke - 4M/Parental	Het-1A-Smoke - 6M/Parental	Het-1A-Smoke - 8M/Parental
																	KSMYEEEINETR	2	1	1	5031877	N-Term(TMT6plex); K1(TMT6plex); M3(Oxidation)	2003.0	1	**1.2**	**1.5**	**1.3**	**1.5**
																	ALDDTAR	4	1	1	5031877	N-Term(TMT6plex)	990.5	0	**1.3**	**1.4**	**1.2**	**1.5**
																	EELMESR	2	2	1	5031877	N-Term(TMT6plex); M4(Oxidation)	1138.6	0	**1.1**	**1.2**	**1.0**	**1.4**
																	VDLENR	1	2	2	5031877;388240801	N-Term(TMT6plex)	974.5	0				
																	ACLER	1	2	1	5031877	N-Term(TMT6plex); C2(Carbamidomethyl)	877.5	0	**1.1**	**1.3**	**1.2**	**1.4**
																	LQEKEELR	1	2	2	5031877;388240801	N-Term(TMT6plex); K4(TMT6plex)	1502.9	1				
																	EMAEIR	1	2	1	5031877	N-Term(TMT6plex); M2(Oxidation)	993.5	0	**1.0**	**1.2**	**1.1**	**1.5**
																	ELTGLK	1	1	1	5031877	N-Term(TMT6plex); K6(TMT6plex)	1118.7	0	**1.1**	**1.1**	**1.0**	**1.5**
32189394	NP_001677.2	**ATP5B**	506	**ATP synthase subunit beta, mitochondrial precursor**	71.83	1	25	25	559	**1.4**	**1.4**	**1.5**	**1.7**	529	56.5	5.40												
																	QFAPIHAEAPEFMEMSVEQEILVTGIK	16	1	1	32189394	N-Term(TMT6plex); M13(Oxidation); M15(Oxidation); K27(TMT6plex)	3534.8	0	**1.2**	**1.2**	**1.3**	**1.8**
																	SLQDIIAILGMDELSEEDKLTVSR	34	1	1	32189394	N-Term(TMT6plex); M11(Oxidation); K19(TMT6plex)	3149.7	1	**1.2**	**1.3**	**1.3**	**1.9**
																	LVLEVAQHLGESTVR	20	1	1	32189394	N-Term(TMT6plex)	1880.1	0	**1.4**	**1.5**	**1.5**	**1.7**
																	EGNDLYHEMIESGVINLK	9	1	1	32189394	N-Term(TMT6plex); M9(Oxidation); K18(TMT6plex)	2535.3	0	**1.2**	**1.3**	**1.4**	**1.5**
																	EGNDLYHEMIESGVINLK	8	1	1	32189394	N-Term(TMT6plex); K18(TMT6plex)	2519.3	0	**1.3**	**1.4**	**1.6**	**1.6**
																	GSITSVQAIYVPADDLTDPAPATTFAHLDATTVLSR	2	1	1	32189394	N-Term(TMT6plex)	3944.0	0	**1.6**	**1.5**	**1.8**	**1.3**
																	VALVYGQMNEPPGAR	20	1	1	32189394	N-Term(TMT6plex); M8(Oxidation)	1847.0	0	**1.6**	**1.6**	**1.5**	**1.7**
																	TIAMDGTEGLVR	4	1	1	32189394	N-Term(TMT6plex)	1491.8	0	**1.6**	**1.6**	**1.9**	**1.6**
																	VVDLLAPYAK	36	1	1	32189394	N-Term(TMT6plex); K10(TMT6plex)	1547.0	0	**1.2**	**1.4**	**1.5**	**1.7**
																	FTQAGSEVSALLGR	31	1	1	32189394	N-Term(TMT6plex)	1664.9	0	**1.5**	**1.2**	**1.4**	**1.5**
																	VALTGLTVAEYFR	24	1	1	32189394	N-Term(TMT6plex)	1669.0	0	**1.4**	**1.2**	**1.5**	**1.4**
																	TVLIMELINNVAK	26	1	1	32189394	N-Term(TMT6plex); K13(TMT6plex)	1916.2	0	**1.7**	**1.6**	**2.2**	**1.9**
																	IMNVIGEPIDER	24	1	1	32189394	N-Term(TMT6plex); M2(Oxidation)	1630.9	0	**1.5**	**1.4**	**1.5**	**1.6**
																	AIAELGIYPAVDPLDSTSR	36	1	1	32189394	N-Term(TMT6plex)	2217.2	0	**1.3**	**1.4**	**1.4**	**1.7**
																	TIAMDGTEGLVR	24	1	1	32189394	N-Term(TMT6plex); M4(Oxidation)	1507.8	0	**1.4**	**1.3**	**1.4**	**1.7**
																	GFQQILAGEYDHLPEQAFYMVGPIEEAVAK	33	1	1	32189394	N-Term(TMT6plex); M20(Oxidation); K30(TMT6plex)	3825.0	0	**1.2**	**1.4**	**1.4**	**2.1**
																	IPSAVGYQPTLATDMGTMQER	20	1	1	32189394	N-Term(TMT6plex); M15(Oxidation); M18(Oxidation)	2527.2	0	**1.6**	**1.6**	**1.7**	**1.6**
																	AHGGYSVFAGVGER	2	1	1	32189394	N-Term(TMT6plex)	1635.8	0	**1.4**	**1.4**	**1.6**	**1.6**
																	SLQDIIAILGMDELSEEDK	26	1	1	32189394	N-Term(TMT6plex); M11(Oxidation); K19(TMT6plex)	2593.4	0	**1.4**	**1.4**	**1.5**	**1.8**
																	SLQDIIAILGMDELSEEDK	6	1	1	32189394	N-Term(TMT6plex); K19(TMT6plex)	2577.4	0	**1.5**	**1.5**	**1.6**	**1.6**
																	QFAPIHAEAPEFMEMSVEQEILVTGIK	4	1	1	32189394	N-Term(TMT6plex); M13(Oxidation); K27(TMT6plex)	3518.8	0	**1.6**	**1.6**	**1.6**	**1.7**
																	IMDPNIVGSEHYDVAR	17	1	1	32189394	N-Term(TMT6plex); M2(Oxidation)	2061.0	0	**1.6**	**1.5**	**1.4**	**1.6**
																	IGLFGGAGVGK	18	1	1	32189394	N-Term(TMT6plex); K11(TMT6plex)	1433.9	0	**1.4**	**1.4**	**1.6**	**1.7**
																	DQEGQDVLLFIDNIFR	37	1	1	32189394	N-Term(TMT6plex)	2151.1	0	**1.3**	**1.3**	**1.6**	**1.4**
																	IPVGPETLGR	6	1	1	32189394	N-Term(TMT6plex)	1267.8	0	**1.4**	**1.4**	**1.4**	**1.7**
																	VALVYGQMNEPPGAR	8	1	1	32189394	N-Term(TMT6plex)	1831.0	0	**1.9**	**1.8**	**1.9**	**1.7**
																	IMDPNIVGSEHYDVAR	4	1	1	32189394	N-Term(TMT6plex)	2045.0	0	**1.3**	**1.4**	**1.5**	**1.4**
																	GFQQILAGEYDHLPEQAFYMVGPIEEAVAK	12	1	1	32189394	N-Term(TMT6plex); K30(TMT6plex)	3809.0	0	**1.4**	**1.9**	**2.2**	**1.9**
																	VLDSGAPIK	18	1	1	32189394	N-Term(TMT6plex); K9(TMT6plex)	1357.8	0	**1.3**	**1.3**	**1.5**	**1.7**
																	FLSQPFQVAEVFTGHMGK	12	1	1	32189394	N-Term(TMT6plex); M16(Oxidation); K18(TMT6plex)	2497.3	0	**1.3**	**1.2**	**1.6**	**1.4**
																	TVLIMELINNVAK	14	1	1	32189394	N-Term(TMT6plex); M5(Oxidation); K13(TMT6plex)	1932.2	0	**1.2**	**1.1**	**1.5**	**1.4**
																	FLSQPFQVAEVFTGHMGK	3	1	1	32189394	N-Term(TMT6plex); K18(TMT6plex)	2481.3	0	**1.5**	**1.8**	**2.0**	**1.5**
																	KGSITSVQAIYVPADDLTDPAPATTFAHLDATTVLSR	3	1	1	32189394	N-Term(TMT6plex); K1(TMT6plex)	4301.3	1	**1.4**	**1.5**	**1.6**	**1.7**
																	ILQDYK	2	1	1	32189394	N-Term(TMT6plex); K6(TMT6plex)	1237.8	0	**1.2**	**1.3**	**1.4**	**1.6**
41393614;41393545	NP_958842.1	**RAB5C**	5878	**ras-related protein Rab-5C isoform a**	71.76	2	7	10	147	**1.3**	**1.2**	**1.3**	**1.3**	216	23.5	8.41												
																	GVDLQENNPASR	16	2	1	41393614;41393545	N-Term(TMT6plex)	1528.8	0	**1.2**	**1.1**	**1.1**	**1.4**
																	NEPQNATGAPGR	12	2	1	41393614;41393545	N-Term(TMT6plex)	1440.7	0	**1.1**	**0.7**	**1.0**	**0.9**
																	AVEFQEAQAYADDNSLLFMETSAK	10	2	1	41393614;41393545	N-Term(TMT6plex); K24(TMT6plex)	3136.6	0	**1.1**	**1.5**	**1.4**	**1.5**
																	TAMNVNEIFMAIAK	2	2	1	41393614;41393545	N-Term(TMT6plex); K14(TMT6plex)	2011.1	0	**1.9**	**1.6**	**2.1**	**1.6**
																	AVEFQEAQAYADDNSLLFMETSAK	18	2	1	41393614;41393545	N-Term(TMT6plex); M19(Oxidation); K24(TMT6plex)	3152.5	0	**1.3**	**1.3**	**1.4**	**1.6**
																	GAQAAIVVYDITNTDTFAR	22	2	1	41393614;41393545	N-Term(TMT6plex)	2255.2	0	**1.5**	**1.5**	**1.5**	**1.5**
																	QASPNIVIALAGNK	8	2	1	41393614;41393545	N-Term(TMT6plex); K14(TMT6plex)	1854.1	0	**1.3**	**1.4**	**1.4**	**1.5**
																	TAMNVNEIFMAIAK	12	2	1	41393614;41393545	N-Term(TMT6plex); M3(Oxidation); M10(Oxidation); K14(TMT6plex)	2043.1	0	**1.2**	**1.1**	**1.1**	**1.2**
																	FEIWDTAGQER	9	5	3	4506371;41393614;41393545;19923262	N-Term(TMT6plex)	1580.8	0				
																	TAMNVNEIFMAIAK	10	2	1	41393614;41393545	N-Term(TMT6plex); M10(Oxidation); K14(TMT6plex)	2027.1	0	**1.3**	**1.2**	**1.4**	**1.2**
																	YHSLAPMYYR	2	5	3	4506371;41393614;41393545;19923262	N-Term(TMT6plex); M7(Oxidation)	1545.8	0				
																	GQFHEYQESTIGAAFLTQTVCLDDTTVK	2	2	1	41393614;41393545	N-Term(TMT6plex); C21(Carbamidomethyl); K28(TMT6plex)	3617.8	0	**2.5**	**1.4**	**1.4**	**1.3**
																	LVLLGESAVGK	24	6	3	4506371;41393614;41393545;19923262	N-Term(TMT6plex); K11(TMT6plex)	1544.0	0				
45439306	NP_001340.2	**DARS**	1615	**aspartate--tRNA ligase, cytoplasmic**	71.66	2	34	34	217	**1.3**	**1.4**	**1.4**	**1.4**	501	57.1	6.55												
																	EAGVEMGDEDDLSTPNEK	23	2	1	45439306	N-Term(TMT6plex); M6(Oxidation); K18(TMT6plex)	2410.1	0	**1.3**	**1.4**	**1.3**	**1.4**
																	ESIVDVEGVVR	8	1	1	45439306	N-Term(TMT6plex)	1430.8	0	**1.4**	**1.5**	**1.4**	**1.4**
																	QMCICADFEK	7	1	1	45439306	N-Term(TMT6plex); M2(Oxidation); C3(Carbamidomethyl); C5(Carbamidomethyl); K10(TMT6plex)	1775.9	0	**1.4**	**1.4**	**1.6**	**1.4**
																	IHDPQLLTER	8	2	1	45439306	N-Term(TMT6plex)	1450.8	0	**1.2**	**1.3**	**1.4**	**1.4**
																	YGISSMIQSQEKPDR	4	1	1	45439306	N-Term(TMT6plex); M6(Oxidation); K12(TMT6plex)	2213.2	0	**1.5**	**1.3**	**1.5**	**1.4**
																	YGISSMIQSQEKPDR	2	1	1	45439306	N-Term(TMT6plex); K12(TMT6plex)	2197.2	0	**2.1**	**2.0**	**2.4**	**1.7**
																	GEEILSGAQR	12	1	1	45439306	N-Term(TMT6plex)	1288.7	0	**1.4**	**1.5**	**1.5**	**1.4**
																	YDTDFYILDK	2	1	1	45439306	N-Term(TMT6plex); K10(TMT6plex)	1750.9	0	**1.3**	**1.2**	**1.3**	**1.3**
																	VTMLFLGLHNVR	4	1	1	45439306	N-Term(TMT6plex); M3(Oxidation)	1644.9	0	**1.4**	**1.4**	**1.4**	**1.4**
																	LEYCEALAMLR	4	1	1	45439306	N-Term(TMT6plex); C4(Carbamidomethyl); M9(Oxidation)	1613.8	0	**1.4**	**1.3**	**1.3**	**1.4**
																	LQSGICHLFR	6	1	1	45439306	N-Term(TMT6plex); C6(Carbamidomethyl)	1459.8	0	**1.3**	**1.2**	**1.3**	**1.3**
																	EIMDAAEDYAK	29	1	1	45439306	N-Term(TMT6plex); M3(Oxidation); K11(TMT6plex)	1729.9	0	**1.5**	**1.5**	**1.5**	**1.6**
																	FGAPPHAGGGIGLER	6	1	1	45439306	N-Term(TMT6plex)	1664.9	0	**1.2**	**1.3**	**1.3**	**1.4**
																	EKYDTDFYILDK	8	1	1	45439306	N-Term(TMT6plex); K2(TMT6plex); K12(TMT6plex)	2237.2	1	**1.3**	**1.4**	**1.4**	**1.4**
																	LLGHLVK	6	1	1	45439306	N-Term(TMT6plex); K7(TMT6plex)	1237.8	0	**1.3**	**1.2**	**1.5**	**1.3**
																	ALHHGIDLEK	2	1	1	45439306	N-Term(TMT6plex); K10(TMT6plex)	1590.9	0	**1.3**	**1.3**	**1.4**	**1.5**
																	ATVNQDTR	4	2	1	45439306	N-Term(TMT6plex)	1133.6	0	**1.6**	**1.5**	**1.6**	**1.5**
																	VTMLFLGLHNVR	8	1	1	45439306	N-Term(TMT6plex)	1629.0	0	**1.4**	**1.3**	**1.4**	**1.3**
																	IYVISLAEPR	8	1	1	45439306	N-Term(TMT6plex)	1389.8	0	**1.3**	**1.2**	**1.3**	**1.2**
																	GFVEIQTPK	12	1	1	45439306	N-Term(TMT6plex); K9(TMT6plex)	1476.9	0	**1.2**	**1.3**	**1.4**	**1.4**
																	LPLQLDDAVRPEAEGEEEGR	14	1	1	45439306	N-Term(TMT6plex)	2452.2	0	**1.5**	**1.5**	**1.5**	**1.5**
																	NNAYLAQSPQLYK	2	1	1	45439306	N-Term(TMT6plex); K13(TMT6plex)	1968.1	0	**1.3**	**1.0**	**1.3**	**1.1**
																	FQTEIQTVNK	8	2	1	45439306	N-Term(TMT6plex); K10(TMT6plex)	1666.0	0	**1.5**	**1.2**	**1.5**	**1.4**
																	QQQFNVQALVAVGDHASK	4	1	1	45439306	N-Term(TMT6plex); K18(TMT6plex)	2398.3	0	**1.3**	**1.4**	**1.4**	**1.4**
																	ADEVVWVR	6	1	1	45439306	N-Term(TMT6plex)	1202.7	0	**1.3**	**1.5**	**1.5**	**1.4**
																	QFPCEPFK	4	1	1	45439306	N-Term(TMT6plex); C4(Carbamidomethyl); K8(TMT6plex)	1510.8	0	**1.4**	**1.4**	**1.4**	**1.3**
																	VFSIGPVFR	2	1	1	45439306	N-Term(TMT6plex)	1250.7	0	**1.4**	**1.0**	**1.3**	**1.1**
																	IGSCTQQDVELHVQK	1	1	1	45439306	N-Term(TMT6plex); C4(Carbamidomethyl); K15(TMT6plex)	2200.2	0	**1.4**	**1.6**	**1.9**	**1.7**
																	QSNSYDMFMR	1	1	1	45439306	N-Term(TMT6plex); M7(Oxidation); M9(Oxidation)	1539.7	0	**1.4**	**1.2**	**1.2**	**1.2**
																	IISAASEGGANVFTVSYFK	1	1	1	45439306	N-Term(TMT6plex); K19(TMT6plex)	2419.3	0	**1.4**	**1.3**	**1.6**	**1.2**
																	FAANINK	3	1	1	45439306	N-Term(TMT6plex); K7(TMT6plex)	1235.8	0	**1.2**	**1.1**	**1.2**	**1.2**
																	QCFLVLR	2	1	1	45439306	N-Term(TMT6plex); C2(Carbamidomethyl)	1164.7	0	**1.5**	**1.7**	**1.9**	**1.5**
																	AYIDSFR	2	1	1	45439306	N-Term(TMT6plex)	1100.6	0	**1.2**	**1.3**	**1.3**	**1.2**
																	ETLINK	1	1	1	45439306	N-Term(TMT6plex); K6(TMT6plex)	1175.7	0	**1.3**	**1.4**	**1.4**	**1.3**
																	DLTIQK	1	1	1	45439306	N-Term(TMT6plex); K6(TMT6plex)	1175.7	0	**1.6**	**1.8**	**1.6**	**1.5**
																	FLEPTLR	2	1	1	45439306	N-Term(TMT6plex)	1104.7	0	**1.4**	**1.2**	**1.3**	**1.3**
23510338	NP_003325.2	**UBA1**	7317	**ubiquitin-like modifier-activating enzyme 1**	71.64	1	50	50	558	**1.2**	**1.1**	**1.1**	**1.1**	1058	117.8	5.76												
																	YDGQVAVFGSDLQEK	35	1	1	23510338	N-Term(TMT6plex); K15(TMT6plex)	2114.1	0	**1.2**	**1.0**	**1.1**	**1.0**
																	DEFEGLFK	22	1	1	23510338	N-Term(TMT6plex); K8(TMT6plex)	1442.8	0	**1.1**	**1.0**	**1.0**	**1.0**
																	AAVATFLQSVQVPEFTPK	33	1	1	23510338	N-Term(TMT6plex); K18(TMT6plex)	2391.4	0	**1.2**	**1.1**	**1.0**	**1.2**
																	NFAMIGLGCGEGGEIIVTDMDTIEK	2	1	1	23510338	N-Term(TMT6plex); M4(Oxidation); C9(Carbamidomethyl); M20(Oxidation); K25(TMT6plex)	3160.6	0	**1.1**	**1.1**	**1.0**	**0.9**
																	MYPIDFEKDDDSNFHMDFIVAASNLR	8	1	1	23510338	N-Term(TMT6plex); M1(Oxidation); K8(TMT6plex); M16(Oxidation)	3580.7	1	**1.1**	**1.1**	**1.0**	**1.1**
																	HQYYNQEWTLWDR	5	1	1	23510338	N-Term(TMT6plex)	2068.0	0	**1.3**	**1.2**	**1.2**	**1.2**
																	KPLLESGTLGTK	14	1	1	23510338	N-Term(TMT6plex); K1(TMT6plex); K12(TMT6plex)	1931.2	0	**1.0**	**1.1**	**1.1**	**1.1**
																	GLGVEIAK	6	1	1	23510338	N-Term(TMT6plex); K8(TMT6plex)	1244.8	0	**1.1**	**1.1**	**1.1**	**1.2**
																	QFLDYFK	12	1	1	23510338	N-Term(TMT6plex); K7(TMT6plex)	1418.8	0	**1.2**	**1.0**	**1.0**	**1.1**
																	IHVSDQELQSANASVDDSR	7	1	1	23510338	N-Term(TMT6plex)	2300.1	0	**1.3**	**1.1**	**1.4**	**1.3**
																	YFLVGAGAIGCELLK	23	1	1	23510338	N-Term(TMT6plex); C11(Carbamidomethyl); K15(TMT6plex)	2069.2	0	**1.3**	**1.0**	**1.1**	**0.9**
																	LDQPMTEIVSR	20	1	1	23510338	N-Term(TMT6plex); M5(Oxidation)	1533.8	0	**1.4**	**1.2**	**1.2**	**1.2**
																	NGSEADIDEGLYSR	3	1	1	23510338	N-Term(TMT6plex)	1754.8	0	**1.4**	**1.0**	**1.1**	**1.1**

Accession	RefSeq Accession	Gene Symbol	Entrez Gene ID	Description	Σ Coverage	Σ# Proteins	Σ# Unique Peptides	Σ# Peptides	Σ# PSMs	Het-1A-Smoke - 2M/Parental	Het-1A-Smoke - 4M/Parental	Het-1A-Smoke - 6M/Parental	Het-1A-Smoke - 8M/Parental	# AAs	MW [kDa]	calc. pI	Sequence	# PSMs	# Proteins	# Protein Groups	Protein Group Accessions	Modifications	MH+ [Da]	# Missed Cleavages	Het-1A-Smoke - 2M/Parental	Het-1A-Smoke - 4M/Parental	Het-1A-Smoke - 6M/Parental	Het-1A-Smoke - 8M/Parental
																	FMPIMQWLYFDALECLPEDK	2	1	1	23510338	N-Term(TMT6plex); M2(Oxidation); M5(Oxidation); C15(Carbamidomethyl); K20(TMT6plex)	3036.5	0	**1.1**	**0.7**	**0.6**	**0.9**
																	AENYDIPSADR	10	1	1	23510338	N-Term(TMT6plex)	1479.7	0	**1.3**	**1.1**	**1.1**	**1.2**
																	FEVQGLQPNGEEMTLK	5	1	1	23510338	N-Term(TMT6plex); M13(Oxidation); K16(TMT6plex)	2294.2	0	**1.2**	**1.3**	**1.3**	**1.2**
																	SDTAAAAVR	7	1	1	23510338	N-Term(TMT6plex)	1090.6	0	**1.2**	**0.9**	**1.0**	**0.9**
																	VLGPYTFSICDTSNFSDYIR	7	1	1	23510338	N-Term(TMT6plex); C10(Carbamidomethyl)	2584.3	0	**1.2**	**1.2**	**1.1**	**1.3**
																	FMPIMQWLYFDALECLPEDKEVLTEDK	19	1	1	23510338	N-Term(TMT6plex); M2(Oxidation); M5(Oxidation); C15(Carbamidomethyl); K20(TMT6plex); K27(TMT6plex)	4080.1	1	**1.1**	**0.8**	**0.8**	**1.1**
																	QLLHNFPPDQLTSSGAPFWSGPK	6	1	1	23510338	N-Term(TMT6plex); K23(TMT6plex)	2982.6	0	**1.3**	**1.3**	**1.3**	**1.4**
																	FMPIMQWLYFDALECLPEDKEVLTEDK	2	1	1	23510338	N-Term(TMT6plex); M5(Oxidation); C15(Carbamidomethyl); K20(TMT6plex); K27(TMT6plex)	4064.1	1	**1.1**	**1.1**	**1.1**	**0.9**
																	EVLTEDK	4	1	1	23510338	N-Term(TMT6plex); K7(TMT6plex)	1291.8	0	**1.0**	**1.0**	**1.0**	**1.0**
																	LAGTQPLEVLEAVQR	20	1	1	23510338	N-Term(TMT6plex)	1853.1	0	**1.4**	**1.2**	**1.3**	**1.1**
																	SLVASLAEPDFVVTDFAK	40	1	1	23510338	N-Term(TMT6plex); K18(TMT6plex)	2367.3	0	**1.1**	**0.9**	**1.0**	**1.0**
																	NFPNAIEHTLQWAR	18	1	1	23510338	N-Term(TMT6plex)	1926.0	0	**1.2**	**1.1**	**1.1**	**1.2**
																	LAYVAAGDLAPINAFIGGLAAQEVMK	23	1	1	23510338	N-Term(TMT6plex); M25(Oxidation); K26(TMT6plex)	3077.7	0	**1.2**	**1.2**	**1.1**	**1.3**
																	IYDDDFFQNLDGVANALDNVDAR	26	1	1	23510338	N-Term(TMT6plex)	2829.4	0	**1.3**	**1.1**	**1.1**	**1.1**
																	LQTSSVLVSGLR	21	1	1	23510338	N-Term(TMT6plex)	1488.9	0	**1.2**	**1.0**	**1.1**	**1.1**
																	ALVLELCCNDESGEDVEVPYVR	20	1	1	23510338	N-Term(TMT6plex); C7(Carbamidomethyl); C8(Carbamidomethyl)	2795.3	0	**1.4**	**1.5**	**1.4**	**1.4**
																	NIILGGVK	9	1	1	23510338	N-Term(TMT6plex); K8(TMT6plex)	1271.8	0	**1.2**	**0.8**	**1.0**	**0.9**
																	QFLFRPWDVTK	3	1	1	23510338	N-Term(TMT6plex); K11(TMT6plex)	1895.1	0	**1.1**	**1.0**	**1.1**	**1.1**
																	ALPAVQQNNLDEDLIR	19	1	1	23510338	N-Term(TMT6plex)	2038.1	0	**1.2**	**1.3**	**1.3**	**1.2**
																	DNPGVVTCLDEAR	8	1	1	23510338	N-Term(TMT6plex); C8(Carbamidomethyl)	1674.8	0	**1.3**	**1.3**	**1.2**	**1.2**
																	AVTLHDQGTAQWADLSSQFYLR	14	1	1	23510338	N-Term(TMT6plex)	2736.4	0	**1.3**	**1.5**	**1.5**	**1.4**
																	LEITMLSQGVSMLYSFFMPAAK	6	1	1	23510338	N-Term(TMT6plex); M5(Oxidation); M12(Oxidation); M18(Oxidation); K22(TMT6plex)	2970.5	0	**1.4**	**1.1**	**1.1**	**1.4**
																	ATLPSPDKLPGFK	7	1	1	23510338	N-Term(TMT6plex); K8(TMT6plex); K13(TMT6plex)	2058.3	1	**1.0**	**1.1**	**1.0**	**1.2**
																	QPAENVNQYLTDPK	16	1	1	23510338	N-Term(TMT6plex); K14(TMT6plex)	2075.1	0	**1.2**	**1.0**	**1.1**	**1.1**
																	LAYVAAGDLAPINAFIGGLAAQEVMK	4	1	1	23510338	N-Term(TMT6plex); K26(TMT6plex)	3061.7	0	**1.5**	**2.0**	**1.6**	**1.5**
																	NEEDAAELVALAQAVNAR	14	1	1	23510338	N-Term(TMT6plex)	2113.1	0	**1.4**	**1.2**	**1.3**	**1.4**
																	QLYVLGHEAMK	4	1	1	23510338	N-Term(TMT6plex); M10(Oxidation); K11(TMT6plex)	1763.0	0	**1.1**	**1.0**	**1.0**	**1.2**
																	GNVQVVIPFLTESYSSSQDPPEK	7	1	1	23510338	N-Term(TMT6plex); K23(TMT6plex)	2979.6	0	**1.1**	**1.4**	**1.1**	**1.3**
																	GGIVSQVK	5	1	1	23510338	N-Term(TMT6plex); K8(TMT6plex)	1245.8	0	**1.3**	**1.1**	**1.1**	**1.1**
																	GLFGQLFCDFGEEMILTDSNGEQPLSAMVSMVTK	2	1	1	23510338	N-Term(TMT6plex); C8(Carbamidomethyl); M14(Oxidation); M28(Oxidation); M31(Oxidation); K34(TMT6plex)	4258.0	0	**1.0**	**0.7**	**0.8**	**0.8**
																	IIPAIATTTAAVVGLVCLELYK	3	1	1	23510338	N-Term(TMT6plex); C17(Carbamidomethyl); K22(TMT6plex)	2774.7	0	**1.4**	**1.1**	**1.6**	**0.9**
																	SIPICTLK	2	1	1	23510338	N-Term(TMT6plex); C5(Carbamidomethyl); K8(TMT6plex)	1389.9	0	**1.2**	**1.1**	**1.2**	**1.1**
																	HGFESGDFVSFSEVQGMVELNGNQPMEIK	1	1	1	23510338	N-Term(TMT6plex); M17(Oxidation); M26(Oxidation); K29(TMT6plex)	3702.8	0				
																	AEVSQPR	4	1	1	23510338	N-Term(TMT6plex)	1015.6	0	**1.2**	**1.0**	**0.9**	**1.0**
																	LKSDTAAAAVR	1	1	1	23510338	N-Term(TMT6plex); K2(TMT6plex)	1560.9	1	**1.2**	**1.5**	**1.5**	**1.5**
																	LVVADTR	2	1	1	23510338	N-Term(TMT6plex)	1002.6	0	**1.3**	**1.1**	**1.2**	**1.4**
																	ATLPSPDK	3	1	1	23510338	N-Term(TMT6plex); K8(TMT6plex)	1286.8	0	**1.1**	**1.0**	**1.2**	**1.0**
																	QLDSYK	3	1	1	23510338	N-Term(TMT6plex); K6(TMT6plex)	1211.7	0	**1.2**	**1.0**	**1.0**	**1.2**
																	CPHPLTFDVNNPLHLDYVMAAANLFAQTYGLTGSQDR	1	1	1	23510338	N-Term(TMT6plex); C1(Carbamidomethyl); M19(Oxidation)	4392.1	0	**1.4**	**1.2**	**1.4**	**0.8**
14165466	NP_114368.1	**PTBP1**	5725	**polypyrimidine tract-binding protein 1 isoform c**	71.56	3	19	23	534	**1.2**	**1.2**	**1.3**	**1.3**	531	57.2	9.17												
																	AQAALQAVNSVQSGNLALAASAAAVDAGMAMAGQSPVLR	29	3	1	14165466	N-Term(TMT6plex); M29(Oxidation); M31(Oxidation)	3942.0	0	**1.5**	**1.4**	**1.4**	**1.5**
																	NFQNIFPPSATLHLSNIPPSVSEEDLK	60	3	1	14165466	N-Term(TMT6plex); K27(TMT6plex)	3452.8	0	**1.2**	**1.0**	**1.2**	**1.1**
																	DYTRPDLPSGDSQPSLDQTMAAAFGLSVPNVHGALAPLAIPSAAAAAAAAGR	6	1	1	14165466	N-Term(TMT6plex); M20(Oxidation)	5303.7	0	**2.0**	**3.8**	**2.2**	**2.2**
																	GQPIYIQFSNHK	1	3	1	14165466	N-Term(TMT6plex); K12(TMT6plex)	1890.1	0	**1.3**	**1.3**	**1.2**	**1.3**
																	IAIPGLAGAGNSVLLVSNLNPER	58	3	1	14165466	N-Term(TMT6plex)	2504.4	0	**1.2**	**1.3**	**1.3**	**1.2**
																	AQAALQAVNSVQSGNLALAASAAAVDAGMAMAGQSPVLR	6	3	1	14165466	N-Term(TMT6plex)	3910.0	0	**1.3**	**1.6**	**1.6**	**1.6**
																	LPIDVTEGEVISLGLPFGK	18	3	1	14165466	N-Term(TMT6plex); K19(TMT6plex)	2442.4	0	**1.0**	**1.1**	**1.3**	**1.5**
																	AQAALQAVNSVQSGNLALAASAAAVDAGMAMAGQSPVLR	5	3	1	14165466	N-Term(TMT6plex); M31(Oxidation)	3926.0	0	**1.2**	**1.3**	**1.3**	**1.3**
																	DYGNSPLHR	19	3	1	14165466	N-Term(TMT6plex)	1287.7	0	**1.7**	**1.3**	**1.2**	**1.3**
																	TDSSPNQAR	7	3	1	14165466	N-Term(TMT6plex)	1204.6	0	**1.1**	**0.8**	**1.0**	**0.9**
																	VTNLLMLK	6	9	3	14165466;255003738;10863997	N-Term(TMT6plex); M6(Oxidation); K8(TMT6plex)	1405.9	0				
																	KLPIDVTEGEVISLGLPFGK	71	3	1	14165466	N-Term(TMT6plex); K1(TMT6plex); K20(TMT6plex)	2799.7	1	**1.1**	**1.2**	**1.3**	**1.3**
																	NQAFIEMNTEEAANTMVNYYTSVTPVLR	16	3	1	14165466	N-Term(TMT6plex); M7(Oxidation)	3451.7	0	**1.4**	**1.5**	**1.6**	**1.4**
																	HQNVQLPR	8	3	1	14165466	N-Term(TMT6plex)	1220.7	0	**1.1**	**1.3**	**1.3**	**1.3**
																	IIVENLFYPVTLDVLHQIFSK	20	3	1	14165466	N-Term(TMT6plex); K21(TMT6plex)	2946.7	0	**1.2**	**1.2**	**1.7**	**1.4**
																	EGQEDQGLTK	36	9	2	255003738;14165466	N-Term(TMT6plex); K10(TMT6plex)	1562.8	0				
																	MALIQMGSVEEAVQALIDLHNHDLGENHHLR	16	3	1	14165466	N-Term(TMT6plex); M1(Oxidation)	3735.9	0	**1.5**	**1.7**	**1.7**	**1.4**
																	VTPQSLFILFGVYGDVQR	27	3	1	14165466	N-Term(TMT6plex)	2268.3	0	**1.5**	**1.3**	**1.7**	**1.4**
																	LSLDGQNIYNACCTLR	31	3	1	14165466	N-Term(TMT6plex); C12(Carbamidomethyl); C13(Carbamidomethyl)	2127.0	0	**1.2**	**1.2**	**1.2**	**1.2**
																	NQAFIEMNTEEAANTMVNYYTSVTPVLR	21	3	1	14165466	N-Term(TMT6plex); M7(Oxidation); M16(Oxidation)	3467.7	0	**1.4**	**1.3**	**1.4**	**1.4**
																	LTSLNVK	17	9	2	255003738;14165466	N-Term(TMT6plex); K7(TMT6plex)	1232.8	0				
																	VTNLLMLK	1	9	3	10863997;14165466;255003738	N-Term(TMT6plex); K8(TMT6plex)	1389.9	0				
																	NNQFQALLQYADPVSAQHAK	29	3	1	14165466	N-Term(TMT6plex); K20(TMT6plex)	2701.4	0	**1.3**	**1.1**	**1.3**	**1.1**
																	DYTRPDLPSGDSQPSLDQTMAAAFGLSVPNVHGALAPLAIPSAAAAAAAAGR	8	1	1	14165466	N-Term(TMT6plex)	5287.7	0	**1.4**	**1.5**	**1.7**	**1.6**
																	VTPQSLFILFGVYGDVQRVK	2	3	1	14165466	N-Term(TMT6plex); K20(TMT6plex)	2724.6	1	**1.3**	**1.0**	**2.0**	**0.6**
																	LHGKPIR	1	3	1	14165466	N-Term(TMT6plex); K4(TMT6plex)	1278.8	0	**0.9**	**1.1**	**1.2**	**1.3**
																	IITFTK	14	10	3	255003738;14165466;10863997	N-Term(TMT6plex); K6(TMT6plex)	1180.8	0				
																	SAGVPSR	1	3	1	14165466	N-Term(TMT6plex)	902.5	0	**1.3**	**1.0**	**1.2**	**1.1**
4885417	NP_005330.1	**UBE2K**	3093	**ubiquitin-conjugating enzyme E2 K isoform 1**	71.50	4	10	10	62	**1.6**	**1.5**	**1.6**	**1.5**	200	22.4	5.44												
																	SWDVETATELLLSN	10	3	1	4885417	N-Term(TMT6plex)	1806.9	0	**1.5**	**1.4**	**1.3**	**1.5**
																	VDLVDENFTELR	10	2	1	4885417	N-Term(TMT6plex)	1678.9	0	**1.9**	**1.5**	**1.8**	**1.7**
																	IPETYPFNPPK	13	2	1	4885417	N-Term(TMT6plex); K11(TMT6plex)	1761.0	0	**1.4**	**1.4**	**1.5**	**1.2**
																	IWHPNISSVTGAICLDILK	1	3	1	4885417	N-Term(TMT6plex); C14(Carbamidomethyl); K19(TMT6plex)	2595.5	0	**1.4**	**1.9**	**1.8**	**1.8**
																	TVLLSLQALLAAAEPDDPQDAVVANQYK	10	2	1	4885417	N-Term(TMT6plex); K28(TMT6plex)	3411.9	0	**1.8**	**1.5**	**1.8**	**1.5**
																	GEIAGPPDTPYEGGR	9	3	1	4885417	N-Term(TMT6plex)	1744.9	0	**1.8**	**1.7**	**2.0**	**1.9**
																	LWAHVYAGAPVSSPEYTK	2	2	1	4885417	N-Term(TMT6plex); K18(TMT6plex)	2434.3	0	**1.3**	**1.2**	**1.4**	**1.3**
																	DQWAAAMTLR	1	3	1	4885417	N-Term(TMT6plex); M7(Oxidation)	1407.7	0	**1.6**	**1.3**	**1.5**	**1.5**
																	NAVIVALSSK	4	2	1	4885417	N-Term(TMT6plex); K10(TMT6plex)	1459.9	0	**1.7**	**1.3**	**1.6**	**1.3**
																	YQLEIK	2	2	1	4885417	N-Term(TMT6plex); K6(TMT6plex)	1251.8	0	**1.7**	**1.5**	**1.8**	**1.6**
10835067;659667952	NP_003133.1	**SSB**	6741	**lupus La protein**	71.32	11	37	37	765	**1.1**	**1.2**	**1.1**	**1.0**	408	46.8	7.12												
																	ETDLLILFK	41	1	1	10835067;659667952	N-Term(TMT6plex); K9(TMT6plex)	1550.0	0	**1.0**	**1.0**	**1.1**	**0.9**
																	GFPTDATLDDIK	12	1	1	10835067;659667952	N-Term(TMT6plex); K12(TMT6plex)	1751.0	0	**1.0**	**1.1**	**1.0**	**1.0**
																	AELMEISEDK	14	1	1	10835067;659667952	N-Term(TMT6plex); M4(Oxidation); K10(TMT6plex)	1638.9	0	**1.1**	**1.2**	**1.1**	**1.1**
																	EETDKEEPASK	12	1	1	10835067;659667952	N-Term(TMT6plex); K5(TMT6plex); K11(TMT6plex)	1950.1	1	**1.0**	**1.1**	**1.1**	**1.0**
																	SKAELMEISEDK	10	1	1	10835067;659667952	N-Term(TMT6plex); K2(TMT6plex); M6(Oxidation); K12(TMT6plex)	2083.2	1	**1.4**	**1.4**	**1.3**	**1.2**
																	LDEGWVPLEIMIK	43	1	1	10835067;659667952	N-Term(TMT6plex); K13(TMT6plex)	2001.2	0	**1.4**	**1.7**	**1.6**	**1.2**
																	LDEGWVPLEIMIK	56	1	1	10835067;659667952	N-Term(TMT6plex); M11(Oxidation); K13(TMT6plex)	2017.1	0	**1.1**	**1.2**	**1.1**	**1.1**
																	EDLHILFSNHGEIK	19	1	1	10835067;659667952	N-Term(TMT6plex); K14(TMT6plex)	2110.2	0	**1.1**	**1.2**	**1.0**	**1.1**
																	GSIFVVFDSIESAK	62	1	1	10835067;659667952	N-Term(TMT6plex); K14(TMT6plex)	1957.1	0	**1.1**	**1.2**	**1.2**	**1.1**
																	LTTDFNVIVEALSK	162	1	1	10835067;659667952	N-Term(TMT6plex); K14(TMT6plex)	2008.2	0	**1.2**	**1.2**	**1.2**	**1.1**
																	GFPTDATLDDIKEWLEDK	20	1	1	10835067;659667952	N-Term(TMT6plex); K12(TMT6plex); K18(TMT6plex)	2780.5	1	**1.0**	**1.3**	**1.2**	**1.1**
																	SPSKPLPEVTDEYK	14	1	1	10835067;659667952	N-Term(TMT6plex); K4(TMT6plex); K14(TMT6plex)	2277.3	0	**1.2**	**1.2**	**1.3**	**1.1**
																	SKAELMEISEDKTK	1	1	1	10835067;659667952	N-Term(TMT6plex); K2(TMT6plex); K12(TMT6plex); K14(TMT6plex)	2525.5	2	**1.3**	**1.5**	**1.3**	**1.3**
																	FSGDLDDQTCR	21	1	1	10835067;659667952	N-Term(TMT6plex); C10(Carbamidomethyl)	1542.7	0	**1.3**	**1.2**	**1.2**	**1.0**
																	EGIILFK	38	1	1	10835067;659667952	N-Term(TMT6plex); K7(TMT6plex)	1277.8	0	**1.0**	**1.2**	**1.1**	**1.1**

Khan et al., 2019. Multi-omics analysis to characterize cigarette smoke induced molecular alterations in esophageal cells
Supplementary Table 6. List of all proteins and peptides identified in untreated and chronically treated Het1A cells with cigarette smoke condensate for 8 months

Accession	RefSeq Accession	Gene Symbol	Entrez Gene ID	Description	Σ Coverage	Σ# Proteins	Σ# Unique Peptides	Σ# Peptides	Σ# PSMs	Het-1A-Smoke - 2M/Parental	Het-1A-Smoke - 4M/Parental	Het-1A-Smoke - 6M/Parental	Het-1A-Smoke - 8M/Parental	# AAs	MW [kDa]	calc. pI	Sequence	# PSMs	# Proteins	# Protein Groups	Protein Group Accessions	Modifications	MH+ [Da]	# Missed Cleavages	Het-1A-Smoke - 2M/Parental	Het-1A-Smoke - 4M/Parental	Het-1A-Smoke - 6M/Parental	Het-1A-Smoke - 8M/Parental
																	AKQEQEAK	9	1	1	10835067;659667952	N-Term(TMT6plex); K2(TMT6plex); K8(TMT6plex)	1619.0	1	0.9	1.3	1.0	1.0
																	AELMEISEDKTK	4	1	1	10835067;659667952	N-Term(TMT6plex); M4(Oxidation); K10(TMT6plex); K12(TMT6plex)	2097.2	1	1.2	1.5	1.2	1.2
																	GQVLNIQMR	9	1	1	10835067;659667952	N-Term(TMT6plex); M8(Oxidation)	1303.7	0	1.1	1.2	1.1	1.2
																	FASDDEHDEHDENGATGPVK	2	1	1	10835067;659667952	N-Term(TMT6plex); K20(TMT6plex)	2628.2	0	1.0	1.3	1.3	1.1
																	SKAELMEISEDK	3	1	1	10835067;659667952	N-Term(TMT6plex); K2(TMT6plex); K12(TMT6plex)	2067.2	1	1.1	1.5	1.4	1.1
																	KFVETPGQK	7	1	1	10835067;659667952	N-Term(TMT6plex); K1(TMT6plex); K9(TMT6plex)	1721.1	1	1.1	1.3	1.1	1.2
																	YKETDLLILFK	11	1	1	10835067;659667952	N-Term(TMT6plex); K2(TMT6plex); K11(TMT6plex)	2070.3	1	1.1	1.4	1.2	1.1
																	ICHQIEYYFGDFNLPR	26	1	1	10835067;659667952	N-Term(TMT6plex); C2(Carbamidomethyl)	2301.1	0	1.2	1.2	1.3	1.1
																	IIEDQQESLNK	34	1	1	10835067;659667952	N-Term(TMT6plex); K11(TMT6plex)	1775.0	0	1.2	1.2	1.1	1.1
																	AELMEISEDK	5	1	1	10835067;659667952	N-Term(TMT6plex); K10(TMT6plex)	1622.9	0	1.4	1.5	1.5	1.2
																	EVTWEVLEGEVEK	27	1	1	10835067;659667952	N-Term(TMT6plex); K13(TMT6plex)	2005.1	0	1.0	1.2	1.1	1.1
																	KIIEDQQESLNK	4	1	1	10835067;659667952	N-Term(TMT6plex); K1(TMT6plex); K12(TMT6plex)	2132.3	1	1.0	1.2	1.1	1.1
																	FVETPGQK	10	1	1	10835067;659667952	N-Term(TMT6plex); K8(TMT6plex)	1363.8	0	1.0	1.1	1.1	1.1
																	AAQPGSGK	4	1	1	10835067;659667952	N-Term(TMT6plex); K8(TMT6plex)	1173.7	0	0.9	1.1	1.0	1.0
																	LEEDAEMK	4	1	1	10835067;659667952	N-Term(TMT6plex); M7(Oxidation); K8(TMT6plex)	1438.7	0	1.1	1.3	1.2	1.1
																	QKLEEDAEMK	13	1	1	10835067;659667952	N-Term(TMT6plex); K2(TMT6plex); M9(Oxidation); K10(TMT6plex)	1924.1	1	1.1	1.3	1.1	1.1
																	QKLEEDAEMK	1	1	1	10835067;659667952	N-Term(TMT6plex); K2(TMT6plex); K10(TMT6plex)	1908.1	1	1.4	1.7	1.7	1.4
																	MAALEAK	2	1	1	10835067;659667952	N-Term(TMT6plex); K7(TMT6plex)	1191.7	0	1.6	1.8	1.8	1.2
																	SPSKPLPEVTDEYKNDVK	2	1	1	10835067;659667952	N-Term(TMT6plex); K4(TMT6plex); K14(TMT6plex); K18(TMT6plex)	2962.7	1	0.9	1.3	1.1	1.0
																	VQFQGK	9	1	1	10835067;659667952	N-Term(TMT6plex); K6(TMT6plex)	1164.7	0	1.1	1.1	1.1	1.1
																	MAALEAK	6	1	1	10835067;659667952	N-Term(TMT6plex); M1(Oxidation); K7(TMT6plex)	1207.7	0	1.0	1.0	1.0	1.0
																	WIDFVR	32	1	1	10835067;659667952	N-Term(TMT6plex)	1064.6	0	1.2	1.2	1.0	1.0
																	QEQEAK	2	11	1	10835067;659667952	N-Term(TMT6plex); K6(TMT6plex)	1190.7	0	1.1	1.0	1.0	0.9
																	DANNGNLQLR	3	1	1	10835067;659667952	N-Term(TMT6plex)	1343.7	0	1.2	1.2	1.2	1.1
																	DDYFAK	8	1	1	10835067;659667952	N-Term(TMT6plex); K6(TMT6plex)	1216.7	0	1.1	1.3	1.2	1.0
																	IGCLLK	2	1	1	10835067;659667952	N-Term(TMT6plex); C3(Carbamidomethyl); K6(TMT6plex)	1161.7	0	1.1	1.1	1.1	1.0
																	EWLEDK	1	1	1	10835067;659667952	N-Term(TMT6plex); K6(TMT6plex)	1277.7	0	1.0	1.1	1.0	0.9
39995082	NP_060225.4	NSUN2	54888	tRNA (cytosine(34)-C(5))-methyltransferase isoform 1	71.32	2	38	38	203	1.5	1.5	1.6	1.6	767	86.4	6.77												
																	ELEDLEVDGQK	3	1	1	39995082	N-Term(TMT6plex); K11(TMT6plex)	1732.9	0	1.4	1.4	1.5	1.6
																	MMGLEVLGEK	4	2	1	39995082	N-Term(TMT6plex); M1(Oxidation); M2(Oxidation); K10(TMT6plex)	1596.9	0	1.4	1.4	1.4	1.7
																	IVPEGEWGQFMDALR	6	2	1	39995082	N-Term(TMT6plex); M11(Oxidation)	1993.0	0	1.5	1.5	1.5	1.4
																	LFEHYYQELK	4	2	1	39995082	N-Term(TMT6plex); K10(TMT6plex)	1828.0	0	1.4	1.5	1.7	1.6
																	ILLTQENPFFR	14	2	1	39995082	N-Term(TMT6plex)	1606.9	0	1.6	1.6	1.7	1.7
																	IITVSMEDVK	6	2	1	39995082	N-Term(TMT6plex); M6(Oxidation); K10(TMT6plex)	1608.9	0	1.5	1.4	1.6	1.5
																	EGVILTNESAASTGQPDNDVTEGQR	8	2	1	39995082	N-Term(TMT6plex)	2817.4	0	1.4	1.4	1.2	1.5
																	SEGALELADVSNELPGLK	12	2	1	39995082	N-Term(TMT6plex); K18(TMT6plex)	2300.3	0	1.6	1.6	1.8	1.8
																	EDPFVFIPEDDPLFPPIEK	32	2	1	39995082	N-Term(TMT6plex); K19(TMT6plex)	2702.4	0	1.3	1.8	1.7	1.8
																	ESTQLSPADLTEGKPTDPSK	5	2	1	39995082	N-Term(TMT6plex); K14(TMT6plex); K20(TMT6plex)	2788.5	0	1.3	1.5	1.6	1.5
																	MVYSTCSLNPIEDEAVIASLLEK	6	2	1	39995082	N-Term(TMT6plex); M1(Oxidation); C6(Carbamidomethyl); K23(TMT6plex)	3056.6	0	1.3	1.4	1.7	1.7
																	LESPSFTGTGDTEIAHATEDLENNGSK	1	2	1	39995082	N-Term(TMT6plex); K27(TMT6plex)	3278.6	0	1.6	1.6	2.0	1.9
																	FYALDPSFPR	4	2	1	39995082	N-Term(TMT6plex)	1441.8	0	1.6	1.3	1.5	1.4
																	LAQEGIYTLYPFINSR	2	2	1	39995082	N-Term(TMT6plex)	2114.1	0	1.5	1.4	1.6	1.4
																	LSSETYSQAK	3	2	1	39995082	N-Term(TMT6plex); K10(TMT6plex)	1571.9	0	1.4	1.3	1.7	1.5
																	LQQQQRPEDAEDGAEGGGK	3	2	1	39995082	N-Term(TMT6plex); K19(TMT6plex)	2471.3	0	1.6	1.5	2.0	1.8
																	GEAGWEGGYPEIVK	2	2	1	39995082	N-Term(TMT6plex); K14(TMT6plex)	1950.0	0	1.7	1.8	1.9	2.0
																	RGEAGWEGGYPEIVK	3	2	1	39995082	N-Term(TMT6plex); K15(TMT6plex)	2106.1	1	1.4	1.4	1.4	1.4
																	FHQFLVSETESGNISR	11	2	1	39995082	N-Term(TMT6plex)	2080.1	0	1.3	1.3	1.5	1.3
																	AGEPNSPDAEEANSPDVTAGCDPAGVHPPR	4	2	1	39995082	N-Term(TMT6plex); C21(Carbamidomethyl)	3243.5	0	1.7	2.8	2.0	2.1
																	YEPDSANPDALQCPIVLCGWR	8	2	1	39995082	N-Term(TMT6plex); C13(Carbamidomethyl); C18(Carbamidomethyl)	2690.3	0	1.5	1.6	1.7	1.7
																	LQIDVDGR	4	2	1	39995082	N-Term(TMT6plex)	1144.7	0	1.4	1.5	1.7	1.7
																	DGQWFTDWDAVPHSR	6	2	1	39995082	N-Term(TMT6plex)	2046.0	0	1.5	1.6	1.5	1.4
																	ILCDVPCSGDGTMR	8	2	1	39995082	N-Term(TMT6plex); C3(Carbamidomethyl); C7(Carbamidomethyl); M13(Oxidation)	1825.8	0	1.5	1.8	1.8	1.8
																	GAEQLAEGGR	6	2	1	39995082	N-Term(TMT6plex)	1216.6	0	1.5	1.7	1.8	1.9
																	IVPEGEWGQFMDALR	6	2	1	39995082	N-Term(TMT6plex)	1977.0	0	1.8	2.1	2.3	1.9
																	DGVCGPPPSK	2	2	1	39995082	N-Term(TMT6plex); C4(Carbamidomethyl); K10(TMT6plex)	1471.8	0	1.3	1.3	1.7	1.5
																	ILDMCAAPGSK	4	2	1	39995082	N-Term(TMT6plex); M4(Oxidation); C5(Carbamidomethyl); K11(TMT6plex)	1636.9	0	1.5	1.5	1.5	1.7
																	VEVPQPLSWYPEELAWHTNLSR	4	1	1	39995082	N-Term(TMT6plex)	2880.5	0	1.2	1.2	1.1	1.2
																	NVLLNNSEK	4	2	1	39995082	N-Term(TMT6plex); K9(TMT6plex)	1488.9	0	1.7	1.3	1.8	1.6
																	ILDMCAAPGSK	2	2	1	39995082	N-Term(TMT6plex); C5(Carbamidomethyl); K11(TMT6plex)	1620.9	0	1.6	1.8	2.0	1.5
																	EILHCLK	2	1	1	39995082	N-Term(TMT6plex); C5(Carbamidomethyl); K7(TMT6plex)	1370.8	0	1.4	1.8	2.0	1.8
																	ELEDLEVDGQKVEVPQPLSWYPEELAWHTNLSR	2	1	1	39995082	N-Term(TMT6plex); K11(TMT6plex)	4365.2	1	1.6	1.6	1.3	1.7
																	EPLPATLR	2	2	1	39995082	N-Term(TMT6plex)	1125.7	0	1.4	1.6	1.7	1.7
																	EILFYDR	3	2	1	39995082	N-Term(TMT6plex)	1184.7	0	1.5	1.7	1.7	1.9
																	TTQLIEMLHADMNVPFPEGFVIANDVDNKR	1	2	1	39995082	N-Term(TMT6plex); M7(Oxidation); M12(Oxidation); K29(TMT6plex)	3905.0	1	1.4	1.1	1.5	1.2
																	WTTLNSLQLHGLQLR	1	2	1	39995082	N-Term(TMT6plex)	2009.1	0	1.1	1.2	1.4	1.3
																	WMPGITQWK	3	2	1	39995082	N-Term(TMT6plex); M2(Oxidation); K9(TMT6plex)	1620.9	0	1.3	1.4	1.4	1.5
																	ILPHHQNTGGFFVAVLVK	1	2	1	39995082	N-Term(TMT6plex); K18(TMT6plex)	2435.4	0	1.4	1.2	1.6	1.2
																	LSSPCIMVVNHDASSIPR	1	2	1	39995082	N-Term(TMT6plex); C5(Carbamidomethyl); M7(Oxidation)	2228.1	0	1.1	1.3	1.3	1.4
4505087	NP_002361.1	MAGOH	4116	protein mago nashi homolog	71.23	2	10	10	86	1.2	1.2	1.3	1.3	146	17.2	6.11												
																	IGSLIDVNQSKDPEGLR	1	2	1	4505087	N-Term(TMT6plex); K11(TMT6plex)	2299.3	1	1.2	1.2	1.2	1.3
																	IGSLIDVNQSK	16	2	1	4505087	N-Term(TMT6plex); K11(TMT6plex)	1632.0	0	1.3	1.1	1.3	1.2
																	FGHEFLEFEFRPDGK	8	2	1	4505087	N-Term(TMT6plex); K15(TMT6plex)	2313.2	0	1.1	1.2	1.4	1.3
																	QELEIVIGDEHISFTTSK	13	2	1	4505087	N-Term(TMT6plex); K18(TMT6plex)	2504.4	0	1.2	1.4	1.4	1.4
																	VFYYLVQDLK	24	2	1	4505087	N-Term(TMT6plex); K10(TMT6plex)	1746.0	0	1.2	1.1	1.3	1.2
																	CLVFSLIGLHFK	4	2	1	4505087	N-Term(TMT6plex); C1(Carbamidomethyl); K12(TMT6plex)	1892.1	0	1.4	1.1	1.7	1.1
																	IIDDSEITKEDDALWPPPDR	14	2	1	4505087	N-Term(TMT6plex); K9(TMT6plex)	2783.4	1	1.3	1.4	1.4	1.4
																	IIDDSEITK	3	2	1	4505087	N-Term(TMT6plex); K9(TMT6plex)	1491.9	0	1.4	1.3	1.3	1.3
																	YYVGHK	2	2	1	4505087	N-Term(TMT6plex); K6(TMT6plex)	1224.7	0				
																	EAYVHK	1	2	1	4505087	N-Term(TMT6plex); K6(TMT6plex)	1204.7	0				
294459921;208022622	NP_001170884.1	RPS27A	6233	ubiquitin-40S ribosomal protein S27a precursor	71.15	3	4	16	603	1.4	1.4	1.4	1.3	156	18.0	9.64												
																	TITLEVEPSDTIENVK	171	6	3	294459921;208022622;601984520;4507761;77539055	N-Term(TMT6plex); K16(TMT6plex)	2246.3	0				
																	ECPSDECGAGVFMASHFDR	23	1	1	294459921;208022622	N-Term(TMT6plex); C2(Carbamidomethyl); C7(Carbamidomethyl); M13(Oxidation)	2417.0	0	1.4	1.5	1.4	1.4
																	EGIPPDQQR	118	5	3	294459921;208022622;601984520;4507761;77539055	N-Term(TMT6plex)	1268.7	0				
																	IQDKEGIPPDQQR	47	4	3	294459921;208022622;601984520;4507761;77539055	N-Term(TMT6plex); K4(TMT6plex)	1982.1	1				
																	LIFAGKQLEDGR	18	4	3	294459921;208022622;601984520;4507761;77539055	N-Term(TMT6plex); K6(TMT6plex)	1805.1	1				
																	ECPSDECGAGVFMASHFDR	1	1	1	294459921;208022622	N-Term(TMT6plex); C2(Carbamidomethyl); C7(Carbamidomethyl)	2401.0	0	1.9	1.9	1.5	2.0
																	ESTLHLVLR	90	5	3	294459921;208022622;601984520;4507761;77539055	N-Term(TMT6plex)	1296.8	0				
																	TLSDYNIQK	83	5	3	294459921;208022622;601984520;4507761;77539055	N-Term(TMT6plex); K9(TMT6plex)	1539.9	0				
																	RECPSDECGAGVFMASHFDR	4	1	1	294459921;208022622	N-Term(TMT6plex); C3(Carbamidomethyl); C8(Carbamidomethyl); M14(Oxidation)	2573.1	1	1.3	1.6	1.5	1.4
																	TLSDYNIQKESTLHLVLR	7	4	3	294459921;208022622;601984520;4507761;77539055	N-Term(TMT6plex); K9(TMT6plex)	2588.5	1				
																	CCLTYCFNKPEDK	5	1	1	294459921;208022622	N-Term(TMT6plex); C1(Carbamidomethyl); C2(Carbamidomethyl); C6(Carbamidomethyl); K9(TMT6plex); K13(TMT6plex)	2422.2	0	1.2	1.2	1.3	1.0
																	MQIFVKTLTGK	1	4	3	294459921;208022622;601984520;4507761;77539055	N-Term(TMT6plex); K6(TMT6plex); K11(TMT6plex)	1953.2	1				
																	MQIFVK	15	4	3	294459921;208022622;601984520;4507761;77539055	N-Term(TMT6plex); K6(TMT6plex)	1223.8	0				
																	LIFAGK	13	5	3	294459921;208022622;601984520;4507761;77539055	N-Term(TMT6plex); K6(TMT6plex)	1106.7	0				
																	AKIQDKEGIPPDQQR	1	4	3	294459921;208022622;601984520;4507761;77539055	N-Term(TMT6plex); K2(TMT6plex); K6(TMT6plex)	2410.4	2				
																	QLEDGR	3	4	3	294459921;208022622;601984520;4507761;77539055	N-Term(TMT6plex)	946.5	0				
																	SYTTPK	1	1	1	294459921;208022622	N-Term(TMT6plex); K6(TMT6plex)	1154.7	0	1.1	1.1	1.2	1.1
																	MQIFVK	2	4	3	294459921;208022622;601984520;4507761;77539055	N-Term(TMT6plex); M1(Oxidation); K6(TMT6plex)	1239.8	0				
50345984;4757810	NP_001001937.1	ATP5A1	498	ATP synthase subunit alpha, mitochondrial isoform a precursor	71.07	3	34	35	848	1.4	1.5	1.6	1.5	553	59.7	9.13												
																	GMSLNLEPDNVGVVVFGNDK	57	3	1	50345984;4757810	N-Term(TMT6plex); M2(Oxidation); K20(TMT6plex)	2578.4	0	1.5	1.7	1.7	1.6
																	EVAAFAQFGSDLDAATQQLLSR	135	3	1	50345984;4757810	N-Term(TMT6plex)	2567.3	0	2.1	1.6	1.6	1.6
																	TSIAIDTIINQK	44	3	1	50345984;4757810	N-Term(TMT6plex); K12(TMT6plex)	1775.1	0	1.3	1.3	1.6	1.4
																	STVAQLVK	18	3	1	50345984;4757810	N-Term(TMT6plex); K8(TMT6plex)	1303.8	0	1.2	1.3	1.5	1.4

Khan et al., 2019. Multi-omics analysis to characterize cigarette smoke induced molecular alterations in esophageal cells
Supplementary Table 6. List of all proteins and peptides identified in untreated and chronically treated Het1A cells with cigarette smoke condensate for 8 months

Accession	RefSeq Accession	Gene Symbol	Entrez Gene ID	Description	Σ Coverage	Σ# Proteins	Σ# Unique Peptides	Σ# Peptides	Σ# PSMs	Het-1A-Smoke - 2M/Parental	Het-1A-Smoke - 4M/Parental	Het-1A-Smoke - 6M/Parental	Het-1A-Smoke - 8M/Parental	# AAs	MW [kDa]	calc. pI	Sequence	# PSMs	# Proteins	# Protein Groups	Protein Group Accessions	Modifications	MH+ [Da]	# Missed Cleavages	Het-1A-Smoke - 2M/Parental	Het-1A-Smoke - 4M/Parental	Het-1A-Smoke - 6M/Parental	Het-1A-Smoke - 8M/Parental
																	YTIVVSATASDAAPLQYLAPYSGCSMGEYFR	5	3	1	50345984;4757810	N-Term(TMT6plex); C24(Carbamidomethyl); M26(Oxidation)	3633.8	0	1.8	1.8	1.6	1.8
																	GYLDKLEPSK	2	3	1	50345984;4757810	N-Term(TMT6plex); K5(TMT6plex); K10(TMT6plex)	1837.1	1	1.4	2.2	1.8	2.2
																	ILGADTSVDLEETGR	53	3	1	50345984;4757810	N-Term(TMT6plex)	1804.9	0	1.5	1.5	1.6	1.6
																	EAYPGDVFYLHSR	20	3	1	50345984;4757810	N-Term(TMT6plex)	1782.9	0	1.4	1.5	1.6	1.5
																	TGAIVDVPVGEELLGR	46	2	1	50345984;4757810	N-Term(TMT6plex)	1854.0	0	1.5	1.3	1.5	1.3
																	VLSIGDGIAR	17	3	1	50345984;4757810	N-Term(TMT6plex)	1229.7	0	1.7	1.4	1.5	1.5
																	AVDSLVPIGR	21	3	1	50345984;4757810	N-Term(TMT6plex)	1255.8	0	1.3	1.4	1.6	1.5
																	TGTAEMSSILEER	40	2	1	50345984;4757810	N-Term(TMT6plex); M6(Oxidation)	1668.8	0	1.9	1.3	1.6	1.3
																	EIVTNFLAGFEA	55	3	1	50345984;4757810	N-Term(TMT6plex)	1539.8	0	1.6	1.6	1.7	1.5
																	TGTAEMSSILEER	2	2	1	50345984;4757810	N-Term(TMT6plex)	1652.8	0	2.1	1.6	2.2	1.4
																	VVDALGNAIDGK	30	2	1	50345984;4757810	N-Term(TMT6plex); K12(TMT6plex)	1630.0	0	1.3	1.5	1.5	1.5
																	FENAFLSHVVSQHQALLGTIR	35	3	1	50345984;4757810	N-Term(TMT6plex)	2596.4	0	1.4	1.3	1.5	1.3
																	NVQAEEMVEFSSGLK	28	3	1	50345984;4757810	N-Term(TMT6plex); K15(TMT6plex)	2126.1	0	1.5	1.6	1.8	1.4
																	QGQYSPMAIEEQVAVIYAGVR	36	3	1	50345984;4757810	N-Term(TMT6plex); M7(Oxidation)	2554.3	0	1.5	1.4	1.4	1.5
																	LKEIVTNFLAGFEA	12	3	1	50345984;4757810	N-Term(TMT6plex); K2(TMT6plex)	2010.2	1	1.4	1.5	1.7	1.4
																	HALIIYDDLSK	26	3	1	50345984;4757810	N-Term(TMT6plex); K11(TMT6plex)	1746.0	0	1.3	1.4	1.5	1.5
																	LTDADAMK	26	3	1	50345984;4757810	N-Term(TMT6plex); M7(Oxidation); K8(TMT6plex)	1338.7	0	1.3	1.4	1.4	1.5
																	FNDGSDEK	17	3	1	50345984;4757810	N-Term(TMT6plex); K8(TMT6plex)	1369.7	0	1.7	1.4	1.6	1.5
																	GMSLNLEPDNVGVVVFGNDK	14	3	1	50345984;4757810	N-Term(TMT6plex); K20(TMT6plex)	2562.4	0	1.7	2.1	2.3	1.6
																	NVQAEEMVEFSSGLK	29	3	1	50345984;4757810	N-Term(TMT6plex); M7(Oxidation); K15(TMT6plex)	2142.1	0	1.4	1.3	1.5	1.5
																	ISEQSDAK	13	3	1	50345984;4757810	N-Term(TMT6plex); K8(TMT6plex)	1335.8	0	1.3	1.4	1.4	1.4
																	QMSLLLR	2	3	1	50345984;4757810	N-Term(TMT6plex)	1089.7	0	1.6	1.4	2.0	1.3
																	QGQYSPMAIEEQVAVIYAGVR	4	3	1	50345984;4757810	N-Term(TMT6plex)	2538.3	0	1.7	1.6	2.0	1.5
																	EPMQTGIK	2	3	1	50345984;4757810	N-Term(TMT6plex); K8(TMT6plex)	1361.8	0	2.2	2.7	3.1	1.7
																	LYCIYVAIGQK	8	3	1	50345984;4757810	N-Term(TMT6plex); C3(Carbamidomethyl); K11(TMT6plex)	1786.0	0	1.4	1.5	1.8	1.4
																	EPMQTGIK	9	3	1	50345984;4757810	N-Term(TMT6plex); M3(Oxidation); K8(TMT6plex)	1377.8	0	1.5	1.7	1.9	1.6
																	ELIIGDR	11	3	1	50345984;4757810	N-Term(TMT6plex)	1044.6	0	1.4	1.7	1.7	1.8
																	LELAQYR	10	3	1	50345984;4757810	N-Term(TMT6plex)	1121.7	0	1.5	1.4	1.5	1.5
																	VGSAAQTR	2	3	1	50345984;4757810	N-Term(TMT6plex)	1018.6	0	1.6	1.2	1.5	1.3
																	KLYCIYVAIGQK	1	3	1	50345984;4757810	N-Term(TMT6plex); K1(TMT6plex); C4(Carbamidomethyl); K12(TMT6plex)	2143.3	1	1.4	1.5	1.6	1.2
																	QVAGTMK	3	3	1	50345984;4757810	N-Term(TMT6plex); M6(Oxidation); K7(TMT6plex)	1208.7	0	1.5	1.4	1.6	1.5
																	APGIIPR	3	3	1	50345984;4757810	N-Term(TMT6plex)	952.6	0	1.4	1.6	1.6	1.6
																	GIRPAINVGLSVSR	1	3	1	50345984;4757810	N-Term(TMT6plex)	1668.0	0	1.2	1.3	1.4	1.2
																	QAVAYR	2	3	1	50345984;4757810	N-Term(TMT6plex)	936.5	0	1.6	1.4	1.5	1.4
																	EGDIVK	3	4	2	50345984;4757810;124494254	N-Term(TMT6plex); K6(TMT6plex)	1118.7	0				
																	LTELLK	6	3	1	50345984;4757810	N-Term(TMT6plex); K6(TMT6plex)	1174.8	0	1.4	1.4	1.6	1.4
5803227	NP_006817.1	**YWHAQ**	10971	**14-3-3 protein theta**	71.02	1	13	19	939	**1.3**	**1.3**	**1.3**	**1.1**	245	27.7	4.78												
																	TAFDEAIAELDTLNEDSYK	95	1	1	5803227	N-Term(TMT6plex); K19(TMT6plex)	2603.3	0	1.4	1.2	1.5	1.1
																	DSTLIMQLLR	298	7	7	21735625;208973244;4507951;5454052;5803227;21464101;4507949;5803225	N-Term(TMT6plex); M6(Oxidation)	1434.8	0				
																	KEMQPTHPIR	12	3	3	21735625;208973244;5803227;4507949	N-Term(TMT6plex); K1(TMT6plex); M3(Oxidation)	1711.0	1				
																	EMQPTHPIR	15	3	3	21735625;208973244;5803227;4507949	N-Term(TMT6plex); M2(Oxidation)	1353.7	0				
																	NLLSVAYK	199	7	7	21735625;208973244;4507951;5454052;5803227;21464101;4507949;5803225	N-Term(TMT6plex); K8(TMT6plex)	1365.9	0				
																	LAEQAER	18	7	7	21735625;208973244;4507951;5454052;5803227;21464101;4507949;5803225	N-Term(TMT6plex)	1045.6	0				
																	AVTEQGAELSNEER	20	1	1	5803227	N-Term(TMT6plex)	1761.9	0	1.2	1.3	1.3	1.2
																	YLAEVACGDDRK	3	1	1	5803227	N-Term(TMT6plex); C7(Carbamidomethyl); K12(TMT6plex)	1855.0	1	1.2	1.3	1.4	1.2
																	DSTLIMQLLR	41	7	7	21735625;208973244;4507951;5454052;5803227;21464101;4507949;5803225	N-Term(TMT6plex)	1418.8	0				
																	SICTTVLELLDK	41	1	1	5803227	N-Term(TMT6plex); C3(Carbamidomethyl); K12(TMT6plex)	1850.1	0	1.3	1.3	1.2	1.1
																	VISSIEQK	57	5	5	4507951;5803227;21464101;4507949;5454052	N-Term(TMT6plex); K8(TMT6plex)	1361.8	0				
																	DNLTLWTSDSAGEECDAAEGAEN	34	1	1	5803227	N-Term(TMT6plex); C15(Carbamidomethyl)	2684.1	0	1.4	1.3	1.3	1.3
																	QTIDNSQGAYQEAFDISK	28	1	1	5803227	N-Term(TMT6plex); K18(TMT6plex)	2473.3	0	1.2	1.3	1.3	1.1
																	YLIANATNPESK	18	1	1	5803227	N-Term(TMT6plex); K12(TMT6plex)	1779.0	0	1.2	1.2	1.3	1.5
																	TAFDEAIAELDTLNEDSYKDSTLIMQLLR	5	1	1	5803227	N-Term(TMT6plex); K19(TMT6plex); M25(Oxidation)	3790.0	1	1.6	1.7	1.4	1.8
																	KQTIDNSQGAYQEAFDISK	12	1	1	5803227	N-Term(TMT6plex); K1(TMT6plex); K19(TMT6plex)	2830.5	1	1.2	1.4	1.3	1.4
																	YDDMATCMK	10	1	1	5803227	N-Term(TMT6plex); M4(Oxidation); C7(Carbamidomethyl); M8(Oxidation); K9(TMT6plex)	1624.7	0	1.2	1.3	1.4	1.0
																	YDDMATCMK	5	1	1	5803227	N-Term(TMT6plex); C7(Carbamidomethyl); M8(Oxidation); K9(TMT6plex)	1608.7	0	1.6	1.9	2.3	1.1
																	YLAEVACGDDR	13	1	1	5803227	N-Term(TMT6plex); C7(Carbamidomethyl)	1497.7	0	1.2	1.2	1.3	1.1
																	EMQPTHPIR	2	3	3	21735625;208973244;5803227;4507949	N-Term(TMT6plex)	1337.7	0				
																	YDDMATCMK	2	1	1	5803227	N-Term(TMT6plex); C7(Carbamidomethyl); K9(TMT6plex)	1592.8	0	2.3	2.1	2.9	1.2
																	TELIQK	7	1	1	5803227	N-Term(TMT6plex); K6(TMT6plex)	1189.8	0	1.1	1.0	1.2	0.9
																	KLQLIK	4	1	1	5803227	N-Term(TMT6plex); K1(TMT6plex); K6(TMT6plex)	1430.0	1	1.0	1.1	1.2	1.0
4506467	NP_002897.1	**RDX**	5962	**radixin isoform 2**	71.01	5	28	48	613	**1.3**	**1.5**	**1.5**	**1.2**	583	68.5	6.37												
																	FFPEDVSEELIQEITQR	107	3	1	4506467	N-Term(TMT6plex)	2309.2	0	1.5	1.5	1.6	1.4
																	NQEQLAAELAEFTAK	33	4	1	4506467	N-Term(TMT6plex); K15(TMT6plex)	2121.2	0	1.3	1.2	1.4	1.1
																	VTTMDAELEFAIQPNTTGK	41	5	3	21614499;4505257;4506467	N-Term(TMT6plex); M4(Oxidation); K19(TMT6plex)	2540.3	0				
																	QLFDQVVK	26	4	3	21614499;4505257;4506467	N-Term(TMT6plex); K8(TMT6plex)	1434.9	0				
																	IDEFEAM	7	2	1	4506467	N-Term(TMT6plex); M7(Oxidation)	1099.5	0	1.3	1.4	1.5	1.4
																	ALELDQER	5	4	1	4506467	N-Term(TMT6plex)	1202.7	0	1.3	1.5	1.4	1.2
																	IAQDLEMYGVNYFEIK	35	3	1	4506467	N-Term(TMT6plex); M7(Oxidation); K16(TMT6plex)	2407.3	0	1.4	1.5	1.5	1.3
																	IGFPWSEIR	31	5	3	21614499;4505257;4506467	N-Term(TMT6plex)	1333.7	0				
																	AFAAQEDLEK	16	4	1	4506467	N-Term(TMT6plex); K10(TMT6plex)	1579.9	0	1.7	1.6	1.5	1.4
																	APDFVFYAPR	36	5	3	21614499;4505257;4506467	N-Term(TMT6plex)	1411.8	0				
																	KPDTIEVQQMK	2	5	3	21614499;4505257;4506467	N-Term(TMT6plex); K1(TMT6plex); K11(TMT6plex)	2004.2	0				
																	KPDTIEVQQMK	8	5	3	21614499;4505257;4506467	N-Term(TMT6plex); K1(TMT6plex); M10(Oxidation); K11(TMT6plex)	2020.2	0				
																	RKPDTIEVQQMK	11	5	3	21614499;4505257;4506467	N-Term(TMT6plex); K2(TMT6plex); M11(Oxidation); K12(TMT6plex)	2176.3	1				
																	RKPDTIEVQQMK	2	5	3	21614499;4505257;4506467	N-Term(TMT6plex); K2(TMT6plex); K12(TMT6plex)	2160.3	1				
																	QLQALSSELAQAR	10	4	1	4506467	N-Term(TMT6plex)	1643.9	0	1.3	1.2	1.3	1.2
																	EVWFFGLQYVDSK	37	2	1	4506467	N-Term(TMT6plex); K13(TMT6plex)	2076.1	0	1.4	1.7	1.7	1.3
																	QIEEQTIK	11	4	1	4506467	N-Term(TMT6plex); K8(TMT6plex)	1446.9	0	1.4	1.5	1.4	1.4
																	ILALCMGNHELYMR	5	4	2	4505257;4506467	N-Term(TMT6plex); C5(Carbamidomethyl); M6(Oxidation); M13(Oxidation)	1982.0	0				
																	KALELDQER	2	4	1	4506467	N-Term(TMT6plex); K1(TMT6plex)	1559.9	1	1.3	1.8	1.6	1.2
																	KAPDFVFYAPR	9	5	3	21614499;4505257;4506467	N-Term(TMT6plex); K1(TMT6plex)	1769.0	1				
																	KENPLQFK	9	4	2	21614499;4506467	N-Term(TMT6plex); K1(TMT6plex); K8(TMT6plex)	1691.0	1				
																	FVIKPIDK	10	5	3	21614499;4505257;4506467	N-Term(TMT6plex); K4(TMT6plex); K8(TMT6plex)	1647.1	0				
																	EIHKPGYLANDR	3	3	1	4506467	N-Term(TMT6plex); K4(TMT6plex)	1871.1	0	1.1	1.3	1.3	1.3
																	LFFLQVK	45	5	3	21614499;4505257;4506467	N-Term(TMT6plex); K7(TMT6plex)	1352.9	0				
																	EEEATEWQHK	4	4	1	4506467	N-Term(TMT6plex); K10(TMT6plex)	1744.9	0	1.3	1.5	1.7	1.4
																	ILALCMGNHELYMR	2	4	2	4505257;4506467	N-Term(TMT6plex); C5(Carbamidomethyl); M13(Oxidation)	1966.0	0				
																	GTELWLGVDALGLNIYEHDDKLTPK	10	3	1	4506467	N-Term(TMT6plex); K21(TMT6plex); K25(TMT6plex)	3484.9	1	1.1	1.8	1.6	1.2
																	ILALCMGNHELYMR	10	4	2	4505257;4506467	N-Term(TMT6plex); C5(Carbamidomethyl)	1950.0	0				
																	GTELWLGVDALGLNIYEHDDK	2	3	1	4506467	N-Term(TMT6plex); K21(TMT6plex)	2816.5	0	1.0	1.5	1.4	1.2
																	ENPLQFK	8	4	2	21614499;4506467	N-Term(TMT6plex); K7(TMT6plex)	1333.8	0				
																	KKEEEATEWQHK	1	4	1	4506467	N-Term(TMT6plex); K1(TMT6plex); K2(TMT6plex); K12(TMT6plex)	2459.4	2	1.6	1.7	1.6	1.3
																	KVTQQDVK	3	2	1	4506467	N-Term(TMT6plex); K1(TMT6plex); K8(TMT6plex)	1633.0	1	1.1	1.5	1.5	1.4
																	KTQNDVLHAENVK	1	5	1	4506467	N-Term(TMT6plex); K1(TMT6plex); K13(TMT6plex)	2183.3	1	1.2	1.5	1.4	1.4
																	IALLEEAK	15	4	1	4506467	N-Term(TMT6plex); K8(TMT6plex)	1344.9	0	1.3	1.5	1.5	1.2
																	TVMSAPPPPPPPPVIPPTENEHDEHDENNAEASAELSNEGVMNHR	2	4	1	4506467	N-Term(TMT6plex); M3(Oxidation); M42(Oxidation)	5111.4	0	1.3	1.2	1.1	0.9
																	VTTMDAELEFAIQPNTTGK	3	5	3	21614499;4505257;4506467	N-Term(TMT6plex); K19(TMT6plex)	2524.3	0				
																	ELEEQTR	4	4	1	4506467	N-Term(TMT6plex)	1133.6	0	1.2	1.4	1.3	1.2
																	IQNWHEEHR	4	3	1	4506467	N-Term(TMT6plex)	1477.8	0	1.1	1.3	1.3	1.2
																	EDSMMEYLK	3	3	1	4506467	N-Term(TMT6plex); M4(Oxidation); M5(Oxidation); K9(TMT6plex)	1635.8	0	1.1	1.2	1.1	1.2
																	FVIKPIDKK	3	5	3	21614499;4505257;4506467	N-Term(TMT6plex); K4(TMT6plex); K8(TMT6plex); K9(TMT6plex)	2004.3	1				
																	AQLENEK	7	5	2	4506467;513788264	N-Term(TMT6plex); K7(TMT6plex)	1289.7	0				
																	KQLQALSSELAQAR	1	4	1	4506467	N-Term(TMT6plex); K1(TMT6plex)	2001.2	1	1.3	1.5	1.3	1.4
																	KFVIKPIDK	3	5	3	21614499;4505257;4506467	N-Term(TMT6plex); K1(TMT6plex); K5(TMT6plex); K9(TMT6plex)	2004.3	1				
																	VLEQHK	4	4	2	4505257;4506467	N-Term(TMT6plex); K6(TMT6plex)	1211.8	0				
																	NISFNDK	7	5	3	21614499;4505257;4506467	N-Term(TMT6plex); K7(TMT6plex)	1295.7	0				
																	VTQQDVK	2	2	1	4506467	N-Term(TMT6plex); K7(TMT6plex)	1275.8	0	1.3	1.4	1.5	1.1
																	GYSTWLK	1	2	1	4506467	N-Term(TMT6plex); K7(TMT6plex)	1312.8	0	1.3	1.4	1.4	1.3

Accession	RefSeq Accession	Gene Symbol	Entrez Gene ID	Description	Σ Coverage	Σ# Proteins	Σ# Unique Peptides	Σ# Peptides	Σ# PSMs	Het-1A-Smoke - 2M/Parental	Het-1A-Smoke - 4M/Parental	Het-1A-Smoke - 6M/Parental	Het-1A-Smoke - 8M/Parental	# AAs	MW [kDa]	calc. pI	Sequence	# PSMs	# Proteins	# Protein Groups	Protein Group Accessions	Modifications	MH+ [Da]	# Missed Cleavages	Het-1A-Smoke - 2M/Parental	Het-1A-Smoke - 4M/Parental	Het-1A-Smoke - 6M/Parental	Het-1A-Smoke - 8M/Parental
																	YGDYNK	2	3	1	4506467	N-Term(TMT6plex); K6(TMT6plex)	1217.7	0	1.5	1.5	1.6	1.1
																	IDEFEAM	1	2	1	4506467	N-Term(TMT6plex)	1083.5	0	1.4	1.4	1.5	1.3
																	VTETQK	2	4	1	4506467	N-Term(TMT6plex); K6(TMT6plex)	1163.7	0	1.2	1.3	1.4	1.0
																	QAADQMK	2	4	1	4506467	N-Term(TMT6plex); M6(Oxidation); K7(TMT6plex)	1265.7	0				
																	AAEEAK	2	6	2	4506467;17402893	N-Term(TMT6plex); K6(TMT6plex)	1076.6	0				
																	EKEELMER	1	4	2	4505257;4506467	N-Term(TMT6plex); K2(TMT6plex); M6(Oxidation)	1537.8	1				
																	EKEELMER	1	4	2	4505257;4506467	N-Term(TMT6plex); K2(TMT6plex)	1521.8	1				
																	NISFNDKK	1	5	3	21614499;4505257;4506467	N-Term(TMT6plex); K7(TMT6plex); K8(TMT6plex)	1653.0	1				
24234688	NP_004125.3	**HSPA9**	3313	**stress-70 protein, mitochondrial precursor**	70.99	1	45	46	867	**1.5**	**1.5**	**1.4**	**1.6**	679	73.6	6.16												
																	EQQIVIQSSGGLSK	30	1	1	24234688	N-Term(TMT6plex); K14(TMT6plex)	1932.1	0	1.4	1.5	1.4	1.5
																	SDIGEVILVGGMTR	47	1	1	24234688	N-Term(TMT6plex); M12(Oxidation)	1691.9	0	1.5	1.5	1.5	1.5
																	DAGQISGLNVLR	32	1	1	24234688	N-Term(TMT6plex)	1471.8	0	1.4	1.5	1.4	1.5
																	LLGQFTLIGIPPAPR	74	1	1	24234688	N-Term(TMT6plex)	1822.1	0	1.6	1.5	1.5	1.5
																	YDDPEVQK	16	1	1	24234688	N-Term(TMT6plex); K8(TMT6plex)	1451.8	0	1.5	1.7	1.5	1.7
																	MEEFKDQLPADECNK	15	1	1	24234688	M1(Oxidation); N-Term(TMT6plex); K5(TMT6plex); C13(Carbamidomethyl); K15(TMT6plex)	2557.3	1	2.1	1.7	1.7	1.6
																	EQQIVIQSSGGLSKDDIENMVK	9	1	1	24234688	N-Term(TMT6plex); K14(TMT6plex); M20(Oxidation); K22(TMT6plex)	3121.7	1	1.2	1.6	1.3	1.7
																	EGSGSSGTGEQKEDQK	4	1	1	24234688	N-Term(TMT6plex); K12(TMT6plex); K16(TMT6plex)	2311.2	1	1.5	1.7	1.5	1.7
																	LKEEISK	8	1	1	24234688	N-Term(TMT6plex); K2(TMT6plex); K7(TMT6plex)	1534.0	1	1.4	1.7	1.5	1.7
																	EGSGSSGTGEQK	24	1	1	24234688	N-Term(TMT6plex); K12(TMT6plex)	1581.8	0	1.7	1.5	1.5	1.6
																	VQQTVQDLFGR	43	1	1	24234688	N-Term(TMT6plex)	1519.8	0	1.7	1.5	1.5	1.5
																	VEAVNMAEGIIHDTETK	11	1	1	24234688	N-Term(TMT6plex); K17(TMT6plex)	2315.2	0	1.7	1.8	1.9	1.7
																	TTPSVVAFTADGER	36	1	1	24234688	N-Term(TMT6plex)	1679.9	0	1.4	1.5	1.4	1.4
																	VEAVNMAEGIIHDTETK	33	1	1	24234688	N-Term(TMT6plex); M6(Oxidation); K17(TMT6plex)	2331.2	0	1.5	1.6	1.5	1.7
																	SQVFSTAADGQTQVEIK	66	1	1	24234688	N-Term(TMT6plex); K17(TMT6plex)	2267.2	0	1.5	1.4	1.4	1.5
																	STNGDTFLGGEDFDQALLR	11	1	1	24234688	N-Term(TMT6plex)	2285.1	0	1.7	1.4	1.4	1.5
																	VLENAEGAR	14	1	1	24234688	N-Term(TMT6plex)	1187.7	0	1.5	1.5	1.5	1.6
																	AMQDAEVSK	35	1	1	24234688	N-Term(TMT6plex); M2(Oxidation); K9(TMT6plex)	1452.8	0	1.7	1.8	1.7	1.7
																	LYSPSQIGAFVLMK	17	1	1	24234688	N-Term(TMT6plex); M13(Oxidation); K14(TMT6plex)	2028.2	0	1.3	1.8	1.5	1.8
																	EGSGSSGTGEQKEDQKEEK	4	1	1	24234688	N-Term(TMT6plex); K12(TMT6plex); K16(TMT6plex); K19(TMT6plex)	2926.5	2	1.6	1.8	1.6	1.9
																	VINEPTAAALAYGLDK	49	1	1	24234688	N-Term(TMT6plex); K16(TMT6plex)	2104.2	0	1.5	1.6	1.5	1.6
																	GVPQIEVTFDIDANGIVHVSAK	3	1	1	24234688	N-Term(TMT6plex); K22(TMT6plex)	2767.5	0	1.4	1.4	1.5	1.5
																	GAVVGIDLGTTNSCVAVMEGK	9	1	1	24234688	N-Term(TMT6plex); C14(Carbamidomethyl); M18(Oxidation); K21(TMT6plex)	2552.3	0	1.6	1.7	1.6	1.7
																	AQFEGIVTDLIR	45	1	1	24234688	N-Term(TMT6plex)	1590.9	0	1.5	1.6	1.5	1.7
																	VIAVYDLGGGTFDISILEIQK	13	1	1	24234688	N-Term(TMT6plex); K21(TMT6plex)	2709.5	0	1.4	1.5	1.5	1.8
																	SDIGEVILVGGMTR	4	1	1	24234688	N-Term(TMT6plex)	1675.9	0	1.6	1.8	1.8	1.6
																	DDIENMVK	25	1	1	24234688	N-Term(TMT6plex); M6(Oxidation); K8(TMT6plex)	1437.8	0	1.6	1.8	1.6	1.8
																	DDIENMVK	2	1	1	24234688	N-Term(TMT6plex); K8(TMT6plex)	1421.8	0	1.7	2.0	1.8	1.8
																	RYDDPEVQK	5	1	1	24234688	N-Term(TMT6plex); K9(TMT6plex)	1607.9	1	1.5	1.7	1.5	1.8
																	QAASSLQQASLK	18	1	1	24234688	N-Term(TMT6plex); K12(TMT6plex)	1690.0	0	1.5	1.4	1.4	1.4
																	NAVITVPAYFNDSQR	15	1	1	24234688	N-Term(TMT6plex)	1924.0	0	1.4	1.4	1.4	1.5
																	QAVTNPNNTFYATK	16	1	1	24234688	N-Term(TMT6plex); K14(TMT6plex)	2027.1	0	1.5	1.4	1.4	1.6
																	KSQVFSTAADGQTQVEIK	8	1	1	24234688	N-Term(TMT6plex); K1(TMT6plex); K18(TMT6plex)	2624.5	1	1.5	1.5	1.3	1.6
																	DQLPADECNK	14	1	1	24234688	N-Term(TMT6plex); C8(Carbamidomethyl); K10(TMT6plex)	1647.8	0	1.4	1.5	1.5	1.5
																	ETGVDLTK	21	1	1	24234688	N-Term(TMT6plex); K8(TMT6plex)	1320.8	0	1.3	1.4	1.4	1.5
																	GAVVGIDLGTTNSCVAVMEGK	7	1	1	24234688	N-Term(TMT6plex); C14(Carbamidomethyl); K21(TMT6plex)	2536.4	0	1.9	2.1	2.1	1.8
																	ETAENYLGHTAK	15	1	1	24234688	N-Term(TMT6plex); K12(TMT6plex)	1792.0	0	1.2	1.5	1.5	1.5
																	AMQDAEVSK	2	1	1	24234688	N-Term(TMT6plex); K9(TMT6plex)	1436.8	0	2.6	2.9	2.9	2.1
																	KDSETGENIR	4	1	1	24234688	N-Term(TMT6plex); K1(TMT6plex)	1606.9	1	1.5	1.5	1.4	1.7
																	ASNGDAWVEAHGK	2	1	1	24234688	N-Term(TMT6plex); K13(TMT6plex)	1799.9	0	1.5	1.4	1.5	1.4
																	CELSSSVQTDINLPYLTMDSSGPK	8	1	1	24234688	N-Term(TMT6plex); C1(Carbamidomethyl); M18(Oxidation); K24(TMT6plex)	3116.6	0	1.6	1.2	1.4	1.3
																	MEEFKDQLPADECNK	1	1	1	24234688	N-Term(TMT6plex); K5(TMT6plex); C13(Carbamidomethyl); K15(TMT6plex)	2541.3	1	1.7	2.0	1.9	1.7
																	VINEPTAAALAYGLDKSEDK	2	1	1	24234688	N-Term(TMT6plex); K16(TMT6plex); K20(TMT6plex)	2792.6	1	1.9	3.0	1.7	2.7
																	LFEMAYK	8	1	1	24234688	N-Term(TMT6plex); M4(Oxidation); K7(TMT6plex)	1375.8	0	1.5	1.5	1.3	1.7
																	NTTIPTK	18	4	3	13676857;5729877;24234688	N-Term(TMT6plex); K7(TMT6plex)	1232.8	0				
																	LVGMPAK	2	1	1	24234688	N-Term(TMT6plex); K7(TMT6plex)	1173.7	0	1.5	1.9	1.7	1.5
																	LFEMAYK	1	1	1	24234688	N-Term(TMT6plex); K7(TMT6plex)	1359.8	0	1.4	1.6	1.6	1.7
																	DNMALQR	2	1	1	24234688	N-Term(TMT6plex)	1076.6	0	1.8	2.3	2.3	1.9
																	LVGMPAK	1	1	1	24234688	N-Term(TMT6plex); M4(Oxidation); K7(TMT6plex)	1189.7	0	1.2	1.3	1.4	1.3
																	DNMALQR	1	1	1	24234688	N-Term(TMT6plex); M3(Oxidation)	1092.6	0	1.4	1.5	1.3	1.5
																	GVFEVK	8	1	1	24234688	N-Term(TMT6plex); K6(TMT6plex)	1136.7	0	1.3	1.6	1.4	1.6
																	TIAPCQK	3	1	1	24234688	N-Term(TMT6plex); C5(Carbamidomethyl); K7(TMT6plex)	1275.7	0	1.7	1.7	1.6	1.7
																	YAEEDR	2	1	1	24234688	N-Term(TMT6plex)	1011.5	0	1.6	1.6	1.6	1.7
																	EMAGDNK	2	1	1	24234688	N-Term(TMT6plex); M2(Oxidation); K7(TMT6plex)	1238.6	0	1.4	1.6	1.4	1.8
																	EDQKEEK	2	1	1	24234688	N-Term(TMT6plex); K4(TMT6plex); K7(TMT6plex)	1592.9	1	1.4	1.4	1.3	1.6
4507651	NP_003281.1	**TPM4**	7171	**tropomyosin alpha-4 chain isoform 2**	70.97	3	10	26	545	**1.3**	**1.4**	**1.6**	**1.2**	248	28.5	4.69												
																	IQLVEEELDR	65	22	9	63252898;63252900;499137518;42476296;4507651;114155144;63252904;114155140;47519616	N-Term(TMT6plex)	1472.8	0				
																	KLVILEGELER	30	6	3	42476296;4507651;47519616	N-Term(TMT6plex); K1(TMT6plex)	1757.1	1				
																	MEIQEMQLK	21	4	3	4507651;42476296;47519616	N-Term(TMT6plex); M1(Oxidation); M6(Oxidation); K9(TMT6plex)	1639.9	0				
																	IQALQQQADEAEDR	64	1	1	4507651	N-Term(TMT6plex)	1843.9	0	1.4	1.3	1.6	1.2
																	LVILEGELER	15	4	3	42476296;4507651;47519616	N-Term(TMT6plex)	1399.8	0				
																	SLEAASEK	31	2	1	4507651	N-Term(TMT6plex); K8(TMT6plex)	1292.7	0	1.4	1.4	1.7	1.1
																	AEVSELK	28	4	1	4507651	N-Term(TMT6plex); K7(TMT6plex)	1233.7	0	1.4	1.4	1.6	1.1
																	EENVGLHQTLDQTLNELNCI	42	2	1	4507651	N-Term(TMT6plex); C19(Carbamidomethyl)	2569.3	0	1.4	1.5	1.7	1.4
																	TIDDLEEK	34	2	1	4507651	N-Term(TMT6plex); K8(TMT6plex)	1420.8	0	1.3	1.3	1.6	1.1
																	EDKYEEEIK	21	12	6	499137518;42476296;4507651;114155144;114155140;47519616	N-Term(TMT6plex); K3(TMT6plex); K9(TMT6plex)	1870.0	1				
																	LATALQK	47	22	9	63252898;63252900;499137518;42476296;4507651;114155144;63252904;114155140;47519616	N-Term(TMT6plex); K7(TMT6plex)	1202.8	0				
																	AADESER	4	18	9	63252898;63252900;499137518;42476296;4507651;114155144;63252904;114155140;47519616	N-Term(TMT6plex)	1006.5	0				
																	CGDLEEELK	21	3	2	42476296;4507651	N-Term(TMT6plex); C1(Carbamidomethyl); K9(TMT6plex)	1550.8	0				
																	MEIQEMQLK	10	4	3	4507651;42476296;47519616	N-Term(TMT6plex); M1(Oxidation); K9(TMT6plex)	1623.9	0				
																	AEGDVAALNR	26	1	1	4507651	N-Term(TMT6plex)	1244.7	0	1.3	1.3	1.4	1.2
																	NVTNNLK	14	6	3	4507651;114155144;114155140	N-Term(TMT6plex); K7(TMT6plex)	1260.8	0				
																	KIQALQQQADEAEDR	24	1	1	4507651	N-Term(TMT6plex); K1(TMT6plex)	2201.2	1	1.2	1.3	1.5	1.2
																	EKAEGDVAALNR	9	1	1	4507651	N-Term(TMT6plex); K2(TMT6plex)	1731.0	1	1.3	1.5	1.6	1.2
																	YSEKEDKYEEEIK	5	2	1	4507651	N-Term(TMT6plex); K4(TMT6plex); K7(TMT6plex); K13(TMT6plex)	2606.4	2	1.2	1.3	1.7	1.0
																	HIAEEADR	6	11	4	499137518;4507651;114155144;114155140	N-Term(TMT6plex)	1169.6	0				
																	KYEEVAR	9	20	9	63252898;63252900;499137518;42476296;4507651;114155144;63252904;114155140;47519616	N-Term(TMT6plex); K1(TMT6plex)	1352.8	1				
																	AEFAER	7	24	9	63252898;63252900;499137518;42476296;4507651;114155144;63252904;114155140;47519616	N-Term(TMT6plex)	951.5	0				
																	LEEAEK	4	20	10	63252898;63252900;499137518;42476296;4507651;114155144;63252904;114155140;47519616;116235456;116235451	N-Term(TMT6plex); K6(TMT6plex)	1176.7	0				
																	YEEVAR	4	24	9	63252898;63252900;499137518;42476296;4507651;114155144;63252904;114155140;47519616	N-Term(TMT6plex)	995.5	0				
																	HIAEEADRK	1	11	4	499137518;4507651;114155144;114155140	N-Term(TMT6plex); K9(TMT6plex)	1526.9	1				
																	YEEEIK	2	19	9	63252898;63252900;499137518;42476296;4507651;114155144;63252904;114155140;47519616	N-Term(TMT6plex); K6(TMT6plex)	1268.7	0				
																	ELDGER	1	1	1	4507651	N-Term(TMT6plex)	947.5	0	1.2	1.3	1.5	1.1
30410792	NP_002809.2	**PSME2**	5721	**proteasome activator complex subunit 2**	70.71	1	13	13	125	**1.5**	**1.3**	**1.5**	**1.4**	239	27.4	5.73												
																	VEAFQTTISK	4	1	1	30410792	N-Term(TMT6plex); K10(TMT6plex)	1581.9	0	1.2	1.1	1.2	1.4
																	AMVLDLR	4	1	1	30410792	N-Term(TMT6plex)	1046.6	0	1.6	1.7	1.9	1.7
																	CGFLPGNEK	2	1	1	30410792	N-Term(TMT6plex); C1(Carbamidomethyl); K9(TMT6plex)	1479.8	0	1.5	1.1	1.3	1.2
																	DEAAYGELR	12	1	1	30410792	N-Term(TMT6plex)	1252.6	0	1.3	1.2	1.3	1.3
																	QNLFQEAEEFLYR	26	1	1	30410792	N-Term(TMT6plex)	1916.0	0	1.8	1.4	1.5	1.4
																	APLDIPIPDPPPKDDEMETDKQEK	3	1	1	30410792	N-Term(TMT6plex); K13(TMT6plex); M17(Oxidation); K21(TMT6plex); K24(TMT6plex)	3651.0	2	1.4	1.4	1.5	1.6
																	AFYAELYHIISSNLEK	25	1	1	30410792	N-Term(TMT6plex); K16(TMT6plex)	2356.3	0	1.5	1.5	1.7	1.5
																	IIYLNQLLQEDSLNVADLTSLR	22	1	1	30410792	N-Term(TMT6plex)	2760.5	0	1.4	1.2	1.7	1.3
																	CILVITWIQHLIPK	8	1	1	30410792	N-Term(TMT6plex); C1(Carbamidomethyl); K14(TMT6plex)	2192.3	0	2.0	1.5	2.4	0.9
																	IEDGNDFGVAIQEK	15	1	1	30410792	N-Term(TMT6plex); K14(TMT6plex)	1993.1	0	1.4	1.3	1.4	1.3

Supplementary Table 6. List of all proteins and peptides identified in untreated and chronically treated Het1A cells with cigarette smoke condensate for 8 months

Accession	RefSeq Accession	Gene Symbol	Entrez Gene ID	Description	Σ Coverage	Σ# Proteins	Σ# Unique Peptides	Σ# Peptides	Σ# PSMs	Het-1A-Smoke - 2M/Parental	Het-1A-Smoke - 4M/Parental	Het-1A-Smoke - 6M/Parental	Het-1A-Smoke - 8M/Parental	# AAs	MW [kDa]	calc. pI	Sequence	# PSMs	# Proteins	# Protein Groups	Protein Group Accessions	Modifications	MH+ [Da]	# Missed Cleavages	Het-1A-Smoke - 2M/Parental	Het-1A-Smoke - 4M/Parental	Het-1A-Smoke - 6M/Parental	Het-1A-Smoke - 8M/Parental
																	VLSLLALVKPEVWTLK	1	1	1	30410792	N-Term(TMT6plex); K9(TMT6plex); K16(TMT6plex)	2496.6	0	**1.4**	**1.0**	**1.5**	**0.9**
																	KQVEVFR	2	1	1	30410792	N-Term(TMT6plex); K1(TMT6plex)	1363.8	1	**1.4**	**1.4**	**1.4**	**1.4**
																	ETHVMDYR	1	1	1	30410792	N-Term(TMT6plex)	1279.6	0	**1.2**	**1.5**	**1.4**	**1.3**
4504445	NP_002127.1	**HNRNPA1**	3178	**heterogeneous nuclear ribonucleoprotein A1 isoform a**	70.63	2	1	19	731	**0.8**	**1.1**	**1.1**	**1.1**	320	34.2	9.23												
																	KLFIGGLSFETTDESLR	20	3	2	4504445;14043070	N-Term(TMT6plex); K1(TMT6plex)	2371.3	1				
																	LFIGGLSFETTDESLR	202	3	2	4504445;14043070	N-Term(TMT6plex)	2014.1	0				
																	DYFEQYGK	109	3	2	4504445;14043070	N-Term(TMT6plex); K8(TMT6plex)	1507.8	0				
																	GFAFVTFDDHDSVDK	72	3	2	4504445;14043070	N-Term(TMT6plex); K15(TMT6plex)	2158.1	0				
																	IFVGGIK	42	7	5	34740329;4504445;14043070;14043072;5803036	N-Term(TMT6plex); K7(TMT6plex)	1191.8	0				
																	IEVIEIMTDR	77	3	2	4504445;14043070	N-Term(TMT6plex); M7(Oxidation)	1463.8	0				
																	EDTEEHHLR	22	5	3	4504445;14043070;14043072	N-Term(TMT6plex)	1394.7	0				
																	KIFVGGIK	21	7	5	14043072;34740329;4504445;14043070;5803036	N-Term(TMT6plex); K1(TMT6plex); K8(TMT6plex)	1549.0	1				
																	SHFEQWGTLTDCVVMR	47	3	2	4504445;14043070	N-Term(TMT6plex); C12(Carbamidomethyl); M15(Oxidation)	2211.1	0				
																	IEVIEIMTDR	29	3	2	4504445;14043070	N-Term(TMT6plex)	1447.8	0				
																	IFVGGIKEDTEEHHLR	13	5	3	4504445;14043070;14043072	N-Term(TMT6plex); K7(TMT6plex)	2338.3	1				
																	SESPKEPEQLR	11	2	2	4504445;14043070	N-Term(TMT6plex); K5(TMT6plex)	1758.0	1				
																	EDSQRPGAHLTVK	23	3	2	4504445;14043070	N-Term(TMT6plex); K13(TMT6plex)	1896.1	0				
																	SHFEQWGTLTDCVVMR	5	3	2	4504445;14043070	N-Term(TMT6plex); C12(Carbamidomethyl)	2195.1	0				
																	NQGGYGGSSSSSSYGSGR	26	2	2	4504445;14043070	N-Term(TMT6plex)	1923.9	0				
																	SSGPYGGGGQYFAKPR	3	2	2	4504445;14043070	N-Term(TMT6plex); K14(TMT6plex)	2087.1	0				
																	GGGFGGNDNFGR	4	2	2	4504445;14043070	N-Term(TMT6plex)	1383.7	0				
																	GGGGYGGSGDGYNGFGNDGSNFGGGGSYNDFGNYNNQSSNFGPMK	2	1	1	4504445	N-Term(TMT6plex); M44(Oxidation); K45(TMT6plex)	4880.0	0	**0.8**	**1.1**	**1.1**	**1.1**
																	QEMASASSSQR	1	3	2	4504445;14043070	N-Term(TMT6plex); M3(Oxidation)	1426.7	0				
																	SGSGNFGGGR	1	2	2	4504445;14043070	N-Term(TMT6plex)	1124.6	0				
																	GGNFGGR	1	4	3	34740329;4504445;14043070	N-Term(TMT6plex)	893.5	0				
7657381	NP_055317.1	**PRPF19**	27339	**pre-mRNA-processing factor 19**	70.63	1	20	20	126	**1.3**	**1.5**	**1.4**	**1.5**	504	55.1	6.61												
																	VTDETSGCSLTCAQFHPDGLIFGTGTMDSQIK	5	1	1	7657381	N-Term(TMT6plex); C8(Carbamidomethyl); C12(Carbamidomethyl); M27(Oxidation); K32(TMT6plex)	3947.9	0	**1.5**	**1.5**	**1.4**	**1.5**
																	TVPEELVKPEELSK	12	1	1	7657381	N-Term(TMT6plex); K8(TMT6plex); K14(TMT6plex)	2285.4	0	**1.3**	**1.5**	**1.5**	**1.5**
																	TLQLDNNFEVK	6	1	1	7657381	N-Term(TMT6plex); K11(TMT6plex)	1779.0	0	**1.5**	**1.5**	**1.4**	**1.6**
																	SSEQILATLK	28	1	1	7657381	N-Term(TMT6plex); K10(TMT6plex)	1547.9	0	**1.4**	**1.5**	**1.5**	**1.6**
																	VTSVVFHPSQDLVFSASPDATIR	10	1	1	7657381	N-Term(TMT6plex)	2702.4	0	**1.3**	**1.3**	**1.3**	**1.3**
																	EALATLKPQAGLIVPQAVPSSQPSVVGAGEPMDLGELVGMTPEIIQK	3	1	1	7657381	N-Term(TMT6plex); K7(TMT6plex); M32(Oxidation); M40(Oxidation); K47(TMT6plex)	5486.0	0	**1.0**	**1.7**	**1.3**	**1.8**
																	ALQDEWDAVMLHSFTLR	4	1	1	7657381	N-Term(TMT6plex); M10(Oxidation)	2277.2	0	**1.4**	**1.8**	**1.3**	**1.7**
																	IWSVPNASCVQVVR	6	1	1	7657381	N-Term(TMT6plex); C9(Carbamidomethyl)	1844.0	0	**1.6**	**1.5**	**1.6**	**1.5**
																	QWTEILHFTEHSGLTTGVAFGHHAK	2	1	1	7657381	N-Term(TMT6plex); K25(TMT6plex)	3262.7	0	**1.2**	**1.2**	**1.4**	**1.2**
																	YIAENGTDPINNQPLSEEQLIDIK	5	1	1	7657381	N-Term(TMT6plex); K24(TMT6plex)	3172.7	0	**1.5**	**1.6**	**1.5**	**1.6**
																	QVASHVGLHSASIPGILALDLCPSDTNK	11	1	1	7657381	N-Term(TMT6plex); C22(Carbamidomethyl); K28(TMT6plex)	3358.8	0	**1.3**	**1.5**	**1.5**	**1.5**
																	VAHPIRPKPPSATSIPAILK	5	1	1	7657381	N-Term(TMT6plex); K8(TMT6plex); K20(TMT6plex)	2780.7	0	**1.2**	**1.5**	**1.5**	**1.6**
																	NVVVFDK	5	1	1	7657381	N-Term(TMT6plex); K7(TMT6plex)	1278.8	0	**1.3**	**1.3**	**1.4**	**1.4**
																	ALQDEWDAVMLHSFTLR	6	1	1	7657381	N-Term(TMT6plex)	2261.2	0	**1.8**	**1.7**	**1.7**	**1.7**
																	QELSHALYQHDAACR	2	1	1	7657381	N-Term(TMT6plex); C14(Carbamidomethyl)	2028.0	0	**1.1**	**1.2**	**1.1**	**1.6**
																	ILTGGADK	2	1	1	7657381	N-Term(TMT6plex); K8(TMT6plex)	1232.8	0	**1.2**	**1.2**	**1.3**	**1.3**
																	AHESAVTGLSLHATGDYLLSSSDDQYWAFSDIQTGR	2	1	1	7657381	N-Term(TMT6plex)	4127.9	0	**1.4**	**1.6**	**1.4**	**1.5**
																	KVTSVVFHPSQDLVFSASPDATIR	1	1	1	7657381	N-Term(TMT6plex); K1(TMT6plex)	3059.7	1	**1.3**	**1.3**	**1.2**	**1.3**
																	ATVLTTER	8	1	1	7657381	N-Term(TMT6plex)	1119.7	0	**1.3**	**1.3**	**1.3**	**1.4**
																	FIASTGMDR	2	1	1	7657381	N-Term(TMT6plex); M7(Oxidation)	1242.6	0	**1.6**	**1.4**	**1.3**	**1.5**
																	QQLQTTR	1	1	1	7657381	N-Term(TMT6plex)	1103.6	0				
39777597	NP_004604.2	**TGM2**	7052	**protein-glutamine gamma-glutamyltransferase 2 isoform a**	70.60	2	35	36	447	**1.7**	**1.9**	**1.9**	**1.3**	687	77.3	5.22												
																	NYEASVDSLTFSVVTGPAPSQEAGTK	16	2	1	39777597	N-Term(TMT6plex); K26(TMT6plex)	3113.6	0	**1.6**	**1.5**	**1.5**	**1.2**
																	YDAPFVFAEVNADVVDWIQQDDGSVHK	18	2	1	39777597	N-Term(TMT6plex); K27(TMT6plex)	3522.8	0	**1.7**	**2.0**	**2.1**	**1.5**
																	LVVNFESDK	32	1	1	39777597	N-Term(TMT6plex); K9(TMT6plex)	1508.9	0	**1.5**	**1.9**	**1.8**	**1.4**
																	DCLTESNLIK	20	1	1	39777597	N-Term(TMT6plex); C2(Carbamidomethyl); K10(TMT6plex)	1650.9	0	**1.6**	**2.1**	**2.0**	**1.4**
																	SEGTYCCGPVPVR	17	2	1	39777597	N-Term(TMT6plex); C6(Carbamidomethyl); C7(Carbamidomethyl)	1710.8	0	**1.9**	**2.0**	**2.1**	**1.6**
																	LAEKEETGMAMR	4	2	1	39777597	N-Term(TMT6plex); K4(TMT6plex); M9(Oxidation); M11(Oxidation)	1856.0	1	**1.6**	**2.3**	**1.9**	**1.3**
																	YLLNLNLEPFSEK	50	1	1	39777597	N-Term(TMT6plex); K13(TMT6plex)	2038.2	0	**2.2**	**2.0**	**2.0**	**1.5**
																	DLYLENPEIK	39	1	1	39777597	N-Term(TMT6plex); K10(TMT6plex)	1692.0	0	**1.5**	**2.1**	**2.0**	**1.3**
																	VVSGMVNCNDDQGVLLGR	13	2	1	39777597	N-Term(TMT6plex); M5(Oxidation); C8(Carbamidomethyl)	2178.1	0	**1.9**	**1.8**	**2.1**	**1.4**
																	SLIVGLK	17	2	1	39777597	N-Term(TMT6plex); K7(TMT6plex)	1187.8	0	**1.6**	**1.9**	**1.8**	**1.3**
																	TVEIPDPVEAGEEVK	23	1	1	39777597	N-Term(TMT6plex); K15(TMT6plex)	2070.1	0	**1.6**	**1.9**	**1.8**	**1.3**
																	TVSYNGILGPECGTK	5	2	1	39777597	N-Term(TMT6plex); C12(Carbamidomethyl); K15(TMT6plex)	2054.1	0	**1.4**	**1.4**	**1.4**	**1.1**
																	WDNNYGDGVSPMSWIGSVDILR	7	2	1	39777597	N-Term(TMT6plex); M12(Oxidation)	2726.3	0	**1.8**	**2.1**	**1.9**	**1.7**
																	NEFGEIQGDK	10	2	1	39777597	N-Term(TMT6plex); K10(TMT6plex)	1594.8	0	**1.7**	**1.9**	**2.0**	**1.2**
																	SVPLCILYEK	21	1	1	39777597	N-Term(TMT6plex); C5(Carbamidomethyl); K10(TMT6plex)	1680.0	0	**1.6**	**2.0**	**2.0**	**1.2**
																	MDLLPLHMGLHK	4	1	1	39777597	N-Term(TMT6plex); M1(Oxidation); K12(TMT6plex)	1879.1	0	**1.5**	**2.1**	**2.1**	**1.3**
																	MDLLPLHMGLHK	12	1	1	39777597	N-Term(TMT6plex); M1(Oxidation); M8(Oxidation); K12(TMT6plex)	1895.1	0	**1.5**	**1.7**	**1.7**	**1.3**
																	WDNNYGDGVSPMSWIGSVDILR	20	2	1	39777597	N-Term(TMT6plex)	2710.3	0	**2.1**	**2.3**	**2.7**	**1.6**
																	MDLLPLHMGLHK	6	1	1	39777597	N-Term(TMT6plex); K12(TMT6plex)	1863.1	0	**2.6**	**3.8**	**4.3**	**1.4**
																	DEREDITHTYK	6	2	1	39777597	N-Term(TMT6plex); K11(TMT6plex)	1865.0	1	**1.4**	**1.8**	**1.6**	**1.3**
																	ALLVEPVINSYLLAER	30	1	1	39777597	N-Term(TMT6plex)	2029.2	0	**1.5**	**1.8**	**1.8**	**1.3**
																	VVTNYNSAHDQNSNLLIEYFR	16	2	1	39777597	N-Term(TMT6plex)	2726.4	0	**1.6**	**1.8**	**1.8**	**1.4**
																	QEYVLTQQGFIYQGSAK	3	2	1	39777597	N-Term(TMT6plex); K17(TMT6plex)	2418.3	0	**1.7**	**1.7**	**1.9**	**1.6**
																	EDITHTYK	6	2	1	39777597	N-Term(TMT6plex); K8(TMT6plex)	1464.8	0	**1.3**	**1.8**	**1.8**	**1.4**
																	LVVNFESDKLK	4	1	1	39777597	N-Term(TMT6plex); K9(TMT6plex); K11(TMT6plex)	1979.2	1	**1.6**	**2.5**	**2.0**	**1.6**
																	VGQSMNMGSDFDVFAHITNNTAEEYVCR	6	2	1	39777597	N-Term(TMT6plex); M5(Oxidation); M7(Oxidation); C27(Carbamidomethyl)	3453.5	0	**1.4**	**1.5**	**1.5**	**1.2**
																	VVSGMVNCNDDQGVLLGR	4	2	1	39777597	N-Term(TMT6plex); C8(Carbamidomethyl)	2162.1	0	**2.2**	**2.3**	**2.9**	**1.6**
																	KLVAEVSLQNPLPVALEGCTFTVEGAGLTEEQK	2	1	1	39777597	N-Term(TMT6plex); K1(TMT6plex); C19(Carbamidomethyl); K33(TMT6plex)	4215.3	1	**1.5**	**1.3**	**1.4**	**1.3**
																	GQPFWLTLHFEGR	3	2	1	39777597	N-Term(TMT6plex)	1817.0	0	**1.3**	**1.3**	**1.4**	**1.2**
																	YPEGSSEER	4	3	2	39777597;156627577	N-Term(TMT6plex)	1282.6	0				
																	YGQCWVFAAVACTVLR	2	2	1	39777597	N-Term(TMT6plex); C4(Carbamidomethyl); C12(Carbamidomethyl)	2130.1	0	**1.1**	**1.5**	**2.0**	**1.3**
																	VKYGQCWVFAAVACTVLR	1	2	1	39777597	N-Term(TMT6plex); K2(TMT6plex); C6(Carbamidomethyl); C14(Carbamidomethyl)	2586.4	1	**1.5**	**1.2**	**2.4**	**1.0**
																	LLLCAR	5	2	1	39777597	N-Term(TMT6plex); C4(Carbamidomethyl)	974.6	0	**2.0**	**2.3**	**2.2**	**1.3**
																	NVIIGPA	9	1	1	39777597	N-Term(TMT6plex)	912.6	0	**1.6**	**1.6**	**1.7**	**1.3**
																	EGDLSTK	4	2	1	39777597	N-Term(TMT6plex); K7(TMT6plex)	1207.7	0	**1.7**	**2.0**	**1.9**	**1.3**
																	SEMIWNFHCWVESWMTRPDLQPGYEGWQALDPTPQEK	1	2	1	39777597	N-Term(TMT6plex); M3(Oxidation); C9(Carbamidomethyl); M15(Oxidation); K37(TMT6plex)	5039.4	0	**1.5**	**1.0**	**1.4**	**1.4**
																	SSPVYVGR	4	2	1	39777597	N-Term(TMT6plex)	1093.6	0	**1.5**	**1.7**	**1.7**	**1.4**
																	CDLELETNGR	1	2	1	39777597	N-Term(TMT6plex); C1(Carbamidomethyl)	1435.7	0	**1.3**	**1.3**	**1.5**	**1.1**
																	VKYGQCWVFAAVACTVLRCLGIPTR	1	2	1	39777597	N-Term(TMT6plex); K2(TMT6plex); C6(Carbamidomethyl); C14(Carbamidomethyl); C19(Carbamidomethyl)	3383.8	2	**1.7**	**1.3**	**2.8**	**0.5**
																	ILGEPK	1	1	1	39777597	N-Term(TMT6plex); K6(TMT6plex)	1114.7	0	**2.0**	**2.3**	**2.4**	**1.3**
222136639	NP_005947.3	**MTHFD1**	4522	**C-1-tetrahydrofolate synthase, cytoplasmic**	70.59	1	49	51	531	**1.3**	**1.2**	**1.3**	**1.4**	935	101.5	7.30												
																	AYIQENLELVEK	24	1	1	222136639	N-Term(TMT6plex); K12(TMT6plex)	1907.1	0	**1.3**	**1.3**	**1.3**	**1.5**
																	GALALAQAVQR	9	1	1	222136639	N-Term(TMT6plex)	1326.8	0	**1.2**	**1.2**	**1.3**	**1.4**
																	AAQAPSSFQLLYDLK	24	1	1	222136639	N-Term(TMT6plex); K15(TMT6plex)	2110.2	0	**1.3**	**1.3**	**1.4**	**1.5**
																	DVDGLTSINAGK	11	1	1	222136639	N-Term(TMT6plex); K12(TMT6plex)	1647.9	0	**1.2**	**1.2**	**1.3**	**1.3**
																	LDIDPETITWQR	13	1	1	222136639	N-Term(TMT6plex)	1715.9	0	**1.4**	**1.2**	**1.2**	**1.5**
																	TAHLDEEVNKGDILVVATGQPEMVK	4	1	1	222136639	N-Term(TMT6plex); K10(TMT6plex); M23(Oxidation); K25(TMT6plex)	3396.9	1	**1.3**	**1.2**	**1.2**	**1.4**
																	ETGVPIAGR	5	1	1	222136639	N-Term(TMT6plex)	1128.7	0	**1.3**	**1.2**	**1.2**	**1.3**
																	GEWIKPGAIVIDCGINYVPDDK	13	1	1	222136639	N-Term(TMT6plex); K5(TMT6plex); C13(Carbamidomethyl); K22(TMT6plex)	3146.7	0	**1.3**	**1.2**	**1.4**	**1.3**
																	GDLNDCFIPCTPK	7	1	1	222136639	N-Term(TMT6plex); C6(Carbamidomethyl); C10(Carbamidomethyl); K13(TMT6plex)	1995.0	0	**1.3**	**1.3**	**1.4**	**1.3**

Accession	RefSeq Accession	Gene Symbol	Entrez Gene ID	Description	Σ Coverage	Σ# Proteins	Σ# Unique Peptides	Σ# Peptides	Σ# PSMs	Het-1A-Smoke - 2M/Parental	Het-1A-Smoke - 4M/Parental	Het-1A-Smoke - 6M/Parental	Het-1A-Smoke - 8M/Parental	# AAs	MW [kDa]	calc. pI	Sequence	# PSMs	# Proteins	# Protein Groups	Protein Group Accessions	Modifications	MH+ [Da]	# Missed Cleavages	Het-1A-Smoke - 2M/Parental	Het-1A-Smoke - 4M/Parental	Het-1A-Smoke - 6M/Parental	Het-1A-Smoke - 8M/Parental
																	ASFITPVPGGVGPMTVAMLMQSTVESAK	6	1	1	222136639	N-Term(TMT6plex); M14(Oxidation); M18(Oxidation); M20(Oxidation); K28(TMT6plex)	3312.7	0	**1.8**	**1.6**	**1.4**	**1.8**
																	LVGPEGFVVTEAGFGADIGMEK	10	1	1	222136639	N-Term(TMT6plex); M20(Oxidation); K22(TMT6plex)	2697.4	0	**1.2**	**1.3**	**1.3**	**1.4**
																	QPSQGPTFGIK	16	1	1	222136639	N-Term(TMT6plex); K11(TMT6plex)	1617.9	0	**1.2**	**1.2**	**1.3**	**1.4**
																	EHGAFDAVK	7	1	1	222136639	N-Term(TMT6plex); K9(TMT6plex)	1431.8	0	**1.1**	**1.2**	**1.2**	**1.3**
																	AAEEIGIK	15	1	1	222136639	N-Term(TMT6plex); K8(TMT6plex)	1288.8	0	**1.3**	**1.2**	**1.3**	**1.4**
																	ASFITPVPGGVGPMTVAMLMQSTVESAK	8	1	1	222136639	N-Term(TMT6plex); M14(Oxidation); M18(Oxidation); K28(TMT6plex)	3296.7	0	**1.2**	**1.2**	**1.5**	**1.2**
																	YVVVTGITPTPLGEGK	14	1	1	222136639	N-Term(TMT6plex); K16(TMT6plex)	2089.2	0	**1.3**	**1.2**	**1.3**	**1.3**
																	TTTESEVMK	10	1	1	222136639	N-Term(TMT6plex); M8(Oxidation); K9(TMT6plex)	1499.8	0	**1.4**	**1.0**	**1.1**	**1.2**
																	TAHLDEEVNK	4	1	1	222136639	N-Term(TMT6plex); K10(TMT6plex)	1613.9	0	**1.2**	**1.2**	**1.2**	**1.3**
																	KGEPVSAEDLGVSGALTVLMK	4	1	1	222136639	N-Term(TMT6plex); K1(TMT6plex); K21(TMT6plex)	2788.6	1	**1.9**	**2.1**	**2.1**	**1.8**
																	ASFITPVPGGVGPMTVAMLMQSTVESAK	2	1	1	222136639	N-Term(TMT6plex); K28(TMT6plex)	3264.7	0	**2.1**	**1.8**	**2.1**	**1.4**
																	EIGLLSEEVELYGETK	29	1	1	222136639	N-Term(TMT6plex); K16(TMT6plex)	2267.2	0	**1.3**	**1.3**	**1.3**	**1.5**
																	CTHWAEGGK	2	1	1	222136639	N-Term(TMT6plex); C1(Carbamidomethyl); K9(TMT6plex)	1503.8	0	**1.7**	**1.1**	**1.3**	**1.1**
																	IVGAPMHDLLLWNNATVTTCHSK	2	1	1	222136639	N-Term(TMT6plex); M6(Oxidation); C20(Carbamidomethyl); K23(TMT6plex)	3052.6	0	**1.6**	**1.4**	**1.2**	**1.4**
																	VLLSALER	17	1	1	222136639	N-Term(TMT6plex)	1129.7	0	**1.4**	**1.2**	**1.3**	**1.4**
																	GVPTGFILPIR	8	1	1	222136639	N-Term(TMT6plex)	1398.9	0	**1.2**	**1.4**	**1.4**	**1.6**
																	TPVPSDIDISR	15	1	1	222136639	N-Term(TMT6plex)	1428.8	0	**1.4**	**1.4**	**1.4**	**1.4**
																	THLSLSHNPEQK	8	1	1	222136639	N-Term(TMT6plex); K12(TMT6plex)	1849.0	0	**1.3**	**1.2**	**1.3**	**1.3**
																	MFGIPVVVAVNAFK	15	1	1	222136639	N-Term(TMT6plex); M1(Oxidation); K14(TMT6plex)	1966.2	0	**1.3**	**1.1**	**1.3**	**1.4**
																	ASFITPVPGGVGPMTVAMLMQSTVESAK	13	1	1	222136639	N-Term(TMT6plex); M18(Oxidation); K28(TMT6plex)	3280.7	0	**1.8**	**1.6**	**1.7**	**1.6**
																	YITSLNEDSTVHGFLVQLPLDSENSINTEEVINAIAPEK	2	1	1	222136639	N-Term(TMT6plex); K39(TMT6plex)	4758.5	0	**1.3**	**1.3**	**1.2**	**1.0**
																	KGEPVSAEDLGVSGALTVLMK	32	1	1	222136639	N-Term(TMT6plex); K1(TMT6plex); M20(Oxidation); K21(TMT6plex)	2804.6	1	**1.1**	**1.2**	**1.2**	**1.4**
																	IYGADDIELLPEAQHK	27	1	1	222136639	N-Term(TMT6plex); K16(TMT6plex)	2270.2	0	**1.3**	**1.3**	**1.3**	**1.3**
																	DDSNLYINVK	12	1	1	222136639	N-Term(TMT6plex); K10(TMT6plex)	1638.9	0	**1.2**	**1.2**	**1.2**	**1.3**
																	MFGIPVVVAVNAFK	12	1	1	222136639	N-Term(TMT6plex); K14(TMT6plex)	1950.2	0	**1.5**	**1.2**	**1.5**	**1.2**
																	IFHELTQTDK	13	1	1	222136639	N-Term(TMT6plex); K10(TMT6plex)	1690.0	0	**1.2**	**1.3**	**1.4**	**1.4**
																	TDPTTLTDEEINR	9	1	1	222136639	N-Term(TMT6plex)	1733.9	0	**1.5**	**1.2**	**1.3**	**1.4**
																	ASVGAGFLYPLVGTMSTMPGLPTRPCFYDIDLDPETEQVNGLF	3	1	1	222136639	N-Term(TMT6plex); M15(Oxidation); M18(Oxidation); C26(Carbamidomethyl)	4937.4	0	**1.4**	**1.2**	**1.4**	**1.3**
																	TDTESELDLISR	9	1	1	222136639	N-Term(TMT6plex)	1607.8	0	**1.3**	**1.0**	**1.2**	**1.2**
																	KVVGDVAYDEAK	3	1	1	222136639	N-Term(TMT6plex); K1(TMT6plex); K12(TMT6plex)	1981.2	1	**1.1**	**1.2**	**1.2**	**1.3**
																	WMIQYNNLNLK	10	1	1	222136639	N-Term(TMT6plex); M2(Oxidation); K11(TMT6plex)	1911.0	0	**1.2**	**1.1**	**1.2**	**1.4**
																	KITIGQAPTEK	6	1	1	222136639	N-Term(TMT6plex); K1(TMT6plex); K11(TMT6plex)	1873.2	1	**1.3**	**1.5**	**1.5**	**1.5**
																	VVGDVAYDEAK	11	1	1	222136639	N-Term(TMT6plex); K11(TMT6plex)	1623.9	0	**1.2**	**1.1**	**1.2**	**1.4**
																	MHGGGPTVTAGLPLPK	9	1	1	222136639	N-Term(TMT6plex); M1(Oxidation); K16(TMT6plex)	2007.1	0	**1.2**	**1.1**	**1.2**	**1.3**
																	QGFGNLPICMAK	4	1	1	222136639	N-Term(TMT6plex); C9(Carbamidomethyl); M10(Oxidation); K12(TMT6plex)	1810.0	0	**1.2**	**1.0**	**1.1**	**1.2**
																	LAILQVGNR	14	1	1	222136639	N-Term(TMT6plex)	1212.8	0	**1.4**	**1.2**	**1.3**	**1.3**
																	ITIGQAPTEK	8	1	1	222136639	N-Term(TMT6plex); K10(TMT6plex)	1515.9	0	**1.2**	**1.1**	**1.2**	**1.4**
																	STTTIGLVQALGAHLYQNVFACVR	4	1	1	222136639	N-Term(TMT6plex); C22(Carbamidomethyl)	2848.5	0	**1.7**	**1.7**	**1.6**	**1.4**
																	EISAQIR	4	1	1	222136639	N-Term(TMT6plex)	1045.6	0	**1.3**	**1.2**	**1.3**	**1.3**
																	FSDIQIR	6	1	1	222136639	N-Term(TMT6plex)	1107.6	0	**1.3**	**1.0**	**1.2**	**1.1**
																	EQVPGFTPR	5	1	1	222136639	N-Term(TMT6plex)	1259.7	0	**1.2**	**1.1**	**1.2**	**1.3**
																	AEVYTK	3	1	1	222136639	N-Term(TMT6plex); K6(TMT6plex)	1168.7	0	**1.2**	**1.3**	**1.3**	**1.4**
																	GFSNLK	2	1	1	222136639	N-Term(TMT6plex); K6(TMT6plex)	1123.7	0	**1.1**	**1.2**	**1.2**	**1.4**
																	MVVASSK	4	1	1	222136639	N-Term(TMT6plex); M1(Oxidation); K7(TMT6plex)	1195.7	0	**1.2**	**1.3**	**1.4**	**1.4**
																	GCLELIK	1	1	1	222136639	N-Term(TMT6plex); C2(Carbamidomethyl); K7(TMT6plex)	1290.8	0	**1.3**	**1.5**	**1.4**	**1.7**
																	LVPSVNGVR	2	1	1	222136639	N-Term(TMT6plex)	1169.7	0				
																	FFNIK	1	1	2	222136639;190194365	N-Term(TMT6plex); K5(TMT6plex)	1126.7	0				
6466450	NP_009294.1	**COMT**	1312	**catechol O-methyltransferase isoform S-COMT**	70.59	2	12	12	69	**1.6**	**1.7**	**1.7**	**1.6**	221	24.4	5.33												
																	EWAMNVGDK	3	2	1	6466450	N-Term(TMT6plex); M4(Oxidation); K9(TMT6plex)	1523.8	0	**1.5**	**1.6**	**1.6**	**1.6**
																	GTVLLADNVICPGAPDFLAHVR	10	2	1	6466450	N-Term(TMT6plex); C11(Carbamidomethyl)	2564.4	0	**1.3**	**1.8**	**1.6**	**1.7**
																	VTLVVGASQDIIPQLK	12	2	1	6466450	N-Term(TMT6plex); K16(TMT6plex)	2139.3	0	**1.4**	**1.4**	**1.6**	**1.4**
																	LITIEINPDCAAITQR	10	2	1	6466450	N-Term(TMT6plex); C10(Carbamidomethyl)	2057.1	0	**1.5**	**1.5**	**1.7**	**1.5**
																	YDVDTLDMVFLDHWK	6	2	1	6466450	N-Term(TMT6plex); M8(Oxidation); K15(TMT6plex)	2371.2	0	**2.0**	**1.8**	**1.8**	**1.7**
																	YLPDTLLLEECGLLR	4	2	1	6466450	N-Term(TMT6plex); C11(Carbamidomethyl)	2034.1	0	**1.5**	**1.6**	**1.5**	**1.6**
																	KYDVDTLDMVFLDHWK	6	2	1	6466450	N-Term(TMT6plex); K1(TMT6plex); M9(Oxidation); K16(TMT6plex)	2728.5	1	**1.1**	**1.5**	**1.4**	**1.5**
																	ILNHVLQHAEPGNAQSVLEAIDTYCEQK	2	2	1	6466450	N-Term(TMT6plex); C25(Carbamidomethyl); K28(TMT6plex)	3635.9	0	**1.4**	**1.3**	**1.4**	**1.3**
																	GSSCFECTHYQSFLEYR	4	2	1	6466450	N-Term(TMT6plex); C4(Carbamidomethyl); C7(Carbamidomethyl)	2400.1	0	**1.7**	**1.8**	**1.7**	**1.6**
																	EVVDGLEK	8	2	1	6466450	N-Term(TMT6plex); K8(TMT6plex)	1346.8	0	**1.5**	**1.7**	**1.7**	**1.6**
																	YDVDTLDMVFLDHWK	1	2	1	6466450	N-Term(TMT6plex); K15(TMT6plex)	2355.2	0				
																	EWAMNVGDKK	2	2	1	6466450	N-Term(TMT6plex); M4(Oxidation); K9(TMT6plex); K10(TMT6plex)	1881.1	1	**1.3**	**1.8**	**1.6**	**1.7**
																	MVDFAGVK	1	2	1	6466450	N-Term(TMT6plex); M1(Oxidation); K8(TMT6plex)	1340.8	0				
5031573	NP_005712.1	**ACTR3**	10096	**actin-related protein 3 isoform 1**	70.57	3	17	21	128	**1.5**	**1.5**	**1.7**	**1.4**	418	47.3	5.88												
																	DITYFIQQLLR	16	5	2	9966913;5031573	N-Term(TMT6plex)	1638.9	0				
																	DYEEIGPSICR	9	2	1	5031573	N-Term(TMT6plex); C10(Carbamidomethyl)	1567.8	0	**1.4**	**1.3**	**1.5**	**1.3**
																	AEPEDHYFLLTEPPLNTPENR	9	2	1	5031573	N-Term(TMT6plex)	2711.4	0	**1.4**	**1.4**	**1.4**	**1.4**
																	HGIVEDWDLMER	6	2	1	5031573	N-Term(TMT6plex); M10(Oxidation)	1744.9	0	**1.4**	**1.5**	**1.5**	**1.5**
																	GVDDLDFFIGDEAIEKPTYATK	11	2	1	5031573	N-Term(TMT6plex); K16(TMT6plex); K22(TMT6plex)	3131.7	0	**1.3**	**1.6**	**1.8**	**1.4**
																	LGYAGNTEPQFIIPSCIAIK	4	1	1	5031573	N-Term(TMT6plex); C16(Carbamidomethyl); K20(TMT6plex)	2650.5	0	**1.5**	**1.5**	**1.7**	**1.4**
																	EVGIPPEQSLETAK	13	5	2	9966913;5031573	N-Term(TMT6plex); K14(TMT6plex)	1956.1	0				
																	FLGPEIFFHPEFANPDFTQPISEVVDEVIQNCPIDVR	5	2	1	5031573	N-Term(TMT6plex); C32(Carbamidomethyl)	4545.3	0	**2.0**	**1.7**	**2.0**	**1.4**
																	FMEQVIFK	6	2	1	5031573	N-Term(TMT6plex); M2(Oxidation); K8(TMT6plex)	1515.9	0	**1.4**	**1.2**	**1.5**	**1.2**
																	KDYEEIGPSICR	7	2	1	5031573	N-Term(TMT6plex); K1(TMT6plex); C11(Carbamidomethyl)	1925.0	1	**1.6**	**1.7**	**1.9**	**1.6**
																	FMEQVIFK	4	2	1	5031573	N-Term(TMT6plex); K8(TMT6plex)	1499.9	0	**1.8**	**1.9**	**2.5**	**1.3**
																	TLTGTVIDSGDGVTHVIPVAEGYVIGSCIK	8	2	1	5031573	N-Term(TMT6plex); C28(Carbamidomethyl); K30(TMT6plex)	3516.9	0	**1.5**	**1.2**	**1.7**	**1.2**
																	YSYVCPDLVK	6	2	1	5031573	N-Term(TMT6plex); C5(Carbamidomethyl); K10(TMT6plex)	1701.9	0	**1.5**	**1.5**	**1.9**	**1.4**
																	QYTGINAISK	4	2	1	5031573	N-Term(TMT6plex); K10(TMT6plex)	1552.9	0	**1.5**	**1.2**	**1.6**	**1.1**
																	NIVLSGGSTMFR	2	2	1	5031573	N-Term(TMT6plex); M10(Oxidation)	1526.8	0	**1.4**	**1.5**	**1.6**	**1.5**
																	HNPVFGVMS	2	4	2	5031573;9966913	N-Term(TMT6plex); M8(Oxidation)	1232.6	0				
																	LPACVVDCGTGYTK	5	1	1	5031573	N-Term(TMT6plex); C4(Carbamidomethyl); C8(Carbamidomethyl); K14(TMT6plex)	1999.0	0	**1.3**	**1.4**	**1.5**	**1.3**
																	LSEELSGGR	5	3	2	5031573;9966913	N-Term(TMT6plex)	1176.6	0				
																	EFSIDVGYER	2	2	1	5031573	N-Term(TMT6plex)	1443.7	0	**1.6**	**1.6**	**1.8**	**1.6**
																	EFNKYDTDGSK	1	2	1	5031573	N-Term(TMT6plex); K4(TMT6plex); K11(TMT6plex)	1991.1	1	**1.4**	**1.8**	**1.7**	**1.7**
																	YAVWFGGSMLASTPEFYQVCHTK	1	2	1	5031573	N-Term(TMT6plex); M9(Oxidation); C20(Carbamidomethyl); K23(TMT6plex)	3153.6	0	**1.2**	**1.6**	**1.6**	**1.5**
																	YDTDGSK	2	2	1	5031573	N-Term(TMT6plex); K7(TMT6plex)	1243.7	0	**1.6**	**1.5**	**1.7**	**1.4**
17402893	NP_478059.1	**PSAT1**	29968	**phosphoserine aminotransferase isoform 1**	70.54	2	21	23	216	**2.0**	**2.0**	**1.5**	**1.6**	370	40.4	7.66												
																	QVVNFGPGPAK	13	2	1	17402893	N-Term(TMT6plex); K11(TMT6plex)	1571.9	0	**1.9**	**1.9**	**1.5**	**1.7**
																	GAVLVCDMSSNFLSKPVDVSK	6	2	1	17402893	N-Term(TMT6plex); C6(Carbamidomethyl); M8(Oxidation); K15(TMT6plex); K21(TMT6plex)	2956.6	0	**2.0**	**2.5**	**1.6**	**1.9**
																	VIFLQGGGCGQFSAVPLNLIGLK	20	2	1	17402893	N-Term(TMT6plex); C9(Carbamidomethyl); K23(TMT6plex)	2846.6	0	**2.4**	**2.3**	**2.0**	**1.8**
																	ECPSVLEYK	12	2	1	17402893	N-Term(TMT6plex); C2(Carbamidomethyl); K9(TMT6plex)	1582.9	0	**2.1**	**2.3**	**1.6**	**1.7**
																	ALELNMLSLK	13	1	1	17402893	N-Term(TMT6plex); M6(Oxidation); K10(TMT6plex)	1606.0	0	**1.9**	**1.9**	**1.5**	**1.6**
																	GVGISVLEMSHR	10	2	1	17402893	N-Term(TMT6plex); M9(Oxidation)	1529.8	0	**1.4**	**1.4**	**1.4**	**1.4**
																	IINNTENLVR	9	2	1	17402893	N-Term(TMT6plex)	1414.8	0	**1.9**	**1.6**	**1.5**	**1.4**
																	DDLLGFALR	15	2	1	17402893	N-Term(TMT6plex)	1248.7	0	**2.8**	**2.5**	**1.6**	**2.0**
																	ALELNMLSLK	13	1	1	17402893	N-Term(TMT6plex); K10(TMT6plex)	1590.0	0	**2.5**	**2.6**	**2.0**	**1.8**
																	NVGSAGVTVVIVR	5	2	1	17402893	N-Term(TMT6plex)	1499.9	0	**1.8**	**1.4**	**1.5**	**1.4**
																	KFGTINIVHPK	3	2	1	17402893	N-Term(TMT6plex); K1(TMT6plex); K11(TMT6plex)	1941.2	1	**3.3**	**1.8**	**1.5**	**1.5**
																	ELLAVPDNYK	16	2	1	17402893	N-Term(TMT6plex); K10(TMT6plex)	1619.9	0	**1.8**	**1.9**	**1.5**	**1.6**
																	LPHSVLLEIQK	20	2	1	17402893	N-Term(TMT6plex); K11(TMT6plex)	1735.1	0	**1.8**	**1.9**	**1.6**	**1.5**
																	IPDPSTWNLNPDASYVYYCANETVHGVEFDFIPDVK	16	2	1	17402893	N-Term(TMT6plex); C19(Carbamidomethyl); K36(TMT6plex)	4631.3	0	**2.7**	**3.0**	**1.7**	**1.9**
																	GVGISVLEMSHR	2	2	1	17402893	N-Term(TMT6plex)	1513.8	0	**1.6**	**1.8**	**1.6**	**1.6**
																	FGVIFAGAQK	14	2	1	17402893	N-Term(TMT6plex); K10(TMT6plex)	1495.9	0	**2.4**	**1.9**	**1.6**	**1.5**

Accession	RefSeq Accession	Gene Symbol	Entrez Gene ID	Description	Σ Coverage	Σ# Proteins	Σ# Unique Peptides	Σ# Peptides	Σ# PSMs	Het-1A-Smoke - 2M/Parental	Het-1A-Smoke - 4M/Parental	Het-1A-Smoke - 6M/Parental	Het-1A-Smoke - 8M/Parental	# AAs	MW [kDa]	calc. pI	Sequence	# PSMs	# Proteins	# Protein Groups	Protein Group Accessions	Modifications	MH+ [Da]	# Missed Cleavages	Het-1A-Smoke - 2M/Parental	Het-1A-Smoke - 4M/Parental	Het-1A-Smoke - 6M/Parental	Het-1A-Smoke - 8M/Parental
																	CADYVVTGAWSAK	2	2	1	17402893	N-Term(TMT6plex); C1(Carbamidomethyl); K13(TMT6plex)	1886.0	0	1.5	2.4	1.5	1.9
																	FGTINIVHPK	10	2	1	17402893	N-Term(TMT6plex); K10(TMT6plex)	1584.0	0	2.1	1.6	1.4	1.3
																	ASLYNAVTIEDVQK	6	2	1	17402893	N-Term(TMT6plex); K14(TMT6plex)	2009.1	0	1.7	1.7	1.4	1.4
																	KFLEMHQL	1	2	1	17402893	N-Term(TMT6plex); K1(TMT6plex); M5(Oxidation)	1519.9	1	1.5	1.6	1.3	1.4
																	ELLDYK	4	2	1	17402893	N-Term(TMT6plex); K6(TMT6plex)	1238.7	0	2.5	2.4	1.5	1.7
																	AAEEAK	2	6	2	4506467;17402893	N-Term(TMT6plex); K6(TMT6plex)	1076.6	0				
																	SSDFAK	1	4	2	17149834;17402893	N-Term(TMT6plex); K6(TMT6plex)	1112.6	0				
																	LGSYTK	2	2	1	17402893	N-Term(TMT6plex); K6(TMT6plex)	1126.7	0	2.1	1.7	1.4	1.4
																	LAAFMK	1	2	1	17402893	N-Term(TMT6plex); M5(Oxidation); K6(TMT6plex)	1154.7	0	2.2	1.9	1.3	1.7
6912594	NP_036531.1	**PITPNB**	23760	**phosphatidylinositol transfer protein beta isoform isoform 1**	70.48	3	16	18	122	**1.4**	**1.5**	**1.6**	**1.4**	271	31.5	6.87												
																	IETWHKPDLGTLENVHGLDPNTWK	1	3	1	6912594	N-Term(TMT6plex); K6(TMT6plex); K24(TMT6plex)	3487.9	0				
																	SQVEPADYK	8	3	1	6912594	N-Term(TMT6plex); K9(TMT6plex)	1494.8	0	1.2	1.2	1.4	1.3
																	QLFCWIDK	10	4	2	5453908;6912594	N-Term(TMT6plex); C4(Carbamidomethyl); K8(TMT6plex)	1567.9	0				
																	MIAPEGSLVFHEK	6	3	1	6912594	N-Term(TMT6plex); K13(TMT6plex)	1916.1	0	1.4	1.6	1.8	1.5
																	ELANSPDCPQMCAYK	4	3	1	6912594	N-Term(TMT6plex); C8(Carbamidomethyl); C12(Carbamidomethyl); K15(TMT6plex)	2242.1	0	1.6	1.6	1.8	1.4
																	ELANSPDCPQMCAYK	11	3	1	6912594	N-Term(TMT6plex); C8(Carbamidomethyl); M11(Oxidation); C12(Carbamidomethyl); K15(TMT6plex)	2258.1	0	1.5	1.5	1.6	1.3
																	SQVEPADYKADEDPALFQSVK	5	3	1	6912594	N-Term(TMT6plex); K9(TMT6plex); K21(TMT6plex)	3024.6	1	1.6	2.1	1.9	1.8
																	NETGGGEGIEVLK	17	3	1	6912594	N-Term(TMT6plex); K13(TMT6plex)	1761.0	0	1.4	1.2	1.4	1.3
																	VENFIQK	3	3	1	6912594	N-Term(TMT6plex); K7(TMT6plex)	1335.8	0	1.4	1.4	1.5	1.3
																	WIDLTMEDIR	4	3	1	6912594	N-Term(TMT6plex); M6(Oxidation)	1536.8	0	1.7	1.7	1.8	1.7
																	MIAPEGSLVFHEK	7	3	1	6912594	N-Term(TMT6plex); M1(Oxidation); K13(TMT6plex)	1932.1	0	1.2	1.4	1.4	1.3
																	TIVTNEYMK	5	3	1	6912594	N-Term(TMT6plex); M8(Oxidation); K9(TMT6plex)	1572.9	0	1.4	1.4	1.6	1.3
																	ADEDPALFQSVK	18	3	1	6912594	N-Term(TMT6plex); K12(TMT6plex)	1778.0	0	1.4	1.5	1.6	1.4
																	WIDLTMEDIR	3	3	1	6912594	N-Term(TMT6plex)	1520.8	0	2.2	2.3	2.5	1.7
																	AWNAYPYCR	2	4	2	5453908;6912594	N-Term(TMT6plex); C8(Carbamidomethyl)	1429.7	0				
																	TVEIVHIDIADR	8	3	1	6912594	N-Term(TMT6plex)	1609.9	0	1.6	1.4	1.4	1.3
																	RMEDETQKELETMR	1	2	1	6912594	N-Term(TMT6plex); M2(Oxidation); K8(TMT6plex); M13(Oxidation)	2286.1	2	1.6	1.7	1.4	1.8
																	VVLPCSVQEYQVGQLYSVAEASK	4	3	1	6912594	N-Term(TMT6plex); C5(Carbamidomethyl); K23(TMT6plex)	3012.6	0	1.5	1.9	1.7	1.7
																	WWGLQSK	2	3	1	6912594	N-Term(TMT6plex); K7(TMT6plex)	1362.8	0	1.5	1.3	1.7	1.4
																	DDFFIK	1	3	1	6912594	N-Term(TMT6plex); K6(TMT6plex)	1242.7	0	1.5	1.6	1.8	1.5
																	TIVTNEYMKDDFFIK	2	3	1	6912594	N-Term(TMT6plex); M8(Oxidation); K9(TMT6plex); K15(TMT6plex)	2567.4	1	1.4	1.9	1.7	1.7
10835049	NP_001655.1	**RHOA**	387	**transforming protein RhoA precursor**	70.47	4	3	10	171	**1.2**	**1.2**	**1.4**	**1.4**	193	21.8	6.10												
																	IGAFGYMECSAK	14	1	1	10835049	N-Term(TMT6plex); M7(Oxidation); C9(Carbamidomethyl); K12(TMT6plex)	1807.9	0	1.3	1.2	1.5	1.3
																	LVIVGDGACGK	13	2	2	111494251;28395033;10835049	N-Term(TMT6plex); C9(Carbamidomethyl); K11(TMT6plex)	1546.9	0				
																	IGAFGYMECSAK	2	1	1	10835049	N-Term(TMT6plex); C9(Carbamidomethyl); K12(TMT6plex)	1791.9	0	1.8	1.8	2.5	1.4
																	DQFPEVYVPTVFENYVADIEVDGK	67	1	1	10835049	N-Term(TMT6plex); K24(TMT6plex)	3231.7	0	1.2	1.2	1.3	1.6
																	TCLLIVFSK	6	3	3	111494251;28395033;4757764;10835049	N-Term(TMT6plex); C2(Carbamidomethyl); K9(TMT6plex)	1538.9	0				
																	HFCPNVPIILVGNK	18	5	2	111494251;28395033;10835049	N-Term(TMT6plex); C3(Carbamidomethyl); K14(TMT6plex)	2066.2	0				
																	LRPLSYPDTDVILMCFSIDSPDSLENIPEK	3	4	2	111494251;28395033;10835049	N-Term(TMT6plex); C15(Carbamidomethyl); K30(TMT6plex)	3923.0	0				
																	QVELALWDTAGQEDYDR	18	4	3	111494251;28395033;4757764;10835049	N-Term(TMT6plex)	2238.1	0				
																	KLVIVGDGACGK	5	2	2	111494251;28395033;10835049	N-Term(TMT6plex); K1(TMT6plex); C10(Carbamidomethyl); K12(TMT6plex)	1904.2	1				
																	EVFEMATR	2	2	2	111494251;28395033;10835049	N-Term(TMT6plex)	1211.6	0				
																	LRPLSYPDTDVILMCFSIDSPDSLENIPEK	8	4	2	111494251;28395033;10835049	N-Term(TMT6plex); M14(Oxidation); C15(Carbamidomethyl); K30(TMT6plex)	3939.0	0				
																	QEPVKPEEGR	7	1	1	10835049	N-Term(TMT6plex); K5(TMT6plex)	1626.9	0	1.2	1.3	1.3	1.3
																	EVFEMATR	8	2	2	111494251;28395033;10835049	N-Term(TMT6plex); M5(Oxidation)	1227.6	0				
28416940	NP_057122.2	**SBDS**	51119	**ribosome maturation protein SBDS**	70.40	1	20	21	130	**1.3**	**1.5**	**1.6**	**1.5**	250	28.7	8.75												
																	GSLEVLNLK	8	1	1	28416940	N-Term(TMT6plex); K9(TMT6plex)	1430.9	0	1.2	1.3	1.5	1.4
																	DLDEVLQTHSVFVNVSK	14	1	1	28416940	N-Term(TMT6plex); K17(TMT6plex)	2388.3	0	1.2	1.4	1.6	1.6
																	EIDELIK	12	1	1	28416940	N-Term(TMT6plex); K7(TMT6plex)	1317.8	0	1.3	1.5	1.6	1.5
																	FILPVNEGK	16	1	1	28416940	N-Term(TMT6plex); K9(TMT6plex)	1474.9	0	1.4	1.5	1.6	1.5
																	DVEEGDEKFE	5	1	1	28416940	N-Term(TMT6plex); K8(TMT6plex)	1654.8	1	1.3	1.3	1.5	1.4
																	GSLEVLNLKDVEEGDEKFE	2	1	1	28416940	N-Term(TMT6plex); K9(TMT6plex); K17(TMT6plex)	2837.5	2	1.3	2.1	1.8	2.0
																	GEVQVSDKER	5	1	1	28416940	N-Term(TMT6plex); K8(TMT6plex)	1604.9	1	1.3	1.6	1.7	1.7
																	DIHYSVK	5	1	1	28416940	N-Term(TMT6plex); K7(TMT6plex)	1319.8	0	1.2	1.4	1.4	1.3
																	RPYTVILIER	7	1	1	28416940	N-Term(TMT6plex)	1488.9	0	1.3	1.3	1.4	1.3
																	HTQLEQMFR	1	1	1	28416940	N-Term(TMT6plex)	1418.7	0	1.4	1.5	1.6	1.6
																	VIESEDYGQQLEIVCLIDPGCFR	27	1	1	28416940	N-Term(TMT6plex); C15(Carbamidomethyl); C21(Carbamidomethyl)	2969.5	0	1.5	1.6	1.6	1.6
																	CVNPETK	4	1	1	28416940	N-Term(TMT6plex); C1(Carbamidomethyl); K7(TMT6plex)	1305.7	0	1.5	1.3	1.5	1.2
																	KEDLISAFGTDDQTEICK	10	1	1	28416940	N-Term(TMT6plex); K1(TMT6plex); C17(Carbamidomethyl); K18(TMT6plex)	2757.5	1	1.4	1.6	1.6	1.4
																	LTNVAVVR	2	1	1	28416940	N-Term(TMT6plex)	1100.7	0	1.6	1.3	1.7	1.4
																	DIATIVADK	3	1	1	28416940	N-Term(TMT6plex); K9(TMT6plex)	1403.9	0	1.3	1.6	1.6	1.6
																	GEVQVSDK	2	1	1	28416940	N-Term(TMT6plex); K8(TMT6plex)	1319.8	0	1.2	1.2	1.4	1.3
																	QQALEVIK	2	1	1	28416940	N-Term(TMT6plex); K8(TMT6plex)	1386.9	0	1.2	1.2	1.5	1.3
																	EIDELIKK	1	1	1	28416940	N-Term(TMT6plex); K7(TMT6plex); K8(TMT6plex)	1675.1	1	1.3	2.1	1.8	2.2
																	RFEIACYK	1	1	1	28416940	N-Term(TMT6plex); C6(Carbamidomethyl); K8(TMT6plex)	1544.9	1	1.3	1.5	1.7	1.5
																	LKPLIK	2	2	3	28416940;13124875;110624781	N-Term(TMT6plex); K2(TMT6plex); K6(TMT6plex)	1399.0	0				
																	FEIACYK	1	1	1	28416940	N-Term(TMT6plex); C5(Carbamidomethyl); K7(TMT6plex)	1388.8	0	1.2	1.3	1.5	1.3
14141159	NP_067676.2	**HNRNPH3**	3189	**heterogeneous nuclear ribonucleoprotein H3 isoform b**	70.39	6	1	14	158	**1.5**	**1.5**	**1.5**	**1.8**	331	35.2	6.87												
																	HNGPNDASDGTVR	4	2	2	14141157;14141159	N-Term(TMT6plex)	1568.8	0				
																	ATENDIANFFSPLNPIR	31	2	2	14141157;14141159	N-Term(TMT6plex)	2148.1	0				
																	DGMDNQGGYGSVGR	14	2	2	14141157;14141159	N-Term(TMT6plex); M3(Oxidation)	1657.7	0				
																	EIAENALGK	13	2	2	14141157;14141159	N-Term(TMT6plex); K9(TMT6plex)	1402.8	0				
																	YIEIFR	8	8	3	14141157;14141159;45935393	N-Term(TMT6plex)	1069.6	0				
																	STGEAFVQFASK	20	2	2	14141157;14141159	N-Term(TMT6plex); K12(TMT6plex)	1730.0	0				
																	ATGEADVEFVTHEDAVAAMSK	25	2	2	14141157;14141159	N-Term(TMT6plex); M19(Oxidation); K21(TMT6plex)	2652.3	0				
																	GGGGSGGYYGQGGMSGGGWR	5	2	2	14141157;14141159	N-Term(TMT6plex); M14(Oxidation)	2049.9	0				
																	YIELFLNSTPGGGSGMGGSGMGGYGR	7	2	2	14141157;14141159	N-Term(TMT6plex); M16(Oxidation); M21(Oxidation)	2783.3	0				
																	GMGGHGYGGAGDASSGFHGGHFVHMR	5	2	2	14141157;14141159	N-Term(TMT6plex); M2(Oxidation); M25(Oxidation)	2804.2	0				
																	VHIDIGADGR	16	2	2	14141157;14141159	N-Term(TMT6plex)	1281.7	0				
																	GLPFGCSK	6	4	4	14141157;14141159;9624998;381342476;5031753	N-Term(TMT6plex); C6(Carbamidomethyl); K8(TMT6plex)	1323.7	0				
																	GSYGGFDDYGGYNNYGYGNDGFDDR	2	1	1	14141159	N-Term(TMT6plex)	2974.2	0	1.5	1.5	1.5	1.8
																	EEIVQFFQGLEIVPNGITLTMDYQGR	2	2	2	14141157;14141159	N-Term(TMT6plex); M21(Oxidation)	3242.7	0				
469608407	NP_001264152.1	**TAGLN2**	8407	**transgelin-2 isoform b**	70.35	3	10	10	36	**1.4**	**1.5**	**1.5**	**1.9**	199	22.4	8.25												
																	DDGLFSGDPNWFPK	3	2	1	469608407	N-Term(TMT6plex); K14(TMT6plex)	2053.0	0	1.3	1.7	1.6	1.9
																	QMEQISQFLQAAER	1	2	1	469608407	N-Term(TMT6plex); M2(Oxidation)	1924.0	0				
																	GASQAGMTGYGMPR	3	3	1	469608407	N-Term(TMT6plex); M7(Oxidation); M12(Oxidation)	1644.8	0	1.9	1.7	1.4	1.9
																	ENFQNWLK	2	2	1	469608407	N-Term(TMT6plex); K8(TMT6plex)	1536.9	0	1.3	1.7	1.7	2.2
																	IQASTMAFK	2	2	1	469608407	N-Term(TMT6plex); M6(Oxidation); K9(TMT6plex)	1470.8	0	1.2	1.5	1.4	1.9
																	YGINTTDIFQTVDLWEGK	11	2	1	469608407	N-Term(TMT6plex); K18(TMT6plex)	2558.4	0	1.4	1.6	1.6	2.1
																	QYDADLEQILIQWITTQCR	2	2	1	469608407	N-Term(TMT6plex); C18(Carbamidomethyl)	2623.3	0	1.3	1.2	1.1	1.5
																	NFSDNQLQEGK	6	2	1	469608407	N-Term(TMT6plex); K11(TMT6plex)	1737.9	0	1.5	1.2	1.5	1.6
																	TLMNLGGLAVAR	2	2	1	469608407	N-Term(TMT6plex); M3(Oxidation)	1460.8	0	1.5	1.5	1.6	1.8
																	DGTVLCELINALYPEGQAPVK	4	2	1	469608407	N-Term(TMT6plex); C6(Carbamidomethyl); K21(TMT6plex)	2745.5	0	1.2	1.2	1.5	1.7
4507951	NP_003396.1	**YWHAH**	7533	**14-3-3 protein eta**	70.33	1	13	18	830	**1.3**	**1.4**	**1.3**	**1.3**	246	28.2	4.84												
																	DSTLIMQLLR	298	7	7	21735625;208973244;4507951;5454052;5803227;21464101;4507949;5803225	N-Term(TMT6plex); M6(Oxidation)	1434.8	0				
																	NLLSVAYK	199	7	7	21735625;208973244;4507951;5454052;5803227;21464101;4507949;5803225	N-Term(TMT6plex); K8(TMT6plex)	1365.9	0				
																	EAFEISK	8	1	1	4507951	N-Term(TMT6plex); K7(TMT6plex)	1281.7	0	1.2	1.4	1.4	1.2
																	LAEQAER	18	7	7	21735625;208973244;4507951;5454052;5803227;21464101;4507949;5803225	N-Term(TMT6plex)	1045.6	0				
																	DSTLIMQLLR	41	7	7	21735625;208973244;4507951;5454052;5803227;21464101;4507949;5803225	N-Term(TMT6plex)	1418.8	0				
																	VISSIEQK	57	5	5	4507951;5803227;21464101;4507949;5454052	N-Term(TMT6plex); K8(TMT6plex)	1361.8	0				

Accession	RefSeq Accession	Gene Symbol	Entrez Gene ID	Description	Σ Coverage	Σ# Proteins	Σ# Unique Peptides	Σ# Peptides	Σ# PSMs	Het-1A-Smoke - 2M/Parental	Het-1A-Smoke - 4M/Parental	Het-1A-Smoke - 6M/Parental	Het-1A-Smoke - 8M/Parental	# AAs	MW [kDa]	calc. pI	Sequence	# PSMs	# Proteins	# Protein Groups	Protein Group Accessions	Modifications	MH+ [Da]	# Missed Cleavages	Het-1A-Smoke - 2M/Parental	Het-1A-Smoke - 4M/Parental	Het-1A-Smoke - 6M/Parental	Het-1A-Smoke - 8M/Parental
																	YDDMASAMK	20	1	1	4507951	N-Term(TMT6plex); M8(Oxidation); K9(TMT6plex)	1505.7	0	1.2	1.3	1.3	1.3
																	DNLTLWTSDQQDEEAGEGN	27	1	1	4507951	N-Term(TMT6plex)	2351.0	0	1.6	1.4	1.5	1.4
																	QAFDDAIAELDTLNEDSYKDSTLIMQLLR	5	1	1	4507951	N-Term(TMT6plex); K19(TMT6plex); M25(Oxidation)	3803.0	1	1.3	1.6	1.3	1.5
																	YLAEVASGEK	12	1	1	4507951	N-Term(TMT6plex); K10(TMT6plex)	1524.9	0	1.3	1.5	1.4	1.3
																	EQMQPTHPIR	2	1	1	4507951	N-Term(TMT6plex); M3(Oxidation)	1481.8	0	1.1	1.2	1.2	1.3
																	QAFDDAIAELDTLNEDSYK	23	1	1	4507951	N-Term(TMT6plex); K19(TMT6plex)	2616.3	0	1.5	1.4	1.4	1.3
																	AVTELNEPLSNEDR	26	1	1	4507951	N-Term(TMT6plex)	1815.9	0	1.3	1.5	1.3	1.3
																	ELETVCNDVLSLLDK	33	1	1	4507951	N-Term(TMT6plex); C6(Carbamidomethyl); K15(TMT6plex)	2206.2	0	1.3	1.5	1.5	1.3
																	NCNDFQYESK	13	1	1	4507951	N-Term(TMT6plex); C2(Carbamidomethyl); K10(TMT6plex)	1762.8	0	1.6	1.2	1.4	1.0
																	NSVVEASEAAYK	18	1	1	4507951	N-Term(TMT6plex); K12(TMT6plex)	1725.9	0	1.4	1.3	1.4	1.2
																	TMADGNEK	2	1	1	4507951	N-Term(TMT6plex); K8(TMT6plex)	1323.7	0	1.9	1.8	2.2	1.2
																	KNSVVEASEAAYK	2	1	1	4507951	N-Term(TMT6plex); K1(TMT6plex); K13(TMT6plex)	2083.2	1	1.2	1.9	1.3	1.5
																	YDDMASAMK	16	1	1	4507951	N-Term(TMT6plex); M4(Oxidation); M8(Oxidation); K9(TMT6plex)	1521.7	0	1.4	1.2	1.3	1.2
																	YDDMASAMK	1	1	1	4507951	N-Term(TMT6plex); K9(TMT6plex)	1489.7	0	2.0	1.8	1.8	1.4
																	TMADGNEK	2	1	1	4507951	N-Term(TMT6plex); M2(Oxidation); K8(TMT6plex)	1339.7	0	1.2	1.1	1.1	1.0
																	NVVGAR	7	4	4	21735625;208973244;4507951;21464101;4507949	N-Term(TMT6plex)	844.5	0				
31621305	NP_573566.2	**LRPPRC**	10128	**leucine-rich PPR motif-containing protein, mitochondrial precursor**	70.23	1	86	86	849	**1.4**	**1.4**	**1.6**	**1.4**	1394	157.8	6.13												
																	YAGEPVPFIEPPESFEFYAQQLR	43	1	1	31621305	N-Term(TMT6plex)	2943.5	0	1.5	1.7	1.5	1.7
																	DAHLLVESK	11	1	1	31621305	N-Term(TMT6plex); K9(TMT6plex)	1469.9	0	1.3	1.3	1.5	1.4
																	DLPVTEAVFSALVTGHAR	10	1	1	31621305	N-Term(TMT6plex)	2112.2	0	1.5	1.5	1.5	1.5
																	GDLSTALEVAIDCYEK	23	1	1	31621305	N-Term(TMT6plex); C13(Carbamidomethyl); K16(TMT6plex)	2242.2	0	1.4	1.4	1.6	1.4
																	SELHLMDR	3	1	1	31621305	N-Term(TMT6plex); M6(Oxidation)	1245.6	0	1.3	1.5	1.4	1.6
																	NNNIDAAIENIENMLTSENK	17	1	1	31621305	N-Term(TMT6plex); M14(Oxidation); K20(TMT6plex)	2721.4	0	1.6	1.3	1.4	1.4
																	GFTLNDAANSR	7	1	1	31621305	N-Term(TMT6plex)	1394.7	0	1.3	1.4	1.4	1.4
																	NNNIDAAIENIENMLTSENK	16	1	1	31621305	N-Term(TMT6plex); K20(TMT6plex)	2705.4	0	1.8	1.6	1.9	1.4
																	SVLELIPELNEK	19	1	1	31621305	N-Term(TMT6plex); K12(TMT6plex)	1842.1	0	1.6	1.5	1.7	1.6
																	VEDALNLKEEFDR	3	1	1	31621305	N-Term(TMT6plex); K8(TMT6plex)	2036.1	1	1.5	1.3	1.6	1.5
																	DAGIEPGPDTYLALLNAYAEK	23	1	1	31621305	N-Term(TMT6plex); K21(TMT6plex)	2679.4	0	1.5	1.4	1.6	1.4
																	ISNQFDWALMR	10	1	1	31621305	N-Term(TMT6plex); M10(Oxidation)	1625.8	0	1.6	1.3	1.5	1.4
																	MVFINNIALAQIK	6	1	1	31621305	N-Term(TMT6plex); K13(TMT6plex)	1933.2	0	1.7	2.2	2.2	2.2
																	LEDVALQILLACPVSK	13	1	1	31621305	N-Term(TMT6plex); C12(Carbamidomethyl); K16(TMT6plex)	2227.3	0	1.4	1.7	1.8	1.6
																	HSLNSSSASTTEPDFQK	11	1	1	31621305	N-Term(TMT6plex); K17(TMT6plex)	2294.2	0	1.3	1.3	1.4	1.4
																	GETDLIQK	5	1	1	31621305	N-Term(TMT6plex); K8(TMT6plex)	1361.8	0	1.2	1.4	1.5	1.3
																	ADAVWNK	8	1	1	31621305	N-Term(TMT6plex); K7(TMT6plex)	1261.7	0	1.3	1.5	1.5	1.5
																	LLAEILR	24	1	1	31621305	N-Term(TMT6plex)	1056.7	0	1.6	1.5	1.7	1.5
																	EEAYNSLMK	3	1	1	31621305	N-Term(TMT6plex); M8(Oxidation); K9(TMT6plex)	1558.8	0	1.4	1.4	1.4	1.6
																	QYFHQLEK	7	1	1	31621305	N-Term(TMT6plex); K8(TMT6plex)	1550.9	0	1.3	1.3	1.5	1.4
																	EDGPSVFGSFFLQHCVTMNTPVEK	7	1	1	31621305	N-Term(TMT6plex); C15(Carbamidomethyl); M18(Oxidation); K24(TMT6plex)	3200.6	0	1.7	1.5	1.8	1.8
																	TVQLTSSELESTLETLK	15	1	1	31621305	N-Term(TMT6plex); K17(TMT6plex)	2337.3	0	1.4	1.3	1.5	1.4
																	IQEENVIPR	10	1	1	31621305	N-Term(TMT6plex)	1326.8	0	1.4	1.4	1.5	1.5
																	GMQELGVHPDQETYTDYVIPCFDSVNSAR	9	1	1	31621305	N-Term(TMT6plex); M2(Oxidation); C21(Carbamidomethyl)	3573.7	0	1.4	1.4	1.5	1.5
																	EGNQEVPFDVPELWYEDEK	19	1	1	31621305	N-Term(TMT6plex); K19(TMT6plex)	2781.4	0	1.4	1.4	1.6	1.4
																	AGDMENAENILTVMR	9	1	1	31621305	N-Term(TMT6plex); M4(Oxidation); M14(Oxidation)	1924.9	0	1.3	1.3	1.2	1.4
																	GPTEAVGYFLYNLIDSMSDSEVQAK	20	1	1	31621305	N-Term(TMT6plex); M17(Oxidation); K25(TMT6plex)	3208.6	0	1.3	1.4	1.6	1.6
																	KVIEEQLEPAVEK	9	1	1	31621305	N-Term(TMT6plex); K1(TMT6plex); K13(TMT6plex)	2199.3	1	1.3	1.5	1.6	1.6
																	LDDLFLK	25	1	1	31621305	N-Term(TMT6plex); K7(TMT6plex)	1321.8	0	1.4	1.4	1.5	1.4
																	QLILVLCSEENMQK	5	1	1	31621305	N-Term(TMT6plex); C7(Carbamidomethyl); M12(Oxidation); K14(TMT6plex)	2179.2	0	1.3	1.3	1.4	1.4
																	EKDIQEESTFSSR	16	1	1	31621305	N-Term(TMT6plex); K2(TMT6plex)	2014.0	1	1.5	1.5	1.6	1.6
																	GAYDIFLNAK	6	1	1	31621305	N-Term(TMT6plex); K10(TMT6plex)	1569.9	0	1.1	1.2	1.4	1.3
																	LQDAINILK	19	1	1	31621305	N-Term(TMT6plex); K9(TMT6plex)	1485.9	0	1.3	1.4	1.6	1.4
																	ALYEHLTAK	2	1	1	31621305	N-Term(TMT6plex); K9(TMT6plex)	1503.9	0	1.4	1.6	1.8	1.6
																	VIQALAMK	2	1	1	31621305	N-Term(TMT6plex); K8(TMT6plex)	1331.8	0	1.8	1.9	2.4	1.5
																	VIQALAMK	9	1	1	31621305	N-Term(TMT6plex); M7(Oxidation); K8(TMT6plex)	1347.8	0	1.2	1.2	1.4	1.3
																	CGAIAEQTPILLLFLLR	3	1	1	31621305	N-Term(TMT6plex); C1(Carbamidomethyl)	2157.3	0	1.2	1.3	1.4	1.3
																	VIEEQLEPAVEK	8	1	1	31621305	N-Term(TMT6plex); K12(TMT6plex)	1842.1	0	1.4	1.6	1.7	1.7
																	LDSSAVLDTGK	18	1	1	31621305	N-Term(TMT6plex); K11(TMT6plex)	1563.9	0	1.4	1.2	1.4	1.3
																	GDVENIEVVQK	8	1	1	31621305	N-Term(TMT6plex); K11(TMT6plex)	1688.0	0	1.4	1.5	1.5	1.6
																	LLMSEDYFTQAMEVK	12	1	1	31621305	N-Term(TMT6plex); M3(Oxidation); M12(Oxidation); K15(TMT6plex)	2295.2	0	1.3	1.3	1.4	1.4
																	EQNIVFNAETYSNLIK	10	1	1	31621305	N-Term(TMT6plex); K16(TMT6plex)	2341.3	0	1.4	1.6	1.7	1.6
																	LIASYCNVGDIEGASK	6	1	1	31621305	N-Term(TMT6plex); C6(Carbamidomethyl); K16(TMT6plex)	2155.1	0	1.7	1.7	2.0	1.7
																	AGYPQYVSEILEK	8	1	1	31621305	N-Term(TMT6plex); K13(TMT6plex)	1955.1	0	1.3	1.5	1.5	1.5
																	SEAANGNLDFVLSFLK	4	1	1	31621305	N-Term(TMT6plex); K16(TMT6plex)	2183.2	0	1.5	1.4	1.7	1.6
																	SCGSLLPELK	7	1	1	31621305	N-Term(TMT6plex); C2(Carbamidomethyl); K10(TMT6plex)	1561.9	0	1.3	1.4	1.6	1.3
																	SGGLGGSHALLLLR	15	1	1	31621305	N-Term(TMT6plex)	1579.9	0	1.5	1.4	1.6	1.4
																	VIEPQYFGLAYLFR	29	1	1	31621305	N-Term(TMT6plex)	1945.1	0	1.6	1.8	1.9	1.7
																	NVQGIIEILK	15	1	1	31621305	N-Term(TMT6plex); K10(TMT6plex)	1585.0	0	1.5	1.4	1.6	1.3
																	NLLESYHVPELIK	17	1	1	31621305	N-Term(TMT6plex); K13(TMT6plex)	2013.2	0	1.4	1.4	1.6	1.4
																	YIPDAMNLILLLVTEK	15	1	1	31621305	N-Term(TMT6plex); M6(Oxidation); K16(TMT6plex)	2320.4	0	1.4	1.3	1.5	1.4
																	MVFINNIALAQIK	20	1	1	31621305	N-Term(TMT6plex); M1(Oxidation); K13(TMT6plex)	1949.2	0	1.3	1.3	1.4	1.4
																	SVLELIPELNEKEEAYNSLMK	22	1	1	31621305	N-Term(TMT6plex); K12(TMT6plex); M20(Oxidation); K21(TMT6plex)	3152.7	1	1.2	1.4	1.3	1.5
																	VYLQNEYK	20	1	1	31621305	N-Term(TMT6plex); K8(TMT6plex)	1514.9	0	1.4	1.4	1.5	1.4
																	KGAYDIFLNAK	6	1	1	31621305	N-Term(TMT6plex); K1(TMT6plex); K11(TMT6plex)	1927.2	1	1.2	1.3	1.4	1.4
																	MLNGLEDSIGLSK	6	1	1	31621305	N-Term(TMT6plex); K13(TMT6plex)	1835.0	0	1.6	1.6	1.8	1.4
																	LFECDRDQMYYNLLK	5	1	1	31621305	N-Term(TMT6plex); C4(Carbamidomethyl); M9(Oxidation); K15(TMT6plex)	2482.2	1	1.4	1.5	1.6	1.5
																	LANQFAIYKPVTDFFLQLVDAGK	3	1	1	31621305	N-Term(TMT6plex); K9(TMT6plex); K23(TMT6plex)	3285.9	0	1.6	1.3	2.1	1.1
																	MEEANIQPNR	10	1	1	31621305	N-Term(TMT6plex); M1(Oxidation)	1446.7	0	1.5	1.5	1.7	1.3
																	LLMSEDYFTQAMEVK	14	1	1	31621305	N-Term(TMT6plex); M3(Oxidation); K15(TMT6plex)	2279.2	0	1.8	1.9	2.1	1.7
																	GMQELGVHPDQETYTDYVIPCFDSVNSAR	4	1	1	31621305	N-Term(TMT6plex); C21(Carbamidomethyl)	3557.7	0	1.8	1.6	2.1	1.4
																	AFAETHIK	12	1	1	31621305	N-Term(TMT6plex); K8(TMT6plex)	1374.8	0	1.3	1.4	1.4	1.4
																	TVLDQQQTPSR	5	1	1	31621305	N-Term(TMT6plex)	1501.8	0	1.5	1.3	1.4	1.4
																	SNTLPISLQSIR	4	1	1	31621305	N-Term(TMT6plex)	1557.9	0	1.4	1.5	1.7	1.3
																	GDIDHVK	6	1	1	31621305	N-Term(TMT6plex); K7(TMT6plex)	1241.7	0	1.2	1.5	1.6	1.5
																	FSPTDFLAK	10	1	1	31621305	N-Term(TMT6plex); K9(TMT6plex)	1483.9	0	1.4	1.4	1.8	1.5
																	SSLLLGFR	8	1	1	31621305	N-Term(TMT6plex)	1121.7	0	1.6	1.4	1.6	1.4
																	EKDVLIK	1	1	1	31621305	N-Term(TMT6plex); K2(TMT6plex); K7(TMT6plex)	1532.0	1	1.2	1.5	1.6	1.6
																	LLMSEDYFTQAMEVK	2	1	1	31621305	N-Term(TMT6plex); K15(TMT6plex)	2263.2	0	1.8	2.0	2.4	1.6
																	YESDMVTGGYAALINLCCR	2	1	1	31621305	N-Term(TMT6plex); M5(Oxidation); C17(Carbamidomethyl); C18(Carbamidomethyl)	2438.1	0	2.1	1.5	1.6	1.6
																	EEAYNSLMK	2	1	1	31621305	N-Term(TMT6plex); K9(TMT6plex)	1542.8	0	2.0	2.0	2.5	1.7
																	ISNQFDWALMR	2	1	1	31621305	N-Term(TMT6plex)	1609.8	0	1.8	1.6	2.0	1.5
																	IHDVLCK	2	1	1	31621305	N-Term(TMT6plex); C6(Carbamidomethyl); K7(TMT6plex)	1342.8	0	1.3	1.3	1.6	1.4
																	SVLELIPELNEKEEAYNSLMK	4	1	1	31621305	N-Term(TMT6plex); K12(TMT6plex); K21(TMT6plex)	3136.7	1	1.9	2.3	2.6	1.7
																	YIPDAMNLILLLVTEK	2	1	1	31621305	N-Term(TMT6plex); K16(TMT6plex)	2304.4	0	1.9	1.7	2.2	1.6
																	LGAVYDVSHYNALLK	4	1	1	31621305	N-Term(TMT6plex); K15(TMT6plex)	2121.2	0	1.2	1.4	1.5	1.4
																	EEGFPIRPHYFWPLLVGR	2	1	1	31621305	N-Term(TMT6plex)	2442.3	0	0.9	0.8	1.5	1.3
																	DIQEESTFSSR	4	1	1	31621305	N-Term(TMT6plex)	1527.7	0	1.5	1.3	1.3	1.4
																	CVANNQVETLEK	2	1	1	31621305	N-Term(TMT6plex); C1(Carbamidomethyl); K12(TMT6plex)	1863.0	0				
																	IIETPGIR	4	1	1	31621305	N-Term(TMT6plex)	1127.7	0	1.4	1.4	1.6	1.4
																	LVELTQK	6	1	1	31621305	N-Term(TMT6plex); K7(TMT6plex)	1288.8	0	1.5	1.6	1.6	1.6
																	EAVTTLK	3	1	1	31621305	N-Term(TMT6plex); K7(TMT6plex)	1219.8	0	1.3	1.4	1.5	1.4
																	IPENIYR	2	1	1	31621305	N-Term(TMT6plex)	1133.7	0	1.7	1.6	1.7	1.6
																	HDKVEDALNLKEEFDR	1	1	1	31621305	N-Term(TMT6plex); K3(TMT6plex); K11(TMT6plex)	2645.5	2	1.1	1.0	1.3	1.2
																	LQWFCDR	1	1	1	31621305	N-Term(TMT6plex); C5(Carbamidomethyl)	1253.6	0	1.4	1.4	1.6	1.4
																	GEIETVK	6	1	1	31621305	N-Term(TMT6plex); K7(TMT6plex)	1233.7	0	1.5	1.5	1.5	1.4
																	DLLQIIFSFSK	1	1	1	31621305	N-Term(TMT6plex); K11(TMT6plex)	1769.1	0	1.6	1.3	2.6	0.9
																	AENQPIR	3	1	1	31621305	N-Term(TMT6plex)	1056.6	0	1.3	1.5	1.5	1.4
																	LDLSVR	4	1	1	31621305	N-Term(TMT6plex)	931.6	0	1.5	1.4	1.5	1.4
																	DILIACR	1	1	1	31621305	N-Term(TMT6plex); C6(Carbamidomethyl)	1089.6	0	1.2	1.2	1.3	1.1
																	ISIMAER	3	1	1	31621305	N-Term(TMT6plex); M4(Oxidation)	1064.6	0	1.4	1.3	1.4	1.5
																	DVTSAK	1	1	1	31621305	N-Term(TMT6plex); K6(TMT6plex)	1078.7	0	1.4	1.6	1.8	1.6
																	NLDFQK	4	1	1	31621305	N-Term(TMT6plex); K6(TMT6plex)	1222.7	0	1.4	1.3	1.6	1.3
																	LTDYCK	3	1	1	31621305	N-Term(TMT6plex); C5(Carbamidomethyl); K6(TMT6plex)	1257.7	0	1.5	1.4	1.7	1.4

Accession	RefSeq Accession	Gene Symbol	Entrez Gene ID	Description	Σ Coverage	Σ# Proteins	Σ# Unique Peptides	Σ# Peptides	Σ# PSMs	Het-1A-Smoke - 2M/Parental	Het-1A-Smoke - 4M/Parental	Het-1A-Smoke - 6M/Parental	Het-1A-Smoke - 8M/Parental	# AAs	MW [kDa]	calc. pI	Sequence	# PSMs	# Proteins	# Protein Groups	Protein Group Accessions	Modifications	MH+ [Da]	# Missed Cleavages	Het-1A-Smoke - 2M/Parental	Het-1A-Smoke - 4M/Parental	Het-1A-Smoke - 6M/Parental	Het-1A-Smoke - 8M/Parental
																	VFNDTCR	2	1	1	31621305	N-Term(TMT6plex); C6(Carbamidomethyl)	1140.6	0	**1.5**	**1.2**	**1.6**	**1.2**
																	LIITQVR	1	1	1	31621305	N-Term(TMT6plex)	1071.7	0	**1.3**	**1.5**	**1.4**	**1.4**
33413400	NP_001975.1	**ESD**	2098	**S-formylglutathione hydrolase**	70.21	1	13	13	62	**1.5**	**1.8**	**1.8**	**1.9**	282	31.4	7.02												
																	MYSYVTEELPQLINANFPVDPQR	6	1	1	33413400	N-Term(TMT6plex); M1(Oxidation)	2969.5	0	**1.5**	**1.6**	**1.6**	**1.9**
																	LQEGYDHSYYFIATFITDHIR	3	1	1	33413400	N-Term(TMT6plex)	2818.4	0	**1.1**	**1.2**	**1.2**	**1.6**
																	FAVYLPPK	4	1	1	33413400	N-Term(TMT6plex); K8(TMT6plex)	1392.9	0	**1.5**	**1.5**	**1.7**	**1.6**
																	CPALYWLSGLTCTEQNFISK	4	1	1	33413400	N-Term(TMT6plex); C1(Carbamidomethyl); C12(Carbamidomethyl); K20(TMT6plex)	2846.5	0	**1.2**	**1.5**	**1.6**	**1.9**
																	KIPVVFR	3	1	1	33413400	N-Term(TMT6plex); K1(TMT6plex)	1316.9	1	**1.3**	**1.8**	**1.8**	**1.9**
																	SYPGSQLDILIDQGKDDQFLLDGQLLPDNFIAACTEK	2	1	1	33413400	N-Term(TMT6plex); K15(TMT6plex); C34(Carbamidomethyl); K37(TMT6plex)	4825.5	1	**2.3**	**4.1**	**3.8**	**2.7**
																	AYDATHLVK	2	1	1	33413400	N-Term(TMT6plex); K9(TMT6plex)	1475.9	0	**1.3**	**1.5**	**1.5**	**1.8**
																	MYSYVTEELPQLINANFPVDPQR	9	1	1	33413400	N-Term(TMT6plex)	2953.5	0	**1.5**	**1.4**	**1.5**	**1.4**
																	DDQFLLDGQLLPDNFIAACTEK	5	1	1	33413400	N-Term(TMT6plex); C19(Carbamidomethyl); K22(TMT6plex)	2981.5	0	**1.6**	**2.2**	**2.1**	**2.2**
																	VFEHDSVELNCK	4	1	1	33413400	N-Term(TMT6plex); C11(Carbamidomethyl); K12(TMT6plex)	1935.0	0	**1.5**	**1.5**	**1.5**	**2.0**
																	AFSGYLGTDQSK	12	1	1	33413400	N-Term(TMT6plex); K12(TMT6plex)	1731.9	0	**1.4**	**1.5**	**1.6**	**1.9**
																	SVSAFAPICNPVLCPWGK	6	1	1	33413400	N-Term(TMT6plex); C9(Carbamidomethyl); C14(Carbamidomethyl); K18(TMT6plex)	2461.3	0	**1.5**	**1.5**	**1.6**	**1.6**
																	GEDESWDFGTGAGFYVDATEDPWK	1	1	1	33413400	N-Term(TMT6plex); K24(TMT6plex)	3137.5	0	**1.7**	**1.7**	**1.6**	**1.8**
																	CFGGLQK	1	1	1	33413400	N-Term(TMT6plex); C1(Carbamidomethyl); K7(TMT6plex)	1267.7	0	**1.7**	**1.5**	**1.8**	**1.6**
32189392	NP_005800.3	**PRDX2**	7001	**peroxiredoxin-2**	70.20	1	10	11	99	**1.4**	**1.7**	**1.6**	**2.0**	198	21.9	5.97												
																	QITVNDLPVGR	15	2	2	32189392;4505591;32455266	N-Term(TMT6plex)	1440.8	0				
																	SVDEALR	6	1	1	32189392	N-Term(TMT6plex)	1018.6	0	**1.5**	**1.6**	**1.5**	**1.8**
																	GLFIIDGK	15	1	1	32189392	N-Term(TMT6plex); K8(TMT6plex)	1320.8	0	**1.4**	**1.7**	**1.6**	**1.9**
																	LSEDYGVLK	6	1	1	32189392	N-Term(TMT6plex); K9(TMT6plex)	1481.9	0	**1.5**	**1.7**	**1.7**	**2.0**
																	LVQAFQYTDEHGEVCPAGWKPGSDTIKPNVDDSK	2	1	1	32189392	N-Term(TMT6plex); C15(Carbamidomethyl); K20(TMT6plex); K27(TMT6plex); K34(TMT6plex)	4705.4	0	**1.3**	**1.7**	**1.6**	**2.0**
																	ATAVVDGAFK	11	1	1	32189392	N-Term(TMT6plex); K10(TMT6plex)	1436.9	0	**1.6**	**2.1**	**1.7**	**2.2**
																	KEGGLGPLNIPLLADVTR	16	1	1	32189392	N-Term(TMT6plex); K1(TMT6plex)	2321.4	1	**1.4**	**1.7**	**1.6**	**1.9**
																	KLGCEVLGVSVDSQFTHLAWINTPR	1	1	1	32189392	N-Term(TMT6plex); K1(TMT6plex); C4(Carbamidomethyl)	3285.8	1	**1.8**	**1.7**	**1.7**	**1.8**
																	IGKPAPDFK	4	1	1	32189392	N-Term(TMT6plex); K3(TMT6plex); K9(TMT6plex)	1660.0	0	**1.3**	**1.9**	**1.6**	**2.2**
																	EGGLGPLNIPLLADVTR	22	1	1	32189392	N-Term(TMT6plex)	1964.1	0	**1.4**	**1.8**	**1.7**	**2.1**
																	TDEGIAYR	1	1	1	32189392	N-Term(TMT6plex)	1153.6	0	**1.4**	**1.3**	**1.4**	**1.6**
372466577	NP_001243222.1	**KRT8**	3856	**keratin, type II cytoskeletal 8 isoform 2**	70.19	6	24	34	600	**1.2**	**1.3**	**1.1**	**1.3**	483	53.7	5.59												
																	LEGLTDEINFLR	17	2	1	372466577	N-Term(TMT6plex)	1648.9	0	**1.2**	**1.3**	**1.3**	**1.2**
																	QLETLGQEK	16	2	1	372466577	N-Term(TMT6plex); K9(TMT6plex)	1503.9	0	**1.1**	**1.3**	**1.0**	**1.2**
																	DVDEAYMNK	26	2	1	372466577	N-Term(TMT6plex); M7(Oxidation); K9(TMT6plex)	1558.8	0	**1.2**	**1.4**	**1.1**	**1.4**
																	TEMENEFVLIK	21	2	1	372466577	N-Term(TMT6plex); M3(Oxidation); K11(TMT6plex)	1827.0	0	**1.2**	**1.3**	**1.1**	**1.1**
																	LKLEAELGNMQGLVEDFK	14	2	1	372466577	N-Term(TMT6plex); K2(TMT6plex); M10(Oxidation); K18(TMT6plex)	2737.5	1	**1.1**	**1.6**	**1.1**	**1.5**
																	LESGMQNMSIHTK	13	2	1	372466577	N-Term(TMT6plex); M5(Oxidation); M8(Oxidation); K13(TMT6plex)	1966.0	0	**1.2**	**1.1**	**1.2**	**1.3**
																	EYQELMNVK	19	5	3	372466577;155969697;119703753	N-Term(TMT6plex); M6(Oxidation); K9(TMT6plex)	1627.9	0				
																	KLLEGEESR	14	6	5	67782365;55749932;372466577;62414289;21264345	N-Term(TMT6plex); K1(TMT6plex)	1518.9	1				
																	SLDMDSIIAEVK	25	2	1	372466577	N-Term(TMT6plex); M4(Oxidation); K12(TMT6plex)	1795.0	0	**1.2**	**1.2**	**1.2**	**1.4**
																	NKYEDEINK	2	8	5	372466577;119395750;155969697;119395754;119703753	N-Term(TMT6plex); K2(TMT6plex); K9(TMT6plex)	1840.0	1				
																	AQYEDIANR	33	3	1	372466577	N-Term(TMT6plex)	1308.7	0	**1.2**	**1.2**	**1.1**	**1.3**
																	FLEQQNK	51	22	9	67782365;372466577;155969697;119395754;62414289;119703753;15431316;109148552;89357932	N-Term(TMT6plex); K7(TMT6plex)	1364.8	0				
																	LVSESSDVLPK	19	2	1	372466577	N-Term(TMT6plex); K11(TMT6plex)	1632.0	0	**1.1**	**1.1**	**1.1**	**1.2**
																	ASLEAAIADAEQR	39	2	1	372466577	N-Term(TMT6plex)	1573.8	0	**1.4**	**1.5**	**1.3**	**1.5**
																	ELQSQISDTSVVLSMDNSR	24	2	1	372466577	N-Term(TMT6plex); M15(Oxidation)	2354.2	0	**1.3**	**1.5**	**1.2**	**1.4**
																	LLEGEESR	28	7	6	67782365;15431316;55749932;372466577;62414289;21264345	N-Term(TMT6plex)	1161.6	0				
																	LEAELGNMQGLVEDFK	20	2	1	372466577	N-Term(TMT6plex); M8(Oxidation); K16(TMT6plex)	2267.2	0	**1.3**	**1.4**	**1.2**	**1.3**
																	LSELEAALQR	29	2	1	372466577	N-Term(TMT6plex)	1358.8	0	**1.3**	**1.3**	**1.2**	**1.4**
																	YEDEINK	3	13	8	372466577;119395750;109148552;155969697;119395754;119703753;47132620;194018511	N-Term(TMT6plex); K7(TMT6plex)	1368.7	0				
																	LEAELGNMQGLVEDFK	4	2	1	372466577	N-Term(TMT6plex); K16(TMT6plex)	2251.2	0	**1.5**	**2.0**	**1.3**	**1.3**
																	ISSSSFSR	2	2	1	372466577	N-Term(TMT6plex)	1099.6	0	**1.2**	**0.8**	**0.8**	**1.1**
																	YEELQSLAGK	49	2	1	372466577	N-Term(TMT6plex); K10(TMT6plex)	1595.9	0	**1.2**	**1.3**	**1.1**	**1.3**
																	LQAEIEGLK	16	2	1	372466577	N-Term(TMT6plex); K9(TMT6plex)	1458.9	0	**1.2**	**1.2**	**1.1**	**1.2**
																	LALDIEIATYR	7	10	4	67782365;372466577;125628632;119395750	N-Term(TMT6plex)	1506.9	0				
																	SLDMDSIIAEVK	6	2	1	372466577	N-Term(TMT6plex); K12(TMT6plex)	1779.0	0	**1.3**	**1.4**	**1.2**	**1.3**
																	YEDEINKR	6	12	8	372466577;119395750;109148552;155969697;119395754;119703753;47132620;194018511	N-Term(TMT6plex); K7(TMT6plex)	1524.8	1				
																	QLYEEEIR	7	2	1	372466577	N-Term(TMT6plex)	1308.7	0	**1.2**	**1.3**	**1.1**	**1.3**
																	SNMDNMFESYINNLR	6	2	1	372466577	N-Term(TMT6plex); M3(Oxidation); M6(Oxidation)	2109.0	0	**1.2**	**1.0**	**1.0**	**1.2**
																	GGLGGGYGGASGMGGITAVTVNQSLLSPLVLEVDPNIQAVR	4	2	1	372466577	N-Term(TMT6plex); M13(Oxidation)	4170.2	0	**1.5**	**0.8**	**1.4**	**1.5**
																	WSLLQQQK	14	2	1	372466577	N-Term(TMT6plex); K8(TMT6plex)	1488.9	0	**1.2**	**1.1**	**1.0**	**1.3**
																	SNMDNMFESYINNLR	6	2	1	372466577	N-Term(TMT6plex); M3(Oxidation)	2093.0	0	**1.4**	**1.5**	**1.5**	**1.4**
																	TEMENEFVLIK	2	2	1	372466577	N-Term(TMT6plex); K11(TMT6plex)	1811.0	0	**1.2**	**1.3**	**1.2**	**1.1**
																	EYQELMNVK	3	5	3	372466577;155969697;119703753	N-Term(TMT6plex); K9(TMT6plex)	1611.9	0				
																	AEAESMYQIK	18	2	1	372466577	N-Term(TMT6plex); M6(Oxidation); K10(TMT6plex)	1643.9	0	**1.2**	**1.3**	**1.1**	**1.4**
																	GGLGGGYGGASGMGGITAVTVNQSLLSPLVLEVDPNIQAVR	2	2	1	372466577	N-Term(TMT6plex)	4154.2	0	**1.3**	**1.8**	**1.3**	**1.7**
																	AEAESMYQIK	2	2	1	372466577	N-Term(TMT6plex); K10(TMT6plex)	1627.9	0	**1.3**	**1.5**	**1.3**	**1.2**
																	FASFIDK	22	20	11	67782365;15431316;226246665;372466577;28173564;109148552;155969697;119395754;119703753;47132620;194018511	N-Term(TMT6plex); K7(TMT6plex)	1285.8	0				
																	SYTSGPGSR	3	2	1	372466577	N-Term(TMT6plex)	1140.6	0	**1.1**	**1.0**	**0.9**	**1.1**
																	TEISEMNR	2	2	1	372466577	N-Term(TMT6plex); M6(Oxidation)	1224.6	0	**1.3**	**1.1**	**1.1**	**1.1**
																	GELAIK	3	2	4	372466577;67782365;119395754;89357932	N-Term(TMT6plex); K6(TMT6plex)	1088.7	0				
																	DVDEAYMNK	1	2	1	372466577	N-Term(TMT6plex); K9(TMT6plex)	1542.8	0	**1.3**	**1.1**	**1.4**	**1.2**
																	TQEKEQIK	1	2	1	372466577	N-Term(TMT6plex); K4(TMT6plex); K8(TMT6plex)	1691.0	1	**1.0**	**1.3**	**0.9**	**1.6**
																	TEISEMNR	1	2	1	372466577	N-Term(TMT6plex)	1208.6	0	**1.5**	**1.4**	**1.6**	**1.5**
499137518	NP_001265118.1	**TPM3**	7170	**tropomyosin alpha-3 chain isoform 7**	70.16	2	3	25	402	**1.2**	**1.4**	**1.4**	**1.2**	248	28.9	4.75												
																	IQVLQQQADDAEER	58	6	2	499137518;114155144	N-Term(TMT6plex)	1872.0	0				
																	IQLVEEELDR	65	22	9	63252898;63252900;499137518;42476296;4507651;114155144;63252904;114155140;47519616	N-Term(TMT6plex)	1472.8	0				
																	AISEELDHALNDMTSI	8	3	3	63252898;114155140;499137518	N-Term(TMT6plex); M13(Oxidation)	2004.0	0				
																	KLVIIEGDLER	11	9	3	499137518;114155144;114155140	N-Term(TMT6plex); K1(TMT6plex)	1743.1	1				
																	MELQEIQLK	12	18	6	63252898;63252900;63252904;499137518;114155144;114155140	N-Term(TMT6plex); M1(Oxidation); K9(TMT6plex)	1605.9	0				
																	KIQVLQQQADDAEER	29	6	2	499137518;114155144	N-Term(TMT6plex); K1(TMT6plex)	2229.2	1				
																	AELAESR	11	5	1	499137518	N-Term(TMT6plex)	1004.6	0	**1.2**	**1.4**	**1.3**	**1.2**
																	EQAEAEVASLNR	55	6	2	499137518;114155144	N-Term(TMT6plex)	1545.8	0				
																	LVIIEGDLER	25	9	3	499137518;114155144;114155140	N-Term(TMT6plex)	1385.8	0				
																	EDKYEEEIK	21	12	6	499137518;42476296;4507651;114155144;114155140;47519616	N-Term(TMT6plex); K3(TMT6plex); K9(TMT6plex)	1870.0	1				
																	LATALQK	47	22	9	63252898;63252900;499137518;42476296;4507651;114155144;63252904;114155140;47519616	N-Term(TMT6plex); K7(TMT6plex)	1202.8	0				
																	TIDDLEDELYAQK	6	2	2	114155140;499137518	N-Term(TMT6plex); K13(TMT6plex)	2011.1	0				
																	AADESER	4	18	9	63252898;63252900;499137518;42476296;4507651;114155144;63252904;114155140;47519616	N-Term(TMT6plex)	1006.5	0				
																	MELQEIQLK	4	18	6	499137518;114155144;114155140;63252898;63252900;63252904	N-Term(TMT6plex); K9(TMT6plex)	1589.9	0				
																	EMDEQIR	2	5	1	499137518	N-Term(TMT6plex)	1149.6	0	**1.7**	**2.1**	**2.5**	**1.2**
																	LMDQNLK	2	5	1	499137518	N-Term(TMT6plex); K7(TMT6plex)	1319.8	0	**1.4**	**1.5**	**1.8**	**1.1**
																	HIAEEADR	6	11	4	499137518;4507651;114155144;114155140	N-Term(TMT6plex)	1169.6	0				
																	KYEEVAR	9	20	9	63252898;63252900;499137518;42476296;4507651;114155144;63252904;114155140;47519616	N-Term(TMT6plex); K1(TMT6plex)	1352.8	1				
																	YSQKEDKYEEEIK	1	8	3	499137518;114155144;114155140	N-Term(TMT6plex); K4(TMT6plex); K7(TMT6plex); K13(TMT6plex)	2605.5	2				
																	AEFAER	5	24	9	63252898;63252900;499137518;42476296;4507651;114155144;63252904;114155140;47519616	N-Term(TMT6plex)	951.5	0				
																	EMDEQIR	2	5	1	499137518	N-Term(TMT6plex); M2(Oxidation)	1165.6	0	**1.0**	**1.1**	**1.0**	**1.3**
																	LMDQNLK	3	5	1	499137518	N-Term(TMT6plex); M2(Oxidation); K7(TMT6plex)	1335.8	0	**0.9**	**1.1**	**1.1**	**1.1**

Accession	RefSeq Accession	Gene Symbol	Entrez Gene ID	Description	Σ Coverage	Σ# Proteins	Σ# Unique Peptides	Σ# Peptides	Σ# PSMs	Het-1A-Smoke - 2M/Parental	Het-1A-Smoke - 4M/Parental	Het-1A-Smoke - 6M/Parental	Het-1A-Smoke - 8M/Parental	# AAs	MW [kDa]	calc. pI	Sequence	# PSMs	# Proteins	# Protein Groups	Protein Group Accessions	Modifications	MH+ [Da]	# Missed Cleavages	Het-1A-Smoke - 2M/Parental	Het-1A-Smoke - 4M/Parental	Het-1A-Smoke - 6M/Parental	Het-1A-Smoke - 8M/Parental
																	LEEAEK	4	20	10	63252898;63252900;499137518;42476296;4507651;114155144;63252904;114155140;47519616;116235456;116235451	N-Term(TMT6plex); K6(TMT6plex)	1176.7	0				
																	YEEVAR	4	24	9	63252898;63252900;499137518;42476296;4507651;114155144;63252904;114155140;47519616	N-Term(TMT6plex)	995.5	0				
																	HIAEEADRK	1	11	4	499137518;4507651;114155144;114155140	N-Term(TMT6plex); K9(TMT6plex)	1526.9	1				
																	EVEGER	1	6	2	499137518;114155144	N-Term(TMT6plex)	947.5	0				
																	ALKDEEK	4	9	3	499137518;114155144;114155140	N-Term(TMT6plex); K3(TMT6plex); K7(TMT6plex)	1519.9	1				
																	YEEEIK	2	19	9	63252898;63252900;499137518;42476296;4507651;114155144;63252904;114155140;47519616	N-Term(TMT6plex); K6(TMT6plex)	1268.7	0				
302191611	NP_001180499.1	**CSRP1**	1465	**cysteine and glycine-rich protein 1 isoform 3**	70.05	2	11	11	106	**1.8**	**2.2**	**2.3**	**2.5**	187	20.0	8.48												
																	TVYFAEEVQCEGNSFHK	4	2	1	302191611	N-Term(TMT6plex); C10(Carbamidomethyl); K17(TMT6plex)	2503.2	0	**1.8**	**2.3**	**2.2**	**2.3**
																	GYGYGQGAGTLSTDK	10	2	1	302191611	N-Term(TMT6plex); K15(TMT6plex)	1933.0	0	**2.1**	**2.7**	**2.8**	**3.1**
																	GLESTTLADK	6	2	1	302191611	N-Term(TMT6plex); K10(TMT6plex)	1492.9	0	**1.8**	**2.0**	**2.3**	**2.2**
																	HEEAPGHRPTTNPNASK	14	2	1	302191611	N-Term(TMT6plex); K17(TMT6plex)	2301.2	0	**2.0**	**2.6**	**2.9**	**3.4**
																	NLDSTTVAVHGEEIYCK	14	2	1	302191611	N-Term(TMT6plex); C16(Carbamidomethyl); K17(TMT6plex)	2394.2	0	**1.9**	**2.0**	**2.2**	**2.1**
																	GYGYGQGAGTLSTDKGESLGIK	16	2	1	302191611	N-Term(TMT6plex); K15(TMT6plex); K22(TMT6plex)	2846.6	1	**1.8**	**2.6**	**2.5**	**2.9**
																	GLESTTLADKDGEIYCK	5	2	1	302191611	N-Term(TMT6plex); K10(TMT6plex); C16(Carbamidomethyl); K17(TMT6plex)	2587.4	1	**2.1**	**3.2**	**3.0**	**3.4**
																	GFGFGQGAGALVHSE	29	2	1	302191611	N-Term(TMT6plex)	1662.8	0	**1.6**	**1.8**	**1.7**	**1.9**
																	SCFLCMVCK	4	2	1	302191611	N-Term(TMT6plex); C2(Carbamidomethyl); C5(Carbamidomethyl); C8(Carbamidomethyl); K9(TMT6plex)	1662.8	0	**2.0**	**2.3**	**2.7**	**2.3**
																	CSQAVYAAEK	3	2	1	302191611	N-Term(TMT6plex); C1(Carbamidomethyl); K10(TMT6plex)	1584.8	0	**1.8**	**1.6**	**1.8**	**1.6**
																	PNWGGGK	1	2	1	302191611	N-Term(TMT6plex); K7(TMT6plex)	1173.7	0	**2.0**	**1.6**	**2.4**	**1.8**
28195394	NP_778235.1	**HIST2H2AB**	317772	**histone H2A type 2-B**	70.00	2	2	8	923	**0.9**	**1.2**	**1.2**	**1.2**	130	14.0	10.89												
																	LLGGVTIAQGGVLPNIQAVLLPK	153	3	2	28195394;4504253	N-Term(TMT6plex); K23(TMT6plex)	2729.7	0				
																	NDEELNK	248	13	4	10645195;28195394;24638446;4504253	N-Term(TMT6plex); K7(TMT6plex)	1319.7	0				
																	AGLQFPVGR	448	17	5	10645195;28195394;24638446;4504253;4504255	N-Term(TMT6plex)	1173.7	0				
																	VGAGAPVYLAAVLEYLTAEILELAGNAAR	66	10	3	10645195;28195394;4504253	N-Term(TMT6plex)	3144.8	0				
																	KGNYAER	2	9	2	28195394;24638446	N-Term(TMT6plex); K1(TMT6plex)	1295.7	1				
																	GNYAER	2	9	2	28195394;24638446	N-Term(TMT6plex)	938.5	0				
																	HLQLAVR	2	1	1	28195394	N-Term(TMT6plex)	1065.7	0	**1.3**	**1.2**	**1.3**	**1.3**
																	KTESHKPGK	2	1	1	28195394	N-Term(TMT6plex); K1(TMT6plex); K6(TMT6plex); K9(TMT6plex)	1928.2	1	**0.7**	**1.1**	**1.1**	**1.2**
213511012	NP_001132913.1	**BCAP31**	10134	**B-cell receptor-associated protein 31 isoform b**	69.92	2	17	17	113	**1.2**	**1.1**	**1.1**	**1.1**	246	28.0	8.44												
																	LDVGNAEVK	14	2	1	213511012	N-Term(TMT6plex); K9(TMT6plex)	1402.8	0	**1.3**	**1.2**	**1.2**	**1.2**
																	QAESASEAAK	13	2	1	213511012	N-Term(TMT6plex); K10(TMT6plex)	1449.8	0	**1.2**	**1.2**	**1.1**	**1.1**
																	LKDELASTK	9	2	1	213511012	N-Term(TMT6plex); K2(TMT6plex); K9(TMT6plex)	1692.1	1	**1.1**	**1.1**	**1.2**	**1.2**
																	LQAAVDGPMDK	16	2	1	213511012	N-Term(TMT6plex); M9(Oxidation); K11(TMT6plex)	1618.9	0	**1.5**	**1.4**	**1.3**	**1.5**
																	LLEEHAK	12	2	1	213511012	N-Term(TMT6plex); K7(TMT6plex)	1297.8	0	**1.1**	**1.1**	**1.0**	**1.1**
																	YMEENDQLK	9	2	1	213511012	N-Term(TMT6plex); M2(Oxidation); K9(TMT6plex)	1643.8	0	**1.1**	**0.9**	**0.9**	**1.1**
																	VNLQNNPGAMEHFHMK	4	2	1	213511012	N-Term(TMT6plex); M10(Oxidation); M15(Oxidation); K16(TMT6plex)	2357.2	0	**1.3**	**1.3**	**1.2**	**1.3**
																	KQAESASEAAK	2	2	1	213511012	N-Term(TMT6plex); K1(TMT6plex); K11(TMT6plex)	1807.1	1	**1.3**	**1.3**	**1.2**	**1.2**
																	KYMEENDQLK	3	2	1	213511012	N-Term(TMT6plex); K1(TMT6plex); M3(Oxidation); K10(TMT6plex)	2001.1	1	**1.1**	**1.0**	**1.0**	**1.1**
																	KGAAVDGGK	3	2	1	213511012	N-Term(TMT6plex); K1(TMT6plex); K9(TMT6plex)	1489.9	1	**1.1**	**1.2**	**1.1**	**1.2**
																	LVTLISQQATLLASNEAFK	10	2	1	213511012	N-Term(TMT6plex); K19(TMT6plex)	2505.5	0	**1.2**	**0.9**	**1.2**	**0.8**
																	GAAVDGGK	4	2	1	213511012	N-Term(TMT6plex); K8(TMT6plex)	1132.7	0	**1.1**	**1.1**	**1.0**	**1.1**
																	SLQWTAVATFLYAEVFVVLLLCIPFISPK	1	1	1	213511012	N-Term(TMT6plex); C22(Carbamidomethyl); K29(TMT6plex)	3784.2	0	**1.8**	**1.2**	**2.5**	**0.5**
																	NLYIAGFSLLLSFLLRR	2	2	1	213511012	N-Term(TMT6plex)	2225.3	1	**1.7**	**0.9**	**1.8**	**0.5**
																	KYDDVTEK	5	2	1	213511012	N-Term(TMT6plex); K1(TMT6plex); K8(TMT6plex)	1685.0	1	**1.1**	**1.1**	**1.1**	**1.2**
																	AENQVLAMR	3	2	1	213511012	N-Term(TMT6plex); M8(Oxidation)	1276.7	0	**1.1**	**1.1**	**1.0**	**1.1**
																	KQSEGLTK	3	2	1	213511012	N-Term(TMT6plex); K1(TMT6plex); K8(TMT6plex)	1578.0	1	**1.1**	**1.1**	**1.1**	**1.1**
83641885	NP_001032726.1	**BTF3**	689	**transcription factor BTF3 isoform A**	69.90	2	12	14	267	**1.2**	**1.3**	**1.4**	**1.4**	206	22.2	9.38												
																	QLTEMLPSILNQLGADSLTSLR	57	2	1	83641885	N-Term(TMT6plex); M5(Oxidation)	2645.4	0	**1.4**	**1.4**	**1.5**	**1.3**
																	LGVNNISGIEEVNMFTNQGTVIHFNNPK	31	2	1	83641885	N-Term(TMT6plex); M14(Oxidation); K28(TMT6plex)	3560.9	0	**1.2**	**1.3**	**1.4**	**1.4**
																	LGVNNISGIEEVNMFTNQGTVIHFNNPK	5	2	1	83641885	N-Term(TMT6plex); K28(TMT6plex)	3544.8	0	**1.5**	**1.5**	**1.6**	**1.5**
																	APLATGEDDDDEVPDLVENFDEASKNEAN	25	2	1	83641885	N-Term(TMT6plex); K25(TMT6plex)	3577.7	1	**1.2**	**1.4**	**1.5**	**1.5**
																	ETIMNQEK	7	2	1	83641885	N-Term(TMT6plex); K8(TMT6plex)	1450.8	0	**1.6**	**1.9**	**2.1**	**1.5**
																	ETIMNQEK	9	2	1	83641885	N-Term(TMT6plex); M4(Oxidation); K8(TMT6plex)	1466.8	0	**1.0**	**1.3**	**1.3**	**1.4**
																	KLGVNNISGIEEVNMFTNQGTVIHFNNPK	2	2	1	83641885	N-Term(TMT6plex); K1(TMT6plex); M15(Oxidation); K29(TMT6plex)	3918.1	1	**0.7**	**1.2**	**1.1**	**1.1**
																	TATADDK	3	4	2	56847620;83641885	N-Term(TMT6plex); K7(TMT6plex)	1179.7	0				
																	APLATGEDDDDEVPDLVENFDEASK	52	2	1	83641885	N-Term(TMT6plex); K25(TMT6plex)	3149.5	0	**1.2**	**1.4**	**1.5**	**1.3**
																	VQASLAANTFTITGHAETK	12	2	1	83641885	N-Term(TMT6plex); K19(TMT6plex)	2418.3	0	**1.2**	**1.2**	**1.5**	**1.4**
																	TGAPAQADSR	4	1	1	83641885	N-Term(TMT6plex)	1202.6	0	**1.6**	**1.2**	**1.7**	**1.4**
																	QLTEMLPSILNQLGADSLTSLR	32	2	1	83641885	N-Term(TMT6plex)	2629.4	0	**1.4**	**1.3**	**1.4**	**1.3**
																	LAEALPK	17	2	1	83641885	N-Term(TMT6plex); K7(TMT6plex)	1199.8	0	**1.1**	**1.4**	**1.4**	**1.4**
																	TATADDKK	5	4	2	56847620;83641885	N-Term(TMT6plex); K7(TMT6plex); K8(TMT6plex)	1536.9	1				
																	KLQFSLK	2	2	1	83641885	N-Term(TMT6plex); K1(TMT6plex); K7(TMT6plex)	1551.0	1	**1.1**	**1.9**	**1.6**	**1.9**
																	LQFSLK	3	2	1	83641885	N-Term(TMT6plex); K6(TMT6plex)	1193.8	0	**1.3**	**1.2**	**1.3**	**1.3**
																	QSVDGK	1	2	1	83641885	N-Term(TMT6plex); K6(TMT6plex)	1091.6	0	**1.1**	**1.1**	**1.2**	**1.1**
4758496;93141020	NP_004884.1	**H2AFY**	9555	**core histone macro-H2A.1 isoform 2**	69.81	2	3	22	295	**1.3**	**1.1**	**1.2**	**1.4**	371	39.5	9.77												
																	GVTIASGGVLPNIHPELLAK	36	3	2	4758496;93141020;20336746	N-Term(TMT6plex); K20(TMT6plex)	2444.5	0				
																	EFVEAVLELR	12	3	2	4758496;93141020;20336746	N-Term(TMT6plex)	1433.8	0				
																	NCLALADDK	8	3	2	4758496;93141020;20336746	N-Term(TMT6plex); C2(Carbamidomethyl); K9(TMT6plex)	1477.8	0				
																	SLFLGQK	15	3	2	4758496;93141020;20336746	N-Term(TMT6plex); K7(TMT6plex)	1250.8	0				
																	NGPLEVAGAAVSAGHGLPAK	8	3	2	4758496;93141020;20336746	N-Term(TMT6plex); K20(TMT6plex)	2274.3	0				
																	HILLAVANDEELNQLLK	17	4	3	4758496;93141020;20336746;8923920	N-Term(TMT6plex); K17(TMT6plex)	2391.4	0				
																	AGVIFPVGR	16	4	3	4758496;93141020;20336746;8923920	N-Term(TMT6plex)	1144.7	0				
																	QTAAQLILK	9	3	2	4758496;93141020;20336746	N-Term(TMT6plex); K9(TMT6plex)	1443.9	0				
																	AASADSTTEGTPADGFTVLSTK	60	3	2	4758496;93141020;20336746	N-Term(TMT6plex); K22(TMT6plex)	2585.3	0				
																	LEAIITPPPAK	8	3	2	4758496;93141020;20336746	N-Term(TMT6plex); K11(TMT6plex)	1608.0	0				
																	IGVGAPVYMAAVLEYLTAEILELAGNAAR	23	3	2	4758496;93141020;20336746	N-Term(TMT6plex); M9(Oxidation)	3220.8	0				
																	GKLEAIITPPPAK	30	3	2	4758496;93141020;20336746	N-Term(TMT6plex); K2(TMT6plex); K13(TMT6plex)	2022.3	1				
																	SIAFPSIGSGR	17	3	2	4758496;93141020;20336746	N-Term(TMT6plex)	1320.7	0				
																	AISSYFVSTMSSSIK	11	3	2	4758496;93141020;20336746	N-Term(TMT6plex); M10(Oxidation); K15(TMT6plex)	2082.1	0				
																	FVIHCNSPVWGADK	6	3	2	4758496;93141020;20336746	N-Term(TMT6plex); C5(Carbamidomethyl); K14(TMT6plex)	2088.1	0				
																	NCLALADDKK	1	3	2	4758496;93141020;20336746	N-Term(TMT6plex); C2(Carbamidomethyl); K9(TMT6plex); K10(TMT6plex)	1835.1	1				
																	LNLIHSEISNLAGFEVEAIINPTNADIDLK	4	2	1	4758496;93141020	N-Term(TMT6plex); K30(TMT6plex)	3722.0	0	**1.4**	**1.2**	**1.2**	**1.4**
																	LNLIHSEISNLAGFEVEAIINPTNADIDLKDDLGNTLEK	4	2	1	4758496;93141020	N-Term(TMT6plex); K30(TMT6plex); K39(TMT6plex)	4936.7	1	**1.1**	**1.2**	**1.2**	**1.4**
																	IGVGAPVYMAAVLEYLTAEILELAGNAAR	2	3	2	4758496;93141020;20336746	N-Term(TMT6plex)	3204.7	0				
																	AISSYFVSTMSSSIK	2	3	2	4758496;93141020;20336746	N-Term(TMT6plex); K15(TMT6plex)	2066.1	0				
																	FVIHCNSPVWGADKCEELLEK	1	3	2	4758496;93141020;20336746	N-Term(TMT6plex); C5(Carbamidomethyl); K14(TMT6plex); C15(Carbamidomethyl); K21(TMT6plex)	3218.7	1				
																	DDLGNTLEK	2	2	1	4758496;93141020	N-Term(TMT6plex); K9(TMT6plex)	1462.8	0	**1.1**	**1.1**	**1.1**	**1.3**
																	KQGEVSK	2	3	2	4758496;93141020;20336746	N-Term(TMT6plex); K1(TMT6plex); K7(TMT6plex)	1462.9	1				
																	QGEVSK	1	3	2	4758496;93141020;20336746	N-Term(TMT6plex); K6(TMT6plex)	1105.7	0				
284795266	NP_067026.3	**SRPRB**	58477	**signal recognition particle receptor subunit beta**	69.74	1	17	17	144	**1.7**	**1.8**	**1.7**	**1.6**	271	29.7	9.04												
																	GDVGSADIQDLEK	6	1	1	284795266	N-Term(TMT6plex); K13(TMT6plex)	1805.0	0	**1.7**	**2.2**	**1.9**	**1.9**
																	DTQTSITDSCAVYR	12	1	1	284795266	N-Term(TMT6plex); C10(Carbamidomethyl)	1845.9	0	**1.8**	**1.9**	**1.7**	**1.7**
																	GNSLTLIDLPGHESLR	2	1	1	284795266	N-Term(TMT6plex)	1951.1	0	**1.4**	**1.6**	**1.7**	**1.4**
																	DVAEFLYQVLIDSMGLK	6	1	1	284795266	N-Term(TMT6plex); M14(Oxidation); K17(TMT6plex)	2415.3	0	**1.4**	**1.5**	**1.5**	**1.5**
																	GKEFEFSQLPLK	2	1	1	284795266	N-Term(TMT6plex); K2(TMT6plex); K12(TMT6plex)	2110.3	1	**1.7**	**2.0**	**1.8**	**1.8**
																	NTPSFLIACNK	2	1	1	284795266	N-Term(TMT6plex); C9(Carbamidomethyl); K11(TMT6plex)	1723.0	0	**1.6**	**1.5**	**1.6**	**1.5**
																	AVLLVGLCDSGK	20	1	1	284795266	N-Term(TMT6plex); C8(Carbamidomethyl); K12(TMT6plex)	1690.0	0	**1.7**	**1.9**	**1.8**	**1.7**

Supplementary Table 6. List of all proteins and peptides identified in untreated and chronically treated Het1A cells with cigarette smoke condensate for 8 months

Accession	RefSeq Accession	Gene Symbol	Entrez Gene ID	Description	Σ Coverage	Σ# Proteins	Σ# Unique Peptides	Σ# Peptides	Σ# PSMs	Het-1A-Smoke - 2M/Parental	Het-1A-Smoke - 4M/Parental	Het-1A-Smoke - 6M/Parental	Het-1A-Smoke - 8M/Parental	# AAs	MW [kDa]	calc. pI	Sequence	# PSMs	# Proteins	# Protein Groups	Protein Group Accessions	Modifications	MH+ [Da]	# Missed Cleavages	Het-1A-Smoke - 2M/Parental	Het-1A-Smoke - 4M/Parental	Het-1A-Smoke - 6M/Parental	Het-1A-Smoke - 8M/Parental
																	VADGGGAGGTFQPYLDTLR	22	1	1	284795266	N-Term(TMT6plex)	2124.1	0	**1.6**	**1.6**	**1.6**	**1.4**
																	SAAPSTLDSSSTAPAQLGK	16	1	1	284795266	N-Term(TMT6plex); K19(TMT6plex)	2247.2	0	**1.7**	**2.2**	**1.7**	**2.1**
																	LIQQQLEK	12	1	1	284795266	N-Term(TMT6plex); K8(TMT6plex)	1457.9	0	**1.7**	**1.9**	**1.7**	**1.8**
																	TLLFVR	5	1	1	284795266	N-Term(TMT6plex)	977.6	0	**1.9**	**1.7**	**1.8**	**1.5**
																	AIVFVVDSAAFQR	18	1	1	284795266	N-Term(TMT6plex)	1651.9	0	**1.5**	**1.6**	**1.7**	**1.7**
																	EFEFSQLPLK	14	1	1	284795266	N-Term(TMT6plex); K10(TMT6plex)	1696.0	0	**1.6**	**1.8**	**1.7**	**1.7**
																	DVAEFLYQVLIDSMGLK	1	1	1	284795266	N-Term(TMT6plex); K17(TMT6plex)	2399.3	0	**1.9**	**1.4**	**2.2**	**1.1**
																	VEFLECSAK	2	1	1	284795266	N-Term(TMT6plex); C6(Carbamidomethyl); K9(TMT6plex)	1540.8	0	**1.8**	**2.0**	**1.9**	**1.6**
																	QDIAMAK	1	1	1	284795266	N-Term(TMT6plex); M5(Oxidation); K7(TMT6plex)	1250.7	0	**1.4**	**1.7**	**1.6**	**1.3**
																	LLTGLYR	1	1	1	284795266	N-Term(TMT6plex)	1064.7	0	**1.8**	**1.5**	**1.6**	**1.5**
																	LQFLER	2	1	1	284795266	N-Term(TMT6plex)	1034.6	0	**2.0**	**2.0**	**1.9**	**1.8**
35493916	NP_002942.2	**RPN2**	6185	**dolichyl-diphosphooligosaccharide--protein glycosyltransferase subunit 2 isoform 1 precursor**	69.73	2	28	28	331	**1.5**	**1.4**	**1.5**	**1.4**	631	69.2	5.69												
																	YHVPVVVVPEGSASDTHEQAILR	22	2	1	35493916	N-Term(TMT6plex)	2732.5	0	**1.7**	**1.6**	**1.7**	**1.7**
																	LMDHVGTEPSIK	5	2	1	35493916	N-Term(TMT6plex); M2(Oxidation); K12(TMT6plex)	1801.0	0	**1.3**	**1.3**	**1.3**	**1.4**
																	TSFTPVGDVFELNFMNVK	34	2	1	35493916	N-Term(TMT6plex); M15(Oxidation); K18(TMT6plex)	2519.3	0	**1.6**	**1.3**	**1.6**	**1.4**
																	LSKEETVLATVQALQTASHLSQQADLR	7	2	1	35493916	N-Term(TMT6plex); K3(TMT6plex)	3395.9	1	**1.3**	**2.0**	**1.6**	**1.9**
																	NPILWNVADVVIK	29	2	1	35493916	N-Term(TMT6plex); K13(TMT6plex)	1939.2	0	**1.4**	**1.2**	**1.5**	**1.3**
																	LQVTNVLSQPLTQATVK	26	2	1	35493916	N-Term(TMT6plex); K17(TMT6plex)	2298.4	0	**1.3**	**1.5**	**1.4**	**1.4**
																	SNLDPSNVDSLFYAAQASQALSGCEISISNETK	5	1	1	35493916	N-Term(TMT6plex); C24(Carbamidomethyl); K33(TMT6plex)	3975.0	0	**1.5**	**1.3**	**1.5**	**1.2**
																	FSSGYYDFLVEVEGDNR	4	2	1	35493916	N-Term(TMT6plex)	2226.0	0	**1.6**	**1.1**	**1.4**	**1.3**
																	YIANTVELR	6	2	1	35493916	N-Term(TMT6plex)	1307.8	0	**1.4**	**1.3**	**1.4**	**1.4**
																	EDQVIQLMNAIFSK	5	2	1	35493916	N-Term(TMT6plex); M8(Oxidation); K14(TMT6plex)	2110.2	0	**1.2**	**1.2**	**1.3**	**1.3**
																	EDQVIQLMNAIFSK	12	2	1	35493916	N-Term(TMT6plex); K14(TMT6plex)	2094.2	0	**1.4**	**1.3**	**1.6**	**1.5**
																	LDELGGVYLQFEEGLETTALFVAATYK	4	2	1	35493916	N-Term(TMT6plex); K27(TMT6plex)	3435.8	0	**1.9**	**1.6**	**1.7**	**1.8**
																	TSFTPVGDVFELNFMNVK	5	2	1	35493916	N-Term(TMT6plex); K18(TMT6plex)	2503.3	0	**1.8**	**2.0**	**2.1**	**1.8**
																	ISTEVGITNVDLSTVDKDQSIAPK	12	2	1	35493916	N-Term(TMT6plex); K17(TMT6plex); K24(TMT6plex)	3217.8	1	**1.4**	**1.3**	**1.4**	**1.5**
																	NFESLSEAFSVASAAAVLSHNR	9	2	1	35493916	N-Term(TMT6plex)	2536.3	0	**1.7**	**1.7**	**1.7**	**1.7**
																	QEIQHLFR	2	2	1	35493916	N-Term(TMT6plex)	1299.7	0	**1.5**	**1.4**	**1.4**	**1.4**
																	LMDHVGTEPSIKEDQVIQLMNAIFSK	4	2	1	35493916	N-Term(TMT6plex); M2(Oxidation); K12(TMT6plex); K26(TMT6plex)	3647.0	1	**1.2**	**1.7**	**1.5**	**1.9**
																	EETVLATVQALQTASHLSQQADLR	20	2	1	35493916	N-Term(TMT6plex)	2838.5	0	**1.4**	**1.2**	**1.5**	**1.2**
																	KNFESLSEAFSVASAAAVLSHNR	6	2	1	35493916	N-Term(TMT6plex); K1(TMT6plex)	2893.6	1	**1.7**	**1.8**	**1.8**	**1.8**
																	ASLDRPFTNLESAFYSIVGLSSLGAQVPDAK	28	2	1	35493916	N-Term(TMT6plex); K31(TMT6plex)	3712.0	0	**1.5**	**1.7**	**1.8**	**1.7**
																	FPEEEAPSTVLSQNLFTPK	30	2	1	35493916	N-Term(TMT6plex); K19(TMT6plex)	2592.4	0	**1.4**	**1.6**	**1.6**	**1.5**
																	TGQEVVFVAEPDNK	8	2	1	35493916	N-Term(TMT6plex); K14(TMT6plex)	1991.1	0	**1.5**	**1.1**	**1.4**	**1.2**
																	FELDTSER	7	2	1	35493916	N-Term(TMT6plex)	1225.6	0	**1.5**	**1.4**	**1.5**	**1.5**
																	SIVEEIEDLVAR	28	2	1	35493916	N-Term(TMT6plex)	1601.9	0	**1.4**	**1.3**	**1.2**	**1.4**
																	KIEFDSASGTYTLYLIIGDATLK	2	2	1	35493916	N-Term(TMT6plex); K1(TMT6plex); K23(TMT6plex)	3206.8	1	**1.7**	**1.4**	**2.0**	**1.1**
																	DLLLAAVSEDSSVTQIYHAVAALSGFGLPLASQEALSALTAR	2	2	1	35493916	N-Term(TMT6plex)	4485.4	0	**1.8**	**1.4**	**2.5**	**0.9**
																	MLAQQAVK	4	2	1	35493916	N-Term(TMT6plex); M1(Oxidation); K8(TMT6plex)	1362.8	0	**1.5**	**1.3**	**1.8**	**1.4**
																	VTYPAK	3	2	1	35493916	N-Term(TMT6plex); K6(TMT6plex)	1136.7	0	**1.2**	**1.4**	**1.4**	**1.4**
																	ACTYIR	1	1	1	35493916	N-Term(TMT6plex); C2(Carbamidomethyl)	1012.5	0	**1.5**	**1.5**	**1.5**	**1.5**
																	ATVLQK	1	2	1	35493916	N-Term(TMT6plex); K6(TMT6plex)	1117.7	0	**1.3**	**1.6**	**1.5**	**1.4**
4758484	NP_004823.1	**GSTO1**	9446	**glutathione S-transferase omega-1 isoform 1**	69.71	3	17	17	141	**1.5**	**1.6**	**1.9**	**2.0**	241	27.5	6.60												
																	GSAPPGPVPEGSIR	14	2	1	4758484	N-Term(TMT6plex)	1549.9	0	**1.7**	**1.7**	**2.0**	**2.2**
																	EDPTVSALLTSEK	17	3	1	4758484	N-Term(TMT6plex); K13(TMT6plex)	1848.0	0	**1.5**	**1.5**	**1.9**	**1.9**
																	LNECVDHTPK	6	3	1	4758484	N-Term(TMT6plex); C4(Carbamidomethyl); K10(TMT6plex)	1670.9	0	**1.4**	**1.5**	**1.9**	**1.9**
																	LLPDDPYEK	11	3	1	4758484	N-Term(TMT6plex); K9(TMT6plex)	1547.9	0	**1.5**	**1.5**	**1.7**	**2.0**
																	KNPFGLVPVLENSQGQLIYESAITCEYLDEAYPGK	4	3	1	4758484	N-Term(TMT6plex); K1(TMT6plex); C25(Carbamidomethyl); K35(TMT6plex)	4630.4	1	**1.5**	**1.9**	**2.3**	**2.6**
																	MILELFSK	13	3	1	4758484	N-Term(TMT6plex); M1(Oxidation); K8(TMT6plex)	1454.9	0	**1.6**	**1.6**	**1.9**	**2.0**
																	VPSLVGSFIR	22	2	1	4758484	N-Term(TMT6plex)	1303.8	0	**1.5**	**1.7**	**2.0**	**2.1**
																	NPFGLVPVLENSQGQLIYESAITCEYLDEAYPGK	13	3	1	4758484	N-Term(TMT6plex); C24(Carbamidomethyl); K34(TMT6plex)	4273.2	0	**1.9**	**1.5**	**2.3**	**1.9**
																	NKPEWFFK	9	3	1	4758484	N-Term(TMT6plex); K2(TMT6plex); K8(TMT6plex)	1783.0	0	**1.5**	**1.5**	**1.9**	**1.9**
																	EDYAGLKEEFR	2	2	1	4758484	N-Term(TMT6plex); K7(TMT6plex)	1815.0	1	**1.6**	**1.6**	**1.9**	**2.0**
																	LEEVLTNK	9	2	1	4758484	N-Term(TMT6plex); K8(TMT6plex)	1403.8	0	**1.5**	**1.4**	**1.8**	**1.9**
																	HEVININLK	6	3	1	4758484	N-Term(TMT6plex); K9(TMT6plex)	1537.9	0	**1.4**	**1.6**	**1.8**	**2.0**
																	MILELFSK	4	3	1	4758484	N-Term(TMT6plex); K8(TMT6plex)	1438.9	0	**1.7**	**2.0**	**2.7**	**2.4**
																	DWQGFLELYLQNSPEACDYGL	6	3	1	4758484	N-Term(TMT6plex); C17(Carbamidomethyl)	2747.3	0	**1.5**	**1.5**	**1.5**	**1.7**
																	KLLPDDPYEK	1	3	1	4758484	N-Term(TMT6plex); K1(TMT6plex); K10(TMT6plex)	1905.1	1	**1.3**	**1.3**	**1.5**	**1.7**
																	SQNKEDYAGLK	1	2	1	4758484	N-Term(TMT6plex); K4(TMT6plex); K11(TMT6plex)	1940.1	1	**1.3**	**1.3**	**1.6**	**1.6**
																	EDYAGLK	2	2	1	4758484	N-Term(TMT6plex); K7(TMT6plex)	1253.7	0				
																	FCPFAER	1	3	1	4758484	N-Term(TMT6plex); C2(Carbamidomethyl)	1155.6	0	**1.5**	**1.4**	**1.7**	**1.8**
63162572	NP_005989.3	**CCT3**	7203	**T-complex protein 1 subunit gamma isoform a**	69.54	3	40	41	506	**1.3**	**1.4**	**1.4**	**1.5**	545	60.5	6.49												
																	ALDDMISTLK	14	2	1	63162572	N-Term(TMT6plex); M5(Oxidation); K10(TMT6plex)	1580.9	0	**1.2**	**1.4**	**1.3**	**1.6**
																	EILSEVER	14	2	1	63162572	N-Term(TMT6plex)	1203.7	0	**1.2**	**1.4**	**1.3**	**1.5**
																	EIQVQHPAAK	13	1	1	63162572	N-Term(TMT6plex); K10(TMT6plex)	1578.9	0	**1.3**	**1.5**	**1.4**	**1.5**
																	IVSRPEELREDDVGTGAGLLEIK	10	2	1	63162572	N-Term(TMT6plex); K23(TMT6plex)	2954.7	1	**1.4**	**1.4**	**1.4**	**1.4**
																	GISDLAQHYLMR	24	2	1	63162572	N-Term(TMT6plex); M11(Oxidation)	1648.9	0	**1.4**	**1.4**	**1.3**	**1.4**
																	ELGIWEPLAVK	29	2	1	63162572	N-Term(TMT6plex); K11(TMT6plex)	1713.0	0	**1.3**	**1.6**	**1.5**	**1.6**
																	TLIQNCGASTIR	12	2	1	63162572	N-Term(TMT6plex); C6(Carbamidomethyl)	1562.9	0	**1.7**	**1.4**	**1.4**	**1.4**
																	ISIPVDISDSDMMLNIINSSITTK	28	2	1	63162572	N-Term(TMT6plex); M12(Oxidation); M13(Oxidation); K24(TMT6plex)	3097.6	0	**1.5**	**1.6**	**1.5**	**1.7**
																	KISIPVDISDSDMMLNIINSSITTK	8	2	1	63162572	N-Term(TMT6plex); K1(TMT6plex); K25(TMT6plex)	3422.9	1	**1.6**	**1.8**	**2.1**	**1.9**
																	AVAQALEVIPR	20	2	1	63162572	N-Term(TMT6plex)	1395.8	0	**1.4**	**1.5**	**1.4**	**1.5**
																	MLLDPMGGIVMTNDGNAILR	15	1	1	63162572	N-Term(TMT6plex); M6(Oxidation); M11(Oxidation)	2392.2	0	**1.4**	**1.4**	**1.4**	**1.6**
																	IGDEYFTFITDCKDPK	11	2	1	63162572	N-Term(TMT6plex); C12(Carbamidomethyl); K13(TMT6plex); K16(TMT6plex)	2636.4	1	**1.3**	**1.6**	**1.6**	**1.6**
																	MLLDPMGGIVMTNDGNAILR	10	1	1	63162572	N-Term(TMT6plex); M6(Oxidation)	2376.2	0	**1.5**	**1.7**	**1.7**	**1.5**
																	VQSGNINAAK	13	2	1	63162572	N-Term(TMT6plex); K10(TMT6plex)	1459.9	0	**1.5**	**1.3**	**1.4**	**1.3**
																	KGESQTDIEITR	18	2	1	63162572	N-Term(TMT6plex); K1(TMT6plex)	1835.0	1	**1.2**	**1.3**	**1.4**	**1.4**
																	TAVETAVLLLR	33	2	1	63162572	N-Term(TMT6plex)	1414.9	0	**1.4**	**1.3**	**1.4**	**1.4**
																	NLQDAMQVCR	17	2	1	63162572	N-Term(TMT6plex); M6(Oxidation); C9(Carbamidomethyl)	1479.7	0	**1.3**	**1.3**	**1.4**	**1.3**
																	IPGGIIEDSCVLR	23	2	1	63162572	N-Term(TMT6plex); C10(Carbamidomethyl)	1657.9	0	**1.5**	**1.6**	**1.7**	**1.5**
																	HTQENCETWGVNGETGTLVDMK	4	2	1	63162572	N-Term(TMT6plex); C6(Carbamidomethyl); M21(Oxidation); K22(TMT6plex)	2980.4	0	**1.2**	**1.2**	**1.4**	**1.2**
																	MLLDPMGGIVMTNDGNAILR	12	1	1	63162572	N-Term(TMT6plex); M1(Oxidation); M6(Oxidation); M11(Oxidation)	2408.2	0	**1.4**	**1.4**	**1.4**	**1.4**
																	ISIPVDISDSDMMLNIINSSITTK	25	2	1	63162572	N-Term(TMT6plex); M12(Oxidation); K24(TMT6plex)	3081.6	0	**1.3**	**1.5**	**1.4**	**1.6**
																	KIGDEYFTFITDCK	2	2	1	63162572	N-Term(TMT6plex); K1(TMT6plex); C13(Carbamidomethyl); K14(TMT6plex)	2424.3	1	**1.3**	**1.3**	**1.4**	**1.4**
																	KVQSGNINAAK	6	2	1	63162572	N-Term(TMT6plex); K1(TMT6plex); K11(TMT6plex)	1817.1	1	**1.2**	**1.5**	**1.4**	**1.5**
																	KISIPVDISDSDMMLNIINSSITTK	4	2	1	63162572	N-Term(TMT6plex); K1(TMT6plex); M13(Oxidation); M14(Oxidation); K25(TMT6plex)	3454.9	1	**1.5**	**1.6**	**1.7**	**1.8**
																	KIGDEYFTFITDCKDPK	2	2	1	63162572	K1(TMT6plex); N-Term(TMT6plex); C13(Carbamidomethyl); K14(TMT6plex); K17(TMT6plex)	2993.6	2	**1.1**	**1.6**	**1.5**	**1.7**
																	IVLLDSSLEYK	22	2	1	63162572	N-Term(TMT6plex); K11(TMT6plex)	1738.0	0	**1.5**	**1.5**	**1.5**	**1.7**
																	WSSLACNIALDAVK	3	2	1	63162572	N-Term(TMT6plex); C6(Carbamidomethyl); K14(TMT6plex)	2006.1	0	**1.7**	**1.6**	**1.6**	**1.6**
																	ALDDMISTLK	11	2	1	63162572	N-Term(TMT6plex); K10(TMT6plex)	1564.9	0	**1.7**	**1.9**	**2.1**	**1.7**
																	QGGAPDAGQE	10	2	1	63162572	N-Term(TMT6plex)	1158.6	0	**1.1**	**1.2**	**1.2**	**1.2**
																	GISDLAQHYLMR	4	2	1	63162572	N-Term(TMT6plex)	1632.9	0	**1.5**	**1.7**	**1.8**	**1.6**
																	AMTGVEQWPYR	8	2	1	63162572	N-Term(TMT6plex); M2(Oxidation)	1582.8	0	**1.3**	**1.2**	**1.1**	**1.4**
																	NVLLDPQLVPGGGASEMAVAHALTEK	6	2	1	63162572	N-Term(TMT6plex); K26(TMT6plex)	3075.7	0	**1.6**	**1.6**	**1.9**	**1.6**
																	NVLLDPQLVPGGGASEMAVAHALTEK	19	2	1	63162572	N-Term(TMT6plex); M17(Oxidation); K26(TMT6plex)	3091.7	0	**1.2**	**1.0**	**1.3**	**1.1**
																	KALDDMISTLK	2	2	1	63162572	N-Term(TMT6plex); K1(TMT6plex); K11(TMT6plex)	1922.2	1	**1.6**	**2.0**	**2.0**	**1.8**
																	KISIPVDISDSDMMLNIINSSITTK	2	2	1	63162572	N-Term(TMT6plex); K1(TMT6plex); M13(Oxidation); K25(TMT6plex)	3438.9	1	**1.0**	**1.5**	**1.3**	**1.6**
																	IGDEYFTFITDCK	4	2	1	63162572	N-Term(TMT6plex); C12(Carbamidomethyl); K13(TMT6plex)	2067.0	0	**0.9**	**1.0**	**1.0**	**1.2**
																	NLQDAMQVCR	5	2	1	63162572	N-Term(TMT6plex); C9(Carbamidomethyl)	1463.7	0	**1.5**	**1.4**	**1.6**	**1.4**
																	IVSRPEELR	3	2	1	63162572	N-Term(TMT6plex)	1327.8	0	**1.9**	**1.3**	**1.3**	**1.4**
																	EDDVGTGAGLLEIK	6	2	1	63162572	N-Term(TMT6plex); K14(TMT6plex)	1875.0	0	**1.2**	**1.2**	**1.4**	**1.4**

Accession	RefSeq Accession	Gene Symbol	Entrez Gene ID	Description	Σ Coverage	Σ# Proteins	Σ# Unique Peptides	Σ# Peptides	Σ# PSMs	Het-1A-Smoke - 2M/Parental	Het-1A-Smoke - 4M/Parental	Het-1A-Smoke - 6M/Parental	Het-1A-Smoke - 8M/Parental	# AAs	MW [kDa]	calc. pI	Sequence	# PSMs	# Proteins	# Protein Groups	Protein Group Accessions	Modifications	MH+ [Da]	# Missed Cleavages	Het-1A-Smoke - 2M/Parental	Het-1A-Smoke - 4M/Parental	Het-1A-Smoke - 6M/Parental	Het-1A-Smoke - 8M/Parental
																	IDDIVSGHK	6	2	1	63162572	N-Term(TMT6plex); K9(TMT6plex)	1441.8	0	1.2	1.4	1.3	1.4
																	KGESQTDIEITREEDFTR	2	2	1	63162572	N-Term(TMT6plex); K1(TMT6plex)	2612.4	2	1.3	1.7	1.5	1.7
																	TIADIIR	10	2	1	63162572	N-Term(TMT6plex)	1030.6	0	1.3	1.3	1.3	1.5
																	ACTILLR	3	2	1	63162572	N-Term(TMT6plex); C2(Carbamidomethyl)	1075.6	0	1.4	1.5	1.5	1.5
																	ANITAIR	2	2	1	63162572	N-Term(TMT6plex)	987.6	0	1.3	1.5	1.5	1.7
																	SMIEISR	3	2	1	63162572	N-Term(TMT6plex)	1064.6	0	1.5	1.7	1.9	1.5
																	LLTSLR	1	3	2	63162572;41393559	N-Term(TMT6plex)	931.6	0				
																	SMIEISR	2	2	1	63162572	N-Term(TMT6plex); M2(Oxidation)	1080.6	0	1.3	1.2	1.2	1.4
																	DVTHPR	1	2	1	63162572	N-Term(TMT6plex)	953.5	0	1.1	1.3	1.3	1.5
																	EEDFTR	1	2	1	63162572	N-Term(TMT6plex)	1025.5	0	1.4	1.4	1.4	1.6
																	TCLGPK	2	1	1	63162572	N-Term(TMT6plex); C2(Carbamidomethyl); K6(TMT6plex)	1133.7	0	1.3	1.2	1.3	1.3
																	KGDDQSR	1	2	1	63162572	N-Term(TMT6plex); K1(TMT6plex)	1263.7	1	1.3	1.4	1.4	1.6
223029410	NP_006280.3	**TLN1**	7094	**talin-1**	69.50	2	116	118	683	**1.4**	**1.6**	**1.6**	**1.6**	2541	269.6	6.07												
																	ASVPTIQDQASAMQLSQCAK	4	1	1	223029410	N-Term(TMT6plex); M13(Oxidation); C18(Carbamidomethyl); K20(TMT6plex)	2608.3	0	1.3	1.5	1.4	1.5
																	DLDQASLAAVSQQLAPR	14	1	1	223029410	N-Term(TMT6plex)	2012.1	0	1.4	1.6	1.5	1.6
																	GAAAHPDSEEQQQR	12	1	1	223029410	N-Term(TMT6plex)	1752.8	0	1.5	1.9	1.5	1.7
																	IPEAPAGPPSDFGLFLSDDDPK	15	1	1	223029410	N-Term(TMT6plex); K22(TMT6plex)	2743.4	0	1.5	1.6	1.7	1.8
																	GSQAQPDSPSAQLALIAASQSFLQPGGK	14	1	1	223029410	N-Term(TMT6plex); K28(TMT6plex)	3212.7	0	1.3	1.6	1.6	1.6
																	VVAPTISSPVCQEQLVEAGR	6	1	1	223029410	N-Term(TMT6plex); C11(Carbamidomethyl)	2369.3	0	1.1	1.3	1.3	1.5
																	ILADATAK	4	1	1	223029410	N-Term(TMT6plex); K8(TMT6plex)	1260.8	0	1.6	1.6	1.8	1.8
																	ASSLIEEAK	12	1	1	223029410	N-Term(TMT6plex); K9(TMT6plex)	1405.8	0	1.5	1.6	1.6	1.6
																	SGASGPENFQVGSMPPAQQQITSGQMHR	7	1	1	223029410	N-Term(TMT6plex); M14(Oxidation); M26(Oxidation)	3188.5	0	1.3	1.2	1.5	1.3
																	KAAGHPGDPESQQR	4	1	1	223029410	N-Term(TMT6plex); K1(TMT6plex)	1936.0	1	2.0	1.9	1.8	2.5
																	QEDVIATANLSR	7	2	1	223029410	N-Term(TMT6plex)	1545.8	0	1.4	1.3	1.4	1.4
																	VAGSVTELIQAAEAMK	16	1	1	223029410	N-Term(TMT6plex); M15(Oxidation); K16(TMT6plex)	2092.2	0	1.3	1.5	1.4	1.5
																	LLAALLEDEGGSGRPLLQAAK	8	1	1	223029410	N-Term(TMT6plex); K21(TMT6plex)	2580.5	0	1.5	1.8	1.9	2.0
																	VLVQNAAGSQEK	12	1	1	223029410	N-Term(TMT6plex); K12(TMT6plex)	1702.0	0	1.4	1.5	1.6	1.6
																	ELLENPVQPINDMSYFGCLDSVMENSK	6	1	1	223029410	N-Term(TMT6plex); M13(Oxidation); C18(Carbamidomethyl); M23(Oxidation); K27(TMT6plex)	3619.7	0	1.5	2.0	1.9	2.1
																	ADQDSEAMK	6	1	1	223029410	N-Term(TMT6plex); M8(Oxidation); K9(TMT6plex)	1468.7	0	1.3	1.4	1.4	1.7
																	AAMEPIVISAK	6	1	1	223029410	N-Term(TMT6plex); K11(TMT6plex)	1588.0	0	1.4	1.5	1.7	1.4
																	GVAALTSDPAVQAIVLDTASDVLDK	24	1	1	223029410	N-Term(TMT6plex); K25(TMT6plex)	2927.6	0	1.5	1.9	1.9	1.9
																	FGQDFSTFLEAGVEMAGQAPSQEDR	10	1	1	223029410	N-Term(TMT6plex); M15(Oxidation)	2962.4	0	1.5	1.4	1.4	1.4
																	QELAVFCSPEPPAK	8	1	1	223029410	N-Term(TMT6plex); C7(Carbamidomethyl); K14(TMT6plex)	2031.1	0	1.3	1.4	1.4	1.5
																	ALDGAFTEENR	4	1	1	223029410	N-Term(TMT6plex)	1451.7	0	1.5	1.7	1.6	1.7
																	GLAGAVSELLR	9	1	1	223029410	N-Term(TMT6plex)	1314.8	0	1.5	1.7	1.8	1.7
																	ALEATTEHIR	4	1	1	223029410	N-Term(TMT6plex)	1369.8	0	1.3	1.6	1.8	1.6
																	NCGQMSEIEAK	6	1	1	223029410	N-Term(TMT6plex); C2(Carbamidomethyl); M5(Oxidation); K11(TMT6plex)	1740.9	0	1.4	1.1	1.5	1.2
																	ILAQATSDLVNAIK	6	1	1	223029410	N-Term(TMT6plex); K14(TMT6plex)	1915.2	0	1.5	1.5	1.6	1.4
																	AGALQCSPSDAYTK	8	1	1	223029410	N-Term(TMT6plex); C6(Carbamidomethyl); K14(TMT6plex)	1927.0	0	1.7	1.6	1.6	1.6
																	AAMEPIVISAK	4	1	1	223029410	N-Term(TMT6plex); M3(Oxidation); K11(TMT6plex)	1604.0	0	1.6	1.8	1.6	1.9
																	TSTPEDFIR	2	1	1	223029410	N-Term(TMT6plex)	1294.7	0	2.0	1.4	1.5	1.4
																	FLPSELRDEH	5	1	1	223029410	N-Term(TMT6plex)	1471.8	1	1.5	1.6	1.5	1.6
																	TMQFEPSTMVYDACR	2	1	1	223029410	N-Term(TMT6plex); M2(Oxidation); M9(Oxidation); C14(Carbamidomethyl)	2096.9	0	1.1	1.3	1.2	1.4
																	ALCGFTEAAAQAAYLVGVSDPNSQAGQQGLVEPTQFAR	2	1	1	223029410	N-Term(TMT6plex); C3(Carbamidomethyl)	4152.1	0	1.7	1.5	1.8	2.3
																	VAGSVTELIQAAEAMK	5	1	1	223029410	N-Term(TMT6plex); K16(TMT6plex)	2076.2	0	1.6	3.6	2.1	4.1
																	EGISQEALHTQMLTAVQEISHLIEPLANAAR	4	1	1	223029410	N-Term(TMT6plex)	3599.9	0	1.2	1.9	1.7	1.9
																	ADAEGESDLENSR	4	1	1	223029410	N-Term(TMT6plex)	1621.8	0	1.5	1.6	1.6	1.8
																	ASVPTIQDQASAMQLSQCAK	2	1	1	223029410	N-Term(TMT6plex); C18(Carbamidomethyl); K20(TMT6plex)	2592.4	0	1.3	1.6	1.4	1.4
																	TLSHPQQMALLDQTK	2	1	1	223029410	N-Term(TMT6plex); M8(Oxidation); K15(TMT6plex)	2185.2	0	1.4	1.6	1.3	1.5
																	AVTDSINQLITMCTQQAPGQK	2	1	1	223029410	N-Term(TMT6plex); M12(Oxidation); C13(Carbamidomethyl); K21(TMT6plex)	2778.5	0	1.3	1.6	1.6	1.6
																	EGTETFADHR	1	1	1	223029410	N-Term(TMT6plex)	1391.7	0	1.2	1.3	1.2	1.4
																	ACEFAGFQCQIQFGPHNEQK	5	1	1	223029410	N-Term(TMT6plex); C2(Carbamidomethyl); C9(Carbamidomethyl); K20(TMT6plex)	2854.4	0	1.5	1.7	1.7	1.6
																	MVGGIAQIIAAQEEMLR	10	1	1	223029410	N-Term(TMT6plex); M1(Oxidation); M15(Oxidation)	2091.1	0	1.2	1.3	1.3	1.4
																	SNTSPEELGPLANQLTSDYGR	7	1	1	223029410	N-Term(TMT6plex)	2478.2	0	1.3	1.4	1.5	1.6
																	GIWLEAGK	2	1	1	223029410	N-Term(TMT6plex); K8(TMT6plex)	1331.8	0	1.3	1.5	1.7	1.7
																	GHMPPLTSAQQALTGTINSSMQAVQAAQATLDDFDTLPPLGQDAASK	2	1	1	223029410	N-Term(TMT6plex); M3(Oxidation); M21(Oxidation); K47(TMT6plex)	5284.7	0	1.6	1.9	1.2	1.9
																	GTQACITAASAVSGIIADLDTTIMFATAGTLNR	2	1	1	223029410	N-Term(TMT6plex); C5(Carbamidomethyl); M24(Oxidation)	3556.8	0	1.8	1.9	1.7	1.6
																	LKPLPGETMEK	1	1	1	223029410	N-Term(TMT6plex); K2(TMT6plex); M9(Oxidation); K11(TMT6plex)	1946.2	0	1.2	1.6	1.5	1.7
																	VKADQDSEAMK	2	1	1	223029410	N-Term(TMT6plex); K2(TMT6plex); M10(Oxidation); K11(TMT6plex)	1925.1	1	1.3	1.8	1.6	1.7
																	CTQDLGNSTK	2	1	1	223029410	N-Term(TMT6plex); C1(Carbamidomethyl); K10(TMT6plex)	1581.8	0	1.5	1.6	2.0	1.5
																	TLAESALQLLYTAK	8	1	1	223029410	N-Term(TMT6plex); K14(TMT6plex)	1980.2	0	1.5	1.7	1.8	1.7
																	ALGDLISATK	2	1	1	223029410	N-Term(TMT6plex); K10(TMT6plex)	1446.9	0	1.3	1.6	1.6	1.6
																	EADESLNFEEQILEAAK	18	1	1	223029410	N-Term(TMT6plex); K17(TMT6plex)	2394.2	0	1.4	1.8	1.6	1.8
																	AVSSAIAQLLGEVAQGNENYAGIAAR	24	1	1	223029410	N-Term(TMT6plex)	2802.5	0	1.5	1.5	1.6	1.4
																	VSQMAQYFEPLTLAAVGAASK	2	1	1	223029410	N-Term(TMT6plex); K21(TMT6plex)	2640.4	0	1.8	1.9	2.5	1.6
																	MATNAAAQNAIK	6	1	1	223029410	N-Term(TMT6plex); M1(Oxidation); K12(TMT6plex)	1677.9	0	1.4	1.4	1.5	1.5
																	AVAEQIPLLVQGVR	24	1	1	223029410	N-Term(TMT6plex)	1722.0	0	1.4	1.6	1.5	1.6
																	DHFGLEGDEESTMLEDSVSPK	8	1	1	223029410	N-Term(TMT6plex); M13(Oxidation); K21(TMT6plex)	2796.3	0	1.3	1.3	1.4	1.5
																	QFVQSAK	7	1	1	223029410	N-Term(TMT6plex); K7(TMT6plex)	1265.8	0	1.3	1.5	1.6	1.7
																	AEASQLGHK	4	1	1	223029410	N-Term(TMT6plex); K9(TMT6plex)	1398.8	0	1.4	1.6	1.7	1.4
																	ADQDSEAMK	4	1	1	223029410	N-Term(TMT6plex); K9(TMT6plex)	1452.7	0	1.7	2.2	2.1	1.7
																	GVGAAATAVTQALNELLQHVK	9	1	1	223029410	N-Term(TMT6plex); K21(TMT6plex)	2549.5	0	1.5	1.6	2.2	1.7
																	AVEDEATK	1	1	1	223029410	N-Term(TMT6plex); K8(TMT6plex)	1320.7	0	1.0	1.0	1.0	1.0
																	SIAAATSALVK	12	2	1	223029410	N-Term(TMT6plex); K11(TMT6plex)	1489.9	0	1.5	1.6	1.6	1.6
																	LGAASLGAEDPETQVVLINAVK	14	1	1	223029410	N-Term(TMT6plex); K22(TMT6plex)	2653.5	0	1.4	1.6	1.7	1.7
																	ASAGPQPLLVQSCK	4	1	1	223029410	N-Term(TMT6plex); C13(Carbamidomethyl); K14(TMT6plex)	1914.1	0	2.0	1.8	1.9	1.7
																	GTEWVDPEDPTVIAENELLGAAAAIEAAAK	11	1	1	223029410	N-Term(TMT6plex); K30(TMT6plex)	3509.8	0	1.5	2.0	1.7	1.8
																	QAAASATQTIAAAQHAASTPK	2	1	1	223029410	N-Term(TMT6plex); K21(TMT6plex)	2453.3	0	1.5	1.4	1.6	1.7
																	LHTDDELNWLDHGR	6	1	1	223029410	N-Term(TMT6plex)	1950.0	0	1.4	1.5	1.4	1.5
																	MVGGIAQIIAAQEEMLR	6	1	1	223029410	N-Term(TMT6plex); M1(Oxidation)	2075.1	0	1.5	1.5	1.5	1.4
																	QNLLQAAGNVGQASGELLQQIGESDTDPHFQDALMQLAK	6	1	1	223029410	N-Term(TMT6plex); M35(Oxidation); K39(TMT6plex)	4610.3	0	1.5	1.4	1.5	1.9
																	VMVTNVTSLLK	7	2	1	223029410	N-Term(TMT6plex); M2(Oxidation); K11(TMT6plex)	1679.0	0	1.2	1.5	1.5	1.5
																	SQLAAAAR	2	1	1	223029410	N-Term(TMT6plex)	1016.6	0	1.3	1.3	1.6	1.5
																	VQELGHGCAALVTK	2	1	1	223029410	N-Term(TMT6plex); C8(Carbamidomethyl); K14(TMT6plex)	1941.1	0	1.2	1.5	1.6	1.4
																	ANQAIQMACQSLGEPGCTQAQVLSAATIVAK	2	1	1	223029410	N-Term(TMT6plex); M7(Oxidation); C9(Carbamidomethyl); C17(Carbamidomethyl); K31(TMT6plex)	3690.9	0	1.6	1.6	1.9	1.6
																	EVIQEWNLTNIK	10	1	1	223029410	N-Term(TMT6plex); K12(TMT6plex)	1945.1	0	1.4	1.6	1.7	1.7
																	DPVQLNLLYVQAR	10	2	1	223029410	N-Term(TMT6plex)	1758.0	0	1.1	1.3	1.3	1.3
																	AIAVTVQEMVTK	2	1	1	223029410	N-Term(TMT6plex); M9(Oxidation); K12(TMT6plex)	1764.0	0	1.3	1.7	1.5	1.8
																	FFYSDQNVDSR	4	2	1	223029410	N-Term(TMT6plex)	1606.8	0	1.5	1.5	1.8	1.6
																	VSHVLAALQAGNR	2	1	1	223029410	N-Term(TMT6plex)	1564.9	0	1.3	1.4	1.4	1.4
																	AQVVSNLK	3	1	1	223029410	N-Term(TMT6plex); K8(TMT6plex)	1316.8	0	1.4	1.6	1.6	1.6
																	AVTQALNR	3	1	1	223029410	N-Term(TMT6plex)	1101.7	0	1.4	1.5	1.5	1.7
																	VSQMAQYFEPLTLAAVGAASK	4	1	1	223029410	N-Term(TMT6plex); M4(Oxidation); K21(TMT6plex)	2656.4	0	1.3	1.4	1.6	1.3
																	ASDNLVK	2	1	1	223029410	N-Term(TMT6plex); K7(TMT6plex)	1204.7	0	1.4	1.7	1.6	1.8
																	ELLENPVQPINDMSYFGCLDSVMENSK	2	1	1	223029410	N-Term(TMT6plex); C18(Carbamidomethyl); M23(Oxidation); K27(TMT6plex)	3603.7	0	1.4	1.3	1.5	1.3
																	KGIWLEAGK	2	1	1	223029410	N-Term(TMT6plex); K1(TMT6plex); K9(TMT6plex)	1689.1	1	1.3	1.7	1.7	1.8
																	KLEQLKPR	2	2	1	223029410	N-Term(TMT6plex); K1(TMT6plex); K6(TMT6plex)	1699.1	1	1.1	1.5	1.4	1.6
																	LNEAAAGLNQAATELVQASR	10	1	1	223029410	N-Term(TMT6plex)	2256.2	0	1.4	1.5	1.5	1.5
																	AATAPLLEAVDNLSAFASNPEFSSIPAQISPEGR	12	1	1	223029410	N-Term(TMT6plex)	3699.9	0	1.5	1.7	1.6	1.5
																	LQAAGNAVK	4	2	1	223029410	N-Term(TMT6plex); K9(TMT6plex)	1329.8	0	1.6	1.7	1.7	1.7
																	VEHGSVALPAIMR	3	1	1	223029410	N-Term(TMT6plex); M12(Oxidation)	1624.9	0	1.0	1.2	1.0	1.1

Supplementary Table 6. List of all proteins and peptides identified in untreated and chronically treated Het1A cells with cigarette smoke condensate for 8 months

Accession	RefSeq Accession	Gene Symbol	Entrez Gene ID	Description	Σ Coverage	Σ# Proteins	Σ# Unique Peptides	Σ# Peptides	Σ# PSMs	Het-1A-Smoke - 2M/Parental	Het-1A-Smoke - 4M/Parental	Het-1A-Smoke - 6M/Parental	Het-1A-Smoke - 8M/Parental	# AAs	MW [kDa]	calc. pI	Sequence	# PSMs	# Proteins	# Protein Groups	Protein Group Accessions	Modifications	MH+ [Da]	# Missed Cleavages	Het-1A-Smoke - 2M/Parental	Het-1A-Smoke - 4M/Parental	Het-1A-Smoke - 6M/Parental	Het-1A-Smoke - 8M/Parental
																	EVANSTANLVK	4	2	1	223029410	N-Term(TMT6plex); K11(TMT6plex)	1603.9	0	**1.2**	**1.5**	**1.5**	**1.7**
																	DKAPGQLECETAIAALNSCLR	2	1	1	223029410	N-Term(TMT6plex); K2(TMT6plex); C9(Carbamidomethyl); C19(Carbamidomethyl)	2775.5	1	**1.7**	**2.2**	**2.1**	**1.7**
																	GITMATAK	2	2	1	223029410	N-Term(TMT6plex); K8(TMT6plex)	1250.8	0	**1.4**	**1.6**	**1.6**	**1.4**
																	TMLESAGGLIQTAR	8	1	1	223029410	N-Term(TMT6plex); M2(Oxidation)	1692.9	0	**1.5**	**1.3**	**1.3**	**1.4**
																	AQEACGPLEMDSALSVVQNLEK	4	1	1	223029410	N-Term(TMT6plex); C5(Carbamidomethyl); K22(TMT6plex)	2847.5	0	**2.0**	**2.9**	**3.3**	**1.9**
																	AVEGCVSASQAATEDGQLLR	4	1	1	223029410	N-Term(TMT6plex); C5(Carbamidomethyl)	2291.2	0	**1.3**	**1.5**	**1.5**	**1.5**
																	STVLQQQYNR	2	1	1	223029410	N-Term(TMT6plex)	1465.8	0	**2.1**	**1.4**	**1.5**	**1.7**
																	YDQATDTILTVTENIFSSMGDAGEMVR	2	1	1	223029410	N-Term(TMT6plex); M25(Oxidation)	3209.5	0	**1.1**	**1.1**	**1.4**	**1.3**
																	GISMSSSK	2	1	1	223029410	N-Term(TMT6plex); K8(TMT6plex)	1254.7	0	**1.5**	**1.9**	**1.9**	**1.6**
																	AAGHPGDPESQQR	8	1	1	223029410	N-Term(TMT6plex)	1578.8	0	**1.2**	**1.6**	**1.2**	**1.4**
																	VGAIPANALDDGQWSQGLISAAR	6	1	1	223029410	N-Term(TMT6plex)	2539.3	0	**1.4**	**1.4**	**1.4**	**1.4**
																	AAAFEEQENETVVVK	4	1	1	223029410	N-Term(TMT6plex); K15(TMT6plex)	2122.1	0	**1.5**	**1.8**	**1.7**	**1.7**
																	EGISQEALHTQMLTAVQEISHLIEPLANAAR	2	1	1	223029410	N-Term(TMT6plex); M12(Oxidation)	3615.9	0	**1.4**	**1.5**	**1.8**	**1.8**
																	ALSTDPAAPNLK	2	1	1	223029410	N-Term(TMT6plex); K12(TMT6plex)	1656.0	0	**1.3**	**1.5**	**1.6**	**1.6**
																	AQEACGPLEMDSALSVVQNLEK	6	1	1	223029410	N-Term(TMT6plex); C5(Carbamidomethyl); M10(Oxidation); K22(TMT6plex)	2863.4	0	**1.6**	**2.1**	**1.8**	**2.3**
																	VGDDPAVWQLK	2	1	1	223029410	N-Term(TMT6plex); K11(TMT6plex)	1686.0	0	**1.3**	**1.6**	**1.4**	**1.6**
																	EAAYHPEVAPDVR	4	1	1	223029410	N-Term(TMT6plex)	1682.9	0	**1.2**	**1.5**	**1.4**	**1.6**
																	QVAASTAQLLVACK	1	2	1	223029410	N-Term(TMT6plex); C13(Carbamidomethyl); K14(TMT6plex)	1918.1	0	**1.5**	**1.6**	**1.6**	**1.5**
																	LAQAAQSSVATITR	1	1	1	223029410	N-Term(TMT6plex)	1645.9	0	**1.2**	**1.5**	**1.4**	**1.4**
																	IFQAHK	8	1	1	223029410	N-Term(TMT6plex); K6(TMT6plex)	1201.7	0	**1.2**	**1.4**	**1.5**	**1.4**
																	VLVEDTK	4	1	1	223029410	N-Term(TMT6plex); K7(TMT6plex)	1261.8	0	**1.5**	**1.9**	**1.7**	**1.9**
																	ELIECAR	4	2	1	223029410	N-Term(TMT6plex); C5(Carbamidomethyl)	1119.6	0	**1.4**	**1.9**	**1.7**	**1.6**
																	AVAAGNSCR	1	2	1	223029410	N-Term(TMT6plex); C8(Carbamidomethyl)	1134.6	0	**0.9**	**1.4**	**1.9**	**1.8**
																	ALDYYMLR	2	1	1	223029410	N-Term(TMT6plex); M6(Oxidation)	1289.7	0	**1.3**	**1.5**	**1.6**	**1.6**
																	DPPSWSVLAGHSR	1	1	1	223029410	N-Term(TMT6plex)	1637.9	0	**1.3**	**1.5**	**1.5**	**1.4**
																	SKDHFGLEGDEESTMLEDSVSPK	1	1	1	223029410	N-Term(TMT6plex); K2(TMT6plex); M15(Oxidation); K23(TMT6plex)	3240.6	1	**1.3**	**1.5**	**1.5**	**1.6**
																	CVSCLPGQR	1	1	1	223029410	N-Term(TMT6plex); C1(Carbamidomethyl); C4(Carbamidomethyl)	1305.7	0	**1.5**	**1.3**	**1.5**	**1.3**
																	TIMVDDSK	5	1	1	223029410	N-Term(TMT6plex); M3(Oxidation); K8(TMT6plex)	1382.8	0	**1.2**	**1.3**	**1.4**	**1.5**
																	GITMATAK	2	2	1	223029410	N-Term(TMT6plex); M4(Oxidation); K8(TMT6plex)	1266.7	0	**1.2**	**1.6**	**1.5**	**1.7**
																	KLLSAAK	1	1	1	223029410	N-Term(TMT6plex); K1(TMT6plex); K7(TMT6plex)	1418.0	1	**1.7**	**1.7**	**1.9**	**1.8**
																	AGFLDLK	4	1	1	223029410	N-Term(TMT6plex); K7(TMT6plex)	1221.8	0	**1.4**	**1.6**	**1.6**	**1.7**
																	TVSDSIK	2	1	1	223029410	N-Term(TMT6plex); K7(TMT6plex)	1207.7	0	**1.4**	**1.6**	**1.6**	**1.6**
																	ELEEAR	2	5	2	367460087;223029410	N-Term(TMT6plex)	975.5	0				
																	GTPQDLAR	2	1	1	223029410	N-Term(TMT6plex)	1086.6	0	**1.3**	**1.5**	**1.6**	**1.5**
																	ALAVNPR	2	1	1	223029410	N-Term(TMT6plex)	969.6	0	**1.5**	**1.6**	**1.6**	**1.6**
																	QAAHTQEALEEAVQMMTEAVEDLTTTLNEAASAAGVVGGMVDSITQAINQLDEGPMGEPEGSFVDYQTTMVR	1	1	1	223029410	N-Term(TMT6plex); M16(Oxidation); M40(Oxidation); M56(Oxidation)	7860.7	0	**1.0**	**1.0**	**1.0**	**1.0**
																	DLQEVK	3	2	1	223029410	N-Term(TMT6plex); K6(TMT6plex)	1189.7	0	**1.3**	**1.5**	**1.6**	**1.5**
																	LADVVK	3	1	1	223029410	N-Term(TMT6plex); K6(TMT6plex)	1102.7	0	**1.3**	**1.6**	**1.6**	**1.7**
																	TYGVSFFLVK	1	2	1	223029410	N-Term(TMT6plex); K10(TMT6plex)	1619.0	0	**1.9**	**1.7**	**2.1**	**1.5**
																	LLSAAK	1	3	2	47132589;223029410	N-Term(TMT6plex); K6(TMT6plex)	1060.7	0				
41872631	NP_004095.4	**FASN**	2194	**fatty acid synthase**	69.49	1	124	126	2493	**1.6**	**1.7**	**1.7**	**1.5**	2511	273.3	6.44												
																	SLYQSAGVAPESFEYIEAHGTGTK	27	1	1	41872631	N-Term(TMT6plex); K24(TMT6plex)	3000.5	0	**1.6**	**2.0**	**1.9**	**1.6**
																	DLAAVNLDSSLADLGLDSLMSVEVR	4	1	1	41872631	N-Term(TMT6plex); M20(Oxidation)	2848.5	0	**1.5**	**1.8**	**1.7**	**1.9**
																	DTVTISGPQAPVFEFVEQLR	81	1	1	41872631	N-Term(TMT6plex)	2462.3	0	**1.5**	**1.7**	**1.7**	**1.5**
																	GNAGQSNYGFANSAMER	22	1	1	41872631	N-Term(TMT6plex); M15(Oxidation)	2018.9	0	**2.3**	**2.2**	**2.1**	**1.6**
																	LHLSGIDANPNALFPPVEFPAPR	56	1	1	41872631	N-Term(TMT6plex)	2701.4	0	**1.5**	**1.5**	**1.5**	**1.4**
																	ACLDTAVENMPSLK	17	1	1	41872631	N-Term(TMT6plex); C2(Carbamidomethyl); M10(Oxidation); K14(TMT6plex)	2023.1	0	**1.5**	**1.8**	**1.6**	**1.5**
																	MVVPGLDGAQIPR	27	1	1	41872631	N-Term(TMT6plex); M1(Oxidation)	1597.9	0	**1.5**	**1.6**	**1.5**	**1.5**
																	VVEVLAGHGHLYSR	13	1	1	41872631	N-Term(TMT6plex)	1766.0	0	**1.4**	**1.5**	**1.5**	**1.4**
																	LTPGCEAEAETEAICFFVQQFTDMEHNR	77	1	1	41872631	N-Term(TMT6plex); C5(Carbamidomethyl); C15(Carbamidomethyl); M24(Oxidation)	3575.6	0	**1.7**	**1.7**	**1.8**	**1.5**
																	DNWVSFMDTMLQMSILGSAK	8	1	1	41872631	N-Term(TMT6plex); M7(Oxidation); M10(Oxidation); M13(Oxidation); K20(TMT6plex)	2780.4	0	**1.5**	**1.9**	**1.6**	**1.7**
																	VLEALLPLK	55	1	1	41872631	N-Term(TMT6plex); K9(TMT6plex)	1454.0	0	**1.7**	**2.0**	**2.0**	**1.5**
																	CTVFHGAQVEDAFR	11	1	1	41872631	N-Term(TMT6plex); C1(Carbamidomethyl)	1865.9	0	**1.5**	**1.5**	**1.5**	**1.3**
																	GYAVLGGER	20	1	1	41872631	N-Term(TMT6plex)	1150.6	0	**1.5**	**1.8**	**1.7**	**1.5**
																	LFDHPESPTPNPTEPLFLAQAEVYK	26	1	1	41872631	N-Term(TMT6plex); K25(TMT6plex)	3298.7	0	**1.4**	**1.8**	**1.8**	**1.7**
																	AINCATSGVVGLVNCLR	17	1	1	41872631	N-Term(TMT6plex); C4(Carbamidomethyl); C15(Carbamidomethyl)	2033.1	0	**1.6**	**1.6**	**1.7**	**1.4**
																	SDEAVKPFGLK	11	1	1	41872631	N-Term(TMT6plex); K6(TMT6plex); K11(TMT6plex)	1878.1	0	**1.6**	**2.2**	**2.0**	**1.7**
																	DNLEFFLAGIGR	50	1	1	41872631	N-Term(TMT6plex)	1580.9	0	**1.8**	**1.9**	**1.9**	**1.5**
																	DGAWGAFR	7	1	1	41872631	N-Term(TMT6plex)	1108.6	0	**1.5**	**1.8**	**1.7**	**1.5**
																	EDGLAQQQTQLNLR	63	1	1	41872631	N-Term(TMT6plex)	1843.0	0	**1.7**	**1.7**	**1.6**	**1.5**
																	DPETLVGYSMVGCQR	19	1	1	41872631	N-Term(TMT6plex); M10(Oxidation); C13(Carbamidomethyl)	1956.9	0	**1.5**	**1.7**	**1.6**	**1.6**
																	DIMLATGK	8	1	1	41872631	N-Term(TMT6plex); M3(Oxidation); K8(TMT6plex)	1322.8	0	**1.3**	**1.6**	**1.6**	**1.4**
																	HSQDLAFLSMLNDIAAVPATAMPFR	24	1	1	41872631	N-Term(TMT6plex); M10(Oxidation); M22(Oxidation)	2977.5	0	**1.5**	**1.5**	**1.5**	**1.5**
																	GVDLVLNSLAEEK	35	1	1	41872631	N-Term(TMT6plex); K13(TMT6plex)	1845.1	0	**1.7**	**2.0**	**2.0**	**1.6**
																	LPEDPLLSGLLDSPALK	155	1	1	41872631	N-Term(TMT6plex); K17(TMT6plex)	2236.3	0	**1.7**	**1.9**	**2.0**	**1.6**
																	QQEQQVPILEK	20	1	1	41872631	N-Term(TMT6plex); K11(TMT6plex)	1798.0	0	**1.6**	**1.8**	**1.9**	**1.6**
																	TLLEGSGLESIISIIHSSLAEPR	48	1	1	41872631	N-Term(TMT6plex)	2651.5	0	**1.7**	**1.8**	**2.1**	**1.5**
																	MVVPGLDGAQIPR	14	1	1	41872631	N-Term(TMT6plex)	1581.9	0	**1.7**	**1.9**	**1.9**	**1.6**
																	ALGLGVEQLPVVFEDVVLHQATILPK	41	1	1	41872631	N-Term(TMT6plex); K26(TMT6plex)	3243.9	0	**1.8**	**2.3**	**2.3**	**1.8**
																	YSGTLNLDR	20	1	1	41872631	N-Term(TMT6plex)	1267.7	0	**1.6**	**1.4**	**1.6**	**1.3**
																	AAEQYTPK	32	1	1	41872631	N-Term(TMT6plex); K8(TMT6plex)	1365.8	0	**1.4**	**1.7**	**1.6**	**1.4**
																	FDASFFGVHPK	22	1	1	41872631	N-Term(TMT6plex); K11(TMT6plex)	1709.9	0	**1.8**	**2.0**	**2.1**	**1.6**
																	EQGVTFPSGDIQEQLIR	31	1	1	41872631	N-Term(TMT6plex)	2146.1	0	**1.5**	**1.6**	**1.7**	**1.4**
																	GTPLISPLIK	21	1	1	41872631	N-Term(TMT6plex); K10(TMT6plex)	1497.0	0	**1.5**	**2.1**	**1.9**	**1.6**
																	IPGLLSPHPLLQLSYTATDR	19	1	1	41872631	N-Term(TMT6plex)	2421.4	0	**1.6**	**1.7**	**1.9**	**1.6**
																	FDLSQNHPLGMAIFLK	33	1	1	41872631	N-Term(TMT6plex); M11(Oxidation); K16(TMT6plex)	2305.3	0	**1.5**	**1.7**	**1.7**	**1.6**
																	FPQLDSTSFANSR	38	1	1	41872631	N-Term(TMT6plex)	1698.9	0	**1.8**	**1.7**	**1.7**	**1.5**
																	LSPDAIPGK	20	1	1	41872631	N-Term(TMT6plex); K9(TMT6plex)	1355.8	0	**1.5**	**1.9**	**1.8**	**1.5**
																	LQELSSK	8	1	1	41872631	N-Term(TMT6plex); K7(TMT6plex)	1262.8	0	**1.6**	**1.8**	**1.9**	**1.5**
																	LGMLSPEGTCK	16	1	1	41872631	N-Term(TMT6plex); M3(Oxidation); C10(Carbamidomethyl); K11(TMT6plex)	1666.9	0	**1.6**	**1.8**	**1.8**	**1.5**
																	VTQQGLK	10	1	1	41872631	N-Term(TMT6plex); K7(TMT6plex)	1231.8	0	**1.8**	**1.8**	**1.9**	**1.4**
																	QELSFAAR	11	1	1	41872631	N-Term(TMT6plex)	1150.6	0	**1.5**	**1.5**	**1.6**	**1.4**
																	VLQGDLVMNVYR	3	1	1	41872631	N-Term(TMT6plex)	1635.9	0	**1.6**	**1.9**	**2.0**	**1.5**
																	SNMGHPEPASGLAALAK	13	1	1	41872631	N-Term(TMT6plex); M3(Oxidation); K17(TMT6plex)	2125.1	0	**1.3**	**1.4**	**1.4**	**1.3**
																	VFTTVGSAEK	18	1	1	41872631	N-Term(TMT6plex); K10(TMT6plex)	1496.9	0	**1.6**	**1.4**	**1.6**	**1.3**
																	ADEASELACPTPK	27	1	1	41872631	N-Term(TMT6plex); C9(Carbamidomethyl); K13(TMT6plex)	1847.0	0	**1.7**	**1.7**	**1.7**	**1.4**
																	EACPELDYFVVFSSVSCGR	9	1	1	41872631	N-Term(TMT6plex); C3(Carbamidomethyl); C17(Carbamidomethyl)	2451.2	0	**1.8**	**1.9**	**1.7**	**1.5**
																	EAHLPPGAMAAVGLSWEECK	12	1	1	41872631	N-Term(TMT6plex); M9(Oxidation); C19(Carbamidomethyl); K20(TMT6plex)	2627.3	0	**1.5**	**1.5**	**1.5**	**1.3**
																	DGLLENQTPEFFQDVCKPK	21	1	1	41872631	N-Term(TMT6plex); C16(Carbamidomethyl); K17(TMT6plex); K19(TMT6plex)	2952.6	0	**1.7**	**2.2**	**2.2**	**1.5**
																	WTSQDSLLGMEFSGR	32	1	1	41872631	N-Term(TMT6plex); M10(Oxidation)	1958.9	0	**1.5**	**1.5**	**1.6**	**1.3**
																	GLKPSCTIIPLMK	6	1	1	41872631	N-Term(TMT6plex); K3(TMT6plex); C6(Carbamidomethyl); M12(Oxidation); K13(TMT6plex)	2161.3	0	**1.4**	**2.0**	**1.8**	**1.5**
																	LYTLQDK	8	1	1	41872631	N-Term(TMT6plex); K7(TMT6plex)	1338.8	0	**1.5**	**1.5**	**1.6**	**1.3**
																	AALQEELQLCK	9	1	1	41872631	N-Term(TMT6plex); C10(Carbamidomethyl); K11(TMT6plex)	1761.0	0	**1.9**	**2.2**	**2.1**	**1.6**
																	VVVQVLAEEPEAVLK	57	1	1	41872631	N-Term(TMT6plex); K15(TMT6plex)	2081.3	0	**1.6**	**1.8**	**1.8**	**1.5**
																	LLLEVTYEAIVDGGINPDSLR	31	1	1	41872631	N-Term(TMT6plex)	2516.4	0	**1.6**	**1.5**	**1.6**	**1.4**
																	DNWVSFMDTMLQMSILGSAK	10	1	1	41872631	N-Term(TMT6plex); M10(Oxidation); M13(Oxidation); K20(TMT6plex)	2764.4	0	**1.4**	**1.7**	**1.6**	**1.6**
																	HSQDLAFLSMLNDIAAVPATAMPFR	20	1	1	41872631	N-Term(TMT6plex); M10(Oxidation)	2961.5	0	**1.7**	**1.8**	**1.6**	**1.6**
																	FDLSQNHPLGMAIFLK	5	1	1	41872631	N-Term(TMT6plex); K16(TMT6plex)	2289.3	0	**1.8**	**2.3**	**2.2**	**1.8**
																	WLSTSIPEAQWHSSLAR	10	1	1	41872631	N-Term(TMT6plex)	2198.2	0	**1.6**	**1.5**	**1.6**	**1.4**
																	VTAIHIDPATHR	2	1	1	41872631	N-Term(TMT6plex)	1559.9	0	**1.2**	**1.3**	**1.4**	**1.2**

Accession	RefSeq Accession	Gene Symbol	Entrez Gene ID	Description	Σ Coverage	Σ# Proteins	Σ# Unique Peptides	Σ# Peptides	Σ# PSMs	Het-1A-Smoke - 2M/Parental	Het-1A-Smoke - 4M/Parental	Het-1A-Smoke - 6M/Parental	Het-1A-Smoke - 8M/Parental	# AAs	MW [kDa]	calc. pI	Sequence	# PSMs	# Proteins	# Protein Groups	Protein Group Accessions	Modifications	MH+ [Da]	# Missed Cleavages	Het-1A-Smoke - 2M/Parental	Het-1A-Smoke - 4M/Parental	Het-1A-Smoke - 6M/Parental	Het-1A-Smoke - 8M/Parental
																	VYQWDDPDPR	20	1	1	41872631	N-Term(TMT6plex)	1519.7	0	1.7	1.6	1.7	1.5
																	YHGNVMLLR	4	1	1	41872631	N-Term(TMT6plex); M6(Oxidation)	1347.7	0	1.1	1.5	1.4	1.4
																	LSIPTYGLQCTR	6	1	1	41872631	N-Term(TMT6plex); C10(Carbamidomethyl)	1637.9	0	1.6	1.6	1.6	1.4
																	SLLVNPEGPTLMR	54	1	1	41872631	N-Term(TMT6plex); M12(Oxidation)	1671.9	0	1.6	1.6	1.6	1.4
																	TGGMAFHSYFMEAIAPPLLQELK	6	1	1	41872631	N-Term(TMT6plex); M4(Oxidation); K23(TMT6plex)	3025.6	0	1.5	2.0	1.8	1.6
																	LNSVQSSERPLFLVHPIEGSTTVFHSLASR	2	1	1	41872631	N-Term(TMT6plex)	3537.9	0	1.4	1.6	1.4	1.5
																	DPSQQELPR	13	1	1	41872631	N-Term(TMT6plex)	1298.7	0	1.7	1.8	1.7	1.5
																	GNAGQSNYGFANSAMER	2	1	1	41872631	N-Term(TMT6plex)	2002.9	0	3.5	4.6	5.5	1.9
																	NVTFHGVLLDAFFNESSADWR	36	1	1	41872631	N-Term(TMT6plex)	2654.3	0	1.6	1.5	1.7	1.3
																	EAHLPPGAMAAVGLSWEECK	3	1	1	41872631	N-Term(TMT6plex); C19(Carbamidomethyl); K20(TMT6plex)	2611.3	0	1.3	1.4	1.4	1.3
																	CPPGVVPACHNSK	2	1	1	41872631	N-Term(TMT6plex); C1(Carbamidomethyl); C9(Carbamidomethyl); K13(TMT6plex)	1881.0	0	2.1	2.5	2.9	1.8
																	LQVVDQPLPVR	33	1	1	41872631	N-Term(TMT6plex)	1492.9	0	1.6	1.7	1.6	1.5
																	EVWALVQAGIR	14	1	1	41872631	N-Term(TMT6plex)	1470.9	0	1.6	1.7	1.7	1.5
																	TGGAYGEDLGADYNLSQVCDGK	18	1	1	41872631	N-Term(TMT6plex); C19(Carbamidomethyl); K22(TMT6plex)	2748.3	0	1.9	1.4	1.6	1.1
																	VAAAVDLIIK	20	1	1	41872631	N-Term(TMT6plex); K10(TMT6plex)	1471.0	0	1.5	1.8	1.9	1.5
																	LPESENLQEFWDNLIGGVDMVTDDDR	6	1	1	41872631	N-Term(TMT6plex); M20(Oxidation)	3252.5	0	1.6	1.5	1.6	1.7
																	VGDPQELNGITR	2	1	1	41872631	N-Term(TMT6plex)	1527.8	0	1.6	1.4	1.6	1.3
																	LGMLSPEGTCK	2	1	1	41872631	N-Term(TMT6plex); C10(Carbamidomethyl); K11(TMT6plex)	1650.9	0	1.8	2.0	2.3	1.5
																	FCFTPHTEEGCLSER	7	1	1	41872631	N-Term(TMT6plex); C2(Carbamidomethyl); C11(Carbamidomethyl)	2098.9	0	1.5	1.7	1.7	1.7
																	TGGMAFHSYFMEAIAPPLLQELK	31	1	1	41872631	N-Term(TMT6plex); M4(Oxidation); M11(Oxidation); K23(TMT6plex)	3041.6	0	1.8	1.7	1.8	1.6
																	DTSFEQHVLWHTGGK	12	1	1	41872631	N-Term(TMT6plex); K15(TMT6plex)	2200.2	0	1.4	1.8	1.7	1.5
																	MASCLEVLDLFLNQPHMVLSSFVLAEK	18	1	1	41872631	M1(Oxidation); N-Term(TMT6plex); C4(Carbamidomethyl); M17(Oxidation); K27(TMT6plex)	3581.9	0	1.9	2.2	2.1	1.9
																	SEGVVAVLLTK	24	1	1	41872631	N-Term(TMT6plex); K11(TMT6plex)	1574.0	0	2.1	1.9	2.4	1.4
																	AAPLDSIHSLAAYYIDCIR	7	1	1	41872631	N-Term(TMT6plex); C17(Carbamidomethyl)	2378.2	0	1.4	1.4	1.8	1.3
																	VYATILNAGTNTDGFK	16	1	1	41872631	N-Term(TMT6plex); K16(TMT6plex)	2143.2	0	1.5	1.4	1.6	1.2
																	GGPEVQQVPAGERPLWFICSGMGTQWR	3	1	1	41872631	N-Term(TMT6plex); C19(Carbamidomethyl); M22(Oxidation)	3288.6	0	1.8	2.4	1.8	2.2
																	VTVAGGVHISGLHTESAPR	4	1	1	41872631	N-Term(TMT6plex)	2117.2	0	1.4	1.6	1.5	1.6
																	QEPLLIGSTK	12	1	1	41872631	N-Term(TMT6plex); K10(TMT6plex)	1543.9	0	1.6	1.9	1.8	1.6
																	LPESENLQEFWDNLIGGVDMVTDDDRR	38	1	1	41872631	N-Term(TMT6plex); M20(Oxidation)	3408.6	1	1.7	1.8	1.7	1.5
																	EGGFLLLHTLLR	41	1	1	41872631	N-Term(TMT6plex)	1598.0	0	2.1	2.4	2.5	1.8
																	HFLLEEDKPEEPTAHAFVSTLTR	8	1	1	41872631	N-Term(TMT6plex); K8(TMT6plex)	3125.7	0	1.4	1.6	1.6	1.5
																	GTHTGVWVGVSGSETSEALSR	28	1	1	41872631	N-Term(TMT6plex)	2346.2	0	1.5	1.7	1.5	1.4
																	VLQGDLVMNVYR	11	1	1	41872631	N-Term(TMT6plex); M8(Oxidation)	1651.9	0	1.5	1.5	1.6	1.4
																	VLFPATGYLSIVWK	23	1	1	41872631	N-Term(TMT6plex); K14(TMT6plex)	2052.2	0	2.1	2.2	2.3	1.6
																	DLVEAVAHILGIR	53	1	1	41872631	N-Term(TMT6plex)	1635.0	0	1.9	2.1	1.6	1.7
																	GYDYGPHFQGILEASLEGDSGR	64	1	1	41872631	N-Term(TMT6plex)	2597.3	0	1.4	1.6	1.6	1.3
																	GLVQALQTK	28	1	1	41872631	N-Term(TMT6plex); K9(TMT6plex)	1415.9	0	1.6	2.0	2.0	1.5
																	VLLSLEHGLWAPNLHFHSPNPEIPALLDGR	9	1	1	41872631	N-Term(TMT6plex)	3571.9	0	1.4	1.6	1.7	1.4
																	WVCSSLR	7	1	1	41872631	N-Term(TMT6plex); C3(Carbamidomethyl)	1136.6	0	1.8	2.1	1.9	1.7
																	QGVQVQVSTSNISSLEGAR	5	1	1	41872631	N-Term(TMT6plex)	2189.2	0	1.8	1.6	1.7	1.4
																	QVQPEGPYR	8	1	1	41872631	N-Term(TMT6plex)	1302.7	0	1.8	2.0	1.9	1.5
																	SFYGSTLFLCR	7	1	1	41872631	N-Term(TMT6plex); C10(Carbamidomethyl)	1579.8	0	1.9	1.8	2.1	1.6
																	TFCPAHK	8	1	1	41872631	N-Term(TMT6plex); C3(Carbamidomethyl); K7(TMT6plex)	1318.7	0	1.4	1.8	1.8	1.4
																	LSFFFDFR	8	1	1	41872631	N-Term(TMT6plex)	1307.7	0	1.9	1.8	1.8	1.3
																	DIMLATGK	4	1	1	41872631	N-Term(TMT6plex); K8(TMT6plex)	1306.8	0	1.5	2.0	2.1	1.3
																	SLLVNPEGPTLMR	4	1	1	41872631	N-Term(TMT6plex)	1655.9	0	1.5	1.8	1.9	1.3
																	VLEALLPLKGLEER	1	1	1	41872631	N-Term(TMT6plex); K9(TMT6plex)	2038.3	1	1.6	1.8	1.9	1.6
																	WTSQDSLLGMEFSGR	8	1	1	41872631	N-Term(TMT6plex)	1943.0	0	3.4	2.5	2.7	1.5
																	LPESENLQEFWDNLIGGVDMVTDDDRR	22	1	1	41872631	N-Term(TMT6plex)	3392.6	1	2.2	2.8	2.4	1.7
																	TGTVSLEVR	7	1	1	41872631	N-Term(TMT6plex)	1190.7	0	1.7	1.4	1.6	1.2
																	AQVADVVVSR	20	1	1	41872631	N-Term(TMT6plex)	1272.7	0	1.5	1.7	1.6	1.6
																	YHGNVMLLR	2	1	1	41872631	N-Term(TMT6plex)	1331.7	0	1.5	1.6	1.6	1.3
																	AGLYGLPR	10	1	1	41872631	N-Term(TMT6plex)	1075.6	0	1.5	1.7	1.7	1.5
																	DNWVSFMDTMLQMSILGSAK	5	1	1	41872631	N-Term(TMT6plex); M7(Oxidation); K20(TMT6plex)	2748.4	0	1.6	1.9	1.9	1.5
																	VSVHVIEGDHR	10	1	1	41872631	N-Term(TMT6plex)	1476.8	0	1.1	1.3	1.5	1.3
																	GGPEVQQVPAGERPLWFICSGMGTQWR	1	1	1	41872631	N-Term(TMT6plex); C19(Carbamidomethyl)	3272.6	0	1.7	2.4	2.1	1.5
																	AFDTAGNGYCR	1	1	1	41872631	N-Term(TMT6plex); C10(Carbamidomethyl)	1460.7	0	1.7	1.6	1.5	1.2
																	QAHTMDPQLR	2	1	1	41872631	N-Term(TMT6plex); M5(Oxidation)	1441.7	0	1.5	1.9	2.0	1.8
																	CVLLSNLSSTSHVPEVDPGSAELQK	11	1	1	41872631	N-Term(TMT6plex); C1(Carbamidomethyl); K25(TMT6plex)	3125.6	0	1.9	1.8	2.2	1.5
																	RPTPQDSPIFLPVDDTSFR	21	1	1	41872631	N-Term(TMT6plex)	2417.3	0	1.6	1.8	1.9	1.6
																	GILADEDSSRPVWLK	6	1	1	41872631	N-Term(TMT6plex); K15(TMT6plex)	2144.2	0	1.7	2.5	2.2	1.8
																	LTPGCEAEAETEAICFFVQQFTDMEHNR	6	1	1	41872631	N-Term(TMT6plex); C5(Carbamidomethyl); C15(Carbamidomethyl)	3559.6	0	1.9	1.9	2.4	1.5
																	MASCLEVLDLFLNQPHMVLSSFVLAEK	4	1	1	41872631	N-Term(TMT6plex); M1(Oxidation); C4(Carbamidomethyl); K27(TMT6plex)	3565.9	0	1.6	2.1	2.1	1.4
																	DPETLVGYSMVGCQR	4	1	1	41872631	N-Term(TMT6plex); C13(Carbamidomethyl)	1940.9	0	1.7	2.1	2.0	1.6
																	GLIAEAAQLGPVGGVFNLAVVLR	3	1	1	41872631	N-Term(TMT6plex)	2493.5	0	1.6	1.5	2.0	1.2
																	GHPLGDIVAFLTSTEPQYGQGILSQDAWESLFSR	3	1	1	41872631	N-Term(TMT6plex)	3949.0	0	1.6	1.3	1.8	1.1
																	SYIIAGGLGGFGLELAQWLIQR	6	1	1	41872631	N-Term(TMT6plex)	2591.4	0	1.6	1.5	2.3	1.1
																	AFEVSENGNLVVSGK	2	1	1	41872631	N-Term(TMT6plex); K15(TMT6plex)	2008.1	0	1.4	1.5	1.5	1.4
																	ELNLVLSVR	2	1	1	41872631	N-Term(TMT6plex)	1271.8	0				
																	LVLTSR	3	1	1	41872631	N-Term(TMT6plex)	917.6	0	1.9	2.2	2.1	1.8
																	VMGLVPAK	13	1	1	41872631	N-Term(TMT6plex); M2(Oxidation); K8(TMT6plex)	1288.8	0	1.6	1.7	1.8	1.4
																	LLEQGLR	8	2	2	41872631;156616275	N-Term(TMT6plex)	1057.7	0				
																	KLQELSSK	4	1	1	41872631	N-Term(TMT6plex); K1(TMT6plex); K8(TMT6plex)	1620.0	1	1.4	1.8	1.8	1.5
																	TPEAVQK	5	1	1	41872631	N-Term(TMT6plex); K7(TMT6plex)	1230.7	0	1.5	1.9	1.7	1.4
																	DGVVRPLK	4	1	1	41872631	N-Term(TMT6plex); K8(TMT6plex)	1341.9	0	1.5	1.7	1.8	1.4
																	VMGLVPAK	2	1	1	41872631	N-Term(TMT6plex); K8(TMT6plex)	1272.8	0	1.7	1.7	2.0	1.2
																	QAHTMDPQLR	1	1	1	41872631	N-Term(TMT6plex)	1425.7	0	1.3	1.7	1.6	1.7
																	LLSAACR	4	1	1	41872631	N-Term(TMT6plex); C6(Carbamidomethyl)	1019.6	0	1.8	1.6	1.8	1.3
																	LMSAISK	5	1	1	41872631	N-Term(TMT6plex); K7(TMT6plex)	1207.7	0	2.1	2.4	2.8	1.5
																	QAHTMDPQLRLLLEVTYEAIVDGGINPDSLR	1	1	1	41872631	N-Term(TMT6plex); M5(Oxidation)	3709.9	1	1.7	1.5	2.1	1.1
																	GDLSSIR	3	1	1	41872631	N-Term(TMT6plex)	976.6	0	1.3	1.6	1.5	1.3
																	LLEASR	4	1	1	41872631	N-Term(TMT6plex)	917.6	0	1.9	1.8	1.8	1.4
																	TGYQAK	2	1	1	41872631	N-Term(TMT6plex); K6(TMT6plex)	1125.7	0	1.8	1.5	1.9	1.2
																	WVESLK	5	1	1	41872631	N-Term(TMT6plex); K6(TMT6plex)	1219.7	0	1.6	1.8	1.7	1.5
																	FLEIGK	5	1	2	41872631;41393573	N-Term(TMT6plex); K6(TMT6plex)	1164.7	0				
																	AYLQAR	1	1	1	41872631	N-Term(TMT6plex)	950.6	0	1.6	1.9	1.8	1.5
																	EGVFAK	4	1	1	41872631	N-Term(TMT6plex); K6(TMT6plex)	1108.7	0	1.7	2.1	2.0	1.7
																	ALCATR	1	1	1	41872631	N-Term(TMT6plex); C3(Carbamidomethyl)	920.5	0	1.4	1.6	1.7	1.4
4758950	NP_000933.1	**PPIB**	5479	**peptidyl-prolyl cis-trans isomerase B precursor**	69.44	1	17	19	467	**1.4**	**1.5**	**1.4**	**1.3**	216	23.7	9.41												
																	TVDNFVALATGEK	100	1	1	4758950	N-Term(TMT6plex); K13(TMT6plex)	1823.0	0	1.4	1.4	1.4	1.3
																	HYGPGWVSMANAGK	32	1	1	4758950	N-Term(TMT6plex); M9(Oxidation); K14(TMT6plex)	1949.0	0	1.5	1.4	1.4	1.3
																	DKPLKDVIIADCGK	6	1	1	4758950	N-Term(TMT6plex); K2(TMT6plex); K5(TMT6plex); C12(Carbamidomethyl); K14(TMT6plex)	2488.5	1	1.2	2.4	1.5	1.8
																	IEVEKPFAIAKE	2	1	1	4758950	N-Term(TMT6plex); K5(TMT6plex); K11(TMT6plex)	2061.3	1	1.4	3.0	1.7	2.3
																	DFMIQGGDFTR	55	1	1	4758950	N-Term(TMT6plex); M3(Oxidation)	1531.7	0	1.5	1.6	1.6	1.4
																	DTNGSQFFITTVK	4	1	1	4758950	N-Term(TMT6plex); K13(TMT6plex)	1916.1	0	1.3	1.3	1.4	1.2
																	IEVEKPFAIAK	17	1	1	4758950	N-Term(TMT6plex); K5(TMT6plex); K11(TMT6plex)	1932.2	0	1.2	1.5	1.3	1.4
																	TAWLDGK	24	1	1	4758950	N-Term(TMT6plex); K7(TMT6plex)	1248.7	0	1.4	1.4	1.4	1.3
																	DFMIQGGDFTR	7	1	1	4758950	N-Term(TMT6plex)	1515.7	0	1.5	1.9	1.7	1.6
																	VLEGMEVVR	30	1	1	4758950	N-Term(TMT6plex); M5(Oxidation)	1276.7	0	1.4	1.4	1.4	1.3
																	VIFGLFGK	64	1	1	4758950	N-Term(TMT6plex); K8(TMT6plex)	1338.8	0	1.7	1.5	1.3	1.3
																	DVIIADCGK	59	1	1	4758950	N-Term(TMT6plex); C7(Carbamidomethyl); K9(TMT6plex)	1448.8	0	1.3	1.5	1.4	1.2
																	VLEGMEVVR	13	1	1	4758950	N-Term(TMT6plex)	1260.7	0	1.8	2.0	2.1	1.5
																	IGDEDVGR	22	1	1	4758950	N-Term(TMT6plex)	1089.6	0	1.4	1.4	1.3	1.4
																	HYGPGWVSMANAGK	2	1	1	4758950	N-Term(TMT6plex); K14(TMT6plex)	1933.0	0	1.6	1.7	2.0	1.1
																	FPDENFK	13	1	1	4758950	N-Term(TMT6plex); K7(TMT6plex)	1354.7	0	1.3	1.5	1.3	1.4

Accession	RefSeq Accession	Gene Symbol	Entrez Gene ID	Description	Σ Coverage	Σ# Proteins	Σ# Unique Peptides	Σ# Peptides	Σ# PSMs	Het-1A-Smoke - 2M/Parental	Het-1A-Smoke - 4M/Parental	Het-1A-Smoke - 6M/Parental	Het-1A-Smoke - 8M/Parental	# AAs	MW [kDa]	calc. pI	Sequence	# PSMs	# Proteins	# Protein Groups	Protein Group Accessions	Modifications	MH+ [Da]	# Missed Cleavages	Het-1A-Smoke - 2M/Parental	Het-1A-Smoke - 4M/Parental	Het-1A-Smoke - 6M/Parental	Het-1A-Smoke - 8M/Parental
																	GDGTGGK	1	1	1	4758950	N-Term(TMT6plex); K7(TMT6plex)	1049.6	0	1.2	1.5	1.3	1.3
																	SIYGER	4	1	2	4758950;44681484	N-Term(TMT6plex)	953.5	0				
																	KVESTK	2	1	1	4758950	N-Term(TMT6plex); K1(TMT6plex); K6(TMT6plex)	1378.9	1	1.1	1.5	1.3	1.3
																	VIKDFMIQGGDFTR	2	1	1	4758950	N-Term(TMT6plex); K3(TMT6plex); M6(Oxidation)	2101.2	1	1.6	2.7	1.5	2.4
																	VYFDLR	6	1	1	4758950	N-Term(TMT6plex)	1041.6	0	1.5	1.5	1.4	1.4
																	HVVFGK	2	7	4	4758950;10863927;4505991;5454154	N-Term(TMT6plex); K6(TMT6plex)	1144.7	0				
13129100	NP_077001.1	**DCTPP1**	79077	**dCTP pyrophosphatase 1**	69.41	1	8	8	50	**1.4**	**1.3**	**1.5**	**1.5**	170	18.7	5.03												
																	LHAEFAAER	6	1	1	13129100	N-Term(TMT6plex)	1272.7	0	1.3	1.5	1.5	1.5
																	VDLPLAVLSK	11	1	1	13129100	N-Term(TMT6plex); K10(TMT6plex)	1513.0	0	1.3	1.1	1.5	1.4
																	DWEQFHQPR	4	1	1	13129100	N-Term(TMT6plex)	1471.7	0	1.3	1.4	1.4	1.4
																	FSFSPEPTLEDIR	10	1	1	13129100	N-Term(TMT6plex)	1766.9	0	1.4	1.1	1.4	1.5
																	AALQEELSDVLIYLVALAAR	6	1	1	13129100	N-Term(TMT6plex)	2387.4	0	1.1	1.4	1.6	1.4
																	KYTELPHGAISEDQAVGPADIPCDSTGQTST	9	1	1	13129100	N-Term(TMT6plex); K1(TMT6plex); C23(Carbamidomethyl)	3703.8	1	1.7	1.4	1.7	1.7
																	TDGEPGPQGWSPR	2	1	1	13129100	N-Term(TMT6plex)	1612.8	0	1.4	1.0	1.5	1.3
																	GDTGGEDTAAPGR	2	1	1	13129100	N-Term(TMT6plex)	1432.7	0	1.2	1.3	1.4	1.6
20336746	NP_613075.1	**H2AFY**	9555	**core histone macro-H2A.1 isoform 1**	69.38	1	1	20	292	**1.4**	**1.6**	**1.6**	**1.6**	369	39.2	9.83												
																	GVTIASGGVLPNIHPELLAK	36	3	2	4758496;93141020;20336746	N-Term(TMT6plex); K20(TMT6plex)	2444.5	0				
																	LQVVQADIASIDSDAVVHPTNTDFYIGGEVGNTLEK	7	1	1	20336746	N-Term(TMT6plex); K36(TMT6plex)	4274.2	0	1.4	1.6	1.6	1.6
																	EFVEAVLELR	12	3	2	4758496;93141020;20336746	N-Term(TMT6plex)	1433.8	0				
																	NCLALADDK	8	3	2	4758496;93141020;20336746	N-Term(TMT6plex); C2(Carbamidomethyl); K9(TMT6plex)	1477.8	0				
																	SLFLGQK	15	3	2	4758496;93141020;20336746	N-Term(TMT6plex); K7(TMT6plex)	1250.8	0				
																	NGPLEVAGAAVSAGHGLPAK	8	3	2	4758496;93141020;20336746	N-Term(TMT6plex); K20(TMT6plex)	2274.3	0				
																	HILLAVANDEELNQLLK	17	4	3	4758496;93141020;20336746;8923920	N-Term(TMT6plex); K17(TMT6plex)	2391.4	0				
																	AGVIFPVGR	16	4	3	4758496;93141020;20336746;8923920	N-Term(TMT6plex)	1144.7	0				
																	QTAAQLILK	9	3	2	4758496;93141020;20336746	N-Term(TMT6plex); K9(TMT6plex)	1443.9	0				
																	AASADSTTEGTPADGFTVLSTK	60	3	2	4758496;93141020;20336746	N-Term(TMT6plex); K22(TMT6plex)	2585.3	0				
																	LEAIITPPPAK	8	3	2	4758496;93141020;20336746	N-Term(TMT6plex); K11(TMT6plex)	1608.0	0				
																	IGVGAPVYMAAVLEYLTAEILELAGNAAR	23	3	2	4758496;93141020;20336746	N-Term(TMT6plex); M9(Oxidation)	3220.8	0				
																	GKLEAIITPPPAK	30	3	2	4758496;93141020;20336746	N-Term(TMT6plex); K2(TMT6plex); K13(TMT6plex)	2022.3	1				
																	SIAFPSIGSGR	17	3	2	4758496;93141020;20336746	N-Term(TMT6plex)	1320.7	0				
																	AISSYFVSTMSSSIK	11	3	2	4758496;93141020;20336746	N-Term(TMT6plex); M10(Oxidation); K15(TMT6plex)	2082.1	0				
																	FVIHCNSPVWGADK	6	3	2	4758496;93141020;20336746	N-Term(TMT6plex); C5(Carbamidomethyl); K14(TMT6plex)	2088.1	0				
																	NCLALADDKK	1	3	2	4758496;93141020;20336746	N-Term(TMT6plex); C2(Carbamidomethyl); K9(TMT6plex); K10(TMT6plex)	1835.1	1				
																	IGVGAPVYMAAVLEYLTAEILELAGNAAR	2	3	2	4758496;93141020;20336746	N-Term(TMT6plex)	3204.7	0				
																	AISSYFVSTMSSSIK	2	3	2	4758496;93141020;20336746	N-Term(TMT6plex); K15(TMT6plex)	2066.1	0				
																	FVIHCNSPVWGADKCEELLEK	1	3	2	4758496;93141020;20336746	N-Term(TMT6plex); C5(Carbamidomethyl); K14(TMT6plex); C15(Carbamidomethyl); K21(TMT6plex)	3218.7	1				
																	KQGEVSK	2	3	2	4758496;93141020;20336746	N-Term(TMT6plex); K1(TMT6plex); K7(TMT6plex)	1462.9	1				
																	QGEVSK	1	3	2	4758496;93141020;20336746	N-Term(TMT6plex); K6(TMT6plex)	1105.7	0				
11559929	NP_057212.1	**COPG1**	22820	**coatomer subunit gamma-1**	69.22	5	38	43	306	**1.5**	**1.6**	**1.6**	**1.4**	874	97.7	5.47												
																	QEIFQEQLAAVPEFR	10	1	1	11559929	N-Term(TMT6plex)	2034.1	0	1.5	1.5	1.5	1.4
																	ELAPAVSVLQLFCSSPK	6	1	1	11559929	N-Term(TMT6plex); C13(Carbamidomethyl); K17(TMT6plex)	2304.3	0	1.5	1.9	1.8	1.7
																	TESTPITAVK	6	1	1	11559929	N-Term(TMT6plex); K10(TMT6plex)	1504.9	0	1.3	1.2	1.4	1.1
																	GLGPLFK	4	1	1	11559929	N-Term(TMT6plex); K7(TMT6plex)	1189.8	0	1.5	1.7	1.7	1.5
																	ILHLLGQEGPK	6	1	1	11559929	N-Term(TMT6plex); K11(TMT6plex)	1663.0	0	1.3	1.3	1.2	1.3
																	EMSCIAEDVIIVTSSLTK	4	1	1	11559929	N-Term(TMT6plex); C4(Carbamidomethyl); K18(TMT6plex)	2454.3	0	3.1	3.1	3.9	1.7
																	FGAQNEEMLPSILVLLK	24	1	1	11559929	N-Term(TMT6plex); M8(Oxidation); K17(TMT6plex)	2376.4	0	1.5	1.3	1.7	1.2
																	AIVDCIISIIEENSESK	6	1	1	11559929	N-Term(TMT6plex); C5(Carbamidomethyl); K17(TMT6plex)	2378.3	0	2.1	2.0	2.0	1.7
																	ILYLINQGEHLGTTEATEAFFAMTK	8	1	1	11559929	N-Term(TMT6plex); M23(Oxidation); K25(TMT6plex)	3272.7	0	1.7	1.8	1.7	1.5
																	ETGLSHLCEFIEDCEFTVLATR	3	1	1	11559929	N-Term(TMT6plex); C8(Carbamidomethyl); C14(Carbamidomethyl)	2856.4	0	1.4	1.4	1.4	1.5
																	HEMVVYEAASAIVNLPGCSAK	4	1	1	11559929	N-Term(TMT6plex); M3(Oxidation); C18(Carbamidomethyl); K21(TMT6plex)	2720.4	0	1.6	1.5	1.7	1.4
																	EMSCIAEDVIIVTSSLTK	16	1	1	11559929	N-Term(TMT6plex); M2(Oxidation); C4(Carbamidomethyl); K18(TMT6plex)	2470.3	0	1.4	1.6	1.7	1.4
																	EEGGFEYK	7	1	1	11559929	N-Term(TMT6plex); K8(TMT6plex)	1416.7	0	1.6	1.6	1.7	1.5
																	VFNETPINPR	4	1	1	11559929	N-Term(TMT6plex)	1415.8	0	1.7	1.4	1.7	1.3
																	SLPYNQPGTCYTLVALPK	3	1	1	11559929	N-Term(TMT6plex); C10(Carbamidomethyl); K18(TMT6plex)	2480.4	0	1.6	1.7	1.9	1.5
																	LLLLDTVTMQVTAR	9	1	1	11559929	N-Term(TMT6plex); M9(Oxidation)	1819.1	0	1.6	1.7	1.7	1.7
																	VVLEHEEVR	6	1	1	11559929	N-Term(TMT6plex)	1338.8	0	1.4	1.6	1.6	1.5
																	FGAQNEEMLPSILVLLK	2	1	1	11559929	N-Term(TMT6plex); K17(TMT6plex)	2360.4	0	1.9	1.9	2.2	1.3
																	SLEELPVDIILASVG	6	1	1	11559929	N-Term(TMT6plex)	1784.0	0	1.4	1.6	1.6	1.6
																	EDPTAVACTFSCMMK	2	1	1	11559929	N-Term(TMT6plex); C8(Carbamidomethyl); C12(Carbamidomethyl); M13(Oxidation); M14(Oxidation); K15(TMT6plex)	2238.0	0	1.8	1.8	1.6	1.8
																	SVPLATAPMAEQR	8	1	1	11559929	N-Term(TMT6plex); M9(Oxidation)	1615.9	0	4.5	1.8	1.7	1.6
																	VVVVQAISALCQK	14	3	2	109134349;11559929	N-Term(TMT6plex); C11(Carbamidomethyl); K13(TMT6plex)	1873.1	0				
																	SIATLAITTLLK	35	3	2	109134349;11559929	N-Term(TMT6plex); K12(TMT6plex)	1703.1	0				
																	VPSVSSSALVSSLHLLK	8	1	1	11559929	N-Term(TMT6plex); K17(TMT6plex)	2182.3	0	1.5	1.6	2.0	1.4
																	ILYLINQGEHLGTTEATEAFFAMTK	8	1	1	11559929	N-Term(TMT6plex); K25(TMT6plex)	3256.7	0	2.1	2.0	2.6	1.5
																	ALCQITDSTMLQAIER	5	1	1	11559929	N-Term(TMT6plex); C3(Carbamidomethyl); M10(Oxidation)	2095.1	0	1.5	1.4	1.5	1.4
																	DCDPTTGETDDEGYEDEYVLEDLEVTVADHIQK	10	1	1	11559929	N-Term(TMT6plex); C2(Carbamidomethyl); K33(TMT6plex)	4258.9	0	1.4	1.7	1.7	1.5
																	ATFYLNVLEQK	6	1	1	11559929	N-Term(TMT6plex); K11(TMT6plex)	1784.0	0	1.5	1.8	1.8	1.6
																	TLEEAVGNIVK	13	1	1	11559929	N-Term(TMT6plex); K11(TMT6plex)	1631.0	0	1.5	1.5	1.5	1.4
																	DSPLFDFIESCLR	11	1	1	11559929	N-Term(TMT6plex); C11(Carbamidomethyl)	1827.9	0	1.6	1.7	1.8	1.5
																	HPSAVTACNLDLENLVTDSNR	6	1	1	11559929	N-Term(TMT6plex); C8(Carbamidomethyl)	2555.3	0	1.6	1.5	1.7	1.6
																	SSPEPVALTESETEYVIR	13	1	1	11559929	N-Term(TMT6plex)	2236.2	0	1.7	1.4	1.4	1.4
																	AGAVSALAK	2	1	1	11559929	N-Term(TMT6plex); K9(TMT6plex)	1245.8	0	1.6	1.8	2.0	1.5
																	QLEEEDGSR	2	1	1	11559929	N-Term(TMT6plex)	1291.6	0	2.1	1.8	1.8	1.8
																	ALQQYTLEPSEKPFDLK	2	1	1	11559929	N-Term(TMT6plex); K12(TMT6plex); K17(TMT6plex)	2694.5	0	1.4	1.7	1.6	1.5
																	QISSFMSEISDEFK	4	1	1	11559929	N-Term(TMT6plex); M6(Oxidation); K14(TMT6plex)	2122.1	0	1.4	1.4	1.4	1.3
																	DEESGGGSNPFQHLEK	3	1	1	11559929	N-Term(TMT6plex); K16(TMT6plex)	2189.1	0	1.2	1.4	1.3	1.3
																	LFQSNDPTLR	2	1	1	11559929	N-Term(TMT6plex)	1419.8	0				
																	SDKVPDNK	4	1	1	11559929	N-Term(TMT6plex); K3(TMT6plex); K8(TMT6plex)	1589.9	1	1.4	1.4	1.6	1.3
																	LLLLDTVTMQVTAR	1	1	1	11559929	N-Term(TMT6plex)	1803.1	0	1.3	1.7	1.6	1.5
																	NTHTLLLAGVFR	2	1	1	11559929	N-Term(TMT6plex)	1570.9	0	1.3	1.5	1.5	1.1
																	LNFEAAWDEVGDEFEKEETFTLSTIK	1	1	1	11559929	N-Term(TMT6plex); K16(TMT6plex); K26(TMT6plex)	3735.9	1	1.4	1.3	1.6	1.1
																	CVMDDDNEVR	2	1	1	11559929	N-Term(TMT6plex); C1(Carbamidomethyl); M3(Oxidation)	1497.7	0	1.4	1.4	1.1	1.3
																	MCYLTIK	2	3	2	109134349;11559929	N-Term(TMT6plex); M1(Oxidation); C2(Carbamidomethyl); K7(TMT6plex)	1402.8	0				
																	SAVLQEAR	2	3	2	109134349;11559929	N-Term(TMT6plex)	1102.6	0				
																	QAIVDK	2	3	2	11559929;109134349	N-Term(TMT6plex); K6(TMT6plex)	1131.7	0				
																	TTNPSK	2	5	1	11559929	N-Term(TMT6plex); K6(TMT6plex)	1105.7	0	1.7	1.4	1.7	1.4
5803076;187960037	NP_006798.1	**CBX1**	10951	**chromobox protein homolog 1**	69.19	1	9	11	53	**1.5**	**1.0**	**1.7**	**1.0**	185	21.4	4.93												
																	IIGATDSSGELMFLMK	16	2	2	15082258;20544151;5803076;187960037	N-Term(TMT6plex); M12(Oxidation); M15(Oxidation); K16(TMT6plex)	2203.2	0				
																	GFSDEDNTWEPEENLDCPDLIAEFLQSQK	13	1	1	5803076;187960037	N-Term(TMT6plex); C17(Carbamidomethyl); K29(TMT6plex)	3884.8	0	2.1	0.9	2.1	0.9
																	NSDEADLVPAK	4	1	1	5803076;187960037	N-Term(TMT6plex); K11(TMT6plex)	1616.9	0	1.5	1.0	1.5	1.1
																	CPQVVISFYEER	1	1	1	5803076;187960037	N-Term(TMT6plex); C1(Carbamidomethyl)	1755.9	0				
																	TAHETDKSEGGK	1	1	1	5803076;187960037	N-Term(TMT6plex); K7(TMT6plex); K12(TMT6plex)	1947.1	1	1.5	0.9	1.9	0.9
																	IIGATDSSGELMFLMK	2	2	2	15082258;20544151;5803076;187960037	N-Term(TMT6plex); M15(Oxidation); K16(TMT6plex)	2187.2	0				
																	KVEEVLEEEEEEYVVEK	7	1	1	5803076;187960037	N-Term(TMT6plex); K1(TMT6plex); K17(TMT6plex)	2796.5	1	1.5	1.3	1.6	1.1
																	VEEVLEEEEEEYVVEK	2	1	1	5803076;187960037	N-Term(TMT6plex); K16(TMT6plex)	2439.2	0	1.5	1.3	1.5	1.3
																	GKVEYLLK	3	1	1	5803076;187960037	N-Term(TMT6plex); K2(TMT6plex); K8(TMT6plex)	1637.1	1	1.4	1.0	1.9	1.1
																	TAHETDK	2	1	1	5803076;187960037	N-Term(TMT6plex); K7(TMT6plex)	1259.7	0	1.3	1.1	1.5	1.0
																	KADSDSEDKGEESKPK	1	1	1	5803076;187960037	N-Term(TMT6plex); K1(TMT6plex); K9(TMT6plex); K14(TMT6plex); K16(TMT6plex)	2895.6	2	1.3	0.7	1.9	0.8
																	EESEKPR	1	1	1	5803076;187960037	N-Term(TMT6plex); K5(TMT6plex)	1332.8	0	1.6	0.8	1.9	0.8
284005463	NP_001164981.1	**MCFD2**	90411	**multiple coagulation factor deficiency protein 2 isoform B**	69.15	3	4	4	21	**1.8**	**1.4**	**2.0**	**1.0**	94	10.7	4.41												

Supplementary Table 6. List of all proteins and peptides identified in untreated and chronically treated Het1A cells with cigarette smoke condensate for 8 months

Accession	RefSeq Accession	Gene Symbol	Entrez Gene ID	Description	Σ Coverage	Σ# Proteins	Σ# Unique Peptides	Σ# Peptides	Σ# PSMs	Het-1A-Smoke - 2M/Parental	Het-1A-Smoke - 4M/Parental	Het-1A-Smoke - 6M/Parental	Het-1A-Smoke - 8M/Parental	# AAs	MW [kDa]	calc. pI	Sequence	# PSMs	# Proteins	# Protein Groups	Protein Group Accessions	Modifications	MH+ [Da]	# Missed Cleavages	Het-1A-Smoke - 2M/Parental	Het-1A-Smoke - 4M/Parental	Het-1A-Smoke - 6M/Parental	Het-1A-Smoke - 8M/Parental
																	EEGSEQAPLMSEDELINIIDGVLR	12	3	1	284005463	N-Term(TMT6plex); M10(Oxidation)	2902.5	0	**1.6**	**1.4**	**1.8**	**1.2**
																	MHDYDGNNLLDGLELSTAITHVHK	1	3	1	284005463	N-Term(TMT6plex); M1(Oxidation); K24(TMT6plex)	3167.6	0	**1.8**	**1.5**	**1.5**	**1.3**
																	DDDKNNDGYIDYAEFAK	6	3	1	284005463	N-Term(TMT6plex); K4(TMT6plex); K17(TMT6plex)	2680.3	1	**2.1**	**1.4**	**2.3**	**0.8**
																	NNDGYIDYAEFAK	2	3	1	284005463	N-Term(TMT6plex); K13(TMT6plex)	1978.0	0	**1.9**	**1.0**	**2.0**	**0.7**
4504297;4504281	NP_003528.1	**HIST1H3B**	8358	**histone H3.1**	69.12	2	2	13	1778	**1.0**	**0.8**	**1.1**	**0.8**	136	15.4	11.12												
																	FQSSAVMALQEACEAYLVGLFEDTNLCAIHAK	363	1	1	4504297;4504281	N-Term(TMT6plex); M7(Oxidation); C13(Carbamidomethyl); C27(Carbamidomethyl); K32(TMT6plex)	4061.0	0	**0.9**	**0.8**	**1.1**	**0.8**
																	STELLIR	368	5	4	4504297;4504281;31742503;183076548;4504299;4885385	N-Term(TMT6plex)	1060.7	0				
																	FQSSAVMALQEACEAYLVGLFEDTNLCAIHAK	107	1	1	4504297;4504281	N-Term(TMT6plex); C13(Carbamidomethyl); C27(Carbamidomethyl); K32(TMT6plex)	4045.0	0	**1.1**	**1.0**	**1.4**	**0.9**
																	EIAQDFK	535	4	4	4504297;4504281;31742503;183076548;4504299;4885385	N-Term(TMT6plex); K7(TMT6plex)	1308.8	0				
																	YRPGTVALR	308	5	4	4504297;4504281;31742503;183076548;4504299;4885385	N-Term(TMT6plex)	1261.8	0				
																	EIAQDFKTDLR	3	4	4	4504297;4504281;31742503;183076548;4504299;4885385	N-Term(TMT6plex); K7(TMT6plex)	1794.0	1				
																	SAPATGGVK	54	3	3	4504297;4504281;31742503;183076548;4504299	N-Term(TMT6plex); K9(TMT6plex)	1245.8	0				
																	KSAPATGGVK	4	3	3	4504297;4504281;31742503;183076548;4504299	N-Term(TMT6plex); K1(TMT6plex); K10(TMT6plex)	1603.0	1				
																	KLPFQR	1	5	4	4504297;4504281;31742503;183076548;4504299;4885385	N-Term(TMT6plex); K1(TMT6plex)	1246.8	1				
																	KQLATK	16	5	4	4504297;4504281;31742503;183076548;4504299;4885385	N-Term(TMT6plex); K1(TMT6plex); K6(TMT6plex)	1375.9	1				
																	VTIMPKDIQLAR	1	5	4	4504297;4504281;31742503;183076548;4504299;4885385	N-Term(TMT6plex); K6(TMT6plex)	1843.1	1				
																	VTIMPK	9	5	4	4504297;4504281;31742503;183076548;4504299;4885385	N-Term(TMT6plex); M4(Oxidation); K6(TMT6plex)	1162.7	0				
																	VTIMPK	5	5	4	4504297;4504281;31742503;183076548;4504299;4885385	N-Term(TMT6plex); K6(TMT6plex)	1146.7	0				
																	DIQLAR	3	5	4	4504297;4504281;31742503;183076548;4504299;4885385	N-Term(TMT6plex)	944.6	0				
																	FQSSAVMALQEACEAYLVGLFEDTNLCAIHAKR	1	1	1	4504297;4504281	N-Term(TMT6plex); M7(Oxidation); C13(Carbamidomethyl); C27(Carbamidomethyl); K32(TMT6plex)	4217.2	1	**0.8**	**0.7**	**0.8**	**0.8**
14141157	NP_036339.1	**HNRNPH3**	3189	**heterogeneous nuclear ribonucleoprotein H3 isoform a**	69.08	6	1	14	159	**1.2**	**1.3**	**1.2**	**1.2**	346	36.9	6.87												
																	HNGPNDASDGTVR	4	2	2	14141157;14141159	N-Term(TMT6plex)	1568.8	0				
																	ATENDIANFFSPLNPIR	31	2	2	14141157;14141159	N-Term(TMT6plex)	2148.1	0				
																	DGMDNQGGYGSVGR	14	2	2	14141157;14141159	N-Term(TMT6plex); M3(Oxidation)	1657.7	0				
																	EIAENALGK	13	2	2	14141157;14141159	N-Term(TMT6plex); K9(TMT6plex)	1402.8	0				
																	YIEIFR	8	8	3	14141157;14141159;45935393	N-Term(TMT6plex)	1069.6	0				
																	STGEAFVQFASK	20	2	2	14141157;14141159	N-Term(TMT6plex); K12(TMT6plex)	1730.0	0				
																	ATGEADVEFVTHEDAVAAMSK	25	2	2	14141157;14141159	N-Term(TMT6plex); M19(Oxidation); K21(TMT6plex)	2652.3	0				
																	GGGGSGGYYGQGGMSGGGWR	5	2	2	14141157;14141159	N-Term(TMT6plex); M14(Oxidation)	2049.9	0				
																	YIELFLNSTPGGGSGMGGSGMGGYGR	7	2	2	14141157;14141159	N-Term(TMT6plex); M16(Oxidation); M21(Oxidation)	2783.3	0				
																	GMGGHGYGGAGDASSGFHGGHFVHMR	5	2	2	14141157;14141159	N-Term(TMT6plex); M2(Oxidation); M25(Oxidation)	2804.2	0				
																	VHIDIGADGR	16	2	2	14141157;14141159	N-Term(TMT6plex)	1281.7	0				
																	GGDGYDGGYGGFDDYGGYNNYGYGNDGFDDR	3	1	1	14141157	N-Term(TMT6plex)	3508.4	0	**1.2**	**1.3**	**1.2**	**1.2**
																	GLPFGCSK	6	4	4	14141157;14141159;9624998;381342476;5031753	N-Term(TMT6plex); C6(Carbamidomethyl); K8(TMT6plex)	1323.7	0				
																	EEIVQFFQGLEIVPNGITLTMDYQGR	2	2	2	14141157;14141159	N-Term(TMT6plex); M21(Oxidation)	3242.7	0				
4827038	NP_005070.1	**TPD52**	7163	**tumor protein D52 isoform 3**	69.02	7	10	10	77	**1.4**	**1.3**	**1.5**	**1.4**	184	19.9	4.96												
																	TDPVPEEGEDVAATISATETLSEEEQEELRR	12	7	1	4827038	N-Term(TMT6plex)	3659.8	1	**1.6**	**1.3**	**1.3**	**1.4**
																	ASAAFSSVGSVITK	4	7	1	4827038	N-Term(TMT6plex); K14(TMT6plex)	1783.0	0	**1.1**	**1.2**	**1.2**	**1.3**
																	LGINSLQELK	7	7	1	4827038	N-Term(TMT6plex); K10(TMT6plex)	1573.0	0	**1.2**	**1.0**	**1.3**	**1.4**
																	TSETLSQAGQK	11	7	1	4827038	N-Term(TMT6plex); K11(TMT6plex)	1607.9	0	**1.3**	**1.0**	**1.2**	**1.2**
																	KLGINSLQELK	2	7	1	4827038	N-Term(TMT6plex); K1(TMT6plex); K11(TMT6plex)	1930.2	1	**1.2**	**1.3**	**1.5**	**1.5**
																	VEEEIQTLSQVLAAK	24	7	1	4827038	N-Term(TMT6plex); K15(TMT6plex)	2116.2	0	**1.4**	**1.4**	**1.9**	**1.4**
																	VGGTKPAGGDFGEVLNSAANASATTEPLPEK	8	6	1	4827038	N-Term(TMT6plex); K5(TMT6plex); K32(TMT6plex)	3774.0	0	**1.2**	**1.2**	**1.4**	**1.4**
																	GWQDVTATSAYK	5	7	1	4827038	N-Term(TMT6plex); K12(TMT6plex)	1785.0	0	**1.2**	**1.2**	**1.3**	**1.5**
																	KTSETLSQAGQK	2	7	1	4827038	N-Term(TMT6plex); K1(TMT6plex); K12(TMT6plex)	1965.2	1	**1.2**	**1.3**	**1.3**	**1.5**
																	TDPVPEEGEDVAATISATETLSEEEQEELR	2	7	1	4827038	N-Term(TMT6plex)	3503.6	0				
47519616	NP_998839.1	**TPM2**	7169	**tropomyosin beta chain isoform 2**	69.01	2	4	27	396	**1.5**	**1.6**	**1.7**	**1.3**	284	33.0	4.67												
																	IQLVEEELDR	65	22	9	63252898;63252900;499137518;42476296;4507651;114155144;63252904;114155140;47519616	N-Term(TMT6plex)	1472.8	0				
																	TIDDLEETLASAK	32	1	2	47519616;42476296	N-Term(TMT6plex); K13(TMT6plex)	1864.0	0				
																	KLVILEGELER	30	6	3	42476296;4507651;47519616	N-Term(TMT6plex); K1(TMT6plex)	1757.1	1				
																	SLMASEEEYSTK	28	1	1	47519616	N-Term(TMT6plex); M3(Oxidation); K12(TMT6plex)	1848.9	0	**1.4**	**1.5**	**1.5**	**1.2**
																	EENVEIHQTLDQTLLELNNL	17	1	1	47519616	N-Term(TMT6plex)	2594.4	0	**1.5**	**1.5**	**1.5**	**1.5**
																	MELQEMQLK	7	4	3	4507651;42476296;47519616	N-Term(TMT6plex); M1(Oxidation); M6(Oxidation); K9(TMT6plex)	1639.9	0				
																	QLEEELR	7	1	1	47519616	N-Term(TMT6plex)	1145.6	0	**1.3**	**1.4**	**1.4**	**1.4**
																	LVILEGELER	15	4	3	42476296;4507651;47519616	N-Term(TMT6plex)	1399.8	0				
																	QLEEEQQALQK	21	2	2	42476296;47519616	N-Term(TMT6plex); K11(TMT6plex)	1802.0	0				
																	EDKYEEEIK	21	12	6	499137518;42476296;4507651;114155144;114155140;47519616	N-Term(TMT6plex); K3(TMT6plex); K9(TMT6plex)	1870.0	1				
																	LATALQK	47	22	9	63252898;63252900;499137518;42476296;4507651;114155144;63252904;114155140;47519616	N-Term(TMT6plex); K7(TMT6plex)	1202.8	0				
																	GTEDEVEK	8	2	2	42476296;47519616	N-Term(TMT6plex); K8(TMT6plex)	1364.7	0				
																	AEQAEADKK	9	9	4	63252898;63252900;42476296;47519616	N-Term(TMT6plex); K8(TMT6plex); K9(TMT6plex)	1677.0	1				
																	AADESER	4	18	9	63252898;63252900;499137518;42476296;4507651;114155144;63252904;114155140;47519616	N-Term(TMT6plex)	1006.5	0				
																	AEQAEADK	6	12	4	63252898;63252900;42476296;47519616	N-Term(TMT6plex); K8(TMT6plex)	1319.7	0				
																	SLMASEEEYSTK	12	1	1	47519616	N-Term(TMT6plex); K12(TMT6plex)	1832.9	0	**2.0**	**2.0**	**2.5**	**1.2**
																	KATDAEADVASLNR	13	6	4	63252898;63252900;42476296;47519616	N-Term(TMT6plex); K1(TMT6plex)	1919.1	1				
																	ATDAEADVASLNR	7	6	4	63252898;63252900;42476296;47519616	N-Term(TMT6plex)	1561.8	0				
																	MELQEMQLK	5	4	3	4507651;42476296;47519616	N-Term(TMT6plex); M6(Oxidation); K9(TMT6plex)	1623.9	0				
																	LKGTEDEVEK	2	2	2	42476296;47519616	N-Term(TMT6plex); K2(TMT6plex); K10(TMT6plex)	1835.1	1				
																	KYEEVAR	9	20	9	63252898;63252900;499137518;42476296;4507651;114155144;63252904;114155140;47519616	N-Term(TMT6plex); K1(TMT6plex)	1352.8	1				
																	TMDQALK	4	1	1	47519616	N-Term(TMT6plex); M2(Oxidation); K7(TMT6plex)	1280.7	0	**1.4**	**1.5**	**1.7**	**1.1**
																	YSESVK	4	2	2	42476296;47519616	N-Term(TMT6plex); K6(TMT6plex)	1170.7	0				
																	LDKENAIDR	1	10	5	63252898;63252900;114155140;42476296;47519616	N-Term(TMT6plex); K3(TMT6plex)	1531.9	1				
																	AEFAER	7	24	9	63252898;63252900;499137518;42476296;4507651;114155144;63252904;114155140;47519616	N-Term(TMT6plex)	951.5	0				
																	LEEAEK	4	20	10	63252898;63252900;499137518;42476296;4507651;114155144;63252904;114155140;47519616;116235456;116235451	N-Term(TMT6plex); K6(TMT6plex)	1176.7	0				
																	YEEVAR	4	24	9	63252898;63252900;499137518;42476296;4507651;114155144;63252904;114155140;47519616	N-Term(TMT6plex)	995.5	0				
																	LEQAEK	5	7	3	42476296;507588248;47519616	N-Term(TMT6plex); K6(TMT6plex)	1175.7	0				
																	YEEEIK	2	19	9	63252898;63252900;499137518;42476296;4507651;114155144;63252904;114155140;47519616	N-Term(TMT6plex); K6(TMT6plex)	1268.7	0				
42822872;21361547	NP_976317.1	**RNH1**	6050	**ribonuclease inhibitor**	68.98	1	20	20	131	**1.5**	**1.5**	**1.6**	**1.7**	461	49.9	4.82												
																	ELSLAGNELGDEGAR	12	1	1	42822872;21361547	N-Term(TMT6plex)	1759.9	0	**1.3**	**1.3**	**1.4**	**1.4**
																	WAELLPLLQQCQVVR	15	1	1	42822872;21361547	N-Term(TMT6plex); C11(Carbamidomethyl)	2082.2	0	**1.4**	**1.4**	**1.5**	**1.4**
																	ELTVSNNDINEAGVR	26	1	1	42822872;21361547	N-Term(TMT6plex)	1860.0	0	**1.5**	**1.5**	**1.6**	**1.8**
																	LGDVGMAELCPGLLHPSSR	5	1	1	42822872;21361547	N-Term(TMT6plex); M6(Oxidation); C10(Carbamidomethyl)	2254.1	0	**1.3**	**1.4**	**1.2**	**1.4**
																	LQLEYCSLSAASCEPLASVLR	4	1	1	42822872;21361547	N-Term(TMT6plex); C6(Carbamidomethyl); C13(Carbamidomethyl)	2596.3	0	**1.5**	**1.6**	**1.7**	**1.7**
																	TLPTLQELHLSDNLLGDAGLQLLCEGLLDPQCR	4	1	1	42822872;21361547	N-Term(TMT6plex); C24(Carbamidomethyl); C32(Carbamidomethyl)	3932.1	0	**1.4**	**1.0**	**2.0**	**1.5**
																	LESCGVTSDNCR	4	1	1	42822872;21361547	N-Term(TMT6plex); C4(Carbamidomethyl); C11(Carbamidomethyl)	1626.7	0	**1.9**	**1.6**	**2.2**	**1.8**
																	ELDLSNNCLGDAGILQLVESVR	9	1	1	42822872;21361547	N-Term(TMT6plex); C8(Carbamidomethyl)	2644.4	0	**2.0**	**1.7**	**1.9**	**2.1**
																	LLCETLLEPGCQLESLWVK	14	1	1	42822872;21361547	N-Term(TMT6plex); C3(Carbamidomethyl); C11(Carbamidomethyl); K19(TMT6plex)	2746.5	0	**1.7**	**1.8**	**2.1**	**2.2**
																	VLWLADCDVSDSSCSSLAATLLANHSLR	4	1	1	42822872;21361547	N-Term(TMT6plex); C7(Carbamidomethyl); C14(Carbamidomethyl)	3290.6	0	**1.3**	**1.1**	**1.4**	**1.7**
																	VNPALAELNLR	5	1	1	42822872;21361547	N-Term(TMT6plex)	1438.9	0	**1.4**	**1.5**	**1.7**	**1.6**
																	LSLQNCCLTGAGCGVLSSTLR	8	1	1	42822872;21361547	N-Term(TMT6plex); C6(Carbamidomethyl); C7(Carbamidomethyl); C13(Carbamidomethyl)	2496.3	0	**1.4**	**1.5**	**1.6**	**1.8**
																	SNELGDVGVHCVLQGLQTPSCK	1	1	1	42822872;21361547	N-Term(TMT6plex); C11(Carbamidomethyl); C21(Carbamidomethyl); K22(TMT6plex)	2856.5	0				
																	DSPCQLEALK	8	1	1	42822872;21361547	N-Term(TMT6plex); C4(Carbamidomethyl); K10(TMT6plex)	1618.9	0	**1.5**	**1.6**	**1.7**	**1.7**
																	LDDCGLTEAR	2	1	1	42822872;21361547	N-Term(TMT6plex); C4(Carbamidomethyl)	1378.7	0	**1.4**	**1.4**	**1.5**	**1.5**

Supplementary Table 6. List of all proteins and peptides identified in untreated and chronically treated Het1A cells with cigarette smoke condensate for 8 months

Accession	RefSeq Accession	Gene Symbol	Entrez Gene ID	Description	Σ Coverage	Σ# Proteins	Σ# Unique Peptides	Σ# Peptides	Σ# PSMs	Het-1A-Smoke - 2M/Parental	Het-1A-Smoke - 4M/Parental	Het-1A-Smoke - 6M/Parental	Het-1A-Smoke - 8M/Parental	# AAs	MW [kDa]	calc. pI	Sequence	# PSMs	# Proteins	# Protein Groups	Protein Group Accessions	Modifications	MH+ [Da]	# Missed Cleavages	Het-1A-Smoke - 2M/Parental	Het-1A-Smoke - 4M/Parental	Het-1A-Smoke - 6M/Parental	Het-1A-Smoke - 8M/Parental
																	DLCGIVASK	2	1	1	42822872;21361547	N-Term(TMT6plex); C3(Carbamidomethyl); K9(TMT6plex)	1420.8	0	**1.5**	**1.5**	**1.8**	**1.9**
																	ELCQGLGQPGSVLR	2	1	1	42822872;21361547	N-Term(TMT6plex); C3(Carbamidomethyl)	1742.9	0	**1.2**	**1.5**	**1.6**	**1.7**
																	ELALGSNK	2	1	1	42822872;21361547	N-Term(TMT6plex); K8(TMT6plex)	1289.8	0	**1.4**	**1.4**	**1.5**	**1.5**
																	LEDAGVR	2	1	1	42822872;21361547	N-Term(TMT6plex)	988.6	0	**1.5**	**1.4**	**1.6**	**1.7**
																	DISSALR	2	1	1	42822872;21361547	N-Term(TMT6plex)	990.6	0	**1.3**	**1.4**	**1.7**	**1.7**
5032007	NP_005850.1	**PURA**	5813	**transcriptional activator protein Pur-alpha**	68.94	1	11	12	68	**1.3**	**1.4**	**1.3**	**1.3**	322	34.9	6.44												
																	LIDDYGVEEEPAELPEGTSLTVDNK	10	1	1	5032007	N-Term(TMT6plex); K25(TMT6plex)	3191.6	0	**1.2**	**1.4**	**1.3**	**1.2**
																	LIDDYGVEEEPAELPEGTSLTVDNKR	4	1	1	5032007	N-Term(TMT6plex); K25(TMT6plex)	3347.7	1	**1.3**	**1.8**	**1.2**	**1.5**
																	DYLGDFIEHYAQLGPSQPPDLAQAQDEPR	8	1	1	5032007	N-Term(TMT6plex)	3499.7	0	**1.6**	**1.7**	**1.7**	**1.6**
																	IAEVGAGGNK	4	1	1	5032007	N-Term(TMT6plex); K10(TMT6plex)	1373.8	0	**1.3**	**1.4**	**1.3**	**1.3**
																	DSGSEQGGAALGSGGSLGHPGSGSGSGGGGGGGGGGGSGGGGGGAPGGLQHETQELASK	4	1	1	5032007	N-Term(TMT6plex); K60(TMT6plex)	5291.5	0	**1.3**	**1.2**	**1.3**	**1.5**
																	GPGLGSTQGQTIALPAQGLIEFR	19	1	1	5032007	N-Term(TMT6plex)	2540.4	0	**1.3**	**1.5**	**1.4**	**1.4**
																	VSEVKPTYR	2	1	1	5032007	N-Term(TMT6plex); K5(TMT6plex)	1536.9	0	**1.1**	**1.2**	**1.2**	**1.2**
																	FFFDVGSNK	9	1	1	5032007	N-Term(TMT6plex); K9(TMT6plex)	1518.8	0	**1.3**	**1.3**	**1.3**	**1.2**
																	NSITVPYK	4	1	1	5032007	N-Term(TMT6plex); K8(TMT6plex)	1379.8	0	**1.2**	**1.2**	**1.3**	**1.2**
																	AACEQLHQQQQQQEETAAATLLLQGEEEGEED	2	1	1	5032007	N-Term(TMT6plex); C3(Carbamidomethyl)	3968.8	0	**1.3**	**1.2**	**1.2**	**1.1**
																	ALKSEFLVR	1	2	2	5032007;15147219	N-Term(TMT6plex); K3(TMT6plex)	1521.0	1				
																	YYMDLK	1	1	1	5032007	N-Term(TMT6plex); K6(TMT6plex)	1290.7	0	**1.3**	**1.6**	**1.7**	**1.3**
4505029	NP_000886.1	**LTA4H**	4048	**leukotriene A-4 hydrolase isoform 1**	68.90	3	30	30	293	**1.4**	**1.5**	**1.5**	**1.6**	611	69.2	6.18												
																	WEDAIPLALK	24	2	1	4505029	N-Term(TMT6plex); K10(TMT6plex)	1614.0	0	**1.4**	**1.5**	**1.5**	**1.7**
																	SSALQWLTPEQTSGK	14	3	1	4505029	N-Term(TMT6plex); K15(TMT6plex)	2091.2	0	**1.4**	**1.3**	**1.5**	**1.4**
																	ELVALMSAIR	8	3	1	4505029	N-Term(TMT6plex)	1331.8	0	**1.7**	**1.9**	**2.1**	**1.8**
																	SAYEFSETESMLK	6	3	1	4505029	N-Term(TMT6plex); K13(TMT6plex)	1980.0	0	**1.7**	**2.0**	**2.1**	**1.7**
																	AILPCQDTPSVK	10	3	1	4505029	N-Term(TMT6plex); C5(Carbamidomethyl); K12(TMT6plex)	1787.0	0	**1.3**	**1.4**	**1.6**	**1.4**
																	DLSSHQLNEFLAQTLQR	14	3	1	4505029	N-Term(TMT6plex)	2229.2	0	**1.3**	**1.5**	**1.4**	**1.5**
																	LTYTAEVSVPK	16	3	1	4505029	N-Term(TMT6plex); K11(TMT6plex)	1666.0	0	**1.4**	**1.4**	**1.4**	**1.5**
																	SAYEFSETESMLK	13	3	1	4505029	N-Term(TMT6plex); M11(Oxidation); K13(TMT6plex)	1996.0	0	**1.4**	**1.5**	**1.4**	**1.6**
																	ASMHPVTAMLVGK	8	2	1	4505029	N-Term(TMT6plex); M3(Oxidation); M9(Oxidation); K13(TMT6plex)	1832.0	0	**1.2**	**1.3**	**1.3**	**1.3**
																	EDDLNSFNATDLK	12	3	1	4505029	N-Term(TMT6plex); K13(TMT6plex)	1940.0	0	**1.4**	**1.5**	**1.6**	**1.6**
																	LVVDLTDIDPDVAYSSVPYEK	20	3	1	4505029	N-Term(TMT6plex); K21(TMT6plex)	2796.5	0	**1.5**	**1.7**	**1.7**	**1.9**
																	GSPMEISLPIALSK	4	3	1	4505029	N-Term(TMT6plex); K14(TMT6plex)	1901.1	0	**1.7**	**1.9**	**2.1**	**1.6**
																	SLSNVIAHEISHSWTGNLVTNK	7	3	1	4505029	N-Term(TMT6plex); K22(TMT6plex)	2865.6	0	**1.3**	**1.4**	**1.6**	**1.6**
																	SITTDDWK	7	3	1	4505029	N-Term(TMT6plex); K8(TMT6plex)	1423.8	0	**1.4**	**1.5**	**1.6**	**1.6**
																	GSPMEISLPIALSK	25	3	1	4505029	N-Term(TMT6plex); M4(Oxidation); K14(TMT6plex)	1917.1	0	**1.3**	**1.6**	**1.6**	**1.7**
																	EHPYLFSQCQAIHCR	9	3	1	4505029	N-Term(TMT6plex); C9(Carbamidomethyl); C14(Carbamidomethyl)	2175.0	0	**1.3**	**1.4**	**1.5**	**1.4**
																	DGETPDPEDPSR	8	3	1	4505029	N-Term(TMT6plex)	1543.7	0	**1.5**	**1.4**	**1.4**	**1.5**
																	DFLYSYFK	25	3	1	4505029	N-Term(TMT6plex); K8(TMT6plex)	1540.8	0	**1.4**	**1.5**	**1.6**	**1.6**
																	APLPLGHIK	4	2	1	4505029	N-Term(TMT6plex); K9(TMT6plex)	1403.9	0	**1.2**	**1.4**	**1.3**	**1.6**
																	VVINGQEVK	2	3	1	4505029	N-Term(TMT6plex); K9(TMT6plex)	1443.9	0	**1.5**	**1.5**	**1.5**	**1.5**
																	PEIVDTCSLASPASVCR	3	1	1	4505029	N-Term(TMT6plex); C7(Carbamidomethyl); C16(Carbamidomethyl)	2091.0	0	**1.5**	**1.3**	**1.5**	**1.2**
																	TWDHFWLNEGHTVYLER	3	3	1	4505029	N-Term(TMT6plex)	2432.2	0	**1.3**	**1.2**	**1.4**	**1.2**
																	TLTGTAALTVQSQEDNLR	4	1	1	4505029	N-Term(TMT6plex)	2147.2	0	**1.6**	**1.3**	**1.5**	**1.4**
																	HFNALGGWGELQNSVK	4	3	1	4505029	N-Term(TMT6plex); K16(TMT6plex)	2215.2	0	**1.4**	**1.5**	**1.5**	**1.4**
																	IAEDLGGPYVWGQYDLLVLPPSFPYGGMENPCLTFVTPTLLAGDK	2	3	1	4505029	N-Term(TMT6plex); M28(Oxidation); C32(Carbamidomethyl); K45(TMT6plex)	5385.8	0	**1.8**	**1.4**	**1.6**	**1.7**
																	TFGETHPFTK	7	3	1	4505029	N-Term(TMT6plex); K10(TMT6plex)	1622.9	0	**1.3**	**1.3**	**1.5**	**1.4**
																	ASMHPVTAMLVGK	2	2	1	4505029	N-Term(TMT6plex); K13(TMT6plex)	1800.0	0	**1.4**	**1.6**	**1.7**	**1.4**
																	GFALLFYLEQLLGGPEIFLGFLK	2	3	1	4505029	N-Term(TMT6plex); K23(TMT6plex)	3043.8	0	**1.8**	**1.6**	**3.0**	**0.8**
																	MQEVYNFNAINNSEIR	2	2	1	4505029	N-Term(TMT6plex); M1(Oxidation)	2187.1	0	**1.5**	**1.5**	**1.5**	**1.5**
																	ELVALMSAIR	4	3	1	4505029	N-Term(TMT6plex); M6(Oxidation)	1347.8	0	**1.3**	**1.4**	**1.3**	**1.4**
																	TLVWSEK	5	3	1	4505029	N-Term(TMT6plex); K7(TMT6plex)	1320.8	0	**1.3**	**1.3**	**1.5**	**1.5**
																	SLVLDTK	12	1	1	4505029	N-Term(TMT6plex); K7(TMT6plex)	1233.8	0	**1.3**	**1.5**	**1.4**	**1.6**
																	TYQEHK	5	2	1	4505029	N-Term(TMT6plex); K6(TMT6plex)	1263.7	0	**1.2**	**1.4**	**1.6**	**1.5**
																	DLAAFDK	2	2	1	4505029	N-Term(TMT6plex); K7(TMT6plex)	1237.7	0	**1.6**	**1.8**	**1.6**	**1.6**
4503571	NP_001419.1	**ENO1**	2023	**alpha-enolase isoform 1**	68.89	2	22	27	2582	**1.3**	**1.4**	**1.4**	**1.6**	434	47.1	7.39												
																	AAVPSGASTGIYEALELR	228	4	3	301897479;4503571;5803011	N-Term(TMT6plex)	2034.1	0				
																	FTASAGIQVVGDDLTVTNPK	284	2	1	4503571	N-Term(TMT6plex); K20(TMT6plex)	2491.4	0	**1.4**	**1.3**	**1.4**	**1.4**
																	VVIGMDVAASEFFR	341	2	1	4503571	N-Term(TMT6plex); M5(Oxidation)	1785.9	0	**1.3**	**1.4**	**1.3**	**1.5**
																	AVEHINK	21	1	1	4503571	N-Term(TMT6plex); K7(TMT6plex)	1268.8	0	**1.2**	**1.4**	**1.3**	**1.7**
																	SGETEDTFIADLVVGLCTGQIK	354	5	3	301897479;4503571;5803011	N-Term(TMT6plex); C17(Carbamidomethyl); K22(TMT6plex)	2811.5	0				
																	KLNVTEQEK	46	1	1	4503571	N-Term(TMT6plex); K1(TMT6plex); K9(TMT6plex)	1776.1	1	**1.2**	**1.3**	**1.3**	**1.6**
																	LMIEMDGTENK	115	1	1	4503571	N-Term(TMT6plex); M2(Oxidation); M5(Oxidation); K11(TMT6plex)	1770.9	0	**1.5**	**1.5**	**1.5**	**1.6**
																	DATNVGDEGGFAPNILENK	85	2	1	4503571	N-Term(TMT6plex); K19(TMT6plex)	2419.3	0	**1.4**	**1.6**	**1.4**	**1.8**
																	SCNCLLLK	23	2	1	4503571	N-Term(TMT6plex); C2(Carbamidomethyl); C4(Carbamidomethyl); K8(TMT6plex)	1465.8	0	**1.4**	**1.4**	**1.5**	**1.5**
																	VVIGMDVAASEFFR	197	2	1	4503571	N-Term(TMT6plex)	1769.9	0	**1.5**	**1.6**	**1.6**	**1.6**
																	DYPVVSIEDPFDQDDWGAWQK	243	2	1	4503571	N-Term(TMT6plex); K21(TMT6plex)	2968.4	0	**1.4**	**1.4**	**1.4**	**1.7**
																	YISPDQLADLYK	149	2	1	4503571	N-Term(TMT6plex); K12(TMT6plex)	1884.1	0	**1.4**	**1.4**	**1.4**	**1.7**
																	VNQIGSVTESLQACK	16	4	2	301897479;4503571	N-Term(TMT6plex); C14(Carbamidomethyl); K15(TMT6plex)	2092.1	0				
																	GNPTVEVDLFTSK	146	1	1	4503571	N-Term(TMT6plex); K13(TMT6plex)	1865.0	0	**1.2**	**1.4**	**1.4**	**1.6**
																	LNVTEQEK	26	1	1	4503571	N-Term(TMT6plex); K8(TMT6plex)	1418.8	0	**1.4**	**1.4**	**1.3**	**1.7**
																	LAMQEFMILPVGAANFR	65	2	1	4503571	N-Term(TMT6plex); M3(Oxidation); M7(Oxidation)	2169.1	0	**1.5**	**1.5**	**1.5**	**1.7**
																	LAMQEFMILPVGAANFR	25	2	1	4503571	N-Term(TMT6plex); M3(Oxidation)	2153.1	0	**1.4**	**1.5**	**1.4**	**1.6**
																	LMIEMDGTENK	17	1	1	4503571	N-Term(TMT6plex); M2(Oxidation); K11(TMT6plex)	1754.9	0	**1.7**	**1.9**	**1.8**	**1.8**
																	TIAPALVSK	38	1	1	4503571	N-Term(TMT6plex); K9(TMT6plex)	1357.9	0	**1.3**	**1.3**	**1.3**	**1.5**
																	IEEELGSK	37	2	1	4503571	N-Term(TMT6plex); K8(TMT6plex)	1362.8	0	**1.3**	**1.3**	**1.3**	**1.6**
																	LAMQEFMILPVGAANFR	23	2	1	4503571	N-Term(TMT6plex)	2137.2	0	**1.6**	**1.8**	**1.6**	**1.4**
																	FGANAILGVSLAVCK	18	5	3	301897479;4503571;5803011	N-Term(TMT6plex); C14(Carbamidomethyl); K15(TMT6plex)	1978.1	0				
																	IGAEVYHNLK	30	2	1	4503571	N-Term(TMT6plex); K10(TMT6plex)	1601.9	0	**1.2**	**1.3**	**1.3**	**1.6**
																	SGKYDLDFK	5	2	1	4503571	N-Term(TMT6plex); K3(TMT6plex); K9(TMT6plex)	1760.0	1	**1.4**	**2.3**	**1.5**	**3.0**
																	LAQANGWGVMVSHR	3	2	1	4503571	N-Term(TMT6plex); M10(Oxidation)	1770.9	0	**1.3**	**1.5**	**1.4**	**1.7**
																	EGLELLK	33	2	1	4503571	N-Term(TMT6plex); K7(TMT6plex)	1259.8	0	**1.2**	**1.4**	**1.3**	**1.6**
																	IDKLMIEMDGTENK	2	1	1	4503571	N-Term(TMT6plex); K3(TMT6plex); M5(Oxidation); M8(Oxidation); K14(TMT6plex)	2356.3	1	**2.1**	**2.8**	**2.3**	**2.9**
																	YNQLLR	3	2	1	4503571	N-Term(TMT6plex)	1035.6	0	**1.5**	**1.0**	**1.2**	**1.2**
																	YDLDFK	4	5	3	301897479;4503571;5803011	N-Term(TMT6plex); K6(TMT6plex)	1258.7	0				
																	EIFDSR	3	1	1	4503571	N-Term(TMT6plex)	995.5	0	**1.4**	**1.4**	**1.3**	**1.6**
																	AGAVEK	2	2	1	4503571	N-Term(TMT6plex); K6(TMT6plex)	1032.6	0	**1.2**	**1.3**	**1.2**	**1.6**
114796642	NP_001041664.1	**RCC1**	1104	**regulator of chromosome condensation isoform c**	68.88	3	16	18	179	**1.3**	**1.3**	**1.4**	**1.4**	421	44.9	7.52												
																	DTSVEGSEMVPGK	35	3	1	114796642	N-Term(TMT6plex); M9(Oxidation); K13(TMT6plex)	1809.9	0	**1.2**	**1.4**	**1.5**	**1.4**
																	VVQVSAGDSHTAALTDDGR	18	3	1	114796642	N-Term(TMT6plex)	2128.1	0	**1.2**	**1.3**	**1.3**	**1.3**
																	LGLGEGAEEK	12	3	1	114796642	N-Term(TMT6plex); K10(TMT6plex)	1460.8	0	**1.2**	**1.2**	**1.4**	**1.4**
																	SHSTEPGLVLTLGQGDVGQLGLGENVMER	6	3	1	114796642	N-Term(TMT6plex); M27(Oxidation)	3238.7	0	**1.2**	**1.4**	**1.3**	**1.6**
																	VASGNDHLVMLTADGDLYTLGCGEQGQLGR	6	3	1	114796642	N-Term(TMT6plex); C22(Carbamidomethyl)	3376.6	0	**2.0**	**1.6**	**1.7**	**1.5**
																	VASGNDHLVMLTADGDLYTLGCGEQGQLGR	14	3	1	114796642	N-Term(TMT6plex); M10(Oxidation); C22(Carbamidomethyl)	3392.6	0	**1.4**	**1.5**	**1.3**	**1.5**
																	DTSVEGSEMVPGK	2	3	1	114796642	N-Term(TMT6plex); K13(TMT6plex)	1793.9	0	**1.6**	**1.9**	**2.0**	**1.4**
																	VFAWGMGTNYQLGTGQDEDAWSPVEMMGK	8	3	1	114796642	N-Term(TMT6plex); M6(Oxidation); M26(Oxidation); K29(TMT6plex)	3695.7	0	**1.5**	**1.6**	**1.6**	**1.4**
																	VFAWGMGTNYQLGTGQDEDAWSPVEMMGK	10	3	1	114796642	N-Term(TMT6plex); M6(Oxidation); M26(Oxidation); M27(Oxidation); K29(TMT6plex)	3711.7	0	**1.1**	**1.1**	**1.2**	**1.3**
																	VVLSVSSGGQHTVLLVK	3	3	1	114796642	N-Term(TMT6plex); K17(TMT6plex)	2181.3	0	**1.7**	**1.7**	**1.8**	**1.4**
																	VPELFANR	8	3	1	114796642	N-Term(TMT6plex)	1174.7	0	**1.3**	**1.4**	**1.3**	**1.5**
																	SPPADAIPK	6	3	1	114796642	N-Term(TMT6plex); K9(TMT6plex)	1353.8	0	**1.2**	**1.4**	**1.3**	**1.6**
																	VFLWGSFR	9	3	1	114796642	N-Term(TMT6plex)	1240.7	0	**1.5**	**1.3**	**1.4**	**1.4**
																	SGQVYSFGCNDEGALGR	7	3	1	114796642	N-Term(TMT6plex); C9(Carbamidomethyl)	2046.0	0	**1.3**	**1.1**	**1.2**	**1.2**
																	LPAVSSVACGASVGYAVTK	4	3	1	114796642	N-Term(TMT6plex); C9(Carbamidomethyl); K19(TMT6plex)	2295.3	0	**1.3**	**1.3**	**1.5**	**1.2**

Accession	RefSeq Accession	Gene Symbol	Entrez Gene ID	Description	Σ Coverage	Σ# Proteins	Σ# Unique Peptides	Σ# Peptides	Σ# PSMs	Het-1A-Smoke - 2M/Parental	Het-1A-Smoke - 4M/Parental	Het-1A-Smoke - 6M/Parental	Het-1A-Smoke - 8M/Parental	# AAs	MW [kDa]	calc. pI	Sequence	# PSMs	# Proteins	# Protein Groups	Protein Group Accessions	Modifications	MH+ [Da]	# Missed Cleavages	Het-1A-Smoke - 2M/Parental	Het-1A-Smoke - 4M/Parental	Het-1A-Smoke - 6M/Parental	Het-1A-Smoke - 8M/Parental
																	SWVGFSGGQHHTVCMDSEGK	2	3	1	114796642	N-Term(TMT6plex); C14(Carbamidomethyl); M15(Oxidation); K20(TMT6plex)	2680.3	0	**1.2**	**1.4**	**1.3**	**1.6**
																	SMVPVQVQLDVPVVK	12	3	1	114796642	N-Term(TMT6plex); M2(Oxidation); K15(TMT6plex)	2112.2	0	**1.1**	**1.2**	**1.3**	**1.5**
																	KKPALVSIPEDVVQAEAGGMHTVCLSK	3	3	1	114796642	N-Term(TMT6plex); K1(TMT6plex); K2(TMT6plex); M20(Oxidation); C24(Carbamidomethyl); K27(TMT6plex)	3797.2	1	**1.2**	**1.4**	**1.5**	**1.6**
																	SMVPVQVQLDVPVVK	8	3	1	114796642	N-Term(TMT6plex); K15(TMT6plex)	2096.3	0	**1.6**	**1.5**	**1.6**	**1.3**
																	VFAWGMGTNYQLGTGQDEDAWSPVEMMGK	2	3	1	114796642	N-Term(TMT6plex); M6(Oxidation); K29(TMT6plex)	3679.7	0	**1.5**	**1.5**	**1.6**	**1.4**
																	AYSLGR	2	4	2	126116596;114796642	N-Term(TMT6plex)	895.5	0				
																	VELQEK	1	4	2	114796642;225690529	N-Term(TMT6plex); K6(TMT6plex)	1203.7	0				
																	SIPTLISR	1	3	1	114796642	N-Term(TMT6plex)	1115.7	0	**1.3**	**1.3**	**1.5**	**1.3**
11056044	NP_066952.1	**PPA1**	5464	**inorganic pyrophosphatase**	68.86	1	15	17	131	**1.5**	**1.6**	**1.7**	**1.8**	289	32.6	5.86												
																	VIAINVDDPDAANYNDINDVKR	4	1	1	11056044	N-Term(TMT6plex); K21(TMT6plex)	2902.5	1	**1.7**	**2.1**	**1.9**	**2.4**
																	HTGCCGDNDPIDVCEIGSK	4	1	1	11056044	N-Term(TMT6plex); C4(Carbamidomethyl); C5(Carbamidomethyl); C14(Carbamidomethyl); K19(TMT6plex)	2592.2	0	**1.3**	**1.2**	**1.4**	**1.3**
																	YKVPDGKPENEFAFNAEFK	1	1	1	11056044	N-Term(TMT6plex); K2(TMT6plex); K7(TMT6plex); K19(TMT6plex)	3146.7	1	**1.4**	**1.8**	**1.9**	**2.0**
																	GQYISPFHDIPIYADK	7	1	1	11056044	N-Term(TMT6plex); K16(TMT6plex)	2322.3	0	**1.4**	**1.5**	**1.7**	**1.7**
																	GQYISPFHDIPIYADKDVFHMVVEVPR	8	1	1	11056044	N-Term(TMT6plex); K16(TMT6plex); M21(Oxidation)	3646.9	1	**1.3**	**1.4**	**1.5**	**1.9**
																	DKDFAIDIIK	11	1	1	11056044	N-Term(TMT6plex); K2(TMT6plex); K10(TMT6plex)	1865.1	1	**1.4**	**1.7**	**1.8**	**2.0**
																	VLGILAMIDEGETDWK	20	1	1	11056044	N-Term(TMT6plex); M7(Oxidation); K16(TMT6plex)	2264.2	0	**1.6**	**1.3**	**1.7**	**1.7**
																	VPDGKPENEFAFNAEFK	5	1	1	11056044	N-Term(TMT6plex); K5(TMT6plex); K17(TMT6plex)	2626.4	0	**1.4**	**1.6**	**1.7**	**1.7**
																	VIAINVDDPDAANYNDINDVK	12	1	1	11056044	N-Term(TMT6plex); K21(TMT6plex)	2746.4	0	**1.2**	**1.3**	**1.4**	**1.4**
																	GYIWNYGAIPQTWEDPGHNDK	14	1	1	11056044	N-Term(TMT6plex); K21(TMT6plex)	2919.4	0	**1.4**	**1.5**	**1.6**	**1.6**
																	LKPGYLEATVDWFR	9	1	1	11056044	N-Term(TMT6plex); K2(TMT6plex)	2153.2	0	**1.4**	**1.7**	**1.8**	**1.9**
																	GQYISPFHDIPIYADKDVFHMVVEVPR	3	1	1	11056044	N-Term(TMT6plex); K16(TMT6plex)	3630.9	1	**1.9**	**2.0**	**2.0**	**1.9**
																	YVANLFPYK	6	3	2	29171702;11056044	N-Term(TMT6plex); K9(TMT6plex)	1572.9	0				
																	VLGILAMIDEGETDWK	14	1	1	11056044	N-Term(TMT6plex); K16(TMT6plex)	2248.2	0	**1.9**	**1.9**	**2.5**	**1.7**
																	DVFHMVVEVPR	2	1	1	11056044	N-Term(TMT6plex)	1556.8	0	**1.6**	**1.7**	**1.6**	**1.6**
																	AIVDALPPPCESACTVPTDVDK	2	1	1	11056044	N-Term(TMT6plex); C10(Carbamidomethyl); C14(Carbamidomethyl); K22(TMT6plex)	2813.5	0	**1.4**	**1.4**	**1.7**	**1.2**
																	GEIIGVK	2	1	1	11056044	N-Term(TMT6plex); K7(TMT6plex)	1173.8	0	**1.4**	**1.7**	**1.8**	**2.1**
																	DPLNPIK	4	1	1	11056044	N-Term(TMT6plex); K7(TMT6plex)	1254.8	0	**1.3**	**1.5**	**1.5**	**1.8**
																	MEIATK	2	3	2	11056044;29171702	N-Term(TMT6plex); K6(TMT6plex)	1150.7	0				
																	MEIATK	1	3	2	29171702;11056044	N-Term(TMT6plex); M1(Oxidation); K6(TMT6plex)	1166.7	0				
156071462	NP_001627.2	**SLC25A6**	293	**ADP/ATP translocase 3**	68.79	2	5	20	537	**1.4**	**1.4**	**1.6**	**1.4**	298	32.8	9.74												
																	AAYFGVYDTAK	18	2	2	156071462;55749577	N-Term(TMT6plex); K11(TMT6plex)	1663.9	0				
																	YFPTQALNFAFK	113	4	3	156071462;156071459;55749577	N-Term(TMT6plex); K12(TMT6plex)	1905.0	0				
																	EQGVLSFWR	45	2	2	156071462;156071459	N-Term(TMT6plex)	1350.7	0				
																	YFAGNLASGGAAGATSLCFVYPLDFAR	84	2	2	156071462;55749577	N-Term(TMT6plex); C18(Carbamidomethyl)	3025.5	0				
																	GAWSNVLR	10	3	3	156071462;156071459;55749577	N-Term(TMT6plex)	1131.6	0				
																	LLLQVQHASK	25	3	3	156071462;156071459;55749577	N-Term(TMT6plex); K10(TMT6plex)	1595.0	0				
																	GNLANVIR	10	4	3	156071462;156071459;55749577	N-Term(TMT6plex)	1085.7	0				
																	DFLAGGIAAAISK	75	1	1	156071462	N-Term(TMT6plex); K13(TMT6plex)	1692.0	0	**1.3**	**1.4**	**1.6**	**1.4**
																	GMGGAFVLVLYDELKK	1	3	3	156071459;55749577;156071462	N-Term(TMT6plex); M2(Oxidation); K15(TMT6plex); K16(TMT6plex)	2443.4	1				
																	GIVDCIVR	4	1	1	156071462	N-Term(TMT6plex); C5(Carbamidomethyl)	1160.7	0	**1.6**	**1.8**	**2.0**	**1.7**
																	GADIMYTGTVDCWR	10	2	2	156071462;55749577	N-Term(TMT6plex); C12(Carbamidomethyl)	1873.9	0				
																	GADIMYTGTVDCWR	8	2	2	156071462;55749577	N-Term(TMT6plex); M5(Oxidation); C12(Carbamidomethyl)	1889.9	0				
																	NTHIVVSWMIAQTVTAVAGVVSYPFDTVR	7	1	1	156071462	N-Term(TMT6plex)	3390.8	0	**1.5**	**1.3**	**2.1**	**0.9**
																	GMGGAFVLVLYDELK	16	3	3	156071462;156071459;55749577	N-Term(TMT6plex); M2(Oxidation); K15(TMT6plex)	2086.2	0				
																	KGADIMYTGTVDCWR	4	1	1	156071462	K1(TMT6plex); N-Term(TMT6plex); M6(Oxidation); C13(Carbamidomethyl)	2247.1	1	**1.3**	**1.3**	**1.3**	**1.3**
																	GLGDCLVK	33	2	2	156071462;156071459	N-Term(TMT6plex); C5(Carbamidomethyl); K8(TMT6plex)	1319.8	0				
																	QIFLGGVDK	17	2	2	156071462;156071459	N-Term(TMT6plex); K9(TMT6plex)	1434.9	0				
																	KGADIMYTGTVDCWR	2	2	2	156071462;55749577	N-Term(TMT6plex); K1(TMT6plex); C13(Carbamidomethyl)	2231.1	1				
																	GMGGAFVLVLYDELKK	1	3	3	156071462;156071459;55749577	N-Term(TMT6plex); K15(TMT6plex); K16(TMT6plex)	2427.4	1				
																	GMLPDPKNTHIVVSWMIAQTVTAVAGVVSYPFDTVR	2	1	1	156071462	N-Term(TMT6plex); K7(TMT6plex); M16(Oxidation)	4374.3	1	**1.7**	**1.8**	**3.6**	**1.4**
																	LAADVGK	27	3	3	156071462;156071459;55749577	N-Term(TMT6plex); K7(TMT6plex)	1131.7	0				
																	TAVAPIER	22	4	3	156071462;156071459;55749577	N-Term(TMT6plex)	1085.7	0				
																	GMLPDPK	2	3	3	156071462;156071459;55749577	N-Term(TMT6plex); M2(Oxidation); K7(TMT6plex)	1231.7	0				
																	GMLPDPK	1	3	3	156071462;156071459;55749577	N-Term(TMT6plex); K7(TMT6plex)	1215.7	0				
4506209	NP_002794.1	**PSMC2**	5701	**26S protease regulatory subunit 7 isoform 1**	68.59	2	29	29	232	**1.4**	**1.5**	**1.5**	**1.5**	433	48.6	5.95												
																	FVVDLSDQVAPTDIEEGMR	16	2	1	4506209	N-Term(TMT6plex); M18(Oxidation)	2366.2	0	**1.5**	**1.5**	**1.5**	**1.5**
																	ALDEGDIALLK	10	2	1	4506209	N-Term(TMT6plex); K11(TMT6plex)	1616.0	0	**1.4**	**1.6**	**1.7**	**1.7**
																	ESDTGLAPPALWDLAADK	12	2	1	4506209	N-Term(TMT6plex); K18(TMT6plex)	2328.2	0	**1.4**	**1.6**	**1.7**	**1.7**
																	TMLELINQLDGFDPR	13	1	1	4506209	N-Term(TMT6plex); M2(Oxidation)	2007.0	0	**1.3**	**1.6**	**1.2**	**1.7**
																	KIEFSLPDLEGR	6	1	1	4506209	N-Term(TMT6plex); K1(TMT6plex)	1862.1	1	**1.3**	**1.6**	**1.4**	**1.5**
																	VIGSELVQK	10	1	1	4506209	N-Term(TMT6plex); K9(TMT6plex)	1430.9	0	**1.4**	**1.5**	**1.6**	**1.6**
																	IDPTVTMMQVEEKPDVTYSDVGGCK	5	1	1	4506209	N-Term(TMT6plex); M7(Oxidation); M8(Oxidation); K13(TMT6plex); C24(Carbamidomethyl); K25(TMT6plex)	3518.8	0	**1.1**	**1.4**	**1.4**	**1.4**
																	SVCTEAGMFAIR	8	1	1	4506209	N-Term(TMT6plex); C3(Carbamidomethyl); M8(Oxidation)	1586.8	0	**1.5**	**1.5**	**1.6**	**1.6**
																	QVEDDIQQLLK	16	2	1	4506209	N-Term(TMT6plex); K11(TMT6plex)	1787.0	0	**1.4**	**1.5**	**1.5**	**1.5**
																	QTLQSEQPLQVAR	11	2	1	4506209	N-Term(TMT6plex)	1727.0	0	**1.3**	**1.5**	**1.6**	**1.5**
																	EVVETPLLHPER	8	1	1	4506209	N-Term(TMT6plex)	1647.9	0	**2.0**	**1.5**	**1.5**	**1.5**
																	TMLELINQLDGFDPR	12	1	1	4506209	N-Term(TMT6plex)	1991.0	0	**1.7**	**1.7**	**1.8**	**1.6**
																	TYGQSTYSR	2	2	1	4506209	N-Term(TMT6plex)	1291.6	0	**1.4**	**1.0**	**1.2**	**1.0**
																	GVLLFGPPGTGK	14	1	1	4506209	N-Term(TMT6plex); K12(TMT6plex)	1601.0	0	**1.6**	**1.5**	**1.5**	**1.5**
																	TKEDEKDDKPIR	1	2	1	4506209	N-Term(TMT6plex); K2(TMT6plex); K6(TMT6plex); K9(TMT6plex)	2390.4	2	**1.5**	**1.6**	**1.6**	**1.6**
																	IEFSLPDLEGR	6	1	1	4506209	N-Term(TMT6plex)	1504.8	0	**1.4**	**1.5**	**1.6**	**1.6**
																	EDEKDDKPIR	2	2	1	4506209	N-Term(TMT6plex); K4(TMT6plex); K7(TMT6plex)	1932.1	1	**1.3**	**1.4**	**1.5**	**1.4**
																	IINADSEDPK	10	2	1	4506209	N-Term(TMT6plex); K10(TMT6plex)	1559.9	0	**1.5**	**1.8**	**1.5**	**2.0**
																	YQIHIPLPPK	11	1	1	4506209	N-Term(TMT6plex); K10(TMT6plex)	1664.0	0	**1.4**	**1.5**	**1.5**	**1.4**
																	FVNLGIEPPK	11	1	1	4506209	N-Term(TMT6plex); K10(TMT6plex)	1572.0	0	**1.5**	**1.4**	**1.6**	**1.5**
																	KINELTGIK	1	2	1	4506209	N-Term(TMT6plex); K1(TMT6plex); K9(TMT6plex)	1703.1	1	**1.3**	**1.2**	**1.5**	**1.3**
																	FDDGAGGDNEVQR	7	1	1	4506209	N-Term(TMT6plex)	1608.7	0	**1.4**	**1.4**	**1.4**	**1.5**
																	FVVDLSDQVAPTDIEEGMR	4	2	1	4506209	N-Term(TMT6plex)	2350.2	0	**1.9**	**1.9**	**2.3**	**1.5**
																	INELTGIK	4	2	1	4506209	N-Term(TMT6plex); K8(TMT6plex)	1345.8	0	**1.3**	**1.4**	**1.6**	**1.4**
																	ACLIFFDEIDAIGGAR	19	1	1	4506209	N-Term(TMT6plex); C2(Carbamidomethyl)	1997.0	0	**1.5**	**1.6**	**1.7**	**1.6**
																	DFLEAVNK	2	1	1	4506209	N-Term(TMT6plex); K8(TMT6plex)	1393.8	0				
																	ELFEMAR	2	1	1	4506209	N-Term(TMT6plex)	1124.6	0	**1.5**	**1.7**	**1.9**	**1.5**
																	ELFEMAR	2	1	1	4506209	N-Term(TMT6plex); M5(Oxidation)	1140.6	0				
																	FELLAR	1	1	1	4506209	N-Term(TMT6plex)	977.6	0	**1.6**	**1.4**	**1.6**	**1.5**
																	YIINVK	4	2	1	4506209	N-Term(TMT6plex); K6(TMT6plex)	1207.8	0	**1.4**	**1.6**	**1.6**	**1.4**
																	TDACFIR	1	1	1	4506209	N-Term(TMT6plex); C4(Carbamidomethyl)	1111.6	0	**1.4**	**1.4**	**1.6**	**1.5**
																	YMTYN	1	1	1	4506209	N-Term(TMT6plex); M2(Oxidation)	936.4	0	**1.0**	**1.2**	**1.1**	**1.2**
10716563	NP_001737.1	**CANX**	821	**calnexin precursor**	68.41	1	36	38	1000	**1.3**	**1.8**	**1.4**	**1.5**	592	67.5	4.60												
																	GTLSGWILSK	82	1	1	10716563	N-Term(TMT6plex); K10(TMT6plex)	1519.9	0	**1.3**	**1.9**	**1.4**	**1.5**
																	THLYTLILNPDNSFEILVDQSVVNSGNLLNDMTPPVNPSR	29	1	1	10716563	N-Term(TMT6plex); M32(Oxidation)	4682.4	0	**1.7**	**1.7**	**1.6**	**1.7**
																	TPELNLDQFHDK	40	1	1	10716563	N-Term(TMT6plex); K12(TMT6plex)	1915.0	0	**1.3**	**1.7**	**1.4**	**1.5**
																	KIPNPDFFEDLEPFR	217	1	1	10716563	N-Term(TMT6plex); K1(TMT6plex)	2322.3	1	**1.4**	**1.8**	**1.5**	**1.5**
																	QKSDAEEDGGTVSQEEEDR	13	1	1	10716563	N-Term(TMT6plex); K2(TMT6plex)	2567.2	1	**1.1**	**1.7**	**1.4**	**1.3**
																	WEVEEMK	25	1	1	10716563	N-Term(TMT6plex); M6(Oxidation); K7(TMT6plex)	1424.7	0	**1.5**	**1.9**	**1.6**	**1.4**
																	CESAPGCGVWQRPVIDNPNYK	2	1	1	10716563	N-Term(TMT6plex); C1(Carbamidomethyl); C7(Carbamidomethyl); K21(TMT6plex)	2905.4	0	**1.5**	**1.9**	**1.5**	**1.4**
																	YDGKWEVEEMK	4	1	1	10716563	N-Term(TMT6plex); K4(TMT6plex); M10(Oxidation); K11(TMT6plex)	2117.1	1	**1.4**	**1.9**	**1.3**	**1.7**
																	TGIYEEK	37	1	1	10716563	N-Term(TMT6plex); K7(TMT6plex)	1297.7	0	**1.4**	**1.4**	**1.3**	**1.2**
																	APVPTGEVYFADSFDR	87	1	1	10716563	N-Term(TMT6plex)	2000.0	0	**1.5**	**1.9**	**1.6**	**1.6**
																	IPDPEAVKPDDWDEDAPAK	42	1	1	10716563	N-Term(TMT6plex); K8(TMT6plex); K19(TMT6plex)	2795.5	0	**1.4**	**1.9**	**1.5**	**1.7**
																	IPNPDFFEDLEPFR	43	1	1	10716563	N-Term(TMT6plex)	1965.0	0	**1.5**	**1.7**	**1.5**	**1.5**
																	DDTDDEIAK	23	1	1	10716563	N-Term(TMT6plex); K9(TMT6plex)	1479.8	0	**1.4**	**1.8**	**1.4**	**1.5**
																	TDAPQPDVK	5	1	1	10716563	N-Term(TMT6plex); K9(TMT6plex)	1428.8	0	**1.3**	**1.2**	**1.3**	**1.2**
																	KPEDWDERPK	17	1	1	10716563	N-Term(TMT6plex); K1(TMT6plex); K10(TMT6plex)	1987.1	0	**1.2**	**1.8**	**1.5**	**1.5**
																	KDDTDDEIAK	41	1	1	10716563	N-Term(TMT6plex); K1(TMT6plex); K10(TMT6plex)	1837.0	1	**1.3**	**1.7**	**1.4**	**1.5**
																	WKPPMIDNPSYQGIWKPR	12	1	1	10716563	N-Term(TMT6plex); K2(TMT6plex); M5(Oxidation); K16(TMT6plex)	2916.6	0	**1.2**	**2.1**	**1.5**	**1.7**

Supplementary Table 6. List of all proteins and peptides identified in untreated and chronically treated Het1A cells with cigarette smoke condensate for 8 months

Accession	RefSeq Accession	Gene Symbol	Entrez Gene ID	Description	Σ Coverage	Σ# Proteins	Σ# Unique Peptides	Σ# Peptides	Σ# PSMs	Het-1A-Smoke - 2M/Parental	Het-1A-Smoke - 4M/Parental	Het-1A-Smoke - 6M/Parental	Het-1A-Smoke - 8M/Parental	# AAs	MW [kDa]	calc. pI	Sequence	# PSMs	# Proteins	# Protein Groups	Protein Group Accessions	Modifications	MH+ [Da]	# Missed Cleavages	Het-1A-Smoke - 2M/Parental	Het-1A-Smoke - 4M/Parental	Het-1A-Smoke - 6M/Parental	Het-1A-Smoke - 8M/Parental
																	THLYTLILNPDNSFEILVDQSVVNSGNLLNDMTPPVNPSR	11	1	1	10716563	N-Term(TMT6plex)	4666.4	0	**1.7**	**1.8**	**1.9**	**1.6**
																	IVDDWANDGWGLK	95	1	1	10716563	N-Term(TMT6plex); K13(TMT6plex)	1947.0	0	**1.4**	**1.7**	**1.5**	**1.4**
																	YDGKWEVEEMK	1	1	1	10716563	N-Term(TMT6plex); K4(TMT6plex); K11(TMT6plex)	2101.1	1	**1.9**	**2.8**	**2.2**	**1.9**
																	EIEDPEDR	10	1	1	10716563	N-Term(TMT6plex)	1231.6	0	**1.4**	**1.7**	**1.4**	**1.6**
																	KTDAPQPDVK	6	1	1	10716563	N-Term(TMT6plex); K1(TMT6plex); K10(TMT6plex)	1786.1	1	**1.0**	**1.4**	**1.2**	**1.3**
																	WKPPMIDNPSYQGIWKPR	1	1	1	10716563	N-Term(TMT6plex); K2(TMT6plex); K16(TMT6plex)	2900.6	0	**1.6**	**2.9**	**2.2**	**1.7**
																	HHAISAK	8	1	1	10716563	N-Term(TMT6plex); K7(TMT6plex)	1221.7	0	**1.2**	**1.5**	**1.3**	**1.3**
																	SDAEEDGGTVSQEEEDR	26	1	1	10716563	N-Term(TMT6plex)	2081.9	0	**1.0**	**1.5**	**1.2**	**1.2**
																	TPYTIMFGPDK	19	1	1	10716563	N-Term(TMT6plex); M6(Oxidation); K11(TMT6plex)	1743.9	0	**1.3**	**1.7**	**1.4**	**1.6**
																	RPDADLK	10	1	1	10716563	N-Term(TMT6plex); K7(TMT6plex)	1272.8	0	**1.3**	**1.8**	**1.4**	**1.4**
																	QTSGMEYK	6	1	1	10716563	N-Term(TMT6plex); K8(TMT6plex)	1401.7	0	**1.8**	**1.9**	**2.0**	**1.2**
																	DKGDEEEEGEEK	6	1	1	10716563	N-Term(TMT6plex); K2(TMT6plex); K12(TMT6plex)	2081.1	1	**1.3**	**2.0**	**1.4**	**1.8**
																	QTSGMEYK	8	1	1	10716563	N-Term(TMT6plex); M5(Oxidation); K8(TMT6plex)	1417.7	0	**1.6**	**1.7**	**1.6**	**1.2**
																	SDAEEDGGTVSQEEEDRKPK	7	1	1	10716563	N-Term(TMT6plex); K18(TMT6plex); K20(TMT6plex)	2893.5	1	**1.4**	**2.3**	**1.6**	**1.7**
																	LNKPFLFDTKPLIVQYEVNFQNGIECGGAYVK	1	1	1	10716563	N-Term(TMT6plex); K3(TMT6plex); K10(TMT6plex); C26(Carbamidomethyl); K32(TMT6plex)	4618.5	0	**1.3**	**2.0**	**1.9**	**1.5**
																	KTHLYTLILNPDNSFEILVDQSVVNSGNLLNDMTPPVNPSR	4	1	1	10716563	N-Term(TMT6plex); K1(TMT6plex); M33(Oxidation)	5039.6	1	**1.3**	**1.6**	**1.6**	**1.5**
																	IPDEEATKPEGWLDDEPEYVPDPDAEKPEDWDEDMDGEWEAPQIANPR	11	1	1	10716563	N-Term(TMT6plex); K8(TMT6plex); K27(TMT6plex); M35(Oxidation)	6268.9	0	**2.5**	**3.3**	**3.1**	**2.7**
																	TPYTIMFGPDK	2	1	1	10716563	N-Term(TMT6plex); K11(TMT6plex)	1727.9	0	**2.0**	**2.5**	**2.1**	**1.7**
																	AKKDDTDDEIAK	2	1	1	10716563	N-Term(TMT6plex); K2(TMT6plex); K3(TMT6plex); K12(TMT6plex)	2265.3	2	**1.4**	**1.9**	**1.5**	**1.7**
																	WEVEEMK	6	1	1	10716563	N-Term(TMT6plex); K7(TMT6plex)	1408.8	0	**1.7**	**2.2**	**2.1**	**1.4**
																	AEEDEILNR	10	1	1	10716563	N-Term(TMT6plex)	1317.7	0	**1.3**	**1.5**	**1.5**	**1.5**
																	IVDDWANDGWGLKK	2	1	1	10716563	N-Term(TMT6plex); K13(TMT6plex); K14(TMT6plex)	2304.3	1	**1.4**	**3.4**	**1.7**	**2.3**
																	IPDEEATKPEGWLDDEPEYVPDPDAEKPEDWDEDMDGEWEAPQIANPR	2	1	1	10716563	N-Term(TMT6plex); K8(TMT6plex); K27(TMT6plex)	6252.9	0	**1.8**	**2.1**	**1.8**	**1.4**
																	GLVLMSR	4	1	1	10716563	N-Term(TMT6plex)	1004.6	0	**1.7**	**2.3**	**2.0**	**1.5**
																	GLVLMSR	4	1	1	10716563	N-Term(TMT6plex); M5(Oxidation)	1020.6	0	**1.3**	**1.9**	**1.5**	**1.6**
																	EIEDPEDRKPEDWDERPK	1	1	1	10716563	N-Term(TMT6plex); K9(TMT6plex); K18(TMT6plex)	2970.5	1	**0.7**	**0.8**	**0.7**	**0.9**
																	TYFTDK	12	1	1	10716563	N-Term(TMT6plex); K6(TMT6plex)	1232.7	0	**1.4**	**1.5**	**1.4**	**1.3**
																	LHFIFR	5	2	2	194578888;10716563	N-Term(TMT6plex)	1061.6	0				
																	CGEDYK	1	2	2	194578888;10716563	N-Term(TMT6plex); C1(Carbamidomethyl); K6(TMT6plex)	1229.6	0				
31742503;183076548	NP_066403.2	**HIST2H3C**	126961	**histone H3.2**	68.38	2	1	12	1718	**1.2**	**1.0**	**1.2**	**1.0**	136	15.4	11.27												
																	STELLIR	368	5	4	4504297;4504281;31742503;183076548;4504299;4885385	N-Term(TMT6plex)	1060.7	0				
																	FQSSAVMALQEASEAYLVGLFEDTNLCAIHAK	294	1	1	31742503;183076548	N-Term(TMT6plex); M7(Oxidation); C27(Carbamidomethyl); K32(TMT6plex)	3988.0	0	**1.1**	**1.0**	**1.2**	**1.0**
																	EIAQDFK	535	4	4	4504297;4504281;31742503;183076548;4504299;4885385	N-Term(TMT6plex); K7(TMT6plex)	1308.8	0				
																	YRPGTVALR	308	5	4	4504297;4504281;31742503;183076548;4504299;4885385	N-Term(TMT6plex)	1261.8	0				
																	EIAQDFKTDLR	3	4	4	4504297;4504281;31742503;183076548;4504299;4885385	N-Term(TMT6plex); K7(TMT6plex)	1794.0	1				
																	SAPATGGVK	54	3	3	4504297;4504281;31742503;183076548;4504299	N-Term(TMT6plex); K9(TMT6plex)	1245.8	0				
																	FQSSAVMALQEASEAYLVGLFEDTNLCAIHAK	117	1	1	31742503;183076548	N-Term(TMT6plex); C27(Carbamidomethyl); K32(TMT6plex)	3972.0	0	**1.3**	**1.2**	**1.7**	**1.0**
																	KSAPATGGVK	4	3	3	4504297;4504281;31742503;183076548;4504299	N-Term(TMT6plex); K1(TMT6plex); K10(TMT6plex)	1603.0	1				
																	KLPFQR	1	5	4	4504297;4504281;31742503;183076548;4504299;4885385	N-Term(TMT6plex); K1(TMT6plex)	1246.8	1				
																	KQLATK	16	5	4	4504297;4504281;31742503;183076548;4504299;4885385	N-Term(TMT6plex); K1(TMT6plex); K6(TMT6plex)	1375.9	1				
																	VTIMPKDIQLAR	1	5	4	4504297;4504281;31742503;183076548;4504299;4885385	N-Term(TMT6plex); K6(TMT6plex)	1843.1	1				
																	VTIMPK	9	5	4	4504297;4504281;31742503;183076548;4504299;4885385	N-Term(TMT6plex); M4(Oxidation); K6(TMT6plex)	1162.7	0				
																	VTIMPK	5	5	4	4504297;4504281;31742503;183076548;4504299;4885385	N-Term(TMT6plex); K6(TMT6plex)	1146.7	0				
																	DIQLAR	3	5	4	4504297;4504281;31742503;183076548;4504299;4885385	N-Term(TMT6plex)	944.6	0				
4504299	NP_003484.1	**HIST3H3**	8290	**histone H3.1t**	68.38	2	1	12	1304	**1.0**	**0.9**	**1.1**	**0.9**	136	15.5	11.12												
																	STELLIR	368	5	4	4504297;4504281;31742503;183076548;4504299;4885385	N-Term(TMT6plex)	1060.7	0				
																	EIAQDFK	534	4	4	4504297;4504281;31742503;183076548;4504299;4885385	N-Term(TMT6plex); K7(TMT6plex)	1308.8	0				
																	YRPGTVALR	306	5	4	4504297;4504281;31742503;183076548;4504299;4885385	N-Term(TMT6plex)	1261.8	0				
																	EIAQDFKTDLR	3	4	4	4504297;4504281;31742503;183076548;4504299;4885385	N-Term(TMT6plex); K7(TMT6plex)	1794.0	1				
																	SAPATGGVK	54	3	3	4504297;4504281;31742503;183076548;4504299	N-Term(TMT6plex); K9(TMT6plex)	1245.8	0				
																	KSAPATGGVK	3	3	3	4504297;4504281;31742503;183076548;4504299	N-Term(TMT6plex); K1(TMT6plex); K10(TMT6plex)	1603.0	1				
																	KLPFQR	1	5	4	4504297;4504281;31742503;183076548;4504299;4885385	N-Term(TMT6plex); K1(TMT6plex)	1246.8	1				
																	KQLATK	16	5	4	4504297;4504281;31742503;183076548;4504299;4885385	N-Term(TMT6plex); K1(TMT6plex); K6(TMT6plex)	1375.9	1				
																	FQSSAVMALQEACESYLVGLFEDTNLCVIHAK	1	1	1	4504299	N-Term(TMT6plex); C13(Carbamidomethyl); C27(Carbamidomethyl); K32(TMT6plex)	4089.0	0	**1.0**	**0.9**	**1.1**	**0.9**
																	VTIMPKDIQLAR	1	5	4	4504297;4504281;31742503;183076548;4504299;4885385	N-Term(TMT6plex); K6(TMT6plex)	1843.1	1				
																	VTIMPK	9	5	4	4504297;4504281;31742503;183076548;4504299;4885385	N-Term(TMT6plex); M4(Oxidation); K6(TMT6plex)	1162.7	0				
																	VTIMPK	5	5	4	4504297;4504281;31742503;183076548;4504299;4885385	N-Term(TMT6plex); K6(TMT6plex)	1146.7	0				
																	DIQLAR	3	5	4	4504297;4504281;31742503;183076548;4504299;4885385	N-Term(TMT6plex)	944.6	0				
212549553	NP_060090.2	**ILF3**	3609	**interleukin enhancer-binding factor 3 isoform d**	68.37	2	3	42	533	**1.2**	**1.4**	**1.4**	**1.3**	898	95.7	8.81												
																	LFPDTPLALDANK	53	5	2	24234750;212549553	N-Term(TMT6plex); K13(TMT6plex)	1873.1	0				
																	HSSVYPTQEELEAVQNMVSHTER	26	5	2	24234750;212549553	N-Term(TMT6plex); M17(Oxidation)	2916.4	0				
																	FVMEVEVDGQK	26	7	3	24234750;212549553;283806705	N-Term(TMT6plex); M3(Oxidation); K11(TMT6plex)	1754.9	0				
																	GSSEQAESDNMDVPPEDDSK	22	5	2	24234750;212549553	N-Term(TMT6plex); M11(Oxidation); K20(TMT6plex)	2611.2	0				
																	LAAFGQLHK	6	5	2	24234750;212549553	N-Term(TMT6plex); K9(TMT6plex)	1442.9	0				
																	YELISETGGSHDKR	12	7	3	24234750;212549553;283806705	N-Term(TMT6plex); K13(TMT6plex)	2050.1	1				
																	NENPVDYTVQIPPSTTYAITPMK	6	5	2	24234750;212549553	N-Term(TMT6plex); M22(Oxidation); K23(TMT6plex)	3053.6	0				
																	NPVMELNEK	26	7	3	24234750;212549553;283806705	N-Term(TMT6plex); M4(Oxidation); K9(TMT6plex)	1547.9	0				
																	VLGMDPLPSK	24	5	2	24234750;212549553	N-Term(TMT6plex); M4(Oxidation); K10(TMT6plex)	1530.9	0				
																	FQGAGSNK	5	5	2	24234750;212549553	N-Term(TMT6plex); K8(TMT6plex)	1266.7	0				
																	EPPLSLTIHLTSPVVR	12	5	2	24234750;212549553	N-Term(TMT6plex)	1988.2	0				
																	QGGYSQSNYNSPGSGQNYSGPPSSYQSSQGGYGR	3	2	2	24234750;212549553	N-Term(TMT6plex)	3732.6	0				
																	EDITQSAQHALR	12	5	2	24234750;212549553	N-Term(TMT6plex)	1597.8	0				
																	VLQDMGLPTGAEGR	40	5	2	24234750;212549553	N-Term(TMT6plex); M5(Oxidation)	1688.9	0				
																	VPTWGPLR	8	5	2	24234750;212549553	N-Term(TMT6plex)	1154.7	0				
																	GDLDLELVLLCK	28	5	2	24234750;212549553	N-Term(TMT6plex); C11(Carbamidomethyl); K12(TMT6plex)	1846.1	0				
																	FNYSGSGGR	4	2	2	24234750;212549553	N-Term(TMT6plex)	1173.6	0				
																	NPVMELNEK	4	7	3	24234750;212549553;283806705	N-Term(TMT6plex); K9(TMT6plex)	1531.9	0				
																	VLQDMGLPTGAEGR	4	5	2	24234750;212549553	N-Term(TMT6plex)	1672.9	0				
																	VLGMDPLPSK	2	5	2	24234750;212549553	N-Term(TMT6plex); K10(TMT6plex)	1514.9	0				
																	SCVIVIR	1	7	3	24234750;212549553;283806705	N-Term(TMT6plex); C2(Carbamidomethyl)	1075.7	0				
																	GWPLELLCEK	14	7	3	283806705;24234750;212549553	N-Term(TMT6plex); C8(Carbamidomethyl); K10(TMT6plex)	1703.0	0				
																	AYAALAALEK	19	5	2	24234750;212549553	N-Term(TMT6plex); K10(TMT6plex)	1478.9	0				
																	LNQLKPGLQYK	7	5	2	24234750;212549553	N-Term(TMT6plex); K5(TMT6plex); K11(TMT6plex)	1989.2	0				
																	CLAALASLR	4	5	2	24234750;212549553	N-Term(TMT6plex); C1(Carbamidomethyl)	1203.7	0				
																	SIGTANRPMGAGEALR	7	5	2	24234750;212549553	N-Term(TMT6plex); M9(Oxidation)	1846.0	0				
																	AEPPQAMNALMR	4	5	2	24234750;212549553	N-Term(TMT6plex); M7(Oxidation); M11(Oxidation)	1589.8	0				
																	EGAGEQK	5	5	2	24234750;212549553	N-Term(TMT6plex); K7(TMT6plex)	1176.7	0				
																	LVSQTGPVHAPIFTMSVEVDGNSFEASGPSK	22	5	2	24234750;212549553	N-Term(TMT6plex); M15(Oxidation); K31(TMT6plex)	3662.9	0				
																	VLAGETLSVNDPPDVLDR	28	5	2	24234750;212549553	N-Term(TMT6plex)	2139.2	0				
																	QQGPILTK	6	2	1	212549553	N-Term(TMT6plex); K8(TMT6plex)	1342.8	0	**1.2**	**1.3**	**1.4**	**1.4**
																	EATDAIGHLDR	16	5	2	24234750;212549553	N-Term(TMT6plex)	1426.8	0				
																	NADHSMNYQYR	2	2	2	24234750;212549553	N-Term(TMT6plex); M6(Oxidation)	1643.7	0				
																	SGGNSYGSGGASYNPGSHGGYGGGSGGGSSYQGK	2	2	2	24234750;212549553	N-Term(TMT6plex); K34(TMT6plex)	3470.6	0				
																	AVSDWIDEQEK	12	5	2	24234750;212549553	N-Term(TMT6plex); K11(TMT6plex)	1777.9	0				
																	VLECLASGIVMPDGSGIYDPCEK	17	5	2	24234750;212549553	N-Term(TMT6plex); C4(Carbamidomethyl); M11(Oxidation); C21(Carbamidomethyl); K23(TMT6plex)	2984.5	0				
																	SIGTANRPMGAGEALR	2	5	2	24234750;212549553	N-Term(TMT6plex)	1830.0	0				
																	DSSKGEDSAEETEAKPAVVAPAPVVEAVSTPSAAFPSDATAENVK	1	2	1	212549553	N-Term(TMT6plex); K4(TMT6plex); K15(TMT6plex); K45(TMT6plex)	5372.8	1	**1.3**	**1.2**	**1.8**	**1.2**
																	VADNLAIQLAAVTEDKYEILQSVDDAAIVIK	6	5	2	24234750;212549553	N-Term(TMT6plex); K16(TMT6plex); K31(TMT6plex)	4016.3	1				
																	EGAGEQKTEHMTR	2	5	2	24234750;212549553	N-Term(TMT6plex); K7(TMT6plex); M11(Oxidation)	1948.0	1				
																	EKPTTALLDK	9	5	2	24234750;212549553	N-Term(TMT6plex); K2(TMT6plex); K10(TMT6plex)	1803.1	0				
																	GSSEQAESDNMDVPPEDDSK	2	5	2	24234750;212549553	N-Term(TMT6plex); K20(TMT6plex)	2595.2	0				
																	YELISETGGSHDK	8	7	3	24234750;212549553;283806705	N-Term(TMT6plex); K13(TMT6plex)	1894.0	0				
																	LVSQTGPVHAPIFTMSVEVDGNSFEASGPSK	2	5	2	24234750;212549553	N-Term(TMT6plex); K31(TMT6plex)	3646.9	0				
																	GEDSAEETEAKPAVVAPAPVVEAVSTPSAAFPSDATAENVK	1	2	1	212549553	N-Term(TMT6plex); K11(TMT6plex); K41(TMT6plex)	4726.4	0	**1.2**	**1.7**	**1.1**	**1.2**

Accession	RefSeq Accession	Gene Symbol	Entrez Gene ID	Description	Σ Coverage	Σ# Proteins	Σ# Unique Peptides	Σ# Peptides	Σ# PSMs	Het-1A-Smoke - 2M/Parental	Het-1A-Smoke - 4M/Parental	Het-1A-Smoke - 6M/Parental	Het-1A-Smoke - 8M/Parental	# AAs	MW [kDa]	calc. pI	Sequence	# PSMs	# Proteins	# Protein Groups	Protein Group Accessions	Modifications	MH+ [Da]	# Missed Cleavages	Het-1A-Smoke - 2M/Parental	Het-1A-Smoke - 4M/Parental	Het-1A-Smoke - 6M/Parental	Het-1A-Smoke - 8M/Parental
																	RPMEEDGEEK	4	5	2	24234750;212549553	N-Term(TMT6plex); M3(Oxidation); K10(TMT6plex)	1693.8	0				
																	IFVNDDR	6	5	2	24234750;212549553	N-Term(TMT6plex)	1107.6	0				
																	GYNHGQGSYSYSNSYNSPGGGGGS DYNYESK	1	2	2	24234750;212549553	N-Term(TMT6plex); K31(TMT6plex)	3711.6	0				
47519490	NP 997403.1	**RTN4**	57142	**reticulon-4 isoform D**	68.37	2	1	13	209	**1.6**	**1.5**	**1.5**	**1.5**	392	42.2	4.74												
																	EPEDEEEEEEEEEDEDEDLEELEVL ER	12	3	3	47519490;24431935;24431933	N-Term(TMT6plex)	3722.5	0				
																	KPAAGLSAAPVPTAPAAGAPLMDFG NDFVPPAPR	28	3	3	47519490;24431935;24431933	N-Term(TMT6plex); K1(TMT6plex); M22(Oxidation)	3746.0	0				
																	GPLPAAPPVAPER	34	3	3	47519490;24431935;24431933	N-Term(TMT6plex)	1500.9	0				
																	AYLESEVAISEELVQK	78	5	3	47519490;24431935;24431933	N-Term(TMT6plex); K16(TMT6plex)	2266.3	0				
																	LFLVDDLVDSLKFAVLMWVFTYVG ALFNGLTLLILALISLFSVPVIYER	1	5	3	47519490;24431935;24431933	N-Term(TMT6plex); K12(TMT6plex)	6005.4	1				
																	SSAVVDLLYWR	6	1	1	47519490	N-Term(TMT6plex)	1537.9	0	**1.6**	**1.5**	**1.5**	**1.5**
																	GVIQAIQK	2	5	3	47519490;24431935;24431933	N-Term(TMT6plex); K8(TMT6plex)	1314.9	0				
																	QPSWDPSPVSSTVPAPSPLSAAAVSP SK	12	3	3	47519490;24431935;24431933	N-Term(TMT6plex); K28(TMT6plex)	3205.7	0				
																	HQAQIDHYLGLANK	12	5	3	47519490;24431935;24431933	N-Term(TMT6plex); K14(TMT6plex)	2066.2	0				
																	LFLVDDLVDSLK	6	5	3	47519490;24431935;24431933	N-Term(TMT6plex); K12(TMT6plex)	1835.1	0				
																	LPEDDEPPARPPPPPPASVSPQAEPV WTPPAPAPAAPPSTPAAPK	6	3	3	47519490;24431935;24431933	N-Term(TMT6plex); K45(TMT6plex)	4950.6	0				
																	YSNSALGHVNCTIK	3	5	3	47519490;24431935;24431933	N-Term(TMT6plex); C11(Carbamidomethyl); K14(TMT6plex)	2022.1	0				
																	KPAAGLSAAPVPTAPAAGAPLMDFG NDFVPPAPR	7	3	3	47519490;24431935;24431933	N-Term(TMT6plex); K1(TMT6plex)	3730.0	0				
																	SDEGHPFR	2	5	3	47519490;24431935;24431933	N-Term(TMT6plex)	1173.6	0				
440309857	NP_001258978.1	**TRAP1**	10131	**heat shock protein 75 kDa, mitochondrial isoform 2**	68.36	2	37	38	529	**1.5**	**1.5**	**1.5**	**1.6**	651	74.2	7.87												
																	ELISNASDALEK	24	2	1	440309857	N-Term(TMT6plex); K12(TMT6plex)	1748.0	0	**1.4**	**1.6**	**1.5**	**1.7**
																	ELLQESALIR	14	2	1	440309857	N-Term(TMT6plex)	1400.8	0	**1.8**	**1.6**	**1.5**	**1.7**
																	LVSDGQALPEMEIHLQTNAEK	20	2	1	440309857	N-Term(TMT6plex); M11(Oxidation); K21(TMT6plex)	2797.5	0	**1.4**	**1.6**	**1.6**	**1.6**
																	GVVDSEDIPLNLSR	143	7	3	154146191;20149594;431822403;440309857	N-Term(TMT6plex)	1742.9	0				
																	ASEPGLAQLLVDQIYENAMIAAGLV DDPR	18	2	1	440309857	N-Term(TMT6plex); M19(Oxidation)	3314.7	0	**1.5**	**1.4**	**1.6**	**1.6**
																	FFEDYGLFMR	12	2	1	440309857	N-Term(TMT6plex); M9(Oxidation)	1569.8	0	**1.6**	**1.4**	**1.4**	**1.6**
																	LDTHPAMVTVLEMGAAR	9	2	1	440309857	N-Term(TMT6plex); M7(Oxidation); M13(Oxidation)	2073.1	0	**1.4**	**1.4**	**1.4**	**1.3**
																	GTITIQDTGIGMTQEELVSNLGTIAR	11	2	1	440309857	N-Term(TMT6plex); M12(Oxidation)	2963.6	0	**1.4**	**1.5**	**1.5**	**1.5**
																	HLAEHSPYYEAMK	14	2	1	440309857	N-Term(TMT6plex); M12(Oxidation); K13(TMT6plex)	2050.0	0	**1.7**	**1.5**	**1.5**	**1.5**
																	YESSALPSGQLTSLSEYASR	21	2	1	440309857	N-Term(TMT6plex)	2375.2	0	**1.7**	**1.4**	**1.6**	**1.5**
																	AQLLQPTLEINPR	25	2	1	440309857	N-Term(TMT6plex)	1722.0	0	**1.6**	**1.6**	**1.6**	**1.6**
																	NIYYLCAPNR	8	2	1	440309857	N-Term(TMT6plex); C6(Carbamidomethyl)	1512.8	0	**2.0**	**1.5**	**1.6**	**1.5**
																	SIFYVPDMKPSMFDVSR	2	2	1	440309857	N-Term(TMT6plex); M8(Oxidation); K9(TMT6plex)	2493.3	0	**1.4**	**1.8**	**1.7**	**1.6**
																	SAAPGSLGYQWLSDGSGVFEIAEAS GVR	44	2	1	440309857	N-Term(TMT6plex)	3040.5	0	**1.3**	**1.3**	**1.3**	**1.5**
																	EWQHEEFYR	8	2	1	440309857	N-Term(TMT6plex)	1552.7	0	**1.3**	**1.4**	**1.4**	**1.5**
																	GTITIQDTGIGMTQEELVSNLGTIAR	4	2	1	440309857	N-Term(TMT6plex)	2947.5	0	**1.1**	**1.5**	**1.5**	**1.3**
																	ASEPGLAQLLVDQIYENAMIAAGLV DDPR	8	2	1	440309857	N-Term(TMT6plex)	3298.7	0	**1.5**	**1.9**	**1.6**	**1.9**
																	DTEVLFCFEQFDELTLLHLR	3	2	1	440309857	N-Term(TMT6plex); C7(Carbamidomethyl)	2754.4	0	**1.8**	**1.8**	**1.9**	**1.5**
																	FFEDYGLFMR	3	2	1	440309857	N-Term(TMT6plex)	1553.8	0	**2.0**	**1.8**	**2.1**	**1.7**
																	EGIVTATEQEVK	14	2	1	440309857	N-Term(TMT6plex); K12(TMT6plex)	1762.0	0	**1.5**	**1.5**	**1.6**	**1.7**
																	YVAQAHDKPR	3	2	1	440309857	N-Term(TMT6plex); K8(TMT6plex)	1642.9	0	**1.3**	**1.6**	**1.5**	**1.8**
																	SIFYVPDMKPSMFDVSR	5	2	1	440309857	N-Term(TMT6plex); M8(Oxidation); K9(TMT6plex); M12(Oxidation)	2509.3	0	**1.4**	**1.5**	**1.4**	**1.8**
																	KLLDIVAR	1	2	1	440309857	N-Term(TMT6plex); K1(TMT6plex)	1385.9	1	**1.6**	**2.2**	**1.9**	**2.3**
																	AFLDALQNQAEASSK	14	2	1	440309857	N-Term(TMT6plex); K15(TMT6plex)	2051.1	0	**1.4**	**1.5**	**1.5**	**1.5**
																	MNTLQAIWMMDPK	5	2	1	440309857	N-Term(TMT6plex); M1(Oxidation); M9(Oxidation); M10(Oxidation); K13(TMT6plex)	2085.1	0	**1.4**	**1.4**	**1.4**	**1.4**
																	ELGSSVALYSR	18	2	1	440309857	N-Term(TMT6plex)	1410.8	0	**1.6**	**1.5**	**1.5**	**1.7**
																	SPAAECLSEK	4	2	1	440309857	N-Term(TMT6plex); C6(Carbamidomethyl); K10(TMT6plex)	1549.8	0	**1.5**	**1.6**	**1.7**	**1.8**
																	FFIDQSK	14	2	1	440309857	N-Term(TMT6plex); K7(TMT6plex)	1342.8	0	**1.6**	**1.5**	**1.6**	**1.7**
																	ATDILPK	4	2	1	440309857	N-Term(TMT6plex); K7(TMT6plex)	1215.8	0	**1.5**	**1.5**	**1.5**	**1.7**
																	EGIVTATEQEVKEDIAK	3	2	1	440309857	N-Term(TMT6plex); K12(TMT6plex); K17(TMT6plex)	2547.4	1	**1.7**	**2.4**	**1.9**	**2.4**
																	LVSDGQALPEMEIHLQTNAEK	4	2	1	440309857	N-Term(TMT6plex); K21(TMT6plex)	2781.5	0	**1.9**	**2.2**	**2.1**	**1.9**
																	MNTLQAIWMMDPK	4	2	1	440309857	N-Term(TMT6plex); M1(Oxidation); M9(Oxidation); K13(TMT6plex)	2069.1	0	**1.2**	**1.0**	**1.2**	**1.1**
																	SIFYVPDMKPSMFDVSR	1	2	1	440309857	N-Term(TMT6plex); K9(TMT6plex)	2477.3	0	**1.8**	**1.8**	**1.9**	**1.6**
																	HLAEHSPYYEAMK	2	2	1	440309857	N-Term(TMT6plex); K13(TMT6plex)	2034.1	0	**1.7**	**1.8**	**2.0**	**1.6**
																	ETEELMAWMR	3	2	1	440309857	N-Term(TMT6plex); M6(Oxidation); M9(Oxidation)	1556.7	0	**1.4**	**1.5**	**1.3**	**1.5**
																	SPAAECLSEKETEELMAWMR	1	2	1	440309857	N-Term(TMT6plex); C6(Carbamidomethyl); K10(TMT6plex)	2826.4	1	**2.5**	**2.9**	**2.7**	**2.3**
																	LISVETDIVVDHYK	2	2	1	440309857	N-Term(TMT6plex); K14(TMT6plex)	2089.2	0	**1.3**	**1.3**	**1.5**	**1.5**
																	HEFQAETK	4	2	1	440309857	N-Term(TMT6plex); K8(TMT6plex)	1447.8	0	**1.4**	**1.5**	**1.5**	**1.6**
																	YTLHYK	4	2	1	440309857	N-Term(TMT6plex); K6(TMT6plex)	1282.8	0	**1.5**	**1.5**	**1.6**	**1.8**
																	LLDIVAR	8	2	1	440309857	N-Term(TMT6plex)	1028.7	0	**1.6**	**1.5**	**1.5**	**1.6**
																	LNELLVK	8	2	1	440309857	N-Term(TMT6plex); K7(TMT6plex)	1286.8	0	**1.5**	**1.5**	**1.5**	**1.6**
																	EFSSEAR	3	2	1	440309857	N-Term(TMT6plex)	1054.5	0	**1.4**	**1.5**	**1.5**	**1.5**
																	TDAPLNIR	2	2	1	440309857	N-Term(TMT6plex)	1128.7	0	**1.9**	**1.9**	**2.0**	**1.7**
																	VLIQTK	5	2	1	440309857	N-Term(TMT6plex); K6(TMT6plex)	1159.8	0	**1.5**	**1.7**	**1.7**	**1.7**
																	SLYSEK	1	2	1	440309857	N-Term(TMT6plex); K6(TMT6plex)	1184.7	0	**1.5**	**1.5**	**1.6**	**1.7**
																	VEVYSR	1	2	1	440309857	N-Term(TMT6plex)	981.6	0	**1.7**	**1.8**	**1.6**	**1.8**
24234750	NP_036350.2	**ILF3**	3609	**interleukin enhancer-binding factor 3 isoform a**	68.23	3	2	41	527	**1.2**	**1.3**	**1.4**	**1.4**	894	95.3	8.76												
																	LFPDTPLALDANK	53	5	2	24234750;212549553	N-Term(TMT6plex); K13(TMT6plex)	1873.1	0				
																	HSSVYPTQEELEAVQNMVSHTER	26	5	2	24234750;212549553	N-Term(TMT6plex); M17(Oxidation)	2916.4	0				
																	FVMEVEVDGQK	26	7	3	24234750;212549553;283806705	N-Term(TMT6plex); M3(Oxidation); K11(TMT6plex)	1754.9	0				
																	GSSEQAESDNMDVPPEDDSK	22	5	2	24234750;212549553	N-Term(TMT6plex); M11(Oxidation); K20(TMT6plex)	2611.2	0				
																	LAAFGQLHK	6	5	2	24234750;212549553	N-Term(TMT6plex); K9(TMT6plex)	1442.9	0				
																	YELISETGGSHDKR	12	7	3	24234750;212549553;283806705	N-Term(TMT6plex); K13(TMT6plex)	2050.1	1				
																	NENPVDYTVQIPPSTTYAITPMK	6	5	2	24234750;212549553	N-Term(TMT6plex); M22(Oxidation); K23(TMT6plex)	3053.6	0				
																	NPVMELNEK	26	7	3	24234750;212549553;283806705	N-Term(TMT6plex); M4(Oxidation); K9(TMT6plex)	1547.9	0				
																	VLGMDPLPSK	24	5	2	24234750;212549553	N-Term(TMT6plex); M4(Oxidation); K10(TMT6plex)	1530.9	0				
																	FQGAGSNK	5	5	2	24234750;212549553	N-Term(TMT6plex); K8(TMT6plex)	1266.7	0				
																	EPPLSLTIHLTSPVVR	12	5	2	24234750;212549553	N-Term(TMT6plex)	1988.2	0				
																	QGGYSQSNYNSPGSGQNYSGPPSSY QSSQGGYGR	3	2	2	24234750;212549553	N-Term(TMT6plex)	3732.6	0				
																	EDITQSAQHALR	12	5	2	24234750;212549553	N-Term(TMT6plex)	1597.8	0				
																	VLQDMGLPTGAEGR	40	5	2	24234750;212549553	N-Term(TMT6plex); M5(Oxidation)	1688.9	0				
																	VPTWGPLR	8	5	2	24234750;212549553	N-Term(TMT6plex)	1154.7	0				
																	GDLDLELVLLCK	28	5	2	24234750;212549553	N-Term(TMT6plex); C11(Carbamidomethyl); K12(TMT6plex)	1846.1	0				
																	FNYSGSGGR	4	2	2	24234750;212549553	N-Term(TMT6plex)	1173.6	0				
																	NPVMELNEK	4	7	3	24234750;212549553;283806705	N-Term(TMT6plex); K9(TMT6plex)	1531.9	0				
																	VLQDMGLPTGAEGR	4	5	2	24234750;212549553	N-Term(TMT6plex)	1672.9	0				
																	VLGMDPLPSK	2	5	2	24234750;212549553	N-Term(TMT6plex); K10(TMT6plex)	1514.9	0				
																	SCVIVIR	1	7	3	24234750;212549553;283806705	N-Term(TMT6plex); C2(Carbamidomethyl)	1075.7	0				
																	GWPLELLCEK	14	7	3	283806705;24234750;212549553	N-Term(TMT6plex); C8(Carbamidomethyl); K10(TMT6plex)	1703.0	0				
																	AYAALAALEK	19	5	2	24234750;212549553	N-Term(TMT6plex); K10(TMT6plex)	1478.9	0				
																	LNQLKPGLQYK	7	5	2	24234750;212549553	N-Term(TMT6plex); K5(TMT6plex); K11(TMT6plex)	1989.2	0				
																	CLAALASLR	4	5	2	24234750;212549553	N-Term(TMT6plex); C1(Carbamidomethyl)	1203.7	0				
																	SIGTANRPMGAGEALR	7	5	2	24234750;212549553	N-Term(TMT6plex); M9(Oxidation)	1846.0	0				
																	AEPPQAMNALMR	4	5	2	24234750;212549553	N-Term(TMT6plex); M7(Oxidation); M11(Oxidation)	1589.8	0				
																	EGAGEQK	5	5	2	24234750;212549553	N-Term(TMT6plex); K7(TMT6plex)	1176.7	0				
																	LVSQTGPVHAPIFTMSVEVDGNSFE ASGPSK	22	5	2	24234750;212549553	N-Term(TMT6plex); M15(Oxidation); K31(TMT6plex)	3662.9	0				
																	VLAGETLSVNDPPDVLDR	28	5	2	24234750;212549553	N-Term(TMT6plex)	2139.2	0				
																	EATDAIGHLDR	16	5	2	24234750;212549553	N-Term(TMT6plex)	1426.8	0				
																	NADHSMNYQYR	2	2	2	24234750;212549553	N-Term(TMT6plex); M6(Oxidation)	1643.7	0				
																	SGGNSYGSGGASYNPGSHGGYGGGS GGGSSYQGK	2	2	2	24234750;212549553	N-Term(TMT6plex); K34(TMT6plex)	3470.6	0				
																	AVSDWIDEQEK	12	5	2	24234750;212549553	N-Term(TMT6plex); K11(TMT6plex)	1777.9	0				
																	VLECLASGIVMPDGSGIYDPCEK	17	5	2	24234750;212549553	N-Term(TMT6plex); C4(Carbamidomethyl); M11(Oxidation); C21(Carbamidomethyl); K23(TMT6plex)	2984.5	0				
																	SIGTANRPMGAGEALR	2	5	2	24234750;212549553	N-Term(TMT6plex)	1830.0	0				

Accession	RefSeq Accession	Gene Symbol	Entrez Gene ID	Description	Σ Coverage	Σ# Proteins	Σ# Unique Peptides	Σ# Peptides	Σ# PSMs	Het-1A-Smoke - 2M/Parental	Het-1A-Smoke - 4M/Parental	Het-1A-Smoke - 6M/Parental	Het-1A-Smoke - 8M/Parental	# AAs	MW [kDa]	calc. pI	Sequence	# PSMs	# Proteins	# Protein Groups	Protein Group Accessions	Modifications	MH+ [Da]	# Missed Cleavages	Het-1A-Smoke - 2M/Parental	Het-1A-Smoke - 4M/Parental	Het-1A-Smoke - 6M/Parental	Het-1A-Smoke - 8M/Parental
																	VADNLAIQLAAVTEDKYEILQSVDDAAIVIK	6	5	2	24234750;212549553	N-Term(TMT6plex); K16(TMT6plex); K31(TMT6plex)	4016.3	1				
																	EGAGEQKTEHMTR	2	5	2	24234750;212549553	N-Term(TMT6plex); K7(TMT6plex); M11(Oxidation)	1948.0	1				
																	DSSKGEDSAEETEAKPAVVAPAPVVEAVSTPSAAFPSDATAEQGPILTK	1	3	1	24234750	N-Term(TMT6plex); K4(TMT6plex); K15(TMT6plex); K49(TMT6plex)	5769.0	1	1.2	1.3	1.4	1.4
																	EKPTTALLDK	9	5	2	24234750;212549553	N-Term(TMT6plex); K2(TMT6plex); K10(TMT6plex)	1803.1	0				
																	GSSEQAESDNMDVPPEDDSK	2	5	2	24234750;212549553	N-Term(TMT6plex); K20(TMT6plex)	2595.2	0				
																	YELISETGGSHDK	8	7	3	24234750;212549553;283806705	N-Term(TMT6plex); K13(TMT6plex)	1894.0	0				
																	LVSQTGPVHAPIFTMSVEVDGNSFEASGPSK	2	5	2	24234750;212549553	N-Term(TMT6plex); K31(TMT6plex)	3646.9	0				
																	RPMEEDGEEK	4	5	2	24234750;212549553	N-Term(TMT6plex); M3(Oxidation); K10(TMT6plex)	1693.8	0				
																	GEDSAEETEAKPAVVAPAPVVEAVSTPSAAFPSDATAEQGPILTK	1	3	1	24234750	N-Term(TMT6plex); K11(TMT6plex); K45(TMT6plex)	5122.7	0				
																	IFVNDDR	6	5	2	24234750;212549553	N-Term(TMT6plex)	1107.6	0				
																	GYNHGQGSYSYSNSYNSPGGGGGSDYNYESK	1	2	2	24234750;212549553	N-Term(TMT6plex); K31(TMT6plex)	3711.6	0				
38202257	NP_938148.1	**GANAB**	23193	**neutral alpha-glucosidase AB isoform 2 precursor**	68.22	3	2	46	578	**1.3**	**1.3**	**1.6**	**1.4**	944	106.8	6.14												
																	AHAHLDTGR	12	5	2	88900491;38202257	N-Term(TMT6plex)	1206.7	0				
																	MMDYLQGSGETPQTDVR	44	5	2	88900491;38202257	N-Term(TMT6plex); M1(Oxidation); M2(Oxidation)	2189.0	0				
																	NPEPELLVR	12	5	2	88900491;38202257	N-Term(TMT6plex)	1295.8	0				
																	VPDVLVADPPIAR	33	3	2	88900491;38202257	N-Term(TMT6plex)	1590.9	0				
																	DAQHYGGWEHR	4	5	2	88900491;38202257	N-Term(TMT6plex)	1584.8	0				
																	LVAIVDPHIK	11	5	2	88900491;38202257	N-Term(TMT6plex); K10(TMT6plex)	1563.0	0				
																	SIRPGLSPYR	4	2	2	88900491;38202257	N-Term(TMT6plex)	1374.8	0				
																	AEKDEPGAWEETFK	20	5	2	88900491;38202257	N-Term(TMT6plex); K3(TMT6plex); K14(TMT6plex)	2324.2	1				
																	LSFQHDPETSVLVLR	22	5	2	88900491;38202257	N-Term(TMT6plex)	1970.1	0				
																	VTEGGEPYR	15	5	2	88900491;38202257	N-Term(TMT6plex)	1236.6	0				
																	DGSDYEGWCWPGSAGYPDFTNPTMR	10	5	2	88900491;38202257	N-Term(TMT6plex); C9(Carbamidomethyl); M24(Oxidation)	3111.3	0				
																	VVIIGAGKPAAVVLQTK	13	5	2	88900491;38202257	N-Term(TMT6plex); K8(TMT6plex); K17(TMT6plex)	2351.5	0				
																	DLGIFWLNAAETWVDISSNTAGK	10	5	2	88900491;38202257	N-Term(TMT6plex); K23(TMT6plex)	2966.5	0				
																	GLLEFEHQR	21	5	2	88900491;38202257	N-Term(TMT6plex)	1357.7	0				
																	DEPGAWEETFK	11	5	2	88900491;38202257	N-Term(TMT6plex); K11(TMT6plex)	1766.9	0				
																	DENSVELTMAEGPYK	38	5	2	88900491;38202257	N-Term(TMT6plex); M9(Oxidation); K15(TMT6plex)	2157.1	0				
																	REPWLLPSQHNDIIR	7	5	2	88900491;38202257	N-Term(TMT6plex)	2103.2	1				
																	EPWLLPSQHNDIIR	6	5	2	88900491;38202257	N-Term(TMT6plex)	1947.1	0				
																	DGDKPEETQGK	16	5	2	88900491;38202257	N-Term(TMT6plex); K4(TMT6plex); K11(TMT6plex)	1891.0	0				
																	DENSVELTMAEGPYK	4	5	2	88900491;38202257	N-Term(TMT6plex); K15(TMT6plex)	2141.1	0				
																	DPAEGDGAQPEETPR	8	5	2	88900491;38202257	N-Term(TMT6plex)	1797.8	0				
																	ALLDSLQLGPDSLTVHLIHEVTK	27	2	2	88900491;38202257	N-Term(TMT6plex); K23(TMT6plex)	2957.7	0				
																	MMDYLQGSGETPQTDVR	8	5	2	88900491;38202257	N-Term(TMT6plex); M1(Oxidation)	2173.0	0				
																	WYQMGAYQPFFR	4	5	2	88900491;38202257	N-Term(TMT6plex); M4(Oxidation)	1838.9	0				
																	QYASLTGTQALPPLFSLGYHQSR	14	5	2	88900491;38202257	N-Term(TMT6plex)	2764.5	0				
																	HHGPQTLYLPVTLSSIPVFQR	6	5	2	88900491;38202257	N-Term(TMT6plex)	2619.5	0				
																	DVHNIYGLYVHMATADGLR	2	5	2	88900491;38202257	N-Term(TMT6plex); M12(Oxidation)	2390.2	0				
																	VSQGSKDPAEGDGAQPEETPR	6	3	1	38202257	N-Term(TMT6plex); K6(TMT6plex)	2613.3	1	1.4	1.4	1.6	1.5
																	FSFSGNTLVSSSADPEGHFETPIWIER	24	5	2	88900491;38202257	N-Term(TMT6plex)	3239.6	0				
																	FGAVWTGDNTAEWDHLK	32	5	2	88900491;38202257	N-Term(TMT6plex); K17(TMT6plex)	2405.2	0				
																	VLLVLELQGLQK	15	2	2	88900491;38202257	N-Term(TMT6plex); K12(TMT6plex)	1811.2	0				
																	YSLLPFWYTLLYQAHR	13	5	2	88900491;38202257	N-Term(TMT6plex)	2300.2	0				
																	DEADVLEVDQGFDDHNLPCDVIWLDIEHADGK	8	5	2	88900491;38202257	N-Term(TMT6plex); C19(Carbamidomethyl); K32(TMT6plex)	4138.0	0				
																	DVHNIYGLYVHMATADGLR	6	5	2	88900491;38202257	N-Term(TMT6plex)	2374.2	0				
																	KPGINVASDWSIHLR	10	5	2	88900491;38202257	N-Term(TMT6plex); K1(TMT6plex)	2151.2	0				
																	LYNLDVFQYELYNPMALYGSVPVLLAHNPHR	9	5	2	88900491;38202257	N-Term(TMT6plex); M15(Oxidation)	3892.0	0				
																	LYNLDVFQYELYNPMALYGSVPVLLAHNPHR	5	5	2	88900491;38202257	N-Term(TMT6plex)	3876.0	0				
																	LDLLEDR	10	5	2	88900491;38202257	N-Term(TMT6plex)	1102.6	0				
																	SLLLSVNAR	8	5	2	88900491;38202257	N-Term(TMT6plex)	1201.7	0				
																	TCEESSFCK	10	2	2	88900491;38202257	N-Term(TMT6plex); C2(Carbamidomethyl); C8(Carbamidomethyl); K9(TMT6plex)	1605.8	0				
																	NLGLYVK	13	5	2	88900491;38202257	N-Term(TMT6plex); K7(TMT6plex)	1264.8	0				
																	AWWANMFSYDNYEGSAPNLFVWNDMNEPSVFNGPEVTMLK	1	5	2	88900491;38202257	N-Term(TMT6plex); M25(Oxidation); M38(Oxidation); K40(TMT6plex)	5161.4	0				
																	SGGMERPFVLAR	1	5	2	88900491;38202257	N-Term(TMT6plex); M4(Oxidation)	1564.8	0				
																	AWWANMFSYDNYEGSAPNLFVWNDMNEPSVFNGPEVTMLK	2	5	2	88900491;38202257	N-Term(TMT6plex); M6(Oxidation); M25(Oxidation); M38(Oxidation); K40(TMT6plex)	5177.4	0				
																	WMSETGIIDVFLLLGPSISDVFR	2	5	2	88900491;38202257	N-Term(TMT6plex); M2(Oxidation)	2840.5	0				
																	IDELEPR	8	3	2	88900491;38202257	N-Term(TMT6plex)	1100.6	0				
																	AFFAGSQR	2	5	2	88900491;38202257	N-Term(TMT6plex)	1112.6	0				
																	YFTWDPSR	6	5	2	88900491;38202257	N-Term(TMT6plex)	1300.7	0				
																	VSQGSK	2	3	1	38202257	N-Term(TMT6plex); K6(TMT6plex)	1063.7	0	1.3	1.2	1.6	1.2
																	GGTIVPR	2	5	2	88900491;38202257	N-Term(TMT6plex)	928.6	0				
																	VDSGYR	1	7	3	4503943;88900491;38202257	N-Term(TMT6plex)	925.5	0				
112382252	NP_842565.2	**SPTBN1**	6711	**spectrin beta chain, non-erythrocytic 1 isoform 2**	68.21	4	2	126	813	**1.3**	**1.3**	**1.3**	**1.4**	2155	251.2	5.53												
																	EVDDLEQWIAER	18	2	2	112382250;112382252	N-Term(TMT6plex)	1731.9	0				
																	VSEEAESQQQWDTSK	10	2	2	112382250;112382252	N-Term(TMT6plex); K15(TMT6plex)	2210.1	0				
																	EIGQSVDEVEK	15	2	2	112382250;112382252	N-Term(TMT6plex); K11(TMT6plex)	1690.9	0				
																	TQTAIASEDMPNTLTEAEK	12	2	2	112382250;112382252	N-Term(TMT6plex); M10(Oxidation); K19(TMT6plex)	2524.3	0				
																	NDSFTTCIELGK	12	2	2	112382250;112382252	N-Term(TMT6plex); C7(Carbamidomethyl); K12(TMT6plex)	1843.0	0				
																	VIESTQDLGNDLAGVMALQR	16	2	2	112382250;112382252	N-Term(TMT6plex); M16(Oxidation)	2375.2	0				
																	LAEISDVWEEMK	13	2	2	112382250;112382252	N-Term(TMT6plex); M11(Oxidation); K12(TMT6plex)	1924.0	0				
																	FQIQDISVETEDNK	6	3	3	112382250;5902122;112382252	N-Term(TMT6plex); K14(TMT6plex)	2124.1	0				
																	LLEVLSGER	6	2	2	112382250;112382252	N-Term(TMT6plex)	1244.7	0				
																	IVSSSDVGHDEYSTQSLVK	7	2	2	112382250;112382252	N-Term(TMT6plex); K19(TMT6plex)	2509.3	0				
																	ELEAENYHDIK	2	2	2	112382250;112382252	N-Term(TMT6plex); K11(TMT6plex)	1819.0	0				
																	GNLEVLLFTIQSK	8	3	3	112382250;5902122;112382252	N-Term(TMT6plex); K13(TMT6plex)	1920.2	0				
																	SAATWDER	2	2	2	112382250;112382252	N-Term(TMT6plex)	1164.6	0				
																	DMGEMVTQGQTDAQYMFLR	5	2	2	112382250;112382252	N-Term(TMT6plex); M2(Oxidation); M5(Oxidation); M16(Oxidation)	2498.1	0				
																	AQQYYFDAAEAEAWMSEQELYMMSEEK	13	2	2	112382250;112382252	N-Term(TMT6plex); M15(Oxidation); M22(Oxidation); M23(Oxidation); K27(TMT6plex)	3784.7	0				
																	MWEVLESTTQTK	11	2	2	112382250;112382252	N-Term(TMT6plex); M1(Oxidation); K12(TMT6plex)	1927.0	0				
																	ENEVLEAWK	12	2	2	112382250;112382252	N-Term(TMT6plex); K9(TMT6plex)	1575.9	0				
																	QNLLSQSHAYQQFLR	4	2	2	112382250;112382252	N-Term(TMT6plex)	2062.1	0				
																	EVVAGSHELGQDYEHVTMLQER	4	2	2	112382250;112382252	N-Term(TMT6plex); M18(Oxidation)	2772.3	0				
																	IDDIFER	8	2	2	112382250;112382252	N-Term(TMT6plex)	1136.6	0				
																	KQEDFMTTMDANEEK	4	2	2	112382250;112382252	N-Term(TMT6plex); K1(TMT6plex); M6(Oxidation); K15(TMT6plex)	2520.3	1				
																	WVNSHLAR	4	6	3	112382250;5902122;112382252	N-Term(TMT6plex)	1211.7	0				
																	AKDEQSAVSMLK	4	2	2	112382250;112382252	N-Term(TMT6plex); K2(TMT6plex); K12(TMT6plex)	1994.2	1				
																	EGMQLISEKPETEAVVK	12	2	2	112382250;112382252	N-Term(TMT6plex); M3(Oxidation); K9(TMT6plex); K17(TMT6plex)	2591.5	0				
																	KQEDFMTTMDANEEK	14	2	2	112382250;112382252	N-Term(TMT6plex); K1(TMT6plex); M6(Oxidation); M9(Oxidation); K15(TMT6plex)	2536.3	1				
																	QIEAQEKPR	4	2	2	112382250;112382252	N-Term(TMT6plex); K7(TMT6plex)	1556.9	0				
																	SQNIVTDSSSLSAEAIR	11	2	2	112382250;112382252	N-Term(TMT6plex)	2007.1	0				
																	DASVAEAWLLGQEPYLSSR	14	2	2	112382250;112382252	N-Term(TMT6plex)	2321.2	0				
																	DLVAIEAK	8	2	2	112382250;112382252	N-Term(TMT6plex); K8(TMT6plex)	1316.8	0				
																	EGEDMIAEEHFGSEK	6	2	2	112382250;112382252	N-Term(TMT6plex); M5(Oxidation); K15(TMT6plex)	2182.0	0				
																	EQWANLEQLSAIR	9	2	2	112382250;112382252	N-Term(TMT6plex)	1787.0	0				
																	DVSSVELLMNNHQGIK	2	2	2	112382250;112382252	N-Term(TMT6plex); M9(Oxidation); K16(TMT6plex)	2258.2	0				
																	FATDGEGYKPCDPQVIR	4	2	2	112382250;112382252	N-Term(TMT6plex); K9(TMT6plex); C11(Carbamidomethyl)	2411.2	0				
																	MLTAQDMSYDEAR	25	2	2	112382250;112382252	N-Term(TMT6plex); M1(Oxidation); M7(Oxidation)	1791.8	0				
																	VIESTQDLGNDLAGVMALQR	10	2	2	112382250;112382252	N-Term(TMT6plex)	2359.3	0				
																	MLTAQDMSYDEAR	3	2	2	112382250;112382252	N-Term(TMT6plex); M1(Oxidation)	1775.8	0				
																	QLWGLLIEETEK	8	2	2	112382250;112382252	N-Term(TMT6plex); K12(TMT6plex)	1917.1	0				
																	VHLENMGSHDIVDGNHR	2	5	3	112382250;5902122;112382252	N-Term(TMT6plex); M6(Oxidation)	2175.1	0				
																	LEEASLLHQFQADADDIDAWMLDILK	8	2	2	112382250;112382252	N-Term(TMT6plex); M21(Oxidation); K26(TMT6plex)	3474.8	0				
																	GVNASAQK	3	2	2	112382250;112382252	N-Term(TMT6plex); K8(TMT6plex)	1232.7	0				

Accession	RefSeq Accession	Gene Symbol	Entrez Gene ID	Description	Σ Coverage	Σ# Proteins	Σ# Unique Peptides	Σ# Peptides	Σ# PSMs	Het-1A-Smoke - 2M/Parental	Het-1A-Smoke - 4M/Parental	Het-1A-Smoke - 6M/Parental	Het-1A-Smoke - 8M/Parental	# AAs	MW [kDa]	calc. pI	Sequence	# PSMs	# Proteins	# Protein Groups	Protein Group Accessions	Modifications	MH+ [Da]	# Missed Cleavages	Het-1A-Smoke - 2M/Parental	Het-1A-Smoke - 4M/Parental	Het-1A-Smoke - 6M/Parental	Het-1A-Smoke - 8M/Parental
																	TQTAIASEDMPNTLTEAEK	2	2	2	112382250;112382252	N-Term(TMT6plex); K19(TMT6plex)	2508.3	0				
																	RLEEASLLHQFQADADDIDAWMLDILK	2	2	2	112382250;112382252	N-Term(TMT6plex); M22(Oxidation); K27(TMT6plex)	3630.9	1				
																	DLMLWMEDVIR	2	2	2	112382250;112382252	N-Term(TMT6plex); M6(Oxidation)	1665.9	0				
																	SQTYQNYK	1	1	1	112382252	N-Term(TMT6plex); K8(TMT6plex)	1489.8	0	**1.2**	**1.2**	**1.4**	**1.6**
																	TSSISGPLSPAYTGQVPYNYNQLEGR	3	1	1	112382252	N-Term(TMT6plex)	3028.5	0	**1.4**	**1.4**	**1.4**	**1.4**
																	LILEVHQFSR	4	2	2	112382250;112382252	N-Term(TMT6plex)	1470.9	0				
																	LEMNLGLQK	4	2	2	112382250;112382252	N-Term(TMT6plex); K9(TMT6plex)	1503.9	0				
																	SGHFEQAIK	2	2	2	112382250;112382252	N-Term(TMT6plex); K9(TMT6plex)	1474.8	0				
																	ALVADSHPESER	4	2	2	112382250;112382252	N-Term(TMT6plex)	1539.8	0				
																	LLTQHENIKNEIDNYEEDYQK	2	2	2	112382250;112382252	N-Term(TMT6plex); K9(TMT6plex); K21(TMT6plex)	3323.7	1				
																	IFQEMLYIMDWMDEMK	1	2	2	112382250;112382252	N-Term(TMT6plex); M12(Oxidation); M15(Oxidation); K16(TMT6plex)	2613.2	0				
																	VDTVNHLADELINSGHSDAATIAEWK	15	2	2	112382250;112382252	N-Term(TMT6plex); K26(TMT6plex)	3264.7	0				
																	QALQDTLALYK	10	2	2	112382250;112382252	N-Term(TMT6plex); K11(TMT6plex)	1722.0	0				
																	DLDDFQSWLSR	8	2	2	112382250;112382252	N-Term(TMT6plex)	1610.8	0				
																	AELFTQSCADLDK	13	2	2	112382250;112382252	N-Term(TMT6plex); C8(Carbamidomethyl); K13(TMT6plex)	1956.0	0				
																	HTLVEADIGIQAER	4	2	2	112382250;112382252	N-Term(TMT6plex)	1781.0	0				
																	HLLGVEDLLQK	8	2	2	112382250;112382252	N-Term(TMT6plex); K11(TMT6plex)	1723.0	0				
																	FQIQDISVETEDNKEK	4	3	3	112382250;5902122;112382252	N-Term(TMT6plex); K14(TMT6plex); K16(TMT6plex)	2610.4	1				
																	IHCLENVDK	4	5	3	112382250;5902122;112382252	N-Term(TMT6plex); C3(Carbamidomethyl); K9(TMT6plex)	1585.9	0				
																	QLWGLLIEETEKR	4	2	2	112382250;112382252	N-Term(TMT6plex); K12(TMT6plex)	2073.2	1				
																	FESLEPEMNNQASR	7	2	2	112382250;112382252	N-Term(TMT6plex); M8(Oxidation)	1896.9	0				
																	KNQTLQK	1	3	3	112382250;5902122;112382252	N-Term(TMT6plex); K1(TMT6plex); K7(TMT6plex)	1547.0	1				
																	HQILEQAVEDYAETVHQLSK	9	2	2	112382250;112382252	N-Term(TMT6plex); K20(TMT6plex)	2796.5	0				
																	QAEAFLNNQEYVLAHTEMPTTLEGAEAAIK	2	2	2	112382250;112382252	N-Term(TMT6plex); K30(TMT6plex)	3747.9	0				
																	FLQDCQELSLWINEK	20	2	2	112382250;112382252	N-Term(TMT6plex); C5(Carbamidomethyl); K15(TMT6plex)	2381.3	0				
																	DLMLWMEDVIR	10	2	2	112382250;112382252	N-Term(TMT6plex)	1649.8	0				
																	LQALDTGWNELHK	16	2	2	112382250;112382252	N-Term(TMT6plex); K13(TMT6plex)	1983.1	0				
																	FMELLEPLNER	10	2	2	112382250;112382252	N-Term(TMT6plex)	1619.9	0				
																	IFQEMLYIMDWMDEMK	4	2	2	112382250;112382252	N-Term(TMT6plex); M9(Oxidation); M12(Oxidation); M15(Oxidation); K16(TMT6plex)	2629.3	0				
																	DLMLWMEDVIR	19	2	2	112382250;112382252	N-Term(TMT6plex); M3(Oxidation); M6(Oxidation)	1681.9	0				
																	KHQILEQAVEDYAETVHQLSK	4	2	2	112382250;112382252	N-Term(TMT6plex); K1(TMT6plex); K21(TMT6plex)	3153.8	1				
																	EVVAGSHELGQDYEHVTMLQER	2	2	2	112382250;112382252	N-Term(TMT6plex)	2756.3	0				
																	MPLATSTDHGHNLQTVQLLIK	6	2	2	112382250;112382252	N-Term(TMT6plex); M1(Oxidation); K21(TMT6plex)	2791.6	0				
																	LTTLELLEVR	12	2	2	112382250;112382252	N-Term(TMT6plex)	1415.9	0				
																	LVSQDNFGFDLPAVEAATK	16	2	2	112382250;112382252	N-Term(TMT6plex); K19(TMT6plex)	2480.3	0				
																	DGLNEAWADLLELIDTR	6	2	2	112382250;112382252	N-Term(TMT6plex)	2173.1	0				
																	VAVVNQIAR	2	2	2	112382250;112382252	N-Term(TMT6plex)	1198.7	0				
																	LVSDGNINSDR	13	2	2	112382250;112382252	N-Term(TMT6plex)	1418.7	0				
																	DLTSVNILLK	14	3	3	112382250;5902122;112382252	N-Term(TMT6plex); K10(TMT6plex)	1574.0	0				
																	EIEELQSQAQALSQEGK	10	2	2	112382250;112382252	N-Term(TMT6plex); K17(TMT6plex)	2346.3	0				
																	LQAAYAGDK	4	2	2	112382250;112382252	N-Term(TMT6plex); K9(TMT6plex)	1394.8	0				
																	DQNTVETLQR	8	2	2	112382250;112382252	N-Term(TMT6plex)	1432.8	0				
																	VLVLSQDYGK	13	2	2	112382250;112382252	N-Term(TMT6plex); K10(TMT6plex)	1579.9	0				
																	VQAVVAVAR	2	2	2	112382250;112382252	N-Term(TMT6plex)	1141.7	0				
																	HYASEEIK	6	2	2	112382250;112382252	N-Term(TMT6plex); K8(TMT6plex)	1434.8	0				
																	KHEAIETDIAAYEER	5	2	2	112382250;112382252	N-Term(TMT6plex); K1(TMT6plex)	2233.2	1				
																	LLQLTEK	7	3	2	112382250;112382252	N-Term(TMT6plex); K7(TMT6plex)	1302.8	0				
																	VAHMEFCYQELCQLAAER	4	2	2	112382250;112382252	N-Term(TMT6plex); M4(Oxidation); C7(Carbamidomethyl); C12(Carbamidomethyl)	2500.2	0				
																	WEDRWEWLR	2	2	2	112382250;112382252	N-Term(TMT6plex)	1604.8	1				
																	QAEAFLNNQEYVLAHTEMPTTLEGAEAAIK	2	2	2	112382250;112382252	N-Term(TMT6plex); M18(Oxidation); K30(TMT6plex)	3763.9	0				
																	TAGYPNVNIHNFTTSWR	2	2	2	112382250;112382252	N-Term(TMT6plex)	2207.1	0				
																	FANSLVGVQQQLQAFNTYR	3	2	2	112382250;112382252	N-Term(TMT6plex)	2413.3	0				
																	QLQEDAAR	4	2	2	112382250;112382252	N-Term(TMT6plex)	1159.6	0				
																	WLHGLESQIQSDDYGK	6	2	2	112382250;112382252	N-Term(TMT6plex); K16(TMT6plex)	2334.2	0				
																	DVEDEILWVGER	8	2	2	112382250;112382252	N-Term(TMT6plex)	1688.9	0				
																	LEMNLGLQK	2	2	2	112382250;112382252	N-Term(TMT6plex); M3(Oxidation); K9(TMT6plex)	1519.9	0				
																	DVSSVELLMNNHQGIK	2	2	2	112382250;112382252	N-Term(TMT6plex); K16(TMT6plex)	2242.2	0				
																	KHYASEEIK	4	2	2	112382250;112382252	N-Term(TMT6plex); K1(TMT6plex); K9(TMT6plex)	1792.1	1				
																	HRPDLIDFDK	4	4	2	112382250;112382252	N-Term(TMT6plex); K10(TMT6plex)	1714.0	0				
																	EAEKLESEHPDQAQAILSR	2	2	2	112382250;112382252	N-Term(TMT6plex); K4(TMT6plex)	2609.4	1				
																	HEAFEK	2	4	2	112382250;112382252	N-Term(TMT6plex); K6(TMT6plex)	1218.7	0				
																	VLDNAIETEK	7	2	2	112382250;112382252	N-Term(TMT6plex); K10(TMT6plex)	1589.9	0				
																	STDEVDSK	8	2	2	112382250;112382252	N-Term(TMT6plex); K8(TMT6plex)	1338.7	0				
																	FYHDAK	7	2	2	112382250;112382252	N-Term(TMT6plex); K6(TMT6plex)	1238.7	0				
																	SLLDACESR	2	2	2	112382250;112382252	N-Term(TMT6plex); C6(Carbamidomethyl)	1279.6	0				
																	MWEVLESTTQTK	3	2	2	112382250;112382252	N-Term(TMT6plex); K12(TMT6plex)	1911.0	0				
																	INAVVETGR	2	2	2	112382250;112382252	N-Term(TMT6plex)	1187.7	0				
																	TQILAASYELHK	6	2	2	112382250;112382252	N-Term(TMT6plex); K12(TMT6plex)	1832.1	0				
																	KQQMLENQMEVR	3	2	2	112382250;112382252	K1(TMT6plex); N-Term(TMT6plex); M4(Oxidation); M9(Oxidation)	2024.1	1				
																	DALLLWCQMK	2	6	3	112382250;5902122;112382252	N-Term(TMT6plex); C7(Carbamidomethyl); K10(TMT6plex)	1736.0	0				
																	QLMHSGHPSEK	2	2	2	112382250;112382252	N-Term(TMT6plex); M3(Oxidation); K11(TMT6plex)	1724.9	0				
																	EIQGHQPR	2	2	2	112382250;112382252	N-Term(TMT6plex)	1193.7	0				
																	DGMAFNALIHK	2	2	2	112382250;112382252	N-Term(TMT6plex); M3(Oxidation); K11(TMT6plex)	1690.9	0				
																	KQEDFMTTMDANEEK	2	2	2	112382250;112382252	N-Term(TMT6plex); K1(TMT6plex); K15(TMT6plex)	2504.3	1				
																	SIITYVVTYYHYFSK	1	2	2	112382250;112382252	N-Term(TMT6plex); K15(TMT6plex)	2342.3	0				
																	EASLGEASK	3	2	2	112382250;112382252	N-Term(TMT6plex); K9(TMT6plex)	1349.8	0				
																	LLDPEDISVDHPDEK	4	2	2	112382250;112382252	N-Term(TMT6plex); K15(TMT6plex)	2180.1	0				
																	NEIDNYEEDYQK	4	2	2	112382250;112382252	N-Term(TMT6plex); K12(TMT6plex)	2018.0	0				
																	LQAAYAGDKADDIQK	1	2	2	112382250;112382252	N-Term(TMT6plex); K9(TMT6plex); K15(TMT6plex)	2294.3	1				
																	MFSEADACELWIDEK	1	2	2	112382250;112382252	N-Term(TMT6plex); M1(Oxidation); C8(Carbamidomethyl); K15(TMT6plex)	2318.1	0				
																	EGMQLISEKPETEAVVK	1	2	2	112382250;112382252	N-Term(TMT6plex); K9(TMT6plex); K17(TMT6plex)	2575.5	0				
																	AKDEQSAVSMLK	1	2	2	112382250;112382252	N-Term(TMT6plex); K2(TMT6plex); M10(Oxidation); K12(TMT6plex)	2010.2	1				
																	ALAVEGK	4	4	3	112382250;5902122;112382252	N-Term(TMT6plex); K7(TMT6plex)	1145.7	0				
																	LISDINK	2	3	3	112382250;5902122;112382252	N-Term(TMT6plex); K7(TMT6plex)	1260.8	0				
																	AFEDEMSGR	2	2	2	112382250;112382252	N-Term(TMT6plex); M6(Oxidation)	1286.6	0				
																	STDEVDSKR	2	2	2	112382250;112382252	N-Term(TMT6plex); K8(TMT6plex)	1494.8	1				
																	VDSIDDR	2	2	2	112382250;112382252	N-Term(TMT6plex)	1048.5	0				
																	KHNLLASK	2	2	2	112382250;112382252	N-Term(TMT6plex); K1(TMT6plex); K8(TMT6plex)	1598.0	1				
																	LVDTGDKFR	1	2	2	112382250;112382252	N-Term(TMT6plex); K7(TMT6plex)	1508.9	1				
																	ALQFLK	1	6	3	112382250;5902122;112382252	N-Term(TMT6plex); K6(TMT6plex)	1177.8	0				
																	FMELLEPLNER	1	2	2	112382250;112382252	N-Term(TMT6plex); M2(Oxidation)	1635.9	0				
																	EVAELTR	2	2	2	112382250;112382252	N-Term(TMT6plex)	1046.6	0				
																	LVDTGDK	5	2	2	112382250;112382252	N-Term(TMT6plex); K7(TMT6plex)	1205.7	0				
																	TVEKPPK	1	5	3	112382250;5902122;112382252	N-Term(TMT6plex); K4(TMT6plex); K7(TMT6plex)	1486.0	0				
																	LFQLNR	1	2	2	112382250;112382252	N-Term(TMT6plex)	1019.6	0				
																	FFWEMAEEEGWIR	1	2	2	112382250;112382252	N-Term(TMT6plex); M5(Oxidation)	1974.9	0				
																	ILSSDDYGK	1	2	2	112382250;112382252	N-Term(TMT6plex); K9(TMT6plex)	1455.8	0				
																	LYAGLK	3	3	3	112382250;5902122;112382252	N-Term(TMT6plex); K6(TMT6plex)	1122.7	0				
																	LQQFLR	1	4	3	164607124;112382250;112382252	N-Term(TMT6plex)	1033.6	0				
																	LTVQTK	2	2	2	112382250;112382252	N-Term(TMT6plex); K6(TMT6plex)	1147.7	0				
																	LPEELGR	2	2	2	112382250;112382252	N-Term(TMT6plex)	1042.6	0				
																	LFDANK	2	6	3	18201905;112382250;112382252	N-Term(TMT6plex); K6(TMT6plex)	1165.7	0				
4759034	NP_004721.1	**ETF1**	2107	**eukaryotic peptide chain release factor subunit 1 isoform 1**	68.19	3	24	24	287	**1.3**	**1.4**	**1.5**	**1.4**	437	49.0	5.71												
																	FHTEALTALLSDDSK	8	3	1	4759034	N-Term(TMT6plex); K15(TMT6plex)	2106.1	0	**1.2**	**1.0**	**1.3**	**1.0**
																	YVLHCQGTEEEK	9	3	1	4759034	N-Term(TMT6plex); C5(Carbamidomethyl); K12(TMT6plex)	1951.0	0	**1.3**	**1.5**	**1.5**	**1.4**
																	VDFQGMEYQGGDDEFFDLDDY	38	3	1	4759034	N-Term(TMT6plex); M6(Oxidation)	2750.1	0	**1.5**	**1.7**	**1.6**	**1.6**
																	TELSQSDMFDQR	31	3	1	4759034	N-Term(TMT6plex); M8(Oxidation)	1701.8	0	**1.6**	**1.3**	**1.5**	**1.3**
																	ILYLTPEQEKDK	7	3	1	4759034	N-Term(TMT6plex); K10(TMT6plex); K12(TMT6plex)	2164.3	1	**1.4**	**1.8**	**1.6**	**1.7**
																	LSVLGAITSVQQR	37	3	1	4759034	N-Term(TMT6plex)	1601.0	0	**1.5**	**1.5**	**1.6**	**1.4**
																	GFGGIGGILR	7	3	1	4759034	N-Term(TMT6plex)	1175.7	0	**1.3**	**1.6**	**1.5**	**1.5**
																	VAETAVQLFISGDK	10	3	1	4759034	N-Term(TMT6plex); K14(TMT6plex)	1936.1	0	**1.3**	**1.4**	**1.4**	**1.5**
																	ILYLTPEQEK	8	3	1	4759034	N-Term(TMT6plex); K10(TMT6plex)	1692.0	0	**1.3**	**1.4**	**1.4**	**1.4**
																	TELSQSDMFDQR	2	3	1	4759034	N-Term(TMT6plex)	1685.8	0	**1.7**	**1.6**	**2.2**	**1.5**

Supplementary Table 6. List of all proteins and peptides identified in untreated and chronically treated Het1A cells with cigarette smoke condensate for 8 months

Accession	RefSeq Accession	Gene Symbol	Entrez Gene ID	Description	Σ Coverage	Σ# Proteins	Σ# Unique Peptides	Σ# Peptides	Σ# PSMs	Het-1A-Smoke - 2M/Parental	Het-1A-Smoke - 4M/Parental	Het-1A-Smoke - 6M/Parental	Het-1A-Smoke - 8M/Parental	# AAs	MW [kDa]	calc. pI	Sequence	# PSMs	# Proteins	# Protein Groups	Protein Group Accessions	Modifications	MH+ [Da]	# Missed Cleavages	Het-1A-Smoke - 2M/Parental	Het-1A-Smoke - 4M/Parental	Het-1A-Smoke - 6M/Parental	Het-1A-Smoke - 8M/Parental
																	VDFQGMEYQGGDDEFFDLDDY	4	3	1	4759034	N-Term(TMT6plex)	2734.1	0	**1.8**	**1.8**	**1.7**	**1.5**
																	ETGQEHELIESMPLLEWFANNYK	5	3	1	4759034	N-Term(TMT6plex); M12(Oxidation); K23(TMT6plex)	3252.6	0	**1.2**	**1.2**	**1.2**	**1.3**
																	ALEMGAVEILIVYENLDIMR	2	3	1	4759034	N-Term(TMT6plex); M4(Oxidation)	2537.4	0	**1.7**	**1.5**	**1.7**	**1.7**
																	FGATLEIVTDK	21	3	1	4759034	N-Term(TMT6plex); K11(TMT6plex)	1652.0	0	**1.3**	**1.3**	**1.5**	**1.3**
																	SQEGSQFVK	15	3	1	4759034	N-Term(TMT6plex); K9(TMT6plex)	1467.8	0	**1.4**	**1.5**	**1.4**	**1.4**
																	LVDISYGGENGFNQAIELSTEVLSNVK	8	3	1	4759034	N-Term(TMT6plex); K27(TMT6plex)	3354.8	0	**1.1**	**1.4**	**1.3**	**1.1**
																	VNVAGLVLAGSADFK	30	3	1	4759034	N-Term(TMT6plex); K15(TMT6plex)	1919.1	0	**1.3**	**1.5**	**1.6**	**1.4**
																	YFDEISQDTGK	10	3	1	4759034	N-Term(TMT6plex); K11(TMT6plex)	1760.9	0	**1.3**	**1.5**	**1.5**	**1.5**
																	YCFGVEDTLK	3	3	1	4759034	N-Term(TMT6plex); C2(Carbamidomethyl); K10(TMT6plex)	1689.9	0	**1.3**	**1.5**	**1.4**	**1.4**
																	VNIDFEPFKPINTSLYLCDNK	2	3	1	4759034	N-Term(TMT6plex); K9(TMT6plex); C18(Carbamidomethyl); K21(TMT6plex)	3214.7	0	**1.2**	**1.4**	**1.5**	**1.4**
																	KVAETAVQLFISGDK	6	3	1	4759034	N-Term(TMT6plex); K1(TMT6plex); K15(TMT6plex)	2293.4	1	**1.3**	**1.5**	**1.4**	**1.5**
																	KVNIDFEPFKPINTSLYLCDNK	3	3	1	4759034	K1(TMT6plex); N-Term(TMT6plex); K10(TMT6plex); C19(Carbamidomethyl); K22(TMT6plex)	3572.0	1	**1.2**	**1.6**	**1.6**	**1.5**
																	FTVDLPK	16	3	1	4759034	N-Term(TMT6plex); K7(TMT6plex)	1277.8	0	**1.2**	**1.5**	**1.4**	**1.4**
																	SLEAAR	1	1	1	4759034	N-Term(TMT6plex)	875.5	0	**1.0**	**1.4**	**1.3**	**1.4**
																	NVEIWK	3	1	1	4759034	N-Term(TMT6plex); K6(TMT6plex)	1246.8	0	**1.2**	**1.3**	**1.4**	**1.3**
																	FGFIVIDGSGALFGTLQGNTR	1	3	1	4759034	N-Term(TMT6plex)	2399.3	0	**1.6**	**1.4**	**1.7**	**1.2**
4885049	NP_005150.1	**ACTC1**	70	**actin, alpha cardiac muscle 1 proprotein**	68.17	6	2	25	1880	**1.5**	**1.9**	**2.0**	**1.6**	377	42.0	5.39												
																	SYELPDGQVITIGNER	184	10	4	4885049;63055057;316659409;4501881	N-Term(TMT6plex)	2020.0	0				
																	GYSFVTTAER	4	5	2	4885049;4501881	N-Term(TMT6plex)	1359.7	0				
																	DSYVGDEAQSK	313	6	3	4885049;316659409;4501881	N-Term(TMT6plex); K11(TMT6plex)	1656.8	0				
																	EITALAPSTMK	216	7	3	4885049;316659409;4501881	N-Term(TMT6plex); M10(Oxidation); K11(TMT6plex)	1635.9	0				
																	DLTDYLMK	52	8	4	4885049;63055057;316659409;4501881	N-Term(TMT6plex); K8(TMT6plex)	1456.8	0				
																	DLTDYLMK	164	8	4	4885049;63055057;316659409;4501881	N-Term(TMT6plex); M7(Oxidation); K8(TMT6plex)	1472.8	0				
																	TTGIVLDSGDGVTHNVPIYEGYALPHAIMR	2	5	2	4885049;4501881	N-Term(TMT6plex); M29(Oxidation)	3441.8	0				
																	HQGVMVGMGQK	118	7	4	4885049;63055057;316659409;4501881	N-Term(TMT6plex); M5(Oxidation); M8(Oxidation); K11(TMT6plex)	1661.9	0				
																	GILTLK	148	10	4	4885049;475808427;316659409;4501881	N-Term(TMT6plex); K6(TMT6plex)	1102.8	0				
																	YPIEHGIITNWDDMEK	90	4	2	4885049;4501881	N-Term(TMT6plex); M14(Oxidation); K16(TMT6plex)	2435.2	0				
																	IIAPPERK	4	8	4	4885049;63055057;316659409;4501881	N-Term(TMT6plex); K8(TMT6plex)	1381.9	1				
																	EITALAPSTMK	42	7	3	4885049;316659409;4501881	N-Term(TMT6plex); K11(TMT6plex)	1619.9	0				
																	HQGVMVGMGQK	15	7	4	4885049;63055057;316659409;4501881	N-Term(TMT6plex); K11(TMT6plex)	1629.9	0				
																	HQGVMVGMGQK	56	7	4	4885049;63055057;316659409;4501881	N-Term(TMT6plex); M5(Oxidation); K11(TMT6plex)	1645.9	0				
																	IWHHTFYNELR	84	8	4	475808427;4885049;316659409;4501881	N-Term(TMT6plex)	1744.9	0				
																	YSVWIGGSILASLSTFQQMWISK	11	5	3	4885049;63055057;316659409	N-Term(TMT6plex); K23(TMT6plex)	3060.7	0				
																	KYSVWIGGSILASLSTFQQMWISK	11	5	3	4885049;63055057;316659409	N-Term(TMT6plex); K1(TMT6plex); M20(Oxidation); K24(TMT6plex)	3433.9	1				
																	YSVWIGGSILASLSTFQQMWISK	12	5	3	4885049;63055057;316659409	N-Term(TMT6plex); M19(Oxidation); K23(TMT6plex)	3076.7	0				
																	AGFAGDDAPR	215	11	4	4885049;475808427;316659409;4501881	N-Term(TMT6plex)	1205.6	0				
																	AVFPSIVGRPR	16	10	4	4885049;475808427;316659409;4501881	N-Term(TMT6plex)	1427.9	0				
																	YPIEHGIITNWDDMEK	26	4	2	4885049;4501881	N-Term(TMT6plex); K16(TMT6plex)	2419.2	0				
																	DSYVGDEAQSKR	3	6	3	4885049;316659409;4501881	N-Term(TMT6plex); K11(TMT6plex)	1813.0	1				
																	LCYVALDFENEMATAASSSSLEK	27	5	2	4885049;4501881	N-Term(TMT6plex); C2(Carbamidomethyl); M12(Oxidation); K23(TMT6plex)	3010.5	0				
																	HQGVMVGMGQKDSYVGDEAQSK	1	6	3	4885049;316659409;4501881	N-Term(TMT6plex); M5(Oxidation); M8(Oxidation); K11(TMT6plex); K22(TMT6plex)	3070.6	1				
																	KYSVWIGGSILASLSTFQQMWISK	4	5	3	4885049;63055057;316659409	N-Term(TMT6plex); K1(TMT6plex); K24(TMT6plex)	3417.9	1				
																	VAPEEHPTLLTEAPLNPK	10	5	2	4885049;4501881	N-Term(TMT6plex); K18(TMT6plex)	2414.4	0				
																	LCYVALDFENEMATAASSSSLEK	4	5	2	4885049;4501881	N-Term(TMT6plex); C2(Carbamidomethyl); K23(TMT6plex)	2994.5	0				
																	DLYANNVLSGGTTMYPGIADR	4	4	1	4885049	N-Term(TMT6plex); M14(Oxidation)	2473.2	0	**1.5**	**1.9**	**2.0**	**1.7**
																	KDLYANNVLSGGTTMYPGIADR	2	4	1	4885049	N-Term(TMT6plex); K1(TMT6plex); M15(Oxidation)	2830.5	1	**1.5**	**2.0**	**2.0**	**1.5**
																	IIAPPERKYSVWIGGSILASLSTFQQMWISK	1	5	3	4885049;63055057;316659409	N-Term(TMT6plex); K8(TMT6plex); K31(TMT6plex)	4194.3	2				
																	IIAPPERKYSVWIGGSILASLSTFQQMWISK	1	5	3	4885049;63055057;316659409	N-Term(TMT6plex); K8(TMT6plex); M27(Oxidation); K31(TMT6plex)	4210.4	2				
																	IIAPPER	29	8	4	4885049;63055057;316659409;4501881	N-Term(TMT6plex)	1024.6	0				
																	LDLAGR	10	15	7	5031569;5031571;4885049;475808427;63055057;316659409;4501881	N-Term(TMT6plex)	873.5	0				
																	ILTER	1	13	6	4885049;475808427;63055057;316659409;4501881;24233517	N-Term(TMT6plex)	860.5	0				
4506189	NP_002783.1	**PSMA7**	5688	**proteasome subunit alpha type-7**	68.15	5	15	15	203	**1.3**	**1.3**	**1.4**	**1.4**	248	27.9	8.46												
																	NYTDEAIETDDLTIK	30	1	1	4506189	N-Term(TMT6plex); K15(TMT6plex)	2199.1	0	**1.4**	**1.2**	**1.4**	**1.3**
																	LYQTDPSGTYHAWK	13	4	1	4506189	N-Term(TMT6plex); K14(TMT6plex)	2125.1	0	**1.2**	**1.2**	**1.3**	**1.3**
																	LTVEDPVTVEYITR	38	5	1	4506189	N-Term(TMT6plex)	1864.0	0	**1.4**	**1.5**	**1.5**	**1.6**
																	ALLEVVQSGGK	26	5	1	4506189	N-Term(TMT6plex); K11(TMT6plex)	1559.0	0	**1.3**	**1.4**	**1.3**	**1.4**
																	RPFGISALIVGFDFDGTPR	9	1	1	4506189	N-Term(TMT6plex)	2294.2	0	**1.3**	**1.3**	**1.6**	**1.2**
																	ILNPEEIEK	19	1	1	4506189	N-Term(TMT6plex); K9(TMT6plex)	1542.9	0	**1.4**	**1.4**	**1.4**	**1.5**
																	NIELAVMR	2	1	1	4506189	N-Term(TMT6plex)	1174.7	0	**1.7**	**1.5**	**2.0**	**1.3**
																	AITVFSPDGHLFQVEYAQEAVK	23	4	1	4506189	N-Term(TMT6plex); K22(TMT6plex)	2907.6	0	**1.3**	**1.4**	**1.6**	**1.5**
																	ICALDDNVCMAFAGLTADAR	8	1	1	4506189	N-Term(TMT6plex); C2(Carbamidomethyl); C9(Carbamidomethyl)	2413.2	0	**1.5**	**1.5**	**1.7**	**1.5**
																	DIVVLGVEK	17	1	1	4506189	N-Term(TMT6plex); K9(TMT6plex)	1429.9	0	**1.1**	**1.3**	**1.3**	**1.3**
																	GSTAVGVR	2	1	1	4506189	N-Term(TMT6plex)	975.6	0	**1.3**	**1.4**	**1.5**	**1.6**
																	YVAEIEK	6	1	1	4506189	N-Term(TMT6plex); K7(TMT6plex)	1309.8	0	**1.3**	**1.3**	**1.4**	**1.5**
																	EKEENEK	1	1	1	4506189	N-Term(TMT6plex); K2(TMT6plex); K7(TMT6plex)	1592.9	1	**1.2**	**1.2**	**1.3**	**1.3**
																	NIELAVMR	4	1	1	4506189	N-Term(TMT6plex); M7(Oxidation)	1190.7	0	**1.4**	**1.2**	**1.3**	**1.4**
																	YVAEIEKEKEENEK	1	1	1	4506189	N-Term(TMT6plex); K7(TMT6plex); K9(TMT6plex); K14(TMT6plex)	2654.5	2	**1.4**	**1.6**	**1.6**	**1.7**
																	YIASLK	4	1	1	4506189	N-Term(TMT6plex); K6(TMT6plex)	1152.7	0	**1.3**	**1.3**	**1.4**	**1.4**
4506787	NP_003861.1	**IQGAP1**	8826	**ras GTPase-activating-like protein IQGAP1**	67.95	1	84	91	1230	**1.2**	**1.2**	**1.4**	**1.3**	1657	189.1	6.48												
																	LEGVLAEVAQHYQDTLIR	26	1	1	4506787	N-Term(TMT6plex)	2284.3	0	**1.3**	**1.1**	**1.3**	**1.0**
																	YMNPAIVAPDAFDIIDLSAGGQLTTDQR	34	1	1	4506787	N-Term(TMT6plex); M2(Oxidation)	3237.6	0	**1.5**	**1.6**	**1.6**	**1.8**
																	NVIFEISPTEEVGDFEVK	41	1	1	4506787	N-Term(TMT6plex); K18(TMT6plex)	2510.3	0	**1.3**	**1.1**	**1.4**	**1.1**
																	YQELINDIAR	17	1	1	4506787	N-Term(TMT6plex)	1463.8	0	**1.3**	**1.2**	**1.3**	**1.3**
																	AQEIQDESAVLWLDEIQGGIWQSNK	27	1	1	4506787	N-Term(TMT6plex); K25(TMT6plex)	3315.7	0	**1.4**	**1.4**	**1.5**	**1.5**
																	QNVAYEYLCHLEEAK	6	1	1	4506787	N-Term(TMT6plex); C9(Carbamidomethyl); K15(TMT6plex)	2325.2	0	**1.3**	**1.1**	**1.4**	**1.2**
																	SWVNQMESQTGEASK	19	1	1	4506787	N-Term(TMT6plex); M6(Oxidation); K15(TMT6plex)	2156.1	0	**1.5**	**1.4**	**1.6**	**1.4**
																	FDVPGDENAEMDAR	11	1	1	4506787	N-Term(TMT6plex); M11(Oxidation)	1810.8	0	**1.3**	**1.3**	**1.4**	**1.3**
																	EEYLLLR	6	1	1	4506787	N-Term(TMT6plex)	1164.7	0	**1.1**	**1.2**	**1.3**	**1.2**
																	SNQQLENDLNLMDIK	19	1	1	4506787	N-Term(TMT6plex); M12(Oxidation); K15(TMT6plex)	2249.2	0	**1.2**	**1.2**	**1.3**	**1.3**
																	EEVITLIR	6	1	1	4506787	N-Term(TMT6plex)	1201.7	0	**1.2**	**1.1**	**1.2**	**1.2**
																	TLINAEDPPMVVVR	21	1	1	4506787	N-Term(TMT6plex); M10(Oxidation)	1799.0	0	**1.2**	**1.2**	**1.3**	**1.2**
																	GVLLEIEDLQVNQFK	37	1	1	4506787	N-Term(TMT6plex); K15(TMT6plex)	2203.3	0	**1.2**	**1.4**	**1.5**	**1.4**
																	EEIQSSISGVTAAYNR	25	1	1	4506787	N-Term(TMT6plex)	1954.0	0	**1.2**	**1.2**	**1.3**	**1.3**
																	ILAIGLINEALDEGDAQK	69	1	1	4506787	N-Term(TMT6plex); K18(TMT6plex)	2341.3	0	**1.3**	**1.3**	**1.4**	**1.3**
																	FALGIFAINEAVESGDVGK	26	1	1	4506787	N-Term(TMT6plex); K19(TMT6plex)	2395.3	0	**1.0**	**1.1**	**1.4**	**1.2**
																	FPDAGEDELLK	16	1	1	4506787	N-Term(TMT6plex); K11(TMT6plex)	1691.9	0	**1.1**	**1.2**	**1.3**	**1.2**
																	IFYPETTDIYDR	22	1	1	4506787	N-Term(TMT6plex)	1761.9	0	**1.3**	**1.4**	**1.5**	**1.3**
																	TALQEEIK	18	2	2	4506787;39753961	N-Term(TMT6plex); K8(TMT6plex)	1389.8	0				
																	LTAEEMDER	4	1	1	4506787	N-Term(TMT6plex); M6(Oxidation)	1338.6	0	**1.0**	**1.0**	**0.9**	**1.2**
																	WMEACLGEDLPPTTELEEGLR	19	1	1	4506787	N-Term(TMT6plex); M2(Oxidation); C5(Carbamidomethyl)	2691.3	0	**1.3**	**1.2**	**1.4**	**1.3**
																	EDSNLTLQEK	22	1	1	4506787	N-Term(TMT6plex); K10(TMT6plex)	1634.9	0	**1.2**	**1.2**	**1.3**	**1.2**
																	NPNAMLVNLEEPLASTYQDILYQAK	17	1	1	4506787	N-Term(TMT6plex); M5(Oxidation); K25(TMT6plex)	3309.8	0	**1.1**	**1.1**	**1.2**	**1.1**
																	ALESGDVNTVWK	15	1	1	4506787	N-Term(TMT6plex); K12(TMT6plex)	1777.0	0	**1.2**	**1.3**	**1.4**	**1.3**
																	FLSAIVSSVDK	20	1	1	4506787	N-Term(TMT6plex); K11(TMT6plex)	1624.0	0	**1.3**	**0.8**	**1.2**	**1.0**
																	ITLQDVVSHSK	3	1	1	4506787	N-Term(TMT6plex); K11(TMT6plex)	1685.0	0	**1.1**	**1.1**	**1.3**	**1.2**
																	EQLWLANEGLITR	20	1	1	4506787	N-Term(TMT6plex)	1772.0	0	**1.2**	**1.3**	**1.3**	**1.3**
																	QSPEHNLTHPELSVAVEMLSSVALINR	2	1	1	4506787	N-Term(TMT6plex); M18(Oxidation)	3216.7	0	**1.7**	**1.3**	**1.4**	**1.3**
																	IIGNLLYYR	14	1	1	4506787	N-Term(TMT6plex)	1353.8	0	**1.4**	**1.3**	**1.5**	**1.3**
																	LIVDVIR	12	1	1	4506787	N-Term(TMT6plex)	1056.7	0	**1.3**	**1.3**	**1.3**	**1.3**
																	RIPADTFAALK	3	1	1	4506787	N-Term(TMT6plex); K11(TMT6plex)	1661.0	1	**1.3**	**1.4**	**1.4**	**1.5**
																	KLTELGTVDPK	3	1	1	4506787	N-Term(TMT6plex); K1(TMT6plex); K11(TMT6plex)	1888.2	1	**1.3**	**1.7**	**1.9**	**1.5**
																	SWVNQMESQTGEASK	2	1	1	4506787	N-Term(TMT6plex); K15(TMT6plex)	2140.1	0	**1.3**	**1.3**	**1.6**	**1.2**
																	LQQTYAALNSK	7	1	1	4506787	N-Term(TMT6plex); K11(TMT6plex)	1695.0	0	**1.3**	**1.2**	**1.4**	**1.2**
																	ATFYGEQVDYYK	22	1	1	4506787	N-Term(TMT6plex); K12(TMT6plex)	1942.0	0	**1.2**	**1.3**	**1.4**	**1.4**

Supplementary Table 6. List of all proteins and peptides identified in untreated and chronically treated Het1A cells with cigarette smoke condensate for 8 months

Accession	RefSeq Accession	Gene Symbol	Entrez Gene ID	Description	Σ Coverage	Σ# Proteins	Σ# Unique Peptides	Σ# Peptides	Σ# PSMs	Het-1A-Smoke - 2M/Parental	Het-1A-Smoke - 4M/Parental	Het-1A-Smoke - 6M/Parental	Het-1A-Smoke - 8M/Parental	# AAs	MW [kDa]	calc. pI	Sequence	# PSMs	# Proteins	# Protein Groups	Protein Group Accessions	Modifications	MH+ [Da]	# Missed Cleavages	Het-1A-Smoke - 2M/Parental	Het-1A-Smoke - 4M/Parental	Het-1A-Smoke - 6M/Parental	Het-1A-Smoke - 8M/Parental
																	FVHLLDQSDQDFQEELDLMK	10	1	1	4506787	N-Term(TMT6plex); M19(Oxidation); K20(TMT6plex)	2924.5	0	**1.1**	**1.0**	**1.1**	**1.1**
																	SKVDQIQEIVTGNPTVIK	12	1	1	4506787	N-Term(TMT6plex); K2(TMT6plex); K18(TMT6plex)	2656.6	1	**1.2**	**1.5**	**1.6**	**1.5**
																	VDFTEEEINNMK	22	1	1	4506787	N-Term(TMT6plex); M11(Oxidation); K12(TMT6plex)	1943.0	0	**1.2**	**1.3**	**1.4**	**1.3**
																	MFLGDNAHLSIINEYLSQSYQK	3	1	1	4506787	N-Term(TMT6plex); M1(Oxidation); K22(TMT6plex)	3045.6	0	**1.3**	**1.2**	**1.2**	**1.4**
																	KLAVGDNNSK	2	1	1	4506787	N-Term(TMT6plex); K1(TMT6plex); K10(TMT6plex)	1733.1	1	**0.9**	**1.2**	**1.2**	**1.2**
																	IPADTFAALK	10	1	1	4506787	N-Term(TMT6plex); K10(TMT6plex)	1504.9	0	**1.2**	**1.4**	**1.4**	**1.3**
																	LGNFFSPK	8	1	1	4506787	N-Term(TMT6plex); K8(TMT6plex)	1367.8	0	**1.2**	**1.1**	**1.3**	**1.1**
																	LIFQMPQNK	2	6	3	550544226;4506787;39753961	N-Term(TMT6plex); K9(TMT6plex)	1576.9	0				
																	LIFQMPQNK	22	6	3	550544226;4506787;39753961	N-Term(TMT6plex); M5(Oxidation); K9(TMT6plex)	1592.9	0				
																	EQLSDMMMINK	12	1	1	4506787	N-Term(TMT6plex); M6(Oxidation); M7(Oxidation); M8(Oxidation); K11(TMT6plex)	1845.9	0	**1.2**	**1.1**	**1.2**	**1.3**
																	MLQHAASNK	6	1	1	4506787	N-Term(TMT6plex); M1(Oxidation); K9(TMT6plex)	1473.8	0	**1.2**	**1.2**	**1.6**	**1.3**
																	SHKDEVVK	7	1	1	4506787	N-Term(TMT6plex); K3(TMT6plex); K8(TMT6plex)	1629.0	1	**1.0**	**1.2**	**1.4**	**1.3**
																	SVKEDSNLTLQEK	2	1	1	4506787	N-Term(TMT6plex); K3(TMT6plex); K13(TMT6plex)	2178.3	1	**1.4**	**1.7**	**1.5**	**1.7**
																	GLQQQNSDWYLK	16	1	1	4506787	N-Term(TMT6plex); K12(TMT6plex)	1938.0	0	**1.1**	**1.3**	**1.4**	**1.3**
																	QSGQTDPLQKEELQSGVDAANSAAQQYQR	4	1	1	4506787	N-Term(TMT6plex); K10(TMT6plex)	3605.8	1	**1.4**	**1.4**	**1.4**	**1.2**
																	TLQALQIPAAK	8	1	1	4506787	N-Term(TMT6plex); K11(TMT6plex)	1612.0	0	**1.2**	**1.1**	**1.3**	**1.1**
																	LTELGTVDPK	12	1	1	4506787	N-Term(TMT6plex); K10(TMT6plex)	1530.9	0	**1.2**	**1.2**	**1.3**	**1.2**
																	EKLEAYQHLFYLLQTNPTYLAK	9	1	1	4506787	N-Term(TMT6plex); K2(TMT6plex); K22(TMT6plex)	3370.9	1	**1.3**	**1.5**	**1.7**	**1.4**
																	SNQQLENDLNLMDIK	2	1	1	4506787	N-Term(TMT6plex); K15(TMT6plex)	2233.2	0	**1.2**	**1.3**	**1.6**	**1.2**
																	LAAVALINAAIQK	31	1	1	4506787	N-Term(TMT6plex); K13(TMT6plex)	1754.1	0	**1.2**	**1.0**	**1.4**	**1.0**
																	VDQIQEIVTGNPTVIK	28	1	1	4506787	N-Term(TMT6plex); K16(TMT6plex)	2212.3	0	**1.2**	**1.2**	**1.4**	**1.2**
																	HTDNVIQWLNAMDEIGLPK	28	1	1	4506787	N-Term(TMT6plex); K19(TMT6plex)	2652.4	0	**1.4**	**1.5**	**1.6**	**1.3**
																	EELQSGVDAANSAAQQYQR	20	1	1	4506787	N-Term(TMT6plex)	2294.1	0	**1.2**	**1.1**	**1.3**	**1.2**
																	VNVNLLIFLLNK	10	2	2	4506787;39753961	N-Term(TMT6plex); K12(TMT6plex)	1858.2	0				
																	LEAYQHLFYLLQTNPTYLAK	10	1	1	4506787	N-Term(TMT6plex); K20(TMT6plex)	2884.6	0	**1.1**	**1.2**	**1.5**	**0.9**
																	LPYDVTPEQALAHEEVK	23	1	1	4506787	N-Term(TMT6plex); K17(TMT6plex)	2397.3	0	**1.2**	**1.4**	**1.5**	**1.3**
																	DHINDIIK	16	1	1	4506787	N-Term(TMT6plex); K8(TMT6plex)	1425.8	0	**1.1**	**1.2**	**1.4**	**1.2**
																	DTQEAQK	10	1	1	4506787	N-Term(TMT6plex); K7(TMT6plex)	1277.7	0	**1.2**	**1.2**	**1.3**	**1.3**
																	TILLNTK	8	1	1	4506787	N-Term(TMT6plex); K7(TMT6plex)	1260.8	0	**1.1**	**1.1**	**1.3**	**1.1**
																	EQLSDMMMINK	7	1	1	4506787	N-Term(TMT6plex); M7(Oxidation); M8(Oxidation); K11(TMT6plex)	1829.9	0	**1.2**	**1.6**	**1.6**	**1.4**
																	FQPGETLTEILETPATSEQEAEHQR	31	1	1	4506787	N-Term(TMT6plex)	3070.5	0	**1.3**	**1.4**	**1.4**	**1.3**
																	NPNAMLVNLEEPLASTYQDILYQAK	12	1	1	4506787	N-Term(TMT6plex); K25(TMT6plex)	3293.8	0	**1.5**	**1.4**	**1.6**	**1.3**
																	SPDVGLYGVIPECGETYHSDLAEAK	20	1	1	4506787	N-Term(TMT6plex); C13(Carbamidomethyl); K25(TMT6plex)	3165.6	0	**1.1**	**1.2**	**1.3**	**1.1**
																	QLSSSVTGLTNIEEENCQR	10	1	1	4506787	N-Term(TMT6plex); C17(Carbamidomethyl)	2394.2	0	**1.3**	**1.2**	**1.3**	**1.3**
																	LGLAPQIQDLYGK	6	1	1	4506787	N-Term(TMT6plex); K13(TMT6plex)	1874.1	0	**1.1**	**1.2**	**1.4**	**1.3**
																	VDFTEEEINNMK	8	1	1	4506787	N-Term(TMT6plex); K12(TMT6plex)	1927.0	0	**1.5**	**1.6**	**1.9**	**1.4**
																	FMDSVIFTLYNYASNQR	8	1	1	4506787	N-Term(TMT6plex); M2(Oxidation)	2314.1	0	**1.4**	**1.4**	**1.5**	**1.5**
																	TCLDNLASK	8	1	1	4506787	N-Term(TMT6plex); C2(Carbamidomethyl); K9(TMT6plex)	1479.8	0	**1.5**	**1.2**	**1.4**	**1.1**
																	LAVGDNNSK	8	1	1	4506787	N-Term(TMT6plex); K9(TMT6plex)	1375.8	0	**1.3**	**1.3**	**1.4**	**1.3**
																	QILAPVVK	6	5	2	4506787;550544226	N-Term(TMT6plex); K8(TMT6plex)	1325.9	0				
																	YGIQMPAFSK	11	1	1	4506787	N-Term(TMT6plex); K10(TMT6plex)	1599.9	0	**1.3**	**1.4**	**1.7**	**1.3**
																	TEVSLTLTNK	9	1	1	4506787	N-Term(TMT6plex); K10(TMT6plex)	1563.9	0	**1.1**	**1.2**	**1.4**	**1.4**
																	QSGQTDPLQK	12	1	1	4506787	N-Term(TMT6plex); K10(TMT6plex)	1559.9	0	**1.1**	**1.0**	**1.2**	**1.0**
																	FDVPGDENAEMDAR	4	1	1	4506787	N-Term(TMT6plex)	1794.8	0	**1.6**	**1.7**	**2.2**	**1.4**
																	QIPAITCIQSQWR	5	1	1	4506787	N-Term(TMT6plex); C7(Carbamidomethyl)	1830.0	0	**1.2**	**1.3**	**1.4**	**1.5**
																	YGIQMPAFSK	5	1	1	4506787	N-Term(TMT6plex); M5(Oxidation); K10(TMT6plex)	1615.9	0	**1.2**	**1.2**	**1.4**	**1.3**
																	HTDNVIQWLNAMDEIGLPK	11	1	1	4506787	N-Term(TMT6plex); M12(Oxidation); K19(TMT6plex)	2668.4	0	**1.3**	**1.4**	**1.4**	**1.3**
																	TDPVDIYK	10	1	1	4506787	N-Term(TMT6plex); K8(TMT6plex)	1408.8	0	**1.2**	**1.1**	**1.3**	**1.1**
																	KFVHLLDQSDQDFQEELDLMK	4	1	1	4506787	N-Term(TMT6plex); K1(TMT6plex); M20(Oxidation); K21(TMT6plex)	3281.7	1	**1.0**	**1.3**	**1.3**	**1.5**
																	DVYEELLTQAEIQGNINK	2	1	1	4506787	N-Term(TMT6plex); K18(TMT6plex)	2535.4	0	**1.2**	**1.4**	**1.6**	**1.4**
																	TVLELMNPEAQLPQVYPFAADLYQK	2	1	1	4506787	N-Term(TMT6plex); M6(Oxidation); K25(TMT6plex)	3352.8	0	**1.2**	**1.4**	**1.3**	**1.6**
																	LTAEEMDER	2	1	1	4506787	N-Term(TMT6plex)	1322.6	0	**1.1**	**1.3**	**1.4**	**1.1**
																	GGYYYYHNLETQEGGWDEPPNFVQNSMQLSR	1	1	1	4506787	N-Term(TMT6plex)	3908.8	0	**0.8**	**1.3**	**1.1**	**1.2**
																	CIYCIHALSLYLFK	4	1	1	4506787	N-Term(TMT6plex); C1(Carbamidomethyl); C4(Carbamidomethyl); K14(TMT6plex)	2259.2	0	**1.4**	**1.0**	**1.8**	**1.0**
																	ALQSPALGLR	3	1	1	4506787	N-Term(TMT6plex)	1254.8	0	**1.2**	**1.3**	**1.3**	**1.3**
																	FMDSVIFTLYNYASNQR	2	1	1	4506787	N-Term(TMT6plex)	2298.1	0	**4.2**	**3.6**	**5.0**	**2.6**
																	AQAHAENNEFITWNDIQACVDHVNLVVQEEHER	2	1	1	4506787	N-Term(TMT6plex); C19(Carbamidomethyl)	4145.0	0	**1.3**	**1.6**	**1.5**	**1.3**
																	FFQTACDVPELQDK	4	1	1	4506787	N-Term(TMT6plex); C6(Carbamidomethyl); K14(TMT6plex)	2156.1	0				
																	YLDELMK	6	1	1	4506787	N-Term(TMT6plex); M6(Oxidation); K7(TMT6plex)	1385.8	0	**1.1**	**1.1**	**1.2**	**1.2**
																	NKEQLSDMMMINK	2	1	1	4506787	N-Term(TMT6plex); K2(TMT6plex); M8(Oxidation); M9(Oxidation); M10(Oxidation); K13(TMT6plex)	2317.2	1	**0.8**	**1.0**	**0.8**	**1.3**
																	YLDELMK	2	1	1	4506787	N-Term(TMT6plex); K7(TMT6plex)	1369.8	0	**1.7**	**1.8**	**2.4**	**1.2**
																	ELATLQR	4	1	1	4506787	N-Term(TMT6plex)	1059.6	0	**1.2**	**1.4**	**1.5**	**1.3**
																	NLGSIAK	4	1	1	4506787	N-Term(TMT6plex); K7(TMT6plex)	1160.7	0	**1.2**	**0.9**	**1.3**	**1.0**
																	KAELVK	2	2	2	4506787;39753961	N-Term(TMT6plex); K1(TMT6plex); K6(TMT6plex)	1374.9	1				
																	IGLLVK	5	8	4	613410201;4506787;550544226;39753961	N-Term(TMT6plex); K6(TMT6plex)	1100.8	0				
																	MREEVITLIR	1	1	1	4506787	N-Term(TMT6plex); M1(Oxidation)	1504.9	1	**1.3**	**1.3**	**1.3**	**1.4**
																	TLINAEDPPMVVVR	1	1	1	4506787	N-Term(TMT6plex)	1783.0	0	**1.4**	**1.4**	**1.6**	**1.4**
																	IQTGLK	1	1	1	4506787	N-Term(TMT6plex); K6(TMT6plex)	1117.7	0	**1.1**	**1.0**	**1.2**	**1.1**
																	MVVSFNR	1	5	2	550544226;4506787	N-Term(TMT6plex); M1(Oxidation)	1097.6	0				
4504425	NP_002119.1	**HMGB1**	3146	**high mobility group protein B1**	67.91	1	12	17	214	**1.2**	**1.2**	**1.4**	**1.4**	215	24.9	5.74												
																	IKGEHPGLSIGDVAK	24	1	1	4504425	N-Term(TMT6plex); K2(TMT6plex); K15(TMT6plex)	2208.3	1	**1.2**	**1.3**	**1.5**	**1.5**
																	HPDASVNFSEFSK	13	1	1	4504425	N-Term(TMT6plex); K13(TMT6plex)	1923.0	0	**1.2**	**1.2**	**1.3**	**1.3**
																	TYIPPKGETK	13	1	1	4504425	N-Term(TMT6plex); K6(TMT6plex); K10(TMT6plex)	1821.1	1	**1.2**	**1.3**	**1.3**	**1.5**
																	LGEMWNNTAADDKQPYEK	19	1	1	4504425	N-Term(TMT6plex); M4(Oxidation); K13(TMT6plex); K18(TMT6plex)	2813.4	1	**1.2**	**1.1**	**1.4**	**1.4**
																	MSSYAFFVQTCR	6	2	2	4504425;194688133	N-Term(TMT6plex); C11(Carbamidomethyl)	1725.8	0				
																	KEEEEDEEDEEDEEEEEDEEDEDEEEDDDDE	2	1	1	4504425	N-Term(TMT6plex); K1(TMT6plex)	4336.6	1	**2.0**	**1.5**	**2.2**	**1.5**
																	MSSYAFFVQTCR	11	2	2	4504425;194688133	N-Term(TMT6plex); M1(Oxidation); C11(Carbamidomethyl)	1741.8	0				
																	KLGEMWNNTAADDK	4	1	1	4504425	N-Term(TMT6plex); K1(TMT6plex); M5(Oxidation); K14(TMT6plex)	2296.2	1	**1.0**	**0.9**	**1.2**	**1.4**
																	GEHPGLSIGDVAK	42	1	1	4504425	N-Term(TMT6plex); K13(TMT6plex)	1738.0	0	**1.1**	**1.1**	**1.3**	**1.3**
																	KHPDASVNFSEFSK	29	1	1	4504425	N-Term(TMT6plex); K1(TMT6plex); K14(TMT6plex)	2280.3	1	**1.1**	**1.3**	**1.4**	**1.4**
																	LGEMWNNTAADDK	17	1	1	4504425	N-Term(TMT6plex); M4(Oxidation); K13(TMT6plex)	1939.0	0	**1.2**	**1.1**	**1.3**	**1.4**
																	RPPSAFFLFCSEYRPK	7	1	1	4504425	N-Term(TMT6plex); C10(Carbamidomethyl); K16(TMT6plex)	2460.3	0	**1.5**	**1.8**	**2.0**	**2.1**
																	FKDPNAPK	9	2	2	4504425;19923236	N-Term(TMT6plex); K2(TMT6plex); K8(TMT6plex)	1604.0	1				
																	LGEMWNNTAADDKQPYEK	5	1	1	4504425	N-Term(TMT6plex); K13(TMT6plex); K18(TMT6plex)	2797.4	1	**1.7**	**1.4**	**2.5**	**1.4**
																	KLGEMWNNTAADDKQPYEK	2	1	1	4504425	K1(TMT6plex); N-Term(TMT6plex); M5(Oxidation); K14(TMT6plex); K19(TMT6plex)	3170.7	2	**0.9**	**1.0**	**1.2**	**1.4**
																	GKPDAAK	2	1	1	4504425	N-Term(TMT6plex); K2(TMT6plex); K7(TMT6plex)	1373.9	0	**1.0**	**1.0**	**1.2**	**1.3**
																	TYIPPK	7	2	2	4504425;19923236	N-Term(TMT6plex); K6(TMT6plex)	1176.7	0				
																	GKFEDMAK	1	2	2	4504425;19923236	N-Term(TMT6plex); K2(TMT6plex); K8(TMT6plex)	1612.9	1				
																	DIAAYR	1	3	3	4504425;194688133;19923236	N-Term(TMT6plex)	937.5	0				
7661920	NP_055555.1	**EIF4A3**	9775	**eukaryotic initiation factor 4A-III**	67.88	1	21	24	309	**1.3**	**1.3**	**1.3**	**1.4**	411	46.8	6.73												
																	VEFETSEEVDVTPTFDTMGLR	25	1	1	7661920	N-Term(TMT6plex); M18(Oxidation)	2647.3	0	**1.3**	**1.3**	**1.3**	**1.4**
																	DIEQYYSTQIDEMPMNVADLI	29	1	1	7661920	N-Term(TMT6plex); M13(Oxidation); M15(Oxidation)	2749.3	0	**1.3**	**1.5**	**1.5**	**1.6**
																	DVIAQSQSGTGK	21	1	1	7661920	N-Term(TMT6plex); K12(TMT6plex)	1648.9	0	**1.1**	**1.1**	**1.2**	**1.2**
																	LDYGQHVVAGTPGR	6	1	1	7661920	N-Term(TMT6plex)	1698.9	0	**1.2**	**1.2**	**1.2**	**1.3**
																	VDWLTEK	11	4	3	83700235;4503529;7661920	N-Term(TMT6plex); K7(TMT6plex)	1348.8	0				
																	KVDWLTEK	12	4	3	83700235;4503529;7661920	N-Term(TMT6plex); K1(TMT6plex); K8(TMT6plex)	1706.0	1				
																	GLLALGDYMNVQCHACIGGTNVGEDIR	7	1	1	7661920	N-Term(TMT6plex); M9(Oxidation); C13(Carbamidomethyl); C16(Carbamidomethyl)	3178.5	0	**1.3**	**1.4**	**1.3**	**1.4**
																	YLPPATQVVLISATLPHEILEMTNK	6	1	1	7661920	N-Term(TMT6plex); K25(TMT6plex)	3236.8	0	**1.4**	**1.6**	**1.8**	**1.3**
																	GIYAYGFEKPSAIQQR	11	4	3	83700235;4503529;7661920	N-Term(TMT6plex); K9(TMT6plex)	2286.3	0				
																	EANFTVSSMHGDMPQK	11	1	1	7661920	N-Term(TMT6plex); M9(Oxidation); M13(Oxidation); K16(TMT6plex)	2269.1	0	**1.3**	**1.3**	**1.3**	**1.3**
																	EQIYDVYR	14	1	1	7661920	N-Term(TMT6plex)	1314.7	0	**1.1**	**1.2**	**1.2**	**1.3**
																	KGVAINFVK	4	1	1	7661920	N-Term(TMT6plex); K1(TMT6plex); K9(TMT6plex)	1663.1	1	**1.0**	**1.1**	**1.1**	**1.1**
																	MLVLDEADEMLNK	7	1	1	7661920	N-Term(TMT6plex); M1(Oxidation); K13(TMT6plex)	1995.1	0	**1.5**	**1.7**	**1.6**	**1.6**
																	DIEQYYSTQIDEMPMNVADLI	12	1	1	7661920	N-Term(TMT6plex); M15(Oxidation)	2733.3	0	**1.6**	**1.7**	**1.7**	**1.6**
																	VLISTDVWAR	4	1	1	7661920	N-Term(TMT6plex)	1388.8	0	**1.3**	**1.3**	**1.5**	**1.4**

Accession	RefSeq Accession	Gene Symbol	Entrez Gene ID	Description	Σ Coverage	Σ# Proteins	Σ# Unique Peptides	Σ# Peptides	Σ# PSMs	Het-1A-Smoke - 2M/Parental	Het-1A-Smoke - 4M/Parental	Het-1A-Smoke - 6M/Parental	Het-1A-Smoke - 8M/Parental	# AAs	MW [kDa]	calc. pI	Sequence	# PSMs	# Proteins	# Protein Groups	Protein Group Accessions	Modifications	MH+ [Da]	# Missed Cleavages	Het-1A-Smoke - 2M/Parental	Het-1A-Smoke - 4M/Parental	Het-1A-Smoke - 6M/Parental	Het-1A-Smoke - 8M/Parental
																	YLPPATQVVLISATLPHEILEMTNK	31	1	1	7661920	N-Term(TMT6plex); M22(Oxidation); K25(TMT6plex)	3252.8	0	1.3	1.2	1.5	1.5
																	EANFTVSSMHGDMPQK	2	1	1	7661920	N-Term(TMT6plex); M13(Oxidation); K16(TMT6plex)	2253.1	0	1.2	1.3	1.6	1.4
																	KLDYGQHVVAGTPGR	12	1	1	7661920	N-Term(TMT6plex); K1(TMT6plex)	2056.2	1	1.7	1.3	1.2	1.3
																	RDELTLEGIK	7	1	1	7661920	N-Term(TMT6plex); K10(TMT6plex)	1632.0	1	1.3	1.3	1.2	1.3
																	EANFTVSSMHGDMPQK	2	1	1	7661920	N-Term(TMT6plex); K16(TMT6plex)	2237.1	0	1.7	1.4	1.6	1.3
																	ETQALILAPTR	13	1	1	7661920	N-Term(TMT6plex)	1441.9	0	1.3	1.3	1.3	1.3
																	MLVLDEADEMLNK	29	1	1	7661920	N-Term(TMT6plex); M1(Oxidation); M10(Oxidation); K13(TMT6plex)	2011.0	0	1.1	1.1	1.0	1.3
																	ELAVQIQK	14	1	1	7661920	N-Term(TMT6plex); K8(TMT6plex)	1386.9	0	1.2	1.2	1.3	1.3
																	GVAINFVK	3	1	1	7661920	N-Term(TMT6plex); K8(TMT6plex)	1305.8	0	1.0	1.1	1.1	1.2
																	GLDVPQVSLIINYDLPNNR	8	1	1	7661920	N-Term(TMT6plex)	2369.3	0	1.4	1.5	1.4	1.5
																	LLKEEDMTK	1	1	1	7661920	N-Term(TMT6plex); K3(TMT6plex); K9(TMT6plex)	1794.1	1	1.5	1.7	1.8	1.4
																	QFFVAVER	4	1	1	7661920	N-Term(TMT6plex)	1224.7	0	1.2	1.1	1.1	1.2
																	FMTDPIR	2	1	1	7661920	N-Term(TMT6plex); M2(Oxidation)	1124.6	0	1.1	0.9	1.0	1.0
																	VFDMIR	1	1	1	7661920	N-Term(TMT6plex)	1009.6	0	1.6	1.5	2.0	1.3
157389005	NP_001739.2	CAPN2	824	calpain-2 catalytic subunit isoform 1	67.86	3	37	37	368	1.5	1.7	1.9	1.6	700	80.0	4.98												
																	LPPGEYILVPSTFEPNK	15	2	1	157389005	N-Term(TMT6plex); K17(TMT6plex)	2359.3	0	1.6	1.4	1.6	1.5
																	KPPPNLFK	12	2	1	157389005	N-Term(TMT6plex); K1(TMT6plex); K8(TMT6plex)	1628.1	0	1.4	1.8	2.0	1.6
																	IMVDMLDSDGSGK	15	2	1	157389005	N-Term(TMT6plex); M2(Oxidation); M5(Oxidation); K13(TMT6plex)	1857.9	0	1.6	1.6	1.7	1.6
																	LFAQLAGEDAEISAFELQTILR	9	2	1	157389005	N-Term(TMT6plex)	2664.4	0	1.2	1.3	1.3	1.3
																	NYPNTFWMNPQYLIK	9	2	1	157389005	N-Term(TMT6plex); K15(TMT6plex)	2387.3	0	1.7	1.8	2.7	1.5
																	LPPGEYILVPSTFEPNKDGDFCIR	19	2	1	157389005	N-Term(TMT6plex); K17(TMT6plex); C22(Carbamidomethyl)	3222.7	1	1.5	2.0	2.1	1.7
																	NYPNTFWMNPQYLIK	6	2	1	157389005	N-Term(TMT6plex); M8(Oxidation); K15(TMT6plex)	2403.3	0	1.6	1.7	2.0	1.6
																	KADYQAVDDEIEANLEEFDISEDDIDDGFR	30	2	1	157389005	N-Term(TMT6plex); K1(TMT6plex)	3934.8	1	1.6	1.8	2.0	1.7
																	ADYQAVDDEIEANLEEFDISEDDIDDGFR	4	2	1	157389005	N-Term(TMT6plex)	3577.6	0	1.8	1.9	1.8	2.0
																	FADDQLIIDFDNFVR	56	2	1	157389005	N-Term(TMT6plex)	2057.1	0	1.5	1.5	1.5	1.6
																	ADYQAVDDEIEANLEEFDISEDDIDDGFRR	4	2	1	157389005	N-Term(TMT6plex)	3733.7	1	1.6	1.8	1.9	1.8
																	ALEEAGFK	5	2	1	157389005	N-Term(TMT6plex); K8(TMT6plex)	1322.8	0	1.3	1.6	1.7	1.5
																	EAAEGLGSHER	4	1	1	157389005	N-Term(TMT6plex)	1384.7	0	1.3	1.7	1.6	1.6
																	SDTFINLR	6	2	1	157389005	N-Term(TMT6plex)	1194.7	0	1.6	1.5	1.8	1.6
																	KADYQAVDDEIEANLEEFDISEDDIDDGFRR	2	2	1	157389005	N-Term(TMT6plex); K1(TMT6plex)	4090.9	2	1.8	2.7	2.7	2.3
																	NPWGEVEWTGR	10	2	1	157389005	N-Term(TMT6plex)	1559.8	0	1.8	1.7	2.0	1.5
																	IMVDMLDSDGSGK	4	2	1	157389005	N-Term(TMT6plex); M2(Oxidation); K13(TMT6plex)	1841.9	0	1.6	2.1	2.7	1.6
																	HEDGEFWMSFSDFLR	4	2	1	157389005	N-Term(TMT6plex); M8(Oxidation)	2148.0	0	1.2	1.2	1.3	1.4
																	LEICNLTPDTLTSDTYK	12	2	1	157389005	N-Term(TMT6plex); C4(Carbamidomethyl); K17(TMT6plex)	2442.3	0	1.8	2.0	2.2	1.5
																	GHAYSVTGAEEVESNGSLQK	4	2	1	157389005	N-Term(TMT6plex); K20(TMT6plex)	2521.3	0	1.3	1.3	1.5	1.3
																	LEICNLTPDTLTSDTYKK	3	2	1	157389005	N-Term(TMT6plex); C4(Carbamidomethyl); K17(TMT6plex); K18(TMT6plex)	2799.5	1	1.5	2.3	2.1	1.9
																	LEEEDEDEEDGESGCTFLVGLIQK	24	2	1	157389005	N-Term(TMT6plex); C15(Carbamidomethyl); K24(TMT6plex)	3199.5	0	1.5	1.7	1.9	1.6
																	RPTEICADPQFIIGGATR	12	1	1	157389005	N-Term(TMT6plex); C6(Carbamidomethyl)	2231.2	0	1.4	1.6	1.7	1.4
																	SDGFSIETCK	11	2	1	157389005	N-Term(TMT6plex); C9(Carbamidomethyl); K10(TMT6plex)	1601.8	0	1.6	1.7	2.3	1.4
																	EFYILWTK	10	2	1	157389005	N-Term(TMT6plex); K8(TMT6plex)	1557.9	0	1.5	1.9	2.1	1.6
																	NECLEAGTLFQDPSFPAIPSALGFK	31	1	1	157389005	N-Term(TMT6plex); C3(Carbamidomethyl); K25(TMT6plex)	3167.7	0	1.6	1.5	2.1	1.4
																	WNDNCPSWNTIDPEER	4	2	1	157389005	N-Term(TMT6plex); C5(Carbamidomethyl)	2262.0	0	1.7	1.8	1.9	1.5
																	GSLLGCSIDITSAADSEAITFQK	4	2	1	157389005	N-Term(TMT6plex); C6(Carbamidomethyl); K23(TMT6plex)	2842.5	0	1.6	2.2	2.8	1.6
																	YLNQDYEALR	7	1	1	157389005	N-Term(TMT6plex)	1513.8	0	1.5	1.5	1.9	1.3
																	MPCQLHQVIVAR	2	2	1	157389005	N-Term(TMT6plex); M1(Oxidation); C3(Carbamidomethyl)	1696.9	0	1.0	1.5	1.4	1.4
																	KALEEAGFK	1	2	1	157389005	N-Term(TMT6plex); K1(TMT6plex); K9(TMT6plex)	1680.0	1	1.5	2.1	2.2	1.8
																	MPCQLHQVIVAR	2	2	1	157389005	N-Term(TMT6plex); C3(Carbamidomethyl)	1680.9	0	1.4	1.6	1.8	1.5
																	DGELLFVHSAEGSEFWSALLEK	1	2	1	157389005	N-Term(TMT6plex); K22(TMT6plex)	2922.5	0				
																	MGEDMHTIGFGIYEVPEELSGQTNIHLSK	2	2	1	157389005	N-Term(TMT6plex); M1(Oxidation); M5(Oxidation); K29(TMT6plex)	3722.8	0				
																	ELGPYSSK	9	1	1	157389005	N-Term(TMT6plex); K8(TMT6plex)	1338.8	0	1.4	1.6	1.7	1.5
																	LETLFK	11	3	1	157389005	N-Term(TMT6plex); K6(TMT6plex)	1208.8	0	1.5	1.3	2.0	1.3
																	DGDFCIR	1	2	1	157389005	N-Term(TMT6plex); C5(Carbamidomethyl)	1111.5	0	1.3	1.3	1.7	1.2
																	INGCYEALSGGATTEGFEDFTGGIAEWYELK	1	2	1	157389005	N-Term(TMT6plex); C4(Carbamidomethyl); K31(TMT6plex)	3843.8	0	1.5	1.4	1.6	1.3
																	AGIAAK	1	1	1	157389005	N-Term(TMT6plex); K6(TMT6plex)	988.7	0	1.1	1.4	1.2	1.3
																	NFFLTNR	1	2	1	157389005	N-Term(TMT6plex)	1140.6	0	1.7	1.7	1.8	1.5
4506605	NP_000969.1	RPL23	9349	60S ribosomal protein L23	67.86	1	8	8	211	1.3	1.4	1.5	1.3	140	14.9	10.51												
																	LPAAGVGDMVMATVK	52	1	1	4506605	N-Term(TMT6plex); M9(Oxidation); M11(Oxidation); K15(TMT6plex)	1950.1	0	1.3	1.4	1.5	1.3
																	IASNAGSIA	7	1	1	4506605	N-Term(TMT6plex)	1032.6	0	1.3	1.4	1.4	1.4
																	ISLGLPVGAVINCADNTGAK	38	1	1	4506605	N-Term(TMT6plex); C13(Carbamidomethyl); K20(TMT6plex)	2428.4	0	1.3	1.3	1.5	1.2
																	LPAAGVGDMVMATVK	2	1	1	4506605	N-Term(TMT6plex); K15(TMT6plex)	1918.1	0	2.0	1.9	2.4	1.6
																	DGVFLYFEDNAGVIVNNK	35	1	1	4506605	N-Term(TMT6plex); K18(TMT6plex)	2472.3	0	1.2	1.2	1.3	1.3
																	LPAAGVGDMVMATVK	25	1	1	4506605	N-Term(TMT6plex); M11(Oxidation); K15(TMT6plex)	1934.1	0	1.4	1.6	1.8	1.4
																	NLYIISVK	17	1	1	4506605	N-Term(TMT6plex); K8(TMT6plex)	1407.9	0	1.2	1.0	1.3	1.1
																	GSAITGPVAK	23	1	1	4506605	N-Term(TMT6plex); K10(TMT6plex)	1358.8	0	1.0	1.2	1.2	1.3
																	ECADLWPR	9	1	1	4506605	N-Term(TMT6plex); C2(Carbamidomethyl)	1275.6	0	1.2	1.2	1.3	1.2
																	GGSSGAK	3	1	1	4506605	N-Term(TMT6plex); K7(TMT6plex)	1021.6	0	1.0	1.1	1.2	1.2
156523968	NP_001609.2	PARP1	142	poly [ADP-ribose] polymerase 1	67.85	1	64	64	646	1.3	1.4	1.4	1.4	1014	113.0	8.88												
																	NTHATTHNAYDLEVIDIFK	6	1	1	156523968	N-Term(TMT6plex); K19(TMT6plex)	2660.4	0	1.2	1.3	1.4	1.4
																	AMVEYEIDLQK	21	1	1	156523968	N-Term(TMT6plex); M2(Oxidation); K11(TMT6plex)	1813.0	0	1.3	1.4	1.4	1.5
																	EELGFRPEYSASQLK	8	1	1	156523968	N-Term(TMT6plex); K15(TMT6plex)	2212.2	0	1.2	1.3	1.4	1.3
																	FYTLIPHDFGMK	13	1	1	156523968	N-Term(TMT6plex); M11(Oxidation); K12(TMT6plex)	1943.1	0	1.4	1.1	1.3	1.3
																	QIQAAYSILSEVQQAVSQGSSDSQILDLSNR	33	1	1	156523968	N-Term(TMT6plex)	3564.8	0	1.5	1.4	2.3	1.4
																	GGSDDSSKDPIDVNYEK	16	1	1	156523968	N-Term(TMT6plex); K8(TMT6plex); K17(TMT6plex)	2513.3	1	1.1	2.1	1.4	1.7
																	LPKPVQDLIK	4	1	1	156523968	N-Term(TMT6plex); K3(TMT6plex); K10(TMT6plex)	1838.2	0	1.3	1.7	1.5	1.6
																	QQVPSGESAILDR	25	1	1	156523968	N-Term(TMT6plex)	1628.9	0	1.5	1.4	1.5	1.4
																	TAEAGGVTGK	17	1	1	156523968	N-Term(TMT6plex); K10(TMT6plex)	1348.8	0	1.3	1.1	1.2	1.1
																	VADGMVFGALLPCEECSGQLVFK	16	1	1	156523968	N-Term(TMT6plex); M5(Oxidation); C13(Carbamidomethyl); C16(Carbamidomethyl); K23(TMT6plex)	3001.5	0	1.4	1.6	1.6	1.7
																	GQDGIGSK	7	1	1	156523968	N-Term(TMT6plex); K8(TMT6plex)	1219.7	0	1.2	1.3	1.3	1.3
																	SANYCHTSQGDPIGLILLGEVALGNMYELK	2	1	1	156523968	N-Term(TMT6plex); C5(Carbamidomethyl); M26(Oxidation); K30(TMT6plex)	3737.9	0	2.3	2.5	1.6	1.7
																	MIFDVESMK	7	1	1	156523968	N-Term(TMT6plex); M8(Oxidation); K9(TMT6plex)	1573.8	0	1.4	1.9	1.8	1.6
																	VVSEDFLQDVSASTK	39	1	1	156523968	N-Term(TMT6plex); K15(TMT6plex)	2083.1	0	1.3	1.4	1.5	1.4
																	VFSATLGLVDIVK	30	1	1	156523968	N-Term(TMT6plex); K13(TMT6plex)	1820.1	0	1.5	1.2	1.5	1.2
																	HASHISK	2	1	1	156523968	N-Term(TMT6plex); K7(TMT6plex)	1237.7	0	1.1	1.4	1.3	1.4
																	VCSTNDLK	9	1	1	156523968	N-Term(TMT6plex); C2(Carbamidomethyl); K8(TMT6plex)	1394.8	0	1.4	1.2	1.4	1.2
																	LTVNPGTK	8	1	1	156523968	N-Term(TMT6plex); K8(TMT6plex)	1287.8	0	1.3	1.4	1.4	1.5
																	IAPPEAPVTGYMFGK	20	1	1	156523968	N-Term(TMT6plex); M12(Oxidation); K15(TMT6plex)	2052.1	0	1.3	1.5	1.5	1.4
																	MAIMVQSPMFDGK	12	1	1	156523968	N-Term(TMT6plex); M1(Oxidation); M4(Oxidation); M9(Oxidation); K13(TMT6plex)	1961.0	0	1.2	1.2	1.1	1.3
																	SDAYYCTGDVTAWTK	8	1	1	156523968	N-Term(TMT6plex); C6(Carbamidomethyl); K15(TMT6plex)	2196.1	0	1.3	1.3	1.5	1.2
																	VEMLDNLLDIEVAYSLLR	4	1	1	156523968	N-Term(TMT6plex)	2335.3	0	1.8	2.2	2.2	1.7
																	TTNFAGILSQGLR	8	1	1	156523968	N-Term(TMT6plex)	1606.9	0	1.6	1.3	1.4	1.1
																	IAPPEAPVTGYMFGK	6	1	1	156523968	N-Term(TMT6plex); K15(TMT6plex)	2036.1	0	1.4	1.6	1.6	1.4
																	VGTVIGSNK	4	1	1	156523968	N-Term(TMT6plex); K9(TMT6plex)	1332.8	0	1.3	1.2	1.4	1.2
																	FYTLIPHDFGMK	4	1	1	156523968	N-Term(TMT6plex); K12(TMT6plex)	1927.1	0	1.6	1.2	1.6	1.2
																	LEQMPSKEDAIEHFMK	5	1	1	156523968	N-Term(TMT6plex); M4(Oxidation); K7(TMT6plex); M15(Oxidation); K16(TMT6plex)	2652.4	1	1.1	1.2	1.3	1.3
																	MIFDVESMK	2	1	1	156523968	N-Term(TMT6plex); K9(TMT6plex)	1557.8	0	2.1	2.3	2.9	1.6
																	GFSLLATEDKEALK	1	1	1	156523968	N-Term(TMT6plex); K10(TMT6plex); K14(TMT6plex)	2209.3	1	1.3	1.5	1.4	1.5
																	FYPLEIDYGQDEEAVK	16	1	1	156523968	N-Term(TMT6plex); K16(TMT6plex)	2374.2	0	1.4	1.4	1.4	1.4
																	GGAAVDPDSGLEHSAHVLEK	11	1	1	156523968	N-Term(TMT6plex); K20(TMT6plex)	2447.3	0	1.2	1.5	1.5	1.4
																	SLQELFLAHILSPWGAEVK	22	1	1	156523968	N-Term(TMT6plex); K19(TMT6plex)	2596.5	0	1.5	1.6	1.8	1.5
																	GTNSYYK	2	1	1	156523968	N-Term(TMT6plex); K7(TMT6plex)	1290.7	0	1.2	1.5	1.6	1.4
																	IFPPETSASVAATPPPSTASAPAAVNSSASADKPLSNMK	5	1	1	156523968	N-Term(TMT6plex); K33(TMT6plex); M38(Oxidation); K39(TMT6plex)	4471.4	0	1.2	1.4	1.3	1.5
																	LQLLEDDKENR	3	1	1	156523968	N-Term(TMT6plex); K8(TMT6plex)	1831.0	1	1.2	1.4	1.5	1.4
																	KFYPLEIDYGQDEEAVK	14	1	1	156523968	N-Term(TMT6plex); K1(TMT6plex); K17(TMT6plex)	2731.5	1	1.4	1.4	1.4	1.4

Accession	RefSeq Accession	Gene Symbol	Entrez Gene ID	Description	Σ Coverage	Σ# Proteins	Σ# Unique Peptides	Σ# Peptides	Σ# PSMs	Het-1A-Smoke - 2M/Parental	Het-1A-Smoke - 4M/Parental	Het-1A-Smoke - 6M/Parental	Het-1A-Smoke - 8M/Parental	# AAs	MW [kDa]	calc. pI	Sequence	# PSMs	# Proteins	# Protein Groups	Protein Group Accessions	Modifications	MH+ [Da]	# Missed Cleavages	Het-1A-Smoke - 2M/Parental	Het-1A-Smoke - 4M/Parental	Het-1A-Smoke - 6M/Parental	Het-1A-Smoke - 8M/Parental
																	VADGMVFGALLPCEECSGQLVFK	12	1	1	156523968	N-Term(TMT6plex); C13(Carbamidomethyl); C16(Carbamidomethyl); K23(TMT6plex)	2985.5	0	1.7	2.0	2.3	1.4
																	GIYFADMVSK	22	1	1	156523968	N-Term(TMT6plex); M7(Oxidation); K10(TMT6plex)	1604.9	0	1.1	1.3	1.3	1.4
																	AEPVEVVAPR	14	1	1	156523968	N-Term(TMT6plex)	1295.8	0	1.4	1.4	1.5	1.5
																	ELLIFNK	8	1	1	156523968	N-Term(TMT6plex); K7(TMT6plex)	1334.8	0	1.3	1.5	1.5	1.5
																	AQNDLIWNIKDELK	16	1	1	156523968	N-Term(TMT6plex); K10(TMT6plex); K14(TMT6plex)	2387.4	1	1.3	1.6	1.5	1.6
																	AQNDLIWNIK	16	1	1	156523968	N-Term(TMT6plex); K10(TMT6plex)	1673.0	0	1.2	1.3	1.4	1.2
																	HPDVEVDGFSELR	18	1	1	156523968	N-Term(TMT6plex)	1728.9	0	1.3	1.4	1.5	1.4
																	WYHPGCFVK	12	1	1	156523968	N-Term(TMT6plex); C6(Carbamidomethyl); K9(TMT6plex)	1651.9	0	1.6	1.4	1.5	1.4
																	EDAIEHFMK	5	1	1	156523968	N-Term(TMT6plex); K9(TMT6plex)	1577.8	0	1.6	2.0	2.0	1.9
																	KLTVNPGTK	7	1	1	156523968	N-Term(TMT6plex); K1(TMT6plex); K9(TMT6plex)	1645.1	1	1.2	1.4	1.3	1.4
																	LTGTANK	4	1	1	156523968	N-Term(TMT6plex); K7(TMT6plex)	1162.7	0	1.3	1.2	1.4	1.2
																	TLGDFAAEYAK	12	1	1	156523968	N-Term(TMT6plex); K11(TMT6plex)	1643.9	0	1.4	1.4	1.4	1.4
																	KGDEVDGVDEVAK	10	1	1	156523968	N-Term(TMT6plex); K1(TMT6plex); K13(TMT6plex)	2048.2	1	1.3	1.5	1.5	1.5
																	EDAIEHFMK	2	1	1	156523968	N-Term(TMT6plex); M8(Oxidation); K9(TMT6plex)	1593.8	0	1.1	1.3	1.4	1.4
																	MAIMVQSPMFDGK	4	1	1	156523968	N-Term(TMT6plex); M4(Oxidation); M9(Oxidation); K13(TMT6plex)	1945.0	0	1.3	1.5	1.5	1.4
																	VVDRDSEEAEIIR	9	1	1	156523968	N-Term(TMT6plex)	1759.9	1	1.4	1.5	1.4	1.4
																	VEMLDNLLDIEVAYSLLR	6	1	1	156523968	N-Term(TMT6plex); M3(Oxidation)	2351.3	0	1.7	1.4	1.5	1.5
																	LEQMPSKEDAIEHFMK	1	1	1	156523968	N-Term(TMT6plex); K7(TMT6plex); M15(Oxidation); K16(TMT6plex)	2636.4	1	1.4	1.7	1.8	1.6
																	GFSLLATEDKEALKK	1	1	1	156523968	N-Term(TMT6plex); K10(TMT6plex); K14(TMT6plex); K15(TMT6plex)	2566.5	2	1.1	1.6	1.5	1.7
																	GFSLLATEDK	7	1	1	156523968	N-Term(TMT6plex); K10(TMT6plex)	1538.9	0	1.2	1.3	1.4	1.4
																	ASLCISTK	7	1	1	156523968	N-Term(TMT6plex); C4(Carbamidomethyl); K8(TMT6plex)	1337.8	0	1.2	1.4	1.4	1.2
																	KPPLLNNADSVQAK	4	1	1	156523968	N-Term(TMT6plex); K1(TMT6plex); K14(TMT6plex)	2182.3	0	1.1	1.3	1.3	1.2
																	KTAEAGGVTGK	6	1	1	156523968	N-Term(TMT6plex); K1(TMT6plex); K11(TMT6plex)	1706.0	1	1.1	1.5	1.5	1.4
																	KCSESIPK	2	1	1	156523968	N-Term(TMT6plex); K1(TMT6plex); C2(Carbamidomethyl); K8(TMT6plex)	1636.0	1	1.3	1.5	1.5	1.5
																	SANYCHTSQGDPIGLILLGEVALGNMYELK	4	1	1	156523968	N-Term(TMT6plex); C5(Carbamidomethyl); K30(TMT6plex)	3721.9	0	1.7	1.8	2.1	1.4
																	VPHWYHFSCFWK	1	1	1	156523968	N-Term(TMT6plex); C9(Carbamidomethyl); K12(TMT6plex)	2152.1	0	1.3	1.2	1.4	1.5
																	DPIDVNYEK	2	1	1	156523968	N-Term(TMT6plex); K9(TMT6plex)	1550.8	0	1.5	1.5	1.6	1.8
																	SGAALSK	2	1	1	156523968	N-Term(TMT6plex); K7(TMT6plex)	1091.7	0	1.3	1.5	1.6	1.6
																	FYPLEIDYGQDEEAVKK	4	1	1	156523968	N-Term(TMT6plex); K16(TMT6plex); K17(TMT6plex)	2731.5	1				
																	MVDPEKPQLGMIDR	2	1	1	156523968	N-Term(TMT6plex); M1(Oxidation); K6(TMT6plex); M11(Oxidation)	2119.1	0	1.3	1.5	1.6	1.5
																	CSESIPK	1	1	1	156523968	N-Term(TMT6plex); C1(Carbamidomethyl); K7(TMT6plex)	1278.7	0	1.2	1.2	1.4	1.0
																	LEQMPSK	2	1	1	156523968	N-Term(TMT6plex); M4(Oxidation); K7(TMT6plex)	1306.7	0	1.2	1.2	1.3	1.3
																	EWVTPK	4	1	1	156523968	N-Term(TMT6plex); K6(TMT6plex)	1217.7	0	1.3	1.5	1.5	1.4
																	EISYLK	3	1	1	156523968	N-Term(TMT6plex); K6(TMT6plex)	1210.7	0	1.2	1.4	1.4	1.4
																	WDDQQK	4	1	1	156523968	N-Term(TMT6plex); K6(TMT6plex)	1277.7	0	1.2	1.4	1.3	1.4
																	ILTLGK	1	1	1	156523968	N-Term(TMT6plex); K6(TMT6plex)	1102.8	0	1.3	1.1	1.4	1.1
																	KMEEVK	1	1	1	156523968	N-Term(TMT6plex); K1(TMT6plex); K6(TMT6plex)	1450.9	1	1.7	2.2	2.3	1.6
83700235	NP_001958.2	EIF4A2	1974	eukaryotic initiation factor 4A-II	67.81	1	9	22	414	1.2	1.4	1.5	1.3	407	46.4	5.48												
																	LQAEAPHIVVGTPGR	10	1	1	83700235	N-Term(TMT6plex)	1774.0	0	1.3	1.3	1.3	1.2
																	DFTVSALHGDMDQK	11	1	1	83700235	N-Term(TMT6plex); M11(Oxidation); K14(TMT6plex)	2038.0	0	1.2	1.3	1.3	1.2
																	MFVLDEADEMLSR	63	3	2	83700235;4503529	N-Term(TMT6plex); M1(Oxidation); M10(Oxidation)	1816.9	0				
																	VDWLTEK	11	4	3	83700235;4503529;7661920	N-Term(TMT6plex); K7(TMT6plex)	1348.8	0				
																	KVDWLTEK	12	4	3	83700235;4503529;7661920	N-Term(TMT6plex); K1(TMT6plex); K8(TMT6plex)	1706.0	1				
																	GIYAYGFEKPSAIQQR	11	4	3	83700235;4503529;7661920	N-Term(TMT6plex); K9(TMT6plex)	2286.3	0				
																	QFYINVER	13	3	2	83700235;4503529	N-Term(TMT6plex)	1297.7	0				
																	ELAQQIQK	37	3	2	83700235;4503529	N-Term(TMT6plex); K8(TMT6plex)	1415.9	0				
																	EHGGPEGMDPDGVIESNWNEIVDNFDDMNLK	11	1	1	83700235	N-Term(TMT6plex); M8(Oxidation); M28(Oxidation); K31(TMT6plex)	3963.8	0	1.2	1.3	1.3	1.3
																	MFVLDEADEMLSR	4	3	2	83700235;4503529	N-Term(TMT6plex)	1784.9	0				
																	EHGGPEGMDPDGVIESNWNEIVDNFDDMNLK	6	1	1	83700235	N-Term(TMT6plex); M28(Oxidation); K31(TMT6plex)	3947.8	0	1.3	1.7	1.9	1.4
																	GIDVQQVSLVINYDLPTNR	100	2	2	83700235;4503529	N-Term(TMT6plex)	2373.3	0				
																	MFVLDEADEMLSR	14	3	2	83700235;4503529	N-Term(TMT6plex); M10(Oxidation)	1800.9	0				
																	GYDVIAQAQSGTGK	35	3	2	83700235;4503529	N-Term(TMT6plex); K14(TMT6plex)	1853.0	0				
																	VLITTDLLAR	18	2	2	83700235;4503529	N-Term(TMT6plex)	1343.8	0				
																	DIETFYNTTVEEMPMNVADLI	4	1	1	83700235	N-Term(TMT6plex); M13(Oxidation); M15(Oxidation)	2706.3	0	1.2	1.4	1.4	1.7
																	LNTSIQVVLLSATMPTDVLEVTK	4	1	1	83700235	N-Term(TMT6plex); K23(TMT6plex)	2930.7	0	1.5	1.6	2.1	1.5
																	DQIYEIFQK	14	1	1	83700235	N-Term(TMT6plex); K9(TMT6plex)	1641.9	0	1.1	1.3	1.5	1.1
																	DIETFYNTTVEEMPMNVADLI	2	1	1	83700235	N-Term(TMT6plex); M15(Oxidation)	2690.3	0	1.4	1.5	1.6	1.7
																	ETQALVLAPTR	5	1	1	83700235	N-Term(TMT6plex)	1427.8	0	1.3	1.3	1.4	1.1
																	KEELTLEGIK	2	1	1	83700235	N-Term(TMT6plex); K1(TMT6plex); K10(TMT6plex)	1847.1	1	1.1	1.5	1.5	1.2
																	LNTSIQVVLLSATMPTDVLEVTK	2	1	1	83700235	N-Term(TMT6plex); M14(Oxidation); K23(TMT6plex)	2946.7	0	1.5	1.3	1.5	1.4
																	GFKDQIYEIFQK	2	1	1	83700235	N-Term(TMT6plex); K3(TMT6plex); K12(TMT6plex)	2203.3	1	1.0	1.8	1.6	1.4
																	LDTLCDLYETLTITQAVIFLNTR	1	3	2	4503529;83700235	N-Term(TMT6plex); C5(Carbamidomethyl)	2942.6	0				
																	VFDMLNR	12	3	2	83700235;4503529	N-Term(TMT6plex); M4(Oxidation)	1139.6	0				
																	VFDMLNR	4	3	2	83700235;4503529	N-Term(TMT6plex)	1123.6	0				
																	AIIPCIK	4	1	2	83700235;4503529	N-Term(TMT6plex); C5(Carbamidomethyl); K7(TMT6plex)	1272.8	0				
																	DIETFYNTTVEEMPMNVADLI	1	1	1	83700235	N-Term(TMT6plex)	2674.3	0	1.3	1.1	1.5	1.3
																	ENYIHR	1	2	2	83700235;4503529	N-Term(TMT6plex)	1060.6	0				
4826964	NP_005044.1	RAD23A	5886	UV excision repair protein RAD23 homolog A isoform 1	67.77	2	1	14	105	1.6	1.8	1.9	2.0	363	39.6	4.58												
																	EDKSPSEESAPTTSPESVSGSVPSSGSSGR	10	3	2	4826964;392996949	N-Term(TMT6plex); K3(TMT6plex)	3380.6	1				
																	TLQQQTFK	11	4	3	4506387;4826964;392996949	N-Term(TMT6plex); K8(TMT6plex)	1451.9	0				
																	AGQGTSAPPEASPTAAPESSTSFPPAPTSGMSHPPPAAR	3	3	2	4826964;392996949	N-Term(TMT6plex); M31(Oxidation)	3944.9	0				
																	SPSEESAPTTSPESVSGSVPSSGSSGR	7	3	2	4826964;392996949	N-Term(TMT6plex)	2779.3	0				
																	QVIQQNPALLPALLQQLGQENPQLLQQISR	14	2	2	4826964;392996949	N-Term(TMT6plex)	3608.0	0				
																	AVEYLLTGIPGSPEPEHGSVQESQVSEQPATEAAGENPLEFLR	2	2	1	4826964	N-Term(TMT6plex)	4792.4	0	1.6	1.8	1.9	2.0
																	DAFPVAGQK	18	5	3	4506387;4826964;392996949	N-Term(TMT6plex); K9(TMT6plex)	1390.8	0				
																	EEDAASTLVTGSEYETMLTEIMSMGYER	10	3	2	4826964;392996949	N-Term(TMT6plex); M17(Oxidation); M24(Oxidation)	3404.5	0				
																	NENLAANFLLSQNFDDE	9	3	2	4826964;392996949	N-Term(TMT6plex)	2183.0	0				
																	ILSDDVPIR	8	3	2	4826964;392996949	N-Term(TMT6plex)	1256.7	0				
																	NFVVVMVTK	3	3	2	4826964;392996949	N-Term(TMT6plex); M6(Oxidation); K9(TMT6plex)	1510.9	0				
																	EEDAASTLVTGSEYETMLTEIMSMGYER	1	3	2	4826964;392996949	N-Term(TMT6plex)	3372.5	0				
																	DQPQFQNMR	2	3	2	4826964;392996949	N-Term(TMT6plex); M8(Oxidation)	1408.7	0				
																	MEPDETVK	5	3	2	4826964;392996949	N-Term(TMT6plex); M1(Oxidation); K8(TMT6plex)	1422.8	0				
																	AGQGTSAPPEASPTAAPESSTSFPPAPTSGMSHPPPAAR	1	3	2	4826964;392996949	N-Term(TMT6plex)	3928.9	0				
																	EKIEAEK	1	3	2	4826964;392996949	N-Term(TMT6plex); K2(TMT6plex); K7(TMT6plex)	1533.9	1				
508083035;21070978	NP_001265233.1	DIABLO	56616	diablo homolog, mitochondrial isoform 2	67.74	5	10	11	79	1.3	1.3	1.3	1.3	186	21.2	4.81												
																	LETTWMTAVGLSEMAAEAAYQTGADQASITAR	23	5	1	508083035;21070978	N-Term(TMT6plex); M6(Oxidation); M14(Oxidation)	3605.7	0	1.3	1.4	1.3	1.6
																	LETTWMTAVGLSEMAAEAAYQTGADQASITAR	14	5	1	508083035;21070978	N-Term(TMT6plex); M6(Oxidation)	3589.7	0	1.4	1.4	1.3	1.3
																	LQVEEVHQLSR	10	5	1	508083035;21070978	N-Term(TMT6plex)	1566.9	0	1.2	1.6	1.3	1.5
																	MNSEEEDEVWQVIIGAR	2	5	1	508083035;21070978	N-Term(TMT6plex)	2234.1	0	0.9	1.0	1.0	0.9
																	LAEAQIEELR	4	5	1	508083035;21070978	N-Term(TMT6plex)	1400.8	0	1.5	1.2	1.2	1.3
																	AESEQEAYLR	4	5	1	508083035;21070978	N-Term(TMT6plex)	1424.7	0	1.3	1.0	1.2	1.1
																	LETTWMTAVGLSEMAAEAAYQTGADQASITAR	4	5	1	508083035;21070978	N-Term(TMT6plex)	3573.7	0	1.6	1.7	1.4	1.5
																	QYTSLLGK	4	5	1	508083035;21070978	N-Term(TMT6plex); K8(TMT6plex)	1367.8	0	1.2	0.9	1.2	1.0
																	AVYTLTSLYR	1	3	1	508083035;21070978	N-Term(TMT6plex)	1415.8	0	1.4	1.3	1.4	1.3
																	NHIQLVK	5	5	1	508083035;21070978	N-Term(TMT6plex); K7(TMT6plex)	1309.8	0	1.2	0.9	1.2	1.0
																	AESEQEAYLRED	2	5	1	508083035;21070978	N-Term(TMT6plex)	1668.8	1	1.5	1.1	1.4	1.3
																	SEPHSLSSEALMR	4	2	1	508083035;21070978	N-Term(TMT6plex); M12(Oxidation)	1688.8	0	1.2	1.2	1.4	1.2
																	HQEYLK	2	6	2	508083035;21070978;13236553	N-Term(TMT6plex); K6(TMT6plex)	1275.7	0				
4505585	NP_002563.1	PAFAH1B2	5049	platelet-activating factor acetylhydrolase IB subunit beta isoform a	67.69	5	8	8	56	1.7	1.3	1.8	1.4	229	25.6	5.92												
																	LANVQLLDTDGGFVHSDGAISCHDMFDFLHLTGGGYAK	2	1	1	4505585	N-Term(TMT6plex); C22(Carbamidomethyl); K38(TMT6plex)	4537.2	0	1.6	1.4	2.3	1.1

Accession	RefSeq Accession	Gene Symbol	Entrez Gene ID	Description	Σ Coverage	Σ# Proteins	Σ# Unique Peptides	Σ# Peptides	Σ# PSMs	Het-1A-Smoke - 2M/Parental	Het-1A-Smoke - 4M/Parental	Het-1A-Smoke - 6M/Parental	Het-1A-Smoke - 8M/Parental	# AAs	MW [kDa]	calc. pI	Sequence	# PSMs	# Proteins	# Protein Groups	Protein Group Accessions	Modifications	MH+ [Da]	# Missed Cleavages	Het-1A-Smoke - 2M/Parental	Het-1A-Smoke - 4M/Parental	Het-1A-Smoke - 6M/Parental	Het-1A-Smoke - 8M/Parental
																	IIVLGLLPR	24	1	1	4505585	N-Term(TMT6plex)	1222.8	0	**1.9**	**1.3**	**1.8**	**1.4**
																	ICKPLHELIMQLLEETPEEK	4	1	1	4505585	N-Term(TMT6plex); C2(Carbamidomethyl); K3(TMT6plex); K20(TMT6plex)	3137.8	0	**1.6**	**1.4**	**2.0**	**1.2**
																	DKEPDVLFVGDSMVQLMQQYEIWR	4	4	1	4505585	N-Term(TMT6plex); K2(TMT6plex)	3384.7	1	**1.9**	**1.4**	**2.1**	**1.5**
																	ELFSPLHALNFGIGGDTTR	14	4	1	4505585	N-Term(TMT6plex)	2274.2	0	**1.4**	**1.4**	**1.6**	**1.4**
																	DKEPDVLFVGDSMVQLMQQYEIWR	4	4	1	4505585	N-Term(TMT6plex); K2(TMT6plex); M13(Oxidation); M17(Oxidation)	3416.7	1	**1.4**	**1.3**	**1.4**	**1.4**
																	ICKPLHELIMQLLEETPEEK	1	1	1	4505585	N-Term(TMT6plex); C2(Carbamidomethyl); K3(TMT6plex); M10(Oxidation); K20(TMT6plex)	3153.7	0	**1.5**	**1.5**	**1.6**	**1.6**
																	WMSQHNR	1	4	1	4505585	N-Term(TMT6plex); M2(Oxidation)	1203.6	0	**1.3**	**1.5**	**1.5**	**1.8**
																	VIVVWVGTNNHENTAEEVAGGIEAIVQLINTR	1	4	1	4505585	N-Term(TMT6plex)	3675.0	0	**1.7**	**1.4**	**2.7**	**1.0**
																	VNQLLK	1	2	1	4505585	N-Term(TMT6plex); K6(TMT6plex)	1172.8	0	**1.5**	**1.2**	**1.5**	**1.4**
392996949	NP_001257291.1	**RAD23A**	5886	**UV excision repair protein RAD23 homolog A isoform 2**	67.68	1	1	14	106	**1.3**	**1.3**	**1.2**	**1.6**	362	39.5	4.58												
																	EDKSPSEESAPTTSPESVSGSVPSSGSSGR	10	3	2	4826964;392996949	N-Term(TMT6plex); K3(TMT6plex)	3380.6	1				
																	TLQQQTFK	11	4	3	4506387;4826964;392996949	N-Term(TMT6plex); K8(TMT6plex)	1451.9	0				
																	AGQGTSAPPEASPTAAPESSTSFPPAPTSGMSHPPPAAR	3	3	2	4826964;392996949	N-Term(TMT6plex); M31(Oxidation)	3944.9	0				
																	SPSEESAPTTSPESVSGSVPSSGSSGR	7	3	2	4826964;392996949	N-Term(TMT6plex)	2779.3	0				
																	QVIQQNPALLPALLQQLGQENPQLLQQISR	14	2	2	4826964;392996949	N-Term(TMT6plex)	3608.0	0				
																	AVEYLLTGIPGSPEPEHGSVQESQVSEQPATEAGENPLEFLR	3	1	1	392996949	N-Term(TMT6plex)	4721.4	0	**1.3**	**1.3**	**1.2**	**1.6**
																	DAFPVAGQK	18	5	3	4506387;4826964;392996949	N-Term(TMT6plex); K9(TMT6plex)	1390.8	0				
																	EEDAASTLVTGSEYETMLTEIMSMGYER	10	3	2	4826964;392996949	N-Term(TMT6plex); M17(Oxidation); M24(Oxidation)	3404.5	0				
																	NENLAANFLLSQNFDDE	9	3	2	4826964;392996949	N-Term(TMT6plex)	2183.0	0				
																	ILSDDVPIR	8	3	2	4826964;392996949	N-Term(TMT6plex)	1256.7	0				
																	NFVVVMVTK	3	3	2	4826964;392996949	N-Term(TMT6plex); M6(Oxidation); K9(TMT6plex)	1510.9	0				
																	EEDAASTLVTGSEYETMLTEIMSMGYER	1	3	2	4826964;392996949	N-Term(TMT6plex)	3372.5	0				
																	DQPQFQNMR	2	3	2	4826964;392996949	N-Term(TMT6plex); M8(Oxidation)	1408.7	0				
																	MEPDETVK	5	3	2	4826964;392996949	N-Term(TMT6plex); M1(Oxidation); K8(TMT6plex)	1422.8	0				
																	AGQGTSAPPEASPTAAPESSTSFPPAPTSGMSHPPPAAR	1	3	2	4826964;392996949	N-Term(TMT6plex)	3928.9	0				
																	EKIEAEK	1	3	2	4826964;392996949	N-Term(TMT6plex); K2(TMT6plex); K7(TMT6plex)	1533.9	1				
4885385	NP_005315.1	**H3F3B**	3021	**histone H3.3**	67.65	2	2	11	1460	**1.0**	**1.0**	**1.2**	**1.0**	136	15.3	11.27												
																	STELLIR	368	5	4	4504297;4504281;31742503;183076548;4504299;4885385	N-Term(TMT6plex)	1060.7	0				
																	EIAQDFK	534	4	4	4504297;4504281;31742503;183076548;4504299;4885385	N-Term(TMT6plex); K7(TMT6plex)	1308.8	0				
																	YRPGTVALR	307	5	4	4504297;4504281;31742503;183076548;4504299;4885385	N-Term(TMT6plex)	1261.8	0				
																	EIAQDFKTDLR	3	4	4	4504297;4504281;31742503;183076548;4504299;4885385	N-Term(TMT6plex); K7(TMT6plex)	1794.0	1				
																	FQSAAIGALQEASEAYLVGLFEDTNLCAIHAK	194	1	1	4885385	N-Term(TMT6plex); C27(Carbamidomethyl); K32(TMT6plex)	3896.0	0	**1.1**	**0.9**	**1.3**	**0.9**
																	SAPSTGGVK	19	1	1	4885385	N-Term(TMT6plex); K9(TMT6plex)	1261.8	0	**1.2**	**1.1**	**1.0**	**1.1**
																	KLPFQR	1	5	4	4504297;4504281;31742503;183076548;4504299;4885385	N-Term(TMT6plex); K1(TMT6plex)	1246.8	1				
																	KQLATK	16	5	4	4504297;4504281;31742503;183076548;4504299;4885385	N-Term(TMT6plex); K1(TMT6plex); K6(TMT6plex)	1375.9	1				
																	VTIMPKDIQLAR	1	5	4	4504297;4504281;31742503;183076548;4504299;4885385	N-Term(TMT6plex); K6(TMT6plex)	1843.1	1				
																	VTIMPK	9	5	4	4504297;4504281;31742503;183076548;4504299;4885385	N-Term(TMT6plex); M4(Oxidation); K6(TMT6plex)	1162.7	0				
																	VTIMPK	5	5	4	4504297;4504281;31742503;183076548;4504299;4885385	N-Term(TMT6plex); K6(TMT6plex)	1146.7	0				
																	DIQLAR	3	5	4	4504297;4504281;31742503;183076548;4504299;4885385	N-Term(TMT6plex)	944.6	0				
18375503;18375501	NP_542379.1	**APEX1**	328	**DNA-(apurinic or apyrimidinic site) lyase**	67.61	1	20	20	169	**1.2**	**1.2**	**1.3**	**1.5**	318	35.5	8.12												
																	GAVAEDGDELR	6	1	1	18375503;18375501	N-Term(TMT6plex)	1360.7	0	**1.2**	**1.3**	**1.2**	**1.6**
																	KPLVLCGDLNVAHEEIDLR	9	1	1	18375503;18375501	N-Term(TMT6plex); K1(TMT6plex); C6(Carbamidomethyl)	2649.5	0	**1.1**	**1.3**	**1.2**	**1.5**
																	ICSWNVDGLR	6	1	1	18375503;18375501	N-Term(TMT6plex); C2(Carbamidomethyl)	1448.7	0	**1.2**	**1.0**	**1.3**	**1.3**
																	QGFGELLQAVPLADSFR	29	1	1	18375503;18375501	N-Term(TMT6plex)	2077.1	0	**1.4**	**1.2**	**1.4**	**1.5**
																	NDKEAAGEGPALYEDPPDQK	2	1	1	18375503;18375501	N-Term(TMT6plex); K3(TMT6plex); K20(TMT6plex)	2831.5	1	**1.3**	**1.3**	**1.2**	**1.4**
																	NAGFTPQER	6	1	1	18375503;18375501	N-Term(TMT6plex)	1248.7	0	**1.3**	**1.0**	**1.2**	**1.4**
																	LDYFLLSHSLLPALCDSK	23	1	1	18375503;18375501	N-Term(TMT6plex); C15(Carbamidomethyl); K18(TMT6plex)	2550.4	0	**1.2**	**1.2**	**1.4**	**1.6**
																	KNDKEAAGEGPALYEDPPDQK	2	1	1	18375503;18375501	N-Term(TMT6plex); K1(TMT6plex); K4(TMT6plex); K21(TMT6plex)	3188.7	2	**1.3**	**1.4**	**1.5**	**2.1**
																	EEAPDILCLQETK	10	1	1	18375503;18375501	N-Term(TMT6plex); C8(Carbamidomethyl); K13(TMT6plex)	2004.1	0	**1.2**	**1.2**	**1.3**	**1.5**
																	VSYGIGDEEHDQEGR	15	1	1	18375503;18375501	N-Term(TMT6plex)	1919.9	0	**1.1**	**1.2**	**1.2**	**1.7**
																	EAAGEGPALYEDPPDQK	16	1	1	18375503;18375501	N-Term(TMT6plex); K17(TMT6plex)	2245.1	0	**1.1**	**1.1**	**1.1**	**1.4**
																	GLDWVKEEAPDILCLQETK	6	1	1	18375503;18375501	N-Term(TMT6plex); K6(TMT6plex); C14(Carbamidomethyl); K19(TMT6plex)	2931.6	1	**1.1**	**1.6**	**1.4**	**2.0**
																	GAVAEDGDELRTEPEAK	5	1	1	18375503;18375501	N-Term(TMT6plex); K17(TMT6plex)	2245.2	1	**1.2**	**1.3**	**1.4**	**1.6**
																	LPAELQELPGLSHQYWSAPSDKEGYSGVGLLSR	2	1	1	18375503;18375501	N-Term(TMT6plex); K22(TMT6plex)	4043.2	1	**1.1**	**1.5**	**1.3**	**2.2**
																	EGYSGVGLLSR	8	1	1	18375503;18375501	N-Term(TMT6plex)	1366.8	0	**1.2**	**1.2**	**1.3**	**1.5**
																	KGAVAEDGDELR	5	1	1	18375503;18375501	N-Term(TMT6plex); K1(TMT6plex)	1717.9	1	**1.4**	**1.2**	**1.2**	**1.8**
																	LPAELQELPGLSHQYWSAPSDK	16	1	1	18375503;18375501	N-Term(TMT6plex); K22(TMT6plex)	2924.6	0	**1.1**	**1.1**	**1.2**	**1.7**
																	VIVAEFDSFVLVTAYVPNAGR	1	1	1	18375503;18375501	N-Term(TMT6plex)	2496.4	0	**1.2**	**1.2**	**1.2**	**1.2**
																	TEPEAK	1	1	1	18375503;18375501	N-Term(TMT6plex); K6(TMT6plex)	1132.7	0	**1.0**	**0.8**	**1.0**	**1.4**
																	ALGSDHCPITLYLAL	1	1	1	18375503;18375501	N-Term(TMT6plex); C7(Carbamidomethyl)	1873.0	0	**2.0**	**1.6**	**1.5**	**1.5**
88900491	NP_938149.2	**GANAB**	23193	**neutral alpha-glucosidase AB isoform 3 precursor**	67.60	2	2	46	585	**1.4**	**1.5**	**1.6**	**1.6**	966	109.4	6.24												
																	AHAHLDTGR	12	5	2	88900491;38202257	N-Term(TMT6plex)	1206.7	0				
																	MMDYLQGSGETPQTDVR	44	5	2	88900491;38202257	N-Term(TMT6plex); M1(Oxidation); M2(Oxidation)	2189.0	0				
																	NPEPELLVR	12	5	2	88900491;38202257	N-Term(TMT6plex)	1295.8	0				
																	VPDVLVADPPIAR	33	3	2	88900491;38202257	N-Term(TMT6plex)	1590.9	0				
																	DAQHYGGWEHR	4	5	2	88900491;38202257	N-Term(TMT6plex)	1584.8	0				
																	LVAIVDPHIK	11	5	2	88900491;38202257	N-Term(TMT6plex); K10(TMT6plex)	1563.0	0				
																	SIRPGLSPYR	4	2	2	88900491;38202257	N-Term(TMT6plex)	1374.8	0				
																	AEKDEPGAWEETFK	20	5	2	88900491;38202257	N-Term(TMT6plex); K3(TMT6plex); K14(TMT6plex)	2324.2	1				
																	LSFQHDPETSVLVLR	22	5	2	88900491;38202257	N-Term(TMT6plex)	1970.1	0				
																	VTEGGEPYR	15	5	2	88900491;38202257	N-Term(TMT6plex)	1236.6	0				
																	DGSDYEGWCWPGSAGYPDFTNPTMR	10	5	2	88900491;38202257	N-Term(TMT6plex); C9(Carbamidomethyl); M24(Oxidation)	3111.3	0				
																	VVIIGAGKPAAVVLQTK	13	5	2	88900491;38202257	N-Term(TMT6plex); K8(TMT6plex); K17(TMT6plex)	2351.5	0				
																	DLGIFWLNAAETWVDISSNTAGK	10	5	2	88900491;38202257	N-Term(TMT6plex); K23(TMT6plex)	2966.5	0				
																	GLLEFEHQR	21	5	2	88900491;38202257	N-Term(TMT6plex)	1357.7	0				
																	DEPGAWEETFK	11	5	2	88900491;38202257	N-Term(TMT6plex); K11(TMT6plex)	1766.9	0				
																	DENSVELTMAEGPYK	38	5	2	88900491;38202257	N-Term(TMT6plex); M9(Oxidation); K15(TMT6plex)	2157.1	0				
																	REPWLLPSQHNDIIR	7	5	2	88900491;38202257	N-Term(TMT6plex)	2103.2	1				
																	EPWLLPSQHNDIIR	6	5	2	88900491;38202257	N-Term(TMT6plex)	1947.1	0				
																	DGDKPEETQGK	16	5	2	88900491;38202257	N-Term(TMT6plex); K4(TMT6plex); K11(TMT6plex)	1891.0	0				
																	DENSVELTMAEGPYK	4	5	2	88900491;38202257	N-Term(TMT6plex); K15(TMT6plex)	2141.1	0				
																	DPAEGDGAQPEETPR	8	5	2	88900491;38202257	N-Term(TMT6plex)	1797.8	0				
																	ALLDSLQLGPDSLTVHLIHEVTK	27	2	2	88900491;38202257	N-Term(TMT6plex); K23(TMT6plex)	2957.7	0				
																	MMDYLQGSGETPQTDVR	8	5	2	88900491;38202257	N-Term(TMT6plex); M1(Oxidation)	2173.0	0				
																	WYQMGAYQPFFR	4	5	2	88900491;38202257	N-Term(TMT6plex); M4(Oxidation)	1838.9	0				
																	QYASLTGTQALPPLFSLGYHQSR	14	5	2	88900491;38202257	N-Term(TMT6plex)	2764.5	0				
																	HHGPQTLYLPVTLSSIPVFQR	6	5	2	88900491;38202257	N-Term(TMT6plex)	2619.5	0				
																	DVHNIYGLYVHMATADGLR	2	5	2	88900491;38202257	N-Term(TMT6plex); M12(Oxidation)	2390.2	0				
																	FSFSGNTLVSSSADPEGHFETPIWIER	24	5	2	88900491;38202257	N-Term(TMT6plex)	3239.6	0				
																	FGAVWTGDNTAEWDHLK	32	5	2	88900491;38202257	N-Term(TMT6plex); K17(TMT6plex)	2405.2	0				
																	VLLVLELQGLQK	15	2	2	88900491;38202257	N-Term(TMT6plex); K12(TMT6plex)	1811.2	0				
																	YSLLPFWYTLLYQAHR	13	5	2	88900491;38202257	N-Term(TMT6plex)	2300.2	0				
																	DEADVLEVDQGFDDHNLPCDVIWLDIEHADGK	8	5	2	88900491;38202257	N-Term(TMT6plex); C19(Carbamidomethyl); K32(TMT6plex)	4138.0	0				
																	DVHNIYGLYVHMATADGLR	6	5	2	88900491;38202257	N-Term(TMT6plex)	2374.2	0				
																	KPGINVASDWSIHLR	10	5	2	88900491;38202257	N-Term(TMT6plex); K1(TMT6plex)	2151.2	0				
																	LYNLDVFQYELYNPMALYGSVPVLLAHNPHR	9	5	2	88900491;38202257	N-Term(TMT6plex); M15(Oxidation)	3892.0	0				
																	VNLTLGSIWDK	10	2	1	88900491	N-Term(TMT6plex); K11(TMT6plex)	1704.0	0	**1.3**	**1.6**	**1.5**	**1.6**
																	LYNLDVFQYELYNPMALYGSVPVLLAHNPHR	5	5	2	88900491;38202257	N-Term(TMT6plex)	3876.0	0				
																	LDLLEDR	10	5	2	88900491;38202257	N-Term(TMT6plex)	1102.6	0				
																	SLLLSVNAR	8	5	2	88900491;38202257	N-Term(TMT6plex)	1201.7	0				

Accession	RefSeq Accession	Gene Symbol	Entrez Gene ID	Description	Σ Coverage	Σ# Proteins	Σ# Unique Peptides	Σ# Peptides	Σ# PSMs	Het-1A-Smoke - 2M/Parental	Het-1A-Smoke - 4M/Parental	Het-1A-Smoke - 6M/Parental	Het-1A-Smoke - 8M/Parental	# AAs	MW [kDa]	calc. pI	Sequence	# PSMs	# Proteins	# Protein Groups	Protein Group Accessions	Modifications	MH+ [Da]	# Missed Cleavages	Het-1A-Smoke - 2M/Parental	Het-1A-Smoke - 4M/Parental	Het-1A-Smoke - 6M/Parental	Het-1A-Smoke - 8M/Parental
																	TCEESSFCK	10	2	2	88900491;38202257	N-Term(TMT6plex); C2(Carbamidomethyl); C8(Carbamidomethyl); K9(TMT6plex)	1605.8	0				
																	NLGLYVK	13	5	2	88900491;38202257	N-Term(TMT6plex); K7(TMT6plex)	1264.8	0				
																	AWWANMFSYDNYEGSAPNLFVWNDMNEPSVFNGPEVTMLK	1	5	2	88900491;38202257	N-Term(TMT6plex); M25(Oxidation); M38(Oxidation); K40(TMT6plex)	5161.4	0				
																	SGGMERPFVLAR	1	5	2	88900491;38202257	N-Term(TMT6plex); M4(Oxidation)	1564.8	0				
																	QGSKDPAEGDGAQPEETPR	5	2	1	88900491	N-Term(TMT6plex); K4(TMT6plex)	2427.2	1	1.5	1.5	1.6	1.5
																	AWWANMFSYDNYEGSAPNLFVWNDMNEPSVFNGPEVTMLK	2	5	2	88900491;38202257	N-Term(TMT6plex); M6(Oxidation); M25(Oxidation); M38(Oxidation); K40(TMT6plex)	5177.4	0				
																	WMSETGIIDVFLLLGPSISDVFR	2	5	2	88900491;38202257	N-Term(TMT6plex); M2(Oxidation)	2840.5	0				
																	IDELEPR	8	3	2	88900491;38202257	N-Term(TMT6plex)	1100.6	0				
																	AFFAGSQR	2	5	2	88900491;38202257	N-Term(TMT6plex)	1112.6	0				
																	YFTWDPSR	6	5	2	88900491;38202257	N-Term(TMT6plex)	1300.7	0				
																	GGTIVPR	2	5	2	88900491;38202257	N-Term(TMT6plex)	928.6	0				
																	VDSGYR	1	7	3	4503943;88900491;38202257	N-Term(TMT6plex)	925.5	0				
12667788	NP_002464.1	**MYH9**	4627	**myosin-9**	67.60	1	117	147	3253	**1.6**	**1.6**	**1.8**	**1.5**	1960	226.4	5.60												
																	DFSALESQLQDTQELLQEENR	111	1	1	12667788	N-Term(TMT6plex)	2722.3	0	**1.6**	**1.5**	**1.7**	**1.5**
																	EQLEEEEEAK	27	1	1	12667788	N-Term(TMT6plex); K10(TMT6plex)	1691.9	0	**1.7**	**1.8**	**2.0**	**1.7**
																	NTDQASMPDNTAAQK	52	5	2	13124875;12667788	N-Term(TMT6plex); M7(Oxidation); K15(TMT6plex)	2066.0	0				
																	NMDPLNDNIATLLHQSSDK	30	1	1	12667788	N-Term(TMT6plex); M2(Oxidation); K19(TMT6plex)	2600.3	0	**1.5**	**1.5**	**1.8**	**1.4**
																	LEEEQIILEDQNCK	25	1	1	12667788	N-Term(TMT6plex); C13(Carbamidomethyl); K14(TMT6plex)	2219.2	0	**1.6**	**1.8**	**1.8**	**1.5**
																	ELEDATETADAMNR	61	1	1	12667788	N-Term(TMT6plex); M12(Oxidation)	1810.8	0	**1.7**	**1.9**	**2.1**	**1.7**
																	KFDQLLAEEK	23	11	4	116284394;367460087;13124875;12667788	N-Term(TMT6plex); K1(TMT6plex); K10(TMT6plex)	1908.1	1				
																	EEILAQAK	24	1	1	12667788	N-Term(TMT6plex); K8(TMT6plex)	1359.8	0	**1.6**	**1.7**	**1.8**	**1.6**
																	VMQEQGTHPK	29	1	1	12667788	N-Term(TMT6plex); M2(Oxidation); K10(TMT6plex)	1628.9	0	**1.6**	**1.7**	**2.0**	**1.6**
																	HSQAVEELAEQLEQTK	46	1	1	12667788	N-Term(TMT6plex); K16(TMT6plex)	2298.2	0	**1.7**	**1.4**	**1.6**	**1.3**
																	HSQAVEELAEQLEQTKR	7	1	1	12667788	N-Term(TMT6plex); K16(TMT6plex)	2454.3	1	**1.3**	**1.6**	**1.6**	**1.4**
																	LEVNLQAMK	21	1	1	12667788	N-Term(TMT6plex); M8(Oxidation); K9(TMT6plex)	1519.9	0	**1.5**	**1.6**	**1.8**	**1.6**
																	LKDVLLQVDDER	16	1	1	12667788	N-Term(TMT6plex); K2(TMT6plex)	1901.1	1	**1.5**	**1.6**	**1.7**	**1.5**
																	MQQNIQELEEQLEEEESAR	72	1	1	12667788	N-Term(TMT6plex); M1(Oxidation)	2578.2	0	**1.6**	**1.7**	**1.9**	**1.5**
																	YEILTPNSIPK	16	1	1	12667788	N-Term(TMT6plex); K11(TMT6plex)	1733.0	0	**1.6**	**1.9**	**1.8**	**1.7**
																	TVGQLYK	18	11	4	116284394;367460087;13124875;12667788	N-Term(TMT6plex); K7(TMT6plex)	1266.8	0				
																	ADFCIIHYAGK	16	4	2	367460087;12667788	N-Term(TMT6plex); C4(Carbamidomethyl); K11(TMT6plex)	1753.0	0				
																	IMGIPEEEQMGLLR	38	1	1	12667788	N-Term(TMT6plex); M2(Oxidation); M10(Oxidation)	1877.0	0	**1.5**	**1.6**	**1.7**	**1.5**
																	MEDSVGCLETAEEVK	6	1	1	12667788	N-Term(TMT6plex); M1(Oxidation); C7(Carbamidomethyl); K15(TMT6plex)	2171.1	0	**1.9**	**1.7**	**2.0**	**1.5**
																	DLEAHIDSANK	31	1	1	12667788	N-Term(TMT6plex); K11(TMT6plex)	1670.9	0	**1.4**	**1.6**	**1.7**	**1.5**
																	IIGLDQVAGMSETALPGAFK	68	1	1	12667788	N-Term(TMT6plex); M10(Oxidation); K20(TMT6plex)	2492.4	0	**1.6**	**1.6**	**1.9**	**1.5**
																	QIATLHAQVADMK	22	1	1	12667788	N-Term(TMT6plex); M12(Oxidation); K13(TMT6plex)	1900.1	0	**1.4**	**1.5**	**1.7**	**1.6**
																	EEVGEEAIVELVENGKK	1	1	1	12667788	N-Term(TMT6plex); K16(TMT6plex); K17(TMT6plex)	2559.4	1	**1.6**	**1.7**	**1.8**	**2.6**
																	VKPLLQVSR	16	1	1	12667788	N-Term(TMT6plex); K2(TMT6plex)	1498.0	0	**1.5**	**1.7**	**1.9**	**1.9**
																	LQEMEGTVK	34	1	1	12667788	N-Term(TMT6plex); M4(Oxidation); K9(TMT6plex)	1508.8	0	**1.9**	**1.7**	**1.8**	**1.6**
																	VAAYDKLEK	7	1	1	12667788	N-Term(TMT6plex); K6(TMT6plex); K9(TMT6plex)	1724.1	1	**1.6**	**2.0**	**1.9**	**1.8**
																	IAEFTTNLTEEEEK	49	1	1	12667788	N-Term(TMT6plex); K14(TMT6plex)	2112.1	0	**1.5**	**1.6**	**1.7**	**1.4**
																	IAQLEEELEEEQGNTELINDR	79	1	1	12667788	N-Term(TMT6plex)	2701.3	0	**1.7**	**1.7**	**1.9**	**1.8**
																	ANLQIDQINTDLNLER	32	1	1	12667788	N-Term(TMT6plex)	2099.1	0	**1.6**	**1.6**	**1.7**	**1.6**
																	TQLEELEDELQATEDAK	94	8	3	367460087;13124875;12667788	N-Term(TMT6plex); K17(TMT6plex)	2420.2	0				
																	VSHLLGINVTDFTR	56	1	1	12667788	N-Term(TMT6plex)	1801.0	0	**1.1**	**1.2**	**1.3**	**1.2**
																	AGVLAHLEEER	33	4	2	367460087;12667788	N-Term(TMT6plex)	1452.8	0				
																	KLEGDSTDLSDQIAELQAQIAELK	67	1	1	12667788	N-Term(TMT6plex); K1(TMT6plex); K24(TMT6plex)	3302.8	1	**1.7**	**1.8**	**2.3**	**1.6**
																	NFINNPLAQADWAAK	32	1	1	12667788	N-Term(TMT6plex); K15(TMT6plex)	2131.2	0	**1.9**	**1.3**	**1.7**	**1.3**
																	LEGDSTDLSDQIAELQAQIAELK	40	1	1	12667788	N-Term(TMT6plex); K23(TMT6plex)	2945.6	0	**1.8**	**1.7**	**1.9**	**1.6**
																	IMGIPEEEQMGLLR	13	1	1	12667788	N-Term(TMT6plex); M10(Oxidation)	1861.0	0	**1.6**	**1.7**	**2.0**	**1.5**
																	QSACNLEK	26	1	1	12667788	N-Term(TMT6plex); C4(Carbamidomethyl); K8(TMT6plex)	1407.8	0	**1.7**	**1.5**	**1.9**	**1.4**
																	LTEMETLQSQLMAEK	51	1	1	12667788	N-Term(TMT6plex); M4(Oxidation); M12(Oxidation); K15(TMT6plex)	2242.2	0	**1.6**	**1.6**	**1.7**	**1.5**
																	THEAQIQEMR	18	1	1	12667788	N-Term(TMT6plex); M9(Oxidation)	1487.7	0	**1.3**	**1.4**	**1.4**	**1.3**
																	LDPHLVLDQLR	49	4	2	367460087;12667788	N-Term(TMT6plex)	1547.9	0				
																	FVSELWK	28	1	1	12667788	N-Term(TMT6plex); K7(TMT6plex)	1366.8	0	**1.8**	**1.6**	**2.1**	**1.5**
																	LVWVPSDK	22	1	1	12667788	N-Term(TMT6plex); K8(TMT6plex)	1401.9	0	**1.6**	**1.6**	**2.0**	**1.6**
																	EQEVNILK	25	1	1	12667788	N-Term(TMT6plex); K8(TMT6plex)	1430.9	0	**1.6**	**1.7**	**1.8**	**1.5**
																	TDLLLEPYNK	29	1	1	12667788	N-Term(TMT6plex); K10(TMT6plex)	1664.0	0	**1.5**	**1.4**	**1.6**	**1.4**
																	VEDMAELTCLNEASVLHNLK	53	4	2	367460087;12667788	N-Term(TMT6plex); M4(Oxidation); C9(Carbamidomethyl); K20(TMT6plex)	2760.4	0				
																	HEMPPHIYAITDTAYR	19	1	1	12667788	N-Term(TMT6plex); M3(Oxidation)	2160.1	0	**1.2**	**1.4**	**1.4**	**1.3**
																	QEEEMMAK	8	1	1	12667788	N-Term(TMT6plex); M6(Oxidation); K8(TMT6plex)	1469.7	0	**2.2**	**2.5**	**3.1**	**1.6**
																	VVFQEFR	21	1	1	12667788	N-Term(TMT6plex)	1153.7	0	**1.6**	**1.6**	**1.8**	**1.5**
																	RGDLPFVVPR	12	1	1	12667788	N-Term(TMT6plex)	1384.8	1	**1.6**	**1.5**	**1.6**	**1.5**
																	KMEDSVGCLETAEEVKR	1	1	1	12667788	N-Term(TMT6plex); K1(TMT6plex); M2(Oxidation); C8(Carbamidomethyl); K16(TMT6plex)	2684.4	2	**1.5**	**2.0**	**2.1**	**1.7**
																	NAEQYKDQADK	10	1	1	12667788	N-Term(TMT6plex); K6(TMT6plex); K11(TMT6plex)	1997.1	1	**1.7**	**1.7**	**1.8**	**1.5**
																	SMEAEMIQLQEELAAAER	55	1	1	12667788	N-Term(TMT6plex); M2(Oxidation); M6(Oxidation)	2310.1	0	**1.5**	**1.5**	**1.5**	**1.6**
																	EDQSILCTGESGAGK	41	11	4	116284394;367460087;13124875;12667788	N-Term(TMT6plex); C7(Carbamidomethyl); K15(TMT6plex)	2010.0	0				
																	DVLLQVDDER	26	1	1	12667788	N-Term(TMT6plex)	1430.8	0	**1.6**	**1.7**	**1.7**	**1.8**
																	VEAQLQELQVK	17	1	1	12667788	N-Term(TMT6plex); K11(TMT6plex)	1743.0	0	**1.5**	**1.4**	**1.7**	**1.4**
																	HEAMITDLEER	10	1	1	12667788	N-Term(TMT6plex); M4(Oxidation)	1588.8	0	**1.3**	**1.3**	**1.3**	**1.3**
																	ALEQQVEEMK	41	1	1	12667788	N-Term(TMT6plex); M9(Oxidation); K10(TMT6plex)	1678.9	0	**1.4**	**1.6**	**1.7**	**1.5**
																	NLPIYSEEIVEMYK	47	1	1	12667788	N-Term(TMT6plex); M12(Oxidation); K14(TMT6plex)	2202.2	0	**1.5**	**1.5**	**1.7**	**1.4**
																	GALALEEK	15	1	1	12667788	N-Term(TMT6plex); K8(TMT6plex)	1288.8	0	**1.4**	**1.6**	**1.7**	**1.4**
																	DELADEIANSSGK	14	1	1	12667788	N-Term(TMT6plex); K13(TMT6plex)	1806.9	0	**1.5**	**1.4**	**1.6**	**1.4**
																	SMEAEMIQLQEELAAAER	24	1	1	12667788	N-Term(TMT6plex); M2(Oxidation)	2294.1	0	**1.5**	**1.6**	**1.6**	**1.5**
																	TLEEEAK	18	1	1	12667788	N-Term(TMT6plex); K7(TMT6plex)	1277.7	0	**1.6**	**1.6**	**1.8**	**1.5**
																	TEMEDLMSSK	26	1	1	12667788	N-Term(TMT6plex); M3(Oxidation); M7(Oxidation); K10(TMT6plex)	1660.8	0	**1.6**	**1.5**	**1.8**	**1.4**
																	ALEEAMEQK	34	1	1	12667788	N-Term(TMT6plex); M6(Oxidation); K9(TMT6plex)	1522.8	0	**1.5**	**1.7**	**1.7**	**1.6**
																	NTNPNFVR	9	4	2	367460087;12667788	N-Term(TMT6plex)	1190.6	0				
																	ELESQISELQEDLESER	46	1	1	12667788	N-Term(TMT6plex)	2263.1	0	**1.5**	**1.5**	**1.7**	**1.6**
																	KLVWVPSDK	2	1	1	12667788	N-Term(TMT6plex); K1(TMT6plex); K9(TMT6plex)	1759.1	1	**1.6**	**1.8**	**2.0**	**1.7**
																	KTLEEEAK	2	1	1	12667788	N-Term(TMT6plex); K1(TMT6plex); K8(TMT6plex)	1635.0	1	**1.5**	**1.8**	**1.7**	**1.6**
																	SMEAEMIQLQEELAAAER	6	1	1	12667788	N-Term(TMT6plex)	2278.1	0	**1.5**	**1.8**	**2.1**	**1.9**
																	NTDQASMPDNTAAQK	6	5	2	13124875;12667788	N-Term(TMT6plex); K15(TMT6plex)	2050.0	0				
																	INFDVNGYIVGANIETYLLEK	3	1	1	12667788	N-Term(TMT6plex); K21(TMT6plex)	2843.6	0	**1.4**	**1.5**	**1.6**	**1.4**
																	ASITALEAK	14	1	1	12667788	N-Term(TMT6plex); K9(TMT6plex)	1361.8	0	**1.5**	**1.6**	**1.8**	**1.5**
																	IAQLEEQLDNETK	46	1	1	12667788	N-Term(TMT6plex); K13(TMT6plex)	1989.1	0	**1.5**	**1.5**	**1.6**	**1.4**
																	DEQSEEK	8	1	1	12667788	N-Term(TMT6plex); K7(TMT6plex)	1322.7	0	**1.6**	**1.7**	**1.9**	**1.6**
																	QLAAENR	2	1	1	12667788	N-Term(TMT6plex)	1030.6	0	**1.9**	**1.6**	**2.0**	**1.4**
																	QQQLTAMK	12	5	2	13124875;12667788	N-Term(TMT6plex); M7(Oxidation); K8(TMT6plex)	1421.8	0				
																	NCAAYLK	7	11	4	116284394;367460087;13124875;12667788	N-Term(TMT6plex); C2(Carbamidomethyl); K7(TMT6plex)	1297.7	0				
																	VEDMAELTCLNEASVLHNLK	7	4	2	367460087;12667788	N-Term(TMT6plex); C9(Carbamidomethyl); K20(TMT6plex)	2744.4	0				
																	LTEMETLQSQLMAEK	28	1	1	12667788	N-Term(TMT6plex); M12(Oxidation); K15(TMT6plex)	2226.2	0	**1.8**	**2.0**	**2.4**	**1.6**
																	IMGIPEEEQMGLLR	4	1	1	12667788	N-Term(TMT6plex)	1845.0	0	**1.8**	**1.8**	**2.2**	**1.5**
																	TEMEDLMSSK	6	1	1	12667788	N-Term(TMT6plex); M7(Oxidation); K10(TMT6plex)	1644.8	0	**1.9**	**1.9**	**2.4**	**1.5**
																	LQQLFNHTMFILEQEEYQR	7	8	3	367460087;13124875;12667788	N-Term(TMT6plex); M9(Oxidation)	2712.4	0				
																	DLEGLSQR	13	1	1	12667788	N-Term(TMT6plex)	1146.6	0	**1.4**	**1.7**	**1.6**	**1.6**
																	LQVELDNVTGLLSQSDSK	75	1	1	12667788	N-Term(TMT6plex); K18(TMT6plex)	2404.3	0	**1.5**	**1.7**	**1.8**	**1.5**
																	QIATLHAQVADMK	4	1	1	12667788	N-Term(TMT6plex); K13(TMT6plex)	1884.1	0	**1.5**	**1.5**	**2.0**	**1.4**
																	VEDMAELTCLNEASVLHNLKER	2	1	1	12667788	N-Term(TMT6plex); M4(Oxidation); C9(Carbamidomethyl); K20(TMT6plex)	3045.6	1	**1.7**	**1.9**	**1.9**	**1.9**
																	KLEEEQIILEDQNCK	38	1	1	12667788	N-Term(TMT6plex); K1(TMT6plex); C14(Carbamidomethyl); K15(TMT6plex)	2576.4	1	**1.6**	**1.8**	**1.9**	**1.5**
																	QLLQANPILEAFGNAK	61	8	3	116284394;13124875;12667788	N-Term(TMT6plex); K16(TMT6plex)	2185.3	0				
																	DLGEELEALKTELEDTLDSTAAQQELR	15	1	1	12667788	N-Term(TMT6plex); K10(TMT6plex)	3475.8	1	**2.2**	**2.2**	**2.5**	**1.8**
																	VISGVLQLGNIVFK	49	1	1	12667788	N-Term(TMT6plex); K14(TMT6plex)	1945.2	0	**1.7**	**1.5**	**1.9**	**1.4**
																	LQQELDDLLVDLDHQR	36	1	1	12667788	N-Term(TMT6plex)	2179.2	0	**1.5**	**1.6**	**1.7**	**1.5**
																	QLEEAEEEAQR	24	1	1	12667788	N-Term(TMT6plex)	1560.8	0	**1.5**	**1.5**	**1.5**	**1.6**
																	TFHIFYYLLSGAGEHLK	24	1	1	12667788	N-Term(TMT6plex); K17(TMT6plex)	2454.3	0	**1.8**	**1.6**	**2.4**	**1.5**
																	VEEEAAQK	43	1	1	12667788	N-Term(TMT6plex); K8(TMT6plex)	1361.8	0	**1.4**	**1.4**	**1.5**	**1.4**
																	ALELDSNLYR	17	1	1	12667788	N-Term(TMT6plex)	1422.8	0	**1.5**	**1.4**	**1.6**	**1.4**
																	EQLEEEEEAKHNLEK	14	1	1	12667788	N-Term(TMT6plex); K10(TMT6plex); K15(TMT6plex)	2542.4	1	**1.5**	**1.7**	**1.8**	**1.5**
																	VIQYLAYVASSHK	14	1	1	12667788	N-Term(TMT6plex); K13(TMT6plex)	1937.1	0	**1.7**	**1.6**	**2.0**	**1.5**
																	KVEAQLQELQVK	23	1	1	12667788	N-Term(TMT6plex); K1(TMT6plex); K12(TMT6plex)	2100.3	1	**1.5**	**1.8**	**2.0**	**1.6**
																	EQADFAIEALAK	56	4	2	12667788;116284394	N-Term(TMT6plex); K12(TMT6plex)	1764.0	0				
																	MEDSVGCLETAEEVK	8	1	1	12667788	N-Term(TMT6plex); C7(Carbamidomethyl); K15(TMT6plex)	2155.1	0	**2.2**	**2.1**	**2.8**	**1.5**
																	ADGAEAKPAE	18	1	1	12667788	N-Term(TMT6plex); K7(TMT6plex)	1416.8	0	**1.5**	**1.5**	**1.7**	**1.5**

Khan et al., 2019. Multi-omics analysis to characterize cigarette smoke induced molecular alterations in esophageal cells
Supplementary Table 6. List of all proteins and peptides identified in untreated and chronically treated Het1A cells with cigarette smoke condensate for 8 months

Accession	RefSeq Accession	Gene Symbol	Entrez Gene ID	Description	Σ Coverage	Σ# Proteins	Σ# Unique Peptides	Σ# Peptides	Σ# PSMs	Het-1A-Smoke - 2M/Parental	Het-1A-Smoke - 4M/Parental	Het-1A-Smoke - 6M/Parental	Het-1A-Smoke - 8M/Parental	# AAs	MW [kDa]	calc. pI	Sequence	# PSMs	# Proteins	# Protein Groups	Protein Group Accessions	Modifications	MH+ [Da]	# Missed Cleavages	Het-1A-Smoke - 2M/Parental	Het-1A-Smoke - 4M/Parental	Het-1A-Smoke - 6M/Parental	Het-1A-Smoke - 8M/Parental
																	EMEAELEDER	10	1	1	12667788	N-Term(TMT6plex); M2(Oxidation)	1495.7	0	1.4	1.5	1.5	1.4
																	QAQQERDELADEIANSSGK	20	1	1	12667788	N-Term(TMT6plex); K19(TMT6plex)	2547.3	1	1.7	1.7	1.9	1.7
																	QACVLMIK	13	1	1	12667788	N-Term(TMT6plex); C3(Carbamidomethyl); M6(Oxidation); K8(TMT6plex)	1436.9	0	1.5	1.4	1.7	1.4
																	GDLPFVVPR	4	1	1	12667788	N-Term(TMT6plex)	1228.7	0	1.5	1.7	1.8	1.6
																	GELANEVK	15	1	1	12667788	N-Term(TMT6plex); K8(TMT6plex)	1317.8	0	1.2	1.4	1.5	1.4
																	FDQLLAEEK	13	11	4	116284394;367460087;13124875;12667788	N-Term(TMT6plex); K9(TMT6plex)	1550.9	0				
																	YYSGLIYTYSGLFCVVINPYK	2	7	3	116284394;367460087;12667788	N-Term(TMT6plex); C14(Carbamidomethyl); K21(TMT6plex)	2978.6	0				
																	KEEELQAALAR	18	8	3	116284394;13124875;12667788	N-Term(TMT6plex); K1(TMT6plex)	1716.0	1				
																	DLGEELEALK	35	5	2	13124875;12667788	N-Term(TMT6plex); K10(TMT6plex)	1574.9	0				
																	LTEMETLQSQLMAEK	10	1	1	12667788	N-Term(TMT6plex); K15(TMT6plex)	2210.2	0	2.1	2.1	2.8	1.5
																	MQQNIQELEEQLEEEESAR	7	1	1	12667788	N-Term(TMT6plex)	2562.2	0	1.4	1.3	1.4	1.4
																	KANLQIDQINTDLNLER	9	1	1	12667788	N-Term(TMT6plex); K1(TMT6plex)	2456.4	1	1.5	1.8	1.9	1.6
																	ALEEAMEQK	4	1	1	12667788	N-Term(TMT6plex); K9(TMT6plex)	1506.8	0	2.3	2.7	3.3	1.6
																	SGFEPASLK	14	1	1	12667788	N-Term(TMT6plex); K9(TMT6plex)	1393.8	0	1.4	1.4	1.7	1.3
																	ADEWLMK	9	4	2	367460087;12667788	N-Term(TMT6plex); K7(TMT6plex)	1350.7	0				
																	KMQQNIQELEEQLEEEESAR	13	1	1	12667788	N-Term(TMT6plex); K1(TMT6plex); M2(Oxidation)	2935.5	1	1.5	1.7	1.6	1.5
																	KKVEAQLQELQVK	2	1	1	12667788	N-Term(TMT6plex); K1(TMT6plex); K2(TMT6plex); K13(TMT6plex)	2457.6	2	1.3	1.8	1.7	1.7
																	KQELEEICHDLEAR	29	1	1	12667788	N-Term(TMT6plex); K1(TMT6plex); C8(Carbamidomethyl)	2228.2	1	1.5	1.5	1.6	1.4
																	THEAQIQEMR	2	1	1	12667788	N-Term(TMT6plex)	1471.8	0	1.5	1.4	1.5	1.3
																	NKHEAMITDLEER	15	1	1	12667788	N-Term(TMT6plex); K2(TMT6plex); M6(Oxidation)	2060.1	1	2.5	1.7	1.6	1.6
																	CIIPNHEK	6	8	3	367460087;13124875;12667788	N-Term(TMT6plex); C1(Carbamidomethyl); K8(TMT6plex)	1468.8	0				
																	MEDSVGCLETAEEVKR	3	1	1	12667788	N-Term(TMT6plex); M1(Oxidation); C7(Carbamidomethyl); K15(TMT6plex)	2327.2	1	1.4	1.4	1.4	1.3
																	ELEDATETADAMNR	11	1	1	12667788	N-Term(TMT6plex)	1794.8	0	1.7	1.8	1.8	1.6
																	QEEEMMAK	2	1	1	12667788	N-Term(TMT6plex); K8(TMT6plex)	1453.7	0	2.1	1.8	2.3	1.4
																	SVHELEK	14	1	1	12667788	N-Term(TMT6plex); K7(TMT6plex)	1299.8	0	1.3	1.4	1.4	1.4
																	CQHLQAEK	9	1	1	12667788	N-Term(TMT6plex); C1(Carbamidomethyl); K8(TMT6plex)	1471.8	0	1.5	1.6	1.7	1.4
																	KMEDSVGCLETAEEVK	13	1	1	12667788	N-Term(TMT6plex); K1(TMT6plex); M2(Oxidation); C8(Carbamidomethyl); K16(TMT6plex)	2528.3	1	1.8	1.6	1.9	1.7
																	LEVNLQAMK	4	1	1	12667788	N-Term(TMT6plex); K9(TMT6plex)	1503.9	0	2.0	2.2	2.6	1.6
																	VMQEQGTHPK	2	1	1	12667788	N-Term(TMT6plex); K10(TMT6plex)	1612.9	0	2.0	2.1	2.8	1.4
																	LQEMEGTVK	6	1	1	12667788	N-Term(TMT6plex); K9(TMT6plex)	1492.8	0	2.3	2.6	3.2	2.2
																	DKADFCIIHYAGK	2	4	2	367460087;12667788	N-Term(TMT6plex); K2(TMT6plex); C6(Carbamidomethyl); K13(TMT6plex)	2225.2	1				
																	EEELQAALAR	2	8	3	116284394;13124875;12667788	N-Term(TMT6plex)	1358.7	0				
																	MEDSVGCLETAEEVKR	1	1	1	12667788	N-Term(TMT6plex); C7(Carbamidomethyl); K15(TMT6plex)	2311.2	1	1.5	1.7	2.0	1.2
																	SGFEPASLKEEVGEEAIVELVENGK	9	1	1	12667788	N-Term(TMT6plex); K9(TMT6plex); K25(TMT6plex)	3347.8	1	1.7	1.5	2.0	1.2
																	HEMPPHIYAITDTAYR	2	1	1	12667788	N-Term(TMT6plex)	2144.1	0	1.5	1.5	1.8	1.6
																	KMEDSVGCLETAEEVK	2	1	1	12667788	N-Term(TMT6plex); K1(TMT6plex); C8(Carbamidomethyl); K16(TMT6plex)	2512.3	1	2.4	2.3	3.0	1.8
																	KMQQNIQELEEQLEEEESAR	4	1	1	12667788	N-Term(TMT6plex); K1(TMT6plex)	2919.5	1	1.6	1.9	1.9	1.7
																	NKHEAMITDLEER	3	1	1	12667788	N-Term(TMT6plex); K2(TMT6plex)	2044.1	1	2.0	2.4	2.8	2.0
																	TEMEDLMSSK	1	1	1	12667788	N-Term(TMT6plex); K10(TMT6plex)	1628.8	0	2.1	1.7	2.7	1.5
																	SGFEPASLKEEVGEEAIVELVENGKK	3	1	1	12667788	N-Term(TMT6plex); K9(TMT6plex); K25(TMT6plex); K26(TMT6plex)	3705.0	2	1.6	1.9	2.3	1.7
																	TELEDTLDSTAAQQELR	18	1	1	12667788	N-Term(TMT6plex)	2149.1	0	1.6	1.6	1.6	1.4
																	DVLLQVDDERR	5	1	1	12667788	N-Term(TMT6plex)	1586.9	1	1.4	1.5	1.5	1.5
																	SMMQDREDQSILCTGESGAGK	2	1	1	12667788	N-Term(TMT6plex); M2(Oxidation); M3(Oxidation); C13(Carbamidomethyl); K21(TMT6plex)	2790.3	1	1.9	2.4	1.6	2.0
																	VNKDDIQK	6	1	1	12667788	N-Term(TMT6plex); K3(TMT6plex); K8(TMT6plex)	1647.0	1	1.4	1.6	1.8	1.4
																	LKDVLLQVDDERR	2	1	1	12667788	N-Term(TMT6plex); K2(TMT6plex)	2057.2	2	1.4	1.6	1.7	1.4
																	DMFQETMEAMR	2	1	1	12667788	N-Term(TMT6plex); M7(Oxidation); M10(Oxidation)	1649.7	0	1.4	1.5	1.8	1.3
																	VEEEEER	2	4	2	367460087;12667788	N-Term(TMT6plex)	1148.6	0				
																	DMFQETMEAMR	1	1	1	12667788	N-Term(TMT6plex); M2(Oxidation); M7(Oxidation); M10(Oxidation)	1665.7	0	1.5	1.2	1.2	1.5
																	QTLENER	2	1	1	12667788	N-Term(TMT6plex)	1118.6	0	1.6	1.3	1.4	1.2
																	ADEWLMK	8	4	2	367460087;12667788	N-Term(TMT6plex); M6(Oxidation); K7(TMT6plex)	1366.7	0				
																	HEAMITDLEER	2	1	1	12667788	N-Term(TMT6plex)	1572.8	0	1.8	1.8	2.0	1.7
																	KDQGELER	3	1	1	12667788	N-Term(TMT6plex); K1(TMT6plex)	1432.8	1	1.8	1.8	2.1	1.6
																	EMEAELEDERK	2	1	1	12667788	N-Term(TMT6plex); M2(Oxidation); K11(TMT6plex)	1852.9	1	1.4	1.6	1.7	1.6
																	EEELVK	8	1	1	12667788	N-Term(TMT6plex); K6(TMT6plex)	1204.7	0	1.5	1.6	1.8	1.4
																	AEAEAR	2	8	3	367460087;13124875;12667788	N-Term(TMT6plex)	875.5	0				
																	VAAYDK	4	1	1	12667788	N-Term(TMT6plex); K6(TMT6plex)	1124.7	0	1.5	1.5	1.7	1.4
																	QEEEMMAKEEELVK	1	1	1	12667788	N-Term(TMT6plex); M5(Oxidation); M6(Oxidation); K8(TMT6plex); K14(TMT6plex)	2442.3	1	2.1	2.5	2.1	2.5
																	NRDEAIK	1	1	1	12667788	N-Term(TMT6plex); K7(TMT6plex)	1303.8	1	1.3	1.3	1.5	1.2
																	YLYVDK	11	1	1	12667788	N-Term(TMT6plex); K6(TMT6plex)	1258.7	0	1.6	1.6	1.9	1.5
																	KLEMDLK	2	1	1	12667788	N-Term(TMT6plex); K1(TMT6plex); K7(TMT6plex)	1564.0	1	1.8	2.2	2.4	1.6
																	QVEDEK	3	1	1	12667788	N-Term(TMT6plex); K6(TMT6plex)	1205.7	0	1.7	1.8	1.8	1.5
																	DQGELER	1	1	1	12667788	N-Term(TMT6plex)	1075.6	0	1.9	1.6	1.7	1.7
																	VLLQGK	2	1	1	12667788	N-Term(TMT6plex); K6(TMT6plex)	1115.8	0	1.7	1.7	1.9	1.5
																	EVSSLK	5	1	1	12667788	N-Term(TMT6plex); K6(TMT6plex)	1120.7	0	1.5	1.7	1.8	1.6
																	KGAGDGSDEEVDGK	1	1	1	12667788	N-Term(TMT6plex); K1(TMT6plex); K14(TMT6plex)	2051.1	1				
																	GDSEHK	1	1	1	12667788	N-Term(TMT6plex); K6(TMT6plex)	1130.6	0	1.6	1.9	2.0	1.6
																	LQQLFNHTMFILEQEEYQR	1	8	3	367460087;13124875;12667788	N-Term(TMT6plex)	2696.4	0				
																	NAEQYK	1	1	1	12667788	N-Term(TMT6plex); K6(TMT6plex)	1210.7	0	1.8	1.4	1.8	1.4
																	VTTEAK	2	4	2	116284394;12667788	N-Term(TMT6plex); K6(TMT6plex)	1106.7	0				
																	TELADK	1	1	1	12667788	N-Term(TMT6plex); K6(TMT6plex)	1134.7	0	1.7	1.3	1.9	1.3
																	GFMDGK	1	11	4	116284394;367460087;13124875;12667788	N-Term(TMT6plex); K6(TMT6plex)	1112.6	0				
																	QEEEMMAKEEELVK	1	1	1	12667788	N-Term(TMT6plex); M5(Oxidation); K8(TMT6plex); K14(TMT6plex)	2426.3	1	1.7	2.1	1.7	1.6
																	QEEEMMAK	1	1	1	12667788	N-Term(TMT6plex); M5(Oxidation); M6(Oxidation); K8(TMT6plex)	1485.7	0	1.6	1.4	1.7	1.6
																	LMATLR	1	4	2	367460087;12667788	N-Term(TMT6plex)	933.6	0				
																	ELDDTR	1	1	1	12667788	N-Term(TMT6plex)	977.5	0	1.4	1.6	1.5	1.5
																	GILTPR	1	1	2	12667788;5730073	N-Term(TMT6plex)	885.6	0				
																	ALSLAR	1	8	3	367460087;13124875;12667788	N-Term(TMT6plex)	859.6	0				
32484973	NP_001114.2	ADK	132	adenosine kinase isoform a	67.54	4	16	16	87	1.4	1.7	1.7	1.6	345	38.7	6.68												
																	DDTIMATESEVTAFAVLDQDQK	6	4	1	32484973	N-Term(TMT6plex); M5(Oxidation); K22(TMT6plex)	2901.4	0	1.3	1.3	1.3	1.5
																	VAHHASENNR	8	3	1	32484973	N-Term(TMT6plex)	1363.7	0	1.9	1.8	1.9	2.5
																	VMPYVDILFGNETEAATFAR	8	3	1	32484973	N-Term(TMT6plex); M2(Oxidation)	2489.3	0	1.3	1.5	1.4	1.4
																	YSLKPNDQILAEDK	7	3	1	32484973	N-Term(TMT6plex); K4(TMT6plex); K14(TMT6plex)	2321.3	0	1.5	1.9	1.9	1.6
																	ELFDELVK	10	3	1	32484973	N-Term(TMT6plex); K8(TMT6plex)	1450.9	0	1.6	1.8	2.0	1.8
																	TGCTFPEKPDFH	4	4	1	32484973	N-Term(TMT6plex); C3(Carbamidomethyl); K8(TMT6plex)	1894.0	0	1.7	1.5	1.7	1.5
																	VMPYVDILFGNETEAATFAR	6	3	1	32484973	N-Term(TMT6plex)	2473.3	0	1.4	1.5	1.5	1.5
																	ENILFGMGNPLLDISAVVDKDFLDK	13	4	1	32484973	N-Term(TMT6plex); M7(Oxidation); K20(TMT6plex); K25(TMT6plex)	3466.9	1	1.4	1.7	1.7	1.9
																	AAEAHVDAHYYEQNEQPTGTCAACITGDNR	2	4	1	32484973	N-Term(TMT6plex); C21(Carbamidomethyl); C24(Carbamidomethyl)	3578.6	0	1.5	2.0	2.5	1.9
																	SLIANLAAANCYK	2	4	1	32484973	N-Term(TMT6plex); C11(Carbamidomethyl); K13(TMT6plex)	1867.1	0	1.4	1.6	1.5	1.7
																	IVIFTQGR	8	4	1	32484973	N-Term(TMT6plex)	1162.7	0	1.7	1.8	2.0	1.8
																	EIIDTNGAGDAFVGGFLSQLVSDKPLTECIR	1	4	1	32484973	N-Term(TMT6plex); K24(TMT6plex); C29(Carbamidomethyl)	3780.9	0	1.3	1.7	1.7	1.6
																	ENILFGMGNPLLDISAVVDK	2	4	1	32484973	N-Term(TMT6plex); M7(Oxidation); K20(TMT6plex)	2619.4	0	1.3	1.2	1.2	1.4
																	VAQWMIQQPHK	2	4	1	32484973	N-Term(TMT6plex); M5(Oxidation); K11(TMT6plex)	1840.0	0	1.3	1.4	1.5	1.4
																	IFTLNLSAPFISQFYK	1	3	1	32484973	N-Term(TMT6plex); K16(TMT6plex)	2347.3	0				
																	HLDLEK	6	4	1	32484973	N-Term(TMT6plex); K6(TMT6plex)	1212.7	0	1.4	1.6	1.8	1.7
																	KTQALPK	1	4	1	32484973	N-Term(TMT6plex); K1(TMT6plex); K7(TMT6plex)	1473.0	1	1.2	1.4	1.5	1.3
24430151	NP_002793.2	PSMC1	5700	26S protease regulatory subunit 4	67.50	2	24	26	270	1.3	1.4	1.5	1.4	440	49.2	6.21												
																	MTLADDVTLDDLIMAK	32	1	1	24430151	N-Term(TMT6plex); M1(Oxidation); M14(Oxidation); K16(TMT6plex)	2255.2	0	1.4	1.5	1.5	1.5
																	KIEFPLPDEK	11	1	1	24430151	N-Term(TMT6plex); K1(TMT6plex); K10(TMT6plex)	1903.2	1	1.3	1.4	1.4	1.4
																	AVANQTSATFLR	8	1	1	24430151	N-Term(TMT6plex)	1507.8	0	1.4	1.3	1.4	1.3
																	ESVELPLTHPEYYEEMGIKPPK	12	1	1	24430151	N-Term(TMT6plex); M16(Oxidation); K19(TMT6plex); K22(TMT6plex)	3289.8	0	1.1	1.3	1.4	1.4
																	VAEEHAPSIVFIDEIDAIGTK	20	1	1	24430151	N-Term(TMT6plex); K21(TMT6plex)	2712.5	0	1.3	1.4	1.5	1.5
																	GVILYGPPGTGK	22	5	3	312596881;157671927;24430151	N-Term(TMT6plex); K12(TMT6plex)	1617.0	0				
																	TMLELLNQLDGFDSR	25	1	1	24430151	N-Term(TMT6plex); M2(Oxidation)	1997.0	0	1.4	1.4	1.4	1.4
																	DDLSGADIK	6	1	1	24430151	N-Term(TMT6plex); K9(TMT6plex)	1391.8	0	1.2	1.4	1.5	1.4
																	DYLLMEEEFIR	5	1	1	24430151	N-Term(TMT6plex); M5(Oxidation)	1702.9	0	1.4	1.4	1.3	1.5

Accession	RefSeq Accession	Gene Symbol	Entrez Gene ID	Description	Σ Coverage	Σ# Proteins	Σ# Unique Peptides	Σ# Peptides	Σ# PSMs	Het-1A-Smoke - 2M/Parental	Het-1A-Smoke - 4M/Parental	Het-1A-Smoke - 6M/Parental	Het-1A-Smoke - 8M/Parental	# AAs	MW [kDa]	calc. pI	Sequence	# PSMs	# Proteins	# Protein Groups	Protein Group Accessions	Modifications	MH+ [Da]	# Missed Cleavages	Het-1A-Smoke - 2M/Parental	Het-1A-Smoke - 4M/Parental	Het-1A-Smoke - 6M/Parental	Het-1A-Smoke - 8M/Parental
																	SKENVLYK	1	1	1	24430151	N-Term(TMT6plex); K2(TMT6plex); K8(TMT6plex)	1668.0	1	1.3	1.5	1.5	1.5
																	TMLELLNQLDGFDSR	8	1	1	24430151	N-Term(TMT6plex)	1981.0	0	1.7	1.4	1.7	1.5
																	VHAVIGVLMDDTDPLVTVMK	4	1	1	24430151	N-Term(TMT6plex); M19(Oxidation); K20(TMT6plex)	2627.5	0	1.6	1.7	1.9	1.6
																	MTLADDVTLDDLIMAK	6	1	1	24430151	N-Term(TMT6plex); M14(Oxidation); K16(TMT6plex)	2239.2	0	1.5	1.6	1.9	1.4
																	VHAVIGVLMDDTDPLVTVMK	36	1	1	24430151	N-Term(TMT6plex); M9(Oxidation); M19(Oxidation); K20(TMT6plex)	2643.4	0	1.3	1.4	1.5	1.5
																	LPLVTPHTQCR	2	1	1	24430151	N-Term(TMT6plex); C10(Carbamidomethyl)	1550.9	0	1.4	1.5	1.5	1.5
																	APQETYADIGGLDNQIQEIK	14	1	1	24430151	N-Term(TMT6plex); K20(TMT6plex)	2661.4	0	1.4	1.5	1.6	1.6
																	IETLDPALIRPGR	2	1	1	24430151	N-Term(TMT6plex)	1680.0	0	1.3	1.3	1.5	1.2
																	IKDYLLMEEEFIR	14	1	1	24430151	N-Term(TMT6plex); K2(TMT6plex)	2157.2	1	1.6	1.8	2.3	1.5
																	VVGSELIQK	6	1	1	24430151	N-Term(TMT6plex); K9(TMT6plex)	1430.9	0	1.3	1.5	1.6	1.5
																	AICTEAGLMALR	7	1	1	24430151	N-Term(TMT6plex); C3(Carbamidomethyl); M9(Oxidation)	1550.8	0	1.1	1.2	1.2	1.4
																	VTNEDFK	4	1	1	24430151	N-Term(TMT6plex); K7(TMT6plex)	1310.7	0	1.3	1.3	1.4	1.3
																	IKDYLLMEEEFIR	7	1	1	24430151	N-Term(TMT6plex); K2(TMT6plex); M7(Oxidation)	2173.2	1	1.3	1.4	1.6	1.5
																	DLLEPGCSVLLNHK	4	1	1	24430151	N-Term(TMT6plex); C7(Carbamidomethyl); K14(TMT6plex)	2053.2	0	1.4	1.3	1.5	1.2
																	IEFPLPDEK	2	1	1	24430151	N-Term(TMT6plex); K9(TMT6plex)	1545.9	0	1.3	1.6	1.6	1.5
																	IFQIHTSR	4	1	1	24430151	N-Term(TMT6plex)	1230.7	0	1.2	1.5	1.5	1.3
																	KYEPPVPTR	3	1	1	24430151	N-Term(TMT6plex); K1(TMT6plex)	1544.9	1	1.5	1.4	1.5	1.4
																	NQEQMKPLEEK	2	1	1	24430151	N-Term(TMT6plex); M5(Oxidation); K6(TMT6plex); K11(TMT6plex)	2077.2	0	1.3	1.1	1.3	1.2
																	KQEGTPEGLYL	1	1	1	24430151	N-Term(TMT6plex); K1(TMT6plex)	1693.0	1	1.3	1.4	1.6	1.5
																	GPDAASK	1	1	1	24430151	N-Term(TMT6plex); K7(TMT6plex)	1103.6	0	1.2	1.5	1.5	1.4
																	VIMATNR	1	5	3	24430151;5729991;312596881	N-Term(TMT6plex); M3(Oxidation)	1049.6	0				
4759302	NP_004729.1	VAPB	9217	vesicle-associated membrane protein-associated protein B/C isoform 1	67.49	2	14	15	144	1.3	1.3	1.4	1.4	243	27.2	7.30												
																	GPFTDVVTTNLK	38	4	2	4759302;94721252	N-Term(TMT6plex); K12(TMT6plex)	1750.0	0				
																	TETPIVSK	11	1	1	4759302	N-Term(TMT6plex); K8(TMT6plex)	1332.8	0	1.3	1.1	1.3	1.2
																	QFKEEDGLR	3	1	1	4759302	N-Term(TMT6plex); K3(TMT6plex)	1579.9	1	1.3	1.5	1.3	1.6
																	EAKPEDLMDSK	5	1	1	4759302	N-Term(TMT6plex); K3(TMT6plex); M8(Oxidation); K11(TMT6plex)	1966.1	0	1.2	1.4	1.4	1.5
																	SLSSSLDDTEVK	10	1	1	4759302	N-Term(TMT6plex); K12(TMT6plex)	1738.9	0	1.4	1.4	1.4	1.6
																	TVQSNSPISALAPTGK	22	1	1	4759302	N-Term(TMT6plex); K16(TMT6plex)	2029.2	0	1.4	1.4	1.4	1.3
																	FMVQSMFAPTDTSDMEAVWK	4	1	1	4759302	N-Term(TMT6plex); M15(Oxidation); K20(TMT6plex)	2795.4	0	1.6	1.7	1.9	1.6
																	VEQVLSLEPQHELK	14	2	1	4759302	N-Term(TMT6plex); K14(TMT6plex)	2107.2	0	1.4	1.4	1.5	1.5
																	CVFELPAENDKPHDVEINK	2	1	1	4759302	N-Term(TMT6plex); C1(Carbamidomethyl); K11(TMT6plex); K19(TMT6plex)	2941.6	0	1.2	1.2	1.5	1.3
																	SLSSSLDDTEVKK	9	1	1	4759302	N-Term(TMT6plex); K12(TMT6plex); K13(TMT6plex)	2096.2	1	1.2	1.3	1.3	1.4
																	IISTTASK	10	1	1	4759302	N-Term(TMT6plex); K8(TMT6plex)	1278.8	0	1.3	1.2	1.4	1.4
																	TVQSNSPISALAPTGKEEGLSTR	2	1	1	4759302	N-Term(TMT6plex); K16(TMT6plex)	2801.5	1	1.2	0.9	1.2	1.1
																	FMVQSMFAPTDTSDMEAVWK	5	1	1	4759302	N-Term(TMT6plex); M2(Oxidation); M6(Oxidation); M15(Oxidation); K20(TMT6plex)	2827.3	0	1.3	1.4	1.5	1.5
																	FMVQSMFAPTDTSDMEAVWK	4	1	1	4759302	N-Term(TMT6plex); M2(Oxidation); M6(Oxidation); K20(TMT6plex)	2811.3	0	1.5	1.5	1.7	1.6
																	LLALVVLFFIVGVIIGKIAL	2	1	1	4759302	N-Term(TMT6plex); K17(TMT6plex)	2569.7	1	1.7	1.3	2.4	1.0
																	EEGLSTRLLALVVLFFIVGVIIGKIAL	1	1	1	4759302	N-Term(TMT6plex); K24(TMT6plex)	3342.1	2	1.9	1.6	3.9	0.7
																	LGNPTDR	2	2	1	4759302	N-Term(TMT6plex)	1001.6	0	1.2	1.1	1.3	1.5
28173554	NP_778225.1	HIST3H2BB	128312	histone H2B type 3-B	67.46	1	3	11	2930	1.0	1.1	1.1	1.1	126	13.9	10.32												
																	AMGIMNSFVNDIFER	984	16	3	28173554;4504263;578846900	N-Term(TMT6plex); M2(Oxidation); M5(Oxidation)	2005.0	0				
																	QVHPDTGISSK	328	17	4	28173554;24586679;4504263;578846900	N-Term(TMT6plex); K11(TMT6plex)	1626.9	0				
																	LAHYNK	122	14	2	28173554;4504263	N-Term(TMT6plex); K6(TMT6plex)	1203.7	0				
																	LLLPGELAK	773	17	4	28173554;24586679;4504263;578846900	N-Term(TMT6plex); K9(TMT6plex)	1411.9	0				
																	AMGIMNSFVNDIFER	390	16	3	28173554;4504263;578846900	N-Term(TMT6plex); M5(Oxidation)	1989.0	0				
																	HAVSEGTK	119	17	4	28173554;24586679;4504263;578846900	N-Term(TMT6plex); K8(TMT6plex)	1286.7	0				
																	AMGIMNSFVNDIFER	34	16	3	28173554;4504263;578846900	N-Term(TMT6plex)	1973.0	0				
																	ESYSIYVYK	89	5	1	28173554	N-Term(TMT6plex); K9(TMT6plex)	1609.9	0	1.0	1.2	1.1	1.1
																	KESYSIYVYK	26	5	1	28173554	N-Term(TMT6plex); K1(TMT6plex); K10(TMT6plex)	1967.1	1	0.9	1.1	1.1	1.1
																	IASEASR	9	3	2	28173554;24586679	N-Term(TMT6plex)	962.5	0				
																	SAPAPK	44	15	2	28173554;578846900	N-Term(TMT6plex); K6(TMT6plex)	1028.7	0				
																	STITSR	10	16	3	28173554;4504263;578846900	N-Term(TMT6plex)	893.5	0				
																	EVQTAVR	2	1	1	28173554	N-Term(TMT6plex)	1031.6	0	0.9	1.0	1.2	1.1
4504263	NP_003512.1	HIST1H2BM	8342	histone H2B type 1-M	67.46	1	1	11	3480	0.9	0.9	0.9	1.0	126	14.0	10.32												
																	AMGIMNSFVNDIFER	984	16	3	28173554;4504263;578846900	N-Term(TMT6plex); M2(Oxidation); M5(Oxidation)	2005.0	0				
																	QVHPDTGISSK	328	17	4	28173554;24586679;4504263;578846900	N-Term(TMT6plex); K11(TMT6plex)	1626.9	0				
																	LAHYNK	122	14	2	28173554;4504263	N-Term(TMT6plex); K6(TMT6plex)	1203.7	0				
																	ESYSVYVYK	228	11	2	4504263;578846900	N-Term(TMT6plex); K9(TMT6plex)	1595.9	0				
																	LLLPGELAK	773	17	4	28173554;24586679;4504263;578846900	N-Term(TMT6plex); K9(TMT6plex)	1411.9	0				
																	EIQTAVR	356	16	3	24586679;4504263;578846900	N-Term(TMT6plex)	1045.6	0				
																	AMGIMNSFVNDIFER	390	16	3	28173554;4504263;578846900	N-Term(TMT6plex); M5(Oxidation)	1989.0	0				
																	HAVSEGTK	119	17	4	28173554;24586679;4504263;578846900	N-Term(TMT6plex); K8(TMT6plex)	1286.7	0				
																	AMGIMNSFVNDIFER	34	16	3	28173554;4504263;578846900	N-Term(TMT6plex)	1973.0	0				
																	KESYSVYVYK	113	11	2	4504263;578846900	N-Term(TMT6plex); K1(TMT6plex); K10(TMT6plex)	1953.1	1				
																	IAGEASR	21	14	2	4504263;578846900	N-Term(TMT6plex)	932.5	0				
																	STITSR	10	16	3	28173554;4504263;578846900	N-Term(TMT6plex)	893.5	0				
																	SAPVPK	2	1	1	4504263	N-Term(TMT6plex); K6(TMT6plex)	1056.7	0	0.9	0.9	0.9	1.0
578846900	XP_006726868.1	LOC102724334	1.03E+08	histone H2B type F-S-like	67.46	2	1	11	3402					126	13.9	10.32												
																	AMGIMNSFVNDIFER	984	16	3	28173554;4504263;578846900	N-Term(TMT6plex); M2(Oxidation); M5(Oxidation)	2005.0	0				
																	QVHPDTGISSK	328	17	4	28173554;24586679;4504263;578846900	N-Term(TMT6plex); K11(TMT6plex)	1626.9	0				
																	ESYSVYVYK	228	11	2	4504263;578846900	N-Term(TMT6plex); K9(TMT6plex)	1595.9	0				
																	LLLPGELAK	773	17	4	28173554;24586679;4504263;578846900	N-Term(TMT6plex); K9(TMT6plex)	1411.9	0				
																	EIQTAVR	356	16	3	24586679;4504263;578846900	N-Term(TMT6plex)	1045.6	0				
																	AMGIMNSFVNDIFER	390	16	3	28173554;4504263;578846900	N-Term(TMT6plex); M5(Oxidation)	1989.0	0				
																	HAVSEGTK	119	17	4	28173554;24586679;4504263;578846900	N-Term(TMT6plex); K8(TMT6plex)	1286.7	0				
																	AMGIMNSFVNDIFER	34	16	3	28173554;4504263;578846900	N-Term(TMT6plex)	1973.0	0				
																	KESYSVYVYK	113	11	2	4504263;578846900	N-Term(TMT6plex); K1(TMT6plex); K10(TMT6plex)	1953.1	1				
																	IAGEASR	21	14	2	4504263;578846900	N-Term(TMT6plex)	932.5	0				
																	SAPAPK	44	15	2	28173554;578846900	N-Term(TMT6plex); K6(TMT6plex)	1028.7	0				
																	LPHYNK	2	1	1	578846900	N-Term(TMT6plex); K6(TMT6plex)	1229.7	0				
																	STITSR	10	16	3	28173554;4504263;578846900	N-Term(TMT6plex)	893.5	0				
19923483	NP_057406.2	RAB14	51552	ras-related protein Rab-14	67.44	8	11	12	71	1.4	1.4	1.5	1.4	215	23.9	6.21												
																	DVTYEEAK	9	1	1	19923483	N-Term(TMT6plex); K8(TMT6plex)	1412.8	0	1.5	1.6	1.6	1.6
																	FMADCPHTIGVEFGTR	1	1	1	19923483	N-Term(TMT6plex); M2(Oxidation); C5(Carbamidomethyl)	2083.0	0	1.4	1.6	1.4	1.3
																	NLTNPNTVIILIGNK	17	1	1	19923483	N-Term(TMT6plex); K15(TMT6plex)	2082.3	0	1.3	1.3	1.6	1.2
																	TGENVEDAFLEAAK	20	1	1	19923483	N-Term(TMT6plex); K14(TMT6plex)	1952.0	0	1.3	1.1	1.3	1.1
																	YIIIGDMGVGK	7	1	1	19923483	N-Term(TMT6plex); M7(Oxidation); K11(TMT6plex)	1640.0	0	1.4	1.4	1.3	1.5
																	SCLLHQFTEK	2	1	1	19923483	N-Term(TMT6plex); C2(Carbamidomethyl); K10(TMT6plex)	1720.9	0	1.4	1.6	1.6	1.5
																	QFAEENGLLFLEASAK	2	1	1	19923483	N-Term(TMT6plex); K16(TMT6plex)	2225.2	0	1.3	1.3	1.4	1.5
																	LQIWDTAGQER	4	31	17	5803135;4758988;13569962;7706563;16933567;256222019;82659107;19923483;19923750;4759000;39930371;38679888;4506367;96975097;106507164;19923260;325910899	N-Term(TMT6plex)	1545.8	0				
																	GAAGALMVYDITR	2	1	1	19923483	N-Term(TMT6plex)	1566.9	0	1.7	1.9	1.9	1.7
																	STYNHLSSWLTDAR	1	1	1	19923483	N-Term(TMT6plex)	1879.9	0	1.4	1.7	1.5	1.4
																	IIEVSGQK	2	1	1	19923483	N-Term(TMT6plex); K8(TMT6plex)	1331.8	0	1.3	1.6	1.5	1.5
																	GAAGALMVYDITR	2	1	1	19923483	N-Term(TMT6plex); M7(Oxidation)	1582.8	0	1.2	1.3	1.5	1.4
																	LTSEPQPQR	2	1	1	19923483	N-Term(TMT6plex)	1284.7	0	1.4	1.5	1.7	1.6
319655561	NP_001188306.1	ALDH7A1	501	alpha-aminoadipic semialdehyde dehydrogenase isoform 2	67.32	4	27	27	251	1.4	1.4	1.5	1.5	511	55.3	6.86												
																	ILVEGVGEVQEYVDICDYAVGLSR	37	3	1	319655561	N-Term(TMT6plex); C16(Carbamidomethyl)	2912.5	0	1.5	1.6	1.3	1.5
																	GSDCGIVNVNIPTSGAEIGGAFGGEK	16	3	1	319655561	N-Term(TMT6plex); C4(Carbamidomethyl); K26(TMT6plex)	2964.5	0	1.4	1.7	1.6	1.6
																	QAVSMFLGAVEEAK	18	2	1	319655561	N-Term(TMT6plex); M5(Oxidation); K14(TMT6plex)	1954.1	0	1.5	1.3	1.5	1.5
																	VLEDNKLPGAICSLTCGGADIGTAMAK	4	3	1	319655561	N-Term(TMT6plex); K6(TMT6plex); C12(Carbamidomethyl); C16(Carbamidomethyl); M25(Oxidation); K27(TMT6plex)	3465.8	1	1.4	1.6	1.7	1.6
																	IQVLGSLVSLEMGK	28	3	1	319655561	N-Term(TMT6plex); M12(Oxidation); K14(TMT6plex)	1948.1	0	1.5	1.4	1.6	1.6
																	VNLLSFTGSTQVGK	18	3	1	319655561	N-Term(TMT6plex); K14(TMT6plex)	1909.1	0	1.4	1.4	1.4	1.6
																	GAPTTSLISVAVTK	10	3	1	319655561	N-Term(TMT6plex); K14(TMT6plex)	1803.1	0	1.3	1.4	1.4	1.5
																	VMDRPGNYVEPTIVTGLGHDASIAHTETFAPILYVFK	3	2	1	319655561	N-Term(TMT6plex); M2(Oxidation); K37(TMT6plex)	4533.4	0	1.1	1.5	1.4	1.7
																	MIGGPILPSER	1	3	1	319655561	N-Term(TMT6plex)	1398.8	0	1.6	1.6	1.8	1.5
																	QASVADYEETVK	12	3	1	319655561	N-Term(TMT6plex); K12(TMT6plex)	1798.0	0	1.4	1.3	1.4	1.5
																	MSTLLINQPQYAWLK	4	1	1	319655561	N-Term(TMT6plex); K15(TMT6plex)	2264.3	0	1.8	1.6	2.0	1.5

Khan et al., 2019. Multi-omics analysis to characterize cigarette smoke induced molecular alterations in esophageal cells
Supplementary Table 6. List of all proteins and peptides identified in untreated and chronically treated Het1A cells with cigarette smoke condensate for 8 months

Accession	RefSeq Accession	Gene Symbol	Entrez Gene ID	Description	Σ Coverage	Σ# Proteins	Σ# Unique Peptides	Σ# Peptides	Σ# PSMs	Het-1A-Smoke - 2M/Parental	Het-1A-Smoke - 4M/Parental	Het-1A-Smoke - 6M/Parental	Het-1A-Smoke - 8M/Parental	# AAs	MW [kDa]	calc. pI	Sequence	# PSMs	# Proteins	# Protein Groups	Protein Group Accessions	Modifications	MH+ [Da]	# Missed Cleavages	Het-1A-Smoke - 2M/Parental	Het-1A-Smoke - 4M/Parental	Het-1A-Smoke - 6M/Parental	Het-1A-Smoke - 8M/Parental
																	VGNPWDPNVLYGPLHTK	15	2	1	319655561	N-Term(TMT6plex); K17(TMT6plex)	2365.3	0	1.2	1.4	1.4	1.5
																	STCTINYSK	7	3	1	319655561	N-Term(TMT6plex); C3(Carbamidomethyl); K9(TMT6plex)	1531.8	0	2.0	1.6	1.6	1.5
																	IWADIPAPK	12	3	1	319655561	N-Term(TMT6plex); K9(TMT6plex)	1468.9	0	1.5	1.4	1.5	1.6
																	QGLSSSIFTK	5	3	1	319655561	N-Term(TMT6plex); K10(TMT6plex)	1525.9	0	1.4	1.4	1.5	1.4
																	FKNEEEVFAWNNEVK	1	3	1	319655561	N-Term(TMT6plex); K2(TMT6plex); K15(TMT6plex)	2570.4	1	1.5	1.5	1.8	1.5
																	KEGGTVVYGGK	6	2	1	319655561	N-Term(TMT6plex); K1(TMT6plex); K11(TMT6plex)	1782.1	1	1.3	1.5	1.5	1.6
																	EGGTVVYGGK	18	2	1	319655561	N-Term(TMT6plex); K10(TMT6plex)	1424.8	0	1.3	1.4	1.4	1.5
																	QVGLMVQER	3	3	1	319655561	N-Term(TMT6plex); M5(Oxidation)	1304.7	0	1.6	1.3	1.3	1.4
																	DLPLAQGIK	14	4	1	319655561	N-Term(TMT6plex); K9(TMT6plex)	1412.9	0	1.3	1.5	1.5	1.5
																	QASVADYEETVKK	3	3	1	319655561	N-Term(TMT6plex); K12(TMT6plex); K13(TMT6plex)	2155.2	1	1.3	1.4	1.5	1.6
																	MIGGPILPSER	5	3	1	319655561	N-Term(TMT6plex); M1(Oxidation)	1414.8	0	1.6	1.4	1.4	1.5
																	STLLINQPQYAWLK	1	1	1	319655561	N-Term(TMT6plex); K14(TMT6plex)	2133.2	0	1.6	1.3	1.6	1.3
																	GEVITTYCPANNEPIAR	2	3	1	319655561	N-Term(TMT6plex); C8(Carbamidomethyl)	2134.1	0	1.5	1.3	1.4	1.4
																	EENEGVYNGSWGGR	2	3	1	319655561	N-Term(TMT6plex)	1782.8	0	1.7	1.3	1.2	1.1
																	ESGSDAWK	3	3	1	319655561	N-Term(TMT6plex); K8(TMT6plex)	1337.7	0	1.2	1.3	1.3	1.4
																	QIGDALR	2	3	1	319655561	N-Term(TMT6plex)	1001.6	0				
																	VLEDNK	1	3	1	319655561	N-Term(TMT6plex); K6(TMT6plex)	1175.7	0	1.5	1.6	1.7	1.6
116063573	NP_001447.2	**FLNA**	2316	**filamin-A isoform 1**	67.30	2	109	124	1498	**1.3**	**1.3**	**1.2**	**1.1**	2639	279.8	6.05												
																	AEAGVPAEFSIWTR	27	2	1	116063573	N-Term(TMT6plex)	1762.9	0	1.3	1.3	1.2	1.2
																	SAGQGEVLVYVEDPAGHQEEAK	20	2	1	116063573	N-Term(TMT6plex); K22(TMT6plex)	2771.4	0	1.3	1.2	1.3	1.1
																	FGGEHVPNSPFQVTALAGDQPSVQPPLR	14	2	1	116063573	N-Term(TMT6plex)	3174.6	0	1.4	1.5	1.5	1.4
																	YWPQEAGEYAVHVLCNSEDIR	7	2	1	116063573	N-Term(TMT6plex); C15(Carbamidomethyl)	2765.3	0	1.3	1.5	1.4	1.3
																	IVGPSGAAVPCK	17	2	1	116063573	N-Term(TMT6plex); C11(Carbamidomethyl); K12(TMT6plex)	1613.9	0	1.3	1.4	1.4	1.2
																	DAGEGLLAVQITDPEGKPK	16	2	1	116063573	N-Term(TMT6plex); K17(TMT6plex); K19(TMT6plex)	2625.5	0	1.2	1.3	1.2	1.2
																	VPVHDVTDASK	11	2	1	116063573	N-Term(TMT6plex); K11(TMT6plex)	1625.9	0	1.1	1.2	1.2	1.1
																	AVPTGDASK	8	2	1	116063573	N-Term(TMT6plex); K9(TMT6plex)	1303.8	0	1.1	1.2	1.1	1.0
																	EAMQQADDWLGIPQVITPEEIVDPNVDEHSVMTYLSQFPK	12	2	1	116063573	N-Term(TMT6plex); M3(Oxidation); M32(Oxidation); K40(TMT6plex)	5060.5	0	1.4	1.4	1.5	1.3
																	VAQPTITDNK	24	2	1	116063573	N-Term(TMT6plex); K10(TMT6plex)	1544.9	0	1.3	1.2	1.2	1.1
																	AEISFEDR	22	2	1	116063573	N-Term(TMT6plex)	1195.6	0	1.2	1.3	1.2	1.2
																	IQQNTFTR	7	8	4	116063573;256222415;256222411;188595687	N-Term(TMT6plex)	1236.7	0				
																	VHGPGIQSGTTNKPNK	8	2	1	116063573	N-Term(TMT6plex); K13(TMT6plex); K16(TMT6plex)	2322.3	0	1.1	1.1	1.2	1.0
																	AGVAPLQVK	8	2	1	116063573	N-Term(TMT6plex); K9(TMT6plex)	1340.9	0	1.2	1.2	1.2	1.1
																	VTANNDK	4	2	1	116063573	N-Term(TMT6plex); K7(TMT6plex)	1219.7	0	1.2	1.0	1.1	0.8
																	IECDDKGDGSCDVR	8	4	2	116063573;188595687	N-Term(TMT6plex); C3(Carbamidomethyl); K6(TMT6plex); C11(Carbamidomethyl)	2084.0	1				
																	VGSAADIPINISETDLSLLTATVVPPSGR	68	2	1	116063573	N-Term(TMT6plex)	3122.7	0	1.5	1.4	1.5	1.4
																	FVPAEMGTHTVSVK	8	2	1	116063573	N-Term(TMT6plex); M6(Oxidation); K14(TMT6plex)	1977.1	0	1.2	1.2	1.3	1.1
																	VDVGKDQEFTVK	5	2	1	116063573	N-Term(TMT6plex); K5(TMT6plex); K12(TMT6plex)	2052.2	1	1.3	1.4	1.3	1.2
																	LVSIDSK	7	8	4	116063573;256222415;256222411;188595687	N-Term(TMT6plex); K7(TMT6plex)	1219.8	0				
																	LDVQFSGLTK	9	2	1	116063573	N-Term(TMT6plex); K10(TMT6plex)	1565.9	0	1.2	1.2	1.2	1.1
																	GTVEPQLEAR	14	2	1	116063573	N-Term(TMT6plex)	1328.7	0	1.2	1.3	1.1	1.1
																	DAGEGGLSLAIEGPSK	26	2	1	116063573	N-Term(TMT6plex); K16(TMT6plex)	1959.1	0	1.2	1.3	1.3	1.1
																	VQVQDNEGCPVEALVK	18	2	1	116063573	N-Term(TMT6plex); C9(Carbamidomethyl); K16(TMT6plex)	2243.2	0	1.3	1.3	1.4	1.0
																	YGGQPVPNFPSK	29	2	1	116063573	N-Term(TMT6plex); K12(TMT6plex)	1749.0	0	1.3	1.2	1.3	1.2
																	YGGDEIPFSPYR	20	2	1	116063573	N-Term(TMT6plex)	1629.8	0	1.3	1.2	1.2	1.1
																	AHVVPCFDASK	12	2	1	116063573	N-Term(TMT6plex); C6(Carbamidomethyl); K11(TMT6plex)	1688.9	0	1.2	1.3	1.4	1.2
																	GAGSYTIMVLFADQATPTSPIR	8	2	1	116063573	N-Term(TMT6plex); M8(Oxidation)	2541.3	0	1.9	1.6	1.8	1.4
																	VTVLFAGQHIAK	12	2	1	116063573	N-Term(TMT6plex); K12(TMT6plex)	1742.1	0	1.2	1.2	1.3	1.1
																	LLGWIQNK	32	8	4	116063573;256222415;256222411;188595687	N-Term(TMT6plex); K8(TMT6plex)	1429.9	0				
																	ALTQTGGPHVK	8	2	1	116063573	N-Term(TMT6plex); K11(TMT6plex)	1566.9	0	1.1	1.2	1.3	1.1
																	WCNEHLK	8	8	4	116063573;256222415;256222411;188595687	N-Term(TMT6plex); C2(Carbamidomethyl); K7(TMT6plex)	1444.8	0				
																	MDCQECPEGYR	10	2	1	116063573	M1(Oxidation); N-Term(TMT6plex); C3(Carbamidomethyl); C6(Carbamidomethyl)	1689.7	0	1.3	1.1	1.1	0.9
																	VKVEPSHDASK	5	2	1	116063573	N-Term(TMT6plex); K2(TMT6plex); K11(TMT6plex)	1884.1	1	1.1	1.4	1.2	1.1
																	TGVAVNKPAEFTVDAK	9	2	1	116063573	N-Term(TMT6plex); K7(TMT6plex); K16(TMT6plex)	2334.4	0	1.2	1.0	1.2	0.9
																	GLVEPVDVVDNADGTQTVNYVPSR	27	2	1	116063573	N-Term(TMT6plex)	2773.4	0	1.3	1.6	1.8	1.3
																	VTAQGPGLEPSGNIANK	20	2	1	116063573	N-Term(TMT6plex); K17(TMT6plex)	2111.2	0	1.3	1.1	1.2	1.1
																	VEPGLGADNSVVR	14	2	1	116063573	N-Term(TMT6plex)	1541.8	0	1.3	1.3	1.2	1.2
																	QMQLENVSVALEFLDR	41	6	3	116063573;256222415;256222411	N-Term(TMT6plex); M2(Oxidation)	2137.1	0				
																	VHSPSGALEECYVTEIDQDK	21	2	1	116063573	N-Term(TMT6plex); C11(Carbamidomethyl); K20(TMT6plex)	2735.4	0	1.3	1.3	1.2	1.3
																	IANLQTDLSDGLR	8	2	1	116063573	N-Term(TMT6plex)	1644.9	0	1.4	1.1	1.2	1.0
																	KDGSCGVAYVVQEPGDYEVSVK	5	2	1	116063573	N-Term(TMT6plex); K1(TMT6plex); C5(Carbamidomethyl); K22(TMT6plex)	3073.6	1	1.4	1.4	1.3	1.4
																	AEFTVETR	9	2	1	116063573	N-Term(TMT6plex)	1181.6	0	1.3	1.3	1.2	1.2
																	YAPSEAGLHEMDIR	23	2	1	116063573	N-Term(TMT6plex); M11(Oxidation)	1833.9	0	1.3	1.3	1.3	1.2
																	EEGPYEVEVTYDGVPVPGSPFPLEAVAPTKPSK	6	2	1	116063573	N-Term(TMT6plex); K30(TMT6plex); K33(TMT6plex)	4173.2	0	1.2	1.2	1.2	1.0
																	YGGPYHIGGSPFK	7	2	1	116063573	N-Term(TMT6plex); K13(TMT6plex)	1838.0	0	1.1	1.2	1.1	1.1
																	VEYTPYEEGLHSVDVTYDGSPVPSSPFQVPVTEGCDPSR	9	2	1	116063573	N-Term(TMT6plex); C35(Carbamidomethyl)	4526.1	0	1.7	1.4	1.5	1.2
																	DVDIIDHHDNTYTVK	7	2	1	116063573	N-Term(TMT6plex); K15(TMT6plex)	2243.2	0	1.2	1.4	1.3	1.3
																	DAPQDFHPDR	6	2	1	116063573	N-Term(TMT6plex)	1426.7	0	1.2	1.4	1.3	1.3
																	KGEITGEVR	5	2	1	116063573	N-Term(TMT6plex); K1(TMT6plex)	1446.9	1	1.2	1.2	1.2	1.1
																	LTVSSLQESGLK	13	2	1	116063573	N-Term(TMT6plex); K12(TMT6plex)	1720.0	0	1.2	1.2	1.2	1.0
																	DAGYGGLSLSIEGPSK	4	2	1	116063573	N-Term(TMT6plex); K16(TMT6plex)	2009.1	0	1.3	1.4	1.4	1.5
																	YAPSEAGLHEMDIR	2	2	1	116063573	N-Term(TMT6plex)	1817.9	0	1.3	1.4	1.4	1.1
																	QMQLENVSVALEFLDR	5	6	3	116063573;256222415;256222411	N-Term(TMT6plex)	2121.1	0				
																	TGVELGKPTHFTVNAK	2	2	1	116063573	N-Term(TMT6plex); K7(TMT6plex); K16(TMT6plex)	2386.4	0	1.2	1.3	1.4	1.3
																	AFGPGLQGGSAGSPAR	16	2	1	116063573	N-Term(TMT6plex)	1658.9	0	1.4	1.4	1.3	1.1
																	VANPSGNLTETYVQDR	24	2	1	116063573	N-Term(TMT6plex)	1993.0	0	1.5	1.2	1.2	1.1
																	CAPGVVGPAEADIDFDIIR	2	2	1	116063573	N-Term(TMT6plex); C1(Carbamidomethyl)	2244.1	0	1.5	1.6	1.5	1.5
																	ASGPGLNTTGVPASLPVEFTIDAK	16	2	1	116063573	N-Term(TMT6plex); K24(TMT6plex)	2800.6	0	1.6	1.4	1.5	1.6
																	SPFSVAVSPSLDLSK	8	2	1	116063573	N-Term(TMT6plex); K15(TMT6plex)	1992.1	0	1.4	1.5	1.4	1.3
																	DGSCGVAYVVQEPGDYEVSVK	11	2	1	116063573	N-Term(TMT6plex); C4(Carbamidomethyl); K21(TMT6plex)	2716.4	0	1.3	1.2	1.3	1.2
																	YTPVQQGPVGVNVTYGGDPIPK	16	2	1	116063573	N-Term(TMT6plex); K22(TMT6plex)	2744.5	0	1.4	1.3	1.3	1.2
																	KTHIQDNHDGTYTVAYVPDVTGR	5	2	1	116063573	N-Term(TMT6plex); K1(TMT6plex)	3045.6	1	1.2	1.2	1.2	1.3
																	AYGPGIEPTGNMVK	16	2	1	116063573	N-Term(TMT6plex); M12(Oxidation); K14(TMT6plex)	1908.0	0	1.3	1.4	1.4	1.2
																	SSFTVDCSK	12	2	1	116063573	N-Term(TMT6plex); C7(Carbamidomethyl); K9(TMT6plex)	1488.8	0	1.3	1.2	1.2	1.2
																	LKPGAPLRPK	8	2	1	116063573	N-Term(TMT6plex); K2(TMT6plex); K10(TMT6plex)	1764.2	0	1.0	1.2	1.2	1.1
																	EGPYSISVLYGDEEVPR	13	2	1	116063573	N-Term(TMT6plex)	2139.1	0	1.2	1.2	1.2	1.1
																	IPEISIQDMTAQVTSPSGK	5	2	1	116063573	N-Term(TMT6plex); M9(Oxidation); K19(TMT6plex)	2476.3	0	1.4	1.3	1.3	1.3
																	ALGALVDSCAPGLCPDWDSWDASKPVTNAR	6	2	1	116063573	N-Term(TMT6plex); C9(Carbamidomethyl); C14(Carbamidomethyl); K24(TMT6plex)	3687.8	0	1.2	1.4	1.3	1.0
																	GAGTGGLGLAVEGPSEAK	26	2	1	116063573	N-Term(TMT6plex); K18(TMT6plex)	2029.1	0	1.2	1.3	1.1	1.0
																	LIALLEVLSQK	22	8	4	116063573;256222415;256222411;188595687	N-Term(TMT6plex); K11(TMT6plex)	1685.1	0				
																	AWGPGLEGGVVGK	33	2	1	116063573	N-Term(TMT6plex); K13(TMT6plex)	1685.0	0	1.2	1.4	1.3	1.2
																	LQVEPAVDTSGVQCYGPGIEGQGVFR	24	2	1	116063573	N-Term(TMT6plex); C14(Carbamidomethyl)	2992.5	0	1.3	1.3	1.4	1.2
																	IPEISIQDMTAQVTSPSGK	12	2	1	116063573	N-Term(TMT6plex); K19(TMT6plex)	2460.3	0	1.6	1.6	1.7	1.3
																	DLAEDAPWK	24	8	4	116063573;256222415;256222411;188595687	N-Term(TMT6plex); K9(TMT6plex)	1502.8	0				
																	LYSVSYLLK	12	2	1	116063573	N-Term(TMT6plex); K9(TMT6plex)	1544.0	0	1.4	1.1	1.3	0.9
																	EAMQQADDWLGIPQVITPEEIVDPNVDEHSVMTYLSQFPK	18	2	1	116063573	N-Term(TMT6plex); M3(Oxidation); K40(TMT6plex)	5044.5	0	1.4	1.6	1.8	1.1
																	VTYTPMAPGSYLISIK	17	2	1	116063573	N-Term(TMT6plex); M6(Oxidation); K16(TMT6plex)	2215.2	0	1.4	1.3	1.6	1.2
																	LVSNHSLHETSSVFVDSLTK	13	2	1	116063573	N-Term(TMT6plex); K20(TMT6plex)	2658.5	0	1.2	1.3	1.2	1.2
																	TFSVWYVPEVTGTHK	7	2	1	116063573	N-Term(TMT6plex); K15(TMT6plex)	2209.2	0	1.3	1.3	1.4	1.2
																	GAGGQGK	3	6	3	116063573;256222415;256222411	N-Term(TMT6plex); K7(TMT6plex)	1032.6	0				
																	DKGEYTLVVK	13	2	1	116063573	N-Term(TMT6plex); K2(TMT6plex); K10(TMT6plex)	1839.1	1	1.1	1.3	1.3	1.2
																	TPCEEILVK	10	2	1	116063573	N-Term(TMT6plex); C3(Carbamidomethyl); K9(TMT6plex)	1546.9	0	1.3	1.3	1.3	1.1
																	ANLPQSFQVDTSK	8	2	1	116063573	N-Term(TMT6plex); K13(TMT6plex)	1893.0	0	1.2	1.3	1.3	1.1
																	AGQSAAGAAPGGGVDTR	10	2	1	116063573	N-Term(TMT6plex)	1671.9	0	2.1	1.8	1.8	1.5
																	EATTEFSVDAR	21	2	1	116063573	N-Term(TMT6plex)	1454.7	0	1.4	1.4	1.4	1.3
																	ATCAPQHGAPGPGPADASK	8	2	1	116063573	N-Term(TMT6plex); C3(Carbamidomethyl); K19(TMT6plex)	2248.2	0	1.8	1.4	1.4	1.2
																	NDNDTFTVK	12	4	2	116063573;188595687	N-Term(TMT6plex); K9(TMT6plex)	1511.8	0				
																	LPQLPITNFSR	24	2	1	116063573	N-Term(TMT6plex)	1514.9	0	1.3	1.4	1.3	1.2
																	AIVDGNLK	6	8	4	116063573;256222415;256222411;188595687	N-Term(TMT6plex); K8(TMT6plex)	1287.8	0				
																	VSGQGLHEGHTFEPAEFIIDTR	18	2	1	116063573	N-Term(TMT6plex)	2669.4	0	1.3	1.3	1.3	1.2
																	MDCQECPEGYR	2	2	1	116063573	N-Term(TMT6plex); C3(Carbamidomethyl); C6(Carbamidomethyl)	1673.7	0	1.4	1.5	1.7	1.0
																	ETGEHLVHVK	2	2	1	116063573	N-Term(TMT6plex); K10(TMT6plex)	1606.9	0	0.9	1.1	1.0	0.9

Khan et al., 2019. Multi-omics analysis to characterize cigarette smoke induced molecular alterations in esophageal cells
Supplementary Table 6. List of all proteins and peptides identified in untreated and chronically treated Het1A cells with cigarette smoke condensate for 8 months

Accession	RefSeq Accession	Gene Symbol	Entrez Gene ID	Description	Σ Coverage	Σ# Proteins	Σ# Unique Peptides	Σ# Peptides	Σ# PSMs	Het-1A-Smoke - 2M/Parental	Het-1A-Smoke - 4M/Parental	Het-1A-Smoke - 6M/Parental	Het-1A-Smoke - 8M/Parental	# AAs	MW [kDa]	calc. pI	Sequence	# PSMs	# Proteins	# Protein Groups	Protein Group Accessions	Modifications	MH+ [Da]	# Missed Cleavages	Het-1A-Smoke - 2M/Parental	Het-1A-Smoke - 4M/Parental	Het-1A-Smoke - 6M/Parental	Het-1A-Smoke - 8M/Parental
																	VSGLGEK	3	2	1	116063573	N-Term(TMT6plex); K7(TMT6plex)	1147.7	0	1.1	1.1	1.1	0.9
																	VDINTEDLEDGTCR	4	2	1	116063573	N-Term(TMT6plex); C13(Carbamidomethyl)	1865.9	0	1.4	1.5	1.5	1.3
																	SPFEVYVDK	11	2	1	116063573	N-Term(TMT6plex); K9(TMT6plex)	1541.9	0	1.3	1.3	1.4	1.1
																	VLPTHDASK	8	2	1	116063573	N-Term(TMT6plex); K9(TMT6plex)	1425.8	0	1.1	1.3	1.2	1.2
																	GKLDVQFSGLTK	2	2	1	116063573	N-Term(TMT6plex); K2(TMT6plex); K12(TMT6plex)	1980.2	1	1.0	1.5	1.2	1.2
																	YNEQHVPGSPFTAR	6	2	1	116063573	N-Term(TMT6plex)	1831.9	0	1.2	1.1	1.2	1.1
																	HTAMVSWGGVSIPNSPFR	4	2	1	116063573	N-Term(TMT6plex); M4(Oxidation)	2188.1	0	1.5	1.6	1.7	1.7
																	AHEPTYFTVDCAEAGQGDVSIGIK	11	2	1	116063573	N-Term(TMT6plex); C11(Carbamidomethyl); K24(TMT6plex)	3023.5	0	1.3	1.4	1.3	1.2
																	DAEMPATEK	6	2	1	116063573	N-Term(TMT6plex); M4(Oxidation); K9(TMT6plex)	1465.8	0	1.7	1.8	1.8	1.4
																	EEPCLLK	5	2	1	116063573	N-Term(TMT6plex); C4(Carbamidomethyl); K7(TMT6plex)	1346.8	0	1.2	1.2	1.2	1.0
																	AGNNMLLVGVHGPR	2	2	1	116063573	N-Term(TMT6plex)	1663.9	0	1.4	1.3	1.4	1.3
																	NGQHVASSPIPVVISQSEIGDASR	1	2	1	116063573	N-Term(TMT6plex)	2677.4	0	0.9	0.9	0.9	0.9
																	VTYCPTEPGNYIINIK	4	2	1	116063573	N-Term(TMT6plex); C4(Carbamidomethyl); K16(TMT6plex)	2340.3	0	1.3	1.3	1.2	1.2
																	FADQHVPGSPFSVK	2	2	1	116063573	N-Term(TMT6plex); K14(TMT6plex)	1974.1	0	1.5	1.5	1.5	1.7
																	SADFVVEAIGDDVGTLGFSVEGPSQAK	56	2	1	116063573	N-Term(TMT6plex); K27(TMT6plex)	3153.7	0	1.4	1.4	1.2	1.4
																	GQHVPGSPFQFTVGPLGEGGAHK	8	4	2	116063573;188595687	N-Term(TMT6plex); K23(TMT6plex)	2762.5	0				
																	EAGAGGLAIAVEGPSK	21	2	1	116063573	N-Term(TMT6plex); K16(TMT6plex)	1885.1	0	1.1	1.2	1.1	1.0
																	VGTECGNQK	8	2	1	116063573	N-Term(TMT6plex); C5(Carbamidomethyl); K9(TMT6plex)	1450.8	0	1.5	1.0	1.2	0.8
																	FNEEHIPDSPFVVPVASPSGDAR	14	2	1	116063573	N-Term(TMT6plex)	2696.4	0	1.2	1.2	1.2	1.0
																	GAGSGELK	5	4	2	116063573;188595687	N-Term(TMT6plex); K8(TMT6plex)	1176.7	0				
																	SPYTVTVGQACNPSACR	7	2	1	116063573	N-Term(TMT6plex); C11(Carbamidomethyl); C16(Carbamidomethyl)	2097.0	0	1.6	1.6	1.6	1.4
																	AGNNMLLVGVHGPR	4	2	1	116063573	N-Term(TMT6plex); M5(Oxidation)	1679.9	0	1.3	1.6	1.5	1.6
																	VAQPTITDNKDGTVTVR	2	2	1	116063573	N-Term(TMT6plex); K10(TMT6plex)	2273.3	1	1.6	1.8	1.3	1.3
																	EAMQQADDWLGIPQVITPEEIVDPNVDEHSVMTYLSQFPK	2	2	1	116063573	N-Term(TMT6plex); K40(TMT6plex)	5028.5	0	2.0	1.9	2.0	1.4
																	VTYTPMAPGSYLISIK	4	2	1	116063573	N-Term(TMT6plex); K16(TMT6plex)	2199.3	0	1.6	1.5	1.8	1.3
																	VEPSHDASK	6	2	1	116063573	N-Term(TMT6plex); K9(TMT6plex)	1427.8	0	1.2	1.2	1.2	1.0
																	AEISCTDNQDGTCSVSYLPVLPGDYSILVK	6	2	1	116063573	N-Term(TMT6plex); C5(Carbamidomethyl); C13(Carbamidomethyl); K30(TMT6plex)	3759.9	0	1.2	1.3	1.1	1.0
																	VNVGAGSHPNK	2	2	1	116063573	N-Term(TMT6plex); K11(TMT6plex)	1537.9	0	1.2	1.1	1.1	1.0
																	CSGPGLSPGMVR	4	2	1	116063573	N-Term(TMT6plex); C1(Carbamidomethyl); M10(Oxidation)	1462.7	0	1.4	1.0	1.1	0.9
																	AGGPGLER	5	6	3	116063573;256222415;256222411	N-Term(TMT6plex)	985.6	0				
																	VTCTVCTPDGSEVDVDVVENEDGTFDIFYTAPQPGK	2	2	1	116063573	N-Term(TMT6plex); C3(Carbamidomethyl); C6(Carbamidomethyl); K36(TMT6plex)	4420.1	0	1.1	1.3	1.2	1.1
																	VTGDDSMR	2	2	1	116063573	N-Term(TMT6plex); M7(Oxidation)	1125.5	0	1.3	1.5	1.2	1.3
																	WGDEHIPGSPYR	5	2	1	116063573	N-Term(TMT6plex)	1642.8	0	1.1	1.5	1.5	1.4
																	VKETADFK	1	2	1	116063573	N-Term(TMT6plex); K2(TMT6plex); K8(TMT6plex)	1625.0	1	1.2	1.3	1.3	1.3
																	DGTVTVR	1	6	3	116063573;256222415;256222411	N-Term(TMT6plex)	976.6	0				
																	YVICVR	2	2	1	116063573	N-Term(TMT6plex); C4(Carbamidomethyl)	1038.6	0	1.4	1.3	1.3	1.1
																	GAIDAK	2	2	1	116063573	N-Term(TMT6plex); K6(TMT6plex)	1032.6	0	1.1	1.2	1.1	1.0
																	YTILIK	1	2	1	116063573	N-Term(TMT6plex); K6(TMT6plex)	1208.8	0	1.2	1.1	1.1	0.9
																	ETADFK	1	2	1	116063573	N-Term(TMT6plex); K6(TMT6plex)	1168.7	0	1.3	1.3	1.3	1.2
4758112;18375623	NP_004631.1	**DDX39B**	7919	**spliceosome RNA helicase DDX39B**	67.29	2	12	23	405	1.3	1.2	1.3	1.3	428	49.0	5.67												
																	VNIAFNYDMPEDSDTYLHR	25	1	1	4758112;18375623	N-Term(TMT6plex); M9(Oxidation)	2545.2	0	1.3	1.2	1.2	1.2
																	CIALAQLLVEQNFPAIAIHR	11	1	1	4758112;18375623	N-Term(TMT6plex); C1(Carbamidomethyl)	2506.4	0	1.6	1.2	1.4	1.1
																	ELAFQISK	25	2	2	21040371;4758112;18375623	N-Term(TMT6plex); K8(TMT6plex)	1393.8	0				
																	DVQEIFR	19	2	2	21040371;4758112;18375623	N-Term(TMT6plex)	1135.6	0				
																	KDEEVLK	8	2	2	21040371;4758112;18375623	N-Term(TMT6plex); K1(TMT6plex); K7(TMT6plex)	1548.0	1				
																	AIVDCGFEHPSEVQHECIPQAILGMDVLCQAK	20	2	2	21040371;4758112;18375623	N-Term(TMT6plex); C5(Carbamidomethyl); C17(Carbamidomethyl); M25(Oxidation); C29(Carbamidomethyl); K32(TMT6plex)	4126.0	0				
																	GSYVSIHSSGFR	15	2	2	21040371;4758112;18375623	N-Term(TMT6plex)	1525.8	0				
																	FMQDPMEIFVDDETK	15	1	1	4758112;18375623	N-Term(TMT6plex); M2(Oxidation); K15(TMT6plex)	2319.1	0	1.5	1.5	1.6	1.5
																	ILVATNLFGR	28	2	2	21040371;4758112;18375623	N-Term(TMT6plex)	1332.8	0				
																	FMQDPMEIFVDDETK	11	1	1	4758112;18375623	N-Term(TMT6plex); M2(Oxidation); M6(Oxidation); K15(TMT6plex)	2335.1	0	1.2	1.7	1.4	1.6
																	ILNDVQDR	15	2	2	21040371;4758112;18375623	N-Term(TMT6plex)	1201.7	0				
																	QVMMFSATLSK	3	1	1	4758112;18375623	N-Term(TMT6plex); M3(Oxidation); M4(Oxidation); K11(TMT6plex)	1732.9	0	1.4	1.5	1.4	1.7
																	HFILDECDK	10	1	1	4758112;18375623	N-Term(TMT6plex); C7(Carbamidomethyl); K9(TMT6plex)	1634.9	0	1.2	1.2	1.2	1.2
																	VNIAFNYDMPEDSDTYLHR	7	1	1	4758112;18375623	N-Term(TMT6plex)	2529.2	0	1.5	1.6	1.5	1.5
																	FMQDPMEIFVDDETK	2	1	1	4758112;18375623	N-Term(TMT6plex); K15(TMT6plex)	2303.1	0	1.8	1.9	2.0	1.3
																	NCPHIVVGTPGR	9	1	1	4758112;18375623	N-Term(TMT6plex); C2(Carbamidomethyl)	1535.8	0	1.0	1.2	1.2	1.3
																	DFLLKPELLR	24	2	2	21040371;4758112;18375623	N-Term(TMT6plex); K5(TMT6plex)	1702.1	0				
																	AIVDCGFEHPSEVQHECIPQAILGMDVLCQAK	2	2	2	21040371;4758112;18375623	N-Term(TMT6plex); C5(Carbamidomethyl); C17(Carbamidomethyl); C29(Carbamidomethyl); K32(TMT6plex)	4110.0	0				
																	KFMQDPMEIFVDDETK	9	1	1	4758112;18375623	N-Term(TMT6plex); K1(TMT6plex); M3(Oxidation); M7(Oxidation); K16(TMT6plex)	2692.4	1	1.3	1.4	1.3	1.5
																	LTLHGLQQYYVK	43	2	2	21040371;4758112;18375623	N-Term(TMT6plex); K12(TMT6plex)	1921.1	0				
																	VAVFFGGLSIK	28	1	1	4758112;18375623	N-Term(TMT6plex); K11(TMT6plex)	1596.0	0	1.3	1.1	1.3	1.3
																	GLAITFVSDENDAK	44	2	2	21040371;4758112;18375623	N-Term(TMT6plex); K14(TMT6plex)	1938.1	0				
																	KFMQDPMEIFVDDETK	4	1	1	4758112;18375623	N-Term(TMT6plex); K1(TMT6plex); K16(TMT6plex)	2660.4	1	1.5	1.7	1.6	1.3
																	TAVFVLATLQQLEPVTGQVSVLVMCHTR	3	1	1	4758112;18375623	N-Term(TMT6plex); C25(Carbamidomethyl)	3326.8	0	1.4	1.3	2.3	1.0
																	FEVNISELPDEIDISSYIEQTR	8	1	1	4758112;18375623	N-Term(TMT6plex)	2826.4	0	2.4	1.1	1.9	1.6
																	TAVFVLATLQQLEPVTGQVSVLVMCHTR	4	1	1	4758112;18375623	N-Term(TMT6plex); M24(Oxidation); C25(Carbamidomethyl)	3342.8	0	1.5	1.2	2.3	1.0
																	MLEQLDMR	2	2	2	21040371;4758112;18375623	N-Term(TMT6plex)	1264.7	0				
																	ILALAR	10	2	1	4758112;18375623	N-Term(TMT6plex)	885.6	0	1.3	1.1	1.2	1.2
																	MTPHEK	1	1	1	4758112;18375623	N-Term(TMT6plex); M1(Oxidation); K6(TMT6plex)	1216.7	0	1.0	1.2	1.2	1.4
14165437;14165439	NP_112553.1	**HNRNPK**	3190	**heterogeneous nuclear ribonucleoprotein K isoform a**	67.24	4	28	28	1189	1.2	1.3	1.3	1.4	464	51.0	5.33												
																	IILDLISESPIK	213	4	1	14165437;14165439	N-Term(TMT6plex); K12(TMT6plex)	1799.1	0	1.2	1.3	1.2	1.4
																	TDYNASVSVPDSSGPER	84	4	1	14165437;14165439	N-Term(TMT6plex)	2010.0	0	1.5	1.2	1.3	1.4
																	AQPYDPNFYDETYDYGGFTMMFDDR	75	4	1	14165437;14165439	N-Term(TMT6plex); M20(Oxidation); M21(Oxidation)	3319.4	0	1.4	1.4	1.4	1.5
																	DLAGSIIGK	35	4	1	14165437;14165439	N-Term(TMT6plex); K9(TMT6plex)	1331.8	0	1.1	1.3	1.3	1.5
																	NTDEMVELR	33	2	1	14165437;14165439	N-Term(TMT6plex); M5(Oxidation)	1351.7	0	1.4	1.3	1.4	1.4
																	RPAEDMEEEQAFK	49	2	1	14165437;14165439	N-Term(TMT6plex); M6(Oxidation); K13(TMT6plex)	2054.0	0	1.2	1.3	1.2	1.5
																	NLPLPPPPPPR	73	2	1	14165437;14165439	N-Term(TMT6plex)	1423.9	0	1.2	1.2	1.3	1.3
																	IITITGTQDQIQNAQYLLQNSVK	60	2	1	14165437;14165439	N-Term(TMT6plex); K23(TMT6plex)	3047.7	0	1.3	1.3	1.4	1.5
																	GSYGDLGGPIITTQVTIPK	66	4	1	14165437;14165439	N-Term(TMT6plex); K19(TMT6plex)	2375.3	0	1.1	1.3	1.3	1.5
																	LLIHQSLAGGIIGVK	50	4	1	14165437;14165439	N-Term(TMT6plex); K15(TMT6plex)	1977.3	0	1.2	1.3	1.3	1.4
																	IDEPLEGSEDR	23	2	1	14165437;14165439	N-Term(TMT6plex)	1488.7	0	1.4	1.3	1.2	1.3
																	NAGAVIGK	25	4	1	14165437;14165439	N-Term(TMT6plex); K8(TMT6plex)	1187.7	0	1.1	1.0	1.2	1.1
																	GGDLMAYDR	44	4	1	14165437;14165439	N-Term(TMT6plex); M5(Oxidation)	1242.6	0	1.2	1.4	1.3	1.4
																	DYDDMSPR	21	4	1	14165437;14165439	N-Term(TMT6plex); M5(Oxidation)	1243.5	0	1.3	1.4	1.4	1.5
																	LFQECCPHSTDR	14	2	1	14165437;14165439	N-Term(TMT6plex); C5(Carbamidomethyl); C6(Carbamidomethyl)	1778.8	0	1.3	1.4	1.4	1.4
																	ILSISADIETIGEILK	119	2	1	14165437;14165439	N-Term(TMT6plex); K16(TMT6plex)	2173.3	0	1.2	1.1	1.3	1.2
																	ENTQTTIK	32	4	1	14165437;14165439	N-Term(TMT6plex); K8(TMT6plex)	1392.8	0	1.0	1.1	1.1	1.2
																	IIPTLEEGLQLPSPTATSQLPLESDAVECLNYQHYK	42	2	1	14165437;14165439	N-Term(TMT6plex); C29(Carbamidomethyl); K36(TMT6plex)	4513.3	0	1.2	1.4	1.3	1.5
																	GSDFDCELR	24	2	1	14165437;14165439	N-Term(TMT6plex); C6(Carbamidomethyl)	1327.6	0	1.2	1.3	1.3	1.4
																	AQPYDPNFYDETYDYGGFTMMFDDR	17	4	1	14165437;14165439	N-Term(TMT6plex); M21(Oxidation)	3303.4	0	1.5	1.3	1.3	1.4
																	DYDDMSPR	2	4	1	14165437;14165439	N-Term(TMT6plex)	1227.6	0	1.6	1.8	1.9	1.5
																	HESGASIK	6	4	1	14165437;14165439	N-Term(TMT6plex); K8(TMT6plex)	1286.7	0	1.1	1.1	1.2	1.3
																	QYADVEGF	9	1	1	14165437;14165439	N-Term(TMT6plex)	1157.6	0	1.3	1.4	1.4	1.5
																	VVLIGGKPDR	36	4	1	14165437;14165439	N-Term(TMT6plex); K7(TMT6plex)	1512.0	0	1.1	1.3	1.2	1.4
																	AQPYDPNFYDETYDYGGFTMMFDDR	2	4	1	14165437;14165439	N-Term(TMT6plex)	3287.4	0	1.4	1.7	1.7	1.7
																	AQPYDPNFYDETYDYGGFTMMFDDRR	2	2	1	14165437;14165439	N-Term(TMT6plex); M20(Oxidation)	3459.5	1	1.8	4.1	2.2	3.0
																	NTDEMVELR	3	2	1	14165437;14165439	N-Term(TMT6plex)	1335.7	0	1.4	1.4	1.6	1.4
																	RPAEDMEEEQAFK	4	2	1	14165437;14165439	N-Term(TMT6plex); K13(TMT6plex)	2038.0	0	2.9	2.1	2.2	1.6
																	ILSISADIETIGEILKK	3	2	1	14165437;14165439	N-Term(TMT6plex); K16(TMT6plex); K17(TMT6plex)	2530.6	1	1.3	1.4	1.6	1.4
																	RPAEDMEEEQAFKR	2	2	1	14165437;14165439	N-Term(TMT6plex); M6(Oxidation); K13(TMT6plex)	2210.1	1	1.2	1.7	1.2	1.8
																	AQPYDPNFYDETYDYGGFTMMFDDRR	2	2	1	14165437;14165439	N-Term(TMT6plex); M20(Oxidation); M21(Oxidation)	3475.5	1	1.8	2.2	1.5	2.7

Supplementary Table 6. List of all proteins and peptides identified in untreated and chronically treated Het1A cells with cigarette smoke condensate for 8 months

Accession	RefSeq Accession	Gene Symbol	Entrez Gene ID	Description	Σ Coverage	Σ# Proteins	Σ# Unique Peptides	Σ# Peptides	Σ# PSMs	Het-1A-Smoke - 2M/Parental	Het-1A-Smoke - 4M/Parental	Het-1A-Smoke - 6M/Parental	Het-1A-Smoke - 8M/Parental	# AAs	MW [kDa]	calc. pI	Sequence	# PSMs	# Proteins	# Protein Groups	Protein Group Accessions	Modifications	MH+ [Da]	# Missed Cleavages	Het-1A-Smoke - 2M/Parental	Het-1A-Smoke - 4M/Parental	Het-1A-Smoke - 6M/Parental	Het-1A-Smoke - 8M/Parental
																	GGDLMAYDR	1	4	1	14165437;14165439	N-Term(TMT6plex)	1226.6	0	**1.4**	**1.4**	**1.5**	**1.4**
																	VVECIK	9	2	1	14165437;14165439	N-Term(TMT6plex); C4(Carbamidomethyl); K6(TMT6plex)	1205.7	0	**1.2**	**1.3**	**1.3**	**1.4**
																	ILLQSK	8	2	1	14165437;14165439	N-Term(TMT6plex); K6(TMT6plex)	1159.8	0	**1.2**	**1.2**	**1.3**	**1.4**
																	RDYDDMSPR	1	2	1	14165437;14165439	N-Term(TMT6plex); M6(Oxidation)	1399.6	1	**1.5**	**1.6**	**1.5**	**1.5**
112382250	NP_003119.2	**SPTBN1**	6711	**spectrin beta chain, non-erythrocytic 1 isoform 1**	67.22	4	11	136	864	**1.1**	**1.2**	**1.2**	**1.3**	2364	274.4	5.57												
																	EVDDLEQWIAER	18	2	2	112382250;112382252	N-Term(TMT6plex)	1731.9	0				
																	VSEEAESQQQWDTSK	10	2	2	112382250;112382252	N-Term(TMT6plex); K15(TMT6plex)	2210.1	0				
																	EIGQSVDEVEK	15	2	2	112382250;112382252	N-Term(TMT6plex); K11(TMT6plex)	1690.9	0				
																	TQTAIASEDMPNTLTEAEK	12	2	2	112382250;112382252	N-Term(TMT6plex); M10(Oxidation); K19(TMT6plex)	2524.3	0				
																	NDSFTTCIELGK	12	2	2	112382250;112382252	N-Term(TMT6plex); C7(Carbamidomethyl); K12(TMT6plex)	1843.0	0				
																	VIESTQDLGNDLAGVMALQR	16	2	2	112382250;112382252	N-Term(TMT6plex); M16(Oxidation)	2375.2	0				
																	LAEISDVWEEMK	13	2	2	112382250;112382252	N-Term(TMT6plex); M11(Oxidation); K12(TMT6plex)	1924.0	0				
																	FQIQDISVETEDNK	6	3	3	112382250;5902122;112382252	N-Term(TMT6plex); K14(TMT6plex)	2124.1	0				
																	LLEVLSGER	6	2	2	112382250;112382252	N-Term(TMT6plex)	1244.7	0				
																	IVSSSDVGHDEYSTQSLVK	7	2	2	112382250;112382252	N-Term(TMT6plex); K19(TMT6plex)	2509.3	0				
																	ELEAENYHDIK	2	2	2	112382250;112382252	N-Term(TMT6plex); K11(TMT6plex)	1819.0	0				
																	GNLEVLLFTIQSK	8	3	3	112382250;5902122;112382252	N-Term(TMT6plex); K13(TMT6plex)	1920.2	0				
																	SAATWDER	2	2	2	112382250;112382252	N-Term(TMT6plex)	1164.6	0				
																	DMGEMVTQGQTDAQYMFLR	5	2	2	112382250;112382252	N-Term(TMT6plex); M2(Oxidation); M5(Oxidation); M16(Oxidation)	2498.1	0				
																	AQQYYFDAAEAEAWMSEQELYMMSEEK	13	2	2	112382250;112382252	N-Term(TMT6plex); M15(Oxidation); M22(Oxidation); M23(Oxidation); K27(TMT6plex)	3784.7	0				
																	MWEVLESTTQTK	11	2	2	112382250;112382252	N-Term(TMT6plex); M1(Oxidation); K12(TMT6plex)	1927.0	0				
																	ENEVLEAWK	12	2	2	112382250;112382252	N-Term(TMT6plex); K9(TMT6plex)	1575.9	0				
																	QNLLSQSHAYQQFLR	4	2	2	112382250;112382252	N-Term(TMT6plex)	2062.1	0				
																	EVVAGSHELGQDYEHVTMLQER	4	2	2	112382250;112382252	N-Term(TMT6plex); M18(Oxidation)	2772.3	0				
																	IDDIFER	8	2	2	112382250;112382252	N-Term(TMT6plex)	1136.6	0				
																	KQEDFMTTMDANEEK	4	2	2	112382250;112382252	N-Term(TMT6plex); K1(TMT6plex); M6(Oxidation); K15(TMT6plex)	2520.3	1				
																	WVNSHLAR	4	6	3	112382250;5902122;112382252	N-Term(TMT6plex)	1211.7	0				
																	AKDEQSAVSMLK	4	2	2	112382250;112382252	N-Term(TMT6plex); K2(TMT6plex); K12(TMT6plex)	1994.2	1				
																	EGMQLISEKPETEAVVK	12	2	2	112382250;112382252	N-Term(TMT6plex); M3(Oxidation); K9(TMT6plex); K17(TMT6plex)	2591.5	0				
																	KQEDFMTTMDANEEK	14	2	2	112382250;112382252	N-Term(TMT6plex); K1(TMT6plex); M6(Oxidation); M9(Oxidation); K15(TMT6plex)	2536.3	1				
																	QIEAQEKPR	4	2	2	112382250;112382252	N-Term(TMT6plex); K7(TMT6plex)	1556.9	0				
																	SQNIVTDSSSLSAEAIR	11	2	2	112382250;112382252	N-Term(TMT6plex)	2007.1	0				
																	DASVAEAWLLGQEPYLSSR	14	2	2	112382250;112382252	N-Term(TMT6plex)	2321.2	0				
																	DLVAIEAK	8	2	2	112382250;112382252	N-Term(TMT6plex); K8(TMT6plex)	1316.8	0				
																	EGEDMIAEEHFGSEK	6	2	2	112382250;112382252	N-Term(TMT6plex); M5(Oxidation); K15(TMT6plex)	2182.0	0				
																	DDEEMNTWIQAISSAISSDK	6	1	1	112382250	N-Term(TMT6plex); M5(Oxidation); K20(TMT6plex)	2714.3	0	**1.0**	**1.0**	**1.1**	**1.2**
																	EQWANLEQLSAIR	9	2	2	112382250;112382252	N-Term(TMT6plex)	1787.0	0				
																	DVSSVELLMNNHQGIK	2	2	2	112382250;112382252	N-Term(TMT6plex); M9(Oxidation); K16(TMT6plex)	2258.2	0				
																	WDVDDWDNENSSAR	3	1	1	112382250	N-Term(TMT6plex)	1937.8	0	**1.2**	**1.3**	**1.3**	**1.4**
																	FATDGEGYKPCDPQVIR	4	2	2	112382250;112382252	N-Term(TMT6plex); K9(TMT6plex); C11(Carbamidomethyl)	2411.2	0				
																	MLTAQDMSYDEAR	25	2	2	112382250;112382252	N-Term(TMT6plex); M1(Oxidation); M7(Oxidation)	1791.8	0				
																	VIESTQDLGNDLAGVMALQR	10	2	2	112382250;112382252	N-Term(TMT6plex)	2359.3	0				
																	MLTAQDMSYDEAR	3	2	2	112382250;112382252	N-Term(TMT6plex); M1(Oxidation)	1775.8	0				
																	QLWGLLIEETEK	8	2	2	112382250;112382252	N-Term(TMT6plex); K12(TMT6plex)	1917.1	0				
																	VHLENMGSHDIVDGNHR	2	5	3	112382250;5902122;112382252	N-Term(TMT6plex); M6(Oxidation)	2175.1	0				
																	LEEASLLHQFQADADDIDAWMLDILK	8	2	2	112382250;112382252	N-Term(TMT6plex); M21(Oxidation); K26(TMT6plex)	3474.8	0				
																	GVNASAQK	3	2	2	112382250;112382252	N-Term(TMT6plex); K8(TMT6plex)	1232.7	0				
																	TQTAIASEDMPNTLTEAEK	2	2	2	112382250;112382252	N-Term(TMT6plex); K19(TMT6plex)	2508.3	0				
																	RLEEASLLHQFQADADDIDAWMLDILK	2	2	2	112382250;112382252	N-Term(TMT6plex); M22(Oxidation); K27(TMT6plex)	3630.9	1				
																	DLMLWMEDVIR	2	2	2	112382250;112382252	N-Term(TMT6plex); M6(Oxidation)	1665.9	0				
																	DDEEMNTWIQAISSAISSDKHEVSASTQSTPASSR	2	1	1	112382250	N-Term(TMT6plex); M5(Oxidation); K20(TMT6plex)	4240.0	1	**1.5**	**1.5**	**1.5**	**1.6**
																	LILEVHQFSR	4	2	2	112382250;112382252	N-Term(TMT6plex)	1470.9	0				
																	LEMNLGLQK	4	2	2	112382250;112382252	N-Term(TMT6plex); K9(TMT6plex)	1503.9	0				
																	SGHFEQAIK	2	2	2	112382250;112382252	N-Term(TMT6plex); K9(TMT6plex)	1474.8	0				
																	ALVADSHPESER	4	2	2	112382250;112382252	N-Term(TMT6plex)	1539.8	0				
																	LLTQHENIKNEIDNYEEDYQK	2	2	2	112382250;112382252	N-Term(TMT6plex); K9(TMT6plex); K21(TMT6plex)	3323.7	1				
																	IFQEMLYIMDWMDEMK	1	2	2	112382250;112382252	N-Term(TMT6plex); M12(Oxidation); M15(Oxidation); K16(TMT6plex)	2613.2	0				
																	VDTVNHLADELINSGHSDAATIAEWK	15	2	2	112382250;112382252	N-Term(TMT6plex); K26(TMT6plex)	3264.7	0				
																	QALQDTLALYK	10	2	2	112382250;112382252	N-Term(TMT6plex); K11(TMT6plex)	1722.0	0				
																	DLDDFQSWLSR	8	2	2	112382250;112382252	N-Term(TMT6plex)	1610.8	0				
																	AELFTQSCADLDK	13	2	2	112382250;112382252	N-Term(TMT6plex); C8(Carbamidomethyl); K13(TMT6plex)	1956.0	0				
																	HTLVEADIGIQAER	4	2	2	112382250;112382252	N-Term(TMT6plex)	1781.0	0				
																	HLLGVEDLLQK	8	2	2	112382250;112382252	N-Term(TMT6plex); K11(TMT6plex)	1723.0	0				
																	FQIQDISVETEDNKEK	4	3	3	112382250;5902122;112382252	N-Term(TMT6plex); K14(TMT6plex); K16(TMT6plex)	2610.4	1				
																	IHCLENVDK	4	5	3	112382250;5902122;112382252	N-Term(TMT6plex); C3(Carbamidomethyl); K9(TMT6plex)	1585.9	0				
																	QLWGLLIEETEKR	4	2	2	112382250;112382252	N-Term(TMT6plex); K12(TMT6plex)	2073.2	1				
																	FESLEPEMNNQASR	7	2	2	112382250;112382252	N-Term(TMT6plex); M8(Oxidation)	1896.9	0				
																	KNQTLQK	1	3	3	112382250;5902122;112382252	N-Term(TMT6plex); K1(TMT6plex); K7(TMT6plex)	1547.0	1				
																	HQILEQAVEDYAETVHQLSK	9	2	2	112382250;112382252	N-Term(TMT6plex); K20(TMT6plex)	2796.5	0				
																	QAEAFLNNQEYVLAHTEMPTTLEGAEAAIK	2	2	2	112382250;112382252	N-Term(TMT6plex); K30(TMT6plex)	3747.9	0				
																	LNDGNEYLFQAK	9	1	1	112382250	N-Term(TMT6plex); K12(TMT6plex)	1870.0	0	**1.1**	**1.2**	**1.2**	**1.3**
																	FLQDCQELSLWINEK	20	2	2	112382250;112382252	N-Term(TMT6plex); C5(Carbamidomethyl); K15(TMT6plex)	2381.3	0				
																	DLMLWMEDVIR	10	2	2	112382250;112382252	N-Term(TMT6plex)	1649.8	0				
																	LQALDTGWNELHK	16	2	2	112382250;112382252	N-Term(TMT6plex); K13(TMT6plex)	1983.1	0				
																	FMELLEPLNER	10	2	2	112382250;112382252	N-Term(TMT6plex)	1619.9	0				
																	IFQEMLYIMDWMDEMK	4	2	2	112382250;112382252	N-Term(TMT6plex); M9(Oxidation); M12(Oxidation); M15(Oxidation); K16(TMT6plex)	2629.3	0				
																	DLMLWMEDVIR	19	2	2	112382250;112382252	N-Term(TMT6plex); M3(Oxidation); M6(Oxidation)	1681.9	0				
																	KHQILEQAVEDYAETVHQLSK	4	2	2	112382250;112382252	N-Term(TMT6plex); K1(TMT6plex); K21(TMT6plex)	3153.8	1				
																	EVVAGSHELGQDYEHVTMLQER	2	2	2	112382250;112382252	N-Term(TMT6plex)	2756.3	0				
																	MPLATSTDHGHNLQTVQLLIK	6	2	2	112382250;112382252	N-Term(TMT6plex); M1(Oxidation); K21(TMT6plex)	2791.6	0				
																	LTTLELLEVR	12	2	2	112382250;112382252	N-Term(TMT6plex)	1415.9	0				
																	LVSQDNFGFDLPAVEAATK	16	2	2	112382250;112382252	N-Term(TMT6plex); K19(TMT6plex)	2480.3	0				
																	DGLNEAWADLLELIDTR	6	2	2	112382250;112382252	N-Term(TMT6plex)	2173.1	0				
																	VAVVNQIAR	2	2	2	112382250;112382252	N-Term(TMT6plex)	1198.7	0				
																	LVSDGNINSDR	13	2	2	112382250;112382252	N-Term(TMT6plex)	1418.7	0				
																	DLTSVNILLK	14	3	3	112382250;5902122;112382252	N-Term(TMT6plex); K10(TMT6plex)	1574.0	0				
																	EIEELQSQAQALSQEGK	10	2	2	112382250;112382252	N-Term(TMT6plex); K17(TMT6plex)	2346.3	0				
																	LQAAYAGDK	4	2	2	112382250;112382252	N-Term(TMT6plex); K9(TMT6plex)	1394.8	0				
																	DQNTVETLQR	8	2	2	112382250;112382252	N-Term(TMT6plex)	1432.8	0				
																	VLVLSQDYGK	13	2	2	112382250;112382252	N-Term(TMT6plex); K10(TMT6plex)	1579.9	0				
																	VQAVVAVAR	2	2	2	112382250;112382252	N-Term(TMT6plex)	1141.7	0				
																	HYASEEIK	6	2	2	112382250;112382252	N-Term(TMT6plex); K8(TMT6plex)	1434.8	0				
																	KHEAIETDIAAYEER	5	2	2	112382250;112382252	N-Term(TMT6plex); K1(TMT6plex)	2233.2	1				
																	LLQLTEK	7	3	2	112382250;112382252	N-Term(TMT6plex); K7(TMT6plex)	1302.8	0				
																	VAHMEFCYQELCQLAAER	4	2	2	112382250;112382252	N-Term(TMT6plex); M4(Oxidation); C7(Carbamidomethyl); C12(Carbamidomethyl)	2500.2	0				
																	HEVSASTQSTPASSR	4	1	1	112382250	N-Term(TMT6plex)	1773.9	0	**1.2**	**1.2**	**1.3**	**1.2**
																	WEDRWEWLR	2	2	2	112382250;112382252	N-Term(TMT6plex)	1604.8	1				
																	QAEAFLNNQEYVLAHTEMPTTLEGAEAAIK	2	2	2	112382250;112382252	N-Term(TMT6plex); M18(Oxidation); K30(TMT6plex)	3763.9	0				
																	DDEEMNTWIQAISSAISSDK	2	1	1	112382250	N-Term(TMT6plex); K20(TMT6plex)	2698.3	0	**1.1**	**1.3**	**1.1**	**1.3**
																	TAGYPNVNIHNFTTSWR	2	2	2	112382250;112382252	N-Term(TMT6plex)	2207.1	0				
																	FANSLVGVQQQLQAFNTYR	3	2	2	112382250;112382252	N-Term(TMT6plex)	2413.3	0				
																	QLQEDAAR	4	2	2	112382250;112382252	N-Term(TMT6plex)	1159.6	0				
																	WLHGLESQIQSDDYGK	6	2	2	112382250;112382252	N-Term(TMT6plex); K16(TMT6plex)	2334.2	0				
																	DVEDEILWVGER	8	2	2	112382250;112382252	N-Term(TMT6plex)	1688.9	0				
																	LEMNLGLQK	2	2	2	112382250;112382252	N-Term(TMT6plex); M3(Oxidation); K9(TMT6plex)	1519.9	0				
																	DVSSVELLMNNHQGIK	2	2	2	112382250;112382252	N-Term(TMT6plex); K16(TMT6plex)	2242.2	0				
																	KHYASEEIK	4	2	2	112382250;112382252	N-Term(TMT6plex); K1(TMT6plex); K9(TMT6plex)	1792.1	1				
																	KHEWEAHNK	1	1	1	112382250	N-Term(TMT6plex); K1(TMT6plex); K9(TMT6plex)	1866.1	1	**1.1**	**1.2**	**1.1**	**1.4**
																	HRPDLIDFDK	4	4	2	112382250;112382252	N-Term(TMT6plex); K10(TMT6plex)	1714.0	0				
																	EAEKLESEHPDQAQAILSR	2	2	2	112382250;112382252	N-Term(TMT6plex); K4(TMT6plex)	2609.4	1				
																	HEAFEK	2	4	2	112382250;112382252	N-Term(TMT6plex); K6(TMT6plex)	1218.7	0				

Supplementary Table 6. List of all proteins and peptides identified in untreated and chronically treated Het1A cells with cigarette smoke condensate for 8 months

Accession	RefSeq Accession	Gene Symbol	Entrez Gene ID	Description	Σ Coverage	Σ# Proteins	Σ# Unique Peptides	Σ# Peptides	Σ# PSMs	Het-1A-Smoke - 2M/Parental	Het-1A-Smoke - 4M/Parental	Het-1A-Smoke - 6M/Parental	Het-1A-Smoke - 8M/Parental	# AAs	MW [kDa]	calc. pI	Sequence	# PSMs	# Proteins	# Protein Groups	Protein Group Accessions	Modifications	MH+ [Da]	# Missed Cleavages	Het-1A-Smoke - 2M/Parental	Het-1A-Smoke - 4M/Parental	Het-1A-Smoke - 6M/Parental	Het-1A-Smoke - 8M/Parental
																	VLDNAIETEK	7	2	2	112382250;112382252	N-Term(TMT6plex); K10(TMT6plex)	1589.9	0				
																	TALPAQSAATLPAR	14	1	1	112382250	N-Term(TMT6plex)	1596.9	0	**1.2**	**1.2**	**1.3**	**1.2**
																	STDEVDSK	8	2	2	112382250;112382252	N-Term(TMT6plex); K8(TMT6plex)	1338.7	0				
																	FYHDAK	7	2	2	112382250;112382252	N-Term(TMT6plex); K6(TMT6plex)	1238.7	0				
																	SLLDACESR	2	2	2	112382250;112382252	N-Term(TMT6plex); C6(Carbamidomethyl)	1279.6	0				
																	MWEVLESTTQTK	3	2	2	112382250;112382252	N-Term(TMT6plex); K12(TMT6plex)	1911.0	0				
																	INAVVETGR	2	2	2	112382250;112382252	N-Term(TMT6plex)	1187.7	0				
																	TQILAASYELHK	6	2	2	112382250;112382252	N-Term(TMT6plex); K12(TMT6plex)	1832.1	0				
																	SWHNVYCVINNQEMGFYK	2	1	1	112382250	N-Term(TMT6plex); C7(Carbamidomethyl); M14(Oxidation); K18(TMT6plex)	2763.3	0	**0.8**	**1.1**	**1.0**	**1.2**
																	KQQMLENQMEVR	3	2	2	112382250;112382252	K1(TMT6plex); N-Term(TMT6plex); M4(Oxidation); M9(Oxidation)	2024.1	1				
																	TQETPSAQMEGFLNR	5	1	1	112382250	N-Term(TMT6plex); M9(Oxidation)	1954.0	0	**1.2**	**1.2**	**1.2**	**1.3**
																	DALLLWCQMK	2	6	3	112382250;5902122;112382252	N-Term(TMT6plex); C7(Carbamidomethyl); K10(TMT6plex)	1736.0	0				
																	QLMHSGHPSEK	2	2	2	112382250;112382252	N-Term(TMT6plex); M3(Oxidation); K11(TMT6plex)	1724.9	0				
																	EIQGHQPR	2	2	2	112382250;112382252	N-Term(TMT6plex)	1193.7	0				
																	DGMAFNALIHK	2	2	2	112382250;112382252	N-Term(TMT6plex); M3(Oxidation); K11(TMT6plex)	1690.9	0				
																	KQEDFMTTMDANEEK	2	2	2	112382250;112382252	N-Term(TMT6plex); K1(TMT6plex); K15(TMT6plex)	2504.3	1				
																	SIITYVVTYYHYFSK	1	2	2	112382250;112382252	N-Term(TMT6plex); K15(TMT6plex)	2342.3	0				
																	EASLGEASK	3	2	2	112382250;112382252	N-Term(TMT6plex); K9(TMT6plex)	1349.8	0				
																	AQTLPTSVVTITSESSPGK	4	1	1	112382250	N-Term(TMT6plex); K19(TMT6plex)	2361.3	0	**1.2**	**1.4**	**1.3**	**1.4**
																	LLDPEDISVDHPDEK	4	2	2	112382250;112382252	N-Term(TMT6plex); K15(TMT6plex)	2180.1	0				
																	NEIDNYEEDYQK	4	2	2	112382250;112382252	N-Term(TMT6plex); K12(TMT6plex)	2018.0	0				
																	EAVCEVALDYK	2	1	1	112382250	N-Term(TMT6plex); C4(Carbamidomethyl); K11(TMT6plex)	1754.9	0	**1.2**	**1.5**	**1.6**	**1.5**
																	LQAAYAGDKADDIQK	1	2	2	112382250;112382252	N-Term(TMT6plex); K9(TMT6plex); K15(TMT6plex)	2294.3	1				
																	MFSEADACELWIDEK	1	2	2	112382250;112382252	N-Term(TMT6plex); M1(Oxidation); C8(Carbamidomethyl); K15(TMT6plex)	2318.1	0				
																	EGMQLISEKPETEAVVK	1	2	2	112382250;112382252	N-Term(TMT6plex); K9(TMT6plex); K17(TMT6plex)	2575.5	0				
																	AKDEQSAVSMLK	1	2	2	112382250;112382252	N-Term(TMT6plex); K2(TMT6plex); M10(Oxidation); K12(TMT6plex)	2010.2	1				
																	ALAVEGK	4	4	3	112382250;5902122;112382252	N-Term(TMT6plex); K7(TMT6plex)	1145.7	0				
																	LISDINK	2	3	3	112382250;5902122;112382252	N-Term(TMT6plex); K7(TMT6plex)	1260.8	0				
																	AFEDEMSGR	2	2	2	112382250;112382252	N-Term(TMT6plex); M6(Oxidation)	1286.6	0				
																	STDEVDSKR	2	2	2	112382250;112382252	N-Term(TMT6plex); K8(TMT6plex)	1494.8	1				
																	VDSIDDR	2	2	2	112382250;112382252	N-Term(TMT6plex)	1048.5	0				
																	KHNLLASK	2	2	2	112382250;112382252	N-Term(TMT6plex); K1(TMT6plex); K8(TMT6plex)	1598.0	1				
																	LVDTGDKFR	1	2	2	112382250;112382252	N-Term(TMT6plex); K7(TMT6plex)	1508.9	1				
																	ALQFLK	1	6	3	112382250;5902122;112382252	N-Term(TMT6plex); K6(TMT6plex)	1177.8	0				
																	FMELLEPLNER	1	2	2	112382250;112382252	N-Term(TMT6plex); M2(Oxidation)	1635.9	0				
																	EVAELTR	2	2	2	112382250;112382252	N-Term(TMT6plex)	1046.6	0				
																	LVDTGDK	5	2	2	112382250;112382252	N-Term(TMT6plex); K7(TMT6plex)	1205.7	0				
																	TVEKPPK	1	5	3	112382250;5902122;112382252	N-Term(TMT6plex); K4(TMT6plex); K7(TMT6plex)	1486.0	0				
																	LFQLNR	1	2	2	112382250;112382252	N-Term(TMT6plex)	1019.6	0				
																	FFWEMAEEEGWIR	1	2	2	112382250;112382252	N-Term(TMT6plex); M5(Oxidation)	1974.9	0				
																	ILSSDDYGK	1	2	2	112382250;112382252	N-Term(TMT6plex); K9(TMT6plex)	1455.8	0				
																	LYAGLK	3	3	3	112382250;5902122;112382252	N-Term(TMT6plex); K6(TMT6plex)	1122.7	0				
																	LQQFLR	1	4	3	164607124;112382250;112382252	N-Term(TMT6plex)	1033.6	0				
																	LTVQTK	2	2	2	112382250;112382252	N-Term(TMT6plex); K6(TMT6plex)	1147.7	0				
																	FSLFGK	1	1	2	112382250;6005882;643374324	N-Term(TMT6plex); K6(TMT6plex)	1156.7	0				
																	LPEELGR	2	2	2	112382250;112382252	N-Term(TMT6plex)	1042.6	0				
																	LFDANK	2	6	3	18201905;112382250;112382252	N-Term(TMT6plex); K6(TMT6plex)	1165.7	0				
5174613	NP_005960.1	**NAP1L4**	4676	**nucleosome assembly protein 1-like 4**	67.20	1	19	21	293	**1.3**	**1.5**	**1.6**	**1.3**	375	42.8	4.69												
																	NVDMLSELVQEYDEPILK	32	1	1	5174613	N-Term(TMT6plex); M4(Oxidation); K18(TMT6plex)	2609.4	0	**1.4**	**1.7**	**1.9**	**1.3**
																	AAATAEEPDPK	20	1	1	5174613	N-Term(TMT6plex); K11(TMT6plex)	1557.9	0	**1.3**	**1.6**	**1.5**	**1.3**
																	QVPNESFFNFFNPLK	51	1	1	5174613	N-Term(TMT6plex); K15(TMT6plex)	2286.2	0	**1.4**	**1.5**	**1.6**	**1.3**
																	NVDMLSELVQEYDEPILK	8	1	1	5174613	N-Term(TMT6plex); K18(TMT6plex)	2593.4	0	**1.7**	**1.9**	**2.2**	**1.4**
																	FYEEVHDLERK	1	2	2	5174613;21327708;4758756	N-Term(TMT6plex); K11(TMT6plex)	1923.0	1				
																	ADPFSFEGPEIVDCDGCTIDWK	8	1	1	5174613	N-Term(TMT6plex); C14(Carbamidomethyl); C17(Carbamidomethyl); K22(TMT6plex)	3016.4	0	**1.6**	**1.8**	**1.8**	**1.5**
																	EFITGDVEPTDAESEWHSENEEEEK	8	1	1	5174613	N-Term(TMT6plex); K25(TMT6plex)	3394.6	0	**1.3**	**1.7**	**1.5**	**1.5**
																	FYEEVHDLER	43	2	2	5174613;21327708;4758756	N-Term(TMT6plex)	1565.8	0				
																	KYAALYQPLFDK	6	1	1	5174613	N-Term(TMT6plex); K1(TMT6plex); K12(TMT6plex)	2144.3	1	**1.2**	**1.3**	**1.5**	**1.3**
																	LDNVPHTPSSYIETLPK	18	1	1	5174613	N-Term(TMT6plex); K17(TMT6plex)	2369.3	0	**1.2**	**1.3**	**1.5**	**1.2**
																	GIPEFWFTIFR	13	1	1	5174613	N-Term(TMT6plex)	1641.9	0	**1.2**	**1.5**	**1.8**	**1.4**
																	FSDPGQPMSFVLEFHFEPNDYFTNSVLTK	8	1	1	5174613	N-Term(TMT6plex); M8(Oxidation); K29(TMT6plex)	3867.9	0	**1.2**	**1.4**	**1.3**	**1.2**
																	LTDQVMQNPR	14	1	1	5174613	N-Term(TMT6plex); M6(Oxidation)	1446.8	0	**1.3**	**1.5**	**1.4**	**1.2**
																	SEPDKADPFSFEGPEIVDCDGCTIDWK	13	1	1	5174613	N-Term(TMT6plex); K5(TMT6plex); C19(Carbamidomethyl); C22(Carbamidomethyl); K27(TMT6plex)	3801.8	1	**1.4**	**2.0**	**1.9**	**1.4**
																	YAALYQPLFDK	21	1	1	5174613	N-Term(TMT6plex); K11(TMT6plex)	1787.0	0	**1.3**	**1.3**	**1.5**	**1.1**
																	VLAALQER	10	1	1	5174613	N-Term(TMT6plex)	1128.7	0	**1.3**	**1.4**	**1.6**	**1.2**
																	LTDQVMQNPR	2	1	1	5174613	N-Term(TMT6plex)	1430.8	0	**1.9**	**2.4**	**2.7**	**1.5**
																	CAHIEAK	2	1	1	5174613	N-Term(TMT6plex); C1(Carbamidomethyl); K7(TMT6plex)	1286.7	0	**1.1**	**1.4**	**1.5**	**1.4**
																	ASGDGESLDEDSEFTLASDFEIGHFFR	2	1	1	5174613	N-Term(TMT6plex)	3207.5	0	**1.0**	**1.5**	**1.1**	**1.6**
																	NASNTEK	2	1	1	5174613	N-Term(TMT6plex); K7(TMT6plex)	1221.7	0	**1.2**	**1.1**	**1.4**	**0.9**
																	HLQDIK	7	1	1	5174613	N-Term(TMT6plex); K6(TMT6plex)	1211.8	0	**1.2**	**1.5**	**1.5**	**1.4**
																	VVVTEK	3	1	1	5174613	N-Term(TMT6plex); K6(TMT6plex)	1132.7	0	**1.4**	**1.5**	**1.5**	**1.3**
																	LAGDMK	1	1	1	5174613	N-Term(TMT6plex); K6(TMT6plex)	1092.6	0	**1.6**	**2.1**	**2.1**	**1.6**
4557809	NP_000265.1	**OAT**	4942	**ornithine aminotransferase, mitochondrial isoform 1 precursor**	67.20	2	20	20	135	**1.4**	**1.3**	**1.3**	**1.2**	439	48.5	7.03												
																	VAIAALEVLEEENLAENADK	21	2	1	4557809	N-Term(TMT6plex); K20(TMT6plex)	2599.4	0	**1.2**	**1.2**	**1.1**	**1.1**
																	TVQGPPTSDDIFER	13	1	1	4557809	N-Term(TMT6plex)	1790.9	0	**1.3**	**1.2**	**1.2**	**1.2**
																	ESIEIINK	9	2	1	4557809	N-Term(TMT6plex); K8(TMT6plex)	1403.8	0	**1.1**	**1.2**	**1.2**	**1.2**
																	ALQDPNVAAFMVEPIQGEAGVVVPDPGYLMGVR	2	2	1	4557809	N-Term(TMT6plex); M11(Oxidation); M30(Oxidation)	3700.9	0	**1.2**	**1.6**	**1.6**	**1.6**
																	VLPMNTGVEAGETACK	10	1	1	4557809	N-Term(TMT6plex); M4(Oxidation); C15(Carbamidomethyl); K16(TMT6plex)	2151.1	0	**1.4**	**1.4**	**1.3**	**1.2**
																	FAPPLVIK	6	2	1	4557809	N-Term(TMT6plex); K8(TMT6plex)	1342.9	0	**1.2**	**1.1**	**1.2**	**1.1**
																	FAPPLVIKEDELR	1	2	1	4557809	N-Term(TMT6plex); K8(TMT6plex)	1985.2	1	**1.4**	**1.6**	**1.3**	**1.7**
																	LPSDVVTAVR	5	2	1	4557809	N-Term(TMT6plex)	1285.8	0	**1.2**	**1.3**	**1.2**	**1.3**
																	KTVQGPPTSDDIFER	2	1	1	4557809	N-Term(TMT6plex); K1(TMT6plex)	2148.2	1	**1.2**	**1.6**	**1.4**	**1.5**
																	ALSGGLYPVSAVLCDDDIMLTIKPGEHGSTYGGNPLGCR	2	2	1	4557809	N-Term(TMT6plex); C14(Carbamidomethyl); M19(Oxidation); K23(TMT6plex); C38(Carbamidomethyl)	4565.3	0	**1.2**	**1.1**	**0.9**	**1.0**
																	ALSGGLYPVSAVLCDDDIMLTIKPGEHGSTYGGNPLGCR	5	2	1	4557809	N-Term(TMT6plex); C14(Carbamidomethyl); K23(TMT6plex); C38(Carbamidomethyl)	4549.3	0	**2.0**	**2.1**	**2.1**	**1.5**
																	AFYNNVLGEYEEYITK	12	1	1	4557809	N-Term(TMT6plex); K16(TMT6plex)	2411.3	0	**1.2**	**1.2**	**1.2**	**1.2**
																	IVFAAGNFWGR	5	2	1	4557809	N-Term(TMT6plex)	1466.8	0	**1.5**	**1.4**	**1.7**	**1.2**
																	GLLNAIVIK	14	2	1	4557809	N-Term(TMT6plex); K9(TMT6plex)	1398.9	0	**1.2**	**1.3**	**1.3**	**1.1**
																	WLAVDYENVRPDIVLLGK	8	2	1	4557809	N-Term(TMT6plex); K18(TMT6plex)	2558.5	0	**1.3**	**1.2**	**1.1**	**1.2**
																	HQVLFIADEIQTGLAR	4	2	1	4557809	N-Term(TMT6plex)	2040.1	0	**1.6**	**1.6**	**1.9**	**1.5**
																	TLSAISSSTDPTSYDGFGPFMPGFDIIPYNDLPALER	4	2	1	4557809	N-Term(TMT6plex)	4221.1	0	**1.9**	**2.4**	**1.6**	**2.0**
																	ALQDPNVAAFMVEPIQGEAGVVVPDPGYLMGVR	2	2	1	4557809	N-Term(TMT6plex); M30(Oxidation)	3684.9	0	**1.4**	**1.5**	**1.6**	**1.7**
																	YGAHNYHPLPVALER	1	1	1	4557809	N-Term(TMT6plex)	1966.1	0	**1.5**	**1.4**	**1.6**	**1.4**
																	KWGYTVK	1	2	1	4557809	N-Term(TMT6plex); K1(TMT6plex); K7(TMT6plex)	1569.0	1	**1.3**	**1.4**	**1.3**	**1.3**
																	LFNYHK	2	1	1	4557809	N-Term(TMT6plex); K6(TMT6plex)	1279.8	0	**1.2**	**1.1**	**1.2**	**1.1**
																	LGIILR	6	2	1	4557809	N-Term(TMT6plex)	913.6	0	**1.4**	**1.1**	**1.1**	**1.0**
574280091	NP_001276032.1	**PRKCSH**	5589	**glucosidase 2 subunit beta isoform 3 precursor**	67.10	2	2	28	548	**1.6**	**1.6**	**1.9**	**1.5**	535	60.2	4.41												
																	ESLQQMAEVTR	56	3	2	48255889;574280091	N-Term(TMT6plex); M6(Oxidation)	1536.8	0				
																	MPPYDEQTQAFIDAAQEAR	40	3	2	48255889;574280091	N-Term(TMT6plex); M1(Oxidation)	2426.1	0				
																	SLEDQVEMLR	39	3	2	48255889;574280091	N-Term(TMT6plex); M8(Oxidation)	1464.8	0				
																	EAPPPLSPPQPASPAEEDKMPPYDEQTQAFIDAAQEAR	4	1	1	574280091	N-Term(TMT6plex); K19(TMT6plex); M20(Oxidation)	4593.3	1	**1.8**	**2.0**	**2.5**	**1.7**
																	CEYLMELMTPAACPEPPPEAPTEDDHDEL	10	3	2	48255889;574280091	N-Term(TMT6plex); C1(Carbamidomethyl); M5(Oxidation); M8(Oxidation); C13(Carbamidomethyl)	3618.5	0				
																	EAPPPLSPPQPASPAEEDK	11	1	1	574280091	N-Term(TMT6plex); K19(TMT6plex)	2415.3	0	**1.4**	**1.3**	**1.3**	**1.2**
																	SEALPTDLPAPSAPDLTEPK	12	3	2	48255889;574280091	N-Term(TMT6plex); K20(TMT6plex)	2507.4	0				
																	TVKEEAEKPER	6	3	2	48255889;574280091	N-Term(TMT6plex); K3(TMT6plex); K8(TMT6plex)	2003.2	1				
																	KLIELQAGK	13	3	2	48255889;574280091	N-Term(TMT6plex); K1(TMT6plex); K9(TMT6plex)	1687.1	1				

Accession	RefSeq Accession	Gene Symbol	Entrez Gene ID	Description	Σ Coverage	Σ# Proteins	Σ# Unique Peptides	Σ# Peptides	Σ# PSMs	Het-1A-Smoke - 2M/Parental	Het-1A-Smoke - 4M/Parental	Het-1A-Smoke - 6M/Parental	Het-1A-Smoke - 8M/Parental	# AAs	MW [kDa]	calc. pI	Sequence	# PSMs	# Proteins	# Protein Groups	Protein Group Accessions	Modifications	MH+ [Da]	# Missed Cleavages	Het-1A-Smoke - 2M/Parental	Het-1A-Smoke - 4M/Parental	Het-1A-Smoke - 6M/Parental	Het-1A-Smoke - 8M/Parental
																	VNDGVCDCCDGTDEYNSGVICENTCK	18	3	2	48255889;574280091	N-Term(TMT6plex); C6(Carbamidomethyl); C8(Carbamidomethyl); C9(Carbamidomethyl); C21(Carbamidomethyl); C25(Carbamidomethyl); K26(TMT6plex)	3499.5	0				
																	AQQEQELAADAFK	50	3	2	48255889;574280091	N-Term(TMT6plex); K13(TMT6plex)	1907.0	0				
																	KILIEDWK	6	3	2	48255889;574280091	N-Term(TMT6plex); K1(TMT6plex); K8(TMT6plex)	1732.1	1				
																	ELDDDMDGTVSVTELQTHPELDTDGDGALSEAEAQALLSGDTQTDATSFYDR	23	3	2	48255889;574280091	N-Term(TMT6plex); M6(Oxidation)	5790.6	0				
																	LGGSPTSLGTWGSWIGPDHDK	28	3	2	48255889;574280091	N-Term(TMT6plex); K21(TMT6plex)	2626.4	0				
																	ELDDDMDGTVSVTELQTHPELDTDGDGALSEAEAQALLSGDTQTDATSFYDR	6	3	2	48255889;574280091	N-Term(TMT6plex)	5774.6	0				
																	LIELQAGK	18	3	2	48255889;574280091	N-Term(TMT6plex); K8(TMT6plex)	1329.9	0				
																	YEQGTGCWQGPNR	33	3	2	48255889;574280091	N-Term(TMT6plex); C7(Carbamidomethyl)	1781.8	0				
																	ETMVTSTTEPSR	24	3	2	48255889;574280091	N-Term(TMT6plex); M3(Oxidation)	1583.8	0				
																	ILIEDWK	43	3	2	48255889;574280091	N-Term(TMT6plex); K7(TMT6plex)	1374.8	0				
																	GVSLTNHHFYDESKPFTCLDGSATIPFDQVNDDYCDCK	3	3	2	48255889;574280091	N-Term(TMT6plex); K14(TMT6plex); C18(Carbamidomethyl); C35(Carbamidomethyl); C37(Carbamidomethyl); K38(TMT6plex)	5140.4	0				
																	SLEDQVEMLR	2	3	2	48255889;574280091	N-Term(TMT6plex)	1448.8	0				
																	CEYLMELMTPAACPEPPPEAPTEDDHDEL	4	3	2	48255889;574280091	N-Term(TMT6plex); C1(Carbamidomethyl); M5(Oxidation); C13(Carbamidomethyl)	3602.6	0				
																	LWEEQLAAAK	54	3	2	48255889;574280091	N-Term(TMT6plex); K10(TMT6plex)	1616.9	0				
																	ETMVTSTTEPSR	9	3	2	48255889;574280091	N-Term(TMT6plex)	1567.8	0				
																	EEAEKPER	3	3	2	48255889;574280091	N-Term(TMT6plex); K5(TMT6plex)	1445.8	0				
																	MPPYDEQTQAFIDAAQEAR	6	3	2	48255889;574280091	N-Term(TMT6plex)	2410.1	0				
																	LGGSPTSLGTWGSWIGPDHDKFSAMK	4	3	2	48255889;574280091	N-Term(TMT6plex); K21(TMT6plex); M25(Oxidation); K26(TMT6plex)	3435.8	1				
																	AQQEQELAADAFKELDDDMDGTVSVTELQTHPELDTDGDGALSEAEAQALLSGDTQTDATSFYDR	2	3	2	48255889;574280091	N-Term(TMT6plex); K13(TMT6plex); M19(Oxidation)	7449.4	1				
																	LVSQKPK	5	3	2	48255889;574280091	N-Term(TMT6plex); K5(TMT6plex); K7(TMT6plex)	1487.0	0				
																	NKFEEAER	3	3	2	48255889;574280091	N-Term(TMT6plex); K2(TMT6plex)	1480.8	1				
																	DMEESIR	4	3	2	48255889;574280091	N-Term(TMT6plex); M2(Oxidation)	1124.5	0				
																	VWAAIR	8	3	3	48255889;574280091;28212272	N-Term(TMT6plex)	944.6	0				
																	EQHQK	1	4	3	48255889;31563507;574280091	N-Term(TMT6plex); K5(TMT6plex)	1127.7	0				
50593002	NP_003081.2	**SNRPA1**	6627	**U2 small nuclear ribonucleoprotein A'**	67.06	1	16	16	247	**1.2**	**1.3**	**1.3**	**1.2**	255	28.4	8.62												
																	NAIANASTLAEVER	11	1	1	50593002	N-Term(TMT6plex)	1687.9	0	**1.2**	**1.1**	**1.3**	**1.1**
																	TLLVNNNR	6	1	1	50593002	N-Term(TMT6plex)	1172.7	0	**1.3**	**1.4**	**1.3**	**1.3**
																	IGEGLDQALPCLTELILTNNSLVELGDLDPLASLK	11	1	1	50593002	N-Term(TMT6plex); C11(Carbamidomethyl); K35(TMT6plex)	4193.3	0	**1.4**	**1.4**	**1.6**	**1.2**
																	LTAELIEQAAQYTNAVR	36	1	1	50593002	N-Term(TMT6plex)	2120.2	0	**1.2**	**1.3**	**1.2**	**1.2**
																	IPVIENLGATLDQFDAIDFSDNEIR	98	1	1	50593002	N-Term(TMT6plex)	3034.6	0	**1.2**	**1.3**	**1.3**	**1.2**
																	GLLQSGQIPGR	14	1	1	50593002	N-Term(TMT6plex)	1354.8	0	**1.3**	**1.4**	**1.4**	**1.4**
																	KLDGFPLLR	22	1	1	50593002	N-Term(TMT6plex); K1(TMT6plex)	1517.0	1	**1.2**	**1.3**	**1.3**	**1.2**
																	SLTYLSILR	14	1	1	50593002	N-Term(TMT6plex)	1294.8	0	**1.3**	**1.1**	**1.3**	**1.1**
																	GGPSPGDVEAIK	12	1	1	50593002	N-Term(TMT6plex); K12(TMT6plex)	1584.9	0	**1.1**	**1.3**	**1.1**	**1.2**
																	KGGPSPGDVEAIK	2	1	1	50593002	N-Term(TMT6plex); K1(TMT6plex); K13(TMT6plex)	1942.2	1	**1.1**	**1.2**	**1.2**	**1.1**
																	TFNPGAGLPTDK	6	1	1	50593002	N-Term(TMT6plex); K12(TMT6plex)	1675.9	0	**1.2**	**1.0**	**1.1**	**1.0**
																	IPVIENLGATLDQFDAIDFSDNEIRK	2	1	1	50593002	N-Term(TMT6plex); K26(TMT6plex)	3391.8	1	**1.6**	**1.7**	**1.9**	**1.4**
																	LDGFPLLR	2	1	1	50593002	N-Term(TMT6plex)	1159.7	0	**1.3**	**1.1**	**1.1**	**1.0**
																	LYVIYK	9	1	1	50593002	N-Term(TMT6plex); K6(TMT6plex)	1256.8	0	**1.2**	**1.3**	**1.3**	**1.3**
																	GAQLAK	1	1	1	50593002	N-Term(TMT6plex); K6(TMT6plex)	1045.7	0	**1.1**	**1.2**	**1.1**	**1.1**
																	VLDFQK	1	1	1	50593002	N-Term(TMT6plex); K6(TMT6plex)	1207.7	0	**1.1**	**1.2**	**1.2**	**1.1**
4885063	NP_005156.1	**ALDOC**	230	**fructose-bisphosphate aldolase C**	67.03	2	15	21	324	**1.4**	**1.6**	**1.5**	**1.6**	364	39.4	6.87												
																	TPSALAILENANVLAR	27	1	1	4885063	N-Term(TMT6plex)	1882.1	0	**1.9**	**1.9**	**2.0**	**1.8**
																	YEGSGEDGGAAAQSLYIANHAY	6	1	1	4885063	N-Term(TMT6plex)	2473.1	0	**1.4**	**1.5**	**1.5**	**1.5**
																	CQYVTEK	14	3	3	342187211;34577112;4557305;4885063	N-Term(TMT6plex); C1(Carbamidomethyl); K7(TMT6plex)	1385.7	0				
																	VLAAVYK	40	4	3	342187211;34577112;4557305;4885063	N-Term(TMT6plex); K7(TMT6plex)	1221.8	0				
																	GVVPLAGTDGETTTQGLDGLSER	109	1	1	4885063	N-Term(TMT6plex)	2502.3	0	**1.3**	**1.5**	**1.5**	**1.6**
																	DNAGAATEEFIK	10	1	1	4885063	N-Term(TMT6plex); K12(TMT6plex)	1723.9	0	**1.3**	**1.5**	**1.4**	**1.6**
																	GILAADESVGSMAK	15	1	1	4885063	N-Term(TMT6plex); M12(Oxidation); K14(TMT6plex)	1823.0	0	**1.2**	**1.5**	**1.3**	**1.7**
																	QVLFSADDR	6	1	1	4885063	N-Term(TMT6plex)	1279.7	0	**1.3**	**1.4**	**1.4**	**1.5**
																	DGADFAK	27	3	3	342187211;34577112;4557305;4885063	N-Term(TMT6plex); K7(TMT6plex)	1181.7	0				
																	PHSYPALSAEQK	1	1	1	4885063	N-Term(TMT6plex); K12(TMT6plex)	1786.0	0	**1.4**	**1.3**	**1.5**	**1.3**
																	LSQIGVENTEENR	5	1	1	4885063	N-Term(TMT6plex)	1717.9	0	**1.5**	**1.4**	**1.6**	**1.6**
																	ALQASALNAWR	6	1	1	4885063	N-Term(TMT6plex)	1429.8	0	**1.4**	**1.5**	**1.5**	**1.4**
																	YTPEEIAMATVTALR	8	1	1	4885063	N-Term(TMT6plex)	1895.0	0	**1.5**	**1.7**	**1.8**	**1.5**
																	YTPEEIAMATVTALR	7	1	1	4885063	N-Term(TMT6plex); M8(Oxidation)	1911.0	0	**1.4**	**1.4**	**1.3**	**1.3**
																	KDGADFAK	7	3	3	342187211;34577112;4557305;4885063	N-Term(TMT6plex); K1(TMT6plex); K8(TMT6plex)	1538.9	1				
																	YASICQQNGIVPIVEPEILPDGDHDLKR	6	3	3	342187211;34577112;4557305;4885063	N-Term(TMT6plex); C5(Carbamidomethyl); K27(TMT6plex)	3634.9	1				
																	LSQIGVENTEENRR	6	1	1	4885063	N-Term(TMT6plex)	1874.0	1	**1.5**	**1.5**	**1.5**	**1.4**
																	YASICQQNGIVPIVEPEILPDGDHDLK	2	3	3	342187211;34577112;4557305;4885063	N-Term(TMT6plex); C5(Carbamidomethyl); K27(TMT6plex)	3478.8	0				
																	GILAADESVGSMAK	6	1	1	4885063	N-Term(TMT6plex); K14(TMT6plex)	1807.0	0	**1.3**	**1.6**	**1.5**	**1.7**
																	GIVVGIK	6	2	1	4885063	N-Term(TMT6plex); K7(TMT6plex)	1143.8	0	**1.2**	**1.7**	**1.4**	**1.7**
																	TVPPAVPGVTFLSGGQSEEEASFNLNAINR	1	1	1	4885063	N-Term(TMT6plex)	3330.7	0				
																	ELSDIALR	7	1	1	4885063	N-Term(TMT6plex)	1145.7	0	**1.4**	**1.5**	**1.4**	**1.7**
																	KELSDIALR	2	1	1	4885063	N-Term(TMT6plex); K1(TMT6plex)	1502.9	1	**1.1**	**1.5**	**1.3**	**1.5**
4758304	NP_004902.1	**PDIA4**	9601	**protein disulfide-isomerase A4 precursor**	66.98	2	43	43	582	**1.4**	**1.5**	**1.4**	**1.1**	645	72.9	5.07												
																	VEGFPTIYFAPSGDK	25	1	1	4758304	N-Term(TMT6plex); K15(TMT6plex)	2086.1	0	**1.5**	**1.5**	**1.4**	**1.0**
																	FAMEPEEFDSDTLR	31	1	1	4758304	N-Term(TMT6plex); M3(Oxidation)	1931.9	0	**1.4**	**1.4**	**1.4**	**1.2**
																	YALPLVGHR	19	1	1	4758304	N-Term(TMT6plex)	1254.8	0	**1.3**	**1.4**	**1.3**	**1.3**
																	QFAPEYEK	8	1	1	4758304	N-Term(TMT6plex); K8(TMT6plex)	1469.8	0	**1.4**	**1.3**	**1.3**	**1.0**
																	IDATSASVLASR	16	1	1	4758304	N-Term(TMT6plex)	1419.8	0	**1.4**	**1.4**	**1.4**	**1.1**
																	FHHTFSTEIAK	7	1	1	4758304	N-Term(TMT6plex); K11(TMT6plex)	1776.0	0	**1.2**	**1.5**	**1.4**	**1.1**
																	DKDPPIPVAK	6	1	1	4758304	N-Term(TMT6plex); K2(TMT6plex); K10(TMT6plex)	1767.1	1	**1.2**	**1.5**	**1.3**	**1.1**
																	KLAPEYEK	2	1	1	4758304	N-Term(TMT6plex); K1(TMT6plex); K8(TMT6plex)	1665.0	1	**1.6**	**1.5**	**1.4**	**1.1**
																	DLGLSESGEDVNAAILDESGKK	22	1	1	4758304	N-Term(TMT6plex); K21(TMT6plex); K22(TMT6plex)	2934.6	1	**1.2**	**1.7**	**1.4**	**1.1**
																	MDATANDVPSDR	16	1	1	4758304	N-Term(TMT6plex); M1(Oxidation)	1536.7	0	**1.4**	**1.4**	**1.3**	**0.9**
																	FEGGDRDLEHLSK	10	1	1	4758304	N-Term(TMT6plex); K13(TMT6plex)	1961.0	1	**1.4**	**1.6**	**1.5**	**1.0**
																	DLGLSESGEDVNAAILDESGK	13	1	1	4758304	N-Term(TMT6plex); K21(TMT6plex)	2577.3	0	**1.2**	**1.4**	**1.3**	**0.9**
																	SHMMDVQGSTQDSAIK	24	1	1	4758304	N-Term(TMT6plex); M3(Oxidation); M4(Oxidation); K16(TMT6plex)	2225.1	0	**1.2**	**1.3**	**1.2**	**1.0**
																	YGIVDYMIEQSGPPSK	16	1	1	4758304	N-Term(TMT6plex); M7(Oxidation); K16(TMT6plex)	2258.2	0	**1.2**	**1.4**	**1.3**	**1.1**
																	FDVSGYPTIK	23	1	1	4758304	N-Term(TMT6plex); K10(TMT6plex)	1584.9	0	**1.3**	**1.3**	**1.3**	**0.9**
																	EVSQPDWTPPPEVTLVLTK	32	1	1	4758304	N-Term(TMT6plex); K19(TMT6plex)	2594.5	0	**1.5**	**1.8**	**1.5**	**1.2**
																	FIEEHATK	9	1	1	4758304	N-Term(TMT6plex); K8(TMT6plex)	1432.8	0	**1.3**	**1.5**	**1.4**	**1.2**
																	KGQAVDYEGSR	5	1	1	4758304	N-Term(TMT6plex); K1(TMT6plex)	1667.9	1	**1.4**	**1.3**	**1.3**	**0.9**
																	EFVTAFK	8	1	1	4758304	N-Term(TMT6plex); K7(TMT6plex)	1299.8	0	**1.3**	**1.6**	**1.5**	**1.0**
																	VSQGQLVVMQPEK	12	1	1	4758304	N-Term(TMT6plex); M9(Oxidation); K13(TMT6plex)	1917.1	0	**1.3**	**1.3**	**1.3**	**1.0**
																	GESDPAYQQYQDAANNLR	20	1	1	4758304	N-Term(TMT6plex)	2269.1	0	**1.4**	**1.4**	**1.4**	**1.2**
																	DGDDVIIIGVFK	16	1	1	4758304	N-Term(TMT6plex); K12(TMT6plex)	1749.0	0	**1.2**	**1.7**	**1.6**	**0.8**
																	DFPEYTFAIADEEDYAGEVK	75	1	1	4758304	N-Term(TMT6plex); K20(TMT6plex)	2767.3	0	**1.4**	**1.7**	**1.5**	**1.3**
																	TFDSIVMDPK	18	1	1	4758304	N-Term(TMT6plex); M7(Oxidation); K10(TMT6plex)	1626.9	0	**1.3**	**1.4**	**1.4**	**1.0**
																	MDATANDVPSDR	4	1	1	4758304	N-Term(TMT6plex)	1520.7	0	**2.4**	**1.9**	**1.9**	**1.1**
																	VEGFPTIYFAPSGDKK	1	1	1	4758304	N-Term(TMT6plex); K15(TMT6plex); K16(TMT6plex)	2443.4	1	**0.9**	**1.0**	**0.9**	**1.0**
																	YGIVDYMIEQSGPPSK	22	1	1	4758304	N-Term(TMT6plex); K16(TMT6plex)	2242.2	0	**1.6**	**2.1**	**1.9**	**1.4**
																	QVQEFLKDGDDVIIIGVFK	13	1	1	4758304	N-Term(TMT6plex); K7(TMT6plex); K19(TMT6plex)	2850.7	1	**1.4**	**1.9**	**1.5**	**1.0**
																	SHMMDVQGSTQDSAIK	4	1	1	4758304	N-Term(TMT6plex); K16(TMT6plex)	2193.1	0	**1.6**	**1.5**	**1.7**	**1.2**
																	KDVLIEFYAPWCGHCK	7	1	1	4758304	N-Term(TMT6plex); K1(TMT6plex); C12(Carbamidomethyl); C15(Carbamidomethyl); K16(TMT6plex)	2710.4	1	**1.5**	**2.1**	**1.9**	**1.5**
																	QLEPVYNSLAK	23	1	1	4758304	N-Term(TMT6plex); K11(TMT6plex)	1720.0	0	**1.5**	**1.5**	**1.4**	**1.1**
																	QVQEFLK	2	1	1	4758304	N-Term(TMT6plex); K7(TMT6plex)	1349.8	0	**1.3**	**1.3**	**1.2**	**0.7**
																	TFDSIVMDPK	2	1	1	4758304	N-Term(TMT6plex); K10(TMT6plex)	1610.9	0	**1.4**	**1.5**	**1.6**	**1.0**
																	GQAVDYEGSR	6	1	1	4758304	N-Term(TMT6plex)	1310.7	0	**1.4**	**1.6**	**1.5**	**1.1**
																	TQEEIVAK	15	1	1	4758304	N-Term(TMT6plex); K8(TMT6plex)	1375.8	0	**1.3**	**1.3**	**1.3**	**1.0**
																	VDATAETDLAK	20	1	1	4758304	N-Term(TMT6plex); K11(TMT6plex)	1591.9	0	**1.2**	**1.3**	**1.3**	**1.1**

Accession	RefSeq Accession	Gene Symbol	Entrez Gene ID	Description	Σ Coverage	Σ# Proteins	Σ# Unique Peptides	Σ# Peptides	Σ# PSMs	Het-1A-Smoke - 2M/Parental	Het-1A-Smoke - 4M/Parental	Het-1A-Smoke - 6M/Parental	Het-1A-Smoke - 8M/Parental	# AAs	MW [kDa]	calc. pI	Sequence	# PSMs	# Proteins	# Protein Groups	Protein Group Accessions	Modifications	MH+ [Da]	# Missed Cleavages	Het-1A-Smoke - 2M/Parental	Het-1A-Smoke - 4M/Parental	Het-1A-Smoke - 6M/Parental	Het-1A-Smoke - 8M/Parental
																	ENAIEDEEEEEEEDDDEEEDDLEVK	2	1	1	4758304	N-Term(TMT6plex); K25(TMT6plex)	3483.5	0	**1.5**	**1.6**	**1.9**	**1.2**
																	DLEHLSK	8	1	1	4758304	N-Term(TMT6plex); K7(TMT6plex)	1299.8	0	**1.2**	**1.3**	**1.3**	**1.0**
																	LAPEYEK	2	1	1	4758304	N-Term(TMT6plex); K7(TMT6plex)	1307.8	0	**1.3**	**1.5**	**1.3**	**0.8**
																	GRPYDYNGPR	1	1	1	4758304	N-Term(TMT6plex)	1423.7	0	**1.1**	**1.3**	**1.3**	**1.2**
																	SPPIPLAK	1	1	1	4758304	N-Term(TMT6plex); K8(TMT6plex)	1280.8	0	**1.3**	**1.6**	**1.4**	**1.5**
																	FEGGDR	1	1	1	4758304	N-Term(TMT6plex)	909.5	0	**1.2**	**1.1**	**1.2**	**0.9**
																	VLEVAK	2	1	1	4758304	N-Term(TMT6plex); K6(TMT6plex)	1116.7	0	**1.3**	**1.6**	**1.4**	**0.9**
																	LKPVIK	6	1	1	4758304	N-Term(TMT6plex); K2(TMT6plex); K6(TMT6plex)	1385.0	0	**1.1**	**1.3**	**1.2**	**0.9**
																	IANILK	5	2	1	4758304	N-Term(TMT6plex); K6(TMT6plex)	1129.8	0	**1.3**	**1.2**	**1.4**	**0.9**
																	VSNDAK	1	1	1	4758304	N-Term(TMT6plex); K6(TMT6plex)	1091.6	0	**1.3**	**1.5**	**1.4**	**1.1**
																	AATQFWR	1	1	1	4758304	N-Term(TMT6plex)	1108.6	0	**1.3**	**1.6**	**1.4**	**1.1**
4503521	NP_001559.1	**EIF3E**	3646	**eukaryotic translation initiation factor 3 subunit E**	66.97	1	26	26	237	**1.5**	**1.4**	**1.6**	**1.5**	445	52.2	6.04												
																	LNMTPEEAER	5	1	1	4503521	N-Term(TMT6plex); M3(Oxidation)	1434.7	0	**1.4**	**1.3**	**1.3**	**1.5**
																	QEYLDTLYR	5	1	1	4503521	N-Term(TMT6plex)	1429.8	0	**1.6**	**1.4**	**1.5**	**1.4**
																	TTVVAQLK	6	1	1	4503521	N-Term(TMT6plex); K8(TMT6plex)	1317.9	0	**1.3**	**1.2**	**1.5**	**1.3**
																	MLFDYLADK	6	1	1	4503521	N-Term(TMT6plex); K9(TMT6plex)	1573.9	0	**1.8**	**2.0**	**2.5**	**1.9**
																	LGHVVMGNNAVSPYQQVIEK	6	1	1	4503521	N-Term(TMT6plex); M6(Oxidation); K20(TMT6plex)	2657.4	0	**1.2**	**1.4**	**1.5**	**1.5**
																	NLYSDDIPHALR	10	1	1	4503521	N-Term(TMT6plex)	1642.9	0	**1.3**	**1.4**	**1.5**	**1.4**
																	QLQAETEPIVK	10	1	1	4503521	N-Term(TMT6plex); K11(TMT6plex)	1714.0	0	**1.3**	**1.3**	**1.6**	**1.5**
																	LASEILMQNWDAAMEDLTR	27	1	1	4503521	N-Term(TMT6plex); M7(Oxidation); M14(Oxidation)	2468.2	0	**1.4**	**1.4**	**1.4**	**1.6**
																	SEAPNWATQDSGFY	13	1	1	4503521	N-Term(TMT6plex)	1801.8	0	**1.5**	**1.5**	**1.5**	**1.6**
																	MLFDYLADK	12	1	1	4503521	N-Term(TMT6plex); M1(Oxidation); K9(TMT6plex)	1589.9	0	**1.3**	**1.4**	**1.4**	**1.6**
																	VIQQESYTYKDPITEFVECLYVNFDFDGAQK	2	1	1	4503521	N-Term(TMT6plex); K10(TMT6plex); C19(Carbamidomethyl); K31(TMT6plex)	4434.2	1	**1.3**	**1.6**	**1.6**	**1.7**
																	NALSSLWGK	2	1	1	4503521	N-Term(TMT6plex); K9(TMT6plex)	1433.9	0	**1.3**	**1.2**	**1.5**	**1.4**
																	LASEILMQNWDAAMEDLTR	4	1	1	4503521	N-Term(TMT6plex)	2436.2	0	**1.6**	**1.1**	**1.3**	**1.1**
																	HLVFPLLEFLSVK	31	1	1	4503521	N-Term(TMT6plex); K13(TMT6plex)	2000.2	0	**1.5**	**1.4**	**1.7**	**1.5**
																	LKETIDNNSVSSPLQSLQQR	6	1	1	4503521	N-Term(TMT6plex); K2(TMT6plex)	2715.5	1	**1.5**	**1.6**	**1.6**	**1.7**
																	WIVNLIR	14	1	1	4503521	N-Term(TMT6plex)	1142.7	0	**1.8**	**1.5**	**1.7**	**1.7**
																	SQMLAMNIEK	5	1	1	4503521	N-Term(TMT6plex); M3(Oxidation); M6(Oxidation); K10(TMT6plex)	1654.9	0	**1.4**	**1.3**	**1.4**	**1.5**
																	FQYECGNYSGAAEYLYFFR	3	1	1	4503521	N-Term(TMT6plex); C5(Carbamidomethyl)	2614.2	0	**1.7**	**1.7**	**1.9**	**1.7**
																	LASEILMQNWDAAMEDLTR	6	1	1	4503521	N-Term(TMT6plex); M7(Oxidation)	2452.2	0	**1.3**	**1.3**	**1.4**	**1.5**
																	LDLLSDTNMVDFAMDVYK	12	1	1	4503521	N-Term(TMT6plex); M14(Oxidation); K18(TMT6plex)	2564.3	0	**1.6**	**1.8**	**1.9**	**1.7**
																	YLTTAVITNK	17	1	1	4503521	N-Term(TMT6plex); K10(TMT6plex)	1582.0	0	**1.3**	**1.2**	**1.5**	**1.3**
																	LDLLSDTNMVDFAMDVYK	8	1	1	4503521	N-Term(TMT6plex); M9(Oxidation); M14(Oxidation); K18(TMT6plex)	2580.3	0	**1.5**	**1.9**	**2.0**	**1.7**
																	LFIFETFCR	6	1	1	4503521	N-Term(TMT6plex); C8(Carbamidomethyl)	1461.8	0	**2.3**	**1.8**	**1.8**	**1.7**
																	SQMLAMNIEK	2	1	1	4503521	N-Term(TMT6plex); M6(Oxidation); K10(TMT6plex)	1638.9	0	**1.4**	**1.7**	**2.1**	**1.7**
																	LNMTPEEAER	2	1	1	4503521	N-Term(TMT6plex)	1418.7	0	**1.3**	**1.3**	**1.6**	**1.4**
																	LDLLSDTNMVDFAMDVYK	2	1	1	4503521	N-Term(TMT6plex); K18(TMT6plex)	2548.3	0	**2.3**	**2.4**	**3.0**	**2.0**
																	IHQCISINMLADK	2	1	1	4503521	N-Term(TMT6plex); C4(Carbamidomethyl); M9(Oxidation); K13(TMT6plex)	2017.1	0	**1.4**	**1.6**	**1.5**	**1.6**
																	ETIDNNSVSSPLQSLQQR	2	1	1	4503521	N-Term(TMT6plex)	2245.2	0	**1.3**	**0.9**	**1.5**	**1.3**
																	VIQQESYTYK	2	1	1	4503521	N-Term(TMT6plex); K10(TMT6plex)	1717.0	0	**1.3**	**1.3**	**1.7**	**1.6**
																	VLVPATDR	4	1	1	4503521	N-Term(TMT6plex)	1099.7	0	**1.4**	**1.5**	**1.6**	**1.6**
																	ELLQGK	2	1	1	4503521	N-Term(TMT6plex); K6(TMT6plex)	1145.7	0	**1.2**	**1.4**	**1.5**	**1.5**
																	IAHFLDR	2	1	1	4503521	N-Term(TMT6plex)	1100.6	0	**1.4**	**1.4**	**1.5**	**1.6**
																	EIYNEK	1	1	1	4503521	N-Term(TMT6plex); K6(TMT6plex)	1253.7	0	**1.4**	**1.8**	**1.9**	**1.9**
4503475	NP_001949.1	**EEF1A2**	1917	**elongation factor 1-alpha 2**	66.95	1	12	24	1182	**1.6**	**2.0**	**2.4**	**1.9**	463	50.4	9.03												
																	THINIVVIGHVDSGK	85	2	2	4503475;4503471	N-Term(TMT6plex); K15(TMT6plex)	2047.2	0				
																	NMITGTSQADCAVLIVAAGVGEFEAGISK	333	2	2	4503475;4503471	N-Term(TMT6plex); M2(Oxidation); C11(Carbamidomethyl); K29(TMT6plex)	3383.8	0				
																	EGNASGVSLLEALDTILPPTRPTDKPLR	23	1	1	4503475	N-Term(TMT6plex); K25(TMT6plex)	3418.9	0	**1.5**	**1.8**	**2.0**	**1.5**
																	VETGILRPGMVVTFAPVNITTEVK	19	1	1	4503475	N-Term(TMT6plex); M10(Oxidation); K24(TMT6plex)	3045.7	0	**1.8**	**2.0**	**3.0**	**1.8**
																	EHALLAYTLGVK	82	2	2	4503475;4503471	N-Term(TMT6plex); K12(TMT6plex)	1773.1	0				
																	STTTGHLIYK	82	2	2	4503475;4503471	N-Term(TMT6plex); K10(TMT6plex)	1578.9	0				
																	SGDAAIVEMVPGKPMCVESFSQYPPLGR	24	1	1	4503475	N-Term(TMT6plex); M9(Oxidation); K13(TMT6plex); M15(Oxidation); C16(Carbamidomethyl)	3512.8	0	**1.5**	**2.0**	**2.1**	**2.0**
																	QTVAVGVIK	75	2	2	4503475;4503471	N-Term(TMT6plex); K9(TMT6plex)	1372.9	0				
																	QLIVGVNK	51	2	2	4503475;4503471	N-Term(TMT6plex); K8(TMT6plex)	1328.9	0				
																	EAAEMGK	21	2	2	4503475;4503471	N-Term(TMT6plex); M5(Oxidation); K7(TMT6plex)	1209.7	0				
																	IGGIGTVPVGR	98	2	2	4503475;4503471	N-Term(TMT6plex)	1254.8	0				
																	GNVCGDSK	6	1	1	4503475	N-Term(TMT6plex); C4(Carbamidomethyl); K8(TMT6plex)	1294.7	0	**1.6**	**2.5**	**3.1**	**2.2**
																	IGYNPATVPFVPISGWHGDNMLEPSPNMPWFK	6	1	1	4503475	N-Term(TMT6plex); M21(Oxidation); M28(Oxidation); K32(TMT6plex)	4089.0	0	**1.5**	**2.4**	**2.7**	**2.3**
																	IGYNPATVPFVPISGWHGDNMLEPSPNMPWFK	4	1	1	4503475	N-Term(TMT6plex); K32(TMT6plex)	4057.1	0	**2.5**	**3.0**	**4.3**	**2.3**
																	LPLQDVYK	164	2	2	4503475;4503471	N-Term(TMT6plex); K8(TMT6plex)	1433.9	0				
																	NMITGTSQADCAVLIVAAGVGEFEAGISK	28	2	2	4503475;4503471	N-Term(TMT6plex); C11(Carbamidomethyl); K29(TMT6plex)	3367.8	0				
																	EAAEMGK	2	2	2	4503475;4503471	N-Term(TMT6plex); K7(TMT6plex)	1193.7	0				
																	VETGILRPGMVVTFAPVNITTEVK	4	1	1	4503475	N-Term(TMT6plex); K24(TMT6plex)	3029.7	0	**2.3**	**2.8**	**4.8**	**2.2**
																	KEGNASGVSLLEALDTILPPTRPTDKPLR	8	1	1	4503475	N-Term(TMT6plex); K1(TMT6plex); K26(TMT6plex)	3776.2	1	**1.4**	**1.8**	**2.0**	**1.9**
																	EVSAYIK	10	1	1	4503475	N-Term(TMT6plex); K7(TMT6plex)	1267.8	0	**1.4**	**1.9**	**2.0**	**1.7**
																	YAWVLDK	18	2	2	4503475;4503471	N-Term(TMT6plex); K7(TMT6plex)	1352.8	0				
																	IGYNPATVPFVPISGWHGDNMLEPSPNMPWFK	6	1	1	4503475	N-Term(TMT6plex); M28(Oxidation); K32(TMT6plex)	4073.0	0	**2.1**	**2.6**	**3.3**	**2.0**
																	SVEMHHEALSEALPGDNVGFNVK	5	2	2	4503475;4503471	N-Term(TMT6plex); M4(Oxidation); K23(TMT6plex)	2954.5	0				
																	MDSTEPAYSEK	2	1	1	4503475	N-Term(TMT6plex); M1(Oxidation); K11(TMT6plex)	1731.9	0	**1.4**	**1.3**	**1.4**	**1.4**
																	RYDEIVK	2	1	1	4503475	N-Term(TMT6plex); K7(TMT6plex)	1380.8	1	**1.5**	**1.5**	**1.7**	**1.7**
																	YYITIIDAPGHR	10	1	1	4503475	N-Term(TMT6plex)	1647.9	0	**1.1**	**1.3**	**1.4**	**1.3**
																	TIEKFEK	3	2	2	4503475;4503471	N-Term(TMT6plex); K4(TMT6plex); K7(TMT6plex)	1582.0	1				
																	SGGAGK	1	1	1	4503475	N-Term(TMT6plex); K6(TMT6plex)	934.6	0	**1.8**	**1.8**	**2.4**	**1.6**
																	YDEIVK	10	1	1	4503475	N-Term(TMT6plex); K6(TMT6plex)	1224.7	0	**1.9**	**2.2**	**2.8**	**2.0**
4759158	NP_004588.1	**SNRPD2**	6633	**small nuclear ribonucleoprotein Sm D2 isoform 1**	66.95	2	9	9	204	**1.2**	**1.3**	**1.3**	**1.4**	118	13.5	9.91												
																	GDSVIVVLR	11	2	1	4759158	N-Term(TMT6plex)	1186.7	0	**1.1**	**1.3**	**1.3**	**1.4**
																	SEMTPEELQK	60	1	1	4759158	N-Term(TMT6plex); M3(Oxidation); K10(TMT6plex)	1665.9	0	**1.3**	**1.3**	**1.4**	**1.4**
																	REEEEFNTGPLSVLTQSVK	40	2	1	4759158	N-Term(TMT6plex); K19(TMT6plex)	2621.4	1	**1.3**	**1.4**	**1.4**	**1.4**
																	EMWTEVPK	31	2	1	4759158	N-Term(TMT6plex); M2(Oxidation); K8(TMT6plex)	1493.8	0	**1.3**	**1.4**	**1.5**	**1.5**
																	EEEEFNTGPLSVLTQSVK	24	2	1	4759158	N-Term(TMT6plex); K18(TMT6plex)	2465.3	0	**1.3**	**1.4**	**1.4**	**1.4**
																	EMWTEVPK	4	2	1	4759158	N-Term(TMT6plex); K8(TMT6plex)	1477.8	0	**1.8**	**2.1**	**2.6**	**1.6**
																	HCNMVLENVK	12	2	1	4759158	N-Term(TMT6plex); C2(Carbamidomethyl); M4(Oxidation); K10(TMT6plex)	1717.9	0	**1.3**	**1.2**	**1.4**	**1.3**
																	SEMTPEELQK	2	1	1	4759158	N-Term(TMT6plex); K10(TMT6plex)	1649.9	0	**1.5**	**1.7**	**2.0**	**1.5**
																	NPLIAGK	11	2	1	4759158	N-Term(TMT6plex); K7(TMT6plex)	1170.8	0	**1.2**	**1.2**	**1.3**	**1.2**
																	NNTQVLINCR	6	2	1	4759158	N-Term(TMT6plex); C9(Carbamidomethyl)	1460.8	0	**1.5**	**1.1**	**1.4**	**1.1**
																	SKPVNK	3	2	1	4759158	N-Term(TMT6plex); K2(TMT6plex); K6(TMT6plex)	1359.9	0	**1.0**	**1.2**	**1.3**	**1.3**
42476296	NP_003280.2	**TPM2**	7169	**tropomyosin beta chain isoform 1**	66.90	3	4	27	348	**1.3**	**1.2**	**1.3**	**1.2**	284	32.8	4.70												
																	IQLVEEELDR	65	22	9	63252898;63252900;499137518;42476296;4507651;114155144;63252904;114155140;47519616	N-Term(TMT6plex)	1472.8	0				
																	KLVILEGELER	30	6	3	42476296;4507651;47519616	N-Term(TMT6plex); K1(TMT6plex)	1757.1	1				
																	MELQEMQLK	7	4	3	4507651;42476296;47519616	N-Term(TMT6plex); M1(Oxidation); M6(Oxidation); K9(TMT6plex)	1639.9	0				
																	LVILEGELER	15	4	3	42476296;4507651;47519616	N-Term(TMT6plex)	1399.8	0				
																	SLEAQADK	15	2	1	42476296	N-Term(TMT6plex); K8(TMT6plex)	1319.8	0	**1.2**	**1.1**	**1.1**	**1.0**
																	QLEEEQQALQK	21	2	2	42476296;47519616	N-Term(TMT6plex); K11(TMT6plex)	1802.0	0				
																	EDKYEEEIK	21	12	6	499137518;42476296;4507651;114155144;114155140;47519616	N-Term(TMT6plex); K3(TMT6plex); K9(TMT6plex)	1870.0	1				
																	LATALQK	47	22	9	63252898;63252900;499137518;42476296;4507651;114155144;63252904;114155140;47519616	N-Term(TMT6plex); K7(TMT6plex)	1202.8	0				
																	GTEDEVEK	8	2	2	42476296;47519616	N-Term(TMT6plex); K8(TMT6plex)	1364.7	0				
																	AEQAEADKK	9	9	4	63252898;63252900;42476296;47519616	N-Term(TMT6plex); K8(TMT6plex); K9(TMT6plex)	1677.0	1				
																	AADESER	4	18	9	63252898;63252900;499137518;42476296;4507651;114155144;63252904;114155140;47519616	N-Term(TMT6plex)	1006.5	0				
																	AEQAEADK	6	12	4	63252898;63252900;42476296;47519616	N-Term(TMT6plex); K8(TMT6plex)	1319.7	0				
																	CGDLEEELK	21	3	2	42476296;4507651	N-Term(TMT6plex); C1(Carbamidomethyl); K9(TMT6plex)	1550.8	0				
																	KATDAEADVASLNR	13	6	4	63252898;63252900;42476296;47519616	N-Term(TMT6plex); K1(TMT6plex)	1919.1	1				
																	ATDAEADVASLNR	7	6	4	63252898;63252900;42476296;47519616	N-Term(TMT6plex)	1561.8	0				

Supplementary Table 6. List of all proteins and peptides identified in untreated and chronically treated Het1A cells with cigarette smoke condensate for 8 months

Accession	RefSeq Accession	Gene Symbol	Entrez Gene ID	Description	Σ Coverage	Σ# Proteins	Σ# Unique Peptides	Σ# Peptides	Σ# PSMs	Het-1A-Smoke - 2M/Parental	Het-1A-Smoke - 4M/Parental	Het-1A-Smoke - 6M/Parental	Het-1A-Smoke - 8M/Parental	# AAs	MW [kDa]	calc. pI	Sequence	# PSMs	# Proteins	# Protein Groups	Protein Group Accessions	Modifications	MH+ [Da]	# Missed Cleavages	Het-1A-Smoke - 2M/Parental	Het-1A-Smoke - 4M/Parental	Het-1A-Smoke - 6M/Parental	Het-1A-Smoke - 8M/Parental
																	AISEELDNALNDITSL	12	1	1	42476296	N-Term(TMT6plex)	1947.0	0	1.4	1.4	1.4	1.4
																	MELQEMQLK	5	4	3	4507651;42476296;47519616	N-Term(TMT6plex); M6(Oxidation); K9(TMT6plex)	1623.9	0				
																	LKGTEDEVEK	2	2	2	42476296;47519616	N-Term(TMT6plex); K2(TMT6plex); K10(TMT6plex)	1835.1	1				
																	KYEEVAR	9	20	9	63252898;63252900;499137518;42476296;4507651;114155144;63252904;114155140;47519616	N-Term(TMT6plex); K1(TMT6plex)	1352.8	1				
																	TIDDLEDEVYAQK	1	1	1	42476296	N-Term(TMT6plex); K13(TMT6plex)	1997.0	0	1.5	1.3	1.4	1.5
																	IVTNNLK	3	1	1	42476296	N-Term(TMT6plex); K7(TMT6plex)	1259.8	0	1.0	1.0	1.2	0.9
																	YSESVK	4	2	2	42476296;47519616	N-Term(TMT6plex); K6(TMT6plex)	1170.7	0				
																	LDKENAIDR	1	10	5	63252898;63252900;114155140;42476296;47519616	N-Term(TMT6plex); K3(TMT6plex)	1531.9	1				
																	AEFAER	7	24	9	63252898;63252900;499137518;42476296;4507651;114155144;63252904;114155140;47519616	N-Term(TMT6plex)	951.5	0				
																	LEEAEK	4	20	10	63252898;63252900;499137518;42476296;4507651;114155144;63252904;114155140;47519616;116235456;116235451	N-Term(TMT6plex); K6(TMT6plex)	1176.7	0				
																	YEEVAR	4	24	9	63252898;63252900;499137518;42476296;4507651;114155144;63252904;114155140;47519616	N-Term(TMT6plex)	995.5	0				
																	LEQAEK	5	7	3	42476296;507588248;47519616	N-Term(TMT6plex); K6(TMT6plex)	1175.7	0				
																	YEEEIK	2	19	9	63252898;63252900;499137518;42476296;4507651;114155144;63252904;114155140;47519616	N-Term(TMT6plex); K6(TMT6plex)	1268.7	0				
4758356	NP_004102.1	**FEN1**	2237	**flap endonuclease 1**	66.84	1	20	21	230	**1.3**	**1.2**	**1.2**	**1.2**	380	42.6	8.62												
																	QHNDECK	3	1	1	4758356	N-Term(TMT6plex); C6(Carbamidomethyl); K7(TMT6plex)	1388.7	0	**1.3**	**1.2**	**1.3**	**1.1**
																	LPIQEFHLSR	3	1	1	4758356	N-Term(TMT6plex)	1468.8	0	**1.3**	**1.2**	**1.2**	**1.3**
																	QLQQAQAAGAEQEVEK	36	1	1	4758356	N-Term(TMT6plex); K16(TMT6plex)	2186.2	0	**1.4**	**1.2**	**1.2**	**1.2**
																	QGGDVLQNEEGETTSHLMGMFYR	19	1	1	4758356	N-Term(TMT6plex); M18(Oxidation); M20(Oxidation)	2860.3	0	**1.4**	**1.2**	**1.2**	**1.3**
																	QGGDVLQNEEGETTSHLMGMFYR	3	1	1	4758356	N-Term(TMT6plex)	2828.3	0	**1.3**	**1.4**	**1.4**	**1.3**
																	HLLSLMGIPYLDAPSEAEASCAALVK	15	1	1	4758356	N-Term(TMT6plex); M6(Oxidation); C21(Carbamidomethyl); K26(TMT6plex)	3230.7	0	**1.4**	**1.3**	**1.3**	**1.3**
																	HLTASEAK	5	1	1	4758356	N-Term(TMT6plex); K8(TMT6plex)	1314.8	0	**1.2**	**1.3**	**1.2**	**1.4**
																	VAIDASMSIYQFLIAVR	9	1	1	4758356	N-Term(TMT6plex); M7(Oxidation)	2142.2	0	**1.5**	**1.4**	**1.2**	**1.5**
																	VYAAATEDMDCLTFGSPVLMR	3	1	1	4758356	N-Term(TMT6plex); C11(Carbamidomethyl); M20(Oxidation)	2592.2	0	**1.4**	**1.3**	**1.3**	**1.3**
																	HLLSLMGIPYLDAPSEAEASCAALVK	6	1	1	4758356	N-Term(TMT6plex); C21(Carbamidomethyl); K26(TMT6plex)	3214.7	0	**1.4**	**1.4**	**1.7**	**1.2**
																	QGGDVLQNEEGETTSHLMGMFYR	4	1	1	4758356	N-Term(TMT6plex); M18(Oxidation)	2844.3	0	**1.5**	**1.6**	**1.6**	**1.5**
																	VYAAATEDMDCLTFGSPVLMR	25	1	1	4758356	N-Term(TMT6plex); M9(Oxidation); C11(Carbamidomethyl); M20(Oxidation)	2608.2	0	**1.3**	**1.3**	**1.3**	**1.3**
																	EAHQLFLEPEVLDPESVELK	24	1	1	4758356	N-Term(TMT6plex); K20(TMT6plex)	2780.5	0	**1.2**	**1.2**	**1.3**	**1.1**
																	KLPIQEFHLSR	11	1	1	4758356	N-Term(TMT6plex); K1(TMT6plex)	1826.1	1	**1.2**	**1.2**	**1.2**	**1.3**
																	LIADVAPSAIR	18	1	1	4758356	N-Term(TMT6plex)	1354.8	0	**1.2**	**1.2**	**1.2**	**1.1**
																	WSEPNEEELIK	16	1	1	4758356	N-Term(TMT6plex); K11(TMT6plex)	1832.0	0	**1.2**	**1.3**	**1.3**	**1.2**
																	VTGSLSSAK	9	1	1	4758356	N-Term(TMT6plex); K9(TMT6plex)	1307.8	0	**1.2**	**1.0**	**1.2**	**1.0**
																	GIQGLAK	1	1	1	4758356	N-Term(TMT6plex); K7(TMT6plex)	1144.7	0	**1.1**	**1.2**	**1.1**	**1.1**
																	LDPNKYPVPENWLHK	2	1	1	4758356	N-Term(TMT6plex); K5(TMT6plex); K15(TMT6plex)	2537.4	1	**1.1**	**1.2**	**1.2**	**1.1**
																	TGAAGK	2	1	1	4758356	N-Term(TMT6plex); K6(TMT6plex)	962.6	0	**1.2**	**0.8**	**1.0**	**0.8**
																	AVDLIQK	9	1	1	4758356	N-Term(TMT6plex); K7(TMT6plex)	1244.8	0	**1.0**	**1.2**	**1.1**	**1.2**
																	KEPEPK	1	2	2	4758356;95147555	N-Term(TMT6plex); K1(TMT6plex); K6(TMT6plex)	1414.9	1				
																	LDDFFK	4	1	1	4758356	N-Term(TMT6plex); K6(TMT6plex)	1242.7	0	**1.2**	**1.2**	**1.2**	**1.2**
																	MMENGIKPVYVFDGKPPQLK	1	1	1	4758356	N-Term(TMT6plex); M1(Oxidation); M2(Oxidation); K7(TMT6plex); K15(TMT6plex); K20(TMT6plex)	3239.8	0	**0.8**	**1.0**	**0.9**	**1.2**
																	SIEEIVR	1	1	1	4758356	N-Term(TMT6plex)	1074.6	0	**1.3**	**1.2**	**1.2**	**1.3**
157649073	NP_001099000.1	**CAP1**	10487	**adenylyl cyclase-associated protein 1**	66.74	2	26	26	358	**1.4**	**1.5**	**1.6**	**1.5**	475	51.6	8.02												
																	VENQENVSNLVIEDTELK	25	1	1	157649073	N-Term(TMT6plex); K18(TMT6plex)	2531.4	0	**1.6**	**1.4**	**1.6**	**1.6**
																	LSDLLAPISEQIK	52	1	1	157649073	N-Term(TMT6plex); K13(TMT6plex)	1885.1	0	**1.4**	**1.4**	**1.5**	**1.5**
																	TDGCHAYLSK	4	1	1	157649073	N-Term(TMT6plex); C4(Carbamidomethyl); K10(TMT6plex)	1609.8	0	**1.4**	**1.4**	**1.4**	**1.4**
																	EFHTTGLAWSK	10	1	1	157649073	N-Term(TMT6plex); K11(TMT6plex)	1735.0	0	**1.4**	**1.5**	**1.6**	**1.6**
																	NSLDCEIVSAK	11	1	1	157649073	N-Term(TMT6plex); C5(Carbamidomethyl); K11(TMT6plex)	1693.9	0	**1.5**	**1.3**	**1.5**	**1.4**
																	AGAAPYVQAFDSLLAGPVAEYLK	70	1	1	157649073	N-Term(TMT6plex); K23(TMT6plex)	2809.6	0	**1.5**	**1.5**	**1.6**	**1.5**
																	ALLVTASQCQQPAENK	7	1	1	157649073	N-Term(TMT6plex); C9(Carbamidomethyl); K16(TMT6plex)	2216.2	0	**1.4**	**1.6**	**1.6**	**1.5**
																	EMNDAAMFYTNR	6	1	1	157649073	N-Term(TMT6plex); M7(Oxidation)	1707.8	0	**1.7**	**1.8**	**1.8**	**1.6**
																	KLGLVFDDVVGIVEIINSK	1	1	1	157649073	N-Term(TMT6plex); K1(TMT6plex); K19(TMT6plex)	2745.7	1	**1.1**	**1.2**	**1.2**	**1.1**
																	SALFAQINQGESITHALK	14	1	1	157649073	N-Term(TMT6plex); K18(TMT6plex)	2386.4	0	**1.4**	**1.4**	**1.6**	**1.4**
																	SSEMNVLIPTEGGDFNEFPVPEQFK	30	1	1	157649073	N-Term(TMT6plex); M4(Oxidation); K25(TMT6plex)	3285.6	0	**1.6**	**1.7**	**1.9**	**1.6**
																	ELSGLPSGPSAGSGPPPPPPGPPPPPVSTSSGSDESASR	15	1	1	157649073	N-Term(TMT6plex)	3819.9	0	**1.7**	**2.0**	**2.0**	**1.4**
																	LVTTVTEIAG	6	1	1	157649073	N-Term(TMT6plex)	1232.7	0	**1.2**	**1.3**	**1.3**	**1.4**
																	SSEMNVLIPTEGGDFNEFPVPEQFK	18	1	1	157649073	N-Term(TMT6plex); K25(TMT6plex)	3269.6	0	**1.6**	**1.9**	**2.0**	**1.6**
																	GYADSPSK	13	1	1	157649073	N-Term(TMT6plex); K8(TMT6plex)	1282.7	0	**1.3**	**1.6**	**1.6**	**1.6**
																	EPAVLELEGK	10	1	1	157649073	N-Term(TMT6plex); K10(TMT6plex)	1542.9	0	**1.3**	**1.4**	**1.4**	**1.4**
																	VPTISINK	9	2	1	157649073	N-Term(TMT6plex); K8(TMT6plex)	1329.9	0	**1.2**	**1.4**	**1.4**	**1.5**
																	EIGGDVQK	19	1	1	157649073	N-Term(TMT6plex); K8(TMT6plex)	1303.8	0	**1.3**	**1.4**	**1.4**	**1.5**
																	LEAVSHTSDMHR	7	1	1	157649073	N-Term(TMT6plex); M10(Oxidation)	1627.8	0	**1.2**	**1.3**	**1.3**	**1.3**
																	HVSDDMK	2	1	1	157649073	N-Term(TMT6plex); K7(TMT6plex)	1289.7	0	**2.0**	**1.7**	**1.7**	**1.5**
																	INSITVDNCK	4	1	1	157649073	N-Term(TMT6plex); C9(Carbamidomethyl); K10(TMT6plex)	1621.9	0	**1.4**	**1.1**	**1.5**	**1.1**
																	EMNDAAMFYTNR	6	1	1	157649073	N-Term(TMT6plex); M2(Oxidation); M7(Oxidation)	1723.8	0	**1.2**	**1.3**	**1.2**	**1.6**
																	LEAVSHTSDMHR	2	1	1	157649073	N-Term(TMT6plex)	1611.8	0	**1.3**	**1.3**	**1.5**	**1.5**
																	LGLVFDDVVGIVEIINSK	2	1	1	157649073	N-Term(TMT6plex); K18(TMT6plex)	2388.4	0	**1.3**	**1.8**	**1.5**	**1.6**
																	CVNTTLQIK	2	1	1	157649073	N-Term(TMT6plex); C1(Carbamidomethyl); K9(TMT6plex)	1534.9	0	**1.6**	**1.3**	**1.7**	**1.3**
																	KEPAVLELEGK	2	1	1	157649073	N-Term(TMT6plex); K1(TMT6plex); K11(TMT6plex)	1900.2	1	**1.3**	**1.4**	**1.3**	**1.4**
																	HVDWVK	4	2	1	157649073	N-Term(TMT6plex); K6(TMT6plex)	1241.7	0	**1.2**	**1.4**	**1.5**	**1.5**
																	AQSGPVR	4	1	1	157649073	N-Term(TMT6plex)	943.6	0	**1.2**	**1.3**	**1.3**	**1.4**
																	EVITFR	3	1	1	157649073	N-Term(TMT6plex)	993.6	0	**1.4**	**1.5**	**1.5**	**1.6**
70995396	NP_001020604.1	**NQO1**	1728	**NAD(P)H dehydrogenase [quinone] 1 isoform b**	66.67	4	16	16	210	**1.9**	**2.1**	**1.7**	**1.7**	240	27.3	8.72												
																	LKDPANFQYPAESVLAYK	24	4	1	70995396	N-Term(TMT6plex); K2(TMT6plex); K18(TMT6plex)	2741.5	1	**1.6**	**2.1**	**1.7**	**1.6**
																	KGWEVVESDLYAMNFNPIISR	19	4	1	70995396	N-Term(TMT6plex); K1(TMT6plex); M13(Oxidation)	2942.6	1	**1.6**	**1.9**	**1.6**	**1.7**
																	IQILEGWK	37	4	1	70995396	N-Term(TMT6plex); K8(TMT6plex)	1444.9	0	**2.1**	**2.6**	**1.9**	**1.9**
																	GWEVVESDLYAMNFNPIISR	17	4	1	70995396	N-Term(TMT6plex); M12(Oxidation)	2585.3	0	**1.8**	**1.6**	**1.6**	**1.7**
																	EGHLSPDIVAEQK	18	4	1	70995396	N-Term(TMT6plex); K13(TMT6plex)	1881.0	0	**1.6**	**2.0**	**1.7**	**1.7**
																	EAAAAALK	10	4	1	70995396	N-Term(TMT6plex); K8(TMT6plex)	1202.8	0	**1.7**	**2.0**	**1.6**	**1.6**
																	SIPTDNQIK	19	4	1	70995396	N-Term(TMT6plex); K9(TMT6plex)	1473.9	0	**2.0**	**2.0**	**1.7**	**1.6**
																	KGWEVVESDLYAMNFNPIISR	6	4	1	70995396	N-Term(TMT6plex); K1(TMT6plex)	2926.5	1	**2.3**	**2.6**	**1.8**	**1.6**
																	TSFNYAMK	2	4	1	70995396	N-Term(TMT6plex); K8(TMT6plex)	1419.8	0	**2.5**	**2.3**	**2.3**	**1.6**
																	TSFNYAMK	6	4	1	70995396	N-Term(TMT6plex); M7(Oxidation); K8(TMT6plex)	1435.8	0	**2.0**	**1.8**	**1.7**	**1.5**
																	ALIVLAHSER	4	4	1	70995396	N-Term(TMT6plex)	1337.8	0	**1.4**	**1.6**	**1.3**	**1.4**
																	FGLSVGHHLGK	32	4	1	70995396	N-Term(TMT6plex); K11(TMT6plex)	1610.0	0	**1.9**	**2.4**	**1.8**	**1.8**
																	GWEVVESDLYAMNFNPIISR	4	4	1	70995396	N-Term(TMT6plex)	2569.3	0	**1.5**	**1.6**	**1.6**	**1.5**
																	KEVQDEEK	4	4	1	70995396	N-Term(TMT6plex); K1(TMT6plex); K8(TMT6plex)	1692.0	1	**1.9**	**2.0**	**1.5**	**1.6**
																	VFIGEFAYTYAAMYDK	2	2	1	70995396	N-Term(TMT6plex); K16(TMT6plex)	2347.2	0	**1.5**	**1.8**	**1.6**	**1.3**
																	LEAADLVIFQFPLQWFGVPAILK	1	2	1	70995396	N-Term(TMT6plex); K23(TMT6plex)	3073.8	0	**2.0**	**1.8**	**3.6**	**0.9**
																	EVQDEEK	2	4	1	70995396	N-Term(TMT6plex); K7(TMT6plex)	1334.7	0	**2.1**	**2.4**	**1.8**	**2.1**
																	KLEAADLVIFQFPLQWFGVPAILK	1	2	1	70995396	N-Term(TMT6plex); K1(TMT6plex); K24(TMT6plex)	3431.0	1	**1.8**	**1.6**	**2.7**	**0.9**
																	KDITGK	2	4	1	70995396	N-Term(TMT6plex); K1(TMT6plex); K6(TMT6plex)	1348.9	1	**2.1**	**2.9**	**1.9**	**1.9**
5803165	NP_006799.1	**SEC61B**	10952	**protein transport protein Sec61 subunit beta**	66.67	1	4	4	21	**1.6**	**1.3**	**1.5**	**1.1**	96	10.0	11.56												
																	PGPTPSGTNVGSSGR	3	1	1	5803165	N-Term(TMT6plex)	1599.8	0	**1.6**	**1.1**	**1.3**	**0.9**
																	TTSAGTGGMWR	4	1	1	5803165	N-Term(TMT6plex); M9(Oxidation)	1369.7	0	**1.6**	**1.1**	**1.2**	**1.1**
																	VGPVPVLVMSLLFIASVFMLHIWGKYTR	2	1	1	5803165	N-Term(TMT6plex); K25(TMT6plex)	3632.1	1	**1.7**	**1.5**	**2.6**	**1.0**
																	FYTEDSPGLK	12	1	1	5803165	N-Term(TMT6plex); K10(TMT6plex)	1614.9	0	**1.7**	**1.5**	**1.4**	**1.3**
189409150	NP_057494.3	**CHMP5**	51510	**charged multivesicular body protein 5 isoform 1**	66.67	2	8	8	117	**1.5**	**1.5**	**1.6**	**1.3**	219	24.6	4.83												
																	IDQIEDLQDQLEDMMEDANEIQEALSR	23	2	1	189409150	N-Term(TMT6plex); M15(Oxidation)	3436.6	0	**1.7**	**1.6**	**1.6**	**1.5**
																	IDQIEDLQDQLEDMMEDANEIQEALSR	22	2	1	189409150	N-Term(TMT6plex); M14(Oxidation); M15(Oxidation)	3452.6	0	**1.5**	**1.5**	**1.6**	**1.4**
																	NKDGVLVDEFGLPQIPAS	7	1	1	189409150	N-Term(TMT6plex); K2(TMT6plex)	2357.3	1	**1.4**	**1.6**	**1.5**	**1.6**
																	DGVLVDEFGLPQIPAS	26	1	1	189409150	N-Term(TMT6plex)	1886.0	0	**1.7**	**1.5**	**1.6**	**1.4**
																	LDAELVK	10	2	1	189409150	N-Term(TMT6plex); K7(TMT6plex)	1245.8	0	**1.3**	**1.2**	**1.3**	**1.2**
																	DNLAQQSFNMEQANYTIQSLK	2	2	1	189409150	N-Term(TMT6plex); K21(TMT6plex)	2901.5	0	**1.6**	**1.8**	**1.9**	**1.9**
																	DNLAQQSFNMEQANYTIQSLK	10	2	1	189409150	N-Term(TMT6plex); M10(Oxidation); K21(TMT6plex)	2917.5	0	**1.6**	**1.5**	**1.7**	**1.4**
																	APPPSLTDCIGTVDSR	8	2	1	189409150	N-Term(TMT6plex); C9(Carbamidomethyl)	1915.0	0	**1.3**	**1.3**	**1.4**	**1.3**

Accession	RefSeq Accession	Gene Symbol	Entrez Gene ID	Description	Σ Coverage	Σ# Proteins	Σ# Unique Peptides	Σ# Peptides	Σ# PSMs	Het-1A-Smoke - 2M/Parental	Het-1A-Smoke - 4M/Parental	Het-1A-Smoke - 6M/Parental	Het-1A-Smoke - 8M/Parental	# AAs	MW [kDa]	calc. pI	Sequence	# PSMs	# Proteins	# Protein Groups	Protein Group Accessions	Modifications	MH+ [Da]	# Missed Cleavages	Het-1A-Smoke - 2M/Parental	Het-1A-Smoke - 4M/Parental	Het-1A-Smoke - 6M/Parental	Het-1A-Smoke - 8M/Parental
																	SYGTPELDEDDLEAELDALGDELLADEDSSYLDEAASAPAIPEGVPTDTK	6	1	1	189409150	N-Term(TMT6plex); K50(TMT6plex)	5710.7	0	1.2	1.2	1.1	1.1
																	AESIDKK	3	2	1	189409150	N-Term(TMT6plex); K6(TMT6plex); K7(TMT6plex)	1477.9	1	1.1	1.3	1.3	1.3
9257257	NP_059830.1	WDR1	9948	WD repeat-containing protein 1 isoform 1	66.67	2	29	29	160	1.5	1.7	1.8	1.6	606	66.2	6.65												
																	NIDNPALADIYTEHAHQVVVAK	6	1	1	9257257	N-Term(TMT6plex); K22(TMT6plex)	2876.6	0	1.5	1.5	1.8	1.6
																	IAVVGEGR	4	1	1	9257257	N-Term(TMT6plex)	1029.6	0	1.9	2.0	2.1	1.8
																	DYSGQGVVK	13	2	1	9257257	N-Term(TMT6plex); K9(TMT6plex)	1410.8	0	1.4	1.7	1.9	1.6
																	IKDIAWTEDSK	6	1	1	9257257	N-Term(TMT6plex); K2(TMT6plex); K11(TMT6plex)	1993.2	1	1.4	1.8	1.8	1.8
																	VFASLPQVER	8	2	1	9257257	N-Term(TMT6plex)	1374.8	0	1.4	1.5	1.6	1.5
																	YAPSGFYIASGDVSGK	20	1	1	9257257	N-Term(TMT6plex); K16(TMT6plex)	2077.1	0	1.6	1.8	2.1	1.7
																	FGAVFLWDSGSSVGEITGHNK	8	1	1	9257257	N-Term(TMT6plex); K21(TMT6plex)	2666.4	0	1.4	1.5	1.7	1.5
																	DIAWTEDSK	4	1	1	9257257	N-Term(TMT6plex); K9(TMT6plex)	1522.8	0	1.5	2.0	2.1	1.9
																	LYSILGTTLK	11	2	1	9257257	N-Term(TMT6plex); K10(TMT6plex)	1567.0	0	1.7	1.3	2.1	1.4
																	VINSVDIK	7	1	1	9257257	N-Term(TMT6plex); K8(TMT6plex)	1345.8	0	1.6	1.8	2.1	1.8
																	MTVDESGQLISCSMDDTVR	10	2	1	9257257	N-Term(TMT6plex); M1(Oxidation); C12(Carbamidomethyl); M14(Oxidation)	2405.1	0	2.5	2.0	2.2	1.8
																	VCALGGSK	1	2	1	9257257	N-Term(TMT6plex); C2(Carbamidomethyl); K8(TMT6plex)	1249.7	0				
																	MTVDESGQLISCSMDDTVR	4	2	1	9257257	N-Term(TMT6plex); C12(Carbamidomethyl); M14(Oxidation)	2389.1	0	1.6	1.9	1.7	1.7
																	AHDGGIYAISWSPDSTHLLSASGDK	7	2	1	9257257	N-Term(TMT6plex); K25(TMT6plex)	3043.6	0	1.5	1.8	1.8	1.6
																	NNPSKPLHVIK	5	2	1	9257257	N-Term(TMT6plex); K5(TMT6plex); K11(TMT6plex)	1934.2	0	1.2	1.3	1.5	1.5
																	GPVTDVAYSHDGAFLAVCDASK	4	2	1	9257257	N-Term(TMT6plex); C18(Carbamidomethyl); K22(TMT6plex)	2738.4	0	1.5	1.8	1.8	1.7
																	KVFASLPQVER	6	2	1	9257257	N-Term(TMT6plex); K1(TMT6plex)	1732.1	1	1.4	1.7	1.8	1.8
																	SYIYSGSHDGHINYWDSETGENDSFAGK	8	2	1	9257257	N-Term(TMT6plex); K28(TMT6plex)	3594.6	0	1.7	1.8	2.0	1.8
																	DHLLSVSLSGYINYLDR	5	2	1	9257257	N-Term(TMT6plex)	2194.2	0	1.4	1.3	1.5	1.3
																	YEYQPFAGK	6	1	1	9257257	N-Term(TMT6plex); K9(TMT6plex)	1560.8	0	1.5	1.8	1.8	1.7
																	FATASADGQIYIYDGK	2	2	1	9257257	N-Term(TMT6plex); K16(TMT6plex)	2178.1	0	1.5	1.6	2.0	1.7
																	LATGSDDNCAAFFEGPPFK	3	1	1	9257257	N-Term(TMT6plex); C9(Carbamidomethyl); K19(TMT6plex)	2502.2	0	1.3	1.6	1.5	1.3
																	SIQCLTVHK	2	2	1	9257257	N-Term(TMT6plex); C4(Carbamidomethyl); K9(TMT6plex)	1543.9	0	1.4	1.7	1.8	1.6
																	CVAVGPGGYAVVVCIGQIVLLK	1	2	1	9257257	N-Term(TMT6plex); C1(Carbamidomethyl); C14(Carbamidomethyl); K22(TMT6plex)	2730.6	0	2.4	2.1	2.5	1.7
																	CFSIDNPGYEPEVVAVHPGGDTVAIGGVDGNVR	2	2	1	9257257	N-Term(TMT6plex); C1(Carbamidomethyl)	3626.8	0	1.7	1.4	1.8	1.2
																	IWDVSVNSVVSTFPMGSTVLDQQLGCLWQK	1	2	1	9257257	N-Term(TMT6plex); M15(Oxidation); C26(Carbamidomethyl); K30(TMT6plex)	3869.0	0	1.3	1.0	1.1	2.3
																	DIAWTEDSKR	1	1	1	9257257	N-Term(TMT6plex); K9(TMT6plex)	1678.9	1	1.2	1.6	1.7	1.5
																	EWTITY	3	2	1	9257257	N-Term(TMT6plex)	1041.5	0	2.1	1.7	1.8	1.8
																	LDVQPK	1	1	1	9257257	N-Term(TMT6plex); K6(TMT6plex)	1157.7	0	1.6	2.1	2.3	2.0
																	YTSLMLR	1	2	1	9257257	N-Term(TMT6plex); M5(Oxidation)	1128.6	0	1.5	1.4	1.6	1.5
23065552	NP_000840.2	GSTM3	2947	glutathione S-transferase Mu 3	66.67	1	15	15	123	1.3	1.1	1.3	1.1	225	26.5	5.54												
																	SQWLDVK	10	1	1	23065552	N-Term(TMT6plex); K7(TMT6plex)	1333.8	0	1.3	1.2	1.4	1.2
																	LLLEFTDTSYEEK	20	1	1	23065552	N-Term(TMT6plex); K13(TMT6plex)	2046.1	0	1.3	1.1	1.2	1.1
																	LDLDFPNLPYLLDGK	23	1	1	23065552	N-Term(TMT6plex); K15(TMT6plex)	2191.2	0	1.3	1.0	1.1	1.0
																	FSWFAGEK	5	1	1	23065552	N-Term(TMT6plex); K8(TMT6plex)	1429.8	0	1.4	1.1	1.3	1.0
																	LCYSSDHEK	2	1	1	23065552	N-Term(TMT6plex); C2(Carbamidomethyl); K9(TMT6plex)	1596.8	0	1.3	1.0	1.1	0.9
																	IAAYLQSDQFCK	4	1	1	23065552	N-Term(TMT6plex); C11(Carbamidomethyl); K12(TMT6plex)	1902.0	0	1.3	0.9	1.2	1.0
																	MAQWGNKPVC	6	1	1	23065552	N-Term(TMT6plex); M1(Oxidation); K7(TMT6plex); C10(Carbamidomethyl)	1664.9	0	1.2	1.1	1.2	1.0
																	KHNMCGETEEEK	7	1	1	23065552	N-Term(TMT6plex); K1(TMT6plex); M4(Oxidation); C5(Carbamidomethyl); K12(TMT6plex)	2195.1	1	1.1	1.0	1.1	1.2
																	YTCGEAPDYDR	11	1	1	23065552	N-Term(TMT6plex); C3(Carbamidomethyl)	1575.7	0	1.5	1.3	1.3	1.1
																	CLDEFPNLK	8	1	1	23065552	N-Term(TMT6plex); C1(Carbamidomethyl); K9(TMT6plex)	1593.9	0	1.4	1.2	1.3	1.1
																	LTFVDFLTYDILDQNR	11	1	1	23065552	N-Term(TMT6plex)	2202.2	0	1.4	1.4	1.6	1.2
																	LKPQYLEELPGQLK	4	1	1	23065552	N-Term(TMT6plex); K2(TMT6plex); K14(TMT6plex)	2343.4	0	1.2	1.1	1.2	1.2
																	VDIIENQVMDFR	6	1	1	23065552	N-Term(TMT6plex); M9(Oxidation)	1723.9	0	1.4	1.4	1.4	1.2
																	VDIIENQVMDFR	2	1	1	23065552	N-Term(TMT6plex)	1707.9	0	1.6	1.5	1.7	1.6
																	FKLDLDFPNLPYLLDGK	2	1	1	23065552	N-Term(TMT6plex); K2(TMT6plex); K17(TMT6plex)	2695.6	1	1.1	1.3	1.1	1.3
																	HNMCGETEEEK	2	1	1	23065552	N-Term(TMT6plex); M3(Oxidation); C4(Carbamidomethyl); K11(TMT6plex)	1837.8	0	1.2	0.8	1.1	0.8
51036603	NP_061329.3	GNG12	55970	guanine nucleotide-binding protein G(I)/G(S)/G(O) subunit gamma-12 precursor	66.67	1	4	4	48	2.1	1.2	1.4	1.3	72	8.0	8.97												
																	ASADLMSYCEEHAR	17	1	1	51036603	N-Term(TMT6plex); M6(Oxidation); C9(Carbamidomethyl)	1884.8	0	2.4	1.4	1.5	1.5
																	SDPLLIGIPTSENPFK	26	1	1	51036603	N-Term(TMT6plex); K16(TMT6plex)	2186.2	0	1.2	1.2	1.4	1.2
																	TASTNNIAQAR	2	1	1	51036603	N-Term(TMT6plex)	1375.7	0	2.2	1.0	1.3	0.9
																	LEASIER	3	1	1	51036603	N-Term(TMT6plex)	1046.6	0	1.3	1.3	1.4	1.3
5453832	NP_006380.1	HYOU1	10525	hypoxia up-regulated protein 1 precursor	66.57	2	56	56	945	1.5	1.7	1.6	1.4	999	111.3	5.22												
																	NINADEAAAMGAVYQAAALSK	25	1	1	5453832	N-Term(TMT6plex); M10(Oxidation); K21(TMT6plex)	2553.3	0	1.6	1.6	1.5	1.3
																	VLQLINDNTATALSYGVFR	41	1	1	5453832	N-Term(TMT6plex)	2324.3	0	1.6	1.6	1.6	1.3
																	LGNTISSLFGGGTTPDAK	24	1	1	5453832	N-Term(TMT6plex); K18(TMT6plex)	2194.2	0	1.5	1.3	1.5	1.2
																	AHFNLDESGVLSLDR	32	1	1	5453832	N-Term(TMT6plex)	1902.0	0	1.4	1.6	1.6	1.6
																	LIPEMDQIFTEVEMTTLEK	93	1	1	5453832	N-Term(TMT6plex); M5(Oxidation); M14(Oxidation); K19(TMT6plex)	2757.4	0	1.7	2.0	1.8	1.5
																	VIPPAGQTEDAEPISEPEKVETGSEPGDTEPLELGGPGAEPEQK	4	1	1	5453832	N-Term(TMT6plex); K19(TMT6plex); K44(TMT6plex)	5198.6	1	1.2	1.4	1.1	1.1
																	EEAEAPVEDGSQPPPPEPK	4	1	1	5453832	N-Term(TMT6plex); K19(TMT6plex)	2461.3	0	1.4	1.4	1.2	1.1
																	EAGMQPQLQIR	30	1	1	5453832	N-Term(TMT6plex); M4(Oxidation)	1515.8	0	1.6	1.8	1.6	1.5
																	VQEVLLK	16	1	1	5453832	N-Term(TMT6plex); K7(TMT6plex)	1286.8	0	1.6	1.8	1.7	1.3
																	TVLSANADHMAQIEGLMDDVDFK	12	1	1	5453832	N-Term(TMT6plex); M10(Oxidation); M17(Oxidation); K23(TMT6plex)	3010.5	0	1.4	1.6	1.5	1.3
																	VKPFVVR	5	1	1	5453832	N-Term(TMT6plex); K2(TMT6plex)	1302.9	0	1.5	2.1	1.8	1.5
																	LSAASTWLEDEGVGATTVMLK	6	1	1	5453832	N-Term(TMT6plex); K21(TMT6plex)	2637.4	0	2.7	3.2	3.1	1.7
																	VESVFETLVEDSAEEESTLTK	93	1	1	5453832	N-Term(TMT6plex); K21(TMT6plex)	2800.4	0	1.5	1.7	1.7	1.3
																	LSAASTWLEDEGVGATTVMLK	33	1	1	5453832	N-Term(TMT6plex); M19(Oxidation); K21(TMT6plex)	2653.4	0	1.6	1.7	1.6	1.4
																	TLGGLEMELR	13	1	1	5453832	N-Term(TMT6plex); M7(Oxidation)	1363.7	0	1.4	1.4	1.4	1.2
																	SLAEDFAEQPIK	18	1	1	5453832	N-Term(TMT6plex); K12(TMT6plex)	1806.0	0	1.5	1.6	1.8	1.3
																	AANSLEAFIFETQDK	26	1	1	5453832	N-Term(TMT6plex); K15(TMT6plex)	2142.1	0	1.4	1.6	1.4	1.4
																	GDATPEGEK	7	1	1	5453832	N-Term(TMT6plex); K9(TMT6plex)	1361.7	0	1.5	2.1	1.7	1.6
																	FPEHELTFDPQR	25	1	1	5453832	N-Term(TMT6plex)	1744.9	0	1.4	1.6	1.5	1.5
																	KYPDYESK	5	1	1	5453832	N-Term(TMT6plex); K1(TMT6plex); K8(TMT6plex)	1717.0	1	1.6	2.3	1.9	1.6
																	YFQHLLGK	13	1	1	5453832	N-Term(TMT6plex); K8(TMT6plex)	1463.9	0	1.5	1.4	1.5	1.1
																	AEPPLNASASDQGEK	6	1	1	5453832	N-Term(TMT6plex); K15(TMT6plex)	1972.0	0	1.7	1.8	1.6	1.5
																	DEPGEQVELKEEAEAPVEDGSQPPPPEPK	4	1	1	5453832	N-Term(TMT6plex); K10(TMT6plex); K29(TMT6plex)	3814.9	1	1.6	2.0	1.3	1.5
																	EVEEEPGIHSLK	6	1	1	5453832	N-Term(TMT6plex); K12(TMT6plex)	1825.0	0	1.5	2.0	1.6	1.4
																	QADNPHVALYQAR	13	1	1	5453832	N-Term(TMT6plex)	1711.9	0	1.5	1.6	1.5	1.4
																	FFGDSAASMAIK	10	1	1	5453832	N-Term(TMT6plex); M9(Oxidation); K12(TMT6plex)	1718.9	0	1.4	1.4	1.4	1.2
																	EQSTGQK	4	1	1	5453832	N-Term(TMT6plex); K7(TMT6plex)	1235.7	0	1.8	2.1	1.7	1.4
																	KTPVIVTLK	5	1	1	5453832	N-Term(TMT6plex); K1(TMT6plex); K9(TMT6plex)	1686.2	1	1.4	1.9	1.6	1.5
																	LYQPEYQEVSTEEQREEISGK	13	1	1	5453832	N-Term(TMT6plex); K21(TMT6plex)	3000.5	1	1.7	1.9	1.6	1.5
																	VAIVKPGVPMEIVLNK	28	1	1	5453832	N-Term(TMT6plex); K5(TMT6plex); M10(Oxidation); K16(TMT6plex)	2410.5	0	1.5	2.1	1.9	1.5
																	LAGLFNEQR	7	1	1	5453832	N-Term(TMT6plex)	1276.7	0	1.7	1.6	1.6	1.4
																	YSHDFNFHINYGDLGFLGPEDLR	6	1	1	5453832	N-Term(TMT6plex)	2955.4	0	1.1	1.3	1.3	1.3
																	LIPEMDQIFTEVEMTTLEK	13	1	1	5453832	N-Term(TMT6plex); M5(Oxidation); K19(TMT6plex)	2741.4	0	1.8	2.2	2.0	1.5
																	DAVVYPILVEFTR	62	1	1	5453832	N-Term(TMT6plex)	1751.0	0	1.7	1.9	1.7	1.4
																	VEFEELCADLFER	24	1	1	5453832	N-Term(TMT6plex); C7(Carbamidomethyl)	1885.9	0	1.8	2.0	1.8	1.6
																	AEAGPEGVAPAPEGEK	33	1	1	5453832	N-Term(TMT6plex); K16(TMT6plex)	1967.1	0	1.4	1.7	1.6	1.4
																	EVQYLLNK	23	1	1	5453832	N-Term(TMT6plex); K8(TMT6plex)	1464.9	0	1.5	1.9	1.7	1.4
																	MVEEIGVELVVLDLPDLPEDK	16	1	1	5453832	N-Term(TMT6plex); K21(TMT6plex)	2810.6	0	2.2	2.3	2.3	1.8
																	LAQSVQK	12	1	1	5453832	N-Term(TMT6plex); K7(TMT6plex)	1231.8	0	1.5	1.6	1.6	1.4
																	LPATEKPVLLSK	12	1	1	5453832	N-Term(TMT6plex); K6(TMT6plex); K12(TMT6plex)	1983.3	0	1.4	1.9	1.7	1.4
																	LCQGLFFR	2	1	1	5453832	N-Term(TMT6plex); C2(Carbamidomethyl)	1269.7	0	1.7	1.6	1.7	1.1
																	TPVIVTLK	10	1	1	5453832	N-Term(TMT6plex); K8(TMT6plex)	1328.9	0	1.4	1.6	1.6	1.3
																	VPGPVQQALQSAEMSLDEIEQVILVGGATR	4	1	1	5453832	N-Term(TMT6plex)	3364.8	0	1.3	1.5	1.5	1.6
																	VPGPVQQALQSAEMSLDEIEQVILVGGATR	5	1	1	5453832	N-Term(TMT6plex); M14(Oxidation)	3380.8	0	1.7	1.9	1.6	1.7

Khan et al., 2019. Multi-omics analysis to characterize cigarette smoke induced molecular alterations in esophageal cells
Supplementary Table 6. List of all proteins and peptides identified in untreated and chronically treated Het1A cells with cigarette smoke condensate for 8 months

Accession	RefSeq Accession	Gene Symbol	Entrez Gene ID	Description	Σ Coverage	Σ# Proteins	Σ# Unique Peptides	Σ# Peptides	Σ# PSMs	Het-1A-Smoke - 2M/Parental	Het-1A-Smoke - 4M/Parental	Het-1A-Smoke - 6M/Parental	Het-1A-Smoke - 8M/Parental	# AAs	MW [kDa]	calc. pI	Sequence	# PSMs	# Proteins	# Protein Groups	Protein Group Accessions	Modifications	MH+ [Da]	# Missed Cleavages	Het-1A-Smoke - 2M/Parental	Het-1A-Smoke - 4M/Parental	Het-1A-Smoke - 6M/Parental	Het-1A-Smoke - 8M/Parental
																	SLAEDFAEQPIKDAVITVPVFFNQAER	22	1	1	5453832	N-Term(TMT6plex); K12(TMT6plex)	3492.9	1	**2.3**	**3.2**	**2.7**	**2.4**
																	DAVITVPVFFNQAER	11	1	1	5453832	N-Term(TMT6plex)	1935.1	0	**1.4**	**1.6**	**1.5**	**1.3**
																	MVEEIGVELVVLDLPDLPEDK	9	1	1	5453832	N-Term(TMT6plex); M1(Oxidation); K21(TMT6plex)	2826.5	0	**1.5**	**1.6**	**1.5**	**1.5**
																	SEAQKPSEK	1	1	1	5453832	N-Term(TMT6plex); K5(TMT6plex); K9(TMT6plex)	1691.0	0	**1.6**	**1.7**	**1.7**	**1.2**
																	YPDYESK	7	1	1	5453832	N-Term(TMT6plex); K7(TMT6plex)	1359.7	0	**1.6**	**1.9**	**1.7**	**1.4**
																	TLGGLEMELR	2	1	1	5453832	N-Term(TMT6plex)	1347.8	0	**1.7**	**1.8**	**1.9**	**1.2**
																	TVLSANADHMAQIEGLMDDVDFK	3	1	1	5453832	N-Term(TMT6plex); M10(Oxidation); K23(TMT6plex)	2994.5	0	**1.8**	**2.7**	**2.3**	**1.7**
																	LYQPEYQEVSTEEQR	13	1	1	5453832	N-Term(TMT6plex)	2128.0	0	**1.6**	**1.6**	**1.6**	**1.3**
																	AEAGPEGVAPAPEGEKK	4	1	1	5453832	N-Term(TMT6plex); K16(TMT6plex); K17(TMT6plex)	2324.3	1	**1.6**	**2.4**	**1.8**	**1.8**
																	VIPPAGQTEDAEPISEPEK	3	1	1	5453832	N-Term(TMT6plex); K19(TMT6plex)	2465.3	0	**1.3**	**1.3**	**1.3**	**1.2**
																	VETGSEPGDTEPLELGGPGAEPEQK	2	1	1	5453832	N-Term(TMT6plex); K25(TMT6plex)	2981.5	0				
																	AVGKEELGK	2	1	1	5453832	N-Term(TMT6plex); K4(TMT6plex); K9(TMT6plex)	1618.0	1	**1.5**	**2.4**	**1.8**	**1.6**
																	KLCQGLFFR	4	1	1	5453832	N-Term(TMT6plex); K1(TMT6plex); C3(Carbamidomethyl)	1627.0	1	**1.7**	**2.1**	**2.0**	**1.7**
																	AVLQAAR	5	1	1	5453832	N-Term(TMT6plex)	957.6	0	**2.1**	**1.6**	**1.5**	**1.3**
																	GVGDSFK	5	1	1	5453832	N-Term(TMT6plex); K7(TMT6plex)	1167.7	0	**1.5**	**2.0**	**1.8**	**1.5**
																	LQDLTLR	6	2	1	5453832	N-Term(TMT6plex)	1087.7	0	**1.8**	**1.9**	**1.7**	**1.5**
																	ATEKENGDKSEAQKPSEK	2	1	1	5453832	N-Term(TMT6plex); K4(TMT6plex); K9(TMT6plex); K14(TMT6plex); K18(TMT6plex)	3121.8	2	**1.6**	**2.9**	**2.0**	**1.9**
																	VITFNR	1	1	1	5453832	N-Term(TMT6plex)	978.6	0	**1.7**	**2.0**	**1.7**	**1.4**
10880989	NP_067075.1	**RAB18**	22931	**ras-related protein Rab-18 isoform 1**	66.50	5	11	11	61	**1.4**	**1.5**	**1.6**	**1.5**	206	23.0	5.24												
																	FTDDTFDPELAATIGVDFK	21	4	1	10880989	N-Term(TMT6plex); K19(TMT6plex)	2560.3	0	**1.4**	**1.5**	**1.6**	**1.5**
																	ILIIGESGVGK	8	5	1	10880989	N-Term(TMT6plex); K11(TMT6plex)	1544.0	0	**1.3**	**1.4**	**1.6**	**1.5**
																	GAQGVILVYDVTR	8	4	1	10880989	N-Term(TMT6plex)	1619.9	0	**1.5**	**1.5**	**1.6**	**1.5**
																	HSMLFIEASAK	1	4	1	10880989	N-Term(TMT6plex); M3(Oxidation); K11(TMT6plex)	1708.0	0				
																	LDNWLNELETYCTR	4	4	1	10880989	N-Term(TMT6plex); C12(Carbamidomethyl)	2056.0	0	**1.5**	**1.5**	**1.7**	**1.5**
																	TCDGVQCAFEELVEK	6	4	1	10880989	N-Term(TMT6plex); C2(Carbamidomethyl); C7(Carbamidomethyl); K15(TMT6plex)	2243.1	0	**1.7**	**1.5**	**1.7**	**1.5**
																	IIQTPGLWESENQNK	4	4	1	10880989	N-Term(TMT6plex); K15(TMT6plex)	2215.2	0	**1.4**	**1.6**	**1.8**	**1.5**
																	LAIWDTAGQER	2	2	1	10880989	N-Term(TMT6plex)	1488.8	0	**1.2**	**1.2**	**1.4**	**1.3**
																	NDIVNMLVGNK	2	4	1	10880989	N-Term(TMT6plex); M6(Oxidation); K11(TMT6plex)	1691.0	0				
																	TISVDGNK	3	4	1	10880989	N-Term(TMT6plex); K8(TMT6plex)	1291.8	0	**1.3**	**1.3**	**1.5**	**1.4**
																	EVDRNEGLK	2	4	1	10880989	N-Term(TMT6plex); K9(TMT6plex)	1517.9	1	**1.2**	**1.4**	**1.4**	**1.6**
25777600	NP_002798.2	**PSMD1**	5707	**26S proteasome non-ATPase regulatory subunit 1 isoform 1**	66.42	2	49	49	415	**1.3**	**1.3**	**1.4**	**1.3**	953	105.8	5.39												
																	EAINLLEPMTNDPVNYVR	14	2	1	25777600	N-Term(TMT6plex); M9(Oxidation)	2333.2	0	**1.6**	**1.6**	**1.7**	**1.5**
																	DSDSMETEEK	5	2	1	25777600	N-Term(TMT6plex); M5(Oxidation); K10(TMT6plex)	1644.8	0	**1.6**	**1.6**	**1.5**	**1.6**
																	EPEPNFQLLDNPAR	6	2	1	25777600	N-Term(TMT6plex)	1869.0	0	**1.4**	**1.3**	**1.3**	**1.3**
																	LEGIVNK	9	2	1	25777600	N-Term(TMT6plex); K7(TMT6plex)	1230.8	0	**1.3**	**1.3**	**1.4**	**1.2**
																	RLDVFEK	7	2	1	25777600	N-Term(TMT6plex); K7(TMT6plex)	1364.8	1	**1.1**	**1.3**	**1.3**	**1.3**
																	SNCKPSTFAYPAPLEVPK	6	1	1	25777600	N-Term(TMT6plex); C3(Carbamidomethyl); K4(TMT6plex); K18(TMT6plex)	2693.5	0	**1.1**	**1.1**	**1.4**	**1.1**
																	DTSEDIEELVEPVAAHGPK	23	2	1	25777600	N-Term(TMT6plex); K19(TMT6plex)	2494.3	0	**1.2**	**1.3**	**1.5**	**1.4**
																	VSTAVLSITAK	11	2	1	25777600	N-Term(TMT6plex); K11(TMT6plex)	1548.0	0	**1.3**	**1.3**	**1.4**	**1.2**
																	QCVENADLPEGEK	11	2	1	25777600	N-Term(TMT6plex); C2(Carbamidomethyl); K13(TMT6plex)	1947.0	0	**1.5**	**1.4**	**1.5**	**1.3**
																	QFAALVASK	15	2	1	25777600	N-Term(TMT6plex); K9(TMT6plex)	1392.9	0	**1.2**	**1.2**	**1.4**	**1.1**
																	EALQLMATYLPK	15	2	1	25777600	N-Term(TMT6plex); M6(Oxidation); K12(TMT6plex)	1852.1	0	**1.4**	**1.3**	**1.3**	**1.4**
																	EFALHK	12	2	1	25777600	N-Term(TMT6plex); K6(TMT6plex)	1202.7	0	**1.1**	**1.3**	**1.3**	**1.2**
																	FTATASLGVIHK	15	2	1	25777600	N-Term(TMT6plex); K12(TMT6plex)	1703.0	0	**1.3**	**1.3**	**1.4**	**1.3**
																	MITSAAGIISLLDEDEPQLK	30	2	1	25777600	N-Term(TMT6plex); M1(Oxidation); K20(TMT6plex)	2618.4	0	**1.4**	**1.4**	**1.6**	**1.3**
																	TVGTPIASVPGSTNTGTVPGSEKDSDSMETEEK	5	2	1	25777600	N-Term(TMT6plex); K23(TMT6plex); M28(Oxidation); K33(TMT6plex)	4012.0	1	**1.4**	**1.3**	**1.4**	**1.3**
																	TVGTPIASVPGSTNTGTVPGSEK	8	2	1	25777600	N-Term(TMT6plex); K23(TMT6plex)	2615.4	0	**1.4**	**1.1**	**1.3**	**1.3**
																	TNLYQDDAVTGEAAGLALGLVMLGSK	19	2	1	25777600	N-Term(TMT6plex); M22(Oxidation); K26(TMT6plex)	3081.7	0	**1.4**	**1.2**	**1.4**	**1.4**
																	TPEASPEPK	20	2	1	25777600	N-Term(TMT6plex); K9(TMT6plex)	1413.8	0	**1.2**	**1.4**	**1.3**	**1.3**
																	MEEADALIESLCR	15	2	1	25777600	N-Term(TMT6plex); M1(Oxidation); C12(Carbamidomethyl)	1781.9	0	**1.3**	**1.2**	**1.3**	**1.3**
																	AAVESLGFILFR	25	2	1	25777600	N-Term(TMT6plex)	1551.9	0	**1.5**	**1.6**	**1.6**	**1.5**
																	NNNTDLMILK	4	2	1	25777600	N-Term(TMT6plex); M7(Oxidation); K10(TMT6plex)	1649.9	0	**1.4**	**1.0**	**1.3**	**1.1**
																	TNLYQDDAVTGEAAGLALGLVMLGSK	12	2	1	25777600	N-Term(TMT6plex); K26(TMT6plex)	3065.7	0	**1.5**	**1.3**	**1.7**	**1.1**
																	QAIGIALETR	2	2	1	25777600	N-Term(TMT6plex)	1300.8	0	**1.2**	**1.0**	**1.1**	**1.0**
																	HGGSLGLGLAAMGTAR	2	2	1	25777600	N-Term(TMT6plex); M12(Oxidation)	1713.9	0	**1.4**	**1.4**	**1.6**	**1.3**
																	VINDKHDDVMAK	1	2	1	25777600	N-Term(TMT6plex); K5(TMT6plex); M10(Oxidation); K12(TMT6plex)	2088.2	1	**1.1**	**1.5**	**1.4**	**1.5**
																	DTSPGSAYQEGGGLYALGLIHANHGGDIIDYLLNQLK	3	2	1	25777600	N-Term(TMT6plex); K37(TMT6plex)	4329.3	0	**1.5**	**1.6**	**2.0**	**1.4**
																	QDVYDLLK	4	2	1	25777600	N-Term(TMT6plex); K8(TMT6plex)	1451.8	0	**1.5**	**1.3**	**1.4**	**1.3**
																	IEEEEQEPEPPEPFEYIDD	10	2	1	25777600	N-Term(TMT6plex)	2563.1	0	**1.4**	**1.7**	**1.7**	**1.6**
																	LNAVVNDFWAEISESVDK	14	2	1	25777600	N-Term(TMT6plex); K18(TMT6plex)	2494.3	0	**1.3**	**1.2**	**1.5**	**1.2**
																	DNLEWLAR	12	2	1	25777600	N-Term(TMT6plex)	1245.7	0	**1.3**	**1.3**	**1.4**	**1.3**
																	YQPFKPLSIGGIIILK	11	2	1	25777600	N-Term(TMT6plex); K5(TMT6plex); K16(TMT6plex)	2474.6	0	**1.3**	**1.4**	**1.6**	**1.3**
																	NAQAIEDMVGYAQETQHEK	9	2	1	25777600	N-Term(TMT6plex); M8(Oxidation); K19(TMT6plex)	2636.3	0	**1.1**	**1.1**	**1.1**	**1.1**
																	NASNDIVR	2	2	1	25777600	N-Term(TMT6plex)	1117.6	0	**1.4**	**1.1**	**1.3**	**1.0**
																	IEVLYEDEGFR	6	2	1	25777600	N-Term(TMT6plex)	1598.8	0	**1.3**	**1.3**	**1.5**	**1.4**
																	TPEQCPSVVSLLSESYNPHVR	4	2	1	25777600	N-Term(TMT6plex); C5(Carbamidomethyl)	2628.3	0	**1.8**	**1.5**	**1.4**	**1.4**
																	YGAAMALGICCAGTGNK	2	2	1	25777600	N-Term(TMT6plex); M5(Oxidation); C10(Carbamidomethyl); C11(Carbamidomethyl); K17(TMT6plex)	2189.1	0	**1.2**	**1.0**	**1.3**	**1.0**
																	IYMNLEKPDFINVCQCLIFLDDPQAVSDILEK	2	2	1	25777600	N-Term(TMT6plex); M3(Oxidation); K7(TMT6plex); C14(Carbamidomethyl); C16(Carbamidomethyl); K32(TMT6plex)	4544.4	0	**1.1**	**1.2**	**1.2**	**1.4**
																	EALQLMATYLPK	2	2	1	25777600	N-Term(TMT6plex); K12(TMT6plex)	1836.1	0	**1.5**	**1.6**	**1.9**	**1.4**
																	SGMYTVAMAYCGSGNNK	2	2	1	25777600	N-Term(TMT6plex); M3(Oxidation); M8(Oxidation); C11(Carbamidomethyl); K17(TMT6plex)	2301.1	0	**1.4**	**1.3**	**1.3**	**1.4**
																	EAINLLEPMTNDPVNYVR	2	2	1	25777600	N-Term(TMT6plex)	2317.2	0	**1.7**	**1.7**	**2.0**	**1.6**
																	TSSAFVGK	10	2	1	25777600	N-Term(TMT6plex); K8(TMT6plex)	1254.7	0	**1.2**	**1.1**	**1.3**	**1.1**
																	KEPEPNFQLLDNPAR	1	2	1	25777600	N-Term(TMT6plex); K1(TMT6plex)	2226.2	1	**1.4**	**1.4**	**1.5**	**1.5**
																	MITSAAGIISLLDEDEPQLK	8	2	1	25777600	N-Term(TMT6plex); K20(TMT6plex)	2602.4	0	**1.7**	**1.7**	**2.3**	**1.2**
																	NSVCHTATVIANSFMHCGTTSDQFLR	1	2	1	25777600	N-Term(TMT6plex); C4(Carbamidomethyl); M15(Oxidation); C17(Carbamidomethyl)	3199.5	0	**1.4**	**1.6**	**1.5**	**1.7**
																	LLHVAVSDVNDDVR	2	2	1	25777600	N-Term(TMT6plex)	1781.0	0	**1.4**	**1.6**	**1.4**	**1.4**
																	CLDDHK	3	2	1	25777600	N-Term(TMT6plex); C1(Carbamidomethyl); K6(TMT6plex)	1245.7	0	**1.2**	**1.2**	**1.3**	**1.2**
																	HDDVMAK	2	2	1	25777600	N-Term(TMT6plex); M5(Oxidation); K7(TMT6plex)	1289.7	0	**1.3**	**1.5**	**1.3**	**1.3**
																	CIDHYTK	1	2	1	25777600	N-Term(TMT6plex); C1(Carbamidomethyl); K7(TMT6plex)	1394.7	0	**1.2**	**1.3**	**1.3**	**1.2**
																	NAQAIEDMVGYAQETQHEK	1	2	1	25777600	N-Term(TMT6plex); K19(TMT6plex)	2620.3	0	**1.2**	**1.1**	**1.5**	**1.3**
																	LCMSLMQNK	1	2	1	25777600	N-Term(TMT6plex); C2(Carbamidomethyl); M3(Oxidation); M6(Oxidation); K9(TMT6plex)	1614.8	0	**1.3**	**1.3**	**1.4**	**1.5**
																	VLTMPETCR	5	2	1	25777600	N-Term(TMT6plex); M4(Oxidation); C8(Carbamidomethyl)	1351.7	0	**1.4**	**1.3**	**1.4**	**1.3**
																	DKDPILR	1	2	1	25777600	N-Term(TMT6plex); K2(TMT6plex)	1314.8	1	**1.2**	**1.4**	**1.4**	**1.3**
																	MEVDEAEK	1	2	1	25777600	N-Term(TMT6plex); M1(Oxidation); K8(TMT6plex)	1424.7	0	**1.2**	**1.1**	**1.1**	**1.2**
																	LDVFEK	1	2	1	25777600	N-Term(TMT6plex); K6(TMT6plex)	1208.7	0	**1.4**	**1.5**	**1.5**	**1.3**
255653002	NP_001157412.1	**PYGL**	5836	**glycogen phosphorylase, liver form isoform 2**	66.42	2	43	52	536	**1.4**	**1.4**	**1.5**	**1.4**	813	93.1	7.30												
																	VIPATDLSEQISTAGTEASGTGNMK	15	2	1	255653002	N-Term(TMT6plex); M24(Oxidation); K25(TMT6plex)	2952.5	0	**1.5**	**1.4**	**1.4**	**1.5**
																	QEYFVVAATLQDIIR	11	5	2	255653002;21361370	N-Term(TMT6plex)	1995.1	0				
																	WPVDLVEK	28	2	1	255653002	N-Term(TMT6plex); K8(TMT6plex)	1443.9	0	**1.4**	**1.4**	**1.5**	**1.5**
																	FSQFLETEYK	8	2	1	255653002	N-Term(TMT6plex); K10(TMT6plex)	1749.9	0	**1.6**	**1.2**	**1.5**	**1.2**
																	DGWQVEEADDWLR	10	2	1	255653002	N-Term(TMT6plex)	1847.9	0	**1.4**	**1.5**	**1.5**	**1.7**
																	MSLIEEEGSK	4	2	1	255653002	N-Term(TMT6plex); M1(Oxidation); K10(TMT6plex)	1596.9	0	**1.9**	**1.7**	**2.3**	**1.6**
																	DFNVGDYIQAVLDR	34	2	1	255653002	N-Term(TMT6plex)	1854.0	0	**1.7**	**1.6**	**1.7**	**1.8**
																	VFADYEAYVK	20	2	1	255653002	N-Term(TMT6plex); K10(TMT6plex)	1662.9	0	**1.5**	**1.4**	**1.6**	**1.5**
																	EYYEALPELK	17	2	1	255653002	N-Term(TMT6plex); K10(TMT6plex)	1713.0	0	**1.3**	**1.4**	**1.4**	**1.4**
																	HLEIIYEINQK	12	2	1	255653002	N-Term(TMT6plex); K11(TMT6plex)	1858.1	0	**1.5**	**1.4**	**1.5**	**1.5**
																	IVALFPK	39	2	1	255653002	N-Term(TMT6plex); K7(TMT6plex)	1245.8	0	**1.6**	**1.4**	**1.5**	**1.4**
																	VEHTNTGTK	4	2	1	255653002	N-Term(TMT6plex); K9(TMT6plex)	1444.8	0	**1.4**	**1.3**	**1.5**	**1.4**
																	LHSFLGDDVFLR	18	2	1	255653002	N-Term(TMT6plex)	1647.9	0	**1.3**	**1.3**	**1.3**	**1.3**
																	EYAQNIWNVEPSDLK	16	2	1	255653002	N-Term(TMT6plex); K15(TMT6plex)	2264.2	0	**1.5**	**1.5**	**1.6**	**1.5**
																	IFVDIEK	16	2	1	255653002	N-Term(TMT6plex); K7(TMT6plex)	1321.8	0	**1.4**	**1.3**	**1.4**	**1.3**
																	DFSELEPDKFQNK	2	2	1	255653002	N-Term(TMT6plex); K9(TMT6plex); K13(TMT6plex)	2284.2	1	**1.3**	**1.4**	**1.5**	**1.4**
																	SRPEFMLPVHFYGK	4	2	1	255653002	N-Term(TMT6plex); M6(Oxidation); K14(TMT6plex)	2182.2	0	**1.4**	**1.3**	**1.6**	**1.5**
																	LVIDQIDNGFFSPK	7	2	1	255653002	N-Term(TMT6plex); K14(TMT6plex)	2051.2	0	**1.5**	**1.4**	**1.7**	**1.5**

Accession	RefSeq Accession	Gene Symbol	Entrez Gene ID	Description	Σ Coverage	Σ# Proteins	Σ# Unique Peptides	Σ# Peptides	Σ# PSMs	Het-1A-Smoke - 2M/Parental	Het-1A-Smoke - 4M/Parental	Het-1A-Smoke - 6M/Parental	Het-1A-Smoke - 8M/Parental	# AAs	MW [kDa]	calc. pI	Sequence	# PSMs	# Proteins	# Protein Groups	Protein Group Accessions	Modifications	MH+ [Da]	# Missed Cleavages	Het-1A-Smoke - 2M/Parental	Het-1A-Smoke - 4M/Parental	Het-1A-Smoke - 6M/Parental	Het-1A-Smoke - 8M/Parental
																	VIPATDLSEQISTAGTEASGTGNMK	2	2	1	255653002	N-Term(TMT6plex); K25(TMT6plex)	2936.5	0	**2.2**	**2.1**	**1.9**	**1.6**
																	TFAYTNHTVLPEALER	2	2	1	255653002	N-Term(TMT6plex)	2091.1	0	**1.6**	**1.7**	**1.6**	**1.6**
																	WLLLCNPGLAELIAEK	16	2	1	255653002	N-Term(TMT6plex); C5(Carbamidomethyl); K16(TMT6plex)	2298.3	0	**1.5**	**1.2**	**1.6**	**1.2**
																	GAGTVFDAFPDQVAIQLNDTHPALAIPELMR	10	2	1	255653002	N-Term(TMT6plex); M30(Oxidation)	3552.8	0	**1.4**	**1.5**	**1.4**	**1.5**
																	INPSSMFDVQVK	18	2	1	255653002	N-Term(TMT6plex); M6(Oxidation); K12(TMT6plex)	1839.0	0	**1.5**	**1.4**	**1.5**	**1.4**
																	VIFLENYR	12	5	2	255653002;21361370	N-Term(TMT6plex)	1282.7	0				
																	INPSSMFDVQVK	3	2	1	255653002	N-Term(TMT6plex); K12(TMT6plex)	1823.0	0	**1.5**	**1.6**	**1.8**	**1.5**
																	IDDVAALDKK	3	2	1	255653002	N-Term(TMT6plex); K9(TMT6plex); K10(TMT6plex)	1775.1	1	**1.4**	**1.5**	**1.6**	**1.6**
																	GIVGVENVAELK	14	2	1	255653002	N-Term(TMT6plex); K12(TMT6plex)	1686.0	0	**1.3**	**1.4**	**1.4**	**1.4**
																	VLYPNDNFFEGK	43	5	2	255653002;21361370	N-Term(TMT6plex); K12(TMT6plex)	1901.0	0				
																	AWELTQK	6	2	1	255653002	N-Term(TMT6plex); K7(TMT6plex)	1333.8	0	**1.4**	**1.4**	**1.5**	**1.5**
																	LKQEYFVVAATLQDIIR	15	5	2	255653002;21361370	N-Term(TMT6plex); K2(TMT6plex)	2465.5	1				
																	IHSDIVK	6	2	1	255653002	N-Term(TMT6plex); K7(TMT6plex)	1269.8	0	**1.3**	**1.2**	**1.4**	**1.2**
																	ISLSNESNK	10	2	1	255653002	N-Term(TMT6plex); K9(TMT6plex)	1449.8	0	**1.3**	**1.2**	**1.4**	**1.3**
																	DIINMLFYHDR	13	2	1	255653002	N-Term(TMT6plex); M5(Oxidation)	1681.9	0	**1.5**	**1.3**	**1.4**	**1.5**
																	DYYFALAHTVR	4	4	1	255653002	N-Term(TMT6plex)	1584.8	0	**1.4**	**1.4**	**1.5**	**1.6**
																	APNDFNLR	7	2	1	255653002	N-Term(TMT6plex)	1175.6	0	**1.4**	**1.4**	**1.5**	**1.4**
																	NLAENISR	7	5	2	255653002;21361370	N-Term(TMT6plex)	1145.6	0				
																	LITSVADVVNNDPMVGSK	6	2	1	255653002	N-Term(TMT6plex); K18(TMT6plex)	2317.3	0	**1.8**	**1.8**	**2.1**	**1.6**
																	LITSVADVVNNDPMVGSK	8	2	1	255653002	N-Term(TMT6plex); M14(Oxidation); K18(TMT6plex)	2333.3	0	**1.4**	**1.4**	**1.5**	**1.5**
																	VSQLYMNPK	6	2	1	255653002	N-Term(TMT6plex); M6(Oxidation); K9(TMT6plex)	1553.9	0	**1.4**	**1.2**	**1.4**	**1.3**
																	WIDTQVVLALPYDTPVPGYMNNTVNTMR	4	2	1	255653002	N-Term(TMT6plex); M20(Oxidation); M27(Oxidation)	3469.7	0	**1.4**	**1.4**	**1.4**	**1.2**
																	IDDVAALDK	4	2	1	255653002	N-Term(TMT6plex); K9(TMT6plex)	1417.8	0	**1.5**	**1.3**	**1.6**	**1.4**
																	DLSQLTK	9	2	1	255653002	N-Term(TMT6plex); K7(TMT6plex)	1262.8	0	**1.3**	**1.3**	**1.4**	**1.3**
																	TQQHYYDK	4	2	1	255653002	N-Term(TMT6plex); K8(TMT6plex)	1540.8	0	**1.2**	**1.2**	**1.3**	**1.3**
																	YEYGIFNQK	8	2	1	255653002	N-Term(TMT6plex); K9(TMT6plex)	1619.9	0	**1.4**	**1.3**	**1.5**	**1.3**
																	YGNPWEK	5	5	2	255653002;21361370	N-Term(TMT6plex); K7(TMT6plex)	1351.7	0				
																	DIINMLFYHDR	4	2	1	255653002	N-Term(TMT6plex)	1665.9	0	**1.8**	**1.4**	**1.6**	**1.4**
																	GIVGVENVAELKK	2	2	1	255653002	N-Term(TMT6plex); K12(TMT6plex); K13(TMT6plex)	2043.3	1	**1.1**	**1.2**	**1.2**	**1.4**
																	DFSELEPDK	3	2	1	255653002	N-Term(TMT6plex); K9(TMT6plex)	1537.8	0	**1.3**	**1.2**	**1.3**	**1.2**
																	IGEDYVK	5	2	1	255653002	N-Term(TMT6plex); K7(TMT6plex)	1281.7	0	**1.4**	**1.4**	**1.5**	**1.5**
																	AWNTMVLK	3	2	1	255653002	N-Term(TMT6plex); M5(Oxidation); K8(TMT6plex)	1436.8	0	**1.4**	**1.3**	**1.3**	**1.3**
																	NIAASGK	2	2	1	255653002	N-Term(TMT6plex); K7(TMT6plex)	1118.7	0	**1.7**	**1.7**	**1.7**	**1.4**
																	TVIIGGK	3	2	1	255653002	N-Term(TMT6plex); K7(TMT6plex)	1145.8	0	**1.4**	**1.3**	**1.4**	**1.3**
																	AAPGYHMAK	1	5	2	255653002;21361370	N-Term(TMT6plex); M7(Oxidation); K9(TMT6plex)	1419.8	0				
																	VKQENK	1	5	2	255653002;21361370	N-Term(TMT6plex); K2(TMT6plex); K6(TMT6plex)	1432.9	1				
																	VSLAEK	1	3	2	255653002;21361370	N-Term(TMT6plex); K6(TMT6plex)	1104.7	0				
																	KLFVPR	1	2	1	255653002	N-Term(TMT6plex); K1(TMT6plex)	1217.8	1	**1.5**	**1.7**	**1.7**	**1.8**
58761500	NP_037473.3	**OLA1**	29789	**obg-like ATPase 1 isoform 1**	66.41	2	22	22	150	**1.2**	**1.4**	**1.4**	**1.2**	396	44.7	7.81												
																	IGIVGLPNVGK	14	1	1	58761500	N-Term(TMT6plex); K11(TMT6plex)	1525.0	0	**1.2**	**1.1**	**1.2**	**1.0**
																	LKPEYDIMCK	4	2	1	58761500	N-Term(TMT6plex); K2(TMT6plex); M8(Oxidation); C9(Carbamidomethyl); K10(TMT6plex)	2000.1	0	**1.2**	**1.3**	**1.3**	**1.2**
																	IPAFLNVVDIAGLVK	22	1	1	58761500	N-Term(TMT6plex); K15(TMT6plex)	2027.3	0	**1.3**	**1.4**	**1.5**	**1.2**
																	IHTDFEK	8	2	1	58761500	N-Term(TMT6plex); K7(TMT6plex)	1347.8	0	**1.2**	**1.3**	**1.3**	**1.2**
																	STFFNVLTNSQASAENFPFCTIDPNESR	10	1	1	58761500	N-Term(TMT6plex); C20(Carbamidomethyl)	3422.6	0	**1.3**	**1.3**	**1.5**	**1.3**
																	YDPGALVIPFSGALELK	4	2	1	58761500	N-Term(TMT6plex); K17(TMT6plex)	2248.3	0	**1.4**	**2.0**	**1.4**	**2.0**
																	YLEANMTQSALPK	11	2	1	58761500	N-Term(TMT6plex); M6(Oxidation); K13(TMT6plex)	1940.1	0	**1.4**	**1.3**	**1.4**	**1.3**
																	GFIMAEVMK	8	2	1	58761500	N-Term(TMT6plex); M4(Oxidation); M8(Oxidation); K9(TMT6plex)	1515.8	0	**1.2**	**1.3**	**1.3**	**1.2**
																	LQELSAEER	8	2	1	58761500	N-Term(TMT6plex)	1303.7	0	**1.3**	**1.3**	**1.3**	**1.3**
																	AFEDDDITHVEGSVDPIRDIEIIHEELQLK	2	1	1	58761500	N-Term(TMT6plex); K30(TMT6plex)	3934.0	1	**1.3**	**1.3**	**1.5**	**1.4**
																	LKPEYDIMCK	1	2	1	58761500	N-Term(TMT6plex); K2(TMT6plex); C9(Carbamidomethyl); K10(TMT6plex)	1984.1	0	**1.4**	**1.5**	**1.7**	**1.2**
																	NYIVEDGDIIFFK	12	2	1	58761500	N-Term(TMT6plex); K13(TMT6plex)	2031.1	0	**1.2**	**1.2**	**1.4**	**1.1**
																	YEDFKEEGSENAVK	10	2	1	58761500	N-Term(TMT6plex); K5(TMT6plex); K14(TMT6plex)	2332.2	1	**1.3**	**1.2**	**1.3**	**1.2**
																	FYHDWNDKEIEVLNK	8	2	1	58761500	N-Term(TMT6plex); K8(TMT6plex); K15(TMT6plex)	2637.4	1	**1.2**	**1.4**	**1.4**	**1.2**
																	YLEANMTQSALPK	4	2	1	58761500	N-Term(TMT6plex); K13(TMT6plex)	1924.1	0	**1.8**	**1.8**	**2.1**	**1.5**
																	GGDGIKPPPIIGR	2	1	1	58761500	N-Term(TMT6plex); K6(TMT6plex)	1735.1	0	**1.1**	**1.3**	**1.2**	**1.4**
																	FNTPQQPK	5	2	1	58761500	N-Term(TMT6plex); K8(TMT6plex)	1417.8	0	**1.3**	**0.9**	**1.1**	**0.9**
																	SWVIDQK	5	2	1	58761500	N-Term(TMT6plex); K7(TMT6plex)	1333.8	0	**1.3**	**1.3**	**1.3**	**1.2**
																	FDFLCQYHKPASK	1	1	1	58761500	N-Term(TMT6plex); C5(Carbamidomethyl); K9(TMT6plex); K13(TMT6plex)	2328.3	0	**1.1**	**1.3**	**1.2**	**1.1**
																	YLEANMTQSALPKIIKAGFAALQLEYFFTAGPDEVR	1	2	1	58761500	N-Term(TMT6plex); M6(Oxidation); K13(TMT6plex); K16(TMT6plex)	4706.5	2	**1.6**	**1.1**	**2.3**	**0.8**
																	AFEDDDITHVEGSVDPIR	2	1	1	58761500	N-Term(TMT6plex)	2244.1	0	**1.4**	**1.3**	**1.3**	**1.5**
																	KGGDGIKPPPIIGR	1	1	1	58761500	N-Term(TMT6plex); K1(TMT6plex); K7(TMT6plex)	2092.3	1	**1.0**	**1.3**	**1.2**	**1.1**
																	APQAAGK	6	2	1	58761500	N-Term(TMT6plex); K7(TMT6plex)	1100.7	0	**1.1**	**1.3**	**1.2**	**1.1**
																	AGFAALQLEYFFTAGPDEVR	1	2	1	58761500	N-Term(TMT6plex)	2431.2	0	**0.8**	**1.6**	**1.5**	**1.5**
25777602	NP_002799.3	**PSMD2**	5708	**26S proteasome non-ATPase regulatory subunit 2 isoform 1**	66.41	3	46	46	434	**1.3**	**1.4**	**1.5**	**1.4**	908	100.1	5.20												
																	LVGSQEELASWGHEYVR	9	2	1	25777602	N-Term(TMT6plex)	2189.1	0	**1.4**	**1.6**	**1.5**	**1.4**
																	LALMLNDMELVEDIFTSCK	20	3	1	25777602	N-Term(TMT6plex); M4(Oxidation); M8(Oxidation); C18(Carbamidomethyl); K19(TMT6plex)	2732.4	0	**1.3**	**1.6**	**1.6**	**1.5**
																	EWQELDDAEK	14	3	1	25777602	N-Term(TMT6plex); K10(TMT6plex)	1720.9	0	**1.4**	**1.5**	**1.6**	**1.4**
																	EDVLTLLLPVMGDSK	37	3	1	25777602	N-Term(TMT6plex); M11(Oxidation); K15(TMT6plex)	2104.2	0	**1.3**	**1.4**	**1.6**	**1.3**
																	APVQPQQSPAAAPGGTDEKPSGK	8	1	1	25777602	N-Term(TMT6plex); K19(TMT6plex); K23(TMT6plex)	2905.6	0	**1.2**	**1.4**	**1.5**	**1.3**
																	NECDPALALLSDYVLHNSNTMR	3	3	1	25777602	N-Term(TMT6plex); C3(Carbamidomethyl); M21(Oxidation)	2778.3	0	**1.7**	**1.6**	**1.7**	**1.4**
																	VQQLLHICSEHFDSK	4	3	1	25777602	N-Term(TMT6plex); C8(Carbamidomethyl); K15(TMT6plex)	2299.2	0	**1.2**	**1.2**	**1.4**	**1.2**
																	EIYENMAPGENK	8	1	1	25777602	N-Term(TMT6plex); M6(Oxidation); K12(TMT6plex)	1868.9	0	**1.2**	**1.2**	**1.2**	**1.3**
																	QLQDELEMLVER	4	1	1	25777602	N-Term(TMT6plex); M8(Oxidation)	1747.9	0	**1.3**	**1.3**	**1.4**	**1.3**
																	VGQAVDVVGQAGKPK	9	3	1	25777602	N-Term(TMT6plex); K13(TMT6plex); K15(TMT6plex)	2140.3	0	**1.2**	**1.3**	**1.5**	**1.2**
																	SSTTSMTSVPKPLK	3	1	1	25777602	N-Term(TMT6plex); M6(Oxidation); K11(TMT6plex); K14(TMT6plex)	2167.3	0	**1.2**	**1.3**	**1.5**	**1.2**
																	FGGSGSQVDSAR	7	3	1	25777602	N-Term(TMT6plex)	1396.7	0	**1.3**	**1.1**	**1.3**	**1.1**
																	LGSIFGLGLAYAGSNR	14	3	1	25777602	N-Term(TMT6plex)	1825.0	0	**1.1**	**1.1**	**1.2**	**1.1**
																	EAPADMGAHQGVAVLGIALIAMGEEIGAEMALR	6	3	1	25777602	N-Term(TMT6plex); M6(Oxidation); M22(Oxidation); M30(Oxidation)	3568.8	0	**1.5**	**2.0**	**1.6**	**1.8**
																	SGALLACGIVNSGVR	4	3	1	25777602	N-Term(TMT6plex); C7(Carbamidomethyl)	1702.9	0	**1.6**	**1.4**	**1.6**	**1.4**
																	DIDENAYAK	23	3	1	25777602	N-Term(TMT6plex); K9(TMT6plex)	1496.8	0	**1.3**	**1.4**	**1.5**	**1.4**
																	HLAGEVAK	4	2	1	25777602	N-Term(TMT6plex); K8(TMT6plex)	1282.8	0	**1.1**	**1.3**	**1.5**	**1.3**
																	DAGDKDKEQELSEEDK	2	1	1	25777602	N-Term(TMT6plex); K5(TMT6plex); K7(TMT6plex); K16(TMT6plex)	2752.5	2	**1.3**	**1.4**	**1.6**	**1.4**
																	QLQDELEMLVER	2	1	1	25777602	N-Term(TMT6plex)	1731.9	0	**1.6**	**1.6**	**2.0**	**1.4**
																	FSHDADPEVSYNSIFAMGMVGSGTNNAR	1	3	1	25777602	N-Term(TMT6plex); M17(Oxidation)	3219.5	0	**1.4**	**1.2**	**1.3**	**1.3**
																	SFANTLVDVCAYAGSGNVLK	4	3	1	25777602	N-Term(TMT6plex); C10(Carbamidomethyl); K20(TMT6plex)	2544.4	0	**1.5**	**1.6**	**1.7**	**1.3**
																	EAPADMGAHQGVAVLGIALIAMGEEIGAEMALR	2	3	1	25777602	N-Term(TMT6plex); M6(Oxidation); M30(Oxidation)	3552.8	0	**1.7**	**1.2**	**1.3**	**1.8**
																	EPLLTLVK	16	3	1	25777602	N-Term(TMT6plex); K8(TMT6plex)	1370.9	0	**1.2**	**1.5**	**1.6**	**1.4**
																	LALMLNDMELVEDIFTSCK	8	3	1	25777602	N-Term(TMT6plex); M4(Oxidation); C18(Carbamidomethyl); K19(TMT6plex)	2716.4	0	**1.5**	**1.6**	**1.9**	**1.3**
																	MNLASSFVNGFVNAAFGQDK	2	3	1	25777602	N-Term(TMT6plex); M1(Oxidation); K20(TMT6plex)	2591.3	0	**1.4**	**1.3**	**1.6**	**1.3**
																	LLTDDGNK	7	3	1	25777602	N-Term(TMT6plex); K8(TMT6plex)	1333.8	0	**1.2**	**1.4**	**1.5**	**1.3**
																	LKEIYENMAPGENK	1	1	1	25777602	N-Term(TMT6plex); K2(TMT6plex); M8(Oxidation); K14(TMT6plex)	2339.3	1	**1.1**	**1.3**	**1.3**	**1.3**
																	GEAIEAILAALEVVSEPFR	17	3	1	25777602	N-Term(TMT6plex)	2243.3	0	**1.4**	**1.5**	**1.7**	**1.5**
																	AELATEEFLPVTPILEGFVILR	16	3	1	25777602	N-Term(TMT6plex)	2686.5	0	**1.3**	**1.6**	**1.7**	**1.5**
																	FSHDADPEVSYNSIFAMGMVGSGTNNAR	4	3	1	25777602	N-Term(TMT6plex); M17(Oxidation); M19(Oxidation)	3235.5	0	**1.6**	**1.7**	**1.4**	**1.5**
																	WLPLGLGLNHLGK	20	3	1	25777602	N-Term(TMT6plex); K13(TMT6plex)	1876.2	0	**1.5**	**1.6**	**1.7**	**1.5**
																	AVPLALALISVSNPR	48	3	1	25777602	N-Term(TMT6plex)	1750.1	0	**1.4**	**1.4**	**1.6**	**1.4**
																	LNILDTLSK	14	3	1	25777602	N-Term(TMT6plex); K9(TMT6plex)	1474.9	0	**1.6**	**1.7**	**2.0**	**1.6**
																	FAADIISVLAMTMSGER	8	1	1	25777602	N-Term(TMT6plex); M13(Oxidation)	2057.1	0	**2.1**	**3.1**	**2.9**	**2.2**
																	YLYSSEDYIK	21	3	1	25777602	N-Term(TMT6plex); K10(TMT6plex)	1738.9	0	**1.3**	**1.3**	**1.5**	**1.3**
																	LAQGLTHLGK	8	3	1	25777602	N-Term(TMT6plex); K10(TMT6plex)	1495.9	0	**1.3**	**1.3**	**1.5**	**1.3**
																	DTSLYRPALEELR	2	1	1	25777602	N-Term(TMT6plex)	1792.0	0	**1.3**	**1.3**	**1.4**	**1.4**
																	TITGFQTHTTPVLLAHGER	8	3	1	25777602	N-Term(TMT6plex)	2308.3	0	**1.4**	**1.6**	**1.6**	**1.5**
																	QLAQYHAK	4	3	1	25777602	N-Term(TMT6plex); K8(TMT6plex)	1416.8	0	**1.2**	**1.3**	**1.5**	**1.3**
																	MLVTFDEELRPLPVSVR	2	3	1	25777602	N-Term(TMT6plex)	2230.2	0	**1.6**	**1.6**	**1.8**	**1.3**

Khan et al., 2019. Multi-omics analysis to characterize cigarette smoke induced molecular alterations in esophageal cells
Supplementary Table 6. List of all proteins and peptides identified in untreated and chronically treated Het1A cells with cigarette smoke condensate for 8 months

Accession	RefSeq Accession	Gene Symbol	Entrez Gene ID	Description	Σ Coverage	Σ# Proteins	Σ# Unique Peptides	Σ# Peptides	Σ# PSMs	Het-1A-Smoke - 2M/Parental	Het-1A-Smoke - 4M/Parental	Het-1A-Smoke - 6M/Parental	Het-1A-Smoke - 8M/Parental	# AAs	MW [kDa]	calc. pI	Sequence	# PSMs	# Proteins	# Protein Groups	Protein Group Accessions	Modifications	MH+ [Da]	# Missed Cleavages	Het-1A-Smoke - 2M/Parental	Het-1A-Smoke - 4M/Parental	Het-1A-Smoke - 6M/Parental	Het-1A-Smoke - 8M/Parental
																	FAADIISVLAMTMSGER	14	1	1	25777602	N-Term(TMT6plex); M11(Oxidation); M13(Oxidation)	2073.1	0	**1.4**	**1.5**	**1.4**	**1.7**
																	VCLYLTSCVNYVPEPENSALLR	1	3	1	25777602	N-Term(TMT6plex); C2(Carbamidomethyl); C8(Carbamidomethyl)	2826.5	0	**1.2**	**1.3**	**1.4**	**1.4**
																	DKAPVQPQQSPAAAPGGTDEKPSGK	2	1	1	25777602	N-Term(TMT6plex); K2(TMT6plex); K21(TMT6plex); K25(TMT6plex)	3377.9	1	**1.1**	**1.4**	**1.6**	**1.3**
																	EQELSEEDKQLQDELEMLVER	1	1	1	25777602	N-Term(TMT6plex); K9(TMT6plex); M17(Oxidation)	3064.5	1	**1.4**	**1.4**	**1.6**	**1.5**
																	VPDDIYK	6	3	1	25777602	N-Term(TMT6plex); K7(TMT6plex)	1307.8	0	**1.3**	**1.4**	**1.4**	**1.3**
																	EIYENMAPGENKR	1	1	1	25777602	N-Term(TMT6plex); M6(Oxidation); K12(TMT6plex)	2025.0	1	**1.1**	**1.1**	**1.4**	**1.3**
																	DPNNLFMVR	2	3	1	25777602	N-Term(TMT6plex)	1334.7	0	**1.5**	**1.8**	**2.0**	**1.4**
																	MLVTFDEELRPLPVSVR	3	3	1	25777602	N-Term(TMT6plex); M1(Oxidation)	2246.2	0	**1.4**	**1.6**	**1.6**	**1.4**
																	LKEIYENMAPGENKR	1	1	1	25777602	N-Term(TMT6plex); K2(TMT6plex); M8(Oxidation); K14(TMT6plex)	2495.4	2	**1.3**	**1.4**	**1.4**	**1.4**
																	ELDIMEPK	4	3	1	25777602	N-Term(TMT6plex); M5(Oxidation); K8(TMT6plex)	1448.8	0	**1.1**	**1.4**	**1.4**	**1.3**
																	YGEPTLR	4	3	1	25777602	N-Term(TMT6plex)	1064.6	0	**1.3**	**1.3**	**1.4**	**1.2**
																	SETELK	1	3	1	25777602	N-Term(TMT6plex); K6(TMT6plex)	1164.7	0	**1.2**	**1.3**	**1.5**	**1.3**
377520143	NP_001243692.1	**FAM49B**	51571	**protein FAM49B**	66.36	2	17	17	126	**1.3**	**1.3**	**1.3**	**1.4**	324	36.7	6.06												
																	EAIQHPADEK	6	1	1	377520143;42734438	N-Term(TMT6plex); K10(TMT6plex)	1595.9	0	**1.0**	**1.3**	**1.3**	**1.5**
																	EIYNQVNVVLK	10	1	1	377520143;42734438	N-Term(TMT6plex); K11(TMT6plex)	1777.1	0	**1.2**	**1.3**	**1.4**	**1.5**
																	QFAEILHFTLR	4	1	1	377520143;42734438	N-Term(TMT6plex)	1603.9	0	**1.5**	**1.5**	**1.5**	**1.6**
																	DAEGILEDLQSYR	28	1	1	377520143;42734438	N-Term(TMT6plex)	1737.9	0	**1.5**	**1.4**	**1.4**	**1.7**
																	VLTCTDLEQGPNFFLDFENAQPTESEK	6	1	1	377520143;42734438	N-Term(TMT6plex); C4(Carbamidomethyl); K27(TMT6plex)	3587.8	0	**1.3**	**1.1**	**1.4**	**1.3**
																	TLSDATTK	8	1	1	377520143;42734438	N-Term(TMT6plex); K8(TMT6plex)	1294.8	0	**1.2**	**1.3**	**1.3**	**1.4**
																	DQPPNSVEGLLNALR	10	1	1	377520143;42734438	N-Term(TMT6plex)	1852.0	0	**1.4**	**1.5**	**1.4**	**1.6**
																	AWGAVVPLVGK	5	1	1	377520143;42734438	N-Term(TMT6plex); K11(TMT6plex)	1555.0	0	**1.1**	**1.2**	**1.1**	**1.3**
																	MTNPAIQNDFSYYR	2	1	1	377520143;42734438	N-Term(TMT6plex)	1948.9	0	**1.4**	**1.5**	**1.7**	**1.4**
																	INNVPAEGENEVNNELANR	13	1	1	377520143;42734438	N-Term(TMT6plex)	2325.2	0	**1.5**	**1.1**	**1.4**	**1.2**
																	MSLFYAEATPMLK	11	2	1	377520143;42734438	N-Term(TMT6plex); M1(Oxidation); M11(Oxidation); K13(TMT6plex)	1992.1	0	**1.1**	**1.2**	**1.1**	**1.3**
																	GLLGALTSTPYSPTQHLER	3	1	1	377520143;42734438	N-Term(TMT6plex)	2270.2	0	**1.2**	**1.0**	**1.3**	**1.1**
																	HLNDETTSK	3	1	1	377520143;42734438	N-Term(TMT6plex); K9(TMT6plex)	1502.8	0	**1.9**	**1.3**	**1.2**	**1.4**
																	FYEFSQR	2	1	1	377520143;42734438	N-Term(TMT6plex)	1205.6	0	**0.9**	**0.8**	**1.0**	**1.0**
																	NLPIENTTDCLSTMASVCR	1	1	1	377520143;42734438	N-Term(TMT6plex); C10(Carbamidomethyl); M14(Oxidation); C18(Carbamidomethyl)	2427.2	0	**1.2**	**1.0**	**1.3**	**1.0**
																	MSLFYAEATPMLK	2	2	1	377520143;42734438	N-Term(TMT6plex); M11(Oxidation); K13(TMT6plex)	1976.1	0	**1.3**	**1.6**	**1.5**	**1.6**
																	VLTCTDLEQGPNFFLDFENAQPTESEKEIYNQVNVVLK	2	1	1	377520143;42734438	N-Term(TMT6plex); C4(Carbamidomethyl); K27(TMT6plex); K38(TMT6plex)	5116.6	1	**1.2**	**1.9**	**1.3**	**1.6**
																	VMLETPEYR	2	2	1	377520143;42734438	N-Term(TMT6plex)	1366.7	0	**1.6**	**1.8**	**1.9**	**1.6**
																	MTNPAIQNDFSYYR	2	1	1	377520143;42734438	N-Term(TMT6plex); M1(Oxidation)	1964.9	0	**1.7**	**1.3**	**1.3**	**1.3**
																	VMLETPEYR	6	2	1	377520143;42734438	N-Term(TMT6plex); M2(Oxidation)	1382.7	0	**1.3**	**1.4**	**1.3**	**1.5**
573014815;4503841	NP_001275905.1	**XRCC6**	2547	**X-ray repair cross-complementing protein 6 isoform 1**	66.34	5	39	41	1214	**1.3**	**1.3**	**1.3**	**1.2**	609	69.8	6.64												
																	DIISIAEDEDLR	37	3	1	573014815;4503841	N-Term(TMT6plex)	1617.9	0	**1.4**	**1.4**	**1.4**	**1.3**
																	DSLIFLVDASK	71	2	1	573014815;4503841	N-Term(TMT6plex); K11(TMT6plex)	1666.0	0	**1.3**	**1.3**	**1.3**	**1.2**
																	NLEALALDLMEPEQAVDLTLPK	122	3	1	573014815;4503841	N-Term(TMT6plex); M10(Oxidation); K22(TMT6plex)	2897.6	0	**1.6**	**1.4**	**1.7**	**1.2**
																	DLLAVVFYGTEK	62	2	1	573014815;4503841	N-Term(TMT6plex); K12(TMT6plex)	1813.1	0	**1.3**	**1.3**	**1.4**	**1.2**
																	TFNTSTGGLLLPSDTK	52	3	1	573014815;4503841	N-Term(TMT6plex); K16(TMT6plex)	2110.2	0	**1.3**	**1.0**	**1.2**	**0.9**
																	HDNEGSGSK	2	3	1	573014815;4503841	N-Term(TMT6plex); K9(TMT6plex)	1388.7	0	**1.1**	**1.2**	**1.1**	**1.3**
																	LGSLVDEFK	47	3	1	573014815;4503841	N-Term(TMT6plex); K9(TMT6plex)	1465.9	0	**1.3**	**1.1**	**1.2**	**1.0**
																	VEYSEEELK	44	3	1	573014815;4503841	N-Term(TMT6plex); K9(TMT6plex)	1583.9	0	**1.3**	**1.3**	**1.2**	**1.2**
																	IQVTPPGFQLVFLPFADDK	58	3	1	573014815;4503841	N-Term(TMT6plex); K19(TMT6plex)	2590.5	0	**1.3**	**1.3**	**1.4**	**1.2**
																	ILELDQFK	61	3	1	573014815;4503841	N-Term(TMT6plex); K8(TMT6plex)	1463.9	0	**1.3**	**1.3**	**1.3**	**1.2**
																	IMLFTNEDNPHGNDSAK	22	3	1	573014815;4503841	N-Term(TMT6plex); M2(Oxidation); K17(TMT6plex)	2377.2	0	**1.4**	**1.4**	**1.3**	**1.3**
																	VHFEESSK	16	3	1	573014815;4503841	N-Term(TMT6plex); K8(TMT6plex)	1420.8	0	**1.2**	**1.2**	**1.3**	**1.3**
																	NIPPYFVALVPQEEELDDQK	59	3	1	573014815;4503841	N-Term(TMT6plex); K20(TMT6plex)	2802.5	0	**1.3**	**1.2**	**1.3**	**1.1**
																	KPGGFDISLFYR	24	3	1	573014815;4503841	N-Term(TMT6plex); K1(TMT6plex)	1858.1	0	**1.2**	**1.3**	**1.3**	**1.2**
																	ETNEPVK	21	3	1	573014815;4503841	N-Term(TMT6plex); K7(TMT6plex)	1274.7	0	**1.2**	**1.3**	**1.2**	**1.2**
																	DKNSVNFK	2	2	1	573014815;4503841	N-Term(TMT6plex); K2(TMT6plex); K8(TMT6plex)	1639.0	1	**1.1**	**1.5**	**1.3**	**1.3**
																	ALKPPPIK	10	3	1	573014815;4503841	N-Term(TMT6plex); K3(TMT6plex); K8(TMT6plex)	1551.1	0	**1.1**	**1.3**	**1.2**	**1.2**
																	KQELLEALTK	17	3	1	573014815;4503841	N-Term(TMT6plex); K1(TMT6plex); K10(TMT6plex)	1860.2	1	**1.2**	**1.2**	**1.3**	**1.1**
																	SDSFENPVLQQHFR	35	3	1	573014815;4503841	N-Term(TMT6plex)	1933.0	0	**1.3**	**1.3**	**1.3**	**1.3**
																	ELVYPPDYNPEGK	57	3	1	573014815;4503841	N-Term(TMT6plex); K13(TMT6plex)	1979.0	0	**1.2**	**1.3**	**1.3**	**1.3**
																	DTGIFLDLMHLK	8	3	1	573014815;4503841	N-Term(TMT6plex); K12(TMT6plex)	1861.1	0	**1.3**	**1.7**	**1.7**	**1.4**
																	FDDPGLMLMGFKPLVLLK	2	3	1	573014815;4503841	N-Term(TMT6plex); K12(TMT6plex); K18(TMT6plex)	2721.6	0	**1.6**	**2.0**	**2.0**	**1.5**
																	KMPFTEK	2	3	1	573014815;4503841	N-Term(TMT6plex); K1(TMT6plex); K7(TMT6plex)	1567.9	1	**1.5**	**1.8**	**1.8**	**1.3**
																	QIILEKEETEELK	7	3	1	573014815;4503841	N-Term(TMT6plex); K6(TMT6plex); K13(TMT6plex)	2289.3	1	**1.3**	**1.3**	**1.3**	**1.3**
																	IMATPEQVGK	48	3	1	573014815;4503841	N-Term(TMT6plex); M2(Oxidation); K10(TMT6plex)	1547.9	0	**1.3**	**1.2**	**1.3**	**1.2**
																	IQVTPPGFQLVFLPFADDKR	38	3	1	573014815;4503841	N-Term(TMT6plex); K19(TMT6plex)	2746.6	1	**1.4**	**1.4**	**1.3**	**1.3**
																	AMFESQSEDELTPFDMSIQCIQSVYISK	22	1	1	573014815;4503841	N-Term(TMT6plex); M2(Oxidation); M16(Oxidation); C20(Carbamidomethyl); K28(TMT6plex)	3773.8	0	**1.3**	**1.4**	**1.3**	**1.4**
																	KHDNEGSGSK	21	3	1	573014815;4503841	N-Term(TMT6plex); K1(TMT6plex); K10(TMT6plex)	1746.0	1	**1.2**	**1.2**	**1.2**	**1.1**
																	NIYVLQELDNPGAK	39	2	1	573014815;4503841	N-Term(TMT6plex); K14(TMT6plex)	2032.1	0	**1.3**	**1.0**	**1.2**	**1.1**
																	QELLEALTK	2	3	1	573014815;4503841	N-Term(TMT6plex); K9(TMT6plex)	1502.9	0	**1.4**	**1.1**	**1.2**	**1.2**
																	NLEALALDLMEPEQAVDLTLPK	6	3	1	573014815;4503841	N-Term(TMT6plex); K22(TMT6plex)	2881.6	0	**2.0**	**1.8**	**2.3**	**1.3**
																	DTGIFLDLMHLK	67	3	1	573014815;4503841	N-Term(TMT6plex); M9(Oxidation); K12(TMT6plex)	1877.0	0	**1.2**	**1.3**	**1.3**	**1.2**
																	FDDPGLMLMGFKPLVLLK	3	3	1	573014815;4503841	N-Term(TMT6plex); M7(Oxidation); K12(TMT6plex); K18(TMT6plex)	2737.6	0	**1.3**	**1.7**	**1.6**	**1.2**
																	DIVISVGIYNLVQK	17	3	1	573014815;4503841	N-Term(TMT6plex); K14(TMT6plex)	2019.2	0	**1.2**	**1.4**	**1.6**	**1.2**
																	TEGDEEAEEEQEENLEASGDYK	64	2	1	573014815;4503841	N-Term(TMT6plex); K22(TMT6plex)	2959.3	0	**1.2**	**1.1**	**1.1**	**1.1**
																	VHFEESSKLEDLLR	2	3	1	573014815;4503841	N-Term(TMT6plex); K8(TMT6plex)	2160.2	1	**1.4**	**1.5**	**1.4**	**1.4**
																	IMLFTNEDNPHGNDSAK	4	3	1	573014815;4503841	N-Term(TMT6plex); K17(TMT6plex)	2361.2	0	**1.4**	**1.5**	**1.6**	**1.3**
																	FDDPGLMLMGFKPLVLLK	6	3	1	573014815;4503841	N-Term(TMT6plex); M7(Oxidation); M9(Oxidation); K12(TMT6plex); K18(TMT6plex)	2753.6	0	**1.4**	**1.4**	**1.4**	**1.5**
																	IMATPEQVGK	6	3	1	573014815;4503841	N-Term(TMT6plex); K10(TMT6plex)	1531.9	0	**1.6**	**1.8**	**1.9**	**1.4**
																	QIILEKEETEELKR	1	3	1	573014815;4503841	N-Term(TMT6plex); K6(TMT6plex); K13(TMT6plex)	2445.5	2	**1.1**	**1.4**	**1.3**	**1.3**
																	QIILEK	2	3	1	573014815;4503841	N-Term(TMT6plex); K6(TMT6plex)	1201.8	0	**1.2**	**1.1**	**1.2**	**1.0**
																	EETEELKR	7	3	1	573014815;4503841	N-Term(TMT6plex); K7(TMT6plex)	1491.8	1	**1.2**	**1.2**	**1.2**	**1.1**
																	SQIYGSR	3	3	1	573014815;4503841	N-Term(TMT6plex)	1039.6	0	**1.2**	**1.0**	**1.1**	**1.0**
																	EETEELK	2	3	1	573014815;4503841	N-Term(TMT6plex); K7(TMT6plex)	1335.7	0	**1.3**	**1.3**	**1.3**	**1.4**
																	IISSDR	2	4	3	573014815;4503841;4504901;34485722	N-Term(TMT6plex)	919.5	0				
																	NSVNFK	5	2	1	573014815;4503841	N-Term(TMT6plex); K6(TMT6plex)	1166.7	0	**1.2**	**0.9**	**1.1**	**0.9**
																	LEDLLR	9	6	2	33356128;573014815;4503841	N-Term(TMT6plex)	987.6	0				
20127454	NP_004035.2	**ATIC**	471	**bifunctional purine biosynthesis protein PURH**	66.22	1	29	29	392	**1.4**	**1.4**	**1.4**	**1.4**	592	64.6	6.71												
																	ALFEEVPELLTEAEK	46	1	1	20127454	N-Term(TMT6plex); K15(TMT6plex)	2176.2	0	**1.5**	**1.4**	**1.5**	**1.5**
																	EALGIPAAASFK	12	1	1	20127454	N-Term(TMT6plex); K12(TMT6plex)	1633.0	0	**1.4**	**1.3**	**1.3**	**1.4**
																	AFTHTAQYDEAISDYFR	8	1	1	20127454	N-Term(TMT6plex)	2264.1	0	**1.5**	**1.7**	**1.6**	**1.5**
																	NLTALGLNLVASGGTAK	8	1	1	20127454	N-Term(TMT6plex); K17(TMT6plex)	2058.2	0	**1.4**	**1.1**	**1.3**	**1.1**
																	DLPESALR	8	1	1	20127454	N-Term(TMT6plex)	1129.6	0	**1.6**	**1.5**	**1.5**	**1.5**
																	VVACNLYPFVK	9	1	1	20127454	N-Term(TMT6plex); C4(Carbamidomethyl); K11(TMT6plex)	1768.0	0	**1.4**	**1.5**	**1.4**	**1.5**
																	HVSPAGAAVGIPLSEDEAK	14	1	1	20127454	N-Term(TMT6plex); K19(TMT6plex)	2306.3	0	**1.3**	**1.2**	**1.3**	**1.3**
																	EVSDGIIAPGYEEEALTILSK	39	1	1	20127454	N-Term(TMT6plex); K21(TMT6plex)	2692.5	0	**1.4**	**1.5**	**1.6**	**1.6**
																	DVSELTGFPEMLGGR	30	1	1	20127454	N-Term(TMT6plex); M11(Oxidation)	1852.9	0	**1.5**	**1.5**	**1.5**	**1.4**
																	DLIVATIAVK	22	1	1	20127454	N-Term(TMT6plex); K10(TMT6plex)	1501.0	0	**1.3**	**1.3**	**1.5**	**1.3**
																	APGQLALFSVSDK	2	1	1	20127454	N-Term(TMT6plex); K13(TMT6plex)	1791.0	0	**1.2**	**1.2**	**1.2**	**1.3**
																	LTEVSISSDAFFPFR	19	1	1	20127454	N-Term(TMT6plex)	1945.0	0	**1.5**	**1.4**	**1.3**	**1.4**
																	MSSFGDFVALSDVCDVPTAK	6	1	1	20127454	N-Term(TMT6plex); M1(Oxidation); C14(Carbamidomethyl); K20(TMT6plex)	2620.3	0	**1.5**	**1.2**	**1.4**	**1.3**
																	SGVAYIAAPSGSAADK	6	1	1	20127454	N-Term(TMT6plex); K16(TMT6plex)	1923.1	0	**1.6**	**1.4**	**1.5**	**1.4**
																	DVSELTGFPEMLGGR	5	1	1	20127454	N-Term(TMT6plex)	1836.9	0	**1.7**	**1.8**	**1.9**	**1.4**
																	YTQSNSVCYAK	4	1	1	20127454	N-Term(TMT6plex); C8(Carbamidomethyl); K11(TMT6plex)	1778.9	0	**1.5**	**1.2**	**1.5**	**1.1**
																	HHPQVLSMK	2	1	1	20127454	N-Term(TMT6plex); M8(Oxidation); K9(TMT6plex)	1550.9	0	**1.1**	**1.4**	**1.3**	**1.3**
																	TLFGLHLSQK	4	1	1	20127454	N-Term(TMT6plex); K10(TMT6plex)	1602.0	0	**1.4**	**1.2**	**1.4**	**1.3**
																	TVASPGVTVEEAVEQIDIGGVTLLR	82	1	1	20127454	N-Term(TMT6plex)	2782.5	0	**1.4**	**1.4**	**1.5**	**1.4**
																	AEISNAIDQYVTGTIGEDEDLIK	17	1	1	20127454	N-Term(TMT6plex); K23(TMT6plex)	2952.5	0	**1.4**	**1.3**	**1.3**	**1.4**
																	SLFSNVVTK	14	1	1	20127454	N-Term(TMT6plex); K9(TMT6plex)	1452.9	0	**1.4**	**1.3**	**1.3**	**1.4**
																	VVIEACDELGIILAHTNLR	8	1	1	20127454	N-Term(TMT6plex); C6(Carbamidomethyl)	2365.3	0	**1.4**	**1.5**	**1.5**	**1.4**
																	TLTPISAAYAR	4	1	1	20127454	N-Term(TMT6plex)	1392.8	0	**1.5**	**1.3**	**1.3**	**1.3**
																	YGMNPHQTPAQLYTLQPK	2	1	1	20127454	N-Term(TMT6plex); M3(Oxidation); K18(TMT6plex)	2561.3	0	**1.3**	**1.3**	**1.4**	**1.4**
																	LDFNLIR	6	1	1	20127454	N-Term(TMT6plex)	1119.7	0	**1.7**	**1.6**	**1.5**	**1.5**
																	TGLVEFAR	5	1	1	20127454	N-Term(TMT6plex)	1121.7	0	**1.6**	**1.1**	**1.3**	**1.1**
																	ANYWWLR	5	1	1	20127454	N-Term(TMT6plex)	1237.7	0	**1.4**	**1.3**	**1.4**	**1.4**
																	VCMVYDLYK	3	1	1	20127454	N-Term(TMT6plex); C2(Carbamidomethyl); M3(Oxidation); K9(TMT6plex)	1664.9	0	**1.4**	**1.3**	**1.4**	**1.4**

Khan et al., 2019. Multi-omics analysis to characterize cigarette smoke induced molecular alterations in esophageal cells

Supplementary Table 6. List of all proteins and peptides identified in untreated and chronically treated Het1A cells with cigarette smoke condensate for 8 months

Accession	RefSeq Accession	Gene Symbol	Entrez Gene ID	Description	Σ Coverage	Σ# Proteins	Σ# Unique Peptides	Σ# Peptides	Σ# PSMs	Het-1A-Smoke - 2M/Parental	Het-1A-Smoke - 4M/Parental	Het-1A-Smoke - 6M/Parental	Het-1A-Smoke - 8M/Parental	# AAs	MW [kDa]	calc. pI	Sequence	# PSMs	# Proteins	# Protein Groups	Protein Group Accessions	Modifications	MH+ [Da]	# Missed Cleavages	Het-1A-Smoke - 2M/Parental	Het-1A-Smoke - 4M/Parental	Het-1A-Smoke - 6M/Parental	Het-1A-Smoke - 8M/Parental
																	KEWVEK	1	1	1	20127454	N-Term(TMT6plex); K1(TMT6plex); K6(TMT6plex)	1505.9	1	**1.5**	**1.6**	**1.6**	**1.5**
																	NIPEDNADMAR	1	1	1	20127454	N-Term(TMT6plex); M9(Oxidation)	1490.7	0	**3.1**	**1.4**	**1.2**	**1.2**
21361621	NP_002624.2	**PGM1**	5236	**phosphoglucomutase-1 isoform 1**	66.19	3	29	29	165	**1.4**	**1.5**	**1.6**	**1.4**	562	61.4	6.76												
																	QEATLVVGGDGR	10	1	1	21361621	N-Term(TMT6plex)	1430.8	0	**1.7**	**1.4**	**1.6**	**1.3**
																	QSVEDILK	5	3	1	21361621	N-Term(TMT6plex); K8(TMT6plex)	1389.8	0	**1.5**	**1.5**	**1.6**	**1.3**
																	TQAYQDQKPGTSGLR	3	1	1	21361621	N-Term(TMT6plex); K8(TMT6plex)	2108.1	0	**1.3**	**1.2**	**1.3**	**1.2**
																	ELLSGPNR	3	3	1	21361621	N-Term(TMT6plex)	1114.6	0	**1.4**	**1.7**	**1.8**	**1.4**
																	YDYEEVEAEGANK	10	3	1	21361621	N-Term(TMT6plex); K13(TMT6plex)	1975.0	0	**1.6**	**1.9**	**2.2**	**1.6**
																	LYIDSYEK	10	3	1	21361621	N-Term(TMT6plex); K8(TMT6plex)	1488.8	0	**1.5**	**1.8**	**1.8**	**1.5**
																	INQDPQVMLAPLISIALK	4	3	1	21361621	N-Term(TMT6plex); K18(TMT6plex)	2422.5	0	**1.7**	**1.8**	**2.2**	**1.4**
																	IALYETPTGWK	7	3	1	21361621	N-Term(TMT6plex); K11(TMT6plex)	1737.0	0	**1.4**	**1.7**	**1.7**	**1.6**
																	LSGTGSAGATIR	9	3	1	21361621	N-Term(TMT6plex)	1319.7	0	**1.3**	**1.2**	**1.4**	**1.2**
																	LSLCGEESFGTGSDHIR	6	3	1	21361621	N-Term(TMT6plex); C4(Carbamidomethyl)	2094.0	0	**1.4**	**1.4**	**1.5**	**1.5**
																	FFGNLMDASK	4	3	1	21361621	N-Term(TMT6plex); K10(TMT6plex)	1587.9	0	**1.9**	**2.0**	**3.0**	**1.4**
																	TIEEYAVCPDLK	15	2	1	21361621	N-Term(TMT6plex); C8(Carbamidomethyl); K12(TMT6plex)	1896.0	0	**1.6**	**1.5**	**1.5**	**1.4**
																	HGFFVNPSDSVAVIAANIFSIPYFQQ TGVR	2	3	1	21361621	N-Term(TMT6plex)	3510.8	0	**1.5**	**1.1**	**2.0**	**1.0**
																	KQSVEDILK	6	3	1	21361621	N-Term(TMT6plex); K1(TMT6plex); K9(TMT6plex)	1747.1	1	**1.4**	**1.9**	**2.0**	**1.4**
																	QQFDLENK	6	2	1	21361621	N-Term(TMT6plex); K8(TMT6plex)	1479.8	0	**1.5**	**1.8**	**1.9**	**1.3**
																	INQDPQVMLAPLISIALK	14	3	1	21361621	N-Term(TMT6plex); M8(Oxidation); K18(TMT6plex)	2438.4	0	**1.2**	**1.3**	**1.3**	**1.4**
																	ADNFEYSDPVDGSISR	10	3	1	21361621	N-Term(TMT6plex)	2000.9	0	**1.3**	**1.4**	**1.5**	**1.5**
																	SMPTSGALDR	4	3	1	21361621	N-Term(TMT6plex); M2(Oxidation)	1279.7	0	**1.4**	**1.5**	**1.5**	**1.4**
																	SIFDFSALK	6	3	1	21361621	N-Term(TMT6plex); K9(TMT6plex)	1485.9	0	**1.3**	**1.5**	**1.4**	**1.4**
																	DLEALMFDR	2	3	1	21361621	N-Term(TMT6plex)	1338.7	0	**1.8**	**2.1**	**2.4**	**1.6**
																	VFQSSANYAENFIQSIISTVEPAQR	2	1	1	21361621	N-Term(TMT6plex)	3028.6	0	**2.2**	**2.3**	**2.6**	**2.2**
																	ILCEELGAPANSAVNCVPLEDFGGH HPDPNLTYAADLVETMK	2	3	1	21361621	N-Term(TMT6plex); C3(Carbamidomethyl); C16(Carbamidomethyl); K42(TMT6plex)	5023.5	0	**2.4**	**3.1**	**3.5**	**1.5**
																	DGLWAVLAWLSILATR	2	3	1	21361621	N-Term(TMT6plex)	2014.2	0	**1.0**	**1.0**	**1.0**	**1.0**
																	SGEHDFGAAFDGDGDR	4	3	1	21361621	N-Term(TMT6plex)	1881.8	0	**1.4**	**1.4**	**1.5**	**1.4**
																	IDAMHGVVGPYVK	1	3	1	21361621	N-Term(TMT6plex); M4(Oxidation); K13(TMT6plex)	1860.0	0	**1.4**	**1.7**	**1.6**	**1.6**
																	DLEALMFDR	3	3	1	21361621	N-Term(TMT6plex); M6(Oxidation)	1354.7	0				
																	ILCEELGAPANSAVNCVPLEDFGGH HPDPNLTYAADLVETMK	1	3	1	21361621	N-Term(TMT6plex); C3(Carbamidomethyl); C16(Carbamidomethyl); M41(Oxidation); K42(TMT6plex)	5039.5	0				
																	EAIQLIAR	4	1	1	21361621	N-Term(TMT6plex)	1142.7	0	**1.4**	**1.5**	**1.5**	**1.5**
																	QFSANDK	1	3	1	21361621	N-Term(TMT6plex); K7(TMT6plex)	1267.7	0	**1.4**	**1.2**	**1.3**	**1.2**
																	FFGNLMDASK	3	3	1	21361621	N-Term(TMT6plex); M6(Oxidation); K10(TMT6plex)	1603.9	0	**1.5**	**1.4**	**1.6**	**1.4**
																	VYTVEK	3	3	1	21361621	N-Term(TMT6plex); K6(TMT6plex)	1196.7	0	**1.3**	**1.5**	**1.6**	**1.3**
																	IFQISK	2	2	1	21361621	N-Term(TMT6plex); K6(TMT6plex)	1193.8	0	**1.4**	**1.6**	**1.9**	**1.3**
																	VASATK	1	3	1	21361621	N-Term(TMT6plex); K6(TMT6plex)	1034.7	0	**1.1**	**1.1**	**1.2**	**1.2**
21914862	NP_060299.4	**CECR5**	27440	**cat eye syndrome critical region protein 5 isoform 1**	66.16	2	15	15	45	**1.3**	**1.3**	**1.4**	**1.3**	393	43.6	6.90												
																	MLVSGQGPVMENAQGLGFR	5	2	1	21914862	N-Term(TMT6plex); M1(Oxidation); M10(Oxidation)	2252.1	0	**1.3**	**1.3**	**1.2**	**1.4**
																	NPQSTEPVLGGGEPPFHGHR	2	2	1	21914862	N-Term(TMT6plex)	2342.2	0	**1.3**	**1.3**	**1.3**	**1.3**
																	LYAVGDNPMSDVYGANLFHQYLQK	2	2	1	21914862	N-Term(TMT6plex); M9(Oxidation); K24(TMT6plex)	3217.6	0	**1.1**	**0.9**	**1.3**	**1.2**
																	LVNSQGQLR	2	2	1	21914862	N-Term(TMT6plex)	1243.7	0	**1.5**	**1.1**	**1.5**	**1.4**
																	VPVVFVTNAGNILQHSK	2	2	1	21914862	N-Term(TMT6plex); K17(TMT6plex)	2281.3	0	**1.4**	**1.6**	**1.6**	**1.6**
																	IEGVLLLGEPVR	6	2	1	21914862	N-Term(TMT6plex)	1523.9	0	**1.3**	**1.1**	**1.2**	**1.1**
																	MAFPLLDMVDLER	4	2	1	21914862	N-Term(TMT6plex); M8(Oxidation)	1794.9	0	**1.7**	**1.8**	**2.0**	**1.6**
																	ATHDGAPELGAGGTR	4	2	1	21914862	N-Term(TMT6plex)	1638.8	0	**1.3**	**1.4**	**1.3**	**1.3**
																	DLCFSPGLMEASHVVNDVNEAVQL VFR	2	2	1	21914862	N-Term(TMT6plex); C3(Carbamidomethyl); M9(Oxidation)	3291.6	0	**1.2**	**1.0**	**1.3**	**1.3**
																	LFSEYHEK	4	2	1	21914862	N-Term(TMT6plex); K8(TMT6plex)	1510.8	0	**1.3**	**1.2**	**1.4**	**1.3**
																	NVVTVDELR	2	2	1	21914862	N-Term(TMT6plex)	1273.7	0	**1.4**	**1.2**	**1.3**	**1.3**
																	AQELSALLGCEVDADQVILSHSPMK	4	2	1	21914862	N-Term(TMT6plex); C10(Carbamidomethyl); M24(Oxidation); K25(TMT6plex)	3185.6	0	**1.3**	**1.2**	**1.5**	**1.4**
																	MAFPLLDMVDLER	3	2	1	21914862	N-Term(TMT6plex); M1(Oxidation); M8(Oxidation)	1810.9	0	**1.2**	**1.4**	**1.3**	**1.3**
																	WETSLQLIMDVLLSNGSPGAGLATP PYPHLPVLASNMDLLWMAEAKMPR	1	2	1	21914862	N-Term(TMT6plex); M42(Oxidation); K46(TMT6plex)	5806.0	1	**1.4**	**1.3**	**1.2**	**1.7**
																	EGWALE	1	2	1	21914862	N-Term(TMT6plex)	933.5	0	**1.2**	**1.2**	**1.2**	**1.3**
																	VIPAALK	1	2	1	21914862	N-Term(TMT6plex); K7(TMT6plex)	1169.8	0	**1.2**	**1.2**	**1.4**	**1.3**
7705618	NP_057134.1	**MRPL11**	65003	**39S ribosomal protein L11, mitochondrial isoform a**	66.15	3	12	12	73	**1.2**	**1.4**	**1.3**	**1.4**	192	20.7	9.91												
																	EVAGLVTLK	4	3	1	7705618	N-Term(TMT6plex); K9(TMT6plex)	1387.9	0	**1.1**	**1.3**	**1.4**	**1.4**
																	IGQPTVSYFLK	12	3	1	7705618	N-Term(TMT6plex); K11(TMT6plex)	1711.0	0	**1.2**	**1.1**	**1.3**	**1.2**
																	AGLAMPGPPLGPVLGQR	9	2	1	7705618	N-Term(TMT6plex); M5(Oxidation)	1876.1	0	**1.4**	**1.5**	**1.4**	**1.5**
																	AIFLAAQK	5	2	1	7705618	N-Term(TMT6plex); K8(TMT6plex)	1319.8	0	**1.2**	**1.4**	**1.4**	**1.4**
																	DLSSEELAAFQK	8	2	1	7705618	N-Term(TMT6plex); K12(TMT6plex)	1796.0	0	**1.2**	**1.3**	**1.3**	**1.3**
																	EADLAAQEEAAK	17	2	1	7705618	N-Term(TMT6plex); K12(TMT6plex)	1703.9	0	**1.4**	**1.4**	**1.3**	**1.4**
																	AQDEAFALQDVPLSSVVR	8	3	1	7705618	N-Term(TMT6plex)	2174.2	0	**1.3**	**1.3**	**1.3**	**1.3**
																	GVSINQFCK	2	3	1	7705618	N-Term(TMT6plex); C8(Carbamidomethyl); K9(TMT6plex)	1510.8	0	**1.2**	**1.4**	**1.4**	**1.4**
																	EGIPLPTK	2	3	1	7705618	N-Term(TMT6plex); K8(TMT6plex)	1312.8	0	**1.2**	**1.4**	**1.3**	**1.3**
																	AAAGIEK	4	3	1	7705618	N-Term(TMT6plex); K7(TMT6plex)	1117.7	0	**1.3**	**1.6**	**1.4**	**1.4**
																	KPEVGGVIR	1	2	1	7705618	N-Term(TMT6plex); K1(TMT6plex)	1412.9	0				
																	ILVKPDR	1	3	1	7705618	N-Term(TMT6plex); K4(TMT6plex)	1298.9	0	**1.1**	**1.3**	**1.2**	**1.3**
4505701	NP_003672.1	**PDXK**	8566	**pyridoxal kinase**	66.03	1	16	16	124	**1.4**	**1.4**	**1.4**	**1.5**	312	35.1	6.13												
																	VLSIQSHVIR	4	1	1	4505701	N-Term(TMT6plex)	1380.9	0	**1.2**	**1.1**	**1.1**	**1.2**
																	WDGEGSMYVPEDLLPVYK	7	1	1	4505701	N-Term(TMT6plex); M7(Oxidation); K18(TMT6plex)	2572.3	0	**1.4**	**1.2**	**1.4**	**1.3**
																	AQAGEGVRPSPMQLELR	2	1	1	4505701	N-Term(TMT6plex); M12(Oxidation)	2084.1	0	**1.2**	**1.3**	**1.3**	**1.4**
																	LVYVCDPVLGDKWDGEGSMYVPE DLLPVYK	4	1	1	4505701	N-Term(TMT6plex); C5(Carbamidomethyl); K12(TMT6plex); M19(Oxidation); K30(TMT6plex)	4160.2	1	**1.4**	**1.7**	**1.3**	**1.9**
																	IHSQEEALR	2	1	1	4505701	N-Term(TMT6plex)	1311.7	0	**1.2**	**1.2**	**1.2**	**1.2**
																	YDYVLTGYTR	2	1	1	4505701	N-Term(TMT6plex)	1479.8	0	**1.5**	**1.4**	**1.4**	**1.6**
																	VVPLADIITPNQFEAELLSGR	47	1	1	4505701	N-Term(TMT6plex)	2511.4	0	**1.4**	**1.3**	**1.4**	**1.2**
																	VMDMLHSMGPDTVVITSSDLPSPQG SNYLIVLGSQR	8	1	1	4505701	N-Term(TMT6plex); M2(Oxidation); M4(Oxidation); M8(Oxidation)	4122.0	0	**1.2**	**1.2**	**1.2**	**1.4**
																	SFLAMVVDIVQELK	20	1	1	4505701	N-Term(TMT6plex); M5(Oxidation); K14(TMT6plex)	2066.2	0	**1.5**	**1.3**	**1.2**	**1.7**
																	GQVLNSDELQELYEGLR	18	1	1	4505701	N-Term(TMT6plex)	2192.1	0	**1.3**	**1.4**	**1.4**	**1.5**
																	LVYVCDPVLGDK	2	1	1	4505701	N-Term(TMT6plex); C5(Carbamidomethyl); K12(TMT6plex)	1836.0	0	**1.5**	**1.6**	**1.7**	**1.7**
																	NPAGSVVMER	1	1	1	4505701	N-Term(TMT6plex); M8(Oxidation)	1304.7	0	**1.1**	**1.2**	**1.1**	**1.2**
																	DIEDPEIVVQATVL	2	1	1	4505701	N-Term(TMT6plex)	1770.0	0	**1.9**	**2.0**	**1.9**	**2.2**
																	LVYVCDPVLGDKWDGEGSMYVPE DLLPVYK	1	1	1	4505701	N-Term(TMT6plex); C5(Carbamidomethyl); K12(TMT6plex); K30(TMT6plex)	4144.2	1	**2.0**	**2.3**	**2.3**	**1.7**
																	KIHSQEEALR	2	1	1	4505701	N-Term(TMT6plex); K1(TMT6plex)	1669.0	1	**1.1**	**1.5**	**1.4**	**1.4**
																	TVSTLHHVLQR	1	1	1	4505701	N-Term(TMT6plex)	1519.9	0	**1.1**	**1.3**	**1.4**	**1.3**
																	TIQCAK	1	1	1	4505701	N-Term(TMT6plex); C4(Carbamidomethyl); K6(TMT6plex)	1178.7	0	**1.4**	**1.2**	**1.4**	**1.4**
117190192;117189975	NP_001070910.1	**HNRNPC**	3183	**heterogeneous nuclear ribonucleoproteins C1/C2 isoform a**	66.01	3	1	26	856	**1.3**	**1.4**	**1.3**	**1.3**	306	33.6	5.08												
																	MIAGQVLDINLAAEPK	174	2	2	117190174;117190192;117189975	N-Term(TMT6plex); M1(Oxidation); K16(TMT6plex)	2157.2	0				
																	GFAFVQYVNER	54	2	2	117190174;117190192;117189975	N-Term(TMT6plex)	1558.8	0				
																	QKVDSLLENLEK	81	6	3	226052117;117190174;117190192;117189975	N-Term(TMT6plex); K2(TMT6plex); K12(TMT6plex)	2103.3	1				
																	SAAEMYGSVTEHPSPSPLLSSSFDLD YDFQR	27	1	1	117190192;117189975	N-Term(TMT6plex); M5(Oxidation)	3678.7	0	**1.3**	**1.3**	**1.2**	**1.2**
																	NDKSEEEQSSSSVK	34	2	2	117190174;117190192;117189975	N-Term(TMT6plex); K3(TMT6plex); K14(TMT6plex)	2241.2	1				
																	SDVEAIFSK	39	6	3	226052117;117190174;117190192;117189975	N-Term(TMT6plex); K9(TMT6plex)	1453.8	0				
																	KSDVEAIFSK	38	6	3	226052117;117190174;117190192;117189975	N-Term(TMT6plex); K1(TMT6plex); K10(TMT6plex)	1811.1	1				
																	VFIGNLNTLVVK	93	6	3	226052117;117190174;117190192;117189975	N-Term(TMT6plex); K12(TMT6plex)	1775.1	0				
																	SEEEQSSSSVK	35	2	2	117190174;117190192;117189975	N-Term(TMT6plex); K11(TMT6plex)	1654.9	0				
																	SAAEMYGSVTEHPSPSPLLSSSFDLD YDFQR	12	1	1	117190192;117189975	N-Term(TMT6plex)	3662.7	0	**1.2**	**1.6**	**1.6**	**1.2**
																	GDDLQAIK	14	6	3	226052117;117190174;117190192;117189975	N-Term(TMT6plex); K8(TMT6plex)	1317.8	0				
																	VDSLLENLEK	76	6	3	226052117;117190174;117190192;117189975	N-Term(TMT6plex); K10(TMT6plex)	1617.9	0				
																	IVGCSVHK	21	5	2	117190174;117190192;117189975	N-Term(TMT6plex); C4(Carbamidomethyl); K8(TMT6plex)	1357.8	0				
																	LKGDDLQAIK	26	6	3	226052117;117190174;117190192;117189975	N-Term(TMT6plex); K2(TMT6plex); K10(TMT6plex)	1788.1	1				
																	AAVAGEDGR	19	6	3	226052117;117190174;117190192;117189975	N-Term(TMT6plex)	1074.6	0				
																	KDETNVK	11	2	2	117190174;117190192;117189975	N-Term(TMT6plex); K1(TMT6plex); K7(TMT6plex)	1520.9	1				
																	GDDQLELIKDDEK	2	2	2	117190174;117190192;117189975	N-Term(TMT6plex); K9(TMT6plex); K13(TMT6plex)	2205.2	1				
																	LKGDDLQAIKK	2	2	2	117190174;117190192;117189975	N-Term(TMT6plex); K2(TMT6plex); K10(TMT6plex); K11(TMT6plex)	2145.4	2				
																	MIAGQVLDINLAAEPK	70	2	2	117190174;117190192;117189975	N-Term(TMT6plex); K16(TMT6plex)	2141.2	0				
																	VSGNTSR	4	2	2	117190174;117190192;117189975	N-Term(TMT6plex)	949.5	0				
																	VPPPPPIAR	9	2	2	117190174;117190192;117189975	N-Term(TMT6plex)	1172.7	0				
																	MYSYPAR	2	2	2	117190174;117190192;117189975	N-Term(TMT6plex)	1116.6	0				

Khan et al., 2019. Multi-omics analysis to characterize cigarette smoke induced molecular alterations in esophageal cells
Supplementary Table 6. List of all proteins and peptides identified in untreated and chronically treated Het1A cells with cigarette smoke condensate for 8 months

Accession	RefSeq Accession	Gene Symbol	Entrez Gene ID	Description	Σ Coverage	Σ# Proteins	Σ# Unique Peptides	Σ# Peptides	Σ# PSMs	Het-1A-Smoke - 2M/Parental	Het-1A-Smoke - 4M/Parental	Het-1A-Smoke - 6M/Parental	Het-1A-Smoke - 8M/Parental	# AAs	MW [kDa]	calc. pI	Sequence	# PSMs	# Proteins	# Protein Groups	Protein Group Accessions	Modifications	MH+ [Da]	# Missed Cleavages	Het-1A-Smoke - 2M/Parental	Het-1A-Smoke - 4M/Parental	Het-1A-Smoke - 6M/Parental	Het-1A-Smoke - 8M/Parental
																	KELTQIK	2	6	2	117190174;117190192;117189975	N-Term(TMT6plex); K1(TMT6plex); K7(TMT6plex)	1547.0	1				
																	AVVPSK	2	2	2	117190174;117190192;117189975	N-Term(TMT6plex); K6(TMT6plex)	1058.7	0				
																	DETNVK	2	2	2	117190174;117190192;117189975	N-Term(TMT6plex); K6(TMT6plex)	1163.7	0				
																	SGFNSK	5	6	3	226052117;117190174;117190192;117189975	N-Term(TMT6plex); K6(TMT6plex)	1097.6	0				
																	AVVPSKR	1	2	2	117190174;117190192;117189975	N-Term(TMT6plex); K6(TMT6plex)	1214.8	1				
																	QAVEMK	1	2	2	117190174;117190192;117189975	N-Term(TMT6plex); K6(TMT6plex)	1163.7	0				
10835057	NP_003482.1	**NAA10**	8260	**N-alpha-acetyltransferase 10 isoform 1**	65.96	4	12	12	69	**1.3**	**1.4**	**1.4**	**1.4**	235	26.4	5.64												
																	HVVLGAIENK	10	3	1	10835057	N-Term(TMT6plex); K10(TMT6plex)	1537.9	0	**1.2**	**1.3**	**1.4**	**1.4**
																	MEEDPDDVPHGHITSLAVK	7	2	1	10835057	N-Term(TMT6plex); M1(Oxidation); K19(TMT6plex)	2564.3	0	**1.2**	**1.4**	**1.3**	**1.5**
																	AMIENFNAK	4	4	1	10835057	N-Term(TMT6plex); M2(Oxidation); K9(TMT6plex)	1511.8	0	**1.2**	**1.4**	**1.3**	**1.4**
																	AALHLYSNTLNFQISEVEPK	3	2	1	10835057	N-Term(TMT6plex); K20(TMT6plex)	2732.5	0	**1.4**	**1.7**	**1.6**	**1.6**
																	YYADGEDAYAMK	2	4	1	10835057	N-Term(TMT6plex); K12(TMT6plex)	1854.9	0	**1.8**	**2.0**	**2.1**	**1.5**
																	DLTQMADELR	4	3	1	10835057	N-Term(TMT6plex); M5(Oxidation)	1436.7	0	**1.3**	**1.3**	**1.2**	**1.5**
																	DLSEVSETTESTDVK	20	3	1	10835057	N-Term(TMT6plex); K15(TMT6plex)	2098.1	0	**1.1**	**1.2**	**1.1**	**1.1**
																	YYADGEDAYAMK	9	4	1	10835057	N-Term(TMT6plex); M11(Oxidation); K12(TMT6plex)	1870.9	0	**1.5**	**1.3**	**1.3**	**1.4**
																	AMIENFNAK	2	4	1	10835057	N-Term(TMT6plex); K9(TMT6plex)	1495.8	0	**1.4**	**1.7**	**1.8**	**1.3**
																	NARPEDLMNMQHCNLLCLPENYQMK	1	3	1	10835057	N-Term(TMT6plex); M8(Oxidation); M10(Oxidation); C13(Carbamidomethyl); C17(Carbamidomethyl); M24(Oxidation); K25(TMT6plex)	3625.7	0	**1.3**	**1.5**	**1.4**	**1.5**
																	GLAAEDSGGDSK	2	3	1	10835057	N-Term(TMT6plex); K12(TMT6plex)	1564.8	0	**1.2**	**1.3**	**1.4**	**1.3**
																	IVGYVLAK	2	3	1	10835057	N-Term(TMT6plex); K8(TMT6plex)	1320.9	0	**1.2**	**1.2**	**1.3**	**1.3**
																	GNSPPSSGEACR	2	3	1	10835057	N-Term(TMT6plex); C11(Carbamidomethyl)	1447.7	0	**1.4**	**1.7**	**1.3**	**1.6**
																	GNSPPSSGEACREEK	1	3	1	10835057	N-Term(TMT6plex); C11(Carbamidomethyl); K15(TMT6plex)	2063.0	1	**1.0**	**1.2**	**1.0**	**1.3**
63252900	NP_001018006.1	**TPM1**	7168	**tropomyosin alpha-1 chain isoform 4**	65.85	5	5	25	273	**1.2**	**1.1**	**1.0**	**1.0**	284	32.9	4.78												
																	IQLVEEELDR	65	22	9	63252898;63252900;499137518;42476296;4507651;114155144;63252904;114155140;47519616	N-Term(TMT6plex)	1472.8	0				
																	MEIQEIQLK	27	18	6	63252898;63252900;63252904;499137518;114155144;114155140	N-Term(TMT6plex); M1(Oxidation); K9(TMT6plex)	1605.9	0				
																	KLVIIESDLER	6	7	3	63252898;63252900;63252904	N-Term(TMT6plex); K1(TMT6plex)	1773.1	1				
																	QLEEQLR	5	3	1	63252900	N-Term(TMT6plex)	1144.7	0	**1.1**	**0.9**	**0.8**	**0.8**
																	EENLSMHQMLDQTLLELNNM	6	5	1	63252900	N-Term(TMT6plex); M6(Oxidation); M9(Oxidation); M20(Oxidation)	2680.2	0	**1.3**	**1.2**	**1.2**	**1.4**
																	QLEDELVSLQK	11	4	2	63252898;63252900	N-Term(TMT6plex); K11(TMT6plex)	1760.0	0				
																	LVIIESDLER	6	7	3	63252898;63252900;63252904	N-Term(TMT6plex)	1415.8	0				
																	SIDDLEEK	9	4	1	63252900	N-Term(TMT6plex); K8(TMT6plex)	1406.8	0	**1.0**	**0.8**	**0.8**	**0.6**
																	EDRYEEEIK	4	7	3	63252898;63252900;63252904	N-Term(TMT6plex); K9(TMT6plex)	1668.9	1				
																	LATALQK	47	22	9	63252898;63252900;499137518;42476296;4507651;114155144;63252904;114155140;47519616	N-Term(TMT6plex); K7(TMT6plex)	1202.8	0				
																	AEQAEADKK	9	9	4	63252898;63252900;42476296;47519616	N-Term(TMT6plex); K8(TMT6plex); K9(TMT6plex)	1677.0	1				
																	AADESER	4	18	9	63252898;63252900;499137518;42476296;4507651;114155144;63252904;114155140;47519616	N-Term(TMT6plex)	1006.5	0				
																	AEQAEADK	6	12	4	63252898;63252900;42476296;47519616	N-Term(TMT6plex); K8(TMT6plex)	1319.7	0				
																	KATDAEADVASLNR	13	6	4	63252898;63252900;42476296;47519616	N-Term(TMT6plex); K1(TMT6plex)	1919.1	1				
																	ATDAEADVASLNR	7	6	4	63252898;63252900;42476296;47519616	N-Term(TMT6plex)	1561.8	0				
																	MEIQEIQLK	5	18	6	499137518;114155144;114155140;63252898;63252900;63252904	N-Term(TMT6plex); K9(TMT6plex)	1589.9	0				
																	EENLSMHQMLDQTLLELNNM	1	5	1	63252900	N-Term(TMT6plex); M20(Oxidation)	2648.3	0	**1.2**	**1.3**	**1.3**	**1.4**
																	GTEDELDKYSEALK	1	5	3	63252898;63252900;114155140	N-Term(TMT6plex); K8(TMT6plex); K14(TMT6plex)	2285.3	1				
																	AELSEGQVR	5	3	1	63252900	N-Term(TMT6plex)	1217.7	0	**1.4**	**1.7**	**1.5**	**1.5**
																	KYEEVAR	9	20	9	63252898;63252900;499137518;42476296;4507651;114155144;63252904;114155140;47519616	N-Term(TMT6plex); K1(TMT6plex)	1352.8	1				
																	LDKENALDR	7	10	5	63252898;63252900;114155140;42476296;47519616	N-Term(TMT6plex); K3(TMT6plex)	1531.9	1				
																	IMDQTLK	2	3	1	63252900	N-Term(TMT6plex); M2(Oxidation); K7(TMT6plex)	1322.8	0	**1.0**	**0.8**	**0.9**	**0.8**
																	AEFAER	7	24	9	63252898;63252900;499137518;42476296;4507651;114155144;63252904;114155140;47519616	N-Term(TMT6plex)	951.5	0				
																	LEEAEK	4	20	10	63252898;63252900;499137518;42476296;4507651;114155144;63252904;114155140;47519616;116235456;116235451	N-Term(TMT6plex); K6(TMT6plex)	1176.7	0				
																	YEEVAR	4	24	9	63252898;63252900;499137518;42476296;4507651;114155144;63252904;114155140;47519616	N-Term(TMT6plex)	995.5	0				
																	AQKDEEK	1	7	3	63252898;63252900;63252904	N-Term(TMT6plex); K3(TMT6plex); K7(TMT6plex)	1534.9	1				
																	YEEEIK	2	19	9	63252898;63252900;499137518;42476296;4507651;114155144;63252904;114155140;47519616	N-Term(TMT6plex); K6(TMT6plex)	1268.7	0				
21361144	NP_002795.2	**PSMC3**	5702	**26S protease regulatory subunit 6A**	65.83	1	20	20	114	**1.3**	**1.4**	**1.3**	**1.9**	439	49.2	5.24												
																	MATVWDEAEQDGIGEEVLK	4	1	1	21361144	N-Term(TMT6plex); M1(Oxidation); K19(TMT6plex)	2594.3	0	**1.2**	**1.2**	**1.0**	**1.7**
																	EKFENLGIQPPK	6	1	1	21361144	N-Term(TMT6plex); K2(TMT6plex); K12(TMT6plex)	2087.2	1	**1.2**	**1.3**	**1.3**	**1.8**
																	APSIIFIDELDAIGTK	12	1	1	21361144	N-Term(TMT6plex); K16(TMT6plex)	2161.2	0	**1.1**	**1.2**	**1.4**	**1.9**
																	TMLELLNQLDGFQPNTQVK	4	1	1	21361144	N-Term(TMT6plex); M2(Oxidation); K19(TMT6plex)	2663.5	0	**1.3**	**1.2**	**1.2**	**1.8**
																	MNVSPDVNYEELAR	7	1	1	21361144	N-Term(TMT6plex); M1(Oxidation)	1881.9	0	**1.3**	**1.1**	**1.1**	**1.7**
																	TMLELLNQLDGFQPNTQVK	6	1	1	21361144	N-Term(TMT6plex); K19(TMT6plex)	2647.5	0	**1.4**	**1.5**	**1.4**	**1.9**
																	LKPGDLVGVNK	4	1	1	21361144	N-Term(TMT6plex); K2(TMT6plex); K11(TMT6plex)	1827.2	0	**1.2**	**1.3**	**1.3**	**1.7**
																	KIEFPMPNEEAR	3	1	1	21361144	N-Term(TMT6plex); K1(TMT6plex); M6(Oxidation)	1935.0	1	**1.0**	**1.2**	**1.3**	**1.4**
																	LAGPQLVQMFIGDGAK	3	1	1	21361144	N-Term(TMT6plex); K16(TMT6plex)	2103.2	0	**1.6**	**1.7**	**1.8**	**1.7**
																	QTYFLPVIGLVDAEK	14	1	1	21361144	N-Term(TMT6plex); K15(TMT6plex)	2151.2	0	**1.3**	**1.3**	**1.3**	**2.1**
																	AMEVDERPTEQYSDIGGLDK	4	1	1	21361144	N-Term(TMT6plex); M2(Oxidation); K20(TMT6plex)	2727.4	0	**1.2**	**1.3**	**1.2**	**2.4**
																	EKAPSIIFIDELDAIGTK	10	1	1	21361144	N-Term(TMT6plex); K2(TMT6plex); K18(TMT6plex)	2647.6	1	**1.1**	**1.5**	**1.3**	**2.1**
																	LAGPQLVQMFIGDGAK	4	1	1	21361144	N-Term(TMT6plex); M9(Oxidation); K16(TMT6plex)	2119.2	0	**1.3**	**1.6**	**1.4**	**2.2**
																	GVLMYGPPGTGK	2	1	1	21361144	N-Term(TMT6plex); K12(TMT6plex)	1634.9	0	**1.5**	**1.4**	**1.3**	**1.6**
																	VDILDPALLR	6	1	1	21361144	N-Term(TMT6plex)	1353.8	0	**1.3**	**1.2**	**1.2**	**2.0**
																	GVLMYGPPGTGK	5	1	1	21361144	N-Term(TMT6plex); M4(Oxidation); K12(TMT6plex)	1650.9	0	**1.2**	**1.3**	**1.4**	**1.8**
																	DSYLILETLPTEYDSR	4	1	1	21361144	N-Term(TMT6plex)	2144.1	0	**1.5**	**1.4**	**1.5**	**1.7**
																	AVCVEAGMIALR	2	1	1	21361144	N-Term(TMT6plex); C3(Carbamidomethyl); M8(Oxidation)	1534.8	0	**1.3**	**1.4**	**1.4**	**1.9**
																	TLPYLVSNVIELLDVDPNDQEEDGANIDLDSQR	2	1	1	21361144	N-Term(TMT6plex)	3928.9	0	**2.5**	**2.1**	**2.2**	**2.0**
																	QIQELVEAIVLPMNHK	2	1	1	21361144	N-Term(TMT6plex); K16(TMT6plex)	2320.3	0	**1.3**	**1.7**	**1.7**	**1.6**
																	QIQELVEAIVLPMNHK	2	1	1	21361144	N-Term(TMT6plex); M13(Oxidation); K16(TMT6plex)	2336.3	0	**1.4**	**1.7**	**1.1**	**2.1**
																	GATELTHEDYMEGILEVQAK	2	1	1	21361144	N-Term(TMT6plex); M11(Oxidation); K20(TMT6plex)	2708.4	0	**1.6**	**1.5**	**1.5**	**1.6**
																	LLDSEIK	4	1	1	21361144	N-Term(TMT6plex); K7(TMT6plex)	1275.8	0	**1.3**	**1.4**	**1.4**	**2.1**
																	VIAATNR	2	1	1	21361144	N-Term(TMT6plex)	973.6	0	**1.4**	**1.4**	**1.3**	**2.0**
514052659	NP_001265481.1	**TUBA4A**	7277	**tubulin alpha-4A chain isoform 2**	65.82	3	5	21	1606	**1.3**	**1.3**	**1.5**	**1.5**	433	48.3	5.01												
																	AVFVDLEPTVIDEIR	21	2	1	514052659	N-Term(TMT6plex)	1945.1	0	**1.7**	**1.7**	**1.9**	**1.8**
																	LISQIVSSITASLR	110	6	3	514052659;14389309;301171345	N-Term(TMT6plex)	1717.0	0				
																	AYHEQLSVAEITNACFEPANQMVK	209	7	2	514052659;17986283	N-Term(TMT6plex); C15(Carbamidomethyl); M22(Oxidation); K24(TMT6plex)	3224.6	0				
																	FDGALNVDLTEFQTNLVPYPR	132	12	4	514052659;14389309;17986283;301171345	N-Term(TMT6plex)	2638.4	0				
																	AFVHWYVGEGMEEGEFSEAR	157	11	4	514052659;14389309;17986283;301171345	N-Term(TMT6plex); M11(Oxidation)	2575.2	0				
																	IHFPLATYAPVISAEK	266	10	3	514052659;14389309;17986283	N-Term(TMT6plex); K16(TMT6plex)	2215.3	0				
																	VGINYQPPTVVPGGDLAK	212	12	4	514052659;14389309;17986283;301171345	N-Term(TMT6plex); K18(TMT6plex)	2283.3	0				
																	QLFHPEQLITGK	67	12	4	514052659;14389309;17986283;301171345	N-Term(TMT6plex); K12(TMT6plex)	1869.1	0				
																	GHYTIGK	34	10	3	514052659;14389309;17986283	N-Term(TMT6plex); K7(TMT6plex)	1233.7	0				
																	DVNAAIAAIK	17	2	1	514052659	N-Term(TMT6plex); K10(TMT6plex)	1443.9	0	**1.5**	**1.6**	**1.9**	**1.7**
																	EDAANNYAR	39	13	5	514052659;14389309;285395246;17986283;301171345	N-Term(TMT6plex)	1252.6	0				
																	AVCMLSNTTAIAEAWAR	16	10	3	514052659;17986283;301171345	N-Term(TMT6plex); C3(Carbamidomethyl)	2094.1	0				
																	EIIDPVLDR	3	2	1	514052659	N-Term(TMT6plex)	1298.8	0	**1.4**	**1.4**	**1.6**	**1.6**
																	FDLMYAK	16	15	5	514052659;14389309;285395246;17986283;301171345	N-Term(TMT6plex); K7(TMT6plex)	1345.8	0				
																	YMACCLLYR	2	8	4	514052659;14389309;285395246;17986283	N-Term(TMT6plex); C4(Carbamidomethyl); C5(Carbamidomethyl)	1478.7	0				
																	NLDIERPTYTNLNR	33	12	4	514052659;14389309;17986283;301171345	N-Term(TMT6plex)	1948.0	0				
																	YMACCLLYR	10	8	4	514052659;14389309;285395246;17986283	N-Term(TMT6plex); M2(Oxidation); C4(Carbamidomethyl); C5(Carbamidomethyl)	1494.7	0				
																	AVCMLSNTTAIAEAWAR	37	10	3	514052659;17986283;301171345	N-Term(TMT6plex); C3(Carbamidomethyl); M4(Oxidation)	2110.1	0				
																	SIQFVDWCPTGFK	45	3	1	514052659	N-Term(TMT6plex); C8(Carbamidomethyl); K13(TMT6plex)	2043.1	0	**1.1**	**1.0**	**1.2**	**1.2**
																	VGINYQPPTVVPGGDLAKVQR	3	10	4	514052659;14389309;17986283;301171345	N-Term(TMT6plex); K18(TMT6plex)	2666.5	1				
																	AFVHWYVGEGMEEGEFSEAR	55	11	4	514052659;14389309;17986283;301171345	N-Term(TMT6plex)	2559.2	0				
																	AYHEQLSVAEITNACFEPANQMVK	18	7	2	514052659;17986283	N-Term(TMT6plex); C15(Carbamidomethyl); K24(TMT6plex)	3208.6	0				
																	LSDQCTGLQGFLVFHSFGGGTGSGFTSLLMER	3	2	1	514052659	N-Term(TMT6plex); C5(Carbamidomethyl); M30(Oxidation)	3651.8	0	**1.4**	**1.3**	**1.6**	**1.2**
																	FDLMYAK	44	15	5	514052659;14389309;285395246;17986283;301171345	N-Term(TMT6plex); M4(Oxidation); K7(TMT6plex)	1361.8	0				
																	LSVDYGK	44	9	3	514052659;14389309;17986283	N-Term(TMT6plex); K7(TMT6plex)	1239.7	0				
																	EDMAALEK	13	6	3	514052659;14389309;17986283	N-Term(TMT6plex); M3(Oxidation); K8(TMT6plex)	1380.7	0				

Accession	RefSeq Accession	Gene Symbol	Entrez Gene ID	Description	Σ Coverage	Σ# Proteins	Σ# Unique Peptides	Σ# Peptides	Σ# PSMs	Het-1A-Smoke - 2M/Parental	Het-1A-Smoke - 4M/Parental	Het-1A-Smoke - 6M/Parental	Het-1A-Smoke - 8M/Parental	# AAs	MW [kDa]	calc. pI	Sequence	# PSMs	# Proteins	# Protein Groups	Protein Group Accessions	Modifications	MH+ [Da]	# Missed Cleavages	Het-1A-Smoke - 2M/Parental	Het-1A-Smoke - 4M/Parental	Het-1A-Smoke - 6M/Parental	Het-1A-Smoke - 8M/Parental
73486658	NP_002071.2	**GOT2**	2806	**aspartate aminotransferase, mitochondrial isoform 1 precursor**	65.81	2	26	26	267	**1.4**	**1.4**	**1.5**	**1.4**	430	47.5	9.01												
																	HFIEQGINVCLCQSYAK	6	2	1	73486658	N-Term(TMT6plex); C10(Carbamidomethyl); C12(Carbamidomethyl); K17(TMT6plex)	2525.3	0	**1.6**	**1.7**	**1.6**	**1.6**
																	NLDKEYLPIGGLAEFCK	24	1	1	73486658	N-Term(TMT6plex); K4(TMT6plex); C16(Carbamidomethyl); K17(TMT6plex)	2654.5	1	**1.4**	**1.5**	**1.7**	**1.5**
																	QWLQEVK	6	2	1	73486658	N-Term(TMT6plex); K7(TMT6plex)	1388.8	0	**1.4**	**1.3**	**1.5**	**1.4**
																	NMGLYGER	3	2	1	73486658	N-Term(TMT6plex); M2(Oxidation)	1184.6	0	**1.6**	**1.1**	**1.3**	**1.1**
																	IGASFLQR	7	2	1	73486658	N-Term(TMT6plex)	1120.7	0	**1.5**	**1.4**	**1.5**	**1.4**
																	EFSIYMTK	14	2	1	73486658	N-Term(TMT6plex); M6(Oxidation); K8(TMT6plex)	1492.8	0	**1.4**	**1.5**	**1.5**	**1.5**
																	EGSTHNWQHITDQIGMFCFTGLKPEQVER	1	2	1	73486658	N-Term(TMT6plex); M16(Oxidation); C18(Carbamidomethyl); K23(TMT6plex)	3919.9	0				
																	TQLVSNLK	22	2	1	73486658	N-Term(TMT6plex); K8(TMT6plex)	1360.9	0	**1.4**	**1.3**	**1.5**	**1.3**
																	TCGFDFTGAVEDISK	18	2	1	73486658	N-Term(TMT6plex); C2(Carbamidomethyl); K15(TMT6plex)	2105.1	0	**1.8**	**1.5**	**1.7**	**1.1**
																	FVTVQTISGTGALR	18	2	1	73486658	N-Term(TMT6plex)	1679.0	0	**1.4**	**1.3**	**1.4**	**1.3**
																	IAAAILNTPDLR	14	2	1	73486658	N-Term(TMT6plex)	1496.9	0	**1.4**	**1.3**	**1.2**	**1.2**
																	NLFAFFDMAYQGFASGDGDKDAWAVR	7	2	1	73486658	N-Term(TMT6plex); M8(Oxidation); K20(TMT6plex)	3373.6	1	**1.5**	**1.3**	**1.3**	**1.4**
																	MNLGVGAYR	2	2	1	73486658	N-Term(TMT6plex)	1209.7	0	**1.5**	**1.4**	**1.7**	**1.3**
																	VGAFTMVCK	4	2	1	73486658	N-Term(TMT6plex); C8(Carbamidomethyl); K9(TMT6plex)	1470.8	0	**1.8**	**2.0**	**2.2**	**1.5**
																	KAEAQIAAK	24	1	1	73486658	N-Term(TMT6plex); K1(TMT6plex); K9(TMT6plex)	1617.0	1	**1.3**	**1.5**	**1.5**	**1.4**
																	KQWLQEVK	7	2	1	73486658	N-Term(TMT6plex); K1(TMT6plex); K8(TMT6plex)	1746.1	1	**1.3**	**1.6**	**1.6**	**1.6**
																	VGAFTMVCK	5	2	1	73486658	N-Term(TMT6plex); M6(Oxidation); C8(Carbamidomethyl); K9(TMT6plex)	1486.8	0	**1.5**	**1.4**	**1.5**	**1.4**
																	ASAELALGENSEVLK	24	1	1	73486658	N-Term(TMT6plex); K15(TMT6plex)	1989.1	0	**1.3**	**1.5**	**1.5**	**1.5**
																	NLFAFFDMAYQGFASGDGDK	6	2	1	73486658	N-Term(TMT6plex); M8(Oxidation); K20(TMT6plex)	2675.3	0	**1.5**	**1.1**	**1.1**	**1.2**
																	EYLPIGGLAEFCK	14	1	1	73486658	N-Term(TMT6plex); C12(Carbamidomethyl); K13(TMT6plex)	1955.1	0	**1.4**	**1.3**	**1.7**	**1.4**
																	EGSTHNWQHITDQIGMFCFTGLKPEQVER	1	2	1	73486658	N-Term(TMT6plex); C18(Carbamidomethyl); K23(TMT6plex)	3903.9	0	**1.5**	**1.7**	**1.8**	**1.6**
																	IPEQSVLLLHACAHNPTGVDPRPEQWK	2	2	1	73486658	N-Term(TMT6plex); C12(Carbamidomethyl); K27(TMT6plex)	3550.9	0	**1.2**	**1.5**	**1.4**	**1.4**
																	MNLGVGAYR	5	2	1	73486658	N-Term(TMT6plex); M1(Oxidation)	1225.7	0	**1.4**	**1.1**	**1.3**	**1.2**
																	DDNGKPYVLPSVR	1	2	1	73486658	N-Term(TMT6plex); K5(TMT6plex)	1918.1	0	**1.3**	**1.5**	**1.5**	**1.5**
																	RVESQLK	2	2	1	73486658	N-Term(TMT6plex); K7(TMT6plex)	1317.8	1	**1.3**	**1.6**	**1.6**	**1.6**
																	DAGMQLQGYR	11	2	1	73486658	N-Term(TMT6plex); M4(Oxidation)	1383.7	0	**1.4**	**1.5**	**1.5**	**1.5**
																	EIATVVK	13	2	1	73486658	N-Term(TMT6plex); K7(TMT6plex)	1217.8	0	**1.4**	**1.5**	**1.4**	**1.4**
																	NMGLYGER	4	2	1	73486658	N-Term(TMT6plex)	1168.6	0	**1.2**	**1.2**	**1.3**	**1.1**
																	DADEAK	1	2	1	73486658	N-Term(TMT6plex); K6(TMT6plex)	1106.6	0	**1.4**	**1.3**	**1.3**	**1.4**
																	KMNLGVGAYR	1	2	1	73486658	N-Term(TMT6plex); K1(TMT6plex); M2(Oxidation)	1582.9	1	**1.4**	**1.6**	**1.6**	**1.8**
4826659	NP_004921.1	**CAPZB**	832	**F-actin-capping protein subunit beta isoform 1**	65.81	5	16	16	175	**1.3**	**1.3**	**1.3**	**1.3**	272	30.6	6.00												
																	STLNEIYFGK	19	3	1	4826659	N-Term(TMT6plex); K10(TMT6plex)	1629.9	0	**1.3**	**1.3**	**1.4**	**1.3**
																	NDLVEALK	9	2	1	4826659	N-Term(TMT6plex); K8(TMT6plex)	1359.8	0	**1.3**	**1.1**	**1.3**	**1.1**
																	SGSGTMNLGGSLTR	10	4	1	4826659	N-Term(TMT6plex); M6(Oxidation)	1582.8	0	**1.4**	**0.9**	**1.0**	**1.0**
																	QMEKDETVSDCSPHIANIGR	3	4	1	4826659	N-Term(TMT6plex); M2(Oxidation); K4(TMT6plex); C11(Carbamidomethyl)	2761.4	1	**1.2**	**1.2**	**1.2**	**1.4**
																	NLSDLIDLVPSLCEDLLSSVDQPLK	53	4	1	4826659	N-Term(TMT6plex); C13(Carbamidomethyl); K25(TMT6plex)	3241.8	0	**1.5**	**1.5**	**1.5**	**1.5**
																	KLEVEANNAFDQYR	14	4	1	4826659	N-Term(TMT6plex); K1(TMT6plex)	2155.2	1	**1.2**	**1.3**	**1.3**	**1.3**
																	RLPPQQIEK	3	4	1	4826659	N-Term(TMT6plex); K9(TMT6plex)	1567.0	1	**1.2**	**1.3**	**1.2**	**1.3**
																	SVQTFADK	6	2	1	4826659	N-Term(TMT6plex); K8(TMT6plex)	1353.8	0	**1.2**	**1.2**	**1.4**	**1.3**
																	SPWSNKYDPPLEDGAMPSAR	2	4	1	4826659	N-Term(TMT6plex); K6(TMT6plex); M16(Oxidation)	2692.3	1	**1.2**	**2.0**	**1.2**	**2.0**
																	LTSTVMLWLQTNK	5	4	1	4826659	N-Term(TMT6plex); K13(TMT6plex)	1993.2	0	**0.9**	**1.0**	**1.2**	**1.2**
																	YDPPLEDGAMPSAR	24	5	1	4826659	N-Term(TMT6plex); M10(Oxidation)	1763.9	0	**1.6**	**1.3**	**1.3**	**1.3**
																	LVEDMENK	9	3	1	4826659	N-Term(TMT6plex); M5(Oxidation); K8(TMT6plex)	1451.8	0	**1.4**	**1.5**	**1.4**	**1.4**
																	GCWDSIHVVEVQEK	3	4	1	4826659	N-Term(TMT6plex); C2(Carbamidomethyl); K14(TMT6plex)	2144.1	0	**1.2**	**1.3**	**1.3**	**1.2**
																	DYLLCDYNR	5	4	1	4826659	N-Term(TMT6plex); C5(Carbamidomethyl)	1460.7	0	**1.3**	**1.4**	**1.5**	**1.3**
																	LVEDMENK	2	3	1	4826659	N-Term(TMT6plex); K8(TMT6plex)	1435.8	0	**1.8**	**2.0**	**2.2**	**1.6**
																	YDPPLEDGAMPSAR	2	5	1	4826659	N-Term(TMT6plex)	1747.9	0	**2.1**	**1.8**	**2.1**	**1.4**
																	KAGDGSK	1	4	1	4826659	N-Term(TMT6plex); K1(TMT6plex); K7(TMT6plex)	1349.8	1	**0.9**	**1.0**	**1.1**	**1.2**
																	LPPQQIEK	5	4	1	4826659	N-Term(TMT6plex); K8(TMT6plex)	1410.9	0	**1.2**	**1.2**	**1.4**	**1.3**
112382377	NP_055316.2	**UBE2S**	27338	**ubiquitin-conjugating enzyme E2 S**	65.77	1	10	10	67	**1.3**	**1.5**	**1.7**	**1.5**	222	23.8	8.38												
																	HVLLTIK	4	1	1	112382377	N-Term(TMT6plex); K7(TMT6plex)	1281.9	0	**1.1**	**1.3**	**1.8**	**1.3**
																	DFPASPPK	9	1	1	112382377	N-Term(TMT6plex); K8(TMT6plex)	1316.8	0	**1.3**	**1.6**	**1.7**	**1.5**
																	DWTAELGIR	3	1	1	112382377	N-Term(TMT6plex)	1289.7	0	**1.6**	**1.6**	**1.6**	**1.5**
																	CLLIHPNPESALNEEAGR	5	1	1	112382377	N-Term(TMT6plex); C1(Carbamidomethyl)	2249.2	0	**1.5**	**1.7**	**1.5**	**1.6**
																	LLLENYEEYAAR	8	1	1	112382377	N-Term(TMT6plex)	1712.9	0	**1.4**	**1.6**	**1.8**	**1.6**
																	VFPNEEDLTDLQVTIEGPEGTPYAGGLFR	6	1	1	112382377	N-Term(TMT6plex)	3393.7	0	**1.5**	**1.5**	**1.6**	**1.5**
																	ALASGTEASSTDPGAPGGPGGAEGPMAK	8	1	1	112382377	N-Term(TMT6plex); M26(Oxidation); K28(TMT6plex)	2915.4	0	**1.4**	**1.8**	**1.7**	**1.7**
																	EVTTLTADPPDGIK	18	1	1	112382377	N-Term(TMT6plex); K14(TMT6plex)	1915.1	0	**1.3**	**1.5**	**1.6**	**1.5**
																	LLTEIHGGAGGPSGR	2	1	1	112382377	N-Term(TMT6plex)	1650.9	0	**1.4**	**1.6**	**1.8**	**1.4**
																	ALASGTEASSTDPGAPGGPGGAEGPMAK	2	1	1	112382377	N-Term(TMT6plex); K28(TMT6plex)	2899.5	0	**1.8**	**1.9**	**2.2**	**1.5**
																	GYFLTK	2	1	1	112382377	N-Term(TMT6plex); K6(TMT6plex)	1186.7	0	**1.5**	**1.5**	**1.6**	**1.5**
4506663	NP_000964.1	**RPL8**	6132	**60S ribosomal protein L8**	65.76	1	16	17	190	**1.2**	**1.3**	**1.3**	**1.3**	257	28.0	11.03												
																	ASGNYATVISHNPETK	42	1	1	4506663	N-Term(TMT6plex); K16(TMT6plex)	2147.1	0	**1.3**	**1.4**	**1.3**	**1.4**
																	TELFIAAEGIHTGQFVYCGK	6	1	1	4506663	N-Term(TMT6plex); C18(Carbamidomethyl); K20(TMT6plex)	2699.4	0	**1.0**	**0.9**	**1.0**	**0.9**
																	VGLIAAR	16	1	1	4506663	N-Term(TMT6plex)	928.6	0	**1.2**	**1.2**	**1.2**	**1.2**
																	AVVGVVAGGGR	31	1	1	4506663	N-Term(TMT6plex)	1170.7	0	**1.2**	**1.3**	**1.3**	**1.3**
																	KAQLNIGNVLPVGTMPEGTIVCCLEEKPGDR	5	1	1	4506663	K1(TMT6plex); N-Term(TMT6plex); M15(Oxidation); C22(Carbamidomethyl); C23(Carbamidomethyl); K27(TMT6plex)	4099.2	1	**1.1**	**1.3**	**1.2**	**1.3**
																	AQLNIGNVLPVGTMPEGTIVCCLEEKPGDR	27	1	1	4506663	N-Term(TMT6plex); M14(Oxidation); C21(Carbamidomethyl); C22(Carbamidomethyl); K26(TMT6plex)	3741.9	0	**1.4**	**1.5**	**1.8**	**1.5**
																	VISSANR	7	1	1	4506663	N-Term(TMT6plex)	975.6	0	**1.3**	**1.3**	**1.3**	**1.3**
																	AQLNIGNVLPVGTMPEGTIVCCLEEKPGDR	2	1	1	4506663	N-Term(TMT6plex); C21(Carbamidomethyl); C22(Carbamidomethyl); K26(TMT6plex)	3725.9	0	**1.5**	**1.8**	**2.1**	**1.2**
																	KVGLIAAR	14	1	1	4506663	N-Term(TMT6plex); K1(TMT6plex)	1285.9	1	**1.0**	**1.4**	**1.3**	**1.4**
																	IDKPILK	11	1	1	4506663	N-Term(TMT6plex); K3(TMT6plex); K7(TMT6plex)	1514.0	0	**1.1**	**1.3**	**1.3**	**1.3**
																	KGAGSVFR	4	1	1	4506663	N-Term(TMT6plex); K1(TMT6plex)	1279.8	1	**1.1**	**1.2**	**1.3**	**1.2**
																	AVDFAER	14	1	1	4506663	N-Term(TMT6plex)	1036.6	0	**1.4**	**1.5**	**1.5**	**1.4**
																	GVAMNPVEHPFGGGNHQHIGKPSTIR	1	1	1	4506663	N-Term(TMT6plex); M4(Oxidation); K21(TMT6plex)	3211.7	0	**1.4**	**1.7**	**1.7**	**1.5**
																	VKLPSGSK	1	1	1	4506663	N-Term(TMT6plex); K2(TMT6plex); K8(TMT6plex)	1503.0	1	**1.0**	**1.6**	**1.3**	**1.5**
																	GAPLAK	5	3	2	4506663;187960109	N-Term(TMT6plex); K6(TMT6plex)	1014.7	0				
																	DIIHDPGR	2	1	1	4506663	N-Term(TMT6plex)	1151.6	0	**1.2**	**1.2**	**1.3**	**1.4**
																	GAGSVFR	1	1	1	4506663	N-Term(TMT6plex)	922.5	0	**1.0**	**1.1**	**1.3**	**1.1**
																	DAPAGR	1	1	1	4506663	N-Term(TMT6plex)	815.5	0	**1.1**	**1.3**	**1.3**	**1.3**
194578885	NP_001124146.1	**CALU**	813	**calumenin isoform b precursor**	65.71	2	3	22	275	**1.8**	**1.8**	**2.0**	**1.3**	315	37.1	4.59												
																	DIVVQETMEDIDK	32	5	2	4502551;194578885	N-Term(TMT6plex); M8(Oxidation); K13(TMT6plex)	2009.1	0				
																	HLVYESDQNK	9	4	2	4502551;194578885	N-Term(TMT6plex); K10(TMT6plex)	1690.9	0				
																	MADKDGDLIATK	8	5	2	4502551;194578885	N-Term(TMT6plex); M1(Oxidation); K4(TMT6plex); K12(TMT6plex)	1981.1	1				
																	IDADKDGFVTEGELK	5	2	1	194578885	N-Term(TMT6plex); K5(TMT6plex); K15(TMT6plex)	2324.3	1	**1.8**	**1.8**	**2.0**	**1.5**
																	EEFTAFLHPEEYDYMK	32	5	2	4502551;194578885	N-Term(TMT6plex); M15(Oxidation); K16(TMT6plex)	2523.2	0				
																	DWILPSDYDHAEAEAR	18	4	2	4502551;194578885	N-Term(TMT6plex)	2117.0	0				
																	EEIVDKYDLFVGSQATDFGEALVR	31	4	2	4502551;194578885	N-Term(TMT6plex); K6(TMT6plex)	3159.7	1				
																	MDKEETKDWILPSDYDHAEAEAR	1	4	2	4502551;194578885	N-Term(TMT6plex); M1(Oxidation); K3(TMT6plex); K7(TMT6plex)	3452.7	2				
																	TFDQLTPEESKER	2	5	2	4502551;194578885	N-Term(TMT6plex); K11(TMT6plex)	2038.1	1				
																	NADGFIDLEEYIGDMYSHDGNTDEPEWVK	32	4	2	4502551;194578885	N-Term(TMT6plex); M15(Oxidation); K29(TMT6plex)	3833.8	0				
																	VHNDAQSFDYDHDAFLGAEEAK	11	5	2	4502551;194578885	N-Term(TMT6plex); K22(TMT6plex)	2937.4	0				
																	DIVVQETMEDIDK	5	5	2	4502551;194578885	N-Term(TMT6plex); K13(TMT6plex)	1993.1	0				
																	DIVVQETMEDIDKNADGFIDLEEYIGDMYSHDGNTDEPEWVK	2	4	2	4502551;194578885	N-Term(TMT6plex); M8(Oxidation); K13(TMT6plex); M28(Oxidation); K42(TMT6plex)	5594.6	1				
																	HLVYESDQNKDGK	10	4	2	4502551;194578885	N-Term(TMT6plex); K10(TMT6plex); K13(TMT6plex)	2220.2	1				
																	TFDQLTPEESK	29	5	2	4502551;194578885	N-Term(TMT6plex); K11(TMT6plex)	1752.9	0				

Accession	RefSeq Accession	Gene Symbol	Entrez Gene ID	Description	Σ Coverage	Σ# Proteins	Σ# Unique Peptides	Σ# Peptides	Σ# PSMs	Het-1A-Smoke - 2M/Parental	Het-1A-Smoke - 4M/Parental	Het-1A-Smoke - 6M/Parental	Het-1A-Smoke - 8M/Parental	# AAs	MW [kDa]	calc. pI	Sequence	# PSMs	# Proteins	# Protein Groups	Protein Group Accessions	Modifications	MH+ [Da]	# Missed Cleavages	Het-1A-Smoke - 2M/Parental	Het-1A-Smoke - 4M/Parental	Het-1A-Smoke - 6M/Parental	Het-1A-Smoke - 8M/Parental
																	EEFTAFLHPEEYDYMK	10	5	2	4502551;194578885	N-Term(TMT6plex); K16(TMT6plex)	2507.2	0				
																	YDLFVGSQATDFGEALVR	12	4	2	4502551;194578885	N-Term(TMT6plex)	2217.1	0				
																	DGFVTEGELK	4	2	1	194578885	N-Term(TMT6plex); K10(TMT6plex)	1552.9	0	1.7	1.6	1.9	1.1
																	MADKDGDLIATK	1	5	2	4502551;194578885	N-Term(TMT6plex); K4(TMT6plex); K12(TMT6plex)	1965.1	1				
																	VHHEPQLSDK	10	5	2	4502551;194578885	N-Term(TMT6plex); K10(TMT6plex)	1647.9	0				
																	LTKEEIVDKYDLFVGSQATDFGEALVR	1	4	2	4502551;194578885	N-Term(TMT6plex); K3(TMT6plex); K9(TMT6plex)	3731.1	2				
																	LGMIVDK	2	2	1	194578885	N-Term(TMT6plex); K7(TMT6plex)	1233.8	0	2.1	2.2	2.7	1.1
																	EQFVEFR	5	4	2	4502551;194578885	N-Term(TMT6plex)	1183.6	0				
																	EEIVDK	2	5	3	4502551;194578885;45439327	N-Term(TMT6plex); K6(TMT6plex)	1190.7	0				
																	DGDLIATK	1	5	2	4502551;194578885	N-Term(TMT6plex); K8(TMT6plex)	1290.8	0				
5730023	NP_006657.1	**RUVBL2**	10856	**ruvB-like 2**	65.66	1	27	27	172	**1.4**	**1.6**	**1.5**	**1.6**	463	51.1	5.64												
																	LLIVSTTPYSEK	19	1	1	5730023	N-Term(TMT6plex); K12(TMT6plex)	1809.1	0	1.4	1.5	1.5	1.7
																	VYSLFLDESR	7	1	1	5730023	N-Term(TMT6plex)	1457.8	0	1.7	1.5	1.8	1.5
																	QASQGMVGQLAAR	6	1	1	5730023	N-Term(TMT6plex); M6(Oxidation)	1561.8	0	1.6	1.6	1.5	1.7
																	TQGFLALFSGDTGEIK	14	1	1	5730023	N-Term(TMT6plex); K16(TMT6plex)	2142.2	0	1.5	1.4	1.5	1.5
																	GTSYQSPHGIPIDLLDR	6	1	1	5730023	N-Term(TMT6plex)	2098.1	0	1.4	1.7	1.6	1.7
																	GTEVQVDDIK	4	1	1	5730023	N-Term(TMT6plex); K10(TMT6plex)	1561.9	0	1.5	1.5	1.6	1.7
																	DYDAMGSQTK	6	1	1	5730023	N-Term(TMT6plex); M5(Oxidation); K10(TMT6plex)	1589.8	0	1.5	1.9	1.9	1.9
																	VQAGDVITIDK	6	1	1	5730023	N-Term(TMT6plex); K11(TMT6plex)	1617.0	0	1.5	1.5	1.5	1.7
																	AAGVVLEMIR	6	1	1	5730023	N-Term(TMT6plex); M8(Oxidation)	1303.8	0	1.3	1.4	1.4	1.5
																	EYQDAFLFNELK	10	1	1	5730023	N-Term(TMT6plex); K12(TMT6plex)	1975.1	0	1.4	1.5	1.5	1.8
																	FVQCPDGELQK	6	1	1	5730023	N-Term(TMT6plex); C4(Carbamidomethyl); K11(TMT6plex)	1779.0	0	1.5	1.5	1.6	1.7
																	IGLETSLR	2	1	1	5730023	N-Term(TMT6plex)	1117.7	0	1.5	1.5	1.4	1.7
																	DKVQAGDVITIDK	3	1	1	5730023	N-Term(TMT6plex); K2(TMT6plex); K13(TMT6plex)	2089.2	1	1.4	1.9	1.8	2.0
																	AAGVVLEMIR	10	1	1	5730023	N-Term(TMT6plex)	1287.8	0	1.8	2.1	2.3	1.8
																	TTEMETIYDLGTK	4	1	1	5730023	N-Term(TMT6plex); K13(TMT6plex)	1960.0	0	2.3	2.4	2.8	2.0
																	EYQDAFLFNELKGETMDTS	11	1	1	5730023	N-Term(TMT6plex); K12(TMT6plex); M16(Oxidation)	2712.3	1	1.5	1.9	1.7	2.1
																	ALESDMAPVLIMATNR	4	1	1	5730023	N-Term(TMT6plex); M6(Oxidation)	1977.0	0	1.3	1.6	1.6	1.5
																	GLGLDDALEPR	10	1	1	5730023	N-Term(TMT6plex)	1384.8	0	1.4	1.4	1.5	1.6
																	TEALTQAFR	6	1	1	5730023	N-Term(TMT6plex)	1265.7	0	1.6	1.4	1.5	1.5
																	TAIAMGMAQALGPDTPFTAIAGSEIFSLEMSK	4	1	1	5730023	N-Term(TMT6plex); M5(Oxidation); M7(Oxidation); M30(Oxidation); K32(TMT6plex)	3762.9	0	1.2	1.4	1.4	1.6
																	TAIAMGMAQALGPDTPFTAIAGSEIFSLEMSK	2	1	1	5730023	N-Term(TMT6plex); M30(Oxidation); K32(TMT6plex)	3730.9	0	0.9	1.5	1.4	1.3
																	IKEETEIIEGEVVEIQIDRPATGTGSK	1	1	1	5730023	N-Term(TMT6plex); K2(TMT6plex); K27(TMT6plex)	3629.0	1	1.4	1.8	1.6	1.9
																	TAIAMGMAQALGPDTPFTAIAGSEIFSLEMSK	1	1	1	5730023	N-Term(TMT6plex); M7(Oxidation); M30(Oxidation); K32(TMT6plex)	3746.9	0	1.2	1.3	1.3	1.2
																	YAIQLITAASLVCR	4	1	1	5730023	N-Term(TMT6plex); C13(Carbamidomethyl)	1808.0	0	1.1	1.2	1.1	1.3
																	AVLIAGQPGTGK	6	1	1	5730023	N-Term(TMT6plex); K12(TMT6plex)	1570.0	0	1.3	1.5	1.4	1.6
																	TTEMETIYDLGTK	2	1	1	5730023	N-Term(TMT6plex); M4(Oxidation); K13(TMT6plex)	1976.0	0	1.3	1.3	1.3	1.5
																	ALESDMAPVLIMATNR	6	1	1	5730023	N-Term(TMT6plex); M6(Oxidation); M12(Oxidation)	1993.0	0	1.3	1.5	1.3	1.6
																	KGTEVQVDDIK	1	1	1	5730023	N-Term(TMT6plex); K1(TMT6plex); K11(TMT6plex)	1919.1	1	1.3	1.5	1.5	1.6
																	MIESLTK	2	1	1	5730023	N-Term(TMT6plex); K7(TMT6plex)	1279.8	0	1.5	1.5	1.6	1.4
																	GTEVQVDDIKR	1	1	1	5730023	N-Term(TMT6plex); K10(TMT6plex)	1718.0	1	1.3	1.5	1.4	1.5
																	EQINAK	1	1	1	5730023	N-Term(TMT6plex); K6(TMT6plex)	1160.7	0	1.1	1.7	1.5	1.7
																	STQYMK	1	1	1	5730023	N-Term(TMT6plex); M5(Oxidation); K6(TMT6plex)	1231.7	0	1.4	1.4	1.4	1.8
4506723	NP_000997.1	**RPS3A**	6189	**40S ribosomal protein S3a isoform 1**	65.53	2	21	21	577	**1.2**	**1.2**	**1.3**	**1.3**	264	29.9	9.73												
																	LMELHGEGSSSGK	22	1	1	4506723	N-Term(TMT6plex); M2(Oxidation); K13(TMT6plex)	1805.9	0	1.1	1.2	1.2	1.3
																	EVQTNDLK	24	1	1	4506723	N-Term(TMT6plex); K8(TMT6plex)	1404.8	0	1.1	1.2	1.3	1.3
																	TSYAQHQQVR	20	1	1	4506723	N-Term(TMT6plex)	1446.8	0	1.1	1.0	1.2	1.1
																	LIPDSIGK	32	1	1	4506723	N-Term(TMT6plex); K8(TMT6plex)	1300.8	0	1.1	1.2	1.2	1.3
																	NCLTNFHGMDLTR	7	1	1	4506723	N-Term(TMT6plex); C2(Carbamidomethyl); M9(Oxidation)	1823.9	0	1.3	1.3	1.3	1.2
																	KDWYDVK	3	2	1	4506723	N-Term(TMT6plex); K1(TMT6plex); K7(TMT6plex)	1641.0	1	1.0	1.1	1.1	1.2
																	TTDGYLLR	19	1	1	4506723	N-Term(TMT6plex)	1167.7	0	1.2	1.0	1.2	1.1
																	KVVDPFSK	10	2	1	4506723	N-Term(TMT6plex); K1(TMT6plex); K8(TMT6plex)	1607.0	1	1.0	1.4	1.3	1.4
																	VFEVSLADLQNDEVAFR	295	1	1	4506723	N-Term(TMT6plex)	2181.1	0	1.3	1.4	1.4	1.3
																	ATGDETGAK	22	1	1	4506723	N-Term(TMT6plex); K9(TMT6plex)	1307.7	0	1.1	1.2	1.2	1.3
																	ADGYEPPVQESV	22	1	1	4506723	N-Term(TMT6plex)	1519.7	0	1.6	1.4	1.3	1.4
																	DWYDVK	5	2	1	4506723	N-Term(TMT6plex); K6(TMT6plex)	1283.7	0	1.0	1.1	1.1	1.2
																	LITEDVQGK	27	1	1	4506723	N-Term(TMT6plex); K9(TMT6plex)	1460.9	0	1.2	1.2	1.2	1.3
																	WQTMIEAHVDVK	1	1	1	4506723	N-Term(TMT6plex); M4(Oxidation); K12(TMT6plex)	1931.0	0	1.2	1.2	1.2	1.3
																	LFCVGFTK	23	1	1	4506723	N-Term(TMT6plex); C3(Carbamidomethyl); K8(TMT6plex)	1429.8	0	1.2	1.2	1.3	1.2
																	KWQTMIEAHVDVK	1	1	1	4506723	N-Term(TMT6plex); K1(TMT6plex); M5(Oxidation); K13(TMT6plex)	2288.3	1	1.1	1.2	1.3	1.2
																	ACQSIYPLHDVFVR	12	1	1	4506723	N-Term(TMT6plex); C2(Carbamidomethyl)	1934.0	0	1.3	1.2	1.3	1.3
																	KTSYAQHQQVR	10	1	1	4506723	N-Term(TMT6plex); K1(TMT6plex)	1804.0	1	1.1	1.3	1.3	1.3
																	APAMFNIR	2	2	1	4506723	N-Term(TMT6plex)	1148.6	0	1.4	1.6	1.9	1.4
																	WQTMIEAHVDVK	1	1	1	4506723	N-Term(TMT6plex); K12(TMT6plex)	1915.1	0	1.2	1.3	1.5	1.2
																	VVDPFSK	8	2	1	4506723	N-Term(TMT6plex); K7(TMT6plex)	1249.7	0	1.1	1.2	1.2	1.3
																	IASDGLK	11	1	1	4506723	N-Term(TMT6plex); K7(TMT6plex)	1161.7	0	1.2	1.1	1.3	1.2
5453790	NP_006160.1	**NNMT**	4837	**nicotinamide N-methyltransferase**	65.53	1	12	12	285	**1.3**	**1.1**	**1.2**	**1.5**	264	29.6	5.74												
																	DTYLSHFNPR	31	1	1	5453790	N-Term(TMT6plex)	1478.8	0	1.2	1.3	1.2	1.3
																	HSAESQILK	23	1	1	5453790	N-Term(TMT6plex); K9(TMT6plex)	1470.9	0	1.1	1.2	1.2	1.4
																	EIVVTDYSDQNLQELEK	53	1	1	5453790	N-Term(TMT6plex); K17(TMT6plex)	2481.3	0	1.3	1.2	1.2	1.6
																	IFCLDGVK	20	1	1	5453790	N-Term(TMT6plex); C3(Carbamidomethyl); K8(TMT6plex)	1409.8	0	1.3	1.1	1.2	1.4
																	NLGSLLKPGGFLVIMDALK	22	1	1	5453790	N-Term(TMT6plex); K7(TMT6plex); M15(Oxidation); K19(TMT6plex)	2689.6	0	1.0	0.8	0.9	1.4
																	FSSLPLGR	10	1	1	5453790	N-Term(TMT6plex)	1105.7	0	1.3	0.9	1.0	1.2
																	CDVTQSQPLGAVPLPPADCVLSTLCLDAACPDLPTYCR	8	1	1	5453790	N-Term(TMT6plex); C1(Carbamidomethyl); C19(Carbamidomethyl); C25(Carbamidomethyl); C30(Carbamidomethyl); C37(Carbamidomethyl)	4460.1	0	1.4	1.5	1.4	1.4
																	SSYYMIGEQK	30	1	1	5453790	N-Term(TMT6plex); M5(Oxidation); K10(TMT6plex)	1679.9	0	1.3	1.1	1.2	1.5
																	GDLLIDIGSGPTIYQLLSACESFK	9	1	1	5453790	N-Term(TMT6plex); C20(Carbamidomethyl); K24(TMT6plex)	3055.6	0	1.2	1.1	1.0	1.4
																	KEPEAFDWSPVVTYVCDLEGNR	28	1	1	5453790	N-Term(TMT6plex); K1(TMT6plex); C16(Carbamidomethyl)	3069.6	1	1.6	1.4	1.3	1.6
																	NLGSLLKPGGFLVIMDALK	14	1	1	5453790	N-Term(TMT6plex); K7(TMT6plex); K19(TMT6plex)	2673.6	0	1.4	1.1	1.4	1.5
																	EPEAFDWSPVVTYVCDLEGNR	23	1	1	5453790	N-Term(TMT6plex); C15(Carbamidomethyl)	2712.3	0	1.3	1.3	1.2	1.6
																	EAVEAAVK	14	1	1	5453790	N-Term(TMT6plex); K8(TMT6plex)	1274.8	0	1.2	1.2	1.1	1.5
4885649	NP_005490.1	**UBA2**	10054	**SUMO-activating enzyme subunit 2**	65.47	1	35	35	281	**1.3**	**1.3**	**1.5**	**1.4**	640	71.2	5.29												
																	DVEFEVVGDAPEK	10	1	1	4885649	N-Term(TMT6plex); K13(TMT6plex)	1892.0	0	1.2	1.1	1.4	1.3
																	MCLAADVPLIESGTAGYLGQVTTIK	15	1	1	4885649	N-Term(TMT6plex); M1(Oxidation); C2(Carbamidomethyl); K25(TMT6plex)	3082.6	0	1.3	1.4	1.4	1.4
																	ESVLQFYPK	16	1	1	4885649	N-Term(TMT6plex); K9(TMT6plex)	1568.9	0	1.2	1.3	1.3	1.4
																	GDGAELIWDKDDPSAMDFVTSAANLR	29	1	1	4885649	N-Term(TMT6plex); K10(TMT6plex); M16(Oxidation)	3268.6	1	1.3	1.4	1.5	1.5
																	ASNEDGDIK	4	1	1	4885649	N-Term(TMT6plex); K9(TMT6plex)	1406.8	0	1.3	1.3	1.3	1.3
																	VLVVGAGGIGCELLK	16	1	1	4885649	N-Term(TMT6plex); C11(Carbamidomethyl); K15(TMT6plex)	1943.2	0	1.3	1.3	1.5	1.3
																	GTILISSEEGETEANNHK	10	1	1	4885649	N-Term(TMT6plex); K18(TMT6plex)	2387.2	0	1.3	1.4	1.6	1.5
																	ADPEAAWEPTEAEAR	13	1	1	4885649	N-Term(TMT6plex)	1871.9	0	1.4	1.4	1.5	1.5
																	FAMVAPDVQIEDGK	12	1	1	4885649	N-Term(TMT6plex); M3(Oxidation); K14(TMT6plex)	1994.1	0	1.3	1.2	1.4	1.2
																	ASNEDGDIKR	5	1	1	4885649	N-Term(TMT6plex); K9(TMT6plex)	1562.9	1	1.1	1.5	1.4	1.5
																	ANIVAYHDSIMNPDYNVEFFR	10	1	1	4885649	N-Term(TMT6plex); M11(Oxidation)	2760.3	0	1.3	1.2	1.2	1.3
																	GTILISSEEGETEANNHKK	6	1	1	4885649	N-Term(TMT6plex); K18(TMT6plex); K19(TMT6plex)	2744.5	1	1.2	1.4	1.6	1.4
																	VTVLTLQDK	9	1	1	4885649	N-Term(TMT6plex); K9(TMT6plex)	1474.9	0	1.3	1.2	1.4	1.2
																	YLFNQLFGEEDADQEVSPDR	18	1	1	4885649	N-Term(TMT6plex)	2601.2	0	1.3	1.2	1.4	1.3
																	NLVLTGFSHIDLIDLDTIDVSNLNR	19	1	1	4885649	N-Term(TMT6plex)	3026.6	0	1.5	1.3	1.6	1.3
																	LQADDFLQDYTLLINILHSEDLGK	10	1	1	4885649	N-Term(TMT6plex); K24(TMT6plex)	3232.8	0	1.2	1.1	1.5	1.1
																	STGYDPVK	4	1	1	4885649	N-Term(TMT6plex); K8(TMT6plex)	1324.8	0	1.1	1.1	1.2	1.1
																	DQQVLDVK	7	1	1	4885649	N-Term(TMT6plex); K8(TMT6plex)	1402.8	0	1.2	1.3	1.4	1.4
																	KPPVPLDWAEVQSQGEETNASDQQNEPQLGLK	10	1	1	4885649	N-Term(TMT6plex); K1(TMT6plex); K32(TMT6plex)	4220.2	0	1.3	1.3	1.4	1.4
																	LQADDFLQDYTLLINILHSEDLGKDVEFEVVGDAPEK	4	1	1	4885649	N-Term(TMT6plex); K24(TMT6plex); K37(TMT6plex)	4876.6	1	1.3	1.6	1.5	1.7
																	NTPSEPIHCIVWAK	2	1	1	4885649	N-Term(TMT6plex); C9(Carbamidomethyl); K14(TMT6plex)	2110.1	0	1.4	1.3	1.5	1.3
																	GVTECYECHPKPTQR	6	1	1	4885649	N-Term(TMT6plex); C5(Carbamidomethyl); C8(Carbamidomethyl); K11(TMT6plex)	2320.2	0	1.2	1.4	1.6	1.6
																	LSEFGIR	4	1	1	4885649	N-Term(TMT6plex)	1050.6	0	1.3	1.0	1.3	1.2
																	KGVTECYECHPKPTQR	1	1	1	4885649	N-Term(TMT6plex); K1(TMT6plex); C6(Carbamidomethyl); C9(Carbamidomethyl); K12(TMT6plex)	2677.4	1	1.9	1.7	1.7	1.8

Supplementary Table 6. List of all proteins and peptides identified in untreated and chronically treated Het1A cells with cigarette smoke condensate for 8 months

Accession	RefSeq Accession	Gene Symbol	Entrez Gene ID	Description	Σ Coverage	Σ# Proteins	Σ# Unique Peptides	Σ# Peptides	Σ# PSMs	Het-1A-Smoke - 2M/Parental	Het-1A-Smoke - 4M/Parental	Het-1A-Smoke - 6M/Parental	Het-1A-Smoke - 8M/Parental	# AAs	MW [kDa]	calc. pI	Sequence	# PSMs	# Proteins	# Protein Groups	Protein Group Accessions	Modifications	MH+ [Da]	# Missed Cleavages	Het-1A-Smoke - 2M/Parental	Het-1A-Smoke - 4M/Parental	Het-1A-Smoke - 6M/Parental	Het-1A-Smoke - 8M/Parental
																	MCLAADVPLIESGTAGYLGQVTTIK	5	1	1	4885649	N-Term(TMT6plex); C2(Carbamidomethyl); K25(TMT6plex)	3066.7	0	**1.5**	**1.6**	**1.8**	**1.4**
																	GDGAELIWDKDDPSAMDFVTSAANLR	2	1	1	4885649	N-Term(TMT6plex); K10(TMT6plex)	3252.6	1	**1.5**	**1.7**	**2.0**	**1.4**
																	QFILVMNALDNR	3	1	1	4885649	N-Term(TMT6plex)	1662.9	0	**1.6**	**1.4**	**1.7**	**1.5**
																	SMAGNIIPAIATTNAVIAGLIVLEGLK	4	1	1	4885649	N-Term(TMT6plex); M2(Oxidation); K27(TMT6plex)	3124.8	0	**1.7**	**1.5**	**2.2**	**1.4**
																	IEQKEELDDVIALD	5	1	1	4885649	N-Term(TMT6plex); K4(TMT6plex)	2088.2	1	**1.4**	**1.2**	**1.4**	**1.4**
																	EELDDVIALD	2	1	1	4885649	N-Term(TMT6plex)	1360.7	0	**2.3**	**1.5**	**1.2**	**1.5**
																	ELAEAVAGGR	6	1	1	4885649	N-Term(TMT6plex)	1201.7	0	**1.4**	**1.5**	**1.5**	**1.6**
																	QAEDAAK	3	1	1	4885649	N-Term(TMT6plex); K7(TMT6plex)	1190.7	0	**1.5**	**1.2**	**1.3**	**1.3**
																	YLLTMDK	3	1	1	4885649	N-Term(TMT6plex); K7(TMT6plex)	1341.8	0	**1.4**	**1.6**	**1.8**	**1.4**
																	YLFNQLFGEEDADQEVSPDRADPEAAWEPTEAEAR	1	1	1	4885649	N-Term(TMT6plex)	4225.0	1	**1.9**	**2.3**	**1.8**	**1.9**
																	TIFLNK	3	1	1	4885649	N-Term(TMT6plex); K6(TMT6plex)	1193.8	0	**1.2**	**1.2**	**1.3**	**1.3**
																	QFLFQK	3	1	1	4885649	N-Term(TMT6plex); K6(TMT6plex)	1268.8	0	**1.3**	**1.3**	**1.5**	**1.4**
																	KLSEFGIR	1	1	1	4885649	N-Term(TMT6plex); K1(TMT6plex)	1407.9	1	**1.4**	**1.3**	**1.5**	**1.4**
10645195	NP_066390.1	**HIST1H2AE**	3012	**histone H2A type 1-B/E**	65.38	4	4	9	1807	**0.6**	**0.8**	**0.7**	**0.9**	130	14.1	11.05												
																	VTIAQGGVLPNIQAVLLPK	987	10	2	10645195;24638446	N-Term(TMT6plex); K19(TMT6plex)	2389.5	0				
																	NDEELNK	248	13	4	10645195;28195394;24638446;4504253	N-Term(TMT6plex); K7(TMT6plex)	1319.7	0				
																	AGLQFPVGR	448	17	5	10645195;28195394;24638446;4504253;4504255	N-Term(TMT6plex)	1173.7	0				
																	HLQLAIR	25	17	4	10645195;4504255;24638446;4504253	N-Term(TMT6plex)	1079.7	0				
																	VGAGAPVYLAAVLEYLTAEILELAGNAAR	66	10	3	10645195;28195394;4504253	N-Term(TMT6plex)	3144.8	0				
																	KTESHHK	25	8	1	10645195	N-Term(TMT6plex); K1(TMT6plex); K7(TMT6plex)	1553.9	1	**0.6**	**0.8**	**0.7**	**0.9**
																	KGNYSER	2	3	1	10645195	N-Term(TMT6plex); K1(TMT6plex)	1311.7	1	**0.7**	**0.7**	**0.7**	**0.7**
																	GNYSER	3	3	1	10645195	N-Term(TMT6plex)	954.5	0	**0.8**	**0.8**	**0.8**	**0.9**
																	TESHHK	3	8	1	10645195	N-Term(TMT6plex); K6(TMT6plex)	1196.7	0	**0.6**	**0.8**	**0.8**	**1.0**
21361399	NP_055040.2	**PPP2R1A**	5518	**serine/threonine-protein phosphatase 2A 65 kDa regulatory subunit A alpha isoform**	65.37	1	23	33	531	**1.2**	**1.2**	**1.3**	**1.2**	589	65.3	5.11												
																	SALASVIMGLSPILGK	47	1	1	21361399	N-Term(TMT6plex); M8(Oxidation); K16(TMT6plex)	2031.2	0	**1.3**	**1.2**	**1.5**	**1.3**
																	NLCSDDTPMVR	13	1	1	21361399	N-Term(TMT6plex); C3(Carbamidomethyl); M9(Oxidation)	1552.7	0	**1.3**	**1.1**	**1.2**	**1.1**
																	EWAHATIIPK	4	1	1	21361399	N-Term(TMT6plex); K10(TMT6plex)	1624.0	0	**0.8**	**0.9**	**0.9**	**1.1**
																	IGPILDNSTLQSEVKPILEK	22	1	1	21361399	N-Term(TMT6plex); K15(TMT6plex); K20(TMT6plex)	2881.7	0	**1.0**	**1.1**	**1.2**	**1.2**
																	TDLVPAFQNLMK	20	1	1	21361399	N-Term(TMT6plex); M11(Oxidation); K12(TMT6plex)	1851.0	0	**1.4**	**1.2**	**1.4**	**1.3**
																	QLSQSLLPAIVELAEDAK	71	6	2	32455246;21361399	N-Term(TMT6plex); K18(TMT6plex)	2383.4	0				
																	TSACGLFSVCYPR	9	5	2	32455246;21361399	N-Term(TMT6plex); C4(Carbamidomethyl); C10(Carbamidomethyl)	1746.9	0				
																	ELVSDANQHVK	14	1	1	21361399	N-Term(TMT6plex); K11(TMT6plex)	1698.0	0	**1.2**	**1.2**	**1.3**	**1.4**
																	AVGPEITK	10	1	1	21361399	N-Term(TMT6plex); K8(TMT6plex)	1272.8	0	**1.1**	**1.2**	**1.3**	**1.3**
																	LAIIEYMPLLAGQLGVEFFDEK	10	6	2	32455246;21361399	N-Term(TMT6plex); M7(Oxidation); K22(TMT6plex)	2970.6	0				
																	VLAMSGDPNYLHR	4	1	1	21361399	N-Term(TMT6plex); M4(Oxidation)	1717.9	0	**1.2**	**1.2**	**1.2**	**1.2**
																	YFAQEALTVLSLA	62	1	1	21361399	N-Term(TMT6plex)	1654.9	0	**1.4**	**1.2**	**1.5**	**1.3**
																	LAIIEYMPLLAGQLGVEFFDEK	3	6	2	32455246;21361399	N-Term(TMT6plex); K22(TMT6plex)	2954.6	0				
																	NLCSDDTPMVR	2	1	1	21361399	N-Term(TMT6plex); C3(Carbamidomethyl)	1536.7	0	**1.6**	**1.3**	**1.9**	**1.2**
																	DNTIEHLLPLFLAQLKDECPEVR	2	1	1	21361399	N-Term(TMT6plex); K16(TMT6plex); C19(Carbamidomethyl)	3208.7	1	**1.1**	**1.4**	**1.3**	**1.5**
																	AISHEHSPSDLEAHFVPLVK	4	1	1	21361399	N-Term(TMT6plex); K20(TMT6plex)	2671.5	0	**1.2**	**1.3**	**1.3**	**1.4**
																	QLSQSLLPAIVELAEDAKWR	3	6	2	32455246;21361399	N-Term(TMT6plex); K18(TMT6plex)	2725.6	1				
																	VLAMSGDPNYLHR	4	1	1	21361399	N-Term(TMT6plex)	1701.9	0	**1.4**	**1.5**	**1.5**	**1.4**
																	SEIIPMFSNLASDEQDSVR	17	1	1	21361399	N-Term(TMT6plex); M6(Oxidation)	2383.2	0	**1.3**	**1.4**	**1.2**	**1.4**
																	DNTIEHLLPLFLAQLK	46	1	1	21361399	N-Term(TMT6plex); K16(TMT6plex)	2323.4	0	**1.1**	**1.0**	**1.3**	**1.1**
																	SALASVIMGLSPILGK	24	1	1	21361399	N-Term(TMT6plex); K16(TMT6plex)	2015.2	0	**1.6**	**1.5**	**1.8**	**1.4**
																	LNSLCMAWLVDHVYAIR	6	6	2	32455246;21361399	N-Term(TMT6plex); C5(Carbamidomethyl)	2290.2	0				
																	VLELDNVK	23	1	1	21361399	N-Term(TMT6plex); K8(TMT6plex)	1387.9	0	**1.2**	**1.1**	**1.2**	**1.2**
																	TDLVPAFQNLMK	8	1	1	21361399	N-Term(TMT6plex); K12(TMT6plex)	1835.0	0	**1.4**	**1.2**	**1.9**	**1.0**
																	LTQDQDVDVK	31	1	1	21361399	N-Term(TMT6plex); K10(TMT6plex)	1618.9	0	**1.3**	**1.1**	**1.3**	**1.3**
																	LSTIALALGVER	15	5	2	32455246;21361399	N-Term(TMT6plex)	1471.9	0				
																	LAGGDWFTSR	6	1	1	21361399	N-Term(TMT6plex)	1338.7	0	**1.4**	**1.2**	**1.3**	**1.2**
																	EFCENLSADCR	6	1	1	21361399	N-Term(TMT6plex); C3(Carbamidomethyl); C10(Carbamidomethyl)	1629.7	0	**1.3**	**1.2**	**1.4**	**1.1**
																	EAATSNLK	4	1	1	21361399	N-Term(TMT6plex); K8(TMT6plex)	1291.8	0	**0.9**	**0.8**	**0.8**	**0.9**
																	LNIISNLDCVNEVIGIR	11	6	2	32455246;21361399	N-Term(TMT6plex); C9(Carbamidomethyl)	2171.2	0				
																	LNSLCMAWLVDHVYAIR	2	6	2	32455246;21361399	N-Term(TMT6plex); C5(Carbamidomethyl); M6(Oxidation)	2306.2	0				
																	ENVIMSQILPCIK	1	1	1	21361399	N-Term(TMT6plex); M5(Oxidation); C11(Carbamidomethyl); K13(TMT6plex)	2019.1	0				
																	HMLPTVLR	10	1	1	21361399	N-Term(TMT6plex); M2(Oxidation)	1211.7	0	**1.2**	**1.3**	**1.3**	**1.3**
																	MAGDPVANVR	3	1	1	21361399	N-Term(TMT6plex); M1(Oxidation)	1274.7	0	**1.1**	**1.0**	**1.3**	**1.1**
																	FTELQK	9	1	1	21361399	N-Term(TMT6plex); K6(TMT6plex)	1223.7	0	**1.1**	**1.0**	**1.3**	**1.1**
																	YMVADK	2	1	1	21361399	N-Term(TMT6plex); K6(TMT6plex)	1184.7	0	**1.5**	**1.6**	**2.0**	**1.4**
																	AVESLR	1	5	2	32455246;21361399	N-Term(TMT6plex)	903.5	0				
																	LGEFAK	1	5	2	32455246;21361399	N-Term(TMT6plex); K6(TMT6plex)	1122.7	0				
																	QAAEDK	1	6	2	32455246;21361399	N-Term(TMT6plex); K6(TMT6plex)	1119.6	0				
217330646	NP_006704.3	**SUB1**	10923	**activated RNA polymerase II transcriptional coactivator p15**	65.35	1	9	9	29	**1.3**	**1.5**	**1.5**	**2.1**	127	14.4	9.60												
																	DDNMFQIGK	5	1	1	217330646	N-Term(TMT6plex); M4(Oxidation); K9(TMT6plex)	1541.8	0	**1.5**	**1.5**	**1.5**	**2.3**
																	EQISDIDDAVR	8	1	1	217330646	N-Term(TMT6plex)	1489.8	0	**1.4**	**1.4**	**1.5**	**2.1**
																	GISLNPEQWSQLK	4	1	1	217330646	N-Term(TMT6plex); K13(TMT6plex)	1958.1	0	**1.3**	**1.4**	**1.5**	**2.3**
																	DDNMFQIGK	2	1	1	217330646	N-Term(TMT6plex); K9(TMT6plex)	1525.8	0	**1.4**	**1.5**	**1.9**	**1.7**
																	ELVSSSSSGSDSDSEVDKK	4	1	1	217330646	N-Term(TMT6plex); K18(TMT6plex); K19(TMT6plex)	2630.4	1	**1.3**	**2.7**	**2.0**	**2.8**
																	KGISLNPEQWSQLK	2	1	1	217330646	N-Term(TMT6plex); K1(TMT6plex); K14(TMT6plex)	2315.4	1	**1.2**	**1.2**	**1.4**	**1.6**
																	EYWMDPEGEMKPGR	1	1	1	217330646	N-Term(TMT6plex); M4(Oxidation); M10(Oxidation); K11(TMT6plex)	2215.1	0	**1.2**	**1.2**	**1.4**	**2.2**
																	QVAPEKPVK	1	1	1	217330646	N-Term(TMT6plex); K6(TMT6plex); K9(TMT6plex)	1683.1	0				
																	QVAPEKPVKK	1	1	1	217330646	N-Term(TMT6plex); K6(TMT6plex); K9(TMT6plex); K10(TMT6plex)	2040.3	1	**1.1**	**1.5**	**1.3**	**2.5**
																	VLIDIR	1	1	1	217330646	N-Term(TMT6plex)	957.6	0	**1.2**	**1.3**	**1.5**	**2.1**
225543215	NP_001139438.1	**MKI67**	4288	**antigen KI-67 isoform 2**	65.33	2	1	155	1154	**1.2**	**1.4**	**1.4**	**1.4**	2896	319.2	9.51												
																	SSPELEDTATSSK	10	2	2	225543215;103472005	N-Term(TMT6plex); K13(TMT6plex)	1809.9	0				
																	ELFQTPGHTEEAVAAGK	10	2	2	225543215;103472005	N-Term(TMT6plex); K17(TMT6plex)	2243.2	0				
																	TSPEMDIQNPDDGAR	9	2	2	225543215;103472005	N-Term(TMT6plex); M5(Oxidation)	1890.9	0				
																	GKSEVPEDLAGFIELFQTPSHTK	6	2	2	225543215;103472005	N-Term(TMT6plex); K2(TMT6plex); K23(TMT6plex)	3217.8	1				
																	KADVEEEFLAFR	10	2	2	225543215;103472005	N-Term(TMT6plex); K1(TMT6plex)	1912.1	1				
																	LDLPGNLPGSK	14	2	2	225543215;103472005	N-Term(TMT6plex); K11(TMT6plex)	1568.9	0				
																	QTPAPAASVTGSR	11	2	2	225543215;103472005	N-Term(TMT6plex)	1471.8	0				
																	EDLSGIAEMFK	7	2	2	225543215;103472005	N-Term(TMT6plex); M9(Oxidation); K11(TMT6plex)	1713.9	0				
																	EEPSAVK	4	2	2	225543215;103472005	N-Term(TMT6plex); K7(TMT6plex)	1217.7	0				
																	SSEPVVIMK	15	2	2	225543215;103472005	N-Term(TMT6plex); M8(Oxidation); K9(TMT6plex)	1463.9	0				
																	KADTEEEFLAFR	6	2	2	225543215;103472005	N-Term(TMT6plex); K1(TMT6plex)	1914.0	1				
																	KADVEEEFLALR	11	2	2	225543215;103472005	N-Term(TMT6plex); K1(TMT6plex)	1878.1	1				
																	CMPAPEEIVEELPASK	8	2	2	225543215;103472005	N-Term(TMT6plex); C1(Carbamidomethyl); M2(Oxidation); K16(TMT6plex)	2274.2	0				
																	QILDPAASVTGSR	8	2	2	225543215;103472005	N-Term(TMT6plex)	1543.9	0				
																	ADTEEEFLAFR	4	2	2	225543215;103472005	N-Term(TMT6plex)	1556.8	0				
																	QKLDPAASVTGSK	2	2	2	225543215;103472005	N-Term(TMT6plex); K2(TMT6plex); K13(TMT6plex)	1989.2	1				
																	SPQPDPVGTPTIFKPQSK	5	2	2	225543215;103472005	N-Term(TMT6plex); K14(TMT6plex); K18(TMT6plex)	2611.5	0				
																	QMLDPANYGTGMER	11	2	2	225543215;103472005	N-Term(TMT6plex); M2(Oxidation); M12(Oxidation)	1843.9	0				
																	KADVEGELLACR	5	2	2	225543215;103472005	N-Term(TMT6plex); K1(TMT6plex); C11(Carbamidomethyl)	1819.0	1				
																	EKAEALEDLVGFK	8	2	2	225543215;103472005	N-Term(TMT6plex); K2(TMT6plex); K13(TMT6plex)	2136.2	1				
																	VEVKEELLAVGK	5	2	2	225543215;103472005	N-Term(TMT6plex); K4(TMT6plex); K12(TMT6plex)	2001.3	1				
																	QESGSEIHVEVK	4	2	2	225543215;103472005	N-Term(TMT6plex); K12(TMT6plex)	1800.0	0				
																	VGVKEEVLPVGK	2	2	2	225543215;103472005	N-Term(TMT6plex); K4(TMT6plex); K12(TMT6plex)	1941.2	1				
																	KKPNPVEEEPSR	8	2	2	225543215;103472005	N-Term(TMT6plex); K1(TMT6plex); K2(TMT6plex)	2097.2	1				
																	ELFSAPGHTEESMTIDK	16	2	2	225543215;103472005	N-Term(TMT6plex); M13(Oxidation); K17(TMT6plex)	2366.2	0				
																	ASQPDLVDTPTSSKPQPK	8	2	2	225543215;103472005	N-Term(TMT6plex); K14(TMT6plex); K18(TMT6plex)	2583.5	0				
																	SQPDPVDTPTSSKPQSK	10	2	2	225543215;103472005	N-Term(TMT6plex); K13(TMT6plex); K17(TMT6plex)	2486.4	0				
																	EIERPFETYK	9	2	2	225543215;103472005	N-Term(TMT6plex); K10(TMT6plex)	1770.0	0				
																	AQPLEDLAGFTELSETSGHTQESLTAGK	27	2	2	225543215;103472005	N-Term(TMT6plex); K28(TMT6plex)	3375.7	0				
																	LDLLGNLPGSK	54	2	2	225543215;103472005	N-Term(TMT6plex); K11(TMT6plex)	1585.0	0				
																	VLNNFISNQK	2	2	2	225543215;103472005	N-Term(TMT6plex); K10(TMT6plex)	1635.0	0				
																	AQALEDLAGFK	31	2	2	225543215;103472005	N-Term(TMT6plex); K11(TMT6plex)	1620.9	0				

Accession	RefSeq Accession	Gene Symbol	Entrez Gene ID	Description	Σ Coverage	Σ# Proteins	Σ# Unique Peptides	Σ# Peptides	Σ# PSMs	Het-1A-Smoke - 2M/Parental	Het-1A-Smoke - 4M/Parental	Het-1A-Smoke - 6M/Parental	Het-1A-Smoke - 8M/Parental	# AAs	MW [kDa]	calc. pI	Sequence	# PSMs	# Proteins	# Protein Groups	Protein Group Accessions	Modifications	MH+ [Da]	# Missed Cleavages	Het-1A-Smoke - 2M/Parental	Het-1A-Smoke - 4M/Parental	Het-1A-Smoke - 6M/Parental	Het-1A-Smoke - 8M/Parental
																	GHTEESMTNDK	13	2	2	225543215;103472005	N-Term(TMT6plex); M7(Oxidation); K11(TMT6plex)	1722.8	0				
																	ESAQAIEDLAGFK	6	2	2	225543215;103472005	N-Term(TMT6plex); K13(TMT6plex)	1837.0	0				
																	MPCQSLQPEPINTPTHTK	8	2	2	225543215;103472005	N-Term(TMT6plex); M1(Oxidation); C3(Carbamidomethyl); K18(TMT6plex)	2553.3	0				
																	TSGETTQTHTEPTGDSK	12	2	2	225543215;103472005	N-Term(TMT6plex); K17(TMT6plex)	2235.1	0				
																	ELFQTPICTDKPTTHEK	7	2	2	225543215;103472005	N-Term(TMT6plex); C8(Carbamidomethyl); K11(TMT6plex); K17(TMT6plex)	2732.5	0				
																	KLTPSAGK	6	2	2	225543215;103472005	N-Term(TMT6plex); K1(TMT6plex); K8(TMT6plex)	1489.0	1				
																	DGSVTGTK	7	2	2	225543215;103472005	N-Term(TMT6plex); K8(TMT6plex)	1222.7	0				
																	ELFQTPGTDKPTTDEK	8	2	2	225543215;103472005	N-Term(TMT6plex); K10(TMT6plex); K16(TMT6plex)	2494.4	0				
																	AMDTPKPAGGDEK	5	2	2	225543215;103472005	N-Term(TMT6plex); M2(Oxidation); K6(TMT6plex); K13(TMT6plex)	2020.1	0				
																	SPPPESVDTPTSTK	17	2	2	225543215;103472005	N-Term(TMT6plex); K14(TMT6plex)	1901.0	0				
																	IEPAEELNSNDMK	6	2	2	225543215;103472005	N-Term(TMT6plex); M12(Oxidation); K13(TMT6plex)	1964.0	0				
																	QFQGTDSGEEPLLPTSESFGGNVFFSAQNAAK	6	2	2	225543215;103472005	N-Term(TMT6plex); K32(TMT6plex)	3818.9	0				
																	SPQSDPADTPTNTK	14	2	2	225543215;103472005	N-Term(TMT6plex); K14(TMT6plex)	1917.0	0				
																	SPQPDPVDTPASTK	21	2	2	225543215;103472005	N-Term(TMT6plex); K14(TMT6plex)	1898.0	0				
																	VEPVGDVVSTR	6	2	2	225543215;103472005	N-Term(TMT6plex)	1386.8	0				
																	ADVEEEFLAFR	6	2	2	225543215;103472005	N-Term(TMT6plex)	1554.8	0				
																	EELSAVER	2	2	2	225543215;103472005	N-Term(TMT6plex)	1161.6	0				
																	LDLAGTLPGSK	9	2	2	225543215;103472005	N-Term(TMT6plex); K11(TMT6plex)	1529.9	0				
																	RIEPAEELNSNDMK	8	2	2	225543215;103472005	N-Term(TMT6plex); M13(Oxidation); K14(TMT6plex)	2120.1	1				
																	LDAEDVIGSR	2	2	2	225543215;103472005	N-Term(TMT6plex)	1303.7	0				
																	SPPPESMDTPTSTR	4	2	2	225543215;103472005	N-Term(TMT6plex); M7(Oxidation)	1747.8	0				
																	AMDTPKPAVSDEK	9	2	2	225543215;103472005	N-Term(TMT6plex); M2(Oxidation); K6(TMT6plex); K13(TMT6plex)	2092.2	0				
																	QILDSAASLTGSK	6	2	2	225543215;103472005	N-Term(TMT6plex); K13(TMT6plex)	1749.0	0				
																	SGASEANLIVAK	4	2	2	225543215;103472005	N-Term(TMT6plex); K12(TMT6plex)	1618.0	0				
																	ELFQTPGHTEELVAAGK	2	2	2	225543215;103472005	N-Term(TMT6plex); K17(TMT6plex)	2285.3	0				
																	GQNLLQTQDHAK	4	2	2	225543215;103472005	N-Term(TMT6plex); K12(TMT6plex)	1811.0	0				
																	SLVMHTPPVLK	2	2	2	225543215;103472005	N-Term(TMT6plex); M4(Oxidation); K11(TMT6plex)	1696.0	0				
																	LTQTSGQSTHTHKEPASGDEGIK	2	2	2	225543215;103472005	N-Term(TMT6plex); K13(TMT6plex); K23(TMT6plex)	3096.6	1				
																	FTQTSGETTDADKEPAGEDK	13	2	2	225543215;103472005	N-Term(TMT6plex); K13(TMT6plex); K20(TMT6plex)	2814.4	1				
																	LDQPGNLPGSNR	13	2	2	225543215;103472005	N-Term(TMT6plex)	1496.8	0				
																	SSEPVVIMK	2	2	2	225543215;103472005	N-Term(TMT6plex); K9(TMT6plex)	1447.9	0				
																	TPKEEAQSLEDLAGFK	2	2	2	225543215;103472005	N-Term(TMT6plex); K3(TMT6plex); K16(TMT6plex)	2450.4	1				
																	AMHTPKPSVGEEK	1	2	2	225543215;103472005	N-Term(TMT6plex); M2(Oxidation); K6(TMT6plex); K13(TMT6plex)	2114.2	0				
																	AMHTPKPAVGEEK	1	2	2	225543215;103472005	N-Term(TMT6plex); M2(Oxidation); K6(TMT6plex); K13(TMT6plex)	2098.2	0				
																	KLDAEDVIGSR	8	2	2	225543215;103472005	N-Term(TMT6plex); K1(TMT6plex)	1661.0	1				
																	AQPLEDLAGLK	22	2	2	225543215;103472005	N-Term(TMT6plex); K11(TMT6plex)	1613.0	0				
																	TEAEQQITEVFVLAER	38	2	2	225543215;103472005	N-Term(TMT6plex)	2092.1	0				
																	EELLAVGK	10	2	2	225543215;103472005	N-Term(TMT6plex); K8(TMT6plex)	1316.8	0				
																	SEETNTEIVECILK	10	2	2	225543215;103472005	N-Term(TMT6plex); C11(Carbamidomethyl); K14(TMT6plex)	2123.1	0				
																	VAEESGGQK	6	2	2	225543215;103472005	N-Term(TMT6plex); K9(TMT6plex)	1362.8	0				
																	AQSLVISPPAPSPR	4	2	2	225543215;103472005	N-Term(TMT6plex)	1649.0	0				
																	KVDVEEEFFALR	8	2	2	225543215;103472005	N-Term(TMT6plex); K1(TMT6plex)	1940.1	1				
																	SNTSLPPLPFK	10	2	2	225543215;103472005	N-Term(TMT6plex); K11(TMT6plex)	1659.0	0				
																	DPAAGHTEESMTDDK	2	2	2	225543215;103472005	N-Term(TMT6plex); M11(Oxidation); K15(TMT6plex)	2078.0	0				
																	ALEDLVDFK	5	2	2	225543215;103472005	N-Term(TMT6plex); K9(TMT6plex)	1507.9	0				
																	DINTFLGTPVQK	8	2	2	225543215;103472005	N-Term(TMT6plex); K12(TMT6plex)	1791.0	0				
																	KAEDNVCVK	3	2	2	225543215;103472005	N-Term(TMT6plex); K1(TMT6plex); C7(Carbamidomethyl); K9(TMT6plex)	1750.0	1				
																	KPVGEVHSQFSTGHANSPCTIIIGK	1	2	2	225543215;103472005	N-Term(TMT6plex); K1(TMT6plex); C19(Carbamidomethyl); K25(TMT6plex)	3351.8	0				
																	SGVDGPHFPLSLSTCLFGR	1	2	2	225543215;103472005	N-Term(TMT6plex); C15(Carbamidomethyl)	2276.2	0				
																	KSGNLPSK	1	2	2	225543215;103472005	N-Term(TMT6plex); K1(TMT6plex); K8(TMT6plex)	1518.0	1				
																	DIVEELSALK	20	2	2	225543215;103472005	N-Term(TMT6plex); K10(TMT6plex)	1574.9	0				
																	EEAQSLEDLAGFK	14	2	2	225543215;103472005	N-Term(TMT6plex); K13(TMT6plex)	1895.0	0				
																	ESAQAIEDLAGFKDPAAGHTEESMTDDK	5	2	2	225543215;103472005	N-Term(TMT6plex); K13(TMT6plex); M24(Oxidation); K28(TMT6plex)	3666.8	1				
																	LQDSVPENK	16	2	2	225543215;103472005	N-Term(TMT6plex); K9(TMT6plex)	1487.8	0				
																	EPASGDEGIK	10	2	2	225543215;103472005	N-Term(TMT6plex); K10(TMT6plex)	1460.8	0				
																	ELFQTPGHTEESMTDDKITEVSCK	6	2	2	225543215;103472005	N-Term(TMT6plex); K17(TMT6plex); C23(Carbamidomethyl); K24(TMT6plex)	3469.7	1				
																	LTQTSGETTHTDKVPGGEDK	7	2	2	225543215;103472005	N-Term(TMT6plex); K13(TMT6plex); K20(TMT6plex)	2788.5	1				
																	LTQTSGETTHTDKEPVGEGK	8	2	2	225543215;103472005	N-Term(TMT6plex); K13(TMT6plex); K20(TMT6plex)	2802.5	1				
																	ELFQTPGHTEESMTDDKITEVSCK	12	2	2	225543215;103472005	N-Term(TMT6plex); M13(Oxidation); K17(TMT6plex); C23(Carbamidomethyl); K24(TMT6plex)	3485.7	1				
																	SSQPDPDKNPASSK	10	2	2	225543215;103472005	N-Term(TMT6plex); K8(TMT6plex); K14(TMT6plex)	2145.2	1				
																	VSFGGHLRPELFDENLPPNTPLK	4	2	2	225543215;103472005	N-Term(TMT6plex); K23(TMT6plex)	3035.7	0				
																	AFMESPK	4	2	2	225543215;103472005	N-Term(TMT6plex); K7(TMT6plex)	1267.7	0				
																	SLPDTELMK	5	2	2	225543215;103472005	N-Term(TMT6plex); M8(Oxidation); K9(TMT6plex)	1507.8	0				
																	MPCESSPPESADTPTSTR	4	2	2	225543215;103472005	N-Term(TMT6plex); C3(Carbamidomethyl)	2179.0	0				
																	ADVEGELLACR	1	2	2	225543215;103472005	N-Term(TMT6plex); C10(Carbamidomethyl)	1461.8	0				
																	SPPPELTDTATSTK	10	2	2	225543215;103472005	N-Term(TMT6plex); K14(TMT6plex)	1903.0	0				
																	DINTFVGTPVEK	10	2	2	225543215;103472005	N-Term(TMT6plex); K12(TMT6plex)	1778.0	0				
																	EDLSGIAEMFK	10	2	2	225543215;103472005	N-Term(TMT6plex); K11(TMT6plex)	1697.9	0				
																	LTQTSGETTHTHTEPTGDGK	6	2	2	225543215;103472005	N-Term(TMT6plex); K20(TMT6plex)	2556.3	0				
																	AFMGTPVQK	2	2	2	225543215;103472005	N-Term(TMT6plex); K9(TMT6plex)	1436.8	0				
																	IPCDSPQSDPVDTPTSTK	13	2	2	225543215;103472005	N-Term(TMT6plex); C3(Carbamidomethyl); K18(TMT6plex)	2403.2	0				
																	ELFQTPGHTEESMTDDK	9	2	2	225543215;103472005	N-Term(TMT6plex); M13(Oxidation); K17(TMT6plex)	2439.2	0				
																	AAVGEEK	4	2	2	225543215;103472005	N-Term(TMT6plex); K7(TMT6plex)	1161.7	0				
																	LDPAASVTGSK	14	2	2	225543215;103472005	N-Term(TMT6plex); K11(TMT6plex)	1503.9	0				
																	SEVPEDLAGFIELFQTPSHTK	18	2	2	225543215;103472005	N-Term(TMT6plex); K21(TMT6plex)	2803.5	0				
																	SQPAASTLESK	8	2	2	225543215;103472005	N-Term(TMT6plex); K11(TMT6plex)	1576.9	0				
																	NINTFVETPVQK	9	2	2	225543215;103472005	N-Term(TMT6plex); K12(TMT6plex)	1848.1	0				
																	DIIIFVGTPVQK	4	2	2	225543215;103472005	N-Term(TMT6plex); K12(TMT6plex)	1788.1	0				
																	ALEDLAGFK	6	2	2	225543215;103472005	N-Term(TMT6plex); K9(TMT6plex)	1421.8	0				
																	KADVEEESLALR	4	2	2	225543215;103472005	N-Term(TMT6plex); K1(TMT6plex)	1818.0	1				
																	KLTQTSGETTHTDKVPGGEDK	2	2	2	225543215;103472005	N-Term(TMT6plex); K1(TMT6plex); K14(TMT6plex); K21(TMT6plex)	3145.7	2				
																	ELFQTPGPSEESMTDEK	18	2	2	225543215;103472005	N-Term(TMT6plex); M13(Oxidation); K17(TMT6plex)	2399.2	0				
																	SPQPDPVK	6	2	2	225543215;103472005	N-Term(TMT6plex); K8(TMT6plex)	1325.8	0				
																	LDLTENLTGSK	9	2	2	225543215;103472005	N-Term(TMT6plex); K11(TMT6plex)	1648.9	0				
																	QLTQTTHTDK	2	2	2	225543215;103472005	N-Term(TMT6plex); K10(TMT6plex)	1630.9	0				
																	GKGEAGNSDSMCLR	6	2	2	225543215;103472005	N-Term(TMT6plex); K2(TMT6plex); M11(Oxidation); C12(Carbamidomethyl)	1956.0	1				
																	IPCESPPLEVVDTTASTK	11	2	2	225543215;103472005	N-Term(TMT6plex); C3(Carbamidomethyl); K18(TMT6plex)	2402.3	0				
																	ELFQTPCTDNPTTDEK	11	2	2	225543215;103472005	N-Term(TMT6plex); C7(Carbamidomethyl); K16(TMT6plex)	2354.2	0				
																	TSDTETEPSK	9	2	2	225543215;103472005	N-Term(TMT6plex); K10(TMT6plex)	1552.8	0				
																	EEVLPVGK	9	2	2	225543215;103472005	N-Term(TMT6plex); K8(TMT6plex)	1328.8	0				
																	IQLPVVSK	6	2	2	225543215;103472005	N-Term(TMT6plex); K8(TMT6plex)	1341.9	0				
																	QLTQTTHTDKVPGDEDK	4	2	2	225543215;103472005	N-Term(TMT6plex); K10(TMT6plex); K17(TMT6plex)	2600.4	1				
																	SWADVVK	4	2	2	225543215;103472005	N-Term(TMT6plex); K7(TMT6plex)	1262.8	0				
																	LKIPLVK	3	2	2	225543215;103472005	N-Term(TMT6plex); K2(TMT6plex); K7(TMT6plex)	1498.1	1				
																	EQPQLTSTCHIAISNSENLLGK	4	2	2	225543215;103472005	N-Term(TMT6plex); C9(Carbamidomethyl); K22(TMT6plex)	2898.5	0				
																	KPNPVEEEPSR	2	2	2	225543215;103472005	N-Term(TMT6plex); K1(TMT6plex)	1740.0	0				
																	GEAGNSDSMCLR	2	2	2	225543215;103472005	N-Term(TMT6plex); M9(Oxidation); C10(Carbamidomethyl)	1541.7	0				
																	SQHDILQMICSK	1	2	2	225543215;103472005	N-Term(TMT6plex); C10(Carbamidomethyl); K12(TMT6plex)	1918.0	0				
																	GHTEESMTNDK	3	2	2	225543215;103472005	N-Term(TMT6plex); K11(TMT6plex)	1706.8	0				
																	EEAQALEDLTGFK	8	2	2	225543215;103472005	N-Term(TMT6plex); K13(TMT6plex)	1909.0	0				
																	ELFQTPDHTEESTTDDK	3	2	2	225543215;103472005	N-Term(TMT6plex); K17(TMT6plex)	2451.2	0				
																	TSGETTHTHR	2	2	2	225543215;103472005	N-Term(TMT6plex)	1355.7	0				
																	VGVKEELLAVGK	1	2	2	225543215;103472005	N-Term(TMT6plex); K4(TMT6plex); K12(TMT6plex)	1929.2	1				
																	AEALEDLVGFK	8	2	2	225543215;103472005	N-Term(TMT6plex); K11(TMT6plex)	1649.9	0				
																	AQALEELTGFR	4	2	2	225543215;103472005	N-Term(TMT6plex)	1463.8	0				
																	HGDVITIIDR	2	2	2	225543215;103472005	N-Term(TMT6plex)	1367.8	0				
																	EEPLAVSK	3	2	2	225543215;103472005	N-Term(TMT6plex); K8(TMT6plex)	1330.8	0				
																	EVKEELSAVER	1	2	2	225543215;103472005	N-Term(TMT6plex); K3(TMT6plex)	1747.0	1				
																	VLMQNQK	1	2	2	225543215;103472005	N-Term(TMT6plex); M3(Oxidation); K7(TMT6plex)	1334.8	0				
																	EQPQPSGK	5	2	2	225543215;103472005	N-Term(TMT6plex); K8(TMT6plex)	1328.8	0				

Accession	RefSeq Accession	Gene Symbol	Entrez Gene ID	Description	Σ Coverage	Σ# Proteins	Σ# Unique Peptides	Σ# Peptides	Σ# PSMs	Het-1A-Smoke - 2M/Parental	Het-1A-Smoke - 4M/Parental	Het-1A-Smoke - 6M/Parental	Het-1A-Smoke - 8M/Parental	# AAs	MW [kDa]	calc. pI	Sequence	# PSMs	# Proteins	# Protein Groups	Protein Group Accessions	Modifications	MH+ [Da]	# Missed Cleavages	Het-1A-Smoke - 2M/Parental	Het-1A-Smoke - 4M/Parental	Het-1A-Smoke - 6M/Parental	Het-1A-Smoke - 8M/Parental
																	AMDTPKPAVSDEK	1	2	2	225543215;103472005	N-Term(TMT6plex); K6(TMT6plex); K13(TMT6plex)	2076.2	0				
																	MPCESSPPESADTPTSTR	1	2	2	225543215;103472005	N-Term(TMT6plex); M1(Oxidation); C3(Carbamidomethyl)	2195.0	0				
																	SSFSSDPDESEGIPLK	2	1	1	225543215	N-Term(TMT6plex); K16(TMT6plex)	2153.1	0	**1.2**	**1.4**	**1.4**	**1.4**
																	CAENPK	2	2	2	225543215;103472005	N-Term(TMT6plex); C1(Carbamidomethyl); K6(TMT6plex)	1176.6	0				
																	SQTEVPK	4	2	2	225543215;103472005	N-Term(TMT6plex); K7(TMT6plex)	1246.7	0				
																	QTPDSGKPLK	2	2	2	225543215;103472005	N-Term(TMT6plex); K7(TMT6plex); K10(TMT6plex)	1758.1	0				
																	SPQPESFK	2	2	2	225543215;103472005	N-Term(TMT6plex); K8(TMT6plex)	1377.8	0				
																	RQPQTPK	3	2	2	225543215;103472005	N-Term(TMT6plex); K7(TMT6plex)	1312.8	1				
																	NLMPSAGK	1	2	2	225543215;103472005	N-Term(TMT6plex); M3(Oxidation); K8(TMT6plex)	1291.7	0				
																	AFMGTPVQK	1	2	2	225543215;103472005	N-Term(TMT6plex); M3(Oxidation); K9(TMT6plex)	1452.8	0				
																	LTQTSGK	2	2	2	225543215;103472005	N-Term(TMT6plex); K7(TMT6plex)	1192.7	0				
																	ENIELK	1	4	3	225543215;190194412;103472005	N-Term(TMT6plex); K6(TMT6plex)	1203.7	0				
																	TAPASSSK	3	2	2	225543215;103472005	N-Term(TMT6plex); K8(TMT6plex)	1206.7	0				
																	ETAGDGK	2	2	2	225543215;103472005	N-Term(TMT6plex); K7(TMT6plex)	1135.6	0				
																	AFMESPK	2	2	2	225543215;103472005	N-Term(TMT6plex); M3(Oxidation); K7(TMT6plex)	1283.7	0				
																	EPAGDGK	3	2	2	225543215;103472005	N-Term(TMT6plex); K7(TMT6plex)	1131.6	0				
																	QILDPAAR	2	2	2	225543215;103472005	N-Term(TMT6plex)	1112.7	0				
																	ESMTNEK	2	2	2	225543215;103472005	N-Term(TMT6plex); M3(Oxidation); K7(TMT6plex)	1312.7	0				
																	ELFQTR	1	2	2	225543215;103472005	N-Term(TMT6plex)	1022.6	0				
																	SINAFR	1	2	2	225543215;103472005	N-Term(TMT6plex)	936.5	0				
																	QMLDPANYGTGMER	1	2	2	225543215;103472005	N-Term(TMT6plex); M12(Oxidation)	1827.9	0				
																	GEAPTK	1	2	2	225543215;103472005	N-Term(TMT6plex); K6(TMT6plex)	1060.6	0				
103472005	NP_002408.3	**MKI67**	4288	**antigen KI-67 isoform 1**	65.33	2	18	173	1249	**1.2**	**1.4**	**1.4**	**1.4**	3256	358.5	9.45												
																	SSPELEDTATSSK	10	2	2	225543215;103472005	N-Term(TMT6plex); K13(TMT6plex)	1809.9	0				
																	ELFQTPGHTEEAVAAGK	10	2	2	225543215;103472005	N-Term(TMT6plex); K17(TMT6plex)	2243.2	0				
																	TSPEMDIQNPDDGAR	9	2	2	225543215;103472005	N-Term(TMT6plex); M5(Oxidation)	1890.9	0				
																	GKSEVPEDLAGFIELFQTPSHTK	6	2	2	225543215;103472005	N-Term(TMT6plex); K2(TMT6plex); K23(TMT6plex)	3217.8	1				
																	KADVEEEFLAFR	10	2	2	225543215;103472005	N-Term(TMT6plex); K1(TMT6plex)	1912.1	1				
																	LDLPGNLPGSK	14	2	2	225543215;103472005	N-Term(TMT6plex); K11(TMT6plex)	1568.9	0				
																	QTPAPAASVTGSR	11	2	2	225543215;103472005	N-Term(TMT6plex)	1471.8	0				
																	EDLSGIAEMFK	7	2	2	225543215;103472005	N-Term(TMT6plex); M9(Oxidation); K11(TMT6plex)	1713.9	0				
																	GSIPTDVEVLPTETEIHNEPFLTLWLTQVER	8	1	1	103472005	N-Term(TMT6plex)	3793.0	0	**1.4**	**1.6**	**1.5**	**1.7**
																	EEPSAVK	4	2	2	225543215;103472005	N-Term(TMT6plex); K7(TMT6plex)	1217.7	0				
																	SSEPVVIMK	15	2	2	225543215;103472005	N-Term(TMT6plex); M8(Oxidation); K9(TMT6plex)	1463.9	0				
																	KADTEEEFLAFR	6	2	2	225543215;103472005	N-Term(TMT6plex); K1(TMT6plex)	1914.0	1				
																	KADVEEEFLALR	11	2	2	225543215;103472005	N-Term(TMT6plex); K1(TMT6plex)	1878.1	1				
																	CMPAPEEIVEELPASK	8	2	2	225543215;103472005	N-Term(TMT6plex); C1(Carbamidomethyl); M2(Oxidation); K16(TMT6plex)	2274.2	0				
																	QILDPAASVTGSR	8	2	2	225543215;103472005	N-Term(TMT6plex)	1543.9	0				
																	ADTEEEFLAFR	4	2	2	225543215;103472005	N-Term(TMT6plex)	1556.8	0				
																	QKLDPAASVTGSK	2	2	2	225543215;103472005	N-Term(TMT6plex); K2(TMT6plex); K13(TMT6plex)	1989.2	1				
																	SPQPDPVGTPTIFKPQSK	5	2	2	225543215;103472005	N-Term(TMT6plex); K14(TMT6plex); K18(TMT6plex)	2611.5	0				
																	QMLDPANYGTGMER	11	2	2	225543215;103472005	N-Term(TMT6plex); M2(Oxidation); M12(Oxidation)	1843.9	0				
																	KADVEGELLACR	5	2	2	225543215;103472005	N-Term(TMT6plex); K1(TMT6plex); C11(Carbamidomethyl)	1819.0	1				
																	EKAEALEDLVGFK	8	2	2	225543215;103472005	N-Term(TMT6plex); K2(TMT6plex); K13(TMT6plex)	2136.2	1				
																	VEVKEELLAVGK	5	2	2	225543215;103472005	N-Term(TMT6plex); K4(TMT6plex); K12(TMT6plex)	2001.3	1				
																	QESGSEIHVEVK	4	2	2	225543215;103472005	N-Term(TMT6plex); K12(TMT6plex)	1800.0	0				
																	VGVKEEVLPVGK	2	2	2	225543215;103472005	N-Term(TMT6plex); K4(TMT6plex); K12(TMT6plex)	1941.2	1				
																	KKPNPVEEEPSR	8	2	2	225543215;103472005	N-Term(TMT6plex); K1(TMT6plex); K2(TMT6plex)	2097.2	1				
																	ELFSAPGHTEESMTIDK	16	2	2	225543215;103472005	N-Term(TMT6plex); M13(Oxidation); K17(TMT6plex)	2366.2	0				
																	ASQPDLVDTPTSSKPQPK	8	2	2	225543215;103472005	N-Term(TMT6plex); K14(TMT6plex); K18(TMT6plex)	2583.5	0				
																	SQPDPVDTPTSSKPQSK	10	2	2	225543215;103472005	N-Term(TMT6plex); K13(TMT6plex); K17(TMT6plex)	2486.4	0				
																	DVESVQTPSK	5	1	1	103472005	N-Term(TMT6plex); K10(TMT6plex)	1547.9	0	**1.3**	**1.4**	**1.4**	**1.4**
																	EIERPFETYK	9	2	2	225543215;103472005	N-Term(TMT6plex); K10(TMT6plex)	1770.0	0				
																	AQPLEDLAGFTELSETSGHTQESLTAGK	27	2	2	225543215;103472005	N-Term(TMT6plex); K28(TMT6plex)	3375.7	0				
																	SGLQTDYATEK	8	1	1	103472005	N-Term(TMT6plex); K11(TMT6plex)	1670.9	0	**1.2**	**1.2**	**1.5**	**1.3**
																	LDLLGNLPGSK	54	2	2	225543215;103472005	N-Term(TMT6plex); K11(TMT6plex)	1585.0	0				
																	VLNNFISNQK	2	2	2	225543215;103472005	N-Term(TMT6plex); K10(TMT6plex)	1635.0	0				
																	AQALEDLAGFK	31	2	2	225543215;103472005	N-Term(TMT6plex); K11(TMT6plex)	1620.9	0				
																	GHTEESMTNDK	13	2	2	225543215;103472005	N-Term(TMT6plex); M7(Oxidation); K11(TMT6plex)	1722.8	0				
																	NAADPISGDFK	2	1	1	103472005	N-Term(TMT6plex); K11(TMT6plex)	1592.9	0	**1.2**	**1.2**	**1.4**	**1.3**
																	ESAQAIEDLAGFK	6	2	2	225543215;103472005	N-Term(TMT6plex); K13(TMT6plex)	1837.0	0				
																	MPCQSLQPEPINTPTHTK	8	2	2	225543215;103472005	N-Term(TMT6plex); M1(Oxidation); C3(Carbamidomethyl); K18(TMT6plex)	2553.3	0				
																	TSGETTQTHTEPTGDSK	12	2	2	225543215;103472005	N-Term(TMT6plex); K17(TMT6plex)	2235.1	0				
																	ELFQTPICTDKPTTHEK	7	2	2	225543215;103472005	N-Term(TMT6plex); C8(Carbamidomethyl); K11(TMT6plex); K17(TMT6plex)	2732.5	0				
																	KLTPSAGK	6	2	2	225543215;103472005	N-Term(TMT6plex); K1(TMT6plex); K8(TMT6plex)	1489.0	1				
																	KSGLQTDYATEK	5	1	1	103472005	N-Term(TMT6plex); K1(TMT6plex); K12(TMT6plex)	2028.2	1	**1.0**	**1.4**	**1.4**	**1.3**
																	DGSVTGTK	7	2	2	225543215;103472005	N-Term(TMT6plex); K8(TMT6plex)	1222.7	0				
																	ELFQTPGTDKPTTDEK	8	2	2	225543215;103472005	N-Term(TMT6plex); K10(TMT6plex); K16(TMT6plex)	2494.4	0				
																	ESADGLQGETQLLVSR	14	1	1	103472005	N-Term(TMT6plex)	1932.0	0	**1.3**	**1.3**	**1.4**	**1.3**
																	AMDTPKPAGGDEK	5	2	2	225543215;103472005	N-Term(TMT6plex); M2(Oxidation); K6(TMT6plex); K13(TMT6plex)	2020.1	0				
																	SPPPESVDTPTSTK	17	2	2	225543215;103472005	N-Term(TMT6plex); K14(TMT6plex)	1901.0	0				
																	IEPAEELNSNDMK	6	2	2	225543215;103472005	N-Term(TMT6plex); M12(Oxidation); K13(TMT6plex)	1964.0	0				
																	QFQGTDSGEEPLLPTSESFGGNVFFSAQNAAK	6	2	2	225543215;103472005	N-Term(TMT6plex); K32(TMT6plex)	3818.9	0				
																	SPQSDPADTPTNTK	14	2	2	225543215;103472005	N-Term(TMT6plex); K14(TMT6plex)	1917.0	0				
																	SPQPDPVDTPASTK	21	2	2	225543215;103472005	N-Term(TMT6plex); K14(TMT6plex)	1898.0	0				
																	VEPVGDVVSTR	6	2	2	225543215;103472005	N-Term(TMT6plex)	1386.8	0				
																	ADVEEEFLAFR	6	2	2	225543215;103472005	N-Term(TMT6plex)	1554.8	0				
																	EELSAVER	2	2	2	225543215;103472005	N-Term(TMT6plex)	1161.6	0				
																	LDLAGTLPGSK	9	2	2	225543215;103472005	N-Term(TMT6plex); K11(TMT6plex)	1529.9	0				
																	RIEPAEELNSNDMK	8	2	2	225543215;103472005	N-Term(TMT6plex); M13(Oxidation); K14(TMT6plex)	2120.1	1				
																	LDAEDVIGSR	2	2	2	225543215;103472005	N-Term(TMT6plex)	1303.7	0				
																	SPPPESMDTPTSTR	4	2	2	225543215;103472005	N-Term(TMT6plex); M7(Oxidation)	1747.8	0				
																	AMDTPKPAVSDEK	9	2	2	225543215;103472005	N-Term(TMT6plex); M2(Oxidation); K6(TMT6plex); K13(TMT6plex)	2092.2	0				
																	QILDSAASLTGSK	6	2	2	225543215;103472005	N-Term(TMT6plex); K13(TMT6plex)	1749.0	0				
																	SGASEANLIVAK	4	2	2	225543215;103472005	N-Term(TMT6plex); K12(TMT6plex)	1618.0	0				
																	ELFQTPGHTEELVAAGK	2	2	2	225543215;103472005	N-Term(TMT6plex); K17(TMT6plex)	2285.3	0				
																	GQNLLQTQDHAK	4	2	2	225543215;103472005	N-Term(TMT6plex); K12(TMT6plex)	1811.0	0				
																	SLVMHTPPVLK	2	2	2	225543215;103472005	N-Term(TMT6plex); M4(Oxidation); K11(TMT6plex)	1696.0	0				
																	LTQTSGQSTHTHKEPASGDEGIK	2	2	2	225543215;103472005	N-Term(TMT6plex); K13(TMT6plex); K23(TMT6plex)	3096.6	1				
																	FTQTSGETTDADKEPAGEDK	13	2	2	225543215;103472005	N-Term(TMT6plex); K13(TMT6plex); K20(TMT6plex)	2814.4	1				
																	LDQPGNLPGSNR	13	2	2	225543215;103472005	N-Term(TMT6plex)	1496.8	0				
																	SSEPVVIMK	2	2	2	225543215;103472005	N-Term(TMT6plex); K9(TMT6plex)	1447.9	0				
																	TPKEEAQSLEDLAGFK	2	2	2	225543215;103472005	N-Term(TMT6plex); K3(TMT6plex); K16(TMT6plex)	2450.4	1				
																	EDSTADDSKDSVAQGTTNVHSSEHAGR	4	1	1	103472005	N-Term(TMT6plex); K9(TMT6plex)	3259.5	1	**0.9**	**1.0**	**1.1**	**1.2**
																	AMHTPKPSVGEEK	1	2	2	225543215;103472005	N-Term(TMT6plex); M2(Oxidation); K6(TMT6plex); K13(TMT6plex)	2114.2	0				
																	AMHTPKPAVGEEK	1	2	2	225543215;103472005	N-Term(TMT6plex); M2(Oxidation); K6(TMT6plex); K13(TMT6plex)	2098.2	0				
																	KLDAEDVIGSR	8	2	2	225543215;103472005	N-Term(TMT6plex); K1(TMT6plex)	1661.0	1				
																	AQPLEDLAGLK	22	2	2	225543215;103472005	N-Term(TMT6plex); K11(TMT6plex)	1613.0	0				
																	TEAEQQITEVFVLAER	38	2	2	225543215;103472005	N-Term(TMT6plex)	2092.1	0				
																	EELLAVGK	10	2	2	225543215;103472005	N-Term(TMT6plex); K8(TMT6plex)	1316.8	0				
																	SEETNTEIVECILK	10	2	2	225543215;103472005	N-Term(TMT6plex); C11(Carbamidomethyl); K14(TMT6plex)	2123.1	0				
																	VAEESGGQK	6	2	2	225543215;103472005	N-Term(TMT6plex); K9(TMT6plex)	1362.8	0				
																	AQSLVISPPAPSPR	4	2	2	225543215;103472005	N-Term(TMT6plex)	1649.0	0				
																	KVDVEEEFFALR	8	2	2	225543215;103472005	N-Term(TMT6plex); K1(TMT6plex)	1940.1	1				
																	SNTSLPPLPFK	10	2	2	225543215;103472005	N-Term(TMT6plex); K11(TMT6plex)	1659.0	0				
																	LGTTAGQMCSGLPGLSSVDINNFGDSINESEGIPLK	2	1	1	103472005	N-Term(TMT6plex); M8(Oxidation); C9(Carbamidomethyl); K36(TMT6plex)	4153.1	0	**1.1**	**1.2**	**1.3**	**1.1**
																	DPAAGHTEESMTDDK	2	2	2	225543215;103472005	N-Term(TMT6plex); M11(Oxidation); K15(TMT6plex)	2078.0	0				
																	ALEDLVDFK	5	2	2	225543215;103472005	N-Term(TMT6plex); K9(TMT6plex)	1507.9	0				
																	DINTFLGTPVQK	8	2	2	225543215;103472005	N-Term(TMT6plex); K12(TMT6plex)	1791.0	0				
																	KAEDNVCVK	3	2	2	225543215;103472005	N-Term(TMT6plex); K1(TMT6plex); C7(Carbamidomethyl); K9(TMT6plex)	1750.0	1				
																	KPVGEVHSQFSTGHANSPCTIIIGK	1	2	2	225543215;103472005	N-Term(TMT6plex); K1(TMT6plex); C19(Carbamidomethyl); K25(TMT6plex)	3351.8	0				

Accession	RefSeq Accession	Gene Symbol	Entrez Gene ID	Description	Σ Coverage	Σ# Proteins	Σ# Unique Peptides	Σ# Peptides	Σ# PSMs	Het-1A-Smoke - 2M/Parental	Het-1A-Smoke - 4M/Parental	Het-1A-Smoke - 6M/Parental	Het-1A-Smoke - 8M/Parental	# AAs	MW [kDa]	calc. pI	Sequence	# PSMs	# Proteins	# Protein Groups	Protein Group Accessions	Modifications	MH+ [Da]	# Missed Cleavages	Het-1A-Smoke - 2M/Parental	Het-1A-Smoke - 4M/Parental	Het-1A-Smoke - 6M/Parental	Het-1A-Smoke - 8M/Parental
																	SGVDGPHFPLSLSTCLFGR	1	2	2	225543215;103472005	N-Term(TMT6plex); C15(Carbamidomethyl)	2276.2	0				
																	KSGNLPSK	1	2	2	225543215;103472005	N-Term(TMT6plex); K1(TMT6plex); K8(TMT6plex)	1518.0	1				
																	DIVEELSALK	20	2	2	225543215;103472005	N-Term(TMT6plex); K10(TMT6plex)	1574.9	0				
																	EEAQSLEDLAGFK	14	2	2	225543215;103472005	N-Term(TMT6plex); K13(TMT6plex)	1895.0	0				
																	ESAQAIEDLAGFKDPAAGHTEESMTDDK	5	2	2	225543215;103472005	N-Term(TMT6plex); K13(TMT6plex); M24(Oxidation); K28(TMT6plex)	3666.8	1				
																	LQDSVPENK	16	2	2	225543215;103472005	N-Term(TMT6plex); K9(TMT6plex)	1487.8	0				
																	AVGASFPLYEPAK	9	1	1	103472005	N-Term(TMT6plex); K13(TMT6plex)	1808.0	0	**1.1**	**1.5**	**1.5**	**1.4**
																	SVPTTQCLDNSK	8	1	1	103472005	N-Term(TMT6plex); C7(Carbamidomethyl); K12(TMT6plex)	1808.0	0	**1.1**	**1.3**	**1.5**	**1.2**
																	EPASGDEGIK	10	2	2	225543215;103472005	N-Term(TMT6plex); K10(TMT6plex)	1460.8	0				
																	ELFQTPGHTEESMTDDKITEVSCK	6	2	2	225543215;103472005	N-Term(TMT6plex); K17(TMT6plex); C23(Carbamidomethyl); K24(TMT6plex)	3469.7	1				
																	LTQTSGETTHTDKVPGGEDK	7	2	2	225543215;103472005	N-Term(TMT6plex); K13(TMT6plex); K20(TMT6plex)	2788.5	1				
																	LTQTSGETTHTDKEPVGEGK	8	2	2	225543215;103472005	N-Term(TMT6plex); K13(TMT6plex); K20(TMT6plex)	2802.5	1				
																	ELFQTPGHTEESMTDDKITEVSCK	12	2	2	225543215;103472005	N-Term(TMT6plex); M13(Oxidation); K17(TMT6plex); C23(Carbamidomethyl); K24(TMT6plex)	3485.7	1				
																	SSQPDPDKNPASSK	10	2	2	225543215;103472005	N-Term(TMT6plex); K8(TMT6plex); K14(TMT6plex)	2145.2	1				
																	VSFGGHLRPELFDENLPPNTPLK	4	2	2	225543215;103472005	N-Term(TMT6plex); K23(TMT6plex)	3035.7	0				
																	AFMESPK	4	2	2	225543215;103472005	N-Term(TMT6plex); K7(TMT6plex)	1267.7	0				
																	SLPDTELMK	5	2	2	225543215;103472005	N-Term(TMT6plex); M8(Oxidation); K9(TMT6plex)	1507.8	0				
																	MPCESSPPESADTPTSTR	4	2	2	225543215;103472005	N-Term(TMT6plex); C3(Carbamidomethyl)	2179.0	0				
																	ADVEGELLACR	1	2	2	225543215;103472005	N-Term(TMT6plex); C10(Carbamidomethyl)	1461.8	0				
																	SPPPELTDTATSTK	10	2	2	225543215;103472005	N-Term(TMT6plex); K14(TMT6plex)	1903.0	0				
																	DINTFVGTPVEK	10	2	2	225543215;103472005	N-Term(TMT6plex); K12(TMT6plex)	1778.0	0				
																	EDLSGIAEMFK	10	2	2	225543215;103472005	N-Term(TMT6plex); K11(TMT6plex)	1697.9	0				
																	LTQTSGETTHTHTEPTGDGK	6	2	2	225543215;103472005	N-Term(TMT6plex); K20(TMT6plex)	2556.3	0				
																	AFMGTPVQK	2	2	2	225543215;103472005	N-Term(TMT6plex); K9(TMT6plex)	1436.8	0				
																	ENVLQYCR	2	1	1	103472005	N-Term(TMT6plex); C7(Carbamidomethyl)	1310.7	0	**1.2**	**1.6**	**1.8**	**1.4**
																	IPCDSPQSDPVDTPTSTK	13	2	2	225543215;103472005	N-Term(TMT6plex); C3(Carbamidomethyl); K18(TMT6plex)	2403.2	0				
																	ELFQTPGHTEESMTDDK	9	2	2	225543215;103472005	N-Term(TMT6plex); M13(Oxidation); K17(TMT6plex)	2439.2	0				
																	AAVGEEK	4	2	2	225543215;103472005	N-Term(TMT6plex); K7(TMT6plex)	1161.7	0				
																	LDPAASVTGSK	14	2	2	225543215;103472005	N-Term(TMT6plex); K11(TMT6plex)	1503.9	0				
																	VSGNPQVHIK	2	1	1	103472005	N-Term(TMT6plex); K10(TMT6plex)	1536.9	0	**1.0**	**1.3**	**1.4**	**1.5**
																	SEVPEDLAGFIELFQTPSHTK	18	2	2	225543215;103472005	N-Term(TMT6plex); K21(TMT6plex)	2803.5	0				
																	SQPAASTLESK	8	2	2	225543215;103472005	N-Term(TMT6plex); K11(TMT6plex)	1576.9	0				
																	NINTFVETPVQK	9	2	2	225543215;103472005	N-Term(TMT6plex); K12(TMT6plex)	1848.1	0				
																	SGGSGHAVAEPASPEQELDQNK	10	1	1	103472005	N-Term(TMT6plex); K22(TMT6plex)	2666.3	0	**1.6**	**1.4**	**1.6**	**1.5**
																	DIIIFVGTPVQK	4	2	2	225543215;103472005	N-Term(TMT6plex); K12(TMT6plex)	1788.1	0				
																	ALEDLAGFK	6	2	2	225543215;103472005	N-Term(TMT6plex); K9(TMT6plex)	1421.8	0				
																	KADVEEESLALR	4	2	2	225543215;103472005	N-Term(TMT6plex); K1(TMT6plex)	1818.0	1				
																	KLTQTSGETTHTDKVPGGEDK	2	2	2	225543215;103472005	N-Term(TMT6plex); K1(TMT6plex); K14(TMT6plex); K21(TMT6plex)	3145.7	2				
																	ELFQTPGPSEESMTDEK	18	2	2	225543215;103472005	N-Term(TMT6plex); M13(Oxidation); K17(TMT6plex)	2399.2	0				
																	SPQPDPVK	6	2	2	225543215;103472005	N-Term(TMT6plex); K8(TMT6plex)	1325.8	0				
																	VEDAADSATKPENLSSK	7	1	1	103472005	N-Term(TMT6plex); K10(TMT6plex); K17(TMT6plex)	2449.3	0	**1.3**	**1.4**	**1.4**	**1.4**
																	LDLTENLTGSK	9	2	2	225543215;103472005	N-Term(TMT6plex); K11(TMT6plex)	1648.9	0				
																	QLTQTTHTDK	2	2	2	225543215;103472005	N-Term(TMT6plex); K10(TMT6plex)	1630.9	0				
																	GKGEAGNSDSMCLR	6	2	2	225543215;103472005	N-Term(TMT6plex); K2(TMT6plex); M11(Oxidation); C12(Carbamidomethyl)	1956.0	1				
																	IPCESPPLEVVDTTASTK	11	2	2	225543215;103472005	N-Term(TMT6plex); C3(Carbamidomethyl); K18(TMT6plex)	2402.3	0				
																	ELFQTPCTDNPTTDEK	11	2	2	225543215;103472005	N-Term(TMT6plex); C7(Carbamidomethyl); K16(TMT6plex)	2354.2	0				
																	TSDTETEPSK	9	2	2	225543215;103472005	N-Term(TMT6plex); K10(TMT6plex)	1552.8	0				
																	EEVLPVGK	9	2	2	225543215;103472005	N-Term(TMT6plex); K8(TMT6plex)	1328.8	0				
																	IQLPVVSK	6	2	2	225543215;103472005	N-Term(TMT6plex); K8(TMT6plex)	1341.9	0				
																	QLTQTTHTDKVPGDEDK	4	2	2	225543215;103472005	N-Term(TMT6plex); K10(TMT6plex); K17(TMT6plex)	2600.4	1				
																	SWADVVK	4	2	2	225543215;103472005	N-Term(TMT6plex); K7(TMT6plex)	1262.8	0				
																	LKIPLVK	3	2	2	225543215;103472005	N-Term(TMT6plex); K2(TMT6plex); K7(TMT6plex)	1498.1	1				
																	EQPQLTSTCHIAISNSENLLGK	4	2	2	225543215;103472005	N-Term(TMT6plex); C9(Carbamidomethyl); K22(TMT6plex)	2898.5	0				
																	KPNPVEEEPSR	2	2	2	225543215;103472005	N-Term(TMT6plex); K1(TMT6plex)	1740.0	0				
																	GEAGNSDSMCLR	2	2	2	225543215;103472005	N-Term(TMT6plex); M9(Oxidation); C10(Carbamidomethyl)	1541.7	0				
																	SQHDILQMICSK	1	2	2	225543215;103472005	N-Term(TMT6plex); C10(Carbamidomethyl); K12(TMT6plex)	1918.0	0				
																	GHTEESMTNDK	3	2	2	225543215;103472005	N-Term(TMT6plex); K11(TMT6plex)	1706.8	0				
																	EEAQALEDLTGFK	8	2	2	225543215;103472005	N-Term(TMT6plex); K13(TMT6plex)	1909.0	0				
																	ELFQTPDHTEESTTDDK	3	2	2	225543215;103472005	N-Term(TMT6plex); K17(TMT6plex)	2451.2	0				
																	TSGETTHTHR	2	2	2	225543215;103472005	N-Term(TMT6plex)	1355.7	0				
																	VGVKEELLAVGK	1	2	2	225543215;103472005	N-Term(TMT6plex); K4(TMT6plex); K12(TMT6plex)	1929.2	1				
																	AEALEDLVGFK	8	2	2	225543215;103472005	N-Term(TMT6plex); K11(TMT6plex)	1649.9	0				
																	AQALEELTGFR	4	2	2	225543215;103472005	N-Term(TMT6plex)	1463.8	0				
																	SSFSSDPDEK	4	1	1	103472005	N-Term(TMT6plex); K10(TMT6plex)	1556.8	0	**1.2**	**1.4**	**1.6**	**1.4**
																	HGDVITIIDR	2	2	2	225543215;103472005	N-Term(TMT6plex)	1367.8	0				
																	EEPLAVSK	3	2	2	225543215;103472005	N-Term(TMT6plex); K8(TMT6plex)	1330.8	0				
																	EVKEELSAVER	1	2	2	225543215;103472005	N-Term(TMT6plex); K3(TMT6plex)	1747.0	1				
																	VLMQNQK	1	2	2	225543215;103472005	N-Term(TMT6plex); M3(Oxidation); K7(TMT6plex)	1334.8	0				
																	EQPQPSGK	5	2	2	225543215;103472005	N-Term(TMT6plex); K8(TMT6plex)	1328.8	0				
																	AMDTPKPAVSDEK	1	2	2	225543215;103472005	N-Term(TMT6plex); K6(TMT6plex); K13(TMT6plex)	2076.2	0				
																	MPCESSPPESADTPTSTR	1	2	2	225543215;103472005	N-Term(TMT6plex); M1(Oxidation); C3(Carbamidomethyl)	2195.0	0				
																	CAENPK	2	2	2	225543215;103472005	N-Term(TMT6plex); C1(Carbamidomethyl); K6(TMT6plex)	1176.6	0				
																	SQTEVPK	4	2	2	225543215;103472005	N-Term(TMT6plex); K7(TMT6plex)	1246.7	0				
																	QTPDSGKPLK	2	2	2	225543215;103472005	N-Term(TMT6plex); K7(TMT6plex); K10(TMT6plex)	1758.1	0				
																	SPQPESFK	2	2	2	225543215;103472005	N-Term(TMT6plex); K8(TMT6plex)	1377.8	0				
																	RQPQTPK	3	2	2	225543215;103472005	N-Term(TMT6plex); K7(TMT6plex)	1312.8	1				
																	NLMPSAGK	1	2	2	225543215;103472005	N-Term(TMT6plex); M3(Oxidation); K8(TMT6plex)	1291.7	0				
																	AFMGTPVQK	1	2	2	225543215;103472005	N-Term(TMT6plex); M3(Oxidation); K9(TMT6plex)	1452.8	0				
																	LTQTSGK	2	2	2	225543215;103472005	N-Term(TMT6plex); K7(TMT6plex)	1192.7	0				
																	ESVNLGK	3	1	1	103472005	N-Term(TMT6plex); K7(TMT6plex)	1204.7	0	**1.2**	**1.5**	**1.5**	**1.4**
																	ENIELK	1	4	3	225543215;190194412;103472005	N-Term(TMT6plex); K6(TMT6plex)	1203.7	0				
																	TAPASSSK	3	2	2	225543215;103472005	N-Term(TMT6plex); K8(TMT6plex)	1206.7	0				
																	ETAGDGK	2	2	2	225543215;103472005	N-Term(TMT6plex); K7(TMT6plex)	1135.6	0				
																	EISSVK	2	1	2	103472005;9910266	N-Term(TMT6plex); K6(TMT6plex)	1120.7	0				
																	AFMESPK	2	2	2	225543215;103472005	N-Term(TMT6plex); M3(Oxidation); K7(TMT6plex)	1283.7	0				
																	EPAGDGK	3	2	2	225543215;103472005	N-Term(TMT6plex); K7(TMT6plex)	1131.6	0				
																	QILDPAAR	2	2	2	225543215;103472005	N-Term(TMT6plex)	1112.7	0				
																	ESMTNEK	2	2	2	225543215;103472005	N-Term(TMT6plex); M3(Oxidation); K7(TMT6plex)	1312.7	0				
																	ELFQTR	1	2	2	225543215;103472005	N-Term(TMT6plex)	1022.6	0				
																	NVKEDSTADDSK	1	1	1	103472005	N-Term(TMT6plex); K3(TMT6plex); K12(TMT6plex)	1996.1	1	**1.3**	**1.6**	**1.4**	**1.4**
																	EDSTADDSK	1	1	1	103472005	N-Term(TMT6plex); K9(TMT6plex)	1425.7	0	**1.0**	**1.1**	**1.3**	**1.0**
																	SINAFR	1	2	2	225543215;103472005	N-Term(TMT6plex)	936.5	0				
																	QMLDPANYGTGMER	1	2	2	225543215;103472005	N-Term(TMT6plex); M12(Oxidation)	1827.9	0				
																	GEAPTK	1	2	2	225543215;103472005	N-Term(TMT6plex); K6(TMT6plex)	1060.6	0				
543173169;11321585	NP_001269468.1	**GNB1**	2782	**guanine nucleotide-binding protein G(I)/G(S)/G(T) subunit beta-1 isoform 1**	65.29	2	7	14	120	**1.3**	**1.5**	**1.4**	**1.5**	340	37.4	6.00												
																	ACADATLSQITNNIDPVGR	14	1	1	543173169;11321585	N-Term(TMT6plex); C2(Carbamidomethyl)	2245.1	0	**1.2**	**1.4**	**1.4**	**1.4**
																	LLVSASQDGK	20	4	4	20357529;11055998;4504053;543173169;11321585	N-Term(TMT6plex); K10(TMT6plex)	1475.9	0				
																	IYAMHWGTDSR	1	2	2	20357529;543173169;11321585	N-Term(TMT6plex)	1565.8	0				
																	LFVSGACDASAK	4	2	1	543173169;11321585	N-Term(TMT6plex); C7(Carbamidomethyl); K12(TMT6plex)	1683.9	0	**1.1**	**1.0**	**1.1**	**1.1**
																	ELAGHTGYLSCCR	2	2	1	543173169;11321585	N-Term(TMT6plex); C11(Carbamidomethyl); C12(Carbamidomethyl)	1752.8	0	**1.0**	**1.5**	**1.1**	**1.6**
																	AGVLAGHDNR	10	4	3	20357529;11055998;543173169;11321585	N-Term(TMT6plex)	1238.7	0				
																	LIIWDSYTTNK	30	3	3	20357529;11055998;543173169;11321585	N-Term(TMT6plex); K11(TMT6plex)	1812.0	0				
																	QTFTGHESDINAICFFPNGNAFATGSDDATCR	2	2	1	543173169;11321585	N-Term(TMT6plex); C14(Carbamidomethyl); C31(Carbamidomethyl)	3750.6	0	**2.3**	**2.9**	**2.2**	**2.7**
																	IYAMHWGTDSR	3	2	2	20357529;543173169;11321585	N-Term(TMT6plex); M4(Oxidation)	1581.8	0				
																	VSCLGVTDDGMAVATGSWDSFLK	13	3	2	20357529;543173169;11321585	N-Term(TMT6plex); C3(Carbamidomethyl); M11(Oxidation); K23(TMT6plex)	2890.4	0				
																	KACADATLSQITNNIDPVGR	5	1	1	543173169;11321585	N-Term(TMT6plex); K1(TMT6plex); C3(Carbamidomethyl)	2602.4	1	**1.2**	**1.3**	**1.4**	**1.4**
																	SSWVMTCAYAPSGNYVACGGLDNICSIYNLK	4	2	2	11055998;543173169;11321585	N-Term(TMT6plex); M5(Oxidation); C7(Carbamidomethyl); C18(Carbamidomethyl); C25(Carbamidomethyl); K31(TMT6plex)	3945.9	0				
																	VSCLGVTDDGMAVATGSWDSFLK	4	3	2	20357529;543173169;11321585	N-Term(TMT6plex); C3(Carbamidomethyl); K23(TMT6plex)	2874.5	0				
																	LLLAGYDDFNCNVWDALK	2	2	1	543173169;11321585	N-Term(TMT6plex); C11(Carbamidomethyl); K18(TMT6plex)	2585.3	0	**1.4**	**1.3**	**1.4**	**1.3**
																	ADQELMTYSHDNIICGITSVSFSK	2	2	1	543173169;11321585	N-Term(TMT6plex); M6(Oxidation); C15(Carbamidomethyl); K24(TMT6plex)	3190.6	0	**1.1**	**1.2**	**1.3**	**1.4**

Accession	RefSeq Accession	Gene Symbol	Entrez Gene ID	Description	Σ Coverage	Σ# Proteins	Σ# Unique Peptides	Σ# Peptides	Σ# PSMs	Het-1A-Smoke - 2M/Parental	Het-1A-Smoke - 4M/Parental	Het-1A-Smoke - 6M/Parental	Het-1A-Smoke - 8M/Parental	# AAs	MW [kDa]	calc. pI	Sequence	# PSMs	# Proteins	# Protein Groups	Protein Group Accessions	Modifications	MH+ [Da]	# Missed Cleavages	Het-1A-Smoke - 2M/Parental	Het-1A-Smoke - 4M/Parental	Het-1A-Smoke - 6M/Parental	Het-1A-Smoke - 8M/Parental
																	QEAEQLK	4	2	2	4504053;543173169;11321585	N-Term(TMT6plex); K7(TMT6plex)	1303.8	0				
111494251;28395033	NP_001036144.1	**RHOC**	389	**rho-related GTP-binding protein RhoC precursor**	65.28	4	2	9	136	**1.2**	**1.4**	**1.5**	**1.3**	193	22.0	6.58												
																	DQFPEVYVPTVFENYIADIEVDGK	43	1	1	111494251;28395033	N-Term(TMT6plex); K24(TMT6plex)	3245.7	0	**1.1**	**1.4**	**1.5**	**1.4**
																	LVIVGDGACGK	13	2	2	111494251;28395033;10835049	N-Term(TMT6plex); C9(Carbamidomethyl); K11(TMT6plex)	1546.9	0				
																	ISAFGYLECSAK	12	1	1	111494251;28395033	N-Term(TMT6plex); C9(Carbamidomethyl); K12(TMT6plex)	1804.0	0	**1.2**	**1.3**	**1.5**	**1.2**
																	TCLLIVFSK	6	3	3	111494251;28395033;4757764;10835049	N-Term(TMT6plex); C2(Carbamidomethyl); K9(TMT6plex)	1538.9	0				
																	HFCPNVPIILVGNK	18	5	2	111494251;28395033;10835049	N-Term(TMT6plex); C3(Carbamidomethyl); K14(TMT6plex)	2066.2	0				
																	LRPLSYPDTDVILMCFSIDSPDSLENIPEK	3	4	2	111494251;28395033;10835049	N-Term(TMT6plex); C15(Carbamidomethyl); K30(TMT6plex)	3923.0	0				
																	QVELALWDTAGQEDYDR	18	4	3	111494251;28395033;4757764;10835049	N-Term(TMT6plex)	2238.1	0				
																	KLVIVGDGACGK	5	2	2	111494251;28395033;10835049	N-Term(TMT6plex); K1(TMT6plex); C10(Carbamidomethyl); K12(TMT6plex)	1904.2	1				
																	EVFEMATR	2	2	2	111494251;28395033;10835049	N-Term(TMT6plex)	1211.6	0				
																	LRPLSYPDTDVILMCFSIDSPDSLENIPEK	8	4	2	111494251;28395033;10835049	N-Term(TMT6plex); M14(Oxidation); C15(Carbamidomethyl); K30(TMT6plex)	3939.0	0				
																	EVFEMATR	8	2	2	111494251;28395033;10835049	N-Term(TMT6plex); M5(Oxidation)	1227.6	0				
20986531;66932916	NP_620407.1	**MAPK1**	5594	**mitogen-activated protein kinase 1**	65.28	11	12	18	98	**1.2**	**1.3**	**1.4**	**1.3**	360	41.4	6.98												
																	HENIIGINDIIR	8	1	1	20986531;66932916	N-Term(TMT6plex)	1635.9	0	**1.2**	**1.2**	**1.3**	**1.2**
																	DVYIVQDLMETDLYK	6	4	2	91718899;20986531;66932916	N-Term(TMT6plex); K15(TMT6plex)	2303.2	0				
																	APTIEQMK	2	1	1	20986531;66932916	N-Term(TMT6plex); M7(Oxidation); K8(TMT6plex)	1391.8	0	**1.3**	**1.2**	**1.3**	**1.3**
																	DVYIVQDLMETDLYK	20	4	2	91718899;20986531;66932916	N-Term(TMT6plex); M9(Oxidation); K15(TMT6plex)	2319.2	0				
																	IEVEQALAHPYLEQYYDPSDEPIAEAPFK	10	1	1	20986531;66932916	N-Term(TMT6plex); K29(TMT6plex)	3820.9	0	**1.2**	**1.3**	**1.5**	**1.3**
																	SIDIWSVGCILAEMLSNRPIFPGK	4	4	2	91718899;20986531;66932916	N-Term(TMT6plex); C9(Carbamidomethyl); M14(Oxidation); K24(TMT6plex)	3177.7	0				
																	LFPNADSK	3	1	1	20986531;66932916	N-Term(TMT6plex); K8(TMT6plex)	1349.8	0	**1.2**	**1.2**	**1.4**	**1.2**
																	YTNLSYIGEGAYGMVCSAYDNVNK	2	1	1	20986531;66932916	N-Term(TMT6plex); M14(Oxidation); C16(Carbamidomethyl); K24(TMT6plex)	3163.5	0	**1.4**	**1.3**	**1.3**	**1.6**
																	APEIMLNSK	5	4	2	91718899;20986531;66932916	N-Term(TMT6plex); M5(Oxidation); K9(TMT6plex)	1476.8	0				
																	DLKPSNLLLNTTCDLK	1	4	2	20986531;66932916;91718899	N-Term(TMT6plex); K3(TMT6plex); C13(Carbamidomethyl); K16(TMT6plex)	2532.5	0				
																	FDMELDDLPK	9	1	1	20986531;66932916	N-Term(TMT6plex); M3(Oxidation); K10(TMT6plex)	1696.9	0	**1.3**	**1.2**	**1.3**	**1.3**
																	GQVFDVGPR	6	1	1	20986531;66932916	N-Term(TMT6plex)	1203.7	0	**1.3**	**1.3**	**1.3**	**1.3**
																	FDMELDDLPK	2	1	1	20986531;66932916	N-Term(TMT6plex); K10(TMT6plex)	1680.9	0	**1.7**	**1.5**	**1.9**	**1.2**
																	NYLLSLPHK	3	1	1	20986531;66932916	N-Term(TMT6plex); K9(TMT6plex)	1542.9	0	**1.3**	**1.4**	**1.5**	**1.4**
																	TQHLSNDHICYFLYQILR	2	1	1	20986531;66932916	N-Term(TMT6plex); C10(Carbamidomethyl)	2550.3	0	**1.0**	**1.4**	**1.5**	**1.6**
																	ELIFEETAR	2	1	1	20986531;66932916	N-Term(TMT6plex)	1336.7	0	**1.2**	**1.2**	**1.3**	**1.3**
																	ALDLLDK	6	1	1	20986531;66932916	N-Term(TMT6plex); K7(TMT6plex)	1245.8	0	**1.3**	**1.4**	**1.5**	**1.3**
																	ISPFEHQTYCQR	2	4	2	91718899;20986531;66932916	N-Term(TMT6plex); C10(Carbamidomethyl)	1794.9	0				
																	ICDFGLAR	2	15	3	91718899;20986531;66932916;4505683	N-Term(TMT6plex); C2(Carbamidomethyl)	1180.6	0				
																	MLTFNPHK	1	1	1	20986531;66932916	N-Term(TMT6plex); M1(Oxidation); K8(TMT6plex)	1461.8	0	**1.2**	**1.3**	**1.4**	**1.4**
																	APEIMLNSK	2	4	2	91718899;20986531;66932916	N-Term(TMT6plex); K9(TMT6plex)	1460.9	0				
4503727	NP_002004.1	**FKBP3**	2287	**peptidyl-prolyl cis-trans isomerase FKBP3**	65.18	1	14	14	188	**1.1**	**1.1**	**1.1**	**1.2**	224	25.2	9.28												
																	DHLVTAYNHLFETK	8	1	1	4503727	N-Term(TMT6plex); K14(TMT6plex)	2146.2	0	**0.8**	**0.9**	**1.0**	**1.0**
																	LTFEVELVDID	28	1	1	4503727	N-Term(TMT6plex)	1521.8	0	**1.3**	**1.4**	**1.3**	**1.5**
																	LEIEPEWAYGK	42	1	1	4503727	N-Term(TMT6plex); K11(TMT6plex)	1793.0	0	**1.0**	**1.0**	**1.0**	**1.2**
																	SEETLDEGPPK	35	1	1	4503727	N-Term(TMT6plex); K11(TMT6plex)	1659.9	0	**1.2**	**1.2**	**1.1**	**1.3**
																	AWTVEQLR	3	1	1	4503727	N-Term(TMT6plex)	1231.7	0	**1.1**	**1.2**	**1.1**	**1.2**
																	FLQEHGSDSFLAEHK	8	1	1	4503727	N-Term(TMT6plex); K15(TMT6plex)	2203.2	0	**1.0**	**1.3**	**1.1**	**1.3**
																	GWDEALLTMSK	22	1	1	4503727	N-Term(TMT6plex); K11(TMT6plex)	1708.9	0	**1.2**	**1.4**	**1.4**	**1.1**
																	KGDVVHCWYTGTLQDGTVFDTNIQTSAK	11	1	1	4503727	N-Term(TMT6plex); K1(TMT6plex); C7(Carbamidomethyl); K28(TMT6plex)	3829.0	1	**0.9**	**1.1**	**1.1**	**1.1**
																	GWDEALLTMSK	10	1	1	4503727	N-Term(TMT6plex); M9(Oxidation); K11(TMT6plex)	1724.9	0	**1.1**	**1.3**	**1.2**	**1.3**
																	NAKPLSFK	5	1	1	4503727	N-Term(TMT6plex); K3(TMT6plex); K8(TMT6plex)	1592.0	0	**0.9**	**0.9**	**0.9**	**0.9**
																	FKGTESISK	2	1	1	4503727	N-Term(TMT6plex); K2(TMT6plex); K9(TMT6plex)	1684.0	1	**1.0**	**1.3**	**1.2**	**1.4**
																	LNEDKPK	4	1	1	4503727	N-Term(TMT6plex); K5(TMT6plex); K7(TMT6plex)	1530.9	0	**1.0**	**1.0**	**1.0**	**1.1**
																	GTESISK	5	1	1	4503727	N-Term(TMT6plex); K7(TMT6plex)	1179.7	0	**0.8**	**1.0**	**0.9**	**1.0**
																	VSEQVK	3	1	1	4503727	N-Term(TMT6plex); K6(TMT6plex)	1147.7	0	**0.9**	**0.8**	**0.9**	**0.9**
																	KGQPDAK	2	1	1	4503727	N-Term(TMT6plex); K1(TMT6plex); K7(TMT6plex)	1430.9	1	**0.8**	**0.9**	**0.8**	**1.0**
38569423	NP_942127.1	**ACLY**	47	**ATP-citrate synthase isoform 2**	65.17	3	1	57	944	**1.1**	**1.7**	**1.5**	**1.3**	1091	119.7	7.33												
																	DLVSSLTSGLLTIGDR	73	2	2	38569423;38569421	N-Term(TMT6plex)	1876.1	0				
																	MIVVLGEIGGTEEYK	12	2	2	38569423;38569421	N-Term(TMT6plex); M1(Oxidation); K15(TMT6plex)	2112.2	0				
																	EEADEYIDIGALNGIFVLGR	9	2	2	38569423;38569421	N-Term(TMT6plex)	2423.3	0				
																	LGLVGVNLTLDGVK	30	2	2	38569423;38569421	N-Term(TMT6plex); K14(TMT6plex)	1856.2	0				
																	EAYPEEAYIADLDAK	30	2	2	38569423;38569421	N-Term(TMT6plex); K15(TMT6plex)	2156.1	0				
																	AFDSGIIPMEFVNK	48	4	2	38569423;38569421	N-Term(TMT6plex); M9(Oxidation); K14(TMT6plex)	2042.1	0				
																	WGDIEFPPPFGR	49	2	2	38569423;38569421	N-Term(TMT6plex)	1646.9	0				
																	LLVGVDEK	8	2	2	38569423;38569421	N-Term(TMT6plex); K8(TMT6plex)	1330.8	0				
																	YQDTPGVK	14	2	2	38569423;38569421	N-Term(TMT6plex); K8(TMT6plex)	1365.8	0				
																	QHFPATPLLDYALEVEK	20	2	2	38569423;38569421	N-Term(TMT6plex); K17(TMT6plex)	2429.4	0				
																	HPWDDISYVLPEHMSM	8	4	2	38569423;38569421	N-Term(TMT6plex); M14(Oxidation); M16(Oxidation)	2218.0	0				
																	AISEQTGK	10	2	2	38569423;38569421	N-Term(TMT6plex); K8(TMT6plex)	1291.8	0				
																	VTPDTDWAR	10	2	2	38569423;38569421	N-Term(TMT6plex)	1289.7	0				
																	VDATADYICK	9	2	2	38569423;38569421	N-Term(TMT6plex); C9(Carbamidomethyl); K10(TMT6plex)	1613.9	0				
																	SAYDSTMETMNYAQIR	36	4	2	38569423;38569421	N-Term(TMT6plex); M7(Oxidation); M10(Oxidation)	2142.0	0				
																	TIAIIAEGIPEALTR	55	4	2	38569423;38569421	N-Term(TMT6plex)	1797.1	0				
																	EEMGIGGVLGLLWFQK	21	4	2	38569423;38569421	N-Term(TMT6plex); M3(Oxidation); K16(TMT6plex)	2251.3	0				
																	AVQGMLDFDYVCSR	23	4	2	38569423;38569421	N-Term(TMT6plex); M5(Oxidation); C12(Carbamidomethyl)	1905.9	0				
																	SGGMSNELNNIISR	5	2	2	38569423;38569421	N-Term(TMT6plex)	1720.9	0				
																	ILIIGGSIANFTNVAATFK	6	2	2	38569423;38569421	N-Term(TMT6plex); K19(TMT6plex)	2408.4	0				
																	EILIPVFK	28	4	2	38569423;38569421	N-Term(TMT6plex); K8(TMT6plex)	1416.9	0				
																	SMGFIGHYLDQK	22	4	2	38569423;38569421	N-Term(TMT6plex); M2(Oxidation); K12(TMT6plex)	1870.0	0				
																	EAGVFVPR	10	4	2	38569423;38569421	N-Term(TMT6plex)	1103.6	0				
																	ADEVAPAK	13	2	2	38569423;38569421	N-Term(TMT6plex); K8(TMT6plex)	1258.7	0				
																	DEPSVAAMVYPFTGDHK	10	2	2	38569423;38569421	N-Term(TMT6plex); M8(Oxidation); K17(TMT6plex)	2338.2	0				
																	GVTIIGPATVGGIKPGCFK	14	2	2	38569423;38569421	N-Term(TMT6plex); K14(TMT6plex); C17(Carbamidomethyl); K19(TMT6plex)	2559.5	0				
																	EGDYVLFHHEGGVDVGDVDAK	6	2	2	38569423;38569421	N-Term(TMT6plex); K21(TMT6plex)	2716.4	0				
																	SINNPDMR	9	2	2	38569423;38569421	N-Term(TMT6plex); M7(Oxidation)	1191.6	0				
																	SGGMSNELNNIISR	12	2	2	38569423;38569421	N-Term(TMT6plex); M4(Oxidation)	1736.9	0				
																	YPGSTFMDHVLR	13	2	2	38569423;38569421	N-Term(TMT6plex); M7(Oxidation)	1667.8	0				
																	EEMGIGGVLGLLWFQK	4	4	2	38569423;38569421	N-Term(TMT6plex); K16(TMT6plex)	2235.3	0				
																	SAYDSTMETMNYAQIR	12	4	2	38569423;38569421	N-Term(TMT6plex); M10(Oxidation)	2126.0	0				
																	KHPEVDVLINFASLR	8	2	2	38569423;38569421	N-Term(TMT6plex); K1(TMT6plex)	2196.3	1				
																	KPASFMTSICDER	1	2	2	38569423;38569421	N-Term(TMT6plex); K1(TMT6plex); C10(Carbamidomethyl)	2000.0	0				
																	AVQGMLDFDYVCSR	7	4	2	38569423;38569421	N-Term(TMT6plex); C12(Carbamidomethyl)	1889.9	0				
																	LGQEATVGK	17	2	2	38569423;38569421	N-Term(TMT6plex); K9(TMT6plex)	1360.8	0				
																	YPGSTFMDHVLR	6	2	2	38569423;38569421	N-Term(TMT6plex)	1651.8	0				
																	TTDGVYEGVAIGGDR	43	2	2	38569423;38569421	N-Term(TMT6plex)	1738.9	0				
																	IGNTGGMLDNILASK	37	4	2	38569423;38569421	N-Term(TMT6plex); M7(Oxidation); K15(TMT6plex)	1978.1	0				
																	LLQDHPWLLSQNLVVKPDQLIK	23	2	2	38569423;38569421	N-Term(TMT6plex); K16(TMT6plex); K22(TMT6plex)	3284.9	0				
																	FICTTSAIQNR	16	2	2	38569423;38569421	N-Term(TMT6plex); C3(Carbamidomethyl)	1539.8	0				
																	FGGALDAAAK	16	2	2	38569423;38569421	N-Term(TMT6plex); K10(TMT6plex)	1378.8	0				
																	EHEVTIFVR	9	2	2	38569423;38569421	N-Term(TMT6plex)	1358.8	0				
																	GQELIYAGMPITEVFK	12	2	2	38569423;38569421	N-Term(TMT6plex); K16(TMT6plex)	2254.3	0				
																	GQELIYAGMPITEVFK	14	2	2	38569423;38569421	N-Term(TMT6plex); M9(Oxidation); K16(TMT6plex)	2270.2	0				
																	DGVYVLDLAAK	16	2	2	38569423;38569421	N-Term(TMT6plex); K11(TMT6plex)	1622.0	0				
																	LYRPGSVAYVSR	2	2	2	38569423;38569421	N-Term(TMT6plex)	1596.9	0				
																	HLLVHAPEDK	11	4	2	38569423;38569421	N-Term(TMT6plex); K10(TMT6plex)	1617.0	0				
																	AFDSGIIPMEFVNK	8	4	2	38569423;38569421	N-Term(TMT6plex); K14(TMT6plex)	2026.1	0				
																	KPASFMTSICDER	4	2	2	38569423;38569421	K1(TMT6plex); N-Term(TMT6plex); M6(Oxidation); C10(Carbamidomethyl)	2016.0	0				
																	DYQGPLK	6	2	2	38569423;38569421	N-Term(TMT6plex); K7(TMT6plex)	1278.7	0				
																	AIVWGMQTR	5	2	2	38569423;38569421	N-Term(TMT6plex); M6(Oxidation)	1306.7	0				
																	MIVVLGEIGGTEEYK	3	2	2	38569423;38569421	N-Term(TMT6plex); K15(TMT6plex)	2096.2	0				
																	HPWDDISYVLPEHMSM	6	4	2	38569423;38569421	N-Term(TMT6plex); M16(Oxidation)	2202.0	0				
																	LTLLNPK	16	2	2	38569423;38569421	N-Term(TMT6plex); K7(TMT6plex)	1256.8	0				
																	LNPEDIK	5	2	2	38569423;38569421	N-Term(TMT6plex); K7(TMT6plex)	1286.8	0				
																	TILSLMTR	5	2	2	38569423;38569421	N-Term(TMT6plex)	1163.7	0				
																	FYWGHK	2	2	2	38569423;38569421	N-Term(TMT6plex); K6(TMT6plex)	1295.7	0				
																	LNPEDIKK	4	2	2	38569423;38569421	N-Term(TMT6plex); K7(TMT6plex); K8(TMT6plex)	1644.0	1				
																	VMGEVGK	2	2	2	38569423;38569421	N-Term(TMT6plex); K7(TMT6plex)	1177.7	0				

Khan et al., 2019. Multi-omics analysis to characterize cigarette smoke induced molecular alterations in esophageal cells
Supplementary Table 6. List of all proteins and peptides identified in untreated and chronically treated Het1A cells with cigarette smoke condensate for 8 months

Accession	RefSeq Accession	Gene Symbol	Entrez Gene ID	Description	Σ Coverage	Σ# Proteins	Σ# Unique Peptides	Σ# Peptides	Σ# PSMs	Het-1A-Smoke - 2M/Parental	Het-1A-Smoke - 4M/Parental	Het-1A-Smoke - 6M/Parental	Het-1A-Smoke - 8M/Parental	# AAs	MW [kDa]	calc. pI	Sequence	# PSMs	# Proteins	# Protein Groups	Protein Group Accessions	Modifications	MH+ [Da]	# Missed Cleavages	Het-1A-Smoke - 2M/Parental	Het-1A-Smoke - 4M/Parental	Het-1A-Smoke - 6M/Parental	Het-1A-Smoke - 8M/Parental
																	KPNLILNVDGLIGVAFVDMLR	1	2	2	38569423;38569421	N-Term(TMT6plex); K1(TMT6plex); M19(Oxidation)	2771.6	0				
																	AKPAMPQGK	1	1	1	38569423	N-Term(TMT6plex); K2(TMT6plex); M5(Oxidation); K9(TMT6plex)	1631.0	0	**1.1**	**1.7**	**1.5**	**1.3**
																	ELGLIR	2	2	2	38569423;38569421	N-Term(TMT6plex)	929.6	0				
																	IWTMVAGGGASVVYSDTICDLGGVNELANYGEYSGAPSEQQTYDYAK	1	2	2	38569423;38569421	N-Term(TMT6plex); M4(Oxidation); C19(Carbamidomethyl); K47(TMT6plex)	5481.6	0				
																	TILSLMTR	2	2	2	38569423;38569421	N-Term(TMT6plex); M6(Oxidation)	1179.7	0				
																	ATGFLK	2	2	2	38569423;38569421	N-Term(TMT6plex); K6(TMT6plex)	1094.7	0				
																	SWLKPR	1	2	2	38569423;38569421	N-Term(TMT6plex); K4(TMT6plex)	1244.8	0				
																	EKHPDGK	1	2	2	38569423;38569421	N-Term(TMT6plex); K2(TMT6plex); K7(TMT6plex)	1497.9	1				
[truncated: 10,498,053 more chars]
